# iSS-PseDNC: Identifying Splicing Sites Using Pseudo Dinucleotide Composition

**DOI:** 10.1155/2014/623149

**Published:** 2014-05-21

**Authors:** Wei Chen, Peng-Mian Feng, Hao Lin, Kuo-Chen Chou

**Affiliations:** ^1^Department of Physics, School of Sciences, Center for Genomics and Computational Biology, Hebei United University, Tangshan 063000, China; ^2^Gordon Life Science Institute, Boston, MA 02478, USA; ^3^School of Public Health, Hebei United University, Tangshan 063000, China; ^4^Key Laboratory for Neuro-Information of Ministry of Education, Center of Bioinformatics, School of Life Science and Technology, University of Electronic Science and Technology of China, Chengdu 610054, China; ^5^Center of Excellence in Genomic Medicine Research (CEGMR), King Abdulaziz University, Jeddah 21589, Saudi Arabia

## Abstract

In eukaryotic genes, exons are generally interrupted by introns. Accurately removing introns and joining exons together are essential processes in eukaryotic gene expression. With the avalanche of genome sequences generated in the postgenomic age, it is highly desired to develop automated methods for rapid and effective detection of splice sites that play important roles in gene structure annotation and even in RNA splicing. Although a series of computational methods were proposed for splice site identification, most of them neglected the intrinsic local structural properties. In the present study, a predictor called “iSS-PseDNC” was developed for identifying splice sites. In the new predictor, the sequences were formulated by a novel feature-vector called “pseudo dinucleotide composition” (PseDNC) into which six DNA local structural properties were incorporated. It was observed by the rigorous cross-validation tests on two benchmark datasets that the overall success rates achieved by iSS-PseDNC in identifying splice donor site and splice acceptor site were 85.45% and 87.73%, respectively. It is anticipated that iSS-PseDNC may become a useful tool for identifying splice sites and that the six DNA local structural properties described in this paper may provide novel insights for in-depth investigations into the mechanism of RNA splicing.

## 1. Introduction

**Figure 1:**
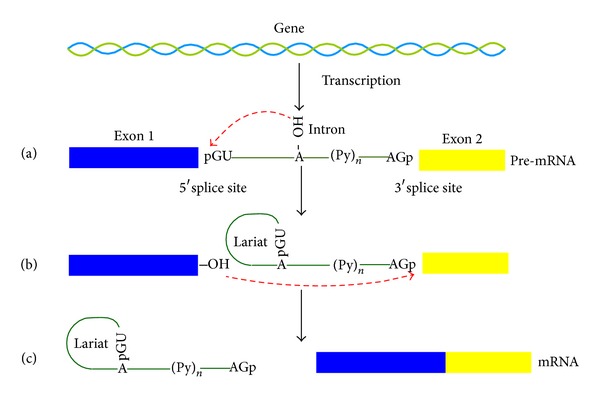
A schematic drawing to show the pathways of RNA splicing. (a) The 2′OH of the branchpoint nucleotide within the intron (solid line) carries out a nucleophilic attack at the first nucleotide of the intron at the 5′ splice site (GU) forming the lariat intermediate. (b) The 3′OH of the released 5′ exon then performs a nucleophilic attack at the last nucleotide of the intron at the 3′ splice site (AG). (c) Joining the exons and releasing the intron lariat.

In eukaryotic genomes, exons that code for proteins are typically interrupted by introns termed as protein noncoding regions. The borders between exons and introns are called splice sites (Figure 1). A splice site can be located at either the upstream or the downstream part of an intron. For the former, it is called the 5′ splice site or donor site; for the latter, it is called the 3′ splice site or acceptor site. The vast majority of the donor and acceptor sites are canonical or regular splice sites that are characterized by the presence of the GT and AG, respectively. During RNA splicing, both the donor and acceptor sites will be recognized by a large macromolecule called spliceosome that is comprised of more than 300 proteins and five small nuclear RNAs (snRNAs U1, U2, U4, U5, and U6) [1]. Once the splice sites are recognized, the spliceosome will remove introns through two sequential transesterification reactions (Figure 1). Removing introns from precursor messenger RNA (pre-mRNA) so that exons can be joined together to form mature mRNA is an essential step of gene expression. Therefore, to better understand the splicing process and mechanism, it is important to accurately detect the splice sites in the genome.

Although biochemical experimental approaches can provide some details about the splice sites, it is both time-consuming and expensive to rely on the biochemical experimental techniques alone. Hence, it is a big challenge and also highly desirable to develop computational methods for timely and effectively identifying the splice sites. In view of this, the present study was initiated in an attempt to develop a computational method for predicting splice sites.

According to a comprehensive review [2] and demonstrated by a series of recent publications [3–9], to establish a really useful statistical predictor for a biological system, we need to consider the following procedures: (i) construct or select a valid benchmark dataset to train and test the predictor; (ii) formulate the biological samples with an effective mathematical expression that can truly reflect their intrinsic correlation with the target to be predicted; (iii) introduce or develop a powerful algorithm to operate the prediction; (iv) properly perform cross-validation tests to objectively evaluate the anticipated accuracy of the predictor. Below, let us describe how to deal with these procedures one by one.

## 2. Materials and Methods

### 2.1. Benchmark Dataset

The human splice site-containing sequences were obtained from the database HS^3^D (http://www.sci.unisannio.it/docenti/rampone/), which contained the sequences of exons, introns, and splice regions extracted from GenBank Rel.123. All the splice site-containing sequences in HS^3^D obey the GT-AG rule; that is, begin with the dinucleotides GT (GU in RNA) and end with the dinucleotides AG, and their lengths are of 140 nucleotides with the splice donor site GT (or acceptor site AG) in the middle positions.

At present, there are 2,796 (2,880) true splice donor (acceptor) site-containing sequences and 271,937 (329,374) false splice donor (acceptor) site-containing sequences in HS^3^D. To balance the number of the true and false splice site-containing sequences and to avoid the overfitting problem in the model-training processes, we randomly selected out 2,800 false splice donor (acceptor) site-containing sequences from the 271,937 (329,374) false splice donor (acceptor) site-containing sequences.

As pointed out in a comprehensive review [10], there is no need to separate a benchmark dataset into a training dataset and a testing dataset for examining the performance of a prediction method if it is tested by the jackknife test or subsampling cross-validation test.

Finally, we obtained two benchmark datasets, one for the splice donor site-containing sequence, while the other for the splice acceptor, as can be formulated by

(1) S1=S1+∪S1−  for  splice  donor,S2=S2+∪S2−  for  splice  acceptor,

where the positive dataset *S*_1_^+^ contains 2,796 true splice donor site-containing sequences while the negative dataset *S*_1_^−^ contains 2,800 false splice donor site-containing sequences; *S*_2_^+^ contains 2,880 true splice acceptor site-containing sequences, while *S*_2_^−^ contains 2,800 false splice acceptor site-containing sequences, and the symbol ∪ means the union in the set theory. The detailed sequences in the two benchmark datasets *S*_1_ and *S*_2_ are given in Supplementary Information S1 and Supplementary Information S2, respectively; see Supplementary Material available online at http://dx.doi.org/10.1155/2014/623149.

### 2.2. DNA Sample Formulation

Given a DNA sample **D** with *L* nucleic acid residues, the most straightforward way to express the sample is to use the following sequential model:

(2) $$\begin{array}{c} D=R_{1}R_{2}R_{3}R_{4}R_{5}R_{6}R_{7}\cdots R_{L}, \end{array}$$

where *R*_1_ represents the first nucleic acid residue at position 1, *R*_2_ represents the second nucleic acid residue at position 2, and so forth. Although the sequential formulation of (2) contains the complete information of the DNA sample, it is difficult to be handled for statistical prediction. This is because all the existing operation engines, such as optimization approach [11], covariance discriminant (CD) [12], neural network [13], support vector machine (SVM) [14–16], random forest [17, 18], conditional random field [8], nearest neighbor (NN) [19], K-nearest neighbor (KNN) [20], OET-KNN [21], fuzzy K-nearest neighbor [22–24], ML-KNN algorithm [25], and SLLE algorithm [26], can only handle vector but not sequence samples. Although some sequence-similarity-search-based tools, such as BLAST [27], can be used to directly search for those sequences with high similarity to the query sample, unfortunately, this kind of straightforward and intuitive approach failed to work when the query sample did not have significant similarity to any of the character-known sequences. Therefore, various nonsequential or discrete models to represent the DNA samples were proposed in hopes of establishing some sort of correlation or cluster manner through which the prediction could be more effectively carried out.

The simplest discrete model used to represent a DNA sample is its nucleic acid composition or NAC, as given below:

(3) $$\begin{array}{c} D={\left[\begin{array}{cccc} f(\text{A}) & f(\text{C}) & f(\text{G}) & f(\text{T}) \end{array}\right]}^{T}, \end{array}$$

where *f*(A), *f*(C), *f*(G), and *f*(T) are the normalized occurrence frequencies of adenine (A), cytosine (C), guanine (G), and thymine (T) in the DNA sequence, respectively; the symbol **T** is the transpose operator. However, as we can see from (3), all its sequence-order information is completely lost if using NAC to represent a DNA sample. Actually, one of the most important but also most difficult problems in computational biology is how to effectively formulate a biological sequence with a discrete model or a vector, yet still keep considerable sequence-order information.

One way to cope with such a problem is to represent the DNA segment with the *k*-tuple nucleotide composition, a vector with 4^*k*^ components; that is,

(4) $$\begin{array}{c} D={\left[\begin{array}{cccccc} f_{1}^{K\text{-tuple}} & f_{2}^{K\text{-tuple}} & \cdots & f_{i}^{K\text{-tuple}} & \cdots & f_{4^{k}}^{K\text{-tuple}} \end{array}\right]}^{T}, \end{array}$$

where *f*_*i*_^*K*-tuple^ is the normalized occurrence frequency of the *i*th *k*-tuple nucleotide in the DNA segment. As we can see from (4), the dimension of the vector is

(5) $$\begin{array}{c} 4^{k}=\{\begin{array}{cc} 64 & k=3,\\ 256 & k=4,\\ 1024 & k=5,\\ 4096 & k=6,\\ 16384 & k=7,\\ \vdots & \vdots \end{array} \end{array}$$

indicating that by increasing the value of *k*, although the coverage scope of sequence order will be gradually increased, the dimension of the vector **D** will be rapidly increased as well. This will cause the high-dimension disaster [28] as reflected by the following disadvantages: (i) the overfitting problem that will make the predictor with a serious bias and extremely low capacity for generalization; (ii) the information redundancy or noise that will bring about the error of misrepresentation resulting in very poor prediction accuracy; and (iii) unnecessarily increasing the computational time.

To avoid the high-dimension disaster, here, the dinucleotide composition (DNC) was used to formulate the DNA sample, as given by

(6) D=[f12-tuplef22-tuple⋯fi2-tuple⋯f162-tuple]T=[f(AA)f(AC⁡)f(AG)f(AT)⋯f(TT)]T,

where *f*_1_^2-tuple^ = *f*(AA) is the normalized occurrence frequency of AA in the DNA sequence, *f*_2_^2-tuple^ = *f*(AC⁡) is that of AC, *f*_3_^2-tuple^ = *f*(AG) is that of AG, and so forth. By doing so, we can only incorporate the local sequence-order information between the most contiguous nucleotides, but none of the global or long-range sequence-order information can be reflected.

Actually, similar problem also occurred in computational proteomics, where, in order to incorporate the global or long-range sequence-order information for proteins, the pseudo amino acid composition [29] or Chou's PseAAC [30] was proposed. Since the concept of PseAAC was proposed in 2001 [29], it has been penetrating into almost all the fields of protein attribute predictions (see, e.g., [31–73]). Because it has been widely used, recently two types of open access software, called “PseAAC-Builder” [51] and “propy” [74], were established for generating various modes of PseAAC.

**Figure 2:**
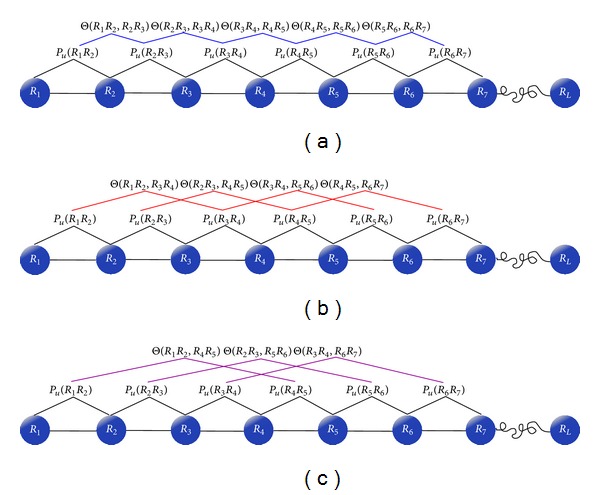
A schematic illustration to show the correlations of dinucleotides along a DNA sequence. (a) The first-tier correlation reflects the sequence-order mode between all the most contiguous dinucleotides. (b) The second-tier correlation reflects the sequence-order mode between all the second-most contiguous dinucleotides. (c) The third-tier correlation reflects the sequence-order mode between all the third-most contiguous dinucleotides.

Encouraged by the successes of introducing the PseAAC approach into computational proteomics, Chen et al. [4] proposed the “pseudo dinucleotide composition” or PseDNC to identify recombination spots of DNA. The formulation of PseDNC is given by

(7) $$\begin{array}{c} D_{\text{PseDNC}}={\left[\begin{array}{ccccccc} d_{1} & d_{2} & \cdots & d_{16} & d_{16+1} & \cdots & d_{16+\lambda } \end{array}\right]}^{T}, \end{array}$$

where

(8) $$\begin{array}{c} d_{u}=\{\begin{array}{cc} \frac{f_{u}^{2\text{-tuple}}}{\sum_{i=1}^{16}f_{i}^{2\text{-tuple}}+w\sum_{j=1}^{\lambda }\theta_{j}}, & 1\leq u\leq 16,\\ \frac{w\theta_{u}}{\sum_{i=1}^{16}f_{i}^{2\text{-tuple}}+w\sum_{j=1}^{\lambda }\theta_{j}}, & \left(16+1\right)\leq u\leq \left(16+\lambda \right), \end{array} \end{array}$$

where *f*_*i*_^2-tuple^ (*i* = 1,2,…, 16) have the same meaning as those in (6), while *θ*_*j*_ is the *j*th tire correlation factor that reflects the sequence-order correlation between all the *j*th most contiguous dinucleotides along a DNA sequence (see Figure 2), as formulated by

(9) θj=1L−j−1∑i=1L−j−1Θ(RiRi+1;Ri+jRi+1+j)(j=1,2,…,λ<L).

In the above two equations, *λ* is the number of the total counted ranks or tiers of the correlations along a DNA sequence, and *w* is the weight factor. Their concrete values as well as the final value for *k* will be further discussed later. The correlation function Θ(*R*_*i*_ *R*_*i*+1_; *R*_*i*+*j*_ *R*_*i*+1+*j*_) in (9) is defined by

(10) $$\begin{array}{c} \Theta \left(R_{i}R_{i+1};R_{i+j}R_{i+1+j}\right)=\frac{1}{\mu }\overset{\mu }{\underset{\nu =1}{\sum }}{\left[P_{\nu }(R_{i}R_{i+1})-P_{\nu }(R_{i+j}R_{i+1+j})\right]}^{2}, \end{array}$$

where *μ* is the number of local DNA structural properties considered that is equal to 6 in the current study as will be explained below, *P*_*ν*_(*R*_*i*_ *R*_*i*+1_) is the numerical value of the *ν*th (*ν* = 1,2,…, *μ*) DNA local structural property for the dinucleotide *R*_*i*_ *R*_*i*+1_ at position *i*, and *P*_*ν*_(*R*_*i*+*j*_ *R*_*i*+1+*j*_) is the corresponding value for the dinucleotide *R*_*i*+*j*_ *R*_*i*+1+*j*_ at position *i* + *j*, as will be given below.

### 2.3. DNA Local Structural Property Parameters

**Figure 3:**
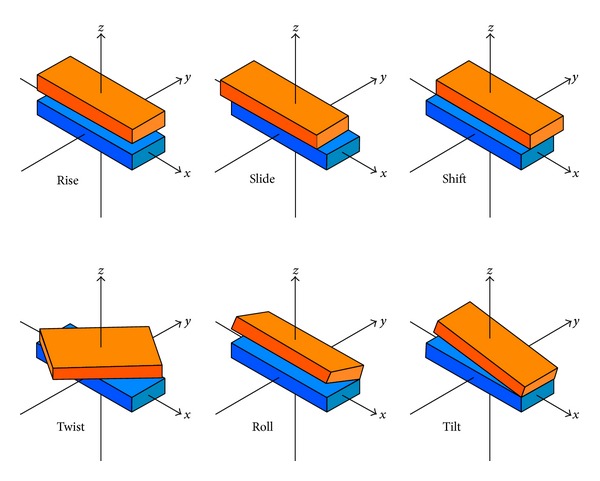
A schematic drawing to illustrate the six spatial arrangements between two neighboring base pairs in DNA. Of the six panels, three are for the local translational arrangements and the other three are for the local angular ones [6].

A lot of evidences have shown that DNA local structural properties play important roles in many biological processes, such as protein-DNA interactions [75], formation of chromosomes [76], and meiotic recombination [4]. Generally speaking, the spatial arrangements of two successive base pairs can be characterized by six parameters, of which three are the local translational ones and the other three are the local angular ones (Figure 3), as formulated by

(11) $$\begin{array}{c} \text{translational}=\{\begin{array}{cc} \text{slide}, & \\ \text{shift}, & \\ \text{rise}, & \end{array}\;\;\text{angular}=\{\begin{array}{cc} \text{roll}, & \\ \text{tilt}, & \\ \text{twist}. & \end{array} \end{array}$$

The six structural parameters of dinucleotides have been calculated by Goñi et al. [75] based on the long atomistic molecular dynamics (MD) simulations in water, and their concrete values are given in Table 1, which will be used to calculate the global or long-range sequence-order effects for the DNA sequences via (9) and (10).

Note that before substituting the values of physicochemical property into (10), they were all subjected to a standard conversion as described by the following equation:

(12) $$\begin{array}{c} P_{\nu }\left(R_{i}R_{i+1}\right)=\frac{P_{\nu }^{0}\left(R_{i}R_{i+1}\right)-\langle P_{\nu }^{0}\left(R_{i}R_{i+1}\right)\rangle }{\text{SD}\langle P_{\nu }^{0}\left(R_{i}R_{i+1}\right)\rangle }, \end{array}$$

where the symbols 〈〉 mean taking the average of the quantity therein over the 16 different combinations of A, C, G, T for *R*_*i*_ *R*_*i*+1_ and SD means the corresponding standard deviation [10]. The converted values obtained by (12) will have a zero mean value over the 16 different dinucleotides and will remain unchanged if going through the same conversion procedure again. Listed in Table 2 are the values of *P*_*ν*_(*R*_*i*_ *R*_*i*+1_) (*v* = 1,2,…, 6) obtained via the standard conversion of (12) from those of Table 1.

### 2.4. Support Vector Machine (SVM)

Support vector machine (SVM) is an effective method for supervised pattern recognition and has been widely used in the realm of bioinformatics [4, 14, 77, 78]. The basic idea of SVM is to transform the data into a high dimensional feature space and then determine the optimal separating hyperplane. A brief introduction about the formulation of SVM has been given in [14]. In this study, the SVM implementation was based on the freely available package LIBSVM 2.84 written by Chang and Lin [79], which can be downloaded from http://www.csie.ntu.edu.tw/~cjlin/libsvm/. Because of its effectiveness and speed in training process, the radial basis kernel function (RBF) was used to obtain the best classification hyperplane. The regularization parameter *C* and the kernel width parameter *γ* were tuned via the grid search method in the 10-fold cross-validation.

The predictor obtained via the above procedures is called iSS-PseDNC, where “i” stands for “identifying,” “SS” for “splice site,” “Pse” for “pseudo,” “D” for “di,” “N” for “nucleotide,” and “C” for “composition.”

### 2.5. Criteria for Performance Evaluation

To provide a more intuitive and easier-to-understand method to measure the prediction quality, the following set of four metrics based on the formulation used by Chou [80] in studying signal peptide prediction was adopted. According to Chou's formulation, the sensitivity (Sn), specificity (Sp), overall accuracy (Acc), and Matthew's correlation coefficient (MCC) can be expressed as follows [4, 7–9]:

(13) $$\begin{array}{c} \text{Sn}=1-\frac{N_{-}^{+}}{N^{+}},\\ \text{Sp}=1-\frac{N_{+}^{-}}{N^{-}},\\ \text{Acc}=1-\frac{N_{-}^{+}+N_{+}^{-}}{N^{+}+N^{-}},\\ \text{MCC}=\frac{1-\left(\left(N_{-}^{+}/N^{+}\right)+\left(N_{+}^{-}/N^{-}\right)\right)}{\sqrt{\left(1+\left(N_{+}^{-}-N_{-}^{+}\right)/N^{+}\right)\left(1+\left(N_{-}^{+}-N_{+}^{-}\right)/N^{-}\right)}}, \end{array}$$

where *N* ^+^ is the total number of the true splice site-containing sequences investigated, while *N*_−_^+^ is the number of true splice site-containing sequences incorrectly predicted as the false splice site-containing sequences; *N* ^−^ is the total number of the false splice site-containing sequences investigated, while *N*_+_^−^ is the number of the false splice site-containing sequences incorrectly predicted as true splice site-containing sequences. From (13), we can easily see the following. When *N*_−_^+^ = 0 meaning that none of the true splice site-containing sequences was incorrectly predicted to be a false splice site-containing sequence, we have the sensitivity Sn = 1. When *N*_−_^+^ = *N* ^+^ meaning that all the true splice site-containing sequences were incorrectly predicted to be the false splice site-containing sequences, we have the sensitivity Sn = 0. Likewise, when *N*_+_^−^ = 0 meaning that none of the false splice site-containing sequences was incorrectly predicted to be a true splice site-containing sequence, we have the specificity Sp = 1, whereas when *N*_+_^−^ = *N* ^−^ meaning that all the false splice site-containing sequences were incorrectly predicted to be the true splice site-containing sequences, we have the specificity Sp = 0. When *N*_−_^+^ = *N*_+_^−^ = 0 meaning that none of the true splice site-containing sequences and none of the false splice site-containing sequences were incorrectly predicted, we have the overall accuracy Acc = 1 and Mathew's correlation coefficient MCC = 1; when *N*_−_^+^ = *N* ^+^ and *N*_+_^−^ = *N* ^−^ meaning that all the false splice site-containing sequences and all the true splice site-containing sequences were incorrectly predicted, we have Acc = 0 and MCC = −1, whereas when *N*_−_^+^ = *N* ^+^/2 and *N*_+_^−^ = *N* ^−^/2, we have Acc = 0.5 and MCC = 0 meaning no better than random prediction. As we can see from the above discussion based on (13), the meanings of the four metrics have become much more intuitive and easier to understand than the conventional formulation often used in the literature, particularly for Mathew's correlation coefficient, which is usually used for measuring the quality of binary (two-class) classifications as in the case of the current study. However, it is instructive to point out that the set of the metrics in (13) is valid only for the single-label systems. For the multilabel systems whose existence has become more frequent in system biology [81–83] and system medicine [24, 84], a completely different set of metrics as defined in [25] is needed.

## 3. Results and Discussions

### 3.1. Graphic Profiles of True and False Splice Site-Containing Sequences

**Figure 4:**
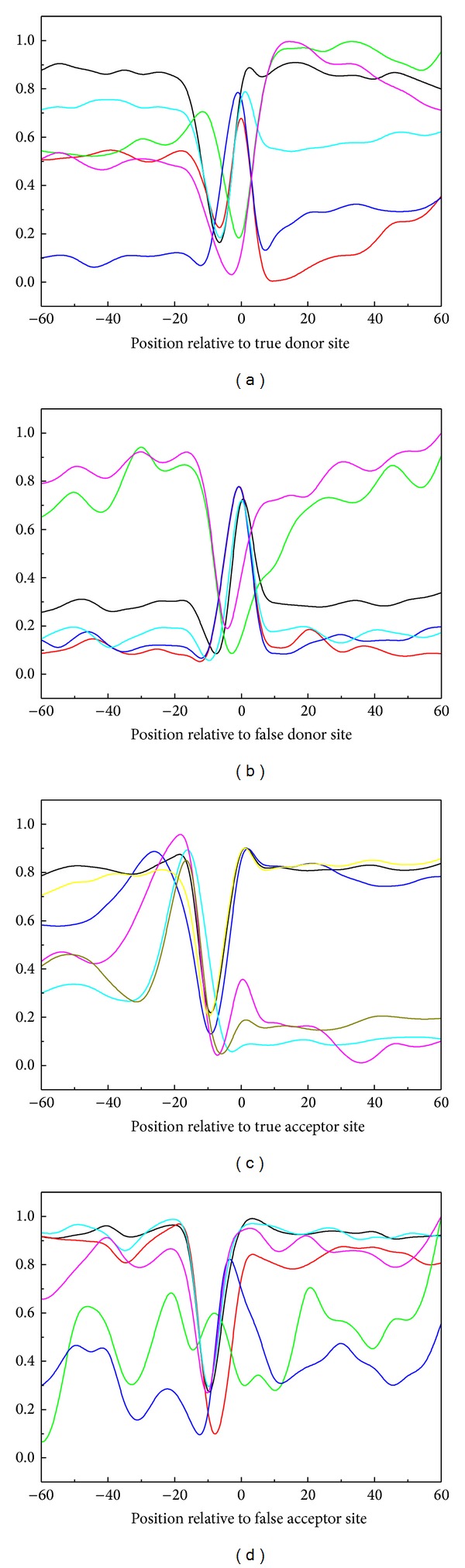
Graphic profiles to show the difference between the true and false splice site-containing sequences. The profiles of six DNA structural properties (i.e., rise (black), slide (red), shift (blue), twist (orange), roll (green) and tilt (purple)) for (a) true splice donor site-containing sequences, (b) false splice donor site- containing sequences, (c) true splice acceptor site-containing sequences, and (d) false acceptor donor site-containing sequences. The profiles are plotted with a window size of 10 bp and a step size of 5 bp.

It has been reported that the DNA local structural properties, that is, angular parameters (twist, tilt, and roll) and translational parameters (shift, slide, and rise), play important roles in prokaryotic transcription initiation, protein-DNA interactions, and meiotic recombination [4, 75, 76, 85]. Accordingly, it is quite natural to ask whether these DNA structural properties may also play some role in regulating RNA splicing. Here, let us use the graphic approach to address this question. This is because using graphical approaches to study biological problems can provide an intuitive picture or useful insights for helping in analyzing complicated relations in these systems [30], as demonstrated by many previous studies on a series of important biological topics, such as enzyme-catalyzed reactions [86–89], inhibition of HIV-1 reverse transcriptase [90–93], inhibition kinetics of processive nucleic acid polymerases and nucleases [94], protein folding kinetics [95], drug metabolism systems [96], protein sequence evolutionary analysis [97], protein remote homology detection [5], and using Wenxiang diagram or graph [98] to study protein-protein interactions [99–102]. Shown in Figure 4 is a comparison of the graphic profiles between the true and false splice site-containing sequences. As we can see there, the divergence between the true and false splice site-containing sequence profiles is remarkable, clearly indicating that the six structural property parameters can indeed play important roles in RNA splicing. That was why we used them to calculate the global sequence-order effects as elaborated in Section 2.3.

### 3.2. Cross-Validation

How to properly evaluate the anticipated accuracy is an important step in developing a new predictor. Generally speaking, to avoid the “memory effect” [10] of the resubstitution test in which a same dataset was used to train and test a predictor, the following three cross-validation methods are often used to examine a predictor for its effectiveness in practical application: independent dataset test, subsampling or *K*-fold (such as 5-fold, 7-fold, or 10-fold) test, and jackknife test. However, as elaborated by a penetrating analysis in [2], considerable arbitrariness exists in the independent dataset test. Also, as demonstrated by (28)–(30) in [2], the subsampling test (or *K*-fold cross-validation) cannot avoid arbitrariness either. Only the jackknife test is the least arbitrary that can always yield a unique result for a given benchmark dataset. Therefore, the jackknife test has been widely recognized and increasingly adopted by investigators to examine the quality of various predictors (see, e.g., [42, 58, 59, 62, 64, 66, 67, 70, 103–107]). Therefore, in this study, the jackknife test was also used to examine the performance of the predictor. During the jackknife test, each sequence in the benchmark dataset *S*_1_ (or *S*_2_) was in turn singled out as an independent test sample and all the rule-parameters were derived based on the remaining data without including the one under the prediction.

### 3.3. Parameter Optimization

As we can see from (8), the predictive accuracy of the present model depends on the two parameters *w* and *λ*, where *w* is the weight factor which was usually within the range from 0 to 1 and *λ* is the number of the correlation tiers to be counted for the global sequence-order information. Generally speaking, the greater the *λ* is, the more global sequence-order information the model will contain. However, if *λ* is too large, it would reduce the cluster-tolerant capacity [108] so as to lower down the cross-validation accuracy due to overfitting or “high dimension disaster” [28] problem. Therefore, our searching for the optimal values of the two parameters was confined in the range

(14) $$\begin{array}{c} 0\leq w\leq 1,\\ 1\leq \lambda \leq 10. \end{array}$$

Furthermore, to reduce the computational time during the search process, the 10-fold cross-validation approach was adopted. Once the optimal values thus obtained for the two parameters were determined, the rigorous jackknife test was utilized to evaluate the anticipated accuracy of the predictor.

Listed in Table 3 are the jackknife test results of the iSS-PseDNC predictor in identifying the splice donor site-containing sequences and the splice acceptor site-containing sequences on the benchmark datasets *S*_1_ and *S*_2_, respectively, where the optimal values for *w* and *λ* are also explicitly given.

To further show the power of the iSS-PseDNC predictor, we also did some comparison calculations as described below.

First, based on the sequence similarity principle, we used BLAST [109] to conduct the jackknife test on the same benchmark dataset as used by the iSS-PseDNC predictor. The results thus obtained are given in Table 4, from which we can see that the percentage rates for Sn, Sp, and Acc by BLAST are about 40% lower than those by iSS-PseDNC and that the rates of MCC by BLAST are about 0.5 lower than those by iSS-PseDNA, for the cases of both donor and acceptor.

Second, rather than pseudo dinucleotide composition (7), we used the dinucleotide compositions (6) to represent the DNA samples for prediction. The corresponding results thus obtained are given in Table 5, from which we can see that the rates for Sn, Sp, Acc, and MCC are all lower than those reported in Table 3, clearly implying that the additional components in the pseudo nucleotide composition did play a role in enhancing the prediction quality.

All these results indicate that the iSS-PseDNC model as proposed in this paper is quite promising and may become a useful tool in identifying splice sites.

## 4. Conclusions

RNA splicing is a complicated biological process that involves interactions among DNA, RNA, and proteins. Hence, it is reasonable to analyze the structural properties that can be used to describe these interactions. In view of this, we firstly plotted the profiles of the six DNA structural properties (twist, tilt, roll, shift, slide, and rise) for splice site-containing sequences and found the differences between true and false splice site-containing sequences. The structural divergences surrounding splice sites may facilitate the removal of the introns by spliceosome.

By defining PseDNC using the above six DNA structural properties, we proposed a model, namely, iSS-PseDNC, for identifying splice sites. The predictive performance demonstrated that our model is helpful for splice site recognitions. Since user-friendly and publicly accessible web-servers represent the direction of developing practically more useful models [110], simulated methods, or predictors, we will make efforts in our future work to provide a web-server for the approach presented in this paper.

It has not escaped our notice that the web-server PseKNC (pseudo *K*-tuple nucleotide composition) developed very recently [111] will be very useful for further improving the prediction quality in identifying the splicing sites.

## Supplementary Material

Supporting Information S1. The benchmark dataset for splice donor sites.
Supporting Information S2. The benchmark dataset for splice acceptor sites.

## Figures and Tables

**Table 1:** The original values for the six DNA dinucleotide physical structures.

Dinucleotide	Physical structures^a^					
	*P*_1_(R_*i*_R_*i*+1_)	*P*_2_(R_*i*_R_*i*+1_)	*P*_3_(R_*i*_R_*i*+1_)	*P*_4_(R_*i*_R_*i*+1_)	*P*_5_(R_*i*_R_*i*+1_)	*P*_6_(R_*i*_R_*i*+1_)
AA	0.026	0.038	0.020	1.69	2.26	7.65
AC	0.036	0.038	0.023	1.32	3.03	8.93
AG	0.031	0.037	0.019	1.46	2.03	7.08
AT	0.033	0.036	0.022	1.03	3.83	9.07
CA	0.016	0.025	0.017	1.07	1.78	6.38
CC	0.026	0.042	0.019	1.43	1.65	8.04
CG	0.014	0.026	0.016	1.08	2.00	6.23
CT	0.031	0.037	0.019	1.46	2.03	7.08
GA	0.025	0.038	0.020	1.32	1.93	8.56
GC	0.025	0.036	0.026	1.20	2.61	9.53
GG	0.026	0.042	0.019	1.43	1.65	8.04
GT	0.036	0.038	0.023	1.32	3.03	8.93
TA	0.017	0.018	0.016	0.72	1.20	6.23
TC	0.025	0.038	0.020	1.32	1.93	8.56
TG	0.016	0.025	0.017	1.07	1.78	6.38
TT	0.026	0.038	0.020	1.69	2.26	7.65

^a^In this table, the following symbols were used to represent the six physical structures of dinucleotide: *P*_1_ for “twist”, *P*_2_ for “tilt”, *P*_3_ for “roll”, *P*_4_ for “shift”, *P*_5_ for “slide”, and *P*_6_ for “rise”. The data was obtained from [75].

**Table 2:** The normalized values for the six DNA dinucleotide physical structures.

Dinucleotide	Physical structures^a^					
	*P*_1_(R_*i*_R_*i*+1_)	*P*_2_(R_*i*_R_*i*+1_)	*P*_3_(R_*i*_R_*i*+1_)	*P*_4_(R_*i*_R_*i*+1_)	*P*_5_(R_*i*_R_*i*+1_)	*P*_6_(R_*i*_R_*i*+1_)
AA	0.06	0.5	0.27	1.59	0.11	−0.11
AC	1.50	0.50	0.80	0.13	1.29	1.04
AG	0.78	0.36	0.09	0.68	−0.24	−0.62
AT	1.07	0.22	0.62	−1.02	2.51	1.17
CA	−1.38	−1.36	−0.27	−0.86	−0.62	−1.25
CC	0.06	1.08	0.09	0.56	−0.82	0.24
CG	−1.66	−1.22	−0.44	−0.82	−0.29	−1.39
CT	0.78	0.36	0.09	0.68	−0.24	−0.62
GA	−0.08	0.5	0.27	0.13	−0.39	0.71
GC	−0.08	0.22	1.33	−0.35	0.65	1.59
GG	0.06	1.08	0.09	0.56	−0.82	0.24
GT	1.50	0.50	0.80	0.13	1.29	1.04
TA	−1.23	−2.37	−0.44	−2.24	−1.51	−1.39
TC	−0.08	0.5	0.27	0.13	−0.39	0.71
TG	−1.38	−1.36	−0.27	−0.86	−0.62	−1.25
TT	0.06	0.5	0.27	1.59	0.11	−0.11

^a^See footnote a of Table 1 for further explanation.

**Table 3:** The prediction quality as measured by metrics of (13) by iSS-PseDNC in identifying the splice donor and acceptor sites, respectively.

Splice sites	Optimal parameters		Metrics			
	*λ*	*w*	Sn (%)	Sp (%)	Acc (%)	MCC
Donor^a^	4	0.3	86.66	84.25	85.45	0.71
Acceptor^b^	2	0.3	88.78	86.64	87.73	0.75

^a^See Supplementary Information S1 for benchmark dataset of donor.

^b^See Supplementary Information S2 for benchmark dataset of acceptor.

**Table 4:** The prediction quality as measured by metrics of (13) by using BLAST [109] and sequence similarity principle in identifying splice acceptor and donor sites, respectively.

Splice sites	Metrics			
	Sn (%)	Sp (%)	Acc (%)	MCC
Acceptor^a^	39.09	40.20	39.62	−0.21
Donor^b^	42.75	37.63	40.23	0.20

^a^See footnote a of Table 3 for further explanation.

^b^See footnote b of Table 3 for further explanation.

**Table 5:** The prediction quality as measured by metrics of (13) by using the dinucleotide composition (6) to formulate the DNA samples in identifying the splice donor and acceptor sites, respectively.

Splice sites	Metrics			
	Sn (%)	Sp (%)	Acc (%)	MCC
Donor^a^	81.23	84.42	82.58	0.67
Acceptor^b^	83.39	85.60	83.78	0.68

^a^See footnote a of Table 3 for further explanation.

^b^See footnote b of Table 3 for further explanation.
