# Supplementary material for: iSS-PseDNC: Identifying Splicing Sites Using Pseudo Dinucleotide Composition
Source: Biomed Res Int. 2014 May 21;2014:623149. doi: 10.1155/2014/623149 (PMC4055483; doi:10.1155/2014/623149)
Supplement: Supplementary file 1 — Supporting Information S1. The benchmark dataset for splice donor sites. Supporting Information S2. The benchmark dataset for splice acceptor sites. [file 623149.f1.zip › Supp1.pdf]

Supporting Information S1. The benchmark dataset for splice donor sites. It consists of a positive dataset $\mathbf{S}_1^+$ and a negative dataset $\mathbf{S}_1^-$. The former contains 2,796 true splice donor site containing sequences, while the latter contains 2,800 false splice donor site containing sequences.

$\mathbf{S}_1^+$: 2,796 true splice donor site containing sequences

>1
CTCCTCTTTGCCTTACTCCTAGCCATGGAGCTCCCATTGGTGGCAGCCAGTGCCACCAT
GCGCGCTCAGTGTAAGTATCATTCCCTCTCACTGTCCTGGAGAGGACGAGAATTCCAC
CTGGGGTGCTGGGGGTCACTGGG
>2
AATGACTTCAACTGTCCCAACATTAGAGTATGTCCGTATCATATTAGGCGCTGTATGACA
ATCTCCATTCGTAAGTACCTCTTGGTCATTTGGACACATTGTAGATTAGTCCCCTACCTG
GGTAGTTTCTGGGGCCAGGG
>3
TGACCAGGAACTGGCGGGTGGGCGCCCTGCAGAGGCTGCTGCAGTTTGGGATCGTGG
TCTATGTGGTAGGGTAAGAGAGAAGAGCTTTTGGCCAGGCTGGAGGGGCAAGGGAAG
AGGTGGGGGGTGGGGCTTGGTCCTGC
>4
TTCCGTCACTCAGATCAAGGAGCTTGGAAACCGGCTGTGGGATGTGGCCGACTTCGTG
AAGCCACCTCAGGTGGGGGCCCTGATGTTGCTGACGGGGGCGCAAGTCCTTTCCCCAC
TGACAGCCTGAACACCCGCCATGC
>5
AGAGAACGTGTTCTTCTTGGTGACCAACTTCCTTGTGACGCCAGCCCAAGTTCAGGGC
AGATGCCCAGAGGTGAGTTTACCCAGGATCCTCCCAGCGGGTCCCTTGTTCCTCCATCA
GCCCCAGGTGGCCACCCGTGTTT
>6
TCCGTCCCACTGGCTAACTGCTGGGTCGACGAGGACTGCCCCGAAGGGGAGGGAGGC
ACACACAGCCACGGTAACTGTGGGCTCTGTCTTCCAGTGCCCCCAGCAGGGTGGGGG
CCGGGCTGGGATCCTGGGTGGCTCCT
>7
TGTTCAATGGGACCCACAGGACCTGTGAGATCTGGAGTTGGTGCCCCGTGGAGAGTG
GCGTTGTGCCCTCGTAAGTGTCCCCACAATCCCCTACCCCAACTGGCGCAGGGCCCCA
GGCCTGGCAGAGGCTGTCACCTCCC
>8
TGGCCCAGGCCCAGAACTTCACACTGTTCATCAAAAACACAGTCACCTTCAGCAAGTT
CAACTTCTCTAAGTAAGCAGAGTGGGTCTCATCTGCCCCAAGACCCTCCTTGTCCCCTA
CCTCATCTGACCTTTCCCACTCC
>9
CTGTCCCGTGTTCCGCATTGGGGACCTCGTGGCCAAGGCTGGAGGGACCTTCGAGGAC
CTGGCGTTGCTGGTGGGTCCCAAGTTGGGGGCAGGGTTCCTAGAGGGCTCTGGGAGA
GGGTCCCGGGCCCACCCACCGGTGG
>10
TGGACACCGGGGACTCTGGCTGCTGGCCTCACTACTCCTTCCAGCTGCAGGAGAAGA

GCTACAACTTCAGGTGAGGCCCCACAGCTCCCAGTGCCCAGATGCTGGGCCCATCGCC
CTCTCCCTGTGGCGGCCAGGACAGA
>11
ACCGGGTGTGGAGGCCCGCACCCTGCTCAAGCTCTATGGAATCCGCTTCGACATCCTC
GTCACCGGGCAGGTAGGCACAGGTAGGGGTCAGGCCGGGGATGGGATGGGGCAGGCA
GACAGGGCTGGAGGAGGCATGAGGC
>12
TTTCTGTGACCTGCTACTGCTGTATGTGGATAGAGAAGCCCATTTCTACTGGAGGACAA
AGTATGAGGAGGTGAGCTGAGGTCGCTCTGCTTGGACCCTGGGTTCTGCCACACTTAG
GAAGATGTTGGCTGGATCCCTGA
>13
GCCTCCTGTCGGACCCGGTTCGCCGGCGCGCTCGCCCGCCTAGTGCTGCGCCTCAA
CGCGCCGTTGTGGTGAGGACAGGGCCCGGGGAGGCGGGGACCCGAGGGCCCTGGGC
TCGCGCCGGCGGCCCCATGCGCGGTC
>14
AGCAGTTTGCGGGCGGAGACCGTGCCCGGGCTTTTGCCCGGGACTTCGCCGCCCACC
GCAAGAAGTCGGGGTGAGCGGCAGAGCAGGGCGTATGGGGCGAGCAGTGGTGGCTA
AGGCCGGGGAGCTCTGCTCAGAGGCTC
>15
AGGGCTGGAGGTCTACACGCAGAGTTTCTCCCGGAAACTGCCCTTCCCAGATGAGACC
CACGAGCGCTATGTACTGGGGGAGTGGGGTGTGCCTGGGCCCAGAAAGCACCTTGGA
GGGAGGGGTCTGGGCTGGGTATCAC
>16
CCCTGTGGCTCTGACTCTACCAACAGCCAGGCTGTGGGGCTGCTGCTGGCACTGGCTG
CCCACTTCCGGGGTGAGTCTCAGGGCTGCTGGCCGTGGAGGGGTTTGGTCAGTGCCCC
AGACCCTGCTGATCCTGCTTCTGG
>17
CTGGTAACAGAACATGACCTTCTGGGCACTGAGGCTTGGCTTGAAGCCTACCACGATG
TCAATGTCACTGGTAGGTTCTCTTGTCCTGCCTGCCCTGGTCCCCCTCTCAGGGTCACT
GTCCTCACCACGTCCTCCATTAG
>18
CCTTGACCTGCTCAATCTCTTCCAGACCTTCTGCCAGAAAGGGGGCCTGTTGTGCACG
CTTCAGGGCAAGGTGGGGCTGCCTGCCTGCCTGAGGGCTGAAGGGCACAGGGTCTGT
GGGAGGGGCTGTCTCATCCTGTCCT
>19
TGGAGGCCCTAACCCTGCGTGGCATCAATAGCTTCCGCCAGTACAAGTATGACCTGGT
GGCAGTGGGCAAGTAAGTAGTCTCTGGTCCCCCCTTTCCATGCCCAGGATGGGGTCTA
GCTGGAGCGGAGTGGGGGATGGCC
>20
TGAGGCTGGAATGGGCCTTGAGGAGCCCGGGGGTGCCCCTGGCCCCAGTGTACCCCTT
CCCCCATCACAGGTGATGGCACCCCCTTCTGTTGTTGGAATGGGCTTCTGGGGTCTGAC
TGGTGTTTAGTGTTGCAACTTAG
>21
CTGAGGCTGTGGTGCTGACACTGCTGGCGATTTATGCAGCTGGCCTGGCCCTGCCCCA

CAATACCCACCGGTAAGAGGCTGGGCTGGTTGTTGGGGGCAGGGGTAGAGGTCCCCT
GGACATGCAGACAGCTTGTGGGTTG
>22
TCTCACTGGGCTTCCTGCTGGCCACCACCATGGTGCCCACTGCTGCGCTTGCCAAGCC
TCATGGGCCCCGGTATGTATGGATCAGCCCCACTTCCCCCAATGCCTGTGCCCTCATCA
CAGACCGCACCCTCATTCAGTAG
>23
TTCGCTTTGACCCAAAGAACATCAAGGAGAGGTCACCTCTGGCTTTTATTCCCTTCTCA
GCAGGGCCCAGGTAAGGGCGGCCTGTGTTTGAGGCGGGGACGGGGAGATAGGTGCAG
GGGTCTGGGCATGACAGTGGGGAA
>24
GCCGTCATGGCGCCCCGAACCCTCGTCCTGCTACTCTCGGGGGCCCTGGCCCTGACCC
AGACCTGGGCGGGTGAGTGCGGGGTCGGGAGGGAAACGGCCTCTGTGGGGAGAAGC
AAGGGGCCCGCCCGGCGGGGGCGCAG
>25
AAGGCCCACTCACAGACTGACCGAGCGAACCTGGGGACCCTGCGCGGCTACTACAAC
CAGAGCGAGGACGGTGAGTGACCCCGGCCCGGGGCGCAGGTCACGACCCCTCATCCC
CCACGGACGGGCCAGGTCGCCCACAG
>26
CTGGAGGGCCGGTGCGTGGAGTGGCTCCGCAGATACCTGGAGAACGGGAAGGAGACG
CTGCAGCGCACGGGTACCAGGGGCCACGGGGCGCCTCCCTGATCGCCTGTAGATCTCC
CGGGCTGGCCTCCCACAAGGAGGGG
>27
GGACAGGAGCAGAGATACACCTGCCATGTGCAGCATGAGGGTCTGCCCAAGCCCCTC
ACCCTGAGATGGGGTAAGGAGGGAGACGGGGGTGTCATGTCTTTTAGGGAAAGCAGG
AGCCTCTCTGACCTTTAGCAGGGTCA
>28
CTGGTTCTCTTTGGAGCTGTGATCGCTGGAGCTGTGGTCGCTGCTGTGATGTGGAGGA
GGAAGAGCTCAGGTGGGGAAGGGATGAAGGGTGGGTCTGAGATTTCTTGTCTCACTG
AGGGTTCCAAGACCCAGGTAGAAGT
>29
CAATAAAAGGCGACGGGATGGCTACCTTTTCCAATTGCTGCGGATTGCTGATGCCCACT
TGGACAGAGTGGTGAGGAATTGCCAATGGCAGAGCTGAGTTGGGAGATTACTCACGG
GGGCAAATGTGACTCTACCCCCTA
>30
CTGTTCGGTCCTATCCAGGAAATACTGGAATGACTGTGAGCCACCTGATTCCAGACGTC
CATCTGAAATAGTAAGTAAAGAGGGCACCTTCACTCTGCTCTTTTCATTCTTATTTTCCA
TCTACTCTGTTCACTCAGCCA
>31
ATAGCTACAAGACATTCCCATGAATCTCAGGACCTCAGAGTGATTGACTTTAACTGCAC
CACAAGTTCTGGTATGGTAATCTGTTAAATTGCTAATTAATTCTTGATTAATACAAATTCA
ATCATACAGTGGTATTTGTC
>32
GAAGTACAAAGAGGAGAATGATGACTTTGCCTCTTTCAGAGTGGACCGAATCGAGAG

AGTTGCAAGAGTGGTGAGTCTCCACTAAGGTTCGGTTGGAGTCTGAAGGCCCAGGCT
CCAACCTGGCAGCAGGAACTGAATGG
>33
AGGAACTGGTTACTTCGTGGACTTCTCTGTGCGGAACTGCCCCAGACACCATTTCCCC
AGACACCCCAATGTGAGTATAAGAAATGTCTGTGATCGTTGACTAGAGTCACAGAGAA
AAAAGAAAGAGAAGAAGGAGAAGG
>34
TGATGTAGAAGCCTTGGACTTGGAAAGCCCGAAAAACCTTGTCATAAACTGTGAAGTC
TTCGACCCTCAGGTGGGTTGTCTAAGCAGACTTTGTCATGGCAGTGCCAGATTAAGTG
ACATACGTACACAAATAGTGTTGT
>35
CCCCTCTACGCCCAGCTTTCTGGCCGAACTTTTCTGCAAGGGGGGGCTGTCGAGGCTA
CACGAGCTGCAGGTAGGAAGGGACGCCTTTCCCGAGACAGAGTGCTGGGGAAACTGG
TTTTGACAGCGTCAGAAAGGACTGA
>36
AGCGCTGCAGCTTCTCGCCCTGGACGGAGCACCGCCGCTGCCGCCAGCGGGCTTGCG
GACTGCTCACTGCGTTGAGTCTTCTCTGCCCCCAGAAGCCCAGACGCCCTGCACCGGC
CCTCTCCTGCTGCGGGTCCCGAAAC
>37
GGACGTGGCACGGGGCCGCCCGCCCTGGTTCGGGACGTGGCGGGGGAATTTTACAAG
TTCGGACTGGAAGGTGAGTCCCAGGACAGAGCTGGGCAGGCGTCGGGGGCGCCCTAC
CAGAGCCTCCCGGAACCCTGACGGCG
>38
TGGCTGCGCCACCTTGTGCCTGGGCCCTGGGGCCGCCTCTGCCGAGACTGGGACCAGA
TGTTTGCATTTGGTAAGGCACAGGTCGAGGTGGAAATGGGGGAATGTAAAGCTGTCCA
GGGGTAGCGAGGTATTCACGTGCC
>39
CCGGGAAGAGTTGCCTGCCCAGTCCATCCTGGGAAATGTGACAGAGTTGCTATTGGCG
GGAGTGGACACGGTGAGGTTCTCCCTCCGTGCTGTGAGCCGGTTCCAGGGCTTAGCCT
CCGCAGACTCCGGCTCCATTTTTC
>40
GTGCCTACCCCTCAGCCACTGTTCTGTCCCAGCTGCCCCTGCTGAAGGCGGTGGTCAA
GGAAGTGCTAAGGTGAGGGGGAAGGAGAGGAGGAACAAGAGGAAATGCCAAGGAA
GGGCTGGGGAAGCAACTAGTGGATGGA
>41
TGTGGTACCTGGAAATTCTCGTGTCCCAGACAAAGACATTCATGTGGGTGACTATATTA
TCCCCAAAAATGTGAGTAAAGCCTATGCACCCTTTCTACTGAGCCATTCCTCACTATCTT
GGGCCCCAACCCTGTTCTCAA
>42
TGGCTTTGGCAAGCGCAGCTGTATGGGGAGACGCCTGGCAGAGCTTGAATTGCAAATG
GCTTTGGCCCAGGTGAGTGCTCTAGATTTTATACCTTCCCCAGACTGGAGAGACCCTAA
CCCTCTAAAGTTGTGAGCTCTTT
>43
GACTTACGGAAAATTGGAAGCTGTGCAGTATAAAACTCAAGTTGTTGCTGGAACAAAT

TACTACATTAAGGTTAGAGTTCAGCACCTACTTTAGCGCCAAAAGATGTATTTCTCATTT
TATGTAAAATATTCCCTGATTT
>44
CCAACAGCTCTGGCACCATCGAGCTCGTGAAGAAGGGCTGCTGGCTAGATGACTTCAA
CTGCTACGATAGGTACCCCAAGACTTGCCCTCCTTTCCTCTTGGACCCACCTGCGCTTAT
ACTGCCCACTGGGCCATTTGGG
>45
TTCTGCTGCTGTGAAGGCAACTTCTGCAACGAGCGCTTCACTCATTTGCCAGAGGCTG
GGGGCCCGGAAGGTAAGGGGGCAGTGTGGAAGTGGGGCCTTGAGTCAGCCGACCTG
GGCTTCTTTGGCTTGGAGCGCTTGGC
>46
CATCGTCCTGCTGGCCTTTTGGATGTACCGGCATCGCAAGCCCCCCTACGGTCATGTGG
ACATCCATGAGGTGAGACAGTGCTGGCTTGGGGCAGGGCAGGATAGAGGTGGGGAGG
ACAGGCCAGACCTTTTTAACCCTT
>47
GCGCTTTGGCTGTGTCTGGAAGGCCCAGCTCATGAATGACTTTGTAGCTGTCAAGATCT
TCCCACTCCAGGTGAGTGTTTGAGGGGCTCCATCCAGGTCCTCTGCCTCCACTGTAGCT
TGAACTGGAGGCTCTCCTGACC
>48
CATTGCTGCCGAGAAGCGAGGCTCCAACCTCGAAGTAGAGCTGTGGCTCATCACGGCC
TTCCATGACAAGGTGAGCCACACCCATCAGAATGGACTCTGAGAGGAGATGGAATGTC
CCCTGGGTGGCTCTCCATAATATG
>49
GCCTCTCATACCTGCATGAGGATGTGCCCTGGTGCCGTGGCGAGGGCCACAAGCCGTC
TATTGCCCACAGGTACCTGGGTCAGCAGGTGCCTTTCCTGCACTTGACCTGGAGCTGT
GGGAAGGGTTGGTGGGCGAGAGCA
>50
CGTGCTGGCTGACTTTGGCTTGGCTGTTCGATTTGAGCCAGGGAAACCTCCAGGGGAC
ACCCACGGACAGGTAACAGGCCTCACAGGGCAGGAAGAGAGGGACAGACCCAGGGT
AGATACTTTGCCCTTTTTGTGCTCAG
>51
CTGCGCATTGACATGTATGCCATGGGGTTGGTGCTGTGGGAGCTTGTGTCTCGCTGCAA
GGCTGCAGACGGTAAGTAGGATGGCAGCCCTGGGCATCCTAGATTGAGTGATAGGGAG
CAGTGGGACCTTAGAGTGATCCT
>52
GGAGCTGCAGGAGGTGGTGGTGCACAAGAAGATGAGGCCCACCATTAAAGATCACTG
GTTGAAACACCCGGTAAGGGGCCTGGTTCAGGCAACTTTGCAGGGGGGTGGAGAAGG
GAAAACCCTTCATGTGTAGCAGGTAA
>53
CGCTACCTTGAGATAGAGTTCTGCTTGAAACATAGGAGCTGCCCTCCTGGATTTGGAGT
GGTGCAAGCTGGTACGTGTCAATGTGCAGCAAAATTAATTAGGATCATGCAAAGTCAG
ATAGTTGTGACAGTTTAGGAGAA
>54
CAGAAAGGAAATGCAACACACGACAACATATGTTCCGGAAACAGTGAATCAACTCAA

AAATGTGGAATAGGTAATTACATTCCAAAATACGTCTTTGTACGATTTTGTAGTATCATC
TCTCTCTCTGAGTTGAACACAAG
>55
ACTTTCCAGCTGCTGAAGTTATGGAAACATCAAAACAAAGACCAAGATATAGTCAAGA
AGATCATCCAAGGTATGATAATCTAAAATAAAAAGATCAATCAGAAATCAAAGACACCT
ATTTATCATAAACCAGGAACAAG
>56
TAGGAATTTCTGATTCATTTAAAGGATTTACAAATTCATCAACCCCTGAAAACTAAAGC
AAATTGAACAGGTATGATAATAAAATAATTTTCAATTTTTTTAAGAATGTATTCCTACTGT
TAAAATTATAAAAGTATATT
>57
AAATTGAAGCAAGCATTCTATATTCCAAGAAATTTGGAACACCTATACCTACAAAATAA
TGAAATAGAAAGTATGTAGACTTTTATTGTGTTTTGAAGAGAGAAAGAATGGAATCCTG
ATCTTTTAAAGAAAAAAATATT
>58
ATCATGAAGGGCCTTGCAGCTGCCCTCCTTGTCCTCGTCTGCACCATGGCCCTCTGCTC
CTGTGCACAAGGTGAGTCTGTCATCCATGTGCTTTGATGGCTCCCTGGGCAGAAGTCA
GGCGACATCTTCCAAGTGCTGTG
>59
GGCAGATTCCACAAAAGTTCATAGTTGACTATTCTGAAACCAGCCCCCAGTGCCCCAA
GCCAGGTGTCATGTAAGTGCCAGTGCTCCTGCCCACCCCTCGGGAGGGAGGATGGGA
GGTTTGGGGTGAGGTCCCCTCAGAG
>60
TTCAGCGTGAGAAGCAGGCCAGGCCTGGGTCTGGAGCCGTCCTGGCCTTCCCAGATG
ACAAGGACCTCAGGTCAGCTTCAGCGTGAGAAGCAGGCCAGGCCTGGGTCTGGAGCC
GGGTGATGTGCACATGGGGCTCACTT
>61
ATGGTCCAGCAGAGTCTCAGAGCGCGGTCTTTGGAGATGTGTGCCCCCTACTCACTTC
TCTCTTGGATGGGTGAGTTAAAATATTTTTAAGGCCAGTCATCATAGTTACATTTGAATAT
AAAGAAGCTTCACTGTGCTGT
>62
GCCCTGTTCTGCCGCTTGACCCACAGAGTGACTTAGGAATTATCCCTAGAGTGGCTGA
GGAGCTCTTCAGGTACTGCCGATGGTGATGAGTGGGGCAGAGAAATATGGCAGGGAA
GCCAGGCGGTCAGCACCTTGGTTAT
>63
TGTCGGGGGTCAAGCGTGAGGTGGTGACAGCCAAGGATGGACGGACAGAGGTTGCGC
TGCTGGCCTCTGAGTGAGTACTGCACCTGCCCCGTGCTGCTGGCCTCTGAGTGAGAAT
GGCACCTGCCCCTCCCACCTGGTCA
>64
GCGGATTCCTCCAGGTCTCACCTGATAATTACGGTGACTCTAACCACAGCCTCCTGCTC
TGACAGCACTGGTAAGTCACCATTTGTGCTTGGTCAGTGGCAAGTAATAGAAAAACCC
ACTTAAGAAGAAAAAAATCAGGC
>65
CCCGCAGGGCATGCGGAGCAGGTGCAGGCTCGACTACAGCTCGTGGACTCGGCCGGC

AGCGAGTGCGTTGGTGAGCAGGGGCAGGCATTTCCCTGGGGGGTGGGTGCTGCAGAA
GCCTATGATCTGATGGAGCTGGATGT
>66
CCGGACCCCTTTGTGCGGCCGGAGGCGGCGGCGGGAACGGCCATGGCGGCCAACATG
TACCGGGTGGGAGGTGAGTACCGGGCGCAGGGATACGGGGCCCGGGGCGGGAGAGGT
TGGGGTAGGGACCTGAGGGGCTCGGC
>67
AAGGTTGTCTGTCTTTTCCGGCGCAGGGACATTTCTAGTAGCCTCAACAGCCTGGCTGA
TAGTAATGCCAGTGAGTATCTTCCTCCCTTTCTTTTTCTCCTACCCATGATCTGAGGCCT
GGGCATGTCCCTGCAGGTATT
>68
GCCATCAACTGAAGCACCGGGAACTTTTTCTTTCTCGGCAATTTGAATCATTACCAGCC
ACCCACATACGGTACAGGACAACTGGGGCCAGCATAGCCTGCGTGCACTTACGTAGAA
GATGGAGAGAAGGAGGTGGTATT
>69
CTCGCTGATCAGGGCGAGATTAGAGTTGGTTGCAAATACCAAGCTGAGATCCCAGATC
GCCTAGTAGAGGGTAAGCAACAGGACAAGACTGTACGGCTACCAGGTTGTCTAATTCC
AAGGGGCACTTTAACATTATAGTC
>70
AGATGAAGGTCTGGGACCCAGACAACCCTCTCACAGACCGGCAGATCGACCAGTTTC
TTGTGGTGGCCCGGTGAGGGGTGGGTGGATAGGGAAGATGGGCTGGGGGAGGTGTGG
ACATTGTTCTGAGTCCCATGGTTCTT
>71
TTGTAGCAGCTCCATTCGGCAGCCAAGCTTGCACATGAGTGCAGCTGCTGCCTCCCGA
GATATCACTCTGGTGAGCAGAAGTTGGTGGGTAGTTGGGCAGGCTGGGTTTTCTGGGG
ACTTGCAGAGCCTGGGAACAGAAG
>72
AGAGGCCATGCTATTTGAGGAGGCCCTAGAGAAGTATGGGAAGGACTTCAATGATATT
CGCCAGGATTTTGTAAGTGGATGGCACTAGGAGGAGAAGTGGGAGAGATGACAGGTG
AGGGAACCAGGACCTGGGGCCAGAT
>73
CTGGAAGTCACTTGCCAGCATAGTCCAGTTTTATTACATGTGGAAAACCACAGACCGGT
ATATTCAGCAGGTATTATCTGGGCTGGGGAGGGTGGACCATAGGCTACCTGCTGACCTC
TGCCTTTGAAGTTGATGACATT
>74
TCTGTGGGTTCAAAACCTGGCATGAATGGGGCTGGATTTCAGAAGGGCCTGACTTGTG
AGAGTTGCCACAGTGAGTTACGGGGGCACTGGGATGGTGGGGATGGGTGGAAGGCCT
GCTGTGGCTCAAGAATCTGAGGGGC
>75
GATCTACTGGAAGAAGTATGGGGGACTGAAGACCCCAACTCAGCTTGAGGGGGCCAC
TCGGGGCACCACGGTAAGGATCAGTGGGAAGAAAAGCAGGGGAGAGCAGTAAGCATA
TTGATGGTGAGCTTAGGTGAAGGCCA
>76
CTATTACAGCCAAGGAGGGCCGCCCGACGGCCTTATGCTCCTATCAATGCCAATGCCAT

CAAAGCAGAGTGTAAGGGCAGGGAGTGGTGGGACTGGTGGTGGGTTTCGGATTGCTC
TGCCCAGAGACCGTTGTTTCTCTG
>77
GCCGCCAAGACTCCATTGAAGATTCACCCTCTGGTGCGGCTGCCCCTGGCAACTATCG
TCAAAGATCTGGGTATGGGATGGGGACCAGTGAGGTTGGCATCAGGCTCCCAGTCCTT
TCCCTGTCTGACATCTCTCCCCTT
>78
GATCAACAGAAACCAGCTGTCCCAGAACCGGGGACTGGGGGGCATTATGGTGAAACG
GGCCTATGAGACTGTGAGTTGACAGAAAGGGGTGGGCCAGATTGAGTTGGGTGGGCT
TTTGTCTCTCATAGGAGTCTGAGAGG
>79
CCAAGCGTCAGAAACTAAACCCAGCTGATGCCCCCAATCCTGTGGTGTTTGTGGCCAC
AAAGGATACCAGGTAAGGCGTCTACCATGGGAGGGGCTGAGAGGGACAGTCCAGGCT
GGTGAGTGTGGGGAGTAGGAGTATC
>80
GATTGAAAATGATCAGTCGATCAGCCGAAATCATGCTGTGTTAACTGCTAACTTTTCTG
TAACCAACCTGGTATGTTACTAATTTTATTTCACTAGTTTCCAGTAAGGGGGTTGAAATC
ACAAAATATTCTCTTGGTTTG
>81
AGAATGGCTTTTCCCGAACTTTGAAGTCGGGGGATGGTATTACTTTTGGAGTGTTTGGA
AGTAAATTCAGGTAAGACATTTTAAAATTGATTTTAAAATGGACAGCTTTGTTTCAGCC
AAATCCTAAAGGAAAATGATAT
>82
ATTTACTGTAAACAATTGGACAGAAGAATGCACTCACCTTGTCATGGTATCAGTGAAAG
TTACCATTAAAGTAGGTTGAATGTCTTGTTCTTATGTTAAAAATAAGTTATTTGAATTTAA
GCTTACCCACTATACACAGA
>83
TAAAGCCAGAATATTTTACTGAATTCCTGAAAGCAGTTGAGTCCAAGAAGCAGCCTCC
ACAAATTGAAAGGTATATTATGTATTTTAAATATAACAAGATGTCACTTATATGATAGCAT
TGATTTTTATTTAAGTTAATT
>84
TCTGTCAGGACGGCAGGAAAGAAAACAAATCTTCAAAGGGAAAACATTTATATTTTTG
AATGCCAAACAGGTAATTATGTTATAAGCTAAATTTTCCTAAAGAATACATTACAAACTA
GGATACATTATTAACTCTTATC
>85
CAAACTCACAGACCTTAATTCCTGACTGTCAGAAGAAATGGATTCAGTCAATAATGGAT
ATGCTCCAAAGGTATAGAATTATTCTTTATTAGTAAAAAACTGCAAGTAGGAGATTTTAT
GTTTAACCTTTTTTGTTGCTT
>86
GAAATTGGATTGGCGGTGATTTTCATGACTACAAAGAATTACTGTGATCCTCAGGGCCA
TCCCAGTACAGGTAATTGAAGCATTATTTTATTGATTCGTTAGCAACTTTATTATGTAAAT
CGATAAGCTAAATATACGTT
>87
TAATGCCAAGCGCCCCAGTGAACACTACAACATACGTAGCTGACACAGAATCAGAGCA

AGCAGATACATGGTAAAGCTTCTTCATTACCGTACTATTGTTATTATTCTATCAATAAATT
AATAGTATATTCAGGAAGTAA
>88
AAGATAGGGCTTCTCAGCAGCAGCAGACCAACTCCATCAGAAACTACTTTCAGCCGTC
TACCAAAAAAAGGTCTGTTTTTGATAACTTCTCTCATGTCTCTGAAAAAAAAATGAAA
GTTTTTTATACTGTTGTAGAATGG
>89
GCCAAGGATGGATATAGAAACAAATGACACTTTCAGTGATGAAGCAGTACCAGAAAGT
AGCAAAATATCTGTAAGTAACATTTTAAATGAGAGGTATAGTACATAGAAGCTTAGAAT
GCTAACAGGTACTTTCGATTTTG
>90
TACTTCCAGAAATCCATCTGGCATAAATGATGATTATGGTCAACTAAAAAATTTCAAGA
AATTCAAAAAGGTAGGTATTTCAACTGTATCACAAGAGGTCAGTGTTTGAAATGTTTTT
AGAGATACCAGTGATGCTAAAC
>91
ATCAGATCTAATAGCTCATCATGCTCGAAAGAATACAGAACTAGAAGAGTGGCTAAGG
CAGGAAATGGAGGTTAGTAGGAAGTGAGGCCCAGAAACTTGCTTAAATTGCACTTTGG
TCCATTATGGTGGCCTTCAAGCAA
>92
CCGGAGGACGAGTCGACCCCGGAAAAGAGGACTGAGAAAATCTTCCGCCAAATGGAC
ACAAACAACGACGGTGAGGGGCAGGGGCGGGACGGGGTGGACGGGGCGGGCGCCTT
TCCCTCCCTCCCTCCCTGCCTCCCTTT
>93
GGCGCTGGCTCCCGGAATATGGAGCCCTGGGCGCGGGGGCGGCGTGTCAGGTGGTTG
CGGAGCTGGTAAGGTGAGGGGAAGGCAAGGTCAGGGGTCACGGCATTCCGCTCGCTG
GCAGGGAGGGGCGCGGGGCGGCTCGA
>94
TCATATTCTAAGAACTCAGCCACTCAGGTATCCACCATGGTGCTGGGTCCTGAACAGAA
GATGTCAGATGGTAATTTACCACTTTGTGTTATTGTCTAATTTTATTTGTAATTAGGTTTAT
GTTGGTAACAGAAATTTAT
>95
GTCCCCTGGGGACTCAGAGGAGTACTTCGCCACTTACTTTAATGAGAAGATCTCCATTC
CTGAGGAGGAGGTTAGTATCAGGTCCTTGGAGGAGAACACTATTGTCTGGGGAAAACC
TGTGAGTGAAAGATCTGCTGGAT
>96
GAGCATTGCCTACCTGGATCCAGGAAATATTGAATCCGATTTGCAGTCTGGAGCAGTGG
CTGGATTTAAGGTGAACATCTAGTCCTACCCCTGTCCTTTTAAGCACATAATACCCTCTC
ACATCCTTTTCTCCACCCTGC
>97
GCTTGCAGCTAGACTGGGAGTGGTTACTGGGCTGCATCTTGCTGAAGTATGTCACCGTC
AGTATCCCAAGGTGAGCAATGTATTGTCCTGTTTGTCTCTCACCCATGCCATAGGAATCT
GTGTGTCTGGCGGTATGTGCT
>98
CTATCATCGGCTCAGACATGCAAGAAGTCATTGGCTCAGCCATTGCTATCAATCTTCTGT

CTGTAGGAAGGTGAGGGGTTTTCCTCTAGAAAGTCATCAGTGTTTTGCTGGATTTCCAA
GCAGAGATAAAAACAAAAACC
>99
ATTCCTCTGTGGGGTGGCGTTCTCATCACCATTGCAGATACTTTTGTATTTCTCTTCTTG
GACAAATATGGTAAGGAGAGCAGGAATGGGAGGGTCAAAGGAGCTGCTCTCTGTCAT
GTGGCAGATGGGGAAGTCTACCT
>100
ACAGGCTGTGGGCATCGTGGGAGCTGTCATCATGCCACACAACATGTACCTGCATTCTG
CCTTAGTCAAGGTGAGCAAGGCCCTTATCTCCTTCACCTCCTAGACAGTTCATGGGATC
TTTTCCTTCAATTGCAAGGGAC
>101
CTTCATCATCAATGTCTTTGTTGTCTCAGTCTTTGCTGAAGCATTTTTTGGGAAAACCA
ACGAGCAGGTGGTGAGTAAGCCAGGGTTATGGGTGGTAGTGAATGTGAGGGTAAAGT
GAAGACTGCAATGGGATGGGGAAG
>102
TACCAGCAGTCCTCATGCTGGCCTCTTTCCTAAAGATAACTCGACACTGGCTGTGGACA
TCTACAAAGGGGTAAGCATGTTTGGATTTGTGATCTTTGTTCCTGAATCAGTATTTTTAG
CATAGTATGTGGTCTTTTTTG
>103
AGTGGGGATCCTGGCTGCAGGACAGAGCTCCACCATGACAGGAACCTATTCTGGCCAG
TTTGTCATGGAGGTACGTGCTGGTATAGGATAGAGGGAGATAACACAACCCTTTTGCTA
CTTCTTCTTCTTCTTCTTCTTTT
>104
TGCTGTCTTCCAAGATGTAGAGCATCTAACAGGGATGAATGACTTTCTGAATGTTCTAC
AGAGCTTACAGGTGAGGAGGAAGCTGAGGAAACACATCCTAGTTATTCAGGTAGCATT
ATGTACTGCCCTTGGGTACCAAG
>105
CCTTTGCTCTCATACCCATCCTCACATTTACGAGCTTGCGGCCAGTAATGAGTGACTTTG
CCAATGGACTGTGAGTGTACTCCTCTTCGTTCCCAAAGGGGATGCTGATATGGGGAAA
GGGTATCTTCATGCAGTAGGCC
>106
AGGGCATGTGGCATTATATGTGGTGGCTGCTGTGGTCAGCGTGGCTTATCTGGGCTTTG
TGTTCTACTTGGTAAGTCTAGCCTGGAAGAGCAGGGGCATAGGTATCAGGGATGGTTG
AGGTGGGGTTCACTGACATTCAG
>107
GGGCCGCGTATGGAGCCCCCGCCCCCCGGAGCTGCCAACATTGCCAACGCCACCGCCA
CGCTACACACAGGTGAGCTCTGGGCCTGGAGGGTGGAGGGCCCAGTCCGTGACCCCA
CGTATCCTTCCCGCCCCCGCGCAGA
>108
ATTCCACCCTTCCTGTACATCGTAGCGAATCAATCCGTGGCGCCGCACTCCTCCGCATC
CCTCTTTAACAGTGAGTCTACCTGAGTTTGTATTCCTGCCTCCTCCAGTCCCCCAATGCA
TCAGTCCATGGCTTTTTTCAA
>109
ACTACGCAAGAACAAGACCAACATGAATTACGACAAGCTCAGCCGGGCCTTGCGGTA

CTACTATGACAAGGTACAGTCTCTCAGACCAGGGCTGGAACCCCCCCTGACATTTATA
TCAGAGATTTTATTTTGTTTTAAT
>110
GGGAAGTGGCTCCGGCCTCCAGGCTCCGGGGCCGGCGCTGACCCCATCCCTGCTTCCT
ACGCATACATTGGTGAGTGCTGCGGAGGGGTGGGGTGTGTGGGCGCCAGCACCAATG
GGATTGGCATGTGGCGGGTGACTGA
>111
CGCGCTGCGCGCTGGGGACCGCCTGGTCGAGGTCAACGGCGTCAACGTGGAGGGCGA
GACGCACCACCAGGTGGGGGCCAGCGCTCGCCCCCGGCCCGCCGCCCCCTCCCCGAG
CGCGTCCCCCTGGGGCGAGCAGGGGT
>112
CCACGACCCCTGGGAGCCGAAGCCAGACTGGGCACACACCGGCAGCCACAGCTCCGA
AGCTGGCAAGAAGGTATGGCGGGCTTGGCCCACAGGGACCACCCTAGCCTCTCCCAG
AGGCTCTCTCAGCTTTTGACGACGAT
>113
CATCCGCTCTGTGGACCCGGGCTCACCTGCCGCCCGCTCTGGCCTCCGCGCCCAGGAC
CGGCTCATTGAGGTACCGGCCCACCAGGGCTGCGGGGTGCCGAGTGCCCCGCACCTGT
CCACACTGGGGCCCTGGGTCCTTG
>114
GTCGTGGACCCCGAGACAGATGAACACTTCAAGCGGCTTCGGGTCACACCCACCGAG
GAGCACGTGGAAGGTGGGCCACGGCCCAGGGCACAGGGTGGGTGCGGTGTGGTGGC
TGAGCAGCCACTGACACGCTGTCCCCA
>115
CCGGGAGGCCTGGCCTCCCCCAGGGCCGCCACCTCTGCTGGTTGCTCTGTGCTTTCAC
CTTAAAGCTCTGGTAAGGAGAGTAAGGGGAGGGAAAAGAGTCACTGGAGGACAGATT
CGTTCCCCGGCCTCCGGGCTGGGGA
>116
TTTGCCATGCTGGCTGGTGGAAGAGTTTGTGGTAGCAGAAGAGTGCTCTCCATGCTCT
AATTTCCGGGCTGTGAGTATCTATTTCTCTACCCTTTTTTTTTTTAAACATCTAAGCAAA
CACTTAAGCAACATCTTAACAT
>117
CTGAGTGTGGTCCCACAGGATATGTAGAGAAAATCACATGCAGCTCATCTAAGAGAAA
TGAGTTCAAAAGGTGAGTGCTGTTTCTCTTCTCTGAGATCACCTCCTTTGCTCCTATGA
CTTTAGATGGATGGTCCCACCTT
>118
GGAGCAGGAGAGCTGTCTGGATGGCTGGGAGTTCAGTCAGGACGTCTACCTGTCCAC
CATTGTGACCGAGGTGGGTGCCGGCCCCTGCTGGGGCTGAGACCAGGGCTCGGAGGA
CCTGTCGCGGTCCTTGAACCCGAGCT
>119
CACTCACAATCTCCTTGTTCTTCGTGGGTGTGCTGTTGGGCTCCTTCATTTCAGGGCAG
CTGTCAGACAGGTAAGGTGTCTGTCTTCTGGAGCACCAGGGGACCTCAGCACTGAGG
AAGAAGCGTGTGCCTGGCCCTTGA
>120
ATGTTTGTCGTGCTGTTTGTCCTTGTAGGCATGGGCCAGATCTCCAACTATGTGGCAGC

ATTTGTCCTGGGTATGGCCATCAGGTTGGAGTTGAGTACTTGATCCTGTATTTCACCATC
ATCCCATCACCTACCTTTCTG
>121
TCATCCGAGACTGGCGGATGCTGCTGGTGGCGCTGACGATGCCGGGGGTGCTGTGCGT
GGCACTCTGGTGGTGAGTGTGACCCTGTGCCCCATGTGCCCACTGGCAGGATGATTTC
TGTCTGGCCTTCACTAGAGGGCAG
>122
GGTGATCATCCGCAAGGCTGCCAAAGCCAATGGGATTGTTGTGCCTTCCACTATCTTTG
ACCCGAGTGAGGTAAGCACCATGTGGGTGTGGGTGAGAGGGACAGACTGACCGTGAT
TTGAGAGCAGCAGCACCCAGCCCT
>123
CCCACAACATTCTGGATCTGCTTCGAACCTGGAATATCCGGATGGTCACCATCATGTCC
ATAATGCTGTGGTATGTAAAAGAGACCTGCCTGAGGCTTCCAGACAAAGCTTCTTGAA
GTGGCCATTGGGCCTCTTGTTTA
>124
CGCTATTCCATGGCCACTGCCCTCTTCCTGGGTGGCAGTGTCCTTCTCTTCATGCAGCT
GGTACCCCCAGGTAGGGACCATGTGCATCTATGGTTTGGGGTCTTCACTGAGTCTCTTA
CTGTCTACCAGGCTGTCTCAAT
>125
ATGGGTGTGGGAGTCAGCTCCACAGCATCCCGCCTGGGCAGCATCCTGTCTCCCTACTT
CGTTTACCTTGGTAAGTCCCATGAGCCAAGGGCACACTAGAGCAACGGGATGGAAGTA
CTAACTGGCTTGAATGTGAGCTG
>126
CCTTGTTTCTCCCAGAGAGCTTCGGTACCCCACTCCCAGACACCATTGACCAGATGCTA
AGAGTCAAAGGGTAAGAAGACCTCCTCTGTCAGTGTTGATGCACTGGGTCTGGGTCTG
GCCAGGTCTCAGGAGCCCCTCAC
>127
CTCGGGGTCGTATGACGCACTTTTCCAGCTCGAGCCCTCACGAGGCCGTGGGTACGAC
CGGAAGCCGCAGGTGGGTGAAGCGGAAACGTTTCAAACCGGGATGTTGCGGGCCGTA
GCGCGTGGGCGGCCGTGGGGTCTTG
>128
GGGTTCAAGCGATTCTCCTGCCTCAGCCTCCCGAGTAGCTGAGATTACAGGCGCCCGC
CACCACGCCCAGGTAATTTTTGTATTTTTGGTAGAGACGGGGTTTCACCATGTTGGCCA
GGCTGGTCTCAAACTCCTGACCT
>129
ATTCTGGAACCCTAACAAGATCACATTTTTGACCAAAGTCAATATCTTGGACTATAGTTA
ATTTATCAAGGTAAATGCCACTTTGATGTGTCTTGGTTAGGAATCTTTGTTCACTTGAGG
GAATACATGGTATATGATAG
>130
GAGTGCTTTAGGAGGCCAAGGCAGGAGGACTGCTTGAGCCCAGGAGTTTGAGACCAG
CCTGGACAACAAAGTGAGATTCATCTCTACAAAAAAAAAAAAAAAAAAAAAAAATAGC
CAGGCGTGGTGGCACATGCTGGCTAA
>131
CCCGGGCTCCCAGAGGGAACTGCACTTCGGCAGAGTTGAATGAATGAAGAGAGACGC

GGAGAACTCCTAAGTGAGTAGATGCAGCCCATGCAGTTCGCCTTCTTTTATGCTTTTTC
CTTCTTTTGCACGTCTCTTCTTTC
>132
GATGGGATCCGGCTCCTCCAGCTACCGGCCCAAGGCCATCTACCTGGACATCGATGGA
CGCATTCAGAAGGTAGCCCCTCCCCCACCCAGACACCCCCTCCTCCCCCCGGGTGACA
GCGCCGGGGCCGGGCGCGGCGGGG
>133
TAATCTTCAGCAAGTACTGCAACTCCAGCGACATCATGGACCTGTTCTGCATCGCCACC
GGCCTGCCTCGGTGAGTGCGCGCTGCGGGCTCTGCCCGGTGACGCCACGCGGCCTCCT
CGCCTTTTCGGGATGGCTGGGAG
>134
CCATCTCCCTGCTGACCACCGACGACGCCATGGTCTCCATCGACCCCACCATGCCCGC
GAATTCAGAACGGTAAGAGGCTCCGGCCGCGCTCCTCGGGGTGTGCCTGGCACTTCTC
TCCATGACATGGGAGGCTTTCTGT
>135
AGTGGACAGGTAGAGCCCAGGCCCAGAGAGCCCCAGGGCTGCTACCAGGAAGGCCA
GCGCATCCCTCCAGGTAACGGGCAGCTCCTCGGCCACAGCCTCCACCCCCCAACACGT
GGGATTCGGGCTGCAATGGCCTACAC
>136
GCATTCAAAATCAATGAACTGAAAGCTGAAGTTGCAAATCACTTGGCTGTCCTAGAGA
AACGCGTGGAATGTGAGTGACGTTTCTGTTTCCTTTTTGCTTCTCTGTCCTTTAACCTCT
ATTCAAATTAACCACTTGGAAC
>137
TGGTGGAGATTGAGAAATGCAAGAGTGACATTAAGAAGATGAGGGAGGAGCTGGCGG
CCAGAAGCAGCAGGTAGGGTCTGCGCTGGGGCCACGGGCGGCCGGGCCTGGGGAGG
GCTCCCCGTACCAGTTGGGCTGGGGCT
>138
CTGTAAGTACAGTTTTTTGGATAACCACAAGAAGTTGACTCCTCGACGCGATGTTCCCA
CTTACCCCAAGGTAAGATGAGATTCCGGCCCAGAAGAAGCTGCAGCTGTGTCCCCAGC
CCCACGCCCAGCCCTGTGGCCCT
>139
GCTCTCTCCAGAGACCATCGAGGCCCTGCGGAAGCCGACCTTTGACGTCTGGCTTTGG
GAGCCCAATGAGGTAAGTGCGGGGCTTGCAGGCACCACGTCCCAGGGGGAGGCAGCT
CAGGATCTTGGACGCCAGTGGGACC
>140
GCACATGTACCACGACCTCGGGCTGGTCAGGGACTTCAGCATCAACCCTGTCACCCTC
AGGAGGTGGCTGGTGAGTGCCAAACCCGCCTTCGGTTCTTCCTGGGCACGTGGTTTCA
TCCAGTTCCACAGGAATGGAGGGA
>141
CCACAACTTCCGGCACTGCTTCTGCGTGGCCCAGATGATGTACAGCATGGTCTGGCTCT
GCAGTCTCCAGGTGGGTCCTGCCCGCTGCACACCCAGACCTCTACTCTCGGGGGTCAG
ACGGAGGCCCCCTTCCAGGGAGC
>142
AAACGGATATCCTGATCCTAATGACAGCGGCCATCTGCCACGATCTGGACCATCCCGGC

TACAACAACACGTATGTACAGGATTTTCTCTTTTTTTCCTTTTAAAAGGCACCCTGGCTA
CTGGAGGGAACCTGTCAGCCC
>143
GATCCTCGCCGAGCCTGAGTGCAACATCTTCTCCAACATCCCACCTGATGGGTTCAAG
CAGATCCGACAGGTGTGTGGGGTGAGGGCCCTCCCACCGGAGTGGGGGCACATTCAG
GGACAGAGCAGCCCCCACTCTCCAT
>144
AGAAATTATGGATTCTTTCAAAGAGAAAATGGAGAATTTTGACTACAGCAACGAGGAG
CACATGACCCTGGTGAGTGGCTTATTCTGCCTGGGTGGGCAGCCAGGCGGTGGGCTGG
CGAAGCAGGTCATCCATCCAGCTC
>145
TAACGAGGTCCGTCCAATGGAAGTCGCAGAGCCTTGGGTGGACTGTTTATTAGAGGAA
TATTTTATGCAGGTAAGAGTCTTGCAGAGCAATCAAGCCTCCAGCCACTCTTATTAGCC
CCACTTAGTAAGGGTTTGCGTTA
>146
GACCAAGGCCACAGCCCAGATTGGGTTCATCAAGTTTGTCCTGATCCCAATGTTTGAA
ACAGTGACCAAGGTGAGTAACTGTCACCACATGTCACACTTGCTTACACTCAGATACA
TGCATGCACACACAGGCACACACA
>147
GCTGCAGCCACTTTGGGAATCCCGAGATCGCTACGAGGAGCTGAAGCGGATAGATGAC
GCCATGAAAGAGGTAAAACACACTGAGAAGAGCCTGCCTTCCTTGCGGCAAGCAGGC
ACGCTGCCCTCCGCACTCCCGACTC
>148
ACTGACAGCTTGACGTCTGGGGCCACCGAGAAGTCCAGAGAGAGAAGCAGAGATGTG
AAAAACAGTGAAGGTAATGCTTGCTCTGCTGAAGTGGCATCTCAGCGCATACAATGAT
TCTGACAAAGGACAGAAGGAAAGAG
>149
ACTGGGCACCCAGTCCAGGAAAACCAACCCAAAAGGAAGCCAAGTATTAAGAAATCA
CAAGGCAGGAAGGGTAAGACAGCATTCGCAGCATATTTTATTTCAGAAATGCATTTTCT
CCTCCCAGCCTCTTAAGGATAGGG
>150
CCCGGGCTCCCAGAGGGAACTGCACTTCGGCAGAGTTGAATGAATGAAGAGAGACGC
GGAGAACTCCTAAGTGAGTAGATGCAGCCCATGCAGTTCGGCCCTTCTTTTATGCTTTT
TCCTTCTTTTGCACGTCTCTTCTT
>151
TTTGTCCTTCATTGAATTACGACACTGTCCACCTTTAATTTCCTCGAAAACGCCTGTAAC
TCGGCTGAAGGTTAGTGCAACTTCATTTCTTTCCTTTACTCTCCAGAGCTCCCCAAACA
TCAAGAAACAGGACAAGGCAA
>152
GTGTGGGGACAACGCGGCCTGCCAACACTACGGCGTGCGCACCTGTGAGGGCTGCAA
AGGCTTCTTTAAGGTGAGCAATGGCGGGAGCGGAGTAGGCAGGTAGGGAGCCCCTAG
TGCCCGGGACCTCGGAGTGTGCCCTC
>153
AAGCGTCGCCGGAATCGCTGTCAGTACTGCCGATTTCAGAAGTGCCTGGCTGTTGGGA

TGGTCAAAGAAGGTAGGCTGAGGGGAGCTGCCGACCCTCCAGTTTGCGCCTTTAGGA
AACCACTGCTCATACTCCAGCATCA
>154
TCTGATCAGTGCCCTCGTCAGGGCCCATGTCGACTCCAACCCGGCTATGACCAGCCTG
GACTATTCCAGGGTAAGAAGCTGGCGGGGGGGATATCATGTGGACAAACCGACAGATG
GGCAGGACCCCTCCCCACATCCGT
>155
CCAAAGCCGACCAAGACCTGCTTTTTGAATCAGCTTTCTTAGAACTGTTTGTCCTTCGA
TTAGCATACAGGTAATAAGGGAGGGAGGAGACAATCCAGGGAGGCTGTGAGAGAAAT
CAAGAAAGGAAAAGAAAGGGAGGA
>156
TCCAACTTGCAGAATATGAACATCGACATTTCTGCCTTCTCCTGCATTGCTGCCCTGGCT
ATGGTCACAGGTCAGTACTGCAGGCGCAGGGCGCTTCCCCTCCAGAACTGCCTAGCAG
GATTTGTCCTGAGTTTCCCTTG
>157
CAGACCGCGGACGTCTGTAATCTCAGAGGCTTGTTTGCTGAGGGTGCCTGCGCAGCTG
CGACGGCTGCTGGTGAGCGCCCCCGACCAGACCGAGGGACAGGGCGAAGGGGGACT
GGAGGAGGGAACCCCCCTTTAGGGAC
>158
AAAATTTGACCAAAATTTGGATACAAAGTGGTACCAGTGGAAGGCAACACACAGAAG
ATTATATGGCGCGGTTGGTAACATCTGAAACTGTCCAGAGGGACTCTGGAGAGAATGG
TCCTCGCTGTTGGGAGTTGATAGCC
>159
CACAATGGGGAATACAGCCAAGGGAAACATGGCTTCACAATGGCCATGAATGCTTTTG
GTGACATGGTGAGTGTGGCAAGGATTGCTGAACTCTGTGCTGCTTCTCAGTTCTTCAC
CAAAATAGTCTTGCTTTTAACATT
>160
GATCTTCCCAAATCTGTGGATTGGAGAAAGAAAGGCTACGTGACGCCAGTGAAGAATC
AGGTGAGACAGCGTCAGATTCAGACCCTGTCTCCACAGGAAAGCCAAGAAGGAATTG
GTGTCATTGCTGTGGTAGATGGTGG
>161
TAGGGCCTTCCAGTATGTCAAGGAGAACGGAGGCCTGGACTCTGAGGAATCCTATCCA
TATGTAGCAGTGGTAAATGGAGCTCCTTATCTTGTCATTCGTGCTCTGCTTTTGGAAAGT
GGAACACTTTCAGAGATAACAG
>162
GTCGCAACTGTGGGGCCCATCTCCGTTGCTATGGATGCAGGCCATTCGTCCTTCCAGTT
CTACAAATCAGGTAAGTGTCATTTTATTATAGAAATTTAGGCAGAATTGGGAAGCATCA
CTGTGATTGATTCTTGGATATG
>163
TTCTGGTGGTTGGCTACGGCTTTGAAGGAGCAAATTCGAATAACAGCAAGTATTGGCT
CGTCAAAAACAGGTATAAAACTCAAAATTGAAAAGGGAATTTTTGTTCCAAATCAGTA
TTTGGATACAAGTTCACAAAAGCT
>164
GGGGGAAAGATGGTGGGGACCAAGGCCTGGGTGTTCTCCTTCCTGGTCCTGGAAGTC

ACATCTGTGTTGGGTATAGGCTAAGTATCTGCTGGCTCCCAAATCTTCCACTTGCCACA
GTGAGGTTGTGTCTGGTCTGGGAC
>165
TCAGTAAGAAGAGTCCAGCCTGGGAAGAAGAACCCCAGCATCTTTGCCAAGCCTGCC
GACACCCTGGAGAGTGAGTTGTGGCTGAAGCAGGGAGGGGCTGGAGGGGCAGCCTTT
AACGAGCTTCCGAAGTGGATCTCTGA
>166
TGCCTCAACAGGGAGCAGCGGCCTGGCCAGAACTGCTCTAATTACACCGTACGCTTCC
TCTGCCCACCAGGTAAGCCAGAACGCCCCCACCCCCACCCGCTACTTCCCACTGGCCT
CTACTCAGGGAGAGGGAGAACCTG
>167
GCAGAGATGGTGTCGCTGTGCAGTGAGGCCAGCGAAGAGGGTCAGCACTGCATGGGC
CAGGACTGTACAGGTACCACCCTCCCTCCTGGCACTGGGCAGGGTGTGGTGGGTGAG
GCTGAAGAATGAGGAGGGAGTTAGAG
>168
CCCATTGTACTCACAATGCCCAAGACTAGCCTGAAGGCAGCCACCATCAAGGCAGAGT
TTGTGAGGGCAGGTAACTAAATTCTTTGGGATAGGGCTGGTGAGAAGGTCCAGATTTTT
AAATAAAGGGATTATAGTGCTAA
>169
GGAGAGCTGGGCAGAGCGTGTCTCTGTGCTGTAAGGCCACAGGGAAGCCCAGGCCAG
ACAAGTATTTTTGGTGCGTATTCTGCTAGCTACCCTGCCCAATTCTCCTCCTGGAAACC
AGAGCTTCTTGCATTGGGTCTGGA
>170
GAGTACTTTTGCAAGGCCCAGAGTGATGCTGGGGCTGTGAAGTCCAAGGTTGCCCAGC
TGATTGTCACAGGTAAGCCTGTCTGGGTCCCTGGGGCCCCTGGGTCTGCAACAGGGTT
GGCTGAGCACCCAGGTGGGCATAA
>171
CCGCTCCTCCTGGAGAGGGGATCTCTGCTGCTCCGACAGTTGAGCCCAGTTCCGGGGA
GGCTGAAGGCGGGTAAGAGGTCCTGCCGCCCGAGGAAGACGCGGGAGGGAGGCCCG
CCCCTCGCGAATCCCGCGGCTCCTGG
>172
GTGAGGTAGAGCTGCAATGTGGGATTTGTACAAAATGGTTCACGGCTGACACATTTGG
CATAGATACCTCGTGAGTACTTTTCATAGTTTTTGTGAGAATTGCTCGGTAAAATAAATC
TGAACATGCTCAACAGTTACTT
>173
ACCAACTACAGTTTTCATTGCAACGTCTGCCATCACAGTGGGAATACCTATTTCCTCCG
GAAGCAAGCAAGTAAGAACAAACTCTGGAGTATTTGAAGATGATTATCCACTGGAAAA
GAAAAGGGATCTGGGCGTAGCAG
>174
TTTGGCCAACCTGACATGGCAGTCCCGAACACAGGATGAACATCCGAAGACAATGTTC
TCCAAAGATAAGGTACAGGTGGAACTAATGTGATTGCAGTTATATTGAAGAGTTAGGTG
GAACTTCTAAATATACTCCCTAA
>175
CTGGGAGTGCATGACAACCAGACAGAGACCTGGGAAAATGACTTGGCCAAATAACAT

TGTTAAAACAATGGTAAGTAGATTAAAATTGATTAGACTTGACATTTAAAAAACCATTG
TTTTTTGTAAGAATTCAATAATAT
>176
AAAGGAACACCCAGATCCAGGCAGTAAAGATCCAGAAGAAGATTACCCCAAATTTGG
ACTTTTGGATCAGGTACATTAATATGTTTTACATTTTCTTTTTGATTTTAAGCCACTCGGG
GATAGGTAATCAGGTTCTGTTT
>177
AGTAACATTGGTCCTGCTTATGACAACCAAAAACAGAGCAGTGCTGTGTCTACTAGTG
GGAATTTAAATGGTAAGTGTTTACATATCTCATTGCTGAAATATTTGGAAGCTATTGAAT
CCAGTTGTGCCTAGAACTGGCC
>178
GAAAAGGACGAGGAGCCAAGCGCAAACAGCAGGATGGAGGGACCACAGGGACCACC
AAGAAGGCCCGGAGGTGGGGAGAGTGCCCACCCTGCCCCCCTCACTATTTAAGTATTA
TTTGGTGCTGGGAAAGTCCAGCAGAG
>179
AAACCTATTCCTGGAGACCTCTACAGAGCCTGCTTGTATGAACGGGTTTTGTTAGCCCT
ACATGATCGAGGTATGTAAAGTATTGGAGATCATATGGATGCAGGGTTACTTTGAACTG
CCATCATTAGCCTTGTCAGTCT
>180
TATTTTGAAATCACTGTGGATGAGATGCCACCAGATACCGCTGCCAGACTGGGTTGGTC
CCAGCCCCTAGGTAAGCTGGGGCCTTAATATGGACATCACAGCAGATAGAGAGGATAG
ACCATCTGGCCAAGGGCTAAGGC
>181
GGGATTTTATATTAATCTTCCTGAAGACACAGAGACAGCCAAGTCATTGCCAGACACAT
ACAAAGATAAGGTGAGTTTGTCCTCTCCCGGCAGATTCCTGGCTTTGAAGGCCTGTGG
CAGCCAGTGCTTATCACTGGCTT
>182
TTTGTATTTTGAGGAAAAAGACTTTGTGGATAAAGCAGAGAAGAGCCTGAAGCAGACT
CCCCATAGTGAGGTGAGTCATGGCCATAAGAACATTAGAATCATAAGGCCTTGAGCGTT
AGGACCCATTCCTTCATTATAAA
>183
AGGTGTGGCTTACAAAGATATTTTTGAGGGGGTTTACTTCCCAGCCATCTCACTGTACA
AGAGCTGCACGGTACGTACATGTTTCCATCCCATGAGCAAAACTTGAGGGAAGCAGAT
GAATGGGTTGTGATGACAAAGTT
>184
GCTGGAAGCCAAGCGGCTGCAGTGCCCCTTTGAGCAGGAGCTGCTGAGACGGCAGCA
GAGGCTGAACCAGGTGGGTGATGGGCACCCAGCAGGGACCACGCATCCTCCAGGGCT
GTCCTCCAGGGAGGAGCTCTGCTGTG
>185
GGCTAAGGTCCTCTGTTGCTTTTTGGTTCCACCTTAGAAGAGGCTCCGCTTGACTAAGA
GTAGCTTGAAGGTAAGCCAGTGGGGAGGAGGGCTCCAGGGCCAGCGGCGGGAGCGG
GAGGCCTGTTGGACATAGGGGCTGG
>186
ATGTTGACAACATTGCCGCCGATACTGCTGCTGTCTGGCTGGGCCTTTTGTAGCCAAGA

CGCCTCAGATGGTGAGTCGGGGGCACATCTCCTGCCTCAGGATGGTTCTGGAGAATCT
CAGTCTATCTGGGCACATGGCAA
>187
GGCCTGCAGTCCTACCTGCTCCAGTTCCACGGCCTCGTGCGCCTGGTGCACCAGGAGC
GGACCTTGGCCTGTGAGTAGGCGCGCAGCGGGGGCGGGGTCTGGGCGGGGCTAGTGG
GGGCGGGGCCTGGCGGGTGGGGGCG
>188
CTGCGGGAATTCCTGGAGGACACCTGTGTGCAGTATGTGCAGAAACATATTTCCGCGG
AAAACACGAAAGGTATGATGGGACGGGGCCCAGGCCTGCAAGCTGGGGAGAGGGCG
GGTTCCAGACAAATGGATGGACCTGA
>189
CAGGCTCAAGCAATCCTCCCGCCTCAGCCTTCCAAGTAACTAGGACTACAGGCATGTG
CCACCACGCCTGGTGAATTTTTTTAAATTTTGTTTTGTAGAGATAGGTTCTCACCATATG
CCCAGGCTGGTCTTGAACTCCT
>190
TCTTCACTAGTTTCCTGCTGCTGGTGTACTCCTATGCCGTGCCCCCGCTGCATGCCGGC
CTGGCCTCCACGTGAGTGGCCCAGCTGGGCAGATGATGGGTGTAGGTGGGGGAGGGA
TGGCACCCCACCCATGGGGCCCTG
>191
TCAGTGCCAGCAGCTTTCACAGAAGCTCTGCACACTGGCCTGGAGTGCACATCCACTA
GTGGGACACATGGTGAGTGGCAGCCCCTGGAGCCTAACAGGAGTCCAGGCTCTCCAA
GGCTGTGGCAGAAGACCGTGACCTT
>192
TACAAATGATGTTCCCCATATCCAGTGTGGAGATGGCTGTGACCCCCAAGGACTCAGG
GACAACAGTCAGGTACCACTGGGATGTGGCTGGGCAATGAAGGAGAGGGGACTGAGA
ACATGGCTGGGTACCATGGTAAACC
>193
TTCTCTGCTCCCTGATAGCCCTGTGGGCCAGCTTCATGCCTCCCTACTGGGCCTCAGCC
AACTCCTGCAGGTATGAAGTAGGGGCGTGGAGGATGGGGGCTTGCAGGTGTCAGAGA
CAGAGGGTTGGGGGTTAAGGGTTT
>194
GATGTGGGAGTCTTCTATGGTTCCACTGCTCCATCTCGCCTGCCCACCTGGGGCACAGG
GGCCTCAGCAGGTGAGGTGCCCAACCGCTGTGGGGAGGCATGAGCAGTCCCAGGAAA
TTCCTGCTGTGAGGGGCCGGGGAA
>195
GTCTCAGATGGGGCCCCTGCACAGCCGTGCTGCCTGCTGATTGCAGAGCGCATCCATT
CGGGTTACGAAGGTGGGCATGTCAGGGCCCTGGCCTGGTGGGGGCTGGGAGGAAGGA
CATCTCCTCATGGCCAGACATGAAT
>196
CTGGGGGCCCCAGTGCTCCTGTTCCTGCATCCTGAGGACCGACCCCTCATGCTGGCTAT
CCACAAGAAGAGTTCCTCTCGCCCTGCTCGCCCTTCCCACTGTCCTGGGCTTTTGAGT
GGCTGCCCTTGTGGTTGTGTCTC
>197
CAGCCCAGCTCCCTCCCTGGACACTGATATCCAGGAGCTGTCAGAGCAGATCCACCGG

CTGCTGCTGCAGGTGAGAGTAGCGGACAGGGAGCCTGGGAGGTGAGAAAAGGTGTG
GGAAGCCGGGTCAAGCCATCTAACCT
>198
TCTCCACAGCCCTGGGTCCTCCAGTGATAGCAACGGGGGTGATGCAGAGGGGCCTGG
GCCTCCTGCGCCAGTGAGTGACCTGCTCCTACCTCACCCTCTAATCGCCCTTTCCCCTC
TCCTTCTTGGAACCAGCACTGCCA
>199
TATACCACCTCCTCAGCCTCTGACGACGACAGGCAGAGGACAGGTCCAGTCTCTGTGG
GGACCAAGAAAGGTAAAGATCCAATGCACCCTGCTCCCACTGCCCCGTCCTGGCCTGG
TGGCCCTGTGTCTTTTCCCTTCTG
>200
AGGATCCCATTTGGCTGCTCATGGCCAATGCTGACCAGCGCGTCATGATGACCTACCAG
GTGCCCTCCAGGTGAGGCATTTCAGAGGCCTTCTTGGCCTTCCTTTCAGAGGTAGTAGT
GGGGGCAGTTTTCTTTTCTGGG
>201
GCGGCGGGAACTGGGTGCTGTGCACTCCTGGGTCCGGAAGGGCCAACTGCCTCGGGC
TCTTGATGTGATGGTGAGAGAAGCCTGGGACGGGGAGAAAAAAGAATTGAGCTCAAT
TCAAGGGGGAGAAAAAAGAATTGAGC
>202
CCTCCTCCCCGCGCTGGTGCTGCTGGCCCGGCTGGGGGCCCCGGCGGTGCCGGCCTGG
AGCGCAGCGCAGGTGAGCGGCGGGGGGGAGGCCAGGTGCCGGGTTGGGGGGGTCCT
CACGTCTGTGGGTCTGGTCTATCGGG
>203
CACGGCGGCATCCACTTCGACGACAGCGAGTACTGGGTCCTGGGCCCCACGCGCTACA
GCTGGAAGAAAGGTGACCGTCCAGGCTCCTCCTGGAGGCCTCTCCTCTGCAGCACAG
TGGGCTGCCGCGGTCGGGCTTTGGG
>204
CTGAACGCCACGCTGCGCGGCTGGAAGGCGTTGTCCCAGGACGAGCTGTGGGGGCTG
CACCGGCTCTACGGTGAGTCCCTTTGTCGGGCGGGAGGGCGGGGACCGGGCGGTCCT
GAGCCAGGCGTGCTCCCCACGCTCCC
>205
AGGGGCTTCTGCGACGCTCGCCGGCGGCTCATGAAGAGGCTCTGCCCCAGCAGCTGC
GACTTCTGCTACGGTGATGCCCACGGGCCGGGACAGGGCTGCGTGGGAGCTGGGCCT
TGGCCATGGCGCAGCTGAGGGGGCAC
>206
TGGTGCCCGAGGGCAGGAACGTGACCTTCCGCTGCGGCCAGAAGATCCTCCACAAGA
AAGGGAAAGTGTAGTGAGTGAGCGCCCCGGGCGGTCCTCGGGGTGGGCAGCCCGCGG
GCGGCCTTGGGGCAGGGGTGCGGGGC
>207
CGTCTTTCAACTGGCAAGAGTCATTTTGACCAGCAGATTAATCAACTGTAAGACGGATC
CTCACACAAAGGTCGGTACTTGGAAATATAAACTGAATATTTTTACTTTGAATATTGAAA
TTTTTCTCAAAGATAACATTT
>208
GAATGTAAGGGGAAGCGAGGTGTGAAGTATATTTATATACATATATACACACATATAGTC

AATGCCAAAAGTTTATCTTCTGTTCCCTCGGCGCCAACTTATTTCAACAACGCGAGCTT
TGCTTAGAAAATCAATTCCAC
>209
TGGAACAGCCAGGTGTACTGCACCAAGGACCAGCCACAGGGGTCCCACTTTTGGAAA
TCTTCCTTTCCAGGTAGGGGCGATGGAGAAAAGGGACCGACACGAGGGAGGGGGAGA
GAGAAGGAGAAAAGAAAGGACTAAGA
>210
GAGTGGCGACCGCGGCTGGAGTGTGGGCCACCTCAAGGCCCACCTGAGCCGCGTCTA
CCCCGAGCGTCCGGTGAGAGCGGCCCCGACTTCCCGCCCCTAGGCTGTGGCCCCCCGC
CCTCTGCTGGGGATGCCACGTCCGG
>211
GGACCAGAGGTTAATTTATTCTGGGAAGCTGTTGTTGGATCACCAATGTCTCAGGGACT
TGCTTCCAAAGGTACATCACTTACACATTAACTTCTGAATGTTTTTAAGCACTCACCAG
GTTAGGTTCAGGTCCTTAAGTA
>212
AAAACGGCATGTTTTGCATCTGGTGTGCAATGTGAAGAGTCCTTCAAAAATGCCAGAA
ATCAACGCCAAGGTGTGTCTGCCTCTTCATGATGGTAACAATTTGTATCATTCAGACTTT
CAGGGGAGTAATAAAAGAAGTT
>213
CAAGTGATGGTTTAAGGCAAAGGGAAGTTCTTCGGAACCTTTCTTCCCCTGGATGGGA
AAACATCTCAAGGTGAGTGTTATAATAAAGATCTTGGCTTATGCAACATGAATGTTCCT
CGTTTGCATCAATTTAAGAATAA
>214
ACACACCCTATGGGTGGCTTCAGCTTTCCTGGTTCCAGCAGATATATGCACGACAGTAC
TACATGCAATAGTGAGTCCTTCCCGCCATGCTGGGTGTGGCCAGGGCTCCCGGGAATT
GAAGGGAATTTTATCCATGTTAC
>215
GTATCCTCTACTTCTACTCCTCCCTGAGCAGATTCCTCATGGTCATGGGGGCCACCGTTG
TTATGTACCTGTAAGCAGATGGTTTCTCTAATATAAATTACACTACACTGTGTTCACACT
AAGCAGATTTTGCTCTTTTT
>216
GGTTCAGAACTTCCCAAATGATGGTCCTCCTCCTGACGTTGTAAATCAGGACCCCAAC
AATAACTTACAGGTATGGAGCCTCCCACGAAGCCCAGGCGAGCTTGACGTGATATGCC
AGGCTCTCCAATCCTCAACCTTTA
>217
CAGCATCACATCAGCATTAGGGATGGTCCTCTTAGGAGCCAAAGACAACACTGCACAA
CAAATTAGCAAGGTAGCTATCAGCATCATTACGTTGTCCTGTTGCAGTTTTTCTCTGGTT
CCGTCGGCTAGCACGCAGATGG
>218
ATCCACTGATGCATATGAGCTGAAGATCGCCAACAAGCTCTTCGGAGAAAAGACGTAT
CAATTTTTACAGGTAATTTCACCTGGCCTACCCACATTTCATTTGCATCCTGATGTCTGT
GTCTCTGAGTGGCCAAATGGAA
>219
ACTGATTTTGCAAATGCTCCAGAAGAAAGTCGAAAGAAGATTAACTCCTGGGTGGAAA

GTCAAACGAATGGTAGGAGAGCCACCCATTATAGAAACACCTTTGAGAAACCTATGCC
AGTGAGCCTTGTGCTTGACACTGC
>220
TTTCAAAGGGCAGTGGGAGAATAAATTTAAAAAAGAAAACACTAAAGAGGAAAAATT
TTGGCCAAACAAGGTATTGTCTATATTTTATTTATATAGTGTAATATGTTAATACATGGAAT
GTTAAACATTTCTGATGGAAT
>221
AATACCATACAAAGGCAAAGATCTAAGCATGATTGTGCTGCTGCCAAATGAAATCGATG
GTCTGCAGAAGGTAAGAACTTGCATCTACAACTCTTCCTTCTACTGCCGGACATTTTTC
CAAAGATACCAAGTTTAAACAA
>222
TTTAACGCTGACAAGTTCTGCATTTCTGCTGTTTCAGTTGTACTACTACAAGCACTATTT
ATCAACAAAGGTAATTTTATTCCTTCTTTCTTATCATTCCTCCTTTCTTATCATTGTATATT
TTCTGATCACACTTCTCT
>223
ATGCAAGTTTTTCTGTGTTCCAAGAGACTTTACTGCATTTGCACTGCAGTATCACCTATG
GAAGAATGAGGTAAGTGACTTGCTTTCAGATAATGGAATGTGTCTCTTTTTACAAAATA
GTATAGGTTTAGGCTAGTTTA
>224
AACACATTGACAGGAAATTTGTTCCCTTCCGAAAGTTACAGTTTGGTCGTTATCCAGGA
GCTTTTGACAGGTAAGTTCAGAGTCAAAACGTGAAATGTGAAATGAGTGTTGTTCAGT
CATAATTCTTGCAGTGTTTTTAC
>225
CTTTGTAGAAGAAGTACCACACTCTAGGTTTATTGAGTGTAGGTATAAAGAAGCTCGAG
CATTCTTTCAGGTTAGAGACAACCAAATGTGTACTTTTAAATTAAAGAAAATGTTGGGA
TTATTTTTAATATTTGAGGGTG
>226
CTGCAACTAGCAGCGAAAACATTAAACAAATTGGGAGTACCATTCTGGCTGAGCAGTG
GAACTTGTCTAGGTAAAATTCTTACGACTTTCCATTTGTCTTTCTGTCATTTTGTCCTCT
GGCTTTAGGGATTACTGCTTGA
>227
AGATTACAAATCTGATATTATTTTAGCATTTCAGGATGCAGGACTTCCGCTCAAACACA
AATTTGGGAAGGTCAGTAACAAAAGTCGGCTTCATTTCATAAGTAACATATCTCACTTG
TAAAGTTTACATTAAGCAACTC
>228
TTTTCTTCTATGAAGAAACTGATCACATGTGGAATGGAGGCACTCAGGCCAAAACAGG
AAAAAAATTCAAGTATGAATCAAATAAGTACTTATTTATAAAGGTACTACAGAAATAATT
TCAGGAGGTAGAAATAAAGAAG
>229
ACCGAAACCCTGCAGAGGGAACCTGGTACATCCAGTCACTTTGCCAGAGCCTGAGAG
AGCGATGTCCTCGGTAAGTTTTGCCTACTCAGCCCTCCTCACTGTTACACTACCTTCCC
CCCCTACTCCATCACACTACTATC
>230
GTGGTGGCCGTCGCTGCCAGGCACAAGTGTCCCGCCGCATCTCCTTCAGCGCGAGCCA

CCGATTGTACAGGTAGGGTGTGCACACAGGTACAGCGGCGGGCGGTGGGCGCCGGGC
CCCGGAACGTCACCGGGGCGGGGCC
>231
AGTGATGAAGAAAACTTGAAACTGTTTGGGAAATGCAACAATCCAAATGGCCATGGGC
ACAATTATAAAGGTGAGAGAAAAACTGATGACATTTCAGCCCTTCAATAAGGATGAAA
GAGTATTCAGCAAATGTAGACATA
>232
GTATTCCCGATCTGCCATGTATTCCAGAAAGGCCATGTACAAGAGGAAGTACTCAGCCG
CTAAATCCAAGGTAAGGGAAGAGGACCACGGACCATTATGTCTTAACCTTAATTGGCAT
TCTCTTACTGTTGATGCATTTG
>233
CGCAACTGTTACAAAACCAGTTGGTGGTGACAAGAACGGCGGTACCCGGGTGGTTAA
ACTTCGCAAAATGGTAAGATGTGGGGACTGTAAATTGGATTTTCTGTTTATGCTTGAAT
ACTGTGAAGATGTTTTTGAATACT
>234
GAGAAAACTGCGAGCCAGCATTACCCCCGGGACCATTCTGATCATCCTCACTGGACGC
CACAGGGGCAAGGTGAGAGTACCTGTGCTTGGGGCGCTTCACTGCAGCTGCCTGGGG
TGCCTGGTGGCAATGCGTTTGCACG
>235
TTACTTCAAGAAGAAGAAGCTGCGGAAGCCCAGACACCAGGAAGGTGAGATCTTCGA
CACAGAAAAAGAGGTAAGTTTCTACTTGTCATCTCCTGTGTTAGCACTGGCCCTTCTAC
CTGGGAGTGAAAAGAAACAGGTTG
>236
TCCCCAGCGCGCAACCACCCCCCGCCTGCCTGCCCAAAGCACCGGGACTGGAGCACG
GAGACCTACCAGGGTAAGTTGGCACTCCTTGGCCCTTCAAGCCTCAACTGCAGCCCCA
TGTTATGCGCCACTCTCAACTTGGA
>237
GATGTACCCTGCCCACATGTACCAAGTGTACAAGTCAAGACGGGGAATAAAGCGGAGC
GAGGACAGCAAGGTAAGCAAGTGCACCCCTAGGGACCCTGCGCTCAGCCCCTCGCGC
GCGCTGAGTTTCCAAGAAAAGTTTT
>238
GAGACGTGACCGGATTAACGAGTGCATCGCCCAGCTGAAGGATCTCCTACCCGAACAT
CTCAAACTTACAGTAAGTGAGAAGCTGGCCCCTTTCCAACCCAGTCCTTTGCCCAGAG
GGCGGGCGGGTCACTCGCCGCCTG
>239
AAAGCACTAACAAACCTAATTGATCAGCAGCAGCAGAAAATCATTGCCCTGCAGAGTG
GTTTACAAGCTGGTGAGTGCTGATTCTGGCTATGCTCTCTTTAAGAGTTTGAGTGCAAA
TAGAGAGCCCGTGGGCTCAACAT
>240
GGCCGGGCTGCAGGAGCTGCGATTCCCTGAGGAGAAGCCGCTGCTCCGGGGCCAGGA
CGCCACCGAGCTGGTGAGTTGGAGTCGCGCCTTTGAGGTCTTAGGGCCGAGCGCGCC
ACCTCGTGGCCAAGAGGGAGACCGCG
>241
GGCCTTTGTTTGTCCTTCCTGGTAGAGGCGGGTTCCCTCCCTCGGCAAGATGCCGGAG

TGCTGGGATGGGGTGAGTGAGGGCGCTGCGGGCATCAAGGTGGGCCGGGAGGATGGT
GCATCCTTATGACCCACCGCAGGGA
>242
GTAGTGATCCGGGGCTCCCCCAAGGGGAACCGCCCAGCCATCCTCACCTACCATGATG
TGGGCCTCAACCGTAAGTGCAGCCCAGCCTCAGTCAGCCCTCCTCTGCCTCCCATCAG
CCAGAGCGGTGAGGCCCCCCACCC
>243
CCAAGCACTTTGTGGTGTGTCACGTGGATGCCCCTGGACAACAGGTGGGGGCGTCGC
AGTTTCCTCAGGGGTAGGTACCCTGAGCCCCCTCTGCCTGTCTCCAGCTCTGCACCTG
AGGCCACACCTGGGCCCTGACCTCC
>244
GGTGGAGGGGCTGGTGCTGGTGAACATCGACCCCAATGGCAAAGGCTGGATAGACTG
GGCTGCCACCAAGGTGTGTGTGGTGACCGGGGGTGGGGTGGGTATACCTAGGGTGGG
GTGAGGGGCGGCACTCACGCTGGCGC
>245
GCTACCGGCAGCAGATTGGGAACGTGGTGAACCAGGCCAACCTGCAGCTCTTCTGGA
ACATGTACAACAGGTGCGGGTGGGATCAGCAGCCCTGGGACCCAGCACACCCCAGTG
GGGGCCCTTGCACACTGCCCCCTGAG
>246
GCTGGGACTTGAACCAGCAGTAAGATTTTCACGACACAGTGCTGTCTGCTTCTCCGTA
AGAAGTTAGAAGGTAAGAAAGCCAGAAATCCCGTGGAGAGGCAAATGTAAGGGATGT
GGCAACGACCACTCTATTCCTACAG
>247
ACCTGACGACTTTGATTCTGAAGACAAAGAGATATTAAGCTGGGATATTAATGATGTGA
AACTGCCACAGGTATGTACTTGCTATAAAAATCTGCCTTCAACATTGGGCTGTGAAGTC
TTGACTATTAACATGCGGAGAC
>248
CTCGCAGAGGAGGGGCGCAAGATCTACCTGAGACCTGCCATCTGTGACAAGGCCCGG
CAGAAGCAGCAGCGTGAGTAGAGCTTCGTGGCACCAGCAAAAAAGCTGACATCCCTC
AGATGCCCCTGCCAGATGGGTCACTG
>249
TTGCCGAGGTCATGGGGGCTTCCCGGTCACCAACTTCTGGAGGCACGACGGACGCTTT
ATATTTTTCCAGGTTGGATTTTTGAGTTAGTTACGTGAGAGTTTCATTACTGAGGAGTTT
CACCAGAGGCTAGAAATGCACC
>250
AGCCATGAAGAAAGTGAACACAGCACCTTCCTCCGTCTCATTGAGCCTGAAGGGGAG
CACAGAGACCAGGGTAATCCTTCATTATGTATGTTCAATACATGCAGTAGCTTGCCACG
CCTGTGCTGGGGGCGGTAGATTTC
>251
CTAGAGCGGTCCTTGGGGAGACGCGGGTCCCAGTCCTGCGGCTCCTACTGGGGAGTG
CGCTGGTCGGAAGGTGAGTGATCCCCTGTCGGGGACCGGGGGACTTGGGAAGGACAG
TTCCCGGACTGGACGGCCAGAACGCT
>252
GACTACGCAGGAAAGCCCCAGCCACCCATCAAATCAGAGAGAAGGAATCCACCTTCTT

ACGCTATGGCAGGTAATGATTCACTATTGTGGAGTAAGACTTTTTTTTTTTGAGATGGG
ATTTTGTTGTATTGCCCAGGCT
>253
TGCACCGTCACAGTGTCTGATTTGTATGCCATGAACTTTGAGCCGAGGGCCACAGACA
AAGATATCACTGGTGAGTCATGGGATAAATGCTCTATTTATAAAAACTATCTTTATGTTTT
TTACTTTAAAAAATGTTGACT
>254
AAGGTCTCTGGCTAGCGACATCACTGATGAGCAGAAAAAGGTTCGGGAGGCTGACCT
AGTGATATTTCAGGTTTGTTTTTCTCTAATTAATATATTGAATCAGATTCATCTTATGTACA
AACTCTTTCTGAATATTGAGT
>255
GATGGATAGGGTGCTGTGCCAGGGCTTTGCCTTTGACATCCCAGGATTCTACGATTCCG
GTTTGCTCCAGGTATGTGCTCTTGGATAAGGATCACTATGGATAGTTGGAGGGAGGGGA
CAGAGGATGCGTCTTCTATCAA
>256
AGGCACGGCCGAGATGTACACGAAGACAGGAGTCAATGGAGATTCTCGATACTTCCTG
TGGCCACTCCAGGTAGACCAGCTGCGAGTGGCTCCCTTGCTTGTTGGCACACGCACAC
ACAGACACACACATGCACACATGC
>257
TCAGAATGACCCTTGGGCCCCCACTGTGGATTTCAGTGACTTCATCAACAATGAGACC
ATTGCTGGAAAGGTCAGCTGGCCGGGGTAGAGGGTACAGGATGAGCCTTGCTTTCTCT
TGGGAGGGTCCCTGAAAACCACCT
>258
GGCAGTGTGACCAAGAACCATGCCTGGTGGATCCAGACATGATCAAAGCCATCAACCA
GGGCAACTATGGGTGAGAGGCCCTAGAGGCACCCTCAGTGGGCACACATGCATACTCA
TGCATGTATACACGCATGCTGTGC
>259
TGAGGGCATTCGCTACCGCCTGGGCACCATCCGCCCATCTTCCTCGGTCATGAACATGC
ATGAAATTTATGTAAGTCCATCCTTCCCCACAATGCTGCCATCTCCCCATGGCTCAGAAC
CTCAGGGATGCTGGCCCTGTG
>260
TGGCCCAACCTGATTCATGAGCCTCTTGACCAAGGCAACTGTGCAGGCTCCTGGGCCT
TCTCCACAGCAGGTAAGCCAAGGGCAAGGGCTGGCGCCTGGGAGAGGAGGGCCAAG
TCCTGAGCCTCCCGACAGCCCCTCTA
>261
ACACCCACCAGCAGCAGGGCTGCCGCGGTGGGCGTCTCGATGGTGCCTGGTGGTTCCT
GCGTCGCCGAGGGTATGCAGCAACAGGGGATGTGGGCAGAGAAGAGGGCAAGGAGC
TCCGTGGGCATGGCCTGGGCCATGAT
>262
CTGCCCCAACAGCTATGTTAATAACAATGACATCTACCAGGTCACTCCTGTCTACCGCC
TCGGCTCCAACGTAAGTCAGCACTTGGGTGAGGGCGCCGAGGCAGGAGGGTTGTGAA
AGCCTGGAGTCTCACCCCTGACGT
>263
ACACGCCAGTGAGCCTTGGGAGGCCAGAGAGATACCGCCGGCATGGACCCACTCAG

TCAAGATCACAGGGTGAGGGGCGTGTGGGCAGAGGGGGTTTGGGACAGCAGGGTTTG
TGCTAGGGGCTCTGGAGCCTGCCTTG
>264
GGTGCTGGTGTACGGCGGCAGGGGCGCTCTGGGTTCTCGATGCGTGCAGGCTTTTCGG
GCTCGCAACTGGGTAATGCTGCGGGCTGGGGCTGCTGGGTCTCCGCGGGGGTGGCCG
GGGGCTTTCGTGTGGAACCCGGCGG
>265
GGAGAATGAAGAGGCCAGCGCTAGCATCATTGTTAAAATGACAGACTCGTTCACTGAG
CAGGCTGACCAGGTAGTTTCAAAAAGGCCCCCAAACATTGTGAGGGGAACCCTGGGG
AGAGGTGACCACTGGCTGTATGTTC
>266
GGTGAAGAGAAGGTGGATGCAATTCTTTGCGTTGCTGGAGGATGGGCCGGGGGCAAT
GCCAAATCCAAGTGTGAGTCCTTTCCTGAGTCACTCATTTGTTCTGCTCTGCTCTTTTTC
CAGCTGACAAGGATTGGGTTTGC
>267
ACCAAGCATCTCAAGGAAGGAGGCCTCCTAACCTTGGCTGGCGCAAAGGCTGCCCTG
GATGGGACTCCTGGTAAGCCTTTATTTCCCTATCTGTGGAGTGGGGTTGCTTACCTTGCT
TGCTGGGGCTGCTGGGCACTTTC
>268
AGCTCTGCCAGAGCCTGGCTGGGAAGAACAGCGGCATGCCGCCCGGGGCAGCCGCCA
TCGCTGTGCTCCCGTAAGTGCTTGGGGTTCCCTGGCTGCTGCCCACCTCTGCCCCACTG
CTGCAGGTGACCGTTTGTCTGACT
>269
CCCCGATGAACAGGAAATCAATGCCTGAGGCTGACTTCAGCTCCTGGACACCCTTAGA
ATTCCTAGTTGAGTGAGTATTACAGACATGTCCATGACTGAGCAGCTTCCAGCCCCTAT
TCTCTGCCTGCTGTGGTCCAGAG
>270
CGCGCTGTTTACATCTTTGCCAAGAAGAACGACATTCCCTTCGAGCTGCGCATCGTGGA
TCTGATTAAAGGTAGGTCCAGCCTCGGGTTTGGGGAACCGAAAAGTCAGGAAGGGGA
CAGGTAGGCATACATAGCTTAGGG
>271
CCTTTGCCCAGGTGAACCCCCTCAAGAAGGTGCCAGCCTTGAAGGACGGGGACTTCA
CCTTGACGGAGAGGTAACTGGGACCCTAGGACTGCTGCCAGGCCTGCTGGAACCATCC
TGTTCTAACCCTCTATTTCATAGAC
>272
TGTGGATGAGTACCTGGCATGGCAGCACACGACTCTGCGGAGAAGCTGCCTCCGGGCC
TTGTGGCATAAGGTGAGGCTGGGAATGTGGGGGGCGGCAGCGAGAGCATTCCCCAAA
GGTGTTCAGGCACCAGTCTCTTCTT
>273
GAACAAGGCCTTCCTTACTGGTCCTCACATCTCCTTAGCTGACCTCGTAGCCATCACGG
AGCTGATGCATGTGAGTGCTGTGGGCAGGTGAACCCACTAGGCAGGGGGCCCTGGCT
AGTTGCTGAAGTCCTGCTTATGCT
>274
TTATATCCTCTGGGCCTGGGTTTCCTGAAGTCACCACACATAGAGGAGAGAGAAAATG

GCTGAGTTAAAGGTAAGAAACCATCTGACAAGTTTGCTATGGCTTCTTCAGCCAAAATT
TATGGAAGTTTCTGAGAACTTCA
>275
CATTTCTGGATTTGGGAATGAGTGTTCTTCAGAGGATCCTCGCTGCCCAGGTTCCCTGC
CAGAAGGACAGGTATGAGCAAATAAAGTGCTTAGAAGAGCTCTGATTCCTATTTCCTG
GATGACTAGCTTTCAGGCCAAAG
>276
CCTACAATCTCTATGCTGAGCAGCTCTCAGGATCGGCTTTCACTTGTCCACGGAGCACC
AATAAGAGAAGGTACAAGGATTAGATGAATTCTGACCTGCAGACTGTGGGTACTATGA
CAGGGACTTTGTGCTGCCTCCTT
>277
GCCCTTTGAATCCATTGACGAAGGCCATGTCACTCACAACTGGGATGAAGTTGATCCT
GATCCTAACCAGGTAACCTGGTCCTGTGAATTGAAGCTTATCATATACCCAAAGCCTTG
ACTAGAAATACCTAAATAGAATA
>278
CTGGAGACATAAAGTCTAACAATGGGCTTGCTATCCACATTTTCCTCTGCAATACCTCCA
TGGAGAACAGGTAAGTGGCTCAGTTTTAGGGAAGGACATGCCCATCTTCTAAGGGATT
TTGGCCAGAAGTCAAACTCCAG
>279
AGGGAACCTTCTCATTTACACCGAGTTTGGCAAGATGCTTGTACAGCCCAATGAGATCT
GCGTCATTCAGGTTGGTGATGTGTCCCCTTCTCTTCCCTGCTTCTCACTAATTTGGGAGC
AGTCAGATGTTTCAGCTGCTG
>280
GAGGAGACCAGGGGCTACATCTTGGAGGTCTATGGTGTCCACTTTGAGTTACCTGACC
TTGGACCAATTGGTAAATCTTCATTACAAGCCTCTAAGCCTCGCTTGAGATGTAGGACT
CAGATAATTGCATGAAAGTTAAA
>281
GGATCGCCAAGTACCAGGTGGTTACACGGTCATTAATAAATACCAGGGCAAGCTGTTTG
CTGCCAAACAGGTAAAGTAAAAGGGTTGGCAGGCAAGGGAGGAAATGAAGAGTGATT
GAGCACTTACTGATTGCCAAGGCG
>282
GAATTATACACCCTACAAGTACAACCTGAAGAATTTCATGGTTATCAACTCAGTGGCCT
TTGACCATGCAGTAAGTCTGAACCCTGTAGGGGCCTTGGGATGAGGCAACGCTTGGGT
GGGAGGGGTCCACAGTAGACCCC
>283
GCTGATTTTGTCATCTTCCCACCTCGATGGGGGGTTGCTGATAAGACCTTCAGGCCTCC
TTATTACCATAGTAAGTCTCTCTTTTACCAAGCCACATTTGCAAGCCAAAGAGACAACA
AAGCAGTGAGCGCTACACATTG
>284
TGATGCTGACTGCTTTGAGAAGGCCAGCAAGGTCAAGCTGGCACCTGAGAGGATTGC
CGATGGCACCATGGTAAGTATGTTAACTGCCACATTCCAGCGGCCTCTGAACTCCCCAC
CCTTACTGAGAAGGGTTGCCGTTT
>285
TCAGGACACAACTGTGGGACGTGCCGTCCTGGCTGGAGAGGAGCTGCCTGTGACCAG

AGGGTTCTCATAGGTAAGTGGAGATATGAATGAGTTCATAAGTCCTGCATGAGACTCAA
GGCTCTTAATAAAATCTTAAATCA
>286
CTCTCATGAGGGACCAGCTTTTCTCACATGGCACAGGTACCACCTCCTGCGTCTGGAG
AAAGACATGCAGGTATGTAAGAAGCATTTCAGTTTGCAGACTCTTTACAGACAAGATG
CCTTGTTTGTAAGTGATTTTAATT
>287
GTCTTTTCTCAATGGCGAGTGGTCTGTGACTCCTTGGAAGATTATGATACCCTGGGAAC
ACTTTGTAACAGTAAGTTCCAAATGATAGCTTGGAGTCAGAATTTCTTTTTAGATAAAG
AGATTAAATATGTTGCCTGAAA
>288
CCTATTTTTGTCCTCCTGCACACCTTCACAGATGCAGTCTTTGATGAATGGCTGAGGAG
ATACAATGCTGGTAAGACATTTTCATATGCTTTTTGCATGCTCAGCTGGGCGGATTGTTT
AGATGGCATAGTTATCAGTTC
>289
GTCACCAACACAGAAATGTTTGTTACTGCTCCAGACAACCTGGGATACACTTATGAAAT
TCAATGGCCAAGTGAGTGTTGAAAGTGTATTTTTACTGTGATAATTTCCAAAAGCAAAT
GTGTTATCTTTCAAGTAGAGTA
>290
CTTTTCCAGCTCCAATCTTTGATGATCAGTGTAAACTACACGATACTTTTCTTTTGTCCT
GGTTTTCCCTGTTAGGAGAGAAAGCATTGCATTTAATCTTTTTCTGCTTCTTGGAGATTG
TTTTAGTTAAAGAAATCATA
>291
ATGCTCAGCTTTGTGGATACGCGGACTTTGTTGCTGCTTGCAGTAACCTTATGCCTAGC
AACATGCCAATGTAAGTGCCTTCAGCTTGTTTGGGGGAGACTGGGTAGAGAGGTTAGA
TGGGAGGGCACCCTGCCCTGAAA
>292
AGATGGTGAAGATGGTCCCACAGGCCCTCCTGGTCCACCTGGTCCTCCTGGCCCCCCT
GGTCTCGGTGGGGTAAGGTGTCTTACGTATTGCTAACTTTTAGCTAACTTCAGTTGAAA
GAAGGTTTATTGTGGAATTTATT
>293
TCCTGCTGGTCCCGCCGGTCCCCGTGGTGAAGTGGGTCTTCCAGGCCTCTCCGGCCCC
GTTGGACCTCCTGTAAGTAGCCACTGTCTTTAAACTTTATTGAGTAAAAGAAAACAAA
GGTGGAGTATGGGGAAGAAGAAGA
>294
CCTCCCTGGACCCCGCGGTATTCCTGGCCCTGTTGGTGCTGCCGGTGCTACTGGTGCCA
GAGGACTTGTTGTAAGTGGTCATGACTGTGGTTCTCATCATCCTGAAATACCACCTCTG
CCATCATTTCATCACTATCTAG
>295
TGAAGAAGGAAAGAGAGGCCCTAATGGGGAAGCTGGATCTGCCGGCCCTCCAGGACC
TCCTGGGCTGAGAGTAGGTTTCAAATGCTCCCAACACCCTAACACACCAGAGGCAGAT
TATGATACCCCTTCATTGGGAATTG
>296
CCCTGCTGGAGTCCGAGGACCTAATGGAGATGCTGGTCGCCCTGGGGAGCCTGGTCTC

ATGGGACCCAGAGTAAGTTTCAAACTGATTCTGAGCAAATCACACCTGGCATTACTTCC
TTCTTTAAAGGGTTGGTTAATAT
>297
CCCAATTGGCCCCGTTGGAGCAAGAGGAGAGCCTGGCAACATTGGATTCCCTGGACCC
AAAGGCCCCACTGTAAGAATCACCACAACTTTCTTACCCTCAGCACTTTCTGTAGCCA
AATTTTACCAAACTCTAGTATTTA
>298
TGCTGCCGGTCCTACTGGTCCTATTGGAAGCCGAGGTCCTTCTGGACCCCCAGGGCCT
GATGGAAACAAGGTAAAATCTTATGTTTTCTATATTGCTGGTTTGGCCCAGTCTGCCTG
GAATAAGTAGACCCTTTACAATA
>299
TCCATCTGGTCCTAGTGGACTCCCAGGAGAGAGGGGTGCTGCTGGCATACCTGGAGGC
AAGGGAGAAAAGGTACGTGTTGACCCCTATTACATATTGTTGATGAACTCTAGTAAAGA
AGGCTGCACAAGGATGCCCAAGT
>300
AGGCAAAGGGCCTAAGGGTGAAAACGGTGTTGTTGGTCCCACAGGCCCCGTTGGAGC
TGCTGGCCCAGCTGTAAGTTGAATTCACTGGTGGTCCACACAGCAGCTACCCATTAGAT
CTTCCAATTAAATATATATCCGTC
>301
AGAAGTAGGTGCAGTTGGTCCCCCTGGCTTCGCTGGTGAGAAGGGTCCCTCTGGAGA
GGCTGGTACTGCTGTAAGTGATTTCCAACTCCTCTTTCTTAATACCTTATGCTGAATTAA
AATAAAGCCCCTACACAGATCTT
>302
TGCTCCTGGTATTCTGGGTCTCCCTGGCTCGAGAGGTGAACGTGGTCTACCTGGTGTTG
CTGGTGCTGTGGTGAGTGCTTGACAGTATTCTGACTCCATTAACATAAGAAAAGATTTT
AAAAGCTGCCACTTCAAATGTG
>303
GGCCCGTGGTCCTCCTGGTGCTGTGGGTAGTCCTGGAGTCAACGGTGCTCCTGGTGAA
GCTGGTCGTGATGTGAGTCCAACACTTGGTTTGTAAAATAAAACTGAGCAGGATTTCAT
TGTGTGAAACTTTATGTCCTGAG
>304
TGCTGCAGGTGCACCTGGTCCTCATGGCCCCGTGGGTCCTGCTGGCAAACATGGAAAC
CGTGGTGAAACTGTAAGTTTGTGAATACCAGTCCCTCAGTGCAGCATTCTCGTGGGCTT
CACTTCTGACTTCCCCACACTTG
>305
TGAAAAGGGGCCCAGAGGTCTTCCTGGCTTCAAGGGACACAATGGATTGCAAGGTCT
GCCTGGTATCGCTGTAAGTAAACTGTAGCCATCTCGCACATAAACTGATCCTGAAGGCC
TTCAGCTCAGAAGGATTTTCATAT
>306
TCGCACTGGACATCCTGGTACGGTTGGACCTGCTGGCATTCGAGGCCCTCAGGGTCAC
CAAGGCCCTGCTGTAAGTATGATTTGGGGAAATAATAAAGAAGATCACGGACCTAAGG
AATGTTTTCTTCAGACTAAACCAA
>307
GAAGGCTCTAGAAAGAACCCAGCTCGCACATGCCGTGACTTGAGACTCAGCCACCCA

GAGTGGAGCAGTGGTAGGTCAAGATGTCCAGACCAGACTGACCCTTCTCACAAGTTG
AGCTTTTCAAAATTAGTTTCCATTGA
>308
GAACTGGTATAGGAGCTCCAAGGACAAGAAACACGTCTGGCTAGGAGAAACTATCAAT
GCTGGCAGCCAGGTGAGGAATCCCACAAACACCTCTCCTTCTGCTAAATAATATTTTGG
TAGGACTGTTTGTTAATTATCTG
>309
TAATGATGTTGAACTTGTTGCTGAGGGCAACAGCAGGTTCACTTACACTGTTCTTGTAG
ATGGCTGCTCTGTAAGTAATAGTGAAATATGGGAATAGCTTTGGGAAGTGGGATGGAGG
GGGTTCTAACTTAGACTGCCCC
>310
CGCCGAGGGCCTGAGTGCTCCAGTAGCCACCGCATCTGGAGAACCAGCGGTTACCATG
GAGGGGATCAGTGTAAGTCCAGTTTCAACCTGCTTTGTCATAAATGTACAAACGTTTGA
ACTTAGAGCGCAGCCCCTCTCCG
>311
GAGGAGTGGAAGGAGTTCGTGTGGAACCCGAGGACGCACCAGTTTATGGGCCGCACC
GGGACCAGCTGGGGTACGCAGGGCCGGCACGCAAGGGGCGGGGGAAAGCCGCGGGG
CGACGCCTCGGGGGCGCAGGGTCCCGC
>312
ACCATGTGGGTGATGCTGCAGACTGTCTCCGACCATACCCCCAAGTACCAGGACCGAC
TGGCCACACCGGGTGAGTGTGGAGGCTCCCCCTGCCAGCTACTCTAACTGCTCTTGTG
CCCCCAAACCTCCAGAAGGAACTC
>313
GTCATTGTCAATGTCAGTGACACTGAAAGCTGGGACCAGCATGTTCAGAAGCTCAACA
AGTTCTTGGAGCGTGAGTGTGGGCCTGGTTATGTGTCAGTTCAAGACTTCGGGCAGGG
GACTGGGGACCTTGGAAGTGGAAC
>314
CGGCATTGGGGACTCCACCCACTATGGTTACAGCACTGGGCAGCCCTGTGTCTTCATCA
AGATGAACCGGGTATCTATGACCTTGGTCCCCAGGGTGAATGGAGGAAGGATCTGGGG
ACACCACCTGCAGACAATTGCAT
>315
CTTCGTCATGTTCCCCGCCAACGGCAACATCGACCTCATGTACTTCCCCTACTATGGCA
AAAAGTTCCACGTAAGTCCCAGGGGAGGCCCAGGCTGGATGGCGGGTGCGGGTGGTG
AGCTAGGGAAGGAGGCCGCGTTGC
>316
AACCAGAGCTGGGAAGATTCGAACACCGACCTCGTCCCGGCCCCTGCAGTCCGGATAC
TCACGCCAGAAGGTAAGTGAAATCTTAGAGATCCCCTCCCACCCCCCAAGCAGCCCCC
ATATCTAATCAGGGATTCCTCATC
>317
CTACGTGCTGCCCTGCGCCCGCAGCCATGTGCCGCACCCTGGCCGCCTTCCCCACCAC
CTGCCTGGAGAGGTAAGGGGAGGTTGTCCAACTGCTTGGGGGAAGTCCCACCAACTC
TTAGGGAGTGGAGCGGCAGGCCTGG
>318
AATCAGAGCTGGGCTGCGATACTGGGAGTACTGGCAAGTTCGAGTGGGGCAGTAAAC

ACAGCAAAGAGAAGTAAGTGTTGCTGACCAGCTCAGGGCTCCGAGAGGGAAGGGAG
TGGGAAGGGAGTCATGAGGAGGGAAAT
>319
AGCTACCAAGCTGGCCTCCAGGGCACACCAGATCTTTGAGGAGTTCATTTGCAGTGAG
GCCCCTAAAGAGGTTAGAGCCCCCATATGCCCAGTGAATCCTCACGACCCTGCCTGCTC
CTTCCCCTCTGAGAATCAGGGCT
>320
TTCGACGGCGAGCCGCAGCCCTACCCAACGCTGCCGCCTGGCACGGGCCGCCGCATCC
ACAGCTACCGAGGTACGGGCCCGGCGCTTAGGCCCGACCCAGCAGGGACGATAGCAC
GGTCTGAAGCCCCTCTACCGCCCCG
>321
CAAACTGAATTATTTGTGCCATCTCTCAATGTTGACGGACAGCCTATTTTTGCCAATATC
ACACTGCCAGGTACTGACGTTTTACTTTTTAAAAAGATAAGGTTGTTGTGGTAAGTACA
GGATAGACCACTTGAAAAATT
>322
TTCAGCCATGCCTCACAGCTCCGACAGCAGTGACTCCAGCTTCAGCCGCTCTCCTCCC
CCTGGCAAACAGGTAGAGTCCTCCTTTTTCTCTCTCCTACCTTCTGATTCTCCTGGGGG
ATGGAAAGAGAGCCAGGCTTCCT
>323
GGAGAAAAATCGTATTGCCGCCCAGAAGAGCCGACAGAGGCAGACACAGAAGGCCG
ACACCCTGCACCTGGTAAGTGTTCAGATCAATCAACATCCACGTGAGCTTTAGGCTTTG
CCCTCCGCCATCTGGGAACCCTTGG
>324
CTCTTAGCGGCCACCGCCCTCCTGACGCACGGCCAAGAGGAAGGCCAAGTCGAGGGC
CAAGACGAAGACAGTAAGTCCCAAACTTTTGGGAGTGCAAGGATACTCTATATCGCGC
CTTGCGCTTGGTCCCGGGGGCCGCG
>325
GAGACCAAGAACTGCCCCGGCGCCGAAGTCCCCGAGGGCGAGTGCTGTCCCGTCTGC
CCCGACGGCTCAGGTGCGGCTGCGCTCGGGGCCTGGGGCCTGGGGCTGGGGCTGGGG
GTGGTCGGCGCTCGCTGGCCCTCCGT
>326
CCCTGGACAGCCTGGACTTCCCGGACCCCCCGGACCCCCCGGACCTCCCGGACCCCCT
GGCCTCGGAGGAGTAAGTGGAGAGGCCTTGTGTGTCCACTCTCCCCTGTTTTGTTTTT
GTTTTTTGGCAGATGACATAATTT
>327
CTTTGCTCCCCAGCTGTCTTATGGCTATGATGAGAAATCAACCGGAGGAATTTCCGTGC
CTGGCCCCATGGTGAGCCAGCAGGGGGAGCATGGATGACAGAAGAGAGAATGGGTAT
CCAGAGGATGTGGGCATACGCGGC
>328
CTCTGAAGGTCCCCAGGGTGTGCGTGGTGAGCCTGGCCCCCCTGGCCCTGCTGGTGCT
GCTGGCCCTGCTGTAAGTGTCCCCGACTCAGTGTCCCCTTTGCCACTTTCTAACCTCAG
AGTCCTTGCCTGTTGCTGACACT
>329
CTTCCCTGGTGCCCGAGGCCCCTCTGGACCCCAGGGCCCCGGCGGCCCTCCTGGTCCC

AAGGGTAACAGCGTGAGTACCAAACTCTCCCTTCTGCCCACCCCATGCACTGGCTCCA
GTGCGGCTCTCATCTGGGGAGCAG
>330
AGAGGAAGGAAAGCGAGGAGCTCGAGGTGAACCCGGACCCACTGGCCTGCCCGGAC
CCCCTGGCGAGCGTGTAAGTGTCCCTGCCCGCCCCCTCCCACTCCACCCTCATTGCCTG
GCTGGTGCCTGTGTGTCGCGGAGTT
>331
CCCCGCTGGCCCCAAAGGATCTCCTGGTGAAGCTGGTCGTCCCGGTGAAGCTGGTCTG
CCTGGTGCCAAGGTGAGGCCCCAGGCTTTCAGCCTGGCTTGGCCAGGCCCTGACCATC
CCGTGTAGGGTCTGGGATGAGGCG
>332
ACCCCCAGGCCCACCTGGTGCCCGTGGTCAGGCTGGTGTGATGGGATTCCCTGGACCT
AAAGGTGCTGCTGTGAGTATTAAGTGAGGATCCATGAAGAGCCAGGGACAAACACAC
CTGAGACTTGAAGGAGTCCTGGGCT
>333
TGTGCAAGGTCCCCCTGGTCCTGCTGGTCCCCGAGGGGCCAACGGTGCTCCCGGCAA
CGATGGTGCTAAGGTGAGGGCAGCGTGGAAGGGGCTCTGGCAAGTGGCCCAGGGACC
AGGTCTCACCCCTCCTGCAGCAGGGG
>334
CGCCCCTGGCCTTCAGGGAATGCCTGGTGAACGTGGTGCAGCTGGTCTTCCAGGGCCT
AAGGGTGACAGAGTAAGTTCAACCTTCCCCCTCCCCTGAGCCCTACATGGCTCCCATC
TCTGCCTGCTTTGAATCTCTCAGC
>335
CAAAGATGGCGTCCGTGGTCTGACCGGCCCCATTGGTCCTCCTGGCCCTGCTGGTGCC
CCTGGTGACAAGGTGAGGTGGCCGCCTCCCCACCTTCTGCCCTAACACATAGCCTCCT
CAGCAGGCCTGGGCACGGTTCCGT
>336
TGATGCTGGTGCTAAAGGCGATGCTGGTCCCCCTGGCCCTGCCGGACCCGCTGGACCC
CCTGGCCCCATTGTGAGTGGCTTGGCCCTCTGTGCCCACGAGGCTGGTGGGCTGGGAC
CCAGGACGGGTCCAGGCTTGATGC
>337
TGAAGTTGGTCCCCCTGGTCCCCCTGGCCCTGCTGGCGAGAAAGGATCCCCTGGTGCT
GATGGTCCTGCTGTAAGTGCCAGCTCAGATCTCTGCAGCTCCGGAGGTGTGCAGAGCT
GGGGAGGGGTCCCTGTGCTGCTGT
>338
ACAGCGTGGTGTGGTCGGCCTGCCTGGTCAGAGAGGAGAGAGAGGCTTCCCTGGTCT
TCCTGGCCCCTCTGTAAGTGCCCCCCTCACCTTGGGGGGCCCTGAGAAAAACCATCAC
AGGACTTGGAGTGGGGCGGAGCCAA
>339
TGAACGTGGTCCCCCTGGTCCCATGGGCCCCCCTGGATTGGCTGGACCCCCTGGTGAA
TCTGGACGTGAGGTGAGCAGTCCCCAGCCCCCATGCCAGTACCCTCAGCATGGCCATT
GTGGCCTTGCCTAAGCCCTCTTCC
>340
TGCTCCTGGTGCTCCTGGTGCCCCTGGCCCCGTTGGCCCTGCTGGCAAGAGTGGTGAT

CGTGGTGAGACTGTAAGTAGCTGGGCTCCAGTTCCCTGTACCTGGTCAGGCCAGGGAC
TCTTCAGGCCTCCTTAGAGGCCTG
>341
CGAACAGGGCGACAGAGGCATAAAGGGTCACCGTGGCTTCTCTGGCCTCCAGGGTCC
CCCTGGCCCTCCTGTAAGTATGCTCAGCCCCTCCCCAGTCCCCATGCTGTGCTGTGGGA
TAGGAGGGGGAGCTTCGCCTCAGT
>342
ACTCAACGGTCTCCCTGGCCCCATTGGGCCCCCTGGTCCTCGCGGTCGCACTGGTGAT
GCTGGTCCTGTTGTATGTAGCCCCTCATCCCCTCTGCTCATGGCCCTCCAGCCCCCATAG
CACTTGGATGCCGGAATCCCCA
>343
GAGGGAAGCCGCAAGAACCCCGCCCGCACCTGCCGTGACCTCAAGATGTGCCACTCT
GACTGGAAGAGTGGTGTGGGCCTGCCCTAGCCTCTCCCTCCCTCCTACTCCTGCCATGC
CAGGGTCCCCATGCCCATATGTGC
>344
GTACATCAGCAAGAACCCCAAGGACAAGAGGCATGTCTGGTTCGGCGAGAGCATGAC
CGATGGATTCCAGGTGCGTGAGCTGGACCTCAGAGCCAGTGTTAGGAGATGGGCTAGC
CCAGTGCTCAGAAGGGACATGAAGT
>345
CAACGAGATCGAGATCCGCGCCGAGGGCAACAGCCGCTTCACCTACAGCGTCACTGTC
GATGGCTGCACGGTGAGTGCCCAGAATCCCCAGGCAGGGCCCCACCTCTCCGGCCTTG
GGCATTTTGGCCAGGCCATAGTGC
>346
CGCCGCTACGGCCTCCCCCCGCAGCCTCCTTGTTCTCCTCCAGGTGGTAGTGCTCGCTC
TGGCGCAGATTGTAAGTTTCGCAGCCCCTCCGCTGCCAGGGTCCAGGGTGCGGGGTCT
GGAGTCCGGGATGGAGGAGCTCT
>347
TCCACCGGGAGAGCGGGGCCCCCCGGGTCCCCCGGGACCGCCGGGAGTGCCTGGATC
CGACGGCATCGACGTAAGTCTCTAACCTGAGACCAGGGCGGGAGGGAGGCGGCAGAC
AAAGGAGAACTTTGTGAGCGCTGTGG
>348
TCCCCCCGGCCCTCGAGGACTGGCCGGGAACCGAGGCGTGCCAGGACAGCCCGGGAG
ACAGGGCGTGGAGGTGAGTCGGGCCCCGGGGTAGGAGGTGCTTCTTCTAGGTAGATCT
GTTCTGGGGTGCGGCTTACCCGCCA
>349
CACCCTGGCCCTCGGGGCGTTCCTGGCATCGTGGGAGCCGTGGGTCAGATCGGCAACA
CGGGGCCCAAGGGTGAGTGCTCCTCTGCGGTGGGCATGGGGGCCAGGCAGTGAGGAT
TTGTCCAGGCCGGCCCTTTCCCCAT
>350
GGAGAGAAGGGTGATCCAGGAGAAGTGGGACGGGGGCACCCCGGGATGCCTGGGCC
CCCAGGGATCCCAGGTAAGCCATTGGCCCTGCCCAGCTGCAGTGTGTTCCTCAGCTTG
GGAATTAATGACGTGTGGACACTGCG
>351
GGCTGGCACAGCTGCCTGCATCGCAGATCTCATCACCTTTCCTCTGGATACTGCTAAAG

TCCGGTTACAGGTGAGGGGATGAAGCCTGGGAGTCTTGATGGTGTCTACTCTGTTCCC
TCCCCAAAGACACAGACCCCTCA
>352
ATGAGCTTTGCCTCTGTCCGCATCGGCCTGTATGATTCTGTCAAACAGTTCTACACCAA
GGGCTCTGAGCGTGAGTATGGAGCAAGGGTGTAGGCCCCTTGGCCCTTTTTTCTCAGT
GATGATTGATCTTAGTTCATTCA
>353
AGATACCAAAGCACCGTCAATGCCTACAAGACCATTGCCCGAGAGGAAGGGTTCCGG
GGCCTCTGGAAAGGTGTGTACCAGTTGTTTTCCCTTCCCCTTTTCCTCCTCCCCGATAC
TCTGGTCTCACCCAGGATCTTCCT
>354
GTCAACTGTGCTGAGCTGGTGACCTATGACCTCATCAAGGATGCCCTCCTGAAAGCCA
ACCTCATGACAGGTGAGTCATGAGGTAGACGGTGCTGGGTCTCACCCTTCCCCCATGC
CAGGAGCAGGTGCGGGGGTCTAGC
>355
AGTACAGTAGCGCTGGCCACTGTGCCCTTACCATGCTCCAGAAGGAGGGGCCCCGAGC
CTTCTACAAAGGGTGAGCCTCTGGTTCCTCCCCACCCAGTTCAGGCCTCTTGGCTATGC
ATGTCTATTGTGGGTGGGAGAGA
>356
CCGCCCCCAATTCCTGCCCCTATTCTCTGCCTGGGAGATGGCTTCCCCGAGCCCCCCGC
CGGAGTCGAAGGTAAGTAAGCACCCCCCCGGGGATCCCGGTTGTCTCGCCGCTACCCC
AGCTAGGCCGCACCACTGCTTTC
>357
CTCCAGGTGAAGCACCATTTTTGACCTTCCCTTGTGTCTTCTGTTCCTGGCACAGTGCC
TGGACCCCAAGGTACACGCTTAGAGAACCTCAAGGAATTGAATTGAATAGAGTCACAA
GCCAGTGCAAATGGAAAATTGAA
>358
CTGGACCCAGCTCAGAGAAGCCTGTATAAAGATGTCATGATGGAGAATTATGGAAACC
TGGTCTCACTGGGTAAGAATCTGCTGTTTCTCTAATTAAAATATCTAAGAAGGTAGAGA
GAAATCAGACTAACAGAACATTT
>359
AGCCGCACTCGCCGATCCTCCAGGCATGCCCCGCTACGAGCTGGCTTTAATCCTGAAA
GCCATGCAGCGGGTAAGTGACCTTCCCTCAGAGCCGGTTTTCCCGCGCGGGCGCCCCC
TGTGCTTGTCATGACCCCTATATT
>360
TCTACTTAACCCCACCCACCAGATCTACCCTGTGACTCAGCCACTTGCCCCAGTTCATA
ACCCCATTAGTGTAAGTCCAAATTTACTGGCTTTGCTGTTTCATTCAAGATGTGTATGTG
ATGGTAGAATAAAAGAATAAA
>361
ACGGACGAGGGCGCGCTGCATGGCGCGGCTGAGGCCAATTTCCGCATCTACCCCTGGA
TGCGGTCTTCAGGTAGGCGCAGTCGCTAGGCGGGCCAGGCTGGCGGAGCGGGACCGG
GAGCGGGGAGCGCAGCGCTGGGGAG
>362
GGCTGGTACCCTGGAGGAAAGCGAGCCCTCAGCTCAGCCCAGGATCCCCAGAATGCC

CTTAGGCCCCCAGGTGGGTGTCTCCCAGCCTCATGGGGAGGAAGAAAGTGATGGCCG
GGGGCTCCCCCACCCTCCTGGAGCCT
>363
GCCCTGGGAGGGCAGGACCACGGCCCAGTGGTCCCTTCACAGGAAGCGACACCTGGC
ACGGACACTGCTGGTGAGTAGGGTGAGAGGTCCCCAGCATCAAGACCAGCCACTGGT
CATCAGAGGCCATTGTGGCTTAGGGT
>364
CAGTCTCTTAAACAAAAGCAAAAGAATAAGCTTCGGGCGCTGTAGTACCTGCCAGCTT
TCGCCACAGGAGGTAAGTGGATCTGGGAGCTGGGGGAACTGAGAAGACTAGCCAGAT
ATTACATGTATTGCCAACTCAAAAC
>365
TTGGATATCACTGATCCCCAGAGCGTCCAGCAGGCAGCCAAGTGGGTGGAGATGCACG
TTAAGGAAGCAGGTAAGTATGGTAGACCACCAGGAATATGGTGTGGGGTGTCCTGATC
CCCACAGTCACCCCAGGAGTCACC
>366
GCCTGGCAGCCAATGGTGGGGGCTACTGTGTCTCCAAATTTGGCCTGGAGGCCTTCTC
TGACAGCCTGAGGTGAGGGGTACAGGGCTCTGGGTTCCAGGACTAACAGCAGCCCAC
TCAACAAACGTGGGCCAGCAGAGGT
>367
CTGCAGGCCTGCTGGGCACGGCTGCCTCCTGCCACACAGGCCCACTATGGGGGGGCCT
TCCTCACCAAGTGTGAGTAGCCAGGCCCACACAGGGGCACATGAAGGGAAACAAGTA
CCAGAAAGGCCAGTCCTGCATAAGC
>368
GCGGTGGAAAAGACCAAGCAGGGGGTGACGGAAGCAGCTGAGAAGACCAAGGAGG
GGGTCATGTATGTGGGTAAGTGGGGCATGGCAGGGTGGGACAGTGTGGTGGCCAAAG
GGTGAGTGCCCAGTTACCTTCGCCAGAC
>369
CAACACTGTGGCCACCAAGACCGTGGAGGAGGCGGAGAACATCGCGGTCACCTCCGG
GGTGGTGCGCAAGGTGAGCCCCGGCCCTCAGACCTGCCCAGTCCTCTCCTGGGCCCA
GAAAGGCTGCTGGGCGGAGGCGGCAT
>370
GGACTTGAGGCCATCTGCCCCCCAACAGGAGGGTGTGGCATCCAAAGAGAAAGAGGA
AGTGGCAGAGGAGGTAGGAGCTGGGCTCCTGGGGTGCACCATGGGGGGTTCCTTGGT
AGGGACCCCATCCCCCACAGACGCAC
>371
TGGATGTGGAATATAATTGAATATTTTCTTGTTTAAGGGGAGCATGAAGAGGTGTTGAG
GTTATGTCAAGGTAAACAAAACCACCATTGTGGTAAAGACCACCAGGAGGGATAACTG
AATATATGGCTTACAAAATGTTT
>372
CTATGAAGGCATTGTCGTTGCAATCGACCCCAATGTGCCAGAAGATGAAACACTCATTC
AACAAATAAAGGTAAGTTCATAGTAATTATCCATACTTTTTTAAAAATAGCATAATGTTG
TGATAGAGAGAACATGAGTGC
>373
CATTTTGATTCCTGAAACATGGAAGACAAAGGCTGACTATGTGAGACCAAAACTTGAG

ACCTACAAAAATGTGAGAATATCAAGTTCTTCTTTAAAATTCCACTTTCTCCTGTAACC
AAGATTTTTTAAAATCAAAATAT
>374
AAGGGTGAAAGGATCCACCTCACTCCTGATTTCATTGCAGGAAAAAAGTTAGCTGAAT
ATGGACCACAAGGTAAGAAATATTCTACCATACTTCTCATACAATTTAACTATTTTATGTT
CAAAGTGTATCAACACTAGCA
>375
GGGGAGTATTTGACGAGTACAATAATGATGAGAAATTCTACTTATCCAATGGAAGAATA
CAAGCAGTAAGGTATGTATATTTTGATTTAACTTGTTCCAAACTATTTTAAATTGCAACC
TTTTTTTCTTGAATTGATTAC
>376
ATGTGAGTTTGTTCTCCAATCCCGCCAGACGGAGAAGGCTTCTATAATGTTTGCACAAC
ATGTTGATTCTGTAAGTACCTTGTTCTCACCCCCTCCCCCAGATTCTTATGAATTTAGTG
AGGCTTTTACTCCAAGTGCTC
>377
CACCTTCTCATTGCTGCAGATTGGACAAAGAATTGTGTGTTTAGTCCTTGACAAATCTG
GAAGCATGGCGGTATGTTCAATGAGTCTTGGTCTTCTGGATGCTGGTCACAGATATGCA
TGTTCTGGACCCTGACTCGGAA
>378
CGCCAAAAGATTACCTGCAGCAGCTTCAGGAGGGACGTCCATCTGCAGCGGGCTTCGA
TCGGCATTTACTGTGAGATGTGTTTTTCTATTGTAGTTTGGAATGTTTGCAATTTATGTTG
CTTAACAAACTTCCATTTCCA
>379
GCCATCATCCACACAGTCGCTTTGGGGCCCTCTGCAGCTCAAGAACTAGAGGAGCTGT
CCAAAATGACAGGTGAGGGATGATTTGCTGAGACCCCCGGTATTGTCTCTCCTACTGG
ACTGTGAGCTCCAGTGAACTGGGG
>380
CAATGGCCTCATTGATGCTTTTGGGGCCCTTTCATCAGGAAATGGAGCTGTCTCTCAGC
GCTCCATCCAGGTTGGAGTTCTTAATCTTTGGTTTTTCATATTTACACATAATCATAGCCA
AGATGGAGAATTTAATGGCT
>381
GAAGCAAGGTGGCTTTGTAGTGGACAAAAACACCAAAATGGCCTACCTCCAAATCCC
AGGCATTGCTAAGGTATGGAGTCAGCTTTTTTTCTTTCTCATAATTCAACAAGATAAAAT
AAAGTTTTTACAGAATAATTGTA
>382
AGTGTCACAGCCCTGATTGAATCAGTGAATGGAAAAACAGTTACCTTGGAACTACTGG
ATAATGGAGCAGGTAATCACCCAAGAAATTGGAAGATATTTATTTCTTTTTCCAAGAAG
CAACCAGGGAATGCCAAGAGGGC
>383
AACGCAGCCAGACGGAGAGTGATACCCCAGCAGAGTGGAGCACTGTACATACCTGGC
TGGATTGAGAATGGTAAGTAATTTGTAATAACATACCTGGCTTGTGCAAAAGCATTGGT
TTCAAGAGGAAACTATTAAGCCTG
>384
GAAATTCACGGGGGCAGTCTCATTAATCTGACTTGGACAGCTCCTGGGGATGATTATGA

CCATGGAACAGGTAAGCTGAACTGGTGTGGACCTGTAAAAGATTTACCAGCCAAGTAG
AAGAGCAGAAGCGGGTGAGGCAG
>385
GTCTCCGAGGCTGCCCTGTCTCTCCTTGTCCTCATCCTTATCATTACTTCGGCTTCTCGC
AGCCAGCCAAGTGAGTCCACTTGTCTCTGTAGGGCAGGAACGAGAAAGGGGGATGGA
GCAGCTGGCAGGGAGGTAGTGCC
>386
AGAAAGTGTTGCCAAGGAGACTAGTGGTGGGATACAGAAAGGCCCTCAACTGTCACC
TGCCAGCAATCATGTAAGCACGTCCTTTTAGATATTTCTGTGGAGAGGGACAGGGAGC
CCAGGGGCAGGAATGAGATACATGG
>387
AGTCAGCTTGCCGCCGGCGGCCAAGCAGCCAACCATGCTCAACTTCGGTGCCTCTCTC
CAGCAGACTGCGGTAAGTCATTTGGGGATGCCCCTGTGCTTCCTCGCCTGGTCTTGTCT
GGGGGGCCAAAGGGGGCGCGAAC
>388
TTTTTACACAATCTCTACTTTGACCAAACGAGTCTGGACAGTTTTCTTTTAATGGAAAAT
AGGAGAAATGGTGAGTAGTACCATTTTTTTTTAGCGTAGTTCAAAGAACTTTACTATAA
AACATTAGGTAGTTCTACAAA
>389
AGTATCTGATTTGTCAGAGGAAGAGAATCTGCTTCCGGGAACACCTGATTTTCATACAA
TCCCAGCATTTGTAAGTATTATTGTTTTTAAGATAGACGTAAATTATATAATTTCTTAAAA
TTTATATAATAAACTGTAGA
>390
AGCCACCCAGGATGTGGCAAGACATACTTTAAAAGTTCCCATCTGAAGGCCCACACGA
GGACGCACACAGGTACCAGCCACTTCTTATATCTTTAGTGTTTAAATGAAGAGATTTTG
GCTGTGATCACACATTTAAACCC
>391
GATCGTCAGCCTCGCAGTGGCCGACCTCCTCGTCGCCACACTGGTCATGCCCTGGGTT
GTCTACCTGGAGGTAGGTGGGCCCCCTGCTTGCTCCAGCACTTTCTCCAGCAGGGCCC
TGCACTGGACACTGGGGACTCTAG
>392
TCTTCGTCACTCTGGACGTCATGATGTGCACGGCGAGCATCCTGAACTTGTGTGCCATC
AGCATCGACAGGTGAGCCCAGCCAGGGTGGAAGAGGTTGCTCTGGCCACTCACCATT
CTGGCTGCCCCAGCTTTTCTCAAA
>393
ATGATCTCCATCGTCTGGGTCCTGTCCTTCACCATCTCCTGCCCACTCCTCTTCGGACTC
AATAACGCAGGTACATTCTGCTTTTGTTTGCCTGAGGCTCAGCTGGCCCTGGGCCCCTG
CTCCCTCGAAGGGCCCTGAGG
>394
ACGCCGCAAGCGAGTCAACACCAAACGCAGCAGCCGAGCTTTCAGGGCCCACCTGAG
GGCTCCACTAAAGGTCTCAAGACACCCCCCAACCAACTCCAAGGGTCCCCACCTAACC
ATTACCAAGAGGGCTCATCTTATGC
>395
CGAGGACATGAAACTCTGCACCGTTATCATGAAGTCTAATGGGAGTTTCCCAGTGAAC

AGGCGGAGAGTGGTAAGTGCTCAGGCCAGGACCCAGAGCCAGGTCTTTCTGCCCCTA
GGGAAGCCCACTGGCCATGGTTCTG
>396
ACCATGAGCCGTAGGAAGCTCTCCCAGCAGAAGGAGAAGAAAGCCACTCAGATGCTC
GCCATTGTTCTCGGTGAGTCGGCCCTGGCTGCTGGCCACAGCCCGGTCTGTGAAAGGT
CCCATTCCCTGCCATGTCCTTCCTA
>397
CAGCATTTCATCTGCTATGGCCATGGTTTTTCTGGGGACCAGAGGTAACACGGCAGCAC
AGCTGTCCAAGGTCAGCAGAAACAAAAAAAAGGGTCTGCTTTCCTCCCATCTCCCTAC
GAATGGACAGTCTGCATGGAAAG
>398
ACGTGGAGCGTCTTATATTCTGAAACTTGCTAATAGATTATATGGAGAGAAAACTTACAA
TTTCCTTCCTGTAAGTACTTTGCCCTTCACATTCTAAAACCCCACTAGACAGTGCAGATA
TTTTCCAGCACAATCAAGTC
>399
GTGGATTTTCAGCATGCCTCTGAAGATGCAAGGAAGACCATAAACCAGTGGGTCAAAG
GACAGACAGAAGGTGAGAGCCTGGGCAGAATGGAATCATTATGCGCTGGTCATGTCAT
TCGTTTCCTCAGTCCGCCTTCCCT
>400
TTTCAAGGGAAACTGGAAGGATAAATTCATGAAAGAAGCCACGACGAATGCACCATTC
AGATTGAATAAGGTGAGGTGAGGTAACTGTACAGAGTCATGCATTGCTCTAAAAGAAT
GCAGAGAATAATTTCAGTGATTTT
>401
AGGCGAGGAGCTCAGCATGGTCATCCTGCTGCCGGATGACATTGAGGACGAGTCCACG
GGCCTGAAGAAGGTATGGCTCCTGGGCTTCATGCTTTGTGGTTCCTCTTGCATGTGTGT
GTCGCATGGTAGGTAGGTGCTTG
>402
CACCTCTCCGCCGCCTCTGGCGCCCGGGCTCTGGCGAAGCTGCTGCCGCTGCTGATGG
CGCAACTCTGGGGTAAGGTGGGGGACAGGGGGCGGGGAGAGGCGCCGGTGGGAGGC
ACGGGCGGGAGGGCAATGTGTTCCCG
>403
TCTCGTTCCACTGCGCGGGTGTCCTGGTGGACCAGAGTTGGGTGCTGACGGCCGCGCA
CTGCGGAAACAAGTAGGAGGAGATCCATCCCCGAGGACGCCACGGGGGGCTGTGGAG
GCGGCCTCCAGGGAGGAGCGGGCAG
>404
CTTCTACCCTGGCGTGGTCACCAACAACATGATATGTGCTGGACTGGACCGGGGCCAG
GACCCTTGCCAGGTAGGGTCTGAACAGGGAGAGTCTCTGACTCCTGGGAGGGAGGAC
AGGGAGGTTATGGGAAAAGAGCAGA
>405
CGGTGCACTTCCTCATCTTCCTCACCATGCACGCCAGCAGCTTCACGCTGGCCGCCGTC
TCCCTGGACAGGTGAGCCAGCGCCTTGGCCTCCCTGGGAGATGGGCATCCACGCGGG
GGATGGAGCGGGAGGCGGGACTGG
>406
GCTCCCGCCGGCACCATGGCCAAGACCGTGGCCTATTTCTACGACCCCGACGTGGGCA

ACTTCCACTACGGTGAGGAAACAGGGCTATGCATGAGGGGTTGGGACACGGAGGTTTT
GAGGCGGGGTTGCAACTCCAGGGG
>407
TATGAAGCCTCATCGCCTGGCATTGACCCATAGCCTGGTCCTGCATTACGGTCTCTATAA
GAAGATGATCGTGAGTTCCGCCGCCTCGGCCGGGGGTTGGGGGGTGGGGCGAGCTGT
GGCCGTAGGGATAGGTGGGCTGG
>408
AGAGAGTCAGCCCCACCAATATGCAAGGCTTCACCAAGAGTCTTAATGCCTTCAACGT
AGGCGATGACTGGTGAGGGAGGACTGGGAGTTTTGATGTCTAACAGTTTAATGCCAAG
CCCTTCAGTGAGGTCTTTTGGCAG
>409
CGGGCTCTTTGAGTTCTGCTCGCGTTACACAGGCGCATCTCTGCAAGGAGCAACCCAG
CTGAACAACAAGGTGACATAGTCCCGAGTCCTGTTCTTCCTTTCCTCTGGATCCCTGGA
CTCGGGATTTAACCCTGATCCTG
>410
TTCTACCTCACTGACCGGGTCATGACGGTGTCCTTCCACAAATACGGAAATTACTTCTT
CCCTGGCACAGGTATGGGAATAGGGATTACTTTCCCCTTTGCACCCTTCAGCTCTTTACT
ATGAGTGCCTCCTTCTCAGCC
>411
GAAGTCGGGGCAGAGAGTGGCCGCTACTACTGTCTGAACGTGCCCCTGCGGGATGGC
ATTGATGACCAGAGTAAGTACCGAAGTCTTCCCTCTGTTCCCTAGGCCCCTTCTTTTTCT
CCTTGAAATAACCTCTTCTGCCT
>412
CAAGCACCTTTTCCAGCCGGTTATCAACCAGGTAGTGGACTTCTACCAACCCACGTGC
ATTGTGCTCCAGGTAATACTGTCAGTTCCTCCTCAGGAGTGTTCTGCCTGGGCTTGGGG
AAAGAGAGCAAGGGAAGAGGAGG
>413
AGAGCTTCAATATCCCTCTACTCGTGCTGGGTGGTGGTGGTTATACTGTCCGAAATGTT
GCCCGCTGCTGGTGAGCACTCTCCTGATTTCCTCCCCTGTTGTCCAGTGTGAGCTAGGG
TGTCCCTCAGATATGGCCACAT
>414
GTACTTTGCCCCAGACTTCACACTTCATCCAGATGTCAGCACCCGCATCGAGAATCAGA
ACTCACGCCAGGTCAGCAGCTCAGAAAAGCTGAGCACAAACTTCTCCTGAAAGGTGG
AGCCCAGGAGAGGTCAAAGATGTG
>415
ACCTCCTGACCTATGACAGGACTGATGAGGCTGATGCAGAGGAGAGGGGTCCTGAGG
AGAACTATAGCAGGTCTGGGAGCAGGGACTTCAGCCTACTTTCAATAGGCTTGCTGGG
GAGGTTTAAAAGGGAAAAAGTAAAT
>416
GGACCATCGGAGAGCAGTGCAGCGTGTGTGGGGTCGAGCTTCCTGATAACGAGGCTG
TGGAGCAGCACAGGTAGGCCCCGCTCCAGCCCCGCACCTGATGTAGGACTTGAGGCC
CTCACACCCCTCCTTCACACCCTGGC
>417
GGGTGCGAGCTCTGCGGGAAGCGGTTCCTGGATAGTTTGCGGCTGAGAATGCACTTAC

TGGCTCATTCAGGTAGGCAAGTTCGCCTTAGTGGCCCGTTCAGATACAGGCAACCATCT
CCTGCTTGCCTTTACCCCTCCTC
>418
GTCTGTGATCAGTGCGGTGCACAGTTTTCGAAGGAGGATGCCCTGGAGACACACAGG
CAGACCCATACTGGTGAGTTGACTTGGATTCCTGTTCTCCAGGTTTTCCACAGTGTTGG
TAGCACATGGACATGAACTGTCTG
>419
ATCTGCAGTGAGTGCAACCGCACCTTCCCCAGCCACACGGCTCTCAAACGCCACCTGC
GCTCACATACAGGTAGGTCAGTCCAGCTGATGGGTGGATCTGGGTCTCTGGGAGCCAG
CGTCTATATTTACCTCCAAGGACG
>420
GAGTGCAATGGCTGTGGCAAGAAGTTCAGCCTCAAGCATCAGCTGGAGACGCACTATA
GGGTGCACACAGGTACCGAAGGCCAGGGAGGGGCCTGAGCTGGCTCTGGGACCTGGG
CGGAGGTGGGAAGCAGATAGTCTCC
>421
TGGGTGTGAGGGACACATACATCCTAAAAGCACCACAGCAGAGGAGGCCCAGGCAGT
GCCAGGAGTCAAGGTGAGTGCACGACCTGACTGTGTACCAAGGGCCGTACCCCCAGA
AACAGTGTCAGACCTGGCAGCACCGG
>422
CTGTGAGGCCCTAGAGCACCACCTTAAGAGAAGAAGAGCTGTAAGCCGGCCTTTGTC
AGAGCCATCATGGGTGAGTTTCTCAGCTGAGGCCACTGGCACTGTCCCTCTCTCCCTC
AGTCCTGTGGGATCCCATCATACCT
>423
GGAGAACAACAAACCCCGGAGCCGGAGCCAGGGGAGGCTGGACGGGACGGGATGGG
CGACAGCGGGCGGGGTGAGTGCCCCAGCAGAAGGGCAGGCGAGCCATGGACCGCTTT
ATCTGCGACCCCTCCCCCACACCCTCC
>424
TTCCATGGGGTCCCCTGGTCTGCCCCCTCCAGCTCCCCCAGGATTCTCCGGGCCTGTCA
GCAGCCCCCAGGTGAGAGGTTGTGACCAACTTACTGCCTGGCCACTTTCATTTTCTTGT
TTTCCTTGTAACCCCAGATCCT
>425
TGTCCACCCCCTCCAGGTGGCCCTGGGGCTGGCAAACGGCTATGTGCAATCTGCGGGG
ACAGAAGCTCAGGTATGTGGCTCAGAGGATGAACAGATAGGGAGAGTCTGGGCCATGT
ATCATCACCTGTGGGATTCCCAGG
>426
AAGCGCCAGCGGAACCGCTGTCAGTACTGCCGCTATCAGAAGTGCCTGGCCACTGGCA
TGAAGAGGGAGGGTAAGGACGAGTCCTGTCCACGCGTCTTAGACCACGTTCCCAGAC
TCCCTATACGCCACTCGGACTGTGT
>427
GCTTGCTGTGGAACAGAAGAGTGACCAGGGCGTTGAGGGTCCTGGGGGAACCGGGG
GTAGCGGCAGCAGCGTGAGTGTTGGGGTCAATCCACTCTCCTTCGTGATGGGGGTTGG
GGGAGGCAGTCTAGGTCTGTTAAACA
>428
GAGTGGGCGAAGAGGATCCCACACTTTTCCTCCTTGCCTCTGGATGATCAGGTCATATT

GCTGCGGGCAGGTCAGTGACCTTGGATCCCTTTGACTTCTTGACATTTGACCCCTCTTT
GACTTCCCGATCTTTAGTGACC
>429
TCCTTGCCACAGGTCTTCACGTGCACCGCAACTCAGCCCATTCAGCAGGAGTAGGAGC
CATCTTTGATCGGTCAGTGGCCCTCGGCTAGGCTGGCATGTAGATAGAGGGGGTGGGG
CTATAGGCTGGTCCGTGTCCAAGG
>430
AAAATGCGTGACATGAGGATGGACAAGACAGAGCTTGGCTGCCTGAGGGCAATCATTC
TGTTTAATCCAGGTAAGAGGAGGATTACCTTTGTCTCTCAAGGCCAAGGCAACCTTGG
AGCCTCCTCTTCTCCGAGACTCCT
>431
TCCTGCGGGAGAAAGTGTATGCATCACTGGAGACCTACTGCAAACAGAAGTACCCTGA
GCAGCAGGGACGGTGAGATGGGGCTGGGGGGCACTGAGGCTCCCTTGGGGGTGGGG
AGTGCTATGAGGATGTGTTCAGGGCC
>432
CCAGGCCCGGAGGACGAGGACTCCAGCCTGGATGAATCTGACCTCTATAGCCTGGCCC
ATTCCTACCTCGGTAAGGCCCACTCAGCCATCTCCACGGTCCTTCCTCCTCTCCCGAAA
TCAGGACCCACCCCTCTTGTTTC
>433
AAACTGGTGACCAAGCTGCAGAATTCAGAGAGGAAGAAGCGAGGGGCACGGCGCTG
AGACAGAGCTGGAGGTAAGGAGTCGGGGGGCCCAGAGAGCTCAAGGTGGTGCTTCTG
CCATGAAGGACAGGCCGGAAGGTGTGT
>434
CACCATGGGGCGCCTGCAACTGGTTGTGTTGGGCCTCACCTGCTGCTGGGCAGTGGCG
AGTGCCGCGAAGGTAAGAGCCCAGCAGAGGGGCAGGTCCTGCTGCTCTCTCGCTCAA
TCAGATCTGGAAACTTCGGGCCAGG
>435
ATCGTGGTCACCTTCAACTACCGTGTCGGCCCCCTTGGGTTCCTCAGCACTGGGGACG
CCAATCTGCCAGGTGCGTGGGTGCCTTCGGCCCTGAGGTGGGGCGACCAGCATGCTGA
GCCCAGCAGGGAGATTTTCCTCAG
>436
CAGTGTCTGAAGGTTACTGATCCCCGAGCCCTGACGCTGGCCTATAAGGTGCCGCTGG
CAGGCCTGGAGTGTGAGTAGCTGCTCGGGTTGGCCCATGGGGTCTCGAGGTGGGGGTT
GAGGGGGGTACTGCCAGGGAGTAC
>437
ACATGGACGGCCACATCTTCGCCAGCATCGACATGCCTGCCATCAACAAGGGCAACAA
GAAAGTCACGGAGTAAGCAGGGGGCACAGGACTCAGGGGCGACCCGTGCGGGAGGG
CCGCCGGGAAAGCACTGGCGAGGGGG
>438
CCCAGCCACCAATGGCTCTGAGCTGGTGACAGTACAGCTTATGGTGTCACTGGCCCAG
CTCATCAGTGTGGTGAGTAGAGGTCCCAGGCTCTCTGCCCAGCTACTGAAATCAGCCC
CGCCAAAATGTGTTAATGCTTGTG
>439
TTGACAACATGAAGAAAGTTCGGCTCCCTTCCAAACACATCTGGCTCCCAGATGTGGT

CCTGTACAACAAGTAGGTGCAATGGGAAGGTTGGGGGAGACCATTGGAGGGCTCAGG
GAGGGAAGGGGTTCTGGGGAAAATA
>440
CCTCCGGGAGGCGGTGGACGGCGTGCGCTTCATCGCAGACCACATGCGGAGCGAGGA
CGATGACCAGAGCGTGAGTGCCGCAGGCTGGGACCCCGGGCGTGAGATATGGGGTCT
GCCAGGGCCCGGGTATCTGGAAAGCA
>441
GTCACTCGGCCAGAGCACTTCGACTTTGTAAAGCCTGAGGACCTGGACGGCATAGGCA
TGGGCCGGCCTGGTGAGGGGACCCTGCCCCGAGGCCCTGGTCTCTCTGTCCACAGCCT
ATCAGTTGCCTTCCCCCACCACAA
>442
CCAGCGCAGACTGTCCGAAGCTCTGAAAAGGCTACGTTCTGGGCCTAAGTCTAAGAAC
TGGGTCTACAAGGTGTGTGTTGTAGGTGGGCAGCTTGGGCCTGGGAATGAGGTGGCTT
GAGGGGCAGGGAGGGGGGCGCAGG
>443
GGAGCTGCTGGGTTCAGGCTGCTTCGGTGTGGTGCACCGAGGGCTGTGGACGCTGCC
CAGTGGCAAGAGTGTGAGTGTCCAGGGAGCCCGCTTCATCCAGGCCAGCTGCCCCCT
CTGTTCTTCATGCCCGCAATGCCTAA
>444
CATGATGAACTTGGAGCACCCACACGTGCTGCGTCTGCACGGCCTTGTACTGGGCCAG
CCTCTGCAGATGGTGAGCAGATCCAGCCGCTGGTTCCCGGGACAGCCGTGCGGCAGG
AGCGTGGGCGGCCAGGGTCCAATGG
>445
TGGTGCGGCCTCTGGGCGGTGCCCGGGGCCGCTACGTCATGGGCGGGCCCCGCCCTAT
CCCCTACGCCTGGTGAGAGCGGGTCCGCGGGCGGTCGGGCTCTGAGCCGGGCGGATC
CGGAGGGCAGCAGCCGAGGGAGATC
>446
CTTGCGCTGCTGGGCCCCCCACCCTGCCGACCGGCCTAGCTTTTCCCACCTGGAGGGG
CTGCTGCAAGAGGTGGGAACCCCCGACCTCACCAGATACACAGTCCCTCTTCCCTCCA
TTCGCTCTCCCAGGGTTCCATGCG
>447
TGAGGGATGTCACAGAACCAGGCGCCCTGAGGATGGAGACTGGTGACCCCATCACAG
TCATCGAGGGCAGGTGACAGTCCCATACCTCCCAAGCACCCTCAGGAGGAGGATACCC
TCCCAGCCTAATTCCTGATCCCTTC
>448
TGCCAGCCACCCGTCCAGTCCACAGAGGCACCCCTGCCCGGGGAGATCAACACCCAG
GAAGCATAGATGGGTGAGGACCTGAAAGGGTGAGGGCAGGGGTTGGCTGGGAGAACT
GATGAGGTCCAGGAGCTTCTCTGGAA
>449
AAGGCAAATCTTTGGGATGCGCCCCCAGCACGGGGCCAGAGGAGGAACATGCCCCTG
GAGAGGATGAAAGGTGGGTGTGGTGGACTCCAGAGTCTCTGAGGAGATCAAGACCAG
TCCTTCAATTCCGACCCCCTGCCCTA
>450
AGCCCGTAAAGCCGCTGCCCTCTCTGGAGGCCTCTTGTCCGATCCTGAGTTGCAGAGG

AAGATTATGGAGGTGAGGTCTCACTGAAATGGCCTGGTGTCCAGAAGGGGCTACAGGC
AGGGGCAGGGGCCTGAGTGAGGCT
>451
CCACCAGGAGTGCCAGACAGCACTAGGAGCCACTGGGGGAGATGTGGTTTCTGCCAT
CCGGAACCTCAAGGTAAAGCCAGCCCCTTCTCTTGGGGTCCCTCCTCTCCTGCCCCTG
CCGCCTGGCAGTCAGCTGCAACCCA
>452
GCTCACACAGCTCCTCCTCAGGCAACGGCAAGGACTCAGCCCTGCTGGAGACCACTG
AGAGCAGCAAGAGGTGTGTATGGATGTGTATGTTGGGTCTAGGAATGGTCTTGTGAGC
AGGCAGAGCTGGGTGACAGGCCTCG
>453
GCAGTTCCATTGCCTACAGCCTCCTGAGTGCCAGCTCAGAGCAGGACAACCCGTCCAC
CAGTGGCTGCAGGTACGTCGGGTGAGGCTGGAGGAGAGGTCTGGGGGCCCAGGCCAC
ACTCTGGGACTAATGTCTTTATCTC
>454
CGGGGCAAGGGCCGCTCTGGGACCCTGGCCACGCTGCAGTACGCACTGGCCTGTGTC
AAGCAGGTGCAGGGTAAGTGGTGTGTCCCAAGAGGGAATGGCCTGGGCCTGGGCTCC
CGTGTTGAGGCAGCCTCCTTACTGAA
>455
CATGGACATGTCCACCTATACCCTGGAGGAGCTGGAGCACATCACGTCTGAGTACACA
CTTCAGAACCAGGTCAGCGGCGGCCTGGCCTCTGCATCCCGACGCACCCCTACTCCTT
CTCCCACAGCTCTTGAATCTGCTC
>456
GATGTGGGAGTCTTCTATGGTTCCACTGCTCCATCTCGCCTGCCCACCTGGGGCACAGG
GGCCTCAGCAGGTGAGGTGCCCAAGCGCTGTGGGGAGGCATGAGCAGTCCCAGGAAA
TTCCTGCTGTGAGGGGCCGGGGAA
>457
GTCTCAGATGGGGCCCCTGCACAGCCGTGCTGCCTGCTGATTGCAGAGCGCATCCATT
CGGGTTACGAAGGTGGGCAGGTCAGGGCCCTGGCCTGGTGGGGGCTGGGAGGAAGG
ACATTTCCTCATGGCCAGACATGAAT
>458
TACCCCCTGACAAGAGGATTTTCACTACGCGGCACACACCCAGCTGCCTCTTCCAGGA
TGTGGATGAAAGGTGAGGATAGGACCTAGAGGAGACGAGGGCAGCCAGGGCTGAGG
CCACGGCCACTCTGATGCCTCCTTCC
>459
CTGGGGGCCCCAGTGCTCCTGTTCCTGCATCCTGAGGACCGACCCCTCATGCTGGCTAT
CCACAAGAAGAGTGAGTTCCTCTCGCCCTGCTCGCCCTTCCCACTGTCCTGGGCTTTT
GAGTGGCTGCCCTTGTGGTTGTG
>460
GCTGGGCTGGCTTTGTGCACCCCTGGAGCCGCAAGGTAGCCTTCGTGTTGGGCCGCCA
CAAAGTACGCACGTAAGTGGGCCATGCCCCCGAGCTGGCGTTGGGGATAGGGCAGTG
CGGTGGGGGACAGGACCGGGCCAGG
>461
CAGCCCAGCTCCCTCCCTGGACACTGATATCCAGGAGCTGTCAGAGCAGATCCACCGG

CTGCTGCTGCAGGTGAGAGTAGCGGAGAGGGAGCCTGGGAGGTGAGAAAAGGTGTG
GGAAGCGGGGTCAAGCCATCTAACCT
>462
TCTCCACAGCCCTGGGTCCTCCAGTGATAGCAACGGGGGTGATGCAGAGGGGCCTGG
GCCTCCTGCGCCAGTGAGTGACCTGCTCCTACCTACCCTCTAATCGCCCTTTCCCCTCT
CCTTCTTGGAACCAGCACTGCCAG
>463
GTGAAGCACCAGGGCCAGCAGCTTTTTATTGAGTCTCGGGCCCGGCCTCAGTCCCGGC
CCCGCCTCCCTGGTGAGTTGATGATGATGTGTGGGTGAAGAGACAGACCTGGGGGGTC
CCCTGACCTTCACCCCCAACCCAT
>464
AGGAGGCCGAGAGGAAAGAAGCCTCCAGCTGCTCCTACCAGCAGATCAACTGCCTGG
ACAGCATCCTCAGGTAAGGCCTGCCAGGCATCTTCTCCACCTCGGGCTCTAACCCTGC
CCACCCCAAGCCCTGCTCTGATGCC
>465
TCCGTCACCAGTCAGTGTAGCTTCAGCTCCACCATCGTCCATGTGGGAGACAAGAAGC
CCCCGGAGTCGGGTATGGGTGTGGAATTGGGGGGCAGTGCTTGGGCTCCACCGGTCTC
CCAGCTAGAGCTCCCTCCTGCCTT
>466
TTCCGAGACCTGGGCAGGCTGCGTGGACTCGACAGCTCTTCCACAGCTCCCTCAGCCC
TTGGCGAGCGAGGTAGCCACCTGGGGCCTCCTGGAGCCTGCCCTCTGCCCAGTCTAGG
ACTGGATTGTTGGGGGGTGGGTCT
>467
AGGAGGCCCTGGGAGCAGTGCCGGGCCCCCACCTCCCAGTGCGGAGGCTGCTGAGCC
AGAGGCCAGACTGGTGAGCACTGACCCCTGCGTCTGCCTGCCAGCCCCCACCCCAGC
CCCGCCCCTCTGCCACCCTGTGCTGC
>468
GGCTCTGGCTTGGGCTCTGGGTCTGGTTCAGGCTCCCATGAAGGGGGCAGCACCTCAG
CCAGCATCACTCGTGAGTACCCCGCCTCCAGCATCTCCCAGGGTAGGGCAGTGATTGG
GGAGCCGGGAGCCCAGGCCCCGTC
>469
AGGATCCCATTTGGCTGCTCATGGCCAATGCTGACCAGCGCGTCATGATGACCTACCAG
GTGCCCTCCAGGTGAGGCATTTCAGAGGCCTTCTTGGCCTTCCTTTCAGAGGTAGAGT
GGGGGCAGTTTTCTTTTCTGGGG
>470
GCGGCGGGAACTGGGTGCTGTGCACTCCTGGGTCCGGAAGGGCCAACTGCCTCGGGC
TCTTGATGTGATGGTGAGAGAAGCCTGGGACGGGGAGAAAAAAGAATTGAGCTCAAG
TTCAAGGGGGAGAAAAAGAATTGAGC
>471
AAAGTTGCTGACCTCGCCGTCCCTAAAGCCCTCTGCAGTTAGTAGTGTGACATCGTCTA
CCTCCTTGTCAGTGAGTATCTGTCATAGCAGCCTGCCCTGCCCCACTCACAGGGGCCTT
GGGGATGAGGTTGCTTGCCTTG
>472
GCAGGGGTCTCCAAGGATGCCATCGTCACAGGCCCTGCCCCCGGGTCCTTCCACCATG

GCCTTGGCCACAGTAAGTGCTTCTTTGCTGCCTCTGGTCACTCAGGAACCTCTAGATTG
TGGCCAGGGGTCCTGCTGTTGTG
>473
TCCAGCCCGCCCCATGCAGCGCCTCTCCCACACGCTGCGGTGCCCACCCATATCCCGC
AGAGTCTGCCAGGTAATCACCCGACGGTCAGTGTGCCACGCGCACCGTGTGCCTTTGC
CCTCTCCACCCCTAGGTGCTTTGC
>474
GCTACCTGGCGGAACTGGATTTCTCTCCCGCCTGCCGGCCTGCCTGCCACAGCCGGAC
TCCGCCACTCCGGTAGGATTCCCCGCCTGTCATTCCCTAGCCCAGCTCTTGGGAAACTG
CAGAGGGGTCCAGAGGATTTGCA
>475
GCTGCCACCTTTGGGGCCGATGACTTGGTACTGACCCTGAGCAACCCCCAGATGTCAT
TGGAGGGTACAGGTGGGTCTCAGCGGGGTGGGATGGGGCACGGAGTGGGAGACAGAT
CCATCTAAGGGCCTGTTAGACAAAT
>476
GCCCTTGAGGAGCTGCGTCTGGTCTTTGGGCCTCTGGGGGACCAACTCCATGCCCAGC
TGCGAGACCTCAGTGAGTCCAGGCCCCTGGAGGCTGGGGAGCAGCTCCACATGTTGA
GCTGAGTCGAGTTCAGTGTGGCCGT
>477
AGTTGGATCATTGAGCTGCTGGAGAAGGATGGCATGGCCTTCCAGGAGGCCCTAGACC
CAGGGCCCTTTGGTGAGAACCCGTTTTCTCCTTCCTTCCCCAGCCTGTCTTGTCCCATC
CCTGCCCCTCCACAGAGTGCTAG
>478
AGCCCCTACCACCCCGGCAGCTGTGGCGCAGGAGCCCCCTCCCCTGGCAGCTCTGACG
TCTCCACCGCAGGTGAGAGCTCTCTCTGGGCCACAACCTCCCTTCCCCGAAGTGTCCC
TTGTTCCCTCTGGCTCCCAGCACC
>479
TCCCACTCCTCAGACTCCGGTGGAAGTGACGTGGACCTGGATCCCACTGATGGCAAGC
TCTTCCCCAGCGGTGAGTCGAGGGAGGTCCCCAAGAGGGCGTCCCATTTAGCAATGCA
CAGGGGGCCCGGCTCTTCCTGCAG
>480
AAACGAGGCCGGCCCCGAAAGCTGAGCAAAGAGTACTGGGACTGTCTCGAGGGCAA
GAAGAGCAAGCACGGTGAGCTCCGGGGGCACGTGGGTCCTCCCTGCGCCGGGCTGAG
CGGCTTCCTGGGGCACTGCGGGTTGTT
>481
TGGCCCAACTATGGGGCCAAAAGAAAAAGAACAGCAACATGACCTACGAGAAGCTGA
GCCGGGCCATGAGGTGAGCTGGCGGCCAGGACCCTCACGATACAGCCGGACATGGGG
ACAGGCGCTCACACTCCCACCGCCCT
>482
ACGAATTGTTCTGCTCGGGCGTCCTGGTGCATCCGCAGTGGGTGCTGTCAGCCGCACA
CTGTTTCCAGAAGTGAGTGCAGAGGTAGGGGGAGTGGGCAGGGCCTGGGTCCGGGGG
CGGGGCCTAATATCAGGCTCATCTT
>483
AGCATTGCTTCGCAGTGCCCTACCGCGGGGAACTCTTGCCTCGTTTCTGGCTGGGGTCT

GCTGGCGAACGGTGAGCTCACGGGTGTGTGTCTGCCCTCTTCAAGGAGGTCCTCTGCC
CAGTCGCGGGGGCTGACCCAGAG
>484
CTATGACCCGCTGTACCACCCCAGCATGTTCTGCGCCGGCGGAGGGCACGACCAGAAG
GACTCCTGCAACGTGAGAGAGGGGAAAGGGGAGGGCAGGCGACTCAGGGAAGGGTG
GAGAAGGGGGAGACAGAGACACACAG
>485
GGGAGGGGAGGCTTCGCGGAACGCTCTCGGCGCCAGGACTCGCGTGCAAAGCCCAG
GCCCGGGCGGCCAGGTGAGGCCAGCGTCGCTCGCGGCATCGGGGCGCCCCGCTCCTT
CCGCAGACCCCGAAGTGGGGCGCAGGG
>486
GTCATTGGCTTTGCTGTGGACCTGGCCAACGTCTGGCGGTTCCCCTACCTGTGCTACAA
AAATGGTGGCGGTAAGTCCCATCTCAGCCTCCCTGAGCATGCTGGCCGGGCGGGGGTC
GTAGGTGCAGCCGGGGCCAAGGC
>487
GAGCTGGCCCTCGGCCAGTTCAACAGGGAAGGGGCCGCTGGTGTCTGGAAGATCTGC
CCCATACTGAAAGGTAATATGCGGGTGCCTCCCTTCACCCTGGCGCAGCGCGGATCATG
GTGGTGACGCTGGAGCTTTCAAAG
>488
GTGACTCCAGTGGAGACAGCTCGGGCCTCAACGACACTTTTGGGACCACACCTGCTG
CCGAGTACTTTGAGTAAGTGGGAGTCGGGTCCTCGGGAACGGGAGAGATGGCGCAGC
CAGGTCCCCCATGGTAGCCCCTTGGT
>489
AGCCTGCCTGGTGCTGGTCATCGTGCTGCTCTACTTCAGCCTCTGGAAGGGCGTGAAG
ACCTCAGGGAAGGTGAGGCTCGGGGGTCACCAATTGGGCCTGTAGACATGGGGCCAC
CAAGTGGACATGTGGCCGTGGCTGG
>490
CCTCCCTGGAGCCATAGACGGCATCAGAGCATACCTGAGCGTTGACTTCTACCGGCTCT
GCGAGGCGTCTGTGAGTACAGTCTGCTCACTGCTTCACAGTCCACAGGGGCTCTGGCA
TTCCCAGGTTTCCATATGCATTG
>491
CCCTGGGCGTGGGGTTCGGGGTGCTGATCGCCTTCTCCAGCTACAACAAGTTCACCAA
CAACTGCTACAGGTGAGCCCCTAGCAGGGCCAGGCAGGGGCCTCATCAATACTAGGGA
GAGGTCGCTGACTTCCAGAAATAG
>492
GTCGTCTTCTCCTTCCTGGGGTACATGGCACAGAAGCACAGTGTGCCCATCGGGGACG
TGGCCAAGGACGGTGAGCCCCTCCTGCTGCACCTGGGCCTGCTCCGTGTAGCACCAGC
GCCGAGCTCTCAGCAAAGCCTTTT
>493
GCTCCCTCTGTCCTCAGCCTGGGCCGTGGTCTTCTTCATCATGCTGCTCACCCTGGGTA
TCGACAGCGCCGTGAGTAACCCGCACCGCCCGGCCCTGTCCTCGGCAGTTGGGTTCCT
TCCACCCGAGGGGGCCCTGCCCT
>494
ACACCGTGAGCTCTTCACGCTCTTCATCGTCCTGGCGACCTTCCTCCTGTCCCTGTTCT

GCGTCACCAACGTACGTACCATCGCCGGCTTCGCCTTCTCCTGGTCATGTCTCCTTAGC
ACGTGGCACGGGGCGCCCTGGC
>495
CATTTTGCAGCCGGCACGTCCATCCTCTTTGGAGTGCTCATCGAAGCCATCGGAGTGGC
CTGGTTCTATGGTGAGTCCGGGGGGAGGCAGCCAAGCCGGCACCCTCTTGTTTGGTGG
CTGTGCCACTCCCCTTCTGAAAA
>496
GATGACCGGGCAGCGGCCCAGCCTGTACTGGCGGCTGTGCTGGAAGCTGGTCAGCCC
CTGCTTTCTCCTGGTAAGGGACCTCGGGCCACCTGCCCTTCCCCCGTCTGCCCACTGCC
CCCACTCTGGCACCTGCCCCGAGG
>497
ACTGGCCTACGCCATTGCACCCGAGAAGGACCGTGAGCTGGTGGACAGAGGGGAGGT
GCGCCAGTTCACGGTGAGGTCGAGGTCCCTGCTGGGCCTCTCTCGGGGGAATTCAGAA
TGATCCCAAGCTCAGTGTTCTTAGC
>498
CGGGGAAGAGGAGAAGGCACCCGCGGAACCGACGGCTCAGGTGCCGGACGCTGGCG
GATGTGCGAGCGAGGTAGGTGCAGAGCGGGAAGCGAGCGAAGTGCGGCGTCTACGCC
GCCGGGAGCCCCCGAGGCTTGTGTCGG
>499
TGATGATGAAAACAAAATAGCAGAGAAACCTGACTGGGAGGCAGAAAAGACCACTGA
ATCTAGAAATGAGGTAAGCCTGTCAGAATATTCAAGCCTTGCCGTTTTGGTTTTAATGTA
GATTTTGTGGCATTGACAGTTTG
>500
GACAGATACTTCTTTCTCTCTGGAAGACTTATTCCAGTTGCTTTCATCACAGCCTGAAA
ATTCACTGGAGGTAATTGGAACTTTGGCTTTTATCCTCGCAGGAACATATCTGCACTGA
CCTTTTGGTGAACAAATACAAC
>501
GAACTGCTGGATTAATGGTAGCAGGGAACTGCCTCCTCTCCCCACCAGCACCATGGCT
GGCATCGCTCAGGTGGGCAGGGTAGAGTAAACAGGAGGCATAGCTGCAGCTTCTGTG
GCAGAGCTTGCCTTAACTTCTCATT
>502
GACTCATGAAGGTGTTCGTCACCCGCAGGATACCCGCCGAGGGTAGGGTCGCGCTCGC
CCGGGCGGCAGAGTAAGAGCCTCGCGCGCCGTGGAGGAGGGAGCAGGGCGGTCCCA
GGGACCGGAGAGCCGGGCGGGGCGTT
>503
GTGGCGGGGGCCCACGGCCTGCTCTGCCTCCTCTCCGACCACGTGGACAAGAGGATC
CTGGATGCTGCAGGTGCACACTGGGTGGGCAGGGGACTTGAGGGTGGCTTGGCCCTG
TGGGCCCCTCAGCCTGTGCTGCTGGC
>504
CCAATCTCAAAGTCATCAGCACCATGTCTGTGGGCATCGACCACTTGGCTTTGGATGAA
ATCAAGAAGCGGTAACTGCAGCTTGGGATCTGGAGGGGGCCTAGAGAGAGGGGTGGC
TATGAGAGAAAGAAGAGCTGTTGA
>505
CCGAACTCGCAGTCTCCCTGCTACTTACCACCTGCCGCCGGTTGCCGGAGGCCATCGA

GGAAGTGAAGAAGTAAGTGAACGCAGACCAGGTGCGGTGGCTCACGGCTGTAATCCC
AGCACTTTGGGAGGCCAAAGTGAGC
>506
AAGCCCCTCTGGCTGTGTGGCTATGGACTCACGCAGAGCACTGTCGGCATCATCGGGC
TGGGGCGCATAGGTGAGGCTCCCACCGGCCCGCTTGCCCGCCCCGGCTCTCACAGCGT
GGTTTGCATCCCTGGCACCACGTG
>507
GTCCAGAGATTTCTGTACACAGGGCGCCAGCCCAGGCCTGAGGAAGCAGCAGAATTC
CAGGCAGAGTTTGGTAATAAGCCTTGATTCCCAGAAGCCTATCTGTGCCCAGTTGAAA
GGCTGTATTTTCTCTCAATGGGGCA
>508
CCGAGGGACTCTGCAACAAGGACTTCTTCCAGAAGATGAAGGAAACAGCTGTGTTCA
TCAACATCAGCAGGTATCCTAGGGCCACCTTACCCAGCAAGCCTGGAGAGGAGCCATC
CCCACTGCTGCCATCTGGAGATTTT
>509
GCTGGACTGGATGTGACGAGCCCAGAACCACTGCCTACAAACCACCCTCTCCTGACCC
TGAAGAACTGTGGTAAGAACTGCACTTTCTGATGCAAACTCCCTGCTGCCCTGCAGGA
CCAGTGTTTTTGAGGGGGTCCCAA
>510
CCCGAGGCCGTTCCGGGTCTGAACTGTAACAGGGAGGGGCCTCGCAGGAGCAGCAGC
GGGCGAGTTAAAGGTGTGTACGTAGTTTTTCTAAATAGGACAGCGCTTTGCAAATTGGC
TGTCATGGACTTGCTGTTTTGACA
>511
CAATGCCACCTTCTGCCTCATCTCTGGCCACCGTCCCGAGGCTGCCTCGCGCTTCCTCC
AAGCCCTGCAGGTACAACCCCAATTTGAGCATCACCCATCCTCTGTCCCCCAGCACTCA
TGAGCTGGCAGGGTACCAACCT
>512
GCTGCCCTTCTCCGAGGTCATCGACTGGACCAAGGCAGCCATCGTAGCTGATGAGAGG
CTCCCACTTCAGGTAGCTCAGAGCCCTGCCCACAGGGTGGGAACTGGACCCAGGACA
GGGGAGGGACCTAGAATCCTGGATC
>513
GGGGCACTCCTGGCCCTGTCTACTTTTTCCACAAGCCCCCAGGACTTCCCCTTCTACTA
CCTGCAACAGGGTATGCCCTGGGGATGGGACAACCTGAAGTCCCCTCAGCTCTACCCA
GGGCTGCCCCAGCCTCATCTGGA
>514
GGTGGGGCCCCCAGGCCAGCCCCCTCTGAAGCTCATCCAGGCGGTGGCAGGCTCCCA
GCACTGTGCCCAGGTCTGCCCCCTTCCCAGCTGGAGTCCTGGGATCCAGGGTGGGGGT
AAGGGACAGGGCAGGGCCCTGATGA
>515
TGAGAGGCCACTCCCATCCAGGTGGCCGGAGACAGCTGTGCCCTTGACAGTCATTGAT
GGGCACAGGAAGGTAAGGGATGAGGAGAGCCATGAAAGGGGTGGGCCCATGCTGGG
AGCTGGGGGAGGGCACCCATGGGGTG
>516
CCCATATAGCACCATCAGAACAGATGCCATCCTCAGCCTCGATGCCCGCAGCAGTCTTT

CCACAAGTGAGGTGAGGGCTGGGCCCAAGAGAAGCCCAGTGTGGGTAGACACAGAG
CCAGAGGGCCCTGGCAGCCCCTCAG
>517
GCTGGGGCTACACTGCTGAGAGGACCAACGAATTCTCCATGGTTCTCACCACAGCCGC
CTTCTACCATAGGTACAGACCCTACCCTGCACAGGGATCAGAGTATCAGAGGCCAGAG
ACCACCCCCACCCCGAACGGAGCA
>518
AGCAGCAGTCACCAAGCTGCCCCCTATCAAGGTGCCCTATGGCAAGCAGCGCCAGGA
GGCTGCTCCACTGGTGAGGGCTGAGGGGGATTGGTCGGAACTGGCACGGATTGGGCG
GGGATGCCGGAGAGATCCCTCTCACT
>519
TGGGGGAATGGACCATCTTGGAGAGGCTGCTAGAAGCCGCGGTGCAGCAGCACTCCA
CTATGATCGGAAGGTAAGGGCGCCAAGCCGGCGGTCAGGGGGCCCCGTCGGGCGGCC
CCCGGGAGTCCCTTCTCACACCTGGA
>520
TTCCTCGTCAGGAAGCCCTCCGACCCCAATCGGAAGCCTAACTACAGCGAGCTGCAGG
ACTCTAATCCAGGTGCGTTGGAGGGGTTCTGGGCTCCAGGAGGTTTGGGGGAGACAG
GCGAAGGCTGCGTGGGGGGCACCTG
>521
GTCTGCGGAACCTGCGGGAAGGCCTTCTCTAGGCCCTGGCTGCTACAAGGCCATGTCC
GGACCCACACTGGTACGTGCCCCTCCAGGCGCCCCCACCGTTGCTCTCTCTGGCAGCT
TTTGTGAATCTGGGCTTGCTGTTC
>522
GCCACTTTCAGTCTTCAAAGAGAAAGATAAGAAATTCTGGATTTTCAAAATCCTTTTGA
AGCCTTTTAAGGTAAGATGAAATATCCTTTTTACTCAGAACCAACTGATTCATTTAGAA
AGAACTTTGAATTTCAAAGATG
>523
ATTATTATTCCTAAAGAAAATGAAGATAATTACATATTTTTGCATTTGGGCAGTAGCATG
GGCCATTCCAGTAAGTATGCCTTTCTTAGAAAACCTCTTCACTTTGTTATCTTTTTTAAC
CTAACATTAATACAAAATGT
>524
ACCACTGGAGAGACATGTCGAAAAATCCATGAATTTGCATCTCCTAGCAAGATCAAAT
GTGTCAGTACAGGTATAGGATGTAATATATTTCATTTTATTTCCTATTTCTGAGTTGCTAC
ATTCCATTAACTTCTCCAAGA
>525
AAGCAAACGCGTAGAAAATAGAATCACCAAAGAATCAGAGACACATGCTGTTGGGAA
GAGCCAAGATAAGGTTAGTTTGTAAAGCTGATTTCTTTCAATGGCAGTTTAAATTCTTC
CCCTCCATCTATTGATGCTAGCAC
>526
GGTGATTGTATCCAACTTCAGTCGCATCTACCACCAGAATCAACGAGCAGACAAACGA
AGGGCACAAAAGGTGCGTATTCAACTCCGTGCAACCATGGTTTAGCACTTCCCACTTT
TTATAATGAGCTTTAAAATTTCTT
>527
AGCCAAAAGCGGAAGCGCAAATGCTTACATGCAGAGCAAACGGAATGGTTTACTCAGT

AATCAGCTGCAGGTACAATCAATTACATCTCTTTTTTAATGTTCAATTTCTTGGTTTAAT
ATTGATTTAGTTCTACTTTCC
>528
TGTTAGCAAATCCGGCTCCAGCTTTGAAACCCAGCACCACCACCTGCTTCACTGCCTG
GAAAAAACCACGGTAAGGAGACAGCATGACTGCCTTCCCTTGCTCTCTGACAGTAATT
CCATTTGCTTTTGTGCATACTTAA
>529
ATCAAGGTAGTATACAAGAACTCAGCACGATTCAGATCAGATGTGTGGAGAGAACACC
TCTGTCTAACAGGTACCTGAGATTAATCTGTGTTGTCTACACACCTGTGCTGGTTCCCA
GGGTGTGTCTTCTGCACTCATGT
>530
CTCGGTGCTCAACAGCCTGGTGGGGCCTGCCTGCATCTTCCTGCGGCCCAGCATTGCC
GCCACCCAACTCGTATGTACGGCCACCCGCCCGCCAGCCTGCCTGCCCTTGCCCCTCTC
CCACCAGCTCACCTGCCCTTGCC
>531
GGTGCCTGCATGCGGACCCTGGGCTACATGCCCACCGAGATGGAGCTCATCGAGATCT
CACAGCAAATCAGTATGTGCCTCGGACCCATCCCCATCCCCAGTCCTCCAGATGCCCCC
GCTGGGCCCATGAAGCCAAGCAG
>532
GGGCCCCAAGCTGCTGGCAGAGACGGCAGACATGATCGGTGTCCGGGAGCTACGGGA
CGCCTTCCGGGAGGTGCGGCCACTGGGGCTGAGGGCGGGCAGGGCTGCGTGTCTGCC
GTGAGTGAGGGGCTGCGCGTGTGAAT
>533
AGCCAGCGGGAGGTGGACGAGATCCTCCAGGACGTGGACCTCAATGGGGACGGTCTG
GTCGACTTCGAAGGTACCCCCTGCCGCACATAGCAACACACGCCCTGGAAGGGTCCTA
GCAGATAAGAAACCGCACAGCAAGA
>534
TATGCTGGGAGCGGTGAAGATGGAAGGGCACGAGCCGTCCGACTGGAGCAGCTACTAT
GCAGAGCCCGAGGTAAGCGCTCGTCTTTTCCAGGGGGAAGTGGAGCGGCGGCGCTCG
CTCCTTCCCCAACCTCAGGACCGCT
>535
CCAGGAAGATGTCATTGCCACAGCCAATCTGAGCCGCCGTGCTATTGCAGATATGCTTC
GGGCTTGCAAGGTAGAAATCCTTGAGGCATTTCCGTGAGGCAGCTTTGGGCCAGAACA
TAGTGGGAGAGAAACTGTAGGGA
>536
TTGACAGGACATTCAAAGCGTGTGGCTGGTTCCGTCACTGAGCTCATCCAGGCTGCTG
AAGCCATGAAGGGTGAGGGGTGACCAGGGACAGGGACGGATGGAAAACCCTTATCAT
GGTGTGATCTCTGCTTCCTGGCTGG
>537
GGGAGCTGCAGCCGCCATTGAGGCTGCAGCCAAAAAGCTAGAGCAGCTGAAGCCCCG
GGCCAAACCCAAGGTCAGTCCTGCTCCCTTCCTCTTCCACCCTCACCATGGCAGTGTC
CCCACCTTCATCTCTGACTTTACAT
>538
TGCAGCAGCCACCAGTGCACTGGTAAAGGCTGCGTCGGCTGCCCAGAGAGAACTAGT

GGCCCAAGGGAAGGTGAGTGCTTAAATACCCAGAAGAATGGGGACAGGTTGGCACTT
TGATGTCCACATCCACATCCATAGCC
>539
CACAGCCCAGCTCCTTGTGGCCTGCAAGGTCAAGGCTGACCAGGACTCGGAGGCAAT
GAAACGACTTCAGGTGAGAACCCTGACCCATCCCATTGTCCCTTGGGCTATACTGCTTC
AAACTGTCACTCCCGAGATGTATG
>540
TGCAGCCTTTGAAGAGCAGGAGAATGAGACAGTGGTGGTGAAAGAGAAGATGGTTGG
CGGCATTGCCCAGGTGAGCTTCACCCCAGATGCTGAGGGACGCTGTCAGGAGGGCAG
TCATGTTGCCTGCGCTGGTCCCAACT
>541
GGAGAAGAAGGGACCCCAGGTCAGAGACTGGAGCCATTACTTCAAGATCATCGAGGA
CCTGAGGGCTCAGGTAAGGGGTAGGAGGGACCTCAACTCCCAGCCTTGTCTGACCCTC
CAATTATACACTCCTTTGCCTCTTT
>542
CTGTGGACAATGCCCGCATCGTTCTGCAGATTGACAATGCCCGTCTTGCTGCTGATGAC
TTTAGAGTCAAGTAAGTTTGGGGGCTAGAGAGCTGGGGGTCCAGGGGTGGAGCTAAG
AAGGACTGCTCCCCAGGCTGGGTA
>543
CATCCGGGCCCAATATGACGAGCTGGCTCGGAAGAACCGAGAGGAGCTAGACAAGTA
CTGGTCTCAGCAGGTGCGTGAGGGGAGGGGATGGCTGCCAAGGTGTGGGAGGGAGG
CAGACGGAATGAGGGGCCTGATGGACT
>544
GACGACGCTCACAGAGCTGAGACGTACAGTCCAGTCCTTGGAGATCGACCTGGACTC
CATGAGAAATCTGGTGAGTGCCTTCACATCACCTGCCCAGCTCCTCCTTCACTTGGCCT
CAGACCCAACCCTGTCTCAACCCA
>545
ACATCAAGGTCAAGCTGGAGGCTGAGATCGCCACCTACCGCCGCCTGCTGGAAGATGG
CGAGGACTTTAAGTGAGTGGGGCTCTCCTACCCACACGTGCTGGGATCAGGAGATCAC
TTCTCCCCAAAGTCTGAGCTTTTG
>546
TATGTTAAATGTCTGTCTCCAGAATATGCTGGAAAGGCTTTTAAAGCATTGCATGGCTCT
TGGTTTGATGGTAAGAAATTTGAGTACTACAAACATTTTGAAAAGATAGTTATAGTTTAA
TTTTAAACTATACCAGATTT
>547
ACCGCTAACGTGCTGCGCGACATGGGCCTGCAGGAGATGGCCGGGCAGCTGCAGGCG
GCCACGCACCAGGGTGAGCCGCCCCCGTTCCCCTCCACCCCGTCTTTCCCCTCCACCC
ACACCAGCGCTTACCCCGCGGGCTC
>548
CGCCTGGAGGAAAAACAAAGACTTTCCAGAAAGAAGAGAGAAGCGGAAGCTATGAA
AGCCACAGAGGATGGTAATGAACTTCCCTTCTACTCTTCTTTCTTGATGCATGGAGAAT
GCCAGCACTAATTTTTTTCTTTCCT
>549
GGCAAGCCGGTGATGGAGCGCTGGGAGACCCCCTATATGCACGCGCTGGCCGCCGCCG

CCTCCTCCAAAGGTAGCCCCGCCCGGCCTGCACCGCCCCCGGGGGCGCAGGGGAAA
CTGAGGCCGGGGCTGCCCCGACCCG
>550
TTGGATCATCGAGTGCAATGACGGCGTCTTCCAGCGGCTCCGGGACTGGGCCCCACGG
CAGACACACAAGGTGCCCCTCTGCCCGCAGGCCCTCCAGGACCCCCACTCCTTGGGG
TGAGGGCACTGCTTCCTGGGGGTTG
>551
GGGGCCGCCTGCTCCCCCAAAGCAGCACAGCAGTACCAGTCCCTTCCCCACCTCCACG
CCCCTCCGCAGAGTGAGTATCTCCCATGGAGTCCCTGCACATCAGTGCTCACGGATCTC
AGAGCGCTGTCAGTCTCCACTGG
>552
ATGAGAAGGAGAAGGCGGAGAAGCAGCGGCTCAAGGAGGAGCGGCGCAAGGAGAG
ACAGGAAGCCCTGGAGTGAGTGTCCTTGGAGGCCATGCTGGGCCCGCCACCCTGCTG
CTGGATTCCGCTCCTGGCCACTCTGATG
>553
TCAAAGGCCGGCAGCCCCTGAGGTCCGGACCCACGCACGTTTCCACCCGGAATGCAG
ATATTTTTAACAGGTCAGAGCCTGAGGAGGTCGGCCTTCACCCACTAGTGATGCTGGA
GGCCAGCATCCTGAACCCTGGGGTC
>554
TGCCTACTGGGGTACCTGGAATAAGAAGACGGCACTCATCCGCGCGCGAGACCCCTGG
GCCCAGGACACGGTGAGCTAGCCCCAGAGTGCCTCCGTCCCCGTACCTCCTCACTGTG
CCCCTTTCCTCCAGCCCCAAAGAC
>555
CTATGAGGTGGACAGTGATGAGGAGTGGGAAGAAGAGGAGCCTGGGGAGTCCCTGTC
CCACAGTGAGGGGGTAAGGATGTGCCCCAGCTGTCTTCACTCACAGACGGCTTGTGTT
TGAGTCTCTGTCCCACAGTGAGGGG
>556
GGATGAAGATGAGGACGATGGTTTCTTTGTGCCCCATGGGTACCTGTCTGAGGACGAA
GGTGTGACAGAGGTGAGGGAGTGAAGGGGGAGGTCACCGGCTCAGCAAAAGTTCATT
TCTGGACTGGTGCTCAGTGGCCTGA
>557
CTGGAGACCCTGCCGGCCCAGGAGGAGCAGACGCCCAAGGCCTCCAAGCGGGAGAG
GAGAGACGAGCAGAGTGAGTGTGGGCGGGGCCAGGCCACCCACCTGTTCCTGGGCCC
AGCTAATTCCAGGGCTGAGGGCAAGAG
>558
GCTGAAGAGGAAGTCAGCGGGCAGCATGTGCATCACCCAATTCATGAAGAAGCGCAG
GCACGACGGCCAGGTGAGGTGGGGTGGCAGGTGGGGGCCTCTGCAGGGCTTTTCTT
TTTTTGTTTGTTTTTTGTTGTTTTGT
>559
CAGGCAGACACGGAGGAGGAGGAAGAGGAGGAGGGCGACTGTATGATCGTGGATGTC
CCGGATGCTGTGGGTGAGAAGGGCTGTAGATAGCAGAACGCACTGGTGAGGTGGGCG
CTGGAGGTAAACCTCAACAGAGAAGG
>560
GCATGCGTACCCTGATGGCCGTAGAGAGGAGCCACAGAGACAGAAAGTGGGAACATC

AAGCAGATACCGGGTAACTCTCACTGTTTTTTTAAAGGAGGGGAAATTCCAAGGTATC
AGTTGAGTCCTGCACATATTGGGGT
>561
CCATTCCCGATCAGCTGGATCCTGGAACTTTGATTGTGATATGTGGGCATGTTCCTAGTG
ACGCAGACAGGTAAAATCACTGTGCTAAAGGAAGGAGCATGAATAGGCTGTCTTTTTG
TGATTGTGGAATGATAACAGAG
>562
CTATGACACGCCTTTCAAAAGAGAAAAGTCTTTTGAGATCGTGATTATGGTGCTAAAGG
ACAAATTCCAGGTAGGTTTTGGAGAGGGACAGGTTGAGTCCTCATTAGTGAGCAGGAG
TGCACAGGGGGGCCTTTCACATT
>563
AGAGAAAATAGACACTCTGGGCATTTATGGCAAAGTGAATATTCACTCAATTGGTTTTA
GCTTCAGCTCGGTGAGTGACCTTCCACAGCTTGGGGTCTTTTATGAGGATGGTTTCTGA
TGAGATGGTAGAAAAAATCTTC
>564
GGTTGAACACCCCCATGGGCCCTGGACGAACTGTCGTCGTTAAAGGAGAAGTGAATG
CAAATGCCAAAAGGTCAGTATCCTTCGGTACCAGTCACAGTGCAGATACTTCCGTGCCT
GTTACCGCCTTCTACCCGTGAACG
>565
TCAGGAGTCCTGGGGAGAAGAAGAGAGAAATATTACCTCTTTCCCATTTAGTCCTGGG
ATGTACTTTGAGGTGAGGTTCCAGTTTTTGAAAATGGGACAGCAATAAGAATCCTGGG
AGCAGGGGTGGGATAAGTGGTCCA
>566
GGGTGCACGTGTGCGGCGGGTCACTGCTCAGCCCCCAGTGGGTGCTCACAGCTGCCC
ACTGCTTCTCCGGGTGAGTGTCCGGGTGGGCAGGGGCAGTCTCAATGTCCAGGCCCCA
GTGGGGCATGCGCCAGCGCAGAGGC
>567
GAGGCCTCAGATGACTTCTGCCCTGGGATCCGGTGCTGGGTGACCGGCTGGGGCTATA
CGCGGGAGGGAGGTGAGGAGCCCCCGTGGGGATGCTGGGATGGAGGAAGGCAAGGG
CGGCCATGGAAGAGAGCTGGCTTGAG
>568
TCCCGGCCCCGGGGGCAGCATCATTCAGCCCGACATGCTGTGTGCCCGGGGCCCCGGG
GATGCCTGCCAGGTGAGCCTGCCAGGCCGTGACAGAGGATGGGGGCGGCTGAAGCCC
AGGGAGGGGCTCCCCATCTTCTCGG
>569
TGAATTTTTAGTTGGGAAGCCTCCTTTTGAGGCAAACACATACCAAGAGACCTACAAA
AGAATATCACGGGTAAGACGCAACTGAAAAGGACCCAGCACATCTGCTGGAATTCTTT
CCTTATCAGCCTGAACTGCAGGCC
>570
CTAATTAAATTTAGGGGATCCTGATGCCACTTAGCAGTGGGATTTCAGAGGACCTTTCA
GAGGAGCTAAGGTAAGATGAGATGGAGGCAGAGTGGGGCAGGAAAGATGGCTTAGGG
GCCTCTTTGGGCTTTGTGACACTC
>571
TTGCCTTGATTTTCTTCAAAAGACAACTATAATCATTCAAAAATTCCAAGTTGGGTCCA

AAATGTCAAAGGTAGGTATTGGACTCTGCCATGTGCCAGGTCATATGCCTGAGTCTGTG
TCCTTCAACAGGCTGTCCTTAT
>572
GGCGATGAGCACCACTACTGCTGCATTGTGCAGCACGCGGGGCTGGCGCAGCCCCTCA
GGGTGGAGCTGGGTGAGGTCCCGCCAGGTGGTGATGCTCCTGGTTTCCCGTTGCCTTG
TCTCACTGCTGCGCCGGTCCTTCC
>573
GTCTTGCTACTCACGGCAGCGGCTGTAGGAGGAGCTCTGTTGTGGAGAAGGATGAGG
AGTGGGCTGCCAGGTGGGGGGCAGCGGGAGGAAGAGCCTCTGAGAGAGGGACAGAG
ACCCCAAGAGAGGGGCACACAGACCTG
>574
CGGAGCAGGTTGAGGACTGTGTGCTGACTGTGGAAGGAAGTGGCCTCTTCAAGAAAC
AGCAGAAAGTCTTGTAAGTGCTGCAAGTGCTCAGCCTTCTCCTGTTTTTCCCCCCTGA
GATTAGCACTATCCTTCCTGAACAT
>575
TGGCTCTCATCCCAGACTTGGCCATGGAAACCTGGCTTCTCCTGGCTGTCAGCCTGGT
GCTCCTCTATCTGTGAGTAACTGTCCAGGCTCCTCTTCTCTGTTTCCTTGGACTTGGGG
CGCTAATCACGCCTCTCTTTCCC
>576
TTTTAAGAAGCTTGGAATTCCAGGGCCCACACCTCTGCCTTTTTTGGGAAATATTTTGT
CCTACCATAAGGTGAGTGTTTTTGAGCTCACTCTTTTGCTTCTTATGATTGCCAAGAGC
AGCTTAGTTCCATCAGTAAAAA
>577
TATCACAGATCCTGACATGATCAAAACAGTGCTAGTGAAAGAATGTTATTCTGTCTTCA
CAAACCGGAGGGTAAGCATTCATGTGTTGAAATTAAAATACTGATTGATTAAATTTATAT
TTTGAAATTCTTATATATTCA
>578
TGAGGATGAAGAATGGAAGAGATTACGATCATTGCTGTCTCCAACCTTCACCAGTGGA
AAACTCAAGGAGGTATGAAAATAACATGAGTTTTAATAAGAAACTTAAAGAATGAATC
TGGTGGGGACAGGTATAAAATAAG
>579
AGTATGGAGATGTGTTGGTGAGAAATCTGAGGCGGGAAGCAGAGACAGGCAAGCCTG
TCACCTTGAAAGAGTAAGTAGAAGCGCAGCCATGGGGTTCTGAGCTGTCATGAACCCC
TCCAGCTGCCTGCCATGGAGCTGAT
>580
GACCCCTTTGTGGAAAACACCAAGAAGCTTTTAAGATTTGATTTTTTGGATCCATTCTT
TCTCTCAATAAGTATGTGGACTACTATTTCCTTTAATTTATCTTTCTCTCTTAAAAATAAC
TGCTTTATTGAGATATAAAT
>581
AGAAGTTACAAATTTTTTAAGAAAATCTGTAAAAAGGATGAAAGAAAGTCGCCTCGAA
GATACACAAAAGGTAAAATGTGGTGGTAGTTATAGGAGGATGTTTAGTTTTTCATAATTT
TTTAGATAATATACATATGATC
>582
TGAACTGGCCACTCACCCTGATGTCCAGCAGAAACTGCAGGAGGAAATTGATGCAGTT

TTACCCAATAAGGTGAGTGGATGGTACATGGAGAAGGAGGGAGGAGGTGAAACCTTA
GCAAAAATGCCTCCTCACCACTTCC
>583
TGATTCCAAGCTATGCTCTTCACCGTGACCCAAAGTACTGGACAGAGCCTGAGAAGTT
CCTCCCTGAAAGGTACAAGGCCCCTGGGAAGGGAGCCCTCCCTGAACCAGCCTGGTT
CAAGCATATTCTGCCTCTCTTAATC
>584
CATGAACATGAAACTTGCTCTAATCAGAGTCCTTCAGAACTTCTCCTTCAAACCTTGTA
AAGAAACACAGGTTAGTCAATTTTCTATAAAAATAATGTTGTATTAATAATTCTTTTAAC
TGAGTGGTCTGTATTTTTTAA
>585
TGTCTTGGCCATGGTCCCCTGGTGCTGCAACTCCTCTCCTTCACGCTCTTGGCTGGGCT
CCTTGTCCAAGGTCAGGGGCAGGTTCTGAGGGTGTGGGGTCCCCACGCCTGGCTTTTG
GCTGTGGACACAGCCTGGGGCTG
>586
GGTGAGCTTCCAGAGAAATCTAAGCAGCAGGAGATCTACCAGGAGCTGACCCAGCTG
AAGGCTGCAGTGGGTGAGTATCTGGAACCAGGCTTCTTGGGCCTGAGATGGTCGTTGT
GTGGTGTGAGTTTGCTTGAGTCACT
>587
GCACGACTCCATCACCGCCTGCAAAGAAGTGGGGGCCCAGCTCGTCGTAATCAAAAG
TGCTGAGGAGCAGGTACACCTGGTGGGGATCCCCGTCCTGGCCTAGGGCATGGCTTCT
GGCCAACTGGGGAGGAGGTGCTCAG
>588
GGATGGGACTTTCAGATCTAAATCAGGAAGGCACGTGGCAATGGGTGGACGGCTCACC
TCTGTTGCCCAGGTAGATCTCAGAAGGGGCACCCTATCCACTGGGGCTGGAATTGTGA
AACTCTGATTTGAACTTTCCCTGG
>589
GGCGCCCTGGTGCTGCAACTCCTCTCCTTCATGCTCTTGGCTGGGGTCCTGGTGGCCAT
CCTTGTCCAAGGTCAGGGGCAGGTTCTGAGGGTCTGGGGTCCCCAGGCCTGGCCTTTT
GGCTATGAACAGAGCCTGGAGTG
>590
GGTGAGTTGCCAGACCAGTCCAAGCAGCAGCAAATCTATCAAGAACTGACCGATTTGA
AGACTGCATTTGGTGAGTTCCTGCACATCAAGGGTCCTTGGGCCTGAGATGGTCTCTG
TGTGATGTGACTTTACTTGAGTTA
>591
GCACGACTCCGTCACCGCCTGCCAGGAAGTGAGGGCCCAGCTCGTCGTAATCAAAAC
TGCTGAGGAGCAGGTACACCTGGTGGGGGTCCTCGTCCTGGCCTGGGGCATGCTTCTG
GCCAACTGGGGAGGAAGTGCTCAGA
>592
GGATGGGACTTTCAGACCTAAATCAGGAAGGCACGTGGCAATGGGTGGACGGCTCAC
CTCTGTCACCCAGGTAGATTCTGGGAAAAAGGGACACTGTATCCAGTCCGGCTGGGTT
CCTGCAACTCTGACTTGAGCTTTCC
>593
TTCAGAGACGAATAGTTGTTTCCCTGCTAGCCTCAGCCTCCATTGTGGTATAGCAGAAC

TTCACCCACTTGTAAGCCAGCGCTTCTTCTCTCCATCCTTGGACCTTCACAAATGCCCT
GAGACGGTTCTCTGTTCGATTT
>594
GGCAGGCCCGCGCCGCTTAGGAGGGAGAGCCCACCCGCGCCAGGAGGCCGAACGCG
GACTCGCCACCCGGGTAAGCCAAACTCGGGCGAGTGGGGGCCCGGCAGGGGACGCGT
GGCCCAAGTCCTGCCGCCCAGCCCTCC
>595
TTGGATCCTTCTTTGACTCATACAATATTTAATCCAGAAGTATTTCAACCACAGATGGCA
CTGCCAACAGGTAAGAAAACTCATCCCTGTTACCCTGTTGTTCTGCTTTCAGTCTTAGT
AAAATGCAGGATTTGTTAATA
>596
AGGCCCATCCCATGGTGGACTACCTGGTGCCTCTAGTGAAAAGAACAAGAAGTCTTAC
CCTCAGGTCAAAGTAAGTTTGTGGTAGCTCTCCTTCTATTTGAATTCTGGAAATTTTGAT
TTCCTACGATTTCCAAGGAATT
>597
GCCTGGTGGGAAAACACTGTGAGGATGGGATCTGCACTGTAACTGCTGGACCCAAGG
ACATGGTGGTCGGGTAAGTAGGGGTATATGATGCTGTGGAAGGTAGGAACAGATAGAA
TATGGGATGCAGGAACTTAGAATGA
>598
CCTGGACTCTTGGTGCACCCTGACCTTGCCTATTTGCAAGCAGAAGGTGGAGGGGACC
GGCAGCTGGGAGGTAAGCATCATTTTCCTGGCCTTGATCCTCCAAGGGGTCCAGGCTT
TGGTTTTCATCTGTATGAATTATA
>599
CTTCCGGATAGCACTGGCAGCTTCACAAGGCGCCTGGAACCCGTGGTATCAGACGCCA
TCTATGACAGTAGTGAGTACTTCACTTCCAACAGGGGGCACACCAAGAATAGACTTCC
AGCCCTGCCCTGCCATTTACTTGC
>600
ATGGACAGGACAGCTGGATGTGTGACTGGAGGGGAGGAAATTTATCTTCTTTGTGACA
AAGTTCAGAAAGGTAAATACATTCTGTGATCTCTGATCTCAAGAGGTGTGATCTTGACA
CACTACAGTTCTGAGTGTGTCTG
>601
TGAAGAGGAAGAAAATGGTGGAGTCTGGGAAGGATTTGGAGATTTTTCCCCCACAGAT
GTTCATAGACAAGTAAGTGATTTATTATTATTATTAATCCTTATTATTTTTAGAGATGGGAT
CTCACTCTGACACCCAGGCT
>602
CAGCTTCGGAGGAAATCTGACTTGGAAACTAGTGAACCAAAACCTTTCCTCTACTATC
CTGAAATCAAAGGTAAGTCAGTTGTTTAAAATCTTATGCTCATATTTTATTTTATTTTATT
TTTGGTTTTTGTTTTGTTTTG
>603
GCTGGAGCTGGAGGCGGAGGCATGTTTGGTAGTGGCGGTGGAGGAGGGGGCACTGGA
AGTACAGGTCCAGGTACAAAAATACTTATTCTTCCTAAAACTTTTTCATATTGGAGAGG
GTAGAATTTTCTTTTCTTTGGTTT
>604
GGATTTCCTACTTATGGTGGGATTACTTTCCATCCTGGAACTACTAAATCTAATGCTGGG

ATGAAGCATGGTAAGTAATGCTTTGTTCTTAATACCAAGAAAGGAAAAAAATAATTAAT
GCTAAAAAGGGTTTTTAAAAT
>605
ACCGTTGGGAATGGTGAGGTCACTCTAACGTATGCAACAGGAACAAAAGAAGAGAGT
GCTGGAGTTCAGGGTAAGTGAGCACACAAATTACGTTCTGTTGGTTGGCTGGGGAGGG
GTCAGTCCTAGGTGCAGAAAGATAT
>606
CAGGAGACGTGAAGATGCTGCTGGCCGTCCAGCGCCATCTCACTGCTGTGCAGGATGA
GAATGGGGACAGGTAAGTCAGAACTTTTGCATGATAGGTTGTCCTGGGTGGGGAAGAA
GAAGAGCATCGTATAATGCATACT
>607
GGATCTACTAGAAGTCACATCTGGTTTGATTTCTGATGACATTATCAACATGAGAAATGA
TCTGTACCAGGTAAGCAGAAATCTCAAGAAAACAACTGAAGAAAAATCTGTAGTTTAC
TTTTTCTTCTGTTATGTTTGGG
>608
GATAAAGTTCTCAGTATCTTACTCAAGCACAAAAAGGCAGCACTACTTCTTGACCACC
CCAACGGGGACGGTAAGAGACAATCACACATCATTGGTGTAACTTTCTCCACCTTCTA
AATATCTACTAGGTATTTGATAAA
>609
CGGGCGCACAGCACTGCACCTGGCTGTGGAGCACGACAACATCTCATTGGCAGGCTG
CCTGCTCCTGGAGGTGAAGGGCACACTTATTTGCTTTTGCATTAAATTTCTGAGGGAGA
TTTAAGGAAATCTTTTCAAAGAAG
>610
GGAACCACACCCCTGCATATAGCAGCTGGGAGAGGGTCCACCAGGCTGGCAGCTCTTC
TCAAAGCAGCAGGTAAGATGGTGATCTGGCGGTCATTAATGAAAAATGTTACCAGGAA
TGCAAACCCAACTTCAATAGAGCA
>611
GGAAAATGCAGGAGAGGATGAAGGAGTTGTGCCTGGAACCACGCCTCTAGATATGGC
CACCAGCTGGCAGGTGAGTGCCGCTCCATCTGTCTGATGGCTGCCCCTGAGGGAGTCA
GAGGTTCAGGAATGTAAGAACAGAT
>612
TCTGGGGATACTTAATAATGCCTTCCGGCTGAGTCCTGCTCCTTCCAAAACACTTATGG
ACAACTATGAGGTAACACCTTACCTTACAGATTTAGCAATTTTCATTTGACTTTACTCTG
TTAACATCTCTGGCCAGGGCA
>613
AGCCCAGTGAAGACCACCTCTCAGGCCCACTCGCTGCCTCTCTCGCCTGCCTCCACAA
GGCAGCAAATAGGTAAAAAAAAAGACAAAAGACAGTGGAGATATTTTCCAGCTCCCC
CAGGCTGGTGTCTTCAGCTCTTTTT
>614
AGTGCAACCAATGGTCAGCCCGACCAACAGGCCGCACCAAAAGCACCCTCAAAGAAG
GAAAAAAAGAAAGGTATGGAGAAATCCACTTGAAGCCAAGGAAGGGAGCTAGAGAT
GGTTACCCCTGGAGAAATGTGAGATTC
>615
GATTGGCATTGATGATGTGCCAGATGCAAGAGGGGATAAAATGAGCCAAGACTCTATG

ATGAAACTAAAGGTAAAAGGGAAATGAATGCTTTATCTGTATATTAAGCAAAAGTCCAA
GAGAGGGAATTATATTAGAAGCT
>616
ACAACACAAACAAAGGATCTGGGTCAACATTTCCCTTTCTGGGATAAAAATAATTGATG
AGAAAACTGGGGTAAGAATTCACATTTGCTGATACCTCTCTGACCACCTGAGTCTGAC
CAACAATTTGCTACTTGAATTTC
>617
CCGGGCATTTGGTTACGTGTGTGGAGGAGAAGGCCAGCATCAGTTTTTTGCCATAAAA
ACCGGGCAACAGGTGAGCTCTAAGTCTTGACATAGACCTGGGGACTTGTCCTCTGTGG
ACATTGGAGTGTCTGTCACTCAAC
>618
ACATGACTCCATAGCCATTATCCCACCTCCACAAAGTACCAAACCAGGAAGAGGCAGA
AGGACTGCTAAGGTGAATTGTCTTCTCCACATATCCATTAGCAGAGTGCATGTTCGGTA
CCAAAGGGTGGTGTGATGCAAGC
>619
CTGCAGCCCAACCCTCTGGATCTCTTCAAAACAAGTGCTCCTGCCCCAGTGGGGCCCC
TGGTGGGTCTAGGTAGGTGCCTAGAGATAAAATTAAGTATGACTCTTTTGCTTTTGCTCA
TAAAATGATGATGCATCGGAAG
>620
GTTGGCATTCCTCAGGAGAATGCAGACCATGATGACTTTGATGCTAATCAACTATTGAA
CAAGATCAATGGTAAGCCACTCACCACTCTGCCTACTGTTCCAACAACTATCTCCACTG
CCACATGGACCTAAATGAGTTC
>621
TACCAAATCTACTGACAATGCATTTGAGAACCCTTTCTTTAAAGATTCTTTTGGTTCATC
ACAAGCCTCTGTAAGTATGAACTACTAGAAGCTCTTCTCCAAAGTACATGCCACATAGA
GCTCCAGCCTCCTGAATTAAC
>622
TGGCTTCTTCTCAACCTGTATCTTCTGAGATGTATAGGGATCCATTTGGAAATCCTTTTG
CCTAAATTCTGTAAGTAGAAGATCCTAATAAGCCTGTCTCAGGAGTTTTTGTGAGAAAG
GGTGACATGGAATAAAGAGTG
>623
GGTGTCCCCACTACCCATGGCTTCCTCGGTACCCTTGGTACCCTAGGCTCCCAGGGCAG
GTGAGGAGCAGGTGAGCATGGGCTTGGCTTGGGGGATGACAGGGAGGTTCTCCCAGT
GGGGGCCCAGCTCTCAGAATCAGA
>624
CTTGGCAACTTTCCTGGCCCTTTTGAAGAGGAAATGAAGGGTATTGCCGCTGTTACTGA
TATACCTTTAGGTAAAGTTCACTTAGAAGCTTTAAAAAAAAAAGTAATTAAATTCTTTTA
CAAGTTTCCATTGAGGTGTGA
>625
ATTATTTCATTCAATATTTTTTATGAATTATTTACCATTTGTACTTCAATAGTAGCAGAAGA
CAAAAAAGGTAATTGTGCGCTGAGGGAGGGTGGGCAGCATGAAGCACTACATAAAGA
AAAATCTTCTAAATGAAATTA
>626
AAACAACAAAACTGTCTTCAAGGCTTCAAGCTTTGCTGGCTATGTGGGCATGTTAACA

GGATTCAAACCAGTAAGGAGTCTGTTGTTACATAATTATACAGGTAGCCTTCTGTCCTG
TTACCGTTATGTAAAAGTTGAAA
>627
GGATTCTGGGAAAGAAAGATGCCATGTGGATAGGGTTCCTCACTAGAACAGTTCTGGA
AAATAGCACAAGGTAAAGTGTTTCGTTGCTTTACTATTAAATATTTATTTTAAATTACTCA
TATTAAAAAATAAAAATATTT
>628
GAAACATCCCTTCTTCCTTGATGATCGCAGAACGCCTGCAAAGATGTGTTTGAACCGC
ACCAGCCAAGAGGTACCTTTGCAGGGTCATCTATAGAAAGAAACTGTTGCCATTAAAG
ATTCCCTTGGTTCTGGCATTTTTC
>629
GGAAGCCATCGCCTGGCCCCGGCTCTCTGGACCTTGTCTCGCTCGGGAGCGGAAACA
GCGGCAGCCAGAGGTGCGCGGGCGGCTTTCTCTGCCGCCTCCTGGGGACTTGGGCGC
GGGAACTGCGCGGAGCCTGCGCTGGG
>630
AATGCTTCTCACCCGGAAACAAACTTGCCAGTTGGGTATCCTCCTCAGTATCCACCGAC
AGCATTCCAAGGTAAAGCAGTTGCTGAAAGAAGAAAAAGTAAAATTAATTGAGAGAC
AGAGAGAGAGAGTGAGAGTGGGAG
>631
AGGGGTACCATGGATGCCAGCGCCACAGCCTCCATTAAACTGTCCACCTGGATTAGAAT
ATTTAAGTCAGGTAATTTCAAAGACACAAAATACTCATAAAAAACAGAACTGTGCTTC
CAGCTTGCTTAACCAGATTAGCA
>632
TCAAGAAGTCATAACTCTGGAGAGACCACTAAGATGTAGCAGCTGTTGTTGTCCCTGC
TGCCTTCAGGAGGTCTGTGATCAATTACCAGGGCACTTTGTAAAATAGTTCAGCAAGAA
TTTCTGCCCTGTCATTGAACCTT
>633
AAGAGAGGATGTACTAAAAATAAGTGGTCCATGTGTTGTGTGCAGCTGTTGTGGAGAT
GTTGATTTTGAGGTAAGAGATGTCATAATGTTTATAAGATTTGCTTTCCTAATATAAGAA
GAATGTAATTTTGCAGGACATT
>634
TGGAATCCAGTTCCCTTTAGACCTTGATGTTAAAATGAAAGCTGTAATGATTGGTGCCT
GTTTCCTCATTGTAAGTCTGTTCTTATTAATCTTGATTATAGATGCTTTCTCTAGGTTCATT
CTGAAATGATTCAGTAAAG
>635
CTGCTTACCCTGAAGCCTGGGGAGGCCATGTTTCTGGAGGCCAACGTACCCCATGCCT
ACCTGAAAGGAGGTGAGCACATTTCAGCAGTGAGCCCACTGCCATCCCTGCTGGGCCC
TGTTTCCCTATCTGGACAAAGGAA
>636
GAGTCAGGAAGACCCCTACCTCTCAATCTATGACCCCCCTGTACCAGACTTCACCATTA
TGAAGACGGAGGTGAGTGAGGGGCTATGATGGGTGTCCTTCGTGTGCCATGAAAATCT
CTGGCAAGGGTCACGATGTGGAG
>637
TCAAGATTATAAAATAACCCAACGGATGGTCTGTGCTGGCTATAAAGAAGGGGGAAAA

GATGCTTGTAAGGTAACTCATGAGATTATGAAAACACAATAGGCTGCTTGAGAAAATTC
ATTTCAAAATATATTTTCCAATA
>638
CCAACTGCTGGTGAAGGCCCACCAGGATGCCCAGCGCCACGTAGCTGCAGGCACACA
GCAGCCCTACACGGTATGTGAGCTGGTGGTGCCTCTGACAGCCTTCCTGGGGGGTCAG
GGTGTCCTCTGTGGGTGTGTGTGTG
>639
CATCCTCGCCCGGGACCCCATGAACCGCATGGAGATCTTCCGGCTCAACACCATTCCCC
TGTTTGTGCAGGTGAGTCTGGGGCAGGTGGGCAGGAGAGGTCCCCCCACCACTGCTT
AAAGGGCAAGGTTAGGAGTCCCAG
>640
GACGCCATTGATGCAGAGGGGGCCTCGGCCCCACTCATGGAGTTGCTGCACTCCCGCA
ACGAGGGCACTGGTGAGCTGGGGGAGGGCAGGAGGCTGGGTCCCACTCCCCCAGCC
ACGATGGAGGCTGGCCGACGTTTAAC
>641
CTACCGGAAGCGCGTGTCCGTGGAGCTCACCAACTCCCTCTTCAAGCATGACCCGGCT
GCCTGGGAGGCTGTGAGTATCCTAGGTTGGACCGCAGTAGTTGGTTGTGCAAGTTGGG
CACTGCCCATGGGCAACACTTTTG
>642
TCTATGGCCATGCAGGCAGCTGCACTGGGTTCCTCTATCGGAACAGCAGTTGTTGGAG
GTCTGATGACAGGTCAGCATAAAAAGTGAACACTTCCCTGTATCTTATGCTCTCAGATT
ATTTACAATAGATAATTAATGAG
>643
AATTCTAAAGTTGCCTCTCTGGGGACAGCCATCTGACCAGAACTGCTATGATGATTCTG
TTTATTGGAAGGTACTGTACAGTATTCTAAACAAGAATGTGAACGATGACTCCATTAGT
GAAAGCTTTACACACTTCAAGC
>644
AAAGGTGAAGTCTCGAGAAAGGGAGCAAATCAAGTCACTCAACAACCAATTTGCCTC
CTTCATTGACAAGGTGAGTTTCTCTCTCATTGCACTGGTAGGGCTGCCGCTGGTCCACT
TGGGATTGGTGCAGTCAAAACACA
>645
AGAGTGATCAATCTCGGTTGGATTCGGAACTGAAGAACATGCAGGACATGGTGGAGGA
TTACCGGAACAAGTAAGGGACCCTGTCTGGGCAGTTCTTAACTTTTGCTGTAAAAGAG
TTCCAGAAAGTAATAAGCTAAGAT
>646
GGTGGACCTTCAGGCCAAACTTGACAACTTGCAGCAGGAAATTGATTTCCTTACAGCA
CTCTACCAAGCAGTAAGTCTTCCAGTTTCAACCAAGTTTATCTAAATGGAGAGTTTTTA
AGCCTGAACCCACAACGATTCAG
>647
GATAGAAATTTCTGAGCTGAATCGTGTGATCCAGAGACTTAGATCTGAAATCGACAATG
TCAAGAAGCAGGTATGTGCTTTCTCCTTCTACCACTCAGCTGTATGGAATGGGGGTAAC
CCTCAGGTAAAGGGCGAGTGCT
>648
TGAACACCAAGCTGGCCCTGGATCTGGAGATTGCCACCTACAGGACCCTCCTGGAGGG

AGAAGAAAGCAGGTGAGGAAGGGACGCTGGGAGTCGAACCTCTTCTCATGGTCTTCC
TTCCTTGCAAGCTGATTGTTGTTGA
>649
GCCCACATTTATGCCACCCGCCTCATCCAAATGAGACCCTTCCTGGAGAACCGGGCCC
AGCAGCACTGGGGTAAGTGAGAGTTTGGGAAGGTGCTTCCCCCACAGCATCCCTGAA
CTTAGAAGTGTTCTGCAAGAGAATG
>650
CTGTGTGGTGGGCACTCTGTTCAAGGCCATGCCGCTGCAGCCCTCCATCCTGCGGGAG
GTCAGCGAGGAGGTGAGGCAGGGTGCTACACAGTGGGGCCGCCAGGCAGACCTGGC
CTCCCACTAGAACACCTCCCTGGAGG
>651
CTGGTCTTGGAAGATGAACTGCAGCGTATCAAACTAAAAGGCACCATTGACGTGTCAA
AGCTGGTTACGGGTAGGGAGCCCAATGAGAGGATGTGGGTGATGCAGGTGAAGAGCC
CAGCGGTGGTGTGTTAGGGATGGTG
>652
TTCTGGTGGAGGACTATTGCTTTGCTGACCTTGCTCCCCAGAAGCCCGCACCCCCACTT
GACACAGATAGGTGAGCAGCAGTTCTCGGGAGCTGGAACCAGCTCATGGTCAGTGGA
ATCTTTGAGTTGCACCTAGGAGGG
>653
CGTCTCCCGGGTTATCCTCGCTGGCAACCTCCTCAGCCACAGCACCCAGAGCAGGGAT
TCTATCAATAAGGTATGGAGCCCACCTGGCTGCATTCAGCCCCAGCCCAGGAGCCTGC
AAGCCTGTAAGACCCTCCTTCCCC
>654
CACCAAGAAAACCCAGGCAGCCAGCGTGGAGGCTGTTAAGATGCTGGATGAGATCCT
CCTGCAGCTGAGCGTGAGCGAGCTGGGGGCTGGAGGGGTGATGGGGATTGCAGTCTT
CAAAGCTGCCACTGGGCAACAGAAGG
>655
CGCTGGCCACTGCCTACTCCACGCTCCAGCTGGTCACCAACCCCTACCAGGCCACCAT
TGATGGAGTCAGGTAGCTGGCACAGCCACACTTCAGTCTGACCCAGCCTTTTGCCTCA
GGAGGCACAAAGAAGGGAGGGGAG
>656
GATCACTTGGAGATCCTGGAGTGGACCCTGCGGGTCCGTCACATCAGCCCCACAGCCC
CTGACACTCTAGGTAACAGGCTCAGCCATACAGGGTGGGAGCAGAGGGCCAGGAGGC
CTGGCAGGACCCTGAAGTGCACAGG
>657
ATCTTCCCAGAGTGCCCGCATGTCTACTTTTGTGGCAACACCCCCAGCTTTGGCTCCAA
AATCATCCGAGGTAATTTTTGTCTTCTGGGGGCCCAGGCTGATTTGCTGATTTGCTCTCA
CCTGGGGACAAGGTTCACAGA
>658
AGAGTACCCTGAATACCAGCGTTGGATGGGCCTCAACGACCGCCTCAGCTCCTGCAGA
GCTGTTCATCTGGTGAGTCTGGGGACTGTGTACTCCCTCTGTTTCCCACGCCCTCTGTC
CGCTTTCTCTTGCTTTTAGGTGA
>659
AGTTAGACTCCTTCGCTGTATCGTCTACTGATTCTTAAAATGTGACAAATCTGATTGGAC

GACTTACATGGTACGGTGCAATTTTTGATGTCCAGACTAAAGTGAGTCTTCTCTAGGCA
AACAGTAATTTTGGTTAAAAC
>660
AGTGCCGGGAGGGCAACGCAGTCGCCGTTCGCCTGTGGCTGGACAACACGGAGAACG
ACCTCAACCAGGGGTGAGCTGAAACGGTTGGTGGATGAGAGGAAGGCTAGAGATCTC
CTGGCTACGTGGAGTGGAGTGCTCAT
>661
AATGAACCGTGGGGATGACACCCCCCTGCATCTGGCAGCCAGTCATGGACACCGTGAT
ATTGTACAGAAGGTACGTACAAACTCCTTCGTCATCCACATCACATACATGCCATGAGG
GTCAGTCACAGGCACTGTAACAT
>662
TGCAGTGAATGAACACGGGAATGTGCCCCTGCACTATGCCTGTTTTTGGGGCCAAGAT
CAAGTGGCAGAGGTGAGTACTCAGCCCTTAATTCCTGAGATGGGTAGGAAGTAAAGTC
TGAGCCTTGGTGGGAGATTTTGGA
>663
AGCATCTGTAACAAGTATGGAGAGATGCCTGTGGACAAAGCCAAGGCACCCCTGAGA
GAGCTTCTCCGAGGTCCATCTCCCCATCCCCTAGCTTGTGTCCTCTCGTCCCTTCCCAC
CTGTCTTCTCCCTCTGTACCACAG
>664
ATGGGCCAGAATCTCAACCGTATTCCATACAAGGACACATTCTGGAAGGGGACCACCC
GCACTCGGCCCCGTGAGTCACCACTGTGGGAAGAAGGGTTGTAAAAGGAAATAATCC
TGGCCTCTTGGGGCTGGGTTAGGGT
>665
CAAACACTCTGGCATTGACTTCAAACAGCTTAACTTCCTGACGAAGCTCAACGAGAAT
CACTCTGGAGAGGTGACCCCTGCCCTTCTTGCCCTTCCCTCACTAAACCCCCATAAATT
ACTTGCTTTGTACCTGTTTTAAG
>666
AGGTGCTGAAGGTTCGAGACTGGAGTACAAGGAAGAGCAGGGACTTCAATGAAGAGT
GTCCCCGGCTCAGGTAGTGCAAGGCGTAACCTGGAAGCTGCTAGTTCCAAGGAACCCT
GAATAGCACTGAAAGAGATCTTTTG
>667
CCTCATCCTACTCTCATCACACACTGGATGCCGTATGGATCCCTCTACAATGTACTACAT
GAAGGCACCAGTGAGTAGGGATGTTGAATTTCCTTGGGGAGGAAATGGCAGAGAGGG
AGCCTCTCTGAACTATTTGACTT
>668
GGGCATGGCCTTCCTACACACACTAGAGCCCCTCATCCCACGACATGCACTCAATAGCC
GTAGTGTAATGGTGAGGCCACAAGCTCACTCCTGGCCCAGGCCCCAAAAGCCCTTTGC
CTATCTATGACTTACCTCCTTCT
>669
ATGGCTGATGTCAAGTTCTCTTTCCAATGTCCTGGTCGCATGTATGCACCTGCCTGGGT
AGCCCCCGAAGGTGAGTGAAGTCATCATGTCGGGAGGTAAAAAAGGACCACCTCAGA
AGTAGTGGAAGGGGGCAGAGACAG
>670
GCTTCTGTGGGAACTGGTGACACGGGAGGTACCCTTTGCTGACCTCTCCAATATGGAG

ATTGGAATGAAGGTGAGAGCACAACAGCATACATTTGTGTTGCGGGAGTGGTTGGTGA
TGGTGAAAATAACTGTAGTGGGCC
>671
TGATAATCCAAATGGAGATGGAAGATGTGTGACATGTATATATTATCTTAATAAAGACTG
GGATGCCAAGGTATGCAACTTAATTGAGCATTTATTTTTTTCTTACAGGTGTCTTTTTCT
CAAAAGCATTAAGTTTCATA
>672
TTGATAGACTGCTGTTTTTCTGGTCTGACCGTCGCAACCCTCATGAAGTACAACCAGCA
TATGCTACAAGGTAGTATTCCTTTTCAGAAAAAATTGGTTTAGAGTATGGTAGGACAGG
AGGGAATCTGTAGAGTAAACGT
>673
CTTTCCAAAGCAATTCACAAGTCCTTCCTAGAGGTTAATGAAGAAGGCTCAGAAGCTG
CTGCTGTCTCAGGTACTGTTTGCTAGAATCTTCTCCCCTCTTCTAGATTTGTATTTTTAGA
GCAATCTTGAGTGGGGAGTGG
>674
ATGGCTGTGCTGTATCCTCAAGTTATTGTCGACCATCCATTTTTCTTTCTTATCAGAAAC
AGGAGAACTGGTAAGTTTATTATGAAAACAAAATTTTATTTAATAGGTCACAAAGAACC
TCTTTTTTAAATGGAGATTTT
>675
GACTGCCTTCGGCTTTTTTTCTGCTGACTAAGATCTCCTATAGAGAGCTACAACAATGC
CCAAAAGAAAGGTAAGTGAAATGGAAAGACAGACGGGGAATGAGCTGCTGAGTTACC
TATTTCCTAATAGGAAGAATTTAT
>676
CAAGCAAGAAGCAGTTGTTGAAGAAGACTACAATGAAAATGCTAAAAATGGAGAAGC
CAAAATTACAGAGGTATTTTCTAATGTTAACAGTTTTATCTATTTGAAAGTTTAACAATG
GGTACAACTGAAAGAAATCCTAT
>677
GGCCTTCAACCAGAGGTTCCTGTGGTTCCTGCAGAAAACAGTCCAGGAGCACTATCAG
GACTTTGACAAGGTGAGCCCGGGGTGCAGGTGGCAAGGGGCACCTTGCAGGGCCTGG
GTGCAGCCCCTCCCTCCCAGCTCCA
>678
CCTAGAGCCAGCGGCAACCTCATCCCACAGGAGAAGATTGTCAACCTTGTCAATGACA
TCTTTGGAGCAGGTAGGAACCAGAACCTTGCCCCTCCATCCAACAATGCCTGCTGTTC
ACCCACAGCCTTGCCCAGCCCCTC
>679
GCCATCTCCTGGAGCCTCATGTACCTTGTGACCAAGCCTGAGATACAGAGGAAGATCC
AGAAGGAGCTGGGTACATGGGGGCCCCCAACCCTATAGCCAGGAGAAGCCTTGAAAC
CCAGGTTGTTTGTTCAGTCTACAAA
>680
CCTACTTGGAGGCCTTCATCCTGGAGACCTTCCGACACTCCTCCTTCTTGCCCTTCACC
ATCCCCCACAGGTGAGGCCTGCCGGTTCTGCCCTCCCACCTCTAAAGTGCTTGCCATGT
TTTCTCTTCCTGGCTTCTCAGC
>681
CGCTGAATGGCTTCTACATCCCCAAGAAATGCTGTGTCTTCGTAAACCAGTGGCAGGT

CAACCATGACCCGTGAGTACATACCCCTCACGAAAAAATGTGTGCAGGTTCAGCAGTC
AGGAAGGCTGTTTGTCCCTGCTAG
>682
AAGGGGAAAGTTGCACATTTATATCCTAGAGGGAAGCGACAGCAGTGCTTCTCCCTGT
GCTGAGGTACAGGTAAGGAGGGTGGTTTGTAAAGTTCACTGGGGAGGGTGATCCCTCT
TTATTGTTCTAATATTATGCTCGC
>683
GAGGTCCGCAGCCAGGGAGGACGACAGCATCCGCCCTTTCAAGGTGGAAACGTCAGA
TGAGGAGATCCACGTAAGGCACCTTGGGCCGGGCCGGGCTGGGCAGTGGAGAGGGTG
GTGTGTCGAAGACAGGGGTTGGGTCT
>684
AATGAATTTGACTGGAAGAAGCAGGTGGAGATTCTCAACAGACACCCTCACTTCAAG
ACTAAGATTGAAGGTATGTTTGCAAAACGCCAGCCAGAGAGGGATGTATGTCATGAGA
ACAGCCTTCTTCCACAATGTGACTT
>685
GAGCACGTTTTTGAAGTCATCTGCCCTTCCATCCCTGGCTATGGCTTCTCAGAGGCATC
CTCCAAGAAGGGTACGGGGCTGCTAGAGGTTCCATAACTGCCCCGTCCTCGCCAAGGG
TGGGCCCGGTGTTCCCACCAGGC
>686
AGGAATTCTACATTCAAGGAGGGGACTGGGGGTCCCTGATCTGCACTAATATGGCCCA
GCTGGTGCCCAGGTGAGGTCACTGTTGGGGTGGTGTGTGTGTGTGTGTGTGTGTGTGT
GTGTGTGTGTGTGTCCTCTAAGAA
>687
AAGGTATTCTACAGCCTGATGAGGGAGAGCGGCTACATGCACATCCAGTGCACCAAGC
CTGACACCGTAGGTGAGTGTGCTCAGGGGTCCTCGCCCACTGCCGGCTCCACTGGGGC
AGGGAGACACCGCGGGGTAACCTC
>688
ATATTCTAGAGAAGTTTTCCACCTGGACCAATACGGAATTCCGATACCTGGAGGATGGA
GGCCTGGAAAGGTGAGGCCCTGGTTTGCCCCCTGCAGTCATGACCCTGGTCCCAGCAG
CCAACCTCCTCACCCTCTTCATC
>689
CCATCATCTCCTCCCAGCGCTTCTACAAGGAGAACCTGGGACAGGGCTGGATGACCCA
GAAGCATGAGCGGTGAGCCTGGCTGAGCCGAGAACAGGGGCCTCTGAGGCTGGAGG
CAGGGGGACGGCCAGTCTTGGGCTCC
>690
AATGATCATAGCACCTTGGATGGGTATTCCAGAAGAACCACCTTGTCTTCAAAAATGTA
TCACACCAAAGGTAAGGATGTTAAGACTCATTCTTAGCACATCAGAAGTGTCTTTTGAA
TTATTTTAGTGAAACGATGCAG
>691
GAGGGTGAGGACCGAGGAGCGCGAGCAGATCAAGACCCTCAACAATAAGTTTGCCTC
CTTCATCGACAAGGTGAGCTACGATCTTTTGTAAAAAATCACTGTGGGTCTGAAATAAA
TGCCAAAGAGAGAGAGAAAGAAGGAA
>692
TGGGGGAACGGGGCCGCCTGGACTCAGAGCTGAGAAACATGCAGGACCTGGTGGAA

GACTTCAAGAACAAGTGAGTTGGGGTGGAGGGTGGACACAGGGGAGGGTGGTGTCTT
CTTGGTACCAGATGGGCTTTGTTACTA
>693
GGTGGAGCTGGAGGCCAAGGTTGATGCACTGATGGATGAGATTAACTTCATGAAGATG
TTCTTTGATGCGGTAAGAAACTTATCTAAATTTTTCACATGGGTGGGTTTTTTTTCCTCA
AGTTGTCATATCACAATGAGTG
>694
GGTCAAGGCCCAGTATGAGGAGATTGCCAACCGCAGCCGGACAGAAGCCGAGTCCTG
GTATCAGACCAAGGTGGGTGCTCTGATGACTGTCTCCTGAGTGAGGGGACACACCCAT
GTCTAGGATTCTAGGCCATGACGAC
>695
GCATGAGATCACAGAGATGAACCGGATGATCCAGAGGCTGAGAGCCGAGATTGACAAT
GTCAAGAAACAGGTAGGGTGAGATTGAAAGAGGGCAAGGAAGGGGCCTGAGTTCTA
AAAGAAACACCTACTTTGTTTTATTT
>696
TGAACACCAAGCTGGCCCTGGACGTGGAGATCGCCACTTACCGCAAGCTGCTGGAGG
GCGAGGAATGCAGGTGAGTAGACAGCATGAACTCAAGATGGCCTTCAGCTGATAAAG
CGAAGCTGCTCTACTGTGGGGTGTAC
>697
GGCCCCCTGATACCCCTCTGCCTGAGGAGGTAAAGAGGTCCCAGCCTCTCCTCATCCC
AACCACCGGCAGGTACGATGGGGCGTGGGGCTTGGGGGAGGTCAGTGCTGGATAATA
CACAGAGGCTTGCAGGCCACTGCTC
>698
ACAGAGCCCAATTCTCGGGGGCCCCTCCAGTGCAAGGGGGCTGCTCCCCCGCGATGCC
AGCCGCCCCCATGTGAGTTGTCCCTCAGAAGGGAAGGGAGGGATGCACGGGTTCTGG
GTCTGTGGGAGATACACAGCCGCTT
>699
GTGCAGCGAGCTCACGCCTTGAGCGACGAGACCTGGGGGCTGGTGGAGTGCCACCCC
CACCTAGCACTGGGTAAGTCAGGTGCATGGAACTATCCGGGCTGGGAGATGATGCCTT
GCATCTTGGGCTAGGCATGTGGCTC
>700
CGTGGGCGGAGATAGCCGCTTCGTCTTCCGGAAAAACTTCGCCAAGTACGAACTGTTC
AAGAGCTCCCCAGTGAGTGCATGAGGGCTGTCTGGGCGCTGGGATGCCCTGATCCTCA
ACCTGGATGCTGGAGCCCTGATCC
>701
GTTCCCAGAAAAAATGGTCTCCAGCTGTCTCGATGCACACACTGGTATATCCCATGAAG
ACCTCATCCAGGTGGGGGGACCCCCCATTTCACTGCAGATTCACGACTCCCCAGCATT
GGCCAGTGCTTCTCCACCCTTAA
>702
GCTTTGGAAACGCTTTTTCTGTTTCTTGCGCCGATCTGGCCTCTATTACTCCACCAAGG
GCACCTCTAAGGTAAGGTCTTGAGGGTACCAGCCCCAGCCCCTCCAGTCCCTGGTCCT
TTTAGAAGTTGCCCCTTCTCTGC
>703
CAACGTGTACGTGGTGACGCAGGGCCGCAAGCTCTACGGGATGCCCACTGACTTCGGT

TTCTGTGTCAAGGTGAAGACCTGGCCAGGCCTGGCCCCTGGCCTGGGGAAGCAGGAA
CTGCTCAGGCCCCTGGATCCTGCCC
>704
GCTTCGGATCTTCTGCAGTGAAGATGAGCAGAGCCGCACCTGCTGGCTGGCTGCCTTC
CGCCTCTTCAAGGTGAGACCCTGGGAGTGGCATGGGGGGCTGGCCTGGCCAGAGGGA
TCCCCAGCTCTGCCCTCAGGAAGTC
>705
GCTGTACAAGAATTACCAGCAGGCACAGTCTCGCCATCTGCATCCATCTTGTTTGGGCT
CCCCACCCTTGGTGAGTGTGCCCAAGGGGATGGGAGGGTGGGTATGCAGGCCCTGTCT
TACGGGTACCTGGGCCCTGTTCT
>706
TGCTGGGCGTGTCATTGAGAACCCCCGGGAGGCTCTGAGTGTGGCCCTGGAGGAGGC
CCAGGCCTGGAGGGTGAGGCCTGCTGTGTGTGTGTGTGTTTGTGCTGGGGACCCACTC
TCTGGGTGGGATCCCTGAAATAGGA
>707
TCTGGTTCCACGGGCGCATTTCCCGTGAGGAGAGCCAGCGGCTTATTGGACAGCAGGG
CTTGGTAGACGGGTAAGGGGCAGGGCCGGGCAACAGACCCAGGGATAAGAGAGACTG
GGGTCCAGGTGGCAGCCATGGTCCT
>708
GCGGAACCCCCAGGGCTTTGTCCTCTCTTTGTGCCACCTGCAGAAAGTGAAGCATTAT
CTCATCCTGCCGGTGAGCTTCCCTGCGTCCCCGGAGTCCTGCAATGAGACACAGGACT
CCCAGCAACCTGTCCTCCTCACCA
>709
GATGGCAGTGGCCGGCTGCTCCAACTGGACCTCCAACTTCCTAGTCGGATTGCTCTTCC
CCTCCGCTGCTGTAAGTAAACTCACCTTATCTTAAAACCAGCCTATGTAAGCTGACATG
AAGATACTCCTTAATAATACAG
>710
TTACCCCGATGGGCGGCTAGCAGAGCCTGAGAAACCTCGGAAACAGCTCAGTTCTCA
AGGCACTGTGAGGGTAAGGACTTTAGCCATCTCTAGTTGGGGACACTTAGCAGCTGTG
GGCATTTCCAGGTACCGTTCCTGGC
>711
GATATGCTTTGTACGATGCCACATACGAAACAAAAGAGTCTAAGAAAGAAGACCTAGT
ATTTATATTCTGGTGTGTAAAAACAAAAGAAAAAAGTACTTTTAAAATGCAAGGATTAT
TATCATAGTCAGTCTTACCTTTG
>712
GAAAGTGCACCTTTAAAAAGCAAGATGATTTATGCTAGCTCTAAAGATGCCATTAAAAA
GAAATTTACAGGTACAAACTTGAATATTTGGGGCAGAGAAATTATTCTTTATTAATTATA
ATAAAGTAATAGGATAATGTT
>713
CACAAGAAGCAGCTGGACTCTCTGCGGGAGAGGCTGCAGCGGAGGCGGAAGCAGGA
CTCGGGGCACTTGGGTGAGGCACTGGGCTGTTGGGGCCGAGGCTCAACCGGGGAGGA
CGGGCGGGATCCCTAGAGAAAAAGCTC
>714
ACACCACCTCTCTGATCTGGCCTTAACATTGCCCACTGATGCTGTGTCCATCTGTCTTGC

CATCTCCACGGTAATGACCTGACAGAGTTAGGATTTACCACTTATTTTCAGTTCACGGT
CAGTGTCTTATTGCCCATACC
>715
CTCAGATGTTGATCTGGAGATAGAGAGCCTTCTGAACCAACAGTCCACTAAGGAACAA
CAGAGCAAGAAGGTAGGGGTCATGTGAAAATACTCTCACAATATGCTCTTAATTATAGC
TTTGTTTGACAGGGATTTACCCC
>716
ACTATGGAACCAAGGAGGAGTGCATGAAAGCCAGTGATGCTGATCGACCCTGTCGCAA
GCTGCACTTCAGGTCTCTTGGAAGGGAATGAGGTGGGATGGGTTTGTCAGAAGCAGC
CATTCCTTTTCAGCATATTCCTTCC
>717
AAGCCAGGAGCTTGCTCTTACACAGAGTGTCGGAGGTGATTCCAGTGCAGACCGACTC
TTCCCACCTCAGGTACCTGTCTGCTCAGAAAACTCGTCTCAACTTACGGACTTATGTTT
GATAGGGCAGCCATACACGTTAA
>718
ATGAGATGCGCAGGCTCAACATACCCGTACTACAGGATGATGGCTTTCTCTTCCTCTGG
GTCACAGGCAGGTAATGCAATTCAGTTATGTAGGTATGGACAGATACAGTACAAGAGT
GAATACTGAATGGGAGATAAAAT
>719
TCAACTGCAACGCATCATTCGGACAGGCCGTACAGGTCACTGGTTGAACCATGGGAAG
GAACACTGCTTGGTGAGCAGCAGTGGGGCCCAATTCAATAGGTGGAGCACAAAGAAG
AATCATTTGATTTCTTACTGAGAAA
>720
TTGAAAGACTATCTCCTGGCACTCGCAAGATTGAGTTATTTGGACGACCACACAATGTG
CAACCCAACTGGTAAGGTGACCAGAGAACAGGCTAGTCCTCAAGGGGCTATTATTTAT
GATTAAGGACAGAACTTAGTAGA
>721
GGCTGGGCCGAGTGGGTGTCTGGTGCCGGCGTTTGGGCTACGGTTGTTGTTGGCGACT
GTGCTTCAAGCGGTGAGTGTAGATCACCTTCGCAGCTGCCACAGGGAGAGTAGGAGT
GATTGCAGCTGAGAAAGACGCAACA
>722
CAACCTGCTTCAGCTGGATCCTGATTGCAGAGGATGCTGTCAGGAGGAAGCACAATTT
GAAACCAAAAAGGTACTGTTTTCTAGTATATTCCTTCCATTGGATGGTTTAGTTGAGAC
AAGGTAAAAATGTAAGTTGCTTA
>723
GCTGCCCCTGAACCCCAGAACAACCAGCTGGATCAGTTCTCACAGGAGCTACAGCGCT
GAGACTGGGTAAGTCAACGATCCCCAGAGCTGGGACAGAAGGGGCAGCAATGGGGCA
GCAACTGAGGGAGAAGAGAGCTGAC
>724
GCTGCCCCTGAACCCCAGAACAACCAGCTGGATCAGTTCTCACAGGAGCTACAGCGC
GGAGACTGGGTAAGTCAACGATCCCCAGAGCTGGGACAGAAGGGGCAGCAATGGGGC
AGCAACTGAGGGAGAAGAGAGCTGAC
>725
ACAGCCCCACCTCCGGCAAGTTCCGCCGCGGGAGCGGCTCGGCCTGCAGCCTTCTCTG

CTGCTGCGGAAGGTTCGAGTCCCGGGTCTGGCACATTCAGATTGGAGGTTAGGGAATG
GGGAAAGGGGAGCGAGCCAGAGAA
>726
GAAGCTGGAGTTTAACAAGGCAGAGAAGCACGTGCACAACTTCATGATGGATATCCAG
TATACCAAAGAGGTGAGATGGGCATATGGCACCCATGGGACACCCCTCTTCCAAGAAG
CAGGAGTCCAGGCGCCCCTGCTTC
>727
AACATACTCGCAGGAAGGAGTCTCATGCTGCCCGCAGGCATCAGCGCAAGCTGCTGGC
CGCCATCAACGCGTGAGGGCCGCTTTGTATGCACACGTGTCCATGTGCACCCATGTCCA
AGTCACCTCTCTGCACGGCTGTG
>728
GTTCCGCCAGGTGCGGCTGAAACACCGGAAGCTCCGGGAACAAGTGAACTCCATGGT
GGACATCTCCAAGGTACTAGGATCCCGTGGGAGGAGGTCTTGAAGAAGGGGGAGGTT
GGGGCCACAGGGAGGGAAATCTGGCC
>729
GACTGAGCTGCTTAGCACTGCCCTGGGGCCGAGGCAGCTTCCAGAACCCAGCCAGCA
GTCCAAGTAGCTGGTGAGGGGGCTGGGACTTGGGCAGGAAGGCATCCTGGAGGAAGG
GTTCCTGTGGCCAGCACCCTATGTGG
>730
CGTGGTAGCCTAGAGTCCTGGGGAGCTTCTGTCCACCTGTCCTGCAGAGGAGTCGTTT
CCAGCCCGGCTGGTGAGTGCTGGCCAGGGTGGGGCAGAGCAGGCGGGGCGGCGAAA
GTCCAGACCTGGCTGTGCTGTCTGGA
>731
CGAGAAGAAGACAACATGTGGCACCGTTTGCCTCAAGTACCTGCTGTTTACCTACAAT
TGCTGCTTCTGGGTGAGGAGGGGTCGCCTTGCCCCCACCCCCACCCCCACCCCTCCCG
GGCCACCATCAGACCTGGGCAGAT
>732
TGTCGTCATGGTGACTGGGGTCTTGGGCTGCTGCGCCACCTTCAAGGAGCGTCGGAAC
CTGCTGCGCCTGGTCAGGAGGGCGCAGGGCCACGGGGTGGGGGTGGTGCAGATGGGC
CCAAGGAGGTTGTCGCTGCAGGCTT
>733
CATCCTGCTCCTCATCATCTTTCTGCTGGAGATCATCGCTGGTATCCTCGCCTACGCCTA
CTACCAGCAGGTGAGGGGCCTGGGCAGGCCATTCAGAGACACAGACATGCACAGGCG
GGGCGGACACACACACATGCACA
>734
CATGACCAGGCGCTACCACCAGTCGGGCCATGAGGCTGTGACCAGCGCTGTGGACCA
GCTGCAGCAGGAGGTGGGTGGGTGGTGCTGGGAGGGCGCGTGCATCCCCAGGGCCTC
CCTGGACCTCTGCAGGAGCCTCTGGC
>735
CAGCTGCTGCAAGACGGTGGTGGCTCTTTGTGGACAGCGAGACCATGCCTCCAACATC
TACAAGGTGGAGGTGGGTGTGCAGCGGGATCATGCCTCCAGTGTCTACGAGGTGGTGG
GGGGGCACCCCAGTGACTGGCTGT
>736
GTTGGAGACCTTCATCCAGGAGCACCTGAGGGTCATTGGGGCTGTGGGGATCGGCATT

GCCTGTGTGCAGGTGAGGGCACATGGGGGTGGCGGTCATCTTGTTGGGGACACGGGG
CAGGGCGGTGGCTGGTGGCCCCATG
>737
GATCCTAAGGACCTTGAGCCCCGAGGAGCTAGAGCAGCTGGACTGCGAACTACAGGA
GATGGATCCTGAGGTAGGGGCTCCAGCACAGGGAACCAGAGGCAGTCCCAGGCATTT
GGGAAGGGTGTTGGGCTCTGGGTGGC
>738
TACTTGGAGCAACAGGCACTAGAAGTCAAAGAGCGTGATGACTTGGTGCCCTTCACA
GGCGAGAAGAAGGGTATGGGGACCCTGACATCCATGCCTCGTAGAACCCAAGTGGCT
GTGCTGTCTCTCACTCGTCCCCACTC
>739
ATCACCCTGGAGCCTGAGCTGGAGGAGGCACTGGCACATGCCACAGATGCTGAAATGT
GTGACATTGCAGGTGGGTAGCTGTGGGAAGTTGAGGGTGAGAGGATCAGGGGCAGGT
GGATTCTAGCCATGACATGGGACTA
>740
CTGATGAGTAACAAGCAATACTATGATGCCCTCTGCAGTGGAGAAATCTGCAACACTG
AAGGCATTAGCAGTGAGTGTGTTTGGGGAGCATGGTCCCCAGGGCTCTGGGCAGGCA
GTCCTAGTGTCTGGGAGAAGGTAGA
>741
TGAGGAGATACTAAAGAGGGTCCGAAGCAATGACAAGGAGCTGGAGGAGGTGAACTT
GAATAATATACAGGTATTTGACTTGCATCCTCCAAACCATAACACCGGATAACCACTGA
CATCCGAGAGTCCCTTAGCCCACC
>742
AATGAAGGCAAATACCTATGTGCGGAGCTTCAGTCTGGTAGCCACGAGGAGTGGTGAC
CCCATTGCCAATGTAAGGCTAGTTGACATCCCCAGCCCTCTCCCCACATCTTACATGCA
CCTGTTGCATGGCTGTCATAAAG
>743
AGGACTCATGGCTGTGCTGAAGGCAGTTCGGGAAAATGCCACACTCACTGAGCTCCGT
GTAGACAATCAGGTCAGCATTCCCTTCCCCTGATGTTAGCATTCAGTCTCATTGAGTCT
CCTCCCTGAGCCAGCCAGTGGGG
>744
GGCTACCACTTTACACAGCAGGGGCCACGAGCTCGGGCAGCCCAGGCCATGACCCGA
AACAATGAACTACGTGAGTAACTGCAGACATGATGTGTGGAGTGGTCAGGGAAGTGC
AGAAATTGGTGGATCCTCCTGAAAGC
>745
GACCAGGAGGAGACCAACACCCTGGTGGCCAATACCAGCAACTCCAACGGGCTGAAA
TTGGACCCCGCAGGTGAGCCGGGAAAGTCGCTGCCGCAGGTGGTTGGGTTTGGGGTG
CCCAGAGCCTCGCCTGGCCTTCGGCG
>746
AAAGCTATATTAGTTCACTAGCACCCCGGAAGCTCAACAGCACAAGTGATATTGAAGA
AAAGGAGAACAGGTAATCCTAAAACCAGCCAAAAGATGGTCCTCATCCTCTTAGGTCT
ACTGGACATAGTCATCTCCTGGGC
>747
ATGAAGCAAAAGGAAATGAAATCCCACAGACAATTGCTCCCTCCACCTATGTATCAAC

TTACTTGAAAAGGTACCAGGCTCAAAGGGGGTGGGAGATGTTTCTTTCACTCTAGCCA
TGGGAAATGGGTAATGATTGTATG
>748
GGGTTACAGCTACTCCTGTGCTCTCCATTACTGGAACAGATTCCTCTGTGGAAGCCAGG
GAGAAGAGGAGGTATGTTGAGTTACTGATTTCATTTGTGGGGCTCTGCTTTGAGGTAGA
CGATAAAGGAATGGGCATAAAG
>749
GGATGAGGAAGCAGAGTCTTTACGGAAAGCACGCTCCAGACAAGCTCGGCAGACACG
AAGGTCTACTCAAGTGAGTGTGGCTTTTTTTAAAATGTCATTTTGTGTATCTACCCTTCT
GTTGGAGGTACAATCAAAACACA
>750
GCCCTCAGCAGTTCCAGCAACAGAAGCTGGGGAGGGCCAGCAGCCCTGGGGCAGGA
GTCTGGATGAAGAGGTGAGCTCATTTTTGCTGGCATGTACTGTGCTAAAAGCAGGGTA
ATTAGTTTGGGGAGTATCTTCTTCAC
>751
TCAGTCCCTGATTCTGAGAGTTCAGAGACTACCACAAACACTACAACTGCAAAGGAAA
TGGACAAAAATGGTATGTAGAACTTCAAAAGACACAGGCCCCTCCACAGTGCACATCC
CCAGATTCTTAAAGTTCATACTTT
>752
GTCCATCCGAGAGAGGAGGCGGCCCAAGGAACGACGAAGAGGCACAGGCATCAATTT
CTGGACAAAGGATGTAAGTGGATTGGTCTGTGCTGAGGCATATCATATTACTCTTGGTC
ACCATTCTTTCAGTTGAGTTTAAA
>753
GGCCCGGGAGGCCCGCCTAGCCACCCTGACCAGCCGTGTAGAAGAAGACAGCAACAG
AGATTATAAAAAAGTAGGGTCTTTATTCAATTTTCCGGTTCTTTGGTGACATGTAAACCT
CTCAGCCTTCCTCCACCACACCC
>754
GAAAACAAAACTTCAGGAAGCCCAGCTAGAGCTAGCAGATATAAAGTCCAAGCTTGA
GAAGGTGGCCCAGGTAAGACGGAAGAAGAAGAAAAAAGATGAGAACCAAGGGTTGA
TGTAAGGAAGCAAACTAGACTAGGGAT
>755
GCAGGGCCCAGGCGCCCTTGGCCTTAGGACCCAACTTCTCTTACCGCCATGGAGTTCG
ACCTGGGAGCAGGTGAGCTCCTGGGGAGTGAGGACTGGAGGTGCAGGGGGCCGGAC
TCAAGCCCAGAAGCTGCCTGCACCAA
>756
GGGAGATCCCAAGCTCAGTCCCCACAAAGTTCAGGGCCGGTCGGAGGCAGGGGCAGG
TCCGGGTCCAAAGGTAAGTCGCCTCATCACCGGCTGCGGAGGGGCGGGAAGGCTGGG
GTTGCCCCTGACCCCAGGGTCCTGCC
>757
GCGCGGCCACAGCATGACCGCTCGCGGCCTGGCCCTTGGCCTCCTCCTGCTGCTACTG
TGTCCAGCGCAGGTGAGCGGTCGCCGGCCACCGGGACGCCCTGTGGGGACGTGGCGG
GGCCGAGAGCCGGGGAAGGCGACGG
>758
CAAGAGGTACAATTGCGAATATTCTGGCCCACCAAAACCATTGCCAAAGGATGGATATG

ACTTAGTGCAGGTAAGTTCATTATCTTAGGCACTGGGAACACAAAATGCTGATCACAA
GATCCTCTGTAATTATTCCTTAA
>759
TCTGTTGTGATGTTCGGCAGCTTCAGACACTAAAAGACAACCTGCAGCTGCCTCTACA
GTTTCTGTCCAGGTAGGTTCTGCTGGGGAAACAGAACAACTGGGCTGTAGATACTTGT
GAACTGGTAATGCTCAGCTTTCCA
>760
CCTGTTACAAACCAGACGAAAACAAATGTGAAAGAGTTACAATACTACGTCGGACAG
AGTTTTGCCAATGGTAAGTAAACTTTTAATTATTCCTCTTTTACAGCTGGCATCAGGACA
GCAGAGCAAATTGTTCCTTTTAG
>761
AACTGGATTGAATACATGTTCAATAAGGACAATGGACAGGCACCTTTTACCATCACTCC
TGTGTTTTCAGGTAGGTATAAAGATTCCAAGTTTGGTGTGTTTATGATATTGTTTTAAAG
CCAAGGAACTGGCTGGGCACA
>762
TCATGTGGATCACCTACATGGCGTTTTTGCTTGTGTTTTTTGGAGCATTTTTTGCAGTGT
GGTGCTACAGGTAAGCAGTTTTGTTTGTCATCCAGGGCAAAAAGAGCAAAATTGCCCA
CTGGCACTTTGAGCTTTCATTT
>763
CGGTATTTTGTCTCCGAGTACACTCCCATCGATAGCAATATAGCTTTTTCTGTTAATGCA
AGTGACAAAGGTAGGCATATTTGTCCATGAATAAAAGGGTTCAGATGATGCTGTCTGTC
ATGAGAGCATTCGTTTCCTCA
>764
TTACCAGCCATACCCTTCGGGAGCTGATGTACCCTTTGGACCTCCGCTTGACATACAGA
TACTGCACCAGGTAACCTGCTGTTTGAAGAAATAAGTCACTCTAGATGACCTTGTGGTT
TGCTGGCTTTTACATGTTGACT
>765
TGGACCACAAGAAAGGGGACGACTTCTTTGTGTATGCCGATTACCACACGCACTTTCT
GTACTGCGTACGGTAAGTGGCAAGAGACAATCATTGGTAGACACCATTCTTGGGTCTG
ACTTAGGGTACATGGGCAAAAGCT
>766
CTCCATGACCCTTGTCTGGGTACGTTTGGTGGACCAGTGTTCCCGTGGCTTGTGTTGGG
AGGCTATGATGGTAAGTAAGAGAAGCTTTGACTTTTCCTTTTAGATAAAGTCATCTCGG
TTTTGTCATTAGCATCAAGTGG
>767
TTACCTTCCCTGTCAATAATTACTATAATGATACAGAGAAGCTCCAGAGGGCCCAGGCC
TGGGAAAAAGAGTGAGTCACTCATGGGTGTGAGCAGACTGTCCTTGCATTGTGGGAA
GCTAGACACTTGCAGCAAGCACTT
>768
TAGCTATGCCATCATGTTTCTATATATTTCCCTAGCCTTGGGGCACATCAAAAGCTGTCG
CAGGCTTCTGGTAAGCCGGGGGGGGGTGGTGTATGTTCTAGAGGTTAACAGCAGTGCA
CAGTGTAACGTCATATTTTTGC
>769
TGAAGTCATCCCGTTCCTGGTGCTGGCTGTTGGAGTGGACAACATCTTCATTCTGGTGC

AGGCCTACCAGGTATACTTCCATATTCTCACAGGGCTCAGCATCTGTTCAAGTTTCAGG
GACTGTGAATGGCTCCTGGGCT
>770
AGGGTCCTAGGAGAAGTGGCTCCCAGTATGTTCCTGTCATCCTTTTCTGAGACTGTAGC
ATTTTTCTTAGGTAATTATGCTTTCAATCCTACCAGTCCTGTAGTCTGCTGAGCCTGGAG
AAAGAAGGAGCTGGCTACCTG
>771
CTTCATTGACTTTCTTCTGCAGATTACCTGTTTCGTGAGTCTCTTGGGGTTAGACATTAA
ACGTCAAGAGGTAAGTTGGTGCCAGGATTATAGTCTATTTAATTTGAGTCTAGAATCTA
GAGGAAGCAGCTAGCGGGTAT
>772
CTGTTTGTTTCGCTTCTTCAAAAACTCCTATTCTCCACTTCTGCTAAAGGACTGGATGA
GACCAATTGTGGTATGCGCTTATCTGTGGTTTTTCTCTTTCCTCTCACTGAAACAGATTA
TCACATGCCTTCTAAGAAGCC
>773
TCTGTCATTCAGCATCGCAGTCCTGAACAAAGTAGATATTGGATTGGATCAGTCTCTTT
CGATGCCAGATGTAAGATGACTTCCTTTTTTTTTTTTTTTAACTTCTTTAGCTGATGATGA
ATACATTTTTCAAAACATGC
>774
GCGGCGGCATGGGCTGCAACAATGATTCCCTGGTGCAGCAGATATTTAACGCGGCGCA
GCTGGACAACTAGTCAGTACCACCTTGTCTTGTACTTCAGAAGCAGCGGAGGAGGCTC
TCAGCCAGGCAGTCAGCTCAGGCC
>775
AGGGGGGAGACTTCATGAGATTCCTGCCCATGTTCCTTTCGGATAACCCTAACCCCAAG
TGTGGCAAAGGGTAAGTGCTGCTGCCATTGCAGATAAGCATCCACTGCAACTTTAATTT
GCAGTAGAAAACTAGGAGAGGA
>776
GACTTATAGCCAGTAATGTCACCGAAACCATGGGCATTAACGGCAGTGCCTACCGAGTA
TTTCCTTACAGGTAAAGCCTGCCCTTTTTCAATGGGGTTTACCCAGCAAAGGGCCTACA
CTGGGTGGGAGTGGGGAGGGTT
>777
CAACATGTTTGGAGTTATGTGGCTCTGGGGCATCAGTCTGAACGCTGTATCCTTGGTCA
ACCTGGTGATGGTGAGTCCTCATACAGTCTCAGTTTGTCCCTAGCTCCTGTTTCACTTG
GGGGAATTGGCTGTCTTAGATT
>778
GTTCACGGTGAGCATGAAAGGCAGCCGCGTGGAGCGCGCGGAAGAGGCACTTGCCCA
CATGGGCAGCTCCGTGAGTACCCTGAGCAGGGCCACGCCGGCAGAGTCGCACCACAA
AGTAATCCTGGTCACTGGCTGAAGAG
>779
TATTTGGCCATGGTCTTACTGGGAGCCACTCACGGATTAATATTTCTCCCTGTCTTACTC
AGTTACATAGGTAAGAGTTCTCATCTTAAAAGGGTGGCACAATGGAAGGACATTACTG
AAAGGGAATTCTATCCTGAGGC
>780
AATTGGCGGGCAACGACCTTTCTTTTATCCACCCAAAGGCCTTGTCTGGGTTGAAAGA

ACTCAAAGTTCTGTAAGTAATGCAATTTTAGAGTTTCACAGCTGGCTGTGTATTTTCTTT
CTGCGTAGTCAACATGCTTGGA
>781
CGCTCCAGAATAATCAGTTGAAAACAGTACCCAGTGAAGCCATTCGAGGGCTGAGTGC
TTTGCAGTCTTTGTAAGTAACTTCTTGGTTTCTTGACTCACCAAGAGACTACTCTGGCT
GTTAAAGATGTAAATAACTAGAA
>782
CCCTGGCTCTCAACAAGATCTCAAGCATCCCTGACTTTGCATTTACCAACCTTTCAAGC
CTGGTAGTTCTGTAAGTATCTATCCCTTTTAATTCCATATATTTGTGTTAATTGGGGTACA
AAAACAGATTTCTTCTGACA
>783
ATCTTCATAACAATAAAATTAGAGGCCTGAGTCAACACTGTTTTGATGGACTAGATAAC
CTGGAGACCTTGTAAGTATATTCATATTTTGGGCAACTGCATTGTGATATTGAAAGTATA
ATTTTATACTTCACTGCAGAA
>784
GATTTCATAGTAATTCTATTTCTGTTATCCCTGATGGAGCATTTGATGGTAATCCACTCTT
AAGAACTATGTAAGTATTTTTGAAAGTTTCTTTTAATGTATAATTATGTGAAGCTGTGTC
CGTACAATGCCAAACACCC
>785
ATTTGTATGATAATCCTCTGTCTTTTGTGGGGAACTCAGCATTTCACAATTTATCTGATCT
TCATTCCCTGTAAGTATGTGAATTTAAAAACAAACCTGGATATTTATACAGTTGTTTTAG
TGGAAAACAAGAATTTATT
>786
CTTTACAGCGTAATCAAATCTACCAAATAAAGGAAGGCACCTTTCAAGGCCTGATATCT
CTAAGGATTCTGTAAGTTTGTCTATGATTATTAAAGACAATAAATTTTTTCCAAACTATGT
TAATATGTTGTTATGATTTC
>787
ATCTGAGTAGAAACCTGATACATGAAATTCACAGTAGAGCTTTTGCCACACTTGGGCCA
ATAACTAACCTGTAAGTGGATGATTATGCAGTAATGTCATGAAAGTAGTAACTAGTGCTA
TAGAAACATCTGTTTTAACAT
>788
ATCAACTGAAACTTGTGGGCAACTTCAAGCTGAAAGAAGCCTTAGCAGCAAAAGACT
TTGTTAACCTCAGGTGGTTTTGCTTTTTCTTTTTTGTTGAAGTTATGGTAGGCTCCAAAA
TTACAGAGTGTCCTGGTTTTTTG
>789
GCAAATGTCACAAGCACTCTTGAAAATGAAGAACATAGTCAAATAATTATCCATTGTAC
ACCTTCAACAGGTATGCCTGGCCTATTTTTTCACAGTTACCCATGTTAAACAAACATTAG
GCAAATATATCTAAATGTCCT
>790
CCCTAAACCCAGTGCAAACAGTGTAACGTCACCCCACTCCAAAGAGAAAAGAATGCC
CTTCTTTAAGAAGGTAACATTAACTTCCAAGCTCCCATTGTCCACCTGCTCAACTGCAC
TGCGTCACATTTCTAGTCCTGTTG
>791
TCCTCCGTATGATGTGGTACCTTCCATGCGACCAGTGGTCCTAGTGGGCCCTTCTCTGA

AGGGCTACGAGGTGGGTAGCAGCCTTCCACAGGAAGCTTAACTTGCATGCTGAACTTC
TTTGTGATGCTGCCTCCTACTCC
>792
AAACGCTCGGTATTAAACAATCCCAGTAAGCACGCAATAATAGAAAGATCCAACACAA
GGTCAAGCTTAGGTAAGTCTGTGCAATGAGCTTAAGCTTTTTAAACTCTCCTCTCCCGA
TGTTGATGATGAGAAGACCTAAC
>793
TCATCCAGCTCAACTCAGTAAAACCTCCTTGGCCCCTATTATAGTATATGTAAAGATTTC
TTCTCCTAAGGTAAGTAGGACTGCTACTGTTTGCTCTATAATCAAACTTTCCTAAAATGT
ATTTTATGTTCTGCTTTCTA
>794
AGGGAAATCTCAAGCTAAACACCTCAACGTCCAGATGGTAGCAGCTGATAAACTGGCT
CAGTGTCCTCCAGTAAGTTATCTCTATATACAGCATAATCCAGTTACAGAGATCAGACCT
TTTTTTTTTTTTTTTTTTTTT
>795
CCCTCTCCTTAGCCGTACATTAGCCACTTCAAGTCTGCCTCTTAGCCCCACCCTAGCCTC
TAATTCACAGGTAAGGGGAGTTTTTATATATATCTATATATACAATCTTCATAGAAAAAAG
GTTGCCTATGGTGACACCT
>796
GGGCCCCTTCCTCCTCCTCCTCCTGCTGCTGCTGCTGCTGCCACGGGGGTTCCAGCCCC
AGCCTGGCGGGGTGAGCACTGACCTTGTCGCAGTGCGCACAGAGGTTCCCAGTGGCT
ACTTGGGAGGAGGCCAGAGGGAGG
>797
GGACCCCAAAGTGGGGGGCTCCCGCCCCCGCCCAGGGCAGTTCCCTCGAGCAGTAGC
CCCCAGGCCCAAGGTGGGTCAGGTGGGCCTGGGAGGAGGTGTCGTGCTTCACCTTAG
GGCTGGGTGGGAGGAGCATGGCTGCG
>798
GGTTCCCCTTCCGCTACGGGGGCCGCATGCTGCATGCCTGCACTTCGGAGGGCAGTGC
ACACAGGAAGTGGTGGGTCCGGGCAGCCGGGGCACCCGAGCTGGGGTCACCTGCCCC
ACGCTGCCTGCTTTTCTGGGAGCCG
>799
ACTCACAACTACGACCGGGACAGGGCCTGGGGCTACTGTGTGGAGGCCACCCCGCCT
CCAGGGGGCCCAGGTGGGTGCTGGGTTGGGTAGCCTGGGGCGAGCAGGGGGCACTGG
GCCAGGCCAGAAGGGCCAAGGGGAGG
>800
AATACCCAGGACCCCCAGTCCTATCACTGCAGCTGCCCCCGGGCCTTCACCGGCAAGG
ACTGCGGCACAGGTGAGCTGGGCCCGGAGGTCCGCAGGGTCCAGGGGCCGGAGCAA
GTCCCGGAGGATGGGAGAACAGGTGG
>801
CGCCAGGGCCACGTGGAACAGTGCGAGTGCTTCGGGGGCCGGACCTGGTGCGAAGGC
ACCCGACATACAGGTGCGCCACGGGGTGTGAGCCGTGCCACTGACCCCTGACGGGTG
TCCCTGTCCACACCCGAGTGGGAGGA
>802
CTGATCGTGGCCACCGGGACCACCGTGTGTGCCTGCCCACCAGGCTTCGCTGGACGGC

TCTGCAACATCGGTGAGTGGGTCAGCCCCCGGGGTGCCCTGGGGCAGTGCCGGGTG
GACCCACCGTGGGCCGGCCTCACTG
>803
ACCAGGAGCTGCACGTGGACTCCGTGGGCGCCGCGGCCCTGCTGGGCCTGGGCCCCC
ATGCCTACTGCCGGTCAGCACCAACGCCGCTCCAGGCCGCCGCACGCGGGGCAGGCA
GGATTTTTCCTGGTGAGAGGCCCCCC
>804
GAGAGGCCCTGGTGCTACGTGGTGAAGGACAGCGCGCTCTCCTGGGAGTACTGCCGC
CTGGAGGCCTGCGGTGCGCGGCTGGCGGGGGGTGCTGCCTTGGGCCCCACCGAGGTC
ACAGCAGTTTTCCCCGTGATCCTCCT
>805
ACAGCTTCTGCGCCGGGAGCCTGGTCCACACCTGCTGGGTGGTGTCGGCCGCCCACTG
CTTCTCCCACAGGTGCACCTCCTCTGGGCCCCAGTCACCTGCCCTGAGGCCCCACACA
CCATCCAGCGTCACTATGCGCCTG
>806
ACCTTCGGCATCGAGAAGTACATCCCGTACACCCTGTACTCGGTGTTCAACCCCAGCG
ACCACGACCTCGGTGAGCTCCGGCGTGTCGTGGCTGCACTCTGGGCAGGTGGGCCCT
GTGCTCCCCAGGCCAGGCCCAGACA
>807
CCCGAGCCCGGCAGCACCTTCCCCGCAGGACACAAGTGCCAGATTGCGGGCTGGGGC
CACTTGGATGAGAGTGAGTTGGGAGGGGGGCGCCCAGCGTCCGCCAGGTGGACAGGT
GGCCAGCCACAGTGTGGTGTCACGCT
>808
CTACGGCGCCGACATCAGCCCCAACATGCTCTGTGCCGGCTACTTCGACTGCAAGTCC
GACGCCTGCCAGGTGAGCTGGTGCCCGCCCCACCAGGACCCGACTGGTGGGGGCTCA
GCTGGTCCTGAGTCTCCGAGATGCT
>809
ACCTCAGCCATGAGACTTCTCATCCTGGCCCTCCTTGGCATCTGCTCTCTCACTGCATA
CATTGTGGAAGGTAAGTGGAGAAGCTGTCTGTGAGATAAAGAATAGGGAGGCAAGGC
AGGTGGGCACACATTTTGGGTTTG
>810
CTACCCAGCGACTGCCAGTTAGCAGAATCAAGACCTACACCATCACGGAAGGCTCCTT
GAGAGCAGTAATGTGAGTCTGCCTCCTCAGAAGTTGGGCTGGGTGGGTACCTAGAGGT
ATAGAAATACACTCTATAGAAATG
>811
ACCTCAGCCATGAGACTTCTCATCCTGGCCCTCCTTGGCATCTGCTCTCTCACTGCATA
CATTGTGGAAGGTAAGTGGAGAAGCTGTCTGTGAGATAAAGAACAGGGAGGCAAGGC
AGGTGGGCACACATTTTGGGTTTG
>812
CTACCCAGCGACTGCCGGTTAGCAGAATCAAGACCTACACCATCACGGAAGGCTCCTT
GAGAGCAGTAATGTGAGTCTGCCTCCTCAGAAGTTGTGCTGGGTGGGTATCTAGAAGT
ATAGAAATACACTCTGTAGAAATG
>813
CCCCAAGCCAAGCCCTCCAACCCCAGTAACCCTCGAGTCTTCTTTGACGTGGACATCG

GAGGGGAGCGAGGTGAGCGGAGTCTGACGCTCGCGGAAAAGCCCCGAGGAGGCGGG
GGTTCCCGGGATTTGGGGGATTCCAA
>814
ACAGGAGAAAAAGGCATTGGACACACGACTGGGAAACCTCTCCATTTCAAAGGATGC
CCTTTTCATCGAAGTATGTGTAAATTTTCTTAATCTTGTTATTCTTCATACAGACAAGTCC
TGTTCTTTAGGATTTGGGGAAG
>815
AAATCAGAATGGGACAGGTGGAGAAAGTATTTATGGTGAAAAATTTGAAGATGAAAAT
TTCCATTACAAGGTAAAGTAGAGAAAATGCATGTTATTGCTGATAAAAAGATTGTTATAT
ATCTGGAATACTTGTTGGATTA
>816
AGTAATTAAAGGAATAGGAGTGGCAAGGATATTGGAAAATGTGGAAGTGAAAGGTGA
AAAACCTGCTAAAGTAAGTAAAAAGTTACAGTGAAATACACTTATTATCTTAGTTGCTA
TCTTTTTGAGACGGAGTCTCGATC
>817
ACATTGGAAATACTTTTTTCAAATCCCAGAACTGGGAGATGGCTATTAAAAAATATGCA
GAAGTTTTAAGGTAATATTATTTCTAACAAATTATTTTTAAGAAATTATAAATTGAATTAT
AAACTAAAATTAAGAATTGC
>818
TGTACTGAATATTGGTGCTTGTAAACTGAAGATGTCAAATTGGCAGGGAGCAATTGACA
GTTGTTTAGAGGTAAGTCTGTTTGATTTTGAACTTTTTGAATAAGTTGGATTAAGACTTA
GTTTGAATAGTAGCAACTTTT
>819
ATCAAATACCAAAGCATTGTACCGCAGAGCTCAAGGATGGCAAGGATTAAAAGAATAT
GATCAAGCATTGGTAAATTTTGTTCCAAATGTTTAATTTTTTAAAATAGACAACTACCTT
TATAAATCATACACCTAACTTA
>820
CAATCAAAACCTTCCTTGGGGATACAAACACACCTTGAACCAGATTGATGAAGTAAAG
GTGTGGCCTCAGGTAAGTGGACCTGCTGTCTATGAGCTGAAATAATGTGTACATGGAGC
TCAATCAGGTGCCTCAAAAAATC
>821
GGAAACCACCTGCCATGTGCTGGACCCCACCCCTGTGGCAAGATGCAGCGTGAGGCA
GCTGAAGGAGCATGTGAGTACCCTTCTTAGGATGACTGTAGGTGGCCCTTCGGCCAGC
TCCACCGATTCACCCAGCGTCTCAG
>822
TGTGATTTCCAGCTGTTGAAACTAGATGGCAAGTTTTCCGTGGTATACGCAAAATGTGA
TTCCAGTCCAGGTACAGATGACTATTCTTATTCTCATTTTTTCCTTGTAGAGAAAGTGGG
GAAGGGATCTGAATAATTTTC
>823
GGCCGCCTTCAACGCTCAGAACAACGGCTCCAATTTTCAGCTGGAGGAAATTTCCCGG
GCTCAGCTTGTGGTAAAGACTGAGATTCTTTTGACAGGTTGGGCAGTTCGGTGGCACT
TCGGGAATGTACTGTACGTGGTGG
>824
AGTGTGTGGCACTGACTGTGTTGCTAAAGAGGCCACAGAGGCAGCCAAGTGTAACCT

GCTGGCAGAAAAGGTGAGTGGGCCGGGACCTTGGGGTGTTACCACTCGGACAGAGCT
GTTTGTGGAACAGAACATCCTTGGTT
>825
TAAGGCAACACTCAGTGAGAAGCTTGGTGGGGCAGAGGTTGCAGTGACCTGCACGGT
GTTCCAAACACAGGTAACAGCTCCGTGAATATTCTTGCCTACACCTTCAGAATACAATG
ACCCCTTCACATTTATGCAGTGCA
>826
TGTCATCAAAGGATATTCCAGATTCTTGACAGCATTCTCGTCATCTCTGAGGACATCAC
CATCATCTCAGGTGAGCACCAGGTGGAGTGCCTCTGGGTGACTGGCCGGTTTGGAGCA
GGGAGGGAGGCTTAGAGTCTCAT
>827
AGCCTTCAACATCCAGACATTTGGGGAGACCAAGATGTCCAATGCCACCCTCGTCAGC
TACATTGTGCAGGTGAGGCCAGGGCAGCCTCCCCCCAAAAGCAGAGGAGCTCTGGAG
TCTAGGGCTGGTGGGCAGGGCCAGC
>828
TCGCCCTGGTCCAGGAGGTCAGAGACAGCCACCTGACTGCCGTGGGGAAGCTGCTGG
ACAACCTCAATCAGTGGGTGACAGTGGCAGGGTCATAGGAAGGTGACATCTCGTCCAC
GGCACAGCCTCACTTCACTTGGGCC
>829
CCTATCACTACGTGGTGAGTGAGCCACTGGGACGGAACAGCTATAAGGAGCGCTACCT
GTTCGTGTACAGGTGGGTGGTCTAGAAAGCCAGGAAGCCCCTCCCTCACCTGGGAGG
GCCCCAACAGAGCAGGGAAGTAGTT
>830
TGCGAGCCCTGCGGGAACGACACCTTCAACCGAGAGCCAGCCATTGTCAGGTTCTTCT
CCCGGTTCACAGGTGGGTGCTGCCTGGGCCAGGGTGGGGCTCGGCTTGGCGCTTATGG
CCTCCACCCCCTCCTAGGGAACCT
>831
GGACGCAGTAGCCGAGATCGACGCTCTCTATGACGTCTACCTGGATGTCCAAGAGAAA
TGGGGCTTGGAGGTGAGGCCCTCCCAGGGGCAGTGGGCACCAGCGGCCTCCGCATGT
CCCAGGGCCACAGGCAGCGTTTCCT
>832
CCACCTTCCAGTGGCTGATCCCCGACAGCGCTGACACCACAGCTACACCCACGCACTG
TGCCTATGACAGGTGAGCAGGGCCTCGCGCTTAGGGCAGACTGAGGGCACCTCCAAG
GGCAGCCGTGACTCATAGGTCCGGC
>833
AGGCGCCGTTGTTCCCGACTCGGCTCTTCCCTTTAACTTCCAGGCTGCCTATGGCCTGA
GTGACCAACTGGTATGTGTCCTCCCTTGCACAGCCACATGAGGATGGGACACAGGAGC
TCAGGTAGGCTCAGCCCAGACCC
>834
CACGAAGAAGAGAAAACTGGGGAAGAACCCAGACGTTGACACAAGCTTCTTGCCTGA
TCGAGACCGTGAGGTAAAGGCTGCCTGGCCCTGACCCCGGCCTCGACTCCAGCCCTG
GTGGAGACCCTTGCTTAGGGAGCCAG
>835
AGGGCAACACCATGCAGCAGTTCCTGCAGAAGGCGCTCGAGATCCTTCGGAAAGACT

TCAGTGAGCTGAGGTGTGAGGTGTGCGTGTGTGCACGGTGGTGTGTGTGGCACCTGTG
GCTCCAGCCCGGGTGCCAGACACCC
>836
ACCACTCTTCAACTTTGATGTTCATGACGATGTGCGGTTGCTCAGTGACGCCACTGTGG
AGAAGGATGAGGTACAGCGTAGGGGGCCGTGTGAGGGGGAACGGGTGTCCCTGGGCT
ATGGAGGAGAAGCCAAGGGAAGGG
>837
GAACAAGCACATCTTTCCCGCCAGCCGCTGGGAACCCTACGACCCTGAAAAGAAGTG
GGACAAGTACACGGTGAGGAGGGGCTGGCAGGGACCCCTCCAAGTTGGGGACGGCA
GCCAGCCCCTGCTCACCCCTCGCCTTC
>838
GCATTTTGACACCCTCTCTAAGGGTGAGCTGAAGCAGCTGCTTACAAAGGAGCTTGCA
AACACCATCAAGGTAGGTGATGCCCCTCTCACTGCAAACTTGCCCATGACCCTGCACT
GAGGGGAGTGGCAAGGCCTGGCAG
>839
CTGGTCATGGCGCCCCGAACCGTCCTCCTGCTGCTCTCGGCGGCCCTGGCCCTGACCG
AGACCTGGGCCGGTGAGTGCGGGTCGGGAGGGAAATGGCCTCTGCCGGGAGGAGCG
AGGGGACCGCAGGCGGGGGCGCAGGA
>840
AAGACCAACACACAGACTGACCGAGAGAGCCTGCGGAACCTGCGCGGCTACTACAAC
CAGAGCGAGGCCGGTGAGTGACCCCGGCCCGGGGCGCAGGTCACGACTCCCCATCCC
CCACGGACGGCCCGGGTCGCCCCGAG
>841
CTGGAGGGCACGTGCGTGGAGTGGCTCCGCAGATACCTGGAGAACGGGAAGGACACG
CTGGAGCGCGCGGGTACCAGGGGCAGTGGGGAGCCTTCCCCATCTCCTATAGGTCGCC
GGGGATGGCCTCCCACGAGAAGAGG
>842
CTGGCTGTCCTAGCAGTTGTGGTCATCGGAGCTGTGGTCGCTGCTGTGATGTGTAGGA
GGAAGAGCTCAGGTAGGGAAGGGGTGAGGGGTGGGGTCTGGGTTTTCTTGTCCCACT
GGGGGTTTCAAGCCCCAGGTAGAAG
>843
CGGGTCATGGCGCCCCGAGCCCTCCTCCTGCTGCTCTCGGGAGGCCTGGCCCTGACCG
AGACCTGGGCCTGTGAGTGCGGGGTTGGGAGGGAAGCGGCCTCTGCGGAGAGGAGC
GAGGGGCCCGCCCGGCGAGGGCGCAG
>844
AAGCGCCAGGCACAGGCTGACCGAGTGAGCCTGCGGAACCTGCGCGGCTACTACAAC
CAGAGCGAGGACGGTGAGTGACCCCGGCCCGGGGCGCAGGTCACGACCCCTCCCCAT
CCCCCACGGACGGCCCGGGTCGCCCC
>845
CTGGAGGGCACGTGCGTGGAGTGGCTCCGCAGATACCTGGAGAACGGGAAGGAGACG
CTGCAGCGCGCAGGTACCAGGGGCAGTGGGGAGCCTTCCCCATCTCCTATAGATCTCC
CGGGATGGCCTCCCACGAGGAGGGG
>846
GGACAAGAGCAGAGATACACGTGCCATATGCAGCACGAGGGGCTGCAAGAGCCCCTC

ACCCTGAGCTGGGGTAAGGAGGGGAATGGGGGGTCACATCTCTTATCAGAGAAAGCA
GAAGTCCTTCTGGAGCCCTTCAGCCG
>847
GCTGTCCTGGTTGTCCTAGCTGTCCTTGGAGCTGTGGTCACCGCTATGATGTGTAGGAG
GAAGAGCTCAGGTAGGGAAGGGGTGAAGAGCGGGGTCTGGGTTTTCTTGTCCCACTG
GGAGTTTCAAGCCCCAGGTAGAAG
>848
GTGTGGCCCTGATGTGGGCCGCCCAGACCGTGGGCGCTTGGGCTTCCAGGTCTGGCTG
AAGAATGGCGTGGTGAGTGGCACCCTGGGCTAGGGCGCTGGGGGGCTGGGGTGTGAC
CCCCTGTGAGTCCTGGGCCAATCCC
>849
TTCCTGAAGGCGGCTGAGGACTATGGGGTCATCAAGACTGACATGTTCCAGACTGTTG
ACCTCTTTGAAGGTAGAGAGGAGAATGCTGGGGGAGGAGGTGGGCAGGAGGACAGG
GTGCTGGGACAGGGAGAGGGTATGAC
>850
TGGCTTTGGGCAGCTTGGCAGTGACCAAGAATGATGGGCACTACCGTGGAGATCCCAA
CTGGTTTATGAAGTATGTGGCCCCCAGGGAGCTTGAGTCTCCGCATGGGGTGGGAGGT
GGCTTGTTCTAAGGAGCTTGCGGG
>851
ATCGCTGAGGCCTACGACGTGCTCAGCGACCCGCGCAAGCGCGAGATCTTCGACCGCT
ACGGGGAGGAAGGTGTGTGCTCGCGGCCAGGGGCGCGGCCCCGCCTTTTGACAGACA
GGGAAACTGAGGCACGCTGGCCCCT
>852
CAAGCCCCACAATATCTTTAAGAGAGATGGCTCTGATGTCATTTATCCTGCCAGGATCA
GCCTCCGGGAGGTAAGGTGCCAGGTGGGGCGGTGTCTGTAGAGGGCGATGGCTGCTT
TTTGAAGCAGTTTAGTATTTGCTG
>853
ACTCCTCCCTGTGAACTCGGGCTCGAAAATGAGACGCTTTTCTGCTTGGATCAGCCCC
GTCCTTCCAAAGGTAAGTGTCGGAAGCTGTTATTTTAATTCAGGAAACTATTGAGTTGT
GGGAGAAATCAGCAAAGGCAAGG
>854
CTCATCCCCAATCTGCTCAAGGATTTCATCTTCGGGAGCGCCGTTTGCAAGACCACCAC
CTACTTCATGGGTAAGTTGCTGCTGGTGACTCTTTCTGGAGCTTGCCCCAGACCCCACT
CAGCCCCAGACCTTCCCAAACC
>855
CCAAAAATAACAACCAGACCGCGAATATGTGCCGCTTTCTACTGCCAAATGATGTTATG
CAGCAGTCCTGGTAAGGTTGCATTGTTTATTTTTAAAAATACAGCTTGATGTAATTTTGC
AATATCATGAAAAACTGAGAT
>856
TATGGATTAATCTCTTTGGAACTCTACCAGGGAATAAAATTTGAGGCTAGCCAGAAGAA
GTCTGCTAAAGGTAACTATCTTGTAGCTGTGTGTTTATGCCAAATGAGCACAAAAGCTT
GTTTTCTATCATTTAAATAGAT
>857
GCGCAGGGTCGCGATGCTGCCCGGTTTGGCACTGCTCCTGCTGGCCGCCTGGACGGCT

CGGGCGCTGGAGGTGGGTGCCGCGCCTCGGAAGGCGGGGGGAGGCTGCACGGTGGG
GACGCGATACCCCCCAAGACCTTAAC
>858
GGATTCAGATCCATCAGGGACCAAAACCTGCATTGATACCAAGGAAGGCATCCTGCAG
TATTGCCAAGAAGTAAGTCCTGTCCGGTGGCTAGCAATTCACGTTGGATCACATGCATT
TGTTTTCAAAAAATTTAACTTCT
>859
AACTGGTGCAAGCGGGGCCGCAAGCAGTGCAAGACCCATCCCCACTTTGTGATTCCCT
ACCGCTGCTTAGGTGAGCCGGCCGGCCGTGGGGCTGGTGTTGATTGGGGGCCTGGTCT
TGAGGGAAGAAAAAGAGGATGCTC
>860
CAAATTCTTACACCAGGAGAGGATGGATGTTTGCGAAACTCATCTTCACTGGCACACC
GTCGCCAAAGAGGTACCAGCCCATAAATTCTTTCTTATTGCAAAGTGAAGATTTCCTGG
GGACGTGCTTAATGGCATTTTAG
>861
CTGCTGATGCGGAGGAGGATGACTCGGATGTCTGGTGGGGCGGAGCAGACACAGACT
ATGCAGATGGGAGGTAAGGTGGCCTTTGTGTTCAGCCTCAGAGATGCTGAAACATCTT
GTATGGAGTATTTGTATCCTGTAAA
>862
ACAGAGAGAACCACCAGCATTGCCACCACCACCACCACCACCACAGAGTCTGTGGAA
GAGGTGGTTCGAGGTAATCCACCATTTGCTTGGATTCCCCCCACCCCCAAGGAAAAGA
AAGCGTAATACCAGAGTTGGAAATA
>863
GGCGGATGTGGCGGCAACCGGAACAACTTTGACACAGAAGAGTACTGCATGGCCGTG
TGTGGCAGCGCCAGTAAGTGGACCCTTCTTCGAGCCTGGCCACCTTTCGTCTCTCTCG
CCACTGACTCTGCTTTTTGTAACAG
>864
GAATGAACATGCCCATTTCCAGAAAGCCAAAGAGAGGCTTGAGGCCAAGCACCGAGA
GAGAATGTCCCAGGTAAGTCTGGCTCTTCCATCATTCAGCCCTACGATATTGGGAAACC
TGAGCTTGCCTCTGCCTCAGTCTC
>865
GAGAGAATGGGAAGAGGCAGAACGTCAAGCAAAGAACTTGCCTAAAGCTGATAAGA
AGGCAGTTATCCAGGTAAAACCTGAACCCATTTCCTACCAAACATCACATGCCAGCAG
TCTTTTTAATGAGTGTCTGCAGGTTT
>866
CATGCTCAATGACCGCCGCCGCCTGGCCCTGGAGAACTACATCACCGCTCTGCAGGCT
GTTCCTCCTCGGGTAGGTCTCGCTGCAGCCGAGTTCACACTTCAGGTCACAGCACAGA
CAGTAAGGGTGGGGCACTGGGAAC
>867
GCACACCCTAAAGCATTTCGAGCATGTGCGCATGGTGGATCCCAAGAAAGCCGCTCAG
ATCCGGTCCCAGGTAAGCGTGGGGTATAATCATCTTCTGCAGCTTTGACAATCCAGGAT
TCTTGCTTCTTGGGTTGGTGACA
>868
CGCATGAATCAGTCTCTCTCCCTGCTCTACAACGTGCCTGCAGTGGCCGAGGAGATTC

AGGATGAAGTTGGTAAGTAAGCTGTTCTTTTGATGCTGCACATGGACATGTATTTTCCC
CCAGAGGAAAATTGAGGAAGTGA
>869
AGCCTGGACGATCTCCAGCCGTGGCATTCTTTTGGGGCTGACTCTGTGCCAGCCAACA
CAGAAAACGAAGGTAAGAGTCCCCTGAGCCAGCAAGGGCGTTCTGGGAGGTATATATA
CACATACATAACGTGTGTGCAAGA
>870
GATCTCTGAAGTGAAGATGGATGCAGAATTCCGACATGACTCAGGATATGAAGTTCATC
ATCAAAAATTGGTACGTAAAATAATTTACCTCTTTCCACTACTGTTTGTCTTGCCAAATG
ACCTATTAACTCTGGTTCATC
>871
AGTGATCGTCATCACCTTGGTGATGCTGAAGAAGAAACAGTACACATCCATTCATCATG
GTGTGGTGGAGGTAGGTAAACTTGACTGCATGTTTCCAAGTGGGAATTAAGACTATGA
GAGAATTAGGCTTAGCTTTTTGC
>872
TTGACATCTGACGACGTGAAGGAGCAGATTTACAAACTGGCCAAGAAGGGCCTTACTC
CTTCACAGATCGGTGAGTGTTTGTGTCTAACATAGCCTATTTCGCCTGTCCTCGTGTGA
CTTGTAGGATCTAGTAGGTGGTA
>873
TCCTGAAGATCTCTACCATTTAATTAAGAAAGCAGTTGCTGTTCGAAAGCATCTTGAGA
GGAACAGAAAGGTAAGCTAATAAATAAAACCTGGTGCTAAGTAGGACTGCTTGTACTA
AATTTTTTCGGTAAAATGTAATG
>874
TAGAGAGCCGGATTCACCGTTTGGCTCGATATTATAAGACCAAGCGAGTCCTCCCTCCC
AATTGGAAATAGTAAGTATCAACTCTTTTGTCGTTGTTATCAAGAATAGGAGTCAGCCA
GTAGTAAAAGTCCTAGTAGTAA
>875
CGCACCTTAGTGCTGCTGGACAACCTCAACGTGCGGGAGACTCATTCGCTTTTCTTCC
GGAGCCTGAAGGGTGAGAGCGGGGTCCGAGGGGGTAGCGTGTGGGGGCCTGGGGTT
GGTACAGACATCGAGGGCATTGACCA
>876
AGCCTGTCTCTCATAAAGTATGGGGAATTCCTCTATGACAATCTCATCATTTTCTCCCCT
TCGGTAGAAGGTAAGGGTTCCGCGTCGCCCCTAGCACTGGAATTCGAGACCCGGGACT
TGATGGATTGCTTTCTTCTGGT
>877
AACGTGGAGACCATCAGTGCCTTTATTGACGGTGGAGGCAGTGTGCTGGTAGCTGCCA
GCTCCGACATTGGTGAGTCCGACCTGGGAGGTTCTGGTGCTTTCCACTTCTCCAGGGA
GTTGGAGAGCATTACAAACAGAAG
>878
GATTGAGTTTGACGAGGAGAAAACGGCTGTCATTGACCATCACAACTATGACATCTCA
GACCTTGGCCAGGTAATCAGGCCTGTCACCTTCCAGGTTTCAGGGGCATATGGCAAAG
CTCTGCTTAGCCAAAAGCCAAGAG
>879
AGAACCTGCTGAAGGCCCCAACCATCGTTGGGAAATCATCTCTAAATCCCATCCTCTTT

CGAGGTGTTGGGTGAGTGAGTGAGCAGGGAGAGGACAGCAGAAACCTGGGGGAAGA
AGGGGTCACTTAATTTTTGTGGGAA
>880
TTTGGTGCTGGACATCCTGACGGGCTCTTCCACCTCTTACTCCTTCTTCCCGGACAAGC
CTATCACCCAGGTAAGGGCTTCACAACCTTGAGGCTTCGAAATAAAAATGAAGCAGAA
CAGCACATTTGGCTGAGGCGAAG
>881
GCTCCCTCGACTTCTTCAGCGACTCCTTCTTCAACTCAGCAGTGCAGAAGGCGGCGCC
CGGCTCCCAGAGGTAGCTGCCAGGAGGCTTAGGGGCCGGGGATCCGGGTGGTGCAGT
GTGTGTATATGGGGAGGACTCAGAA
>882
GGGGCCTGTGTCCCATCATCGGGTGGGCGAGACAGCCCCACCCAATGCCTACACTGTC
ACTGACCTAGTGGTAAGAGATGTTTGGGGTGGGAGGGCCCAAAGGGTTTGCCAAGCT
GACTGAGCTCAGCTCAGAAGCACTT
>883
GATGGCGATGACATTCAGCTGGAGTTTGTCCGCATTGATCCTTTTGTGAGGACCTTCCT
GAAGAAGAAAGGTGAGTGTAGTTCCTGCACGAGGATGCTGCCAGAAAACGGCCCCAG
GCCTCACTTGCTCTCGAGGCCCAA
>884
GTATGGTGTATTCCAGTTTAAAGTGGATTACAACCGGCTAGGCTACACACACCTGTACT
CTTCCACTCAGGTAAGCACAGGTGACAGCTTCTGTTTTCAGCTAGCATGTGCCCTGCCT
GGGTTCTGCAGTGGGCAGGTCC
>885
TGTCTTTCAACTGGCAAGAGTCATTTTGACCAGCAGGTTAATCAACTCTAAGACAGATC
CTCACGCAAAGGTATGTACCTGGAAATATTAACTGAAGAAATTTTTTCTCAGACTTTTC
ATGAGCTTTTTATAGGACATTC
>886
CCACCATCCACGCCAGCCGCACCATGCCGCTCCTAAGCCTGCACAGCCGCGGCGGCAG
CAGCAGTGAGAGGTAAGTGCCCCGCCCCCGTCCGAAGCTTCCAGAAGCGCCGGCCGC
GCAGGCCTGCGGGGGGCCGTCCTCC
>887
CCTGAAGATCCCAGGTGGGCGAGGGAATAGTCAGAGGGATCACAATCTTTCAGCTAAC
TTATTCTACTCCGTGAGTATGGGGCCGGGCCTGCTGGGGCTGCTCTGGGGCCACAGGT
ACTTCTTGTGCACCTTGCTGCCCG
>888
CAGTGCTGAGTGGCGGCAGCCTCTACATCGAGATCCCGGGCGGCGCGCTGCCCGAGG
GGAGCAAGGACAGGTGAGGGGCAAGGACAGGCAGGGAGTGCTCACCCAGGGGAGCA
GGGACCAGGTGAGGGGCAAGGACAGGC
>889
GAGTTCGCTGAGGAGCAGCTGCGAGCCGACCATGTCTTCATTTGCTTCCACAAGAACC
GCGAGGACAGAGGTAGGTGACCCGTATCGCCTGCAGTAGGGTGCGTGGGGGTGCCTG
CGCCGCCCCTTCATTCTCAGCCCGC
>890
CCCTCTTGAACACGACCCTCAGCAGCAGCAGCTTTGAGATTTACCAGTGCTGCCGTGA

GGCCTTCCAGAGGTGAGGGCCTGGGCTGTGGGACCTGCAGGGCACCCACAGTGATAG
ACAGAATGCTTGAGCGCTGGGGGGG
>891
GTGGGTGATCATTTTAGCAGTTCTAGCAGGATTGTTGCTACTGGCTGTTTTGGTATTTGT
AATGTACAGGGTAAGTAACGGACTTAGAAAGAAGGAGAGAGGGAAAGCAGAAGAAA
AGGGAAAGGGAAGAAGGAAAAGAA
>892
TGATAGAAAAACAAATCAGTCATGGCAACTTCAGCACCCAGGCCCGGGCCAAGACAG
AGAACCCGGGCAGGTGAGTGAGCGTGTGTAGATAGAATGGGGCCAGGGAGGAGGGG
AGAAGGAGGGACAGAGCTCTGTAGAAC
>893
CTGGGGAATTGTCACTTATGGCAGCATTGGCAGCAGGACATCTTGTCAAAAGTCACAT
GATTCACAACAGGTAAGACTCAAAGATATATTTAACATGTTCCCCCTATACTTCAAAAA
ATATGCAGTGTAAAAACTTACTA
>894
ATCACCTTTGGTGCCTTCCTCATCGGGGCCCTGCTCACTGCTGCACTCTGGTACATCTA
CTCGCACACGCGTGAGTACCCCAGGCCCCCACAGTGAGCATGCCGGGCCCCTCCATCC
ACCCGGGGGAGCCCAGTGAAGCC
>895
GAGCAGGAGCACCAGTCCAAGCCCCTTGGAGGCCTGTCTGGAGGCTGAAGAGGCCGG
CTTGCCTCTGCAGGTAAGGACTGGAATATAGAAATTCAGTAAGAGGAGGCAGTAAAGA
GGAGGGATGTGTAATCCCCGGATGA
>896
GGGAAGTGGCTCCGGCCTCCAGGCTCCGGGGCCGGCGCTGACCCCATCCCTGCTTCCT
ACGCATACATTGGTGAGTGCCTGCGGAGGGGTGGGGTGTGTGGGCGCCAGACCAATG
GGATTGGCATGTGGCGGGTGACTGA
>897
TCCTAGCATTCACTTCTGGAGCACCCTGAGTCCCATTGCGCCCCGTAGCCCGGCCAAG
CTCTCCTTCCAGGTAGGATCCTCTTTCCCCGTTTTGGACAGGGTGAGAGACCCAAGAA
TGGCCCCCATCTGTGACCTCTCCC
>898
CCTGGGCCCTGCGTGTGATCTCACCCTCTTCGACTTCAAACAAGGCATCTTGTGCTATC
CAGCCCCCAAGGTACTTCACAACATGAGATACCTAGGTAGGGGTGGATGTGCCACCCA
ACACCCAACCACTGACCCATATT
>899
GGAAGGAGGCCCCGAGGGATGAGACCAAGGCTGACACTGACACAGCCCCAGCTTCAT
TCCCTGCTCCTGGGTGAGTGTTGGTCTAAGGTGAGCCAGATGGGTGAGGGTTGTGATT
GGGGGTGCTCTTGGAGCCCCACACC
>900
TGTGGAATCAGCTTCTCAGGGGATGGCACCCAAAGGCAGCGGCTCAAAACGTTGTGC
AAAGACTGTCGAGGTGAGTGGCCTCCAGAGGATGGGGCATCGGGAGATAGGAGCTGG
AGCAGGTCCAGTGTCATGCTACGCTG
>901
GCCCCATGATGTGGCATCGGGGCTGTTCTGCAAGTGTGAACGGAGACGCTGCCTCCGG

ATTGTGGAAAGGGTGAGTTGGGCAAGTGGAAAGAGCCCAAGGCTCACCTCGGCCCCT
GGCCCCCATGGGCTTGGCCCCATGC
>902
CATCTGCCTTCGCCCTCCCCGCCCTGGTCTCAGGCGCCAGTGGAAATGTGTCCAGCGA
CGTTGCCTACGGGTGAGTTGGGCTGGAGGGAGCAGAGCTCATTCTGCTGTTAAAATCG
TCGCTGCCTCTGCTTCCTTTCAAC
>903
CAGGCGGCGCCCCGGAGCCCAGCCACTGCCTCCACCACCCCCATCACAGTCCCCAGA
GCCCACAGAGCCGGTGAGGCCCCAGTGGGTGGAGGGAAGGCACAACCCTGACCTTAC
CCAGTACCTGCCCTGACCCCACCCCA
>904
GCCCAAATTCGGGGGCAGCAACCAGAAGCGCCAGAAGTGTCGTTGGCGCCAATGCCT
GCAGTTTGCCATGGTGGGTGGGGCAGGAAGGGTCAGGTGGACGGGATAGGTTGGGCC
AGGCGCCCCAGTGCCTGGAAGTGGTG
>905
ACGGGAAGGCGCAAGCAGTCCTGTGCAGGTGCCGGGCCCTGTTGCAGCTTCCACAGA
AGCCCTGTTGCAGGTGAGGGCCCCACCCTGTCCTGCCTTCCCAGCCTCACCCAGCTGC
ATGCCAGGGAGCTGAGACCAAGTCT
>906
CCAGGACGAGTGGACACCAGGCACAGCTGTCCTGACTTCTCCCGTATTGGTGCCTGGC
TGCCCTAGCAAGGTGGGGGCAGTGATGGATGTTCTGTTGGTGCCGTAGCGGAGTGGTC
TGTAAGCAGAGTGATGATGAGCCA
>907
GGAGAACAAGGATGATTCTGCCTCCAAATTGGCCCCAGAGGAAGAGGCAGGAGGGGC
TGGCACACCCGTGGTCAGTGCTGGGGATGCCCCACCCTGCCCTGACCCTGCTGTAGCC
CCAACAACCACATTGACCTATGGTG
>908
CCGCGGAGAGAACCCGGGCACCCCTGCTCTCTCCATAGGCTTCTCCGTGGCCCTGGGC
CACCTCCTTGGGGTAGGTCATGGCCATGGGTTCCAGCCTCCCTGGAGAAACAGACACA
CAGACCACTCCAGAGACAGACACA
>909
CGGCTGCTCTATGAATCCTGCCCGCTCCCTGGCTCCAGCTGTCGTCACTGGCAAATTTG
ATGACCACTGGGTAATGGCTGAAACCCCCTGCCCTCCCCTTCTCTAGAAACCCATTTTA
GAGGGAGAACAAGAGCTGGAAT
>910
AAATCATTCCATTCCGAGTGGCTGCCTACAACAAAGCCAAAAAAGCCATGGACTTCTA
TGATCCAGCAAGGTAGGTTTTCAGAGGAAAATTCTTTTATCAACATCTGTATAAAAGAA
ATGATGAGGCAGAATTTGGGCCC
>911
ACCTGCAAAAGTCAGGGCAAGACGTGTCCCAGGCCCAACGCCAGATCAAGCGGGGG
GCGCCGCCCCGCAGGTAAGCGGGGGTCCCCGGGGCTGGGCGGGGTCGAGCGGGGGC
GCACCACGGGTTCGCTCTGTCTAGGCCA
>912
AGCGTCCGTTTTCTGCAGCAGCGACGCCGCCAGGCGCGGCCGGGACCGCAGAGCCCC

GGGAGCCCGCTCGGTGAGTGCAGCGGAGAGGGCAGGAAGGGCAAGCCGTAGAGGCG
GAGTCAGCGGGAGAGGCGGGGCCAGAG
>913
ACCATCCCACGAACACAGATAGAAAGAAGGAAAACAAGCCTGTATTTTTCCAGCAAAT
ATAACAAAGAAGGTATCCCTTTCCCAATCAGAACAGCAAATTCTAATTCCATTTTGGGT
TTTCAATTCTGATGCACTATGTT
>914
GTTCAAGAAACACTTTTTCATGATCCATGGAAGCTTCTCATCGCTACTATATTTCTCAAT
CGGACCTCAGGTTTGGGGATTATTATCATCTTTGTCTTAGTAGAGACAGTGTGGTAGGG
AGAAAGCACTGAATTGAGGCC
>915
GATGTGTCAGAACTTCTTAAACCTCTTGGTCTCTACGATCTTCGGGCAAAAACCATTGT
CAAGTTCTCAGGTATTTTCCTATATACCCAAAGGAAAAACATAATACATTGTGCTTATTT
AAGAGAGCCACACCTTAAACT
>916
TGAGCTTCATGGGATTGGTAAATATGGCAACGACTCTTACCGAATTTTTTGTGTCAATG
AGTGGAAGCAGGTGAGGCTCACTCCCATCCATAATTCAGCACATTTGGTCTCTGAGGC
AAAATAAGTCCACCATTATGGTT
>917
AGTTGGGGAAGCTCCAGATGGACCAGCCAGTGCCTACTTGGCCGACACCACCCATACC
TCCTTGTACATGGTGAGGCCCCAAGGATGGGTGTAGACCAGCGCAGGGGGTAGAAAC
GATTTTTAGAAACGAAAGCTTTTTA
>918
TGTCTCCTCCAGGCCTGGAGGCCTTGGTGTCTGAACTCTGTGCTGTCCTGAAGCCTCG
CCTCCAGCCAGGGTGAGTGAGGTCATCACCCCAGGGTGTGAGAAAAGCCTGCTTAGA
ACATCCCTGGAGGAGCAGCAGCCAG
>919
CCCCCCAGGCTGTGGGAAGACCACAGTAGTTGCTGCTGCCTGTAGTCACCTTGGGCTC
CACTTACTGAAGGTGAGGGTCTCTGGAGAGTAGAGCCCCAGCTACTTCCCCTCCCCAG
CTCATGTTCTCCCTCTCTGCTGCT
>920
GGCTGGGTGAGGATGCCCGTGTGATGGCTGTGCTGCGTCACCTCCTCCTCAATGAGGA
CCCCCTCAACAGGTACTTATACAGAGCTGGGCCTCTCAAAGCTGGGGGGGCCGGCTGG
GCGCGGTGGCTCACACTTATAATC
>921
GCGGGCCCTCACTGCCCACCTTCCCCTGGGCCAGGAGGTGAACTTGGCACAGCTAGC
ACGGCGGTGTGCAGTGAGTATCAAGGGAGAGTACTGGCCCTCCCTAGGAGGTCCCTGG
CTTTTGTTACAAGAACTCAGTCCTG
>922
TGGTAGGGGATCTCTATGCCCTTCTGACCCACAGCAGCCGGGCAGCCTGCACCAGGAT
CAAGAACTCAGGGTAAGAGGACGTGGGCACCGGAAAGGCGGAAAGAGGAAATGGAT
GTAGATGGGTGGGTGGTGAGGAAGGG
>923
GGCTGAGGACTTTGGGCAGGCACTGGAGCAACTGCAGACAGCTCACTCCCAGGCCGT

TGGAGCCCCCAAGGTGGAGACCAAGTCCTTGGAGGTGGGACAGGAGCCCCTCCAAGA
GCATATCCCAACCACGCACTTTCCAT
>924
GGCCCCCTGGCACCGGCAAGACCCTTCTGGCCAAGGCAGTAGCCACTGAGTGCAGCC
TTACCTTCCTCAGGTGGCCAGGGGGCCTGGAAGGGATGGTGGGTGGATCAGGGGCTG
CAGCAGGCTGGGGAGCAGGAGGGGCT
>925
TCTTTGATGAACTGGACTCTTTGGCCCCAAGCCGGGGGCGAAGTGGAGATTCTGGAGG
AGTGATGGACAGGTAGGGGTCTGAGTGGGAATGAAAGCTGAGGGGTCCAGGGTGGGA
AGGATATGAGAAAGCTCACAGCAGT
>926
AGGATGTGTTTGTGATTGGAGCCACCAACAGACCAGATCTCCTGGACCCTGCCCTTCT
GCGGCCTGGCAGGTGGGCCCCTTGTACTGCTCATCTCCACCCTCATTTTGTGCCTCCTT
CACCCCTCATGGCTGGGCTCCCT
>927
AGCTGGTGTTTGTGGGGGCAAATGAGGACCGGGCCTCCCAGCTACGCGTTCTAAGTGC
CATCACACGCAAGTATGCTCTGTTAGAGGAAGACTGCGAAGGGCTTGGGCCTTGGGAT
GCTTAGGGGATGCATGCAACATGC
>928
GACCTCTACTCTCTCTGCTCTGATGCTATGACAGCTGCCCTCAAACGCAGGGTTCATGA
CCTGGAGGAAGGTGAGCCACTTGCCGGTCTGAGAGGTCAACCAAAAAGCTAGTGGGG
AGCTGGGTGCACCTGAGTCCCAGC
>929
TGGGCCGCGACACTAAGAAACATCTAGTGGTGCCGGGGGATACAATCACTACGGACAC
AGGATTCATGCGGTACGTGGGGACTTGGGGGAGTCGAGGCTTCAGAGAGCGGCTTCA
GGGCTCTCTGCACGTGGTCCAGGCC
>930
TCATTGCATCTGTTGCTGGCTCTGTGGAGAGAGTAAACAAGTTGATCTGTGTGAAAGC
TTTGAAAACCAGGTGAGAACAAAAGGTGTGTATTCCCTTTCTTGCAGCATCAGGTTGT
ACAACCAGGCTTTTCCTGCCCCCT
>931
CTTCATCTGGATTTACCCAACACCTGAGCACAAAGAAGAGGAAGCAGGGGGCTTCATT
GCAAACCTGGAGGTGAGCAAACACTGTGGCCATTTTCAGTGGGATGGAGGGGGCTCA
GTCTTTGCTGTGTTTTTGTGGCCAG
>932
GGTAACTCAGAGGATGATGCTGTATGATACCAGCATCCTGTACTGCTATGAAGCATCCC
TTCCACATCAGGTACTCTCCCCAGGGCCTCTCCCTTCTTCACTGATCTGTGAGCTGCTC
TGTTGTTTGTTCAGAGACAATA
>933
ATCTGCCTGAAGCTGGTGGGCTGCAAATCCAAGAAGGGGCTGTCCTCGTCCTCCAGCT
GTTATCTGGAAGGTAAGCCCGGGCCGCACGGGTTGGGCTGAGTAGCCGCGCGCCCTCC
CGCTGCTGCTGGGCCCTTCCTAGG
>934
GACCCCAACCTTTTCGTTGCACTGTATGATTTTGTGGCCAGTGGAGATAACACTCTAAG

CATAACTAAAGGTAAAAGGGTTGTGGGCAGCTAGTGGTGGTTGCAGGAGATAGAAATC
TGGGAATTGCGGTTTGACCTACC
>935
GAGGTCCATCTCGCTGAGATACGAAGGGAGGGTGTACCATTACAGGATCAACACTGCT
TCTGATGGCAAGGTAGGGGACCCTTGGCAGGGGGCGCTGATGGGCCCAGGGCAGGGG
AACCAGAGGTCCTGCTGTCGGATTG
>936
CCAGTACGGGGAGGTGTACGAGGGCGTGTGGAAGAAATACAGCCTGACGGTGGCCGT
GAAGACCTTGAAGGTAGGCTGGGACTGCCGGGGGTGCCCAGGGTACGTGGGGCAAGG
CGTCTGCTGGCATTAGGCGATGCATC
>937
GTGGAAGAGTTCTTGAAAGAAGCTGCAGTCATGAAAGAGATCAAACACCCTAACCTG
GTGCAGCTCCTTGGTGAGTAAGCCCGGGGCTCTGAAGAGAGGGTCTCGCGCCGCACC
CCCAGGGTGACACAGGCGCTGGGGAA
>938
TGCTGCTGTACATGGCCACTCAGATCTCGTCAGCCATGGAGTACCTGGAGAAGAAAAA
CTTCATCCACAGGTAGGGGCCTGGCCAGGCAGCCTGCGCCATGGAGTCACAGGGCGT
GGAGCCGGGCAGCCTTTTACAAAAA
>939
TTCCCCATCAAATGGACTGCACCCGAGAGCCTGGCCTACAACAAGTTCTCCATCAAGT
CCGACGTCTGGGGTAAGGGCTGCTGCTGCACTGAAGTGGTCCTTCCTGACTACAGGAG
GGTTTTTTTCTGCCTCTTTCTTGC
>940
CTAGAGAAGGACTACCGCATGGAGCGCCCAGAAGGCTGCCCAGAGAAGGTCTATGAA
CTCATGCGAGCATGTAAGCCTTCCTCAGCCTGTTCTCACGAGTATATGTGGGCATTCCA
GGAAATTCAACTGTGCAGGAGTGT
>941
GACCGGCCCTCCTTTGCTGAAATCCACCAAGCCTTTGAAACAATGTTCCAGGAATCCA
GTATCTCAGACGGTAAAGTACCCATCCCGGGGTACCTGCAGTGGGGTGAAAGGGCAGC
CATGTGGGACTGCAGCCTGGGTCA
>942
AGAGCTGCAGAGCACAGAGACACCACTGACGTGCCTGAGATGCCTCACTCCAAGGGC
CAGGGAGAGAGCGGTAAGTCCCCCGCTTCCCCCAACCCCACTGCTCTTCCCTTCCCTG
CCAGAGGCTACATTCAGGCCATCAT
>943
CAAAGCTAAAATTGAACTACTTGGGAGCTATGATCCACAAAAACAACTTATTATTGAAG
ATCCCTATTATGTAAGTACAGTTCACGTTTTAGGGCTAATATGAAGACCCAACACATTTG
TATCCTGCCATATTAAATAAC
>944
ACTATAAGCTCCAGGTTGACTTCTCCTCAGACCACCTGGGCTGTTTCACCAAGTGCCG
GAAGCCATGCAGGTTAGTGTCCCCTTCCCCGGGTACAGGTTGGGTAGAATACAGGTTA
CCGAGCCAAGCATTTGGGAGAGCT
>945
AGTGGCCAAAGTCAACATCTTCTTCAAGGAGCTGAACTACAAAACCAATTCTGAGTCT

CCCTCTGTCACGGTAGGTCGTGCCTGGATGGAGCCTGTGATGCTCTGGGGGTCTTACTT
AGCAGGGCGGCTGTCGGGGAAAA
>946
GCGCCATCTACAACAACATGAAGCAGTTCATCCGCTACCTCATTTCCTCCAACGTGGGC
GAGGTGGTCTGGTGAGCAGCTGGGTGGGCGTCCAGGAGGAAGCCGGGGTTAGGGTGG
GGTGGCTGCAGGTCTGGGAGGCAG
>947
CGCCCCCCCCGGAGCCCCAAGGAGCCCCTCATCAGTGGCTGGCTCTTCTTCCGCTACA
TGGCAATCGGGGGTGAGCTGGAGGGGTTCCTCGATCCTCCCCACCCCTTGGGACTAAC
CCCCTCTCTGGGACACCAGCTCCC
>948
CACCGTGGGAGCAGCTGCCTGGTGGTTCCTGTACGCTGAGGATGGGCCTCATGTCAAC
TACAGCCAGCTGGTAGGGGGAGGCCACAAAGGAGGGGACCAGGAGGGGGGGGGGAT
GCAGGAGGGTACCAGGAGGGTGGCAT
>949
AGGCCCCCGAGCCCATGACCATGGCCCTGTCCGTGCTGGTGACCATCGAGATGTGCAA
TGCACTGAACAGGTGGGGGCCCCCCAGCTACACCCACCACCCTCCCCTGAGGCCACT
GCCCACATCCTCCACTGTGCCGCCC
>950
CTGGCTGCTGGGCTCCATCTGCCTCTCCATGTCCCTGCACTTCCTCATCCTCTATGTTGA
CCCCCTGCCGGTGAGGTTTCTTCCGCCCAGGGCCGCCCACCCCAGCACTGGGGAGCCC
ACGGGCGCCCATGACCACTCCC
>951
CTCAAGATCTCACTGCCAGTCATTGGGCTCGACGAAATCCTCAAGTTCGTTGCTCGGA
ACTACCTAGAGGGTAAGGAGTGCCCTCTCTGTCCCAAGCCCTGGCCCCACCACAGCCC
CTTCCCCATGACGTCCGTCCCCCG
>952
ATGTGAACAGGAAGAAAAGTATGAAATTCCTGAAGGTCCACGCAGAAGCAGGCTCAA
CAGAGAACAGCTGGTATTTTTCTTTTAATACAACTTTCATTGTTCTTATTATGACATACTA
TTATTATCACCATCAGGAAGAA
>953
TGTGCACCGGCTGGTCAACCTCTTCCAGCAGGGGGGCCGGGAGCTCGACATCTACAAC
AACCTACGGCAGGTGAGGCTCGGTCTGAGGGAGGAGGGCTGGGGCCTGGACTCCTGG
GTCTGACAGAGGAGGGGCTGGGGGC
>954
ACCTGCCTGCGAGCCCTGCTGGACAGCGCAGCTCCGGGCACGTTGGACCTGGAGGCC
CGCAATTATGACGGTAAGCATTTACCGCGGGGCACCGCTGGGCTGTCCAGCGGACCTG
GAGTCCATCAGCGGCCGCAAAGCCC
>955
GGCAGTGAACACCGAGTGCCAAGAAACCGTGCAGCTCTTGCTAGAGCGCGGTGCCGA
CATCGACGCAGTGGTGAGCGTGCACTAGGAGCTGGGAGGGAGCGGGGCCTTAGCAGG
GGCGGGGTCTTGGCGGGGGCGGGGCC
>956
GAGCGGCCGCTCCCCGCTCATCCACGCCGTGGAAAACAACAGCCTTAGCATGGTGCAG

CTGCTGCTGCAGGTGCGTACAGCCCCCCTGAGCCTCGCGCCCACCCTATCCTCTGACC
CCAACCCGGCTCTGGCCTCAGCCC
>957
CAGCGGCGCTGACAGCAGCCTCAAGAACTGCCACAACGACACGCCGCTCATGGTGGC
GCGCAGCCGCAGGGTGAGCCGGGGCAGCTGTGGACATGCCCCTTGCACACGTGTGTC
CGCGGGCTGGTTGCAGGCGAGTGTGC
>958
CAGCCAGACCCCTCCCCTGACCGGAGCGCCAACACCTCCCCCGAGAGCAGCAGCCGC
CTCAGCTCCAATGGTGAGAAACCGTTCCCAACCTCCAGCCCTGGCGCCTCCTCCCTCA
GACCCAGGAATCCCAACCCACAGCC
>959
AGCTGCTGAAGAACCACTGATTGAGCCCCTGATGGAGCCAGAAGGGGAGAGTTATGA
GGACCCACCCCAGGTGAGGGGGCAGCAGGGCTGGGCGGGACTTTGGGAATCCAGGAT
GATTGCTGCCGTCCCTAGCTGGGGTG
>960
CTTCATGCCGTCTCCCCAGGTGGTCAGGTGGAACCCATGCCTGGAGCCTCACCGCTTC
AATGACACAGAGGTGATTCCTGCCTTTTTCCTCAGTAGGCTGAGGGTACACAGAGGTG
ATCTAAGTCAGGGACCAGAAGCTG
>961
CAGTATCTCTGCTGCTCTCAGACAGCCAAAAATGCTATGGGCTGCCAAATTTTGGAGA
ACAGGAATGGAAGTAAGCAATCTGATCGATATAATTTCTTTCTCCTTTGACTGTTTCCAT
CACTCTCCAAGGGTCTGTCAAG
>962
GGAAGGGTGATGAATATTTTATCTTGGAGGAAAGCAACTTACCATGGTGGAGAGCACG
AGATAAAAATGGGTGAGTCCACACCAGCCCTTCCTGAGCCTGGTGCCTGCCCACTCCC
TACACGCAAAAAGTGCTGCGGCAT
>963
GAGGTTTCATTGTCAGAGACTCCAGCAAAGCTGGCAAATATACAGTGTCTGTGTTTGCT
AAATCCACAGGGTGAGTGCTACTATTCCAAGGCCCTGAGGACAAAGAACAGGGGTAC
CCTCCTAATAGCTCCTTGATGCTG
>964
TACCTGGCTGAGAAGCACCTTTTCAGCACCATCCCTGAGCTCATTAACTACCATCAGCA
CAACTCTGCAGGTGAGTACCAGGGGCAACTGAGAAGGGAGTTACAGACACCAGGATA
AGCAATGCAGGACAAGGTGATAAG
>965
ATATCCAGGCTCAAATATCCAGTGTCTCAACAAAACAAGAATGCACCTTCCACTGCAG
GCCTGGGATACGGTAACTCCTTATTTCTCTGGGGTAGGGTGGACTGGCCAGTTGCAAA
AACTACCTTTGCTGGCCTTGCTTA
>966
TGGCCATCAAGATGATCAAAGAAGGCTCCATGTCTGAAGATGAATTCATTGAAGAAGC
CAAAGTCATGATGTGAGTTATAGCCCAAACTCAACTCTCAATCTATTTGCTGGAGTCTA
GGAATTCACACAACAACCCACTG
>967
AGAGATGTGCAAGGATGTCTGTGAAGCCATGGAATACCTGGAGTCAAAGCAGTTCCTT

CACCGAGACCTGGTGGGACCTTAGAAGGATTGGCCTAGGACAAAGGGCTGATGGGGT
GGAAGTAGCAGTGGAAGATATATCA
>968
GTCCGGTGGTCCCCACCGGAAGTCCTGATGTATAGCAAGTTCAGCAGCAAATCTGACA
TTTGGGCTTTTGGTAAGTGGATAAGATTACACAGATTATACAGCTCAAAGGATAAGAAA
TGCAATGGGGAAAATTCACACTT
>969
CCTACGTCTCTACAGGCCTCATCTGGCTTCAGAGAAGGTATATACCATCATGTACAGTTG
CTGGCATGAGGTAAGTGCTTTATTAGGATCTCTTAAATTATTTCCTTGAATCTACTTGCC
CATTTAGCTGGCCATTCAGC
>970
ATGAGATCTCTTGGGCAGAATCCCACAGAAGCAGAGTTACAGGACATGATTAATGAAG
TAGATGCTGATGGTAAGTCTTCAATTTTGTGGGGCTGGGAGGGGTTGAAGAAGTGATA
CTTAGATGTTTCCACTAAACCTTT
>971
GATGGCAAGAAAAATGAAAGACACAGACAGTGAAGAAGAAATTAGAGAAGCATTCCG
TGTGTTTGATAAGGTACGTCAAGGACTGGATATGTTAAATTTTGAGGATGAAATGAAGA
TACCAGGACTCATTATGAAGACTT
>972
ACAGATGAAGAAGTTGATGAAATGATCAGGGAAGCAGATATTGATGGTGATGGTCAAG
TAAACTATGAAGGTAAATGTTTCGGTCTCACGCCTCATTCTTCACTTTGATTTTTAAGAT
AAGAGGCTCTTTGTTAGGGAAC
>973
ATGAGGTCACTGGGTCAGAACCCAACAGAAGCTGAATTGCAGGATATGATCAATGAAG
TGGATGCTGATGGTAAGAGCTTTAAAACCATGAATGAGGGCCATTGTTGTGTAATTCAA
GTTCAGACATGTTACAGGATTGT
>974
GATGGCTAGAAAAATGAAAGATACAGATAGTGAAGAAGAAATCCGTGAGGCATTCCGA
GTCTTTGACAAGGTAATCCAGCATCTACATAGCAGATGGTACTTAAGTATGGCTTCTTCC
GCTTTCACTTCTAAAAGCTATA
>975
ACAGATGAAGAAGTAGATGAAATGATCAGAGAAGCAGATATTGATGGAGACGGACAA
GTCAACTATGAAGGTAAAACTAAATTCTCTGAGCTCAGTGTTTCATAGTCTTACCTTTA
GATCTGTAAGCAAGCCAACTGCTT
>976
TCAAAGAACAGGACATAAAAACAAAGTCACAATGACATTCCATAGTAAATTTGGAATC
AGAACTCCAAATGTGAGTATAATTTCTTCTAGGTTTAAATGCTCTGTAGGTTGGCACTGT
TACACTGCATGTATGCAAAGAA
>977
GACACGGACGCCTTCCGCAGCTGCAAGTTCCCCACCAAGAGGTCCAAGAAAGCTGGC
CGCCATTAAACAGGTGGGGGCCTAGGTAAACAGGTCCCCTATGTCCCTCTGGGTGATTC
CAGCAAGTGAGCTGGTATCTTCCA
>978
GCCCCCCGGTCCTCTGGGCCTGCAGGGCGTCCCGGGTGTTCCTGGCATCACGGGGAAG

CCGGGAGTTCCGGTACGTCGCTTTTCCGGCTTTTCCAGCTTTCACAGGGTTGAGATCGT
GTTTTTTCCGGAAGGAAGTTACT
>979
CACCCTGGCGCTCGAGGACCCCCCGGATACCGCGGTCCCACTGGGGAGCTGGGAGAC
CCCGGGCCCAGAGGTGAGTGTTTGACCCCATGACACGGTCACCCTGCTGTAAAAATCC
CTGAGACTGACTTGTTAGTAGGCGC
>980
GGTGACAGAGGAGACAAAGGCGCGGCAGGAGCAGGGCTGGACGGGCCTGAAGGAG
ACCAGGGGCCCCAAGGTACGAGTCCACGGCCAGCAAGGCTTCACTGGGTGACATCTC
TTCCGCCATCTTGTAGCTTTATGTGGGG
>981
AGGACCGTGTACCACGCAGAAGAGGTGCAGTGTGATGGCAGGAGCTTCCACCGCTGC
TGCTTTCTCTGCAGTAAGTAGCTCTCCATGCTCCACCTTTGACGGAATTTGTATGTACAG
AGCTTCAATATGTCTATTTAAGG
>982
ACGGTTATGGCCAGGGCGCTGGCACGCTTAACATGGACCGTGGCGAGAGGCTGGGCAT
CAAACCAGAGAGGTGAGTTGGGAGACCGTTGGTTACCTTCAGAAATGAGTTGCGACA
GTTAGTGCCGCTTGCTGAACAGGAA
>983
AGGTGCTGAGAAGTGTTCCAGATGTGGGGATTCTGTATATGCTGCCGAGAAGATAATTG
GAGCTGGAAAGGTAAAGTGCTATTTGGAATTAATAACAGCAAATGACTTCTCTCTGCTC
CCTTTCTCCCCTAACCCTTCCA
>984
TGTGCAAAGTGTGGGAAGAGTCTTGAATCAACAACTCTGACTGAAAAAGAAGGTGAA
ATCTATTGTAAAGGTAAAATTCATTTCAGTTACTGCTGTCCATATGAATATCCACACTGG
TTCTTGTTTAATGAAATGTCACT
>985
AGTATCTCAGCGCCCTGGAGCCACAGTACCCACTGACTTTGCCACCTTCCCTTCATCAG
CCTTCCTCAGGGTGAGGGGGAGCATGGGGTGGAGGAGGTGGGACACGTGTGGAGAG
CTCAGGCGGCCCCCCTCTCCTCCAG
>986
TGCAGAGCAGATGGTTATGGTGATCAAAGCCCCTCCTGAGACCCAGCTCCAAGCCGTG
GACTCTTCGGAGGTGAGATCTGGGAACTCCGGGCCCAACCTGGGCTGGGCTGGGCTG
GGCTGGTGGTCACCTGGGCCTCAGT
>987
CCACCACCATCATCTCCCCCCTCATCCCTCACCACAGATCCCAGCCAGTCTCTACTCAG
CCTGGAGCAAGGTGGGTGATGGGTAGGTGGGTGGGGTGGGGCAGGGCCCCTCTTCTG
GGGGGTGGGCAGGCACGACAGCCC
>988
AGGTGGACGTGGGTTTGGCTGCCGATGTAGGAACACTGGAGCGCCTGCCCAAGGTCAT
CGGGAACCAGAGGTGGCGTGCAGGGGGTGTGGGGGTGTAAACAGAGACCCCAGAGC
ATCTCCCAGCCCCGGGGTGGCAGCCT
>989
TGGTCAACGAGCTGGCCTTCACCGCCCACAAGATGATGGCTGACGAGGCCCTGGACA

GTGGGCTGGTCAGGTAGGGCCATGGGCTGGTAGCTCAGTGCTGGGGGCAGCCAGGCC
TGGAGGGAAGGAGGCCTCTCCGGGTC
>990
GGTGCAGAGCACCAAGGTCAACCTGCTGTATTCCCGCGACCATTCGGTGGCCGAGAGC
CTCAACTACGTGGTAAGGTGCACGCTCCGACCAATCACAGCCCTCCTCTAACCCCAGG
CGACCAACCAGGGTGCAGGGTGCT
>991
ACCGGGTGCGCATCAATGTACCTCCACACAGAGGGCTTCTCTGGGCCCTCTCCAGGTG
ACGGGGCCATGGGTAAGGCAGCCCCCCTTCCCCTGCCAAGCCCTCCATGTTGGGGGAG
GGAGCGCTGCCATGGGGGAGGGTC
>992
CCCAACAAATGCCCATTTCATTGCCTGGACGGGCCGGGGAATGGAGTTCAAGCTCATT
GAGCCTGAGGAGGTGGGCCTCTCGGATGTTCCCAGCCCTCCTTTCCAAAGTTTACAGC
CTGGAGGTGGGAGAACCTGGGAAA
>993
GCCAGCCATGAATTACGACAAGCTGAGCCGCTCGCTCCGATACTATTATGAGAAAGGCA
TCATGCAGAAGGTGGGGGCTGTGGGTCTAGGGACAAGGTGGTGGGGGGCAGTGGCTG
TGAGAAGCTGACTGGGGAGAGGGT
>994
ATAGCCAACGAGAGTGCCCCTCCCAACTTGTGTGGCACCTCCCACGCCATTGTGGGAC
TCATGGCCAATGGTAAGTGTTAACTTCTAGAGTCTCTCCCTCACCCCCACCTGTGCTTT
GCATATAGACCAGTGAGAAGCTG
>995
AGCATGAGATCACCATGCCCAACCAGATGGTCCGCATCTACAAGGATGGCAAGGTGTT
GTACACAATTAGGTATGTCAAGCCTCTGGAGTCTCACTTCCTGGAATTCTCTCTCCCCTT
CTGATAATTTTAGCTAAAGATC
>996
GCCGGATGCTCACTCCACATGCTCAGATTTCCAATGGATTCTCACTCTTGCCCTCTATCT
TTCTCTAGCTGTGAGTACCTTCTTAAGTTTCTGGGGCCCCAGAAACATGCTGGGCTCCT
TCTTTTTCTCATCCTTGCCAT
>997
TCCTGGAAGCTCTTCCAGTTTGATTTTACAGGAGTGAGCAACAAAACTGAAATAATCA
CAACCCCAGTTGGTAAGCGTGCCAGGGCTTGGCGGAAGTCCAGGAAGGTGGTAGGGA
TGATTGGAGATGGCCATCCATACAA
>998
GTGACCACGATGCTCTCCTGGGTTTCCTTTTGGATCAAGACAGAGTCTGCTCCAGCCC
GGACCTCTCTAGGTAAGAGGAGAAACAGGTATACGCATAGGCACATGGCTGGGAGTTG
GCTGGGCCAGGGCAGAGTTGCCTT
>999
GGAGTTTGCTGTGCTCAACTTCCTGATCTACAACCAGACAAAAGCCCATGCTTCTCCTA
AACTCCGCCATGTATGAGCTGGGTATGGGAGTGGTGGCAAGGCTTTGGAGTGTAGAGA
CATGCTAGCAAGGGTACTGGGGT
>1000
CAAGACCAGCTGCTCACACCCGAGGAGGTCGTGGACAGGATCTTCCTCCTGGTGGATG

AGAATGGAGATGGTAAGAGGGGCAGAGATGGGGAGAGTGCTGTCCACTCTGCATCATC
GCCACTTTCTGGCCGCACGTCCTT
>1001
GATGGCATTTCCCATGCACCTAATGCTGTGAAAACCGCATTTTATTTAGCAGAGTTTTTT
GTTAATGAAGGTAATATGAGGGTACATAATTTTGTTATTCTGGGGTAGTTTTGAAGAAAA
TTGCCCTTATCTGAACTTTT
>1002
ATCATGTCTTTTCCCTCTACTTCATGATACCCTTTTTTTTCACCACTTTGGGATTGCTCTA
CCACAACTGGTAAGTAGGCCTGTGGATAAGGGGACAACTACCTGAACACATGGCAAA
GATGGCCCTTATCATAACCCAC
>1003
TGTTCAACTTCCTCTACTCACTGCCTCAGCTCCTGCATATCATCCCCTGCCCTCGCCACC
GCATACCCAGGTAGCCGCTTTTTGGCTTGAAATGGACATCATAGCCTTTTCACTTGGGA
TATCTAATGCCAGCCTATACA
>1004
CAAACTGGAGATGAGCTATTCCAAGTTCAAGACCAAGAGCCTCTCTTTCTTGGGCACC
TTTATTTTAAAGGTAACAGGGTAACAAGGAGGTAAGGCCCTAGGCTGCCATCCTGACC
TTGAGGAATGGGGAACCTAGTCCT
>1005
CAACTTGCTACTTAAAGTCCTTGGGCCCATACATGAGAGAAACCTCACATTGCTCCTGC
TGCTGCTGCAGGTGAGGATGGGAATCGAGTTTATACCTCCGTGTCTCCCTTTCTGCGTG
ATTCTTACTCCAGTCCATTTCT
>1006
TCCTACTGGGGAGCCCTGCCCAGAGCCCCTGCGTACCTATGGGCTTCCTTGCCACTGTC
CCTTCAAAGAAGTAAGTACTTAGGGAGGAGAGAGCGTTACCCCTGTGGCTAAAGAGAT
GGGGTTTGGAGAGAAGGGTCTTT
>1007
AATTCACCACCTCGGACTCCTGCGACCGCATCACAGACGAATTTCAGCTACTGCAAGC
TCAGTACCACAGGTGAGCTGGGGGACTGGGGGTCCCTGCGTCCCGAGAATGTGAGTC
CCCCCATTTCTTCCTGCGCAAGAGG
>1008
GCAGCAGGTCTTGGGAGCCATTGAGAGGGCCAAGCAGGTCACCGCTCCCGAGCTGAA
CTCTATCATCCGAGTATGTATGGGGACCTGGGCGGGTGGGCGGGGGACAGGTGGGCCC
CAGGCAGCTAATGTTCCCCTCCCCC
>1009
CCCCTGCCCGACGTCAGGCTGTTCAAGTACCTCCCCCATCATTCTGCACACTCCTCCCA
ACTGGACGCTGGTAAGGACTTTGGAAGAAAAAGTTTACCTTCAGTATTTGAGCAGGTG
AGCACTTTGTGCGAGAGGTGATA
>1010
GTTCAACAAGTACCTGTCGCGGCCCAAGCGCTTCGAGGTGGCCACCTCGCTCATGCTC
ACCGAGACCCAGGTGCGCGCCCTCCCCGCCCCCGACGCATCCCGGCCTCCCAGGGGA
CACTGTTACCTTTGCACTTGTGACC
>1011
GAACTACGTATCCAGGCCGCGGAGATGTGAGCTGGCGGCCGCCCTAAACCTGCCGGAA

ACCACCATCAAGGTATGCGGGGTCCAGGCTGGGGAGGCGGGTGTGCACCTATTTAGCG
GGAAGTAAATGCCAACTGCCAACT
>1012
GAAGTGTTACTTTCGGAAGGAGAACCTCCTGGAGCATGAAGCCCGGAATTGCATGAAC
CGCTCGGAACAGGTACTTGGGAGCTGGCCCAGGTACTTGTGGGCAGGGCACACTGGC
CTCTCTGTCTTCGTGCCATCCGGGA
>1013
GGAGACATTCCGCCGAAGGATGGAGCTGCGGGTGCACATGGTGTCTCACACAGGGGA
GATGCCCTACAAGGTCAGGCTTGGCCTGTCTCCAGGGCCAGGGTTGGGTGGCCCCAGG
ATCCTTCCTGCTGAGCCCTTTACGT
>1014
GCAGTTCATGCAGAAGAAGGACTTGCAGAGCCACATGATCAAACTTCATGGAGCCCCC
AAGCCCCATGCAGTAAGTGACAGGGAGGGCTGGGCATGCTTTGATGCTGGCATGAGCA
AGAGTGAGCTGTGCCCAGAGTGGG
>1015
TGTGTGAGGAGTGTGGGCACCGGGCCTCGAGCCGGAATGGCCTGCAGATGCACATCA
AGGCCAAGCACAGGTGCGTGTCGCCCGTTCTCTCTTGGGGCCCAGTCCTGCTGCCAGC
CCAGGCTTGCACCGGCAGGGAAGAC
>1016
CACCTGCGCACACACACGGGTGAGAAGCCCTTCCAGTGCCACCTCTGTGGCAAGACC
TTCCGAACCCAAGGTGAGGTACGCCCTGCCCCTCCCCTCGTCTCCCCATCCTGAGGTC
AAGGCCACAGGCTGAGCTCTTGCCT
>1017
AGGCACGTGGCCAGCCGCCATCAGGAGGGCCGGCCCCACTTCTGCCAGATATGCGGCA
AGACCTTCAAAGGTACCTGGGCGGCCCTGGGAGAGCCATTTCCTGCTCATCCGAGTTG
GAGGGTCTCTGAGGAGGAAACGCT
>1018
GCACGTCAGACGGCACAAGGGGGTGAGGAAGTTTGAGTGCACCGAGTGTGGCTACAA
GTTTACCCGACAGGTAGGCCAGGGCCTGGCCCCCTTCCCCTACCCTAGGATCCCCCAA
AGTTCTGAGCTCACCCTCCCCGCCA
>1019
GTCAGGTTTAAGATTCGGGACATGGACTCAGGCACTGTCCTCTTTGAAATCAAGAAGC
CCCCAGTCTCAGGTGAGTGGCTGGGTGGGCCATTGATGGGGAGGCAGATAGGAGCTG
CGGAGTGGGCTGCCCAGGGAATCCT
>1020
CCAATGCTGGGCGCTTTGTCCGCTACCAGTTCACGCCTGCCTTCCTCCGCCTGAGGCA
GGTGGGAGCCACGTGAGTCGCTGGGCGATGGGTGGTGGTGGGGGTGAGGGAGAGGG
TGTGAGTGGGCCCTTCTCAGCCTCTC
>1021
TTCTGCATCCCCAGCAGCAAGAACACCTGCGAGCACATTTACGACTTCCCCCCTCTCTC
CGAGGAGCTGAGTGCGCGGGCAGGGTCTTCTGGGAGTGGGGAAGTGGGGGCGTCTAG
AGACTGAGCTGCGGGAGGGAGAGG
>1022
TGGATCGCGTTGTCCCGCCTGGCGCGCCCGCAGCGCCTGCCGGTGGCCACTCGCGCGG

TGCTCATCACCGGTGAGTGCGCGGGTCGCGGAGCGCGGGGACTCCAGGCTCGAGGGC
GGGACTGGACACTCAACAGGACTGA
>1023
ATGGACCTGACCAAACCAGGAGACATTAGCCGCTTTCTAGAGTTCACCAAGGCCCACA
CCACCAGCACCGGTCAGTGGCAAGTGTCCACCAGGCAAGGGCGTGGCAGGGGAGTG
GGAAGGACACGGGGACTGGAAGTTTG
>1024
GCTGACCAAGGGCCTCCTGCCCCTGCTGCGCAGCTCAAGGGGCCGCATCGTGACTGTG
GGGAGCCCAGCGGTGAGTGCCCCCCCCCACTGGAGCAAAAAGGAGCCCCCTGGGGTG
GGGGAGGGCTTAGGGAGCCCCTTGC
>1025
ACATTCAGCTGTGAACTCCTTCCCTGGGGGGTCAAGGTCAGCATCATCCAGCCTGGCT
GCTTCAAGACAGGTGGGAATCAGGGCTGGGGGTGGGGTGGGGGTGGGGAATGGGGCT
GGGAATGGTCTTATGGGGGCAGGTC
>1026
CCAAGAGTGTGGCCCGCCGGGTCCTCCAAGCCCTGGAGGGGCTGAAAATGGTGGAAA
AGGACCAAGATGGGTAAGCAGGGTAGGGGGGGCTGCATTGATGGAGTAGCCTTGAGG
CCCGGTCATCAATTCCCCAACGAATG
>1027
GCCTATCGTATGGCCCAGTCTCCCCGATCATAAGAAGACTCTGGAACATCTTTGTAAGA
AACCAAGAAAAGTGAGTGTTTTTGGTGCTTAAAAAGTGTTGTGTTGGCAACATCCCAG
TGGCCAAGAATGATATTCCAGGA
>1028
TGCTCCCCGCCGCTTCCGTTTCTCGAGGGAAAGGCTGCTGCCTCCTGCTCTGTCCTCAT
CCCCGGCTTAGGTAACACGTTTTCCTCCTTACGACACCCACCACAGGGTCCCCGCGCC
GCTGCCCCTCCTCCTTGGATCTG
>1029
TGCAGTTGACTTCAAGACGCTGGCTGATGTACTGGTACAGGAAGTTATAAAACAGAAT
ATGGAGAACAAGGTAAGAAAGGTCATAGCCAAGGTAACTGCAAAATAGTTGCATCTGT
GGCTTTCTCCATCATAGAATAGCA
>1030
GACCCAACTCTGGATTCCACAGAGATCAATGTTCCACAGGACATTTTGGGAATTTGGG
TGGACCCCATAGGTAAGTATAGGAAAGTATGTTTGTTCTTATTTGCAATCTTTTTTTTGG
TGGTGGGGTTGGGGATGGGTGT
>1031
TACAGACAGGGGTTCCCCTGATGGGAGTCATCAATCAACCTTTTGTGTCACGAGATCC
AAACACCCTCAGGTAAAAGGCAAATATTTTTGGTATATTATGGTTGTTAGAACTTACGA
CCTCAGCTTTGCTTGTTGTTAGG
>1032
ATCCATGCTCAGCCATCTGATAAAGAGAGGCTTACTTCTGATGCATAGTATTGATCTACT
TCTGTAATTGGTAATTGATCAATGTTTTTTAATTGCTAGCTGTGGGACCCGCTATGGTTG
TGGTGGCTAACTTTAAGAAC
>1033
CAGGGCCTGGGGATCTGGCCGAGGAGAGGACTGAGTTCCTGCACAGCCAGAACTCGC

TGTCACCACGCAGGTGCACACTCTTGGCCTTCCCTCTCCTCCAGGCCTCTGGGAAAGC
GGCTTCCCTTTCTGAGCCACCCCTG
>1034
GGACCTCCTCCTGGCCACCACAGCCAAGCCATCAGTACCCAGTGCTGACAGTGAGAC
ACCCCTGACCCAGGTGAGCAGAAACCAGGCTCTGGAGCCCAACAGCACAGTGGTGAC
AGTGGGCATCACTGAATCTGTATCCA
>1035
AACCCGGCGCCGGAGCTCAGGGCCTCCTTTCTGCCACGTACCTTGTCTCTGCGGAACT
CCATCAGCAGGAGTGAGTCTGGACCCAGGAGAGCAGGGGAGGGGTTGGGGGGTGCT
CCTGGGCTTCCCTAGCTCAGCCTGCT
>1036
GACGAGTGGCAGGCCATCTCGGAGATTGCCTCTACTTGCAACACCATTCTGGAGTCGC
TGTCCCGAGAGGGTAGGCCACCAGGGTGCTGCGGTAAGCCTGGGCAGTGCTCTTGCC
CCCTTTCTGACCTGCCCACCTGATC
>1037
TGGGAACCTCTCAGAGAAGGTCTCTCACTTGGAGTCCATGCTCAGGAAGCTGCAGGA
GGACCTGCAGAAGGTGAGGGGTGGGCTCAGCTTGGGGGGAGTCACAGGGCTGCCCC
AGGAAAGGGGCTGGCGGCTCTGACTCC
>1038
TCAGTGGTCATCATCCCCTTTGCTATCCTCCTCTTCTTTCTCCTTCATCGCTGGTGTGCC
AACAAAAAGAGTAAGTCTCACGAAGCAGAAGCCAGAGAGCTCAGGGCCATGTGGGG
AAGCAGGATGGGAGCACTCAGGTG
>1039
TCAGTGGCCATCATCCTCTTTACCATCCTTCCCTTCTTTCTCCTTCATCGCTGGTGCTCC
AAAAAAAAAAGTAAGCCTCACGAAGCAGAGGCCAGAGAACTCAGGGCCCTGTGCGG
AAGCAGGATGGGAGCACGCAGGTA
>1040
GGCAGCAGCGACCCCTTCCAGCAGGGCCTCACGCCGCCCCAAATGCCAGGTGACCAC
ATGAACCCCTATGGTAAGCCGCCCTACCCCCACCCGCCCGCCCCAGCACAGCCCCTGC
CCCCTGCCAGGCCAGGCAGGCCTGG
>1041
GTCAACCTGACAGGGGGCCTGAGCCACGCCTCTGACGTGGTCCTTTACCATCAGCACA
AGCAGCCCGAAGGTGGGGGCAGAGGGGAGCCTGGGCTGGGGAAGGGAGGCCTGTGG
GCATTGAGTCTCCAAGCTGTGGCCCT
>1042
TGTCCCAATGGGAAGCGGCTGGACAACGGCACATGCGTGCCTGTGCCCTCTCCAACGC
CCCCCCCAGATGGTATGCTTATGCCCTCCCAGTCCCAGCCATGCCTCAGGACCATCTCC
TTCCTGTGGCTCCTGACTTCCCC
>1043
GACAAGTGTGAACTGGACCAGTGCTGGGAGCACTGTCGCAATGGGGGCACCTGTGCT
GCCTCCCCCTCTGGTATGCCCCCTCATCCCGCCACGCCTGCTCCTTGCCCCTGGGCCCC
AGCCCCGCCACCCACCACCCCACC
>1044
ACTGTCAACCAGGGCAACCAGCCCCAGTGCCGATGCCTACCCGGCTTCCTGGGCGACC

GCTGCCAGTACCGTGAGTGAGCCATCCCTGGGCCCCAGGGCATGCGGGAGGGTGACG
GGGGACCCCAGAGCATGGGGTGATG
>1045
TGAACAAGTGCAGCCGCTGTCTCGAAGGGGCCTGTGTGGTCAACAAGCAGAGTGGGG
ATGTCACCTGCAAGTGAGTGGGGCCCTCCTCCACAGTTCCACCCAGCCGGGCCCCTGC
CCTGTCCTAGCCCTGCCCTGCCCCT
>1046
TGACCTGCGTCGGCCACTGCAGCAATGGCGGCTCCTGTACCATGAACAGCAAAATGAT
GCCTGAGTGCCAGTGAGTTGGGCCCGGGCTTCACCCAGGCATAGATCATCGCTCCCTT
CCCCAGATTTGAGCAGGAGGACCG
>1047
CTCTGCTGTTGCTGCTGCTGCTGGTTCTGGTGGCCGGAGTGGTATTCTGGTATAAGCGG
CGAGTCCAAGGGTGAGTCACAGGGATTCTGGAACATTCTGGTCATTATTTGCCATCCT
AGCCTTCCCCCCCAATAATCTC
>1048
GTACGAAGGCGGAGAGCCTGATGATGTGGGAGGCCTACTGGACGCTGACTTTGCCCTG
GACCCTGACAAGGTGGGCTGGGAGGCGGGCAGGGTCAATTGCCAAAAGGCCGTGGGT
GGCCTAACCAAAGGTGTTGGGTTAG
>1049
CACTTTTATTCTCTTGGACTCTGTTTCTCAATGTTGGACCTAAGATATTGAAGACAGGCT
GGAGTCCAGAGTGAGAGACTTCAACTATTCTTTGTGTGTGTCTGTCGTGTGAGCAAAA
ATGATGTAGTTAAGAGGCTCCC
>1050
TGAATGAGGCCTTGGAACTCAAGGATGCCCAGGCTGGGAAGGAGCCAGGGGGGAGC
AGGGCTCACTCCAGGTGAGTGACCTCAGCCCCTTCCTGGCCCTTCTCCCCTGCCTTCCT
AGGTTGGAAAGCCATAGGATTCCAT
>1051
GCTCTTTCGGGACATTGCCCAAGAGTTCTCTGATGACCTGCACCATATCGCCAGCCTCA
TTGGGAAAGTAGTGAGTAGAAGGAAAAAGGGAGTGCACCCAGGGAGGTCAGGGAGA
GAGAATGCAGTGTGCAAGATGGGGA
>1052
GACTTTGAGGGCAGCCTTGCTGAAAATCGCTTCACAGTCCTCCCCAACATAGATCCTG
AAATTGATGAGAGTGAGTGTTGGGTGTGGATGGGCCTGTGAGCCCTGCGCAGTGATGG
AGTACCATCCTTGGCAGGTGGTCA
>1053
GGTTGCCCGCAAGGAGCTGGAGAATCTGGACTCCCGTATTCCTTCATGCAGTGTCATCT
ACATCCCTCTGGTGAGGGCAGGAGAGTGGGTGTAGCCTTCAGATGTCTTTTGGGGGAG
ATATTAGGCTTATGAAAGACATA
>1054
CCCGGGACTATGGCTACTCAAGGCCGCGTTACTCCCCACAAGTCCTTGGGGTACGAAT
CCAGAATGGCAGGTAAGAATAGAGGCGGGTGGAGGAATAGACATGAGGGGCCCAAAG
GCTACATCTTCTGGGGGTTCATCTA
>1055
CAGACTTGATCCGCAGTGGAAAACCCATCAAGCCTGTCAAGGATTTGCTAAAGAAGAA

CCAAATGGAAAAGTGCGTATATGGCCCCAGTGTCTTTACCCTCTCTGCATCTTCTCCTG
CAACTCTTCTCCCCTTTTCAGGG
>1056
TCTTTCTATTCCCCGCCTGCCTTCCATGGTAGAGGCCAGTGACTTTGAGATTAATGGACT
GGACTTCATGGTAAGACCCTCAACCTCTGTAAGGTGAGTGATGAGGAAAATGAGTCAG
CAGCTGAGGAAGAGCGTTACTC
>1057
CTGCACTATCGTAGTGCCCGAACCAAGGAGCTGGATGCATTGCTGGGGGACCTGCACT
GCGAGATCCGGGGTGAGGAAAAGCCAGAGGTTATATGCATTGTAAGATGTTTAAAAAA
AGCAGCAGCCAGGGGAAGGAGGGG
>1058
CCCGGGACTATGGCTACTCAAGGCCGCGTTACTCCCCACAAGTCCTTGGGGTACGAAT
CCAGAATGGCAGGTAAGAATAGAGGCGGGTGGAGGAATACACATGAGGGGCCCAAAG
GCTACATCTTCTGGGGGTTCATCTA
>1059
TGGGGACAAAGGGAGGGTCAAAGTCATCACTGGACCCAACTCATCAGGGAAGAGCAT
ATACCTCAAACAGGTGAGGAGAAGCCCTGCAGCCTGGGCCTCTGGCGTCTCCTGCATC
TACTCCACCCCTACTTGCCAGCCAA
>1060
CTTCACACGAATTCATAGCTGCGAATCCATCTCCCTTGGCCTCTCCACCTTCATGATCGA
CCTCAACCAGGTCAAAGGGAACAAAGGGAGGTGGGATTGAGGAAGGGGATAATGGG
AAAGGAACCCCTGAAAATGCTCAT
>1061
GAAAGCAGTGAACAATGCCACTGCACAGTCGCTGGTCCTTATTGATGAATTTGGAAAG
GGAACCAACACGGTGAGGGGAGAAACTGATGAGGGGAGAAACTAAGGAGGGGAAAA
TGGAGGAGGATGAAGGAGCATGACAG
>1062
TGTGGCCACCAACTTTCTGAGCCTTGTTCAGCTACAACTGCTGCCACAAGGGCCCCTG
GTGCAGTATTTGGTGAGGAGACCAATCTAGCTCCTCGGGGACCCCCAGGCTGGGCATT
TCCCAGAGGTGGGGATTGGCTCCT
>1063
GGCCAGCCATGCCTCCCACACAGCTGCCCAGGCTGGGCTTCCTGACAAGCTTGTGGCT
CGTGGCAAGGAGGTGATGAGATCCAAATGTGCAACCACCTCCACATCAGAGCTCCCTT
TCATTCCTAGTCCTACTGGGCCTG
>1064
CAGATTTGATCCGCAGTGGAAAACCCATCAAGCCTGTCAAGGATTTGCTAAAGAAGAA
CCAAATGGAAAAGTGCGTATATGGCCCCAGTGTCTTTACCCTCTCTGCATCTTCTCCTG
CAACTCTTCTCCCCTTTTCAGGG
>1065
CTGCCTCCCACATTCTTCCCTGTCTTTTTGGGTTTTGTTTTTTGTTGTTGTGGTGGTTTTT
TAACACAAAGTCTCACTCTGTTGCCCAGGCTGGAGTGCAGTGGCATGATCTTGGCTCA
CTGCAACCTCTGCCTTCCAGG
>1066
TCCACTAGGACCAGACTGCACCCGGCACACAACACTTGGTTTGCTCAACAAAGCCAA

AGCACACATCAAGGTGAGAATTTTTACTTTCAGATTTGCACAATTCCCTCAGTTCTGGT
TTAATTATTGGCACTGATTCCTAT
>1067
GAGATGGAACGAATACGAATGGACAGCATTGGATCAACTATTTCTTCAGATCGTTCTGA
TTCAGAGCGAGGTAGGCAGCTTGCCTCTTCTCTAATGAAATACTAAACATCTCTGTATC
TGGATTTAGGGAAGGTATGTTG
>1068
CATCAAGATGAAGCAGGGGGAGACCCTGGCCCTGAACGAGCGCATCATGCTCTCGCTC
GTCAGCACTGGGGTGAGCTGGGTGGGCCGGCGTGCCGCTGAGGCTCTGCAACCCCGG
GCGCCCCTGCTAACTGCCCGCCCCC
>1069
CTATGCGGCCGAGATCATCCTGGGCCTGGAGCACATGCACAACCGCTTCGTGGTCTAC
CGGGACCTGAAGGTGAGCATGCCCCTGCTGTCCCCAGGCTGGACCTCCGTGGCTGTCC
TCTCCTTCCTCTCGACATCCCGGC
>1070
AGGGGAGGTGAACGCGGCCGACGCCTTCGACATTGGCTCCTTCGATGAGGAGGACAC
AAAAGGAATCAAGGTACTGGCCTTGCCTGGCCTCTTGTACCTAGGCTGTGATCCTGGC
CTGGGGAAGGATCCCTCTCCCTTCT
>1071
CCAGTGGCAGCGGCGGTACTTCTACCTGTTCCCCAACCGCCTCGAGTGGCGGGGCGAG
GGCGAGGCCCCGGTAAGGAGCCCATGCGGGGGTCCGGGAGCCGGGCTTCCGGGTGGC
TGGCCGGCCCGCTCTTCACCTGCAC
>1072
GCAGATCAAGGAGCGCAAGTGCCTGCTCCTCAAGATCCGCGGTGGGAAACAGTTCATT
TTGCAGTGCGATGTGAGTGGGGGCTGAGCCAGGGATGGGAGGCCGAGGGGCCAGGCC
AGGCAGGCTGGGGAGAGCCCCAGCT
>1073
CAGCCAGGAGACAGAGGCTCTAGGGCGGTCCCAGGGCCGCTTGGAGGCCAAGAAGA
GAGAGCTGCTGCTGGTGACAGCGGGGGAAGCTGCTTCCATCCACTGAATCTCTGGCTG
CCCGTGTCCGTGTCCCCATGGGTGTC
>1074
CAGCAGCAGCAAGGCCACAAGGCCCCGGCTGCCCACCCTGAGGGGCAGCTCAAGTTC
CACCCAGACACAGGTGCTGGCCCTAGTGCAGGGAGGAGGGGCTGCAGGGCTGGACCC
CTGGGTCTGAGGTTGGAGGGGCTGGG
>1075
GGAAAAGAGCCAAGAGAAGTGCTTGTCTTTGGATGCCAAGTTGCTGAAAATTAATAGC
ACAGCTGATCTGGTGAGTGTTCATGGATATTTGTTGGGGTGGAGTATTGGTTGATAGGG
AAATTAATCTGGTTTAAACTGTC
>1076
GGCTGGCAACGAGCAGCGCACGCTCATCAAGGCCTATGGCATCCGCTTCGACATCATT
GTGTTTGGGAAGGTAGCTCGCCGCCACTGGCTCCCCTCCGTCACTCCCTGCAGGGACA
AGGGGCCTCTCCCTGCCCCTGCAG
>1077
CCTCTACTGCATGAAGAAAAGACTCTACTATCGGGAGAAGAAATATAAATATGTGGAAG

ATTACGAGCAGGTAGGCCCCTCCTGGCCCCCAGCAGGCACAGGCCTCTCATCTCTTGG
GTGTGGGAGCCCTGGGCGTGGGC
>1078
CGAGCGTGAGTCGGGCCTGGACATCAGTCCCATGTGTGACAAGCATACGGCCTCAGTG
GAGAAGTCCCAGGTTGGCAGGGGACTGGGCTGAACCGGTAGACACTGGGAGGAAGT
AAAAGGAGGGGCTGAGCCAACCCCAC
>1079
GATGGTGGACCTTGCCCATCTGGCTCTTGGTCTCAGAAGAGAAGCTTTGTTTATGTCTG
GAAGACCTGGGGTGAGGGACTCCCTTCTCAGCCTATCATCCACACTTGTGTTTACTTCT
TTCTACCTGATCACCTTTCTTT
>1080
GTCTACCATCGCCGGGGATCCCGGAGCTATGTGCCTCTTGCTCATTCCAGCTCAGCCTT
CACCATTACTGGTAAGGGTTCAGGAAGGGCAAGGCCAGTTGTAGGGCAAAGAGAAGG
CAGGGAGGCTTGGATGGACTGCAA
>1081
TACTGCCTCAATGTGTCTCTGGCTGATACCAACAGCCTGGCAGTGGTCAGCACCCAGC
TTATCATGCCTGGTAGGTCCTTGGACAGAGACTAAGTGAGGAGGGAAGTGGATAGAGG
GGACAGCTGGCAAGCAGCAGACAT
>1082
CCAGTTGCAGCCTGCGATGACACAGCGAATCTATGACAAGTTTATAGCTCAGTTGCAG
ACATCTATCCGGGTGAGTGGCGGGAAGCCTGGCAGGTGCTGTTGACTTGGGTTCTGTC
TCCAGATTGTCAGTCCCCTGAGGG
>1083
ACCTAGAAGCTGTCTTGAATGCCTTGGATAAAATTGTGGAAGAAGGCAAAGTCCGCAA
AGAGCCAGCCTGGTGAGAGTGGGGTGGGGAGGTGAGAAGGTACAGGAAAGAGGCAG
CAATTAAGCTTTTTTTTTTTTTTTTT
>1084
CGTCCTGGCAGGGCGGAGGCAGGTGGAGGAGCTGCAGCTACAGGTCCAGGCCCAGCA
GCAGGCCTGGCAGGTCAGTGTCCCAGCCTGCCTCTTCCTCTTCCTTCTCTAATGGGCCC
TCTGAGATCCGCAAATTGGTGGCT
>1085
GGTGAAGGAGACCAGCATGCCTGTGTACTGCACGTGCGCGGGAGACTTCGCCCTCAC
CATCCACACCCAGGTGGGCTCTCCCTCATCTGCCTCGGCTCAGCCTGGCCTCCTTGGCC
TCCTCTCTGAGTGGACTGGGGTCT
>1086
CCTGAACCAGAAGTATGGCCCTGACTTCAAGGACCGGTACCAGATTGAGCTGCTGCTG
CGGGAGCTGGATGTGAGCGATGGCCCAGGACCCCTGCTGGCCCGGGGGGCGGGGGAC
CCCTCTACACCATGCCTGCCCTCAG
>1087
CAAGTACCGCCGGGAGACTCCGCTCAAGCCCATCCCCGTGGAGGCACTCTGTGACTTT
GAGGGGGAGCAGGTGCGTGGGGCCAGCTCCTCTGTTCACATCACTACAGAGTTTTTGG
GGTAACATGGTTGGATCCCGGGCC
>1088
AGCTGATTGCTCCGGCCGTCTGTTTTGTGATCCCCCCCACAGACCCTGAGGCCCTGGCT

CTGGCTGACAGGTACAAGGGCAAAGGCGGGAGTCTGCATGGACCAGTCCCACCCCCA
GGGCTTCTCAGGCCCCATTCATAG
>1089
CAGAAGGCAGCTGGGAGCAAACGCACGCTGCAGCAGCGGTATGAGGTGCTGAAGACC
GAGAATCCCGGAGGTGGGTGCCAGGGCCACTCGCCTGAGGCTCTGGGGGTACCGGGG
AGGAGGGGCTGGACCAGGGTCTGTGC
>1090
GATCCTGCGGCCACCACTGGAGCAAGGCCGGGCTGTGCAGGACAGTGCCGAGCGGGC
CAAGGACCTCAAGGTACCTGCGGGTCAGGAGCACAGGTGGGGCCGGGGGAGCCCTGC
ACTGGCCCCCTGGGGCCCCTATTTGA
>1091
GGACCCGGGTGGAGGACACCAACCGGAAATACGAGCACCTCCTGCAGCTGCTGGACT
TGGCCCAGGAGAAGTGGGTGGCAGACAGGCAGCATGGAGGTTGGGGTGGGGTCAGG
GGGCCAGGTGGGCATCCCCCGACGCCT
>1092
CCATCTGAATCAGGATGACACAGTGCCTGAGAGCAGCCGTGTCCTGGACAGCAAGGG
GCAGGAGCTGGCGGTGAGTCTAGGGCCCAAGTCCTGGGAGTCCTTCTCTGAGCAGGT
GAGAGCATCTGGCACATCCACCCGCC
>1093
AGCGCCAGGAGGCCGAGGTGCACAAGCTGGGCCAGCGTTTCAACAACCTGCGCCAGC
AGGTGGAACGCAGGTGAGGGTGCGGTGGGTGGTGGCCATTTTTGTTTTCTGAGAATGC
GATCTAATTCCCTCTAAGCAATGCT
>1094
CATCCCCAGTTACGAGCCCCAGGAGACAGACAGCCTCAGCCAGATGGAGACCAAGCT
GAAGAACCAGAAGGTGAGATGGCCGTTTCCGCAGCCCCAGGCACACACTGGCAAGGG
AGGGTCAGAGGTCAACAAGCCCCTTT
>1095
GATAGCAAGTAGGGAGCAGGAAGTACAGAAGATCTGTGCCAATTCCCAGCAGTACCA
GCAAGCTGTAAAGGTGAGCCGGGAAACTTCCGCCCAAAGCACAAACAGCAGGGGTCT
TATTCCACCGTGCCCTGGTCAGAGAA
>1096
GAATGGAAGGAGCAGCCACGTGAGCAAGAGAGCCAGGCTCCAATCTCCTGCCACCAA
AGTGAAGGAAGAGGTGAGTTCCATGGGCCAGGCGGCCTCGGGACTGAGGTGGAGGC
CAAGCTGCCCTGCTCCCCAGGGAGCAT
>1097
CACTGAAGTTTATGCCATCAACAGACAGAGGCTGCAGAATCTGGAGTTTGCTCTGAAT
CTCCTCAGACAGGTAACTGATCTTAGTAAGAAATTCTACTTCAGGCTGTTCTAACAGGT
GACAAATACAGACTCTGTCTTAG
>1098
ACCGGCACCTGGCCCAACAACCAGTTTGACACAGAGATGCTGCAAGCCATGATCTTGG
CGTCCGCCAGTGGTAAGTGGTGTCAGTGTGTGTATGGAAGAGTGGGAGACCTGGGGTT
CTGGGGTGCATCTCACAGCCACCA
>1099
CCACGTCCGTGAGCAGATTCTCATGAAGAGGTTCGAGATCTCTTGCAACCTGTCTCTG

GACGCCATGGAGGTGAGCCGCCAGCGCGGGGCCGGATGGATGTTGCTTCCAATGCAG
GTGGAATGCGTCGGGCTCCTGCGTT
>1100
CTGGCACTAATTGATCAGGATGCTGGGCAGGAGGATGGAGTCGCGATACCACTGCCAG
GCGTCAGGAAAGGTGAGCAGAGGTTCCTCCCGCATCTGCCCACCACTCACCGGTCCTG
GGTGACCACGCATTCATGCCCCTG
>1101
CTCATGGCCCTCCGGCTCAACGAGAGCAAACTGGTGCAGGAGGCCCTGGAGGCGGTG
CCCAGGGGCGAGAGTGAGTTGGGGCTTCGGTGTCGGGCGCCGGGGTCATCCTCTCTTC
CCCTGGTTTGAGTTGGTGGCAGAAT
>1102
GCCACCTGGAATTCTACCTCCTCTGGACTCACAAACTGCTCATGTTGCACGGACAGAA
GCTGAAGTCCAGGTAGAGGGTCTCCCCCGCAGTTCATCGGTGGCTCAGGTCACTGGAC
CAGCTTGTCCTGCTGGATAAAAGG
>1103
GGACGCTGCTGCCTGTCATTCAGTTCCTCCAGAAGAGCATCCAGCGGCACCTGGACGA
CCTGTCGAAACTGTACGTGTGGGTGCAGGGCCTGGGGGTGGGTGGGGTGCACGGTCC
CCTGCTGGTCCACACTGTGGAGTGC
>1104
GTGGCTTCTTCTCCCCCAAACAGCCCGACCTGTTCAAGGACATTGTCAATATGCTCATG
CACCATGACCGGTGAGCTGGTTGGCCCGGGAATCACTCAGGTGGGGCCGCAGGGCAG
ATCGAGGCTCAGGTGTGTGGTGGG
>1105
AGGAGGCAGTGGAGGAAGTGGGGGTTATGGTGGGAGGAGCCGATACTGAGCTTCTTC
CTATTTGCCATGGGTAAGTAGCTTTTGAGTTTTACAATTATTATTATCTTGGGGAGACATA
GCTGCAGGAGTAAAAGCTTTTT
>1106
CCAGGCCCTACAGGAGCTCATCTCAGAGCTAGATCGAAGGTGCCACAGCTCAGCACTG
GAACTGCTGCAGGTGAGACAGGGAGGGGTTCCCTTCTACAATTCAGGGAATAACTGA
AAAAGACCAGAGCTATCTGGAACTG
>1107
ATTACCTCTCCAGAACTCAGGAGTGTGTGCCATGTGCCAGGGCTGAAGAAGATGCTGA
GGACATGTGCAGGTGAGGCAAGTTCTAGTTTTGCGGGGGATAATGGGGTGCAGAGTAG
ATCCCAGGGTCAGGGAGCCTGGAT
>1108
CTACGCCATCGCCATGGAGGAGATCAGCCGTGGCTGCGCCTCCACCGGAGTCATCATG
AGTGTCAACAACGTGAGCCCCCTCCCAGGCCCCTGGGACACACGGGTGGAGGGAGGC
TCCCGTGAGCGGGCAGGCTCTGGCC
>1109
CAGAAGCAGGCGTGGGTCACGCCTTTCACCAGTGGTGACAAAATTGGCTGCTTTGCCC
TCAGCGAACCAGGTACCTGCCCTGTCCCCTCACCTGTCCTTAGGGTGACAGGCCCAGA
GGGGAGGAGAGGAAGGTGCTAGGC
>1110
GATCACCAATGCCTGGGAGGCTTCGGCTGCCGTGGTCTTTGCCAGCACGGACAGAGCC

CTGCAAAACAAGGTGGGCCCACCCAGAGAGGGGTTCAGCCGGATCCTGGGCTGCTGT
CATTTCTGTTTCTAGGGCCTTCTCT
>1111
CTTTGAGGACTGTCGCATCCCCAAGGACAGCATCCTGGGGGAGCCAGGGATGGGCTTC
AAGATAGCCATGGTGAGCCCGGCAGTGGGGGTGGCACCTTGAGGCCAGGCCCGCGCC
CCGGCTGGCGGGCCACTGACCAGGG
>1112
TTGTGCTGTGAACTACGCTGAGAATCGCATGGCCTTCGGGGCGCCCCTCACCAAGCTC
CAGGTCATCCAGGTAATGGTGGCAGCTTTAGGAGCTGGGCCTAGAGGCTGGACAGCGG
GCGGAGAGTGTGGCCTCCTGACTG
>1113
CCTGGAGAGTGCCCGGCTGCTGACCTGGCGCGCTGCCATGCTGAAGGATAACAAGAA
GCCTTTCATCAAGGTGCCCACAGGGGTCCCCGAGCCATGGCCCAGAATGTGGTGGGCC
CAGGGACGGGGAGAGGTTGGGGCGG
>1114
CACAGTGAACCACAAACAGTTCTCCCTGAACTGGACTTACCAGGAGTGCAACAACTG
CTCTGAGGAGATGGTGAGTCCTGGGCTGAAGGCAGGGGCAGGGGAGGAGGTAGGAG
CCCCATGGCATGAAGCCCCTGGTCCCA
>1115
TGCTACATCATGAACCCCCCTGACCGCCACCGTGGCCATGGCAAGATCCATCTGCAGGT
CCTCATGGAAGGTGAAGGCTGGGGTGCCAGCTGCGGGGCCCTGACCTTTCCCACCCA
CCTACTCTCTGCGGCATTTGCTCA
>1116
GTATCTCAGAAAAGAAGACATGGAATATGCCCTGCGTAAACTGGATGACACCAAATTC
CGCTCTCATGAGGTGGGTCCTTGCCTTCTGTTGAGGAGCTTGTACTCAAATCACTCCAC
TTGAACCTGACCTAGCAGAGGTA
>1117
GCACCAGGACATGCACAGCTACATCGCGGCCTTCGGGGCCGTCACGGGCCTCTGCACG
CTCTTCACCCTGGTCAGCCGCGGGAGGGCAGGCCCCGGGGGCCCTCAGCCTGGAACG
TGGGAAGAGAGCCAGAGGGAAGGGG
>1118
AGTTCATGGATGGTGCCCGCCGAGAGATCGTCTGCCGTGCAGATGGCACCATGAGGCT
TGGGGAGCCCACGTAGGTGTCTTGGGGACCCAGAGGTGAAGGTACAGGGAGGAAGG
CTGAAGTGTGCGTTTACCCCAAAGGG
>1119
CTCCTACTTCCACCTGCTCACCTGGTCACTCCCCTTTGTCCTCACTGTGGCAATCCTTG
CTGTGGCGCAGGTATAGTGACTGGTAGGAACGGGAGACCTGGATGGGGTGAGTTTGA
GGGAGGGGGCCAGTAACCCACCTT
>1120
TACCGTGCGGGCTTCGTGCTGGCCCCAATCGGCCTGGTGCTCATCGTGGGAGGCTACT
TCCTCATCCGAGGTGAGTGAAGACCAGGCCAGGACCAGTTGGGCAACAAAATGTACT
GGGCACTTGCTGCCAGTACTGGGAG
>1121
AAGAGCAACCACCCCGGGCTGCTGAGTGAGAAGGCTGCCAGCAAGATCAACGAGAC

CATGCTGCGCCTGGGTGAGTGGCCCCGGGGGACTTCGGTCTGAGGTCCTGGCCAGCCC
AACACTGCACCCTCCTGGGGCTATGC
>1122
CCTTCAGCTGCCACTTCTACGACTTCTTCAACCAGGCTGAGTGGGAGCGCAGCTTCCG
GGACTATGTGCTGTGAGTGAGGGGCATGGAGGCGGCAGTGCTGGGAGCTGGAGCCAA
GGCCATAAAGACACCCACCTCCACC
>1123
CTGGCATCGCCATGAGCACCTGGGTCTGGACCAAGGCCACGCTGCTCATCTGGAGGCG
TACCTGGTGCAGGTGGGCATGGCAGCCAGCCCCTCCTGCCCTGCCCGCCTCACCCTCA
GCCTTGGGACCCCATCTTTAGGTT
>1124
GAGCTCCTGCAGAACCCAGGCCAGGAGCTGTCCTTCAGCATGCACACTGTGTCCCACG
ACGGGCCCGTGGGTGAGCCTCACCCCTCCTCTACCGGAGCCGCCTGGCCCCGCGCTGC
CCATGTGCTAGTCTCTCCCAGCCT
>1125
ACCAAGATGGTGGCTCGGAGAGGAGCCATACTGCCCCAGGATATTTCTGTCACCCCTG
TGGCAACTCCAGGTATGAGAGTTCAAGCTTCTGGAGGAAGGTGGGGGGAGCACAGAG
GCTGGGGGCTTCTGGGACTGGAGTA
>1126
CCACCGTACCCCTACTGCATGCAGCCATCCGGGAGGTGGATATCAAGGGCGTGTTTCGA
TACTGCAACACGTGAGTATGCTGTGGGTGAAGCCGGGATGCCCAGCCTCCAGCAAGAC
CATGGCAGGCCCCACTCAGCCTC
>1127
CCTTTTGGCAGTGGTTTGATGGTGTCCTGGACCTCACCAAACGCTGTCTCCGGAGCTA
CTGGTCTGACCGGTGAGTCCCCACCCTGGGTAGTTTGAGCAGCCATACACCAGTCACC
TCCATACTCACTGCCCATGCCCCA
>1128
TTTCTCCTCCGCTTCAGCGACTCAGAGATTGGGGGCATCACCATTGCCCATGTCATCCG
GGGCCAGGATGGTGAGGCCACCCCAGCCAGTCCTCTGTCTCTGTGCCTGTGCCCTCTG
GGGTTTCTTCTGGGAATGAAATG
>1129
GATCTTGCTCAGCTCAAAAATCTCTATCCCAAGAAGCCCAAGGATGAGGCTTTCCGGA
GCCACTACAAGCGTGAGCTGGAACTGGCAGCTCTGATTCCTTCCTGTCACCCACTTCC
TGCCATGCTCCCCGCTGCCATCCT
>1130
TGGTGCCTTCTTATGACCTTGGAATGGCCCCTGATTCCTCCATGAGCATGCAGCTTGGC
CCAGATATGGTGTAAGGAGCTGGAAAGACAGGAATGGGAGTGGTCTGTGCAGATGGG
CTAATCTTAGCATGGGCAGCTGGG
>1131
CTCACTCCATCCCCCCGTATCAAGGCCTCTCCCCAGAAGAATCAGTCAACGTGTTGTCA
GCCTTCCAGGAGTAAGTGAAAAACCTCATGGGGATACCATCCCACTCTAAGGGGGTGG
GCATTTGAATTGTTAGAAGAGGC
>1132
GCTCAGATGTGACCATGGTGGAAGACAGCTGCCTGAGCCAGCCAGTGACAGCGTTTC

CTCAGGGCACTTGGTGAGTGGCAGCTTGGGAGTGGAGGCTGGGTGGCATCTAGGGGA
GTGGGCGCCATGCCTACTCCACTGCT
>1133
TTACTGGTGTCATCCCCGGAGCACCCAAAACGCTGGGGCCAAGTCAGCGAGGCCTAC
GTGAGCCTCCTAGGTATGCCTTTGTCTGTCTCCGTCAGGAAAGGTGTGGGAGTGGATC
TGGGACGGGGCCATGACCTGTCAGA
>1134
CGCCATCACCCGCAAGAGAGAAGCTGACCAGGCCGGCCGCGTCCCCTGCTGTGGGCG
AGAAGCTGAAAAAGTGAGCGGGGCGGGTCTGGAGGAAGAGGGCTGCCAATCTCGGG
TGGGGAGGGTGAACATCACTGTTTTCT
>1135
CGAGAGAAGGAGAGAGAAGAGTGGGAACGCCAGTACAGCCGGCAGAGCCGCTCACC
CTCCCCCCGATACAGTGAGTGTCCCCACCAGGCTGCAGGGCTCTGGGGCTTTAAAGAA
AAGTGTCAAGCAGGGTTACCAAAAAA
>1136
TTTTTGTGTTGTTTGGAGCAGAAATACTAAAGAAGATTCCGGGCCGAGTATCCACAGA
AGTAGACGCAAGGTAAGGATGCTTGCTCCTGCACTGGATGGGCTGGTCAGGTGTCCAC
AGTTGCACACGTGGATGCCAACAT
>1137
AAGCTGGGATCAGCAAGGACCGAATTCTTATAAAGCTGTCATCAACCTGGGAAGGAAT
TCAGGCTGGAAAGTAAGTGTCCCCCACAAGGAAGGAGCTCTCAGAGATTTTTCCTCTG
TTGGAGCAGGGTCTACATGGCATC
>1138
TTGGGCGCATCCTTGATTGGCATGTGGCAAACACCGACAAGAAATCCTATGAGCCCCT
GGAAGACCCTGGGTGAGGGTCCCTCTGTGGTAATGGGGTAAGGGGAGCAGCCTCAGC
AGCACCTGAGGCAGGAAGAGCCCGA
>1139
TCACCCAAGCTCCTGGGAGAGCTGCTGCAGGACAACGCCAAGCTGGTGCCTGTGCTC
TCAGCCAAGGCGGGTGAGGCCCCACTGCCCAGCTGTGGCTCCTGCTCGGGCAAGGCC
AGCACTGCCCTGCCACGCTGCCCTGT
>1140
TGTGGAGAAGCTCTCTGACGGGATCCGCAAGTTTGCCGCTGATGCAGTGAAGCTGGA
GCGGATGCTGACAGTGAGTGTTGTGTGTGGGTACCTACATATGCCAACCCCTATGAGAG
CCCGGGCCTGGGCGTCGGGGTTCA
>1141
GAAGAAGAAAGCAAAAAAGGCCTCAACCAAAGATTCTGAGTCACCGTCCCAGAAGA
AAAAGAAGAAAAAGGTAGAGAGTTCCTGGGGTGTCTCAGGCCAGAAAACAGACCCA
AACCCAAGCCCCCTCCTACACACCCAGA
>1142
GTACATGCGACAGTTCGTGGCTCACCTGCAGGAGACCAGCCCGCTGAGGGATGCCGTC
GTCATCGAGCAGGTCTGGGCACTGCCCTGCAGGGTTGGGCACGGACTCCCAGCAGTG
GGTCCTCCCCTGGGCAATCACTGGG
>1143
AGGCCAGCAGTGGCCTCTTCGACGTCTTCCTACGCTTCATGTGCCACCACGCCGTGCG

CATCAGGGGCAAGTGAGTCAGGTGGCCAGGTGCCATTGCCCTGCGGGTGGCTGGGCG
GGCTGGCAGGGCTTCTGCTCACCTC
>1144
CCACGCTGCTCTGCAGCCTGTGCTACGGCGACATGGAGAACAAGCTGTTTGCGGGGAT
TCGGCGGGACGGGTGAGGCCTCCTCTTCCCCAGGGGGGCTTGGGTGGGGGTTGATTTG
CTTTTGATGCATTCAGTGTTAATA
>1145
TGCTCCTGCGTTTGGTGGATGATTTCTTGTTGGTGACACCTCACCTCACCCACGCGAAA
ACCTTCCTCAGGTGAGGCCCGTGCCGTGTGTCTGTGGGGACCTCCACAGCCTGTGGGC
TTTGCAGTTGAGCCCCCCGTGTC
>1146
ACGGCCTATTCCCCTGGTGCGGCCTGCTGCTGGATACCCGGACCCTGGAGGTGCAGAG
CGACTACTCCAGGTGAGCGCACCTGGCCGGAAGTGGAGCCTGTGCCCGGCTGGGGCA
GGTGCTGCTGCAGGGCCGTTGCGTC
>1147
GAGGAACATGCGTCGCAAACTCTTTGGGGTCTTGCGGCTGAAGTGTCACAGCCTGTTT
CTGGATTTGCAGGTGAGCAGGCTGATGGTCAGCACAGAGTTCAGAGTTCAGGAGGTG
TGTGCGCAAGTATGTGTGTGTGTGT
>1148
ACATTTTTCCTGCGCGTCATCTCTGACACGGCCTCCCTCTGCTACTCCATCCTGAAAGC
CAAGAACGCAGGTATGTGCAGGTGCCTGGCCTCAGTGGCAGCAGTGCCTGCCTGCTG
GTGTTAGTGTGTCAGGAGACTGAG
>1149
GAAGCCATCCACTTCTGATGATTCTGACTCTAATTTTGAGAAAATTGTTTCGAAAGCAG
TCACAAGCAAGGTGAGTGTTGATCCTAGTCAGTCCTTTTGCTGTAGATGTTCTGAAACA
CGTAACTAAGCCATTGTTCTTA
>1150
GTGGCCCTCACTTCCAGACCTGCACCGGGTCCTAGGCCAGTACCTTAGGGACACTGCA
GCCCTGAGCCCGGTGAGTGTGCTTCCCTCCCCTGTGCCCACCACCAACCCTGCCTGGT
ACTGGATCCTTGCCCCAACAATAC
>1151
GCTGGACCTGGACTTTGACCCCCTCCCGCCCGTGACGAGCCCTGTGAAGGCACCCAC
GCCCTCTGGTCAGGTTGGTTGTGCCCACCACTGCCCATGGGCCCACCAGCTTCCAGGT
GCCCAACCCTGGGCTCATGTTGCCT
>1152
GCCCAGCGCTGCCGAGGGCACCTTTGCTGTGTCCTGGCCCAGCCAGACGGCCGAGCC
GGGGCCTGCCCAAGTAAGTGCCCACCTCCAGCCCCTGTCTGGCTTGTCCCCAGTCTCT
AGGGGTGCAGCATGGAAGGAGAGCC
>1153
GGCGGGTGGGACCCAACCTGCGGCTGGAGCCCAGGAGCCAGGGGAGACGGCGGCAA
GTGAAGCAGCCTCCGTAAGACAGCAGGGACAAAGCCCTGCCTTTTCCTCCCTGCCGCC
CGCCTGCCTGTCCGGGGCTCCCCTGT
>1154
TGTGAATGGCACCGTGGAGGGCGGCTGTGGGGCCGGGCGCTTGGACCTGCCCCCAGG

TTTCATGTTCAAGGTGAGCCCACAGCCTCTGACTGCTGCAGTCCCTCGGTGCCCTGGT
GGGCAGATGACAACCCTGAGCCTCA
>1155
GGCGGACTCTGTGAGGAAACAAGAAGAGAGGCCCAAGATGGAGACGGCGGCGGCTG
TAGCGGCGTGACAGGTGAGGGCGGGCCCGGGAGGGCTCGGTTTCTGGAGCGGCTGCC
GGGCACGGGCAGGGAGCCCGGACCGAA
>1156
CAATGGGGCCGAGCCATCCCGGGCAGTGGGCACCGTCAAAGTATACCTGCCCAACAA
GCAACGCACGGTGGTGAGTCATGGAAGCGAAATGGCAGGGGCTGTGGATGGACCCAG
TTGTAACTCTGGGATCAAAAGGGTGA
>1157
CTCTAGACAAGGCCCTGAAGGTGCGGGGTCTAAATCAGGACTGCTGTGTGGTCTACCG
ACTCATCAAGGGGTGAGTGTGGCAGCCCCACGCCCACCCACTGGGTGTCCCACCTCCC
ATCCCCTCCTCAGTCATTTGATTC
>1158
TGCTCCCCTGGATGGCGAGGAGCTCATTGTCGAGGTCCTTGAAGATGTCCCGCTGACC
ATGCACAATTTTGTGAGTGCAGGGTGGACGGTGGGGGTGGACCATGGTTGGGGGTGTC
CTTGACCAGGTCTCAAACTTCCCT
>1159
ACAAGTTCCACCAGCATTGTTCCTCCAAGGTCCCCACAGTCTGTGTTGACATGAGTAC
CAACCGCCAACAGTGAGCCCAGCCTGGGGTGGGTGGGGGGATATGGGGAGCACAGAG
GCCCAGCCACGAGGCCCTTACAGAC
>1160
GAGGCTCCAGACAGCATGAGGCTCCCTCGAACCGCCCCCTGAATGAGTTGCTAACCCC
CCAGGGTCCCAGGTAGGGATGCCTTAGCTGAAGAGCTGCTGGGGGAGAAAAAGATCT
TGGGGCTCTTGTAGACCACGAGCCA
>1161
CCGCTCCACGTCCACTCCCAACGTCCATATGGTCAGCACCACGGCCCCCATGGACTCC
AACCTCATCCAGGTTGGTGCTGTGGGGGACAATGCCGGGGACCACAGGGCAGAGGGT
AGAGCCATCCCTGTTGGCCTCCATG
>1162
GTCCCCACATTCCAAGTCACCAGCAGAGCAGCGCGAGCGGAAGTCCTTGGCCGATGA
CAAGAAGAAAGTGGTATGCTCGAGGGGGATCCTTTTGGGGCCACCAAGGCTGAGTGA
CGTGGGATGAGGGATGTGGGCAGGCC
>1163
AGGTGCTCAAGGTGTCCCAGCCCACAGCTGAGCAGGCCCAGGCTTTCAAGAATGAGA
TGCAGGTGCTCAGGTGAGGTGGCATGTGTGCGTGGATGGGGGTGGCAGGCCCGGCCT
TGGCATCTCTCTGGGAAAAGGCCATG
>1164
ATGTGGCCGACACACGCTTCGACATGGTCCAGCTCATCGACGTGGCCCGGCAGACTGC
CCAGGGCATGGAGTGAGCCTCCCAGCCTGGGGAGGGGTGGGGGAAGGGTGGGGGAA
ATGAGGTAACCCCCAGCCAATCCGCC
>1165
GGCCACAGTGAAGACTCGATGGAGCGGGGCCCAGCCCTTGGAGCAGCCCTCAGGATC

TGTGCTGTGGATGGTGAGTTGGGCCAGGCTGAATGGGGGGCACATGGGGACGTGGGC
TCCGGGATTGGGGAGTGTGGGGCCAA
>1166
CTACGGGGTTGTGCTCTACGAGCTTATGACTGGCTCACTGCCTTACAGCCACATTGGCT
GCCGTGACCAGGTGAGCCCCACACCTTACCCACAGTTCCCTGGCCTGAGGGATCCCCT
CAGGCATCCATACCCCACATCCC
>1167
CAAGGCCATGCGGCGCCTGCTGTCTGACTGCCTCAAGTTCCAGCGGGAGGAGCGGCC
CCTCTTCCCCCAGGTGGGCTGGGTAGGGGGCTGGACACCTTGGGTGGGTGACTCTGGG
TTAGAGGAAGACTGAGATGGAGGCA
>1168
TGAGCACCTGGTTACCACTGCCACCTTCTCCATCGGCTCCACTGGCCTCGTGGTGTATG
ACTACCAGCAGGTGGGTATGCCAGACCTCCTGACCTGGACCAATGACAAGGCTCTGCT
AGAGCCACCCAGCTGGGCCACTT
>1169
CATCATGAAGATAGCTCCAGAGAGCATCCCCAGTCTTGAGGCTCTCACTAGAAAAGTC
CACAACTTCCAGGTGTGTGTGTGTGGGTGAAAAGAGTGGGCTGTCTCCCTCCCAGGG
CTGCTGGGAGGAGTGTCCGAATGGT
>1170
TACGTGTGCCGGGTCCAGGAGGGCAACGAGTCATACCAGCAGTCCTGCGGCACCTACC
TCCGCGTGCGCCGTGAGTGGCCCAGCCCTGGCCCCTACTCCCACTGTCCCGCTGGGCA
CTCGGTTTATCTTTGAAGTGGGGA
>1171
AATCATCACAGCCGAGGGGATCATCCTCCTGTTCTGCGCGGTGGTGCCTGGGACGCTG
CTGCTGTTCAGGGTGAGCCCCCTCGGACCTCTGAGTCAGCCGGGCGAGGGCTGGGCG
AGGGACCCCCAATACCCAGGTAGCC
>1172
AGGAACCCTGCGGAGGGACTTCAATCACATCAATGTAGAACTCAGCCTTCTTGGAAAG
AAAAAAAAGAGGGTGAGGGTTTTTCTTCTGATAATTCAGTTGCTCGTTGGAAGGTGGT
TTATAACAGCATCACATTTGGTTG
>1173
CAGAAAGGAACTGGCTACCGTTCGGACTATTTGTAGTCATGTACAGAACATGATCAAG
GGTGTTACACTGGTAAGCAGATGTATCAGACTTCCTTGTTTTGGAAAGGGAGGTTTCTC
AAACCTGTGCTTTATACAGCGTA
>1174
AATGGGTCTCTTGTTGAAATCCGAAATTTCTTGGGTGAAAAATACATCCGCAGGGTTCG
GATGAGACCAGGTATGTGCATACCCTCAGACAGAATTAAGAAATTTTTCCTTCATATTTT
TTTCCTCCTTTTCCACTCCCA
>1175
TCAGTATCTCAAGCCCAGAAAGATGAATTAATCCTTGAAGGAAATGACATTGAGCTTGT
TTCAAATTCAGGTTTGTATGTTTACTATGTCTAACTATGCCTACAAGATTTTGTCTAAATC
TTGTTTAAAATACAGTATTT
>1176
AAATTTTTGGATGGTATCTATGTCTCTGAAAAAGGAACTGTTCAGCAGGCTGATGAATA

AGATCTAAGAGGTAAGTTCTTACAGTGTCTTAAGTTTTATTACTGCTCTAGTTGAATGAA
GCTCTTAATTTACTAAAAGAC
>1177
TCAGGGGACAGGGAGAGCAAGAGGTCAAGAGCTGTGGGACACCACAGAGCAGCACT
GAAGGAGAAGACCTGTAAGTTGGCCTTTGTTAGAACCTCCAGGGTGTGGTTCTCAGCT
GTGGCCACTTACACCCTCCCTCTCTC
>1178
TTAGAGAACAGGCCAACCTGGAGGACAGGAGTCCCAGGAGAACCCAGAGGATCACTG
GAGGAGAACAAGTGTAAGTAGGCCTTTGTTAGATTCTCCATGGTTCATATCTCATCTGA
GTCTGTTCTCACGCTCCCTCTCTC
>1179
AGCTTCTGCGTCTGACTCGCAGCTTGAGACTGGCGGAGGGAAGCCCGCCCAGGCTCTA
TAAGGAGACAAGGTGAGATGCTGAGGGAGGACTCAGGAGGACCCCCACCCCACATAG
ACGACCACAAAAAATCCAACACCAC
>1180
GAGCTGCTCTGTCTGACCAGCAGCTTGGGATTGGCGGAGGGAAGCGGGCCAGGCCCT
GTGAGGAGTCAAGGTGAGACGCTGAGGGAGGACTCAGGAGGCCCCCACCCCAGATAG
ATGACCCCAAATAATCCCGCACCACT
>1181
GAGCTGCTCTGTCTGACCAGCAGCTTGGGATTGGCGGAGGGAAGCGGGCCAGGCCCT
GTGAGGAGTCAAGGTGAGACGCTGAGGGAGGACTCAGGAGGCCCCCACCCCAGATAG
ACGACCCCAAATAATCCCGCACCACT
>1182
AAGCGAGCTGCTCTGACCAGCCGCTTGGGATTGGCGGAGGGAAGCGGGCCAGGCCCT
GTGAGGAGTCAAGGTGAGATGCTGAGGGAGGACTCAGGAGGCCCCCACCCCAGATAG
ACGACCCCAAATAATCCCGCACCACT
>1183
GCGAGCTGCTGTCTGACCAGCAGCTTGGGATTGGTGGAAGGAAGCAGGCCAGGCCCT
GTGAGGAGTCAAGGTGAGACGCTGAGGAGGACTCAGGAGGCCACCACCCCAGATAG
AAGACCCCAAATAATCCCGCACCACTC
>1184
GAGCTGCTCTGTCTGACCAGCAGCTTGGGACTGGTGGAGGGAAGCGGGCCAGGCCCT
GTGAGGAGTCAAGGTGAGACGCTGAGGGAGGACTCAGGAGGCCCCCACCGCAGATA
GACAATCCCAAATAATCCCGAACCACT
>1185
TCTGAGCAGACAGGCCAACCGGAGGACAGGATTCCCTGGAGGCCACAGAGGAGCAC
CAAGGAGAAGATCTGTAAGTAAGCCTTTGTTAGAGCCTCTAAGATTTGGTTCTCAGCTG
AGGTCTCTCACATGCTCCCTCTCTC
>1186
TGACCAGGATCACCAGGAAGCTCCAGAGGATCCCCAGGAGGCCCTAGAGGAGCACCA
AAGGAGAAGATCTGTAAGTAAGCCTTTGTTAGAGCCTCCAAGGTTCAGTTTTTAGCTG
AGGCTTCTCACATGCTCCCTCTCTC
>1187
TCAGCGGACAGGCCGGCCAGGAGGTCAGAAGCCCCAGGAGGCCCCAGAGGAGCACC

GAAGGAGAAGATCTGTAAGTAGGCCTTTGTTAGGGCCTCCAGGGCGTGGTTCACAAAT
GAGGCCCCTCACAAGCTCCTTCTCTC
>1188
CTGTGAGCGAGGACCCGGTTCCGCCCGAAGGATGAAGACTTGTCTTTAAAGATGACAA
CGTACAGGAAAGGTCCATCAGCCGATCTCCCCCTGCCTGGGCCCACAGCGCCCCCCAA
ACCCTCACCACCCTCTCTCACTGC
>1189
TCTTCCTACCCATCTGCTCCCCAGAGGGCTGCCTGCTGTGCACTTGGGTCCTGGAGCCC
TTCTCCACCCGGTGAGTGGCCAGCAGGGTGTGGGGTTATGTGAGGGTAGAAAGGACA
GCAAAGAGAAATGGGCTCCCAGCT
>1190
GGCGGCACGGCTCGGAGGCAGCGGTTGGGCTCGCGGCGAGGACGGGGTCGAGTCAG
TGCGTTCGCGCGAGGTGAGAGCGGGCAGGGCGCGTGTGCGGTACCTTGGCTTGGGCC
CGGGAGAAGATGGAACTGCGCGGGGGT
>1191
GCTCAAAGGCCGGCCATGTAAGATCGTCGAGATGTCTACTTCGAAGACTGGCAAGCAC
GGCCACGCCAAGGTTAGAATTTCACCTCCGCATCTTGCCTTCCCCATGCCTCCAGTATC
TTTGGCCCCTCCCAGCAGCAAGC
>1192
GAAATATGAAGATATCTGCCCGTCAACTCATAATATGGATGTCCCCAACATCAAAAGGA
ATGACTTCCAGGTATGTAGATGGTCTGGATGAGGATGGGTTAGCGGTTTATGGTGGAGG
GAGGGGTTGGGGGATAGGGACT
>1193
CCTTCGTCTCCCTGAGGGAGACCTTGGCAAGGAGATTGAGCAGAAGTACGACTGTGG
AGAAGAGATCCTGGTATGGTGCCTCCCTCCCTGCTTTTGTGCTCAGCTTTGTTCTGTAC
GTTTCTTCCTGAGCTCAGACATCT
>1194
CGGTGCTGTCTGCCATGACAGAGGAGGCAGCTGTTGCAATCAAGGCCATGGCAAAATA
ACTGGCTCCCAGGTGAGTGTGACAAATCCCTCACTGTCCCCTTCACATTTTGTTGTCCT
CAGCAGAGCTGCTGTAGTCTTAA
>1195
TTTACTCGCACAGCCTCCTGCCTGTGGACATTCGACAGTACTTGGCTGTCTGGATTGAA
GACCAGAACTGGTGAGGCCTTCAGGAAGTTGGGGGAATGAAAAAGGTGGCCTTCCAC
TTCTGGGCCCCCGGGATCCTGGAA
>1196
CCGTTGCAGCCAGGACCCAGAGTCCTTGTTGCTGCAGCACAATTTGCGGAAATTCTGC
CGGGACATTCAGGTACTTGGAACGGTTGGGAGTGATGGGGTAGCACTGGGAGCAGAG
CATAGAGGAGTAAGGTTTGGAGAAT
>1197
GGCTGAGATGATCTTTAACCTCCTTCTGGAAGAAAAAAGAATTTTGATCCAGGCTCAG
AGGGCCCAATTGGTGAGGACAATTCAGTGGTAATGTTGGAAACTCCTGAAGTAGAGAG
GAACCATGGAAAGGACTCAGGGAG
>1198
CGAAACACCTGTGGAGAGCCAGCAACATGAGATTGAATCCCGGATCCTGGATTTAAGG

GCTATGATGGAGGTTAGTAGATGTGGTAGGAGTTAGGGTTGACAGTGTTCAGCCTAAC
ACCTCCCTGAGAAGCAGCCTCATC
>1199
GTAAAATCCATCAGCCAACTGAAAGACCAGCAGGATGTCTTCTGCTTCCGATATAAGAT
CCAGGCCAAAGGTAGGAAGCACATTGAGGGGCTGGAGAAAGATAAGTGCCTGCTGAG
AAGCCGGAGCTGGAAGTGAACAGG
>1200
GGACCCCCATCAGACCAAAGAGCAGAAGATTCTGCAGGAAACTCTCAATGAACTGGA
CAAAAGGAGAAAGGTGGGAGGCAGCAGAACAGAACATGTGGGCAACAAGGACCTGA
AAAAATGAGGGATGTTGGGAACCCTGG
>1201
GGAAGGCCCAGCAGCAAAAAGCCTGCATCAGAGCTCCCATTGACCACGGGTTGGAAC
AGCTGGAGACATGGTGAGAGGTACCACCCCAACCCTCGTCCTCGCCATGCGCTGTGAT
TTGTAAGTTGCAGTGCCCTGCATAT
>1202
ACCCTCTGACCAAAGGGGTGGACCTACGCAACGCCCAGGTCACAGAGTTGCTACAGC
GTCTGCTCCACAGGTCTAGAGGCCAGGCAGGAACCCTGGGGGAAAGAAGGAACAAG
GGAAGCCATTCTTACACATACTGAGCT
>1203
CCTGCATGCCCCAAACTCCCCATCGACCCCTCATCCTCAAGACTGGCAGCAAGTTCAC
CGTCCGAACAAGGTTGGCATTCCAGAACTCATTCCCACTTCCTTTTTCCAACCCTGCCA
CTGTGTATTTTCTGGCTTTACAG
>1204
TTCAAACCAGAAAACTTTGACCCCCGAGAAGGGGCAGAGTCAGGGTTTGATTTGGGA
CTTTGGTTACCTGGTAAGAATAGTTTGTGACCTATGCTTTTATTACTATTTTTATTTTTTC
GAGACGGAGTCTCACTCTGTCC
>1205
GACAGAGGAACTGCACATCATCAGCTTCACGGTCAAATATACCTACCAGGGTCTGAAG
CAGGAGCTGAAAGTGAGTGAAAATGGAGGGCAAGGAGAGAGAAAGCAGCTTTGGAA
GAAGGCATAAGAAGGGGATAAACAGA
>1206
CAACATGAACCAGCTCTCAATTGCCTGGGCTTCAGTTCTCTGGTTCAATTTGCTCAGCC
CAAACCTTCAGGTAGGGGAGTGGGGCCGACAGGTCCCGGCGCGAGAGCAGGGGTGT
GGAAGCTTGGTGTGATAGGTTGCTT
>1207
CAGTTCTCCTCCTATGTTGGCCGAGGCCTCAACTCAGACCAGCTGAGCATGCTGAGAA
ACAAGCTGTTCGGTACAGATTTCCTTTTCTCTCAGCCTTTCCCCAGCCTTAGTCTTTTCT
GTCCCTCTGTCCTATCTATCCC
>1208
CATTCTGGACATGGCTGGACAAAATTCTGGAGTTGGTACATGACCACCTGAAGGATCT
CTGGAATGATGGGTAAGGCCTTGGTCACCCTTCCCTCATGGGCTTGTGCTTCCGGGCTT
GAGAGTGGAGTCTCTGCACCCTC
>1209
TTTCTACTGCGCTTCAGTGAATCGTCAGAAGGGGGCATTACCTGCTCCTGGGTGGAGC

ACCAGGATGATGGTAGCTGCTCTGCCCTGCCATTCCCACAGCCTCTCCTTTCTGCCTGG
CTCTCCTCTGGCCCCTCTGCCTG
>1210
AACCCACTGCGCTTCCTCTATCCCCGAATCCCCCGGGATGAAGCTTTTGGGTGCTACTA
CCAGGAGAAAGGTGGGAATCGTTGACATACTTCATTGCTAGATTGCAGAGATCTACCA
GACATCCATAGATCCCACTCCTT
>1211
GTATCACAGCCAGTGCCAGAGCCAGATTTGCCCTGTGATCTGAGACATTTGAACACTG
AGCCAATGGAAAGTAAGTGATGAGATGGAGTGGCACACATTCCCTTTCCTACCTCTTCT
CCCTCTCCCATTACAGAAAAAGC
>1212
GGTCACCCGTGTATGGCACCCTGCCATACCAGCTCACCCTGCCCTGTGACTGCTTGTAA
AGCTAAGGTGGGTATTTCTGGCCACAGATGCAGCATTGACTGTAGTTCTGAGGCTACTA
GTGAATCCAGAACTGTCTACAG
>1213
AGTGTGAATGTGGACGAAGAAAAGAGATGGTGATTTGCTCTGAAGCATCTAGTACTTA
TCAAAGGTTAGTGTTACTTAAATGTTAACAACTGATGGCCTAGGTGAAACAGATACATG
TGTTTTGTTTTGTTTTTAAAGCT
>1214
CAGACATGCAGCTTGGAGGTTCAGTGGAGATCAGCAAGTTAATTACCAAAAAGGAAG
TTCATCAAGCCAGGTAATTTTTAAAATGCATATATGTGCCTTCTTTCTTCAATTAGACTTC
AATTAGAGGGCAGAGATTATTC
>1215
ACCTTGCGAGCCGTCTTCCCCAGGCCTGCGTCCGAATCTCCGCCGCTGCGGGCCCGCT
CCGACGCGGAAGGTGAGGGCTGGGGGAGGGGACCGGCGCTGACGGAGCCGCAGTGC
GGGGTCGGGTCTGTGGCGGACAGAGA
>1216
TGTCCTACTGGTGGAGGCCGTGGTCGTGGAATGAGAAGCCGTGGCAGAGGTGGTTTTA
CCTCGGATAGAGGTATTTTGTCGAATAGAAAAATTTGAAGTACTTCAGTATTTGTTAGTA
TCAAGACTGGTCTGACTAGCCG
>1217
CTTCCAGATATTTGTTATCGCTGTGGTGAGTCTGGTCATCTTGCCAAGGATTGTGATCTT
CAGGAGGATGGTAAGTATTTAACACTTCCTTTTCATACCCCTCTAGAGCTTGGAGAGGT
GAGCACATGCAACTGTGTATA
>1218
AGCAGAAATGCTATTCTTGTGGAGAATTCGGACACATTCAAAAAGACTGCACCAAAGT
GAAGTGCTATAGGTAAGGTGTCAGAATGTTGTTAGAAGAAAACTCATTGCAGAGATTC
TTCCAGAGATGAATTAGCTATAAA
>1219
GACACCATGAGGAACAGCGCCTCTGGCCCCGGATGGTACGGCGCCAACCCAGACCCG
CGCTTCCCCGCCAGTAAGTGAGGCCGCCCCACTGCGGGGCCGCGGGCTGAGCTCAGG
AGGTGCGGCGAGAGGCTCCAGAAGGC
>1220
CGCCGAGCTCCAGCCCCGAGCCCTGGACATCTACTCTGCCGTGGATGATGCCTCCCAC

GAGAAGGAGCTGGTCGGTATTCCCCTCGCTCTCGACCCCCTTGACGTGTCGCCTTGTCT
CTTCTCTTGCACGCCTCCCTCCT
>1221
AGAAGTCTTGAAGAAGCTCAAGAGTAAACGTGTTCCCATCTATGAGAAGAAGTATGGC
CAAGTCCCCATGGTAAGGTTTGTGGTCACTCCCTTCCCGTGTTTTTCCAAGAGAAAGTA
CACCGCCTTGAATCGTACACACA
>1222
GAATAAGGCAAGGGGGAGTGTAGAGCAGAGCAGAAGCCTGAGCCAGACGGAGAGCC
ACCTCCTCTCCCAGGTATGTGACACTCCCCATCCCCCTTCAGAGGCCACACACCCTATG
GCATTCCCACCATGTGTTAAGGATT
>1223
GCAGGAACTGAACCCAAAGGATCACCTGGTATTCCCTGAGAGTACAGATTTCTCCGGC
GTGGCCCTCAAGGTTAGTGAGTGAGCAGGTCCACAGGGGCATGATTGGATCCTGGAAT
GAATGAATCAACCATGAGAGAGTG
>1224
TCTCAATGTCTGCATGAACGCCAAGCACCACAAGGAAAAGCCAGGCCCCGAGGACAA
GTTGCATGAGCAGGTGGGCCAGGGGGTGATCTGGGGTGGTGAGGGACTGGCTCAGGA
AGAGGAAACGAGGACATGGAAATGCC
>1225
CAAACGGCATTTCATCCAGGACACCTGCCTCTACGAGTGCTCCCCCAACTTGGGGCCC
TGGATCCAGCAGGTATGCATGGCTTCCTGCAGGTACAAGACCTAGCGGAGCAGCTGAG
CTTTCCAGGCATCTCTGCAGGCTG
>1226
TGGTGGGAAGATTGTCGCACCTCCTACACCTGCAAGAGCAACTGGCACAAGGGCTGG
AACTGGACTTCAGGTGAGGGCTGGGGTGGGCAGGAATGGAGGGATTTGGAAGTGGAG
GTGTGTGGGTGTGGAACAGGTATGTG
>1227
TCTGCCTGGCAGAGGAGTTTCTTCAGGAAGAACCAGGGCAGCTTCTGCCCCTAGAGG
GCCAATGCCCTTGGTGAGTGCAGTCCCCTGGCCCCAGCCTGGTCCACCTCTGGGAAGA
GGGTGCCCAGTTGTGCAATCCAGGC
>1228
TTGGGGAGACCTGATCTGGCTGTGCCAGATGGACACTGAGAAAGAAGTAGAAGACTC
AGAATTAGAAGAGGTGAGTGGGCTTTGGTGGCGGGCTCCCTACCCCACTCCCTGCCCT
GGGCTGCCTGTGACCACACTGCTTG
>1229
CTCCCTACACCACACCCATACTCAGCCCCTGCAGGCAAGGAGAGAACAGGTCAGGTTC
CCGAGAGCTCAGGTGAGTGACACGTTGGAATGGCCCAGGGCACCTTCACCCTGCTCA
GCTTGTGGCTCCAACATTCTAGAAG
>1230
GCACTGGGGCCCCTGCAGAGCTTCCAAGCCCGACCTGATGACCTGCTCATCAACACCT
ACCCCAAGTCTGGTAAGTGAGGAGGGCCACCCACCCTCTCCCAGGCGGCAGTCCCCA
CCTTGGTCAGCAAGGTCGTGCCCTC
>1231
AAGTGTAACCGGGCTCCCATCTACGTACGGGTGCCCTTCCTTGAGGTCAATGATCCAGG

GGAACCCTCAGGTGCATGGCTGGGTCCTGGGGGTAAGGGAAGTGGAGGAAGACAGG
GCTGGGGCTTCAGCTCACCAGACCT
>1232
CCCACGGCTCATCAAGTCACACCTGCCCCTGGCTCTGCTCCCTCAGACTCTGTTGGATC
AGAAGGTCAAGGTGAGGCCGGCCTCAATGGTTCACACCTGTCATCCCAGTTTGAGACT
GAGGAGGGAGGATCCCTTGAAGG
>1233
CGTATGGAAAAGGCGCACCCTGAGCCTGGGACCTGGGACAGCTTCCTGGAAAAGTTC
ATGGCTGGAGAAGGTGGGCTTGACTGGAGGAAGGAGGGTGTGAAGCCGAGGGGTGG
TGGCTATAACGTACAGCAACCCTGTGT
>1234
CGTGCAGGAGTGGTGGGAGCTGAGCCGCACCCACCCTGTTCTCTACCTCTTCTATGAA
GACATGAAGGAGGTGAGACCGACTGTGATGCTTCCCCCCATGTGACACCTGGGGGCA
GGCACCTCACAGGGACCCACCAAGG
>1235
CCTATGACCAACTACACCACCGTCCCCCAGGAGCTCATGGACCACAGCATCTCCCCCTT
CATGAGGAAAGGTGGGTGCTGGCCAGCACGGGGGTTTGGGGCGGGTGGGAGCAGCA
GCTGCAGCCTCCCCATAGGCACTTG
>1236
AGAGACAGCATCGTCGGGACCAGACTCGTCTCAGGCCAGTTGCAGCCTTCTCAGCCA
AACGCCGACCAAGGTACAGCTTCAGTTTGCTACTGGGTTGTGCATTCAGCTGAATTTCA
TGGGGAAGTCCAAATTCTAAGGAA
>1237
ATACCCAGATGCTGTGGCCACATGGCTAAACCCTGACCCATCTCAGAAGCAGAATCTC
CTAGCCCCACAGGTATTTTTAAACTTCTCATAATTAAACTACAGTGATGAAAGATAGCC
ACACTCAGGCCATTTGGGCTGCT
>1238
TGGCCGAGGTGATAGTGTGGTTTATGGACTGAGGTCAAAATCTAAGAAGTTTCGCAGA
CCTGACATCCAGGTAAATCCTTTAACAGACACACCTGATGGTTCTGACTAGCGCTCAAG
TCTAGGAAACCACAGTTTGCATA
>1239
ATGCACGGGCAAGGCGTGTGCATGGAGCTGGCGGAGATCGAGGCCATCCAGGAAAGC
CTGCAGCCCTCTGGTAAGGTACCCCTGCCTCCCAATTCCCTCCTGAGTAGCGCTCCCTT
CCCAGCGGCTTCTTCCCCATTCCA
>1240
TCGAGACCGGAGCACCAGTGGGGGCAAGATGAAGGTCAATGGGGCGCCCCGGGAGG
ATGCCCGGCCTGTGGTAAGGACCTCCGATGCACAAATGTGCATGTGCATAGACACACA
CACACACACATGCCCCCTGCCCCCCA
>1241
CACCCACGAGGACCTCTACTTCATCCCCATCCCCAACTGCGACCGCAACGGCAACTTC
CACCCCAAGCAGGTGGGTCTCTGTCTCCCGCTGGCTTGGCCCTGGACTCAGCTCTGGG
GCATGGTCTCTTTTCCTGCGTCGG
>1242
CACCCCATTGCCCTTCATTGGAAACTACCTGCAGCTGAACACAGAGCAGATGTACAAC

TCCCTCATGAAGGTGTCCCAAGACAGGGAGATGGGTGTCTCGGGGTGGGGGCTGCCTA
GTTGGCTGGGGCTTTGTGGCAGGG
>1243
GTGGACCAGGCTGAGGAGTTCAGCGGGCGAGGCGAGCAAGCCACCTTCGACTGGGTC
TTCAAAGGCTATGGTGCCCAAGAGGGGGAAGGTGGGCAGGTGGACACGAAGGTCTCA
GTGTTCCCAGCCTTCTCCCTGACTCT
>1244
AAGCGAGGCATCGAGGAGCGCATCCAGGAGGAGTCGGGCTTCCTCATCGAGGCCATC
CGGAGCACGCACGGTGAGCAGGGGACCCCGAGTGCGGGGGCAGGAGAAGGAAAACA
CCCAGGACGAGGAACCCGCGCGCGTTC
>1245
CAAAGAGTTCCTGTCACTGTTGCGCATGATGCTAGGAATCTTCCAGTTCACGTCAACCT
CCACGGGGCAGGTAATGGTTGCAGCCCGGCCCGTGAAGGCCCTTACCAAAACCGGCA
AATTGTTCCCCTACCGGGGGAAGG
>1246
CAACCAGCGCACGCTGGATCCCAATTCCCCACGGGACTTCATTGACTCCTTTCTCATCC
GCATGCAGGAGGTACACCCCAGCAGCCACTGCGGGGAGATGCAAAGCCAGGCAGAGG
GAAATCAGTCTGGGAGTGGGGCAG
>1247
GGCACCGAGACGGTCAGCACCACCCTGCACTATGGCTTCTTACTGCTCATGAAGCACC
CAGAGGTGGAGGGTAAGGCTGGAGGGGGACGGAAGTGGAGGGCCCCAGACCCTCAA
AATTCCCCTTCGACTGGTGCAATGTC
>1248
CGTGATCCCCATGAGTTTGGCCCGCAGAGTCAAAAAGGACACCAAGTTTCGGGATTTC
TTCCTCCCTAAGGTGCTATCCGCCCCCACCCCCCAGACTACGGGGACTCCAGCCCCTCT
CTGTGTCCCCAGCATCCCACCCA
>1249
AATCCCCAGCACTTCCTGGGTGAGAAGGGGCAGTTTAAGAAGCGTGATGCTTTTGTGC
CCTTCTCCATCAGTAAGAGACCACTGTTTGGTGCCAGGCTTACTACTCACACCAGCAG
GGGCCTCCCTTACCCAGTTCCCCT
>1250
CACCCCATTGCCCTTCATTGGAAACTACCTCCAGCTGAACACAGAGCAGATGTACAAC
TCCCTCATGAAGGTGTCCTAAGGCAGGAGATGGGTGGCACGGGGTGGGGGCTGCCCA
GTTGGCTGGGGCTTAGTGGCAGGGG
>1251
GTGGACCAGGCTGAGGAGTTCAGCGGGCGAGGCGAGCAGGCCACCTTCGACTGGCTC
TTCAAAGGCTATGGTGAGGGGGTGCCAAGAGGGGGAAGGTGGTCAGGTGGATGCAAT
GGTCTCCGTGTCCCCAGCCTTCTCCC
>1252
AAGCGCGGCATCGAGGAACGCATCCAGGAGGAGGCGGGCTTCCTCATCGACGCCCTC
CGGGGCACGCACGGTGAGTAGGGGACCCCGAGTGCGAGGGCGGGAACCCGCGCTTTC
TGCCTGGGGATGGGGACTAGGTGGGG
>1253
CAAAGAGTTCCTGTCACTGTTGCGCATGATGCTGGGAAGGTTCCAGTTCACGGGAACC

TCCACGGGGCAGGTAACTGGATGCAGCCCGCCAGTGACGCCCCTACCACAACCTGCC
AACTGCTCCCCTACCTGGAGACAGG
>1254
CAACCAGCGCACGCTGGATCCCAATTCCCCACGGGACTTCATCGACTCCTTTCTCATCC
GCATGCAGGAGGTACATCCCAGCAGCCAGTGCAGGCAGGTGCAAAGCCAGGGAGAGG
GAAATCAGGATGGGAGTGGGGTGG
>1255
GGCACTGAGACCGTGAGCACCACCCTGCGCTACGGTTTCCTGCTGCTCATGAAGCACC
CAGAGGTGGAGGGTAAGACTGGAAAGGGAGGAAAGTGAAGGGCCCCAGACCCTCAA
AACTCCCCTGAGCCTGGTGCAGTGTA
>1256
CATGCTCCCCATGGGTTTGGCCCACAGGGTCAACAAGGACACCAAGTTTCGGGATTTC
TTCCTCCCTAAGGTGCTGTCTCCCCTCCACCACCACCACTCAGACTACGGGGACTTCC
AGCCTCTCTCTGTGTCCCCAGAAT
>1257
AGTCCCCAGCACTTCCTGGATGAGAAGGGGCAGTTTAAGAAGAGTGATGCTTTTGTGC
CCTTTTCCATCGGTAAGAGACACTGTTTGCTGCCAGGCCACGGCTCACACCAGCAGGG
GCCTCTCTCACCCACCTCCCCTCT
>1258
CACGCCGAACTGCATGCCGAGCCCCGGGGTGATGACTGGAGGGTCAGCCTGGAAGAC
CACAGCAGCCAAGGTGAGCATTAAGCAGGGCAGCTTTGCCCCTGGGTGGTTGAAGCG
CCAGGCTGGAATGAGTAAGGTCTCCA
>1259
ACCCGCCACCGGTCCTTATGGAGCCCAGGAAGAAACTCCGTGTAGACAAAGCCCCAC
TGACTCCCACTGGGTAAGTGGAGTCCTCACTTGGCCCTCTCAGTGTTTTACTGCTTTTC
GATTCCTTGTATCCCTAGGCTGTG
>1260
GCTGCAGTTGGAGCCGCCCACCGTGGTGGAGACCCTGCGGCGCGGGAGTAAGTTCAT
CAAATGGGACGAGGTAAGCGCGCGGGCCCCTGCTCCGCCCAAATCCCGGGACTCTTTC
AGTCAAGCTCGACCAGACGCTGGGG
>1261
CAGTCGGAACCTGGTGACCCTGCGTGTGGACCCCAATGGCTTCTTCTTGTACTGGACG
GGCCCCAACATGGTGAGGGTGGGCGCTGGTGCAGCTCGCTCAGGCCAAGGACCCCTG
TCCCAGACCCCTGCCCTGCCCGTGG
>1262
GGTGTCTGGGCCAGACCCGGTGAACACAGTGTTCTTGAACTTCATGGCCGTGCAGGAT
GACACAGCCAAGGTGGGCTGGCACCAAGGGGACGAAGGGGGAGTCACTGTCTTATTC
TGTGAGTCGGCCGTCACTTACCGAA
>1263
AGGAGCTATTCAAGCTGGCTATGAACATCCTGGCTCAGAACGCCTCCCGGAACACCTT
CCTGCGCAAAGCGTGAGCCCCAGGCCACCCGAGGGGGAGCCGGGGGGTTCACGTGG
CCGTTTTCAGGGTGTGACCTCTTCAT
>1264
GAAGATGTTCTCAGCAGACAAGAAGCGGGTGGAGACTGCGCTGGAATCCTGTGGCCT

CAAATTCAACCGGGTGTGTGGGGTGGGGACAGGGGCGGGGTGTGGTGTGTCACGGTG
GACACCCACCCTTACGGGGGTGCCCG
>1265
TGGAAATCTTTGAGCGGTTCCTGAACAAGCTGTGTCTGCGGCCGGACATTGACAAGAT
CCTGCTGGAGATGTGAGTGGGCCCGGCCTGCCTCACAACCCCTGTGCTCTGTGTTTAG
CTTCTGCTTAGCGTGCCTCTAGCT
>1266
CCCCTGCGGCCCTCCCAGGCCCGGCTGCTCATCGAAAAGTATGAGCCCAACCAGCAGT
TTCTGGAGCGAGGTGAGCTGGCTGGGGTGCAGGTGGGTGGGGGCAGGTGGGACACC
AGCTGACCCTGCTGATCCTCTCCCAC
>1267
CTGAGCACGGACATGACCCAGCCACTGAGTGCCTACTTCATCAACTCCTCGCATAACA
CCTATCTCACTGGTGAGTGGGGAGCAAGGGTGGCACCCGTGACCTCTGCCCTGTGACC
CTTGGACAGCCCCACTCCTCCTGG
>1268
CCATTGCCGAGACTGCCTTCAAGACCTCGCCCTACCCCGTCATCCTCTCCTTCGAGAAC
CATGTGGACTCGTGAGTGAGCCCCTGGCATGAAACCCCATGGACTGGGGGACAGTCTT
CCAGCTTCAGTGCTGTTGGACTG
>1269
AAAGATGGCTGAGTACTGCCGCTCCATCTTTGGAGACGCGCTACTCATCGAGCCTCTG
GACAAGTACCCGGTACGGGAGCTCGGAGCGAGGGGGGTGGCATGGGGGCCAGGGTG
CTGCGGACCCGGTCCAGGGCCCTGAC
>1270
CCCCTGGAGCAGAGCAATTCTGCCCTGAGCGAGAGCTCCGCGGCCACCGAGCCCTCC
TCCCCGCAGCTGGGTAGGCCCCAGCCCGGCCCGCCACCCTGATCGACCCTAGCCTCTG
GCCTCATGTTCCAGGCGCCGTGACA
>1271
CCGTGAGGATGAGGAGGAAGATGAGGAAGAGGAGGAACAGACAGACCCCAAAAAGC
CAACTACAGATGAGGTCAGGCCCACAGGGTGGGCAGGTCGGGGAGGTAGCATCTATTT
ACCTCCCAGGGGGCTGGGTCTAACCA
>1272
ACTGAGGAGATGTCCACGCTTGTCAACTACATCGAACCTGTCAAGTTCAAGTCCTTTG
AGGCTGCTCGAAGTGAGTGGGGGTGGGTGGCAGGCATGGGAGCTTGCCCAGCTCAGC
CCTGCCAGGTCTGACGCCCTTTCTT
>1273
TCGAGATGTCGTCCTTTGTGGAGACCAAGGCCATGGAGCAACTGACCAAGAGCCCCAT
GGAGTTTGTGGAGTATCCTTTGAAGGTGCTGTGGGCGGACAGGCCAGGGTGGGGCGC
TCGGAGGCCGGCTCCGGTGTTCTAT
>1274
TCCAACTACATGCCCCAGCTCTTCTGGAACGTAGGGTGCCAGCTTGTTGCGCTCAACTT
CCAGACCCTCGGTGAGCCCTGGCCCCCTCCATCTTGACCCCGACCCTCAGTCTTACTG
ACTTCTGACCCACGATCCTGCTG
>1275
GGACAAGTCCTTCGACCCCTTCACTGAGGTCATCGTGGATGGCATCGTGGCCAATGCC

TTGCGGGTCAAGGTGGGGCTTGCGGGCGGCTCAGGCCAGGGGTGTCCTGGGGGCAGG
ACTCTCAGCATCCGCCTCACCCTCC
>1276
CCGCACCCGGACCTCTCAGGGGAACTCGTTCAACCCCGTGTGGGACGAAGAGCCCTT
CGACTTCCCCAAGGTGAGCCTGGCCCCTGCACCCGCCCAGGCACAGGCAGATCCAGC
CCAGACCGCCCCGACTCTCAGGTGGG
>1277
ATTGCAGCCTTTGAGGAGGGGGGTAAATTCGTAGGGCACCGGATCCTGCCTGTCTCTG
CCATCCGCTCCGGTGAGGCCTTGGTGGGCTCTGGGCAGCACAGCGGGCAGTGGGTAG
GGCCCCCACCTGGCCCAGCAACCCA
>1278
CAACCGCTGTGCCTGCCGGCCCTGCTCATCTACACCGAAGCCTCGGACTACATTCCTG
ACGACCACCAGGGTGAGCTGGGGGTGGGCGGGGCCTGCCTGGCCAGGGAGTGTGAG
GGACAAGGGCCACCCCCAGGGCCTGG
>1279
CCCCATTAAGCACGTCAGCCTGATGGACCAGAGGGCCCGGCAGCTGGCCGCCCTCATT
GGGGAGAGTGAGGTGAGCCGGGGCAGGGCAGGGCTCAGGCTCACTGTGACCCAGGG
GGCCGCTGGACATGACTGCTGTGGCC
>1280
CTGGATGCCTCCCCCCGCCGGCCCCCTGGCCCCACCACCTCCCCTGCCAGCACCTCCC
TCAGCAGCCCAGGTAAGGAGTGGCCTGGGTCGGGGGTGGGTTGCAGGGAGGCACCCC
CCACCCTGTCCTCCAGCTCCTCACA
>1281
CCCGCCGCCTGCTGGATGGCCTGGCTCAGGCACAGGCTGAGGGCAGGTGCCGGCTGC
GGCCAGGTGCCCTGTGAGTGTCTGGGCCGCCTGTGTGCTATGTGTGCTGGGTGTGCTG
ATGTGAACATGGGGGTCGGTGGGCA
>1282
GAGCCTGCTGGAGCTGCGGGAGGCCCAGGTGGACGCAGAGGCCCAGCGGAGGCTGG
AACACCTGAGACAGGTAGGGGGCCTGCAGTGGCCAGGGAAAGCCTGCTGGATAGACC
CGTCGTCAGCCCGGCATCACCTGTCAG
>1283
AGCGGCTCAGGGAGGTCGTCCTTGATGCAAACACAACTCAGTTCAAGAGGCTGAAAG
AGATGAACGAGAGGTGAAAGCCGAGGATTGTCTATGGGAAGGGCTGGGGACTTCTAG
TACCAGAAGGAGGGCAGAGTCTGTGC
>1284
AGATCCTGGACAGAAAGCGCCATAACAGCATCTCGGAGGCCAAGATGAGGGACAAGC
ATAAGAAGGAGGCGTAAGGGCACCGGGACCGGGGGCCATCTGGGTACTGGGGAGGCA
GGGCAGGTGTCTGGGCCCCGAGCCAT
>1285
GCAGCGGCATGACCGTCTTGTGGCTGGGCAGCAGCAGGTCCTGCAACAGCTGGCAGA
AGAGGAGCCCAAGGTGAGGCCATGGGCGAACAGGTGGGCAGACGGTGTGCAAGGCA
GCCAGGCCTCGCCTGTGATGCCCATCC
>1286
AAGCCGCACGGCCCTGCCCCACAGGCCCCTACAGCCTCCACAGCCACTAAGCCTGCA

CCCCCAAGTGAAGGTGACTGAAGGTGGGGCAGGGGGAGGAGGGAGGCAGGCATGTC
AGGGGTGGAGGGGAGCTCTTGGCCATG
>1287
TGGGAGCCCCCAGAGAGGCTGGGGAGGCTGGGCCTCCAGGGCTATGTGCTGGAGCTC
TGCAGAGAGGGAGGTGAGTCGTCTGGGCTAAGTCTTGCATACATCCAGGGGAGGTTGT
CATGGAGGGCTGCAGGGACAAGTGC
>1288
GCAGTTCGTCCTGCAGGGGCTGGCCCGCCGGCCATGCTGGACCAGCCCATCCACATCC
GAGAGAACATTGGTAAAGCCCATCCTGGCCTTCAGCTACCCAGCCCTTCCCAAACACC
TTATCTAATGGAGAAACTGAGGCC
>1289
CCCCCGCCACCTCCGTCAGACCTACATCCGCCAGGTGGGAGAGACGGTCAACCTGCA
AATCCCCTTCCAGGTGGGTATTACCCCCACCAAGGGTAACAGCCTGGGTGGGAACCAG
GGCCCCCATTTAAGGGGAGCCTGGG
>1290
GGCCGCTACGAGCTCACTGTGCGCGTGGAAGACCTGGAGGCCAAGGCAGTCATTGAC
ATCCTGGTGATTGGTATGGAGCCAGGGGAACAGTGCTGTCTCATGGGGGACCTGGAGC
CCCCAACCTCCTATCCCAGAGCTGA
>1291
GCCACCCCAGGACACAGGCAACACAGAGCTCCTGGGCTACATGGTGCAGAAGGCAGA
CAAAAAGACAGGGGTGAGGCTGGCTGAGGCAAAGTGGGGTCGGGGTACGAAGTGGT
CAAGGTGGTGGGGAAGGCCCATCCCAC
>1292
TCAGAAAACCTGTGTGGACTCAGCACCTCGGCCACCGTCACCAAGGAGCTCGCCCAC
ATCCAGAAGGCAGGTGGGTTTGGGCCCGGAGCTGGCTGTGCTAACTCTCTCCCTGTCT
CTGCTTCTTCCTCCGCGTCTCTGCA
>1293
CCTGGCTGACCACACCTCCACCCCTGGCTACAGCACCCAGTTGTTCTGCAGTGTCCGA
GCTTCACCCAAGGTGAGGGGCCAGGCCCTGTTGTGCCCTGGGCTGGTGGGGGCAGGT
GGGGACAGCAGGGCTGAGGAATCCT
>1294
GGGGTCTACACCTGCAAGGCCATAAATGTGCTGGGGGAGGCATCTGTGGACTGCCGGC
TGGAGGTCAAAGGTGAGAGTTTGAGGACGGGAGAACAGGAGTAAGCACCTCTCTGGA
ACTGGACTAGAGGCAGCAGACAGCT
>1295
GGCCTTGGCAGGAGGATGTGGCAGATGCTGAAGAGTGTGCTGGTCGCTGTGGGCTCTT
AATGGACTGCTGGTGAGTGGCCACTGGGCCTAGATAAGACTGGGGGCAGGGGAGCCT
GGGCCGTGGCGTTACCCTGTGCCTT
>1296
ACACCATGGCCACGACCGTGGGTGGCCTGTCCTGCCAGGCTTGGAGCCACAAGTTCCC
GAATGATCACCAGTGAGACAAACACCTTCCCTCCATCCCGGCCTGGGACCTTCCCCCA
GCACACACTATAGTGATGCTCTGG
>1297
GGTCCTTGGTGCCACACAACAGACCCTGCCGTGCGCTTCCAGAGCTGCGGCATCAAAT

CCTGCCTGGTGGGTAAGCGGCGCCGGGTCAAGCTGGGAGAGTGGAGGGACAAGCCCA
CGCCCATCCACGAACCCACTGGCTC
>1298
CCGAGTCAGGGCGCGAGTGCCAGCGCTGGGATCTTCAGCACCCGCACCAGCACCCCT
TCGAGCCGGGCAAGTACGCGTAGGCGGTATCGGCGCCCTGGGGGCCGGGCTAGGGAA
GGTCCAGGACTCCAGGGGCAGGGCTC
>1299
TCCCAGCGGCCATGGTGCTACACTACGGATCCGCAGATCGAGCGAGAGTTCTGTGACC
TCCCCCGCTGCGGTAGGCGGCGGGGACCAGGCCTGGGAGGGTACCTGGGAACCTTGG
GGAGGGGCGTGGCTTGGCCGGGAGG
>1300
TACCTTGCCAGCGTTGGGACGCGCAAATCCCGCATCAGCACCGATTTACGCCAGAAAA
ATACGCGTGCAAGTGAGGTGGGGGGGCGGGCGTTGGGACGTGCTGCTGCGGGTGAGA
CGGGAGGAGGGTAGTCACAGGCTTA
>1301
CTGCGGCCCGGCACGCGCGTGGGCTTTTGCTACCAGATCCGGCGTTGTACAGACGACG
TGCGGCCCCAGGGTGAGGCCCAAGCTTGGGGGCTACAGAGCCGGGGCTGGAAGCCTG
GAACCGAAGGGCCGGGGCGAGGTCT
>1302
GCACGGTCAGCAAGACCCGCAAGGGTGTCCAGTGCCAGCGCTGGTCCGCTGAGACGC
CGCACAAGCCGCAGTGAGTCCCTGGTGCTCCCGGCCCCGCCAGGGCCCTAACCCTGG
GGCGGCATGCTTTGATGTCTGGGACC
>1303
AGCCATGGGCCCTGGTGCTACACGATGGACCCAAGGACCCCATTCGACTACTGTGCCC
TGCGACGCTGCGGTGAGCACTAGTGACGCTTGCCCCATGACCCTGCCTCAGCCCCCAC
CACCAAAGGCTGGCTCCCTTAACC
>1304
GTCGTTCCAAGCTGCGCGTGGCTGGGGGCCATCCGGGCAACTCACCCTGGACAGTCA
GCTTGGGGAATCGGTGAGGCACAACTGCCTGTCTCCCACAGAGAGGAGCTGAGGTTG
TGTCCTCTGTGGTTATGCCACTGGGG
>1305
AGCATTTCTGCGGGGGGTCTCTAGTGAAGGAGCAGTGGATACTGACTGCCCGGCAGTG
CTTCTCCTCCTGGTGAGCCTCCCTTGTGTTTGGGACCCAGTCTCATCCCACCTTCCCCT
TTCCCCAGGCAAGCTAACAAGTG
>1306
AGCGGGTCCCAGTAGCCAAGATGCTGTGTGGGCCCTCAGGCTCCCAGCTTGTCCTGCT
CAAGCTGGAGAGGTATGTGGACAACCTGGGAGGGTGTGAGGTGGGGCTGAGCCTTGT
GGCCTCAGACCCTGAGTGCCCCCAT
>1307
CTGCCGCCTGAATGATATGTGGTGCCTCCAGGGACCAAGTGTGAGATTGCAGGCTGGG
GTGAGACCAAAGGTAAGAGCATAGTGCACAGGACTGCTGGCGGCCAGGAGCCCAGCC
CTGGATCTTCCTCCAGGACCGTCTC
>1308
GCACCGAGGACATGTGCGGGAGAGCGAGATGTGCACTGAGGGACTGTTGGCCCCTGT

GGGGGCCTGTGAGGTTGGTAGCAGGGCCCTGGGCCAGCCCTGGAAGGGTATGGGGGG
CTAGAAATGAACTATTTTATCATGAA
>1309
GCCTGTCCTCCGAGCCAGTCGCTGACAGCCGCGGCGCCGCGAGCTTCTCCTCTCCTCA
CGACCGAGGCAGGTAAACGCCCGGGGTGGGAGGAACGCGGGCGGGGGCAGGGGAGC
CGCGGGGGCCGAGTGAGGACCCCGGG
>1310
ATTTCAGCCATGTCTGAGCTTTCCGTCTTCCTGGAGGCACAAATCTAGTTTAGCTGAAC
CACAACAGATTGTACATATCCTGCAGAACCTCTGTGGTCTTAGGAAGGTTGAAAGTCA
CCAAATGTCACAGAAAATGAGGG
>1311
CTGCTGCCATCCTCCAGCAAGATGCTAGGGTCTTTGGGGCTTTGGGCATTACTTCCCAC
AGCTGTGGAAGGTAAGTGTCTACAGGGAGGGGAAGGGTCTCTCCATCCATCCAGCAA
GGGAAAGGGGCGCTTGAAGCAAGA
>1312
TGCCTCTACAGCCGCTGCTGCTTTGGGATCTGGAACCTGACCCAAGACCGGGCACAGG
TGGAAATGCAAGGTGAATGGCAAAGTATATGGCAGGTGATGGCTAGGGTGGGAGACA
GACACATCCTGGGGTGTGGGTGGCA
>1313
AATTACAGCCATCTGCCTCCTCCAGGGAGCCCTGGGACTCCTGGCTCCCAGGGTCCCC
AGGCTGCCCCAGGTAGCCACCCAAGGGTACTGAAGCCTGATGGGGGCTGGGGCCCAG
GTTAGGATGAGAGGTGGAACCAGGG
>1314
ATCTGGATGGCACTGGTGCTGCTGGGGCTGTTCCTCCTCCTCCTGCTGCTGCTGGGCAG
CATCATCTTGGGTACTAATCCACCCCATCCCTCCCTTGTGACCCCAAGACATTGCCCCA
AAAGCTCTGACCCCTCTCCAGG
>1315
GCCAAGGCCAGACTCAGGCAGGGACTGGAGTGTGGAGCTGCAGGAGCTGCCTGAGCT
GTGTTTCTCCCAGGTGCCCCAGGGAGGGAGAGAAGGGCTCCTCTGGGCACTCCTGGA
GGTTGTGCTGGGGAGGAATCCTGGCC
>1316
CACTGCCAGCCGGGGGGGTCCTGGCCGCCTGCTCTCTGGGCCCCTGCTGGTACTGGAA
CTGCATCCCAAGGTGAGCACCAAGGAGTGTATATGTGTGTGTGTGTGCCTGTGTGTATG
TATAGAGGTGGGGGCTACATGGC
>1317
AGTTCCCTGCGGATGGCACTGTCCCTGGCCCAGGGCCTGGCATTTCTCCATGAGGAGC
GCTGGCAGAATGGTGGGTGAGCTGGGCATAGGAAGTCAAGGGAGCCACAGTGCTATG
TTTGTGATTCTGCCTTAGTTTGGAG
>1318
GCTCCCTGGCCTCACTCAGCCCCCTGCCTGGACCCCTACTCAACCACAAGGCCCAGCT
GCCATCATGGAAGTGAGTTCTCTGGATAACTGGTGAGGCCCAGGATGATGTTGGTGCT
GCTGATGGCAATGCAGCCATTGTG
>1319
CGAGCTGATATTTACTCTTTGGCTCTGCTCCTGTGGGAGATACTGAGCCGCTGCCCAGA

TTTGAGGCCTGGTAAGGATGGGTGGTACAGTCCCCTCTCCTGGGCTCCCCCCGCCCAT
TCTAGGTTCACCCCAACCTGAC
>1320
GCTATGGGCCTTGGCAGTGCAGGAGAGGAGGCGTCCCTACATCCCATCCACCTGGCGC
TGCTTTGCCACAGTAAGAGGCCTAGGCTGTTGGTCTGGGAACCTGGAGAGTGGGGGC
TGGGCATGGGCTTCAAGGACGTCTC
>1321
ACCTCTAGCCTACAGGGGCCTGGAGACCGGTTCCCGGGAGGTGGTCAGCCATGTAATC
AAACAAGGGAAGGTGAGTCACCACCCCCAGGACAGACCCCAACCCCTGCCTGGCCAA
CATGGTGAAACCCCATCTCTATTAA
>1322
CAAAATCATGCGGGAGCCCTGGGTAGAGCAAGACAAGTTTGGGAAGGTGAAGTTTGC
TGTGCTGCAGACGGTGAGTTCCCTTTGGTGCCCCCACCCCTCCTGCTGTCGGCACCAC
TGAGATGTCAGGAGCCCCGCAGATG
>1323
TGAACTACATCGGCCAATTCTTGCCTGGATATGAGCCCCCAGCGTTCATGGACCCCCTA
CTTCCTAAACTGTGAGTGTCCCTCAGGGCAGAGGTGGGTGGAAACCCCTGAGGGACT
GTGAAAAGTAATGCCCCTTCCTCC
>1324
GCAGTCTGGAGATGATCGACCACATTGTGGGAAACCAGCCTGATCAGGAGATGGTGTC
CGCCTCCGAATGGTGAGACCTTGGCCCTCTCCCTCTGCGTGAAGATGACGGGTCCTCA
CGGTGGACTGGCCCACTCCCTGGT
>1325
CAACTATGAAGAGTCCATCAAGATGCCCATCAATGAGCCAGCGCCTGGCAAGAAGAAG
TCCCAGATCCAGGTGAGGCCGCCCCTGGCTGGGGGAGAGGGGAGCAGGGAAGAGGT
CTGGGGGCTCTTGTAGGGTCAGGGAT
>1326
ATATGTGGACTATAACGGGGGCGCTGGGGTCCAGCACATCGCTCTCAAGACCGAAGAC
ATCATCACAGCGGTTAGAACCCCCTTCCCTGTCCGAATTCCTCTCTCATTCAGAGCCAG
GGACGGTCTCTGCAAAATTGAGA
>1327
CTACAAACAACTGCGGGAGAAGCTGAAGACGGCCAAGATCAAGGTGAAGGAGAACA
TTGATGCCCTGGAGGTGAGGCCCAGGCAGGATCCGCAGCCTTGTGGGGGGAGTATGA
GCTCTGCATTGCATCTTTTCAGAAATA
>1328
GATCTTCACCAAACCGGTGCAGGACCGGCCCACGCTCTTCCTGGAAGTCATCCAGCGC
CACAACCACCAGGTACTGCTTGTCCCCGGCAGGCCCGAGGGGACAGGCAGCTAGCAC
ACTCGGTTTTCTGGGTCCTTGGCTC
>1329
TGGCTGGAGCCCCCTCAGTGTGCAGGCTTAGAGGGACTAGGCTGGGTGTGGAGCTGC
AGCGTATCCACAGGTAAAGCAGCTCCCTGGCTGCTCTGATGCCAGGGACGGCGGGAG
AGGCTCCCCTGGGCTGGGGGGACAGG
>1330
GCTACTCCTCTGCATTGGAGCCCTCCTCGGGCACAGCAGCTGCCAGAACCCTGCCAGC

CCCCCGGAGGAGGTCAGTAGGCAGGCGGGGAGGGCGTGGTCAGCATTCCCCGCCCCT
CCTTGGCAGGCAGCACGGGAAACAG
>1331
ATGAGCCCCACGACCAACGTGCTCCTGTCTCCTCTCAGTGTGGCCACGGCCCTCTCGG
CCCTCTCGCTGGGTGAGTGCTCAGATGCAGGAAGCCCCAGGCAGACCTGGAGAGGCC
CCCTGTGGCCTCTGCGTAAACGTGG
>1332
GAGCTCCTTGACACGGTCACTGCCCCCCAGAAGAACCTCAAGAGTGCCTCCCGGATC
GTCTTTGAGAAGAGTGAGTCGCCTTTGCAGCCCAAGTTGCCTGAGGCATGTGGGCTCC
ATGCTGCAGGCTGGGGGGGTCTTTT
>1333
CTCGCCAGGTCCACAAAGGAAATTCCCGATGAGATCAGCATTCTCCTTCTCGGTGTGG
CGCACTTCAAGGGTGAGCGCGTCTCCAATTCTTTTTCATTTATTTTACTGTATTTTAACT
AATTAATTAATTCGATGGAGTC
>1334
GAGGGTCCCCATGATGTCGGACCCTAAGGCTGTTTTACGCTATGGCTTGGATTCAGATC
TCAGCTGCAAGGTCTGTGGGGATAGGGGCAGGGTGGGGGGTGGATGGAGGGAGAGGA
TAGAGAAGCAAAACAGGGTAGTGG
>1335
GCGGTCCTCACTGTCCCCAAGCTGAAGCTGAGTTACGAAGGCGAAGTCACCAAGTCC
CTGCAGGAGATGAGTATGTCTGAAGACCCTTTCGCTCTTGGTGGGTGGATGGGGTGGG
GCAGGGTCTTTGGGCCTTCCACTGT
>1336
CCCTGTGCCTCCCTCTACAGCCCTCAGGGAGCTCATTGAGGAGCTGGTCAACATCACC
CAGAACCAGAAGGTGAGTGTCGGCTAGCCAGGGTCCTAGCTATGAGGGCTCCAGGGT
GGGTGATTCCCAAGATGAGGTCATG
>1337
AGGCTGCAGTGCCATCGAGAAGACCCAGAGGATGCTGAGCGGATTCTGCCCGCACAA
GGTCTCAGCTGGGGTAAGGCATCCCCCACCCTCTCACACCCACCCTGCACCCCCTCCT
GCCAACCCTGGGCTCGCTGAAGGGA
>1338
GTCCTGGTGGCCGTGGCTACAGCCCTGGTGTCTGCCTCCTCCCCCTGCCCCCAGGCCT
GGGGCCCCCCAGGTGAGAAGAACCCTGCTCTACAACCTCCCAACTCTGACTTGTGACT
TGCCACCCTCACTGTGGCCCCCTT
>1339
GATGAGGGCACCTACATCTGCCAGACCCTGGATGGTGCACTTGGGGGCACAGTGACCC
TGCAGCTGGGCTGTGAGTTGGGGAGGGTGGCACTGATGACACATAGGGATCCTGAGG
GAGTATGGGACCTAAGTAAGCCCTC
>1340
AGAACTTCTCTTGCACTTGGAGTCCCAGCCAGATCAGCGGTTTACCCACCCGCTACCT
CACCTCCTACAGGTGTGTGTGTGATTGGGTGTGGATGCCTACACATTTGGGTATGCTGG
TGCAATGTGGGTGTGGGAGGCAG
>1341
ATTAATGTGACTGAGGTGAACCCACTGGGTGCCAGCACACGCCTGCTGGATGTGAGCT

TGCAGAGCATCTGTGAGTACCCACCCCAGACCTCCCCCAGACCCCCATCACAGAGACC
CCAAATGGACTCAGATCTCTCCAC
>1342
GTGCCAGCCCCACTTCCTGCTCAAGTTCCGTTTGCAGTACCGTCCGGCGCAGCATCCA
GCCTGGTCCACGGTGAGGCCTGGAGTGCGTCCCAACCCACGGCTGTGGTCCTGTCTCT
GATTTCACGATCCTGGGTGTTCTG
>1343
GCCCGGGACTTTCTAGATGCTGGCACCTGGAGCACCTGGAGCCCGGAGGCCTGGGGA
ACTCCGAGCACTGGTGAGAGACAAAGCCAAAGAAAAGGGCAGAGGCCCCATCCCAA
AGCATCTATCAGCTGAACCAGTTCCAA
>1344
GAGGTGGAGCCTCAGGTGGACAGCCCTGCTCCTCCAAGGCCCTCCCTCCAACCACAC
CCTCGGCTACTTGGTGAGCTTGGGCAGATGGGCAAGACTTGTAAAGGACTGAAAACC
CCAGAGAAAAGGGGACACCGAGCTTG
>1345
CGGGGTGGGAAGGATGGATCCCCAAAGCCTGGGTTCTTGGCCTCAGTGATTCCAGTGG
ACAGGCGTCCAGGTGAGTAGGACATCCAGAAGATTTGGACTTGGAGATGTTTGCCCCT
ATTTTGAGTGTCCAGATTAAGAGC
>1346
GTCCTGGGGCCTGGGCAGCTGGGGGCTGTGGCCATTCTGCTCTATCTTGGATTACTCCG
GTCGGGGACAGGTAAGTGAGGACGTGGGGGCAGGGTGGGGACAATGCACTGGAGGG
AGCCGGCGTGATCATTTTCTGGGGT
>1347
GCGTCCATGTGTGTGGTGGCTCTCTCGTGTCTGAGCAGTGGGTGCTGTCAGCTGCTCA
CTGCTTCCCCAGGTACCAAATGGTGAAAGTCAAAAGTGGGCTGGAGGTCAAAGTTAAT
GGGTCAATCCAGGTCAGAGGTTGG
>1348
GCAGCCAACGCCTCCTTCCCCAACGGCCTCCACTGCACTGTCACTGGCTGGGGTCATG
TGGCCCCCTCAGGTGAGGTGGGACGTGGGTGCCTAGAGGTGTGGAGGGGCACCTGAC
TTGGGGAGGGGCCCAGGGTAAGCCT
>1349
GGAGCCGCACTTTGTCCAAGAGGACATGGTGTGTGCTGGCTATGTGGAGGGGGGCAA
GGACGCCTGCCAGGTAAGCACAGGCCCGGGGGCAGATGACCAGTGCAACTTCGGAAA
GGAGGCCTGGCCCGGTCCTGATGGCT
>1350
GCGCCAGCAAGTTCGCGCTCGAAGGCTTATGCGAGAGTCTGGCGGTTCTGCTGCTGCC
CTTTGGGGTCCAGTGAGTCAACACCCCCGTTCCCCGAACCCTCTTAACTCTGACCTAG
AGACGCCGAGCACCCTGTCCTGCG
>1351
CTACCAATACCTCGCCCACAGCAAGCAAGTCTTTCGCGAGGCGGCGCAGAACCCTGA
GGAGGTGGCGGAGGTGAGCGCCGGGCTGGACTCCAGGAGTGGGGGCGGTGCGTCCT
CCGGCGCGCAGCGGTGGCCACAGCTCT
>1352
CAGCCTCCAGCCAGAAGGATGGGGTGGCTCCCACTCCTGCTGCTTCTGACTCAATGCT

TAGGGGTCCCTGGTAAGTGCCCCCAACCCTGATCCCCATCTGCCTTCAGGAGGGGGTT
GGCCCCATTCTCCTATTCTAGGAT
>1353
GGCCTTGGCAGGAGGATGTGGCAGATGCTGAAGAGTGTGCTGGTCGCTGTGGGCCCTT
AATGGACTGCCGGTGAGTGGCCACTGGGCTAGATAAGACTGGGGGCAGGGAAGCCTG
GGCTGTGGCGTTACCCTGTGCCTTC
>1354
GCACCATGGCCACGACCGTGGGTGGCCTGCCCTGCCAGGCTTGGAGCCACAAGTTCC
CGAATGATCACAAGTGAGACAAACACCTTCCCTCCGTCCCGGCCTGGGGCTTCCCCCA
GCACACACTATAGTGATGCTCTGGG
>1355
GGTCCTTGGTGCTACACAACAGACCCTGCTGTGCGCTTCCAGAGCTGCGGCATCAAAT
CCTGCCGGGAGGGTAAGCGGCGCCGGGTCAAGCTGGGAGAGTGGAGACAAGCCCAC
GTCCATCCACGAACCCACTGGCTCTT
>1356
CGGAGTCAGGGCGCGAGTGCCAGCGCTGGGATCTTCAGCACCCGCACCAGCACCCCT
TCGAGCCGGGCAAGTACGCGTAGGCGGTATCGGCGTCCTGGGGGCCGGGCTAGGGAA
GGTCCAGGACTCCAGGGGCAGGGCTC
>1357
TCCGAGCGGCCATGGTGCTACACTACGGATCCGCAGATCGAGCGAGAGTTCTGTGACC
TCCCCCGCTGCGGTAGGCGGCGGGGACCAGGCCTGGGAGGGTACCTGGGAACCTTGG
GGAGGGGCGTGGCTTGGCCGGGGAG
>1358
TACCTTGCCAGCGTTGGGACGCGCAAATCCCTCATCAGCACCGATTTACGCCAGAAAA
ATACGCGTGCAAGTGAGGTGGGGGGGGGGGCGGGCGTTGGGACGTGCTGCTGCGGG
TGAGACGGGAGGAAGGTAGTCACGG
>1359
CTGCGGCCCGGCATGCGCGCGGCCTTTTGCTACCAGATCCGGCGTTGTACAGACGACG
TGCGGCCCCAGGGTGAGGCCCAAGCTTGGGGGCTACAGAGCCGGGCTGGAAGCTGGA
ACCGGAGGCCGGGGCGAGGTCTCGG
>1360
GCACGGTCAGCAAGACCCGCAAGGGTGTCCAGTGCCAGCGCTGGTCCGCTGAGACGC
CGCACAAGCCGCAGTGAGTCCCTGGTGCTCCCGGCCCCGCCAGGGCCCTAACCCTGG
GGCGGCATGCTTTGGTGTCTGGGACC
>1361
AGCCATGGGCCCTGGTGCTACACGATGGACCCAAGGACCCCATTCGACTACTGTGCCC
TGCGACGCTGCGGTGAGCACTAGTGACGCTTCCCCCATGACCCTGCCTCAGCCCCCAC
CCAAAGGCTGGCTCCCTTAACCCC
>1362
GGCGTTCCAAGCTGCGCGTGGTTGGGGGCCATCCGGGCAACTCACCCTGGACAGTCA
GCTTGCGGAATCGGTGAGGCACAACTGCCTGTCTCCCACAGAGAGGAGCTGAGGTTG
TGTCCTCTGTGGTTATCCACTGGGGC
>1363
AGCATTTCTGCGGGGGGTCTCTAGTGAAGGAGCAGTGGATACTGACTGCCCGGCAGTG

CTTCTCCTCCTGGTGAGCCTCCCTTGTGTTTGGGGACCCAGTCTCATCCCACCTTCCCC
CTTCCCCAGGCAAGCTAACAAGT
>1364
AGCGGGTCCCAGTAGCCAAGATGGTGTGTGGGCCCTCAGGCTCCCAGCTTGTCCTGCT
CAAGCTGGAGAGGTATGTGGACAACCTGGGAGGGTGTGAGGTGGGGCTGGGCCTTGT
GGCCTCAGACCCTGAGTGCCCCCAT
>1365
CTGCCCCCTGAATGGTATGTGGTGCCTCCAGGGACCAAGTGTGAGATTGCAGGCTGGG
GTGAGACCAAAGGTAAGAGCACAGTGCACAGGACTGCTGGTGGCCAGGAGGCCAGC
CCTGGATCTTCCTGCAGGACCCTCTC
>1366
GCACCGAGGACGTGTGCGGGAGAGTGAGATGTGCACTGAGGGACTGTTGGCCCCTGT
GGGGGCCTGTGAGGTTGGTGGCAGGGCCTGGGCAGCCCTGGAAGTATGGGGGGCTAG
AAATGAACTATTTTATCATGAAGCAG
>1367
TGCCTGCGGACAGGCCCCCAGGGCTAGCGGCTTTGTGGAGGGAACACTGGGTATCCTC
TCCACAGTCCAGGTAGGTGGGACCCCCACTCCTGGCTCTCAGCCTTTAGGAACAGGAG
CTTCTCTACTTGGAGCTTCTAGAC
>1368
CTCCCTGCCAGTGAGTTTCGCTGCCTCACCCCGGAGGACGCTGTCAGCGCCTTTGAGA
TCGAGCGTGAAGGTGAGTGGCCCCGCACAGGGTCAGAGGGATGCTCCACTCTGGTCC
AGTTATCCTGTGGGGAGGAGACCCT
>1369
CTGGTTCGAGGAGGGCTGCCTTGTGGCCTTCATCATCGGCTCGCTCTGGGACAAGGAG
AGACTCATGCAGGTGAGGACAGGCCTGCGACGCCCAGCTCCAGGGAGGCCTCTGAAG
ACAGAGGTCAGCCAGATGGCGGGGA
>1370
AAATTCGTCATTCGAAACATAGTGGAGGCCGCAGCAGTCAGGGACATTTCTGAAGCGA
GCGTCTTCGATGGTAAGTGGGTCACCGGCGCGAACTGTGTGAGGATCCAGTATCTTAA
AGCCTTCGCCCAACTTCGCCCTTT
>1371
CAAAGTAGTCAGGAATCGATCTCGTGAAGCCCGCAAGGACCGAACACCCCCACCCCG
ATTTAGACCTGCGGTGAGTATTTTAAAAGGAGAATGGAAGCCAGAGGAGTGATGGTTA
AAATTTCATCCTGGAGGGTCAGGGT
>1372
AGGTTCACTGCAACCGTTCAGAGGTCCCCAGGAGCTGCTGCTGGCGAGCCCGCTACTG
CAGGGACCTATGGTGAGCAAGGCTACCTGGTGAGGGGAGACAGGCAGAGGGGGTCTA
GGAGCCTCCTTGGGGGGAAGAAGCT
>1373
TTCCAGCAAGTTTGTTCAAGATTGGCTGTCAAGAATCATGGACTGTTATTATATGCCTTG
TTTTCTGTCAGTGAGTAGACACCTCTTCCTTCCCCCTCTCCGGAATTCACTCTGCCCTC
ACCACCCCTGCTCGCCGGCTG
>1374
AGCACTGGTCTTGAGTATCCTGAGCGCCCGGCCAGCCGGGCCAACACCGTGAGGAGC

TTCCACCACGAAGGTCAGTCTCTTCCCCCAGTCTGCGTGGGGGAGGGCTGGTGGGACT
GGCTAGAGGGGCAGTGAAAGCCCTG
>1375
GGAACCCACTTAGGTGGCACCAATCTTGACTTCCAGATGGAACAGTACATCTATAAAA
GGAAAAGTGATGGTTAGTCATTGCTTTAATTTTTTGTTACTCCAGCTGTAAGTACAAATT
TTGAGCTTGCTATTCTCGTGGT
>1376
AGCTCGTGCAATTGTTGCCATTGAAAACCCTGCTGATGTCAGTGTTATATCCTCCAGGA
ATACTGGCCAGGTTTGTGGAACAGTGGTTAGTTTTTATATTATAGAAATAAAGCCTTACA
GACATTGTGAGACATATAGAA
>1377
TACCATTGCGCTGTGTAACACAGATTCTCCTCTGCGCTATGTGGACATTGCCATCCCATG
CAACAACAAGGTAATGATTTTAGGATCTAGAGTTTGTGAATGCGTGCTCTAGAAAAAA
CATTCCTGTGCACATTGTTAGA
>1378
TGGCACCATTTCCCGTGAACACCCATGGGAGGTCATGCCTGATCTGTACTTCTACAGAG
ATCCTGAAGAGGTAAGCTTCTCCAAAGGCTTGTGGTTACATAAGCAAATTGGACGACT
TGGACTGCTGTCTAGGAAGCAAA
>1379
CAGCCTGAGGTTGCAGACTGGTCTGAAGGTGTACAGGTGCCCTCTGTGCCTATTCAGC
AATTCCCTACTGGTATGTATCAGGATAGAGGTGAATCAAGCTGATATTTTGCAACTTCTC
AGTTTTATTCTAACTTTAATGA
>1380
CATGGAGCTGTTCTGCTGGTCAGGGGGCTGGGGGCTGCCGTCAGTGGACCTGGACAG
CCTGGCCGTGCTGGTGAGGGGTGGCGCCGGCGCCCTCTGCTGTGCCCTGATGACTGGG
GAGGGGGCCGAACTAGCCAAGCATC
>1381
ACCTATGCCAGATTTACTGGTGCTCCACTGAAGGTACACAAGATCAGCAACCCCTGGC
AGAGCCCTTCAGGTACCCAGTATCCCTTGGGGGTAAGGAAGGGTGTGTACAAGGGGA
GAAGCAAGAAATAGGCAGGAATGTG
>1382
TGCCCTTCGGACCAGTCATGGAGAGGTCATCTCAGTTCCACACAAGATCATCACCCAC
CTTCGAAAAGAGGTAGGTGACTTGGATAGAGGGGGCTGCCAGTGAGAGAAGTTCAGT
CAATTCTATACAATACACATTTATT
>1383
GTCAGCTCGGCAAGGGGCAGACACCCTGGCCTTCATGTCTCTCCTGGAGGAGAAGTT
GCTCCCGGTGCTGGTGAGTGTGCCCAGACCTCCCAGCATCCATGGCCAGCCGGGGAG
GGTTCGGGAACACACAGACCCACACA
>1384
GTACATGGAACGGCTACAGCTGCTGACTGGGGAGCACAGGCCTGAGGACGAGGAAGA
GCTGGAGAAGGAGGTAGCTCTGAGACCGGGGGCTATTGTATGAGATGAGCCCCAAGGA
TGCTGGCCAGGAATGGGAGTGCTTA
>1385
GAGAGGCTCGGGAGTGTCTGACCCTGCTCTCTCAGCGCCTGGGCTCTCAAAAGTTCTT

CTTTGGAGATGCGTGAGTCTGACTCCAAGAGGGTAATGGGTGGCTTGGAAGAAGATAC
AGGTTCAGATGGAGCAGCTGGAGC
>1386
CTGCGTGGGCTGCACAACCTCTGTGCCTATTGTACCCACATTCTCAGTCTCTACTTCCC
CTGGGATGGAGGTAAGGGGCAGATGGGAGGGGCAGCCCTGGGGAGAGTGGGCAGGG
ATCCAAGAACTAGTTCTCCTAACAC
>1387
GGACCATCCCAGACAGTGACATCTCTGCTTCCAGCTCCTGGTCAGATTCCACTGCCGC
CCGCCACAGCAGGTACTTGGCACACCTGGCACACTTGTAGCTGCCCCGAGAGGAGCT
CCTGGGACCTCTACTTCCCCTCCAA
>1388
TTCTACCCCCGGGCTGACCGGGTCATGAGTGTCTGTCTGCGGGTAGAGCTCTATGGCTG
CCTCTGGAGGGGTGAGTGGCTCAGCTTCCTGGGAATCTGTTTCCTGAGCAGGGGACTG
GAGGGTGGGGAGTGTGGCGAATG
>1389
CAGCTTCTCCAGTGGCTATGTGGAGATGGAGTTTGAGTTTGACCGGCTGAGGGCCTTC
CAGGCTATGCAGGTGAGTGAGTCCGGCTCTCGAGGAGGGCTCTGAAGCCATGCAGGG
TGCCGTTGGGGTGCCCCCACCACTC
>1390
CGCTTTCTGCAGTGCCGCTTCCTCTTTGCGGGGCCCTGGTTACTCTTCAGCGAAATCTC
CTTCATCTCTGGTAAGCCCTGGAGTAGCCCAGTCTCCAGTCCCTGAAATTGACAACTG
AGTTCATTCCTAACCCTGCAGTG
>1391
CTGGGAGGCACCTTCCCGCCAGCCCCCTGGTGGCCGCCTGGCCCACCTCCCACCAACT
TCAGCAGCTTGGGTGAGCAATCTTGGGTGGGCGTGTGGACCCTCTGCACCCTTCTCCC
TGGGCCTCCCCCTCGGCTAGGGTG
>1392
CATCCTGCTCCTGCTGCTCATCATTGCCCTCATGCTCTGGCGGCTGCACTGGCGCAGGC
TCCTCAGCAAGGTGGGCACAGCCGTGGCATGTGGAGTGGCGGGGGGAGGCCAGGCCC
CAGCACGAGCCAGCGTCCAGTGGG
>1393
CCCCCGTACCAGGAGCCCCGGCCTCGTGGGAATCCGCCCCACTCCGCTCCCTGTGTCC
CCAATGGCTCTGGTAAGACCTGCCTTGTTCCAGTCGCACCTCTGTCCTCTCTGCTGTTT
TCTTATTGTATCCCTTTCCCATT
>1394
ACTTACGCCCGTCCCCCTCGAGGCCCGGGCCCCCCACACCCGCCTGGGCCAAACCCA
CCAACACCCAGGGTAAGCCCCTCTGCCCCTGGGCTCCGCCAGGCTCCCCATACCTCTA
CTGGGGCAGGGAAAAGCCCTCACA
>1395
CCCCAGAGTGGATTTCCCTCGATCTCGACTCCGCTTCAAGGAGAAGCTTGGCGAGGGC
CAGTTTGGGGAGGTAAGGAGGGTGCCTACCCAGTGTCTGGCCCTATTGTGTGCTCTGA
TGCCATGCCTGCGCATCCCCCTAG
>1396
ATGTGCGTAAGGGACACCCTTTGCTGGTAGCTGTCAAGATCTTACGGCCAGATGCCAC

CAAGAATGCCAGGTGAGGACCAGGGATGGCATCTGGAAGAAGGGAGGGGAGGCCGC
GAAGAGTGGCGAGCCATCTAGAGAGA
>1397
ACCAGCTGGAGGACAAGGCAGCCGAGGGGGCCCCTGGGGACGGGCAGGCTGCGCAG
GGGCCCACCATCAGGTACCTGTTTACCCAGGCTGGGCCATGCTCAGAATTCCCCCAGG
GGATCTCCTCCTCTCCCCTCGCTTCA
>1398
GGACTATTACCGTGTGCAGGGCCGGGCAGTGCTGCCCATCCGCTGGATGGCCTGGGAG
TGCATCCTCATGGTGAGCAGCCCGAGGACAGCCAGGTTGGAGCAGGGCAGGTGGGAG
AACACTGGCCGCCACTCACAGCCCT
>1399
TGGCATCAACCAGCGGTCCTCGGATGCTAACCTCTACCGCCTCCTGGACCTGGACCCC
AGGCCCACGATGGTGAGCTTTGGGGGACATTCTGCTCTGCTCTGGCTGGGCTTGGCAC
GTGTTGTTCCTTCTGCTCCTGCTG
>1400
AGTGAAGGAGACAGTGTGCCCCAGGACGACACAGCAGTCACCAGAGGATTGTGACTT
CAAGAAGGACGGGGTGAGGCTGGGGGCTGGGGGTGTTGGTGGGTGCCTCCCAAGGA
GCTGAACAGGGGGCACCTGGGGAATAT
>1401
GGTGAAGCGGTGTATGGGGACAGTGACCCTCAACCAGGCCAGGGGCTCCTTTGACATC
AGTTGTGATAAGGTGAGTGGGCTGTTCTGGGATGCAGGGGCTGATGGGGGCATAGAGT
GTGGACCATCCAATGGGTCAATTA
>1402
TCTCTGCATGGATACTTCGTCTTCGGGCCCACAGGATGCAATTTGGAGGGCTTCTTTGC
CACCCTGGGCGGTATGAGCCGGGTGTGGGTGGGGTGTGCAGGAGCCCGGGAGCATGG
AGGGGTCTGGGAGAGTCCCGGGCT
>1403
TCATGGGCGTTGCCTTCACCTGGGTCATGGCGCTGGCCTGCGCCGCACCCCCACTCGC
CGGCTGGTCCAGGTAATGGCACTGAGCAGAAGGGAAGAAGCTCCGGGGGCTCTTTGT
AGGGTCCTCCAGTCAGGACTCAAAC
>1404
GGTCCACTTCACCATCCCCATGATTATCATCTTTTTCTGCTATGGGCAGCTCGTCTTCAC
CGTCAAGGAGGTACGGGCCGGGGGGTGGGCGGCCTCACGGCTCTGAGGGTCCAGCCC
CCAGCATGCATCTGCGGCTCCTG
>1405
CATCCCAGCGTTCTTTGCCAAGAGCGCCGCCATCTACAACCCTGTCATCTATATCATGAT
GAACAAGCAGGTGCCTACTGCGGGTGGGAGGGCCCCAGTGCCCCAGGCCACAGGCGC
TGCCTGCCAAGGACAAGCTACTC
>1406
ACAGGTTTTATTTTTTTCTTGTTTTTTTTGACTGAATCCCTAACCCTGCCCACACAACCT
CGGGATATAGGTAAGAAATTTACTTGTATATCAGCTAAAGCATGACCAGTTAGGATAATT
TCTATTTTTAAAATTTTGTT
>1407
GAAAGACATGGTAGAATACAAAGACAGATGTATGGCTGACAAGACGCTCCCAGAGTGT

TCAAAATTACCTGTAAGTAAATTGCTTGTGTTTCTTTTCCTTTTATCTTATGTCTTTCTTT
CTCTTTCTTTCTTTCTTTCTT
>1408
CTTTCCCTACCCTGGATCCCGAAGAGAAATGCCAGGCTTATGAAAGTAACAGAGAATC
CCTTTTAAATCAGTAAGTTTAATCTTAGTAAAAAATGATCCAGTTGAAGAATTAGTCTTA
GGCTGAATCTAATATCTATCTC
>1409
TGTTCATTTTGAGGAGGTGGCCAAATCATGTTGTGAAGAACAAAACAAAGTCAACTGC
CTTCAAACAAGGGTGGGTATAGCATTTGTTCCATGAAGAGGATAAGAAATCACTCAATA
CCACAGATCCAGACCCTGACTTA
>1410
CATTTTCTTCTTATCAAAAACATGTCTGTGGGGCACTTTTGAAATTTGGAACCAAAGTT
GTACACTTTATGTGAGTTTTATACTATATGTCTTGTCTCTGCTTGCATTTCCTTGTCTGTG
TCCCATTTTTGTTAAATGGT
>1411
TGTAGAAGATGTTTCTTCCAACTATGATGGATGCTGTGAAGGGGATGTTGTGCAGTGCA
TCCGTGACACGGTGAATATTCTCTAAAACCAAGTTAAAATAGTGATTTTTGGCAATTATC
ATTTTTTTATTCTCAAGTTGC
>1412
AAGGAAAATTTACTGACAGTGAAAATGTGTGTCAAGAACGAGATGCTGACCCAGACA
CCTTCTTTGCGAAGTAATATAACTCTTTATTGAATTTATAAGGGAAACAGAAAGAAACT
TGTAAGATTTGACTTTTGTTGCTA
>1413
TCAAATATACAAAGATCTCCTGAGAAATTGCTGCAACACAGAAAACCCTCCAGGTTGT
TACCGTTACGCGGTAGGTTCCATTGTTGTAGGTTCAGAAAATCAAAAAAGAACAACTA
GAAAACACTTAAAAATTGATATCA
>1414
AAAGCCTCAAGATGGTACAACAAGAATGTAAACATTTCCAGAATTTGGGGAAGGATGG
TTTGAAATACCAGTATGTTGTTTGCACAAGTGGGCTAACACTTGCCACTAGAATTGAAT
ACATAATCCCTCGAAGCGTAAAA
>1415
CGAGAAAATGGTGACAGCTTTCACTACTTGCTGTACGCTAAGTGAAGAGTTTGCCTGT
GTTGATAATTTGGTGAGCATGGCCTGTGTACCAGACTACTCTTTTTTTTTTTTTTTAAAA
AAAAGAATATTTGCACTCATTG
>1416
AAGATTTATTTACCTTTCACGCAGACATGTGTCAATCTCAGAATGAGGAGCTTCAGAGG
AAGACAGACAGGTACAAATAATCTCTTCCATTCTTCTTTTTCTTTGGTTTGAAGACACA
CCATGTATTAGTGAGAAGGTTT
>1417
TACAAATTTCGCAAATGTAGTGGATAAGTGCTGCAAAGCAGAGAGTCCTGAAGTCTGC
TTTAATGAAGAGGTAGTTTTATTTCTTTTCTACTGAAATTCAACTGGTTTTCCTGGTCAA
ATAAAATAAAACAGAACCCTGC
>1418
ACAACCACAGTTGCAGTTACTCCACCTACGGCATAAAAACACCAAGGAAATATCAAAG

AACACAACGCAGGTAAATTAACAGTATATAAAATGAGTAATTCCGACAAGAAGCATGG
ATTTATGAATACAACCATAAATTC
>1419
GTGGATTCCCAGGGACTGTCGGAGGTGTGGACTCTGCCTGCCTACCTGGTCTGGGAAG
ATGTTCTACCATGTGAGCAGGGCTCAGGGTGGCGGCAAGGGCTGGGTGGAAAGGAAG
AGCAAGGCCAGGCGCCGGCGCGAGA
>1420
TCGGCCCCAACTTGCTCAACACGGTGAAGCAGAAGCTCTTCACCGAGGTGGAGGGGA
CCTGCACAGGGAAGTGAGTGTCGAGCTCACCGCACCGCCAGATCGTTCGATGCGCAC
ACGGGGCGCTATACCTGCAACCCCTC
>1421
GTACAAGGCCATTGTTTTCCGGCCATTTAAAGGGGAGGTCGTGGATGCTGTTGTCACTC
AGGTCAACAAGGTGAGACCATACATAGGGGAGGCAGTGGGGTAGTCTCTCGGAAGAT
CTGGGTTGGGTTCTGGCTCCACTT
>1422
TATTGTGATTCAGCAGGACGATGAGATCCGCTTAAAGATTGTGGGGACCCGTGTGGAC
AAGAATGACATTGTGATCTTTTCCCCACCTCTCTACCTATACCAGGTTGAGCTGAGAAC
TGTCGATTTCTTTTTTCTTTTTT
>1423
GCACTGGGGCCCCTGCAGAGCTTCCAGGCCCGGCCTGATGACCTGCTCATCAGCACCT
ACCCCAAGTCCGGTAAGTGAGGAGGGCCACCCACCCTCTCCCAGGTGGCAGTCCCCA
CCTTGGCCAGCGAGGTCGTGCCCTC
>1424
AAGTGTCACCGAGCTCCCATCTTCATGCGGGTGCCCTTCCTTGAGTTCAAAGCCCCAG
GGATTCCCTCAGGTGTGTGAGTGTGTCCTGGGTGCAAGGGGAGTGGAGGAAGACAGG
GCTGGGGCTTCAGCTCACCAGACCT
>1425
CCCACGACTCCTGAAGACACACCTGCCCCTGGCTCTGCTCCCCCAGACTCTGTTGGAT
CAGAAGGTCAAGGTGAGGCAGGGCACAGTGTTTCACATCCATAATCCCAGCACTTTGG
GAGGCTGAGGCAGGCAGATCACCT
>1426
CACATGGCCAAGGTGCACCCTGAGCCTGGGACCTGGGACAGCTTCCTGGAGAAGTTC
ATGGTCGGAGAAGGTGGGTTTGATGGGAGGAAGGAAAGTGTGGAGCCGAGGGGTGGT
GGCTACAACGCACAGCAACCCTGTGT
>1427
CGTGCAGGAGTGGTGGGAGCTGAGCCGCACCCACCCTGTTCTCTACCTCTTCTATGAA
GACATGAAGGAGGTGAGACCACCTGTGAAGCTTCCCTCCATGTGACACCTGGGGGCC
GGCACCTCACAGGGACCCACCAGGG
>1428
CCTATGACCAACTACACCACCGTCCCCCAGGAGTTCATGGACCACAGCATCTCCCCCTT
CATGAGGAAAGGTGGGTGCTGGCCAGTACGGGGGTTTGGGGCGGGTGGGAGCAGCAG
CTGCAGCCTCCCCATAGGCACTCG
>1429
AGCTGTAACGGATACTTCGTCTTCGGTCGCCATGTTTGTGCTTTGGAGGGCTTCCTGGG

CACTGTAGCAGGTACTGCAGGGGAAAAAGGGGTTAGGGGAAGGCAAAGGTTGCTACT
CCGCTGGAGGGGGTTCCTAAAGAG
>1430
TGACGGTGGTCCTGGCTACCTGGACCATTGGTATTGGCGTCTCCATCCCACCCTTCTTT
GGCTGGAGCCGGTGAGAGTGCAGGGCAGTGGTGCTGAGTTAACTAGGAGCTCAGGTT
GATGTGGGTGGAAAGAGAGCTTGG
>1431
CTTCTGCTTCATTGTGCCTCTCTCCCTCATCTGCTTCTCCTACACTCAGCTGCTGAGGGC
CCTGAAAGCTGTGAGTGGCATTTGATAGTCAGGGAAGAAGGGGTTCGGGGCTCCACAT
GAGAAGGAAGAGTGCTCTGAAA
>1432
CATTCCTTCATTCTTCTCCAAGAGTGCTTGCATCTACAATCCCATCATCTACTGCTTCAT
GAATAAGCAGGTAAAGCTCTTTATTCACATTCCTATGGTCCAGAAGACCCTGGTTCTTT
TCTCACCATTGACTTTTAACT
>1433
GTGGACGCTTTCCTGGGCACCTGGAAGCTAGTGGACAGCAAGAATTTCGATGACTACA
TGAAGTCACTCGGTGAGCAAGCCGCGGGGCTCAGGATGTTGGCTTGGGGACTGGCTG
GTGGCGTGCCTAGCCCCACGCAGCA
>1434
GAACACAGAGATCAGCTTTAAGTTGGGGGTGGAGTTCGATGAGACAACAGCAGATGA
CAGGAAGGTCAAGGTAAGTCAGGGAAACAGGGGTGGGGAATGGAGAGTGCTGAGAC
TCTAAAAGAGAATAGGCTGGTAGTCTT
>1435
TCACCTGCAGAAATGGGACGGGCAAGAGACCACACTTGTGCGGGAGCTAATTGATGG
AAAACTCATCCTGGTAAGATGGGCAACTTTGGAGCTATATCTGATTGGTTATTACTACTG
CTCTTTCAGCCAAGCCTGTTCTA
>1436
GGGCCGAGGGCTGCCGGTCCGGGCTCCGCTCAGCACCCTCAACGGCGAGATCAGCGC
CCTGACGGCCGAGGTGAGATCCAGATCCGACCACTAGATCATCCTTATACCGACGGGG
AAACGGAGGCCAGAGAGGGCGTGGG
>1437
ACCGACGGTACCATGAAGGACGAGGTAGCTCTACTGGCTGCTGTCACCCTCCTGGGAG
TCCTGCTGCAAGGTGGGCTGGTTCCTATCTAGGAAGAGGGTGGGCCTTAGATCCCTAC
AGCTTGCCCTCTGCCCCCTAGGCC
>1438
GCAGGGCCTTCCGCGTGTCGCCGCCGCTCACCACCGGCCCACCCGAGTTCGAGCGCG
TCTACCGAGCCCAGTGAGGCGCGGCGGGAGGGCGCGGGGCGGGGAGCGAGCCCCAG
GCGGGTCCGGGTCGCAGGACCATCCCG
>1439
GTGAACTGCAGCGAGTACTTCCCGCTGTTCCTCGCCACGCTCTGGGTCGCCGGCATCT
TCTTTCATGAAGGTCGGGGTGTGGGGCAGGGGCGCACGCGCTGGACCCCCGGGACCC
GCGCAGGGCGCTCACCAGGCCCGTG
>1440
TGTGCGGCCTGGTCTACCTGTTCGCGCGCCTCCGCTACTTCCAGGGCTACGCGCGCTCC

GCGCAGCTCAGGTGAGGGCCGGGCGGGGAGCGGGGCGGGGCCGGGGAAAGATCGCG
GGCGGGCGGGGCTCCTGGGGAGCGG
>1441
ATGGGGCTGGGCCGGGTCCTGCTGTTTCTGGCCGTCGCCTTCCCTTTTGCACCCCCGGC
AGCCGCCGCTGGTGAGTGGGGTTCCTGGCGGTCCCCGGCGGAGCGGGAGCGGCGGGG
CGTTTCCGGGGGTCCGGGTGGGTT
>1442
ACCGAGGACTTGACAGAGAATGGGCAAGACCTCAGGAGGACCCTGACTCATATCAAG
GACCAGAAAGGAGGTGAGAGTCGGCAGGGGCAAGAGTAATGGGAGGCCTTCTCCAG
GAAAGTTGGAGACAGAGAGCAGGGACC
>1443
GCTATGCAGGCAGACTGCCTGCAGAAACTACAGCGATATCTGAAATCCGGGGTGGCCA
TCAGGAGAACAGGTACCGACCCTGGCCAGGGGCTCTACTGTTCCCGCAATTCTGCTAG
AGTTGCCTCGCCTCCCAGCTCTGT
>1444
GGAGAGGAGCAGAGGTTCACCTGCTACATGGAACACAGCGGGAATCACGGCACTCAC
CCTGTGCCCTCTGGTGAGCCTGGGGTGACCCTGGAGAGGGTCAGGCCAGGGTAGGAA
CAGCAGGGACGGCTGTGGCTCTCTGC
>1445
TGTTTTGTTATTATTATTATTCTCTGTGTCCCTTGTTGCAAGAAGAAAACATCAGCGGCA
GAGGGTCCAGGTGAGAAAAGGGGACAGTTTCTGGAGATGGGAAAGCTCCTTTCTAGG
CAGTAGGGTCTCCTCATTGCTCC
>1446
CAGCCGCGGTCCAGAGCGCGCGAGGTTCGGGGAGCTCCGCCAGGCTGCTGGTACCTG
CGTCCGCCCGGCGGTGAGTCCGCGCGGCCCCGGCGGGACGCCCCCGCCACCTGCGCG
CACCGTCAGACCCGGCGGCCTCGGCT
>1447
ACGGCCATCCTCTCCAGAATGAAGATCTTCTTGCCAGTGCTGCTGGCTGCCCTTCTGGG
TGTGGAGCGAGGTGAGGTGCCCTTGGGGACCCCAGACCTTTGTCCAGCTGTGCCCTGC
TCCACTCCCTCTCCACCCCTCTC
>1448
TGCCTGAAGCCGACCATCTGCTCCGACCAGGACAACTACTGCGTGACTGTGTCTGCTA
GTGCCGGCATTGGTGAGTGCCAGGCTCAGACCGTGCCTTCCTCCCCTGGCCATCTCCCT
AGCCCGGGCCGGGGCTCAGCAGA
>1449
GCTTTGCCTCTGAGGCCCCTGACCCGGGGCTTGGCCAGCGCTGCCAAAGGAGGCCAC
GGAGGAGCAGGAGGTGAGTGGGGAACGGGCGGATCCGGGGGCTCCCCTACCCTGCCC
ACCTGTTCACAGGCCCGCCGCCCCAG
>1450
CTATCTCCACTCGGGCCACCGCCCGCGCCCCGAGTTCCGTCCCTACCAACACCTCCGCA
TCCGCACCAAGGTACGCGGGACGGGCGCGCGGGCGGCACGGGGGTGCTGCGGGGGC
GGGGGGGGGTGCTGCGGGGGCGGGG
>1451
CTGAAGGGGCTGAACCATACACTCCTTTTTCTACAACCAGCTTGCATTTTTTCTGCCCA

CAATGAGCGGGGTAAGATTTTTATTTTTGGCAAGGGGTATAATTTGGGTTCACTGTGGC
TACTTGAACACTACACTGCAGC
>1452
CAGCGACTATCTTTCTCCTCTGGGGACCACTTGGCTGAGGACCTGTGCGTGCAGGCTG
CCAAGGCCAGCGGTGAGTGCATCCCTAGTGGATCGGGCCAGAGGGAAGGATGGGGCT
GTGTGGGGCCAAGATTGGAAGCTGG
>1453
GCTGGTTCCCCCCGAGCCACATCTTCTCCGTGGAGGATGCCAGCACCCAAGTCCTGCT
GTACAGGATTCGGTCAGCCCCCGCACAAGGCGGATAGGACAGTTAGTATCAGGGTCCT
CTGCATCTCTGCTGGGACCCACAC
>1454
CTTCGGGCTACGCAAGGATTTGGCCAGTGCTATCCTTGACCTGCCAGTCCTGGAGCAC
CTCTTTGCCCAGGTGGGGTTCTGCCTGGGGTTTGACCAGGGGGTTGGGGGTCCAATGG
GCAACATGAGGACTGGCATGCAAT
>1455
CCGTGTTGGACCTGGCCCGGATGGCGCGAGAGCAGGCCCAGCGGCCGGGAGAGCTGC
TGAAGACTGTCAGGTGAGAGCCACCAGGCTGTGGGGACGGGCTCTGTTTGGGAGTGA
GCAACGTGGGCTCCATCGGGGGTTTG
>1456
GGCCACGACGGGCTGGGGCTGTTCCGCGTGGCTGGTGACGGCGGCATCGCCTGGACC
CAGGGAGAACAGGGTGAGGACGGCTTCCCCCCTGGCGGGGCCAAATGGGGGGGGGT
TTGGGGAGGGGCCGGAGATTTGTAGGG
>1457
GCCCCGCGCGTTGGCCCGGCCGGAGAGCACCGCCTGGTCACTGTTACCAGGACAGAC
AACCAGATTTTAGGTGGTGCAGGATTCCCCTCCCCCTTCAGCCTTACCCCGAAGGGCG
GGACCGGCACCCTCGGGTTTCACTG
>1458
TCCTGGCTCCTATGTTCTCCGCCGCAGCCCCCAGGACTTTGACAGCTTCCTCCTCACTG
TCTGTGTCCAGGTCGGTCTACTGCTAGGGTGGGTAGTGGAGGGCTGCCTGGACAAGAG
GTTACCTCTGAATTGAGATTTAC
>1459
TGGGATGGGGGGCTGCACGTAGATGGGGTGGCAGTGACCCTCACTTCCTGCTGTATCC
CCAGACCCAAAGGTGAGGCCCCTTGGTCCCCTGGAATGAGTGGCTGATCTGGGACCCT
GGTTTCCTATGTCTGATAACAGCT
>1460
TCAGCCCCAATCCCAATACCAGCTGAGTCAGATGACATTTCACAAGATCCCTGCTGACA
GCCTGGAGTGGGTAAGAGGCCCTGGGAAATGAGGCGATACCTCAGTCTGGGGTCCAG
AGACTCAGATGCGTGGCCTCAGGC
>1461
GGATGGGGAGGCCCGAAAGACAGAAGTGCTGCTGAAGGTCATGGATGCCAAGCACAA
GAACTGCATGGAGGTGAGAGGCAATGTGGACCAGACTTTTGGAGTCGGGGGCTGGCT
TGAGCGGGGCTCGTGGATGCAGAGAC
>1462
CGAGCTTGATGAGCCAAGTGTCGTACCGGCATCTCGTGCTGCTCCACGGCGTGTGCAT

GGCTGGAGACAGGTGAGTGCTCCTAGGGGGTGTTAAAGGGGAGAGGGAGGAAAAGA
GGGCGAGGGGGGAAAGAGAGCTAGGG
>1463
TGGCCACCTGGTGCCAGCCAGCTGGAAGCTGCAGGTGGTCAAACAGCTGGCCTACGC
CCTCAACTATCTGGTGAGTGCTCCTCCCCCAGGGGATTTGGTTTGGGGGGAGGGGGG
GGGTTTAAAAAAACCCAACCCCCTA
>1464
GGCTGATGGGAGCCCGCCCTTCATCAAGCTGAGTGACCCTGGGGTCAGCCCCGCTGTG
TTAAGCCTGGAGGTAAGTTCCTGGAGGTGGAGGAGGGAGGGGCTGAACAGGGCAAG
GAAGTGGATCCCTGATCCCACTTTCA
>1465
CTTCGGCGCCACGGTCTGGGAAGTGTTTAGTGGCGTCACCATGCCCATCAGTGCCCTG
GATCCTGCTAAGGTCAGAGCCCCTCACCCGGCATCGGTCTCCGAACCCCCACTTTGAC
AGCCGGGCAGACTGACATCCAGTC
>1466
GCCTATGAGCCGGTCCAGAGGCCCTCCTTCCGAGCCGTCATTCGTGACCTCAATAGCCT
CATCTCTTCAGGTGCCCGCTGGGACGGGTTGGGTGGGGAGGGCTGTGATGTCATATTG
GGCCCAGTGGAAGGAGCGTGGTT
>1467
GCTCTATGCCTGCCAAGACCCCACGATCTTCGAGGAGAGACACCTCAAGTACATCTCA
CAGCTGGGCAAGGTAAGGTGGGCAGGGCCAGGGTGGGTTGGAGAGGCAGGGCAGCA
TCCAGGTACCTGGACATCAGTGCCGC
>1468
CTTCCTGCAGCGGCACCGCGCGCGCCTCGATGCCAGCCGCCTCCTTCTCTATTCCTCGC
AGATCTGCAAGGTGAGAGGGGGCGCCCCGGGACTTGTGGGGATTCAGCTGGCACGGC
CTGGGCAGGGGTCTGCTTGGAGGT
>1469
CTGTGGGCAGACAGCCATGCACCTGCCCCCAACTCTGGCAGCACCCGGCCAGCCTCTG
CGCGTTCTCAAGGTAAGAGCAGGCCAGCCCTCCTCCCTCCTGCCCGGGGCTGGAGAG
GCTGGTGTGGACAGGACCCTGTGCC
>1470
CCGCTCCTTCAGCTCCACCGCCCGGAACCGCTTTCAGAACCGAGTGCGCGAGAAACA
GAAGCTCTTCCAGGTGGGGGGTCGGGGGGGGTGGGGATCCGACGCGCCAGCCGGGA
GCGCGCCGAGCCGGGGCAGGCGGGGCG
>1471
CCGTTGTACCTGAAGGGCGGCATCGTTGACAACATCCTGTACCGAGTGACAATGACGC
TGTGTCTGGGCGGTGAGCGCAGGGCCCGTCTGGGCTGCGGGGGAGGCGGGGCTGGAC
CCAGAGTAAGAGGTGGCTGGTTTCT
>1472
TCCTGCCTTGGCCATGAGGGAAATCGTGCTCACGCAGACCGGGCAGTGCGGGAACCA
GATCGGGGCCAAGGTTGGCAGCCGGGGCTCTGAGGGCCCAGCCCGGCCTGCCGGGTG
GCCGGGGAAGATGTTGGCAGCGGCGG
>1473
GCTGGCACCTACCACGGGGACAGCCACCTGCAGCTGGAGCGCATCAACGTGCACCAC

CACGAGGCCAGCGGTGCGACCCCCGTCCTTCCCCCACCGCCCTCCTGGGAACGCGGC
CCTCCCCTCGCTCATGCCCTCCCGCC
>1474
CCGGGCACCATGGACTCTGTGCGCTCGGGGCCCTTCGCGGAGGTCTTCAGGCCAGAC
AACTTCATTTCCCGTGAGCTGCGGGCGAGGACTGGGGTGCGGCTCCTTAGCCAGGGCA
GCTCGAAATCCAGGAACGCTCCAAG
>1475
CGCTGCAAGCCCTTCTACTGCGACAGGCCATGGCAGCGGGCCACTGCCCGGGAATCCC
ACGCCTGCCTCGGTGAGGCCTTGGAGGGTGGCCTGGGGACCTTGGACACAACCAGCC
TGCCCCTGACCCATCCCTCCCTGCA
>1476
TGCCACTACTGCCGGGAGGGCTTCTATCGAGACCCTGGCCGTGCCCTGAGTGACCGTC
GGGCTTGCAGGGGTGAGCCACCACCGGCCACCTGCAGGCCCTCACCCTCTGACTTCCC
AGATCCCCAGACAGGCTTCTGACC
>1477
ACTGGCCTCACCTGCAACCGCTGCGCGCCTGGCTTCCAGCAAAGCCGCTCCCCAGTGG
CGCCCTGTGTTAGTGAGTGACCCTGCCCCGCCTCAGCCACCAAGCCAAGGCCACCCCA
GCTCCCTGCTGTTGTCCCGTCTAT
>1478
GACTCGCACTGCAAACCTGCCCGTGGCAGCTACCGCATCAGCCTAAAGAAGTTCTGCA
AGAAGGACTATGGTAGGTGCCCTCAGGCCTCCCGCGGACCTTCCCACCTTCCTCCTCT
CCCTACCTTCCCTCCTCCGCCAGC
>1479
GCGGCGGGTGGCAGCGATCCGCGGGCTGGCGACGTAGAGGAGGACGCCTCACAGCTC
ATCTTTCCTAAAGGTTGGGCTCGGGGCTGCCAAACTCCCCCGCGCCACTTCGCGTGGT
CGCCGCAGGCCTGGCGTTATGCGCG
>1480
TGAAAACATTAAACTACACAGCCCGTTTCAGTCGTTTCAAAAACAGAGAGACCATTGC
CAGTGTTCGTAGGTGAGTGCTAAAAGAAAGTTTTTATTAATCCAAACCATTGGACAACT
GCATGTAGAATGCTGTCCCCTCC
>1481
AGTTTGAGTTGGCCTGTTTGGCCAACCTTTGCCCAGAGACTGCTGAGGAGTCCAAGGC
TCTAATCCCAAGGTTAGTACCAGTTGTGATAAGTTCTCTGTATACTGGATAAATTCCTAC
AAATAGAATTGGTGTGTCTAAA
>1482
GCTCCGGGGACACTTTGCGTTCGGGCTGGGAGCGTGCTTTCCACGACGGTGACACGCT
TCCCTGGATTGGGTAAGCTCCTGACTGAACTTGATGAGTCCTCTCTGAGTCACGGGCTC
TCGGCTCCGTGTATTTTCAGCTC
>1483
AGGAGCCGCAGTCAGATCCTAGCGTCGAGCCCCCTCTGAGTCAGGAAACATTTTCAGA
CCTATGGAAACTGTGAGTGGATCCATTGGAAGGGCAGGCCACCACCCCGACCCCAACC
CCAGCCCCCTAGCAGAGACCTGTG
>1484
CTACCAGGGCAGCTACGGTTTCCGTCTGGGCTTCTTGCATTCTGGGACAGCCAAGTCT

GTGACTTGCACGGTCAGTTGCCCTGAGGGGCTGGCTTCCATGAGACTTCAATGCCTGG
CCGTATCCCCCTGCATTTCTTTTG
>1485
AAGCAGTCACAGCACATGACGGAGGTTGTGAGGCGCTGCCCCCACCATGAGCGCTGC
TCAGATAGCGATGGTGAGCAGCTGGGGCTGGAGAGACGACAGGGCTGGTTGCCCAGG
GTCCCCAGGCCTCTGATTCCTCACTG
>1486
GCGTGTGGAGTATTTGGATGACAGAAACACTTTTCGACATAGTGTGGTGGTGCCCTATG
AGCCGCCTGAGGTCTGGTTTGCAACTGGGGTCTCTGGGAGGAGGGGTTAAGGGTGGT
TGTCAGTGGCCCTCCGGGTGAGCA
>1487
GTAACAGTTCCTGCATGGGCGGCATGAACCGGAGGCCCATCCTCACCATCATCACACT
GGAAGACTCCAGGTCAGGAGCCACTTGCCACCCTGCACACTGGCCTGCTGTGCCCCA
GCCTCTGCTTGCCGCTGACCCCTGG
>1488
ACAGAGGAAGAGAATCTCCGCAAGAAAGGGGAGCCTCACCACGAGCTGCCCCCAGG
GAGCACTAAGCGAGGTAAGCAAGCAGGACAAGAAGCGGTGGAGGAGACCAAGGGTG
CAGTTATGCCTCAGATTCACTTTTATCA
>1489
GCCCAACAACACCAGCTCCTCTCCCCAGCCAAAGAAGAAACCACTGGATGGAGAATA
TTTCACCCTTCAGGTACTAAGTCTTGGGACCTCTTATCAAGTGGAAAGTTTCCAGTCTA
ACACTCAAAATGCCGTTTTCTTCT
>1490
TGAATGAGGCCTTGGAACTCAAGGATGCCCAGGCTGGGAAGGAGCCAGGGGGGAGC
AGGGCTCACTCCAGGTGAGTGACCTCAGCCCCTTCCTGGCCCTACTCCCCTGCCTTCCT
AGGTTGGAAAGCCATAGGATTCCAT
>1491
TGCGCCTGCTGCTGCTGCTGCGGCCCCCGCGCGGCGCCCTGCGGGCCCCCAGAGGCG
GCCGCCGGGCTGGGTGAGTGGGCGCGGAGCGGGGCGCGGGGCCCGGCGGAGCCCGG
GGCGCGGCGCAGTGGGTGCCGAGCTTG
>1492
CCAGTATCCTGACGTGAGTACGCGCGAGCGCCTGGCCGGCCGCATCCGCCTTCGCGAG
GAGCGCGTGGAGGTGAGTGCCCCGCCCAGCCTTTCCCCGGAGCGCGCGGGCCGCGGC
TACACTGGACTGGGGTCCTGGCGGG
>1493
CCCGAGGAGGTGATCGCCACCATCTTTTCCTCCGCCTGGTGGGTCCCTCCCTGCTGCG
GGACAGCAGCTGGTGAGTACCCCTCCTGGTTCTTCTGGAAGTAAAAAGGGTGTCTTTT
CGGTTGAAAGTACTTTTCAGCCTA
>1494
ATTTAAGAGAGAATGGGCCAGTGTCATGCGCTGCATAGCAGGTTTTGGTGGCATTAACC
ACGCCAGTGCTGTATCCTTAATTTTCTGTGCTACGTCCAGAGTATCTTCTTAGGTTATATA
TTGAAGCATTTTGTTTTGTT
>1495
GGCTGGGGATCACCATAGCTTTTCTAGCTACGCTGATCACGCAGTTTCTCGTGTATAATG

GTGTCTATCAGTATACAGTAAGTGTGTGTTTTCAAATATTGGCTTTGGAAAGCTTAGATA
TTTTCATATTTTATTTGTTT
>1496
TTCTTGGCTCCCTTGTATATTTTTCTCAGGAGGCGTCACGGTGGGGAACATAGGACGAC
AGTTAGCTATGGTAAGTGAAATGATCATATTATCTTCTAAAACTTGCTTCTCTTTACCTTG
ATAGAATGACTTTACATGAT
>1497
GGCCGGAAGATGAAACTGAGCTTCACCCTACTCTGAATGCAGATGACCAAGAATGCAA
ACGAAATCTCAGGTGAGCAGTATTTTAAGAAGGTCCTGTTGTCAGTTTTTCAGATTTTC
TTATTCCCAAGGCCTTTAAACTG
>1498
ACAGAGCACCTGTGGGAGGACTAGGAAAATTAAAGATGATTATAGCCAAAAATGGCCC
AGACACAGAAAGGTAGGTAATTATTAACTTGTGACTGTATACCTACCGAAAACCTTGCA
TTCCTCGTCACATACATATGAAC
>1499
AGGTCAGGAGGAGACTGTGAGAATTGTTGCTCTGGATCGAAGTTTTCACATTGGCTGT
TACAAGTGCGAGGTCAGGGGCCCCCAGCACGTGCAAGGGGCTGGCAGTGTCTAGGGT
GCTGGGTAGAGCATGAGGGGGAACA
>1500
CTGCCCTTCTTCCCCGAGAGCCCCAGGTACCTGCTGATTCAGAAGAAAGACGAAGCGG
CCGCCAAGAAAGGTAACGAGGGCTCTGGCGGGGGCGGCCTCCAGGTTCTGCTCCCCA
GCCCTGGTCCGACGGTGCCGCTTCT
>1501
CTCGCTGCGCTGGCAGCTGCTGTCCATCATCGTCCTCATGGGCGGCCAGCAGCTGTCG
GGCGTCAACGCTGTATGGGCCGGGCTGCGGCTCGGGAGCTGGGGAGGGCTCGGGGGG
CGCGGGGCGCAGGTCCTGGGGCGCC
>1502
GCCGGAGGAGCACGTGCAGTACGTGACGGCCGGCACCGGGGCCGTGAACGTGGTCAT
GACCTTCTGCGCCGTGAGTGTCCTGGGAGCTGCCCACAGCCTCCACCCAAGGCCCCTC
GGGCCCCACGCCCCAGCCTCGGCGC
>1503
GCTGCTGCTGCTGGGCTTCTCCATCTGCCTCATAGCCTGCTGCGTGCTCACTGCAGCTC
TGGCACTGCAGGTGAGGACAGGCCAGGGTCCCAGGAGCCCGGAGCTGCACCAGGAA
GACCAGTTGCCCCTCCCATCCTGCG
>1504
GTGTCCTGGATGCCATACATCAGCATCGTCTGTGTCATCTCCTACGTCATAGGACATGCC
CTCGGGCCCAGTACGTACCCAAAGCTGGCCTCAGGGTGTCACAACCCCCCCTGGTCCC
AGAAGCTCCGGGCTGGAGCTCC
>1505
CTTCATGGTGGGGGGCAGTGTGCACTGGCTCTCCAACTTCACCGTGGGCTTGATCTTC
CCGTTCATCCAGGTGAGTGGGGCCCGGGGCCCACGCTCACCACCAGATCCCCATGCAG
TACAGTGCGAGCCCCTGGGCAAAC
>1506
GGCTGTTCCTCAGGTATTGGCCTGCACTTGGCAATACATCTGGCTTTGGACCCATCCCA

GAGCTTCAAAGGTATAGATAGGTAGGGACAGGGAGGGAGAGAAGGGAAAAGCCCTTG
GAGGCCAGAAGAGAAGTCAGATCT
>1507
GATGTAAGGAACTCAAGCTCCCTGGCCGCTGCCCGGGAACGCGTGACCGAGGGCCGT
GTGGATGTGCTGGGTGAGCCTCCTGGAAGCATATGGGCTCCTAGGAGCCTTCTCCGCC
CTGCGTTGAAACCAACATGTCCCCA
>1508
TTCCTGCCAGACATGAAGCGGCCGGGTTCGGGACGCGTGTTGGTGACTGGGAGCATG
GGAGGATTGATGGGTGAGTGGCAGGGACCGGGCCCCGAGCTCCAGATTCTTTGTGTGC
AGAGCTGAGCCTTGAAGGCAGGCTC
>1509
GCGCCAGCAAGTTCGCGCTCGAAGGCTTATGCGAGAGTCTGGCGGTTCTGCTGCTGCC
CTTTGGGGTCCAGTGAGTCAACACCCCCGTCTCCTCAACCCTCTTAACTCTGACCTAG
AGATGCCGAGCACCCTGTCCTGCG
>1510
TTATGACCTCACCCGGGAGCGCAAAGCACGCGACATGGTGGGGCAGGTGGCCATCAC
AAGGATTGAGCAGGTGAGGGCAGGTGGTGCCATCAGGGTGTCCCCCCAGCGGGGTTC
AGGGCTCTGGTTCCTTCACAGAACAG
>1511
AGAACCAAGGCTACTATGACTACGTGAAGCCAAGACTTCGGACCACCATCAGCCGCGC
CAAGCCCGTCTGGTAAGGCTTCAGTCCCTGCCAGGAAGGCTGTGGGACCCTCCCCTCA
GGCCAGTAGGCAGTTAGCCAGGCA
>1512
ATTGTGGATTTGGTCAAGGAGCTTGACAGAGACACAGTTTTTGCTCTGGTGAATTACAT
CTTCTTTAAAGGTAAGGTTGCTCAACCAGCCTGAGCTGTTTCCCATAGAAACAAGCAA
AAATATTTCTCAAACCATCAGTT
>1513
AACTACAGCACCTGGTAAATGAACTCACCCACGATATCATCACCAAGTTCCTGGAAAAT
GAAGACAGAAGGTGATTCCCCAACCTGAGGGTGACCAAGAAGCTGCCCACACCTCTT
AGCCATGTTGGGACTGAGGCCCAT
>1514
CACTAAGGTCTTCAGCAATGGGGCTGACCTCTCCGGGGTCACAGAGGAGGCACCCCT
GAAGCTCTCCAAGGTGAGATCACCCTGACGACCTTGTTGCACCATGGTATCTGTAGGG
AAGAATGTGTGGGGGCTGCAGCACT
>1515
GGCCGGCTTCGCGGGCGACGATGCCCCCCGGGCCGTCTTCCCCTCCATCGTGGGGCGC
CCCAGGCACCAGGTAGGGGAGCTGGCTGGGTGGGGCAGCCCCGGGAGCGGGCGGGA
GGCAAGGGCGCTTTCTCTGCACAGGA
>1516
CGAGGAGCACCCCGTGCTGCTGACCGAGGCCCCCCTGAACCCCAAGGCCAACCGCGA
GAAGATGACCCAGGTGAGTGGCCCGCTACCTCTTCTGGTGGCCGCCTCCCTCCTTCCT
GGCCTCCCGGAGCTGCGCCCTTTCT
>1517
GGCCAGGTCATCACCATTGGCAATGAGCGGTTCCGCTGCCCTGAGGCACTCTTCCAGC

CTTCCTTCCTGGGTGAGTGGAGACTGTCTCCCGGCTCTGCCTGACATGAGGGTTACCC
CTCGGGGCTGTGCTGTGGAAGCTA
>1518
GTACCCTGGCATTGCCGACAGGATGCAGAAGGAGATCACTGCCCTGGCACCCAGCACA
ATGAAGATCAAGGTGGGTGTCTTTCCTGCCTGAGCTGACCTGGGCAGGTCAGCTGTGG
GGTCCTGTGGTGTGTGGGGAGCTG
>1519
AGCTGGTTTTGCTGGGGACGACGCTCCCCGAGCCGTGTTTCCTTCCATCGTCGGGCGC
CCCAGACACCAGGTGAGTGGATGGCGCCGCGGGGCTCCTGGGTTCTGCGTTGCGGGG
TGGGTTCGGTGTCGCCGGCGAGGCT
>1520
GGAGGAGCACCCAGTGCTGCTGACCGAGGCCCCCCTGAACCCCAAGGCCAACAGAG
AGAAGATGACTCAGGTGAGGCTCGGCCGACGCCCGTGCTCCTCCCGTCCTTTCCCCAG
TCATTTTCTCGCCCGGCTTGATTTCT
>1521
GGCCAGGTCATCACCATTGGCAATGAGCGGTTCCGGTGTCCGGAGGCGCTGTTCCAGC
CTTCCTTCCTGGGTAGGTGTTGTGAGCTAAAGGTTTCTACTCTTTCATCCTCGGTGACA
CAGCATCACTAAGGGAGGGCTCT
>1522
GTACCCGGGCATTGCCGACAGGATGCAGAAGGAGATCACCGCCCTGGCGCCCAGCAC
CATGAAGATCAAGGTGAGTCGAGGGGTTGGTGGCCCTCTGCCTGGCTCGGGAGAGCT
GACTGGGGGGCGCTCTGCGAGCTGAA
>1523
TTGGCATGGACAAGCCGCTCACCCTTCCAGACTTCCTGGCCAAGTTTGACTACTACATG
CCTGCTATCGCGTGAGTTGCCCCCAACCCACAGGTCCTAGGGCAGCATTGATCCCTATG
ACTAGGACCAGGCCTGTCCCTC
>1524
TGGAGGTGCGGTACAGTCCGCACCTGCTGGCCAACTCCAAAGTGGAGCCAATCCCCTG
GAACCAGGCTGAGTGAGTGATGGGCCTGGAAGGGGCCATGCTGAGGGTGTGGCTGGG
AGGCTCAGCTCTGAGACTGGAAGGG
>1525
CTGCAGGAGGGGGAGCGAGACTTCGGGGTCAAGGCCCGGTCCATCCTGTGCTGCATG
CGCCACCAGCCCAGTGAGTAGGATCACCGCCCTGCCCAGGGCCGCCCGTCTCACCCTG
GCCCTGACCTCCTGGCCTAGCAGTG
>1526
CATTGACCTGGCTGGAGATGAGACCATCCCAGGAAGCAGCCTCTTGCCTGGACATGTC
CAGGCCTACCAGGTGGGTCCTGTGAGAAGGAATGGAGAGGCTGGCCCTGGGTGAGCT
TGTCTCCCACCCATAGTTGGGAGAA
>1527
GGCTGTGAAGAGCGGCATTCACCGTACTGTCCACGCCGGGGAGGTGGGCTCGGCCGA
AGTAGTAAAAGAGGTGAGGGCCTGGGCTGGCCATGGGGTCCCTCCTCACTGCCTCCTC
CCATACTTGGCTCTATTCTGCTTCT
>1528
ACACGGCTACCACACCCTGGAAGACCAGGCCCTTTATAACAGGCTGCGGCAGGAAAA

CATGCACTTCGAGGTAAGCGGGCCAGGGAGTGGGGAGGAACCATCCCCGGCTGTCCC
AACTTCCTGTATAGAGAGGCAGAAAG
>1529
CACCCTGGACACTGATTACCAGATGACCAAACGGGACATGGGCTTTACTGAAGAGGAG
TTTAAAAGGCTGGTGAGTGGGTGTGAGCCATACTGGCCTTGACTCGGGTTTGGGAGTA
TGGTATCTACAGGTCCAGTCCGGG
>1530
GAAGATGAAAAGAGGGAGCTTCTCGACCTGCTCTATAAAGCCTATGGGATGCCACCTT
CAGCCTCTGCAGGTAGGTTCCTGTCTGGGCTTCTGGGCAGTTGCCTGTCCTGGCCCCA
GTGTGGCTTTCTGTGGGACTTCTA
>1531
AATTTGTGACTCACAATCTGTCTTTTGATGAAATCAACAAAGCCTTTGAACTGATGCAT
TCTGGAAAGAGGTAGGCTTTCTCTTTATATATCATAGAATGTAATAATGATGTTGAGTTT
GAGGGGATGGAGAGTCGAAAA
>1532
TCAATTTTTTTAATTTTCCTACTAAATTTTACTGAATCCAGAACACTGCATAGAAATGAA
TATGGAATAGGTGAGATATTTTGTGTTTTTCTTGTCTTTTCTCTATATCAAAATTTTTTAA
ATTATAAAATTTGCATTAA
>1533
GAAAGATGCATTGACTGCAATTGAGAAACCCACTGGAGATGAACAGTCTTCAGGGTGT
TTAGAAAACCAGGTGAGTGAATAATTTTAAAAAAGCATTGTGATATTTGACAAAAATTT
AGCATGCTGAAGAGAAGATACAA
>1534
TTTTCCAAGTTCCAGAACCTGTCACAAGCTGTGAAGCATATGAAGAAGACAGGGAGA
CATTCATGAACAAGTAAGGATCCAGTTTAAAGGTAGATGCAAACCTCAGAAACACAGC
AATGGCAAGCCTAATTTAGTATTTT
>1535
TGCTCGCTATGACAAAATAATTCCATCTTGCTGCAAAGCTGAAAATGCAGTTGAATGCT
TCCAAACAAAGGTATCATATTTGCGTGGATATCTGAACCAGTACTGTAGTCTATGACTCA
TTAAAACAAAACAAAGTTAAA
>1536
AAAGCAGCTTGTTAAATCAACATGCATGTGCAGTAATGAAAAATTTTGGGACCCGAAC
TTTCCAAGCCATGTAAGTTCAAGTTCTATCTAGGGAAGAGGGTGAGAGCTACAGAACT
ACCATTTTGCAATTTGGGTTCGTT
>1537
AGTCCTGGATGTGGCCCATGTACATGAGCACTGTTGCAGAGGAGATGTGCTGGATTGT
CTGCAGGATGGGGTGAGGAGTCTTGCTTCTTAAAATAGAAGATTTTCACTCCCTTTTCT
TTCTTTTTGTCTCATTCTAAAAG
>1538
TAAACAGGTTTTTAGGAGATAGAGATTTTAACCAATTTTCTTCAGGGGAAAAAAATATC
TTCTTGGCAAGGTAACACACTCTGTAAATGCATGTTCATGCAAGTAAAAATGATTATGT
GGCTGACAGATTTGCGTTGTTG
>1539
TAAAGGATACCAGGAGTTATTGGAGAAGTGTTTCCAGACTGAAAACCCTCTTGAATGC

CAAGATAAAGGAGTAAGTTGCTCTAGAATTTTAGGGGAGTATGAAAAACTGGATTGATA
TCATCTGTTAAAAATGCTGTTTG
>1540
AGAGCCAAGCATTGGCAAAGCGAAGCTGCGGCCTCTTCCAGAAACTAGGAGAATATTA
CTTACAAAATGCGTATGTTTTTGTAAACAGTATTTTTAGTGAATTAAAATTATTAAAGAG
AATGTAGCCTTCCCCAATTCTC
>1541
AATGGCAGCCACAGCAGCCACTTGTTGCCAACTCAGTGAGGACAAACTATTGGCCTGT
GGCGAGGGAGCGGTGAGTGTCTGCTTGGTTTGGTCCCATCTCATTTCTGCCCTGTTTGA
CTTGAAATAGCCTCATAATTCCC
>1542
ATGACAAGTTCATTTTCCATAAGGATCTGTGCCAAGCTCAGGGTGTAGCGCTGCAAAC
GATGAAGCAAGAGTAAGAAACTGTTACTTGCTAGCATGGAAAAGAATGACAACCCCA
AAGAGTAACTGAGACTTCTACCTCG
>1543
TGCAGATTTCTCAGGCCTGTTGGAGAAATGCTGCCAAGGCCAGGAACAGGAAGTCTG
CTTTGCTGAAGAGGTACATGCAGCTCATTTCATACTCAAAATACTTGCTATGGAATTTTC
TGTAGTGGATAATGAAAGGAAGA
>1544
GCTGATACACGCAGACCAGATCTGGTCAGGTCCTCGGAAGCTGAGTCCAGAGCGATGC
TGCTGAAGACAGGTACCTACACCGGCTGGGGTGGCTTGGAAGGGTAGGGGTGAGTGC
TACAAGGAGTGCTTTGGCCCTTCTT
>1545
TGGGCTGGCTGGCCTGGGAACGCTTCCGCTGCAACATTAACTGTGATGAGGACCCAAA
GAACTGCATAAGGTGAGTAAGGTCCACACTCACCTGATGAGCCTTGCCCAGGGCCCTG
CAGATTCGGGGGCAGGAAGTGGAG
>1546
CGATGCCAGTGGCCGCCTGATGCCAGATCCCAAGCGCTTCCCTCATGGCATTCCTTTCC
TGGCTGACTACGTGAGCCGCACCCAGCCCTGCCCTTGCGCTCAGGGCTAGACCACTCC
TTTCACCCTACCTCGCTTAGGGC
>1547
TTCGCCGAGTGGAAGGTAGACATGCTCAAGCTGGATGGCTGCTTCTCCACCCCCGAGG
AGCGGGCCCAGGGTGAGTTACGCAGCTGGCTGGGGCACGTAGGCAAACGGGGAGGG
TGGCCATGGAGACCCACCCAGCGCCC
>1548
GAATGCCACAGGCCGCCCCATCGCCTTCTCCTGCAGCTGGCCAGCCTATGAAGGCGGC
CTCCCCCCAAGGGTAAGCCATTCTCCCCCCTGCCTGATGGCGCTTCCCACACCCAGGC
CTCCTTACCTCCTGAGTGCCAGGC
>1549
TTGGTTCGTGGAGCACCAGGACATACTGCAGCCAGTGGCCGGCCCTGGGCACTGGAAT
GACCCTGACATGGTACCAGGATGGAGGGGGATGTCCTAGGCCCACCATCTGTGACGAC
CCTGCTCCCCACTGCCCTGTGCTT
>1550
TCTGCAGAATCCACTCATGATCAAAATCAACCAGGATCCCTTAGGCATCCAGGGACGC

AGGATTCACAAGGTACTAGGGTGTGGAGGGAAGGAAGGGGAGGGCTGAGGAACTGG
GTTCTCCTGAGAGAAAGGCTGCCAGC
>1551
GACCGATATGCCTTATCGCTACCACTCCTCCCTTGGCCAGCTGAACTTCACCGGGTCTG
TGATATATGAGGTGAGCACCCGGCTGCCTGCCAGCCCGCTGGGCTTCAGATTTCTGTGG
GAGGGTATTATGGGGGGTGGTT
>1552
GGTCAGCTCAGGCTCGGCCAGGGGCAAGAAGCTGTCGGAAATCATGGAGAAGGGGCA
GCTGGTTCCACTGGTGAGTGGGCCCTGGTGGGGTGAGAGGCAGGGGATGACAGTAGC
CCTGAGTGGGTGTCCCACCAGCAGCA
>1553
TACTTCCAAAGGCTTCCTGATTGATGGCTACCCGCGGGAGGTGCAGCAAGGAGAAGA
GTTTGAGCGACGGGTAAGGCACTGACCCAAGTGGGGATCCTGGTGGGCTGGGGCAGG
ATAAGACTCCCATCCCCATGGGCGGG
>1554
GCTGGAGACCTATTACAAGGCCACAGAACCCGTCATCGCCTTCTATGAGAAACGTGGC
ATTGTGCGCAAGGTGGGCCCCGCGGGACGGGGCAGCTCCGGGAAGAACGGGTTCCTA
TGTGGCTCCGCCTCAGCTCACTGTG
>1555
TTGTGCTGCTATCTACGGCAATGAGCCTGAGATTGGGGAGGCCCTGAAGGAGGACGTG
GGACCAGGCAAGGTAAGGACTGGGGTTGTAAATAGAGGTGGATAAGAGAACTTAGAA
GCTGAAGCTAGGCTGGGGCCCAGCT
>1556
GGAAGACTCTGGCTGACCTCCAGCTGGAGTATCTGGACCTGTACCTGATGCACTGGCC
TTATGCCTTTGAGTGAGCCTTGCCAGAGCCTCATCTGGGGAATCAGGGGGTTGAGCAG
GATGGTGTTAGTAACTTATTGTAA
>1557
GTCCAACTTCAACAGTCGGCAGATTGATGACATACTCAGTGTGGCCTCCGTGCGTCCA
GCTGTCTTGCAGGTAAGACAGCAAGCAGATGAGTGGTTTAGGGGTTGTCTGCTCAAGA
GCATGAGGGAGCAGACGATGGATC
>1558
TGCTGGAGGAACCAGTAGTCCTGGCATTGGCTGAAAAGTATGGCCGATCTCCAGCTCA
GATCTTGCTCAGGTATGGGGCAGTCTTAGGGAGAGGGCCCTGGGTTGGGAGGCAAGG
GTTAAGGGATTTCTTATTTCAGTGT
>1559
GCAGGTCCAGCGGAAAGTGATCTGCATCCCCAAAAGTATCACTCCTTCTCGAATCCTTC
AGAACATCAAGGTACTTGGTAATGGGTTCTATCTTACTTTAGCTCTTTGGGACATTTTCT
TGGCCCTGACTCTACCTGGCT
>1560
TAGCCCAGAAGAGATGAAGCAGCTAAATGCCCTGAACAAAAATTGGAGATATATTGTG
CCTATGCTTACGGTGAGGATGTATCAGCCTCCTAGACTTGGGGAATGTGAGATTTGGGG
TGGGATTCTGGCCCAGGTGTGAC
>1561
AACTGCCGGCATCTGAGGTTTCCTCCAAGGCCCTCTGAAGTGCAGCCCATAATGAAGG

TCTTGGCGGCAGGTAAATACACCCGCCCCGCGCCGGCTTCGCGTCCCCGCTGCGGGGC
GCGGCGGCAACTTGGGGCGCTTGG
>1562
CCTCATGAACCAGATCAGGAGCCAACTGGCACAGCTCAATGGCAGTGCCAATGCCCTC
TTTATTCTCTATGTAAGTTACCCCTGGGATACTGACAGGAGATGGCAGGGAGGGGGCTT
GTAAATATCATTAGGGGCTGTCC
>1563
GACATGCAGGGGCCCTGGGTGCTGCTCCTGCTGGGCCTGAGGCTACAGCTCTCCCTGG
GCATCATCCCAGGTAATGAGGCTCCCCAGCCTGCCCTACACACACACACACACACACA
CCACCCCCCAGCCCAGGCTGACCT
>1564
CTGGGTGCCGCCAAGAAGCTGCAGCCTGCACAGACAGCCGCCAAGAACCTCATCATG
TTCCTGGGTGACGGTGAGTGAGCCAGCCTTCCAGCCCCGCAGCCCTCACAGCCCCGG
CGCCCGGACCCTCAGTGGTTCCAGGA
>1565
GAAGAAGGACAAACTGGGGCCTGAGACCTTCCTGGCCATGGACCGCTTCCCGTACGT
GGCTCTGTCCAAGGTAAGTGCTGGGCTACCTTAGAGTCCTCCAAGCAGAGAAGGGGA
ATCCTGGCTATGGAGTGTGGTAGGAG
>1566
CGCTTTAACCAGTGCAACACGACACGCGGCAACGAGGTCATCTCCGTGGTGAATCGGG
CCAAGAAAGCAGGTGGAGCTGGGGCCCGGCTGTGGGGTCAGGGCCAGTGACAGACC
TCTATCGCATATCCTGACCTCTATCA
>1567
GCCTGCCTCGGCCCGCCAGGAGGGGTGCCAGGACATCGCCACGCAGCTCATCTCCAA
CATGGACATTGATGTGCGACCCCCGGGCCAAGGGTGGGGCTGGGCAGAGAGTAGCAG
GGAGGGGGCACCAGCTCAGACCAGGC
>1568
TGACTACAGCCAAGGTGGGACCAGGCTGGACGGGAAGAATCTGGTGCAGGAATGGCT
GGCGAAGCACCAGGTGATGGGGGCTGGTGGGTGTGCTGGGCACAGCAGGGGGAGGG
CAGAGGTGTGGGGCTCGGGGCTGTGGG
>1569
GCCCGGTACGTGTGGAACCGCACTGAGCTCCTGCAGGCTTCCCTGGACCCGTCTGTGA
CCCATCTCATGGGTAATGACCCCCTTCCTGCCCTGGCATCCTCAGATGGCCTCAGATGG
CACTTCTGAGCCTGTGTGCACAT
>1570
ATGGAGATGACAGAGGCTGCCCTGCTCCTGCTGAGCAGGAACCCCCGCGGCTTCTTCC
TCTTCGTGGAGGGTGCGTGGTGGCCCTGGGAGTGGGGGGTTGGGGGTTGGAGCAGGG
CAGGCTCAGCATCTCCCCCCTCTGG
>1571
CTCGTCACTGCCGACCACTCCCACGTCTTCTCCTTCGGAGGCTACCCCCTGCGAGGGA
GCTCCATCTTCGGTAGGCCTGGGGATGAGTGGCAGGTGCTGCTGCAGCAATTAAGTGG
GTGAAATCTGAGCCTCAGTCTCCT
>1572
CTATACGGAAACGGTCCAGGCTATGTGCTCAAGGACGGCGCCCGGCCGGATGTTACGG

AGAGCGAGAGCGGTGAGTGCCGTGGGGTGGCCTGAGGGGGACCAGGGTGCCAAGGA
TGGGGGGCTGGCGGGAAGGGGTCACC
>1573
GACATGCAGGGGCCCTGGGTGCTGCTGCTGCTGGGCCTGAGGCTACAGCTCTCCCTGG
GCGTCATCCCAGGTAATGAGGCTCCCCAAGCTGTTCCACACACAGGGCACCCCCTCAG
CCAGGCTGACCTGATCTCTACTCT
>1574
CTGGATGCTGCCAAGAAGCTGCAGCCCATCCAGAAGGTCGCCAAGAACCTCATCCTCT
TCCTGGGCGATGGTGAGTGAGCAAGGCCTGTCCAGCCCCGTAGTCCTCACAGCCCCGG
CACCCGGGACCTTCAGTGGTTCCA
>1575
GAAGAATGGCAAACTGGGGCCTGAGACGCCCCTGGCCATGGACCGCTTCCCATACCTG
GCTCTGTCCAAGGTAAGGGCTGGGCCACCTCAGAGTCCTCCAAGCAGAGGAGAGGGA
TCAAGGATATGGAGTGTGGCAGGAG
>1576
CGCTTTAACCAGTGCAACACGACACGCGGCAATGAGGTCATCTCCGTGATGAACCGGG
CCAAGCAAGCAGGTGAGCTGGGGCCCGCTGTGGGGTCAGGACCAGGCCCAAGATCTC
GGTCACCGATCCTGACCTCTGTCAC
>1577
GCCTGCCTCAGCCCGCCAGGAGGGGTGCCAGGACATCGCCACTCAGCTCATCTCCAAC
ATGGACATTGACGTGCGACCCCCGGGCCAAGGGCTGGGGCTGGGCAGAGGGGAAGGT
GGCACAGGCTCAGATCCAGGCAACC
>1578
TGATGCCAGCCAGAATGGAATCAGGCTGGACGGGAAGAACCTGGTGCAGGAATGGCT
GGCAAAGCACCAGGTGATGGGGGCTGGTGGGTGTGGGAGGCACGGCAGGGGGAGGC
CAAGTGTGTGGGTCTCAGGGCTGTGGG
>1579
GCCTGGTATGTGTGGAACCGCACTGAGCTCATGCAGGCGTCCCTGGACCAGTCTGTGA
CCCATCTCATGGGTAATGACCCCCTTCCTGCCCTGGCATTCCTCAGACAACCTCAGAGG
GTGCCATCCGAGCCTGTGTGCCC
>1580
ATGGAGATGACAGAGGCTGCCCTGCGCCTGCTGAGCAGGAACCCCCGCGGCTTCTACC
TCTTTGTGGAGGGTGCGTGGTGGCCCCTGGGGAGTGGAGGAAGGCGGGGCGCGGCAG
GGCAGGTTCAAGCATCACCCCCCTC
>1581
CTCGTCACCGCTGACCACTCCCATGTCTTCTCCTTTGGTGGCTACACCTTGCGAGGGAG
CTCCATCTTCGGTAGGCCTGGGGAGAGTGGCAGGTGCTGCTGCATCAATTATGAGGGT
GAAGTTTGAGCCTCAGTTTCCTC
>1582
CTGTACGGCAATGGCCCGGGCTACGTGTTCAACTCAGGCGTGCGACCAGACGTGAATG
AGAGCGAGAGCGGTGAGTGAGGCTGAATGGCCCGTGCAGGGGGACCAGGGTGCCAG
GGATGGGGGCATTCGCGGGAGGGGGA
>1583
GGGCCCTGCATGCTGCTGCTGCTGCTGCTGCTGGGCCTGAGGCTACAGCTCTCCCTGG

GCATCATCCCAGGTAATGAGGCTCCCCGAGCTGCCCCTACACAACACACACACAGGGC
ACCCCCCAGCCCAGGCTGACCTGA
>1584
CTGGGTGCCGCCAAGAAGCTGCAGCCTGCACAGACAGCCGCCAAGAACCTCATCATC
TTCCTGGGCGATGGTGAGTGAGCCAGGCCTTCCAGCCCTGCAGCCCTCACAGCCCCGG
CGCCCGGACCCTCAGTGGTTCCAGG
>1585
GAAGAAGGACAAACTGGGGCCTGAGATACCCCTGGCCATGGACCGCTTCCCATATGTG
GCTCTGTCCAAGGTAAGTGCTGGGCTACCTTAGAGTCCTCCAAGCACAGAAGGGGAAT
CCTGGCTATGGAGTGTGGTAGGAG
>1586
CGCTTTAACCAGTGCAACACGACACGCGGCAACGAGGTCATCTCCGTGATGAATCGGG
CCAAGAAAGCAGGTGAGCTGGGGCCCGCTGCTGGGTCACGGCCAGGTCACAGACGTT
GGTCACATATACTGACCTCTGACAC
>1587
GCCTGCCTCGGCCCGCCAGGAGGGGTGCCAGGACATCGCTACGCAGCTCATCTCCAAC
ATGGACATTGACGTGCGACCCCCAGGACCAAGGGCTGGGGCTGGGCAGAGAGTAGCA
GGGAGGGGGCACTAGCTCAGACCCA
>1588
TGACTACAGCCAAGGTGGGACCAGGCTGGACGGGAAGAATCTGGTGCAGGAATGGCT
GGCGAAGCGCCAGGTGATGGGGGCTGGCGGGTGCAGGGGGCACAGCAGGGGGAGGG
CAGAGGTGTGGGGCTCAGGGCTGTGGG
>1589
GCCCGGTACGTGTGGAACCGCACTGAGCTCATGCAGGCTTCCCTGGACCCGTCTGTGA
CCCATCTCATGGGTAATGACCCCCTTCCTGCCCTGGCATCCCTCAGATGGCCTCAGATG
GCACCTTCTGAGCCTGTGTGCAC
>1590
ATGGAGATGACAGAGGCTGCCCTGCGCCTGCTGAGCAGGAACCCCCGCGGCTTCTTCC
TCTTCGTGGAGGGTGCGTGGTGGCCCCTGGGGAGTGGGGGTTGGGGGTTGGAGCAGG
GCAGGCTCAGCATCTCCCCCCTCTG
>1591
CTCGTCACTGCCGACCACTCCCACGTCTTCTCCTTCGGAGGCTACCCCCTGCGAGGGA
GCTCCATCTTCGGTAGGCCTGGGGAGAGTGGCAGGTGCTGCTGCAGCAATTAAGTGGG
TGAAATCTGAGCCTCAGTCTCCTC
>1592
CTATACGGAAACGGTCCAGGCTATGTGCTCAAGGACGGCGCCCGGCCGGATGTTACCG
AGAGCGAGAGCGGTGAGTGCCGCGGGGTGGCCCCCTGAGGGGGGACCAGGGTGCCA
AGGATGGGGGGCTGGCGGGAAGGGGT
>1593
GGATTTCCTTCAGAAAGGGCTAATGATCTCACTGTCTACAGATGACCCAATGCAATTCC
ACTTTACCAAGGTATAGTATGGACTTGGGCAATACAAGCATATTTTGCTGGACTGTTGTT
TCCCGTCCAGAATTTGAAAGT
>1594
CAAGCTGAGCACCTGTGATATGTGCGAAGTGGCAAGGAACAGTGTCTTGCAGTGTGG

AATTTCTCATGAGGTAGACCTGTCTTCAAATGGCTTCCCTTCTGAGTATAGGGTCTGCTA
ACTTGAATACCGAAAAAGTAGTT
>1595
GCTCCTAGGTCAGACCAGAGCTAATCCCATGTACAATGCCGTGTCCAACGCAGACCTG
ATGGATTTCAAGGTAGGGCCAGGAAAGCGGGTGCAGTCTGGGGCCAGGGGGCTTTCT
GATGCTGTGCTCACTCCTCTTGATT
>1596
AGGTGGAGTGGCCGCAGCCATCTCCAAGACGGCGGTAGCGCCCATCGAGCGGGTCAA
GCTGCTGCTGCAGGTACGTCTGGGATCCAGAGCCCAACCAGGAAGTGGGGGAAGGGT
CGCACAGAAGGCGGGCGCCGAGGGGT
>1597
TTTAACGTGTCTGTGCAGGGTATTATCATCTACCGAGCCGCCTACTTCGGTATCTATGAC
ACTGCAAAGGGTAAGTTTGCTGTGGGCTTTAACGTTGTGTTCTTAGGAGACAGTTTAA
AAGAGCATTGTACCAACCTAAC
>1598
GCCGGGTTGACTTCCTATCCATTTGACACCGTTCGCCGCCGCATGATGATGCAGTCAGG
GCGCAAAGGAAGTAAGTTCCACTTGAGACAGAAGACAAAGTTGTAGTCGTGGGGCAA
TCTGCTGCCACAAACTGGTGATAC
>1599
GCCAGCTGAGCAACAATGCCAAGGAGGCCGTGGAACATCTCCAGAAATCTGAACTCA
CCCAGCAACTCAAGTAAGAGGGACTACAGTGTGTGGTGGTGACGGGGAATTCTTAAA
GGCCATGCAATGTACTGGCAAGGGTT
>1600
ACACACTGGAGGACAAGGCTCGGGAACTCATCAGCCGCATCAAACAGAGTGAACTTT
CTGCCAAGATGCGGTTAGAACCCTTCCCAGGGCACGGGAGAGCTGGGGTGTGTTTTTG
GGTGGAGCCCTGGCAGATGGTCCAA
>1601
AGACCCTGGAGGACTACACTCGGGAATTCATCAACCGCATCACACAGAGTGAACTTCC
TGCCAAGATGTGGTTAGAACCCTTCCCAGGGCACGGGAGGGCTGGGGTGTGTTTGTGT
GGAGCCCTGGAGGATGTCCAAGAT
>1602
GCTGGGTGCAGACACTGTCTGAGCAGGTGCAGGAGGAGCTGCTCAGCTCCCAGGTCA
CCCAGGAACTGAGGTGAGTGTCCCCATCCTGGCCCTTGACCCTCCTGGTGGGCGGCTA
TACCTCCCCAGGTCCAGGTTTCATT
>1603
CCGAGCTGCAGCTGGTTGAGCAGCGGATCCGCAGCTTCCCCGACTTCCCCACCCCAGG
CGTGGTATTCAGGTGCACGCACAGGCCGCCCTCGTGGCCCCGACCTGCGGGCCTACGG
ATGGGAGCGCGTGCCCGCGACCTC
>1604
GCTCATCCGAAAGCGGGGGAAGCTGCCAGGCCCCACTCTGTGGGCCTCCTATTCCCTG
GAGTACGGGAAGGTAAGAGGGCTGGGGTGGCCGGAGGAAGGGCAGGGTCGGGTCAC
CGTGACCACTCTCCCCCAGTTCTAAA
>1605
GAGATTCAGAAAGACGCCCTGGAGCCAGGACAGAGGGTGGTCGTCGTGGATGATCTG

CTGGCCACTGGTGGTAAGGGTCTCCCCGCAGCCAACTCTGTGCTCCAAGGGCCTGGTG
GGAGTGGGACAGGACCTCGCTGTGT
>1606
GCTTGGGATCCTCCTGGTGACCTCTCCCGCTAAGGTCCCTCAGCCACTCTGCCCCAAGA
TGGGCCGTGGGGTGAGTATCCCTAAAGAGCAGGGGTCGCAGTCTAGAGGGTGAGGGA
GGCTGCTGAGGAAGGATGAAGTGG
>1607
AGAGAATGGGGGCGGCAAGAAGAAACAGAAGGAGAAGGAACTGGATGAGCTGAAGA
AGGAGGTGGCAATGGTGAGGGAACTGCTGGGCCATGGAGGAGGGGCCCCATGCTGGG
AGAGCTGTCCCTGCAGCCCATTGCACT
>1608
TGGGGCTATCCTCTGCTTCCTGGCCTACGGCATCCAGGCTGCCATGGAGGATGAACCAT
CCAACGACAATGTGAGCCCACACGCCTGACCCGGGAACAGCCCGTGACTGTCCTCCA
ACCCTGAACCCCCAACACAGTGGG
>1609
CTGCTTCTCCTACTACCAGGAGGCCAAGAGCTCCAAGATCATGGATTCCTTCAAGAAC
ATGGTACCTCAGGTAAGATGGCAGGGCTGGGCTCTGGGCTAGGCTGTAAGGTTTTGGC
AAGAGTCCAGCTCATCTTCTGTCA
>1610
CCTGGTGGAGGTGAAGGGTGGAGACCGCGTCCCTGCTGACCTCCGGATCATCTCTTCT
CATGGCTGTAAGGTGAGGAGGTCATACCAGAGCAAGCAGTTGAGTCTAAGGAGAAGG
CTGTGTGCAGAGCTGAGAGGGGCCC
>1611
CCCGAGTTCACCCATGAGAACCCCCTGGAGACCCGCAATATCTGTTTCTTCTCCACCAA
CTGTGTTGAAGGTGAGAAGCCAGGCTGCCCCCTGTAGGAAAGAGTCTGAATCCTGAAT
CCATAGTCAGGATGAAGGGCTCT
>1612
GGAGGCAGTCATCTTCCTCATCGGCATCATAGTGGCCAACGTGCCTGAGGGGCTTCTG
GCCACTGTCACTGTGAGTGGGTCAGGCTGAGGTGCCACCAGGGGAGGGTCTCACTAC
TCTTTCCTCAGAGTGATAGAGGCAC
>1613
CGCATGACCGTCGCCCACATGTGGTTCGACAACCAAATCCATGAGGCTGACACCACCG
AAGATCAGTCTGGTGATTGGGTGCTCCAGAGGGGGTGGATAGGATTAGAGGAGGCTGA
GGGCAGTGGCGTGGTGGGGTGAGT
>1614
GTCTCGAATTGCTGGTCTCTGCAACCGCGCCGTCTTCAAGGCAGGACAGGAGAACATC
TCCGTGTCTAAGGTAGGGGGTCAGGACACACACCAGGTATGTTTTGGGGGTGTCTCCA
AAGCCTCTTGCTGGCCCCAGCTTT
>1615
AGTGAGGAAAATGAGAGACAGAAACCCCAAGGTGGCAGAGATTCCTTTCAACTCTAC
CAACAAGTACCAGGTCTGCTTGGGTTGCCAGGACAGAGGAAGAGAGAGGGATATAAA
TGGGTGAGGGTGGACAAAGCCAAGGG
>1616
GACAAGGAGATGCAAGATGCCTTTCAAAATGCCTACATGGAGCTGGGGGGACTTGGG

GAGCGTGTGCTGGGTGAGAGGCCAGAAACAGGAGGCTCAGAAGGGGATTCCCAAGC
CTCTGCGGCATCCCTGGGGTGGGGGAC
>1617
GTCTATGATTGACCCTCCCCGGGCTGCTGTGCCAGATGCTGTGGGCAAGTGCCGAAGC
GCAGGCATCAAGGTACTGGCCTCCCATCCTCCCCTCCATTCTAGCCTCCCCCATGCCAG
AGTTCAAGGAGCTGCAGTGGCTG
>1618
CAGAGGGTAACGAGACTGTGGAGGACATTGCAGCCCGGCTCAACATTCCCATGAGTCA
AGTCAACCCCAGGTGAGGCCTCTGCAGGAAGCCCCTGTGCCCTAATCAACACGTCCTC
TTGCACAGAAGGCTTGGGTGTCCC
>1619
CACAGAGATCGTCTTTGCTCGAACGTCTCCCCAGCAGAAGCTCATCATTGTGGAGGGA
TGTCAGAGGCAGGTGAGCACAGCCACGGGAGGCAGATGACAGGCAGGGACCGGGGA
GGCAGGGACAGGGCCAAGACAAGCAT
>1620
TCTAAGCAGGCAGCCGACATGATCCTGCTGGATGACAACTTTGCCTCCATCGTCACGG
GGGTGGAGGAGGGTGAGGAGGCTGCATGGGTTGGGATGGTTTGCAGGATACTGAAGC
CGGCACCTCTGTTCCCTGTCCCTTT
>1621
CATCATTGCCAACATCCCCCTACCTCTGGGCACTGTGACCATCCTTTGCATTGACCTGG
GCACAGATATGGTGAGCGCAGGAGGTGGAGGAGGGGACAGGCAAGGCAATCGTGATG
GCACAGTGGCAGGGAGGAGAGGTG
>1622
CAGCCACGAAACTCCCAGACGGACAAGCTGGTGAATGAGAGGCTCATCAGCATGGCC
TACGGACAGATCGGTGCGCCAAGCCCCGGGCCTCGGGAGGGAACCCCAACAGGGTTC
TTTTCCCAGCTTTCAGAGGAATGAGC
>1623
ACTGGGAATCCGCCTCGACTGGGATGACCGGACCATGAATGATCTGGAGGACAGCTAT
GGACAGGAGTGGGTGAGTGGTGCTGTGTAAACACAGCGCACATGTGTGAAGGTACGG
GAAGCTGAATGCCTGGCAGGAGTGG
>1624
TGGTGGTGCAGTGGGCTGACCTCATCATCTGCAAGACCCGCCGCAACTCAGTCTTCCA
GCAGGGCATGAAGTGAGTGCCCACCCCCATGGCACCTACCCACCCAGGCTCTGGGCAC
ACTCACCAACCCACACAGGCCAAG
>1625
AGACGGCGTTGGCTGCCTTTCTCTCTTACTGCCCAGGCATGGGTGTAGCCCTCCGCATG
TACCCGCTCAAGTGAGTGTCTCTTTCGGGCGGCCTGAGTAGTCATACGGGGGGCCTTC
AGCCCCCTCCAGGATCCAAGTTC
>1626
TTCCCCTACAGCCTCCTCATCTTCATCTATGATGAGGTCCGAAAGCTCATCCTGCGGCG
GTATCCTGGTGGTAAGCCCCTCCACATTCCCCCCAGCAAAGTGCAAGCCCCACCACCA
GCTCCTCCCTCCAGGACCACACG
>1627
GTGCCGCACGCCATCCTGATCTGGGTGTATGATGAGGTGCGGAAGCTCTTCATCAGGCT

CTACCCTGGAAGTGAGTAGCCTATGATTTTAGAGGCTCTGTTTACCCACTTCAACAGAC
TCTCCATTTCTGATGTACTTTT
>1628
CGCATGACTGTGTCCCATCTTTGGTTTGACAACCACATCCACACAGCTGACACCACGG
AAGACCAGTCAGGTGTGGGGGAGGGAGGGCACAGGCGCCAGGGCAGGATCTGGTGG
ATGGTGGAGACCTGAAGAGGGATAGG
>1629
CATCACCGTCAGCGTGCCCCTGCCCCTCGGGTGCATCACCATCCTCTTCATCGAACTCT
GCACTGACATTGTAAGTCCACAGCCCAGGGTACCCATCCACAGGGTGCCCAGAGACAC
TTGCTTGCAGATGGACAGGAGGG
>1630
AACTGCAAAGATGCTGTCCGTGCGCGTTGCTGCGGCCGTGGTCCGCGCCCTTCCTCGG
CGGGCCGGACTGGTGAGCACCGAAGGCCGGCATGATGCAGGCGGCCGGGTGGGGCTG
CAGGGTGGTGGTGCGCCGGCTCGGG
>1631
AATGCTTTGGGTTCATCTTTCATTGCTGCAAGGAACTTCCATGCCTCTAACACTCATCTT
CAAAAGACTGGTAAGTTATTATTTCTCAGTCTACGCCGCACTTACTAGATGAAGATATAA
ATTACATACATCGTATAACT
>1632
TATTGCCCGCGTACATGGGCTGAGGAATGTTCAAGCAGAAGAAATGGTAGAGTTTTCTT
CAGGCTTAAAGGTAAATTATAAAATAAGATTTTGGTGTTTTAGGATCTAAGTCTTCTCAG
TTATTCATTTAGCGATTCAAA
>1633
GGACGTTCCAGTTGGTGAGGAGCTGTTGGGTCGTGTAGTTGATGCCCTTGGTAATGCTA
TTGATGGAAAGGTAGGTTTAATGTTCGTTAGATATAGTTGCAGCTTCTAACAAACATCA
AAACTGATTATGCTTAGGGTTT
>1634
AGGCTGTGGATAGCTTGGTGCCAATTGGTCGTGGTCAGCGTGAACTGATTATTGGTGAC
CGACAGACTGGGTAAAGACTCAAAAGCATATTAAAAGTTCTAACTAAAATTTGCTCAAT
GGAAGGATTATATTTTAGTCTT
>1635
TGTATTTATGTTGCTATTGGTCAAAAGAGATCCACTGTTGCCCAGTTGGTGAAGAGACT
TACAGATGCAGGTATTAAAAGAAATTTAGTCCCATTGCTATTATCTTAAGTCCCATTGCT
GCTGATTGTAGAATAGCTTCT
>1636
TTCCATGGGAGAGTATTTTAGAGACAATGGCAAACATGCTTTGATCATCTATGACGACT
TATCCAAACAGGTCAAAGGAAATAAATTTTTAGAATCCATTTATTTGTACTGAAGTAAA
AGTTCACATATGCAACTTCTAT
>1637
CATAGAAACACAGGCTGGTGATGTGTCTGCTTACATTCCAACAAATGTCATTTCCATCA
CTGACGGACAGGTATTATTTTAATGATTAAAAGAAATGTTAAAAAAGATTTGGATAATTC
TAAGGCATTGGTTGAGACTAA
>1638
CCCTGCAATTAACGTTGGTCTGTCTGTATCTCGTGTCGGATCCGCTGCCCAAACCAGGG

CTATGAAGCAGGTAATTTTGCATCACTTTGGCAGTAAGGGGGCTATCATTAGATTAGCAT
CTTAAGGTATTAATATATTAA
>1639
GATGCTGCCACTCAACAACTTTTGAGTCGTGGCGTGCGTCTAACTGAGTTGCTGAAGC
AAGGACAGTATTGTAAGTTGTTCTTTTCTTCATTTGGTCAGGTTCCTATTGTGTATCAGG
GTCTTCCCAGGCCTACAGAGCT
>1640
TTACAAAGTTTGAGAATGCTTTCTTGTCTCATGTCGTCAGCCAGCACCAAGCCTTGTTG
GGCACTATCAGGTATGAATGCAATTGTTGGCATCTTTTTTTAAAGTTATGTTTAAGATAT
GAAGTTAAAATTATTTTCAAA
>1641
CTCACCCCTTCAGCGTCGCTGCCCCCAGCTCAGCTCTTACTGCGGGCCGCTCCGACGG
CGGTCCATCCTGGTAAGTGCTTTTCTCTAGGAGCTAACGTTCCATTTTGCCGCCCCATG
ACCTTGAGCCGGGGAACGATGGT
>1642
CCAATTCTAAATGCCCTGGAAGTGCAAGGCAGGGAGACCAGACTGGTTTTGGAGGTG
GCCCAGCATTTGGGTAAGTAGAGTTTGTTAGGAATAGTAAAGACCTTGTGTAGCCCAAA
TATCCCCAAAACCAAGCTGTCTGC
>1643
AGACTTTGGGCAGAATCATGAATGTCATTGGAGAACCTATTGATGAAAGAGGTCCCATC
AAAACCAAACAGTAAGTTGCTCTTATTGCATCTATGCACAAAGGAACTTCTGTTATTTG
CAGTTTTACACATTAAGGTGAC
>1644
CAGGAAATTCTGGTGACTGGTATCAAGGTTGTCGATCTGCTAGCTCCCTATGCCAAGGG
TGGCAAAATTGGTATGTTATTAAGGGGTACATATTTTCCAAACCTAATATGGGAGGAAA
AACTCATACAATGTTATGAGTG
>1645
TGAAGGCAATGATTTATACCATGAAATGATTGAATCTGGTGTTATCAACTTAAAAGATGC
CACCTCTAAGGTAAGCATTGAGCTAATTGATGTCCACGTGGTAGTTACGCCAGGAAACT
ATTGCCAACTGAAGCCATGTT
>1646
AGACCAAGAAGGTCAAGATGTACTGCTATTTATTGATAACATCTTTCGCTTCACCCAGG
CTGGTTCAGAGGTAAGAGGGAAGGCTTGAGAGGACCTGGTTCATCTGGCCTTTCTTCG
GATGAGGCTAAGGATGTAGACAC
>1647
GGCCACTGACATGGGTACTATGCAGGAAAGAATTACCACTACCAAGAAGGGATCTATC
ACCTCTGTACAGGTAAGAAAAATTACATAGATGAAGATCTGATTTGTATAAAGGCAGGG
TGCAGTGGTGCATCTCAGCTACT
>1648
TATCATGGATCCCAACATTGTTGGCAGTGAGCATTACGATGTTGCCCGTGGGGTGCAAA
AGATCCTGCAGGTGAGTATATTACTATGTGGGATCAGTGTCCGGAAAGTCAAAAAGAG
GGCCTGTGGGGACTATATGAGGA
>1649
TTCACAGGTCATATGGGGAAGCTGGTACCCCTGAAGGAGACCATCAAAGGATTCCAGC

AGATTTTGGCAGGTGAGATTTTGAGTACAAATCTTGAATGTTTACTGTGCTGTGGTCCC
ATTCCAACAACCGTCAAGCAATA
>1650
GGCACTTCTGCGGGGGTGCCCTGATCCATGCCCGCTTCGTGATGACCGCGGCCAGCTG
CTTCCAAAGCCAGTGAGGGGTCCTGGGGAGGGGGCCTAGGGGGCATTGGGGCTCAGA
GAAGGGGCTTGGGGGGCTTAGGCAT
>1651
TTCCATCAGCAGCATGAGCGAGAATGGCTACGACCCCCAGCAGAACCTGAACGACCTG
ATGCTGCTTCAGGTGAGAGGATGGTGCCACCTGTGATCCCAGCACCTCGGGAGGCCGA
CGTTAGCCAGGGAAACAAGTCCAA
>1652
CCCCGAGGACCAGTGTCGCCCCAACAACGTGTGCACCGGTGTGCTCACCCGCCGCGG
TGGCATCTGCAATGTGAGTGCTCCCTGTGGCGGGAGGAGGGGTCCTGAGAGGTACTGA
GCTCTCCGTGGCAGGAGAAAGCAAG
>1653
CAAGGGCACCTTTGCCACACTGAGTGAGCTGCACTGTGACAAGCTGCACGTGGATCCT
GAGAACTTCAGGGTGAGTCTATGGGACGCTTGATGTTTTCTTTCCCCTTCTTTTCTATGG
TTAAGTTCATGTCATAGGAAGG
>1654
GAAAGTGGAAGACAAGAAGAGCAAACCACGTGTACAACAGAAGGCTGGTCTCCAGA
GCCAAGGTGCTTCAGTAAGTCAGCTGGATATGTCACTCAATGTTTCAATACTCAAAGAA
ATTTGTATATAAAAATAGGGGTCCA
>1655
AAGGATGAAGAAGTGGTTCAATGTCTCTCTGATGGATGGTCTTCTCAACCAACCTGTA
GGAAAGAACATGGTATAAGAATCATTTCCTAAACTGAAGATAAGTGCTTATCAAGCTTA
TATTGAATATATTGAAGCTTATA
>1656
GCTGGAGGAAAGAAGACAGAGGAGGTAGAATGTCTCACATACGGATGGTCTCTCACA
CCAAAATGTACCAGTAGGTTCTCATTTGGAATAAAATCTACTCTCACTCTGAAAGCTTT
TTCTCATAGAGAAGTGTATATAAG
>1657
TATCTAAGTGGATCTGATTTAATTCAATGCTATAACTTTGGTTGGTACCCAGAATCTCCT
GTATGCGAAGGTAAATTTTTAAATTTAATACTCGCAAAATGCCATGTCTTTATCAGCTTT
AATCTCTATTAGCTCTGACA
>1658
TTTGAGATCCATGGGTCAGCAGAAATACGTTGTGAAGATGGAAAATCGACAGAACCTC
CAAAATGCATTGGTTAGTAACACCTTGAAGAAGCTTTGCTAAAATGAAATCTGCATGTG
TAGCTAAATGGTCAGTTCTCTTT
>1659
TACCTTCTCCATGGATCGAATGAGATAACTTGTAATCGTGGAAAATGGACACTTCCTCC
TGAGTGTGTTGGTATGTATGCTACATTTACCATCAGTAGCTAAACTTATGGTGTAAAATT
TTCCATTCTGTTACTTTAATC
>1660
TACTTACTGAGGGGATCAAAAATATCTCGTTGCGAACAAGGAAAATGGTCATCCCCAC

CTGTTTGCTTGGGTAAGAAAGAGAACACATGGAATGTCTACGTTTGTACTTTTATGTGA
TTTTCTTACAGTGTTTTATATCA
>1661
ACCCCATTGTCTGAATTATCTGTGCAGTGCAACAGAGGAGAAGTGAAATATCCTTTATG
TACTAGAAAAGGTAAAATAATAATGTTCTCTGCAAGTATCTTTACTTGACCAATATCTGC
TACCTCGGTTGAAAATTTATT
>1662
CATTTCCTAGAAGGATCTAGGGAGGCCTATTGTTTAGATGGAATGTGGACTACACCACC
ATTGTGTTTAGGTATGTACTACTAAATATGCCTCTAACAAAGTAAAACTATATATTTTTAA
TTTGCTGTCATTTTTGAGTT
>1663
CTGGATCTATACTTAGAATGCAATGTGACAGAGGGCAGTTAAAATATCCAAGATGTATT
CCAAGACAAAGGTAAGAAGTTTTTTTTTTTTGGTCAGATTATTTTTTTTGGTCAGATTGT
TATTCAACATTTCAGAAATGT
>1664
GAGAAGGAGCTGAAGGAGATCAGGGTCTTGGACAAGGCCTTCGGACCAGAATTGAGA
GAGGAATTTTCTAGTAAGTAAACTTGGGTCATGGGTGTGTGGTTCCATCTTAAACACTG
CATGTATGTGGGGGGAGATGGACC
>1665
CACACCGCCTGTATCATTGATGTCTTCGGTGTCACTCACAGAGAGTCTATCATGAATGT
CAATGTGAAAGGTATGGTAGGCTGGGGAGGAGATGCAGCAAGGTGGGGAATTAAGGA
TCACAAAGAAGGGCCAGGAAGGGA
>1666
GGAGTTTGTCAAGGCCAAGATCGTGTCTCGAGAGGGTGGCAAAGTCACTGCCGAGAC
TGAGTATGGCAAGGTGGGTGTCAGGCTGATGTGAGAGTCCACCCTGGCCACCTGTACA
CCTGGGTGGACAGAGAGGGGTACAC
>1667
GCTGACCTTCCTGCATGAGCCCGCGGTGCTCTACAACCTCAAGGATCGCTACGGCTCC
TGGATGATCTACGTGAGTGCTGCACCTGGCCCTACGTTGGGATCTCTGTTCTTGCTCCA
TCCATGTCCACCCCAGGAGCCAA
>1668
AAGAAGAGGAGCGAGGCCCCGCCCCACATCTTCTCCATCTCCGACAACGCCTATCAGT
ACATGCTGACAGGTGAGAGGCCCTGGAAGGTCTTCCTGAAGGGAACTGGGATAGGCC
GGGAGGGAGAGGGAGAAGGAAGGGA
>1669
GGTCATCCAGTACTTTGCTGTTATTGCAGCCATTGGGGACCGCAGCAAGAAGGACCAG
AGCCCGGGCAAGGTAGGCCTGCTGCCCTCCAAGGTCCTGTACCGCAGAAGGGAGGGA
GAAGAGCTCTCACCTGCCTCCTTCT
>1670
CCAGGCCAACCCTGCTCTGGAGGCCTTTGGCAATGCCAAGACCGTCCGGAACGACAA
CTCCTCCCGCTTCGTGAGTGGTCCCTGACCTTGGCCTTGGGACTTGGACTGGTGGAGG
AATGGTCTCAGATATGAGCCTTCCC
>1671
CAGCTGAAAGCAGAGAGAGATTATCACATTTTCTACCAAATCCTGTCTAACAAAAAGC

CTGAGCTGCTGGGTGAGTCAGAGCCACCGACTGAGACCAACTATCTCCATGGCAACCT
GGTCCCCTCTGCTTGTGGCTGGAC
>1672
TGCATTCATCTCCCAAGGAGAGACCACCGTGGCCTCCATTGATGACGCTGAGGAGCTC
ATGGCCACTGATGTGAGTGTGTGAGGACCCAGCCAGGGGGTGGGAGGATTGGCAGTG
AGGGGCAAACAGGGGTCCAAAAGCA
>1673
ATGCACTTTGGAAACATGAAGTTCAAGCTGAAGCAGCGGGAGGAGCAGGCGGAGCCA
GACGGCACTGAAGGTGGGAGGCAGGATTCTTGGGGCAGCTGTAAGTCATGAGGCAGT
CTGCTCAGCAGTATCTCTCTTCTCTT
>1674
CAAGGGGCTGTGCCACCCTCGGGTGAAAGTGGGCAATGAGTACGTCACCAAGGGGCA
GAATGTCCAGCAGGTGGGTCCATCTTCAGATGATAATGGGTGGGCAGGGTAGGGAGAC
TGGCATGGTGGGATGAGAGTTTTCA
>1675
CCTGGAGACCAAGCAGCCACGCCAGTACTTCATAGGAGTCCTGGACATCGCTGGCTTC
GAGATCTTCGATGTGAGTTGGGACCCCTGGGAGTGGGAGAACAATCACTCACTCGCTC
CCACATTCAACAGCTATTTGCTTA
>1676
GGAGGGCATCGAGTGGACATTCATTGACTTTGGCATGGACCTGCAGGCCTGCATTGAC
CTCATCGAGAAGGTGCCTCTTTGGCCTTACCACCTGAATTCTCCCTGCACACCCAACAA
GAACACCATAGACAAAATAGCAG
>1677
GGCTTGTATCAGAAGTCTTCCCTCAAGCTGCTCAGCACCCTGTTTGCCAACTATGCTGG
GGCTGATGCGCGTAAGTAGGGACTGAGGCTCCCGGTACAGAGGAGCAGGGATTCTGC
CAAGGTTCTGAGCCAGGTCAATTG
>1678
ATGACCAACTTGCGCTCCACCCATCCCCACTTTGTACGTTGTATCATCCCTAATGAGAC
AAAGTCTCCAGGTGAGGCCACAAACTAGGGCCAACCCACTGCTGGGCATACACCGCC
CTGGGAACAGGACCTCCTAGGACA
>1679
TGCTGGAGGGCATCCGCATCTGCAGGAAAGGCTTCCCCAACCGCATCCTCTACGGGGA
CTTCCGGCAGAGGTGGGTATGAGGGTGCACCAGAGCTCATAGAACAGGGGGAGCCAG
GCTGCCCTGATGGGAATGGGATCTG
>1680
GAAGGGGGCAGAGAAGCTGCTCAGCTCCCTGGACATTGATCACAACCAGTACAAGTT
TGGCCACACCAAGGTGAGGAAAGGAGACTAATTAATTAAAGGAAGACATCTCTTTTCC
ATTGACTCCTCTGATGCTTTTCCTG
>1681
TCACGCGTATCCAGGCCCAGTCCCGAGGTGTGCTCGCCAGAATGGAGTACAAAAAGCT
GCTGGAACGTAGGTGAGAGATCTCAAGAGGAGGTTTCCCGCTTCTCTGAGGCCCAGG
CTGGTTCAGGGGCAGTGTCAGGAAA
>1682
CAAGGAGCTGGAGGAGAAGATGGTGTCCCTGCTGCAGGAGAAGAATGACCTGCAGCT

CCAAGTGCAGGCGGTGAGGCTCCTGGGCTACTGTTGGCTCTTCCACCCTGCTCTGCCT
TCACTTCCCACAACCCTGCACCTCC
>1683
AAGGGACATCGATGATCTGGAGCTGACACTGGCCAAAGTGGAGAAGGAGAAACACGC
AACAGAGAACAAGGTAAGGGCAGCTCCCTTTGGCTCCAGCCCGGGTCTCATCAGGAC
TCTCAGACCATACTGACCTTGACCCA
>1684
TCAGGCCGAGGAGGACAAGGTCAACACCCTGACTAAGGCCAAAGTCAAGCTGGAGC
AGCAAGTGGATGATGTGAGTAGATTGAGAGTTGTGGGGCCTAGATATGCCATGTCTATC
TGTGCCCAGAGCTCTGTGTGTGTGT
>1685
TGAAGCTGACCCAGGAGAGCATCATGGACCTGGAGAATGACAAGCAGCAGCTGGATG
AGCGGCTGAAAAAGTACTGTGTCCCCTCCCTGCTGCCCTTCCTCCTCCCCAGCCCATAA
CAGTACAAGCAGACCCAAGACAGC
>1686
TCTCAACGCAAGGATTGAGGATGAACAGGCCCTCGGCAGCCAGCTGCAGAAGAAGCT
CAAGGAGCTTCAGGTGAGGTCTGGACACCCACGCGGGATGCTGAGTCCCTGGGCTGG
TTCTGTTTCCCCTGCCCCCTCCTCTA
>1687
AAGGCGGAGGAGACCCAGCGTTCTGTCAACGACCTCACCAGCCAGCGGGCCAAGTTG
CAAACCGAGAATGGTGAGCCTAGAGCAGGGGCTCCATGGTTCCCACCACAGTCTCCCC
AACCTCCTGAGCCACTCCAGCCTCG
>1688
GACCCGAGGCAAGCTCACCTACACCCAGCAGCTGGAGGACCTCAAGAGGCAGCTGGA
GGAGGAGGTTAAGGTAAGGGCTAGACTCGGGCCACCTGGCCCAAGCAAGGAGCACAC
TGACTAGCCTTGCATCAAATCACTTC
>1689
AGGTGGCCCAGTGGAGGACCAAGTATGAGACGGACGCCATTCAGCGGACTGAGGAGC
TCGAGGAGGCCAAGTGAGTTCTGAGCAGCCTGACTTCTGGCTGAGGCCCCTTTGCAG
GCAGGACTCAGCCCAGCCCCAGCCTC
>1690
GATGGTGGACGTAGAGCGCTCCAATGCTGCTGCTGCAGCCCTGGACAAGAAGCAGAG
GAACTTCGACAAGGTGGGCCCTGGGTGGGGGCCGCAGCCAGCATGCAGGGCAAGGG
GGCATGAGGGGTTCAGTGAGAGGCCAG
>1691
CTCAAGAACGCCTATGAGGAGTCCCTGGAACATCTGGAGACCTTCAAGCGGGAGAAC
AAAAACCTGCAGGGTGTGCTGGGGGCCCAAGAGGCTGGGGAGGGGCTGCACTGGCA
GTGTTCCCATATGGTGCCACCCAAGGG
>1692
GCTGGAGAAGGTCCGAAAGCAGCTGGAGGCCGAGAAGATGGAGCTGCAGTCAGCCC
TGGAGGAGGCCGAGGTGTGTGTGTGTGCAGGGCACGGGGTGCAGGGAGCTGAGCTCC
AGGATTTTGGCTCCCTGTTCTCATCCC
>1693
GCTCAGCCACGCCAACCGCATGGCCGCCGAGGCCCAGAAGCAAGTCAAGAGCCTCCA

GAGCTTGTTGAAGGTACTCACCCAGAGGGGACTGGCCTCCACGTGGCCTGGCGAAGC
AGTAGTGTCTTGATACAGGCACCAGA
>1694
GCGGTCCCGGAAGCTGGCGGAGCAGGAGCTGATTGAGACTAGTGAGCGGGTGCAGCT
GCTGCATTCCCAGGTGAGCAGCTCCCCTGCTCATTCCTGAAGGGAGCACAGGCTGGGG
CTCAGCAAGCAAGGCTTGAGAGCTA
>1695
GACTGAAGTGGAGGAGGCAGTGCAGGAGTGCAGGAATGCTGAGGAGAAGGCCAAGA
AGGCCATCACGGATGTAAGTCCCCCACTCCACCGACCCGATCCAGACCAGTGTCTCTC
CGTGGGCTGGGCAGCAAGTGTGTGAG
>1696
GAAGCGCAACGCAGAGTCGGTGAAGGGCATGAGGAAGAGCGAGCGGCGCATCAAGG
AGCTCACCTACCAGGTGCGACGGGCGGTGACTCCAGGCAGAGCCCTGGCACCATAGC
CACAGTGACAACCAGCTGAGGAGAATG
>1697
GCGGCTGCAGGACCTGGTAGACAAGCTGCAGCTAAAGGTCAAGGCCTACAAGCGCCA
GGCCGAGGAGGCGGTGAGTGACCCTGCTGGGGACTAGGCCCAGGGGAGGCATAGGA
GAGCTCGTCCCCCAAGCCAGGAGTCTG
>1698
GGAGCGGGCGGACATCGCCGAGTCCCAGGTCAACAAGCTGCGGGCCAAGAGCCGTG
ACATTGGCACGAAGGTGGGTCCCTCTTTTGGGCTTTGCTAGTCACCCCCACAGCAGGC
ATACCCAGACAGAGCACCCTCAAACC
>1699
CCTGGGAGGTCGTTCCCACCCGCTGGGCAGCCCCGGTTCAGCCTCGGACTTGGAAAC
GTCCGGGTTACAGGTGAGAGCGGAGGGCAGCTCAGGGGGATTGGACAGCAGCAATGA
AAGGGTCCTCACCTGCTGTCCCAAGA
>1700
GTGCAAGGGTCTGGCTGCTTTGGGAGGAAGATGGACCGGATCAGCTCCTCCAGTGGC
CTGGGCTGCAAAGGTAAGCACCCCCTGCCACCCCGGCCGCCTTCCCCCATTCCAGTGT
GTGACACTGTTAGAGTCACTTTGGG
>1701
ATCTTAAACCAGCTTGGTGCGGATAGTCTGACTAGTTTAAGGAGACTGGCCGAAGCTC
TGCCCAAACAATGTGAGTTTCCTAGTAATGGTTTTACCAGGGAATTACTCATTTAGCAG
CTGATTTCTGATCTCAGGGCTCA
>1702
GATGGTGAGAGGCATGCCTTCATTCTGCAGGACACAAAGGCTCTGCACCAGGTCTTTG
AACATATGCTGGGTGAGTGAGCTTTGCCCTCCTTGGTGTGGGGAGGATGGTGAGGAGC
CCGCCAAAGGCCCGTTTTGGGAAC
>1703
CTGCGGGGTGGGGAACATGTCAGCAAACGCCTCTGACCAGGAGAGGACACCCTGGCA
TGTCACTATTAAGGTACCAGGAAGGAGGGGCAGGGCTTGGATTCCAGAGGTAAAAGC
GGCCATGGGCCAGACATACTGCAATC
>1704
CAATGGGTCCTGACAGCAGCTCATTGCTTCCGCGATGGCAACGACCACTCCCTGTGGA

GGGTCAATGTGGGTAAGGCAGGGGATGCACCAGCCTCCTGATCCGTGAAGCCACAGAT
CCTACCACCTCACCCAGCCTCTGG
>1705
TGGAGTTCTATGGTGATGACATAGCTCTGCTGAAGCTGGCCCAGAAAGTAAAGATGTC
CACCCATGCCAGGTGCCTGGAGTCTTGGATGGGAGGGTGCCCTGCAGGGAAGAGTGC
TCTGGAGATCCCTGGAAGAGACTAC
>1706
TGCCTTCCCTGCACGATGGAGGCCAATCTGGCTCTGCGGAGACCTCAAGGCAGCACCT
GTAGGGACCATGGTGAGTGCTGGGACTTATGGTGCTTGAGAGCTGGGGCCGGGGTTTG
GGGGTGATAACAAGGACTAGGCTG
>1707
GAGTGTTCCTGCTCATTTTGTCGCCTTGAATGGGAGCAAACTGAACATTAACCTTAAGA
TGGGAGTGGAGGTGAGGGTCTCAGGTTGGGGATGCTGGGATCCCCCTGTGACAGCTC
CCAGAATGTCTCTCTTCCTTCTCC
>1708
GATGTCAGGGAGGTGGTGACAGACCAGTTCCTATGCAGTGGGACCCAGGAGGATGAG
AGTCCCTGCAAGGGTGAGTCCCTCACCATGCCTGGATTCCCAAGGGGAAGGCCACCTG
TGTCTCTGTGGCCAGCATGCATGCC
>1709
GGGTGACAAGCACACCCTGAGCAAGAAGGAGCTGAAGGAGCTGATCCAGAAGGAGC
TCACCATTGGCTCGGTGAGTGGCCTCCTCCCCAGGACCCCTTTTCCCACCCTTGTCCTT
TGGAAGCAAGGATTAGGGGAGAGAG
>1710
AGGGCAGAAAGTGAAGGGCACCGTCCGCCGTGTGGTCCTGCGAGGGGAGGTTGCCTA
TATCGATGGGCAGGTACGCAAGTAGCCCCTGCCTGATCTCAGTAGTGCCCTCTTCTGCA
CCACGTTCATTTCTTCCCTACCCA
>1711
ACGGAAGTGGCCACAGGGGGCTGTTCCTCAGCTCCCACCCTCAGCCCCTGCCACTAGT
GAGATGACCACGGTATCCACACTACTGGGCTAGGGGGCTGGGAGGTGTTAGTCTCAGG
GCAGGATGAACAGCTTGAACATTT
>1712
CCAGGGCTTCCTGATGGCCGCTTCCATCTGCCGCCCCGAATCCATCGAGCCTCCGACCC
AGGTTTGCCAGGTAAGAGTGGGGTTCCTGTGACTCAGAGACTGTGTAGGGACAGGAT
CCACTTCTTCCCAGTGCCTCGCCT
>1713
GACCTCACCCCTGCTGCACTCATTAGTGGGCCAACATATCCTGTCCGTCCAGCAGTTCA
CCAAGGATCAGGTGCCTGGGGCAGGGAGGATGGGACCACCCAGAGCTTTGAGGATTT
GGAAAGCTGGGGCTAGGACCTTTC
>1714
TCACCTGTTCAATGTGGCACACACACTGCGTATGATGGTGCAGAAGGAGCGGAGCCTC
GACATCCTGAAGGTCAGGATCAGGGCCGGGGGTAGGGTCCAGGCCATCGCCTGCCCTT
GGGCCGCATCAGCCAGGGCCGCGC
>1715
CTCCGTGCAGACCATGAGCTGCTATGCCGACGTCGTCGTGCTCCGGCACCCCCAGCCT

GGAGCAGTGGAGGTGAGGCCAGCCTGGGTACTGAGATGGGGTTAAGAAGGCTGGACC
CAGGGGCATGAGAACCCTTCTGCCC
>1716
GCACCCCACCCAGGCCCTGCTGGACATCTTCACCATCCGTGAGGAGCTGGGAACTGTC
AATGGCATGACGGTGAGGGTGGTGGCAGGGTTTGGATCCCTGCCAGGGGACGATCTAG
AGAGGGAGGCAGGAAGTGGTTACC
>1717
CGTGGCACCTCCCAGCCTGCGCATGCCACCCACTGTGCGGGCCTTCGTGGCCTCCCGC
GGCACCAAGCAGGTGAGACCCTCACAGCCCTGCCTGGAAGCCATGGAGATGTGGGTT
GGGCAGTCAGAGCCCAGCGCTGCAT
>1718
TGACACTGATGTGCTCTACATGACTCGAATCCAGAAGGAACGATTTGGCTCTACCCAG
GAGTACGAAGCTGTGAGTGCTGGGCTTGAGGAAGAAGCCAGGGCTGCTGCCGTAGGG
CATCAGATATGAGGACAGAAAGGCT
>1719
CCCACATCATGACCCGGGCCAAGAAGAAGATGGTGGTGATGCACCCGATGCCCCGTGT
CAACGAGATAAGGTGGTGCAGCATCAGAGTCAGAGACTGCCTCGGGGCTGGTGGACG
GGAGGAGACTTAGTCCTGGACAAGC
>1720
GTCAGAGCAGATGTGGAGGGTTCCTGGTGCGAGAAGACTTTGTGCTGACAGCAGCTC
ATTGCTGGGGAAGGTGAGGAGCTAAGGAACTTCCTGGCCAGCCAGGAACACAGCCCT
GCGGAGCTCTTCGGTGGAAGAGCCAT
>1721
TGCGCGCAGAGCCATCCGCCACCCTCAATATAATCAGCGGACCATCCAGAATGACATCA
TGTTATTGCAGGTACCACCTACCTGGCCCTCTGGCTCCTTCCTAGTGTGTCCGGGGACA
ATGGAGGAGGAAGTGAGGGCAA
>1722
CTTCGGTTCCTACGACCCCCGAAGGCAGATTTGTGTGGGGGACCGGCGGGAACGGAA
GGCTGCCTTCAAGGTAAGGCATGGGCATTGGCCAACACACCCCGGGAGAGAGGGGCC
CGTGCAGAGCCAGGCAGTGCGAACAG
>1723
GAAGTACAGCACTTTTGGGGAGGCGGCCTCAGCACCTGATGGCCTGGCTGTGGTTGGT
GTTTTTTTGGAGGTGAGTGGTGGTTTGCTGGGGCCTGGAGGGGCATGGGAGGCCTGCA
CTGCCCCTAGTTCTAGAACACAGT
>1724
GACTCCCCCACTCAGTGAGAGTGTCACCTGGATTGTGCTCCGGGAGCCCATCTGCATC
TCTGAAAGGCAGGTGAGTCCTCTCAGAGGACCAGATGGAGGGACATGGCACTCAGGG
CTCACCCCAGGCAGTGTGACTCTCC
>1725
CGCGACTTCCTGCGCAAGGAGTACGGGGGCCTGGACGTGCTGGTCAACAACGCGGGC
ATCGCCTTCAAGGGTATGGGGAGGGGACGTGGCCTCCCCGAAGAAGAACCGATGCAC
TGGGGCTCCTGGCGTCTGCGGGGTCC
>1726
ACGATGAAAACAAATTTCTTTGGTACCCGAGATGTGTGCACAGAATTACTCCCTCTAAT

AAAACCCCAAGGTGAGTCTGATGGGAAACAGCGCACCTTCTCTTTGGGGCTTGATTTG
GCCCCTGCTTGCCTGGGATTTCT
>1727
CTCTTCTTCCTCCTCTTCCTCACCCCCATGGAAGTCAGGCCCGAGGAACCTCTAGTGGT
GAAGGTGGAAGGTATGTCCAAAGGGCAGAAAGGGAAGGGATTGAGGCTGGAAACTT
GAGTTGTGGCTGGGTGTCCTTGGCT
>1728
CTGTGCCAGCCGGGGCCCCCCTCTGAGAAGGCCTGGCAGCCTGGCTGGACAGTCAAT
GTGGAGGGCAGCGGTGAGGGCCGGGCTGGGGCAGGGGCAGGAGGAGAGAAGGGAG
GCCACCATGGACAGAAGAGGTCCGCGGC
>1729
CCTGAGATCTGGGAGGGAGAGCCTCCGTGTGTCCCACCGAGGGACAGCCTGAACCAG
AGCCTCAGCCAGGGTATGGTGATGACTGGGGAGATGCCGGGAAGCGGGGGTCCAGAG
ACAGAGGGGAGGGGAAACTGAAGAGG
>1730
GCTGGAAAGTATTATTGTCACCGTGGCAACCTGACCATGTCATTCCACCTGGAGATCAC
TGCTCGGCCAGGTAGAGTTTCTCTCAACTGGGAGGCATCTGTGTGGGGGTACTGGGAA
GAAGTGGAAGCCAGTCAATCTTA
>1731
TCAGCTGTGACTTTGGCTTATCTGATCTTCTGCCTGTGTTCCCTTGTGGGCATTCTTCAT
CTTCAAAGAGGTGAGTCATGTCCCCAGTGGGTCTGTCCAAACCCTACTCCATCTTCCCC
AGGATAAGCCGGCTCTGGCCA
>1732
CCCCCAGGAAGCGGGCCCCAGAACCAGTACGGGAACGTGCTGTCTCTCCCCACACCC
ACCTCAGGCCTCGGTAAGAGGCACCGCCCCTCCAGCCTATAGCTCCGCCCCAGATCCG
GGGCTCCACCCCCACTCTCCTCATC
>1733
TCTTATGGAAACCCGAGCAGCGACGTCCAGGCGGATGGAGCCTTGGGGTCCCGGAGC
CCGCCGGGAGTGGGTGAATGACTGGGAGAGGGAAGGGTCGTTCCCCACATGGAGGGG
GTTGGAGCGGTCTGTGGCCCGAATAG
>1734
CCTGACAGTGAGGAGGACTCCGAGTTCTATGAGAACGACTCCAACCTTGGGCAGGAC
CAGCTCTCCCAGGGTAAGGCTGCCCTCCCCCGTGGCCCCCCACCTCTGCGGTGGCCTG
TGGACTCCCATGGACACCCCTCCTT
>1735
AGAGGAATCCTGTATGCAGCCCCCCAGCTCCGCTCCATTCGGGGCCAGCCTGGACCCA
ATCATGAGGAAGGTGGGTGCTTCTGCCGCTGTCCCCTGCTGTCCCCTGGGCTGACTTT
GCCTTCCAGCCTACTTCCAGTGCC
>1736
GCCAGACCCAGCCTGGGGAGGAGGGGGCCGCATGGGCACCTGGAGCACCAGGTGATC
CTCAGGTGGCCAGGTGAGCTGGGACTGCCCCTAGGGAAAGCGGGGAGGGAGGGAGAT
AGGCACGGATGGCAGTGGCTGCTGGC
>1737
AGTCTTGCCCCAGCCTCGGGAGGTGGTGGAGTGACCTGGCCCCAGTGCTGCGTCCTTA

TCAGCCGAGCCGGTAAGAGGGTGAGACTTGGTGGGGTAGGGGCCTCAGTGGGCCTGG
GAATGTGCCTGTGGCTTGAAAAGAC
>1738
GGTCCAGGAGTCCTCCAAGCTCTGCCTGCCACCATCTTCCTCCTCTTCCTGCTGTCTGC
TGTCTACCTGGGTATGTGGCCAAAGGGCAGGAACTGGCGGGAGGTGGGGGAAGCTGT
GGAGGCTGCAGAGAGGGCACAGGC
>1739
TACGTGTGCCGGGTCCAGGAGGGCAACGAGTCATACCAGCAGTCCTGCGGCACCTACC
TCCGCGTGCGCCGTGAGTGGCCCAGCCCTGGCCCCTACTCCCACTGTCCCGCTGGGGA
CACTCGGTTTATCTTTGAAGTGGG
>1740
AATCATCACAGCCGAGGGGATCATCCTCCTGTTCTGCGCGGTGGTGCCTGGGACGCTG
CTGCTGTTCAGGGTGAGCCCCCTCGGACCTCTGAGTCAGCCGGGCGAGGGCCTGGGC
CCGAGGGACCCCCAATACCCAGGTA
>1741
AAACGATGGCAGAACGAGAAGCTCGGGTTGGATGCCGGGGATGAATATGAAGATGAA
AACCTTTATGAAGGTGAGTGAAGGGTGGGGATGGGGTAGGGGCAGTTTTGTTAGGGGT
GGGGGTGTTCCCTCTGGGGGTGGCT
>1742
GAGACCATGGAGTCTCCCTCGGCCCCTCCCCACAGATGGTGCATCCCCTGGCAGAGGC
TCCTGCTCACAGGTGAAGGGAGGACAACCCCTGGGAGAGGGTGGGAGGAGGGAGCA
CAGAGACTGGCTGGGGTCTCCTGGGT
>1743
CACCATGGGGCGCCTGCAACTGGTTGTGTTGGGCCTCACCTGCTGCTGGGCAGTGGCG
AGTGCCGCGAAGGTAAGAGCCCAGCAGAGGGGCAGGTCCTGCTGCTCTCTCGCTCAA
TCAGATCTGGAACTTCGGGCCAGGC
>1744
TTCAAGGGCATCCCCTTCGCAGCTCCCACCAAGGCCCTGGAAAATCCTCAGCCACATC
CTGGCTGGCAAGGTGGGAGTGGGTGGTGCCGGACTGGCCCTGCGGCGGGGCGGGTGA
GGGCGGCTGCCTTCCTCATGCCAAC
>1745
CAGGACAGCACCTACGGGGATGAAGACTGCCTGTACCTCAACATTTGGGTGCCCCAGG
GCAGGAAGCAAGGTCTGCCTCCCCTCTACTCCCCAAGGGACCCTCCCATGCAGCCACT
GCCCCGGGTCTACTCCTGGCTTGA
>1746
ATCGTGGTCACCTTCAACTACCGTGTCGGCCCCCTTGGGTTCCTCAGCACTGGGGACG
CCAATCTGCCAGGTGCGTGGTGTCGGCCCTGAGGTGGGCGGACCAGCATGCTGAGCC
CAGCAGGGAGATTTTCCTCAGCACC
>1747
CTTCGGGGGGGACCCCAACAACATCACGCTCTTCGGGGAGTCTGCTGGAGGTGCCAG
CGTCTCTCTGCAGGTCTCGGGATCCCTGTGGGGAGGGCCTGCCCCACAGGTTGAGAGG
AAGCTCAAACGGGAAGGGGAGGGTG
>1748
CATCAGCCAGAGCGGCGTGGCCCTGAGTCCCTGGGTCATCCAGAAAAACCCACTCTTC

TGGGCCAAAAAGGTAAACGGAGGAGGGCAGGGCTGGGCGGGGTGGGGGCTGTCCAC
ATTTCCGTTCTTTATCCTGGACCCCA
>1749
CAGTGTCTGAAGGTTACTGATCCCCGAGCCCTGACGCTGGCCTATAAGGTGCCGCTGG
CAGGCCTGGAGTGTGAGTAGCCTGCTGGGTTGGCCCATGGGGTCTCGAGGTGGGGGTT
GAGGGGGGTACTGCCAGGGAGTAC
>1750
ACATGGACGGCCACATCTTCGCCAGCATCGACATGCCTGCCATCAACAAGGGCAACAA
GAAAGTCACGGAGTAAGCAGGGGGCACAGGACTCAGGGGCGACCCGTGCGGGAGGG
CCGCCGGGAAAGGCACTGGCGAGGGG
>1751
ACTTTGAGACCGATGTCCTCTTCCTGGTGCCCACCGAGATTGCCCTAGCCCAGCACAG
AGCCAATGCCAAGTGAGGATCTGGGCAGCGGGTGGCTCCTGGGGGCCTTCCTGGGGT
GCTGCACCTTCCAGCCGAGGCCTCG
>1752
GCTACCGGCCCCAAGACAGGACAGTCTCTAAGGCCATGATCGCCTACTGGACCAACTT
TGCCAAAACAGGGTAAGACGTGGGTTGAGTGCAGGGCGGAGGGCCACAGCCGAGAA
GGGCCTCCCACCACGAGGCCTTGTTC
>1753
GATGCGTGACCAGGACAAGAAACTGGTGGAGAAGAGCTGCAAGGATGCCCAGGGCT
GGTTCTTCAGCGTGGTGGGTGGCCGTGTGTAAGCAGGTGTGCACACGTGTGGGCCACA
TACGCCGTGTGCTGGTGCAGTTGGAA
>1754
CAGAGCGGCAGGATGGAGATGTGGAGCTCTACATCTCTGTGCAGAACCTGAGCCGTCC
CAGCTCAGCAAGGTAGGGCCCCTGTCCCCTCCCCACCCTGTACCTGTCACCCCAGGGT
GAGCGCACGCTTATAGACCCCTGC
>1755
TGGACGTGAAGACGCGGCTGGAGCAGGAGATCGCCACCTACAGCCGCTTGCTAGAGG
TTGAGGACGCCCGGTGAGTGTGCACCCAGGTACCTGCTGGGGCGGGCTGGGGGCCAC
TTCACCCAGGGAGAAGTTGGTGTCTG
>1756
AGGAAGGATTAAGGGTGGAGGGAAGGGACAGCCCCTTGTATTGGGAAAAGTTTCAAG
AATCAGCCCCTGAGTCCCTGTCACCTGGTGGCTGGTCCCTGGGAGGGCCACACATACT
TTCCAAGGACCACACCTGTTGGGGA
>1757
AGAGGCAGGCCCCGGGGCCGCCCGTGACTACAGCCAGTACTACAGGATAATCGAGGA
GCTGCAGAACAAGGTAGGGCCTGCTGGTGGGAGGGGTCTCCGGGGGGCATGACTTTT
TCCCCCCAACTCCTGCCCTGGCCAAA
>1758
CCGTGGACAATGCCAACATCCTGCTACAGAATGACAATGCCCATCTGGCTGCTGCTGA
CTTCCGCACCAAGTGAGTCCTAGCTGTGGGCTTGGGCAGCCTGGGTCAGCTGGGGGA
GGATCTCAGGTACCCCTCCTGACCC
>1759
GCGACCCGGAGATGCACATTGAGAACCTCAAGGAGGAGCTGGCCTACCTGAAGAAGA

ACCACGAGGTGAGGTGGCTGGGGCAGAAGGTCAAAGATGCTGAGGAGTGGGTGCAG
AGCCCTGGGGTTGGGCCATGGCTGAGG
>1760
GATGCGTGAGCAGTATGAGAAGATGGCAGAGAAGAACCGCAAGGATGCCGAGGATTG
GTTCTTCAGCAAGGTGGGAGCTGCTGCAGGCCAGAGGTCTCTCTTCGGGGCTGGGCTC
AGGGGCCTTAGCACTGACAGTAGGC
>1761
TGGACATGAAGATGCGGCTGGAGCTGGAGATCACCACCTACCACCGCCTGCTGGAGG
GCGAGGATGCCCAGTGAGTGGGGGAGCCTGGGGTCAGGGCTGGGGGCCTCTTGGCAG
GGGTGGGGCTCTCAGACTCACATCTA
>1762
CCTCAAAATTTTGTGGTGGTTTCCTTATAAGACGGAACTTTGTGCTGACGGCTGCTCAT
TGTGCAGGAAGGTGAGACAACAGGGTCTATTTATCTCCAAATGGGAGATGAACAACCA
GAGTAGCATCCAGGAATACACCT
>1763
CCTCAAAATTTTGTGGTGGTTTCCTTATAAGACGGAACTTTGTGCTGACGGCTGCTCAT
TGTGCAGGAAGGTGAGACAACAGGGTCTATTTATCTCCAAATGGGAGATGAACAACCA
GGTAGCATCCAGGATACACCTGC
>1764
GGTTATAAAGCAATTCCGTCATCCAAAATATAACACTTCTACTCTTCACCACGATATCAT
GTTACTAAAGGTGACAACACCTCTCTTCTCCCTTTCCACTTCCCATTCTCCTAAGCTTCT
CCTTCAGGTCCTCATTGCCC
>1765
AGCGTAGCCGCCCACGCTACGCCCTGGACTGAGGAACCGCGACACCTCACCACGGAG
GCCCAGGCTGCGGGTGAGCTTGCTCCCGCCCCTACCTCCTGGCTTTGCTGTCCTGGAG
GCCTGTGGTAGAACCTGTGGCCTGG
>1766
TCTTGGTCAGCGTCCCCATCACTCTGGCCCTGGCTGCCGCTGCCGCCACTGCCAGCAG
TCTCTTGATCTGGTAGGTTGAGTGAGGCTGGGTGGCAGAGGGAGCCTGAGGGAACCC
CCACTCCAGCCCCCTCCCTGACAGG
>1767
GCTCCCCAGTCGCTGGTGCTGCTGACGCTGCTCGTCGCCGCTGTCCTTCGGTGTCAGG
GCCAGGATGTCCGTAAGTCTTCCCCCGCCGCTGCCTGCCTGCCTGCTTTCCATGCGTCC
CTCAGCATCCTTCTCCCCGGCCC
>1768
TGCCTCAGCCCTGAGATCCCCTTCGGAGAGTGCTGCCCCATCTGCCCAACTGACCTCG
CCACTGCCAGTGGTTGTAATTTATTTATTTCCTGTTCAACATAAATAAATTACTTGCAAG
CACTGCAAACACGCTCCCATAG
>1769
ACCTGGGACCCCTGGAAATCCTGGCCCCCCTGGTCCTCCCGGCCCCCCTGGTCCCCCT
GGTCTTGGTGGAGTAAGTATCCTTACTTCCCATTCCTTCAGGCTGTCCCTCCAGAAATG
TGGCTTTTAAATTGCTGCTTGCA
>1770
TGCCCAGATGGCTGGAGGATTTGATGAAAAGGCTGGTGGCGCCCAGTTGGGAGTAATG

CAAGGACCAATGGTAAGAAAAGACACTAGTTCTTTGCAGCCAAAATGGCAGGAGGTG
GCCCTTAGCAGAGCCAGAGAGTCTG
>1771
TCCTGAAGGTGCTCAAGGTCCTCGCGGTGAACCTGGTACTCCTGGGTCCCCTGGGCCT
GCTGGTGCCTCCGTAAGTGCAGCTTCTCTTTGGCCTGGGGGGGTCTGGGGTCTGTGGC
TTTGGAACTCTTGACTCTGTACTT
>1772
CTTCCCTGGGCCACGGGGTCCTCCTGGCCCTCAAGGTGCAACTGGTCCTCTGGGCCCG
AAAGGTCAGACGGTAAGAGCCCAAAGTGACCCCCAAGTTCCACTGACATCTCTGGAG
TCAAACCCCATCACCCCTCTTTCCC
>1773
TGAAGAAGGCAAGAGAGGTGCCCGTGGAGAGCCTGGTGGCGTTGGGCCCATCGGTCC
CCCTGGAGAAAGAGTTAAGTGAATGTGGAGGCTCCATCCCATGGGGCCTGTGACCTCG
AGAGGGAAGTGGAGTCCTTGTGGTC
>1774
TCTTGCTGGCCCCAAGGGAGCCAACGGTGACCCTGGCCGTCCTGGAGAACCTGGCCT
TCCTGGAGCCCGGGTAAGTAGCAGAGCTGCTGTTGCCCTTGGCTTCAGACCCTCAGGC
CCTTCCTGGCTGGCTCCTTCCAGCC
>1775
ACCTCCAGGTCCTCAGGGGGCTCGTGGGCAGCCTGGTGTCATGGGTTTCCCTGGCCCC
AAAGGTGCCAACGTAAGTAATAATTTGCTCTTCTATTTCCTTCCATGTGGTGCTACCTAC
CTCCCTGCCCTCTTGGGGAAAG
>1776
CTCTCCCGGTGCCCAGGGCCTCCAGGGTCCCCGTGGCCTCCCCGGCACTCCTGGCACT
GATGGTCCCAAAGTAAGTGAGGCTGCATCCAGTAGGGGTCTTCGTGGTAGCCTGGAGT
CCCACTGAGCAGGAGAGAGGAGCG
>1777
CCCTCCAGGTCTTCAGGGAATGCCTGGCGAGAGGGGAGCAGCTGGTATCGCTGGGCCC
AAAGGCGACAGGGTAAGTACTGAGGTTACAGCCTCCTCACCAAAGCTGTGGCTTTGCC
AATGTCCTGCCCCTTGTGATCGCT
>1778
AGAGGCCGGCCAGAAAGGCGATGCTGGTGCCCCTGGTCCTCAGGGCCCCTCTGGAGC
ACCTGGGCCTCAGGTGGGTAACGCTGCACTCCAAGAATTGTTCCCTCAAGGAAGGGCT
CCTGGCGTGCAGATGGGAAGGCCCC
>1779
TGAACCCGGCCTCCAAGGTCCTGCTGGACCCCCTGGCGAGAAGGGAGAGCCTGGAGA
TGACGGTCCCTCTGTAAGTCCCTCACCAGGCCCATGCCAAGGTCCCTGGGAGCAGGGT
CGTGAGTGGCTTCTGAGCTCACAGA
>1780
TCAGAGAGGCATCGTCGGTCTGCCTGGGCAACGTGGTGAGAGAGGATTCCCTGGCTTG
CCTGGCCCATCGGTGAGTGTGGGGTATCCCTCCCTCCATCCAAGCTGGCCCTGCCTGCC
AAGGCTTCTACCTCCCTCAGCAC
>1781
AGACAGAGGTCCTCCTGGCCCCGTGGGTCCTCCTGGCCTGACGGGTCCTGCAGGTGA

ACCCGGACGAGAGGTGAGCAGTGAGACCCCCTGGGGTGGCCCTGATTGGGGAGAGG
GGCCCTGTGAGTCTCTGTGCTGGGTCA
>1782
AGCCCCTGGGCCCCCTGGCTCCCCTGGCCCCGCTGGTCCAACTGGCAAGCAAGGAGA
CAGAGGAGAAGCTGTAAGTATCCTGGAATTCAGTAAAAGCCGCCTTCCCCTGCGCGGT
GGGGCTGAGGCAGTCCCTGGGTTTC
>1783
AGAGCCTGGCGAGAGAGGCCTGAAGGGACACCGTGGCTTCACTGGTCTGCAGGGTCT
GCCCGGCCCTCCTGTGAGTGTCACTGCCTGCGTGGGACTTCCCGAGGCCTCCTGCCAC
ACAGAGCCCACTTGAGCTCCCTGTG
>1784
TGCTAATGGAATCCCTGGCCCCATTGGGCCTCCTGGTCCCCGTGGACGATCAGGCGAA
ACCGGTCCTGCTGTAAGTGTCCTGACTCCTTCCCTGCTGTCGAGGTGTCCCTACCATCC
GGGAGGCTTGAGCTCTTTTTTGC
>1785
GAGGGCTCCCGCAAGAACCCTGCTCGCACCTGCAGAGACCTGAAACTCTGCCACCCT
GAGTGGAAGAGTGGTAAGCTTGGAGAACAGGATCCCCTGCCCCGGGAAGCAGGGAGT
CATCCCTTAGGCCTAGCAGCAAGGGA
>1786
CTGGTGGAGCAGCAAGAGCAAGGAGAAGAAACACATCTGGTTTGGAGAAACCATCAA
TGGTGGCTTCCATGTGAGTACCTGGGTGCCCTAGATGATGAGCAGAGATGGCTCCTCA
AACTCTTTCTTTTCTTTCTCCCTGG
>1787
CAATGACGTGGAGATCCGGGCAGAGGGCAATAGCAGGTTCACGTACACTGCCCTGAA
GGATGGCTGCACGGTGAGTGGGGCTGCCAGAGAGAAGAGCTGCCTGTGCCCAAACTG
CCTGGAGCAGGGCTGAGGGTTGGCCC
>1788
CTGGTGGCCGCGCTCTGCGCCGGGATCCTGGCAGAGGCGCCCCGAGTGCGAGCCCAG
CACAGGGAGAGAGGTGGGCACCGCAAGGGAGGACCCGGCCCGGGGCACTGCACCGT
GCCACCCTCCGCTCCACTCGGCCTTCA
>1789
TGCCTTTCTCTGGAGCAGCCAGTGCACAGGGTGTGCGCTTTGCCACAGTGCAGTACAG
CGATGACCCACGGTGAGTAGGGGCCCTGGGGGCTGGGGACCACGAGATCCCCACCAA
GACTCCTTCCCCAGAGAGGGAGGAG
>1790
AGGGGCTGCAATTCTCCATGTGGCTGACCATGTCTTCCTGCCCCAGCTGGCCCGACCT
GGTGTCCCCAAGGTGATCCCTACCCCTACCATGCCTCCCAAGATGACCCCAAATGAAG
TGTCCAGGGGAACCGTGATTTGAC
>1791
AAGTCCCAGGACCTGGTGGACACAGCTGCCCAAAGGCTGAAGGGGCAGGGGGTCAA
GCTATTTGCTGTGGGTAAGGACCGAGCAGGAGTGACAGGTCAGCTGGGGGGTGGGGG
CAGTCAGAGAGCATGTGGGTGACTGAG
>1792
AGGACACTACTGCCCCTCGTTTCCCGGAGAGTGTGCACGACTGCTGGTGGCGTGCCTG

TGACCCGACCTCGTGAGTTCCTGCCCACACGGTGTACCCTGACCTAGACCCCGGACCC
CAATCCCCACTTGGCAGTGCTGAT
>1793
GACTGGCTACAAGGTCCAGTACACTCCTCTGACGGGGCTGGGACAGCCACTGCCGAG
TGAGCGGCAGGAGGTAGGATGTCAGGAGTATAGGTGGTGGCTGGGGACTTGGCTGGG
CAAGATAAAGTGACCTCTTGCCCTGG
>1794
TACCAAGTGACTGTGATTGCCCTCTACGCCAACAGCATCGGGGAGGCTGTGAGCGGGA
CAGCTCGGACCAGTGAGCAATTCTGCCAGCCTCTGACCCCATTCACCTAACCCCCGAC
CCCAGTACCCCCTCCCACTTCTGA
>1795
AGCCTCCTGGTGGCCTGGCGGAGTGTGCCAGGTGCCACTGGCTACCGTGTGACATGGC
GGGTCCTCAGTGGTGAGTGAGAGATGTGGCTGAGGGAGTCCCCGCGCCTCAGACAAG
GCTGTAGAGTCCTGAGTCTGCAAGC
>1796
TATGAGGTGACCGTGAGCACCCTATTTGGCCGCAGTGTGGGGCCCGCCACTTCCCTGA
TGGCTCGCACTGGTGAGAAGGCTGGGCACTTTCTTCAGGCTGGGACGGGCAGGCAGG
GCAAGGCCCGGAGGCCACTGACATC
>1797
TCCATCCTCCTTTCCTGGAACTTGGTGCCTGAGGCCCGTGGCTACCGGTTGGAATGGCG
GCGTGAGACTGGTCAGTGCGGGGGAAGGGATGGACAGGCAAGGGTCAGGGCATGGC
CTCTGCTGGTCTGACCCTGTTATCT
>1798
TACCGCCTCACACTCTACACTCTGCTGGAGGGCCACGAGGTGGCCACCCCTGCAACCG
TGGTTCCCACTGGTGAGGGCTCTGTGGGCTGGGCCAGTGAGTGGGGGAGGTGTCGGA
GCCCCAGGCTGCCTCTATGCTGTGC
>1799
CGGGTGCGAGTGTCCTGGAGCCCAGTCCCTGGTGCCACCCAGTACCGCATCATTGTGC
GCAGCACCCAGGGTGAGGTGGACGCAGCCAGCACCCCCACCACACACACTGAAGTTC
CAGCCTGAGGGGTCTGAGTGTACCT
>1800
TACACTGTGCGGGTGTCTGCTCGAGTGGGTCCCCGTGAGGGCAGTGCCAGTGTCCTCA
CTGTCCGCCGGGGTGAGTACTGCAGGAGGCTTGTGGAGGACAGCCTGCCTGCCTCACT
CTGGTCCTGGTTCTGACTTCTGAC
>1801
CGAGTGAGGGTGGCCTGGGGACCCGTCCCTGGAGCCAGTGGATTTCGGATTAGCTGGA
GCACAGGCAGTGGTCAGTGTGGGGTGTGTGGGGGGACTGCCAAAGGGACCAATGAG
GGTATGGGTGCCAGAGGGGACAGGCA
>1802
TACCAGGTGGCTGTGTCGGTACTGCGAGGCAGAGAGGAGGGCCCTGCTGCAGTCATC
GTGGCTCGAACGGGTCAGGCCCTGCCCCCGTCCCTTGGCTCTCTGCCTCCATTGCTCTT
TCAGACCCCCATGCCTTCCCTTGC
>1803
TCTGTCACCATTACCTGGACCAGGGTTCCTGGCGCCACAGGATACAGGGTTTCCTGGC

ACTCAGCCCACGGTGGGGACTGGGGTTTGGGAGGGGGCAGGTCAGGGTGGAATGGG
GGCTGGGGGTTTATTGGGGGTTCAGG
>1804
TATACGGTGCATGTGAGGGCCCATGTGGCTGGCGTGGATGGGCCCCCTGCCTCTGTGGT
TGTGAGGACTGGTGAGTGGACCCTGGCCAGCTACTAGCCACACCGCATTAGCTACCCT
GCCCTGCTGTGTGTTCCTGATTG
>1805
GTTCTACGGATCACCTGGGTAGGGGTCACTGGAGCCACAGCTTACAGACTGGCCTGGG
GCCGGAGTGAAGGTATGGCTCCCTGACGCCACCCCTGTCCTTCCTGGCTGGGACTGCT
CACCCCTAACCATTGCTGTATGCC
>1806
TACTCAGTGCGAGTGACTGCACTTGTCGGGGACCGCGAGGGCACACCTGTCTCCATTG
TTGTCACTACGCGTAGGCAGAGCATGCGCTGGAGAGCTTCGGATGGGTGGTGTGGATG
GTTTGGGGATCCGGGCTTGTGCTC
>1807
CACTCGCTGAGGCTGCGCTGGGAGCCGGTGCCCAGAGAGCAGGGCTTCCTTCTGCAC
TGGCAACCTGAGGGTGAGAGTGTCCCCAGAGAAGTTAGGACCATTGGGGCAGGCTGT
GGCAGACTCCGCAAGGTAGGCGAGAC
>1808
TACCGCGTGAGGCTGAGTGTCCTAGGGCCAGCTGGAGAAGGGCCCTCTGCAGAGGTG
ACTGCGCGCACTGGTAAGCCTCACCTTGGCGTGCTCCTCCCCTGCTGATGACCCACCC
TGACTTCCTGCACCCCGACTCTGAG
>1809
GCCTGGACTCCAGTGTCCAGGGCATCCAGCTACATCCTATCCTGGCGGCCACTCAGAG
GCCCTGGCCAGGGTGAGGGGGAGGCCAGGATTTGGGTGGGCTGGCAGTTGGGGCTCT
GTGGAGACAGCTTTTTAATCAAGTT
>1810
TACATCTTCTCCCTGACGCCTGTCCTGGATGGTGTGCGGGGTCCTGAGGCATCTGTCAC
ACAGACGCCAGGTATAGTGGGCATAGTGGGAAGGGCAGAAAGGTGTGTCTGGGTGGG
CTGCCCGCTTCAGTAACTTGTTCC
>1811
GGAGGCTACGAGGAGGGTCCTGGAGCGTCTGGTGTTGGCACTTGGGCCTCTTGGGCC
ACAGGCAGTTCAGGTTTGGCCCTGGGGCAGCCAGATTCTACCTCTCCCTGAGGCCCCC
CACCCAGGCTCCATACCAGTTACCA
>1812
CATGACCTTGGCATTATCTTGCAAAGGATCCGTGACATGCCCTACATGGACCCAAGTGG
GAACAACCTGGGTGAGGACTGCAGCAGGCATGGACTCCTGGGGCTATCCACTGGGAG
CTTGGTGCCCCAGGGTTCTGACAT
>1813
ATGGTTCTGCTAGTGGATGAACCCTTGAGAGGTGACATATTCAGCCCCATCCGTGAGGC
CCAGGCTTCTGGTAAGGAGTAGGCTGATGGGGAAGGGGTCTGGGAATAGGGGTGGCC
CTGAAAAGGCTATCATGCAGCCAC
>1814
TGGGCCAAGCCTGGACCAGGCAGTCAGTGGTCTGGCCACAGCCCTGTGTCAGGCATC

CTTCACTACTCAGGTTTGGAAGAGGCCTCTGGGGGACTGGGTGGTAGAATATAAGGGG
TCTGGGGGTTCTTGGTAGAAATGTT
>1815
AGCAGATGGGCGTCCAGGCAGCCCTGGCCGCGCCGGGAATCCTGGGACCCCTGGAGC
CCCTGGCCTAAAGGTGAGCAAGCCTTGTCCTGCAGGTCAGGGTGGGCGCTGCTGAGT
GGGTGGGGTGGCTCCGACTGTTCTGC
>1816
GGCTCCCGGACAAGTCATCGGAGGTGAAGGACCTGGGCTTCCTGGGCGGAAAGGGGA
CCCTGGACCATCGGTAAGTGCAGGGTATGTGGAGGCAAGTGATGTGTAGTGGGGGGAC
CAACACCAGGGGGGCGAGAGTGAGG
>1817
TGGACCTCGTGGACCACTGGGGGACCCAGGACCCCGTGGCCCCCCAGGGCTTCCTGG
AACAGCCATGAAGGTGACAGCCTCATGAGTGCCATGTGATGCGGAGACCTGGTGACCC
CATTTGAACCCACATAACCCCTGCC
>1818
GCCTCCTGGAGAGAAGGGAGACCCTGGGCGGCCTGGCCCCCCAGGACCTGTTGGCCC
CCGAGGACGAGATGTAAGAGGCTGGAGTCGGGGGAGTCATGGCGGGTAAGGGAGTAG
GGCTGTTGCCAGCATCATGGGGGTTC
>1819
GGCACCTGGAGTTCGGGGGCCTGTGGGTGAAAAGGGAGACCAGGGAGATCCTGGAG
AGGATGGACGAAATGTGAGTCCCAGCCTATGACTCCTCACCCCAACCTTAACCCTCCA
ACCAGCCAATTCCCAATACCTGATCC
>1820
GGACCGCGGACAAGAGGGTCCTCGAGGGCCCAAGGGTGATCCTGGCCTCCCTGGAGC
CCCTGGGGAAAGGGTGAGTGTGATTGGTCCTCGGGGGTGCAGGCATGGGAGGGCCTG
CTCTGATTTCTTCCTCCCCTATCCTC
>1821
TCCCCCTGGGCTGGATGGCCGGAGCGGACTGGATGGGAAACCAGGAGCCGCTGGGCC
CTCTGGGCCGAATGTGAGTCTTGGTAGTCCTGCCTGGTTGTCCCCTTCCCCTACCCCTT
CACATGAGGACCCTAGACCCCAGC
>1822
TGGAGCTCCTGGTATCCTTGGACCCCAGGGGCCTCCAGGCCTCCCAGGGCCAGTGGGC
CCTCCTGGCCAGGTGAGTGTCCTGGGTCATTCTGGGACTTCAGAGCATTAGAAGCCAT
GATGTTTCAATGGGCAACCCTCCT
>1823
CCTGCCTGTGCCCGAACGGCGTCGAGGCCCCAAGGGGGACTCAGGCGAACAGGGCCC
CCCAGGCAAGGAGGTGAGCAGAAGTGGCTCAGTGGGTTGTGCCCCGTGGAGTGGGGT
GTAGCTGTACAGCCACCAGCATTCTC
>1824
AAAGCCTGGTATTCCCGGGCTCCCAGGCAGGGCTGGGGGTGTGGGAGAGGCAGGAAG
GCCAGGAGAGAGGGTGAGGCTGGGGGCTGGCCAGGAGAGTGAGGGAAGAGGGGTTG
GGAGGGGTGGGACCCCCCATGGGCTTG
>1825
GAAGCCGGGAGCCCCAGGTCGAGATGGTGCCAGTGGAAAAGATGGAGACAGAGGGA

GCCCTGGTGTGCCAGTATGTGTTCTGGGGGCAGCTCGCTAGGGTGTGGTGCCCAGCTG
TGGGCCTGAAATATGAGGAGTGGGGC
>1826
TGTTGTTGGGTTCCCGGGTCAGACAGGCCCTCGAGGAGAGATGGGTCAGCCAGGCCCT
AGTGGAGAGCGGGTGAGGGGCTGGGAACAGCATGTAGGGGCATGGTGAATCCATGGT
GGGTGAGGAGGGGGCCCTGCCTTGG
>1827
TCTGGCAGGCCCCCCAGGGAGAGAAGGAATCCCAGGACCCCTGGGGCCACCTGGACC
ACCGGGGTCAGTGGTGAGTAGAGGTCCCTAAACCCCACGTATTTGATTTCCTGTCCTCG
TGAGGACTTAGGATGGGGCGGGCC
>1828
TGAACGAGGGCCTCGGGGCTTGGATGGTGACAAAGGACCTCGGGGAGACAATGGGGA
CCCTGGTGACAAGGTACAGGGAAGAGGGTGGTGTACTCTCTGTGGGGTCCTGTGGCT
GTGGTGGTTCCACAGCTACTGTGAGG
>1829
AAAGGATGGAGTGCCTGGTATCCGAGGAGAAAAAGGAGATGTTGGCTTCATGGGTCCC
CGGGGCCTCAAGGTAGGAAAGAAACAAGATTGGTTTTTTTCTGGTCAGGAAGGCCTAA
TGTTGAAGGGCGAGGGACCTGGGG
>1830
CTCTGCTGGTCCCCCAGGGCCACCTGGCAGTGTTGGTCCCAGAGGCCCCGAAGGACTT
CAGGGCCAGAAGGTAAGGGGCCCCAGCTCTGACTCCTGATCCCTGAACCTTCCCTACC
CTTCCAACCTCATAACCATCTTCC
>1831
AGGTCCCCCCGGAGAGAGAGTGGTGGGGGCTCCTGGGGTCCCTGGAGCTCCTGGCGA
GAGAGGGGAGCAGGTGAGTAGGGATTCCAAGGCTTGGGTCAGAGATCGGGGTGACTT
CTGTTGTCCCTGAGGTCAGAGGTCAC
>1832
GCTGCAGACACTGCCGGCTCCCAGCTCCATGCTGTGCCTGTGCTCCGCGTCTCTCATGC
AGAGGAGGAAGGTGAGGACAGCTGAACCCGTGGGGCAGCTATGGGTGGGGCCGAGA
CACGCACATGGGTGTCCATGAATGC
>1833
GAGTACTCTGAATACTCCGAGTATTCTGTGGAGGAGTACCAGGACCCTGAAGCTCCTT
GGGATAGTGATGGTGAGAATGGGGGGCTGCGCAGCGGGGTCTGGGGAGGGGCAGGCA
GGGCTGAGCCCTGCTGACCTCCCCC
>1834
AACCGTTTTGGGACCCGTGAGGCCTGCGAGCGCCGCTGCCCACCCCGGGTGGTCCAG
AGCCAGGGGACAGGTATGGGCTGAGCCCCCACCGTGGGGAACTGGGCACTGAGCCTG
CCTGGATCGGGTTCTGGGGGAGGAGT
>1835
GATCCTCCTGCTTGTCGCCCTCTCTGTGGCCTATGCAGCTCCTGGCCCCCGGGGGATCA
TTATCAACCTGGTAAGAGGGTCTTCAGGGGAGGCGCCTCCTGAGTCCCCAGATGAAGA
CTTCCTAAGCTTGGACCTTGACC
>1836
GCATTCAAGTGCGCTGGGCCTGGCCCGCTGCACATCCATGGCCAGCGAGAACAGCGA

GTGCTCTGTCAAGGTGGGTGCCTGAGGACTTGGCGTGGGTGGGGGAGAATGGGGAGT
AGGGCCCTGAACACTTGTGAGTGGGA
>1837
CTGGCCGCGCAGGCCCTGAGGGCTCGCGGCCCCAGTGGCGCGGCCGCGATGCGCTCC
ATGGCATCTGGAGGTACTCGGGTCTCCGGGCGTGCCAGGGACCAGAGTGTTGCCCTCC
CAGGGTGGTCCCAGGGCGGCAAAGC
>1838
TGTTCCCACTGATGAAGAGCAGGCGACTGGGTTGGAGAGGGAGATCATGCTGGCTGC
AAAGAAGGGACTGGTAAGAGAAACTCCCTTCTGTCTTCTGTGTAACTTATGGCCTTGG
ATGTGTTCATAGTGGTCTCCTCTCT
>1839
GCTTCAGGCACCAGGGAAGACCCTAATTTAGTCCCCTCCATCTCCAACAAGAGAATAG
TAGGCTGCATCTGTAAGTACCTCACCTCTATTTTTTATCCACTTGCTTAATATATCCTACA
ATAGTGTGTAAGCTGCCTCAA
>1840
CTCCCAATCTATCTGCTTGGCCTGACTCAGAAATTCGGGCCCATCTACAGGCTCCACCT
TGGGCTGCAAGGTGAGAGGCTGATCTCGCTCTGGCCCTCACCATAGGAGGGGGCGGA
GGTGACGGAGAGGGTCCTCTCTCC
>1841
AAGAGGACCATTGAGGAAGCCATGGTCAAAAAGTGGGCAGACTTTGCTGGCAGACCT
GAGCCACTTACCTGTAAGGGCTGGGGGCATTTTTTCTTTCTTAAAAAAATTTTTTTTTAA
GAGATGGGTTCTTGCTATGCTGC
>1842
AGCCCTGCTGCTGGGCATCCGTGACTCCATGGAGCCAGTGGTGGAGCAGCTGACCCA
GGAGTTCTGTGAGGTAAGGCTGGGCTCCTGAGGCCACCTCGGGTCAGCCTTGCCTCTC
ACAGTAGCCCCCGCCCTGCCCGCTG
>1843
TCTCCGGAGGCTGAAGCAGGCCATAGAGAAGAGGGATCACATCGTGGAGATGCAGCT
GAGGCAGCACAAGGTGGGGACTGTACGTGGACGGCCTCCCCTCGGCCCACAGCCAGT
GATGCTACCGGCCTCAGCATTGCTAT
>1844
AAGAGGACCATTGAGGAAGCCATGGTCAAAAAGTGGGCAGACTTTGCTGGCAGACCT
GAGCCACTTACCTGTAAGGGCCGGGGGCATTTTTTCTTTCTTAAACAAATTTTTTTTTTG
TTAGAGATGGGGTCTTGCTATGT
>1845
AGCCCTGCTGCTGGGCATCCGTGACTCCATGGAGCCAGTGGTGGAGCAGCTGACCCA
GGAGTTCTGTGAGGTAAGGCTGGGCTCCTGAGGCCACCTCGGGTCAGCCTCACCTCTC
ACAGTAGCCCCCGCCCTGCCCGCTG
>1846
CCATTGAGGAGGAATTCTCTCTCCTCACCTGCAGCATCAATGTTACCTCACCTTCGGAG
ACAAGATCAAGGTGCCTCACAGCCCCTCAGGCCCACCCCCAGCCCCTCCCTGAGCCTC
TCCTTGTCCTGAACTGAAAGTAC
>1847
GTATCCAGGAGGTGTTAAAAACCTGGAGCCACTGGTCCATCCAAATTGTGGACAGATT

CCCTTTCTCAGGGTGAGGACCTGGAGCCTAGACACCCCTGGGTTGTGGGGGAGAGGC
TGGGGTGGAGGGAGAGGCTCCTTCC
>1848
AGGTCTCCGGAGGCTGAAGCAGGCCATAGAGAAGAGGGACCACAAGAGAGAACAGC
TGAGGCAGCACAAGGTGGGGACTGTGTGTGGACGGCCTCCCCTCGGCCCACAGCCAG
TGATGCTACCGGCCTCAGCATTGCTAT
>1849
GATCGGTGGCACTGAGACCACAGCAAACACCCTCTCCTGGGCCGTGGTTTTTTTGCTT
CACCACCCTGAGGTGCGTCCTGCGGACAAGCAAAAGGCTCCTTCCCAGCAACCTGGC
CAGGGCGGTGGCCACCCTCACTCAG
>1850
CACCACAGAGATTAACTTCAAGGTTGGGGAGGAGTTTGAGGAGCAGACTGTGGATGG
GAGGCCCTGTAAGGTGAGTGCCAGAAGGGGCTCCAGGGTCATGGCGTCATTGCCCTGC
CTCTCAACCTGCCATTTTCCAGGCT
>1851
GCTCCTGAAGGGAGAGGGCCCCAAGACCTCGTGGACCAGAGAACTGACCAACGATGG
GGAACTGATCCTGGTAAGTCCTGCCTCCTCCCCACTAATAGCAAACCCAGTGCTACCTT
CCAAGATTCTCTGGGAGACCCCAG
>1852
GACGTGACCATGGAGAAGCTGTTGTGTTTCTTGGTCTTGACCAGCCTCTCTCATGCTTT
TGGCCAGACAGGTAAGGGCCACCCCAGGCTATGGGAGAGTTTTGATCTGAGGTATGGG
GGTGGGGTCTAAGACTGCATGAA
>1853
GCTTTTTGACCACGCTATGCTCCAAGCCCATCGCGCGCACCAGCTGGCCATTGACACCT
ACCAGGAGTTTGTAAGTTCTTGGGGAATGGGTGCGGGTCAGGGGTGGCAAGAAGGGG
TGACTTTCCCCCACTGGGGAAGTA
>1854
CCAGACCTCCTTCTGCTTCTCAGACTCTATTCCGACACCCTCCAACATGGAGGAAACG
CAACAGAAATCCGTGAGTGGATGCCGTCTCCCCTAGGCGGGGATGGGGAGACCTGTG
GTCAGGGCTCCCGGGCAGCACAGCC
>1855
TGACACCTCGGACAGCGATGACTATCACCTCCTAAAGGACCTAGAGGAAGGCATCCAA
ACGCTGATGGGGGTGAGGGTGGCGCCAGGGGTCACCAATCTTGGAACCCCACTGGCT
TCGAGGGCTGGGGGAGAGAAATACT
>1856
GGAGCATGTGAATGCCATCCAGGAGGCCCGGCGTCTCCTGAACCTGAGTAGAGACACT
GCTGCTGAGATGGTAAGTGAGAGAATGTGGGCCTGTGCCTAGGCCACCCAGCTGGCCC
CTGACTGGCCACGCCTGTCAGCTT
>1857
CCTCACCAAGCTCAAGGGCCCCTTGACCATGATGGCCAGCCACTACAAGCAGCACTGC
CCTCCAACCCCGGTGAGTGCCTACGGCAGGGCCTCCAGCAGGAATGTCTTAATCTAGG
GGGTGGGGTCGACATGGGGAGAGA
>1858
AGCCTTCCTGAGAAGATGCAACCAATCCTGCTTCTGCTGGCCTTCCTCCTGCTGCCCAG

GGCAGATGCAGGTGAGTGACCGTCTCCACCCTCGGGGGCCCAACCCGATCCCACAGG
TCTCCTGCCCTTTCTCCACATTCC
>1859
ACGCCATTATTACGACAGTACCATTGAGTTGTGCGTGGGGGACCCAGAGATTAAAAAG
ACTTCCTTTAAGGTAAGACTATGCACCTGCCTGGATTGGCTCTTGGGAGAAAGATCTTT
GGGGAATATCTGAGACCTGGAGA
>1860
AGCCTTCCTGAGAAGATGCAACCAATCCTGCTTCTGCTGGCCTTCCTCCTGCTGCCCAG
GGCAGATGCAGGTGAGTGACCGTCTCCACCCTCGGGGGCCCAACCCCATCCCACAGG
TCTCCTGCCCTTTCTCCACATTCC
>1861
TGTGAAAAGACCCATCCCCCATCCAGCCTATAATCCTAAGAACTTCTCCAACGACATCA
TGCTACTGCAGGTGAGGCACACTCCTGCCACTCTTGCTCTTCTTGGTCCAGTTGGTTCC
ACTCCCCCTGGAATGCCGGCCC
>1862
CTCTGAAGAGGTGCGGTGGCTTCCTGATACAAGACGACTTCGTGCTGACAGCTGCTCA
CTGTTGGGGAAGGTGAGGAGCAGAAAACAGCCCACACCCTCCTGGAAACACTCCACA
GAGACCCCTGCCTTCTTCCCAAGGA
>1863
TGTGAAAAGAGCCATCCCCCATCCAGCCTATAATCCTAAGAACTTCTCCAATGACATCA
TGCTACTGCAGGTGAGGCACACTCCTGCCACTCTTGCTCTTCTTGGTCCAGTTGGTTCC
ACTCCCCCTGGAATGCCGGCCC
>1864
ACGCCATTATTACGACAGTACCATTGAGTTGTGCGTGGGGGACCCAGAGATTAAAAAG
ACTTCCTTTAAGGTAAGACTATGCACCTGCCTGGATTGGCTCTTGGGAGAAAGATGTTT
GGGGAATATCTGAGACCTGGAGA
>1865
CATGGTCTCTTTGGGCGGAAGACAGGTCAGGCCCCTGGATACTCTTACACAGCCGCCA
ATAAGAACAAAGGTAAGAGTCACTTGTTAAATAAAACAACACAAAATGCAGGAATATA
ACATGTGGCAAACTATCAGGAGTG
>1866
CATCGTCCCCCTGGGTGTGACCCATATGACATCCCGTGACATCGAAGTACAGGGCTTCC
GCATCCCTAAGGTAGGCCTGGCGCCCTCCTCACCCCAGCTCAGCACCAGCACCTGGTG
ATAGCCCCAGCATGGCTACTGCC
>1867
CCTGCCACTGCCCGGGCTGGGCAACCTGCTGCATGTGGACTTCCAGAACACACCATAC
TGCTTCGACCAGGTGAGGGAGGAGGTCCTGGAGGGCGGCAGAGGTGCTGAGGCTCCC
CTACCAGAAGCAAACATGGATGGTG
>1868
CGGCCGACCGCCCGCCTGTGCCCATCACCCAGATCCTGGGTTTCGGGCCGCGTTCCCA
AGGCAAGCAGCGGTGGGGACAGAGACAGATTTCCGTGGGACCCGGGTGGGTGATGAC
CGTAGTCCGAGCTGGGCAGAGAGGG
>1869
AAGAAGTCGCTGGAGCAGTGGGTGACCGAGGAGGCCGCCTGCCTTTGTGCCGCCTTC

GCCAACCACTCCGGTGGGTGATGGGCAGAAGGGCACAAAGCGGGAACTGGGAAGGC
GGGGGACGGGGAAGGCGACCCCTTACC
>1870
CCCTCGCTTCCTCAGGCTGCTGGACCTAGCTCAGGAGGGACTGAAGGAGGAGTCGGG
CTTTCTGCGCGAGGTGCGGAGCGAGAGACCGAGGAGTCTCTGCAGGGCGAGCTCCCG
AGAGGTGCCGGGGCTGGACTGGGGCC
>1871
CAGGATGACCTGGGACCCAGCCCAGCCCCCCCGAGACCTGACTGAGGCCTTCCTGGC
AGAGATGGAGAAGGTGAGAGTGGCTGCCACGGTGGGGGGCAAGGGTGGTGGGTTGA
GCGTCCCAGGAGGAATGAGGGGAGGCT
>1872
GGGATGGTGACCACCTCGACCACGCTGGCCTGGGGCCTCCTGCTCATGATCCTACATCC
GGATGTGCAGCGTGAGCCCATCTGGGAAACAGTGCAGGGGCCGAGGGAGGAAGGGTA
CAGGCGGGGGCCCATGAACTTTGC
>1873
CATCGTCCCCCTGGGTGTGACCCATATGACATCCCGTGACATCGAAGTACAGGGCTTCC
GCATCCCTAAGGTAGGCCTGGCGCCCTCCTCACCCCAGCTCAGCACCAGCCCCTGGTG
ATAGCCCCAGCATGGCTACTGCC
>1874
CACCCCGAACACTTCCTGGATGCCCAGGGCCACTTTGTGAAGCCGGAGGCCTTCCTGC
CTTTCTCAGCAGGTGCCTGTGGGGAGCCCGGCTCCCTGTCCCCTTCCGTGGAGTCTTG
CAGGGGTATCACCCAGGAGCCAGG
>1875
GGGAGCAGGGTTATGAGCACCTGCACCTGGAGATGCACCAGACCTTCCAGGAGCTGG
GGCCCATTTTCAGGTAAAGCCCTCCCTGGCCCTCGCTGGGAACACCCAGATCCCTGCC
CCTGCTGCCCAGGACCCTGCCAGGC
>1876
GCAGGATGATCCTGGAGCCCTGGGTGGCCTACAGACAACATCGTGGGCACAAATGTGG
CGTGTTCTTGTTGTAAGCGGCGAGTTGGGAGCTGAGAGCTGGGAGCAGGGTGGGCAG
CCTGGGTGTAGGGGGGAGGCGAGAG
>1877
GTGCTGCAGAACGCCCGGGGGAGCCTGACCCTGGACGTCCAGCCCAGCATCTTCCACT
ACACCATAGAAGGTGTGGGCCATGCGGGAAGGTCCAGCCCCAGAGACCCTGGAGTGG
CCAGGGATGGGGATGGAGGACTGAA
>1878
CTGTCTCGCTGGATCAGCCCCAAGGTGTGGAAGGAGCACTTTGAGGCCTGGGACTGCA
TCTTCCAGTACGGTGAGGCCAGGGACCCGGGCAGTGCTATGGGGAAGGGACACCATG
GGGGCCCAATTTCTCCTTCTCCACC
>1879
GAAGGCGGAACTGTCACTAGAAGCCATCAAGGCCAACTCTATGGAACTCACTGCAGG
GAGCGTGGACACGGTCAGGCCAGCAACCAGCCCCACCCAGAGAGGGTGATGCCAAG
CCTGCCTCCCAGGCACTGCCTGCCAAT
>1880
TCAGTGAACATCCCCAGAAGGCAACCACCGAGCTGCCCTTGCTGCGGGCGGCCCTCA

AGGAGACCTTGAGGTGGGTGCTGGCTGAGGCCTCCCTGTGGCCCTGGCCCCCTGCTG
GAGAGCAGCCCCCACTGGGTGGTGGC
>1881
TGTGGGTCTGTTTTTGGAGCGAGTGGTGAGCTCAGACTTGGTGCTTCAGAACTACCAC
ATCCCAGCTGGGGTGAGTGAGCCCCACACCCCTCGAGCTGAGAACCTCCCTCCCCAGT
CATTCCCTGATCCCTGCTCTGCAC
>1882
TGGCTTTGGCATGCGCCAGTGCCTCGGGCGGCGCCTGGCAGAGGCAGAGATGCTGCT
GCTGCTGCACCACGTAAGCAGGCCTGGGGGCGGGGGCGGGACCTGGGCAGCAGAGG
CGGGACCTGCACACTGGGGGCGGGGCT
>1883
TTTCCCGCTTCCCATCATCGGGAACCTCTTCCAGTTGGAATTGAAGAATATTCCCAAGT
CCTTCACCCGGGTAAGAGAAATAGTGTTGATTTTAGGGAGAATAACTCAGCAATTGGAT
CTGGTATGTGTGTATTCAACTC
>1884
CTGCTGGACTACAAGGACGAGTTCTCGGGCAGAGGCGACCTCCCCGCGTTCCATGCGC
ACAGGGACAGGGGTGAGTCCGCGTCCCTGGCACGGAGCGGGGGGTGCATAACACGCC
CCGGGACAGTTACGGGCGCTAGCCA
>1885
AAACAGGGCAATGAGAGCCGGATCCAGAGGGAGGCCCACTTCCTGCTGGAAGCACTC
AGGAAGACCCAAGGTGCGTATCTGCTGCCTAGCAGGGCCCAGTCCTCTTGCAGACCAG
CGGTGTGGGGAGCCCTGGCTGGGAC
>1886
TGAGAAGTTTCTAAGGCTGATGTATTTGTTTAATGAGAACTTCCACCTACTCAGCACTC
CCTGGCTCCAGGTGAAGCCACTTTCCTCTTTCATCAGTCATCAACTGTAGAGTTTACGT
TAGAAAAAGAAGGAAAATTTGG
>1887
GCACCATCAATCTCTGGACCCCAACTGTCCCCGGGACCTCACCGACTGCCTGCTCGTG
GAAATGGAGAAGGTAGGCTCGGCCTCCCATGATGTGGGCTCTCCGGGGTGGGCAGAG
AATGCACAATTTCAGATTTACAGAG
>1888
GGGACAGAGACCACCAGCACAACTCTGAGATATGGGCTCCTGATTCTCATGAAATACC
CTGAGATCGAAGGTAGGCAAGTGACTGAAGGGACACCGTGCGTGCGGCTGCATCTCC
CTGGATGGCCAGCCTTGCACATTTT
>1889
CCTCGTGCCCTCCAACCTGCCCCATGAAGCAACCCGAGACACCATTTTCAGAGGATAC
CTCATCCCCAAGGTTAAGCAATGAGCCTGCAGCACACAGCATGAACACCATCCTATCA
CTAATCGCCTTCCTGCCAGGGAGC
>1890
AAGCCAGAACACTTCCTGAATGAAAATGGAAAGTTCAAGTACAGTGACTATTTCAAGC
CATTTTCCACAGGTGAGAAAGATCAGAGGCAGTACCTTCCCTTGAGGAGCAGCCCACA
CTCCTCATCTCCCCTCCACATGTG
>1891
TCTGGGGAAGACAACCAGGACCTTGCTATCTCCTTTGCAGGAAATGGACTCTCTGCTC

TTAGAACCTCAGGTAGGAGACATCAATCTTGCACATCTGCAAAATCTAGAAAAAAAGG
ATTGGAGAAAGGATCTGGAGTCAA
>1892
CTAACCAGCGCGCGCGCGTCGACGTCGAGCGCGACAACCTGCTCGACGACCTGCAGC
GGCTCAAGGCCAAGTGAGGGCCCGGCACCCCAGACTCCTCTTTCTGCGGGCAGGGCA
CAGGAGGCTAGGCCTGGGGGCTGGGG
>1893
CATTGACCTGGAGCGCAGAATTGAATCTCTCAACGAGGAGATCGCGTTCCTTAAGAAA
GTGCATGAAGAGGTATACTTGGCCCCTCTTCCTGGGGTCACTGGGCCATTGGGGAAAG
CAGCCGGAAAGTGGGGTTTGGGGT
>1894
TATCCGGGCTCAGTATGAGACCATCGCGGCTAAGAACATTTCTGAAGCTGAGGAGTGG
TACAAGTCGAAGGTGGGTGGCCTCGCCCGGGGACTGGGATCTCCGTCCCCCTGGAATC
CCAAGGCCTTTGGATTGTTGCTTG
>1895
GCAGGAGATGATGGAATACCGACACCAGATCCAGTCCTACACCTGCGAGATTGACGCC
CTCAAGGGCACTGTGAGTCCCTGCCCACCTGGGCCAGGCCCTGCCCCTTCTCTGTCTG
CAGTTCACACCCTCACTTTGTGAC
>1896
TCAACGTGAAGATGGCCCTGGATGTGGAGATTGCCACCTACCGGAAGCTGCTGGAGGG
AGAGGAGAGCCGGTGAGGGGCCAGGCAGGAGCCGAGTGGGAGGTGCGGGGTGCTGG
GTGGTCCATTTCTGTCCCCAGGAGGC
>1897
GCAAAGGGGTTCTGAGGTCCATACCAAGAAGACGGTGATGATCAAGACCATCGAGAC
ACGGGATGGGGAGGTAAGTGGTCTGTCTGGGCTCCTTACCCTTGGTTGGGGCTATGGA
TGTGTCTGGGGGGACTGTCTTCCAC
>1898
CCAGTGTGTGAAGCTGAATGATGGTCACTTCATGCCTGTCCTGGGATTTGGCACCTATG
CGCCTGCAGAGGTAACAATTAATATTTTAGTGTTGAGAGTTCAAAGGAGCTAGAGTAA
GTGGAAGCTGACCAGGTTGTCAG
>1899
AAGGTCACTGAAAAATCTTCAATTGGACTATGCTGACCTCTATCTTATTCATTTTCCAGT
GTCTGTAAAGGTAGGCAGCTTGTGTGATCAAATTAATTTCACTTTTGTTCTCAGCATAAA
TATTGTTTTCATGGAGATTT
>1900
GGAAGTGATCCCAAAAGATGAAAATGGAAAAATACTATTTGACACAGTGGATCTCTGT
GCCACATGGGAGGTGAGTGTTTGGAGGTGAGAGAACGGATAAGAAAGATGAGGTAGG
ATACATCTGTTTCCTATCTTCTTAG
>1901
CTTCAACCACAGGCTGCTGGAGATGATCCTCAACGAGCCAGGGCTCAAGTACGAGCCT
GTCTGCAACCAGGTGAGGCGCCCTCAGCCTCCTCTGCCTTTCTGTTCTTCATGCCCCTG
CTTTCTGTCCTATTGGCCAAGTA
>1902
ATTTCTGCAAGTCAAAAGACATTGTTCTGGTTGCCTATAGTGCTCTGGGATCCCATCGA

GAAGAACCATGGTAATAAGAGATACAGGAAGTTTGCCGAAAACACTGGTATGATAAAA
AAGCTGGGAGAAAAAGGGATCAT
>1903
CCAGCTGCAGCGTGGGGTTGTGGTCCTGGCCAAGAGCTACAATGAGCAGCGCATCAG
ACAGAACGTGCAGGTGAGGAGCGGGGCTGTGGGCCTCAGGTCTCCTGCACAGTGTCC
TTCACACGTGTGCTTCTTGTAAGGCT
>1904
AGTTGACTTCAGAGGAGATGAAAGCCATAGATGGCCTAAACAGAAATGTGCGATATTT
GACCCTTGATATGTAAGTGATTTTGGAGATGGTTGTTCTAATTTATTTCAGAGGAGGAA
CGTAGGATGGGTGTTGAGAGTGT
>1905
GGCCGTGCGCACCCAGGAGAAGGAGCAGATCAAGACCCTCAACAACAAGTTTGCCTC
CTTCATAGACAAGGTGAGGGTCCCCTGCGTGGCTGACTGTGCCCCGCAGCCCCTTTCT
CCTGGTAGTCCCGGTCCCTATGCAC
>1906
GCCAGGAGAAGCTGAAGCTGGAGGCGGAGCTTGGCAACATGCAGGGGCTGGTGGAG
GACTTCAAGAACAAGTGAGCAACTCCACCCTCCACCCAACTGAAGTCACCTGCTCTCC
TCCACCCCTTGACCTTGGGACTAAGT
>1907
GGTAGAGCTGGAGTCTCGCCTGGAAGGGCTGACCGACGAGATCAACTTCCTCAGGCA
GCTATATGAAGAGGTATGTTCCTGGTCGCAGGAGAGTGAGGGTCCCCAGCCTTGTCAG
CGCCTCCACCCTGAGACTCAACCAG
>1908
GACTGAGATCTCTGAGATGAACCGGAACATCAGCCGGCTCCAGGCTGAGATTGAGGG
CCTCAAAGGCCAGGTATGGGCCGGGTTGGGGGTGGGAGGGTTCCTTGGACACAATCCT
GGTGAGAGGAGATAATGTAGGAAGA
>1909
TGAACGTCAAGCTGGCCCTGGACATCGAGATCGCCACCTACAGGAAGCTGCTGGAGG
GCGAGGAGAGCCGGTGGGTGTGGGTACCTCTGACCGGACCTGCTTCCCTATCCCTGGG
ACCTGGGGTGGGGACGGTGGGAGCC
>1910
CCACTCACTGGTCCAGAAGCAGCTGTAGGTGCCCACCAAGCCCATGACGACGCTGCTG
GCCAGGGTCCAGGTGAGCGGCGGCACCGCGGGGAACGGCCACTTTCACGTGCAGAG
GCCACCCCACCCGACCGCCGGCCGGG
>1911
AGCTGCTCTTCTGGGGTATCGCGATCCACTTAAGGATGAGGCAGACTTGGTGACAAGC
TGGTCTGAGCAGGTATGGGAGCCCCCTGGGGAGACGGAAGAAGGGAGGAAGTTGCCT
TCTGCCTGGGGAGGGTTTGAGAGGG
>1912
CGCCTTCAATGCCCAGCGGCTGACACTGGCCAAGGTGGCCAGGGAGCAGGTGATGGA
CACCTTAGTTCGGGTAAGTTCATCACTTCAGGGAAGCCGTGCCCCTAACCACCATGAG
CCAAAGCCCCCATCCCTGCCTGACC
>1913
TCATGGTGCTGCAGGAGGTGGTGGACTCTTCCGGCAGCGCCATCCCGCTCCTGCTTCG

AGAACTCAATCGGTGAGGAAGATTCTGTGGGTCTAATCAGGGGCACACCCAGAGGCTT
GAGGCCCTGACCCCGCCTCCCCTT
>1914
CCTACAGCACCCTGAGCAGCCCCCAGCTGGGGCGCAGCACCTACATGGAGACGTATGT
GTACTTCTATCGGTGAGCACGGAGCTGGCAGCGCAGCAGGGTGGGGGGCGGTCCTTG
GCCCTAATGTGAGGTAGGCTCATTA
>1915
GTGTACAACGATGAGGATGACGTCTTTGCCCGGGAGCCATTTGTGGCCCAGTTCTCTTT
GCCCAGCAATGGTAGGAAGGGAAGGGTGGGCTTGGTATGTGGGAGCAGAAGGGGAGT
TCTGGCTGGCTCCACTGGGGGCTT
>1916
GGCCGTAGAGAAGGAGCTGAACGCCCTCTACGATGTGTTTCTGGAGGTCTCCCAGCAC
TGGCAGAGCAAGGTAGGCCTGGGCAGTTTGTACAGGAGGGACAAGGATAGGGTGGCC
CAGCGGCCAGCCTGATGGCTGCTGT
>1917
CTGCCGGAGTCTGCTGCACACTGCGGCTGCCTTTGACTTCCCCACGAGCTTCCAGCTC
ACCGAGGAGGAGGTGAAGGGATGGGGCTGGCATGAGGAGGGAGGGCAGCAGCAGGG
TCACAGGGAAGCCCCTCGGCCCCGGG
>1918
GGAATGGCCAGAGTATTTTATGGCTGTGGCCTTCTTATCAGCACAGAGAAGCAAAGATC
CAAATTCCCAGGTAAATGAATTTCACGGGAGAATGCTTAGATACTCACAGGCAAAGAG
ATTACTGCACATGAGCAGAAAGG
>1919
GGGTGCAGTGATGACGTGTTGCCTTGGAGAAGGACAGCAGAGAATAAGCTGGACACC
AAATACCCGTACGGTAGGGCAAGTTTTATGTCCCATTCTGCTTAGTGACATAGCCTGGT
GAGTGCCTAATTAAGAAAGTTGGT
>1920
GTGAAAGGCTGTAGTATGTATGTTGCCTTGTTCCCTTGTAATGAATGCGCTAAGCTCATC
ATCCAGGCAGGTAGGAACCGGGTCTTTCCATTTGTCACATTTGCATTGCTGCCTGCTCG
TCAGTAGCTCATTGCTGATGT
>1921
ATAAATACCATGATAGTGACGAGGCAACTGCTGCGAGGCTCCTGTTTAATATGGCCGGG
GTGACATTCCGGTAAGTGGGGAGAATGCGGGCGGGGGGACGGGGTAGGGTGAAGGCT
GATGAAGAGATGTATTTGACCTTG
>1922
CGCCGTGATGCTGTCCCGATCCCGCTGTGTGTCTCGGGCGTTCAGCGCTCCGCTCTCCG
CCTTCCAGAAGGTACGGTCTGGCCGAGCCGGGGCCCCGACGGGTGAGGAGTCTGTTG
GCGGGCAGAGAGGCGGCCTGGAAG
>1923
GACTTGGTTACAGTCAAAACCCCAGCGTTTGCAGAATCTGTCACAGAGGGAGATGTCA
GGTGGGAGAAAGGTAAGATTTAGTTTCCTATTTTTTTTTTTTTTTTTTTGAGACAGAGT
CTTGCTCTGTTCCCAGCCTGGA
>1924
GCTCTTTTGGTACCTGATGGGGGAAAAGTCGAAGGAGGCACTCCACTTTTCACACTCA

GGAAAACTGGTGGTAAAGAAGTTCTCCTGGTGGTCAAGGTCTCCAGTGTTCCCTCTTG
GGATTGGGACTGAGCATAATGTGC
>1925
CCTGCAGCACCCATACCCACTCAGATGCCACCGGTGCCCTCGCCCTCACAGCCTCCTTC
TGGCAAACCTGGTAGGCTTCCAACTCCCACATGTCATGTGGGAGAACATCTCGTCTAAT
ATGTTCCTTCAAAGGGTAAACT
>1926
AGTAAAACCCACTGTTGCCCCACCACTAGCTGACGCAGGAGCTGGCAAAGGTCTGCG
TTCAGAACATCGGGTAAGCCTCTGAGGACCACTTTCAGGAAAGAGGCAGGGGGCAGT
GTTGAGATTAGTGGAGTATGGGTGTG
>1927
TTGCTCAGCGTCTGAAGGAGGCCCAGAATACATGTGCAATGCTGACAACTTTTAATGA
GATTGACATGAGGTAGTGTCTCTAGTCCCTCTTATCCCCTAGGCCCCTTTTTCTTAGAGA
ACACGAACTTGCCCCACTCCTT
>1928
CTAGGCTTCATGTCGGCATTTGTGAAGGCCTCAGCCTTTGCCTTGCAGGAACAGCCTGT
TGTAAATGCAGGTGAGTTGCTTGTGGCTGGAATTGGAGAGGTCCTGGGAGGTAGGTGT
ATGAGCACAGACCTGCTCCCACA
>1929
TGACGACACAACCAAAGAGGTGGTGTATACGGATTATATTGACATCAGTGTTGCAGTG
GCCACCCCACGGGTATGTTGGGGCAGGAGGTGGGGAATGTTGGTCTTAGACCCTCACC
TTATCTGTGTGAAGGAGATCACAC
>1930
AGTCATCAGGAATGTGGAAGCTATGAATTTTGCAGATATTGAACGGACCATCACTGAAC
TGGGAGAGAAGGTAAAGTAGAAAGATGTATACAAGCTGCTAAGCAGGCGAGGGAAGA
GAGCCTTCAGAAGGCTGGGCTCAC
>1931
CAACCCCCCTCAGTCTGCCATCCTGGGGATGCATGGCATCTTTGACAGGCCAGTGGCTA
TAGGAGGCAAGGTAGGAACCGTCACTTCTAAGGTCCTAGTGGCTAGGTCTCGATGAAA
GGGAAATCCAACTAAATGCTAAT
>1932
AGAATGGTGCCTGTCCTGCTGTCTCTGCTGCTGCTTCTGGGTCCTGCTGTCCCCCAGGA
GAACCAAGATGGTGAGTGGGGAAAGCAAGGGATGGGTGCTGGAGAGGACTGGAAGG
AGGTGAGGAACAGGACATGTGGCTG
>1933
CAGAAGGCCAGGGAGGACATCTTTATGGAGACCCTGAAAGACATCGTGGAGTATTACA
ACGACAGTAACGGTCAGTGAATAACAGACCACAGGGGTGGAAGGTCTAACCCAAGAG
GCAGCCCCCCCAGTGTGAGTGGCAA
>1934
CTGGAGGAGGAGTGCCCTGCGACTCTGCGGAAATACCTGAAATACAGCAAAAATATCC
TGGACCGGCAAGGTACTCACTGCTTCCTGCTCCCCAGTACTGAGCCCAGAATAAAAGA
CGATCTCAGGCTAGGAGCTCAGGC
>1935
GGCTGTTCCTCGGGCATCGGCCTGCACTTGGCCGTACGTCTGGCTTCAGATCCATCCCA

GAGCTTCAAAGGTATAGATAGGCAGGGACAGGGAGGGAGAGAAGGGAGGAGCCCTTG
GAGGCTAGAAGGGAAGTCAGATCT
>1936
GACGTAAGGGACTCAAAATCCGTGGCCGCTGCCCGGGAACGCGTGACTGAGGGCCGC
GTGGACGTGCTGGGTGAGCCTCCTGGAAGCATATGGGCTCCTAGGAGCCTTCTCCGCC
CTGCGTTGAAACCAACATGTTCCCA
>1937
TTCCTGCCAGACATGAAGAGGCGCGGTTCGGGACGCGTGTTGGTGACCGGGAGCGTG
GGAGGATTGATGGGTGAGTGGTAGGGAGTGGCCTCGGCAGCTCCAGATTCTTTGTGTG
CGGAGCTGAGCCTTGAAGGCAGGTT
>1938
GCGCCAGCAAGTTCGCGCTCGAAGGCTTATGCGAGAGTCTGGCGGTTCTGCTGCTGCC
CTTTGGGGTCCAGTGAGTCAACACCCCCGTTCCCCGAACCCTCTTAACTCTGACCTAG
AGATGCCGAGCACCCTGTCCTGCG
>1939
CTACCAATACCTCGCCCACAGCAAGCAAGTCTTTCGCGAGGCGGCGCAGAACCCTGA
GGAGGTGGCGGAGGTGAGCGCCGGGCTGGACTCCAGGAGTGGGGGCGGTGCGTCCT
CCGGCCGGCAGCGGTGGCCACAGCTCT
>1940
TTCAGAATGGATTATTTGCTCATGATTTTCTCTCTGCTGTTTGTGGCTTGCCAAGGAGCT
CCAGAAACAGGTAGGCACGCTCGTTGACTTGTAAGTCTCGGAATTACAAGTTAGTGTG
TTCTTATCCACCTTCATGCTTT
>1941
GCTCGTCCCTGATGGATAAAGAGTGTGTCTACTTCTGCCACCTGGACATCATTTGGGTC
AACACTCCCGAGTAAGTCTCTAGAGGGCATTGTAACCCTATTCATTCATTAGCGCTGGC
TCCACTGGAGCCCAGTTTTAGA
>1942
GTATTTATCAGCAGTTAGTGAGAGGAAGAAAAATCAGAAGAAGTTCAGAGGAACACCT
AAGACAAACCAGGTAAGAGGGAAGGAAGAAAAATTAGGTAAGAGGTTCACAAGAAC
AACTAGCCCCAGTCAGTGATGCCAGC
>1943
CCATCTGATCTATAAATGCGGTGGCATCGACAAAAGAACCATTGAAAAATTTGAGAAG
GAGGCTGCTGAGGTATGTTTAATACCAGAAAGGGAAAGATCAACTAAAATGAGTTTTA
CCAGCAGAATCATTAGGTGATTTC
>1944
CTATGTGACTATCATTGATGCCCCAGGACACAGAGACTTTATCAAAAACATGATTACAG
GGACATCTCAGGTTGGGATTAATAATTCTAGGTTTCTTTATCCCAAAAGGCTTGCTTTGT
ACACTGGTTTTGTCATTTGGA
>1945
CCCCGACACAGTAGCATTTGTGCCAATTTCTGGTTGGAATGGTGACAACATGCTGGAG
CCAAGTGCTAACGTAAGTGGCTTTCAAGACCATTGTTAAAAAGCTCTGGGAATGGCGA
TTTCATGCTTACACAAATTGGCAT
>1946
ATCCTACCACCAACTCGTCCAACTGACAAGCCCTTGCGCCTGCCTCTCCAGGATGTCTA

CAAAATTGGTGGTAAGTTGGCTGTAAACAAAGTTGAATTTGAGTTGATAGAGTACTGT
CTGCCTTCATAGGTATTTAGTAT
>1947
TCGTCGTGGCAACGTTGCTGGTGACAGCAAAAATGACCCACCAATGGAAGCAGCTGG
CTTCACTGCTCAGGTAACAATTTAAAGTAACATTAACTTATTGCAGAGGCTAAAGTCAT
TTGAGACTTTGGATTTGCACTGAA
>1948
GCTGCCATTGTTGATATGGTTCCTGGCAAGCCCATGTGTGTTGAGAGCTTCTCAGACTA
TCCACCTTTGGGTAAGGATGACTACTTAAATGTAAAAAAGTTGTGTTAAAGATGAAAAA
TACAACTGAACAGTACTTTGGG
>1949
AGCAGCTTCTTGATCGTGGTGGTGTTCCTCATCGCTGGGACGCTGGTTCTAGAGGCAG
CTGTCACGGGAGGTGAGTGAACAGGTGACCTGCTGGGCTGGGTTGGACTAAGGGGAG
ACCCTCTGGGACACCCTGGGCCAGG
>1950
GGGCATCCCTGGGTGCCAGACCTGCCGCTACCTCCTGGTGAGAAGTCTTCAGACGTTT
AGTCAAGCTTGGGTGAGTGGCCTATGGCTGAGGCTGAGGTGGGAGCATGGAACGGGT
GTGGGATATGCCCCCAGCATTGCTA
>1951
TCGAATCCAGTGTTCTGTCAGCGCGCTCAACCAGGGTCAAGTCTGGATTGGAGGCAGG
ATCACAGGCTCGGTAAGAGAAGTGTGAACACTAAATGGGGTGCACCTGCTGATCTCAG
CCAGCACTCAGCTTGCATCAGATT
>1952
AACTTTGCGTACTGGGCTGCTCACCAGCCCTGGTCCCGCGGTGGTCACTGCGTGGCCC
TGTGTACCCGAGGTGAGGTGGGGCTGGGGATGAACGATGGAAAGGTCTGGGAGATGG
GAAGTGCCCCAAGGAGGAGATGCTA
>1953
CCTTCGAGCTCCTTGTGCGCGCTGTTGGTGCTGCTGCTGCTGCTGACGCAGCCAGGGC
CCATCGCCAGCGGTGAGAGCGCATGGCGCGCGGGACGCACTCGCACTCGGGCACAGA
GGTGCATCCCAGCCTCTGCGGGGTC
>1954
TTCATCCCAAAATGATCAGTAATCTGCAAGTGTTCGCCATAGGCCCACAGTGCTCCAAG
GTGGAAGTGGTGTAAGTTCTGTGCTGCTGTGTCCGCTGTGACCTTGGCAAGAGAGAAA
TCCCGCAGCCTGGGTCTTCAACC
>1955
ACGGGAAGGAAATTTGTCTTGATCCAGAAGCCCCTTTTCTAAAGAAAGTCATCCAGAA
AATTTTGGACGGGTACTTGTCACTTTGATCTTTGTGGTTTCTAAATCTGATCTAGGGAGA
CCATAGACTTCACAAGGTCTTT
>1956
ACGCCTCATCTGTGACAGCCGAGTCCTGGAGAGGTACCTCTTGGAGGCCAAGGAGGC
CGAGAATATCACGGTGAGACCCCTTCCCCAGCACATTCCACAGAACTCACGCTCAGGG
CTTCAGGGAACTCCTCCCAGATCCA
>1957
CTGCAGCTTGAATGAGAATATCACTGTCCCAGACACCAAAGTTAATTTCTATGCCTGGA

AGAGGATGGAGGTGAGTTCCTTTTTTTTTTTTTTTCCTTTCTTTTGGAGAATCTCATTTG
CGAGCCTGATTTTGGATGAAA
>1958
GCTGCATGTGGATAAAGCCGTCAGTGGCCTTCGCAGCCTCACCACTCTGCTTCGGGCT
CTGGGAGCCCAGGTGAGTAGGAGCGGACACTTCTGCTTGCCCTTTCTGTAAGAAGGG
GAGAAGGGTCTTGCTAAGGAGTACA
>1959
ATAGATGCCAATGTGCTAGCCAAAAAGACAAGAAGTGCTGGAATTTTTGCCAAGCAGG
AAAAGAACTCAGGTGAGCAGAAACACCTTTGCTTTTCAATCAGTTTAACAGCCTCCTG
AACTCCTTCCTATCATGGTACTGC
>1960
AAACATTGAAGTTGTTTTTGAACTTGGTGTCACCTTTAATTACAACCTAGCAGACGGAA
CTGAACTCAGGGTAAGAATTTTTTTTTTTATGAGCAATGCATTCTTGATTTTTCTACCCA
ATATTAAAATGATTTCTGCTC
>1961
ATTCAAACGGACAGACAATGGAAACGAACTGAATACTGTCCGAGAAATTATAGGTGAT
GAACTAGTCCAGGTGAGTTGTCAAATTTATAGCTATTTTCAAAAGGCAAAAATTACTAC
AAAACAATAATTTTTGTCACTGC
>1962
CTGCTATATCCTGGATGCCATCCTGTTTCTGTATGGAATTGTCCTCACCCTCCTCTACTGT
CGACTGAAGGTAGCGCTGGGCAGGGTGGGGTAAGGGCTGGAAGGGGAAGTGGGAGG
AGGGCAGCAGCAAGGATTCGAAG
>1963
ATCATGGCAGAATCACCAGGCCTCATCACCATCTGCCTTTTAGGATATCTACTCAGTGCT
GAATGTACAGGTTTGTTTCCTTTTTTAAAATACATTGAGTATGCTTGCCTTTTAGATATAG
AAATATCTGATGCTGTCTT
>1964
GAGAGAATGTATGGAAGAAAAGTGTAGTTTTGAAGAAGCACGAGAAGTTTTTGAAAA
CACTGAAAGAACAGTGAGTATTTCCACATAATACCCTTCAGATGCAGAGCATAGAATAG
AAAATCTTTAAAAAGACACTTCTC
>1965
TGCAAGGATGACATTAATTCCTATGAATGTTGGTGTCCCTTTGGATTTGAAGGAAAGAA
CTGTGAATTAGGTAAGTAACTATTTTTTGAATACTCATGGTTCAAAGTTTCCCTCTGAAA
CAAGTTGAAACTGGAAAATGC
>1966
AACAAGGTGGTTTGCTCCTGTACTGAGGGATATCGACTTGCAGAAAACCAGAAGTCCT
GTGAACCAGCAGGTCATAATCTGAATAAGATTTTTTAAAGAAAATCTGTATCTGAAACT
TCAGCATTTTAACAAACCTACAT
>1967
CCAATCATTTAATGACTTCACTCGGGTTGTTGGTGGAGAAGATGCCAAACCAGGTCAAT
TCCCTTGGCAGGTACTTTATACTGATGGTGTGTCAAAACTGGAGCTCAGCTGGCAAGA
CACAGGCCAGGTGGGAGACTGAG
>1968
GTTAATGAAAAATGGATTGTAACTGCTGCCCACTGTGTTGAAACTGGTGTTAAAATTAC

AGTTGTCGCAGGTAAATACACAGAAAGAATAATAATCTGCAGCACCACTAGCTCTTTAA
TATGATTGGTACACCATATTTT
>1969
GGATAGCCTCTCTTACTACCACTCACCCGCAGACTCCTTCTCCAGCATGGGCTCGCCTG
TCAACGCGCAGGTAAGGCTGGCTTCCCGTCGCCGCGGGGCCGGGGGCTTGGGGTCGC
GGAGGAGGAGACACCGGGCGGGAC
>1970
GGCTGGCGTTGTGAAGACCATGACAGGAGGCCGAGCGCAGAGCATTGGCAGGAGGG
GCAAGGTGGAACAGGTGAGGAACTCTAGCGTACTCTTCCTGGGAATGTGGGGGCTGG
GTGGGAAGCAGCCCCGGAGATGCAGGA
>1971
GGAAAGGAATAAGATGGCTGCAGCCAAATGCCGCAACCGGAGGAGGGAGCTGACTGA
TACACTCCAAGCGGTAGGTACTCTGTGGGTTGCTCCTTTTTAAAACTTAAGGGAAAGTT
GGAGATTGAGCATAAGGGCCCTTG
>1972
GAGTCACTCTCCCCAGGAGACCCAGACCTAGAACTACCCAGAGCAAGACCACAGCTG
GTGAACAGTCCAGGTAAGAAACATATTCTGGGGGACTGGGTAGCTTGACATAGACACA
CTTAATACACTGGTGAACACAATAG
>1973
CATTTTTCTGCACTGGCCAGAAAATGGAGTCTTAAACCTTGAATCCCCCATAACTACCT
CAACTACAAAGGTAAAGAGGCTTAGTCAGTCTTAAGATGTAACTTTCTGTAAAGGAAT
TGGCAGATCCTTCCTTTCTTCCT
>1974
GTCTCCACTGCTGCCCTTGCCGTCCTCCTCTGCACCATGGCTCTCTGCAACCAGGTCCT
CTCTGCACCACGTGAGTCCATGTTGTTGTTGTGGGTATCACCACTCTCTGGCCATGGTT
AGACCACATCAGTCTTTTTTTG
>1975
GACAGATTCCACAGAATTTCATAGCTGACTACTTTGAGACGAGCAGCCAGTGCTCCAA
GCCCAGTGTCATGTAAGTGCCAGTCTTCCTGCTCACCTCTAGGGAGGTAGGGAGTGTC
AGGGTGGGGGCAGAAACAGGCCAG
>1976
GCGCTCCCACTCTCGGCCGACACCCCTCATGGCCAACCGTTACACCATGGATCTGACT
GCCATCTACGAGGTGAGTCCCCGCCGCACGGCATCCCCGGTACCTGCATGCCTGAGTC
CGAGTCCCCACCTCTCTAGCGCCG
>1977
GACCCATGGACAAGAGCGCAGGCAGTGGCCACAAGAGCGAGGAGAAGCGAGAAAAG
ATGAAACGGACCCTGTGAGTATGGCTTTCTTCCCTCTCCCGCCACCCCCTGCCCCACAC
TGCAAGCTGCAAACGCGGTACTTTC
>1978
TCTTACAAAATTCCTCTACTCCTGGGAAGCCCAAAACCGGCAAAAAAAGCAAACAGC
AAGCTTTCATCAAGTAAGTTGAGAATCCTGTGCTTGCAAATATCAATAGTTAGCTGCTG
AACTGAAAAGGGGAACTCTGATGT
>1979
ACCCCAAAAGCTGTCCTCAAAAGCAAGGAAAATATATACTGACTTCATAGAAAAGGAA

GCTCCAAAAGAGGTAAGGAAACAAGTTCCTAATTTCAGCACAATCTGGACATCTTTAG
CACAAAAGTGAAACAGTAATCAAG
>1980
GTCAGCGCCGGGCCGCGTCAACCTCATCGGGGAACACACGGACTACAACCAGGGCCT
GGTGCTGCCTATGGTGAGGGGCTGCACGGGGAGCCCCTAGCCCGCCGCCGCCTGTCCC
GGTCGCCGAGGAGGGCGGGCCTCGG
>1981
CAGCGCTCGCTGGAGCCTGGGACTCCTCGGTGGGCCAACTATGTCAAGGGAGTGATTC
AGTACTACCCAGGTATGGGGCCCAGGCCTGAGCCAAGTCCTCACTGATACTAGGAGTG
CCACCTCACAGCCACAGAGCCCAT
>1982
GGGGGTGGCCTGTCCAGCTCAGCATCCTTGGAAGTGGCCACGTACACCTTCCTCCAGC
AGCTCTGTCCAGGTACCAGCTAGGCCCCAGCCCTGACCCAGCCCTCCTTCCCTGAGGT
CTCCAGGTGGTCCCAGCTTCTACT
>1983
CCTGTGGCATCATGGACCAGTTCATCTCACTTATGGGACAGAAAGGCCACGCGCTGCT
CATTGACTGCAGGTTGGGCTCGCTCCCCTCGTCCCCTCCCGCCCTGCACTCAGCAGCT
CCTGGGTGGAGTGTGCCCACTGCC
>1984
CAATGTGAAGAAGTGGCCCGGGCGCTGGGCAAGGAAAGCCTCCGGGAGGTACAACTG
GAAGAGCTAGAGGGTGAGAACTGCCAGGGTGCTCTATCCTGGAGGCGGCTGTGCTCC
CTGCTGGCGCCTCAGTGTGGCCTTGA
>1985
CGGCCGCCCTGAGACGTGGCGACTACAGAGCCTTTGGCCGCCTCATGGTGGAGAGCC
ACCGCTCACTCAGGTGAGGCCCTCTGGGCGCCCCGCTCCTGCCGGGCACAGGCCGGC
CCAGGCCCACCCCTTCAATATCCTCT
>1986
TGGCTTCGGTGGCTGCACGGTGACACTGCTGGAGGCCTCCGCTGCTCCCCACGCCATG
CGGCACATCCAGGTGGGCGGGCACCAGGGCCTGGGCGGGCAGGAGCGGCAGCTTCCC
GGGGCCCTGCCACTCACCCCCAGCC
>1987
GGAACCGATCCTCAGCAACGCCAGCAGGCGTCAGAGGCGGACGCCGCAGCAGCAAC
CTTCCGGGCAAACGGTAACTGCACCGCGGCAGGGACTCGCTGGGGCGCGGAGCCGAG
CCCTCCCCTTCCTTAGGAAGCTTTCGT
>1988
TCTGAAGACAGTGCCCCGCCATGACCCTCTCAACCCTCTGTGTCCTGGGGCCATCCGA
GCCAACGGAGAGGTAAGCCTGTAGAGCCCTGCATCTGCAGGCTGGGCCACGGGGAGT
AGTTCCCTCTTAGAACTGTCCTCCA
>1989
CCCCAGTACGATAGCACCTTCCTGTTTGACAACGACTTCCCAGCTCTGCAGCCTGATGC
CCCCAGTCCAGGTAACCTGGCTCCAACTGCTGCTGGGGAGGAGGGTGGCTAGACCTCT
TGAGGGACTTCTGCTGCAGAGAT
>1990
GATCCGGGCTGTTGTTGATGCATGGGCCTCAGTCACAGAGGAGCTGGGTGCCCAGTAC

CCTTGGGTGCAGGTTTGTGAGGTCGCCCCTTCCCCTGGATGGGCAGGGAGGGGGTGAT
GAAGCTTTGGTTCTGGGGAGTAAC
>1991
GCAGGCCTATAAGAGTCAGCATGGAGAGCCCCTGCTAATGGAGTACAGCCGCCAGGAG
CTACTCAGGAAGGTGGGAGAGAGCCAAGCCCTGTGTCCCCAAGGAGTCCCTAACTTT
CTTATCCCATGAGAGAGGTGTGTAA
>1992
TACCAGACACTGCTGCTGCCCCGTCGGCATGTGCGGCGGCTACCTGAGCTGACCCCTG
CTGAGCGTGATGGTCAGTCTCCCAAGTAGGATCCTGGGGCTAGGCACTGGATGGAGGT
TGCTCCCAGTAGGGTCAGCATCTG
>1993
ATGAAGAAGCTCTTGACCAAGTATGACAACCTCTTTGAGACGTCCTTTCCCTACTCCAT
GGGCTGGCATGGTGAGGCTTTTCAAGTACCTATATTTAGCCCCAACACCATTTCTGGGC
TCCTGGGCTCAGCCTAGTGAAC
>1994
CACTGTCCGGAAATTCATGGTTGGCTACGAAATGCTTGCTCAGGCTCAGAGGGACCTC
ACCCCTGAGCAGGTCAGGACTCAGAACAGTCTGGCGTCTCCAGACTCTCACATGCAGT
ATGTGCAGGCACCTGATACTTCTG
>1995
ACAAGGCTACTATCACAAGCCTGTGGGGCAAGGTGAATGTGGAAGATGCTGGAGGAG
AAACCCTGGGAAGGTAGGCTCTGGTGACCAGGACAAGGGAGGGAAGGAAGGACCCT
GTGCCTGGCAAAAGTCCAGGTCGCTTC
>1996
ACAAGGCTACTATCACAAGCCTGTGGGGCAAGGTGAATGTGGAAGATGCTGGAGGAG
AAACCCTGGGAAGGTAGGCTCTGGTGACCAGGACAAGGGAGGGAAGGAAGGACCCT
GTGCCTGGCAAAAGTCCAGGTCGCTTC
>1997
CAAGGGCACCTTTGCCCAGCTGAGTGAACTGCACTGTGACAAGCTGCATGTGGATCCT
GAGAACTTCAAGGTGAGTCCAGGAGATGTTTCAGCACTGTTGCCTTTAGTCTCGAGGC
AACTTAGACAACTGAGTATTGATC
>1998
GGCTGCTTTTAACTCTGGTAAAGTGGATATTGTTGCCATCAATGACCCCTTCATTGACCT
CAACTACATGGTGAGTGCTACATGGTGAGCCCCAAAGCTGGTGTGGGAGGAGCCACCT
GGCTGATGGGCAGCCCCTTCAT
>1999
TCCATGGCACCGTCAAGGCTGAGAACGGGAAGCTTGTCATCAATGGAAATCCCATCAC
CATCTTCCAGGAGTGAGTGGAAGACAGAATGGAAGAAATGTGCTTTGGGGAGGCAAC
TAGGATGGTGTGGCTCCCTTGGGTA
>2000
GTGGGGCGATGCTGGCGCTGAGTACGTCGTGGAGTCCACTGGCGTCTTCACCACCATG
GAGAAGGCTGGGGTGAGTGCAGGAGGGCCCGCGGGAGGGGAAGCTGACTCAGCCCT
GCAAAGGCAGGACCCGGGTTCATAAC
>2001
CTGCTGATGCCCCCATGTTCGTCATGGGTGTGAACCATGAGAAGTATGACAACAGCCTC

AAGATCATCAGGTGAGGAAGGCAGGGCCCGTGGAGAAGCGGCCAGCCTGGCACCCTA
TGGACACGCTCCCCTGACTTGCGC
>2002
CACCACCAACTGCTTAGCACCCCTGGCCAAGGTCATCCATGACAACTTTGGTATCGTG
GAAGGACTCATGGTATGAGAGCTGGGGAATGGGACTGAGGCTCCCACCTTTCTCATCC
AAGACTGGCTCCTCCCTGCTGGGG
>2003
CCCACTCCTCCACCTTTGACGCTGGGGCTGGCATTGCCCTCAACGACCACTTTGTCAA
GCTCATTTCCTGGTATGTGGCTGGGGCCAGAGACTGGCTCTTAAAAAGTGCAGGGTCT
GGCGCCCTCTGGTGGCTGGCTCAG
>2004
CTCCTCAACAGCAGCAGTGTGGGCAACCTCAGCTGCGAGCCCCCTCGCATTCGCGGA
GCCGGGACACGAGGTGGGTGCCTCCCTCAGCCCCCCCACAAGCTATTTCTCACTGTA
CCCCAGAACACTAATAGAGTCCCCC
>2005
CTCCTGCCCAATCTCATGGGCACATTCATCTTTGGCACCGTCATCTGCAAGGCGGTTTC
CTACCTCATGGGTGGGTGAGACAACCACCCACCCCCACTTGCCACTCTCCCCGCCTAA
AGCTCTTGTGGATGTAGGGGTCT
>2006
TGCAACCAGTGGGGCCTCGTGTGCTGCAGTGCGTGCATCGCTGGCCCAGTGCGCGGGT
CCGCCAGACCTGGTGAGCTTGCCCATAAACTATCCTAGGAATTCCTTTCTCACCCCTATT
AGATGCTTACGACCATTGCCCA
>2007
GGGCTTCGCTTTGACGGCGACAGTGACAGCGACAGCCAAAGCAGGGTCCGAAACCAA
GGCGGGCTGCCAGGTGGGGCTGGACCACGTGAGCAAAATCTGGGCGAGGCGGACGTT
TGGAGGGCGACGGGGCCTGCTGGAGT
>2008
GAGCAGCAGGGCCCAGCCTCTCATCATCGAAGGCAGCTGGGACCCCAGGGTCCCCCA
CACCTCGTGGCAGGTAGGAGCTGCTGACTGCCCTGCTTGCCTCACTTGGCCATGTTTG
GCCAAGGTCTCCCCAGACTGGCTCT
>2009
CCTCAAGAACCTGAGCCCGTCGGCCAGCCAGTACGTGGAACAGATGTTTGAGACTTTT
GACTTCAACAAGGTGAGCAGGGGCCCAGTGGCAGGGAGGGGAAGTGCTGGAGGGAC
CCCTCTGGAAGCCTGACCAGCTGGGG
>2010
GTACTTCAAGCTCTATGATGTAGATGGCAACGGCTGCATTGACCGCGATGAGCTGCTCA
CCATCATCCAGGTGCAGAGGGCCCGGCACTGGGGGGCAGCGGTCTGGGGTGGGACCC
GGAACTGAGAGCCCAGGGTTAGAA
>2011
AGCGATACCACCATGACTGCAGAGGAGTTCACCGATACAGTGTTCTCCAAGATTGACG
TCAACGGGGATGGTGAGGGGGCCGAGGAGGGGCTCCCCAGCGGAGGGGTCACCATG
GATGTGGGGTCACCAGGGGTGGAAGG
>2012
TGGAACCCCTGTGGTCTTCTCTTCATCTAATGACCCTGAGGGGATGGAGTTTTCAAGTC

CTTCCAGAGAGGTAAGAGAGAGAGCTCCCAATCAGCATTGTCACAGTGCTTCTGGAAT
CCTGGCACTGGAATTTAATGAAT
>2013
AGTAGGGTAAGCATCATGGCTGGCAGCCTCACAGGATTGCTTCTACTTCAGGCAGTGT
CGTGGGCATCAGGTGAGTGAGTCAAGGCAGTGGGGAGGTAGCACAGAGCCTCCCTTC
TGCCTCATAGTCCTTTGGTAGCCTT
>2014
GAGAGTACACGCAGTGGGCGACGGATGGAGCTGAGTATGGGGCCCATCCAGGCTAATC
ACACGGGCACAGGTAACCATTACACCCCTCACCCCCTGGGCCAGGCTGGGTCCTCCTA
GAGGTAAATGGTGTCAGTGATCAC
>2015
GCTCTCAACATCCTTGCCCTGTCACCCCCTGCCCAAAATTTGCTACTTAAATCGTACTTC
TCTGAAGAAGGTGAGGAGGAAGGGGACAAGATGACATAGAGCCATTGAAACTTTTCG
TTTTTCTTTTCTTTTTTTAAAAT
>2016
ATGGGAAGGGGTCACTCAAGGGACAGCCCGGAGACATCTACCACCAGACCTGGGCCA
GATACTTTGTGAAGTAAGGGATCAGCAAGGATGTGGGATCAGGACTGGCCTCCCATTT
AGCCATGCTGATCTGTGTCCCAACC
>2017
TACTCACCACAATGTCCGCCTACTCATGCTGGATGACCAACGCTTGCTGCTGCCCCACT
GGGCAAAGGTGGTAAGGCCTGGACCTCCATGGTGCTCCAGTGACCTTCAAATCCAGCA
TCCAAATGACTGGCTCCCAAACT
>2018
GTTCTGGGAGCAGAGTGTGCGGCTAGGCTCCTGGGATCGAGGGATGCAGTACAGCCA
CAGCATCATCACGGTAAGCCACCCCAGTCTCCCTTCCTGCAAAGCAGACCTCAGACCT
CTTACTAGTTTCACCAAAGACTGAC
>2019
CCATCATTGTAGACATCACCAAGGACACGTTTTACAAACAGCCCATGTTCTACCACCTT
GGCCACTTCAGGTGAGTGGAGGGCGGGCACCCCCATTCCATACCAGGCCTATCATCTC
CTACATCGGATGGCTTACATCAC
>2020
AGAAGAACGACCTGGACGCAGTGGCACTGATGCATCCCGATGGCTCTGCTGTTGTGGT
CGTGCTAAACCGGTGAGGGCAATGGTGAGGTCTGGGAAGTGGGCTGAAGACAGCGTT
GGGGGCCTTGGCAGGATCACACTCT
>2021
TGGAACCCCTGTGCTCTTCTCCTCATCTAGTGACCCTGAGGTGATGGAGTTTTCAAGTC
CTTCCAGAGAGGTAAGAGAGAGAGCTCCCAATCAGCATTGTCACAGTGCTTCTGGAAT
CCTGGCACTGGAATTTAATGAAT
>2022
CAAGAGCAGAAGCAGTGGGCATTGGATGGAGCTGAGTACAGGACCATACAGGCTAATT
GCACCGGCACAGGTAACCATTACACCCTTCACCCCCCGGGCCAGGCTGGGTCCTCCTA
GAGGTAAACGGTGTCAGTGATCAC
>2023
CTATGCAGACACCCCTGATGATTTCCAGTTGCACAACTTCAGCCTCCCAGAGGAAGAT

ACCAAGCTCAAGGTAGGCATTCTAGCTTTTTCAGGCCCTGAGGGCCCTGATGTCTGGG
GGTTGAGAAACTGTAGGGTAGGTC
>2024
ATGGGAAGGGGCCACTCAAGGGACAGCCCAGAGACATCTACCACCAGACCTGGGCCA
GATACATTGTGAAGTAAGGGATCAACAAGGATGTGGGATCAGGACTGGCCTCCCCTTT
GGCCATGCTGATCTGTGTCCCAACC
>2025
TACTCACCACAATGTCCGCCTACTCATGCTGGATGACCAACGCTTGCTGCTGCCCCACT
GGGCAAAGGTGGTAAGGCCTGGACCTCCATGGTGCTCCAGTGACCTTCAAATCCAGCA
TCCAAATGATTGGCTCCCAAACT
>2026
GTTCTGGGAGCAGAGTGTGCGGCTAGGCTCCTGGGATCGAGGGATGCAGTACAGCCA
CAGCATCATCACAGTAAGCCACCCCAGTCTCCCTTCCTGCAAAGGAGACCTCAGACCC
ATTAGTAGTCTCACCAAAGACTGAT
>2027
CCATCATTGTAGACATCACCAAGCACACGTTTTACAAACAGCCCATGTTCTACCACCTT
GGCCACTTCAGGTGAGTGGAGGGCGGGCACCCCCATTCCATACCAGGCCTATCATCTC
CTACATCGGATGGCTTACATCAC
>2028
AGAAGAACGACCCGGACGCAGTGGCACTGATGCATCCCGATGGCTCTCCTGTTGTGGT
CGTCCTAAACCGGTGAGGGCAATGGTGAGGTCTGGGAAGTGGGCTGAAGACAGCGTT
GGGGGCCTTGGCAGGATCACACTCT
>2029
AGCCTTCCTGAGAAAATGCAGCCATTCCTCCTCCTGTTGGCCTTTCTTCTGACCCCTGG
GGCTGGGACAGGTAAGTGACTATCCCTATTCCAGAGGCCTGAACCCATCTTATAAGATA
CCCTGTACCCATGAGCACTGGT
>2030
GATCCCAAGGCCCAACTCCCCGAACCACTCAGGGTCCTGTGGACAGCTCACCTAGCTG
CAATGGCTACAGGTAAGCGCCCCTAAAATCCCTTTGGCACAATGTGTCCTGAGGGGAG
AGGCAGCGACCTGTAGATGGGACG
>2031
GCTTTTTGACAACGCTATGCTCCGCGCCCATCGTCTGCACCAGCTGGCCTTTGACACCT
ACCAGGAGTTTGTAAGCTCTTGGGGAATGGGTGCGCATCAGGGGTGGCAGGAAGGGG
TGACTTTCCCCCGCTGGAAATAAG
>2032
CCAGACCTCCCTCTGTTTCTCAGAGTCTATTCCGACACCCTCCAACAGGGAGGAAACA
CAACAGAAATCCGTGAGTGGATGCCTTCTCCCCAGGCGGGGATGGGGGAGACCTGTA
GTCAGAGCCCCCGGGCAGCACAGCC
>2033
CGGCGCCTCTGACAGCAACGTCTATGACCTCCTAAAGGACCTAGAGGAAGGCATCCAA
ACGCTGATGGGGGTGAGGGTGGCGCCAGGGGTCCCCAATCCTGGAGCCCCACTGACT
TTGAGAGACTGTGTTAGAGAAACAC
>2034
GGATCCCAAGGCCCAACTCCCCGAACCACTCAGGGTCCTGTGGACAGCTCACTAGCG

GCAATGGCTGCAGGTAAGCGCCCCTAAAATCCCTTTGGCACAATGTGTCCTGAGGGGA
GAGGCGGCGTCCTGTAGATGGGACG
>2035
GCTTTTTGACAACGCTATGCTCCGCGCCCGTCGCCTGTACCAGCTGGCATATGACACCT
ATCAGGAGTTTGTAAGCTCTTGGGTAATGGGTGCGCTTCAGAGGTGGCAGGAAGGGGT
GAATTTCCCCCGCTGGGAAGTAA
>2036
CCAGACCTCCCTCTGCTTCTCAGAGTCTATTCCAACACCTTCCAACAGGGTGAAAACG
CAGCAGAAATCTGTGAGTGGATGCCTTCTCCCCAGGTGGGATGGGGTAGACCTGTGGT
CAGAGCCCCCGGGCAGCACAGCCA
>2037
TGGCGCCTCGGACAGCAACGTCTATCGCCACCTGAAGGACCTAGAGGAAGGCATCCA
AACGCTGATGTGGGTGAGGGTGGCACCAGGATCCAATCCTGGGGCCCCACTGGCTTCC
AGGGACTGGGGAGAGAAACACTGCT
>2038
GCTGCAGCGGCGCCTCGGACCCCGGGGCCTGGTGGTGCTCGGCTTCCCGTGCAACCA
GTTTGGGCATCAGGTGCGCCGGGCGGAGCGGGGCGGGGCGGGGGCGGACGTGCAGTA
GTGGCTGGGGGCGCCGGCGGTGTGCT
>2039
TTCGCCGCCCCTGCGCGTCCAGCTCTTCTAAGACGAGATGCCGTCGGGCTTCCAACAG
ATAGGCTCCGAAGTAGGATTCATCATGAGGGGGCGGGGCGGGGGGGCACGGGTCCCG
CTTTTCTTGGGCTGGGGTCGCGGTT
>2040
TGTGTTCTCTGCGGTGCTTGGCTCCCTGCAGTTTGGGTACAACATTGGGGTCATCAATG
CCCCTCAGAAGGTGAGGGCCTGCAGCTGGCAGGGTGGGGGTACCCAAACGAGGAGG
ACAGGTGTCTCGGGGGTGGTGGAAA
>2041
TGGCCATCTTTTCCGTGGGCGGCATGATTTCCTCCTTCCTCATTGGTATCATCTCTCAGT
GGCTTGGAAGGTTCGCAGCTGGAGGGCAGGGGTGGGGGAAACAGGAAGGGAGCCAC
TGCTGGGTGCCCTCACCCTCACAG
>2042
GGCCTGGCCAACGCTGCTGCCTCCTATGAAATGCTCATCCTTGGACGATTCCTCATTGG
CGCCTACTCAGGTACTCACGGGCACCACAGCCCTGCCTAGCGCCCTGTTCTCTTTCACC
ATGCCTGGGCTTTCAGATGGGA
>2043
CACTCACCTGCGGGGCGCCCTGGGGACGCTCAACCAACTGGCCATTGTTATCGGCATT
CTGATCGCCCAGGTGACCGGAGCAAGCCTCATGGGTGCCTGGGCAGTGGTTAGAGTG
GGGCTCTGGAGAATATGGTGGGCTT
>2044
CTGCCCTTCTGTCCCGAGAGCCCCCGCTACCTCTACATCATCCAGAATCTCGAGGGGCC
TGCCAGAAAGAGTAAGCTCTCCCGCTGCAGCCTGGCCCAGGCCCATGCCTCCGCCTCA
TCTTGCTAGCACCTGGCTTCCTC
>2045
TACCCACCGGCAGCCCCTGATCATTGCGGTCGTGCTGCAGCTGAGCCAGCAGCTCTCT

GGCATCAATGCTGTATGTGTGGAGCAGCCTCCAGGCAGGGCACAGCCCCGGGAGGGT
AGACGAGAGTGGGGAGCAAACCCCC
>2046
AGGGGTAGGCCAGCCTGCCTATGCCACCATAGGAGCTGGTGTGGTCAACACAGTCTTC
ACCTTGGTCTCGGTAACTGCTCACCTCTGGAATGGCCCGAGCCACTGGCTTCACCTCC
CTGGGTGTCCCGGAGGTCCTGCTC
>2047
GCTCCATCTCCTGGGCCTGGCGGGCATGTGTGGCTGTGCCATCCTGATGACTGTGGCTC
TGCTCCTGCTGGTAAGGCCTGGAGGCTAGGAGGGGCTAGCAGCCCACCCCATGGGAAT
GGTCCTGTGAGTCTCTGTGACCA
>2048
CATGGCTGTGGCTGGTTTCTCCAACTGGACGAGCAACTTCATCATTGGCATGGGTTTCC
AGTATGTTGCGGTAGGTCCCCCCGCCCCAGCCTCCCACACCGTAGGCCAGAGGTGGGC
ATCACACAGCTAGCCCACCTGCT
>2049
GCCCAGCCACCCCGGCCCCTGAGCCCAGCCGGGCCCCAGCATCCCTACTCCCACCAGC
GCCAGAACACAGGTAAGGGCCAGGCAGCTAGGAGCAGGTGGGCAACAAGGGTGGTG
TCAAGGCCTGAAGCCTGGGGCTGGGA
>2050
GTTCCCTCGTGTGAAGAACTGGGAGGTGGGGAGCATCACCTATGACACCCTCAGCGCC
CAGGCGCAGCAGGTAAGGCCGGCATGCCCTGTCCCCATCGTCTCCAGGGAAAGGGTG
GGTAAGGCCTGGCCTCAGATGGGGC
>2051
CCCCGGCCCCTGAGCAGCTGCTGAGTCAGGCCCGGGACTTCATCAACCAGTACTACAG
CTCCATTAAGAGGTGACAGCTTCCCGGACGCCACAGCCTCCCTTGTCCCACTGAGGCC
CCAGAAACCCCGTGACGACCTTCC
>2052
GTTCGGGGCTAAGCAGGCCTGGCGCAACGCTCCCCGCTGCGTGGGCCGGATCCAGTG
GGGGAAGCTGCAGGTGCGGCTGGCCAGCGACTGAGAGACCCGGGCGCTACCAAAAG
GGGAGCGGGGTGGCGGGGCAGTTCCTA
>2053
GGTCTGCACAGGAAATGTTCACCTACATCTGCAACCACATCAAGTATGCCACCAACCG
GGGCAACCTTCGGTGAGTGCCCCCCACCATGCCAGGCCCCAGCCTTCTTCCCCAAGGC
AGGGAAGGCGGGGCTCTGACCAGC
>2054
GCGCTACGCGGGCTACCGGCAGCAGGACGGCTCTGTGCGGGGGGACCCAGCCAACGT
GGAGATCACCGAGGTGGGCACCGAGGGCCACCCATGAGGGTGTCCCCAAGGTGGAGA
ATGAGGAAACCAGTGGGAGAAGGCTC
>2055
CAGATGAGCCCCCAGAACTCTTCCTTCTGCCCCCCGAGCTGGTCCTTGAGGTGCCCCT
GGAGCACCCCACGTGAGCACCAAAGGGATTGACTGGGTGGGATGGAGGGGGCCATCC
CTGAGCCTCTCAAGAAGGGCCTGCA
>2056
TGGCTGGTACATGAGCACTGAGATCGGCACGAGGAACCTGTGTGACCCTCACCGCTAC

AACATCCTGGAGGTGAGGTGCGGGATGGGGCTCGGGCACCGAATGCACCTGTCCAAG
GCAGGAGTCTGGCTCTCACTCCATC
>2057
GACCACCTCGTCCCTGTGGAAAGACAAGGCAGCAGTGGAAATCAACGTGGCCGTGCT
GCACAGTTACCAGGTGCAGAGGCCCAGACTGGCCAGGAAGGCAAAGGGTTTGCATAC
GGGGGCAGCAGGGGCGGGGGATGGAG
>2058
GGGCAGCCTCACTCCTGTTTTCCATCAGGAGATGGTCAACTATTTCCTGTCCCCGGCCT
TCCGCTACCAGGTGCCCACCCTAATGGCTCTGCCAGCCTGGGCCCAGCTCTAATTCTAA
GCAGCCCCTGGGGACCTCTAAC
>2059
ACCCCTGGAAGGGGAGTGCCGCCAAGGGCACCGGCATCACCAGGAAGAAGACCTTTA
AAGAAGTGGCCAAGTGGGTCCCCTGGGAGCCCCGCTCTCCCACACACACCCTGGGGG
CCCCACTCTCCCCCACACACCCTGGG
>2060
CGAGACCGGCCGGGCCCAGAGCTACGCACAGCAGCTGGGGAGACTCTTCCGGAAGGC
TTTTGATCCCCGGGTAGGGCTGAGCCCAGGGGAGCAGGGAGCTAGAAAGAGGGGGCT
CTATCAGCATCTTCAGGGGTGCCCTG
>2061
CGAACACGAGACGCTGGTGCTGGTGGTAACCAGCACATTTGGGAATGGGGATCCCCCG
GAGAATGGAGAGGTGAGAACTTCCAGGAAAGGGGCTGCTGGGAATGAGGAGAGACT
CAGAATTGGAGTGACTGGGCAGGAAC
>2062
TGTCCTCTTGGCGGCGGAAGAGGAAGGAGTCCAGTAACACAGACAGTGCAGGGGCCC
TGGGCACCCTCAGGTCAGGGCCTCACCAAGAGGGGTGCAACGGGTGGGCAAGCTGCC
TGGGCAAACGTGGCCTGCAAAGGGAG
>2063
GCTGGGCCAGGGCGACGAGCTGTGCGGCCAGGAGGAGGCCTTCCGAGGCTGGGCCC
AGGCTGCCTTCCAGGTGAGCCCAGCCCAGCCCCTGCTCTGACTCCTGCCCCCTGGGAT
GCCTCCTCCTGCCTCACTCTGCCCTG
>2064
CCCAAACGGAGCTGGAAGCGCCAGAGGTACCGGCTGAGCGCCCAGGCCGAGGGCCT
GCAGTTGCTGCCAGGTGGGCCCTGCCTCACCCTAACCCGGCTGGTTCTCTGAGGCCCC
CACACCCCGGGACTAAAGCACTCTGG
>2065
ACGTGCACAGGCGGAAGATGTTCCAGGCTACAATCCGCTCAGTGGAAAACCTGCAAA
GCAGCAAGTCCACGTGAGGACGACGGCTTTACCGCCCCCCAACCCCTGTCCTGAACA
CCCTGACCCTGGACCCTCCTCCTCCC
>2066
AGCCGCGTGGAGGACCCGCCGGCGCCCACTGAGCCCGTGGCAGTAGAGCAGCTGGAG
AAGGGCAGCCCTGGTGAGGGGCAGCCTGGGAAGCAACAGGGCACACCAGCCCCATG
CCCAGCCCCACCCCCGGCCCCAGGCCT
>2067
GCTCTTGCGGCTGCTCAGCACCTTGGCAGAAGAGCCCAGGGAACAGCAGGAGCTGGA

GGCCCTCAGCCAGGTTGGGGGCCACCCCAATGAGGCACAGGGGCTAGAGAGACGGGA
TGAGCTGGGGGGACCCCAGTGGCAGG
>2068
AGCTCGGCACCCAGCACCCACCCAGGAGAGATCCACCTCACTGTAGCTGTGCTGGCAT
ACAGGACTCAGGGTGAGGCAACAAGCAGGAGCAGGCCTGGCCACAGCAGGGTTGGG
ACCGGCCCCTCTCTGGCCCCTCACCG
>2069
ACTATGGAGTCTGCTCCACGTGGCTAAGCCAGCTCAAGCCCGGAGACCCTGTGCCCTG
CTTCATCCGGGGGTAAGTGAGATGGAGGACTTGGTGGGGAGCTGCCCAGGGTCAGGG
TGGCAGCTTTGGTGAGGAGTGTCAC
>2070
GGTCCAGGCACTGGCATTGCCCCCTTCCGGGGATTCTGGCAGGAGCGGCTGCATGACA
TTGAGAGCAAAGGTGAGGCTGGTGACTAAAGGACTGCCTGAAGGGAGTCACACAATC
TAGGGACAGAGGGGTGGGGCTGGAA
>2071
GAACGCCCAGCAGCGCGGGGTGTTTGGCCGAGTCCTCACCGCCTTCTCCCGGGAACC
TGACAACCCCAAGGTGTGAGACCCTGAGGGCGCAATGGTAACCTGAAGATAGGGAGA
GAGGGGAGGACTCGCGCTCTCCAGCG
>2072
AGAGTTGCCACCCAGGCCTCAGCCAGGACCTTTCAGGCCAGACAGGAGCACCTGACC
AAAGGCTTCACAGGTAAGTGGCCCTCCGTATTACCCAGCATGGCCTCCCACAGGGAGA
GAGGAGGTCAGTTCCTGTCCCTTTA
>2073
TGGGCACACTCCACCTTCCCTAGTCACCAGCTGGTTTCCCAGAGGAGAAGGCTGAGAC
CCGAGAAGGGAGGTGAGTGCACCCCGTCCCATGTCAGGCTCCGCTACATCCCCAGTGC
TTGCCGTCCCTGAGGATCGGTCCA
>2074
CCGAGGAGGAGGACAAGAAGGAGGACGTGGGCACGGTGGTCGGCATCGACTTGGGG
ACCACCTACTCCTGGTAAGTGGGGTTGCGGATGAGGGGGACGGGGCGTGGCGCTGGC
TGGCGTGAGAAGTGCGGTGCTGATGTC
>2075
CAAGCGGCTCATCGGCCGCACGTGGAATGACCCGTCTGTGCAGCAGGACATCAAGTTC
TTGCCGTTCAAGGTTCGACCGGTTTTCCTCATCCAGTTAGAGAACGGGTGGGTGGTGG
GAGTATTTAGAGTTATAAGTCTCT
>2076
TCCTGAAGAAATTTCTGCCATGGTTCTCACTAAAATGAAAGAAACCGCTGAGGCTTATT
TGGGAAAGAAGGTAAATATTTCTAGAACAATGTTAAGTATTTTTTGATCATTAGTATTCT
CGGTTGGCTGTTATGTATAGA
>2077
CCCAACGCCAAGCAACCAAAGACGCTGGAACTATTGCTGGCCTAAATGTTATGAGGAT
CATCAACGAGCCGTAAGTATGAAATTCAGGGATACGGCATATTTGCCAAATAGTGGAAA
TGTGAAGTACTGACAAAACTTTT
>2078
TGAGTCCTTCTATGAAGGAGAAGACTTTTCTGAGACCCTGACTCGGGCCAAATTTGAA

GAGCTCAACATGGTATGTTCCTTGTTTTCTGCTTTGCTAATGAGATCTCCTTAGACTCTG
AATTCAGGACATTGCATCTAGA
>2079
AACCCAGATGAAGCTGTAGCGTATGGTGCTGCTGTCCAGGCTGGTGTGCTCTCTGGTG
ATCAAGATACAGGTAGGTCATCATCGCAGCATCTTTCTTAGTGATTCAGTAGCTTGATGG
AAGAGCTCGGTACCCCTATTGC
>2080
ACCAAGAACTCTCAGATCTTTTCTACAGCTTCTGATAATCAACCAACTGTTACAATCAA
GGTCTATGAAGGTAATTACCTTAAGTTTGGTTAATATCATGGCTTTTTTTTTGAGATGAA
GTCTTGCTCTGTTGCCCAGGC
>2081
AGGCCTGTCTGCAGAATCGACACCAACCAGCATCATGTCCATGACACTGGGGTACTGG
GACATCCGCGGGGTGAGTGAGGGTCCGCTGCACTGTGGGACCGGGCGCGTGGGCGGG
AAGTGCCGAGCGGCTGGGGACCGGC
>2082
CACGCCATGCGCCTGCTCCTGGAATACACAGACTCAAGCTACGAGGAAAAGAAGTATA
CGATGGGGGACGGTAATGACACCCTTGTGTCCGGGCTCTGCCCACTCACGCTGAGTTG
GCACCAAGCAACCCATGGTGGCCA
>2083
ATTGATGGGGCTCACAAGATCACCCAGAGCAACGCCATCCTGTGCTACATTGCCCGCA
AGCACAACCTGTGTGAGTGTGGTTGGCTGCAGTGTGTGGGGGGAAGGTGGCATCCTC
CTTGGCTGGATTGGGGTGCTATGCT
>2084
GGACATTTTGGAGAACCAGGCTATGGACGTCTCCAATCAGCTGGCCAGAGTCTGCTAC
AGCCCTGACTTTGTGAGTCCCTCCCTGGTCTGGACCAGAAGCCAGGCTTGTATTCCCAT
CTACTCTGGTCCTATTCACAATT
>2085
GGAACTTCCTACAATGATGCAGCACTTCTCACAGTTCCTGGGGAAGAGGCCATGGTTT
GTTGGAGACAAGGTAATGGGGGCATGTGATGAGGACACTAGAGATTTGCCATACATCC
TATGTTACAGAGATTCCAGCCCAC
>2086
CCACCGTATATTTGAGCCCAACTGCTTGGACGCCTTCCCAAATCTGAAGGACTTCATCT
CCCGCTTTGAGGTGATGCCCCCATCCTCCTTTCTCTTTGATGCCCCTTGTTCCGTTACCT
CCTTTCAGATGCTTTCCCAGT
>2087
GGGCCAGAGCTGGAAGGAGGAGGTGGTGACCGTGGAGACGTGGCAGGAGGGCTCAC
TCAAAGCCTCCTGCGTAAGTGACCATGCCCGGGCAAGGGGAGGGGGTGCTGGGCCTT
AGGGGGCTGTGACTAGGATCGGGGGAC
>2088
AAGTTCCAGGACGGAGACCTCACCCTGTACCAGTCCAATACCATCCTGCGTCACCTGG
GCCGCACCCTTGGTGAGTCTTGAACCTCCAAGTCCAGGGCAGGCATGGGCAAGCCTCT
GCCCCCGGAGCCCTTTTGTTTAAA
>2089
GGTGGACATGGTGAATGACGGCGTGGAGGACCTCCGCTGCAAATACATCTCCCTCATC

TACACCAACTATGTGAGCATCTGCACCAGGGTTGGGCACTGGGGGCTGAACAAAGAA
AGGGGCTTCTTGTGCCCTCACCCCC
>2090
GCAACTGAAGCCTTTTGAGACCCTGCTGTCCCAGAACCAGGGAGGCAAGACCTTCATT
GTGGGAGACCAGGTGAGCATCTGGCCCCATGCTGTTCCTTCCTCGCCACCCTCTGCTTC
CAGATGGACACAGGTGTGAGCCA
>2091
GGATCTCACCGTGGGTCCGATTAGCCTTTTCTCTGCCTTGCTTGCTTGAGCTTCAGCGG
AATTCGAAATGGTAAACTTCGCAGAGACGCTCGATGACTCCGCGGGTTTTTGCCGGCG
AGAGAAAAACCCGTTACACAGAG
>2092
TAAGGCTGGAAAGGACTCCGGAAAGGCCAAGACAAAGGCGGTTTCCCGCTCGCAGAG
AGCCGGCTTGCAGGTCAGTAGTTGTTCGTGCAGTCTGGGAGTCATGATGTTTTTCTTTG
CATTTCCTTTTTTGTTATTCATCG
>2093
CAGTCATGGACGTGTGGGCGCGACTGCCGCTGTGTACAGCGCAGCCATCCTGGAGTAC
CTCACCGCAGAGGTAAATGGGTGAACGCCTATAATCCGGAGAAGGTTTGGGGGGCGG
GGAGGCGGCAAGAGGGAAGGAGGAA
>2094
CACTTGCAACTTGCTATTCGTGGAGATGAAGAATTGGATTCTCTCATCAAGGCTACAAT
TGCTGGTGGTGGTATGTTAACTTCTAACATTTTAAAAAATTTCTTCAGAGGAAGGAATT
TTTTGCTGCTTTTAATTAGTTT
>2095
AACGGCAAGCTTGAGTCAGGACCCTTAATTAAGATCCTCAATTGGCTGGAGGGCAGAT
CTCGCGAGTAGGGTACAAGGCACTATGAAATGATCTAGTTTCGTGGGTGAGGGGCTGA
AGGGCCTATGATGCACGGAGGCGG
>2096
TTCGTAACGGGAATGCCGAAGCGTGGGAAAAAGGGAGCGGTGGCGGAAGACGGGGA
TGAGCTCAGGACAGGTAAGGGAATGAAATCAGCCCTTCTTCCTAGAAGCTGCGGCGG
GGGTGTTTGTCATTCCCTTGATGTACG
>2097
TGCCACACTCAAGATCTGCTCTTGGAATGTGGATGGGCTTCGAGCCTGGATTAAGAAG
AAAGGATTAGATGTGAGTGGAATTTGAGGGAAAGAGACATTTTTTAGTATTGAATGGTC
TTAGGGTTTAGTCACCCCTTTTC
>2098
GACAAGGAAGGGTACAGTGGCGTGGGCCTGCTTTCCCGCCAGTGCCCACTCAAAGTT
TCTTACGGCATAGGTGAGACCCTATTGATGCCTAATGCCTGAACTCTTCAAAACCAATT
GCTAATTCTCTATCTCTGCCCCAC
>2099
GCCCAACGCGCTGTCCGCCCTGAGCGACCTGCACGCGCACAAGCTTCGGGTGGACCC
GGTCAACTTCAAGGTGAGCGGCGGGCCGGGAGCGATCTGGGTCGAGGGGCGAGATGG
CGCCTTCCTCTCAGGGCAGAGGATCA
>2100
AGAAGTCTGCCGTTACTGCCCTGTGGGGCAAGGTGAACGTGGATGAAGTTGGTGGTG

AGGCCCTGGGCAGGTTGGTATCAAGGTTACAAGACAGGTTTAAGGAGACCAATAGAA
ACTGGGCATGTGGAGACAGAGAAGAC
>2101
CAAGGGCACCTTTGCCACACTGAGTGAGCTGCACTGTGACAAGCTGCACGTGGATCCT
GAGAACTTCAGGGTGAGTCTATGGGACCCTTGATGTTTTCTTTCCCCTTCTTTTCTATGG
TTAAGTTCATGTCATAGGAAGG
>2102
CAAGGGCACCTTTGCCCAGCTGAGTGAACTGCACTGTGACAAGCTGCATGTGGATCCT
GAGAACTTCAAGGTGAGTCCAGGAGATGTTTCAGCCCTGTTGCCTTTAGTCTCGAGGC
AACTTAGACAACTGAGTATTGATC
>2103
ACAAGGCTACTATCACAAGCCTGTGGGGCAAGGTGAATGTGGAAGATGCTGGAGGAG
AAACCCTGGGAAGGTAGGCTCTGGTGACCAGGACAAGGGAGGGAAGGAAGGACCCT
GTGCCTGGCAAAAGTCCAGGTTGCTTC
>2104
CGCTGGTGCGCGCCCTGTGGAAGAAGCTGGGCAGCAACGTCGGCGTCTACACGACAG
AGGCCCTGGAAAGGTGCGGCAGGCTGGGCGCCCCCGCCCCCAGGGGCCCTCCCTCCC
CAAGCCCCCCGGACGCGCCTCACCCA
>2105
ACCCCACGCGCTGTCCGCGCTGAGCCACCTGCACGCGTGCCAGCTGCGAGTGGACCC
GGCCAGCTTCCAGGTGAGCGGCTGCCGTGCTGGGCCCCTGTCCCCGGGAGGGCCCCG
GCGGGGTGGGTGCGGGGGGCGTGCGG
>2106
TGAAGCTGCAAGTATTTCTCATTGTGCTCTCTGTTGCATTGAACCATCTGAAAGCTACA
CCCATTGAAAGGTTGGTAACGTTTAAAATCCTGTTTCTTTGTAACTTTTGTAAAGTGTG
AGAAAATTAGCATTAAATACTG
>2107
GAAGAAGGCGGGTGGCGGGGGTGGCAAGAGGAAGGAGAAGCTGGAGAACATGAAG
AAGGAGATGGAGATTGTGAGCTCTGACTGGGGAGGGGCGGGAGCTGGAGGGTAGGCT
GGGAACCAGGCAGTGGGCGGGGAGGGGG
>2108
TGCCGCCGCCATCTGCCTCATCGCCTTTGCCATCCAGGCTAGTGAGGGGGACCTCACC
ACCGACGACAATGTGAGCCATGTTGTGGGGACAGGCTCAGGACATGGGGACCTGGGG
ATGGAAGACACTGAGCCTGGGACTT
>2109
CTGCTTTGGCTACTACCAGGAATTCAAGAGCACCAACATCATCGCCAGCTTTAAGAAC
CTTGTGCCACAGGTGGGTTCCCCAGCACCCAGCCACTCCAGATCCCAGAGACTCCAAA
CTGCTCCTGTAGCCTCCTTCCATC
>2110
CCCGAGTGCACGCACGAGAGCCCTCTGGAGACCCGCAACATCGCCTTCTTCTCCACCA
TGTGCCTTGAGGGTCTGTGAAGCCCCCGCCTGCCTGCCCAGTAACTGCTGCCTCCACT
GGCCTCTCTCCTGCTATCTTCTAG
>2111
GCGGGCCATGGTCTTCTTCATGGCCATCGTGGTGGCCTATGTGCCTGAGGGGCTGCTGG

CCACTGTCACAGTGAGTGGGGGCAGGCGGGGATGCAGGGAGCTGAGGGGCCTGCTCA
ACCATCTGTTCTTTCCTCCACATT
>2112
CGCATGACTGTGTCCCATCTGTGGTTTGACAACCACATCCACACAGCTGACACCACGG
AAGACCAGTCAGGTGTGGGGGAGGGAGGGCACAGGCGCCAGGGCAGGATCTGGTGG
ATGGTGGAGACCTGAAGAGGGATAGG
>2113
GTGCCGGGTGCTCACCCTGTGCAACCGCGCCGCCTTCAAGTCCGGCCAGGATGCAGTG
CCTGTGCCCAAGGTGAGAGCCAGGGGGCCTCCAGGGAATCCCGGGAGGCTGGTGTAG
CCGGCTTCGCCCCTGACACCGGTCC
>2114
CGCCATGGGCTACCGGGACCGCTTCCCAAAAGTCTGCGAGATACCCTTCAACTCCACC
AACAAGTTCCAGGTGCGCAGCCGGCCCCGCCCACGGCCTGGGCCCGCCCACCTCGGG
CCCCGCCCCAGGCCACACCCACATC
>2115
GACGAGCAGTGGCGCGAGGCCTTCCAGACCGCCTACCTCAGCCTGGGAGGCCTGGGC
GAACGCGTGCTCGGTGAGACCCCTGGGCAGGGGAGGAGACTCCAGGTGCGAGGAGG
AGCTAGCACAGACCCTGCCCACCACTC
>2116
ATCCATGATTGACCCACCCCGGGCCACCGTCCCTGATGCTGTGCTCAAGTGTCGCACC
GCAGGCATCCGGGTATGCCACTGTGGGGAGGAACAAGTGGAGTGAGGACAGAGGATG
GCAGCAGGATTTGGGTTGAAGAACC
>2117
CGGAAGGCAGCGAGACAGTGGAGGACATCGCTGCCCGCCTCCGTGTGCCCGTAGACC
AGGTTAATCGCAAGTAAGCCCCCCCAGACCTCCCCGGGGTTCTCCCCACATCTGGCAC
AGACCGAGGTCCCAGCGAGGTCCTC
>2118
CCCCGAGATGGTGTTTGCGCGCACCAGCCCCCAGCAGAAGCTGGTGATCGTGGAGAG
CTGCCAGCGGCTGGTGAGCCCTGCTGATGAGGGTGGGCAGGCGGGCAGACAGGCGGG
GCTGGTCTTGGACCGGCCTCTCACTG
>2119
GCCAAAAATGCAGCTGACATGATCCTGCTGGATGACAACTTTGCCTCCATTGTGACAG
GCGTGGAGCAGGGTCCGAGCCACGCCAGGGGGAGGGCAGGCAGTGTGGGCACAGGA
GGGAGAGGGCTGTGAGGAGGCTGAGG
>2120
CATCACCGTCAGCGTGCCCCTGCCCCTCGGGTGCATCACCATCCTCTTCATCGAACTCT
GCACTGACATTGTAAGTCCACAGCCCAGGGTACCCATCCACAGGGTGCCCAGAGACAC
TTGCCTGCAGATGGACAGGAGGG
>2121
CGTCCACGCAACCCAAAGCGTGACAGATTGGTCAACGAGCCCCTGGCTGCCTACTCCT
ACTTCCAGATTGGTGGGTGCCCGTGGGCCGGGGAGCCTGGAGGTGGGGGGCTCTGCG
CTGGGCTGCTCGCCTGCCCCAGGCC
>2122
CGTGGGGCTGCGGGCGCAGTGGGAGGACCACCACCTACAAGATCTGCAGGACAGCTA

CGGCCAGGAGTGGGTGAGCCCTGCCCCGCCCCATCCCATCCCTGGGCTCCCTCCTTCC
CAGGGCAACTTGCCTTGTGTGCGGC
>2123
TGTGCCAGATCGCCGATGTCCTCATCCGCAAGACGCGCCGTCTCTCTGCCTTCCAGCA
AGGCTTCTTCAGGTGTCCCTGGCTGGCCCTCTTCCTGCCCTCCAACCCCTGGTCCCAAC
TCCCATCAGATCCTGACTGTGGT
>2124
AGGTCTGCATCGGCTGCTTCCTGTGCTACTGCCCCGGCATGCCCAACATCTTCAACTTC
ATGCCCATTCGGTGAGAGGCTGTGAGGGGCAAGGACAGGGAGGGGGCTGTAGGGCAC
AGGCCTGGGATGGCCTTCACCTTG
>2125
CTGCCCTACGGCATCCTCATCTTCGTCTATGATGAGATCCGGAAGCTTGGAGTTCGCTG
TTGCCCAGGGAGTGAGTGTGGAGACTGGGAGCCTGGGGGCAGGGGACTGAAACGTTT
GAGCAGCTGCACCCACCTTTGATC
>2126
CTCGGGTGGAGTCTTCTGACAGCTGGTGCGCCTGCCCGGGAACATCCTCCTGGACTCA
ATCATGGCTTGTGTGAGTGTGGGGACCCCCCCCAAGGTCCAGGGGATAGGGCAGGA
ACTGATGGCCAGAGGAGAGCTGGGC
>2127
CCAGCAACCTGAATCTCAAACCTGGAGAGTGCCTTCGAGTGCGAGGCGAGGTGGCTC
CTGACGCTAAGAGGTGAGAAGTGAAGTCGGGGTGGTGGGCGGCAGGGACGGGCTTG
GTCCAGCAGGGAGGGCGTGGCCGGCCA
>2128
GGACGGCGGGGCCTGGGGGACCGAGCAGCGGGAGGCTGTCTTTCCCTTCCAGCCTGG
AAGTGTTGCAGAGGTGGGCTGCAGACCGGAACCGGGGACCAGGGACAGGGGCTGGG
TGGGCTGGGGCGGGGCTGGGTTAGTGA
>2129
GGCCGAAGTGGCTAACCAAGAAACTAAAGAAGACTTACCTGCGGAAAACGGGGAAA
CGAAGACTGAGGAGGTCAGAAGTGTGTGTGTGTGTGTGTGTGTGTGTGTGTGTGTGTG
TGATTGCTTTGACTCTGAACAGTAGT
>2130
CAAGAAGAAACACCCGGACTCTTCCGTCAATTTCGCGGAATTCTCCAAGAAGTGTTCG
GAGAGATGGAAGGTGAGGCAGGAGAGGGACGGAGCTCAGGGGGTGCAGGTGTGGTT
TTCGGCTAGGAGGGCCTTAGGCAGGT
>2131
AAAATTACGTTCCTCCCAAAGGTGATAAGAAGGGGAAGAAAAAGGACCCCAATGCTC
CTAAAAGGCCACCGTAAGTTTAAAATAACCCAAATTGCTCCTTGGATTTTTCCTTCAGT
TTATTAAACTCTGTTGCTTCCTTT
>2132
TGAGCAGTCAGCCAAAGATAAACAACCATATGAACAGAAAGCAGCTAAGCTAAAGGA
GAAATATGAAAAGGTACAGTGTCATCTTTTTTAAAGCCGTGGATAAGACTAGGTATAGG
TAATAACTGTAGAAAACCTGGGAA
>2133
AGCCCAGGGACAACCTGCCGCCCCAGCGCCTCAGAAGAGAGGACGCGGCCGCCCCA

GGAAGCAGCAGCAAGTCAGTACGAGGGCGCGGTGGGGGCACCAGCCCACCCCGTCCC
CACTGCCGGGGCCCAGACACGCGCGGG
>2134
CTCTCCTAAGAGACCCAGGGGAAGACCCAAAGGCAGCAAAAACAAGAGTCCCTCTAA
AGCAGCTCAAAAGGTGAGATTTCTCAAGTCAAGCTCTCCTAACTTCATCAATGACTGA
CTACAGGAGCCTGCCTGTAACTTTC
>2135
GCCGGTGCTGCGCTCCTCTAATTGGGACTCCGAGCCGGGGCTATTTCTGGCGCTGGCG
CTCCAAGAAGGCGTGAGTTCGCGGCCGCTCCGGTGGCTTCTTTTTTTTATATCTATAATT
TAATTAAATTATTTATTTATTG
>2136
TCCTCAGCGCCCAGCACCGCCGCTCCCGGCAACCCGGAGCGCGCACCGCAGGCCGGC
GGCCGAGCTCGCGGTGAGTCGTCCCCGGGGCCCGCGGCGGCGGCGGCGGAGGGGGG
CGCCCGCGCCCCGCCCGACGTCGCCCC
>2137
GGCGGGGAGAGACACGGGCTGCGGCGCGGCAGGAGAGTGGGGGGTCGGGCGCCCCC
CGCAGCTCAGGGAAGTTCTGGAAGTGAGGGAGATGGGGAGGAACCCCCAAATTCGGC
CCTACGCCCTCTCGGCCTTTCTCTCGC
>2138
GGTCTCGAACTCCTGACCTCAAGCAGTCCTCCTGCTTTGGCATCCCAAAGTGCTGGGA
TTACAGGCATGAGTCACCTCACCTGGCCCATAAATCATTTTCAAATGGTCTAGTTCTTAT
TTTGGTTAGAGTGAAGATTTCT
>2139
GAAGCGGGGCCGGGGCAGGCCGCGCAAGCAGCCTCCGGTGAGTCCCGGGACAGCGC
TGGTAGGGAGTCAGGTGGGTGTCCAAACCTTTGCTTCACTTGGTTTACCCTTTGGGGCT
AGGGAGGTGCCTGGAGTTTCATTCA
>2140
AGTGCCAACACCTAAGAGACCTCGGGGCCGACCAAAGGGAAGCAAAAACAAGGGTG
CTGCCAAGACCCGGGTGAGACTTGAGATGGGACTACCCCTGGGTGGATGACTAGCAG
AGGATCCTCTTGTTGGCACCATGGCCA
>2141
GAGCCGAGCCCGGCGCCGCCCGCCCAACCCCAGATCTACCCCTGGATGCGCAAGCTGC
ACATAAGTCATGGTAAAGCCAGCCTTTTTCTAAATCCACGCGACCGCGGGGACGCGGC
TCTCGGTCCCCCCTCTCTTCTCTG
>2142
AAGCAGATCTTCCCCTGGATGAAAGAGTCTCGACAGAACTCCAAGCAGAAGAACAGC
TGTGCCACTGCAGGTAGCTCCCTGAGGTGGCCTACTGCCAGACCAAGCCCCCTCCAGA
TTGACCCAAGGAAGCCTAGTCAGGG
>2143
AAGACATCGGGGACATTCTGCAGCAGATAATGACCATCACCGACCAGAGCCTGGACGA
GGCCCAGGCCAAGTGAGTGCCCCCACTCCGGGACCCCACACAGACCCAGCAAACCCC
GTTCACATGTTCTGAATCTTCTGGG
>2144
CACGCCCTAAACTGCCACCGAATGAAGCCTGCTCTCTTTAGCGTCCTGTGTGAAATCA

AGGAGAAAACTGGTATGTGGGCGCCCCCGGATTGCTCAACTCTGGGAACAGAACCC
TGTTCATTATAGGGCTAGAGTGTGA
>2145
CTCGGACTATCGCAGCAAACTTGCCCAGATCCGTCACATATACCACTCGGAGCTGGAG
AAGTATGAGCAGGTAAGGAGAGGAGGCTTGGGTGGGTGGAGGGAAGGGCTCTTGCAG
GGGAATCCCATGGTCAAAGGGCTCC
>2146
TCCAGATGCAGCTGAAGCAGAGCACCTGCGAGGCTGTGATGATCCTGCGCTCCCGTTT
CCTGGATGCCAGGTGGGCCCAGGGACCCCAGGCTGGCCCCCAGCACTGGGCTCCTTC
CCATTCCTCTCCAAGACCCTGAGCT
>2147
GAGTAACCCATATCCTAGTGAGGAGGCCAAGGAGGAGCTTGCCAAGAAGTGTGGCATC
ACCGTGTCTCAGGTATTATGGAGGTTGCGGGAGGAGTTGTCAGGCAAAGTGCACGCAT
CTCAGCTAGGTGCAGTGGTGTGTT
>2148
AAGACCGCCGTGTCAGTCACCCAGGGGGGCCACAGCCGCACCAGCTCCCCGACACCC
CCTTCCTCTGCAGGTGGATCCCACTGTCACCCCGGCTGTTTTGCACACTTCCTGCTTTT
GTTCCCACTTCCTATCTAGGCAGG
>2149
TCTCTCAGGATCTGGAGACATGTTTCTGGGGATGCCTGGGCTCAACGGAGATTCCTATT
CTGCTTCCCAGGTCAGATGCCCATCTCCTCTCGAATAGGGCTTTCCCCAACTCCATTTC
CTCTACTTTAGGATACAAGACC
>2150
CTCGATGGGGCCAGGGGGCTATGGGGATAACCTCGGGGGAGGCCAGATGTACAGCCCA
CGGGAAATGAGGGTGAGTGGATCCTGAAGCTCCTCTCTGTCCAGTTCTCACAGGACAG
AGGGGCATTTTCCCTAGTAATGTT
>2151
TGGCGAGCCACTGGTGCTGAAGTGTAAGGGGGCCCCCAAGAAACCACCCCAGCGGCT
GGAATGGAAACTGGTAAGCGGGGCTCCTGTTGCAGCCTCCCAACTTCCAGGGAGACC
AGCAATGATTTGGATCCCCGTCACTC
>2152
ATTTTCCGGTGCCAGGCAATGAACAGGAATGGAAAGGAGACCAAGTCCAACTACCGA
GTCCGTGTCTACCGTAAGAATTCCAGGGTCTTCTCCAAGGCCTCCCTCTTACCTAAGAA
AAAGCCTTCAACCCCAGCCTTGGC
>2153
GAGGGAAGCTACCCTGCAGGGACTCTTAGCTGGCACTTGGATGGGAAGCCCCTGGTG
CCTAATGAGAAGGGTGAGTCCTAAGGTGCCCCCCAAGCTGCCTTCTCCCTGATCTCAC
TCCCACACCCACCCTGGGATAATTT
>2154
TGTAGCTTCAGCCCAGGCCTTCCCCGACACCGGGCCTTGCGCACAGCCCCCATCCAGC
CCCGTGTCTGGGGTGAGCATAGGTGGGGAGGGCCCCAAGCTCACGTGAGCACGTTCT
GGAAGTCTGACCCTTAGGGAAAGAG
>2155
TGGTGGAACCGTAACCCTGACCTGTGAAGTCCCTGCCCAGCCCTCTCCTCAAATCCAC

TGGATGAAGGATGTGAGTGACCTGGAGAGAGGGGCTGGGAGGTAGGGTGAACCATAA
CTAGCAACAGGGAGGGCAGAGGGCT
>2156
TACAGCTGTGTGGCCACCCATTCCAGCCACGGGCCCCAGGAAAGCCGTGCTGTCAGCA
TCAGCATCATCGGTGAGACCTCTCCCCAAGCCCTACAGACCCTGGGACTAGGGTGCAG
GACAGCACAGGCTCTAATTTCCTG
>2157
GCCTGGGGACAGCCGCCCTGCTCATTGGGGTCATCTTGTGGCAAAGGCGGCAACGCC
GAGGAGAGGAGAGGTGAGTGGAGAAAGCCAGACCCCTCAGACCTAGGGCTTCCAGG
CAGCAAGCGAAGAGGGGTCGGGGGGTG
>2158
GTCATTGCCCTCCTGCTCTGGGGACAGCTTTTTGCAGTGGACTCAGGCAATGATGTCAC
GGATATCGCAGGTCAGTCTTTGGTTGGGTAGGAGTGTGCATCCCACTCTGACCCTCTCG
GGTCTGCACTCTCTCTGAGAAC
>2159
CATGGCTATGTGGAGCACTCGGTTCGCTACCAGTGTAAGAACTACTACAAACTGCGCA
CAGAAGGAGATGGTAAGATGTGGACAACTGTCTCCATGCCCTACATACAACCCCCTTCT
CTGACATTTCCATGATGGGTGGT
>2160
TACACCTTAAATGATAAGAAGCAGTGGATAAATAAGGCTGTTGGAGATAAACTTCCTGA
ATGTGAAGCAGGTGGGTGCTGAGCACTGAGCACTTAAGAGAGCAGGCAGGCGTCCAG
CGGGGAACGTCCTAGAGGCACAGC
>2161
CATGGCTATGTGGAGCACTCGGTTCGCTACCAGTGTAAGAACTACTACAAACTGCGCA
CAGAAGGAGATGGTAAGATGTGGACAACTGTCTCCATGCCCTACATACAACCCCCTTCT
CTGACATTTCCATGATGGGTGGT
>2162
TACACCTTAAACAATGAGAAGCAGTGGATAAATAAGGCTGTTGGAGATAAACTTCCTG
AATGTGAAGCAGGTGGGTGCTGAGCACTTAAGAGAGCAGGCAGGCGTCCAGCGGGGA
ACGTCCTAGAGGCACAGCCTTCCAG
>2163
TTTGCATACCTAATCATTATGCTGAGGATTTGGAAAGGGTGTTTATTCCTCATGGACTAA
TTATGGACAGGTAAGTAAGATCTTAAAATGAGGTTTTTTACTTTTTCTTGTGTTAATTTC
AAACATCAGCAGCTGTTCTG
>2164
AGCACTGAATAGAAATAGTGATAGATCCATTCCTATGACTGTAGATTTTATCAGACTGAA
GAGCTATTGTGTGAGTATATTTAATATATGATTCTTTTTAGTGGCAACAGTAGGTTTTCTT
ATATTTTCTTTGAATCTCT
>2165
CTGGCAAAACAATGCAGACTTTGCTTTCCTTGGTCAGGCAGTATAATCCAAAGATGGTC
AAGGTCGCAAGGTATGTATGACATTTTGACACAGAATATTTTCCTCATTTGAAGGGGGA
TTAAGTGATTGCTTCTTTTTAA
>2166
ATTTGAAATTCCAGACAAGTTTGTTGTAGGATATGCCCTTGACTATAATGAATACTTCAG

GGATTTGAATGTAAGTAATTGCTTCTTTTTCTCACTCATTTTTCAAAACACGCATAAAAA
TTTAGGAAAGAGAATTGTTT
>2167
GAGAAGGAACTGAAGGAGATCAGGGCCTTGGACAAGGCCTTCAGACCAGAATTGAGA
GAGGAATTTTCTAGTAAGTAAACTTGAGTCATGGGTCTGTGGCTCCATCTTAAACTCTG
CATGGGTGTGGGGAGGTTGACCTT
>2168
CACACCGCCTGTATCATTGATGTCTTTGGTGTCACTCACAGAGAGTCCATCATGAATGT
CAATGTGAAAGGTACAGTAGCCTGGGGAGGAGATAAAACAAGTTGGTTAAATGAGGAT
CAGAAAGAAGGACAAGAAAGGGA
>2169
GCAAAAGTGGATGAGTTTCCGCTTTGTGGCCACATGGTGTCAGATGAATATGAGCAGC
TGTCCTCTGAAGGTAAGGCAGGATTCTTTGTTCGTCACCCCCCAGTCCTTCCTCCGTGC
TCCCTCAACCCCACCCACATACA
>2170
TCCGGGTGCGGCTCCACCCCTTCCACGTCATCCGCATCAACAAGATGTTGTCCTGTGCT
GGGGCTGACAGGTGAGCTTGGTCTGGGCCTTTTAAGGCAGTTGGAGTCTCTACATTATT
AGGCTTGCATTATTTTATCAGA
>2171
GCTGCAGAACAAGGAGCATGTGATTGAGGCCCTGCGCAGGGCCAAGTTCAAGTTTCC
TGGCCGCCAGAAGGTATGTAGTGCTGCAGCCCCCTTCTCCCACCTTTGCCCCAGCCTCC
TGACTCAGTTCTTTCCATTGCTCC
>2172
ATTGATCATCAATACTTTCTACTCGAACAAAGAGATCTTTCTGAGAGAGCTCATTTCAA
ATTCATCAGATGTAAGTCACTTATTAACCCAGAATCGGATTTTGGTTTCAGTGTGAACTT
CTTGGGGGTGCTGTATGCTTA
>2173
CATAACGATGATGAGCAGTACGCTTGGGAGTCCTCAGCAGGGGGATCATTCACAGTGA
GGACAGACACAGGTAGGCACCTGAACATTCACTGGTTAAGTGAGCGGTGGAAGGGTG
GGGTGCTGCAACCCTTGGATCCCTG
>2174
GGAGGAACGAAGAATAAAGGAGATTGTGAAGAAACATTCTCAGTTTATTGGATATCCC
ATTACTCTTTTTGTAAGTTTTTATGTAATTGCAGAGTGAATTTCTGTCTGTAGGTGATTG
GGGTGACTGTACTACATCCTTA
>2175
TACTAATGAGGAGTACGGAGAATTCTATAAGAGCTTGACCAATGACTGGGAAGATCAC
TTGGCAGTGAAGGTGAGTGACTGATGGGTGCTTCAAGCTTGTCCTTAGATTATCATCTT
TCTCCACCACCCCAAATATCTTC
>2176
AACATCAAATTGTATGTACGCAGAGTTTTCATCATGGATAACTGTGAGGAGCTAATCCC
TGAATATCTGAGTAAGTATAGATAGGAAAAATAATCACTGTCACTGATTAAAGAAGTAC
TTTCTGGGTGGGCATGGTGGCT
>2177
CTTTACTGAACTGGCGGAAGATAAAGAGAACTACAAGAAATTCTATGAGCAGTTCTCT

AAAAACATAAAGGTTGGTGTAAATAACCATTAGTTTTCCAATTGGCCTCTTTAGTTTTTT
TTTTTTTTTTTTAATTCAGAAA
>2178
GAGATGGTTTCTCTCAAGGACTACTGCACCAGAATGAAGGAGAACCAGAAACATATCT
ATTATATCACAGGTAAGAGAACACTATGTTACAGTCATACACGTCGTTCTTACAACCTTG
TAGGCTCTGTGGGTGTGTTTTC
>2179
AGAGAAAAAAACAAAGTTTGAGAACCTCTGCAAAATCATGAAAGACATATTGGAGAA
AAAAGTTGAAAAGGTATGTGAATACAGCATTTCCTGATCATTGATACTTCTAAGGTGCT
TTCAAGCTTAGTCATACATAGCCC
>2180
TCTGGCTTCAGTCTGGAAGATCCCCAGACACATGCTAACAGGATCTACAGGATGATCA
AACTTGGTCTGGGTAAGCCTTATACTATGTAATGTTAAAAAGAAAATAAACACACGTGA
CATTGAAGAAAATGGGTAAACTT
>2181
CCTCATCATCAATACCTTCTATTCCAACAAGGAGATTTTCCTTCGGGAGTTGATCTCTAA
TGCTTCTGATGTAGGTGCTCTGGTTTCCACATTTGGCATGGTTTTTTTTTTTGATACTCTA
GAAGGAGGGGAAAGGAGTG
>2182
AGCTGATCTCATAAATAATTTGGGAACCATTGCCAAGTCTGGTACTAAAGCATTCATGG
AGGCTCTTCAGGTATTGCAGTTCTGTAGGCATTCATACTTATCTGTGTTCTTTGGTTTTTT
GCTTCTTTAAAACTTGTGAT
>2183
CACAACGATGATGAACAGTATGCTTGGGAGTCTTCTGCTGGAGGTTCCTTCACTGTGC
GTGCTGACCATGGTAAGTTAGCTTTTCTGTTACAAGGTAGTTGGGTTAGGATTTTCTGG
GCTCACACCAGTAGCAGAAATTT
>2184
AGAAGAGAGGCGGGTCAAAGAAGTAGTGAAGAAGCATTCTCAGTTCATAGGCTATCCC
ATCACCCTTTATGTGAGTATGGACTTTTAAATCTTTTACACTTAACGTGCAGGATGTTTC
CTGTTCTGGAGAATCTCATTGT
>2185
CACCCAAGAGGAGTATGGAGAATTCTACAAGAGCCTCACTAATGACTGGGAAGACCAC
TTGGCAGTCAAGGTGTGAGAAGCCTTTGCATGTTGGCTCAACATGCACATATGGAGAG
GAATGAGTTAGGTGGAAGAGTGTT
>2186
AACATCAAACTCTATGTCCGCCGTGTGTTCATCATGGACAGCTGTGATGAGTTGATACC
AGAGTATCTCAGTGAGTATCTCCTTGGCCTAATTTAGTTGGGTGAAGTCTTGGGAGGTT
TTAGGCATTCTGCTAGGATATT
>2187
CTTCTCTGAGCTGGCAGAAGACAAGGAGAATTACAAGAAATTCTATGAGGCATTCTCT
AAAAATCTCAAGGTAAAAAGGCAAATAATGCTTATTCCCTTTACCACTTTCTTAGTAATA
ACAATAAATTATTCCATTCACA
>2188
GAGATGACATCTCTGTCAGAGTATGTTTCTCGCATGAAGGAGACACAGAAGTCCATCTA

TTACATCACTGGTGCGTTGACTCTGATTGAAGCCTTTTTGGAGGAGTGGGGAGCACAA
TTAGGGCTTCCTGGGAACTGGCA
>2189
AGAGAGCAAGGCAAAGTTTGAGAACCTCTGCAAGCTCATGAAAGAAATCTTAGATAA
GAAGGTTGAGAAGGTAAGCCATTCTGGGGCTAGGATATATTTTGTAACATCTTCGAGGT
GGGCTCCCTCACAAGCATGTTTCT
>2190
TCTGGCTTTTCCCTTGAGGATCCCCAGACCCACTCCAACCGCATCTATCGCATGATCAA
GCTAGGTCTAGGTAAGTAGCTTTGGTACTTGGTGTGGCAAGGAGTTTGTGCAACTCGT
CTCCTCTATGGATTTGACTTAAT
>2191
GGCATCGGCTTCCACCTCCAGGCGCTCCCCGACGGCCGCATCGGCGGCGCGCACGCG
GACACCCGCGACAGTGAGTGGCGCGGCCAGGCGCGAAGGGGCGGGGGCGGGGGGCA
ACGGCCGCCGGGCCAACCCGCTCAGTC
>2192
GGTGAGCATCTTCGGCGTGGCCAGCCGGTTCTTCGTGGCCATGAGCAGCAAGGGCAA
GCTCTATGGCTCGGTGAGTACCGCAGGGGTCTGGCTAGGCACCTAGTTGGGAACAGCG
GACATGGCTAGCAGGCTCGTGGCTT
>2193
ATCATCACCACAGCCCTGGTGTGCTTGCTGCTAGCTGGGATGTGGCCGGAAGATGTGG
ACAGCAAGAGCAGTGAGTGTGGCAGGCATCATTTTGCTTCTCTCTGGGGAGGGCAGA
AACGTGGTCAGCCACTCTGGGGTTG
>2194
AAGAGATTCCCCTGAGGGCAATCCTGTGTTACAGAAATACCAGCTCCATCTGCTCCAAT
GAGGGCTTAATGTAAGTGATCACCTGCTCAATCTCTCCCTAGAGAACAGAACCCCGCC
AGCCTGGAATTACAAGAGTAGAC
>2195
GGGCTCTGCGTCAACGCTAGTGCCGTCAGCCGCCTGCGCGCCTACCTGCTGCCAGCGC
CGCCAGCTCCAGGTGAGCCGCCCGCCAGGTGCGCTGCGTGCAGCACCGCCACTGGCG
CCGAAGGGCCTGGGGGTTGCTGGGT
>2196
AGTTGACTACGAGTCTCAGAGCACAGATACCCAGAACTTCTCCTCCGAGTCCAAGCGG
GAGACAGAATATGTGAGAGCTTTTCCTCTTGTTAAAGGAGGAGGGCAAGACCTGCCAA
GCCTGGGTACTCAGAGCCTCTTGA
>2197
CAATGTGCTGAGTCCCAGGGGTGTACACATTCCCAACTGTGACAAGAAGGGATTTTAT
AAGAAAAAGCAGGTGAGTGAGGTCCTCAGTGTGTTTTCTTCCTCTTCTGTTGACACAG
AGGAGAAACCCATGTCACCAGCGC
>2198
GCGCATGAGACCCTGGCTAGAGATGCAGATTAATTCCAACCAAATCCCGGGGCTCATCT
GGATTAATAAAGTGAGTGTAACTCTTTGGGTTTTCCTGCCACTGTTTTAACCCATGTACT
TCTGGAGGGACCAAAGCTTCA
>2199
CATGCTGCCAAGCATGGCTGGGACATCAACAAGGATGCCTGTTTGTTCCGGAGCTGGG

CCATTCACACAGGTGTGTGCCTGGGACTCAGGCCTAGGAAGCCCAGGGTAGAGACAA
GAGGAGGCACTCACGTTAACACAGA
>2200
AGGAACAAGGGCAGCTCAGCTGTGCGAGTGTACCGGATGCTTCCACCTCTCACCAAG
AACCAGAGAAAAGGTATCCAAGGACTCTGGGTCCTTGGGAAGCCCTCAGGGAGGGAG
GGTAGAAGGAGGTCAGCTGGGGCTGG
>2201
GATGACCACAGCAGCTACACAGTTCCAGGCTACATGCAGGACTTGGAGGTGGAGCAG
GCCCTGACTCCAGGTGAGCTGGTCCAGGTCTGGCAGGAGACCCCACAGGTCAGTGGG
ATGACTCTTTCTCTTGGAGGCATGGT
>2202
GAAGTTGTGCCGGACAGCACCAGTGATCTGTACAACTTCCAGGTGTCACCCATGCCCT
CCACCTCTGAAGGTTGGTGCTCCTGGGGCCTGGCCTGCTGCTTGACTGTCTGGGTCTG
TGAAGGGCTTCCTGAGAGAGAAAA
>2203
GGAGTCCAGCCCACCTCTGTCTATGGAGACTTTAGCTGTAAGGAGGAGCCAGAAATTG
ACAGCCCAGGGGGTAAGAAGGCCCTGGATCCTTATGGCTTCTTAGATGAGGGAGAACC
ACGTAGGGATGGAGAAAGCTTGGG
>2204
GAGTCCATGAAGAAGATGGCTCCTGCCATGGAATCCCCTACTCTACTGTGTGTAGCCTT
ACTGTTCTTCGGTAAGTAGAGATTCAATTACCCCTCCCAGGGAGGCCCAAATGAATTTG
GGGAGCAGCTGGGGTAGGAACC
>2205
AGTGGAGAATACAAATGTCAGCACCAACAAGTTAATGAGAGTGAACCTGTGTACCTGG
AAGTCTTCAGTGGTAAGTTCCAGGGATATGGAAATACAGATCTCTCATGTGAGGGATGG
CTCATCTGAAGATGGGAAAAAAC
>2206
TACTACTGTACGGGCAAAGTGTGGCAGCTGGACTATGAGTCTGAGCCCCTCAACATTA
CTGTAATAAAAGGTGAGTTGGTAAAGGAAAGGAAAAGCATCCATAGCAGGGGAAGGA
AGAGAGAACTTCTGAGCCTGAGCAG
>2207
CTGCACGCCCTCACCCGCGGCCAAGGCGCCTGCGTGCAGGAGTCTGACGCCTCCGCT
CCCCATGCTGCAGGTACCACAGTCCCGCCCCTGCTCCAGCACCTCCTGCCGCCCGCCC
CCGCCCGGCACCTCCCGCCCCCACT
>2208
CCTTTGGGACGCCATCAGTACCTATGATGGCTCGAAGGCTCTCCATGTCACCAACATCA
AAAAATGGAAGGTGAGGCCCAGCAGGTGGGTGGTGGGAACTCTCCTGTCAGAGGATG
TAGCTTGAGTCGGCTGTGACAAGC
>2209
ATCAGGAGAAGAAATTTCCAAATTTTACCTGCCAAACTGCAACAAGAATGGATTTTATC
ACAGCAGACAGGTAGGTGGCCTTGCCAGTGTGCGTCGTCAGGGTGAAAGGGACTACT
GCCCTACATTCCTGCCAAGCCACG
>2210
CTGGTTCCCTGCCCACAGACCTTCCAGGAGAATGACCTGAGCACCTTCTTTCCCTTCAT

CTTTGAAGAAGGTAGTTAGCCAAGAGCAGGCAGTAGATCTCCACTTGTGTCCTCTTGG
AAGTCATCAAGCCCCAGCCAACT
>2211
AGCTTGGTGATGTCTGGTCCATATGAACTGAAAGCTCTCCACCTCCAGGGACAGGATAT
GGAGCAACAAGGTAAATGGAAACATCCTGGTTTCCCTGCCTGGCCTCCTGGCAGCTTG
CTAATTCTCCATGTTTTAAACAA
>2212
GGGCCTCAAGGAAAAGAATCTGTACCTGTCCTGCGTGTTGAAAGATGATAAGCCCACT
CTACAGCTGGAGGTAAGTGAATGCTATGGAATGAAGCCCTTCTCAGCCTCCTGCTACCA
CTTATTCCCAGACAACCACCTTC
>2213
AAGCAAAAACTTTCACTTAAGACCCAGGGACTTAATCAGCAATATCAACGTAATAGTTC
TGGAACTAAAGGTAAGGCATTACTTTATTTGCTCTCCTGGAAATAAAAAAAAAAAAGT
CAGGGGGAAAAGTACCACATTTT
>2214
TACAAAGAAAACACAGCTACAACTGGAGCATTTACTGCTGGATTTACAGATGATTTTGA
ATGGAATTAATGTAAGTATATTTCCTTTCTTACTAAAATTATTACATTTAGTAATCTAGCTG
GAGATCATTTCTTAATAAC
>2215
AAGCAAAAACTTTCACTTAAGACCCAGGGACTTAATCAGCAATATCAACGTAATAGTTC
TGGAACTAAAGGTAAGGCATTACTTTATTTGCTCTCCTGGAAATAAAAAAAAAAAAGT
AGGGGGAAAAGTACCACATTTTA
>2216
CTGCCCCTGCTGGGAGTGGGGCTGAACACGACAATTCTGACGCCCAATGGGAATGAA
GACACCACAGCTGGTGGGAAATCTGGGACTGGAGGGGGCTGGTGAGAAGGGTGGCT
GTGGGAAGGGGCCGTACAGAGATCTGG
>2217
TCGAGTACATGAATTGCACTTGGAACAGCAGCTCTGAGCCCCAGCCTACCAACCTCAC
TCTGCATTATTGGTATGAGAAGGGACGAGGGGGAGGGGATGAAGAAGAGGTGGGTTG
GATCAGAGACCAAGAGAGAGGGTAG
>2218
GTTGTTCAGCTCCAGGACCCACGGGAACCCAGGAGACAGGCCACACAGATGCTAAAA
CTGCAGAATCTGGGTAATTTGGAAAGAAAGGGTCAAGAGACCAGGGATACTGTGGGA
CATTGGAGTCTACAGAGTAGTGTTCT
>2219
CAACAGATTCTTGAACCACTGTTTGGAGCACTTGGTGCAGTACCGGACTGACTGGGAC
CACAGCTGGACTGTGAGTGACTAGGGACGTGAATGTAGCAGCTAAGGCCAAGAAAGT
AGGGCTAAAGGATTCAACCAGACAG
>2220
CTCTGTGGAAGTGCTCAGCATTGGAGTGAATGGAGCCACCCAATCCACTGGGGGAGC
AATACTTCAAAAGGTAAAATGGGCCCACATGACCCAATCCATGAGCCCAACACCCCAG
CCTTTCTAACACCACTGTCTTTTGC
>2221
CCGTGGTTATCTCTGTTGGCTCCATGGGATTGATTATCAGCCTTCTCTGTGTGTATTTCTG

GCTGGAACGGTGAGATTTGGAGAAGCCCAGAAAAATGAGGGGAACGGTAGCTGACAA
TAGCAGAGGAGGGTTTTGCAGG
>2222
GACGATGCCCCGAATTCCCACCCTGAAGAACCTAGAGGATCTTGTTACTGAATACCAC
GGGAACTTTTCGGTGAGAACGCTGTCATAAGCATGCTGCAGTCTATCAACTGCCAACT
GCCTGCCAGCAAGACAGACAGAGT
>2223
CCACGGACACAAGTGCGATATCACCTTACAGGAGATCATCAAAACTTTGAACAGCCTC
ACAGAGCAGAAGGTGAGTACCTATCTGGCACCATCTCTCCAGATGTTCTGGTGATGCTC
TCAGTATTTCTAGGCATGAAAAC
>2224
CCACAGGCACAAGCAGCTGATCCGATTCCTGAAACGGCTCGACAGGAACCTCTGGGG
CCTGGCGGGCTTGGTAAGCTGCACTGTATTCCTGGCAAGCCGGCCGCGTGGCTCCTGG
TGGACAGCAGCCTCACTTCTAAACA
>2225
GCAAGGGGGTACTGTGGAAAGACTATTCAAAAACTTGTCCTTAATAAAGAAATACATT
GACGGCCAAAAAGTAAGTTACACACATTCAATGGAAGCTATATTTGTCCTGGCTGTGCC
TATTTCTATGGAATTGACAGTTT
>2226
CACAAGTGCATTGGTGAAAGAGACCTTGGCACTGCTTTCTACTCATCGAACTCTGCTG
ATAGCCAATGAGGTAATTTTCTTTATGATTCCTACAGTCTGTAAAGTGCATAGGTAATCA
TTTGTGATGGTTCCTTTACTAT
>2227
GCAAGGGGGTACTGTGGAAAGACTATTCAAAAACTTGTCCTTAATAAAGAAATACATT
GACGGCCAAAAAGTAAGTTACACACATTCAATGGAAGCTATATTTGTCTGGCTGTGCCT
ATTTCTATGGAATTGACAGTTTC
>2228
CGTGGCAGGCCAGGGGTGTCCAACCTTGGCGGGGATCCTGGACATCAACTTCCTCATC
AACAAGATGCAGGTAGGCTGCAGGGGGAGCCCATGGGAAAGACAGCTACTGACAAAG
TGAAATATGTATGAGGATGAAAAAA
>2229
AACAAGATACCCACTGATTTTCAGTCGGGTGAAAAAATCAGTTGAAGTACTAAAGAAC
AACAAGTGTCCAGTAAGTTTGTTTTCATATGTGATATGTTCCTGTTGGTGATTTCTATGT
GAATGGTGATGCCAACCCTGTT
>2230
GGTGACAAATCACCTCCAGGTTGGGGATGCCTCAGACTTGTGATGGGACTGGGCAGAT
GCATCTGGGAAGGTGAGTCTGTGCTTTGGGCTTCCCAACCTCTCAAGTCAGCATGAAA
TTCAGAAGGCAGAGAGGGACATGT
>2231
GCATCCCAGAGCCCCGCTCCTGGGTACCCACAACATGAACACCGTCCAGAAGCAAGG
GCAGCCTCTGCAGGTGGGGCGGGGGTTGGAAAACATTTATCAAACGGTTGAGTTGGGT
GCAGGGGATGCAACATGATCAAACA
>2232
CAGAGAACTTGCCACGGTGTTTCATGCTGTGGCTGGTGGTTCCAGGCTGCACGCTCCA

TTCTAGGAAAGGGTGAGGCTTCCTGATCATCAGTCTTAACAGGGGACTGTCCTATGGGT
ACCTGTATACGCTGCCGGGAGTG
>2233
TGGCTCCTGGCCTGCATCTGCATCTGCACCTGTGTCTGCTTGGGAGTCTCTGTCACAGG
GGAAGGACAAGGTGAGGGCTGGGCACTAATGTCTGTATGAGGTGGGTGGAGAACTAG
GGCATGTTTGGGGGACTGGGTTGT
>2234
TCAGGATCGATTGCCACTGGTCTGCCCCAGAGCTGGGACAGGGCTCCAGCCCCTGGCT
CCTCTTCACCAGGTGAGCATGGAGGGCCATGCCCACCTGGACAGGGATGAGGGTGAG
TTCCCCAGGATTGAAGCAGCTATGC
>2235
TTCCACCACTGCATGTCTGGGAGGGAGCAGGTCAGCCTGGTGGACCCGGAGTACCTGC
CCCGGAGACACGGTGAGCAGCAGCTATAGGTCTGGGGCGGGGCCGCTTGGCAAGAAC
ATCCTGGCTGCTTGGGGGTTTGGAG
>2236
TGCCTTGGAGCCAATGACCACACTTCTCAGCTATGAGCTGGCCTTCAAGAAGCAGGAA
GAGGCCTGGGAGGTAACACTTTGGCTGGCTTTCCCTGGGGGCCTCTCTCCTGGGAACA
GCAGTCCAGGGTAGACTCCCCACT
>2237
GAGGAGCGTTATACAGGCCAGTGGAGTGAGTGGAGCCAGCCTGTGTGCTTCCAGGCT
CCCCAGAGACAAGGTGGGCACTGCTGTGGCTGCTGCACTTCCAGCGGAGTCTGGGCT
GGGCGTCTTCTCCCCTGTTCACCTCA
>2238
CCCTTGTTGCTGTGTCCATCTTTCTCCTGCTGACTGGCCCGACCTACCTCCTGTTCAAG
CTGTCGCCCAGGTAGGTGGCTGATGTGTGCGTGTGTGTACATGTGTGAGCGGGCAAGA
GTGTGCATGTTAGTGTATGTGTG
>2239
CTACCAGAACGTGCCCTCTCCAGCGATGTTCTTCCAGCCCCTCTACAGTGTACACAATG
GGAACTTCCAGGTGTGTGCAGAGACCACAGAAGGACATGGGGGGCAGGGGTTGCCCA
GAGCTCTGGCCTGCCCAAGATGTT
>2240
CGTCCTACGTGCCAGACGACGGACTCACAGCACAGCAGCTCTTCAACTGCGGAGACG
GCCTCACCTACAAGTGCGGGCCTATGGGGCTCACCTGGGCGAAGAGAGGGGCGGCAC
CTGGGGAAAAGGCCAGATGGGGGCTT
>2241
TCTTAAGACCCCACTGGTTTCCTCTCCCATGGACACAGTCACAGAGGCTGGGATGGCC
ATAGCAATGGCGGTGAGCCCCATGGGGTGTGGGGGAAGGAGCAAGGACTCCATCGCC
TTTCCCCAAAGGCATTCAGTCCTGC
>2242
AGGCGGTATTGGCTTCATCCACCACAACTGTACACCTGAATTCCAGGCCAATGAAGTTC
GGAAAGTGAAGGTCAGAAGGGCAACGATCATTAGCAAGCGCTCCTGGGAATTGCACT
GAGGTGGGGTGGGGTGGGAGTAGG
>2243
GGTGGGCATCATCTCCTCCAGGGACATTGATTTTCTCAAAGAGGAGGAACATGACTGT

TTCTTGGAAGAGGTGGGTGCCACTGGCAGAGTAGATCCAAGCTCTAGCAGCCCAGGTT
GAAGACAAGGGGCTTCTGTTGTCT
>2244
GACTTGGTGGTAGCCCCTGCAGGCATCACACTGAAGGAGGCAAATGAAATTCTGCAGC
GCAGCAAGAAGGGTAAGTCCTAGAGCTGGGAAAGGGCCTGGAACTAATGCCTAGGGT
CCTGATTCATGTCCTGCCCTGACCA
>2245
GGCACTCATGAGGATGACAAGTATAGGCTGGACTTGCTCGCCCAGGCTGGTGTGGATG
TAGTGGTTTTGGGTGAGCCTGCTACACAGGTGGGTTGGGGCAATAGGGGCAGCTGCCA
CATGACAGTCTGAGACTTACTTCT
>2246
ATCTTCCAGATCAATATGATCAAGTACATCAAAGACAAATACCCTAATCTCCAAGTCATT
GGAGGCAATGGTAAGGCAAGATTGTGCCCTGAAGGATGTGTGGTGGGAGTGGGTAAG
CTGGGCTCCCACCTTAACCTTCA
>2247
CTCATTGATGCAGGTGTGGATGCCCTGCGGGTGGGCATGGGAAGTGGCTCCATCTGCA
TTACGCAGGAAGGTAAGAATATACTTTGATAGGGCCCACAGAGACCCCAGTTTCAGGC
CCCACATACCTTGGAACCAAGCAC
>2248
AGAAATATCGCGGTATGGGTTCTCTCGATGCCATGGACAAGCACCTCAGCAGCCAGAA
CAGATATTTCAGGTGGGACAGGCAAGCCAGTTACCCCACCCTAATCCTGCACTGACAG
TCTCATCTCTGCTGTGACCTTGCC
>2249
TCCCTTACCTGATTGCTGGCATCCAACACTCATGCCAGGACATTGGTGCCAAGAGCTTG
ACCCAAGTCCGGTGAGCTTGGGGAGCTGGGACATGGGTAGAGGGGCTGGTCCAGGGC
CAGCTACCCACATTTGACCTCTGC
>2250
CTGGGGAGCTTAAGTTTGAGAAGAGAACGTCCTCAGCCCAGGTGGAAGGTGGCGTCC
ATAGCCTCCATTCGTAAGTCACCCTGTCCTTGGTGGGGCCTCTGCCATACCTCATGCCTC
CTTTCTCCTGCCCTCACCTCACA
>2251
CGAGTATAACAAGGCCACCAAAGATGACTACTACAGACGTCCGCTGCGGGTACTAAGA
GCCAGGCAACAGGTAGGTGCTCCCTCCACCCCAGGGGTCCTGGGTCCCAGCCTGGTTT
GTTCCCCAACCCCCAAGAGCATTC
>2252
AACCATATGTACCAAGTCCCAGCCCAACTTGGACACCTGTGCCTTCCATGAACAGCCA
GAACTGCAGAAGGTACGTTCCTGATGCAGGTCCCGGGCCAGTCATGCACTGCAGAGG
GGTGCGTATGTGTCAGCCTCTGCCC
>2253
CCCACACCTGAAGGCAGCCCATATCCCTGAGAATGCCAAGGACCGCATTCCTGGAATT
GTGCCCATGCAGGTGAGACCCAAGAGAGACCTGGCTGAACCCAGTCCCGGGAGTGAG
TTGACCCATTGTCCGCACATGCAGG
>2254
TCTCCAGGCTGCTGGTGGAGCACATCTGGAAGAAGATCATGCACACGGATGCCATGAT

CATCGACATGAGGTCAGTGGCCAGGGGTCAGTGCTTCCTAGCCAGGACGCAGGGCTG
CCAGGGGACAGTCAAAGCTATGGGC
>2255
CTGCTGGACAAGATCTACAGCCGGCCTGATGACTCTGTCAGTGAACTCTGGACACACG
CCCAGGTTGTAGGTACGTGGAGAAGCTTTCTCCTTTCTGCTGTCATTCTAGAAGCTTCT
GGGAAACCAGGGAAAGACAGCTT
>2256
GCACTTTCCAGTGTGGGGGCATCCTGGTGCACCGCCAGTGGGTGCTCACAGCTGCTCA
TTGCATCAGCGAGTGAGTAGGGGCCTGGGGTCTGGGAGGAGGGCATCTATGCTGCCAC
AGGATTGACAGGCCAGTGGCATTC
>2257
CCCACCCAGGAACCCGAAGTGGGGAGCACCTGTTTGGCTTCCGGCTGGGGCAGCATC
GAACCAGAGAATTGTATGTGGGGGCAGACTGTGTAGCCCAAGGCGGGGATGGGGACT
CCTGCGTCCAAGGGAGAAAGGGCCAG
>2258
CCACGTCCAGAAGGTGACAGACTTCATGCTGTGTGTCGGACACCTGGAAGGTGGCAA
AGACACCTGTGTGGTGAGGCAGCCCTGCCCCCAGGGTCTGGAAGGGCTGAGGGAGGG
GACTCAGCCTCTGAACTGGCTTCTGA
>2259
GATGGGCACACTGTGGGGGTGTCCTGGTGCACCCCCAGTGGGTGCTCACAGCTGCCCA
TTGCCTAAAGAAGTAAGTAGGACCCTGGGATCTGGGGAGGGAATGGCTGTGTCCCACA
GGAATAACAGCGGGATGCTTCCCC
>2260
CCCACCCAGGAGCCAGCACTGGGGACCACCTGCTACGCCTCAGGCTGGGGCAGCATC
GAACCAGAGGAGTGTACGCCTGGGCCAGATGGTGTAGCTGGGAGCCCAGATGCCTGG
GTCTGAGGGAAGTGGGGCCAAAGAAC
>2261
TTACTCTGAGAAGGTGACAGAGTTCATGTTGTGTGCTGGGCTCTGGACAGGTGGTAAA
GACACTTGTGGGGTGAGTCATCCCTACTCCCAACATCTGGAGGGGAAAGGTGAGTGA
AGACCCTAATTCTGGGCTGCAATCT
>2262
ATTGTGGATTTGGTCAGTGAGCTCAAGAAGGACGTCTTGATGGTGCTGGTGAATTACAT
TTACTTCAAAGGTGAGAGTCAGATCATTGGTATATGCTAAATCCACACCACCGCCTCCA
ACCCACTAATTTGTTGACTGGT
>2263
AAATGAGGGAGATTGAAGAGGTTCTGACTCCAGAGATGCTAATGAGGTGGAACAACTT
GTTGCGGAAGAGGTAATCAGTGTGCTATGGGGGCTGAATCTACAGTACTATCCATCAAA
TGTAACTTTCTAATGAAAGACAG
>2264
CACGGATCTGTTCTCCAAGTGGGCTGACTTATCCGGCATCACCAAACAGCAAAAACTG
GAGGCATCCAAAGTAAGTCGTCAACAGTCAGCAATCCCTAGAGAACTCGGTAGGGCA
GTGCCCATGGGAGCTGCCAGGCGAT
>2265
GAGGCAGCGGCCTGCTGAGATCAAAGACTACAGTCCCTACTTCAAGACCATTGAGGAC

CTGAGGAACAAGGTGGGTGAATGGGCAGCAGAAGGCACCATTCCAGCTAGCTCCTTC
TGGGAACAATTCATGCCCCAGGCCG
>2266
CAGTGGACAATGCCAATGTCCTTCTGCAGATTGACAATGCCCGTCTGGCCGCGGATGA
CTTCCGCACCAAGTGAGTTTGAAATGGTGGGCCAGAACATCCAGTGTCCCCAGAGTAG
GGCATTTTTGGAGCAGTGTTTCCC
>2267
AGCTGACCTGGAGATGCAGATTGAGAGCCTGAAGGAGGAGCTGGCCTACCTGAAGAA
GAACCACGAGGAGGTGAGAACTATATGGAAAAGTCAGCTTAAAAGAAATGCAGGGAG
GCTGGGTACAGTGGTGCGTGCCCATA
>2268
GATGCGTGACCAGTATGAGAAGATGGCAGAGAAGAACCGCAAGGATGCCGAGGAATG
GTTCTTCACCAAGGTGGGTGTCATTTGAGGTGGAAGGAACCCAGACCACCTGCCTTCT
GGGGCCTTCTGGTGTGAATGGCATT
>2269
GAGCGAGATCTCGGAGCTCCGGCGCACCATGCAGAACCTGGAGATTGAGCTGCAGTC
CCAGCTCAGCATGGTAGGAATAGTGCCAGGAAGGGTGGTGCACCCAGGACTGGCAGG
GAGAGAACGGCCACACTCACTAATCG
>2270
TGGACGTGAAGACGCGGCTGGAGCAGGAGATCGCCACCTACCGCCGCCTGCTGGAGG
GCGAGGACGCCCAGTGAGTCTTGGCCCTCCCCTTAGTCCGCCCCCCCCATGGCACTCT
CACGGCCCCACCATGTATCTAATGA
>2271
AAAGGTGAAGTCTCGAGAAAGGGAGCAAATCAAGTCACTCAACAACCAATTTGCCTC
CTTCATTGACAAGGTGAGTTTCTCTCTCATTGCACTGGTAGCTGCGCCGGTCCACTCGG
GATCGGTGCAGTCAAAACACACGC
>2272
AGAGTGATCAATCTCGGTTGGATTCGGAACTGAAGAACATGCAGGACATGGTGGAGGA
TTACCGGAACAAGTAAGGGACCCTGTCTGGGCAGTTCTTAACTTTTGCTGTAAAAAAG
AGTTCCAAGAAAAGTAATAAGCTA
>2273
GGTGGACCTTCAGGCCAAACTTGACAACTTGCAGCAGGAAATTGATTTCCTTACAGCA
CTCTACCAAGCAGTAAGTCTTCCAGTTTCAACCAAGTTTATCTAAATGGAGAGTTTTTA
AGCCGGAACCCACAACGATCTAG
>2274
GGTCAAGGCCCAGAACGAGGATATAGCCCAGAAGAGCAAAGCTGAGGCCGAGTCCTT
GTACCAGAGCAAGGTGAGTGGCTGAAAGCCTAGCCAGTTTCCCTGAAATGGCTTGTCT
TGCTATCCTGTGTTATCTCATGTAT
>2275
GATAGAAATTTCTGAGCTGAATCGTGTGATCCAGAGACTTAGATCTGAAATCGACAATG
TCAAGAAGCAGGTATGTGCTTTCTCCTTCTACCACTCAGCTGTATGGAATGGGGGTAAC
CCTACAGGTAAAGGGCGAGTGC
>2276
TGAACACCAAGCTGGCCCTGGATCTGGAGATTGCCACCTACAGGACCCTCCTGGAGGG

AGAAGAAAGCAGGTGAGGAAGGGACGCTGGGAGTACTCGCCCCCTTCCCCTGGTCCT
TCCTTCCTTGCCACCGAATGATGAT
>2277
GTAATCTTCCGAGGTGCAAATTCTCAGAAAAATGCATTCCGGAAAAATCCCAGTGATCC
AAAGCTTGCACGTAAGTATTGATTTATGTTTATATTATATATATTACATGAAGGTTTTATTT
TTCTTTTTGTCTCGACATC
>2278
TGGGGCTTTGTTCGCCACCCCAATTACTTGGGTGATCTCATCATGGCCTTGGCGTGGTC
CCTCCCATGTGGTAAGCATTTCAGAAGAGTGCTAGTTTGACCCTTTTAGCCGCCAGAAA
GATGTGTGTGTGTGTCCTTTGT
>2279
TACTCGTTCCCAGAAAGGAAGTTCTGGAGACCAGGAAATGACAGCTACACTTCTCACA
GCAGGGTTCCAGGTGAGTTGTGCTCCTTCTCACTGAAACGCCATAGCTGTGCTTGTCA
GTGTTTGTGGCATCTGCCCTATAT
>2280
CTTGATCCATCACAGAAGGATCTGTGTAGAGATAACAGGCCAGAAAATTTCAGAAACA
TGTTCTCCCTGGGTAAGGAGAGGTGTGGTTATTATTGTCATTTTAGATTTTTTGTTTGTT
TGTTGTTTATGTGTATAGTAGC
>2281
CAGGTCTCCACTGCTGCCCTTGCTGTCCTCCTCTGCACCATGGCTCTCTGCAACCAGTT
CTCTGCATCACGTGAGTCTGAGTTTCGTTGTGGGTATCACCACTCTCTGGCCATGGTTA
GACCACATCAATCTTTTCTTGT
>2282
GGCAGATTCCACAGAATTTCATAGCTGACTACTTTGAGACGAGCAGCCAGTGCTCCAA
GCCCGGTGTCATGTAAGTGCCAGTCTTCCTGCTCACCTCTATGGAGGTAGGGAGGGTC
AGGGTTGGGGCAGAGACAGGCCAG
>2283
GCGTGTCCATCTGTGAGAAGGAGCCAGAGCCCAAGGGAGATGATGGAGCCTGAGGAG
TACAGAGAGAGAGGTGAGCAACGGGCATCCCTGGCCCTCTTCCCTGCTGCTACTCCTG
CAGGAACGGCTGCTGGTGGGTGGCC
>2284
GAGTGCTCCTGAGGACCCCGACAGCTGGGACAGCATCTTTGGGGACATTGAACGAATC
ATCATGCCTGGGGTGAGACACAGTGACCACAAGGGCAGTTCTGGATTTCAGGGCACA
GAAGGGTGGGTGGTGGCCTGGAGAA
>2285
CCTCACCTCTTGGCCCTCCCTGCTAGGAGACATGCTGGCTGATGCCATCAACTGCTTGG
GATTCACCTGGGTGAGTAGCAACGGCTGTAACTACTCGCAATGGGGAACGTAGCAGGG
AGTGGTATCCTAGGGCAGACATA
>2286
AAAAATGCTGGGACTTCCAGAGCACTTCTTGCACCACCACCCCAGCAGCCAGGGCGG
AGGCGTCCTGCAGGTACCTCCCTTGGCAAAGCTTCACCTAGCTTGGGGGCTAAGTAGC
GAAAGACAAGCCAAACCCTAACCTG
>2287
GAAAACGTCTGAGCCCGATGCTGATGAGTCCTGCCTAAATGCCCGACTCGTGGCCTAT

GCCTCTGACCAGGTGAGTTGCCCGACGCAGGACAGCCCTTCGGGTTGGTGCTTGGCTT
CTGGAATCTTGGTCCTTGAGCAAT
>2288
CTCACTCCGAGGGGAAGCTCTTCAGAAGGCCATCGAGGAAGACAAGCAGCGGGGCTT
GGTGCCCGTCTTTGTAAGTCAGGATATATGCCTAAGCTTAGAATGATAGAACTTGGATG
TGAGGAAGGAATTTTGGCAGAATT
>2289
AGTATGCCGACTCCTTCACCTTTAATCCTTCCAAGTGGATGATGGTGCATTTTGACTGTA
CTGGGTTCTGGTGAGTGTAGCAGCCCAGCTCCGAGCACGCAGGAACGCCTTGCCTCCT
CTGGAGAGACCTCAGCCACTAA
>2290
GCAGCAGACCTTCAGTGTGAATCCCATCTACCTCAGGCATGCCAACTCAGGCGTGGCC
ACCGACTTCATGGTGAGTGGCCAGGGACGGGCAGCCTGGTGGGCTCGGGTGGCCAGT
GGGAACTGTAGGCGTCTGACCCCCC
>2291
TCGCTCTGTTAAACTCTGGTTCGTGATTCGGTCCTTCGGGGTGAAGAATCTTCAAGCAC
ATGTCAGACATGTATGTAGCATGCTGCTGTGGGGGCTGAGCCTGTGTTCCTCTGTTTTC
TCATGGCGAGGAAGGCTGGTTT
>2292
GGTCAGAAACGACCCTTCCTTTGAAATTCCTGCCAAGAGGCACCTTGGCCTGGTGGTT
TTTCGTCTAAAGGTAATACCATCTTCCAAGCCCCTCTGTGAATGATGTCTTGTGGTGCTC
CAGAGCCTCTCGGAAACACAAA
>2293
GTAAACATGACTTCCAAGCTGGCCGTGGCTCTCTTGGCAGCCTTCCTGATTTCTGCAGC
TCTGTGTGAAGGTAAGCACATCTTTCTGACCTACAGCGTTTTCCTATGTCTAAATGTGAT
CCTTAGATAGCAAAGCTATTC
>2294
TCCACCCCAAATTTATCAAAGAACTGAGAGTGATTGAGAGTGGACCACACTGCGCCAA
CACAGAAATTATGTAAGTACTTTAAAAAAGATTAGATATTTTGTTTTAGCAAACTTAAAA
TTAAGGAAGGTGGAAATATTTA
>2295
ATGGAAGAGAGCTCTGTCTGGACCCCAAGGAAAACTGGGTGCAGAGGGTTGTGGAGA
AGTTTTTGAAGAGGTAAGTTATATATTTTTTAATTTAAATTTTTCATTTATCCTGAGACAT
ATAATCCAAAGTCAGCCTATAA
>2296
AACCACACCCTGGCCCAGACTGCCAGCCCTGACGGGACAGAGCCAGGGCACTCACCA
GGCTGCAAGAACAGTGCTGGGGTAAGAGGGGAGCGGGGGATCCCGGGCCTGGGACC
CAGCCTGCATTCCTTTGTTCATTCCTT
>2297
AACTCTCCACCCTGGGAACTGCCCCGCCCACTTTGGCCCTGCACTACCACCCTCACCC
CTTCCTCAACAGGTAGTGGGGATCTGGGGTGGGGGGCAGTGGGGATTGGGGGCCAGG
GTCCTTGCCCACAAGGACTTAGTGA
>2298
CCTTTTAGCATCTTCCCTAGCAGCCGGTTGAAGCGGAGACCAAGCCACTGTGAGCTGG

ACCTGGCTGAGGGTGAGTGTGGGTTTGTGTGCCTTGTGGGTTTGTATGCCTGTATGTGC
ACTTGTGGGTGCACAGAAGGCCT
>2299
CTTGTTGCTGGCGGTGCCTATCACTGTCCTGGCTGTGCTGGCCTTAGTGCCCCAGGATC
AGGGAGGACTGGTGAGTGGCTGCAACAGGCCCTGGTGGAGAGTTGTATCTTGCGGAT
GCTTGGCTCCCTCTGGTTGTGCCT
>2300
TTTCAGAAGCTGCCAGAGGAGGAGCCAGAAACAGATCTCAGCCCCGGGCTCCCAGCT
GCCCACCTCATAGGTAAGGACCTCCAAGACCTGAATAAGAGTGTAAATAATCCGAAGG
TTCCAGTTCTGCTCGCCCAGAGTCC
>2301
TTCAGGGGACAGGCCAACCCAGAGGACAGGATTCCCTGGAGGCCACAGAGGAGCAC
CAAGGAGAAGATCTGTAAGTAGGCCTTTGTTAGAGTCTCCAAGGTTCAGTTCTCAGCT
GAGGCCTCTCACACACTCCCTCTCTC
>2302
AGATTCTCGCCCTGAGCAACGGCCTGACGTCGGCGGAGGGAAGCAGGCGCAGGCTCC
GTGAGGAGGCAAGGTAAGATGCCGAGGGAGGACTGAGGCGGGCCTCACCCCAGACA
GAGGGCCCCCAATAATCCAGCGCTGCC
>2303
ACTTCTCAGGCTCAGTCGCCACCACCTCACCCCGCCACCCCCCGCCGCTTTAACCGCA
GGGAACTCTGGCGTAAGAGCTTTGTGTGACCAGGGCAGGGCTGGTTAGAAGTGCTCA
GGGCCCAGACTCAGCCAGGAATCAA
>2304
CAGGGGCCCCATCTGGTCGACAGATGCAGTGGTTCTAGGATCTGCCAAGCATCCAGGT
GGAGAGCCTGAGGTAGGATTGAGGGTACCCCTGGGCCAGAATGCAGCAAGGGGGCCC
CATAGAAATCTGCCCTGCCCCTGCG
>2305
GACGAAACCATGAAGAGCCTGATCCTTCTTGCCATCCTGGCCGCCTTAGCGGTAGTAA
CTTTGTGTTATGGTGAGAAACTTTTCTCCCATTTCTCTGTGTTTACTTTTCTGCCTCTGA
CTTTGGCTTACTTCTATTTTTC
>2306
TCATTAACAGGAGAAATGCAAATACCTTCATATCCCCTCAGCAGAGATGGAGAGCTAA
AGTCCAAGAGAGGTCAGTAACAAAACTTCATGAGGAGTGGTCATTTTTCCCAGTGTAG
ATCACAGATCTGAATTGGAGTGGG
>2307
GTGGTCATGGCGCCCCGAACCCTCTTCCTGCTGCTCTCGGGGGCCCTGACCCTGACCG
AGACCTGGGCGGGTGAGTGCGGGGTCAGGAGGGAAACGGCCCCTGCGCGGAGGAGG
GAGGGGCCCGCCTGGCGGGGGCGCAG
>2308
AAGGCCCACGCACAGACTGACAGAATGAACCTGCAGACCCTGCGCGGCTACTACAAC
CAGAGCGAGGCCAGTGAGTAACTCCGGCCCAGGGAGCAGATCACGACCCCCACCTCC
ATGCCCCACGGACGGCCCGGGTACTC
>2309
CTGGAGGGCACGTGCGTGGAGTGGCTCCACAGATACCTGGAGAACGGGAAGGAGATG

CTGCAGCGCGCGGGTACCAGGGGCAGTGGGGCGCCTCCCTGATCTCCTGTAGACCTCT
CAGCCTGGCCTAGCACAAGGAGAGG
>2310
GGAGAGGAGCAGAGATACACGTGCCATGTGCAGCATGAGGGGCTGCCGGAGCCCCTC
ATGCTGAGATGGAGTAAGGAGGGAGATGGAGGCATCATGTCTGTTAGGGAAAGCAGG
AGCCTCTCTGAAGACCTTTAACAGGG
>2311
CTGGTTGTCCTTGCAGCTGTAGTCACTGGAGCTGCGGTCGCTGCTGTGCTGTGGAGAA
AGAAGAGCTCAGGTAAGGAAGGGGTGACAAGTGGGGTCTGAGTTTTCTTGTCCCACT
GGGGGTTTCAAGCCCCAGGTAGAAG
>2312
GCCGTCATGGCGCCCCGAACCCTCGTCCTGCTACTCTCGGGGGCTCTGGCCCTGACCC
AGACCTGGGCGGGTGAGTGCGGGGTCGGGAGGGAAACGGCCTCTGTGGGGAGAAGC
AACGGGCCGCCTGGCGGGGGCGCAGG
>2313
AAGGCCCACTCACAGACTCACCGAGTGGACCTGGGGACCCTGCGCGGCTACTACAAC
CAGAGCGAGGCCGGTGAGTGACCCCGGCCCGGGGCGCAGGTCACGACCTCTCATCCC
CCACGGACGGGCCAGGTCGCCCACAG
>2314
CTGGAGGGCACGTGCGTGGAGTGGCTCCGCAGATACCTGGAGAACGGGAAGGAGACG
CTGCAGCGCACGGGTACCAGGGGCCACGGGGCGCCTCCCTGATCGCCTGTAGATCTCC
CGGGCTGGCCTCCCACAAGGAGGGG
>2315
GGACAGGAGCAGAGATACACCTGCCATGTGCAGCATGAGGGTTTGCCCAAGCCCCTCA
CCCTGAGATGGGGTAAGGAGGGAGACGGGGGTGTCATGTCTTTTAGGGAAAGCAGGA
GCCTCTCTGACCTTTAGCAGGGTCA
>2316
CTGGTTCTCTTTGGAGCTGTGATCACTGGAGCTGTGGTCGCTGCTGTGATGTGGAGGA
GGAAGAGCTCAGGTGGGGAAGGGGTGAAGGGTGGGTCTGAGATTTCTTGTCTCACTG
AGGGTTCCAAGACCCAGGTAGAAGT
>2317
GTGCTCATGGCGCCCCGAACCCTCCTCCTGCTGCTCTCAGGGGCCCTGGCCCTGACCC
AGACCTGGGCGCGTGAGTGCAGGGTCTGCAGGGAAATGGTCGGGAGGAGCGAGGGG
CCCGCCCGGCGGGGGCGCAGGACCCA
>2318
AAGGCCCAGGCACAGACTGAACGAGAGAACCTGCGGATCGCGCTCCGCTACTACAAC
CAGAGCGAGGGCGGTGAGTGACCCCGGCCCGGGACGCAGGTCACGACCCCTCCCCAT
CCCCCACGGAGGGCCGGGTCGCCTCG
>2319
CTGGAGGGCGAGTTCGTGGAGTGGCTCCGCAGATACCTGGAGAACGGGAAGGAGACG
CTGCAGCGCGCGGGTACCAGGGGCCACAGGGCGCCTCCCGGATGGCCTGTAGATCTCC
GGGGCTGGCCTCCCACAAGAAAGGG
>2320
GGAGAGGAGCAGAGATACACCTGCCATGTGCAGCATGAGGGTCTGCCAGAGCCCCTC

ACCCTGAGATGGGGTAAGGAGGGAGATGGGGGTGTCATGTCCCTTAGGAAAAGCCGG
AGCCTCTCTGGAGAGCTTTAGCAGGG
>2321
CTGGTTCTACTTGTAGCTGTGGTCACTGGAGCTGTGGTCGCTGCTGTAATGTGGAGGA
AGAAGAGCTCAGGTAAGGAAGGGGTGAGGAGTGTGGTCTGAGATTTCTTGTCTCACT
GAGAGTTCCAAGCCCCAGGTAGAAG
>2322
CGGGTCACGGCGCCCCGAACCCTCCTCCTGCTGCTCTGGGGGGCAGTGGCCCTGACC
GAGACCTGGGCTGGTGAGTGCGGGGTCAGGCAGGGAAATGGCCTCTGTGGGGAGGA
GCGAGGGGACGCAGGCGGGGGCGCAGG
>2323
AAGGCCAAGGCACAGACTGACCGAGAGGACCTGCGGACCCTGCTCCGCTACTACAAC
CAGAGCGAGGCCGGTGAGTGACCCCGGCCCGGGCGCAGGTCACGACTCCCCATCCCC
CACGTACGGCCCGGGTCGCCCCGAGT
>2324
GGAGAAGAGCAGAGATACACATGCCATGTACAGCATGAGGGGCTGCCGAAGCCCCTC
ACCCTGAGATGGGGTAAGGAGGGGGATGAGGGGTCATATCTCTTCTCAGGGAAAGCA
GGAGCCCTTCAGCAGGTCAGGGCCCC
>2325
CGGGTCACGGCGCCCCGAACCCTCCTCCTGCTGCTCTGGGGGGCAGTGGCCCTGACC
GAGACCTGGGCTGGTGAGTGCGGGGTCGGCAGGGAAATGGCCTCTGTGGGGAGGAGC
GAGGGGACCGCAGCGGGGCGCAGGAC
>2326
AAGGCCAAGGCACAGACTGACCGAGAGGACCTGCGGACCCTGCTCCGCTACTACAAC
CAGAGCGAGGCCGGTGAGTGACCCCGGCCCGGGGCGCAGGTCACGACTCCCCATCCC
CCACGTACGGCCCGGGTCGCCCCGAG
>2327
CTGGAGGGCGAGTGCGTGGAGTGGCTCCGCAGATACCTGGAGAACGGGAAGGAGAC
GCTGCAGCGCGCGGGTACCAGGGGCAGTGGGGAGCCTTCCCCATCTCCTATAGGTCGC
CGGGGATGGCCTCCCACGAGAAGAGG
>2328
GGAGAAGAGCAGAGATACACATGCCATGTACAGCATGAGGGGCTGCCGAAGCCCCTC
ACCCTGAGATGGGGTAAGGAGGGGGATGAGGGGTCATATCTCTTCTCAGGGAAAGCA
GGAGCCCTTCAGCAGGGTCAGGGCCC
>2329
CTGGCTGTCCTAGCAGTTGTGGTCATCGGAGCTGTGGTCGCTGCTGTGATGTGTAGGA
GGAAGAGCTCAGGTAGGGAAGGGGTGAGGGGTGGGGTCTGAGTTTTCTTGTCCCACT
GGGGGTTTCAAGCCCCAGGTAGAAG
>2330
AAGGCCTCCGCGCAGACTTACCGAGAGAACCTGCGGATCGCGCTCCGCTACTACAACC
AGAGCGAGGCCGGTGAGTGACCCCGGCCCGGGGCGCAGGTCACGACTCCCCATCCCC
CACGTACGGCCCGGGGTCGCCCCGA
>2331
GCGCCCACCATCGCGTCGCAGCCCCTGTCCCTGCGCCCAGAGGCGTGCCGGCCAGCG

GCGGGGGGCGCAGGTGAGGCGTGCGGGGGCCCGGGGCGGGGGCGGCACGCACCGCG
GGGAGAGCCCATGCCAGGGACAGCCCT
>2332
TGGGCGCCCCTGGCCGGGACTTGTGGGGTCCTTCTCCTGTCACTGGTTATCACCCTTTA
CTGCAACCACAGTAAGTCCCGAGGAATCGCGGCGGGAAGGAGTCGGCCCCGCTCCTG
CCCTTGGCTCTGCTAGCTCCAGGC
>2333
CGGGTCACGGCGCCCCGAACCGTCCTCCTGCTGCTCTGGGGGGCAGTGGCCCTGACC
GAGACCTGGGCCGGTGAGTGCGGGGTCGGGAGGGAAATGGCCTCTGTGGGGAGGAG
CGAGGGGACCGCAGGCGGGGGCGCAGG
>2334
AAGACCAACACACAGACTTACCGAGAGAGCCTGCGGAACCTGCGCGGCTACTACAAC
CAGAGCGAGGCCGGTGAGTGACCCCGGCCCGGGGCGCAGGTCACGACTCCCCATCCC
CCACGTACGGCCCGGGGTCGCCCCGA
>2335
CTGGAGGGCCTGTGCGTGGAGTGGCTCCGCAGATACCTGGAGAACGGGAAGGAGACG
CTGCAGCGCGCGGGTACCAGGGGCAGTGGGGAGCCTTCCCCATCTCCTATAGGTCGCC
GGGGATGGCCTCCCACGAGAAGAGG
>2336
GGAGAAGAGCAGAGATACACATGCCATGTACAGCATGAGGGGCTGCCGAAGCCCCTC
ACCCTGAGATGGGGTAAGGAGGGGGATGAGGGGTCATATCTCTTCTCAGGGAAAGCA
GGAGCCCTTCTGGAGCCCTTCAGCAG
>2337
CTGGCTGTCCTAGCAGTTGTGGTCATCGGAGCTGTGGTCGCTACTGTGATGTGTAGGAG
GAAGAGCTCAGGTAGGGAAGGGGTGAGGGGTGGGGTCTGGGTTTTCTTGTCCCACTG
GGGGTTTCAAGCCCCAGGTAGAAG
>2338
CTCCCCATCTATCTGCTTGGCCTGACTCAGAAATTCGGGCCCATCTACAGGCTCCACCT
TGGGCTGCAAGGTGAGAGGCTGATCTCGCTCTGGCCCTCACCATAGGAGGGGGCGGA
GGTGACGGAGAGGGTCCTCTCTCC
>2339
AAGAGGACCATTGAGGAAGCCATGGTCAAAAAGTGGGCAGACTTTGCTGGCAGACCT
GAGCCACTTACCTGTAAGGGCTGGGGGCATTTTTTCTTTCTTAAACAAATTTTTTTTTAA
GAGATGGGTTCTTGCTATGCTGC
>2340
AGCCCTGCTGCTGGGCATCCGTGACTCCATGGAGCCAGTGGTGGAGCAGCTGACCCA
GGAGTTCTGTGAGGTAAGGCTGGGCTCCTGAGGCCACCTCGGGTCAGCCTCGCCTCTC
ACAGTAGCCCCCGCCCTGCCCGCTG
>2341
CATTGAGGAGGAATTCTCTCTCCTCACCTGCAGCATCATCTGTTACCTCACCTTCGGAG
ACAAGATCAAGGTGCCTCACAGCCCCTCAGGCCCACCCCCAGCCCCTCCCTGAGCCTC
TCCTTGTCCTGAACTGAAAGTAC
>2342
TATCCAGGAGGTGTTAAAAACCTGGAGCCACTGGTCCATCCAAATTGTGGACGTGATT

CCCTTTCTCAGGGTGAGGACCTGGAGCCTAGACACCCCTGGGTTGTAGGGGAGAGGC
TGGGGTGGAGGGAGAGGCTCCTTCC
>2343
TCTCCGGAGGCTGAAGCAGGCCATAGAGAAGAGGGATCACATCGTGGAGATGCAGCT
GAGGCAGCACAAGGTGGGGACTCTACGTGGACGGCCTCCCCTCGGCCCACAGCCAGT
GATGCTACCGGCCTCAGCATTGCTAT
>2344
GATCGGTGGCACTGAGACCACAGCAAACACCCTCTCCTGGGCCGTGGTTTTTTTGCTT
CACCACCCTGAGGTGCGTCCTGGGGACAAGCAAAAGGCTCCTTCCCAGCAACCTGGC
CAGGGCGGTGGGCACCCTCACTCAG
>2345
TCGCCGAGGTGCTGCGCCTGCGGCCCGTTGTGCCCTTAGCCTTGCCCCACCGCACCAC
ACGGCCCAGCAGGTGACTCCCGAGGGTTGGGGATGAGTGAGGAAAGCCCGAGCCCA
GGGAGGTCCTGGCCAGCCTCTAACTC
>2346
ATCATTCCGAACCTCCAAGGCGCCCACCTGGATGAGACGGTCTGGGAGAGGCCACATG
AGTTCTGGCCTGGTATGTGGGGGGCCGGGGGCCTGCCGTGAAAATGTGGTGGAGGCT
GGTCCCCGCTGCCGCTGAACGCCTC
>2347
CGGGTCATGGCGCCCCGAACCCTCATCCTGCTGCTCTCGGGAGCCCTGGCCCTGACCG
AGACCTGGGCCTGTGAGTGCGGGGTTGGGAGGGAAACGGCCTCTGCGGAGAGGAAC
GAGGTGCCCGCCCGGCAGGCGCAGGA
>2348
AAGCGCCAGGCACAGACTGACCGAGTGAGCCTGCGGAACCTGCGCGGCTACTACAAC
CAGAGCGAGGCCGGTGAGTGACCCCGGCCCGGGGCGCAGGTCACGACCCCTCCTCAT
CCCCCACGGACGGCCCGGGTCGCCCC
>2349
CTGGAGGGCACGTGCGTGGAGTGGCTCCGCAGATACCTGGAGAACGGGAAGGAGAGC
CTGCAGCGCGCGGGTACCAGGGGCAGTGGGGAGCCTTCCCCATCTCCCGTAGATCTCC
CGGCATGGCCTCCCACGAGGAGGGG
>2350
GGAGAAGAGCAGAGATACACGTGCCATGTGCAGCACGAGGGGCTGCCGGAGCCCCTC
ACCCTGAGATGGGGTAAGGAGGGGGATGAGGGGTGATGTGTCTTCTCAGGGAAAGCA
GAAGTCCTGGAGCCCTTCACGCGGGT
>2351
GCTGTCCTGGCTGTCCTAGCTGTCCTAGGAGCTGTGGTGGCTGTTGTGATGTGTAGGAG
GAAGAGCTCAGGTAGGGAAGGGGTGAGGAGTGGGGTCTGGGTTTTCTTGTTCCACTG
GGAGTTTCAAGCCCCAGGTAGAAG
>2352
CGGGTCATGGCGCCCCGAACCCTCATCCTGCTGCTCTCGGGAGCCCTGGCCCTGACCG
AGACCTGGGCCTGTGAGTGCGGGGTTGGGAGGGAAACGGCCTCTGCGGAGAGGAGC
GAGGGGCCCGCCCGGCGAGGGCCAGG
>2353
AAGCGCCAGGCACAGACTGACCGAGTGAACCTGCGGAAACTGCGCGGCTACTACAAC

CAGAGCGAGGCCGGTGAGTGACCCCGGCCCGGGCGCAGGTCACGACCCCTCCCCATC
CCCCACGGACGGCCCGGGTCGCCCCG
>2354
CTGGAGGGCGAGTGCGTGGAGTGGCTCCGCAGATACCTGGAGAACGGGAAGGAGAA
GCTGCAGCGCGCGGGTACCAGGGGCAGTGGGGAGCCTTCCCCATCTCCTGTAGATCTC
CCGGGATGGCCTCCCACGAGGAGGGG
>2355
GGAGAAGAGCAGAGATACACGTGCCATGTGCAGCACGAGGGGCTGCCGGAGCCCCTC
ACCCTGAGATGGGGTAAGGAGGGGGATGAGGGGTACTGTGTCTTCTCAGGGAAAGCA
GAAGTCCTGGAGCCCTTCACGTGGGT
>2356
GCTGTCCTGGCTGTCCTAGCTGTCCTAGGAGCTGTGGTGGCTGTTGTGATGTGTAGGAG
GAAGAGCTCAGGTAGGGAAGGGGTGAGGAGTGGGGTCTGGGTTTTCTTGTCCCACTG
GGAGTTTCAAGCCCCAGGTAGAAG
>2357
CGGGTAGCAACTGTGACCTTGATGCTGGCGATGCTGAGCACCCCGGTGGCTGAGGGCA
GAGACTCTCCCGGTAAGTGCAGGGCCACTGCTCTCCAGAGCCGCCACTCTGGGAACA
GGCTCTCCTTGGGCTGGGGTAGGGG
>2358
CGGGCGGAGTTGGACACGGTGTGCAGACACAACTACCAGTTGGAGCTCCGCACGACC
TTGCAGCGGCGAGGTGAGCGTCGTCGCCCGTCTGCGAGGCCCATCCTTGGCAGGGGC
CCAGAGTCTCTGCCGCAGGAGGGGCG
>2359
CAGCGTGGAGACGTCTACACCTGCCACGTGGAGCACCCCAGCCTCCAGAACCCCATCA
TCGTGGAGTGGCGTAAGGGGATATTGAGTTTCTGTTACTATGGGCCCCACAAGACAAA
GGGCAGAGCTCCTTCTGACCCATT
>2360
ATTGGAGGCTTCGTGCTGGGGCTGATCTTCCTCGGGCTGGGCCTTATTATCCATCACAG
GAGTCAGAAAGGTGAGGAACCCCAAGGGAAAAGGGGAAGATGGGCTGTGACCCAGA
CCCTCTGTTCAGGGAGGTCCTGTCT
>2361
AGGGACACCGCACAGATTTTCCGAGTGAACCTGCGGACGCTGCGGCGCTACTACAATC
AGAGCGAGGCCGGTGAGTGACCCCGGCCAGGAGCAGGTCACGACCCCTCCCCATCCC
CCACGGACGGCGCGGGTCCCCTCGA
>2362
CTGGAAGACACATGCGTGGAGTGGCTCCACAAATACCTGGAGAAGGGGAAGGAGACG
CTGCTTCACCTGGGTAAGAGGGTCCACAGGGCTACTCTCCCATCTCCTTCTTGGGCTAG
GACTGTGCCCACAGCTGACAGACC
>2363
GGAGAGGAGCAGAGATACACGTGCCATGTGCAGCATGAGGGGCTACCCGAGCCCGTC
ACCCTGAGATGGAGTAAGGAGGGGGATGGGAGGTCATGTCTCTTCTCAGGGAAAGCG
GGAGCCCTTCTGGAGCCCTTCCGCAG
>2364
CTGGTTCTCCTTGGATCTGTGGTCTCTGGAGCTGTGGTTGCTGCTGTGATATGGAGGAA

GAAGAGCTCAGGTGGGGAAGGGAGAAGGGTGGGGTCTGAGTTTTCTTGTCCCACTGG
GTGTTTCAAGCCCTAGGTAAAAGT
>2365
TGGGATCATGGTAGTAGGAACCCTCCTTTTACTCCTCTCGGAGGCCCGGCCCTTACCCA
GACCTGGGCGGGTGATGCGGGGTCGGGATGGAAACGGCTTTACCGGGAGTTACACAG
GGGCCGGCCGGCGGGGGCGAAGGA
>2366
AGGGAAACCGCAAAGATTTTCCGAGTGAATCTGCGGACCGTGCGGGCTTACTACAATC
AGAGCGAGGCCGGTGAGTGACCCCGGCCAAGGGAGCAGGTCACGACCCCTCCCCATC
CCCCACGGACGGCGCGGGTCCCCTC
>2367
CTGGAAGACACATGCGTGGAGTGGCTCCACAAATACCTGGAGAAGGGGAAGGAGACG
CTGCTTCACCTGGGTAAGAGGGTCCACAGGGCTACTCTCCATCTCCTTCTTGGGCTAGG
ACTGTGCCCACAGCTGACACACCT
>2368
GGAGAGGAGCAGAGATACACGTGCCATGTGCAGCATGAGGGGCTACCCGAGCCCGTC
ACCCTGAGATGGAGTAAGGAGGGGGATGGGAGGTCATGTCTCTTCTCAGGGAAAGCG
GGAACCCTTCTGGAGCCCTTCCGCAG
>2369
CTGGTTCTCCTTGGATCTGTGGTCTCTGGAGCTGTGGTTGCTGCTGTGATATGGAGGAA
GAAGAGCTCAGGTGGGGAAGGGAGAAGGGTGGGGTCTGAGTTTTCTTGTCCCACTGG
TGTTTCAAGCCCTAGGTAAAAGTG
>2370
GGGGTCATGGCGCCCCGAACCCTCCTCCTGCTGCTCTCGGGGACCCTGGCCCTGGCCG
AGACCTGGGCGGGTGAGTGCGGGGTCAGGAGGGAAACGGCCTCTGCCGTGAGGAGC
GAAAGGTCCGCCTGGCTGGGGCGCAG
>2371
GGAGAGGAACAGAGATACACATGCCATGTGCAGCACAAGGGGCTGCCCAAGCCCCTC
ATCCTGAGATGGGGTAAGGAGAGAGATGGGGGCGGTCATGTCTCTTAGGGAAAGCAG
GAGCCCCTCTGGAGACCTTTAGCAGG
>2372
GGTATCATTGCTGGCCTGGTTCTCCTTGGAGCTGTGGTCACTGCTGTGATGTGGAGGAA
GAAGAGCTCAGGTGGGGAAGGGGTGAGGAGTGGGGTTTGAGTTTTCTTGTCCCACTG
GGGGTTTCAAGCTCCAGGTAGAAA
>2373
GGGGTCATGGCGCCCCGAACCCTCCTCCTGCTGCTCTCGGGGACCCTGGCCCTGGCCG
AGACCTGGGCGGGTGAGTGCGGGGTCAGGAGGGAAACGGCCTCTGCCGTGAGGAGC
GAAAGGTCCACCTGGCTGGGGCGCAG
>2374
AAGGCCCAGGCACAGACTGACCGAGTGAACCTGCGGACCCTGCTCCGCTACTACAAC
CAGAGCGAGGCGGGTGAGTGACCCCGGCCCGGGGCGCAGATCACTTACTCCCCGCTC
CATGCCTCACGGACGGCCCTGGTCCC
>2375
CTGGAGGGCACCTGCATGGAGTGGCTCCGCAGACACCTGGAGAACGGGAAGGAGAC

GCTGCAGCGCGCGGGTACCAGGGGCCATGGGGAGCCTGCTCGATCTCCTGTAGATCTC
CCGGGCTGGCCTCGCACAAGGAGGGG
>2376
GGAGAGGAACAGAGATACACATGCCATGTGCAGCACAAGGGGCTGCCCAAGCCCCTC
ATCCTGAGATGGGGTAAGGAGAGAGATGGGGGCGGCCATGTCTCTTAGGGAAAGCAG
GAGCCCCTCTGGAGACCTTTAGCAGG
>2377
CTGGTTCTCCTTGGAGCTGTGGTCACTGGAGCTGTGGTCACTGCTGTGATGTGGAGGA
AGAAGAGCTCAGGTGGGGAAGGGGTGAGGAGTCGGGTTTGAGTTTTCTTGTCCCACT
GGGGGTTTCAAGCTCCAGGTAGAAA
>2378
GCCAGGGGCTTCATGCTGGGGCTCATCATCTGTGGAGTGGACATCTTCACGCACAGAA
GGAGCAAGAAAGGTGAGAAATCCTGTGAGGTGACCGATACCCACCTTTCTCCTGACTT
GCTCACCCTTCTTCCATGATGAGG
>2379
CTCCGTGCCGGACGGGTTCCTCTCCGAGCTCACCCAGCAGCTGGCGCAGGCCACCGG
CAAGCCCCCCCAGGTTTGCCGGGAGGGGACAGGAAGAGGGGGGTGCCCACCGGACG
AGGGGTTCCGCGCTGGGAGCTGGGGAG
>2380
CGCAGAACCGCTCCTACAGCAAGCTGCTGTGCGGCCTGCTGGCCGAGCGCCTGCGCAT
CAGCCCGGACAGGTACGCGGAGTCGCGGAGGGGCGGGGGAGGGGCGGCGGCGCG
GCCAGGCCCGGGACTGAGCCACCCGC
>2381
CTACGGCGGCTGGGGGCCTGGCTGCGGGACCCTGGGGGGCAGCGCCTGGTGGTCCTA
CACCTGGAGGAAGGTATGTGGGGCCCAGCCCCAAGCTTGGCACCGCCGTCTTCCTTCA
GGTGGGCCGGGTCCTCCTAGGGAAG
>2382
GCTGCTGGTGCTGTACCCTGGGCCTGGCCCTGAGGTCACTGTGACGAGGGCTGGGCTG
CCGGGTGCCCAGGTACCAGGGAGTTGCATGGGGCAGTGCCCGGGCCGTGGCGGGGGG
CATGAATTTGTTGCAGGGTCTGCAG
>2383
GCGGTGGACCGCCCTGCGGGGGCCTGGCGCGGCTCCGGGCTGGCCTTGACCCTGCAG
CCCCGCGGAGAGGGTAGGTCCGCGTGGAGAGGGACGGGGAGCCGGGTCGACTGCCC
CCGGGCCCCCAGCCCCTGAGCCAGCCG
>2384
TGCCGCGGTCCGAGCCCGCGCCGCTGCCTGCGCACGGCCAGCTGGACACCGTGCCCT
TCCCGCCGCCCAGGTGCGCGCAGGCACCGGGACACGGGGCAGGAGCGGGCGGGGGC
GGCGTGGCCTCGTGGCCGCTCTCAACT
>2385
CACCGAGGCTTCCTCCTCCTCACCCTCCTCGCCCTGCTGGCGCTCACCTCCGCGGTCG
CCAAAAAGAAAGGTTATGGGGGATGATCGAAGGAGGGCTGGGGACGGGCAGGCGAG
GCCCCTCCACTTCTGGGCTGGGCCGC
>2386
GGCACCTGCGGGGCCCAGACCCAGCGCATCCGGTGCAGGGTGCCCTGCAACTGGAAG

AAGGAGTTTGGAGGTGAGGCGGGGGGCAGTCAGAGGGCAGGAGACGGGGGCACAGC
CTCGCCGAAGCCTGGGGGGACCCTTGG
>2387
AATGCTCAGTGCCAGGAGACCATCCGCGTCACCAAGCCCTGCACCCCCAAGACCAAA
GCAAAGGCCAAAGGTCAGCGAAAGGAGAAGGGGGTGGGGCTGTCGCGGGGGGCTGC
CCCCCCCCCCCCCGCCTGTGAGGGGAC
>2388
CACCGAGGCTTCCTCCTCCTCACCCTCCTCGCCCTACTGGCGCTCACCTCCGCGGTCGC
CAAAAAGAAAGGTGATGGGGGATGATCGAAGGAGGGCTGGGGACGGGCAGGCAGGC
CCCTCCACTTCTGGCTGGCCGCCTG
>2389
GGCACCTGCGGGGCCCAGACCCAGCGCATCCGGTGCAGGGTGCCCTGCAACTGGAAG
AAGGAGTTTGGAGGTAGGCGGGCGCAGTCAGAGGGCAGAGACGGGGGCACAGCCTC
GCCGAAGCCTGGGCGGACCCTTGGCGG
>2390
AATGCTCAGTGCCAGGAGACCATCCGCGTCACCAAGCCCTGCACCCCCAAGACCAAA
GCAAAGGCCAAAGGTCAGCGAAAGGAGAAGGGGGTGGGGCTGTCGCGGGGGGCTGC
CCCCCCCCCCCCCGCCTGTGAGGGGACA
>2391
GCACCCAGACACCCTGAACCAGGGGGAATTCAAAGAGCTGGTGCGAAAAGATCTGCA
AAATTTTCTCAAGGTAGGGCTGGACTCTGGCAGGTCTGACCCAGCCTCACCGCAGTTT
GGGTTGACAAGGGAGGATGGGAGTA
>2392
GAATTTCCATGCCGTCTACAGGGATGACCTGAAGAAATTGCTAGAGACCGAGTGTCCT
CAGTATATCAGGGTGAGGAGGGGCTGGGTGTGGCGGGGGCTCTCTGCCTGGTCCTGGG
GCTGCCCTGGGCCAGCGGTCCTCC
>2393
GAATTAAGTGAAACTGCCAGCATACTCATGCATGCAACAGCACATTCTCTGGTGCTTGT
GGATGAATTAGGTAAGACATTAAACTTCTCATTTGAAGACTATCTTAAAAACATTTGTAC
AAATAACTATTTTTATAGAAG
>2394
ATTTTCAACTCACTACCATTCATTAGTAGAAGATTATTCTCAAAATGTTGCTGTGCGCCT
AGGACATATGGTATGTGCAAATTGTTTTTTTCCACAAATTCGGTTTTTTGAGAGGGCAC
TTCTGTTGCTAGCACATGTAT
>2395
AAGTTATTCAAAAGGGACATAGAAAAGCAAGAGAATTTGAGAAGATGAATCAGTCACT
ACGATTATTTCGGTAACTAACTAACTATAATGGAATTATAACTAACTGACCTTAAGTTTC
AAAGAAACAGTAAAAGGGGAAG
>2396
TTCCCTTCTCGCTTGGGAACTCTAGTCTCGCCTCGGGTTGCAATGGACCCCAACTGCTC
CTGTGCCGCTGGTAAGGGACGCCCGGGTTCTGTGCCTTGGAATGCCAAATTCCCAGAC
ACCATAGAGAGTGTCCCTGGGTT
>2397
CCCGCTTCTCTGAAAGGCTCTCCTTGCAGCTGCTTAGATACTGAATTTTTTTCGGGAAG

TGGAAAACCAGGTAAGCATCGAAGTCCACTTGTCTTTTAATTTACTTTTTATCAGTTTA
ATTCTGAGATGAGTCGAATGC
>2398
GCAGCGCATCCTGGCGACGGAGGGCGACATGGAGCTGGACGAGGCCGGCGACGTCAT
CGGCGTGCTGCGGGTGCGGAGGGGCGGGCCGGGCCTGAGCGTGCGGGGTTCCTGCTA
AGGTCTCCGAGTCGGGTTCTGATCCA
>2399
CTCAGCGGCTGCCCCACCATGGCTACCTGGGCCCTCCTGCTCCTTGCAGCCATGCTCCT
GGGCAACCCAGGTAAGGCCTTCCCCTCGGGATCGATCCTGATGGCCCACCCAGCCTCG
CACTCTCAGGCTGGCTGAACCTG
>2400
CGACCTGGCAAGAGCCCACCTGCGTGATGAGGAGAAATCCTGCCCGTGCCTGGCCCA
GGAGGGCCCCCAGGTACGTGTTGGCTCTCTGCTCACCTGCCACAGTCCCTCTCCTTTCC
CTCCTCCCTGGTGGCTCCTGGGGT
>2401
GGGCCGTGACTACAGGACCTGTCTGACGATAGTCCAAAAACTGAAGAAGATGGTGGAT
AAGCCCACCCAGGTGAGGCCAAGGGGCTACAGAGCCTCCTGTCTGCTGCTCAATGGA
GGGGCCAGCCTGTGACCAGGTCGGG
>2402
CAGGGCCTCGTGGCCGGAGAAACTGCCCAGCAGATCTGTGAGGACCTCAGGTTGTGTA
TACCTTCTACAGGTGAGTGCAGAGGTGACAGCAGGGATACCTCCTGAGGGTTGGAGAC
AGCTTCCCCCAGGATATATCAAAG
>2403
ATTGCAGCGGACATCCCAGAAGTGGTTGTTTCCCTTGCATGGGACGAAAGCTTGGCTC
CAAAGCATCCAGGTGAGAGAGGCAGGCATGCAGAGCTGCTAAGTCTAGAGGGAAGGA
CGGGAGAGAGGTTCCAGAGTTGGGT
>2404
CTTCTTCTGGACTCATCTGCGCCACTTGTCCGCTTCACACTCCGCCGCCATCATGGTGA
AGCTCGCGAAGGTAAACGGCCTTGAGCGCGACGCAGACGTGTAGGCCTGCTTCCGAG
GGGCGAGCGCGGCGCCGCGGGGAG
>2405
GGAGGTAGAAGAAGATAGTGAAGATGAGGAAATGTCAGAAGATGAAGAAGATGATAG
CAGTGGAGAAGAGGTAATTTTATCCAACTTAATGCAGAATTATGTTAAAACTACAAAAT
GGAGAGTTAAGACATGAAATTGGA
>2406
CCTGCCTCAGAGGATGAGGACGATGAGGATGACGAAGATGATGAGGATGACGATGAC
GATGAGGAAGATGGTAAGGAGTTGTCTTGGTAGTTACTGGGCTTCTGATTACAAGGTAT
CTTGAGATTCTGGGATCACATATT
>2407
GACGACGACGAAGATGATGAAGATGATGATGATGAAGATGATGAGGAGGAGGAAGAA
GAGGAGGAGGAAGGTACTTAAATTAGATTCTGACATACGACATGAGTTATGTTTAAAGG
AGGCACTTAAGTGTTTGTGGCTAC
>2408
CCTGGAAAACGAAAGAAGGAAATGGCCAAACAGAAAGCAGCTCCTGAAGCCAAGAA

ACAGAAAGTGGAAGGTAACTTGCAGAATTAGGGGATATGGGGGAGATAAACAGCACA
AATGATGAATAACAAAGGGACTTAATA
>2409
AAACTGGTATCAGCGATGTTTTTGCTAAAAATGATCTTGCTGTTGTGGATGTCAGAATT
GGTATGACTAGGTAGCTGCTTCACTGCACGTTACATACCGTGGGTCTGTTAATTTTTCCT
TCCCCTGTTAGCACAGTTACT
>2410
CTCACTGGTTTGAAAGTCTTTGGCAATGAAATTAAACTAGAGAAACCAAAAGGAAAA
GACAGTAAGAAAGGTATGTAAGGCTTTATGAGTTATGCAATGAACTCAGGAGCTAGAC
TGCTAGGGAAAATGCTTTGTAACCC
>2411
ATGAATTGAAAGAAGTGTTTGAAGATGCTGCGGAGATCAGATTAGTCAGCAAGGATGG
GAAAAGTAAAGGGTATGTTCTTCTATTGAAATGTAAGGGTTTTATTAACATTAATGCACT
TCCTGCTTTATAAAAGAAATAT
>2412
TCCCTGTACTATACTGGAGAGAAAGGTCAAAATCAAGACTATAGAGGTGGAAAGAATA
GCACTTGGAGTGGTAAGAAATTAGGCTTGTTCCAAGGTTTTCAGAATTGGTTGAGGGA
ACTCTTCTAGTCTTTGTATTTCAT
>2413
CTCTTCAGGAAGTATTTGAGAAAGCAACTTTTATCAAAGTACCCCAGAACCAAAATGG
CAAATCTAAAGGGTAAGATAATACCTTTGTATCATCAGTTATAGGCCTATATATGTCTTAG
AGGTCTAAGGACGTAAGGTCA
>2414
AGGGAAATTGAGGGCAGAGCAATCAGGCTGGAGTTGCAAGGACCCAGGGGATCACCT
AATGCCAGAAGCCGTAAGTTCACCTGGTTAGGGTGCTGTGGTTGGGGGTAGCACTCTC
GGTGCTTTGTTTATTTTTGCACAAA
>2415
TAAAGGAGTCATTTGACGGCTCCGTTCGGGCAAGGATAGTTACTGACCGGGAAACTGG
GTCCTCCAAAGGGTAAGGGAAGGAAGCGTGAGTGCTGCTTCCACTTGAAGGGGTTTT
TGTTCTGTGCAGACCTTGAGTCTAA
>2416
TTTGGAGGACGAGGTGGTGGTAGAGGAGGCCGAGGAGGATTTGGTGGCAGAGGCCGG
GGAGGCTTTGGAGGTAAGGCACGCAGAGATAATGACACCACATAGCATGTGCTCTTCA
GACCCTGTGCCCTGTCACGGTTCCT
>2417
CGGCTGCGACTCAGGCTCCGGCGTCTGCGCTTCCCCATGGGGCTGGCCTGCGGCGCCT
GGGCGCTCTGAGGTGAGGGACTCCCCGGCCGCGGAGGAAGGGAGGGAGCGAGGGCG
GGAGCGGGGCGGGCTGCGGGCCCCGG
>2418
GAATTCCTGGAGAGTTGCCTTTGTGAGAAGCTGGAAATATTTCTTTCAATTCCATCTCTT
AGTTTTCCATGTAAGTATTCAGTTTACATTTATGTTGCAGGTTAATCTTAAGAATTGTATT
GCTAAGGCTTCTAAGTGAA
>2419
TCCTTATCCCAGTTGATTGTGCAGAATACACTGCCTGTCGCTTGTCTTCTATTCACCATG

GCTTCTTCTGGTAGGTAATCTATTTGGAAAATCTGAAATTGTAATGGGCTTATGATTTTA
GATTGAGATGGCTCAGGTCT
>2420
TCCAAAGAAGAAGGATCTTTCCCTGGAGGAAATTCAGAAGAAATTAGAAGCTGCAGA
AGAAAGACGCAAGGTAAACGAAGCAATTCACAGAAAGCAGGATATTAATTTATGTAAT
GGGCAGATCAATTTTATTTCTATAA
>2421
CAAAATGGAAGCTAATAAAGAGAACCGAGAGGCACAAATGGCTGCCAAACTGGAACG
TTTGCGAGAGAAGGTTGGTTTCTTACTTTGTAAAAGGGTTGAGCTTGGAGTTTGATGC
ACCAATGAGTTGGCTTGAACTAAGT
>2422
GAGCACAGCATCGTCGGGACCAGACTCGTCTCAGGCCAGTTGCAGCCTTCTCAGCCA
AACGCCGACCAAGGTACAGCTTCAGTTTGCTACTGGGTTGTGCATTCAGCTGAATTTCA
TGGGGAAGTCCAAATTCTAAGGAA
>2423
CTCCGCCTGCTACATCCAGAACTGCCCCCTGGGAGGCAAGAGGGCCGCGCCGGACCT
CGACGTGCGCAAGGTGAGTCCCCAGCCCTGGTCCCGCGGCGCTCCGGGGAGGGAGGG
ACCCGCAGCCACAGGGGCGCGCCCCG
>2424
CCCTGCCAGTCCGGCCAGAAGGCGTGCGGGAGCGGGGGCCGCTGCGCCTTGGGCCTC
TGCTGCAGCCCGGGTGAGCGGGGCAAGGCGCTCCGGGGCCAGGGGGAGGCGGGCGG
GGGTGCGGCCGGGATTCCCCTGACTCC
>2425
CACACCAGTTGAAGCAGCATGTCATCGATGGAGAAAAAACCATTATCCAGAATCCCAC
AGACCAGCAGAAGTAGGTGCCAACCCTACTTATTACCTTCTACCACACAAGACTAGTC
ATCAGGTTTTTTTCTATACTCCCA
>2426
TGAAAAAGCTGAATTTGAGGTACATGAAGTATATGCTGTGGATGTTCTCGTCAGCTCAG
GAGAGGGCAAGGTGAGGAGAGTACCAGAGTTGGCAAAGAGGGGTGACTGAGAGTGT
TCACCAGACCAAATGTTACTTAAAT
>2427
AAATGAAAACTTCACGTGCCTTCTTCAGTGAGGTGGAAAGGCGTTTTGATGCCATGCC
GTTTACTTTAAGGTAACTAAGCAAATGATAGTACTTGGAACCAGTCTGGACTCACAGAC
CATACACCCAGGAACACTTTTTC
>2428
CGGATGGGTGTGGTGGAGTGCGCCAAACATGAACTGCTGCAACCATTTAATGTTCTCTA
TGAGAAGGAGGGTGAGTTCCAAAAAGAGCTTCACTTTGGATTCCCTGATTATAAGATAT
CCTTCCTGTAAGTTCAGAAGAG
>2429
GATAACCAGTGGTCCCTTCGAGCCTGACCTCTACAAGTCTGAGATGGAGGTCCAGGAT
GCAGAGCTAAAGGTTAGTATGGAATAGAAGGTGGTGAGTATGTACATGGTATCCTGCTT
GGGAGTGAGGGCTAAATGCCAGC
>2430
CGGCCACCACCAGCTGGCCAAGGAGGTGCTTATTAAGAAGGGCAAGGACTTCTCTGG

GCGGCCTCAAATGGTAAGTGGTGCCCATCTCCTCCCTGCCCCCTTCACCACCCCCTGGG
ATTGGTTCAGGTCTTCAGACCATG
>2431
CTGCATCGAAGGCTGGCGATGGCCACCTTTGCCCTGTTCAAGGATGGCGATCAGAAGC
TGGAGAAGATCAGTGAGTGCCAGGCTGGCCCCTGGGGCTGGGGCTGGATCCCACAGG
AGCTGCTGGAGGGAGAGGAGGGTTG
>2432
GAATTACAATGAAGGCATCATAGACAACCTGAGCAAAGACAGCCTGGTGGACCTAGTC
CCCTGGTTGAAGGTGAGATGCTGCCAGCCCTGCCTTCAGGTTCTAGTAGACCCTGACA
TTGTCCCCAATCTTCCTTCCTTTT
>2433
CCTGGAAAAATTAAAGAGCCATGTTAAAATACGAAATGATCTGCTGAATAAAATACTTG
AAAATTACAAGGTAGGTGATAGAGCAGAAGAGAATATGAGTTAGGCTAAAAGGAATCA
CAAGAGCAGGGTGGAGTCCATTC
>2434
TGGGGCTGGCGTGGAGACCACCACCTCTGTGGTTAAATGGACCCTGGCCTTCCTGCTG
CACAATCCTCAGGTGTGCTTCCCCCTCATTGATCCTAGACCCCAGCCAGCCCAATCTCT
GGGCTCCAGAGAAAGGGAGAGCC
>2435
TCCGAGAGGTGCTTCGCCTCAGGCCCGTGGCCCCTATGCTCATCCCCCACAAGGCCAA
CGTTGACTCCAGGTGTGCCTGCCCTCCCAGTGACATCTAGCCCCATGATGCATTCACTG
CTTGCCAGCCCACCTGGCTCCCC
>2436
GTTATCATCAATCTGTGGGCGCTGCATCACAATGAGAAGGAGTGGCACCAGCCGGATC
AGTTCATGCCTGGTGAGTCTGTCCTGTCCTGCGCCCTGGGCCACACAGCGAGCCTGGA
CTCTGCTCCAACCACCCCAGTACC
>2437
GATGGCTTCTCATCGTCTGCTCCTCCTCTGCCTTGCTGGACTGGTATTTGTGTCTGAGGC
TGGCCCTACGGTGAGTGTTTCTGTGACATCCCATTCCTACATTTAAGATTCACGCTAAAT
GAAGTAGAAGTGACTCCTTC
>2438
CTGCCATCAATGTGGCCGTGCATGTGTTCAGAAAGGCTGCTGATGACACCTGGGAGCC
ATTTGCCTCTGGGTAAGTTGCCAAAGAACCCTCCCACAGGACTTGGTTTTATCTTCCCG
TTTGCCCCTCACTTGGTAGAGAG
>2439
CAAAGTGGAAATAGACACCAAATCTTACTGGAAGGCACTTGGCATCTCCCCATTCCAT
GAGCATGCAGAGGTGAGTATACAGACCTTCGAGGGTTGTTTTGGTTTTGGTTTTTGCTT
TTGGCATTCCAGGAAATGCACAG
>2440
CCTCCCATGGCCCTGCCCAGTGTATCTTGGATGCTGCTTTCCTGCCTCATGCTGCTGTCT
CAGGTTCAAGGTGAGATTGCTTTGCCTCTAGCACTGGGTTCCCTATGAATCCTCAGAGC
TAACAAGAGGAGGAAGGCTCC
>2441
AGGCTCCAAGGCCTATGGCTCCCACTGCTATGCCTTGTTTTTGTCACCAAAATCCTGGA

CAGATGCAGATGTGAGTGGTTAGATGTGGTGTTGGAGGTGACCGGTCTCAGGGGGAG
GAGGGTCTCCATTCAGGAGAGTTC
>2442
GTCCTCCCTGGTGAAGAGCATTGGTAACAGCTACTCATACGTCTGGATTGGGCTCCATG
ACCCCACACAGGTGCCAGTATATCCTCCCTCTCTGTTACCTCTCAAGGTGCTATTGTTGC
CCAGGCCCACTCCCTGTCCCC
>2443
TTTGCATGGGAGAGAAATCCCTCCACCATCTCAAGCCCCGGCCACTGTGCGAGCCTGT
CGAGAAGCACAGGTAAGAAACAGAGGAGCTGCCTCTTCCCAGTGTTTTCCATCTCATC
CCCCATTCCTGGGTCTGACCTTCA
>2444
TGTGGGGTGGAATGAGCTGGAAGGCCGTGATGCCATCTTCAAGCAGTTTCATTTCAAA
GACTTCAACAGGGTATGGCACCAGGGTGGGACAGAGTTTTCTGAGACGGGCTGATGG
GGTCACCTTACAAAGCATGTTCTCT
>2445
CATGACAAGAGTGGCCCTGCAGGCTGAGAAACTGGACCACCATCCTGAATGGTTTAAC
GTGTACAACAAGGTGAGTGATGCTGTGTGCCTCTGTTGTGCTCTGCCCCCATCCAAATA
CTGCGAACTCTGACACATTCTGA
>2446
GAGTGGGTCCCCCGAGCTCTGTGACCTTATGCTCCACACTAACTCTGGCAGAGCCTCC
GTTTCCTCATAGGTAAGATGGAAATAATTACACCCTCTGGATGGTGTGACTGAAGATTA
AATACAGCGGGTGCTCTCACTCA
>2447
ATTGTGGACTTGCTTAAGAACCTCGATAGCAATGCGGTCGTGATCATGGTGAATTACAT
CTTCTTTAAAGGTAAGGCCCTTGGGCCCAAACCTGCACTTTCTTTGGCTTTTCTGCTGC
TTTTATCTAAAGAATACCCAAT
>2448
AGATGCAGCAGGTGGAGAATGGACTGAGTGAGAAAACGCTGAGGAAGTGGCTTAAGA
TGTTCAAAAAGAGGTACTTTCAGACTACCCCAGGGCCAGCCTAAACCCACACAGCCCC
AGGGAGACACACACGCCCTACCAGG
>2449
CAGTAACGTCTTCACCTCCCATGCTGATCTGTCCGGCATCAGCAACCACTCAAATATCC
AGGTGTCTGAGGTGGGTTCAGAAGCTCCTATGCATCTGCTTCCCAAGATCTATTCTGTT
CTATTCTTTCTATTCTACTCTA
>2450
CCCTGCGGTCTGAGGGCTTCGACACCTACCGCTGCGACCGCAACCTGGCCATGGGCGT
GAACCTCACCAGGTGAGCCTCGCGCCCCGGGAAGCCGCCCCGGCCCGCCTGCACCTC
CGGCTGTGGCGAGCGCTTCGAGCCT
>2451
AATGAAGATATCATTACACTAAGGGCCGAAGATAACGCGGATACCTTGGCGCTAGTATT
TGAAGCACCAAGTAAGTCGTACCTTTTTACCGAGTCACGAAGCTACAGGAAAATCAAA
ACTCTGTGTGAGTAGAAACTCAA
>2452
AAGTGGAGAACTTGGAAATGGAAACATTAAATTGTCACAGACAAGTAATGTCGATAAA

GAGGAGGAAGCTGTAAGTAGTTTTTAAGTAAAAAGAAAATAGTTTGAAGAGAATTATA
ATACTGCTTATTAGGTTAATTGCT
>2453
AACTTCTTTACAAAAGCCACTCCACTCTCTTCAACGGTGACACTCAGTATGTCTGCAGA
TGTACCCCTTGGTAAGATAATAAATTTGAACCTTGTTTTGATGGTAGTCATATGTGATAC
ATACTCCTCAGTAATTAACCA
>2454
TGTCCATGTGCAAGTCTGTGGTGGCCCCTGCCACGGATGGTGGCCTCAACCTGACCTC
CACCTTCCTCAGGTGGGACAGCGGGCAGGTGGGTTTCTGGGACGGAGAGGTCCAGGG
ACACCCTCGGGCCTAACCCCTGACC
>2455
CAGTGTGAGACCCGAACCATGCTGCTGCAGCCCGCGGGGTCCCTCGGCTCCTACAGCT
ACCGGAGTCCCCGTGAGTGGGGCCTCCACCGGCCCTGGGAGCCTGGGGGACACTTGC
CGGGACGACTCTGGGCCAGCCCCCT
>2456
CAGTACGCGCTGCTGTACAGCCAGGGCAGCAAGGGCCCTGGCGAGGACTTCCGCATG
GCCACCCTCTACAGTACGTGCCCCGTGGACGCCGCCCACACGCAGGCCTGACCAGAG
GGGGGCTCCCCAAGACCCAGGGCAGG
>2457
GAGAAATTCACCGCCTTCTGCAAGGCCCAGGGCTTCACAGAGGATACCATTGTCTTCC
TGCCCCAAACCGGTGAGGGGTCCTAACTGATGGGGAGAGGATCAAGGTCAGATTAGA
GGCAGGCAGGCAGTCTCCTGACTGG
>2458
TTCACCACCACCATGACACCGGGCACCCAGTCTCCTTTCTTCCTGCTGCTGCTCCTCAC
AGTGCTTACAGGTGAGGGGCACGAGGTGGGGAGTGGGCTGCCCTGCTTAGGTGGTCT
TCGTGGTCTTTCTGTGGGTTTTGC
>2459
GTTTAATTCCTCTCTGGAAGATCCCAGCACCGACTACTACCAAGAGCTGCAGAGAGAC
ATTTCTGAAATGGTGAGTATCGGCCTTTCCTTCCCCATGCTCCCCTGAAGCAGCCATCA
GAACTGTCCACACCCTTTGCATC
>2460
GAGACACAGTTCAATCAGTATAAAACGGAAGCAGCCTCTCGATATAACCTGACGATCT
CAGACGTCAGCGGTGAGGCTACTTCCCTGGCTGCAGCCAGCACCATGCCGGGGCCCCT
CTCCTTCCAGTGTCTGGGTCCCCG
>2461
CTGGGGCATCGCGCTGCTGGTGCTGGTCTGTGTTCTGGTTGCGCTGGCCATTGTCTATC
TCATTGCCTTGGTGAGTGCAGTCCCTGGCCCTGATCAGAGCCCCCCGGTAGAAGGCAC
TCCATGGCCTGCCATAACCTCCT
>2462
GTACCCCACCTACCACACCCATGGGCGCTATGTGCCCCCTAGCAGTACCGATCGTAGCC
CCTATGAGAAGGTGAGATTGGCCCCACAGGCCAGGGGAAGCAGAGGGTTTGGCTGGG
CAAGGATTCTGAAGGGGGTACTTG
>2463
TGCGGCAGTGCAGCCCTTGGGACTTCCCTCGCCTTCCACCTCCTGCTCGTCTGCTTCAC

AAGCTATCGCTGTGAGTGGCTGGGGGCTCAAGCCTGGGGAAACCCAGGCTGTTTTGTG
GGGAAACTGAGGCTGGGATGGTG
>2464
CCTCGCCGGAGGAGCTGAACCGCTACTACGCCTCCCTGCGCCACTACCTCAACCTGGT
CACCCGGCAGCGGTGAGCGCAGGCGCTGAGCGGACCCCTGGTGCTCTCACCCTGCG
GCCCCGCTCCACCTGGGGGCGTGGC
>2465
GAGACGGCCCGGACACGCTTCTTTCCAAAACGTTCTTCCCCGACGGCGAGGACCGCC
CCGTCAGGTCGCGGTAAAAGCGCCCGTTACCACACATCCTGCATCCGAGAGAGCGGCC
TGGCCCTACCCTGGCAACATCATTT
>2466
CGCGCCACCCGCCAGGAGATGCTGTTCTTGGCGTTGCTGCTCCTGCCAGTTGTGGTCG
CCTTCGCCAGAGGTGAGAGCAGAAACCAGGCTGGGAGGGCCAGCAGCGGCGAGGGG
GAGTCCGGGAAGCCCTGGGGCTGGGA
>2467
TCCGTCCCAGGCACATCACCAGCCTGGAGGTGATCAAGGCCGGACCCCACTGCCCCAC
TGCCCAACTCATGTGAGTCCTCGCACTGCATCAGTTAGTGCTCCCGCTCCGTGCCTCCT
CTGCCCATCCCTCCCCCTTCTAA
>2468
GAGGCGCTCCTTCGACATCCCGCCGCCCCCGATGGACGAGAAGCACCCCTACTACAAC
TCCATTAGCAAGGTGGGCTGCCTTTGCTGGGAAGGCCTCTGGGAAGCTGCAGAGTGG
GGAGTCGGGTGGGGGCCCACTGGCT
>2469
AAGGCCGGCAAGCGAGTGCTCATTGCAGCCCACGGGAACAGCCTGCGGGGCATTGTC
AAGCACCTGGAAGGTAGGCCACCTTCAGGAGCCTGGGCAGGGTGGGTGGGCAGCAGC
CAGCTGGCTTCTCATCTCAGCAAAGT
>2470
AGAATGGGCGGCTTCTGGGCCAGATGGAGGAAGAGGGCATCCTCAGGCGGCTGAAGA
AGTATGACAACTGGTAAGCTCGGCCCCCGCTGCCTGTCCCAGCACCCTGCAGGCAGGG
CTCCCCTGCGTCTCCTGGGAGTTGG
>2471
CTCGGTCGCTGGATGTCAGAGGAGGATTTTGAGAAAGCGTTCAATGCCAGGTTCCCAG
GGTGCATGAAAGGTGAGCGGAACATTGATTTGATTGGGTAAAACAGCAGAGAGCCTTT
TCTTATTTACATCTATCCTAATGG
>2472
CTGGAAGCACTGGGCGATGGGGAGTTTGTCAAATGCCTCCATTCTGTGGGGTGCCCTC
TGCCTTTACAAAGTAAGTGTATTATTTCAGAATCAAAAGTCAAAATAAAAAAGAAAGC
TGAACGCAAACCCCAGTGAGTGCC
>2473
GAAGTGCTTTGCTCTCAGGAATGCCAGCCGGCTGGCCAAGGAGGAAGGGTGGCTGGC
AGAGCACATGCTGGTGAGCTGCAGGAAGCCCTGATGTGCAGATGAGAGGCCTGGGGG
GTGGCAGAAACAAACAGCATTACAGT
>2474
CCCAGCCTCCCCGGGTGGAAGGTTGAGTGCGTCGGGGATGACATTGCCTGGATGAAGT

TTGACGCACAAGGTGACTCTTTTAGACCCAACTCTTGGTAACGATTGGACTCAAGCGA
ATCGTTGGCCTTCGAAACATGTCA
>2475
ATTGATGAGCCGCTAGCTTCAGGCGTCACCATCACGTCCTGGAAGAATAAGGAGTGGA
GCTCAGAGGATGGTGTGTCCCTGCCAGAGGCCTGTGTGTGCCGGGCTGCAGGGACTG
CCTGTTTTGAGCCAGGCACTCACGA
>2476
GATGCTGCCTGGGAGTCTCCGGAAGGTGTTCCCATTGAAGGCATTATCTTTGGAGGCC
GTAGACCTGCTGGTGAGGCTCTCCTTCATTTAGGCTGGGAACATGGGTGTGCTGGGTA
CCGAAGGCATCTGTGAAATCTCTC
>2477
AGCTGGCAACATGGAGTCTTTGTGGGGGCGGCCATGAGATCAGAGGCCACAGCGGCT
GCAGAACATAAAGGTAAATCAAAGTCCTGATCTGAAACCACAGAGAAGTGGGATTAG
AGCACTCTTCGTCACTCTTATGTCTC
>2478
AAAATCAACTCGCTTGCCCACCTGCGCGCCGCGCCCTGCAACGACCTGCACGCCACCA
AGCTGGCGCCCGGTGAGAGCACCCCCCGCTCCGGCCGGGGATGCGGGGCGGCGGCGG
GATCTCCTGGGTGGGGAGCTGGCGG
>2479
GGGCCCGCTACTGGGCAGCGGCGGCTTCGGCTCGGTCTACTCAGGCATCCGCGTCTCC
GACAACTTGCCGGTGAGTGGGCGCCCCGCGGTGGGGAGGGCGCGCCGGGCGGGGGG
CGCACGGGCGTGCTTTAGCCCGGACG
>2480
GGCGAGCTCAAGCTCATCGACTTCGGGTCGGGGGCGCTGCTCAAGGACACCGTCTAC
ACGGACTTCGATGGTGAGCCAGGCCCGGGAGGGAGCTGCCCAGGTGACTCGGCCCGG
CCCGGCCCAGTCCGGAGGCCTCGGCC
>2481
GATATTCCTTTCGAGCATGACGAAGAGATCATCAGGGGCCAGGTTTTCTTCAGGCAGA
GGGTCTCTTCAGGTAACTGATGGAAACCCCTGGCCATGGGGTTATTGGTCTTAATGGGG
CTATTAGTCTTCATGGGACAGTC
>2482
TCAACTGCTCGGGCCGCGGGCTGCGGACGCTCGGTCCCGCGCTGCGCATCCCCGCGG
ACGCCACAGCGCTGTGAGTAGCGGGCCCAGCGGCACCCGGGAGAGGCCGCGGGACG
GGCGGGCGTGGGCGGGTTCCCTGGCCC
>2483
ACGTCTCCCACAACCTGCTCCGGGCGCTGGACGTTGGGCTCCTGGCGAACCTCTCGGC
GCTGGCAGAGCTGTGAGTGTCCCCCAGTCGTGCCAGCATGCGGGGCTCACTCCGGGT
GGGCTGGCGGCACCGCCTCTTGCTG
>2484
ATATAAGCAACAACAAGATTTCTACGTTAGAAGAAGGAATATTTGCTAATTTATTTAATT
TAAGTGAAATGTAAGTTGTGGTTCTTTGGGTGGGGTCCTGGCTGGACCCCAGGCCCCC
AATATCCCTTCTGCCCTCCCAG
>2485
ACGTGTGCTGGGCCTGGCTCCCTGGCTGGCCAGCCTCTGCTTGGCATCCCCTTGCTGG

ACAGTGGCTGTGGTGAGTGCCGGTGGGTGGGGCCAGCTCTGTCCTTCCCAGCCAGGT
GGGACCTGGGCCCTGCAGACACTGG
>2486
CAGAACCGCGGTGGTTCAGGCCTGGAGGCCGCCTACAGCATCGTGGCCCTGGGCGAG
GAGCCGGCCCGAGGTGAGTGTCTGCTGCCCACTCCCCTTCCTCCCCAGGGCCATCCAG
ATGGGGCAGAGCCTGGTACCCCCGT
>2487
GGGCCGGGGCCGCCCTGGCAATGGTGGACAGTCCCGCCGTGCAGCGCTTCCTGGTCTC
CCGGGTCACCAGGTGCCTGCCCCCACCCCCCGAGGGGCCATAGGTTGGGAGATCTCTG
AAGCACTGGGGCAGAGACTGCGGC
>2488
CCCACCGGGTGGTGTAACACCGACCTGTGCTCAGCGCCGCACAGCTACGTCTGCGAGC
TGCAGCCCGGAGGTGTGCGGGGGGCCAGGCAGGGGCCTGAGACGCTGGCTGTGGTTA
GGGGCCTGCCGAGCGCCCGCGGTGG
>2489
GGACCTGCAGGGACCCCTGACGCCTCTGGCACAGCAGGACGGCCTCTCAGCCCCGCA
CGAGCCCGTGGAGGTAGTCGGCCCCCCACGTTCTACAACCTGCCCTCCTGCCTGCCCC
TGGAGGCCTTGCCTGCCCTGCCCAC
>2490
TTTGGGACCCAGGAGCTCCGGCGGCCCGCCCAGCTGCGGCTGCAGGTGTACCGGCTC
CTCAGCACAGCAGGTGGGACTCTGGGTGGTGGGTGGTGGGTGGTGGGCGCCGCAGGA
CTCGGGGTGGCCTCTCTGAGCTTTCA
>2491
GCCCTGGAGCCAACATCTGCTTGCCGCTGGACGCCTCCTGCCACCCCCAGGCCTGCGC
CAATGGCTGCACGTCAGGGCCAGGGCTACCCGGGGCCCCCTATGCGCTATGGAGAGAG
TTCCTCTTCTCCGTTCCCGCGGGG
>2492
GGAGGAGCCCATCTGTGGCCTCCGCGCCACGCCCAGCCCCGAGGCCCGTGTACTGCA
GGGAGTCCTAGTGGTGAGTATGGCCGAGGCTCCACCACCAGCCCCCAGGCAGGTGCC
TGCAGACAGGGTGCTCACACAGGGCG
>2493
GCAGTCCCTGACCTTCCAGAACGTGGTCTTCAATGTCATTTATCAGAGCGCGGCGGTCT
TCAAGCTCTCAGTAGGTGGGCGGGGGTGGGGAGGGGAGGGGATGGGGCGGGGCAGG
GCGGGGGCGGGCTCCACCTTCACCT
>2494
TGTCCCCCAATGCCACGCTAGCACTGACGGCGGGCGTGCTGGTGGACTCGGCCGTGGA
GGTGGCCTTCCTGTGAGTGACTCGGGGGCCGGTTTGGGGTGGGCACCAGGCTCTTGTC
CCAGCCCCAGCCTCAGCCGAGGGA
>2495
CCGGTTCCAGACCCCTCGGTGGCCCAGGTGCTGGTGGAGCACAATGTCATGCACACCT
ACGCTGCCCCAGGTGAGGGATGAGGGGGTGAGGGGGCCACTGCCTTTCAGGCTCTGA
GCACGGGTCCCCCCAGCTCCCCAGT
>2496
GCAGCAGGTGCCTGTGAGCGTGCGCGCCTCCCTGCCCTCCGTGGCTGTGGGTGTGAGT

GACGGCGTCCTGGTGGCCGGCCGGCCCGTCACCTTCTACCCGCACCCGCTGCCCTCGC
CTGGGGGTGTTCTTTACACGTGGG
>2497
TCTGGGGGACGGTGGGTGGCTCGAGGGTCCGGAGGTCACCCACGCTTACAACAGCAC
AGGTGACTTCACCGTTAGGTGGCCGGCTGGAATGAGGTGAGCCGCAGCGAGGCCTGG
CTCAATGTGACGGTGAAGCGGCGCGT
>2498
GGCGCCCGGGCAGCTGGTCCATTTTCAGATCCTGCTGGCTGCCGGCTCAGCTGTCACC
TTCCGCCTGCAGGTCGGCGGGGCCAACCCCGAGGTGCTCCCCGGGCCCCGTTTCTCCC
ACAGCTTCCCCCGCGTCGGAGACC
>2499
CGACCCCAACCTGGAGGACGGCGACCAGACGCCGCTCAGTTTCCACTGGGCCTGTGT
GGCTTCGACACAGGTCAGTGCGTGGCAGGGCCGTCCTCCATGCCCCTCACCCGTCCAC
ACCCATGAGCCCAGAGAACACCCAG
>2500
TGGCGTGGAGTACACCTTCAGCCTGACCGTGTGGAAGGCCGGCCGCAAGGAGGAGGC
CACCAACCAGACGGTGGGTGCCGCCCGCCCCTCGGCCACTTGCCTTGGACAGCCCAG
CCTCCCTGGTCATCTACTGTTTTCCG
>2501
AGTGAGCCGCAGCTCCTACGTGTACTTGGAGGGCCGCTGCCTCAATTGCAGCAGCGGC
TCCAAGCGAGGGGTGAGTGTTGAGCGGGGTGTGGGCGGGCTGGGGATGGGTCCCATG
GCCGAGGGGACGGGGCCTGCAGGCA
>2502
GGCTCTTGCCGCCTCTTCCCACTGGGCGCTGTGCACGCCCTCACCACCAAGGTGCACT
TCGAATGCACGGGTGAGTGCAGGCCTGCGTGGGGGGAGCAGCGGGATCCCCCGACTC
TGTGACGTCACGGAGCCCTCCCGTG
>2503
GCTGCTGCGGCAGGCCGATCCCCAGCACGTCATCGAGTACTCGTTGGCCCTGGTCACC
GTGCTGAACGAGGTGAGTGCAGCCTGGGAGGGGACGTCACATCTGCTGCATGCGTGC
TTGGGACCAAGACCTGTACCCCTGC
>2504
TCTGGTGTCCCTGAGGGTCCACACTGTGGATGACATCCAGCAGATCGCTGCTGCGCTG
GCCCAGTGCATGGTAGGATGGCCCCACCTGCTCACCCTGCCCCGCATGCCTGCCAGGG
CACTGGGTTCAGCCCCCCAGGGCA
>2505
CTGCAGGCAGAGACCACCGCGGGCACCGTGACGCCCACCGCCATCGGAGACAGCATC
CTCAACATCACAGGTGCCGCGGCCCGTGCCCCATGCCACCCGCCCGCCCCGTGCGGCC
CTTTCCTCTGCCTCCCTCCTCCCCC
>2506
GTGGTCACCCTGGACAGCAGCAACCCTGCGGCCGGGCTGCATCTGCAGCTCAACTATA
CGCTGCTGGACGGTGCGTGCAGCGGGTGGGGCACACGCGGCCCCCTGGCCTTGTTCTT
GGGGGGAAGGCGTTTCTCGTAGGG
>2507
GCAGGAGGATCCGCCCAGAGTCACTCCAGGGTGCTGACCACCGGCCCTACACCTTCTT

CATTTCCCCGGGGTGAGCTCTGCGGGCCAGCCTGGCAGGGCAGGGCAGGGCATCATG
GGTCAGCATTGCCTGGGTTACTGGC
>2508
CACCCGCCACCTCACCGCCTTCGGCGCCAGCCTCTTCGTGCCCCCAAGCCATGTCCGC
TTTGTGTTTCCTGTGAGTGACCCTGTGCTCCTGGGAGCCTCTGCAGAGTCGAGGAGGG
CCTGGGTGGGCTCGGCTCTATCCT
>2509
CCTTTCTGTGGGCAGCGGGGCCGCTTCAAGTACGAGATCCTCGTCAAGACAGGCTGGG
GCCGGGGCTCAGGTGAGGGGCGCAGCGGGGTGGCAGGGCCTCCCCTGCTCTCACTGG
CTGTGCTGGTTGCACCCTCTGGGAG
>2510
ATCTTCCGGATCGCCACCCCGCACAGCCTGGGTAGCGTGTGGAAGATCCGAGTGTGGC
ACGACAACAAAGGTTTGTGCGGACCCTGCCAAGCTCTGCCCCTCTGCCCCCGCATTGG
GGCGCCCTGCGAGCCTGACCTCCC
>2511
GTCAATGACTGGCTTTCGGTGGAGACGGAGGCCAACGGGGGCCTGGTGGAGAAGGAG
GTGCTGGCCGCGAGTAAGGCCTCGTTCCATGGTCCCACTCCGTGGGAGGTTGGGCAGG
GTGGTCCTGCCCCGTGGCCTCCTGC
>2512
TTCTCCTCATCTGCCTCTTCCTGGGCGCCAACGCCGTGTGGTACGGGGCTGTTGGCGAC
TCTGCCTACAGGTGGGTGCCGTAGGGGTCGGGGCAGCCTCTTCCTGCCCAGCCCTTCC
TGCCCCTCAGCCTCACCTGTGTG
>2513
GGTGTCCAGCGTGGTTGTCTATCCCGTCTACCTGGCCATCCTTTTTCTCTTCCGGATGTC
CCGGAGCAAGGTGGGCTGGGGCTGGGGACCCGGGAGTACTGGGAATGGAGCCTGGGC
CTCGGCACCATGCCTAGGGCCGC
>2514
CATCGACAGCTGCCTGGACTCGTCCGTGCTGGACAGCTCCTTCCTCACGTTCTCAGGC
CTCCACGCTGAGGTGAGGACTCTACTGGGGGTCCTGGGCTGGGCTGGGGGTCCTGCC
GCCTTGGCGCAGCTTGGACTCAAGA
>2515
CTGGGCCCAGAGGAGGACGGCTTCTCCCTGGCCAGCCCCTACTCGCCTGCCAAATCCT
TCTCAGCATCAGGTGAGCTGGGGTGAGAGGAGGGGGCTCTGAAGCTCACCCTTGCAG
CTGGGCCCACCCTATGCCTCCTGTA
>2516
TTGCCGAGGGGGTCAGCAGCCCAGCCCCTACCCAAGACACCCACATGGAAACGGACC
TGCTCAGCAGCCTGTGAGTGTCCGGCTCTCGGGGGAGGGGGGATTGCCAGAGGAGGG
GCCGGGACTCAGGCCAGGCAGCCGTG
>2517
GGGGAGCTGGGGCCACCCAGCCCAGGCCTGAACTGGGAACAGCCCCAGGCAGCGAG
GCTGTCCAGGACAGGTGTGCTTGCGTAGCCCCGGGATGCCCCTAGCCCCTCCCTGTGA
GCTGCCTCTCACAGGTCTGTCTCTGC
>2518
GAGTGTTGCGTGGCTCCTGTCCAGCAGCGCCAGCTTCCTGGCCTCATTCCTCGGCTGG

GAGCCACTGAAGGTGAGGGGGCTGCCAGGGGTAGGCTACAGGCCTCCATCACGGGGG
ACCCCTCTGAAGCCACCCCCTCCCC
>2519
CCACGGCTTTGCACTCTTCCTGGCCAAGGAAGAAGCCCGCAAGGTCAAGAGGCTACA
TGGCATGCTGCGGGTGAGCCTGGGTGCGGCCTGTGCCCCTGCCACCTCCGTCTCTTGT
CTCCCACCTCCCACCCATGCACGCA
>2520
GGCACGCCTACCGTCTGCAAAGCGCCATCAAGCAGGAGCTGCACAGCCGGGCCTTCC
TGGCCATCACGCGGTACGGGCATCCGGTGCACTGGTCTGTCTTCTGGGCTTTAGTTTTG
CCTTTAGTCCAGCCAGACCCTAGG
>2521
TACGTCCACGGGAACCAGTCCAGCCCAGAGCTGGGGCCCCCACGGCTGCGGCAGGTG
CGGCTGCAGGAAGGTGAGCTGGCAGGGCGTGCCCCAAGACTTAAATCGTTCCTCTTGT
TGAGAGAGCAGCCTTTAGCGGAGCT
>2522
ACGACGTTGGCTGGGAGAGTCCTCACAATGGCTCGGGGACGTGGGCCTATTCAGCGCC
GGATCTGCTGGGGTGAGCAGAGCGAGGGCCCCGGGCGTCTACGCCAAGGACAAGGGA
GTAGTTCTCCAGGAGTGCCGCGGCC
>2523
TGGGCCTGAGCCTGGAGGAGAGCCGCGACCGGCTGCGCTTCCTGCAGCTGCACAACT
GGCTGGACAACAGGTGGGAGCTCCCTCCCCTGCCCTCTCCGGGGTGGCCGCAGTCAC
CAGCCAGGAGCCCACCCTCACTCCTC
>2524
CGCCCTCAGCGTCCGCCCCTTTGCGCTGCGCCGCCTCAGCGCGGGCCTCTCGCTGCCT
CTGCTCACCTCGGTACGCCCGTCCCCGGCCAGACCCCGCGCCTCCCACCGGCAGCGTC
CCGCCCCCTCGCGGGGCCCCGCCC
>2525
CCAGGTGGCGCAGCTGAGCTCCGCAGCCCGTGGCCTGGCGGCCTCGCTGCTCTTCCTG
CTTTTGGTCAAGGTGAGGGCTGGGCCGGTGGGCGCGGGGCTGGGCGCACACCCCAGG
GCTGCAAGCAGACAGATTTCTCGTC
>2526
GCCAGAGCTCCTGGGGGTCACCTTGGGCCTGGTGGTGCTCGGGGTAGCCTACGCCCAG
CTGGCCATCCTGGTAGGTGACTGCGCGGCCGGGGAGGGCGTCTTAGCTCAGCTCAGCT
CAGCTGTACGCCCTCACTGGTGTC
>2527
GGACTACGAGATGGTGGAGTTGTTCCTGCGCAGGCTGCGCCTCTGGATGGGCCTCAGC
AAGGTCAAGGAGGTGGGTACGGCCCAGTGGGGGGGAGAGGGACACGCCCTGGGCTC
TGCCCAGGGTGCAGCCGGACTGACTG
>2528
GATCCCAAGGCCCAACTCCCCGAACCACTCAGGGTCCTGTGGACAGCTCACCTAGTGG
CAATGGCTGCAGGTAAGCGCCCCTAAAATCCCTTTGGCACAACGTGTCCTGAGGGGAG
AGGCAGCGCCCTGTAGATGGGACG
>2529
GCTTTTTGACCACGCTATGCTCCAAGCCCATCGCGCGCACCAGCTGGCCATTGACACCT

ACCAGGAGTTTGTAAGTTCTTGGGGAATGGGTGCGGGTCAGGGGTGGCAAGAAGGGG
TGACTTTCCCCCACTGGAAGTAAT
>2530
CCAGACCTCCTTCTGCTTCTCAGACTCTATTCCGACACCCTCCAACATGGAGGAAACG
CAACAGAAATCCGTGAGTGGATTCCGTCTCCCTAGGCGGGGATGGGGGAGACCTGTGG
TCAGGGCTCCCGGGCAGCACAGCC
>2531
TGACACCTCGGACAGCGATGACTATCACCTCCTAAAGGACCTAGAGGAAGGCATCCAA
ACGCTGATGGGGGTGAGGGTGGCGCCAGGGGTCCGCAATCCTGGAACCCCACTGGCT
TAGAGGGCTGGGGGAGAGAAACACT
>2532
GGGGACCTGTGCAACCCGAACGGCGTCGGGCCGTGGAAGCTGCGCTGCTTGGCGCAG
ACCTTCGCCACCGGTGAGCGGGGGAAACTGAGGCACGAGGGACAAGAGGTCGTCGG
GGAGTGAAAGCAGGCGCAGGGAAATAA
>2533
TGCAGGAGGAGCCGGGGGCCTTCAACTGGAGCATGTACAGCCAACATGCCTGCCTCAT
TGAGGGCAAGGGGTAAGGACTGGGGGGTGAGGGTTGGGAGGAGGCTTCCCATAGAGT
GGCTGGTTGGGGCAACAGAGGCCTG
>2534
CGTGGCCCTGGTCTGTGGTGTCCCGGCCATGGACATCCCCCAGACCAAGCAGGACCTG
GAGCTCCCAAAGGTTTGAGGCTGGGGGAGCGGGCACTTTACTGTGGGAGGCCTGGGG
CGGGTGGGAGCTGCGGGCAGGCGGG
>2535
CTCTGAGGGTCCACATCACCTCACTGTTGCCCACCCCCGAGGACAACCTGGAGATCGT
TCTGCACAGATGGTGGGTTTCTCATCATTGAGACGGGCTGGGCGGGGGCTCAGTCTCC
CCCCTCAGGGGTCCAGGACTGGGT
>2536
AACAACAGCTGTGTTGAGAAGAAGGTCCTTGGAGAGAAGACTGAGAATCCAAAGAA
GTTCAAGATCAACTGTGAGTGTCCCCAGGCCCCAAGGGCTGGCTCAGTGCTGGCATGC
TAGCCACGCTCTCCCAGAGGCGGCTC
>2537
GACAATTTCCTGTTTCTCTGCCTACAGGACACCACCACCCCCATCCAGAGCATGATGTG
CCAGTACCTGGGTGGGTCTCACAGCACATGAGCTCAACGTGGGTGAGAGGCAGCAGC
TACGTCCATGGCTGGGAACCCTGG
>2538
GGATTCATCAGGGCTTTCAGGCCCCTGCCCAGGCACCTATGGTACTTGCTGGACTTGAA
ACAGATGGAAGGTGAGCTCTGCCTAGGACACGCCCAGCCTCAGCTGGAGGAGAAGCT
GCCTCTTTCTTAGCCCGAGCCCCC
>2539
CCACACCAGAGCAGATGGCCCAGTATGCAGCTGATCTCCGTAGATACATCAACATGCTG
ACCAGGCCTAGGTGTGTGCCACAGTTGGGGAGAGAGATCCCAGCCCCTGGGACCCTG
GGCCCACTCCACATTCCTGGCCAC
>2540
ATGGGAAAAGACACAAAGAGGACACGCTGGCCTTCTCGGAGTGGGGGTCCCCGCATG

CTGCTGTCCCCAGGTGAGTTTGACTCCCTGCCCTGTCTGTCCAGGCTCCCTGGGGCTG
AAATGGGGGTGGTGGGACTGAATCA
>2541
ATGTGGCAGCTCACAAGCCTCCTGCTGTTCGTGGCCACCTGGGGAATTTCCGGCACAC
CAGCTCCTCTTGGTAAGGCCACCCCACCCCTACCCCGGGACCCTTGTGGCCTCTACAA
GGCCCTGGTGGCATCTGCCCAGGC
>2542
GCGGGAGTGCATAGAGGAGATCTGTGACTTCGAGGAGGCCAAGGAAATTTTCCAAAAT
GTGGATGACACAGTAAGGCCACCATGGGTCCAGAGGATGAGGCTCAGGGGCGAGCTG
GTAACCAGCAGGGGCCTCGAGGAGC
>2543
TGCATCGACGGCATCGGCAGCTTCAGCTGCGACTGCCGCAGCGGCTGGGAGGGCCGC
TTCTGCCAGCGCGGTGAGGGGGAGAGGTGGATGCTGGCGGGCGGCGGGGCGGGGCT
GGGGCCGGGTTGGGGGCGCGGCACCAG
>2544
GGCTGGCGGCGCTGTAGCTGTGCGCCTGGCTACAAGCTGGGGGACGACCTCCTGCAG
TGTCACCCCGCAGGTGAGAAGCCCCCAATACATCGCCCAGGAATCACGCTGGGTGCGG
GGTGGGCAGGCCCCTGACGGGCGCG
>2545
CCAAGAAGACCAAGTAGATCCGCGGCTCATTGATGGGAAGATGACCAGGCGGGGAGA
CAGCCCCTGGCAGGTGGGAGGCGAGGCAGCACCGGCTCGTCACGTGCTGGGTCCGGG
ATCACTGAGTCCATCCTGGCAGCTAT
>2546
ATCCACCCCTCCTGGGTGCTGACAGCGGCCCACTGCATGGATGAGTCCAAGAAGCTCC
TTGTCAGGCTTGGTATGGGCTGGAGCCAGGCAGAAGGGGGCTGCCAGAGGCCTGGGT
AGGGGGACCAGGCAGGCTGTTCAGG
>2547
AGCAGCTTCTTGATCGTGGTGGTGTTCCTCATCGCTGGGACGCTGGTTCTAGAGGCAG
CTGTCACGGGAGGTGAGTGAACAGGTGACCTGCTGGGCTGGGTTGGACTAAGGGGAG
ACCCTCTGGACACCCTGGGCCAGGA
>2548
ACTGACTGCCCAGGAATCAAGAAGTGCTGTGAAGGCTCTTGCGGGATGGCCTGTTTCG
TTCCCCAGTGAGGTGAGCACTAGCTGGAGAACGAGGAGACCCCTGAAGACACAAAAG
AAGGCTGAGCGGTGGGGAAGCATCC
>2549
GTTTTGCCATTGCCAGTGGGACGTCTGAGACTTTCTCCTTCAAGTACTTGGCAGATCAC
TCTCTTAGCAGGTAGGTGCCGCAGACCCTGCGGGTTAAGAGGTGGGGTGGGGGGCAG
TGCTTGCCAAGGCCCTAAACTGGG
>2550
ACCTGGGTTCGCTCGCCTTCCTAGGCGCTGACACCGCTCGGTTGGATGTCGCGTCGGA
GTTTCGAAAGAAGTGAGTCCGGGCAGCGCCTTCCCCCTTGCTGGTACCTGGCAGGCAA
GGGGAACTGACCGTTGGTCCCGAA
>2551
AGGCCGGGCCTGCCCAGACCCTTATTCGGCCCCAGGACATGAAGGGTGCCTCTCGAAG

CCCCGAAGACAGGTAACTACGCCCTGTGCTGTCCAGGGACGGGAGGGAAGGAAGGTG
TGCGGGAGGAGTTCTCTGTCTCCAC
>2552
TTGCAGCCCAGAAGACCCACCAGGACCAGTACAGCTTCAGCACTGACACGGTGGAGT
GCCGCTTCTACAGGTGAGAGCTGGGGCTAGGGGTGGGGGGCTGGAAAAGGCGCGGG
AAACTCTGGGTGGTCTAAGAGCCCTGG
>2553
TGCAAGATGCTTCTGATTCTGCTGTCAGTGGCCCTGCTGGCCTTCAGCTCAGCTCAGGA
TTTAAATGAAGGTAAGATGAATTGGGGGAAGATATTGTGACTCTGATTGGGGTTTACAG
GCAAATGCTATAGAGGAGGAAA
>2554
TCCCCAAGGGGGCCGCCCACAAGGACCTCCACAGGGGCAGTCTCCTCAGTAATCTAG
GATTCAATGACAGGTACGATTCCAGTTTATTATCCATCAAAGGCTCCAACTGCTACAGT
TCTCCAACTTCATTGTGCCAATGA
>2555
TGCAAGATGCTTCTGATTCTGCTGTCAGTGGCCCTGCTGGCCTTCAGCTCAGCTCAGG
ACTTAGATGAAGGTAAGCCGAATTGGGGGAAGATATTGTGACTCTGATTGGGGTTTACG
GGCGAATGCTATAGAGGGGGAAA
>2556
TCCCCAAGGGGGCCGCCCACAAGGACCTCCACAGGGGCAGTCTCCTCAGTAATCTAG
GATTCAATGACAGGTATGATTCCAGTTTATTATCCATCAAAGGCTCCAACTGCTACAGTT
CTCCAACTTCATTGTGCCAGTGA
>2557
GCCGCGAGCGTGACCGGGTGCAGGTGGAGCGCGACGGGCTGGCGGAGGACCTGGCG
GCGCTCAAGCAGAGGTCAGGGGGCAGGGCTGGGCCGCTGCCGTCGAGGCGAGGTCG
AAGCGGCGTCGAGGCGGCTGCTCTTGCC
>2558
CCTGGAACTAGAGCGCAAGATTGAGTCTCTGATGGATGAGATTGAGTTCCTCAAGAAG
CTGCACGAGGAGGTAAGTGGGCCCGGTATCAGGGGCGGTTTCTGAGGTTGTGGGGTG
GTCTCGCTGGAGCTGGCGGGTGGAG
>2559
CATCCGCGCGCAGTACGAGAGCATCGCCGCGAAGAACCTGCAGGAGGCGGAGGAGTG
GTACAAGTCCAAGGTGCAAGAGCCGGGAGGGCCTGCGAGGCGGGACGCTGGGGTGG
TGTCGCGCGTCCCAGCCGACTAAAGCC
>2560
GCAGGAGATGAACGAGTCCCGACGCCAGATCCAGAGTCTAACGTGCGAGGTGGACGG
GCTGCGCGGCACGGTGAGTACGAAGCTGCGCGCTCGGGCCCGGGGAGCGGACGATGA
AATGTTCTGCAACTGGCCCCTTCCAC
>2561
TCAACGTCAAGATGGCCCTGGACATCGAGATCGCCACCTACCGCAAGCTGCTGGAGGG
CGAGGAGAGCCGGTGAGGGTGGAGCTGCTGGGGCGGGGCAGGGCGGGGTCGGGACT
GGGCCGGGCAGGGCGGGGCCTGGGCA
>2562
GGAGCCTCCCCAGGACAGCCACAGCCGGAAGACGGTTCTGATCAAGACCATTGAGAC

CCGGAATGGGGAGGTGAGGCAGGTCCCCTAATGCCAGGACCCCACCATTTCCCATATTA
TTTCTGAGCCCCAGCCTGGCTGTC
>2563
TATTACCGCCAGAGACAAAGAAGTCGCAGACGAAGGAGGCGGAGCTGCCAGACACG
GAGGAGAGCCATGAGTAAGTGGGCCCAGCTGAGGGTGGGCTGGGGCTGAGGCTGGG
AGCTCTCAGGGCCCAGCCTTCCTCTCAC
>2564
GCAGGGCAGTCTGCGGCGGTGTTCTGGTGCACCCCCAGTGGGTCCTCACAGCTGCCC
ACTGCATCAGGAAGTGAGTAGGGGCCTGGGGTCTGGGGAGCAGGTGTCTGTGTCCCA
GAGGAATAACAGCTGGGCATTTTCCC
>2565
CCCACCCAGGAGCCAGCACTGGGGACCACCTGCTACGCCTCAGGCTGGGGCAGCATT
GAACCAGAGGAGTGTACGCCTGGGCCAGATGGTGCAGCCGGGAGCCCAGATGCCTGG
GTCTGAGGGAGGAGGGGACAGGACTC
>2566
TCACCCTCAGAAGGTGACCAAGTTCATGCTGTGTGCTGGACGCTGGACAGGGGGCAA
AAGCACCTGCTCGGTGAGTCATCCCTACTCCCAAGATCTTGAGGGGAAAGGTGAGTGG
GGACCTTAATTCTGGGCTGGGGTCT
>2567
ACTCCTGGATCCCACGGCCTGCCAGGCAGGCACGGGAGAGATGGTCTCAAAGGAGAC
CTGGGCCCTCCAGGTACTGTGCTGCAGACCCCACCCTCAGCTGAGGACACAGACCCCT
TTTCAGGAGGCCCATCTGTCCAGGC
>2568
CTGCCTGGAGCCCCTGGTATCCCTGGAGAGTGTGGAGAGAAGGGGGAGCCTGGCGAG
AGGGGCCCTCCAGGTGAGCAGGGTGGGGCAGGTGGGCAGTGGAAACATGGGCACAG
CGACCCTGAAGTCAGTTACACGGGGAT
>2569
GCTCATCTAGATGAGGAGCTCCAAGCCACACTCCACGACTTTAGACATCAAATCCTGC
AGACAAGGGGAGGTAAGGGGACCCCCTGGGCTCACGGGGTAGGAGTTTCCCACAAAT
TCCCCTCATTCTCAGCACCAGCTTC
>2570
GCCCTGCCCGCCGCTGCGATGCTCGCCCGCGCCCTGCTGCTGTGCGCGGTCCTGGCGC
TCAGCCATACAGGTGAGTACCTGGCGCCGCGCACCGGGGACTCCGGTTCCACGCACCC
GGGCAGAGTTTCCGCTCTGACCTC
>2571
AGTGTGGGATTTGACCAGTATAAGTGCGATTGTACCCGGACAGGATTCTATGGAGAAA
ACTGCTCAACACGTAAGTTTGTCCTTTGGTTGCCTCATTAGGAGTGGGGCTGGATACAG
TTATCATTGTATAGATTTGTGTC
>2572
AAGGGATTTTGGAACGTTGTGAATAACATTCCCTTCCTTCGAAATGCAATTATGAGTTAT
GTGTTGACATGTAAGTACAAGTGTCTTTCTAAGGTTTTTAGCCTTCTCAAAGAAAAATA
TGCTTTATAATACTGTAAGCC
>2573
AACCTCTCCTATTATACTAGAGCCCTTCCTCCTGTGCCTGATGATTGCCCGACTCCCTTG

GGTGTCAAAGGTGAGTAAGAAGAATCCATTAGAGATGTATTAACTATAAGACGGGCTG
CATTGCTGCCAAAAAAAAAAAT
>2574
CTTCACGCATCAGTTTTTCAAGACAGATCATAAGCGAGGGCCAGCTTTCACCAACGGG
CTGGGCCATGGGGTAAGATAGAGTTAATATCTTAGAGTTAGTAAAATTATACCAAATCAT
AGTCAAGGGCTAACATTAAAGG
>2575
TATTTACGGTGAAACTCTGGCTAGACAGCGTAAACTGCGCCTTTTCAAGGATGGAAAA
ATGAAATATCAGGTATGCTTCCTTTGACTATTAAGACTTAGTTATTACCGCTTATACCCAT
ATTTTAAAATCCCTAAAAATG
>2576
GTGCTTAAACAGGAGCATCCTGAATGGGGTGATGAGCAGTTGTTCCAGACAAGCAGGC
TAATACTGATAGGTAAACAAGAAAATGATTTATATAAAACCCTCTTCCCCAGGGAAAAT
TAGTGTGCTATCTTTGTTATGTT
>2577
CAACTCTATATTGCTGGAACATGGAATTACCCAGTTTGTTGAATCATTCACCAGGCAAA
TTGCTGGCAGGGTAAGCATTATTATTGAAAACCAAAACAAAAGACTAGTCAGTAACTTT
AGAATTTCTGCCACGGAAATTA
>2578
TACCAGTCTTTTAATGAGTACCGCAAACGCTTTATGCTGAAGCCCTATGAATCATTTGAA
GAACTTACAGGTAAGAAACAGTTTCTAAACTTCTTCGTTTTTTGTTTGTTTGTTTGTTTT
TGTTGTTTTTGGTTTTCTTT
>2579
AGGCGATTATGCAGCCCCTTGATCATAAGAATCTGGATATGGATGTGCCATACTTTGCAG
ATGTGGTGAGGTGGGTGGCACTGTATCTTGCCTTATGTGGATTGTAAAACAAGAATTGA
TTTGAATACTTTGATTGTTGT
>2580
AGACGATCTGCACTTCGCATTTTGGCATTGACATTTAATTTTAGGGTCCTTTATATAGAA
GGGAGAGTAGGTAAACTGATTTTTTTTTTTAACAGGGAGGGTTTGACAATCTTTGGCAG
ACTTGGAGCAAAAGATTGAGG
>2581
GTTGGAGAAATTTCACAGTACATCATCCAAAAGAGGAGTCCATGATGGAGGCAGAGGT
AAACTTGGAGAGGTAAGAAACCCTGAAGACAGGGGAGTGCTTTGTGGCAGGCTCTGC
ATATAAGAATTCAGCCTGGCCAACA
>2582
GCGCATGCTCTGGGGCAGTTCGCGGCCCGGCGGGGAGCGCCGGAGTTCCTTGTGGCC
GACGTGCACCAAGGTAGGTCTCGCCTGGGACGCGCGGAGGGTCCGGGCAGAGGGCG
GTAACGAGCGGGCCACAGCGGAGCACG
>2583
TCACCCAAGCGCATAGCTAAAAGAAGGTCCCCCCCAGCAGATGCCATCCCCAAAAGCA
AGAAGGTGAAGGGTAAGTTGGCCTTGGCCTCTTTGTGGGTACAGGTGGCCCCTTGAAA
CCCTAAGAACCCGGACTGGGCTCC
>2584
CATTCCGGAGGATGTTGTGCAGGCTGAGGCTGGGGGCATGCACACCGTGTGTCTAAGC

AAAAGTGGCCAGGTAGGTGTTGGGGACTGGCACAGGGTTGGACAAGGCCTGGGGTTG
GGTGGCTTGGGGCAGGGCTTTTGAA
>2585
GTCAGCAGGAGACAGTCACACAGCAGCCCTCACCGATGATGGCCGTGTCTTCCTCTGG
GGCTCCTTCCGGGTAAGGCTGGGTCTGAAAGTCTGCATGGTCCCGTGAAAGACAGAAT
TAATTGCGGGGCCCCAAAGATAAT
>2586
GAGCCCATGAAGAAGAGCATGGTGCCTGTGCAGGTGCAGCTGGATGTGCCTGTGGTAA
AGGTGGCCTCAGGTGGGTCTGGGGGCACTTGCTCAGGGCAGGAGTTGGAGGACCTTG
TTCTGGGGCTGGCCTAGCCTTGGGC
>2587
TGCGGGGAACAGGGCCAGCTAGGCCGTGTGCCTGAGTTATTTGCCAACCGTGGTGGCC
GGCAAGGCCTCGGTAAGTGGCCTTGGTACCTCCAGCAGGGCAAATTGGCAGGCCACC
CCCACAGTGAAGGCCAAACGGAGGA
>2588
TATTTCACCTTTGCCATCTCCCATGAGGGCCACGTGTACGGCTTCGGCCTCTCCAACTA
CCATCAGCTTGGTGAGCCCCGAGCCCAGCTTCAGGCATGACCCAGTGGCCTGCGTTCC
TGTCCTGGCTCTGCACTCATTCA
>2589
AAGAATTCCACCAAGTCCTGGGTGGGCTTCTCTGGTGGCCAGCACCATACAGTCTGCA
TGGATTCGGAAGGTAGGGCCTTTACGTCCTTCTCTAGTTTGGGGGTGGAGTGTTCCCTG
GCCTAGGCCTAGCCAGATTCCTG
>2590
ATCTCCAGGCTGCCTGCTGTCTCCTCGGTGGCTTGTGGGGCCTCTGTGGGGTATGCTGT
GACCAAGGATGGTGAGTGGGGCTGCCTACACTCTGTCTAGTTGGGACCTGGGGGTCAT
GGTTCTTACCCAATTCCCCAATA
>2591
CCGCGGTGCCATGGCCCCCGCCCGTCTGTTCGCGCTGCTGCTGTTCTTCGTAGGCGGA
GTCGCCGAGTCGGTGGGTGCTTGGAGGTTCCCGGGCTGGGGGCGAAGCGGGGGCGCA
GGCCGGTGCCTCCTTTGTTCGTCGG
>2592
GACGATGAGGATGTAGTGGGGCCCGGGCAGGAATCTGATGACTTTGAGCTGTCTGGCT
CTGGAGATCTGGGTACGGAAGGTGTGCTGGGCAGGCGTAGGCACAAAGCTGGAGGGA
GTGGTGGCTTCACCAGCCAGGAGGG
>2593
TCCAACAAGGTGTCAATGTCCAGCACTGTGCAGGGCAGCAACATCTTTGAGAGAACG
GAGGTCCTGGCAGGTAAGTCCCATGCTGCTTATAAGATGCCTTGAAGGTGGAATGGGG
CTCAGCGGGGGAGAGCACCTGCAGG
>2594
AGGCACCAATGCCTATCGCTCCTACTGCTACTACTTTAATGAAGACCGCGAGACCTGGG
TTGATGCAGATGTGAGTGAGGAGAGCAGTGTGGGAAGGGAGACTCATGAAGGGAGGG
GAAGCTGCCACTCTCCAGTGTGTT
>2595
GGCCTCACTGATTAAGGAGAGTGGCACTGATGACTTCAATGTCTGGATTGCCCTCCATG

ACCCCAAAAAGGTAGGCTGCAGCCTTCTTTATCTCCTAATGATCAGGTTTGAGAAGTAA
GAAGGAGGTTCAAGTTCTGGTC
>2596
AAGTCCTGGGGCATTGGAGCCCCAAGCAGTGTTAATCCTGGCTACTGTGTGAGCCTGA
CCTCAAGCACAGGTGAGAGGCAGAGAATCCATCCACCTGTTTCTGTTCTCTCCTGCTTA
GCTCCAGGGATGGAACTGGGACT
>2597
CTAAGCATGGCTCAGACCAACTCGTTCTTCATGCTGATCTCCTCCCTGATGTTCCTGTCT
CTGAGCCAAGGTGAGATTTTCCCCCACACTTCCCACAACCCCAACTCTGAATTCTTCCC
TCCATCCTCATGTATAAGGTT
>2598
AGGCACCAATGCCTATCGCTCCTACTGCTACTACTTTAATGAAGACCCTGAGACCTGGG
TTGATGCAGATGTGAGTGAGGAGAGCAGCAGGGGAAGGGAGGCTTATGAAGGTAGAG
GCAGCTGCTAATTTGCAGTGTGTT
>2599
GGCCTCACTGATTAAGGAGAGTAGCACTGATGACAGCAATGTCTGGATTGGCCTCCAT
GACCCAAAAAAGGTCAGTCTGCAGCCACCTCTATCTCCTTATAAACATTTTTGAGAGGT
AAGAGGGACGTTTAAGGTCTGGC
>2600
AAGTCCTGGGACACTGGATCCCCGAGCAGTGCTAATGCTGGCTACTGTGCAAGCCTGA
CTTCATGCTCAGGTGAGAGGCAGACAATCTATCCACCTGTTGCCATTTCCTTCCCACTT
ATCTCTGGGGATGAACATGGGGA
>2601
CGCCGCCGCCGCCCCCTCCTGAGGAGGACCCAGAGCAGGACAGCGGCCCGGAGGAC
CTGCCTCTCGTCAGGTGAGCGAGCAGAGCCGCCGTCGCCTCACGCGGGAAGGGCGCC
CCGGGTGTGCGTAGGGCGGGCGCAAGG
>2602
GATACCAGATCATGTCAGAGAGAGAGCTTGGTTAACTTGGGAGAAAGTTTCATCTGTG
GATGGAGTATTGGTAAGGATTTTCTTAAAACGTTTTGAAATTTTTTTTTCTCATTTTAAA
AACAACTTCAAATCACTATACA
>2603
TTATTGCAGCAGTTGACCTAGATGAGATGTCGTTCACTTTTACTGAGCTACAGAAAAAC
ATAGAAATCAGGTAAAGTTTCTTGTATAAATATAAGCCTCTGCCATAAAAGGAAATGAA
TTCTGGATTTTCCTCTCAATAG
>2604
TTGATAATGCTATGTCAAGACTGTTGAAGAAGTATGATGTATTGTTTGCACTCTTCAGCA
AATTGGAAAGGTAAAGTAAACATTTTATTAGGGTTACACTCTGATTTTTTATGTCATTGT
TCACAATTAGATTCTGGGAA
>2605
CAGTTAATGCTATGTGTCCTTGACTATTTTATTAAACTCTCACCTCCCATGTTGCTCAAA
GAACCATATAGTAAGTATTTAATTTATGCCCCTTTTACTTTCTCATTCAGCAGTTGCTTAT
TGAATGTCTAGTGGGTACC
>2606
AAAACAACTAGAAAATGATACAAGAATTATTGAAGTTCTCTGTAAAGAACATGAATGTA

ATATAGATGAGGTAATTTAACTTCATGATTTCTTTAAAACAGTTAAAGTAGATTTAGATGT
AAGTTCTCCCTAACAATATT
>2607
TGTTTATTTCAAAAATTTTATACCTTTTATGAATTCTCTTGGACTTGTAACATCTAATGGA
CTTCCAGAGGTAATCTGAAAGGAAATTTAATAAAATATTAATGTTTTGAGACTGTGGAG
GGAGGATAATTGTCTAACTT
>2608
AAAATAAAGATCTAGATGCAAGATTATTTTTGGATCATGATAAAACTCTTCAGACTGATT
CTATAGACAGGTATTGCACATGGTATATTTGATTGATTTGCTTTAGATATAGGTTGATACT
GATATAGGTAGATTATATA
>2609
CACAGAGAACACCACGAAAAAGTAACCTTGATGAAGAGGTGAATGTAATTCCTCCAC
ACACTCCAGTTAGGTATGAATTTTCCTACTTTTAATTATATTATAATTTTGTTATTCATGGC
TTTATAGTGTTTCAGATTTGT
>2610
CCAACAATTAATGATGATTTTAAATTCAGCAAGTGATCAACCTTCAGAAAATCTGATTT
CCTATTTTAACGTAAGCCATATATGAAACATTATTTATTGTAATATCTTGGCAAAGAAACT
TGAAATTAAAAGTTAAAGTA
>2611
TATAGGATACATCTTTAAAGAGAAATTTGCTAAAGCTGTGGGACAGGGTTGTGTCGAAA
TTGGATCACAGGTAACTTGAATTCATTGTAATTCGTGGTACTATAGAGTAATAATATTAA
AAGCAGCATCTTTCCAGTTCG
>2612
CTGAATGACAACATTTTTCATATGTCTTTATTGGCGTGCGCTCTTGAGGTTGTAATGGCC
ACATATAGCAGTAAGTTAAATTTTCATAAATAAACACTTTTGTTCAATTTAAAGTTAAAA
TGTGGTGTGTTTCTTTGGTC
>2613
GACAAGAGAAATGATAAAACATTTAGAACGATGTGAACATCGAATCATGGAATCCCTT
GCATGGCTCTCAGTAAGTAGCTAAATAATTGAAGAAATTCATTCATGTGCATATGGCTAA
CAAATTATTGTTAGTGAGAGGT
>2614
CAACTGATCACCTTGAATCTGCTTGTCCTCTTAATCTTCCTCTCCAGAATAATCACACTG
CAGCAGATATGTAAGCAAAATATATGTTATGTTGACCATTCAAACTGCAAATAGATTTTA
AGCATAAGTGCAATGTAACA
>2615
CAAGCAACCTCAGCCTTCCAGACCCAGAAGCCATTGAAATCTACCTCTCTTTCACTGTT
TTATAAAAAAGGTTAGTAGATGATTATTTTCAAGAGCATGGACTCTGAAACTAGGCTGA
CTGGGTTCAAATCATGTTTCTT
>2616
CATCTGGACCCTTTTCCAGCACACCCTGCAGAATGAGTATGAACTCATGAGAGACAGG
CATTTGGACCAAGTAAGAAAATCAAGCACTTCACCTTCTCTCCTCCCTACTTACTTGTT
AACTGATTTCTTTCTTTCTTTCT
>2617
GAAGAATATAGACCTTAAATTCAAAATCATTGTAACAGCATACAAGGATCTTCCTCATG

CTGTTCAGGAGGTAGGTAATTTTCCATAGTAAGTTTTTTTGATAAATCCATATCCATAACA
TAACATAGGTAATTCATTTG
>2618
TATAGTATTCTATAACTCGGTCTTCATGCAGAGACTGAAAACAAATATTTTGCAGTATGC
TTCCACCAGGGTAGGTCAAAAGTATCCTTTGATTGGAAAAATCTAATGTAATGGGTCCA
CCAAAACATTAAATAAATAAT
>2619
CACCCCTGAAGAGTCCATATAAAATTTCAGAAGGTCTGCCAACACCAACAAAAATGAC
TCCAAGATCAAGGTGTGTGTTTTCTCTTTAGGGAAGTAGTAAAGAATGAGAGGGGGAT
TATTTTGATCCAAGAATAAAAAAT
>2620
GAAGCAACCCTCCTAAACCACTGAAAAAACTACGCTTTGATATTGAAGGATCAGATGA
AGCAGATGGAAGGTAGGAACCAGTTTTGAATGTTTTCCAGTAGCTGAGATGGTCATCT
GGGGAATCCAGAGTCTCAGCACTG
>2621
CGGTTAATGAAAAAATGGACACAGAAAGTAATAGGAGAGCAAATCTTGCTCTCCCACA
GGAGCCTTCCAGGTAGGTACAAGGTATTATTTTTTTCTACCCTCAGTCACTTGTGGCAG
GGGAAGTCATAGTCACGGTGCTT
>2622
GAAGTCGGCCTCATCCCCACCACTGCATACATGGCTGACAGTTTTGAAAAAAGAGCAG
GAGTTCCTGGGGGTGAGTGAGCCTCCTCCAACTTTGACTAGAGTAAGGGTTGGGTCTA
GAAAAGAATATTGAGTTGCATCAA
>2623
TATTTCACACATTGAGGGAGACATTTTTTCATCATTTAAAGCAGGTTATCCATTCTGGGG
AGCCATATTTGTGAGTATATATCTATAATTGTTTCTGAAATAACACTGAACATAGGTTTTT
CTCTTTCTCAGATCTAACC
>2624
GGCCTATATCCACATCCACAGTTGCCAGAAATTTTTTGAGACCAAGTGCTTTATGGCTT
CCTTTTCCACTGTATGTATTTTTTTTTGTGTGGGAAGACTAAGATTCTGGGTCCTAATGT
AAGTAAGAAGCCCTCTTCTCC
>2625
CATTCTGGGACTTGGTAGTGCTGTGTCACTCACAATCTGTGGAGCTGGGGAAGAACTC
AAAGGAAACAAGGTAGATAGAAGCCCGATATAAAATCTTGAATGACAGGTTAACGAAT
TGGAGCTTTATTCCTTAAAATATG
>2626
ACAAGGATTGGTTTGGGGTCCAGTGGGTCAGCAGCCGTTACCTGGATCCCGGTGACCG
GGATGTGGCTGAGTGAGTGATTTGCGTCTCCCTTCCTCCTCCTCCTCCCTGGACAG
GCTCCCTCCTGCTGCCTTGAATC
>2627
GCTGCTGGAGCACTTGCAGGACTTGGCAGGCACACTGGGTAGCATGCTGGCTGTCACC
TTCCTACTGCAGGTGAGTCAGCAAAGCATCTGACACCTCCTCCCACCCGGGACTCCTC
CCTGCCTCCAACCCTGGGCCTCTT
>2628
ACACGAACAAGCGCGTGTGCGAGGAGATCGCCATTATCCCCAGCAAAAAGCTCCGCA

ACAAGATAGCAGGGTGAGTCGGGCCTTCCTCGGCCTCCGGGCGTCCGCGATCTGTTTG
TGTGCTCTGCCGCCCTCCGACTCGG
>2629
CCCAGTAAGAGGTATCTCCATCAAGCTGCAGGAGGAGGAGAGAGAAAGGAGAGACAA
TTATGTTCCTGAGGTAAACTTTCTGGATATTTGGGCTTCTGGCTAATCCTCAAATGATAA
AGCTATGGGTTCTTGGCTTCCAA
>2630
ACTTCGTACTTTCTATGAGAAGCGTATGGCCACAGAAGTTGCTGCTGACGCTCTGGGTG
AAGAATGGAAGGTAAAAGTTGACAAATTGTTGCAGGTATTTAAGTCAGAGACGGTAAA
CGCCATTGGTAACTGGTATTTGG
>2631
AGAAAATCAGTTCGTGGTTGCATTGTGGATGCAAATCTGAGCGTTCTCAACTTGGTTAT
TGTAAAAAAAGGTGAGGGTTACTTGTGTTCAATTTTCGTTGAAATTGATTTAAAGCCAG
TTGTCCAAATGCTTATTCATTT
>2632
AAACTTTTCAATCTCTCTAAAGAAGATGATGTCCGCCAGTATGTTGTAAGAAAGCCCTT
AAATAAAGAAGGTAGGAGGGATTATGCAATTAGGGCTTGCTTAATTTTGGTAATTTGTCT
ATCATTTTGTGTGCATATCAG
>2633
GAAGCAGCGTACCAAGAAAAATAAAGAAGAGGCTGCAGAATATGCTAAACTTTTGGC
CAAGAGAATGAAGGTTAGTCTAAGATGATTTGAGGGGGTGGGAGGAGTTCGACCTGG
CTTTGGATTTGCGTCTTTAACCCAGA
>2634
ACTACTTTGATACGACACCGCATGGCTTCGAGACTCACACGCTGGAGGAGCACAACCT
GGCCAATTACATGTGAGCAGACACACTGGCCAGTCAGGAGGGTGGGGGGCATGGCTG
CCAATAGCCAGCAGTGGGGTACTTA
>2635
GTTTTCTGCTCCTTGGTCCTGGGTGTCAGCAGCCGAAGCTTCTTTTCGTTCCTTGGCGA
GGCTTTTGATGGTAAGGCTTCAGAAGGTTTGCAGGATTTCTAAGAGAAACATCGCCCT
GGACCTATAAACTGGGGAAAATG
>2636
TCCATGCTCGGGGGAACTATGATGCTGCCAAAAGGGGACCTGGGGGTGCCTGGGCTGC
AGAAGTGATCAGGTAACTGAGCTCCTGGGACGTTAGGGCTGGGTGAGCAGAGCTTGC
CTGCCTTGGACAGTCAGGAGGGAGA
>2637
AGCCGGCTTCGCCGGGGATGACGCCCCTAGGGCCGTGTTCCCGTCCATCGTGGGCCGC
CCCCGACACCAGGTCAGGCTGCCCCTCCGCAGAGGGAGCCGGCTCGGGGTCCCCGCG
TAAGCCAGCCTGGTGCCACCCGGAG
>2638
TTCAACGTGCCCGCCATGTACGTGGCCATCCAGGCCGTGCTGTCCCTCTACGCCTCCGG
AAGGACCACCGGTGAGTGCCCGCTGGCCCCCAGTCCCCTCGTCCCGCCCCCGCCCCCG
CCCCCGCCCCCGGCCGCTAGCGC
>2639
GCGGGCCGCGATCTCACCGACTACCTGATGAAGATCCTCACTGAGCGTGGCTACTCCT

TCGTGACCACAGGTGCGCGGCGCCCCTGCACCCGGGCGGAGGGCCGCGGCGGCCTGA
GTGAGGGCTCCTCTCCTGCTTCTGC
>2640
GGGCAGGTCATCACCATCGGCAACGAGCGCTTCCGCTGCCCGGAGACGCTCTTCCAGC
CCTCCTTCATCGGTGAGCCCCGCTCGCCCTCGCCCCGGCCCCCAGGCCCGCGCCCCCC
GGCCCGAGCTTCTGCTCACGCTCC
>2641
GTACCCTGGGATCGCTGACCGCATGCAGAAAGAGATCACCGCGCTGGCACCCAGCACC
ATGAAGATCAAGGTGGGTGGTGGCCTGCGCGGGCTGTCGGCGGGGTGGGCTCCAGGG
TGAGGTCTCCCCACCTCACGCGCTG
>2642
AGGAATATGAACAAGCCGCTGCTTTGGATCTCTGTCCTCACCAGCCTCCTGGAAGCCTT
TGCTCACACAGGTAAGGAGGTGAAGGAATGGTCAAGAATCATAAAGTGAGAAAATAG
GTTGAAGCTGAGATATCTTTTCCC
>2643
CCCAACATCATCTTTGTACTTTCCCTGCTCCTCATCTTGGAGAAGCAAGCAGCTGTGAT
GGGACAAAAAGGTGAGTGGAGAGGGTAAGCCTTGGGGAAAGCTGCTCAGACAGCTA
ATAATCTAAGATTATTTGGGGTGCA
>2644
ACGACCGAAACCCATTATTTACATAAACCTACCATTCGGTAACCATGTGAAAGGATGGA
CCAATATCAAGGTAATTTTTTTTTAGCAAATAGGGGAGATATCTCTCTCATTGTTTAGAA
TTGTTGGGGACTCTCCAGGAC
>2645
TCCATCATCCTCTTTGTCCTTTCCCTGCTCCTTATCTTGGAGAAGCAAGCAGCTGTGATG
GGACAAAAAGGTGAGTGGAGAGGGTAAGCCTTGGGGAAAGCTACTTTAAAAAAATGG
CCTCTAAGGATATTCAGGGTGCA
>2646
AAGACAGAAATCCAATATCTACATAGCCCTGTTGCTTAGCAACCACTTGAAAAGCTGG
ACCAATAGCAAGGTAAGTTTGCTTTTCTTACCAAATAGGAGAGGTGCCTGTCCCAAAG
TTGGGGACTCTCCAGGAACATGGT
>2647
ACTCCTGGATCCCACGGCCTGCCAGGCAGGGACGGGAGAGATGGTGTCAAAGGAGAC
CCTGGCCCTCCAGGTACTGTGCTGCAGACCCCACCCTCAGCTGAGGGACACAGACCCC
TTTTCAGGAGGCCCATCTGTCCAGG
>2648
CTGCCTGGAGCCCCTGGTGTCCCTGGAGAGCGTGGAGAGAAGGGGGAGGCTGGCGA
GAGAGGCCCTCCAGGTGAGCAGGGCGGGCAGGTGGGCAGTGGAAACATGGGCACAG
CGACCTGAAGTCAGTTACACAGGGGTGA
>2649
GCTCATCTAGATGAGGAGCTCCAAGCCACACTCCACGACTTCAGACATCAAATCCTGC
AGACAAGGGGAGGTAAGGGGACCCCCTGGGCCTCACGGGGTAGGAGTTTCCCACAAA
TTCCCCTCATTCTCAGCACCACCTT
>2650
GTGTGGCCCTGATGTGGGCCGCCCAGACCGTGGGCGCTTGGGCTTCCAGGTCTGGCTG

AAGAATGGCGTGGTGAGTGGCACCCTGGGCTAGGGCGCTGGGGGGCTGGGTGTGCCA
CCCTGTGAGTCCTGGGCCAATCCCT
>2651
TTCCTGAAGGCGGCTGAGGACTATGGGGTCATCAAGACTGACATGTTCCAGACTGTTG
ACCTCTTTGAAGGTAGAGAGGAAGAGGCTGGGGGAGGAGGTGGGCAGGAGGACAGG
GTGCTGGGACAGGGAGAGGGAATGAC
>2652
TGGCTTTGGGCAGCTTGGCAGTGACCAAGAATGATGGGCACTACCGTGGAGATCCCAA
CTGGTTTATGAAGTATGTGGCCCCCAGGGAGCTTGGGTCTCCGCATGGGGTGGGAGGT
GGCTTGTTCTAAGGAGCTTGCGGG
>2653
CTTTGGGTGGGGGAACCTCACCTGCCCAATCTGCAAAGGTCTATTCACCGCCATCAAC
CTCGGGCTGAAGGTGAGCACTGAAGGGGCTGCAGTGGAGGAGGCCGAAAGGAGTGC
TGGGGCTGGGGGCTGGGGCTGATGCT
>2654
GCTGGCTCTATGAAGCGATGGCCAAGGCTTGGGAGCCCTGGCTGCCTGCCGAAGCCCT
GCGCACCCTCAGGTACTTATCGTCCGTGGAAACCCAGGAAGGGAAAAGAAAGGTGAA
TGAAAGTGAAGGGAGAAGGGAACCT
>2655
CACGGATCCCGCAGGACAGCTCCAGTGGCTGGTGGGGGAGCTTCAGGCTGCTGAGGA
TCGAGGAGACAAAGTGAGGGCCAGTAGTGGGAACACGGTGGTGCTGGGGGACAAGC
AGGCTCCTGTTGAGCTGGAGCACCTCT
>2656
TAATTGGCCACATTCCCCCAGGGCACTGTCTGAAGAGCTGGAGCTGGAATTATTACCGA
ATTGTAGCCAGGTAGGACGGAGATGAGGGTGGGAATAGGGACAGGGTGAGTGTCTGA
AGGCTGAAAATTCCCTTGAGCATC
>2657
ACTCTGAGCCGGCCGCTGGCTGTAGCCTTCCTGGCACCCAGTGCAACTACCTACATCG
GCCTTAATCCTGGTGAGTGAGGCAGAAGGGAGCCTCCCTTATCCTGGAGTTGGTGGGA
TAGGGGAAGGAGGTTGGAGCCAGA
>2658
CCACTTCCTCGATGAAGGTTTTACTGCCAAGGACATTCTGGACCAGAAAATTAATGAA
GTTTCTTCTTCTGTAAGTATATGAGGCCCATGCTGGCAGTGCAGCTGAGAGTGCCAGGC
AAGTGGAAAACTTTGGCAAGGTC
>2659
ATGTAATGATAGCAAAGCCATCGTGAAGACCCTTGCTGCTACCGGGACAGGATTTGACT
GTGCTAGCAAGGTAAGCGATAGCAGCAGGCCTCAAAAGCGTTGTATAAAATGGGCCTG
GTATTCCCCACGAGGCAGATACA
>2660
GAGTCCAGATGATGACTTTTGATAGTGAAGTTGAGTTGATGAAAGTTGCCAGAGCACA
TCCCAAAGCAAAGTGAGTTATTCCCCCATCTGAGGGCAAGATCGGGAGCATAAGATAT
GTGGATTCTTATCAAACAAACTTA
>2661
CCACGCTCAGAACCAGCAGGCTCCTTTTGGAACGGGCGAAAGAGCTAAATATCGATGT

TGTTGGTGTCAGGTGAGATTTTGGTGGGATAGCTAGAGGTCAAGACATTGAACAGTTT
GAGTTTTACAGGCTTTCTCCTAGT
>2662
AAGCGGCTGTACCGATCCTGAGACCTTCGTGCAGGCAATCTCTGATGCCCGCTGTGTTT
TTGACATGGGGGTGAGTATACGTGACCCTGTTAGGGAAGGGCGGGACACAACTGACAA
TAACTAGTCTTAATTCTAGAGTT
>2663
CAGCATGTATCTGCTTGATATTGGCGGTGGCTTTCCTGGATCTGAGGATGTGAAACTTA
AATTTGAAGAGGTAATTTAGAACAAAACTGTAATACTCAGTAGCCGTTCTAATAAATTC
CTTTTTGGAATATTTCAAAATT
>2664
TCAGCTTTCACGCTTGCAGTTAATATCATTGCCAAGAAAATTGTATTAAAGGAACAGAC
GGGCTCTGATGGTATGTATAAAGGACGAATCACTTCATGTATAACTGAAAGCTGATGCA
AAAAGTCATTAAGATTGTTGAT
>2665
TGATGGCGTCTATGGATCATTTAATTGCATACTCTATGACCACGCACATGTAAAGCCCCT
TCTGCAAAAGGTAATTTCTGAGCATACTGTATAAAACAATTAAGAGGACTGGTCACAAC
ACGTGTAATTAAGTAGTACTT
>2666
TTGCTGCTGCCTCTACGTTCAATGGCTTCCAGAGGCCGACGATCTACTATGTGATGTCA
GGGCCTGCGTGGTAAGTAAGCCATGCATGTTGATGGTGCTGCCAAGAATAGGCACCTT
CTTGGATGTGTGCTTCTTGTCTA
>2667
GCCATGGCTGAGTCACACCTGCTGCAGTGGCTGCTGCTGCTGCTGCCCACGCTCTGTG
GCCCAGGCACTGGTGAGTCTCCCCCAGCCTCCCCTCTCCTAGGCAGCTCCACCACTCA
CTGAGCACTGCTTTGTGCTAGGCA
>2668
CCTGGAGCAAGCATTGCAGTGCAGAGCCCTAGGGCATTGCCTACAGGAAGTCTGGGG
ACATGTGGGAGCCGTGAGTACCACCAAGGATGCATGGCAACTGGGGGTCTGAAATGA
AGGGTGCTGGGTGGGCTCTGGATGGG
>2669
TGACCTATGCCAAGAGTGTGAGGACATCGTCCACATCCTTAACAAGATGGCCAAGGAG
GCCATTTTCCAGGTAATGATGCCCAGATCCTGGATGAAGGTTGGGGCCCAAGAGATGA
GGGACAGAGCAGGGAAGAGCTGAG
>2670
CATGCCCCAGTGCAACCAAGTGCTTGACGACTACTTCCCCCTGGTCATCGACTACTTCC
AGAACCAGATTGTGAGGGCTGCAAGCTCACCTCCTGCCTGCCTCCCCACGCAGGCCCC
TGTGCCCACCCATGGGGGAGCCA
>2671
TCTGCTGGACAAGCTCGTCCTCCCTGTGCTGCCCGGGGCCCTCCAGGCGAGGCCTGGG
CCTCACACACAGGTGAGGGAGGCCCCCACAGCCAGTAAAGTGGAGATCCAGAGGGCT
AGAGCCACCTCCGAAGCCCATGGGC
>2672
CCCCATTCCTCTCCCCTATTGCTGGCTCTGCAGGGCTCTGATCAAGCGGATCCAAGCCA

TGATTCCCAAGGTGAGGCATCCAGGGCCTCAAGAGCCCAGGAGCACACGCATACCTGT
AGCTCCCTGCAGCTCCCACCTCT
>2673
GGCCGCATGCTGCCCCAGCTGGTCTGCCGCCTCGTCCTCCGGTGCTCCATGGATGACA
GCGCTGGCCCAAGTGAGCCCACTGCCCCCTCCTTAGCCCAATGCCCGCTCTCCTCCTCC
CCCTACCCTGCCACTGCATGACC
>2674
CAGCAGCGAGCAGGCCATACCACAGGCAATGCTCCAGGCCTGTGTTGGCTCCTGGCTG
GACAGGGAAAAGGTATGGGCTGGGCACATGGGGACTCATGGTCAGGGCCCGTTCAAG
GCAGAAGGCTGAGCCCAGGAAAGGC
>2675
TGTGGAGCAGCACACGCCCCAGCTGCTGACCCTGGTGCCCAGGGGCTGGGATGCCCA
CACCACCTGCCAGGTACACCCAACCCCTCCCAAGTTGGTCCTAGGACTTCCCTTGGCT
CCCAGAGCCCCCACCCTTTGGGCCC
>2676
TGTCGCTGCAGGTGGAGCAGCTGCTCCACCACCGGCGCTCGCGCTACCAGGACATCCT
CGTCTTCCGCAGGTACCGCCGCTGCCCGCAGGCGCCTGCCCCCTAGGCTCAGCCCGGG
CCGCCTGCTGCCCGCCTCACGGGC
>2677
GAGAGACGAGTTCTCCTACCAGGAGATGATCGCCAACCTGCCTCTCTGCAGCCACCCC
AACCCGCGAAAGGTACCCCAGTGTCCCCTGGAACAGTGCCGGACGAGGGGCGGCCCC
AGGTGTGCTCCGGGCTCTTCCCAGA
>2678
TGGAGGTGTCCTGCGGGAGGTGGTGAAGCACCCCTCCGTGGAGTCCGTGGTCCAGTG
TGAGATCGACGAGGTGAGTGCCGGCGTAGAGCCAGGTTTGAGTCCTGGTTCTCCCAGC
GGCCAGCTGTGCCCTGAAATGGCTG
>2679
GGTTTTGAGTTCATGAAACAGAATCAGGATGCCTTCGACGTGATCATCACTGACTCCTC
AGACCCCATGGGTAAGCAGTGGATGGGCCCCAGGGTTTTCTGGCAGCTGCAGGTCTGG
AGGTCAGCCTCCCCCAGGCCTTC
>2680
CTCTTCAAGGAGTCCTATTACCAGCTCATGAAGACAGCCCTCAAGGAAGATGGTGTCC
TCTGCTGCCAGGGTGAGCCACAGGCCTGGAGCACTGGGGCGGGGCGGGGTGGGGCA
GGGCAGGCCCTGCCGGATGCTGATGC
>2681
CTATGCCTACTGCACCATCCCCACCTACCCCAGCGGCCAGATCGGCTTCATGCTGTGCA
GCAAGAACCCGGTGAGATGGGGGTGTCTGGGGGTGGGGGTTGGGGGGAAGGTGGGC
ATAAATAGAGATCCCTGCCCCTGCC
>2682
GATGCAGCTGAAGTACTACAACTCCGACGTGCACCGCGCCGCCTTTGTGCTGCCCGAG
TTTGCCCGCAAGGTGGGTGGCCTGCGGGGCTGGGTGGTGGGACCCAGGGACCCAGAG
CGCCCTCCTGACTGGCCTCATGTCC
>2683
GTCGGAAGAGGTGTGGCGGCATCCTAGTGAGAAAGGACTTTGTGCTGACAGCTGCTC

ACTGCCAGGGAAGGTAAGGAGCAGCACCAGCTCACCTCCTGAGTTCCCCCTACAGGG
ACCCTTGTTTTCTCCTGGGGACTGCA
>2684
TGTGAAAAGACCCATCCCCCATCCAGCCTATAATCCTAAGAACTTCTCCAACGACATCA
TGCTACTGCAGGTGAGGCACACTCCTGCCACTCTTGCTCTTCTTGGTCCAGTTGGTTCC
ACTCCCACTGGGATGCCGGCCC
>2685
CCATGGCAATTACAGCAGAGCCACTGAGATTTGTGTGGGGGATCCAAAGAAGACACA
GACCGGTTTCAAGGTTGGGTTTCCAGCCTCTACCCAGAAAGACCACAGGGAGAGAGG
AACTTGAGGGAGTTCTGGGGTATGAC
>2686
GCAGATCTTTCGGATACCGAGCTGACCACCTTGCTGCGCCGGTACAACATCCCGCACG
GGCCTGTAGTAGGTACGCGGCGGCGGGCGGGACCCCTTCCGGGCCCCCTTCCTTCGTG
CTCCGCCTTCGCGACCTCCCCGCT
>2687
GAGTACGAGACCCAGAGGCGGCGGCTCTCGCCCCCCAGCTCGTCCGCCGCCTCCTCTT
ATAGCTTCTCTGGTGAGAGCCTCGCCTGTGGGGTCAGCCTGGGACGCGGGGAGGATGG
GGTCGCGAGGGTGTGGCAGGGGGG
>2688
TCGACTAGAGGGGATGCAGATATGTATGATCTTCCCAAGAAAGAGGACGCTTTACTCTA
CCAGAGCAAGGGTAAGGCAGGGGTTGGGTGGGCACGCTGGCACCTTCACCCGACTTC
GTCAGGGACCCCGCTCACAGGGAG
>2689
TGCCGGCCCGTCCAGGGCTGTCCGCCAGTCAGTGACTTCATTCCCAGATGCTGACGCT
TTCCATCACCAGGTGAGCTGGCTGGCAGGCGTCCTGTACTTGGGTACAACCTAGGGGA
TCGCGGCTGTGTTTGGATAAATCC
>2690
GTTGTGGGTCTAATTCAAGACCTCAAGCCAAACACCATCATGGTCTTAGTGAACTATAT
TCACTTTAAAGGTAAGACTTTCAGATGTCAATTTTCAGTCTAGGGCTCAGATACTTGGG
TGGAGTGCTGAGGGGAGCTCAT
>2691
AGATGGAGTCAGTGGAAGCTGCCATGTCATCTAAAACACTGAAGAAGTGGAACCGCTT
ACTACAGAAGGGGTAAATGCCTTAGAGGATGTGTGGGTGGAAGGTGGGCATAAAAGT
GCAGGTGAGACAGAGTTCAGCAGAA
>2692
TCAGCATGCCTATTCTGAAAATGCTGATTTTTCTGGACTCACAGAGGACAATGGTCTGA
AACTTTCCAATGTAAGTTGATAAACTAGAGTTCTTAAATGTATGAACTGGAAGAGCAAT
TAGGAGTCAATTGATTCAACTT
>2693
GCTAACGCCTCTTTTCCCCCTAATTGTTAAAAGCTATGGACTGCAGGAAGATGGCCCGC
TTCTCTTACAGGTATGAGCTAATCTTAGAATAGTGAACTTTTTTTGATTGCTAGAGATTG
CCAGCTTAGGAAGTAATGTTC
>2694
TGGCCCCAGGAGGAGCCTGCAATTCGGCCTCGGTCTTCCCAGCGTGTGCCGCCCATGG

GGATACAGCACAGTAAGAACTGCCTGACTTCGATGCTTCTGCCCTGGCCCTTCATGTGT
CTCCTGACTATCTTTCCAACACT
>2695
TGCTGGGGTCCTTTTGTGCCTGCCCTCCCTCCTTCTACGGACGGAACTGTGAGCACGAT
GTGCGCAAAGAGTAAGCAATTCAGAGGGGCGGGGAGCCGTGGAGAGGAGAGAGAAA
GGGAAGTGGAAATTTCAGACCCAAG
>2696
AAGTGTTCCCTGTGTAAATGCTGGCACGGTCAGCTCCGCTGCTTTCCTCAGGCATTTCT
ACCCGGCTGTGGTAAGCGGAGGTTCTCCTCTTTCTTTTGCCCTTTGAAGTTACGTAGTT
GCCTTGGGGGGTGCTTAGTTAG
>2697
CGCCCCGAGACCGCCGTCGCTCGGACGCTCCTGCTCGGCTGGGTCTTCGCCCAGGTGG
CCGGCGCTTCAGGTGAGTGGCACCAGCCCCTGGAAGCCCGGGGCGCGCCACACGCAG
GAGGGAGGCGACAGTCCTGGCTGGC
>2698
CAACTAATTTCAAGACAATTTTGGAGTGGGAACCCAAACCCGTCAATCAAGTCTACAC
TGTTCAAATAAGGTAAGCTGGGTACAGAAAAAGAAAATTAAGGTCTTTGATGTTTCTAC
TGTCCTATGCTGAACAAGAATGT
>2699
GTGGAGAGCACCGGTTCTGCTGGGGAGCCTCTGTATGAGAACTCCCCAGAGTTCACAC
CTTACCTGGAGAGTAAGTGGCTTGGGCTGTAATACCGTTCATTCTTGTTAGAAACGTCT
GAACATTCTCGTGATCTTGTGCC
>2700
CCTCCGGGATGTTTTTGGCAAGGACTTAATTTATACACTTTATTATTGGAAATCTTCAAG
TTCAGGAAAGGTGAGCATTTTTTAATTTGTTTTTATGACCTGTTTTAAATTGTGAATACT
TGGTTTTACAACCCATTTCT
>2701
CGAACAGTTAACCGGAAGAGTACAGACAGCCCGGTAGAGTGTATGGGCCAGGAGAAA
GGGGAATTCAGAGGTGAGTGGCTCTGCCAGCCATTTGCCTGGGGGTATGGGTGCTGTG
GGTGACTTCTGGAGGAGTAGCTCCA
>2702
GTCCGAGGCTTGCAGCTGCCTGGCTGCCTGGCCCTGGCTGCCCTGTGTAGCCTTGTGC
ACAGCCAGCATGGTAAGGGAGTGCTTGCAGGCTGGAACAGGCTGGAGGACTGGGGTG
TGGGCCCATGGGCTGGGGTCTCCTG
>2703
GCGAGAGTGCGTGGAGGAGACGTGCAGCTACGAGGAGGCCTTCGAGGCTCTGGAGTC
CTCCACGGCTACGGTGAGCCTGGGCTGCTCGGACGGTGCCGGGGCCTCAGACCGGGC
CCAACTCTAGACACTTCCACAGAGAA
>2704
GGCATGTGAACATCACCCGGTCAGGCATTGAGTGCCAGCTATGGAGGAGTCGCTACCC
ACATAAGCCTGAGTGAGTGAGGGGTCGGCCTTCCCACCATGGGCTGAGAACAGGGAG
CAAGCGTACCTCAAGTTCAACAGCC
>2705
ACCACGGGACCCTGGTGCTACACTACAGACCCCACCGTGAGGAGGCAGGAATGCAGC

ATCCCTGTCTGTGGTAGGCTGGGGGCAGTGGGGCGACCCATGACCAAGCCCGGGGGC
TTCATGGGGCCTGGCAGCCTGGGATG
>2706
GAGGAGGGCGTGTGGTGCTATGTGGCCGGGAAGCCTGGCGACTTTGGGTACTGCGAC
CTCAACTATTGTGGTGAGCTGCCTGGGTAGGGGGCCTGAGTTGCAGGGACAAATCCTA
GTGGGAATAACAACAGCCGCTTCTG
>2707
GAAGGGCGTACCGCCACCAGTGAGTACCAGACTTTCTTCAATCCGAGGACCTTTGGCT
CGGGAGAGGCAGGTGAGGTAGTGGGCATCCGAGGGGATGCGGGGCTGCGGGGCTGGT
GGCCAGGACTTGCCCCTCACTGCTT
>2708
AGCTCCTGGAATCCTACATCGACGGGCGCATTGTGGAGGGCTCGGATGCAGAGATCGG
CATGTCACCTTGGTGTGTCCTGGAGCCCTGCGCTACCATTCACTCCTGGGGGCAGGTGT
GCTGCTGGACCCCCACCCTCAGG
>2709
ACCCGCCCTGGGACAAGAACTTCACCGAGAATGACCTTCTGGTGCGCATTGGCAAGC
ACTCCCGCACAAGGTACAGAACTGGTGGCCCGTGGGTGTCTGGCAGGGGTCTGAGTC
CTCCAAAGCGATCATGAGGGGCCTTG
>2710
TGAAGAAGCCTGTTGCCTTCAGTGACTACATTCACCCTGTGTGTCTGCCCGACAGGGA
GACGGCAGCCAGGTGGGCCACCAGATGCTTGTTAGCTGAGGGGCAGAAGCCAAGTTC
TGGGCCTGGCTCTGATACCAAGTAG
>2711
TTACAAGCCTGATGAAGGGAAACGAGGGGATGCCTGTGAAGGTGACAGTGGGGGACC
CTTTGTCATGAAGGTAAGCTTCTCTAAAGCCCAGGGCCTGGTGAACACATCTTCTGGG
GGTGGGGAGAAACTCTAGTATCTAG
>2712
TCTTCCTACCAATCTGCTCCCCAGAGGGCTGCCTGCTGTGCACTTGGGTCCTGGAGCCC
TTCTCCACCCGGTGAGTGGCCAGCAGGGTGTGGGGTTATGTGAGGGTAGAAAGGACA
GCAAAGAGAAATGGCCTCCCAGCT
>2713
GGATTCAGGGGAGAGGCCCCAAACAGGGAGCCACGCCAGCCAGACACCCCGGCCAG
AATGGAGCTGACTGGTGAGAACACACCTGAGGGGCTAGGGCCATATGGAAACATGAC
AGAAGGGGAGAGAGAAAGGAGACACGC
>2714
TCCTCCTGCTTGTGACCTCCGAGTCCTCAGTAAACTGCTTCGTGACTCCCATGTCCTTC
ACAGCAGACTGGTGAGAACTCCCAACATTATCCCCTTTATCCGCGTAACTGGTAAGAC
ACCCATACTCCCAGGAAGACACC
>2715
TCACCCTTTGCCTACACCTGTCCTGCTGCCTGCTGTGGACTTTAGCTTGGGAGAATGGA
AAACCCAGATGGTAAGAAAGCCATCCCTAACCTTGGCTTCCCTAAGTCCTGTCTTCAGT
TTCCCACTGCTTCCCATGGATT
>2716
CCTCCTGGGGCAGCTTTCTGGACAGGTCCGTCTCCTCCTTGGGGCCCTGCAGAGCCTC

CTTGGAACCCAGGTAAGTCCCCAGTCAAGGGATCTGTAGAAACTGTTCTTTTCTGACT
CAGTCCCCCTAGAAGACCTGAGGG
>2717
TACACGTGTGCACTCCACCACTCTGGCCATTCCCCACCCATCTCCTCCCAGAACGTCAC
AGTGCTCAGAGGTGAGACAAGCCCCTAACAAGGTCAAGTGAGCTGGGAGAGCCAGG
CTCGGGGACAGCAGGCAGTTCCCTT
>2718
AGTACAAGTGCCTGGTGATCAAGTATGCCAAAGACACTCGCTACAGCAGCAGCTTCTG
CACACATGACCGGTCAGTCCCTGCCCCCTGCAGTCCTGTCCAGTGGAAAATCACAAGG
CACAGGACACACTGTTAGGACTCT
>2719
CTGCCTGCTCCGAGACGTGGCCCAGGAGGCCCTGGGCGTGGCTGTCATAGGCATCGAC
GAGGGGCAGTTTGTAAGTTGGCTTGTCTTGGCATCACTCTTCCTGCCTTCCGCTGTGTC
CTCCCGTTTTCCCTCGCTGACTT
>2720
CTGCGAGGCCATGGCCAACGCCGGGAAGACCGTAATTGTGGCTGCACTGGATGGGAC
CTTCCAGAGGAAGGTAAGGCGTCTGATCCAGGTCTGGAGCTGGGATTGAGGAGGGCA
AGAGGCTTCTGGATGGGCACAGAGAC
>2721
GCTGACGGCGGTGTGCATGGAGTGCTTCCGGGAAGCCGCCTATACCAAGAGGCTCGGC
ACAGAGAAGGAGGTAGCTCCACCTGCCTTCCCTGCAGGCCGGCGGGGTGGGGGTATG
GCTCTGCCTCCTTCCTGTCCTGGCC
>2722
ATGAGGATGCTGGGCCAGACACCCACCAAGGAGGAGCTGGACGCCATCATCGAGGAG
GTGGATGAGGACGGTGAGCGGGTGTCCCTCGGAGGCAGGGGATGGTGGGGAGAGGC
GGCAGCGGCTGGGCTCAGGCTCAGTCT
>2723
GCCAGATGAAAGAGGACGCGAAAGGGAAGAGCGAGGAGGAGCTGGCCGAGTGCTTC
CGCATCTTCGACAGGTGCGCTGGGGGCCCGGGAGCCGAGGGAGGGGCTTAGCAGTCA
GAACCCGGCCGGCAGCGCTGGAAGCTT
>2724
ACGGACGAGGAGATCGAATCTCTGATGAAAGACGGCGACAAGAACAACGACGGCCGC
ATTGACTTCGACGGTGAGGGCCAACGGGAGCTTGGGAAGAGCGGGTGGGAGCCCCAG
AGGACGGCGGGCACTGGTGCCTGGCT
>2725
CCTCTGCCTGGGCCTCGGTCCCTCCTGCACCTGCTGCCTGGATCCCCGGCCTGCCTGG
GCCTGGGCCTTGGTGGGTTTGGTTTTGGTTTCCTTCTCTGTCTCTGACTCTCCATCTGTC
AGTCTCATTGTCTCTGTCACAC
>2726
AAGGGTGTGTGGCACCACCCTACACCTCCTCCTTCTGGGGCTGCTGCTGGTTCTGCTG
CCTGGGGCCCAGGTGAGGCAGCAGGAGAATGGGGGCTGCTGGGGTGGCTCAGCCAA
ACCTTGAGCCCTAGAGCCCCCCTCAA
>2727
CAGACTGCCCGTCAGCACCCCAAGATGCATCTTGCCCACAGCACCCTCAAACCTGCTG

CTCACCTCATTGGTAAACATCCACCTGACCTCCCAGACATGTCCCCACCAGCTCTCCTC
CTACCCCTGCCTCAGGAACCCAA
>2728
GATCGTGGCAGGCGCCACCACGCTCTTCTGCCTGCTGCACTTTGGAGTGATCGGCCCC
CAGAGGGAAGAGGTGAGTGCCTGGCCAGCCTTCATCCACTCTCCCACCCAAGGGGAA
ATGGAGACGCAAGAGAGGGAGAGAG
>2729
GGCAAGCTGAAAAAGAAAAAAATGGCCAAGAGGATCCAGCAGGTGTACAAAACCAA
GACGCGGAGCTCAGGTACCCTTTAAGGAGGTGGGGAAGGGCCACCGAGCCCACAGAT
GATGGAGAGCAGACCTTGGGGGCAGTG
>2730
AGCTGAGATCCTACAGGAGTCCAGGGCTGGAGAGAAAACCTCTGCGAGGAAAGGGA
AGGAGCAAGCCGTGGTAGGTCGGGTTTCTGTACCTTGGGGTCTGTCTCCTCTTCTTTCT
CTTAAAAGTCTTTCCAGCAAGCTGA
>2731
GGATGCAATGAAGAGAGGGCTCTGCTGTGTGCTGCTGCTGTGTGGAGCAGTCTTCGTT
TCGCCCAGCCAGGTTGGTGTGCAGGATCCCTGTGTCCCGCCCCTCAAGGCTGTGATGC
TTCCGGAGGCTCCAGGGGTCTGTG
>2732
AGAAGCAACCGGGTGGAATATTGCTGGTGCAACAGTGGCAGGGCACAGTGCCACTCA
GTGCCTGTCAAAAGTATGTGCTGAGGCTGGAAGGTGGTGCATGCCTGTGATCCCAGCA
CTTTAGGAGGCCAAGGTGGGAGGGT
>2733
CAGGCCCTGTACTTCTCAGATTTCGTGTGCCAGTGCCCCGAAGGATTTGCTGGGAAGT
GCTGTGAAATAGGTGAGTAGGTGAGAGCACGTGAAACGAGCTGAAATCCCACCTCTC
CAGTCTCCCCCGCCCCCGGAAGGAA
>2734
CCCAGAAGCCCTACAGCGGGCGGAGGCCAGATGCCATCAGGCTGGGCCTGGGGAACC
ACAACTACTGCAGGTGAGGGCACATCCCCAAGGAGGGATTCCCCCTGTGAGAAGCTG
CTACAGCTGGGGAAAGATTCTGTCAG
>2735
CCCTGGTGCTACGTCTTTAAGGCGGGGAAGTACAGCTCAGAGTTCTGCAGCACCCCTG
CCTGCTCTGAGGGTAAGTGGCAGCCACCCCGCCTTTCCCTGAATGTCCCTGGGGGAGG
AAAGTCCAGGAGGACAGGCCGATA
>2736
TAGGCAAGGTTTACACAGCACAGAACCCCAGTGCCCAGGCACTGGGCCTGGGCAAAC
ATAATTACTGCCGGTAGGTAGCACAGGGGTGGGGGGTTCAGGTCTTGGCAGAACGTGG
GATTAGGGTGTGAGACGGGGGAAGA
>2737
GCCAAGCCCTGGTGCCACGTGCTGAAGAACCGCAGGCTGACGTGGGAGTACTGTGAT
GTGCCCTCCTGCTGTAAGGGCTGGGCCCCGGCTGCCTCCCTGCACCTGTCTCCCTTCTT
CACCATCTCCTCGCCACTTTCTAG
>2738
GGTTCCTGTGCGGGGGCATACTCATCAGCTCCTGCTGGATTCTCTCTGCCGCCCACTGC

TTCCAGGAGAGGTAGGGGCTCGGAAACCCAGTTGGTTTTTCCACCCTCTAACGCCTAG
AAACCAAGCCCTTTAGGGGAAAT
>2739
CAGAAATTTGAAGTCGAAAAATACATTGTCCATAAGGAATTCGATGATGACACTTACGA
CAATGACATTGGTAAGAGCTCGTCATTCCCGGCTCCAGTTCATACAGATCACACAACAC
ACCCACATGCACTCACCTATGT
>2740
CCCCCGGCGGACCTGCAGCTGCCGGACTGGACGGAGTGTGAGCTCTCCGGCTACGGC
AAGCATGAGGCCTGTAAGTGGAAGGAAGCCTCGGCCCCATCCTGTCTGCGGGACAGC
AGGGGAGGCTGCGGTCCACCAGAGGA
>2741
CGACAACATGCTGTGTGCTGGAGACACTCGGAGCGGCGGGCCCCAGGCAAACTTGCA
CGACGCCTGCCAGGTAACCAGGAGTGGCGGCCCATGCTGGGCACAACCCAGGGGAGA
ATGCCCCTGGGACAACAGGTTCTTCA
>2742
CGTCAGCCTTGGCCTCATTGCTGCCCTGCAGGCCCACCACCTCCTGGCCTCAGACGAG
GAGATTCAGGATGTGAGGCCCGGATGGAAGGCTGGGCTGGAGGGGGCAAGGGGCGA
GGCTGAGACTGGATGGAGACCCCATG
>2743
TGGAATCGGTGACACCCATGACCCTCACGACCCTGGAAGGGGGCAACCTGGAAGCCA
AGGTCACCATGCTGTGAGTGTCTGCCAGCCGGCCGGGCAGCCTGCAACCTGGTCTAGG
GCCTTCCCTTTCCCCACCCAGGAGA
>2744
ATAAGTGGCCGGTGCCAGGAGGTGAAGGCCGTCCTGGAGAAAACTGACGAGCCGGGA
AAATACACGGCCGGTGAGTCCCGGGGCCTGAGCCAGAGCCTGAGCTTGAACACACGC
TGGATGTGGGGGGGCAGCCAGTGCCT
>2745
AAGGACCACTACATCTTTTACTGTGAGGGCGAGCTGCACGGGAAGCCGGTCCGAGGG
GTGAAGCTCGTGGGTGGGTCCCGCACCCTCACCCTGCAACCCATGCCTCCACCTGCCC
TCCCTCCTCCCTCGCCCTCCTGCAC
>2746
GAGGACTTTGAGAAAGCCGCAGGAGCCCGCGGACTCAGCACGGAGAGCATCCTCATC
CCCAGGCAGAGCGGTAGGAGGCATGGCCCTGCAGAGCCCCCCATGTCCCCGCGTGGG
GACATCAGCAGAGCTGCATTGCACGG
>2747
TGATGGAGCAGCATTAACTAAGAAAGACCTGAAGAACCTCCTTGAAAGGGAATTTGG
AGCTGTGCTTCGGGTAAGAACTAACAAGAAAATGAGATCTATTGACTTGAGGCTATGA
GATTTATTCTCAGAGGAGACCAGAG
>2748
ATGAGGATGCTGGGCCAGAACCCCACCCCTGAGGAGCTGCAGGAGATGATCGATGAG
GTGGACGAGGACGGTGAGCCCCCCTCCTCCCCAGGCTCCAGAAGAACCCCAGCTGGC
TGGGGGCTGGAATGCTGGCTCTGTTT
>2749
GGTGCATGAAGGACGACAGCAAAGGGAAATCTGAGGAGGAGCTGTCTGACCTCTTCC

GCATGTTTGACAAGTGAGCACGTGACCCTTGACCTCTGACCCTGACCCACACTCAAGC
CGAGCTGTACAGGAGGGCAGTCTCA
>2750
ACGGAGGACGACATCGAGGAGCTCATGAAGGACGGAGACAAGAACAACGACGGCCG
CATCGACTATGATGGTAAGCGGGTGGGTGGGCTGATCTCCTGCCTCCATGCCCTGCCCA
GCCCCTACCCTCAACCCACACCTGC
>2751
GTTTAACTGGGTGTCCCGGCTGGCAAACCTCACGCAAGGCGAAGACCAGTACTATCTG
CGGGTCACCACGGTGAGCTGTGTCCCGGCCACATGCTGTCGGTCGGAGCCCGACGTGT
GATCGAGCAGGGGCATGTGTGCTT
>2752
CCAGGAAGAACCCTAAATTTATGGAGACCGTGGCGGAGAAAGCGCTGCAGGAATACC
GCAAAAAGCACCGGTAAGCAGGCGGGCCTTTCCTGCGCGTGCAGGGCCCAGTGAGTC
TCTGGGAGCCACAAAAAAACAAACAA
>2753
AGGAAGGACGACCGCACGGGCACCGGCACCCTGTCGGTATTCGGCATGCAGGCGCGC
TACAGCCTGAGAGGTGACGCCGCGGGCCCCTGCGGGACGGGTGGCGGGAAGGAGGG
AGGCGCGGCTGGGGAGAGCGCTCGGGA
>2754
ATTCCCTCTGCTGACAACCAAACGTGTGTTCTGGAAGGGTGTTTTGGAGGAGTTGCTG
TGGTTTATCAAGGTAAAGAAGTCGCTGCTATTAGAAGTCAGTAGTCTGTTCTCAACACA
GCAGCCAGTGAGATCCTTTCAAA
>2755
GGGGACTTGGGCCCAGTTTATGGCTTCCAGTGGAGGCATTTTGGGGCAGAATACAGAG
ATATGGAATCAGGTGAGGAGATAGAACAATGCCTTCCATTTCCGGGTGCCCTTCCTAGC
ACGTGTTTGCTCCGTTGTTTTAG
>2756
AGAGTGATTGACACCATCAAAACCAACCCTGACGACAGAAGAATCATCATGTGCGCTT
GGAATCCAAGAGGTTGAAAGAACCCCGTCGTCTTCATTTATACTAACCATACTCTTAGA
GGGAAGCAATCTGGTTTTGTGCA
>2757
CGGTGTGCCTTTCAACATCGCCAGCTACGCCCTGCTCACGTACATGATTGCGCACATCA
CGGGCCTGAAGGTGGGCTGTCTCGGGAAGGGTGACTTGCCAGCCTACCACATGAGCT
CTTCAGTTCTTTAATATGGGAAAA
>2758
AGGTGACTTTATACACACTTTGGGAGATGCACATATTTACCTGAATCACATCGAGCCAC
TGAAAATTCAGGTAAGAATTAGATGTTATACTTTTGGGTTTGGTACCTTCTCTTGATAAA
AGGTTGACTGTGGAACAGGTA
>2759
CTGGTCTCCGAGAAGCTGAACGTCCCAGTGCGCCAGCAGCGGCTGCTGTTCAAGGGC
AAGGCCCTGGCAGGTACCCAGGGAGAGGAGACGCCCGGGGAGCCCCGCAGGAAGCG
GGACCGGGGTGCGCGGGCCGAGGCCAG
>2760
GATCTCCAAAGTCTTGGCCCGCCACTTCAGTGCGGCAGATGCCAGCAGGGTCCTGGAA

CAGCTACAGAGGGTGAGAAGGGTAACCCTGGGCATCCTCTTCAGGCCCAGTTGCTCCC
AGCCCCTACCTCTGTTGCAGTGTA
>2761
CGCACATCACCTCCAGGCCCTCAGAACACCTGCCCCAGCCCCACCATGCTCATGGCGT
CCACCACTTCCGGTAAGGCTTGCCCCTCCATGAGTCCGGTGGGCAGAGTGGGTTTGAC
GATTCAGGGAAGCCCCTCTTTCTA
>2762
GTGCTGTGCTGGGCACCCTTCTTCCTGGTGCAGCTGTGGGCCGCGTGGGACCCGGAGG
CACCTCTGGAAGGTGGGTGTAGCCGTGGCTAGGGCTGACGGGGCCACTTGGGCTTGG
CCGCATGCCCCTGTGCCCCACCAGC
>2763
TCTGGTAGAAAAATGAAGAGGGTCCTGGTACTACTGCTTGCTGTGGCATTTGGACATG
CTTTAGAGAGAGGTAAGATTTCTTTTGTTGTGACCATTTACAGGAATTCTTACTAGTTTA
ATTTTATATTATCACTTAAAAA
>2764
GCCGGGATTATGAAAAGAATAAAGTCTGCAAGGAATTCTCCCATCTGGGAAAGGAGGA
CTTCACATCTCTGTAAGTGTGCAGCAGCCACTCACTCTGTTGGTGATTTGATCTTGAAC
AAAACTGAGTGTGAGGTAACTTT
>2765
GAAGGAAGTTGTCTCCTTGACCGAAGCCTGCTGTGCGGAAGGGGCTGACCCTGACTG
CTATGACACCAGGGTAGGTTTCTGTGGCTGGCCGTCTCTGTGGCAGCCCAGAGAAGGA
AGCCAAAATAGGCCTTTATACCACG
>2766
CCTACGTGGAACCCACAAATGATGAAATCTGTGAGGCGTTCAGGAAAGATCCAAAGG
AATATGCTAATCAGTGAGTGCCTTCATCATAAATAGAACTTTAGGACCTAAAGTATCAGA
AATGACTCTAATCTAACCCCCTA
>2767
CAAGAGTTATCTTTCTATGGTAGGGTCCTGCTGTACCTCTGCAAGCCCAACTGTATGCTT
TTTGAAAGAGGTATGTCCCATTTTACTTATTATATGTTTACGTTTTTTTCTTCTAATAGCAT
TCCTTTTAGATAATGGTA
>2768
TTCTCACCACTCTGTCAAATAGAGTCTGCTCACAATATGCTGCTTATGGGGAGAAGAAA
TCAAGGCTCAGGTAAAGATTAAGTGCTATTGTCATATTTAGATGTTTGGTTCTGCACTGT
CTATCCATAATATTAGGGTTT
>2769
AGCTGAAGATATTACTAACATCCTCTCCAAATGCTGTGAGTCTGCCTCTGAAGATTGCA
TGGCCAAAGAGGTAAGACAAGCTTTATTATCATTATCACCATGTGCCAGGTATTGAGTG
CCAAGTACTGAGTGGCAGGTAC
>2770
AGCTTCCAGATGTAGAGTTGCCCACAAACAAAGATGTGTGTGATCCAGGAAACACCAA
AGTCATGGATAAGTAAGTAGAGGTGATGTGAAAACGTGTTCCCATTTTAAGTTCATCTG
GACAAAAAGTGGGGGCCAGCCAG
>2771
TGAGCCAACCCTAAAAAGCCTTGGTGAATGCTGTGATGTTGAAGACTCAACTACCTGT

TTTAATGCTAAGGTATATTTGTTGGATTTTCTTTATCAAGCACATAGATCAAGGGTTGGA
AAATTATAGTTCATGGGCCAAA
>2772
CTTTCATTGACAAGGGACAAGAACTATGTGCAGATTATTCAGAAAATACATTTACTGAG
TACAAGAAAAAGTAAGAAACTTGTTCTGGCTGTATCCTCCAAATTTATCAATAATATTTT
CATAGTACTATGAATTGAAAG
>2773
TAACAAGCGCTCAGACTTTGCCTCCAACTGCTGTTCCATAAACTCACCTCCTCTTTACT
GTGATTCAGAGGTAGGAAAATGTAACCCTCCACTTAACATGGCAGAATCTTTTAAGAA
CGTATGCACTCCAATCTACTCAT
>2774
CTCCGCGTGCTACTTCCAGAACTGCCCGAGGGGCGGCAAGAGGGCCATGTCCGACCT
GGAGCTGAGACAGGTACTTCCCACTGTGGGCCATCTCAGGGCAGCCATAGCGGGCAGT
GCTGACACCCTGGGTCAGGGGCTAG
>2775
TGCCAGTCCGGCCAGAAGGCGTGCGGGAGCGGGGGCCGCTGCGCCGCCTTCGGCGTT
TGCTGCAACGACGGTGCGCGGCGGGGGCGGGCCTGGGGGGGGGCGCAGACGCTTGG
GTGGGGGGGACGCGGGCCTGCGGCGGG
>2776
CGTGGGCCAGAAGACCAGGACATATCTATTTCCTTTGCATGGGATAAAAGCTCTGCTCT
TCAGGTTTCAGGTGAGAGAGGCCAGCATAAAAAAGCTACCGAGTCTAGAGAGACGGA
TGGGAGATGGGCTCTGGAATCACA
>2777
TGGGTGGTGGCTCTGCTAGTGAATCTGACCCAACTGGATTCCTCCATGACTCAAGGCA
CAGACTCTCCAGGTAAGAACAGAGCAATTGTTTTTTTCCAGTGTGTATGCAAGAATTG
GCATGGGGGAGTGATGCCTTTCTT
>2778
AGACAGGCCGTGGATGGGGTCTGTAGACACAACTACAGGCTGGGCGCACCCTTCACT
GTGGGGAGAAAAGGTGAGCTGGAAGCTGAGGTCTGGCGGGGCTCAGGAATGTCCCCC
ATGTGAACCATGGCTCTTCTTTCTTA
>2779
GAACTTGGACATGTCTACACCTGCCTTGTCGATCACTCCAGCCTGCTGAGCCCTGTTTC
TGTGGAGTGGAGTGAGAATTAGTTTCTAGTACTCTCTGGGCCTGACTCAGGACTATACT
GACTCAATACAGAGCCTGTGTC
>2780
ATTGCAGCCTTCCTACTTGGGCTAATCTTCCTTCTGGTGGGAATCGTCATCCAGCTAAG
GGCTCAGAAAGGTAATGAGCCTGTGAGGAGTGCCCTGCCACCTGTCCCAGACCTTCCC
CACTCCCACCTTCCCTAACGTCA
>2781
GCCGTCATGGCGCCCCGAACCCTCGTCCTGCTACTCTCGGGGGCCCTGGCCCTGACCC
AGACCTGGGCAGGTGAGTGCGGGGTCGGGAGGGAAACGGCCTCTGCGGGGAGAAGC
AAGGGGCCCGCCTGGCGGGGGCGCAA
>2782
AAGGCCCACTCACAGACTGACCGAGAGAACCTGCGGATCGCGCTCCGCTACTACAAC

CAGAGCGAGGCCGGTGAGTGACCCCGGCCCGGGGCGCAGGTCACGACCCCTCATCCC
CCACGGACGGGCCGGGTCGCCCACAG
>2783
CTGGAGGGCACGTGCGTGGACGGGCTCCGCAGATACCTGGAGAACGGGAAGGAGAC
GCTGCAGCGCACGGGTACCAGGGGCCACGGGGCGCCTACCTGATCGCCTGTAGGTCTC
CCGGGCTGGCCTCCCACAAGGAGGGG
>2784
GGAGAGGAGCAGAGATACACCTGCCATGTGCAGCATGAGGGTCTGCCCAAGCCCCTC
ACCCTGAGATGGGGTAAGGAGGGAGATGGGGGTGTCATGTCTCTTAGGGAAAGCAGG
AGCCTCTCTGGAGACCTTTAGCAGGG
>2785
CTGGTTCTCCTTGGAGCTGTGATCACTGGAGCTGTGGTCGCTGCTGTGATGTGGAGGA
GGAACAGCTCAGGTGGAGAAGGGGTGAAGGGTGGGGTCTGAGATTTCTTGTCTCACT
GAGGGTTCCAAGCCCCAGCTAGAAA
>2786
GGATGCACTTCTGCGGGGGCTCCCTCATCCACCCCCAGTGGGTGCTGACCGCAGCGCA
CTGCGTGGGACCGTGAGTCTCCCGGGGCCTGGAGGGGTGGGGAAGGGCTGGATGTGA
GCCCTGGCTCCCGGGTGCTCCTGGG
>2787
CCTGCCTCAGAGACCTTCCCCCCGGGGATGCCGTGCTGGGTCACTGGCTGGGGCGATG
TGGACAATGATGGTGGGTCTGGGGACAGTGGAGGTGGGGCCAGGGTCTTAGCCACAG
CCCAGCCCCTGGGTCCCTCTGGGCT
>2788
GGGAGACGACGTCCGCATCGTCCGTGACGACATGCTGTGTGCCGGGAACACCCGGAG
GGACTCATGCCAGGTGGGCCCCGCCTGTCCCCCGCCCCCCGCCCCCCAACCCCCACTC
CCAGGCCTGTTCGGCGAGCGCTGAC
>2789
CTGAAGATCTCTGAGGAGGATGAGCTGGACACCAAGCTAACCCGTATCCCCAGTGCCA
AGAAGTACAAAGGTAAGCGGCCACTCCTTTAACTAGCTGCACCTCTGCCTCGTCCCGT
TGACTATCCTTGGAGTACTTGAGT
>2790
AGACCAGTGGCACTGTTGTTTCACAAGATGATGTTTGAAACTATTCCAATGTTCAGTGG
CGGAACTTGCAGTAAGTGCTTGAAATTCTCATCCTTCCATGTATTGGAACAGTTTTCTTA
ACCATATCTAGAAGTTTACAT
>2791
TCAGCCGTTACCTGTGTGTGGTGATATCAAAGTAGAGTTCTTCCACAAACAGAACAAG
ATGCTAAAAAAGGTTTGTACTTTACTTTCATTGGGAGAAATATCCAAAATAAGGACAGA
TTAAAAGCTATATTTTATTTTAT
>2792
TTTAACAAAAAATGATCTTGACAAAGCAAATAAAGACAAAGCCAACCGATACTTTTCT
CCAAATTTTAAGGTCAGTTAAATTAAACATTTTGTGGGGGTTGGTGACTTGTATGTATGT
GATGTGTGTTTAATTCTAGGAG
>2793
AGCAAGGACTAGAGTTCCTGACCTCCAGGCCAGTCCCTGATCCCTGACCTAATGTTATC

GCGGAATGATGGTAAGTAAAGTGTCTCTTGCATCTGCATAGAGAGAGTCCTGGGAGCT
TAGGAAGTGATGGGGAACAGTGA
>2794
AAGAAAAGCCTGGAGTGCACGAAGTTGTGCCTACCCCAGATTGAGAATGTTAAGGGC
ACTGAGGACTCAGGTGAGGAGAGGTGACCTGGTGCCCATGCTCACCTGCCCTCTCCCT
CTTCTTGCCCCCACCCGTCCATCCA
>2795
CTTTTATCCCTCCTCTTCATTGGTTTAATGTATCGCTACCAACGGTGGAAGTCCAAGCTC
TACTCCATTGGTGAGTGGGGGCTTTGGGAGGGAGAGGGAGCTGGTGGGGGTGAGGGA
GGACATGGGTGGGTGCGATGGAC
>2796
GCCTCCGACCCCATCCCCAACCCCCTTCAGAAGTGGGAGGACAGCGCCCACAAGCCA
CAGAGCCTAGACAGTGCGTTTCTCCCGCGGCTGGAGACGAGGAGGCTGGGGGAGGGC
CGGGGGAGCGCGGGAGGCGTCAGAGG

$\mathbf{S}_1^-$: 2,800 false splice donor site containing sequences

>1
ACTGTGTCCTCATTCATGTCATTCGTCCTCAGCTGAAGGATGAAAGAGATTGGCAAGG
AAGCAGGAATGAGTCAGCTTTGGGGGAATATTACCTGGTGGCTCCCATCATTTATTGCT
TTAGGACATACCCTTTTACCTAC
>2
TCCTCAGCTGAAGGATGAAAGAGATTGGCAAGGAAGCAGGAATGAGTCAGCTTTGGG
GGAATATTACCTGGTGGCTCCCATCATTTATTGCTTTAGGACATACCCTTTTACCTACTTG
CTATCATGCTGGACCTCAGCAT
>3
ACCTCAGCATTTCAGGGACCAGAAAAGATGGGGACACCAATGCAAGCCCAAGACTAC
CATCCAGAGGACCGTTCCTGCCCATGCTGCTTTCTCTAGTTTTGCTTTCAGAGTGGTGT
CACCCCACTTGCTAACTCAAGAGT
>4
AGATGGGGACACCAATGCAAGCCCAAGACTACCATCCAGAGGACCGTTCCTGCCCATG
CTGCTTTCTCTAGTTTTGCTTTCAGAGTGGTGTCACCCCACTTGCTAACTCAAGAGTGC
ATCACTAGGCTTCCTGAGCAGGT
>5
CAAGCCCAAGACTACCATCCAGAGGACCGTTCCTGCCCATGCTGCTTTCTCTAGTTTTG
CTTTCAGAGTGGTGTCACCCCACTTGCTAACTCAAGAGTGCATCACTAGGCTTCCTGA
GCAGGTTTGATCAGGATTTGGCC
>6
AGCCCAAGACTACCATCCAGAGGACCGTTCCTGCCCATGCTGCTTTCTCTAGTTTTGCT
TTCAGAGTGGTGTCACCCCACTTGCTAACTCAAGAGTGCATCACTAGGCTTCCTGAGC
AGGTTTGATCAGGATTTGGCCTG
>7
CCGTTCCTGCCCATGCTGCTTTCTCTAGTTTTGCTTTCAGAGTGGTGTCACCCCACTTG
CTAACTCAAGAGTGCATCACTAGGCTTCCTGAGCAGGTTTGATCAGGATTTGGCCTGG

GTCTGTTGGTCAGGGAGGCAGAG
>8
TAGTTTTGCTTTCAGAGTGGTGTCACCCCACTTGCTAACTCAAGAGTGCATCACTAGGC
TTCCTGAGCAGGTTTGATCAGGATTTGGCCTGGGTCTGTTGGTCAGGGAGGCAGAGGT
GTGGGGAGAAGGGTGAAAGGTGA
>9
TCACCCCACTTGCTAACTCAAGAGTGCATCACTAGGCTTCCTGAGCAGGTTTGATCAG
GATTTGGCCTGGGTCTGTTGGTCAGGGAGGCAGAGGTGTGGGGAGAAGGGTGAAAGG
TGACTGAGCCAGGGTTCACAGGCTC
>10
CCCACTTGCTAACTCAAGAGTGCATCACTAGGCTTCCTGAGCAGGTTTGATCAGGATTT
GGCCTGGGTCTGTTGGTCAGGGAGGCAGAGGTGTGGGGAGAAGGGTGAAAGGTGAC
TGAGCCAGGGTTCACAGGCTCCTCA
>11
GTGCATCACTAGGCTTCCTGAGCAGGTTTGATCAGGATTTGGCCTGGGTCTGTTGGTCA
GGGAGGCAGAGGTGTGGGGAGAAGGGTGAAAGGTGACTGAGCCAGGGTTCACAGGC
TCCTCACTCACGGGGCTGAAGCTGG
>12
GATTTGGCCTGGGTCTGTTGGTCAGGGAGGCAGAGGTGTGGGGAGAAGGGTGAAAGG
TGACTGAGCCAGGGTTCACAGGCTCCTCACTCACGGGGCTGAAGCTGGGCATCGTCA
GGCTCAGAGCCCCTAGACGTGCGGCA
>13
GGGAGAAGGGTGAAAGGTGACTGAGCCAGGGTTCACAGGCTCCTCACTCACGGGGCT
GAAGCTGGGCATCGTCAGGCTCAGAGCCCCTAGACGTGCGGCAGCGGTAGCCCTTCTT
CTTGAGCACGTGGCAGATCATGAAG
>14
GAGCCAGGGTTCACAGGCTCCTCACTCACGGGGCTGAAGCTGGGCATCGTCAGGCTC
AGAGCCCCTAGACGTGCGGCAGCGGTAGCCCTTCTTCTTGAGCACGTGGCAGATCATG
AAGCCTACAGGCCCATGAGGAAGAA
>15
CACAGGCTCCTCACTCACGGGGCTGAAGCTGGGCATCGTCAGGCTCAGAGCCCCTAG
ACGTGCGGCAGCGGTAGCCCTTCTTCTTGAGCACGTGGCAGATCATGAAGCCTACAGG
CCCATGAGGAAGAAGACCAGCACAA
>16
GCTGAAGCTGGGCATCGTCAGGCTCAGAGCCCCTAGACGTGCGGCAGCGGTAGCCCTT
CTTCTTGAGCACGTGGCAGATCATGAAGCCTACAGGCCCATGAGGAAGAAGACCAGC
ACAAGCAGGAAGAGCATATATAGCC
>17
GAAGCCTACAGGCCCATGAGGAAGAAGACCAGCACAAGCAGGAAGAGCATATATAGC
CCATGTTGGGGCGGTTCCAGGTCAGGCTGTGGTTCCGACATGAGGCCCTGGGGGGCG
AGCGCGGGCCAGGGCGCCTCCTGGGT
>18
ACAGGCCCATGAGGAAGAAGACCAGCACAAGCAGGAAGAGCATATATAGCCCATGTT
GGGGCGGTTCCAGGTCAGGCTGTGGTTCCGACATGAGGCCCTGGGGGGCGAGCGCGG

GCCAGGGCGCCTCCTGGGTTTAGGGG
>19
AGGAAGAAGACCAGCACAAGCAGGAAGAGCATATATAGCCCATGTTGGGGCGGTTCC
AGGTCAGGCTGTGGTTCCGACATGAGGCCCTGGGGGGCGAGCGCGGGCCAGGGCGCC
TCCTGGGTTTAGGGGGCTGCAGCTGG
>20
CGGTTCCAGGTCAGGCTGTGGTTCCGACATGAGGCCCTGGGGGGCGAGCGCGGGCCA
GGGCGCCTCCTGGGTTTAGGGGGCTGCAGCTGGGAATGGGGGAGGGGTAAGAATGCC
GTCCTCAGGATCTGAAACAAGCACGT
>21
GCCCTGGGGGGCGAGCGCGGGCCAGGGCGCCTCCTGGGTTTAGGGGGCTGCAGCTGG
GAATGGGGGAGGGGTAAGAATGCCGTCCTCAGGATCTGAAACAAGCACGTCTAAGAC
GTCCGGCTGAGGTCCGGCCCCACCCC
>22
CGAGCGCGGGCCAGGGCGCCTCCTGGGTTTAGGGGGCTGCAGCTGGGAATGGGGGAG
GGGTAAGAATGCCGTCCTCAGGATCTGAAACAAGCACGTCTAAGACGTCCGGCTGAG
GTCCGGCCCCACCCCCTACCCAACCA
>23
GGGTTTAGGGGGCTGCAGCTGGGAATGGGGGAGGGGTAAGAATGCCGTCCTCAGGAT
CTGAAACAAGCACGTCTAAGACGTCCGGCTGAGGTCCGGCCCCACCCCCTACCCAAC
CATGGGAGCTGTCCGGGCCAACAGTG
>24
GGGCTGCAGCTGGGAATGGGGGAGGGGTAAGAATGCCGTCCTCAGGATCTGAAACAA
GCACGTCTAAGACGTCCGGCTGAGGTCCGGCCCCACCCCCTACCCAACCATGGGAGCT
GTCCGGGCCAACAGTGCTGTTTCGC
>25
GATCTGAAACAAGCACGTCTAAGACGTCCGGCTGAGGTCCGGCCCCACCCCCTACCCA
ACCATGGGAGCTGTCCGGGCCAACAGTGCTGTTTCGCCTTTCGGCAAGAGGTCAGCG
ACAACGCAAGCTCTCTACCACGACC
>26
CACGTCTAAGACGTCCGGCTGAGGTCCGGCCCCACCCCCTACCCAACCATGGGAGCTG
TCCGGGCCAACAGTGCTGTTTCGCCTTTCGGCAAGAGGTCAGCGACAACGCAAGCTC
TCTACCACGACCATGGTCCGCTTCC
>27
CCGGCCCCACCCCCTACCCAACCATGGGAGCTGTCCGGGCCAACAGTGCTGTTTCGCC
TTTCGGCAAGAGGTCAGCGACAACGCAAGCTCTCTACCACGACCATGGTCCGCTTCCC
CGACGCCCCGAGCGTTCCCCTTTC
>28
CGGGCCAACAGTGCTGTTTCGCCTTTCGGCAAGAGGTCAGCGACAACGCAAGCTCTCT
ACCACGACCATGGTCCGCTTCCCCGACGCCCCGAGCGTTCCCCTTTCCAGCTTGTTCG
CGCTTCTGTCCCTCCCAAGGACAC
>29
TTCGGCAAGAGGTCAGCGACAACGCAAGCTCTCTACCACGACCATGGTCCGCTTCCCC
GACGCCCCGAGCGTTCCCCTTTCCAGCTTGTTCGCGCTTCTGTCCCTCCCAAGGACAC

TGCAGCACGAGATGGGACGGCATT
>30
GACAACGCAAGCTCTCTACCACGACCATGGTCCGCTTCCCCGACGCCCCGAGCGTTCC
CCTTTCCAGCTTGTTCGCGCTTCTGTCCCTCCCAAGGACACTGCAGCACGAGATGGGA
CGGCATTCTTAGGCGGCGGGAGCA
>31
TCTCTACCACGACCATGGTCCGCTTCCCCGACGCCCCGAGCGTTCCCCTTTCCAGCTTG
TTCGCGCTTCTGTCCCTCCCAAGGACACTGCAGCACGAGATGGGACGGCATTCTTAGG
CGGCGGGAGCAGAAATCCTGCCT
>32
CCCTCCCAAGGACACTGCAGCACGAGATGGGACGGCATTCTTAGGCGGCGGGAGCAG
AAATCCTGCCTGAGTCAGCGCCCGCACCTCCTCCACGCCGGCCTGAGCCAGGCCTCTG
CAGCCCCTTCCTCTCAGTACTCCCG
>33
CCTGCCTGAGTCAGCGCCCGCACCTCCTCCACGCCGGCCTGAGCCAGGCCTCTGCAGC
CCCTTCCTCTCAGTACTCCCGCACCCCTCTCCCTTTTTGGTTTCTCCCACCCTGACCACC
TGGCCGACCCGGTACCGGTGAT
>34
TCCACGCCGGCCTGAGCCAGGCCTCTGCAGCCCCTTCCTCTCAGTACTCCCGCACCCC
TCTCCCTTTTTGGTTTCTCCCACCCTGACCACCTGGCCGACCCGGTACCGGTGATTTGG
TTCTGGTTCCGGCTTCTGCTGGG
>35
CCTTCCTCTCAGTACTCCCGCACCCCTCTCCCTTTTTGGTTTCTCCCACCCTGACCACCT
GGCCGACCCGGTACCGGTGATTTGGTTCTGGTTCCGGCTTCTGCTGGGGTTCCGGCTCC
AGGGGCGTCCTAGTGCGGCTC
>36
TCTCAGTACTCCCGCACCCCTCTCCCTTTTTGGTTTCTCCCACCCTGACCACCTGGCCG
ACCCGGTACCGGTGATTTGGTTCTGGTTCCGGCTTCTGCTGGGGTTCCGGCTCCAGGG
GCGTCCTAGTGCGGCTCAGGGCC
>37
GTTTCTCCCACCCTGACCACCTGGCCGACCCGGTACCGGTGATTTGGTTCTGGTTCCGG
CTTCTGCTGGGGTTCCGGCTCCAGGGGCGTCCTAGTGCGGCTCAGGGCCCCCGACGCC
CTTCCGGCGCTCATCCCTTGATT
>38
CACCTGGCCGACCCGGTACCGGTGATTTGGTTCTGGTTCCGGCTTCTGCTGGGGTTCCG
GCTCCAGGGGCGTCCTAGTGCGGCTCAGGGCCCCCGACGCCCTTCCGGCGCTCATCCC
TTGATTCATCCCCTCCCCCTTCG
>39
CCCGACGCCCTTCCGGCGCTCATCCCTTGATTCATCCCCTCCCCCTTCGCCGCCTCACC
GGCCCCGGGCTGTGGCTGCGGATACCCGCGCCGTGGCAGGCGGGGGGAGGGAAGAAC
AGAGAAGGATGCGGGCTGGCTGAG
>40
ATCCCTTGATTCATCCCCTCCCCCTTCGCCGCCTCACCGGCCCCGGGCTGTGGCTGCGG
ATACCCGCGCCGTGGCAGGCGGGGGGAGGGAAGAACAGAGAAGGATGCGGGCTGGC

TGAGAGCGGTGGCAGGCTCCGGGCA
>41
CTGCGGATACCCGCGCCGTGGCAGGCGGGGGGAGGGAAGAACAGAGAAGGATGCGG
GCTGGCTGAGAGCGGTGGCAGGCTCCGGGCAACTCCGGCCCTGGGTCAGGGAGATTG
CCTCCGCAGCCGCCCTGGCTTCCCAAA
>42
GGAGGGAAGAACAGAGAAGGATGCGGGCTGGCTGAGAGCGGTGGCAGGCTCCGGGC
AACTCCGGCCCTGGGTCAGGGAGATTGCCTCCGCAGCCGCCCTGGCTTCCCAAATGCC
TTCACCCGTCGCGGGCCCAGGACCAG
>43
CGGGCAACTCCGGCCCTGGGTCAGGGAGATTGCCTCCGCAGCCGCCCTGGCTTCCCAA
ATGCCTTCACCCGTCGCGGGCCCAGGACCAGGGGGCGGAGCGCAGGTGTGCGGGGCG
GAGAGCGTGAGCTCTGCGCGCGCGT
>44
CTCCGCAGCCGCCCTGGCTTCCCAAATGCCTTCACCCGTCGCGGGCCCAGGACCAGGG
GGCGGAGCGCAGGTGTGCGGGGCGGAGAGCGTGAGCTCTGCGCGCGCGTGACTGCG
CCGGGCCCCCCCGGGGCAATGTGCGG
>45
CCGCAGCCGCCCTGGCTTCCCAAATGCCTTCACCCGTCGCGGGCCCAGGACCAGGGG
GCGGAGCGCAGGTGTGCGGGGCGGAGAGCGTGAGCTCTGCGCGCGCGTGACTGCGCC
GGGCCCCCCCGGGGCAATGTGCGGCG
>46
TTCCCAAATGCCTTCACCCGTCGCGGGCCCAGGACCAGGGGGCGGAGCGCAGGTGTG
CGGGGCGGAGAGCGTGAGCTCTGCGCGCGCGTGACTGCGCCGGGCCCCCCCGGGGCA
ATGTGCGGCGCCGAGTTTGGGTCCGG
>47
GCGCAGGTGTGCGGGGCGGAGAGCGTGAGCTCTGCGCGCGCGTGACTGCGCCGGGCC
CCCCCGGGGCAATGTGCGGCGCCGAGTTTGGGTCCGGGTAGGGGACAGGCGGTCCGG
CGTGGGTGTTGTCCACTGGTCTATCC
>48
GGGGCGGAGAGCGTGAGCTCTGCGCGCGCGTGACTGCGCCGGGCCCCCCCGGGGCAA
TGTGCGGCGCCGAGTTTGGGTCCGGGTAGGGGACAGGCGGTCCGGCGTGGGTGTTGT
CCACTGGTCTATCCGGTACGCCTGGC
>49
CGCGTGACTGCGCCGGGCCCCCCCGGGGCAATGTGCGGCGCCGAGTTTGGGTCCGGG
TAGGGGACAGGCGGTCCGGCGTGGGTGTTGTCCACTGGTCTATCCGGTACGCCTGGCG
CAGCTCCTGCCCTCAGATGCCCCGA
>50
GCCGGGCCCCCCCGGGGCAATGTGCGGCGCCGAGTTTGGGTCCGGGTAGGGGACAGG
CGGTCCGGCGTGGGTGTTGTCCACTGGTCTATCCGGTACGCCTGGCGCAGCTCCTGCC
CTCAGATGCCCCGACCGAGCGGAGA
>51
CGGGCCCCCCCGGGGCAATGTGCGGCGCCGAGTTTGGGTCCGGGTAGGGGACAGGCG
GTCCGGCGTGGGTGTTGTCCACTGGTCTATCCGGTACGCCTGGCGCAGCTCCTGCCCTC

AGATGCCCCGACCGAGCGGAGAGT
>52
GGGGCAATGTGCGGCGCCGAGTTTGGGTCCGGGTAGGGGACAGGCGGTCCGGCGTGG
GTGTTGTCCACTGGTCTATCCGGTACGCCTGGCGCAGCTCCTGCCCTCAGATGCCCCGA
CCGAGCGGAGAGTCAGGAATCTTC
>53
GTGTTGTCCACTGGTCTATCCGGTACGCCTGGCGCAGCTCCTGCCCTCAGATGCCCCGA
CCGAGCGGAGAGTCAGGAATCTTCTCCGAGGGCTGGGGGAAGCCGGCTTTCCGCACC
TTTGAGGGACGGAGTGGCGGGATC
>54
CCGAGCGGAGAGTCAGGAATCTTCTCCGAGGGCTGGGGGAAGCCGGCTTTCCGCACC
TTTGAGGGACGGAGTGGCGGGATCTTCGGAGCTTCCTAGAGAAGTGACCACGGCCTG
CGCCCGGTTTGGGGCAAGCGGCTGGA
>55
GGCTGGGGGAAGCCGGCTTTCCGCACCTTTGAGGGACGGAGTGGCGGGATCTTCGGA
GCTTCCTAGAGAAGTGACCACGGCCTGCGCCCGGTTTGGGGCAAGCGGCTGGATGGG
ATGGCTCGGCTTCCCGAGGATCTGAC
>56
CCGCACCTTTGAGGGACGGAGTGGCGGGATCTTCGGAGCTTCCTAGAGAAGTGACCA
CGGCCTGCGCCCGGTTTGGGGCAAGCGGCTGGATGGGATGGCTCGGCTTCCCGAGGAT
CTGACCCCCGGCCGGAGACAAATGG
>57
CTCGGCTTCCCGAGGATCTGACCCCCGGCCGGAGACAAATGGCCCTCGCATCCTAGGA
GCTATGCTGCCAGTTCCCTGCCCAACCCGGGACGCTCAAAGCCCCAGATACCGGGAGA
GCTGAGCCGGGCGAGGGGCGGAGC
>58
ACCCGGGACGCTCAAAGCCCCAGATACCGGGAGAGCTGAGCCGGGCGAGGGGCGGA
GCTCCCCTGGGGGCGTGGCCTGGGTGTGGATGGAGGCGGGGCCGCAACGCCCAGTTC
CGCAGAGGCCGGCTACTTGCTTGCGCA
>59
GCTCAAAGCCCCAGATACCGGGAGAGCTGAGCCGGGCGAGGGGCGGAGCTCCCCTGG
GGGCGTGGCCTGGGTGTGGATGGAGGCGGGGCCGCAACGCCCAGTTCCGCAGAGGCC
GGCTACTTGCTTGCGCAGCACGCACG
>60
GCCGGGCGAGGGGCGGAGCTCCCCTGGGGGCGTGGCCTGGGTGTGGATGGAGGCGG
GGCCGCAACGCCCAGTTCCGCAGAGGCCGGCTACTTGCTTGCGCAGCACGCACGGCC
GGGGAGGGGCTGGCGGCGGCCGCGGGC
>61
CGGCTACTTGCTTGCGCAGCACGCACGGCCGGGGAGGGGCTGGCGGCGGCCGCGGGC
GGCGGGCGGCGGAGTCGCGGGGCCTGCTCCCGCCGGCACCATGGCCAAGACCGTGGC
CTATTTCTACGACCCCGACGTGGGCA
>62
CTGGCGGCGGCCGCGGGCGGCGGGCGGCGGAGTCGCGGGGCCTGCTCCCGCCGGCAC
CATGGCCAAGACCGTGGCCTATTTCTACGACCCCGACGTGGGCAACTTCCACTACGGT

GAGGAAACAGGGCTATGCATGAGGG
>63
CGGCGGAGTCGCGGGGCCTGCTCCCGCCGGCACCATGGCCAAGACCGTGGCCTATTTC
TACGACCCCGACGTGGGCAACTTCCACTACGGTGAGGAAACAGGGCTATGCATGAGG
GGTTGGGACACGGAGGTTTTGAGGC
>64
GTGGCCTATTTCTACGACCCCGACGTGGGCAACTTCCACTACGGTGAGGAAACAGGGC
TATGCATGAGGGGTTGGGACACGGAGGTTTTGAGGCGGGGTTGCAACTCCAGGGGTG
AGAGCTGACGAACTCTCTTCTCCCC
>65
CGACCCCGACGTGGGCAACTTCCACTACGGTGAGGAAACAGGGCTATGCATGAGGGG
TTGGGACACGGAGGTTTTGAGGCGGGGTTGCAACTCCAGGGGTGAGAGCTGACGAAC
TCTCTTCTCCCCACCCCCAGGAGCTG
>66
GGCAACTTCCACTACGGTGAGGAAACAGGGCTATGCATGAGGGGTTGGGACACGGAG
GTTTTGAGGCGGGGTTGCAACTCCAGGGGTGAGAGCTGACGAACTCTCTTCTCCCCAC
CCCCAGGAGCTGGACACCCTATGAA
>67
GGTGAGGAAACAGGGCTATGCATGAGGGGTTGGGACACGGAGGTTTTGAGGCGGGGT
TGCAACTCCAGGGGTGAGAGCTGACGAACTCTCTTCTCCCCACCCCCAGGAGCTGGA
CACCCTATGAAGCCTCATCGCCTGGC
>68
TCTCTTCTCCCCACCCCCAGGAGCTGGACACCCTATGAAGCCTCATCGCCTGGCATTGA
CCCATAGCCTGGTCCTGCATTACGGTCTCTATAAGAAGATGATCGTGAGTTCCGCCGCC
TCGGCCGGGGGTTGGGGGGTGG
>69
CCCCCAGGAGCTGGACACCCTATGAAGCCTCATCGCCTGGCATTGACCCATAGCCTGG
TCCTGCATTACGGTCTCTATAAGAAGATGATCGTGAGTTCCGCCGCCTCGGCCGGGGGT
TGGGGGGTGGGGCGAGCTGTGGC
>70
CCTGGTCCTGCATTACGGTCTCTATAAGAAGATGATCGTGAGTTCCGCCGCCTCGGCCG
GGGGTTGGGGGGTGGGGCGAGCTGTGGCCGTAGGGATAGGTGGGCTGGACTGAGTGC
ATGAAGTGCGACCCGCCCCTAGAG
>71
TTACGGTCTCTATAAGAAGATGATCGTGAGTTCCGCCGCCTCGGCCGGGGGTTGGGGG
GTGGGGCGAGCTGTGGCCGTAGGGATAGGTGGGCTGGACTGAGTGCATGAAGTGCGA
CCCGCCCCTAGAGTATCGGGGAGCT
>72
AAGATGATCGTGAGTTCCGCCGCCTCGGCCGGGGGTTGGGGGGTGGGGCGAGCTGTG
GCCGTAGGGATAGGTGGGCTGGACTGAGTGCATGAAGTGCGACCCGCCCCTAGAGTAT
CGGGGAGCTGGATAGAAGTCACCAG
>73
GGTGGGGCGAGCTGTGGCCGTAGGGATAGGTGGGCTGGACTGAGTGCATGAAGTGCG
ACCCGCCCCTAGAGTATCGGGGAGCTGGATAGAAGTCACCAGGAGTACCTTGAAAGTG

CAGGGTGTTGGGGGTGGCGTCCTCT
>74
AGGGATAGGTGGGCTGGACTGAGTGCATGAAGTGCGACCCGCCCCTAGAGTATCGGGG
AGCTGGATAGAAGTCACCAGGAGTACCTTGAAAGTGCAGGGTGTTGGGGGTGGCGTC
CTCTGCTTAATGGAAAGTACTTTCT
>75
AGTGCATGAAGTGCGACCCGCCCCTAGAGTATCGGGGAGCTGGATAGAAGTCACCAGG
AGTACCTTGAAAGTGCAGGGTGTTGGGGGTGGCGTCCTCTGCTTAATGGAAAGTACTT
TCTCACCTCAGGTCGGCCACCCTC
>76
GAAGTGCGACCCGCCCCTAGAGTATCGGGGAGCTGGATAGAAGTCACCAGGAGTACCT
TGAAAGTGCAGGGTGTTGGGGGTGGCGTCCTCTGCTTAATGGAAAGTACTTTCTCACC
TCAGGTCGGCCACCCTCTGACTGT
>77
AGTGCGACCCGCCCCTAGAGTATCGGGGAGCTGGATAGAAGTCACCAGGAGTACCTTG
AAAGTGCAGGGTGTTGGGGGTGGCGTCCTCTGCTTAATGGAAAGTACTTTCTCACCTC
AGGTCGGCCACCCTCTGACTGTAG
>78
CCCGCCCCTAGAGTATCGGGGAGCTGGATAGAAGTCACCAGGAGTACCTTGAAAGTGC
AGGGTGTTGGGGGTGGCGTCCTCTGCTTAATGGAAAGTACTTTCTCACCTCAGGTCGG
CCACCCTCTGACTGTAGGCACACT
>79
TGGATAGAAGTCACCAGGAGTACCTTGAAAGTGCAGGGTGTTGGGGGTGGCGTCCTCT
GCTTAATGGAAAGTACTTTCTCACCTCAGGTCGGCCACCCTCTGACTGTAGGCACACTA
GCCCTTGGCCTAGGGTTAGCGTG
>80
GGGTGTTGGGGGTGGCGTCCTCTGCTTAATGGAAAGTACTTTCTCACCTCAGGTCGGC
CACCCTCTGACTGTAGGCACACTAGCCCTTGGCCTAGGGTTAGCGTGATGTGGGGCTG
GTTTGAAGACAGAAGGTGTGTTCC
>81
TAATGGAAAGTACTTTCTCACCTCAGGTCGGCCACCCTCTGACTGTAGGCACACTAGC
CCTTGGCCTAGGGTTAGCGTGATGTGGGGCTGGTTTGAAGACAGAAGGTGTGTTCCTC
CTCTGCATTTTAGAGTGTGGATAG
>82
CCTCTGACTGTAGGCACACTAGCCCTTGGCCTAGGGTTAGCGTGATGTGGGGCTGGTTT
GAAGACAGAAGGTGTGTTCCTCCTCTGCATTTTAGAGTGTGGATAGGGCCTTGGCCTC
AGGAATTAGGGTTAGGGGAGCGG
>83
TCTGACTGTAGGCACACTAGCCCTTGGCCTAGGGTTAGCGTGATGTGGGGCTGGTTTG
AAGACAGAAGGTGTGTTCCTCCTCTGCATTTTAGAGTGTGGATAGGGCCTTGGCCTCA
GGAATTAGGGTTAGGGGAGCGGAT
>84
TTGGCCTAGGGTTAGCGTGATGTGGGGCTGGTTTGAAGACAGAAGGTGTGTTCCTCCT
CTGCATTTTAGAGTGTGGATAGGGCCTTGGCCTCAGGAATTAGGGTTAGGGGAGCGGAT

ATCACTGTATCAGGGTGTGAGGT
>85
TTGAAGACAGAAGGTGTGTTCCTCCTCTGCATTTTAGAGTGTGGATAGGGCCTTGGCCT
CAGGAATTAGGGTTAGGGGAGCGGATATCACTGTATCAGGGTGTGAGGTTTACCCTCA
GTTCCCACTCCTCTTTTACCACC
>86
CTCCTCTGCATTTTAGAGTGTGGATAGGGCCTTGGCCTCAGGAATTAGGGTTAGGGGAG
CGGATATCACTGTATCAGGGTGTGAGGTTTACCCTCAGTTCCCACTCCTCTTTTACCACC
TTTCTTTACCTCCAAGGAGCT
>87
GAGTGTGGATAGGGCCTTGGCCTCAGGAATTAGGGTTAGGGGAGCGGATATCACTGTAT
CAGGGTGTGAGGTTTACCCTCAGTTCCCACTCCTCTTTTACCACCTTTCTTTACCTCCA
AGGAGCTAGGGCTCCAGGGTGG
>88
TGAGGTTTACCCTCAGTTCCCACTCCTCTTTTACCACCTTTCTTTACCTCCAAGGAGCTA
GGGCTCCAGGGTGGAACGATGCTGCCGTCTTTGTAAATGTGTATGTGCGTGTGGAACA
GAGGCGATATTTTAGATATTCC
>89
TTCCCACTCCTCTTTTACCACCTTTCTTTACCTCCAAGGAGCTAGGGCTCCAGGGTGGA
ACGATGCTGCCGTCTTTGTAAATGTGTATGTGCGTGTGGAACAGAGGCGATATTTTAGA
TATTCCTTTTTCAGCCTGGAAT
>90
CTCCTCTTTTACCACCTTTCTTTACCTCCAAGGAGCTAGGGCTCCAGGGTGGAACGATG
CTGCCGTCTTTGTAAATGTGTATGTGCGTGTGGAACAGAGGCGATATTTTAGATATTCCT
TTTTCAGCCTGGAATACCTCT
>91
CACCTTTCTTTACCTCCAAGGAGCTAGGGCTCCAGGGTGGAACGATGCTGCCGTCTTT
GTAAATGTGTATGTGCGTGTGGAACAGAGGCGATATTTTAGATATTCCTTTTTCAGCCTG
GAATACCTCTACATGGTTCTGC
>92
TGTGTATGTGCGTGTGGAACAGAGGCGATATTTTAGATATTCCTTTTTCAGCCTGGAATA
CCTCTACATGGTTCTGCTTAATCGCTATCCAGTTTTTCATCTACCAGACATACACATGCT
GTCTTCCCAGCTTGGAATTT
>93
GAGGCGATATTTTAGATATTCCTTTTTCAGCCTGGAATACCTCTACATGGTTCTGCTTAAT
CGCTATCCAGTTTTTCATCTACCAGACATACACATGCTGTCTTCCCAGCTTGGAATTTAT
GGCTGTGATATGGGATAGT
>94
GCCTGGAATACCTCTACATGGTTCTGCTTAATCGCTATCCAGTTTTTCATCTACCAGACA
TACACATGCTGTCTTCCCAGCTTGGAATTTATGGCTGTGATATGGGATAGTAACAGTTAA
GCCACGCCAGGCGTGGTGGC
>95
CTTAATCGCTATCCAGTTTTTCATCTACCAGACATACACATGCTGTCTTCCCAGCTTGGA
ATTTATGGCTGTGATATGGGATAGTAACAGTTAAGCCACGCCAGGCGTGGTGGCCCATG

CCTGTAATCCCAGCACTTTGG
>96
TTCATCTACCAGACATACACATGCTGTCTTCCCAGCTTGGAATTTATGGCTGTGATATGG
GATAGTAACAGTTAAGCCACGCCAGGCGTGGTGGCCCATGCCTGTAATCCCAGCACTT
TGGGAGGTTGAAATGGGGGGGA
>97
CACATGCTGTCTTCCCAGCTTGGAATTTATGGCTGTGATATGGGATAGTAACAGTTAAGC
CACGCCAGGCGTGGTGGCCCATGCCTGTAATCCCAGCACTTTGGGAGGTTGAAATGGG
GGGGATCGCTTGAGGCCAGGAG
>98
AGCTTGGAATTTATGGCTGTGATATGGGATAGTAACAGTTAAGCCACGCCAGGCGTGGT
GGCCCATGCCTGTAATCCCAGCACTTTGGGAGGTTGAAATGGGGGGGATCGCTTGAGG
CCAGGAGTTGGAGACCAGCTTGG
>99
CGTGGTGGCCCATGCCTGTAATCCCAGCACTTTGGGAGGTTGAAATGGGGGGGATCGC
TTGAGGCCAGGAGTTGGAGACCAGCTTGGGCCACAGTGGGACCCACATCTACAAAAA
ATAAAAATAAAAAAAGTAGCCATGT
>100
CCAGCACTTTGGGAGGTTGAAATGGGGGGGATCGCTTGAGGCCAGGAGTTGGAGACC
AGCTTGGGCCACAGTGGGACCCACATCTACAAAAAATAAAAATAAAAAAAGTAGCCAT
GTGCGGTGGGTGTGCCTGTAGTCCC
>101
GAGGCCAGGAGTTGGAGACCAGCTTGGGCCACAGTGGGACCCACATCTACAAAAAAT
AAAAATAAAAAAAGTAGCCATGTGCGGTGGGTGTGCCTGTAGTCCCAACTACTTGGGA
GGCCGAGGCGAGAGGATGGCTTGGG
>102
GAGTTGGAGACCAGCTTGGGCCACAGTGGGACCCACATCTACAAAAAATAAAAATAA
AAAAAGTAGCCATGTGCGGTGGGTGTGCCTGTAGTCCCAACTACTTGGGAGGCCGAGG
CGAGAGGATGGCTTGGGCCCAGGAG
>103
TGTGCGGTGGGTGTGCCTGTAGTCCCAACTACTTGGGAGGCCGAGGCGAGAGGATGG
CTTGGGCCCAGGAGTTTAAAGTTACAGTGATCTATGATCAGCAGCCTGAGTTGACAGG
GAGGACCTTGTCTCAAAAGAAAAAA
>104
TGGGTGTGCCTGTAGTCCCAACTACTTGGGAGGCCGAGGCGAGAGGATGGCTTGGGC
CCAGGAGTTTAAAGTTACAGTGATCTATGATCAGCAGCCTGAGTTGACAGGGAGGACC
TTGTCTCAAAAGAAAAAATAAGACA
>105
TGCCTGTAGTCCCAACTACTTGGGAGGCCGAGGCGAGAGGATGGCTTGGGCCCAGGA
GTTTAAAGTTACAGTGATCTATGATCAGCAGCCTGAGTTGACAGGGAGGACCTTGTCTC
AAAAGAAAAAATAAGACAAAACAG
>106
GAGGCCGAGGCGAGAGGATGGCTTGGGCCCAGGAGTTTAAAGTTACAGTGATCTATGA
TCAGCAGCCTGAGTTGACAGGGAGGACCTTGTCTCAAAAGAAAAAATAAGACAAAAC

AGCTAAACCCAGGCTTTATGTGTTG
>107
TGGCTTGGGCCCAGGAGTTTAAAGTTACAGTGATCTATGATCAGCAGCCTGAGTTGAC
AGGGAGGACCTTGTCTCAAAAGAAAAAATAAGACAAAACAGCTAAACCCAGGCTTTA
TGTGTTGCTAGGGAAACTAGTGAAC
>108
GCCTGAGTTGACAGGGAGGACCTTGTCTCAAAAGAAAAAATAAGACAAAACAGCTAA
ACCCAGGCTTTATGTGTTGCTAGGGAAACTAGTGAACAATATGAATGAATGTTCATTGC
TTGTGCCAAGGCACAGACAAATGG
>109
CTGAGTTGACAGGGAGGACCTTGTCTCAAAAGAAAAAATAAGACAAAACAGCTAAAC
CCAGGCTTTATGTGTTGCTAGGGAAACTAGTGAACAATATGAATGAATGTTCATTGCTT
GTGCCAAGGCACAGACAAATGGAC
>110
AAATAAGACAAAACAGCTAAACCCAGGCTTTATGTGTTGCTAGGGAAACTAGTGAACA
ATATGAATGAATGTTCATTGCTTGTGCCAAGGCACAGACAAATGGACTGGTCATAGAAA
AGACTGAGGTGATGTTAGTGCAG
>111
AACAGCTAAACCCAGGCTTTATGTGTTGCTAGGGAAACTAGTGAACAATATGAATGAAT
GTTCATTGCTTGTGCCAAGGCACAGACAAATGGACTGGTCATAGAAAAGACTGAGGTG
ATGTTAGTGCAGGAATCTATATC
>112
TGCTAGGGAAACTAGTGAACAATATGAATGAATGTTCATTGCTTGTGCCAAGGCACAG
ACAAATGGACTGGTCATAGAAAAGACTGAGGTGATGTTAGTGCAGGAATCTATATCCCT
TGAGATTGTTTGCCTGTATGTAC
>113
ACAATATGAATGAATGTTCATTGCTTGTGCCAAGGCACAGACAAATGGACTGGTCATAG
AAAAGACTGAGGTGATGTTAGTGCAGGAATCTATATCCCTTGAGATTGTTTGCCTGTAT
GTACCTGTGTGCATTTTCTGGG
>114
ATGAATGAATGTTCATTGCTTGTGCCAAGGCACAGACAAATGGACTGGTCATAGAAAA
GACTGAGGTGATGTTAGTGCAGGAATCTATATCCCTTGAGATTGTTTGCCTGTATGTACC
TGTGTGCATTTTCTGGGAGAAG
>115
ATGAATGTTCATTGCTTGTGCCAAGGCACAGACAAATGGACTGGTCATAGAAAAGACT
GAGGTGATGTTAGTGCAGGAATCTATATCCCTTGAGATTGTTTGCCTGTATGTACCTGTG
TGCATTTTCTGGGAGAAGAAAA
>116
ACAGACAAATGGACTGGTCATAGAAAAGACTGAGGTGATGTTAGTGCAGGAATCTATA
TCCCTTGAGATTGTTTGCCTGTATGTACCTGTGTGCATTTTCTGGGAGAAGAAAAGCTC
TTTCTTGTGCAGTTTTCAAAGAG
>117
CATAGAAAAGACTGAGGTGATGTTAGTGCAGGAATCTATATCCCTTGAGATTGTTTGCC
TGTATGTACCTGTGTGCATTTTCTGGGAGAAGAAAAGCTCTTTCTTGTGCAGTTTTCAA

AGAGGTGTGACACATCCCCCTG
>118
CTATATCCCTTGAGATTGTTTGCCTGTATGTACCTGTGTGCATTTTCTGGGAGAAGAAAA
GCTCTTTCTTGTGCAGTTTTCAAAGAGGTGTGACACATCCCCCTGACCTTCCAAAGATA
ATAAATCTTTGATTTTAATTA
>119
TCCCTTGAGATTGTTTGCCTGTATGTACCTGTGTGCATTTTCTGGGAGAAGAAAAGCTC
TTTCTTGTGCAGTTTTCAAAGAGGTGTGACACATCCCCCTGACCTTCCAAAGATAATAA
ATCTTTGATTTTAATTAGAAAG
>120
AGTTTTCAAAGAGGTGTGACACATCCCCCTGACCTTCCAAAGATAATAAATCTTTGATT
TTAATTAGAAAGTTGACATATATCCCATATATTTGATCCAGAAAGAAGATCCAGAGGAA
AGAGGACAGAATCAGAGCTTGG
>121
AGAATCAGAGCTTGGGGAGGAGATGAGCATGGAGAGAACTGGGAATAGGAGATTCCA
AGGAACAGGGCAGGTGGATCAGGGCTCAAAGTTGAGAAGGTACGATAGGCTGAGCTG
GAGGTGGAAAGGAAAAGAAGTGGTCT
>122
AGGAGATGAGCATGGAGAGAACTGGGAATAGGAGATTCCAAGGAACAGGGCAGGTGG
ATCAGGGCTCAAAGTTGAGAAGGTACGATAGGCTGAGCTGGAGGTGGAAAGGAAAAG
AAGTGGTCTTCAGGGGGCTCATTTGT
>123
GCATGGAGAGAACTGGGAATAGGAGATTCCAAGGAACAGGGCAGGTGGATCAGGGCT
CAAAGTTGAGAAGGTACGATAGGCTGAGCTGGAGGTGGAAAGGAAAAGAAGTGGTCT
TCAGGGGGCTCATTTGTAATAACTTA
>124
GGAGATTCCAAGGAACAGGGCAGGTGGATCAGGGCTCAAAGTTGAGAAGGTACGATA
GGCTGAGCTGGAGGTGGAAAGGAAAAGAAGTGGTCTTCAGGGGGCTCATTTGTAATA
ACTTACCTGGTTAATCGGAGTCTCTC
>125
AAGTTGAGAAGGTACGATAGGCTGAGCTGGAGGTGGAAAGGAAAAGAAGTGGTCTTC
AGGGGGCTCATTTGTAATAACTTACCTGGTTAATCGGAGTCTCTCTACTGACTTAAGGA
TTGTGGGATGAGGGGAATGCTATG
>126
GATAGGCTGAGCTGGAGGTGGAAAGGAAAAGAAGTGGTCTTCAGGGGGCTCATTTGT
AATAACTTACCTGGTTAATCGGAGTCTCTCTACTGACTTAAGGATTGTGGGATGAGGGG
AATGCTATGTTTGGCTTCATATAG
>127
GTGGTCTTCAGGGGGCTCATTTGTAATAACTTACCTGGTTAATCGGAGTCTCTCTACTG
ACTTAAGGATTGTGGGATGAGGGGAATGCTATGTTTGGCTTCATATAGTCTCCAGGACT
GGTTCAGTATGGGTGGTCTGAG
>128
TGTAATAACTTACCTGGTTAATCGGAGTCTCTCTACTGACTTAAGGATTGTGGGATGAG
GGGAATGCTATGTTTGGCTTCATATAGTCTCCAGGACTGGTTCAGTATGGGTGGTCTGA

GCCTGTGGAGTGGGGCTGACCC
>129
GGTTAATCGGAGTCTCTCTACTGACTTAAGGATTGTGGGATGAGGGGAATGCTATGTTT
GGCTTCATATAGTCTCCAGGACTGGTTCAGTATGGGTGGTCTGAGCCTGTGGAGTGGG
GCTGACCCTGCCTGGGTTCATCT
>130
CTTAAGGATTGTGGGATGAGGGGAATGCTATGTTTGGCTTCATATAGTCTCCAGGACTG
GTTCAGTATGGGTGGTCTGAGCCTGTGGAGTGGGGCTGACCCTGCCTGGGTTCATCTT
GTGTATCCATCCCGAGGTCTTCA
>131
AAGGATTGTGGGATGAGGGGAATGCTATGTTTGGCTTCATATAGTCTCCAGGACTGGTT
CAGTATGGGTGGTCTGAGCCTGTGGAGTGGGGCTGACCCTGCCTGGGTTCATCTTGTG
TATCCATCCCGAGGTCTTCAAGC
>132
GGATGAGGGGAATGCTATGTTTGGCTTCATATAGTCTCCAGGACTGGTTCAGTATGGGT
GGTCTGAGCCTGTGGAGTGGGGCTGACCCTGCCTGGGTTCATCTTGTGTATCCATCCCG
AGGTCTTCAAGCCATACCAGGC
>133
TTCATATAGTCTCCAGGACTGGTTCAGTATGGGTGGTCTGAGCCTGTGGAGTGGGGCTG
ACCCTGCCTGGGTTCATCTTGTGTATCCATCCCGAGGTCTTCAAGCCATACCAGGCCTC
CCAGCATGACATGTGCCGCTTC
>134
AGTATGGGTGGTCTGAGCCTGTGGAGTGGGGCTGACCCTGCCTGGGTTCATCTTGTGTA
TCCATCCCGAGGTCTTCAAGCCATACCAGGCCTCCCAGCATGACATGTGCCGCTTCCAC
TCCGAGGACTACATTGACTTCC
>135
CCCTGCCTGGGTTCATCTTGTGTATCCATCCCGAGGTCTTCAAGCCATACCAGGCCTCC
CAGCATGACATGTGCCGCTTCCACTCCGAGGACTACATTGACTTCCTGCAGAGAGTCA
GCCCCACCAATATGCAAGGCTTC
>136
GCCATACCAGGCCTCCCAGCATGACATGTGCCGCTTCCACTCCGAGGACTACATTGACT
TCCTGCAGAGAGTCAGCCCCACCAATATGCAAGGCTTCACCAAGAGTCTTAATGCCTT
CAACGTAGGCGATGACTGGTGAG
>137
TTCCACTCCGAGGACTACATTGACTTCCTGCAGAGAGTCAGCCCCACCAATATGCAAG
GCTTCACCAAGAGTCTTAATGCCTTCAACGTAGGCGATGACTGGTGAGGGAGGACTGG
GAGTTTTGATGTCTAACAGTTTAA
>138
CATTGACTTCCTGCAGAGAGTCAGCCCCACCAATATGCAAGGCTTCACCAAGAGTCTT
AATGCCTTCAACGTAGGCGATGACTGGTGAGGGAGGACTGGGAGTTTTGATGTCTAAC
AGTTTAATGCCAAGCCCTTCAGTG
>139
AATATGCAAGGCTTCACCAAGAGTCTTAATGCCTTCAACGTAGGCGATGACTGGTGAG
GGAGGACTGGGAGTTTTGATGTCTAACAGTTTAATGCCAAGCCCTTCAGTGAGGTCTT

TTGGCAGCAGTGGGAAGATCTCAA
>140
CCAAGAGTCTTAATGCCTTCAACGTAGGCGATGACTGGTGAGGGAGGACTGGGAGTTT
TGATGTCTAACAGTTTAATGCCAAGCCCTTCAGTGAGGTCTTTTGGCAGCAGTGGGAA
GATCTCAAGGGGGGCCTACTTGTC
>141
AACGTAGGCGATGACTGGTGAGGGAGGACTGGGAGTTTTGATGTCTAACAGTTTAATG
CCAAGCCCTTCAGTGAGGTCTTTTGGCAGCAGTGGGAAGATCTCAAGGGGGGCCTACT
TGTCAATGCATCATAATTCATTTT
>142
AGGCGATGACTGGTGAGGGAGGACTGGGAGTTTTGATGTCTAACAGTTTAATGCCAAG
CCCTTCAGTGAGGTCTTTTGGCAGCAGTGGGAAGATCTCAAGGGGGGCCTACTTGTCA
ATGCATCATAATTCATTTTATTTG
>143
ACAGTTTAATGCCAAGCCCTTCAGTGAGGTCTTTTGGCAGCAGTGGGAAGATCTCAAG
GGGGGCCTACTTGTCAATGCATCATAATTCATTTTATTTGTTTATTCAATAAATATTTATTG
GTCACTTGTTCTAGGCAGGC
>144
GGTCTTTTGGCAGCAGTGGGAAGATCTCAAGGGGGGCCTACTTGTCAATGCATCATAAT
TCATTTTATTTGTTTATTCAATAAATATTTATTGGTCACTTGTTCTAGGCAGGCCCTGTGC
TAAGTGCTGGGAATGCAGTG
>145
ATCTCAAGGGGGGCCTACTTGTCAATGCATCATAATTCATTTTATTTGTTTATTCAATAAA
TATTTATTGGTCACTTGTTCTAGGCAGGCCCTGTGCTAAGTGCTGGGAATGCAGTGATG
AACAAAAACAAAATTTTAAT
>146
GGGGGGCCTACTTGTCAATGCATCATAATTCATTTTATTTGTTTATTCAATAAATATTTATT
GGTCACTTGTTCTAGGCAGGCCCTGTGCTAAGTGCTGGGAATGCAGTGATGAACAAAA
ACAAAATTTTAATATCGTAT
>147
AATGCATCATAATTCATTTTATTTGTTTATTCAATAAATATTTATTGGTCACTTGTTCTAGG
CAGGCCCTGTGCTAAGTGCTGGGAATGCAGTGATGAACAAAAACAAAATTTTAATATC
GTATTTTTGCTAAGTCTTAT
>148
CATAATTCATTTTATTTGTTTATTCAATAAATATTTATTGGTCACTTGTTCTAGGCAGGCCC
TGTGCTAAGTGCTGGGAATGCAGTGATGAACAAAAACAAAATTTTAATATCGTATTTTT
GCTAAGTCTTATAAAGAGG
>149
ACTTGTTCTAGGCAGGCCCTGTGCTAAGTGCTGGGAATGCAGTGATGAACAAAAACAA
AATTTTAATATCGTATTTTTGCTAAGTCTTATAAAGAGGAGGTGTATGACAGATTATGAG
AGTGTATACGTGGAGGATTGTT
>150
AGGCCCTGTGCTAAGTGCTGGGAATGCAGTGATGAACAAAAACAAAATTTTAATATCG
TATTTTTGCTAAGTCTTATAAAGAGGAGGTGTATGACAGATTATGAGAGTGTATACGTGG

AGGATTGTTTAGCTTGGTGGAG
>151
GCTGGGAATGCAGTGATGAACAAAAACAAAATTTTAATATCGTATTTTTGCTAAGTCTTA
TAAAGAGGAGGTGTATGACAGATTATGAGAGTGTATACGTGGAGGATTGTTTAGCTTGG
TGGAGTGGCTGGTCGGTGGGG
>152
AAAACAAAATTTTAATATCGTATTTTTGCTAAGTCTTATAAAGAGGAGGTGTATGACAGA
TTATGAGAGTGTATACGTGGAGGATTGTTTAGCTTGGTGGAGTGGCTGGTCGGTGGGG
CCTTCCTAGAGGAAGTAAAGAT
>153
AAATTTTAATATCGTATTTTTGCTAAGTCTTATAAAGAGGAGGTGTATGACAGATTATGA
GAGTGTATACGTGGAGGATTGTTTAGCTTGGTGGAGTGGCTGGTCGGTGGGGCCTTCCT
AGAGGAAGTAAAGATTGAGCT
>154
ATCGTATTTTTGCTAAGTCTTATAAAGAGGAGGTGTATGACAGATTATGAGAGTGTATAC
GTGGAGGATTGTTTAGCTTGGTGGAGTGGCTGGTCGGTGGGGCCTTCCTAGAGGAAGT
AAAGATTGAGCTGAGATCTGAA
>155
AGTCTTATAAAGAGGAGGTGTATGACAGATTATGAGAGTGTATACGTGGAGGATTGTTT
AGCTTGGTGGAGTGGCTGGTCGGTGGGGCCTTCCTAGAGGAAGTAAAGATTGAGCTG
AGATCTGAAAAAAGAGTAGAAGTA
>156
ATGAGAGTGTATACGTGGAGGATTGTTTAGCTTGGTGGAGTGGCTGGTCGGTGGGGCC
TTCCTAGAGGAAGTAAAGATTGAGCTGAGATCTGAAAAAAGAGTAGAAGTAATCAGAT
GAAAGATGGTTGGAGGGTGAGGGG
>157
CTTGGTGGAGTGGCTGGTCGGTGGGGCCTTCCTAGAGGAAGTAAAGATTGAGCTGAGA
TCTGAAAAAAGAGTAGAAGTAATCAGATGAAAGATGGTTGGAGGGTGAGGGGAGAAG
TATAGCAGGCAGTAGGGACAAGTGC
>158
GGAGTGGCTGGTCGGTGGGGCCTTCCTAGAGGAAGTAAAGATTGAGCTGAGATCTGA
AAAAAGAGTAGAAGTAATCAGATGAAAGATGGTTGGAGGGTGAGGGGAGAAGTATAG
CAGGCAGTAGGGACAAGTGCTAAGGG
>159
TAGAGGAAGTAAAGATTGAGCTGAGATCTGAAAAAAGAGTAGAAGTAATCAGATGAA
AGATGGTTGGAGGGTGAGGGGAGAAGTATAGCAGGCAGTAGGGACAAGTGCTAAGGG
TCTGTGGTAGGACTGAGTATGACTCA
>160
AGATTGAGCTGAGATCTGAAAAAAGAGTAGAAGTAATCAGATGAAAGATGGTTGGAG
GGTGAGGGGAGAAGTATAGCAGGCAGTAGGGACAAGTGCTAAGGGTCTGTGGTAGGA
CTGAGTATGACTCATTTGAGGAACAG
>161
GATCTGAAAAAAGAGTAGAAGTAATCAGATGAAAGATGGTTGGAGGGTGAGGGGAGA
AGTATAGCAGGCAGTAGGGACAAGTGCTAAGGGTCTGTGGTAGGACTGAGTATGACTC

ATTTGAGGAACAGAAAAAAGGCCAA
>162
AGATGAAAGATGGTTGGAGGGTGAGGGGAGAAGTATAGCAGGCAGTAGGGACAAGTG
CTAAGGGTCTGTGGTAGGACTGAGTATGACTCATTTGAGGAACAGAAAAAAGGCCAA
GTGCAGAGTATGAAGAGGAGGTGAAC
>163
TGGTTGGAGGGTGAGGGGAGAAGTATAGCAGGCAGTAGGGACAAGTGCTAAGGGTCT
GTGGTAGGACTGAGTATGACTCATTTGAGGAACAGAAAAAAGGCCAAGTGCAGAGTA
TGAAGAGGAGGTGAACTGAATTGAGG
>164
GTAGGGACAAGTGCTAAGGGTCTGTGGTAGGACTGAGTATGACTCATTTGAGGAACAG
AAAAAAGGCCAAGTGCAGAGTATGAAGAGGAGGTGAACTGAATTGAGGCTAAAGAAA
TAGGACCTTTTAAAAGCAAACAGCT
>165
CAAGTGCTAAGGGTCTGTGGTAGGACTGAGTATGACTCATTTGAGGAACAGAAAAAA
GGCCAAGTGCAGAGTATGAAGAGGAGGTGAACTGAATTGAGGCTAAAGAAATAGGAC
CTTTTAAAAGCAAACAGCTCTATTAT
>166
AGGAGGTGAACTGAATTGAGGCTAAAGAAATAGGACCTTTTAAAAGCAAACAGCTCTA
TTATGAATTGGGGTTTTTACCCGTAGAGTATAGGGAAGCTATCAAAGTGTTTTAAAAAG
CAAGGGATCTTAAGAGGGCTATG
>167
CTGAATTGAGGCTAAAGAAATAGGACCTTTTAAAAGCAAACAGCTCTATTATGAATTGG
GGTTTTTACCCGTAGAGTATAGGGAAGCTATCAAAGTGTTTTAAAAAGCAAGGGATCTT
AAGAGGGCTATGGGCAAGTTAA
>168
ACCTTTTAAAAGCAAACAGCTCTATTATGAATTGGGGTTTTTACCCGTAGAGTATAGGG
AAGCTATCAAAGTGTTTTAAAAAGCAAGGGATCTTAAGAGGGCTATGGGCAAGTTAAA
AAAACAAAAAAAACAACGAAGGA
>169
TACCCGTAGAGTATAGGGAAGCTATCAAAGTGTTTTAAAAAGCAAGGGATCTTAAGAG
GGCTATGGGCAAGTTAAAAAAACAAAAAAAACAACGAAGGAGTGGTTTGATATCATTA
GGTTTACATTGAGCTTGTATCATC
>170
GTGTTTTAAAAAGCAAGGGATCTTAAGAGGGCTATGGGCAAGTTAAAAAAACAAAAA
AAACAACGAAGGAGTGGTTTGATATCATTAGGTTTACATTGAGCTTGTATCATCCATAG
AAGAGTCAGGGTTAGACCCTCTGG
>171
GATCTTAAGAGGGCTATGGGCAAGTTAAAAAAACAAAAAAAACAACGAAGGAGTGGT
TTGATATCATTAGGTTTACATTGAGCTTGTATCATCCATAGAAGAGTCAGGGTTAGACCC
TCTGGGGAAGAGAGTGGACAACA
>172
ATGGGCAAGTTAAAAAAACAAAAAAAACAACGAAGGAGTGGTTTGATATCATTAGGTT
TACATTGAGCTTGTATCATCCATAGAAGAGTCAGGGTTAGACCCTCTGGGGAAGAGAG

TGGACAACAAAGATAAGCCACACT
>173
ACAAAAAAAACAACGAAGGAGTGGTTTGATATCATTAGGTTTACATTGAGCTTGTATCA
TCCATAGAAGAGTCAGGGTTAGACCCTCTGGGGAAGAGAGTGGACAACAAAGATAAG
CCACACTCTACTCTATATCCATGA
>174
ATATCATTAGGTTTACATTGAGCTTGTATCATCCATAGAAGAGTCAGGGTTAGACCCTCT
GGGGAAGAGAGTGGACAACAAAGATAAGCCACACTCTACTCTATATCCATGAAAAAAT
AGTAACCATCACAGCTACCAGT
>175
TTAGACCCTCTGGGGAAGAGAGTGGACAACAAAGATAAGCCACACTCTACTCTATATC
CATGAAAAAATAGTAACCATCACAGCTACCAGTTGCTCAATTCTACTTACTTTTGTTTTT
AAACTTTTTATTAGGGAAAATT
>176
GAGTGGACAACAAAGATAAGCCACACTCTACTCTATATCCATGAAAAAATAGTAACCAT
CACAGCTACCAGTTGCTCAATTCTACTTACTTTTGTTTTTAAACTTTTTATTAGGGAAAA
TTTCAAAAATTTCTGAAAGTG
>177
CACTCTACTCTATATCCATGAAAAAATAGTAACCATCACAGCTACCAGTTGCTCAATTCT
ACTTACTTTTGTTTTTAAACTTTTTATTAGGGAAAATTTCAAAAATTTCTGAAAGTGAAG
TAGTGCAGTGACCTCCCATA
>178
CCAGTTGCTCAATTCTACTTACTTTTGTTTTTAAACTTTTTATTAGGGAAAATTTCAAAA
ATTTCTGAAAGTGAAGTAGTGCAGTGACCTCCCATACGTCCATCACCTAGCTTCAACGC
TTATGAGCAAATGGCCACTCT
>179
TGCTCAATTCTACTTACTTTTGTTTTTAAACTTTTTATTAGGGAAAATTTCAAAAATTTCT
GAAAGTGAAGTAGTGCAGTGACCTCCCATACGTCCATCACCTAGCTTCAACGCTTATGA
GCAAATGGCCACTCTTTTTT
>180
CCATCACCTAGCTTCAACGCTTATGAGCAAATGGCCACTCTTTTTTTTTTTTTTTTTTTT
TGAGACAGAGTCTCACTCTGTAGCCCAGGCTGGAGTGCAGTGGTGCAGTCTTGGCTCA
CTGCAACCTCTGCCTCCTGGG
>181
GCTTCAACGCTTATGAGCAAATGGCCACTCTTTTTTTTTTTTTTTTTTTTTTGAGACAGA
GTCTCACTCTGTAGCCCAGGCTGGAGTGCAGTGGTGCAGTCTTGGCTCACTGCAACCT
CTGCCTCCTGGGTTCAAGCGAT
>182
AGCAAATGGCCACTCTTTTTTTTTTTTTTTTTTTTTTGAGACAGAGTCTCACTCTGTAGC
CCAGGCTGGAGTGCAGTGGTGCAGTCTTGGCTCACTGCAACCTCTGCCTCCTGGGTTC
AAGCGATTCTCATGCCTCAGAC
>183
TCTTTTTTTTTTTTTTTTTTTTTTGAGACAGAGTCTCACTCTGTAGCCCAGGCTGGAGTG
CAGTGGTGCAGTCTTGGCTCACTGCAACCTCTGCCTCCTGGGTTCAAGCGATTCTCATG

CCTCAGACTCCCGAGTAGCTG
>184
AGTCTCACTCTGTAGCCCAGGCTGGAGTGCAGTGGTGCAGTCTTGGCTCACTGCAACC
TCTGCCTCCTGGGTTCAAGCGATTCTCATGCCTCAGACTCCCGAGTAGCTGGGATTACA
GGCGCCTGCCACCATGCCTGGCT
>185
TGGTGCAGTCTTGGCTCACTGCAACCTCTGCCTCCTGGGTTCAAGCGATTCTCATGCCT
CAGACTCCCGAGTAGCTGGGATTACAGGCGCCTGCCACCATGCCTGGCTAATTTTTGTA
TGTTTAGTAGAGATGGGGTTTC
>186
CGATTCTCATGCCTCAGACTCCCGAGTAGCTGGGATTACAGGCGCCTGCCACCATGCCT
GGCTAATTTTTGTATGTTTAGTAGAGATGGGGTTTCACTATGTTGGTCAAGCTGGTTTTG
AACTCCCGACCTCAGGTGATT
>187
TCTCATGCCTCAGACTCCCGAGTAGCTGGGATTACAGGCGCCTGCCACCATGCCTGGCT
AATTTTTGTATGTTTAGTAGAGATGGGGTTTCACTATGTTGGTCAAGCTGGTTTTGAACT
CCCGACCTCAGGTGATTCACC
>188
ATTACAGGCGCCTGCCACCATGCCTGGCTAATTTTTGTATGTTTAGTAGAGATGGGGTTT
CACTATGTTGGTCAAGCTGGTTTTGAACTCCCGACCTCAGGTGATTCACCAGCCTCAGC
CTCCAAAAGTGCTGGGATTAC
>189
GCCTGCCACCATGCCTGGCTAATTTTTGTATGTTTAGTAGAGATGGGGTTTCACTATGTT
GGTCAAGCTGGTTTTGAACTCCCGACCTCAGGTGATTCACCAGCCTCAGCCTCCAAAA
GTGCTGGGATTACAGACGTGAG
>190
ATTTTTGTATGTTTAGTAGAGATGGGGTTTCACTATGTTGGTCAAGCTGGTTTTGAACTC
CCGACCTCAGGTGATTCACCAGCCTCAGCCTCCAAAAGTGCTGGGATTACAGACGTGA
GCCATCGCACTCAGCTGAGCAA
>191
TTTCACTATGTTGGTCAAGCTGGTTTTGAACTCCCGACCTCAGGTGATTCACCAGCCTC
AGCCTCCAAAAGTGCTGGGATTACAGACGTGAGCCATCGCACTCAGCTGAGCAAGTG
GCCACTCTTACTTCATCTATACTC
>192
AGCTGGTTTTGAACTCCCGACCTCAGGTGATTCACCAGCCTCAGCCTCCAAAAGTGCT
GGGATTACAGACGTGAGCCATCGCACTCAGCTGAGCAAGTGGCCACTCTTACTTCATCT
ATACTCCCTCATGTTAGATTGTT
>193
GTGATTCACCAGCCTCAGCCTCCAAAAGTGCTGGGATTACAGACGTGAGCCATCGCAC
TCAGCTGAGCAAGTGGCCACTCTTACTTCATCTATACTCCCTCATGTTAGATTGTTTTAA
AGCAGATCCTAGATGTTATATC
>194
GGATTACAGACGTGAGCCATCGCACTCAGCTGAGCAAGTGGCCACTCTTACTTCATCTA
TACTCCCTCATGTTAGATTGTTTTAAAGCAGATCCTAGATGTTATATCCTTTTATCCATAA

ATATTTTATTATGTATCTCT
>195
GACGTGAGCCATCGCACTCAGCTGAGCAAGTGGCCACTCTTACTTCATCTATACTCCCT
CATGTTAGATTGTTTTAAAGCAGATCCTAGATGTTATATCCTTTTATCCATAAATATTTTAT
TATGTATCTCTAAATGATA
>196
CTGAGCAAGTGGCCACTCTTACTTCATCTATACTCCCTCATGTTAGATTGTTTTAAAGCA
GATCCTAGATGTTATATCCTTTTATCCATAAATATTTTATTATGTATCTCTAAATGATACTCT
TTTAAAAAAACATAGTA
>197
TCCCTCATGTTAGATTGTTTTAAAGCAGATCCTAGATGTTATATCCTTTTATCCATAAATAT
TTTATTATGTATCTCTAAATGATACTCTTTTAAAAAAACATAGTATCATTATCAAACCTAA
AAAGTTAACAGTGATTC
>198
GATGTTATATCCTTTTATCCATAAATATTTTATTATGTATCTCTAAATGATACTCTTTTAAAA
AAACATAGTATCATTATCAAACCTAAAAAGTTAACAGTGATTCCTTAATGTCATCAAATA
TGGATCAATATTCAAAT
>199
AAATATTTTATTATGTATCTCTAAATGATACTCTTTTAAAAAAACATAGTATCATTATCAA
ACCTAAAAAGTTAACAGTGATTCCTTAATGTCATCAAATATGGATCAATATTCAAATTTC
TGCAGTTGTCTCATAATTA
>200
TTATTATGTATCTCTAAATGATACTCTTTTAAAAAAACATAGTATCATTATCAAACCTAAA
AAGTTAACAGTGATTCCTTAATGTCATCAAATATGGATCAATATTCAAATTTCTGCAGTT
GTCTCATAATTATTTTGTA
>201
CTAAATGATACTCTTTTAAAAAAACATAGTATCATTATCAAACCTAAAAAGTTAACAGTG
ATTCCTTAATGTCATCAAATATGGATCAATATTCAAATTTCTGCAGTTGTCTCATAATTAT
TTTGTAGCTAATTTAAATC
>202
TATCAAACCTAAAAAGTTAACAGTGATTCCTTAATGTCATCAAATATGGATCAATATTCA
AATTTCTGCAGTTGTCTCATAATTATTTTGTAGCTAATTTAAATCCAGATCTAAACAAGG
TCTATACAGTGCATTTGGTT
>203
CAAACCTAAAAAGTTAACAGTGATTCCTTAATGTCATCAAATATGGATCAATATTCAAAT
TTCTGCAGTTGTCTCATAATTATTTTGTAGCTAATTTAAATCCAGATCTAAACAAGGTCTA
TACAGTGCATTTGGTTGAT
>204
ACAGTGATTCCTTAATGTCATCAAATATGGATCAATATTCAAATTTCTGCAGTTGTCTCAT
AATTATTTTGTAGCTAATTTAAATCCAGATCTAAACAAGGTCTATACAGTGCATTTGGTT
GATATGTCTCTTAAGACTT
>205
ATCAATATTCAAATTTCTGCAGTTGTCTCATAATTATTTTGTAGCTAATTTAAATCCAGAT
CTAAACAAGGTCTATACAGTGCATTTGGTTGATATGTCTCTTAAGACTTTTAATTTGTAG

CTTTTCTGTCACCCCCTTG
>206
CAAATTTCTGCAGTTGTCTCATAATTATTTTGTAGCTAATTTAAATCCAGATCTAAACAA
GGTCTATACAGTGCATTTGGTTGATATGTCTCTTAAGACTTTTAATTTGTAGCTTTTCTGT
CACCCCCTTGAATTTATTT
>207
GCAGTTGTCTCATAATTATTTTGTAGCTAATTTAAATCCAGATCTAAACAAGGTCTATAC
AGTGCATTTGGTTGATATGTCTCTTAAGACTTTTAATTTGTAGCTTTTCTGTCACCCCCTT
GAATTTATTTCTTGAAGAA
>208
CTCATAATTATTTTGTAGCTAATTTAAATCCAGATCTAAACAAGGTCTATACAGTGCATTT
GGTTGATATGTCTCTTAAGACTTTTAATTTGTAGCTTTTCTGTCACCCCCTTGAATTTATT
TCTTGAAGAAACCAGGTT
>209
ATTTAAATCCAGATCTAAACAAGGTCTATACAGTGCATTTGGTTGATATGTCTCTTAAGA
CTTTTAATTTGTAGCTTTTCTGTCACCCCCTTGAATTTATTTCTTGAAGAAACCAGGTTG
TTGATCCTGTAGAATTTCTT
>210
TATGTCTCTTAAGACTTTTAATTTGTAGCTTTTCTGTCACCCCCTTGAATTTATTTCTTGA
AGAAACCAGGTTGTTGATCCTGTAGAATTTCTTATGATAGCTACCATTTACTGAGTGCTT
ACTCTGCTAGGTATTATGC
>211
GTCTCTTAAGACTTTTAATTTGTAGCTTTTCTGTCACCCCCTTGAATTTATTTCTTGAAG
AAACCAGGTTGTTGATCCTGTAGAATTTCTTATGATAGCTACCATTTACTGAGTGCTTAC
TCTGCTAGGTATTATGCCAA
>212
TGAATTTATTTCTTGAAGAAACCAGGTTGTTGATCCTGTAGAATTTCTTATGATAGCTAC
CATTTACTGAGTGCTTACTCTGCTAGGTATTATGCCAAGGGTTGCTGGTAAATCCACTTA
ATTCTCATACCAATCCTTAG
>213
AGAAACCAGGTTGTTGATCCTGTAGAATTTCTTATGATAGCTACCATTTACTGAGTGCTT
ACTCTGCTAGGTATTATGCCAAGGGTTGCTGGTAAATCCACTTAATTCTCATACCAATCC
TTAGATGTATTATAATAGAC
>214
GTAGAATTTCTTATGATAGCTACCATTTACTGAGTGCTTACTCTGCTAGGTATTATGCCAA
GGGTTGCTGGTAAATCCACTTAATTCTCATACCAATCCTTAGATGTATTATAATAGACCTT
TTTCTGATGTTATATATA
>215
GCTTACTCTGCTAGGTATTATGCCAAGGGTTGCTGGTAAATCCACTTAATTCTCATACCA
ATCCTTAGATGTATTATAATAGACCTTTTTCTGATGTTATATATAAGTAAATTGAGGTTTG
GGAATATCTAGGTAACTTA
>216
AGGGTTGCTGGTAAATCCACTTAATTCTCATACCAATCCTTAGATGTATTATAATAGACCT
TTTTCTGATGTTATATATAAGTAAATTGAGGTTTGGGAATATCTAGGTAACTTAGCCGTAG

TCACATAACTGGTCATTG
>217
TAAATCCACTTAATTCTCATACCAATCCTTAGATGTATTATAATAGACCTTTTTCTGATGTT
ATATATAAGTAAATTGAGGTTTGGGAATATCTAGGTAACTTAGCCGTAGTCACATAACTG
GTCATTGGAGGGGGCATG
>218
TAATTCTCATACCAATCCTTAGATGTATTATAATAGACCTTTTTCTGATGTTATATATAAGT
AAATTGAGGTTTGGGAATATCTAGGTAACTTAGCCGTAGTCACATAACTGGTCATTGGA
GGGGGCATGATCCAGATCC
>219
CCTTAGATGTATTATAATAGACCTTTTTCTGATGTTATATATAAGTAAATTGAGGTTTGGG
AATATCTAGGTAACTTAGCCGTAGTCACATAACTGGTCATTGGAGGGGGCATGATCCAG
ATCCCGGGTTTTTAAACCAC
>220
TTCTGATGTTATATATAAGTAAATTGAGGTTTGGGAATATCTAGGTAACTTAGCCGTAGT
CACATAACTGGTCATTGGAGGGGGCATGATCCAGATCCCGGGTTTTTAAACCACTAATC
TGTTTTCCTGAAAGATCTTAA
>221
TGGGAATATCTAGGTAACTTAGCCGTAGTCACATAACTGGTCATTGGAGGGGGCATGAT
CCAGATCCCGGGTTTTTAAACCACTAATCTGTTTTCCTGAAAGATCTTAATGACAGAAG
CAGCCAGGGCTGTAGGATAGAG
>222
TAGCCGTAGTCACATAACTGGTCATTGGAGGGGGCATGATCCAGATCCCGGGTTTTTAA
ACCACTAATCTGTTTTCCTGAAAGATCTTAATGACAGAAGCAGCCAGGGCTGTAGGATA
GAGGCCAACTCATTAGGCTGGC
>223
CCAGATCCCGGGTTTTTAAACCACTAATCTGTTTTCCTGAAAGATCTTAATGACAGAAG
CAGCCAGGGCTGTAGGATAGAGGCCAACTCATTAGGCTGGCATTGGCACCTTTCCAAC
TTTATCCCTACACCTCAGCTGCA
>224
TTCAAGGCCTTCTCAGCCTTCACACCATCTCCATGAAACCTTTCTTGACCACACCAGCC
CATAGAGCTCTGTCTTGCACACACTGCACTGCTCATTTGGTGTTCAGTCACATGCTGTT
TGGGGAGGTTTCTTGTTTTTTT
>225
CTCCATGAAACCTTTCTTGACCACACCAGCCCATAGAGCTCTGTCTTGCACACACTGCA
CTGCTCATTTGGTGTTCAGTCACATGCTGTTTGGGGAGGTTTCTTGTTTTTTTGTTAGTA
TTCCTGCTGTTATTTCTGAGC
>226
AAACCTTTCTTGACCACACCAGCCCATAGAGCTCTGTCTTGCACACACTGCACTGCTCA
TTTGGTGTTCAGTCACATGCTGTTTGGGGAGGTTTCTTGTTTTTTTGTTAGTATTCCTGC
TGTTATTTCTGAGCTCACTTT
>227
TGACCACACCAGCCCATAGAGCTCTGTCTTGCACACACTGCACTGCTCATTTGGTGTTC
AGTCACATGCTGTTTGGGGAGGTTTCTTGTTTTTTTGTTAGTATTCCTGCTGTTATTTCT

GAGCTCACTTTCTAGCTCTGT
>228
AGCCCATAGAGCTCTGTCTTGCACACACTGCACTGCTCATTTGGTGTTCAGTCACATGC
TGTTTGGGGAGGTTTCTTGTTTTTTTGTTAGTATTCCTGCTGTTATTTCTGAGCTCACTTT
CTAGCTCTGTGAATACAGGG
>229
GTCTTGCACACACTGCACTGCTCATTTGGTGTTCAGTCACATGCTGTTTGGGGAGGTTT
CTTGTTTTTTTGTTAGTATTCCTGCTGTTATTTCTGAGCTCACTTTCTAGCTCTGTGAATA
CAGGGTCAGCCCTTACGCCA
>230
CTGTTTGGGGAGGTTTCTTGTTTTTTTGTTAGTATTCCTGCTGTTATTTCTGAGCTCACTT
TCTAGCTCTGTGAATACAGGGTCAGCCCTTACGCCACACTGGGGTCCCGTAGATGCTCT
TTGTGCGATTAGGGAATGCT
>231
GGTTTCTTGTTTTTTTGTTAGTATTCCTGCTGTTATTTCTGAGCTCACTTTCTAGCTCTGT
GAATACAGGGTCAGCCCTTACGCCACACTGGGGTCCCGTAGATGCTCTTTGTGCGATTA
GGGAATGCTAATTAGAATAC
>232
TTCCTGCTGTTATTTCTGAGCTCACTTTCTAGCTCTGTGAATACAGGGTCAGCCCTTACG
CCACACTGGGGTCCCGTAGATGCTCTTTGTGCGATTAGGGAATGCTAATTAGAATACTAT
CTCTGGGCTATTTATGCTGC
>233
AGCTCACTTTCTAGCTCTGTGAATACAGGGTCAGCCCTTACGCCACACTGGGGTCCCGT
AGATGCTCTTTGTGCGATTAGGGAATGCTAATTAGAATACTATCTCTGGGCTATTTATGCT
GCATCAGTCTTCCTGTCAAA
>234
CGTAGATGCTCTTTGTGCGATTAGGGAATGCTAATTAGAATACTATCTCTGGGCTATTTAT
GCTGCATCAGTCTTCCTGTCAAACAAGTTACAGTTGTGCTTATCTGTATTTCCGATGAG
AATCAGGAATTTTTCTTCTG
>235
CTCTTTGTGCGATTAGGGAATGCTAATTAGAATACTATCTCTGGGCTATTTATGCTGCATC
AGTCTTCCTGTCAAACAAGTTACAGTTGTGCTTATCTGTATTTCCGATGAGAATCAGGA
ATTTTTCTTCTGTTTAATGA
>236
CGATTAGGGAATGCTAATTAGAATACTATCTCTGGGCTATTTATGCTGCATCAGTCTTCCT
GTCAAACAAGTTACAGTTGTGCTTATCTGTATTTCCGATGAGAATCAGGAATTTTTCTTC
TGTTTAATGAATTTCTGAT
>237
AGTCTTCCTGTCAAACAAGTTACAGTTGTGCTTATCTGTATTTCCGATGAGAATCAGGA
ATTTTTCTTCTGTTTAATGAATTTCTGATTGGTTTCAAAGTATTCTAACATAAGTAGCCAA
ATTGTACTATAGGCCTTTAA
>238
TACAGTTGTGCTTATCTGTATTTCCGATGAGAATCAGGAATTTTTCTTCTGTTTAATGAAT
TTCTGATTGGTTTCAAAGTATTCTAACATAAGTAGCCAAATTGTACTATAGGCCTTTAAA

TGAAAAAAATAAAAAAGTA
>239
TCCGATGAGAATCAGGAATTTTTCTTCTGTTTAATGAATTTCTGATTGGTTTCAAAGTAT
TCTAACATAAGTAGCCAAATTGTACTATAGGCCTTTAAATGAAAAAAATAAAAAAGTAG
TCCCAAGCCGGGCGCAGTGGC
>240
TCAGGAATTTTTCTTCTGTTTAATGAATTTCTGATTGGTTTCAAAGTATTCTAACATAAGT
AGCCAAATTGTACTATAGGCCTTTAAATGAAAAAAATAAAAAAGTAGTCCCAAGCCGG
GCGCAGTGGCTCACGCCTGTA
>241
TTGGTTTCAAAGTATTCTAACATAAGTAGCCAAATTGTACTATAGGCCTTTAAATGAAAA
AAATAAAAAAGTAGTCCCAAGCCGGGCGCAGTGGCTCACGCCTGTAATCCCAGCACTT
TCGGAGGCCGAGGCGGGTGGAC
>242
CATAAGTAGCCAAATTGTACTATAGGCCTTTAAATGAAAAAAATAAAAAAGTAGTCCCA
AGCCGGGCGCAGTGGCTCACGCCTGTAATCCCAGCACTTTCGGAGGCCGAGGCGGGT
GGACCACCTAAGGTCAGGAGTTCA
>243
ATTGTACTATAGGCCTTTAAATGAAAAAAATAAAAAAGTAGTCCCAAGCCGGGCGCAG
TGGCTCACGCCTGTAATCCCAGCACTTTCGGAGGCCGAGGCGGGTGGACCACCTAAGG
TCAGGAGTTCAAGACCAGCCTGGC
>244
AAAAAAGTAGTCCCAAGCCGGGCGCAGTGGCTCACGCCTGTAATCCCAGCACTTTCGG
AGGCCGAGGCGGGTGGACCACCTAAGGTCAGGAGTTCAAGACCAGCCTGGCCAACAT
GGTGAAACCCCATCTCTACTAAAAA
>245
AAGCCGGGCGCAGTGGCTCACGCCTGTAATCCCAGCACTTTCGGAGGCCGAGGCGGG
TGGACCACCTAAGGTCAGGAGTTCAAGACCAGCCTGGCCAACATGGTGAAACCCCAT
CTCTACTAAAAATACAAAAATTAGTC
>246
GCGCAGTGGCTCACGCCTGTAATCCCAGCACTTTCGGAGGCCGAGGCGGGTGGACCA
CCTAAGGTCAGGAGTTCAAGACCAGCCTGGCCAACATGGTGAAACCCCATCTCTACTA
AAAATACAAAAATTAGTCATGTGTG
>247
CAGCACTTTCGGAGGCCGAGGCGGGTGGACCACCTAAGGTCAGGAGTTCAAGACCAG
CCTGGCCAACATGGTGAAACCCCATCTCTACTAAAAATACAAAAATTAGTCATGTGTGG
TGGCACGTGCCTGTAGTCCCAGCT
>248
AAGGTCAGGAGTTCAAGACCAGCCTGGCCAACATGGTGAAACCCCATCTCTACTAAAA
ATACAAAAATTAGTCATGTGTGGTGGCACGTGCCTGTAGTCCCAGCTACTCCAGGAGG
CTGAGGCAGGAGAATTGCTTGAAC
>249
CAGGAGTTCAAGACCAGCCTGGCCAACATGGTGAAACCCCATCTCTACTAAAAATACA
AAAATTAGTCATGTGTGGTGGCACGTGCCTGTAGTCCCAGCTACTCCAGGAGGCTGAG

GCAGGAGAATTGCTTGAACCCGGG
>250
ACCAGCCTGGCCAACATGGTGAAACCCCATCTCTACTAAAAATACAAAAATTAGTCATG
TGTGGTGGCACGTGCCTGTAGTCCCAGCTACTCCAGGAGGCTGAGGCAGGAGAATTGC
TTGAACCCGGGAGTTGGAGGTTG
>251
TGTGGTGGCACGTGCCTGTAGTCCCAGCTACTCCAGGAGGCTGAGGCAGGAGAATTGC
TTGAACCCGGGAGTTGGAGGTTGCAGTGACCTGAGATTGCACCACTGCACTCCAGCCT
GGGCAACAGAGTGAGACTCTGTCT
>252
GCACGTGCCTGTAGTCCCAGCTACTCCAGGAGGCTGAGGCAGGAGAATTGCTTGAACC
CGGGAGTTGGAGGTTGCAGTGACCTGAGATTGCACCACTGCACTCCAGCCTGGGCAA
CAGAGTGAGACTCTGTCTTAAAAAA
>253
GCTTGAACCCGGGAGTTGGAGGTTGCAGTGACCTGAGATTGCACCACTGCACTCCAG
CCTGGGCAACAGAGTGAGACTCTGTCTTAAAAAAAAAAAAAAAAAAAAAATTCCAGAC
CTCTCCTTTTCACCCATAAATCCCTC
>254
GGGAGTTGGAGGTTGCAGTGACCTGAGATTGCACCACTGCACTCCAGCCTGGGCAAC
AGAGTGAGACTCTGTCTTAAAAAAAAAAAAAAAAAAAAAATTCCAGACCTCTCCTTTTC
ACCCATAAATCCCTCTGCCCAGTGA
>255
CTCTGTCTTAAAAAAAAAAAAAAAAAAAAAATTCCAGACCTCTCCTTTTCACCCATAAA
TCCCTCTGCCCAGTGACAGCCACTTTCAGCTCCTTTCCCCTATGTCTTCTGGTATTTACC
TTTGGATTTCTAAATAATAATA
>256
TCCAGACCTCTCCTTTTCACCCATAAATCCCTCTGCCCAGTGACAGCCACTTTCAGCTC
CTTTCCCCTATGTCTTCTGGTATTTACCTTTGGATTTCTAAATAATAATAATATTAATTGAC
TGTTATTACTAATCTGTAA
>257
TCTCCTTTTCACCCATAAATCCCTCTGCCCAGTGACAGCCACTTTCAGCTCCTTTCCCCT
ATGTCTTCTGGTATTTACCTTTGGATTTCTAAATAATAATAATATTAATTGACTGTTATTAC
TAATCTGTAATCCTTAGG
>258
CTATGTCTTCTGGTATTTACCTTTGGATTTCTAAATAATAATAATATTAATTGACTGTTATT
ACTAATCTGTAATCCTTAGGTAATACCTGTTATATACTAGGTACTGTTCTAAGTGCTTTAC
AGCCATTAGCTTATTTA
>259
GGTATTTACCTTTGGATTTCTAAATAATAATAATATTAATTGACTGTTATTACTAATCTGTA
ATCCTTAGGTAATACCTGTTATATACTAGGTACTGTTCTAAGTGCTTTACAGCCATTAGCT
TATTTAATCTCAACAAC
>260
AAATAATAATAATATTAATTGACTGTTATTACTAATCTGTAATCCTTAGGTAATACCTGTTA
TATACTAGGTACTGTTCTAAGTGCTTTACAGCCATTAGCTTATTTAATCTCAACAACCCCT

ATGAAGCAGGGTCTATT
>261
ATACTAGGTACTGTTCTAAGTGCTTTACAGCCATTAGCTTATTTAATCTCAACAACCCCT
ATGAAGCAGGGTCTATTATTGTGTCCTTTTACCGATGAGGACACTTAAGCACAGAAAGG
TTAAACAGTTTGTACAACCTC
>262
CTGTTCTAAGTGCTTTACAGCCATTAGCTTATTTAATCTCAACAACCCCTATGAAGCAGG
GTCTATTATTGTGTCCTTTTACCGATGAGGACACTTAAGCACAGAAAGGTTAAACAGTT
TGTACAACCTCACTATCATTA
>263
GTTCTAAGTGCTTTACAGCCATTAGCTTATTTAATCTCAACAACCCCTATGAAGCAGGGT
CTATTATTGTGTCCTTTTACCGATGAGGACACTTAAGCACAGAAAGGTTAAACAGTTTG
TACAACCTCACTATCATTAGT
>264
TCAACAACCCCTATGAAGCAGGGTCTATTATTGTGTCCTTTTACCGATGAGGACACTTA
AGCACAGAAAGGTTAAACAGTTTGTACAACCTCACTATCATTAGTAGGTGTTAAAGAA
CCTAGGTAGTCTAGCTCCAGAAT
>265
CCCTATGAAGCAGGGTCTATTATTGTGTCCTTTTACCGATGAGGACACTTAAGCACAGA
AAGGTTAAACAGTTTGTACAACCTCACTATCATTAGTAGGTGTTAAAGAACCTAGGTAG
TCTAGCTCCAGAATCGGTGCTT
>266
GTGTCCTTTTACCGATGAGGACACTTAAGCACAGAAAGGTTAAACAGTTTGTACAACC
TCACTATCATTAGTAGGTGTTAAAGAACCTAGGTAGTCTAGCTCCAGAATCGGTGCTTG
TAATCATACTCCCCTCCTGTATT
>267
CCTTTTACCGATGAGGACACTTAAGCACAGAAAGGTTAAACAGTTTGTACAACCTCAC
TATCATTAGTAGGTGTTAAAGAACCTAGGTAGTCTAGCTCCAGAATCGGTGCTTGTAAT
CATACTCCCCTCCTGTATTGTCT
>268
ACACTTAAGCACAGAAAGGTTAAACAGTTTGTACAACCTCACTATCATTAGTAGGTGTT
AAAGAACCTAGGTAGTCTAGCTCCAGAATCGGTGCTTGTAATCATACTCCCCTCCTGTA
TTGTCTCTCATTGCTATTTCTT
>269
GTTTGTACAACCTCACTATCATTAGTAGGTGTTAAAGAACCTAGGTAGTCTAGCTCCAG
AATCGGTGCTTGTAATCATACTCCCCTCCTGTATTGTCTCTCATTGCTATTTCTTCGTCTG
TCAATTTTAGGCATTATCTG
>270
CATTAGTAGGTGTTAAAGAACCTAGGTAGTCTAGCTCCAGAATCGGTGCTTGTAATCAT
ACTCCCCTCCTGTATTGTCTCTCATTGCTATTTCTTCGTCTGTCAATTTTAGGCATTATCT
GTTAAGCTTTTAGTATGGGA
>271
GTAGGTGTTAAAGAACCTAGGTAGTCTAGCTCCAGAATCGGTGCTTGTAATCATACTCC
CCTCCTGTATTGTCTCTCATTGCTATTTCTTCGTCTGTCAATTTTAGGCATTATCTGTTAA

GCTTTTAGTATGGGATATGA
>272
TAGTCTAGCTCCAGAATCGGTGCTTGTAATCATACTCCCCTCCTGTATTGTCTCTCATTG
CTATTTCTTCGTCTGTCAATTTTAGGCATTATCTGTTAAGCTTTTAGTATGGGATATGAGC
ATTTAGTCTTCTTACACCA
>273
TGTAATCATACTCCCCTCCTGTATTGTCTCTCATTGCTATTTCTTCGTCTGTCAATTTTAG
GCATTATCTGTTAAGCTTTTAGTATGGGATATGAGCATTTAGTCTTCTTACACCATCACAT
TGGCTTCCTTTCCCCTTT
>274
CCCCTCCTGTATTGTCTCTCATTGCTATTTCTTCGTCTGTCAATTTTAGGCATTATCTGTT
AAGCTTTTAGTATGGGATATGAGCATTTAGTCTTCTTACACCATCACATTGGCTTCCTTT
CCCCTTTCAGCTTCATAGT
>275
ATTGCTATTTCTTCGTCTGTCAATTTTAGGCATTATCTGTTAAGCTTTTAGTATGGGATAT
GAGCATTTAGTCTTCTTACACCATCACATTGGCTTCCTTTCCCCTTTCAGCTTCATAGTA
ATTTAAAAATAAATCAGTA
>276
TAGTATGGGATATGAGCATTTAGTCTTCTTACACCATCACATTGGCTTCCTTTCCCCTTTC
AGCTTCATAGTAATTTAAAAATAAATCAGTATTCTGTGTTTACATTATTATAACTCTGAAA
GATTCATTTACTATCAAG
>277
TTAGTCTTCTTACACCATCACATTGGCTTCCTTTCCCCTTTCAGCTTCATAGTAATTTAAA
AATAAATCAGTATTCTGTGTTTACATTATTATAACTCTGAAAGATTCATTTACTATCAAGC
CAAATAGTGTAGTGTGAT
>278
AAAAATAAATCAGTATTCTGTGTTTACATTATTATAACTCTGAAAGATTCATTTACTATCA
AGCCAAATAGTGTAGTGTGATTTAATCTATTTTCTTCTAAAGCTTTTTGTTTTTCCTGGA
GCTGACAATTGCATTATTT
>279
AAATAAATCAGTATTCTGTGTTTACATTATTATAACTCTGAAAGATTCATTTACTATCAAG
CCAAATAGTGTAGTGTGATTTAATCTATTTTCTTCTAAAGCTTTTTGTTTTTCCTGGAGCT
GACAATTGCATTATTTTT
>280
CTGAAAGATTCATTTACTATCAAGCCAAATAGTGTAGTGTGATTTAATCTATTTTCTTCTA
AAGCTTTTTGTTTTTCCTGGAGCTGACAATTGCATTATTTTTTCATTTGCTTAGTTTTCTG
GTATTAATCACTAATTCT
>281
TAATCTATTTTCTTCTAAAGCTTTTTGTTTTTCCTGGAGCTGACAATTGCATTATTTTTTC
ATTTGCTTAGTTTTCTGGTATTAATCACTAATTCTTTCCAAGCCTTTTGAGAAACTGTAA
AACCCTACAATTCTTGCTT
>282
TTTCTTCTAAAGCTTTTTGTTTTTCCTGGAGCTGACAATTGCATTATTTTTTCATTTGCTT
AGTTTTCTGGTATTAATCACTAATTCTTTCCAAGCCTTTTGAGAAACTGTAAAACCCTAC

AATTCTTGCTTGAACCCGG
>283
TGCATTATTTTTTCATTTGCTTAGTTTTCTGGTATTAATCACTAATTCTTTCCAAGCCTTTT
GAGAAACTGTAAAACCCTACAATTCTTGCTTGAACCCGGGAGGCGGAGGCTGCAGTG
AGCCGAGATCGCACCATTGCA
>284
TCTTTCCAAGCCTTTTGAGAAACTGTAAAACCCTACAATTCTTGCTTGAACCCGGGAG
GCGGAGGCTGCAGTGAGCCGAGATCGCACCATTGCACCCCAGCCTGGGCGACAGAGC
AAGACCCCATCTCAAAAAACAAACA
>285
CATTGCACCCCAGCCTGGGCGACAGAGCAAGACCCCATCTCAAAAAACAAACAAACA
AAAAACCCCTACAGTTCTGTTTTTCACATGGTCAAATGTATCAGCTGCACTATTTTTTTT
TTTTGAGACCTAAAACTAGGGAT
>286
CACCCCAGCCTGGGCGACAGAGCAAGACCCCATCTCAAAAAACAAACAAACAAAAA
ACCCCTACAGTTCTGTTTTTCACATGGTCAAATGTATCAGCTGCACTATTTTTTTTTTTTG
AGACCTAAAACTAGGGATTGCCT
>287
GGCGACAGAGCAAGACCCCATCTCAAAAAACAAACAAACAAAAAACCCCTACAGTTC
TGTTTTTCACATGGTCAAATGTATCAGCTGCACTATTTTTTTTTTTTGAGACCTAAAACT
AGGGATTGCCTAAGCCTTTCCTT
>288
TATCAGCTGCACTATTTTTTTTTTTTGAGACCTAAAACTAGGGATTGCCTAAGCCTTTCC
TTTTTAAAAAGTCAGGTTTATATAGGTATAACTTGCATACAGTAAAATTAACCTTTTTTA
GGTGTAAGAGTTCTGAGAGG
>289
GCTGCACTATTTTTTTTTTTTGAGACCTAAAACTAGGGATTGCCTAAGCCTTTCCTTTTT
AAAAAGTCAGGTTTATATAGGTATAACTTGCATACAGTAAAATTAACCTTTTTTAGGTGT
AAGAGTTCTGAGAGGTTTGA
>290
CTAAAACTAGGGATTGCCTAAGCCTTTCCTTTTTAAAAAGTCAGGTTTATATAGGTATAA
CTTGCATACAGTAAAATTAACCTTTTTTAGGTGTAAGAGTTCTGAGAGGTTTGATAGAAT
ATATCAAGGTATAGGGCCAG
>291
AGCCTTTCCTTTTTAAAAAGTCAGGTTTATATAGGTATAACTTGCATACAGTAAAATTAA
CCTTTTTTAGGTGTAAGAGTTCTGAGAGGTTTGATAGAATATATCAAGGTATAGGGCCA
GCGCCGATGGCTCACACCTGT
>292
AGTCAGGTTTATATAGGTATAACTTGCATACAGTAAAATTAACCTTTTTTAGGTGTAAGA
GTTCTGAGAGGTTTGATAGAATATATCAAGGTATAGGGCCAGCGCCGATGGCTCACACC
TGTAGTCCCAACAATTTGGGA
>293
AACTTGCATACAGTAAAATTAACCTTTTTTAGGTGTAAGAGTTCTGAGAGGTTTGATAG
AATATATCAAGGTATAGGGCCAGCGCCGATGGCTCACACCTGTAGTCCCAACAATTTGG

GAGGCCAAGGCGGGAGGATCAC
>294
AGGTGTAAGAGTTCTGAGAGGTTTGATAGAATATATCAAGGTATAGGGCCAGCGCCGAT
GGCTCACACCTGTAGTCCCAACAATTTGGGAGGCCAAGGCGGGAGGATCACTTAGGCC
CAGGAGTTTGAGACCAGCTTGGG
>295
CGCCGATGGCTCACACCTGTAGTCCCAACAATTTGGGAGGCCAAGGCGGGAGGATCAC
TTAGGCCCAGGAGTTTGAGACCAGCTTGGGCAACATAGTGAGACCCTGTCTCTACAAA
AAAAAAAAAAAAAAAAGGTATCAAG
>296
CAACAATTTGGGAGGCCAAGGCGGGAGGATCACTTAGGCCCAGGAGTTTGAGACCAG
CTTGGGCAACATAGTGAGACCCTGTCTCTACAAAAAAAAAAAAAAAAAAAGGTATCAA
GGTACAGGAAATTTCCACTACCCAAC
>297
GGAGGCCAAGGCGGGAGGATCACTTAGGCCCAGGAGTTTGAGACCAGCTTGGGCAAC
ATAGTGAGACCCTGTCTCTACAAAAAAAAAAAAAAAAAAAGGTATCAAGGTACAGGAA
ATTTCCACTACCCAACAAAGGTCACT
>298
GCCCAGGAGTTTGAGACCAGCTTGGGCAACATAGTGAGACCCTGTCTCTACAAAAAA
AAAAAAAAAAAAAGGTATCAAGGTACAGGAAATTTCCACTACCCAACAAAGGTCACTC
ACCCTTTTGTAGTTAATAACTTCTCT
>299
GTTTGAGACCAGCTTGGGCAACATAGTGAGACCCTGTCTCTACAAAAAAAAAAAAAAA
AAAAGGTATCAAGGTACAGGAAATTTCCACTACCCAACAAAGGTCACTCACCCTTTTG
TAGTTAATAACTTCTCTGCAACCTA
>300
GACCCTGTCTCTACAAAAAAAAAAAAAAAAAAAGGTATCAAGGTACAGGAAATTTCCA
CTACCCAACAAAGGTCACTCACCCTTTTGTAGTTAATAACTTCTCTGCAACCTAGATCC
AGCCAACTACTGATAAGATGTCTG
>301
AAAAAAAAAAAAAAAAAAGGTATCAAGGTACAGGAAATTTCCACTACCCAACAAAGGT
CACTCACCCTTTTGTAGTTAATAACTTCTCTGCAACCTAGATCCAGCCAACTACTGATAA
GATGTCTGTCCTTGTATTTTCCA
>302
CAAAGGTCACTCACCCTTTTGTAGTTAATAACTTCTCTGCAACCTAGATCCAGCCAACT
ACTGATAAGATGTCTGTCCTTGTATTTTCCAGAATGTACTTAAATGGAGTCATACAGTAA
GTAGGCTTTTGTGTCTGGCTT
>303
GGTCACTCACCCTTTTGTAGTTAATAACTTCTCTGCAACCTAGATCCAGCCAACTACTG
ATAAGATGTCTGTCCTTGTATTTTCCAGAATGTACTTAAATGGAGTCATACAGTAAGTAG
GCTTTTGTGTCTGGCTTCAAG
>304
TTAATAACTTCTCTGCAACCTAGATCCAGCCAACTACTGATAAGATGTCTGTCCTTGTAT
TTTCCAGAATGTACTTAAATGGAGTCATACAGTAAGTAGGCTTTTGTGTCTGGCTTCAA

GCCTTTACTTTTTATAAAGAA
>305
CCAGCCAACTACTGATAAGATGTCTGTCCTTGTATTTTCCAGAATGTACTTAAATGGAGT
CATACAGTAAGTAGGCTTTTGTGTCTGGCTTCAAGCCTTTACTTTTTATAAAGAATAACA
ATGGTAATAATTATAGCTGA
>306
ACTGATAAGATGTCTGTCCTTGTATTTTCCAGAATGTACTTAAATGGAGTCATACAGTAA
GTAGGCTTTTGTGTCTGGCTTCAAGCCTTTACTTTTTATAAAGAATAACAATGGTAATAA
TTATAGCTGACATTTATTAA
>307
ATGGAGTCATACAGTAAGTAGGCTTTTGTGTCTGGCTTCAAGCCTTTACTTTTTATAAAG
AATAACAATGGTAATAATTATAGCTGACATTTATTAAAGGCTGAGCGGATACTGTATACT
GTGCTACAGGCTTTTCGTGG
>308
CTTTACTTTTTATAAAGAATAACAATGGTAATAATTATAGCTGACATTTATTAAAGGCTGA
GCGGATACTGTATACTGTGCTACAGGCTTTTCGTGGGTTATCTCATCACTGTTTCACATA
TGTAGGTATACAGTTCTTT
>309
TTTTATAAAGAATAACAATGGTAATAATTATAGCTGACATTTATTAAAGGCTGAGCGGAT
ACTGTATACTGTGCTACAGGCTTTTCGTGGGTTATCTCATCACTGTTTCACATATGTAGG
TATACAGTTCTTTCTTTAAA
>310
AATGGTAATAATTATAGCTGACATTTATTAAAGGCTGAGCGGATACTGTATACTGTGCTA
CAGGCTTTTCGTGGGTTATCTCATCACTGTTTCACATATGTAGGTATACAGTTCTTTCTTT
AAAATATGTGACTGAAAAA
>311
GTAATAATTATAGCTGACATTTATTAAAGGCTGAGCGGATACTGTATACTGTGCTACAGG
CTTTTCGTGGGTTATCTCATCACTGTTTCACATATGTAGGTATACAGTTCTTTCTTTAAAA
TATGTGACTGAAAAATATT
>312
TGACATTTATTAAAGGCTGAGCGGATACTGTATACTGTGCTACAGGCTTTTCGTGGGTTA
TCTCATCACTGTTTCACATATGTAGGTATACAGTTCTTTCTTTAAAATATGTGACTGAAA
AATATTATTGAACATTATGC
>313
AAAGGCTGAGCGGATACTGTATACTGTGCTACAGGCTTTTCGTGGGTTATCTCATCACT
GTTTCACATATGTAGGTATACAGTTCTTTCTTTAAAATATGTGACTGAAAAATATTATTGA
ACATTATGCTAAAATTTGTT
>314
TATGTAGGTATACAGTTCTTTCTTTAAAATATGTGACTGAAAAATATTATTGAACATTATG
CTAAAATTTGTTACACTGAGAAGACTTAAGAAAAACAGAGCTCATGTTTTCTTTCAACT
GATTTCCTTTTTACATTGTA
>315
CTGAAAAATATTATTGAACATTATGCTAAAATTTGTTACACTGAGAAGACTTAAGAAAA
ACAGAGCTCATGTTTTCTTTCAACTGATTTCCTTTTTACATTGTACTTGAGTACCTGGAA

GCTGCTTGCTTCTTTCCGCAG
>316
TTTGTTACACTGAGAAGACTTAAGAAAAACAGAGCTCATGTTTTCTTTCAACTGATTTC
CTTTTTACATTGTACTTGAGTACCTGGAAGCTGCTTGCTTCTTTCCGCAGCTTAGGATAC
AAACTCCCTGACAGTTAAGTG
>317
TTTACATTGTACTTGAGTACCTGGAAGCTGCTTGCTTCTTTCCGCAGCTTAGGATACAA
ACTCCCTGACAGTTAAGTGCTTCTTTACTTCCCATCCAGTGCCTTGCACAGGGCTTGGT
ACATGGTTTGGTCCAAAATATT
>318
ATTGTACTTGAGTACCTGGAAGCTGCTTGCTTCTTTCCGCAGCTTAGGATACAAACTCC
CTGACAGTTAAGTGCTTCTTTACTTCCCATCCAGTGCCTTGCACAGGGCTTGGTACATG
GTTTGGTCCAAAATATTTGTTG
>319
CTGCTTGCTTCTTTCCGCAGCTTAGGATACAAACTCCCTGACAGTTAAGTGCTTCTTTA
CTTCCCATCCAGTGCCTTGCACAGGGCTTGGTACATGGTTTGGTCCAAAATATTTGTTG
AATAAATTTGCAGATAGCCCAC
>320
GCTTAGGATACAAACTCCCTGACAGTTAAGTGCTTCTTTACTTCCCATCCAGTGCCTTG
CACAGGGCTTGGTACATGGTTTGGTCCAAAATATTTGTTGAATAAATTTGCAGATAGCC
CACGTGTGTAGGAGAGGGAGAG
>321
TTAAGTGCTTCTTTACTTCCCATCCAGTGCCTTGCACAGGGCTTGGTACATGGTTTGGT
CCAAAATATTTGTTGAATAAATTTGCAGATAGCCCACGTGTGTAGGAGAGGGAGAGAA
GGTTCTTGAAAAATGGAGATTGG
>322
GTGCCTTGCACAGGGCTTGGTACATGGTTTGGTCCAAAATATTTGTTGAATAAATTTGC
AGATAGCCCACGTGTGTAGGAGAGGGAGAGAAGGTTCTTGAAAAATGGAGATTGGAG
GAATCTAGGATGGGGAGGGAGATA
>323
GCCTTGCACAGGGCTTGGTACATGGTTTGGTCCAAAATATTTGTTGAATAAATTTGCAG
ATAGCCCACGTGTGTAGGAGAGGGAGAGAAGGTTCTTGAAAAATGGAGATTGGAGGA
ATCTAGGATGGGGAGGGAGATATG
>324
GAAAAATGGAGATTGGAGGAATCTAGGATGGGGAGGGAGATATGGGAAGGGCCCAGA
CAGAGGCAGGAGGGTTCCAACTTGTTCCTCTTCCCTGCAGCCCAGTGTTTCCCGGGCT
CTTTGAGTTCTGCTCGCGTTACACA
>325
GATTGGAGGAATCTAGGATGGGGAGGGAGATATGGGAAGGGCCCAGACAGAGGCAGG
AGGGTTCCAACTTGTTCCTCTTCCCTGCAGCCCAGTGTTTCCCGGGCTCTTTGAGTTCT
GCTCGCGTTACACAGGCGCATCTC
>326
GGAGGGAGATATGGGAAGGGCCCAGACAGAGGCAGGAGGGTTCCAACTTGTTCCTCT
TCCCTGCAGCCCAGTGTTTCCCGGGCTCTTTGAGTTCTGCTCGCGTTACACAGGCGCAT

CTCTGCAAGGAGCAACCCAGCTGA
>327
CCCAGACAGAGGCAGGAGGGTTCCAACTTGTTCCTCTTCCCTGCAGCCCAGTGTTTCC
CGGGCTCTTTGAGTTCTGCTCGCGTTACACAGGCGCATCTCTGCAAGGAGCAACCCAG
CTGAACAACAAGGTGACATAGTCC
>328
GCAGGAGGGTTCCAACTTGTTCCTCTTCCCTGCAGCCCAGTGTTTCCCGGGCTCTTTG
AGTTCTGCTCGCGTTACACAGGCGCATCTCTGCAAGGAGCAACCCAGCTGAACAACA
AGGTGACATAGTCCCGAGTCCTGTT
>329
TTGAGTTCTGCTCGCGTTACACAGGCGCATCTCTGCAAGGAGCAACCCAGCTGAACAA
CAAGGTGACATAGTCCCGAGTCCTGTTCTTCCTTTCCTCTGGATCCCTGGACTCGGGAT
TTAACCCTGATCCTGGGCTCCCA
>330
CTGCTCGCGTTACACAGGCGCATCTCTGCAAGGAGCAACCCAGCTGAACAACAAGGT
GACATAGTCCCGAGTCCTGTTCTTCCTTTCCTCTGGATCCCTGGACTCGGGATTTAACC
CTGATCCTGGGCTCCCAGCTTGAG
>331
CGCGTTACACAGGCGCATCTCTGCAAGGAGCAACCCAGCTGAACAACAAGGTGACAT
AGTCCCGAGTCCTGTTCTTCCTTTCCTCTGGATCCCTGGACTCGGGATTTAACCCTGAT
CCTGGGCTCCCAGCTTGAGGGGTG
>332
CCTGTTCTTCCTTTCCTCTGGATCCCTGGACTCGGGATTTAACCCTGATCCTGGGCTCCC
AGCTTGAGGGGTGGGCAGGAAGGACTGTGACTTAGGTGTTTGTCTTTCAGATCTGTGA
TATTGCCATTAACTGGGCTGGT
>333
TCTGGATCCCTGGACTCGGGATTTAACCCTGATCCTGGGCTCCCAGCTTGAGGGGTGG
GCAGGAAGGACTGTGACTTAGGTGTTTGTCTTTCAGATCTGTGATATTGCCATTAACTG
GGCTGGTGGTCTGCACCATGCCA
>334
CTGATCCTGGGCTCCCAGCTTGAGGGGTGGGCAGGAAGGACTGTGACTTAGGTGTTTG
TCTTTCAGATCTGTGATATTGCCATTAACTGGGCTGGTGGTCTGCACCATGCCAAGAAG
TTTGAGGTGAGTGAGGAGGTGAT
>335
GGGTGGGCAGGAAGGACTGTGACTTAGGTGTTTGTCTTTCAGATCTGTGATATTGCCAT
TAACTGGGCTGGTGGTCTGCACCATGCCAAGAAGTTTGAGGTGAGTGAGGAGGTGAT
GGGAAAGACAGTGGCCATCCTAGG
>336
TGGGCAGGAAGGACTGTGACTTAGGTGTTTGTCTTTCAGATCTGTGATATTGCCATTAA
CTGGGCTGGTGGTCTGCACCATGCCAAGAAGTTTGAGGTGAGTGAGGAGGTGATGGG
AAAGACAGTGGCCATCCTAGGGTA
>337
GTCTTTCAGATCTGTGATATTGCCATTAACTGGGCTGGTGGTCTGCACCATGCCAAGAA
GTTTGAGGTGAGTGAGGAGGTGATGGGAAAGACAGTGGCCATCCTAGGGTAGGTGTTT

AGGATGATGGTGGGGGGCAGCTG
>338
GATCTGTGATATTGCCATTAACTGGGCTGGTGGTCTGCACCATGCCAAGAAGTTTGAGG
TGAGTGAGGAGGTGATGGGAAAGACAGTGGCCATCCTAGGGTAGGTGTTTAGGATGAT
GGTGGGGGGCAGCTGGGAGGGGA
>339
CATTAACTGGGCTGGTGGTCTGCACCATGCCAAGAAGTTTGAGGTGAGTGAGGAGGTG
ATGGGAAAGACAGTGGCCATCCTAGGGTAGGTGTTTAGGATGATGGTGGGGGGCAGCT
GGGAGGGGAATTGCTCTTCTCTTT
>340
GTGGTCTGCACCATGCCAAGAAGTTTGAGGTGAGTGAGGAGGTGATGGGAAAGACAG
TGGCCATCCTAGGGTAGGTGTTTAGGATGATGGTGGGGGGCAGCTGGGAGGGGAATTG
CTCTTCTCTTTATGAGACCACTGTC
>341
AGGGTAGGTGTTTAGGATGATGGTGGGGGGCAGCTGGGAGGGGAATTGCTCTTCTCTT
TATGAGACCACTGTCTTGCCATAGGCCTCTGGCTTCTGCTATGTCAACGACATTGTGATT
GGCATCCTGGAGCTGCTCAAGT
>342
CAGCTGGGAGGGGAATTGCTCTTCTCTTTATGAGACCACTGTCTTGCCATAGGCCTCTG
GCTTCTGCTATGTCAACGACATTGTGATTGGCATCCTGGAGCTGCTCAAGTAAGTAGCC
CAGGAGGAGATGGGGGCAGTTA
>343
GAATTGCTCTTCTCTTTATGAGACCACTGTCTTGCCATAGGCCTCTGGCTTCTGCTATGT
CAACGACATTGTGATTGGCATCCTGGAGCTGCTCAAGTAAGTAGCCCAGGAGGAGATG
GGGGCAGTTACGGGATTCTTGG
>344
CTGTCTTGCCATAGGCCTCTGGCTTCTGCTATGTCAACGACATTGTGATTGGCATCCTGG
AGCTGCTCAAGTAAGTAGCCCAGGAGGAGATGGGGGCAGTTACGGGATTCTTGGCTGA
GGAGGTCTCTCAGTCTCAACCC
>345
CTATGTCAACGACATTGTGATTGGCATCCTGGAGCTGCTCAAGTAAGTAGCCCAGGAG
GAGATGGGGGCAGTTACGGGATTCTTGGCTGAGGAGGTCTCTCAGTCTCAACCCTGAC
CTTGACCTCCCTCCTCCCTGATTA
>346
CATCCTGGAGCTGCTCAAGTAAGTAGCCCAGGAGGAGATGGGGGCAGTTACGGGATTC
TTGGCTGAGGAGGTCTCTCAGTCTCAACCCTGACCTTGACCTCCCTCCTCCCTGATTAG
CTTCTCCCAAGTTCTTGATGCTT
>347
CTTGGCTGAGGAGGTCTCTCAGTCTCAACCCTGACCTTGACCTCCCTCCTCCCTGATTA
GCTTCTCCCAAGTTCTTGATGCTTTATTCAGGCTGCCTCTTCCACTGCTGCAGATTCCCT
ACTGCCTGCTCACATTTCAGC
>348
TGCCTCTTCCACTGCTGCAGATTCCCTACTGCCTGCTCACATTTCAGCCTTCTCTGCATA
TCCCTCTCCTGTCCCTGCCTCTAGCCTCTAGGCTGGAGCCCCCAGTTGCCTTTAGGGAA

CTGGTGACATTAAGTTTCAGG
>349
GCTCACATTTCAGCCTTCTCTGCATATCCCTCTCCTGTCCCTGCCTCTAGCCTCTAGGCT
GGAGCCCCCAGTTGCCTTTAGGGAACTGGTGACATTAAGTTTCAGGTGACCAGGGCAG
AAGCAGACTCTTCACAATTCGA
>350
TCTGCATATCCCTCTCCTGTCCCTGCCTCTAGCCTCTAGGCTGGAGCCCCCAGTTGCCTT
TAGGGAACTGGTGACATTAAGTTTCAGGTGACCAGGGCAGAAGCAGACTCTTCACAAT
TCGAGACCCGGTGTTTGAGAGG
>351
CCTCTCCTGTCCCTGCCTCTAGCCTCTAGGCTGGAGCCCCCAGTTGCCTTTAGGGAACT
GGTGACATTAAGTTTCAGGTGACCAGGGCAGAAGCAGACTCTTCACAATTCGAGACCC
GGTGTTTGAGAGGAGTGGGTTGG
>352
TGTCCCTGCCTCTAGCCTCTAGGCTGGAGCCCCCAGTTGCCTTTAGGGAACTGGTGACA
TTAAGTTTCAGGTGACCAGGGCAGAAGCAGACTCTTCACAATTCGAGACCCGGTGTTT
GAGAGGAGTGGGTTGGGGGGTTC
>353
TTTAGGGAACTGGTGACATTAAGTTTCAGGTGACCAGGGCAGAAGCAGACTCTTCACA
ATTCGAGACCCGGTGTTTGAGAGGAGTGGGTTGGGGGGTTCACTTCTAGCTCAGACCC
TGGCTTTGGCTTCCCTCTAGGTAC
>354
TAGGGAACTGGTGACATTAAGTTTCAGGTGACCAGGGCAGAAGCAGACTCTTCACAAT
TCGAGACCCGGTGTTTGAGAGGAGTGGGTTGGGGGGTTCACTTCTAGCTCAGACCCTG
GCTTTGGCTTCCCTCTAGGTACCA
>355
TGACATTAAGTTTCAGGTGACCAGGGCAGAAGCAGACTCTTCACAATTCGAGACCCGG
TGTTTGAGAGGAGTGGGTTGGGGGGTTCACTTCTAGCTCAGACCCTGGCTTTGGCTTC
CCTCTAGGTACCACCCTCGGGTGC
>356
TCAGGTGACCAGGGCAGAAGCAGACTCTTCACAATTCGAGACCCGGTGTTTGAGAGG
AGTGGGTTGGGGGGTTCACTTCTAGCTCAGACCCTGGCTTTGGCTTCCCTCTAGGTACC
ACCCTCGGGTGCTCTACATTGACA
>357
CCCGGTGTTTGAGAGGAGTGGGTTGGGGGGTTCACTTCTAGCTCAGACCCTGGCTTTG
GCTTCCCTCTAGGTACCACCCTCGGGTGCTCTACATTGACATTGACATCCACCATGGTG
ACGGGGTTCAAGAAGCTTTCTAC
>358
AGGAGTGGGTTGGGGGGTTCACTTCTAGCTCAGACCCTGGCTTTGGCTTCCCTCTAGG
TACCACCCTCGGGTGCTCTACATTGACATTGACATCCACCATGGTGACGGGGTTCAAGA
AGCTTTCTACCTCACTGACCGGG
>359
AGACCCTGGCTTTGGCTTCCCTCTAGGTACCACCCTCGGGTGCTCTACATTGACATTGA
CATCCACCATGGTGACGGGGTTCAAGAAGCTTTCTACCTCACTGACCGGGTCATGACG

GTGTCCTTCCACAAATACGGAAA
>360
GGTGCTCTACATTGACATTGACATCCACCATGGTGACGGGGTTCAAGAAGCTTTCTACC
TCACTGACCGGGTCATGACGGTGTCCTTCCACAAATACGGAAATTACTTCTTCCCTGGC
ACAGGTATGGGAATAGGGATTA
>361
CATTGACATTGACATCCACCATGGTGACGGGGTTCAAGAAGCTTTCTACCTCACTGACC
GGGTCATGACGGTGTCCTTCCACAAATACGGAAATTACTTCTTCCCTGGCACAGGTATG
GGAATAGGGATTACTTTCCCCT
>362
CTTCTTCCCTGGCACAGGTATGGGAATAGGGATTACTTTCCCCTTTGCACCCTTCAGCT
CTTTACTATGAGTGCCTCCTTCTCAGCCTTCTAGTCTCATCTCAGGTGTCCCCTCCTCAG
AGAAGCCTTTCCTTACCACTC
>363
GGAATAGGGATTACTTTCCCCTTTGCACCCTTCAGCTCTTTACTATGAGTGCCTCCTTCT
CAGCCTTCTAGTCTCATCTCAGGTGTCCCCTCCTCAGAGAAGCCTTTCCTTACCACTCC
AGTTATCATAATCACCTCCTT
>364
GCCTCCTTCTCAGCCTTCTAGTCTCATCTCAGGTGTCCCCTCCTCAGAGAAGCCTTTCC
TTACCACTCCAGTTATCATAATCACCTCCTTCCCCATTCCTCTGTTACATCAGCCTCATTG
TTTCCCTCAGAACACTCACT
>365
GTGTCCCCTCCTCAGAGAAGCCTTTCCTTACCACTCCAGTTATCATAATCACCTCCTTCC
CCATTCCTCTGTTACATCAGCCTCATTGTTTCCCTCAGAACACTCACTGCTATTTGTGTT
TATTGTGGTTTTTTGTTTTG
>366
AAGCCTTTCCTTACCACTCCAGTTATCATAATCACCTCCTTCCCCATTCCTCTGTTACATC
AGCCTCATTGTTTCCCTCAGAACACTCACTGCTATTTGTGTTTATTGTGGTTTTTTGTTT
TGTTTTGTTTACTGCCTCC
>367
TAATCACCTCCTTCCCCATTCCTCTGTTACATCAGCCTCATTGTTTCCCTCAGAACACTC
ACTGCTATTTGTGTTTATTGTGGTTTTTTGTTTTGTTTTGTTTACTGCCTCCCTCCTCCTC
ATGGCTATTCCCTAAGAAT
>368
TCCTCTGTTACATCAGCCTCATTGTTTCCCTCAGAACACTCACTGCTATTTGTGTTTATT
GTGGTTTTTTGTTTTGTTTTGTTTACTGCCTCCCTCCTCCTCATGGCTATTCCCTAAGAAT
ATAAGCGTGGTGGGATATC
>369
TGTTACATCAGCCTCATTGTTTCCCTCAGAACACTCACTGCTATTTGTGTTTATTGTGGT
TTTTTGTTTTGTTTTGTTTACTGCCTCCCTCCTCCTCATGGCTATTCCCTAAGAATATAAG
CGTGGTGGGATATCGTAAA
>370
CATCAGCCTCATTGTTTCCCTCAGAACACTCACTGCTATTTGTGTTTATTGTGGTTTTTT
GTTTTGTTTTGTTTACTGCCTCCCTCCTCCTCATGGCTATTCCCTAAGAATATAAGCGTG

GTGGGATATCGTAAATTTCA
>371
GTGGTTTTTTGTTTTGTTTTGTTTACTGCCTCCCTCCTCCTCATGGCTATTCCCTAAGAAT
ATAAGCGTGGTGGGATATCGTAAATTTCATTAGTTCCATTCGTTGCTGCATCCCTACTGC
CTAGTACACAGTTGGCTCA
>372
GTTTTGTTTTGTTTACTGCCTCCCTCCTCCTCATGGCTATTCCCTAAGAATATAAGCGTG
GTGGGATATCGTAAATTTCATTAGTTCCATTCGTTGCTGCATCCCTACTGCCTAGTACAC
AGTTGGCTCAGAGTGGATCC
>373
TACTGCCTCCCTCCTCCTCATGGCTATTCCCTAAGAATATAAGCGTGGTGGGATATCGTA
AATTTCATTAGTTCCATTCGTTGCTGCATCCCTACTGCCTAGTACACAGTTGGCTCAGAG
TGGATCCCCCAAAACTATTT
>374
TAAGAATATAAGCGTGGTGGGATATCGTAAATTTCATTAGTTCCATTCGTTGCTGCATCC
CTACTGCCTAGTACACAGTTGGCTCAGAGTGGATCCCCCAAAACTATTTGCCCAATGAA
TGCAAGCTGCAAGAGATTTAG
>375
ATAAGCGTGGTGGGATATCGTAAATTTCATTAGTTCCATTCGTTGCTGCATCCCTACTGC
CTAGTACACAGTTGGCTCAGAGTGGATCCCCCAAAACTATTTGCCCAATGAATGCAAG
CTGCAAGAGATTTAGTGGCTCT
>376
GGGATATCGTAAATTTCATTAGTTCCATTCGTTGCTGCATCCCTACTGCCTAGTACACAG
TTGGCTCAGAGTGGATCCCCCAAAACTATTTGCCCAATGAATGCAAGCTGCAAGAGAT
TTAGTGGCTCTAGGGAGTTTAT
>377
AGTACACAGTTGGCTCAGAGTGGATCCCCCAAAACTATTTGCCCAATGAATGCAAGCT
GCAAGAGATTTAGTGGCTCTAGGGAGTTTATGGTGAAGCTACATAGATGGAATGAGAG
GCTCCCAGAGTGCAGATTGGGGCT
>378
CTCAGAGTGGATCCCCCAAAACTATTTGCCCAATGAATGCAAGCTGCAAGAGATTTAG
TGGCTCTAGGGAGTTTATGGTGAAGCTACATAGATGGAATGAGAGGCTCCCAGAGTGC
AGATTGGGGCTGAAACAGCTGGGT
>379
TGGATCCCCCAAAACTATTTGCCCAATGAATGCAAGCTGCAAGAGATTTAGTGGCTCTA
GGGAGTTTATGGTGAAGCTACATAGATGGAATGAGAGGCTCCCAGAGTGCAGATTGGG
GCTGAAACAGCTGGGTTGTGGGT
>380
GCTGCAAGAGATTTAGTGGCTCTAGGGAGTTTATGGTGAAGCTACATAGATGGAATGAG
AGGCTCCCAGAGTGCAGATTGGGGCTGAAACAGCTGGGTTGTGGGTGCTTCCCATGTG
TGGCTACCACCACCCACTTCCCT
>381
GAGTTTATGGTGAAGCTACATAGATGGAATGAGAGGCTCCCAGAGTGCAGATTGGGGC
TGAAACAGCTGGGTTGTGGGTGCTTCCCATGTGTGGCTACCACCACCCACTTCCCTAC

AGGTGACATGTATGAAGTCGGGGC
>382
TTTATGGTGAAGCTACATAGATGGAATGAGAGGCTCCCAGAGTGCAGATTGGGGCTGA
AACAGCTGGGTTGTGGGTGCTTCCCATGTGTGGCTACCACCACCCACTTCCCTACAGG
TGACATGTATGAAGTCGGGGCAGA
>383
AGATTGGGGCTGAAACAGCTGGGTTGTGGGTGCTTCCCATGTGTGGCTACCACCACCC
ACTTCCCTACAGGTGACATGTATGAAGTCGGGGCAGAGAGTGGCCGCTACTACTGTCT
GAACGTGCCCCTGCGGGATGGCAT
>384
GGCTGAAACAGCTGGGTTGTGGGTGCTTCCCATGTGTGGCTACCACCACCCACTTCCC
TACAGGTGACATGTATGAAGTCGGGGCAGAGAGTGGCCGCTACTACTGTCTGAACGTG
CCCCTGCGGGATGGCATTGATGAC
>385
GGGTGCTTCCCATGTGTGGCTACCACCACCCACTTCCCTACAGGTGACATGTATGAAGT
CGGGGCAGAGAGTGGCCGCTACTACTGTCTGAACGTGCCCCTGCGGGATGGCATTGAT
GACCAGAGTAAGTACCGAAGTCT
>386
CACCACCCACTTCCCTACAGGTGACATGTATGAAGTCGGGGCAGAGAGTGGCCGCTAC
TACTGTCTGAACGTGCCCCTGCGGGATGGCATTGATGACCAGAGTAAGTACCGAAGTC
TTCCCTCTGTTCCCTAGGCCCCTT
>387
GAGAGTGGCCGCTACTACTGTCTGAACGTGCCCCTGCGGGATGGCATTGATGACCAGA
GTAAGTACCGAAGTCTTCCCTCTGTTCCCTAGGCCCCTTCTTTTTCTCCTTGAAATAACC
TCTTCTGCCTGGTATTGTCTGC
>388
TAAGTACCGAAGTCTTCCCTCTGTTCCCTAGGCCCCTTCTTTTTCTCCTTGAAATAACCT
CTTCTGCCTGGTATTGTCTGCTGTTGGGCCAGTTGTCCTTAAGGATGAATGGCCTTCAG
AGGGACCATGAATCTCTGGAA
>389
ACCGAAGTCTTCCCTCTGTTCCCTAGGCCCCTTCTTTTTCTCCTTGAAATAACCTCTTCT
GCCTGGTATTGTCTGCTGTTGGGCCAGTTGTCCTTAAGGATGAATGGCCTTCAGAGGGA
CCATGAATCTCTGGAAATTGT
>390
TGTTCCCTAGGCCCCTTCTTTTTCTCCTTGAAATAACCTCTTCTGCCTGGTATTGTCTGC
TGTTGGGCCAGTTGTCCTTAAGGATGAATGGCCTTCAGAGGGACCATGAATCTCTGGA
AATTGTATGCAACATATCATGT
>391
TCCCTAGGCCCCTTCTTTTTCTCCTTGAAATAACCTCTTCTGCCTGGTATTGTCTGCTGT
TGGGCCAGTTGTCCTTAAGGATGAATGGCCTTCAGAGGGACCATGAATCTCTGGAAAT
TGTATGCAACATATCATGTGTA
>392
CAGTTGTCCTTAAGGATGAATGGCCTTCAGAGGGACCATGAATCTCTGGAAATTGTATG
CAACATATCATGTGTATGTGCATTTTTCTGGGAAAAGTCATTGGCTTTCATCAGTCACTC

ATAGCAGTCTGGGATCCCTTC
>393
GTTGTCCTTAAGGATGAATGGCCTTCAGAGGGACCATGAATCTCTGGAAATTGTATGCA
ACATATCATGTGTATGTGCATTTTTCTGGGAAAAGTCATTGGCTTTCATCAGTCACTCAT
AGCAGTCTGGGATCCCTTCCA
>394
TTCAGAGGGACCATGAATCTCTGGAAATTGTATGCAACATATCATGTGTATGTGCATTTT
TCTGGGAAAAGTCATTGGCTTTCATCAGTCACTCATAGCAGTCTGGGATCCCTTCCAGA
AGTTTCAGACCAGTGATTTGA
>395
TATGCAACATATCATGTGTATGTGCATTTTTCTGGGAAAAGTCATTGGCTTTCATCAGTC
ACTCATAGCAGTCTGGGATCCCTTCCAGAAGTTTCAGACCAGTGATTTGATTCTTTTGG
GTGGCCCCTTTCCTGGGCTAC
>396
TGTGCATTTTTCTGGGAAAAGTCATTGGCTTTCATCAGTCACTCATAGCAGTCTGGGAT
CCCTTCCAGAAGTTTCAGACCAGTGATTTGATTCTTTTGGGTGGCCCCTTTCCTGGGCT
ACATGCTCGCTCAACCCCCTTT
>397
CCAGTGATTTGATTCTTTTGGGTGGCCCCTTTCCTGGGCTACATGCTCGCTCAACCCCC
TTTTTCCTTTTGTTCCCTGTCTCCCCATCCCCTTGGGGTGCCAGGTGCCCATCTTTGGCC
CTCTCCCAGGTTACAAGCACC
>398
TTTGATTCTTTTGGGTGGCCCCTTTCCTGGGCTACATGCTCGCTCAACCCCCTTTTTCCT
TTTGTTCCCTGTCTCCCCATCCCCTTGGGGTGCCAGGTGCCCATCTTTGGCCCTCTCCC
AGGTTACAAGCACCTTTTCCA
>399
CCCTTTCCTGGGCTACATGCTCGCTCAACCCCCTTTTTCCTTTTGTTCCCTGTCTCCCCA
TCCCCTTGGGGTGCCAGGTGCCCATCTTTGGCCCTCTCCCAGGTTACAAGCACCTTTTC
CAGCCGGTTATCAACCAGGTA
>400
CTGGGCTACATGCTCGCTCAACCCCCTTTTTCCTTTTGTTCCCTGTCTCCCCATCCCCTT
GGGGTGCCAGGTGCCCATCTTTGGCCCTCTCCCAGGTTACAAGCACCTTTTCCAGCCG
GTTATCAACCAGGTAGTGGACT
>401
CTTTTTCCTTTTGTTCCCTGTCTCCCCATCCCCTTGGGGTGCCAGGTGCCCATCTTTGGC
CCTCTCCCAGGTTACAAGCACCTTTTCCAGCCGGTTATCAACCAGGTAGTGGACTTCTA
CCAACCCACGTGCATTGTGCT
>402
CCCCATCCCCTTGGGGTGCCAGGTGCCCATCTTTGGCCCTCTCCCAGGTTACAAGCACC
TTTTCCAGCCGGTTATCAACCAGGTAGTGGACTTCTACCAACCCACGTGCATTGTGCTC
CAGGTAATACTGTCAGTTCCTC
>403
GGGGTGCCAGGTGCCCATCTTTGGCCCTCTCCCAGGTTACAAGCACCTTTTCCAGCCG
GTTATCAACCAGGTAGTGGACTTCTACCAACCCACGTGCATTGTGCTCCAGGTAATACT

GTCAGTTCCTCCTCAGGAGTGTT
>404
GCCCTCTCCCAGGTTACAAGCACCTTTTCCAGCCGGTTATCAACCAGGTAGTGGACTTC
TACCAACCCACGTGCATTGTGCTCCAGGTAATACTGTCAGTTCCTCCTCAGGAGTGTTC
TGCCTGGGCTTGGGGAAAGAGA
>405
TTTTCCAGCCGGTTATCAACCAGGTAGTGGACTTCTACCAACCCACGTGCATTGTGCTC
CAGGTAATACTGTCAGTTCCTCCTCAGGAGTGTTCTGCCTGGGCTTGGGGAAAGAGAG
CAAGGGAAGAGGAGGTTCTTTAG
>406
CCAGCCGGTTATCAACCAGGTAGTGGACTTCTACCAACCCACGTGCATTGTGCTCCAG
GTAATACTGTCAGTTCCTCCTCAGGAGTGTTCTGCCTGGGCTTGGGGAAAGAGAGCAA
GGGAAGAGGAGGTTCTTTAGGTTG
>407
ACCAGGTAGTGGACTTCTACCAACCCACGTGCATTGTGCTCCAGGTAATACTGTCAGTT
CCTCCTCAGGAGTGTTCTGCCTGGGCTTGGGGAAAGAGAGCAAGGGAAGAGGAGGTT
CTTTAGGTTGGTGGTGGAAGGGGT
>408
GGTAATACTGTCAGTTCCTCCTCAGGAGTGTTCTGCCTGGGCTTGGGGAAAGAGAGCA
AGGGAAGAGGAGGTTCTTTAGGTTGGTGGTGGAAGGGGTATCTGTGATGTTCTGATCA
GTTCTGATCACTGCACTTTCCTGG
>409
GTCAGTTCCTCCTCAGGAGTGTTCTGCCTGGGCTTGGGGAAAGAGAGCAAGGGAAGA
GGAGGTTCTTTAGGTTGGTGGTGGAAGGGGTATCTGTGATGTTCTGATCAGTTCTGATC
ACTGCACTTTCCTGGGGTTTTGCT
>410
GAGTGTTCTGCCTGGGCTTGGGGAAAGAGAGCAAGGGAAGAGGAGGTTCTTTAGGTT
GGTGGTGGAAGGGGTATCTGTGATGTTCTGATCAGTTCTGATCACTGCACTTTCCTGGG
GTTTTGCTTTTTGCAGTGTGGAGC
>411
TTCTTTAGGTTGGTGGTGGAAGGGGTATCTGTGATGTTCTGATCAGTTCTGATCACTGC
ACTTTCCTGGGGTTTTGCTTTTTGCAGTGTGGAGCTGACTCTCTGGGCTGTGATCGATT
GGGCTGCTTTAACCTCAGCATC
>412
GTGGAAGGGGTATCTGTGATGTTCTGATCAGTTCTGATCACTGCACTTTCCTGGGGTTT
TGCTTTTTGCAGTGTGGAGCTGACTCTCTGGGCTGTGATCGATTGGGCTGCTTTAACCT
CAGCATCCGAGGGCATGGGTGA
>413
CTGATCAGTTCTGATCACTGCACTTTCCTGGGGTTTTGCTTTTTGCAGTGTGGAGCTGA
CTCTCTGGGCTGTGATCGATTGGGCTGCTTTAACCTCAGCATCCGAGGGCATGGGTGAG
ACTTGCTCTCCTTCTTCTCCCC
>414
TGCAGTGTGGAGCTGACTCTCTGGGCTGTGATCGATTGGGCTGCTTTAACCTCAGCATC
CGAGGGCATGGGTGAGACTTGCTCTCCTTCTTCTCCCCAAGGTTTTTAAGTGGCCTTGG

ACTTCAGTACGTGGGTGGGGGT
>415
ATCGATTGGGCTGCTTTAACCTCAGCATCCGAGGGCATGGGTGAGACTTGCTCTCCTTC
TTCTCCCCAAGGTTTTTAAGTGGCCTTGGACTTCAGTACGTGGGTGGGGGTAGTTGGG
GAATGGGCCTCACCTCTTCTTGG
>416
GGCTGCTTTAACCTCAGCATCCGAGGGCATGGGTGAGACTTGCTCTCCTTCTTCTCCCC
AAGGTTTTTAAGTGGCCTTGGACTTCAGTACGTGGGTGGGGGTAGTTGGGGAATGGGC
CTCACCTCTTCTTGGAAATCAAA
>417
GCATCCGAGGGCATGGGTGAGACTTGCTCTCCTTCTTCTCCCCAAGGTTTTTAAGTGGC
CTTGGACTTCAGTACGTGGGTGGGGGTAGTTGGGGAATGGGCCTCACCTCTTCTTGGA
AATCAAAAATAGCTACAAATTGT
>418
CAGTACGTGGGTGGGGGTAGTTGGGGAATGGGCCTCACCTCTTCTTGGAAATCAAAAA
TAGCTACAAATTGTACTTGAACTAGAAATGTCATCTTGAACAGGGTTCCCTAGATGGTC
ATTCATTCACGATATATTTTTAT
>419
TAGTTGGGGAATGGGCCTCACCTCTTCTTGGAAATCAAAAATAGCTACAAATTGTACTT
GAACTAGAAATGTCATCTTGAACAGGGTTCCCTAGATGGTCATTCATTCACGATATATTT
TTATTAAGCACCTGTTATGTT
>420
CCTCACCTCTTCTTGGAAATCAAAAATAGCTACAAATTGTACTTGAACTAGAAATGTCA
TCTTGAACAGGGTTCCCTAGATGGTCATTCATTCACGATATATTTTTATTAAGCACCTGTT
ATGTTCCAGGCATCATACCA
>421
TTGGAAATCAAAAATAGCTACAAATTGTACTTGAACTAGAAATGTCATCTTGAACAGGG
TTCCCTAGATGGTCATTCATTCACGATATATTTTTATTAAGCACCTGTTATGTTCCAGGCA
TCATACCACATCATGGGGAT
>422
CTAGAAATGTCATCTTGAACAGGGTTCCCTAGATGGTCATTCATTCACGATATATTTTTAT
TAAGCACCTGTTATGTTCCAGGCATCATACCACATCATGGGGATGGAATAAGGAGTCTC
CCATGGAGTTTACAGTTTAG
>423
AATGTCATCTTGAACAGGGTTCCCTAGATGGTCATTCATTCACGATATATTTTTATTAAGC
ACCTGTTATGTTCCAGGCATCATACCACATCATGGGGATGGAATAAGGAGTCTCCCATG
GAGTTTACAGTTTAGGAAAC
>424
CACGATATATTTTTATTAAGCACCTGTTATGTTCCAGGCATCATACCACATCATGGGGATG
GAATAAGGAGTCTCCCATGGAGTTTACAGTTTAGGAAACAGACAAAAAATAAATAATC
AAATGAAATAAATAGATTCTT
>425
GCACCTGTTATGTTCCAGGCATCATACCACATCATGGGGATGGAATAAGGAGTCTCCCA
TGGAGTTTACAGTTTAGGAAACAGACAAAAAATAAATAATCAAATGAAATAAATAGATT

CTTATAAATTCTGTGAAAGAAA
>426
GGAGTTTACAGTTTAGGAAACAGACAAAAAATAAATAATCAAATGAAATAAATAGATTC
TTATAAATTCTGTGAAAGAAAAAAAGAAAGCTGAGACACAGGATACAATGGGGGACCT
ACTTGAGATAGCATGGTTTCGGA
>427
TAAATTCTGTGAAAGAAAAAAAGAAAGCTGAGACACAGGATACAATGGGGGACCTAC
TTGAGATAGCATGGTTTCGGAAGTTTTTCTGAGGAAGTAAAATTTGAGTTGAGACCTG
GAGACTAAGAAGGAGTGAGTCATAA
>428
TGAAAGAAAAAAAGAAAGCTGAGACACAGGATACAATGGGGGACCTACTTGAGATAG
CATGGTTTCGGAAGTTTTTCTGAGGAAGTAAAATTTGAGTTGAGACCTGGAGACTAAG
AAGGAGTGAGTCATAAGAGGAGCAG
>429
AAAGCTGAGACACAGGATACAATGGGGGACCTACTTGAGATAGCATGGTTTCGGAAGT
TTTTCTGAGGAAGTAAAATTTGAGTTGAGACCTGGAGACTAAGAAGGAGTGAGTCATA
AGAGGAGCAGAGAGAAGCCTGACC
>430
GAGATAGCATGGTTTCGGAAGTTTTTCTGAGGAAGTAAAATTTGAGTTGAGACCTGGA
GACTAAGAAGGAGTGAGTCATAAGAGGAGCAGAGAGAAGCCTGACCCAAGCATGGA
CAAAGGCCCACAAAAGGCTCTGAGGG
>431
TAGCATGGTTTCGGAAGTTTTTCTGAGGAAGTAAAATTTGAGTTGAGACCTGGAGACT
AAGAAGGAGTGAGTCATAAGAGGAGCAGAGAGAAGCCTGACCCAAGCATGGACAAA
GGCCCACAAAAGGCTCTGAGGGCTGG
>432
AAGAGGAGCAGAGAGAAGCCTGACCCAAGCATGGACAAAGGCCCACAAAAGGCTCT
GAGGGCTGGGCCCAGTGGCACACACCTATAATCCCAACACTTCCAGAGGCCAAGGTG
GGAGAATCGCCTGAGGCTAGGAGTTTG
>433
GCCCACAAAAGGCTCTGAGGGCTGGGCCCAGTGGCACACACCTATAATCCCAACACTT
CCAGAGGCCAAGGTGGGAGAATCGCCTGAGGCTAGGAGTTTGAGGCCAGCCTGGGCA
ACATAGTGAGAGCCATCTCTACAGA
>434
GCCCAGTGGCACACACCTATAATCCCAACACTTCCAGAGGCCAAGGTGGGAGAATCGC
CTGAGGCTAGGAGTTTGAGGCCAGCCTGGGCAACATAGTGAGAGCCATCTCTACAGAA
AATAAAAAACAAAATCAGCTGGGC
>435
CAACACTTCCAGAGGCCAAGGTGGGAGAATCGCCTGAGGCTAGGAGTTTGAGGCCAG
CCTGGGCAACATAGTGAGAGCCATCTCTACAGAAAATAAAAAACAAAATCAGCTGGGC
ATGATGGTGCATGCCTATAGTCCTA
>436
GGCCAGCCTGGGCAACATAGTGAGAGCCATCTCTACAGAAAATAAAAAACAAAATCA
GCTGGGCATGATGGTGCATGCCTATAGTCCTAGCTACTCAGGAAGCTGAGGCAGGAGG

ATCACTTGAGCCTAGGAGTTTGAGG
>437
AACATAGTGAGAGCCATCTCTACAGAAAATAAAAAACAAAATCAGCTGGGCATGATGG
TGCATGCCTATAGTCCTAGCTACTCAGGAAGCTGAGGCAGGAGGATCACTTGAGCCTA
GGAGTTTGAGGTTACAGTGAACTA
>438
GCATGATGGTGCATGCCTATAGTCCTAGCTACTCAGGAAGCTGAGGCAGGAGGATCAC
TTGAGCCTAGGAGTTTGAGGTTACAGTGAACTATGATTGTGCCACTGGACTTCAGTCTG
TTGAGGTGAGGTCCGAGAAGCTG
>439
GGTGCATGCCTATAGTCCTAGCTACTCAGGAAGCTGAGGCAGGAGGATCACTTGAGCC
TAGGAGTTTGAGGTTACAGTGAACTATGATTGTGCCACTGGACTTCAGTCTGTTGAGGT
GAGGTCCGAGAAGCTGGCCATGG
>440
TGCCTATAGTCCTAGCTACTCAGGAAGCTGAGGCAGGAGGATCACTTGAGCCTAGGAG
TTTGAGGTTACAGTGAACTATGATTGTGCCACTGGACTTCAGTCTGTTGAGGTGAGGTC
CGAGAAGCTGGCCATGGTGAAGA
>441
AGCTACTCAGGAAGCTGAGGCAGGAGGATCACTTGAGCCTAGGAGTTTGAGGTTACA
GTGAACTATGATTGTGCCACTGGACTTCAGTCTGTTGAGGTGAGGTCCGAGAAGCTGG
CCATGGTGAAGATTGTGCATGAGCC
>442
GAGGCAGGAGGATCACTTGAGCCTAGGAGTTTGAGGTTACAGTGAACTATGATTGTGC
CACTGGACTTCAGTCTGTTGAGGTGAGGTCCGAGAAGCTGGCCATGGTGAAGATTGTG
CATGAGCCTTTAAACCCTGGTAAG
>443
GGTTACAGTGAACTATGATTGTGCCACTGGACTTCAGTCTGTTGAGGTGAGGTCCGAG
AAGCTGGCCATGGTGAAGATTGTGCATGAGCCTTTAAACCCTGGTAAGGAGTTTGAATT
ATATTCCAAAGATGATGGGAAAC
>444
GAACTATGATTGTGCCACTGGACTTCAGTCTGTTGAGGTGAGGTCCGAGAAGCTGGCC
ATGGTGAAGATTGTGCATGAGCCTTTAAACCCTGGTAAGGAGTTTGAATTATATTCCAA
AGATGATGGGAAACCATTGAAGG
>445
CTTCAGTCTGTTGAGGTGAGGTCCGAGAAGCTGGCCATGGTGAAGATTGTGCATGAGC
CTTTAAACCCTGGTAAGGAGTTTGAATTATATTCCAAAGATGATGGGAAACCATTGAAG
GATTTTAAGCAGGAAAGTGACAT
>446
AACCCTGGTAAGGAGTTTGAATTATATTCCAAAGATGATGGGAAACCATTGAAGGATTT
TAAGCAGGAAAGTGACATGATCCAATTTGTGTTTGAAAGTGACAATTCTGACTGCTGA
ATAGAGAAAGGTTTGGGTAGGGG
>447
TGAATTATATTCCAAAGATGATGGGAAACCATTGAAGGATTTTAAGCAGGAAAGTGACA
TGATCCAATTTGTGTTTGAAAGTGACAATTCTGACTGCTGAATAGAGAAAGGTTTGGGT

AGGGGGGATGGAGATGGAAGTA
>448
TCCAAAGATGATGGGAAACCATTGAAGGATTTTAAGCAGGAAAGTGACATGATCCAAT
TTGTGTTTGAAAGTGACAATTCTGACTGCTGAATAGAGAAAGGTTTGGGTAGGGGGGA
TGGAGATGGAAGTAGAAGAGACCT
>449
TTTAAGCAGGAAAGTGACATGATCCAATTTGTGTTTGAAAGTGACAATTCTGACTGCTG
AATAGAGAAAGGTTTGGGTAGGGGGGATGGAGATGGAAGTAGAAGAGACCTAGTTAG
GAAGCCATGGTGTTGGTTAGGTGC
>450
TTTGTGTTTGAAAGTGACAATTCTGACTGCTGAATAGAGAAAGGTTTGGGTAGGGGGG
ATGGAGATGGAAGTAGAAGAGACCTAGTTAGGAAGCCATGGTGTTGGTTAGGTGCCCA
GACTAAGGTGGAGGGGGTGGCAAT
>451
TGACAATTCTGACTGCTGAATAGAGAAAGGTTTGGGTAGGGGGGATGGAGATGGAAGT
AGAAGAGACCTAGTTAGGAAGCCATGGTGTTGGTTAGGTGCCCAGACTAAGGTGGAG
GGGGTGGCAATGGAGAGAAGTAGAT
>452
GCTGAATAGAGAAAGGTTTGGGTAGGGGGGATGGAGATGGAAGTAGAAGAGACCTAG
TTAGGAAGCCATGGTGTTGGTTAGGTGCCCAGACTAAGGTGGAGGGGGTGGCAATGG
AGAGAAGTAGATGATCTGAGAGGTGT
>453
GGGGGATGGAGATGGAAGTAGAAGAGACCTAGTTAGGAAGCCATGGTGTTGGTTAGG
TGCCCAGACTAAGGTGGAGGGGGTGGCAATGGAGAGAAGTAGATGATCTGAGAGGTG
TGTGTGTGTGTGTGTGTATATATATA
>454
AGATGGAAGTAGAAGAGACCTAGTTAGGAAGCCATGGTGTTGGTTAGGTGCCCAGACT
AAGGTGGAGGGGGTGGCAATGGAGAGAAGTAGATGATCTGAGAGGTGTGTGTGTGTG
TGTGTGTATATATATATATATACAC
>455
GACCTAGTTAGGAAGCCATGGTGTTGGTTAGGTGCCCAGACTAAGGTGGAGGGGGTG
GCAATGGAGAGAAGTAGATGATCTGAGAGGTGTGTGTGTGTGTGTGTGTATATATATATA
TATACACACACACACACACATAT
>456
CATGGTGTTGGTTAGGTGCCCAGACTAAGGTGGAGGGGGTGGCAATGGAGAGAAGTA
GATGATCTGAGAGGTGTGTGTGTGTGTGTGTGTATATATATATATATACACACACACACA
CACATATATATGTTTTTTTTTTT
>457
ATGATCTGAGAGGTGTGTGTGTGTGTGTGTGTGTATATATATATATATACACACACACACAC
ACATATATATGTTTTTTTTTTTCTTTTTTGAGATGGAGTCTTGCTCTGTCACCCAGGCTGG
AGTGCAGTGGCACGATCTT
>458
TGTGTATATATATATATATACACACACACACACACATATATATGTTTTTTTTTTTCTTTTTTG
AGATGGAGTCTTGCTCTGTCACCCAGGCTGGAGTGCAGTGGCACGATCTTGGCTCAGT

GCAAGCTCTGCCTCCTGGG
>459
TATATATATACACACACACACACACATATATATGTTTTTTTTTTTCTTTTTGAGATGGAGT
CTTGCTCTGTCACCCAGGCTGGAGTGCAGTGGCACGATCTTGGCTCAGTGCAAGCTCT
GCCTCCTGGGTTCAAGCATT
>460
ACACACACACATATATATGTTTTTTTTTTTCTTTTTGAGATGGAGTCTTGCTCTGTCACC
CAGGCTGGAGTGCAGTGGCACGATCTTGGCTCAGTGCAAGCTCTGCCTCCTGGGTTCA
AGCATTTCTCCTGCCTCAGCC
>461
TTTTTTCTTTTTTGAGATGGAGTCTTGCTCTGTCACCCAGGCTGGAGTGCAGTGGCACG
ATCTTGGCTCAGTGCAAGCTCTGCCTCCTGGGTTCAAGCATTTCTCCTGCCTCAGCCTC
CTGAGTAGCTGGGACTACTACA
>462
AGTCTTGCTCTGTCACCCAGGCTGGAGTGCAGTGGCACGATCTTGGCTCAGTGCAAGC
TCTGCCTCCTGGGTTCAAGCATTTCTCCTGCCTCAGCCTCCTGAGTAGCTGGGACTACT
ACAAAGGCGTGAGCACCGCGCCG
>463
TGGCACGATCTTGGCTCAGTGCAAGCTCTGCCTCCTGGGTTCAAGCATTTCTCCTGCCT
CAGCCTCCTGAGTAGCTGGGACTACTACAAAGGCGTGAGCACCGCGCCGGGCGTGAG
TTATATTTTCAAGGCTAGAATTGA
>464
AGCTCTGCCTCCTGGGTTCAAGCATTTCTCCTGCCTCAGCCTCCTGAGTAGCTGGGACT
ACTACAAAGGCGTGAGCACCGCGCCGGGCGTGAGTTATATTTTCAAGGCTAGAATTGA
CAGAACTTGGGTTGATGAGAATA
>465
CAAGCATTTCTCCTGCCTCAGCCTCCTGAGTAGCTGGGACTACTACAAAGGCGTGAGC
ACCGCGCCGGGCGTGAGTTATATTTTCAAGGCTAGAATTGACAGAACTTGGGTTGATG
AGAATAGTTGATTAGCAGGCAGGG
>466
CTACTACAAAGGCGTGAGCACCGCGCCGGGCGTGAGTTATATTTTCAAGGCTAGAATT
GACAGAACTTGGGTTGATGAGAATAGTTGATTAGCAGGCAGGGTGCCAGGGAGGGTG
TTGAGCAGAACAGAGGGGGGACTGT
>467
GTGAGCACCGCGCCGGGCGTGAGTTATATTTTCAAGGCTAGAATTGACAGAACTTGGG
TTGATGAGAATAGTTGATTAGCAGGCAGGGTGCCAGGGAGGGTGTTGAGCAGAACAG
AGGGGGGACTGTGCTTTATTTTAAA
>468
CGTGAGTTATATTTTCAAGGCTAGAATTGACAGAACTTGGGTTGATGAGAATAGTTGAT
TAGCAGGCAGGGTGCCAGGGAGGGTGTTGAGCAGAACAGAGGGGGGACTGTGCTTTA
TTTTAAAATTTTTATTGAGACACA
>469
GGGTTGATGAGAATAGTTGATTAGCAGGCAGGGTGCCAGGGAGGGTGTTGAGCAGAA
CAGAGGGGGGACTGTGCTTTATTTTAAAATTTTTATTGAGACACAGCATATAAAGTATAC

CCAAATCTTAAGGGTATAACATA
>470
AGGGTGTTGAGCAGAACAGAGGGGGGACTGTGCTTTATTTTAAAATTTTTATTGAGAC
ACAGCATATAAAGTATACCCAAATCTTAAGGGTATAACATAATGAATTTCACTATCAAGA
TAAAGATTTAGAACATTTATAG
>471
AGGGGGGACTGTGCTTTATTTTAAAATTTTTATTGAGACACAGCATATAAAGTATACCCA
AATCTTAAGGGTATAACATAATGAATTTCACTATCAAGATAAAGATTTAGAACATTTATA
GTTTTCTTGTGCTCCTTTCA
>472
AGTATACCCAAATCTTAAGGGTATAACATAATGAATTTCACTATCAAGATAAAGATTTAG
AACATTTATAGTTTTCTTGTGCTCCTTTCAGGCAATACCTAAAGGTATTTACCTTGTCTTT
TGATAACCTCCTCCACATC
>473
CAAATCTTAAGGGTATAACATAATGAATTTCACTATCAAGATAAAGATTTAGAACATTTA
TAGTTTTCTTGTGCTCCTTTCAGGCAATACCTAAAGGTATTTACCTTGTCTTTTGATAAC
CTCCTCCACATCTTCCTCTG
>474
ATTTCACTATCAAGATAAAGATTTAGAACATTTATAGTTTTCTTGTGCTCCTTTCAGGCA
ATACCTAAAGGTATTTACCTTGTCTTTTGATAACCTCCTCCACATCTTCCTCTGGTTCCTT
TTAACAGGGAATGCGTTGA
>475
GTGCTCCTTTCAGGCAATACCTAAAGGTATTTACCTTGTCTTTTGATAACCTCCTCCACA
TCTTCCTCTGGTTCCTTTTAACAGGGAATGCGTTGAATATGTCAAGAGCTTCAATATCCC
TCTACTCGTGCTGGGTGGTG
>476
TAAAGGTATTTACCTTGTCTTTTGATAACCTCCTCCACATCTTCCTCTGGTTCCTTTTAAC
AGGGAATGCGTTGAATATGTCAAGAGCTTCAATATCCCTCTACTCGTGCTGGGTGGTGG
TGGTTATACTGTCCGAAATG
>477
ACATCTTCCTCTGGTTCCTTTTAACAGGGAATGCGTTGAATATGTCAAGAGCTTCAATAT
CCCTCTACTCGTGCTGGGTGGTGGTGGTTATACTGTCCGAAATGTTGCCCGCTGCTGGT
GAGCACTCTCCTGATTTCCTC
>478
CCTCTGGTTCCTTTTAACAGGGAATGCGTTGAATATGTCAAGAGCTTCAATATCCCTCTA
CTCGTGCTGGGTGGTGGTGGTTATACTGTCCGAAATGTTGCCCGCTGCTGGTGAGCACT
CTCCTGATTTCCTCCCCTGTT
>479
CGTTGAATATGTCAAGAGCTTCAATATCCCTCTACTCGTGCTGGGTGGTGGTGGTTATAC
TGTCCGAAATGTTGCCCGCTGCTGGTGAGCACTCTCCTGATTTCCTCCCCTGTTGTCCA
GTGTGAGCTAGGGTGTCCCTC
>480
TGGGTGGTGGTGGTTATACTGTCCGAAATGTTGCCCGCTGCTGGTGAGCACTCTCCTGA
TTTCCTCCCCTGTTGTCCAGTGTGAGCTAGGGTGTCCCTCAGATATGGCCACATGGGCC

CTTCAGCCCACTTGGTCAGCCT
>481
GGTGGTTATACTGTCCGAAATGTTGCCCGCTGCTGGTGAGCACTCTCCTGATTTCCTCC
CCTGTTGTCCAGTGTGAGCTAGGGTGTCCCTCAGATATGGCCACATGGGCCCTTCAGCC
CACTTGGTCAGCCTGCTCCCCT
>482
CCTCCCCTGTTGTCCAGTGTGAGCTAGGGTGTCCCTCAGATATGGCCACATGGGCCCTT
CAGCCCACTTGGTCAGCCTGCTCCCCTCTGCTGGTTGGTTTCAGGACATATGAGACATC
GCTGCTGGTAGAAGAGGCCATT
>483
GCTAGGGTGTCCCTCAGATATGGCCACATGGGCCCTTCAGCCCACTTGGTCAGCCTGCT
CCCCTCTGCTGGTTGGTTTCAGGACATATGAGACATCGCTGCTGGTAGAAGAGGCCATT
AGTGAGGAGCTTCCCTATAGTG
>484
CCTTCAGCCCACTTGGTCAGCCTGCTCCCCTCTGCTGGTTGGTTTCAGGACATATGAGA
CATCGCTGCTGGTAGAAGAGGCCATTAGTGAGGAGCTTCCCTATAGTGGTAAGGACAC
CCACAACACCCCATAACAGGAAG
>485
TCAGCCTGCTCCCCTCTGCTGGTTGGTTTCAGGACATATGAGACATCGCTGCTGGTAGA
AGAGGCCATTAGTGAGGAGCTTCCCTATAGTGGTAAGGACACCCACAACACCCCATAA
CAGGAAGGCCATTCTCACCAGAT
>486
CTGGTTGGTTTCAGGACATATGAGACATCGCTGCTGGTAGAAGAGGCCATTAGTGAGG
AGCTTCCCTATAGTGGTAAGGACACCCACAACACCCCATAACAGGAAGGCCATTCTCA
CCAGATTCCAGAATGACCTGGTTT
>487
GTTGGTTTCAGGACATATGAGACATCGCTGCTGGTAGAAGAGGCCATTAGTGAGGAGC
TTCCCTATAGTGGTAAGGACACCCACAACACCCCATAACAGGAAGGCCATTCTCACCA
GATTCCAGAATGACCTGGTTTGAA
>488
TATAGTGGTAAGGACACCCACAACACCCCATAACAGGAAGGCCATTCTCACCAGATTC
CAGAATGACCTGGTTTGAAGGCACATGGGAAATTTTACACTCTCAGAGCTCTGCAGAC
TAAACCCCTGGGCTCAGAGCAGGG
>489
TTTACACTCTCAGAGCTCTGCAGACTAAACCCCTGGGCTCAGAGCAGGGAAGAGATTG
GGCAGCTATTATGTAGGTGCTCTGGATGGGAAGGAAAAAGATTGGGAAGTCTTGTTAG
CTGCCTTTCTCAGTCTGAGCCATA
>490
CACTCTCAGAGCTCTGCAGACTAAACCCCTGGGCTCAGAGCAGGGAAGAGATTGGGC
AGCTATTATGTAGGTGCTCTGGATGGGAAGGAAAAAGATTGGGAAGTCTTGTTAGCTG
CCTTTCTCAGTCTGAGCCATACTTT
>491
GCTCAGAGCAGGGAAGAGATTGGGCAGCTATTATGTAGGTGCTCTGGATGGGAAGGAA
AAAGATTGGGAAGTCTTGTTAGCTGCCTTTCTCAGTCTGAGCCATACTTTCTCCTCTCC

TCCCCAAGAATACTTCGAGTACT
>492
GAGCAGGGAAGAGATTGGGCAGCTATTATGTAGGTGCTCTGGATGGGAAGGAAAAAG
ATTGGGAAGTCTTGTTAGCTGCCTTTCTCAGTCTGAGCCATACTTTCTCCTCTCCTCCCC
AAGAATACTTCGAGTACTTTGCC
>493
GGCAGCTATTATGTAGGTGCTCTGGATGGGAAGGAAAAAGATTGGGAAGTCTTGTTAG
CTGCCTTTCTCAGTCTGAGCCATACTTTCTCCTCTCCTCCCCAAGAATACTTCGAGTACT
TTGCCCCAGACTTCACACTTCA
>494
GGGAAGTCTTGTTAGCTGCCTTTCTCAGTCTGAGCCATACTTTCTCCTCTCCTCCCCAA
GAATACTTCGAGTACTTTGCCCCAGACTTCACACTTCATCCAGATGTCAGCACCCGCAT
CGAGAATCAGAACTCACGCCAG
>495
CCATACTTTCTCCTCTCCTCCCCAAGAATACTTCGAGTACTTTGCCCCAGACTTCACACT
TCATCCAGATGTCAGCACCCGCATCGAGAATCAGAACTCACGCCAGGTCAGCAGCTCA
GAAAAGCTGAGCACAAACTTCT
>496
CCGCATCGAGAATCAGAACTCACGCCAGGTCAGCAGCTCAGAAAAGCTGAGCACAAA
CTTCTCCTGAAAGGTGGAGCCCAGGAGAGGTCAAAGATGTGGGAAGTAGGAAAAACC
AAAGAGAAGGAAGTTCTAAGCCATCA
>497
AACTCACGCCAGGTCAGCAGCTCAGAAAAGCTGAGCACAAACTTCTCCTGAAAGGTG
GAGCCCAGGAGAGGTCAAAGATGTGGGAAGTAGGAAAAACCAAAGAGAAGGAAGTT
CTAAGCCATCACAGTCACTGTTATTGA
>498
GCAGCTCAGAAAAGCTGAGCACAAACTTCTCCTGAAAGGTGGAGCCCAGGAGAGGTC
AAAGATGTGGGAAGTAGGAAAAACCAAAGAGAAGGAAGTTCTAAGCCATCACAGTCA
CTGTTATTGACTAGGAGGCAGTCCAG
>499
ACTTCTCCTGAAAGGTGGAGCCCAGGAGAGGTCAAAGATGTGGGAAGTAGGAAAAAC
CAAAGAGAAGGAAGTTCTAAGCCATCACAGTCACTGTTATTGACTAGGAGGCAGTCCA
GTTTAAAGTAGAGGCAGTTGGGGTT
>500
GGAGCCCAGGAGAGGTCAAAGATGTGGGAAGTAGGAAAAACCAAAGAGAAGGAAGT
TCTAAGCCATCACAGTCACTGTTATTGACTAGGAGGCAGTCCAGTTTAAAGTAGAGGC
AGTTGGGGTTGGTGGGGAGGGAGAGC
>501
TGGGAAGTAGGAAAAACCAAAGAGAAGGAAGTTCTAAGCCATCACAGTCACTGTTAT
TGACTAGGAGGCAGTCCAGTTTAAAGTAGAGGCAGTTGGGGTTGGTGGGGAGGGAGA
GCTATCAGTTGGGGGACCTGAACTAG
>502
GAGAAGGAAGTTCTAAGCCATCACAGTCACTGTTATTGACTAGGAGGCAGTCCAGTTT
AAAGTAGAGGCAGTTGGGGTTGGTGGGGAGGGAGAGCTATCAGTTGGGGGACCTGAA

CTAGAGGTACCACTGAGATGAAAAC
>503
GAAGTTCTAAGCCATCACAGTCACTGTTATTGACTAGGAGGCAGTCCAGTTTAAAGTA
GAGGCAGTTGGGGTTGGTGGGGAGGGAGAGCTATCAGTTGGGGGACCTGAACTAGAG
GTACCACTGAGATGAAAACCAGTAT
>504
TGTTATTGACTAGGAGGCAGTCCAGTTTAAAGTAGAGGCAGTTGGGGTTGGTGGGGAG
GGAGAGCTATCAGTTGGGGGACCTGAACTAGAGGTACCACTGAGATGAAAACCAGTAT
GGTAGGGGTAAAAGGCAGCTAGGA
>505
TGGGGTTGGTGGGGAGGGAGAGCTATCAGTTGGGGGACCTGAACTAGAGGTACCACT
GAGATGAAAACCAGTATGGTAGGGGTAAAAGGCAGCTAGGAGATTCCATGTCCTGGGA
ATCTCTGGAAACTGCTCACTCTGTA
>506
TTGGTGGGGAGGGAGAGCTATCAGTTGGGGGACCTGAACTAGAGGTACCACTGAGAT
GAAAACCAGTATGGTAGGGGTAAAAGGCAGCTAGGAGATTCCATGTCCTGGGAATCTC
TGGAAACTGCTCACTCTGTATTCCT
>507
ACCTGAACTAGAGGTACCACTGAGATGAAAACCAGTATGGTAGGGGTAAAAGGCAGCT
AGGAGATTCCATGTCCTGGGAATCTCTGGAAACTGCTCACTCTGTATTCCTCATTTCCA
ACCAGTATCTGGACCAGATCCGC
>508
CCAGTATGGTAGGGGTAAAAGGCAGCTAGGAGATTCCATGTCCTGGGAATCTCTGGAA
ACTGCTCACTCTGTATTCCTCATTTCCAACCAGTATCTGGACCAGATCCGCCAGACAAT
CTTTGAAAACCTGAAGATGCTGA
>509
GGCAGCTAGGAGATTCCATGTCCTGGGAATCTCTGGAAACTGCTCACTCTGTATTCCTC
ATTTCCAACCAGTATCTGGACCAGATCCGCCAGACAATCTTTGAAAACCTGAAGATGCT
GAACCATGCACCTAGTGTCCAG
>510
TCCAACCAGTATCTGGACCAGATCCGCCAGACAATCTTTGAAAACCTGAAGATGCTGA
ACCATGCACCTAGTGTCCAGATTCATGACGTGCCTGCAGACCTCCTGACCTATGACAGG
ACTGATGAGGCTGATGCAGAGGA
>511
CAACCAGTATCTGGACCAGATCCGCCAGACAATCTTTGAAAACCTGAAGATGCTGAAC
CATGCACCTAGTGTCCAGATTCATGACGTGCCTGCAGACCTCCTGACCTATGACAGGAC
TGATGAGGCTGATGCAGAGGAGA
>512
CCAGATCCGCCAGACAATCTTTGAAAACCTGAAGATGCTGAACCATGCACCTAGTGTC
CAGATTCATGACGTGCCTGCAGACCTCCTGACCTATGACAGGACTGATGAGGCTGATG
CAGAGGAGAGGGGTCCTGAGGAGA
>513
CAGATTCATGACGTGCCTGCAGACCTCCTGACCTATGACAGGACTGATGAGGCTGATG
CAGAGGAGAGGGGTCCTGAGGAGAACTATAGCAGGTCTGGGAGCAGGGACTTCAGCC

TACTTTCAATAGGCTTGCTGGGGAG
>514
GTCCTGAGGAGAACTATAGCAGGTCTGGGAGCAGGGACTTCAGCCTACTTTCAATAGG
CTTGCTGGGGAGGTTTAAAAGGGAAAAAGTAAATGGGAAGGTATTAGTTTAATTGCCT
CTGGCTTACCTCCTTTAGTCTGCT
>515
TAGCAGGTCTGGGAGCAGGGACTTCAGCCTACTTTCAATAGGCTTGCTGGGGAGGTTT
AAAAGGGAAAAAGTAAATGGGAAGGTATTAGTTTAATTGCCTCTGGCTTACCTCCTTTA
GTCTGCTACAATTTCCTCACTCT
>516
GGACTTCAGCCTACTTTCAATAGGCTTGCTGGGGAGGTTTAAAAGGGAAAAAGTAAAT
GGGAAGGTATTAGTTTAATTGCCTCTGGCTTACCTCCTTTAGTCTGCTACAATTTCCTCA
CTCTTTTCTTTGTTATTTTTAA
>517
TGGGGAGGTTTAAAAGGGAAAAAGTAAATGGGAAGGTATTAGTTTAATTGCCTCTGGC
TTACCTCCTTTAGTCTGCTACAATTTCCTCACTCTTTTCTTTGTTATTTTTAATGACAAAA
GTAACACGTATTTATTGCAAA
>518
GGAAGGTATTAGTTTAATTGCCTCTGGCTTACCTCCTTTAGTCTGCTACAATTTCCTCAC
TCTTTTCTTTGTTATTTTTAATGACAAAAGTAACACGTATTTATTGCAAAATTTTAAAAC
AGCACCAAAATATGGTGTTG
>519
GCCTCTGGCTTACCTCCTTTAGTCTGCTACAATTTCCTCACTCTTTTCTTTGTTATTTTTA
ATGACAAAAGTAACACGTATTTATTGCAAAATTTTAAAACAGCACCAAAATATGGTGTT
GGCGTTCACACAATCCCAGA
>520
GCTTACCTCCTTTAGTCTGCTACAATTTCCTCACTCTTTTCTTTGTTATTTTTAATGACAA
AAGTAACACGTATTTATTGCAAAATTTTAAAACAGCACCAAAATATGGTGTTGGCGTTC
ACACAATCCCAGACCTATTC
>521
TTCTTTGTTATTTTTAATGACAAAAGTAACACGTATTTATTGCAAAATTTTAAAACAGCA
CCAAAATATGGTGTTGGCGTTCACACAATCCCAGACCTATTCCACAGGAGTAACCACTG
TTAATAGTTTTTATTGTTTTG
>522
TTGCAAAATTTTAAAACAGCACCAAAATATGGTGTTGGCGTTCACACAATCCCAGACCT
ATTCCACAGGAGTAACCACTGTTAATAGTTTTTATTGTTTTGGATAGACCTTTTTCTATAG
ATTGCAAATATACTATTCTG
>523
TTTAAAACAGCACCAAAATATGGTGTTGGCGTTCACACAATCCCAGACCTATTCCACAG
GAGTAACCACTGTTAATAGTTTTTATTGTTTTGGATAGACCTTTTTCTATAGATTGCAAAT
ATACTATTCTGTATGTACAT
>524
AGTAACCACTGTTAATAGTTTTTATTGTTTTGGATAGACCTTTTTCTATAGATTGCAAATA
TACTATTCTGTATGTACATATAAACAAACACATATACACTCATTTACAGTTTGTGTGGTTT

TTGGTTTTTCCGTAAATA
>525
ACCACTGTTAATAGTTTTTATTGTTTTGGATAGACCTTTTTCTATAGATTGCAAATATACT
ATTCTGTATGTACATATAAACAAACACATATACACTCATTTACAGTTTGTGTGGTTTTTGG
TTTTTCCGTAAATACAAA
>526
CTTTTTCTATAGATTGCAAATATACTATTCTGTATGTACATATAAACAAACACATATACAC
TCATTTACAGTTTGTGTGGTTTTTGGTTTTTCCGTAAATACAAATAGGGTCATTCTACGT
ATGCTGCTTTACATCATAT
>527
TTCTATAGATTGCAAATATACTATTCTGTATGTACATATAAACAAACACATATACACTCAT
TTACAGTTTGTGTGGTTTTTGGTTTTTCCGTAAATACAAATAGGGTCATTCTACGTATGC
TGCTTTACATCATATAGCT
>528
CTATTCTGTATGTACATATAAACAAACACATATACACTCATTTACAGTTTGTGTGGTTTTT
GGTTTTTCCGTAAATACAAATAGGGTCATTCTACGTATGCTGCTTTACATCATATAGCTGT
TTTCAGCTAGCTAATATA
>529
ACACATATACACTCATTTACAGTTTGTGTGGTTTTTGGTTTTTCCGTAAATACAAATAGG
GTCATTCTACGTATGCTGCTTTACATCATATAGCTGTTTTCAGCTAGCTAATATAGATCTG
CTTCAGTATTTTATCAGCT
>530
GTGTGGTTTTTGGTTTTTCCGTAAATACAAATAGGGTCATTCTACGTATGCTGCTTTACA
TCATATAGCTGTTTTCAGCTAGCTAATATAGATCTGCTTCAGTATTTTATCAGCTGTATAAT
AATTTACTATGTGGATAG
>531
TAGGGTCATTCTACGTATGCTGCTTTACATCATATAGCTGTTTTCAGCTAGCTAATATAGA
TCTGCTTCAGTATTTTATCAGCTGTATAATAATTTACTATGTGGATAGATTATTTTCCATTT
TTTTGGTATTACAATAA
>532
GTATGCTGCTTTACATCATATAGCTGTTTTCAGCTAGCTAATATAGATCTGCTTCAGTATT
TTATCAGCTGTATAATAATTTACTATGTGGATAGATTATTTTCCATTTTTTTGGTATTACAA
TAATAATGTGGCAATGA
>533
ATATAGCTGTTTTCAGCTAGCTAATATAGATCTGCTTCAGTATTTTATCAGCTGTATAATAA
TTTACTATGTGGATAGATTATTTTCCATTTTTTTGGTATTACAATAATAATGTGGCAATGA
ACCCCCTTACATTATAC
>534
AGATCTGCTTCAGTATTTTATCAGCTGTATAATAATTTACTATGTGGATAGATTATTTTCCA
TTTTTTTGGTATTACAATAATAATGTGGCAATGAACCCCCTTACATTATACATCTTTGCAT
TCTTGTTTGAGTGGGTT
>535
TTTATCAGCTGTATAATAATTTACTATGTGGATAGATTATTTTCCATTTTTTTGGTATTACA
ATAATAATGTGGCAATGAACCCCCTTACATTATACATCTTTGCATTCTTGTTTGAGTGGG

TTAGATAGTAGAATGGTT
>536
TTCCATTTTTTTGGTATTACAATAATAATGTGGCAATGAACCCCCTTACATTATACATCTT
TGCATTCTTGTTTGAGTGGGTTAGATAGTAGAATGGTTGCTGCTAGGTCAAAGGCTGTG
TGCATTAATTTTTTTTTTTT
>537
TTTTTTGGTATTACAATAATAATGTGGCAATGAACCCCCTTACATTATACATCTTTGCATT
CTTGTTTGAGTGGGTTAGATAGTAGAATGGTTGCTGCTAGGTCAAAGGCTGTGTGCATT
AATTTTTTTTTTTTTTTTTT
>538
TTGGTATTACAATAATAATGTGGCAATGAACCCCCTTACATTATACATCTTTGCATTCTTG
TTTGAGTGGGTTAGATAGTAGAATGGTTGCTGCTAGGTCAAAGGCTGTGTGCATTAATT
TTTTTTTTTTTTTTTTTTTT
>539
ACAATAATAATGTGGCAATGAACCCCCTTACATTATACATCTTTGCATTCTTGTTTGAGT
GGGTTAGATAGTAGAATGGTTGCTGCTAGGTCAAAGGCTGTGTGCATTAATTTTTTTTT
TTTTTTTTTTTTGAGATGGA
>540
GAACCCCCTTACATTATACATCTTTGCATTCTTGTTTGAGTGGGTTAGATAGTAGAATGG
TTGCTGCTAGGTCAAAGGCTGTGTGCATTAATTTTTTTTTTTTTTTTTTTTTGAGATGG
AGTCTCACTCTGTTGCCAGG
>541
GTAGAATGGTTGCTGCTAGGTCAAAGGCTGTGTGCATTAATTTTTTTTTTTTTTTTTTTT
TGAGATGGAGTCTCACTCTGTTGCCAGGCTGGAGTGCAGTGGCTAATCCCGGCTCACT
GCAACCTCCGCCTCCCAGGTT
>542
TGCTGCTAGGTCAAAGGCTGTGTGCATTAATTTTTTTTTTTTTTTTTTTTTGAGATGGA
GTCTCACTCTGTTGCCAGGCTGGAGTGCAGTGGCTAATCCCGGCTCACTGCAACCTCC
GCCTCCCAGGTTCAAGCGATTC
>543
GGAGTCTCACTCTGTTGCCAGGCTGGAGTGCAGTGGCTAATCCCGGCTCACTGCAACC
TCCGCCTCCCAGGTTCAAGCGATTCTCCTGCCTCAGCCTCCTCAGTAGCTGAGATTACA
GGCATGTGCCACCACACCCGGCT
>544
GTGGCTAATCCCGGCTCACTGCAACCTCCGCCTCCCAGGTTCAAGCGATTCTCCTGCCT
CAGCCTCCTCAGTAGCTGAGATTACAGGCATGTGCCACCACACCCGGCTAACTTTAGTA
TTTTTAGTAGAGACGGGGTTTC
>545
GCAACCTCCGCCTCCCAGGTTCAAGCGATTCTCCTGCCTCAGCCTCCTCAGTAGCTGA
GATTACAGGCATGTGCCACCACACCCGGCTAACTTTAGTATTTTTAGTAGAGACGGGGT
TTCACCATGTTGGCCAGGATGGT
>546
CGATTCTCCTGCCTCAGCCTCCTCAGTAGCTGAGATTACAGGCATGTGCCACCACACCC
GGCTAACTTTAGTATTTTTAGTAGAGACGGGGTTTCACCATGTTGGCCAGGATGGTCTT

GATCTCTTGACCTTGTGATCCA
>547
CCTCAGTAGCTGAGATTACAGGCATGTGCCACCACACCCGGCTAACTTTAGTATTTTTA
GTAGAGACGGGGTTTCACCATGTTGGCCAGGATGGTCTTGATCTCTTGACCTTGTGATC
CACCTGCCTTGGCCTCCCAAAG
>548
TGAGATTACAGGCATGTGCCACCACACCCGGCTAACTTTAGTATTTTTAGTAGAGACGG
GGTTTCACCATGTTGGCCAGGATGGTCTTGATCTCTTGACCTTGTGATCCACCTGCCTT
GGCCTCCCAAAGTGCTGGGATT
>549
ATGTGCCACCACACCCGGCTAACTTTAGTATTTTTAGTAGAGACGGGGTTTCACCATGT
TGGCCAGGATGGTCTTGATCTCTTGACCTTGTGATCCACCTGCCTTGGCCTCCCAAAGT
GCTGGGATTACAGGTGTGAGCT
>550
GGTTTCACCATGTTGGCCAGGATGGTCTTGATCTCTTGACCTTGTGATCCACCTGCCTT
GGCCTCCCAAAGTGCTGGGATTACAGGTGTGAGCTACCACGCCCGGCTTTTGAGAATA
CTTTTTCAAAAAGAAAGAAACCA
>551
GCCAGGATGGTCTTGATCTCTTGACCTTGTGATCCACCTGCCTTGGCCTCCCAAAGTGC
TGGGATTACAGGTGTGAGCTACCACGCCCGGCTTTTGAGAATACTTTTTCAAAAAGAA
AGAAACCAATATTGAGTACCTGT
>552
GATTACAGGTGTGAGCTACCACGCCCGGCTTTTGAGAATACTTTTTCAAAAAGAAAGA
AACCAATATTGAGTACCTGTTGGGCCCTGTTTTGCTTTCACATCCATTCTTTCACTATGG
GTTTATAATACCACGTCACTCT
>553
AGGTGTGAGCTACCACGCCCGGCTTTTGAGAATACTTTTTCAAAAAGAAAGAAACCAA
TATTGAGTACCTGTTGGGCCCTGTTTTGCTTTCACATCCATTCTTTCACTATGGGTTTATA
ATACCACGTCACTCTTCTTAA
>554
AAAAGAAAGAAACCAATATTGAGTACCTGTTGGGCCCTGTTTTGCTTTCACATCCATTC
TTTCACTATGGGTTTATAATACCACGTCACTCTTCTTAAAATTAAAACTCTCCTTTACCTT
GCACTTAGAAGAAAATCCAA
>555
AATATTGAGTACCTGTTGGGCCCTGTTTTGCTTTCACATCCATTCTTTCACTATGGGTTTA
TAATACCACGTCACTCTTCTTAAAATTAAAACTCTCCTTTACCTTGCACTTAGAAGAAA
ATCCAAAATTTTGACTGTGG
>556
CCACGTCACTCTTCTTAAAATTAAAACTCTCCTTTACCTTGCACTTAGAAGAAAATCCA
AAATTTTGACTGTGGCTTAAAAAGGTCCTGCATGCTAGCCCTTACCTACATCCAGGATC
TCATCTCTAACTACTCTCCTCC
>557
CTTAAAATTAAAACTCTCCTTTACCTTGCACTTAGAAGAAAATCCAAAATTTTGACTGT
GGCTTAAAAAGGTCCTGCATGCTAGCCCTTACCTACATCCAGGATCTCATCTCTAACTA

CTCTCCTCCATGCACAGTGCTC
>558
AAAAAGGTCCTGCATGCTAGCCCTTACCTACATCCAGGATCTCATCTCTAACTACTCTCC
TCCATGCACAGTGCTCCCGGCTGTCCTGGCCTCCTTGTTCTTACTGGAATACATCAGTC
TCTTTCCATTTCATGACCTCT
>559
CATGCTAGCCCTTACCTACATCCAGGATCTCATCTCTAACTACTCTCCTCCATGCACAGT
GCTCCCGGCTGTCCTGGCCTCCTTGTTCTTACTGGAATACATCAGTCTCTTTCCATTTCA
TGACCTCTGTAGTTGGTTTT
>560
CCTACATCCAGGATCTCATCTCTAACTACTCTCCTCCATGCACAGTGCTCCCGGCTGTCC
TGGCCTCCTTGTTCTTACTGGAATACATCAGTCTCTTTCCATTTCATGACCTCTGTAGTT
GGTTTTCTTGCAGCTTGGAA
>561
TCTAACTACTCTCCTCCATGCACAGTGCTCCCGGCTGTCCTGGCCTCCTTGTTCTTACTG
GAATACATCAGTCTCTTTCCATTTCATGACCTCTGTAGTTGGTTTTCTTGCAGCTTGGAA
TGGCCTTCCTGAAGTCTTTG
>562
GTGCTCCCGGCTGTCCTGGCCTCCTTGTTCTTACTGGAATACATCAGTCTCTTTCCATTT
CATGACCTCTGTAGTTGGTTTTCTTGCAGCTTGGAATGGCCTTCCTGAAGTCTTTGCCT
GCTTAGCTCCTCCTCAGACTT
>563
CTCCCGGCTGTCCTGGCCTCCTTGTTCTTACTGGAATACATCAGTCTCTTTCCATTTCAT
GACCTCTGTAGTTGGTTTTCTTGCAGCTTGGAATGGCCTTCCTGAAGTCTTTGCCTGCT
TAGCTCCTCCTCAGACTTTAG
>564
TACATCAGTCTCTTTCCATTTCATGACCTCTGTAGTTGGTTTTCTTGCAGCTTGGAATGG
CCTTCCTGAAGTCTTTGCCTGCTTAGCTCCTCCTCAGACTTTAGGTCTTATGTCCCAATA
GTCCTGTCTGAAATTTCACC
>565
GTTGGTTTTCTTGCAGCTTGGAATGGCCTTCCTGAAGTCTTTGCCTGCTTAGCTCCTCC
TCAGACTTTAGGTCTTATGTCCCAATAGTCCTGTCTGAAATTTCACCCTTTTCCTCTCAT
CACCTTCAGTCCTATCACTTC
>566
TTCTTGCAGCTTGGAATGGCCTTCCTGAAGTCTTTGCCTGCTTAGCTCCTCCTCAGACT
TTAGGTCTTATGTCCCAATAGTCCTGTCTGAAATTTCACCCTTTTCCTCTCATCACCTTC
AGTCCTATCACTTCATTTTAT
>567
CCTCAGACTTTAGGTCTTATGTCCCAATAGTCCTGTCTGAAATTTCACCCTTTTCCTCTC
ATCACCTTCAGTCCTATCACTTCATTTTATTAATTGTACTCTCCATCATCTGAAATTATTAT
ATTTATTTACTTTGTTAG
>568
AATAGTCCTGTCTGAAATTTCACCCTTTTCCTCTCATCACCTTCAGTCCTATCACTTCAT
TTTATTAATTGTACTCTCCATCATCTGAAATTATTATATTTATTTACTTTGTTAGTTACTAGT

CTCATTTATTAGGTAAG
>569
CTTCAGTCCTATCACTTCATTTTATTAATTGTACTCTCCATCATCTGAAATTATTATATTTA
TTTACTTTGTTAGTTACTAGTCTCATTTATTAGGTAAGCTTTTTGAGGATAGACAACCTT
GTCCAGCTTGTTACTCTA
>570
AGTCCTATCACTTCATTTTATTAATTGTACTCTCCATCATCTGAAATTATTATATTTATTTA
CTTTGTTAGTTACTAGTCTCATTTATTAGGTAAGCTTTTTGAGGATAGACAACCTTGTCC
AGCTTGTTACTCTACTGT
>571
ATTATATTTATTTACTTTGTTAGTTACTAGTCTCATTTATTAGGTAAGCTTTTTGAGGATAG
ACAACCTTGTCCAGCTTGTTACTCTACTGTATTGCTAAGGTTTATGACAGTGTCTGTATA
GAGTAGTTCCTTAATGTT
>572
ATTTACTTTGTTAGTTACTAGTCTCATTTATTAGGTAAGCTTTTTGAGGATAGACAACCTT
GTCCAGCTTGTTACTCTACTGTATTGCTAAGGTTTATGACAGTGTCTGTATAGAGTAGTT
CCTTAATGTTTATTTGATA
>573
CTCATTTATTAGGTAAGCTTTTTGAGGATAGACAACCTTGTCCAGCTTGTTACTCTACTG
TATTGCTAAGGTTTATGACAGTGTCTGTATAGAGTAGTTCCTTAATGTTTATTTGATACGT
GAATGGGTGAATGAATAGC
>574
GTAAGCTTTTTGAGGATAGACAACCTTGTCCAGCTTGTTACTCTACTGTATTGCTAAGG
TTTATGACAGTGTCTGTATAGAGTAGTTCCTTAATGTTTATTTGATACGTGAATGGGTGA
ATGAATAGCAGTCTCTACCAA
>575
GCTTTTTGAGGATAGACAACCTTGTCCAGCTTGTTACTCTACTGTATTGCTAAGGTTTAT
GACAGTGTCTGTATAGAGTAGTTCCTTAATGTTTATTTGATACGTGAATGGGTGAATGAA
TAGCAGTCTCTACCAACAAG
>576
GAGGATAGACAACCTTGTCCAGCTTGTTACTCTACTGTATTGCTAAGGTTTATGACAGT
GTCTGTATAGAGTAGTTCCTTAATGTTTATTTGATACGTGAATGGGTGAATGAATAGCAG
TCTCTACCAACAAGAGGAGGA
>577
CTTGTCCAGCTTGTTACTCTACTGTATTGCTAAGGTTTATGACAGTGTCTGTATAGAGTA
GTTCCTTAATGTTTATTTGATACGTGAATGGGTGAATGAATAGCAGTCTCTACCAACAAG
AGGAGGAGTGGTTCTCAACG
>578
TTTATGACAGTGTCTGTATAGAGTAGTTCCTTAATGTTTATTTGATACGTGAATGGGTGA
ATGAATAGCAGTCTCTACCAACAAGAGGAGGAGTGGTTCTCAACGGGGCCTGAGAAA
TGGCTTTGGAAAAGGGTGCTGGG
>579
GTAGTTCCTTAATGTTTATTTGATACGTGAATGGGTGAATGAATAGCAGTCTCTACCAAC
AAGAGGAGGAGTGGTTCTCAACGGGGCCTGAGAAATGGCTTTGGAAAAGGGTGCTGG

GAGAAGATTAGAACTTAGAGTGA
>580
GTTCCTTAATGTTTATTTGATACGTGAATGGGTGAATGAATAGCAGTCTCTACCAACAAG
AGGAGGAGTGGTTCTCAACGGGGCCTGAGAAATGGCTTTGGAAAAGGGTGCTGGGAG
AAGATTAGAACTTAGAGTGAGAT
>581
GAATAGCAGTCTCTACCAACAAGAGGAGGAGTGGTTCTCAACGGGGCCTGAGAAATG
GCTTTGGAAAAGGGTGCTGGGAGAAGATTAGAACTTAGAGTGAGATCCTGACCTCTCT
CCCCTTGCTTTGCAGGCCAGAGGCA
>582
AGGAGTGGTTCTCAACGGGGCCTGAGAAATGGCTTTGGAAAAGGGTGCTGGGAGAAG
ATTAGAACTTAGAGTGAGATCCTGACCTCTCTCCCCTTGCTTTGCAGGCCAGAGGCAC
CCAATGAGTTCTATGATGGAGACCA
>583
AGAAGATTAGAACTTAGAGTGAGATCCTGACCTCTCTCCCCTTGCTTTGCAGGCCAGA
GGCACCCAATGAGTTCTATGATGGAGACCATGACAATGACAAGGAAAGCGATGTGGAG
ATTTAAGAGTGGCTTGGGATGCTG
>584
CTTGCTTTGCAGGCCAGAGGCACCCAATGAGTTCTATGATGGAGACCATGACAATGAC
AAGGAAAGCGATGTGGAGATTTAAGAGTGGCTTGGGATGCTGTGTCCCAAGGAATTTC
TTTTCACCTCTTGGTTGGGCTGGA
>585
CAGAGGCACCCAATGAGTTCTATGATGGAGACCATGACAATGACAAGGAAAGCGATGT
GGAGATTTAAGAGTGGCTTGGGATGCTGTGTCCCAAGGAATTTCTTTTCACCTCTTGGT
TGGGCTGGAGGGAAAAGGAGTGG
>586
AGTTCTATGATGGAGACCATGACAATGACAAGGAAAGCGATGTGGAGATTTAAGAGTG
GCTTGGGATGCTGTGTCCCAAGGAATTTCTTTTCACCTCTTGGTTGGGCTGGAGGGAA
AAGGAGTGGCTCCTAGAGTCCTGG
>587
AAGAGTGGCTTGGGATGCTGTGTCCCAAGGAATTTCTTTTCACCTCTTGGTTGGGCTG
GAGGGAAAAGGAGTGGCTCCTAGAGTCCTGGGGGTCACCCCAGGGCTTTTGCTGACT
CTGGGAAAGAGTCTGGAGACCACAT
>588
GGATGCTGTGTCCCAAGGAATTTCTTTTCACCTCTTGGTTGGGCTGGAGGGAAAAGGA
GTGGCTCCTAGAGTCCTGGGGGTCACCCCAGGGCTTTTGCTGACTCTGGGAAAGAGTC
TGGAGACCACATTTGGTTCTCGAA
>589
CTGGAGGGAAAAGGAGTGGCTCCTAGAGTCCTGGGGGTCACCCCAGGGCTTTTGCTG
ACTCTGGGAAAGAGTCTGGAGACCACATTTGGTTCTCGAACCATCTACCTGCTTTTCCT
CTCTCTCCCAAGGCCTGACAATGG
>590
GCTCCTAGAGTCCTGGGGGTCACCCCAGGGCTTTTGCTGACTCTGGGAAAGAGTCTGG
AGACCACATTTGGTTCTCGAACCATCTACCTGCTTTTCCTCTCTCTCCCAAGGCCTGAC

AATGGTACCTATTAGGGATGAGA
>591
AGTCTGGAGACCACATTTGGTTCTCGAACCATCTACCTGCTTTTCCTCTCTCTCCCAAG
GCCTGACAATGGTACCTATTAGGGATGAGATACAGACAAGGATAGCTATCTGGGACATT
ATTGGCAGTGGGCCCTGGAGGC
>592
CAAGGCCTGACAATGGTACCTATTAGGGATGAGATACAGACAAGGATAGCTATCTGGG
ACATTATTGGCAGTGGGCCCTGGAGGCCAGTCCCTAGCCCCCCTTGCCCCTTATTTCTT
CCCTGCTTCCCTCGAACCCAGAG
>593
ACCTATTAGGGATGAGATACAGACAAGGATAGCTATCTGGGACATTATTGGCAGTGGGC
CCTGGAGGCCAGTCCCTAGCCCCCCTTGCCCCTTATTTCTTCCCTGCTTCCCTCGAACC
CAGAGATTTTTGAGGGATGAAC
>594
CCCTAGCCCCCCTTGCCCCTTATTTCTTCCCTGCTTCCCTCGAACCCAGAGATTTTTGAG
GGATGAACGGGTAGACAAGGACTGAGATTGCCTCTGACTTCCTCCTCCCCTGGGTTCT
GACTTCTTCCTCCCCTTGCTTC
>595
ACCCAGAGATTTTTGAGGGATGAACGGGTAGACAAGGACTGAGATTGCCTCTGACTTC
CTCCTCCCCTGGGTTCTGACTTCTTCCTCCCCTTGCTTCCAGGGAAGATGAAGAGAGA
GAGATTTGGAAGGGGCTCTGGCTC
>596
GGGAAGATGAAGAGAGAGAGAGATTTGGAAGGGGCTCTGGCTCCCTAACACCTGAATCC
CAGATGATGGGAAGTATGTTTTCAAGTGTGGGGAGGATATGAAAATGTTCTGTTCTCAC
TTTTGGCTTTATGTCCATTTTACC
>597
AGATGAAGAGAGAGAGAGATTTGGAAGGGGCTCTGGCTCCCTAACACCTGAATCCCAGAT
GATGGGAAGTATGTTTTCAAGTGTGGGGAGGATATGAAAATGTTCTGTTCTCACTTTTG
GCTTTATGTCCATTTTACCACTG
>598
TCTGGCTCCCTAACACCTGAATCCCAGATGATGGGAAGTATGTTTTCAAGTGTGGGGA
GGATATGAAAATGTTCTGTTCTCACTTTTGGCTTTATGTCCATTTTACCACTGTTTTTATC
CAATAAACTAAGTCGGTATTT
>599
GGTGAAATCCCAGCTCAGGTGTTATATCCTTGCTGCTTCTGACAAATGAAACCAAGGTG
TATTAATTAAGGTAATGCTAGCTACTACAATAAGCTGAAAAATATATAATAGCTAAAAGC
TAGGGTGCCAGATTTAGGAAA
>600
AAGGTGTATTAATTAAGGTAATGCTAGCTACTACAATAAGCTGAAAAATATATAATAGCT
AAAAGCTAGGGTGCCAGATTTAGGAAAATAAAAACAAACAAAAAACAATTGAAATTT
GAATTTCAGATAAACAATTTTTT
>601
GCCAGATTTAGGAAAATAAAAACAAACAAAAAACAATTGAAATTTGAATTTCAGATAA
ACAATTTTTTTAGTATAATTATGTCCCATGCAATATTTGAGACATACTAAAAGGTTATTCA

TTGTTTATCTGATATTCAAAT
>602
GGAAAATAAAAACAAACAAAAAACAATTGAAATTTGAATTTCAGATAAACAATTTTTTT
AGTATAATTATGTCCCATGCAATATTTGAGACATACTAAAAGGTTATTCATTGTTTATCTG
ATATTCAAATTTAACATTTT
>603
ATTTGAATTTCAGATAAACAATTTTTTTAGTATAATTATGTCCCATGCAATATTTGAGACA
TACTAAAAGGTTATTCATTGTTTATCTGATATTCAAATTTAACATTTTCATTAATTTATTTT
ATTTGGCAGCCCTATGG
>604
CAGATAAACAATTTTTTTAGTATAATTATGTCCCATGCAATATTTGAGACATACTAAAAG
GTTATTCATTGTTTATCTGATATTCAAATTTAACATTTTCATTAATTTATTTTATTTGGCAG
CCCTATGGAAGACATCAA
>605
TTATCTGATATTCAAATTTAACATTTTCATTAATTTATTTTATTTGGCAGCCCTATGGAAGA
CATCAAAAGTTTATTTCTTATTTATATAAAGTCCAAAATAGATGTTTTTTAGTAGGCAGG
CAGCTCTTCTCTAAGCGT
>606
ATTTTCATTAATTTATTTTATTTGGCAGCCCTATGGAAGACATCAAAAGTTTATTTCTTAT
TTATATAAAGTCCAAAATAGATGTTTTTTAGTAGGCAGGCAGCTCTTCTCTAAGCGTAAT
TCAGGGCTCTGGGTTCTGT
>607
TATTTTATTTGGCAGCCCTATGGAAGACATCAAAAGTTTATTTCTTATTTATATAAAGTCC
AAAATAGATGTTTTTTAGTAGGCAGGCAGCTCTTCTCTAAGCGTAATTCAGGGCTCTGG
GTTCTGTCCATCCTGGCCCT
>608
TTGGCAGCCCTATGGAAGACATCAAAAGTTTATTTCTTATTTATATAAAGTCCAAAATAG
ATGTTTTTTAGTAGGCAGGCAGCTCTTCTCTAAGCGTAATTCAGGGCTCTGGGTTCTGT
CCATCCTGGCCCTGACGTCTT
>609
AAGTTTATTTCTTATTTATATAAAGTCCAAAATAGATGTTTTTTAGTAGGCAGGCAGCTC
TTCTCTAAGCGTAATTCAGGGCTCTGGGTTCTGTCCATCCTGGCCCTGACGTCTTTCATT
GCTCAGCTTCTAAGATAGCT
>610
TTTTAGTAGGCAGGCAGCTCTTCTCTAAGCGTAATTCAGGGCTCTGGGTTCTGTCCATC
CTGGCCCTGACGTCTTTCATTGCTCAGCTTCTAAGATAGCTCTATGCTCATGGGGTCAA
GTCACAGGAAAGAAAAGGGAAT
>611
CTGGGTTCTGTCCATCCTGGCCCTGACGTCTTTCATTGCTCAGCTTCTAAGATAGCTCTA
TGCTCATGGGGTCAAGTCACAGGAAAGAAAAGGGAATGTGGAGTTCCACTTGGGAGT
TTTTATGGGCCAGTCCTGGAAGC
>612
TTCTGTCCATCCTGGCCCTGACGTCTTTCATTGCTCAGCTTCTAAGATAGCTCTATGCTC
ATGGGGTCAAGTCACAGGAAAGAAAAGGGAATGTGGAGTTCCACTTGGGAGTTTTTAT

GGGCCAGTCCTGGAAGCGGCAC
>613
GTCTTTCATTGCTCAGCTTCTAAGATAGCTCTATGCTCATGGGGTCAAGTCACAGGAAA
GAAAAGGGAATGTGGAGTTCCACTTGGGAGTTTTTATGGGCCAGTCCTGGAAGCGGCA
CAGAGGATTTCTGCTCACAATCT
>614
TCTAAGATAGCTCTATGCTCATGGGGTCAAGTCACAGGAAAGAAAAGGGAATGTGGAG
TTCCACTTGGGAGTTTTTATGGGCCAGTCCTGGAAGCGGCACAGAGGATTTCTGCTCA
CAATCTATTGGCCAGAACTCAATC
>615
GCGGCACAGAGGATTTCTGCTCACAATCTATTGGCCAGAACTCAATCACATGGCCTGC
ACCTAACTGCAAGTTAAGCCAGAAAAATGTAGGTGTGCCCAGGAAGAAGTTTGGGGA
ACAGCTAGCCAGTCTCTGCCACACC
>616
CTGCTCACAATCTATTGGCCAGAACTCAATCACATGGCCTGCACCTAACTGCAAGTTAA
GCCAGAAAAATGTAGGTGTGCCCAGGAAGAAGTTTGGGGAACAGCTAGCCAGTCTCT
GCCACACCTGGTAACAGCAGAGTG
>617
AGAACTCAATCACATGGCCTGCACCTAACTGCAAGTTAAGCCAGAAAAATGTAGGTGT
GCCCAGGAAGAAGTTTGGGGAACAGCTAGCCAGTCTCTGCCACACCTGGTAACAGCA
GAGTGAATCTGTCAGTACTCGTGCC
>618
GCACCTAACTGCAAGTTAAGCCAGAAAAATGTAGGTGTGCCCAGGAAGAAGTTTGGG
GAACAGCTAGCCAGTCTCTGCCACACCTGGTAACAGCAGAGTGAATCTGTCAGTACTC
GTGCCTAATGCGTGTCTCATATGCT
>619
TAAGCCAGAAAAATGTAGGTGTGCCCAGGAAGAAGTTTGGGGAACAGCTAGCCAGTC
TCTGCCACACCTGGTAACAGCAGAGTGAATCTGTCAGTACTCGTGCCTAATGCGTGTCT
CATATGCTAGTGCTGGATTCAGTC
>620
TGTGCCCAGGAAGAAGTTTGGGGAACAGCTAGCCAGTCTCTGCCACACCTGGTAACA
GCAGAGTGAATCTGTCAGTACTCGTGCCTAATGCGTGTCTCATATGCTAGTGCTGGATT
CAGTCAGCAGTCCTACATCCGCTG
>621
GGAACAGCTAGCCAGTCTCTGCCACACCTGGTAACAGCAGAGTGAATCTGTCAGTACT
CGTGCCTAATGCGTGTCTCATATGCTAGTGCTGGATTCAGTCAGCAGTCCTACATCCGCT
GGGGTCCCTAGGAGGGGTCAGA
>622
AACAGCTAGCCAGTCTCTGCCACACCTGGTAACAGCAGAGTGAATCTGTCAGTACTCG
TGCCTAATGCGTGTCTCATATGCTAGTGCTGGATTCAGTCAGCAGTCCTACATCCGCTG
GGGTCCCTAGGAGGGGTCAGATG
>623
TCTCTGCCACACCTGGTAACAGCAGAGTGAATCTGTCAGTACTCGTGCCTAATGCGTGT
CTCATATGCTAGTGCTGGATTCAGTCAGCAGTCCTACATCCGCTGGGGTCCCTAGGAGG

GGTCAGATGCTCTCCTCTTTGC
>624
CAGTACTCGTGCCTAATGCGTGTCTCATATGCTAGTGCTGGATTCAGTCAGCAGTCCTA
CATCCGCTGGGGTCCCTAGGAGGGGTCAGATGCTCTCCTCTTTGCCCTGAACTGGTTAG
CTACTTCGGACTGGGATGACAA
>625
TAATGCGTGTCTCATATGCTAGTGCTGGATTCAGTCAGCAGTCCTACATCCGCTGGGGT
CCCTAGGAGGGGTCAGATGCTCTCCTCTTTGCCCTGAACTGGTTAGCTACTTCGGACTG
GGATGACAAGGCCATTGTGAGA
>626
TCAGTCAGCAGTCCTACATCCGCTGGGGTCCCTAGGAGGGGTCAGATGCTCTCCTCTTT
GCCCTGAACTGGTTAGCTACTTCGGACTGGGATGACAAGGCCATTGTGAGAGTGATAA
GCAATGTGATTCAAACAAATTCA
>627
GGAGGGGTCAGATGCTCTCCTCTTTGCCCTGAACTGGTTAGCTACTTCGGACTGGGAT
GACAAGGCCATTGTGAGAGTGATAAGCAATGTGATTCAAACAAATTCATTTCACTTCAA
TTCAATGCAGGTTTTTTTTTTTT
>628
GTCAGATGCTCTCCTCTTTGCCCTGAACTGGTTAGCTACTTCGGACTGGGATGACAAGG
CCATTGTGAGAGTGATAAGCAATGTGATTCAAACAAATTCATTTCACTTCAATTCAATG
CAGGTTTTTTTTTTTTTTTGAG
>629
TGACAAGGCCATTGTGAGAGTGATAAGCAATGTGATTCAAACAAATTCATTTCACTTCA
ATTCAATGCAGGTTTTTTTTTTTTTTTGAGACGGGCTGTCATGCTGTTGCCAACCCTGG
AGAGCAGTGGCGCCATTACGGC
>630
GCAATGTGATTCAAACAAATTCATTTCACTTCAATTCAATGCAGGTTTTTTTTTTTTTTT
GAGACGGGCTGTCATGCTGTTGCCAACCCTGGAGAGCAGTGGCGCCATTACGGCTCAC
TGCAGCCTCCTGGGCTCAAGCA
>631
CTTCAATTCAATGCAGGTTTTTTTTTTTTTTTGAGACGGGCTGTCATGCTGTTGCCAACC
CTGGAGAGCAGTGGCGCCATTACGGCTCACTGCAGCCTCCTGGGCTCAAGCAATTCTC
CCTCCTCAGCTTCCCAAGTAGC
>632
GAGCAGTGGCGCCATTACGGCTCACTGCAGCCTCCTGGGCTCAAGCAATTCTCCCTCC
TCAGCTTCCCAAGTAGCTGGGACTACAGGTGCATGCCACCATGTTCAGCTCATTTATTT
TTTATTTTTTTGTAGACAGGAGG
>633
ACGGCTCACTGCAGCCTCCTGGGCTCAAGCAATTCTCCCTCCTCAGCTTCCCAAGTAG
CTGGGACTACAGGTGCATGCCACCATGTTCAGCTCATTTATTTTTTATTTTTTTGTAGAC
AGGAGGTCTCACTATGTTACCC
>634
TCAGCTTCCCAAGTAGCTGGGACTACAGGTGCATGCCACCATGTTCAGCTCATTTATTT
TTTATTTTTTTGTAGACAGGAGGTCTCACTATGTTACCCAGGCTGCTGTCCAAGTCCTG

GGCTCACGTGATTCTCCCGCCT
>635
AGTAGCTGGGACTACAGGTGCATGCCACCATGTTCAGCTCATTTATTTTTATTTTTTTG
TAGACAGGAGGTCTCACTATGTTACCCAGGCTGCTGTCCAAGTCCTGGGCTCACGTGA
TTCTCCCGCCTCAGCCTCCCAA
>636
CACCATGTTCAGCTCATTTATTTTTATTTTTTTGTAGACAGGAGGTCTCACTATGTTACC
CAGGCTGCTGTCCAAGTCCTGGGCTCACGTGATTCTCCCGCCTCAGCCTCCCAAAATG
CTGGGATTACAGGCGTAAGCC
>637
ATTTTTATTTTTTTGTAGACAGGAGGTCTCACTATGTTACCCAGGCTGCTGTCCAAGTC
CTGGGCTCACGTGATTCTCCCGCCTCAGCCTCCCAAAATGCTGGGATTACAGGCGTAA
GCCACCATGCCCAGACAATTTA
>638
GGCTGCTGTCCAAGTCCTGGGCTCACGTGATTCTCCCGCCTCAGCCTCCCAAAATGCT
GGGATTACAGGCGTAAGCCACCATGCCCAGACAATTTAATAAATATTTATTAAAACTTCT
AGGATATTGAGAAAAGCTTGGT
>639
TAAAACTTCTAGGATATTGAGAAAAGCTTGGTTCTAGGCCACTGGGGAAGACATCAAC
TGAAACAGCCCAGTTATACTCTCAATTAGCTGTATACAATTAAATTCACAGATATCCTCA
ACCCTTATCCATATCCCATTCA
>640
AGAAAAGCTTGGTTCTAGGCCACTGGGGAAGACATCAACTGAAACAGCCCAGTTATAC
TCTCAATTAGCTGTATACAATTAAATTCACAGATATCCTCAACCCTTATCCATATCCCATT
CATCTTTCAAAGGTTGGTGAA
>641
TAGCTGTATACAATTAAATTCACAGATATCCTCAACCCTTATCCATATCCCATTCATCTTT
CAAAGGTTGGTGAAAACCTCATAGCACAAAGAATTCTGCCCTAAATGTTTGAATACAG
AATGGTCCCCTCTCTCCCTAA
>642
CTTATCCATATCCCATTCATCTTTCAAAGGTTGGTGAAAACCTCATAGCACAAAGAATTC
TGCCCTAAATGTTTGAATACAGAATGGTCCCCTCTCTCCCTAATTCCTAAAATATTTGGT
CTCTATGTTCATTCATTCAT
>643
TCATCTTTCAAAGGTTGGTGAAAACCTCATAGCACAAAGAATTCTGCCCTAAATGTTTG
AATACAGAATGGTCCCCTCTCTCCCTAATTCCTAAAATATTTGGTCTCTATGTTCATTCAT
TCATTCATGCATGCATGCTT
>644
CACAAAGAATTCTGCCCTAAATGTTTGAATACAGAATGGTCCCCTCTCTCCCTAATTCCT
AAAATATTTGGTCTCTATGTTCATTCATTCATTCATGCATGCATGCTTTCAGTTAGTTCAT
CGACAAACATTTACATCTA
>645
ATTCTGCCCTAAATGTTTGAATACAGAATGGTCCCCTCTCTCCCTAATTCCTAAAATATTT
GGTCTCTATGTTCATTCATTCATTCATGCATGCATGCTTTCAGTTAGTTCATCGACAAAC

ATTTACATCTACTCTGTTT
>646
CCCTCTCTCCCTAATTCCTAAAATATTTGGTCTCTATGTTCATTCATTCATTCATGCATGC
ATGCTTTCAGTTAGTTCATCGACAAACATTTACATCTACTCTGTTTGACAGTGTGCTAGG
CTCTGGGGCTTGGGAGTGG
>647
CTCTCCCTAATTCCTAAAATATTTGGTCTCTATGTTCATTCATTCATTCATGCATGCATGC
TTTCAGTTAGTTCATCGACAAACATTTACATCTACTCTGTTTGACAGTGTGCTAGGCTCT
GGGGCTTGGGAGTGGAAAT
>648
CTATGTTCATTCATTCATTCATGCATGCATGCTTTCAGTTAGTTCATCGACAAACATTTAC
ATCTACTCTGTTTGACAGTGTGCTAGGCTCTGGGGCTTGGGAGTGGAAATACTAAATTG
AACAAGACTGAGGCCTTGCC
>649
TTCAGTTAGTTCATCGACAAACATTTACATCTACTCTGTTTGACAGTGTGCTAGGCTCTG
GGGCTTGGGAGTGGAAATACTAAATTGAACAAGACTGAGGCCTTGCCTTCCAGAAACT
CACAAAGGAGTGGGTGGTCAGA
>650
CTGGGGCTTGGGAGTGGAAATACTAAATTGAACAAGACTGAGGCCTTGCCTTCCAGAA
ACTCACAAAGGAGTGGGTGGTCAGAATGCACATGACTCAGTCAGATATAAGGCAGATG
GTGGTAAATACTATGATAAATGTT
>651
GGCTTGGGAGTGGAAATACTAAATTGAACAAGACTGAGGCCTTGCCTTCCAGAAACTC
ACAAAGGAGTGGGTGGTCAGAATGCACATGACTCAGTCAGATATAAGGCAGATGGTGG
TAAATACTATGATAAATGTTCAGA
>652
TTGAACAAGACTGAGGCCTTGCCTTCCAGAAACTCACAAAGGAGTGGGTGGTCAGAA
TGCACATGACTCAGTCAGATATAAGGCAGATGGTGGTAAATACTATGATAAATGTTCAG
ATAGTGCTAAGGGGCTTATGGAGA
>653
CTTCCAGAAACTCACAAAGGAGTGGGTGGTCAGAATGCACATGACTCAGTCAGATATA
AGGCAGATGGTGGTAAATACTATGATAAATGTTCAGATAGTGCTAAGGGGCTTATGGAG
AATGGGAGAAATTAAATCTGACT
>654
GGAGTGGGTGGTCAGAATGCACATGACTCAGTCAGATATAAGGCAGATGGTGGTAAAT
ACTATGATAAATGTTCAGATAGTGCTAAGGGGCTTATGGAGAATGGGAGAAATTAAATC
TGACTCAAGGGTGGGTGGAGAAG
>655
GGTCAGAATGCACATGACTCAGTCAGATATAAGGCAGATGGTGGTAAATACTATGATAA
ATGTTCAGATAGTGCTAAGGGGCTTATGGAGAATGGGAGAAATTAAATCTGACTCAAG
GGTGGGTGGAGAAGATTACATCC
>656
TACTATGATAAATGTTCAGATAGTGCTAAGGGGCTTATGGAGAATGGGAGAAATTAAAT
CTGACTCAAGGGTGGGTGGAGAAGATTACATCCAAGATCCAGCAATGACTTCCAGAAG

AGACAAATGAACCTGGCCTGGCA
>657
ATGATAAATGTTCAGATAGTGCTAAGGGGCTTATGGAGAATGGGAGAAATTAAATCTGA
CTCAAGGGTGGGTGGAGAAGATTACATCCAAGATCCAGCAATGACTTCCAGAAGAGA
CAAATGAACCTGGCCTGGCAAGGG
>658
AGATTACATCCAAGATCCAGCAATGACTTCCAGAAGAGACAAATGAACCTGGCCTGGC
AAGGGAGGCAGAGTCAAAGTGTCTGGCTCAAAGCCCACCACAGCCTTGGCTCACAGG
CATCTCCCGGTCTTCCACTCACTCA
>659
CATCCAAGATCCAGCAATGACTTCCAGAAGAGACAAATGAACCTGGCCTGGCAAGGG
AGGCAGAGTCAAAGTGTCTGGCTCAAAGCCCACCACAGCCTTGGCTCACAGGCATCT
CCCGGTCTTCCACTCACTCAGTCCTT
>660
TGGCAAGGGAGGCAGAGTCAAAGTGTCTGGCTCAAAGCCCACCACAGCCTTGGCTCA
CAGGCATCTCCCGGTCTTCCACTCACTCAGTCCTTTCAGGGAAAGGCACTGAGCTTTT
CTCAAGGGAGAACTCCAAAGACGTC
>661
GTCAAAGTGTCTGGCTCAAAGCCCACCACAGCCTTGGCTCACAGGCATCTCCCGGTCT
TCCACTCACTCAGTCCTTTCAGGGAAAGGCACTGAGCTTTTCTCAAGGGAGAACTCCA
AAGACGTCTTTCATTTCATCTTAC
>662
CCGGTCTTCCACTCACTCAGTCCTTTCAGGGAAAGGCACTGAGCTTTTCTCAAGGGAG
AACTCCAAAGACGTCTTTCATTTCATCTTACAAATCCACACAAATTTGCAATTCCACTC
CACAAGATACGAGCATTTGTCTT
>663
AAGACGTCTTTCATTTCATCTTACAAATCCACACAAATTTGCAATTCCACTCCACAAGA
TACGAGCATTTGTCTTGCTATAATTTTAATATGATTGGCATAAATTACCTGTTATCAAGTA
AAATAGTTTCTTCTTATGGC
>664
TGCAATTCCACTCCACAAGATACGAGCATTTGTCTTGCTATAATTTTAATATGATTGGCAT
AAATTACCTGTTATCAAGTAAAATAGTTTCTTCTTATGGCTTGTGTGCCCCTGACATACC
ACTGCTATTTGAAAAGAGG
>665
CACTCCACAAGATACGAGCATTTGTCTTGCTATAATTTTAATATGATTGGCATAAATTACC
TGTTATCAAGTAAAATAGTTTCTTCTTATGGCTTGTGTGCCCCTGACATACCACTGCTAT
TTGAAAAGAGGCTTCTCCA
>666
CTTGCTATAATTTTAATATGATTGGCATAAATTACCTGTTATCAAGTAAAATAGTTTCTTC
TTATGGCTTGTGTGCCCCTGACATACCACTGCTATTTGAAAAGAGGCTTCTCCAGAAAC
TCAGTGTCTGAGAGTACGTG
>667
GTTTCTTCTTATGGCTTGTGTGCCCCTGACATACCACTGCTATTTGAAAAGAGGCTTCTC
CAGAAACTCAGTGTCTGAGAGTACGTGACTGGAAGGTGAGTGTTTGCTTACCCCTTGC

GTTTTCTCCCTGTCAGGATAAA
>668
TTCTTCTTATGGCTTGTGTGCCCCTGACATACCACTGCTATTTGAAAAGAGGCTTCTCCA
GAAACTCAGTGTCTGAGAGTACGTGACTGGAAGGTGAGTGTTTGCTTACCCCTTGCGT
TTTCTCCCTGTCAGGATAAATG
>669
CTTGTGTGCCCCTGACATACCACTGCTATTTGAAAAGAGGCTTCTCCAGAAACTCAGT
GTCTGAGAGTACGTGACTGGAAGGTGAGTGTTTGCTTACCCCTTGCGTTTTCTCCCTGT
CAGGATAAATGGAATATTCTGCT
>670
CTGACATACCACTGCTATTTGAAAAGAGGCTTCTCCAGAAACTCAGTGTCTGAGAGTA
CGTGACTGGAAGGTGAGTGTTTGCTTACCCCTTGCGTTTTCTCCCTGTCAGGATAAATG
GAATATTCTGCTTTAAGGACCCT
>671
CATACCACTGCTATTTGAAAAGAGGCTTCTCCAGAAACTCAGTGTCTGAGAGTACGTG
ACTGGAAGGTGAGTGTTTGCTTACCCCTTGCGTTTTCTCCCTGTCAGGATAAATGGAAT
ATTCTGCTTTAAGGACCCTAAGT
>672
AAGAGGCTTCTCCAGAAACTCAGTGTCTGAGAGTACGTGACTGGAAGGTGAGTGTTT
GCTTACCCCTTGCGTTTTCTCCCTGTCAGGATAAATGGAATATTCTGCTTTAAGGACCCT
AAGTCTGGCAATATCATGTTTTG
>673
GAGTGTTTGCTTACCCCTTGCGTTTTCTCCCTGTCAGGATAAATGGAATATTCTGCTTTA
AGGACCCTAAGTCTGGCAATATCATGTTTTGTGAACAGCCAACACAGTAACTATGGTTG
AGGCCTGTATTCGATGAATGA
>674
CCTTGCGTTTTCTCCCTGTCAGGATAAATGGAATATTCTGCTTTAAGGACCCTAAGTCTG
GCAATATCATGTTTTGTGAACAGCCAACACAGTAACTATGGTTGAGGCCTGTATTCGAT
GAATGAGGTTTTCATATGCTT
>675
GGATAAATGGAATATTCTGCTTTAAGGACCCTAAGTCTGGCAATATCATGTTTTGTGAAC
AGCCAACACAGTAACTATGGTTGAGGCCTGTATTCGATGAATGAGGTTTTCATATGCTT
CTTTCACATCAGGAGAGAGAG
>676
GAATATTCTGCTTTAAGGACCCTAAGTCTGGCAATATCATGTTTTGTGAACAGCCAACA
CAGTAACTATGGTTGAGGCCTGTATTCGATGAATGAGGTTTTCATATGCTTCTTTCACAT
CAGGAGAGAGAGAGAGAAAAA
>677
CTTTAAGGACCCTAAGTCTGGCAATATCATGTTTTGTGAACAGCCAACACAGTAACTAT
GGTTGAGGCCTGTATTCGATGAATGAGGTTTTCATATGCTTCTTTCACATCAGGAGAGA
GAGAGAGAAAAAAAAATAATAA
>678
TCTGGCAATATCATGTTTTGTGAACAGCCAACACAGTAACTATGGTTGAGGCCTGTATT
CGATGAATGAGGTTTTCATATGCTTCTTTCACATCAGGAGAGAGAGAGAGAAAAAAAA

ATAATAAACTTGGTAGAGGACGT
>679
CGATGAATGAGGTTTTCATATGCTTCTTTCACATCAGGAGAGAGAGAGAGAGAAAAAAAA
ATAATAAACTTGGTAGAGGACGTGGAGTTGTATTTCTGGACTATGGTGGCACCTCTAAG
GAGCATGGTTTTCCTTTTGCATG
>680
AGGTTTTCATATGCTTCTTTCACATCAGGAGAGAGAGAGAGAGAAAAAAAAATAATAAAC
TTGGTAGAGGACGTGGAGTTGTATTTCTGGACTATGGTGGCACCTCTAAGGAGCATGGT
TTTCCTTTTGCATGGCTTTGTCC
>681
TCAGGAGAGAGAGAGAGAGAAAAAAAAATAATAAACTTGGTAGAGGACGTGGAGTTGTA
TTTCTGGACTATGGTGGCACCTCTAAGGAGCATGGTTTTCCTTTTGCATGGCTTTGTCCA
TTACTATAACACTCCTGTTATAA
>682
AAAAATAATAAACTTGGTAGAGGACGTGGAGTTGTATTTCTGGACTATGGTGGCACCTC
TAAGGAGCATGGTTTTCCTTTTGCATGGCTTTGTCCATTACTATAACACTCCTGTTATAA
AATAGCTGCCGTGGATTGAAA
>683
GGACGTGGAGTTGTATTTCTGGACTATGGTGGCACCTCTAAGGAGCATGGTTTTCCTTT
TGCATGGCTTTGTCCATTACTATAACACTCCTGTTATAAAATAGCTGCCGTGGATTGAAA
TCCATAATGTTAACTCAAAAG
>684
GACTATGGTGGCACCTCTAAGGAGCATGGTTTTCCTTTTGCATGGCTTTGTCCATTACTA
TAACACTCCTGTTATAAAATAGCTGCCGTGGATTGAAATCCATAATGTTAACTCAAAAG
TTAAGCAGTCCGCTATTAAAC
>685
TAAGGAGCATGGTTTTCCTTTTGCATGGCTTTGTCCATTACTATAACACTCCTGTTATAA
AATAGCTGCCGTGGATTGAAATCCATAATGTTAACTCAAAAGTTAAGCAGTCCGCTATT
AAACAGCATAAGAAACTGTTA
>686
TTTGCATGGCTTTGTCCATTACTATAACACTCCTGTTATAAAATAGCTGCCGTGGATTGA
AATCCATAATGTTAACTCAAAAGTTAAGCAGTCCGCTATTAAACAGCATAAGAAACTGT
TACCTATCCCCTCTTCCTGGC
>687
ACTATAACACTCCTGTTATAAAATAGCTGCCGTGGATTGAAATCCATAATGTTAACTCAA
AAGTTAAGCAGTCCGCTATTAAACAGCATAAGAAACTGTTACCTATCCCCTCTTCCTGG
CCCCCCAACAAAGAAACAGCA
>688
TGCCGTGGATTGAAATCCATAATGTTAACTCAAAAGTTAAGCAGTCCGCTATTAAACAG
CATAAGAAACTGTTACCTATCCCCTCTTCCTGGCCCCCCAACAAAGAAACAGCAAACT
GTTAAAAAAGCCCATGTCTCCCC
>689
GCTATTAAACAGCATAAGAAACTGTTACCTATCCCCTCTTCCTGGCCCCCCAACAAAGA
AACAGCAAACTGTTAAAAAAGCCCATGTCTCCCCATGAGGACTTGGGGTTAATTTTCA

GCATGGTGTCTGGGGATTCATCT
>690
AAGAAACTGTTACCTATCCCCTCTTCCTGGCCCCCCAACAAAGAAACAGCAAACTGTT
AAAAAAGCCCATGTCTCCCCATGAGGACTTGGGGTTAATTTTCAGCATGGTGTCTGGG
GATTCATCTGTGGGACTCCTATTA
>691
ACAAAGAAACAGCAAACTGTTAAAAAAGCCCATGTCTCCCCATGAGGACTTGGGGTT
AATTTTCAGCATGGTGTCTGGGGATTCATCTGTGGGACTCCTATTACCCATAATAGGACT
CATGCTGCTTCATTTCATGCACA
>692
AAAGAAACAGCAAACTGTTAAAAAAGCCCATGTCTCCCCATGAGGACTTGGGGTTAAT
TTTCAGCATGGTGTCTGGGGATTCATCTGTGGGACTCCTATTACCCATAATAGGACTCAT
GCTGCTTCATTTCATGCACACG
>693
GTTAAAAAAGCCCATGTCTCCCCATGAGGACTTGGGGTTAATTTTCAGCATGGTGTCTG
GGGATTCATCTGTGGGACTCCTATTACCCATAATAGGACTCATGCTGCTTCATTTCATGC
ACACGATATGGAAAATGCACC
>694
CCTATTACCCATAATAGGACTCATGCTGCTTCATTTCATGCACACGATATGGAAAATGCA
CCCTTCCCTAGTGATGAAATTTTAGTTTTGAAATTGAGGCTAGCTTTTGCAGAACAAAG
CAGATTCCCAGAAACACCAAA
>695
TAGGACTCATGCTGCTTCATTTCATGCACACGATATGGAAAATGCACCCTTCCCTAGTGA
TGAAATTTTAGTTTTGAAATTGAGGCTAGCTTTTGCAGAACAAAGCAGATTCCCAGAA
ACACCAAAAACTATTCAATGTT
>696
TTAGTTTTGAAATTGAGGCTAGCTTTTGCAGAACAAAGCAGATTCCCAGAAACACCAA
AAACTATTCAATGTTGGCCTAGAGCCTGTGTGTCCGTGCTTAGGTGAGTATGTGTATCT
GAAAGGGAGAAAATGCCTTCCTC
>697
AGGCTAGCTTTTGCAGAACAAAGCAGATTCCCAGAAACACCAAAAACTATTCAATGTT
GGCCTAGAGCCTGTGTGTCCGTGCTTAGGTGAGTATGTGTATCTGAAAGGGAGAAAAT
GCCTTCCTCTAAGTGCGTATGCCC
>698
AACAAAGCAGATTCCCAGAAACACCAAAAACTATTCAATGTTGGCCTAGAGCCTGTGT
GTCCGTGCTTAGGTGAGTATGTGTATCTGAAAGGGAGAAAATGCCTTCCTCTAAGTGCG
TATGCCCCTCTCAAAGACAATAT
>699
GGCCTAGAGCCTGTGTGTCCGTGCTTAGGTGAGTATGTGTATCTGAAAGGGAGAAAAT
GCCTTCCTCTAAGTGCGTATGCCCCTCTCAAAGACAATATTCAAATATCATGCCCGATAG
CTGACAGCGGTCAATGGTTGCT
>700
TAGAGCCTGTGTGTCCGTGCTTAGGTGAGTATGTGTATCTGAAAGGGAGAAAATGCCTT
CCTCTAAGTGCGTATGCCCCTCTCAAAGACAATATTCAAATATCATGCCCGATAGCTGAC

AGCGGTCAATGGTTGCTATGC
>701
TGCCTTCCTCTAAGTGCGTATGCCCCTCTCAAAGACAATATTCAAATATCATGCCCGATA
GCTGACAGCGGTCAATGGTTGCTATGCTTCATCCCAAAGCTTTGTTAGGAACCCCATAA
AACCTGGCACATGGTTTGTGA
>702
CTCTAAGTGCGTATGCCCCTCTCAAAGACAATATTCAAATATCATGCCCGATAGCTGACA
GCGGTCAATGGTTGCTATGCTTCATCCCAAAGCTTTGTTAGGAACCCCATAAAACCTGG
CACATGGTTTGTGACACCCAG
>703
GACAATATTCAAATATCATGCCCGATAGCTGACAGCGGTCAATGGTTGCTATGCTTCATC
CCAAAGCTTTGTTAGGAACCCCATAAAACCTGGCACATGGTTTGTGACACCCAGTGAA
ACACCGTGCTTCTTCCATAATA
>704
TGACAGCGGTCAATGGTTGCTATGCTTCATCCCAAAGCTTTGTTAGGAACCCCATAAAA
CCTGGCACATGGTTTGTGACACCCAGTGAAACACCGTGCTTCTTCCATAATATCCTGAG
CCCTGCTGCCTGTCATTAAGGG
>705
AGCGGTCAATGGTTGCTATGCTTCATCCCAAAGCTTTGTTAGGAACCCCATAAAACCTG
GCACATGGTTTGTGACACCCAGTGAAACACCGTGCTTCTTCCATAATATCCTGAGCCCT
GCTGCCTGTCATTAAGGGACCT
>706
CCTGGCACATGGTTTGTGACACCCAGTGAAACACCGTGCTTCTTCCATAATATCCTGAG
CCCTGCTGCCTGTCATTAAGGGACCTCTCTTATTAAGCATCTCTTTTTTAAATGGCTGCC
ATCATCTGGTCCCAGTGTACA
>707
AGCCCTGCTGCCTGTCATTAAGGGACCTCTCTTATTAAGCATCTCTTTTTTAAATGGCTG
CCATCATCTGGTCCCAGTGTACATTAAATCCCATTATAATTATAAACACACTTCCCCATAA
CATTCGTTGCAGATCTCAC
>708
GCTGCCTGTCATTAAGGGACCTCTCTTATTAAGCATCTCTTTTTTAAATGGCTGCCATCA
TCTGGTCCCAGTGTACATTAAATCCCATTATAATTATAAACACACTTCCCCATAACATTCG
TTGCAGATCTCACCCTCAG
>709
GCTGCCATCATCTGGTCCCAGTGTACATTAAATCCCATTATAATTATAAACACACTTCCC
CATAACATTCGTTGCAGATCTCACCCTCAGTGCCTACCCCCTTAGGGCATCTTTGCACTA
ATAACAACTCAGATTTGCTA
>710
AGTGTACATTAAATCCCATTATAATTATAAACACACTTCCCCATAACATTCGTTGCAGATC
TCACCCTCAGTGCCTACCCCCTTAGGGCATCTTTGCACTAATAACAACTCAGATTTGCTA
TCTGAGTTGACAACATTAG
>711
AGATCTCACCCTCAGTGCCTACCCCCTTAGGGCATCTTTGCACTAATAACAACTCAGAT
TTGCTATCTGAGTTGACAACATTAGGTCCCAACACCATTTGCAATATGCTGGGGAAAGA

GACAGAGAGAGAAGAAAAAGCT
>712
GTGCCTACCCCCTTAGGGCATCTTTGCACTAATAACAACTCAGATTTGCTATCTGAGTTG
ACAACATTAGGTCCCAACACCATTTGCAATATGCTGGGGAAAGAGACAGAGAGAGAA
GAAAAAGCTTTTTCTTCCTTAAT
>713
GCAATATGCTGGGGAAAGAGACAGAGAGAGAAGAAAAAGCTTTTTCTTCCTTAATCAC
GAAGATCTTGGGGTCACAGTGACCCAAGGCCTCAGGAGCTAAAAAAAAAAAATCCCT
CCTCCTACCCTTTTGAAAAGTCACT
>714
TGCTGGGGAAAGAGACAGAGAGAGAAGAAAAAGCTTTTTCTTCCTTAATCACGAAGA
TCTTGGGGTCACAGTGACCCAAGGCCTCAGGAGCTAAAAAAAAAAAATCCCTCCTCCT
ACCCTTTTGAAAAGTCACTCTCTAC
>715
CTTGGGGTCACAGTGACCCAAGGCCTCAGGAGCTAAAAAAAAAAAATCCCTCCTCCT
ACCCTTTTGAAAAGTCACTCTCTACTCTACAGATGAGGGAAGGGCATACTTGGCACCT
GGGAGAGAAACTCAAGTTTGTAAAC
>716
CTTTTGAAAAGTCACTCTCTACTCTACAGATGAGGGAAGGGCATACTTGGCACCTGGG
AGAGAAACTCAAGTTTGTAAACACCACTAACCAGGTCTCTGTTGGTTGGGGCGGTGAT
GGTGGTGGTAGTGGTGGGGTGGGG
>717
TGAAAAGTCACTCTCTACTCTACAGATGAGGGAAGGGCATACTTGGCACCTGGGAGAG
AAACTCAAGTTTGTAAACACCACTAACCAGGTCTCTGTTGGTTGGGGCGGTGATGGTG
GTGGTAGTGGTGGGGTGGGGGATG
>718
TCTACAGATGAGGGAAGGGCATACTTGGCACCTGGGAGAGAAACTCAAGTTTGTAAAC
ACCACTAACCAGGTCTCTGTTGGTTGGGGCGGTGATGGTGGTGGTAGTGGTGGGGTGG
GGGATGAGGGATGATGGCAATCAC
>719
CACCTGGGAGAGAAACTCAAGTTTGTAAACACCACTAACCAGGTCTCTGTTGGTTGGG
GCGGTGATGGTGGTGGTAGTGGTGGGGTGGGGGATGAGGGATGATGGCAATCACTAAT
CAAAGAAGCTGTCTTATTTGGTTT
>720
CTGGGAGAGAAACTCAAGTTTGTAAACACCACTAACCAGGTCTCTGTTGGTTGGGGCG
GTGATGGTGGTGGTAGTGGTGGGGTGGGGGATGAGGGATGATGGCAATCACTAATCAA
AGAAGCTGTCTTATTTGGTTTGCT
>721
GAGAAACTCAAGTTTGTAAACACCACTAACCAGGTCTCTGTTGGTTGGGGCGGTGATG
GTGGTGGTAGTGGTGGGGTGGGGGATGAGGGATGATGGCAATCACTAATCAAAGAAG
CTGTCTTATTTGGTTTGCTGAGCCT
>722
GGGCGGTGATGGTGGTGGTAGTGGTGGGGTGGGGGATGAGGGATGATGGCAATCACTA
ATCAAAGAAGCTGTCTTATTTGGTTTGCTGAGCCTCAACTAACCGGAATGCATACATCA

CCTGATTCTTTCCCCCTCTGCTT
>723
GGTGGTGGTAGTGGTGGGGTGGGGGATGAGGGATGATGGCAATCACTAATCAAAGAA
GCTGTCTTATTTGGTTTGCTGAGCCTCAACTAACCGGAATGCATACATCACCTGATTCTT
TCCCCCTCTGCTTTTTGGAGGGG
>724
TAACCGGAATGCATACATCACCTGATTCTTTCCCCCTCTGCTTTTTGGAGGGGGCATCTA
AGGACATAATGTAAAAGAATTAAAATTTAAAAACAAAAACAAAATGTCAACATATGCA
GGAGTAAATTCCACTCTGCCTA
>725
CTCTGCTTTTTGGAGGGGGCATCTAAGGACATAATGTAAAAGAATTAAAATTTAAAAAC
AAAAACAAAATGTCAACATATGCAGGAGTAAATTCCACTCTGCCTAATTTTTTTTTTTTT
TTGAAATGAGATCTTGTTCTG
>726
GGGCATCTAAGGACATAATGTAAAAGAATTAAAATTTAAAAACAAAAACAAAATGTCA
ACATATGCAGGAGTAAATTCCACTCTGCCTAATTTTTTTTTTTTTTTGAAATGAGATCTT
GTTCTGTCACCCAGGCTGGAGG
>727
CAAAATGTCAACATATGCAGGAGTAAATTCCACTCTGCCTAATTTTTTTTTTTTTTTGAA
ATGAGATCTTGTTCTGTCACCCAGGCTGGAGGGCAGTGGTGCAATCTCAGCTCACTGC
AGCCACCACCTCCCAGGTTCAA
>728
TGTCAACATATGCAGGAGTAAATTCCACTCTGCCTAATTTTTTTTTTTTTTTGAAATGAG
ATCTTGTTCTGTCACCCAGGCTGGAGGGCAGTGGTGCAATCTCAGCTCACTGCAGCCA
CCACCTCCCAGGTTCAAGCGAT
>729
AATTCCACTCTGCCTAATTTTTTTTTTTTTTTGAAATGAGATCTTGTTCTGTCACCCAGG
CTGGAGGGCAGTGGTGCAATCTCAGCTCACTGCAGCCACCACCTCCCAGGTTCAAGC
GATTCTCCCGCCTCAGCCCCCTA
>730
GATCTTGTTCTGTCACCCAGGCTGGAGGGCAGTGGTGCAATCTCAGCTCACTGCAGCC
ACCACCTCCCAGGTTCAAGCGATTCTCCCGCCTCAGCCCCCTAAGTAGCTGGTATTACA
GGCACGTGCCACCACGCCTGGCT
>731
TGGTGCAATCTCAGCTCACTGCAGCCACCACCTCCCAGGTTCAAGCGATTCTCCCGCC
TCAGCCCCCTAAGTAGCTGGTATTACAGGCACGTGCCACCACGCCTGGCTAATTTTTGT
ATTTTTAGTAGAGATAGGGTTTC
>732
GCAGCCACCACCTCCCAGGTTCAAGCGATTCTCCCGCCTCAGCCCCCTAAGTAGCTGG
TATTACAGGCACGTGCCACCACGCCTGGCTAATTTTTGTATTTTTAGTAGAGATAGGGTT
TCACCATGTTGGCCAGGCTGGT
>733
CGATTCTCCCGCCTCAGCCCCCTAAGTAGCTGGTATTACAGGCACGTGCCACCACGCCT
GGCTAATTTTTGTATTTTTAGTAGAGATAGGGTTTCACCATGTTGGCCAGGCTGGTCTCA

AACTCCTGACCTCAAGTGATC
>734
CCTAAGTAGCTGGTATTACAGGCACGTGCCACCACGCCTGGCTAATTTTTGTATTTTTAG
TAGAGATAGGGTTTCACCATGTTGGCCAGGCTGGTCTCAAACTCCTGACCTCAAGTGA
TCCGCCTGCCTCGGCCTCCTAA
>735
TGGTATTACAGGCACGTGCCACCACGCCTGGCTAATTTTTGTATTTTTAGTAGAGATAGG
GTTTCACCATGTTGGCCAGGCTGGTCTCAAACTCCTGACCTCAAGTGATCCGCCTGCCT
CGGCCTCCTAAAGTGCTGGGA
>736
ACGTGCCACCACGCCTGGCTAATTTTTGTATTTTTAGTAGAGATAGGGTTTCACCATGTT
GGCCAGGCTGGTCTCAAACTCCTGACCTCAAGTGATCCGCCTGCCTCGGCCTCCTAAA
GTGCTGGGAGTACAGGCATGAG
>737
ATTTTTGTATTTTTAGTAGAGATAGGGTTTCACCATGTTGGCCAGGCTGGTCTCAAACTC
CTGACCTCAAGTGATCCGCCTGCCTCGGCCTCCTAAAGTGCTGGGAGTACAGGCATGA
GCCACTGCGCCCAGCCTACTCT
>738
TTTCACCATGTTGGCCAGGCTGGTCTCAAACTCCTGACCTCAAGTGATCCGCCTGCCTC
GGCCTCCTAAAGTGCTGGGAGTACAGGCATGAGCCACTGCGCCCAGCCTACTCTGCCC
AATTGTTGACATCACCTACAGCT
>739
GTTGGCCAGGCTGGTCTCAAACTCCTGACCTCAAGTGATCCGCCTGCCTCGGCCTCCT
AAAGTGCTGGGAGTACAGGCATGAGCCACTGCGCCCAGCCTACTCTGCCCAATTGTTG
ACATCACCTACAGCTCTATAGTAG
>740
CCTGCCTCGGCCTCCTAAAGTGCTGGGAGTACAGGCATGAGCCACTGCGCCCAGCCTA
CTCTGCCCAATTGTTGACATCACCTACAGCTCTATAGTAGATGTCTGGAATGATGATCTG
AAGGACTGTAAGACTGTAAGAT
>741
GGGAGTACAGGCATGAGCCACTGCGCCCAGCCTACTCTGCCCAATTGTTGACATCACC
TACAGCTCTATAGTAGATGTCTGGAATGATGATCTGAAGGACTGTAAGACTGTAAGATT
TTTTGACTCCAGAAATGGACACA
>742
ACAGGCATGAGCCACTGCGCCCAGCCTACTCTGCCCAATTGTTGACATCACCTACAGC
TCTATAGTAGATGTCTGGAATGATGATCTGAAGGACTGTAAGACTGTAAGATTTTTTGA
CTCCAGAAATGGACACAATAGGC
>743
CTACTCTGCCCAATTGTTGACATCACCTACAGCTCTATAGTAGATGTCTGGAATGATGAT
CTGAAGGACTGTAAGACTGTAAGATTTTTTGACTCCAGAAATGGACACAATAGGCTTG
CGGTGTCTTCCCCTAACAGTCC
>744
CCCAATTGTTGACATCACCTACAGCTCTATAGTAGATGTCTGGAATGATGATCTGAAGG
ACTGTAAGACTGTAAGATTTTTTGACTCCAGAAATGGACACAATAGGCTTGCGGTGTCT

TCCCCTAACAGTCCCTGGCAGG
>745
GAATGATGATCTGAAGGACTGTAAGACTGTAAGATTTTTTGACTCCAGAAATGGACAC
AATAGGCTTGCGGTGTCTTCCCCTAACAGTCCCTGGCAGGGTAAAGGTCTGATGCCCT
GGTACGTTCCCCAGTGGCAATCTG
>746
ATGATGATCTGAAGGACTGTAAGACTGTAAGATTTTTTGACTCCAGAAATGGACACAAT
AGGCTTGCGGTGTCTTCCCCTAACAGTCCCTGGCAGGGTAAAGGTCTGATGCCCTGGT
ACGTTCCCCAGTGGCAATCTGGA
>747
GTAAGATTTTTTGACTCCAGAAATGGACACAATAGGCTTGCGGTGTCTTCCCCTAACAG
TCCCTGGCAGGGTAAAGGTCTGATGCCCTGGTACGTTCCCCAGTGGCAATCTGGAGTT
CTCAACTGCAGAGAATACTTTTC
>748
TTTTTTGACTCCAGAAATGGACACAATAGGCTTGCGGTGTCTTCCCCTAACAGTCCCTG
GCAGGGTAAAGGTCTGATGCCCTGGTACGTTCCCCAGTGGCAATCTGGAGTTCTCAAC
TGCAGAGAATACTTTTCCTCTAC
>749
GAAATGGACACAATAGGCTTGCGGTGTCTTCCCCTAACAGTCCCTGGCAGGGTAAAGG
TCTGATGCCCTGGTACGTTCCCCAGTGGCAATCTGGAGTTCTCAACTGCAGAGAATACT
TTTCCTCTACTCAGGGGCGTCAC
>750
CCCTGGTACGTTCCCCAGTGGCAATCTGGAGTTCTCAACTGCAGAGAATACTTTTCCTC
TACTCAGGGGCGTCACTACCTGCATCTCTCCCTGAACATGCTTCATTTTCCACTCATCAC
AAGTTTTTGGGGAAATGCAAA
>751
TTTTCCTCTACTCAGGGGCGTCACTACCTGCATCTCTCCCTGAACATGCTTCATTTTCCA
CTCATCACAAGTTTTTGGGGAAATGCAAAGTGGCATCTTCTCCCCAAAAGGTAGGGAT
CTTTGATTTTTTTAATCACGAT
>752
GTCACTACCTGCATCTCTCCCTGAACATGCTTCATTTTCCACTCATCACAAGTTTTTGGG
GAAATGCAAAGTGGCATCTTCTCCCCAAAAGGTAGGGATCTTTGATTTTTTTAATCACG
ATAGAAAAGTTTACTTTGAAA
>753
TGAACATGCTTCATTTTCCACTCATCACAAGTTTTTGGGGAAATGCAAAGTGGCATCTT
CTCCCCAAAAGGTAGGGATCTTTGATTTTTTTAATCACGATAGAAAAGTTTACTTTGAA
ATAAAAAAATTAAATATATGTT
>754
GGGGAAATGCAAAGTGGCATCTTCTCCCCAAAAGGTAGGGATCTTTGATTTTTTTAATC
ACGATAGAAAAGTTTACTTTGAAATAAAAAAATTAAATATATGTTCCAAATTAAGAGTT
GGAACTTAAAGAGTTGCAACAT
>755
AAGGTAGGGATCTTTGATTTTTTTAATCACGATAGAAAAGTTTACTTTGAAATAAAAAA
ATTAAATATATGTTCCAAATTAAGAGTTGGAACTTAAAGAGTTGCAACATTCCATTCATA

CTCAGATACTCAAAACACCAC
>756
TGATTTTTTTAATCACGATAGAAAAGTTTACTTTGAAATAAAAAAATTAAATATATGTTC
CAAATTAAGAGTTGGAACTTAAAGAGTTGCAACATTCCATTCATACTCAGATACTCAAA
ACACCACTGTATTCTTCTTCC
>757
CGATAGAAAAGTTTACTTTGAAATAAAAAAATTAAATATATGTTCCAAATTAAGAGTTG
GAACTTAAAGAGTTGCAACATTCCATTCATACTCAGATACTCAAAACACCACTGTATTC
TTCTTCCTATCTCTTTAGTTGA
>758
TTCCAAATTAAGAGTTGGAACTTAAAGAGTTGCAACATTCCATTCATACTCAGATACTC
AAAACACCACTGTATTCTTCTTCCTATCTCTTTAGTTGAAGTTTTTTTTTGTAATTCAAA
GCCCCCTCCCCAATTTCTGCC
>759
AAAGAGTTGCAACATTCCATTCATACTCAGATACTCAAAACACCACTGTATTCTTCTTC
CTATCTCTTTAGTTGAAGTTTTTTTTTGTAATTCAAAGCCCCCTCCCCAATTTCTGCCTG
AGATTCTGAGGGAGGAGGAAA
>760
CCATTCATACTCAGATACTCAAAACACCACTGTATTCTTCTTCCTATCTCTTTAGTTGAA
GTTTTTTTTTGTAATTCAAAGCCCCCTCCCCAATTTCTGCCTGAGATTCTGAGGGAGGA
GGAAAAGTGTAAGATCCTGGG
>761
TTGAAGTTTTTTTTTGTAATTCAAAGCCCCCTCCCCAATTTCTGCCTGAGATTCTGAGG
GAGGAGGAAAAGTGTAAGATCCTGGGGGATCTAAGTCTCTTTACCTGGTGACCAAGCT
GAGAACCATTTGGTCCTCAGCTT
>762
AAGCCCCCTCCCCAATTTCTGCCTGAGATTCTGAGGGAGGAGGAAAAGTGTAAGATCC
TGGGGGATCTAAGTCTCTTTACCTGGTGACCAAGCTGAGAACCATTTGGTCCTCAGCTT
GGCTGGCTGCTGCACAGATCTTG
>763
AATTTCTGCCTGAGATTCTGAGGGAGGAGGAAAAGTGTAAGATCCTGGGGGATCTAAG
TCTCTTTACCTGGTGACCAAGCTGAGAACCATTTGGTCCTCAGCTTGGCTGGCTGCTGC
ACAGATCTTGGCCTCAGCAGAGT
>764
GAGGAGGAAAAGTGTAAGATCCTGGGGGATCTAAGTCTCTTTACCTGGTGACCAAGCT
GAGAACCATTTGGTCCTCAGCTTGGCTGGCTGCTGCACAGATCTTGGCCTCAGCAGAG
TAAGAGAAGAAGGAAAATTAAAAG
>765
TGGTGACCAAGCTGAGAACCATTTGGTCCTCAGCTTGGCTGGCTGCTGCACAGATCTT
GGCCTCAGCAGAGTAAGAGAAGAAGGAAAATTAAAAGCCCTTGCTTCCTTCATCTATT
TCACTAGAGGCTACATAATCTAGA
>766
TAAGAGAAGAAGGAAAATTAAAAGCCCTTGCTTCCTTCATCTATTTCACTAGAGGCTAC
ATAATCTAGAAGTTAAGAGCATGAGCTTTGCAGTCAGAATGGTCTGATTTAAAGTTTCA

GCTCTGATGTTTAATAGATCTA
>767
AAGCCCTTGCTTCCTTCATCTATTTCACTAGAGGCTACATAATCTAGAAGTTAAGAGCAT
GAGCTTTGCAGTCAGAATGGTCTGATTTAAAGTTTCAGCTCTGATGTTTAATAGATCTAC
AGTCTGCAGTAAGGCACTTA
>768
CTTCCTTCATCTATTTCACTAGAGGCTACATAATCTAGAAGTTAAGAGCATGAGCTTTGC
AGTCAGAATGGTCTGATTTAAAGTTTCAGCTCTGATGTTTAATAGATCTACAGTCTGCA
GTAAGGCACTTAATCTCTCTG
>769
TACATAATCTAGAAGTTAAGAGCATGAGCTTTGCAGTCAGAATGGTCTGATTTAAAGTT
TCAGCTCTGATGTTTAATAGATCTACAGTCTGCAGTAAGGCACTTAATCTCTCTGAACCT
CAGTTTTCATGTCCACAGAAA
>770
TAAGAGCATGAGCTTTGCAGTCAGAATGGTCTGATTTAAAGTTTCAGCTCTGATGTTTA
ATAGATCTACAGTCTGCAGTAAGGCACTTAATCTCTCTGAACCTCAGTTTTCATGTCCA
CAGAAATTGGGAAAATAATAGC
>771
ATGAGCTTTGCAGTCAGAATGGTCTGATTTAAAGTTTCAGCTCTGATGTTTAATAGATCT
ACAGTCTGCAGTAAGGCACTTAATCTCTCTGAACCTCAGTTTTCATGTCCACAGAAATT
GGGAAAATAATAGCACCTGCA
>772
TTAAAGTTTCAGCTCTGATGTTTAATAGATCTACAGTCTGCAGTAAGGCACTTAATCTCT
CTGAACCTCAGTTTTCATGTCCACAGAAATTGGGAAAATAATAGCACCTGCATTAATAG
GATTGTTATGTGGATTAAATA
>773
AATCTCTCTGAACCTCAGTTTTCATGTCCACAGAAATTGGGAAAATAATAGCACCTGCA
TTAATAGGATTGTTATGTGGATTAAATAAAACAACATAGGTAATGTGTTGAGTTTCGTAG
TCAGTGCTCAGAATGAGTTAT
>774
CTCTGAACCTCAGTTTTCATGTCCACAGAAATTGGGAAAATAATAGCACCTGCATTAAT
AGGATTGTTATGTGGATTAAATAAAACAACATAGGTAATGTGTTGAGTTTCGTAGTCAG
TGCTCAGAATGAGTTATTATTT
>775
CACAGAAATTGGGAAAATAATAGCACCTGCATTAATAGGATTGTTATGTGGATTAAATAA
AACAACATAGGTAATGTGTTGAGTTTCGTAGTCAGTGCTCAGAATGAGTTATTATTTTCG
CTGTCTCCTTCCTGTATGAT
>776
AAATTGGGAAAATAATAGCACCTGCATTAATAGGATTGTTATGTGGATTAAATAAAACA
ACATAGGTAATGTGTTGAGTTTCGTAGTCAGTGCTCAGAATGAGTTATTATTTTCGCTGT
CTCCTTCCTGTATGATCTCTT
>777
ATTGGGAAAATAATAGCACCTGCATTAATAGGATTGTTATGTGGATTAAATAAAACAACA
TAGGTAATGTGTTGAGTTTCGTAGTCAGTGCTCAGAATGAGTTATTATTTTCGCTGTCTC

CTTCCTGTATGATCTCTTAG
>778
TAATAGCACCTGCATTAATAGGATTGTTATGTGGATTAAATAAAACAACATAGGTAATGT
GTTGAGTTTCGTAGTCAGTGCTCAGAATGAGTTATTATTTTCGCTGTCTCCTTCCTGTAT
GATCTCTTAGAACTGCTATA
>779
GGATTGTTATGTGGATTAAATAAAACAACATAGGTAATGTGTTGAGTTTCGTAGTCAGT
GCTCAGAATGAGTTATTATTTTCGCTGTCTCCTTCCTGTATGATCTCTTAGAACTGCTATA
CCCTCCTGTTCCACTAACTG
>780
AACATAGGTAATGTGTTGAGTTTCGTAGTCAGTGCTCAGAATGAGTTATTATTTTCGCTG
TCTCCTTCCTGTATGATCTCTTAGAACTGCTATACCCTCCTGTTCCACTAACTGAGCTCT
GAAAGGCTGCCAGTGGTGGG
>781
GTGCTCAGAATGAGTTATTATTTTCGCTGTCTCCTTCCTGTATGATCTCTTAGAACTGCTA
TACCCTCCTGTTCCACTAACTGAGCTCTGAAAGGCTGCCAGTGGTGGGAGGTACATGG
GTAAAGAGGGAGATGAGGGAG
>782
TCCTTCCTGTATGATCTCTTAGAACTGCTATACCCTCCTGTTCCACTAACTGAGCTCTGA
AAGGCTGCCAGTGGTGGGAGGTACATGGGTAAAGAGGGAGATGAGGGAGTAAGATTT
AGCAATGGAATTCCATGCAAAGG
>783
TTCCTGTATGATCTCTTAGAACTGCTATACCCTCCTGTTCCACTAACTGAGCTCTGAAAG
GCTGCCAGTGGTGGGAGGTACATGGGTAAAGAGGGAGATGAGGGAGTAAGATTTAGC
AATGGAATTCCATGCAAAGGCCA
>784
ATGATCTCTTAGAACTGCTATACCCTCCTGTTCCACTAACTGAGCTCTGAAAGGCTGCC
AGTGGTGGGAGGTACATGGGTAAAGAGGGAGATGAGGGAGTAAGATTTAGCAATGGA
ATTCCATGCAAAGGCCAGCTCATC
>785
TGTTCCACTAACTGAGCTCTGAAAGGCTGCCAGTGGTGGGAGGTACATGGGTAAAGAG
GGAGATGAGGGAGTAAGATTTAGCAATGGAATTCCATGCAAAGGCCAGCTCATCTATTC
CTCTGTCTCTAGCATGATCTCCC
>786
TAAAGAGGGAGATGAGGGAGTAAGATTTAGCAATGGAATTCCATGCAAAGGCCAGCTC
ATCTATTCCTCTGTCTCTAGCATGATCTCCCCCTAAGATTGAGATCTCCTGTATTTTTAGA
ACTGATACCCAGGTTTCCCTT
>787
TTCCATGCAAAGGCCAGCTCATCTATTCCTCTGTCTCTAGCATGATCTCCCCCTAAGATT
GAGATCTCCTGTATTTTTAGAACTGATACCCAGGTTTCCCTTAAAAGACGCTTCAAGAA
AAAATGTAAAAGAAAAATCAC
>788
TATTCCTCTGTCTCTAGCATGATCTCCCCCTAAGATTGAGATCTCCTGTATTTTTAGAACT
GATACCCAGGTTTCCCTTAAAAGACGCTTCAAGAAAAAATGTAAAAGAAAAATCACTC

TAAATACACGAACATGAGAGA
>789
AAGATTGAGATCTCCTGTATTTTTAGAACTGATACCCAGGTTTCCCTTAAAAGACGCTT
CAAGAAAAAATGTAAAAGAAAAATCACTCTAAATACACGAACATGAGAGAGACAGAA
AGAGAGCGGGCATGTGCGCGCGAG
>790
CAAGAAAAAATGTAAAAGAAAAATCACTCTAAATACACGAACATGAGAGAGACAGAA
AGAGAGCGGGCATGTGCGCGCGAGAGAGATTGAGTATTCTCTTGTAATTTTCCATCCTG
CTTGTTTTTAGGTGCTGCCATATG
>791
AAATCACTCTAAATACACGAACATGAGAGAGACAGAAAGAGAGCGGGCATGTGCGCG
CGAGAGAGATTGAGTATTCTCTTGTAATTTTCCATCCTGCTTGTTTTTAGGTGCTGCCAT
ATGAAATCTCTGAACTGAGGAGT
>792
AAATACACGAACATGAGAGAGACAGAAAGAGAGCGGGCATGTGCGCGCGAGAGAGA
TTGAGTATTCTCTTGTAATTTTCCATCCTGCTTGTTTTTAGGTGCTGCCATATGAAATCTC
TGAACTGAGGAGTCTCATCAGCC
>793
AGAGAGCGGGCATGTGCGCGCGAGAGAGATTGAGTATTCTCTTGTAATTTTCCATCCTG
CTTGTTTTTAGGTGCTGCCATATGAAATCTCTGAACTGAGGAGTCTCATCAGCCTTCCC
AGGACCCAAGGACATAGCTCTG
>794
GAGTATTCTCTTGTAATTTTCCATCCTGCTTGTTTTTAGGTGCTGCCATATGAAATCTCTG
AACTGAGGAGTCTCATCAGCCTTCCCAGGACCCAAGGACATAGCTCTGGCTATAAATG
GTGCCAGTTTTGGTGGGGGAG
>795
ATGAAATCTCTGAACTGAGGAGTCTCATCAGCCTTCCCAGGACCCAAGGACATAGCTC
TGGCTATAAATGGTGCCAGTTTTGGTGGGGGAGCTGCTGCTGCTTTATCTGGGCCCAAT
AAACCTTATCAAACTGCTGTGCA
>796
TCTCTGAACTGAGGAGTCTCATCAGCCTTCCCAGGACCCAAGGACATAGCTCTGGCTAT
AAATGGTGCCAGTTTTGGTGGGGGAGCTGCTGCTGCTTTATCTGGGCCCAATAAACCTT
ATCAAACTGCTGTGCAGCCAGC
>797
AAATGGTGCCAGTTTTGGTGGGGGAGCTGCTGCTGCTTTATCTGGGCCCAATAAACCTT
ATCAAACTGCTGTGCAGCCAGCCTTCAGCGCTAAGTGGAAATGCTTGGAGCCCAATGC
TGGCATTCAGCCCACAGCCTCGC
>798
GAGCTGCTGCTGCTTTATCTGGGCCCAATAAACCTTATCAAACTGCTGTGCAGCCAGCC
TTCAGCGCTAAGTGGAAATGCTTGGAGCCCAATGCTGGCATTCAGCCCACAGCCTCGC
CTGCCTTCTTCCTTTTTGTTACC
>799
CGCTAAGTGGAAATGCTTGGAGCCCAATGCTGGCATTCAGCCCACAGCCTCGCCTGCC
TTCTTCCTTTTTGTTACCTTCTCCACCTCTTAAATTCAGGCATTAATCTCTCTTTAACACT

TACTAACAAGGGCTGGGCTTT
>800
CACCTCTTAAATTCAGGCATTAATCTCTCTTTAACACTTACTAACAAGGGCTGGGCTTTT
AATGGGCAGCGTTTATGAAGCAAGGGAGATTTTCACATAGAGTGAATATGGCACGGGG
GTGGGGAGGCCAGGGCCATAGA
>801
TAACACTTACTAACAAGGGCTGGGCTTTTAATGGGCAGCGTTTATGAAGCAAGGGAGA
TTTTCACATAGAGTGAATATGGCACGGGGGTGGGGAGGCCAGGGCCATAGAGGATTGT
GCTTTCTCTCCCATTGTTTCCAGA
>802
GGCTGGGCTTTTAATGGGCAGCGTTTATGAAGCAAGGGAGATTTTCACATAGAGTGAAT
ATGGCACGGGGGTGGGGAGGCCAGGGCCATAGAGGATTGTGCTTTCTCTCCCATTGTT
TCCAGAGATTCTCTCTTTCTCTG
>803
TGAAGCAAGGGAGATTTTCACATAGAGTGAATATGGCACGGGGGTGGGGAGGCCAGG
GCCATAGAGGATTGTGCTTTCTCTCCCATTGTTTCCAGAGATTCTCTCTTTCTCTGATTT
GAGGATTAATAGGAGATTAGGAT
>804
TCACATAGAGTGAATATGGCACGGGGGTGGGGAGGCCAGGGCCATAGAGGATTGTGCT
TTCTCTCCCATTGTTTCCAGAGATTCTCTCTTTCTCTGATTTGAGGATTAATAGGAGATT
AGGATCAGGGGTTAATTGTGTG
>805
TTCTCTCCCATTGTTTCCAGAGATTCTCTCTTTCTCTGATTTGAGGATTAATAGGAGATT
AGGATCAGGGGTTAATTGTGTGCTTCTCCTGCTACTGCACTGAGGAGAGGCTGGTAGG
TGGATGTGGACAGCAAAGCGGA
>806
CCATTGTTTCCAGAGATTCTCTCTTTCTCTGATTTGAGGATTAATAGGAGATTAGGATCA
GGGGTTAATTGTGTGCTTCTCCTGCTACTGCACTGAGGAGAGGCTGGTAGGTGGATGT
GGACAGCAAAGCGGAAACCTAC
>807
AGGATTAATAGGAGATTAGGATCAGGGGTTAATTGTGTGCTTCTCCTGCTACTGCACTG
AGGAGAGGCTGGTAGGTGGATGTGGACAGCAAAGCGGAAACCTACAGCAGGCACTAT
CTAGGCAGAAGCTCAACAAGTGTA
>808
TTAATAGGAGATTAGGATCAGGGGTTAATTGTGTGCTTCTCCTGCTACTGCACTGAGGA
GAGGCTGGTAGGTGGATGTGGACAGCAAAGCGGAAACCTACAGCAGGCACTATCTAG
GCAGAAGCTCAACAAGTGTAGTGA
>809
GGCTGGTAGGTGGATGTGGACAGCAAAGCGGAAACCTACAGCAGGCACTATCTAGGC
AGAAGCTCAACAAGTGTAGTGATTCTTTCTTCTGTTTCCCTGGTGAGGCACCAGGAGG
GTCTTTTCTCCTCTCCTTACATCCC
>810
CTGGTAGGTGGATGTGGACAGCAAAGCGGAAACCTACAGCAGGCACTATCTAGGCAG
AAGCTCAACAAGTGTAGTGATTCTTTCTTCTGTTTCCCTGGTGAGGCACCAGGAGGGT

CTTTTCTCCTCTCCTTACATCCCTC
>811
GGAAACCTACAGCAGGCACTATCTAGGCAGAAGCTCAACAAGTGTAGTGATTCTTTCT
TCTGTTTCCCTGGTGAGGCACCAGGAGGGTCTTTTCTCCTCTCCTTACATCCCTCCACT
CTTGCTCTCCTTGCAGCCCAGTT
>812
CACTATCTAGGCAGAAGCTCAACAAGTGTAGTGATTCTTTCTTCTGTTTCCCTGGTGAG
GCACCAGGAGGGTCTTTTCTCCTCTCCTTACATCCCTCCACTCTTGCTCTCCTTGCAGC
CCAGTTCTTTCCTAACTTTCTT
>813
CTGGTGAGGCACCAGGAGGGTCTTTTCTCCTCTCCTTACATCCCTCCACTCTTGCTCTC
CTTGCAGCCCAGTTCTTTCCTAACTTTCTTTAAATCCCTTTCCTCTAACAGGGTGTATAG
ACCTTAGTTAGAAAAACAGGT
>814
CCCTCCACTCTTGCTCTCCTTGCAGCCCAGTTCTTTCCTAACTTTCTTTAAATCCCTTTC
CTCTAACAGGGTGTATAGACCTTAGTTAGAAAAACAGGTAGTCTCTAAATGGGATTGCT
CTTTATTGTTAATGAAATGAA
>815
TCTCCTTGCAGCCCAGTTCTTTCCTAACTTTCTTTAAATCCCTTTCCTCTAACAGGGTGT
ATAGACCTTAGTTAGAAAAACAGGTAGTCTCTAAATGGGATTGCTCTTTATTGTTAATGA
AATGAATACCCAGGGACTGG
>816
CAGTTCTTTCCTAACTTTCTTTAAATCCCTTTCCTCTAACAGGGTGTATAGACCTTAGTT
AGAAAAACAGGTAGTCTCTAAATGGGATTGCTCTTTATTGTTAATGAAATGAATACCCA
GGGACTGGGCTTCCCCTCCGC
>817
TTTCCTCTAACAGGGTGTATAGACCTTAGTTAGAAAAACAGGTAGTCTCTAAATGGGAT
TGCTCTTTATTGTTAATGAAATGAATACCCAGGGACTGGGCTTCCCCTCCGCTTGCCCT
GGGTTTGATGTGGTTGTATCCC
>818
AAATGGGATTGCTCTTTATTGTTAATGAAATGAATACCCAGGGACTGGGCTTCCCCTCC
GCTTGCCCTGGGTTTGATGTGGTTGTATCCCGTGCTATCAGAGGAGCCCTTCCTTCACT
CAAGTGTGTTCCCCTGCCCAGC
>819
ATTGCTCTTTATTGTTAATGAAATGAATACCCAGGGACTGGGCTTCCCCTCCGCTTGCCC
TGGGTTTGATGTGGTTGTATCCCGTGCTATCAGAGGAGCCCTTCCTTCACTCAAGTGTG
TTCCCCTGCCCAGCTCTCTCC
>820
TCCCCTCCGCTTGCCCTGGGTTTGATGTGGTTGTATCCCGTGCTATCAGAGGAGCCCTT
CCTTCACTCAAGTGTGTTCCCCTGCCCAGCTCTCTCCGCAGACTCCTGCTGGGCTGAG
CTTTCCCTGCTCTTAAGAGTCAG
>821
CCCTCCGCTTGCCCTGGGTTTGATGTGGTTGTATCCCGTGCTATCAGAGGAGCCCTTCC
TTCACTCAAGTGTGTTCCCCTGCCCAGCTCTCTCCGCAGACTCCTGCTGGGCTGAGCTT

TCCCTGCTCTTAAGAGTCAGGA
>822
CTCAAGTGTGTTCCCCTGCCCAGCTCTCTCCGCAGACTCCTGCTGGGCTGAGCTTTCCC
TGCTCTTAAGAGTCAGGAGTGGCTCTTGCTGGGATGGAATGACCCGTCTTTGGGGCTG
CCTCATGAGCGGCTCTTGTGAAC
>823
GTGTTCCCCTGCCCAGCTCTCTCCGCAGACTCCTGCTGGGCTGAGCTTTCCCTGCTCTT
AAGAGTCAGGAGTGGCTCTTGCTGGGATGGAATGACCCGTCTTTGGGGCTGCCTCATG
AGCGGCTCTTGTGAACCCGGATC
>824
GACTCCTGCTGGGCTGAGCTTTCCCTGCTCTTAAGAGTCAGGAGTGGCTCTTGCTGGG
ATGGAATGACCCGTCTTTGGGGCTGCCTCATGAGCGGCTCTTGTGAACCCGGATCAGT
TCCGATGTGTAAACTCTACCGCCT
>825
TTAAGAGTCAGGAGTGGCTCTTGCTGGGATGGAATGACCCGTCTTTGGGGCTGCCTCA
TGAGCGGCTCTTGTGAACCCGGATCAGTTCCGATGTGTAAACTCTACCGCCTGGCCTTC
AGCGAACAGATACAGATTTCTGC
>826
TGGCTCTTGCTGGGATGGAATGACCCGTCTTTGGGGCTGCCTCATGAGCGGCTCTTGTG
AACCCGGATCAGTTCCGATGTGTAAACTCTACCGCCTGGCCTTCAGCGAACAGATACA
GATTTCTGCCACCTTCCATGACC
>827
TGGGATGGAATGACCCGTCTTTGGGGCTGCCTCATGAGCGGCTCTTGTGAACCCGGAT
CAGTTCCGATGTGTAAACTCTACCGCCTGGCCTTCAGCGAACAGATACAGATTTCTGCC
ACCTTCCATGACCCTACAGTTCA
>828
GATGTGTAAACTCTACCGCCTGGCCTTCAGCGAACAGATACAGATTTCTGCCACCTTCC
ATGACCCTACAGTTCATGGGACTGGGTCTGGGGCAGTGCCAGAGGCACGCATGGAGGT
GTGATTCTAGGTGAGTCCTGCGG
>829
ACCGCCTGGCCTTCAGCGAACAGATACAGATTTCTGCCACCTTCCATGACCCTACAGTT
CATGGGACTGGGTCTGGGGCAGTGCCAGAGGCACGCATGGAGGTGTGATTCTAGGTG
AGTCCTGCGGAAAACCTCTGGCCC
>830
CTTCAGCGAACAGATACAGATTTCTGCCACCTTCCATGACCCTACAGTTCATGGGACTG
GGTCTGGGGCAGTGCCAGAGGCACGCATGGAGGTGTGATTCTAGGTGAGTCCTGCGG
AAAACCTCTGGCCCACCCGTGAGT
>831
CCATGACCCTACAGTTCATGGGACTGGGTCTGGGGCAGTGCCAGAGGCACGCATGGAG
GTGTGATTCTAGGTGAGTCCTGCGGAAAACCTCTGGCCCACCCGTGAGTCACGGACAG
AACATGCAGACTCGGGCCTTGGTG
>832
GACCCTACAGTTCATGGGACTGGGTCTGGGGCAGTGCCAGAGGCACGCATGGAGGTG
TGATTCTAGGTGAGTCCTGCGGAAAACCTCTGGCCCACCCGTGAGTCACGGACAGAAC

ATGCAGACTCGGGCCTTGGTGACAT
>833
GGGGCAGTGCCAGAGGCACGCATGGAGGTGTGATTCTAGGTGAGTCCTGCGGAAAAC
CTCTGGCCCACCCGTGAGTCACGGACAGAACATGCAGACTCGGGCCTTGGTGACATAA
GCTCCGCATTGCTAAAACCGCGTGA
>834
TAGGTGAGTCCTGCGGAAAACCTCTGGCCCACCCGTGAGTCACGGACAGAACATGCA
GACTCGGGCCTTGGTGACATAAGCTCCGCATTGCTAAAACCGCGTGACCTCGAGGGCT
GACTGGCCTGAGAACCCTGGATGGC
>835
ACCCGTGAGTCACGGACAGAACATGCAGACTCGGGCCTTGGTGACATAAGCTCCGCAT
TGCTAAAACCGCGTGACCTCGAGGGCTGACTGGCCTGAGAACCCTGGATGGCGCTCTC
GGCCACCCCCACCTCCCACCCCAA
>836
TGACTGGCCTGAGAACCCTGGATGGCGCTCTCGGCCACCCCCACCTCCCACCCCAACC
TCCTGGGCTTCGGTCAGAATCCACAGCCCGTGCCCGAAGAGCGCTTCCCGCCTCTGGC
ACCCTACCTTCGCTCAGCTCCAGG
>837
CTGGATGGCGCTCTCGGCCACCCCCACCTCCCACCCCAACCTCCTGGGCTTCGGTCAG
AATCCACAGCCCGTGCCCGAAGAGCGCTTCCCGCCTCTGGCACCCTACCTTCGCTCAG
CTCCAGGGAAAAGGGGAGAGGGCA
>838
AGCGCTTCCCGCCTCTGGCACCCTACCTTCGCTCAGCTCCAGGGAAAAGGGGAGAGG
GCAGCTTTCTGCAGTCAGAGGAAGAGTACATTTTCTTTGGCTGCTCTACCCTCTGAAGT
AGGGCGGCCAGCTGAAGGAGGACA
>839
CTCTGGCACCCTACCTTCGCTCAGCTCCAGGGAAAAGGGGAGAGGGCAGCTTTCTGC
AGTCAGAGGAAGAGTACATTTTCTTTGGCTGCTCTACCCTCTGAAGTAGGGCGGCCAG
CTGAAGGAGGACACACTTTTGAGGG
>840
AAAAGGGGAGAGGGCAGCTTTCTGCAGTCAGAGGAAGAGTACATTTTCTTTGGCTGCT
CTACCCTCTGAAGTAGGGCGGCCAGCTGAAGGAGGACACACTTTTGAGGGGCCCAGA
GGTTGTCCAAGCTTCCCCCTGCCCC
>841
TCTTTGGCTGCTCTACCCTCTGAAGTAGGGCGGCCAGCTGAAGGAGGACACACTTTTG
AGGGGCCCAGAGGTTGTCCAAGCTTCCCCCTGCCCCCTGAAGACTGTGCACTGAGCT
GGGCGCAGTTCTCGGGAACTGTTTC
>842
TTGGCTGCTCTACCCTCTGAAGTAGGGCGGCCAGCTGAAGGAGGACACACTTTTGAGG
GGCCCAGAGGTTGTCCAAGCTTCCCCCTGCCCCCTGAAGACTGTGCACTGAGCTGGGC
GCAGTTCTCGGGAACTGTTTCCAC
>843
CCAGCTGAAGGAGGACACACTTTTGAGGGGCCCAGAGGTTGTCCAAGCTTCCCCCTG
CCCCCTGAAGACTGTGCACTGAGCTGGGCGCAGTTCTCGGGAACTGTTTCCACCCAGA

TTGCTGGGGGGCGGGGGGGGGTGGG
>844
CTTTTGAGGGGCCCAGAGGTTGTCCAAGCTTCCCCCTGCCCCCTGAAGACTGTGCACT
GAGCTGGGCGCAGTTCTCGGGAACTGTTTCCACCCAGATTGCTGGGGGGCGGGGGGG
GGTGGGATGAGGGCAGAGCCGAGAG
>845
CAGAGGTTGTCCAAGCTTCCCCCTGCCCCCTGAAGACTGTGCACTGAGCTGGGCGCA
GTTCTCGGGAACTGTTTCCACCCAGATTGCTGGGGGGCGGGGGGGGGTGGGATGAGG
GCAGAGCCGAGAGGCTGTCCAAGGTT
>846
AGACTGTGCACTGAGCTGGGCGCAGTTCTCGGGAACTGTTTCCACCCAGATTGCTGGG
GGGCGGGGGGGGGTGGGATGAGGGCAGAGCCGAGAGGCTGTCCAAGGTTTGGGAGA
GAGAAAAGTTTCTCCCAGGACTCGAC
>847
CTCGGGAACTGTTTCCACCCAGATTGCTGGGGGGCGGGGGGGGGTGGGATGAGGGCA
GAGCCGAGAGGCTGTCCAAGGTTTGGGAGAGAGAAAAGTTTCTCCCAGGACTCGACC
TTGGCCTCCAGCAATCGCGACAGCTA
>848
ACTGTTTCCACCCAGATTGCTGGGGGGCGGGGGGGGGTGGGATGAGGGCAGAGCCGA
GAGGCTGTCCAAGGTTTGGGAGAGAGAAAAGTTTCTCCCAGGACTCGACCTTGGCCT
CCAGCAATCGCGACAGCTAAAAACGG
>849
TGCTGGGGGGCGGGGGGGGGTGGGATGAGGGCAGAGCCGAGAGGCTGTCCAAGGTT
TGGGAGAGAGAAAAGTTTCTCCCAGGACTCGACCTTGGCCTCCAGCAATCGCGACAG
CTAAAAACGGGTGTCTCGCTTCGACAA
>850
GTTTGGGAGAGAGAAAAGTTTCTCCCAGGACTCGACCTTGGCCTCCAGCAATCGCGA
CAGCTAAAAACGGGTGTCTCGCTTCGACAATAGATCCCCGCGGACCTTCTGGCACCTG
GTTCACTAGCGCCCGCGAACTCTGC
>851
TTGGGAGAGAGAAAAGTTTCTCCCAGGACTCGACCTTGGCCTCCAGCAATCGCGACA
GCTAAAAACGGGTGTCTCGCTTCGACAATAGATCCCCGCGGACCTTCTGGCACCTGGT
TCACTAGCGCCCGCGAACTCTGCCT
>852
CAGCAATCGCGACAGCTAAAAACGGGTGTCTCGCTTCGACAATAGATCCCCGCGGACC
TTCTGGCACCTGGTTCACTAGCGCCCGCGAACTCTGCCTCGGGAGACTTATTGAAATCC
GGATGCTCAAGCCGGGAGGCGCG
>853
TCACTAGCGCCCGCGAACTCTGCCTCGGGAGACTTATTGAAATCCGGATGCTCAAGCC
GGGAGGCGCGCAGTAACCAGGAGGATGAGAGGGCCGGGTTTGGGCTAGGAAAGCGG
CCTTTTAAAACAGATGTCAGGGGGAC
>854
CGGGAGACTTATTGAAATCCGGATGCTCAAGCCGGGAGGCGCGCAGTAACCAGGAGG
ATGAGAGGGCCGGGTTTGGGCTAGGAAAGCGGCCTTTTAAAACAGATGTCAGGGGGA

CTGCAGCCCCGAGCCATGAGAAAAAA
>855
GGAGGCGCGCAGTAACCAGGAGGATGAGAGGGCCGGGTTTGGGCTAGGAAAGCGGC
CTTTTAAAACAGATGTCAGGGGGACTGCAGCCCCGAGCCATGAGAAAAAAGTTAAAG
GCGAGATGACACGCACTGAATTGGGGC
>856
GTTTGGGCTAGGAAAGCGGCCTTTTAAAACAGATGTCAGGGGGACTGCAGCCCCGAG
CCATGAGAAAAAAGTTAAAGGCGAGATGACACGCACTGAATTGGGGCAAACATTGGA
AGAGGAGACAAAACTGCGTGCTTGAG
>857
GAGAAAAAAGTTAAAGGCGAGATGACACGCACTGAATTGGGGCAAACATTGGAAGAG
GAGACAAAACTGCGTGCTTGAGCACCGGGGTGCGGGGAGGGGGGGACAAAACCCGT
ATCCAGTGCAAATTAAAATCTTGGGAG
>858
GCGAGATGACACGCACTGAATTGGGGCAAACATTGGAAGAGGAGACAAAACTGCGTG
CTTGAGCACCGGGGTGCGGGGAGGGGGGGACAAAACCCGTATCCAGTGCAAATTAAA
ATCTTGGGAGTAGGTGGGGGCTGCTG
>859
GCAAACATTGGAAGAGGAGACAAAACTGCGTGCTTGAGCACCGGGGTGCGGGGAGG
GGGGGACAAAACCCGTATCCAGTGCAAATTAAAATCTTGGGAGTAGGTGGGGGCTGCT
GCGCGCCCTTCACCCTCAGTTCCCCT
>860
CGTGCTTGAGCACCGGGGTGCGGGGAGGGGGGGACAAAACCCGTATCCAGTGCAAAT
TAAAATCTTGGGAGTAGGTGGGGGCTGCTGCGCGCCCTTCACCCTCAGTTCCCCTATTA
AGGATTCTGAGTCCCCATGCACTC
>861
CTTGAGCACCGGGGTGCGGGGAGGGGGGGACAAAACCCGTATCCAGTGCAAATTAAA
ATCTTGGGAGTAGGTGGGGGCTGCTGCGCGCCCTTCACCCTCAGTTCCCCTATTAAGGA
TTCTGAGTCCCCATGCACTCCTGT
>862
CAAAACCCGTATCCAGTGCAAATTAAAATCTTGGGAGTAGGTGGGGGCTGCTGCGCGC
CCTTCACCCTCAGTTCCCCTATTAAGGATTCTGAGTCCCCATGCACTCCTGTCCTCTGGC
TCCTTCCTTCCTCTCCGCTCGG
>863
TTAAAATCTTGGGAGTAGGTGGGGGCTGCTGCGCGCCCTTCACCCTCAGTTCCCCTATT
AAGGATTCTGAGTCCCCATGCACTCCTGTCCTCTGGCTCCTTCCTTCCTCTCCGCTCGG
CCGGTGAGAGGCGGCCGCCGGC
>864
AGGTGGGGGCTGCTGCGCGCCCTTCACCCTCAGTTCCCCTATTAAGGATTCTGAGTCCC
CATGCACTCCTGTCCTCTGGCTCCTTCCTTCCTCTCCGCTCGGCCGGTGAGAGGCGGCC
GCCGGCGCCCCAGCAGCAGCTA
>865
CCCCTATTAAGGATTCTGAGTCCCCATGCACTCCTGTCCTCTGGCTCCTTCCTTCCTCTC
CGCTCGGCCGGTGAGAGGCGGCCGCCGGCGCCCCAGCAGCAGCTAGATGTCAGGCGA

AGCCCGGAGCGCGGAGCGCGGGG
>866
CTCTGGCTCCTTCCTTCCTCTCCGCTCGGCCGGTGAGAGGCGGCCGCCGGCGCCCCAG
CAGCAGCTAGATGTCAGGCGAAGCCCGGAGCGCGGAGCGCGGGGAAGGGAGGGGAA
GGGAGGGGAGGGGAAAGGAGGGGTGG
>867
AGATGTCAGGCGAAGCCCGGAGCGCGGAGCGCGGGGAAGGGAGGGGAAGGGAGGG
GAGGGGAAAGGAGGGGTGGGGCGGGGAGCGCGCGCGGGCTCGGCCAGTGCGGGGTG
GTGGGCGGGCTGAGCCCGGGGACGGCGCG
>868
CGGGGAAGGGAGGGGAAGGGAGGGGAGGGGAAAGGAGGGGTGGGGCGGGGAGCGC
GCGCGGGCTCGGCCAGTGCGGGGTGGTGGGCGGGCTGAGCCCGGGGACGGCGCGGG
AGGGGGGAGGGAAGGGGGGCAGCTGTGGG
>869
GGGAGGGGAAGGGAGGGGAGGGGAAAGGAGGGGTGGGGCGGGGAGCGCGCGCGGG
CTCGGCCAGTGCGGGGTGGTGGGCGGGCTGAGCCCGGGGACGGCGCGGGAGGGGGG
AGGGAAGGGGGGCAGCTGTGGGCAGGGAG
>870
GGCCAGTGCGGGGTGGTGGGCGGGCTGAGCCCGGGGACGGCGCGGGAGGGGGGAGG
GAAGGGGGGCAGCTGTGGGCAGGGAGCCGGGCTCGGCCGCCAGCACTAAAGATGGA
GAGGCGCCGGGGCCTTGCAGGGGAGGGG
>871
GGGAGCCGGGCTCGGCCGCCAGCACTAAAGATGGAGAGGCGCCGGGGCCTTGCAGGG
GAGGGGGCTCGGCGTTGACGTGGGACGCGGCGGAGGCGCAGCAGCCGGTGGTGATTT
GCTAACCTCGCAGCAGAGAGGAGTTG
>872
CGGGCTCGGCCGCCAGCACTAAAGATGGAGAGGCGCCGGGGCCTTGCAGGGGAGGG
GGCTCGGCGTTGACGTGGGACGCGGCGGAGGCGCAGCAGCCGGTGGTGATTTGCTAA
CCTCGCAGCAGAGAGGAGTTGAGGGCG
>873
AGAGGCGCCGGGGCCTTGCAGGGGAGGGGGCTCGGCGTTGACGTGGGACGCGGCGG
AGGCGCAGCAGCCGGTGGTGATTTGCTAACCTCGCAGCAGAGAGGAGTTGAGGGCGA
TGAGAGCGGGTACTGCGAACTGCCGGG
>874
GGCGCCGGGGCCTTGCAGGGGAGGGGGCTCGGCGTTGACGTGGGACGCGGCGGAGG
CGCAGCAGCCGGTGGTGATTTGCTAACCTCGCAGCAGAGAGGAGTTGAGGGCGATGA
GAGCGGGTACTGCGAACTGCCGGGCGA
>875
CGGCGTTGACGTGGGACGCGGCGGAGGCGCAGCAGCCGGTGGTGATTTGCTAACCTC
GCAGCAGAGAGGAGTTGAGGGCGATGAGAGCGGGTACTGCGAACTGCCGGGCGATGC
TGTCGCTGCCGCCGTGATACGGAGAG
>876
GCGGAGGCGCAGCAGCCGGTGGTGATTTGCTAACCTCGCAGCAGAGAGGAGTTGAGG
GCGATGAGAGCGGGTACTGCGAACTGCCGGGCGATGCTGTCGCTGCCGCCGTGATACG

GAGAGCAACAGTTCCCCAGCAACAC
>877
TTTGCTAACCTCGCAGCAGAGAGGAGTTGAGGGCGATGAGAGCGGGTACTGCGAACT
GCCGGGCGATGCTGTCGCTGCCGCCGTGATACGGAGAGCAACAGTTCCCCAGCAACA
CCCCTCCCCGACACAGGCACACACCC
>878
GCAGCAGAGAGGAGTTGAGGGCGATGAGAGCGGGTACTGCGAACTGCCGGGCGATGC
TGTCGCTGCCGCCGTGATACGGAGAGCAACAGTTCCCCAGCAACACCCCTCCCCGACA
CAGGCACACACCCCCCGACAGGCAC
>879
CAGTTCCCCAGCAACACCCCTCCCCGACACAGGCACACACCCCCCGACAGGCACGCA
CACCCACCCCACAGTGCCCGGCTCGGCTGCGGTGAGTGAGGGGCCGGGAAGAGGCGC
ACCGCCCAGCGAGCGCCGCGCGCCGG
>880
CCTCCCCGACACAGGCACACACCCCCCGACAGGCACGCACACCCACCCCACAGTGCC
CGGCTCGGCTGCGGTGAGTGAGGGGCCGGGAAGAGGCGCACCGCCCAGCGAGCGCC
GCGCGCCGGGGCTTCCCGGGGCTGGAG
>881
GAGTGAGGGGCCGGGAAGAGGCGCACCGCCCAGCGAGCGCCGCGCGCCGGGGCTTC
CCGGGGCTGGAGAGGTCTGGGGGGCGCGCGCTCGCTTCGGCCACTCGGCCGCTGGGC
TTGTGCCTTTTTTATTTGGCGTGTTCC
>882
GCCGGGGCTTCCCGGGGCTGGAGAGGTCTGGGGGGCGCGCGCTCGCTTCGGCCACTC
GGCCGCTGGGCTTGTGCCTTTTTTATTTGGCGTGTTCCCTTCCTGCCGCTGCAGCCGTC
GCCGCCACCGGTTGGGGGTCGGCT
>883
TGGAGAGGTCTGGGGGGCGCGCGCTCGCTTCGGCCACTCGGCCGCTGGGCTTGTGCC
TTTTTTATTTGGCGTGTTCCCTTCCTGCCGCTGCAGCCGTCGCCGCCACCGGTTGGGGG
TCGGCTAGAAGGGGGAGCCCCGGC
>884
CGCTTCGGCCACTCGGCCGCTGGGCTTGTGCCTTTTTTATTTGGCGTGTTCCCTTCCTG
CCGCTGCAGCCGTCGCCGCCACCGGTTGGGGGTCGGCTAGAAGGGGGAGCCCCGGCT
GTCAGCCTGGGCGCAGCTGCCTCC
>885
CGGCCGCTGGGCTTGTGCCTTTTTTATTTGGCGTGTTCCCTTCCTGCCGCTGCAGCCGT
CGCCGCCACCGGTTGGGGGTCGGCTAGAAGGGGGAGCCCCGGCTGTCAGCCTGGGCG
CAGCTGCCTCCCCAGCCCTTCCTC
>886
TGGGCTTGTGCCTTTTTTATTTGGCGTGTTCCCTTCCTGCCGCTGCAGCCGTCGCCGCC
ACCGGTTGGGGGTCGGCTAGAAGGGGGAGCCCCGGCTGTCAGCCTGGGCGCAGCTGC
CTCCCCAGCCCTTCCTCTTCAGGG
>887
TGTTCCCTTCCTGCCGCTGCAGCCGTCGCCGCCACCGGTTGGGGGTCGGCTAGAAGGG
GGAGCCCCGGCTGTCAGCCTGGGCGCAGCTGCCTCCCCAGCCCTTCCTCTTCAGGGCA

CGGTCGGGGTGAGAGGTGGGGGGC
>888
GCTAGAAGGGGGAGCCCCGGCTGTCAGCCTGGGCGCAGCTGCCTCCCCAGCCCTTCCT
CTTCAGGGCACGGTCGGGGTGAGAGGTGGGGGGCGTAGCGGTGTGCGGGGGCTGAG
GGCGTGAGCAGAGGGGTCGGGGATCC
>889
AGGGGGAGCCCCGGCTGTCAGCCTGGGCGCAGCTGCCTCCCCAGCCCTTCCTCTTCAG
GGCACGGTCGGGGTGAGAGGTGGGGGGCGTAGCGGTGTGCGGGGGCTGAGGGCGTG
AGCAGAGGGGTCGGGGATCCGGAGGC
>890
GTCAGCCTGGGCGCAGCTGCCTCCCCAGCCCTTCCTCTTCAGGGCACGGTCGGGGTGA
GAGGTGGGGGGCGTAGCGGTGTGCGGGGGCTGAGGGCGTGAGCAGAGGGGTCGGGG
ATCCGGAGGCTTTGTACCGCCAGGGG
>891
CTGGGCGCAGCTGCCTCCCCAGCCCTTCCTCTTCAGGGCACGGTCGGGGTGAGAGGT
GGGGGGCGTAGCGGTGTGCGGGGGCTGAGGGCGTGAGCAGAGGGGTCGGGGATCCG
GAGGCTTTGTACCGCCAGGGGCTGGCG
>892
CAGCCCTTCCTCTTCAGGGCACGGTCGGGGTGAGAGGTGGGGGGCGTAGCGGTGTGC
GGGGGCTGAGGGCGTGAGCAGAGGGGTCGGGGATCCGGAGGCTTTGTACCGCCAGGG
GCTGGCGGAGCAACAGAGCCCGTGGG
>893
TTCAGGGCACGGTCGGGGTGAGAGGTGGGGGGCGTAGCGGTGTGCGGGGGCTGAGG
GCGTGAGCAGAGGGGTCGGGGATCCGGAGGCTTTGTACCGCCAGGGGCTGGCGGAGC
AACAGAGCCCGTGGGTGCTCTTATGTA
>894
AGGGCGTGAGCAGAGGGGTCGGGGATCCGGAGGCTTTGTACCGCCAGGGGCTGGCGG
AGCAACAGAGCCCGTGGGTGCTCTTATGTATGCGGACCGGTGCGCGGGCGCAAGATAA
GGTTGTGGTTTATTTATTTGTGTGT
>895
CGTGAGCAGAGGGGTCGGGGATCCGGAGGCTTTGTACCGCCAGGGGCTGGCGGAGCA
ACAGAGCCCGTGGGTGCTCTTATGTATGCGGACCGGTGCGCGGGCGCAAGATAAGGTT
GTGGTTTATTTATTTGTGTGTTTAT
>896
GGGGCTGGCGGAGCAACAGAGCCCGTGGGTGCTCTTATGTATGCGGACCGGTGCGCG
GGCGCAAGATAAGGTTGTGGTTTATTTATTTGTGTGTTTATTCGCCGGCCGGCTGGGAA
GCTAGAATCGGAGGAGCTGACGAG
>897
GCTGGCGGAGCAACAGAGCCCGTGGGTGCTCTTATGTATGCGGACCGGTGCGCGGGC
GCAAGATAAGGTTGTGGTTTATTTATTTGTGTGTTTATTCGCCGGCCGGCTGGGAAGCT
AGAATCGGAGGAGCTGACGAGTAG
>898
CCGTGGGTGCTCTTATGTATGCGGACCGGTGCGCGGGCGCAAGATAAGGTTGTGGTTTA
TTTATTTGTGTGTTTATTCGCCGGCCGGCTGGGAAGCTAGAATCGGAGGAGCTGACGA

GTAGATCTGGGGGCGGAGGGGAG
>899
GGTTGTGGTTTATTTATTTGTGTGTTTATTCGCCGGCCGGCTGGGAAGCTAGAATCGGA
GGAGCTGACGAGTAGATCTGGGGGCGGAGGGGAGCAGGACTGGGACTGCTTACGTTT
TGTTTCTCTTTGAGAAAACGTGGT
>900
GGGAAGCTAGAATCGGAGGAGCTGACGAGTAGATCTGGGGGCGGAGGGGAGCAGGA
CTGGGACTGCTTACGTTTTGTTTCTCTTTGAGAAAACGTGGTGGGCTTTTTCTTGATTG
GACTTGATCCCCACCCCCCTTTTGC
>901
GCTAGAATCGGAGGAGCTGACGAGTAGATCTGGGGGCGGAGGGGAGCAGGACTGGG
ACTGCTTACGTTTTGTTTCTCTTTGAGAAAACGTGGTGGGCTTTTTCTTGATTGGACTT
GATCCCCACCCCCCTTTTGCAGGGG
>902
GACGAGTAGATCTGGGGGCGGAGGGGAGCAGGACTGGGACTGCTTACGTTTTGTTTCT
CTTTGAGAAAACGTGGTGGGCTTTTTCTTGATTGGACTTGATCCCCACCCCCCTTTTGC
AGGGGAGGGAGGGAAGCTCCAGA
>903
GGTGGGCTTTTTCTTGATTGGACTTGATCCCCACCCCCCTTTTGCAGGGGAGGGAGGG
AAGCTCCAGAGGGTCTGCAGCGCTGCGGGCCCTCCTCGGCTCTCGGCGGGACCGGCG
GTGACACCGGAGCTCGCCGTGCGCT
>904
AGGGGAGGGAGGGAAGCTCCAGAGGGTCTGCAGCGCTGCGGGCCCTCCTCGGCTCTC
GGCGGGACCGGCGGTGACACCGGAGCTCGCCGTGCGCTCCCGGCCGCTCTCGGTGGG
TGCCGGTCTCTGCACCTGATGCGTTC
>905
CCAGAGGGTCTGCAGCGCTGCGGGCCCTCCTCGGCTCTCGGCGGGACCGGCGGTGAC
ACCGGAGCTCGCCGTGCGCTCCCGGCCGCTCTCGGTGGGTGCCGGTCTCTGCACCTGA
TGCGTTCGGGATGCCTTTCCCACCC
>906
GGGCCCTCCTCGGCTCTCGGCGGGACCGGCGGTGACACCGGAGCTCGCCGTGCGCTC
CCGGCCGCTCTCGGTGGGTGCCGGTCTCTGCACCTGATGCGTTCGGGATGCCTTTCCC
ACCCTGGCGCGCCCGCCGCTAGCTC
>907
CCTCCTCGGCTCTCGGCGGGACCGGCGGTGACACCGGAGCTCGCCGTGCGCTCCCGG
CCGCTCTCGGTGGGTGCCGGTCTCTGCACCTGATGCGTTCGGGATGCCTTTCCCACCCT
GGCGCGCCCGCCGCTAGCTCGCAC
>908
GGCGGTGACACCGGAGCTCGCCGTGCGCTCCCGGCCGCTCTCGGTGGGTGCCGGTCT
CTGCACCTGATGCGTTCGGGATGCCTTTCCCACCCTGGCGCGCCCGCCGCTAGCTCGC
ACAGCGCCTCGCACACTCCCGCACG
>909
CACCCTGGCGCGCCCGCCGCTAGCTCGCACAGCGCCTCGCACACTCCCGCACGCGCTT
GAAATGCGCACGGTCCCGCCGGCCCGCGGAACCACCCGGACGCACGGAGCGCTCCGC

ACCGACTCGCTCGCCGCCTCCCCGA
>910
CGCGGAACCACCCGGACGCACGGAGCGCTCCGCACCGACTCGCTCGCCGCCTCCCCG
AGACGCTCGCACCGTGCTTGGGCCGGGCGCGCTGGCCGCTGGCGCCGCTGGCCAGAG
GCCTGGGACCCAGCCGGTCGCTCCCA
>911
CGCTCGCACCGTGCTTGGGCCGGGCGCGCTGGCCGCTGGCGCCGCTGGCCAGAGGCC
TGGGACCCAGCCGGTCGCTCCCAGGGGGTCACGGCCCTGGGTCGGAGAGGGAGGGC
GGGCAGACCCCTTCTCGCCTTTCCTCC
>912
TTGGGCCGGGCGCGCTGGCCGCTGGCGCCGCTGGCCAGAGGCCTGGGACCCAGCCGG
TCGCTCCCAGGGGGTCACGGCCCTGGGTCGGAGAGGGAGGGCGGGCAGACCCCTTCT
CGCCTTTCCTCCCACAACTCGCTGCG
>913
CCTGGGTCGGAGAGGGAGGGCGGGCAGACCCCTTCTCGCCTTTCCTCCCACAACTCG
CTGCGGGGCTTTTGTGCTTCCCCTTCGCCGCGGGGCGGGTCCGCCTCCCCTGCCGCTC
TCGCCGCGGAGTCCAGCCCGCCCGG
>914
AGACCCCTTCTCGCCTTTCCTCCCACAACTCGCTGCGGGGCTTTTGTGCTTCCCCTTCG
CCGCGGGGCGGGTCCGCCTCCCCTGCCGCTCTCGCCGCGGAGTCCAGCCCGCCCGGA
CTGTCGCCGTTCCTCCCCGTCTCT
>915
CGCTGCGGGGCTTTTGTGCTTCCCCTTCGCCGCGGGGCGGGTCCGCCTCCCCTGCCGC
TCTCGCCGCGGAGTCCAGCCCGCCCGGACTGTCGCCGTTCCTCCCCGTCTCTTTCGCTT
TCCCTCGTCCCTAGCTCAGCTCT
>916
CTTCCCCTTCGCCGCGGGGCGGGTCCGCCTCCCCTGCCGCTCTCGCCGCGGAGTCCAG
CCCGCCCGGACTGTCGCCGTTCCTCCCCGTCTCTTTCGCTTTCCCTCGTCCCTAGCTCA
GCTCTCCTTCTTTCAGGAGTCTA
>917
CTTCGCCGCGGGGCGGGTCCGCCTCCCCTGCCGCTCTCGCCGCGGAGTCCAGCCCGCC
CGGACTGTCGCCGTTCCTCCCCGTCTCTTTCGCTTTCCCTCGTCCCTAGCTCAGCTCTC
CTTCTTTCAGGAGTCTAGCTCCT
>918
GGGCGGGTCCGCCTCCCCTGCCGCTCTCGCCGCGGAGTCCAGCCCGCCCGGACTGTCG
CCGTTCCTCCCCGTCTCTTTCGCTTTCCCTCGTCCCTAGCTCAGCTCTCCTTCTTTCAGG
AGTCTAGCTCCTCGGGAAAAGT
>919
GGACTGTCGCCGTTCCTCCCCGTCTCTTTCGCTTTCCCTCGTCCCTAGCTCAGCTCTCCT
TCTTTCAGGAGTCTAGCTCCTCGGGAAAAGTTGCTTCCCCAAGTTTGCTGAAGTCGTC
TCCAAGTCTCGGTGGGGGTCGC
>920
CCGTCTCTTTCGCTTTCCCTCGTCCCTAGCTCAGCTCTCCTTCTTTCAGGAGTCTAGCTC
CTCGGGAAAAGTTGCTTCCCCAAGTTTGCTGAAGTCGTCTCCAAGTCTCGGTGGGGGT

CGCTGGGAACTGGGGGGGTGTG
>921
CCCTAGCTCAGCTCTCCTTCTTTCAGGAGTCTAGCTCCTCGGGAAAAGTTGCTTCCCCA
AGTTTGCTGAAGTCGTCTCCAAGTCTCGGTGGGGGTCGCTGGGAACTGGGGGGGTGT
GAGAGCGCGGTCGATCCCCGGAGC
>922
CTCTCCTTCTTTCAGGAGTCTAGCTCCTCGGGAAAAGTTGCTTCCCCAAGTTTGCTGAA
GTCGTCTCCAAGTCTCGGTGGGGGTCGCTGGGAACTGGGGGGGTGTGAGAGCGCGGT
CGATCCCCGGAGCTCGGGCGGGTT
>923
TTCTTTCAGGAGTCTAGCTCCTCGGGAAAAGTTGCTTCCCCAAGTTTGCTGAAGTCGT
CTCCAAGTCTCGGTGGGGGTCGCTGGGAACTGGGGGGGTGTGAGAGCGCGGTCGATC
CCCGGAGCTCGGGCGGGTTATCGCC
>924
GAAAAGTTGCTTCCCCAAGTTTGCTGAAGTCGTCTCCAAGTCTCGGTGGGGGTCGCTG
GGAACTGGGGGGGTGTGAGAGCGCGGTCGATCCCCGGAGCTCGGGCGGGTTATCGCC
GGCTCTTCCCCGCGCCGCCTGGACT
>925
CAAGTCTCGGTGGGGGTCGCTGGGAACTGGGGGGGTGTGAGAGCGCGGTCGATCCCC
GGAGCTCGGGCGGGTTATCGCCGGCTCTTCCCCGCGCCGCCTGGACTGGACGCTGCAG
CCAGCGCCAGCCGGATCGCGGGCGC
>926
GCTCTTCCCCGCGCCGCCTGGACTGGACGCTGCAGCCAGCGCCAGCCGGATCGCGGG
CGCCGAGCGGCGGGTGGGGGAGACGGCTGCCCCGTGGGCGCCCAGATTTCCCTCAGG
ACCCAAAAAGCTTGTTTCAGGGCGCC
>927
GACTGGACGCTGCAGCCAGCGCCAGCCGGATCGCGGGCGCCGAGCGGCGGGTGGGG
GAGACGGCTGCCCCGTGGGCGCCCAGATTTCCCTCAGGACCCAAAAAGCTTGTTTCAG
GGCGCCAAGCTTCCATTCTTTTGGCT
>928
CGCCGAGCGGCGGGTGGGGGAGACGGCTGCCCCGTGGGCGCCCAGATTTCCCTCAGG
ACCCAAAAAGCTTGTTTCAGGGCGCCAAGCTTCCATTCTTTTGGCTGTTCCCTCCCTAG
TTCTCAGAAACCCCGATGTCTGCG
>929
GTGGGCGCCCAGATTTCCCTCAGGACCCAAAAAGCTTGTTTCAGGGCGCCAAGCTTCC
ATTCTTTTGGCTGTTCCCTCCCTAGTTCTCAGAAACCCCGATGTCTGCGGGAAAGCACG
CGGTGGCTCGGCTCGGCTGAATG
>930
ATTTCCCTCAGGACCCAAAAAGCTTGTTTCAGGGCGCCAAGCTTCCATTCTTTTGGCTG
TTCCCTCCCTAGTTCTCAGAAACCCCGATGTCTGCGGGAAAGCACGCGGTGGCTCGGC
TCGGCTGAATGGTCATCCCAACC
>931
AAAGCTTGTTTCAGGGCGCCAAGCTTCCATTCTTTTGGCTGTTCCCTCCCTAGTTCTCA
GAAACCCCGATGTCTGCGGGAAAGCACGCGGTGGCTCGGCTCGGCTGAATGGTCATCC

CAACCTTGACGGCCTCCGTCCCC
>932
GTTCCCTCCCTAGTTCTCAGAAACCCCGATGTCTGCGGGAAAGCACGCGGTGGCTCGG
CTCGGCTGAATGGTCATCCCAACCTTGACGGCCTCCGTCCCCACCCCGGGGCGGGCAG
CAAAAATGTCTTGACCAGCCGTCC
>933
CCCGATGTCTGCGGGAAAGCACGCGGTGGCTCGGCTCGGCTGAATGGTCATCCCAACC
TTGACGGCCTCCGTCCCCACCCCGGGGCGGGCAGCAAAAATGTCTTGACCAGCCGTCC
TGTGAATCCTTTCTGCGTGAGCTT
>934
CTCGGCTCGGCTGAATGGTCATCCCAACCTTGACGGCCTCCGTCCCCACCCCGGGGCG
GGCAGCAAAAATGTCTTGACCAGCCGTCCTGTGAATCCTTTCTGCGTGAGCTTGGTGG
CTCCTGAACTGATAGAGCTCCTTC
>935
AATGGTCATCCCAACCTTGACGGCCTCCGTCCCCACCCCGGGGCGGGCAGCAAAAATG
TCTTGACCAGCCGTCCTGTGAATCCTTTCTGCGTGAGCTTGGTGGCTCCTGAACTGATA
GAGCTCCTTCTGGGGGTGGAGGC
>936
CGGCCTCCGTCCCCACCCCGGGGCGGGCAGCAAAAATGTCTTGACCAGCCGTCCTGTG
AATCCTTTCTGCGTGAGCTTGGTGGCTCCTGAACTGATAGAGCTCCTTCTGGGGGTGG
AGGCAAGCAAGCAGTGTTCGAAGA
>937
GACCAGCCGTCCTGTGAATCCTTTCTGCGTGAGCTTGGTGGCTCCTGAACTGATAGAG
CTCCTTCTGGGGGTGGAGGCAAGCAAGCAGTGTTCGAAGAGTGAGGGAGAGACCTTG
CTGTTCCTTGCTCTCAGTGCATTCA
>938
ATCCTTTCTGCGTGAGCTTGGTGGCTCCTGAACTGATAGAGCTCCTTCTGGGGGTGGA
GGCAAGCAAGCAGTGTTCGAAGAGTGAGGGAGAGACCTTGCTGTTCCTTGCTCTCAG
TGCATTCACTGCAATATTGGTCGCT
>939
AACTGATAGAGCTCCTTCTGGGGGTGGAGGCAAGCAAGCAGTGTTCGAAGAGTGAGG
GAGAGACCTTGCTGTTCCTTGCTCTCAGTGCATTCACTGCAATATTGGTCGCTGTAAGA
CAGACACGGGGAGGGTCCTGGGCC
>940
CTTCTGGGGGTGGAGGCAAGCAAGCAGTGTTCGAAGAGTGAGGGAGAGACCTTGCTG
TTCCTTGCTCTCAGTGCATTCACTGCAATATTGGTCGCTGTAAGACAGACACGGGGAG
GGTCCTGGGCCTGCGCGGTCTTTCT
>941
CAAGCAGTGTTCGAAGAGTGAGGGAGAGACCTTGCTGTTCCTTGCTCTCAGTGCATTC
ACTGCAATATTGGTCGCTGTAAGACAGACACGGGGAGGGTCCTGGGCCTGCGCGGTCT
TTCTAACACTCCTTTAAGCGAGGT
>942
AGACCTTGCTGTTCCTTGCTCTCAGTGCATTCACTGCAATATTGGTCGCTGTAAGACAG
ACACGGGGAGGGTCCTGGGCCTGCGCGGTCTTTCTAACACTCCTTTAAGCGAGGTTTG

GAGTCTAGGAGCCCTCTTATTGA
>943
TGCTCTCAGTGCATTCACTGCAATATTGGTCGCTGTAAGACAGACACGGGGAGGGTCC
TGGGCCTGCGCGGTCTTTCTAACACTCCTTTAAGCGAGGTTTGGAGTCTAGGAGCCCT
CTTATTGATCCCGGACAGGCTCTG
>944
TGGTCGCTGTAAGACAGACACGGGGAGGGTCCTGGGCCTGCGCGGTCTTTCTAACACT
CCTTTAAGCGAGGTTTGGAGTCTAGGAGCCCTCTTATTGATCCCGGACAGGCTCTGGG
AGAGGCCTTTGTTTCCCAGCCCTG
>945
CTCCTTTAAGCGAGGTTTGGAGTCTAGGAGCCCTCTTATTGATCCCGGACAGGCTCTGG
GAGAGGCCTTTGTTTCCCAGCCCTGTCTTCCCTTCGTACTGGTTGTAACTCACACCACA
GGTGCTGGTGCCAATATCACTT
>946
TAGGAGCCCTCTTATTGATCCCGGACAGGCTCTGGGAGAGGCCTTTGTTTCCCAGCCCT
GTCTTCCCTTCGTACTGGTTGTAACTCACACCACAGGTGCTGGTGCCAATATCACTTCC
AGCGCCTCTGAGCCATGTCCAG
>947
CCCTCTTATTGATCCCGGACAGGCTCTGGGAGAGGCCTTTGTTTCCCAGCCCTGTCTTC
CCTTCGTACTGGTTGTAACTCACACCACAGGTGCTGGTGCCAATATCACTTCCAGCGCC
TCTGAGCCATGTCCAGCATCCC
>948
CAGGCTCTGGGAGAGGCCTTTGTTTCCCAGCCCTGTCTTCCCTTCGTACTGGTTGTAAC
TCACACCACAGGTGCTGGTGCCAATATCACTTCCAGCGCCTCTGAGCCATGTCCAGCAT
CCCTTGCTGCCCCTGGTCACAG
>949
CCCTTCGTACTGGTTGTAACTCACACCACAGGTGCTGGTGCCAATATCACTTCCAGCGC
CTCTGAGCCATGTCCAGCATCCCTTGCTGCCCCTGGTCACAGGACTTGCAGACTCCTTG
GAGCCTGTGGGACACACGTGGA
>950
ACCACAGGTGCTGGTGCCAATATCACTTCCAGCGCCTCTGAGCCATGTCCAGCATCCCT
TGCTGCCCCTGGTCACAGGACTTGCAGACTCCTTGGAGCCTGTGGGACACACGTGGA
GTTCACACACCCAGCCCAGCATGA
>951
AGCGCCTCTGAGCCATGTCCAGCATCCCTTGCTGCCCCTGGTCACAGGACTTGCAGAC
TCCTTGGAGCCTGTGGGACACACGTGGAGTTCACACACCCAGCCCAGCATGAGCACT
CCTGGCTGTAGGGGACCATATTTAA
>952
GCCATGTCCAGCATCCCTTGCTGCCCCTGGTCACAGGACTTGCAGACTCCTTGGAGCC
TGTGGGACACACGTGGAGTTCACACACCCAGCCCAGCATGAGCACTCCTGGCTGTAG
GGGACCATATTTAACTATGGTCTCT
>953
GCAGACTCCTTGGAGCCTGTGGGACACACGTGGAGTTCACACACCCAGCCCAGCATG
AGCACTCCTGGCTGTAGGGGACCATATTTAACTATGGTCTCTCTCCATACCTTAATGACC

CATCCTTCTTCGTTTTTTAGATC
>954
ACACACGTGGAGTTCACACACCCAGCCCAGCATGAGCACTCCTGGCTGTAGGGGACC
ATATTTAACTATGGTCTCTCTCCATACCTTAATGACCCATCCTTCTTCGTTTTTTAGATCC
TCCTGTCCACCGGGCAGTTGTG
>955
GCACTCCTGGCTGTAGGGGACCATATTTAACTATGGTCTCTCTCCATACCTTAATGACCC
ATCCTTCTTCGTTTTTTAGATCCTCCTGTCCACCGGGCAGTTGTGGGCAGGAGGGTAGG
TACGACACCTTACGTAAGTGT
>956
GGACCATATTTAACTATGGTCTCTCTCCATACCTTAATGACCCATCCTTCTTCGTTTTTTA
GATCCTCCTGTCCACCGGGCAGTTGTGGGCAGGAGGGTAGGTACGACACCTTACGTAA
GTGTGTGTGTGTAAACACACA
>957
ACTATGGTCTCTCTCCATACCTTAATGACCCATCCTTCTTCGTTTTTTAGATCCTCCTGTC
CACCGGGCAGTTGTGGGCAGGAGGGTAGGTACGACACCTTACGTAAGTGTGTGTGTGT
AAACACACACACACTTTTTTT
>958
CCTTAATGACCCATCCTTCTTCGTTTTTTAGATCCTCCTGTCCACCGGGCAGTTGTGGGC
AGGAGGGTAGGTACGACACCTTACGTAAGTGTGTGTGTGTAAACACACACACACTTTT
TTTTTTTTTTTCTGAGACGGAG
>959
CCTTCTTCGTTTTTTAGATCCTCCTGTCCACCGGGCAGTTGTGGGCAGGAGGGTAGGTA
CGACACCTTACGTAAGTGTGTGTGTGTAAACACACACACACTTTTTTTTTTTTTTTCTG
AGACGGAGTTTTGCTCCTGTTG
>960
CTTCGTTTTTTAGATCCTCCTGTCCACCGGGCAGTTGTGGGCAGGAGGGTAGGTACGA
CACCTTACGTAAGTGTGTGTGTGTAAACACACACACACTTTTTTTTTTTTTTTCTGAGA
CGGAGTTTTGCTCCTGTTGCCCA
>961
TCGTTTTTTAGATCCTCCTGTCCACCGGGCAGTTGTGGGCAGGAGGGTAGGTACGACA
CCTTACGTAAGTGTGTGTGTGTAAACACACACACACTTTTTTTTTTTTTTTCTGAGACG
GAGTTTTGCTCCTGTTGCCCAGG
>962
TAGATCCTCCTGTCCACCGGGCAGTTGTGGGCAGGAGGGTAGGTACGACACCTTACGT
AAGTGTGTGTGTGTAAACACACACACACTTTTTTTTTTTTTTTCTGAGACGGAGTTTTG
CTCCTGTTGCCCAGGCTGGAGTG
>963
GGTACGACACCTTACGTAAGTGTGTGTGTGTAAACACACACACACTTTTTTTTTTTTTT
TCTGAGACGGAGTTTTGCTCCTGTTGCCCAGGCTGGAGTGCAGTGGCGCAATCTCGGC
TCACGGCAACCTCCGCCTCCCGG
>964
TTACGTAAGTGTGTGTGTGTAAACACACACACACTTTTTTTTTTTTTTTCTGAGACGGA
GTTTTGCTCCTGTTGCCCAGGCTGGAGTGCAGTGGCGCAATCTCGGCTCACGGCAACC

TCCGCCTCCCGGTTTCAAGTGAT
>965
TGTGTAAACACACACACACTTTTTTTTTTTTTTTCTGAGACGGAGTTTTGCTCCTGTTG
CCCAGGCTGGAGTGCAGTGGCGCAATCTCGGCTCACGGCAACCTCCGCCTCCCGGTTT
CAAGTGATTCTCCTGCCTCAGCC
>966
AGTTTTGCTCCTGTTGCCCAGGCTGGAGTGCAGTGGCGCAATCTCGGCTCACGGCAAC
CTCCGCCTCCCGGTTTCAAGTGATTCTCCTGCCTCAGCCTCCCGAGTAGCTGGGATTAC
AGGCGGGTGCCACCGCGCCTGGC
>967
CTCCTGTTGCCCAGGCTGGAGTGCAGTGGCGCAATCTCGGCTCACGGCAACCTCCGCC
TCCCGGTTTCAAGTGATTCTCCTGCCTCAGCCTCCCGAGTAGCTGGGATTACAGGCGG
GTGCCACCGCGCCTGGCTAATTTT
>968
TGGCGCAATCTCGGCTCACGGCAACCTCCGCCTCCCGGTTTCAAGTGATTCTCCTGCCT
CAGCCTCCCGAGTAGCTGGGATTACAGGCGGGTGCCACCGCGCCTGGCTAATTTTGTAT
TTTTTGTAGAGACGGGGCTTCT
>969
GCAACCTCCGCCTCCCGGTTTCAAGTGATTCTCCTGCCTCAGCCTCCCGAGTAGCTGG
GATTACAGGCGGGTGCCACCGCGCCTGGCTAATTTTGTATTTTTTGTAGAGACGGGGCT
TCTCCATGTTGGTCAGGCTGGTC
>970
GTGATTCTCCTGCCTCAGCCTCCCGAGTAGCTGGGATTACAGGCGGGTGCCACCGCGC
CTGGCTAATTTTGTATTTTTTGTAGAGACGGGGCTTCTCCATGTTGGTCAGGCTGGTCTC
GAACTCCCGACCTCAGGTGATC
>971
CTGCCTCAGCCTCCCGAGTAGCTGGGATTACAGGCGGGTGCCACCGCGCCTGGCTAAT
TTTGTATTTTTTGTAGAGACGGGGCTTCTCCATGTTGGTCAGGCTGGTCTCGAACTCCC
GACCTCAGGTGATCCGCCTGCCT
>972
GATTACAGGCGGGTGCCACCGCGCCTGGCTAATTTTGTATTTTTTGTAGAGACGGGGCT
TCTCCATGTTGGTCAGGCTGGTCTCGAACTCCCGACCTCAGGTGATCCGCCTGCCTGG
GCCTCCCAAAGTGCTGGGATTAG
>973
CGGGTGCCACCGCGCCTGGCTAATTTTGTATTTTTTGTAGAGACGGGGCTTCTCCATGT
TGGTCAGGCTGGTCTCGAACTCCCGACCTCAGGTGATCCGCCTGCCTGGGCCTCCCAA
AGTGCTGGGATTAGAGGCGTGAG
>974
AATTTTGTATTTTTTGTAGAGACGGGGCTTCTCCATGTTGGTCAGGCTGGTCTCGAACT
CCCGACCTCAGGTGATCCGCCTGCCTGGGCCTCCCAAAGTGCTGGGATTAGAGGCGTG
AGCCACCGCGCCCTACCCCACAC
>975
CTTCTCCATGTTGGTCAGGCTGGTCTCGAACTCCCGACCTCAGGTGATCCGCCTGCCTG
GGCCTCCCAAAGTGCTGGGATTAGAGGCGTGAGCCACCGCGCCCTACCCCACACACTC

TTTCTGTCTGTCTCTGGCTGGTG
>976
GGCTGGTCTCGAACTCCCGACCTCAGGTGATCCGCCTGCCTGGGCCTCCCAAAGTGCT
GGGATTAGAGGCGTGAGCCACCGCGCCCTACCCCACACACTCTTTCTGTCTGTCTCTG
GCTGGTGTACATACACACAGTTCA
>977
CTGCCTGGGCCTCCCAAAGTGCTGGGATTAGAGGCGTGAGCCACCGCGCCCTACCCCA
CACACTCTTTCTGTCTGTCTCTGGCTGGTGTACATACACACAGTTCAGATTTTATATTAT
ACATACAATAACTTTGAGGCTT
>978
AGTGCTGGGATTAGAGGCGTGAGCCACCGCGCCCTACCCCACACACTCTTTCTGTCTG
TCTCTGGCTGGTGTACATACACACAGTTCAGATTTTATATTATACATACAATAACTTTGA
GGCTTCCGTGGGTAGAAATGAC
>979
GAGGCGTGAGCCACCGCGCCCTACCCCACACACTCTTTCTGTCTGTCTCTGGCTGGTG
TACATACACACAGTTCAGATTTTATATTATACATACAATAACTTTGAGGCTTCCGTGGGT
AGAAATGACCCAAGTTAGTGTG
>980
CTGTCTCTGGCTGGTGTACATACACACAGTTCAGATTTTATATTATACATACAATAACTTT
GAGGCTTCCGTGGGTAGAAATGACCCAAGTTAGTGTGTGGGTTGTGTGTGTTCCTGTT
AGTACCTAGGGATGGGGACAG
>981
CTCTGGCTGGTGTACATACACACAGTTCAGATTTTATATTATACATACAATAACTTTGAG
GCTTCCGTGGGTAGAAATGACCCAAGTTAGTGTGTGGGTTGTGTGTGTTCCTGTTAGTA
CCTAGGGATGGGGACAGGCCT
>982
ATACACACAGTTCAGATTTTATATTATACATACAATAACTTTGAGGCTTCCGTGGGTAGA
AATGACCCAAGTTAGTGTGTGGGTTGTGTGTGTTCCTGTTAGTACCTAGGGATGGGGAC
AGGCCTTGGGTCTGCCAGTGC
>983
ACACAGTTCAGATTTTATATTATACATACAATAACTTTGAGGCTTCCGTGGGTAGAAATG
ACCCAAGTTAGTGTGTGGGTTGTGTGTGTTCCTGTTAGTACCTAGGGATGGGGACAGG
CCTTGGGTCTGCCAGTGCTTTG
>984
ACATACAATAACTTTGAGGCTTCCGTGGGTAGAAATGACCCAAGTTAGTGTGTGGGTT
GTGTGTGTTCCTGTTAGTACCTAGGGATGGGGACAGGCCTTGGGTCTGCCAGTGCTTTG
CAGCCCTGAGAACAGTAGAAGGC
>985
GAAATGACCCAAGTTAGTGTGTGGGTTGTGTGTGTTCCTGTTAGTACCTAGGGATGGG
GACAGGCCTTGGGTCTGCCAGTGCTTTGCAGCCCTGAGAACAGTAGAAGGCCAGAGG
CCAAGGCTGATGGTGAGAGGATAGC
>986
CCAAGTTAGTGTGTGGGTTGTGTGTGTTCCTGTTAGTACCTAGGGATGGGGACAGGCC
TTGGGTCTGCCAGTGCTTTGCAGCCCTGAGAACAGTAGAAGGCCAGAGGCCAAGGCT

GATGGTGAGAGGATAGCCTTTGCGG
>987
TGTGTTCCTGTTAGTACCTAGGGATGGGGACAGGCCTTGGGTCTGCCAGTGCTTTGCA
GCCCTGAGAACAGTAGAAGGCCAGAGGCCAAGGCTGATGGTGAGAGGATAGCCTTTG
CGGAGGAGAGCCCAGTGTCCATAGA
>988
GGACAGGCCTTGGGTCTGCCAGTGCTTTGCAGCCCTGAGAACAGTAGAAGGCCAGAG
GCCAAGGCTGATGGTGAGAGGATAGCCTTTGCGGAGGAGAGCCCAGTGTCCATAGATT
TCTGTGGCCATGGCTACAGGCTCCT
>989
CCCTGAGAACAGTAGAAGGCCAGAGGCCAAGGCTGATGGTGAGAGGATAGCCTTTGC
GGAGGAGAGCCCAGTGTCCATAGATTTCTGTGGCCATGGCTACAGGCTCCTGGCCTCC
AATGAGTTTGACTCTGCTTCTGGGG
>990
AGGCCAGAGGCCAAGGCTGATGGTGAGAGGATAGCCTTTGCGGAGGAGAGCCCAGTG
TCCATAGATTTCTGTGGCCATGGCTACAGGCTCCTGGCCTCCAATGAGTTTGACTCTGC
TTCTGGGGCTCCTGTAGTGAGGCA
>991
CCTTTGCGGAGGAGAGCCCAGTGTCCATAGATTTCTGTGGCCATGGCTACAGGCTCCT
GGCCTCCAATGAGTTTGACTCTGCTTCTGGGGCTCCTGTAGTGAGGCAGAGTTTGGAA
AAACTGTTTAAAGATAAGGCTTGA
>992
CATAGATTTCTGTGGCCATGGCTACAGGCTCCTGGCCTCCAATGAGTTTGACTCTGCTT
CTGGGGCTCCTGTAGTGAGGCAGAGTTTGGAAAAACTGTTTAAAGATAAGGCTTGACA
TCCAGGGGGCTGGCGTGAAGCTG
>993
AGATTTCTGTGGCCATGGCTACAGGCTCCTGGCCTCCAATGAGTTTGACTCTGCTTCTG
GGGCTCCTGTAGTGAGGCAGAGTTTGGAAAAACTGTTTAAAGATAAGGCTTGACATCC
AGGGGGCTGGCGTGAAGCTGACA
>994
GGCCATGGCTACAGGCTCCTGGCCTCCAATGAGTTTGACTCTGCTTCTGGGGCTCCTGT
AGTGAGGCAGAGTTTGGAAAAACTGTTTAAAGATAAGGCTTGACATCCAGGGGGCTG
GCGTGAAGCTGACAAGGAAGGGGC
>995
GGCTCCTGGCCTCCAATGAGTTTGACTCTGCTTCTGGGGCTCCTGTAGTGAGGCAGAG
TTTGGAAAAACTGTTTAAAGATAAGGCTTGACATCCAGGGGGCTGGCGTGAAGCTGAC
AAGGAAGGGGCTAAAGTCTTGCTA
>996
GGGGCTCCTGTAGTGAGGCAGAGTTTGGAAAAACTGTTTAAAGATAAGGCTTGACATC
CAGGGGGCTGGCGTGAAGCTGACAAGGAAGGGGCTAAAGTCTTGCTAATTTTAAGTA
AGTGCACTTCATCATAATGAATGTT
>997
GGAAAAACTGTTTAAAGATAAGGCTTGACATCCAGGGGGCTGGCGTGAAGCTGACAA
GGAAGGGGCTAAAGTCTTGCTAATTTTAAGTAAGTGCACTTCATCATAATGAATGTTGG

CCTTGCCTAGGGAGCCCCTACTCT
>998
GATAAGGCTTGACATCCAGGGGGCTGGCGTGAAGCTGACAAGGAAGGGGCTAAAGTC
TTGCTAATTTTAAGTAAGTGCACTTCATCATAATGAATGTTGGCCTTGCCTAGGGAGCCC
CTACTCTCAGACACAGAGAAAGC
>999
GGCGTGAAGCTGACAAGGAAGGGGCTAAAGTCTTGCTAATTTTAAGTAAGTGCACTTC
ATCATAATGAATGTTGGCCTTGCCTAGGGAGCCCCTACTCTCAGACACAGAGAAAGCT
GAGGACTTATAGGACTTCACTACA
>1000
CTCAGACACAGAGAAAGCTGAGGACTTATAGGACTTCACTACAATTTTCATTTCACTTC
ATCTAACAGATGTCATAGAGTAGTGAATTAGGCATCTGCTGTGGCAGAACCTTCTTTTT
ACTCTCAGCATCCCTGGCTTGA
>1001
CAGAGAAAGCTGAGGACTTATAGGACTTCACTACAATTTTCATTTCACTTCATCTAACA
GATGTCATAGAGTAGTGAATTAGGCATCTGCTGTGGCAGAACCTTCTTTTTACTCTCAG
CATCCCTGGCTTGAAAGGCAGA
>1002
AGGACTTCACTACAATTTTCATTTCACTTCATCTAACAGATGTCATAGAGTAGTGAATTA
GGCATCTGCTGTGGCAGAACCTTCTTTTTACTCTCAGCATCCCTGGCTTGAAAGGCAGA
TTTGTAGATTTCTAGGAAGCT
>1003
GTGAATTAGGCATCTGCTGTGGCAGAACCTTCTTTTTACTCTCAGCATCCCTGGCTTGA
AAGGCAGATTTGTAGATTTCTAGGAAGCTACTTCAATTAAAATCTCTAAGATCCTCTTGC
TAAGGGCTTGGCAACAGGGAG
>1004
AGGAAGCTACTTCAATTAAAATCTCTAAGATCCTCTTGCTAAGGGCTTGGCAACAGGG
AGGAGGGGGCATGTTGTAGTGGTTGAATTCTTACTTTTAGGGACACTGATGAATTTGTC
TTTTGTTCTCTCATCTCTTTTGC
>1005
AAGCTACTTCAATTAAAATCTCTAAGATCCTCTTGCTAAGGGCTTGGCAACAGGGAGG
AGGGGGCATGTTGTAGTGGTTGAATTCTTACTTTTAGGGACACTGATGAATTTGTCTTTT
GTTCTCTCATCTCTTTTGCTTC
>1006
GCTTGGCAACAGGGAGGAGGGGGCATGTTGTAGTGGTTGAATTCTTACTTTTAGGGAC
ACTGATGAATTTGTCTTTTGTTCTCTCATCTCTTTTGCTTCTTCCCCTCTTCTTTCTCCTA
GCCTCCTCTATTGGCCCAGGA
>1007
AACAGGGAGGAGGGGGCATGTTGTAGTGGTTGAATTCTTACTTTTAGGGACACTGATG
AATTTGTCTTTTGTTCTCTCATCTCTTTTGCTTCTTCCCCTCTTCTTTCTCCTAGCCTCCT
CTATTGGCCCAGGAAGCCCAC
>1008
GGAGCACCATGGATCTGACAAAAATGGGCATGATCCAGCTGCAGAACCCTAGCCACCC
CACGGGGCTACTGTGCAAGGCCAACCAGATGCGGCTGGCCGGGACTTTGTGCGATGTG

GTCATCATGGTGGACAGCCAGGAG
>1009
AGCTGCAGAACCCTAGCCACCCCACGGGGCTACTGTGCAAGGCCAACCAGATGCGGC
TGGCCGGGACTTTGTGCGATGTGGTCATCATGGTGGACAGCCAGGAGTTCCACGCCCA
CCGGACGGTGCTGGCCTGCACCAGC
>1010
GAACCCTAGCCACCCCACGGGGCTACTGTGCAAGGCCAACCAGATGCGGCTGGCCGG
GACTTTGTGCGATGTGGTCATCATGGTGGACAGCCAGGAGTTCCACGCCCACCGGACG
GTGCTGGCCTGCACCAGCAAGATGT
>1011
CCCCACGGGGCTACTGTGCAAGGCCAACCAGATGCGGCTGGCCGGGACTTTGTGCGAT
GTGGTCATCATGGTGGACAGCCAGGAGTTCCACGCCCACCGGACGGTGCTGGCCTGC
ACCAGCAAGATGTTTGAGATCCTCT
>1012
GCGGCTGGCCGGGACTTTGTGCGATGTGGTCATCATGGTGGACAGCCAGGAGTTCCAC
GCCCACCGGACGGTGCTGGCCTGCACCAGCAAGATGTTTGAGATCCTCTTCCACCGCA
ATAGTCAACACTATACTTTGGACT
>1013
ATGTGGTCATCATGGTGGACAGCCAGGAGTTCCACGCCCACCGGACGGTGCTGGCCTG
CACCAGCAAGATGTTTGAGATCCTCTTCCACCGCAATAGTCAACACTATACTTTGGACT
TCCTCTCGCCAAAGACCTTCCAG
>1014
GAGTTCCACGCCCACCGGACGGTGCTGGCCTGCACCAGCAAGATGTTTGAGATCCTCT
TCCACCGCAATAGTCAACACTATACTTTGGACTTCCTCTCGCCAAAGACCTTCCAGCAG
ATTCTGGAGTATGCATATACAGC
>1015
TCTTCCACCGCAATAGTCAACACTATACTTTGGACTTCCTCTCGCCAAAGACCTTCCAG
CAGATTCTGGAGTATGCATATACAGCCACGCTGCAAGCCAAGGCGGAGGACCTGGATG
ACCTGCTGTATGCGGCCGAGATC
>1016
TCCAGCAGATTCTGGAGTATGCATATACAGCCACGCTGCAAGCCAAGGCGGAGGACCT
GGATGACCTGCTGTATGCGGCCGAGATCCTGGAGATCGAGTACCTGGAGGAACAGTGC
CTGAAGATGCTGGAGACCATCCAG
>1017
CAGCCACGCTGCAAGCCAAGGCGGAGGACCTGGATGACCTGCTGTATGCGGCCGAGA
TCCTGGAGATCGAGTACCTGGAGGAACAGTGCCTGAAGATGCTGGAGACCATCCAGG
CCTCAGACGACAATGACACGGAGGCC
>1018
CCAAGGCGGAGGACCTGGATGACCTGCTGTATGCGGCCGAGATCCTGGAGATCGAGTA
CCTGGAGGAACAGTGCCTGAAGATGCTGGAGACCATCCAGGCCTCAGACGACAATGA
CACGGAGGCCACCATGGCCGATGGC
>1019
GAGGAAGAAGAGGACCGCAAGGCTCGGTACCTCAAGAACATCTTCATCTCGAAGCAT
TCCAGCGAGGAGAGTGGGTATGCCAGTGTGGCTGGACAGAGCCTCCCTGGGCCCATG

GTGGACCAGAGCCCTTCAGTCTCCAC
>1020
AAGAAGAGGACCGCAAGGCTCGGTACCTCAAGAACATCTTCATCTCGAAGCATTCCAG
CGAGGAGAGTGGGTATGCCAGTGTGGCTGGACAGAGCCTCCCTGGGCCCATGGTGGA
CCAGAGCCCTTCAGTCTCCACTTCA
>1021
CATCTCGAAGCATTCCAGCGAGGAGAGTGGGTATGCCAGTGTGGCTGGACAGAGCCTC
CCTGGGCCCATGGTGGACCAGAGCCCTTCAGTCTCCACTTCATTTGGTCTTTCAGCCAT
GAGTCCCACCAAGGCTGCAGTGG
>1022
CGAGGAGAGTGGGTATGCCAGTGTGGCTGGACAGAGCCTCCCTGGGCCCATGGTGGA
CCAGAGCCCTTCAGTCTCCACTTCATTTGGTCTTTCAGCCATGAGTCCCACCAAGGCTG
CAGTGGACAGTTTGATGACCATAG
>1023
GCCAGTGTGGCTGGACAGAGCCTCCCTGGGCCCATGGTGGACCAGAGCCCTTCAGTCT
CCACTTCATTTGGTCTTTCAGCCATGAGTCCCACCAAGGCTGCAGTGGACAGTTTGATG
ACCATAGGACAGTCTCTCCTGCA
>1024
CAGAGCCTCCCTGGGCCCATGGTGGACCAGAGCCCTTCAGTCTCCACTTCATTTGGTC
TTTCAGCCATGAGTCCCACCAAGGCTGCAGTGGACAGTTTGATGACCATAGGACAGTC
TCTCCTGCAGGGAACTCTTCAGCC
>1025
CATGGTGGACCAGAGCCCTTCAGTCTCCACTTCATTTGGTCTTTCAGCCATGAGTCCCA
CCAAGGCTGCAGTGGACAGTTTGATGACCATAGGACAGTCTCTCCTGCAGGGAACTCT
TCAGCCACCTGCAGGGCCCGAGG
>1026
ATAGGACAGTCTCTCCTGCAGGGAACTCTTCAGCCACCTGCAGGGCCCGAGGAGCCA
ACTCTGGCTGGGGGTGGGCGGCACCCTGGGGTGGCTGAGGTGAAGACGGAGATGATG
CAGGTGGATGAGGTGCCCAGCCAGGA
>1027
GCAGGGAACTCTTCAGCCACCTGCAGGGCCCGAGGAGCCAACTCTGGCTGGGGGTGG
GCGGCACCCTGGGGTGGCTGAGGTGAAGACGGAGATGATGCAGGTGGATGAGGTGCC
CAGCCAGGACAGCCCTGGGGCAGCCG
>1028
TCTTCAGCCACCTGCAGGGCCCGAGGAGCCAACTCTGGCTGGGGGTGGGCGGCACCC
TGGGGTGGCTGAGGTGAAGACGGAGATGATGCAGGTGGATGAGGTGCCCAGCCAGGA
CAGCCCTGGGGCAGCCGAGTCCAGCA
>1029
AACTCTGGCTGGGGGTGGGCGGCACCCTGGGGTGGCTGAGGTGAAGACGGAGATGAT
GCAGGTGGATGAGGTGCCCAGCCAGGACAGCCCTGGGGCAGCCGAGTCCAGCATCTC
AGGAGGGATGGGGGACAAGGTTGAGG
>1030
TGGCTGAGGTGAAGACGGAGATGATGCAGGTGGATGAGGTGCCCAGCCAGGACAGCC
CTGGGGCAGCCGAGTCCAGCATCTCAGGAGGGATGGGGGACAAGGTTGAGGAAAGA

GGCAAAGAGGGGCCTGGGACCCCGACT
>1031
GGATGAGGTGCCCAGCCAGGACAGCCCTGGGGCAGCCGAGTCCAGCATCTCAGGAGG
GATGGGGGACAAGGTTGAGGAAAGAGGCAAAGAGGGGCCTGGGACCCCGACTCGAA
GCAGCGTCATCACCAGTGCTAGGGAGC
>1032
CTCAGGAGGGATGGGGGACAAGGTTGAGGAAAGAGGCAAAGAGGGGCCTGGGACCC
CGACTCGAAGCAGCGTCATCACCAGTGCTAGGGAGCTACACTATGGGCGAGAGGAGA
GTGCCGAGCAGGTGCCACCCCCAGCTG
>1033
ATGGGGGACAAGGTTGAGGAAAGAGGCAAAGAGGGGCCTGGGACCCCGACTCGAAG
CAGCGTCATCACCAGTGCTAGGGAGCTACACTATGGGCGAGAGGAGAGTGCCGAGCA
GGTGCCACCCCCAGCTGAGGCTGGCCA
>1034
GGGCCTGGGACCCCGACTCGAAGCAGCGTCATCACCAGTGCTAGGGAGCTACACTATG
GGCGAGAGGAGAGTGCCGAGCAGGTGCCACCCCCAGCTGAGGCTGGCCAGGCCCCC
ACTGGCCGACCTGAGCACCCAGCACC
>1035
CCCGACTCGAAGCAGCGTCATCACCAGTGCTAGGGAGCTACACTATGGGCGAGAGGA
GAGTGCCGAGCAGGTGCCACCCCCAGCTGAGGCTGGCCAGGCCCCCACTGGCCGACC
TGAGCACCCAGCACCCCCGCCTGAGA
>1036
TGGCCAGGCCCCCACTGGCCGACCTGAGCACCCAGCACCCCCGCCTGAGAAGCATCT
GGGCATCTACTCCGTGTTGCCCAACCACAAGGCTGACGCTGTATTGAGCATGCCGTCTT
CCGTGACCTCTGGCCTCCACGTGC
>1037
GCCAGGCCCCCACTGGCCGACCTGAGCACCCAGCACCCCCGCCTGAGAAGCATCTGG
GCATCTACTCCGTGTTGCCCAACCACAAGGCTGACGCTGTATTGAGCATGCCGTCTTCC
GTGACCTCTGGCCTCCACGTGCAG
>1038
GCACCCAGCACCCCCGCCTGAGAAGCATCTGGGCATCTACTCCGTGTTGCCCAACCAC
AAGGCTGACGCTGTATTGAGCATGCCGTCTTCCGTGACCTCTGGCCTCCACGTGCAGC
CTGCCCTGGCTGTCTCCATGGACT
>1039
GAAGCATCTGGGCATCTACTCCGTGTTGCCCAACCACAAGGCTGACGCTGTATTGAGC
ATGCCGTCTTCCGTGACCTCTGGCCTCCACGTGCAGCCTGCCCTGGCTGTCTCCATGGA
CTTCAGCACCTATGGGGGGCTGC
>1040
CTCCGTGTTGCCCAACCACAAGGCTGACGCTGTATTGAGCATGCCGTCTTCCGTGACCT
CTGGCCTCCACGTGCAGCCTGCCCTGGCTGTCTCCATGGACTTCAGCACCTATGGGGG
GCTGCTGCCCCAGGGCTTCATCC
>1041
CAAGGCTGACGCTGTATTGAGCATGCCGTCTTCCGTGACCTCTGGCCTCCACGTGCAG
CCTGCCCTGGCTGTCTCCATGGACTTCAGCACCTATGGGGGGCTGCTGCCCCAGGGCT

TCATCCAGAGGGAGCTGTTCAGCA
>1042
CCCTGGCTGTCTCCATGGACTTCAGCACCTATGGGGGGCTGCTGCCCCAGGGCTTCATC
CAGAGGGAGCTGTTCAGCAAGCTGGGGGAGCTGGCTGTGGGCATGAAGTCAGAGAGC
CGGACCATCGGAGAGCAGTGCAGC
>1043
CACCTATGGGGGGCTGCTGCCCCAGGGCTTCATCCAGAGGGAGCTGTTCAGCAAGCTG
GGGGAGCTGGCTGTGGGCATGAAGTCAGAGAGCCGGACCATCGGAGAGCAGTGCAGC
GTGTGTGGGGTCGAGCTTCCTGATA
>1044
GGCTGCTGCCCCAGGGCTTCATCCAGAGGGAGCTGTTCAGCAAGCTGGGGGAGCTGG
CTGTGGGCATGAAGTCAGAGAGCCGGACCATCGGAGAGCAGTGCAGCGTGTGTGGGG
TCGAGCTTCCTGATAACGAGGCTGTG
>1045
GGGAGCTGTTCAGCAAGCTGGGGGAGCTGGCTGTGGGCATGAAGTCAGAGAGCCGGA
CCATCGGAGAGCAGTGCAGCGTGTGTGGGGTCGAGCTTCCTGATAACGAGGCTGTGG
AGCAGCACAGGTAGGCCCCGCTCCAG
>1046
GAAGTCAGAGAGCCGGACCATCGGAGAGCAGTGCAGCGTGTGTGGGGTCGAGCTTCC
TGATAACGAGGCTGTGGAGCAGCACAGGTAGGCCCCGCTCCAGCCCCGCACCTGATGT
AGGACTTGAGGCCCTCACACCCCTC
>1047
GGGGTCGAGCTTCCTGATAACGAGGCTGTGGAGCAGCACAGGTAGGCCCCGCTCCAG
CCCCGCACCTGATGTAGGACTTGAGGCCCTCACACCCCTCCTTCACACCCTGGCTGCT
CCCAAGTTAGGGGCCTGGCTCCCCT
>1048
GCTCCAGCCCCGCACCTGATGTAGGACTTGAGGCCCTCACACCCCTCCTTCACACCCT
GGCTGCTCCCAAGTTAGGGGCCTGGCTCCCCTGTCTTGGCAATTTGTGAAAAAACCAG
AACACTTCTTCTAAAGTTCTGGCG
>1049
GTAGGACTTGAGGCCCTCACACCCCTCCTTCACACCCTGGCTGCTCCCAAGTTAGGGG
CCTGGCTCCCCTGTCTTGGCAATTTGTGAAAAAACCAGAACACTTCTTCTAAAGTTCTG
GCGGGGAGGGGAGCAGGTTTTTT
>1050
CCCTCACACCCCTCCTTCACACCCTGGCTGCTCCCAAGTTAGGGGCCTGGCTCCCCTGT
CTTGGCAATTTGTGAAAAAACCAGAACACTTCTTCTAAAGTTCTGGCGGGGAGGGGA
GCAGGTTTTTTAAAGTGTAGTGAG
>1051
TGCTCCCAAGTTAGGGGCCTGGCTCCCCTGTCTTGGCAATTTGTGAAAAAACCAGAAC
ACTTCTTCTAAAGTTCTGGCGGGGAGGGGAGCAGGTTTTTTAAAGTGTAGTGAGGGGG
CCTAGGATCTTTCCAAAAAGCACC
>1052
CTCCCCTGTCTTGGCAATTTGTGAAAAAACCAGAACACTTCTTCTAAAGTTCTGGCGG
GGAGGGGAGCAGGTTTTTTAAAGTGTAGTGAGGGGGCCTAGGATCTTTCCAAAAAGC

ACCAGGGGACACTCATGGGCGCAGC
>1053
TTGGCAATTTGTGAAAAAACCAGAACACTTCTTCTAAAGTTCTGGCGGGGAGGGGAG
CAGGTTTTTTAAAGTGTAGTGAGGGGGCCTAGGATCTTTCCAAAAAGCACCAGGGGAC
ACTCATGGGCGCAGCTTCTTAATAG
>1054
GGCCTAGGATCTTTCCAAAAAGCACCAGGGGACACTCATGGGCGCAGCTTCTTAATAG
GCCCTTCCCTTTGTTTTTTGCTTTTGGGCCCCTGTTTGTTTTTTCTGCTGTTTGGTCTGTT
CTCCTTTGCTTGGAGTGACTG
>1055
GCACCAGGGGACACTCATGGGCGCAGCTTCTTAATAGGCCCTTCCCTTTGTTTTTTGCT
TTTGGGCCCCTGTTTGTTTTTTCTGCTGTTTGGTCTGTTCTCCTTTGCTTGGAGTGACTG
ATAAAATGGAGAGAAGGAGAA
>1056
CAGGGGACACTCATGGGCGCAGCTTCTTAATAGGCCCTTCCCTTTGTTTTTTGCTTTTG
GGCCCCTGTTTGTTTTTTCTGCTGTTTGGTCTGTTCTCCTTTGCTTGGAGTGACTGATAA
AATGGAGAGAAGGAGAAAAGG
>1057
ATGGGCGCAGCTTCTTAATAGGCCCTTCCCTTTGTTTTTTGCTTTTGGGCCCCTGTTTGT
TTTTTCTGCTGTTTGGTCTGTTCTCCTTTGCTTGGAGTGACTGATAAAATGGAGAGAAG
GAGAAAAGGAGTGGGATGAGG
>1058
TTTTTTCTGCTGTTTGGTCTGTTCTCCTTTGCTTGGAGTGACTGATAAAATGGAGAGAA
GGAGAAAAGGAGTGGGATGAGGGTCTCTTGGGCCCTGCATCTGACCATGTTACTAGGT
TTCAGGTCCTCGGGTTCCCATGC
>1059
GTTTGGTCTGTTCTCCTTTGCTTGGAGTGACTGATAAAATGGAGAGAAGGAGAAAAGG
AGTGGGATGAGGGTCTCTTGGGCCCTGCATCTGACCATGTTACTAGGTTTCAGGTCCTC
GGGTTCCCATGCAGTGGTCCCTG
>1060
GTGACTGATAAAATGGAGAGAAGGAGAAAAGGAGTGGGATGAGGGTCTCTTGGGCCC
TGCATCTGACCATGTTACTAGGTTTCAGGTCCTCGGGTTCCCATGCAGTGGTCCCTGTG
AAGACAGCCTGTCCCTTTCTGCAG
>1061
TAAAATGGAGAGAAGGAGAAAAGGAGTGGGATGAGGGTCTCTTGGGCCCTGCATCTG
ACCATGTTACTAGGTTTCAGGTCCTCGGGTTCCCATGCAGTGGTCCCTGTGAAGACAG
CCTGTCCCTTTCTGCAGCTTGGGAG
>1062
GAGAGAAGGAGAAAAGGAGTGGGATGAGGGTCTCTTGGGCCCTGCATCTGACCATGT
TACTAGGTTTCAGGTCCTCGGGTTCCCATGCAGTGGTCCCTGTGAAGACAGCCTGTCC
CTTTCTGCAGCTTGGGAGAATGGGA
>1063
TGGGATGAGGGTCTCTTGGGCCCTGCATCTGACCATGTTACTAGGTTTCAGGTCCTCGG
GTTCCCATGCAGTGGTCCCTGTGAAGACAGCCTGTCCCTTTCTGCAGCTTGGGAGAAT

GGGAGGGTCTGGGGGCTGCTTAA
>1064
CCTCGGGTTCCCATGCAGTGGTCCCTGTGAAGACAGCCTGTCCCTTTCTGCAGCTTGG
GAGAATGGGAGGGTCTGGGGGCTGCTTAAGCCACAAGAGCAGCTGCAGGTTCTGAAC
CTTATTCCTGGTGAAAGTTAATCTC
>1065
CCTGTCCCTTTCTGCAGCTTGGGAGAATGGGAGGGTCTGGGGGCTGCTTAAGCCACAA
GAGCAGCTGCAGGTTCTGAACCTTATTCCTGGTGAAAGTTAATCTCCTTGCTCTGTCTC
CCTTCTCATTCTCTGATCCTGCA
>1066
TGGGAGAATGGGAGGGTCTGGGGGCTGCTTAAGCCACAAGAGCAGCTGCAGGTTCTG
AACCTTATTCCTGGTGAAAGTTAATCTCCTTGCTCTGTCTCCCTTCTCATTCTCTGATCC
TGCACATGCATTTAGGGGGAATT
>1067
GCTGCTTAAGCCACAAGAGCAGCTGCAGGTTCTGAACCTTATTCCTGGTGAAAGTTAA
TCTCCTTGCTCTGTCTCCCTTCTCATTCTCTGATCCTGCACATGCATTTAGGGGGAATTG
ATTTAAATGCAAGGGGAAAGGA
>1068
TCCCTTCTCATTCTCTGATCCTGCACATGCATTTAGGGGGAATTGATTTAAATGCAAGGG
GAAAGGATGGGTATAAGAGTCTTGGTCTTGCAGCCTGTGAAGTCTCATTCTTATCCCAT
CTCTGCTCACACTGAACTAAA
>1069
CATTCTCTGATCCTGCACATGCATTTAGGGGGAATTGATTTAAATGCAAGGGGAAAGGA
TGGGTATAAGAGTCTTGGTCTTGCAGCCTGTGAAGTCTCATTCTTATCCCATCTCTGCTC
ACACTGAACTAAATGCCGCTT
>1070
CTGATCCTGCACATGCATTTAGGGGGAATTGATTTAAATGCAAGGGGAAAGGATGGGTA
TAAGAGTCTTGGTCTTGCAGCCTGTGAAGTCTCATTCTTATCCCATCTCTGCTCACACT
GAACTAAATGCCGCTTCAGGCT
>1071
ATGCATTTAGGGGGAATTGATTTAAATGCAAGGGGAAAGGATGGGTATAAGAGTCTTG
GTCTTGCAGCCTGTGAAGTCTCATTCTTATCCCATCTCTGCTCACACTGAACTAAATGC
CGCTTCAGGCTAGCATTGGCTTC
>1072
TCTTATCCCATCTCTGCTCACACTGAACTAAATGCCGCTTCAGGCTAGCATTGGCTTCCC
TCCGGCTTCCGTTTCCTTCTCTATGAAATAACCATTCATTGAGCTCTTATGACATGTTTG
GTTCAGTACTAAGCACTTTC
>1073
TAGCATTGGCTTCCCTCCGGCTTCCGTTTCCTTCTCTATGAAATAACCATTCATTGAGCT
CTTATGACATGTTTGGTTCAGTACTAAGCACTTTCCATGAGTTGCATTTTCATTACAACA
GCCCTTTGAGATAGGTAATG
>1074
TTGGCTTCCCTCCGGCTTCCGTTTCCTTCTCTATGAAATAACCATTCATTGAGCTCTTAT
GACATGTTTGGTTCAGTACTAAGCACTTTCCATGAGTTGCATTTTCATTACAACAGCCCT

TTGAGATAGGTAATGTCCTT
>1075
CTTCTCTATGAAATAACCATTCATTGAGCTCTTATGACATGTTTGGTTCAGTACTAAGCA
CTTTCCATGAGTTGCATTTTCATTACAACAGCCCTTTGAGATAGGTAATGTCCTTCTTTT
TTTTTTGGATGTGAAGTTTC
>1076
TGACATGTTTGGTTCAGTACTAAGCACTTTCCATGAGTTGCATTTTCATTACAACAGCCC
TTTGAGATAGGTAATGTCCTTCTTTTTTTTTTGGATGTGAAGTTTCGCTCTCCTTGCCCA
GGCTGGAGTGCAATGGTGTG
>1077
TGTTTGGTTCAGTACTAAGCACTTTCCATGAGTTGCATTTTCATTACAACAGCCCTTTGA
GATAGGTAATGTCCTTCTTTTTTTTTTGGATGTGAAGTTTCGCTCTCCTTGCCCAGGCTG
GAGTGCAATGGTGTGATCTC
>1078
CTTTCCATGAGTTGCATTTTCATTACAACAGCCCTTTGAGATAGGTAATGTCCTTCTTTT
TTTTTTGGATGTGAAGTTTCGCTCTCCTTGCCCAGGCTGGAGTGCAATGGTGTGATCTC
TGCTCACCGCAACCTCTGCAT
>1079
CATGAGTTGCATTTTCATTACAACAGCCCTTTGAGATAGGTAATGTCCTTCTTTTTTTTT
GGATGTGAAGTTTCGCTCTCCTTGCCCAGGCTGGAGTGCAATGGTGTGATCTCTGCTCA
CCGCAACCTCTGCATCCCGG
>1080
CCCTTTGAGATAGGTAATGTCCTTCTTTTTTTTTTGGATGTGAAGTTTCGCTCTCCTTGC
CCAGGCTGGAGTGCAATGGTGTGATCTCTGCTCACCGCAACCTCTGCATCCCGGGTTA
AGGTGATTCTCCTGCCTCAGCC
>1081
GTTTCGCTCTCCTTGCCCAGGCTGGAGTGCAATGGTGTGATCTCTGCTCACCGCAACCT
CTGCATCCCGGGTTAAGGTGATTCTCCTGCCTCAGCCTCCCGAGTAGCTCGGATTACAG
GCACGCACCACCATACCTGGCT
>1082
CTCTCCTTGCCCAGGCTGGAGTGCAATGGTGTGATCTCTGCTCACCGCAACCTCTGCAT
CCCGGGTTAAGGTGATTCTCCTGCCTCAGCCTCCCGAGTAGCTCGGATTACAGGCACG
CACCACCATACCTGGCTAATTTT
>1083
TGGTGTGATCTCTGCTCACCGCAACCTCTGCATCCCGGGTTAAGGTGATTCTCCTGCCT
CAGCCTCCCGAGTAGCTCGGATTACAGGCACGCACCACCATACCTGGCTAATTTTGAAT
TTTTAGTAGAGACGGGCTTTCA
>1084
CTGCCTCAGCCTCCCGAGTAGCTCGGATTACAGGCACGCACCACCATACCTGGCTAATT
TTGAATTTTTAGTAGAGACGGGCTTTCACCGTGTTGGTCAGGATGGTCTCGAACTCCCG
ACCTCAGGTGATCGGCCTGCTT
>1085
AGCTCGGATTACAGGCACGCACCACCATACCTGGCTAATTTTGAATTTTTAGTAGAGAC
GGGCTTTCACCGTGTTGGTCAGGATGGTCTCGAACTCCCGACCTCAGGTGATCGGCCT

GCTTCGGCCTCCCAAAGTGCTGG
>1086
CTCGGATTACAGGCACGCACCACCATACCTGGCTAATTTTGAATTTTTAGTAGAGACGG
GCTTTCACCGTGTTGGTCAGGATGGTCTCGAACTCCCGACCTCAGGTGATCGGCCTGC
TTCGGCCTCCCAAAGTGCTGGAT
>1087
GATTACAGGCACGCACCACCATACCTGGCTAATTTTGAATTTTTAGTAGAGACGGGCTT
TCACCGTGTTGGTCAGGATGGTCTCGAACTCCCGACCTCAGGTGATCGGCCTGCTTCG
GCCTCCCAAAGTGCTGGATTACA
>1088
CACGCACCACCATACCTGGCTAATTTTGAATTTTTAGTAGAGACGGGCTTTCACCGTGT
TGGTCAGGATGGTCTCGAACTCCCGACCTCAGGTGATCGGCCTGCTTCGGCCTCCCAA
AGTGCTGGATTACAGGTATGAGC
>1089
AATTTTGAATTTTTAGTAGAGACGGGCTTTCACCGTGTTGGTCAGGATGGTCTCGAACT
CCCGACCTCAGGTGATCGGCCTGCTTCGGCCTCCCAAAGTGCTGGATTACAGGTATGA
GCCACCGTGTCCAGCTGGTAATG
>1090
TTTCACCGTGTTGGTCAGGATGGTCTCGAACTCCCGACCTCAGGTGATCGGCCTGCTTC
GGCCTCCCAAAGTGCTGGATTACAGGTATGAGCCACCGTGTCCAGCTGGTAATGTCCTT
CTTTTACATGGAACAATACTAA
>1091
TCAGGATGGTCTCGAACTCCCGACCTCAGGTGATCGGCCTGCTTCGGCCTCCCAAAGT
GCTGGATTACAGGTATGAGCCACCGTGTCCAGCTGGTAATGTCCTTCTTTTACATGGAA
CAATACTAAGGCTCAGATGTCAA
>1092
CGAACTCCCGACCTCAGGTGATCGGCCTGCTTCGGCCTCCCAAAGTGCTGGATTACAG
GTATGAGCCACCGTGTCCAGCTGGTAATGTCCTTCTTTTACATGGAACAATACTAAGGC
TCAGATGTCAAGTCTCTTTTCTA
>1093
TACAGGTATGAGCCACCGTGTCCAGCTGGTAATGTCCTTCTTTTACATGGAACAATACT
AAGGCTCAGATGTCAAGTCTCTTTTCTAGGGTCACATAGATAGTAAGAGGCAGAGCTG
GATTCAAATTTAGGTCCTTACCC
>1094
GTATGAGCCACCGTGTCCAGCTGGTAATGTCCTTCTTTTACATGGAACAATACTAAGGC
TCAGATGTCAAGTCTCTTTTCTAGGGTCACATAGATAGTAAGAGGCAGAGCTGGATTCA
AATTTAGGTCCTTACCCCCTGG
>1095
ATGTCCTTCTTTTACATGGAACAATACTAAGGCTCAGATGTCAAGTCTCTTTTCTAGGGT
CACATAGATAGTAAGAGGCAGAGCTGGATTCAAATTTAGGTCCTTACCCCCTGGTACTT
TGCTGCCTCTTGGAGTCACTT
>1096
AGGCTCAGATGTCAAGTCTCTTTTCTAGGGTCACATAGATAGTAAGAGGCAGAGCTGG
ATTCAAATTTAGGTCCTTACCCCCTGGTACTTTGCTGCCTCTTGGAGTCACTTAGACTTG

AAGAGGATCATGTCTATGGAAC
>1097
AGTCTCTTTTCTAGGGTCACATAGATAGTAAGAGGCAGAGCTGGATTCAAATTTAGGTC
CTTACCCCCTGGTACTTTGCTGCCTCTTGGAGTCACTTAGACTTGAAGAGGATCATGTC
TATGGAACAGAGCGTTTTGAAG
>1098
TTCAAATTTAGGTCCTTACCCCCTGGTACTTTGCTGCCTCTTGGAGTCACTTAGACTTG
AAGAGGATCATGTCTATGGAACAGAGCGTTTTGAAGATTGCTTATAGGAAGTGGAGTA
AATAACAGGGGGGATAAGTAGCG
>1099
TACCCCCTGGTACTTTGCTGCCTCTTGGAGTCACTTAGACTTGAAGAGGATCATGTCTA
TGGAACAGAGCGTTTTGAAGATTGCTTATAGGAAGTGGAGTAAATAACAGGGGGGATA
AGTAGCGAGTTTGTTTTTTTTTT
>1100
CTTGGAGTCACTTAGACTTGAAGAGGATCATGTCTATGGAACAGAGCGTTTTGAAGAT
TGCTTATAGGAAGTGGAGTAAATAACAGGGGGGATAAGTAGCGAGTTTGTTTTTTTTTT
CCTTGCTTCTGTCATAAACCACG
>1101
AGTCACTTAGACTTGAAGAGGATCATGTCTATGGAACAGAGCGTTTTGAAGATTGCTTA
TAGGAAGTGGAGTAAATAACAGGGGGGATAAGTAGCGAGTTTGTTTTTTTTTTCCTTGC
TTCTGTCATAAACCACGTGCCC
>1102
GATCATGTCTATGGAACAGAGCGTTTTGAAGATTGCTTATAGGAAGTGGAGTAAATAAC
AGGGGGGATAAGTAGCGAGTTTGTTTTTTTTTTCCTTGCTTCTGTCATAAACCACGTGC
CCATCATTTTATCCTCTAGTTT
>1103
AGTGGAGTAAATAACAGGGGGGATAAGTAGCGAGTTTGTTTTTTTTTTCCTTGCTTCTG
TCATAAACCACGTGCCCATCATTTTATCCTCTAGTTTCTGGGTCAGATGAATGGAGTCAA
GAAATCCAGCTTTCTGTTGTC
>1104
ATAAGTAGCGAGTTTGTTTTTTTTTTCCTTGCTTCTGTCATAAACCACGTGCCCATCATT
TTATCCTCTAGTTTCTGGGTCAGATGAATGGAGTCAAGAAATCCAGCTTTCTGTTGTCA
TTGACGACAGCATTTGGCTGC
>1105
TTTTCCTTGCTTCTGTCATAAACCACGTGCCCATCATTTTATCCTCTAGTTTCTGGGTCA
GATGAATGGAGTCAAGAAATCCAGCTTTCTGTTGTCATTGACGACAGCATTTGGCTGCA
TACCCTGTGGTCTGCTGTTTT
>1106
AACCACGTGCCCATCATTTTATCCTCTAGTTTCTGGGTCAGATGAATGGAGTCAAGAAA
TCCAGCTTTCTGTTGTCATTGACGACAGCATTTGGCTGCATACCCTGTGGTCTGCTGTTT
TGCTGATCTTGGGTGGATTCT
>1107
CACGTGCCCATCATTTTATCCTCTAGTTTCTGGGTCAGATGAATGGAGTCAAGAAATCC
AGCTTTCTGTTGTCATTGACGACAGCATTTGGCTGCATACCCTGTGGTCTGCTGTTTTG

CTGATCTTGGGTGGATTCTCCA
>1108
GGTCAGATGAATGGAGTCAAGAAATCCAGCTTTCTGTTGTCATTGACGACAGCATTTG
GCTGCATACCCTGTGGTCTGCTGTTTTGCTGATCTTGGGTGGATTCTCCACTTCCTCCCC
CTTCACCCCCATTCCCCTGTTG
>1109
ATGGAGTCAAGAAATCCAGCTTTCTGTTGTCATTGACGACAGCATTTGGCTGCATACCC
TGTGGTCTGCTGTTTTGCTGATCTTGGGTGGATTCTCCACTTCCTCCCCCTTCACCCCCA
TTCCCCTGTTGCTCGTTCAGG
>1110
CAGCTTTCTGTTGTCATTGACGACAGCATTTGGCTGCATACCCTGTGGTCTGCTGTTTT
GCTGATCTTGGGTGGATTCTCCACTTCCTCCCCCTTCACCCCCATTCCCCTGTTGCTCGT
TCAGGTACGGGGGATGTGAGT
>1111
CCCTGTGGTCTGCTGTTTTGCTGATCTTGGGTGGATTCTCCACTTCCTCCCCCTTCACCC
CCATTCCCCTGTTGCTCGTTCAGGTACGGGGGATGTGAGTGAGAGGGGTTGGGGTGGG
GATATCTGGAAGTTGATCTCAT
>1112
GTCTGCTGTTTTGCTGATCTTGGGTGGATTCTCCACTTCCTCCCCCTTCACCCCCATTCC
CCTGTTGCTCGTTCAGGTACGGGGGATGTGAGTGAGAGGGGTTGGGGTGGGGATATCT
GGAAGTTGATCTCATCACCCCT
>1113
TGTTTTGCTGATCTTGGGTGGATTCTCCACTTCCTCCCCCTTCACCCCCATTCCCCTGTT
GCTCGTTCAGGTACGGGGGATGTGAGTGAGAGGGGTTGGGGTGGGGATATCTGGAAG
TTGATCTCATCACCCCTAGCCGG
>1114
TCTTGGGTGGATTCTCCACTTCCTCCCCCTTCACCCCCATTCCCCTGTTGCTCGTTCAGG
TACGGGGGATGTGAGTGAGAGGGGTTGGGGTGGGGATATCTGGAAGTTGATCTCATCA
CCCCTAGCCGGAATCTGCTCTG
>1115
TTCCTCCCCCTTCACCCCCATTCCCCTGTTGCTCGTTCAGGTACGGGGGATGTGAGTGA
GAGGGGTTGGGGTGGGGATATCTGGAAGTTGATCTCATCACCCCTAGCCGGAATCTGC
TCTGACCCTGCCTCCTCGACAGC
>1116
AGTTGATCTCATCACCCCTAGCCGGAATCTGCTCTGACCCTGCCTCCTCGACAGCTCAG
AGGCTTAACTTGTGGAAAGTGTGGCAGAGCTAAACGTAGCCGACTTTTTCCAGTGGGT
GCGAAAAATAACCGCCACTAAGG
>1117
CTCATCACCCCTAGCCGGAATCTGCTCTGACCCTGCCTCCTCGACAGCTCAGAGGCTTA
ACTTGTGGAAAGTGTGGCAGAGCTAAACGTAGCCGACTTTTTCCAGTGGGTGCGAAA
AATAACCGCCACTAAGGTGTGTCT
>1118
GAATCTGCTCTGACCCTGCCTCCTCGACAGCTCAGAGGCTTAACTTGTGGAAAGTGTG
GCAGAGCTAAACGTAGCCGACTTTTTCCAGTGGGTGCGAAAAATAACCGCCACTAAGG

TGTGTCTTCTTGATGCTTGTACCG
>1119
CCTCGACAGCTCAGAGGCTTAACTTGTGGAAAGTGTGGCAGAGCTAAACGTAGCCGA
CTTTTTCCAGTGGGTGCGAAAAATAACCGCCACTAAGGTGTGTCTTCTTGATGCTTGTA
CCGCTCAGAAATAGACTGAGGTTA
>1120
TGTGGAAAGTGTGGCAGAGCTAAACGTAGCCGACTTTTTCCAGTGGGTGCGAAAAATA
ACCGCCACTAAGGTGTGTCTTCTTGATGCTTGTACCGCTCAGAAATAGACTGAGGTTAT
GTTTGTTTTTGCTCAAACTTTCC
>1121
TGGAAAGTGTGGCAGAGCTAAACGTAGCCGACTTTTTCCAGTGGGTGCGAAAAATAAC
CGCCACTAAGGTGTGTCTTCTTGATGCTTGTACCGCTCAGAAATAGACTGAGGTTATGT
TTGTTTTTGCTCAAACTTTCCTA
>1122
CTAAACGTAGCCGACTTTTTCCAGTGGGTGCGAAAAATAACCGCCACTAAGGTGTGTC
TTCTTGATGCTTGTACCGCTCAGAAATAGACTGAGGTTATGTTTGTTTTTGCTCAAACTT
TCCTAAAGAGGAAACCCGCTCC
>1123
TGCGAAAAATAACCGCCACTAAGGTGTGTCTTCTTGATGCTTGTACCGCTCAGAAATAG
ACTGAGGTTATGTTTGTTTTTGCTCAAACTTTCCTAAAGAGGAAACCCGCTCCCCAAA
GTTGACCAGATAGATGTATCATC
>1124
AAAAATAACCGCCACTAAGGTGTGTCTTCTTGATGCTTGTACCGCTCAGAAATAGACTG
AGGTTATGTTTGTTTTTGCTCAAACTTTCCTAAAGAGGAAACCCGCTCCCCAAAGTTGA
CCAGATAGATGTATCATCTTTC
>1125
GCTCAGAAATAGACTGAGGTTATGTTTGTTTTTGCTCAAACTTTCCTAAAGAGGAAACC
CGCTCCCCAAAGTTGACCAGATAGATGTATCATCTTTCCGGATGTGAATTATTTCGGCTG
TGTGCTATGAGAACTGGGTAT
>1126
GAGGTTATGTTTGTTTTTGCTCAAACTTTCCTAAAGAGGAAACCCGCTCCCCAAAGTTG
ACCAGATAGATGTATCATCTTTCCGGATGTGAATTATTTCGGCTGTGTGCTATGAGAACT
GGGTATGGTGGGAAGTAATTG
>1127
TGCTCAAACTTTCCTAAAGAGGAAACCCGCTCCCCAAAGTTGACCAGATAGATGTATC
ATCTTTCCGGATGTGAATTATTTCGGCTGTGTGCTATGAGAACTGGGTATGGTGGGAAG
TAATTGTTTTTCTGGCTCTCATC
>1128
AAGAGGAAACCCGCTCCCCAAAGTTGACCAGATAGATGTATCATCTTTCCGGATGTGA
ATTATTTCGGCTGTGTGCTATGAGAACTGGGTATGGTGGGAAGTAATTGTTTTTCTGGCT
CTCATCAGGTCCTGGTTCCTGG
>1129
GATAGATGTATCATCTTTCCGGATGTGAATTATTTCGGCTGTGTGCTATGAGAACTGGGT
ATGGTGGGAAGTAATTGTTTTTCTGGCTCTCATCAGGTCCTGGTTCCTGGTTGCTTTTCT

TTACCCGAGAGGGCCACAAA
>1130
TGTATCATCTTTCCGGATGTGAATTATTTCGGCTGTGTGCTATGAGAACTGGGTATGGTG
GGAAGTAATTGTTTTTCTGGCTCTCATCAGGTCCTGGTTCCTGGTTGCTTTTCTTTACCC
GAGAGGGCCACAAAAGATAC
>1131
GAATTATTTCGGCTGTGTGCTATGAGAACTGGGTATGGTGGGAAGTAATTGTTTTTCTG
GCTCTCATCAGGTCCTGGTTCCTGGTTGCTTTTCTTTACCCGAGAGGGCCACAAAAGAT
ACCACTCTCCTGAGACTTCCAA
>1132
GTCCTGGTTCCTGGTTGCTTTTCTTTACCCGAGAGGGCCACAAAAGATACCACTCTCCT
GAGACTTCCAAGTTCCCCTTCAGCAAGACCCTCATTCCTCTGGGGAGAGAGGGAGAG
TTGGCCATGTTATATTCGAAAGAG
>1133
GATACCACTCTCCTGAGACTTCCAAGTTCCCCTTCAGCAAGACCCTCATTCCTCTGGGG
AGAGAGGGAGAGTTGGCCATGTTATATTCGAAAGAGCCTGGGTATTGGAATTGGCCATA
TTTAGTGGTGAGAGCCTGTGAT
>1134
CTCCTGAGACTTCCAAGTTCCCCTTCAGCAAGACCCTCATTCCTCTGGGGAGAGAGGG
AGAGTTGGCCATGTTATATTCGAAAGAGCCTGGGTATTGGAATTGGCCATATTTAGTGGT
GAGAGCCTGTGATACTTATTTA
>1135
TCTGGGGAGAGAGGGAGAGTTGGCCATGTTATATTCGAAAGAGCCTGGGTATTGGAAT
TGGCCATATTTAGTGGTGAGAGCCTGTGATACTTATTTAACCTTTCTGGGCCTTGTTTCA
CTCACTACATTTTTTTTTTTTT
>1136
GGGGAGAGAGGGAGAGTTGGCCATGTTATATTCGAAAGAGCCTGGGTATTGGAATTGG
CCATATTTAGTGGTGAGAGCCTGTGATACTTATTTAACCTTTCTGGGCCTTGTTTCACTC
ACTACATTTTTTTTTTTTTTTT
>1137
GCCTGGGTATTGGAATTGGCCATATTTAGTGGTGAGAGCCTGTGATACTTATTTAACCTT
TCTGGGCCTTGTTTCACTCACTACATTTTTTTTTTTTTTTTTTTTTGAGACGGAGTCTCG
CTCTGTCACCGGGCTGGAGT
>1138
ATACTTATTTAACCTTTCTGGGCCTTGTTTCACTCACTACATTTTTTTTTTTTTTTTTTT
GAGACGGAGTCTCGCTCTGTCACCGGGCTGGAGTGCAATGGCGTGATCTCAGCTCACT
GCAGTCTCCACCTCCCGGGT
>1139
AACCTTTCTGGGCCTTGTTTCACTCACTACATTTTTTTTTTTTTTTTTTTTTGAGACGGA
GTCTCGCTCTGTCACCGGGCTGGAGTGCAATGGCGTGATCTCAGCTCACTGCAGTCTC
CACCTCCCGGGTTCAAGCGATT
>1140
TTGTTTCACTCACTACATTTTTTTTTTTTTTTTTTTTGAGACGGAGTCTCGCTCTGTCAC
CGGGCTGGAGTGCAATGGCGTGATCTCAGCTCACTGCAGTCTCCACCTCCCGGGTTCA

AGCGATTCTCCTGCCTCAGCC
>1141
CACTACATTTTTTTTTTTTTTTTTTTTTGAGACGGAGTCTCGCTCTGTCACCGGGCTGGA
GTGCAATGGCGTGATCTCAGCTCACTGCAGTCTCCACCTCCCGGGTTCAAGCGATTCTC
CTGCCTCAGCCTCCTGAGTAG
>1142
TTTTTTTTTGAGACGGAGTCTCGCTCTGTCACCGGGCTGGAGTGCAATGGCGTGATCTC
AGCTCACTGCAGTCTCCACCTCCCGGGTTCAAGCGATTCTCCTGCCTCAGCCTCCTGA
GTAGCTGGGACTACAGGCACCTG
>1143
TGGCGTGATCTCAGCTCACTGCAGTCTCCACCTCCCGGGTTCAAGCGATTCTCCTGCCT
CAGCCTCCTGAGTAGCTGGGACTACAGGCACCTGTCACCCCGCCCAGCTAATTTTTTGT
ATTTTTAGTAGAGACAGGGTTT
>1144
AGTCTCCACCTCCCGGGTTCAAGCGATTCTCCTGCCTCAGCCTCCTGAGTAGCTGGGA
CTACAGGCACCTGTCACCCCGCCCAGCTAATTTTTTGTATTTTTAGTAGAGACAGGGTT
TCACCATGTTGGCCAGAATGGTC
>1145
GATTCTCCTGCCTCAGCCTCCTGAGTAGCTGGGACTACAGGCACCTGTCACCCCGCCC
AGCTAATTTTTTGTATTTTTAGTAGAGACAGGGTTTCACCATGTTGGCCAGAATGGTCTC
GATCTCTTGACCTTGCGATCCA
>1146
GCCTCAGCCTCCTGAGTAGCTGGGACTACAGGCACCTGTCACCCCGCCCAGCTAATTT
TTTGTATTTTTAGTAGAGACAGGGTTTCACCATGTTGGCCAGAATGGTCTCGATCTCTTG
ACCTTGCGATCCATCCACCTCG
>1147
GGGACTACAGGCACCTGTCACCCCGCCCAGCTAATTTTTTGTATTTTTAGTAGAGACAG
GGTTTCACCATGTTGGCCAGAATGGTCTCGATCTCTTGACCTTGCGATCCATCCACCTC
GGCCTCCCAAAGTGCTGGGATT
>1148
GGTTTCACCATGTTGGCCAGAATGGTCTCGATCTCTTGACCTTGCGATCCATCCACCTC
GGCCTCCCAAAGTGCTGGGATTACAGCTGTGAGCCACCCCGCCCGGCCTTCACTCACT
TCTTAAAATGGAGACGGTCAACA
>1149
CAGAATGGTCTCGATCTCTTGACCTTGCGATCCATCCACCTCGGCCTCCCAAAGTGCTG
GGATTACAGCTGTGAGCCACCCCGCCCGGCCTTCACTCACTTCTTAAAATGGAGACGG
TCAACAGGGTTGGTGCTGAATTA
>1150
TCCCAAAGTGCTGGGATTACAGCTGTGAGCCACCCCGCCCGGCCTTCACTCACTTCTT
AAAATGGAGACGGTCAACAGGGTTGGTGCTGAATTAATGAAGTAAGATCCAGAAATCA
CTAGAGCTGTGATGGGCACATAGT
>1151
GCTGGGATTACAGCTGTGAGCCACCCCGCCCGGCCTTCACTCACTTCTTAAAATGGAG
ACGGTCAACAGGGTTGGTGCTGAATTAATGAAGTAAGATCCAGAAATCACTAGAGCTG

TGATGGGCACATAGTCACCGCAGC
>1152
CCACCCCGCCCGGCCTTCACTCACTTCTTAAAATGGAGACGGTCAACAGGGTTGGTGC
TGAATTAATGAAGTAAGATCCAGAAATCACTAGAGCTGTGATGGGCACATAGTCACCG
CAGCGGTGGAGAACTGACCATCTG
>1153
TCTTAAAATGGAGACGGTCAACAGGGTTGGTGCTGAATTAATGAAGTAAGATCCAGAA
ATCACTAGAGCTGTGATGGGCACATAGTCACCGCAGCGGTGGAGAACTGACCATCTGT
CCCTTGTGTTGTTTAGGCCTCATC
>1154
CGGTCAACAGGGTTGGTGCTGAATTAATGAAGTAAGATCCAGAAATCACTAGAGCTGT
GATGGGCACATAGTCACCGCAGCGGTGGAGAACTGACCATCTGTCCCTTGTGTTGTTT
AGGCCTCATCTCTGCATGGTCCTC
>1155
TTGGTGCTGAATTAATGAAGTAAGATCCAGAAATCACTAGAGCTGTGATGGGCACATAG
TCACCGCAGCGGTGGAGAACTGACCATCTGTCCCTTGTGTTGTTTAGGCCTCATCTCTG
CATGGTCCTCATTGGCTCTGAG
>1156
AGTAAGATCCAGAAATCACTAGAGCTGTGATGGGCACATAGTCACCGCAGCGGTGGAG
AACTGACCATCTGTCCCTTGTGTTGTTTAGGCCTCATCTCTGCATGGTCCTCATTGGCTC
TGAGTTCTAGTGTGTAGTGTCC
>1157
TCCAGAAATCACTAGAGCTGTGATGGGCACATAGTCACCGCAGCGGTGGAGAACTGAC
CATCTGTCCCTTGTGTTGTTTAGGCCTCATCTCTGCATGGTCCTCATTGGCTCTGAGTTC
TAGTGTGTAGTGTCCACAGTGC
>1158
CACATAGTCACCGCAGCGGTGGAGAACTGACCATCTGTCCCTTGTGTTGTTTAGGCCTC
ATCTCTGCATGGTCCTCATTGGCTCTGAGTTCTAGTGTGTAGTGTCCACAGTGCCACCC
TCCATGAGGGTTGTGTTGACTG
>1159
GGTGGAGAACTGACCATCTGTCCCTTGTGTTGTTTAGGCCTCATCTCTGCATGGTCCTC
ATTGGCTCTGAGTTCTAGTGTGTAGTGTCCACAGTGCCACCCTCCATGAGGGTTGTGTT
GACTGTGAGGTGGTGCAGAGAA
>1160
GAACTGACCATCTGTCCCTTGTGTTGTTTAGGCCTCATCTCTGCATGGTCCTCATTGGCT
CTGAGTTCTAGTGTGTAGTGTCCACAGTGCCACCCTCCATGAGGGTTGTGTTGACTGTG
AGGTGGTGCAGAGAATGTCCC
>1161
CCTTGTGTTGTTTAGGCCTCATCTCTGCATGGTCCTCATTGGCTCTGAGTTCTAGTGTGT
AGTGTCCACAGTGCCACCCTCCATGAGGGTTGTGTTGACTGTGAGGTGGTGCAGAGAA
TGTCCCTGTCCTTGTGAGCATA
>1162
GGTCCTCATTGGCTCTGAGTTCTAGTGTGTAGTGTCCACAGTGCCACCCTCCATGAGGG
TTGTGTTGACTGTGAGGTGGTGCAGAGAATGTCCCTGTCCTTGTGAGCATATGACAGC

AAGACACTGGCCCTGGAATCCTC
>1163
TCATTGGCTCTGAGTTCTAGTGTGTAGTGTCCACAGTGCCACCCTCCATGAGGGTTGTG
TTGACTGTGAGGTGGTGCAGAGAATGTCCCTGTCCTTGTGAGCATATGACAGCAAGAC
ACTGGCCCTGGAATCCTCCGTGT
>1164
TTCTAGTGTGTAGTGTCCACAGTGCCACCCTCCATGAGGGTTGTGTTGACTGTGAGGT
GGTGCAGAGAATGTCCCTGTCCTTGTGAGCATATGACAGCAAGACACTGGCCCTGGAA
TCCTCCGTGTCACATGTGGGCTGC
>1165
TGAGGTGGTGCAGAGAATGTCCCTGTCCTTGTGAGCATATGACAGCAAGACACTGGCC
CTGGAATCCTCCGTGTCACATGTGGGCTGCTGACAGGAAGAATGTGCTTTTGTTGTTGT
TGTTGTTGTTGAGGCAGAGTCTT
>1166
AGGTGGTGCAGAGAATGTCCCTGTCCTTGTGAGCATATGACAGCAAGACACTGGCCCT
GGAATCCTCCGTGTCACATGTGGGCTGCTGACAGGAAGAATGTGCTTTTGTTGTTGTTG
TTGTTGTTGAGGCAGAGTCTTAC
>1167
TGAGCATATGACAGCAAGACACTGGCCCTGGAATCCTCCGTGTCACATGTGGGCTGCT
GACAGGAAGAATGTGCTTTTGTTGTTGTTGTTGTTGTTGAGGCAGAGTCTTACTCTGTC
GCCCAGGCTGCAGTGCAGTGGCA
>1168
GACACTGGCCCTGGAATCCTCCGTGTCACATGTGGGCTGCTGACAGGAAGAATGTGCT
TTTGTTGTTGTTGTTGTTGTTGAGGCAGAGTCTTACTCTGTCGCCCAGGCTGCAGTGCA
GTGGCATGATCTTGGCTCACTGC
>1169
ACTGGCCCTGGAATCCTCCGTGTCACATGTGGGCTGCTGACAGGAAGAATGTGCTTTT
GTTGTTGTTGTTGTTGTTGAGGCAGAGTCTTACTCTGTCGCCCAGGCTGCAGTGCAGT
GGCATGATCTTGGCTCACTGCAAC
>1170
CCTCCGTGTCACATGTGGGCTGCTGACAGGAAGAATGTGCTTTTGTTGTTGTTGTTGTT
GTTGAGGCAGAGTCTTACTCTGTCGCCCAGGCTGCAGTGCAGTGGCATGATCTTGGCT
CACTGCAACCTCTACTTCCCGGG
>1171
ACATGTGGGCTGCTGACAGGAAGAATGTGCTTTTGTTGTTGTTGTTGTTGTTGAGGCA
GAGTCTTACTCTGTCGCCCAGGCTGCAGTGCAGTGGCATGATCTTGGCTCACTGCAAC
CTCTACTTCCCGGGTTCAAGTGAT
>1172
AGTCTTACTCTGTCGCCCAGGCTGCAGTGCAGTGGCATGATCTTGGCTCACTGCAACCT
CTACTTCCCGGGTTCAAGTGATTCTCCTGCCTCAGCCTCCTGAGCAGCTGGGACTACA
GATGTGTACCACCATGCCCGACT
>1173
ACTCTGTCGCCCAGGCTGCAGTGCAGTGGCATGATCTTGGCTCACTGCAACCTCTACTT
CCCGGGTTCAAGTGATTCTCCTGCCTCAGCCTCCTGAGCAGCTGGGACTACAGATGTG

TACCACCATGCCCGACTAATTTT
>1174
CTGCAACCTCTACTTCCCGGGTTCAAGTGATTCTCCTGCCTCAGCCTCCTGAGCAGCTG
GGACTACAGATGTGTACCACCATGCCCGACTAATTTTTGTATTTTTAGTAGAGATGGGGT
TTCACCATGTTGGTCAGGCTG
>1175
GCAACCTCTACTTCCCGGGTTCAAGTGATTCTCCTGCCTCAGCCTCCTGAGCAGCTGG
GACTACAGATGTGTACCACCATGCCCGACTAATTTTTGTATTTTTAGTAGAGATGGGGTT
TCACCATGTTGGTCAGGCTGGC
>1176
TGATTCTCCTGCCTCAGCCTCCTGAGCAGCTGGGACTACAGATGTGTACCACCATGCCC
GACTAATTTTTGTATTTTTAGTAGAGATGGGGTTTCACCATGTTGGTCAGGCTGGCCTG
GAACTCCTGGCCTTAAGTGATC
>1177
CCTGAGCAGCTGGGACTACAGATGTGTACCACCATGCCCGACTAATTTTTGTATTTTTA
GTAGAGATGGGGTTTCACCATGTTGGTCAGGCTGGCCTGGAACTCCTGGCCTTAAGTG
ATCCACCTGTCTCGGCCTCCCAT
>1178
TGGGACTACAGATGTGTACCACCATGCCCGACTAATTTTTGTATTTTTAGTAGAGATGGG
GTTTCACCATGTTGGTCAGGCTGGCCTGGAACTCCTGGCCTTAAGTGATCCACCTGTCT
CGGCCTCCCATAGTGCTGGGA
>1179
ATTTTTGTATTTTTAGTAGAGATGGGGTTTCACCATGTTGGTCAGGCTGGCCTGGAACT
CCTGGCCTTAAGTGATCCACCTGTCTCGGCCTCCCATAGTGCTGGGATTATAGGTGTGT
GCCACCCTGCTGGGCCGAGAAT
>1180
TTTAGTAGAGATGGGGTTTCACCATGTTGGTCAGGCTGGCCTGGAACTCCTGGCCTTAA
GTGATCCACCTGTCTCGGCCTCCCATAGTGCTGGGATTATAGGTGTGTGCCACCCTGCT
GGGCCGAGAATGTGCTTTTGAC
>1181
TTTCACCATGTTGGTCAGGCTGGCCTGGAACTCCTGGCCTTAAGTGATCCACCTGTCTC
GGCCTCCCATAGTGCTGGGATTATAGGTGTGTGCCACCCTGCTGGGCCGAGAATGTGCT
TTTGACTGAGGGTGGAAAGTCA
>1182
CAGGCTGGCCTGGAACTCCTGGCCTTAAGTGATCCACCTGTCTCGGCCTCCCATAGTGC
TGGGATTATAGGTGTGTGCCACCCTGCTGGGCCGAGAATGTGCTTTTGACTGAGGGTG
GAAAGTCAGCAGCTGCAGCCCAA
>1183
GGCCTCCCATAGTGCTGGGATTATAGGTGTGTGCCACCCTGCTGGGCCGAGAATGTGCT
TTTGACTGAGGGTGGAAAGTCAGCAGCTGCAGCCCAAGAAGTTAGAGCCAGGGGCAG
TGCCTTTGATGGGAAGCTTGGGGG
>1184
CATAGTGCTGGGATTATAGGTGTGTGCCACCCTGCTGGGCCGAGAATGTGCTTTTGACT
GAGGGTGGAAAGTCAGCAGCTGCAGCCCAAGAAGTTAGAGCCAGGGGCAGTGCCTTT

GATGGGAAGCTTGGGGGTCTAAAG
>1185
TGTGCCACCCTGCTGGGCCGAGAATGTGCTTTTGACTGAGGGTGGAAAGTCAGCAGCT
GCAGCCCAAGAAGTTAGAGCCAGGGGCAGTGCCTTTGATGGGAAGCTTGGGGGTCTA
AAGAAATCAGAAAGGGGAGAAAATA
>1186
GCCGAGAATGTGCTTTTGACTGAGGGTGGAAAGTCAGCAGCTGCAGCCCAAGAAGTT
AGAGCCAGGGGCAGTGCCTTTGATGGGAAGCTTGGGGGTCTAAAGAAATCAGAAAGG
GGAGAAAATAATCCTGCGTGTGCTCA
>1187
GGTGGAAAGTCAGCAGCTGCAGCCCAAGAAGTTAGAGCCAGGGGCAGTGCCTTTGAT
GGGAAGCTTGGGGGTCTAAAGAAATCAGAAAGGGGAGAAAATAATCCTGCGTGTGCT
CAGCCCTCACTCAGATCAACAGCCCC
>1188
CCAGGGGCAGTGCCTTTGATGGGAAGCTTGGGGGTCTAAAGAAATCAGAAAGGGGAG
AAAATAATCCTGCGTGTGCTCAGCCCTCACTCAGATCAACAGCCCCTCGGGTCCCAGC
TAGATGTACTTTGTAGAAGGCTTTT
>1189
AGGGGCAGTGCCTTTGATGGGAAGCTTGGGGGTCTAAAGAAATCAGAAAGGGGAGAA
AATAATCCTGCGTGTGCTCAGCCCTCACTCAGATCAACAGCCCCTCGGGTCCCAGCTA
GATGTACTTTGTAGAAGGCTTTTTT
>1190
AAAGAAATCAGAAAGGGGAGAAAATAATCCTGCGTGTGCTCAGCCCTCACTCAGATCA
ACAGCCCCTCGGGTCCCAGCTAGATGTACTTTGTAGAAGGCTTTTTTTCTCCCCCTCAC
CCTCAAAACTCCAGGATTAATGT
>1191
AGGGGAGAAAATAATCCTGCGTGTGCTCAGCCCTCACTCAGATCAACAGCCCCTCGGG
TCCCAGCTAGATGTACTTTGTAGAAGGCTTTTTTTCTCCCCCTCACCCTCAAAACTCCA
GGATTAATGTGCTATTCCCTTTT
>1192
GGGTCCCAGCTAGATGTACTTTGTAGAAGGCTTTTTTTCTCCCCCTCACCCTCAAAACT
CCAGGATTAATGTGCTATTCCCTTTTTTCTACTGTTCTCTGTCTCCCCCCAACCCATCAG
CAGTCTGAGAAATGCTATGGT
>1193
GTAGAAGGCTTTTTTTCTCCCCCTCACCCTCAAAACTCCAGGATTAATGTGCTATTCCCT
TTTTTCTACTGTTCTCTGTCTCCCCCCAACCCATCAGCAGTCTGAGAAATGCTATGGTG
GAAAAAAAAAAACAACAGCCC
>1194
GCTTTTTTTCTCCCCCTCACCCTCAAAACTCCAGGATTAATGTGCTATTCCCTTTTTTCT
ACTGTTCTCTGTCTCCCCCCAACCCATCAGCAGTCTGAGAAATGCTATGGTGGAAAAA
AAAAAACAACAGCCCTATAACC
>1195
TCAAAACTCCAGGATTAATGTGCTATTCCCTTTTTTCTACTGTTCTCTGTCTCCCCCCAA
CCCATCAGCAGTCTGAGAAATGCTATGGTGGAAAAAAAAAAACAACAGCCCTATAACC

AGTACAAACAAAAAGCCAAGAC
>1196
TCTCCCCCCAACCCATCAGCAGTCTGAGAAATGCTATGGTGGAAAAAAAAAAAACAAC
AGCCCTATAACCAGTACAAACAAAAAGCCAAGACAAAAAAAAAAAAAAAAAAAAATC
AGAAGGGGAGATTTTGAGGCCAGGAAG
>1197
AAAAAAAAAAAAAAAATCAGAAGGGGAGATTTTGAGGCCAGGAAGCCATGGCATTCAC
TGGAAATATATCAGTGATAAAATAACAGGCTACAGGGTTTATTTTATTTTATTTTTAAGTC
AATAGGCCTGAGTGTTGGTAAA
>1198
GGAGATTTTGAGGCCAGGAAGCCATGGCATTCACTGGAAATATATCAGTGATAAAATAA
CAGGCTACAGGGTTTATTTTATTTTATTTTTAAGTCAATAGGCCTGAGTGTTGGTAAAGA
CTAGATACCCAGCAATCCTTC
>1199
CATGGCATTCACTGGAAATATATCAGTGATAAAATAACAGGCTACAGGGTTTATTTTATT
TTATTTTTAAGTCAATAGGCCTGAGTGTTGGTAAAGACTAGATACCCAGCAATCCTTCTT
GCATTTCTTCTTGGGGTCGG
>1200
GAAATATATCAGTGATAAAATAACAGGCTACAGGGTTTATTTTATTTTATTTTTAAGTCAA
TAGGCCTGAGTGTTGGTAAAGACTAGATACCCAGCAATCCTTCTTGCATTTCTTCTTGG
GGTCGGGGGGCAGAAGGGAA
>1201
AATATATCAGTGATAAAATAACAGGCTACAGGGTTTATTTTATTTTATTTTTAAGTCAATA
GGCCTGAGTGTTGGTAAAGACTAGATACCCAGCAATCCTTCTTGCATTTCTTCTTGGGG
TCGGGGGGCAGAAGGGAATG
>1202
TATCAGTGATAAAATAACAGGCTACAGGGTTTATTTTATTTTATTTTTAAGTCAATAGGCC
TGAGTGTTGGTAAAGACTAGATACCCAGCAATCCTTCTTGCATTTCTTCTTGGGGTCGG
GGGGCAGAAGGGAATGGAGA
>1203
TTTAAGTCAATAGGCCTGAGTGTTGGTAAAGACTAGATACCCAGCAATCCTTCTTGCAT
TTCTTCTTGGGGTCGGGGGGCAGAAGGGAATGGAGAGGAAATGAAGGCGGATTGCTG
GTAAGTGAGGCAGGAAGGCAGGGA
>1204
ATCCTTCTTGCATTTCTTCTTGGGGTCGGGGGGCAGAAGGGAATGGAGAGGAAATGAA
GGCGGATTGCTGGTAAGTGAGGCAGGAAGGCAGGGAGTTTATTATGGAATGTCATCGG
TAAGTTTCCCCATGATTTGATCCC
>1205
TTCTTGCATTTCTTCTTGGGGTCGGGGGGCAGAAGGGAATGGAGAGGAAATGAAGGC
GGATTGCTGGTAAGTGAGGCAGGAAGGCAGGGAGTTTATTATGGAATGTCATCGGTAA
GTTTCCCCATGATTTGATCCCAAGC
>1206
GTCGGGGGGCAGAAGGGAATGGAGAGGAAATGAAGGCGGATTGCTGGTAAGTGAGG
CAGGAAGGCAGGGAGTTTATTATGGAATGTCATCGGTAAGTTTCCCCATGATTTGATCC

CAAGCTGGTGTGGAGTGAAGGGTAA
>1207
GGGAATGGAGAGGAAATGAAGGCGGATTGCTGGTAAGTGAGGCAGGAAGGCAGGGA
GTTTATTATGGAATGTCATCGGTAAGTTTCCCCATGATTTGATCCCAAGCTGGTGTGGAG
TGAAGGGTAAGCTGTTGGCTCAAC
>1208
GAGAGGAAATGAAGGCGGATTGCTGGTAAGTGAGGCAGGAAGGCAGGGAGTTTATTA
TGGAATGTCATCGGTAAGTTTCCCCATGATTTGATCCCAAGCTGGTGTGGAGTGAAGGG
TAAGCTGTTGGCTCAACAGATAGT
>1209
GAGGCAGGAAGGCAGGGAGTTTATTATGGAATGTCATCGGTAAGTTTCCCCATGATTTG
ATCCCAAGCTGGTGTGGAGTGAAGGGTAAGCTGTTGGCTCAACAGATAGTGAGAATCA
AACTTAGTGGAGTTGTAGATCCT
>1210
GAAGGCAGGGAGTTTATTATGGAATGTCATCGGTAAGTTTCCCCATGATTTGATCCCAA
GCTGGTGTGGAGTGAAGGGTAAGCTGTTGGCTCAACAGATAGTGAGAATCAAACTTAG
TGGAGTTGTAGATCCTGTTTCCT
>1211
GGGAGTTTATTATGGAATGTCATCGGTAAGTTTCCCCATGATTTGATCCCAAGCTGGTGT
GGAGTGAAGGGTAAGCTGTTGGCTCAACAGATAGTGAGAATCAAACTTAGTGGAGTTG
TAGATCCTGTTTCCTGCAGCGG
>1212
TATTATGGAATGTCATCGGTAAGTTTCCCCATGATTTGATCCCAAGCTGGTGTGGAGTGA
AGGGTAAGCTGTTGGCTCAACAGATAGTGAGAATCAAACTTAGTGGAGTTGTAGATCC
TGTTTCCTGCAGCGGACAGCAA
>1213
CGGTAAGTTTCCCCATGATTTGATCCCAAGCTGGTGTGGAGTGAAGGGTAAGCTGTTG
GCTCAACAGATAGTGAGAATCAAACTTAGTGGAGTTGTAGATCCTGTTTCCTGCAGCG
GACAGCAAGATGTACAAGCCGTCC
>1214
GTGTGGAGTGAAGGGTAAGCTGTTGGCTCAACAGATAGTGAGAATCAAACTTAGTGGA
GTTGTAGATCCTGTTTCCTGCAGCGGACAGCAAGATGTACAAGCCGTCCACTGCAGGA
ATGACGTGCTGTAAAAGGGATGCA
>1215
GGCTCAACAGATAGTGAGAATCAAACTTAGTGGAGTTGTAGATCCTGTTTCCTGCAGC
GGACAGCAAGATGTACAAGCCGTCCACTGCAGGAATGACGTGCTGTAAAAGGGATGC
AGTGTCAAGTGTAGTCATGGATGGG
>1216
GATAGTGAGAATCAAACTTAGTGGAGTTGTAGATCCTGTTTCCTGCAGCGGACAGCAA
GATGTACAAGCCGTCCACTGCAGGAATGACGTGCTGTAAAAGGGATGCAGTGTCAAGT
GTAGTCATGGATGGGTGGGAAGAC
>1217
GAGTTGTAGATCCTGTTTCCTGCAGCGGACAGCAAGATGTACAAGCCGTCCACTGCAG
GAATGACGTGCTGTAAAAGGGATGCAGTGTCAAGTGTAGTCATGGATGGGTGGGAAG

ACCAGACAAAGGCATCCAATGGGAC
>1218
GTTTCCTGCAGCGGACAGCAAGATGTACAAGCCGTCCACTGCAGGAATGACGTGCTGT
AAAAGGGATGCAGTGTCAAGTGTAGTCATGGATGGGTGGGAAGACCAGACAAAGGCA
TCCAATGGGACTGACTTACCTAAAC
>1219
TTCCTGCAGCGGACAGCAAGATGTACAAGCCGTCCACTGCAGGAATGACGTGCTGTAA
AAGGGATGCAGTGTCAAGTGTAGTCATGGATGGGTGGGAAGACCAGACAAAGGCATC
CAATGGGACTGACTTACCTAAACAA
>1220
TGTACAAGCCGTCCACTGCAGGAATGACGTGCTGTAAAAGGGATGCAGTGTCAAGTGT
AGTCATGGATGGGTGGGAAGACCAGACAAAGGCATCCAATGGGACTGACTTACCTAA
ACAAACCAGAAGGAAGTCAGAACAG
>1221
TCATGGATGGGTGGGAAGACCAGACAAAGGCATCCAATGGGACTGACTTACCTAAACA
AACCAGAAGGAAGTCAGAACAGTGGGAAGATCTGATAGATAAGCAGTATTCTGCAGG
CAGATGAAGGAGCAAGCTTTGGGCC
>1222
GGTGGGAAGACCAGACAAAGGCATCCAATGGGACTGACTTACCTAAACAAACCAGAA
GGAAGTCAGAACAGTGGGAAGATCTGATAGATAAGCAGTATTCTGCAGGCAGATGAAG
GAGCAAGCTTTGGGCCCCCAAATCT
>1223
CCAATGGGACTGACTTACCTAAACAAACCAGAAGGAAGTCAGAACAGTGGGAAGATC
TGATAGATAAGCAGTATTCTGCAGGCAGATGAAGGAGCAAGCTTTGGGCCCCCAAATC
TTCACCAGTGCATCCACATTACAGA
>1224
AGATCTGATAGATAAGCAGTATTCTGCAGGCAGATGAAGGAGCAAGCTTTGGGCCCCC
AAATCTTCACCAGTGCATCCACATTACAGAGGATAATGCCTGAGGGAGGGGAAGATCA
TCTCAGGATTCTGGAACACTGGGC
>1225
GGGAGGGGAAGATCATCTCAGGATTCTGGAACACTGGGCCCTCCCTTTCTTATTCCAA
AAGGCTGGAGCAGTTCTGCATGTAGGCTTCTCTGGAAATGGTCTAGGAACCAGCTCAT
CCAGGCCAGGAGCACCTGATCATT
>1226
AGATCATCTCAGGATTCTGGAACACTGGGCCCTCCCTTTCTTATTCCAAAAGGCTGGAG
CAGTTCTGCATGTAGGCTTCTCTGGAAATGGTCTAGGAACCAGCTCATCCAGGCCAGG
AGCACCTGATCATTCTCAGAGTT
>1227
GAACACTGGGCCCTCCCTTTCTTATTCCAAAAGGCTGGAGCAGTTCTGCATGTAGGCTT
CTCTGGAAATGGTCTAGGAACCAGCTCATCCAGGCCAGGAGCACCTGATCATTCTCAG
AGTTACGTACATATGCGATAAGA
>1228
AGGCTTCTCTGGAAATGGTCTAGGAACCAGCTCATCCAGGCCAGGAGCACCTGATCAT
TCTCAGAGTTACGTACATATGCGATAAGATTGCAGGGAACAGTATAGTTCTATAGACTTA

AGTGTGAACATAGTCCTCATTT
>1229
GCTCATCCAGGCCAGGAGCACCTGATCATTCTCAGAGTTACGTACATATGCGATAAGAT
TGCAGGGAACAGTATAGTTCTATAGACTTAAGTGTGAACATAGTCCTCATTTATAGTCCC
TAGTCTCCCATGGCTCACGCA
>1230
CCTGATCATTCTCAGAGTTACGTACATATGCGATAAGATTGCAGGGAACAGTATAGTTCT
ATAGACTTAAGTGTGAACATAGTCCTCATTTATAGTCCCTAGTCTCCCATGGCTCACGCA
GCTTGGTCTTTATGTGTTTC
>1231
TCAGAGTTACGTACATATGCGATAAGATTGCAGGGAACAGTATAGTTCTATAGACTTAA
GTGTGAACATAGTCCTCATTTATAGTCCCTAGTCTCCCATGGCTCACGCAGCTTGGTCTT
TATGTGTTTCTGAATTTAGTG
>1232
CATATGCGATAAGATTGCAGGGAACAGTATAGTTCTATAGACTTAAGTGTGAACATAGT
CCTCATTTATAGTCCCTAGTCTCCCATGGCTCACGCAGCTTGGTCTTTATGTGTTTCTGA
ATTTAGTGCTGCAGAGGCACA
>1233
GATAAGATTGCAGGGAACAGTATAGTTCTATAGACTTAAGTGTGAACATAGTCCTCATTT
ATAGTCCCTAGTCTCCCATGGCTCACGCAGCTTGGTCTTTATGTGTTTCTGAATTTAGTG
CTGCAGAGGCACAATGGGGT
>1234
GTTCTATAGACTTAAGTGTGAACATAGTCCTCATTTATAGTCCCTAGTCTCCCATGGCTC
ACGCAGCTTGGTCTTTATGTGTTTCTGAATTTAGTGCTGCAGAGGCACAATGGGGTCTG
GTTTCTGGATCTTATACAGAC
>1235
ATAGTCCTCATTTATAGTCCCTAGTCTCCCATGGCTCACGCAGCTTGGTCTTTATGTGTTT
CTGAATTTAGTGCTGCAGAGGCACAATGGGGTCTGGTTTCTGGATCTTATACAGACAAG
CACTTAGGTCAGCTGCAGTG
>1236
TAGTCTCCCATGGCTCACGCAGCTTGGTCTTTATGTGTTTCTGAATTTAGTGCTGCAGA
GGCACAATGGGGTCTGGTTTCTGGATCTTATACAGACAAGCACTTAGGTCAGCTGCAG
TGGATGTCCTGCACAGACACCGT
>1237
GTTTCTGAATTTAGTGCTGCAGAGGCACAATGGGGTCTGGTTTCTGGATCTTATACAGA
CAAGCACTTAGGTCAGCTGCAGTGGATGTCCTGCACAGACACCGTCGCAACTCTTGCC
TGGTTCCTTTTTGTTTTTGGAGA
>1238
TTAGTGCTGCAGAGGCACAATGGGGTCTGGTTTCTGGATCTTATACAGACAAGCACTTA
GGTCAGCTGCAGTGGATGTCCTGCACAGACACCGTCGCAACTCTTGCCTGGTTCCTTT
TTGTTTTTGGAGATGGAGTCTCG
>1239
GGGTCTGGTTTCTGGATCTTATACAGACAAGCACTTAGGTCAGCTGCAGTGGATGTCCT
GCACAGACACCGTCGCAACTCTTGCCTGGTTCCTTTTTGTTTTTGGAGATGGAGTCTCG

CTCTGTCTCCCAGGCTGGAGTG
>1240
CAAGCACTTAGGTCAGCTGCAGTGGATGTCCTGCACAGACACCGTCGCAACTCTTGCC
TGGTTCCTTTTTGTTTTTGGAGATGGAGTCTCGCTCTGTCTCCCAGGCTGGAGTGCAGT
GGCGCGATCTCAGCTCACTGCAA
>1241
GCTGCAGTGGATGTCCTGCACAGACACCGTCGCAACTCTTGCCTGGTTCCTTTTTGTTT
TTGGAGATGGAGTCTCGCTCTGTCTCCCAGGCTGGAGTGCAGTGGCGCGATCTCAGCT
CACTGCAAGCTCTGCCTCCCAGG
>1242
ATGTCCTGCACAGACACCGTCGCAACTCTTGCCTGGTTCCTTTTTGTTTTTGGAGATGG
AGTCTCGCTCTGTCTCCCAGGCTGGAGTGCAGTGGCGCGATCTCAGCTCACTGCAAGC
TCTGCCTCCCAGGTTCACGCTAT
>1243
AGTCTCGCTCTGTCTCCCAGGCTGGAGTGCAGTGGCGCGATCTCAGCTCACTGCAAGC
TCTGCCTCCCAGGTTCACGCTATTCTCCTGCCTCAGCCTCCCAAGTAGTTGGGACTACA
GGCACCCGCCACCACGCCCGGCT
>1244
TGGCGCGATCTCAGCTCACTGCAAGCTCTGCCTCCCAGGTTCACGCTATTCTCCTGCCT
CAGCCTCCCAAGTAGTTGGGACTACAGGCACCCGCCACCACGCCCGGCTAATTTTTTG
TATTTTTAGTAGAGACAGGGTTT
>1245
CGCGATCTCAGCTCACTGCAAGCTCTGCCTCCCAGGTTCACGCTATTCTCCTGCCTCAG
CCTCCCAAGTAGTTGGGACTACAGGCACCCGCCACCACGCCCGGCTAATTTTTTGTATT
TTTAGTAGAGACAGGGTTTCAC
>1246
TATTCTCCTGCCTCAGCCTCCCAAGTAGTTGGGACTACAGGCACCCGCCACCACGCCC
GGCTAATTTTTTGTATTTTTAGTAGAGACAGGGTTTCACCGTGTTAGCCAGTATGGTCTC
GATCTCCTGAACTCGTGATCCA
>1247
CCAAGTAGTTGGGACTACAGGCACCCGCCACCACGCCCGGCTAATTTTTTGTATTTTTA
GTAGAGACAGGGTTTCACCGTGTTAGCCAGTATGGTCTCGATCTCCTGAACTCGTGATC
CACCTGCCTCGGCCTCCCAAAG
>1248
TTGGGACTACAGGCACCCGCCACCACGCCCGGCTAATTTTTTGTATTTTTAGTAGAGAC
AGGGTTTCACCGTGTTAGCCAGTATGGTCTCGATCTCCTGAACTCGTGATCCACCTGCC
TCGGCCTCCCAAAGTGCTGGGA
>1249
CCCGCCACCACGCCCGGCTAATTTTTTGTATTTTTAGTAGAGACAGGGTTTCACCGTGT
TAGCCAGTATGGTCTCGATCTCCTGAACTCGTGATCCACCTGCCTCGGCCTCCCAAAGT
GCTGGGATTACAGGTGTGAGCC
>1250
GGTTTCACCGTGTTAGCCAGTATGGTCTCGATCTCCTGAACTCGTGATCCACCTGCCTC
GGCCTCCCAAAGTGCTGGGATTACAGGTGTGAGCCACCACGCCCGGCCTATTGCCTGG

TTCTTGAAGGGATTCTCAGGAAC
>1251
GCCAGTATGGTCTCGATCTCCTGAACTCGTGATCCACCTGCCTCGGCCTCCCAAAGTGC
TGGGATTACAGGTGTGAGCCACCACGCCCGGCCTATTGCCTGGTTCTTGAAGGGATTCT
CAGGAACCACAGGGAATGTCTG
>1252
ATCCACCTGCCTCGGCCTCCCAAAGTGCTGGGATTACAGGTGTGAGCCACCACGCCCG
GCCTATTGCCTGGTTCTTGAAGGGATTCTCAGGAACCACAGGGAATGTCTGAGCCAGC
TTTCTGGATCCGTGTGTACAGCCC
>1253
TACAGGTGTGAGCCACCACGCCCGGCCTATTGCCTGGTTCTTGAAGGGATTCTCAGGA
ACCACAGGGAATGTCTGAGCCAGCTTTCTGGATCCGTGTGTACAGCCCTTCCCAGAGG
CTTTGTGGAGCCAGCTGTACGGTG
>1254
GGCCTATTGCCTGGTTCTTGAAGGGATTCTCAGGAACCACAGGGAATGTCTGAGCCAG
CTTTCTGGATCCGTGTGTACAGCCCTTCCCAGAGGCTTTGTGGAGCCAGCTGTACGGT
GCATGAGCTTTGTGGCTAAGATAT
>1255
CCTATTGCCTGGTTCTTGAAGGGATTCTCAGGAACCACAGGGAATGTCTGAGCCAGCT
TTCTGGATCCGTGTGTACAGCCCTTCCCAGAGGCTTTGTGGAGCCAGCTGTACGGTGC
ATGAGCTTTGTGGCTAAGATATCA
>1256
TCTCAGGAACCACAGGGAATGTCTGAGCCAGCTTTCTGGATCCGTGTGTACAGCCCTT
CCCAGAGGCTTTGTGGAGCCAGCTGTACGGTGCATGAGCTTTGTGGCTAAGATATCAG
AGTTCTGCCTGGCCCTTGGCCTTG
>1257
CAGGGAATGTCTGAGCCAGCTTTCTGGATCCGTGTGTACAGCCCTTCCCAGAGGCTTT
GTGGAGCCAGCTGTACGGTGCATGAGCTTTGTGGCTAAGATATCAGAGTTCTGCCTGG
CCCTTGGCCTTGGTCTCTCTCAGA
>1258
GTACAGCCCTTCCCAGAGGCTTTGTGGAGCCAGCTGTACGGTGCATGAGCTTTGTGGC
TAAGATATCAGAGTTCTGCCTGGCCCTTGGCCTTGGTCTCTCTCAGATGGTCAGCTGCT
CTGTTTTTAGTGGTTGATCATGT
>1259
GTGGAGCCAGCTGTACGGTGCATGAGCTTTGTGGCTAAGATATCAGAGTTCTGCCTGG
CCCTTGGCCTTGGTCTCTCTCAGATGGTCAGCTGCTCTGTTTTTAGTGGTTGATCATGTA
GAAGGCCAACAGGTGTTGACCT
>1260
CTTTGTGGCTAAGATATCAGAGTTCTGCCTGGCCCTTGGCCTTGGTCTCTCTCAGATGG
TCAGCTGCTCTGTTTTTAGTGGTTGATCATGTAGAAGGCCAACAGGTGTTGACCTTGGT
TTAGGCTACTTTTGGGATTTTT
>1261
GCTAAGATATCAGAGTTCTGCCTGGCCCTTGGCCTTGGTCTCTCTCAGATGGTCAGCTG
CTCTGTTTTTAGTGGTTGATCATGTAGAAGGCCAACAGGTGTTGACCTTGGTTTAGGCT

ACTTTTGGGATTTTTGAGGTTT
>1262
GAGTTCTGCCTGGCCCTTGGCCTTGGTCTCTCTCAGATGGTCAGCTGCTCTGTTTTTAG
TGGTTGATCATGTAGAAGGCCAACAGGTGTTGACCTTGGTTTAGGCTACTTTTGGGATT
TTTGAGGTTTCCTGAGACTCTT
>1263
CTCTCTCAGATGGTCAGCTGCTCTGTTTTTAGTGGTTGATCATGTAGAAGGCCAACAGG
TGTTGACCTTGGTTTAGGCTACTTTTGGGATTTTTGAGGTTTCCTGAGACTCTTGGTTTT
CTTTCATCATCCCGTCCAGAA
>1264
TTTAGTGGTTGATCATGTAGAAGGCCAACAGGTGTTGACCTTGGTTTAGGCTACTTTTG
GGATTTTTGAGGTTTCCTGAGACTCTTGGTTTTCTTTCATCATCCCGTCCAGAACCTCTG
ATGTCAAACTAAATTCTGTTT
>1265
TAGAAGGCCAACAGGTGTTGACCTTGGTTTAGGCTACTTTTGGGATTTTTGAGGTTTCC
TGAGACTCTTGGTTTTCTTTCATCATCCCGTCCAGAACCTCTGATGTCAAACTAAATTCT
GTTTGGAAAAGGGTATGCTGT
>1266
TACTTTTGGGATTTTTGAGGTTTCCTGAGACTCTTGGTTTTCTTTCATCATCCCGTCCAG
AACCTCTGATGTCAAACTAAATTCTGTTTGGAAAAGGGTATGCTGTAGAATAATGATAG
CCACAGCAATGATATTAATAA
>1267
TGAGGTTTCCTGAGACTCTTGGTTTTCTTTCATCATCCCGTCCAGAACCTCTGATGTCA
AACTAAATTCTGTTTGGAAAAGGGTATGCTGTAGAATAATGATAGCCACAGCAATGATA
TTAATAATACCTTTTATCGGTT
>1268
AGACTCTTGGTTTTCTTTCATCATCCCGTCCAGAACCTCTGATGTCAAACTAAATTCTGT
TTGGAAAAGGGTATGCTGTAGAATAATGATAGCCACAGCAATGATATTAATAATACCTTT
TATCGGTTGCAGCATTTTCT
>1269
TCTGTTTGGAAAAGGGTATGCTGTAGAATAATGATAGCCACAGCAATGATATTAATAATA
CCTTTTATCGGTTGCAGCATTTTCTTCTTTGCAAAGCACCTCTAATGACTTTATCTCTCTT
GCCCTCACAGCCTTCCTTG
>1270
TTGCAGCATTTTCTTCTTTGCAAAGCACCTCTAATGACTTTATCTCTCTTGCCCTCACAG
CCTTCCTTGAGTGGGTACAGGTCCATAATCCCATATCTGTGATAATGAAGTCTGAAAAG
CTCTGAAAACTAAAAAGTTTG
>1271
AGCATTTTCTTCTTTGCAAAGCACCTCTAATGACTTTATCTCTCTTGCCCTCACAGCCTT
CCTTGAGTGGGTACAGGTCCATAATCCCATATCTGTGATAATGAAGTCTGAAAAGCTCT
GAAAACTAAAAAGTTTGCAGT
>1272
CTCTAATGACTTTATCTCTCTTGCCCTCACAGCCTTCCTTGAGTGGGTACAGGTCCATAA
TCCCATATCTGTGATAATGAAGTCTGAAAAGCTCTGAAAACTAAAAAGTTTGCAGTAA

ACTCATTTAGTGGCAAAACCTG
>1273
TTATCTCTCTTGCCCTCACAGCCTTCCTTGAGTGGGTACAGGTCCATAATCCCATATCTG
TGATAATGAAGTCTGAAAAGCTCTGAAAACTAAAAAGTTTGCAGTAAACTCATTTAGT
GGCAAAACCTGATCTGAACTGA
>1274
CTTGAGTGGGTACAGGTCCATAATCCCATATCTGTGATAATGAAGTCTGAAAAGCTCTG
AAAACTAAAAAGTTTGCAGTAAACTCATTTAGTGGCAAAACCTGATCTGAACTGATGT
GAGGCTATATTTATCCTGCTTAG
>1275
TAATCCCATATCTGTGATAATGAAGTCTGAAAAGCTCTGAAAACTAAAAAGTTTGCAGT
AAACTCATTTAGTGGCAAAACCTGATCTGAACTGATGTGAGGCTATATTTATCCTGCTTA
GTTTGAATATTCATATGTTTT
>1276
TCTGAAAAGCTCTGAAAACTAAAAAGTTTGCAGTAAACTCATTTAGTGGCAAAACCTG
ATCTGAACTGATGTGAGGCTATATTTATCCTGCTTAGTTTGAATATTCATATGTTTTGCTG
CAAAAATATTAATGTGTTTGA
>1277
AGTTTGCAGTAAACTCATTTAGTGGCAAAACCTGATCTGAACTGATGTGAGGCTATATT
TATCCTGCTTAGTTTGAATATTCATATGTTTTGCTGCAAAAATATTAATGTGTTTGATTAC
AGAGTGCTGCCCTAGACTCT
>1278
ATTTAGTGGCAAAACCTGATCTGAACTGATGTGAGGCTATATTTATCCTGCTTAGTTTGA
ATATTCATATGTTTTGCTGCAAAAATATTAATGTGTTTGATTACAGAGTGCTGCCCTAGA
CTCTGCTGGGGCTGTTAAAC
>1279
GAACTGATGTGAGGCTATATTTATCCTGCTTAGTTTGAATATTCATATGTTTTGCTGCAAA
AATATTAATGTGTTTGATTACAGAGTGCTGCCCTAGACTCTGCTGGGGCTGTTAAACAC
GATAGGTGTCAGAGTACCTT
>1280
TATATTTATCCTGCTTAGTTTGAATATTCATATGTTTTGCTGCAAAAATATTAATGTGTTTG
ATTACAGAGTGCTGCCCTAGACTCTGCTGGGGCTGTTAAACACGATAGGTGTCAGAGT
ACCTTTCTTTTTTTTTTTTT
>1281
TTCATATGTTTTGCTGCAAAAATATTAATGTGTTTGATTACAGAGTGCTGCCCTAGACTC
TGCTGGGGCTGTTAAACACGATAGGTGTCAGAGTACCTTTCTTTTTTTTTTTTTTTTTTT
TTTTTGAGACGGAGTCTCGC
>1282
TGCAAAAATATTAATGTGTTTGATTACAGAGTGCTGCCCTAGACTCTGCTGGGGCTGTT
AAACACGATAGGTGTCAGAGTACCTTTCTTTTTTTTTTTTTTTTTTTTTTTTGAGACGGA
GTCTCGCTCTGTCGCCCAGGT
>1283
TGGGGCTGTTAAACACGATAGGTGTCAGAGTACCTTTCTTTTTTTTTTTTTTTTTTTTTTT
TGAGACGGAGTCTCGCTCTGTCGCCCAGGTCGGACTGCGGACTGCAGTGGCGCAATCT

CGGCTCACTGCAAGCTCCGCT
>1284
AAACACGATAGGTGTCAGAGTACCTTTCTTTTTTTTTTTTTTTTTTTTTTTTGAGACGGA
GTCTCGCTCTGTCGCCCAGGTCGGACTGCGGACTGCAGTGGCGCAATCTCGGCTCACT
GCAAGCTCCGCTTCCCGGGTTC
>1285
CTTTTTTTTTTTTTTTTTTTTTTTTGAGACGGAGTCTCGCTCTGTCGCCCAGGTCGGACT
GCGGACTGCAGTGGCGCAATCTCGGCTCACTGCAAGCTCCGCTTCCCGGGTTCATGCC
ATTCTCCTGCCTCAGCCTCCCG
>1286
CTCTGTCGCCCAGGTCGGACTGCGGACTGCAGTGGCGCAATCTCGGCTCACTGCAAGC
TCCGCTTCCCGGGTTCATGCCATTCTCCTGCCTCAGCCTCCCGAGTAGCTGGGACTACA
GGCGCCCGCCACCGCGCCCGGCT
>1287
TGGCGCAATCTCGGCTCACTGCAAGCTCCGCTTCCCGGGTTCATGCCATTCTCCTGCCT
CAGCCTCCCGAGTAGCTGGGACTACAGGCGCCCGCCACCGCGCCCGGCTAATTTTTTG
TATTTTTAGTAGAGACGGGGTTT
>1288
CATTCTCCTGCCTCAGCCTCCCGAGTAGCTGGGACTACAGGCGCCCGCCACCGCGCCC
GGCTAATTTTTTGTATTTTTAGTAGAGACGGGGTTTCACCTTGTTAGCCAGGATGGTCTC
GATCTCCTGACCTCATGATCCA
>1289
GCCTCAGCCTCCCGAGTAGCTGGGACTACAGGCGCCCGCCACCGCGCCCGGCTAATTT
TTTGTATTTTTAGTAGAGACGGGGTTTCACCTTGTTAGCCAGGATGGTCTCGATCTCCTG
ACCTCATGATCCACCCGCCTCG
>1290
CCGAGTAGCTGGGACTACAGGCGCCCGCCACCGCGCCCGGCTAATTTTTTGTATTTTTA
GTAGAGACGGGGTTTCACCTTGTTAGCCAGGATGGTCTCGATCTCCTGACCTCATGATC
CACCCGCCTCGGCCTCCCAAAG
>1291
GGGACTACAGGCGCCCGCCACCGCGCCCGGCTAATTTTTTGTATTTTTAGTAGAGACGG
GGTTTCACCTTGTTAGCCAGGATGGTCTCGATCTCCTGACCTCATGATCCACCCGCCTC
GGCCTCCCAAAGTGCTGGGATT
>1292
GGTTTCACCTTGTTAGCCAGGATGGTCTCGATCTCCTGACCTCATGATCCACCCGCCTC
GGCCTCCCAAAGTGCTGGGATTACAGGCGTGAGCACCGCGCCCGGCCCAGAGTACCTT
TCTAAAGTCTGAACAGTTTTGGT
>1293
CAGGATGGTCTCGATCTCCTGACCTCATGATCCACCCGCCTCGGCCTCCCAAAGTGCTG
GGATTACAGGCGTGAGCACCGCGCCCGGCCCAGAGTACCTTTCTAAAGTCTGAACAGT
TTTGGTCGTTGAAGCACCTGAAG
>1294
CTCATGATCCACCCGCCTCGGCCTCCCAAAGTGCTGGGATTACAGGCGTGAGCACCGC
GCCCGGCCCAGAGTACCTTTCTAAAGTCTGAACAGTTTTGGTCGTTGAAGCACCTGAA

GGTTTTGGATAAGGGATTATGAAC
>1295
CGCCTCGGCCTCCCAAAGTGCTGGGATTACAGGCGTGAGCACCGCGCCCGGCCCAGA
GTACCTTTCTAAAGTCTGAACAGTTTTGGTCGTTGAAGCACCTGAAGGTTTTGGATAAG
GGATTATGAACGATTATCATTCTC
>1296
CTCCCAAAGTGCTGGGATTACAGGCGTGAGCACCGCGCCCGGCCCAGAGTACCTTTCT
AAAGTCTGAACAGTTTTGGTCGTTGAAGCACCTGAAGGTTTTGGATAAGGGATTATGA
ACGATTATCATTCTCTATTGACAA
>1297
GTGAGCACCGCGCCCGGCCCAGAGTACCTTTCTAAAGTCTGAACAGTTTTGGTCGTTG
AAGCACCTGAAGGTTTTGGATAAGGGATTATGAACGATTATCATTCTCTATTGACAAAT
GAGGAAAATGAGATTTGGAGCAA
>1298
GTTTTGGATAAGGGATTATGAACGATTATCATTCTCTATTGACAAATGAGGAAAATGAGA
TTTGGAGCAAGTAAGTGACTTCCCATGGTCACACAGCTATAAATGGGTAGAACCAGGC
ACTCAGCTTGCCTTCTAATTCA
>1299
TGGATAAGGGATTATGAACGATTATCATTCTCTATTGACAAATGAGGAAAATGAGATTTG
GAGCAAGTAAGTGACTTCCCATGGTCACACAGCTATAAATGGGTAGAACCAGGCACTC
AGCTTGCCTTCTAATTCACAGT
>1300
ATGAACGATTATCATTCTCTATTGACAAATGAGGAAAATGAGATTTGGAGCAAGTAAGT
GACTTCCCATGGTCACACAGCTATAAATGGGTAGAACCAGGCACTCAGCTTGCCTTCTA
ATTCACAGTCCTGGGCGCTCTG
>1301
TATTGACAAATGAGGAAAATGAGATTTGGAGCAAGTAAGTGACTTCCCATGGTCACAC
AGCTATAAATGGGTAGAACCAGGCACTCAGCTTGCCTTCTAATTCACAGTCCTGGGCGC
TCTGCACCAGGCCATATAAGAAC
>1302
AAGTGACTTCCCATGGTCACACAGCTATAAATGGGTAGAACCAGGCACTCAGCTTGCC
TTCTAATTCACAGTCCTGGGCGCTCTGCACCAGGCCATATAAGAACATGAGCATTGCTC
TTCTGGGTCAAATCCATCTGACC
>1303
TTGCCTTCTAATTCACAGTCCTGGGCGCTCTGCACCAGGCCATATAAGAACATGAGCAT
TGCTCTTCTGGGTCAAATCCATCTGACCTAGTCTTTGGTTGCCATCACCATGCCTAAGAT
ACCACCATTGGAGGCTACAAC
>1304
CCTGGGCGCTCTGCACCAGGCCATATAAGAACATGAGCATTGCTCTTCTGGGTCAAATC
CATCTGACCTAGTCTTTGGTTGCCATCACCATGCCTAAGATACCACCATTGGAGGCTAC
AACCATCTTCCTTGACGTCAAT
>1305
GCTCTGCACCAGGCCATATAAGAACATGAGCATTGCTCTTCTGGGTCAAATCCATCTGA
CCTAGTCTTTGGTTGCCATCACCATGCCTAAGATACCACCATTGGAGGCTACAACCATC

TTCCTTGACGTCAATCCTAAAG
>1306
GACCTAGTCTTTGGTTGCCATCACCATGCCTAAGATACCACCATTGGAGGCTACAACCA
TCTTCCTTGACGTCAATCCTAAAGGTCAGGTGTAGCTTCTCTTAGTAGCTATTTATGGCT
GCCTTGTCATTCACAGATTTG
>1307
GTTGCCATCACCATGCCTAAGATACCACCATTGGAGGCTACAACCATCTTCCTTGACGT
CAATCCTAAAGGTCAGGTGTAGCTTCTCTTAGTAGCTATTTATGGCTGCCTTGTCATTCA
CAGATTTGCCAAAATCCTTTT
>1308
AACCATCTTCCTTGACGTCAATCCTAAAGGTCAGGTGTAGCTTCTCTTAGTAGCTATTTA
TGGCTGCCTTGTCATTCACAGATTTGCCAAAATCCTTTTTGAATCCATTTCCATTTCCAG
GAAGTATCCTTTTTAGGGGT
>1309
CTATTTATGGCTGCCTTGTCATTCACAGATTTGCCAAAATCCTTTTTGAATCCATTTCCAT
TTCCAGGAAGTATCCTTTTTAGGGGTGATGAATGACAAGTTTATAATCTCCTGGGCTCCT
GGAAATCTGGTCTTTTGGA
>1310
TTGTCATTCACAGATTTGCCAAAATCCTTTTTGAATCCATTTCCATTTCCAGGAAGTATC
CTTTTTAGGGGTGATGAATGACAAGTTTATAATCTCCTGGGCTCCTGGAAATCTGGTCTT
TTGGAGAGAGGTTGTTGGTT
>1311
TTTGCCAAAATCCTTTTTGAATCCATTTCCATTTCCAGGAAGTATCCTTTTTAGGGGTGA
TGAATGACAAGTTTATAATCTCCTGGGCTCCTGGAAATCTGGTCTTTTGGAGAGAGGTT
GTTGGTTGTGAAGATAGGAAG
>1312
TTTCCAGGAAGTATCCTTTTTAGGGGTGATGAATGACAAGTTTATAATCTCCTGGGCTCC
TGGAAATCTGGTCTTTTGGAGAGAGGTTGTTGGTTGTGAAGATAGGAAGATGGCAGAA
GGCTTTGTAGTGGTGAGGCCAG
>1313
CTTTTTAGGGGTGATGAATGACAAGTTTATAATCTCCTGGGCTCCTGGAAATCTGGTCTT
TTGGAGAGAGGTTGTTGGTTGTGAAGATAGGAAGATGGCAGAAGGCTTTGTAGTGGTG
AGGCCAGGTCTAGACCTAGCTC
>1314
GGCTCCTGGAAATCTGGTCTTTTGGAGAGAGGTTGTTGGTTGTGAAGATAGGAAGATG
GCAGAAGGCTTTGTAGTGGTGAGGCCAGGTCTAGACCTAGCTCTGCGTTTTTCAGCTC
TGTCACCATTGACATCGTTTTTTT
>1315
TCCTGGAAATCTGGTCTTTTGGAGAGAGGTTGTTGGTTGTGAAGATAGGAAGATGGCA
GAAGGCTTTGTAGTGGTGAGGCCAGGTCTAGACCTAGCTCTGCGTTTTTCAGCTCTGTC
ACCATTGACATCGTTTTTTTCAT
>1316
GTCTTTTGGAGAGAGGTTGTTGGTTGTGAAGATAGGAAGATGGCAGAAGGCTTTGTAG
TGGTGAGGCCAGGTCTAGACCTAGCTCTGCGTTTTTCAGCTCTGTCACCATTGACATCG

TTTTTTTCATCGTAAGCCTTAAC
>1317
GTTGGTTGTGAAGATAGGAAGATGGCAGAAGGCTTTGTAGTGGTGAGGCCAGGTCTAG
ACCTAGCTCTGCGTTTTTCAGCTCTGTCACCATTGACATCGTTTTTTTCATCGTAAGCCT
TAACTTCTTCATTTTTAAAACT
>1318
AAGGCTTTGTAGTGGTGAGGCCAGGTCTAGACCTAGCTCTGCGTTTTTCAGCTCTGTCA
CCATTGACATCGTTTTTTTCATCGTAAGCCTTAACTTCTTCATTTTTAAAACTAGGCACT
TCAGTGAGCTCTTTCTGGATT
>1319
TGGTGAGGCCAGGTCTAGACCTAGCTCTGCGTTTTTCAGCTCTGTCACCATTGACATCG
TTTTTTTCATCGTAAGCCTTAACTTCTTCATTTTTAAAACTAGGCACTTCAGTGAGCTCT
TTCTGGATTGTTGCGCTCTTT
>1320
TCTGTCACCATTGACATCGTTTTTTTCATCGTAAGCCTTAACTTCTTCATTTTTAAAACTA
GGCACTTCAGTGAGCTCTTTCTGGATTGTTGCGCTCTTTAAAAAATTCTTTTATTCTTTG
TGTACCCACTTATATCTTC
>1321
GTTTTTTTCATCGTAAGCCTTAACTTCTTCATTTTTAAAACTAGGCACTTCAGTGAGCTC
TTTCTGGATTGTTGCGCTCTTTAAAAAATTCTTTTATTCTTTGTGTACCCACTTATATCTT
CATGAAGTCCTTTTGGTTA
>1322
TTTTAAAACTAGGCACTTCAGTGAGCTCTTTCTGGATTGTTGCGCTCTTTAAAAAATTC
TTTTATTCTTTGTGTACCCACTTATATCTTCATGAAGTCCTTTTGGTTAATTGAATTTTGC
TGTTTTGGAATCTGGATTGG
>1323
CTCTTTCTGGATTGTTGCGCTCTTTAAAAAATTCTTTTATTCTTTGTGTACCCACTTATAT
CTTCATGAAGTCCTTTTGGTTAATTGAATTTTGCTGTTTTGGAATCTGGATTGGCAGTTC
AGCCAGTTAAGTAGGGGGT
>1324
GATTGTTGCGCTCTTTAAAAAATTCTTTTATTCTTTGTGTACCCACTTATATCTTCATGAA
GTCCTTTTGGTTAATTGAATTTTGCTGTTTTGGAATCTGGATTGGCAGTTCAGCCAGTTA
AGTAGGGGGTCAGTTTGAT
>1325
AAAAATTCTTTTATTCTTTGTGTACCCACTTATATCTTCATGAAGTCCTTTTGGTTAATTG
AATTTTGCTGTTTTGGAATCTGGATTGGCAGTTCAGCCAGTTAAGTAGGGGGTCAGTTT
GATTTGATTCTGTGTGTTCT
>1326
GTACCCACTTATATCTTCATGAAGTCCTTTTGGTTAATTGAATTTTGCTGTTTTGGAATCT
GGATTGGCAGTTCAGCCAGTTAAGTAGGGGGTCAGTTTGATTTGATTCTGTGTGTTCTG
ACTTTTTTTTTTTTTTTAAA
>1327
TATATCTTCATGAAGTCCTTTTGGTTAATTGAATTTTGCTGTTTTGGAATCTGGATTGGCA
GTTCAGCCAGTTAAGTAGGGGGTCAGTTTGATTTGATTCTGTGTGTTCTGACTTTTTTTT

TTTTTTTAAATACTTTAAC
>1328
CTTCATGAAGTCCTTTTGGTTAATTGAATTTTGCTGTTTTGGAATCTGGATTGGCAGTTC
AGCCAGTTAAGTAGGGGGTCAGTTTGATTTGATTCTGTGTGTTCTGACTTTTTTTTTTTT
TTTAAATACTTTAACCTGCT
>1329
AATTTTGCTGTTTTGGAATCTGGATTGGCAGTTCAGCCAGTTAAGTAGGGGGTCAGTTT
GATTTGATTCTGTGTGTTCTGACTTTTTTTTTTTTTTTAAATACTTTAACCTGCTTAGGGT
TACTGAGCCATCTGGGATCT
>1330
GGGGTCAGTTTGATTTGATTCTGTGTGTTCTGACTTTTTTTTTTTTTTTAAATACTTTAAC
CTGCTTAGGGTTACTGAGCCATCTGGGATCTAGACTGAACTCTTGGAACTTTTGAAGAT
GATTTCCTTTATTTTATTTT
>1331
TCTGGGATCTAGACTGAACTCTTGGAACTTTTGAAGATGATTTCCTTTATTTTATTTTCA
ATGGAGGAAAGTTGCCCACTCTTTCATCTCAGTGAGAAGTCCCATTTTAATTTGCCATC
ACTATTGGATGGAGCCGCTAA
>1332
TTGGAACTTTTGAAGATGATTTCCTTTATTTTATTTTCAATGGAGGAAAGTTGCCCACTC
TTTCATCTCAGTGAGAAGTCCCATTTTAATTTGCCATCACTATTGGATGGAGCCGCTAAT
TGCTGGGAGGGAGAAAGCTA
>1333
TTTTGAAGATGATTTCCTTTATTTTATTTTCAATGGAGGAAAGTTGCCCACTCTTTCATC
TCAGTGAGAAGTCCCATTTTAATTTGCCATCACTATTGGATGGAGCCGCTAATTGCTGG
GAGGGAGAAAGCTATAGTTTA
>1334
GAGAAGTCCCATTTTAATTTGCCATCACTATTGGATGGAGCCGCTAATTGCTGGGAGGG
AGAAAGCTATAGTTTAGCAACATGTTTTCCTCCTGCTCGTTCTAGGAAAACTATTATGAA
TCTTTAAAATAGTGATTATCC
>1335
TTTAATTTGCCATCACTATTGGATGGAGCCGCTAATTGCTGGGAGGGAGAAAGCTATAG
TTTAGCAACATGTTTTCCTCCTGCTCGTTCTAGGAAAACTATTATGAATCTTTAAAATAG
TGATTATCCTAAACTGTTAAA
>1336
GAAAGCTATAGTTTAGCAACATGTTTTCCTCCTGCTCGTTCTAGGAAAACTATTATGAAT
CTTTAAAATAGTGATTATCCTAAACTGTTAAAATCAAATTCTATTCACAGCTCTTTGATTT
CTTGCTAATTCAGGAGAGA
>1337
CAACATGTTTTCCTCCTGCTCGTTCTAGGAAAACTATTATGAATCTTTAAAATAGTGATT
ATCCTAAACTGTTAAAATCAAATTCTATTCACAGCTCTTTGATTTCTTGCTAATTCAGGA
GAGAAACTTTTCCAATCACT
>1338
CAGGAGAGAAACTTTTCCAATCACTTTAATTTTTTTATTAATTTAAAGCAAGCCCTGCT
GAAAGCCCAAGTATATATGACTTAGAGTCTTACTAAATGAATACAGGTGTGCCTCAGTT

TGTCCATTGGAGGGTTTCTGA
>1339
CCAATCACTTTAATTTTTTTTATTAATTTAAAGCAAGCCCTGCTGAAAGCCCAAGTATAT
ATGACTTAGAGTCTTACTAAATGAATACAGGTGTGCCTCAGTTTGTCCATTGGAGGGTT
TCTGAATTTTCCATAACTAAA
>1340
TATTAATTTAAAGCAAGCCCTGCTGAAAGCCCAAGTATATATGACTTAGAGTCTTACTAA
ATGAATACAGGTGTGCCTCAGTTTGTCCATTGGAGGGTTTCTGAATTTTCCATAACTAA
AGATTTTCTATTCTGTTTTGG
>1341
TTAATTTAAAGCAAGCCCTGCTGAAAGCCCAAGTATATATGACTTAGAGTCTTACTAAAT
GAATACAGGTGTGCCTCAGTTTGTCCATTGGAGGGTTTCTGAATTTTCCATAACTAAAG
ATTTTCTATTCTGTTTTGGCT
>1342
AAGCCCAAGTATATATGACTTAGAGTCTTACTAAATGAATACAGGTGTGCCTCAGTTTGT
CCATTGGAGGGTTTCTGAATTTTCCATAACTAAAGATTTTCTATTCTGTTTTGGCTGGGT
GTGTGGGGTGAAAGCTCTTA
>1343
AATACAGGTGTGCCTCAGTTTGTCCATTGGAGGGTTTCTGAATTTTCCATAACTAAAGA
TTTTCTATTCTGTTTTGGCTGGGTGTGTGGGGTGAAAGCTCTTACTCTCTGGCAGTTATT
TAAGTAGTAAGAAATTTGCTA
>1344
GCCTCAGTTTGTCCATTGGAGGGTTTCTGAATTTTCCATAACTAAAGATTTTCTATTCTG
TTTTGGCTGGGTGTGTGGGGTGAAAGCTCTTACTCTCTGGCAGTTATTTAAGTAGTAAG
AAATTTGCTACAACTCTTCCC
>1345
TTTCCATAACTAAAGATTTTCTATTCTGTTTTGGCTGGGTGTGTGGGGTGAAAGCTCTTA
CTCTCTGGCAGTTATTTAAGTAGTAAGAAATTTGCTACAACTCTTCCCCACCCCCACGT
TTTACAGATAGGAAATGAGCA
>1346
CTAAAGATTTTCTATTCTGTTTTGGCTGGGTGTGTGGGGTGAAAGCTCTTACTCTCTGG
CAGTTATTTAAGTAGTAAGAAATTTGCTACAACTCTTCCCCACCCCCACGTTTTACAGAT
AGGAAATGAGCACTTTGACTG
>1347
AAGATTTTCTATTCTGTTTTGGCTGGGTGTGTGGGGTGAAAGCTCTTACTCTCTGGCAG
TTATTTAAGTAGTAAGAAATTTGCTACAACTCTTCCCCACCCCCACGTTTTACAGATAGG
AAATGAGCACTTTGACTGTCC
>1348
GTGAAAGCTCTTACTCTCTGGCAGTTATTTAAGTAGTAAGAAATTTGCTACAACTCTTC
CCCACCCCCACGTTTTACAGATAGGAAATGAGCACTTTGACTGTCCAGGTCAGACTTC
TGATTTTGCTACTTACTAACTGT
>1349
AGTAGTAAGAAATTTGCTACAACTCTTCCCCACCCCCACGTTTTACAGATAGGAAATGA
GCACTTTGACTGTCCAGGTCAGACTTCTGATTTTGCTACTTACTAACTGTGACAGTGGT

TGTGTTACTTAACTTCTGTGTG
>1350
AAGAAATTTGCTACAACTCTTCCCCACCCCCACGTTTTACAGATAGGAAATGAGCACTT
TGACTGTCCAGGTCAGACTTCTGATTTTGCTACTTACTAACTGTGACAGTGGTTGTGTT
ACTTAACTTCTGTGTGCCTGTT
>1351
ACGTTTTACAGATAGGAAATGAGCACTTTGACTGTCCAGGTCAGACTTCTGATTTTGCT
ACTTACTAACTGTGACAGTGGTTGTGTTACTTAACTTCTGTGTGCCTGTTTGCTCATTTG
TGAAACAGAAATAATAGTAGT
>1352
AGATAGGAAATGAGCACTTTGACTGTCCAGGTCAGACTTCTGATTTTGCTACTTACTAA
CTGTGACAGTGGTTGTGTTACTTAACTTCTGTGTGCCTGTTTGCTCATTTGTGAAACAG
AAATAATAGTAGTACCTACTTC
>1353
TAGGAAATGAGCACTTTGACTGTCCAGGTCAGACTTCTGATTTTGCTACTTACTAACTG
TGACAGTGGTTGTGTTACTTAACTTCTGTGTGCCTGTTTGCTCATTTGTGAAACAGAAA
TAATAGTAGTACCTACTTCATT
>1354
TGACTGTCCAGGTCAGACTTCTGATTTTGCTACTTACTAACTGTGACAGTGGTTGTGTT
ACTTAACTTCTGTGTGCCTGTTTGCTCATTTGTGAAACAGAAATAATAGTAGTACCTACT
TCATTGGGTTGTTATAAGGAT
>1355
CTGATTTTGCTACTTACTAACTGTGACAGTGGTTGTGTTACTTAACTTCTGTGTGCCTGT
TTGCTCATTTGTGAAACAGAAATAATAGTAGTACCTACTTCATTGGGTTGTTATAAGGAT
TCATCAGAAAATATTTATCC
>1356
CTGTGACAGTGGTTGTGTTACTTAACTTCTGTGTGCCTGTTTGCTCATTTGTGAAACAG
AAATAATAGTAGTACCTACTTCATTGGGTTGTTATAAGGATTCATCAGAAAATATTTATCC
TGTGTGAGGAACGGTGGCAC
>1357
GTTACTTAACTTCTGTGTGCCTGTTTGCTCATTTGTGAAACAGAAATAATAGTAGTACCT
ACTTCATTGGGTTGTTATAAGGATTCATCAGAAAATATTTATCCTGTGTGAGGAACGGTG
GCACATAGTAAGTGCTCCAT
>1358
ACTTAACTTCTGTGTGCCTGTTTGCTCATTTGTGAAACAGAAATAATAGTAGTACCTACT
TCATTGGGTTGTTATAAGGATTCATCAGAAAATATTTATCCTGTGTGAGGAACGGTGGC
ACATAGTAAGTGCTCCATAAG
>1359
TGAAACAGAAATAATAGTAGTACCTACTTCATTGGGTTGTTATAAGGATTCATCAGAAA
ATATTTATCCTGTGTGAGGAACGGTGGCACATAGTAAGTGCTCCATAAGTGTTTGCTAG
GGTATTTTTCTTGTCATTGTGT
>1360
AATAGTAGTACCTACTTCATTGGGTTGTTATAAGGATTCATCAGAAAATATTTATCCTGTG
TGAGGAACGGTGGCACATAGTAAGTGCTCCATAAGTGTTTGCTAGGGTATTTTTCTTGT

CATTGTGTGAAGTAGGTCAT
>1361
CCTACTTCATTGGGTTGTTATAAGGATTCATCAGAAAATATTTATCCTGTGTGAGGAACG
GTGGCACATAGTAAGTGCTCCATAAGTGTTTGCTAGGGTATTTTTCTTGTCATTGTGTGA
AGTAGGTCATGCTAGCCATG
>1362
TGTTATAAGGATTCATCAGAAAATATTTATCCTGTGTGAGGAACGGTGGCACATAGTAA
GTGCTCCATAAGTGTTTGCTAGGGTATTTTTCTTGTCATTGTGTGAAGTAGGTCATGCTA
GCCATGCAGGGTGAAAAATGT
>1363
TATTTATCCTGTGTGAGGAACGGTGGCACATAGTAAGTGCTCCATAAGTGTTTGCTAGG
GTATTTTTCTTGTCATTGTGTGAAGTAGGTCATGCTAGCCATGCAGGGTGAAAAATGTG
TTCATGTTGGTCCAGATCCTGC
>1364
CTGTGTGAGGAACGGTGGCACATAGTAAGTGCTCCATAAGTGTTTGCTAGGGTATTTTT
CTTGTCATTGTGTGAAGTAGGTCATGCTAGCCATGCAGGGTGAAAAATGTGTTCATGTT
GGTCCAGATCCTGCTTTCTCCC
>1365
TGAGGAACGGTGGCACATAGTAAGTGCTCCATAAGTGTTTGCTAGGGTATTTTTCTTGT
CATTGTGTGAAGTAGGTCATGCTAGCCATGCAGGGTGAAAAATGTGTTCATGTTGGTCC
AGATCCTGCTTTCTCCCTGAGA
>1366
GAACGGTGGCACATAGTAAGTGCTCCATAAGTGTTTGCTAGGGTATTTTTCTTGTCATTG
TGTGAAGTAGGTCATGCTAGCCATGCAGGGTGAAAAATGTGTTCATGTTGGTCCAGATC
CTGCTTTCTCCCTGAGACCGT
>1367
GTGCTCCATAAGTGTTTGCTAGGGTATTTTTCTTGTCATTGTGTGAAGTAGGTCATGCTA
GCCATGCAGGGTGAAAAATGTGTTCATGTTGGTCCAGATCCTGCTTTCTCCCTGAGACC
GTTCTTCCGCATAGGCGTCAA
>1368
GGGTATTTTTCTTGTCATTGTGTGAAGTAGGTCATGCTAGCCATGCAGGGTGAAAAATG
TGTTCATGTTGGTCCAGATCCTGCTTTCTCCCTGAGACCGTTCTTCCGCATAGGCGTCA
ATCCGTACAGTGCTGTAAAACT
>1369
AGGTCATGCTAGCCATGCAGGGTGAAAAATGTGTTCATGTTGGTCCAGATCCTGCTTTC
TCCCTGAGACCGTTCTTCCGCATAGGCGTCAATCCGTACAGTGCTGTAAAACTGCTGCC
TCGGGCACCTCATGTTTGGGGC
>1370
GCAGGGTGAAAAATGTGTTCATGTTGGTCCAGATCCTGCTTTCTCCCTGAGACCGTTCT
TCCGCATAGGCGTCAATCCGTACAGTGCTGTAAAACTGCTGCCTCGGGCACCTCATGTT
TGGGGCTGAGACATTGCTTCTG
>1371
CCTGAGACCGTTCTTCCGCATAGGCGTCAATCCGTACAGTGCTGTAAAACTGCTGCCTC
GGGCACCTCATGTTTGGGGCTGAGACATTGCTTCTGCGTCTTCCCCAGAAGCTGATCTC

TTGTACTGTGGTGGCCAGGGTA
>1372
TCAATCCGTACAGTGCTGTAAAACTGCTGCCTCGGGCACCTCATGTTTGGGGCTGAGA
CATTGCTTCTGCGTCTTCCCCAGAAGCTGATCTCTTGTACTGTGGTGGCCAGGGTACTC
TTTGTCCCATTGACCTGAGGCCT
>1373
TGCTGCCTCGGGCACCTCATGTTTGGGGCTGAGACATTGCTTCTGCGTCTTCCCCAGAA
GCTGATCTCTTGTACTGTGGTGGCCAGGGTACTCTTTGTCCCATTGACCTGAGGCCTGG
TGAGTCATTCCAGTTCTCGTTT
>1374
CCTCGGGCACCTCATGTTTGGGGCTGAGACATTGCTTCTGCGTCTTCCCCAGAAGCTGA
TCTCTTGTACTGTGGTGGCCAGGGTACTCTTTGTCCCATTGACCTGAGGCCTGGTGAGT
CATTCCAGTTCTCGTTTAAATT
>1375
CATGTTTGGGGCTGAGACATTGCTTCTGCGTCTTCCCCAGAAGCTGATCTCTTGTACTG
TGGTGGCCAGGGTACTCTTTGTCCCATTGACCTGAGGCCTGGTGAGTCATTCCAGTTCT
CGTTTAAATTTAGCCGGGCAGC
>1376
TCTTCCCCAGAAGCTGATCTCTTGTACTGTGGTGGCCAGGGTACTCTTTGTCCCATTGA
CCTGAGGCCTGGTGAGTCATTCCAGTTCTCGTTTAAATTTAGCCGGGCAGCGCTCCACT
GCCATCGGGTCCAAATGCCGGG
>1377
CTGATCTCTTGTACTGTGGTGGCCAGGGTACTCTTTGTCCCATTGACCTGAGGCCTGGT
GAGTCATTCCAGTTCTCGTTTAAATTTAGCCGGGCAGCGCTCCACTGCCATCGGGTCCA
AATGCCGGGGAGGCTCACTGAT
>1378
TCTTGTACTGTGGTGGCCAGGGTACTCTTTGTCCCATTGACCTGAGGCCTGGTGAGTCA
TTCCAGTTCTCGTTTAAATTTAGCCGGGCAGCGCTCCACTGCCATCGGGTCCAAATGCC
GGGGAGGCTCACTGATGCGGTG
>1379
TGACCTGAGGCCTGGTGAGTCATTCCAGTTCTCGTTTAAATTTAGCCGGGCAGCGCTCC
ACTGCCATCGGGTCCAAATGCCGGGGAGGCTCACTGATGCGGTGTCCTGTTGCCTGTG
CACGTGGCCAGGCGTGCACATCA
>1380
CTCGTTTAAATTTAGCCGGGCAGCGCTCCACTGCCATCGGGTCCAAATGCCGGGGAGG
CTCACTGATGCGGTGTCCTGTTGCCTGTGCACGTGGCCAGGCGTGCACATCACTGTGC
TTATGAGAGAGATGAAGAGCACAG
>1381
CGTTTAAATTTAGCCGGGCAGCGCTCCACTGCCATCGGGTCCAAATGCCGGGGAGGCT
CACTGATGCGGTGTCCTGTTGCCTGTGCACGTGGCCAGGCGTGCACATCACTGTGCTTA
TGAGAGAGATGAAGAGCACAGAT
>1382
CTGCCATCGGGTCCAAATGCCGGGGAGGCTCACTGATGCGGTGTCCTGTTGCCTGTGC
ACGTGGCCAGGCGTGCACATCACTGTGCTTATGAGAGAGATGAAGAGCACAGATACCA

CCTTACTGATGGGGGTCCTGGGGC
>1383
CCAAATGCCGGGGAGGCTCACTGATGCGGTGTCCTGTTGCCTGTGCACGTGGCCAGGC
GTGCACATCACTGTGCTTATGAGAGAGATGAAGAGCACAGATACCACCTTACTGATGG
GGGTCCTGGGGCTGGGCTTGTGGG
>1384
GTGGCCAGGCGTGCACATCACTGTGCTTATGAGAGAGATGAAGAGCACAGATACCACC
TTACTGATGGGGGTCCTGGGGCTGGGCTTGTGGGTGTTGGGGGTTCCAGAGCTCCTTC
CCTTTCCTGCCTGTCAGTGCAGGA
>1385
TCACTGTGCTTATGAGAGAGATGAAGAGCACAGATACCACCTTACTGATGGGGGTCCT
GGGGCTGGGCTTGTGGGTGTTGGGGGTTCCAGAGCTCCTTCCCTTTCCTGCCTGTCAG
TGCAGGATGCTTCTAAAATGTGGA
>1386
TGTGCTTATGAGAGAGATGAAGAGCACAGATACCACCTTACTGATGGGGGTCCTGGGG
CTGGGCTTGTGGGTGTTGGGGGTTCCAGAGCTCCTTCCCTTTCCTGCCTGTCAGTGCA
GGATGCTTCTAAAATGTGGACTCA
>1387
TTACTGATGGGGGTCCTGGGGCTGGGCTTGTGGGTGTTGGGGGTTCCAGAGCTCCTTC
CCTTTCCTGCCTGTCAGTGCAGGATGCTTCTAAAATGTGGACTCAGCAGACATTGTTTT
GAGTGCTTGCTACATGTCAGATT
>1388
GGCTTGTGGGTGTTGGGGGTTCCAGAGCTCCTTCCCTTTCCTGCCTGTCAGTGCAGGA
TGCTTCTAAAATGTGGACTCAGCAGACATTGTTTTGAGTGCTTGCTACATGTCAGATTC
TGTGCTAAGTGCACTCACAGCCA
>1389
GTTCCAGAGCTCCTTCCCTTTCCTGCCTGTCAGTGCAGGATGCTTCTAAAATGTGGACT
CAGCAGACATTGTTTTGAGTGCTTGCTACATGTCAGATTCTGTGCTAAGTGCACTCACA
GCCAGCATCTCTGTAAGACCTC
>1390
TCCTGCCTGTCAGTGCAGGATGCTTCTAAAATGTGGACTCAGCAGACATTGTTTTGAGT
GCTTGCTACATGTCAGATTCTGTGCTAAGTGCACTCACAGCCAGCATCTCTGTAAGACC
TCTAGCTGTCCTGTAAGGTGGG
>1391
CAGTGCAGGATGCTTCTAAAATGTGGACTCAGCAGACATTGTTTTGAGTGCTTGCTACA
TGTCAGATTCTGTGCTAAGTGCACTCACAGCCAGCATCTCTGTAAGACCTCTAGCTGTC
CTGTAAGGTGGGTATCAGTTTC
>1392
GGATGCTTCTAAAATGTGGACTCAGCAGACATTGTTTTGAGTGCTTGCTACATGTCAGA
TTCTGTGCTAAGTGCACTCACAGCCAGCATCTCTGTAAGACCTCTAGCTGTCCTGTAAG
GTGGGTATCAGTTTCACATTCT
>1393
AGCAGACATTGTTTTGAGTGCTTGCTACATGTCAGATTCTGTGCTAAGTGCACTCACAG
CCAGCATCTCTGTAAGACCTCTAGCTGTCCTGTAAGGTGGGTATCAGTTTCACATTCTC

AGATGGGCGGAGAGAAGCCCAA
>1394
GAGTGCTTGCTACATGTCAGATTCTGTGCTAAGTGCACTCACAGCCAGCATCTCTGTAA
GACCTCTAGCTGTCCTGTAAGGTGGGTATCAGTTTCACATTCTCAGATGGGCGGAGAG
AAGCCCAAGAGGTTAGTGGACTT
>1395
TGTCAGATTCTGTGCTAAGTGCACTCACAGCCAGCATCTCTGTAAGACCTCTAGCTGTC
CTGTAAGGTGGGTATCAGTTTCACATTCTCAGATGGGCGGAGAGAAGCCCAAGAGGTT
AGTGGACTTTTCTCTAGTAAAGA
>1396
ATTCTGTGCTAAGTGCACTCACAGCCAGCATCTCTGTAAGACCTCTAGCTGTCCTGTAA
GGTGGGTATCAGTTTCACATTCTCAGATGGGCGGAGAGAAGCCCAAGAGGTTAGTGGA
CTTTTCTCTAGTAAAGAGTTGTA
>1397
AGACCTCTAGCTGTCCTGTAAGGTGGGTATCAGTTTCACATTCTCAGATGGGCGGAGA
GAAGCCCAAGAGGTTAGTGGACTTTTCTCTAGTAAAGAGTTGTACAGGCCGGGCGCAG
TGGCTCATGCCTGTAATCCCAGCA
>1398
AAGGTGGGTATCAGTTTCACATTCTCAGATGGGCGGAGAGAAGCCCAAGAGGTTAGTG
GACTTTTCTCTAGTAAAGAGTTGTACAGGCCGGGCGCAGTGGCTCATGCCTGTAATCCC
AGCACTTTGGGAGGCCGAGGCGG
>1399
GTATCAGTTTCACATTCTCAGATGGGCGGAGAGAAGCCCAAGAGGTTAGTGGACTTTT
CTCTAGTAAAGAGTTGTACAGGCCGGGCGCAGTGGCTCATGCCTGTAATCCCAGCACT
TTGGGAGGCCGAGGCGGGCGGATC
>1400
AGATGGGCGGAGAGAAGCCCAAGAGGTTAGTGGACTTTTCTCTAGTAAAGAGTTGTAC
AGGCCGGGCGCAGTGGCTCATGCCTGTAATCCCAGCACTTTGGGAGGCCGAGGCGGG
CGGATCACGAGGTCAGGAGATCGAG
>1401
ACAGGCCGGGCGCAGTGGCTCATGCCTGTAATCCCAGCACTTTGGGAGGCCGAGGCG
GGCGGATCACGAGGTCAGGAGATCGAGACCATCCTGGCTAACACAGTGAAACCCCGT
CTCTACTAAAAATACAAAAAATTAGC
>1402
CCCAGCACTTTGGGAGGCCGAGGCGGGCGGATCACGAGGTCAGGAGATCGAGACCAT
CCTGGCTAACACAGTGAAACCCCGTCTCTACTAAAAATACAAAAAATTAGCCGGGCGT
GGTGGGGGGCGCCTGTAGTCCCAGC
>1403
TGGGAGGCCGAGGCGGGCGGATCACGAGGTCAGGAGATCGAGACCATCCTGGCTAAC
ACAGTGAAACCCCGTCTCTACTAAAAATACAAAAAATTAGCCGGGCGTGGTGGGGGGC
GCCTGTAGTCCCAGCTACTCGGGAG
>1404
GAGATCGAGACCATCCTGGCTAACACAGTGAAACCCCGTCTCTACTAAAAATACAAAA
AATTAGCCGGGCGTGGTGGGGGGCGCCTGTAGTCCCAGCTACTCGGGAGGCTGAGGC

AGGAGAATGGCGTGAACCCGGGAGG
>1405
TGGCTAACACAGTGAAACCCCGTCTCTACTAAAAATACAAAAAATTAGCCGGGCGTGG
TGGGGGGCGCCTGTAGTCCCAGCTACTCGGGAGGCTGAGGCAGGAGAATGGCGTGAA
CCCGGGAGGCAGAGCTTGCAGTGAG
>1406
AAAATTAGCCGGGCGTGGTGGGGGGCGCCTGTAGTCCCAGCTACTCGGGAGGCTGAG
GCAGGAGAATGGCGTGAACCCGGGAGGCAGAGCTTGCAGTGAGCCGAGATCATGCCA
CTCACTACACTCCAGCCTGGGCAAAG
>1407
CGCCTGTAGTCCCAGCTACTCGGGAGGCTGAGGCAGGAGAATGGCGTGAACCCGGGA
GGCAGAGCTTGCAGTGAGCCGAGATCATGCCACTCACTACACTCCAGCCTGGGCAAA
GGAGTGAGACTGTCTCAAAAAAAAAA
>1408
GAACCCGGGAGGCAGAGCTTGCAGTGAGCCGAGATCATGCCACTCACTACACTCCAG
CCTGGGCAAAGGAGTGAGACTGTCTCAAAAAAAAAAAAAAAAAAAAAAAGAGTTGTAC
CAGGATTTATGTTCAGGTCTTCTGGCT
>1409
GAGGCAGAGCTTGCAGTGAGCCGAGATCATGCCACTCACTACACTCCAGCCTGGGCA
AAGGAGTGAGACTGTCTCAAAAAAAAAAAAAAAAAAAAAAAGAGTTGTACCAGGATTTA
TGTTCAGGTCTTCTGGCTCCATGCTC
>1410
ATGCCACTCACTACACTCCAGCCTGGGCAAAGGAGTGAGACTGTCTCAAAAAAAAAAAAA
AAAAAAAAAAAAGAGTTGTACCAGGATTTATGTTCAGGTCTTCTGGCTCCATGCTCTCTG
CAGCTGGGTGACCAGGGGGCTCAT
>1411
CCACTCACTACACTCCAGCCTGGGCAAAGGAGTGAGACTGTCTCAAAAAAAAAAAAA
AAAAAAAAGAGTTGTACCAGGATTTATGTTCAGGTCTTCTGGCTCCATGCTCTCTGCAG
CTGGGTGACCAGGGGGCTCATGGC
>1412
AAAAAAAAAAAAAAAGAGTTGTACCAGGATTTATGTTCAGGTCTTCTGGCTCCATGCT
CTCTGCAGCTGGGTGACCAGGGGGCTCATGGCTTCACCAGCTCTGGCCTCCTCAGTTC
CTCTGGTGGCTTCTTGCGAGGAGG
>1413
CCATGCTCTCTGCAGCTGGGTGACCAGGGGGCTCATGGCTTCACCAGCTCTGGCCTCC
TCAGTTCCTCTGGTGGCTTCTTGCGAGGAGGAAGGTTACTGCAGAGGGTTCAGCTCTT
TAGGGGCCCAGGCCGCATGGATCC
>1414
ACCAGGGGGCTCATGGCTTCACCAGCTCTGGCCTCCTCAGTTCCTCTGGTGGCTTCTTG
CGAGGAGGAAGGTTACTGCAGAGGGTTCAGCTCTTTAGGGGCCCAGGCCGCATGGAT
CCAAGGACTAGTACGTCATCTCTT
>1415
TGGCTTCACCAGCTCTGGCCTCCTCAGTTCCTCTGGTGGCTTCTTGCGAGGAGGAAGG
TTACTGCAGAGGGTTCAGCTCTTTAGGGGCCCAGGCCGCATGGATCCAAGGACTAGTA

CGTCATCTCTTACCAACACAGAGA
>1416
TTGCGAGGAGGAAGGTTACTGCAGAGGGTTCAGCTCTTTAGGGGCCCAGGCCGCATG
GATCCAAGGACTAGTACGTCATCTCTTACCAACACAGAGAGATCAGCTTCTAGTGATAG
AGGCCTTTTATGCTGACTTGAAAA
>1417
GAGGAGGAAGGTTACTGCAGAGGGTTCAGCTCTTTAGGGGCCCAGGCCGCATGGATC
CAAGGACTAGTACGTCATCTCTTACCAACACAGAGAGATCAGCTTCTAGTGATAGAGG
CCTTTTATGCTGACTTGAAAAACAG
>1418
AGGGGCCCAGGCCGCATGGATCCAAGGACTAGTACGTCATCTCTTACCAACACAGAGA
GATCAGCTTCTAGTGATAGAGGCCTTTTATGCTGACTTGAAAAACAGAAAAAGAGAGA
AAAAAGAAACCCATTTACAAAAAT
>1419
TTATGCTGACTTGAAAAACAGAAAAAGAGAGAAAAAAGAAACCCATTTACAAAAATA
AAAATCCCATGCTGTTAATTTCATGCCAGCATAATCAGAAATTCCCCTTTCTACTGTTTC
CCCTTAAAAGCCCCGGTGTCCTT
>1420
CCATTTACAAAAATAAAAATCCCATGCTGTTAATTTCATGCCAGCATAATCAGAAATTCC
CCTTTCTACTGTTTCCCCTTAAAAGCCCCGGTGTCCTTATTATGCTGGTGCAGTCTGGAA
AAACTATGGTTGGGTTTTGC
>1421
CCCATGCTGTTAATTTCATGCCAGCATAATCAGAAATTCCCCTTTCTACTGTTTCCCCTT
AAAAGCCCCGGTGTCCTTATTATGCTGGTGCAGTCTGGAAAAACTATGGTTGGGTTTTG
CCTACGTGAGGCTTATCTCTG
>1422
ATGCCAGCATAATCAGAAATTCCCCTTTCTACTGTTTCCCCTTAAAAGCCCCGGTGTCCT
TATTATGCTGGTGCAGTCTGGAAAAACTATGGTTGGGTTTTGCCTACGTGAGGCTTATCT
CTGCAGCTGCAAGGAGGGGA
>1423
CCCCTTTCTACTGTTTCCCCTTAAAAGCCCCGGTGTCCTTATTATGCTGGTGCAGTCTGG
AAAAACTATGGTTGGGTTTTGCCTACGTGAGGCTTATCTCTGCAGCTGCAAGGAGGGG
ATCCGGTACTTTATTGTCAGCA
>1424
TTCTACTGTTTCCCCTTAAAAGCCCCGGTGTCCTTATTATGCTGGTGCAGTCTGGAAAA
ACTATGGTTGGGTTTTGCCTACGTGAGGCTTATCTCTGCAGCTGCAAGGAGGGGATCCG
GTACTTTATTGTCAGCATGGAA
>1425
CCCCTTAAAAGCCCCGGTGTCCTTATTATGCTGGTGCAGTCTGGAAAAACTATGGTTGG
GTTTTGCCTACGTGAGGCTTATCTCTGCAGCTGCAAGGAGGGGATCCGGTACTTTATTG
TCAGCATGGAAACTTCCGCTAA
>1426
AGTCTGGAAAAACTATGGTTGGGTTTTGCCTACGTGAGGCTTATCTCTGCAGCTGCAAG
GAGGGGATCCGGTACTTTATTGTCAGCATGGAAACTTCCGCTAAATACTTAGAAAAACC

TTAATTCTAAATTCCTAGCTCA
>1427
AACTATGGTTGGGTTTTGCCTACGTGAGGCTTATCTCTGCAGCTGCAAGGAGGGGATCC
GGTACTTTATTGTCAGCATGGAAACTTCCGCTAAATACTTAGAAAAACCTTAATTCTAAA
TTCCTAGCTCACTTTCAACTG
>1428
TGTCAGCATGGAAACTTCCGCTAAATACTTAGAAAAACCTTAATTCTAAATTCCTAGCT
CACTTTCAACTGTGTCCTCGGGACCTGATTTTCTTCGGTGTGAACAGATTGTGTTCAGC
ATTGCAGTAGAAAGGGTCCAAG
>1429
TCAGCATGGAAACTTCCGCTAAATACTTAGAAAAACCTTAATTCTAAATTCCTAGCTCA
CTTTCAACTGTGTCCTCGGGACCTGATTTTCTTCGGTGTGAACAGATTGTGTTCAGCAT
TGCAGTAGAAAGGGTCCAAGTT
>1430
ACTTAGAAAAACCTTAATTCTAAATTCCTAGCTCACTTTCAACTGTGTCCTCGGGACCT
GATTTTCTTCGGTGTGAACAGATTGTGTTCAGCATTGCAGTAGAAAGGGTCCAAGTTA
GGTAAGAGAAAGGACTTCCTGAG
>1431
TTAATTCTAAATTCCTAGCTCACTTTCAACTGTGTCCTCGGGACCTGATTTTCTTCGGTG
TGAACAGATTGTGTTCAGCATTGCAGTAGAAAGGGTCCAAGTTAGGTAAGAGAAAGG
ACTTCCTGAGACACTGGGGTGTT
>1432
AATTCTAAATTCCTAGCTCACTTTCAACTGTGTCCTCGGGACCTGATTTTCTTCGGTGTG
AACAGATTGTGTTCAGCATTGCAGTAGAAAGGGTCCAAGTTAGGTAAGAGAAAGGAC
TTCCTGAGACACTGGGGTGTTAC
>1433
TAGCTCACTTTCAACTGTGTCCTCGGGACCTGATTTTCTTCGGTGTGAACAGATTGTGT
TCAGCATTGCAGTAGAAAGGGTCCAAGTTAGGTAAGAGAAAGGACTTCCTGAGACAC
TGGGGTGTTACTGAGAAAGTTGTT
>1434
TTCAACTGTGTCCTCGGGACCTGATTTTCTTCGGTGTGAACAGATTGTGTTCAGCATTG
CAGTAGAAAGGGTCCAAGTTAGGTAAGAGAAAGGACTTCCTGAGACACTGGGGTGTT
ACTGAGAAAGTTGTTAGAAGACTG
>1435
AGATTGTGTTCAGCATTGCAGTAGAAAGGGTCCAAGTTAGGTAAGAGAAAGGACTTCC
TGAGACACTGGGGTGTTACTGAGAAAGTTGTTAGAAGACTGTCACAGACATTTTGATG
AAGGGGGGACATTCTGGGCCATTT
>1436
ATTGTGTTCAGCATTGCAGTAGAAAGGGTCCAAGTTAGGTAAGAGAAAGGACTTCCTG
AGACACTGGGGTGTTACTGAGAAAGTTGTTAGAAGACTGTCACAGACATTTTGATGAA
GGGGGGACATTCTGGGCCATTTGA
>1437
ATTGCAGTAGAAAGGGTCCAAGTTAGGTAAGAGAAAGGACTTCCTGAGACACTGGGG
TGTTACTGAGAAAGTTGTTAGAAGACTGTCACAGACATTTTGATGAAGGGGGGACATT

CTGGGCCATTTGAAGTGGTTTTTAG
>1438
TTACTGAGAAAGTTGTTAGAAGACTGTCACAGACATTTTGATGAAGGGGGGACATTCT
GGGCCATTTGAAGTGGTTTTTAGAGTTCTTTCCTCTTTAGGGCCATTCGGTTCCCCCAG
AAATAAGAAGAAAAGACATATAC
>1439
CTGAGAAAGTTGTTAGAAGACTGTCACAGACATTTTGATGAAGGGGGGACATTCTGGG
CCATTTGAAGTGGTTTTTAGAGTTCTTTCCTCTTTAGGGCCATTCGGTTCCCCCAGAAAT
AAGAAGAAAAGACATATACACG
>1440
TTGATGAAGGGGGGACATTCTGGGCCATTTGAAGTGGTTTTTAGAGTTCTTTCCTCTTT
AGGGCCATTCGGTTCCCCCAGAAATAAGAAGAAAAGACATATACACGCACATAAACCA
CCACCACCAGGCTTGCTGAGAAC
>1441
TCCCCCAGAAATAAGAAGAAAAGACATATACACGCACATAAACCACCACCACCAGGCT
TGCTGAGAACAAGTCAAATGTGCCACCAAACCATCGTCTTAGTGGGAGATGGGGTGGC
ACTGCAGGGGCAGAAAGACTGACG
>1442
GAAATAAGAAGAAAAGACATATACACGCACATAAACCACCACCACCAGGCTTGCTGAG
AACAAGTCAAATGTGCCACCAAACCATCGTCTTAGTGGGAGATGGGGTGGCACTGCAG
GGGCAGAAAGACTGACGTCTGATG
>1443
ACATATACACGCACATAAACCACCACCACCAGGCTTGCTGAGAACAAGTCAAATGTGC
CACCAAACCATCGTCTTAGTGGGAGATGGGGTGGCACTGCAGGGGCAGAAAGACTGA
CGTCTGATGGACTTGGGTTTGAATA
>1444
AGTCAAATGTGCCACCAAACCATCGTCTTAGTGGGAGATGGGGTGGCACTGCAGGGGC
AGAAAGACTGACGTCTGATGGACTTGGGTTTGAATACTAGTGCTCCATGGTGAATTTG
GGCAAATGACTTCACCTCTGTGAA
>1445
CAAACCATCGTCTTAGTGGGAGATGGGGTGGCACTGCAGGGGCAGAAAGACTGACGT
CTGATGGACTTGGGTTTGAATACTAGTGCTCCATGGTGAATTTGGGCAAATGACTTCAC
CTCTGTGAAGCAGCAGTATCCTCT
>1446
ATGGGGTGGCACTGCAGGGGCAGAAAGACTGACGTCTGATGGACTTGGGTTTGAATAC
TAGTGCTCCATGGTGAATTTGGGCAAATGACTTCACCTCTGTGAAGCAGCAGTATCCTC
TTCTGTGAAGTGAGGTTAGCCCC
>1447
CTGACGTCTGATGGACTTGGGTTTGAATACTAGTGCTCCATGGTGAATTTGGGCAAATG
ACTTCACCTCTGTGAAGCAGCAGTATCCTCTTCTGTGAAGTGAGGTTAGCCCCTACCTC
TCAATGTGGTTTTTAAAGTATG
>1448
TGGACTTGGGTTTGAATACTAGTGCTCCATGGTGAATTTGGGCAAATGACTTCACCTCT
GTGAAGCAGCAGTATCCTCTTCTGTGAAGTGAGGTTAGCCCCTACCTCTCAATGTGGTT

TTTAAAGTATGTTAATGAGATC
>1449
CAAATGACTTCACCTCTGTGAAGCAGCAGTATCCTCTTCTGTGAAGTGAGGTTAGCCC
CTACCTCTCAATGTGGTTTTTAAAGTATGTTAATGAGATCACTTGTAACCTAAAGCACAC
AGAGTGTGAGGCCACCTCCCTA
>1450
ATGACTTCACCTCTGTGAAGCAGCAGTATCCTCTTCTGTGAAGTGAGGTTAGCCCCTAC
CTCTCAATGTGGTTTTTAAAGTATGTTAATGAGATCACTTGTAACCTAAAGCACACAGA
GTGTGAGGCCACCTCCCTACTT
>1451
CCTCTTCTGTGAAGTGAGGTTAGCCCCTACCTCTCAATGTGGTTTTTAAAGTATGTTAAT
GAGATCACTTGTAACCTAAAGCACACAGAGTGTGAGGCCACCTCCCTACTTCCTGCTT
CTCTCATGACCCCAGCCTGACA
>1452
TTAGCCCCTACCTCTCAATGTGGTTTTTAAAGTATGTTAATGAGATCACTTGTAACCTAA
AGCACACAGAGTGTGAGGCCACCTCCCTACTTCCTGCTTCTCTCATGACCCCAGCCTG
ACAAGGGAGCCGCTGAGCCCCC
>1453
AGCCCCTACCTCTCAATGTGGTTTTTAAAGTATGTTAATGAGATCACTTGTAACCTAAAG
CACACAGAGTGTGAGGCCACCTCCCTACTTCCTGCTTCTCTCATGACCCCAGCCTGAC
AAGGGAGCCGCTGAGCCCCCTG
>1454
TACTTCCTGCTTCTCTCATGACCCCAGCCTGACAAGGGAGCCGCTGAGCCCCCTGCCA
CCCTCACCCGGCGTTGTGCTCTAAGTGGATTAGCTCAGCGTTGTGATCTTAATGCCATT
AGGCCTGTTGGCACGGCTCTCCT
>1455
TTCCTGCTTCTCTCATGACCCCAGCCTGACAAGGGAGCCGCTGAGCCCCCTGCCACCC
TCACCCGGCGTTGTGCTCTAAGTGGATTAGCTCAGCGTTGTGATCTTAATGCCATTAGG
CCTGTTGGCACGGCTCTCCTTGC
>1456
GACAAGGGAGCCGCTGAGCCCCCTGCCACCCTCACCCGGCGTTGTGCTCTAAGTGGAT
TAGCTCAGCGTTGTGATCTTAATGCCATTAGGCCTGTTGGCACGGCTCTCCTTGCCATA
GTGAGAAGCAGATGTGGCCGACC
>1457
TGCCACCCTCACCCGGCGTTGTGCTCTAAGTGGATTAGCTCAGCGTTGTGATCTTAATG
CCATTAGGCCTGTTGGCACGGCTCTCCTTGCCATAGTGAGAAGCAGATGTGGCCGACC
ATAATGGCCCTTAGCGTGATCCA
>1458
TCTAAGTGGATTAGCTCAGCGTTGTGATCTTAATGCCATTAGGCCTGTTGGCACGGCTC
TCCTTGCCATAGTGAGAAGCAGATGTGGCCGACCATAATGGCCCTTAGCGTGATCCATC
GGGCCCACAGTGCTGGGGCAAC
>1459
GCTCAGCGTTGTGATCTTAATGCCATTAGGCCTGTTGGCACGGCTCTCCTTGCCATAGT
GAGAAGCAGATGTGGCCGACCATAATGGCCCTTAGCGTGATCCATCGGGCCCACAGTG

CTGGGGCAACCATTATTAGTGTT
>1460
TTAGGCCTGTTGGCACGGCTCTCCTTGCCATAGTGAGAAGCAGATGTGGCCGACCATAA
TGGCCCTTAGCGTGATCCATCGGGCCCACAGTGCTGGGGCAACCATTATTAGTGTTAAT
TTGGGGGAGCTTTTTATGGTAG
>1461
TCTCCTTGCCATAGTGAGAAGCAGATGTGGCCGACCATAATGGCCCTTAGCGTGATCCA
TCGGGCCCACAGTGCTGGGGCAACCATTATTAGTGTTAATTTGGGGGAGCTTTTTATGG
TAGCTGGGGGAAATGTGGAGGG
>1462
CAGATGTGGCCGACCATAATGGCCCTTAGCGTGATCCATCGGGCCCACAGTGCTGGGG
CAACCATTATTAGTGTTAATTTGGGGGAGCTTTTTATGGTAGCTGGGGGAAATGTGGAG
GGATTGTTGGAAATTTATTTAAG
>1463
GATGTGGCCGACCATAATGGCCCTTAGCGTGATCCATCGGGCCCACAGTGCTGGGGCA
ACCATTATTAGTGTTAATTTGGGGGAGCTTTTTATGGTAGCTGGGGGAAATGTGGAGGG
ATTGTTGGAAATTTATTTAAGCT
>1464
TAGCGTGATCCATCGGGCCCACAGTGCTGGGGCAACCATTATTAGTGTTAATTTGGGGG
AGCTTTTTATGGTAGCTGGGGGAAATGTGGAGGGATTGTTGGAAATTTATTTAAGCTGT
TCAGCAGGTTAGGTGCTGCTGG
>1465
GTTAATTTGGGGGAGCTTTTTATGGTAGCTGGGGGAAATGTGGAGGGATTGTTGGAAAT
TTATTTAAGCTGTTCAGCAGGTTAGGTGCTGCTGGGGTCTACTATGGCAAAACCCTCAG
CTCCCCCAACCCATCTCTTTAA
>1466
GGGGAGCTTTTTATGGTAGCTGGGGGAAATGTGGAGGGATTGTTGGAAATTTATTTAAG
CTGTTCAGCAGGTTAGGTGCTGCTGGGGTCTACTATGGCAAAACCCTCAGCTCCCCCA
ACCCATCTCTTTAAGGAACGTCC
>1467
GCAGGTTAGGTGCTGCTGGGGTCTACTATGGCAAAACCCTCAGCTCCCCCAACCCATC
TCTTTAAGGAACGTCCCTCCCCAACACCGAAGCCCAGGCCTGATTACTTCCAGAGTTG
GAGAGACAACTTAGCAGGCTCTTG
>1468
GCTCCCCCAACCCATCTCTTTAAGGAACGTCCCTCCCCAACACCGAAGCCCAGGCCTG
ATTACTTCCAGAGTTGGAGAGACAACTTAGCAGGCTCTTGTTTCTTGCCGATGTACCTT
ATTAGTAGCACCTGGCATCATAA
>1469
CGTCCCTCCCCAACACCGAAGCCCAGGCCTGATTACTTCCAGAGTTGGAGAGACAACT
TAGCAGGCTCTTGTTTCTTGCCGATGTACCTTATTAGTAGCACCTGGCATCATAACCCAC
TAATGGGAACCAGTGCTCTCCT
>1470
AGGCCTGATTACTTCCAGAGTTGGAGAGACAACTTAGCAGGCTCTTGTTTCTTGCCGAT
GTACCTTATTAGTAGCACCTGGCATCATAACCCACTAATGGGAACCAGTGCTCTCCTCC

TGCGTGGCCAGTGACAAGTAGC
>1471
GCAGGCTCTTGTTTCTTGCCGATGTACCTTATTAGTAGCACCTGGCATCATAACCCACTA
ATGGGAACCAGTGCTCTCCTCCTGCGTGGCCAGTGACAAGTAGCCCTGTCAGCCGGAG
CTCGCCGTGCGAGGAAGTTCCT
>1472
TTGCCGATGTACCTTATTAGTAGCACCTGGCATCATAACCCACTAATGGGAACCAGTGC
TCTCCTCCTGCGTGGCCAGTGACAAGTAGCCCTGTCAGCCGGAGCTCGCCGTGCGAGG
AAGTTCCTATTAGCTTTATGGTC
>1473
TGTACCTTATTAGTAGCACCTGGCATCATAACCCACTAATGGGAACCAGTGCTCTCCTC
CTGCGTGGCCAGTGACAAGTAGCCCTGTCAGCCGGAGCTCGCCGTGCGAGGAAGTTC
CTATTAGCTTTATGGTCAGGAGAA
>1474
TATTAGTAGCACCTGGCATCATAACCCACTAATGGGAACCAGTGCTCTCCTCCTGCGTG
GCCAGTGACAAGTAGCCCTGTCAGCCGGAGCTCGCCGTGCGAGGAAGTTCCTATTAGC
TTTATGGTCAGGAGAACTTGTCC
>1475
GCACCTGGCATCATAACCCACTAATGGGAACCAGTGCTCTCCTCCTGCGTGGCCAGTG
ACAAGTAGCCCTGTCAGCCGGAGCTCGCCGTGCGAGGAAGTTCCTATTAGCTTTATGG
TCAGGAGAACTTGTCCTGTTCAGG
>1476
GAACCAGTGCTCTCCTCCTGCGTGGCCAGTGACAAGTAGCCCTGTCAGCCGGAGCTCG
CCGTGCGAGGAAGTTCCTATTAGCTTTATGGTCAGGAGAACTTGTCCTGTTCAGGAAAT
GAATGTTGCCGCTAAAGGTCCGC
>1477
TGCGTGGCCAGTGACAAGTAGCCCTGTCAGCCGGAGCTCGCCGTGCGAGGAAGTTCC
TATTAGCTTTATGGTCAGGAGAACTTGTCCTGTTCAGGAAATGAATGTTGCCGCTAAAG
GTCCGCGGCCCTGACCCTGGCGGC
>1478
ACAAGTAGCCCTGTCAGCCGGAGCTCGCCGTGCGAGGAAGTTCCTATTAGCTTTATGG
TCAGGAGAACTTGTCCTGTTCAGGAAATGAATGTTGCCGCTAAAGGTCCGCGGCCCTG
ACCCTGGCGGCATAACCTGTGCGG
>1479
GAGCTCGCCGTGCGAGGAAGTTCCTATTAGCTTTATGGTCAGGAGAACTTGTCCTGTTC
AGGAAATGAATGTTGCCGCTAAAGGTCCGCGGCCCTGACCCTGGCGGCATAACCTGTG
CGGGCCGGGGAGACCCTGTGCAG
>1480
GAACTTGTCCTGTTCAGGAAATGAATGTTGCCGCTAAAGGTCCGCGGCCCTGACCCTG
GCGGCATAACCTGTGCGGGCCGGGGAGACCCTGTGCAGGATGCTTGGGATTTCAGCCC
CTCTCTGGTTCTTGTCATATGTAA
>1481
ATGAATGTTGCCGCTAAAGGTCCGCGGCCCTGACCCTGGCGGCATAACCTGTGCGGGC
CGGGGAGACCCTGTGCAGGATGCTTGGGATTTCAGCCCCTCTCTGGTTCTTGTCATATG

TAAAGAGGGAATCATTGTGGTAT
>1482
CCCTGGCGGCATAACCTGTGCGGGCCGGGGAGACCCTGTGCAGGATGCTTGGGATTTC
AGCCCCTCTCTGGTTCTTGTCATATGTAAAGAGGGAATCATTGTGGTATGTTGCAGCCTT
GAACTTTTGATTTATGCAGTGA
>1483
CGGCATAACCTGTGCGGGCCGGGGAGACCCTGTGCAGGATGCTTGGGATTTCAGCCCC
TCTCTGGTTCTTGTCATATGTAAAGAGGGAATCATTGTGGTATGTTGCAGCCTTGAACTT
TTGATTTATGCAGTGAGGGCGA
>1484
AGACCCTGTGCAGGATGCTTGGGATTTCAGCCCCTCTCTGGTTCTTGTCATATGTAAAG
AGGGAATCATTGTGGTATGTTGCAGCCTTGAACTTTTGATTTATGCAGTGAGGGCGACT
TAACCGTTTCCATGCCTGGACG
>1485
TCTGGTTCTTGTCATATGTAAAGAGGGAATCATTGTGGTATGTTGCAGCCTTGAACTTTT
GATTTATGCAGTGAGGGCGACTTAACCGTTTCCATGCCTGGACGGCTGTGCAGTGACA
GAGCCGTGTTGTGAGCGTATGT
>1486
GTAAAGAGGGAATCATTGTGGTATGTTGCAGCCTTGAACTTTTGATTTATGCAGTGAGG
GCGACTTAACCGTTTCCATGCCTGGACGGCTGTGCAGTGACAGAGCCGTGTTGTGAGC
GTATGTGCCTAGGTACATGTGTG
>1487
TTGCAGCCTTGAACTTTTGATTTATGCAGTGAGGGCGACTTAACCGTTTCCATGCCTGG
ACGGCTGTGCAGTGACAGAGCCGTGTTGTGAGCGTATGTGCCTAGGTACATGTGTGTG
TGGGGTGGTCAAGGGGAAGGAGA
>1488
AACTTTTGATTTATGCAGTGAGGGCGACTTAACCGTTTCCATGCCTGGACGGCTGTGCA
GTGACAGAGCCGTGTTGTGAGCGTATGTGCCTAGGTACATGTGTGTGTGGGGTGGTCA
AGGGGAAGGAGAGACCCCCACGT
>1489
TTGATTTATGCAGTGAGGGCGACTTAACCGTTTCCATGCCTGGACGGCTGTGCAGTGAC
AGAGCCGTGTTGTGAGCGTATGTGCCTAGGTACATGTGTGTGTGGGGTGGTCAAGGGG
AAGGAGAGACCCCCACGTGCTAG
>1490
TAACCGTTTCCATGCCTGGACGGCTGTGCAGTGACAGAGCCGTGTTGTGAGCGTATGT
GCCTAGGTACATGTGTGTGTGGGGTGGTCAAGGGGAAGGAGAGACCCCCACGTGCTA
GCAGGGGCTGTTGCTGGGAGCGGGT
>1491
CCGTGTTGTGAGCGTATGTGCCTAGGTACATGTGTGTGTGGGGTGGTCAAGGGGAAGG
AGAGACCCCCACGTGCTAGCAGGGGCTGTTGCTGGGAGCGGGTGGATTTCATGGGAG
GATTCCTTTTCTTTTTTCTTTTTCT
>1492
ATGTGCCTAGGTACATGTGTGTGTGGGGTGGTCAAGGGGAAGGAGAGACCCCCACGT
GCTAGCAGGGGCTGTTGCTGGGAGCGGGTGGATTTCATGGGAGGATTCCTTTTCTTTTT

TCTTTTTCTTGCATTCTTGCAGCA
>1493
ATTTCATGGGAGGATTCCTTTTCTTTTTTCTTTTTCTTGCATTCTTGCAGCATCTAAATTT
CCTGCGATTGTTGGCTGCAAAGTGGTAAGAAGCTGGAGAGCATTGTGTCTGGAATTGA
ACCTGGGTTCGAATTAATCTT
>1494
GATTCCTTTTCTTTTTTCTTTTTCTTGCATTCTTGCAGCATCTAAATTTCCTGCGATTGTT
GGCTGCAAAGTGGTAAGAAGCTGGAGAGCATTGTGTCTGGAATTGAACCTGGGTTCG
AATTAATCTTCTGTCTCTTAGA
>1495
CTTGCATTCTTGCAGCATCTAAATTTCCTGCGATTGTTGGCTGCAAAGTGGTAAGAAGC
TGGAGAGCATTGTGTCTGGAATTGAACCTGGGTTCGAATTAATCTTCTGTCTCTTAGAG
GCTGTGTGACCGTGGCAAGATT
>1496
AAATTTCCTGCGATTGTTGGCTGCAAAGTGGTAAGAAGCTGGAGAGCATTGTGTCTGG
AATTGAACCTGGGTTCGAATTAATCTTCTGTCTCTTAGAGGCTGTGTGACCGTGGCAAG
ATTCCTCACCACTCACTTCCTCA
>1497
TGGCTGCAAAGTGGTAAGAAGCTGGAGAGCATTGTGTCTGGAATTGAACCTGGGTTCG
AATTAATCTTCTGTCTCTTAGAGGCTGTGTGACCGTGGCAAGATTCCTCACCACTCACT
TCCTCATCTGTGAGATGAAGTTA
>1498
TAAGAAGCTGGAGAGCATTGTGTCTGGAATTGAACCTGGGTTCGAATTAATCTTCTGTC
TCTTAGAGGCTGTGTGACCGTGGCAAGATTCCTCACCACTCACTTCCTCATCTGTGAGA
TGAAGTTAATAACTTCTGATTC
>1499
CGAATTAATCTTCTGTCTCTTAGAGGCTGTGTGACCGTGGCAAGATTCCTCACCACTCA
CTTCCTCATCTGTGAGATGAAGTTAATAACTTCTGATTCATACTGGTGGTAGAGCAGTG
TTGTTAAGTAGGTGATGTATAG
>1500
TTCTGTCTCTTAGAGGCTGTGTGACCGTGGCAAGATTCCTCACCACTCACTTCCTCATC
TGTGAGATGAAGTTAATAACTTCTGATTCATACTGGTGGTAGAGCAGTGTTGTTAAGTA
GGTGATGTATAGAGAATACATG
>1501
CCGTGGCAAGATTCCTCACCACTCACTTCCTCATCTGTGAGATGAAGTTAATAACTTCT
GATTCATACTGGTGGTAGAGCAGTGTTGTTAAGTAGGTGATGTATAGAGAATACATGGC
CCAGCGTCCGGCCAGTGGTGTG
>1502
CCTCACCACTCACTTCCTCATCTGTGAGATGAAGTTAATAACTTCTGATTCATACTGGTG
GTAGAGCAGTGTTGTTAAGTAGGTGATGTATAGAGAATACATGGCCCAGCGTCCGGCC
AGTGGTGTGGCCTCAGGTTGTA
>1503
CACCACTCACTTCCTCATCTGTGAGATGAAGTTAATAACTTCTGATTCATACTGGTGGTA
GAGCAGTGTTGTTAAGTAGGTGATGTATAGAGAATACATGGCCCAGCGTCCGGCCAGT

GGTGTGGCCTCAGGTTGTATAC
>1504
CTCACTTCCTCATCTGTGAGATGAAGTTAATAACTTCTGATTCATACTGGTGGTAGAGC
AGTGTTGTTAAGTAGGTGATGTATAGAGAATACATGGCCCAGCGTCCGGCCAGTGGTGT
GGCCTCAGGTTGTATACTTGAA
>1505
TCATCTGTGAGATGAAGTTAATAACTTCTGATTCATACTGGTGGTAGAGCAGTGTTGTTA
AGTAGGTGATGTATAGAGAATACATGGCCCAGCGTCCGGCCAGTGGTGTGGCCTCAGG
TTGTATACTTGAAAGGATGTGT
>1506
ACTTCTGATTCATACTGGTGGTAGAGCAGTGTTGTTAAGTAGGTGATGTATAGAGAATA
CATGGCCCAGCGTCCGGCCAGTGGTGTGGCCTCAGGTTGTATACTTGAAAGGATGTGTA
TACTTCAGTATTTTTTCCATTG
>1507
AGCAGTGTTGTTAAGTAGGTGATGTATAGAGAATACATGGCCCAGCGTCCGGCCAGTG
GTGTGGCCTCAGGTTGTATACTTGAAAGGATGTGTATACTTCAGTATTTTTTCCATTGTA
AAATTAAGGGTTTTTTTTCTTT
>1508
AGTGTTGTTAAGTAGGTGATGTATAGAGAATACATGGCCCAGCGTCCGGCCAGTGGTGT
GGCCTCAGGTTGTATACTTGAAAGGATGTGTATACTTCAGTATTTTTTCCATTGTAAAAT
TAAGGGTTTTTTTTCTTTTTT
>1509
TGATGTATAGAGAATACATGGCCCAGCGTCCGGCCAGTGGTGTGGCCTCAGGTTGTATA
CTTGAAAGGATGTGTATACTTCAGTATTTTTTCCATTGTAAAATTAAGGGTTTTTTTTCTT
TTTTTCTTGAGACAGAGTCC
>1510
CGTCCGGCCAGTGGTGTGGCCTCAGGTTGTATACTTGAAAGGATGTGTATACTTCAGTA
TTTTTTCCATTGTAAAATTAAGGGTTTTTTTTCTTTTTTTCTTGAGACAGAGTCCTACTTT
GTTGCCCAGGCTTGAGTGCA
>1511
GGTGTGGCCTCAGGTTGTATACTTGAAAGGATGTGTATACTTCAGTATTTTTTCCATTGT
AAAATTAAGGGTTTTTTTTCTTTTTTTCTTGAGACAGAGTCCTACTTTGTTGCCCAGGCT
TGAGTGCAGTGGCGCGATCT
>1512
GGATGTGTATACTTCAGTATTTTTTCCATTGTAAAATTAAGGGTTTTTTTTCTTTTTTTCT
TGAGACAGAGTCCTACTTTGTTGCCCAGGCTTGAGTGCAGTGGCGCGATCTCAGCTCA
CTGCAACTTCCGCCTCCTGGG
>1513
ACTTCAGTATTTTTTCCATTGTAAAATTAAGGGTTTTTTTTCTTTTTTTCTTGAGACAGA
GTCCTACTTTGTTGCCCAGGCTTGAGTGCAGTGGCGCGATCTCAGCTCACTGCAACTTC
CGCCTCCTGGGTCCAAGTGAT
>1514
AGTCCTACTTTGTTGCCCAGGCTTGAGTGCAGTGGCGCGATCTCAGCTCACTGCAACT
TCCGCCTCCTGGGTCCAAGTGATTCTCGTGCCTCAGCCTCCCAAGTACCTGGGACCAC

AGGGGTGCACCACCACACCTGGCT
>1515
ACTTTGTTGCCCAGGCTTGAGTGCAGTGGCGCGATCTCAGCTCACTGCAACTTCCGCC
TCCTGGGTCCAAGTGATTCTCGTGCCTCAGCCTCCCAAGTACCTGGGACCACAGGGGT
GCACCACCACACCTGGCTAATTTT
>1516
TGGCGCGATCTCAGCTCACTGCAACTTCCGCCTCCTGGGTCCAAGTGATTCTCGTGCCT
CAGCCTCCCAAGTACCTGGGACCACAGGGGTGCACCACCACACCTGGCTAATTTTTGT
ATATATATATATATATATATATTTA
>1517
CTGCAACTTCCGCCTCCTGGGTCCAAGTGATTCTCGTGCCTCAGCCTCCCAAGTACCTG
GGACCACAGGGGTGCACCACCACACCTGGCTAATTTTTGTATATATATATATATATATATT
TAGTAGAGATGGGGTTTCGC
>1518
TGATTCTCGTGCCTCAGCCTCCCAAGTACCTGGGACCACAGGGGTGCACCACCACACC
TGGCTAATTTTTGTATATATATATATATATATATTTAGTAGAGATGGGGTTTCGCCATGTTG
GCCAGGCTGGTCTTGAACTC
>1519
GTACCTGGGACCACAGGGGTGCACCACCACACCTGGCTAATTTTTGTATATATATATATA
TATATATTTAGTAGAGATGGGGTTTCGCCATGTTGGCCAGGCTGGTCTTGAACTCCCGA
CCTCAGGTGATCTGCCTGTCT
>1520
CACAGGGGTGCACCACCACACCTGGCTAATTTTTGTATATATATATATATATATATTTAGTA
GAGATGGGGTTTCGCCATGTTGGCCAGGCTGGTCTTGAACTCCCGACCTCAGGTGATC
TGCCTGTCTCAGCCTCCTAA
>1521
GGCTAATTTTTGTATATATATATATATATATATTTAGTAGAGATGGGGTTTCGCCATGTTGG
CCAGGCTGGTCTTGAACTCCCGACCTCAGGTGATCTGCCTGTCTCAGCCTCCTAAAGT
GCTGGGATTATAGGTGTGAG
>1522
ATATATATATATTTAGTAGAGATGGGGTTTCGCCATGTTGGCCAGGCTGGTCTTGAACTC
CCGACCTCAGGTGATCTGCCTGTCTCAGCCTCCTAAAGTGCTGGGATTATAGGTGTGAG
CCACTGCGCCTGGCCTAAGTT
>1523
TTTAGTAGAGATGGGGTTTCGCCATGTTGGCCAGGCTGGTCTTGAACTCCCGACCTCA
GGTGATCTGCCTGTCTCAGCCTCCTAAAGTGCTGGGATTATAGGTGTGAGCCACTGCGC
CTGGCCTAAGTTTTTAAAAATTA
>1524
CAGGCTGGTCTTGAACTCCCGACCTCAGGTGATCTGCCTGTCTCAGCCTCCTAAAGTG
CTGGGATTATAGGTGTGAGCCACTGCGCCTGGCCTAAGTTTTTAAAAATTAAAAAGAAT
GGCATAGTGCTTTTTTGCTCTTT
>1525
CAGGTGATCTGCCTGTCTCAGCCTCCTAAAGTGCTGGGATTATAGGTGTGAGCCACTGC
GCCTGGCCTAAGTTTTTAAAAATTAAAAAGAATGGCATAGTGCTTTTTTGCTCTTTAAC

AGATCAAGACCAAAAGTTTTCC
>1526
AAGTGCTGGGATTATAGGTGTGAGCCACTGCGCCTGGCCTAAGTTTTTAAAAATTAAAA
AGAATGGCATAGTGCTTTTTTGCTCTTTAACAGATCAAGACCAAAAGTTTTCCCCCCAT
CATAGCCCAAAGTCCTAAAGAA
>1527
GGCCTAAGTTTTTAAAAATTAAAAAGAATGGCATAGTGCTTTTTTGCTCTTTAACAGAT
CAAGACCAAAAGTTTTCCCCCCATCATAGCCCAAAGTCCTAAAGAAAGGCTACTTAAG
TTGTTCGTTTTCTTTTGTTTCTC
>1528
AGAATGGCATAGTGCTTTTTTGCTCTTTAACAGATCAAGACCAAAAGTTTTCCCCCCAT
CATAGCCCAAAGTCCTAAAGAAAGGCTACTTAAGTTGTTCGTTTTCTTTTGTTTCTCCC
ATGAGTAATGTTTCTAAGTGTG
>1529
CTCTTTAACAGATCAAGACCAAAAGTTTTCCCCCCATCATAGCCCAAAGTCCTAAAGA
AAGGCTACTTAAGTTGTTCGTTTTCTTTTGTTTCTCCCATGAGTAATGTTTCTAAGTGTG
ATTTTTGCAGAGAACAGCAGTG
>1530
TTTAACAGATCAAGACCAAAAGTTTTCCCCCCATCATAGCCCAAAGTCCTAAAGAAAG
GCTACTTAAGTTGTTCGTTTTCTTTTGTTTCTCCCATGAGTAATGTTTCTAAGTGTGATTT
TTGCAGAGAACAGCAGTGTCT
>1531
ACCAAAAGTTTTCCCCCCATCATAGCCCAAAGTCCTAAAGAAAGGCTACTTAAGTTGTT
CGTTTTCTTTTGTTTCTCCCATGAGTAATGTTTCTAAGTGTGATTTTTGCAGAGAACAGC
AGTGTCTGACCTCATTCTTGA
>1532
AAAGTCCTAAAGAAAGGCTACTTAAGTTGTTCGTTTTCTTTTGTTTCTCCCATGAGTAA
TGTTTCTAAGTGTGATTTTTGCAGAGAACAGCAGTGTCTGACCTCATTCTTGAGGGGA
ACTTTTCTGCCTGCCTCTTCTTG
>1533
TAAGTTGTTCGTTTTCTTTTGTTTCTCCCATGAGTAATGTTTCTAAGTGTGATTTTTGCA
GAGAACAGCAGTGTCTGACCTCATTCTTGAGGGGAACTTTTCTGCCTGCCTCTTCTTG
AGAAAAAAATGTTTGTGAGTTGT
>1534
AGTTGTTCGTTTTCTTTTGTTTCTCCCATGAGTAATGTTTCTAAGTGTGATTTTTGCAGA
GAACAGCAGTGTCTGACCTCATTCTTGAGGGGAACTTTTCTGCCTGCCTCTTCTTGAG
AAAAAAATGTTTGTGAGTTGTCT
>1535
GCAGAGAACAGCAGTGTCTGACCTCATTCTTGAGGGGAACTTTTCTGCCTGCCTCTTC
TTGAGAAAAAAATGTTTGTGAGTTGTCTGGCAAATGGATGCGTTACTTTTGCTGCTGTTG
CTGCCGCTGCCGTTTTAAAGGCC
>1536
AGAACAGCAGTGTCTGACCTCATTCTTGAGGGGAACTTTTCTGCCTGCCTCTTCTTGA
GAAAAAAATGTTTGTGAGTTGTCTGGCAAATGGATGCGTTACTTTTGCTGCTGTTGCTGC

CGCTGCCGTTTTAAAGGCCCCTG
>1537
CTTGAGGGGAACTTTTCTGCCTGCCTCTTCTTGAGAAAAAATGTTTGTGAGTTGTCTGG
CAAATGGATGCGTTACTTTTGCTGCTGTTGCTGCCGCTGCCGTTTTAAAGGCCCCTGGT
GACAAAGGAGTGAACTGCACTT
>1538
TTGAGAAAAAATGTTTGTGAGTTGTCTGGCAAATGGATGCGTTACTTTTGCTGCTGTTG
CTGCCGCTGCCGTTTTAAAGGCCCCTGGTGACAAAGGAGTGAACTGCACTTTCGGGGA
AAGCATGCCGGAATCTGGCCCAG
>1539
GTGAGTTGTCTGGCAAATGGATGCGTTACTTTTGCTGCTGTTGCTGCCGCTGCCGTTTT
AAAGGCCCCTGGTGACAAAGGAGTGAACTGCACTTTCGGGGAAAGCATGCCGGAATC
TGGCCCAGAAAAGAGGGGCCGCTG
>1540
GGAGTGAACTGCACTTTCGGGGAAAGCATGCCGGAATCTGGCCCAGAAAAGAGGGGC
CGCTGGGGGAAGGGTTTGGAGATGACGCCTCCTCAGGAACTTTAAAAGAAATCTGTCC
ATCTGTCTCTGGCATTTATGGCATC
>1541
CCCAGAAAAGAGGGGCCGCTGGGGGAAGGGTTTGGAGATGACGCCTCCTCAGGAACT
TTAAAAGAAATCTGTCCATCTGTCTCTGGCATTTATGGCATCGTTGACAGCAGTTCAAG
GAAAAATAAAAATAAGTTCTTATC
>1542
AGAGGGGCCGCTGGGGGAAGGGTTTGGAGATGACGCCTCCTCAGGAACTTTAAAAGA
AATCTGTCCATCTGTCTCTGGCATTTATGGCATCGTTGACAGCAGTTCAAGGAAAAATA
AAAATAAGTTCTTATCTGATTGCG
>1543
GACGCCTCCTCAGGAACTTTAAAAGAAATCTGTCCATCTGTCTCTGGCATTTATGGCAT
CGTTGACAGCAGTTCAAGGAAAAATAAAAATAAGTTCTTATCTGATTGCGGGGCTGATT
GCAAAGCCGAAGGACTAGAGTG
>1544
AAGAAATCTGTCCATCTGTCTCTGGCATTTATGGCATCGTTGACAGCAGTTCAAGGAAA
AATAAAAATAAGTTCTTATCTGATTGCGGGGCTGATTGCAAAGCCGAAGGACTAGAGT
GGCCAGCGAGTCGGGCGGCTGGA
>1545
GCAGTTCAAGGAAAAATAAAAATAAGTTCTTATCTGATTGCGGGGCTGATTGCAAAGC
CGAAGGACTAGAGTGGCCAGCGAGTCGGGCGGCTGGATCTGGAGTTTTGGAGAAAGG
GCCACTGGCTCATTTCTGGGCACGC
>1546
AAAAATAAAAATAAGTTCTTATCTGATTGCGGGGCTGATTGCAAAGCCGAAGGACTAG
AGTGGCCAGCGAGTCGGGCGGCTGGATCTGGAGTTTTGGAGAAAGGGCCACTGGCTC
ATTTCTGGGCACGCACGCTCACGGA
>1547
ATCTGATTGCGGGGCTGATTGCAAAGCCGAAGGACTAGAGTGGCCAGCGAGTCGGGC
GGCTGGATCTGGAGTTTTGGAGAAAGGGCCACTGGCTCATTTCTGGGCACGCACGCTC

ACGGAGGCAAGTTCTCACCTCCTCG
>1548
GCGGCTGGATCTGGAGTTTTGGAGAAAGGGCCACTGGCTCATTTCTGGGCACGCACGC
TCACGGAGGCAAGTTCTCACCTCCTCGTGGGCTTCTCTTCACTTTACGTGGAAAAACT
GTGTACGTCTCAAATTAAAAGCAG
>1549
AGTTTTGGAGAAAGGGCCACTGGCTCATTTCTGGGCACGCACGCTCACGGAGGCAAG
TTCTCACCTCCTCGTGGGCTTCTCTTCACTTTACGTGGAAAAACTGTGTACGTCTCAAA
TTAAAAGCAGCTCTTGCCCCGCTT
>1550
GGCTCATTTCTGGGCACGCACGCTCACGGAGGCAAGTTCTCACCTCCTCGTGGGCTTC
TCTTCACTTTACGTGGAAAAACTGTGTACGTCTCAAATTAAAAGCAGCTCTTGCCCCGC
TTAGAGCCGGTGAAAGCTGCTTC
>1551
GGGCACGCACGCTCACGGAGGCAAGTTCTCACCTCCTCGTGGGCTTCTCTTCACTTTA
CGTGGAAAAACTGTGTACGTCTCAAATTAAAAGCAGCTCTTGCCCCGCTTAGAGCCGG
TGAAAGCTGCTTCGAAGGCGTTTG
>1552
TCTCTTCACTTTACGTGGAAAAACTGTGTACGTCTCAAATTAAAAGCAGCTCTTGCCCC
GCTTAGAGCCGGTGAAAGCTGCTTCGAAGGCGTTTGTAGAGTAGGGATTGGAAATGTT
CAATGCAAAAGTTAAATCGTGAC
>1553
AAACTGTGTACGTCTCAAATTAAAAGCAGCTCTTGCCCCGCTTAGAGCCGGTGAAAGC
TGCTTCGAAGGCGTTTGTAGAGTAGGGATTGGAAATGTTCAATGCAAAAGTTAAATCG
TGACAGTTGTCCCCTAAATGATCT
>1554
AGCAGCTCTTGCCCCGCTTAGAGCCGGTGAAAGCTGCTTCGAAGGCGTTTGTAGAGTA
GGGATTGGAAATGTTCAATGCAAAAGTTAAATCGTGACAGTTGTCCCCTAAATGATCTG
CTAAATTAAGGATCCGCTAACAT
>1555
CCGCTTAGAGCCGGTGAAAGCTGCTTCGAAGGCGTTTGTAGAGTAGGGATTGGAAATG
TTCAATGCAAAAGTTAAATCGTGACAGTTGTCCCCTAAATGATCTGCTAAATTAAGGAT
CCGCTAACATTTGTGAGTCTACC
>1556
AGCCGGTGAAAGCTGCTTCGAAGGCGTTTGTAGAGTAGGGATTGGAAATGTTCAATGC
AAAAGTTAAATCGTGACAGTTGTCCCCTAAATGATCTGCTAAATTAAGGATCCGCTAAC
ATTTGTGAGTCTACCTCCCTGCA
>1557
TCAATGCAAAAGTTAAATCGTGACAGTTGTCCCCTAAATGATCTGCTAAATTAAGGATC
CGCTAACATTTGTGAGTCTACCTCCCTGCATTCCCCCCACCCCCACCCCCGCCCCCTTG
GCAGAAGGGATAAAGATGGTAC
>1558
TGCAAAAGTTAAATCGTGACAGTTGTCCCCTAAATGATCTGCTAAATTAAGGATCCGCT
AACATTTGTGAGTCTACCTCCCTGCATTCCCCCCACCCCCACCCCCGCCCCCTTGGCAG

AAGGGATAAAGATGGTACCTAA
>1559
ATTTGTGAGTCTACCTCCCTGCATTCCCCCCACCCCCACCCCCGCCCCCTTGGCAGAAG
GGATAAAGATGGTACCTAAAAACAATGTGAAGCGGCTGCTAAATCGAGTGACTCTAAA
AACATTTTTCTTTCCTTGAAGTT
>1560
TCCCTGCATTCCCCCCACCCCCACCCCCGCCCCCTTGGCAGAAGGGATAAAGATGGTA
CCTAAAAACAATGTGAAGCGGCTGCTAAATCGAGTGACTCTAAAAACATTTTTCTTTCC
TTGAAGTTCAGTGTGGAAAACCA
>1561
CACCCCCGCCCCCTTGGCAGAAGGGATAAAGATGGTACCTAAAAACAATGTGAAGCGG
CTGCTAAATCGAGTGACTCTAAAAACATTTTTCTTTCCTTGAAGTTCAGTGTGGAAAAC
CAGACTGGACTTGTCAGGGATGC
>1562
ATGGTACCTAAAAACAATGTGAAGCGGCTGCTAAATCGAGTGACTCTAAAAACATTTTT
CTTTCCTTGAAGTTCAGTGTGGAAAACCAGACTGGACTTGTCAGGGATGCAGACCTAT
ATAGGGCCCATGTGCCTGGCCCC
>1563
ACCTAAAAACAATGTGAAGCGGCTGCTAAATCGAGTGACTCTAAAAACATTTTTCTTTC
CTTGAAGTTCAGTGTGGAAAACCAGACTGGACTTGTCAGGGATGCAGACCTATATAGG
GCCCATGTGCCTGGCCCCTTTCC
>1564
TGCTAAATCGAGTGACTCTAAAAACATTTTTCTTTCCTTGAAGTTCAGTGTGGAAAACC
AGACTGGACTTGTCAGGGATGCAGACCTATATAGGGCCCATGTGCCTGGCCCCTTTCCT
CACTTTCTAGCACAATGGTTTT
>1565
TCTTTCCTTGAAGTTCAGTGTGGAAAACCAGACTGGACTTGTCAGGGATGCAGACCTA
TATAGGGCCCATGTGCCTGGCCCCTTTCCTCACTTTCTAGCACAATGGTTTTCATTGCCA
CCTCTCCCGCGCCTTTCCCCCC
>1566
GACTTGTCAGGGATGCAGACCTATATAGGGCCCATGTGCCTGGCCCCTTTCCTCACTTT
CTAGCACAATGGTTTTCATTGCCACCTCTCCCGCGCCTTTCCCCCCTTCATAATTATCCA
CTATTTTACTTTCTGTGTCCT
>1567
CACAATGGTTTTCATTGCCACCTCTCCCGCGCCTTTCCCCCCTTCATAATTATCCACTATT
TTACTTTCTGTGTCCTGACAGCTACAAGAGTAGTTTTTGTTTTTTTGAGCTGAGTAGCT
GCTAAATATTCCCCAGCAGC
>1568
CAATGGTTTTCATTGCCACCTCTCCCGCGCCTTTCCCCCCTTCATAATTATCCACTATTTT
ACTTTCTGTGTCCTGACAGCTACAAGAGTAGTTTTTGTTTTTTTGAGCTGAGTAGCTGC
TAAATATTCCCCAGCAGCCA
>1569
CCTCTCCCGCGCCTTTCCCCCCTTCATAATTATCCACTATTTTACTTTCTGTGTCCTGACA
GCTACAAGAGTAGTTTTTGTTTTTTTGAGCTGAGTAGCTGCTAAATATTCCCCAGCAGC

CAGATTCAACGGCTGGGGGG
>1570
CTCCCGCGCCTTTCCCCCCTTCATAATTATCCACTATTTTACTTTCTGTGTCCTGACAGCT
ACAAGAGTAGTTTTTGTTTTTTTGAGCTGAGTAGCTGCTAAATATTCCCCAGCAGCCAG
ATTCAACGGCTGGGGGGGAG
>1571
CATAATTATCCACTATTTTACTTTCTGTGTCCTGACAGCTACAAGAGTAGTTTTTGTTTTT
TTGAGCTGAGTAGCTGCTAAATATTCCCCAGCAGCCAGATTCAACGGCTGGGGGGGAG
AATAGGCCAAACCTTGGCATC
>1572
CTGCTAAATATTCCCCAGCAGCCAGATTCAACGGCTGGGGGGGAGAATAGGCCAAACC
TTGGCATCCCCTGTTGCAAAAGTGAAAAATATTTCTGTTTGCAGTTAATTTTAGTGGCA
AGCAGATCATGTTAACTGGATGT
>1573
ATTCCCCAGCAGCCAGATTCAACGGCTGGGGGGGAGAATAGGCCAAACCTTGGCATCC
CCTGTTGCAAAAGTGAAAAATATTTCTGTTTGCAGTTAATTTTAGTGGCAAGCAGATCA
TGTTAACTGGATGTAATTGCTTC
>1574
GATTCAACGGCTGGGGGGGAGAATAGGCCAAACCTTGGCATCCCCTGTTGCAAAAGTG
AAAAATATTTCTGTTTGCAGTTAATTTTAGTGGCAAGCAGATCATGTTAACTGGATGTAA
TTGCTTCCAGATATGGCCGAGG
>1575
CGGCTGGGGGGGAGAATAGGCCAAACCTTGGCATCCCCTGTTGCAAAAGTGAAAAATA
TTTCTGTTTGCAGTTAATTTTAGTGGCAAGCAGATCATGTTAACTGGATGTAATTGCTTC
CAGATATGGCCGAGGGTTTTGT
>1576
CTTGGCATCCCCTGTTGCAAAAGTGAAAAATATTTCTGTTTGCAGTTAATTTTAGTGGC
AAGCAGATCATGTTAACTGGATGTAATTGCTTCCAGATATGGCCGAGGGTTTTGTGTGT
TGAGATGAAGGGTCTGGTAAGT
>1577
CTGTTGCAAAAGTGAAAAATATTTCTGTTTGCAGTTAATTTTAGTGGCAAGCAGATCAT
GTTAACTGGATGTAATTGCTTCCAGATATGGCCGAGGGTTTTGTGTGTTGAGATGAAGG
GTCTGGTAAGTGCTTACTTCCT
>1578
GTTTGCAGTTAATTTTAGTGGCAAGCAGATCATGTTAACTGGATGTAATTGCTTCCAGAT
ATGGCCGAGGGTTTTGTGTGTTGAGATGAAGGGTCTGGTAAGTGCTTACTTCCTGTGTA
AATTAGCCTATTCCATTCAGT
>1579
CAGTTAATTTTAGTGGCAAGCAGATCATGTTAACTGGATGTAATTGCTTCCAGATATGGC
CGAGGGTTTTGTGTGTTGAGATGAAGGGTCTGGTAAGTGCTTACTTCCTGTGTAAATTA
GCCTATTCCATTCAGTCTTTC
>1580
GTAATTGCTTCCAGATATGGCCGAGGGTTTTGTGTGTTGAGATGAAGGGTCTGGTAAGT
GCTTACTTCCTGTGTAAATTAGCCTATTCCATTCAGTCTTTCATTCTGGCCAGCCCTGGA

TTCTGTCATTCTGCTCCCATT
>1581
AATTGCTTCCAGATATGGCCGAGGGTTTTGTGTGTTGAGATGAAGGGTCTGGTAAGTGC
TTACTTCCTGTGTAAATTAGCCTATTCCATTCAGTCTTTCATTCTGGCCAGCCCTGGATT
CTGTCATTCTGCTCCCATTTT
>1582
GGGTTTTGTGTGTTGAGATGAAGGGTCTGGTAAGTGCTTACTTCCTGTGTAAATTAGCC
TATTCCATTCAGTCTTTCATTCTGGCCAGCCCTGGATTCTGTCATTCTGCTCCCATTTTCC
CCCTCCCCAGCCTGCAGCCT
>1583
GTAAGTGCTTACTTCCTGTGTAAATTAGCCTATTCCATTCAGTCTTTCATTCTGGCCAGC
CCTGGATTCTGTCATTCTGCTCCCATTTTCCCCCTCCCCAGCCTGCAGCCTGGTGTGAG
AAGCCGCTTGCTATGTTCCTC
>1584
TCTTTCATTCTGGCCAGCCCTGGATTCTGTCATTCTGCTCCCATTTTCCCCCTCCCCAGC
CTGCAGCCTGGTGTGAGAAGCCGCTTGCTATGTTCCTCTAAGTGTAATTCTTTTGTCAC
TTGGAGCCACTCTCCTCTTGG
>1585
GGATTCTGTCATTCTGCTCCCATTTTCCCCCTCCCCAGCCTGCAGCCTGGTGTGAGAAG
CCGCTTGCTATGTTCCTCTAAGTGTAATTCTTTTGTCACTTGGAGCCACTCTCCTCTTGG
TTCTTGATGTGAGTTTGCCCT
>1586
ATTCTGCTCCCATTTTCCCCCTCCCCAGCCTGCAGCCTGGTGTGAGAAGCCGCTTGCTA
TGTTCCTCTAAGTGTAATTCTTTTGTCACTTGGAGCCACTCTCCTCTTGGTTCTTGATGT
GAGTTTGCCCTTAAATAATGA
>1587
TTTCCCCCTCCCCAGCCTGCAGCCTGGTGTGAGAAGCCGCTTGCTATGTTCCTCTAAGT
GTAATTCTTTTGTCACTTGGAGCCACTCTCCTCTTGGTTCTTGATGTGAGTTTGCCCTTA
AATAATGAGAAGGGTTTGAAA
>1588
AGCCGCTTGCTATGTTCCTCTAAGTGTAATTCTTTTGTCACTTGGAGCCACTCTCCTCTT
GGTTCTTGATGTGAGTTTGCCCTTAAATAATGAGAAGGGTTTGAAAAGATGCCACCTG
GACCCCAGGCCACCTCCCGCCT
>1589
GCTTGCTATGTTCCTCTAAGTGTAATTCTTTTGTCACTTGGAGCCACTCTCCTCTTGGTT
CTTGATGTGAGTTTGCCCTTAAATAATGAGAAGGGTTTGAAAAGATGCCACCTGGACC
CCAGGCCACCTCCCGCCTCTTT
>1590
ATTCTTTTGTCACTTGGAGCCACTCTCCTCTTGGTTCTTGATGTGAGTTTGCCCTTAAAT
AATGAGAAGGGTTTGAAAAGATGCCACCTGGACCCCAGGCCACCTCCCGCCTCTTTCC
CAGCAAAGGCAGATCTGAAGAG
>1591
GATGCCACCTGGACCCCAGGCCACCTCCCGCCTCTTTCCCAGCAAAGGCAGATCTGAA
GAGCATCTCCTGGTCATGGGGGTTGCATCTTGGAACCCAGAACTCCTTGTTTTCTATGC

ATTTTTTTTTCCTACTGCCCTCT
>1592
TGGACCCCAGGCCACCTCCCGCCTCTTTCCCAGCAAAGGCAGATCTGAAGAGCATCTC
CTGGTCATGGGGGTTGCATCTTGGAACCCAGAACTCCTTGTTTTCTATGCATTTTTTTTT
CCTACTGCCCTCTGACCCCCGA
>1593
TCCCAGCAAAGGCAGATCTGAAGAGCATCTCCTGGTCATGGGGGTTGCATCTTGGAAC
CCAGAACTCCTTGTTTTCTATGCATTTTTTTTTCCTACTGCCCTCTGACCCCCGACTCTC
ACCTGGGAGAACACCTTCTCTT
>1594
ATTTTTTTTTCCTACTGCCCTCTGACCCCCGACTCTCACCTGGGAGAACACCTTCTCTTT
CTCTCAATTAGTCCGACTCCTCCTCAGCCTGAACTAAGCACGTTCCAGTGGGTCCGAA
ATCTCAGAAAAAATGAAACGGC
>1595
ACTCTCACCTGGGAGAACACCTTCTCTTTCTCTCAATTAGTCCGACTCCTCCTCAGCCT
GAACTAAGCACGTTCCAGTGGGTCCGAAATCTCAGAAAAAATGAAACGGCTGTTGCG
CAGAGGAGAAGGTTGTGGCCACAG
>1596
ACCTGGGAGAACACCTTCTCTTTCTCTCAATTAGTCCGACTCCTCCTCAGCCTGAACTA
AGCACGTTCCAGTGGGTCCGAAATCTCAGAAAAAATGAAACGGCTGTTGCGCAGAGG
AGAAGGTTGTGGCCACAGAAAGGA
>1597
TCCGACTCCTCCTCAGCCTGAACTAAGCACGTTCCAGTGGGTCCGAAATCTCAGAAAA
AATGAAACGGCTGTTGCGCAGAGGAGAAGGTTGTGGCCACAGAAAGGAGTTGCCTCA
TCAGACGTGAAGCCGCCACCTTGCC
>1598
CTGAACTAAGCACGTTCCAGTGGGTCCGAAATCTCAGAAAAAATGAAACGGCTGTTGC
GCAGAGGAGAAGGTTGTGGCCACAGAAAGGAGTTGCCTCATCAGACGTGAAGCCGCC
ACCTTGCCTTGCAAAGCAGGCTGCA
>1599
AACTAAGCACGTTCCAGTGGGTCCGAAATCTCAGAAAAAATGAAACGGCTGTTGCGC
AGAGGAGAAGGTTGTGGCCACAGAAAGGAGTTGCCTCATCAGACGTGAAGCCGCCAC
CTTGCCTTGCAAAGCAGGCTGCACCT
>1600
CAGAAAAAATGAAACGGCTGTTGCGCAGAGGAGAAGGTTGTGGCCACAGAAAGGAG
TTGCCTCATCAGACGTGAAGCCGCCACCTTGCCTTGCAAAGCAGGCTGCACCTCCTGA
CACACCTGCCCCTCCCTGCAAGAAAG
>1601
ACCTCCTGACACACCTGCCCCTCCCTGCAAGAAAGGGAGGGGAAATCCCACCCAAGA
GAACCTTCTCCAGGTTCCTGTGGGTTTCCAGGGTTGCAACTGAAGAAAAGTGTTAAGA
TTCATGTTCTCCTAAATTCTGCTTT
>1602
TGACACACCTGCCCCTCCCTGCAAGAAAGGGAGGGGAAATCCCACCCAAGAGAACCT
TCTCCAGGTTCCTGTGGGTTTCCAGGGTTGCAACTGAAGAAAAGTGTTAAGATTCATG

TTCTCCTAAATTCTGCTTTTGTCCT
>1603
GAGGGGAAATCCCACCCAAGAGAACCTTCTCCAGGTTCCTGTGGGTTTCCAGGGTTGC
AACTGAAGAAAAGTGTTAAGATTCATGTTCTCCTAAATTCTGCTTTTGTCCTGTGGCCA
GTCCCACATTCTTAGTGCTATTT
>1604
GGGGAAATCCCACCCAAGAGAACCTTCTCCAGGTTCCTGTGGGTTTCCAGGGTTGCAA
CTGAAGAAAAGTGTTAAGATTCATGTTCTCCTAAATTCTGCTTTTGTCCTGTGGCCAGT
CCCACATTCTTAGTGCTATTTGC
>1605
TTCCTGTGGGTTTCCAGGGTTGCAACTGAAGAAAAGTGTTAAGATTCATGTTCTCCTAA
ATTCTGCTTTTGTCCTGTGGCCAGTCCCACATTCTTAGTGCTATTTGCATAAGAGGTTGC
TGTTTATCTTCCTATATCAGC
>1606
TGAAGAAAAGTGTTAAGATTCATGTTCTCCTAAATTCTGCTTTTGTCCTGTGGCCAGTC
CCACATTCTTAGTGCTATTTGCATAAGAGGTTGCTGTTTATCTTCCTATATCAGCCCCACT
GATAACCCCTGTGGTCTGTT
>1607
TTCATGTTCTCCTAAATTCTGCTTTTGTCCTGTGGCCAGTCCCACATTCTTAGTGCTATTT
GCATAAGAGGTTGCTGTTTATCTTCCTATATCAGCCCCACTGATAACCCCTGTGGTCTGT
TTAACAACCTATGAGGAGT
>1608
CACATTCTTAGTGCTATTTGCATAAGAGGTTGCTGTTTATCTTCCTATATCAGCCCCACTG
ATAACCCCTGTGGTCTGTTTAACAACCTATGAGGAGTAGATTACTTGATAAATGCCAGT
TTTTCCAATATGCAAAGATA
>1609
ATTCTTAGTGCTATTTGCATAAGAGGTTGCTGTTTATCTTCCTATATCAGCCCCACTGATA
ACCCCTGTGGTCTGTTTAACAACCTATGAGGAGTAGATTACTTGATAAATGCCAGTTTTT
CCAATATGCAAAGATAAAC
>1610
AGGTTGCTGTTTATCTTCCTATATCAGCCCCACTGATAACCCCTGTGGTCTGTTTAACAA
CCTATGAGGAGTAGATTACTTGATAAATGCCAGTTTTTCCAATATGCAAAGATAAACTGT
TGGGGTCAGGGTGAGGGGTG
>1611
ATCAGCCCCACTGATAACCCCTGTGGTCTGTTTAACAACCTATGAGGAGTAGATTACTT
GATAAATGCCAGTTTTTCCAATATGCAAAGATAAACTGTTGGGGTCAGGGTGAGGGGT
GGTGTGTTATGAAGGAGGGGACA
>1612
TCTGTTTAACAACCTATGAGGAGTAGATTACTTGATAAATGCCAGTTTTTCCAATATGCA
AAGATAAACTGTTGGGGTCAGGGTGAGGGGTGGTGTGTTATGAAGGAGGGGACAGGT
GGGGAGCCAGGACCCTCTTTTAG
>1613
TAACAACCTATGAGGAGTAGATTACTTGATAAATGCCAGTTTTTCCAATATGCAAAGATA
AACTGTTGGGGTCAGGGTGAGGGGTGGTGTGTTATGAAGGAGGGGACAGGTGGGGAG

CCAGGACCCTCTTTTAGTGGGCT
>1614
TTTTTCCAATATGCAAAGATAAACTGTTGGGGTCAGGGTGAGGGGTGGTGTGTTATGAA
GGAGGGGACAGGTGGGGAGCCAGGACCCTCTTTTAGTGGGCTGGAGAAGTGTGAAAG
AGCCTGATGAAAGAAAACAGGTAG
>1615
TGTTGGGGTCAGGGTGAGGGGTGGTGTGTTATGAAGGAGGGGACAGGTGGGGAGCCA
GGACCCTCTTTTAGTGGGCTGGAGAAGTGTGAAAGAGCCTGATGAAAGAAAACAGGT
AGCTTGTCACATACAACAGCTGCATT
>1616
GAGGGGTGGTGTGTTATGAAGGAGGGGACAGGTGGGGAGCCAGGACCCTCTTTTAGT
GGGCTGGAGAAGTGTGAAAGAGCCTGATGAAAGAAAACAGGTAGCTTGTCACATACA
ACAGCTGCATTTATCATACGGTTGAT
>1617
ACAGGTGGGGAGCCAGGACCCTCTTTTAGTGGGCTGGAGAAGTGTGAAAGAGCCTGA
TGAAAGAAAACAGGTAGCTTGTCACATACAACAGCTGCATTTATCATACGGTTGATCAG
CTTCCTGCCCTCACCTACTTTTCT
>1618
GGGAGCCAGGACCCTCTTTTAGTGGGCTGGAGAAGTGTGAAAGAGCCTGATGAAAGA
AAACAGGTAGCTTGTCACATACAACAGCTGCATTTATCATACGGTTGATCAGCTTCCTG
CCCTCACCTACTTTTCTCCTCCTT
>1619
AGAAGTGTGAAAGAGCCTGATGAAAGAAAACAGGTAGCTTGTCACATACAACAGCTG
CATTTATCATACGGTTGATCAGCTTCCTGCCCTCACCTACTTTTCTCCTCCTTCATGTGG
ACATGCAGAGTCTCTGAGAACAT
>1620
ACATACAACAGCTGCATTTATCATACGGTTGATCAGCTTCCTGCCCTCACCTACTTTTCT
CCTCCTTCATGTGGACATGCAGAGTCTCTGAGAACATTTCATCAAGTGGGTTGCCTATG
CCCACATTAATACAAGATGGG
>1621
GCATTTATCATACGGTTGATCAGCTTCCTGCCCTCACCTACTTTTCTCCTCCTTCATGTG
GACATGCAGAGTCTCTGAGAACATTTCATCAAGTGGGTTGCCTATGCCCACATTAATAC
AAGATGGGAAAGGGTGGAGGT
>1622
GCTTCCTGCCCTCACCTACTTTTCTCCTCCTTCATGTGGACATGCAGAGTCTCTGAGAA
CATTTCATCAAGTGGGTTGCCTATGCCCACATTAATACAAGATGGGAAAGGGTGGAGGT
TATCTTTGCTTTTTAAGTTCCC
>1623
CCTGCCCTCACCTACTTTTCTCCTCCTTCATGTGGACATGCAGAGTCTCTGAGAACATT
TCATCAAGTGGGTTGCCTATGCCCACATTAATACAAGATGGGAAAGGGTGGAGGTTATC
TTTGCTTTTTAAGTTCCCATCT
>1624
CATGCAGAGTCTCTGAGAACATTTCATCAAGTGGGTTGCCTATGCCCACATTAATACAA
GATGGGAAAGGGTGGAGGTTATCTTTGCTTTTTAAGTTCCCATCTTTTTCTCCCTTGGTC

CTCTCCCACCCCTACAAGTTG
>1625
CATCAAGTGGGTTGCCTATGCCCACATTAATACAAGATGGGAAAGGGTGGAGGTTATCT
TTGCTTTTTAAGTTCCCATCTTTTTCTCCCTTGGTCCTCTCCCACCCCTACAAGTTGTTG
GCTGGTAAGCAATTGATGCTC
>1626
CACATTAATACAAGATGGGAAAGGGTGGAGGTTATCTTTGCTTTTTAAGTTCCCATCTTT
TTCTCCCTTGGTCCTCTCCCACCCCTACAAGTTGTTGGCTGGTAAGCAATTGATGCTCA
GAAAAATGATGTTCATCTGAA
>1627
GGTGGAGGTTATCTTTGCTTTTTAAGTTCCCATCTTTTTCTCCCTTGGTCCTCTCCCACC
CCTACAAGTTGTTGGCTGGTAAGCAATTGATGCTCAGAAAAATGATGTTCATCTGAAAT
CTTAAATGAGTGGACTTTTTC
>1628
TTATCTTTGCTTTTTAAGTTCCCATCTTTTTCTCCCTTGGTCCTCTCCCACCCCTACAAGT
TGTTGGCTGGTAAGCAATTGATGCTCAGAAAAATGATGTTCATCTGAAATCTTAAATGA
GTGGACTTTTTCTACCTTCC
>1629
TTTCTCCCTTGGTCCTCTCCCACCCCTACAAGTTGTTGGCTGGTAAGCAATTGATGCTC
AGAAAAATGATGTTCATCTGAAATCTTAAATGAGTGGACTTTTTCTACCTTCCCTGACT
TTGCAGGTGCTTCTTGAAGAGC
>1630
CCCCTACAAGTTGTTGGCTGGTAAGCAATTGATGCTCAGAAAAATGATGTTCATCTGAA
ATCTTAAATGAGTGGACTTTTTCTACCTTCCCTGACTTTGCAGGTGCTTCTTGAAGAGC
CAAGGAGAAAAGAAAACTTCTG
>1631
TGCTCAGAAAAATGATGTTCATCTGAAATCTTAAATGAGTGGACTTTTTCTACCTTCCCT
GACTTTGCAGGTGCTTCTTGAAGAGCCAAGGAGAAAAGAAAACTTCTGACCTCATCA
CCTAGTCAATAAGCTGTCTGAGA
>1632
ACCTTCCCTGACTTTGCAGGTGCTTCTTGAAGAGCCAAGGAGAAAAGAAAACTTCTG
ACCTCATCACCTAGTCAATAAGCTGTCTGAGAAACAAACAAAAGAGTTTAGACTGGCT
GCTAAAGCTAGGAACCCAGCTGATA
>1633
CTTTGCAGGTGCTTCTTGAAGAGCCAAGGAGAAAAGAAAACTTCTGACCTCATCACCT
AGTCAATAAGCTGTCTGAGAAACAAACAAAAGAGTTTAGACTGGCTGCTAAAGCTAG
GAACCCAGCTGATAGCTTTGTTTTG
>1634
TAAGCTGTCTGAGAAACAAACAAAAGAGTTTAGACTGGCTGCTAAAGCTAGGAACCC
AGCTGATAGCTTTGTTTTGATGTCCTTGTAATGTTTGCGGATCGCAGAGGGTGCTATTTA
GAGCAGGGGGCATCTACTGGGAC
>1635
CTGAGAAACAAACAAAAGAGTTTAGACTGGCTGCTAAAGCTAGGAACCCAGCTGATA
GCTTTGTTTTGATGTCCTTGTAATGTTTGCGGATCGCAGAGGGTGCTATTTAGAGCAGG

GGGCATCTACTGGGACAGACTCAC
>1636
GCTGCTAAAGCTAGGAACCCAGCTGATAGCTTTGTTTTGATGTCCTTGTAATGTTTGCG
GATCGCAGAGGGTGCTATTTAGAGCAGGGGGCATCTACTGGGACAGACTCACTGGGAA
GTTGAAACAGCTAGCTCAAAGTC
>1637
GTAATGTTTGCGGATCGCAGAGGGTGCTATTTAGAGCAGGGGGCATCTACTGGGACAG
ACTCACTGGGAAGTTGAAACAGCTAGCTCAAAGTCCAAGAGATGCTAAAGATAGTGA
AATACAGCCCAGGAATTGAGGGGGA
>1638
AGGGTGCTATTTAGAGCAGGGGGCATCTACTGGGACAGACTCACTGGGAAGTTGAAAC
AGCTAGCTCAAAGTCCAAGAGATGCTAAAGATAGTGAAATACAGCCCAGGAATTGAGG
GGGAGGGGGTTCCTCACTCAAGAA
>1639
GGCATCTACTGGGACAGACTCACTGGGAAGTTGAAACAGCTAGCTCAAAGTCCAAGA
GATGCTAAAGATAGTGAAATACAGCCCAGGAATTGAGGGGGAGGGGGTTCCTCACTCA
AGAAGGAGACTTTCTAGGAGGGGGC
>1640
AAACAGCTAGCTCAAAGTCCAAGAGATGCTAAAGATAGTGAAATACAGCCCAGGAATT
GAGGGGGAGGGGGTTCCTCACTCAAGAAGGAGACTTTCTAGGAGGGGGCGGGCGTG
GTATTTTCTAGGGACAGTATGGAATG
>1641
AATACAGCCCAGGAATTGAGGGGGAGGGGGTTCCTCACTCAAGAAGGAGACTTTCTA
GGAGGGGGCGGGCGTGGTATTTTCTAGGGACAGTATGGAATGAAGGGAAGAAGGTTG
GCTTTAGAAGGAGGCACCCTGTGGGA
>1642
ACAGCCCAGGAATTGAGGGGGAGGGGGTTCCTCACTCAAGAAGGAGACTTTCTAGGA
GGGGGCGGGCGTGGTATTTTCTAGGGACAGTATGGAATGAAGGGAAGAAGGTTGGCTT
TAGAAGGAGGCACCCTGTGGGAGGC
>1643
GGGGGAGGGGGTTCCTCACTCAAGAAGGAGACTTTCTAGGAGGGGGCGGGCGTGGTA
TTTTCTAGGGACAGTATGGAATGAAGGGAAGAAGGTTGGCTTTAGAAGGAGGCACCCT
GTGGGAGGCTGAGACAGGTGGGTCA
>1644
AAGAAGGAGACTTTCTAGGAGGGGGCGGGCGTGGTATTTTCTAGGGACAGTATGGAAT
GAAGGGAAGAAGGTTGGCTTTAGAAGGAGGCACCCTGTGGGAGGCTGAGACAGGTG
GGTCACCTGAGGTCAGGAGTTTGAGA
>1645
GCGGGCGTGGTATTTTCTAGGGACAGTATGGAATGAAGGGAAGAAGGTTGGCTTTAGA
AGGAGGCACCCTGTGGGAGGCTGAGACAGGTGGGTCACCTGAGGTCAGGAGTTTGAG
ACCAGCCTGGCCAACATGGTGAAAC
>1646
TAGGGACAGTATGGAATGAAGGGAAGAAGGTTGGCTTTAGAAGGAGGCACCCTGTGG
GAGGCTGAGACAGGTGGGTCACCTGAGGTCAGGAGTTTGAGACCAGCCTGGCCAACA

TGGTGAAACCCAGTCTCTACTAAAAA
>1647
GACAGTATGGAATGAAGGGAAGAAGGTTGGCTTTAGAAGGAGGCACCCTGTGGGAGG
CTGAGACAGGTGGGTCACCTGAGGTCAGGAGTTTGAGACCAGCCTGGCCAACATGGT
GAAACCCAGTCTCTACTAAAAATACA
>1648
GCACCCTGTGGGAGGCTGAGACAGGTGGGTCACCTGAGGTCAGGAGTTTGAGACCAG
CCTGGCCAACATGGTGAAACCCAGTCTCTACTAAAAATACAAAAATTAGCTGGGTGGC
AGGTGCCTGTAATCCCAGCTAGTCG
>1649
GGAGGCTGAGACAGGTGGGTCACCTGAGGTCAGGAGTTTGAGACCAGCCTGGCCAAC
ATGGTGAAACCCAGTCTCTACTAAAAATACAAAAATTAGCTGGGTGGCAGGTGCCTGT
AATCCCAGCTAGTCGGGGGAGGCTG
>1650
CAGGAGTTTGAGACCAGCCTGGCCAACATGGTGAAACCCAGTCTCTACTAAAAATACA
AAAATTAGCTGGGTGGCAGGTGCCTGTAATCCCAGCTAGTCGGGGGAGGCTGAGGCA
GGAAAATCGCTTGGGATTACAGGTG
>1651
CATGGTGAAACCCAGTCTCTACTAAAAATACAAAAATTAGCTGGGTGGCAGGTGCCTG
TAATCCCAGCTAGTCGGGGGAGGCTGAGGCAGGAAAATCGCTTGGGATTACAGGTGCC
TGTAATCCCAGCTAGTCGGGAGAG
>1652
TGGGTGGCAGGTGCCTGTAATCCCAGCTAGTCGGGGGAGGCTGAGGCAGGAAAATCG
CTTGGGATTACAGGTGCCTGTAATCCCAGCTAGTCGGGAGAGGCTGAGGCAGGAAAAT
TGCTTGAAGGAGGCGGAGGTTGCAG
>1653
ATCCCAGCTAGTCGGGGGAGGCTGAGGCAGGAAAATCGCTTGGGATTACAGGTGCCTG
TAATCCCAGCTAGTCGGGAGAGGCTGAGGCAGGAAAATTGCTTGAAGGAGGCGGAGG
TTGCAGTGAGCCCAGATCCTGCCGT
>1654
ATTACAGGTGCCTGTAATCCCAGCTAGTCGGGAGAGGCTGAGGCAGGAAAATTGCTTG
AAGGAGGCGGAGGTTGCAGTGAGCCCAGATCCTGCCGTTGCACTCCAGCCCAAGACT
TTGTCTCATTAAAAAAAAAAAAAAGA
>1655
GGTGCCTGTAATCCCAGCTAGTCGGGAGAGGCTGAGGCAGGAAAATTGCTTGAAGGA
GGCGGAGGTTGCAGTGAGCCCAGATCCTGCCGTTGCACTCCAGCCCAAGACTTTGTCT
CATTAAAAAAAAAAAAAAGAAGAAGA
>1656
TAGTCGGGAGAGGCTGAGGCAGGAAAATTGCTTGAAGGAGGCGGAGGTTGCAGTGAG
CCCAGATCCTGCCGTTGCACTCCAGCCCAAGACTTTGTCTCATTAAAAAAAAAAAAAAG
AAGAAGAAGAAGAAGGAGGCACCCT
>1657
AAAATTGCTTGAAGGAGGCGGAGGTTGCAGTGAGCCCAGATCCTGCCGTTGCACTCC
AGCCCAAGACTTTGTCTCATTAAAAAAAAAAAAAAGAAGAAGAAGAAGAAGGAGGCA

CCCTGGATGAAGACTCAGGCTCCAGAA
>1658
AAAAAAAAAAAAGAAGAAGAAGAAGAAGGAGGCACCCTGGATGAAGACTCAGGCTCC
AGAATTTACTATCTGTATGTCTTGGGCAAGTGGCTTAGCTTCTCTGAGCCCCTGTTTACT
TTTCTTTTGAAAATGACCACCTTG
>1659
AAAAAAAGAAGAAGAAGAAGAAGGAGGCACCCTGGATGAAGACTCAGGCTCCAGAA
TTTACTATCTGTATGTCTTGGGCAAGTGGCTTAGCTTCTCTGAGCCCCTGTTTACTTTTC
TTTTGAAAATGACCACCTTGTCCA
>1660
AAGAAGAAGAAGGAGGCACCCTGGATGAAGACTCAGGCTCCAGAATTTACTATCTGTA
TGTCTTGGGCAAGTGGCTTAGCTTCTCTGAGCCCCTGTTTACTTTTCTTTTGAAAATGA
CCACCTTGTCCATGGTAATGAGG
>1661
ATGAAGACTCAGGCTCCAGAATTTACTATCTGTATGTCTTGGGCAAGTGGCTTAGCTTC
TCTGAGCCCCTGTTTACTTTTCTTTTGAAAATGACCACCTTGTCCATGGTAATGAGGATA
AAAAACGTGTAAAGTGCATAG
>1662
TGTATGTCTTGGGCAAGTGGCTTAGCTTCTCTGAGCCCCTGTTTACTTTTCTTTTGAAAA
TGACCACCTTGTCCATGGTAATGAGGATAAAAAACGTGTAAAGTGCATAGCAGAGTGC
AGGTGTACTGGGTAGACATGTA
>1663
CTTGGGCAAGTGGCTTAGCTTCTCTGAGCCCCTGTTTACTTTTCTTTTGAAAATGACCA
CCTTGTCCATGGTAATGAGGATAAAAAACGTGTAAAGTGCATAGCAGAGTGCAGGTGT
ACTGGGTAGACATGTATGATTGC
>1664
CTTCTCTGAGCCCCTGTTTACTTTTCTTTTGAAAATGACCACCTTGTCCATGGTAATGAG
GATAAAAAACGTGTAAAGTGCATAGCAGAGTGCAGGTGTACTGGGTAGACATGTATGA
TTGCGCTGTAGCCCTGTTATGG
>1665
CTTTTGAAAATGACCACCTTGTCCATGGTAATGAGGATAAAAAACGTGTAAAGTGCATA
GCAGAGTGCAGGTGTACTGGGTAGACATGTATGATTGCGCTGTAGCCCTGTTATGGCCC
GGGGAGAAACTATGAAAGGAAC
>1666
TTTGAAAATGACCACCTTGTCCATGGTAATGAGGATAAAAAACGTGTAAAGTGCATAG
CAGAGTGCAGGTGTACTGGGTAGACATGTATGATTGCGCTGTAGCCCTGTTATGGCCCG
GGGAGAAACTATGAAAGGAACCA
>1667
ATGACCACCTTGTCCATGGTAATGAGGATAAAAAACGTGTAAAGTGCATAGCAGAGTG
CAGGTGTACTGGGTAGACATGTATGATTGCGCTGTAGCCCTGTTATGGCCCGGGGAGAA
ACTATGAAAGGAACCAGTGAAAT
>1668
CTTGTCCATGGTAATGAGGATAAAAAACGTGTAAAGTGCATAGCAGAGTGCAGGTGTA
CTGGGTAGACATGTATGATTGCGCTGTAGCCCTGTTATGGCCCGGGGAGAAACTATGAA

AGGAACCAGTGAAATCTTTTGTT
>1669
AAAAAACGTGTAAAGTGCATAGCAGAGTGCAGGTGTACTGGGTAGACATGTATGATTG
CGCTGTAGCCCTGTTATGGCCCGGGGAGAAACTATGAAAGGAACCAGTGAAATCTTTT
GTTTTGGGCACTGCTAAATTGATG
>1670
GTACTGGGTAGACATGTATGATTGCGCTGTAGCCCTGTTATGGCCCGGGGAGAAACTAT
GAAAGGAACCAGTGAAATCTTTTGTTTTGGGCACTGCTAAATTGATGATAGTACTAACC
CTGTGCTAGCAGCCAGCCGGTG
>1671
CATGTATGATTGCGCTGTAGCCCTGTTATGGCCCGGGGAGAAACTATGAAAGGAACCA
GTGAAATCTTTTGTTTTGGGCACTGCTAAATTGATGATAGTACTAACCCTGTGCTAGCA
GCCAGCCGGTGAAATAATAGGTG
>1672
ATGGCCCGGGGAGAAACTATGAAAGGAACCAGTGAAATCTTTTGTTTTGGGCACTGCT
AAATTGATGATAGTACTAACCCTGTGCTAGCAGCCAGCCGGTGAAATAATAGGTGAATA
ATGTTAGTACATTGTGAATATAA
>1673
AGAAACTATGAAAGGAACCAGTGAAATCTTTTGTTTTGGGCACTGCTAAATTGATGATA
GTACTAACCCTGTGCTAGCAGCCAGCCGGTGAAATAATAGGTGAATAATGTTAGTACAT
TGTGAATATAAAAGTTTCCATT
>1674
CCAGTGAAATCTTTTGTTTTGGGCACTGCTAAATTGATGATAGTACTAACCCTGTGCTAG
CAGCCAGCCGGTGAAATAATAGGTGAATAATGTTAGTACATTGTGAATATAAAAGTTTC
CATTTGCAAAGTGCTACTGCT
>1675
TTTGTTTTGGGCACTGCTAAATTGATGATAGTACTAACCCTGTGCTAGCAGCCAGCCGG
TGAAATAATAGGTGAATAATGTTAGTACATTGTGAATATAAAAGTTTCCATTTGCAAAGT
GCTACTGCTGTTCTAAAGAAG
>1676
ACTAACCCTGTGCTAGCAGCCAGCCGGTGAAATAATAGGTGAATAATGTTAGTACATTG
TGAATATAAAAGTTTCCATTTGCAAAGTGCTACTGCTGTTCTAAAGAAGCCACATTTCT
AATAAATGATTGCTTAATCCTG
>1677
GCAGCCAGCCGGTGAAATAATAGGTGAATAATGTTAGTACATTGTGAATATAAAAGTTT
CCATTTGCAAAGTGCTACTGCTGTTCTAAAGAAGCCACATTTCTAATAAATGATTGCTTA
ATCCTGTGATGCTGCCACAGC
>1678
GTGAAATAATAGGTGAATAATGTTAGTACATTGTGAATATAAAAGTTTCCATTTGCAAAG
TGCTACTGCTGTTCTAAAGAAGCCACATTTCTAATAAATGATTGCTTAATCCTGTGATGC
TGCCACAGCCCCACAGAGTT
>1679
AGTTTCCATTTGCAAAGTGCTACTGCTGTTCTAAAGAAGCCACATTTCTAATAAATGATT
GCTTAATCCTGTGATGCTGCCACAGCCCCACAGAGTTTGGGAATGTTTGGGAATTCTTT

CTGAAGTGTGTGCCGCATTCT
>1680
GCTGTTCTAAAGAAGCCACATTTCTAATAAATGATTGCTTAATCCTGTGATGCTGCCACA
GCCCCACAGAGTTTGGGAATGTTTGGGAATTCTTTCTGAAGTGTGTGCCGCATTCTTTT
GCTTTGCCTTTTGGGAGGGTA
>1681
ATGATTGCTTAATCCTGTGATGCTGCCACAGCCCCACAGAGTTTGGGAATGTTTGGGAA
TTCTTTCTGAAGTGTGTGCCGCATTCTTTTGCTTTGCCTTTTGGGAGGGTAGATGTGACT
ATTGGAAATGGTCAAAATAAT
>1682
GATTGCTTAATCCTGTGATGCTGCCACAGCCCCACAGAGTTTGGGAATGTTTGGGAATT
CTTTCTGAAGTGTGTGCCGCATTCTTTTGCTTTGCCTTTTGGGAGGGTAGATGTGACTAT
TGGAAATGGTCAAAATAATGC
>1683
AGAGTTTGGGAATGTTTGGGAATTCTTTCTGAAGTGTGTGCCGCATTCTTTTGCTTTGC
CTTTTGGGAGGGTAGATGTGACTATTGGAAATGGTCAAAATAATGCAGTAGCAAATCTG
TTGACTATAGTGAGTGATAAGC
>1684
TTCTTTCTGAAGTGTGTGCCGCATTCTTTTGCTTTGCCTTTTGGGAGGGTAGATGTGACT
ATTGGAAATGGTCAAAATAATGCAGTAGCAAATCTGTTGACTATAGTGAGTGATAAGCA
CTGTCGGCTGCTGTGTCTGAC
>1685
TGTGCCGCATTCTTTTGCTTTGCCTTTTGGGAGGGTAGATGTGACTATTGGAAATGGTC
AAAATAATGCAGTAGCAAATCTGTTGACTATAGTGAGTGATAAGCACTGTCGGCTGCTG
TGTCTGACTCGGAAGGCAGATT
>1686
CTTTTGCTTTGCCTTTTGGGAGGGTAGATGTGACTATTGGAAATGGTCAAAATAATGCA
GTAGCAAATCTGTTGACTATAGTGAGTGATAAGCACTGTCGGCTGCTGTGTCTGACTCG
GAAGGCAGATTCTGGAAGGGGT
>1687
TTGGAAATGGTCAAAATAATGCAGTAGCAAATCTGTTGACTATAGTGAGTGATAAGCAC
TGTCGGCTGCTGTGTCTGACTCGGAAGGCAGATTCTGGAAGGGGTGTGTGGGGAAGG
CTTGTTGGCTCAGCACTGGGGTGA
>1688
GGAAATGGTCAAAATAATGCAGTAGCAAATCTGTTGACTATAGTGAGTGATAAGCACTG
TCGGCTGCTGTGTCTGACTCGGAAGGCAGATTCTGGAAGGGGTGTGTGGGGAAGGCT
TGTTGGCTCAGCACTGGGGTGAGT
>1689
CTGTTGACTATAGTGAGTGATAAGCACTGTCGGCTGCTGTGTCTGACTCGGAAGGCAG
ATTCTGGAAGGGGTGTGTGGGGAAGGCTTGTTGGCTCAGCACTGGGGTGAGTGTTGG
CCTCACTTCTTAGGTGGGAAGTGGA
>1690
TGATAAGCACTGTCGGCTGCTGTGTCTGACTCGGAAGGCAGATTCTGGAAGGGGTGTG
TGGGGAAGGCTTGTTGGCTCAGCACTGGGGTGAGTGTTGGCCTCACTTCTTAGGTGGG

AAGTGGACAAGGCCAAGGGGCTTT
>1691
TGCTGTGTCTGACTCGGAAGGCAGATTCTGGAAGGGGTGTGTGGGGAAGGCTTGTTG
GCTCAGCACTGGGGTGAGTGTTGGCCTCACTTCTTAGGTGGGAAGTGGACAAGGCCA
AGGGGCTTTTGATTTCCAGCTACTAT
>1692
GTGTCTGACTCGGAAGGCAGATTCTGGAAGGGGTGTGTGGGGAAGGCTTGTTGGCTC
AGCACTGGGGTGAGTGTTGGCCTCACTTCTTAGGTGGGAAGTGGACAAGGCCAAGGG
GCTTTTGATTTCCAGCTACTATGGCT
>1693
ATTCTGGAAGGGGTGTGTGGGGAAGGCTTGTTGGCTCAGCACTGGGGTGAGTGTTGG
CCTCACTTCTTAGGTGGGAAGTGGACAAGGCCAAGGGGCTTTTGATTTCCAGCTACTAT
GGCTGGACTCCCAGTGAGGAAGGA
>1694
TCACTTCTTAGGTGGGAAGTGGACAAGGCCAAGGGGCTTTTGATTTCCAGCTACTATG
GCTGGACTCCCAGTGAGGAAGGAGTCGCTGCCATGGATTGCCTGGGACTGGGAAGAC
TCCTTTTAACTGCTGTAATGTTTCT
>1695
GTGGGAAGTGGACAAGGCCAAGGGGCTTTTGATTTCCAGCTACTATGGCTGGACTCCC
AGTGAGGAAGGAGTCGCTGCCATGGATTGCCTGGGACTGGGAAGACTCCTTTTAACTG
CTGTAATGTTTCTGCATTTGTGGT
>1696
CTGGACTCCCAGTGAGGAAGGAGTCGCTGCCATGGATTGCCTGGGACTGGGAAGACT
CCTTTTAACTGCTGTAATGTTTCTGCATTTGTGGTTTGGCCTCCTGTGAGTTTGGGCTCC
GGGGCGGCACTTCTGTTGTTAAA
>1697
CTCCCAGTGAGGAAGGAGTCGCTGCCATGGATTGCCTGGGACTGGGAAGACTCCTTTT
AACTGCTGTAATGTTTCTGCATTTGTGGTTTGGCCTCCTGTGAGTTTGGGCTCCGGGGC
GGCACTTCTGTTGTTAAAAGAAC
>1698
TGGATTGCCTGGGACTGGGAAGACTCCTTTTAACTGCTGTAATGTTTCTGCATTTGTGG
TTTGGCCTCCTGTGAGTTTGGGCTCCGGGGCGGCACTTCTGTTGTTAAAAGAACAGAA
AAGAAAACAAAACAAAACAAAAA
>1699
TTGCCTGGGACTGGGAAGACTCCTTTTAACTGCTGTAATGTTTCTGCATTTGTGGTTTG
GCCTCCTGTGAGTTTGGGCTCCGGGGCGGCACTTCTGTTGTTAAAAGAACAGAAAAG
AAAACAAAACAAAACAAAAAACCA
>1700
TTAACTGCTGTAATGTTTCTGCATTTGTGGTTTGGCCTCCTGTGAGTTTGGGCTCCGGG
GCGGCACTTCTGTTGTTAAAAGAACAGAAAAGAAAACAAAACAAAACAAAAAACCA
GGACTGTCACTGTTAGATGTTGGTT
>1701
GGCTCCGGGGCGGCACTTCTGTTGTTAAAAGAACAGAAAAGAAAACAAAACAAAAC
AAAAAACCAGGACTGTCACTGTTAGATGTTGGTTGAGGCTAGAAAGAATGGATTGAGT

TTTGACCCCCTACACCCCAGGGAGTA
>1702
GGGGCGGCACTTCTGTTGTTAAAAGAACAGAAAAGAAAACAAAACAAAACAAAAAA
CCAGGACTGTCACTGTTAGATGTTGGTTGAGGCTAGAAAGAATGGATTGAGTTTTGAC
CCCCTACACCCCAGGGAGTAGGGAGG
>1703
AAACAAAACAAAACAAAAAACCAGGACTGTCACTGTTAGATGTTGGTTGAGGCTAGA
AAGAATGGATTGAGTTTTGACCCCCTACACCCCAGGGAGTAGGGAGGCCTGGGAGGC
ACAGTGGCCTGAGTTTGAATTCTGAC
>1704
ACTGTCACTGTTAGATGTTGGTTGAGGCTAGAAAGAATGGATTGAGTTTTGACCCCCTA
CACCCCAGGGAGTAGGGAGGCCTGGGAGGCACAGTGGCCTGAGTTTGAATTCTGACT
CTGCCTCTTTGGAAGGCTCTGGAA
>1705
TGAGGCTAGAAAGAATGGATTGAGTTTTGACCCCCTACACCCCAGGGAGTAGGGAGGC
CTGGGAGGCACAGTGGCCTGAGTTTGAATTCTGACTCTGCCTCTTTGGAAGGCTCTGG
AAGGTTAAAGGAGCTTGTGTGTTC
>1706
AAAGAATGGATTGAGTTTTGACCCCCTACACCCCAGGGAGTAGGGAGGCCTGGGAGG
CACAGTGGCCTGAGTTTGAATTCTGACTCTGCCTCTTTGGAAGGCTCTGGAAGGTTAA
AGGAGCTTGTGTGTTCAAATCTTAG
>1707
TAGGGAGGCCTGGGAGGCACAGTGGCCTGAGTTTGAATTCTGACTCTGCCTCTTTGGA
AGGCTCTGGAAGGTTAAAGGAGCTTGTGTGTTCAAATCTTAGCCTGGTACCTGGCATAT
ACTCAGCATGTATTTTTTCCCCT
>1708
GAGGCACAGTGGCCTGAGTTTGAATTCTGACTCTGCCTCTTTGGAAGGCTCTGGAAGG
TTAAAGGAGCTTGTGTGTTCAAATCTTAGCCTGGTACCTGGCATATACTCAGCATGTATT
TTTTCCCCTTTCCTGACAATGT
>1709
CACAGTGGCCTGAGTTTGAATTCTGACTCTGCCTCTTTGGAAGGCTCTGGAAGGTTAA
AGGAGCTTGTGTGTTCAAATCTTAGCCTGGTACCTGGCATATACTCAGCATGTATTTTTT
CCCCTTTCCTGACAATGTTCCT
>1710
GAATTCTGACTCTGCCTCTTTGGAAGGCTCTGGAAGGTTAAAGGAGCTTGTGTGTTCA
AATCTTAGCCTGGTACCTGGCATATACTCAGCATGTATTTTTTCCCCTTTCCTGACAATGT
TCCTTGCCCCGAACACTTGAA
>1711
GAAGGCTCTGGAAGGTTAAAGGAGCTTGTGTGTTCAAATCTTAGCCTGGTACCTGGCA
TATACTCAGCATGTATTTTTTCCCCTTTCCTGACAATGTTCCTTGCCCCGAACACTTGAA
TAGTGGAGAGAGGAATGTTTGC
>1712
TTGTGTGTTCAAATCTTAGCCTGGTACCTGGCATATACTCAGCATGTATTTTTTCCCCTTT
CCTGACAATGTTCCTTGCCCCGAACACTTGAATAGTGGAGAGAGGAATGTTTGCTTTTC

CTCTCCATTGAACCAACACT
>1713
ACCTGGCATATACTCAGCATGTATTTTTTCCCCTTTCCTGACAATGTTCCTTGCCCCGAA
CACTTGAATAGTGGAGAGAGGAATGTTTGCTTTTCCTCTCCATTGAACCAACACTCGCC
CATCCACTGAGCCCAAGATTC
>1714
CAGCATGTATTTTTTCCCCTTTCCTGACAATGTTCCTTGCCCCGAACACTTGAATAGTGG
AGAGAGGAATGTTTGCTTTTCCTCTCCATTGAACCAACACTCGCCCATCCACTGAGCCC
AAGATTCACCTTAGGATTGTC
>1715
AATGTTTGCTTTTCCTCTCCATTGAACCAACACTCGCCCATCCACTGAGCCCAAGATTC
ACCTTAGGATTGTCTGTTGGACTCGGCTCTTTTGTGACCTAACAGTGCAGGGGCCTTGT
GCTGAGCTGCACATGCTCGCTC
>1716
TTTGCTTTTCCTCTCCATTGAACCAACACTCGCCCATCCACTGAGCCCAAGATTCACCT
TAGGATTGTCTGTTGGACTCGGCTCTTTTGTGACCTAACAGTGCAGGGGCCTTGTGCTG
AGCTGCACATGCTCGCTCTCGC
>1717
TGAACCAACACTCGCCCATCCACTGAGCCCAAGATTCACCTTAGGATTGTCTGTTGGA
CTCGGCTCTTTTGTGACCTAACAGTGCAGGGGCCTTGTGCTGAGCTGCACATGCTCGC
TCTCGCTGGTGCTCCCTCTCTCTC
>1718
TCGCCCATCCACTGAGCCCAAGATTCACCTTAGGATTGTCTGTTGGACTCGGCTCTTTT
GTGACCTAACAGTGCAGGGGCCTTGTGCTGAGCTGCACATGCTCGCTCTCGCTGGTGC
TCCCTCTCTCTCTCTCTCTCTGT
>1719
TGGACTCGGCTCTTTTGTGACCTAACAGTGCAGGGGCCTTGTGCTGAGCTGCACATGC
TCGCTCTCGCTGGTGCTCCCTCTCTCTCTCTCTCTGTCTGCATTATTTATAGTAAACCA
TATTAGAACAGAGTGTTAAAG
>1720
CAGTGCAGGGGCCTTGTGCTGAGCTGCACATGCTCGCTCTCGCTGGTGCTCCCTCTCT
CTCTCTCTCTCTGTCTGCATTATTTATAGTAAACCATATTAGAACAGAGTGTTAAAGAGG
AAGGATGAGTCATTCCCATTGA
>1721
TGCTGAGCTGCACATGCTCGCTCTCGCTGGTGCTCCCTCTCTCTCTCTCTCTCTGTCTG
CATTATTTATAGTAAACCATATTAGAACAGAGTGTTAAAGAGGAAGGATGAGTCATTCC
CATTGAAACCATTGATGACATA
>1722
CTCTCGCTGGTGCTCCCTCTCTCTCTCTCTCTCTGTCTGCATTATTTATAGTAAACCATAT
TAGAACAGAGTGTTAAAGAGGAAGGATGAGTCATTCCCATTGAAACCATTGATGACAT
AAATAAAAGCAAAATGCTTAC
>1723
CTCGCTGGTGCTCCCTCTCTCTCTCTCTCTCTGTCTGCATTATTTATAGTAAACCATATTA
GAACAGAGTGTTAAAGAGGAAGGATGAGTCATTCCCATTGAAACCATTGATGACATAA

ATAAAAGCAAAATGCTTACTA
>1724
CTCTCTCTCTCTCTGTCTGCATTATTTATAGTAAACCATATTAGAACAGAGTGTTAAAGA
GGAAGGATGAGTCATTCCCATTGAAACCATTGATGACATAAATAAAAGCAAAATGCTTA
CTATGTGTTTTGTGCAGTATT
>1725
TTAAAGAGGAAGGATGAGTCATTCCCATTGAAACCATTGATGACATAAATAAAAGCAA
AATGCTTACTATGTGTTTTGTGCAGTATTTAAGGCCAGGTTGTGCTAATTTGAATGCTCC
CAAACTGGATCTGTGAAAGCAT
>1726
AAAGAGGAAGGATGAGTCATTCCCATTGAAACCATTGATGACATAAATAAAAGCAAAA
TGCTTACTATGTGTTTTGTGCAGTATTTAAGGCCAGGTTGTGCTAATTTGAATGCTCCCA
AACTGGATCTGTGAAAGCATAC
>1727
ATTGAAACCATTGATGACATAAATAAAAGCAAAATGCTTACTATGTGTTTTGTGCAGTAT
TTAAGGCCAGGTTGTGCTAATTTGAATGCTCCCAAACTGGATCTGTGAAAGCATACATA
TGTGGGAAAAAATTCAGGTTT
>1728
TGCTTACTATGTGTTTTGTGCAGTATTTAAGGCCAGGTTGTGCTAATTTGAATGCTCCCA
AACTGGATCTGTGAAAGCATACATATGTGGGAAAAAATTCAGGTTTAACTAGAGGGAA
GTTATTTATTGAAAATCCATTT
>1729
TGTGCAGTATTTAAGGCCAGGTTGTGCTAATTTGAATGCTCCCAAACTGGATCTGTGAA
AGCATACATATGTGGGAAAAAATTCAGGTTTAACTAGAGGGAAGTTATTTATTGAAAAT
CCATTTCAGTATCTGCTTTCTC
>1730
TGAATGCTCCCAAACTGGATCTGTGAAAGCATACATATGTGGGAAAAAATTCAGGTTTA
ACTAGAGGGAAGTTATTTATTGAAAATCCATTTCAGTATCTGCTTTCTCCCTTCTATTTAC
TTCAGATCCATATTCATATC
>1731
GAAAGCATACATATGTGGGAAAAAATTCAGGTTTAACTAGAGGGAAGTTATTTATTGAA
AATCCATTTCAGTATCTGCTTTCTCCCTTCTATTTACTTCAGATCCATATTCATATCATGCT
TGTTGTTCTGGCTAACGAG
>1732
TATTGAAAATCCATTTCAGTATCTGCTTTCTCCCTTCTATTTACTTCAGATCCATATTCATA
TCATGCTTGTTGTTCTGGCTAACGAGAGGGACTGGCTTATTCACTAGCTAAGGTTTGTT
TATTTTCGGAAGGTTGAGG
>1733
TGAAAATCCATTTCAGTATCTGCTTTCTCCCTTCTATTTACTTCAGATCCATATTCATATCA
TGCTTGTTGTTCTGGCTAACGAGAGGGACTGGCTTATTCACTAGCTAAGGTTTGTTTATT
TTCGGAAGGTTGAGGAAT
>1734
TTCAGATCCATATTCATATCATGCTTGTTGTTCTGGCTAACGAGAGGGACTGGCTTATTC
ACTAGCTAAGGTTTGTTTATTTTCGGAAGGTTGAGGAATAGCACAGAGAGGTTGTCTAA

GTGTCCCTGTCTCCATTCCTC
>1735
GATCCATATTCATATCATGCTTGTTGTTCTGGCTAACGAGAGGGACTGGCTTATTCACTA
GCTAAGGTTTGTTTATTTTCGGAAGGTTGAGGAATAGCACAGAGAGGTTGTCTAAGTG
TCCCTGTCTCCATTCCTCCTCT
>1736
CATGCTTGTTGTTCTGGCTAACGAGAGGGACTGGCTTATTCACTAGCTAAGGTTTGTTT
ATTTTCGGAAGGTTGAGGAATAGCACAGAGAGGTTGTCTAAGTGTCCCTGTCTCCATTC
CTCCTCTTGCCACCTGTTGAGG
>1737
GAGGGACTGGCTTATTCACTAGCTAAGGTTTGTTTATTTTCGGAAGGTTGAGGAATAGC
ACAGAGAGGTTGTCTAAGTGTCCCTGTCTCCATTCCTCCTCTTGCCACCTGTTGAGGAA
GGCACTCTCCACCTGCTTTGCA
>1738
TTCACTAGCTAAGGTTTGTTTATTTTCGGAAGGTTGAGGAATAGCACAGAGAGGTTGTC
TAAGTGTCCCTGTCTCCATTCCTCCTCTTGCCACCTGTTGAGGAAGGCACTCTCCACCT
GCTTTGCAGGCGTGGGCTCTCA
>1739
TCGGAAGGTTGAGGAATAGCACAGAGAGGTTGTCTAAGTGTCCCTGTCTCCATTCCTC
CTCTTGCCACCTGTTGAGGAAGGCACTCTCCACCTGCTTTGCAGGCGTGGGCTCTCAA
TGTGTCCAAGAATCACAGTCAGAG
>1740
TAAGTGTCCCTGTCTCCATTCCTCCTCTTGCCACCTGTTGAGGAAGGCACTCTCCACCT
GCTTTGCAGGCGTGGGCTCTCAATGTGTCCAAGAATCACAGTCAGAGCTGAAGGGCCC
TTGGAATCATTTATTGCAGCATG
>1741
CTCCATTCCTCCTCTTGCCACCTGTTGAGGAAGGCACTCTCCACCTGCTTTGCAGGCGT
GGGCTCTCAATGTGTCCAAGAATCACAGTCAGAGCTGAAGGGCCCTTGGAATCATTTA
TTGCAGCATGCTCATTTGGGAGG
>1742
GCCACCTGTTGAGGAAGGCACTCTCCACCTGCTTTGCAGGCGTGGGCTCTCAATGTGT
CCAAGAATCACAGTCAGAGCTGAAGGGCCCTTGGAATCATTTATTGCAGCATGCTCATT
TGGGAGGAGTAAGTGGAGGTCCA
>1743
TGTCCAAGAATCACAGTCAGAGCTGAAGGGCCCTTGGAATCATTTATTGCAGCATGCTC
ATTTGGGAGGAGTAAGTGGAGGTCCAGAGAGGTTCTGTGATTTTCCCTAGGTCACACA
GCAAGCTAGAGGCAAAACCAGGA
>1744
CAAGAATCACAGTCAGAGCTGAAGGGCCCTTGGAATCATTTATTGCAGCATGCTCATTT
GGGAGGAGTAAGTGGAGGTCCAGAGAGGTTCTGTGATTTTCCCTAGGTCACACAGCA
AGCTAGAGGCAAAACCAGGACCCT
>1745
TCATTTATTGCAGCATGCTCATTTGGGAGGAGTAAGTGGAGGTCCAGAGAGGTTCTGT
GATTTTCCCTAGGTCACACAGCAAGCTAGAGGCAAAACCAGGACCCTCACTCCCAGCC

CACTTTAATTCTTCCTTCACAAGC
>1746
CAGCAAGCTAGAGGCAAAACCAGGACCCTCACTCCCAGCCCACTTTAATTCTTCCTTC
ACAAGCCTGCCTGTGACTGCCCAGGTCTGTTCTCTGCTTCTACCAAGGGGTAGGCAAG
ACTAGAATCTGACTTGAGAGCAGT
>1747
GGCAAAACCAGGACCCTCACTCCCAGCCCACTTTAATTCTTCCTTCACAAGCCTGCCT
GTGACTGCCCAGGTCTGTTCTCTGCTTCTACCAAGGGGTAGGCAAGACTAGAATCTGA
CTTGAGAGCAGTTTCAGATCTTTG
>1748
GCCCACTTTAATTCTTCCTTCACAAGCCTGCCTGTGACTGCCCAGGTCTGTTCTCTGCT
TCTACCAAGGGGTAGGCAAGACTAGAATCTGACTTGAGAGCAGTTTCAGATCTTTGTC
TGTGAGGGATGCTGGGGGGTTTC
>1749
CTGTGACTGCCCAGGTCTGTTCTCTGCTTCTACCAAGGGGTAGGCAAGACTAGAATCT
GACTTGAGAGCAGTTTCAGATCTTTGTCTGTGAGGGATGCTGGGGGGTTTCTGGCATG
GTGACTTGCCTCCTGGGACACTCA
>1750
GGTCTGTTCTCTGCTTCTACCAAGGGGTAGGCAAGACTAGAATCTGACTTGAGAGCAG
TTTCAGATCTTTGTCTGTGAGGGATGCTGGGGGGTTTCTGGCATGGTGACTTGCCTCCT
GGGACACTCATTAACCTCTCTGC
>1751
AAGGGGTAGGCAAGACTAGAATCTGACTTGAGAGCAGTTTCAGATCTTTGTCTGTGAG
GGATGCTGGGGGGTTTCTGGCATGGTGACTTGCCTCCTGGGACACTCATTAACCTCTCT
GCTGCTGAGCCTGAGCAGACTAA
>1752
AGACTAGAATCTGACTTGAGAGCAGTTTCAGATCTTTGTCTGTGAGGGATGCTGGGGG
GTTTCTGGCATGGTGACTTGCCTCCTGGGACACTCATTAACCTCTCTGCTGCTGAGCCT
GAGCAGACTAATGTACATCAACT
>1753
TTTCTGGCATGGTGACTTGCCTCCTGGGACACTCATTAACCTCTCTGCTGCTGAGCCTG
AGCAGACTAATGTACATCAACTCCTTAGGATTTACAGACAATGCCCTATGTTGGGGGTT
TTGAGACCCAAAGATTGCTAAG
>1754
ACCTCTCTGCTGCTGAGCCTGAGCAGACTAATGTACATCAACTCCTTAGGATTTACAGA
CAATGCCCTATGTTGGGGGTTTTGAGACCCAAAGATTGCTAAGACATGGGACTTTTCCT
TGAAGAGCTTATACTTTGAGTT
>1755
TGCTGCTGAGCCTGAGCAGACTAATGTACATCAACTCCTTAGGATTTACAGACAATGCC
CTATGTTGGGGGTTTTGAGACCCAAAGATTGCTAAGACATGGGACTTTTCCTTGAAGA
GCTTATACTTTGAGTTAAAATAT
>1756
TATGTTGGGGGTTTTGAGACCCAAAGATTGCTAAGACATGGGACTTTTCCTTGAAGAG
CTTATACTTTGAGTTAAAATATGTATGTTGATATCTATGACAGAGAATAGGAAATGGAAA

GACTATGATATATATATTCAGA
>1757
GTTTTGAGACCCAAAGATTGCTAAGACATGGGACTTTTCCTTGAAGAGCTTATACTTTG
AGTTAAAATATGTATGTTGATATCTATGACAGAGAATAGGAAATGGAAAGACTATGATAT
ATATATTCAGAGTTGAAATGA
>1758
TGAGACCCAAAGATTGCTAAGACATGGGACTTTTCCTTGAAGAGCTTATACTTTGAGTT
AAAATATGTATGTTGATATCTATGACAGAGAATAGGAAATGGAAAGACTATGATATATATA
TTCAGAGTTGAAATGACTTC
>1759
GTTAAAATATGTATGTTGATATCTATGACAGAGAATAGGAAATGGAAAGACTATGATATA
TATATTCAGAGTTGAAATGACTTCTGTCTGGAGTGATTCATTTTAAATTTTAAAACCTTG
TTGAGCACTTACTGTCTGCT
>1760
TTGATATCTATGACAGAGAATAGGAAATGGAAAGACTATGATATATATATTCAGAGTTGA
AATGACTTCTGTCTGGAGTGATTCATTTTAAATTTTAAAACCTTGTTGAGCACTTACTGT
CTGCTGGGCACCTGGGTAGG
>1761
ACTATGATATATATATTCAGAGTTGAAATGACTTCTGTCTGGAGTGATTCATTTTAAATTT
TAAAACCTTGTTGAGCACTTACTGTCTGCTGGGCACCTGGGTAGGCCCTGGGAATACT
GTGATGAGCAAGATAGACAAG
>1762
ATTCAGAGTTGAAATGACTTCTGTCTGGAGTGATTCATTTTAAATTTTAAAACCTTGTTG
AGCACTTACTGTCTGCTGGGCACCTGGGTAGGCCCTGGGAATACTGTGATGAGCAAGA
TAGACAAGACGTCTACCCTCCT
>1763
CTTCTGTCTGGAGTGATTCATTTTAAATTTTAAAACCTTGTTGAGCACTTACTGTCTGCT
GGGCACCTGGGTAGGCCCTGGGAATACTGTGATGAGCAAGATAGACAAGACGTCTACC
CTCCTGGGGCTTTCCTAATGGG
>1764
CATTTTAAATTTTAAAACCTTGTTGAGCACTTACTGTCTGCTGGGCACCTGGGTAGGCC
CTGGGAATACTGTGATGAGCAAGATAGACAAGACGTCTACCCTCCTGGGGCTTTCCTAA
TGGGGAAGAAAGATCCTTACTT
>1765
TGAGCACTTACTGTCTGCTGGGCACCTGGGTAGGCCCTGGGAATACTGTGATGAGCAA
GATAGACAAGACGTCTACCCTCCTGGGGCTTTCCTAATGGGGAAGAAAGATCCTTACTT
AGCTAGCAGTCCTGTCCGTAACT
>1766
CAAGATAGACAAGACGTCTACCCTCCTGGGGCTTTCCTAATGGGGAAGAAAGATCCTT
ACTTAGCTAGCAGTCCTGTCCGTAACTGTGTAAGGGTGATTTTGATACATGTTGGGAGC
CTGGATGAAGCAGCACATGAGTA
>1767
TAGACAAGACGTCTACCCTCCTGGGGCTTTCCTAATGGGGAAGAAAGATCCTTACTTAG
CTAGCAGTCCTGTCCGTAACTGTGTAAGGGTGATTTTGATACATGTTGGGAGCCTGGAT

GAAGCAGCACATGAGTAGGATA
>1768
GTCTACCCTCCTGGGGCTTTCCTAATGGGGAAGAAAGATCCTTACTTAGCTAGCAGTCC
TGTCCGTAACTGTGTAAGGGTGATTTTGATACATGTTGGGAGCCTGGATGAAGCAGCAC
ATGAGTAGGATATTAAAGGATA
>1769
AATGGGGAAGAAAGATCCTTACTTAGCTAGCAGTCCTGTCCGTAACTGTGTAAGGGTG
ATTTTGATACATGTTGGGAGCCTGGATGAAGCAGCACATGAGTAGGATATTAAAGGATA
AGTAGGTTTTGAAGACGGGGACA
>1770
GCAGTCCTGTCCGTAACTGTGTAAGGGTGATTTTGATACATGTTGGGAGCCTGGATGAA
GCAGCACATGAGTAGGATATTAAAGGATAAGTAGGTTTTGAAGACGGGGACAGAAAGA
AGGAAAATTGTGGTCAGGGCCAG
>1771
TGTAAGGGTGATTTTGATACATGTTGGGAGCCTGGATGAAGCAGCACATGAGTAGGATA
TTAAAGGATAAGTAGGTTTTGAAGACGGGGACAGAAAGAAGGAAAATTGTGGTCAGG
GCCAGGGGAGAAAACAAGGAGTAA
>1772
GAAGCAGCACATGAGTAGGATATTAAAGGATAAGTAGGTTTTGAAGACGGGGACAGAA
AGAAGGAAAATTGTGGTCAGGGCCAGGGGAGAAAACAAGGAGTAACTTTCTTTGGCA
GGAATGAGGGTGATTCATGCTGGAA
>1773
GCAGCACATGAGTAGGATATTAAAGGATAAGTAGGTTTTGAAGACGGGGACAGAAAGA
AGGAAAATTGTGGTCAGGGCCAGGGGAGAAAACAAGGAGTAACTTTCTTTGGCAGGA
ATGAGGGTGATTCATGCTGGAATGG
>1774
ATAAGTAGGTTTTGAAGACGGGGACAGAAAGAAGGAAAATTGTGGTCAGGGCCAGGG
GAGAAAACAAGGAGTAACTTTCTTTGGCAGGAATGAGGGTGATTCATGCTGGAATGGT
AGGCTGGGGTCAGACTGTGGTGCTA
>1775
AGAAAGAAGGAAAATTGTGGTCAGGGCCAGGGGAGAAAACAAGGAGTAACTTTCTTT
GGCAGGAATGAGGGTGATTCATGCTGGAATGGTAGGCTGGGGTCAGACTGTGGTGCTA
TAAATGCGGGGAGAGAGATTAGAGA
>1776
GGTCAGGGCCAGGGGAGAAAACAAGGAGTAACTTTCTTTGGCAGGAATGAGGGTGAT
TCATGCTGGAATGGTAGGCTGGGGTCAGACTGTGGTGCTATAAATGCGGGGAGAGAGA
TTAGAGAAGGCAGCATGAAGAATGG
>1777
AGGGGAGAAAACAAGGAGTAACTTTCTTTGGCAGGAATGAGGGTGATTCATGCTGGA
ATGGTAGGCTGGGGTCAGACTGTGGTGCTATAAATGCGGGGAGAGAGATTAGAGAAGG
CAGCATGAAGAATGGAGAGAAAATG
>1778
TCAGACTGTGGTGCTATAAATGCGGGGAGAGAGATTAGAGAAGGCAGCATGAAGAATG
GAGAGAAAATGAGTTTGAAGTTGAGCAGACCCGGGTTCAGATTTTCATGCTGCCACTT

GCAAACTGTGTGATGTTGGCCATC
>1779
GTGGTGCTATAAATGCGGGGAGAGAGATTAGAGAAGGCAGCATGAAGAATGGAGAGA
AAATGAGTTTGAAGTTGAGCAGACCCGGGTTCAGATTTTCATGCTGCCACTTGCAAAC
TGTGTGATGTTGGCCATCAAGGACT
>1780
CGGGGAGAGAGATTAGAGAAGGCAGCATGAAGAATGGAGAGAAAATGAGTTTGAAGT
TGAGCAGACCCGGGTTCAGATTTTCATGCTGCCACTTGCAAACTGTGTGATGTTGGCCA
TCAAGGACTGTGAACTGGCTGGGT
>1781
GAATGGAGAGAAAATGAGTTTGAAGTTGAGCAGACCCGGGTTCAGATTTTCATGCTGC
CACTTGCAAACTGTGTGATGTTGGCCATCAAGGACTGTGAACTGGCTGGGTGCAGTGG
CTCACACCTGTCATCCCAGTGCTT
>1782
GAGAAAATGAGTTTGAAGTTGAGCAGACCCGGGTTCAGATTTTCATGCTGCCACTTGC
AAACTGTGTGATGTTGGCCATCAAGGACTGTGAACTGGCTGGGTGCAGTGGCTCACAC
CTGTCATCCCAGTGCTTTGGGAGG
>1783
GTTGAGCAGACCCGGGTTCAGATTTTCATGCTGCCACTTGCAAACTGTGTGATGTTGG
CCATCAAGGACTGTGAACTGGCTGGGTGCAGTGGCTCACACCTGTCATCCCAGTGCTT
TGGGAGGCCAAGGCAGGAGGATTG
>1784
GGGTTCAGATTTTCATGCTGCCACTTGCAAACTGTGTGATGTTGGCCATCAAGGACTGT
GAACTGGCTGGGTGCAGTGGCTCACACCTGTCATCCCAGTGCTTTGGGAGGCCAAGG
CAGGAGGATTGCGTGAGGCCAGAA
>1785
CAGATTTTCATGCTGCCACTTGCAAACTGTGTGATGTTGGCCATCAAGGACTGTGAACT
GGCTGGGTGCAGTGGCTCACACCTGTCATCCCAGTGCTTTGGGAGGCCAAGGCAGGA
GGATTGCGTGAGGCCAGAAGTTTG
>1786
CAAACTGTGTGATGTTGGCCATCAAGGACTGTGAACTGGCTGGGTGCAGTGGCTCACA
CCTGTCATCCCAGTGCTTTGGGAGGCCAAGGCAGGAGGATTGCGTGAGGCCAGAAGT
TTGAGACTGGCTTGGGCAATATAGC
>1787
TGAACTGGCTGGGTGCAGTGGCTCACACCTGTCATCCCAGTGCTTTGGGAGGCCAAGG
CAGGAGGATTGCGTGAGGCCAGAAGTTTGAGACTGGCTTGGGCAATATAGCGAGACG
ATCTTTCCAAAAAAAAAAAAAAAAA
>1788
GTGCAGTGGCTCACACCTGTCATCCCAGTGCTTTGGGAGGCCAAGGCAGGAGGATTGC
GTGAGGCCAGAAGTTTGAGACTGGCTTGGGCAATATAGCGAGACGATCTTTCCAAAAA
AAAAAAAAAAAAAATTAAAAATGC
>1789
GAGACTGGCTTGGGCAATATAGCGAGACGATCTTTCCAAAAAAAAAAAAAAAAAAAATT
AAAAATGCAGCTGTGCATGCCCATAATCCCAGCTTCTCGGGTGGCTGAAGAGGGAGGA

TGGCTTGAACTTATAAGTTCAAGG
>1790
GATCTTTCCAAAAAAAAAAAAAAAAAAAATTAAAAATGCAGCTGTGCATGCCCATAATC
CCAGCTTCTCGGGTGGCTGAAGAGGGAGGATGGCTTGAACTTATAAGTTCAAGGCTGT
GGTGAGCTATGATCACGCCACTGC
>1791
ATGCAGCTGTGCATGCCCATAATCCCAGCTTCTCGGGTGGCTGAAGAGGGAGGATGGC
TTGAACTTATAAGTTCAAGGCTGTGGTGAGCTATGATCACGCCACTGCACTCCAGCCTG
GGCAACAAAGTGAGACCCTATCT
>1792
GCATGCCCATAATCCCAGCTTCTCGGGTGGCTGAAGAGGGAGGATGGCTTGAACTTATA
AGTTCAAGGCTGTGGTGAGCTATGATCACGCCACTGCACTCCAGCCTGGGCAACAAAG
TGAGACCCTATCTCTATTTCAAA
>1793
GCTTGAACTTATAAGTTCAAGGCTGTGGTGAGCTATGATCACGCCACTGCACTCCAGCC
TGGGCAACAAAGTGAGACCCTATCTCTATTTCAAAACAACAACAACAACAACAGCAA
CACACACCATGAACTGTAGCTTCT
>1794
GGCAACAAAGTGAGACCCTATCTCTATTTCAAAACAACAACAACAACAACAGCAACA
CACACCATGAACTGTAGCTTCTTCATTCCTACAGTGCAGTTATTGTGTGGGATAGCCTG
AGGATTTGGGGTCGTGTACTTAAC
>1795
TCTCTATTTCAAAACAACAACAACAACAACAGCAACACACACCATGAACTGTAGCTTC
TTCATTCCTACAGTGCAGTTATTGTGTGGGATAGCCTGAGGATTTGGGGTCGTGTACTTA
ACACACTCTGCACAGTGCCTGT
>1796
ATTTCAAAACAACAACAACAACAACAGCAACACACACCATGAACTGTAGCTTCTTCAT
TCCTACAGTGCAGTTATTGTGTGGGATAGCCTGAGGATTTGGGGTCGTGTACTTAACAC
ACTCTGCACAGTGCCTGTAACAG
>1797
AAACAACAACAACAACAACAGCAACACACACCATGAACTGTAGCTTCTTCATTCCTAC
AGTGCAGTTATTGTGTGGGATAGCCTGAGGATTTGGGGTCGTGTACTTAACACACTCTG
CACAGTGCCTGTAACAGTGTAGT
>1798
ACACACCATGAACTGTAGCTTCTTCATTCCTACAGTGCAGTTATTGTGTGGGATAGCCT
GAGGATTTGGGGTCGTGTACTTAACACACTCTGCACAGTGCCTGTAACAGTGTAGTTG
CTCAATAAATATAACTGCTGTTT
>1799
TTCCTACAGTGCAGTTATTGTGTGGGATAGCCTGAGGATTTGGGGTCGTGTACTTAACA
CACTCTGCACAGTGCCTGTAACAGTGTAGTTGCTCAATAAATATAACTGCTGTTTGTTTT
AACTGCTGTTTCTTTTTGGAC
>1800
CAGTGCAGTTATTGTGTGGGATAGCCTGAGGATTTGGGGTCGTGTACTTAACACACTCT
GCACAGTGCCTGTAACAGTGTAGTTGCTCAATAAATATAACTGCTGTTTGTTTTAACTG

CTGTTTCTTTTTGGACTTTATC
>1801
AGTTATTGTGTGGGATAGCCTGAGGATTTGGGGTCGTGTACTTAACACACTCTGCACAG
TGCCTGTAACAGTGTAGTTGCTCAATAAATATAACTGCTGTTTGTTTTAACTGCTGTTTC
TTTTTGGACTTTATCCCGGGA
>1802
TGGGGTCGTGTACTTAACACACTCTGCACAGTGCCTGTAACAGTGTAGTTGCTCAATAA
ATATAACTGCTGTTTGTTTTAACTGCTGTTTCTTTTTGGACTTTATCCCGGGAGAGTTGG
AGCCTTGGAAGGCATTTGAAA
>1803
GTAGTTGCTCAATAAATATAACTGCTGTTTGTTTTAACTGCTGTTTCTTTTTGGACTTTAT
CCCGGGAGAGTTGGAGCCTTGGAAGGCATTTGAAAAGGAGAGTGACGTGACTGGAGT
GCTCCATACTGATGTGACATCC
>1804
TTTAACTGCTGTTTCTTTTTGGACTTTATCCCGGGAGAGTTGGAGCCTTGGAAGGCATT
TGAAAAGGAGAGTGACGTGACTGGAGTGCTCCATACTGATGTGACATCCACTAGGCAT
GTGTCTGACTGCCCCCTGCTCCT
>1805
CTTTTTGGACTTTATCCCGGGAGAGTTGGAGCCTTGGAAGGCATTTGAAAAGGAGAGT
GACGTGACTGGAGTGCTCCATACTGATGTGACATCCACTAGGCATGTGTCTGACTGCCC
CCTGCTCCTTCCCTGCCTTCACG
>1806
CCCGGGAGAGTTGGAGCCTTGGAAGGCATTTGAAAAGGAGAGTGACGTGACTGGAGT
GCTCCATACTGATGTGACATCCACTAGGCATGTGTCTGACTGCCCCCTGCTCCTTCCCT
GCCTTCACGAGCCTGTCTGAAAAG
>1807
TTGGAAGGCATTTGAAAAGGAGAGTGACGTGACTGGAGTGCTCCATACTGATGTGACA
TCCACTAGGCATGTGTCTGACTGCCCCCTGCTCCTTCCCTGCCTTCACGAGCCTGTCTG
AAAAGGAGTTTAGTGCGTGATCA
>1808
CCATACTGATGTGACATCCACTAGGCATGTGTCTGACTGCCCCCTGCTCCTTCCCTGCC
TTCACGAGCCTGTCTGAAAAGGAGTTTAGTGCGTGATCATCAGCTCCTAAGGAGACCT
CTCCCCTGTATCTTGTACCTGGC
>1809
GACATCCACTAGGCATGTGTCTGACTGCCCCCTGCTCCTTCCCTGCCTTCACGAGCCTG
TCTGAAAAGGAGTTTAGTGCGTGATCATCAGCTCCTAAGGAGACCTCTCCCCTGTATCT
TGTACCTGGCACTCTACCAAGA
>1810
CTGCCTTCACGAGCCTGTCTGAAAAGGAGTTTAGTGCGTGATCATCAGCTCCTAAGGA
GACCTCTCCCCTGTATCTTGTACCTGGCACTCTACCAAGATTTGATGAAAGGACATGAA
ATGGACCCTTGCCACCTCTCGGT
>1811
CACGAGCCTGTCTGAAAAGGAGTTTAGTGCGTGATCATCAGCTCCTAAGGAGACCTCT
CCCCTGTATCTTGTACCTGGCACTCTACCAAGATTTGATGAAAGGACATGAAATGGACC

CTTGCCACCTCTCGGTGGGCGGG
>1812
CTGTATCTTGTACCTGGCACTCTACCAAGATTTGATGAAAGGACATGAAATGGACCCTT
GCCACCTCTCGGTGGGCGGGGAAGCTGGGAGAGGGGCCTGGGGAGAGGGGGTCTTC
AGCTGTCAGGATCAAAGGAAGTGAC
>1813
AGGACATGAAATGGACCCTTGCCACCTCTCGGTGGGCGGGGAAGCTGGGAGAGGGGC
CTGGGGAGAGGGGGTCTTCAGCTGTCAGGATCAAAGGAAGTGACACTCTGAAGCTGG
TCTGCCGGACTGTTACCCGTCTCTCT
>1814
ATGGACCCTTGCCACCTCTCGGTGGGCGGGGAAGCTGGGAGAGGGGCCTGGGGAGAG
GGGGTCTTCAGCTGTCAGGATCAAAGGAAGTGACACTCTGAAGCTGGTCTGCCGGAC
TGTTACCCGTCTCTCTAACTCTCTTT
>1815
TCTCGGTGGGCGGGGAAGCTGGGAGAGGGGCCTGGGGAGAGGGGGTCTTCAGCTGTC
AGGATCAAAGGAAGTGACACTCTGAAGCTGGTCTGCCGGACTGTTACCCGTCTCTCTA
ACTCTCTTTTAATAGAAACCCAGTC
>1816
GAGAGGGGGTCTTCAGCTGTCAGGATCAAAGGAAGTGACACTCTGAAGCTGGTCTGC
CGGACTGTTACCCGTCTCTCTAACTCTCTTTTAATAGAAACCCAGTCTCCCCTCTTGCCT
CCCTGATAGTGAACAGCGACGCT
>1817
GAAGTGACACTCTGAAGCTGGTCTGCCGGACTGTTACCCGTCTCTCTAACTCTCTTTTA
ATAGAAACCCAGTCTCCCCTCTTGCCTCCCTGATAGTGAACAGCGACGCTATCTCCCGC
GGGCCAGTCTCGGCTGTGGTCG
>1818
GCCGGACTGTTACCCGTCTCTCTAACTCTCTTTTAATAGAAACCCAGTCTCCCCTCTTG
CCTCCCTGATAGTGAACAGCGACGCTATCTCCCGCGGGCCAGTCTCGGCTGTGGTCGT
AGCACTACGCAATGTCTGCAAAC
>1819
TTTTAATAGAAACCCAGTCTCCCCTCTTGCCTCCCTGATAGTGAACAGCGACGCTATCT
CCCGCGGGCCAGTCTCGGCTGTGGTCGTAGCACTACGCAATGTCTGCAAACCGACAGT
GGCATGGGGAGATAAGGGCCCCA
>1820
AAACCCAGTCTCCCCTCTTGCCTCCCTGATAGTGAACAGCGACGCTATCTCCCGCGGG
CCAGTCTCGGCTGTGGTCGTAGCACTACGCAATGTCTGCAAACCGACAGTGGCATGGG
GAGATAAGGGCCCCAACGTTGTGT
>1821
CTCCCTGATAGTGAACAGCGACGCTATCTCCCGCGGGCCAGTCTCGGCTGTGGTCGTA
GCACTACGCAATGTCTGCAAACCGACAGTGGCATGGGGAGATAAGGGCCCCAACGTT
GTGTAACGAGGGGCATGGGCTGTTT
>1822
CAGCGACGCTATCTCCCGCGGGCCAGTCTCGGCTGTGGTCGTAGCACTACGCAATGTC
TGCAAACCGACAGTGGCATGGGGAGATAAGGGCCCCAACGTTGTGTAACGAGGGGCA

TGGGCTGTTTGCCCGAGTGGCTCCT
>1823
CTCGGCTGTGGTCGTAGCACTACGCAATGTCTGCAAACCGACAGTGGCATGGGGAGAT
AAGGGCCCCAACGTTGTGTAACGAGGGGCATGGGCTGTTTGCCCGAGTGGCTCCTGGC
CCCTGGGTAACTGCTAACCTCAAC
>1824
CAATGTCTGCAAACCGACAGTGGCATGGGGAGATAAGGGCCCCAACGTTGTGTAACG
AGGGGCATGGGCTGTTTGCCCGAGTGGCTCCTGGCCCCTGGGTAACTGCTAACCTCAA
CGGAGGAGCGGGCCGGCCTTGGAAA
>1825
AAACCGACAGTGGCATGGGGAGATAAGGGCCCCAACGTTGTGTAACGAGGGGCATGG
GCTGTTTGCCCGAGTGGCTCCTGGCCCCTGGGTAACTGCTAACCTCAACGGAGGAGCG
GGCCGGCCTTGGAAACGCCGTTCCA
>1826
GGAGATAAGGGCCCCAACGTTGTGTAACGAGGGGCATGGGCTGTTTGCCCGAGTGGCT
CCTGGCCCCTGGGTAACTGCTAACCTCAACGGAGGAGCGGGCCGGCCTTGGAAACGC
CGTTCCAGGGCCCCTCGCTGGTTCC
>1827
GCCCGAGTGGCTCCTGGCCCCTGGGTAACTGCTAACCTCAACGGAGGAGCGGGCCGG
CCTTGGAAACGCCGTTCCAGGGCCCCTCGCTGGTTCCACGGATGCCTTTTTGTGGTTG
GATAAAGGAAGCTCATGTAGGCCGC
>1828
CCTGGGTAACTGCTAACCTCAACGGAGGAGCGGGCCGGCCTTGGAAACGCCGTTCCA
GGGCCCCTCGCTGGTTCCACGGATGCCTTTTTGTGGTTGGATAAAGGAAGCTCATGTAG
GCCGCCCACAGCGCAGCATGGCCT
>1829
CGGAGGAGCGGGCCGGCCTTGGAAACGCCGTTCCAGGGCCCCTCGCTGGTTCCACGG
ATGCCTTTTTGTGGTTGGATAAAGGAAGCTCATGTAGGCCGCCCACAGCGCAGCATGG
CCTTGGGCTACGGGTGTTTGGTTGT
>1830
GGAAACGCCGTTCCAGGGCCCCTCGCTGGTTCCACGGATGCCTTTTTGTGGTTGGATA
AAGGAAGCTCATGTAGGCCGCCCACAGCGCAGCATGGCCTTGGGCTACGGGTGTTTGG
TTGTTTTGTTTTTCTCTTTTCTTT
>1831
TGCCTTTTTGTGGTTGGATAAAGGAAGCTCATGTAGGCCGCCCACAGCGCAGCATGGC
CTTGGGCTACGGGTGTTTGGTTGTTTTGTTTTTCTCTTTTCTTTGTCTTTTTTAAAAAAA
GAAGTTGAAAGGGGGATAGTCT
>1832
CCTTTTTGTGGTTGGATAAAGGAAGCTCATGTAGGCCGCCCACAGCGCAGCATGGCCT
TGGGCTACGGGTGTTTGGTTGTTTTGTTTTTCTCTTTTCTTTGTCTTTTTTAAAAAAAGA
AGTTGAAAGGGGGATAGTCTTG
>1833
TTGTGGTTGGATAAAGGAAGCTCATGTAGGCCGCCCACAGCGCAGCATGGCCTTGGGC
TACGGGTGTTTGGTTGTTTTGTTTTTCTCTTTTCTTTGTCTTTTTTAAAAAAAGAAGTTG

AAAGGGGGATAGTCTTGCTTGG
>1834
GGATAAAGGAAGCTCATGTAGGCCGCCCACAGCGCAGCATGGCCTTGGGCTACGGGTG
TTTGGTTGTTTTGTTTTTCTCTTTTCTTTGTCTTTTTTAAAAAAAGAAGTTGAAAGGGG
GATAGTCTTGCTTGGCACCTACC
>1835
GTAGGCCGCCCACAGCGCAGCATGGCCTTGGGCTACGGGTGTTTGGTTGTTTTGTTTTT
CTCTTTTCTTTGTCTTTTTTAAAAAAAGAAGTTGAAAGGGGGATAGTCTTGCTTGGCAC
CTACCACCTTTAACCCAAAACC
>1836
ACGGGTGTTTGGTTGTTTTGTTTTTCTCTTTTCTTTGTCTTTTTTAAAAAAAGAAGTTGA
AAGGGGGATAGTCTTGCTTGGCACCTACCACCTTTAACCCAAAACCAAATAAAACAAA
CAGTTCCTAAATATAAATTAAA
>1837
AAGAAGTTGAAAGGGGGATAGTCTTGCTTGGCACCTACCACCTTTAACCCAAAACCAA
ATAAAACAAACAGTTCCTAAATATAAATTAAACTTCAACCTGCAGTATAGACATCCTCCT
TCCCTGCCCGAAACCCACTGGC
>1838
ACCTACCACCTTTAACCCAAAACCAAATAAAACAAACAGTTCCTAAATATAAATTAAAC
TTCAACCTGCAGTATAGACATCCTCCTTCCCTGCCCGAAACCCACTGGCCACTGGAGG
CTAGCTTTAAGAAATTGGTTTTG
>1839
CCTGCAGTATAGACATCCTCCTTCCCTGCCCGAAACCCACTGGCCACTGGAGGCTAGCT
TTAAGAAATTGGTTTTGGAGACTCTGGCTTGAAAAGGAGCCATGCCCACTTGTCTCAC
GTCCCAGTCGCTGGCTCCAGGGG
>1840
TGGCCACTGGAGGCTAGCTTTAAGAAATTGGTTTTGGAGACTCTGGCTTGAAAAGGAG
CCATGCCCACTTGTCTCACGTCCCAGTCGCTGGCTCCAGGGGGCCGTGGTAGAACATAT
ATTGTACTTCAGCTGCCTTTCCC
>1841
TGGAGGCTAGCTTTAAGAAATTGGTTTTGGAGACTCTGGCTTGAAAAGGAGCCATGCC
CACTTGTCTCACGTCCCAGTCGCTGGCTCCAGGGGGCCGTGGTAGAACATATATTGTAC
TTCAGCTGCCTTTCCCCTCCAAG
>1842
TTGGAGACTCTGGCTTGAAAAGGAGCCATGCCCACTTGTCTCACGTCCCAGTCGCTGG
CTCCAGGGGGCCGTGGTAGAACATATATTGTACTTCAGCTGCCTTTCCCCTCCAAGCTT
CCTCAGCCACCCACAGCCCTTTC
>1843
AAAAGGAGCCATGCCCACTTGTCTCACGTCCCAGTCGCTGGCTCCAGGGGGCCGTGGT
AGAACATATATTGTACTTCAGCTGCCTTTCCCCTCCAAGCTTCCTCAGCCACCCACAGC
CCTTTCAGATGAGCTGAGCCCTA
>1844
TCAGCTGCCTTTCCCCTCCAAGCTTCCTCAGCCACCCACAGCCCTTTCAGATGAGCTG
AGCCCTAGCACTGTGCCCTCTCCCCTCTGCCCTCTTAGGGTGATAGAATCCACTACCCT

GACATCTGGGGTTGCCCTGTTCT
>1845
TCAGCCACCCACAGCCCTTTCAGATGAGCTGAGCCCTAGCACTGTGCCCTCTCCCCTCT
GCCCTCTTAGGGTGATAGAATCCACTACCCTGACATCTGGGGTTGCCCTGTTCTGCTGT
GGTGGGAGATGGGCTGGAAGGG
>1846
GAGCCCTAGCACTGTGCCCTCTCCCCTCTGCCCTCTTAGGGTGATAGAATCCACTACCC
TGACATCTGGGGTTGCCCTGTTCTGCTGTGGTGGGAGATGGGCTGGAAGGGACTTCGG
AGGCTCTCCCGTGTTCTGTACCA
>1847
GCACTGTGCCCTCTCCCCTCTGCCCTCTTAGGGTGATAGAATCCACTACCCTGACATCT
GGGGTTGCCCTGTTCTGCTGTGGTGGGAGATGGGCTGGAAGGGACTTCGGAGGCTCTC
CCGTGTTCTGTACCACCCAGCTT
>1848
CCTGACATCTGGGGTTGCCCTGTTCTGCTGTGGTGGGAGATGGGCTGGAAGGGACTTC
GGAGGCTCTCCCGTGTTCTGTACCACCCAGCTTCCCCTTGGATCCCTCCACCCCCGCAG
CCAGGAGAGGGAAGTAACTGTTC
>1849
TGACATCTGGGGTTGCCCTGTTCTGCTGTGGTGGGAGATGGGCTGGAAGGGACTTCGG
AGGCTCTCCCGTGTTCTGTACCACCCAGCTTCCCCTTGGATCCCTCCACCCCCGCAGCC
AGGAGAGGGAAGTAACTGTTCTT
>1850
AGGCTCTCCCGTGTTCTGTACCACCCAGCTTCCCCTTGGATCCCTCCACCCCCGCAGCC
AGGAGAGGGAAGTAACTGTTCTTTGAGCAGGACATTTCATTTTCAAAGTATTCTTGATG
CCGCCCCCCCGCCCCACCGCCA
>1851
TCCCGTGTTCTGTACCACCCAGCTTCCCCTTGGATCCCTCCACCCCCGCAGCCAGGAG
AGGGAAGTAACTGTTCTTTGAGCAGGACATTTCATTTTCAAAGTATTCTTGATGCCGCC
CCCCCGCCCCACCGCCAAGAATT
>1852
TGGATCCCTCCACCCCCGCAGCCAGGAGAGGGAAGTAACTGTTCTTTGAGCAGGACAT
TTCATTTTCAAAGTATTCTTGATGCCGCCCCCCCGCCCCACCGCCAAGAATTCTGCTCT
GTGGGTGATACTTATAAGGTAAC
>1853
GAGCAGGACATTTCATTTTCAAAGTATTCTTGATGCCGCCCCCCCGCCCCACCGCCAAG
AATTCTGCTCTGTGGGTGATACTTATAAGGTAACTATATGGCTTCCTTATTTAGGCAGCA
GTTTCTCTTCTATGATTTTAG
>1854
AGGACATTTCATTTTCAAAGTATTCTTGATGCCGCCCCCCCGCCCCACCGCCAAGAATT
CTGCTCTGTGGGTGATACTTATAAGGTAACTATATGGCTTCCTTATTTAGGCAGCAGTTT
CTCTTCTATGATTTTAGTTCC
>1855
CACCGCCAAGAATTCTGCTCTGTGGGTGATACTTATAAGGTAACTATATGGCTTCCTTAT
TTAGGCAGCAGTTTCTCTTCTATGATTTTAGTTCCTCTCATGATTCTTATAACCCTCTCTC

AGATACTTCCAGGATGATA
>1856
TGTGGGTGATACTTATAAGGTAACTATATGGCTTCCTTATTTAGGCAGCAGTTTCTCTTCT
ATGATTTTAGTTCCTCTCATGATTCTTATAACCCTCTCTCAGATACTTCCAGGATGATAAA
TCTTCTATCACTGTTTCA
>1857
ATTTTAGTTCCTCTCATGATTCTTATAACCCTCTCTCAGATACTTCCAGGATGATAAATCT
TCTATCACTGTTTCATCTGATCACATCAGGGTGATTATATTTCGTGACATAACCAGGTGG
CTTTAATCCGTTGGTGGAA
>1858
CTTATAACCCTCTCTCAGATACTTCCAGGATGATAAATCTTCTATCACTGTTTCATCTGAT
CACATCAGGGTGATTATATTTCGTGACATAACCAGGTGGCTTTAATCCGTTGGTGGAAG
AACCAGGCTCTCACCTCCAT
>1859
CTCAGATACTTCCAGGATGATAAATCTTCTATCACTGTTTCATCTGATCACATCAGGGTG
ATTATATTTCGTGACATAACCAGGTGGCTTTAATCCGTTGGTGGAAGAACCAGGCTCTC
ACCTCCATTGTTTAAGATGTT
>1860
AGGATGATAAATCTTCTATCACTGTTTCATCTGATCACATCAGGGTGATTATATTTCGTGA
CATAACCAGGTGGCTTTAATCCGTTGGTGGAAGAACCAGGCTCTCACCTCCATTGTTTA
AGATGTTATGTTTCTACTAA
>1861
TGATTATATTTCGTGACATAACCAGGTGGCTTTAATCCGTTGGTGGAAGAACCAGGCTC
TCACCTCCATTGTTTAAGATGTTATGTTTCTACTAATCTGTCCACTGAGTGTCTCCATCCT
ATTGTTTCCATTGCATGCTC
>1862
TTCGTGACATAACCAGGTGGCTTTAATCCGTTGGTGGAAGAACCAGGCTCTCACCTCC
ATTGTTTAAGATGTTATGTTTCTACTAATCTGTCCACTGAGTGTCTCCATCCTATTGTTTC
CATTGCATGCTCTCAGGTTAT
>1863
CGTTGGTGGAAGAACCAGGCTCTCACCTCCATTGTTTAAGATGTTATGTTTCTACTAATC
TGTCCACTGAGTGTCTCCATCCTATTGTTTCCATTGCATGCTCTCAGGTTATTTCCTTTGT
TCTTGGCTGTCTGATTGCT
>1864
AGGCTCTCACCTCCATTGTTTAAGATGTTATGTTTCTACTAATCTGTCCACTGAGTGTCT
CCATCCTATTGTTTCCATTGCATGCTCTCAGGTTATTTCCTTTGTTCTTGGCTGTCTGATT
GCTCCCAGAGGCTGCAGTT
>1865
AAGATGTTATGTTTCTACTAATCTGTCCACTGAGTGTCTCCATCCTATTGTTTCCATTGCA
TGCTCTCAGGTTATTTCCTTTGTTCTTGGCTGTCTGATTGCTCCCAGAGGCTGCAGTTAT
TTTATTTTATTTATTTCAT
>1866
CTGTCCACTGAGTGTCTCCATCCTATTGTTTCCATTGCATGCTCTCAGGTTATTTCCTTTG
TTCTTGGCTGTCTGATTGCTCCCAGAGGCTGCAGTTATTTTATTTTATTTATTTCATTTTT

TGGTGGGAGTAGGGGATT
>1867
ATTGTTTCCATTGCATGCTCTCAGGTTATTTCCTTTGTTCTTGGCTGTCTGATTGCTCCCA
GAGGCTGCAGTTATTTATTTATTTATTTCATTTTTTGGTGGGAGTAGGGGATTGATCTC
TCTCTTGCCTTTAGCTTG
>1868
TCCTTTGTTCTTGGCTGTCTGATTGCTCCCAGAGGCTGCAGTTATTTATTTATTTATTT
CATTTTTTGGTGGGAGTAGGGGATTGATCTCTCTCTTGCCTTTAGCTTGAATGGGTGGT
CATTTCCAGGCTTACACATT
>1869
TTTATTTATTTATTTCATTTTTTGGTGGGAGTAGGGGATTGATCTCTCTCTTGCCTTTAG
CTTGAATGGGTGGTCATTTCCAGGCTTACACATTGCTCCATGCCCACTGTAGGGGTCTG
GCTTAGAGATGGTCGGGGGA
>1870
ATTTATTTATTTCATTTTTTGGTGGGAGTAGGGGATTGATCTCTCTCTTGCCTTTAGCTT
GAATGGGTGGTCATTTCCAGGCTTACACATTGCTCCATGCCCACTGTAGGGGTCTGGCT
TAGAGATGGTCGGGGGAGAG
>1871
TTGATCTCTCTCTTGCCTTTAGCTTGAATGGGTGGTCATTTCCAGGCTTACACATTGCTC
CATGCCCACTGTAGGGGTCTGGCTTAGAGATGGTCGGGGGAGAGAAGGTGGTGATTTA
AAAGATTTGGAGGCGGTGGCTT
>1872
TCTCTCTTGCCTTTAGCTTGAATGGGTGGTCATTTCCAGGCTTACACATTGCTCCATGCC
CACTGTAGGGGTCTGGCTTAGAGATGGTCGGGGGAGAGAAGGTGGTGATTTAAAAGAT
TTGGAGGCGGTGGCTTACATTG
>1873
CTTGAATGGGTGGTCATTTCCAGGCTTACACATTGCTCCATGCCCACTGTAGGGGTCTG
GCTTAGAGATGGTCGGGGGAGAGAAGGTGGTGATTTAAAAGATTTGGAGGCGGTGGC
TTACATTGATTTTAGCTTATCTAA
>1874
GCCCACTGTAGGGGTCTGGCTTAGAGATGGTCGGGGGAGAGAAGGTGGTGATTTAAA
AGATTTGGAGGCGGTGGCTTACATTGATTTTAGCTTATCTAACCCTTTAGCTAGAGGCTG
CTCTTCCCTCAGAGTGTCCAACT
>1875
TTTGGAGGCGGTGGCTTACATTGATTTTAGCTTATCTAACCCTTTAGCTAGAGGCTGCTC
TTCCCTCAGAGTGTCCAACTAGCTGTAACTTAAAGCAACAGAGAAATGTATATCATCCC
TTTTCTTGCATTAATTAGGGC
>1876
TGGAGGCGGTGGCTTACATTGATTTTAGCTTATCTAACCCTTTAGCTAGAGGCTGCTCTT
CCCTCAGAGTGTCCAACTAGCTGTAACTTAAAGCAACAGAGAAATGTATATCATCCCTT
TTCTTGCATTAATTAGGGCTG
>1877
AACCCTTTAGCTAGAGGCTGCTCTTCCCTCAGAGTGTCCAACTAGCTGTAACTTAAAGC
AACAGAGAAATGTATATCATCCCTTTTCTTGCATTAATTAGGGCTGAGAAATTCATTATTT

AGAGGGATAAGAGCTCCGGA
>1878
TCCCTTTTCTTGCATTAATTAGGGCTGAGAAATTCATTATTTAGAGGGATAAGAGCTCCG
GAGGAGGAGGGTTTTTTGAACTAATGTAGAGATCTGTGGCCAGCTGTGTGATTCTCAG
GTAGCAGGTGAGGGGGTGGGGG
>1879
TAATTAGGGCTGAGAAATTCATTATTTAGAGGGATAAGAGCTCCGGAGGAGGAGGGTT
TTTTGAACTAATGTAGAGATCTGTGGCCAGCTGTGTGATTCTCAGGTAGCAGGTGAGG
GGGTGGGGGTTGATGAGAGGGGTG
>1880
ATTATTTAGAGGGATAAGAGCTCCGGAGGAGGAGGGTTTTTTGAACTAATGTAGAGATC
TGTGGCCAGCTGTGTGATTCTCAGGTAGCAGGTGAGGGGGTGGGGGTTGATGAGAGG
GGTGAGAAGGAGCAGGTGCCAGGG
>1881
CTCCGGAGGAGGAGGGTTTTTTGAACTAATGTAGAGATCTGTGGCCAGCTGTGTGATT
CTCAGGTAGCAGGTGAGGGGGTGGGGGTTGATGAGAGGGGTGAGAAGGAGCAGGTG
CCAGGGTCAGGCAGGGGAGGGAGCCC
>1882
GAGGAGGGTTTTTTGAACTAATGTAGAGATCTGTGGCCAGCTGTGTGATTCTCAGGTAG
CAGGTGAGGGGGTGGGGGTTGATGAGAGGGGTGAGAAGGAGCAGGTGCCAGGGTCA
GGCAGGGGAGGGAGCCCCCACTCCG
>1883
AATGTAGAGATCTGTGGCCAGCTGTGTGATTCTCAGGTAGCAGGTGAGGGGGTGGGGG
TTGATGAGAGGGGTGAGAAGGAGCAGGTGCCAGGGTCAGGCAGGGGAGGGAGCCCC
CACTCCGTGCATCATCCCAGAGCCCT
>1884
GGTGGGGGTTGATGAGAGGGGTGAGAAGGAGCAGGTGCCAGGGTCAGGCAGGGGAG
GGAGCCCCCACTCCGTGCATCATCCCAGAGCCCTGTTCCTCCAAGCCCAGATTAGACA
CCCTTCTCCACGTGGGGCCTCAAGTT
>1885
GTGAGAAGGAGCAGGTGCCAGGGTCAGGCAGGGGAGGGAGCCCCCACTCCGTGCAT
CATCCCAGAGCCCTGTTCCTCCAAGCCCAGATTAGACACCCTTCTCCACGTGGGGCCT
CAAGTTAGAAATCCTGGGAGGAGGCC
>1886
GGGAGCCCCCACTCCGTGCATCATCCCAGAGCCCTGTTCCTCCAAGCCCAGATTAGAC
ACCCTTCTCCACGTGGGGCCTCAAGTTAGAAATCCTGGGAGGAGGCCTTATTGCTCAG
GATCATCTGGGAATGTTGCTTTCT
>1887
TCCGTGCATCATCCCAGAGCCCTGTTCCTCCAAGCCCAGATTAGACACCCTTCTCCACG
TGGGGCCTCAAGTTAGAAATCCTGGGAGGAGGCCTTATTGCTCAGGATCATCTGGGAA
TGTTGCTTTCTGGGTTTAAAATA
>1888
CCTTCTCCACGTGGGGCCTCAAGTTAGAAATCCTGGGAGGAGGCCTTATTGCTCAGGA
TCATCTGGGAATGTTGCTTTCTGGGTTTAAAATACAGTCAATGTTTAGATGAGCTGCTGT

TCAGGCACATGGTCTGGTTAGC
>1889
GGGGCCTCAAGTTAGAAATCCTGGGAGGAGGCCTTATTGCTCAGGATCATCTGGGAAT
GTTGCTTTCTGGGTTTAAAATACAGTCAATGTTTAGATGAGCTGCTGTTCAGGCACATG
GTCTGGTTAGCAAAGGGTTAAAT
>1890
TATTGCTCAGGATCATCTGGGAATGTTGCTTTCTGGGTTTAAAATACAGTCAATGTTTAG
ATGAGCTGCTGTTCAGGCACATGGTCTGGTTAGCAAAGGGTTAAATCCTGCATCCCGTT
TTTAGTCATCAAGCTAGCTCA
>1891
CATCTGGGAATGTTGCTTTCTGGGTTTAAAATACAGTCAATGTTTAGATGAGCTGCTGTT
CAGGCACATGGTCTGGTTAGCAAAGGGTTAAATCCTGCATCCCGTTTTTAGTCATCAAG
CTAGCTCAGATCTGCCCACCT
>1892
TTTCTGGGTTTAAAATACAGTCAATGTTTAGATGAGCTGCTGTTCAGGCACATGGTCTG
GTTAGCAAAGGGTTAAATCCTGCATCCCGTTTTTAGTCATCAAGCTAGCTCAGATCTGC
CCACCTTGGAGCCCTGTGCTTT
>1893
TGTTTAGATGAGCTGCTGTTCAGGCACATGGTCTGGTTAGCAAAGGGTTAAATCCTGCA
TCCCGTTTTTAGTCATCAAGCTAGCTCAGATCTGCCCACCTTGGAGCCCTGTGCTTTCC
TGCCTTAGTGGGCAGAGTCTAG
>1894
GCAAAGGGTTAAATCCTGCATCCCGTTTTTAGTCATCAAGCTAGCTCAGATCTGCCCAC
CTTGGAGCCCTGTGCTTTCCTGCCTTAGTGGGCAGAGTCTAGGGGACCTGTGGCATGT
TCAATGATTGAGGACTCTTGTCC
>1895
TGCATCCCGTTTTTAGTCATCAAGCTAGCTCAGATCTGCCCACCTTGGAGCCCTGTGCT
TTCCTGCCTTAGTGGGCAGAGTCTAGGGGACCTGTGGCATGTTCAATGATTGAGGACT
CTTGTCCAGATTCCAAAAACCTA
>1896
TTTTTAGTCATCAAGCTAGCTCAGATCTGCCCACCTTGGAGCCCTGTGCTTTCCTGCCTT
AGTGGGCAGAGTCTAGGGGACCTGTGGCATGTTCAATGATTGAGGACTCTTGTCCAGA
TTCCAAAAACCTACTGTGATGA
>1897
AGCTAGCTCAGATCTGCCCACCTTGGAGCCCTGTGCTTTCCTGCCTTAGTGGGCAGAG
TCTAGGGGACCTGTGGCATGTTCAATGATTGAGGACTCTTGTCCAGATTCCAAAAACCT
ACTGTGATGATGCTGTTCTGAAG
>1898
TCAGATCTGCCCACCTTGGAGCCCTGTGCTTTCCTGCCTTAGTGGGCAGAGTCTAGGG
GACCTGTGGCATGTTCAATGATTGAGGACTCTTGTCCAGATTCCAAAAACCTACTGTGA
TGATGCTGTTCTGAAGCTGTGGT
>1899
CCCTGTGCTTTCCTGCCTTAGTGGGCAGAGTCTAGGGGACCTGTGGCATGTTCAATGAT
TGAGGACTCTTGTCCAGATTCCAAAAACCTACTGTGATGATGCTGTTCTGAAGCTGTGG

TAGGCGGTGATTGAGTGGAGTG
>1900
GGGCAGAGTCTAGGGGACCTGTGGCATGTTCAATGATTGAGGACTCTTGTCCAGATTC
CAAAAACCTACTGTGATGATGCTGTTCTGAAGCTGTGGTAGGCGGTGATTGAGTGGAG
TGTGTAGTGACCCTCAGGAATTCT
>1901
AGGGGACCTGTGGCATGTTCAATGATTGAGGACTCTTGTCCAGATTCCAAAAACCTAC
TGTGATGATGCTGTTCTGAAGCTGTGGTAGGCGGTGATTGAGTGGAGTGTGTAGTGAC
CCTCAGGAATTCTTCAGAAGTTAG
>1902
GGCATGTTCAATGATTGAGGACTCTTGTCCAGATTCCAAAAACCTACTGTGATGATGCT
GTTCTGAAGCTGTGGTAGGCGGTGATTGAGTGGAGTGTGTAGTGACCCTCAGGAATTC
TTCAGAAGTTAGGATTTTGACGA
>1903
CTTGTCCAGATTCCAAAAACCTACTGTGATGATGCTGTTCTGAAGCTGTGGTAGGCGGT
GATTGAGTGGAGTGTGTAGTGACCCTCAGGAATTCTTCAGAAGTTAGGATTTTGACGAT
ACCTCTGATAAGTTGGGTTTTT
>1904
ATGCTGTTCTGAAGCTGTGGTAGGCGGTGATTGAGTGGAGTGTGTAGTGACCCTCAGG
AATTCTTCAGAAGTTAGGATTTTGACGATACCTCTGATAAGTTGGGTTTTTAATTTGCTG
GTTTATCTTAGAGTGGAGGCTG
>1905
GATTGAGTGGAGTGTGTAGTGACCCTCAGGAATTCTTCAGAAGTTAGGATTTTGACGAT
ACCTCTGATAAGTTGGGTTTTTAATTTGCTGGTTTATCTTAGAGTGGAGGCTGTGGCCC
ACAGCAAGTAGTACATTTTCCA
>1906
GACCCTCAGGAATTCTTCAGAAGTTAGGATTTTGACGATACCTCTGATAAGTTGGGTTT
TTAATTTGCTGGTTTATCTTAGAGTGGAGGCTGTGGCCCACAGCAAGTAGTACATTTTC
CATCTCAGCCCAGTGCATGGAC
>1907
TTCTTCAGAAGTTAGGATTTTGACGATACCTCTGATAAGTTGGGTTTTTAATTTGCTGGT
TTATCTTAGAGTGGAGGCTGTGGCCCACAGCAAGTAGTACATTTTCCATCTCAGCCCAG
TGCATGGACAAACGTACCTCA
>1908
CGATACCTCTGATAAGTTGGGTTTTTAATTTGCTGGTTTATCTTAGAGTGGAGGCTGTGG
CCCACAGCAAGTAGTACATTTTCCATCTCAGCCCAGTGCATGGACAAACGTACCTCAA
ACCAAAGGTTCAATGAAGCTCT
>1909
TAATTTGCTGGTTTATCTTAGAGTGGAGGCTGTGGCCCACAGCAAGTAGTACATTTTCC
ATCTCAGCCCAGTGCATGGACAAACGTACCTCAAACCAAAGGTTCAATGAAGCTCTAT
TTATCTTTACTATATGCTCTGGT
>1910
ATCTTAGAGTGGAGGCTGTGGCCCACAGCAAGTAGTACATTTTCCATCTCAGCCCAGTG
CATGGACAAACGTACCTCAAACCAAAGGTTCAATGAAGCTCTATTTATCTTTACTATATG

CTCTGGTATCTTCTCTTCCAT
>1911
TGTGGCCCACAGCAAGTAGTACATTTTCCATCTCAGCCCAGTGCATGGACAAACGTAC
CTCAAACCAAAGGTTCAATGAAGCTCTATTTATCTTTACTATATGCTCTGGTATCTTCTCT
TCCATTCTATCTTATTTCATT
>1912
CAGTGCATGGACAAACGTACCTCAAACCAAAGGTTCAATGAAGCTCTATTTATCTTTAC
TATATGCTCTGGTATCTTCTCTTCCATTCTATCTTATTTCATTTAAGAACGTTAATGAAGA
CCCACTAATGGATTGCCACC
>1913
GAAGCTCTATTTATCTTTACTATATGCTCTGGTATCTTCTCTTCCATTCTATCTTATTTCATT
TAAGAACGTTAATGAAGACCCACTAATGGATTGCCACCCATGGTTCGAGGAGGTCCCC
CAAACAGTGAGGCCTTAGC
>1914
CTTCTCTTCCATTCTATCTTATTTCATTTAAGAACGTTAATGAAGACCCACTAATGGATTG
CCACCCATGGTTCGAGGAGGTCCCCCAAACAGTGAGGCCTTAGCCATAGGTATTCAATT
CAAAAAGCATTTACTGAGCA
>1915
ATTCTATCTTATTTCATTTAAGAACGTTAATGAAGACCCACTAATGGATTGCCACCCATG
GTTCGAGGAGGTCCCCCAAACAGTGAGGCCTTAGCCATAGGTATTCAATTCAAAAAGC
ATTTACTGAGCATCACTGTGTA
>1916
TGAAGACCCACTAATGGATTGCCACCCATGGTTCGAGGAGGTCCCCCAAACAGTGAGG
CCTTAGCCATAGGTATTCAATTCAAAAAGCATTTACTGAGCATCACTGTGTACTAGGTG
GTTGCTAAATGCTATGGGGGTAA
>1917
AGGAGGTCCCCCAAACAGTGAGGCCTTAGCCATAGGTATTCAATTCAAAAAGCATTTA
CTGAGCATCACTGTGTACTAGGTGGTTGCTAAATGCTATGGGGGTAATGCAGAGAAATT
AATAAATCCCTGTAAGAGTTCAC
>1918
AAACAGTGAGGCCTTAGCCATAGGTATTCAATTCAAAAAGCATTTACTGAGCATCACTG
TGTACTAGGTGGTTGCTAAATGCTATGGGGGTAATGCAGAGAAATTAATAAATCCCTGT
AAGAGTTCACTGTAAAACAGAA
>1919
ATAGGTATTCAATTCAAAAAGCATTTACTGAGCATCACTGTGTACTAGGTGGTTGCTAA
ATGCTATGGGGGTAATGCAGAGAAATTAATAAATCCCTGTAAGAGTTCACTGTAAAACA
GAAATGACAGCCATATGTGTAT
>1920
CTGAGCATCACTGTGTACTAGGTGGTTGCTAAATGCTATGGGGGTAATGCAGAGAAATT
AATAAATCCCTGTAAGAGTTCACTGTAAAACAGAAATGACAGCCATATGTGTATGACTT
TGTGTGTGTGTGTATATATATA
>1921
ATGGGGGTAATGCAGAGAAATTAATAAATCCCTGTAAGAGTTCACTGTAAAACAGAAA
TGACAGCCATATGTGTATGACTTTGTGTGTGTGTGTATATATATATATCAGACTGTATGATT

AAATATTATATAAGTGAGGT
>1922
GGGGGTAATGCAGAGAAATTAATAAATCCCTGTAAGAGTTCACTGTAAAACAGAAATG
ACAGCCATATGTGTATGACTTTGTGTGTGTGTGTATATATATATATCAGACTGTATGATTA
AATATTATATAAGTGAGGTAT
>1923
CACTGTAAAACAGAAATGACAGCCATATGTGTATGACTTTGTGTGTGTGTGTATATATAT
ATATCAGACTGTATGATTAAATATTATATAAGTGAGGTATGGGGGAGCAGAGGAGAGGA
ATTGAATTTTGAATTTTGATT
>1924
GCCATATGTGTATGACTTTGTGTGTGTGTGTATATATATATATCAGACTGTATGATTAAATA
TTATATAAGTGAGGTATGGGGGAGCAGAGGAGAGGAATTGAATTTTGAATTTTGATTGA
AGATTCAGGAGGGCTTCAT
>1925
ATGTGTATGACTTTGTGTGTGTGTGTATATATATATATCAGACTGTATGATTAAATATTATA
TAAGTGAGGTATGGGGGAGCAGAGGAGAGGAATTGAATTTTGAATTTTGATTGAAGAT
TCAGGAGGGCTTCATGGAGG
>1926
ATGGGGGAGCAGAGGAGAGGAATTGAATTTTGAATTTTGATTGAAGATTCAGGAGGGC
TTCATGGAGGAGGTAGCATTTGGCCAGGTTTTAGAGAATGGCTAGGCTTTGGACAAGT
GGAGACAGGAACTGCCATTTGGAG
>1927
AGAGGAATTGAATTTTGAATTTTGATTGAAGATTCAGGAGGGCTTCATGGAGGAGGTA
GCATTTGGCCAGGTTTTAGAGAATGGCTAGGCTTTGGACAAGTGGAGACAGGAACTGC
CATTTGGAGGTAGGAGATGAGCTA
>1928
AGATTCAGGAGGGCTTCATGGAGGAGGTAGCATTTGGCCAGGTTTTAGAGAATGGCTA
GGCTTTGGACAAGTGGAGACAGGAACTGCCATTTGGAGGTAGGAGATGAGCTAATGT
GTGGCAGTGTATGGGTGGGGGCAGG
>1929
GTAGCATTTGGCCAGGTTTTAGAGAATGGCTAGGCTTTGGACAAGTGGAGACAGGAAC
TGCCATTTGGAGGTAGGAGATGAGCTAATGTGTGGCAGTGTATGGGTGGGGGCAGGTG
CCTGTATACAAGGTAGTGTGCTGG
>1930
TTTAGAGAATGGCTAGGCTTTGGACAAGTGGAGACAGGAACTGCCATTTGGAGGTAGG
AGATGAGCTAATGTGTGGCAGTGTATGGGTGGGGGCAGGTGCCTGTATACAAGGTAGT
GTGCTGGAATGGGGCGATGGGCAT
>1931
TAGAGAATGGCTAGGCTTTGGACAAGTGGAGACAGGAACTGCCATTTGGAGGTAGGA
GATGAGCTAATGTGTGGCAGTGTATGGGTGGGGGCAGGTGCCTGTATACAAGGTAGTG
TGCTGGAATGGGGCGATGGGCATGG
>1932
GCTTTGGACAAGTGGAGACAGGAACTGCCATTTGGAGGTAGGAGATGAGCTAATGTGT
GGCAGTGTATGGGTGGGGGCAGGTGCCTGTATACAAGGTAGTGTGCTGGAATGGGGCG

ATGGGCATGGGGTAGGATGGGAGA
>1933
TGCCATTTGGAGGTAGGAGATGAGCTAATGTGTGGCAGTGTATGGGTGGGGGCAGGTG
CCTGTATACAAGGTAGTGTGCTGGAATGGGGCGATGGGCATGGGGTAGGATGGGAGAT
CAAGCAGGGAAAGTGGATTGGGGC
>1934
TGGCAGTGTATGGGTGGGGGCAGGTGCCTGTATACAAGGTAGTGTGCTGGAATGGGGC
GATGGGCATGGGGTAGGATGGGAGATCAAGCAGGGAAAGTGGATTGGGGCCAGGTCC
TAGAATGATTTGAATACCAAGCTAG
>1935
CCTGTATACAAGGTAGTGTGCTGGAATGGGGCGATGGGCATGGGGTAGGATGGGAGAT
CAAGCAGGGAAAGTGGATTGGGGCCAGGTCCTAGAATGATTTGAATACCAAGCTAGCT
TTCTGGACATTTGTAAGGCGCACT
>1936
GTGTGCTGGAATGGGGCGATGGGCATGGGGTAGGATGGGAGATCAAGCAGGGAAAGT
GGATTGGGGCCAGGTCCTAGAATGATTTGAATACCAAGCTAGCTTTCTGGACATTTGTA
AGGCGCACTTGATAGGGGTTGGAT
>1937
CAAGCAGGGAAAGTGGATTGGGGCCAGGTCCTAGAATGATTTGAATACCAAGCTAGCT
TTCTGGACATTTGTAAGGCGCACTTGATAGGGGTTGGATCATTGCGATATTATTGGAAG
ACAAAACTGGGGAGGGTGTGAGA
>1938
GGGCCAGGTCCTAGAATGATTTGAATACCAAGCTAGCTTTCTGGACATTTGTAAGGCGC
ACTTGATAGGGGTTGGATCATTGCGATATTATTGGAAGACAAAACTGGGGAGGGTGTG
AGACCATGTGGCTGCTCAGAATG
>1939
GGACATTTGTAAGGCGCACTTGATAGGGGTTGGATCATTGCGATATTATTGGAAGACAA
AACTGGGGAGGGTGTGAGACCATGTGGCTGCTCAGAATGCAGAGCCCCTCTGATGTCC
TCAGCCTTCTACGTATTTAGTGA
>1940
GGCGCACTTGATAGGGGTTGGATCATTGCGATATTATTGGAAGACAAAACTGGGGAGG
GTGTGAGACCATGTGGCTGCTCAGAATGCAGAGCCCCTCTGATGTCCTCAGCCTTCTAC
GTATTTAGTGATGGGGGTGGTGG
>1941
TATTATTGGAAGACAAAACTGGGGAGGGTGTGAGACCATGTGGCTGCTCAGAATGCAG
AGCCCCTCTGATGTCCTCAGCCTTCTACGTATTTAGTGATGGGGGTGGTGGTGGTAAAG
AGCCTTGCAGACAGCCTGGCTTG
>1942
AACTGGGGAGGGTGTGAGACCATGTGGCTGCTCAGAATGCAGAGCCCCTCTGATGTCC
TCAGCCTTCTACGTATTTAGTGATGGGGGTGGTGGTGGTAAAGAGCCTTGCAGACAGC
CTGGCTTGGGTGCTTGCTTGAAGT
>1943
AGACCATGTGGCTGCTCAGAATGCAGAGCCCCTCTGATGTCCTCAGCCTTCTACGTATT
TAGTGATGGGGGTGGTGGTGGTAAAGAGCCTTGCAGACAGCCTGGCTTGGGTGCTTGC

TTGAAGTTCTTACGCCCACTAAC
>1944
CCATGTGGCTGCTCAGAATGCAGAGCCCCTCTGATGTCCTCAGCCTTCTACGTATTTAG
TGATGGGGGTGGTGGTGGTAAAGAGCCTTGCAGACAGCCTGGCTTGGGTGCTTGCTTG
AAGTTCTTACGCCCACTAACTCC
>1945
TGTGGCTGCTCAGAATGCAGAGCCCCTCTGATGTCCTCAGCCTTCTACGTATTTAGTGA
TGGGGGTGGTGGTGGTAAAGAGCCTTGCAGACAGCCTGGCTTGGGTGCTTGCTTGAA
GTTCTTACGCCCACTAACTCCTGC
>1946
TCCTCAGCCTTCTACGTATTTAGTGATGGGGGTGGTGGTGGTAAAGAGCCTTGCAGAC
AGCCTGGCTTGGGTGCTTGCTTGAAGTTCTTACGCCCACTAACTCCTGCCCTGCTCTGT
GGAGCCTGCTGAATGGGGCATTA
>1947
GAGCCTTGCAGACAGCCTGGCTTGGGTGCTTGCTTGAAGTTCTTACGCCCACTAACTC
CTGCCCTGCTCTGTGGAGCCTGCTGAATGGGGCATTAGGCTAGTCACACACCTGTCCTT
TTTCACATTCATGCTGTACCCTT
>1948
TGCTTGAAGTTCTTACGCCCACTAACTCCTGCCCTGCTCTGTGGAGCCTGCTGAATGGG
GCATTAGGCTAGTCACACACCTGTCCTTTTTCACATTCATGCTGTACCCTTTGCAAACAT
TACGCAGCAGTGCAAACTCAG
>1949
CTTACGCCCACTAACTCCTGCCCTGCTCTGTGGAGCCTGCTGAATGGGGCATTAGGCTA
GTCACACACCTGTCCTTTTTCACATTCATGCTGTACCCTTTGCAAACATTACGCAGCAG
TGCAAACTCAGCCCTGGGCCTC
>1950
CCTGCTCTGTGGAGCCTGCTGAATGGGGCATTAGGCTAGTCACACACCTGTCCTTTTTC
ACATTCATGCTGTACCCTTTGCAAACATTACGCAGCAGTGCAAACTCAGCCCTGGGCCT
CAGAGGAGCCAGGCGTGGCCAG
>1951
GGCATTAGGCTAGTCACACACCTGTCCTTTTTCACATTCATGCTGTACCCTTTGCAAACA
TTACGCAGCAGTGCAAACTCAGCCCTGGGCCTCAGAGGAGCCAGGCGTGGCCAGGAG
AGTGGGATTTGGAACGGAAGTGC
>1952
TTCATGCTGTACCCTTTGCAAACATTACGCAGCAGTGCAAACTCAGCCCTGGGCCTCA
GAGGAGCCAGGCGTGGCCAGGAGAGTGGGATTTGGAACGGAAGTGCTTTTGTTTTTG
GCATTCACTACTCCCCCACCCCCAC
>1953
CCTTTGCAAACATTACGCAGCAGTGCAAACTCAGCCCTGGGCCTCAGAGGAGCCAGG
CGTGGCCAGGAGAGTGGGATTTGGAACGGAAGTGCTTTTGTTTTTGGCATTCACTACT
CCCCCACCCCCACTGGCACTTGGAT
>1954
AAACTCAGCCCTGGGCCTCAGAGGAGCCAGGCGTGGCCAGGAGAGTGGGATTTGGAA
CGGAAGTGCTTTTGTTTTTGGCATTCACTACTCCCCCACCCCCACTGGCACTTGGATTG

TCCTTCTTTTTCTCCTTGGTGTCT
>1955
TGGGATTTGGAACGGAAGTGCTTTTGTTTTTGGCATTCACTACTCCCCCACCCCCACTG
GCACTTGGATTGTCCTTCTTTTTCTCCTTGGTGTCTCTCATCCTGTCCAGCCTTCAAGAC
CTCCTCAAGTCAGGCTTCTCT
>1956
GCTTTTGTTTTTGGCATTCACTACTCCCCCACCCCCACTGGCACTTGGATTGTCCTTCTT
TTTCTCCTTGGTGTCTCTCATCCTGTCCAGCCTTCAAGACCTCCTCAAGTCAGGCTTCT
CTGGGAAGCGCTGTCTGTCTG
>1957
TTTTGTTTTTGGCATTCACTACTCCCCCACCCCCACTGGCACTTGGATTGTCCTTCTTTT
TCTCCTTGGTGTCTCTCATCCTGTCCAGCCTTCAAGACCTCCTCAAGTCAGGCTTCTCT
GGGAAGCGCTGTCTGTCTGTC
>1958
TGGCACTTGGATTGTCCTTCTTTTTCTCCTTGGTGTCTCTCATCCTGTCCAGCCTTCAAG
ACCTCCTCAAGTCAGGCTTCTCTGGGAAGCGCTGTCTGTCTGTCTTGGTGCTTGCTGG
CTTCCTCTTCTGTCTCCTGTGC
>1959
TTCTCCTTGGTGTCTCTCATCCTGTCCAGCCTTCAAGACCTCCTCAAGTCAGGCTTCTC
TGGGAAGCGCTGTCTGTCTGTCTTGGTGCTTGCTGGCTTCCTCTTCTGTCTCCTGTGCC
ATCTCCAGTTTGCACCATTTGA
>1960
CCTTGGTGTCTCTCATCCTGTCCAGCCTTCAAGACCTCCTCAAGTCAGGCTTCTCTGGG
AAGCGCTGTCTGTCTGTCTTGGTGCTTGCTGGCTTCCTCTTCTGTCTCCTGTGCCATCTC
CAGTTTGCACCATTTGAGTAT
>1961
GACCTCCTCAAGTCAGGCTTCTCTGGGAAGCGCTGTCTGTCTGTCTTGGTGCTTGCTG
GCTTCCTCTTCTGTCTCCTGTGCCATCTCCAGTTTGCACCATTTGAGTATAAGCTCTGCT
GCCTCATTTGAGTTTAAGTTTT
>1962
TCTCTGGGAAGCGCTGTCTGTCTGTCTTGGTGCTTGCTGGCTTCCTCTTCTGTCTCCTG
TGCCATCTCCAGTTTGCACCATTTGAGTATAAGCTCTGCTGCCTCATTTGAGTTTAAGTT
TTATCCCCCCAGCCACAACCT
>1963
GTCTGTCTGTCTTGGTGCTTGCTGGCTTCCTCTTCTGTCTCCTGTGCCATCTCCAGTTTG
CACCATTTGAGTATAAGCTCTGCTGCCTCATTTGAGTTTAAGTTTTATCCCCCCAGCCAC
AACCTAAGTTCCATGAGGAT
>1964
CTTCCTCTTCTGTCTCCTGTGCCATCTCCAGTTTGCACCATTTGAGTATAAGCTCTGCTG
CCTCATTTGAGTTTAAGTTTTATCCCCCCAGCCACAACCTAAGTTCCATGAGGATAGTG
ATTGTGGTTTATCTTCTCTCC
>1965
TTGCACCATTTGAGTATAAGCTCTGCTGCCTCATTTGAGTTTAAGTTTTATCCCCCCAGC
CACAACCTAAGTTCCATGAGGATAGTGATTGTGGTTTATCTTCTCTCCCTCAGCTGTCTG

CTGACACCATGCTGAATACA
>1966
TATAAGCTCTGCTGCCTCATTTGAGTTTAAGTTTTATCCCCCCAGCCACAACCTAAGTTC
CATGAGGATAGTGATTGTGGTTTATCTTCTCTCCCTCAGCTGTCTGCTGACACCATGCTG
AATACATAGTAGGTACTCAG
>1967
TTTTATCCCCCCAGCCACAACCTAAGTTCCATGAGGATAGTGATTGTGGTTTATCTTCTC
TCCCTCAGCTGTCTGCTGACACCATGCTGAATACATAGTAGGTACTCAGTAAATAATAG
CTAATTGAGCATTTACTATGG
>1968
TCCATGAGGATAGTGATTGTGGTTTATCTTCTCTCCCTCAGCTGTCTGCTGACACCATGC
TGAATACATAGTAGGTACTCAGTAAATAATAGCTAATTGAGCATTTACTATGGACAAAGC
ACTTTACTTGCATTAACACA
>1969
AGTGATTGTGGTTTATCTTCTCTCCCTCAGCTGTCTGCTGACACCATGCTGAATACATAG
TAGGTACTCAGTAAATAATAGCTAATTGAGCATTTACTATGGACAAAGCACTTTACTTGC
ATTAACACATCCCATCCTCA
>1970
AGCTAATTGAGCATTTACTATGGACAAAGCACTTTACTTGCATTAACACATCCCATCCTC
ACAACCACCTGTGAGGTGAATCCTATTAAAAGGAATACTTGGGTGCTTTCAGAGTTATT
CTCTCCAGCTTTGGACTTCTT
>1971
ATTGAGCATTTACTATGGACAAAGCACTTTACTTGCATTAACACATCCCATCCTCACAAC
CACCTGTGAGGTGAATCCTATTAAAAGGAATACTTGGGTGCTTTCAGAGTTATTCTCTC
CAGCTTTGGACTTCTTGTTTC
>1972
TTTACTTGCATTAACACATCCCATCCTCACAACCACCTGTGAGGTGAATCCTATTAAAA
GGAATACTTGGGTGCTTTCAGAGTTATTCTCTCCAGCTTTGGACTTCTTGTTTCATTTGC
CCTGAGAGCTCCTCAACTCTG
>1973
TAACACATCCCATCCTCACAACCACCTGTGAGGTGAATCCTATTAAAAGGAATACTTGG
GTGCTTTCAGAGTTATTCTCTCCAGCTTTGGACTTCTTGTTTCATTTGCCCTGAGAGCTC
CTCAACTCTGTTGATGCTGTT
>1974
GTGAGGTGAATCCTATTAAAAGGAATACTTGGGTGCTTTCAGAGTTATTCTCTCCAGCT
TTGGACTTCTTGTTTCATTTGCCCTGAGAGCTCCTCAACTCTGTTGATGCTGTTCTTTCA
AAGGTTGCTTGTGACCTCTTA
>1975
GGTGCTTTCAGAGTTATTCTCTCCAGCTTTGGACTTCTTGTTTCATTTGCCCTGAGAGCT
CCTCAACTCTGTTGATGCTGTTCTTTCAAAGGTTGCTTGTGACCTCTTAGTTCTCAAGA
GCACTGTATTAGGTTGGTGCA
>1976
TCCAGCTTTGGACTTCTTGTTTCATTTGCCCTGAGAGCTCCTCAACTCTGTTGATGCTG
TTCTTTCAAAGGTTGCTTGTGACCTCTTAGTTCTCAAGAGCACTGTATTAGGTTGGTGC

AAAAGTAATTGCTATTTTAGAC
>1977
TTGGACTTCTTGTTTCATTTGCCCTGAGAGCTCCTCAACTCTGTTGATGCTGTTCTTTCA
AAGGTTGCTTGTGACCTCTTAGTTCTCAAGAGCACTGTATTAGGTTGGTGCAAAAGTAA
TTGCTATTTTAGACATTACTT
>1978
GTTTCATTTGCCCTGAGAGCTCCTCAACTCTGTTGATGCTGTTCTTTCAAAGGTTGCTT
GTGACCTCTTAGTTCTCAAGAGCACTGTATTAGGTTGGTGCAAAAGTAATTGCTATTTT
AGACATTACTTTCAATGGCAAA
>1979
CTCAACTCTGTTGATGCTGTTCTTTCAAAGGTTGCTTGTGACCTCTTAGTTCTCAAGAG
CACTGTATTAGGTTGGTGCAAAAGTAATTGCTATTTTAGACATTACTTTCAATGGCAAAA
ACTGCAATTACTTTTGCGCCA
>1980
TTTTAGACATTACTTTCAATGGCAAAAACTGCAATTACTTTTGCGCCAACTAATACTTTC
CCAGACTCCAGTATTGGCTCCATTGACCCCTCCTGTTCCTGCCAACTTCTCCCCCTCCT
CCTCTGGTATTTCCTGGAACT
>1981
AAAACTGCAATTACTTTTGCGCCAACTAATACTTTCCCAGACTCCAGTATTGGCTCCATT
GACCCCTCCTGTTCCTGCCAACTTCTCCCCCTCCTCCTCTGGTATTTCCTGGAACTCCCT
CCCTAGGGTCAGTGCCATGT
>1982
CTTTCCCAGACTCCAGTATTGGCTCCATTGACCCCTCCTGTTCCTGCCAACTTCTCCCCC
TCCTCCTCTGGTATTTCCTGGAACTCCCTCCCTAGGGTCAGTGCCATGTGGTGCCTGGA
ACTCCCTGGGATCTGTGTTTG
>1983
ATTGACCCCTCCTGTTCCTGCCAACTTCTCCCCCTCCTCCTCTGGTATTTCCTGGAACTC
CCTCCCTAGGGTCAGTGCCATGTGGTGCCTGGAACTCCCTGGGATCTGTGTTTGAATCC
TGACATTGCCACTTTCTAAGT
>1984
TTCCTGCCAACTTCTCCCCCTCCTCCTCTGGTATTTCCTGGAACTCCCTCCCTAGGGTCA
GTGCCATGTGGTGCCTGGAACTCCCTGGGATCTGTGTTTGAATCCTGACATTGCCACTT
TCTAAGTGGCAAACACTTTGG
>1985
TCCTCTGGTATTTCCTGGAACTCCCTCCCTAGGGTCAGTGCCATGTGGTGCCTGGAACT
CCCTGGGATCTGTGTTTGAATCCTGACATTGCCACTTTCTAAGTGGCAAACACTTTGGC
CACCTCCCCCTCTTCCTGAGTC
>1986
CTCTGGTATTTCCTGGAACTCCCTCCCTAGGGTCAGTGCCATGTGGTGCCTGGAACTCC
CTGGGATCTGTGTTTGAATCCTGACATTGCCACTTTCTAAGTGGCAAACACTTTGGCCA
CCTCCCCCTCTTCCTGAGTCTG
>1987
GGGTCAGTGCCATGTGGTGCCTGGAACTCCCTGGGATCTGTGTTTGAATCCTGACATTG
CCACTTTCTAAGTGGCAAACACTTTGGCCACCTCCCCCTCTTCCTGAGTCTGTTTCATC

ATCTGCAAAATGCTGGTAGTTT
>1988
TCTGTGTTTGAATCCTGACATTGCCACTTTCTAAGTGGCAAACACTTTGGCCACCTCCC
CCTCTTCCTGAGTCTGTTTCATCATCTGCAAAATGCTGGTAGTTTCTCTTCTCACAGGA
AAGATGGTGGTTGTTCATCGAT
>1989
TGTTTGAATCCTGACATTGCCACTTTCTAAGTGGCAAACACTTTGGCCACCTCCCCCTC
TTCCTGAGTCTGTTTCATCATCTGCAAAATGCTGGTAGTTTCTCTTCTCACAGGAAAGA
TGGTGGTTGTTCATCGATGTCT
>1990
TTTCTAAGTGGCAAACACTTTGGCCACCTCCCCCTCTTCCTGAGTCTGTTTCATCATCT
GCAAAATGCTGGTAGTTTCTCTTCTCACAGGAAAGATGGTGGTTGTTCATCGATGTCTG
TTGAAACCGTGACCCTCTCTGC
>1991
CTCCCCCTCTTCCTGAGTCTGTTTCATCATCTGCAAAATGCTGGTAGTTTCTCTTCTCAC
AGGAAAGATGGTGGTTGTTCATCGATGTCTGTTGAAACCGTGACCCTCTCTGCTGAAT
GTAGAAGAAGATGACCTTACCA
>1992
CCCCTCTTCCTGAGTCTGTTTCATCATCTGCAAAATGCTGGTAGTTTCTCTTCTCACAGG
AAAGATGGTGGTTGTTCATCGATGTCTGTTGAAACCGTGACCCTCTCTGCTGAATGTAG
AAGAAGATGACCTTACCAGTT
>1993
GTTTCATCATCTGCAAAATGCTGGTAGTTTCTCTTCTCACAGGAAAGATGGTGGTTGTT
CATCGATGTCTGTTGAAACCGTGACCCTCTCTGCTGAATGTAGAAGAAGATGACCTTAC
CAGTTCTCCTGTCTCTCCAGTG
>1994
TTCTCTTCTCACAGGAAAGATGGTGGTTGTTCATCGATGTCTGTTGAAACCGTGACCCT
CTCTGCTGAATGTAGAAGAAGATGACCTTACCAGTTCTCCTGTCTCTCCAGTGTCTTTC
TTGACAATCTTGTGGTTTCCAC
>1995
GTGGTTGTTCATCGATGTCTGTTGAAACCGTGACCCTCTCTGCTGAATGTAGAAGAAGA
TGACCTTACCAGTTCTCCTGTCTCTCCAGTGTCTTTCTTGACAATCTTGTGGTTTCCACA
AGCAACCCTCTGTGGGTGATT
>1996
TCATCGATGTCTGTTGAAACCGTGACCCTCTCTGCTGAATGTAGAAGAAGATGACCTTA
CCAGTTCTCCTGTCTCTCCAGTGTCTTTCTTGACAATCTTGTGGTTTCCACAAGCAACC
CTCTGTGGGTGATTTCCAGATC
>1997
CTCTGCTGAATGTAGAAGAAGATGACCTTACCAGTTCTCCTGTCTCTCCAGTGTCTTTC
TTGACAATCTTGTGGTTTCCACAAGCAACCCTCTGTGGGTGATTTCCAGATCTCTACCT
CTAGTTGTTTTTCGTTTTTTGT
>1998
TGCTGAATGTAGAAGAAGATGACCTTACCAGTTCTCCTGTCTCTCCAGTGTCTTTCTTG
ACAATCTTGTGGTTTCCACAAGCAACCCTCTGTGGGTGATTTCCAGATCTCTACCTCTA

GTTGTTTTTCGTTTTTTGTCTT
>1999
GACCTTACCAGTTCTCCTGTCTCTCCAGTGTCTTTCTTGACAATCTTGTGGTTTCCACA
AGCAACCCTCTGTGGGTGATTTCCAGATCTCTACCTCTAGTTGTTTTTCGTTTTTTGTCT
TTTCTTGTGTTTCACACATTC
>2000
TGTCTTTCTTGACAATCTTGTGGTTTCCACAAGCAACCCTCTGTGGGTGATTTCCAGAT
CTCTACCTCTAGTTGTTTTTCGTTTTTTGTCTTTTCTTGTGTTTCACACATTCTTTTCCAG
TAGCCTCACGGGCACACCAG
>2001
GACAATCTTGTGGTTTCCACAAGCAACCCTCTGTGGGTGATTTCCAGATCTCTACCTCT
AGTTGTTTTTCGTTTTTTGTCTTTTCTTGTGTTTCACACATTCTTTTCCAGTAGCCTCAC
GGGCACACCAGTCCTGCAAGG
>2002
TTGTGGTTTCCACAAGCAACCCTCTGTGGGTGATTTCCAGATCTCTACCTCTAGTTGTT
TTTCGTTTTTTGTCTTTTCTTGTGTTTCACACATTCTTTTCCAGTAGCCTCACGGGCACA
CCAGTCCTGCAAGGATCTAGA
>2003
CACAAGCAACCCTCTGTGGGTGATTTCCAGATCTCTACCTCTAGTTGTTTTTCGTTTTTT
GTCTTTTCTTGTGTTTCACACATTCTTTTCCAGTAGCCTCACGGGCACACCAGTCCTGC
AAGGATCTAGAATGTGATGGT
>2004
ATTTCCAGATCTCTACCTCTAGTTGTTTTTCGTTTTTTGTCTTTTCTTGTGTTTCACACAT
TCTTTTCCAGTAGCCTCACGGGCACACCAGTCCTGCAAGGATCTAGAATGTGATGGTTT
GAGCTAGTTCAACACCTTCC
>2005
AGTTGTTTTTCGTTTTTTGTCTTTTCTTGTGTTTCACACATTCTTTTCCAGTAGCCTCAC
GGGCACACCAGTCCTGCAAGGATCTAGAATGTGATGGTTTGAGCTAGTTCAACACCTT
CCTCTCGAATCGTCTCCTGTGT
>2006
CTTTTCTTGTGTTTCACACATTCTTTTCCAGTAGCCTCACGGGCACACCAGTCCTGCAA
GGATCTAGAATGTGATGGTTTGAGCTAGTTCAACACCTTCCTCTCGAATCGTCTCCTGT
GTCCTCATGTTTTGTGGTCTCT
>2007
TTGTGTTTCACACATTCTTTTCCAGTAGCCTCACGGGCACACCAGTCCTGCAAGGATCT
AGAATGTGATGGTTTGAGCTAGTTCAACACCTTCCTCTCGAATCGTCTCCTGTGTCCTC
ATGTTTTGTGGTCTCTGCCCTC
>2008
CGGGCACACCAGTCCTGCAAGGATCTAGAATGTGATGGTTTGAGCTAGTTCAACACCT
TCCTCTCGAATCGTCTCCTGTGTCCTCATGTTTTGTGGTCTCTGCCCTCTTCCCAGGCAC
CCAACTGCAGACTTTGGCATCA
>2009
ACCAGTCCTGCAAGGATCTAGAATGTGATGGTTTGAGCTAGTTCAACACCTTCCTCTCG
AATCGTCTCCTGTGTCCTCATGTTTTGTGGTCTCTGCCCTCTTCCCAGGCACCCAACTG

CAGACTTTGGCATCATCTGTGG
>2010
CAGTCCTGCAAGGATCTAGAATGTGATGGTTTGAGCTAGTTCAACACCTTCCTCTCGAA
TCGTCTCCTGTGTCCTCATGTTTTGTGGTCTCTGCCCTCTTCCCAGGCACCCAACTGCA
GACTTTGGCATCATCTGTGGCT
>2011
ATCTAGAATGTGATGGTTTGAGCTAGTTCAACACCTTCCTCTCGAATCGTCTCCTGTGT
CCTCATGTTTTGTGGTCTCTGCCCTCTTCCCAGGCACCCAACTGCAGACTTTGGCATCA
TCTGTGGCTCCTGTCTTCCCTT
>2012
TCCTGTGTCCTCATGTTTTGTGGTCTCTGCCCTCTTCCCAGGCACCCAACTGCAGACTT
TGGCATCATCTGTGGCTCCTGTCTTCCCTTTGCCTTCCATATCCACCATTTGCCTCTCCA
GCCTACTTAACCAGATGGCTG
>2013
CTCATGTTTTGTGGTCTCTGCCCTCTTCCCAGGCACCCAACTGCAGACTTTGGCATCAT
CTGTGGCTCCTGTCTTCCCTTTGCCTTCCATATCCACCATTTGCCTCTCCAGCCTACTTA
ACCAGATGGCTGTCCTGATTT
>2014
TGTGGCTCCTGTCTTCCCTTTGCCTTCCATATCCACCATTTGCCTCTCCAGCCTACTTAA
CCAGATGGCTGTCCTGATTTCCTCACCAGCCTTCCTGCAGTGCACCCCTGCAAGGCTCA
TCACCCCACTTCCCTGAGCAG
>2015
TATCCACCATTTGCCTCTCCAGCCTACTTAACCAGATGGCTGTCCTGATTTCCTCACCAG
CCTTCCTGCAGTGCACCCCTGCAAGGCTCATCACCCCACTTCCCTGAGCAGAGTAGGG
CTCTGATGGGGTTGCTCTCCTG
>2016
TCCTGATTTCCTCACCAGCCTTCCTGCAGTGCACCCCTGCAAGGCTCATCACCCCACTT
CCCTGAGCAGAGTAGGGCTCTGATGGGGTTGCTCTCCTGCTCTGAACCCTGCAGTGGC
TCACTATGGCCTATTCTAGAGAA
>2017
AGCCTTCCTGCAGTGCACCCCTGCAAGGCTCATCACCCCACTTCCCTGAGCAGAGTAG
GGCTCTGATGGGGTTGCTCTCCTGCTCTGAACCCTGCAGTGGCTCACTATGGCCTATTC
TAGAGAAGAAACGAGAGAATTCT
>2018
GGCTCATCACCCCACTTCCCTGAGCAGAGTAGGGCTCTGATGGGGTTGCTCTCCTGCTC
TGAACCCTGCAGTGGCTCACTATGGCCTATTCTAGAGAAGAAACGAGAGAATTCTTAG
CGTGGCATTCAAAGCCATCCGGG
>2019
CTCTCCTGCTCTGAACCCTGCAGTGGCTCACTATGGCCTATTCTAGAGAAGAAACGAG
AGAATTCTTAGCGTGGCATTCAAAGCCATCCGGGATCCTAGATTCCAGCTACATTTTCA
CTCTTCATCCCTTTCTCTGCATA
>2020
AGCCATCCGGGATCCTAGATTCCAGCTACATTTTCACTCTTCATCCCTTTCTCTGCATATT
TCTTAGAATGTACACCCCTGAAGGCAGGGACCATCTTTTTCACATCTGTGTTACAACCT

TTTAGTCCACTCTCTAGCAT
>2021
CTTCATCCCTTTCTCTGCATATTTCTTAGAATGTACACCCCTGAAGGCAGGGACCATCTT
TTTCACATCTGTGTTACAACCTTTTAGTCCACTCTCTAGCATATGGCAACTCAGCAGATG
CTGCTGTTGAGTAAACTCTT
>2022
TCATCCCTTTCTCTGCATATTTCTTAGAATGTACACCCCTGAAGGCAGGGACCATCTTTT
TCACATCTGTGTTACAACCTTTTAGTCCACTCTCTAGCATATGGCAACTCAGCAGATGCT
GCTGTTGAGTAAACTCTTAT
>2023
GCATATTTCTTAGAATGTACACCCCTGAAGGCAGGGACCATCTTTTTCACATCTGTGTTA
CAACCTTTTAGTCCACTCTCTAGCATATGGCAACTCAGCAGATGCTGCTGTTGAGTAAA
CTCTTATGCTTCCCTTCCCAT
>2024
ATCTTTTTCACATCTGTGTTACAACCTTTTAGTCCACTCTCTAGCATATGGCAACTCAGC
AGATGCTGCTGTTGAGTAAACTCTTATGCTTCCCTTCCCATGCCATATCCTTCTCTGGTC
ATCGCAACAGGGCCAAATCT
>2025
TTTCACATCTGTGTTACAACCTTTTAGTCCACTCTCTAGCATATGGCAACTCAGCAGATG
CTGCTGTTGAGTAAACTCTTATGCTTCCCTTCCCATGCCATATCCTTCTCTGGTCATCGC
AACAGGGCCAAATCTCTCAT
>2026
ATGGCAACTCAGCAGATGCTGCTGTTGAGTAAACTCTTATGCTTCCCTTCCCATGCCATA
TCCTTCTCTGGTCATCGCAACAGGGCCAAATCTCTCATGCTTCAGTGTAAATGCCCTCT
CCTTGCAGCATCCTTTCCTGG
>2027
TCTTATGCTTCCCTTCCCATGCCATATCCTTCTCTGGTCATCGCAACAGGGCCAAATCTC
TCATGCTTCAGTGTAAATGCCCTCTCCTTGCAGCATCCTTTCCTGGGGCATTCTTCCTCC
TTGCATCCAGTTGGAAATAA
>2028
TTATGCTTCCCTTCCCATGCCATATCCTTCTCTGGTCATCGCAACAGGGCCAAATCTCTC
ATGCTTCAGTGTAAATGCCCTCTCCTTGCAGCATCCTTTCCTGGGGCATTCTTCCTCCTT
GCATCCAGTTGGAAATAATA
>2029
CTCATGCTTCAGTGTAAATGCCCTCTCCTTGCAGCATCCTTTCCTGGGGCATTCTTCCTC
CTTGCATCCAGTTGGAAATAATATGTCCCTCCATTGGAGCTCCCACACCAGAAGAGGTA
TAGATAAAGCCAGATACTTCT
>2030
TAAATGCCCTCTCCTTGCAGCATCCTTTCCTGGGGCATTCTTCCTCCTTGCATCCAGTTG
GAAATAATATGTCCCTCCATTGGAGCTCCCACACCAGAAGAGGTATAGATAAAGCCAGA
TACTTCTTTTTTTTTTTTTTT
>2031
GGGCATTCTTCCTCCTTGCATCCAGTTGGAAATAATATGTCCCTCCATTGGAGCTCCCAC
ACCAGAAGAGGTATAGATAAAGCCAGATACTTCTTTTTTTTTTTTTTTTTTTGATACAGA

GTCTCACTCTGTTGCCCAGG
>2032
GAGCTCCCACACCAGAAGAGGTATAGATAAAGCCAGATACTTCTTTTTTTTTTTTTTTTT
TTGATACAGAGTCTCACTCTGTTGCCCAGGCTGGAGTGTAGTGGCATGAACTTGGCTC
ACTGCAGCCTCTGCCTCTTGGG
>2033
ACCAGAAGAGGTATAGATAAAGCCAGATACTTCTTTTTTTTTTTTTTTTTTTGATACAGA
GTCTCACTCTGTTGCCCAGGCTGGAGTGTAGTGGCATGAACTTGGCTCACTGCAGCCT
CTGCCTCTTGGGTTCAAGTGAT
>2034
GATAAAGCCAGATACTTCTTTTTTTTTTTTTTTTTTTGATACAGAGTCTCACTCTGTTGCC
CAGGCTGGAGTGTAGTGGCATGAACTTGGCTCACTGCAGCCTCTGCCTCTTGGGTTCA
AGTGATTCTTGCGTCTCAGCC
>2035
AGCCAGATACTTCTTTTTTTTTTTTTTTTTTTGATACAGAGTCTCACTCTGTTGCCCAGG
CTGGAGTGTAGTGGCATGAACTTGGCTCACTGCAGCCTCTGCCTCTTGGGTTCAAGTG
ATTCTTGCGTCTCAGCCTCCCG
>2036
AGTCTCACTCTGTTGCCCAGGCTGGAGTGTAGTGGCATGAACTTGGCTCACTGCAGCC
TCTGCCTCTTGGGTTCAAGTGATTCTTGCGTCTCAGCCTCCCGAGTAGCTGAGATTACA
GACATGCGCTACCATGCCTGGCT
>2037
ACTCTGTTGCCCAGGCTGGAGTGTAGTGGCATGAACTTGGCTCACTGCAGCCTCTGCC
TCTTGGGTTCAAGTGATTCTTGCGTCTCAGCCTCCCGAGTAGCTGAGATTACAGACATG
CGCTACCATGCCTGGCTAATTTT
>2038
CAGGCTGGAGTGTAGTGGCATGAACTTGGCTCACTGCAGCCTCTGCCTCTTGGGTTCA
AGTGATTCTTGCGTCTCAGCCTCCCGAGTAGCTGAGATTACAGACATGCGCTACCATGC
CTGGCTAATTTTTGTATTTTTAG
>2039
TGGCATGAACTTGGCTCACTGCAGCCTCTGCCTCTTGGGTTCAAGTGATTCTTGCGTCT
CAGCCTCCCGAGTAGCTGAGATTACAGACATGCGCTACCATGCCTGGCTAATTTTTGTA
TTTTTAGTAGAGATGCGGTTTC
>2040
TGATTCTTGCGTCTCAGCCTCCCGAGTAGCTGAGATTACAGACATGCGCTACCATGCCT
GGCTAATTTTTGTATTTTTAGTAGAGATGCGGTTTCACCATGCTGGCCAGGCTGGTCCCC
AACTCCTCGCCTCAAGTGATC
>2041
CGTCTCAGCCTCCCGAGTAGCTGAGATTACAGACATGCGCTACCATGCCTGGCTAATTT
TTGTATTTTTAGTAGAGATGCGGTTTCACCATGCTGGCCAGGCTGGTCCCCAACTCCTC
GCCTCAAGTGATCTTCCTGCCT
>2042
ATGCGCTACCATGCCTGGCTAATTTTTGTATTTTTAGTAGAGATGCGGTTTCACCATGCT
GGCCAGGCTGGTCCCCAACTCCTCGCCTCAAGTGATCTTCCTGCCTTAGCCTCCCAGA

GTGCTAGGATTACAGGTGTGAG
>2043
ATTTTTGTATTTTTAGTAGAGATGCGGTTTCACCATGCTGGCCAGGCTGGTCCCCAACTC
CTCGCCTCAAGTGATCTTCCTGCCTTAGCCTCCCAGAGTGCTAGGATTACAGGTGTGAG
TCACTGTGCCCGGCCTGGATC
>2044
TTTCACCATGCTGGCCAGGCTGGTCCCCAACTCCTCGCCTCAAGTGATCTTCCTGCCTT
AGCCTCCCAGAGTGCTAGGATTACAGGTGTGAGTCACTGTGCCCGGCCTGGATCTTTTA
TTGTTTGTATTTCTGTTCATTT
>2045
CAGGCTGGTCCCCAACTCCTCGCCTCAAGTGATCTTCCTGCCTTAGCCTCCCAGAGTGC
TAGGATTACAGGTGTGAGTCACTGTGCCCGGCCTGGATCTTTTATTGTTTGTATTTCTGT
TCATTTCTTCCCTAAGAAACC
>2046
GGCTGGTCCCCAACTCCTCGCCTCAAGTGATCTTCCTGCCTTAGCCTCCCAGAGTGCTA
GGATTACAGGTGTGAGTCACTGTGCCCGGCCTGGATCTTTTATTGTTTGTATTTCTGTTC
ATTTCTTCCCTAAGAAACCCC
>2047
CAACTCCTCGCCTCAAGTGATCTTCCTGCCTTAGCCTCCCAGAGTGCTAGGATTACAGG
TGTGAGTCACTGTGCCCGGCCTGGATCTTTTATTGTTTGTATTTCTGTTCATTTCTTCCCT
AAGAAACCCCACGTTTTTGT
>2048
TCCTGCCTTAGCCTCCCAGAGTGCTAGGATTACAGGTGTGAGTCACTGTGCCCGGCCT
GGATCTTTTATTGTTTGTATTTCTGTTCATTTCTTCCCTAAGAAACCCCACGTTTTTGTGG
GCAGGACCTGGAATCCTATTC
>2049
GAGTCACTGTGCCCGGCCTGGATCTTTTATTGTTTGTATTTCTGTTCATTTCTTCCCTAA
GAAACCCCACGTTTTTGTGGGCAGGACCTGGAATCCTATTCAGTTATACAGCCCCGCTT
CGTCCACTGCTCCCCTTCAGA
>2050
CTGTGCCCGGCCTGGATCTTTTATTGTTTGTATTTCTGTTCATTTCTTCCCTAAGAAACC
CCACGTTTTTGTGGGCAGGACCTGGAATCCTATTCAGTTATACAGCCCCGCTTCGTCCA
CTGCTCCCCTTCAGAGTCTAG
>2051
TTTGTATTTCTGTTCATTTCTTCCCTAAGAAACCCCACGTTTTTGTGGGCAGGACCTGG
AATCCTATTCAGTTATACAGCCCCGCTTCGTCCACTGCTCCCCTTCAGAGTCTAGTGCCC
TGGACATCATAGGTATTCAGT
>2052
TCTTCCCTAAGAAACCCCACGTTTTTGTGGGCAGGACCTGGAATCCTATTCAGTTATAC
AGCCCCGCTTCGTCCACTGCTCCCCTTCAGAGTCTAGTGCCCTGGACATCATAGGTATT
CAGTACCTCTTGCTGGCTTAGG
>2053
GTTTTTGTGGGCAGGACCTGGAATCCTATTCAGTTATACAGCCCCGCTTCGTCCACTGC
TCCCCTTCAGAGTCTAGTGCCCTGGACATCATAGGTATTCAGTACCTCTTGCTGGCTTAG

GAACAGGAGGATGCCTGATGG
>2054
TCCTATTCAGTTATACAGCCCCGCTTCGTCCACTGCTCCCCTTCAGAGTCTAGTGCCCTG
GACATCATAGGTATTCAGTACCTCTTGCTGGCTTAGGAACAGGAGGATGCCTGATGGCC
AGGGTGTTCCTCTGAAGCAGT
>2055
GTGCCCTGGACATCATAGGTATTCAGTACCTCTTGCTGGCTTAGGAACAGGAGGATGCC
TGATGGCCAGGGTGTTCCTCTGAAGCAGTGGTGGCTGAGCTTTTTGAATATCTGCATTG
TGCTAAGGGTTAACTACCCAGG
>2056
GCCCTGGACATCATAGGTATTCAGTACCTCTTGCTGGCTTAGGAACAGGAGGATGCCTG
ATGGCCAGGGTGTTCCTCTGAAGCAGTGGTGGCTGAGCTTTTTGAATATCTGCATTGTG
CTAAGGGTTAACTACCCAGGCA
>2057
AGGTATTCAGTACCTCTTGCTGGCTTAGGAACAGGAGGATGCCTGATGGCCAGGGTGT
TCCTCTGAAGCAGTGGTGGCTGAGCTTTTTGAATATCTGCATTGTGCTAAGGGTTAACT
ACCCAGGCAGTACTGGTGGGGAA
>2058
TATTCAGTACCTCTTGCTGGCTTAGGAACAGGAGGATGCCTGATGGCCAGGGTGTTCCT
CTGAAGCAGTGGTGGCTGAGCTTTTTGAATATCTGCATTGTGCTAAGGGTTAACTACCC
AGGCAGTACTGGTGGGGAAGGG
>2059
CAGGAGGATGCCTGATGGCCAGGGTGTTCCTCTGAAGCAGTGGTGGCTGAGCTTTTTG
AATATCTGCATTGTGCTAAGGGTTAACTACCCAGGCAGTACTGGTGGGGAAGGGCCCC
AGAAGCAGGCCCAGACTCAGGTAT
>2060
GCCTGATGGCCAGGGTGTTCCTCTGAAGCAGTGGTGGCTGAGCTTTTTGAATATCTGCA
TTGTGCTAAGGGTTAACTACCCAGGCAGTACTGGTGGGGAAGGGCCCCAGAAGCAGG
CCCAGACTCAGGTATTGTTAAAGC
>2061
CTGAAGCAGTGGTGGCTGAGCTTTTTGAATATCTGCATTGTGCTAAGGGTTAACTACCC
AGGCAGTACTGGTGGGGAAGGGCCCCAGAAGCAGGCCCAGACTCAGGTATTGTTAAA
GCAGAGTCATCTTTGCTCTGAAGT
>2062
CATTGTGCTAAGGGTTAACTACCCAGGCAGTACTGGTGGGGAAGGGCCCCAGAAGCA
GGCCCAGACTCAGGTATTGTTAAAGCAGAGTCATCTTTGCTCTGAAGTTTTTTCTTTTAT
CTTTTCTTTTCTTTTTTAATTGG
>2063
TGCTAAGGGTTAACTACCCAGGCAGTACTGGTGGGGAAGGGCCCCAGAAGCAGGCCC
AGACTCAGGTATTGTTAAAGCAGAGTCATCTTTGCTCTGAAGTTTTTTCTTTTATCTTTT
CTTTTCTTTTTTAATTGGGGGAA
>2064
TGGTGGGGAAGGGCCCCAGAAGCAGGCCCAGACTCAGGTATTGTTAAAGCAGAGTCA
TCTTTGCTCTGAAGTTTTTTCTTTTATCTTTTCTTTTCTTTTTTAATTGGGGGAAACCTTG

TCCTAATTGGCATCTTTATTGT
>2065
AGCAGAGTCATCTTTGCTCTGAAGTTTTTTCTTTTATCTTTTCTTTTCTTTTTTAATTGGG
GGAAACCTTGTCCTAATTGGCATCTTTATTGTGGCAGGAATAGGCCAGGCAGGGCTGG
GGGAGTGTGAAGTCTGTGAAG
>2066
AAGTTTTTTCTTTTATCTTTTCTTTTCTTTTTTAATTGGGGGAAACCTTGTCCTAATTGGC
ATCTTTATTGTGGCAGGAATAGGCCAGGCAGGGCTGGGGGAGTGTGAAGTCTGTGAAG
GGAGGTGGCCTGGGGGCCCCC
>2067
TAATTGGGGGAAACCTTGTCCTAATTGGCATCTTTATTGTGGCAGGAATAGGCCAGGCA
GGGCTGGGGGAGTGTGAAGTCTGTGAAGGGAGGTGGCCTGGGGGCCCCCCACACAA
TGCCTGATTGTGCAGGCATAGGTA
>2068
AACCTTGTCCTAATTGGCATCTTTATTGTGGCAGGAATAGGCCAGGCAGGGCTGGGGG
AGTGTGAAGTCTGTGAAGGGAGGTGGCCTGGGGGCCCCCCACACAATGCCTGATTG
TGCAGGCATAGGTATGAACACATTG
>2069
TAATTGGCATCTTTATTGTGGCAGGAATAGGCCAGGCAGGGCTGGGGGAGTGTGAAGT
CTGTGAAGGGAGGTGGCCTGGGGGCCCCCCACACAATGCCTGATTGTGCAGGCATAG
GTATGAACACATTGCAGCCTGTGT
>2070
GGCAGGGCTGGGGGAGTGTGAAGTCTGTGAAGGGAGGTGGCCTGGGGGCCCCCCA
CACAATGCCTGATTGTGCAGGCATAGGTATGAACACATTGCAGCCTGTGTGCACCCAC
AGGGTGACCACGAGCAAGGCCTGGGG
>2071
GGAGGTGGCCTGGGGGCCCCCCACACAATGCCTGATTGTGCAGGCATAGGTATGAAC
ACATTGCAGCCTGTGTGCACCCACAGGGTGACCACGAGCAAGGCCTGGGGACGAGGA
CTACGAGGCCTGCAGCTGGGTCCTC
>2072
AGGTGGCCTGGGGGCCCCCCACACAATGCCTGATTGTGCAGGCATAGGTATGAACAC
ATTGCAGCCTGTGTGCACCCACAGGGTGACCACGAGCAAGGCCTGGGGACGAGGACT
ACGAGGCCTGCAGCTGGGTCCTCTT
>2073
CAGCCTGTGTGCACCCACAGGGTGACCACGAGCAAGGCCTGGGGACGAGGACTACGA
GGCCTGCAGCTGGGTCCTCTTTTCTCCCTCCTCACCCCCACCCAGTACCCACGTGATTG
GGCCACATCCCAGTAACAAGCAGT
>2074
GCAAGGCCTGGGGACGAGGACTACGAGGCCTGCAGCTGGGTCCTCTTTTCTCCCTCCT
CACCCCCACCCAGTACCCACGTGATTGGGCCACATCCCAGTAACAAGCAGTCCTTGTG
GAAGAGCCTTCATGAAGACCTTTG
>2075
TGGGGACGAGGACTACGAGGCCTGCAGCTGGGTCCTCTTTTCTCCCTCCTCACCCCCA
CCCAGTACCCACGTGATTGGGCCACATCCCAGTAACAAGCAGTCCTTGTGGAAGAGCC

TTCATGAAGACCTTTGCTGGGAGT
>2076
GCCTGCAGCTGGGTCCTCTTTTCTCCCTCCTCACCCCCACCCAGTACCCACGTGATTGG
GCCACATCCCAGTAACAAGCAGTCCTTGTGGAAGAGCCTTCATGAAGACCTTTGCTGG
GAGTGGCGGGCAGGGATCTGCGG
>2077
GGGTCCTCTTTTCTCCCTCCTCACCCCCACCCAGTACCCACGTGATTGGGCCACATCCC
AGTAACAAGCAGTCCTTGTGGAAGAGCCTTCATGAAGACCTTTGCTGGGAGTGGCGG
GCAGGGATCTGCGGGGAGCGTTGC
>2078
ACGTGATTGGGCCACATCCCAGTAACAAGCAGTCCTTGTGGAAGAGCCTTCATGAAGA
CCTTTGCTGGGAGTGGCGGGCAGGGATCTGCGGGGAGCGTTGCTGGTTCAAGCTGGG
GCTTTCCTATGGATGATGTCCTGTG
>2079
AAGCAGTCCTTGTGGAAGAGCCTTCATGAAGACCTTTGCTGGGAGTGGCGGGCAGGG
ATCTGCGGGGAGCGTTGCTGGTTCAAGCTGGGGCTTTCCTATGGATGATGTCCTGTGAA
GGGTAGATCCTGGAGGGACAGACA
>2080
TGCTGGGAGTGGCGGGCAGGGATCTGCGGGGAGCGTTGCTGGTTCAAGCTGGGGCTT
TCCTATGGATGATGTCCTGTGAAGGGTAGATCCTGGAGGGACAGACAGGTGTCTGATC
TTTGGCAGGCAGGGATGTCTCATTT
>2081
GGAGTGGCGGGCAGGGATCTGCGGGGAGCGTTGCTGGTTCAAGCTGGGGCTTTCCTAT
GGATGATGTCCTGTGAAGGGTAGATCCTGGAGGGACAGACAGGTGTCTGATCTTTGGC
AGGCAGGGATGTCTCATTTGTACA
>2082
TTGCTGGTTCAAGCTGGGGCTTTCCTATGGATGATGTCCTGTGAAGGGTAGATCCTGGA
GGGACAGACAGGTGTCTGATCTTTGGCAGGCAGGGATGTCTCATTTGTACATTATAGGT
ATTCAGTATCTCTTGCTGGCTT
>2083
ATGGATGATGTCCTGTGAAGGGTAGATCCTGGAGGGACAGACAGGTGTCTGATCTTTG
GCAGGCAGGGATGTCTCATTTGTACATTATAGGTATTCAGTATCTCTTGCTGGCTTAGGC
TTCATAATGTCTCATTTGTACA
>2084
GTCCTGTGAAGGGTAGATCCTGGAGGGACAGACAGGTGTCTGATCTTTGGCAGGCAG
GGATGTCTCATTTGTACATTATAGGTATTCAGTATCTCTTGCTGGCTTAGGCTTCATAATG
TCTCATTTGTACATCATAGCCT
>2085
CCTGGAGGGACAGACAGGTGTCTGATCTTTGGCAGGCAGGGATGTCTCATTTGTACATT
ATAGGTATTCAGTATCTCTTGCTGGCTTAGGCTTCATAATGTCTCATTTGTACATCATAGC
CTTCAGTACCTCTTGCTGAT
>2086
TGGCAGGCAGGGATGTCTCATTTGTACATTATAGGTATTCAGTATCTCTTGCTGGCTTAG
GCTTCATAATGTCTCATTTGTACATCATAGCCTTCAGTACCTCTTGCTGATTTGGTTAACC

CAGTTGTTATACCACGATA
>2087
GGGATGTCTCATTTGTACATTATAGGTATTCAGTATCTCTTGCTGGCTTAGGCTTCATAAT
GTCTCATTTGTACATCATAGCCTTCAGTACCTCTTGCTGATTTGGTTAACCCAGTTGTTAT
ACCACGATATTGATTTTT
>2088
CATTATAGGTATTCAGTATCTCTTGCTGGCTTAGGCTTCATAATGTCTCATTTGTACATCA
TAGCCTTCAGTACCTCTTGCTGATTTGGTTAACCCAGTTGTTATACCACGATATTGATTTT
TCATGCCATTTTCTTTAG
>2089
GGCTTAGGCTTCATAATGTCTCATTTGTACATCATAGCCTTCAGTACCTCTTGCTGATTTG
GTTAACCCAGTTGTTATACCACGATATTGATTTTTCATGCCATTTTCTTTAGGAAGATTGG
AAGATCATTGAGGATAGG
>2090
TTAGGCTTCATAATGTCTCATTTGTACATCATAGCCTTCAGTACCTCTTGCTGATTTGGTT
AACCCAGTTGTTATACCACGATATTGATTTTTCATGCCATTTTCTTTAGGAAGATTGGAA
GATCATTGAGGATAGGGAC
>2091
TTATACCACGATATTGATTTTTCATGCCATTTTCTTTAGGAAGATTGGAAGATCATTGAG
GATAGGGACTGTGACCTTTTCTTTTTTTCTACCCCAGTACCCAACCTGGCAGGTAGCAC
AGCGCAATGATCCACGATGTT
>2092
CCATTTTCTTTAGGAAGATTGGAAGATCATTGAGGATAGGGACTGTGACCTTTTCTTTTT
TTCTACCCCAGTACCCAACCTGGCAGGTAGCACAGCGCAATGATCCACGATGTTTAATA
GGTTGAATTGTAGAGGGGGCC
>2093
GATTGGAAGATCATTGAGGATAGGGACTGTGACCTTTTCTTTTTTTCTACCCCAGTACC
CAACCTGGCAGGTAGCACAGCGCAATGATCCACGATGTTTAATAGGTTGAATTGTAGA
GGGGGCCTCTGATTCCAAAGATG
>2094
ACTGTGACCTTTTCTTTTTTTCTACCCCAGTACCCAACCTGGCAGGTAGCACAGCGCAA
TGATCCACGATGTTTAATAGGTTGAATTGTAGAGGGGGCCTCTGATTCCAAAGATGGGG
GCAGAATATCAGTCCTCTCCAG
>2095
TTTTCTTTTTTTCTACCCCAGTACCCAACCTGGCAGGTAGCACAGCGCAATGATCCACG
ATGTTTAATAGGTTGAATTGTAGAGGGGGCCTCTGATTCCAAAGATGGGGGCAGAATAT
CAGTCCTCTCCAGACTGCCTGG
>2096
TGATCCACGATGTTTAATAGGTTGAATTGTAGAGGGGGCCTCTGATTCCAAAGATGGGG
GCAGAATATCAGTCCTCTCCAGACTGCCTGGGAATCCTCCTCTCTCCAGCCTGGGCCTG
GCCAGTGGGAGACAGAGGCTGC
>2097
GATGGGGGCAGAATATCAGTCCTCTCCAGACTGCCTGGGAATCCTCCTCTCTCCAGCCT
GGGCCTGGCCAGTGGGAGACAGAGGCTGCTGAAGTAGCTGGCAAGGAGGCAAAGAA

GATGATGACCTAGCTACGGTCCTCA
>2098
TCTCCAGACTGCCTGGGAATCCTCCTCTCTCCAGCCTGGGCCTGGCCAGTGGGAGACA
GAGGCTGCTGAAGTAGCTGGCAAGGAGGCAAAGAAGATGATGACCTAGCTACGGTCC
TCATGTGCCCACTGGGGAATCATCT
>2099
CTGGCCAGTGGGAGACAGAGGCTGCTGAAGTAGCTGGCAAGGAGGCAAAGAAGATG
ATGACCTAGCTACGGTCCTCATGTGCCCACTGGGGAATCATCTTTTTGCATCTGAGTTA
GTCAATGTGCTGTGCATTCTCTGCA
>2100
TGGGAGACAGAGGCTGCTGAAGTAGCTGGCAAGGAGGCAAAGAAGATGATGACCTAG
CTACGGTCCTCATGTGCCCACTGGGGAATCATCTTTTTGCATCTGAGTTAGTCAATGTG
CTGTGCATTCTCTGCAGAATTTGT
>2101
GAGGCAAAGAAGATGATGACCTAGCTACGGTCCTCATGTGCCCACTGGGGAATCATCT
TTTTGCATCTGAGTTAGTCAATGTGCTGTGCATTCTCTGCAGAATTTGTGAACCTGGTAT
AGGTATGAGGTGAGAGAGGCCA
>2102
CAAAGAAGATGATGACCTAGCTACGGTCCTCATGTGCCCACTGGGGAATCATCTTTTTG
CATCTGAGTTAGTCAATGTGCTGTGCATTCTCTGCAGAATTTGTGAACCTGGTATAGGTA
TGAGGTGAGAGAGGCCAGGAG
>2103
AGATGATGACCTAGCTACGGTCCTCATGTGCCCACTGGGGAATCATCTTTTTGCATCTG
AGTTAGTCAATGTGCTGTGCATTCTCTGCAGAATTTGTGAACCTGGTATAGGTATGAGG
TGAGAGAGGCCAGGAGGTCTGG
>2104
ATGACCTAGCTACGGTCCTCATGTGCCCACTGGGGAATCATCTTTTTGCATCTGAGTTA
GTCAATGTGCTGTGCATTCTCTGCAGAATTTGTGAACCTGGTATAGGTATGAGGTGAGA
GAGGCCAGGAGGTCTGGCTGCT
>2105
ATGTGCCCACTGGGGAATCATCTTTTTGCATCTGAGTTAGTCAATGTGCTGTGCATTCTC
TGCAGAATTTGTGAACCTGGTATAGGTATGAGGTGAGAGAGGCCAGGAGGTCTGGCTG
CTCCCCCTCTAGACTTCGCAGG
>2106
TTTTTGCATCTGAGTTAGTCAATGTGCTGTGCATTCTCTGCAGAATTTGTGAACCTGGTA
TAGGTATGAGGTGAGAGAGGCCAGGAGGTCTGGCTGCTCCCCCTCTAGACTTCGCAGG
TAAAACCCAAGGTGTGTGCCAC
>2107
GTCAATGTGCTGTGCATTCTCTGCAGAATTTGTGAACCTGGTATAGGTATGAGGTGAGA
GAGGCCAGGAGGTCTGGCTGCTCCCCCTCTAGACTTCGCAGGTAAAACCCAAGGTGT
GTGCCACGTAAAGGTTCAGAGGTT
>2108
TGTGAACCTGGTATAGGTATGAGGTGAGAGAGGCCAGGAGGTCTGGCTGCTCCCCCTC
TAGACTTCGCAGGTAAAACCCAAGGTGTGTGCCACGTAAAGGTTCAGAGGTTTTATAG

GCATCCAGATAGCACATGTGGGTC
>2109
ATAGGTATGAGGTGAGAGAGGCCAGGAGGTCTGGCTGCTCCCCCTCTAGACTTCGCAG
GTAAAACCCAAGGTGTGTGCCACGTAAAGGTTCAGAGGTTTTATAGGCATCCAGATAG
CACATGTGGGTCTCTGCCCCTGCT
>2110
GTGAGAGAGGCCAGGAGGTCTGGCTGCTCCCCCTCTAGACTTCGCAGGTAAAACCCA
AGGTGTGTGCCACGTAAAGGTTCAGAGGTTTTATAGGCATCCAGATAGCACATGTGGG
TCTCTGCCCCTGCTTGCAGCCTCCC
>2111
GAGGCCAGGAGGTCTGGCTGCTCCCCCTCTAGACTTCGCAGGTAAAACCCAAGGTGT
GTGCCACGTAAAGGTTCAGAGGTTTTATAGGCATCCAGATAGCACATGTGGGTCTCTGC
CCCTGCTTGCAGCCTCCCTGGGGC
>2112
GAGGTCTGGCTGCTCCCCCTCTAGACTTCGCAGGTAAAACCCAAGGTGTGTGCCACGT
AAAGGTTCAGAGGTTTTATAGGCATCCAGATAGCACATGTGGGTCTCTGCCCCTGCTTG
CAGCCTCCCTGGGGCAGCCAGCC
>2113
TTCGCAGGTAAAACCCAAGGTGTGTGCCACGTAAAGGTTCAGAGGTTTTATAGGCATC
CAGATAGCACATGTGGGTCTCTGCCCCTGCTTGCAGCCTCCCTGGGGCAGCCAGCCAG
GCTCAGGGCAGGAAGCTGCTGATT
>2114
GTCTCTGCCCCTGCTTGCAGCCTCCCTGGGGCAGCCAGCCAGGCTCAGGGCAGGAAG
CTGCTGATTCTGGGTCACTTGGCTGCTCTTGCTTGCTGCTGCTGTGATTGTTCCTACAGT
TAAGATCTTAGCTCCATAAGGGA
>2115
GCAGCCAGCCAGGCTCAGGGCAGGAAGCTGCTGATTCTGGGTCACTTGGCTGCTCTTG
CTTGCTGCTGCTGTGATTGTTCCTACAGTTAAGATCTTAGCTCCATAAGGGACATACGG
AGGTCATCTAGGTCATCTCATGG
>2116
AGCCAGGCTCAGGGCAGGAAGCTGCTGATTCTGGGTCACTTGGCTGCTCTTGCTTGCT
GCTGCTGTGATTGTTCCTACAGTTAAGATCTTAGCTCCATAAGGGACATACGGAGGTCA
TCTAGGTCATCTCATGGCTCTCA
>2117
CTGCTCTTGCTTGCTGCTGCTGTGATTGTTCCTACAGTTAAGATCTTAGCTCCATAAGGG
ACATACGGAGGTCATCTAGGTCATCTCATGGCTCTCAGGCAAGAAAAGTAGCATTATTG
CTCTCATGCCCACTTTATGGA
>2118
CTTGCTGCTGCTGTGATTGTTCCTACAGTTAAGATCTTAGCTCCATAAGGGACATACGG
AGGTCATCTAGGTCATCTCATGGCTCTCAGGCAAGAAAAGTAGCATTATTGCTCTCATG
CCCACTTTATGGATAAGTGACC
>2119
TTAAGATCTTAGCTCCATAAGGGACATACGGAGGTCATCTAGGTCATCTCATGGCTCTC
AGGCAAGAAAAGTAGCATTATTGCTCTCATGCCCACTTTATGGATAAGTGACCGAGGCC

TGCAGAGGCAGAGTGGCTATTC
>2120
ATCTAGGTCATCTCATGGCTCTCAGGCAAGAAAAGTAGCATTATTGCTCTCATGCCCAC
TTTATGGATAAGTGACCGAGGCCTGCAGAGGCAGAGTGGCTATTCCAGCCATTAAATGT
GGCTCCCTGCTATTCTGTTCCC
>2121
GGCAAGAAAAGTAGCATTATTGCTCTCATGCCCACTTTATGGATAAGTGACCGAGGCCT
GCAGAGGCAGAGTGGCTATTCCAGCCATTAAATGTGGCTCCCTGCTATTCTGTTCCCTG
CCAGGATATTCTGTGGATGGTT
>2122
CTCTCATGCCCACTTTATGGATAAGTGACCGAGGCCTGCAGAGGCAGAGTGGCTATTCC
AGCCATTAAATGTGGCTCCCTGCTATTCTGTTCCCTGCCAGGATATTCTGTGGATGGTTA
GGGGAGGGGTCTTACCTCAGC
>2123
GGATAAGTGACCGAGGCCTGCAGAGGCAGAGTGGCTATTCCAGCCATTAAATGTGGCT
CCCTGCTATTCTGTTCCCTGCCAGGATATTCTGTGGATGGTTAGGGGAGGGGTCTTACC
TCAGCTAACGAGGTGCATTTTCT
>2124
CAGAGGCAGAGTGGCTATTCCAGCCATTAAATGTGGCTCCCTGCTATTCTGTTCCCTGC
CAGGATATTCTGTGGATGGTTAGGGGAGGGGTCTTACCTCAGCTAACGAGGTGCATTTT
CTGTGGGGTGAGGCCATTCTTG
>2125
AGAGTGGCTATTCCAGCCATTAAATGTGGCTCCCTGCTATTCTGTTCCCTGCCAGGATAT
TCTGTGGATGGTTAGGGGAGGGGTCTTACCTCAGCTAACGAGGTGCATTTTCTGTGGG
GTGAGGCCATTCTTGTCACTTA
>2126
CCAGCCATTAAATGTGGCTCCCTGCTATTCTGTTCCCTGCCAGGATATTCTGTGGATGGT
TAGGGGAGGGGTCTTACCTCAGCTAACGAGGTGCATTTTCTGTGGGGTGAGGCCATTC
TTGTCACTTAGGTGTCTGAGGC
>2127
CCTGCTATTCTGTTCCCTGCCAGGATATTCTGTGGATGGTTAGGGGAGGGGTCTTACCT
CAGCTAACGAGGTGCATTTTCTGTGGGGTGAGGCCATTCTTGTCACTTAGGTGTCTGAG
GCCTGATGGTGTTGGGGAATGT
>2128
CTGCCAGGATATTCTGTGGATGGTTAGGGGAGGGGTCTTACCTCAGCTAACGAGGTGC
ATTTTCTGTGGGGTGAGGCCATTCTTGTCACTTAGGTGTCTGAGGCCTGATGGTGTTGG
GGAATGTCCTGGTTCTATTCCCA
>2129
TGTGGATGGTTAGGGGAGGGGTCTTACCTCAGCTAACGAGGTGCATTTTCTGTGGGGT
GAGGCCATTCTTGTCACTTAGGTGTCTGAGGCCTGATGGTGTTGGGGAATGTCCTGGTT
CTATTCCCAGGGCTGTAACGTGG
>2130
TTAGGGGAGGGGTCTTACCTCAGCTAACGAGGTGCATTTTCTGTGGGGTGAGGCCATT
CTTGTCACTTAGGTGTCTGAGGCCTGATGGTGTTGGGGAATGTCCTGGTTCTATTCCCA

GGGCTGTAACGTGGGGGAAGGAT
>2131
CCTCAGCTAACGAGGTGCATTTTCTGTGGGGTGAGGCCATTCTTGTCACTTAGGTGTCT
GAGGCCTGATGGTGTTGGGGAATGTCCTGGTTCTATTCCCAGGGCTGTAACGTGGGGG
AAGGATGGAGGAAGACAGAATGG
>2132
ATTTTCTGTGGGGTGAGGCCATTCTTGTCACTTAGGTGTCTGAGGCCTGATGGTGTTGG
GGAATGTCCTGGTTCTATTCCCAGGGCTGTAACGTGGGGGAAGGATGGAGGAAGACA
GAATGGCCTCTGGAACTTTTGGAT
>2133
GCCATTCTTGTCACTTAGGTGTCTGAGGCCTGATGGTGTTGGGGAATGTCCTGGTTCTA
TTCCCAGGGCTGTAACGTGGGGGAAGGATGGAGGAAGACAGAATGGCCTCTGGAACT
TTTGGATCTTTCTGAGTTGAGGCC
>2134
TCTTGTCACTTAGGTGTCTGAGGCCTGATGGTGTTGGGGAATGTCCTGGTTCTATTCCC
AGGGCTGTAACGTGGGGGAAGGATGGAGGAAGACAGAATGGCCTCTGGAACTTTTGG
ATCTTTCTGAGTTGAGGCCTTGTC
>2135
CCCAGGGCTGTAACGTGGGGGAAGGATGGAGGAAGACAGAATGGCCTCTGGAACTTT
TGGATCTTTCTGAGTTGAGGCCTTGTCTGGCCAACTCCGGGAGGCTGCTGGCATGGCA
AGCAGGGTAGGGATGCCTGGCCTAG
>2136
AACGTGGGGGAAGGATGGAGGAAGACAGAATGGCCTCTGGAACTTTTGGATCTTTCT
GAGTTGAGGCCTTGTCTGGCCAACTCCGGGAGGCTGCTGGCATGGCAAGCAGGGTAG
GGATGCCTGGCCTAGCCAGGGGAGCT
>2137
AACTTTTGGATCTTTCTGAGTTGAGGCCTTGTCTGGCCAACTCCGGGAGGCTGCTGGC
ATGGCAAGCAGGGTAGGGATGCCTGGCCTAGCCAGGGGAGCTCCCTCTCTCCCCTCTG
CTGCCCCTCTTTCCACCCCCATCA
>2138
TGCCTGGCCTAGCCAGGGGAGCTCCCTCTCTCCCCTCTGCTGCCCCTCTTTCCACCCCC
ATCATAAACACGTGTGGACTTTCTGGTGGTGAGAAGATGGTTTTGGAGGGCTGGGCTG
AGGTCTAGGCAGGCAAGAGCTTC
>2139
CCTGGCCTAGCCAGGGGAGCTCCCTCTCTCCCCTCTGCTGCCCCTCTTTCCACCCCCAT
CATAAACACGTGTGGACTTTCTGGTGGTGAGAAGATGGTTTTGGAGGGCTGGGCTGAG
GTCTAGGCAGGCAAGAGCTTCCA
>2140
GGAGCTCCCTCTCTCCCCTCTGCTGCCCCTCTTTCCACCCCCATCATAAACACGTGTGG
ACTTTCTGGTGGTGAGAAGATGGTTTTGGAGGGCTGGGCTGAGGTCTAGGCAGGCAA
GAGCTTCCAAATGTGTGGCTTTGA
>2141
TTCCACCCCCATCATAAACACGTGTGGACTTTCTGGTGGTGAGAAGATGGTTTTGGAG
GGCTGGGCTGAGGTCTAGGCAGGCAAGAGCTTCCAAATGTGTGGCTTTGATTCTCAGC

CTTTTCATCCCTCAATCCTGGGAG
>2142
GACTTTCTGGTGGTGAGAAGATGGTTTTGGAGGGCTGGGCTGAGGTCTAGGCAGGCA
AGAGCTTCCAAATGTGTGGCTTTGATTCTCAGCCTTTTCATCCCTCAATCCTGGGAGAC
TCATTTTAAAACTTAAAAAAGGAC
>2143
CTTTTCATCCCTCAATCCTGGGAGACTCATTTTAAAACTTAAAAAAGGACTTCATTCTAT
TGGATGCTATGTGGGCAGGCCCTGGGCTGTTGTAGAGAGGCCTGAAACCCACTTCAGT
TTCCTGGACAGGCTGGGAGTGT
>2144
TGGGAGACTCATTTTAAAACTTAAAAAAGGACTTCATTCTATTGGATGCTATGTGGGCA
GGCCCTGGGCTGTTGTAGAGAGGCCTGAAACCCACTTCAGTTTCCTGGACAGGCTGGG
AGTGTGTACCCATGGGTTCCCTG
>2145
GAGACTCATTTTAAAACTTAAAAAAGGACTTCATTCTATTGGATGCTATGTGGGCAGGC
CCTGGGCTGTTGTAGAGAGGCCTGAAACCCACTTCAGTTTCCTGGACAGGCTGGGAGT
GTGTACCCATGGGTTCCCTGCTG
>2146
GGACTTCATTCTATTGGATGCTATGTGGGCAGGCCCTGGGCTGTTGTAGAGAGGCCTGA
AACCCACTTCAGTTTCCTGGACAGGCTGGGAGTGTGTACCCATGGGTTCCCTGCTGCC
CTATTCCTGGAAGACACTAATAT
>2147
CTATGTGGGCAGGCCCTGGGCTGTTGTAGAGAGGCCTGAAACCCACTTCAGTTTCCTG
GACAGGCTGGGAGTGTGTACCCATGGGTTCCCTGCTGCCCTATTCCTGGAAGACACTA
ATATCCCTCCCAAACTCTTGGTTG
>2148
ATGTGGGCAGGCCCTGGGCTGTTGTAGAGAGGCCTGAAACCCACTTCAGTTTCCTGGA
CAGGCTGGGAGTGTGTACCCATGGGTTCCCTGCTGCCCTATTCCTGGAAGACACTAATA
TCCCTCCCAAACTCTTGGTTGGT
>2149
CCTGGGCTGTTGTAGAGAGGCCTGAAACCCACTTCAGTTTCCTGGACAGGCTGGGAGT
GTGTACCCATGGGTTCCCTGCTGCCCTATTCCTGGAAGACACTAATATCCCTCCCAAAC
TCTTGGTTGGTTTCTGCTTTTTT
>2150
GGGAGTGTGTACCCATGGGTTCCCTGCTGCCCTATTCCTGGAAGACACTAATATCCCTC
CCAAACTCTTGGTTGGTTTCTGCTTTTTTTCTGTAGCATTTTGTCTTTGTGGGAAATTTT
AAGAAACATATGTATGGCTTT
>2151
GTGTGTACCCATGGGTTCCCTGCTGCCCTATTCCTGGAAGACACTAATATCCCTCCCAA
ACTCTTGGTTGGTTTCTGCTTTTTTTCTGTAGCATTTTGTCTTTGTGGGAAATTTTAAGA
AACATATGTATGGCTTTTTGG
>2152
CCCTGCTGCCCTATTCCTGGAAGACACTAATATCCCTCCCAAACTCTTGGTTGGTTTCTG
CTTTTTTTCTGTAGCATTTTGTCTTTGTGGGAAATTTTAAGAAACATATGTATGGCTTTTT

GGTTTCCACTTATTTATTT
>2153
CAAACTCTTGGTTGGTTTCTGCTTTTTTTCTGTAGCATTTTGTCTTTGTGGGAAATTTTA
AGAAACATATGTATGGCTTTTTGGTTTCCACTTATTTATTTTCCCCTCTGAGTTTCAGATT
TCATCCTTCTCCACCCCTT
>2154
GGTTTCTGCTTTTTTTCTGTAGCATTTTGTCTTTGTGGGAAATTTTAAGAAACATATGTAT
GGCTTTTTGGTTTCCACTTATTTATTTTCCCCTCTGAGTTTCAGATTTCATCCTTCTCCAC
CCCTTCCCCCTGCCCTAA
>2155
GTCTTTGTGGGAAATTTTAAGAAACATATGTATGGCTTTTTGGTTTCCACTTATTTATTTT
CCCCTCTGAGTTTCAGATTTCATCCTTCTCCACCCCTTCCCCCTGCCCTAAGGTGGGGC
TAGTGCAAATCGCAGTGTGT
>2156
TTTCCACTTATTTATTTTCCCCTCTGAGTTTCAGATTTCATCCTTCTCCACCCCTTCCCCC
TGCCCTAAGGTGGGGCTAGTGCAAATCGCAGTGTGTGTGTAGTGGGATGGTTAAAGAG
CCGTACATCCTCACACCTGGT
>2157
ATTTATTTTCCCCTCTGAGTTTCAGATTTCATCCTTCTCCACCCCTTCCCCCTGCCCTAA
GGTGGGGCTAGTGCAAATCGCAGTGTGTGTGTAGTGGGATGGTTAAAGAGCCGTACAT
CCTCACACCTGGTGACGCAGAC
>2158
GTTTCAGATTTCATCCTTCTCCACCCCTTCCCCCTGCCCTAAGGTGGGGCTAGTGCAAA
TCGCAGTGTGTGTGTAGTGGGATGGTTAAAGAGCCGTACATCCTCACACCTGGTGACG
CAGACTAAGGCAATTAACCTTCT
>2159
TTCAGATTTCATCCTTCTCCACCCCTTCCCCCTGCCCTAAGGTGGGGCTAGTGCAAATC
GCAGTGTGTGTGTAGTGGGATGGTTAAAGAGCCGTACATCCTCACACCTGGTGACGCA
GACTAAGGCAATTAACCTTCTTT
>2160
AGATTTCATCCTTCTCCACCCCTTCCCCCTGCCCTAAGGTGGGGCTAGTGCAAATCGCA
GTGTGTGTGTAGTGGGATGGTTAAAGAGCCGTACATCCTCACACCTGGTGACGCAGAC
TAAGGCAATTAACCTTCTTTAGT
>2161
TCCTTCTCCACCCCTTCCCCCTGCCCTAAGGTGGGGCTAGTGCAAATCGCAGTGTGTGT
GTAGTGGGATGGTTAAAGAGCCGTACATCCTCACACCTGGTGACGCAGACTAAGGCAA
TTAACCTTCTTTAGTAAAGACCC
>2162
CCCTTCCCCCTGCCCTAAGGTGGGGCTAGTGCAAATCGCAGTGTGTGTGTAGTGGGAT
GGTTAAAGAGCCGTACATCCTCACACCTGGTGACGCAGACTAAGGCAATTAACCTTCT
TTAGTAAAGACCCTTTCAGCTGGG
>2163
TAGTGGGATGGTTAAAGAGCCGTACATCCTCACACCTGGTGACGCAGACTAAGGCAAT
TAACCTTCTTTAGTAAAGACCCTTTCAGCTGGGTGCGGTGGGAGGCACTTCGGGAGGC

CGAGGTGGAAGGATTGCTTGAGCC
>2164
CGTACATCCTCACACCTGGTGACGCAGACTAAGGCAATTAACCTTCTTTAGTAAAGACC
CTTTCAGCTGGGTGCGGTGGGAGGCACTTCGGGAGGCCGAGGTGGAAGGATTGCTTG
AGCCCTGGAGTTGGAGACCAGCTT
>2165
AAGGCAATTAACCTTCTTTAGTAAAGACCCTTTCAGCTGGGTGCGGTGGGAGGCACTT
CGGGAGGCCGAGGTGGAAGGATTGCTTGAGCCCTGGAGTTGGAGACCAGCTTGGGCA
GCATGGTGAAACCACCTCTCTAAAA
>2166
GACCCTTTCAGCTGGGTGCGGTGGGAGGCACTTCGGGAGGCCGAGGTGGAAGGATTG
CTTGAGCCCTGGAGTTGGAGACCAGCTTGGGCAGCATGGTGAAACCACCTCTCTAAAA
AAATATAAAAATTAACGGGACGTTG
>2167
AGGCACTTCGGGAGGCCGAGGTGGAAGGATTGCTTGAGCCCTGGAGTTGGAGACCAG
CTTGGGCAGCATGGTGAAACCACCTCTCTAAAAAAATATAAAAATTAACGGGACGTTG
TGGCATGCACCTATAGTCCCAGCTA
>2168
TGGAGTTGGAGACCAGCTTGGGCAGCATGGTGAAACCACCTCTCTAAAAAAATATAAA
AATTAACGGGACGTTGTGGCATGCACCTATAGTCCCAGCTACTTGGGAGGCTGGGGCG
CGAGAAGTGCTTGAACCTGAGAGA
>2169
AGTTGGAGACCAGCTTGGGCAGCATGGTGAAACCACCTCTCTAAAAAAATATAAAAAT
TAACGGGACGTTGTGGCATGCACCTATAGTCCCAGCTACTTGGGAGGCTGGGGCGCGA
GAAGTGCTTGAACCTGAGAGATGG
>2170
TATAAAAATTAACGGGACGTTGTGGCATGCACCTATAGTCCCAGCTACTTGGGAGGCTG
GGGCGCGAGAAGTGCTTGAACCTGAGAGATGGAGACTGCGGTGAGGCGAGATTGCAC
CACTGCACTTCAGCTAGGTGACAG
>2171
CACCTATAGTCCCAGCTACTTGGGAGGCTGGGGCGCGAGAAGTGCTTGAACCTGAGAG
ATGGAGACTGCGGTGAGGCGAGATTGCACCACTGCACTTCAGCTAGGTGACAGAGTG
GGACCCTGTCTCAAAACAAAACAAA
>2172
GCGAGAAGTGCTTGAACCTGAGAGATGGAGACTGCGGTGAGGCGAGATTGCACCACT
GCACTTCAGCTAGGTGACAGAGTGGGACCCTGTCTCAAAACAAAACAAAACAACAAC
AAAAAAAGCATGTTTTCCTGTCTTTC
>2173
TGCTTGAACCTGAGAGATGGAGACTGCGGTGAGGCGAGATTGCACCACTGCACTTCA
GCTAGGTGACAGAGTGGGACCCTGTCTCAAAACAAAACAAAACAACAACAAAAAAA
GCATGTTTTCCTGTCTTTCCCTTCCTC
>2174
CTGCACTTCAGCTAGGTGACAGAGTGGGACCCTGTCTCAAAACAAAACAAAACAACA
ACAAAAAAAGCATGTTTTCCTGTCTTTCCCTTCCTCTCCTCCACCCCCTGGCATGAACT

CTCAACTCTTAGCCTGTGGATGTC
>2175
CAGCTAGGTGACAGAGTGGGACCCTGTCTCAAAACAAAACAAAACAACAACAAAAA
AAGCATGTTTTCCTGTCTTTCCCTTCCTCTCCTCCACCCCCTGGCATGAACTCTCAACTC
TTAGCCTGTGGATGTCCGTTTCTA
>2176
AAAAAGCATGTTTTCCTGTCTTTCCCTTCCTCTCCTCCACCCCCTGGCATGAACTCTCA
ACTCTTAGCCTGTGGATGTCCGTTTCTATAAATGCTGGTGCTCGGTGGTCACCGTAGAC
GTCCGAGCAGCCCCTGGGGCTT
>2177
CATGTTTTCCTGTCTTTCCCTTCCTCTCCTCCACCCCCTGGCATGAACTCTCAACTCTTA
GCCTGTGGATGTCCGTTTCTATAAATGCTGGTGCTCGGTGGTCACCGTAGACGTCCGAG
CAGCCCCTGGGGCTTTGGCTT
>2178
CCTCCACCCCCTGGCATGAACTCTCAACTCTTAGCCTGTGGATGTCCGTTTCTATAAATG
CTGGTGCTCGGTGGTCACCGTAGACGTCCGAGCAGCCCCTGGGGCTTTGGCTTGTGTT
TTCTCTGTACTTGTGAGGGTGG
>2179
CCACCCCCTGGCATGAACTCTCAACTCTTAGCCTGTGGATGTCCGTTTCTATAAATGCT
GGTGCTCGGTGGTCACCGTAGACGTCCGAGCAGCCCCTGGGGCTTTGGCTTGTGTTTT
CTCTGTACTTGTGAGGGTGGTTG
>2180
CCTGGCATGAACTCTCAACTCTTAGCCTGTGGATGTCCGTTTCTATAAATGCTGGTGCTC
GGTGGTCACCGTAGACGTCCGAGCAGCCCCTGGGGCTTTGGCTTGTGTTTTCTCTGTA
CTTGTGAGGGTGGTTGCCACTT
>2181
ATGAACTCTCAACTCTTAGCCTGTGGATGTCCGTTTCTATAAATGCTGGTGCTCGGTGG
TCACCGTAGACGTCCGAGCAGCCCCTGGGGCTTTGGCTTGTGTTTTCTCTGTACTTGTG
AGGGTGGTTGCCACTTCTGGCC
>2182
GTCCGTTTCTATAAATGCTGGTGCTCGGTGGTCACCGTAGACGTCCGAGCAGCCCCTGG
GGCTTTGGCTTGTGTTTTCTCTGTACTTGTGAGGGTGGTTGCCACTTCTGGCCTTCTTT
GTGCTTTCTTTTTCTTTCCTTT
>2183
CTCGGTGGTCACCGTAGACGTCCGAGCAGCCCCTGGGGCTTTGGCTTGTGTTTTCTCT
GTACTTGTGAGGGTGGTTGCCACTTCTGGCCTTCTTTGTGCTTTCTTTTTCTTTCCTTTC
CCCAATCTTTTGTTCCTCCCTC
>2184
GGTGGTCACCGTAGACGTCCGAGCAGCCCCTGGGGCTTTGGCTTGTGTTTTCTCTGTA
CTTGTGAGGGTGGTTGCCACTTCTGGCCTTCTTTGTGCTTTCTTTTTCTTTCCTTTCCCC
AATCTTTTGTTCCTCCCTCCCT
>2185
GCAGCCCCTGGGGCTTTGGCTTGTGTTTTCTCTGTACTTGTGAGGGTGGTTGCCACTTC
TGGCCTTCTTTGTGCTTTCTTTTTCTTTCCTTTCCCCAATCTTTTGTTCCTCCCTCCCTCC

CTCCCTCCCTTCCTCCCTTC
>2186
TACTTGTGAGGGTGGTTGCCACTTCTGGCCTTCTTTGTGCTTTCTTTTTCTTTCCTTTCC
CCAATCTTTTGTTCCTCCCTCCCTCCCTCCCTCCCTTCCTCCCTTCCTTCCTTCTGCTTTC
CTGTTTAAAGTATATGGCT
>2187
CCTTTCCCCAATCTTTTGTTCCTCCCTCCCTCCCTCCCTCCCTTCCTCCCTTCCTTCCTTC
TGCTTTCCTGTTTAAAGTATATGGCTGCACCTAGCATGACACAGTTCCTATTTATTCCTC
AGACTTGCTGGGACAGTCT
>2188
CCAATCTTTTGTTCCTCCCTCCCTCCCTCCCTCCCTTCCTCCCTTCCTTCCTTCTGCTTTC
CTGTTTAAAGTATATGGCTGCACCTAGCATGACACAGTTCCTATTTATTCCTCAGACTTG
CTGGGACAGTCTCCTCTGT
>2189
TCCCTCCCTTCCTCCCTTCCTTCCTTCTGCTTTCCTGTTTAAAGTATATGGCTGCACCTA
GCATGACACAGTTCCTATTTATTCCTCAGACTTGCTGGGACAGTCTCCTCTGTATGCGG
CGACTGAGGGTGGGTTCCAGT
>2190
TCCTGTTTAAAGTATATGGCTGCACCTAGCATGACACAGTTCCTATTTATTCCTCAGACT
TGCTGGGACAGTCTCCTCTGTATGCGGCGACTGAGGGTGGGTTCCAGTGCCCCAGCCC
ACTCCCCTTCCCCTTGTTTTTG
>2191
AAGTATATGGCTGCACCTAGCATGACACAGTTCCTATTTATTCCTCAGACTTGCTGGGAC
AGTCTCCTCTGTATGCGGCGACTGAGGGTGGGTTCCAGTGCCCCAGCCCACTCCCCTT
CCCCTTGTTTTTGTCCACTTGG
>2192
TAGCATGACACAGTTCCTATTTATTCCTCAGACTTGCTGGGACAGTCTCCTCTGTATGCG
GCGACTGAGGGTGGGTTCCAGTGCCCCAGCCCACTCCCCTTCCCCTTGTTTTTGTCCA
CTTGGCTGGACAAAGAGAACAA
>2193
TGGGACAGTCTCCTCTGTATGCGGCGACTGAGGGTGGGTTCCAGTGCCCCAGCCCACT
CCCCTTCCCCTTGTTTTTGTCCACTTGGCTGGACAAAGAGAACAAAACCAAATGGCCT
CCCCTCCCCCTCTCCTAAACCGGT
>2194
AGTCTCCTCTGTATGCGGCGACTGAGGGTGGGTTCCAGTGCCCCAGCCCACTCCCCTT
CCCCTTGTTTTTGTCCACTTGGCTGGACAAAGAGAACAAAACCAAATGGCCTCCCCTC
CCCCTCTCCTAAACCGGTCAAGCA
>2195
TTGTTTTTGTCCACTTGGCTGGACAAAGAGAACAAAACCAAATGGCCTCCCCTCCCCC
TCTCCTAAACCGGTCAAGCAGTGGTATTCTGGGATGATTTTCCCTGCAACCATTTAAAC
AAGTGCTTTGATGCTTACGAGTC
>2196
GTCCACTTGGCTGGACAAAGAGAACAAAACCAAATGGCCTCCCCTCCCCCTCTCCTAA
ACCGGTCAAGCAGTGGTATTCTGGGATGATTTTCCCTGCAACCATTTAAACAAGTGCTT

TGATGCTTACGAGTCATTGGAAT
>2197
CCCTCCCCCTCTCCTAAACCGGTCAAGCAGTGGTATTCTGGGATGATTTTCCCTGCAAC
CATTTAAACAAGTGCTTTGATGCTTACGAGTCATTGGAATGACCAGGCCTGTGCGCCCC
TTCTCACTGACACCCTTAGACC
>2198
CCGGTCAAGCAGTGGTATTCTGGGATGATTTTCCCTGCAACCATTTAAACAAGTGCTTT
GATGCTTACGAGTCATTGGAATGACCAGGCCTGTGCGCCCCTTCTCACTGACACCCTTA
GACCAGGCTGGGCCTCACCCTT
>2199
GGGATGATTTTCCCTGCAACCATTTAAACAAGTGCTTTGATGCTTACGAGTCATTGGAA
TGACCAGGCCTGTGCGCCCCTTCTCACTGACACCCTTAGACCAGGCTGGGCCTCACCC
TTTTGTCTTGCTGCCCCTCGGTG
>2200
CATTGGAATGACCAGGCCTGTGCGCCCCTTCTCACTGACACCCTTAGACCAGGCTGGG
CCTCACCCTTTTGTCTTGCTGCCCCTCGGTGCCCAGTGGGCGTGGGCAGTAGGACTGA
GCAGAGGGGCCGAGGCTGTGCTGG
>2201
CCTGTGCGCCCCTTCTCACTGACACCCTTAGACCAGGCTGGGCCTCACCCTTTTGTCTT
GCTGCCCCTCGGTGCCCAGTGGGCGTGGGCAGTAGGACTGAGCAGAGGGGCCGAGGC
TGTGCTGGCCGGCAGCTCTGTTAC
>2202
TCTCACTGACACCCTTAGACCAGGCTGGGCCTCACCCTTTTGTCTTGCTGCCCCTCGGT
GCCCAGTGGGCGTGGGCAGTAGGACTGAGCAGAGGGGCCGAGGCTGTGCTGGCCGGC
AGCTCTGTTACAGTAGAGTGGAAG
>2203
GACACCCTTAGACCAGGCTGGGCCTCACCCTTTTGTCTTGCTGCCCCTCGGTGCCCAG
TGGGCGTGGGCAGTAGGACTGAGCAGAGGGGCCGAGGCTGTGCTGGCCGGCAGCTCT
GTTACAGTAGAGTGGAAGGAGGTCC
>2204
CCCTTTTGTCTTGCTGCCCCTCGGTGCCCAGTGGGCGTGGGCAGTAGGACTGAGCAGA
GGGGCCGAGGCTGTGCTGGCCGGCAGCTCTGTTACAGTAGAGTGGAAGGAGGTCCCG
GCCAGAGGGGCAGGTCAAACAGGCC
>2205
CCTCGGTGCCCAGTGGGCGTGGGCAGTAGGACTGAGCAGAGGGGCCGAGGCTGTGCT
GGCCGGCAGCTCTGTTACAGTAGAGTGGAAGGAGGTCCCGGCCAGAGGGGCAGGTCA
AACAGGCCTCTTTTATTTACAGTGCT
>2206
AGTGGGCGTGGGCAGTAGGACTGAGCAGAGGGGCCGAGGCTGTGCTGGCCGGCAGCT
CTGTTACAGTAGAGTGGAAGGAGGTCCCGGCCAGAGGGGCAGGTCAAACAGGCCTCT
TTTATTTACAGTGCTCTCTTTCACGG
>2207
GGCAGTAGGACTGAGCAGAGGGGCCGAGGCTGTGCTGGCCGGCAGCTCTGTTACAGT
AGAGTGGAAGGAGGTCCCGGCCAGAGGGGCAGGTCAAACAGGCCTCTTTTATTTACA

GTGCTCTCTTTCACGGCCTCACTGAA
>2208
GGGGCCGAGGCTGTGCTGGCCGGCAGCTCTGTTACAGTAGAGTGGAAGGAGGTCCCG
GCCAGAGGGGCAGGTCAAACAGGCCTCTTTTATTTACAGTGCTCTCTTTCACGGCCTC
ACTGAAACAGGAGACCCGAGAGGCT
>2209
GCTCTGTTACAGTAGAGTGGAAGGAGGTCCCGGCCAGAGGGGCAGGTCAAACAGGCC
TCTTTTATTTACAGTGCTCTCTTTCACGGCCTCACTGAAACAGGAGACCCGAGAGGCTC
CGGTGAAAGGGCACTGTGTTTGTG
>2210
AAACAGGCCTCTTTTATTTACAGTGCTCTCTTTCACGGCCTCACTGAAACAGGAGACC
CGAGAGGCTCCGGTGAAAGGGCACTGTGTTTGTGCATTCAGGCCTTCCAGGGCTGGCT
ACAAAGGTGGGGGCACCCGGAGCA
>2211
ATTTACAGTGCTCTCTTTCACGGCCTCACTGAAACAGGAGACCCGAGAGGCTCCGGTG
AAAGGGCACTGTGTTTGTGCATTCAGGCCTTCCAGGGCTGGCTACAAAGGTGGGGGC
ACCCGGAGCACTGAAGGGGCTTTTT
>2212
ACAGTGCTCTCTTTCACGGCCTCACTGAAACAGGAGACCCGAGAGGCTCCGGTGAAA
GGGCACTGTGTTTGTGCATTCAGGCCTTCCAGGGCTGGCTACAAAGGTGGGGGCACCC
GGAGCACTGAAGGGGCTTTTTGTCA
>2213
GAGACCCGAGAGGCTCCGGTGAAAGGGCACTGTGTTTGTGCATTCAGGCCTTCCAGG
GCTGGCTACAAAGGTGGGGGCACCCGGAGCACTGAAGGGGCTTTTTGTCATTGGCAG
TTTGGCTTAGGGGAGAAGCGAGGGAG
>2214
GTTTGTGCATTCAGGCCTTCCAGGGCTGGCTACAAAGGTGGGGGCACCCGGAGCACTG
AAGGGGCTTTTTGTCATTGGCAGTTTGGCTTAGGGGAGAAGCGAGGGAGAGCCAGTG
TGGGAGCCGCCTCTAGGATTGTGAG
>2215
TCAGGCCTTCCAGGGCTGGCTACAAAGGTGGGGGCACCCGGAGCACTGAAGGGGCTT
TTTGTCATTGGCAGTTTGGCTTAGGGGAGAAGCGAGGGAGAGCCAGTGTGGGAGCCG
CCTCTAGGATTGTGAGCAAGTGCCTG
>2216
GGCACCCGGAGCACTGAAGGGGCTTTTTGTCATTGGCAGTTTGGCTTAGGGGAGAAGC
GAGGGAGAGCCAGTGTGGGAGCCGCCTCTAGGATTGTGAGCAAGTGCCTGTTTAGAC
AGCTCCACGCTGCTCCTTAAAACCT
>2217
TTTTTGTCATTGGCAGTTTGGCTTAGGGGAGAAGCGAGGGAGAGCCAGTGTGGGAGC
CGCCTCTAGGATTGTGAGCAAGTGCCTGTTTAGACAGCTCCACGCTGCTCCTTAAAAC
CTATTTTGTGGTCTGGGGTTGGGGC
>2218
ATTGGCAGTTTGGCTTAGGGGAGAAGCGAGGGAGAGCCAGTGTGGGAGCCGCCTCTA
GGATTGTGAGCAAGTGCCTGTTTAGACAGCTCCACGCTGCTCCTTAAAACCTATTTTGT

GGTCTGGGGTTGGGGCGGGGGCAC
>2219
GGAGCCGCCTCTAGGATTGTGAGCAAGTGCCTGTTTAGACAGCTCCACGCTGCTCCTT
AAAACCTATTTTGTGGTCTGGGGTTGGGGCGGGGGCACACTGCCTCGATCCTCTTGTT
GTGTGGAGCAAAGTGATTCCTCTC
>2220
GCCGCCTCTAGGATTGTGAGCAAGTGCCTGTTTAGACAGCTCCACGCTGCTCCTTAAA
ACCTATTTTGTGGTCTGGGGTTGGGGCGGGGGCACACTGCCTCGATCCTCTTGTTGTGT
GGAGCAAAGTGATTCCTCTCTTC
>2221
TCCACGCTGCTCCTTAAAACCTATTTTGTGGTCTGGGGTTGGGGCGGGGGCACACTGC
CTCGATCCTCTTGTTGTGTGGAGCAAAGTGATTCCTCTCTTCTTGTGTTCTCCAGATGG
TTCCGTTGTCCTCACATCCATTG
>2222
AAAACCTATTTTGTGGTCTGGGGTTGGGGCGGGGGCACACTGCCTCGATCCTCTTGTT
GTGTGGAGCAAAGTGATTCCTCTCTTCTTGTGTTCTCCAGATGGTTCCGTTGTCCTCAC
ATCCATTGTGGTTGAGGATACAG
>2223
CTGGGGTTGGGGCGGGGGCACACTGCCTCGATCCTCTTGTTGTGTGGAGCAAAGTGAT
TCCTCTCTTCTTGTGTTCTCCAGATGGTTCCGTTGTCCTCACATCCATTGTGGTTGAGGA
TACAGTGCAGAGAAGACCCCGC
>2224
GGGGCACACTGCCTCGATCCTCTTGTTGTGTGGAGCAAAGTGATTCCTCTCTTCTTGTG
TTCTCCAGATGGTTCCGTTGTCCTCACATCCATTGTGGTTGAGGATACAGTGCAGAGAA
GACCCCGCTGTCCTCTGCTCCC
>2225
TGTTGTGTGGAGCAAAGTGATTCCTCTCTTCTTGTGTTCTCCAGATGGTTCCGTTGTCC
TCACATCCATTGTGGTTGAGGATACAGTGCAGAGAAGACCCCGCTGTCCTCTGCTCCC
CCTCCTTTTCTCTCCAAAGAGAA
>2226
TGTGTGGAGCAAAGTGATTCCTCTCTTCTTGTGTTCTCCAGATGGTTCCGTTGTCCTCA
CATCCATTGTGGTTGAGGATACAGTGCAGAGAAGACCCCGCTGTCCTCTGCTCCCCCTC
CTTTTCTCTCCAAAGAGAAGTT
>2227
AGTGATTCCTCTCTTCTTGTGTTCTCCAGATGGTTCCGTTGTCCTCACATCCATTGTGGT
TGAGGATACAGTGCAGAGAAGACCCCGCTGTCCTCTGCTCCCCCTCCTTTTCTCTCCAA
AGAGAAGTTTCAGGGACAGCA
>2228
TGTTCTCCAGATGGTTCCGTTGTCCTCACATCCATTGTGGTTGAGGATACAGTGCAGAG
AAGACCCCGCTGTCCTCTGCTCCCCCTCCTTTTCTCTCCAAAGAGAAGTTTCAGGGAC
AGCAATAGACTTCTGTGGGGCCT
>2229
GTGGTTGAGGATACAGTGCAGAGAAGACCCCGCTGTCCTCTGCTCCCCCTCCTTTTCTC
TCCAAAGAGAAGTTTCAGGGACAGCAATAGACTTCTGTGGGGCCTTTGTTCCTTGTAG

CTTTCATTTTTCAATTCTTCTTC
>2230
GACCCCGCTGTCCTCTGCTCCCCCTCCTTTTCTCTCCAAAGAGAAGTTTCAGGGACAG
CAATAGACTTCTGTGGGGCCTTTGTTCCTTGTAGCTTTCATTTTTCAATTCTTCTTCTCC
CTGTATTATCTTGCTAGGCCTT
>2231
TCCCCCTCCTTTTCTCTCCAAAGAGAAGTTTCAGGGACAGCAATAGACTTCTGTGGGG
CCTTTGTTCCTTGTAGCTTTCATTTTTCAATTCTTCTTCTCCCTGTATTATCTTGCTAGGC
CTTAGTTTGTGCATCTCTAAA
>2232
AGGGACAGCAATAGACTTCTGTGGGGCCTTTGTTCCTTGTAGCTTTCATTTTTCAATTCT
TCTTCTCCCTGTATTATCTTGCTAGGCCTTAGTTTGTGCATCTCTAAAATGGGCTGGGAG
GTGCTAGCCCATACCTAATA
>2233
TGGGGCCTTTGTTCCTTGTAGCTTTCATTTTTCAATTCTTCTTCTCCCTGTATTATCTTGC
TAGGCCTTAGTTTGTGCATCTCTAAAATGGGCTGGGAGGTGCTAGCCCATACCTAATATA
TAGGGCCGTCGTAAGGCTT
>2234
TTTCAATTCTTCTTCTCCCTGTATTATCTTGCTAGGCCTTAGTTTGTGCATCTCTAAAATG
GGCTGGGAGGTGCTAGCCCATACCTAATATATAGGGCCGTCGTAAGGCTTAGGTCCCAT
AACTCATGGGGTTGGCTTGC
>2235
TGCTAGGCCTTAGTTTGTGCATCTCTAAAATGGGCTGGGAGGTGCTAGCCCATACCTAA
TATATAGGGCCGTCGTAAGGCTTAGGTCCCATAACTCATGGGGTTGGCTTGCACAGGGC
TCTCTCCTTTCAGACTGGTCAG
>2236
GGGCTGGGAGGTGCTAGCCCATACCTAATATATAGGGCCGTCGTAAGGCTTAGGTCCCA
TAACTCATGGGGTTGGCTTGCACAGGGCTCTCTCCTTTCAGACTGGTCAGGCCATGGTC
CTTTTATGAGTCTGAAGAGCCA
>2237
GGGCCGTCGTAAGGCTTAGGTCCCATAACTCATGGGGTTGGCTTGCACAGGGCTCTCT
CCTTTCAGACTGGTCAGGCCATGGTCCTTTTATGAGTCTGAAGAGCCAAAATGAAGAC
CTCACTTTCTAGTATCCCCAATCT
>2238
AGGCTTAGGTCCCATAACTCATGGGGTTGGCTTGCACAGGGCTCTCTCCTTTCAGACTG
GTCAGGCCATGGTCCTTTTATGAGTCTGAAGAGCCAAAATGAAGACCTCACTTTCTAGT
ATCCCCAATCTTCCTCCCACCT
>2239
CATAACTCATGGGGTTGGCTTGCACAGGGCTCTCTCCTTTCAGACTGGTCAGGCCATGG
TCCTTTTATGAGTCTGAAGAGCCAAAATGAAGACCTCACTTTCTAGTATCCCCAATCTT
CCTCCCACCTTTCCCTGGCTCT
>2240
TCCTTTCAGACTGGTCAGGCCATGGTCCTTTTATGAGTCTGAAGAGCCAAAATGAAGA
CCTCACTTTCTAGTATCCCCAATCTTCCTCCCACCTTTCCCTGGCTCTTGAGGGTTCTTT

GGGCATGTTCCCCTCTGGTCTC
>2241
AAGAGCCAAAATGAAGACCTCACTTTCTAGTATCCCCAATCTTCCTCCCACCTTTCCCT
GGCTCTTGAGGGTTCTTTGGGCATGTTCCCCTCTGGTCTCCATTATTATATAGGGAAGAG
AAATTTTCCAGAAGAATATTT
>2242
AAGACCTCACTTTCTAGTATCCCCAATCTTCCTCCCACCTTTCCCTGGCTCTTGAGGGTT
CTTTGGGCATGTTCCCCTCTGGTCTCCATTATTATATAGGGAAGAGAAATTTTCCAGAAG
AATATTTGAAGGATTAAAGT
>2243
ATGTTCCCCTCTGGTCTCCATTATTATATAGGGAAGAGAAATTTTCCAGAAGAATATTTG
AAGGATTAAAGTTTGAGGTTGTAGAATATGTGAAAACACACACACACCACACCACACC
CCAAAATCACCCTTTTAAAGAA
>2244
CCTCTGGTCTCCATTATTATATAGGGAAGAGAAATTTTCCAGAAGAATATTTGAAGGATT
AAAGTTTGAGGTTGTAGAATATGTGAAAACACACACACACCACACCACACCCCAAAAT
CACCCTTTTAAAGAAGATTAAA
>2245
ATTATTATATAGGGAAGAGAAATTTTCCAGAAGAATATTTGAAGGATTAAAGTTTGAGG
TTGTAGAATATGTGAAAACACACACACACCACACCACACCCCAAAATCACCCTTTTAA
AGAAGATTAAAAAACCACCAGTG
>2246
TATGTGAAAACACACACACACCACACCACACCCCAAAATCACCCTTTTAAAGAAGATT
AAAAAACCACCAGTGCCTGTCCTACATTGGTAAACTCTGGCAAGCCTCATAATGTTGA
CTTTACTGACCTGCCTGACTTCAG
>2247
AAAACACACACACACCACACCACACCCCAAAATCACCCTTTTAAAGAAGATTAAAAA
ACCACCAGTGCCTGTCCTACATTGGTAAACTCTGGCAAGCCTCATAATGTTGACTTTAC
TGACCTGCCTGACTTCAGTGGCCG
>2248
CCCTTTTAAAGAAGATTAAAAAACCACCAGTGCCTGTCCTACATTGGTAAACTCTGGC
AAGCCTCATAATGTTGACTTTACTGACCTGCCTGACTTCAGTGGCCGAGTCTACATGGG
GTGTTAAGGCCCCACGTGTTTTT
>2249
AGTGCCTGTCCTACATTGGTAAACTCTGGCAAGCCTCATAATGTTGACTTTACTGACCT
GCCTGACTTCAGTGGCCGAGTCTACATGGGGTGTTAAGGCCCCACGTGTTTTTGCCTGT
GCCCTTTAATTGTATCTTCAGG
>2250
TCCTACATTGGTAAACTCTGGCAAGCCTCATAATGTTGACTTTACTGACCTGCCTGACT
TCAGTGGCCGAGTCTACATGGGGTGTTAAGGCCCCACGTGTTTTTGCCTGTGCCCTTTA
ATTGTATCTTCAGGACCATATT
>2251
CTCATAATGTTGACTTTACTGACCTGCCTGACTTCAGTGGCCGAGTCTACATGGGGTGT
TAAGGCCCCACGTGTTTTTGCCTGTGCCCTTTAATTGTATCTTCAGGACCATATTTTCAG

AACCAGAAATCACAGCCACAG
>2252
ACTTTACTGACCTGCCTGACTTCAGTGGCCGAGTCTACATGGGGTGTTAAGGCCCCAC
GTGTTTTTGCCTGTGCCCTTTAATTGTATCTTCAGGACCATATTTTCAGAACCAGAAATC
ACAGCCACAGTTTGGCAGTGGC
>2253
GCCTGACTTCAGTGGCCGAGTCTACATGGGGTGTTAAGGCCCCACGTGTTTTTGCCTGT
GCCCTTTAATTGTATCTTCAGGACCATATTTTCAGAACCAGAAATCACAGCCACAGTTT
GGCAGTGGCACAGAATGGTTCA
>2254
CGTGTTTTTGCCTGTGCCCTTTAATTGTATCTTCAGGACCATATTTTCAGAACCAGAAAT
CACAGCCACAGTTTGGCAGTGGCACAGAATGGTTCAATTTAGGCCTATCCCAGAGAGG
CTTGGGTTGTCTGTGAAGTTCT
>2255
TGCCTGTGCCCTTTAATTGTATCTTCAGGACCATATTTTCAGAACCAGAAATCACAGCC
ACAGTTTGGCAGTGGCACAGAATGGTTCAATTTAGGCCTATCCCAGAGAGGCTTGGGT
TGTCTGTGAAGTTCTGGGTGGTC
>2256
TAATTGTATCTTCAGGACCATATTTTCAGAACCAGAAATCACAGCCACAGTTTGGCAGT
GGCACAGAATGGTTCAATTTAGGCCTATCCCAGAGAGGCTTGGGTTGTCTGTGAAGTT
CTGGGTGGTCTGTATTGTGGAGA
>2257
AAATCACAGCCACAGTTTGGCAGTGGCACAGAATGGTTCAATTTAGGCCTATCCCAGA
GAGGCTTGGGTTGTCTGTGAAGTTCTGGGTGGTCTGTATTGTGGAGAGGTGAACACCG
TATTGCTGGGGGTGTGGAGGGGGG
>2258
CACAGCCACAGTTTGGCAGTGGCACAGAATGGTTCAATTTAGGCCTATCCCAGAGAGG
CTTGGGTTGTCTGTGAAGTTCTGGGTGGTCTGTATTGTGGAGAGGTGAACACCGTATTG
CTGGGGGTGTGGAGGGGGGATGT
>2259
CCACAGTTTGGCAGTGGCACAGAATGGTTCAATTTAGGCCTATCCCAGAGAGGCTTGG
GTTGTCTGTGAAGTTCTGGGTGGTCTGTATTGTGGAGAGGTGAACACCGTATTGCTGG
GGGTGTGGAGGGGGGATGTTGCTG
>2260
TGGCACAGAATGGTTCAATTTAGGCCTATCCCAGAGAGGCTTGGGTTGTCTGTGAAGT
TCTGGGTGGTCTGTATTGTGGAGAGGTGAACACCGTATTGCTGGGGGTGTGGAGGGGG
GATGTTGCTGTTTCCCTGCTACAG
>2261
AGGCTTGGGTTGTCTGTGAAGTTCTGGGTGGTCTGTATTGTGGAGAGGTGAACACCGT
ATTGCTGGGGGTGTGGAGGGGGGATGTTGCTGTTTCCCTGCTACAGAAAGGCCAGGCT
TCCTTTTCAGCCTTTTGTTCCCCG
>2262
CTGTGAAGTTCTGGGTGGTCTGTATTGTGGAGAGGTGAACACCGTATTGCTGGGGGTG
TGGAGGGGGGATGTTGCTGTTTCCCTGCTACAGAAAGGCCAGGCTTCCTTTTCAGCCT

TTTGTTCCCCGATTCCCCTCTCTT
>2263
AGTTCTGGGTGGTCTGTATTGTGGAGAGGTGAACACCGTATTGCTGGGGGTGTGGAGG
GGGGATGTTGCTGTTTCCCTGCTACAGAAAGGCCAGGCTTCCTTTTCAGCCTTTTGTTC
CCCGATTCCCCTCTCTTCCTCCA
>2264
CTGGGGGTGTGGAGGGGGGATGTTGCTGTTTCCCTGCTACAGAAAGGCCAGGCTTCCT
TTTCAGCCTTTTGTTCCCCGATTCCCCTCTCTTCCTCCACTACACTCAGCCCTGCTGCTG
CTTCCAAGGGACAACTCCCTCT
>2265
CCCCGATTCCCCTCTCTTCCTCCACTACACTCAGCCCTGCTGCTGCTTCCAAGGGACAA
CTCCCTCTCTTGTCCTTGCCCATCTTTGAGAGAGGCGGTGAATAAACACCAAGGCAAG
AAGAAGCAACGGGACCCCTAGAA
>2266
ACACTCAGCCCTGCTGCTGCTTCCAAGGGACAACTCCCTCTCTTGTCCTTGCCCATCTT
TGAGAGAGGCGGTGAATAAACACCAAGGCAAGAAGAAGCAACGGGACCCCTAGAAG
GAGATGAGTGTGTGGCCCAAGGATG
>2267
CCATCTTTGAGAGAGGCGGTGAATAAACACCAAGGCAAGAAGAAGCAACGGGACCCC
TAGAAGGAGATGAGTGTGTGGCCCAAGGATGGTGGAGCATTGTCCTGAAAGGTAGAA
CCAAGACTATAGCCTCCGAGGCTCTG
>2268
ATCTTTGAGAGAGGCGGTGAATAAACACCAAGGCAAGAAGAAGCAACGGGACCCCTA
GAAGGAGATGAGTGTGTGGCCCAAGGATGGTGGAGCATTGTCCTGAAAGGTAGAACC
AAGACTATAGCCTCCGAGGCTCTGTT
>2269
ACCAAGGCAAGAAGAAGCAACGGGACCCCTAGAAGGAGATGAGTGTGTGGCCCAAG
GATGGTGGAGCATTGTCCTGAAAGGTAGAACCAAGACTATAGCCTCCGAGGCTCTGTT
TACCATATTCTTGGCTCTTGTGCCAC
>2270
GAAGAAGCAACGGGACCCCTAGAAGGAGATGAGTGTGTGGCCCAAGGATGGTGGAGC
ATTGTCCTGAAAGGTAGAACCAAGACTATAGCCTCCGAGGCTCTGTTTACCATATTCTT
GGCTCTTGTGCCACTTCTGTGTCC
>2271
AGTGTGTGGCCCAAGGATGGTGGAGCATTGTCCTGAAAGGTAGAACCAAGACTATAGC
CTCCGAGGCTCTGTTTACCATATTCTTGGCTCTTGTGCCACTTCTGTGTCCAGCAGCTG
GAGCTTGTAGAAGAAAGATGGGA
>2272
GAGCATTGTCCTGAAAGGTAGAACCAAGACTATAGCCTCCGAGGCTCTGTTTACCATAT
TCTTGGCTCTTGTGCCACTTCTGTGTCCAGCAGCTGGAGCTTGTAGAAGAAAGATGGG
AAAGACATAGTACAGGAAATGTT
>2273
TGAAAGGTAGAACCAAGACTATAGCCTCCGAGGCTCTGTTTACCATATTCTTGGCTCTT
GTGCCACTTCTGTGTCCAGCAGCTGGAGCTTGTAGAAGAAAGATGGGAAAGACATAGT

ACAGGAAATGTTCCCATTCAGCC
>2274
ATAGCCTCCGAGGCTCTGTTTACCATATTCTTGGCTCTTGTGCCACTTCTGTGTCCAGCA
GCTGGAGCTTGTAGAAGAAAGATGGGAAAGACATAGTACAGGAAATGTTCCCATTCAG
CCAGCACTGATTTATCTGCTGC
>2275
TATTCTTGGCTCTTGTGCCACTTCTGTGTCCAGCAGCTGGAGCTTGTAGAAGAAAGATG
GGAAAGACATAGTACAGGAAATGTTCCCATTCAGCCAGCACTGATTTATCTGCTGCCTG
GTTTCTCAGGAGGCCTAATCTG
>2276
CTTGTGCCACTTCTGTGTCCAGCAGCTGGAGCTTGTAGAAGAAAGATGGGAAAGACAT
AGTACAGGAAATGTTCCCATTCAGCCAGCACTGATTTATCTGCTGCCTGGTTTCTCAGG
AGGCCTAATCTGTAAGAACAGTC
>2277
GAAGAAAGATGGGAAAGACATAGTACAGGAAATGTTCCCATTCAGCCAGCACTGATTT
ATCTGCTGCCTGGTTTCTCAGGAGGCCTAATCTGTAAGAACAGTCGAGAACTTTATTTC
TGCCCTTTAACCTATGTCCATTT
>2278
AGTACAGGAAATGTTCCCATTCAGCCAGCACTGATTTATCTGCTGCCTGGTTTCTCAGG
AGGCCTAATCTGTAAGAACAGTCGAGAACTTTATTTCTGCCCTTTAACCTATGTCCATTT
TCTAGATAGTTTCTAAAAAAG
>2279
AATGTTCCCATTCAGCCAGCACTGATTTATCTGCTGCCTGGTTTCTCAGGAGGCCTAAT
CTGTAAGAACAGTCGAGAACTTTATTTCTGCCCTTTAACCTATGTCCATTTTCTAGATAG
TTTCTAAAAAAGGCATCACAC
>2280
GCTGCCTGGTTTCTCAGGAGGCCTAATCTGTAAGAACAGTCGAGAACTTTATTTCTGCC
CTTTAACCTATGTCCATTTTCTAGATAGTTTCTAAAAAAGGCATCACACCAGCTCTTTGA
GACAACATGTCATATGCAACT
>2281
GGAGGCCTAATCTGTAAGAACAGTCGAGAACTTTATTTCTGCCCTTTAACCTATGTCCA
TTTTCTAGATAGTTTCTAAAAAAGGCATCACACCAGCTCTTTGAGACAACATGTCATAT
GCAACTGTTGGTTTTGGGTGGA
>2282
CCCTTTAACCTATGTCCATTTTCTAGATAGTTTCTAAAAAAGGCATCACACCAGCTCTTT
GAGACAACATGTCATATGCAACTGTTGGTTTTGGGTGGAATGAAACTGAATTGTCAAC
GGACATTTTTTCTTTCTGTATT
>2283
GTCCATTTTCTAGATAGTTTCTAAAAAAGGCATCACACCAGCTCTTTGAGACAACATGT
CATATGCAACTGTTGGTTTTGGGTGGAATGAAACTGAATTGTCAACGGACATTTTTTCT
TTCTGTATTCATGTGTTTGGAA
>2284
ATTTTCTAGATAGTTTCTAAAAAAGGCATCACACCAGCTCTTTGAGACAACATGTCATA
TGCAACTGTTGGTTTTGGGTGGAATGAAACTGAATTGTCAACGGACATTTTTTCTTTCT

GTATTCATGTGTTTGGAATAGT
>2285
AGATAGTTTCTAAAAAAGGCATCACACCAGCTCTTTGAGACAACATGTCATATGCAACT
GTTGGTTTTGGGTGGAATGAAACTGAATTGTCAACGGACATTTTTTCTTTCTGTATTCAT
GTGTTTGGAATAGTGACAACG
>2286
GCATCACACCAGCTCTTTGAGACAACATGTCATATGCAACTGTTGGTTTTGGGTGGAAT
GAAACTGAATTGTCAACGGACATTTTTTCTTTCTGTATTCATGTGTTTGGAATAGTGACA
ACGTCATTGGTTCAGACTTTG
>2287
AACATGTCATATGCAACTGTTGGTTTTGGGTGGAATGAAACTGAATTGTCAACGGACAT
TTTTTCTTTCTGTATTCATGTGTTTGGAATAGTGACAACGTCATTGGTTCAGACTTTGAG
CCCCCTTCTGGTTAGGCTCTT
>2288
ATATGCAACTGTTGGTTTTGGGTGGAATGAAACTGAATTGTCAACGGACATTTTTTCTT
TCTGTATTCATGTGTTTGGAATAGTGACAACGTCATTGGTTCAGACTTTGAGCCCCCTT
CTGGTTAGGCTCTTCAGTATAA
>2289
ATGCAACTGTTGGTTTTGGGTGGAATGAAACTGAATTGTCAACGGACATTTTTTCTTTC
TGTATTCATGTGTTTGGAATAGTGACAACGTCATTGGTTCAGACTTTGAGCCCCCTTCT
GGTTAGGCTCTTCAGTATAAGC
>2290
TGGTTTTGGGTGGAATGAAACTGAATTGTCAACGGACATTTTTTCTTTCTGTATTCATGT
GTTTGGAATAGTGACAACGTCATTGGTTCAGACTTTGAGCCCCCTTCTGGTTAGGCTCT
TCAGTATAAGCCCTTAAAAAT
>2291
GGTGGAATGAAACTGAATTGTCAACGGACATTTTTTCTTTCTGTATTCATGTGTTTGGAA
TAGTGACAACGTCATTGGTTCAGACTTTGAGCCCCCTTCTGGTTAGGCTCTTCAGTATA
AGCCCTTAAAAATATTTTTGA
>2292
TTTTTCTTTCTGTATTCATGTGTTTGGAATAGTGACAACGTCATTGGTTCAGACTTTGAG
CCCCCTTCTGGTTAGGCTCTTCAGTATAAGCCCTTAAAAATATTTTTGACTTTTCAGGGC
CAGGCGCGGTGGCTCACGCC
>2293
ATTCATGTGTTTGGAATAGTGACAACGTCATTGGTTCAGACTTTGAGCCCCCTTCTGGT
TAGGCTCTTCAGTATAAGCCCTTAAAAATATTTTTGACTTTTCAGGGCCAGGCGCGGTG
GCTCACGCCTGTAATCCCAGCA
>2294
AGCCCCCTTCTGGTTAGGCTCTTCAGTATAAGCCCTTAAAAATATTTTTGACTTTTCAGG
GCCAGGCGCGGTGGCTCACGCCTGTAATCCCAGCACTTTGGGAGGCCGAGGCAGGTG
GATCACGAGGTCAGGAGATCGAG
>2295
TTAGGCTCTTCAGTATAAGCCCTTAAAAATATTTTTGACTTTTCAGGGCCAGGCGCGGT
GGCTCACGCCTGTAATCCCAGCACTTTGGGAGGCCGAGGCAGGTGGATCACGAGGTC

AGGAGATCGAGACCATCCTGGCTA
>2296
TTTTTGACTTTTCAGGGCCAGGCGCGGTGGCTCACGCCTGTAATCCCAGCACTTTGGG
AGGCCGAGGCAGGTGGATCACGAGGTCAGGAGATCGAGACCATCCTGGCTAACACGG
TGAAAGCCCGTCTCTACTAAAAATA
>2297
CAGGGCCAGGCGCGGTGGCTCACGCCTGTAATCCCAGCACTTTGGGAGGCCGAGGCA
GGTGGATCACGAGGTCAGGAGATCGAGACCATCCTGGCTAACACGGTGAAAGCCCGT
CTCTACTAAAAATACAAAAAATTAGC
>2298
CCCAGCACTTTGGGAGGCCGAGGCAGGTGGATCACGAGGTCAGGAGATCGAGACCAT
CCTGGCTAACACGGTGAAAGCCCGTCTCTACTAAAAATACAAAAAATTAGCCAGGTGT
GGTGGCGGGCGCCTGTAGTCCCAGC
>2299
TGGGAGGCCGAGGCAGGTGGATCACGAGGTCAGGAGATCGAGACCATCCTGGCTAAC
ACGGTGAAAGCCCGTCTCTACTAAAAATACAAAAAATTAGCCAGGTGTGGTGGCGGGC
GCCTGTAGTCCCAGCTACTCGAGAG
>2300
GAGATCGAGACCATCCTGGCTAACACGGTGAAAGCCCGTCTCTACTAAAAATACAAAA
AATTAGCCAGGTGTGGTGGCGGGCGCCTGTAGTCCCAGCTACTCGAGAGGCTGAGGCT
GGAGAATGGCGTGAACCCGGAGGC
>2301
ATCGAGACCATCCTGGCTAACACGGTGAAAGCCCGTCTCTACTAAAAATACAAAAAAT
TAGCCAGGTGTGGTGGCGGGCGCCTGTAGTCCCAGCTACTCGAGAGGCTGAGGCTGG
AGAATGGCGTGAACCCGGAGGCAGA
>2302
TGGCTAACACGGTGAAAGCCCGTCTCTACTAAAAATACAAAAAATTAGCCAGGTGTGG
TGGCGGGCGCCTGTAGTCCCAGCTACTCGAGAGGCTGAGGCTGGAGAATGGCGTGAA
CCCGGAGGCAGAGTTTGCAGTGAGC
>2303
AAAATTAGCCAGGTGTGGTGGCGGGCGCCTGTAGTCCCAGCTACTCGAGAGGCTGAG
GCTGGAGAATGGCGTGAACCCGGAGGCAGAGTTTGCAGTGAGCTGAGGTTGTGCCAC
TGCACTCCAGCCTGGGAGACAGAGCA
>2304
GTGGCGGGCGCCTGTAGTCCCAGCTACTCGAGAGGCTGAGGCTGGAGAATGGCGTGA
ACCCGGAGGCAGAGTTTGCAGTGAGCTGAGGTTGTGCCACTGCACTCCAGCCTGGGA
GACAGAGCAAGACTCCGTCTCAAAAA
>2305
GCGCCTGTAGTCCCAGCTACTCGAGAGGCTGAGGCTGGAGAATGGCGTGAACCCGGA
GGCAGAGTTTGCAGTGAGCTGAGGTTGTGCCACTGCACTCCAGCCTGGGAGACAGAG
CAAGACTCCGTCTCAAAAAAATAAAA
>2306
CGGAGGCAGAGTTTGCAGTGAGCTGAGGTTGTGCCACTGCACTCCAGCCTGGGAGAC
AGAGCAAGACTCCGTCTCAAAAAAATAAAAAATAAAAAACATTAAAAAATTGACTTTT

TGAGTAAAACAAAAACCAAACGAGT
>2307
CTGGGAGACAGAGCAAGACTCCGTCTCAAAAAAATAAAAAATAAAAAACATTAAAAA
ATTGACTTTTTGAGTAAAACAAAAACCAAACGAGTCCGTGTAAAGTCTAAGATGTTGG
TTCATCTCAACATTGGGATATTGGA
>2308
CCGTCTCAAAAAAATAAAAAATAAAAAACATTAAAAAATTGACTTTTTGAGTAAAACA
AAAACCAAACGAGTCCGTGTAAAGTCTAAGATGTTGGTTCATCTCAACATTGGGATATT
GGAATAGGCCACTTTGGAAGGTT
>2309
CTCAAAAAAATAAAAAATAAAAAACATTAAAAAATTGACTTTTTGAGTAAAACAAAAA
CCAAACGAGTCCGTGTAAAGTCTAAGATGTTGGTTCATCTCAACATTGGGATATTGGAA
TAGGCCACTTTGGAAGGTTTCTC
>2310
CGAGTCCGTGTAAAGTCTAAGATGTTGGTTCATCTCAACATTGGGATATTGGAATAGGC
CACTTTGGAAGGTTTCTCTAATTGATGAAGGGCATGGGCCACCCCATGGATTGCAATGC
TGTGGCTGTTTGGGTGGCATCC
>2311
TGGAATAGGCCACTTTGGAAGGTTTCTCTAATTGATGAAGGGCATGGGCCACCCCATG
GATTGCAATGCTGTGGCTGTTTGGGTGGCATCCAGCTTCTAGGGGATGGTGGCAAACTT
GGGTGTGCAGGCAGTAGGAAGGA
>2312
AGGCCACTTTGGAAGGTTTCTCTAATTGATGAAGGGCATGGGCCACCCCATGGATTGC
AATGCTGTGGCTGTTTGGGTGGCATCCAGCTTCTAGGGGATGGTGGCAAACTTGGGTG
TGCAGGCAGTAGGAAGGAGGAGGT
>2313
GAAGGGCATGGGCCACCCCATGGATTGCAATGCTGTGGCTGTTTGGGTGGCATCCAGC
TTCTAGGGGATGGTGGCAAACTTGGGTGTGCAGGCAGTAGGAAGGAGGAGGTAGCTT
CCAGAGAGAGCCATTGCTGGGGCTC
>2314
CACCCCATGGATTGCAATGCTGTGGCTGTTTGGGTGGCATCCAGCTTCTAGGGGATGGT
GGCAAACTTGGGTGTGCAGGCAGTAGGAAGGAGGAGGTAGCTTCCAGAGAGAGCCAT
TGCTGGGGCTCAAGAAGTTTCTTT
>2315
CCCCATGGATTGCAATGCTGTGGCTGTTTGGGTGGCATCCAGCTTCTAGGGGATGGTGG
CAAACTTGGGTGTGCAGGCAGTAGGAAGGAGGAGGTAGCTTCCAGAGAGAGCCATTG
CTGGGGCTCAAGAAGTTTCTTTCT
>2316
TTGCAATGCTGTGGCTGTTTGGGTGGCATCCAGCTTCTAGGGGATGGTGGCAAACTTG
GGTGTGCAGGCAGTAGGAAGGAGGAGGTAGCTTCCAGAGAGAGCCATTGCTGGGGCT
CAAGAAGTTTCTTTCTGGGCTCAAG
>2317
AAACTTGGGTGTGCAGGCAGTAGGAAGGAGGAGGTAGCTTCCAGAGAGAGCCATTGC
TGGGGCTCAAGAAGTTTCTTTCTGGGCTCAAGCATGGTTGCTAGTGGTCCACACAGTG

CTTTCGTGTGAATTAGAAAAAGCAC
>2318
GAAGGAGGAGGTAGCTTCCAGAGAGAGCCATTGCTGGGGCTCAAGAAGTTTCTTTCT
GGGCTCAAGCATGGTTGCTAGTGGTCCACACAGTGCTTTCGTGTGAATTAGAAAAAGC
ACCCTTGTTGTGGATGGATGTGGCT
>2319
AGAGAGAGCCATTGCTGGGGCTCAAGAAGTTTCTTTCTGGGCTCAAGCATGGTTGCTA
GTGGTCCACACAGTGCTTTCGTGTGAATTAGAAAAAGCACCCTTGTTGTGGATGGATG
TGGCTCTTAACTAGGGTCTATCCC
>2320
ATTGCTGGGGCTCAAGAAGTTTCTTTCTGGGCTCAAGCATGGTTGCTAGTGGTCCACA
CAGTGCTTTCGTGTGAATTAGAAAAAGCACCCTTGTTGTGGATGGATGTGGCTCTTAAC
TAGGGTCTATCCCACAGTGAGCT
>2321
CTTTCTGGGCTCAAGCATGGTTGCTAGTGGTCCACACAGTGCTTTCGTGTGAATTAGAA
AAAGCACCCTTGTTGTGGATGGATGTGGCTCTTAACTAGGGTCTATCCCACAGTGAGCT
GGTTGGGGCTGGATCTCAAACC
>2322
TCTGGGCTCAAGCATGGTTGCTAGTGGTCCACACAGTGCTTTCGTGTGAATTAGAAAA
AGCACCCTTGTTGTGGATGGATGTGGCTCTTAACTAGGGTCTATCCCACAGTGAGCTGG
TTGGGGCTGGATCTCAAACCTCT
>2323
CTTTCGTGTGAATTAGAAAAAGCACCCTTGTTGTGGATGGATGTGGCTCTTAACTAGGG
TCTATCCCACAGTGAGCTGGTTGGGGCTGGATCTCAAACCTCTTTGCCCCTCAATCAGA
GGCAGGAGGTCCCTGGCCCAAG
>2324
TGAATTAGAAAAAGCACCCTTGTTGTGGATGGATGTGGCTCTTAACTAGGGTCTATCCC
ACAGTGAGCTGGTTGGGGCTGGATCTCAAACCTCTTTGCCCCTCAATCAGAGGCAGGA
GGTCCCTGGCCCAAGGGGCTTCC
>2325
GGGTCTATCCCACAGTGAGCTGGTTGGGGCTGGATCTCAAACCTCTTTGCCCCTCAATC
AGAGGCAGGAGGTCCCTGGCCCAAGGGGCTTCCATGGTTGGAGTGGTCGTCAGCCGA
AAGCTGTTCTGGTGAGATGTTTTG
>2326
GGGGCTGGATCTCAAACCTCTTTGCCCCTCAATCAGAGGCAGGAGGTCCCTGGCCCAA
GGGGCTTCCATGGTTGGAGTGGTCGTCAGCCGAAAGCTGTTCTGGTGAGATGTTTTGA
GCTATCAGTTCCAAGTTTCCAAAG
>2327
GGATCTCAAACCTCTTTGCCCCTCAATCAGAGGCAGGAGGTCCCTGGCCCAAGGGGCT
TCCATGGTTGGAGTGGTCGTCAGCCGAAAGCTGTTCTGGTGAGATGTTTTGAGCTATCA
GTTCCAAGTTTCCAAAGGCCAAG
>2328
CAGGAGGTCCCTGGCCCAAGGGGCTTCCATGGTTGGAGTGGTCGTCAGCCGAAAGCT
GTTCTGGTGAGATGTTTTGAGCTATCAGTTCCAAGTTTCCAAAGGCCAAGGCCTCTGG

GATCAGTTTCACTGATCCTTCAACC
>2329
CCCAAGGGGCTTCCATGGTTGGAGTGGTCGTCAGCCGAAAGCTGTTCTGGTGAGATGT
TTTGAGCTATCAGTTCCAAGTTTCCAAAGGCCAAGGCCTCTGGGATCAGTTTCACTGAT
CCTTCAACCAGCTTATGTGCCCT
>2330
GGCTTCCATGGTTGGAGTGGTCGTCAGCCGAAAGCTGTTCTGGTGAGATGTTTTGAGC
TATCAGTTCCAAGTTTCCAAAGGCCAAGGCCTCTGGGATCAGTTTCACTGATCCTTCAA
CCAGCTTATGTGCCCTGTTAGCC
>2331
GAAAGCTGTTCTGGTGAGATGTTTTGAGCTATCAGTTCCAAGTTTCCAAAGGCCAAGG
CCTCTGGGATCAGTTTCACTGATCCTTCAACCAGCTTATGTGCCCTGTTAGCCCAGATA
CCCCTCCAGAGTGCTTCGCACTG
>2332
GCTATCAGTTCCAAGTTTCCAAAGGCCAAGGCCTCTGGGATCAGTTTCACTGATCCTTC
AACCAGCTTATGTGCCCTGTTAGCCCAGATACCCCTCCAGAGTGCTTCGCACTGGGCTG
CAGGTCACTGTGGCCCCACACC
>2333
GTTCCAAGTTTCCAAAGGCCAAGGCCTCTGGGATCAGTTTCACTGATCCTTCAACCAG
CTTATGTGCCCTGTTAGCCCAGATACCCCTCCAGAGTGCTTCGCACTGGGCTGCAGGTC
ACTGTGGCCCCACACCATAGAAG
>2334
GCCTCTGGGATCAGTTTCACTGATCCTTCAACCAGCTTATGTGCCCTGTTAGCCCAGAT
ACCCCTCCAGAGTGCTTCGCACTGGGCTGCAGGTCACTGTGGCCCCACACCATAGAAG
CTGGGGGCCTGTCTGCATGTTCT
>2335
GATCCTTCAACCAGCTTATGTGCCCTGTTAGCCCAGATACCCCTCCAGAGTGCTTCGCA
CTGGGCTGCAGGTCACTGTGGCCCCACACCATAGAAGCTGGGGGCCTGTCTGCATGTT
CTGGCATCCATAAAGCTGCCTGC
>2336
TCAACCAGCTTATGTGCCCTGTTAGCCCAGATACCCCTCCAGAGTGCTTCGCACTGGGC
TGCAGGTCACTGTGGCCCCACACCATAGAAGCTGGGGGCCTGTCTGCATGTTCTGGCA
TCCATAAAGCTGCCTGCAAACGT
>2337
ATACCCCTCCAGAGTGCTTCGCACTGGGCTGCAGGTCACTGTGGCCCCACACCATAGA
AGCTGGGGGCCTGTCTGCATGTTCTGGCATCCATAAAGCTGCCTGCAAACGTGAGAGG
GGGTTTATGGCACCCTTTTAGCAT
>2338
CTGTGGCCCCACACCATAGAAGCTGGGGGCCTGTCTGCATGTTCTGGCATCCATAAAG
CTGCCTGCAAACGTGAGAGGGGGTTTATGGCACCCTTTTAGCATGACTGTCTCTTGTGA
CCAAGGCCAAGAAAAATAGCACA
>2339
ACACCATAGAAGCTGGGGGCCTGTCTGCATGTTCTGGCATCCATAAAGCTGCCTGCAA
ACGTGAGAGGGGGTTTATGGCACCCTTTTAGCATGACTGTCTCTTGTGACCAAGGCCA

AGAAAAATAGCACAAAGATGTAGG
>2340
GCATGTTCTGGCATCCATAAAGCTGCCTGCAAACGTGAGAGGGGGTTTATGGCACCCT
TTTAGCATGACTGTCTCTTGTGACCAAGGCCAAGAAAAATAGCACAAAGATGTAGGAG
TGGTGAGGAATGAAGATGCACGTG
>2341
CTGGCATCCATAAAGCTGCCTGCAAACGTGAGAGGGGGTTTATGGCACCCTTTTAGCAT
GACTGTCTCTTGTGACCAAGGCCAAGAAAAATAGCACAAAGATGTAGGAGTGGTGAG
GAATGAAGATGCACGTGCACGCGC
>2342
AGGGGGTTTATGGCACCCTTTTAGCATGACTGTCTCTTGTGACCAAGGCCAAGAAAAA
TAGCACAAAGATGTAGGAGTGGTGAGGAATGAAGATGCACGTGCACGCGCCCTGCAG
TCTCAGTCAATGCGCTCTTTAGGGT
>2343
ACTGTCTCTTGTGACCAAGGCCAAGAAAAATAGCACAAAGATGTAGGAGTGGTGAGG
AATGAAGATGCACGTGCACGCGCCCTGCAGTCTCAGTCAATGCGCTCTTTAGGGTTTA
GTTTGGAAAACCACAGTGTCGTGTC
>2344
AAGGCCAAGAAAAATAGCACAAAGATGTAGGAGTGGTGAGGAATGAAGATGCACGTG
CACGCGCCCTGCAGTCTCAGTCAATGCGCTCTTTAGGGTTTAGTTTGGAAAACCACAG
TGTCGTGTCTTATGTAACCAATTTC
>2345
ATGTAGGAGTGGTGAGGAATGAAGATGCACGTGCACGCGCCCTGCAGTCTCAGTCAAT
GCGCTCTTTAGGGTTTAGTTTGGAAAACCACAGTGTCGTGTCTTATGTAACCAATTTCT
TTTCTTTTCTTTTCTTTTCTTTT
>2346
GAAGATGCACGTGCACGCGCCCTGCAGTCTCAGTCAATGCGCTCTTTAGGGTTTAGTTT
GGAAAACCACAGTGTCGTGTCTTATGTAACCAATTTCTTTTCTTTTCTTTTCTTTTCTTT
TTTTTTTTTTTTTTTTGTGAT
>2347
AGATGCACGTGCACGCGCCCTGCAGTCTCAGTCAATGCGCTCTTTAGGGTTTAGTTTGG
AAAACCACAGTGTCGTGTCTTATGTAACCAATTTCTTTTCTTTTCTTTTCTTTTCTTTTTT
TTTTTTTTTTTTTGTGATGG
>2348
ACGCGCCCTGCAGTCTCAGTCAATGCGCTCTTTAGGGTTTAGTTTGGAAAACCACAGT
GTCGTGTCTTATGTAACCAATTTCTTTTCTTTTCTTTTCTTTTCTTTTTTTTTTTTTTTTTT
TGTGATGGAGTCTCGTGTCT
>2349
CCACAGTGTCGTGTCTTATGTAACCAATTTCTTTTCTTTTCTTTTCTTTTCTTTTTTTTTTT
TTTTTTTTGTGATGGAGTCTCGTGTCTTGCTCTGTCACCCAGGCTGGAGTGCAGTGGCT
CGATCTCGGCTCACTGCAA
>2350
TCGTGTCTTATGTAACCAATTTCTTTTCTTTTCTTTTCTTTTCTTTTTTTTTTTTTTTTTTT
GTGATGGAGTCTCGTGTCTTGCTCTGTCACCCAGGCTGGAGTGCAGTGGCTCGATCTC

GGCTCACTGCAACCTCCGCC
>2351
AATTTCTTTTCTTTTCTTTTCTTTTCTTTTTTTTTTTTTTTTTTTTGTGATGGAGTCTCGTGT
CTTGCTCTGTCACCCAGGCTGGAGTGCAGTGGCTCGATCTCGGCTCACTGCAACCTCC
GCCTCCCAGGTTCAAGCGAC
>2352
TGTCTTGCTCTGTCACCCAGGCTGGAGTGCAGTGGCTCGATCTCGGCTCACTGCAACC
TCCGCCTCCCAGGTTCAAGCGACTATCCTGCCTCAGCCTCCTGAGTAGCTGGGACTAC
AGGCGCCCGCCACCACGCCCAGCT
>2353
TGGCTCGATCTCGGCTCACTGCAACCTCCGCCTCCCAGGTTCAAGCGACTATCCTGCCT
CAGCCTCCTGAGTAGCTGGGACTACAGGCGCCCGCCACCACGCCCAGCTAATTTTTTG
TGTATTTAGTAGAGACGGGGTTT
>2354
GACTATCCTGCCTCAGCCTCCTGAGTAGCTGGGACTACAGGCGCCCGCCACCACGCCC
AGCTAATTTTTTGTGTATTTAGTAGAGACGGGGTTTCACCGTGTTAGCCAGGATGGTCA
CAATCTCCTGACCTCGTGGTCCG
>2355
GCCTCAGCCTCCTGAGTAGCTGGGACTACAGGCGCCCGCCACCACGCCCAGCTAATTT
TTTGTGTATTTAGTAGAGACGGGGTTTCACCGTGTTAGCCAGGATGGTCACAATCTCCT
GACCTCGTGGTCCGCCCGCCTTG
>2356
CTGAGTAGCTGGGACTACAGGCGCCCGCCACCACGCCCAGCTAATTTTTTGTGTATTTA
GTAGAGACGGGGTTTCACCGTGTTAGCCAGGATGGTCACAATCTCCTGACCTCGTGGT
CCGCCCGCCTTGGCCTCCCACAG
>2357
CTGGGACTACAGGCGCCCGCCACCACGCCCAGCTAATTTTTTGTGTATTTAGTAGAGAC
GGGGTTTCACCGTGTTAGCCAGGATGGTCACAATCTCCTGACCTCGTGGTCCGCCCGC
CTTGGCCTCCCACAGTGCTGGGA
>2358
AATTTTTTGTGTATTTAGTAGAGACGGGGTTTCACCGTGTTAGCCAGGATGGTCACAAT
CTCCTGACCTCGTGGTCCGCCCGCCTTGGCCTCCCACAGTGCTGGGATTACAGGCGTG
AGCCACCACGCCCGGCCACCAAT
>2359
TTTTTGTGTATTTAGTAGAGACGGGGTTTCACCGTGTTAGCCAGGATGGTCACAATCTC
CTGACCTCGTGGTCCGCCCGCCTTGGCCTCCCACAGTGCTGGGATTACAGGCGTGAGC
CACCACGCCCGGCCACCAATTTC
>2360
GGTTTCACCGTGTTAGCCAGGATGGTCACAATCTCCTGACCTCGTGGTCCGCCCGCCTT
GGCCTCCCACAGTGCTGGGATTACAGGCGTGAGCCACCACGCCCGGCCACCAATTTCT
TATACATAGGTTATGGAGCAGGG
>2361
CAGGATGGTCACAATCTCCTGACCTCGTGGTCCGCCCGCCTTGGCCTCCCACAGTGCT
GGGATTACAGGCGTGAGCCACCACGCCCGGCCACCAATTTCTTATACATAGGTTATGGA

GCAGGGAGAAAGAGACATGCAAA
>2362
CTTGGCCTCCCACAGTGCTGGGATTACAGGCGTGAGCCACCACGCCCGGCCACCAATT
TCTTATACATAGGTTATGGAGCAGGGAGAAAGAGACATGCAAACAAGGCAGAATTGGG
ACCTTTGCAAGAAGACCTTCTTCA
>2363
ATGCAAACAAGGCAGAATTGGGACCTTTGCAAGAAGACCTTCTTCACTCGCCCACTTT
CTCATACTTTTAGTCACCAAGGCAGGGTCTTTTTAAACAACTTCGAGGCTCAGGCCTAG
GGGGTTAGGAGAGTAAACCCTAA
>2364
GAATTGGGACCTTTGCAAGAAGACCTTCTTCACTCGCCCACTTTCTCATACTTTTAGTC
ACCAAGGCAGGGTCTTTTTAAACAACTTCGAGGCTCAGGCCTAGGGGGTTAGGAGAGT
AAACCCTAAATCCCTTTCACCCT
>2365
CCCACTTTCTCATACTTTTAGTCACCAAGGCAGGGTCTTTTTAAACAACTTCGAGGCTC
AGGCCTAGGGGGTTAGGAGAGTAAACCCTAAATCCCTTTCACCCTAGCATGCTTGGGT
GCCGTGTTGACAAGGTCTCTGAA
>2366
CAACTTCGAGGCTCAGGCCTAGGGGGTTAGGAGAGTAAACCCTAAATCCCTTTCACCC
TAGCATGCTTGGGTGCCGTGTTGACAAGGTCTCTGAAGTGCCCATGGGAAGCAGACCA
GGCTCTGAGAATAATTTCCATGGA
>2367
TCGAGGCTCAGGCCTAGGGGGTTAGGAGAGTAAACCCTAAATCCCTTTCACCCTAGCA
TGCTTGGGTGCCGTGTTGACAAGGTCTCTGAAGTGCCCATGGGAAGCAGACCAGGCT
CTGAGAATAATTTCCATGGAATTAA
>2368
GACAAGGTCTCTGAAGTGCCCATGGGAAGCAGACCAGGCTCTGAGAATAATTTCCATG
GAATTAATTATTGTTCTTACTCCAATAGATTTGAAATCTATTTAACTTTTCAGGGTCCCCT
AGCTTTTTAAATGTTTCAAGT
>2369
GAGAATAATTTCCATGGAATTAATTATTGTTCTTACTCCAATAGATTTGAAATCTATTTAA
CTTTTCAGGGTCCCCTAGCTTTTTAAATGTTTCAAGTGTATTCTCTCTGATAGGTTGCCT
TTTCTCTCCCTGTAGGCTA
>2370
TTAATTATTGTTCTTACTCCAATAGATTTGAAATCTATTTAACTTTTCAGGGTCCCCTAGC
TTTTTAAATGTTTCAAGTGTATTCTCTCTGATAGGTTGCCTTTTCTCTCCCTGTAGGCTAT
AATTAGTGTCCTTTACAC
>2371
GTTCTTACTCCAATAGATTTGAAATCTATTTAACTTTTCAGGGTCCCCTAGCTTTTTAAAT
GTTTCAAGTGTATTCTCTCTGATAGGTTGCCTTTTCTCTCCCTGTAGGCTATAATTAGTGT
CCTTTACACAAAATTGGC
>2372
ATTTGAAATCTATTTAACTTTTCAGGGTCCCCTAGCTTTTTAAATGTTTCAAGTGTATTCT
CTCTGATAGGTTGCCTTTTCTCTCCCTGTAGGCTATAATTAGTGTCCTTTACACAAAATT

GGCTTAGAATTGGATTTAG
>2373
TTTTCAGGGTCCCCTAGCTTTTTAAATGTTTCAAGTGTATTCTCTCTGATAGGTTGCCTT
TTCTCTCCCTGTAGGCTATAATTAGTGTCCTTTACACAAAATTGGCTTAGAATTGGATTT
AGAGAAGATAAGAATCAAAT
>2374
CTATAATTAGTGTCCTTTACACAAAATTGGCTTAGAATTGGATTTAGAGAAGATAAGAAT
CAAATTACCAGTATTTATCTTGTAAATTCCAAAAGATCTACTTAAGACACTGTATTAAAA
AAGGAGTATAAAAGAAAAGT
>2375
GTCCTTTACACAAAATTGGCTTAGAATTGGATTTAGAGAAGATAAGAATCAAATTACCA
GTATTTATCTTGTAAATTCCAAAAGATCTACTTAAGACACTGTATTAAAAAAGGAGTATA
AAAGAAAAGTTTATTCTATGA
>2376
ATTTAGAGAAGATAAGAATCAAATTACCAGTATTTATCTTGTAAATTCCAAAAGATCTAC
TTAAGACACTGTATTAAAAAAGGAGTATAAAAGAAAAGTTTATTCTATGAAAAAGTAAA
TTCCTCATGGTCTGAGCAACC
>2377
AGAATCAAATTACCAGTATTTATCTTGTAAATTCCAAAAGATCTACTTAAGACACTGTAT
TAAAAAAGGAGTATAAAAGAAAAGTTTATTCTATGAAAAAGTAAATTCCTCATGGTCTG
AGCAACCAGGGGTATTTTAAT
>2378
ATTCCAAAAGATCTACTTAAGACACTGTATTAAAAAAGGAGTATAAAAGAAAAGTTTAT
TCTATGAAAAAGTAAATTCCTCATGGTCTGAGCAACCAGGGGTATTTTAATATCAAATCT
CCAGACTTCCCTTGGAGGCAC
>2379
ACTTAAGACACTGTATTAAAAAAGGAGTATAAAAGAAAAGTTTATTCTATGAAAAAGTA
AATTCCTCATGGTCTGAGCAACCAGGGGTATTTTAATATCAAATCTCCAGACTTCCCTTG
GAGGCACCTAAAATTGCCTTT
>2380
TAAAAAAGGAGTATAAAAGAAAAGTTTATTCTATGAAAAAGTAAATTCCTCATGGTCTG
AGCAACCAGGGGTATTTTAATATCAAATCTCCAGACTTCCCTTGGAGGCACCTAAAATT
GCCTTTTCCCTTGTCGCTCGTT
>2381
GCAACCAGGGGTATTTTAATATCAAATCTCCAGACTTCCCTTGGAGGCACCTAAAATTG
CCTTTTCCCTTGTCGCTCGTTTTTATCTCCAGTTGAGCTTCTGAAATATGTTGCCAGGCT
TTGGATATTGGAGAGCTGGTG
>2382
GGGGTATTTTAATATCAAATCTCCAGACTTCCCTTGGAGGCACCTAAAATTGCCTTTTCC
CTTGTCGCTCGTTTTTATCTCCAGTTGAGCTTCTGAAATATGTTGCCAGGCTTTGGATAT
TGGAGAGCTGGTGTGGTTTC
>2383
ATCAAATCTCCAGACTTCCCTTGGAGGCACCTAAAATTGCCTTTTCCCTTGTCGCTCGT
TTTTATCTCCAGTTGAGCTTCTGAAATATGTTGCCAGGCTTTGGATATTGGAGAGCTGGT

GTGGTTTCAGCTTTCATTAAA
>2384
CCTTGGAGGCACCTAAAATTGCCTTTTCCCTTGTCGCTCGTTTTTATCTCCAGTTGAGCT
TCTGAAATATGTTGCCAGGCTTTGGATATTGGAGAGCTGGTGTGGTTTCAGCTTTCATTA
AAATTCGGAGGTTTTGACTT
>2385
CTTGTCGCTCGTTTTTATCTCCAGTTGAGCTTCTGAAATATGTTGCCAGGCTTTGGATAT
TGGAGAGCTGGTGTGGTTTCAGCTTTCATTAAAATTCGGAGGTTTTGACTTATCTATTAT
TTCTGATTTGTGGTAAGACA
>2386
TCTGAAATATGTTGCCAGGCTTTGGATATTGGAGAGCTGGTGTGGTTTCAGCTTTCATTA
AAATTCGGAGGTTTTGACTTATCTATTATTTCTGATTTGTGGTAAGACATAAGCCTCTAA
AATATGCTATCTTAAAGAGA
>2387
TTGGAGAGCTGGTGTGGTTTCAGCTTTCATTAAAATTCGGAGGTTTTGACTTATCTATTA
TTTCTGATTTGTGGTAAGACATAAGCCTCTAAAATATGCTATCTTAAAGAGATACACACA
AATTTTATCTTAGCATGTTA
>2388
ATTTGTGGTAAGACATAAGCCTCTAAAATATGCTATCTTAAAGAGATACACACAAATTTT
ATCTTAGCATGTTAAGTTTTTAAGCCGTTGAGAGATGGCAGAATAGACCTCTGTGGTCT
CCTGTGTGCTTCTTCGTCCTA
>2389
TGGTAAGACATAAGCCTCTAAAATATGCTATCTTAAAGAGATACACACAAATTTTATCTT
AGCATGTTAAGTTTTTAAGCCGTTGAGAGATGGCAGAATAGACCTCTGTGGTCTCCTGT
GTGCTTCTTCGTCCTAAAATG
>2390
AAGCCTCTAAAATATGCTATCTTAAAGAGATACACACAAATTTTATCTTAGCATGTTAAG
TTTTTAAGCCGTTGAGAGATGGCAGAATAGACCTCTGTGGTCTCCTGTGTGCTTCTTCG
TCCTAAAATGTCAGCATGCTG
>2391
GAGATACACACAAATTTTATCTTAGCATGTTAAGTTTTTAAGCCGTTGAGAGATGGCAG
AATAGACCTCTGTGGTCTCCTGTGTGCTTCTTCGTCCTAAAATGTCAGCATGCTGGTTTT
CATGTGCTGCCTCGTACAGTA
>2392
TAGCATGTTAAGTTTTTAAGCCGTTGAGAGATGGCAGAATAGACCTCTGTGGTCTCCTG
TGTGCTTCTTCGTCCTAAAATGTCAGCATGCTGGTTTTCATGTGCTGCCTCGTACAGTAA
GTCCCCAGCAGATTTAAATAA
>2393
AGTTTTTAAGCCGTTGAGAGATGGCAGAATAGACCTCTGTGGTCTCCTGTGTGCTTCTT
CGTCCTAAAATGTCAGCATGCTGGTTTTCATGTGCTGCCTCGTACAGTAAGTCCCCAGC
AGATTTAAATAAGGTTGTGTCA
>2394
GTTGAGAGATGGCAGAATAGACCTCTGTGGTCTCCTGTGTGCTTCTTCGTCCTAAAATG
TCAGCATGCTGGTTTTCATGTGCTGCCTCGTACAGTAAGTCCCCAGCAGATTTAAATAA

GGTTGTGTCAAAACATATGCCT
>2395
AGACCTCTGTGGTCTCCTGTGTGCTTCTTCGTCCTAAAATGTCAGCATGCTGGTTTTCAT
GTGCTGCCTCGTACAGTAAGTCCCCAGCAGATTTAAATAAGGTTGTGTCAAAACATATG
CCTATGTTTTTTTAACATCG
>2396
TCCTAAAATGTCAGCATGCTGGTTTTCATGTGCTGCCTCGTACAGTAAGTCCCCAGCAG
ATTTAAATAAGGTTGTGTCAAAACATATGCCTATGTTTTTTTAACATCGTTGTCTCATAA
GTGTGCAGTAATGGCATATT
>2397
TAAAATGTCAGCATGCTGGTTTTCATGTGCTGCCTCGTACAGTAAGTCCCCAGCAGATT
TAAATAAGGTTGTGTCAAAACATATGCCTATGTTTTTTTAACATCGTTGTCTCATAAGT
GTGCAGTAATGGCATATTGAG
>2398
TTTCATGTGCTGCCTCGTACAGTAAGTCCCCAGCAGATTTAAATAAGGTTGTGTCAAAA
CATATGCCTATGTTTTTTTAACATCGTTGTCTCATAAGTGTGCAGTAATGGCATATTGAG
ACTGTTCATAGACTGGTCAG
>2399
CCCCAGCAGATTTAAATAAGGTTGTGTCAAAACATATGCCTATGTTTTTTTAACATCGT
TGTCTCATAAGTGTGCAGTAATGGCATATTGAGACTGTTCATAGACTGGTCAGCTGCAT
TTCCTGCAGCCGAGGAACCCT
>2400
CCAGCAGATTTAAATAAGGTTGTGTCAAAACATATGCCTATGTTTTTTTAACATCGTTG
TCTCATAAGTGTGCAGTAATGGCATATTGAGACTGTTCATAGACTGGTCAGCTGCATTTC
CTGCAGCCGAGGAACCCTTT
>2401
AGATTTAAATAAGGTTGTGTCAAAACATATGCCTATGTTTTTTTAACATCGTTGTCTCAT
AAGTGTGCAGTAATGGCATATTGAGACTGTTCATAGACTGGTCAGCTGCATTTCCTGCA
GCCGAGGAACCCTTTATTAA
>2402
TCAAAACATATGCCTATGTTTTTTTAACATCGTTGTCTCATAAGTGTGCAGTAATGGCA
TATTGAGACTGTTCATAGACTGGTCAGCTGCATTTCCTGCAGCCGAGGAACCCTTTATT
AAGGTGCATTTCTGGGGTTGA
>2403
TAATGGCATATTGAGACTGTTCATAGACTGGTCAGCTGCATTTCCTGCAGCCGAGGAAC
CCTTTATTAAGGTGCATTTCTGGGGTTGAACTAAAACTGCTCACCCCCATCAAATGAGG
AAACTTGAGGGGTTTGCAGCCC
>2404
AGACTGTTCATAGACTGGTCAGCTGCATTTCCTGCAGCCGAGGAACCCTTTATTAAGGT
GCATTTCTGGGGTTGAACTAAAACTGCTCACCCCCATCAAATGAGGAAACTTGAGGGG
TTTGCAGCCCAGCATTTTACGGG
>2405
CCTTTATTAAGGTGCATTTCTGGGGTTGAACTAAAACTGCTCACCCCCATCAAATGAGG
AAACTTGAGGGGTTTGCAGCCCAGCATTTTACGGGCACTTTTGCGACTGTGTACCCTT

GCGTGGCTTCCCGCTTCAGCGAC
>2406
TGCTCACCCCCATCAAATGAGGAAACTTGAGGGGTTTGCAGCCCAGCATTTTACGGGC
ACTTTTGCGACTGTGTACCCTTGCGTGGCTTCCCGCTTCAGCGACATTGTTGACTTCAG
TTTTCTAATCTAATTCTTTTTTT
>2407
TCAAATGAGGAAACTTGAGGGGTTTGCAGCCCAGCATTTTACGGGCACTTTTGCGACT
GTGTACCCTTGCGTGGCTTCCCGCTTCAGCGACATTGTTGACTTCAGTTTTCTAATCTAA
TTCTTTTTTTCTTTTTTGGTTT
>2408
TGCAGCCCAGCATTTTACGGGCACTTTTGCGACTGTGTACCCTTGCGTGGCTTCCCGCT
TCAGCGACATTGTTGACTTCAGTTTTCTAATCTAATTCTTTTTTTCTTTTTTGGTTTTGAT
GAGAGTGTTGGATACCAAAG
>2409
CATTTTACGGGCACTTTTGCGACTGTGTACCCTTGCGTGGCTTCCCGCTTCAGCGACAT
TGTTGACTTCAGTTTTCTAATCTAATTCTTTTTTTCTTTTTTGGTTTTGATGAGAGTGTTG
GATACCAAAGCACAAACGAT
>2410
TTGCGTGGCTTCCCGCTTCAGCGACATTGTTGACTTCAGTTTTCTAATCTAATTCTTTTT
TTCTTTTTTGGTTTTGATGAGAGTGTTGGATACCAAAGCACAAACGATGGATTCCTTTT
GAAAGAACAAAAACATATTTT
>2411
CCGCTTCAGCGACATTGTTGACTTCAGTTTTCTAATCTAATTCTTTTTTTCTTTTTTGGTT
TTGATGAGAGTGTTGGATACCAAAGCACAAACGATGGATTCCTTTTGAAAGAACAAAA
ACATATTTTGTGAGCAGTTTT
>2412
GTTTTGATGAGAGTGTTGGATACCAAAGCACAAACGATGGATTCCTTTTGAAAGAACA
AAAACATATTTTGTGAGCAGTTTTACTTCTGGGAGTGTTATGTGTATACACACACACAC
ACACACCCTAAATAATTGAAAGC
>2413
TGAGAGTGTTGGATACCAAAGCACAAACGATGGATTCCTTTTGAAAGAACAAAAACAT
ATTTTGTGAGCAGTTTTACTTCTGGGAGTGTTATGTGTATACACACACACACACACACC
CTAAATAATTGAAAGCAGGAACT
>2414
TGTATACACACACACACACACACACCCTAAATAATTGAAAGCAGGAACTTGAACAGATATT
TGCACACCCATGTTCATAGCAGCATTAGCCAAAATAGCCAAAAGGTGGAAATAATTCA
AATGTTCATTGACCCATGGATAA
>2415
TGAAAGCAGGAACTTGAACAGATATTTGCACACCCATGTTCATAGCAGCATTAGCCAA
AATAGCCAAAAGGTGGAAATAATTCAAATGTTCATTGACCCATGGATAAATTGACAAAA
TGTGGTATATACATATAGTGGAA
>2416
ACAGATATTTGCACACCCATGTTCATAGCAGCATTAGCCAAAATAGCCAAAAGGTGGA
AATAATTCAAATGTTCATTGACCCATGGATAAATTGACAAAATGTGGTATATACATATAGT

GGAATATTATTCAGCCTTAAC
>2417
CATTAGCCAAAATAGCCAAAAGGTGGAAATAATTCAAATGTTCATTGACCCATGGATAA
ATTGACAAAATGTGGTATATACATATAGTGGAATATTATTCAGCCTTAACAAGGAAGGAA
ATTCTGACACATGGTACAACA
>2418
TAGCCAAAATAGCCAAAAGGTGGAAATAATTCAAATGTTCATTGACCCATGGATAAATT
GACAAAATGTGGTATATACATATAGTGGAATATTATTCAGCCTTAACAAGGAAGGAAATT
CTGACACATGGTACAACACAG
>2419
GACAAAATGTGGTATATACATATAGTGGAATATTATTCAGCCTTAACAAGGAAGGAAATT
CTGACACATGGTACAACACAGATTAACTTTGAGGGCATTATACTAGGTGAAATAAGCTA
GATAGCAAAGGACAGATAGTG
>2420
TCAGCCTTAACAAGGAAGGAAATTCTGACACATGGTACAACACAGATTAACTTTGAGG
GCATTATACTAGGTGAAATAAGCTAGATAGCAAAGGACAGATAGTGTATGGTGCCATCT
ATATGAGGCATCTGTGCTTATAT
>2421
ATGGTACAACACAGATTAACTTTGAGGGCATTATACTAGGTGAAATAAGCTAGATAGCA
AAGGACAGATAGTGTATGGTGCCATCTATATGAGGCATCTGTGCTTATATAAAGCAAATT
TGTAGAAACACGAACTAGAAT
>2422
AACACAGATTAACTTTGAGGGCATTATACTAGGTGAAATAAGCTAGATAGCAAAGGAC
AGATAGTGTATGGTGCCATCTATATGAGGCATCTGTGCTTATATAAAGCAAATTTGTAGA
AACACGAACTAGAATAGATGTT
>2423
ATTATACTAGGTGAAATAAGCTAGATAGCAAAGGACAGATAGTGTATGGTGCCATCTATA
TGAGGCATCTGTGCTTATATAAAGCAAATTTGTAGAAACACGAACTAGAATAGATGTTG
CCAGGAGCTGGGGAGAGAGGG
>2424
TAGATAGCAAAGGACAGATAGTGTATGGTGCCATCTATATGAGGCATCTGTGCTTATATA
AAGCAAATTTGTAGAAACACGAACTAGAATAGATGTTGCCAGGAGCTGGGGAGAGAG
GGGAATGGGGAGTTAAGGTTGAG
>2425
ATGGTGCCATCTATATGAGGCATCTGTGCTTATATAAAGCAAATTTGTAGAAACACGAAC
TAGAATAGATGTTGCCAGGAGCTGGGGAGAGAGGGGAATGGGGAGTTAAGGTTGAGT
GGGTACAGAGTTTCAGTTGGGGG
>2426
TAAAGCAAATTTGTAGAAACACGAACTAGAATAGATGTTGCCAGGAGCTGGGGAGAG
AGGGGAATGGGGAGTTAAGGTTGAGTGGGTACAGAGTTTCAGTTGGGGGAGATGAAA
AAGTTCTGTGGCTGGATGGTAGTAAT
>2427
AAATTTGTAGAAACACGAACTAGAATAGATGTTGCCAGGAGCTGGGGAGAGAGGGGA
ATGGGGAGTTAAGGTTGAGTGGGTACAGAGTTTCAGTTGGGGGAGATGAAAAAGTTC

TGTGGCTGGATGGTAGTAATGGTTAC
>2428
GCTGGGGAGAGAGGGGAATGGGGAGTTAAGGTTGAGTGGGTACAGAGTTTCAGTTGG
GGGAGATGAAAAAGTTCTGTGGCTGGATGGTAGTAATGGTTACACAACATCAGGAGTG
TACTAAATGACACTGATCTGTACAC
>2429
GGAGAGAGGGGAATGGGGAGTTAAGGTTGAGTGGGTACAGAGTTTCAGTTGGGGGAG
ATGAAAAAGTTCTGTGGCTGGATGGTAGTAATGGTTACACAACATCAGGAGTGTACTA
AATGACACTGATCTGTACACTTAGA
>2430
AATGGGGAGTTAAGGTTGAGTGGGTACAGAGTTTCAGTTGGGGGAGATGAAAAAGTT
CTGTGGCTGGATGGTAGTAATGGTTACACAACATCAGGAGTGTACTAAATGACACTGAT
CTGTACACTTAGAATCTTTAAAAT
>2431
CAGAGTTTCAGTTGGGGGAGATGAAAAAGTTCTGTGGCTGGATGGTAGTAATGGTTAC
ACAACATCAGGAGTGTACTAAATGACACTGATCTGTACACTTAGAATCTTTAAAATGGT
AAATTTTAGTTACATAGTTTTAC
>2432
GAAAAAGTTCTGTGGCTGGATGGTAGTAATGGTTACACAACATCAGGAGTGTACTAAA
TGACACTGATCTGTACACTTAGAATCTTTAAAATGGTAAATTTTAGTTACATAGTTTTAC
TACAATAAAAATGTATTTTATG
>2433
TAGTAATGGTTACACAACATCAGGAGTGTACTAAATGACACTGATCTGTACACTTAGAA
TCTTTAAAATGGTAAATTTTAGTTACATAGTTTTACTACAATAAAAATGTATTTTATGTTA
ATTGCAATACTTGGCATTTG
>2434
ATCAGGAGTGTACTAAATGACACTGATCTGTACACTTAGAATCTTTAAAATGGTAAATTT
TAGTTACATAGTTTTACTACAATAAAAATGTATTTTATGTTAATTGCAATACTTGGCATTT
GAGAAAAAATTCTTAGAAA
>2435
ACACTGATCTGTACACTTAGAATCTTTAAAATGGTAAATTTTAGTTACATAGTTTTACTA
CAATAAAAATGTATTTTATGTTAATTGCAATACTTGGCATTTGAGAAAAAATTCTTAGAA
AATACTGAGAATATCATACT
>2436
TGTACACTTAGAATCTTTAAAATGGTAAATTTTAGTTACATAGTTTTACTACAATAAAAA
TGTATTTTATGTTAATTGCAATACTTGGCATTTGAGAAAAAATTCTTAGAAAATACTGAG
AATATCATACTGATAAAAAC
>2437
AAAATACTGAGAATATCATACTGATAAAAACCCTCTGCTAAATTTTTTTAAAAAAAAC
ACACATCGCCTGTACTCCTGAGTATCAGCGATCAGTCTAGCGTTGCCCTTTAACTATCAT
TATTGTTATTGTTAAAGTGAT
>2438
GAATATCATACTGATAAAAACCCTCTGCTAAATTTTTTTAAAAAAAACACACATCGCCT
GTACTCCTGAGTATCAGCGATCAGTCTAGCGTTGCCCTTTAACTATCATTATTGTTATTGT

TAAAGTGATTTTTATTAAG
>2439
ATAAAAACCCTCTGCTAAATTTTTTTAAAAAAAACACACATCGCCTGTACTCCTGAGT
ATCAGCGATCAGTCTAGCGTTGCCCTTTAACTATCATTATTGTTATTGTTAAAGTGATTTT
TATTAAGTGCTATCTCTCAA
>2440
AAAAACACACATCGCCTGTACTCCTGAGTATCAGCGATCAGTCTAGCGTTGCCCTTTAA
CTATCATTATTGTTATTGTTAAAGTGATTTTTATTAAGTGCTATCTCTCAAGTGGTATGATT
ACTTGTTGAGGTCTTGTGT
>2441
ACACATCGCCTGTACTCCTGAGTATCAGCGATCAGTCTAGCGTTGCCCTTTAACTATCAT
TATTGTTATTGTTAAAGTGATTTTTATTAAGTGCTATCTCTCAAGTGGTATGATTACTTGT
TGAGGTCTTGTGTTGGTAT
>2442
CGCCTGTACTCCTGAGTATCAGCGATCAGTCTAGCGTTGCCCTTTAACTATCATTATTGT
TATTGTTAAAGTGATTTTTATTAAGTGCTATCTCTCAAGTGGTATGATTACTTGTTGAGGT
CTTGTGTTGGTATTTGTCA
>2443
AGTATCAGCGATCAGTCTAGCGTTGCCCTTTAACTATCATTATTGTTATTGTTAAAGTGAT
TTTTATTAAGTGCTATCTCTCAAGTGGTATGATTACTTGTTGAGGTCTTGTGTTGGTATTT
GTCATGTGATTCTAGAAA
>2444
TTAACTATCATTATTGTTATTGTTAAAGTGATTTTTATTAAGTGCTATCTCTCAAGTGGTAT
GATTACTTGTTGAGGTCTTGTGTTGGTATTTGTCATGTGATTCTAGAAAAATCTCCCCCT
TTTTGGCTCTGAGACATT
>2445
ATCATTATTGTTATTGTTAAAGTGATTTTTATTAAGTGCTATCTCTCAAGTGGTATGATTA
CTTGTTGAGGTCTTGTGTTGGTATTTGTCATGTGATTCTAGAAAAATCTCCCCCTTTTTG
GCTCTGAGACATTCTCTAG
>2446
TATTGTTATTGTTAAAGTGATTTTTATTAAGTGCTATCTCTCAAGTGGTATGATTACTTGT
TGAGGTCTTGTGTTGGTATTTGTCATGTGATTCTAGAAAAATCTCCCCCTTTTTGGCTCT
GAGACATTCTCTAGTTGAC
>2447
TATTGTTAAAGTGATTTTTATTAAGTGCTATCTCTCAAGTGGTATGATTACTTGTTGAGGT
CTTGTGTTGGTATTTGTCATGTGATTCTAGAAAAATCTCCCCCTTTTTGGCTCTGAGACA
TTCTCTAGTTGACCTCTTC
>2448
GGTCTTGTGTTGGTATTTGTCATGTGATTCTAGAAAAATCTCCCCCTTTTTGGCTCTGAG
ACATTCTCTAGTTGACCTCTTCCCAGAGAGAAGTTAAGGAAAATCTTCCGCTCTGATTT
TTTTTTCTTTCTGCACTTAAA
>2449
TGTGATTCTAGAAAAATCTCCCCCTTTTTGGCTCTGAGACATTCTCTAGTTGACCTCTTC
CCAGAGAGAAGTTAAGGAAAATCTTCCGCTCTGATTTTTTTTTCTTTCTGCACTTAAAT

AATTAAATAATATCTGTGGCC
>2450
AGAGAAGTTAAGGAAAATCTTCCGCTCTGATTTTTTTTTCTTTCTGCACTTAAATAATTA
AATAATATCTGTGGCCTTACAACACAGGGAGGTTTCAGTGAGGAAGAAAATTCAACTG
CATTTGCAAAATGTCTGAAAAT
>2451
TGATTTTTTTTTCTTTCTGCACTTAAATAATTAAATAATATCTGTGGCCTTACAACACAGG
GAGGTTTCAGTGAGGAAGAAAATTCAACTGCATTTGCAAAATGTCTGAAAATTATGGG
AGAGATGTTTCTGCTTTTAGA
>2452
AATAATATCTGTGGCCTTACAACACAGGGAGGTTTCAGTGAGGAAGAAAATTCAACTG
CATTTGCAAAATGTCTGAAAATTATGGGAGAGATGTTTCTGCTTTTAGACTTGGTACGTA
TGTGGTCAGGTTTTCAGAGGTT
>2453
CACAGGGAGGTTTCAGTGAGGAAGAAAATTCAACTGCATTTGCAAAATGTCTGAAAAT
TATGGGAGAGATGTTTCTGCTTTTAGACTTGGTACGTATGTGGTCAGGTTTTCAGAGGT
TTTCTATTCACCATCTGTGACGG
>2454
GGAAGAAAATTCAACTGCATTTGCAAAATGTCTGAAAATTATGGGAGAGATGTTTCTG
CTTTTAGACTTGGTACGTATGTGGTCAGGTTTTCAGAGGTTTTCTATTCACCATCTGTGA
CGGGCCTTGTCTCCTCCTGCAA
>2455
GAAAATTCAACTGCATTTGCAAAATGTCTGAAAATTATGGGAGAGATGTTTCTGCTTTT
AGACTTGGTACGTATGTGGTCAGGTTTTCAGAGGTTTTCTATTCACCATCTGTGACGGG
CCTTGTCTCCTCCTGCAACTGG
>2456
AATGTCTGAAAATTATGGGAGAGATGTTTCTGCTTTTAGACTTGGTACGTATGTGGTCA
GGTTTTCAGAGGTTTTCTATTCACCATCTGTGACGGGCCTTGTCTCCTCCTGCAACTGG
TGGCAGATGTAGCCAGAGATTT
>2457
GAGAGATGTTTCTGCTTTTAGACTTGGTACGTATGTGGTCAGGTTTTCAGAGGTTTTCTA
TTCACCATCTGTGACGGGCCTTGTCTCCTCCTGCAACTGGTGGCAGATGTAGCCAGAG
ATTTTGTGAGCTTCTCAACAGC
>2458
TGCTTTTAGACTTGGTACGTATGTGGTCAGGTTTTCAGAGGTTTTCTATTCACCATCTGT
GACGGGCCTTGTCTCCTCCTGCAACTGGTGGCAGATGTAGCCAGAGATTTTGTGAGCT
TCTCAACAGCATCTTTTTCCTG
>2459
CGTATGTGGTCAGGTTTTCAGAGGTTTTCTATTCACCATCTGTGACGGGCCTTGTCTCCT
CCTGCAACTGGTGGCAGATGTAGCCAGAGATTTTGTGAGCTTCTCAACAGCATCTTTTT
CCTGCTCTTCTTCCCTTCCTC
>2460
TTTTCTATTCACCATCTGTGACGGGCCTTGTCTCCTCCTGCAACTGGTGGCAGATGTAG
CCAGAGATTTTGTGAGCTTCTCAACAGCATCTTTTTCCTGCTCTTCTTCCCTTCCTCTTT

GTTTGGTTTTTATTCACATGA
>2461
GCAGATGTAGCCAGAGATTTTGTGAGCTTCTCAACAGCATCTTTTTCCTGCTCTTCTTC
CCTTCCTCTTTGTTTGGTTTTTATTCACATGAAAATTTAAAGACCCATAAGGAAATTCAA
AGTGAAAATCTATGCCCAAAT
>2462
TGTAGCCAGAGATTTTGTGAGCTTCTCAACAGCATCTTTTTCCTGCTCTTCTTCCCTTCC
TCTTTGTTTGGTTTTTATTCACATGAAAATTTAAAGACCCATAAGGAAATTCAAAGTGA
AAATCTATGCCCAAATAAGTC
>2463
CTCTTCTTCCCTTCCTCTTTGTTTGGTTTTTATTCACATGAAAATTTAAAGACCCATAAG
GAAATTCAAAGTGAAAATCTATGCCCAAATAAGTCAGGATCTCAAGATGAAAATATAAG
TTCTGGAAATGGAAGTTTCCT
>2464
TTGGTTTTTATTCACATGAAAATTTAAAGACCCATAAGGAAATTCAAAGTGAAAATCTA
TGCCCAAATAAGTCAGGATCTCAAGATGAAAATATAAGTTCTGGAAATGGAAGTTTCCT
CTGCCCCCGTGGTGGTATCATA
>2465
AAGACCCATAAGGAAATTCAAAGTGAAAATCTATGCCCAAATAAGTCAGGATCTCAAG
ATGAAAATATAAGTTCTGGAAATGGAAGTTTCCTCTGCCCCCGTGGTGGTATCATAGCC
AGCAGCCTCGTTCTGTGCTTTTG
>2466
ATTCAAAGTGAAAATCTATGCCCAAATAAGTCAGGATCTCAAGATGAAAATATAAGTTC
TGGAAATGGAAGTTTCCTCTGCCCCCGTGGTGGTATCATAGCCAGCAGCCTCGTTCTGT
GCTTTTGAAGGTTTAATGGCCT
>2467
AGATGAAAATATAAGTTCTGGAAATGGAAGTTTCCTCTGCCCCCGTGGTGGTATCATAG
CCAGCAGCCTCGTTCTGTGCTTTTGAAGGTTTAATGGCCTGCATGTGAAGGTAGGGGC
GCACTATATATATAGTACCAGGC
>2468
AAAATATAAGTTCTGGAAATGGAAGTTTCCTCTGCCCCCGTGGTGGTATCATAGCCAGC
AGCCTCGTTCTGTGCTTTTGAAGGTTTAATGGCCTGCATGTGAAGGTAGGGGCGCACTA
TATATATAGTACCAGGCGTGTG
>2469
CCTCTGCCCCCGTGGTGGTATCATAGCCAGCAGCCTCGTTCTGTGCTTTTGAAGGTTTA
ATGGCCTGCATGTGAAGGTAGGGGCGCACTATATATATAGTACCAGGCGTGTGGGCGCA
CTGGCACGGATCTGAAAAAGCG
>2470
AGCAGCCTCGTTCTGTGCTTTTGAAGGTTTAATGGCCTGCATGTGAAGGTAGGGGCGC
ACTATATATATAGTACCAGGCGTGTGGGCGCACTGGCACGGATCTGAAAAAGCGTAAGT
TTGGCATTTCTAGGTTTATTTTA
>2471
GTTCTGTGCTTTTGAAGGTTTAATGGCCTGCATGTGAAGGTAGGGGCGCACTATATATAT
AGTACCAGGCGTGTGGGCGCACTGGCACGGATCTGAAAAAGCGTAAGTTTGGCATTTC

TAGGTTTATTTTAAACTTACAA
>2472
TGTGAAGGTAGGGGCGCACTATATATATAGTACCAGGCGTGTGGGCGCACTGGCACGGA
TCTGAAAAAGCGTAAGTTTGGCATTTCTAGGTTTATTTTAAACTTACAACCTTTAACGC
CAGACACAGAGCTTCTTTCCCA
>2473
AAGGTAGGGGCGCACTATATATATAGTACCAGGCGTGTGGGCGCACTGGCACGGATCTG
AAAAAGCGTAAGTTTGGCATTTCTAGGTTTATTTTAAACTTACAACCTTTAACGCCAGA
CACAGAGCTTCTTTCCCACTTA
>2474
TATATATATAGTACCAGGCGTGTGGGCGCACTGGCACGGATCTGAAAAAGCGTAAGTTT
GGCATTTCTAGGTTTATTTTAAACTTACAACCTTTAACGCCAGACACAGAGCTTCTTTCC
CACTTAATGTAACTATACATT
>2475
TTGGCATTTCTAGGTTTATTTTAAACTTACAACCTTTAACGCCAGACACAGAGCTTCTTT
CCCACTTAATGTAACTATACATTGATTGCTTTTAATTAGTGTTGGATCTTTTTTGGTGTTT
TGCAGCTTGGGATATATAA
>2476
ACAACCTTTAACGCCAGACACAGAGCTTCTTTCCCACTTAATGTAACTATACATTGATT
GCTTTTAATTAGTGTTGGATCTTTTTTGGTGTTTTGCAGCTTGGGATATATAAAAAGTCT
GACTTAAATTTAAAAAAAAAA
>2477
ACACAGAGCTTCTTTCCCACTTAATGTAACTATACATTGATTGCTTTTAATTAGTGTTGG
ATCTTTTTTGGTGTTTTGCAGCTTGGGATATATAAAAAGTCTGACTTAAATTTAAAAAAA
AAAAAAGCTAACTCCTGACA
>2478
ACAGAGCTTCTTTCCCACTTAATGTAACTATACATTGATTGCTTTTAATTAGTGTTGGATC
TTTTTTGGTGTTTTGCAGCTTGGGATATATAAAAAGTCTGACTTAAATTTAAAAAAAAA
AAAAGCTAACTCCTGACATT
>2479
ACTATACATTGATTGCTTTTAATTAGTGTTGGATCTTTTTTGGTGTTTTGCAGCTTGGGAT
ATATAAAAAGTCTGACTTAAATTTAAAAAAAAAAAAAGCTAACTCCTGACATTCTCACG
AGTCTTGCAAAAACCAGCAC
>2480
AGCTTGGGATATATAAAAAGTCTGACTTAAATTTAAAAAAAAAAAAAGCTAACTCCTG
ACATTCTCACGAGTCTTGCAAAAACCAGCACGGGAGCCTTAGAAATTCTAACCTCCTC
GTCCTCTTTATTTTTATCTTGAGT
>2481
AGCTAACTCCTGACATTCTCACGAGTCTTGCAAAAACCAGCACGGGAGCCTTAGAAAT
TCTAACCTCCTCGTCCTCTTTATTTTTATCTTGAGTCAGGTGGGCCAAGAAGCAAGGGA
AGAGTCCAATATAACATACTTAA
>2482
GAGTCTTGCAAAAACCAGCACGGGAGCCTTAGAAATTCTAACCTCCTCGTCCTCTTTAT
TTTTATCTTGAGTCAGGTGGGCCAAGAAGCAAGGGAAGAGTCCAATATAACATACTTAA

TGGATGGAGGTAATTTCCTTGG
>2483
TTAGAAATTCTAACCTCCTCGTCCTCTTTATTTTTATCTTGAGTCAGGTGGGCCAAGAAG
CAAGGGAAGAGTCCAATATAACATACTTAATGGATGGAGGTAATTTCCTTGGACTGTTT
TGGCAGAAATTAAGCATCTGT
>2484
ATTTTTATCTTGAGTCAGGTGGGCCAAGAAGCAAGGGAAGAGTCCAATATAACATACTT
AATGGATGGAGGTAATTTCCTTGGACTGTTTTGGCAGAAATTAAGCATCTGTATTTAGAT
AAAGGTAGTGGTGGGTTGGAT
>2485
AGGTGGGCCAAGAAGCAAGGGAAGAGTCCAATATAACATACTTAATGGATGGAGGTAA
TTTCCTTGGACTGTTTTGGCAGAAATTAAGCATCTGTATTTAGATAAAGGTAGTGGTGG
GTTGGATTTTCATAGCATAAGCA
>2486
GAGTCCAATATAACATACTTAATGGATGGAGGTAATTTCCTTGGACTGTTTTGGCAGAA
ATTAAGCATCTGTATTTAGATAAAGGTAGTGGTGGGTTGGATTTTCATAGCATAAGCATA
GTTGCTGAAGATCAATAATAT
>2487
ATACTTAATGGATGGAGGTAATTTCCTTGGACTGTTTTGGCAGAAATTAAGCATCTGTAT
TTAGATAAAGGTAGTGGTGGGTTGGATTTTCATAGCATAAGCATAGTTGCTGAAGATCA
ATAATATCAAATAATGGGTTC
>2488
GATGGAGGTAATTTCCTTGGACTGTTTTGGCAGAAATTAAGCATCTGTATTTAGATAAA
GGTAGTGGTGGGTTGGATTTTCATAGCATAAGCATAGTTGCTGAAGATCAATAATATCAA
ATAATGGGTTCTTGGTGAGGG
>2489
AAAGGTAGTGGTGGGTTGGATTTTCATAGCATAAGCATAGTTGCTGAAGATCAATAATA
TCAAATAATGGGTTCTTGGTGAGGGGGAAAGGAAATGGGGGAGGGGTCTTTTCACTCA
ACAGTTTGGAAATGCTAATGATT
>2490
GTGGTGGGTTGGATTTTCATAGCATAAGCATAGTTGCTGAAGATCAATAATATCAAATAA
TGGGTTCTTGGTGAGGGGGAAAGGAAATGGGGGAGGGGTCTTTTCACTCAACAGTTT
GGAAATGCTAATGATTATTTGTG
>2491
TCAATAATATCAAATAATGGGTTCTTGGTGAGGGGGAAAGGAAATGGGGGAGGGGTCT
TTTCACTCAACAGTTTGGAAATGCTAATGATTATTTGTGGTTTTCAGCCCAGAATTCTTC
ATATTTGCTTGCAAGCTTCTAT
>2492
TTGGTGAGGGGGAAAGGAAATGGGGGAGGGGTCTTTTCACTCAACAGTTTGGAAATG
CTAATGATTATTTGTGGTTTTCAGCCCAGAATTCTTCATATTTGCTTGCAAGCTTCTATGG
CACTGCCGAATTCTACAGTAAA
>2493
TATTTGTGGTTTTCAGCCCAGAATTCTTCATATTTGCTTGCAAGCTTCTATGGCACTGCC
GAATTCTACAGTAAATATTTCGGACATCCTCAGCCCTGTTCTCCTTTCCCTTCAGTAAAT

TACATATTAAAGTAGCCTGG
>2494
TCATATTTGCTTGCAAGCTTCTATGGCACTGCCGAATTCTACAGTAAATATTTCGGACAT
CCTCAGCCCTGTTCTCCTTTCCCTTCAGTAAATTACATATTAAAGTAGCCTGGTAACTGA
GCACTGGTTGCTGTGAGATA
>2495
CTTCTATGGCACTGCCGAATTCTACAGTAAATATTTCGGACATCCTCAGCCCTGTTCTCC
TTTCCCTTCAGTAAATTACATATTAAAGTAGCCTGGTAACTGAGCACTGGTTGCTGTGA
GATAAGGGCTGGGAAATCAAT
>2496
AATTCTACAGTAAATATTTCGGACATCCTCAGCCCTGTTCTCCTTTCCCTTCAGTAAATT
ACATATTAAAGTAGCCTGGTAACTGAGCACTGGTTGCTGTGAGATAAGGGCTGGGAAA
TCAATATGTTTGCCATGTGGCT
>2497
ACATCCTCAGCCCTGTTCTCCTTTCCCTTCAGTAAATTACATATTAAAGTAGCCTGGTAA
CTGAGCACTGGTTGCTGTGAGATAAGGGCTGGGAAATCAATATGTTTGCCATGTGGCTG
CTTGTTTATGACACCTGCCTC
>2498
TCAGCCCTGTTCTCCTTTCCCTTCAGTAAATTACATATTAAAGTAGCCTGGTAACTGAGC
ACTGGTTGCTGTGAGATAAGGGCTGGGAAATCAATATGTTTGCCATGTGGCTGCTTGTT
TATGACACCTGCCTCTAGAAC
>2499
AAATTACATATTAAAGTAGCCTGGTAACTGAGCACTGGTTGCTGTGAGATAAGGGCTGG
GAAATCAATATGTTTGCCATGTGGCTGCTTGTTTATGACACCTGCCTCTAGAACAGTTG
AGCAATGTTCTGTTTGTTAAAG
>2500
TGAGATAAGGGCTGGGAAATCAATATGTTTGCCATGTGGCTGCTTGTTTATGACACCTG
CCTCTAGAACAGTTGAGCAATGTTCTGTTTGTTAAAGCTGCATTTCAGAAGCTTGTATC
CTCCTAAAACAACCGTGATCTT
>2501
GCTGGGAAATCAATATGTTTGCCATGTGGCTGCTTGTTTATGACACCTGCCTCTAGAAC
AGTTGAGCAATGTTCTGTTTGTTAAAGCTGCATTTCAGAAGCTTGTATCCTCCTAAAAC
AACCGTGATCTTACTGGGGGAG
>2502
TTGTTTATGACACCTGCCTCTAGAACAGTTGAGCAATGTTCTGTTTGTTAAAGCTGCAT
TTCAGAAGCTTGTATCCTCCTAAAACAACCGTGATCTTACTGGGGGAGAGAGAGGTGC
TACAACAGATATTGATTTTAGTT
>2503
CTAGAACAGTTGAGCAATGTTCTGTTTGTTAAAGCTGCATTTCAGAAGCTTGTATCCTC
CTAAAACAACCGTGATCTTACTGGGGGAGAGAGAGGTGCTACAACAGATATTGATTTT
AGTTTTTCTAAGTATGGAATTAC
>2504
TTTGTTAAAGCTGCATTTCAGAAGCTTGTATCCTCCTAAAACAACCGTGATCTTACTGG
GGGAGAGAGAGGTGCTACAACAGATATTGATTTTAGTTTTTCTAAGTATGGAATTACAC

TTCTAAACAGTGGTCTTACAGC
>2505
CTTGTATCCTCCTAAAACAACCGTGATCTTACTGGGGGAGAGAGAGGTGCTACAACAG
ATATTGATTTTAGTTTTTCTAAGTATGGAATTACACTTCTAAACAGTGGTCTTACAGCCT
GTGATTTTTTCCCCATGAGGAG
>2506
CCTAAAACAACCGTGATCTTACTGGGGGAGAGAGAGGTGCTACAACAGATATTGATTTT
AGTTTTTCTAAGTATGGAATTACACTTCTAAACAGTGGTCTTACAGCCTGTGATTTTTTC
CCCATGAGGAGGAGATGTAGA
>2507
GGGGGAGAGAGAGGTGCTACAACAGATATTGATTTTAGTTTTTCTAAGTATGGAATTAC
ACTTCTAAACAGTGGTCTTACAGCCTGTGATTTTTTCCCCATGAGGAGGAGATGTAGAT
ACTCTGAATATATATATACACA
>2508
GCTACAACAGATATTGATTTTAGTTTTTCTAAGTATGGAATTACACTTCTAAACAGTGGT
CTTACAGCCTGTGATTTTTTCCCCATGAGGAGGAGATGTAGATACTCTGAATATATATATA
CACATACACACATATATAA
>2509
TCTAAGTATGGAATTACACTTCTAAACAGTGGTCTTACAGCCTGTGATTTTTTCCCCATG
AGGAGGAGATGTAGATACTCTGAATATATATATACACATACACACATATATAAAATAAATC
TATTTTATAATGTTTATGA
>2510
GAGGAGATGTAGATACTCTGAATATATATATACACATACACACATATATAAAATAAATCTA
TTTTATAATGTTTATGATGTGGTCAGTTAAAAAAGGAGTGTAGTTTATAATAAGGAAGTG
TAGGTATTATTTAGATTTA
>2511
TAGATACTCTGAATATATATATACACATACACACATATATAAAATAAATCTATTTTATAATG
TTTATGATGTGGTCAGTTAAAAAAGGAGTGTAGTTTATAATAAGGAAGTGTAGGTATTAT
TTAGATTTATCTCCTCTC
>2512
TACACATACACACATATATAAAATAAATCTATTTTATAATGTTTATGATGTGGTCAGTTAA
AAAAGGAGTGTAGTTTATAATAAGGAAGTGTAGGTATTATTTAGATTTATCTCCTCTCTT
GGCACCTGGCTTAAAAAAA
>2513
ACATACACACATATATAAAATAAATCTATTTTATAATGTTTATGATGTGGTCAGTTAAAAA
AGGAGTGTAGTTTATAATAAGGAAGTGTAGGTATTATTTAGATTTATCTCCTCTCTTGGC
ACCTGGCTTAAAAAAAAAA
>2514
TAAAATAAATCTATTTTATAATGTTTATGATGTGGTCAGTTAAAAAAGGAGTGTAGTTTAT
AATAAGGAAGTGTAGGTATTATTTAGATTTATCTCCTCTCTTGGCACCTGGCTTAAAAAA
AAAAAAAGTAGTAACAAGA
>2515
TATAATAAGGAAGTGTAGGTATTATTTAGATTTATCTCCTCTCTTGGCACCTGGCTTAAA
AAAAAAAAAAGTAGTAACAAGAAACATAGCAGCTGCTGGGAAACATGCATTTTAATCT

AAGTCTCCAGTGTGGTTTTAGT
>2516
AATAAGGAAGTGTAGGTATTATTTAGATTTATCTCCTCTCTTGGCACCTGGCTTAAAAAA
AAAAAAAGTAGTAACAAGAAACATAGCAGCTGCTGGGAAACATGCATTTTAATCTAAG
TCTCCAGTGTGGTTTTAGTTTA
>2517
CTGGCTTAAAAAAAAAAAAAGTAGTAACAAGAAACATAGCAGCTGCTGGGAAACATG
CATTTTAATCTAAGTCTCCAGTGTGGTTTTAGTTTAACCAGTGGATGATACTGTAGTCTC
AAGGCTTAGCCTGCTGGTGAGTC
>2518
AAAAAAAAAAAAAGTAGTAACAAGAAACATAGCAGCTGCTGGGAAACATGCATTTTA
ATCTAAGTCTCCAGTGTGGTTTTAGTTTAACCAGTGGATGATACTGTAGTCTCAAGGCTT
AGCCTGCTGGTGAGTCAATGGTT
>2519
AAGTAGTAACAAGAAACATAGCAGCTGCTGGGAAACATGCATTTTAATCTAAGTCTCC
AGTGTGGTTTTAGTTTAACCAGTGGATGATACTGTAGTCTCAAGGCTTAGCCTGCTGGT
GAGTCAATGGTTCTTAAAGCTGT
>2520
CTGCTGGGAAACATGCATTTTAATCTAAGTCTCCAGTGTGGTTTTAGTTTAACCAGTGG
ATGATACTGTAGTCTCAAGGCTTAGCCTGCTGGTGAGTCAATGGTTCTTAAAGCTGTGT
GGTGTCAGCTCAAGTGAGCGAA
>2521
AATCTAAGTCTCCAGTGTGGTTTTAGTTTAACCAGTGGATGATACTGTAGTCTCAAGGC
TTAGCCTGCTGGTGAGTCAATGGTTCTTAAAGCTGTGTGGTGTCAGCTCAAGTGAGCG
AAGCAGTGTGTGGTGTGTGTGCA
>2522
TAAGTCTCCAGTGTGGTTTTAGTTTAACCAGTGGATGATACTGTAGTCTCAAGGCTTAG
CCTGCTGGTGAGTCAATGGTTCTTAAAGCTGTGTGGTGTCAGCTCAAGTGAGCGAAGC
AGTGTGTGGTGTGTGTGCATTGG
>2523
TAGTTTAACCAGTGGATGATACTGTAGTCTCAAGGCTTAGCCTGCTGGTGAGTCAATGG
TTCTTAAAGCTGTGTGGTGTCAGCTCAAGTGAGCGAAGCAGTGTGTGGTGTGTGTGCA
TTGGAGGGTGAGGGGTATGAGGA
>2524
TCAAGGCTTAGCCTGCTGGTGAGTCAATGGTTCTTAAAGCTGTGTGGTGTCAGCTCAA
GTGAGCGAAGCAGTGTGTGGTGTGTGTGCATTGGAGGGTGAGGGGTATGAGGAGGTA
GTCAGCCTTGGAGGGAGAAGGGAGA
>2525
AAGGCTTAGCCTGCTGGTGAGTCAATGGTTCTTAAAGCTGTGTGGTGTCAGCTCAAGT
GAGCGAAGCAGTGTGTGGTGTGTGTGCATTGGAGGGTGAGGGGTATGAGGAGGTAGT
CAGCCTTGGAGGGAGAAGGGAGACA
>2526
TTAGCCTGCTGGTGAGTCAATGGTTCTTAAAGCTGTGTGGTGTCAGCTCAAGTGAGCG
AAGCAGTGTGTGGTGTGTGTGCATTGGAGGGTGAGGGGTATGAGGAGGTAGTCAGCC

TTGGAGGGAGAAGGGAGACAGGCTA
>2527
AATGGTTCTTAAAGCTGTGTGGTGTCAGCTCAAGTGAGCGAAGCAGTGTGTGGTGTGT
GTGCATTGGAGGGTGAGGGGTATGAGGAGGTAGTCAGCCTTGGAGGGAGAAGGGAGA
CAGGCTAGGTCTTCTTCACGTGGCC
>2528
GGTGTCAGCTCAAGTGAGCGAAGCAGTGTGTGGTGTGTGTGCATTGGAGGGTGAGGG
GTATGAGGAGGTAGTCAGCCTTGGAGGGAGAAGGGAGACAGGCTAGGTCTTCTTCAC
GTGGCCTTCCATTTTGGAAAACAGAA
>2529
TGTGTGTGCATTGGAGGGTGAGGGGTATGAGGAGGTAGTCAGCCTTGGAGGGAGAAG
GGAGACAGGCTAGGTCTTCTTCACGTGGCCTTCCATTTTGGAAAACAGAAGCGATGAT
ACAACTTCTCTTCTTGCCCAAGGGA
>2530
TGGAGGGTGAGGGGTATGAGGAGGTAGTCAGCCTTGGAGGGAGAAGGGAGACAGGCT
AGGTCTTCTTCACGTGGCCTTCCATTTTGGAAAACAGAAGCGATGATACAACTTCTCTT
CTTGCCCAAGGGATTCAGTAGAAA
>2531
TCTTCACGTGGCCTTCCATTTTGGAAAACAGAAGCGATGATACAACTTCTCTTCTTGCC
CAAGGGATTCAGTAGAAAAAAATACATTTCCAAACACATTGCGCTACAGTCATTGAAC
TTCAAAGTGTAAAGCATGGATTG
>2532
TGATACAACTTCTCTTCTTGCCCAAGGGATTCAGTAGAAAAAAATACATTTCCAAACAC
ATTGCGCTACAGTCATTGAACTTCAAAGTGTAAAGCATGGATTGTGTTGGTGCACTTGT
GGGTGCCCATGACACACACTCT
>2533
CTTGCCCAAGGGATTCAGTAGAAAAAAATACATTTCCAAACACATTGCGCTACAGTCAT
TGAACTTCAAAGTGTAAAGCATGGATTGTGTTGGTGCACTTGTGGGTGCCCATGACAC
ACACTCTTCAATGCTCCTGTGCC
>2534
AGTAGAAAAAAATACATTTCCAAACACATTGCGCTACAGTCATTGAACTTCAAAGTGT
AAAGCATGGATTGTGTTGGTGCACTTGTGGGTGCCCATGACACACACTCTTCAATGCTC
CTGTGCCTTCCTAAGAGGTGTCT
>2535
TCCAAACACATTGCGCTACAGTCATTGAACTTCAAAGTGTAAAGCATGGATTGTGTTGG
TGCACTTGTGGGTGCCCATGACACACACTCTTCAATGCTCCTGTGCCTTCCTAAGAGGT
GTCTGTCTTTCTCAACCCCAGT
>2536
TCAAAGTGTAAAGCATGGATTGTGTTGGTGCACTTGTGGGTGCCCATGACACACACTC
TTCAATGCTCCTGTGCCTTCCTAAGAGGTGTCTGTCTTTCTCAACCCCAGTGTGTGTCC
TCTGACGTCTAGTGGATTGCTGC
>2537
TGGATTGTGTTGGTGCACTTGTGGGTGCCCATGACACACACTCTTCAATGCTCCTGTGC
CTTCCTAAGAGGTGTCTGTCTTTCTCAACCCCAGTGTGTGTCCTCTGACGTCTAGTGGA

TTGCTGCATACACAGTCAACCC
>2538
GGGTGCCCATGACACACACTCTTCAATGCTCCTGTGCCTTCCTAAGAGGTGTCTGTCTT
TCTCAACCCCAGTGTGTGTCCTCTGACGTCTAGTGGATTGCTGCATACACAGTCAACCC
TCCTTAAAAGGTTTAAAAAGAA
>2539
GTGCCCATGACACACACTCTTCAATGCTCCTGTGCCTTCCTAAGAGGTGTCTGTCTTTC
TCAACCCCAGTGTGTGTCCTCTGACGTCTAGTGGATTGCTGCATACACAGTCAACCCTC
CTTAAAAGGTTTAAAAAGAAAC
>2540
CACTCTTCAATGCTCCTGTGCCTTCCTAAGAGGTGTCTGTCTTTCTCAACCCCAGTGTG
TGTCCTCTGACGTCTAGTGGATTGCTGCATACACAGTCAACCCTCCTTAAAAGGTTTAA
AAAGAAACATGAGCTCCCTTTT
>2541
CCTAAGAGGTGTCTGTCTTTCTCAACCCCAGTGTGTGTCCTCTGACGTCTAGTGGATTG
CTGCATACACAGTCAACCCTCCTTAAAAGGTTTAAAAAGAAACATGAGCTCCCTTTTA
GAGCACCCCAAAGCAGCTGCATT
>2542
TTCTCAACCCCAGTGTGTGTCCTCTGACGTCTAGTGGATTGCTGCATACACAGTCAACC
CTCCTTAAAAGGTTTAAAAAGAAACATGAGCTCCCTTTTAGAGCACCCCAAAGCAGCT
GCATTCTTGAGTTGGTGCCCTGC
>2543
CCCTCCTTAAAAGGTTTAAAAAGAAACATGAGCTCCCTTTTAGAGCACCCCAAAGCAG
CTGCATTCTTGAGTTGGTGCCCTGCACTGGGATTCCCATGGACTGGGAACATGATCTCT
TAATGACACTAATATATTCTGAG
>2544
CCTTAAAAGGTTTAAAAAGAAACATGAGCTCCCTTTTAGAGCACCCCAAAGCAGCTGC
ATTCTTGAGTTGGTGCCCTGCACTGGGATTCCCATGGACTGGGAACATGATCTCTTAAT
GACACTAATATATTCTGAGATCT
>2545
CACTGGGATTCCCATGGACTGGGAACATGATCTCTTAATGACACTAATATATTCTGAGAT
CTGATTTTTTGTTTGTGTGTTTTTTAACCCAGGTTGGCAGGTTCTCCTTAATCAGTAGTG
GCAAAATGTCTCTTTCCTTT
>2546
GGGATTCCCATGGACTGGGAACATGATCTCTTAATGACACTAATATATTCTGAGATCTGA
TTTTTTGTTTGTGTGTTTTTTAACCCAGGTTGGCAGGTTCTCCTTAATCAGTAGTGGCAA
AATGTCTCTTTCCTTTCTCC
>2547
TAATATATTCTGAGATCTGATTTTTTGTTTGTGTGTTTTTTAACCCAGGTTGGCAGGTTCT
CCTTAATCAGTAGTGGCAAAATGTCTCTTTCCTTTCTCCACGTAATGCTAAATGGCTTAT
CCAGTCATCTCTCTCTGCT
>2548
TATATTCTGAGATCTGATTTTTTGTTTGTGTGTTTTTTAACCCAGGTTGGCAGGTTCTCCT
TAATCAGTAGTGGCAAAATGTCTCTTTCCTTTCTCCACGTAATGCTAAATGGCTTATCCA

GTCATCTCTCTCTGCTCAT
>2549
GTGTTTTTTAACCCAGGTTGGCAGGTTCTCCTTAATCAGTAGTGGCAAAATGTCTCTTT
CCTTTCTCCACGTAATGCTAAATGGCTTATCCAGTCATCTCTCTCTGCTCATGGTCAGCC
CGAGGTGACAGTTAAAATTGG
>2550
AGGTTCTCCTTAATCAGTAGTGGCAAAATGTCTCTTTCCTTTCTCCACGTAATGCTAAAT
GGCTTATCCAGTCATCTCTCTCTGCTCATGGTCAGCCCGAGGTGACAGTTAAAATTGGA
TGGAGGTAGCTTGGGGTAGGA
>2551
TGGCAAAATGTCTCTTTCCTTTCTCCACGTAATGCTAAATGGCTTATCCAGTCATCTCTC
TCTGCTCATGGTCAGCCCGAGGTGACAGTTAAAATTGGATGGAGGTAGCTTGGGGTAG
GAAGACTTGGTGATTTTGGCGG
>2552
CTCTTTCCTTTCTCCACGTAATGCTAAATGGCTTATCCAGTCATCTCTCTCTGCTCATGGT
CAGCCCGAGGTGACAGTTAAAATTGGATGGAGGTAGCTTGGGGTAGGAAGACTTGGT
GATTTTGGCGGCACTGGGTCAA
>2553
TATCCAGTCATCTCTCTCTGCTCATGGTCAGCCCGAGGTGACAGTTAAAATTGGATGGA
GGTAGCTTGGGGTAGGAAGACTTGGTGATTTTGGCGGCACTGGGTCAAACCCAGGGA
AACTGAAGCTGTGTCTGCACAGCT
>2554
CTCTCTGCTCATGGTCAGCCCGAGGTGACAGTTAAAATTGGATGGAGGTAGCTTGGGG
TAGGAAGACTTGGTGATTTTGGCGGCACTGGGTCAAACCCAGGGAAACTGAAGCTGT
GTCTGCACAGCTGGTTGAAAGGCTG
>2555
CCGAGGTGACAGTTAAAATTGGATGGAGGTAGCTTGGGGTAGGAAGACTTGGTGATTT
TGGCGGCACTGGGTCAAACCCAGGGAAACTGAAGCTGTGTCTGCACAGCTGGTTGAA
AGGCTGTGTTGTGATCCAGGGATTT
>2556
GGAGGTAGCTTGGGGTAGGAAGACTTGGTGATTTTGGCGGCACTGGGTCAAACCCAG
GGAAACTGAAGCTGTGTCTGCACAGCTGGTTGAAAGGCTGTGTTGTGATCCAGGGATT
TAAGTAAAAATATTCTTTCCTTACC
>2557
AGGTAGCTTGGGGTAGGAAGACTTGGTGATTTTGGCGGCACTGGGTCAAACCCAGGG
AAACTGAAGCTGTGTCTGCACAGCTGGTTGAAAGGCTGTGTTGTGATCCAGGGATTTA
AGTAAAAATATTCTTTCCTTACCAC
>2558
TAGGAAGACTTGGTGATTTTGGCGGCACTGGGTCAAACCCAGGGAAACTGAAGCTGT
GTCTGCACAGCTGGTTGAAAGGCTGTGTTGTGATCCAGGGATTTAAGTAAAAATATTCT
TTCCTTACCACCCTCTATGCCCCC
>2559
TGATTTTGGCGGCACTGGGTCAAACCCAGGGAAACTGAAGCTGTGTCTGCACAGCTG
GTTGAAAGGCTGTGTTGTGATCCAGGGATTTAAGTAAAAATATTCTTTCCTTACCACCC

TCTATGCCCCCTACTCCCCAGTAA
>2560
CTGTGTTGTGATCCAGGGATTTAAGTAAAAATATTCTTTCCTTACCACCCTCTATGCCCC
CTACTCCCCAGTAATATAGTAGGCATGGGGGTTGACTTATTTTGAGGGGACTAGTATGTA
TTTAATATTTCATCTAATTT
>2561
TGATCCAGGGATTTAAGTAAAAATATTCTTTCCTTACCACCCTCTATGCCCCCTACTCCC
CAGTAATATAGTAGGCATGGGGGTTGACTTATTTTGAGGGGACTAGTATGTATTTAATAT
TTCATCTAATTTTTGTAGCC
>2562
ACCACCCTCTATGCCCCCTACTCCCCAGTAATATAGTAGGCATGGGGGTTGACTTATTTT
GAGGGGACTAGTATGTATTTAATATTTCATCTAATTTTTGTAGCCCTGGCCTGGCTGGAT
GACAAAAGTGTTTAAACTAA
>2563
CCCTCTATGCCCCCTACTCCCCAGTAATATAGTAGGCATGGGGGTTGACTTATTTTGAGG
GGACTAGTATGTATTTAATATTTCATCTAATTTTTGTAGCCCTGGCCTGGCTGGATGACA
AAAGTGTTTAAACTAAGACC
>2564
AATATAGTAGGCATGGGGGTTGACTTATTTTGAGGGGACTAGTATGTATTTAATATTTCAT
CTAATTTTTGTAGCCCTGGCCTGGCTGGATGACAAAAGTGTTTAAACTAAGACCGATTT
GAGCAAGTATTTTATCCTCC
>2565
TTTGAGGGGACTAGTATGTATTTAATATTTCATCTAATTTTTGTAGCCCTGGCCTGGCTGG
ATGACAAAAGTGTTTAAACTAAGACCGATTTGAGCAAGTATTTTATCCTCCCTCTCGGG
AGACTATGGGCATAGAACGG
>2566
TGAGGGGACTAGTATGTATTTAATATTTCATCTAATTTTTGTAGCCCTGGCCTGGCTGGAT
GACAAAAGTGTTTAAACTAAGACCGATTTGAGCAAGTATTTTATCCTCCCTCTCGGGAG
ACTATGGGCATAGAACGGTG
>2567
TTCATCTAATTTTTGTAGCCCTGGCCTGGCTGGATGACAAAAGTGTTTAAACTAAGACC
GATTTGAGCAAGTATTTTATCCTCCCTCTCGGGAGACTATGGGCATAGAACGGTGTGGG
GATTTGGAGGTGCTGGCAAGTC
>2568
AGTGTTTAAACTAAGACCGATTTGAGCAAGTATTTTATCCTCCCTCTCGGGAGACTATG
GGCATAGAACGGTGTGGGGATTTGGAGGTGCTGGCAAGTCTGGCCCCTTTCCTCTTGC
GTTTACTCAGGCACAGATGGAAA
>2569
TGTTTAAACTAAGACCGATTTGAGCAAGTATTTTATCCTCCCTCTCGGGAGACTATGGG
CATAGAACGGTGTGGGGATTTGGAGGTGCTGGCAAGTCTGGCCCCTTTCCTCTTGCGT
TTACTCAGGCACAGATGGAAAAA
>2570
CGGGAGACTATGGGCATAGAACGGTGTGGGGATTTGGAGGTGCTGGCAAGTCTGGCCC
CTTTCCTCTTGCGTTTACTCAGGCACAGATGGAAAAAGATGAAACTCAATGAGGACCC

TTTTGTGAGATGCGGCACATTTCG
>2571
TCTGGCCCCTTTCCTCTTGCGTTTACTCAGGCACAGATGGAAAAAGATGAAACTCAAT
GAGGACCCTTTTGTGAGATGCGGCACATTTCGCTTTGTGGAATTATTAACCTTTTATAAG
ACTGGGATACTTACAAACTTAC
>2572
ACTCAGGCACAGATGGAAAAAGATGAAACTCAATGAGGACCCTTTTGTGAGATGCGG
CACATTTCGCTTTGTGGAATTATTAACCTTTTATAAGACTGGGATACTTACAAACTTACC
GCAGGACCAGACAGTGACTATCA
>2573
ATTTCGCTTTGTGGAATTATTAACCTTTTATAAGACTGGGATACTTACAAACTTACCGCA
GGACCAGACAGTGACTATCAGAATCCTCTCTTCTAAAGATCAGCGATCTACAGCTTTCG
CAAAACTTAAGGCTGACTTGG
>2574
TCTTCTAAAGATCAGCGATCTACAGCTTTCGCAAAACTTAAGGCTGACTTGGGAATCTA
GACAGCCCCTAGTATGGTGAGTGACCCTAGAGTGGTTTTTAATGAGGAGATACTCACG
GGAGCCAGCATCTCGGGGGTCCC
>2575
TAAAGATCAGCGATCTACAGCTTTCGCAAAACTTAAGGCTGACTTGGGAATCTAGACA
GCCCCTAGTATGGTGAGTGACCCTAGAGTGGTTTTTAATGAGGAGATACTCACGGGAG
CCAGCATCTCGGGGGTCCCCGGGG
>2576
GATCAGCGATCTACAGCTTTCGCAAAACTTAAGGCTGACTTGGGAATCTAGACAGCCC
CTAGTATGGTGAGTGACCCTAGAGTGGTTTTTAATGAGGAGATACTCACGGGAGCCAG
CATCTCGGGGGTCCCCGGGGGGTT
>2577
TACAGCTTTCGCAAAACTTAAGGCTGACTTGGGAATCTAGACAGCCCCTAGTATGGTG
AGTGACCCTAGAGTGGTTTTTAATGAGGAGATACTCACGGGAGCCAGCATCTCGGGGG
TCCCCGGGGGGTTCATGGCCAGGA
>2578
CCCTAGTATGGTGAGTGACCCTAGAGTGGTTTTTAATGAGGAGATACTCACGGGAGCC
AGCATCTCGGGGGTCCCCGGGGGGTTCATGGCCAGGAGCGTTGCAAGCTGGTTTGTTT
GTTTGTTTGTTTTGAGACAGGGTC
>2579
TGAGTGACCCTAGAGTGGTTTTTAATGAGGAGATACTCACGGGAGCCAGCATCTCGGG
GGTCCCCGGGGGGTTCATGGCCAGGAGCGTTGCAAGCTGGTTTGTTTGTTTGTTTGTTT
TGAGACAGGGTCTTACTCTGTTG
>2580
GGTTTTTAATGAGGAGATACTCACGGGAGCCAGCATCTCGGGGGTCCCCGGGGGGTTC
ATGGCCAGGAGCGTTGCAAGCTGGTTTGTTTGTTTGTTTGTTTTGAGACAGGGTCTTAC
TCTGTTGCTCAGGCTGGAGTGCA
>2581
CTCACGGGAGCCAGCATCTCGGGGGTCCCCGGGGGGTTCATGGCCAGGAGCGTTGCA
AGCTGGTTTGTTTGTTTGTTTGTTTTGAGACAGGGTCTTACTCTGTTGCTCAGGCTGGA

GTGCAGTGGCATAATCTCGGCTCA
>2582
GGGGTCCCCGGGGGGTTCATGGCCAGGAGCGTTGCAAGCTGGTTTGTTTGTTTGTTTG
TTTTGAGACAGGGTCTTACTCTGTTGCTCAGGCTGGAGTGCAGTGGCATAATCTCGGCT
CACTGCAACCTTGCTTGCTCTCC
>2583
GGGGGTTCATGGCCAGGAGCGTTGCAAGCTGGTTTGTTTGTTTGTTTGTTTTGAGACA
GGGTCTTACTCTGTTGCTCAGGCTGGAGTGCAGTGGCATAATCTCGGCTCACTGCAAC
CTTGCTTGCTCTCCCTGGCTCAAG
>2584
GGAGCGTTGCAAGCTGGTTTGTTTGTTTGTTTGTTTTGAGACAGGGTCTTACTCTGTTG
CTCAGGCTGGAGTGCAGTGGCATAATCTCGGCTCACTGCAACCTTGCTTGCTCTCCCTG
GCTCAAGCAATTCTCCCACATT
>2585
ATAATCTCGGCTCACTGCAACCTTGCTTGCTCTCCCTGGCTCAAGCAATTCTCCCACATT
AGCCTCCCAAGTAGCTGGGATTACAGGTGTATGCCACCACAGCCTGCTAATTTTTAAGT
TTTTTGTAGAGATGAGGTTTC
>2586
GCAACCTTGCTTGCTCTCCCTGGCTCAAGCAATTCTCCCACATTAGCCTCCCAAGTAGC
TGGGATTACAGGTGTATGCCACCACAGCCTGCTAATTTTTAAGTTTTTTGTAGAGATGA
GGTTTCACTGCATTGCCTAGGC
>2587
AACCTTGCTTGCTCTCCCTGGCTCAAGCAATTCTCCCACATTAGCCTCCCAAGTAGCTG
GGATTACAGGTGTATGCCACCACAGCCTGCTAATTTTTAAGTTTTTTGTAGAGATGAGG
TTTCACTGCATTGCCTAGGCTG
>2588
ATTCTCCCACATTAGCCTCCCAAGTAGCTGGGATTACAGGTGTATGCCACCACAGCCTG
CTAATTTTTAAGTTTTTTGTAGAGATGAGGTTTCACTGCATTGCCTAGGCTGATCTTGAA
CTCCTGGGCTCAAACAATCTT
>2589
TAGAGATGAGGTTTCACTGCATTGCCTAGGCTGATCTTGAACTCCTGGGCTCAAACAAT
CTTCCCTCCTTGTCCTCTTAAAGTGCTGGGATTACAGGTGTGAGCCACAGCGCGAGGC
CTTGTTTGGTGTTTTTGTTTGTT
>2590
TTTCACTGCATTGCCTAGGCTGATCTTGAACTCCTGGGCTCAAACAATCTTCCCTCCTT
GTCCTCTTAAAGTGCTGGGATTACAGGTGTGAGCCACAGCGCGAGGCCTTGTTTGGTG
TTTTTGTTTGTTTGTTTGTTTGT
>2591
TAGGCTGATCTTGAACTCCTGGGCTCAAACAATCTTCCCTCCTTGTCCTCTTAAAGTGC
TGGGATTACAGGTGTGAGCCACAGCGCGAGGCCTTGTTTGGTGTTTTTGTTTGTTTGTT
TGTTTGTTTTATGGATCATTAC
>2592
TCAAACAATCTTCCCTCCTTGTCCTCTTAAAGTGCTGGGATTACAGGTGTGAGCCACAG
CGCGAGGCCTTGTTTGGTGTTTTTGTTTGTTTGTTTGTTTGTTTTATGGATCATTACCTAT

AAAGTAATTTAGAATACCCA
>2593
CCTCCTTGTCCTCTTAAAGTGCTGGGATTACAGGTGTGAGCCACAGCGCGAGGCCTTG
TTTGGTGTTTTTGTTTGTTTGTTTGTTTGTTTTATGGATCATTACCTATAAAGTAATTTAG
AATACCCACTCATTTATGATC
>2594
CTTGTCCTCTTAAAGTGCTGGGATTACAGGTGTGAGCCACAGCGCGAGGCCTTGTTTG
GTGTTTTTGTTTGTTTGTTTGTTTGTTTTATGGATCATTACCTATAAAGTAATTTAGAATA
CCCACTCATTTATGATCCAGC
>2595
TCCTCTTAAAGTGCTGGGATTACAGGTGTGAGCCACAGCGCGAGGCCTTGTTTGGTGT
TTTTGTTTGTTTGTTTGTTTGTTTTATGGATCATTACCTATAAAGTAATTTAGAATACCCA
CTCATTTATGATCCAGCCTTC
>2596
CCACAGCGCGAGGCCTTGTTTGGTGTTTTTGTTTGTTTGTTTGTTTGTTTTATGGATCAT
TACCTATAAAGTAATTTAGAATACCCACTCATTTATGATCCAGCCTTCCCTGCATTTTCTA
GCGTTGAGGTCCTTTGGGG
>2597
GGATCATTACCTATAAAGTAATTTAGAATACCCACTCATTTATGATCCAGCCTTCCCTGCA
TTTTCTAGCGTTGAGGTCCTTTGGGGCCATAGAAGTATGTTTTTCCAAGACTGATTGCTT
CGTGTATTTAACCAGGTTT
>2598
TTACCTATAAAGTAATTTAGAATACCCACTCATTTATGATCCAGCCTTCCCTGCATTTTCT
AGCGTTGAGGTCCTTTGGGGCCATAGAAGTATGTTTTTCCAAGACTGATTGCTTCGTGT
ATTTAACCAGGTTTTTTGTG
>2599
ACCCACTCATTTATGATCCAGCCTTCCCTGCATTTTCTAGCGTTGAGGTCCTTTGGGGCC
ATAGAAGTATGTTTTTCCAAGACTGATTGCTTCGTGTATTTAACCAGGTTTTTTGTGAAG
GGTTTGATATAAACTGAGTG
>2600
TTCCCTGCATTTTCTAGCGTTGAGGTCCTTTGGGGCCATAGAAGTATGTTTTTCCAAGA
CTGATTGCTTCGTGTATTTAACCAGGTTTTTTGTGAAGGGTTTGATATAAACTGAGTGAC
TGAAGAGAGGAACATGGTGTG
>2601
TAGCGTTGAGGTCCTTTGGGGCCATAGAAGTATGTTTTTCCAAGACTGATTGCTTCGTG
TATTTAACCAGGTTTTTTGTGAAGGGTTTGATATAAACTGAGTGACTGAAGAGAGGAA
CATGGTGTGATTTGGTAGAAAGA
>2602
TATGTTTTTCCAAGACTGATTGCTTCGTGTATTTAACCAGGTTTTTTGTGAAGGGTTTGA
TATAAACTGAGTGACTGAAGAGAGGAACATGGTGTGATTTGGTAGAAAGATCTGTAAT
TTTGGAGTCAAGACTGGCCTAG
>2603
GCTTCGTGTATTTAACCAGGTTTTTTGTGAAGGGTTTGATATAAACTGAGTGACTGAAG
AGAGGAACATGGTGTGATTTGGTAGAAAGATCTGTAATTTTGGAGTCAAGACTGGCCT

AGATGGACAGCTTGGCCACTTTT
>2604
TTTTGTGAAGGGTTTGATATAAACTGAGTGACTGAAGAGAGGAACATGGTGTGATTTG
GTAGAAAGATCTGTAATTTTGGAGTCAAGACTGGCCTAGATGGACAGCTTGGCCACTT
TTAAAATTTTGTGTCCTTAGCCCC
>2605
GTTTGATATAAACTGAGTGACTGAAGAGAGGAACATGGTGTGATTTGGTAGAAAGATC
TGTAATTTTGGAGTCAAGACTGGCCTAGATGGACAGCTTGGCCACTTTTAAAATTTTGT
GTCCTTAGCCCCATAAACATTTT
>2606
TGGTAGAAAGATCTGTAATTTTGGAGTCAAGACTGGCCTAGATGGACAGCTTGGCCAC
TTTTAAAATTTTGTGTCCTTAGCCCCATAAACATTTTGAGATTCAGTTTTCTCTCCTGTG
AATTGGGGACGATATTGCCTAA
>2607
TGGCCTAGATGGACAGCTTGGCCACTTTTAAAATTTTGTGTCCTTAGCCCCATAAACAT
TTTGAGATTCAGTTTTCTCTCCTGTGAATTGGGGACGATATTGCCTAACTTGTAAGGTTC
TTGCAAAGATCAAATGAAAAA
>2608
ACAGCTTGGCCACTTTTAAAATTTTGTGTCCTTAGCCCCATAAACATTTTGAGATTCAGT
TTTCTCTCCTGTGAATTGGGGACGATATTGCCTAACTTGTAAGGTTCTTGCAAAGATCA
AATGAAAAAATACACATAGAT
>2609
TCCTTAGCCCCATAAACATTTTGAGATTCAGTTTTCTCTCCTGTGAATTGGGGACGATAT
TGCCTAACTTGTAAGGTTCTTGCAAAGATCAAATGAAAAAATACACATAGATCTCCTGG
CTATTATTGGAGTGCCCAGTA
>2610
AGCCCCATAAACATTTTGAGATTCAGTTTTCTCTCCTGTGAATTGGGGACGATATTGCCT
AACTTGTAAGGTTCTTGCAAAGATCAAATGAAAAAATACACATAGATCTCCTGGCTATT
ATTGGAGTGCCCAGTAAATGC
>2611
TGCCTAACTTGTAAGGTTCTTGCAAAGATCAAATGAAAAAATACACATAGATCTCCTGG
CTATTATTGGAGTGCCCAGTAAATGCTAGTTGCCTTATCCTCCATTTTCTTTTTGAGAGC
TGAAGACCTGATTTTATTTCC
>2612
CTTGTAAGGTTCTTGCAAAGATCAAATGAAAAAATACACATAGATCTCCTGGCTATTATT
GGAGTGCCCAGTAAATGCTAGTTGCCTTATCCTCCATTTTCTTTTTGAGAGCTGAAGAC
CTGATTTTATTTCCTTGCATT
>2613
TCTTGCAAAGATCAAATGAAAAAATACACATAGATCTCCTGGCTATTATTGGAGTGCCC
AGTAAATGCTAGTTGCCTTATCCTCCATTTTCTTTTTGAGAGCTGAAGACCTGATTTTAT
TTCCTTGCATTTTTCATGTCA
>2614
GCTAGTTGCCTTATCCTCCATTTTCTTTTTGAGAGCTGAAGACCTGATTTTATTTCCTTG
CATTTTTCATGTCATTGCCTATAGGGAGATACATTCATATATTTGTTGCTCATTTTTGTATG

TGTTACAGTGGTGATAAA
>2615
TTTTATTTCCTTGCATTTTTCATGTCATTGCCTATAGGGAGATACATTCATATATTTGTTGC
TCATTTTTGTATGTGTTACAGTGGTGATAAAGAAACTGATGGTATCCCAGATGGGGAGG
GGCAGGTAAGGTTTTATCA
>2616
ATTTCCTTGCATTTTTCATGTCATTGCCTATAGGGAGATACATTCATATATTTGTTGCTCAT
TTTTGTATGTGTTACAGTGGTGATAAAGAAACTGATGGTATCCCAGATGGGGAGGGGCA
GGTAAGGTTTTATCAGTGG
>2617
ATAGGGAGATACATTCATATATTTGTTGCTCATTTTTGTATGTGTTACAGTGGTGATAAAG
AAACTGATGGTATCCCAGATGGGGAGGGGCAGGTAAGGTTTTATCAGTGGCTTTTAATT
TCTTTATGAAATTGGATGTG
>2618
TGTTGCTCATTTTTGTATGTGTTACAGTGGTGATAAAGAAACTGATGGTATCCCAGATGG
GGAGGGGCAGGTAAGGTTTTATCAGTGGCTTTTAATTTCTTTATGAAATTGGATGTGGC
TCTAGGGAATAGATGTCTCTG
>2619
CTCATTTTTGTATGTGTTACAGTGGTGATAAAGAAACTGATGGTATCCCAGATGGGGAG
GGGCAGGTAAGGTTTTATCAGTGGCTTTTAATTTCTTTATGAAATTGGATGTGGCTCTAG
GGAATAGATGTCTCTGGGATG
>2620
ATGGTATCCCAGATGGGGAGGGGCAGGTAAGGTTTTATCAGTGGCTTTTAATTTCTTTAT
GAAATTGGATGTGGCTCTAGGGAATAGATGTCTCTGGGATGGTCATGTTTTCCCTTGCC
TTTTGGCTCCTGCAATGAGGA
>2621
GGGGCAGGTAAGGTTTTATCAGTGGCTTTTAATTTCTTTATGAAATTGGATGTGGCTCTA
GGGAATAGATGTCTCTGGGATGGTCATGTTTTCCCTTGCCTTTTGGCTCCTGCAATGAG
GACCTCTTTCTGTTGGTGTGG
>2622
GTTTTATCAGTGGCTTTTAATTTCTTTATGAAATTGGATGTGGCTCTAGGGAATAGATGT
CTCTGGGATGGTCATGTTTTCCCTTGCCTTTTGGCTCCTGCAATGAGGACCTCTTTCTGT
TGGTGTGGGTCTTGTCTGAG
>2623
GGGAATAGATGTCTCTGGGATGGTCATGTTTTCCCTTGCCTTTTGGCTCCTGCAATGAG
GACCTCTTTCTGTTGGTGTGGGTCTTGTCTGAGCATGCCAGGGATGGCAGTGAACACA
TCGATGGTGGTTGGGACGTCCTC
>2624
ATAGATGTCTCTGGGATGGTCATGTTTTCCCTTGCCTTTTGGCTCCTGCAATGAGGACCT
CTTTCTGTTGGTGTGGGTCTTGTCTGAGCATGCCAGGGATGGCAGTGAACACATCGAT
GGTGGTTGGGACGTCCTCATTC
>2625
CCTTTTGGCTCCTGCAATGAGGACCTCTTTCTGTTGGTGTGGGTCTTGTCTGAGCATGC
CAGGGATGGCAGTGAACACATCGATGGTGGTTGGGACGTCCTCATTCCCAGACCTCTG

GGACATACTAATTCCCAGACCTC
>2626
AATGAGGACCTCTTTCTGTTGGTGTGGGTCTTGTCTGAGCATGCCAGGGATGGCAGTG
AACACATCGATGGTGGTTGGGACGTCCTCATTCCCAGACCTCTGGGACATACTAATTCC
CAGACCTCTGAAACATGGGTCTC
>2627
GAGGACCTCTTTCTGTTGGTGTGGGTCTTGTCTGAGCATGCCAGGGATGGCAGTGAAC
ACATCGATGGTGGTTGGGACGTCCTCATTCCCAGACCTCTGGGACATACTAATTCCCAG
ACCTCTGAAACATGGGTCTCAGC
>2628
CTTTCTGTTGGTGTGGGTCTTGTCTGAGCATGCCAGGGATGGCAGTGAACACATCGAT
GGTGGTTGGGACGTCCTCATTCCCAGACCTCTGGGACATACTAATTCCCAGACCTCTGA
AACATGGGTCTCAGCTGCTTTGC
>2629
CGATGGTGGTTGGGACGTCCTCATTCCCAGACCTCTGGGACATACTAATTCCCAGACCT
CTGAAACATGGGTCTCAGCTGCTTTGCCCTGCAGACTTGTTCTCAGTAGCTATTTACCT
TTAGGAGAAAAAAAAAATCCTT
>2630
CAGACCTCTGGGACATACTAATTCCCAGACCTCTGAAACATGGGTCTCAGCTGCTTTGC
CCTGCAGACTTGTTCTCAGTAGCTATTTACCTTTAGGAGAAAAAAAAAATCCTTAACCA
AATGCTTTAGGAAGGCTTTCTT
>2631
CTCAGTAGCTATTTACCTTTAGGAGAAAAAAAAAATCCTTAACCAAATGCTTTAGGAAG
GCTTTCTTATCGTGGCTGTAAGGTGCTGGTGGGTTGTTGGCAGCCAAGTTTGGGTTTAA
ATCTCACTGGGGGTTGAGGCTG
>2632
AGCTATTTACCTTTAGGAGAAAAAAAAAATCCTTAACCAAATGCTTTAGGAAGGCTTTC
TTATCGTGGCTGTAAGGTGCTGGTGGGTTGTTGGCAGCCAAGTTTGGGTTTAAATCTCA
CTGGGGGTTGAGGCTGCTGAAC
>2633
TTTACCTTTAGGAGAAAAAAAAAATCCTTAACCAAATGCTTTAGGAAGGCTTTCTTATC
GTGGCTGTAAGGTGCTGGTGGGTTGTTGGCAGCCAAGTTTGGGTTTAAATCTCACTGG
GGGTTGAGGCTGCTGAACTGCTA
>2634
TTTAGGAGAAAAAAAAAATCCTTAACCAAATGCTTTAGGAAGGCTTTCTTATCGTGGCT
GTAAGGTGCTGGTGGGTTGTTGGCAGCCAAGTTTGGGTTTAAATCTCACTGGGGGTTG
AGGCTGCTGAACTGCTATTCCTA
>2635
GGAGAAAAAAAAAATCCTTAACCAAATGCTTTAGGAAGGCTTTCTTATCGTGGCTGTA
AGGTGCTGGTGGGTTGTTGGCAGCCAAGTTTGGGTTTAAATCTCACTGGGGGTTGAGG
CTGCTGAACTGCTATTCCTAACAT
>2636
CCTTAACCAAATGCTTTAGGAAGGCTTTCTTATCGTGGCTGTAAGGTGCTGGTGGGTTG
TTGGCAGCCAAGTTTGGGTTTAAATCTCACTGGGGGTTGAGGCTGCTGAACTGCTATTC

CTAACATGCTCTTGTGTTTTTA
>2637
CTTTCTTATCGTGGCTGTAAGGTGCTGGTGGGTTGTTGGCAGCCAAGTTTGGGTTTAAA
TCTCACTGGGGGTTGAGGCTGCTGAACTGCTATTCCTAACATGCTCTTGTGTTTTTATGC
ATAGACCGTCTTCTGGATCCT
>2638
GGCAGCCAAGTTTGGGTTTAAATCTCACTGGGGGTTGAGGCTGCTGAACTGCTATTCCT
AACATGCTCTTGTGTTTTTATGCATAGACCGTCTTCTGGATCCTTGGTGAGTTTTTTTA
CTCTGGGGTTTTCAGGAGAAA
>2639
CAGCCAAGTTTGGGTTTAAATCTCACTGGGGGTTGAGGCTGCTGAACTGCTATTCCTAA
CATGCTCTTGTGTTTTTATGCATAGACCGTCTTCTGGATCCTTGGTGAGTTTTTTTACTC
TGGGGTTTTCAGGAGAAAGG
>2640
TGAGGCTGCTGAACTGCTATTCCTAACATGCTCTTGTGTTTTTATGCATAGACCGTCTTC
TGGATCCTTGGTGAGTTTTTTTACTCTGGGGTTTTCAGGAGAAAGGTTTGTCATTCAT
CATCACTCTTCTCCTTCATCA
>2641
GCTGCTGAACTGCTATTCCTAACATGCTCTTGTGTTTTTATGCATAGACCGTCTTCTGGA
TCCTTGGTGAGTTTTTTTACTCTGGGGTTTTCAGGAGAAAGGTTTGTCATTCATCATCA
CTCTTCTCCTTCATCACAGC
>2642
CCTAACATGCTCTTGTGTTTTTATGCATAGACCGTCTTCTGGATCCTTGGTGAGTTTTTT
TTACTCTGGGGTTTTCAGGAGAAAGGTTTGTCATTCATCATCACTCTTCTCCTTCATCAC
AGCAGTGGTAACAGTGGTTA
>2643
TGTTTTTATGCATAGACCGTCTTCTGGATCCTTGGTGAGTTTTTTTACTCTGGGGTTTT
CAGGAGAAAGGTTTGTCATTCATCATCACTCTTCTCCTTCATCACAGCAGTGGTAACAG
TGGTTAGAGGGAGGAGGTAGA
>2644
TTTTTTTACTCTGGGGTTTTCAGGAGAAAGGTTTGTCATTCATCATCACTCTTCTCCTT
CATCACAGCAGTGGTAACAGTGGTTAGAGGGAGGAGGTAGAATCAGTTGTTCAAGTTC
TTTTTTCTTCCAGGTAAGTCCC
>2645
TTTTTACTCTGGGGTTTTCAGGAGAAAGGTTTGTCATTCATCATCACTCTTCTCCTTCAT
CACAGCAGTGGTAACAGTGGTTAGAGGGAGGAGGTAGAATCAGTTGTTCAAGTTCTTT
TTTCTTCCAGGTAAGTCCCCCC
>2646
GAAAGGTTTGTCATTCATCATCACTCTTCTCCTTCATCACAGCAGTGGTAACAGTGGTT
AGAGGGAGGAGGTAGAATCAGTTGTTCAAGTTCTTTTTTCTTCCAGGTAAGTCCCCCC
CACCCCCCGGCTGCCCCTGCCCT
>2647
CATCACTCTTCTCCTTCATCACAGCAGTGGTAACAGTGGTTAGAGGGAGGAGGTAGAA
TCAGTTGTTCAAGTTCTTTTTTCTTCCAGGTAAGTCCCCCCCACCCCCCGGCTGCCCCT

GCCCTTCCGCATTTTCTGGGAAG
>2648
ATCACAGCAGTGGTAACAGTGGTTAGAGGGAGGAGGTAGAATCAGTTGTTCAAGTTCT
TTTTTCTTCCAGGTAAGTCCCCCCCACCCCCCGGCTGCCCCTGCCCTTCCGCATTTTCT
GGGAAGTGAATCTATTGGGAAAT
>2649
CAGCAGTGGTAACAGTGGTTAGAGGGAGGAGGTAGAATCAGTTGTTCAAGTTCTTTTT
TCTTCCAGGTAAGTCCCCCCCACCCCCCGGCTGCCCCTGCCCTTCCGCATTTTCTGGGA
AGTGAATCTATTGGGAAATGCAT
>2650
AGTTCTTTTTTCTTCCAGGTAAGTCCCCCCCACCCCCCGGCTGCCCCTGCCCTTCCGCA
TTTTCTGGGAAGTGAATCTATTGGGAAATGCATGGGCCTGTGTTTCTGGGATGATTTTA
GCATATGTAGTAAGAGGGGTGC
>2651
CCCACCCCCCGGCTGCCCCTGCCCTTCCGCATTTTCTGGGAAGTGAATCTATTGGGAAA
TGCATGGGCCTGTGTTTCTGGGATGATTTTAGCATATGTAGTAAGAGGGGTGCTTTTTGC
TGATAGCCCAGGCGTTGGTCC
>2652
CCGCATTTTCTGGGAAGTGAATCTATTGGGAAATGCATGGGCCTGTGTTTCTGGGATGA
TTTTAGCATATGTAGTAAGAGGGGTGCTTTTTGCTGATAGCCCAGGCGTTGGTCCTGTA
ATCCTGAGGTGTGGTTTAATAA
>2653
CATTTTCTGGGAAGTGAATCTATTGGGAAATGCATGGGCCTGTGTTTCTGGGATGATTTT
AGCATATGTAGTAAGAGGGGTGCTTTTTGCTGATAGCCCAGGCGTTGGTCCTGTAATCC
TGAGGTGTGGTTTAATAAGAG
>2654
GGAAGTGAATCTATTGGGAAATGCATGGGCCTGTGTTTCTGGGATGATTTTAGCATATGT
AGTAAGAGGGGTGCTTTTTGCTGATAGCCCAGGCGTTGGTCCTGTAATCCTGAGGTGT
GGTTTAATAAGAGCTGAACAGT
>2655
ATGGGCCTGTGTTTCTGGGATGATTTTAGCATATGTAGTAAGAGGGGTGCTTTTTGCTGA
TAGCCCAGGCGTTGGTCCTGTAATCCTGAGGTGTGGTTTAATAAGAGCTGAACAGTCAT
TTAATTATTTATTGGGTGCCT
>2656
ATTTTAGCATATGTAGTAAGAGGGGTGCTTTTTGCTGATAGCCCAGGCGTTGGTCCTGT
AATCCTGAGGTGTGGTTTAATAAGAGCTGAACAGTCATTTAATTATTTATTGGGTGCCTG
GAGAATTTTATAGGCAGGAGA
>2657
TTAGCATATGTAGTAAGAGGGGTGCTTTTTGCTGATAGCCCAGGCGTTGGTCCTGTAAT
CCTGAGGTGTGGTTTAATAAGAGCTGAACAGTCATTTAATTATTTATTGGGTGCCTGGA
GAATTTTATAGGCAGGAGAGCC
>2658
GGGTGCTTTTTGCTGATAGCCCAGGCGTTGGTCCTGTAATCCTGAGGTGTGGTTTAATA
AGAGCTGAACAGTCATTTAATTATTTATTGGGTGCCTGGAGAATTTTATAGGCAGGAGA

GCCTAGAGCTATTCCAGAAAAA
>2659
CCAGGCGTTGGTCCTGTAATCCTGAGGTGTGGTTTAATAAGAGCTGAACAGTCATTTAA
TTATTTATTGGGTGCCTGGAGAATTTTATAGGCAGGAGAGCCTAGAGCTATTCCAGAAA
AAAGGCCAATCCTAGAGCTTTT
>2660
TGCCTGGAGAATTTTATAGGCAGGAGAGCCTAGAGCTATTCCAGAAAAAAGGCCAATC
CTAGAGCTTTTTGTGTGGCTTTCTTCTTGGGCTTGAAGTTTTTGATGCTCGCCAACATTC
ATAGTAGCCCCCAATGCCACCC
>2661
CCTGGAGAATTTTATAGGCAGGAGAGCCTAGAGCTATTCCAGAAAAAAGGCCAATCCT
AGAGCTTTTTGTGTGGCTTTCTTCTTGGGCTTGAAGTTTTTGATGCTCGCCAACATTCAT
AGTAGCCCCCAATGCCACCCAC
>2662
GAGCCTAGAGCTATTCCAGAAAAAAGGCCAATCCTAGAGCTTTTTGTGTGGCTTTCTTC
TTGGGCTTGAAGTTTTTGATGCTCGCCAACATTCATAGTAGCCCCCAATGCCACCCACA
CATTGGGAAGGGACAATAGAGC
>2663
GCCAATCCTAGAGCTTTTTGTGTGGCTTTCTTCTTGGGCTTGAAGTTTTTGATGCTCGCC
AACATTCATAGTAGCCCCCAATGCCACCCACACATTGGGAAGGGACAATAGAGCCAGC
TAAAGCTCTTTCTTTGTGTGTG
>2664
ATTCATAGTAGCCCCCAATGCCACCCACACATTGGGAAGGGACAATAGAGCCAGCTAA
AGCTCTTTCTTTGTGTGTGCCCATATTAACCCATCTAGTCAAATGTGGGTACCCTTCCAA
ACCATTATGTCTTGTTCCTCTC
>2665
TCATAGTAGCCCCCAATGCCACCCACACATTGGGAAGGGACAATAGAGCCAGCTAAAG
CTCTTTCTTTGTGTGTGCCCATATTAACCCATCTAGTCAAATGTGGGTACCCTTCCAAAC
CATTATGTCTTGTTCCTCTCGG
>2666
CACACATTGGGAAGGGACAATAGAGCCAGCTAAAGCTCTTTCTTTGTGTGTGCCCATAT
TAACCCATCTAGTCAAATGTGGGTACCCTTCCAAACCATTATGTCTTGTTCCTCTCGGCT
GCTGGGAACATTAAAAGAACC
>2667
TGGGAAGGGACAATAGAGCCAGCTAAAGCTCTTTCTTTGTGTGTGCCCATATTAACCCA
TCTAGTCAAATGTGGGTACCCTTCCAAACCATTATGTCTTGTTCCTCTCGGCTGCTGGG
AACATTAAAAGAACCTTATTTG
>2668
AAAGCTCTTTCTTTGTGTGTGCCCATATTAACCCATCTAGTCAAATGTGGGTACCCTTCC
AAACCATTATGTCTTGTTCCTCTCGGCTGCTGGGAACATTAAAAGAACCTTATTTGAAG
GCAAACATTACTGTATCAGTG
>2669
AACCATTATGTCTTGTTCCTCTCGGCTGCTGGGAACATTAAAAGAACCTTATTTGAAGG
CAAACATTACTGTATCAGTGATGCTGAAACTGGAGGTCTGTCAATGGCACTTCATTGCA

TTGATAATTGTTTCTGCTTTGA
>2670
TATGTCTTGTTCCTCTCGGCTGCTGGGAACATTAAAAGAACCTTATTTGAAGGCAAACA
TTACTGTATCAGTGATGCTGAAACTGGAGGTCTGTCAATGGCACTTCATTGCATTGATAA
TTGTTTCTGCTTTGAACCACA
>2671
GCTGCTGGGAACATTAAAAGAACCTTATTTGAAGGCAAACATTACTGTATCAGTGATGC
TGAAACTGGAGGTCTGTCAATGGCACTTCATTGCATTGATAATTGTTTCTGCTTTGAAC
CACAACACTGTGATTATTGAGG
>2672
CTGGGAACATTAAAAGAACCTTATTTGAAGGCAAACATTACTGTATCAGTGATGCTGAA
ACTGGAGGTCTGTCAATGGCACTTCATTGCATTGATAATTGTTTCTGCTTTGAACCACA
ACACTGTGATTATTGAGGTTCG
>2673
GGCAAACATTACTGTATCAGTGATGCTGAAACTGGAGGTCTGTCAATGGCACTTCATTG
CATTGATAATTGTTTCTGCTTTGAACCACAACACTGTGATTATTGAGGTTCGACTGTAAG
GGCTGAGGGAGCCTTGAGGCC
>2674
GCTGAAACTGGAGGTCTGTCAATGGCACTTCATTGCATTGATAATTGTTTCTGCTTTGA
ACCACAACACTGTGATTATTGAGGTTCGACTGTAAGGGCTGAGGGAGCCTTGAGGCCA
TTGAAAGCATTCTCCTGCCTTTA
>2675
GGTCTGTCAATGGCACTTCATTGCATTGATAATTGTTTCTGCTTTGAACCACAACACTGT
GATTATTGAGGTTCGACTGTAAGGGCTGAGGGAGCCTTGAGGCCATTGAAAGCATTCT
CCTGCCTTTAGGCCAGGCCTAT
>2676
AATGGCACTTCATTGCATTGATAATTGTTTCTGCTTTGAACCACAACACTGTGATTATTG
AGGTTCGACTGTAAGGGCTGAGGGAGCCTTGAGGCCATTGAAAGCATTCTCCTGCCTT
TAGGCCAGGCCTATGTTATAAG
>2677
GTTCGACTGTAAGGGCTGAGGGAGCCTTGAGGCCATTGAAAGCATTCTCCTGCCTTTA
GGCCAGGCCTATGTTATAAGTGAGAGACAGGTGTGGAGTGTGCAGGGAGGGAATTAA
GATAGATGGTGACCTGGAATTCAGA
>2678
TGTAAGGGCTGAGGGAGCCTTGAGGCCATTGAAAGCATTCTCCTGCCTTTAGGCCAGG
CCTATGTTATAAGTGAGAGACAGGTGTGGAGTGTGCAGGGAGGGAATTAAGATAGATG
GTGACCTGGAATTCAGAAGGTGGA
>2679
CTTTAGGCCAGGCCTATGTTATAAGTGAGAGACAGGTGTGGAGTGTGCAGGGAGGGAA
TTAAGATAGATGGTGACCTGGAATTCAGAAGGTGGACCCCCACCACCCCCCAGCCTGA
GACCAAAGCACAGTTTAACTCTGT
>2680
TATAAGTGAGAGACAGGTGTGGAGTGTGCAGGGAGGGAATTAAGATAGATGGTGACCT
GGAATTCAGAAGGTGGACCCCCACCACCCCCCAGCCTGAGACCAAAGCACAGTTTAA

CTCTGTCATGGGTCATGTGCAGAGG
>2681
TTAAGATAGATGGTGACCTGGAATTCAGAAGGTGGACCCCCACCACCCCCCAGCCTGA
GACCAAAGCACAGTTTAACTCTGTCATGGGTCATGTGCAGAGGGAAGGGACTGCCCC
AGAATTAGCAGTTGGTTGAACACCC
>2682
TGGTGACCTGGAATTCAGAAGGTGGACCCCCACCACCCCCCAGCCTGAGACCAAAGC
ACAGTTTAACTCTGTCATGGGTCATGTGCAGAGGGAAGGGACTGCCCCAGAATTAGCA
GTTGGTTGAACACCCTGCTTAAAAA
>2683
TGAGACCAAAGCACAGTTTAACTCTGTCATGGGTCATGTGCAGAGGGAAGGGACTGC
CCCAGAATTAGCAGTTGGTTGAACACCCTGCTTAAAAACTTTTTCTTTTCCTTTTTTTGT
GAGTACCGGTCCGCTTGGACTAA
>2684
ACCAAAGCACAGTTTAACTCTGTCATGGGTCATGTGCAGAGGGAAGGGACTGCCCCA
GAATTAGCAGTTGGTTGAACACCCTGCTTAAAAACTTTTTCTTTTCCTTTTTTTGTGAGT
ACCGGTCCGCTTGGACTAAAGAA
>2685
GGGACTGCCCCAGAATTAGCAGTTGGTTGAACACCCTGCTTAAAAACTTTTTCTTTTCC
TTTTTTTGTGAGTACCGGTCCGCTTGGACTAAAGAAGTTTTGGTCGTTGTTTTCTTAATT
TTGGGCTACAGAGTCTCTACC
>2686
GCCCCAGAATTAGCAGTTGGTTGAACACCCTGCTTAAAAACTTTTTCTTTTCCTTTTTTT
GTGAGTACCGGTCCGCTTGGACTAAAGAAGTTTTGGTCGTTGTTTTCTTAATTTTGGGC
TACAGAGTCTCTACCTACATT
>2687
GTTGAACACCCTGCTTAAAAACTTTTTCTTTTCCTTTTTTTGTGAGTACCGGTCCGCTTG
GACTAAAGAAGTTTTGGTCGTTGTTTTCTTAATTTTGGGCTACAGAGTCTCTACCTACAT
TTGTGTTTTGTGTTTTGGTA
>2688
TTTTTGTGAGTACCGGTCCGCTTGGACTAAAGAAGTTTTGGTCGTTGTTTTCTTAATTTT
GGGCTACAGAGTCTCTACCTACATTTGTGTTTTGTGTTTTGGTAACCACATGTAACAGC
CGGTGGAATCTTTTTTCTCTT
>2689
TCCGCTTGGACTAAAGAAGTTTTGGTCGTTGTTTTCTTAATTTTGGGCTACAGAGTCTC
TACCTACATTTGTGTTTTGTGTTTTGGTAACCACATGTAACAGCCGGTGGAATCTTTTTT
CTCTTTGCTTTCTCTGAACTT
>2690
GGACTAAAGAAGTTTTGGTCGTTGTTTTCTTAATTTTGGGCTACAGAGTCTCTACCTAC
ATTTGTGTTTTGTGTTTTGGTAACCACATGTAACAGCCGGTGGAATCTTTTTTCTCTTTG
CTTTCTCTGAACTTTCCAAAG
>2691
CTTAATTTTGGGCTACAGAGTCTCTACCTACATTTGTGTTTTGTGTTTTGGTAACCACAT
GTAACAGCCGGTGGAATCTTTTTTCTCTTTGCTTTCTCTGAACTTTCCAAAGAGTAGGC

CCCTATCTCTCGGGGGATTAA
>2692
TGTTTTGGTAACCACATGTAACAGCCGGTGGAATCTTTTTTCTCTTTGCTTTCTCTGAAC
TTTCCAAAGAGTAGGCCCCTATCTCTCGGGGGATTAACTCCTCGTGGCCCCTGGGTCGT
TGGTGCTCCCCCACCCTGTGT
>2693
TCTTTTTTCTCTTTGCTTTCTCTGAACTTTCCAAAGAGTAGGCCCCTATCTCTCGGGGGA
TTAACTCCTCGTGGCCCCTGGGTCGTTGGTGCTCCCCCACCCTGTGTCAGCATCTAAAC
CTCTGCACACAGGATGTTTTC
>2694
TTTGCTTTCTCTGAACTTTCCAAAGAGTAGGCCCCTATCTCTCGGGGGATTAACTCCTC
GTGGCCCCTGGGTCGTTGGTGCTCCCCCACCCTGTGTCAGCATCTAAACCTCTGCACA
CAGGATGTTTTCTCTAGCGCTTA
>2695
AAGAGTAGGCCCCTATCTCTCGGGGGATTAACTCCTCGTGGCCCCTGGGTCGTTGGTGC
TCCCCCACCCTGTGTCAGCATCTAAACCTCTGCACACAGGATGTTTTCTCTAGCGCTTA
TCAGATGCTTTAGAAGATGCGG
>2696
CTCCTCGTGGCCCCTGGGTCGTTGGTGCTCCCCCACCCTGTGTCAGCATCTAAACCTCT
GCACACAGGATGTTTTCTCTAGCGCTTATCAGATGCTTTAGAAGATGCGGGGTGGAGAT
AGAGGTAACGTTTAACCCATCC
>2697
TGTCAGCATCTAAACCTCTGCACACAGGATGTTTTCTCTAGCGCTTATCAGATGCTTTAG
AAGATGCGGGGTGGAGATAGAGGTAACGTTTAACCCATCCTGGATTCCTTTCATGAACT
CAGCCACAGAGCAGAGGGAGG
>2698
AACCTCTGCACACAGGATGTTTTCTCTAGCGCTTATCAGATGCTTTAGAAGATGCGGGG
TGGAGATAGAGGTAACGTTTAACCCATCCTGGATTCCTTTCATGAACTCAGCCACAGAG
CAGAGGGAGGCTTGGCTGGAGC
>2699
CTGCACACAGGATGTTTTCTCTAGCGCTTATCAGATGCTTTAGAAGATGCGGGGTGGAG
ATAGAGGTAACGTTTAACCCATCCTGGATTCCTTTCATGAACTCAGCCACAGAGCAGAG
GGAGGCTTGGCTGGAGCAAACA
>2700
TTAACCCATCCTGGATTCCTTTCATGAACTCAGCCACAGAGCAGAGGGAGGCTTGGCT
GGAGCAAACAATGTTGGGCCTTCTATTTCAAACAGAGGTGTGGGGGCTTGTGGTGGCA
TCAGTGGTGCACTGAACAACCCTC
>2701
GAACTCAGCCACAGAGCAGAGGGAGGCTTGGCTGGAGCAAACAATGTTGGGCCTTCT
ATTTCAAACAGAGGTGTGGGGGCTTGTGGTGGCATCAGTGGTGCACTGAACAACCCTC
CCTGACCCTAGCGTGTGGGTTTGGA
>2702
AGAGCAGAGGGAGGCTTGGCTGGAGCAAACAATGTTGGGCCTTCTATTTCAAACAGA
GGTGTGGGGGCTTGTGGTGGCATCAGTGGTGCACTGAACAACCCTCCCTGACCCTAGC

GTGTGGGTTTGGAGCCTGGGGAATC
>2703
GCAGAGGGAGGCTTGGCTGGAGCAAACAATGTTGGGCCTTCTATTTCAAACAGAGGT
GTGGGGGCTTGTGGTGGCATCAGTGGTGCACTGAACAACCCTCCCTGACCCTAGCGTG
TGGGTTTGGAGCCTGGGGAATCAAA
>2704
GGCTTGGCTGGAGCAAACAATGTTGGGCCTTCTATTTCAAACAGAGGTGTGGGGGCTT
GTGGTGGCATCAGTGGTGCACTGAACAACCCTCCCTGACCCTAGCGTGTGGGTTTGGA
GCCTGGGGAATCAAAGAGAAATCT
>2705
ATTTCAAACAGAGGTGTGGGGGCTTGTGGTGGCATCAGTGGTGCACTGAACAACCCTC
CCTGACCCTAGCGTGTGGGTTTGGAGCCTGGGGAATCAAAGAGAAATCTAACAAACTA
TTCTTTTTCACGTGTGGGCTGAAT
>2706
CCCTGACCCTAGCGTGTGGGTTTGGAGCCTGGGGAATCAAAGAGAAATCTAACAAACT
ATTCTTTTTCACGTGTGGGCTGAATGAATGTGTGGGTTATCCACATCAGTTTCTTCTCTC
TTCTCATCCTCCCTCCTTCCTC
>2707
CTGACCCTAGCGTGTGGGTTTGGAGCCTGGGGAATCAAAGAGAAATCTAACAAACTAT
TCTTTTTCACGTGTGGGCTGAATGAATGTGTGGGTTATCCACATCAGTTTCTTCTCTCTT
CTCATCCTCCCTCCTTCCTCGC
>2708
GGGTTTGGAGCCTGGGGAATCAAAGAGAAATCTAACAAACTATTCTTTTTCACGTGTG
GGCTGAATGAATGTGTGGGTTATCCACATCAGTTTCTTCTCTCTTCTCATCCTCCCTCCT
TCCTCGCAGACCAGCATTTTGG
>2709
GTTTGGAGCCTGGGGAATCAAAGAGAAATCTAACAAACTATTCTTTTTCACGTGTGGG
CTGAATGAATGTGTGGGTTATCCACATCAGTTTCTTCTCTCTTCTCATCCTCCCTCCTTC
CTCGCAGACCAGCATTTTGGCC
>2710
GGAGCCTGGGGAATCAAAGAGAAATCTAACAAACTATTCTTTTTCACGTGTGGGCTGA
ATGAATGTGTGGGTTATCCACATCAGTTTCTTCTCTCTTCTCATCCTCCCTCCTTCCTCG
CAGACCAGCATTTTGGCCAGGG
>2711
TCAAAGAGAAATCTAACAAACTATTCTTTTTCACGTGTGGGCTGAATGAATGTGTGGGT
TATCCACATCAGTTTCTTCTCTCTTCTCATCCTCCCTCCTTCCTCGCAGACCAGCATTTT
GGCCAGGGTGCCCAGTTTGAG
>2712
GGTTATCCACATCAGTTTCTTCTCTCTTCTCATCCTCCCTCCTTCCTCGCAGACCAGCAT
TTTGGCCAGGGTGCCCAGTTTGAGCTCATTTACCAAAGGTGAATAGGGAAGGGAGTAG
AAACCAAATTAGGCACCTGTCT
>2713
CACATCAGTTTCTTCTCTCTTCTCATCCTCCCTCCTTCCTCGCAGACCAGCATTTTGGCC
AGGGTGCCCAGTTTGAGCTCATTTACCAAAGGTGAATAGGGAAGGGAGTAGAAACCA

AATTAGGCACCTGTCTGACAACA
>2714
CTCATCCTCCCTCCTTCCTCGCAGACCAGCATTTTGGCCAGGGTGCCCAGTTTGAGCTC
ATTTACCAAAGGTGAATAGGGAAGGGAGTAGAAACCAAATTAGGCACCTGTCTGACAA
CACAAGGATCTTTTCTACAATGG
>2715
CAGGGTGCCCAGTTTGAGCTCATTTACCAAAGGTGAATAGGGAAGGGAGTAGAAACC
AAATTAGGCACCTGTCTGACAACACAAGGATCTTTTCTACAATGGTGCATTTGGATTAT
GCAGAGTTTTAGATTTTCTGTTTT
>2716
GGTGAATAGGGAAGGGAGTAGAAACCAAATTAGGCACCTGTCTGACAACACAAGGAT
CTTTTCTACAATGGTGCATTTGGATTATGCAGAGTTTTAGATTTTCTGTTTTATCCCAGG
GTGAACGTGTGTAGAAAACTGGG
>2717
GAAACCAAATTAGGCACCTGTCTGACAACACAAGGATCTTTTCTACAATGGTGCATTTG
GATTATGCAGAGTTTTAGATTTTCTGTTTTATCCCAGGGTGAACGTGTGTAGAAAACTG
GGACCTAACCATGTTCTTCTCT
>2718
CACCTGTCTGACAACACAAGGATCTTTTCTACAATGGTGCATTTGGATTATGCAGAGTT
TTAGATTTTCTGTTTTATCCCAGGGTGAACGTGTGTAGAAAACTGGGACCTAACCATGT
TCTTCTCTTCAGTCTTATGGTC
>2719
GGATCTTTTCTACAATGGTGCATTTGGATTATGCAGAGTTTTAGATTTTCTGTTTTATCCC
AGGGTGAACGTGTGTAGAAAACTGGGACCTAACCATGTTCTTCTCTTCAGTCTTATGGT
CATTGCTACTTTGAAGGCAG
>2720
ATTATGCAGAGTTTTAGATTTTCTGTTTTATCCCAGGGTGAACGTGTGTAGAAAACTGG
GACCTAACCATGTTCTTCTCTTCAGTCTTATGGTCATTGCTACTTTGAAGGCAGAAAGG
ATAAAAGTAGCATAGGGAGTGG
>2721
TTAGATTTTCTGTTTTATCCCAGGGTGAACGTGTGTAGAAAACTGGGACCTAACCATGT
TCTTCTCTTCAGTCTTATGGTCATTGCTACTTTGAAGGCAGAAAGGATAAAAGTAGCAT
AGGGAGTGGTTTACCATAACTG
>2722
ACTGGGACCTAACCATGTTCTTCTCTTCAGTCTTATGGTCATTGCTACTTTGAAGGCAG
AAAGGATAAAAGTAGCATAGGGAGTGGTTTACCATAACTGCAAGTAAATACCAAGCAT
GTGCGAGCCTGTAGACATTGCCT
>2723
CCATGTTCTTCTCTTCAGTCTTATGGTCATTGCTACTTTGAAGGCAGAAAGGATAAAAG
TAGCATAGGGAGTGGTTTACCATAACTGCAAGTAAATACCAAGCATGTGCGAGCCTGTA
GACATTGCCTGCTATTGGGGAA
>2724
TTATGGTCATTGCTACTTTGAAGGCAGAAAGGATAAAAGTAGCATAGGGAGTGGTTTAC
CATAACTGCAAGTAAATACCAAGCATGTGCGAGCCTGTAGACATTGCCTGCTATTGGGG

AAAGACGGAGTTAGCTGGGGAA
>2725
AGAAAGGATAAAAGTAGCATAGGGAGTGGTTTACCATAACTGCAAGTAAATACCAAGC
ATGTGCGAGCCTGTAGACATTGCCTGCTATTGGGGAAAGACGGAGTTAGCTGGGGAAA
TACATCAGTCTTCACTCCCAGCAC
>2726
ACCATAACTGCAAGTAAATACCAAGCATGTGCGAGCCTGTAGACATTGCCTGCTATTGG
GGAAAGACGGAGTTAGCTGGGGAAATACATCAGTCTTCACTCCCAGCACCCAAGATGT
AAACATTTCTTACTCTTCCTGGA
>2727
CAAGCATGTGCGAGCCTGTAGACATTGCCTGCTATTGGGGAAAGACGGAGTTAGCTGG
GGAAATACATCAGTCTTCACTCCCAGCACCCAAGATGTAAACATTTCTTACTCTTCCTG
GATCAGGCTATCTCTGCTTTGCC
>2728
TTGCCTGCTATTGGGGAAAGACGGAGTTAGCTGGGGAAATACATCAGTCTTCACTCCC
AGCACCCAAGATGTAAACATTTCTTACTCTTCCTGGATCAGGCTATCTCTGCTTTGCCTT
TTCTTTCACCTACCTCCTTCTT
>2729
GATCAGGCTATCTCTGCTTTGCCTTTTCTTTCACCTACCTCCTTCTTTCATGGCAGGAAG
GGAAAGGAAAGTACCCCCAAGCAGCCAGCAGTCAGCACTGGGGCCTCTAGGGAAGA
GTGGATTGGTGTTGTTTTGCTGTG
>2730
GCCTTTTCTTTCACCTACCTCCTTCTTTCATGGCAGGAAGGGAAAGGAAAGTACCCCC
AAGCAGCCAGCAGTCAGCACTGGGGCCTCTAGGGAAGAGTGGATTGGTGTTGTTTTGC
TGTGGTTTGCTGTGCGTTTTCCAG
>2731
TTCATGGCAGGAAGGGAAAGGAAAGTACCCCCAAGCAGCCAGCAGTCAGCACTGGGG
CCTCTAGGGAAGAGTGGATTGGTGTTGTTTTGCTGTGGTTTGCTGTGCGTTTTCCAGAA
TCTCTCTTGGATGACTATCAGATT
>2732
AAAGTACCCCCAAGCAGCCAGCAGTCAGCACTGGGGCCTCTAGGGAAGAGTGGATTG
GTGTTGTTTTGCTGTGGTTTGCTGTGCGTTTTCCAGAATCTCTCTTGGATGACTATCAGA
TTCAGTGAAGGGATGGACCTTCA
>2733
GTACCCCCAAGCAGCCAGCAGTCAGCACTGGGGCCTCTAGGGAAGAGTGGATTGGTG
TTGTTTTGCTGTGGTTTGCTGTGCGTTTTCCAGAATCTCTCTTGGATGACTATCAGATTC
AGTGAAGGGATGGACCTTCAGGA
>2734
CAAGCAGCCAGCAGTCAGCACTGGGGCCTCTAGGGAAGAGTGGATTGGTGTTGTTTTG
CTGTGGTTTGCTGTGCGTTTTCCAGAATCTCTCTTGGATGACTATCAGATTCAGTGAAG
GGATGGACCTTCAGGAAGCTGAG
>2735
GGATTGGTGTTGTTTTGCTGTGGTTTGCTGTGCGTTTTCCAGAATCTCTCTTGGATGACT
ATCAGATTCAGTGAAGGGATGGACCTTCAGGAAGCTGAGAGATAGGAGATCCTGTGGC

CTGGGAACCCAGAGGCCTGGGC
>2736
ATCTCTCTTGGATGACTATCAGATTCAGTGAAGGGATGGACCTTCAGGAAGCTGAGAG
ATAGGAGATCCTGTGGCCTGGGAACCCAGAGGCCTGGGCCCTGGGAACAACTCACCA
CAGGACTCAGCCTGCGAAATAGGAA
>2737
GGCCTGGGAACCCAGAGGCCTGGGCCCTGGGAACAACTCACCACAGGACTCAGCCTG
CGAAATAGGAAACGTCATTGTAGCTTCTGCATCATGTTTTCCATGTTCCACTGGAAAAC
CATCTGTTAAATGGACATAGACCA
>2738
GGAACCCAGAGGCCTGGGCCCTGGGAACAACTCACCACAGGACTCAGCCTGCGAAAT
AGGAAACGTCATTGTAGCTTCTGCATCATGTTTTCCATGTTCCACTGGAAAACCATCTG
TTAAATGGACATAGACCAATTCTT
>2739
GGCCCTGGGAACAACTCACCACAGGACTCAGCCTGCGAAATAGGAAACGTCATTGTA
GCTTCTGCATCATGTTTTCCATGTTCCACTGGAAAACCATCTGTTAAATGGACATAGAC
CAATTCTTGCTTTTCTTCTTTCTG
>2740
AACAACTCACCACAGGACTCAGCCTGCGAAATAGGAAACGTCATTGTAGCTTCTGCAT
CATGTTTTCCATGTTCCACTGGAAAACCATCTGTTAAATGGACATAGACCAATTCTTGCT
TTTCTTCTTTCTGGTACCAAAG
>2741
AGCCTGCGAAATAGGAAACGTCATTGTAGCTTCTGCATCATGTTTTCCATGTTCCACTG
GAAAACCATCTGTTAAATGGACATAGACCAATTCTTGCTTTTCTTCTTTCTGGTACCAA
AGAGAACGGTGGTGTCTCTTGT
>2742
GTTTTCCATGTTCCACTGGAAAACCATCTGTTAAATGGACATAGACCAATTCTTGCTTTT
CTTCTTTCTGGTACCAAAGAGAACGGTGGTGTCTCTTGTCATCTAGATGTTCTTGTAGG
GGCACCAGAGTCATTGCAACC
>2743
CTGGAAAACCATCTGTTAAATGGACATAGACCAATTCTTGCTTTTCTTCTTTCTGGTACC
AAAGAGAACGGTGGTGTCTCTTGTCATCTAGATGTTCTTGTAGGGGCACCAGAGTCAT
TGCAACCATTTTGAGTAAAGAT
>2744
ACATAGACCAATTCTTGCTTTTCTTCTTTCTGGTACCAAAGAGAACGGTGGTGTCTCTT
GTCATCTAGATGTTCTTGTAGGGGCACCAGAGTCATTGCAACCATTTTGAGTAAAGATT
GAGTCCACTGGGGCATCAGAAG
>2745
ACCAATTCTTGCTTTTCTTCTTTCTGGTACCAAAGAGAACGGTGGTGTCTCTTGTCATCT
AGATGTTCTTGTAGGGGCACCAGAGTCATTGCAACCATTTTGAGTAAAGATTGAGTCC
ACTGGGGCATCAGAAGGGGAGT
>2746
TTCTTCTTTCTGGTACCAAAGAGAACGGTGGTGTCTCTTGTCATCTAGATGTTCTTGTA
GGGGCACCAGAGTCATTGCAACCATTTTGAGTAAAGATTGAGTCCACTGGGGCATCAG

AAGGGGAGTGTGTCATTGCTGTT
>2747
AGAGAACGGTGGTGTCTCTTGTCATCTAGATGTTCTTGTAGGGGCACCAGAGTCATTGC
AACCATTTTGAGTAAAGATTGAGTCCACTGGGGCATCAGAAGGGGAGTGTGTCATTGC
TGTTGTTACCACACAGGCCGTGG
>2748
TTGTAGGGGCACCAGAGTCATTGCAACCATTTTGAGTAAAGATTGAGTCCACTGGGGC
ATCAGAAGGGGAGTGTGTCATTGCTGTTGTTACCACACAGGCCGTGGTACTTGCCTTTT
CCTGACCCATTTTGCAGAGCAGG
>2749
GTAGGGGCACCAGAGTCATTGCAACCATTTTGAGTAAAGATTGAGTCCACTGGGGCAT
CAGAAGGGGAGTGTGTCATTGCTGTTGTTACCACACAGGCCGTGGTACTTGCCTTTTC
CTGACCCATTTTGCAGAGCAGGCC
>2750
GTCATTGCAACCATTTTGAGTAAAGATTGAGTCCACTGGGGCATCAGAAGGGGAGTGT
GTCATTGCTGTTGTTACCACACAGGCCGTGGTACTTGCCTTTTCCTGACCCATTTTGCA
GAGCAGGCCAGCTACTTAACCCC
>2751
GGTACTTGCCTTTTCCTGACCCATTTTGCAGAGCAGGCCAGCTACTTAACCCCTCGCCA
CCCACTCACCTGTGGAAGGCACATTTGCAAAACAATAGACCAGTTGGTAATTTGGCAC
TTGTTAGGTAGACATAGTGGTAC
>2752
AGCAGGCCAGCTACTTAACCCCTCGCCACCCACTCACCTGTGGAAGGCACATTTGCAA
AACAATAGACCAGTTGGTAATTTGGCACTTGTTAGGTAGACATAGTGGTACATTGAGGA
AGTTCCTCCTGTTAACTAACCTA
>2753
GGCCAGCTACTTAACCCCTCGCCACCCACTCACCTGTGGAAGGCACATTTGCAAAACA
ATAGACCAGTTGGTAATTTGGCACTTGTTAGGTAGACATAGTGGTACATTGAGGAAGTT
CCTCCTGTTAACTAACCTAGTTT
>2754
CTCACCTGTGGAAGGCACATTTGCAAAACAATAGACCAGTTGGTAATTTGGCACTTGTT
AGGTAGACATAGTGGTACATTGAGGAAGTTCCTCCTGTTAACTAACCTAGTTTTTCTTG
CTGTAATCCGAATATACTTCTC
>2755
ACATTTGCAAAACAATAGACCAGTTGGTAATTTGGCACTTGTTAGGTAGACATAGTGGT
ACATTGAGGAAGTTCCTCCTGTTAACTAACCTAGTTTTTCTTGCTGTAATCCGAATATAC
TTCTCTGATTTTTTGAAAAAG
>2756
AAACAATAGACCAGTTGGTAATTTGGCACTTGTTAGGTAGACATAGTGGTACATTGAGG
AAGTTCCTCCTGTTAACTAACCTAGTTTTTCTTGCTGTAATCCGAATATACTTCTCTGATT
TTTTGAAAAAGCCTGTGAGT
>2757
GCACTTGTTAGGTAGACATAGTGGTACATTGAGGAAGTTCCTCCTGTTAACTAACCTAG
TTTTTCTTGCTGTAATCCGAATATACTTCTCTGATTTTTTGAAAAAGCCTGTGAGTGGAG

CCTGGCTCGACAGCCCAAAAC
>2758
CCTCCTGTTAACTAACCTAGTTTTTCTTGCTGTAATCCGAATATACTTCTCTGATTTTTG
AAAAAGCCTGTGAGTGGAGCCTGGCTCGACAGCCCAAAACGCTGAATTAATTCTGTCT
CTCTCTCTCTCTCTTTTTTTT
>2759
CTGTTAACTAACCTAGTTTTTCTTGCTGTAATCCGAATATACTTCTCTGATTTTTGAAAA
AGCCTGTGAGTGGAGCCTGGCTCGACAGCCCAAAACGCTGAATTAATTCTGTCTCTCT
CTCTCTCTCTTTTTTTTTTTT
>2760
CTTCTCTGATTTTTGAAAAAGCCTGTGAGTGGAGCCTGGCTCGACAGCCCAAAACGC
TGAATTAATTCTGTCTCTCTCTCTCTCTCTTTTTTTTTTTTGAGACGTGGAGTCTCGCTCT
GTCACCCAGGCTGGAATGCGC
>2761
GCCTGGCTCGACAGCCCAAAACGCTGAATTAATTCTGTCTCTCTCTCTCTCTCTTTTTTT
TTTTTGAGACGTGGAGTCTCGCTCTGTCACCCAGGCTGGAATGCGCTGGCACGATCTC
GGCTCACTGCAACTTCCACCTC
>2762
AGTCTCGCTCTGTCACCCAGGCTGGAATGCGCTGGCACGATCTCGGCTCACTGCAACT
TCCACCTCCTGGGTCCAAGCAATTCTCCTGCCTCAGCCTCCCAAGTAGCTGGGACTAC
AGGCATTGCCACTACGCCCAGCTA
>2763
TGGCACGATCTCGGCTCACTGCAACTTCCACCTCCTGGGTCCAAGCAATTCTCCTGCCT
CAGCCTCCCAAGTAGCTGGGACTACAGGCATTGCCACTACGCCCAGCTAATTTTTGTAT
TTTTAATAGAGACGGGGTTTCG
>2764
GCAATTCTCCTGCCTCAGCCTCCCAAGTAGCTGGGACTACAGGCATTGCCACTACGCCC
AGCTAATTTTTGTATTTTTAATAGAGACGGGGTTTCGCCGTGTTGGCCAGGCTGGTCTC
AAACTCCTGATCTCAGGTGATC
>2765
TCCCAAGTAGCTGGGACTACAGGCATTGCCACTACGCCCAGCTAATTTTTGTATTTTTAA
TAGAGACGGGGTTTCGCCGTGTTGGCCAGGCTGGTCTCAAACTCCTGATCTCAGGTGA
TCCACCAGCCTTGGCATCCCAA
>2766
CATTGCCACTACGCCCAGCTAATTTTTGTATTTTTAATAGAGACGGGGTTTCGCCGTGTT
GGCCAGGCTGGTCTCAAACTCCTGATCTCAGGTGATCCACCAGCCTTGGCATCCCAAA
ATGCTGGGACTGCAGGAATGAG
>2767
ATTTTTGTATTTTTAATAGAGACGGGGTTTCGCCGTGTTGGCCAGGCTGGTCTCAAACT
CCTGATCTCAGGTGATCCACCAGCCTTGGCATCCCAAAATGCTGGGACTGCAGGAATG
AGCCACCGCGCCCGACCCAATTC
>2768
CACCAGCCTTGGCATCCCAAAATGCTGGGACTGCAGGAATGAGCCACCGCGCCCGAC
CCAATTCTCTTGAGTGACATGCTGAGTGGGGCTGCTTTTTGGTGGAGGTAACAAAAAA

AGAGTTGAGGTATTGATTTTAGTTT
>2769
CATCCCAAAATGCTGGGACTGCAGGAATGAGCCACCGCGCCCGACCCAATTCTCTTGA
GTGACATGCTGAGTGGGGCTGCTTTTTGGTGGAGGTAACAAAAAAAGAGTTGAGGTAT
TGATTTTAGTTTCATTTGAGACAG
>2770
GACTGCAGGAATGAGCCACCGCGCCCGACCCAATTCTCTTGAGTGACATGCTGAGTGG
GGCTGCTTTTTGGTGGAGGTAACAAAAAAAGAGTTGAGGTATTGATTTTAGTTTCATTT
GAGACAGCGATCTGTCCCCTCCC
>2771
AGGAATGAGCCACCGCGCCCGACCCAATTCTCTTGAGTGACATGCTGAGTGGGGCTGC
TTTTTGGTGGAGGTAACAAAAAAAGAGTTGAGGTATTGATTTTAGTTTCATTTGAGACA
GCGATCTGTCCCCTCCCTGAGTT
>2772
GCGCCCGACCCAATTCTCTTGAGTGACATGCTGAGTGGGGCTGCTTTTTGGTGGAGGT
AACAAAAAAAGAGTTGAGGTATTGATTTTAGTTTCATTTGAGACAGCGATCTGTCCCCT
CCCTGAGTTCCCTCGTTTGTTTG
>2773
TTGAGTGACATGCTGAGTGGGGCTGCTTTTTGGTGGAGGTAACAAAAAAAGAGTTGA
GGTATTGATTTTAGTTTCATTTGAGACAGCGATCTGTCCCCTCCCTGAGTTCCCTCGTTT
GTTTGTAAACTGCTTAAATGAAC
>2774
CTGCTTTTTGGTGGAGGTAACAAAAAAAGAGTTGAGGTATTGATTTTAGTTTCATTTGA
GACAGCGATCTGTCCCCTCCCTGAGTTCCCTCGTTTGTTTGTAAACTGCTTAAATGAAC
TTTTTCCCCTGTTCCTCCATCA
>2775
GAGGTAACAAAAAAAGAGTTGAGGTATTGATTTTAGTTTCATTTGAGACAGCGATCTGT
CCCCTCCCTGAGTTCCCTCGTTTGTTTGTAAACTGCTTAAATGAACTTTTTCCCCTGTTC
CTCCATCACTCTGCTGTTCTC
>2776
AAAAAAAGAGTTGAGGTATTGATTTTAGTTTCATTTGAGACAGCGATCTGTCCCCTCCC
TGAGTTCCCTCGTTTGTTTGTAAACTGCTTAAATGAACTTTTTCCCCTGTTCCTCCATCA
CTCTGCTGTTCTCTTCTCCTC
>2777
AGACAGCGATCTGTCCCCTCCCTGAGTTCCCTCGTTTGTTTGTAAACTGCTTAAATGAA
CTTTTTCCCCTGTTCCTCCATCACTCTGCTGTTCTCTTCTCCTCCTCTTATCCTAACAGTC
TCATAGGTAAACTTAGAAGG
>2778
CCCTGAGTTCCCTCGTTTGTTTGTAAACTGCTTAAATGAACTTTTTCCCCTGTTCCTCCA
TCACTCTGCTGTTCTCTTCTCCTCCTCTTATCCTAACAGTCTCATAGGTAAACTTAGAAG
GAAGACAAATACTTACAAAT
>2779
TGCTTAAATGAACTTTTTCCCCTGTTCCTCCATCACTCTGCTGTTCTCTTCTCCTCCTCTT
ATCCTAACAGTCTCATAGGTAAACTTAGAAGGAAGACAAATACTTACAAATTGTCACCG

AGGGTACCATAAAAGAGACA
>2780
TTCTCTTCTCCTCCTCTTATCCTAACAGTCTCATAGGTAAACTTAGAAGGAAGACAAAT
ACTTACAAATTGTCACCGAGGGTACCATAAAAGAGACATTTGGACAGTAGTTCTGGGG
AGATCCTTGTCTTTGCTTCATGT
>2781
CTCCTCTTATCCTAACAGTCTCATAGGTAAACTTAGAAGGAAGACAAATACTTACAAAT
TGTCACCGAGGGTACCATAAAAGAGACATTTGGACAGTAGTTCTGGGGAGATCCTTGT
CTTTGCTTCATGTTAATACAGAG
>2782
GGTAAACTTAGAAGGAAGACAAATACTTACAAATTGTCACCGAGGGTACCATAAAAGA
GACATTTGGACAGTAGTTCTGGGGAGATCCTTGTCTTTGCTTCATGTTAATACAGAGGG
ATTGGGGTAGCCCAGATCACCAG
>2783
AAACTTAGAAGGAAGACAAATACTTACAAATTGTCACCGAGGGTACCATAAAAGAGAC
ATTTGGACAGTAGTTCTGGGGAGATCCTTGTCTTTGCTTCATGTTAATACAGAGGGATT
GGGGTAGCCCAGATCACCAGGGG
>2784
AAATACTTACAAATTGTCACCGAGGGTACCATAAAAGAGACATTTGGACAGTAGTTCTG
GGGAGATCCTTGTCTTTGCTTCATGTTAATACAGAGGGATTGGGGTAGCCCAGATCACC
AGGGGAAAGTGGTTTTTCTTCA
>2785
AAAGAGACATTTGGACAGTAGTTCTGGGGAGATCCTTGTCTTTGCTTCATGTTAATACA
GAGGGATTGGGGTAGCCCAGATCACCAGGGGAAAGTGGTTTTTCTTCATTTAGCTTTTA
ATTGCTTTCTTTCACTTTTATG
>2786
CTGGGGAGATCCTTGTCTTTGCTTCATGTTAATACAGAGGGATTGGGGTAGCCCAGATC
ACCAGGGGAAAGTGGTTTTTCTTCATTTAGCTTTTAATTGCTTTCTTTCACTTTTATGTAT
AAAAGATTGTCCATTTAATT
>2787
GGGAGATCCTTGTCTTTGCTTCATGTTAATACAGAGGGATTGGGGTAGCCCAGATCACC
AGGGGAAAGTGGTTTTTCTTCATTTAGCTTTTAATTGCTTTCTTTCACTTTTATGTATAAA
AGATTGTCCATTTAATTAAC
>2788
GGTAGCCCAGATCACCAGGGGAAAGTGGTTTTTCTTCATTTAGCTTTTAATTGCTTTCTT
TCACTTTTATGTATAAAAGATTGTCCATTTAATTAACTCAAGAATGCAATTTCAAAAACA
CTAATGCTTTACACATGACG
>2789
CACCAGGGGAAAGTGGTTTTTCTTCATTTAGCTTTTAATTGCTTTCTTTCACTTTTATGTA
TAAAAGATTGTCCATTTAATTAACTCAAGAATGCAATTTCAAAAACACTAATGCTTTAC
ACATGACGGCTTTTAAAATA
>2790
CATTTAATTAACTCAAGAATGCAATTTCAAAAACACTAATGCTTTACACATGACGGCTT
TTAAAATAAAAGTAGCATTCTAATATTGCATAATGCTTTTCTATCCCCTTCCTCTCCCCGC

GTTCACCCCCACCCCTCCAG
>2791
TGACGGCTTTTAAAATAAAAGTAGCATTCTAATATTGCATAATGCTTTTCTATCCCCTTCC
TCTCCCCGCGTTCACCCCCACCCCTCCAGACTGTATTAACCTTTCATAGCGGAGGGTTG
TTAGGAGGAAAACGCTGTTC
>2792
GCATTCTAATATTGCATAATGCTTTTCTATCCCCTTCCTCTCCCCGCGTTCACCCCCACCC
CTCCAGACTGTATTAACCTTTCATAGCGGAGGGTTGTTAGGAGGAAAACGCTGTTCTG
GATGGTGCCTCTCTGCCTCTC
>2793
TTTCTATCCCCTTCCTCTCCCCGCGTTCACCCCCACCCCTCCAGACTGTATTAACCTTTC
ATAGCGGAGGGTTGTTAGGAGGAAAACGCTGTTCTGGATGGTGCCTCTCTGCCTCTCC
TTCCCACCAACCAAGTACAGCA
>2794
CTATCCCCTTCCTCTCCCCGCGTTCACCCCCACCCCTCCAGACTGTATTAACCTTTCATA
GCGGAGGGTTGTTAGGAGGAAAACGCTGTTCTGGATGGTGCCTCTCTGCCTCTCCTTC
CCACCAACCAAGTACAGCAGCA
>2795
CCGCGTTCACCCCCACCCCTCCAGACTGTATTAACCTTTCATAGCGGAGGGTTGTTAGG
AGGAAAACGCTGTTCTGGATGGTGCCTCTCTGCCTCTCCTTCCCACCAACCAAGTACA
GCAGCACGACAGCCGCAGGCCTT
>2796
AGCGGAGGGTTGTTAGGAGGAAAACGCTGTTCTGGATGGTGCCTCTCTGCCTCTCCTT
CCCACCAACCAAGTACAGCAGCACGACAGCCGCAGGCCTTTCTGAAGTGTTCCGTAG
CTTCTGTAAGAAAGAGACTACTCAT
>2797
GATGGTGCCTCTCTGCCTCTCCTTCCCACCAACCAAGTACAGCAGCACGACAGCCGCA
GGCCTTTCTGAAGTGTTCCGTAGCTTCTGTAAGAAAGAGACTACTCATCCCTTCCCCCT
TGGCCGGTTTCATGCCTATATTT
>2798
CTCTCCTTCCCACCAACCAAGTACAGCAGCACGACAGCCGCAGGCCTTTCTGAAGTGT
TCCGTAGCTTCTGTAAGAAAGAGACTACTCATCCCTTCCCCCTTGGCCGGTTTCATGCC
TATATTTACCCTCTTTTGGGGGT
>2799
CCGCAGGCCTTTCTGAAGTGTTCCGTAGCTTCTGTAAGAAAGAGACTACTCATCCCTTC
CCCCTTGGCCGGTTTCATGCCTATATTTACCCTCTTTTGGGGGTCACTGTGGCTCCTACT
CCCATTGCTGCCTTTTAAGAA
>2800
CTGTAAGAAAGAGACTACTCATCCCTTCCCCCTTGGCCGGTTTCATGCCTATATTTACCC
TCTTTTGGGGGTCACTGTGGCTCCTACTCCCATTGCTGCCTTTTAAGAAGCATCATTATG
ATGGATTACATGGGAAAATT
