# Supplementary material for: iSS-PseDNC: Identifying Splicing Sites Using Pseudo Dinucleotide Composition
Source: Biomed Res Int. 2014 May 21;2014:623149. doi: 10.1155/2014/623149 (PMC4055483; doi:10.1155/2014/623149)
Supplement: Supplementary file 1 — Supporting Information S1. The benchmark dataset for splice donor sites. Supporting Information S2. The benchmark dataset for splice acceptor sites. [file 623149.f1.zip › Supp2.pdf]

Supporting Information S2. The benchmark dataset for splice acceptor sites. It consists of a positive dataset $\mathbf{S}_2^+$ and a negative dataset $\mathbf{S}_2^-$. The former contains 2,880 true splice acceptor site containing sequences, while the latter contains 2,800 false splice acceptor site containing sequences.

$\mathbf{S}_2^+$: 2,880 true splice acceptor site containing sequences

>1
GGCCAGGGGCATAGAGCTGGCCAAGGAGCCATGGCTCACTAACGTGTTGTATGGGGCT
CCTTCCCTTCAGGTCCAGGCTCCTGCGTGAAGTGATGCTCCTCTTTGCCTTACTCCTAG
CCATGGAGCTCCCATTGGTGGCA
>2
GAGTGAGCTGGTAATGGGTGGAAAAGGCGTAGTGGAGCAGAAGCCTGAAGCCTGCTT
TCTCCCCTCTCAGGGACTTACAGTTTGAGATGCCATGACTGTGCGGTCATAAATGACTT
CAACTGTCCCAACATTAGAGTATG
>3
TGTATGTGCCTCAATATTTACAAGCAGAAAATGTGAAATCAATTATTTTCATTGCTGCTT
CTTTTTTTAGGCATAAATTCTCGTGAACTACTTGTTTATAAGAACTGTACAAACAACTGC
ACATTTGTATATGCAGCTGA
>4
GTAGGGCTCAGCTCCGCCCCTGTCACTACACGCTGGGGACACACCACACTGCCCGACT
TCTCCTCCCCAGGTGGGCGCTCCTCGCCAAAAAAGGCTACCAGGAGCGGGACCTGGA
ACCCCAGTTTTCCATCATCACCAAA
>5
AGGCTGCCGGCTTCCGGCCTTTCCAGTCAACACGAGCCCAGCCAGGCCAACCTTGAG
ACTTGCCTCCTAGGGAGAGAACGTGTTCTTCTTGGTGACCAACTTCCTTGTGACGCCA
GCCCAAGTTCAGGGCAGATGCCCAG
>6
CCTTTGAGACTGGGCTGTGGAGGGGAGACATCCCCTGTGCCATTGGTGACTGCTCTCT
CTCCCACCTCAGCACCCGTCCGTCCCACTGGCTAACTGCTGGGTCGACGAGGACTGCC
CCGAAGGGGAGGGAGGCACACACA
>7
ACCCCCTGGCTGAAGGCCTCCCCAGGCCTGCAGAGATTTGAAGGTCTGGAGTTCATCT
TTTGTTTTCTAGGTGTAAAAACAGGCCAGTGTGTGGTGTTCAATGGGACCCACAGGAC
CTGTGAGATCTGGAGTTGGTGCCC
>8
ACAATCCCCTACCCCAACTGGCGCAGGGCCCCAGGCCTGGCAGAGGCTGTCACCTCCC
TTCCACCTGCAGGAGGCCCCTGCTGGCCCAGGCCCAGAACTTCACACTGTTCATCAAA
AACACAGTCACCTTCAGCAAGTTC
>9
AGAGTGGGTCTCATCTGCCCCAAGACCCTCCTTGTCCCCTACCTCATCTGACCTTTCCC
ACTCCTCCCAGGTCCAATGCCTTGGAGACCTGGGACCCCACCTATTTTAAGCACTGCC
GCTATGAACCACAATTCAGCCCC
>10
GGTTCCTAGAGGGCTCTGGGAGAGGGTCCCGGGCCCACCCACCGGTGGAAAAGCTAT

GTGCTATGTGCAGGGTGGCTCTGTAGGCATCAGAGTTCACTGGGATTGTGACCTGGAC
ACCGGGGACTCTGGCTGCTGGCCTC
>11
GGTCTGGCAGGGCCAGCCCCACCTGGGGGTGGGCAAAGCAGGTCACCAGAGCCTTCT
TTCCTGCCCACAGGACAGCCACTCACTGGTGGGAGCAACCGGGTGTGGAGGCCCGCA
CCCTGCTCAAGCTCTATGGAATCCGC
>12
GTGCAGGCTGGGGAGGTGGCAGGAAAGCAGGCTCGGGGGCTGGAACATGGGTTGGC
CCTGCCTCTCCCAGGTCACCTTTTTCTGTGACCTGCTACTGCTGTATGTGGATAGAGAA
GCCCATTTCTACTGGAGGACAAAGT
>13
TTGGACCCTGGGTTCTGCCACACTTAGGAAGATGTTGGCTGGATCCCTGACCTGCTGTC
CTCATCTGCAGGCCAAGGCCCCGAAAGCAACCGCCAACTCTGTGTGGAGGGAGCTGG
CCCTTGCATCCCAAGCCCGACTGG
>14
GAGGTTGTGGCCTCGGGTCGGCGTCTGAGGACCCGCTGACGCTCCGGTGCCCCTCCGT
CCTCTCTCACAGCGTGCTGAGCTACGTGGCGGGCATCGCCTGGTTCTTGGCGCTGGTTT
TCCCGCCGCTGACCCAGCGCACT
>15
GGGCGTATGGGGCGAGCAGTGGTGGCTAAGGCCGGGGAGCTCTGCTCAGAGGCTCCC
TTCGTGTCTGCAGGGCTCTGCCAGTGGCCTGGCTTGAACGGACGATGCGGTCAGTAGG
GCTGGAGGTCTACACGCAGAGTTTC
>16
GTTGTGGGGGGCCCTTCAGCCCCCTACCACAAAGTTACACTGAGGCTCCCCCCACCGA
TGCTGCATACAGATGGTGTCGGGCACCAACGTGTACGGCATCCTGCGGGCCCCGCGTG
CTGCCAGCACCGAGTCGCTTGTGC
>17
TCAGGGCTGCTGGCCGTGGAGGGGTTTGGTCAGTGCCCCAGACCCTGCTGATCCTGCT
TCTGGCCTCCAGGGCAGATTTATTGGGCCAAAGATATCGTCTTCCTGGTAACAGAACAT
GACCTTCTGGGCACTGAGGCTTG
>18
CCTGGTCCCCCTCTCAGGGTCACTGTCCTCACCACGTCCTCCATTAGCACTCATTCACT
TGCTCCTACAGGCATGCAGTCGTCTCCCCTGCAGGGCCGAGCTGGGGCCATTCAGGCA
GCCGTGGCCCTGGAGCTGAGCAG
>19
GCCTGCCTGAGGGCTGAAGGGCACAGGGTCTGTGGGAGGGGCTGTCTCATCCTGTCCT
GCACCTCTAAAGCTGCAGCCCGAGGACTGGACATCATTGGATGGACCGCTGCAGGGCC
TGCAGACACTGCTGCTCATGGTTC
>20
TCTAGCTGGAGCGGAGTGGGGGATGGCCTGGTGGCTCTGGCCAGCCGTGAACCTGCC
CCACACCTGACAGGGCTTTGGAGGGCATGTTCCGCAAGCTCAACCACCTCCTGGAGC
GCCTGCACCAGTCCTTCTTCCTCTAC
>21
TGGGATGGTGGTGGTCTTTGGGGATCCCCTGGCCCCAGGGTCCATCTCCAAGAGCCTG

TGTGCCCTGCAGGGTGTGGGGCTGGCCTCGCTCGTGGCACCTCTGCTGATCTCACAGG
CCATGGGACTGGCCCTCTATGTCC
>22
GGGGCAGGGGTAGAGGTCCCCTGGACATGCAGACAGCTTGTGGGTTGCCTCTGAGTC
CTTTGTCTTACAGGGTGGTAAGCACACAGGCCCCAGACAGGGGCTGGATGGCACTGA
AGCTGGTAGCCCTGATCTACCTAGCA
>23
AGCCCTTTCCCCCAACCTGGTGCCCATGCCCCCACCATGGAGCCTACCTTGATGTTGCT
TCTCCACCCAGGACCCTCTATGCTGCCCTGCTGGTGCTGACCAGCCCGGCAGCCACGC
TCCTTGGCAGCCTGTTCCTGTGG
>24
AGGTTCCCGGCTTGATGGGGCCAGGATGGGGCTCCTGGGTGCAGTCAGAGTTCCCACC
TCCCTCCCCTAGGAACTGCATCGGGCAGGCGTTCGCGATGGCGGAGATGAAGGTGGTC
CTGGGGCTCACGCTGCTGGCCTTC
>25
GGGGGCGCAGGACCCGGGAAGCCGCGCCTGGAGGAGGGTCGGGCGGGTCTCAGCCA
CTCCTCGCCCCCAGGCTCCCACTCCATGAGGTATTTCTACACCTCCGTGTCCCGGCCCG
GCCGCGGGGAGCCCCGCTTCATCGC
>26
AAAATCCCCCCGGGTTGGTCGGGGCGGGGCGGGGCTCGGGGGACCGGGCTGACCTCG
GGGTCCGGGCCAGGTTCTCACACCATCCAGAGGATGTATGGCTGCGACGTGGGGCCGG
ACGGGCGCTTCCTCCGCGGGTACCA
>27
CCACATGTGTGCTGGAGGAGTGTCCCATGACAGATGCAAAATGCCTGAATGTTCTGAC
TCTTCCTGACAGACGCCCCCAAGACGCATATGACTCACCACGCTGTCTCTGACCATGA
GGCCACCCTGAGGTGCTGGGCCCT
>28
TTTAATACATAAAAAAAAAACCCGCTAGGTTTCCATGTGCTACTCACATGTTGCTTTTG
GCATTCCTCAGGAAAATACAACTGTATATTACTTAGTCTTAGATGTGCAAGAATCGGAC
TGTTCGGTCCTATCCAGGAAAT
>29
CTTTCATATCTGACCTGAGGAAGGCAGGATGCAATGACTCAGCAAGTCCTCAAGAGCC
TCTTTCTTACAGGTGATCGGACAATGTAAGGTAATAGCTACAAGACATTCCCATGAATC
TCAGGACCTCAGAGTGATTGACT
>30
CATTCTTGCCACCAAGCCACCATTAACATTTCCAGCCCTTTACTGTGACACTGCTATCTT
CTTTCTCCAGTCTCTTCAGCACTGGCCAATACCAAAGATAGTCCGGTCCTCATAGATTT
CTTTGAGGATACTGAGCGCTA
>31
AGTTATCTACTGCTTTTTTTTCCTTTTTCTTTGTCTGTTCTTGAAACTATTTTGATCCCAT
CTGTTCTAGAGAGGAGGGGAAGGAACTGGTTACTTCGTGGACTTCTCTGTGCGGAACT
GCCCCAGACACCATTTCCCCA
>32
AAGCTAACTCTGGCTGGTCATCTTCACACCACCTGGACACACACACTAACAGCTCCTC

ATTCCTTTGTAGGTCTTTGGATTCTGCAGAGCAGATTTGTTCTATGATGTAGAAGCCTTG
GACTTGGAAAGCCCGAAAAACC
>33
ATCAAGTCAAGTATCTAACATCTTCTTTTATGTTACATGATGATAGGCACTTTTCTGTGA
CCTTTTCCAGGAACATGAGAACATCAATGGTGTACCGCCTCATTTGGGACATCCCTTCC
ACTGGGGTGGGCATGAGCGTT
>34
GGCGTGGGCACAGGTCAAGCTGAAAGTCCCCGCCCAGGTATCCAAGTGTCCGCTGTGT
CCGCTCCCCCAGGTGCAGGGCGCCGCGCACTTCGGGCCGGTGTGGCTAGCCAGCTTTG
GGACAGTGCGCACCGTGTACGTGG
>35
CCTTCACTGCAGCCAGTCCCGTCGAGCCGTCCCCACCTTCCCGATGCGCACTCTCTCCT
CAACCCTGCAGGGAAGGCGAAGAATGGCAAAGGCTCCGCAGTCTCCTGGCCCCGCTC
CTCCTCCGGCCTCAAGCGGCCGCC
>36
GAGCTGGGCAGGCGTCGGGGGCGCCCTACCAGAGCCTCCCGGAACCCTGACGGCGCC
CCCTCCCGACGAGGCATCGCCGCGGTTCTGCTCGGCTCGCGCTTGGGCTGCCTGGAGG
CTCAAGTGCCACCCGACACGGAGAC
>37
GAGGTGGAAATGGGGGAATGTAAAGCTGTCCAGGGGTAGCGAGGTATTCACGTGCCTT
CTACCCACGCAGCTCAGAGGCACGTGGAGCGGCGAGAGGCAGAGGCAGCCATGAGG
AACGGAGGACAGCCCGAGAAGGACCT
>38
TCTTCCATAATCTGCACCCTCTGCTGGGTTCTCACACCCAACACCTCTCTTGCTTTCACA
TGTTTTTCAGGTGTCCAACACGCTCTCTTGGGCTCTGTATGAGCTCTCCCGGCACCCCG
AAGTCCAGACAGCACTCCACT
>39
GAAACAATGAGAAGGGGGCTGCAGCCCCTAGCCTCATCTTGTTGTCTCCATTTTGTGCT
TTGCAACCTAGACTGTACCCTGTGGTACCTGGAAATTCTCGTGTCCCAGACAAAGACA
TTCATGTGGGTGACTATATTATC
>40
TGAATATCCTCTTCTTCATGCCTGCCCTATTCTGAGCCCAAACTGACAAGTTTGTTTTCC
TTTGCACCAGACGCTGGTCACTCTGTGTCACTATGCCACTTCAAGGGACCCTGCCCAG
TTCCCAGAGCCAAATTCTTTTC
>41
ATTGGATTACCAATGAATCCCCCCATTATAGATATCTTTCATAGTAATGCTCACCTTCTTC
CCTTTCCAGATCCTAACACATTTTGAGGTGCAGCCTGAGCCAGGTGCGGCCCCAGTTA
GACCCAAGACCCGGACTGTCC
>42
GCTCTCTCACTTGATGTAGACCCATTTGAATGAATCTCCTTTTGCTTTCTCTTTCTTTAAT
ATTTTTCAGGTACGAGCAGGTGATAATAAATATATGCACTTGAAAGTATTCAAAAGTCTT
CCCGGACAAAATGAGGACT
>43
GACTGGGATTAGGGGTTGGGGTGGTGGTCCCAGGGGCATGCTCAGTGGTTCTCTTTTC

TCTGGGGCACAGGCTCTGGGCGTGGGGAGGCTGAGACACGGGAGTGCATCTACTACA
ACGCCAACTGGGAGCTGGAGCGCAC
>44
TGGCAGGGCAGGCCTGGGGGAGTCTTGCATCCCCCAGGTGAGGGGTATATGGAAAATT
CTGTGCCTCCAGGCAGGAGTGTGTGGCCACTGAGGAGAACCCCCAGGTGTACTTCTGC
TGCTGTGAAGGCAACTTCTGCAAC
>45
CCCTGTATTCTGGCTCTGGAAGTGTGAGGGTGGGCAGACTGTTTGACACAGGGCTCTG
TGTGTCCCCCAGTCACGTACGAGCCACCCCCGACAGCCCCCACCCTGCTCACGGTGCT
GGCCTACTCACTGCTGCCCATCGG
>46
TGGCTTGGGGCAGGGCAGGATAGAGGTGGGGAGGACAGGCCAGACCTTTTTAACCCT
TGCTCTCCCCCAGGACCCTGGGCCTCCACCACCATCCCCTCTGGTGGGCCTGAAGCCA
CTGCAGCTGCTGGAGATCAAGGCTC
>47
GTAGGGCAATGTGACTGCAGCAGGGGTAAGCCAGCCACTTGTCCCCCCAACCCCTCGC
CCCCGGCCTCAGGACAAGCAGTCGTGGCAGAGTGAACGGGAGATCTTCAGCACACCT
GGCATGAAGCACGAGAACCTGCTAC
>48
TGCCAGGGCTGGGCTGGGTCCTGTCCTGTACCCAGAATCTGTGCTCAAGTTCTGTGCC
GTCTTCTCTCAGGGCTCCCTCACGGATTACCTCAAGGGGAACATCATCACATGGAACG
AACTGTGTCATGTAGCAGAGACGA
>49
GTATCTTGGGAACCAAGGTGGGAGTTGGATCATGATGTTAAGCTTTATCTCTGCCCACT
TGTTTCCACAGGGACTTTAAAAGTAAGAATGTATTGCTGAAGAGCGACCTCACAGCCG
TGCTGGCTGACTTTGGCTTGGCT
>50
AACTCCCATGTCCCAGCTGTGTGTGTATGGCCAGTCACTGTAAATCCTGCCCTCCTCTG
TCCTCACATAGGTAGGCACGAGACGGTACATGGCTCCTGAGGTGCTCGAGGGAGCCAT
CAACTTCCAGAGAGATGCCTTCC
>51
CATCCTAGATTGAGTGATAGGGAGCAGTGGGACCTTAGAGTGATCCTGCCAGGCTCTCT
CTTTCTCCTAGGACCCGTGGATGAGTACATGCTGCCCTTTGAGGAAGAGATTGGCCAG
CACCCTTCGTTGGAGGAGCTGCA
>52
TCCCCCAAAGCTTTTCCTCACTGAAGGGTCCTAACAAAGGTGTCTTTCCTGTCTGCCCT
ATGCTGCTTAGGGCCTGGCCCAGCTTTGTGTGACCATCGAGGATTGCTGGGACCATGAT
GCAGAGGCTCGCTTGTCCGCGG
>53
TGTGTTAAGAGGGCATCTGCTGGGAAACGATTTGAGGAGAAGGTACTAAATTGCTTGG
TATTTTCCGTAGGAACCCCAGAGCGAAATACAGTTTGCAAAAGATGTCCAGATGGGTT
CTTCTCAAATGAGACGTCATCTAA
>54
TTTGTATTGAATAGACTCTCAGAAATCCAATTGTTGAGTAAATCTTCTGGGTTTTCTAAC

CTTTCTTTAGATGTTACCCTGTGTGAGGAGGCATTCTTCAGGTTTGCTGTTCCTACAAA
GTTTACGCCTAACTGGCTTAG
>55
GCTTGTTTTATGATGGCATTGGATGAATATAAATGATGTGAACACTTATCTGGGCTTTTG
CTTTATGCAGATATTGACCTCTGTGAAAACAGCGTGCAGCGGCACATTGGACATGCTAA
CCTCACCTTCGAGCAGCTTCG
>56
GAGAATTTATACCAAGCGTATTCTGAGTTTACTCACTTTCTCCACAATGTTTCCTTTTTT
CCTTGAACAGGAAAAAAAAAAAAGAAGATGGGTTTTTTAAGTCCAATATATGTTATTTTC
TTCTTTTTTGGAGTCAAAGTA
>57
ACATAGAATTATGGCTATAACTATGGATTATACTATAATACAAGATTAATTTTTAATTTATT
TCTTTCAGAGATGAATCTTACAGTGATGTGTCCTTCTATTGACCCACTACATTACCACCA
TTTAACATACATTCGTGT
>58
AAAGGGACCAGGAGCAGCTGGCTTGCCTTGTGAACAATGACTTGGGATCTTTCTGTCC
TGTCTCTTGCAGTTGGTACCAACAAAGAGCTCTGCTGCCTCGTCTATACCTCCTGGCAG
ATTCCACAAAAGTTCATAGTTGA
>59
AAGGACGCAGGGGCCACAGGATTCCCCTGATGAATTCGTCAGTTCTTAACTCTTCCTCC
CTTCTCCACAGCCTCCTAACCAAGAGAGGCCGGCAGATCTGTGCTGACCCCAATAAGA
AGTGGGTCCAGAAATACATCAGC
>60
GCGCTGAGTGGGGCGTGTGGATGTCCCTGAGCCCAAGTGACAGAGCCTCCCCTCCCTC
GTGCTGCTGCAGGGCACGGCCCCACCCGGAGGCTGCGAGGCTTGGGGGTCCTGGCCG
GGGTGGCTCTGCTCGCTGCCCTCTG
>61
TTCTGAGTCTGCATGCTAGAGCCGCTTTCTGCTGCTTCTATTAATACAGCATTTTCACTT
TGTCTCTCAGGGTTTATGGTCCAGCAGAGTCTCAGAGCGCGGTCTTTGGAGATGTGTG
CCCCCTACTCACTTCTCTCTTG
>62
GAAAATTTTTCCTGGAATCTTCTTGGCACCCACGTGTGTTTTGCTGGACTGAATCTGCT
CTGAATTTTAGGTACAATGTTTGTGTTATGGCGTATGGACAGACGGGCAGCGGAAAGA
GCTATACCATGCTGGGACGCCAT
>63
TATGATTGGCTTACACTGAAAATCACTCTGTGAGACTTTCTGTAAGTTTCTCTTGTGTGT
TTTCCCGCAGGCTCATTTTGGAAAATACCTCAAGAAGCCCAAAGGTTGAAGTCTCCAT
AGTGGAAGTTTACAATAATGAC
>64
AGTCTTGGAAGTCGCCTTAGGTAAGGGGGGCCGCCCTCACTGTGTTCCCCACTGCTTG
CCTTGTCTGTAGGGCTGTCGGCAGCGCCTCGAAACTGATGGAGCTCGTTCATGGAGGT
CTGCAGCTCAGGGCGAAGCACCCC
>65
CTGAGGCAAAGCACAGCCTCATGGCTTCATGCAACTGTTTTCCTCCTCGTCGCTCCTTG

GTCCCTTGCAGCAGACCAAGCCTGCAGTGCCACCCTCCCCAGGGAGCAAACAGAGGC
AGGAAGGGCAGGAAGGAGCCGCAG
>66
GGAGCCGGTTTTGCTTTTCCTGCCTCCTTTCTTGGTGCAAACGGTGATGGTGCGTGGCG
GTCCTGTCCAGGTGTGTCTGGAGTGACCGGGTTGGCCCTGAGGGAGATGGCGTGCATC
AGCCGCAGCCTTGCGGCCCTGGC
>67
GCATTTCTGTTCCCAGATGATCAATTTCCACTGCTACGTTGTTTTGTTTTTGTTTTTTTCC
TCCCCTTAGACTGCAAATGGAAATGTGGAGGCAAAGGTTGTCTGTCTTTTCCGGCGCA
GGGACATTTCTAGTAGCCTCA
>68
GTTAGAGTTTCCAGGGTGGTGTTTTGCTGGATCCATTTCTGACTTGGTTTCTTCCTCTTT
AATCTCCCAGGGGAGTTTGAAGAGGAATCAAAGCAGCCAGGGGTGTCTGAGCAGCAG
CGCCATCAACTGAAGCACCGGGA
>69
CAGATACACCTCCCTCCCTGCCGCCTATGTGGATTGCTGCCTTGCTTACCTTGTCTCTCC
CCAATTCCAGGACTGCTTTTTTTACTCACTGGTGTTTGACCCCGTGCAGAAGACACTTC
TCGCTGATCAGGGCGAGATTA
>70
TTGTGAATGAAAAGACACCAACTGTTCCCCCCTGCCCTCTCCCTGTTGATGCCATTGTT
CTTTTCCCCAGGAGAATCTGATAATCGGAACCAGCAGAAGATGGAGATGAAGGTCTGG
GACCCAGACAACCCTCTCACAGA
>71
GGCTTAATTTCCCAGCCCACTTGGGCTTTTGACTTTTCTTTCGCTTTTGACTTCTCAATT
TCTCCCTTAGAGCTGTGGGAACCTTTGCAAGAGCCCTAGATTGTAGCAGCTCCATTCGG
CAGCCAAGCTTGCACATGAGT
>72
GTTTTAAGGAGCAGGAAGGGAGTGGTGCCAAAAGTTTCAGGTCCTCAGTTGCTCTCCA
TCTTCCCCATAGTTTCACGCCATGGATACCTTGCAAAGGAACGGCTACGACCTGGCTAA
GGCCATGTCGACCCTGGTACCCC
>73
GATGACAGGTGAGGGAACCAGGACCTGGGGCCAGATGCCAGCTCATTCTTCCTCCCTC
TGCCCCCCTTAGCTACCCTGGAAGTCACTTGCCAGCATAGTCCAGTTTTATTACATGTG
GAAAACCACAGACCGGTATATTC
>74
AGAGTGGAAATTGATTCTTTTCCTTGACCAGTTTCTTGTTTTTTTCCCTTCTCCTCGCCT
TCCCCGTTAGCACTAAGCCAAACCCTAACCAGATCATTTCTGTGGGTTCAAAACCTGGC
ATGAATGGGGCTGGATTTCAG
>75
GTGGCTCAAGAATCTGAGGGGCTTAGGTAGTATGCTTCTTCCCTGTCCCCCTCCTCTCC
GTACTCTACAGCCACACAGTCTGCTCAGTGGTATGCCTGGGGCCCACCTAACATGCAG
TGCCGCCTCTGTGCTTCCTGTTG
>76
GGGAATGGAAGAAAGAAGGGAGCCTCAGTGCTGATTTCTGCTCTCTCTCTTTCCTATG

CTCTTCTTCTAGGAGCCACACTCAAGGGGTCATTTATCCAGACCTGAAGCTCAAAGTCT
CTCTCCTTACACAACCAGCGCCA
>77
AGTTGGCTTCTGGCCTTAGAGGAGACCTGAATGGTGAAGCCCTTCAGAAGCTTTCTTC
TTTGTTCTCCAGGCTCCATTCGACTTCCTAAGGCCGCCAAGACTCCATTGAAGATTCAC
CCTCTGGTGCGGCTGCCCCTGGC
>78
AGAGAGATGGAAGTAACAAAGGGGAACTGCGTGGGAAGAAAAGGTGCGAAAGTTTT
CTGTTTTTTTCCAGTGGCCCAGGCACCCCTGAAACCAAAAACACCTCGGGGTACCAAG
ACACCGATCAACAGAAACCAGCTGTC
>79
TGGGCTTTTGTCTCTCATAGGAGTCTGAGAGGGTCCCTTCCCTCATTGTGCCATCCTAC
CCCTTTTCCAGATGGCAGGGGCAGGGGTTCCTTTCTCTGCCAATGGAAGGCCTCTGGC
TTCAGGGATTCGTTCAAGCTCAC
>80
GTGGGGCTTCACCTGGAGGTCTTAATGTTCTCTAAGCCTTTTCTGTTTCCCACTTGCTCT
TTCTGGCCAGGGCCCTACGGAAGGCTCTGACCCATCTGGAAATGCGGCGAGCTGCTCG
CCGACCCAACTTGCCCCTGAAG
>81
TATGTGTGTGTTCGTGTACATGTGTATGTGTCTATCAACTTATCTTTTTTTAAAAAAAAAT
TTATCTCAGGAGAACCATACAGACTTTTGACTGGCGTTGAGTACGTTGTTGGAAGGAA
AAACTGTGCCATTCTGATTGA
>82
GAAAACTTTTTCTCTGAGAAGTGAATGTACTGAGTGGTAAAAGACTTTAAATTATTTGC
TAACTTTTCAGAGTCAAACAGATGAAATCCCTGTATTGACATTAAAAGATAATTCTAAG
TATGGTACCTTTGTTAATGAGG
>83
TAAAGTACTAAAAATTGCCATCTCTGCAACTCTGATACTATGACTTTATTTAACTTATTCT
CATTTACAGAATAGAGTATGAGCCTTTGGTTGCATGCTCTTCTTGTTTAGATGTCTCTGG
GAAAACTGCTTTAAATCAA
>84
TAACTTAAAACTTTTCTTTAAAAAATGCTAGCATGTGAATATATATATTCACATGTTTTCT
TCATTGTAGACAATATGTGCACTCATTTGTGGACGTCCAATTGTAAAGCCAGAATATTTT
ACTGAATTCCTGAAAGCAG
>85
TCACTCCGTTTACAATTTAATAGCAAACATAGTAGTATATAATTAGAATATACTTTAATTAT
TTTTACAGTTTTTACCCACCTCTTGATGAACCATCTATTGGAAGTAAAAATGTTGATCTG
TCAGGACGGCAGGAAAGA
>86
CAAATCAAATTCTTATGTGTTCAAAAAATTACTACATTTTACTTTAAAAAATGTTTCCTT
CCATTTTTAGCATAAGAAATTGAGTTCCGCAGTTGTCTTTGGAGGTGGGGAAGCTAGGT
TGATAACAGAAGAGAATGAAG
>87
CTTGACATTATCTGAATAAAAGTTGCTAGTTTTAATATATTATCATACTTAAACTATTTTAT

TCTAATAGGCAAGGTCTTAGACCTATTCCTGAAGCAGAAATTGGATTGGCGGTGATTTT
CATGACTACAAAGAATTAC
>88
TTTAAGTGACCAGATGTTCATCTTCTATCTAGCAGTTGTGATTCTTTCTTTCTACTTGTGT
GATTTACAGGATTAAAGACAACAACTCCAGGACCAAGCCTTTCACAAGGCGTGTCAGT
TGATGAAAAACTAATGCCAAG
>89
GATCTTTGTTTCTCTATTAAAGTTGCTGTAAACTTGATTATTTACCTTGCATTCTTTTCTT
TTTCTACAGGGATTTGAGTGAAAGGCCAAAAGAAATCAAAGTCTCCAAAATGGAACA
AAAATTCAGAATGCTTTCACAA
>90
GCCAAACTAAATGGTTACTTAGCTGTGTTCATTTTCTTGTTTTGTTTTCTTCTTTTTTTTT
AAATCTAAGGGAAAGGGATGAAGAAAATCAAGAAATGTCTTCATGCAAATCAGCAAG
AATAGAAACGTCTTGTTCTCTT
>91
CCCAAATGACAAGTGACTTTTCTTTGTTATTTCTTCTCTTTCTTACCTATCCATCTTAACC
CCATTTAAGAACAATGACAAACTTCAGGATGATAGTGAGATGCTTCCAAAAAAGCTGT
TATTGACTGAATTTAGATCAC
>92
AATCTTTTTAAAAAAATGGTCATTTTTGTTTGTTAATATGATTTACTGTACTACTTTTATTA
TTCAATAGGTCACATATCCTGGAGCAGGAAAACTTCCACACATCATTGGAGGATCAGAT
CTAATAGCTCATCATGCTC
>93
AGTTCTTTACCTTTAAAAGAAATACCATCCCTTATCTAAATATCATTTAACCCCATTTCTT
TCTTTGCAGATACAATCCTTATTTAAAAAGGAGAAGATAACTGAGGATTTTAAAAAGAA
GCCATGGAAAAACTTCCTAG
>94
CCTTTCCCGCTCCGCCTCCCCTCGCTAGGCTGCCGCCTCCTCCCCCATCACCGCTCCCC
TCGCCCTGCAGGCAAGCTGTCCTTGGAGGAGTTCATCCGCGGGGCCAAAAGCGACCC
GTCCATCGTGCGTCTGCTGCAGTG
>95
ACAAACTGCTGGTTCTGAACTTGTTAGTTTGTTCTTCCTAATTCTTCTCCCTCACTTTTC
TCCTTTCCAGAATCATATTCTAAGAACTCAGCCACTCAGGTATCCACCATGGTGCTGGG
TCCTGAACAGAAGATGTCAGA
>96
ATACTAAGGATGAATTGTTAGTGTATTTTGTTCATTTTTTACTAATTTATTTTCAAATCTGT
TAATTCAGACAGTGTTTCTGGAGATCATGGGGAGTCTGCCAGTCTTGGTAACATCAACC
CTGCCTATAGTAATCCCTC
>97
GATAACAGACCAATTATATCAGTAGTTCAATGGTGATGTTCAGTTTAATCTCTTTTCCTC
TCTTGTACAGTACTCTTGTTTTAGCTTTCGTAAACTCTGGGCTTTCACCGGACCAGGTT
TTCTTATGAGCATTGCCTACC
>98
CAACTAAACAAACAAACAGACAAAAACTCAGTTGTTCAATGAGTGTTACAGGAGATTT

TGTTCTTCTTAGTTGCTCTGGATCCTTCTGTTGGCCACCCTTGTGGGGCTGCTGCTCCA
GCGGCTTGCAGCTAGACTGGGAG
>99
TTCCAAATTGTGGAGGAATTTTTTAGAAATGATTAATCAGAACCGTGTCTTTCTACTGTT
TGATGTTTAGGTCCCACGAGTCATCCTGTGGCTGATGGTGGAGTTGGCTATCATCGGCT
CAGACATGCAAGAAGTCATTG
>100
GGAAGAGAGTGTTTTTCAATGTTTCCTTTCTTTCCGTCTCTCTGACTTCCATATCTCATC
TCTGACCTAGAATTCCTCTGTGGGGTGGCGTTCTCATCACCATTGCAGATACTTTTGTAT
TTCTCTTCTTGGACAAATAT
>101
GTTCCCTGGTACTATGTTACTCCCGTTTCACGTTCCTGTAAAGGCTAATCATTGTTATGT
TGGTATGTAGTATGTTACAGTGAAACCCAGCCAGAGCCAGGTACTCAAGGGCATGTTC
GTACCATCCTGTTCAGGCTGTC
>102
AACTCCTGAAATGTCACAAGGAAACTTGTTCTAACTGGTCAAAAAGTGACTGCTGTTG
TTATCTTTCTAGTCTAGACAGGTAAACCGGAACAATAAGCAGGAAGTTCGAGAAGCCA
ATAAGTACTTTTTCATTGAATCCT
>103
ATGTAGGAGAGCTCTTATTTGGTCCTTGCTTCTGAAACAAAAGTTGCTGTTGAATTGCT
TTGGGTTCTAGGTTGAAGTCTGTACAAATACCAGCAGTCCTCATGCTGGCCTCTTTCCT
AAAGATAACTCGACACTGGCTG
>104
AACTGCTGTTTTGGATATTGTCTGAGCAGATTATTGCCACAATCATATCCTTCCTCTTGG
CCTCTCACAGGGTGTTGTGCTGGGATGTTACTTTGGGCCTGCTGCACTCTACATTTGGG
CAGTGGGGATCCTGGCTGCAG
>105
CCTGAACCTCCCCAAAACTTTTTTTTGTTTCCTGTTGCAGACAGTCATCTGCTCCTATGT
TTTCTTCCAGGGATTCCTGAACCTAAAGTGGTCACGCTTTGCCCGAGTGGTTCTGACTC
GCTCTATTGCCATCATCCCCA
>106
TTAATTGTGCTGTTCTCTAGGGTGATACAAGGACCCATAGTAATCCCATCTGATCTTCTT
TCTTTTCCAGCTTCCCTTTGCTCTCATACCCATCCTCACATTTACGAGCTTGCGGCCAGT
AATGAGTGACTTTGCCAATG
>107
TCCTGTTTCCTTTTCTGTCTGTGGCCAAGTTGGATTATTCCATCAGTGACTGTGCTGATT
TCTTTTACAGAGGCTGGCGGATTGCAGGAGGAATCTTGGTCCTTATCATCTGTTCCATC
AATATGTACTTTGTAGTGGTT
>108
GGGAGCTAGCCTGTGACAGTGTTCAGCCCCCTTAATAGTAGGTGTATCTGAGTGTTTGG
ATTTCTCCTAGCCTCAACTTTCAGGAGACCCGTCCGTGGCCTTATTTATTCCACCCTTCC
TGTACATCGTAGCGAATCAAT
>109
GAATCCTCCTCCCGCTGACCTCTGCCCCTCCTATGTCCACAGCCTCTCAACTCAGCTGT

TTGCTCTCCAGGTACCCCTGGGATGGCGTGAGCACTCCCCCAGCGATGGACCCATCTG
TGACGCTGTGGCAGTTTCTGCTG
>110
GTGGCCCACAGGAGAATCCTGTGGTCCCAACAATCCTTACACACCCTCTTTGTGCCTG
CTCCTCTACCAGGTCATCCTGACCCCGCCCGAGGCCCCAAACCTGAAATCGGAAGAGC
TTAATGTGGAGCCGGGTTTGGGCC
>111
GACCAAGTCCCTTCCAGCATCCCACATCTACTTTCTACTCACTTGAGCTCCGACCACCC
TCTCCCCACAGACCCCGGTGCTGCTGACACCCAGCTCGCTGCCTCCTAGCATTCACTTC
TGGAGCACCCTGAGTCCCATTG
>112
GTGGTGCCACTGCCCATGTGCTGCCCGGAATGTGGGCCACTGACCCGCTCCTATCGCC
CATTCGCCCCAGGTGGTGCAAAGGATCAAGGCTGTGGAGGGGCAGACTCGGCTGCTG
GTGGTGGACCAGGAGACAGATGAGG
>113
ACCACCGGGGAGTGGCCTCTGGAGGCGGGAGGCTGGAGCAAGAGACTGACCCTGTCC
GTTGGGCCTGCAGGATGTCAGTGGGCCCCTGAGGGAGCTGCGCCCTCGGCTCTGCCAC
CTGCGAAAGGGACCTCAGGGCTATG
>114
CACACCTGGCCACGCGGCGTTGGGGGCTGTCTGGGCCCATGGCGGGGCTGATACATGC
TGGTGGCGGCAGGTGAACGGGCAGAATGTGGAGGGACTGCGCCATGCTGAGGTGGTG
GCCAGCATCAAGGCACGGGAGGACG
>115
GGAGGGGCCACCGTCTGGGGTGTGGGACTAGGGCTCACTCCAGACACCCCACCCACC
GTGCTCACCCCAGGATGGCAGTGCCTGGAAGCAAGATCCCTTCCAGGAGAGCGGCCT
CCACCTGAGCCCCACGGCGGCCGAGG
>116
GTTGGATGGGCAGAGCCTGGGAGAAATGTTTCTGAATGGCCTGGGGCTGGCATTTAT
CTTTCCTTTCAGCAAGCACCTCAAATTTGCCATGCTGGCTGGTGGAAGAGTTTGTGGTA
GCAGAAGAGTGCTCTCCATGCTC
>117
TGAATGGCAGCCTTTTTGTTTATCCTGTTGCCCCTTCCTAACATGTACTTTGCTCCTTTTC
TCCTGGCAGAAAACTACCCCTGAGTGTGGTCCCACAGGATATGTAGAGAAAATCACAT
GCAGCTCATCTAAGAGAAATG
>118
AGTAGAAGGGAAATTAGAGGGAGAAGCTGTCTGGCCTTCCTTGGGAATAACTATTCCT
TCCTGCCCTCAGCTGCCGCTCAGCTTTGATGGAACAACGCTTATTTTGGAAGTTCGAAG
GGGCTGTCGTGTGTGTGGCCCTG
>119
TCCAGGATGCCTTTGCTTTAAAACCTTTTAAAAAGAAGTGAATGATACACCCCCTTTGC
TCATCTTGCAGTGGAACCTGGTGTGTGAGGACGACTGGAAGGCCCCACTCACAATCTC
CTTGTTCTTCGTGGGTGTGCTGT
>120
CCCACTTGGTGGAGCCCATTCCTGCTGCCCTTTTCCAGCTGGTTATCTGTCACTCTCCTT

TTCTTCCCAGGTTTGGCCGGAAGAATGTGCTGTTCGTGACCATGGGCATGCAGACAGG
CTTCAGCTTCCTGCAGATCTTC
>121
ATACCATAAAAAATTAATAAGGAAGGAACCCAAATTAAACTGCTAACTCGACCTCCCTT
GTTTTGAACAGGGACAGAAATTCTTGGCAAGTCAGTTCGTATAATATTCTCTACGTTAG
GAGTGTGCATATTTTATGCATT
>122
GTGCTCTACCTGGTCTGTGGGTCTGCTGTTGGCAGGGAGGCCTCACTGAGATTGGACC
TTGTACTGCCAGGTTCATCCCTGAGTCCCCCCGATGGCTCATCTCTCAGGGACGATTTG
AAGAGGCAGAGGTGATCATCCGC
>123
GTATGTTATTGTGTGCTCTGAGTCTCTGACCACCTCTTCTTCCCATACACTTATGATGTT
GTTCCTGCAGTTACAAGACCTAAGTTCCAAGAAGCAGCAGTCCCACAACATTCTGGAT
CTGCTTCGAACCTGGAATATCC
>124
TGTAAAAGTTGTACAGGTTGGGAAAGATGTGGATACTGCTTTTCCAGCTTTCTTCTGCA
CTCTGTTTCAGGATGACCATATCAGTGGGCTATTTTGGGCTTTCGCTTGATACTCCTAAC
TTGCATGGGGACATCTTTGTG
>125
TGTTTTGCTCTCAATAGCTGCATGCCATGGGTTGGTACCTACTCCTACCCTCTTTCCTTT
GCTTCTCCAGACTTGTATTATTTGGCTACAGTCCTGGTGATGGTGGGCAAGTTTGGAGT
CACGGCTGCCTTTTCCATGGT
>126
ATAAAGGGGTAGATGAGAGACCAAGTCTAACTGCAGCCCTGGGCCTGAGGCTCCGTCT
GCTTTGCCATAGGTGCCTACGACCGCTTCCTGCCCTACATTCTCATGGGAAGTCTGACC
ATCCTGACAGCCATCCTCACCTT
>127
TTGTTTGTTTGGAGACTGGGAGGCATCTTTTTAAAATGTGTTACTGACATATTTTTGCTT
GTTTTTATAGAATGAAACACAGAAAAACTCCAAGTCACACAAGGATGTTAAAAGATGG
TCAAGAAAGGCCCACAATCCTT
>128
TGCAATGTAAGTTTTCTCACATACACAGTTACAATCCTCCCTTTATCAGCTCTACATTTTT
TTTTAATAGACTGAATCTCGCTCTGTTGCCCAGCCCATCTCGGCCCACTGAAACCTCCA
CCTCCCGGGTTCAAGCGATT
>129
ATGAAAACTTTGTTGAATTAATACCTTTGTTGTGTTTTATTCACACTGTATATACAATACC
TGGATGAAGAGAGATATTTTGAGCACTATTAAAATACATGCATTGGCTCAATACATGAAT
TGGATCAATTCTGGAACCC
>130
CTTCTCTTTCTCTCACCCTAATTCAACCTATTGCCAAGTATTGTACATTCTCCATGCTTAA
AATCTCTAGGCGGTGTCCCTGAAGTATAAATCCAAAGCCTTCATGCCTGTAATCCGAGT
GCTTTAGGAGGCCAAGGCAG
>131
ATAGTTAGAAAATAGAATTTAATGTTCCTTTTCTCGATGAATTAATTTGTTTGTCTTCCAT

TTTTAAAAGCATGCTGATCCCATTTTCAATGAAGAATTGCTTCCAGTTACTTTGTAACTG
CCAGGTCCCAGCAGCTGGC
>132
GTACCCGAGCCCGGGAGAAAGTGTTCAGTTGACCCAGGCTGAGTGTGTTATCACCCTG
TTTCATTTCCAGCCATGCCTTGTGTTCAGGCGCAGTATGGGTCCTCGCCTCAAGGAGCC
AGCCCCGCTTCTCAGAGCTACAG
>133
ACGTGGCGTCTGACGTCTCCAGGGAGACCCGCCGCCCTCGCTGCCGCCTCACCGCGCT
TCTGTTTTGCAGGTAATCTTCAGCAAGTACTGCAACTCCAGCGACATCATGGACCTGTT
CTGCATCGCCACCGGCCTGCCTC
>134
ATGTGTACATTCAAGTGTACATCCCAGAGCACACTATGGGCGAAAACACGGGCTTGGG
TGTGTTTTCCAGGAACACGACCATCTCCCTGCTGACCACCGACGACGCCATGGTCTCC
ATCGACCCCACCATGCCCGCGAAT
>135
ATAAAATAGACCTGGACACTAAGAGGAAACTTCCCATATTTGGAAACCCAGTCCTGCC
TGCTTTTTATAGCTGGTGTCGAGGACAAGAGAACCACAAGCCGTGGCCAGTCTGCTGA
GAGACCACTGAGGGACAGACGGGT
>136
TGCTTAACCGGTTTTGGCTCTGAGAATTTTAAGGGTCATATTTACTCTCTCTTTTCTTTTT
CATTCCTAGAGCATTCAAAATCAATGAACTGAAAGCTGAAGTTGCAAATCACTTGGCT
GTCCTAGAGAAACGCGTGGAA
>137
TTACCAGCCTTGAGAGATCCTCCACATTCGTCCGTGGTAACCCCCTTGCTGTCTCTCTC
TCTGTCACCAGTGGAAGGACTAAAAGTGGTGGAGATTGAGAAATGCAAGAGTGACAT
TAAGAAGATGAGGGAGGAGCTGGC
>138
TTTGCTGTGCTGAGGGCTTCACCAGACTGAAACAGCAGGACCACAGCTCATCTCTGCT
CCTTCTCTCTAGGACCAACTGCCCCTGTAAGTACAGTTTTTTGGATAACCACAAGAAGT
TGACTCCTCGACGCGATGTTCCC
>139
TATCTCAGCATGCACATCACTGTCACAGGCCCACGGCGCCTGGTTAACACGGAACTCC
TGTCCTTTCTAGTACCTGCTCTCTCCAGAGACCATCGAGGCCCTGCGGAAGCCGACCTT
TGACGTCTGGCTTTGGGAGCCCA
>140
GGAAGGAAGCCAGGGGCTGGGGAGCCGGCCACACAACTGCCCAGTGCCTATCCTTCT
CCTGTGCCCACAGATGCTGAGCTGCCTGGAGCACATGTACCACGACCTCGGGCTGGTC
AGGGACTTCAGCATCAACCCTGTCA
>141
CTGGGTGGCTTTGGGAGGAGAGGTGGGCGGGCCCAGGCACAGGGTGACTCGGACCCC
CTGCCTCCCGCAGTTCTGCGTCCACGACAACTACAGAAACAACCCCTTCCACAACTTC
CGGCACTGCTTCTGCGTGGCCCAGA
>142
CACCCCCCCTCACCCCATCCCACCCTCCGAGTGAAGAGAGCAAACACCTACGCCCTGT

TTTCCAATCCAGGAGAAGTTCTCACAAACGGATATCCTGATCCTAATGACAGCGGCCAT
CTGCCACGATCTGGACCATCCCG
>143
GTGTTGCTCCCCCGTGCAGGTACCTGGTGAGGGCGCCTGAGTCTCCCCTCACTCTCTC
CTTGCCTCCCAGGTACCAGATCAATGCCCGCACAGAGCTGGCGGTCCGCTACAATGAC
ATCTCACCGCTGGAGAACCACCAC
>144
CACAGTAGGCCTCCGCTGAATAATTGTTCAGACTATACCCGTGTTAACACGTTGTTCTC
TCGCTATTTAGGGAATGATCACATTAATCTTGGCCACTGACATGGCAAGACATGCAGAA
ATTATGGATTCTTTCAAAGAGA
>145
AGACCCGAGCAGCAGCAGCAGGCCCGTGTTCTACCTCCTGTTACCTCAATTCCAAAAT
CTCTTCTTTCAGCTGAAGATGATTTTGATAAAATGCTGTGATATCTCTAACGAGGTCCGT
CCAATGGAAGTCGCAGAGCCTT
>146
CTCTGAGAACAGCGATCTGGTTATGGGGCATTTCTGTCTCTAATGTCACTGTCTGCTGC
ATTCCCTGCAGAGCGACCGTGAGAAGTCAGAAGGCCTTCCTGTGGCACCGTTCATGGA
CCGAGACAAAGTGACCAAGGCCA
>147
TGTTTTTCTCCGACGTTTCCCATTGCCTTAAGAACGAGGGACTCTGAATAAATCCGTGT
GTCTCTCCCAGCTCTTCCCCATGGTTGAGGAGATCATGCTGCAGCCACTTTGGGAATCC
CGAGATCGCTACGAGGAGCTGA
>148
TGGGAGAGAGGCAGACACTCCCCTGCTGTCTCCTGCTCCCTGACTTGGCCTTCTCTGT
ACTCTGTTCCAGTTACAGAAGAAGACTGACAGCTTGACGTCTGGGGCCACCGAGAAG
TCCAGAGAGAGAAGCAGAGATGTGA
>149
GATTAAAGGTGTGAGCCACCGCGCCCTAATTCTAACAAAAACAAATCAGTCATCGTTTC
CCTTCTTCCAGGAGACTGTGCCTGAGGAAAGCGGGGGGCGTGGCTGCAGTTCTGGAC
GGGCTGGCCGAGCTGCGCGGGATC
>150
TCCCAGTAGCAAACCCCCAGCAGGCCACTTTCCTTATTTATCACTAAAGCCCACATTAA
CCTTGTTACAGGACAAGAGGGAGAAAGTTGTCTGAGAACTTTTGACTGTGGCCCTGG
ACTTTGCTGTGCTCGTCATTTTTG
>151
TGAGTCTACATATGCCCAGCTGAATCTCTGTCGGGAAGAAACCCTGAAGCTTCCTGTGT
CTGTATTTCAGGGAGGAGATTGGACAGGCTGGACTCCCCATTGCTTTTCTAAAAATCTT
GGAAACTTTGTCCTTCATTGAA
>152
TGTACCCGAGCCCGGGAGAAAGTGTTCAGTTGACCAGGCTGAGTGTGTTATCACCCTG
TTTCATTTCCAGCCATGCCTTGTGTTCAGGCGCAGTATGGGTCCTCGCCTCAAGGAGCC
AGCCCCGCTTCTCAGAGCTACAG
>153
AATTTTCTCCGAGTTGCCTGATTTTCAAAAGAAGAAAAAAAATGTTGGTCTATATTGTC

TCCTTTTGCAGCGCACAGTGCAAAAAAATGCAAAATACGTGTGTTTAGCAAATAAAAA
CTGCCCAGTGGACAAGCGTCGCC
>154
GGGGAGGGACCGAGGCAGCAGCTGGGCCTGGGCTCGGAAAAGCGGCGCTAACAGGG
CTCTTCCTTTGCAGTGGTTCGCACAGACAGTTTAAAAGGCCGGAGAGGTCGTTTGCCC
TCGAAACCGAAGAGCCCACAGGAGCC
>155
GGGGTTAGCAATTTCACGGGTTATATATTTTAGAGAACCTCATTAAGTGCTTTTTAAAAT
GAAATTCCAGTTCCAGGCGAACCCTGACTATCAAATGAGTGGAGATGACACCCAGCAT
ATCCAGCAATTCTATGATCTCC
>156
TCAAGTAATTAGTTGTTTAGCTTTTCTCTCTCTCTTTTTCTCCCCTCTCTCTCTTTGGTAT
TAATTGCAGGTCCAACCCAGTGGAGGGTAAACTCATCTTTTGCAATGGGGTGGTCTTG
CACAGGTTGCAATGCGTTCGT
>157
CTCCTTGGTTTTGCCAACTAGCTAACTGTCTTGTACATTCTTCTTTTGTTTCTGATTATGT
TTTCTGCAGAGAGACACGGGCTCAAGGAACCCAAGAGAGTGGAAGAACTGCAAAAC
AAGATTGTAAATTGTCTCAAAGA
>158
TTCCACGGGGGCTGTAAGATGGCTTTTTAGGATTGGTCTAATCAGATACTCATTTCTTTT
CTCTTTCTAGGTTTTGAAACATGAATCTTTCGCTCGTCCTGGCTGCCTTTTGCTTGGGAA
TAGCCTCCGCTGTTCCAAAA
>159
GCAAATCGGCTTGGTGAACATGGGTTGGGTAAGTAGACATAAAGTCCACCAGCTATATG
TTTGCCTCTAGAATGAAGAAGGATGGAGGAGAGCAGTGTGGGAAAAGAATATGAAAA
TGATTGAACTGCACAATGGGGAAT
>160
CTCTGTGCTGCTTCTCAGTTCTTCACCAAAATAGTCTTGCTTTTAACATTTTATTTACTTC
TCCTTGAAGACCAATGAAGAATTCAGGCAGATGATGGGTTGCTTTCGAAACCAGAAAT
TCAGGAAGGGGAAAGTGTTCC
>161
AATTCAATTATGGGGTACTGTATTTGTATTGGTCTTGTAAATTTACAGCTTTTTTTTTCTC
CCTTTTTAGAAACAGTGTGGTTCTTGTTGGGCTTTTAGTGCGACTGGTGCTCTTGAAGG
ACAGATGTTCCGGAAAACTG
>162
GGTTCTAGAACTAATGTTCCCCAGGAGGTAGATGGTAGTGATCAAGTCTCTCCCCCTTT
AACTTTAAAAGGATGAAATCTGTAAGTACAGACCTGAGAATTCTGTTGCTAATGACACT
GGCTTCACAGTGGTCGCACCTG
>163
GGTGGTAGGGTGGGTTACTGTCACATGTCGCTTGGAGCTTCTCACCCCAGCATTAATTT
TACTCTCCCAGGCATTTATTTTGAACCAGACTGCAGCAGCAAAAACCTGGATCATGGTG
TTCTGGTGGTTGGCTACGGCTT
>164
CTATTATTATTAATGGAGCCAATTTTCCCTTGATTCCTTGTATAAAATGGAAAACCTGCTC

CTCTTTCAGCTGGGGTCCAGAATGGGGCTCGAATGGCTATGTAAAAATAGCCAAAGAC
AAGAACAACCACTGTGGAATC
>165
AAATATTTCTGCTTGAGCCATAAGCGGTGATTCAGAAATATGAAACCTGACAAGCTTTG
TTTCTTTACAGGTCACTGGATCTGAGAAACTTCCCAGGGGACCGCATTCCAGAGTCAG
TGACTCTGTGAAGCACCCACATC
>166
CCCTGCATCTTGAGGAATGCTGTAACTTCCAGTCCTGGCCTCTCACTTAGCTTCTCCTT
CTGGCCCTCAGGGAGACAGACGATGCTCACCCAGTCAGTAAGAAGAGTCCAGCCTGG
GAAGAAGAACCCCAGCATCTTTGC
>167
GGTGAGAGGGAAAGGAGAACTGGAGCGCACCAGGATCACGGCTCCTCCGCAATTTGG
CTATGTCTCTCAGGCCCTGGTGAGTGGACAACATTGTTCAACATCGACTACCCAGGCG
GGAAGGGCGACTATGAGCGGCTGGA
>168
TCAGGGTCAGATTTCCTGACAGAGTCCCCTCAAGAGGGAAATCAGACCGTCTGCTGAT
CTCACTTTGCAGGATCCCTGCGCCGAGACACAGAGCGCATCTGGAGCCCATGGTCTCC
CTGGAGCAAGTGCTCAGCTGCCTG
>169
CTAGGCTCTGTAGAACCTGTCACACACACACACAAAAACCCAAAATGCTATCTCTCAT
CTTCTGTCCCAGAGACTCCATACATGGTGATGAACCCTGAGACAAAAGCACGGAGAGC
TGGGCAGAGCGTGTCTCTGTGCTG
>170
TCCTGGCACTTTTCTAGGAATACCTGCCCTCCCACTGCCCATTCAGGTTCTACCTCTCCA
TCCCTTGCAGGTATCATAATGACACATTGCTGGATCCTTCCCTCTACAAGCATGAGAGC
AAGCTGGTGCTGAGGAAACTG
>171
GTGAGGTTGAGGTACTCTTTGTGTAGGATGTAAACTTACATCTTAATTTCCACACCATTT
CCCTACACAGCATCTGATGAGACTCCTTGCAACCCAGTTCCTGAGAGCTATCTTATCCG
GCTGCCCCATGATTGCTTTCA
>172
CTTAAAAAAATAAAATAAAGTAAAATACATTCAAGTCAACTGATTTGAGTCTCGAACTG
CTCTGACACAGGAAGGTGCTGGGGATACATCAGAGGTGATGGATACTCAGGCGGGCTC
CGTGGATGAAGAGAATGGCCGAC
>173
CTTTAAAAAAAGAAAAAGAAGTGTGTGTGTTCACAAGTTCACTAATTCGTATAATTTGG
CTCTTCGGCAGATCCTGTCTACCTTTCATGACCAACTACAGTTTTCATTGCAACGTCTG
CCATCACAGTGGGAATACCTAT
>174
ATCGAGTATTCCCTTGGTAGTAGAGATGTAGTACTACAGATAACTGTTTCTTCCTTTTTG
TTTTTGATAGACTTGAAGGAAATGTGCCTTAGTGCTTTGGCCAACCTGACATGGCAGTC
CCGAACACAGGATGAACATCC
>175
GCTGTTTTCTTTCTTTGTCAGCATATCTTTGATTGCAGCAGTAATAGTCTTAATTTATTTT

GAAAATTAGGATATTATACCATTTATTGATAAATACTGGGAGTGCATGACAACCAGACAG
AGACCTGGGAAAATGACTT
>176
TGGTTGATTATTAATTCCATACTTTTAAGTATAAAATGTTCATTAATAGTAAAACACTACT
TCTTTTTAGAGTAAAGAAAGAGATGTATTCTTGGTAAAGGAACACCCAGATCCAGGCA
GTAAAGATCCAGAAGAAGATT
>177
TCTTGTTTTATGAGCATCCAGATTGACTGACTTACGTAGACTCCTTTTTTAATTGGATGTA
TTTTTGCAGGACCTTAGTAACATTGGTCCTGCTTATGACAACCAAAAACAGAGCAGTG
CTGTGTCTACTAGTGGGAATT
>178
TATGGAATTTCAGTATCCACATGGTGTTTCCCTTGTGGCTCATTGAAAGCAATCTGCCTT
CTCTTCAAAGGGGGAATTGCAGCAGGAAGCAGCGGAAAAGGACGAGGAGCCAAGCG
CAAACAGCAGGATGGAGGGACCAC
>179
CTTACACAGTTGAATGCATTTGAGCTAAAGGGGGTTTTAGTTTTTTGATGTTATTTTTTC
CTTTCTGCAGTGACCCTTTGTTTTCTGCTCAGCGCCTTCCCCCTCATGGCTACCCATTGG
AACACCCGTTTAACAAAGAT
>180
GTTTTTGTGGCAATTGTCCCCAAGAACCTCAAATAAGTTGCAGGCCTTCTTTTAATCTC
TGTCACCGCAGCTCCCCAGTTAAAGATCTCAGATGACCGGCTGACTGTGGTTGGAGAG
AAGGGCTACTCTATGGTGAGGGC
>181
CTTTTTCCTTCAGCAAACTAGATTTGACTTGCAATCTTCTGACTTCCTGTGTATGTTTTTA
TTGGGACAGGAAACCTTCAAGCTCCTTTAGGTTATGATAAATTTAGCTATTCTTGGCGG
AGCAAAAAGGGAACCAAGTT
>182
CGGATGCGTTTTTTTCATGATGATGGAGCTGATGCTTTATTGTGCCTTTTTTTCTTTCTTG
GTTTTCAAGGCTTTGATAAAATTCAAGAGTTATTTGTATTTTGAGGAAAAAGACTTTGT
GGATAAAGCAGAGAAGAGCC
>183
TTCCTAGAGAGTTCTTTTTTTGTTTGTTCTTTTTTACAGTTCTGAAGTAAAACCATGGTT
TTTGTTTCAGATAATATTTTATAAAAATGGTGTCAATCAAGGTGTGGCTTACAAAGATAT
TTTTGAGGGGGTTTACTTCC
>184
GGCTGCATGTGTCCTCAGCATCTCCGTGGCTGCTGTAGTCACCGTGTTCTGTTTCTCATT
CCTCCTGCAGATGAGTGACATGGGCTGGGGCGCCGTGGTAGAGCACACCCTGGCTGAC
GTCTTGTATCACGTGGAGACAG
>185
TAAGGGGTGAGTGATTTGAACCAGGCTGCACAAGGAGAATCAGTCTCATGCTCTGTGC
CCCAGCTACCAGCTGGAAAAACCACCAGAGAAGGAAGAGGATCACGCCCCCGAGTTT
ATTAAAGTCAGGGAAAACCTGCGGA
>186
GGCTTGTGGGAGGAATGAGTCCCTTTGGCAGGTTCTCGGGATCCCCTGGGTGACATGC

CTTTCTCTGCAGGAGGCACCATGCAGGAGCTGCATCTGCTCTGGTGGGCGCTTCTCCT
GGGCCTGGCTCAGGCCTGCCCTGA
>187
GGGTTATTATCTTCCGCTGCCCGCCGCCCCTCCCACGCCGGCCCAGGCTGAAGTTGAC
TCTGCCCGCAGGCCTCCAAAGACTTCATATGCTCCAGATCTCCTACTTCCGCGACCCCT
ATCACGTGTGGTACCAGGGCGC
>188
CTTGGTGGGCCTCGCCCCACACCTGGCACCCTCTCTGCACAGTCCCCTGATCCTGACT
GTCTATCCACAGTTCCTCTGACCATCCGCTGCTTCCTGGGCTGTGAGCTGCCTCCCGAG
GGCTCTAGAGCCCATGTCTTCTT
>189
TGGGGTTTGACTCAAATCATGGACTCCTTGGGGGCCTATTCTTCGGGCTAACTCTTTGC
ATGTTCTGCAGGGAGCCAAACAAGCCGCTCCTACACTTCGCTGGTCCTGGGCGTCCTG
GTGGGCAGTTTCATCATTGCTGG
>190
CCTGGGCAGCCTTGGGGGCTCCCTGGGGCCAGCTTCCTGACACTTGCCCTACTCTGTG
CTGCCCCTGCAGGTGTCTGAGATGCTGCCACCGCAGAAGAAGCCCTGGGAGTCCATG
GCTAAGGGGCTGGTGCTGGGCGCGC
>191
CTCTAACTACCAGAGTCCTGAGGGCCCCAGCACCCCCCTGAGGCTCTCCCCACCTCTC
TGTCCCCAGCAGGACCCCGGAGGCCGCAGCGTCCTGCTCTCCACCTGCACTCGAGCC
AGAGGCAGTGATCCGGGCCAACGGC
>192
TGAGGGTCCAGGTTGGCTTCAGAAGTACTATCTTACTTCTTCATTCTTTCCACCTCTTCC
TTCATTCCAGGATCTAAGAGAAGAGGGAGATGAAGAGACTACAAATGATGTTCCCCAT
ATCCAGTGTGGAGATGGCTGTG
>193
AACTGGGTGAGTCTTCAGTGAATGGAGTAGGAAGAGGGTGTCCTCTTTCATTGCTTTC
TTTTCTCCCTAGTTCTGCTTGCAAAGGATCCACCAGGGTCTGATTTTTTATGAGAAGCT
GCTAGGATCGGATATTTTCACAG
>194
GGTGTCAGAGACAGAGGGTTGGGGGTTAAGGGTTTAGAGTCTTCTCTGACTGTGTCCT
ATGTCCTTTCAGCCTGAGGGTCACCACTGGGAGACTCAGCAGATTCCAAGCCTCAGTC
CCAGCCAGCCATGGCAGCGTCTCC
>195
GTGAAGCCAGCAGGTGCTCTGGAATTAAACCAGCTTTCTGCAAGCCCTGCTTCCTGGT
CTCCCTCTCCAGGTACTGGCTGTGATCGAACTTCTCAACCCTCAGAGACTTAGATCTTC
CACCTCACTCCCTCAGCCAAGCC
>196
TAGTGGCATGGCCTCCCTCTTACTCAGCTCTCCCTCCCTTTGACCGCTCCTCCTTTCCCA
CTTCCATCAGTTCTGCAGTTGGCGGGCCAGCCCTTTGACCACTCCCCTATCCGCTTCTG
TGCCCGCAACGGGGAGTATGT
>197
GAGCCTGGGAGGTGAGAAAAGGTGTGGGAAGCCGGGTCAAGCCATCTAACCTGCCCT

CTCCCTGCTGCAGCCCGTCCACAGCCCCAGCCCCACGGGACTCTGTGGAGTCGGCGCC
GTGACATCCCCAGGCCCTCTCCACA
>198
TATTGTGCCCTTGGCCCCTGACACTGGCGACACCCCCTGGTTCTGCTCACTGTGGTGCT
GTGTCCCACAGGTGACTTTCCAGCAGATCTGTAAGGATGTGCATCTGGTGAAGCACCA
GGGCCAGCAGCTTTTTATTGAGT
>199
AGCCTCCTGGTCTCCAGACTGTTCCCTCTGTTCCCTACCACCATCCCCTTCCCCTTTTCC
CTGGGCACAGATCCGCCGTCAGCAGCGCTGTCTGGGGAGGGGGCCACCCCACGGAAG
GAGCCAGTGGTGGGAGGCACCCT
>200
GAGGGTGTGGTATGGATGTGTTGACCCCTGAAGTGGTTCTGATGACCTTTCCCTATCTC
ACTCTCCTCAGACATCATCATGATGGAGGACCTGCCTGGCCTAGCCCCAGGCCCAGCC
CCCAGCCCAGCCCCCAGCCCCAC
>201
ATTTTCCGCGGCAGTTTTTGGTCCTGGGGACCGTCACCGATGCCTCCCACGCACGCTTT
CTTCCCTGAAGGGAGACGTCGCTGCGCTGGGCCTCTCGGCGGTCCCCCCCACCCGGGT
CCCGGGCCCACTGGCCCCCCGCA
>202
TCGGGGAGCTGGCCCACGGGCGGCGGGGCTGGGCCCGGGGCTCCCAGGCGCTGACCC
CCGGGGCCCGCAGGCGTGTGGCTCACGGACCTGGTGCACGTGGCGGCCCACGAGATC
GGCCACGCGCTGGGCCTGATGCACTC
>203
TCCCTTTGTCGGGCGGGAGGGCGGGGACCGGGCGGTCCTGAGCCAGGCGTGCTCCCC
ACGCTCCCGATAGGATGCCTCGACAGGCTGTTCGTGTGCGCGTCCTGGGCGCGGAGGG
GCTTCTGCGACGCTCGCCGGCGGCT
>204
CGTGGGAGCTGGGCCTTGGCCATGGCGCAGCTGAGGGGGCACTGACGGAGCTTTTCC
CCCACCGGAGCAGAATTCCCCTTCCCCACGGTGGCCACCACCCCACCGCCCCCCAGGA
CCAAAACCAGGCTGGTGCCCGAGGG
>205
GGGGCAGGGGTGCGGGGCAGGCAGCCGGGGGGGGGGCTGTGCCTGCAGGAGACGCC
CCGCCCCCCTGCAGCTGGTACAAGGACCAGGAGCCCCTGGAGTTCTCCTACCCCGGCT
ACCTGGCCCTGGGCGAGGCGCACCTG
>206
AGAAAGCATTCACCCTTATATTTAACTGTGAAATATCTGATTTGAAATTATTTACTTCTTT
TTTCCACAGCCTGGTGAGAGTCAAAGAGGCCCCAGGGGACCATATCCACCTGGACCGC
TGGCTCCTCCTCAACCTTTTG
>207
CACACACAATCCTCAGCTAGGTGCTCCGAATATCCCAGCCTAATTTTTCTTGTGCTTTTG
TTTGTATCAGGGGATGTGGCTCTAAATCAGCCGGACATGTGCGTCTACCGAAGAGGGA
GGAAGAAGAGAGTGCCTTACAC
>208
GTTTATTCTGTGTTTAGGAACTTGTAGGGGTGAAATGCTGACCTGTGTTACTCTTTCTTT

CCATGATCAGCGTCCAGAGGACCAGAGGTTAATTTATTCTGGGAAGCTGTTGTTGGATC
ACCAATGTCTCAGGGACTTGC
>209
AACTTCTGAATGTTTTTAAGCACTCACCAGGTTAGGTTCAGGTCCTTAAGTACCTTCGT
ATTACTTTTAGCAGGAAAAACGGCATGTTTTGCATCTGGTGTGCAATGTGAAGAGTCCT
TCAAAAATGCCAGAAATCAACG
>210
AATTCCTTTTGCTTGATTTCAGACAGTTGCATCAGCTCCTTTGTTCTGTGTTCATGTTAC
ACTTATTTAGGTGGCTGAATCCACAGAGGAGCCTGCTGGTTCTAATCGGGGACAGTATC
CTGAGGATTCCTCAAGTGATG
>211
TTTAAACTTAGTTTTCATTTGTTCATACTCAACCCACTCAAAATGAGTGTTTTTTCAAAT
CTATGTATAGGCCTGAAGCTGCCCAGCAGGCATTCCAAGGCCTGGGTCCTGGTTTCTCC
GGTTACACACCCTATGGGTGG
>212
GTGTAAATAAGCCACAGACTTCACGGTGCTTTGGTCTCAACAGTATCTGCCTCTGTCGT
TTTTATTTTAGTTTAGCAGCCACTGCTGCATCAGGGGCTTTTGTTCCACCACCAAGTGC
ACAAGAGATACCTGTGGTCTCT
>213
AGCCTTGGATTCTTGAGTAAGTCTCACTGTGACATCAGCTAAGATGGACTTTTATGTGC
TTCCTTTGAAGGCATCACGTTGGGTGGTTTCCATTTAGACCGAGGCCGGTTCAGAACTT
CCCAAATGATGGTCCTCCTCCT
>214
TATTTGGTGTTCATCATAGCCCTAATTGTTTTCTCATTGTTTCATTACCTCATTGTTTCATT
ACCTGTAGGAAGGCACTGATCCTGAAACTGAAGACCCCAACCACCTCCCTCCAGACA
GGGATGTACTAGATGGCGAGC
>215
AGAAAATTGTACTACAAAATACCATTCCATTTATTAAAGTCATTCTGACAGGAATCTGAT
GCTTTTCCAGGAGTTCCAGATCACATCGAGTTCACCATGAATTCACTCAGTGAAGCCA
ACACCAAGTTCATGTTCGATCT
>216
CAGACTTAGCATGGGTTTAGTATTTACATTGATACAGCAATTGAATGATCTCCTTTTTTG
ATGTTTGAAGGTTGATAGGTCAGGAAATGTTCATCACCAGTTTCAAAAGCTTCTGACTG
AATTCAACAAATCCACTGATG
>217
TAAGTAAGTTTGGTAATATATAGTTACATAAATTACTCCTAATTCCTACTTCTTCCTTCATA
TCTCAAAGGAATATTTAGATGCCATCAAGAAATTTTACCAGACCAGTGTGGAATCTACT
GATTTTGCAAATGCTCCAG
>218
TTTGCAAAGCATCTTCCAGACAAGCCATTTGTAGCTTGTGTAAAAGACTCTTTTATTCTT
TCCCTTGCAGAAAAAATTAAAAACCTATTTCCTGATGGGACTATTGGCAATGATACGAC
ACTGGTTCTTGTGAACGCAAT
>219
ATCTCAATAATATTATCTTTTTTGTCTTGTGTTTCACGTGTTATTTGTTGGACACATTGAT

TTATTGCAGAATACATACAAATCTGTACAGATGATGAGGCAATACAATTCCTTTAATTTT
GCCTTGCTGGAGGATGTAC
>220
TCAATTCATTGTTAAGGTGTATTAGAGATCGACAGTTAGTCATATCAGTTTCTTTTTTCCA
TTTGTATAGCTTGAAGAGAAACTCACTGCTGAGAAATTGATGGAATGGACAAGTTTGC
AGAATATGAGAGAGACATGTG
>221
ATTGTTGGTTATTACAGGTACAAAATTAAAGAGGTTTTTGTTTCTTTAAAAATATCTTTT
TTGTTCACAGAAAACAAAATTATCTTCCTTTCCAAATCCAAAAAGATGAAAACGACTG
AGATACTTTCAAAAGACAACCA
>222
TTAAAATGTTATAAAATAGACTGTTGTGTTGGCTTACTGGAATTACGAGAATTCTTTTTC
TCTCAAACAGCGTGCAGTTAAAAAATTTATTATGTTAACATCCAACCAAAATGTACCAG
TGTTTCTTATTGATCCTTTGA
>223
CTAGTATTTGGCTTTAAATATTCAATGTTTAAGTGTTGTTTCTTGATGTTTGATGCTTCTT
TGGTTCTAGGAAGGCTGGTTTCGGATAGCTGAGAATATGGGATTTCAGTGCCTAAAGAT
TGAGAGTAAAGATCCCCGGC
>224
TATTTGTCCTAATGTTCTCCCTCCCTGTTTCCCCCACCCCTCATTAATAAATCTTAACTTT
TGTTTTCAGGCCAGAGTTACAGCAAGTTACTGTTGATGGACTGGAAGTTCTCATTCCAA
AGGATCCAATGCACTTTGTA
>225
CAGATGCCACAGAAAGGTTCCTAGCAATTTAGAAATGGCTGTAATAGCTGACTATTTATT
ATATCTGTAGCAGTACCTTGATGATAACACTGTGGAAGCTGTGGCCTTTCGGAAGAGTG
CAAAGGAATTACTGCAACTAG
>226
TATAATTTGTTATAATAACTAATTTTTTCCAAACCTAAATTTTGTTAAAAAAATTTAATCT
TCTTTTTAGGATGGTATCGACAATGCAACATTATTCCTTATAGCAAAGATGTTGACCTAG
GAATTTTTATACAAGATTA
>227
TTTTAAAAAAAGGTTTTTAAAAATGTATTTCCTTTGTTTCAGTGTGTGAAGGTTTTCATC
TTCCCCATAGGTAGAAGACAGCTTGGAACTATCCTTCCAGGGAAAAGATGATGTAAAA
CTTGATGTTTTTTTCTTCTATG
>228
CACTAGCAGCTAGATTCCTTAGCTAGACCTTCTTCCACTGTTGAAGCCTAATCCCTCTGT
TTTGCTGCAGATACCTGTTTCCGAAGTTTACACTGTGCTGGACTGAGTTTGTAGACATG
AAGGTCCATGTACCCTGTGAA
>229
ACAGCAGAGGAGACAGTTCATCAGTTGCGTTCCCCCACAGACAGTCACAATGTTATGT
GATGTATTTCAGAGGCGATGATATTCTCACCATCCTGACTGAAGTGAACTATGAAGTAA
GCAACAAGGATGACAAGAAAAAC
>230
TAAGGAACAGAGAAGGGGGTTTGAATGTGATACTTGTGTCATGCTGACTTTTTTTTTTT

TTTTTGGTCAGTAAATTTCTAAGTGATGAAGAAAACTTGAAACTGTTTGGGAAATGCA
ACAATCCAAATGGCCATGGGCAC
>231
ATAAGTGGAACAATTTGGAATTTGAGTCGTAAATGGAGTCAATGATATTTTCCCTTGGTT
TTGTCTCTAGGAGGCGATTATGCAGCCCCTTGATCATAAGAATCTGGATATGGATGTGCC
ATACTTTGCAGATGTGGTGA
>232
GTTCATTATTGTCTTGTTAGAGAGATGACTGGTGAAATGCCTTTTTTTATCTACAAAAAA
ATATTTTTAGATGGCGGGTGAAAAAGTTGAGAAGCCAGATACTAAAGAGAAGAAACCC
GAAGCCAAGAAGGTTGATGCTG
>233
GGACCACGGACCATTATGTCTTAACCTTAATTGGCATTCTCTTACTGTTGATGCATTTGT
GTCCTTGTAGGTTGAAAAGAAAAAGAAGGAGAAGGTTCTCGCAACTGTTACAAAACC
AGTTGGTGGTGACAAGAACGGCG
>234
GCTGACATAGCTTGCTTCTAGTAATCTGAATGGCTTATTTAAATAAGTTGGATCTATGGA
CTCTCCACAGCCTAGATATTATCCTACTGAAGATGTGCCTCGAAAGCTGTTGAGCCACG
GCAAAAAACCCTTCAGTCAGC
>235
AATGGTCACCTAAATTGCAGGATGATGATTCTTCCCCCTTTTCCTTGCTAAATTAATTTTT
CCCCCTTAGGACCTCTGGTCCTCAATCGAGTTCCTCTACGAAGAACACACCAGAAATT
TGTCATTGCCACTTCAACCAA
>236
ACTCCTTAGTTGACCTACAAGTAATTTGGTATGTGCCTGTTTTAAAATGTTTGATTTTCT
CTTTATTTAGAAATATGAGATTACGGAGCAGCGCAAGATTGATCAGAAAGCTGTGGACT
CACAAATTTTACCAAAAATCA
>237
TTCTTGCCCGGCAGAACGCCTGCAGCCGTGCGCAAGCAGTCACGACCCTTCCTGGTCT
CTCTTCCTGCAGGATGTACCCTGCCCACATGTACCAAGTGTACAAGTCAAGACGGGGA
ATAAAGCGGAGCGAGGACAGCAAG
>238
TGCGGGGCTGGGAGGATAGGCTTCTCATCTCCTTCCCCAAGCGCCCACCGCCTCCCCG
TGCGTCTTGCAGGAGACCTACAAATTGCCGCACCGGCTCATCGAGAAAAAGAGACGT
GACCGGATTAACGAGTGCATCGCCC
>239
ATTGCTAATAAATGTCCCAAAGGTGGGACTTCTCTGACCTCACCGCTGACTAAGGCTCT
TTTCCTTCCAGACTTTGGGTCACTTGGAAAAAGCAGTGGTTCTTGAACTTACCTTGAA
GCATGTGAAAGCACTAACAAACC
>240
TTTGGAGTATAGTTTCCAGGTGCGCCACAGGCCTTCTGAAACGTTTTCCTTCGGATTTT
TCTTTCCCCAGGTGAGCTGTCAGGGAGAAATGTCGAAACAGGTCAAGAGATGTTCTGC
TCAGGTTTCCAGACATGTGCCCG
>241
TCACCTCATTCCCCAGAGGAGCCAAGAGCGGAGGCTGCCCAGAGTGTCCAGCACGGT

CTCTCCCCTTCAGGAACATGACATCGAGACACCCTACGGCCTTCTGCATGTAGTGATCC
GGGGCTCCCCCAAGGGGAACCGCC
>242
CTGGGGGCCCGGCCTTCCTTCCAGTCCCCCGGCCCCTCTGCTCAGCCATAGTGGAAGT
GTGTCTTTGCAGACAAACTATGCTTCAACACCTTCTTCAACTTCGAGGACATGCAGGA
GATCACCAAGCACTTTGTGGTGTG
>243
CTTTACAGAGTGTTTCTGCCATCTGCACCCATCCTGGCCCCGCCCGGCCCTGTTTCCCC
TCTTACTGCAGCTCATCTTCCCCGACCTGGTGGAGGGGCTGGTGCTGGTGAACATCGA
CCCCAATGGCAAAGGCTGGATAG
>244
CCAGCCCCCTCTAAGCCTGCGTCCCTCTGTCTGCCCCTCTGCATGCCTCCATCCATCTCC
CTGGGCCTAGGAGGAGCTGGTGAACAACACAGAGTTGGTGCAGAGCTACCGGCAGCA
GATTGGGAACGTGGTGAACCAGG
>245
CCTACTTTTCCCACCAGAGGCACCTGGCCCTGCCCGCCAGTCCTCAGGCCCATCCTGTC
TCTGTCCACAGTGCCCTCAGCCAGCATGACCCGCCTGGCACGCTCCCGCACTGCATCC
CTCACCAGTGCCAGCTCGGTGGA
>246
GGCAGAGGAGCATTGGGGTTTTTTTTTAAATCTCTTTAATGGCCAATACTAATATAACTT
ATTTTTTTAGCCCTAGAAAACAATCTCCTGGTCCAAGGTGCTTGAGTGGGCCGATCCAG
CTATATCAAGAACCTTTGAGA
>247
TCACTCCACAGTGGGTGACAGCTGTGTTTTCTAGCTTAAAGTATAGTCGTATCTTTTGTG
TTTCTATCAGAACGTGAAAAAAACCGACTGGTTCCAGGAGTGGCCAGATTCCTATGCC
AAACACATCTACAGCTCGGAGG
>248
AAGACTGCACAGGAGTCAACAACCGACATGGTTCAGCCAGGTGTGGTCATTTTCCTCT
GCTCTTTTGTAGGGAAACGCTGTCCCAACTGTGACGGGCCTCTGAAGCTCATCCCTTG
CCGAGGTCATGGGGGCTTCCCGGT
>249
CTGATACACCATGAAACACAGTCTAGTTAGTGTTATATCATTTCCTTTCTACTTCCTTGTC
TCATTTAAGTCAAAGGGAGAGCATGATCATCCAAAACCAGAAACCAAGTTAGAAGCTG
AGGCAAGAAGAGCCATGAAGA
>250
CATGGGTGCCTATGGCAGTGGTTTTGATTTGCTTAACTTATTAACACGAGCATTTCACTT
TCCTTTCCAGTCTCTTCCAGGTGAAACACAAAGTCAGGGGAGTTTACCTTTAACTTGG
TCTTTCCAGGAAGGCGTCCAAT
>251
GCCTAGATGTGGTACATTCGACCTCACCTATGCCTCTCCCCACCCCCTCTGGGTTCGTTT
TGTCTTCCAGATTGCTGGACTCGCTGAAGAGAGACTACGCAGGAAAGCCCCAGCCAC
CCATCAAATCAGAGAGAAGGAAT
>252
TCTTAGTCTTCATTGAATTTGGCAGAGAGAGGTTGTTCTTCAAGAGGAACTGTTTCTTA

TCCTGATTTAGGTAAGAAAGTACTCATTGTCTATGCACACCAGGAACCCAAGTCTTTCA
ACGGATCCTTGAAGAATGTGGC
>253
TACTGGGATGGTTGGGATGGGCTGTGGATGCCCACCTAGGAGTGAGAATGTTTGGCCT
CTTCCCCGACAGGTACTCTTTCTAATCCTGAGGTTTTCAATTATGGAGTGGAAACCCAC
GAAGCCTACAAGCAAAGGTCTCT
>254
CAGATAAACTAAACTAGGAAACCTGAGGCGAGCCGCAGCCTCAGTTTCTCTTTGGTGT
TGCCGCCCACAGTTCCCGCTGTACTGGTTCAGCGTGCCAGCCATCCTGAAGGGCTGGA
TGGATAGGGTGCTGTGCCAGGGCT
>255
GCTGGAGGCTCAGCAACATTTCTGTCCTCTTCTGGAGTCCACACAAATGCATCTGCTTT
CTCCCTTGCAGGGTAAACTAGCGCTCCTTTCCGTAACCACGGGAGGCACGGCCGAGAT
GTACACGAAGACAGGAGTCAATG
>256
AATCATTTAACTGAATGGTATGTAACAGGTGTAGTTTCTAATGAGTTCTTTTCTTCCCCT
GTGGCTTTAGCATGGCACATTACACTTCTGTGGATTTAAAGTCCTTGCCCCTCAGATCA
GCTTTGCTCCTGAAATTGCAT
>257
GCTGAATCCCTACCAGGGCATGTCCTCAGCAAAGCCAGACCTCGTGATCCTCTTTCTTC
TCCCCTGCTAGGATTTGGTGGCCTGGGTGACAGCTGGTTTTCTGCATATCCCACATGCA
GAGGACATTCCTAACACAGTGA
>258
CACATGCACCCACGCCAAGGACCCTGAGCCTCGGCAGATCTGTGACTTCCTTCCGTCC
CCTCTCCTTCAGCTGGCAGGCTGGGAACCACAGCGCCTTCTGGGGCATGACCCTGGAT
GAGGGCATTCGCTACCGCCTGGGC
>259
GTGCCAATGGGCACCTGAGGGGCAGGCCAGGGCAGAGCAGGAGGCAGACAGGGCAA
CCTTTATCTTGCAGACAGTGCTGAACCCAGGGGAGGTGCTTCCCACAGCCTTCGAGGC
CTCTGAGAAGTGGCCCAACCTGATTC
>260
AGGGCTGGCGCCTGGGAGAGGAGGGCCAAGTCCTGAGCCTCCCGACAGCCCCTCTAT
CTCACCCCACCAGCTGTGGCATCCGATCGTGTCTCAATCCATTCTCTGGGACACATGAC
GCCTGTCCTGTCGCCCCAGAACCT
>261
TCCGTGGGCATGGCCTGGGCCATGATGCACTGAGTCTTTCTGCCTTTGCTCCCTCTTGC
TGCCTTTGCAGGGTGGTGTCTGACCACTGCTACCCCTTCTCGGGCCGTGAACGAGACG
AGGCTGGCCCTGCGCCCCCCTGT
>262
CTCAATAGACTCAGGCTCCAGTGCCTGTGCCAACGGGCTGAGTGGACCCTACCTTGAC
ATCTGCCCACAGCCCTCATGGAGGTGCATGAGGACTTCTTCCTATACAAGGGAGGCATC
TACAGCCACACGCCAGTGAGCCT
>263
TTTCTATGTGTGACTCTTTCATGGAATAACAACTCACTGCCTCTTAACCAAAGCTGTTTT

CTCCTTCCAGTGGGTTGCCAGCGTTGATGTGGTGGAGAATGAAGAGGCCAGCGCTAGC
ATCATTGTTAAAATGACAGACT
>264
TATCCGTAAAGATGCTAGCCTGTGTTGGCTTACCTTACAGTTTATACATTTTTTTGTTCTT
GTGTTGAAGGTGACTGCTGAGGTTGGAAAGCTCTTGGGTGAAGAGAAGGTGGATGCA
ATTCTTTGCGTTGCTGGAGGAT
>265
CTGCATGAATTGAGCTCACCTTTTGGTTTCTTTCCTGGGAATGCTGAGCCCACATGTTT
TTTTCACTCAGCTCTCTTTAAGAACTGTGACCTGATGTGGAAGCAGAGCATATGGACAT
CGACCATCTCCAGCCATCTGGC
>266
GAAGATTGCATGTCCTTGCTGTCCCCCTGGCCGCCAGTGGTCACTGAGCCATCTTCTCC
CCATTTCCCAGGTATGATCGGGTACGGCATGGCCAAGGGTGCTGTTCACCAGCTCTGCC
AGAGCCTGGCTGGGAAGAACAG
>267
CAAGGGGGACTGGGCACCTGTTGTTAGAAAGGAGCGACATGAATGACGTGCTTATCTG
TGTCCATTGCAGGGTTACCCTGGATACCCCGATGAACAGGAAATCAATGCCTGAGGCT
GACTTCAGCTCCTGGACACCCTTA
>268
CTGTGAAGGTCAGCATGTGCCCAGATTTTTGCTTTGAGAATATAAAAAAAACTTTTATT
TTCTGTTCCAGAACTTTCCATGACTGGATCACAGGGAAAAACCGACCGAGCTCAGGAA
GCCTAATCCAGGTGGTAACCACA
>269
AGGGAGGGGCACAGGCCATGGCGGGCTCAGCGTTCTCCTCCCACCACCCACCATGCT
GGACTCTTCCCAGGTCAGCACTTAAGCGATGCCTTTGCCCAGGTGAACCCCCTCAAGA
AGGTGCCAGCCTTGAAGGACGGGGA
>270
AAAAGCGACTATGTATGAAATACCCAGCACAGTGCCCTTCCCTTACCCATCATGACCCC
CACACCCACAGTGTGGCCATCCTGCTCTACCTGACGCGCAAATATAAGGTCCCTGACTA
CTGGTACCCTCAGGACCTGCAG
>271
ATTAGGGAAATGCAAATCAAAACTAACTCCCTACTCCAGTAACTCCCGACTTTGCCTGC
CCAATCCCCAGGTGATGTTCCCTGTTTTCCTGGGTGAGCCAGTATCTCCCCAGACACTG
GCAGCCACCCTGGCAGAGTTGG
>272
CCCAAATCAGATGCTGCCCATCCCTGCCCTCACAACCATCCATCCCCAGTCTGTACCCT
TTTCCCCACAGCCCGTGGGTGCTGGCTGCCAAGTCTTCGAAGGCCGACCCAAGCTGGC
CACATGGCGGCAGCGCGTGGAGG
>273
AAACTGCCAATTTTCTGAAATGCTCTTTAAATCCTGCTAATGGTGGCATTTGTGTCTTTG
TCACCTACAGTACATTTCTGGATTTGGGAATGAGTGTTCTTCAGAGGATCCTCGCTGCC
CAGGTTCCCTGCCAGAAGGAC
>274
AGTTGGTTGGTGGGAAGGTGGGATGCTTTTCGGATGGGAGTAATATTGTTTATTGCATC

CTTTGTTTCAGAATAATCCTCAGGTCTGCCCCTACAATCTCTATGCTGAGCAGCTCTCAG
GATCGGCTTTCACTTGTCCAC
>275
CTGAGCCATTCTGTGTATCACTCAGAACATTACTCTAAGGCTTGTATATCTTGTATGTTTT
TCCCTCTAGCTGGCTGTATAGGATTCTACCTTCAGTTTCTCACAAGCCCTTTGAATCCAT
TGACGAAGGCCATGTCACT
>276
GAACAGTGGAAAGGCTGCCTCTTGGGCACCGTTCACATTAACCTTTCCCTCTTCTGTG
GGTCTCTCCCAGGGCCTGCATACCTTGTGTGGAGCTGGAGACATAAAGTCTAACAATG
GGCTTGCTATCCACATTTTCCTCT
>277
CCTTTCCTGTTCATGAAATGGTTATCACACCAAAAGAATTGCTCACCACTGCCCTGTCT
TTTTTTGTCAGTTCCGCAGAAAGGGAACCTTCTCATTTACACCGAGTTTGGCAAGATGC
TTGTACAGCCCAATGAGATCTG
>278
CTCTGGGCAGCTTCTTTATAACTACAGCTGTGATTTCAGAGGGCACACCAGTGTCTGCT
TTCCATTCCAGAGAGGAATGCGGTTCAGCATAGATGTCTTTGAGGAGACCAGGGGCTA
CATCTTGGAGGTCTATGGTGTCC
>279
TCATGCAACCATGGGCATCTTTCCTATGTTTTGGAAGTTTCTAAAAGACTTTTGGGTTAC
TGTTTTCTAGGGGCCAATGGCTTGGCCAATCCTCGTGATTTCTTGATACCCATTGCCTGG
TATGAGGATCGCCAAGTACC
>280
ACATATTGTGGGATCACAGCTACAAAAGCATTAGATGGTAAAATTATCCTTTCCTGCTTT
TTGTGTTTAGGATGTCTCCCCGTTCAATGTTGTGGCCTGGCACGGGAATTATACACCCT
ACAAGTACAACCTGAAGAATT
>281
ATGAAATGTGTGTCATTGTCCAGTCACTTCCCTTGCATCATGCGTTCAGTCTCTCCTTGT
GTGTTCACAGGACCCATCCATTTTCACAGTATTGACTGCTAAGTCTGTCCGCCCTGGAG
TGGCCATTGCTGATTTTGTCA
>282
GTTTAAGGGGCTTTGTGTTTTACTGGTCTTGCCTTGGATAATAAAAATAATACTGCTTCA
ACCTTCCCAGGGAACTGCATGAGTGAGTTCATGGGACTCATCCGAGGTCACTATGAGG
CAAAGCAAGGTGGGTTCCTGCC
>283
GACATCTAATCAAATGTGTTTATTATCTATCTCACCTCTTTTTTCTCATCCCCATCGTCAC
TTCCTCCAGGCATTTATGTTTGAATCATCTTTAAGTCTGGCGGTCACAAAGTGGGGACT
CAAGGCCTCCAGGTGTTTGG
>284
GGGAAGGGGGCATACCATTTTAAGTACCAAGAAAAACTTGCATAATCTCATTTTACTTT
CTCTTTTTCAGCTGGATTTTCCTCTACGTGCTTCAGTCTTCTCTACACAAAGAGCTGCA
AACCAGGTCTTTGTTTTGCACT
>285
TGATTATGCTCTTTCTCTACCCATCCCCGCAAGGCAGATGTTTTCATGCTTGAATTTTGTA

TCCCTAAAGTCAGGAGAAATCTTCTGGACTTAAGTAAAGAAGAAAAGAACCACTTTGT
CCGGGCCCTGGATATGGCAAA
>286
GAGTAGACCAAACAGAAATGAATAATTGTAAACAGAAGCAGAGAGTATTAATGTGGTT
TCTGTGATCTAGGAAATGTTGCAAGAGCCTTCTTTCTCCCTTCCTTACTGGAATTTTGCA
ACGGGGAAAAATGTCTGTGATA
>287
AGTTTTAAAGAGCGACAATAAGAACTCCAAACATTGTGTAAATGTTTCCACATCCCATT
TTTTTCTGCAGGCACCGAGGATGGGCCAATTAGGAGAAATCCAGCTGGAAATGTGGCC
AGACCAATGGTGCAACGTCTTCC
>288
CATTGCTATTACCTGGAAAAGTGAAATATTTGCAATAGTTTTACTATTCTCCTCCTTACCA
TGTGTCTAGGTTACAGTGACCCCACGGGAAAGTATGACCCTGCTGTTCGAAGTCTTCA
CAATTTGGCTCATCTATTCCT
>289
TAGAACTCAATAATGATAGGAATATTAATTTTATTATGTTTATTAATACGTTGTCTTTGGAA
TAATTTAGATATATCCACATTTCCATTGGAAAATGCCCCTATTGGACATAATAGACAATAC
AACATGGTGCCATTCTG
>290
ATGTGTCCACTTTTTGGTGATAACTATTTTAATATTTGTCTTTTTATTTTTAATCTTCCTTT
CCAAATAGGTCGGGAGTTTAGTGTACCTGAGATAATTGCCATAGCAGTAGTTGGCGCTT
TGTTACTGGTTGCACTCAT
>291
TTACCACATATAATTCTTAGGTTTCTACAGGGCCTGTCTAACCTGACCTTACTCACTTTTT
ACATAACAGGGTCCACCAGGCCCCCCAGGCAGAGATGGTGAAGATGGTCCCACAGGC
CCTCCTGGTCCACCTGGTCCTC
>292
GGCATCTTAAAAACAGATATGCTGTTTCATTATTTGCTGGTTAATTCCTTGGTTTAATTTC
CTCTTTTAGGGTGAAATTGGAGCTATTGGTAACGCTGGTCCTGCTGGTCCCGCCGGTCC
CCGTGGTGAAGTGGGTCTTC
>293
GGCATTTAATGTGTGCTGCCTCTACAGCCCATCACCTCCCTAATGGACCACACTGCATTT
TCCTTCATAGGGCCTTCCCGGCGTTGCTGGGGCTCCCGGCCTCCCTGGACCCCGCGGTA
TTCCTGGCCCTGTTGGTGCTG
>294
TTCTCCAGCTGGACATAGTATTAAAATTATTTTTTTTACTCCCTCTTCTTTTGTTCTTTTCA
TTAAACAGGGCTCTGCTGGGCCCCAAGGTCCTCCTGGTCCCAGTGGTGAAGAAGGAA
AGAGAGGCCCTAATGGGGAAG
>295
TCTGGTGGTGGGTGTGTCATTAGCTTTAGCATCCTCCTCCTCTATCTGTTTTTTTTTTTTT
TTTGAATAGGGCCCTCCTGGTAGTCGTGGTGCAAGTGGCCCTGCTGGAGTCCGAGGAC
CTAATGGAGATGCTGGTCGCC
>296
GCAGCATCATAAGCTTGAGGTTGTGAGAATATGTTGACACTGAGTAAACTTGAAATAAC

TCTGCTTTCAGGGCCTCCCTGGCATCGACGGCAGGCCTGGCCCAATTGGCCCCGTTGG
AGCAAGAGGAGAGCCTGGCAACA
>297
CTCTCATATGTAAAACAGTATCACTGAAAGTGATGAATGGTGCAACACTTCTTCTAATC
ACTTTTTTCAGGGGGAACGCGGTCCCCCAGGTGAGAGTGGTGCTGCCGGTCCTACTGG
TCCTATTGGAAGCCGAGGTCCTT
>298
TCTTCTAGAGTTTGATTCTTCATTTTCTTCTTTCTCCACTAAAATTGATTTCACATGTGTT
TGACTCAAGGGTGAACCTGGTGTGGTTGGTGCTGTGGGCACTGCTGGTCCATCTGGTC
CTAGTGGACTCCCAGGAGAGA
>299
CTGAAAACATCCTAAGTTGGGGAGTAGAGTGGGTCGGAATACCAGAGCTGTAACTGTT
TATTTCCAACAGGGTGCTGCTGGTCAACCGGGTGCTAAAGGAGAAAGAGGAGGCAAA
GGGCCTAAGGGTGAAAACGGTGTTG
>300
ATCTTTGCTGCTCTCTTCCAGGCCCTTGGTGATTAACAGAAAGGAAATGACCTTGTACA
TTTGCTCATAGGGTATTTCTGGCCCTCCTGGTCCCCCTGGTCCTGCTGGGAAAGAAGGG
CTTCGTGGTCCTCGTGGTGACC
>301
AATCTTCAAGCCAACCTGTGTTATCACCTAGGGTCTTACCCATAATACTCAGTATTTTTT
CTCTATTTAGGGACCTCCTGGCACTCCAGGTCCTCAGGGTCTTCTTGGTGCTCCTGGTA
TTCTGGGTCTCCCTGGCTCGA
>302
TGTTTTGGAGGGGAAGGTTAGCATTCCATCGAATAAGGGGAATGTCATTTTATCTTCTCT
GCCTGTTTAGGGTGAACCTGGTCCTCTTGGCATTGCCGGCCCTCCTGGGGCCCGTGGT
CCTCCTGGTGCTGTGGGTAGTC
>303
TCTTTCTCTAATTCAAAAGTGATTAAAATGCAACCCAGATTGATGCTAAGCTTCATTTTG
CCTTTGGTAGGGAGAGCGCGGTTACCCTGGCAATATTGGTCCCGTTGGTGCTGCAGGT
GCACCTGGTCCTCATGGCCCCG
>304
GTATTTGTGGTGAAGTGAGTGCCATTTTTTTAAAACGGTAAGTCTTATCCATCCTTCTGT
TTCTTTATAGGGCCCACAAGGCATTCGTGGCGATAAGGGAGAGCCCGGTGAAAAGGGG
CCCAGAGGTCTTCCTGGCTTCA
>305
TGAGCCCCAGGCTGCTGCTCCCTTGGTGGGATTCACCAGCTCACATGTACCTGGTGTCT
GTCTTCCTTAGGGCCCTGCTGGTCCTTCTGGCCCTGCTGGAAAAGATGGTCGCACTGG
ACATCCTGGTACGGTTGGACCTG
>306
TCTGCTGCCATGGATGTCTCTCACTGTAAAAAAATATAAAGCCTCTCCTATCTAACTTTC
ACCTTTGCAGGGCCCCCCTGGTCCCCCTGGCCCTCCTGGACCTCCAGGTGTAAGCGGT
GGTGGTTATGACTTTGGTTACG
>307
TTAGTTATTATTAGAATCTGTGTTCTGCTCAATGAGAAGTTTCATGATCTGAATGTTATTT

TCTTAAAAGGTTACTACTGGATTGACCCCAACCAAGGATGCACTATGGAAGCCATCAA
AGTATACTGTGATTTCCCTAC
>308
TTTTTCATGTTTGACTCTTAGTATCTGAGTCCTTCTCCACTTAACTGGAATTTCATCCTAT
TTTCTGTAGTTTGAATATAATGTTGAAGGAGTGACTTCCAAGGAAATGGCTACCCAACT
TGCCTTCATGCGCCTGCTGG
>309
CAAAGGTTCAGATATTATCAGATTCAGAAATAGTGATGCTTTGTGTATCTATTTTCTTCTC
TTTAAACAGAAAAAGACAAATGAATGGGGAAAGACAATCATTGAATACAAAACAAATA
AGCCATCACGCCTGCCCTTCC
>310
AAATTTTTTTTAACTGGGTTAATGCTTGCTGAATTGGAAGTGAATGTCCATTCCTTTGCC
TCTTTTGCAGATATACACTTCAGATAACTACACCGAGGAAATGGGCTCAGGGGACTATG
ACTCCATGAAGGAACCCTGTT
>311
GGGTGTGTGGGGATAGTTGAGGTTATGGTGGGAACCTTGGGCCCTGCTGACCCTGTTT
CCTCCTCCCTAGCCTTTATCCTCCTCTTCTACCTCGTTTTTTATGGGTTCCTCACCGCCAT
GTTCACCCTCACCATGTGGGT
>312
CCATTTTCTTCCCTCTGTGTGCAGTCCCTCATCTTATAGATCCCCCAACTTCTGCCTTTG
TTGGCTGTAGGCTTGATGATTCGCCCCAAGACTGAGAACCTTGATGTCATTGTCAATGT
CAGTGACACTGAAAGCTGGGA
>313
TCGGGCAGGGGACTGGGGACCTTGGAAGTGGAACATCTGGCCCCTGAGTCTCTCCCTC
CCACCTCTTTAGCTTACAACGACTCTATCCAAGCCCAAAAGAATGATGTCTGCCGCCCT
GGGCGCTATTACGAACAGCCAGA
>314
AAGTCTGGTGAGCTCCTGGGTGCCTGCCATCCCTAACTGGCTAACCCCCTATCTTCCTG
CACCCCCACAGCGAGATGAAGATGCTGAGAATCTCGGCAACTTCGTCATGTTCCCCGC
CAACGGCAACATCGACCTCATGT
>315
TGCGGGTGGTGAGCTAGGGAAGGAGGCCGCGTTGCCCCAGGCCTAGACCCTGCACTG
CTCTCCGGCCCAGGTGAACTACACACAGCCCCTGGTGGCTGTGAAGTTCCTGAATGTG
ACCCCCAACGTGGAGGTGAATGTAG
>316
AATGGGCACCCTCGTTCCTGGAAAACGGTAGGCCTGTGGGTGACCAGCTTCCCTATCT
GTCTTCCCACAGTGCGGCTGGGATCCGGCGGCCACCTGCACCTGCGTATCTCTCGGGC
CGCCCTTCCCGAGGGGCTCCCCGA
>317
GACCCATAAACCTCTCCACACTGGCTGAGCAAATGAGAGAATGCTTTCATGTTCCTGAT
TTGTTTTTCAGAGCCAAAGAGTTCAAGACACGTCTGGGGATCTTTCTTCACAAATCAG
AGCTGGGCTGCGATACTGGGAGT
>318
TTTCCTTTCTTCTTGTATTCCCTGCTGTCCCTCCAGCTGACCTATTTCCTCTTCTTTCCCA

CAACCCCAGATGGAGTGGCTGCCTTCCACGCTTTCCTGAAGACAGAGTTCAGTGAGG
AGAACCTGGAGTTCTGGCTGGC
>319
CCGTTGGCCCTCCCTCAGCCATGCCCACCCTCTTCCCCAACTCACCCTGTGTGTGTCCT
CCTTCCCGCAGGTCAACATTGACCATGAGACCCGCGAGCTGACGAGGATGAACCTGCA
GACTGCCACAGCCACATGCTTTG
>320
TTTGCATTCATGGAGGGCAACTAAATACATTCTAGGACTTTATAAAAGATCACTTTTTAT
TTATGCACAGGGTGGAACAAGATGGATTATCAAGTGTCAAGTCCAATCTATGACATCAA
TTATTATACATCGGAGCCCTG
>321
GATTACAGGTGTGGGCCACCGTGCCCAGCCACCGGTGTGGCTCTTTAACAACCTTTGC
TTGTCCCGATAGGTCACCTTTGGCTCTTCAGAGATGCAGGGACACACGATGGGCTTCT
GGTTAACCAAACTGAATTATTTGT
>322
AAAGCCTCTTGTTCGTTCCTTGTACTGAGACCCTAGTCTGTCACTGAGGATTTGGTTTT
TGCCCTTCCAGTGTATACTCTGAAAGAGCGATGCCTCCAGGTTGTCCGGAGCCTAGTCA
AGCCTGAGAATTACAGGAGACT
>323
CCACCAGAGCCTGCTTTCACTCAGAAGGGAGATGCAGACCCATGTAATCCTCCCCGTC
TCTGCTTCCCAGGACTCATCTGATGATGTGAGAAGAGTTCAGAGGAGGGAGAAAAATC
GTATTGCCGCCCAGAAGAGCCGAC
>324
CCCTCCGCACCCCACCCACCTTGCCTCCTTCCTAGACACTAACCTCCGGTGCTGATCCC
CACCCCTACAGGAGAGCGAAGACCTGGAGAAACAGAACGCGGCTCTACGCAAGGAG
ATCAAGCAGCTCACAGAGGAACTGA
>325
CCCGGGGGAAAGGTGGTTAAGCTGGGGGTTGCAGTCACTACTGACAACGCCCCTCTTC
CGCCTGTCCCAGTCCCACCAATCACCTGCGTACAGAACGGCCTCAGGTACCATGACCG
AGACGTGTGGAAACCCGAGCCCTG
>326
TGTGGGGGGCTGCAGGTGGGCATGGCTCTCGGCCCCACGCTCACCCCGGCCCCGCCCT
CTCCCCCTGCAGGGACCCGCAGGCCCCCCTGGCCGAGATGGCATCCCTGGACAGCCTG
GACTTCCCGGACCCCCCGGACCCC
>327
TTCCATCCTCAGTGCCGTCTTCTGCCTTTCAATTCACTGTCCTCACTCTGACTTCTCTTG
TTTGTTCTAGAACTTTGCTCCCCAGCTGTCTTATGGCTATGATGAGAAATCAACCGGAG
GAATTTCCGTGCCTGGCCCCA
>328
TCCTGCCATCCCGAGAGGCAAGGTTGGGTTTCCCAGGGTGGCTTCTGATATGTCCTTTC
TTCTGATTCAGGGTGAAGCTGGTCCCCAAGGGCCCCGAGGCTCTGAAGGTCCCCAGG
GTGTGCGTGGTGAGCCTGGCCCCC
>329
GCCGCCTGCAGCCTGCCTGCCCCTTTCCCTCTGCTCCTAGGCTCACGCCCTGGCTGTCT

GCCTCCCACAGGGTGCTCCTGGTATTGCTGGTGCTCCTGGCTTCCCTGGTGCCCGAGG
CCCCTCTGGACCCCAGGGCCCCG
>330
CAGCCGGCTCTGAGGCTGGCACAGGATGGCCCCTCACCACAGGCCGCCTCCTCCTCTC
GGCCCTCTCCAGGGCCCTGTTGGTGTTCAAGGACCCCCTGGCCCTGCTGGAGAGGAA
GGAAAGCGAGGAGCTCGAGGTGAAC
>331
CCTCTTTTGGCCCGTGGCAAAGAGCCACAAACTTGAGACCCTAACTGTTCCTGTGACT
TCCCCCAACCAGGGTCCCGCTGGTGAACGTGGTTCTCCTGGCCCCGCTGGCCCCAAAG
GATCTCCTGGTGAAGCTGGTCGTC
>332
GAACCCCCTGCCATTGTCCTGTCTGCCTCCCTGCTGTCCTCACTGCTGCTTTCGTGCCT
CCCATCCTTAGGGTCCCGCCGGTCAAGATGGTCGCCCCGGACCCCCAGGCCCACCTGG
TGCCCGTGGTCAGGCTGGTGTGA
>333
TCCGTCAAGGTGCGTTGTACTTGGCCCTATCTCCAGAGCAGCCTTCACATGCCCTGTCC
TTCCCTTCTAGGGCGAGAGAGGTTTCCCTGGCGAGCGTGGTGTGCAAGGTCCCCCTGG
TCCTGCTGGTCCCCGAGGGGCCA
>334
GCGGGGCTTAGGGCTGTGACCCACTCTTGGGGCCCCAGGCCTCACTCCAGTCTTCTTG
GTTGTCACATAGGGTGATGCTGGTGCCCCTGGAGCTCCCGGTAGCCAGGGCGCCCCTG
GCCTTCAGGGAATGCCTGGTGAAC
>335
ACACAAGCAGAACACTATAGGTCACCTCCTTGCCCCACACTGGAAATCTCAAGCTTAT
CCATGTCTTTAGGGTGATGCTGGTCCCAAAGGTGCTGATGGCTCTCCTGGCAAAGATG
GCGTCCGTGGTCTGACCGGCCCCA
>336
CTGCTCCTCCCCCAGGTAGTGGAAACTCCTGCCTCCTTCCCTCCATTCACCGCCCTGCT
TCCTCCCCCAGGGTGCTGACGGCCAACCTGGTGCTAAAGGCGAACCTGGTGATGCTGG
TGCTAAAGGCGATGCTGGTCCCC
>337
GGGCTGACTGAGGACCCAATGATGCACCAGAGCCCCCTGGAGTCTGACAGCCCCTCCT
ATCCTCATCCAGGGAAATGCTGGACCCCCTGGCCCTCCTGGTCCTGCTGGCAAAGAAG
GCGGCAAAGGTCCCCGTGGTGAGA
>338
GAGGTGTGCAGAGCTGGGGAGGGGTCCCTGTGCTGCTGTCTGGCACCTCACCCCTGTT
TGCCTCCCAAAGGGTGCTCCTGGTACTCCCGGGCCTCAAGGTATTGCTGGACAGCGTG
GTGTGGTCGGCCTGCCTGGTCAGA
>339
GAGATGACTCCAGCGGACTCAAGGGTCCTCCCAGACCCTATCTCTGGCCTGACTCTTT
CTTCTCCCTTAGGGTGAACCTGGCAAACAAGGTCCCTCTGGAGCAAGTGGTGAACGTG
GTCCCCCTGGTCCCATGGGCCCCC
>340
CTGCTGCTTCCCTGCCCCATCCTGGCCCTTCACCCGGGGCTGACCCATATTCCCCTGCT

CTCCCCGCCAGGGTGACCGTGGTGAGACCGGCCCCGCTGGACCCCCTGGTGCTCCTG
GTGCTCCTGGTGCCCCTGGCCCCG
>341
GATGCAGACAGTGCCCCACTCGATGCCAATGGAACTTCCGCCTGACAGTTTGTCCCTT
TCTCTCTTCTAGGGACCCCAAGGCCCCCGTGGTGACAAGGGTGAGACAGGCGAACAG
GGCGACAGAGGCATAAAGGGTCACC
>342
GGCCTCTCCTCCAGAGCTGGGGTTGTTCGGGCTTCTGGCAGCCTTTCTCAAACCATTTC
CCCCACTCCAGGGTCCCCCTGGCTCTGCTGGTGCTCCTGGCAAAGATGGACTCAACGG
TCTCCCTGGCCCCATTGGGCCCC
>343
CTGCTCATGGCCCTCCAGCCCCCATAGCACTTGGATGCCGGAATCCCCACTCTCTTCCC
TCTCTGTGCAGGGTCCCCCCGGCCCTCCTGGACCTCCTGGTCCCCCTGGTCCTCCCAG
CGCTGGTTTCGACTTCAGCTTCC
>344
ATATGTGCCCCTACCATATGGTGCTGGCTGCTCCCTTTCCCTGACTCCATCTTGCCCTGC
CCTACCACAGGAGAGTACTGGATTGACCCCAACCAAGGCTGCAACCTGGATGCCATCA
AAGTCTTCTGCAACATGGAGAC
>345
CCTGGACAGGAGGCCAGCAGGACTAGAGGTTCCCGCATAGCTCACTCTTCCCTCTCTC
TCCTCCCTGCAGTTCGAGTATGGCGGCCAGGGCTCCGACCCTGCCGATGTGGCCATCC
AGCTGACCTTCCTGCGCCTGATGT
>346
GGCCATAGTGCCCTCTCTCCATCACTCCCACGTGGTAATGCCCCCTCCCGTTGTCTCCG
CCCCACCCCAGAGTCACACCGGAGCCTGGGGCAAGACAGTGATTGAATACAAAACCA
CCAAGACCTCCCGCCTGCCCATCA
>347
GCTAAGGTGGGCAGGGCGTCATATCCAGGCCCCTCATTCCATTTATTCCTTTGGTTTCTT
TTCTCCTCAGGGCCCTAAGGGGGAGCAGGGCCCCCCGGAATTCCAGGGCCCCAAGG
CTTGCCAGGCGTCAAAGGAGACA
>348
CCCACCCCCCACTCTCGCCCACCCGGGCGCCTTCTCACCCGGCTCTGCTCCCACCCCC
ATCCCCCCGCAGCAAGGAGTACGTGGAGAACCCGGCTACCCTGGCCCCAGCGGGGAT
GCGGGCGCCCCAGGGGTTCAGGGCT
>349
AACATAGCAAAAAATAGAGGAAAAGCATTTTCCAATTCTGAAAAATGAAGATTTCCTT
TTTTGTCTACAGGAAAACGTGGAGAGAAGGGTGATCCAGGAGAAGTGGGACGGGGGC
ACCCCGGGATGCCTGGGCCCCCAGG
>350
TCCCCACCTTCAAAATTCTGGGCCTGAGACAAGAGGTGCCCCTAATGTCTTGACTTTCT
CCTGTTCACAGGACTCCCTGGCCGGCCTGGCCAGGCAATCAACGGCAAGGATGGAGA
TCGAGGGTCCCCAGGGGCTCCAGG
>351
GCATCGAGATGACTGGAGGTGGGAAGGGCAACATGCTTATCCCTGTAGCTACCCTGTC

TTGGCCTTGCAGATCCAAGGAGAAAGTCAGGGGCCAGTGCGCGCTACAGCCAGCGCC
CAGTACCGCGGTGTGATGGGCACCA
>352
TTGGTGAGGAGAGATGAGGTAGAAAATGAGTGCAAGCCCGCTGGCCACTGACCCCAT
GGCTCGCCCACAGATGCCAGCATTGGGAGCCGCCTCCTAGCAGGCAGCACCACAGGT
GCCCTGGCTGTGGCTGTGGCCCAGCC
>353
GTTGTTTTCCCTTCCCCTTTTCCTCCTCCCCGATACTCTGGTCTCACCCAGGATCTTCCT
CCTCCTACAGGGACCTCTCCCAATGTTGCTCGTAATGCCATTGTCAACTGTGCTGAGCT
GGTGACCTATGACCTCATCAA
>354
AAGTAATTCTCCTGCCTTCTGGAATGATGGGTGAAGATCTTGACTGCCTTGCCTGCTCC
TCCCTTGGCAGATGACCTCCCTTGCCACTTCACTTCTGCCTTTGGGGCAGGCTTCTGCA
CCACTGTCATCGCCTCCCCTGT
>355
TGAATACCAGGCCCAGTGAGGGAAGTGGGAGGTGGAGGTGCCCCCATGACCTGTGAT
TTTTCTCCTCTAGGTTCATGCCCTCCTTTCTCCGCTTGGGTTCCTGGAACGTGGTGATGT
TCGTCACCTATGAGCAGCTGAAA
>356
CCACTAGTGCATTGGGGTGCGGTTCGCGAACAAACTGCCCACTGGCTCCCTTTTTTTTT
TCTTTTTCTAGAGAGACTTGATAAATCGGCCTGAGAAAATAAGTAATGCTCCAGACTAG
CTCTCTCATTTGTGAGTTCTCC
>357
TCTGTCACTTTTAGTGGTAGAATTGTCCTTGAGACAACAGGAACTGACTACAGAAATCA
TACTATTTCAGGGGTTGCTGACATTTGAGGATGTGGCTGTGTTTTTACCCAGGAGGAG
TGGGATTATCTGGACCCAGCTC
>358
CCATCTTACTTTGTTTCTCCTTGCTTCAAAGACCTTGGTTGATCTTTGTATTTCCTTTTTA
TTATTCCAGATGTTTTGAACAGAGATAAGGATGAGGAGCCAACTGTAAAACAAGAGAT
TGAAGAAATTGAGGAAGAAGT
>359
AAAGTTTCCTCATCACGTGGCGAGACCTCCTCTACTTCAATCTTTGTCTTTTGTCTCTGC
TGCCTTGCAGGGTTGGTACAGTAGGCTTCACTAGACTTAGCTGCAACTCAGAATTTCTC
CTCCAGCACCTGAGTAAATGC
>360
AAATGAATTCATTATGAATGGCAATTATAGCTTAATCAAGGACTCAAAGATTCTTTTTCC
TTCTTTCCAGGATGAACACCAGGATAAAATCTACCCCTCTTTCCAGCCACAGCCTCTGA
TCTATCCATTCGTTGAACCTA
>361
AAAAGTGAAGAGAGTTGAATAGTTACGTGTTATAACATAACTAATTATATATTTGCTCTC
TATTCCACAGAATTGACTGAGACTGGAAATATGATGCCTTTTCCGTCTTTGTATCACGTT
ACCCCAAATTAAGTATGTTT
>362
ACAGCAGCCTAACGAGTGCCCTGTCTACTTGTCTGTCCTTGTGTTCATGTGTCTGCATG

TCTGTTCTCAGGACCTGACAGGAAGCGGGGCCGCCAGACCTACACGCGCTACCAGAC
GCTGGAGCTGGAGAAGGAGTTCCA
>363
TGGCCCTGGAGGAAGTAGGGGGATGTGGGGGTGAGGTAAGTCTCCTTGAATGCTGTAC
CCTGTCCATTAGAGCAGCCATGGCCAGCTCCAGGCGAGGCCTCCTGCTCCTGCTGCTG
CTGACTGCCCACCTTGGACCCTCA
>364
GCCGGGGGCTCCCCCACCCTCCTGGAGCCTGAGGTCGGGGTAGGGAGGACAGCATCA
GTTCCCTTCTAAGGAAGGGCCCTGGACACTGCAGCAGGCAGCCCAGTCCAGACTGCC
CATGGCCTCCCAAGTGATGCCCTGGC
>365
CTAGGGAGGGTATTAGGGGAAAGGGCTTGAGGGCCACAGTAAACTGGACAAGTTTTT
CTGCCCAGCCTAGGCTGCCACCTGTAGGTCACTTGGGCTCCAGCTATGTGGCTGCCTCT
TCTGCTGGGTGCCTTACTCTGGGC
>366
GCTCCCAGGAAGAAGAGGGAGCTGTGGGAGTGCCTCACCTACCCCCAGCATCCTTTTC
ATCTCCCCACAGGGCTTTTTGGTCTGGTGAATAATGCTGGTGTGGCTGGTATCATCGGA
CCCACACCATGGCTGACCCGGGA
>367
AACCCATGTCCCTCAAAGTCCCCTCCCTATAGGGCAAGAACCCAGCAACTTCGCTCTG
CCCCGACTCTAGGCGGGATGTAGCTCATTTTGGGATACGAGTCTCCATCGTGGAGCCTG
GCTTCTTCCGAACCCCTGTGACC
>368
CGAGCCTGACTCCAGCAGGCCTGCCTTGGGGCTGGGGCTGGGGTGGAGGCCAGCCAG
TGTCCTCCCATAGTGGCCGAGAAGACCAAGGAGCAGGCCAACGCGGTGAGCGAGGCT
GTGGTGAGCAGCGTCAACACTGTGGC
>369
CCTGGTTGCAGGGCAGCTAGGGGTCTCTGCATTCTCCACATGGTCTCATGCCCCCTTTT
GTCCCCTACAGGAGGACTTGAGGCCATCTGCCCCCCAACAGGAGGGTGTGGCATCCAA
AGAGAAAGAGGAAGTGGCAGAGG
>370
TCAGCCCACTCAGGGTGGCCCATTAAGGCTGGGGCCTGGAGCTGGGTGTGCAGGTCAT
TCTCTCTCCCAGGCCCAGAGTGGGGGAGACTAGAGGGCTACAGGCCAGCGTGGATGA
CCTGAAGAGCGCTCCTCTGCCTTGG
>371
CGGCCAAGCTGCTTGTCACCTGTGAGTGCTTCCAAGGAACCGGATGTGGGCTCCCCCA
CCCTTACCCCAGCATCTGGCACAGCTGAAGGCAGATGGAAATATTTACAAGTACGCAAT
TTGAGACTAAGATATTGTTATCA
>372
TGAATTTGAATGACTGATAATTTAATTTATTTTCAGTCAATTGTTACGTATGTTTTCTTTTT
CTTCCCAGGACATGGTGACCCAGGCATCTCTGTATCTGTTTGAAGCTACAGGAAAGCG
ATTTTATTTCAAAAATGTTG
>373
CTCCAGTACGAATATGGTTAATTCATTTGCTGAAACTTTGTGATGATAAAAACTTTTTCT

TAAATTTCAGGCTGATGTTCTGGTTGCTGAGTCTACTCCTCCAGGTAATGATGAACCCT
ACACTGAGCAGATGGGCAACT
>374
TTTCTTCCAGTGTGAGAAGGTAATTTTGCCATTCTTATTGTAATCAGTTTTTTAAAATACT
TTCTCACAGGTAAGGCATTTGTCCATGAGTGGGCTCATCTACGATGGGGAGTATTTGAC
GAGTACAATAATGATGAGAA
>375
AACTGAGCATAGACTCTGACCCTTTGAAATGACGACATCACCTAAACATCTAACCTTCC
TATATTTTCAGATGTTCAGCAGGTATTACTGGTACAAATGTAGTAAAGAAGTGTCAGGG
AGGCAGCTGTTACACCAAAAGA
>376
GTCTAACAAATTATTAAATATTCTACCACATAGTTTACCATTATCTATATAATTTCATTGAC
AATTTCAGATAGTTGAATTCTGTACAGAACAAAACCACAACAAAGAAGCTCCAAACAA
GCAAAATCAAAAATGCAATC
>377
CCTATGGCCTTTTCTGTTATTAGTCTTTCCATCTAAACGTAACCTGTTTTTCTTTTGCTTC
TCCATTTAGACTGGTAACCGCCTCAATCGACTGAATCAAGCAGGCCAGCTTTTCCTGCT
GCAGACAGTTGAGCTGGGGT
>378
TAAGCAGAATTTTCATAAACACTTTGGCCACCCTAAGTCTGATAACTCGTGCCCTAAAT
TCTGTCCTTAGGTGATTAGGAAGAAATATCCAACTGATGGATCTGAAATTGTGCTGCTG
ACGGATGGGGAAGACAACACTA
>379
AACAACAGCTAAAATGTTGCAAATAGTTGCTTTCTGGAAAATAATTTCTGAAAATGTAA
TTGCATTTTAGGAGGTTTACAGACATATGCTTCAGATCAAGTTCAGAACAATGGCCTCA
TTGATGCTTTTGGGGCCCTTTC
>380
GGGCAGAAGGGAAAAGACCATGTGATGGGAGCTGTATTCTCCAGAAAGTAAGAGCTG
TTTTTCTTAACAGCTTGAGAGTAAGGGATTAACCCTCCAGAACAGCCAGTGGATGAAT
GGCACAGTGATCGTGGACAGCACCG
>381
CGTGACATTGAAGTCAGGTCTGTGTCACCTGAAGTTATTCATTGGAAATGTTTACATGT
GTTTTGGTCAGGTTGGCACTTGGAAATACAGTCTGCAAGCAAGCTCACAAACCTTGAC
CCTGACTGTCACGTCCCGTGCGT
>382
ACATATGAGTTGGAAATATACAAATACCCTCCCCGTTACCTATTTATTAATTCCTTCATTC
TTTATCAAGGTGCTGATGCTACTAAGGATGACGGTGTCTACTCAAGGTATTTCACAACT
TATGACACGAATGGTAGGTA
>383
AAGAAAACAGACGGAGGTGTCACCATTTAGTGATGTTGCTTACCATATTTTGAGTATTT
TTTTAATGCAGATGAAATACAATGGAATCCACCAAGACCTGAAATTAATAAGGATGATG
TTCAACACAAGCAAGTGTGTTT
>384
AAGCTCTACTGCTTAAGAAGTTTCTTTAATATTTCAGAATTAAAGTCACATTCTTCTTTT

TTCTTTTTAGCTCACAAGTATATCATTCGAATAAGTACAAGTATTCTTGATCTCAGAGAC
AAGTTCAATGAATCTCTTCA
>385
GGAGACAGAGGTGGGCAGCTGTGGTCTCCCCTCTGCTTCCCTGGCTCAGCTTGCAATG
TGATTCCTTCAGAAGTTCCTGAGTGGGTGAACACCCCATCCACCTGCTGCCTGAAGTAT
TATGAGAAAGTGTTGCCAAGGAG
>386
AGAGGTGGGGGTGGGCACCGGACTCCTGCCCGAGTCTGCAGCCCATGACGGTCTAATT
CTGCCTCTGCAGCTTCGTCACCAAGAGGAACCGAGAAGTCTGCACCAACCCCAATGA
CGACTGGGTCCAAGAGTACATCAAG
>387
GCTCATTTTTCTGTGTGTCATTTGGATGACAAAAAATATGCTTATAAGCTTTCCTCTTTT
GTTTTTATAGGAGGAAAGAATGGAAATGATTTCTGAAAGGCCAAAAGAGAGTATGTAT
TCCTGGAACAAAACTGCAGAGA
>388
TATATAATTTCTTAAAATTTATATAATAAACTGTAGATTTCTCATGCTTATGTATTTCCCTTT
TCTTTAGTGTTTGACTCCACCTTACAGTCCTTCTGACTTTGAACCCTCTCAAGTGTCAA
ATCTGATGGCACCAGCGC
>389
TTAGACAGAATTGAACATATAATTTACGGCAATCTTATGCATAATAATGATTTGTATGTCT
TTGTTTTAGGAGAAAAGCCTTTCAGCTGTAGCTGGAAAGGTTGTGAAAGGAGGTTTGC
CCGTTCTGATGAACTGTCCAG
>390
GTACCAGGCTACAGGACCTAAGCTAATCTCCCACTCCTGCTGTCCATCCATTATGTTGCT
TTGTCCCCAGGTGGTAGGTGAGTGGAAATTCAGCAGGATTCACTGTGACATCTTCGTC
ACTCTGGACGTCATGATGTGCA
>391
GTGTGTGCATATTGTGGAGTGAGGGGTCCCTGGGCCTGCACCCCAGATTCAGGGTCCC
CCGCCCTTGCAGGTACACAGCTGTGGCCATGCCCATGCTGTACAATACGCGCTACAGCT
CCAAGCGCCGGGTCACCGTCATG
>392
ATGGGCTCTTGTGGAATTATGGCCGGTGGGCCCGCTGACTCCCTGCCTGCCCGGGCTCT
CCCTCCCCCAGACCAGAACGAGTGCATCATTGCCAACCCGGCCTTCGTGGTCTACTCC
TCCATCGTCTCCTTCTACGTGCC
>393
GCACTTTGGGTTTCTGTCCCCCCTCCATCCCCACCACTTTGTGTGACCCATGTGCTGGC
TCACTCCACAGGGCAACTGTACTCACCCCGAGGACATGAAACTCTGCACCGTTATCAT
GAAGTCTAATGGGAGTTTCCCAG
>394
CCAGTGAGATGGGTGGCTGATGCCTGGGAACTTGTCCGGCTTTACCCAGAGCCCTCTG
CCTCTGGTGCAGGAGGCTGCCCGGCGAGCCCAGGAGCTGGAGATGGAGATGCTCTCC
AGCACCAGCCCACCCGAGAGGACCC
>395
CTGGGTGTGGGTGTTCCCAGCCGTGCCTCCCCGGCTCTGGGGACCAGCCTGACCATGC

CCTCTCCCCCAGGCGTGTTCATCATCTGCTGGCTGCCCTTCTTCATCACACACATCCTG
AACATACACTGTGACTGCAACAT
>396
GGAGAAATAGTGCTGCCTAATCTGCTGGCGTGGGCAGTGGGCACCATGAGGACCCTGT
TCTGTGTTGCAGTTTTCACCATGGAGCAGCTGAGCTCAGCAAACACCCGCTTCGCCTT
GGACCTGTTCCTGGCGTTGAGTGA
>397
GGAAACACCAAGGAATCACTTTATAGTTTCAAAATAAATGGTTGTAGAAAGAAGATTTT
CTTTATTTTAGACTTTCCATTTCAACACGGTTGAAGAGGTTCATTCAAGATTCCAGAGT
CTGAATGCTGATATCAACAAAC
>398
GGATTTTAGAAAACAATTTCTATATGTAGTAGGAGTTATATATATATTTCCTTTTCCTTTTT
TTTAATAGGAGTTCTTGGTTTCGACTCAGAAAACATATGGTGCTGACCTGGCCAGTGTG
GATTTTCAGCATGCCTCTG
>399
TAATATATTGTCAGTTCAAATTTGGAATTCCGATTTGGATTTTTTTTACGCTAACTTGATT
TTTTTTTAGGAAAAATTCCGGAACTGTTGGCTTCGGGCATGGTTGATAACATGACCAAA
CTTGTGCTAGTAAATGCCAT
>400
CTGTACAGAGTCATGCATTGCTCTAAAAGAATGCAGAGAATAATTTCAGTGATTTTTTA
AAACTGTTCAGAAAGACAGAAAAACTGTGAAAATGATGTATCAGAAGAAAAAATTTG
CATATGGCTACATCGAGGACCTTA
>401
AATAATACTTTATTGCCTTACTTATGTAAAATTGATATCACTTCCTCTTTCGCCTCATTTTT
AATTGCAGATTGAGGAACAGTTGACTTTGGAAAAGTTGCATGAGTGGACTAAACCTGA
GAATCTCGATTTCATTGAAG
>402
ATGAGCGCGGCGAGGTGGGGGTCTCTAACGGAGCATCTGTTTTAACCCGCCCCTGCAC
ACACCCCAGCAGATCCTGGCCATGAGAGCTCCGCACCTCCACCTCTCCGCCGCCTCTG
GCGCCCGGGCTCTGGCGAAGCTGC
>403
AGCCCAGCTCCAACCCCAAACCCAGACCCTGCCCTCCGTGCTTTGATTCCGTCCCCTTT
CTTTCTCCCAGCCGCAGAGGCGGCGCTGCTCCCCCAAAACGACACGCGCTTGGACCC
CGAAGCCTATGGCGCCCCGTGCGC
>404
GCTTCACTGCCCACCTGCTTTCCCAGTCCCTGTGAAGGGTTTGCCTTCACATGCCTCTT
CCTTCCCCCAGGCCACTGTGGGCTCGAGTAGGGGATGACCACCTGCTGCTTCTTCAGG
GCGAGCAGCTCCGCCGGACGACT
>405
GTATCTGGACTCCTGGCTCCCCAAGGATTGGGGGCTGGACCCAGGGATTACTGGCATAT
TCTCCCTTCAGTGAAGTACAACAAGGGCCTGACCTGCTCCAGCATCACTATCCTGAGC
CCTAAAGAGTGTGAGGTCTTCTA
>406
GCCCCAGATCCCAAAGTGACAGCTCTCACGTTGGCACATTTATTTGATCTCTCCTTTCT

GCCACCCCCAGAGTGACTCTGGAGGCCCCCTGGTCTGTGACGAGACCCTCCAAGGCA
TCCTCTCGTGGGGTGTTTACCCCT
>407
GCAGGGGTCGCGGCCCTCCAGCATGAATGTGCCCGCTCAGCCGACGTCTCCCTTCCCG
GTCTGACCGCAGGTATCTGGCCATCCGCTACCCGCTGCACTCCCGCGAGCTGCGCACG
CCTCGAAACGCGCTGGCAGCCATC
>408
ACGGAGGTTTTGAGGCGGGGTTGCAACTCCAGGGGTGAGAGCTGACGAACTCTCTTC
TCCCCACCCCCAGGAGCTGGACACCCTATGAAGCCTCATCGCCTGGCATTGACCCATA
GCCTGGTCCTGCATTACGGTCTCTA
>409
AGTATGGGTGGTCTGAGCCTGTGGAGTGGGGCTGACCCTGCCTGGGTTCATCTTGTGTA
TCCATCCCGAGGTCTTCAAGCCATACCAGGCCTCCCAGCATGACATGTGCCGCTTCCAC
TCCGAGGACTACATTGACTTCC
>410
ATGGGGAGGGAGATATGGGAAGGGCCCAGACAGAGGCAGGAGGGTTCCAACTTGTTC
CTCTTCCCTGCAGCCCAGTGTTTCCCGGGCTCTTTGAGTTCTGCTCGCGTTACACAGGC
GCATCTCTGCAAGGAGCAACCCAG
>411
CCCGGTGTTTGAGAGGAGTGGGTTGGGGGGTTCACTTCTAGCTCAGACCCTGGCTTTG
GCTTCCCTCTAGGTACCACCCTCGGGTGCTCTACATTGACATTGACATCCACCATGGTG
ACGGGGTTCAAGAAGCTTTCTAC
>412
AGATTGGGGCTGAAACAGCTGGGTTGTGGGTGCTTCCCATGTGTGGCTACCACCACCC
ACTTCCCTACAGGTGACATGTATGAAGTCGGGGCAGAGAGTGGCCGCTACTACTGTCT
GAACGTGCCCCTGCGGGATGGCAT
>413
CTTTTTCCTTTTGTTCCCTGTCTCCCCATCCCCTTGGGGTGCCAGGTGCCCATCTTTGGC
CCTCTCCCAGGTTACAAGCACCTTTTCCAGCCGGTTATCAACCAGGTAGTGGACTTCTA
CCAACCCACGTGCATTGTGCT
>414
CAATACCTAAAGGTATTTACCTTGTCTTTTGATAACCTCCTCCACATCTTCCTCTGGTTC
CTTTTAACAGGGAATGCGTTGAATATGTCAAGAGCTTCAATATCCCTCTACTCGTGCTG
GGTGGTGGTGGTTATACTGTC
>415
GAAAAAGATTGGGAAGTCTTGTTAGCTGCCTTTCTCAGTCTGAGCCATACTTTCTCCTC
TCCTCCCCAAGAATACTTCGAGTACTTTGCCCCAGACTTCACACTTCATCCAGATGTCA
GCACCCGCATCGAGAATCAGAA
>416
GCAGCTAGGAGATTCCATGTCCTGGGAATCTCTGGAAACTGCTCACTCTGTATTCCTCA
TTTCCAACCAGTATCTGGACCAGATCCGCCAGACAATCTTTGAAAACCTGAAGATGCT
GAACCATGCACCTAGTGTCCAGA
>417
TTGGAAAAGGGTGCTGGGAGAAGATTAGAACTTAGAGTGAGATCCTGACCTCTCTCCC

CTTGCTTTGCAGGCCAGAGGCACCCAATGAGTTCTATGATGGAGACCATGACAATGAC
AAGGAAAGCGATGTGGAGATTTAA
>418
CAGGTAGGGGATGGCCCTGCCTCTTGTCCAGCCAGAGCAGCAGCAACCTCTCTTTTCC
TTTCCCTTACAGGAAGCTGCACAGTGGGATGAAGACGTACGGGTGCGAGCTCTGCGG
GAAGCGGTTCCTGGATAGTTTGCGG
>419
TGTGGCCCAGGACCTCCCCGCTCACCAGTGGACTATTGCTCTAGTGGTAACTGCCTCTC
CTTTCTTTCAGCGGGTGCCAAAGCCTTTGTCTGTGATCAGTGCGGTGCACAGTTTTCGA
AGGAGGATGCCCTGGAGACACA
>420
CTCCAGATGGGTAAAGAATGCACCTTGTATTCCCACCTTTCTGAGGCACCCCCTCTCCT
GTCTCCCACAGGCACTGACATGGCCGTCTTCTGTCTGCTGTGTGGGAAGCGCTTCCAG
GCGCAGAGCGCACTGCAGCAGCA
>421
TGTGCCCTACTGTCCCCTGAAGAGGGGGCAGGGAGAGGCAAAGGCCTGATCCAGCCC
CTTGTCTCCACAGGCGACCACCCCTACGAGTGTGAGTTCTGTGGCAGCTGCTTCCGGG
ATGAGAGCACACTCAAGAGCCACAA
>422
AGGGTGACATGGTGCCCTCACTGCCTTCTGTCTGTCCTCACTTTCTCCTGCCCTGTCCC
TCCGCCCTCAGGTGAGAAGCCCTTTGAGTGTAAGCTCTGCCACCAGCGCTCCCGGGAC
TACTCGGCCATGATCAAGCACCT
>423
GGGACTCTGGGAGTCTGGCCAGCCCCAGCTGCCGTCTTAGCTGGAGGGGCTCCCTCCC
TTCCTCTTGCAGGTGCTCCAGAAAGCAGGAGTTGAAGACCTGGGTGTGAGGGACACA
TACATCCTAAAAGCACCACAGCAGA
>424
GTCATTCCTGGTGCCTCATGGCTCTGCCTGCCAGCTGTGCCCCGAGGTGCTTTCTCGCG
TCCTTCTACAGGTTCCCAGAAGACAAACCCCCTAGGAAGACAGGCGACCTGTGAGGC
CCTAGAGCACCACCTTAAGAGAAG
>425
TTATTCCCCTCGTCCTCCTCCTGTTTTTCTTTCTCATCCTCATCCTCCTCTCTGCTTCTGC
GTTCTCCAGGGGACAAGGATATGCCTACTGCTGGGATGCCGAGTCTTCTCCAGAGTTC
CTCTGAGAGTCCTCAGAGTTA
>426
AGACTCATTTTCTTTTTTTGTCTCTTCTGTCTCTTGTGTGTCTGTGTGCCTGTACATACCC
CTCCCTCAGACTCCCGAAGCCCAGACAGCTCCTCCCCAAATCCCCTTCCCCAGGGAGT
CCCTCCCCCTTCTCCTCCTGG
>427
TTCCATCACTTCTGTGACATTCTGCTTCCCAATAAGTCTTCCTGTGACTTCCCTATTTCC
CCCATCCCAGATTAACTCAACAGTGTCACTCCCTGGGGGTGGGTCTGGCCCCCCTGAA
GATGTGAAGCCACCAGTCTTAG
>428
ATTGGAAACAGACTAAATGGTGAAGGTGTCTCCATGCAACCTTCTTATTGCTCCTCCCC

TTTCCCTGTAGGCAAACACTACGGGGTTTACAGCTGTGAGGGTTGCAAGGGCTTCTTC
AAACGCACCATCCGCAAAGACCT
>429
GGATATTTGTGGAACCCCTTCATGGGTGGATGCTGACTGTACATTTTTTCTGTCCCCCTT
CCCCCTGCAGCGGTACAGGAGGAGCGTCAGCGGGGAAAGGACAAGGATGGGGATGG
GGAGGGGGCTGGGGGAGCCCCCGA
>430
AAAGCCTCTTACAAGGGGGCTCCCAGCATATCTCAAAATCTTCCATAACTCTTACCCCC
GTCCCCTGCAGCCAAATGACCCTGTGACTAACATCTGTCAGGCAGCTGACAAACAGCT
ATTCACGCTTGTTGAGTGGGCGA
>431
TCTCTTCCACTGATGTGCTTTGAATCCCTTGGCCTGATTTCTGGCTCCTGACCCTTGCTG
CCCCACCCAGGCTGGAATGAACTCCTCATTGCCTCCTTTTCACACCGATCCATTGATGT
TCGAGATGGCATCCTCCTTGC
>432
GCTATAGGCTGGTCCGTGTCCAAGGCTGGCTGAGCTGTGACCTTTGAGTGACCTGCAG
GTCCCTCTCCAGGGTGCTGACAGAGCTAGTGTCCAAAATGCGTGACATGAGGATGGAC
AAGACAGAGCTTGGCTGCCTGAGG
>433
CACATCTGCATGGCCATCCTGATTTGGTTTGTCTTCATTTCTCTTCCTCCTCTCTTCCCTG
CCATCCCAGATGCCAAGGGCCTCTCCAACCCTAGTGAGGTGGAGGTCCTGCGGGAGA
AAGTGTATGCATCACTGGAGAC
>434
GTGCTATGAGGATGTGTTCAGGGCCCGTGTGCTTCCAGCTCTCAATCCCCTTCTCCCAC
CTCCACCCCAGGTTTGCCAAGCTGCTGCTACGTCTTCCTGCCCTCCGGTCCATTGGCCT
TAAGTGTCTAGAGCATCTGTTT
>435
GCCTCTCCTCTGACAGGGCCTCTCATCATGCCTATGCCTACTTCACCTCTGACTCCTGCC
TTGGTTACAGGAGGTGGAGGCCGGAAAGGTCGCACCAAGAGAGAAGCTGCTGCCAAC
ACCAACCGCCCCAGCCCTGGCGG
>436
TGAGTGGGAGAGGCAGAATTAGAACCCAGATCTCTGTCCCTTTTACCACCTGCTTTTCC
TCACCCCCCAGATGAGGCCAGACCATGGACACTACACCCAGCAATAGAGACGGGACT
GCGGAGGAAGGAGGACCCAGGACA
>437
GCCTTGCCCCCTCCGGATTCAGGCCGATGGGGCTTGAGCCCCCCTGACCCTGCCCGTG
TCTCCCTCGCAGCTGGGCGCCGTGTACACAGAAGGTGGGTTCGTGGAAGGCGTCAATA
AGAAGCTCGGCCTCCTGGGTGACT
>438
GGGACAGGGGACCGGCTGGAGACAGGGCCAGGCGGGGCGTCTGGGGTCACCAGCCG
CTCCCCCATCTCAGTCTCCCGGGACCTGCCCGTTATGATCTGGATCTATGGAGGCGCCT
TCCTCATGGGGTCCGGCCATGGGGC
>439
TGATCTCCCCTCCTGGAGGCCAGGCCTGGGCCACTGGTCTCTAGCACCCCCTCCCCTG

CCCTGCCCCCAGGTAACTATGGCCTTCGGGATCAGCACATGGCCATTGCTTGGGTGAA
GAGGAATATCGCGGCCTTCGGGGG
>440
GAGGCAAGGGGCGGCTCAGGGGAAACTGGGAGGTACAAGAACCTGCTAACCTGCTG
GCTCTCCCACCCAGACCCCATGCTGCACTATGTGGGCTTCGTCCCTGTCATTGATGGAG
ACTTCATCCCCGCTGACCCGATCAA
>441
GCAGGTGGAGAGCAGGGCTTCAGCCCCCTGGGAGTCCCCAGCCCCTGCACAGCCTCT
TCTCACTCTGCAGGGACCCCAACATGGGCGACTCGGCTGTGCCCACACACTGGGAAC
CCTACACTACGGAAAACAGCGGCTAC
>442
CCTGGGGAGGGTGGGATGCTGGGTGGGCTCTCCTTGCCTGCTTACTTTCGTCCTCCTTT
CCCCTGCCCAGGGGTGTGGGGTACGGATACAGAGGAGCGGCTGGTGGAGCATCTCCT
GGATCCTTCCCGCTACAACAAGCT
>443
AGTTCGTTTCCTTAACCTTGAGCCCCACCCAGGGCACAAGTTGGTACTGCCTCCCTCTC
TCATTTCCCAGGAGTGGGAAGATTATCGCCTCACCTGGAAGCCTGAAGAGTTTGACAA
CATGAAGAAAGTTCGGCTCCCTT
>444
AGGGGCTGGGTTGATGGGTAAGGAGGAAGGAACGCTTAGGCCAGGGCTGACTGTGCC
CATCCTTTGGCAGTGCTGACGGCATGTACGAGGTGTCCTTCTATTCCAATGCCGTGGTC
TCCTATGATGGCAGCATCTTCTGG
>445
ACCCGCTTATACTCGTTTGTCTCCCATCCTGCATCATGTGACCTGGGCCTCCTCCGTCTC
CTCCATCCAGGTGAGTGAGGACTGGAAGTACGTCGCCATGGTGATCGACCGCCTCTTC
CTCTGGATCTTTGTCTTTGTCT
>446
AGTGGCAGGGATCCTGGCTGTCTCTGCTGTGTCCCTGCCTCTGTACCTGAGTGTTTCTA
ATGACTTGCAGGTGGAGCTGGAGACCTGGTCTCTCTAGGGCCTACCCTGAGCTCACCA
TCTGAAGGAGAGTGCCATCATCC
>447
TCCCTGTAAGTCTCTCCGCACTCTTCCCCACACCCACCTCCACTCCAGCCCTGATTCTG
GCCTCCCACAGCCCAGCGCAGACTGTCCGAAGCTCTGAAAAGGCTACGTTCTGGGCCT
AAGTCTAAGAACTGGGTCTACAA
>448
ATGAGGTGGCTTGAGGGGCAGGGAGGGGGGCGCAGGGCTCTGCATACCGGATTTCCC
ATCCCTGTTTCAGATCCTTGGAGGTTTTGCCCCTGAGCACAAGGAGCCCACCCTGCCC
TCGGACAGCCCACGGCACCTCCCTG
>449
TCATCCAGGCCAGCTGCCCCCTCTGTTCTTCATGCCCGCAATGCCTAAAGGCGCTTCCC
CCCACCTCCAGGTCCCAGTGGCTGTCAAGTCCCTCCGGGTAGGTCCCGAAGGCCCGAT
GGGCACAGAACTGGGGGACTTCC
>450
GTGCGGCAGGAGCGTGGGCGGCCAGGGTCCAATGGGTCCGGCTCACGCGGCGCGGTG

TTCCCTCCTGCAGGTGATGGAGCTGGCGCCACTGGGCTCCCTGCACGCGCGCCTAACG
GCCCCGGCCCCGACACCCCCGCTGC
>451
GGTGCTCCGTGGAGGGTGGGCCTCCAAGGAGCACGTGTCCCGCCCCTTTCAGCTCCAC
TTCCTTCGGCAGGTGTGCCCCAGAGAGCCTGCGCCACGGAGCCTTCTCGTCTGCCTCG
GACGTGTGGATGTTTGGGGTGACG
>452
ATTCCTGCTCCCATATTACCTATCTGACTCAGGCCACAAGCCCAGCCACCCTTTCCTCTT
GTCTCCACAGGCCGGGCCTTCGGAAGCATGTTGTGTGAGGGATGTCACAGAACCAGG
CGCCCTGAGGATGGAGACTGGTG
>453
CTGTGGGCCAGGGCCCAGCCCTAACTCTTGCCCTTATTCCCATCCTATTTACCAGCTCCT
CTTTCCACAGCCCCGACTCCACAATCTGGAAGGGCCAGAATGGTCGCACCTTCAAAGT
GGGCAGCTTCCCAGCCTCGGCA
>454
TGGGAGGGTATATTGATCTGTGGGGTGGGGAGAGTTGGTGTGGAGGATGAACTGGATC
CCTCTCCGACAGAGACAGAAAGAAGGCAAATCTTTGGGATGCGCCCCCAGCACGGGG
CCAGAGGAGGAACATGCCCCTGGAG
>455
GATCGCTTGAGCCCGGGAGGCGGAGGCTGCAGCCAGCCGAGTTCAAGTTGTTTCCTTC
TCAACTGTGCAGGCATTTCCAGGAGTCTGGAGTCAGTTCTGTCCCTCGGTCCTCGTCC
CACAGGGGGTGGTTCAAGCCCCCC
>456
GAAATGGCCTGGTGTCCAGAAGGGGCTACAGGCAGGGGCAGGGGCCTGAGTGAGGCT
TTGTCTGTCACAGATGGAGCTGAGTGTGCATGGGGTCACCCACCAGGAGTGCCAGAC
AGCACTAGGAGCCACTGGGGGAGATG
>457
TCTTGGGGTCCCTCCTCTCCTGCCCCTGCCGCCTGGCAGTCAGCTGCAACCCACCCTC
CTGCTTCCACAGGTAGATCAGCTCTTCCACCTGAGTAGCCGGTCCAGAGCTGACTGCT
GGCGCATCCTGGAGCATTACCAGT
>458
GTGAAGCCAGCAGGTGCTCTGGAGTTAAACCAGCTTTCTGCAAGCCCTGCTTCCTGGT
CTCCCTCTCCAGGTACTGGCTGTGATCGAACTTCTCAACCCTCAGAGACTTAGATCTTC
CACCTCACTCCCTCAGCCAAGCC
>459
CAGGCAGAGCTGGGTGACAGGCCTCGCACTGCTGGCCCTACCTGTAACTGGGCCTGTT
GCTGTCTCCTAGCACAAACTCTCAGAGCCCATCCCCACCCAGCAGTTCCATTGCCTACA
GCCTCCTGAGTGCCAGCTCAGAG
>460
GAGGTCTGGGGGCCCAGGCCACACTCTGGGACTAATGTCTTTATCTCTTTCTTCCCTGT
GGGCCCCGCAGCAGTGAACAGTCAGCCCGGGCAAGGACTCAGAAGGAACTCATGAC
AGCACTTCGAGAGCTCAAGCTTCGA
>461
ATGGCCTGGGCCTGGGCTCCCGTGTTGAGGCAGCCTCCTTACTGAATGCCTCACTGCCT

CCACTCTGCAGCCAACCAGGAATACTACCAGCAGTGGAGCCTGGAGGAGGGCGAGCC
TTGCTCCATGGACATGTCCACCTA
>462
GCATCCCGACGCACCCCTACTCCTTCTCCCACAGCTCTTGAATCTGCTCTTGCTCTCCC
CCTCCTGCCAGGATACCTTCTCAGTGGCTGTCTCCTTCCTGACGGGCCGAATCGTCTAC
ATTTCGGAGCAGGCAGCCGTCC
>463
GTGACCTTATTGTTGGAGGAGTGGGTGGCTCTCTGTTGAAAGTCCCTAATGCTCCTCTC
TCCTTTGCTAGAGGAGGTCCTGACCGGGATCCAGGGCCTCGGTACCAGCCATTCCGCC
TAACCCCGTATGTGACCAAGATC
>464
AGGCAGAGTGGAGGGCTGAGGAGTTGGGCATTCTGGACGAAACCCAGTGACCACACT
CTCTGTGCCTCAGCTCCCCGGATACCCCCTGACAAGAGGATTTTCACTACGCGGCACA
CACCCAGCTGCCTCTTCCAGGATGT
>465
TAGGACCTAGAGGAGACGAGGGCAGCCAGGGCTGAGGCCACGGCCACTCTGATGCCT
CCTTCCCGTTCAGGGCTGCCCCCCTGCTGGGCTACCTGCCCCAGGACCTCCTGGGGGC
CCCAGTGCTCCTGTTCCTGCATCCT
>466
GCGTTGGGGATAGGGCAGTGCGGTGGGGGACAGGACCGGGCCAGGGCTGGATTCACT
CTTCACTCTACAGGGCCCCCCTGAATGAGGACGTGTTCACTCCCCCGGCCCCCAGCCC
AGCTCCCTCCCTGGACACTGATATC
>467
GAGCCTGGGAGGTGAGAAAAGGTGTGGGAAGCGGGGTCAAGCCATCTAACCTGCCCT
CTCCCTGCTGCAGCCCGTCCACAGCCCCAGCCCCACGGGACTCTGTGGAGTCGGCGCC
GTGACATCCCCAGGCCCTCTCCACA
>468
CCAACCCATTTGCTGTTTTTTTCCATGTGAGCTTGAGAAGGCTATTTTCCTCTTTCTCTC
TTTGCCCCAGCTACAGGCACGTTCAAGGCCAAGGCCCTTCCCTGCCAATCCCCAGACC
CAGAGCTGGAGGCGGGTTCTGC
>469
CTCCACCTCGGGCTCTAACCCTGCCCACCCCAAGCCCTGCTCTGATGCCCTGTGCTGTG
TCCATCCCCAGGTACCTGGAGAGCTGCAACCTCCCCAGCACCACTAAGCGTAAATGTG
CCTCCTCCTCCTCCTATACCACC
>470
TCCCTGGGATCCTCTCTCCTGACTTCCCAGTGGGGGTGGGGATGGTAACTGGCACCATC
TCTCTGCACAGATCCGCCGTCAGCAGCGCTGTCTGGGGAGGGGGCCACCCCACGGAA
GGAGCCAGTGGTGGGAGGCACCCT
>471
GAGGGTGTGGTATGGATGTGTTGACCCCTGAAGTGGTTCTGATGACCTTTCCCTATCTC
ACTCTCCTCAGACATCATCATGATGGAGGACCTGCCTGGTCTAGCCCCAGGCCCAGCC
CCCAGCCCAGCCCCCAGCCCCAC
>472
AGGGCAGTGTGGGTAGCAGGGGTACTAACCCCAGGTTGAGGTCCTTGCTAACCCTAGT

CTCTCCCCACAGGCTGCCACCACGGCCCCGCACCCCCAAGCCGCCGACACCACTGCC
GATCCAAAGCCAAGCGCTCACGCCA
>473
GTAAGAGCAGTTGAAGGGAGGCCTAGGTGCTGACCCCTCCATCCCTCCTTGCCCCCCT
CCCCTCCTCCAGGCGGAGGTCACTGAGTCCTCCAATCAGGACGCACTTTCCGGCTCCA
GTGACCTGCTCGAACTTCTGCTGC
>474
GGCCTTGTGAGGTCCCAGGAGTGGGCATGCAGCCGGCCTGACTCCCATTGGTCTGCCC
CCCACTTCACAGGCAGCAGCCAGAGCAGCCACACAAGCAAATACTTTGGCAGCATCG
ACTCTTCCGAGGCTGAGGCTGGGGC
>475
TGCCAGCTTCATGGGTCCTGGTCCGGGTCCTGGATCCCAGCCTCTGCCTTCTGAACCCC
TTCTTGGGCAGGGACATGACCTCTGTGCTGAAGCAGGATCGGGAGCGGCTCCGAGCCA
TGCAGAAGCAGCAGCCTCGGTTT
>476
GGGAAGCTGCGCTGGCAGGTTAGCAGTGAGAACCCTGTCTGACTCTCTCATGTCCATT
TCTCTCACCAAGGCCTGTGTGGACTGTGGGAGCAGCACCCAAGATCCTGGTCACCCTG
ATGACCCACTCTTCTCAGAGCTGG
>477
CACTTGGGAGGGGGCAGGGGAGCCCAGGCAGGCCTGGTGGGGAGCTGACCCACCGC
CGTTTCTCTGGCAGGTTCTGACGCGATGAGGAAGCACCTGAGCTGGTGGTGGCTGGCC
ACTGTCTGCATGCTGCTCTTCAGCCA
>478
TATCCTCACAACAGGCCAATGTTGGCATCTTTGGACCTTTCTAAACATCAACATTTCTGT
TTTCCTTTAGGAATCGTCTACGAAGCCTGATAAAAAGGTTTCTGTCCCATCAGGACAGA
TTGGTGGCCCCATTGCTTTGC
>479
TGCCAGGTTTGGTGTGAGTGAGCCACGAACAGGACTGGAGTTCTTACCGCTTGCTGTG
TTTCTTCCCTAGAAAGGAGCGAGTGGGACTGTGCTGCTGGCCGGCTCCTCTTTGATGG
CTTCACCCTACAAATCCAGCAGCC
>480
GGAGCTGCTTTCCTGTTCCTGTCTTCAATGTGTGTGTTTTGTTCTTTTCTCCCCTCCTGT
TTTCTCTCAGGTCTTCTGGCTGGCTTGCACTCCAGCCCGCCCCATGCAGCGCCTCTCCC
ACACGCTGCGGTGCCCACCCA
>481
GTTACTACTGCCTTAAGGAAATCAGAGAGCATGAAAAACTTGCGTTCTAATTTTTCTTA
CTCTTTTCCAGGTGCTTCTCAGCTTCATGGGAAAGGGCCTGCTGTACCACGGAAATTGT
GACCGCTTCAGAGGCAAGGCTT
>482
AGTAGCTGGGAGTGTAAGGAGAGGACCCTCGTCCCCTCACCAACCTCATCCTCTCTCC
CCCTACCCACAGGTAGCCTCATGGCTGCAACCTGTGAGATTAGCAACATTTTTAGCAAC
TACTTCAGTGCGATGTACAGCTC
>483
AAAAAAAATGGGGCTGTAAGGTCTGCTGGGTGGCCTGAGCTGAGCCTGTTTCCCTGCC

TGGCCCTTGCAGAGAAGGCCAGCTGGTTGGGGGAACAGCCCCAGTTCTGGTCGAAGA
CGCAGGTTCTGGACTGGATCAGCTA
>484
GCTGCACAGCCAGAGAGAGCCCTTGAGGGAGGGATTAGGGGAGTGTGACCCTTCCTT
CCTTCCTTGTCAGCTTCCAGCTCTTCTGATGAGCTCAGTTGGATCATTGAGCTGCTGGA
GAAGGATGGCATGGCCTTCCAGGA
>485
CCATGGGCAGCATCACCTGTCCTGGTCTGGTCCCCTGAGCCCTCTCTGAGTTCTCACCT
CCTCTTCCCAGACCAGGGCAGCCCCTTTGCCCAGGAGCTGCTGGACGACGGTCAGCA
AGCCAGCCCCTACCACCCCGGCAG
>486
CCTGAGAGCCAGCAGCGTGGTTGAACAGAAGGTGGGCCGGCAGGGGACTTACTCTGA
CCCCGCCCCCCAGGGACTGGTGCTTCTCGGAGCTCCCACTCCTCAGACTCCGGTGGAA
GTGACGTGGACCTGGATCCCACTGA
>487
TCCTGTAGAGGGGCTACTCTCCCTAACTCCCCTCTTGCCCCTCCTTGACCTTCCACCAC
CGTCCCCACAGATGGTTTTCGTGACTGCAAGAAGGGGGATCCCAAGCACGGGAAGCG
GAAACGAGGCCGGCCCCGAAAGCT
>488
GGGCAGGGCTGGCTGGCCTTGGGTGAGAGGGGACACCTGGATGGCAAACTGATGGAG
GCTGGCCTTGCAGCGCCCAGAGGCACCCACCTGTGGGAGTTCATCCGGGACATCCTCA
TCCACCCGGAGCTCAACGAGGGCCT
>489
GGTGGGCGGGCTGGCAGCCTGGGGCCCATTTCCTGCCAAAGCACCTCTGACCATCCTT
CTCTTCACCCAGGTACTACTACAAACGGGAGATCCTGGAACGGGTGGATGGCCGGCGA
CTCGTCTACAAGTTTGGCAAAAAC
>490
CGGTAAGCCCACCCCCAGGAACCCTGGGCCCGCCTCCTGGTCCCGATCCCATCCCTGA
CTCCGCCCTCAGGATCGCTCGTCTCTGGTAGCTGCAGCCAAATCATAAACGGCGAGGA
CTGCAGCCCGCACTCGCAGCCCTG
>491
GGGGTTGAAGATGAGAATGGAGGTAAGGATGTAGTTGCCACAAAACTGACCTGCCCTC
CGTGGCCCACAGCTCCTACACCATCGGGCTGGGCCTGCACAGTCTTGAGGCCGACCAA
GAGCCAGGGAGCCAGATGGTGGAG
>492
TGTGTGTCTGCCCTCTTCAAGGAGGTCCTCTGCCCAGTCGCGGGGGCTGACCCAGAGC
TCTGCGTCCCAGGCAGAATGCCTACCGTGCTGCAGTGCGTGAACGTGTCGGTGGTGTC
TGAGGAGGTCTGCAGTAAGCTCTA
>493
CCTTTCTGAGCCCACATCCATTTCCACCTCTCTGTGTTTTGAATTCTGACTCTCCCTCTC
TTCACAACAGGGTGACTCTGGGGGGCCCCTGATCTGCAACGGGTACTTGCAGGGCCTT
GTGTCTTTCGGAAAAGCCCCGT
>494
GTATGTCCCTAGCCCTGGCTGCTGAATTGTTGCGTTCCTGTTGTGTGTTTATTTTTCATAT

TGGCTGAAGACCAAGAGGGAAGAAGCACAGAATTCCTCAACTCCCAGTGTGCCCATG
AGTAAGAGCAAATGCTCCGTGG
>495
GCCCAAGCTCCCACGAGGAGAGATGGGCCCTTCCGAGGCCCCAAACTAAAGGTGCTT
TTCTGCCCCACAGGTGCCTTCCTGGTCCCCTACCTGCTCTTCATGGTCATTGCTGGGAT
GCCACTTTTCTACATGGAGCTGGC
>496
AGGGACACGTTGCTGATGGTGGCTCTGTGCTCCACCTGCGTGGGCTCCATGGCCTCCC
CCTTCCCGCTAGGTGTGGGCTTCACGGTCATCCTCATCTCACTGTATGTCGGCTTCTTCT
ACAACGTCATCATCGCCTGGGC
>497
AAGCTCAGCCGTCCAGTTCCAGGTGGGTTGACAGCCACCCACAGAGTCCCCCGCTGA
CTCCCCTCTGCAGACGTGGCGTGCTGCACCTCCACCAGAGCCATGGCATCGACGACCT
GGGGCCTCCGCGGTGGCAGCTCACA
>498
CCTTGTGCCAGTGTCTGCTCCCACCAAGGGCCCTGCCTGTGCAGCCTGGTGACACTGT
CCTCTCCTGCAGGTGGTATGGATCACAGCCACCATGCCATACGTGGTCCTCACTGCCCT
GCTCCTGCGTGGGGTCACCCTCC
>499
GGTGCTCAGGTCCTTTGCCTGTGGCCTGCGTTCTCCTGGGACAGCCTCTCACCGCCCT
CTGGCTCTGCAGGTTTGGATTGACGCGGCCACCCAGGTGTGCTTCTCCCTGGGCGTGG
GGTTCGGGGTGCTGATCGCCTTCT
>500
AATAATGACTGGAAAATTGCAGCTGCTGCAGCTCAGCTGGTTCCTGAGACGGCGTGTC
TTGTTCTTACAGGGACGCGATTGTCACCACCTCCATCAACTCCCTGACGAGCTTCTCCT
CCGGCTTCGTCGTCTTCTCCTTC
>501
CGGGGTGGGGCAGGATGGGCGGGAGAGCACAGCGTGGGCTCTGTGGCAAGCAGGGC
GGGTTCTGTTTCAGGGCCAGGGCTGATCTTCATCATCTACCCGGAAGCCATCGCCACGC
TCCCTCTGTCCTCAGCCTGGGCCGT
>502
GTAGGTCTTGGCCCAGGCTGCGGTCAGGAGCGCCGCCAGCCTGCAGCCCGACCCTGT
GCTCTGTGTGCAGATGGGTGGTATGGAGTCAGTGATCACCGGGCTCATCGATGAGTTCC
AGCTGCTGCACAGACACCGTGAGC
>503
TGAATCCCCAGGCTGGGTTTACCTCTGGGGAAAGCCCTTCTGTAGGTCCACAGGCTCC
CCTCTCTTCCAGGGTGGCATCTACGTCTTCACGCTCCTGGACCATTTTGCAGCCGGCAC
GTCCATCCTCTTTGGAGTGCTCA
>504
TGCCCGCTCCCTGCTTTGTCCTGGCACCGCCGGCTGAGAGCTGCCTGACCTCCGTATCT
GCTGGTTGCAGTTCGTGGTCGTGGTCAGCATTGTGACCTTCAGACCCCCCCACTACGG
AGCCTACATCTTCCCCGACTGGG
>505
GTGAGTCAGTGGTGGGTGCTGGCAGTGGGTACTGGTCTGCACTGACCCCTGCACTCTC

TTTCTCTTTCAGAAACTGGCCTACGCCATTGCACCCGAGAAGGACCGTGAGCTGGTGG
ACAGAGGGGAGGTGCGCCAGTTCA
>506
TAGCCACCTTCAGCTGCTCTTAAATGGGGCATCGCCCCAGGGTTTCTGACTAAACCTGT
TTTCTTTCCAGCTCCGCCACTGGCTCAAGGTGTAGAGGGAGCAGAGACGAAGACCCC
AGGAAGTCATCCTGCAATGGGAGA
>507
CCCTGAACAATAAGCACAATGCAGTTTTAAAGACTGTTTGCTTATTTGATGAAATTTCG
TACTTTTATAGGAGAATGGGGTACTAAGAGAAAAGCACGAAGCTGTGGATCATAGTTC
CCAGCATGAGGAAAATGAAGAAA
>508
AGATAAAGCCTAAAGCCCCAACCAGTTCAGTCTTTTATGTGAAAGATGCACTTTTTGTG
TTTCTCTACAGAGACATCTGAATGGGACAGATACTTCTTTCTCTCTGGAAGACTTATTCC
AGTTGCTTTCATCACAGCCTG
>509
TTGTTAGGTAATAGAACTGCTTCAGAAATAGGGCTATTCATGTTTGAAGTGTTTCTCCTT
CGTTTTTCAGGGCATCTCATTGGGAGATATTCCTCTTCCAGGCAGTATCAGTGATGGCAT
GAATTCTTCAGCACATTATC
>510
AGAGTAAACAGGAGGCATAGCTGCAGCTTCTGTGGCAGAGCTTGCCTTAACTTCTCAT
TCTCTTCTTTAGCCCCCAGCCCAATTGCCATCAAGACTCCTGAAGCCAGCTGTGCTTGA
CCAAGGATGGGCCATAAACAATG
>511
TTCCCAGCTGGGAGGGGCGGGGACAGGTGTGCGGCTCCTGCTTCTCCTGAGGGCCTC
CCTTTCCCCGCAGCTGTGAGGTGGAGCAGTGGGACTCGGATGAGCCCATCCCTGCCAA
GGAGCTAGAGCGAGGTGTGGCGGGG
>512
GATAGTCCCCAGTGCTGTGGCTTTGAGTTCCTCGAGGAAAGATACTAACAATGCACTTT
CTGCACAACAGGGGCCAATCTCAAAGTCATCAGCACCATGTCTGTGGGCATCGACCAC
TTGGCTTTGGATGAAATCAAGAA
>513
AAATGTCCCAGCAGTAGACATTGGTAAATATGGTTGAGGCTGATGTTACCACCAGATGT
TTGATTCGTAGTGGGATCCGAGTTGGCTACACCCCAGATGTCCTGACAGATACCACCGC
CGAACTCGCAGTCTCCCTGCTA
>514
TGCCGGGTTCTCAGCGGCCCCATCTTGGTCCAAGGCTGGGCCTCTCACCTGCCCCTCT
CTCTCCCCTCAGTGGTGGCTGGACCTCGTGGAAGCCCCTCTGGCTGTGTGGCTATGGA
CTCACGCAGAGCACTGTCGGCATC
>515
TCCGGAAATGCTGGGTGGTGTCCCTACCCTTTGCGGGACGGGAACGAGACATGGACTC
TCCTTGCTCTAGGCCAGGCCATTGCTCGGCGTCTGAAACCATTCGGTGTCCAGAGATTT
CTGTACACAGGGCGCCAGCCCAG
>516
GCTGGTCTCCCGCCATCTGGTTGTCCCTAGCCTGGGACTCAGTGCCTGATGGAGTCCTG

CCCTCCCTCAGTGTCTACCCCTGAGCTGGCTGCCCAATCTGATTTCATCGTCGTGGCCT
GCTCCTTAACACCTGCAACCGA
>517
GTTGTTCTGCCTCAAAGGTGGCCTGGGCGGAGGGATCTTCGGGGTACCCATGTCACCA
CTGTCATTCCAGGGGCGACGTCGTAAACCAGGACGACCTGTACCAGGCCTTGGCCAGT
GGTAAGATTGCAGCTGCTGGACTG
>518
GAAAACAGCCCAGCTGAAGGCTGCTGAACCACCCTTCTTATCTCCCTCTCTCTCTCTCT
CTCCTTTCCAGTGATTCTGCCCCACATTGGCAGTGCCACCCACAGAACCCGCAACACC
ATGTCCTTGTTGGCAGCTAACAA
>519
TTGGAACTGGGCGGGGGGTGAGTATGTGCTGGGTGGGGCTGGGCTCCCCAGTGACCT
CCCCGCCCCCCAGGCCGGCTGCATCCCAGTGCTTCTCAGCCCCCGCTGGGAGCTGCCC
TTCTCCGAGGTCATCGACTGGACCA
>520
CCCTACTCAGGTCATTCTTCATGGGGTTCTGTTCCCTCCCACTCTAATAGCCTGTGTCTC
ATTCCCCCAGGCTCCCGCCCTGAGGGCAGATTCAGCGCCCTGATCTGGGTGGGGCCCC
CAGGCCAGCCCCCTCTGAAGCT
>521
GCCTCCATTTCCCTGGATGTTCTAGGCATTTAGGAGTCCAACCACACAGCTCACTTGAG
TCCCTCCCCAGGTGGACTTTGCCTTTCTGGTGTGGCAGAGCTTCCCAGAGCGGATGGT
GGGCTTCCTGACGTCGAGCCATT
>522
AGGAGGGAGGAGAATGGGGCCTGGGGATGGATTTGGCTGCAGCCTCTCCCTGTCTTTT
CCTCTTGCCCAGGTATTACCACACTCTCTTCACCCACTCCCTGCCCAAGGCTCTGAGGA
CCCTGGCAGATGAGGCACCCACC
>523
CTTGCTGCACGGGCGTGGGGAGTCGGAAGGGCAAAGAAGCCCGTTAATGCATTGCTTC
TTTCCCTCTTAGGCGCCTGGGGGCCCGGGGCCCAGGCCAAAGCCGCCTGCCCCAGCCC
CCGACTGCATCAACCAGATAGCGG
>524
GAGGAGCAGGGGTTTCCTGTCAGTGACGCAGCCCGTGAGAACCTCTCTCCCTCCCTCT
TCCTCCGTTCAGGATCCTGTTGACTGTGGTGGTGATCTTCCGGATCCTCATTGTGGCCA
TTGTGGGGGAGACGGTGTACGAT
>525
GCGGTGGGCTCATGTTTGTTGATTGAGTGAATGATTTAATTAACGCCTGACTCTGCTTTT
TCTCCCTCAGAGTTTACCTTCCAGCAGCCCTACGACCAGGCCCACCTGCTGGCAGCCA
TCCCACCTCCGGAGATCCTCAA
>526
GGGAGGGATTCCCATCACTGCCAGCCGTTGTCCCACGGCTCACTCGGCCTTTCTGGCG
TTCTCTCCCCAGGCGAGAAGCCCTTCTCCTGTCCCCACTGCAGCCGTGCCTTCGCTGA
CCGCTCCAACCTGCGGGCCCACCT
>527
CAAAATAATATAGTACTATGATTTTCCCTTTACTTTATAAGTAATTTTGTGCTGTTCCTTTT

TTATACAGCCATTGATTATTATTATTCCTAAAGAAAATGAAGATAATTACATATTTTTGCA
TTTGGGCAGTAGCATGG
>528
GTGTGTGCACGCTCACACACATATTCACAAATAAGAACCTTTTCAATAGCCAGTATTTT
CTACTTGGCAGGTTCCTCAAAGCAAACCACTGGAGAGACATGTCGAAAAATCCATGAA
TTTGCATCTCCTAGCAAGATCAA
>529
TCCAAGATTGCAATTTGCTTTCCTTCAAGATCATTGACACTCATAATTGATTGAATTGTT
TCTTTTTCAGGATGAGTTAAATGCCAGTGGAACCATCAAAGAAAGTGGTGTCCTGGTG
CATGAAGGTGATAGAGGAAGGC
>530
TTCCTCAGATCAATGCAATTTTGCAGCAAATATTCACTAGTTAATCATTCTTTCCTCCATC
CTTCCATAGGGAATAGAAATCAAGGGTCCCAGCAGTGGCAACAGAAATATTACCAAAG
AAGTTGGGAAAGGCAACGAAG
>531
AAGGGTTCATTCACTGTTTTAAGGAAACGATTTATAAATGTTAATGGGATGTTTATTTTT
TCTCCTATAGAAAGCTAGACTGGCCAGGATCCGGGCAGCCAAAAGCGGAAGCGCAAA
TGCTTACATGCAGAGCAAACGGA
>532
AATATGTAGCCGAGGTTCGAGTTGTTTGCAAATAAAATAGAAAGCTCTTCTGTCTTCTC
TTTGTTAACAGTCCTCAGAGGATGAGCAGGCTTTTGTTAGCAAATCCGGCTCCAGCTTT
GAAACCCAGCACCACCACCTGC
>533
ATAAAGCTATGTATTTCGTTTTTAAAAATGTGCTTCTCTGTTTCTTCATTTACTTACAACT
GTGGAACAGAATCACGAGTTTGTGGACGAACAAGTCTTTGAAGAAAGCTGCATGGAA
GTTGCAACTGTTAATCGTCCTT
>534
TATTCTTCAGTTGTATGAAAAACATTTTAAGTAAACAACTTACTTTCCTAAATATTTTTTT
TTCTATCAGCCGATCCAGTTTAAATGCCAAAATGGAAGAGTGTGTTAAACTAAACTGTG
AACAACCTTATGTGACTACA
>535
GGAGTGGGGAGGGCTATCAGGCCCAGAGCTCAGCGGCCACTGCCCCAGCTCACGGCC
GGCTCTGCTGCAGGACCCCTTGCAGTGGCTCGGCTCCCCACCAAGGGGCTCCTGCCCC
AGCCCCAGCTCCAGCCCCAAGGAGC
>536
AGAAGGGTAGGCGGGTCCAGGCCAGGATCAGGGGGTTGTGGGGGAAGGACTGACCC
CACCCTGCCTGCAGAGCTGCAGGTCGCCTTCCAGGAGTTTGACCGAGACCAGGACGG
CTACATTGGCTGCCGGGAGCTGGGTGC
>537
AGAGGAGGGAGGGCCAGGGAGGGGCAGGGGTCTCTGATGCCCTGGGTCGTGTAATGG
ACGGGACCCCCAGGTGGCGGAAAGGTGGACTTTGAAGACTTCGTGGAGCTGATGGGC
CCCAAGCTGCTGGCAGAGACGGCAGA
>538
TGCCGTGAGTGAGGGGCTGCGCGTGTGAATGACTGTCAGTGACCAGACGCCACAACC

AACGCCGCCACAGTTCGACACCAATGGGGACGGCCGCATCAGCGTGGGCGAGCTCCG
GGCGGCCCTCAAGGCCCTGCTGGGGG
>539
GGGTGGGGGGTGATTGCTGGTCGTTTGTTGTGGCTGTTAAATTTTAAACTGCCATGCAC
TCGGCTTCCAGTATGCTGGGAGCGGTGAAGATGGAAGGGCACGAGCCGTCCGACTGG
AGCAGCTACTATGCAGAGCCCGAG
>540
GTGGGAGGTCTGGGCACCCTCCAGCCGCGGTGCTAACGCTGTCGCCTCCCTCCCGGGG
CTCTGTCCGCAGGGCTACTCCTCCGTGAGCAACATGAACGCCGGCCTGGGGATGAACG
GCATGAACACGTACATGAGCATGT
>541
CCAGAACATAGTGGGAGAGAAACTGTAGGGATCTGCGGGGCCTAAAGCTAACATATTA
CTCTGGGGACAGGAAGCAGCTTACCACCCAGAAGTGGCCCCTGATGTGCGGCTTCGA
GCCCTGCACTATGGCCGGGAGTGTG
>542
GCCCTATTTCATAGATCAGGGTAGACCCTAAGATGTGATGCTCTTTGTCCCCTGCCCGCT
TCTTGCCCAGACCCTGCAGAAGCCAAGCCCAGAACTGAAGCAGCAGTTGACAGGACA
TTCAAAGCGTGTGGCTGGTTCCG
>543
ACCATGCTCCCTGGTGCTTTTGACCTCCCCACTCTGCCTTAGCCTCTTTCCTCTTCCTTC
ATCCCACCAGGAACAGAATGGGTAGACCCAGAGGACCCCACAGTCATTGCTGAGAAT
GAGCTCCTGGGAGCTGCAGCCGC
>544
CTCACCATGGCAGTGTCCCCACCTTCATCTCTGACTTTACATCTCTTTCTGCAACCTTCG
CTTATTGCAGGAGGCAGATGAGTCCTTGAACTTTGAGGAGCAGATACTAGAAGCTGCC
AAGTCCATTGCAGCAGCCACCA
>545
CTTGAGAAGGCTGGAGTGGGGATGGACCAGGGCAAGGGGCATATTTTTAAGCAAACT
CTCCCTGCTTTAGGCCCGGATGGTGGCTGCGGCCACCAACAATCTGTGTGAGGCAGCC
AATGCAGCTGTACAAGGCCATGCCA
>546
AGAATTGAGGTACTGTCTCAACTGGGACCGTACCTGAGTTCTCAACCTAGGCCACTTT
CTCTCTGCCCAGGCTGCTGGCAACGCAGTGAAGCGAGCCTCAGATAATCTGGTGAAAG
CAGCACAGAAGGCTGCAGCCTTTG
>547
GCTGGTCCCAACTACCCTATGCCCCTGCTGTGCATCCTCACTGAACTAAGTCCCCGATC
TCCACCCTCAGATCATCGCAGCACAGGAAGAAATGCTTCGGAAGGAACGAGAGCTGG
AAGAGGCGCGGAAGAAACTGGCCC
>548
TTTTACACACTCACCCCACCCATCCCTGGCTTTCTATTCATGGAACAACCTCTCTCTACA
ATCCCTCCAGATCTTCGCAAATACTGTGGACAATGCCCGCATCGTTCTGCAGATTGACA
ATGCCCGTCTTGCTGCTGATG
>549
GTAGGTGGCATGAGTTGAGAAGGTTCTGGATCAGAGATAGGGGCCCCTCTGATCACCT

CCACTCCTATAGGTATGAGACAGAGCTGGCCATGCGCCAGTCTGTGGAGAACGACATC
CATGGGCTCCGCAAGGTCATTGAT
>550
ATCACAGAAGAAAGGCCTTGTTGGAGCTCTGACCCTGAACCCTCCTCACTTTTGCCCC
TGTCACCTTTAGGAAGTAAAAGGCCTACAAGCCCAGATTGCCAGCTCTGGGTTGACCG
TGGAGGTAGATGCCCCCAAATCTC
>551
GGATGGCTGCCAAGGTGTGGGAGGGAGGCAGACGGAATGAGGGGCCTGATGGACTGT
CCCCATCCTGCAGATTGAGGAGAGCACCACAGTGGTCACCACACAGTCTGCTGAGGTT
GGAGCTGCTGAGACGACGCTCACAG
>552
CCAAAAGTGAAGGGATGAGCAGTCCTGGGACTCTGGGCTCACCCTGCCCCTCCTCTCT
GTGCCCCTGCAGAAGGCCAGCTTGGAGAACAGCCTGAGGGAGGTGGAGGCCCGCTAC
GCCCTACAGATGGAGCAGCTCAACG
>553
CCTCTACCTTTCTTGTCTCCCTTCTACTCCACGGGGCTGTTTATAACTTGGGCTTGGTCT
TCTGTTACAGTCTTGGTGATGCCTTGGACAGCAGCAACTCCATGCAAACCATCCAAAA
GACCACCACCCGCCGGATAGTG
>554
ACCACAGTTAATTTTCTGCAAATATTAAACAGTTTTACTGATCTAAAATATTATTTTTATC
TTACAACAGGGAAATTGGTTACAGTAAAATATTTACGACTAGATAGATACCACCATCGC
TTTCCCCAGGCTCTCACTTC
>555
AAGAACTCAAGTTCAGCAGGGACAGGCCCCACACCCTGGCTGCTGGCTCATGTTCTCC
TCCCACCCCCAGGCCTGCACTTTATAGACCAGCACCGGGCTGCGCTTATCGCGAGGGT
CACAAACGTTGAGTGGCTGCTGGA
>556
TCTACTCTTCTTTCTTGATGCATGGAGAATGCCAGCACTAATTTTTTTCTTTCCTCTCTTT
TCCTTTTAGGCACACCATATGATCCCTATAAGGCACTGTGGTTTGAGCGGAAGAAGGAC
CCTGTTACCAAGGAGTTAAC
>557
GTGCCCCTCCTCCAGGCAGCCTCCTAAGCCCGCCCCACCTAGTGATGCCAGGCGCCCT
GTGTCTGCCCAGGGGGCCGGGTCCTGGAGGTGGGCTTTGGCATGGCCATCGCAGCGTC
AAAGGTGCAGGAGGCGCCCATTGA
>558
GAGGGGTGGGCGCCAGGGTGCAAGTGACCCCCATGGCCCTCAGAGGTGACCTGGTGC
CATCCCCGACCAGGAGACGCAGGTGCCCGCGCTGCTGGAGGCCGGCTTCCGGAGGGA
GAACATCCGTACGGAGGTGATGGCGC
>559
TAATTTTCAAGCTCATGAGTGGTTGTAACTTTAAACGTTCACTAGAGATGGCTTTCTGT
CTTTGTTTCAGCCCGTCTGCCGTTTAAGCGCCTGAATCTTGTCCCAAAGGGGAAAGCC
GATGACATGTCAGACGATCAGGG
>560
TGCTGTGAACCGAGCTTCCTCCTGGGAGTTGGAGGGAGGGCCACCTGTCACTTGCCAC

ACTGTCTTGTAGGATCAGGAGCGTCTGGGCAAGCAGCTCAAGTTACGTGCAGAAAGG
GAAGAAAAGGAGAAGCTGAAAGAGG
>561
CTGTGTGCGTCCATGGTGCCTCCTTTCTCCCATTGCTCGAAGACCCCATCGGGTCTCTT
CTTGATTTCAGACCCTGGCCGGCTCCTGTGGGAAGTTTGCCCCCTTTGAAATTAAAGA
GCACATGGTCCTGGCCCCTCGGC
>562
AGGTTCCTGGGAGGTTTGTGAGCAGGTCTGTAAGGCAGCGTGATCCTGAGCCTGGGGT
TTTCCTTCTCAGTGATGTCGTCATCGTGGAGCGTGGGAAGGGCGACGGTGTTCCCGAG
AGGAGGAAGTTTGGCAGGATGAAG
>563
TACCTCCTCACTGTGCCCCTTTCCTCCAGCCCCAAAGACAGTTGTGAGTCCCATGTGTT
TCTTTTCTCAGAAGCTCCTGGACTATGAGGTGGACAGTGATGAGGAGTGGGAAGAAG
AGGAGCCTGGGGAGTCCCTGTCCC
>564
ACTTTGTTTTTAATAAGGCACACACTCTGCTTTTCTATCTGATGAACTCTTTTTTTTTCT
CCTTTTGAGGATGATGATGACGACATGGGAGAGGATGAAGATGAGGACGATGGTTTCT
TTGTGCCCCATGGGTACCTGT
>565
TTCCTCAAAAGAGTGCCTGGGCATAGGGGAGCAAGAACCCCAAATAAACTTCTGCTTT
CTAAACCACAAGGAGTGTGCCGACCCTGAGAACCATAAGGTCCGCCAGAAACTGAAG
GCCAAGGAGTGGGACGAGTTCCTGG
>566
TTTTGTTTTTTGGCCTGTGGTGATGGGTGGCTCCCCAAGCCTCATGCCCACCCATGTTC
CCTCCTGCCAGTCCTGGCCCAGCTGCTGCCGCTCCTGCACGGCAATGTGAACGGGAGC
AAGGTCATCATCCGGGAGTTCCA
>567
CCCGCTACCGCACTGGCACCAGGGTGGCTGCTGCCTGCAGTGCTGATGGCCGTGTACC
CTGTCTGTCCAGATTGGTGCTGAAGACATGGACGGCTTCCAGGCAGACACGGAGGAG
GAGGAAGAGGAGGAGGGCGACTGTA
>568
GGGTGGGGTCTTCCCCCAGGGAGCCCAGAGGGCCCAGCCAGCTCAAGACCCTCCCTC
TCTTGCCCTGCAGAGGTCCAAGCCCCGTGTGGAGCCGCTTCCGGAGCTGGGGGTGGT
GTGGGGGTGGACACCGGCAAGGCCAC
>569
GCCTTATAAACAACAGGATAGAAGATACTGCTCGACAGTGCCATCCCTATCATGGCCTT
GTTTTTTACAGGCCCAACGAAGGAACCACTTCTGGGAACTCATTGAGGAAAGAGAGA
ACAGCAACCCCTTTGACAACGATG
>570
CCTGCTGGGCCTTTACTGGTGGCTTTCCTTTCTGAGCACTCATGAGGCCCCTGTGTCTT
CCCTCATATAGATTCCAGGTGGATCTGCAGAATGGCAGCAGTGTGAAACCTCGAGCCG
ATGTGGCCTTTCATTTCAATCCT
>571
GGATAATAAGTTAATTTATTAACTGTTTTTTTTTGGTGGCGGGGGGGCTCTGTCTTCTGT

ATCTCTCTAGGTGGCTGTAAATGGAAAACATACTCTGCTCTATGGCCACAGGATCGGCC
CAGAGAAAATAGACACTCTGG
>572
TTTGGACACGAGTTTTCCCTGGAGATCGCTTTCTGCAGGCCTCTTGGTCCTGACTGTGG
CTTCTTTTCAGAGCCTGCCATTCGCTGCAAGGTTGAACACCCCCATGGGCCCTGGACG
AACTGTCGTCGTTAAAGGAGAAG
>573
CATTGCTGAAATTGCCACTACTCTGTCCTACTTGGTTAATTAAGGTTTTTTTTTCTTTCTT
TCTCAAAAGCTTTAATGTTGACCTACTAGCAGGAAAATCAAAGGATATTGCTCTACACT
TGAACCCACGCCTGAATATT
>574
GTTAGCATGTAACAAACACAAAATTTTAAACTAAGGGGAACCACTAATGGCATGTTTCC
TTTCCTTTCAGATGATAATTTACTGTGATGTTAGAGAATTCAAGGTTGCAGTAAATGGCG
TACACAGCCTGGAGTACAAAC
>575
AGGCTTCATGGAGGAAGTGCAGTGGGTTTCTTGGGATCCCCACCAGGCACCCCTTCCT
CCTTCGACTTAGGGTGTGGCCGGCCGCAGGTTTCGGATGCAGGCGGCCGGATCGTGGG
GGGTCACGCTGCCCCGGCCGGCGC
>576
CTGGGCGGGGCCGACACTGACCTCCAGCCTCCGCAGCTGGCCCCTCTCACCCAGCCA
CCTTCCCTCCCAGGTCCCTGAACTCATCCGACTACCAGGTGCACCTGGGGGAACTGGA
GATCACTTTGTCTCCCCACTTCTCC
>577
CAAGGTCTGCCATGCCCCCAGTGCACGGCCTGGTGCCCCAACCTTCTCTGAAGCCTGT
TCCCCCAACCAGAGCCTCTGCCACCTCCGTACAGCCTGCGGGAGGTGAAAGTCTCCGT
GGTGGACACAGAGACCTGCCGCCG
>578
GGGGCGGCTGAAGCCCAGGGAGGGGCTCCCCATCTTCTCGGCTCCCAATGCTCGCCTT
CTCTGTCCTCAGGACGACTCCGGGGGGCCTCTGGTCTGCCAGGTGAACGGTGCCTGG
GTGCAGGCTGGCATTGTGAGCTGGG
>579
TCCGAACATCTATACTATTTGATCTCTCTAACCTGTGTTTTGTGATATTTTATCATTTGTTG
TACTCCAGGTTGAATTCACATTCCCTGACTTTGTAACAGAGGGAGCCAGGGACCTCATT
TCAAGACTGTTGAAGCATA
>580
GGAAATTAGTTTGCTCTGTATGGGTCAAATATAGGCATATTCTCATGATTGTCATGTTTCT
TTCTCCCAGCATTTATCAGCTGCCGCTTATTCATTGCCTTGATTTTCTTCAAAAGACAAC
TATAATCATTCAAAAATTC
>581
CTCTTGAGAAATCCTGTGTCTCAGAAACCTTCGGAGACATTTAAGCCCTCTCACCCTCT
CTTTAAACCAGGACTCTGCATGCTGCTGAAAGCAAATGAATGTGGAGGGTTTCAAACA
AGACAAAAGGCATTCCTCCCTGC
>582
CCCAGTTCTGTGTCCTGACTGGGTGGGGTGGAGGGGCTGCTCCACATCTCACAGCGTT

CTCTGGCTGCAGAATCTCCAGCCAAGTCCTCCGTGCTCGTGGTGGGAATCGTCATCGG
TGTCTTGCTACTCACGGCAGCGGC
>583
TGGGGGACGCCAGTCAGACCCAGAGCGCCTCAGAGATTCTGATGACCTCTCCCTCTCT
CTCTCTCCTCAGCCCCTTGGATCTCCCTTCGTGGAGACGACACCGGGGTCCTCCTGCCC
ACCCCAGGGGAGGCCCAGGATGC
>584
TGGGTGGAAAAGGGCTCCAGGGTCATTATCATGCTTTTGGTTTTCTGACATGCTCCATT
CTCTGTTGCAGCCTTGGAGTCCTCAAACCCCAACACCAAGCAAGCATCATTCTGGAGA
CCGTCCCCTTCAAGAGTGGACAA
>585
CAATGACTCATCAACCCCTATATCTATAAAGTCACAATCGCTGTGACTTGATTTCTGTTT
CACTTTGTAGATATGGAACCCATTCACATGGACTTTTTAAGAAGCTTGGAATTCCAGGG
CCCACACCTCTGCCTTTTTTG
>586
CTTCCTGGGTTGGGCTCCAGCTGTAGAATAAGGCTGTTGATGTTTAATCAACTCTGTTT
TTTTCACACAGCTTTTATGATGGTCAACAGCCTGTGCTGGCTATCACAGATCCTGACAT
GATCAAAACAGTGCTAGTGAAA
>587
ACACATTTTCTACAACCATGGAGACCTCCACAACTGATGTAGGACAAAATGTTTCTGCT
TTGAACTCTAGCCTTTTGGTCCAGTGGGATTTATGAAAAGTGCCATCTCTATAGCTGAG
GATGAAGAATGGAAGAGATTAC
>588
GGAAAGCCATGTCCTTCTGGGACTAGAGTCTGCACATTTAACTATGGGTGGTGTTGTGT
TTTGTGCTTAGATGGTCCCTATCATTGCCCAGTATGGAGATGTGTTGGTGAGAAATCTG
AGGCGGGAAGCAGAGACAGGCA
>589
CTGTTGCATGCATAGAGGAAGGATGGTAAAAAGGTGCTGATTTTAATTTTCCACATCTT
TCTCCACTCAGCGTCTTTGGGGCCTACAGCATGGATGTGATCACTAGCACATCATTTGG
AGTGAACATCGACTCTCTCAAC
>590
ATTGCTCCAGGTAAATTTTGCACATTCACAATGAATTTCTTTTTCTGTTTTTGTTTTGTTT
TTCCTACAGCAGTCTTTCCATTCCTCATCCCAATTCTTGAAGTATTAAATATCTGTGTGTT
TCCAAGAGAAGTTACAAA
>591
TTCTCTTCATCTAAACTGTGATGCCCTACATTGATCTGATTTACCTAAAATGTCTTTCCTC
TCCTTTCAGCTCTGTCCGATCTGGAGCTCGTGGCCCAATCAATTATCTTTATTTTTGCTG
GCTATGAAACCACGAGCAG
>592
GTACTGCATGGACTGAGTTAAAAGTTAATTCAAAATCTCAATTTATCCAAATCTGTTTCG
TTCTTTCCAGGCACCACCCACCTATGATACTGTGCTACAGATGGAGTATCTTGACATGG
TGGTGAATGAAACGCTCAGAT
>593
AAACCCTAACATGTAACTCTTAGGGGTATTATGTCATTAACTTTTTAAAAATCTACCAAC

GTGGAACCAGATTCAGCAAGAAGAACAAGGACAACATAGATCCTTACATATACACACC
CTTTGGAAGTGGACCCAGAAAC
>594
TCACTTTGATGCTATACTTTCTACTTTTGTTTATTTAATGCTTCTCAATATGCTTGTTTAAC
TGTTGCAGATCCCCCTGAAATTAAGCTTAGGAGGACTTCTTCAACCAGAAAAACCCGT
TGTTCTAAAGGTTGAGTCAA
>595
TTAGACCAGGCTCTCCCTGGGCCAGGCTCCGGTGGGACACTGGCAGGCTGACGCATGT
ATCTTCTCTCAGGGTGTCTTGGCCATGGTCCCCTGGTGCTGCAACTCCTCTCCTTCACG
CTCTTGGCTGGGCTCCTTGTCCA
>596
GGGGCTCAGTTCAAGGCTTGGCACACAGTAGGTGTCTAATAATTGCAGTTCCTTTTTTA
TTTTGGCCCAGTGTCCAAGGTCCCCAGCTCCATAAGTCAGGAACAATCCAGGCAAGAC
GCGATCTACCAGAACCTGACCCA
>597
GAAGGCTGGGCAGGTACAGGAATATGGACAACCTAATCTAAGCCCAGCCCTGACCAGC
CTCCCCCAACAGAACGCCTGTGCCACCCCTGTCCCTGGGAATGGACATTCTTCCAAGG
AAACTGTTACTTCATGTCTAACTC
>598
AAGAGATGGGGAGACAGAGCCTGGAAAATGGGAAGCAGAGCTCCCTCCTGACTCCTC
CTGTACCCTCCAGAACTTCCTACAGCTGCAGTCTTCCAGAAGTAACCGCTTCACCTGGA
TGGGACTTTCAGATCTAAATCAGG
>599
GACAGAAGGTAGAAACACAGCTCATGTAGCTGGCGGGGACAGAGGCTCACATTCCGC
ATGGGCTTTGCAGCTTCAAGCAGTATTGGAACAGAGGAGAGCCCAACAACGTTGGGG
AGGAAGACTGCGCGGAATTTAGTGGC
>600
TGGGGCTCAGTTCAGGGCTTGGCACACAGTAGGTGTTTAATAATTGCAGTTCCTTTTCT
TCTTGGCCCAGTGTCCAAGGTCCCCAGCTCCCTAAGTCAGGAACAATCCGAGCAAGAC
GCAATCTACCAGAACCTGACCCA
>601
GAAGGCTGGGCAGATATGGGAATATGGACAACCTAATCTGAGCCCAGCCCTGACCACC
CTCCCCCAACAGAACGCCTGTGCCGCCACTGTCCCAAGGACTGGACATTCTTCCAAGG
AAACTGTTACTTCATGTCTAACTC
>602
CAGAGACGGGGAGGTGGAACCTGGAAAATGGGAAGCAGAGCTCCCTCCTGACTCCTC
CTGTACCCTCCAGAACTTCCTACAGCTGCAGACTTCCAGGAGTAACCGCTTCTCCTGGA
TGGGACTTTCAGACCTAAATCAGG
>603
TTACAGAAGGTAGGAGTGTGGCTCATCTACTGGGCAGGGCAGAGGCTCACATTCTGCA
TGGGCTTTGCAGCTTCCAGCGGTACTGGAACAGTGGAGAACCCAACAATAGCGGGAA
TGAAGACTGTGCGGAATTTAGTGGC
>604
TCATGTGTAGCTGCCCTGTCCCCTGGGGCTTTATCTCATCCATGCAAACTACCATCTGCT

CAACTTCCAGCTACACCCCGTGCACCCTTTTGACTGGGGACTTGCTGGTTGAAGGAGC
TCATCTTGCAGGCTGGAAGCAC
>605
CCCAACTTCAAATTTTTCATATAATATAGGAAAAATAATGATTGAAACATTTAAATGTTC
TTCTTTACAGATGTTTCATTTGGATCCTTCTTTGACTCATACAATATTTAATCCAGAAGTA
TTTCAACCACAGATGGCAC
>606
TGTACCTTTGTTGCCGTAATTTTTAATAAGTATTTCTCAAACTTAATTGGCTTAACGTTCA
CCTTTGCAGAGAGGATTTCGTTTCCGTTATGTATGTGAAGGCCCATCCCATGGTGGACT
ACCTGGTGCCTCTAGTGAAA
>607
TATCATAAACATGTTTCCATGTTGCTGGAGAGTCAGATATGCTAACTTTTGGAATGTGCT
TCTTATATAGATCTGCAACTATGTGGGACCAGCAAAGGTTATTGTTCAGTTGGTCACAA
ATGGAAAAAATATCCACCTGC
>608
CAGATTCCCTAGACATTAAGTGCCTAACTTTTGGTTGTTGTTGCTGCTGCTGTTACTGTT
TTTTCTCCAGCTTCGCAAACCTGGGTATACTTCATGTGACAAAGAAAAAAGTATTTGAA
ACACTGGAAGCACGAATGACA
>609
TTATAAAAGCATGGTCTTTTAAATGTTCACACTTCCCTGGGCATGAATGGACTGTGCTGT
ATGGCCCTAGATCGGGAAAAAGAGCTAATCCGCCAAGCAGCTCTGCAGCAGACCAAG
GAGATGGACCTCAGCGTGGTGCG
>610
CCTACCTAAAGGACTGGCTTGTTGAGCTGAGGATTAATCATGTTATTTGTTGTTTTTCCC
CTGTGAACAGAAGCCCCCAATGCATCCAACTTGAAAATTGTAAGAATGGACAGGACAG
CTGGATGTGTGACTGGAGGGGA
>611
ATTTTTCAGCATGTTTATAAATACTATTTACACTATGTGAAATTACACACTTCAATGTGAT
TGTTTGCAGATGACATCCAGATTCGATTTTATGAAGAGGAAGAAAATGGTGGAGTCTG
GGAAGGATTTGGAGATTTTTC
>612
TGAGTAGCATTACTGCAAAAAAAAAAAAAAATGTAGTCCTACACCAACATGTGGTTCTT
CGTATCCTGCAGTTTGCCATTGTCTTCAAAACTCCAAAGTATAAAGATATTAATATTACA
AAACCAGCCTCTGTGTTTGTCC
>613
GGTCTTTCTGTGGCTAGTGGTGGGACCAAATCAATCTTTTCTCCTCTGGTTTCTCTTCCT
TTAAATACAGATAAAGAAGAAGTGCAGAGGAAACGTCAGAAGCTCATGCCCAATTTTT
CGGATAGTTTCGGCGGTGGTAG
>614
GACACTAAGTTGTGCTGAGTCTTTGTAAAATATCTTCATGATGTTTCATTTGTTTTACTT
GCCGTTTCAGGGTATAGCTTCCCACACTATGGATTTCCTACTTATGGTGGGATTACTTTC
CATCCTGGAACTACTAAATC
>615
CATTGGAATAGCGAATTATCACTAAATACATTTTACTGAGAAAAATCTGATGTTTTTGCA

TTTATCTTAGGAACCATGGACACTGAATCTAAAAAGGACCCTGAAGGTTGTGACAAAA
GTGATGACAAAAACACTGTAAA
>616
CCTTAAGCATGCCATACCCATAAAACAAAAGTAGGAACACTCAACTATGATTGTGGTCA
TTGCCTTACAGATAACCTCTTTCTAGAGAAGGCTATGCAGCTTGCAAAGAGGCATGCCA
ATGCCCTTTTCGACTACGCGGT
>617
TCACATCTTGGGAATCTTTTCTTTTTCATTAACATTTTAGTTAATTATTGCCACTGTGTTT
TCATTCCAGTGTCTTACACTTAGCAATCATCCACCTTCATTCTCAACTTGTGAGGGATCT
ACTAGAAGTCACATCTGGT
>618
CTACCAAGCTGTGAGTTGTAAGTAAGTATTCACTGCTGACCAGTGAATCTCCTGCCCTT
TCACTTTCCAGACGCCCTTGCACTTGGCAGTGATCACTAAGCAGGAAGATGTGGTGGA
GGATTTGCTGAGGGCTGGGGCCG
>619
TCAGCTTCACAAGGGCCATCTTGTGAGTTAGCCATCCCATCCTGTGACTGTCCCTTTGC
TTGGACTCTAGGTCTGAATGCCATTCATCTAGCCATGATGAGCAATAGCCTGCCATGTTT
GCTGCTGCTGGTGGCCGCTGG
>620
CACTGGGAGGTGGGGACAAAAGGGCAAAAGAACCTTCCACTAACGCTTTCTTGTGTG
GGCTGGATTGTAGGGTGATGCCCATGTGGACAGTACTACCTACGATGGAACCACACCC
CTGCATATAGCAGCTGGGAGAGGGT
>621
TGACCTTTGCAGGCAGTTGGAAGTTGACAAGATCTGGGTTTACCTAATGCTGTGTAATC
TAACTTCGCAGGAGCAGATCCCCTGGTGGAGAACTTTGAGCCTCTCTATGACCTGGAT
GACTCTTGGGAAAATGCAGGAGA
>622
GCAATGATCAGTCCCTCCAGAGTGTCTATGGCATGTTAGAACAAGTGTGTTCCTTCTTC
ATTTCCTTCAGGAGACATGAAACAGCTGGCTGAAGATGTGAAGCTGCAGCTGTATAAG
TTACTAGAAATTCCTGATCCAGA
>623
TGTCAGTGCTTACAGCCTGCACTGGGACTCGAACACAAGAACATGCTCCTCCTTCCTT
TCTTTCTCACAGGTCTCTGGGGGTACAGTCAGAGAGCTGGTGGAGGCCCTGAGACAAA
TGGGCTACACCGAAGCAATTGAAG
>624
CCACAGAATAGTCCATGAGCTTTTTAGAGCCCGGCCATTCCCCCAGTGAATTTGTGCTT
TCTCCCCTCAGACGAGCTCCGAGACAGTGACAGTGTCTGCGACAGCGGCGTGGAGAC
ATCCTTCCGCAAACTCAGCTTTAC
>625
GAGAGTCTAGAGCATATTTGGTCATAATTAGAACTAGCATTCTTCATCCTGTATTCCTTT
CTTTTCTCAGGCCCTGAAAAGACAGATGAATATCTCTTAGCAAGGTTCAAAGGCGATG
GTGTAAAATATAAGGCCAAGCT
>626
ATTTTAATATTAAAACCATTGTAGGATGTTTTTAAAAATTCAACTTCTCTTGTTTTTCCAC

CCTCATCAGGGAATGGCGGCACGTGGTCGGTCTCAGGGACAACACAAACAAAGGATC
TGGGTCAACATTTCCCTTTCTG
>627
GCGTCGGTGTGCTAGCCTGCAGATAGATTCTAGTTTTCTTAATAGATTACTCGTTATGTT
CTTTTGTGAGGTAATAGAGCATGAACATCCAGTAAATAAGATTTCTTTCATTGCCCGTGA
TGTGACAGACAACCGGGCAT
>628
ATTGATAGCAGACAAAGGAAATAAAATACTAAAATTAAAATACTGATAACAGTGCTTTC
CCTTACTGCAGGCTGAACCATTAGTTGTTGATCTTAAAGACCTTTTTCAAGTTATCTATA
ATGTAAAGAAAAAGGAAGAAG
>629
ATGTAATTTTCTCATTTGAAGTCACAACATGGACTAACACTTTCTTTTTTTTTCTTTCCA
AATGTGATAGGAAAGCAAAGATATCCTGTTAGTGGATCTAAACTCTGAAATCGACACCA
ATCAGAATTCTTTAAGAGAAA
>630
AGACAAAATGAGGCTGTATTTCACTGTTAGTGAGTAACTAAGGCTTCTCCCTCCCTCCC
TGTCCTGTCAGTCTTCAGCCAATGACTTGCTTGCATCAGACATCTTTGCTCCTCCCGTC
TCAGAACCTTCAGGCCAGGCGT
>631
TGCTCTCTGAAATTCAAGAATCTTCTTCAAGCTTGATTCCTGATAAGTATTTTTCTTCCT
CTTACTTCAGGTGGTGTAACTGTCACACTCCCTCAGGCAGGACCATGGAACACAGCAT
CTTTGGTCTTCAATCAGTCCCC
>632
GCTGACTCGGGGACTGGCCTATATTTGGGGAAAGCTTGTCTACTGATTGCCTGGATTTT
TTACCTTCTAGAACCACCAAAGCCAGCTCCCAGACAAGTTTCCCTGCCAGTTACCAAA
TCTACTGACAATGCATTTGAGAA
>633
AAGTCTTCATTGCGATTTTTTTCTTATGACTGTAACTGTATTTATTGACTAGATGATTTTA
TCTTCTTAGGTGGCTTCTTCTCAACCTGTATCTTCTGAGATGTATAGGGATCCATTTGGA
AATCCTTTTGCCTAAATTC
>634
ATATTTAATACATTGATATTCAGTTCAAAAATTCTAACCACAAGTTTCTTTCTTCTCCATA
CCTCTCTAGGAACTTGGTCTGCAGACCATCCAGAGGAATAAAAAGGTTGGCCTTAGTA
GTCAAAAACAAAGCTGATAGC
>635
AGAAAATGGTGCCTGAGAGGCTACAGCAGGACCCGGCAGTGACCCGCTGCTCTTGGC
TGTCTCCCTGCAGGCTCCACAGACTCTCCTGGGGCCCAGAGGAGCTGGTGCTGACCTA
GCTAGGGATGCAAGAGTGAGCAAGC
>636
TTTCTTAAAACAAATTTTTCTCCCATTGTCATGGTTCTGTATATTAACACAAAATTTTCTT
TTCTCATAGGAGAGATTATTTCATTCAATATTTTTTATGAATTATTTACCATTTGTACTTCA
ATAGTAGCAGAAGACAA
>637
GCCCTCATCATCATGTTGATCATTACTATCACTGTGTTTCTGAAATGTCACTGAAACATG

TCTTTTATAGGTGGAACATAAATAATGATACCTGGGTCATAACTGAGCAACTAAAACCTT
TAACAGTGAATTTGGATTTC
>638
TCTTCCGGGTCCTGACTGGTTACATAGTGTCACCTCTTCCGGTTCCTGACATTGTGTTCA
TCTCTTCCAGGTATTCTAGAATGGATTCTGGGAAAGAAAGATGCCATGTGGATAGGGTT
CCTCACTAGAACAGTTCTGGA
>639
AAAAATAAATGAAGTTATTTACTAAAATTAAAAAAAAGTTTCATTTTTCTTTTTAAATGT
TTCTATATAGTTATGAAGAAGCCAAGAATTTATTGACCAAGACCAAGATATTGGCCCCA
GCCTACTTTATCCTGGGAGGC
>640
AAATGCTAATATATATCAGAACATCTCTGAATTGAAAACGTCCTTAAAGTAAAAATAACT
TTTCTTTCAGCTGACCGTATACACAACCTTGATAGATGTTACCAAAGGTCAATTCGAAA
CTTACCTGCGGGACTGCCCTG
>641
CAATACATTGTTTCTTTCTAATATATATTTTATTTAATCAGACATCTATTTACCTCTCCTTG
AATTGCAGACTCACAGATGAATGCTTCTCACCCGGAAACAAACTTGCCAGTTGGGTAT
CCTCCTCAGTATCCACCGAC
>642
ATAACCCTAAAAAGGTTGACAATTCCATTTTTGGTGCAGTTACAAATCATTTCACTTGTT
TGTATTTCAGGACCTCCAGGATATAGTGGCTACCCTGGGCCCCAGGTCAGCTACCCACC
CCCACCAGCCGGCCATTCAGG
>643
TTATAATATCTGGATAAATTCAATATTTTTAACATAGAGGTGATTATGATTTCGTCTTTTAT
GTTTTTAGTTTTAACAGGTTTTGAAACTAATAACAAATATGAAATTAAGAACAGCTTTG
GACAGAGGGTTTACTTTGC
>644
GTCATTGAACCTTTCATCGAACCTAGGCATTATGATTATTACATTTACATATATTATTTTGC
CTTTGTAGATAGAAATCCAAGCTCCTCCTGGTGTACCAATAGGTTATGTTATTCAGACTT
GGCACCCATGTCTACCAA
>645
ACTTCAAATTTGAAAGTACTTTTGCTATAAGTTTCCTAAAAGTATTTAATACTTTTTTTTT
TCAATTTAGATTAAATCTCTTGATGAACAGTGTGTGGTTGGCAAAATTTCCAAGCACTG
GACTGGAATTTTGAGAGAGG
>646
TTTCTGCATCTGATAATTGATCTCTGTTTTGTTGTTGAAAATTCATTTGTATACTTTTGTT
TTTATCTAGGACTTCATGTTTTTTGAAAGCACTGGCAGCCAGGAACAAAAATCAGGAG
TGTGGTAGTGGATTAGTGAAA
>647
GTTAGGAGGCCCAGAACTGGAGAGCTAAGGCATACTTCATCAGCTTAGCACATGACGA
CTGTCTCTCCAGACTGCGTGGAGTGCATGGCGTGTTCAGACAACACAGTTCGTGCTGG
CCTGACACCCAAGTTCATTGATGT
>648
TCCTGGGCCCATGAGCTGATGAGAGCCAGAGTCTGAGCTGCACTGCCTTATCATTTCTT

CCTTGCCCCAGGTCCCTGGCTCTGTCACTGAATACAAGGTCTTGGCACTGGACTCTGC
CAGCATCCTCCTGATGGTACAGG
>649
TAGAGGCAGTCACTTATTTGAATCCCATTGTCGTAACTTTCTACTATTTTATTTTTCCACT
GTGACTCAGGGAGATTCAGGTGGTCCCTTAGTTTGCAAACACAACGGAATGTGGCGTT
TGGTGGGCATCACAAGCTGGG
>650
GCTGTCATGGGGAGGAGTTGTGGCCATGTCCAAGGCACCTGTCCCATGCCCCCTGCCT
CCTCTCACACAGGATGGTGTGAGGATGGAGGAGATTGTGGAGGGCTGCACCGGAGCA
CTGCACATCCTCGCCCGGGACCCCA
>651
TGGGGTGTGTTGTCCGGCCTGCCTCACCCCTTTCCAACTCTCCCCTGCTTCCCACGTCC
CCTCTCCCCAGCTCCTGTACTCGTCGGTGGAGAACATCCAGCGCGTGGCTGCCGGGGT
GCTGTGTGAGCTGGCCCAGGACA
>652
GTCCCACTCCCCCAGCCACGATGGAGGCTGGCCGACGTTTAACCTGGCTTTTCCTTTCC
TCTCTGCTCAGCCACCTACGCTGCTGCCGTCCTGTTCCGCATCTCCGAGGACAAGAAC
CCAGACTACCGGAAGCGCGTGTC
>653
AGGCTGTGCAGACTGCTCCCTGCAGGGTAGCTTGCAGACCCTAGGACCGAGCCCCACT
TTTTGTCCCCAGACATGGATGCCACCTACCGCCCCATGTACTCCAGCGATGTGCCCCTT
GACCCGCTGGAGATGCACATGGA
>654
CCCTTTCCCCGCCCCCTCTTCCCCTCGCCCCACTCCCATTCCCCTTCTCACCACCCCTCC
TTATTCCCAGACTGCGGCCAACTCCTGGGGCCCAGCCTGGGGCGAGAGGGGCCACTTC
CGCATCGTGCGCGGCGTCAATG
>655
TAAGCTACTATGCAAGTAGATTTTCCTCTTCAGCGTCACAACATGACTCATTTTCCCTCT
ACCGTCACAGGTTTAATTCTAAAGTTGCCTCTCTGGGGACAGCCATCTGACCAGAACT
GCTATGATGATTCTGTTTATTG
>656
TTCAATACTAATTTCTGACAAGAACAGTGAGAATAAACTATTAAGTGCCCTCAAACTTG
TTTTTTTAAAGGTCCCTAAGACGAGATAACTTGACAATCAGTTCCATGGACATGGTGAC
CACAGCCAGCTGGAACCTGAAG
>657
TGAGGCACAGTTCTCCATGGCTATGAGACTCCATTTATGCATCAGGAGTAAAGGGGTCT
TGTGTTTTTAGGTGAGGTTCCTGGAGCAGCAGAACCAGGTACTGCAAACAAAATGGG
AGCTGCTGCAGCAGGTAGATACCT
>658
CCCCTCACAGGTAATGAGAGGGACCTGCCCTGGAGAGAACGTGCCTTCATGATGTCCC
TTGTTCCTCTAGGATGTGGATGGTGCTTATATGACCAAGGTGGACCTTCAGGCCAAACT
TGACAACTTGCAGCAGGAAATTG
>659
ACTGCTTTCTCTGTCTAAGTTGTTTTTTGTTTTAGTGCTGTAAGAGTCACTAACCTATTA

TGTCTTGCAGGAGTTGTCTCAGATGCAGACTCAAATCAGTGAAACTAATGTCATCCTCT
CTATGGACAACAACCGCAGTC
>660
GGAGACCAATTGGGATGAGGAGGCCTAGTGGGGAATGCATATTATGAGAGGGTGTCAT
ATTCTTTTTCAGATCTCCAACTTGCAGCAGTCCATCAGTGATGCAGAGCAGCGTGGCG
AGAATGCCCTCAAGGATGCCAAGA
>661
TGAGACACCAGGAGAGCTGCTGCTTTGGGGGATGGACAGGGGCAGCAGGAATGCCTT
TGTGTTTTCGCAGTGAACCTCCTTGGCCTGGGCGAAGCTGTGTGGACCAAGCAAGTCA
GGAGTGTGGCCATGTTTTCTGAGCA
>662
CAGGGCCTCCATGGCCTCTGTCGGGCGGGGGGGCTACTTCATCTCTGAGTCTGTACCC
CTCGTGTCCCAGGCAGTGGAGTGGGAGTGAAGAAGCTGTGTGAACTGCAGCCTGAGG
AGAAGTGCTGTGTGGTGGGCACTCT
>663
CCAGCTCACTAACACCAGAGCTTTGGTGGGCTCTGGCCCCAAGATTATACCTCCTGTCT
CTGCATTCCAGCACAACCTGCTCCCCCAGCCTCCTCGGAGTAAATACATACACCCAGAT
GACGAGCTGGTCTTGGAAGATG
>664
CGTTGGGGTGACTGGAAGGAGTGGTAGCACCTGGGGCTCCCTGCTCAGGCCTGATGCC
ACTGCTCCCCAGGGACTGTCCTGGCTGTGTTTGGCTCCGTGAGAGACGACGGGAAGTT
TCTGGTGGAGGACTATTGCTTTGC
>665
TCTTTGAGTTGCACCTAGGAGGGGCTGCCTCCCTTCTCGGCACCCTGGAGGACCCCAC
CTTCTCCCGCAGGTTTGTGCTACTGGTGTCCGGCCTGGGCCTGGGTGGCGGTGGAGGC
GAGAGCCTGCTGGGCACCCAGCTG
>666
GGTGGGGCTCTACTCTCCTCTGAGCCTGGGACTCGCCTGGGCCTGATGGCCTTCTGGG
CTTCTATTCCAGGCCAAATACCTCACCAAGAAAACCCAGGCAGCCAGCGTGGAGGCTG
TTAAGATGCTGGATGAGATCCTCC
>667
GGAGTGGCCGGAGTTGGTGTAGGGGGCTCCTTCGGGGCCCTGTGAGCTCTCCCTGCCC
TGTGCCTTCCAGGCCTCAGTGCCCGTGGACGTGATGCCAGGCGAGTTTGATCCCACCA
ATTACACGCTCCCCCAGCAGCCCC
>668
CAGATGAGCTCTGGGCTGACCACTATGGGTGGCACCCAAAGCCAAGAGTCAGCTGAG
CTTTGCCTTGCAGATTTTTGGGGACATCAGGACAGAACGTGAGTGACATTTTCCGATAC
AGCAGCATGGAGGATCACTTGGAG
>669
AGCCCCACATCCTGTGCAGAGTGCTAGTGCTGAGGCGGAACCCTCCTCAGAGCTGCCC
CTTCTCCTCCAGGTTGTTACCCCTTCTACAAAACTGACCCGTTCATCTTCCCAGAGTGC
CCGCATGTCTACTTTTGTGGCAA
>670
TTCTGGCAGCTTCTCTTTGAGGCAGGACTTACTTGGAACCTACAGTGGGTCCTATGTGA

CTTCTTTGCAGGTCCTGAGGACCAGACAGTGCTGTTGGTGACTGTCCCTGACTTCAGT
GCCACGCAGACCGCCTGCCTTGT
>671
TTCTCTAAGTTCTTGACCTGCTGGTGATTTCCATAATGGTTTCTGTTCATTTTCTCCTGTG
TCTTTTCAGCCTAGTGGAGGCCAGTATAAGATTCAGATCTTTGAGAAAGGGGATTTTAG
TGGTCAGATGTATGAAACCA
>672
CTAGGCAAACAGTAATTTTGGTTAAAACCAAAATACGTGTTCTGAATTACTGTTCTGTG
TTTATTTTCAGGCTTCTGGAGTTACAGTGAATGATGAAGTCATCAAAGTTTTTAATGATA
TGAAAGTAAGGAAATCTTCTA
>673
CACAGACCCCCACTAGCCGGGGACGCAGCTCAGGCCCCCTACCCCCAACACAAACAC
TTCTCTCCTGTAGAGGATAAAGCTTGGGGTTCATCCTCCTTCCCTGGATCACTCCACAG
TCCTCAGGCTTCCCCAATCCAGGG
>674
AGTTTTTCAGGAATCAAAACCTTTGCCCCATCCCACCTCCAGCTCAATGACCATTGCCC
CTTCCTCAAAGGGACGATCATGGCTTCTCCCCCTTGCACTGGGCCTGCCGAGAGGGCC
GCTCTGCTGTGGTTGAGATGTTG
>675
TGACACCAGTATCTCATTTGGAACTGACTGTACTTTCTGCCTCTTCTTTTTGTCTGGCCA
TGGGGTCCAGCTATTGCAGTACAAGGCAGACATCAATGCAGTGAATGAACACGGGAAT
GTGCCCCTGCACTATGCCTGTT
>676
ATTCATGGTTGGTTCAGTGACTGCCAGCGAGGTAGCAGTGGCTCTCATCATAATGGCCT
TTTCATTCCAGGACCTGGTGGCAAATGGGGCCCTTGTCAGCATCTGTAACAAGTATGGA
GAGATGCCTGTGGACAAAGCCA
>677
TGTGTCCTCTCGTCCCTTCCCACCTGTCTTCTCCCTCTGTACCACAGCTTAGGTTGTTTT
TCTTCCCTAGAGCGGGCAGAGAAGATGGGCCAGAATCTCAACCGTATTCCATACAAGG
ACACATTCTGGAAGGGGACCAC
>678
AGGAAATAATCCTGGCCTCTTGGGGCTGGGTTAGGGTGAAGCTGGGTACCTGACCTGC
CCACACTCTTAGGAAATGGAACCCTGAACAAACACTCTGGCATTGACTTCAAACAGCT
TAACTTCCTGACGAAGCTCAACGA
>679
TTTCCTCCAGTTAGTGGGCAAGGAAGTGGCAGCAACATTTCAAGCCTCCTAACCCCTA
CCTGTCCTGCAGCTATGGAAGGGCCGCTGGCAGGGCAATGACATTGTCGTGAAGGTGC
TGAAGGTTCGAGACTGGAGTACAA
>680
CCAAGGAACCCTGAATAGCACTGAAAGAGATCTTTTGTACTGGGTCTCAACCACTCCC
TCCCTCTTCTAGGATTTTCTCGCATCCAAATGTGCTCCCAGTGCTAGGTGCCTGCCAGT
CTCCACCTGCTCCTCATCCTACT
>681
TGAATTTCCTTGGGGAGGAAATGGCAGAGAGGGAGCCTCTCTGAACTATTTGACTTTT

GCCTCCTCTCAGATTTCGTCGTGGACCAGAGCCAGGCTGTGAAGTTTGCTTTGGACAT
GGCAAGGGGCATGGCCTTCCTACA
>682
CTGGCCCAGGCCCCAAAAGCCCTTTGCCTATCTATGACTTACCTCCTTCTATCTGTTTTC
TCTTCCTCAGATTGATGAGGACATGACTGCCCGAATTAGCATGGCTGATGTCAAGTTCT
CTTTCCAATGTCCTGGTCGCA
>683
CAGAAGTAGTGGAAGGGGGCAGAGACAGGACAGGCAAGGGGGCCAGAACAGACAA
GCCCTATCTCTCCAGCTCTGCAGAAGAAGCCTGAAGACACAAACAGACGCTCAGCAG
ACATGTGGAGTTTTGCAGTGCTTCTGTG
>684
CAAGGGCCAGTGGCTTCTCTCTACATGACAGACTCAAATTGTGAGGCTGCTTTTTTTCT
TGTATTCGCAGGTGGCATTGGAAGGCCTTCGGCCTACCATCCCACCAGGTATTTCCCCT
CATGTGTGTAAGCTCATGAAGA
>685
TTTTTCCCCCTAAATTTAATCTTGTAGCATTTTTGGTTACTCCTGCTTACATATTTGGCTC
TTTTCCTAGGTAAGTGGAGGTATACTTCGAATTTTTCCAGAAGGCAAAGCCCAGTTTGC
TGACATTGAACCCAAATTTG
>686
GGAATGCAGTAGCAGAAGCTCTCTGGTGCTTAGTGAATGAATGAAAATTTTTTTTTTT
TTACCTTGCAGGTGAAAAAGGTGTGAGGGTTGAACTCAATAAACCTTCAGATTCGGTC
GGTAAAGACGTCTTCTAGAGCCT
>687
GGTTTTGAGGAATTACTCATTAGTCTCTGCACATCCACAGACATATAATTACTTTTTATCT
GCACTGTAGGAATGATTGCAATTAGTAGGATGGCTGTGCTGTATCCTCAAGTTATTGTCG
ACCATCCATTTTTCTTTCT
>688
AGCAAGAAGGAAGATAAATATTTTATTATTTTGTTCACCCCCTCTTTTGTTTACTTCTGA
ACCAATACAGGTACAATTCTATTCATGGGACGAGTCATGCATCCTGAAACAATGAACAC
AAGTGGACATGATTTCGAAGA
>689
ATTGTGACCATTTAATCAACACAGCAGTTAAATTGTATATTTACTTTCATTGTTTTATATT
GCTTGGCAGAAAATGAAGACAAAAAGTGATATGATGGAAGAAAACATAGATACAAGT
GCCCAAGCAGTTGCTGAAACCA
>690
GTAAATATTTAGGGGAAATGGTTCTAAATTTACAGACAGAATCTTGTTTTTCTTTTCACT
ATACTTACAGGCACCAGCTTCTGAAAAAGAAATTGTGGAAGTAAAAGAAGAAAATATT
GAAGATGCCACAGAAAAGGGAG
>691
GACTGAGGAAATGAATGAATGAATGTCTCCATCTCAACCCTCAGCCTGGTCCCTCCTTT
TTTCCCTGCAGTTGGTACAGATGGCATTGTCCCAGTCTGTTCCCTTCTCGGCCACAGAG
CTTCTCCTGGCCTCTGCCATCT
>692
GGGAGTGGAGCAACGTTCAGCCTTTGACCTTGGAAGTGCCAGAGGTGCCCCTAAGCT

TGTGCCCCCTCAGAACAGTGTCCGGGACATCACGGGTGCCCTGTTCAAGCACAGCAA
GAAGGGGCCTAGAGCCAGCGGCAACC
>693
GATACTTGGGAAATGATGGTTTCCTTGTTTCTGTCTTCCTTCTTTCCTCACCTTACACTA
CACGGTTCAGGATTTGACACAGTCACCACAGCCATCTCCTGGAGCCTCATGTACCTTGT
GACCAAGCCTGAGATACAGAG
>694
TATAAGAGGGGATAATTCATGGGGCAGTTAGGGCAGCCCCTGAGCTCTGCTTGTCCTCT
GTGTTCTACAGACACTGTGATTGGCAGGGAGCGGCGGCCCCGGCTCTCTGACAGACCC
CAGCTGCCCTACTTGGAGGCCTT
>695
GTGGAGGTAGGAGCAACACATGCCCCAGCTTTCCAGCCCTGAGCCTCACAGTGCCCTC
TTCCCTCCTCAGCACAACAAGGGACACAACGCTGAATGGCTTCTACATCCCCAAGAAA
TGCTGTGTCTTCGTAAACCAGTGG
>696
CCCTCCCCCAGGCACCTCCTCCCAGGGCCTCTCCAGCCCTGAGGTCCCATCTCCTCTGT
TCCTCTTGCAGAGAGCTGTGGGAGGACCCCTCTGAGTTCCGGCCTGAGCGGTTCCTCA
CCGCCGATGGCACTGCCATTAAC
>697
AGTCTCTGGGCTGTCAGGGCTCGGGCTGGGCTGGGCTCTCGTTGTTAATGGTCTTCCCC
TCATCTTGCAGGAGCCATGTGGCTAGAAATCCTCCTCACTTCAGTGCTGGGCTTTGCCA
TCTACTGGTTCATCTCCCGGGA
>698
GAAACAGACTTTGCTCTTGTGCTCTGTCCTTCCCATCCCTCTCAACTTGGGGTCCTGAA
TTTTGCTCCAGGACTTACACCAGAGGATCGATAAGTTCCGTTTCACCCCACCTTTGGAG
GACAGCTGCTTCCACTATGGCT
>699
GGGGGTGCCAGAGCCTGACCGTGCAGGGTCTTCTCTCTCCCTCCACCCTGACTGTGCT
CTGTCCCCCCAGGGCTGGACATCCACTTCATCCACGTGAAGCCCCCCCAGCTGCCCGC
AGGCCGTACCCCGAAGCCCTTGCT
>700
AACACCAGAGGGCCCAAGGAGCAATCTGCCTGTGACTCCGTGACTCCATGCCTTTCCC
CATCACTGCCAGGGTTCAACTCGGTGGCCACCGCCAGGATCTTTTACAAGCTGATGCT
GCGGCTGGGCTTCCAGGAATTCTA
>701
CGCCATCTCTCCTCTCTCCCTGTCCTTGACACCAGCCCAGCCTCACCCCGGCCCCTCTC
TCTGCCTTCAGCCACGTGAAAGGCCTGCACTTGAACATGGCTTTGGTTTTAAGCAACT
TCTCTACCCTGACCCTCCTCCTG
>702
CATCACACCTGAAGCTCCAGCTCTCTGGTCCCCAGGCCTGAGTCTCTCCCTTGCTCCCC
GCTCCCGCCAGGCTCTGCTCTGAATGACTCTCCTGTGGGTCTGGCTGCCTATATTCTAG
AGAAGTTTTCCACCTGGACCAA
>703
TTTCTGGCTGCCCTTTGTCACACAACTGCATGTGGCACTGAGAGTGGGGCTTTGTGTTC

TGCGTTCCCAGGAAGTTCTCCCTGGACGACCTGCTGACCAATGTCATGCTCTACTGGA
CAACAGGCACCATCATCTCCTCC
>704
ACCCCCTGCTGTGCAGACGGAGGGGCGCAGGGCCACAGCCTCACCCCTCCGTCGGCT
CTTTCACTTCCAGGATGAAGGTCTATGTGCCCACTGGCTTCTCTGCCTTCCCTTTTGAG
CTATTGCACACGCCTGAAAAGTGG
>705
TTATTTTAGTGAAACGATGCAGGTTTAACAGTAACTATGTACTTTTTTCCTACTGTCTTC
TCCTTCGTAGGACAAGAAGGTTCTGTTTGTCTCCGGTCATCAGACTGTGCCTCAGGATT
GTGTTGTGCTAGACACTTCTG
>706
GCACCTTAGTGAGTTGATCATAGAACATGAAATCCAAGTTTCTCTATCTTCAAACCCTG
CTCCCCTCCAGGTGCGGTTCCTGGAGCAGCAGAACAAGGTTCTGGACACCAAGTGGA
CCCTGCTGCAGGAGCAGGGCACCA
>707
CTCTCTAAGCGTGGAGCTCACTTGAGTAGGGTGACGGTGTGTGCAGTGCCAATCATCC
CTGTTTCCCCAGGATGTAGATGCTGCCTACATGAACAAGGTGGAGCTGGAGGCCAAGG
TTGATGCACTGATGGATGAGATTA
>708
AGATTTGAATGGAACTGACATTAAATAACACAACACAGAACCAGATGACCGACTCCAA
ATCTCCCTGCAGGAGCTGTCCCAGATGCAGACGCATGTCTCTGACACCTCAGTGGTCC
TCTCCATGGACAACAACCGCAACC
>709
CTGTGAACTTTGGGAAAGTTCTTCCCATTCTCATATAAACAGAATCTTTCCTCTTTCCTG
GCTGCATCAGTATGAGGAGCTGCAGCAGACAGCTGGCCGGCATGGCGATGACCTCCGC
AACACCAAGCATGAGATCACAG
>710
CTGACCCAGCCCCTCAGAAGGAGACACCTCAGGTCCCAAGCAACCCCACTCTCCTCCT
TTCTATCTGTAGTGCGCCAATCTGCAGAACGCCATTGCGGATGCCGAGCAGCGTGGGG
AGCTGGCCCTCAAGGATGCCAGGA
>711
GATCAAATAAATGGGCCATGCAGGATCAGCCTGGCAGATGGTCTCACTGAGTCCTCCTC
CTTTCCTGCAGCTGTTGTCACAAGCAGTGTTTCCTCTGGATATGGCAGTGGCAGTGGCT
ATGGCGGTGGCCTCGGTGGAGG
>712
ATAATACACAGAGGCTTGCAGGCCACTGCTCCCTTCCCCACACCTCTCTCCCTTTTTCT
TCTTGCCACAGGAAACTTCGAGAGGAGGAGAGGCGTGCCACCTCCCTCCCCTCTATCC
CCAACCCCTTCCCTGAGCTCTGC
>713
TAGCAGGTGCGGAAAGGGAATGAATCACCCTTTGCCTCCCCTCAAGTCGTGTGCAATT
CCTGGGGCGCAGGTAGTAAAGGTGTACAGTGAGGATGGGGCCTGCAGGTCTGTGGAG
GTGGCAGCAGGTGCCACAGCTCGCC
>714
CTAGGCATGTGGCTCATCCAGGAGATCTGGTTGATCTCCAATAACCCCTGCTTTTTGCC

CTTGTCCCCAGAGCGGGGTTTGGAGGACCACGAGTCCGTGGTGGAAGTGCAGGCTGC
CTGGCCCGTGGGCGGAGATAGCCG
>715
GGGCGCTGGGATGCCCTGATCCTCAACCTGGATGCTGGAGCCCTGATCCCTGACACTT
GTCTACCCACAGCACTCCCTGTTCCCAGAAAAAATGGTCTCCAGCTGTCTCGATGCAC
ACACTGGTATATCCCATGAAGACC
>716
AACATCCTGGTGGTAGAGGGGAAGGGAGGAGGTCAGACCTGTCACTCTCCTCTGCTCT
CCTCTGGCTCAGAACTTCCTGAATGCTGGCAGCTTTCCTGAGATCCAGGGCTTTCTGCA
GCTGCGGGGTTCAGGACGGAAGC
>717
TGGCTTGTGGGGGGAGGTAATAGTGGGCGGGATCTACCTGTACCAAGTCCTGCTCACT
CATGCTGCTTAGGATCCGAGGCACCTGCAGTACGTGGCAGATGTGAACGAGTCCAACG
TGTACGTGGTGACGCAGGGCCGCA
>718
TATGTGGAGCAGGGGCAGACATGTGGTCCTAAAGGCAGATATGGGACCAGTCAATTTC
CTCTCTCTGCAGCCCAACAAGCTTCGAAATGGACACAAGGGGCTTCGGATCTTCTGCA
GTGAAGATGAGCAGAGCCGCACCT
>719
TGAGGGGGGCGTCACAGCCACGCCCCCAGGACCTCTCGACCTCAAGCTCTCTTTCTCT
CCCCACCCCCAGTACGGGGTGCAGCTGTACAAGAATTACCAGCAGGCACAGTCTCGCC
ATCTGCATCCATCTTGTTTGGGCT
>720
GGGTATGCAGGCCCTGTCTTACGGGTACCTGGGCCCTGTTCTGACCTCTCTCCTCTCCT
TCCATCTCCAGAGAAGTGCCTCAGATAATACCCTGGTGGCCATGGACTTCTCTGGCCAT
GCTGGGCGTGTCATTGAGAACC
>721
ATTCCCAGTGGGGAGTAGATGGTATAGGGGTCCCCTCCCAAAGTGACCGCCCATGTCCT
TCCCACCACAGCCATCCACCGCACCCAACTCTGGTTCCACGGGCGCATTTCCCGTGAG
GAGAGCCAGCGGCTTATTGGACA
>722
AGAGACTGGGGTCCAGGTGGCAGCCATGGTCCTTGGGTAGTAATGCTGCCCATCTCCT
GTCTTCTGGCAGCCTGTTCCTGGTCCGGGAGAGTCAGCGGAACCCCCAGGGCTTTGTC
CTCTCTTTGTGCCACCTGCAGAAA
>723
CTGGCCCAGGAGGGAGCTTCCCAAGCCTCGGGCCCCTCCCTGAACTTCCACCCCCTTT
ACTGTACCCCAGAGCGAGGAGGAGGGTCGCCTGTACTTCAGCATGGATGATGGCCAGA
CCCGCTTCACTGACCTGCTGCAGC
>724
GTGAAGGAAATTGACTTTGTCAATGACCAGATTTTTATATCAACCTTCCTTTTTTCTGTC
CTTTCTCTAGCACTATTTAGGAGCCTACGTTTTTATTATCTTCACCGGCTTCCTCATTACC
TTCTTGGCTTTTACCTTCT
>725
ACGATCAAGGAACGCAGCCTTCCTGTTGCTCTTAGCAGTAACCAGGCATGTCCACTGG

CTGTTTTTGCAGTTCTTTAATTCAGTGCGGCTCTGGGATTTCATCGAGAACCGAGAAAG
CCGGCATGTCCCCAACAATGAAG
>726
CTAGGCAACCAGTAATTTTGGTTAAAACCAAAATACGTGTTCTGAATTACTGTTCTGTG
TTTATTTTCAGGCTTCTGGAGTTACAGTGAATGATGAAGTCATCAAAGTTTTTAATGATA
TGAAAGTAAGGAAATCTTCTA
>727
TATTATCATAGTCAGTCTTACCTTTGTTTTTCTTATAAATGGGATTCAATAATTTTTTTTT
TTCTGTAGGGCTCCTGAAAGTGCACCTTTAAAAAGCAAGATGATTTATGCTAGCTCTAA
AGATGCCATTAAAAAGAAA
>728
ATATTTGGGGCAGAGAAATTATTCTTTATTAATTATAATAAAGTAATAGGATAATGTTTTT
TCTTTTTAGGTATTAAACATGAGTGGCAAGTAAATGGCTTGGATGATATTAAGGACCGTT
CGACACTTGGAGAGAAATT
>729
TTTGACTTCTCAGAAATATATAGACCAACAGTGGTTGCCTTTTTTCATTTTCACTTCTCT
TCATTCTTAGATCTACGGAATCCAGAGGCAGCATTGTCTCCAACCTTCCGTAGTGACAG
CCCAGTGCCTACTGCACCCAC
>730
GTTAGGATTTACCACTTATTTTCAGTTCACGGTCAGTGTCTTATTGCCCATACCTCCCCT
CGTTCTCTAGCCAGATGCTCCTGCCACTCAAGATGGGGTAGAAAGCCTCCTGCAGAAG
TTTGCAGCTCAGGAGTTGATTG
>731
TTTGCGGAGTTGGTCTAAGAGCCAGTTTGATCTATAAGACATTTTAAGTCCAAGGCTAC
TTCTGTCACAGGTCAGTCAGGAGATCCTAGAGCTATTAAATACTACAACAGCCAAGGA
ACAATCCATTGTTGAAAAATTTC
>732
TCATTTGGAATTTGAACTGTTGGATCTACATCTGGAGCACCATCTTGATTCTCCCTTTTT
ATTTTCCCAGACGAATTATCAATAAACACACTGATGAGTCTTTAGGTGACTGCTCTTTCC
TTAATACATGTTTCCACATG
>733
GAAAAGAGGGACTCGGGCCATGAATCTCAGAGTTAAAGTAGGGAAGATGACCAAATA
CCACTTTATGCAGTGGATCTGTTGTGATATCCGCTACCTGGACGTCAGTATCTTGGGCA
AGTTTGCAGTTGTGATGGCTGACC
>734
TCTATAAATGGTACTACCCCTAAACTGTAAAAGTTTGGCTAAAATTCTAGTTCTTTTTTA
TGCCCCACAGGTATGAACGGGTAGATGAAATTATTTGGGTGAAGACAAATCAACTGCA
ACGCATCATTCGGACAGGCCGT
>735
TAAACATAATAGAAAGGGCTAGAATTGCAATCTTGTGTAACTTAAAAAGCAGCTAGTTT
TTATTTCCTAGGTTCGTTCCACCAGTCATAAACCAGATGAAATCTATGGCATGATTGAAA
GACTATCTCCTGGCACTCGCA
>736
CATTTTTTAACAATATTAAACCCTCTAATTCCAGGATTTCTGGTTTGCCCCTCCTTTTACA

CTGTTTCAGGATCACCCTTGGAAACCAACTGGATGGGATCCACCTACTAGACCCAGAT
GTGGTTGCACGGTTCAAGCAA
>737
TGTTCTCTTTTTGGAAAGCTTTGTAATATAAATATGGAATTGTGTTTTATTTCCCATTTTTT
ATTTTCAGGTGTCTGCTTTTGGGGCAGAGTTTTCATCGGAGGCATGCAGAGAGTTAGG
CTTTTCTAGCAACTTGCTTT
>738
TTTGTCTTAGATGAGGCTGAGAAGGTTGTTTCTGAACAGAAAGTAATGATGACGTCCC
TCTTTTTTGTAGTATGTCCGTGGTTCAGACCCTGTATTAAAGCTTTTGGACGACAATGG
GAACATTGCTGAAGAACTGAGCA
>739
TAAGACACTACAGTGTGTGTCATAACCAAGACCTGATCAGGGAGTAGTTACTTCTCTTC
TTTTCTTACAGGAAACATGGTTCCAAAACTGTTCACTTCCCAAATTTGTCTGCTTCTTCT
GTTGGGGCTTCTGGCTGTGGA
>740
TAAGACACTACAGTGTGTGTCATAACCAAGACCCGATCAGGGAGTAGTTACTTCTCTTC
TTTTCTTACAGGAAACATGGTTCCAAAACTGTTCACTTCCCAAATTTGTCTGCTTCTTCT
GTTGGGGCTTCTGGCTGTGGA
>741
TGAGACACTATAGAGTGTGTCATAACCGAGACCGGATAGGGGAGTAGTTACTTCTCTTC
TTTTCTTACAGGAAACATGGTTCCAAAACTGTTCACTTCCCAAATTTGTCTGCTTCTTCT
GTTGGGGCTTATGGGTGTGGA
>742
TGAGACACTATAGAGTGTGTCATAACCGAGACTGGATCGGGGAGTAGTTACTTCTCTTC
TTTTCTTACAGGAAACATGGTTCCAAAACTGTTCACTTCCCAAATTTGTCTGCTTCTTCT
GTTGGGGCTTATGGGTGTGGA
>743
TGAGACACTATAGAGTGTGTCATAACCGAGACCGGATCGGGGAGTAGTTACTTCTCTTC
TTTTCTTACAGGAAACATGGTTCCAAAACTGTTCACTTCCCAAATTTGTCTGCTTCTTCT
GTTGGGGCTTATGGGTGTGGA
>744
GTTAGGGAATGGGGAAAGGGGAGCGAGCCAGAGAAAACTGACGCCCCTCTTCCCTGC
TTCCTTCCTCCAGGGACCCCTCGGAGGAGCATTCGCTGCTGGTGAATTGATTCGACCTG
ACTGCCGTTGGACCGTAGGCCCTG
>745
CCAGCCCTAGCCCTGCCTTCCCAGAACCGTAAGGGTCTATCTCCACGGACACTGTGCC
ACCCACCCCCAGATGAAGGAGTCCGCTGCCCGAGTGCTACAAGAAGCCTGGATGTTCT
ACAAACATACTCGCAGGAAGGAGT
>746
TTCCTCCCCTGAGCTACAGCTACATTCAGGGACCAGGCTTGTAAAACTGACTCCCCACT
TGTTCCCACAGGTTCCGCCAGGTGCGGCTGAAACACCGGAAGCTCCGGGAACAAGTG
AACTCCATGGTGGACATCTCCAAG
>747
TGCCTTACGCTATGGAGTAGGTGGGGGTCGACCCCAGCCCCGACACCTCTGATCCTCTC

CTCCCACCCAGATGCACATGATCCTGTATGACCTGCAGCAGAATCTGAGCAGCTCACA
CCGGGCCCTGGAGAAACAGATTG
>748
CCTCGGTGTGTCCCACTCTCACTCTCCCTCTTACTGGCCTGCCTCTCCATCAGTCTCTCT
GTCTCTCCAGGGACCCACGAGGAGGGAACCAAGGCTACTTTCCCCAAGTACTGAGGT
GGTGGACATCGTCTCTGCCACTC
>749
CCCCTCCTATCCGTCTCCCAGTCAGGGGCCTGGTGCTGTGGCCCCGCTGACCCCTCCCC
TGCCTCCTCAGCCCCAGGATGGGTGAGTTCAACGAGAAGAAGACAACATGTGGCACC
GTTTGCCTCAAGTACCTGCTGTTT
>750
CCCCACCCCCACCCCTCCCGGGCCACCATCAGACCTGGGCAGATGCGGTGACCTTTGT
GACTGCTTGTAGCTGGCTGGCCTGGCTGTCATGGCAGTGGGCATCTGGACGCTGGCCC
TCAAGAGTGACTACATCAGCCTGC
>751
CTGCTCTGAGGTGCACTAGGTCTAGGCACTAGGCCTCAGAACAAGGGTGCCCTTGTGC
TGCCCCCCCCAGTACTTCATCCTGCTCCTCATCATCTTTCTGCTGGAGATCATCGCTGGT
ATCCTCGCCTACGCCTACTACC
>752
CTGGGGCTCTGCCAGCCCCACCTTGGAAGGTCTTAGACTGAGGCTGAAGTTTCCTGCA
CCCCAACCCCAGCTGAACACGGAGCTCAAGGAGAACCTGAAGGACACCATGACCAGG
CGCTACCACCAGTCGGGCCATGAGG
>753
CAGGGCCTCCCTGGACCTCTGCAGGAGCCTCTGGCCCCAGGTCTCAACCCCAGCCTTG
TTCTTGATGCAGTTCCACTGCTGTGGCAGCAACAACTCACAGGACTGGCGAGACAGTG
AGTGGATCCGCTCACAGGAGGCCG
>754
GCTGGAGGCAGTAGGGGCCAGTGGGAGGTGCCCCCTGGGCCCGCCTTCAACACCCAT
CCGCGCCCCGCAGGGCGGCTGCATCACCAAGTTGGAGACCTTCATCCAGGAGCACCT
GAGGGTCATTGGGGCTGTGGGGATCG
>755
GGGACACGGGGCAGGGCGGTGGCTGGTGGCCCCATGACGTCTGCTTACGCCCACCCG
GCTCTGCACACAGGTCTTTGGCATGATCTTCACGTGCTGCCTGTACAGGAGTCTCAAG
CTGGAGCACTACTGACCCTGCCTTG
>756
TTTTAGGGAGCCCTAGCAGTACCTAGGTCAGAGGGGAGGTCTGACTTGCTCCCCAAAA
CTCATCCCTAAGAACATGCTCCTGCCAGCTGGACTAAGACAACGTGACCAGACAAAGA
AGAGCCCAACGGGGCCACTGGACC
>757
AAGGAAACCTGGAGGCCAGGATCTTGGGAGAAATTACCTCTACCTGAGCCATCCTTCA
TCTCCCCATCAGGGAAACCCTATATTCAGCCCAAGAGGGAAATCCCAGCAGAGGAGCA
GATCACCCTGGAGCCTGAGCTGGA
>758
AATAGGTAGGAGGGAGGCTTGGAACAGGTTCCTTCTCATTCCCTGTGACTCCTCTTCTT

ACCCTCTCCAGCAATTCTGGACATGTACACACTGATGAGTAACAAGCAATACTATGATG
CCCTCTGCAGTGGAGAAATCTG
>759
GAAAAGAAAACCAATGGGGAGGAGATTATGGGGAACATGTTGGGGTTCCCTTCCTGCC
CTCATTCACCAGGTGTGGTACAGCCTGACAAGTATAAGCCAGTGCCGGATGAACCCCC
AAATCCCACAAACATTGAGGAGAT
>760
ACCATAACACCGGATAACCACTGACATCCGAGAGTCCCTTAGCCCACCATCTCCTATTC
CCCTCCGATAGGACATCCCAATACCCATGCTAAGTGAGCTGTGTGAGGCAATGAAGGC
AAATACCTATGTGCGGAGCTTCA
>761
TCCTGTTCTTTCTTTCCTCAGTCATCTGTGAAACTGTTCCCTAATCCTAACCCAAGTCCC
TACTACCCAGGCAGTGGCTGACATGTTGCGTGAGAATCGTAGCCTCCAGAGCCTAAAC
ATCGAATCCAACTTCATTAGCA
>762
GGGTCTGTGATGTGGGATGGTAAACAGCCCAGAGAGGGTGACTGTCAGCTTTTGCCTC
TCCCTCCCCCAGCGCCAGTGGCCTGGTGATGCAGTGGAGATGGAGATGGCCACCGTGC
TAGAGCAGTGTCCCTCTATTGTCC
>763
AAGAATTAGACAAGGATGTTTATATAAACACTTTAAATGTATTTGATATAAAATTTTTATT
TCTTTGTAGACCCAGAAAACCCCAGAAGTGGTGATACAGTGGAAGTACAAGTGAATGG
AAATCTTGTCAGAGAACCTGA
>764
TCTGCATTGTATCATGGACAAAAACTGGTGGTAGGAATCAGTGTTTTGTTTTCCTTTCAT
GCTTCTACAGAGAATCAGCTGTTAATCTAGTGAGGAGTGGCTCCTATACCCGGCAGCTA
TGGAGGGATGAAGCAAAAGGA
>765
TCTGTGCCAGTGCTTTGTTGTATAGCATGCCTAACCATTTCCTTTCTGCTTGTATCTTTTT
AAATTTCAGGACTCCTCACAAATCCCAGGCCGACACAACAGCAGAGAAAACAGCAGA
CAATGTCTCTTCTAGCACCCCG
>766
AACACTTTGTCATTCCTCAAGTATATGCAGCAAAGATCTTGCTTTTGCTATTACTTTTTTT
TCTCTGCAGGTCCTATCTGACTCCTGTACGGGATGAGGAAGCAGAGTCTTTACGGAAA
GCACGCTCCAGACAAGCTCGG
>767
TATTCCTGATCAATCAAGAGTTATTCCATGGTTAGTGTGAAACAGCCCTGATCTTGTGAT
ATTTTCCCAGGGTGTCACCCTAACAGACCTTCAAGAAGCAGAAAGGACATTCAGCCGG
TCGAGGGCAGAGAGGCAAGCTC
>768
TATAAACCTGCTGTCCTTACTCTTTAGATGGACTTGATTTCTAATATCTCATATTGTGGAT
TATTTCCAGCCTATCTGTCATCGCCTGAGGTGCCCAGCTCAGCCAGACAAACCCACCAC
GCCAGCATCTCCTTCTACGT
>769
AGACACAGGCCCCTCCACAGTGCACATCCCCAGATTCTTAAAGTTCATACTTTATTTTAT

CTCTGGCCAGAGAATGAAGAAGCAGATTTGGATGAGCAGTCCTCTAAGAGGCTGTCCA
TCCGAGAGAGGAGGCGGCCCAA
>770
CTGAGCATTACCTTCTCCCCACTACTTTGTTCTCAAAACCAATTTTTATCTTTAATATTAT
CTCCCACAGGTTGGAATCGGGAGGTAGTAATCCTACAACCAGTGATTCTTACGGTGAC
CGGGCTTCAGCAAGAGCCCGT
>771
GCATTCTCATGTGATGAAATGGTGTGAGTTCTAACGCGAACGCTGATCCTCTTTTTTCC
CTCTCCTCTAGCTCTATGAGAGTGCTCTGACTGAAAACCAAAAACTGAAAACAAAACT
TCAGGAAGCCCAGCTAGAGCTAG
>772
GGCCTGGTCAGGGAGGGCCAAGGAGGATCCTGCCAGGCTCTAACTCACCTTTCCCTCT
GTCACCCTACAGGTGTTAACAGAACTGAAATCCGACAACCAGAGGCTGAAAGATGAA
AATGGTGCCCTCATCAGAGTCATCA
>773
ACTGGAGGTGCAGGGGGCCGGACTCAAGCCCAGAAGCTGCCTGCACCAACCACCCAA
CTTCTCTCCACAGCCCTGGAGCCCACCTCCCAGAAGCCCGGTGTGGGGGCGGGCCAC
GGGGGAGATCCCAAGCTCAGTCCCCA
>774
AGTGGTCCCCCTGTCTCGCTTCCAGCCCAGAACCATCTCTTCTCTCCCATCCCTGCCCT
CGGCCCCACAGTCCCACGCTGCTGGCTCCAAGCAGCACGAGAGCATCCCGGGCAAGG
CCAAGAAGCCCAAAGTGAAGAAGA
>775
AGTGGGCACTTCTTGTTAAATTTACCATTGAGACCCTGGTAACACTGAAGTTTGTGTTG
ACTTTCTGTAGGTGTTTTCACAGTCCTGTGTTTGGTATGGAGAGTGTGGAATTGCATAT
GGGGACAAGAGGTACAATTGCG
>776
GTTCACTGAGGAATGTTGACCTTACTCTAACTGTTGCCTACTCCCGTGTCTGTGCTCTTT
CACCTGAAAGGAACTCTGTCCAGGATTCTTCTTTGGCAATGTCAGTCTCTGTTGTGATG
TTCGGCAGCTTCAGACACTAA
>777
TATTTAAAATCGTTCTTGCTGGCCCTATTATGTGTGAGATCATGCAGAATATAATTTACTT
TTCCTTTAGATGTCCATCCTGTTTTTATAACCTACTGAACCTGTTTTGTGAGCTGACATG
TAGCCCTCGACAGAGTCAG
>778
TTGTAAGATCTTGCCTCGTGAATTACAGCAAGCATCTTGTCTCCTTTTTCCTTTGGGTTT
CCTCTTCTAGCAATGTACAATGCCTGCCGGGATGTGGAGGCCCCCTCAAGTAATGACAA
GGCCCTGGGACTCCTGTGTGG
>779
GTTTAAGTATAATACAAAATTTTGGATGTTTTTAATTGAGATTTGTACTCAACACAATTC
CTTTCTGTAGATTTTCCAGTCCATGGGATGGAGCCCATGAACAATGCCACCAAAGGCTG
TGACGAGTCTGTGGATGAGGT
>780
TCCCTGTTCCGACTTTCAGGAACGGCTTGGCTCTTAACCTCCACCTTCCCTTCCTCTCC

CGTTCTTCCAGGAGAGGCGTCCTGCTGTGACCCTGTCAGCGCAGCATTTGAGGGCTGC
TTGAGGCGGCTGTTCACACGCTG
>781
CTGATTAATCAAGATCTGAGAGAAATGTGACGTGTTTCTGGGTTTGCTTATTTTTAAAAT
CTTCTTTCAGGTTCTTGACTTACAAATAGCCATCGAAAACATTACTGCCTCTTATGACAA
TGAGACTGTGACACTTCAAG
>782
TGTAAATGTTTAGGTCCCTGATGTCTTGAGGCCCTTCTAACTGGGAGGTGTTTTCTAAA
CTTTTTGGCAGGGCTCCTGCCTCTCTGAATGATACAAGTTTGCTCCATGACCCTTGTCT
GGGTACGTTTGGTGGACCAGTG
>783
TCCCCTGGTATGTGTCTAATTTTCTGCATGCTTGTAATCTGCTTTTTGATGTCACATTTTT
CCTTTCTAGATCAAAACTACAATAACGCCACTGCCCTTGTGATTACCTTCCCTGTCAATA
ATTACTATAATGATACAGA
>784
TTAGGGAGAAGTTTCTTACTTAGCTGTCAGTTAGTTTAACTAAAAATATAACATTTTGCT
TACCTTTTAGGTTTATTAATTTTGTGAAAAACTACAAGAATCCCAATCTGACCATTTCCT
TCACTGCTGAACGAAGTATT
>785
GACCTTTTAGTAACAAGTGGGACAGACAACCCTGTAACTAATTGGTGATTGTGTCTGTC
GCCTCTCTCAGGTGGATTCGAAGGTCTCACTAGGCATCGCGGGCATCTTGATCGTGCTG
AGCTCGGTGGCTTGCTCCTTGG
>786
CAAGGCAGCAAGAAATGGCGTTGTTATTGAGAAATTTTAATGTCGCATAATTTTTTTTTT
TTTTTTTAAGAGAGATGAACGTCTTCAAGGGGAAACCCTGGATCAGCAGCTGGGCAGG
GTCCTAGGAGAAGTGGCTCCCA
>787
TGAACATAAGACCTGCAGAGAGCCCCCGGCTGTAAACAGAAGTGACGCAGAGCCCTT
CTCTCCCCGACAGGAGCATTGTCCGTGATGCCAGCCGTGCACACCTTCTCTCTCTTTGC
GGGATTGGCAGTCTTCATTGACTT
>788
TTTAATTTGAGTCTAGAATCTAGAGGAAGCAGCTAGCGGGTATAACCTTTGTTTCCCTC
TGTCCCCTCAGAAAAATCGGCTAGACATCTTTTGCTGTGTCAGAGGTGCTGAAGATGG
AACAAGCGTCCAGGCCTCAGAGA
>789
AAGATTTTATTTCTGGTCGTAAGCTTATTTACTAAGCAGTCTCTGTCTTATGATTCATTTG
TTGTTCCAGATAGCAATATTTGTGGGTGTTCTGTCATTCAGCATCGCAGTCCTGAACAA
AGTAGATATTGGATTGGATC
>790
AAGAAAAAGTACTGAAACTAAAGACTTCCTCCCTGTGGAGCAGGTCAGTAACCCTGTC
TGTCCTCTCTAGTACCCGAATAGGCTTCGCCCCCTCGTCCTGGATCGACGATTATTTCGA
CTGGGTGAAGCCACAGTCGTCT
>791
ATTTTAAAATGTTTTACAAAAAATCTGGAGACCTATTCTTCTAACAGTCCTCCCTGCATG

TCTCCGCCAGTGGTTGACCCTGCCTGCGTTCGCTGCAGGCCTCTGACTCCGGAAGGCA
AACAGAGGCCTCAGGGGGGAGA
>792
TCAAGTGCTCAGATTCTGCTGATATTTTGCAAGACCTGGACTCTCTTGACACCCAGGAT
TCTTTCCTCAGGGGACATGCTGCCTATAGTTCTGCAGTTAACATCCTCCTTGGCCATGGC
ACCAGGGTCGGAGCCACGTAC
>793
GTGACAGGATGAACACGCAGTGTTCGGGAGTGAGAGCGAGCTTTAATGAGGCCTCCC
CTCTCCCCTCCAGTGTGTTTTATGTCTTCTACGAACAGTACCTGACCATCATTGACGAC
ACTATCTTCAACCTCGGTGTGTCC
>794
TCAGAACGGGGTGGCTGCCTAGGGTCTTCAACAGGAAGAGAGCCATCCTAAAGGAAG
TGCTTTATTTCAGAGCTGTGGCATCTCCGTGGAGTTCTGCAGCCACATAACCAGAGCGT
TCACGGTGAGCATGAAAGGCAGCC
>795
TGTAAGGAAGTGTTTTTGTTCAATTACAGGTTGGTAAAAGTGGTTTCTAACACAGTATC
TCTTCTTTTAGGTGTTCAGTGGAATCACACTTACAAAATTTGGAGGGATTGTGGTGTTG
GCTTTTGCCAAATCTCAAATTT
>796
GTGAGCCACTATGCCCAGCCAACTAGCTCCCTTTCTCCTGCAGAAATAAGAAAAAGTC
TCTCTCTCATAGGGCCATCAGTAAATAAAGCCAAAAGTTGTGCCACTGAAGAGCGATA
CAAAGGAACAGAGCGCGAACGGCT
>797
GGCTCTGAATTTTTCTAAGAAAAAATGGTAACTATCACTTGTTACTCACTAACCAAAAA
TTTTCTTTCAGAACGCTCCAGAATAATCAGTTGAAAACAGTACCCAGTGAAGCCATTC
GAGGGCTGAGTGCTTTGCAGTCT
>798
GTTGTGAGACTTGAACTGACTCATTTCACTAGAATTTGTTACTGAACATGGTTGTTTTT
GTTTCTGTTAGGCGTTTAGATGCCAACCATATTACCTCAGTCCCCGAGGACAGTTTTGA
AGGACTTGTTCAGTTACGGCAT
>799
CATATAAAACAATGATAATAGTTGCCAATGGGAGATGGAAAAGTCATTTTATGCTTTTTT
TCCCCCTTAGGCATCTTCATAACAATAAAATTAGAGGCCTGAGTCAACACTGTTTTGAT
GGACTAGATAACCTGGAGACC
>800
CAGTAGATACTTGGGTTTTATTTCGCAAGAGGATAATGCATTTCAAAATACTTTTCTCCT
ATCTTTCTAGAGGATTTCATAGTAATTCTATTTCTGTTATCCCTGATGGAGCATTTGATGG
TAATCCACTCTTAAGAACT
>801
TGAACATAGAGTTAATATTAGAGTGCTTGTGAACATATGAAAATTTTACTTTTTTTGGAT
ATTTTCACAGACATTTGTATGATAATCCTCTGTCTTTTGTGGGGAACTCAGCATTTCACA
ATTTATCTGATCTTCATTCC
>802
TATATGGGAATAAATTTTGCTTACTATCTTGAGTTGAATTATTTCTTACATGAATAATGTTT

TTCCGCAGGACTTTGACAGGTACAAAGATAAGCAGCATACCTAATAATTTGTGTCAAGA
ACAAAAGATGCTTAGGACT
>803
TCTTTCCTCCTCCTCCTGTTCTCTTCTTTGTCTTTGTCTTCGTCTTCTTTCTTCTTCTTTTT
TTATTTAGTTCTTTACAGCGTAATCAAATCTACCAAATAAAGGAAGGCACCTTTCAAGG
CCTGATATCTCTAAGGATT
>804
AGTATAGTCTAAGTAAGTGGCATTCTGAGTGTTTATTTGAAAGATTAACATGTTACTCTT
ATAATTGCAGAGATCTGAGTAGAAACCTGATACATGAAATTCACAGTAGAGCTTTTGCC
ACACTTGGGCCAATAACTAAC
>805
AATACGTGTACAGATGTTTTTATGTGATTTATATATTGGGCATTAACTTGAAGTCTCATTC
ATATTTTAGAGATGTAAGTTTCAATGAATTAACTTCCTTTCCTACGGAAGGCCTGAATGG
GCTAAATCAACTGAAACTT
>806
TACTTTTACAAAACAAAACCATAACTGCGGAGTTGCTGTTCAGTGTAGATTTTACAACT
TTTTTTTCCAGGTCTTTATCAGTACCATATGCTTATCAGTGCTGTGCATTTTGGGGTTGTG
ACTCTTATGCAAATTTAAAC
>807
TTGCAGGAAAATATGGTTCTTTTGAGTTGCATAAGGTTTTAATTAAACATGCTTTGTGTT
ATATGTTCAGGTACTGCTGATGCAGCAAATGTCACAAGCACTCTTGAAAATGAAGAAC
ATAGTCAAATAATTATCCATTG
>808
GCCATATCATTTTCTAAATTGTTTTCTAAGTTATCTTTTATTTCTCTCTCTCTGTGTGTGTT
TTTTTTAGGTGCTTTTAAGCCCTGTGAATATTTACTGGGAAGCTGGATGATTCGTCTTAC
TGTGTGGTTCATTTTCTT
>809
TACCTTATACACAAAATATTTATGTTCTACCTGCCTGTGAAACGTCTAAAAGCCTCTCCT
CTCTCTGCAGACAGAGCACACTCCTCCGTATGATGTGGTACCTTCCATGCGACCAGTGG
TCCTAGTGGGCCCTTCTCTGA
>810
GGCATACACTTCTATCCCCTTTGATTAGACCAATAACCTTAAGGTCTTCTGTGACTTTTT
CCTCCAACAGGATATCCATCACAAGGGTCACCGCTGACATCTCGCTTGCCAAACGCTC
GGTATTAAACAATCCCAGTAAG
>811
AGAAACAGTATACCATTTTACTTTATCTTAAAAATACTGCACTTTAACTGAATTGTTTCG
CCCTTTACAGCGGAAGTTCAGAGTGAAATCGAAAGGATTTTTGAACTTGCAAGAACAT
TGCAGTTGGTGGTCCTTGACGC
>812
GTCAATAATGTACCTTGTACTTTACCTGGATGTAGTTATTACTCTGTTAAAAACTCATTAT
CTTTTACAGGTTTTACAAAGGTTAATAAAATCTCGAGGGAAATCTCAAGCTAAACACCT
CAACGTCCAGATGGTAGCAG
>813
TCTTATGGAGGTGGGAAGGGGGTGAAAACTGGGCTGGCATCAATGTGGTCTGGAATGT

CTGCCTCTGCAGGAGCTGTTCGATGTGATCTTGGATGAGAACCAGCTTGAGGATGCCT
GTGAGCACCTTGCCGACTATCTGG
>814
CACTTGCTCTGGGACATGTTCTTTACACACTGACCTTGGTTAACGCCTGGTGTGCTCCT
TTCGCTGCCAGGGTTCTCAAGGTGATCAGAGGACTGATCGCTCCGCTCCTATCCGTTCT
GCTTCCCAAGCTGAAGAAGAAC
>815
CGGCCGCCGCCCCCGCCCTCTGGCGGCAGCGCGGGGTACGGCGCCGCCTCTGAGCGC
CATCGTCTCACAGGTGCAGTCGGAGATCCGCCGTGGCTGGCACCACTGCCGCCTGCGC
CGCAGCCTGGGCGAGGAGCAACGCC
>816
TGACCTCCTGCCCGGCAGGACCTGAGTGTGAGGGTCTGTCCCACACTGACACCCTTTC
TGCTCCTCCTAGAACCGTACGGAGTCCCCAGAACCTAATGCCACAGCGACCCCTGCGA
TCCCCACTATCCTGGTGACCTCTG
>817
GCGGCTGGAGGTCCTGAGGGGCTCGGGTGCCCCTCGAGGGAGCCCTGACCCTGCCAC
CCCCTCCCCACAGCACTCACCGAGGACGGGAGGCCCTGCAGGTTCCCCTTCCGCTACG
GGGGCCGCATGCTGCATGCCTGCAC
>818
CTCCAGGGTGGTGGGGTGGGGTGGGGGGCCTCTGCCTGGGACCCCCATGCACGCAGG
TGTCGCCCCCCAGGTGTGCCACAACTCACAACTACGACCGGGACAGGGCCTGGGGCT
ACTGTGTGGAGGCCACCCCGCCTCCA
>819
GCAGAGAATGTCACAGAGGGACCCTGAGCCTCCCAGTCCGCCCCTCACATCCCCTCCC
GCATGTCCCCAGCTGCCCTGGATCCCTGTGCCTCCGGCCCCTGCCTCAATGGAGGCTCC
TGCTCCAATACCCAGGACCCCCA
>820
AGTGCAGGGCAGGGGCCCTGCACGGGGACAGCAGGTGGCAGGGTGTGAGGGCTTACT
GTGCACCCCTCAGAGAAATGCTTTGATGAGACCCGCTACGAGTACCTGGAGGGGGGC
GACCGCTGGGCCCGCGTGCGCCAGGG
>821
CCCCGGCCCCGACTCCGCTGTCGTGGGGCACTGCGCGGCCCCTGGCCCAGCTCCTCGG
CCCTGCCCCCAGCTTGTCTGAGCAGCCCTTGCCTGAACGGGGGCACCTGCCACCTGAT
CGTGGCCACCGGGACCACCGTGTG
>822
CCCCGGGGTGCCCTGGGGCAGTGCCGGGTGGACCCACCGTGGGCCGGCCTCACTGCC
CCTCTGCCCGCAGAGCCTGATGAGCGCTGCTTCTTGGGGAACGGCACTGGGTACCGTG
GCGTGGCCAGCACCTCAGCCTCGGG
>823
GATGCCCCCTCCCCATGCCCTCTTCGAGGGGCAGTGTGACTCCCTGCCAGCCCCCACTT
ATGCACCGCAGGAATCCGGACAATGACGAGAGGCCCTGGTGCTACGTGGTGAAGGAC
AGCGCGCTCTCCTGGGAGTACTGC
>824
AGGGCCTGTGTGTGGAAGGGGGCTGGCCCTGTGAACCCCAGGGGTGTGACCCGGTGA

CCTTTGCTCCCAGAATCCCTCACCAGAGTCCAACTGTCACCGGATCTCCTGGCGACCC
TGCCTGAGCCAGCCTCCCCGGGGCG
>825
GGGTGGGGAGAGGTGAGCCCGGTGGCTGGGGGAGGGCTGGTCCATGCAGCCTCCAGC
CCCCCTTGCACAGCCCCCCCAGGGACAGCGTCTCCGTGGTGCTGGGCCAGCACTTCTT
CAACCGCACGACGGACGTGACGCAG
>826
ACCACGGGCCCCGACAGCCTCCCGTTCAGCCCGCACACCACAGGCTGACCCTGGCCA
CTCTTCTGATCAGTCCTGATCCGGCTGAAGAAGAAAGGGGACCGCTGTGCCACACGCT
CGCAGTTCGTGCAGCCCATCTGCCT
>827
CCCAGCGTCCGCCAGGTGGACAGGTGGCCAGCCACAGTGTGGTGTCACGCTGAGGGC
ATTGTGTCCACAGACGTGAGCGGCTACTCCAGCTCCCTGCGGGAGGCCCTGGTCCCCC
TGGTCGCCGACCACAAGTGCAGCAG
>828
CACTTGGGGTAGAGGGGTGTCCCTGAACCAGGCCCCTGGGAGGGTGGCTCTGACCAA
CGTCTCTGCCCAGGGGGACTCAGGGGGGCCCCTGGCCTGCGAGAAGAACGGCGTGGC
TTACCTCTACGGCATCATCAGCTGGG
>829
AGACGATTGTTCCTGTGATCTGGGTAATTGCTTTATTTTTAATTGTCTGTTGTTTTTTTTT
CCTCATCAGGTGTAGGGAGTGAAGTCTCACATAGGAGGACCTGTGTGAGCCTCACTAC
CCAGCGACTGCCAGTTAGCAG
>830
TGATCTTAACTCCTGACCCTGAGGCTTTGAGAACGTGGCTAACTTCATCTGTCTTTTCC
TTGCGTTACAGTTTTATTACCAAACGTGGCCTAAAAGTCTGTGCTGATCCACAAGCCAC
GTGGGTGAGAGACGTGGTCAGG
>831
AGATGATTGTTCCTGTGATCTGGATAATTGCTTTATTTTTAATTGTCTGTTGTTTTTTTTTC
CTCACCAGGTGTAGGGAGTGAAGTCTCAGATAAGAGGACCTGTGTGAGCCTCACTACC
CAGCGACTGCCGGTTAGCAG
>832
TGATCTTAACTCCTGACCCTGAGGCTTTGAGAATGTGGCTAACTTCGTCTGTCTTTTCC
TTGCGTTACAGTTTTATTACCAAACGTGGCCTAAAAGTCTGTGCTGATCCACAAGCCAC
ATGGGTGAGAGACGTGGTCAGG
>833
TACCAACCTTCTTTTTATCATTTGTCTATTTGGTTGTTTTGCATACTGACAAGATTTTCTT
TTCTTTTAGTTGGTCGAATTGTCTTAGAATTGTTTGCAGATATCGTACCCAAAACTGCGG
AAAATTTTCGTGCACTGTG
>834
GTATCCATTAGTAAGCATTGCTAAAGGCATCCATGTCTCAGTCACTAATAGCCATTTTCC
TTTTTTGTAGTTATTAAGAAATTTATGATTCAGGGTGGAGACTTCTCAAATCAGAATGGG
ACAGGTGGAGAAAGTATTTA
>835
TTCAGACTAACACACAATTCAGAAAAGAACACATCTAACCTTGGTTTTCTTTTAATTTT

TATTTCTACAGCATGATCGGGAGGGTTTACTGAGCATGGCAAATGCAGGCCGCAACAC
AAACGGTTCTCAGTTTTTTATCA
>836
CATATGATAGAATCTAGCATTAAACCACATTACTTACTAAAATCCAACATATTTGTATTTT
TTTATACAGGTAGATAAAATTTTATTAATAACAGAAGACTTAAAAAACATTGGAAATACT
TTTTTCAAATCCCAGAACT
>837
TCTGGACATAGAGAAGAGTAATTCCTGTTTATGTGGTCTTTCATACTGCTTTTATTAATG
CATTCTGCAGATACGTGGACAGTTCAAAGGCTGTTATTGAGACAGCAGATAGAGCCAA
GCTGCAACCTATAGCTTTAAGC
>838
GCAATGAATTGAAATCTGGCATATAAGCTTAATACTTTGATATAAATACTAGCTTTATACT
TTTTTGTAGGCTCTTGAACTAGACCCATCAAATACCAAAGCATTGTACCGCAGAGCTCA
AGGATGGCAAGGATTAAAAG
>839
AATAGTTTGTTAGTCACCATAATTTTTGGATATTTTGAGAAATAACTCACATTGCATATTT
ATTTTACAGCTATCCAGGCAGAATTGCTGAAAGTCAAACAAAAGATAAAGGCACAGAA
AGATAAAGAGAAGGCAGTATA
>840
CGCATACCGTGGACCGCACCACAGGATCAGGGGAATAGGTTGCTCGCGGCTTCACTCT
TTGTCTCCACAGCAGCCCTCCGGAGAGCTGTTTGAGATTGAAATAGACACCCTGGAAA
CCACCTGCCATGTGCTGGACCCCA
>841
TGCCTGGAGGGAGCCTGCCCGGGGTGCGAAGGGGAAGGGCAGCCATCCTCACGTGGG
TTTCTTTCTCCAGGCTGTCGAAGGAGACTGTGATTTCCAGCTGTTGAAACTAGATGGCA
AGTTTTCCGTGGTATACGCAAAAT
>842
TAACGCAGCAGAGCTGGGGCCATGCCAGGGAGAATGGCTGCCCACATCCTGGTTTCCT
CTCTCCGAGCAGACTCAGCCGAGGACGTGCGCAAGGTGTGCCAAGACTGCCCCCTGC
TGGCCCCGCTGAACGACACCAGGGT
>843
CGCTCTCCTTCTCTGCCCTTTTCATTGTAAGTCATCTTTCCTCAAGAGCATTTTCATGTA
CTCTTCTCAGCCCCTCCCACCTTCTACCTATGTGGAGTTTACAGTGTGTGGCACTGACT
GTGTTGCTAAAGAGGCCACAG
>844
CGCGTCAATGAAATTGGGGGGGATCCATTTTTGAAATTAGTTAAAATAAATCCTCTTTCT
CTGTGGGCAGCAATATGGCTTTTGTAAGGCAACACTCAGTGAGAAGCTTGGTGGGGCA
GAGGTTGCAGTGACCTGCACGG
>845
CCTGGGCCTGTGGGTTGTCTTGCCTGGGAGGAGGAAGCAAACTAACTGAAGGAAATG
GTCCTTTTTCCAGCCCGTGACCTCACAGCCCCAACCAGAAGGTGCCAATGAAGCAGTC
CCCACCCCCGTGGTGGACCCAGATG
>846
GAAGGCTGGAGTGCTGTGGCAGGGATGACGTCTCACTTCTGTTATGTCTCTGTGCCCT

GTGCTCTCCCAGGATGAGGGGCATGAAGCTGCTGGGGGCGCTGCTGGCACTGGCGGC
CCTACTGCAGGGGGCCGTGTCCCTG
>847
CCCTTTGTGGCGCTGTAGGGTCCCTGGGTGGCACCAGCCCTGCTCAGTACCACTGTGG
CCCTGCCCCCAGATCCTGAGCCGCTATGACATCGCCCTGGTCCAGGAGGTCAGAGACA
GCCACCTGACTGCCGTGGGGAAGC
>848
CCTCGCTATCGGCAGCCAGAGGGGTCCCCTATGGCCCCCGCCACTGGGACCTTTTGTTT
CTTCAATCCAGGGATGCACCAGACACCTATCACTACGTGGTGAGTGAGCCACTGGGAC
GGAACAGCTATAAGGAGCGCTAC
>849
TGCCTCCTGGGAAGCAGGAGTGGGGAGCTTCCAGCCTGGGGTCACCTCCTCCTGCCC
GGCCTTCCCGCAGGCCTGACCAGGTGTCTGCGGTGGACAGCTACTACTACGATGATGG
CTGCGAGCCCTGCGGGAACGACACC
>850
GTTCCAGCTGACATGGTGACTGAACCTGCCCCCAGGGAGTGTGCCTCACACGACGTG
GCTGTCTCCACAGAGGTCAGGGAGTTTGCCATTGTTCCCCTGCATGCGGCCCCGGGGG
ACGCAGTAGCCGAGATCGACGCTCT
>851
GCCCTCCCAGGGGCAGTGGGCACCAGCGGCCTCCGCATGTCCCAGGGCCACAGGCAG
CGTTTCCTGGTAGGACGTCATGTTGATGGGCGACTTCAATGCGGGCTGCAGCTATGTGA
GACCCTCCCAGTGGTCATCCATCC
>852
GTTCCTGCGGGTGCTGAGCCAGGCCCATGTGTGAAAGGGGAACCTACTTTCTCTTCCC
AACACCCATCAGGATCGTGGTTGCAGGGATGCTGCTCCAAGGCGCCGTTGTTCCCGAC
TCGGCTCTTCCCTTTAACTTCCAG
>853
CAGCCACATGAGGATGGGACACAGGAGCTCAGGTAGGCTCAGCCCAGACCCTGTGCC
CACTTGCCTGCAGGCCCAAGCCATCAGTGACCACTATCCAGTGGAGGTGATGCTGAAG
TGAGCAGCCCCCTCCCCACACCAGT
>854
CTCTGCCTTGTTGAGAGCCCTTCTGCAGGCTTCCGCCTTCCCTTCCCAACTCCTGGATT
CCCGGATACAGATGAGAAAGGGCAACACCATGCAGCAGTTCCTGCAGAAGGCGCTCG
AGATCCTTCGGAAAGACTTCAGTG
>855
ATAGCACCTTGCCGCCGATGTTGTCACTGGGGCAGCAGCGGGACATTGGGCTGTCACT
TCTCCCCTGCAGGACCACTCTTCAACTTTGATGTTCATGACGATGTGCGGTTGCTCAGT
GACGCCACTGTGGAGAAGGATGA
>856
CTGGGCTATGGAGGAGAAGCCAAGGGAAGGGGAGGTCAGCAGGCGGCCCTGTGCCCT
CTTCCTCCCTTAGTCCCATGCAGGCAAGGTGGTGCTGAGGAGCTGGTACGAGAAGAAC
AAGCACATCTTTCCCGCCAGCCGCT
>857
GGGACGGCAGCCAGCCCCTGCTCACCCCTCGCCTTCCTTGTCTCCTCTGCCCACCTTGT

CCTCACACTAGATCCGCTGAGCATCCAGGAGCTGCGCGGCCCCGGCTCCTCAGCTCCC
TCAGTGTGCCCCGTGGTGTCACC
>858
AAGAGAGGGGAGAGTGGAGGTTGAGGGGTGGGGGAGAAACTGCCTCTAGACTTCCCT
TGTTTCCTTGAAGGTTAACATTAGGCTGGGAAGATGACAAAACTTGAAGAGCATCTGG
AGGGAATTGTCAATATCTTCCACCA
>859
ATAAGAGGCCTAAGAAAGGGTCTTGTGTGGAGGTTCTGAACTCAAGGTCTCTGTTGTC
CTTTTTTCCTAGAATATCAAAGATAAAGCTGTCATTGATGAAATATTCCAAGGCCTGGAT
GCTAATCAAGATGAACAGGTCG
>860
GGGGGCGCAGGACCTGAGGAGCCGCGCCGGGAGGAGGGTCGGGCGGGTCTCAGCCC
CTCCTCGCCCCCAGGCTCCCACTCCATGAGGTATTTCGACACCGCCATGTCCCGGCCCG
GCCGCGGGGAGCCCCGCTTCATCTC
>861
AAATCCCCGCGGGTTGGTCGGGGCGGGGCGGGGCTCGGGGGGACGGGGCTGACCGC
GGGGCCGGGGCCAGGGTCTCACACCCTCCAGAGCATGTACGGCTGCGACGTGGGGCC
GGACGGGCGCCTCCTCCGCGGGCATAA
>862
TCACATGGGTGGTCCTAGGGTGTGCCATGAGAGATGCAAAGCGCCTGAATTTTCTGAC
TCTTCCCATCAGACCCCCCAAAGACACACGTGACCCACCACCCCATCTCTGACCATGA
GGCCACCCTGAGGTGCTGGGCCCT
>863
TATCTCTTCTCAGGGAAAGCAGGAGCCCTTCAGCAGGGTCAGGGCCCCTCATCTTCCC
CTCCTTTCCCAGAGCCGTCTTCCCAGTCCACCGTCCCCATCGTGGGCATTGTTGCTGGC
CTGGCTGTCCTAGCAGTTGTGGT
>864
GTGGGCTGTTTAGAGTGTCATCACTTACCATGACTAACCAGAATTTGTTCATGACTGTT
GTTTTCTGTAGCCTGAGACAGCTGTCTTGTGAGGGACTGAGATGCAGGATTTCTTCAC
GCCTCCCCTTTGTGACTTCAAGA
>865
GAGGGCGCAGGACCCGGGGAGCCGCGCAGGGAGGTGGGTCGGGCGGGTCTCAGCCC
CTCCTCGCCCCCAGGCTCCCACTCCATGAGGTATTTCGACACCGCCGTGTCCCGGCCCG
GCCGCGGAGAGCCCCGCTTCATCTC
>866
AAAATCCCCGCGGGTTGGTCGGGGCGGGGCGGGGCTCGGGGGACTGGGCTGACCGCG
GGGGCGGGGCCAGGGTCTCACACCCTCCAGAGGATGTATGGCTGCGACCTGGGGCCC
GACGGGCGCCTCCTCCGCGGGTATGA
>867
TCACATGGGCGCTGCTGGAGTGTCCCAAGAGAGATGCAAAGTGTCTGAATTTTCTGAC
TCTTCCCGTCAGAACCCCCAAAGACACACGTGACCCACCACCCCCTCTCTGACCATGA
GGCCACCCTGAGGTGCTGGGCCCT
>868
TGGAGCCCTTCAGCCGGGTCAGGGCTGAGGCTTGGGGGTCAGGGCCCCTCACCTTCTC

CTCCTTTCCCAGAGCCATCTTCCCAGCCCACCATCCCCATCATGGGCATCGTTGCTGGC
CTGGCTGTCCTGGTTGTCCTAGC
>869
GTGGGCCGTTCAGAGTGTCATCACTTACCATGACTGACCTGAATTTGTTCATGACTATT
GTGTTCTGTAGCCTGAGACAGCTGCCTGTGTGGGACTGAGATGCAGGATTTCTTCACA
CCTCTCCTTTGTGACTTCAAGAG
>870
TGACCCCCTGTGAGTCCTGGGCCAATCCCTGAGGACTGCTAAGCTGCGTCCTATGCCCT
ATGCCTGGTAGATTCTGAGCAAGCTGGTGAACAGCCTGTACCCTGATGGCTCCAAGCC
GGTGAAGGTGCCCGAGAACCCAC
>871
AGAGGGATCAGAAAACGGGTGGAAAACACACAGTTCTACCAAGTCTTTATCCTGCTTC
CTCCTCTTCTAGGAAAGCGCAGGAGCATAAGAGGGAATTCACAGAGAGCCAGCTGCA
GGAGGGAAAGCATGTCATTGGCCTT
>872
CCCCTGTAAGGGAAGCTCGAGAGAGAGTCTGCTGTTCAGCCATCTGACTGCTTCTAAA
TCTGCTTTTCAGGCCTAAAGGGGAGTGGCCCCAGTGGCGGTAGCGGCGGTGGTGCCAA
TGGTACCTCTTTCAGCTACACATT
>873
GACGGCCTCTGGGCAAGAGCTGAGCCTCTTGAGCCAGCTTCCATCTAATGCTGTCCTTT
TGCTTTTCAAGGCTCTGTGTGGCTGCACAGTGAACGTCCCCACTCTGGACGGCAGGAC
GATACCCGTCGTATTCAAAGATG
>874
TTGGCTTCGGTTTTAGCATTCTGCTGTCATCACCTCCATCCCTCCGTCTTGCTCCTGTTT
CTCTCTGCAGAGTGGCAGCCAGCGGTGCAGATTCTCTTGTACTCCTTGATATTCCTGCT
CAGCGTGCTGGGAAACACGCT
>875
TTCAAGCTAATGTGCAAATAAACTTTGGATTTCAGAGTCAACATAACCGGCTTCTTTGT
TCCTTTCCCAGGCACCTCTGTGAGTGTATCTACCTTTAATCTGGTAGCCATATCTCTAGA
GAGATATGGTGCGATTTGCAA
>876
ATTCATTCCTTCCCATTTGAGTTTGAAGGTCTAAAGTTGCTGGTTATTGGATTTCTTTTG
ATTTCATTAGGCACACATTCCTGTTACTCATCCTCTTTCTTATTCCTGGAATTGTGATGAT
GGTGGCATATGGATTAATC
>877
GTGGGGGCTCCATGCCTTCTACCGGGCATGACCCATCCCCAGCAATGAGTGATGTCTCT
TGCCCACCCAGAAAGGAAACCTAGCACCACCAGCAGCGGCAAATATGAGGACAGCGA
TGGGTGTTACCTGCAAAAGACCAG
>878
CCCTGTTCTTCCTCCAAGCCTCTGCCTTGGAGCTATGGATACTATAACTAACTGAAGCTT
CTTCTTTCAGGTACCCACTGATGGTAATGCTGGCCTGCTGGCTGAACCCCAGATTGCCA
TGTTCTGTGGCAGACTGAACA
>879
CTTGTTAGATGCTTGTAAATGCCAGCCCCTGCCTCAAGTAACAATTGATTCTTTTTGTGT

GCTCTCCCAGGTCTACCCTGAACTGCAGATCACCAATGTGGTAGAAGCCAACCAACCA
GTGACCATCCAGAACTGGTGCA
>880
TTGATGTCTTCTGCGGTAAGAACACTGTGATACAGATGGAATGACGGGAAGTGGTTTT
CCTTTCTTTCAGTTGGTGAGTTTGTAAGTGATGCCCTTCTCGTTCCTGACAAGTGCAAA
TTCTTACACCAGGAGAGGATGGA
>881
TTATGTGGAGTTTTCTTACAATGAAACACATCTACTCTACCACTCACTGTTTCTCCTTA
CACTTTGTAGACATGCAGTGAGAAGAGTACCAACTTGCATGACTACGGCATGTTGCTG
CCCTGCGGAATTGACAAGTTCC
>882
ACGACTTTTCTTTTCCTTCCCTGAAATGTGTTTAATTGACTTTTTCTGTTATTTGTGTTTG
CCTTCACAGTGAAGACAAAGTAGTAGAAGTAGCAGAGGAGGAAGAAGTGGCTGAGGT
GGAAGAAGAAGAAGCCGATGAT
>883
CGTGTATACATGAACAGAGAGACAGTGCCTTTTCATGCTAAATGTGGTTCCCCACATCT
CCTCTGATTAGAGGTGTGCTCTGAACAAGCCGAGACGGGGCCGTGCCGAGCAATGATC
TCCCGCTGGTACTTTGATGTGAC
>884
ATTAAACGAGTGGATTATTCTGTTGTTGTTGGCTTTTTTTTCTCAAACCTCCTTCTCTTCT
ACTTTATAGTTCCTACAACAGCAGCCAGTACCCCTGATGCCGTTGACAAGTATCTCGAG
ACACCTGGGGATGAGAATGA
>885
CCATCCTATTAAGTCTGTATTCAAAGGATGAACTGATGATTTTAAATTCAAATGTTTCCTT
AATTTATAGGTCATGAGAGAATGGGAAGAGGCAGAACGTCAAGCAAAGAACTTGCCTA
AAGCTGATAAGAAGGCAGTTA
>886
TAGAAGACTTGAAATTGCTGCTTCATCCTACTTATTCAGTCCCCATGGACATATGTGTTT
ATGATGGCAGCATTTCCAGGAGAAAGTGGAATCTTTGGAACAGGAAGCAGCCAACGA
GAGACAGCAGCTGGTGGAGACAC
>887
ACCCATCGCCAATGGAAGAAGCAGTGTTTTGCACAAACTTGAAAAAGAGTTTTTCATT
TTCCTCCCACAGCCTCGTCACGTGTTCAATATGCTAAAGAAGTATGTCCGCGCAGAACA
GAAGGACAGACAGCACACCCTAA
>888
AAAAAGAAAGAAAAAGAACCATTCCTACCCCCAGACATTGTTGACCTGGAGTTGTCAT
CCTTTGATGCAGGTTATGACACACCTCCGTGTGATTTATGAGCGCATGAATCAGTCTCT
CTCCCTGCTCTACAACGTGCCTG
>889
TGGTGGCTGCTGCTGCTGCTGCTGTTGTGATTGTTGTTACTCACCAAAGAGATGGTTTT
GTTTGGTTTAGATGAGCTGCTTCAGAAAGAGCAAAACTATTCAGATGACGTCTTGGCC
AACATGATTAGTGAACCAAGGAT
>890
CTTACAGTGTTATTATTTATGAGTAAAACTAATTGGTTGTCCTGCATACTTTAATTATGAT

GTAATACAGGTTCTGGGTTGACAAATATCAAGACGGAGGAGATCTCTGAAGTGAAGAT
GGATGCAGAATTCCGACATGA
>891
ATTGCGTTTATAAATTGTAAATTATATTGCATTTAGAAATTAAAATTCTTTTTCTTAATTTG
TTTTCAAGGTGTTCTTTGCAGAAGATGTGGGTTCAAACAAAGGTGCAATCATTGGACT
CATGGTGGGCGGTGTTGTCA
>892
ACTGCTTCTCCATGTTCACCCTTAAAAGATTGATTTTTATTTTTTACTCAGCTCTCCTCTT
GTTTTTCAGGTTGACGCCGCTGTCACCCCAGAGGAGCGCCACCTGTCCAAGATGCAGC
AGAACGGCTACGAAAATCCAA
>893
AGGAGAGACCGCTGTTCTGCGGCGCCATTCCTGGGTTCTCATCCTAAGGCTGCTTTCTA
TTCCATAACAGTGGTTGAAGTTGACATCTGACGACGTGAAGGAGCAGATTTACAAACT
GGCCAAGAAGGGCCTTACTCCTT
>894
GAGCCTTGACATCATCACATTCTCCAGTTTGTGTTTCGTGTTGAAAGTTAAAACGTGAT
TCCCTTTTCAGGTGTAATCCTGAGAGATTCACATGGTGTTGCACAAGTACGTTTTGTGA
CAGGCAATAAAATTTTAAGAAT
>895
CTTATTAATGTTTGATAATGTTAGGTCATTTTGGGTGGTTTTCTTGAATTGCACCAAATTT
TATTTTTAGGATAAGGATGCTAAATTCCGTCTGATTCTAATAGAGAGCCGGATTCACCGT
TTGGCTCGATATTATAAGA
>896
ATTCTGTTAATGAATGTGGAGGGACCAGTATTAATCCTGATTTTCTTTCTTTCCCCTTTTG
CTTTTCCAGTGAATCATCTACAGCCTCTGCCCTGGTCGCATAAATTTGTCTGTGTACTCA
AGCAATAAAATGATTGTTT
>897
CCGCGATGTCTGCAGCAGGGGTTTCCCTTCGCGTCCCGTCTTCCTCAACTACCTGTTTT
TTCTTGTCCAGACCGGGGCTTTGAGCTCACATTCAAGACCGCTGATGACCCCAGCCTG
TCTCTCATAAAGTATGGGGAATT
>898
CCTCCCTGCCAGTGTCAGAGGAGCAGGGAGAGGAGACCTCTGGGTCACTGTGTCTGG
TGCCTGCTTTCAGATTTTGGAGGCAACATCAACGTGGAGACCATCAGTGCCTTTATTGA
CGGTGGAGGCAGTGTGCTGGTAGC
>899
GGAAGCCAAGGACACTTGGCCCAAGGCCCCCCTGTAAGGGCTCAGCTAGGTGTCTCTT
CGGTCTGTGCAGGTGACCCTCTTCGAGAGCTGGGCAGTGAGTGCGGGATTGAGTTTGA
CGAGGAGAAAACGGCTGTCATTGA
>900
GTGTGAGGAGGTTCTCCCTGGCTTGGGGAACCTTTTCCTGACCACCCACCTTTCCATCT
ACTCTTTGCAGCATACGCTCATCGTGGCTGACACTGAGAACCTGCTGAAGGCCCCAAC
CATCGTTGGGAAATCATCTCTAA
>901
CTCTCGGGGATCCCATGTCCTTGGGCTCAGTTCCTGGGGCCAGTGCTGGGCTGCTCTC

GTCCTGCTGCAGGATGGTGGCCGATCCTGATAACCCTTTGGTGCTGGACATCCTGACGG
GCTCTTCCACCTCTTACTCCTTC
>902
CTTGTTCGGGAGGCGTGCAGCTTTTCCCTTACTTGGAACTGCTTGGGTCACAGCTTCGT
TTTGTTCCCAGTATCCACATGCGGTGGGGAAGAACACCCTCCTCATTGCTGGGCTCCAG
GCCAGGAACAATGCCCGCGTCA
>903
GTCCCTGCCCATCAGGCAACACTGGAAAGGTGGCTCTGGTGGCCAAGCTGCTCCTGTT
TCCCTCCTGCAGGTATTCCCAGACAGGCAACTATGAACTAGCTGTGGCCCTCTCCCGCT
GGGTGTTCAAGGAGGAGGGTGTC
>904
AGCTCTGCCTGCAGGTGGTGGAAGGCCTCCAGTCATGGCCACCCCTGGCTAACCTGCC
CTTTTATCTCAGGAGTATAGCATCGTGATCCAGCAGCTCTCAAATGCCAAATGGGTCCC
CTTTGATGGCGATGACATTCAGC
>905
TGCTCTCGAGGCCCAACCAGGAAAAGCTCTGGGAAACCCCTCCCGGTTATTCTTGTTG
GTTCTTCCGCAGGTGGCAAATACAGTGTTCAGTTCAAGTTGCCCGACGTGTATGGTGTA
TTCCAGTTTAAAGTGGATTACAA
>906
GTTTTCAGCTAGCATGTGCCCTGCCTGGGTTCTGCAGTGGGCAGGTCCTGCCACCTCTG
TCCTCCTGCAGGTATCCGTGCGGCCACTCCAGCACACGCAGTATGAGCGCTTCATCCCC
TCGGCCTACCCCTACTACGCCA
>907
GAAACCATTCACCCTTATATTTAACTGTGAAATATCTGATTTAAAATTATTTACTTCTTTG
TTTCCACAGCCTGGTGAGAGTCAAAGAGGCCCCAGGGGACCATATCCACCTGGACCAC
TGGCTCCTCCTCCTCCACCAC
>908
GCCGCCCGGGCGCCCTGGAGGGGTCTCCGCCCGGAGCCTGGCGCTGCCTCGTGACCT
GCATCATCTTCAGTTCCAGGGTCTCCCTCCACTGCTGTAGTAACCCGGGTCCGGGGCCT
CGGTGGTGCTCCTGATGCCCCTCA
>909
AGCGCTGCCGGGTTCTTGGAGTCCTGTGGGATGAGTCAGGGTTCTGAATTGGGCTGCA
CCCCCTGGACAGGATGATCGGCTGAATGTAACAGAGGAACTAACGTCCAACGACAAG
ACGAGGATTCTCAACGTCCAGTCCA
>910
AGGGTAGCTGCTAAGGCCCAGGAGGTAGGCAGGTGGGTCGGGCCAGCCGAGACGCTG
ACACTCGCTGCAGCTTTGCAGTTCTCCTGGAGTTCGCTGAGGAGCAGCTGCGAGCCGA
CCATGTCTTCATTTGCTTCCACAAG
>911
CCCTGCGGGGGTGGGGGGTGCGGGCGGGGGGTTCTGGTCGGCAGGGGTTCTGACGGC
TTCTCCCCCGCAGCCGCCTTGCTCCGAACCTTCAGCTTTTTGGGCTTTGAGATTGTGAG
ACCGGGGCATCCCCTTGTCCCCAA
>912
TTGCACAGCCTGATGGTGGCCCGCAGAACCAGGCCTGGAGCTCCCACTGCGATCTTTG

CTTTCTTTTTAGCGTCAATCCAGAGTTCAGCCCAGAGGCCCTGGACAACTGCTGTCGG
TTTTACAACCACACGATCTGTGCA
>913
GTCACCCAAAAAATACTTTTCGTATTCTACACTGATGTGACAGTAATAATGGTTTTTCTT
CAACTGACAGATGGGCTTTTTAAACGGGTCCGGCCACCTCAAGAAGAACAAGAAAG
GGAGCAGCTTCAACCTCGTGAAA
>914
GGCTCTGTGAAGGGTGGGCCACAACACAGCTGTTCCCAGCACAACTCAGTGTTTCCA
ACCTTCTTTTCAGTATTAGGATATCCAAGCCGCCTAGCCCGAAGCCCATGCCTGTCATCC
GAGTGGTGGAAACCTGAGCTGCC
>915
CTGTAGTTCCAAGTTAAAATTCTACACTCCTGATATTTATATATTGCTACTTTGTCATTTT
CTACCATAGGTCGAAGATCAATTTACAAGACCTCCAAGGAGCTTGCACCAAGAAGACA
GCCTGAATAGCCCGACAGTTC
>916
CACTGATTTTAAAAACTGTACAGTGATCAAACCTGGCCGTTTTATCACAAAACAATTTT
TATATTTTCAGTACGTGATGGTTCAAGACATGCTCTCTCCATCCCCCATGGAACGACCTG
AAGCTATAAACATCATTGAAA
>917
AAGCCTCTGAGGGATTGAGGGGCCCTGGCAGGACCCTGACCTCCGCCCCTGCCCCCG
CTCCCGCTCCCAGGTTCCCCCAGCAAGCGGGAGCCCGTGGTGGCGGTGGCTGCCCCG
GCCTCCTCGGAGAGCAGCAGCACCAA
>918
CCCCCCTGACACCCTGCCTCCAGCCTGCCCCTTTCCTCTAAGGCCCTCTGTTCCTCTCC
TCCCTTCCTAGAACATCATCCGCAAGGTGAGCGGCCAGAAGTTCGTCTACAAGTTTGT
GTCCTACCCTGAGGTCGCAGGGT
>919
GGTGGCCACAGGAGAATCCTGTGGTCCCAACAATCCTTACACACCCTCTTTGTGCCTG
CTCCTCTACCAGGTCATCCTGACCCCGCCCGAGGCCCCAAACCTGAAATCGGAAGAGC
TTAATGTGGAGCCGGGTTTGGGCC
>920
CCCGTTTTGGACAGGGTGAGAGACCCAAGAATGGCCCCCATCTGTGACCTCTCCCTCT
TTTTGCCCCCAGTTTCCATCCAGTGGCAGCGCCCAGGTGCACATCCCTTCTATCAGCGT
GGATGGCCTCTCGACCCCCGTGG
>921
CTAGGTAGGGGTGGATGTGCCACCCAACACCCAACCACTGACCCATATTCCTCCTTCCC
TTACTTTCCAGGCCCATCCCGTGGCGGTTGCCAGCAAGAAGCGAAAGAAGCCTTCAAG
GCCAGCCAAGACTCGGAAACGTC
>922
GTGAGCCAGATGGGTGAGGGTTGTGATTGGGGGTGCTCTTGGAGCCCCACACCTGCCT
TTCCCATCCCAGGTGCTGTGAGAACTGTGGAATCAGCTTCTCAGGGGATGGCACCCAA
AGGCAGCGGCTCAAAACGTTGTGC
>923
GGTCAGTAGACATGGGAGGACCAATGCTTCTGGAGCAGGCCCGTGATCTCGTCCCTAC

CATTCCTGGCAGCGTGTGGGCTGTGGGGAGTGTGCAGCCTGCCAGGTAACAGAAGAC
TGTGGGGCCTGCTCCACCTGCCTCC
>924
TCGGCCCCTGGCCCCCATGGGCTTGGCCCCATGCTTCATGCCTCTGCTCCCACCTGCCC
CCCGCCAACAGAGCCGAGGGTGTGGAGTATGCCGGGGCTGTCAGACCCAAGAGGATT
GTGGCCATTGCCCCATCTGCCTTC
>925
TAGGGTGTGGGGAGTTTCTGTGTCTGCCTGGTGCCACCAAGCTGAAACCACCCTTTCT
GATCTTTCCCAGGGTAAACATGCCCGCCGCAAGGGAGGCTGTGACTCCAAGATGGCTG
CCAGGCGGCGCCCCGGAGCCCAGC
>926
ACTGGAATAGGCGTTGAGGATGGCCTCTCCTAGTCCTAGTTGACAGCACCACCCATTCC
CCCCACCCCAGCAGCCCTACACGAACCGCCGGCAGAACCGCAAGTGCGGGGCCTGTG
CAGCCTGCCTACGGCGGATGGACT
>927
TGGAGCCGGGCAGGGTGGATGTGATTGGTCAGGACACCAGGTAGCCATAGCACCCTAT
CTTTCCCCATAGAAGCGGCTGCTGCCCAGTGTCTGGTCAGAGTCTGAGGATGGGGCAG
GATCGCCCGCACCTTACCGTCGTC
>928
CCTTCCCAGCCTCACCCAGCTGCATGCCAGGGAGCTGAGACCAAGTCTGCTGCAATCC
TCCCTCACGCAGGAGGCCCAGTGCTCTGGCCTGAGTTGGGTTGTGGCCTTACCCCAGG
TGAAGCAAGAGAAGGCGGATACCC
>929
TTGGCTGATAGAGACTTTCCTCAGGTGAACTCTGCCCTTCTAATGGTTGCCTGTTTCCT
ACTTCTCCCAGGCAGTAGACCCAGGCCTGCCTTCTGTGAAGCAAGAGCCACCTGACCC
AGAGGAGGACAAGGAGGAGAACA
>930
GGTTTTGTAATGCTCCATTGCGCACATAGCAAACCTCATTCAACATACAGACCTGCTCC
CTTTTCCCTAGGTCCAAAGACCTTAAAAAACCTGGAGCTAGAAAGCAGTAGACTGGAG
GCTTCTACAGACTGTAGGATTCA
>931
GGTGCGGGACAAGGACTTCCTGCCCTGTCCTCACCTCCCTTCTCTCTTTGATGCCCTCC
TCCCACTGCAGATCCATTACACCGGCTGCTCTATGAATCCTGCCCGCTCCCTGGCTCCA
GCTGTCGTCACTGGCAAATTTG
>932
GCCGGGGCCTGAGGGCTCCGCGTGCCGGTGCGGGCGCGGGTGCCAAGCCGCCCTCTC
CGCTCGCCCCCAGGTCTTCTGGATCGGACCCCTGGTGGGCGCCATCCTGGGCTCCCTC
CTCTACAACTACGTGCTGTTTCCGC
>933
CCTACTCCAGGAATCCTTAAGGCAGATATCATTTACCTACACTGAGTTCTTTGTTGCCAC
CCTGTAACAGGCACAATGAGTTTGACTTCATCTCAGGAACTCGAATGAGGAAGCTCGC
CCGGGAAGGAGAGAATCCCCCA
>934
GGTGCCCATCTGAGTCCCTGGGGGCAGGGGCGCCCTCGGGCTTTGACGACGCCCCCTC

CCGCTGGGCCAGGTCGTCCATCCGCAACGTGCACAGCATCCACCAGCGGTCGCGGAA
GCGCCTCAGCCAGGACGCCTATCGC
>935
CAGAGAGCGCTGTGGGCGGGGCCAGTGTGCGGGGCGGGGCGTCTGACTCGGCCCCGC
TCTCTGCCCGCAGAGGAGGAGCGGCAGACGCAGCGCTCTAAACCGCAGCCGGCGGTG
CCCCCGCGGCCGAGCGCCGACCTCAT
>936
TTTCCCAATCAGAACAGCAAATTCTAATTCCATTTTGGGTTTTCAATTCTGATGCACTAT
GTTTGTTTAGCTCTTAGCCCCCCACGACGTAAAGCCTTTAAGAAATGGACACCTCCTCG
GTCACCTTTTAATCTCGTTCA
>937
GAGAAACTACTTGAACGTGAAATCAGCCCACCTGGAGTCTTGTAATCATTCAGTTACTT
TTACTTCCCAGGCAAAATGGCAATACCTGTGCTTTGGAAGTTTCTGGAGAAGTATCCTT
CAGCTGAGGTAGCAAGAACCGC
>938
CCATCACCACACATTTTGGGAGGGTGTCTTTAGAAGCTGACCTGATAATGTGGGATGTT
GTATTCTTCAGATGAATACCTGACAAAGCAGTGGAAGTATCCAATTGAGCTTCATGGGA
TTGGTAAATATGGCAACGACTC
>939
AATTTTAAAAAATGTATTTCTGACTAAGTTACATTAAGGTCTCTGCCTCTGTATCTTATGT
TTCTTCCAGGTGCACCCTGAAGACCACAAATTAAATAAATATCATGACTGGCTTTGGGA
AAATCATGAAAAATTAAGTC
>940
GATGGGTGTAGACCAGCGCAGGGGGTAGAAACGATTTTTAGAAACGAAAGCTTTTTAT
TACCTTTCACAGGTGGGTTCTACCCTGAGCCCTGTTCCATGGCTCCCTTCAGAGGAATC
CACTCTCTGGAGCAGTTTGTCTC
>941
ATCTTGTGTTACTGGGGACTCTAATGTTTGGGCAAAATGAAGGAGACGAATGTCTGTTC
TCTGGTTGCAGGGGTGCCCTGCTGACAGGAACTAGCAGTGTCCTTCTACGGGGCCCCC
CAGGCTGTGGGAAGACCACAGTA
>942
CCTCCCCTGATTCTCCTGCCCTTGTCATGCTTTCCCCTTGCTCACAGACCCATTGGGATC
CCCTCCCCAGGTGCCCTGCTCCAGCCTCTGTGCAGAAAGTAGTGGGGCTGTGGAGACA
AAACTGCAGGCCATCTTCTCCC
>943
TTGGGACAAGGCAGTCCACAGGAGCTGCCAGCCATGTTCTCACCCTCAGTGCCCACTC
CTCTGTCTGCAGCTGCCCTCCCCTCATGGTTGTGGCCACCACAAGCCGGGCCCAGGAC
CTGCCTGCTGATGTGCAGACAGCA
>944
CTGGGGGACGGTCCCTGAAGGTTCCTGCCTACTGTGCCCTGCTCATGTGTCCTTTCCTG
TGGTTCCCTAGGGCTTTGTGGTAGGGGATCTCTATGCCCTTCTGACCCACAGCAGCCGG
GCAGCCTGCACCAGGATCAAGA
>945
GGGTGGTGAGGAAGGGAGAAGGGAATGGGACGCTGATGGTGAGGAGGCTTCCCTGTG

TTCCTCTTTGCAGTTTGGCAGGTGGCTTGACTGAGGAGGATGAGGGGGAGCTGTGTGC
TGCCGGCTTTCCTCTCCTGGCTGAG
>946
CCAAGTCCTTGGAGGTGGGACAGGAGCCCCTCCAAGAGCATATCCCAACCACGCACTT
TCCATCTCCTAGATCCCCTCAGTGTCCTGGCATGATGTGGGTGGGCTGCAGGAGGTGA
AGAAGGAGATCCTGGAGACCATTC
>947
CTGCTCTCACCTAACTTCAACCCCCCACAAGCCGAGCTTTGCCCTCCTGTGGGCCCCTC
TCTCTCTTCAGTGTTTGCCAGGGCCAGGGCTGCAGCTCCATGCATTATCTTCTTTGATG
AACTGGACTCTTTGGCCCCAAG
>948
AGGTACCACAGGCAGGGGCAGAGTCTAAGGGGGACACCAAGGACTCTTGCCCTTGCT
CCCTTCTCTCCAGGGTGGTGTCTCAGCTCCTTGCCGAGCTAGATGGGCTGCACAGCAC
TCAGGATGTGTTTGTGATTGGAGCC
>949
CTCATTTTGTGCCTCCTTCACCCCTCATGGCTGGGCTCCCTGAAACTTATACCTGCCTAT
CTCCCTAAAGATTTGACAAGCTGGTGTTTGTGGGGGCAAATGAGGACCGGGCCTCCCA
GCTACGCGTTCTAAGTGCCATC
>950
GAGCTGGTCAGCACTGGCTTTCGAGGGAGGAGGTGATGCCAACTCTGTTTCTTCCTGT
ATCCCACAACAGATTCAAGCTAGAGCCATCTGTGAGCCTGGTAAACGTGCTAGATTGCT
GCCCTCCCCAGCTGACGGGCGCG
>951
GGAAGCCACCTTCGCAGGGCTCTCCCCTGCCTGGGCATAGACCACAGCCTCCCCACCT
TGTCATCTCCAGGGCTGGAGCCAGGTAGCTCAGCACTGATGCTCACCATGGAGGACTT
GCTGCAGGCTGCCGCCCGGCTGCA
>952
GTTCTTGAAGGGAGAGAAGTCATTTCGAGTTACCCTCGTATCCTTTCTTTGTATCTTCGC
CTTGCATCAGGGGCCATGGAACGTATATGGGAGAAGAGAAGCTCATTGCATCTGTTGCT
GGCTCTGTGGAGAGAGTAAAC
>953
ATTCTGTAGTCTCCTTTTCATTCATCCCGCCAATCTTGCAGTGACCTAGCTTCGTGATATA
TTTCTTTAGCTAGGTCAGGGGGTTTTGGTCCAGGTTTCCCCCTCCCTGGTGAAACGGCA
GAAGACCCACTTTCATGATT
>954
AATTTCTGTGGCTGGTCACGTATTTTGGTCCTGTTTGTATTTCCCTTCCCCTCTCTGTGT
CTCCTTATAGCCTGTCTCTCTTGCTGATCGAGAGGTGATATCCCGGCTTCGGAACTGCAT
CATCTCGCTGGTAACTCAGA
>955
GTTTGGATTGGCTTGGTGGTCTGTTTATGGTTGGTTTCTTTTCTGAACAAATGCCTTTTC
CCTTTTTCAGATCAAAGACATCTTAAAGCCAGAAATAATGGAGGAGATTGTGATGGAA
ACACGCCAGAGGCTTTTGGAAC
>956
AATAAAACTAATTTTTTCTCCCAATTTTCTCTTCCTTTTTCTTTTTTCTGTTCCCCCCTTT

CTCTTCCAGAAGCCCTTCAGCGGCCAGTAGCATCTGACTTTGAGCCTCAGGGTCTGAG
TGAAGCCGCTCGTTGGAACTC
>957
AAGAATCAATGAAAAAGAACGAAGCTGGTTTCCAAAGCTGATATGTCTGATTTGGTTC
CTTTCTTCTCAGGTGAAAAGCTCCGGGTCTTAGGCTATAATCACAATGGGGAATGGTGT
GAAGCCCAAACCAAAAATGGCCA
>958
AACTGGCTCACGTGAGCTCTTTGAGCTTGCCTGTCTCTGTGGGCTGAAGGCTGTTCCC
TGTTTCCTTCAGCTCTACGTCTCCTCCGAGAGCCGCTTCAACACCCTGGCCGAGTTGGT
TCATCATCATTCAACGGTGGCCG
>959
CTAGTCAAGTACTTACCCACTGAAAAGCACTTCCTGAAATAATTTCACCTTCGTTTTTTT
CCTTCTGCAGGAGGACACCATGGAGGTGGAAGAGTTCTTGAAAGAAGCTGCAGTCAT
GAAAGAGATCAAACACCCTAACC
>960
TTCTTTAGACAGTTGTTTGTTCAGTTGGGAGCGGAGCCACGTGTTGAAGTCCTCGTTGT
CTTGTTGGCAGGGGTCTGCACCCGGGAGCCCCCGTTCTATATCATCACTGAGTTCATGA
CCTACGGGAACCTCCTGGACTA
>961
CAGCAGTGGTGGATTTGTGAAGTGGAAGGTTGGCCAGGAGCTCTCATGGGTGAACATT
TTCCTTTCTTAGAGATCTTGCTGCCCGAAACTGCCTGGTAGGGGAGAACCACTTGGTG
AAGGTAGCTGATTTTGGCCTGAGC
>962
CTGATTTTAAATGTAGTGTAGTGAAATGCTACACATCTTGAACAGCCTTTCTCTTTCGGT
TTTCTTTCAGCATTTGGAGTATTGCTTTGGGAAATTGCTACCTATGGCATGTCCCCTTAC
CCGGGAATTGACCTGTCCCA
>963
TTGAGAACTGCTAGCCCCGTATTGCTAGCCAGATCTCATGGATGATCTGACTTGGGTTT
CATCTGTCCAGGTTGGCAGTGGAATCCCTCTGACCGGCCCTCCTTTGCTGAAATCCACC
AAGCCTTTGAAACAATGTTCCA
>964
CCACCACACCAAGCCAACACCAGTACTGATGGCTGCTGGATTTTTGTTTCTGTCCCTGT
ATGATTCTTAGAAGTGGAAAAGGAGCTGGGGAAACAAGGCGTCCGTGGGGCTGTGAG
TACCTTGCTGCAGGCCCCAGAGCT
>965
GTTCAAGCGATTCTCCTCTGTCAGCCTCTAGAGTTGTCTGGAGTTGTCAGCTCTTCCCC
TTGCGTTTCAGATCCTCTGGACCATGAGCCTGCCGTGTCTCCATTGCTCCCTCGAAAAG
AGCGAGGTCCCCCGGAGGGCGG
>966
GATGAGATTGTGTTAAGGATGTTTTTGTTATGCAGGTTTTGCCATTTTCTTCTTTTTCCTG
TCCATTTAGGGGAATGACTCTGACTTTGAGACGGTGTACCAGCAGTGTGTCAGGTGCT
GCAGAGCGTTCTTGGAGAAGG
>967
GGTTGATTAGAGGAAGGTCCAGGAAATTCATCCTCTACCATTTCTTCCCACCCTCTGCC

CCACACCTAAGAAATGGAGTGGCCAAAGTCAACATCTTCTTCAAGGAGCTGAACTACA
AAACCAATTCTGAGTCTCCCTCT
>968
AAGGGGGCTCCTCTGCCAGAGTCCATCCAGAACCCTCTGTCCCATCGTCAATGTACCTC
TCCTCTCACAGATGGTCACCCTCCTGTCCAACCTGGGCAGCCAGTGGAGCCTGTGGTT
CGGCTCCTCGGTGTTGTCTGTGG
>969
ATGATTCTCATGTCCTCTGAGGCTTGAGCCCCTTAGTCCAGCCTTTGATCATTTCCACCT
TGTTCCTTAGGATGGAGTGATGGTGCTCAGCGCAACCCACCGGTACAAGAAGAAGTAC
GTCACCACCTTGTTATACAAGC
>970
CGGGGTTAGGGTGGGGTGGCTGCAGGTCTGGGAGGCAGGACAGAGGCGTGACCACCT
CCTTCCCCACCAGTATCTTCCTGACCGCTGCCCTGGGGCTGCCTGAGGCCCTGATCCCG
GTGCAGCTGCTATGGGTGAACTTG
>971
TGAGGCCCTCAACCCTCGATGCCCCCTATCTCCCCAGCCCTGACCCCCGACTCCCCTCT
CTCCACCACAGGCTATGTGGGTGCAGCCACCGTGGGAGCAGCTGCCTGGTGGTTCCTG
TACGCTGAGGATGGGCCTCATGT
>972
ATGCAGGAGGGTACCAGGAGGGTGGCATGGAGGTGGCCCTGGACCTCAGTCTCCCGT
ACCTTCCCTGCAGACTCACTTCATGCAGTGCACCGAGGACAACACCCACTTTGAGGGC
ATAGACTGTGAGGTCTTCGAGGCCC
>973
CCACATCCTCCACTGTGCCGCCCACCTCCTTCTCCTCACTGTTCCTTCTCCCTCCCCTTC
CCCTCTGCAGCCTGTCCGAGAACCAGTCCCTGCTGCGGATGCCACCCTGGGTGAACAT
CTGGCTGCTGGGCTCCATCTGC
>974
GGGCTGGGCAGTGCTGGTCTCTGGCTCCCTCCCCACCCCCTCCTGAGAGGGCGCTTGT
CCCCTGCCCCAGATGATCTTCAAGCTCCGGGCCCTGGACCTCACCCAGTGGCTCATGG
TCCTCAAGATCTCACTGCCAGTCA
>975
CATTCATATACAGTGTTTTTGAATACAGTATTTTGATCTGCCTTTAACAAATGTTTTCTCA
TTATTTCAGTTGCCAAAGCTGTTTGATGGATGCTACTTCTATTTGTGGGGAACCTTCAAA
CACCATCCAAAGGACAACC
>976
AGAAAACCTGGGGGAGGCAGGACTAGAGAGCAGGGCTGGACACAGGTCCCTCACAG
TCACTGTTCCCCAGACACCGCTCCACCTGGCTGTGATCACCACATTACCGTCTGTGGTC
CGGCTCCTGGTGACAGCTGGTGCCA
>977
AGCGGACCTGGAGTCCATCAGCGGCCGCAAAGCCCGGGCCTAGGTTTCACCGAGCCC
TCCTCTCCCTCAGGGCTCACCGCCCTGCACGTGGCAGTGAACACCGAGTGCCAAGAA
ACCGTGCAGCTCTTGCTAGAGCGCGG
>978
GGACGGGATGCGGGTCGCCGGCTGCTGGAGCAGAGCTTGGAGAAACTAAGACCTTCC

CTCCCCGCCGCAGGACATTAAGAGCGGCCGCTCCCCGCTCATCCACGCCGTGGAAAAC
AACAGCCTTAGCATGGTGCAGCTGC
>979
GCCTCCAGCCTCTGTTCCCTTACCCCGGGTGGCCCGCGCGCCCTCCTGACCCGGCCCT
CCCGTCCCGCAGCACGGCGCCAACGTGAACGCGCAAATGTACTCCGGCAGCTCCGCC
CTGCACTCAGCGTCCGGCCGCGGGC
>980
GAGCTAGGAGGGTCGGGAGAGAGGACTGTGAGGCATGGGTGGCTCTATGGTCACGCC
CATCTTCCTACAGGTCATCGACATCCTGAGGGGGAAGGCCACCCGGCCTGCTTCCACC
TCCCAGCCAGACCCCTCCCCTGACC
>981
TAGGCCCCAGCCCCTGCTCTCTGGGTCTAGCCTCTCACCTCCCACCCCTCTGTCTCTCT
TCCTTCCTCAGGTCTTCTCTCCGCATCACCATCCTCCTCACCCTCCCAGTCTCCCCCCA
GGGACCCCCCTGGATTCCCCAT
>982
GACAGGTTGGTTTGGCTCATAGGCTGTAGTCTGCCACTTCCTGTTTTATTCTACCTTCTG
TTCATTTCAGGTCACTGTGCATGGCATCATCCTGGCCCCCTCTAGAGCTCCAATCCTCC
AACCAGAGCCAGCTCTTCCCT
>983
TTGGCCACTTGGTCTCAAACTCCTCCTCCTTTTTGCTCTTCTCTACTCCACCCCCATCCC
GGTGATGCAGGAGGAATATCAGGAGTATGAGCCAGAGGCGTAGGGGCCCAGGAGAGC
CCCCACCAGCAGCACAATTCTGT
>984
AGAATGAATGAATGCAGCGGTTCTTCCTGCCATCTGATAACAGACTATTCTTTGATGTTT
TCATTTTCAGGATGTACAGATTATAGTGTTTCCAGAAGATGGCATTCATGGATTCAACTT
TACAAGAACATCCATTTATC
>985
CGCCTCAGCCTCCCACAGTGCTGGGATTACAGGCAAAAACCTCATTTATTTACACCTTT
TTTTCCTCTAGGTGCTCCAGCGCCTGAGTTGTATGGCCATCAGGGGAGATATGTTCTTG
GTGGCCAATCTTGGGACAAAGG
>986
GAGTTGGGAGAGAAGAGAAGAGTGCTTGGTATCTTGACAAACCCTCCTACCTTTTCTC
CTAACTACATAGATCTTGAAAAAGCCACTACCGCCTGAGCCAGCAGCAGCACCAGTCT
CCACAAGTGAGCTGAAAAAGGTTG
>987
GTTTGGGCAGGTGGGATGCAGGTGTGAGCACCACTTCCTCCTACAGACAGCTTCTTTT
TCGTTGTTTCAGGGGAAAGAAGGAGGTTTCATTGTCAGAGACTCCAGCAAAGCTGGC
AAATATACAGTGTCTGTGTTTGCTA
>988
ATCTCACTTCACTTCCTGGGTTCTGTTGACCTTTGTGCCCAAGTTACTGACTAAGCATC
CACTTCTTCAGGGACCCTCAAGGGGTGATACGTCATTATGTTGTGTGTTCCACACCTCA
GAGCCAGTATTACCTGGCTGAG
>989
TAAGTGAGGATGTGTGAGGCATCCCACCTCCTACACCACACCAACAGCATGACCTCTC

TCTCTGTTTCAGGACTCATATCCAGGCTCAAATATCCAGTGTCTCAACAAAACAAGAAT
GCACCTTCCACTGCAGGCCTGGG
>990
GGATTTTTCGTCTATTGTAAGAATTATACATTCATGACCCCAAAGAATCACACCAAGACT
TTATTGTTAGGATCATGGGAAATTGATCCAAAGGACCTGACCTTCTTGAAGGAGCTGGG
GACTGGACAATTTGGGGTAGT
>991
CCTCCCCAGCCCCTTTATTGCTATTATGCAGAGCCAAAAGGAGAAGACTAGTTCCTTGC
CTTTCCTGTAGGAATCTTTCCCATGAGAAGCTGGTGCAGTTGTATGGCGTCTGCACCAA
GCAGCGCCCCATCTTCATCATC
>992
CGTGCCAAGAAAACGTAAGCAAATATCAAGCCTCCAAATCCTAATGCAACAAGTCCTG
AATCCCTTGCAGGTATGTCCTGGATGATGAATACACAAGCTCAGTAGGCTCCAAATTTC
CAGTCCGGTGGTCCCCACCGGAA
>993
CTAGGAAGACTAGGACCCCTGCTATCCAAAAAGACTGCAAACCAATTTAATAATTTTTT
TCACCTTCTAGGGGTTTTGATGTGGGAAATTTACTCCCTGGGGAAGATGCCATATGAGA
GATTTACTAACAGTGAGACTGC
>994
TTACTCATTGCATTTCCCTTGAATTCTGGATTCTAGCCACTCTAACACTTTACTTTTTCTT
TGGTTTTAGAAAGCAGATGAGCGTCCCACTTTCAAAATTCTTCTGAGCAATATTCTAGA
TGTCATGGATGAAGAATCCT
>995
TCCACTAAACCTTTGCTGTTTGCTTATATGTTTCCACTAAAGCTTTGATGTTTCCACTAA
ACCATTTCAGGTAATGGCACAATTGACTTCCCTGAATTTCTGACAATGATGGCAAGAAA
AATGAAAGACACAGACAGTGA
>996
TGAGTAAAGTGTATTTTTTTCTTGGTCTTTTGCTATTGACTTACTCTTTTGTGTACTTCTT
CTTTTTCAGAGTTTGTACAAATGATGACAGCAAAGTGAAGACCTTGTACAGAATGTGTT
AAATTTCTTGTACAAAATTG
>997
CCCGGCAAGTGTGTATTGTGACTGGGTTGGATAAAGACATTGATTCTAACGGGTCAACT
TTTGTTTTCAGAATTCAAGGAAGCCTTCTCCCTATTTGATAAAGATGGCGATGGCACCA
TCACAACAAAGGAACTTGGAAC
>998
GAAATGATTACTGCAGAGCAGCGTTAATGGAGTGCTTACTTTACATGAGCTTTTTGTTT
TTTAATTCGAGGTAATGGCACCATTGACTTCCCCGAATTTTTGACTATGATGGCTAGAAA
AATGAAAGATACAGATAGTGA
>999
GCATAGCTAAGTCAAAGACCTACTTCCCCACACTACATCATTAGCAATAACAATTGCTG
AATGTTCACAGGATGGCAATGGTTATATCAGTGCAGCAGAACTACGTCACGTCATGACA
AACTTAGGAGAAAAACTAACAG
>1000
TAAAGATGTTGTGTTCATTAAAGCTGTTTTCAAAGATAACCAAAAGTTACTATTATATTT

GTCTTTTCAGAATTCGTACAGATGATGACTGCAAAATGAAGACCTACTTTCAACTCCTT
TTTCCCCCCTCTAGAAGAATC
>1001
TATTTAGAAGGAGTTAAAATGTCTTTTGTAAAGTTTCTTACACAGCTTTCTTTTTTTCCC
TTTTTTACAGGCAACTTCGGGCTCGCTGGAGAACAACTAAGGGGCACCAAAGCCCTCT
GAGGTTTTACTTTAAGGTTCGC
>1002
GACTTCCACCATTTTCTTCAAGCATCATCTGTGTTGTTGGAGACTGACATCGATCTTTGC
CTCTTTGCAGGTTCTGACCCAGCCAGTCCTGGTGACTGGCCTCTGCCTTCCACCGGAG
AGACTACTGACCTTCTCACCTC
>1003
AAAGAGCACGTCGGGGTGGCTCTGGGCTCCTGGGCTGGCCCCTGACCCACCTTCCTCT
GTTCCTCTGCAGTCTGGCAGTCGAGGGGAGCTGGGCCCCAAAGGCACCCAGGGTCCC
AACGGCACCAGCGGTGTTCAGGGTG
>1004
TATGCTTTACGTAACAATACTTCTGATGATCCTCTCTCGAGTAAACGCCTGCACCCTTGT
TTTCCCAAAGAACAAATTGCACAGTTAGCCGCGCACCTAAGGAAGCCTTTGGCACCCG
GGTCCATTGGTCGGCCCGGTCC
>1005
ATCAGACACCGCTGTGGTGTGGCTGCAACAGATACTCTAACCATATGTCTGTGTCCACA
CCTGGTGACAGGAAACCAGGGTGACAGAGGAGACAAAGGCGCGGCAGGAGCAGGGC
TGGACGGGCCTGAAGGAGACCAGGG
>1006
CAGCTGGATGTCAAGTCCCCCTGCTTTCAGTCCGGGCTGCAGCTGAACTCACCTTTCT
GCTCTGCCCAAGGACCCCAAGGCGTGCCCGGCACCAGCAAGGACGGCCAGGACGGT
GCTCCCGGCGAGCCTGGGCCTCCCGG
>1007
TGTGCCACCATTGTTCTAAGGAAAAAGGCATCTTTGGAACATGTGTAAAGTCTAACTTT
TTCCCCTGCAGTGGTTTGCAGGAAAAATTTAGATAGCACAACAGTGGCAATTCACGAT
GAAGAGATCTACTGCAAATCCTG
>1008
TTCCTGCCTTGTGTACTTAAAAGGGCCCTGTGGAAACTCTTGCCTACTTTTTTTTTCCTC
TTTTCACAAGTGTTCAGCCTCACAGGCCTACAACAAATCCAAACACTTCTAAATTTGCT
CAGAAATATGGAGGTGCTGAG
>1009
TTGCCATACATCTAAATTGAACTGTAGGAAACAGGCCTCTCAAACATGCTGACACATTC
ATCTCTTCCAGCCCTGGCACAAAAACTGTTTCCGATGTGCAAAGTGTGGGAAGAGTCT
TGAATCAACAACTCTGACTGAAA
>1010
TGGGTCTTAAGCAGTGCTAGCAAAGCTTAATGGACTTGCACTAACTATCCTTCCTCCCT
TTCCTTTGTAGGATGCTATGCAAAGAACTTTGGGCCCAAGGGATTTGGCTATGGCCAAG
GAGCAGGGGCTCTTGTTCATGC
>1011
GACCCCTTCAGTGGCCCACAGCTCAAGTGAGCCCCTGACCTGGAGTCTTTTTCTCCTC

ATCAACCACCAGGCCAAGGAGGAGCAGCAGGATGACACAGTCTACATGGGCAAAGTG
ACCTTCTCATGTGCGGCTGGTTTTG
>1012
CCTGGGCTGGGCTGGGCTGGGCTGGTGGTCACCTGGGCCTCAGTTTACCCTGCCTGCT
GCTTCCACCCAGAACTTTCAGATCTCCCTTAAGAGCAAACAAGGCCCGATCGATGTTTT
CCTGTGCCCTGAGGAGACCGTAG
>1013
CAGGCACGACAGCCCCTGCCTTCCTCCCTGCTGGGGCATCCCCGGCCTGTGATGCTCC
CCGTCTCCCCAGAACCGCTGTTGTCCCGGATGGGCAGCCTGCGGGCTCCCGTGGACGA
GGACCGCCTGTCCCCGCTGGTGGC
>1014
GTAAACAGAGACCCCAGAGCATCTCCCAGCCCCGGGGTGGCAGCCTCCTGATGCACG
CTTGCCTTTGCAGCCTGGTCAACGAGCTGGCCTTCACCGCCCACAAGATGATGGCTGA
CGAGGCCCTGGACAGTGGGCTGGTC
>1015
CTTCATCTGCGGGAGGCTGGAGGAGAGAGCCCTTCCCAGCCCACCCGGTCCCTGATCT
CTTTCACTGCAGCCGGGTGTTCCCAGACAAAGAGGTCATGCTGGATGCTGCCTTACCC
CTGGCGCCCGAGATTTCCAGCAAG
>1016
TCTCATGCTTTATCCTGATTGGCCCCTGCTAATCTCTCTCCTCGTTCTCTCTCTTCACCCT
CGGTACCAGGCGTCCTGGAACATGAGCATGCTGCAGACCCAAGACCTCGTGAAGTCG
GTCCAGCCCACGACTGAGAACA
>1017
CGGGTGGGAGGCGGTTGCTTGGGGGACCAAAAGACACAAACCCCACACCTCCTATTT
TCGTGTTCCCCAGGAGACATCAAGCAGGAAGGGGTCGGTGCATTTCGAGAGGGGCCG
CCCTACCAGCGCCGGGGTGCCCTGCA
>1018
TTTCTGACCTTACCCCCATTTTTTCCTCTGCCAAGGTAGCATCTCTACCCCAAAACTGTT
TTGTTCCCAGGTCGCCAGGCTCTGGGGCATCCAGAAGAACCGGCCAGCCATGAATTAC
GACAAGCTGAGCCGCTCGCTCC
>1019
ACTCCCCCACCTTACCCCATACAAAGGCTGGGCGGCTAGCAGGCTGACCAGACCTTCT
CTCTCCCCATAGGTGGCTGGTGAGCGTTACGTGTACAAGTTTGTGTGTGAGCCCGAGG
CCCTCTTCTCTTTGGCCTTCCCGG
>1020
TTTGCATATAGACCAGTGAGAAGCTGGAGCTAGCACCACTGGCTTAGGTTCTTCCCTTT
CCCCCTGACAGTGGGCGGCTTTCTGGCTGGGCTGCCCTTCAGCACCATTGCCAAGCAC
TACAGTTGGAGCACAGCCTTCTG
>1021
CCTAGACCAAAGCTGGGTGGATACGGAAGTGCTTAGGGCCAGCCTGATGAGGCTCCTT
TCTCCCTTCCAGGATGACCATTGATGCCGGATGCTCACTCCACATGCTCAGATTTCCAA
TGGATTCTCACTCTTGCCCTCTA
>1022
TTCATGCTATTGGAGACCTCAGGTGGGCTTCCAAGTCTCATAGGACCCTCTTTCTCACA

TTCCTTTCCAGTTTCCTATCCTGAGAATGAGATGATCTACAAGTGGGAAAATTTCAAGC
TTGAAATCAATGAGAAGAACTC
>1023
TGTTCACGTCCTGCTAAGGGGGACCGTCGTGTCAGCACTGTAATGCAGTGATGTTTTTT
GTGTCTTTCAGGTGACTTCATGGTCATGACGATTTTCTTCAATGTGAGCAGGCGGTTTG
GCTATGTTGCCTTTCAAAACTA
>1024
GTCATGGAGTCTTTTAACCAATGTCGCACATAGGTCAGGAGCTGAGCCCATCACTCTTG
TGCTCTTGCAGGGATCACCTCTGTTCTGACCATGACCACGTTGGGCACCTTTTCTCGTA
AGAATTTCCCGCGTGTCTCCTA
>1025
CCGGAGGCAGCACTTAGACTTGGGTTATCTTCCCTTGACTTTTCCCCACTTCTTTCCTT
GTCCATTTTAGCCTCGTATCAATAGCCGTGCCCATGCCCGTACCCGTGCACGTTCCCGA
GCCTGTGCCCGCCAACATCAGG
>1026
TCTCTCCTCCTGCCCCCAGACCCAGGTCTCCCCTTCTTCTACATGACCACCTCTCCTCC
CCCTTGCTCAGGCCAGCTGTCTCTGAACGAGTTTGTTGAAGGTGCCCGTCGGGACAAG
TGGGTGATGAAGATGCTGCAGAT
>1027
AATATTATAAAATAGTCTCTTGAAGATTTTGTCATCTGATCTTTTTAAAAATTAACTTTTT
TTCTTGCAGCTCGGAAAAACAACCATCATTTTAAATCTGAATCTGAAGAGGAGAAAGC
ATTGATTTATCAGTTCAGTCC
>1028
AGGGTGTGATGTGAGAGAAGGGATTGGTAATGCCTCTTGCCACTGGCTCGGATCCTCT
TCCCCCACACAGGTACCCATCACGGGTGTTTGTGGGAGATACCTTCTGTTACTTTGCTG
GCATGACCTTTGCCGTGGTGGGC
>1029
CTTCCTATTAGCCACAGGGAGGAGGGTTCAAATAGTGGCCCAGTCACATGAAGCTATCT
TCCCCCCGCAGACTCAATATCAAGACAGGCAAACTGGAGATGAGCTATTCCAAGTTCA
AGACCAAGAGCCTCTCTTTCTTG
>1030
GCATTAAATAGATCCACATTCCTAAAGCATAGGTATTAGCTGAGGTTCTCTTCACCTCTG
GTCCCTCCAGGTGGCAGAGAGCCTCCAGCTGGTGACAGTACACCAGAGTGAGACTGA
AGATGGTGAATTCACTGAATGTA
>1031
GGGAATCGAGTTTATACCTCCGTGTCTCCCTTTCTGCGTGATTCTTACTCCAGTCCATTT
CTCCTTGCAGATCCTGGGCAGTGCCATCACCTTCTCCATTCGATATCAGCTCGTTCGACT
CTTCTATGATGTCTGAGTCC
>1032
GCAGTGGCTCTAGGAGAAAGACCCCATCAGTAGGCTCCCACTGACTGGCGGTCCACTG
GCTTTCCCGCAGGGAACCTACTCACTGCCCAAGAGCGAATTCGTTGTGCCTGACCTGG
AGCTGCCCAGTTGGCTCACCACCG
>1033
GGCCGTCCCCCTCTCCTCTCCCAGGATCAGGAGGAATCACTGCCTCTCCTCTCCCCCGA

CCCCCTTCCAGCACCAGCAGCAGGTCTTGGGAGCCATTGAGAGGGCCAAGCAGGTCA
CCGCTCCCGAGCTGAACTCTATCA
>1034
GGGACCTGGGCGGGTGGGCGGGGGACAGGTGGGCCCCAGGCAGCTAATGTTCCCCTC
CCCCGCCCTGCAGCAGCAGCTCCAAGCCCACCAGCTGTCCCAGCTGCAGGCCCTGGC
CCTGCCCTTGACCCCACTACCCGTGG
>1035
CCGTGTGGCTTTGCCCGTCCCCAGGTGAGTGTCGGCGCAGCTTGGGTCTAACTTCCTG
TGTGTTTTGCAGACAGCTGGATAGAAAACGACAGCCTGTCAGTAGACAAGCACGGGG
ACGACCAGCCGGACAGCAGCAGCCT
>1036
GGGGGGACTCCGGGGACAGGAAGCCCTGGGGTGGCGGTGGCCGGTTTCTCACGCCCG
CTTGTGCCCGCAGGTGAAGATTTGGTTCCAGAACCGGCGGATGAAATGGAAACGCAG
CAAAAAGGCCAAAGAGCAGGCGGCGC
>1037
GGACCAGACTACTGGCCTCTGAGCATCGGGCCAAGTCCAGCTACTGAACCTGCTCCGC
TCCTCTCCCCAGGTGTGGTTCCAGAACCGGCGCATGAAGGACAAGCGGCAGCGCCTG
GCCATGACGTGGCCGCACCCGGCGG
>1038
CGGGCAGAGGCTGCTGTCATAGGCAGAGGCCCCCGCTGATGCCGGCCCTGCTTGCCCC
TCACACTGCCAGGTCTTCACGTGCTCTGTGTGCCAGGAGACATTCCGCCGAAGGATGG
AGCTGCGGGTGCACATGGTGTCTC
>1039
AGCCCAAGGGTGGGTGGCAGGGTTTCATGGGTGCAGGCCGCCCTGGAGGTGACTGTG
GCCTCTTTTTCAGTGTTCCTCCTGCTCCCAGCAGTTCATGCAGAAGAAGGACTTGCAG
AGCCACATGATCAAACTTCATGGAG
>1040
TCCCAGGCAGCCCTTCCCTGCTCTCACCCTGGCCCTGGTCCCTCCCTCTGCCTGCCTGG
TCTGCCTGCAGTGCCCCACCTGTGCCAAGTGCTTCCTGTCTCGGACAGAGCTGCAGCT
GCATGAAGCTTTCAAGCACCGTG
>1041
CTAGCTGTAGCAGAGCAAGGGGGTCACTTCCCTTGGTGATGGCCTCTGCCCCATGTCC
CCACCTTAACAGGAATGAGAGGCCACACGTATGTGAGTTCTGCAGCCACGCCTTCACC
CAAAAGGCCAATCTCAACATGCAC
>1042
CCTGCCCCTCCCCTCGTCTCCCCATCCTGAGGTCAAGGCCACAGGCTGAGCTCTTGCC
TTGTGCCTGCAGCCAGCCTGGACAAGCACAACCGCACCCACACCGGGGAAAGGCCCT
TCAGTTGCGAGTTCTGTGAACAGCG
>1043
CCTCCTGCTGGGTTTCCTCGAGGGTCCCGGGGCTCCTGCATGATCCCCCACGGTGTTCT
CCCTCTTGCAGCCGTGGAGCAACTGCGTGTGCACGTCAGACGGCACAAGGGGGTGAG
GAAGTTTGAGTGCACCGAGTGTGG
>1044
TAGGCCAGGGCCTGGCCCCCTTCCCCTACCCTAGGATCCCCCAAAGTTCTGAGCTCAC

CCTCCCCGCCAGGCCCACCTGCGGAGGCACATGGAGATCCACGACCGGGTAGAGAAC
TACAACCCGCGGCAGCGCAAGCTCC
>1045
AAGGGTAGAACCCTGGGTGGACAAGGGCCCAGCCTGAAGCCCACTGAGCCCCAGCTG
GGGTACATTGCAGAACGGTTGCCCATCAACCGGCGGGACCTGGACCCCAATGCTGGGC
GCTTTGTCCGCTACCAGTTCACGCC
>1046
GCCTGTCCTGAGCCTGGCTGGCTAGTTGACTTCAAGGACAGACTTAGCCCTGCCTCCT
TTTCTTGCTCAGGGTGGAGTTCACAGTGGGAGACAAGCCTGTCAACAACTTCCGCATG
ATCGAGAGGCACTACTTCCGCAAC
>1047
CCTGAAGCCCCTGCAGTGGCGGGACTCAAGCTCCTGACCTTTGCCCGGCCTGGCTGGG
CCTCTCTTGCAGTCAGCGAGATGATCCGCCACCCGTATGAGACCCAGTCTGACAGCTT
CTACTTCGTGGATGACCGGCTGGT
>1048
AGGACACGGGGACTGGAAGTTTGCTGCTGGGCTGACCTAAGGCTTCCCTCCTCTGCCT
CTGTGACCCCAGGCCTGTGGGGCCTCGTCAACAACGCAGGCCACAATGAAGTAGTTG
CTGATGCGGAGCTGTCTCCAGTGGC
>1049
CCTGGGGTGGGGGAGGGCTTAGGGAGCCCCTTGCCAAAGCTGAGCTGCCCCACTCCC
AATCCATCCGCAGGGGGACATGCCATATCCGTGCTTGGGGGCCTATGGAACCTCCAAA
GCGGCCGTGGCGCTACTCATGGACA
>1050
GGTTAAACAGTCCTAATTGGCTTTGGCTCCCCTACGTGATCCCAGTCTGACCTTGCCAC
TCCTTCCCCAGAGTCAGTGAGAAACGTGGGTCAGTGGGAAAAGCGCAAGCAATTGCT
GCTGGCCAACCTGCCTCAAGAGCT
>1051
CCACCCTGACTACTGAGGTTCTGTGCACACGTCTCTACCTTCCTGCTCAAGACACTGG
GTCTTTCCACAGCCTGAAGATGACCATGGACAAGACGGGTCTCCTGCTGCTCAGTGAC
CCAGACAAAGTGACCATCGGCCTG
>1052
CCTGAACTGAACATTTGATGGTGTGTCTCTCTGGTGCCATCTTAATACCCTTTCTCCTTT
TTCTGTGCAGAATTTAAATGTGAGTTTCAATCCTGAAAGTTTCCTGGACTGCCAGATTC
ATAGGGTGGATGACATTCAAG
>1053
AGTAGTGCCAGAGGCACCCTGACATCACTCTCTGAACTGCTTTCTCTTTCACATTCTTG
TATTTTTCCAGCTGACGGCCCAGAGGGTGGGTGCCAATTCCACCAGCAGCTGCAACTG
AAAAGCAAGGTTCAGAAATGTCA
>1054
CTCTCTCTCTCTGTTCTTTCTCCACTGAAACTCTTGAAATGTAATGGCTTATCGTGTGGC
TTTACCCTAGGGGAAAAGATTACCTTGAGGTTGTGTTCAACAGAGGAGGAAACAGCA
GAGCTTCTTAGCAAAGTCCTCAA
>1055
GAAGTCATATGGAACAGAGTCTTCTAAATGATCTTTACCTGAATCCAAACACATTTGTT

TCCTTTTTCAGATTCAACTTATCAGTATATAAAAGGTTCTGCTGACATTAAATCCAACCA
GGGAATCTTCCCCTGTGGACT
>1056
GTTTGAAAGTGACATGAGTAATGAAAAACAAATGTGATAATCATCACCAATTGAAATTT
TTCTTCTACAGGTGGAAAGGACAGTGCTATTGGGGCCTTTCTTACATGGGGACCAACAT
GCATTCACTACAGCTCACCATC
>1057
CATTCCCATTTAGTGTATTTGCTTCTTTGTGAGATTAATTTGTTTCTCTTTCTCTTTCCCTC
TTCTCTAGTGTGGATTCTTTAAACGCTCTAGGTACGATGACAGTGTTCCCCGATACCATG
CTGTAAGGATCCGGAAAG
>1058
TTGGCCTTCCCTCTCCTCCAGGCCTCTGGGAAAGCGGCTTCCCTTTCTGAGCCACCCCT
GCTCTCCCCAGCTCTCTGTCGGATGAGGCCCCAGTCCTGCCCAACACCACCCCGGACC
TCCTCCTGGCCACCACAGCCAAG
>1059
GGCTCTGGAGCCCAACAGCACAGTGGTGACAGTGGGCATCACTGAATCTGTATCCACT
TCTGTGGCACAGGACAGGCCAGGCAGTCCCAGTGGCTCTGAGGACAAGGGCAACCCG
GCGCCGGAGCTCAGGGCCTCCTTTC
>1060
AGGAGAGCAGGGGAGGGGTTGGGGGGTGCTCCTGGGCTTCCCTAGCTCAGCCTGCTC
CTTGTGCCCACAGTCATGTCGGAGGCGGGCAGTGGGACCCTGGAGGACGAGTGGCAG
GCCATCTCGGAGATTGCCTCTACTTG
>1061
GGGGCTGGGAGTCAGCTGGTGCACTGCCCCCTGCCTTGGTCCTGCTGAGTCTTGACCC
CCATGTCTTCAGGCCAGAGCCTGGGAACCTCTCAGAGAAGGTCTCTCACTTGGAGTCC
ATGCTCAGGAAGCTGCAGGAGGAC
>1062
TGGGGGGAGTCACAGGGCTGCCCCAGGAAAGGGGCTGGCGGCTCTGACTCCTGTCTC
CCCGGCACCCCAGGAGAAGGCGGACAGGGCGGCCCTGGAGGAGGAGGTGCGGAGCC
TGAGACACAACAACCGGCGGCTGCAGG
>1063
CTAGTCTTATTCCCAAAGAGTCCTGGAAAATGTGAGCACCCTCCCTCACTCAGCATTTC
CCTCCCTCCAGGACTCTGATGAACAAGACCCTCAGGAGGTGACATACGCACAGTTGAA
TCACTGCGTTTTCACACAGAGAA
>1064
ACAGTCTTATTCCGAAATAGTCCTGAAAAATGTGAACACCCTCCCTCACTCAGGATTTC
CCTCTCTCCAGGACTCTGATGAACAAGACCCTCAGGAGGTGACATACGCACAGTTGGA
TCACTGCATTTTCACACAGAGAA
>1065
ACAGGGCAAACAGGGGGCCCCCAGGAGCCCCAGCCTGTACCCCGGTCCTGACACCCC
TTCTGCCCCCCAGGGAACGACTCCATCTTCCATGACATCGACAGCGATACCTCCTTAAC
CAGCCTCAGCGACTGCTTCCTCGG
>1066
TCACCCTGGGCTCACAGGGCTCAGAGAAGGGTCCTGCTCAGCATCCTCCCCACCCCAC

CCATACCTGCAGTGACCAACCCATGTGACCGCAAGAAATGCGAGTGGCTCTGCCTGCT
GAGCCCCAGTGGGCCTGTCTGCAC
>1067
CCCTGACCTTGTTGCCTGTCCCTCTCCTCCTATTCCCCTGGCTCTGCCCCTTGACGGGC
CCTTCCTGCAGCTCCCCGGCCTGGAACCTGTAACCTGCAGTGCTTCAACGGTGGCAGC
TGTTTCCTCAATGCACGGAGGCA
>1068
CCCATTCCTCTGCTATAGGGCCAGGTGGTCTCGAGGGCCACAGTCGCGCTCACACATC
CTCTCCCACCAGGCATGCCCACGTGCCGGTGCCCCACGGGCTTCACGGGCCCCAAATG
CACCCAGCAGGTGTGTGCGGGCTA
>1069
CTCTGTTCAACCTATGGAGAGCCCTCATGAGGGTGGGGCTTGAGGCACTTCTCTCCCTC
CCCAACCACAGGGCAGTGCTCTGGCTACTGTGAGAACTTTGGCACATGCCAGATGGCT
GCTGATGGCTCCCGACAATGCCG
>1070
CCCTCCCTTCCTCAGCATCCCAGGCACGCCTCTGCCAGCCCCAGCCCCAGCCTCTGATT
CCTTCCTGCAGCTGCACGGATGGCCGGGTGGCCCCCAGCTGTCTGACCTGCGTCGGCC
ACTGCAGCAATGGCGGCTCCTGT
>1071
GAGCAGGGGGACCGTGTGCCTCCTGCTTCCCTGAGCCTTGGTGACTCAGTGTCCCACC
TCTTCCCTCCAGATATAGCCTCCATCCTAATCCCTCTGCTGTTGCTGCTGCTGCTGGTTC
TGGTGGCCGGAGTGGTATTCTG
>1072
GTCATTATTTTGCCATCCTAGCCTTCCCCCCCAATAATCTCTGTCTCCTTATACTCCTGCC
TTTCCCCAGGGCTAAGGGCTTCCAGCACCAACGGATGACCAACGGGGCCATGAACGT
GGAGATTGGAAACCCCACCTAC
>1073
GTGGGGGTGGGGTAACCTGGGCTACAGGCCCAGCTCCTGAGCCCTACCTGAACCCTCT
GTCACCCTGCAGCCCACCAACTTCACCAACCCCGTGTATGCCACACTCTACATGGGGG
GCCATGGCAGTCGCCACTCCCTGG
>1074
ACCTGTCCTTGTTCTCACTCATCGAACAGATGGGAATTTAGTCACCTAATATGTTTTGTT
TCTATTATAGGCCTTCATTCAATCTCAGATTTATGAAAATAATTACTGGATTGGATTATCAT
ATGATGAAAGGGAAAGTA
>1075
GGGGCCCAAGGAGAGCTGAGGAACAGGACAGAGGGTGCCAGGTCCTAAGAAACAGT
ACTTATCTCCTCAGGTGGACTTTGAGGGCAGCCTTGCTGAAAATCGCTTCACAGTCCTC
CCCAACATAGATCCTGAAATTGATG
>1076
GCTGGGGATCTTCATAGCAACCAGGGCAGGAGACTCACTTTTGATAACCACGTGTCTT
CCACCCTCGTAGAAAAGCGAAGACTGATGGGACTTCCCAGTTTCCTTACTGAGGTTGC
CCGCAAGGAGCTGGAGAATCTGGA
>1077
TCTAGGGTGGGGAGGTGTCCAGTAAGTCTCCAAGCAGGAGAGTAGAGTATCTCCTCTT

TACTCTCCCCAGATTGGCTTCCTTCTTTCTATTCCCCGCCTGCCTTCCATGGTAGAGGCC
AGTGACTTTGAGATTAATGGAC
>1078
AGGCCAACTGTGGTCAGTGCGTTACGGGCTTCCAATACTAACTTTCCCTTGTCCACCTT
ATACCCAGCAGGTGGCGAAAGCAGTGAACAATGCCACTGCACAGTCGCTGGTCCTTAT
TGATGAATTTGGAAAGGGAACCA
>1079
ATTTCTGACCCTTATTTCCCCTTCTCTTCCCCACTCCCCTTACTCCTCCCACCTTCTTGCT
TGTTCCTAGGTCTCAGACTTGATCCGCAGTGGAAAACCCATCAAGCCTGTCAAGGATT
TGCTAAAGAAGAACCAAATGG
>1080
GTTAGAAAGATGGGGAAGGAGAGGAGGACCAAGAGATGCAAAGTCCACAGCTTTGA
ACCCCTGTACCCAGTTTCTCTCAGAGGAGAAGCTGCACTATCGTAGTGCCCGAACCAA
GGAGCTGGATGCATTGCTGGGGGACC
>1081
TAGGGGCCTCTGCCTCTCCTTCACTTTCCCCTGGAACTGACCTCCAGCTCCCTTCCTCA
CCCACTCCCAGACCAGGAGACGCTGCTGATGTACCAGCTACAGTGCCAGGTGCTGGCA
CGAGCAGCTGTCTTAACCCGAGT
>1082
GGCTACATCTTCTGGGGGTTCATCTATCTTGATCCACAAGCCATGCGAGGTGCCTCTCC
GCCCACTGCAGACATCCTCTGATGGAACTCTGTGCCCGAACCTTTGTGCCCAACTCCA
CAGAATGTGGTGGGGACAAAGGG
>1083
ATGCCTTGAGCCTCACTTTCACCTCAGCCCACGGCACCAGGCCCCAGGCCCTGTCTCC
TTCCCTATTCAGGTAGGCTTGATCACATTCATGGCCCTGGTAGGCAGCTTTGTGCCAGC
AGAGGAGGCCGAAATTGGGGCAG
>1084
AGGCCAACTGTGGTCAGTGCGTTACGGGCTTCCAATACTAACTTCCCCTTGTCCACCTT
ATACCCAGCAGGTGGCGAAAGCAGTGAACAATGCCACTGCACAGTCGCTGGTCCTTAT
TGATGAATTTGGAAAGGGAACCA
>1085
GGCAGTGCAAGTGCAGAGGGGCATATGGGGTCCCCATGGCTCCGAATGCTAACCTCTG
CCCTCTTTGCAGGTGGATGGGCTCGCGCTTCTGGCCGCTGTGCTCCGACACTGGCTGG
CACGTGGACCCACATGCCCCCACA
>1086
AGGATTGGCCAAGGGTTTCAGGACAGGAAGGAGGTGATTGATGATACACTGTCTTTTA
TTCTCTTTTAAGACCATGGAGACCTGTGAGGATGGCAACGATCTTGTCTTCTTCTATCA
GGTTTGCGAAGGTGTTGCGAAGG
>1087
GATTTCTGACCCTTATTTCCCCTTCTCTTCCCCACTCCCCTTACTCCTCCCACCTTCTTGC
TTGTCCTAGGTCTCAGATTTGATCCGCAGTGGAAAACCCATCAAGCCTGTCAAGGATTT
GCTAAAGAAGAACCAAATGG
>1088
CCTCTCCCCTCTGCCCAGGGATTTGGTTGTCCATTCTGCCATAAATCTTGCGATTTTCTC

TCTTCTTCAGTTGCCAGACATTAGTGGATAAGTTTATGAAACTGGATTTGGAAGATCCT
AACCTGGACTTGAACGTTTTC
>1089
AGGCAAGAGAATCTCTTGAACCTGTGAGCCAGAGGTTGCAGTGAGCCGAGATTCCAC
CACTGCACTCCAGCCTGGGCAACAGAGCAAAACTCTGTCTCAAAAAAAAAAAAAAA
AAGGCAAAATTAAAGAATTTCAAGTCC
>1090
TAAAGGAGGAATCAGTTTTATTGTTTGTACTGGACTATACACAAATGTAAAAATCATTTC
ATCATTTCAGAAACTTGAAGAAGCTGAAAGAAAAAGCCAGCACCAGCTCGAGAATTT
GGAACGAGAACAGAGATTTTTAA
>1091
TGTTAGTTTTTGAAGGTGCGCTATACTCGATAAATAATGTTCTTCTTCCTTTTTTTTTTC
CTTTGGCAGAGGAGATTGAAGTGGATGTTGAAAGCACAGAGTTCTCCCATGGAGAAG
TGGACAATATAAGTACCACCAG
>1092
GCCCAGGTGCCTCTGCCCCAGGGCTGGGCAGAGGCAGCCTGTGGTGACCGCAGCTGT
CGCTGCCCCTCAGCTGACCATGAATGACTTCAGCGTGCATCGCATCATTGGGCGCGGG
GGCTTTGGCGAGGTCTATGGGTGCC
>1093
GCCGCTGAGGCTCTGCAACCCCGGGCGCCCCTGCTAACTGCCCGCCCCCTCCCCTCCT
CTCCCCTCCTAGGACTGCCCATTCATTGTCTGCATGTCATACGCGTTCCACACGCCAGA
CAAGCTCAGCTTCATCCTGGACC
>1094
CCAGCTGGCATCTTGCCTGGCTGGGCCCCATCCTGAGCTGCCCAGGCAGCTCACTGGG
CTTCCTTCACAGCCAGCCAACATCCTTCTGGACGAGCATGGCCACGTGCGGATCTCGG
ACCTGGGCCTGGCCTGTGACTTCT
>1095
CCTCTCTCCCTGAGCTGGGATGGGGTCAAGGGCTCTTCCTAAGCCCTGCAATGTCCACT
CCTCTCTAAAGGCCGTGGAGCTGCCCGACTCCTTCTCCCCTGAACTACACTCCCTGCTG
GAGGGGTTGCTGCAGAGGGATG
>1096
TGCGGCAGGGACTGGGGGTGCTCTGCAGCCCCACCCCCGAGCTAATGCCCATGACCCT
GTTCTGCTGCAGTACCCTCCCCCGCTGATCCCCCCACGAGGGGAGGTGAACGCGGCCG
ACGCCTTCGACATTGGCTCCTTCG
>1097
TGGGCAGCCTGTGGCTGATGGTATTCCGGCATCTCTGTCCACCCATGTGCCCCTGCCCC
ACCTGGTAAAGTTACTGGACAGTGATCAGGAGCTCTACCGCAACTTCCCCCTCACCAT
CTCGGAGCGGTGGCAGCAGGAGG
>1098
CCGGGAGCCGGGCTTCCGGGTGGCTGGCCGGCCCGCTCTTCACCTGCACCACCCATCC
CTGGCCCTGCAGCAGAGCCTGCTGACCATGGAGGAGATCCAGTCGGTGGAGGAGACG
CAGATCAAGGAGCGCAAGTGCCTGC
>1099
GGAGGCCGAGGGGCCAGGCCAGGCAGGCTGGGGAGAGCCCCAGCTGACAGAGCTGG

GCTTGGCTGTGCAGAGCGACCCTGAGCTGGTGCAGTGGAAGAAGGAGCTGCGCGACG
CCTACCGCGAGGCCCAGCAGCTGGTGC
>1100
GACTGAGGCCTGAGCACTTGCAATGCCAGCATCACACCCTCTTGTCTGTGTGACCCCC
CACCTCCCACAGGCTGTGCTGCACATGGAGCAGCAGAAGCAGCAGCAGCAGCAGCAG
CAAGGCCACAAGGCCCCGGCTGCCC
>1101
GGGCCTCTGGGTTCGTTTTGTAGGAGGGGTTCGGATGTCCTGTGCCACCATTCCCTCCC
TCCATTTCCAGACGATGTACCTGTCCCAGCTCCAGCCGGTGACCAGAAGGAGGTGGAC
ACTTCAGAAAAGAAACTTCTCGA
>1102
CACTAACTGACTTTCAGATTCTAAAACCACCATGTGGATTCCACAAACTCAGAAACATT
ACTCCCCACAGGACTTCATCCAGCAAGCAATTTCCTATTCCAGTTTTCCATTCTGGATG
GGGCTGTCTCGGAGGAACCCCA
>1103
AAGTTTCTGAAACAAGGTGGCAGTAAACCTAAGTTTTTTTGTTGGTTTGTTTTGTTTTT
CCTACCTGCAGATTTAGAGTCCGAGGCGCTGTCTCCCAGACATACCCTTCAGGTACCTG
TGCATATATACAACGAGGAGCT
>1104
CACGCAAAGAATAAGATGGGTTGATGGGTTGCAAGCATCCTGGCTCACTCTCACCCTAT
GCTAAACTCAGGCGACCGTGCTGTGTGACATCATAGTCCTCTACTGCATGAAGAAAAG
ACTCTACTATCGGGAGAAGAAAT
>1105
GCGCAACTCCCTCCCGCCTGCCACAAGGGGTCCCAGGGGCACCTTGATCTGCTTGTGT
CCTTCTTTGCAGGGTCTTGCTAGTGAGCTGGACCAGTGAGGCCTACCCCACACCTGGG
CTCTCCACAGCCCCATCAAAGAAC
>1106
CTGGAGGCTGGATTGAGTCTAGGGTCACTGTAACTCACCAAATGCCGACATGCCCCAC
CCCTCGCCACAGGTGGGTTTCATTGACTACATTGCTCACCCACTGTGGGAGACTTGGG
CTGACCTGGTCCACCCAGATGCAC
>1107
AAGCCTGTCCCTCAGTCCCCTTTGACCAGTAATCTCTTCTTCCTTGCTTTTCATTCCAAA
AATGCTTCAGGCCAATACTGGCAAGTTCTAGGGGGCCCAGTGTCTGGGCTGAGCATTG
GGACAGGCAGGGCAATGCTGGG
>1108
GAGGGTGTGGCTGTGAAAGGAGAAGCTGTGGCTCAGGCCTGGTTCTCACCTTTTCTGG
CTCCAATCCCAGACCAGGTGCCTTTCTCCGTGAGCGTGTCCCAGTTGCGGGCCTTGGA
TGGAGGGAACAAGCACTTCCTGAG
>1109
GAAGTGGATAGAGGGGACAGCTGGCAAGCAGCAGACATGAGTGAAGCAGTGCCTGG
GATTCTTCTCACAGGTCAAGAAGCAGGCCTTGGGCAGGTTCCGCTGATCGTGGGCATC
TTGCTGGTGTTGATGGCTGTGGTCCT
>1110
CAGCAGTTTAGCATATTTTTGCCATGAGCTCATCTCGTGTCATTACAGCTCTTTCCTCCT

TTCTCTTCAGGAGGAAATCTCTGACATCAAAGAGGAGGGGAACCTAGAAGCTGTCTTG
AATGCCTTGGATAAAATTGTGG
>1111
TGGAAGCTGAACCTTCTTCCACAAGGGCCTTCCGCCTCCTTCATTCCTTGTGCCCCGTG
TGGCCCTCCAGGCGCCCCAGCGGGATCCCAGAGAAGGATCTGCACAGTGTTATAGCAC
CCTACTTCCTGCAGCAACGGGAC
>1112
GGGACAGTGGAGGGCAAGGATTTGGGTTTTCTTAGATCCCAGTTTGTCTGTTGTCTCCC
ATTGATTTCAGGCTCTACACAGAGAACAGAGGGAGCTGGTTGCTGTGCTGAGGGAGCC
TGAGTGAGGAGACCGCCAGCCCC
>1113
GCTCAGCCTGGCCTCCTTGGCCTCCTCTCTGAGTGGACTGGGGTCTCACTGTGCCTGTT
TATTCCTGCAGGTCTTCATGGAACAGATCGGAATATTCCGGAACATTGCCCAGCACTAC
GGCATGTCGTACCTCCTGGAGA
>1114
CTGCTCTGCGTCCTCCCACCAGGGGGAAGGCTGTTGGTCTCACCTGGGCCCCTGACCC
CGTTCTCTCCAGGACCAGGAGAAGGTGCTGGACAAGTATGAGGACGTGGTGCAGGGG
CTGCAGAAGCGAGGCCAGCAGGTGG
>1115
GAGATGGCAACCGGGCTCCCAGGCCCTCAGGCCCTGCCGGGATCCTGACTGTCCAGCC
TGCCCCCGCCAGGGCCTGATCTCGCGGGGCTACAGCTACACCCTGCAGAAGAACAAC
GGGGAGAGCTGGGAGCTCATGGACA
>1116
GTCCGCAGTCCCGCCTCTGTGGGTGCAGGGGGTGGTGGGCTGCCCAGTTCCCAGCTCA
TTCGTCTCACAGCCTGGGCAGCCAGTACCGGAGCGTGCGGCAGAAGGCAGCTGGGAG
CAAACGCACGCTGCAGCAGCGGTAT
>1117
TAGGTCCATCCCTGGGCCTCACACCCCTGGCCCGTTGTCACACCTGCCTGTGTCCTCCC
TCCCTCCACAGATGCCTCTGACCTACAGGGGCGGCAGCTGCTGGCTGGCTTGGACAAG
GTGGCCAGCGACCTGGACCGGCA
>1118
CCCTGGGCACATCCTCAGGGGAAGGGGCTGCCTGGCTGTGTCCAAAACTGAGGGGCC
CTGGTTCCCACAGAACATCACCAACGAGCTACTGCGGATTGAACCTGAGAAGACGCG
GAGCACGGCTGAGGGCGAAGCCTTCA
>1119
GCAGCATGGAGGTTGGGGTGGGGTCAGGGGGCCAGGTGGGCATCCCCCGACGCCTGA
TTGGTGCTCTCAGGGTTGATGTGGCCAACCGCCTGGAGAAGAGCCTGCAGCAGAGCT
GGGAGTTGCTGGCCACACACGAGAAC
>1120
CAGGGGGTGTCATCGCCTGGCTCTGGGAGCTCTGGCCGTAGCACCGCCGTCTCCTCCT
CTGCCCCGCCAGCCCATGGCCTGTGAGTTACAGGCCCAGAAGTCCCTCCTGGGTGAGG
TGGAGCAGAACTTGCAGGCGGCCA
>1121
AAAAAAAAAATGAGGGCTCGGTGAGGCATCTGTTTGCTGTGGCTGAGGCCTGAACGC

TTTGCCTTGACAGGGCGCAGAGCCTACAGAGCGCCAAGGCAGCCTACGAGCACTTCC
ACCGCGGCCATGACCACGTGCTGCAG
>1122
TACTGCATCCATGGTTGCTTTTTTAAATTTTTTTTCTGGTTAGTTTTGATTTTTAAACTCT
GTCTTACAGAACCTGCTAGATGAGATAGCAAGTAGGGAGCAGGAAGTACAGAAGATCT
GTGCCAATTCCCAGCAGTACC
>1123
GGTACAGGAACATGTCTGTCTCTTCTCAGCTGTTTTAATCTCCCCTCTGCCATGTCTCTC
CCTCATGCAGGACTATGAGTTAGAAGCAGAAAAACTAAGGTCTCTTCTCGACTTGGAG
AATGGAAGGAGCAGCCACGTGA
>1124
GTCTAGCTCTCTGTTGCTTACCCTGTTGACACCTGTTTGTTGACCGCTCTCCTAATCCTC
TTCTCTGCAGGAAGCAGCACTTGCCGCCAAGTTCACTGAAGTTTATGCCATCAACAGA
CAGAGGCTGCAGAATCTGGAGT
>1125
CAGGTGGGGACAGGCCCTACATGTGCTGCCAGAGCCTGGCTTTAACCTTGTCTTGTCT
TCTCCATCACAGCAGCCGGAAGTAGAAGTGACCCATGAGACCCTGCAAAGGAATAGG
CCGGACTCTGGAGTGGAGGAGGCGT
>1126
GGGGATATAAGACAGTGACTGTTGAGGACCCTAAGTTTAGCTCCCACCTGATCTTCCTC
TGTCTCTGCAGAAGCTGCTGATGGGAGCTCCACCCTGGGAGGGGGTGCCGGCACCAT
GGGATTGAGCGCCCGCTACGGACC
>1127
AAGGGTAGGAGACGAGGGCCAGGCGGCCAGGCCATCAGAAGCAAGGCGTCCACTGC
TTTTGCTGTTCTAGGAATTTTTGAACCGAAGAAAAATGACAGAGTTTGGCAACCTGGC
ACTAATTGATCAGGATGCTGGGCAGG
>1128
AAAAAAAAAGAAAAAAAATGTTGGGAGGAATGAGCTTAAATTTTTAGGAAAGCACTG
CTTAATTTCCCAGGGCGCTGCTGGGCGGCCACCACCACGGAGGGACTCCTCATCTACT
CCCTGGACACCCGCGTGCTCTTTGA
>1129
CCGGGGTCATCCTCTCTTCCCCTGGTTTGAGTTGGTGGCAGAATAAGCATGATTTTTTC
TCCTTTTGCAGTTGAAGTGGTCACCTCCTCCCTTCCTGAACTGTATGTGGAGAAAGTGC
TGGAGTTTTTAGCTTCCTCCTT
>1130
CAATGCAGCATGAAGTGAGCCCACTGTACCACGGGGTGAGCCCCGCACGCTTAGGGA
GTCTCCCTTTCAGAGCCGGGACGCTGCTGCCTGTCATTCAGTTCCTCCAGAAGAGCAT
CCAGCGGCACCTGGACGACCTGTCG
>1131
AGGTGGTTGGGTGCAGGACAGGCCAGGGCCTGGGCGTTGCTGGCATTGACCATCCCT
GTGTGATTGACAGCTGTAGCTGGAACCACTATAACATGCAGTACGCACTAGCAGTTTCC
AAGCAGCGGGGCACAAAACGCTCC
>1132
CTGGTTGGGATGAGGCAGAAGGTGGAGATCCTGCCAGCAGAGTGAACCAGAGCTTCC

CTTTGACCTGCAGAACCCAAGAGAGTGGACGCGGATGGTGATCCGGAACATAGCCAC
CTCTGGCAAGTTCTCCAGTGACCGCA
>1133
TCCTTAAAATCTGGTTAGATGGATAATTTCATACCTATTTTTTTTTACTCTTTTCTTCTGT
TGAAACAGGCTTCACACTGTATAAATAGGAGAGGATGAGAGCCCAGAGGTAACAGAA
CAGCTTCAGGTTATCGAAATA
>1134
AATGGAGGTTATAGTGTTAGGGTTTGGGATAGGAGTAGGAGACAGGAGTCTCAAACTC
TCTTTCCCCCAGGAGTGAGTCCTGGAACCTGAAGGACCTGGATATTACCTCTCCAGAA
CTCAGGAGTGTGTGCCATGTGCCA
>1135
CCCCCCACCACAGGCACAGACTTAGTGAACTCCCCCCATGCAAGGCCTGACTGTGGTC
CTCTCTCTGCAGTCCACATCACTCTGGATCCAGACACAGCCAATCCGTGGCTGATACTT
TCAGAAGATCGGAGACAAGTGAG
>1136
GGTGCAGCCCGCAGGTGGGCAGGATGCGCCTGGGCCTGGGGCCTCCGACCGCTCCCC
GCTGTCCTCCTAGTCTCTCTACCTGGGGCCCATCTTGAAGTTTGGCTCCAAGGAGCAG
AAGCAGGCGTGGGTCACGCCTTTCA
>1137
GGGAGTGAGGCTGGTGCCCTTAGGTTGTGTGGGGTGGGGCGTGCGCTGAGCCCTGGG
TCTGTGTGGGCAGGGAACGGCAGTGATGCAGGAGCTGCGTCCACCACCGCCCGGGCC
GAGGGCGACTCATGGGTTCTGAATGG
>1138
TGTTGGGTGTGCTGGTGTGAGGGTGTGGCTGAGGGGCAGCTCTGAGAAAACCACCCG
CCTCTCCTTTCAGGGCATCAGTGCCTTCCTGGTCCCCATGCCAACGCCTGGGCTCACGT
TGGGGAAGAAAGAAGACAAGCTGG
>1139
GTGGGGGTGGCACCTTGAGGCCAGGCCCGCGCCCCGGCTGGCGGGCCACTGACCAGG
GCGGTCCCCACAGCAAACCCTGGACATGGGCCGCATCGGCATCGCCTCCCAGGCCCTG
GGCATTGCCCAGACCGCCCTCGATT
>1140
CCTCCTCCCCCTCCCTTCTGTCCCTGGAGGGGCAGCTGCTGACCTGTGGTGTGGGGTG
GGGCTATTGCAGTTCAAGTTGGCAGACATGGCCCTGGCCCTGGAGAGTGCCCGGCTGC
TGACCTGGCGCGCTGCCATGCTGA
>1141
CCAGCTGCCCCTTCTCCAGCTTTCCCCACGCCGGGGTCTTCTCCCTCCTGAGCCACTGT
TCTCATCTCAGGCCATCCAGATCCTGGGCGGCATGGGCTACGTGACAGAGATGCCGGC
AGAGCGGCACTACCGCGACGCCC
>1142
GGGCATCCTCACTGTCCTTGTAGTTCCTTGCTCATCCAGCCACCCATCCTGTCCCTTGCT
CCAACCCCAGTTCCTCCAGTTCCGCATGAAGATCATTAACCTGAAGCTGGAGCGGTTT
CAAGACCGCGTGGAGTTCTCAG
>1143
ACGGGTAGTGGGGTGATGGGGGAGGCCCCTTTCCCAGCCCTGGTGCTCAGTGGCTCTG

CTTCCTTTCCAGAGCCCCCTGAGCGGGACTCCACGGTGGCCGTGATTGTGGGTGCCTC
CGTCGGGGGCTTCCTGGCTGTGGT
>1144
GTTGGTTTGCTTATTCTTCTGGCTCTGATTTTTTTCCCCTTTACTCTTTTTGTTTTTGTTT
GCATTTCAGGGTGAAACTTCCTACATCCGAGTTTATCCTGAGAGAAGCACCAGCTATGG
CTACTCACGGTCTCGGTCTG
>1145
ACTGGTGTCCACAGAGGACATGGTTTAACTGGAATTCGTCAAGCCTCTGGTTCTAATTT
CTCATTTGCAGGAAATGCTGGCATAGAGCAGCACTAAATGACACCACTAAAGAAACGA
TCAGACAGATCTGGAATGTGAAG
>1146
GCTCAGTTAAGGGTGTCTGTGTCAGCAGAATAACCCTAACCTCACAGAATGGCCCACT
TCTCTCTTCTAGGCCACATTCGTGGCTGACTGGCGGAACTCGAATCGCTACCCTGCTGT
TATTCTCTTCTACGTCAATGCGT
>1147
CAGGGTGGGGAGACCAGGTAGAGGGAGTACAGAGTGACCGCCTCAAGTGACACCTCA
CCTCTCTGCACAGCTCCAATGAGACTCTGTCCTGCGTCATCATCTTTGTCATCGTGTACT
ACGCCCTGATGGCTGGTGTGGTT
>1148
GACCTGGATGGGGTGAGTTTGAGGGAGGGGGCCAGTAACCCACCTTCTGTCCCACCCC
TTCCTGCTGCAGGTGGATGGGGACTCTGTGAGTGGCATTTGTTTTGTGGGCTACAAGA
ACTACCGATACCGTGCGGGCTTCG
>1149
CCCACTCACCCATCCTTCCCAGCAGGGCAGCCTCACCCCTGCTAATGTCTGAGGTCCCC
CTTCTGTTCAGGAGTCATGACTCTGTTCTCCATCAAGAGCAACCACCCCGGGCTGCTG
AGTGAGAAGGCTGCCAGCAAGAT
>1150
ACTCTCTCCTCCCCACTGCTGCTGCGGGACTGGGCTTGGTAACGTCCTGTCCTGCCCCT
GTCCTCCACAGGCATTTTTGGCTTCCTGGCCTTTGGCTTTGTGCTCATTACCTTCAGCTG
CCACTTCTACGACTTCTTCAA
>1151
AGATCCTATCTCAAAAAAAGAGAGAGGAAAAGAAAGGAAAGCCTCACCTGTCTACGT
TCCCTCACTGTAGATGTCAGGCCAATGTGACCATCGGGCTGCCCACCAAGCAGCCCAT
CCCTGACTGTGAGATCAAGAATCGC
>1152
TGGCCCTTCCCAAGATTTGATGGGAAGTGGCAGCTTCTTCACGCTCCTTCCCTATCCCT
TCTGCTCTCAGGTTGACTGGGCAGAGTGACGATGAGCCAAAGCGGATCAAGAAGAGC
AAGATGATTGCCAAGGCCTTCTCT
>1153
ACTATGGGAGGCACTGCCAGGGACCGGGAAGTCACTATTCCTTCTCCTTTCCTTCCTTC
CATTCCCACAGCGGGCTTGGCCTTTGACCTCAATGAGCCCTCAGCTGATGTCTCCTCTG
CCTGGGCCCAGCATGTCACCAA
>1154
CTCCCAGGGGAGCGGGGGTGGCATGGACAGAGCCAGGGCCCCAGGCTCGTGTTGTCT

CTCCTCCTGTCAGTGCCCCCAGAGGAACAAGCCAACCTGTGGCTGGTTGAGGCAGAG
ATCTCCCCAGAGCTGCAGAAGCGCCT
>1155
TGCTTGGTAGTTTGGGGCACCTGGCTCTTTCCTCTTGAAGGTTGAATATAATGCTCGTG
CTCTTTTACAGGTGGCCAGTGGCGATTTCGATGCTTGCGTCCAAGTCTGTGAATGTAAA
ACCCCTCGTCACCCATAGGTTT
>1156
TTGAGCAGCCATACACCAGTCACCTCCATACTCACTGCCCATGCCCCATCCTCTCCTTC
ATCCCGGCCAGGCTGATCATTGGCTTCATCAGCAAACAGTACGTTACTAGCCTTCTTCT
CAATGAGCCCGACGGAACCTTT
>1157
CTTATGGTAGGGGTTGGGGAAGGGGAAGAAATCCGGACAGAGGGATCTCAGGGTCTC
CTTCCTACCATAGGCTCTCCACAGATAGAGAACATCCAGCCATTCTCTGCCAAAGACCT
GTCCATTCGCTCACTGGGGGACCG
>1158
TGCTTATCTGCAGGAAGGAGGGGTGGCATCAACCCTTGGTCAGTCACATGTACCTCCTT
CCCTCCTCCAGGGACCAACCACTTCCTACCCCAGAGCTCCAGATGCCTACCATGGTGC
CTTCTTATGACCTTGGAATGGCC
>1159
TGTTCAAAAGCAGGGTGTGGTGGTGTCCAGGAGAAGGGCTGGCATCAGGGGGTCTGT
TTTCTTTCCCCAGGCCCCAGGTGTACCCACCACACTCTCACTCCATCCCCCCGTATCAA
GGCCTCTCCCCAGAAGAATCAGTC
>1160
CCTGACCCATGTGCCTCTTCTTTCCTGGGCCTTGCCCGCTCTCCTTATTTCCATTGCTGG
TTCCTGGCAGGGCCTGCTGCCGTGCCAGCCTCAGGAGCATGCTGTGTCCAGCCCTGAC
CCCCTGCTCTGCTCAGATGTGA
>1161
GTGGAGGCTGGGTGGCATCTAGGGGAGTGGGCGCCATGCCTACTCCACTGCTTCTCCC
ATCTCCTTGCAGGATTGGTGAAGACATATTCCCTCCTCTGCTGCCTCCCACTGAACAGG
ACCTCACTAAGCTTCTCCTGGAG
>1162
TTCGGCCCTTCCGAGGGCAGCTCCTGTGTGCTGAGGGCTCTGCTGCTCTGAGATGCCG
CGTCCCTTCCAGACCTCACGCCCACCATCTTGGATTGGTTCTCGATCCCGTACCCCAGC
TACGCCATCTTTGGCTCGAAGAC
>1163
GAAGCACCTGCCCTCCCTGAGTTCAGTGTGTCCAGTGTTCCCGGCCAGATGCTCATGC
CATGCTTCGCAGGGAGCGGGAACGCCGAGAGAAGGAGAGAGAAGAGTGGGAACGCC
AGTACAGCCGGCAGAGCCGCTCACCC
>1164
GCATCCACGGCCCCAGGTGTGTTGATGTGTGTATGCCCCATCCTCACGGCCCCTCCCTT
TCTCTTTCCAGGCGCTCAAGGTCCCGATCCCGAAGCCCCCATTACCGACATTAGGCAG
AAGAGTGGGGGGTGGGGAGGACA
>1165
CCTTTGCCATAGTCCCACCCCACCTGCCGCCAACACTGTGTGAGGCCTCTCCTGCCCGC

TTCCCATGCAGAAGAAAATCATGATCATCATCTGCTGTGTGATCCTGGGCATCGTCATC
GCCTCCACTGTTGGGGGCATCT
>1166
GCCTCCAGCTTGCTCTGCGTGGTGGGCAACCTGCGTGATCTGAGGGGATGGGTTCTTC
TTGCTCCTACAGGCTCTCCTTTGATAAAGATGCGATGGTGGCCAGAGCCAGGCGGCTC
ATCGAGCTCTACAAGGAAGCTGGG
>1167
CGTCACCCGCCCCCGCCCTCACCCGTCCCCGCCCTCACCTGCCCCGCCCTCACCTGTC
CCCGCCCCGCAGGGAGCTCGAGGAGCAGCACGGCATCCACTGCAACATGACGTTACT
CTTCTCCTTCGCCCAGGCTGTGGCC
>1168
GGCACTGGGAGGGCAGGTGGGGTGGTACCTCTGCCGAAGCCTTCCTGGTCACAGCTT
GGTCTCTTTCCAGGGTAAAGAGTGTCACTAAAATCTACAACTACTACAAGAAGTTTAG
CTACAAAACCATTGTCATGGGCGCC
>1169
CCCTGAGCCCAAGGGGCCAAGGTGGGCAGGGACATGGAGCAGGCATGGAAGGCTGG
TTCTTGTCCCCCAGCCCAAGCCAGTGACCTGGAAAAAATCCACCTGGATGAGAAGTCT
TTCCGTTGGTTGCACAACGAGGACCA
>1170
GTGCCTTGTGAGGCTTCAAGGTGCAGAAGGTAGGGTGGGGAGACACAGCTCGTGCTC
TGTTTGTTTCTAGGAACGAATGTTCAATGCAGAGAATGGAAAGTAGCGCATCCCTGAG
GCTGGACTCCAGATCTGCACCGCCG
>1171
TGGCCACGTGGTCCTGCCTGTCTCAGCACCCACCGGCTCACTCCCATGTGTCTCCCGTC
TGCTTTCGCAGAGCTCCTCCCTGAATGAGGCCAGCAGTGGCCTCTTCGACGTCTTCCTA
CGCTTCATGTGCCACCACGCCG
>1172
GGCTCGGTTTGAGTGTACGCATGTCCAGCACATGCCCTGCCCGTCTCTCACCTGTGTCT
TCCCGCCCCAGGTCCTACGTCCAGTGCCAGGGGATCCCGCAGGGCTCCATCCTCTCCA
CGCTGCTCTGCAGCCTGTGCTAC
>1173
AGTCCCTCGTAGACAGATACTACGTAAAAAGTGTAAAGTTAACCTTGCTGTGTATTTTC
CCTTATTTTAGGCTGCTCCTGCGTTTGGTGGATGATTTCTTGTTGGTGACACCTCACCTC
ACCCACGCGAAAACCTTCCTC
>1174
ACAAGGGTGTCAGGTTACCTCCTGGGTGACGGCCCCGCATCCTGGGGCTGACATTGCC
CCTCTGCCTTAGGACCCTGGTCCGAGGTGTCCCTGAGTATGGCTGCGTGGTGAACTTG
CGGAAGACAGTGGTGAACTTCCCT
>1175
AGCAAGCTTTGCAGGAGGGGGCTGGGTGTGGGGCAGGCACCTGTGTCTGACATTCCC
CCCTGTGTCTCAGCTATGCCCGGACCTCCATCAGAGCCAGTCTCACCTTCAACCGCGG
CTTCAAGGCTGGGAGGAACATGCGT
>1176
CCATGCCCACACCCACGAGCACCGTCTGATTAGGAGGCCTTTCCTCTGACGCTGTCCG

CCATCCTCTCAGGTTTCACGCATGTGTGCTGCAGCTCCCATTTCATCAGCAAGTTTGGA
AGAACCCCACATTTTTCCTGCGC
>1177
AGCTTTCCGGTGTCTCCTGGGAGGGGAGCTGGGCTGGGCCTGTGACTCCTCAGCCTCT
GTTTTCCCCCAGGGATGTCGCTGGGGGCCAAGGGCGCCGCCGGCCCTCTGCCCTCCGA
GGCCGTGCAGTGGCTGTGCCACCA
>1178
ACGTCCTAGGAGGGTTGGAGGATGCCACCTCTGGCCTCTTCTGGAACGGAGTCTGATT
TTGGCCCCGCAGCCCAGACGCAGCTGAGTCGGAAGCTCCCGGGGACGACGCTGACTG
CCCTGGAGGCCGCAGCCAACCCGGC
>1179
ACATTTTTTTACATTACAATTATGTTGTCAGATTTACAATGTGCAAGTTACCTGGGCTTTT
CTCTTTTAGAAATCCAAGGGGGAGAGTGATGACTTCCATATGGACTTTGACTCAGCTGT
GGCTCCTCGGGCAAAATCTG
>1180
TTCCTGTACAGTCCAGCCCCTCCTCCCACAGGATCTGCTTTAATCCAGCGCCTCTCCTC
ATCTCTCCCAGCCCAAGGCCACAGTCTCAGATACCTGTGAAGAAGTGGAACCCAGCCT
CCTTGAAATCCTCCCCAAGTCCT
>1181
TCCTGCCCATTGCCTTTCCACCCCGGCCTCCCCGCCCCCTCCCTGTTCTCTCCTCGTGG
CCTGTTACCAGCCCACAGAGAGTCCAGCCGGCAGCCTGCCTTCCGGGGAGCCCAGCG
CTGCCGAGGGCACCTTTGCTGTGT
>1182
GTGGCCTGTTTCCTGTGTCCTGGCTGTGTCCTGTCCTGTGTCTGACCCCAAGCCGGCAT
TTATGTTGCAGCCAGCAGAGGCCTCGGAGGTGGCGGGTGGGACCCAACCTGCGGCTG
GAGCCCAGGAGCCAGGGGAGACGG
>1183
TTCTCCTCGGTGGCCCTGGCTGTCCTTGGACCACCTTCCCTGCTCAGCTGACCCGTACC
TCTGCCACCAGAGCTCTCTTCCTGCTGTCGTGGTGGAGACCTTCCCAGCAACTGTGAA
TGGCACCGTGGAGGGCGGCTGTG
>1184
CAAGGCCCGTGCTCTGTGCAGGGCTGGAGGTGGGACGGAAGGTCTGACTTGCGATCC
GCATCCTCTGCAGGTACAGGCCCACCACGACTACACGGCCACTGACACAGACGAGCT
GCAGCTCAAGGCTGGTGATGTGGTGC
>1185
TTTGTCCCCTGCCACTTTGCTTCTAGACTATCATTATTGGACCTCTGAATTCTTGTCTGC
CTTCTTGTAGGAGCCCCATGGCACCTGCCCAGCCCCACCTCAGCCCATCTTGACAAAAT
CTAAGGCTCCATGGAGCCACC
>1186
CAGAAGGATGGGAACATCAGCTGCGCTTCTGTTGGGCATTGAGGACCCCTACATCTGC
ACATACACACAGGTGACTGTCCGGGATGGCATGAGTGTCTACGACTCTCTAGACAAGG
CCCTGAAGGTGCGGGGTCTAAATC
>1187
TGTGGGGGTTCCCTGCCTCCCCATGGCCCCTACCCAACCTCCCACTCATTCCTTTCCAT

GCCCCCTGCAGACGAAAGACGGTCACTGCCTGGGACACAGCCATTGCTCCCCTGGATG
GCGAGGAGCTCATTGTCGAGGTC
>1188
ACGGTGGGGGTGGACCATGGTTGGGGGTGTCCTTGACCAGGTCTCAAACTTCCCTGCT
CTGTGGCATCAGGTACGGAAGACCTTCTTCAGCCTGGCGTTCTGTGACTTCTGCCTTAA
GTTTCTGTTCCATGGCTTCCGTT
>1189
GGCCCAGCCACGAGGCCCTTACAGACAGCTGACCTGTGTCCCCTTGCTTTATACCCTTC
ATGCCCTCAAGGTTCTACCACAGTGTCCAGGATTTGTCCGGAGGCTCCAGACAGCATG
AGGCTCCCTCGAACCGCCCCCTG
>1190
GGGGGTGGGGTGGGGGGCTTTCTCGGTTCTCTGATTCCTGGCAGTGATTTCACAGCCT
TTCCCCTGGCAGCCCCCGCACCCAGCACTGTGACCCGGAGCACTTCCCCTTCCCTGCC
CCAGCCAATGCCCCCCTACAGCGC
>1191
GTCCCCTGTGCCTGCACCCTGACCCCCGCTGCCCCACTTACCTCCACTCACATCCTCTC
TGCAACTGCAGCTGCCGGTAGTAGAGGAGGTAGTGATGGAACCCCCCGGGGGAGCCC
CAGCCCAGCCAGCGTGTCCTCGGG
>1192
CACCAAGGCTGAGTGACGTGGGATGAGGGATGTGGGCAGGCCTCTTAGATGCCACCCA
TCTCGCCCACAGAAGAACCTGGGGTACCGGGACTCAGGCTATTACTGGGAGGTACCAC
CCAGTGAGGTGCAGCTGCTGAAGA
>1193
TCCTGATGCTCGGTAAGTGACAGTTGCTATTTTTCCTCCCCACCTCTGACACTGCCCTC
CCTGCCTGCAGGAAGACGCGACATGTCAACATCTTGCTGTTTATGGGCTTCATGACCCG
GCCGGGATTTGCCATCATCACA
>1194
GGGATCTGGGACTGTGGACCAGCCATTTGCTGACCTCCGTGGTCCCCCTGCCTGGTCT
GGCCTGTTGTAGGCAGCTGAGGTGATCCGTATGCAGGACCCGAACCCCTACAGCTTCC
AGTCAGACGTCTATGCCTACGGGG
>1195
ATACAAAATTCCTTGGGCCTCCCAGCCCCTGACCCCAGATCACCCCTTTCCTGCCCCCG
TCTGCCCCCAGATTATCTTTATGGTGGGCCGTGGCTATCTGTCCCCGGACCTCAGCAAA
ATCTCCAGCAACTGCCCCAAGG
>1196
CCATGAGGCTGGGGCTGTTGGGATGCCCACAGCGGTCTGGACCCCCCTCCTCCCCCCC
ACCTGCCCTCAGATCCTGGCCACAATTGAGCTGCTGCAACGGTCACTCCCCAAGATTG
AGCGGAGTGCCTCGGAACCCTCCT
>1197
GTGGCTAAGGACCTGGGTCAGGGAGAGAGCAGGGCAGGACCCCGAATGATCTCCAGC
ATTCTGTGCCTAGCTGCTGATCGCCTACAAGCCAGCCCCTGGCACCTGCTGCTACATCA
TGAAGATAGCTCCAGAGAGCATCC
>1198
TTGCCTGATATACCTGAAGTCCCACAATAAGGGCTGCACATGGGATAGAAACTCACTTC

CTACATTCCAGATGGAATGCTCTCTGCAGGCCAAGCCCGCAGTGCCTACGTCTAAGCTG
GGCCAGGCAGAGGGGCGAGATG
>1199
GAGGTGGGCGGGGCAGGAGGCTAGGGAGGGCAAGAGGGGCCAGGGCTCTGAGCATA
CTACCTCCTTGCAGAGCCGCCCCCCAGGCCCTTCCTGGACATGGGGGAGGGCACCAAG
AACCGAATCATCACAGCCGAGGGGAT
>1200
CTGGGGGTGGCTGGGGGCAGGGACCCCAGGTGTCAGGGTGCTGATGTTCGCTGCCTC
ATTTCCATCCCAGGGCCTGAACCTGGACGACTGCTCCATGTATGAGGACATCTCCCGGG
GCCTCCAGGGCACCTACCAGGATG
>1201
GTCTTTTACTTACATCTTTACTGCACGTTCCAAGCGTTGTGTGGCCTGACGAGTGTGTT
CTCTCTTCTAGTCGACATTACTCTGAAGGGACGCACAGTTATCGTGAAGGGCCCCAGA
GGAACCCTGCGGAGGGACTTCAA
>1202
TCGGTAAGTGGTCTGTTGTCTTTTAAGCCTGCATATTTCTGGAAAGAATACATTTTTTGT
TCTGTTACAGCTCCGGGTTGACAAATGGTGGGGTAACAGAAAGGAACTGGCTACCGTT
CGGACTATTTGTAGTCATGTAC
>1203
TTAGAAAATTTTAAAACTATTAAAAATATTCCTCAGCATTGCTAATAGCTTGCTTATTTAT
TTAACATAGGGCTTCCGTTACAAGATGAGGTCTGTGTATGCTCACTTCCCCATCAACGT
TGTTATCCAGGAGAATGGGT
>1204
TTAGTGCCTCTGCAATTTAAATATTTTTTACACAGATTTGATGCTGTGCAAATGCCCTCT
CCCCTTTTAGGTGTTGCTTGTTCAGTATCTCAAGCCCAGAAAGATGAATTAATCCTTGA
AGGAAATGACATTGAGCTTGT
>1205
CATAATGAAATACTTCCTTTTTAAGATGGGTTAGGCTGATGTATTAATTGCTTTATCTTCA
CTCCTATAGCGGCTTTGATTCAGCAAGCCACAACAGTTAAAAACAAGGATATCAGGAA
ATTTTTGGATGGTATCTATGT
>1206
AAAAAAGATTAGTACTTTCCTGGGCAAGTGTATTGATGGGGATTTACAGATTTTTTTCT
TTTTTTACAGTTACCTGGCTACAGAAAGAAGATGCCAGATGACACTTAAGACCTACTTG
TGATATTTAAATGATGCAATA
>1207
TTGGCCTTTGTTAGAACCTCCAGGGTGTGGTTCTCAGCTGTGGCCACTTACACCCTCCC
TCTCTCCCCAGGCCTGTGGGTCCCCATCGCCCAAGTCCTGCCCACACTCCCACCTGCTA
CCCTGATCAGAGTCATCATGCC
>1208
TAGGCCTTTGTTAGATTCTCCATGGTTCATATCTCATCTGAGTCTGTTCTCACGCTCCCT
CTCTCCCCAGGCTGTGGGGCCCCATCACCCAGATATTTCCCACAGTTCGGCCTGCTGAC
CTAACCAGAGTCATCATGCCT
>1209
CCTCCACCGACCTCACTCCTCCCACCCCCATCCACGCTGAATCGGGTTCTGCTTCCGCT

TTCAACCCAAGAAAGCCGCAGGTGCCGGAATGTGATGCCACTGACTTGCGCATTGGGG
GTTAGAGAAAAGCGAGCTGCTCT
>1210
CATCAATCCTGCAGAATCGACCTCTGCTGGCCGGCTATACCCTGAGGTGCTCTCTCACT
TCCTCCTTCAGGTTCTGAGCAGACAGGCCAACCGGAGGACAGGATTCCCTGGAGGCC
ACAGAGGAGCACCAAGGAGAAGAT
>1211
TAAGCCTTTGTTAGAGCCTCTAAGATTTGGTTCTCAGCTGAGGTCTCTCACATGCTCCC
TCTCTCCGTAGGCCTGTGGGTCCCCATTGCCCAGCTTTTGCCTGCACTCTTGCCTGCTG
CCCTGACCAGAGTCATCATGTC
>1212
TAAGCCTTTGTTAGAGCCTCTAAGATTTGGTTCTCAGCTGAGGTCTCTCACATGCTCCC
TCTCTCCGTAGGCCTGTGGGTCCCCATTGCCCAGCTTTTGCCTGCACTCTTGCCTGCTG
CCCTGAGCAGAGTCATCATGTC
>1213
CTGTCAGTCCTGCAGAATCAGCCTCTGCTTGCTTGTGTACCCTGAGGTGCCCTCTCACT
TTTTCCTTCAGGTTCTCAGGGGACAGGCTGACCAGGATCACCAGGAAGCTCCAGAGGA
TCCCCAGGAGGCCCTAGAGGAGC
>1214
TAAGCCTTTGTTAGAGCCTCCAAGGTTCAGTTTTTAGCTGAGGCTTCTCACATGCTCCC
TCTCTCTCCAGGCCAGTGGGTCTCCATTGCCCAGCTCCTGCCCACACTCCTGCCTGTTG
CGGTGACCAGAGTCGTCATGTC
>1215
CTTTGTTAGAGCCTCCAAGGTTCCATTCAGTACTCAGCTGAGGTCTCTCACATGCTCCC
TCTCTCCCCAGGCCAGTGGGTCTCCATTGCCCAGCTCCTGCCCACACTCCCGCCTGTTG
CCCTGACCAGAGTCATCATGCC
>1216
TAGGCCTTTGTTAGGGCCTCCAGGGCGTGGTTCACAAATGAGGCCCCTCACAAGCTCC
TTCTCTCCCCAGATCTGTGGGTTCCTCCCCATCGCCCAGCTGCTGCCCGCACTCCAGCC
TGCTGCCCTGACCAGAGTCATCA
>1217
TAGACCTTTGTTAGGGCATCCAGGGTGTAGTACCCAGCTGAGGCCTCTCACACGCTTCC
TCTCTCCCCAGGCCTGTGGGTCTCAATTGCCCAGCTCCGGCCCACACTCTCCTGCTGCC
CTGACCTGAGTCATCATGCTTC
>1218
TCAGCCTTTGTTAGAACCTCCAAGGTTCGGTTCTCAGCTGAAGTCTCTCACACACTCCC
TCTCTCCCCAGGCCTGTGGGTCTCCATCGCCCAGCTCCTGCCCACGCTCCTGACTGCTG
CCCTGACCAGAGTCATCATGTC
>1219
CTCACATCATTGATGAGAATGCCTTGAATCCGAGCTACTAAATCACATTTCCTTCCTTCT
AACCTTCCAGTTAGATCAAACCATTGCTGAAACTGAAGAGGACATGTCAAATATTACA
GATCCACAGATGTGGGATTTTG
>1220
GGGGCTGTGACTAGGATCGGGGGACGCCCCAAGCTCAGTGCCCCTCCCTGAGCCATGC

CTCCCCCAACAGCTATACGGGCAGCTCCCCAAGTTCCAGGACGGAGACCTCACCCTGT
ACCAGTCCAATACCATCCTGCGTC
>1221
GGCCCATCCCCAGTGACTGTGTTGATCAGGCGCCCAGTCACGCGGCCTGCTCCCCTCC
ACCCAACCCCAGGGCTCTATGGGAAGGACCAGCAGGAGGCAGCCCTGGTGGACATGG
TGAATGACGGCGTGGAGGACCTCCG
>1222
TGGGGCAGACGGGGGTGTCTCAGGGGCTGGGAGGGATGAGAGTAGGATGATACATGG
TGTGTGCTGGCAGGAGGCGGGCAAGGATGACTATGTGAAGGCACTGCCCGGGCAACT
GAAGCCTTTTGAGACCCTGCTGTCCC
>1223
AGGCCCTCTGCCCCCAATTCCTCTCCAGCCTGCTCCCGCTGGCTGAGTCCCTAGCCCCC
CTGCCCTGCAGATCTCCTTCGCTGACTACAACCTGCTGGACTTGCTGCTGATCCATGAG
GTCCTAGCCCCTGGCTGCCTGG
>1224
CTTCCCCAGCAACACAAATGTCCTGCCAGATTCCTCCTGGAAAAAAACTTCTGCTCCT
GTCCCCCTCCAGGTCCCAGGTTGCCCATGTCCAGGAAAAGATGGATCCCCCTATCCAAA
TCTTCTCCGTGGTGTGTGTGGGT
>1225
GAAATGGGATAGATTGGAGGTCCTGTTGACCTCCATGCTTATTCACTTCAGTTCTCTTCT
TGGCTCTTAGTTGGATCGAAGCCTCTTAAAATGGCAGATGACTTGGACTTCGAGACAG
GAGATGCAGGGGCCTCAGCCAC
>1226
GTCAATTTGAGCCTTTATTTCCTCCGTTTTAGAGTTTGGTTGGGTTTCTCTTTGTGATGC
ATACATACAGGTCCATCTGGTTGGTATTGACATCTTTACTGGGAAGAAATATGAAGATAT
CTGCCCGTCAACTCATAATA
>1227
CAGAGCCTTTACTGTCATGTCAGCCTCCCTGGGCCTACTACCTTCAGCCTCCTTCCCTAT
CTGCCCCCAGCTGATTGGCATCCAGGATGGGTACCTATCACTGCTCCAGGACAGCGGG
GAGGTACGAGAGGACCTTCGTC
>1228
TGCTCAGCTTTGTTCTGTACGTTTCTTCCTGAGCTCAGACATCTCTTGGCTATCCCTCTT
GCTTCTCCAGATCACGGTGCTGTCTGCCATGACAGAGGAGGCAGCTGTTGCAATCAAG
GCCATGGCAAAATAACTGGCTC
>1229
TGGCTCTGTCCTCCCTATCCTTCCTGTTGAAGGTATTATCCTGTCTTACTAATTTCTCTCT
CCTACCTAGGATGGCGGTGGTGGCAGCAGTGATCCTCTGAACCTGCAGAGGCCCCCTC
CCCCAGCCTGGCCTGGCTCTG
>1230
CTCTACGCGTGGGGAAGTTTCCTGGCAGCCTGAAGTGTACACCTGACGCCTGCACTCC
CTCCCTATGCAGGGTGCAAATCCGGCCGCGATGAACGCGAGCGCCGCCTCGCTCAACG
ACTCCCAGGTGGTGGTAGTGGCGG
>1231
GAACAATCATGAAACTATGGATGATTTAGTCCAGCAAAATATCCAATTGGGAACCCTCA

TCCTTCTGCAGAGCCCAAATGGCGCAGTGGGAAATGCTGCAGAATCTTGACAGCCCCT
TTCAGGATCAGCTGCACCAGCTT
>1232
AAGGGGTTGGAAAGCCTCAGGACTACAGTAACACTGCAGAGACACTAATACTTCTTAT
TCCTGGTCCCAGGCAGGAAGCTGCACTTGGGAGTGATGATTCCAAGGCTACCATGCTA
TTCTTCCACTTCTTGGATCAGCTG
>1233
AGGGTCTAGGGCAATAGGTCATTGAGAGTGGTTGAATTGGATCAATCCTTTCTGTTTAC
CTTTCTGTTAGCCCTTTTCCCAGGATCCTACCCAGTTGGCTGAGATGATCTTTAACCTCC
TTCTGGAAGAAAAAAGAATTT
>1234
TATAATTTCAGGCCTGAACTTAAGGCATGAAAGGCCAGAGTTAAAACGTGCTCAGAGC
CTCTTTTTTCAGGAACAAGGAGAGCCAGTTCTCGAAACACCTGTGGAGAGCCAGCAA
CATGAGATTGAATCCCGGATCCTGG
>1235
TAGGGTTGACAGTGTTCAGCCTAACACCTCCCTGAGAAGCAGCCTCATCGGGGTCCTC
TCCCCTCTGCAGAAGCTGGTAAAATCCATCAGCCAACTGAAAGACCAGCAGGATGTCT
TCTGCTTCCGATATAAGATCCAGG
>1236
CTCTGACTGAGACCCCAGTCAGGAAGGAGAGGAAAGAACCCCTGGACTGACTCCTGT
TCCCACCATCCAGGGAAGACACCCTCTCTGGACCCCCATCAGACCAAAGAGCAGAAG
ATTCTGCAGGAAACTCTCAATGAACT
>1237
CTTCATCCCCAGTTGGGTGGTGGAGGGTGAAAGGGAGAGATGCTCAACACTCACATTA
TCTCTTTCCCAGGAGGTGCTGGATGCCTCCAAAGCACTGCTAGGCCGATTAACTACCCT
AATCGAGCTACTGCTGCCAAAGT
>1238
AGATCTTGGAACCTGGATGGGGGAATCCAGGAAACTCATGGATTCCTTCTTCCTGAATT
TTATCACCCAGGTTCACAGCTGGAGCAAAGCTGTTGTTTCACCTGAGGCAGCTGCTGA
AGGAGCTGAAGGGACTGAGTTGC
>1239
TTTTTTTTTTTCTGCTGAGTTTTTACAATAATTCCATTCCTTGTCTCCATGTATCTTCCTCC
TGGAACAGCTTCCGGAAGTTCAACATTCTGACTTCAAACCAGAAAACTTTGACCCCCG
AGAAGGGGCAGAGTCAGGGT
>1240
ACAGAGGACTCGAGGCAGGGGGAGCTTGCCAAAGAGCCTTCTGATGACTATGTCTTTG
CCTGTCCCAGAGGGGCCACTAGGTGTGACAGAGGAACTGCACATCATCAGCTTCACGG
TCAAATATACCTACCAGGGTCTGA
>1241
GAGCAGGGGTGTGGAAGCTTGGTGTGATAGGTTGCTTCTGAGCCAGCCTACACTGCTC
CCACCCCTGCAGAACCAGCAGTTCTTCTCCAACCCCCCCAAGGCCCCCTGGAGCTTGC
TGGGCCCTGCTCTCAGTTGGCAGT
>1242
CTTCCCACGTGCCCTCCTTCCCTACACCAGAGGCAACTCCTCAGCTTTTGCTACCTTTC

CATTCCTCCAGCGAGAGAGCCCTCCTGGCAAGTTACCATTCTGGACATGGCTGGACAA
AATTCTGGAGTTGGTACATGACC
>1243
GGAGCAGGGAGGCAGGGGCCTGGGGGGGCAGGGTGTTGGGCGGTGTCACACGTGAC
ACACTGTGCTCCAGACGCATCATGGGCTTTGTGAGTCGGAGCCAGGAGCGCCGGCTGC
TGAAGAAGACCATGTCTGGCACCTTT
>1244
CAGAGAGTCAGAGAGCTCCAAGGCCCGGCCTCTTCCCTCAAGCCCGCCTGTTCCTGCA
TTCACTCTCCAGACAAGGTGCTCATCTACTCTGTGCAACCGTACACGAAGGAGGTGCT
GCAGTCACTCCCGCTGACTGAAAT
>1245
TCCCTTGGGCTGCTGGGTTGGGAACTCCTAACTAAGATCGGGGCCTCACTTTTCTCTCT
GGATTACCTAGTCTTCAGAAACTGTGTAAAGATTGAAGAAATCATGCCGAATGGTGAC
CCACTGTTGGCTGGCCAGAACAC
>1246
GTATCCCAAGTATAAGGAGGTTCTTGTGAAACTGCAATGCCTCTTTTTCCCTTTCTTTTT
TATATTTCAGAATAGCTGCAATCTCCATGGCCTCTAAGATAACAGACATGCAGCTTGGA
GGTTCAGTGGAGATCAGCAAG
>1247
AAACATTTAATAGAGAATAAAAGAAAAGAACTTTCAGTGGTTTAATGCTGTTACTTTTA
ATATTTCAAAGATCTGACTGCAGCCATGAGCAGCAATGAGTGCTTCAAGTGTGGACGA
TCTGGCCACTGGGCCCGGGAATG
>1248
AAGTACTTCAGTATTTGTTAGTATCAAGACTGGTCTGACTAGCCGAATTCTTTGTTTTTG
CTCAAAACAGGTTTCCAGTTTGTTTCCTCGTCTCTTCCAGATATTTGTTATCGCTGTGGT
GAGTCTGGTCATCTTGCCAA
>1249
ATAAGTTAAAACGTAACACTTTGTAAAGGTATTATAGTACATGGCCTGTTTCTTTTCCTT
ATTGTTGAAGCCTGCTATAACTGCGGTAGAGGTGGCCACATTGCCAAGGACTGCAAGG
AGCCCAAGAGAGAGCGAGAGCA
>1250
ATGGTGACTGTTGCTTTGGTAGACAGTTTTTTCTGAGGCCCATTGTGAAAACTTTTAAT
TTCTTTTTTAGGTGTGGTGAAACTGGTCATGTAGCCATCAACTGCAGCAAGACAAGTG
AAGTCAACTGTTACCGCTGTGGC
>1251
ATGGCGCGGAAAACAGGGGTGGCCTGGCTCGGCCTGGCCCCGGCCGACGCTGTGCGT
TTGTCGCTTACAGTCTCCCGCTTCATGGGCCCGGCGAGCGGCATGAACATGAGCGGCA
TGGGCGGCCTGGGCTCGCTGGGGGA
>1252
CAGAGAAGACTCTTGGGTTTCTGATAGGCACTGACTCTCTCTGCCTATTGGTCTATTTTC
CCACCCTTAGGCTGCTGGTGGTCTACCCTTGGACAGAGGTTCTTTGAGTCCTTTGGGGA
TCTGTCCACTCCTGATGCTGT
>1253
GCTGGAAGTGCGCACCTGGGCTGGGCTCGCAGCAAGGCGCAACTTCAGGCTCCGAAG

CGGTGTGTTGCAGATCGAAGCGCTGCAAGAAGTCTTGAAGAAGCTCAAGAGTAAACG
TGTTCCCATCTATGAGAAGAAGTATG
>1254
GACTGTGAGACTTGCCTGTTGGGAACCTGGGTTTGTTCATACTCGATGACCACACATTT
TGTTGTTTCAGTGTGACGCCGGTGAGCAGTGTGCAGTGAGGAAAGGGGCAAGGATCG
GGAAGCTGTGTGACTGTCCCCGAG
>1255
GCATGTGGAATATTCTGGCTGTGCTGGCTGTGCTGGCTGTGGACTGAGTCCTCTGTCTT
CCCCCATCCAGTGTCGACCCTGGAGGAAGAATGCCTGCTGTTCTACCAACACCAGCCA
GGAAGCCCATAAGGATGTTTCCT
>1256
CAGTTCTATTCGGGGCTGAGTTGCTGGGATTCTTGAACCTGAGCCCTTCTTTTGTATCA
AAATCACCCAGGTGGATCAGAGCTGGCGCAAAGAGCGGGTACTGAACGTGCCCCTGT
GCAAAGAGGACTGTGAGCAATGGT
>1257
AGTTGTAGGGCTGGCAGACCTCAAGATAGTTCCGGGCCCAGTGGCTAAAGGTCTTCCC
TCCTCTCTACAGGGTTTAACAAGTGCGCAGTGGGAGCTGCCTGCCAACCTTTCCATTTC
TACTTCCCCACACCCACTGTTCT
>1258
GCAGGGCGGATACTGGAGGGGGCTTGTGGCATCTGACTCTGTATCTCCTACCTGCCCCT
CTCCTTGGTAGCTGTGAGAAGTCACTGCTTTGGGGAGACCTGATCTGGCTGTGCCAGA
TGGACACTGAGAAAGAAGTAGAA
>1259
GAGAGAATCCCACTTTCTTGCTGTTGTGATGGTGGTAAGGGAACGGGCCTGGCTCTGG
CCCCTGATGCAGGAACATGGAGCTGATCCAGGACACCTCCCGCCCGCCACTGGAGTAC
GTGAAGGGGGTCCCGCTCATCAAG
>1260
GGCAGTCCCCACCTTGGTCAGCAAGGTCGTGCCCTCAGCCTGCTCACCTCCTATCTCCC
TCCCTCTCCAGGCACCACCTGGGTGAGCCAGATACTGGACATGATCTACCAGGGCGGC
GACCTAGAGAAGTGTAACCGGGC
>1261
GGGTAAGGGAAGTGGAGGAAGACAGGGCTGGGGCTTCAGCTCACCAGACCTTCCCTG
ACCCACTACTCAGGGCTGGAGACTCTGAAAGACACACCGCCCCCACGGCTCATCAAG
TCACACCTGCCCCTGGCTCTGCTCCC
>1262
TCAACAGATGAGCTGGCCTGGGGTGGGCTGTGTGGTGATGGTGCTGGGGCTGGGTCCT
CTGCCCCTGCAGGTGGTCTATGTTGCCCGAAACCCAAAGGACGTGGCGGTCTCCTACT
ACCATTTCCACCGTATGGAAAAGG
>1263
TGTGAAGCCGAGGGGTGGTGGCTATAACGTACAGCAACCCTGTGTCGGTGCCCCCTGC
CCGCTTCTCTAGTGTCCTACGGGTCCTGGTACCAGCACGTGCAGGAGTGGTGGGAGCT
GAGCCGCACCCACCCTGTTCTCTA
>1264
AGATCCCCTCTGAGGTTAGAGAGGGGGACCCGTTTTGTTTTGCTCCACTGAGGAGCCC

TCTGCTGCTCAGAACCCCAAAAGGGAGATTCAAAAGATCCTGGAGTTTGTGGGGCGCT
CCCTGCCAGAGGAGACCATGGACT
>1265
CCTCCCCATAGGCACTTGGGGCCTCCCCTGGGATGAGACTCCAGCTTTGCTCCCTGCCT
TCCTCCCCCAGGCATGGCTGGGGACTGGAAGACCACCTTCACCGTGGCGCAGAATGA
GCGCTTCGATGCGGACTATGCGGA
>1266
AAATGCTAATGATTCAAGGATGTCCTCAGCTCTGTATGTGTTCTAAGAGTTCTATGTTTT
TTCTCCACAGAAGGCATAAGAACTAGGAGCTGCTGACATTTCAATATGAAGGGCAACT
CCACCCTTGCCACTACTAGCAA
>1267
GGAGGCTGGGTTGGTGTGACGCTCTGACCTCTTCCGGTGCTGACCTCTCCTTATCGCTA
CCTGAATACAGACAAGGACGAGGGTGACCACCCCAACAACAGCTTCAGCCCCTGTAG
CGCCCATGACCGCAGGTGCCTGCA
>1268
CTCTGACCCCAGGCCTGGGCCTCCTGCCTCTCTTCCTTCTGCTGAGCAATTTTGTCTTC
CCCTCCTCCAGCCCCAGGGCTCCTGCCAGAGCGAGCTGCACCGGGCGCTGGAGCGGC
TGGCCGCTTCACAGAGCCGCACCC
>1269
TGGACTCAGCTCTGGGGCATGGTCTCTTTTCCTGCGTCGGAACTGACCCCTCATGTCCT
TCTCTTGGCAGTGTCACCCAGCTCTGGATGGGCAGCGTGGCAAGTGCTGGTGTGTGGA
CCGGAAGACGGGGGTGAAGCTTC
>1270
ACATCTCGGTGCTGGGCCCCATTCAGAGTGGAGGGTTCTCCCTCTAACCACTCCCACC
CACCTCCATCAGATCAGTGAGCGCTATGGCCCCGTGTTCACCATTCACTTGGGGCCCCG
GCGGGTCGTGGTGCTGTGTGGAC
>1271
GGAGCTAATGCCGTGAAGCTATGTGCATCTCTCTGTCTGGCCGTACCTGGGTAATAACC
TGATCGACTAGGCGTGGTATTCAGCAACGGGGAGCGCGCCAAGCAGCTCCTGCGCTTT
GCCATCGCCACCCTGAGGGACTT
>1272
GGAATTCTGACTCTCCTCAGACCTCTGAGTTGACTCTCTCCCCAACCCCCTTCTCCCGA
CATACCCGGAGGCGCCAATATCGATCCCACCTTCTTCCTGAGCCGCACAGTCTCCAATG
TCATCAGCTCCATTGTCTTTGG
>1273
ATCCCATTCCCATCAGCTCCTGCCCCGTGACAGCTGTCCTTCCCTTCCCATCCTCTCTCT
GCAACCCCAGCTCTATGAGATGTTCTCTTCGGTGATGAAACACCTGCCAGGACCGCAG
CAACAGGCCTTTCAGTTGCTGC
>1274
GGTGAACCCCTAGATGGTGCCCTGAGGTCAAGCAGGAGTGAGGTTGTCCTAAAGCCC
CCTCTCCCTTCAGGAGGAGAAGAACCCCAACACGGAGTTCTACTTGAAGAACCTGATG
ATGAGCACGTTGAACCTCTTCATTG
>1275
GCCTATCCGGACTATCATTTCCTGCTCTGAGACCCCTAGATACCTAAACACATTCCCCCT

CCTCCCCCAGCCAAGGTCCATGAGGAGATTGACAGAGTGATCGGCAAGAACCGGCAG
CCCAAGTTTGAGGACCGGGCCAA
>1276
TCCAGCCCCTGTGTACTCTCAACAATCCCCCAACCCGCCTCATCACATACACCTTCCTC
CTCCCTCCCAGGGCATAGAAGTGTTCCCTATGTTGGGCTCCGTGCTGAGAGACCTCAG
GTTCTTCTCCAACCCCCGGGACT
>1277
AGGTCGGTACTGGGGCGAGGCTGCACTGAGAGTGGGCTTCACCTCCACCCCTCCCGCC
TCTCCTCCTCAGGAAAGCGGAACTGTTTCGGAGAAGGCCTGGCCAGAATGGAGCTCTT
TCTCTTCTTCACCACCGTCATGCA
>1278
GATATCTCAGCGCTGGGCCCATTCAGAGTGGGGGCTGCTCCCTCTAACCACTCCCACCT
GCCTCCAACAGATCAGTGAGCGCTATGGCCCTGTGTTCACCATTCACTTGGGGCCCCG
GCGGGTCGTGGTGCTGTGCGGAC
>1279
CCTTGGAACTGGTCCGCTCCTGCTACCACCACCCCCTGACCTCTCTCCACCCCCGCGTT
CACCTCCCCAGGCGTGGCGTTCAGCAACGGGGAGCGCGCCAAGCAGCTCCGGCGCTT
CTCCATCGCCACCCTAAGGGGTTT
>1280
GAATTCTGACTCTCCTCAGACCTCTGAGTTGACTCTCTCCCCAACCCCCCTTCTCCCGC
CACACCTGTAGGCGCCAATATCGATCCCACCTTCTTCCTGAGCCGCACAGTCTCCAATG
TCATCAGCTCCATTGTCTTTGG
>1281
TCCCCATTCCCATCAGCTCCTGCCCCGTGACAGCTGTCCTTCCCTTCCCATCCTCTCTCT
GCAACCCCAGCTCTATGAGATGTTCTCTTCGGTGATGAAACACCTGCCAGGACCACAG
CAACAGGCCTTTAAGGAGCTGC
>1282
AAGGAAAAGCCCTAGAAGGGCCCCAAGAGCATGGAGAGTGAGCTTGGTCTAAACCGC
CCTCTCCCTGCAGGAGGAGAAGAACCCCAACACAGAGTTCTACTTGAAGAACCTGGT
GATGACCACCCTGAACCTCTTCTTTG
>1283
CTAGGGGGGGCGATACATGCCCTGCTCTAAGACCCCTAGAGAGGGTAAAGATATTCCC
CTCCTCCGCCAGCCAAGGTCCATGAGGAGATTGACAGAGTGATCGGCAAGAACCGGC
AGCCCAAGTTTGAGGACCGGGCCAA
>1284
GGAGCCCCTGTGTACTTTCACCAATCCCCCCAACCTGGCTCATAACACACACCTTCCTC
CTCCCTCCCAGGGCACTGAAGTGTTCCCTATGCTGGGCTCCGAGCTGAGAGACCCCAG
GTTCTTCTCCAACCCCCAGGACT
>1285
GGGGTCAGTAGGGGTTGAGGCTGCACTGAGAGTGGGCTTCACCTTCACCCCTCCTGCC
TCTCCTCCTCAGGAAAGCGGTACTGTTTTGGAGAAGGCCTGGCCAGAATGGAGCTCTT
TCTCTTCTTCACCACCATCATGCA
>1286
GCCATAGGGTGGCAAGGTCTAGGCCTTCTCAGATTTCCGGTGACCCTTGTGTCTCTCTC

ACTTCCTTCAGAAATGCACGTGGCCGTCCTCGGAAGTACCCAGTGAGCGCTCCCATGG
CTCCCCCTGCAGTTGGGGCGGGG
>1287
GGGCCCCAGGGGCCAGGCCTGCACCACAGCCCTCACTTCCTGACCCCTGCTGTCCCTG
TGCTGTCCTCAGGAGACCTCCAGTCGGAACCTGGTGACCCTGCGTGTGGACCCCAATG
GCTTCTTCTTGTACTGGACGGGCC
>1288
GCCTGGGAGTGAAGACCACAGCGAGACTGTGCGGGAGCCCCCTCTTCACCCTTCGCT
GTCACCTACTCAGGACCCCAAGATCCGGGAAGTTCTGGGCTTTGGGGGTCCCGATGCC
CGGCTGGAGGAGAAGCTGATGACGG
>1289
GACCTGGGTTGTGGCTGGGCAGCCCCTGTGTCCCCCACTCACCGCCTCCCCGTGTATA
CTGGCCCCCCAGGTCTGGTCTGAGGAGCTATTCAAGCTGGCTATGAACATCCTGGCTC
AGAACGCCTCCCGGAACACCTTCC
>1290
ACCTTCCTCCTGCCCTGACCTTGGTGACCTTTGTCCTCCACTGACCCTGAACCCCTCCT
GCCCGCATCAGCATCCTGAAGATGTTCTCAGCAGACAAGAAGCGGGTGGAGACTGCG
CTGGAATCCTGTGGCCTCAAATTC
>1291
TCACGGTGGACACCCACCCTTACGGGGGTGCCCGCCCCTGGTTTCTCACCCCACACTC
CTCGACCTCCAGAGTGAGTCCATCCGGCCTGATGAGTTTTCCTTGGAAATCTTTGAGCG
GTTCCTGAACAAGCTGTGTCTGC
>1292
GGCAAGGGCTTGGCGACGGGGTGGGAGCCCTGCCAGGCTCCATCCTGACCCAGCTTC
CTGTCCCCTCCAGAGGCGCCAAGGGCAAGCCATACCTGACGCTGGAGCAGCTCATGG
ACTTCATCAACCAGAAGCAACGCGAC
>1293
CTGGCTGGGGTGCAGGTGGGTGGGGGCAGGTGGGACACCAGCTGACCCTGCTGATCC
TCTCCCACCCCAGACCAGATGTCCATGGAGGGCTTTAGCCGCTACCTGGGAGGCGAGG
AGAATGGCATCCTGCCCCTGGAAGC
>1294
CCAGGTGGGACCGGGGTGGTGAGGAGCTGGGCTGGTGGGCGGCCTCGGTGACAGAG
CCTCGCCGCCCCAGCGGGGCAGCTGGCTGGGACCTCGTCGGTGGAGATGTACCGCCA
GGCACTACTATGGGGCTGCCGCTGCGT
>1295
TGACATGTCCCGTAGACCTCAGCTTCCTCTCTGGGGGAGGGATCTCTGACCTCTGACCT
CGGTTCCGCAGGGCAAAGCAACAGGCAAAGATGGCTGAGTACTGCCGCTCCATCTTTG
GAGACGCGCTACTCATCGAGCCT
>1296
AGGGGGGTGGCATGGGGGCCAGGGTGCTGCGGACCCGGTCCAGGGCCCTGACTCGTC
CATGCCTGCCCAGCTGGCCCCAGGCGTTCCCCTGCCCAGCCCCCAGGACCTGATGGGC
CGTATCCTGGTGAAGAACAAGAAGC
>1297
AAAACCCCTCCAGACTTCCTAAGCAGCTGTGTGGTCCTGACCCCTCACCCTGTGGCCC

TGTCCTCTGCAGGGTCTCCCAGCTCTGACAGCTGCCCAGGCCTGAGCAATGGGGAGG
AGGTAGGGCTTGAGAAGCCCAGCCT
>1298
TGGGGGGCAGGTGAGGGAATGGCCAGCTGTGGCGGGAATGGCACTGCTGACCCTGGG
TTGGGGCCCACAGGGCACAGCCAGCAGCGAGGTGAATGCCACTGAGGAGATGTCCAC
GCTTGTCAACTACATCGAACCTGTCA
>1299
GTGGCAGGCATGGGAGCTTGCCCAGCTCAGCCCTGCCAGGTCTGACGCCCTTTCTTGG
CTCACCCCTAAGAGAGGAACAAATGCTTCGAGATGTCGTCCTTTGTGGAGACCAAGGC
CATGGAGCAACTGACCAAGAGCCC
>1300
GCTGAGGCCGGAGGTGGAGGCTGATGGGGTGGGCCCTGGCACCTGTGTGGCCCCTGA
CCACCAATCTCAGATACAACAAGCAGCAGCTCAGCCGCATCTACCCCAAGGGCACCCG
CGTGGACTCCTCCAACTACATGCCC
>1301
TCTCCCATTCCCCACATGGCACCGTCCTGCCTGATCCCTGCCCCTGCTCGACGTGCCCG
TGGCACCCCAGATGTGGCGATGCAGCTCAACGCGGGCGTTTTTGAGTACAACGGGCGC
AGCGGGTACCTGCTCAAGCCGGA
>1302
GGCTCAGGCCAGGGGTGTCCTGGGGGCAGGACTCTCAGCATCCGCCTCACCCTCCTTG
GCCCACCCCCAGGTGATCTCAGGGCAGTTCCTGTCCGACAGGAAGGTGGGCATCTACG
TGGAGGTGGACATGTTTGGCCTCC
>1303
GTTAGCTGTGGCCTGGTAGCCACAGTGGCCCAGGGTCAGCCCTGCGGCTCAGCTCCAG
TCTGGCCCACAGGTGGTGCTGCCCACGCTGGCTTCACTTCGCATTGCAGCCTTTGAGG
AGGGGGGTAAATTCGTAGGGCACC
>1304
CAGCACAGCGGGCAGTGGGTAGGGCCCCCACCTGGCCCAGCAACCCAACGTTGCCTT
CCTGACCACCCAGGATACCACTACGTCTGCCTGCGGAACGAGGCCAACCAACCGCTGT
GCCTGCCGGCCCTGCTCATCTACAC
>1305
ACCAGAGGTGGCGGGTGGGGGTGGCCTGGGGGCTCTGTCTCTGAGACCTTGGCCTTC
TGCCTCCCCCCAGACTATGCGGAGGCCCTGATCAACCCCATTAAGCACGTCAGCCTGAT
GGACCAGAGGGCCCGGCAGCTGGC
>1306
GTGGTAGAGGACAGAAGGGTCTGGAGGGAACAGCATTCTCTGAAAAGAGCATTCTCC
TTTCTCCCTATAGGCTCAGGCTGGCCAAGAGACGTGCCAGGACACCCAGTCTCAGCAG
CTGGGGTCTCAGCCGTCCTCAAACC
>1307
GTACCTGGAGGCAGGGGGTTGCCTTGGAGGTTCCCCCACCCCCCGCCCACCTTTGGTC
TGCCGCTCCCAGAGGTGGCCCCCACCCCGCTGGATGAGCTCCGAGGTCACAAGGCTCT
GGTCAAGCTCCGGAGCCGGCAAGA
>1308
CCCTACACACCCTCCCCATCCCAGTCCATGTGACGGGCCCAGGCATCACCCAGAGGGG

TTCTCCTCGCAGAGGTGGGGCCGCTGATGTGGAGGACACGAAGGAGGGGGAGGACG
AGGCAAAGCGGTATCAGGAGTTCCAG
>1309
GGCCAGGGAAAGCCTGCTGGATAGACCCGTCGTCAGCCCGGCATCACCTGTCAGCTCC
CTGTGTCCACAGGCTCTGCAGCGGCTCAGGGAGGTCGTCCTTGATGCAAACACAACTC
AGTTCAAGAGGCTGAAAGAGATGA
>1310
AGTACCAGAAGGAGGGCAGAGTCTGTGCTTCTGCCGCTGACCCCTCCTCTTCCCCTGC
CGGCTTCTCCAGGGAGAAGAAGGAGCTGCAGAAGATCCTGGACAGAAAGCGCCATAA
CAGCATCTCGGAGGCCAAGATGAGG
>1311
CCAGCCAGAGCCTCGAGGCTCTAGCCCCTCCCTCAGGGCCCCTCAGCTGAGCCCTTGC
CCACGCCTGTAGCTGGAGGAGGCCCAGAAGCAGCGGCATGACCGTCTTGTGGCTGGG
CAGCAGCAGGTCCTGCAACAGCTGG
>1312
GGCGAACAGGTGGGCAGACGGTGTGCAAGGCAGCCAGGCCTCGCCTGTGATGCCCAT
CCTTCTCCCACAGCTGCTGGCCCAGCTGGCCCAGGAGTGTCAGGAGCAGCGGGCGAG
GCTCCCCCAGGAGATCCGCCGGAGCC
>1313
GGGGAGGAGGGAGGCAGGCATGTCAGGGGTGGAGGGGAGCTCTTGGCCATGAACCTC
TTCATCTCCCCAGATGTCCCCAGTGCCCCACTGCTGCTGACCCTGGATGATGTGAGCAG
CAGCTCTGTGACTGTGAGCTGGGA
>1314
AAAGAAGCCCCATGGAGAGGGGCGCCTGTGGCCACATTGCAAAGACCTCACACTCTC
TCCATCCCCTCAGGCTCGGAGTGGGTGCCTGTGAGTGCCCGGCCCATGATGGTGACCC
AGCAGACTGTGCGGAACCTGGCTCT
>1315
TGCCCCATCTCAGAAACCCCAATCAGCCACCCCACTGCACTGCTAAGCCCAGACTCTT
GCTTTGATCCAGAGGCCCCCAAGATCCGTGTCCCCCGCCACCTCCGTCAGACCTACATC
CGCCAGGTGGGAGAGACGGTCAA
>1316
TCCTGGAGTTAAGGAGGTGTGGGGACTCTGGCACCTCCCTACACTCACCCTGGGGGCT
CCTCCCTGCCAGGGGAAGCCTAAGCCTCAGGCCACATGGACCCACAACGGCCATGCCC
TGGACAGCCAGCGGGTGAGCATGC
>1317
ATTTTGGGTGTGGGGGCTCTGTGCCAGCCTGGCCCTCACACTCACTTCCTCTCACACTC
TGGGCCTACAGAGAAACCTGGACCCCCCTGCTGCATCAGGCTCCTGGACGTCTGGGGC
TGCAATGCTGCTCTTCAGTGGAC
>1318
AGGTCTCCAGGGTAAGCAGGGAGGGTGAGGACAGGGGTGAGAAATGAGGGTGACAC
CCGGGGCCCTGCAGCAATGGTTCACAGTGCTGGAGCGCTACCACCCAACCACCTGCAC
CATCTCTGACCTCATCATCGGCAACT
>1319
TAGTCAGTCCTGACAGGTCTCCCTCACTCCTGTAAGCTCAGATGCACCCGACTTTCTCC

CCTGAACCCAGATATTGCTGCCAAACCTAAAGGGTTTATTGAGCGAGACTTCTCAGAA
GCCCCCTCATTCACCCAGCCCCT
>1320
CTGGGGATAGCTGTTGAGAGCCCCGCCCCAGTCCATACCTCCCCAGCTCTCTTCTGACC
ATGTCTCCCAGCCCAAGATCATCTGGATGAAAAACAAGATGGAGATCCAGGGCAACCC
CAAATACCGCGCCCTCTCTGAGC
>1321
CTCTGCACCTCTCTGGCCTGCAGCCGAGCTCTGCTCACACCAGGGGCTCAGGAAGCAT
GTCCTCCTCCAGGTGCTGCAGGCCTGAGGGGCCAGGTCCACCAGGCCTACAGTCAAA
CTCCAGAGATGCCCAAGACCCCCGC
>1322
TGGACATGCCAATACACATTACTGACATGTATGCCCACCTGACCTGCACCCACTCATGC
CCACTCTGCAGGGCAGCGCTCGCCATTGAATGACTTCCAGGTGCTCCGGGGCACAGAG
CTACCTGCTACATGCGGTGGTGC
>1323
CTGGGCCTAGATAAGACTGGGGGCAGGGGAGCCTGGGCCGTGGCGTTACCCTGTGCCT
TCTTCTCTCCAGGGCCTTCCACTACAATGTGAGCAGCCATGGTTGCCAACTGCTGCCAT
GGACTCAACACTCGCCGCACTCG
>1324
GGGGTGGAGAGGGGCAGGGTGGGAGACAGGGGACCTCAGCCCAAGTTGATCTTCTGT
CTCTTGCTCCCAGACTACATACGGACCTGCATCATGAACAATGGGGTTGGGTACCGGG
ACACCATGGCCACGACCGTGGGTGG
>1325
AACACCTTCCCTCCATCCCGGCCTGGGACCTTCCCCCAGCACACACTATAGTGATGCTC
TGGGCCCTCAGGTACATGCCCACGCTCCGGAATGGCCTGGAAGAGAACTTCTGCCGTA
ACCCTGATGGCGACCCCGGAGGT
>1326
CGGGTCAAGCTGGGAGAGTGGAGGGACAAGCCCACGCCCATCCACGAACCCACTGGC
TCTTTGTCTCCAGCCGCGTGTGTCTGGTGCAATGGCGAGGAATACCGCGGCGCGGTAG
ACCGCACCGAGTCAGGGCGCGAGTG
>1327
TAGGGCAACTGGGCGGGGCCAGATAAGCCAGAGTCCCAGGGTCTTCTTCACGCCCCAT
TACCGCCCCCAGGTTCCTCGACCAAGGTCTGGACGACAACTATTGCCGGAGTCCTGAC
GGCTCCCAGCGGCCATGGTGCTAC
>1328
CGGGAGGAGGGTAGTCACAGGCTTAGGGCTGGAGGCTGGCGGGCTAGGGCTGAGTGC
AGCGCCTGCTTAGAGACCTTCGGGAGAACTTCTGCCGGAACCCCGACGGCTCAGAGG
CGCCCTGGTGCTTCACACTGCGGCCC
>1329
GGGGCTGGAAGCCTGGAACCGAAGGGCCGGGGCGAGGTCTCGGCCTGATGGCTGCCT
GCACCCGCCGCAGACTGCTACCACGGCGCGGGGGAGCAGTACCGCGGCACGGTCAGC
AAGACCCGCAAGGGTGTCCAGTGCCA
>1330
TATGGGTCAGGCCCTTTAGCCCACGACATCCACACAGTCTGGGTTTCATCCAGCCCACC

CCATCCTACAGACCAGGTGCAGTTTGAGAAGTGTGGCAAGAGGGTGGATCGGCTGGAT
CAGCGTCGTTCCAAGCTGCGCGT
>1331
AGCCAGAAGATGGCAGGTCTAGCATCTGTCCCAGGAGTCTGTTCCCTGTCCTAATTCCC
CACTCCTCTAGGCAGGGCCAGCATTTCTGCGGGGGGTCTCTAGTGAAGGAGCAGTGGA
TACTGACTGCCCGGCAGTGCTTC
>1332
CAAGCTAACAAGTGAGCCTTGGGGCAATGGACTGAGAGTCACAAATGACCTAGCAGA
GCTTCTCTCCCAGCCATATGCCTCTCACGGGCTATGAGGTATGGTTGGGCACCCTGTTC
CAGAACCCACAACATGGAGAGCCA
>1333
ACCTGGGAGGGTGTGAGGTGGGGCTGAGCCTTGTGGCCTCAGACCCTGAGTGCCCCC
ATTCTTGCTAAAGATCTGTGACCCTGAACCAGCGTGTGGCCCTGATCTGCCTGCCGCCT
GAATGATATGTGGTGCCTCCAGGG
>1334
TGGCGGCCAGGAGCCCAGCCCTGGATCTTCCTCCAGGACCGTCTCCTTCTCCCCATTCC
CCTCACTGCAGGTACGGGTAATGACACAGTCCTAAATGTGGCCTTGCTGAACGTCATCT
CCAACCAGGAGTGTAACATCAA
>1335
AACTATTTTATCATGAAGCAGGCTAGTCATGGCTGTGGCCCGGGGCCTCATCAGTTCTC
CTACCTGCCAGAGTGACTACGGGGGCCCACTTGCCTGCTTTACCCACAACTGCTGGGT
CCTGAAAGGAATTAGAATCCCCA
>1336
TATTGGGTTTTTTTAAGAATCAGTTCTTAGATTCATTTATCAATTCTAGTTTTTTGTTGTT
GTTTTTAAGGACTCCTGAATATTTTTCAAAACTGAACAATTTCAGCCATGTCTGAGCTT
TCCGTCTTCCTGGAGGCACA
>1337
ATTATGCAGGAAACATTTAGTAATTTCAACATAAATATGGGACTCTGACGTTCTCCTCTT
CATTTTGCAGAGCAGTCATTATGGCGAACCTTGGCTGCTGGATGCTGGTTCTCTTTGTG
GCCACATGGAGTGACCTGGGC
>1338
TTTGGCCAGTTTTTTGCCTCTGCATTCACTCCCACCTTGAATCTTTTCCTTTCCCCACCC
TGGGCCTCAGCACCCCCAAACAGGCGAACCTGTGTGTTCTTTGAGGCCCCTGGAGTGC
GGGGAAGCACAAAGACACTGGG
>1339
TTCCACACCCCATTGTGCTTTCTTCCTTGCCCCCCCTTTCTCTCCTCTTCCCCTAATCCCA
TCCCATCAGGATGCCGAGACAGTGATGAGCCAGGCTGTGAGTCCCTCCACTGTGACCC
AAGTCCCCGAGCCCACCCCAG
>1340
CAGGAGGGGAAGAGCAGAGAGCAGGTTTGGGTCAGTGCTCTCCAGCCTGCATTCTTG
CCTTGATGTCCAGGTGAGTCCATCTGGATGGCACTGGTGCTGCTGGGGCTGTTCCTCCT
CCTCCTGCTGCTGCTGGGCAGCAT
>1341
CTTTCCACGAAGTCCCTTTTCCTGTCCCTATGCATTTGCACCCTGACCCTAAGGCTCTTG

TCTGTTCCAGCCCTGCTACAGCGAAAGAACTACAGAGTGCGAGGTGAGCCAGTGCCA
GAGCCAAGGCCAGACTCAGGCAG
>1342
GTTGTGCTGGGGAGGAATCCTGGCCCTGTTATAGCTCAGAGGCCCACACTCAGCACAG
TGTCCCCAGCAGGTAATCCGGGAAGGAGGTCATGCAGTGGTTTGGGCCGGGCAGCTG
CAAGGAAAACTGGTTGCCATCAAGG
>1343
CTCTGCTCCCTGGGATGGATCAGCCGTCTCCAGCTTTGTGTACCATCCTTTTCTCTCTGC
GTTTCCCAAGGGCTCCCTGTGCCACTACTTGACCCAGTACACCAGTGACTGGGGAAGT
TCCCTGCGGATGGCACTGTCCC
>1344
CGCCGACTCAAAAAAAAAAAAAGAGGGAGGAAGAAAATCCATGTTCCTTCAACCTTG
GATTCCCCCACAGGCCAATATAAACCAGGTATTGCCCACCGAGATCTGAGCAGCCAGA
ATGTGCTCATTCGGGAAGATGGATC
>1345
TGCATGGACCATTGCTGCAATGAGGATTGCCACAGAGATGATTCTTGGCCCTTCGTGCC
TTGCTCTCCAGGCTGGCACCCAGAGGTACATGGCACCAGAGCTCTTGGACAAGACTCT
GGACCTACAGGATTGGGGCATGG
>1346
TGTCTTGCCCTTTCTACATGGTAGGCACCCCTAGGACTAACTGATACCCAGCCCCTCTA
CCTTCCTCCAGACAGCAGTCCACCACCCTTCCAACTGGCCTATGAGGCAGAACTGGGC
AATACCCCTACCTCTGATGAGCT
>1347
AGATGGCTTTTAACCCTGGGGCCCACTCAAGATCCTAGGGTCAACCCTTCCTCCCTGTC
ATTCCCCCCAGGACCCTGATGGGCTGAGGGAGCTCCTAGAAGACTGTTGGGATGCAGA
CCCAGAAGCACGGCTGACAGCTG
>1348
GCTCTCTTGCGGAAGTCCCCCATGCTGGCCCCTTGACCCTCATGAACCCTGCTGCAAC
CCTGTGTTTCAGGCCGCGTCATTCTACTGCAGCAAGATGGGCTTTGAACCTCTAGCCTA
CAGGGGCCTGGAGACCGGTTCCC
>1349
GGAGACTAAAAGCCAGGTGAGGGGACCTAGACTCCAGACTCCAGTGCCATCCTCTCC
CTGCCGGCCACAGAGATGGGCGATCACCTGGTGAAACACGGTGACGGAGTGAAGGAC
ATTGCGTTCGAGGTGGAAGATTGTGA
>1350
TATGAAATGTGCTCTGTCCTCGGCGGGGATGGGTGCCCAGGGGCCGAGCCAGGCTGTT
TTCCACCCGTAGTATGGGGACACCACACACACCCTGGTGGAGAAGATGAACTACATCG
GCCAATTCTTGCCTGGATATGAGC
>1351
GAGGGGTCATGGAGAAGGGAGCAATCTGGGGGGTAGCCACCTTCTCACCCCCATCTTC
TTCCCTTCCCAGGCCCAAATGCAGTCTGGAGATGATCGACCACATTGTGGGAAACCAG
CCTGATCAGGAGATGGTGTCCGCC
>1352
GTGGAAATGGGTGATGGAGGCCGTCCCTGGAGGCGCTCAGAGCATATGTGTTCCACCG

CCCACCCTACAGGTACCTGAAAAACCTGCAGTTCCACCGCTTCTGGTCCGTGGATGAC
ACGCAGGTGCACACGGAATATAGC
>1353
GGGCTCTTGTAGGGTCAGGGATGTGGGTGATGGGGCAAATGACAGTGGTCTCCTTGTT
TCCCTCCCCCAGGAATATGTGGACTATAACGGGGGCGCTGGGGTCCAGCACATCGCTCT
CAAGACCGAAGACATCATCACAG
>1354
AGCACCTCCACCTGGCTAGGGGAGTTGAACTTGGAGGCCGAAGGCTGACCTCAGCTT
TGCTCTGAAACAGATTCGCCACTTGAGAGAGAGAGGCCTGGAGTTCTTATCTGTTCCC
TCCACGTACTACAAACAACTGCGGG
>1355
GCCCCTGTGGACTTTCCAAGGATCTCACCGTGCCAGCCTCCCCTAGCAGAGCCCCCTG
TCTCCCGCCTAGGAGCTGAAAATCCTGGTGGACTACGACGAGAAAGGCTACCTCCTGC
AGATCTTCACCAAACCGGTGCAGG
>1356
CGAGGGGACAGGCAGCTAGCACACTCGGTTTTCTGGGTCCTTGGCTCATCTCCTCTCTT
TCCCGCCACAGGGTTTTGGAGCCGGCAACTTCAACTCACTGTTCAAGGCTTTCGAGGA
GGAGCAGAACCTGCGGGGTAACC
>1357
GCCAGGAAGGGGTAGCGGGGCAGTGCAGTGTCGGGGGAGAGCGGCTTGCTGCCTCGT
TCTTTTCTTGCAGGCCCCAGGATGCAGGCCCTGGTGCTACTCCTCTGCATTGGAGCCCT
CCTCGGGCACAGCAGCTGCCAGAA
>1358
TGAGGAGACAGTCCCTGTGCATCTCTGCGAGTCCCTGAACTCAAACCCAAGACTTCCT
GTCTCCTGCCAGGGCTCCCCAGACCCCGACAGCACAGGGGCGCTGGTGGAGGAGGAG
GATCCTTTCTTCAAAGTCCCCGTGA
>1359
GTTCTGGGAGGGGGCTTGATTTGGGGCCCTGGTGTGCAGTTATCAACGTCCACATCCTT
GTCTCTGGCAGGAGCGGAGCAGCGAACAGAATCCATCATTCACCGGGCTCTCTACTAT
GACTTGATCAGCAGCCCAGACAT
>1360
GGCAGGCTCTCAAAGACGGGATGCTTGTCGTCGAGTCTCATACGCTAACCTCTGCTCC
GCCTCTTCTCAGAGCTGCGCATAAAATCCAGCTTTGTGGCACCTCTGGAAAAGTCATAT
GGGACCAGGCCCAGAGTCCTGAC
>1361
GGGACACAGCATGGCGCCACTGTCTTTCTGGTCTCCTGGGGCTCAGACTATGTCATACA
CTTCTTTCCAGGGCAGTGGGTAACAAAGTTTGACTCCAGAAAGACTTCCCTCGAGGAT
TTCTACTTGGATGAAGAGAGGAC
>1362
TCCTGGCTGTGTCTGTCCCCCGGCTTTTGGGCTCTGAAGGACTAACCACATGCTTTCTC
ACTTGTCTCAGATTGCCCAGCTGCCCTTGACCGGAAGCATGAGTATCATCTTCTTCCTG
CCCCTGAAAGTGACCCAGAATT
>1363
CAAAGGGATCCCTTGGTTGGGGTGTTGGGGAAGGCAGGGTTTTAACGGAAATCTCTCT

CCATCTCTACAGAGCTGCAATCCTTGTTTGATTCACCAGACTTTAGCAAGATCACAGGC
AAACCCATCAAGCTGACTCAAGG
>1364
ATCTTCCTGCAGACTCACAAAAGGCAGCTGCCCAAGCAGGGCCTGACCCCTCGGTGTC
CCCTCCCCACAGTACTGTGCAGCCCTGGAATCCCTGATCAACGTGTCAGGCTGCAGTG
CCATCGAGAAGACCCAGAGGATGC
>1365
AGCCCCTGGTTTGTGCGAGTCGTCCCGGCCTCTGGCGTTCTACTCACGTGCTGACCTCT
TTGTCCTGCAGCAGTTTTCCAGCTTGCATGTCCGAGACACCAAAATCGAGGTGGCCCA
GTTTGTAAAGGACCTGCTCTTAC
>1366
GGAGAGGAAGAGCCAGGCTTTAGCTCCCATCTCAGGGGTGGGGGATTTTTGACTCTAC
CTCTCCCCACAGATGAGCAGCAGCTGCTCAGGGCTGAGCAGGGTCCTGGTGGCCGTG
GCTACAGCCCTGGTGTCTGCCTCCT
>1367
GAGCTGTGGCATGGTCTGACTTATGCTTGACATCTTGTCTTTGTCCATTGTCACCTCATC
TCACTTCCAGGGACCCAGTGTCCTGGTTTCGGGATGGGGAGCCAAAGCTGCTCCAGG
GACCTGACTCTGGGCTAGGGCAT
>1368
CTAAGTAAGCCCTCTGGGACCAGGAGGACCTGAGGACTGCTCAGTCTCCAGGTGTACC
CTCTGCCTCTAGACCCTCCAGCCCGCCCTGTTGTCTCCTGCCAAGCAGCCGACTATGAG
AACTTCTCTTGCACTTGGAGTCC
>1369
GCATGTGGGGGCTCCAGCTGGGTCCCACAGTAGGCATTTTCAAAGCCAAAGACCACAT
CCTCCCTTCTAGGAGGAGTCCATCCACAGGGCCCTGGCCATGCCCACAGGATCCCCTA
GGGGCTGCCCGCTGTGTTGTCCAC
>1370
ACTTTGGGAAGTGGTGGGGAAGTGATGGAGACCCATAGCCTACCCTGACTTTGTGTCT
TGATGCCCTTAGTGCGCCCTGACCCACCCCAGGGCCTGCGGGTAGAGTCAGTACCAGG
TTACCCCCGACGCCTGCGAGCCAG
>1371
CTTTGTAGATGGTGGCTGGGAAGGCCCTGCACTTACAAGCTGGGTAACAGTGAGTCAT
GTTTACCCCCAGGTGGAGCCAGCTGGACTGGAGGAGGTGATCACAGATGCTGTGGCTG
GGCTGCCCCATGCTGTACGAGTCA
>1372
CCTTGAGCACAGGACGTGACCCCCGTCCCCACCAGCGTATGGACACTTATTGGGTCTT
GCCTTCCTTTAGGGACCATACCAAAGGAGATACCAGCATGGGGCCAGCTACACACGCA
GCCAGAGGTGGAGCCTCAGGTGGA
>1373
AACCCCAGAGAAAAGGGGACACCGAGCTTGGTCAGGCTTGCTCTCAGTGACATGTGG
CCCTCCCCCTCAGATCACAGGGACTCTGTGGAGCAGGTAGCTGTGCTGGCGTCTTTGG
GAATCCTTTCTTTCCTGGGACTGGT
>1374
TAATAAACCCTTTGGCGGAGACTGAGATAACCCACATTGTTGGAGAGACAGCTGCCTT

TCTATGCCCCAGGCTGAGGCTGAGACGGGGTGGGAAGGATGGATCCCCAAAGCCTGG
GTTCTTGGCCTCAGTGATTCCAGTG
>1375
TCATTCTCAGGGTGTCTGGGCCAAGATCCGGTCTTACTGTCTCTCCTGATTTGCCTCCT
GCTTCTTCTAGGAGCTCCAAACCTGTAGAGGACCCAGGAGGGCTTCGGCAGATTCCAC
CTATAATTCTGTCTTGCTGGTGT
>1376
ATCAGAGCTCCTGGGCTGCATAGGAGTGGCCAGGCTAACAACCTGACCCCTTCCCTTC
CTCCCTGGGCAGCTCCCTGCGGTGTGGCCCCCCAAGCACGCATCACAGGTGGCAGCA
GTGCAGTCGCCGGTCAGTGGCCCTG
>1377
GGGGTTGGCAGTGGGGGTTGGGGAACAAGGAGAGGTCTCCTGGGTCTCAGCCTTTGC
CCCCTGCCTGCAGCGAGCACCACAAGGAAGCCTATGAGGTCAAGCTGGGGGCCCACC
AGCTAGACTCCTACTCCGAGGACGCC
>1378
GGTGCCTAGAGGTGTGGAGGGGCACCTGACTTGGGGAGGGGCCCAGGGTAAGCCTCT
TTTACCCCCACAGTGAGCCTCCTGACGCCCAAGCCACTGCAGCAACTCGAGGTGCCTC
TGATCAGTCGTGAGACGTGTAACTG
>1379
ATGACCAGTGCAACTTCGGAAAGGAGGCCTGGCCCGGTCCTGATGGCTGCTGTGTGGC
TTCTCTCCTCAGGGTGACTCTGGGGGCCCACTCTCCTGCCCTGTGGAGGGTCTCTGGT
ACCTGACGGGCATTGTGAGCTGGG
>1380
ACTCCAGGAGTGGGGGCGGTGCGTCCTCCGGCGCGCAGCGGTGGCCACAGCTCTCCT
CCCGCCGCCGCAGGTCTTCCTCACCGCTTTGCGCGCCCCGAAGCCGACCCTGCGCTAC
TTCACCACCGAGCGCTTCCTGCCCC
>1381
TGGACATGCCAATACACATTACTGACATGTATGCCCACCTGACCTGCACCCACTCATGC
CTACTCTGCAGGGCAGCGCTCGCCATTGAATGACTTCCAAGTGCTCCGGGGCACAGAG
CTACAGCACCTGCTACATGCGGT
>1382
ACTGGGCTAGATAAGACTGGGGGCAGGGAAGCCTGGGCTGTGGCGTTACCCTGTGCCT
TCTTCTCTCCAGGGCCTTCCACTACAACGTGAGCAGCCATGGTTGCCAACTGCTGCCAT
GGACTCAACACTCGCCCCACACG
>1383
GGGGTGGAGAGGGGCAGGGTGGGAGACAGGGGACCTCAGCCCAAGTTGATCTTCTGT
CTCTTGCTCCCAGACTACGTACGGACCTGCATCATGAACAATGGGGTTGGGTACCGGG
GCACCATGGCCACGACCGTGGGTGG
>1384
AAACACCTTCCCTCCGTCCCGGCCTGGGGCTTCCCCCAGCACACACTATAGTGATGCTC
TGGGCCCTCAGGTACACGCCCACTCTCCGGAATGGCCTGGAAGAGAACTTCTGCCGTA
ACCCTGATGGCGACCCCGGAGGT
>1385
GCCGGGTCAAGCTGGGAGAGTGGAGACAAGCCCACGTCCATCCACGAACCCACTGGC

TCTTTGTCTCCAGCCGCGTGTGTCTGGTGCAATGGCGAGGAATACCGCGGCGCGGTAG
ACCGCACGGAGTCAGGGCGCGAGTG
>1386
TAGGGCAATTGGGCGGGGCCAGATAAGCCAGAGTCCCAGGGTCTTGTTCACGCCCCAT
TACCGCCCCCAGGTTCCTCGACCAAGGTCTGGACGACAACTATTGCCGGAATCCTGAC
GGCTCCGAGCGGCCATGGTGCTAC
>1387
CGGGTTTGGTCCCAGCCCCAGAGGGATCTCAGCTCTCGCTCGGGGCCCGACCTATCTC
GGTCCATCTAAGGGTCCGAGGCACAGCCCCGCCAAGAGGCCACAACTGTCAGCTGCT
TCCGCGGGAAGGGTGAGGGCTACCG
>1388
CGGGAGGAAGGTAGTCACGGGCTCAAGGCTGGAGGCTGGCGGGCTAGGGCTGAGTG
GAGCGCCTGCTTAGAGACCTTCGGGAGAACTTCTGCCGGAACCCCGACGGCTCAGAG
GCGCCCTGGTGCTTCACACTGCGGCCC
>1389
GCCGGGCTGGAAGCTGGAACCGGAGGCCGGGGCGAGGTCTCGGCCTGATGGCTGCCC
GCACCCGCCACAGACTGCTACCACGGCGCAGGGGAGCAGTACCGCGGCACGGTCAGC
AAGACCCGCAAGGGTGTCCAGTGCCA
>1390
GGTGTCTGGGACCAGAGCCTGGAAATGGTTGAGACTACCCTGCCACGATTTTGCTCCC
GCTTCCGCCTAGGTTCACGTTTACCTCCGAACCGCATGCACAACTGGAGGAGAACTTC
TGCCGGAACCCAGATGGGGATAGC
>1391
TATGGGTCAGGCCCTTTAGCCCACGACATCCACACAGTCTGGGTTTCATCCAGCCCACC
CCATCCTACAGACCAGGTGCAGTTTGAGAAGTGTGGCAAGAGGGTGGATCGGCTGGAT
CAGCGGCGTTCCAAGCTGCGCGT
>1392
CAAGAAGATGGCAGGTCTTACGAATCTGTCCCAGGAGTCTGTTACCTGTCCTAATTCCC
CACTCCTCTAGGCAGGGCCAGCATTTCTGCGGGGGGTCTCTAGTGAAGGAGCAGTGGA
TACTGACTGCCCGGCAGTGCTTC
>1393
CAAGCTAACAAGTGAGCCTTGGGGCAATGGACTGAGAGTCACAAATGACCTAGCAGA
GCTTCTCTCCCAGCCATATGCCTCTCACGGGCTATGAGGTATGGTTGGGCACCCTGTTC
CAGAACCCACAGCATGGAGAGCCA
>1394
ACCTGGGAGGGTGTGAGGTGGGGCTGGGCCTTGTGGCCTCAGACCCTGAGTGCCCCC
ATTCTTGCTAAAGATCTGTGACCCTGAACCAGCGTGTGGCCCTGATCTGCCTGCCCCCT
GAATGGTATGTGGTGCCTCCAGGG
>1395
TGGTGGCCAGGAGGCCAGCCCTGGATCTTCCTGCAGGACCCTCTCCCTCTCCCCATTC
CCCTCACTGCAGGTACGGGTAATGACACAGTCCTAAATGTGGCCTTGCTGAATGTCATC
TCCAACCAGGAGTGTAACATCAA
>1396
AACTATTTTATCATGAAGCAGGCTAGTCATTGCTGTGGCCCGGGGCCTCATCAGTTCTC

CTACCTGCCAGGGTGACTACGGGGGCCCACTTGCCTGCTTTACCCACAACTGCTGGGT
CCTGGAAGGAATTATAATCCCCA
>1397
GATACATACTCTGGGGCCTGCCTCCCTGGAGATGAGGATGAGACCCCTGTCCCTTGCTG
TTCTCCAACAGGTGCTGGGAGGCCCTCCTTGGCTTAGGAGGACACTTCCAAAGCTGGG
GCGCCCCAAGGAGGCACCAGTGG
>1398
TCGGGGTGCAGCAGACAGTGGACGCGAGGCACAGCGACTACCAGTCACCCACCTGAG
CCTCCTGCCACAGCCTTCATCTCCGTCTTGGGCGTCTGCCCCCTGTACCTGGATGAGAT
CCGGCACTTCCTGACCCTATGTCC
>1399
GGGTTGGTGGTTGGGGGGGAGCACGTGTCAGCAGAAGTGACCTGGGATCTCATCCCTT
GCTCGCTCCCAGGAGTCACTGACGCTGCACAGGTCTGGGGGCCACATAGCCCACCTGC
ATGTGCTGGCCGTGCACCGCGCCT
>1400
TGTGGCCGGAAAGGGACTGAGGCTGGGTGAGTTGCGCCGTTTTCCTAACAGTTTTCCC
ATCCTGTCGCAGACAAAGAAAAGAAGGAACAATGGTCGTGCCAAAAAGGGCCGCGG
CCACGTGCAGCCTATTCGCTGCACTA
>1401
TCTTCATTCATGTAACAAAGAAGCAAGGTAGGTATATAATACCAGTATAACTCTATTTTC
TATTCCTTAGCCTATGTGCTTCCCAAGCTGTATGTGAAGCTACATTACTGTGTGAGTTGT
GCAATTCACAGCAAAGTAGT
>1402
AAGTTCTTTGGGGGAAGGGAGTCTTGGATCCATGGGTTTTAATTTACTCTTTTGTTTCTT
TGTCTTTCAGGGTGCTGCCCCACGTCCCCCACCAAAGCCCATGTAAGGAGCTGAGTTC
TTAAAGACTGAAGACAGGCTAT
>1403
CATAAGGCAAATCTCAAAAGTTGTTCATTATGCTTTCACATAACCTTGCACTTCTCTCTC
ATAATTTCAGGATCCAGTGCAGATATTGTGATGACCCAGACTCCACTCTCTCTGTCCGT
CACCCCTGGACAGCCGGCCTC
>1404
CATAAGGCAAATCTCAAAAGTTGTTCATTATGCTTTCACATAACCTTGCACTTCTCTCTC
ATAATTTCAGGATCCAGTGCGGATATTGTGATGACCCAGACTCCACTCTCTCTGTCCGT
CACCCCTGGACAGCCGGCCTC
>1405
TCCGAGGGGCTGGAAGAAAAACAGAGCCTGTCTGCGGTGGAGTCTCATTATATTCAAA
TATTCCTTTTAGGAGCCATTCCGTAGTGCCATCCCGAGCAACGCACTGCTGCAGCTTCC
CTGAGCCTTTCCAGCAAGTTTGT
>1406
CATCTCTATTTCTCCCTATTTATTGGTGTTTGTTTATTCTTTAACCTTCCACTCCCCTCCCC
CTCCCCAGAGACACCATGATTCCTGGTAACCGAATGCTGATGGTCGTTTTATTATGCCA
AGTCCTGCTAGGAGGCGCG
>1407
CTTGACTCTTCCATCTTTGAAAAAAGTTCATATGCTTTTTCTTTTCCCCTTCCTTCCTAA

CTGTGCCTAGAACATCTGGAGAACATCCCAGGGACCAGTGAAAACTCTGCTTTTCGTT
TCCTCTTTAACCTCAGCAGCAT
>1408
ATAGAGTAAACTTGAGAAGAATGTTTGCACAGCCAGGTTAAGTGTTACAAATCCTTCT
GCCCTCACTTAGGCATCTATATCATAAATCTCAAGAGGACCTGGGAGAAGCTTCTGCTG
GCAGCTCGTGCAATTGTTGCCAT
>1409
AAGTGCTTACTGGGTGCCAGGATTGTTGCTGTGGAATATCGAGTACCACTAACTTTTAA
ATTCTTCAAAGAGGGCTGTGCTGAAGTTTGCTGCTGCCACTGGAGCCACTCCAATTGC
TGGCCGCTTCACTCCTGGAACCT
>1410
GAGCCAGGAAGGTGCTTGCTGTTTGGGTTTGACCAAGTGTCACTTTTTAATAATCTGCC
ACTCTTGGCAGGGAGCTCACTCAGTGGGTTTAATGTGGTGGATGCTGGCTCGGGAAGT
TCTGCGCATGCGTGGCACCATTT
>1411
GGACTGCTGTCTAGGAAGCAAAACTTGTCAGTCCCTGTAAGTCTCTTCCTCTTTTTTTT
TTGTAACCCAGATTGAAAAAGAAGAGCAGGCTGCTGCTGAGAAGGCAGTGACCAAGG
AGGAATTTCAGGGTGAATGGACTG
>1412
GGAGTGGGGTAAGGAAAAATACTACATTGAGGACAGAGCTGATGGGCTTTTTTTGGTA
TTCTCTTAACAGAAGACTGGAGCGCTCAGCCTGCCACGGAAGACTGGTCTGCAGCTCC
CACTGCTCAGGCCACTGAATGGGT
>1413
TATTTCTCTTCTTGATACCACTGTGGGGGACATGGCTCTGCCTCTCTGACTTCTGCTTCC
TTCCCTGCAGACCTATGCCAGATTTACTGGTGCTCCACTGAAGGTACACAAGATCAGCA
ACCCCTGGCAGAGCCCTTCAG
>1414
GGAATGTGTTGCAACTATGTATGACTAGGCAGCTAAGGGTCTGGTTGGTATACTTCCCT
CTCAATATCAGGAACTCTGCCTGCCCTTCGGACCAGTCATGGAGAGGTCATCTCAGTTC
CACACAAGATCATCACCCACCT
>1415
GACTCCTTCCTCCCTGGTGAAGTGCCCTTGCAGCTCCCTAGTGTCCACGCCATGGATAT
TTCCTCCACAGAAGTACAATGCTGATTATGATCTGTCAGCTCGGCAAGGGGCAGACAC
CCTGGCCTTCATGTCTCTCCTGG
>1416
GAAGCCCACCTTGAATCAGACAGGTGCACTGGCTCAGACCTACCTGTTTCTTCCTGCC
CACCCAATCCAGGTACATACTTTTTGGATAGACACCAAGAACTACGTGGAAGTGACCC
GGAAGTGGTATGCAGAGGCTATGC
>1417
AATGGGAGTGCTTAGGTGGGGAGGTGGCACTGTTCCCACAGCTGCAAGCCTACCTGTG
TCGCCCCTACAGCTGTACCGAGAGGCTCGGGAGTGTCTGACCCTGCTCTCTCAGCGCC
TGGGCTCTCAAAAGTTCTTCTTTG
>1418
CTCCAGCTCCATCTTTACCCCTTCTACCCCAGTGGTTCTCCTCCATCCCACCCTTCTCTC

TCTGCTCCAGCCCTGCCTCCTTGGACGCCTTCGTCTTCAGCTACTTGGCCCTGCTGCTG
CAGGCAAAGCTGCCCAGTGGG
>1419
AGCCCTGGGGAGAGTGGGCAGGGATCCAAGAACTAGTTCTCCTAACACACCTTCCTTC
CTTGACCCTCAGCTGAGGTACCACCGCAACGCCAGACACCAGCAGGCCCAGAGACTG
AGGAGGAGCCATACCGGCGCCGGAA
>1420
TGGGGTTTAAATACTGGAGATGGAGGCAGACCTGGGGCCAGATGTTCTCTGTGCCCCT
CTTCACCCTCAGGTTGGAGAGCAGTGACGGGGATGGGGCCTGGTGCCCCGCAGGGTC
GGTGTTTCCCAAGGAGGAGGAGTAC
>1421
CCAGGCCTGATGAAGGGATGGGGGATGGAGTCTTAGTGCCTCTGACCCCCACCCTCTT
ATTTTTTCCCAGATGGACTCCTGTCTTACACCGCCCCTGTGGGGCAGACAATGTATTTAT
CTGAGGCCGTGTACCTCAACGA
>1422
ATGCAGGGTGCCGTTGGGGTGCCCCCACCACTCCTAGCCTTGACCCTGTGCCCTCTTCC
CTTCCCCCCAGGTCCACTGTAACAACATGCACACGCTGGGAGCCCGTCTGCCTGGCGG
GGTGGAATGTCGCTTCCGGCGTG
>1423
CACTGCCTCTGCCTCCCTTGGGTCTCCTCCTCATTTACCTCCCTCCTTTCTTTTTGTTCCT
TCTCCCCAGATGTGGTGAACAATTCCTCTCCGGCACTGGGAGGCACCTTCCCGCCAGC
CCCCTGGTGGCCGCCTGGCCC
>1424
CTTCTCCCTGGGCCTCCCCCTCGGCTAGGGTGGGACCCTCCTGTGGTGCTGACCCTGCT
GCCTCCACCAGAGCTGGAGCCCAGAGGCCAGCAGCCCGTGGCCAAGGCCGAGGGGA
GCCCGACCGCCATCCTCATCGGCTG
>1425
ATGGACACAGCAGAGGGCCAGGCCGTGTGTGCTGAGCAACACGGGTGATGCCTCCCA
TCCCTATGACAAGGCTGAACGGAGGGTGTTGGAAGAGGAGCTGACGGTTCACCTCTCT
GTCCCTGGGGACACTATCCTCATCA
>1426
AGGTCTCCCTGTCTGTCTAGCCTTGAGTCTCATCCCTTCCCCGTGTTTCCCCTCCTCCTT
CTCCCGACAGCGTTGCTGCTCTCCAATCCAGCCTACCGCCTCCTTCTGGCCACTTACGC
CCGTCCCCCTCGAGGCCCGGG
>1427
GTGTGGGGAACTATAGCTCTTGGGCTGTTCCTGATGCCTCGTCCTGTCTTCTTTCCCCTC
ACCCCTGCAGCCTACAGTGGGGACTATATGGAGCCTGAGAAGCCAGGCGCCCCGCTTC
TGCCCCCACCTCCCCAGAACAG
>1428
CTTAGTCATTTGTAACCGTGTTAATCCGTTTGACCCTGTGACCGCCTAGCAAACGAACT
TCTTTCTCCAGGTGCACCTGTGTGAGGTCGACAGCCCTCAAGATCTGGTTAGTCTTGAT
TTCCCCCTTAATGTGCGTAAGG
>1429
CTCTCTCTCCTCTCCTGGATGGGAATCTGCGAAGCTGCCCCCAGTGACCTTCTGTCGGT

TCCCTTCTCAGCTACCCAATGCTGCTGCATGTGGCAGCCCAGATCGCCTCCGGCATGCG
CTATCTGGCCACACTCAACTTT
>1430
AGGAAGGGAGGGGGGTCAACCGTTGCCTGATGTCCCCTGTCTGTTTTTGCTGCCTTCC
TCTGCATCCCAGGGGAAGTTCACGACTGCGAGTGACGTGTGGGCCTTTGGTGTGACCG
TGTGGGAGGTGCTGATGCTCTGTA
>1431
TTGGTGGGGAGGTCATGGACACAAATCAGAAAATACAAGAATGGGCCTCCCCATTTCC
TCCTCTGACTAGGATGGGGACCCAGACACGCCAAAGCCTGTGAGCTTCACAGTGAAG
GAGACAGTGTGCCCCAGGACGACAC
>1432
TCCCACTGGGATGTGGCTGGGAGGTCATGGCAAATGGTTTCAAGTTTGACCTTGAGCT
TCTCCTTTCCAGCTGGTGAAGCGGTGTATGGGGACAGTGACCCTCAACCAGGCCAGGG
GCTCCTTTGACATCAGTTGTGATA
>1433
GGAGGGGTCTTGGATTGACCCTGGGTACATCCCCGACAAGGAACCTGTTTCTTCCTGT
ACACAACCCCAGGATAACAAGAGATTTGCCCTGCTGGGTGATTTCTTCCGGAAATCTA
AAGAGAAGATTGGCAAAGAGTTTA
>1434
TTCCCGCCCGCCTCCGCTCTGCCTCCTGGCCAGCCCTCACCTCTCCGTCTCTCCGTGCC
TTCCCCTCCAGGCCCCTTTGGCCACTGCCGCACCCCCCTGCAGTCCCCTCCTGAAGGC
GCCCTCCCCGGCTGGCCCCTTAC
>1435
CTGTCTAGGGGGGAGTGCACCCTCCTTAGGCAGTGGGGTCTGTGCTGACCGCCTGCTG
ACTGCCTTGCAGGTGAAATTGCCCTGTGGTCCTTGGTGGTCCTGGCCATCGAGCGGTA
CGTGGTGGTGTGTAAGCCCATGAG
>1436
GCGCTCGGCAGCCACCTTGGCTGTTCCCAAGTCCCTCACAGGCAGGGTCTCCCTACCT
GCCTGTCCTCAGGTACATCCCCGAGGGCCTGCAGTGCTCGTGTGGAATCGACTACTAC
ACGCTCAAGCCGGAGGTCAACAAC
>1437
CCCAGCATGCATCTGCGGCTCCTGCTCCCTGGAGGAGCCATGGTCTGGACCCGGGTCC
CGTGTCCTGCAGGCCGCTGCCCAGCAGCAGGAGTCAGCCACCACACAGAAGGCAGAG
AAGGAGGTCACCCGCATGGTCATCA
>1438
CTCACTAACGTGCCAGTTCCAAGCACACTGTGGGCAGCCCTGGCCCTGACTCAAGCCT
CTTGCCTTCCAGTTCCGGAACTGCATGCTCACCACCATCTGCTGCGGCAAGAACCCAC
TGGGTGACGATGAGGCCTCTGCTA
>1439
GTGACTTTTTCTTGACCATAAATGGAAACTCTGCAACTGATCTTTGTATACCCCATGTGA
ACTGTTGCAGCACCATCATTGCATTTGCTCAGTATGTTCAGGAAGCAACCTTTGAAGAA
ATGGAAAAGCTGGTGAAAGAC
>1440
TTTTCTCAGTGAGTACAGAAGCTTTACTTTACCATGACTAAAAATTGTCCTTTTCTTCTC

TGTTGTATAGAATAATGTTTTACAGGAAAAAATATGTGCTATGGAGGGGCTGCCACAAA
AGCATAATTTCTCACACTGCT
>1441
CTTGTCTACATTCATTGCTTCCTGTTTCTTCCCTCACCCGTCTTCTCTCTTTCATTTTTATT
TTTTATAGCTTTTTATATGAAGTTGCCAGAAGGAACCCATTTGTCTTCGCCCCTACACTT
CTAACTGTTGCTGTTCAT
>1442
TATGACTAAAAAATAAGAGAATAGTGAGATTTTGCTATTGTTTCAAAAGAATTTTCTCTT
TCTTCTTCAGGCAATACCTGTCACACAATATTTAAAAGCATTTTCTTCTTATCAAAAACA
TGTCTGTGGGGCACTTTTGA
>1443
TAGCAAATTACTCCCACTTGTTCTACTTGCTGTTCAAATTATTTTTGTGGTTTATCAATTC
TCTTTCCAGATATATTGCGATACTCAGTCAAAAATTCCCCAAGATTGAATTTAAGGAGCT
TATTTCTCTTGTAGAAGAT
>1444
TGCCATCATTCCATAATGGAAGAAAACCCAGCCTGAAAGTAAAATAATCTCTCATTCTT
ATTTGGTACAGAGCAAGGTTATGAACCATATTTGTTCAAAACAAGATTCTATCTCCAGC
AAAATCAAAGAGTGCTGTGAAA
>1445
TACAAAATAGTGGAATTGGGTTTTTTGCTCTTTCTCTAATTTCTAATATACAAAATTTTAC
ACATTGCAGGTTTACTTTTGAATACTCAAGGAGACATCCAGACCTGTCTATACCAGAGC
TTTTAAGAATTGTTCAAATA
>1446
CACATTGATGTAAAGCTTATGGTTAAATTTTAGATAAAATGTTTGTAACCATGATTATTCT
TATTTTCAGGAAGACAAATTCAATGAGACAACTGAGAAAAGCCTCAAGATGGTACAAC
AAGAATGTAAACATTTCCAGA
>1447
TTCAGCAGTAATTCAAGGGAAAATGGGTATCTGCTTTCATTCAGAACCAAACAGACAA
ATGTTCTTTCAGTTACCTCATCAGGCTCACGAAGATAGCTCCCCAACTCTCCACTGAAG
AACTGGTGTCTCTTGGCGAGAAA
>1448
CTAACCATATTTTTAGAAATTCCATTCAAGTTTTAAGATCGTATCTCAGTTGCAACTCTT
GTTGGTACAGGCAGATTTAGTTTTTGGAGAGTTATGTGGAGTAAATGAAAATCGAACTA
TCAACCCTGCTGTGGACCACT
>1449
AGCATTTTGGAGTAGAGACGCTTCAGAAAGAAAGCGTTAATTAATTTTATTTGACATCT
TTTGGCCACAGGTTTCTTGTCAACTTAGTGAAGCTGAAGCATGAACTCACAGATGAAG
AGCTGCAGTCTTTGTTTACAAAT
>1450
GACTTTCTATCACCCAACTTTCAACATTTCATGTTTTTTTGATGCATGAAATACTATCTTC
TCTTTCCAGGGAAGGCTTCCTATCTGTGTGGTGATGAATCGCATTTCCTGAGAACAAAA
TAAAAGGATTTTTCTGTAAC
>1451
TGCAGATTTAAGGTATTTCCACATTTGGGTCTATAATAATAATATTCTGTATAATTTATTTT

TTTTGCAGTGCCATGAGAATGATGAAAGACCATTCTATCAGAAAACAGCTCCATATGTC
CCAATGTATTATGTGCCAA
>1452
AATAAATATATATCATGAGAACTGTTAACATACTATGAATAAATTTCTAAACTATCATTATT
TTTTATAGGACTTGCTGAAACCAAATTACTACTTCACACTCTCCTTCAGCCATTTGTCTG
CCTTCAGTCAACAGAAAA
>1453
CGCGAGACTCCCCTTGTCGTCCAATAACCTGCCAGCGCCTGGCTGGTCGGCATCCCAC
TTTTCTCCGCAGATCTCCCTAGAGCACGAAATCCTGCTGCACCCGCGCTACTTCGGCCC
CAACTTGCTCAACACGGTGAAGC
>1454
ACTTTTATTGCAGATGTCTTTAGAGGCTCTTGGACACACAGTGTCTTCCTGTTTCTCATC
CTCCTTGCAGGTATGGCTTTGTAATTGCTGTCACCACCATTGACAATATTGGTGCTGGTG
TGATCCAGCCAGGCCGAGGC
>1455
AGTGAAGTGATTGTGATGTAACTTCTCATAAACTGAGGAGCATCTGTACACTGTTCCCT
CCTCTCCTCAGGATATTGTGATTCAGCAGGACGATGAGATCCGCTTAAAGATTGTGGGG
ACCCGTGTGGACAAGAATGACA
>1456
GATGAGGAGTACATGGTTGTGGCTACCCTAAATTCCGGTTCTAACTGATATGCTTTTTCT
GGTTTCGCAGGGCTTGTAAGCTGAGCCTGGTGGCCTCCTACCCTTGGTCCTACTCTAGG
AAGTGTGATTGTCACACTTAT
>1457
GGCAGTCCCCACCTTGGCCAGCGAGGTCGTGCCCTCAGCCTGCTCACCCCCCATCTCC
CTCCCTCTCCAGGCACCACCTGGGTGAGCCAGATTCTGGACATGATCTACCAGGGTGG
TGACCTGGAGAAGTGTCACCGAGC
>1458
GTGCAAGGGGAGTGGAGGAAGACAGGGCTGGGGCTTCAGCTCACCAGACCTTCCCTG
ACCCACTGCTCAGGGATGGAGACTCTGAAAGACACACCGGCCCCACGACTCCTGAAG
ACACACCTGCCCCTGGCTCTGCTCCC
>1459
CCAAGAGATGAGCTGGCCTGGGGCAGGCTGTGTGGTGATGGTGCTGGGGTTGAGTCTT
CTGCCCCTGCAGGTGGTCTATGTTGCCCGCAACGCAAAGGATGTGGCAGTTTCCTACTA
CCACTTCTACCACATGGCCAAGG
>1460
TGTGGAGCCGAGGGGTGGTGGCTACAACGCACAGCAACCCTGTGTTGGCACCCCTTG
CCTGCTTCTCCAGTGTCCTACGGATCCTGGTACCAGCACGTGCAGGAGTGGTGGGAGC
TGAGCCGCACCCACCCTGTTCTCTA
>1461
GAAGGGGACCCCTTTTACTTTTCCTGAATCAGCAATCCGAGCCTCCACTGAGGAGCCC
TCTGCTGCTCAGAACCCCAAAAGGGAGATTCAAAAGATCCTGGAGTTTGTGGGGCACT
CCCTGCCAGAGGAGACCGTGGACT
>1462
CCTCCCCATAGGCACTCGGGGCCTCCCCTGGGATGAGACTCCAGCCTTGCTCCCTGCCT

TCCCCCCCCAGGCATGGCTGGGGACTGGAAGACCACCTTCACCGTGGCGCAGAATGA
GCGCTTCGATGCGGACTATGCGGA
>1463
TCTGTTTGCCACTCTGCCAGCCAGGCTGGTGGGGCTCTGTCTAGCCCATTATTCTCACA
TTTCACCACAGGTCTGGTTACAGGATGGTCACTGGCCTTCCTGGCCTTTGAGCGCTACA
TTGTCATCTGTAAGCCCTTCGG
>1464
AATCCAGGCATCTCAGCTCCCACCGCACTCTTGTGGAGAGTCCAGTGAGCAAGTGTTT
GGTCCTTTGCAGGTTCATCCCTGAGGGCCTGCAGTGTTCCTGTGGCCCTGACTGGTAC
ACCGTGGGCACCAAATACCGCAGC
>1465
AAAAAAAAAAAAAAAAGCATCCAGAGGGACAGGAAAAAGAGAGATGTGATGCTTTCC
GTGCTCCACCCCAGGTTGCAGCTCAGCAGCAGGAGTCAGCTACGACCCAGAAGGCTG
AACGGGAGGTGAGCCGCATGGTGGTTG
>1466
GAGAATAAGGTCTTTTTTCCCATACTTCCTCATCTGCCAAGGTTATCTCCAATTGTACCT
TTCTCTCCAGTTCCAAGCTTGCATCATGAAGATGGTGTGTGGGAAGGCCATGACAGAT
GAATCCGACACATGCAGCTCCC
>1467
TTCATTGAGTTTCTTGTACACTGCTTCTCTACCCCAGCTCATATACTCATAACCTTCCCCC
TACCCTCAGGTGTGGGTTTTGCTACCAGGCAGGTGGCCAGCATGACCAAGCCTACCAC
AATCATCGAAAAGAATGGGGA
>1468
GTCCAGACCCCACCCTGCCCCTCAATTCCTGACTAAGATCACAGCTCAGGCCTCTACCC
TCTTTCCACAGTCCATTGTGACACTGGATGGAGGGAAACTTGTTCACCTGCAGAAATG
GGACGGGCAAGAGACCACACTTG
>1469
AGGGAGGGTAGAATGCTCGCAATGGTGTTCCTGGCTCCCACCCCCCATCTCACTCTGTC
TTTCCTTCCAGACACTCACCCACGGCACTGCAGTTTGCACTCGCACTTACGAGAAAGA
GGCATGACCTGACTGCACTGTTG
>1470
GATCCCTCCCACTCCCATCTCTGGGGCTTCGGGTGTCCAGACCTGACTCCCGCTCCCCC
TCCTCCCCCAGCCTACTTCTCCCTGCAGGTGATCTCGGCGCGCAGGGCCTTCCGCGTGT
CGCCGCCGCTCACCACCGGCCC
>1471
GCGAGCCCCAGGCGGGTCCGGGTCGCAGGACCATCCCGGCCGGCGCGCTCATCCCAC
CCGCCCACCGCAGGGTGAACTGCAGCGAGTACTTCCCGCTGTTCCTCGCCACGCTCTG
GGTCGCCGGCATCTTCTTTCATGAA
>1472
CAGGGGCGCACGCGCTGGACCCCCGGGACCCGCGCAGGGCGCTCACCAGGCCCGTGC
GTACCTCTCGCAGGGGCGGCGGCCCTGTGCGGCCTGGTCTACCTGTTCGCGCGCCTCC
GCTACTTCCAGGGCTACGCGCGCTC
>1473
CCTGTGTGTTAAACATCAATGTGAAGTTATTTCCAGGAAGAAGTTTCACCTGTGATTTC

CTCTTCCCCAGAGCCCCACAGTCTTCGTTACAACCTCATGGTGCTGTCCCAGGATGGAT
CTGTGCAGTCAGGGTTTCTCGC
>1474
GCTGAGTTCCTCACTTGGGTGGAAAGGTGATGGGTTCGGGAATGGAGAAGTCACTGCT
GGGTGGGGGCAGGCTTGCATTCCCTCCAGGAGATTAGGGTCTGTGAGATCCATGAAGA
CAGCAGCACCAGGGGCTCCCGGCA
>1475
AGCAGCCCTGTTCCCTGCATCTCCCTTAGAGGGGAGCAGGGCTTCACTGGCTCTGCCC
TTTCTTCTCCAGTGCCCCCCATGGTGAATGTCACCTGCAGCGAGGTCTCAGAGGGCAA
CATCACCGTGACATGCAGGGCTTC
>1476
AGGCCAGGGTAGGAACAGCAGGGACGGCTGTGGCTCTCTGCCCAGTGTATAACAAGT
CCCTTTTTTTCAGGGAAGGCGCTGGTGCTTCAGAGTCAACGGACAGACTTTCCATATGT
TTCTGCTGCTATGCCATGTTTTGT
>1477
AGAAAAGTCCTTAGGGAATAAACACAACACTGCACCCAGTGGAGCATTTACCCGTTTC
CCTCTTCTCCAGAGCTTGTGAGCCTGCAGGTCCTGGATCAACACCCAGTTGGGACAGG
AGACCACAGGGATGCAGCACAGCT
>1478
GTGGGGGTGCATGGTCTTGGGGAGGAGTGAGGCACCACCCCGGGCCCCCGTAACCAG
TGTGTCTCTCCAGAGCAGGACAGGCTGCTTTGGTTTGTGACCTCCAGGCAGGACGGCC
ATCCTCTCCAGAATGAAGATCTTCT
>1479
AATGTCCACTGGGGTCAGGCCTGGGAGGGACACTGGAGGCTTCCCTGAACAGGTGTG
TCCTCCTTCGCAGCCAGCTCGCTGATGTGCTTCTCCTGCTTGAACCAGAAGAGCAATCT
GTACTGCCTGAAGCCGACCATCTG
>1480
CTCAGCAGAGGCCATTGCTGTCTGTCTGCAGCCGTCTGTCTCTCCCCTGACAGCCTCAT
TTCCCATGCAGGGAATCTCGTGACATTTGGCCACAGCCTGAGCAAGACCTGTTCCCCG
GCCTGCCCCATCCCAGAAGGCGT
>1481
CCCTACCCTGCCCACCTGTTCACAGGCCCGCCGCCCCAGGACCGCCGCGCTCACCCCG
CTCCGTCCGCAGCTCGTACCTGGCGTCTGCTGACCTTCGTGCTGGCGCTGCCCAGCGT
GGCCCTCTGCACCTTCAACTCCTA
>1482
GGCGCGCGGGGCGGGGCGCGGACTCCGGACTCACGGCTCACGCGAGCTCCCTCCCCC
GCTCTCTCCACAGCCCTACCCCTGGGGGGACGGCAACCACACTCTGTTCCACAATAGC
CACGTGAACCCTCTGCCCACGGGCT
>1483
TATAATTTGGGTTCACTGTGGCTACTTGAACACTACACTGCAGCTAACTCTATCTTTGTT
TCCTTTCCAGGAATCAATGAATTTCAGCGATGTTTTCGACTCCAGTGAAGATTATTTTGT
GTCAGTCAATACTTCATATT
>1484
GATGCTGGCACTCCTGAAGGGCACAGATGGGGTGACTCAGGAGGGACCTGATGGGAC

CATCCCCCTGTAGGCATCCTGCCTGTGTACCACTCCCTCTTTGCTCTGGCCACGGAGGA
CCTGTCCTGCTGGTTCCCCCCGAG
>1485
CCACCATAATGTCACTCCTACTGAGGGTGGGTTGCACTTTTCATCCCAGGGTTCTCTCC
TCTCCTCACAGCTCTTTTTACTTCCCCAATTGGTTTGGGCTGGAGAAGTGCCACCGCTT
CGGGCTACGCAAGGATTTGGCC
>1486
GGTCTGCAGGTTAACAACAGGGCTTGAAGTTGGGTGGCCTCAGCTGATGCTCCCTGTG
GCGGCCCCCCAGCACCGCAGTGACCTGGTGAGTGGGCGCCTCCCCGTGGGCCTCAGT
CTCAAGGAGCAGGGTGAGTGTCTCA
>1487
TTTGTGTGTGTACCCCGCGGGGACCCCTCCCGACGCTGAGGACCGGGTCCCTCCCCTC
CAACCCCTGCAGCTACAAGGCCTGCCTACCCCCAAGCCTGCGCGACCTGATCCAGGGC
CTGAGCTTCGTGACGCGGAGGGCT
>1488
TCAGCCTTACCCCGAAGGGCGGGACCGGCACCCTCGGGTTTCACTGGGCTCTGACGCT
TGTCCCTCGCAGAGGCCGAGTTCCCAGGGCTGCCCGAGGCTCTGTCGTTCGTGGCGCT
CGTGGACGGCTACTTCCGGCTGAC
>1489
AAAAGGAAGAAGGACTGAGAAGGAGAGTGTCTGTCGCTCAGTCCCACTCAGGGGCC
ACTCTTCTTTGCAGAACCCCCTTGGTCCTGATTATAAGGGCTGCCTCATCCGGCGCAGC
CCCACAGGAACCTTCCTTCTGGTTG
>1490
TGAGTTAGCTAACCCTTGGGGACTTTTCACCTCTGATTTCTGGTTTTTCTCCCTCATCCT
CTCCCCATAGAAAAGTCCAACCTGATCGTGGTCCAGAGAGGTCACAGCCCACCCACAT
CATCCTTGGTTCAGCCCCAATC
>1491
TGTCTCGCTTGGGCTGGGATCCTGCAGGAACCCCCTCACTGGCCTCTTCTGCTGTCCCC
TCACCATTCAGCATGAGAACCTGGGCCATGGGTCCTTCACCAAGATTTACCGGGGCTG
TCGCCATGAGGTGGTGGATGGGG
>1492
GAGCACAAAATTTGGGATTATTGGAGTGGAAGAAACCCACGCATCTTCTCTCCCTTCCC
ACCTTCCCCAGTCATTCCTGGAAGCAGCGAGCTTGATGAGCCAAGTGTCGTACCGGCA
TCTCGTGCTGCTCCACGGCGTGT
>1493
GATGCCCCCAGATATATTTCGGGAGTGCGCAAAGAACTAAACTTGGAATACTGAGCAA
ATATGGGCTGAGCACCATGGTGCAGGAATTTGTACACCTGGGGGCCATAGACATGTATC
TGCGAAAACGTGGCCACCTGGTG
>1494
AGGAGTTTGCCAAACAGACTCTTCATTTTATCAAACCTCCCGGGCATTTTCGTGTGTTT
GGCCCCCTTAGGAGGACAAAGGCCTGCCCCATGGCAATGTCTCTGCCCGGAAGGTGCT
CCTGGCTCGGGAGGGGGCTGATG
>1495
TGGAGGTGGAGGAGGGAGGGGCTGAACAGGGCAAGGAAGTGGATCCCTGATCCCACT

TTCATTCCCTCAGATGCTCACCGACAGGATCCCCTGGGTGGCCCCCGAGTGTCTCCGG
GAGGCGCAGACACTTAGCTTGGAAG
>1496
ACATCCAGTCTGGGGAGATTGGGGTGGGTCTATTGGGTTGGGGATTACCGACTGCTCCT
CTCACCCTCAGAAACTCCAATTTTATGAGGACCGGCAGCAGCTGCCGGCCCCCAAGTG
GACAGAGCTGGCCCTGCTGATTC
>1497
CAGGGCCAGGGTGGGTTGGAGAGGCAGGGCAGCATCCAGGTACCTGGACATCAGTGC
CGCTATCCTCGAGGGCAACTTTGGCAGCGTGGAGCTGTGCCGCTATGACCCGCTAGGC
GACAATACAGGTGCCCTGGTGGCCG
>1498
GGTCACGCTTGGGGGTACCTGCCGGATTATCCTGGGATCCTCTCTTCAAGATCACAACG
ACCCCCCCCAGGGCATGGAGTACCTGGGCTCCCGCCGCTGCGTGCACCGCGACCTGGC
CGCCCGAAACATCCTCGTGGAGA
>1499
AGAGAGCGGGGTCCCACTTCAGGACACCCCTCCAGGTGCCTCCAATACCCGCCCACCT
CCCACCCCGCAGGTGTCCCAGGCGCTGATCCGCTCCTTCAGCTCCACCGCCCGGAACC
GCTTTCAGAACCGAGTGCGCGAGA
>1500
GCCGGGGCAGGCGGGGCGCGCTCTAAGGAGCAGCCAGCACCCCTTTCTCATCAGACA
CCCCCACATCCAGGAGGACAATGACATCCCGTTGTACCTGAAGGGCGGCATCGTTGAC
AACATCCTGTACCGAGTGACAATGA
>1501
CTGTCAAAAGCCTGGGATGTCCAGGGAGGGGATTAGGTCTCATTCTGTCTCCAACGCT
TCCCCTTTCTAGGCACTGTCTACAGCTTGTACTCCCTTGGCTGGGCCTCCTTCCCCAGG
AATTAAGACCAAGAAGCCTGGGG
>1502
GGGGTGGGAGAGCGGCTGGGGCGCCTCCGTGACTCAGCCCCGGCCTGTCTGGCCCCT
CCCGTCTCCGCAGTTCTGGGAGGTGATCTCTGATGAACATGCCATCGACTCCGCTGGC
ACCTACCACGGGGACAGCCACCTGC
>1503
CTGCATTATGGTGGTGACCACTGATGACCGTATACCTGGCCGTCGAGTGACCGGCTGTA
CTGTCTTACAGGTCAGTGTGGGGCCGGAAACAACTGGGCCAAGGGACGCTACACCGA
AGGCGCGGAGCTGATGGAGTCAGT
>1504
TGAGGCCTTGGAGGGTGGCCTGGGGACCTTGGACACAACCAGCCTGCCCCTGACCCA
TCCCTCCCTGCAGCTTGCTCCTGCAACGGCCATGCCCGCCGCTGCCGCTTCAACATGG
AGCTGTACCGACTGTCCGGCCGCCG
>1505
GCCCTCACCCTCTGACTTCCCAGATCCCCAGACAGGCTTCTGACCAGGCCCTTCCCAC
CTCTGTCCTCAGCCTGCGACTGTCACCCGGTTGGTGCTGCTGGCAAGACCTGCAACCA
GACCACAGGCCAGTGTCCCTGCAA
>1506
TGGGGGAGGGGGCTCTGGGAGGAGAGGGTGGGGAAAGGGAGTCTGTGCCAGCCTCC

CACCTTCTACCCAGACTGTGACTCGCACTGCAAACCTGCCCGTGGCAGCTACCGCATC
AGCCTAAAGAAGTTCTGCAAGAAGGA
>1507
TCCCCGTGCCTCCCCCTACCGCGGGCAGGCCGCCCCTTCCTGACCCCGCCCCCTCTCG
CTCTCCCCGCAGCGGTGCAGGTGGCGGTGGGTGCGCGCGGCGAGGCGCGCGGCGCGT
GGACACGCTTCCCGGTGGCGGTGCT
>1508
CACAGTGAACATTTAGAAGCAAACTTTTGAAGGAGTATTTTCAGCTGCCATTTATGCTT
CTTACAAATAGAGTTTGAAACAGCTGAGACACTTCTAAATTCAGAAGTTCATATGCTTC
TGGAACATCGAAAGCAGCAGAA
>1509
GATTACTCTTTGATTTTTGTTGTTTATTTGTTTTTGATTATTTTGATTTTTTTTTTTTCCTTT
TGATTAGCTTGCTACTCCAGAAAAAGCTTCATAAGTTTGAGTTGGCCTGTTTGGCCAAC
CTTTGCCCAGAGACTGCT
>1510
CACGCCTCAAAGAAGAGTGCGGGGAAATTGCCAGTCGCCAAATCACTCATTTCTTTTC
ATTTTCTTGTAGCTTGGAGGGACGGTTTGAAGATGAGGAGCTGCAGCAGATTCTTGAT
GATATCCAGACAAAGCGCAGCTTT
>1511
CTTCTCTGCAGGCCCAGGTGACCCAGGGTTGGAAGTGTCTCATGCTGGATCCCCACTT
TTCCTCTTGCAGCAGCCAGACTGCCTTCCGGGTCACTGCCATGGAGGAGCCGCAGTCA
GATCCTAGCGTCGAGCCCCCTCTG
>1512
GACCTGGAGGGCTGGGGGGCTGGGGGGCTGAGGACCTGGTCCTCTGACTGCTCTTTT
CACCCATCTACAGTCCCCCTTGCCGTCCCAAGCAATGGATGATTTGATGCTGTCCCCGG
ACGATATTGAACAATGGTTCACTG
>1513
CGTGTTCCAGTTGCTTTATCTGTTCACTTGTGCCCTGACTTTCAACTCTGTCTCCTTCCT
CTTCCTACAGTACTCCCCTGCCCTCAACAAGATGTTTTGCCAACTGGCCAAGACCTGCC
CTGTGCAGCTGTGGGTTGATT
>1514
GGGCTGGAGAGACGACAGGGCTGGTTGCCCAGGGTCCCCAGGCCTCTGATTCCTCAC
TGATTGCTCTTAGGTCTGGCCCCTCCTCAGCATCTTATCCGAGTGGAAGGAAATTTGCG
TGTGGAGTATTTGGATGACAGAAA
>1515
GGCCTCCCCTGCTTGCCACAGGTCTCCCCAAGGCGCACTGGCCTCATCTTGGGCCTGT
GTTATCTCCTAGGTTGGCTCTGACTGTACCACCATCCACTACAACTACATGTGTAACAG
TTCCTGCATGGGCGGCATGAACC
>1516
TTTTTTAAATGGGACAGGTAGGACCTGATTTCCTTACTGCCTCTTGCTTCTCTTTTCCTA
TCCTGAGTAGTGGTAATCTACTGGGACGGAACAGCTTTGAGGTGCGTGTTTGTGCCTG
TCCTGGGAGAGACCGGCGCACA
>1517
GGTGGAGGAGACCAAGGGTGCAGTTATGCCTCAGATTCACTTTTATCACCTTTCCTTGC

CTCTTTCCTAGCACTGCCCAACAACACCAGCTCCTCTCCCCAGCCAAAGAAGAAACCA
CTGGATGGAGAATATTTCACCCT
>1518
GTAACTTGAACCATCTTTTAACTCAGGTACTGTGTATATACTTACTTCTCCCCCTCCTCT
GTTGCTGCAGATCCGTGGGCGTGAGCGCTTCGAGATGTTCCGAGAGCTGAATGAGGCC
TTGGAACTCAAGGATGCCCAGG
>1519
CAGGGAAAAGGGGCACAGACCCTCTCACTCATGTGATGTCATCTCTCCTCCCTGCTTCT
GTCTCCTACAGCCACCTGAAGTCCAAAAAGGGTCAGTCTACCTCCCGCCATAAAAAAC
TCATGTTCAAGACAGAAGGGCCT
>1520
CCCGGGGCGCGGCGCAGTGGGTGCCGAGCTTGGCCCCAGCCCCGCGCCTCACCGCGC
CCTCGCTCCGCAGGCGCTCGTCTGGCGTGGCCGCTGAGGCTGGGACCGGCGGTGCCC
TTGTCTCTGGGTGCGCCAGCCGGAGG
>1521
CTGTGGGACCCTCGGGAGCCGGTGGGACGCAGGACCTGGGGCTAGGGCTGAGCATTC
CCCCCATCCCCAGGTCTGGTTCAAGAACCGCCGGGCCAAATGGCGACACCAGAAGCG
CGCGTCGGCTTCCGCGAGGCTCCTGC
>1522
ATGACCACGTTGGAAATTCTGGAACTTGGAATCTGCTGTATTCTAACATTGGCAACTTT
TTTTAAAACAGCTGTTGTTGGCCTACTGTACCCCTGTATCGACAGTCACCTCGGAGAAC
CCCACAAATTTAAGAGAGAATG
>1523
CCCTTGTGGTTTTACGTTTTTTTATCTTAATAAGAGTCACCGAACTTTTCCTTAACTTTTA
TATCACCAGAAATTGGATTTTGCCAATAATGTCCAGCTGTCCTTGACTTTAGCAGCCCTA
TCTTTGGGCCTTTGGTGGA
>1524
GAGAATCTGTGATGTAGAATTGAGCCAGGTGAAATTGACTGCTAAGGTACAGTTTCTTT
TCTCGCTCCAGGTATACGTCCCCAGATTTCCTCTATATTCGTTCTTGGCTCCCTTGTATAT
TTTTCTCAGGAGGCGTCACG
>1525
TGGTAATTAAAATATTTATACATTTAGTGCTAATTTTCTCTTTCCTTTTTTTTTTCCTTTCT
ACACATAGGGTGTTCCTGAAAAGCCCCATAGTGATTGAGTCTTCAAAACCACCGATTCT
GAGAGCAAGGAAGATTTTG
>1526
TTCAGATTTTCTTATTCCCAAGGCCTTTAAACTGTTCACCTCACTGAAACCTTTGTGTTT
TTGTCCTTAGTGATATTGACCAGAGTTTCGACAAAGTAGCTGAACGTGTCTTAATGAGA
CTACAAGAGAAACTGAAAGGA
>1527
AGCATTTAATTCTAAGTGTATTTCCCATGACTTACAGTTTTCCTGTTTTTTTCCCCTTTTC
TCTATTTAGGTTACCTACATCTCATACTTGCTTTAATGTGCTTTTACTTCCGGAATACTCA
AGCAAAGAAAAACTTAAA
>1528
GCTTCTCAAAGTGCTGGGATTACAGGTGTGAGCCATCGCGCCCGGCCAATTGTTGCTTC

TTTTTCAACAGGAGTGTGGGCTGCTGCTCTCCTCTGAGGGCGAGTGTCAGGGCTGCTA
CCCGCTGGATGGGCACATCTTGT
>1529
AGGGCTCTGGCGGGGGCGGCCTCCAGGTTCTGCTCCCCAGCCCTGGTCCGACGGTGC
CGCTTCTCCCCAGCCCTACAGACGCTGCGCGGCTGGGACTCTGTGGACAGGGAGGTG
GCCGAGATCCGGCAGGAGGATGAGGC
>1530
GCCGGGCGTGGGTCCTGGCGCCGAGGCCGCCTCCTCCGGGCAGCCGCCCAGCCCCGC
GCTTGCCTTGCAGATCTACTACTACGCGGACCAGATCTACCTGAGCGCCGGCGTGCCG
GAGGAGCACGTGCAGTACGTGACGG
>1531
CCCCATTGGAGGGGGCTGGAGGGAGGTGAAGCTGGGCCTCTCCCGCCTGAGCCCTGC
CACCCTCCTGTAGGTGTTCGTGGTGGAGCTCCTGGGTCGGAGGCTGCTGCTGCTGCTG
GGCTTCTCCATCTGCCTCATAGCCT
>1532
GGCCGCCCCCTGGGGTCCCCGTCCTACGCTTTGTCTGCTCCAGGTGAACCAACTGCTT
TCTCTCCGACAGGACACAGTGTCCTGGATGCCATACATCAGCATCGTCTGTGTCATCTC
CTACGTCATAGGACATGCCCTCG
>1533
TCTGCATGGCCAGGCCTGGGATGCTCGGGCCCGCTCTGCCTTCAGGGAGGCTGCTCTC
CTCCCCCTACAGGTCCCATACCCGCGCTGCTCATCACTGAGATCTTCCTGCAGTCCTCT
CGGCCATCTGCCTTCATGGTGGG
>1534
CCCCATGCAGTACAGTGCGAGCCCCTGGGCAAACCACGTCCCACAGTGTCACCTGCCC
CTCTTCCCGTAGGAGGGCCTCGGCCCGTACAGCTTCATTGTCTTCGCCGTGATCTGCCT
CCTCACCACCATCTACATCTTCT
>1535
GGGAGAGAAGGGAAAAGCCCTTGGAGGCCAGAAGAGAAGTCAGATCTTCCTCCTCTC
CCAAAACCTCCAGTGTATGCCACGTTGAGGGACCTGAAAACACAGGGCCGGCTGTGG
GAGGCGGCCCGGGCCCTGGCATGCCC
>1536
TGGAGCATGAGGGGACAGGCCGTGCTGAGGGTGATGCTGAGGCGGGCTGGTTGGGCC
TCTGTCCCCGCAGTGTGTAACACAGGCCTGGGCCTGCTGGGGCCGCTGGGGGAGGAC
GCCGTGGCCTCTGTACTGGATGTGAA
>1537
GAGGGGCACTGCCTGCCGGGGGATGACCCCCTGGCCGCTGCGCCTCAGGAACCTCAT
CTTCCCACCCAAGGGCTGCCTTTCAATGACGTTTATTGCGCCAGCAAGTTCGCGCTCGA
AGGCTTATGCGAGAGTCTGGCGGT
>1538
CTGGGGCAGGAGATGGGACTTGGGCCTGGGTCGCTCCGTCCCTGCCCACTTGCGGCTC
TCGGGCCAGCAGCGTGAGCCTGATCGAGTGCGGCCCTGTGGACACCGCCTTCATGCAG
AAGGTGTTGGGCGGTCCCGACCAG
>1539
TTTCCCTTTGCTGGATCTTGCCTGCCCTCTGAGTTTCCTCTTTTTTGATCTGACCCCTGC

TGTCTCCTAGCTGTCGCCATTCCCCTTTGACCTCCTGCTGAAGGAGGTGCAGAAGTACC
CCAATGCTGAGCTGGCCTGGT
>1540
TCAATCCCTGATCACTGGGAGTCATCATGTGCCTTGACTCGGGCCTGGCCCCCCATCT
CTGTCTTGCAGGACAATGCCGTCTTCTGTCTCGTGGGGCATCCTCCTGCTGGCAGGCCT
GTGCTGCCTGGTCCCTGTCTCC
>1541
TTTCTTATTCTGCTACACTCTTCCAAACCTTCACTCACCCCTGGTGATGCCCACCTTCCC
CTCTCTCCAGGCAAATGGGAGAGACCCTTTGAAGTCAAGGACACCGAGGAAGAGGAC
TTCCACGTGGACCAGGTGACCAC
>1542
CACTGTGGTGGGTCCCAGAAGAACAAGAGGAATGCTGTGCCATGCCTTGAATTTCTTT
TCTGCACGACAGGTCTGCCAGCTTACATTTACCCAAACTGTCCATTACTGGAACCTATG
ATCTGAAGAGCGTCCTGGGTCAA
>1543
GTGTCCACGTGAGCCTTGCTCGAGGCCTGGGATCAGCCTTACAACGTGTCTCTGCTTCT
CTCCCCTCCAGGCCGTGCATAAGGCTGTGCTGACCATCGACGAGAAAGGGACTGAAG
CTGCTGGGGCCATGTTTTTAGAGG
>1544
GCACAGGAGCCTCCCGGTTTCCGGGGTGGGCTGCGCCCGTGCTCAGGGCTTCTTGTCC
TTTCCTTCCCAGGGCGTGATGGTGGGCATGGGTCAGAAGGATTCCTATGTGGGCGACG
AGGCCCAGAGCAAGAGAGGCATCC
>1545
ATGTCCCCCGTCTGGCCTGGCTGTCCCCAGTGGCTTCCCCAGTGTGACATGGTGCATCT
CTGCCTTACAGATCATGTTTGAGACCTTCAACACCCCAGCCATGTACGTTGCTATCCAG
GCTGTGCTATCCCTGTACGCCT
>1546
TGCCTGACATGAGGGTTACCCCTCGGGGCTGTGCTGTGGAAGCTAAGTCCTGCCCTCA
TTTCCCTCTCAGGCATGGAGTCCTGTGGCATCCACGAAACTACCTTCAACTCCATCATG
AAGTGTGACGTGGACATCCGCAA
>1547
GTGGGGTCCTGTGGTGTGTGGGGAGCTGTCACATCCAGGGTCCTCACTGCCTGTCCCC
TTCCCTCCTCAGATCATTGCTCCTCCTGAGCGCAAGTACTCCGTGTGGATCGGCGGCTC
CATCCTGGCCTCGCTGTCCACCT
>1548
GGGCTCCTGGGTTCTGCGTTGCGGGGTGGGTTCGGTGTCGCCGGCGAGGCTGACGGAT
CGTCCCCTGCAGGGCGTCATGGTGGGCATGGGCCAGAAGGACTCCTACGTGGGCGAC
GAGGCCCAGAGCAAGCGTGGCATCC
>1549
TTGTGGCGTGTGAGCATGGGGTGGCCGTGGGTCTCTGTCCCTGACTAAGCCGCCCCTT
GTCCCTTCTCAGATTATGTTTGAGACCTTCAACACCCCGGCCATGTACGTGGCCATCCA
GGCCGTGCTGTCCCTCTACGCCT
>1550
GTGAGCTAAAGGTTTCTACTCTTTCATCCTCGGTGACACAGCATCACTAAGGGAGGGC

TCTGTCCCCTAGGTATGGAATCTTGCGGCATCCACGAGACCACCTTCAACTCCATCATG
AAGTGTGACGTGGACATCCGCAA
>1551
TCTGCCTGGCTCGGGAGAGCTGACTGGGGGGCGCTCTGCGAGCTGAAGCCGTGCCTG
GCTGTCTTTGCAGATCATCGCACCCCCAGAGCGCAAGTACTCAGTGTGGATCGGCGGC
TCCATCCTGGCCTCACTGTCCACCT
>1552
CCATCCTGGCCTGTCAGGCCTGCCTGGATCCTGCCCCGGGTTGGTCCACCACTCACTGT
TTTGTTTCCAGGAGGAGAGGGATCGCCCTCCCAGCTAACACAGCAGAGGGGCTGCTG
AACGTCATTGGCATGGACAAGCCG
>1553
TGTCATTTGCTCCTGGAGCTCCCAAGGGACTTGGGGAAGGTTGTTCCCAACCCCTTTC
TTCCCTTCCCAGGGGCTGCCGGGAGGCTATCAAAAGGATCGCCTATGAGTTTGTAGAG
ATGAAGGCCAAAGAGGGCGTGGTG
>1554
CCCAGTCCCAGAGCTGCCCTTTAGGCCTTCCTTTTGCCTCAGGCCCATCCCTACTCCTC
TCCTCACACAGAGGGGACCTCACCCCAGACGAGGTGGTGGCCCTAGTGGGCCAGGGC
CTGCAGGAGGGGGAGCGAGACTTC
>1555
AGCAGGCAGGTAGGCTGGGAGGTCTCTCCATGGTGCTGGTGACAGAGCCTGGGTGGG
CATCTGCCCACAGACTGGTCCCCCAAGGTGGTGGAGCTGTGTAAGAAGTACCAGCAG
CAGACCGTGGTAGCCATTGACCTGGC
>1556
CAGCATGGTCAAGGCAGGCTGCCGGGGCCCTCCCGCTAGGGCACCCATGACCTGGCTC
TCCCCCTTCCAGGAGGCTGTGAAGAGCGGCATTCACCGTACTGTCCACGCCGGGGAGG
TGGGCTCGGCCGAAGTAGTAAAAG
>1557
GCCTGGGCTGGCCATGGGGTCCCTCCTCACTGCCTCCTCCCATACTTGGCTCTATTCTG
CTTCTCTACAGGCTGTGGACATACTCAAGACAGAGCGGCTGGGACACGGCTACCACAC
CCTGGAAGACCAGGCCCTTTATA
>1558
CGTCAATGTGTTCTGACTATTTATGTAAGAGTAAAAGGAGGCTGATTCTCTCCTCCTCC
CTCTTCTGCAGGCTCAAAAATGACCAGGCTAACTACTCGCTCAACACAGATGACCCGC
TCATCTTCAAGTCCACCCTGGAC
>1559
TCACTACATTGCTAAGAGCTGCCTTCTTTGTTCTCTGGTTCCATGTTGTCTGCCATTCTG
GCCTTTCCAGAACATCAATGCGGCCAAATCTAGTTTCCTCCCAGAAGATGAAAAGAGG
GAGCTTCTCGACCTGCTCTATA
>1560
AGTCGAAAAACACAATTTTAGTTGTCTTAAAGGTATCATTTAAAACTTAGTATGTTGCTT
CCTTTTACAGCATTCGAACTGTTGTAAAGATTTAATTCAAAAGAGAAAATAATGTCCAT
CCTGTCGTGATGTGATAGGAG
>1561
AACTATACTTGAGAAAAATAAGCTTGCTGCAGGTCTGTTCCTTAAGGATTCACACGTAT

TTTTGTTTCAGGGCTACCATATTTTTTGCCCAGTTTGTTCAAGAAGCCACTTACAAGGA
AGTAAGCAAAATGGTGAAAGAT
>1562
TTTCATGTGTCTTATAGATTTTTAAAGCAAAGTTAGTTGTCTTTCTCCAAACATAAAATA
TTTCTTATAGCTACCTGCCTTTCTGGAAGAACTTTGCCATGAGAAAGAAATTTTGGAGA
AGTACGGACATTCAGACTGCT
>1563
TCCAGTTCCAAGCAGTAGTAGTCCTATTTTTAGGTGTTTATAAATCTTCTAGCTCTATTTT
ATTTCACAGATTCATTTATGAGATAGCAAGAAGGCATCCCTTCCTGTATGCACCTACAAT
TCTTCTTTGGGCTGCTCGC
>1564
CTAAATATTAGAGCTTGTAAAGAAAATGTTAGTATATGCTTTCATGACATTTTGTTTCCTC
TACATCTAGGCAGCAACAGTTACAAAAGAATTAAGAGAAAGCAGCTTGTTAAATCAAC
ATGCATGTGCAGTAATGAAAA
>1565
ACTCTATAAATTCTATTTTATTTTGACAGATAACCAAGAAATTAATTTCTAATTTCTTTTTT
TTCCCTAGAACTGTTACTAAACTGAGTCAGAAGTTTACCAAAGTTAATTTTACTGAAAT
CCAGAAACTAGTCCTGGAT
>1566
AAAGCTGGCTTTGAGATCCTTTATTAAAGAATAAATCTTTAAAACTTATACTTTATTTTCC
CTGTTGCAGGAAAAAATCATGTCCTACATATGTTCTCAACAAGACACTCTGTCAAACA
AAATAACAGAATGCTGCAAAC
>1567
CTGGAGAAGTGATGGCTCCTTTTGTCTCTTAGTTCCAATAACTTGAAATATTTTTCTCCA
CATATTTCAGTTTTGTTCATGAATATTCAAGAAGACATCCTCAGCTTGCTGTCTCAGTAA
TTCTAAGAGTTGCTAAAGGA
>1568
ATCAATTTTATATACAAAGTTATGCATCCAAGAAAAGAAAAATGTATATGTAATAATTCTT
CATTTTCAGGAAGAAGAATTACAGAAATACATCCAGGAGAGCCAAGCATTGGCAAAGC
GAAGCTGCGGCCTCTTCCAGA
>1569
GAGAAGAAGCAAGGCGGCTAAAAACTCATGAATGACTCAGCAGGACTTAGTTAAAAA
ATGCTTCTTTCAGGTTTCTCGTTGCTTACACAAAGAAAGCCCCCCAGCTGACCTCGTCG
GAGCTGATGGCCATCACCAGAAAA
>1570
CTGAAAAACTGAACCAACTTTGTGACTAATGCCCAATCTCCTTACTTTTTTTTCTCATTC
TCCTAACCAGGCTGACATTATTATCGGACACTTATGTATCAGACATGAAATGACTCCAGT
AAACCCTGGTGTTGGCCAGT
>1571
TAGGAAGGCTGTAGAAAAATAGCATTGCATAACAGACTTCTCTTGTATTTTGTTTTGTTT
TAAATCACAGGTTTCTCATTAACCTTGTGAAGCAAAAGCCACAAATAACAGAGGAACA
ACTTGAGGCTGTCATTGCAGAT
>1572
TAATAGAACCAGTTACTTACTGCACCTATTAGTTTAATTAGTATTTAATATATTTTTGCTCA

TATTGCAGGGGAAGAGAAGACAAAACGAGTCTTTCATTCGGTGTGAACTTTTCTCTTT
AATTTTAACTGATTTAACAC
>1573
GGGGTGGGATGAGGGCTGGAGCTGTGGGGGCAACTGATAGTCACCATCCCCTCCGTCC
GTTGCCCGGTAGTGCTCTTGCTGGGACATGTGGCCCAGGTGCTGATGCTGGACAATGG
GCTCCTGCAGACACCACCCATGGG
>1574
AGCTGAGGCAGGGTGTGGGTGAGGGCCGGGCTGGGTCCCTGAGCTACCCCATCCCTCT
GCCCCTCACTAGTGAACAGCTCTTCATGGAGATGGCTGACCGGATGGCACAGGATGGA
TGGCGGGACATGGGCTACACATAC
>1575
GGTCAGATATGCCTTGAGGAGGCTCCTTGAGGGTCCTGCTTGAGCCCACTGTGTCCTC
AAACCCCCACAGGTTCACTCCCTGGGCCTGAAGTTGGGTATCTACGCGGACATGGGCA
ACTTCACCTGCATGGGTTACCCAG
>1576
AAAGGTCTGCTAAGGCCCCAGACTGCCTCCTGCACTTCCTCAGGACTGAAACAGGTTG
GGCTTTCTCTAGGGTACCCCAAGATGGCTGCTGCCCTGAATGCCACAGGCCGCCCCAT
CGCCTTCTCCTGCAGCTGGCCAGC
>1577
CTCCTGTGTGGGTCCCTGGGCTGCAGCGTGTGATTCTGGTGCTGGACTCTGCCTTTCCC
TCGACATCCAGGTGAACTACAGTCTGCTGGCGGACATCTGCAACCTCTGGCGTAACTAT
GATGACATCCAGGACTCCTGGT
>1578
TTTGGGGATGGTCCCTGTGTCTACCTCCTTGAGGAGATGAGAGCTGACTACTCCTCTTC
TCTGCCCCCAGCTGCTCATTGGGAACTTTGGTCTCAGCTTAGAGCAATCCCGGGCCCA
GATGGCCCTGTGGACGGTGCTGG
>1579
GGGCCAGGGAGGCAATGGTAGGTCCTGTCTGAGCAAGGGACCCCAGCCAGTAGCCAC
CTTCTGTGCCCAGGAAAAATCTCTCATCGAAGTGTACATGCGGCCTCTGTCCAACAAG
GCTAGCGCCTTAGTCTTCTTCAGCT
>1580
ATCCTCTAGCGGGGGGGGTCTCTGTGCCCCACTCTCATGGTGGCATTGGTGATGTCCCT
CCCCACTGCAGGCCCAGGACGTCTACTCAGGTGACATCATCAGTGGCCTCCGAGATGA
AACCAACTTCACAGTGATCATCA
>1581
AGACCCTGGCAGCCCCTGTTCCTCCTGGGACCCTGTGTCTCGGGGTTTATGAATGGGT
GGGCGTTTGCAGGTGGGCCTGGCTCAGGGAAGGGCACCCAGTGTGAGAAGATCGTGC
AGAAGTATGGCTACACCCACCTCTC
>1582
GTGACGAGAGGCTGGGAGAAGGGATGGGGAGGAGAGGCTCATGGCCCCTTCCTCCTT
TGCTGTGCCCCAGGAGACAGTGTTGGACATGCTCCGGGATGCCATGGTGGCCAAAGTC
AATACTTCCAAAGGCTTCCTGATTG
>1583
TGGGAAGATGGGGAGGACATGGCCCTAGGGGGCAGGAATGGGGTCCCTGTGACATGG

TGCCCGCCTGCAGATTGGACAGCCCACACTGCTGCTGTATGTGGACGCAGGCCCTGAG
ACCATGACCCAGCGGCTCTTGAAAC
>1584
TGGGGCCTGCCATGGTGAGGCCCACCCCACTCCACCTGGTGACTCAGCCTCACGGCCC
ATCTCCCCACAGGTCAACGCTGAGGGCTCCGTGGACAGTGTCTTCTCCCAGGTCTGCA
CCCACCTGGACGCCCTAAAGTAGC
>1585
TCCCAGAGTTGAGGGTGGGTGAGACCACGTGCTCATGGCTCTTCTCACTGTGCCCTGC
CCCCTGCACTAGGCGGTGCCTCGGGAGGAGCTGTTTGTGACATCCAAGCTGTGGAACA
CCAAGCACCACCCCGAGGATGTGG
>1586
GGCTGGATGGGCAGGTAGGGTAGAAGCCTGGCATTTGTGTCCACACTTGGTGGGGCTG
TCTCTCACTCAGGCGGGGAGACAACCCCTTCCCCAAGAATGCTGATGGGACTATATGCT
ACGACTCCACCCACTACAAGGAG
>1587
ATGCATGTCTGGATGGGCCAAGCAAGTCTGGGTGTCACCACTTCATGGTGATGGGTTAT
CTTTGGCTCAGGTGGAATGCCACCCATACTTGGCTCAAAATGAGCTAATTGCCCACTGC
CAAGCACGTGGCTTGGAGGTAA
>1588
TGCATGGGGAACAAAATAGTGCTTATGAATACTGACCCCTTTTCCTCATCTGTCTAATCC
CCCAACTTAGGTGGCAGGTCCAGCGGAAAGTGATCTGCATCCCCAAAAGTATCACTCC
TTCTCGAATCCTTCAGAACATC
>1589
AGGCAGTGTTGTGGAACCCCAGCTTCTGCTCACAAAGCTGGCTTTCTTGAACCCCACT
CTCCATCCTCAGGTGTTTGACTTCACCTTTAGCCCAGAAGAGATGAAGCAGCTAAATG
CCCTGAACAAAAATTGGAGATATA
>1590
GGGAAGATGCCTGGAGTCTTTGGAATGGCAACTCCTGCTGATGGAGTAATCTATCTGTC
TCTCTTTCCAGGTGGATGGGAAGAGAGTCCCAAGGGATGCAGGGCATCCTCTGTACCC
CTTTAATGACCCGTACTGAGACC
>1591
CCGGTGTGCCATGACCCCAGGCCACCCTTTCCTGCCTTTCTACTCATGGCTTCTTCCTG
ACTGTCCCCAGGAGTTGTGCCCCTGCTGTTGGTTCTGCACTGGAAACATGGGGCGGGG
AGCCCCCTCCCCATCACCCCTGT
>1592
CCCAAAGGGTTGGCGGCAGGGCTGGGACACTGACCCCTGACTCCCACGTCACCTCCC
TTCTGCCCCTCAGTACACAGCCCAGGGGGAGCCGTTCCCCAACAACCTGGACAAGCTA
TGTGGCCCCAACGTGACGGACTTCC
>1593
TACACACACACACACACACACACCACCCCCCAGCCCAGGCTGACCTGATCTTTGCTCT
CCCCCTGGCCAGTTGAGGAGGAGAACCCGGACTTCTGGAACCGCCAGGCAGCCGAGG
CCCTGGGTGCCGCCAAGAAGCTGCA
>1594
CGGCGCCCGGACCCTCAGTGGTTCCAGGACAGCCCTGGGGAGCAAGCCTCACACACT

TCTGCTCCTTCAGGGATGGGGGTGTCTACGGTGACAGCTGCCAGGATCCTAAAAGGGC
AGAAGAAGGACAAACTGGGGCCTGA
>1595
TCCCAGAGGACAGAGATCAGGGTCTTGTTTGTCTGCCCCAGAGAAGAGCTCAGAGTG
TCTCTGTCCCCAGACATACAGTGTAGACAAGCATGTGCCAGACAGTGGAGCCACAGCC
ACGGCCTACCTGTGCGGGGTCAAGG
>1596
TGGGGCCCGGCTGTGGGGTCAGGGCCAGTGACAGACCTCTATCGCATATCCTGACCTC
TATCACCCTCAGGAAAGTCAGTGGGAGTGGTAACCACCACACGGGTGCAGCATGCCTC
GCCAGCCGGCACCTACGCCCACAC
>1597
CTCTGCATGAGGAGGGGACACGGGGCCTAGCCATGGCCCAAAGTCCACCTGCCCCATC
CTCTGTTCCCAGGTGATCCTAGGTGGAGGCCGAAAGTACATGTTTCCCATGGGGACCC
CAGACCCTGAGTACCCAGATGACT
>1598
CAGCAGGGGGAGGGCAGAGGTGTGGGGCTCGGGGCTGTGGGCTGAGGCCTGGCTCTC
TCCCTCCCCGCAGGGTGCCCGGTACGTGTGGAACCGCACTGAGCTCCTGCAGGCTTCC
CTGGACCCGTCTGTGACCCATCTCA
>1599
CTGTGTGCACATCCGCCAGCACCCTCCCACCCCCAGCCTGCCAGTCACCACAGGACCC
CTTGTCCCACAGGTCTCTTTGAGCCTGGAGACATGAAATACGAGATCCACCGAGACTC
CACACTGGACCCCTCCCTGATGGA
>1600
CCCTGGGAGTGGGGGGTTGGGGGTTGGAGCAGGGCAGGCTCAGCATCTCCCCCCTCT
GGCCTTCCTGCAGGTGGTCGCATCGACCATGGTCATCATGAAAGCAGGGCTTACCGGG
CACTGACTGAGACGATCATGTTCGA
>1601
TGGCGGGAAGGGGTCACCTCTTGTCTGCCTGGAACTGAAACTTCCTACTGAAACTGAA
CCCTCCAACCAGGGAGCCCCGAGTATCGGCAGCAGTCAGCAGTGCCCCTGGACGGAG
AGACCCACGCAGGCGAGGACGTGGC
>1602
CCCAAGCTGTTCCACACACAGGGCACCCCCTCAGCCAGGCTGACCTGATCTCTACTCT
CCCCCTGGCCAGCTGAGGAGGAGAACCCGGCCTTCTGGAACCGCCAGGCAGCTGAGG
CCCTGGATGCTGCCAAGAAGCTGCA
>1603
GGCACCCGGGACCTTCAGTGGTTCCAGGACAACCCTGGGGCCCAGGACTCACACATT
TCTGCTCCTTCAGGGTTGGGGGTGCCCACGGTGACAGCCACCAGGATCCTAAAGGGGC
AGAAGAATGGCAAACTGGGGCCTGA
>1604
CAGAACCAGGTCCTTGGTTGGGGTCTGGGTGTCCGCCCCGAAGTAGAGCTCAGGGTG
TCTCCGTTCGCAGACATACAATGTGGACAGACAGGTGCCAGACAGCGCAGCCACAGC
CACGGCCTACCTGTGCGGGGTCAAGG
>1605
TGGGGCCCGCTGTGGGGTCAGGACCAGGCCCAAGATCTCGGTCACCGATCCTGACCTC

TGTCACCCTCAGGAAAGTCAGTAGGAGTGGTGACCACCACACGGGTGCAGCACGCCT
CGCCAGCCGGCACCTACGCACACAC
>1606
GGCTATGCATGAGGAGGGGGCACGGGGCCAGCCAGGCCCCAAACCACCTGCCCCAT
CCATTGTCCTCAGGTGATCCTTGGCGGAGGCCGCAAGTACATGTTTCCCATGGGGACCC
CAGACCCTGAGTACCCAGCTGATG
>1607
GGCACGGCAGGGGGAGGCCAAGTGTGTGGGTCTCAGGGCTGTGGGCTGAAGCCTGGC
TCTGTCCCTGCAGGGTGCCTGGTATGTGTGGAACCGCACTGAGCTCATGCAGGCGTCC
CTGGACCAGTCTGTGACCCATCTCA
>1608
CTGTGTGCCCATTTGCCAGCACCCTCCCGCTCACAGCCTGCCAATCACCACCAAGCTC
CTTGTCCCACAGGCCTCTTTGAGCCCGGAGACACGAAATATGAGATCCACCGAGACCC
CACACTGGACCCCTCCCTGATGGA
>1609
CTGGGGAGTGGAGGAAGGCGGGGCGCGGCAGGGCAGGTTCAAGCATCACCCCCCTCT
GGCCTTCCTGCAGGCGGCCGCATCGACCATGGTCATCATGAGGGTGTGGCTTACCAGG
CACTCACTGAGGCGGTCATGTTCGA
>1610
TGCCTGGCACATAGGAGGCACTCCCACAGCTGTGGTCAGCTCAACTACAGGGACCCGC
ATCTCCCTACAGGGTTGGCCCCCAGCAAGGCTCAGGACAGCAAAGCCTACACGTCCAT
CCTGTACGGCAATGGCCCGGGCTA
>1611
CAGGGATGGGGGCATTCGCGGGAGGGGGACGCCGCCTGCCTGCCCTGAAGTGCACTC
ACCCTCCTACCAGGGAGCCCCGATTACCAGCAGCAGGCGGCGGTGCCCCTGTCGTCCG
AGACCCACGGAGGCGAAGACGTGGC
>1612
CCCTACACAACACACACACACAGGGCACCCCCCAGCCCAGGCTGACCTGATCTTTGCTCT
CCCCCTGGCCAGTTGAGGAGGAGAACCCGGACTTCTGGAACCGCGAGGCAGCCGAGG
CCCTGGGTGCCGCCAAGAAGCTGCA
>1613
CGGCGCCCGGACCCTCAGTGGTTCCAGGAGAGCCCTGGGGCCCAAGCCTCACACATT
TCTGTTCCTTCAGGGATGGGGGTGTCTACGGTGACAGCTGCCAGGATCCTAAAAGGGC
AGAAGAAGGACAAACTGGGGCCTGA
>1614
GAGGACAGAGATCAGGGTCTGGGTCTCCGTGTCTGCCCCAGAGAAGAGCTCAGAGTG
TCTCTGTCCCCAGACATACAATGTAGACAAACATGTGCCAGACAGTGGAGCCACAGCC
ACGGCCTACCTGTGCGGGGTCAAGG
>1615
TGGGGCCCGCTGCTGGGTCACGGCCAGGTCACAGACGTTGGTCACATATACTGACCTC
TGACACCCTTAGGGAAGTCAGTGGGAGTGGTAACCACCACACGAGTGCAGCACGCCT
CGCCAGCCGGCACCTACGCCCACAC
>1616
GCTGTGCATGAGGAGGGGGCACGGGGCCAGCCAGGGCCCCAAATCCACCTGCCCCAT

CCTCTGTTCCCAGGTGATCCTAGGTGGAGGCCGAAAGTACATGTTTCGCATGGGAACC
CCAGACCCTGAGTACCCAGATGACT
>1617
CAGCAGGGGGAGGGCAGAGGTGTGGGGCTCAGGGCTGTGGGCTGAGGCCTGGCTCTC
TCCCTCCCCACAGGGTGCCCGGTACGTGTGGAACCGCACTGAGCTCATGCAGGCTTCC
CTGGACCCGTCTGTGACCCATCTCA
>1618
CTGTGTGCACATCCGCCAGCACCCGCCCACCCCCAGCCTGCCAGTCACCACAGGACCC
CTTGTCCCACAGGTCTCTTTGAGCCTGGAGACATGAAATACGAGATCCACCGAGACTC
CACACTGGACCCCTCCCTGATGGA
>1619
CCCTGGGGAGTGGGGGTTGGGGGTTGGAGCAGGGCAGGCTCAGCATCTCCCCCCTCT
GGCCTTCCTGCAGGTGGTCGCATCGACCATGGTCATCATGAAAGCAGGGCTTACCGGG
CACTGACTGAGACGATCATGTTCGA
>1620
CGGTGCCTAGCACGTGGGAGACACTCCACAGCTGTGTTCAGCTCAACCACAGGGACC
CCTCTCTCTGCAGGGCTGGCCCCTGGCAAGGCCCGGGACAGGAAGGCCTACACGGTC
CTCCTATACGGAAACGGTCCAGGCTA
>1621
CGGGAAGGGGTCACCTCCTGTCTGCCTGGAACTGAATGAACCCTCCTACCGGAACTGA
ACCCTCCAACAGGGAGCCCCGAGTATCGGCAGCAGTCAGCAGTGCCCCTGGACGAAG
AGACCCACGCAGGCGAGGACGTGGC
>1622
ATTTTTTCAGGCAGGATGCATATTATTTATTAATTCACTCCTCTTTTTTCCTCATTTAATAT
AACTGCAGGAGCCCCTAATGGAAGAATATGCTATTGCTGCACAAGTCTTCAAGCTGAG
CACCTGTGATATGTGCGAAG
>1623
AGAATGCAATTATTGTCTATACCAGGGTGAAAAGAGACAACAATCATTCTGCCATCCTT
TTCTCTTATAGGAGAAAGTAAAGTTTCTGGGCGACAATTACCTTGAGGAAGGCCCTGC
TGGAAATGATATCCGGAGGACAA
>1624
TGCTCACTCCTCTTGATTTCCTCCAAGTCAGTGAGGTTTATCCCTTTCCCTGTATTTTCC
TTTTCTAAAGAATTTGCTGGACCATTTGGAAGAAAAGATGCCTTTAGAAGATGAGGTC
GTGCCCCCACAAGTGCTCAGTG
>1625
AGATGGCATAAGCTCTTCTCTTTCCCTTCCCCATGGTTATAACTGTCCCTGTTGGCTTCC
TTCCTGTCAGGTGCAGCATGCCAGCAAGCAGATCACTGCAGATAAGCAATACAAAGGC
ATTATAGACTGCGTGGTCCGTA
>1626
TTAGGGGATGTGGGGAAAGGAAGTCAGTAAAACTTCTGCTTTTTGGTAAAGATCTCTT
TCCTATTCCTAGGAATGCTTCCGGATCCCAAGAACACTCACATCGTCATCAGCTGGATG
ATCGCACAGACTGTCACTGCTGT
>1627
TTTAGCCTAGTGAATCTTAGGATTTTTCATCGGCCTTCAGTCACTAACTCCACGTCTTTA

TTCTTTGCAGCTGACATCATGTACACAGGCACGCTTGACTGCTGGCGGAAGATTGCTC
GTGATGAAGGAGGCAAAGCTTT
>1628
TCTGCCCCACCTCTTGATTGCTTTCCCTTTTCTTATAGTTTTTTATGCTAATTCTGTTTCAT
TTCTATAGGCGAGGAGGAGCATGATCAGGAAGGCCGGGTGATTGTGGCTGAATTTGAC
TCGTTTGTGCTGGTAACAGC
>1629
AGCTCAGAGCTGGAGCAGAGGGGCCACACAGGAGACGGGGCCTCATGAATTGCTCTC
TGTTACCACCCAGGAGCCAGGGCTGAGGTCAGTGCTGACCAGGTGGCCACAGTGATG
TGGGACTACTTCAGCCAGCTGAGCAA
>1630
ATACAAGCAGACAAAGTCTTGCCGTGTAAATGCCAAATGTAACGTGGCCTCCTTGTGC
CCTTCCCCACAGTGCCCTCTTCCAGGACAAACTTGGAGAAGTGAACACTTACGCAGGT
GACCTGCAGAAGAAGCTGGTGCCC
>1631
GAGCAAGACTCCATCTCCAAAAAAAAAAAAAAAAAAAACAAATTTTGAACCCCTGCCC
ATCTTCCTGGCAGGCCCAGCCCCAGCCCAGGGGACCCCAGACGTCTCCAGTGCCTTGG
ATAAGCTGAAGGAGTTTGGAAACAC
>1632
CCGTCTCAAAACTACAACAACAACAACAACAACAACCAAATTTTGCACCCCTGCCTCA
TCTTCCTGGCAGGCCCAGCCCCAGCCTAGGGGGCTCCAGAAGTCTCCAACCCCTTTGA
TGGCCTGGAGGAGTTAGGAAAGAC
>1633
CCCCTAGGTAGCTAGATGCCTGGACGGGGTCAGAAGGACCCTGACCCGACCTTGAACT
TGTTCCACACAGGATGCCAGGCCAAGGTGGAGCAAGCGGTGGAGACAGAGCCGGAG
CCCGAGCTGCGCCAGCAGACCGAGTG
>1634
GCCCGCCCCATCCCAGCCCTTCTCCCCCGCCTCCCCACTGTGCGACACCCTCCCGCCCT
CTCGGCCGCAGGGCGCTGATGGACGAGACCATGAAGGAGTTGAAGGCCTACAAATCG
GAACTGGAGGAACAACTGACCCCG
>1635
GAACCCTCGTCTTTCGCCCCCGGGCCCTGCTCCTTCGGCCCCGCGTCACCAGGCCTGT
CCTTGGGTCCAGGGACATCTCGCCCGTCCTGAAGGACCCCGCCTCCTTCCGCGCCGCC
ATCGGCCTCCTGGCGCGACACCTG
>1636
TTGCCACCGCCGAACCCTGCCCCTGGGGATTCCCATGCTGCCGAGTCACTCCTGTCAC
TTACCCTGACAGGCCTAGACTCCCGAGGCTTCCTCTTTGGCCCCTCCCTGGCCCAGGA
GCTTGGACTGGGCTGCGTGCTCAT
>1637
AGCTGCCAAGCTGTGCAACAAAGCTGTTTTCTGCGGGAGGCTGAGGACCACACACCA
CTTCCCACTCCAGGCTGAGCTGGAGATTCAGAAAGACGCCCTGGAGCCAGGACAGAG
GGTGGTCGTCGTGGATGATCTGCTGG
>1638
CAGGGGCTGGCCTGGAGCACCTGCTCTCTGCAGCCCAGGCCAACTGGGGACCTCACC

CTCCCATCCCCAGGAACCATGAACGCTGCCTGTGAGCTGCTGGGCCGCCTGCAGGCTG
AGGTCCTGGAGTGCGTGAGCCTGGT
>1639
CCTGGCTGAGTGGTGGGAATGGAGGCCCCAGCCCCTCTCTTCCCTGACTCTCCGGCTC
TCCCTCCCTCAGGCTGGCCGTGAGTACTCACCTGCCGCCACCACGGCAGAGAATGGGG
GCGGCAAGAAGAAACAGAAGGAGA
>1640
AGGGATGGGCATGGTGACTGGCTGGGTTGGCTCCGGATGCGTGCCCCTACGCCTCTCC
TTGCTCCCTCAGGGCCTCACCAACCAGCGGGCTCAGGACGTTCTGGCTCGAGATGGGC
CCAACGCCCTCACACCACCTCCCA
>1641
TCCCAAGTGGCAGCTGCCCCTTTAGGGTTGGGGGGAAGGTCAGGTCCCTGAAACTCTT
TCTCCTTACCAGCTATATCTGGGTGTGGTGCTGGCAGCTGTGGTCATTGTCACTGGCTG
CTTCTCCTACTACCAGGAGGCCA
>1642
GGGAGACCAGCAGGAGAAGAAGGCAGGGGCAGAGACAAGCATTTCATGAGCTGCCT
GTGGCTCCCCACAGCAAGCCCTTGTGATCCGGGAGGGAGAGAAGATGCAGATCAACG
CAGAGGAAGTGGTGGTGGGAGACCTGG
>1643
GTTCAGGAGACAGCTGTGTGCATACAAGTGGCTCTGCCAGTCTGATGACTATGCACTC
CTTCCTCCTCAGGTGGATAACTCATCCTTAACAGGAGAGTCGGAGCCCCAGACCCGCT
CCCCCGAGTTCACCCATGAGAACC
>1644
AGGAAATAGGATGGGACTGCAGTCCCTGGGAGCCACAAGGCACCCAACCTGATGCCC
CACCATGTTGCAGGCACTGCCAGGGGCATTGTGATTGCCACAGGAGACCGGACGGTGA
TGGGCCGCATAGCTACTCTCGCCTC
>1645
GTCTAGGGTAAGGTTATGGCCATCTCCGGCTTCAGCCTTAACCTTTTTATTCTCCTCTT
TCTCTACCAGGTGTGCCTGACCCTGACAGCCAAGCGCATGGCACGGAAGAACTGCCTG
GTGAAGAACCTGGAGGCGGTGG
>1646
GTGGGGTGAGTGGTTGAGATAAAGGCTCTAAAGGGAGCCACGCTCCTGGTTCCCCCTC
ATTTCCTCCCAGGGGCCACTTTTGACAAACGATCCCCTACGTGGACGGCCCTGTCTCG
AATTGCTGGTCTCTGCAACCGCGC
>1647
TGCCGCCTTCACCTGATCCTCCACTCCCTTCCCTCCCATGCTGACACTGAATTCTTGTCT
CTTCTGGCAGCGGGACACAGCTGGTGATGCCTCTGAGTCAGCTCTGCTCAAGTGCATT
GAGCTCTCCTGTGGCTCAGTGA
>1648
CAGCTGCTGCTCTATGCCGCGCTACCAAGACAAGTATGGCCCTCTCTGTAACTACCTGT
TGTCTCTCCAGCTGTCTATCCACGAGCGAGAAGACAGCCCCCAGAGCCACGTGCTGGT
GATGAAGGGGGCCCCAGAGCGCA
>1649
GGTGGGGGACTGTGGGGGCGTCCAGGAAGCCACTCTGCGGATCTCACTGATCCCTTCT

GCCCCCCTTTAGGATTCTGTCAACTGAATCTGCCATCTGGAAAGTTTCCTCGGGGCTTC
AAATTCGACACGGATGAGCTGAA
>1650
TTGGGAGAGGAACAGGAGGGGGATAAACCCTTAATACTACTTTCCTGTTGTCTCCTCTC
CTTCCCACTAGGTGATCATGGTAACCGGGGATCACCCTATCACAGCCAAGGCCATTGCC
AAAGGCGTGGGCATCATATCAG
>1651
GAGGCAGGGGGCAGGAGGGGCTGGTACAGGTGCCAGGGGTCAGCTGTCTCTGTCCCC
ACCCACCCTCCAGAGAAGCCAAGGCATGCGTGGTGCACGGCTCTGACCTGAAGGACA
TGACATCGGAGCAGCTCGATGAGATC
>1652
ATGGAGTGAGAGGCGAGGAGCCAGGCTTGGGAAGGGGTTTCGTCCTCAAGTGTGGCC
GTCTTCCCTCCAGGGAGCCATTGTGGCCGTGACGGGTGACGGGGTGAACGACTCCCCT
GCATTGAAGAAGGCTGACATTGGCA
>1653
CATGCTTCAGGGGCTGGGGGTGGGGAAGAGTCCCTCTGACCTCCCTGATGCCCTCAGA
ATCTCCCCACAGGCCGCCTGATCTTTGACAACTTGAAGAAATCCATCGCCTACACCCTG
ACCAGCAACATCCCCGAGATCAC
>1654
GATCAATTGCTTCTGTCATCTCCTACGTCCCTTCAAATGCCCTCCCTGCCCCATTTCCTA
CCCCACACAGGTCCCTGCCATCTCCTTGGCCTATGAGGCAGCTGAGAGTGATATCATGA
AGCGGCAGCCACGAAACTCCC
>1655
GCCCTGAGGGGCTGTGCCCCTTCTGCTTCCTGCTCTGACCCTGCCCCTGCCTTTGGCCC
TCTACCCACAGGGATGATCCAGGCACTGGGTGGCTTCTTCACCTACTTTGTGATCCTGG
CAGAGAACGGTTTCCTGCCATC
>1656
AACCATGCTACCTTCTGGTCCTACCCTTTCCTCCGACACTCTCATCTGTCTCTGCCCACC
CTCCCTCCAGACCTATGAGCAGCGGAAGGTGGTGGAGTTCACGTGCCACACGGCATTC
TTTGCCAGCATCGTGGTGGTGC
>1657
CCAGGGGTATCCCAGGATCTCTGCCTCCATGATCCCCCTTCACCTGCCACCTCCTTTCTT
TGCCTTTCAGGAACAAGATCCTGATTTTTGGGCTCCTGGAGGAGACGGCGTTGGCTGC
CTTTCTCTCTTACTGCCCAGGC
>1658
AATGCTCCTTTATGTGACAGCCACCAAGCCAACCTCTGATGCTGCTGACACTCTCCTCC
ATTGCTTTCAGAGTCACCTGGTGGTTCTGCGCCTTCCCCTACAGCCTCCTCATCTTCATC
TATGATGAGGTCCGAAAGCTC
>1659
CCCTAGGAAATTGATCTCTTGCCTCCTTTTAAGCTCATGCTGCAATCTCCACTCCCAATC
TCTCTAACAGGCTGGGTGGAGAAGGAGACATACTACTGACCCCATTGGAAGAAGAAC
CAGGCATGGAAAGATGGGGAGCT
>1660
ATTTTAGAGGCTCTGTTTACCCACTTCAACAGACTCTCCATTTCTGATGTACTTTTTTCT

ACCTCTACAGGCTGGTGGGATAAGAACATGTATTATTAAGACCACCTCCCTTCCTATGTC
TCTCAGCAGCACGTTGGGGC
>1661
CCCCGTTCCCTCGTCCACAGCCCCGCCCTGTTCCCCCGTCCACAGCCTCGCCTCTCCCC
CCCGCCCACAGCTGTCCATACATACGCTGGAGGACCCGCGGGACCCGCGACACTTGCT
GGTGATGAAGGGCGCCCCCGAGC
>1662
GTCCCTCTCTTCCTGTGTCCCTGTCTCTGTGTCCATCTCTGTCTCCCTGTGTCCCCTTGT
CTCTCTCCAGGTGCCATTCAGTCCTTTGCTGGCTTCACTGACTACTTCACGGCAATGGC
CCAGGAGGGCTGGTTCCCACT
>1663
TTTGTTTAAGCTTGTAAGTTTTATAAAGGCTTTGTTAAAAATCAAATTTTTAAGAATACT
TTTGCACTAGGTCTCCAGAAATGCTTTGGGTTCATCTTTCATTGCTGCAAGGAACTTCC
ATGCCTCTAACACTCATCTTC
>1664
GGATTACAGCTGTGAGCCACTGTGCCTGGCCCAGATTATTTTTTAATTGAATTGAATTG
TCTTCCATAGGGACTGCTGAGATGTCCTCTATTCTTGAAGAGCGTATTCTTGGAGCTGAT
ACCTCTGTTGATCTTGAAGA
>1665
ATGTAGTATTGGTGTATGGGGAGGAGTGCCCATTCAGCTTCATTGATGTTCAATTGTGTC
TTTTGTGCAGGGTATGTCCTTGAACTTGGAACCTGACAATGTTGGTGTTGTCGTGTTTG
GAAATGATAAACTAATTAAGG
>1666
ATATAGTTGCAGCTTCTAACAAACATCAAAACTGATTATGCTTAGGGTTTTTCTTTTTATT
TTTTAACAGGGTCCAATTGGTTCCAAGACGCGTAGGCGAGTTGGTCTGAAAGCCCCCG
GTATCATTCCTCGAATTTCAG
>1667
TCTCTTTCTTTCCCCACAGGGAAGTCCTTTTAAGTTTAGGAAGATTTATATTTACTTTCT
CTTTTTAAAGGAAAACCTCAATTGCTATTGACACAATCATTAACCAGAAACGTTTCAAT
GATGGATCTGATGAAAAGAAG
>1668
AGGTAATTTTTATAGTAGCCTCCAATTTAAAACCACAATATTGATAAAATTGTAAAATATA
TTTTCTCAGATGCCATGAAGTACACCATTGTGGTGTCGGCTACGGCCTCGGATGCTGCC
CCACTTCAGTACCTGGCTCC
>1669
CTGAAGTAAAAGTTCACATATGCAACTTCTATTTAATAGGTTAACTTCACAAACCTATTC
TGTACCATAGGCTGTTGCTTACCGTCAGATGTCTCTGTTGCTCCGCCGACCCCCTGGTC
GTGAGGCCTATCCTGGTGATG
>1670
TGACTTAAATTTGAAGATAGTTGATTTCTTACTTTAAGCTTGCTAGCGTGCGTATTTCCTT
ACATTATAGATCTTCTTGGAAACAGAATTGTTCTACAAAGGTATCCGCCCTGCAATTAAC
GTTGGTCTGTCTGTATCTC
>1671
TAATATATTAACTATATTTGTATAGTGCTCTGTGTCACTGAGTTACAGTACAGTAATTTCT

GTATTGCAGGTAGCAGGTACCATGAAGCTGGAATTGGCTCAGTATCGTGAGGTTGCTGC
TTTTGCCCAGTTCGGTTCTG
>1672
TCCTGACCTTGGTAACATGGCATTCATTTCTTTGAGCAACTCACTTGGCATTCTTCTTGT
ATCTTTTCAGCTCCCATGGCTATTGAAGAACAAGTGGCTGTTATCTATGCGGGTGTAAG
GGGATATCTTGATAAACTGGA
>1673
TCTGTAGTTAGGCTAGTCATTAAAACTTTTTCCAGGTCAGAACTTACGACCTGCTTTTAT
TTCCAAATAGGGCTGATGGAAAGATCTCAGAACAATCAGATGCAAAGCTGAAAGAGAT
TGTAACAAATTTCTTGGCTGGA
>1674
TTAAGTCCTGGCTTCCGATGACCTTTAACTGGCCTGACCCCAGCTCCTTCCAATATCTTT
TGTCTGTTAGTCAGGGACTATGCGGCGCAAACATCTCCTTCGCCAAAAGCAGGCGCCG
CCACCGGGCGCATCGTGGCGGT
>1675
GTCTTGGTCGATTTAGGTCTTGATACCAATTTATCCTTATGTATAACCCTGCGATGCCTGT
ATCTAACAGGTGAGAGCACAGTAAGGACTATTGCTATGGATGGTACAGAAGGCTTGGT
TAGAGGCCAGAAAGTACTGGA
>1676
AGGGATATATAAGATTATCATTTGCTTTCAACAAGGTGAATAGATACTATCCTCTCTTCTA
CCTTTACAGATTTGCTCCCATTCATGCTGAGGCTCCAGAGTTCATGGAAATGAGTGTTG
AGCAGGAAATTCTGGTGACT
>1677
ACTTTTTTTGAAGTTTGCTTATTTAGGAAAGCAGAACTCTTAAATATTCTTTCTTTTCAA
CCTCTTATAGGGCTTTTTGGTGGTGCTGGAGTTGGCAAGACTGTACTGATCATGGAGTT
AATCAACAATGTCGCCAAAGC
>1678
TTTGGGAACCTTCAGTATGTGGCTTCTCTACTCCTTTGTCAGGTTTTACGATTATGTATG
ATTACTGCAGGTAGCGCTGGTATATGGTCAAATGAATGAACCACCTGGTGCTCGTGCCC
GGGTAGCTCTGACTGGGCTGA
>1679
ACCTGGTTCATCTGGCCTTTCTTCGGATGAGGCTAAGGATGTAGACACCTAAGACCTTT
TTTTCTTTTAGGTGTCTGCATTATTGGGCCGAATCCCTTCTGCTGTGGGCTATCAGCCTA
CCCTGGCCACTGACATGGGTA
>1680
AGCGAGACCCTCTCAAAAAAAAAAAAAAAAATTTACTTTGGTTCCTGTTCTTCATGGG
ATCTCACTTTAGGCTATCTATGTGCCTGCTGATGACTTGACTGACCCTGCCCCTGCTACT
ACGTTTGCCCATTTGGATGCTA
>1681
TTTCCCTTTGTTAGCTTGTCCCAAATTAAAGGAATGAGAATACTTAACTCAGTCTTCTTT
TTTCTCATAGGACTACAAATCCCTCCAGGATATCATTGCCATCCTGGGTATGGATGAACT
TTCTGAGGAAGACAAGTTGA
>1682
TGTTACATGATGCAGAAAGTTGATATCCCTCCGCTTCTTACTCTTTTTTTTTTTCTCCCCC

ATCATACAGGTGAATATGACCATCTCCCAGAACAGGCCTTCTATATGGTGGGACCCATT
GAAGAAGCTGTGGCAAAAGC
>1683
AAGCCGGCTTGCTCTGTGCCCAGGCCCCAGCCTGGTGTCCTCCCTCTGCCCTTTCCTCC
GCTACTCTCAGGAACCCCGGGGTTAGCACCGTGGTGCTGGGTGCCTATGACCTGAGGC
GGCGGGAGAGGCAGTCCCGCCAG
>1684
TGCTCCCAGGGGTCCCCATGAGGCTCCAGTCCCCAGGGCCACCCTCCCCTGACTCCAT
TTCCTTCCCCAGCTGGACCGTGAGGCCAACCTCACCAGCAGCGTGACGATACTGCCAC
TGCCTCTGCAGAACGCCACGGTGG
>1685
GCCTCTGCAGTTCTGGGGTGGCGTGGGAGCCAGGCCCTGGGACGCCCTGACACAGCT
GCTGCCTGCCCAGGGGGACGGGGGCACCCCCCTCGTCTGCGAGGGCCTGGCCCACGG
CGTGGCCTCCTTTTCCCTGGGGCCCT
>1686
CTGGATTATTCTGAGTCCAAGCTAGGCCCTTTTGCTAATCATGTTCATACCTCTTATCTTC
CTCCCACAGCTCCTGGGCAACGTGCTGGTCTGTGTGCTGGCCCATCACTTTGGCAAAG
AATTCACCCCACCAGTGCAGG
>1687
GTCACCTGATTACAAATTTATGTTTTTAGATTTGTTTTTCCATTTTCAGCTTGACATTTAC
TCATTTCAGAGAAACCCTGTGGTTTTCCTCATGTGGAAAATGGAAGAATTGCCCAATAT
TACTATACTTTTAAAAGCTT
>1688
CTTAATTTTACAATTTAGTAAAAGACAAGCTTAGTTTCATCATTAAGTTAAATAATTTTTT
TTCCCATAGAAAAATGCACTAAGCCTGACCTGAGTAATGGTTACATCTCTGATGTAAAG
TTATTGTATAAAATTCAAGA
>1689
CATATTTTGAGATTTTTTATAATCTAGGTTATTAAAAAGTTTATAAAATAAATAATTTTTAT
AATTTTAGAAACGTGTTTGGCTCCTGAATTATATAATGGAAATTATTCCACAACACAGAA
AACATTCAAAGTGAAGGA
>1690
AATAGTGCATTTTGTTGTAGCAGAAAAAATAAACATTTGTTTGTAATTTTATTCATTCTTT
TATTTGCAGAATTAAAGTGCTCTTCTTTAAGATTAATTGAAAATGGTTATTTTCATCCTGT
AAAGCAAACCTATGAAGA
>1691
TATTTTAATTTAATCTTTATTATTTTTTGTAATTATATGACTAAAGGTGCTGATTAATTTTTT
CCATAAGGAAGAAGAAACAGATGTCCTCCTCCACCTCTGCCCATAAACTCCAAAATTC
AAACACATTCAACAACTTA
>1692
GAAGCTTTGCTAAAATGAAATCTGCATGTGTAGCTAAATGGTCAGTTCTCTTTCAACTG
TACCTTTTCAGAAGGACAGGAGAAGGTAGCCTGTGAGGAACCACCCTTCATTGAAAAT
GGTGCAGCAAATTTACACTCTAA
>1693
ACTATAGTATGAGTTACTATAGTATATTGTTATGAGCTCATATTAATTTTAAAACAACCTC

TTTTCTTAGAAAATAATGAGAATTGTAAGCATCCTCCTGTTGTAATGAATGGGGCTGTTG
CAGACGGGATATTGGCAAG
>1694
TGTTTTAAAAAATATCACTTTTATATTTGTCAGACAAGTAAAGAATTTAAAAGAGCATTT
TGTTTTACAGAACCATGTACTGTTAATGTGGATTACATGAACAGAAATAACATAGAAAT
GAAGTGGAAATATGAAGGGAA
>1695
TGTAGCACTTATAAAGTACTGGATGTTCATTATAGCAATTCATTGTATACTTTAAAACTTA
TTTTTGCAGAATCTAAAGGAATGTGCACATCTCCTCCTCTTATTAAACATGGAGTCATTA
TTAGTTCAACAGTAGACAC
>1696
TGTTCTTACCAAGATGTAACAAGATAGTTTTCTTCTTTTTCTTTCTTTTTTTTTAAATTGT
TGTTCCCAGAGCCATGCACATTATCTTTTACTGAAATGGAAAAGAATAATTTACTTCTGA
AATGGGATTTTGACAACAG
>1697
GGCTGAGATTGTAATTAACACCTGACTGCAATTGATGCTTATTTCAAAAATCTCTCTTTT
TCCCCTCAAGCACTCTGTCTTATCAAGAACCCTTAAGAACATAGAAATGAATGGCAGA
AAGAGGAGTCATATTTCAATAC
>1698
GCAAAAAAAATGGGGTGGAGGAAAATGAGGCATCTGTGTGAGTATATAACCATTTGAC
ATCTCTTTTTAGCCCTCTCCAGGGTCACCCTAGAATCAGATCTGCTCCCCAGCATCTTCT
GTTTCCTGGTGAGTGATTCCTG
>1699
TCACCTTTATCAGAAAACTTCTCAGCCAGATACAGAAATCATTCCAATGACCTGACCTG
TGTTCACACAGAACTCCAGAACAAGACCAAGCTGACAGTGCTGGAAGGAGACATTCT
GGATGAGCCATTCCTGAAGAGAGC
>1700
AGATCTGTGTTCGTGGTTGGCACCTCTTAGGGATATATCCTGACAGTGACAATATGCTCT
TCATGGACAGGTACCCAGCTCCTGTTAGAGGCCTGTGTCCAAGCTAGTGTGCCAGTCT
TCATCTACACCAGTAGCATAGA
>1701
AAGGGGAGAGCCCAGGGAGGGAAGGGAAGAATGGGCAGATGGGAGGGCAGCCAGCT
TCTGCTCACTCCAGGCACAGCCATGGGAGATTCGGAGATGGCAGTCTTTGGGGCTGCC
GCCCCCTACCTGCGCAAGTCAGAGAA
>1702
ACTCCTAACCCTCTTGAGGAAGGAGGGAAAGCCCAGGCTGACAGGAGGGCTTGGGTG
GGGGCTCTTGCAGACAGTGACCGTGAAGGAGGACCAGGTGATGCAGCAGAACCCACC
CAAGTTCGACAAAATCGAGGACATGG
>1703
CTCTAACTCCCAAAATCACCAGCCCTCCCCCTTCGCAACTGGCAAGTCACTGCTCCTTT
TCTATCCCCAGACCTACTCGGGCCTCTTCTGTGTCACCGTCAACCCTTACAAGTGGCTG
CCGGTGTACACTCCTGAGGTGG
>1704
CTCTCCTGATTTGAGGCTTGCTGGTCTCCAGTAGTATTGTTCACTGCCCAATAAGCCCCT

GTCTTCACAGCGGAGAATCCGGAGCAGGGAAGACAGTCAACACCAAGAGGGTCATCC
AGTACTTTGCTGTTATTGCAGCC
>1705
CCCTCCAAGGTCCTGTACCGCAGAAGGGAGGGAGAAGAGCTCTCACCTGCCTCCTTCT
TGGCCTCTGCAGGGCACCCTGGAGGACCAGATCATCCAGGCCAACCCTGCTCTGGAG
GCCTTTGGCAATGCCAAGACCGTCC
>1706
TATCATTAATTTTCCTTGTGCCCAAACCCTAACTTTTCTTTCTCTCCTTCTTCTCCCCACC
TGTTCACAGATCTTCTGGAAAAATCCAGAGTTATTTTCCAGCTGAAAGCAGAGAGAGA
TTATCACATTTTCTACCAAAT
>1707
GTGCTGTGGCGAGCAGCCTCCATGAGAGCCGGGGGCTTGTGTCCCACCCTAACCATGT
TTTTTCCCCTAGACATGCTGCTGATCACCAACAACCCCTACGATTATGCATTCATCTCCC
AAGGAGAGACCACCGTGGCCTC
>1708
CTTACAAGGGATCTCACTTACCCATCATACTTCTTTTTCTGGGGTCCGCCAATATGGGGC
CTCCCTACAGAACGCTTTTGATGTGCTGGGCTTCACTTCAGAGGAGAAAAACTCCATG
TATAAGCTGACAGGCGCCATCA
>1709
AGGCATGAACCACCACACCTGGCCAGCAGTCATCTCTTTACCAACTTTGCTACTTGCCT
TTTCCTTCCAGAGGCTGACAAGTCTGCCTACCTCATGGGGCTGAACTCAGCCGACCTG
CTCAAGGGGCTGTGCCACCCTCG
>1710
GGGATGAGAGTTTTCAAGTTCACTCTTCCCAACAACCCTGCTCAATATGGGTCTCTCCT
CCACCTTGCAGGTGATATATGCCACTGGGGCACTGGCCAAGGCAGTGTATGAGAGGAT
GTTCAACTGGATGGTGACGCGCA
>1711
GGACGGGCACAGCCCCAATGGCCACTCACACCCACTTTCTGACTGCTCCCACCCCTCA
TGCCCCCTGCAGTTCAACAGCTTTGAGCAGCTCTGCATCAACTTCACCAACGAGAAGC
TGCAGCAGTTCTTCAACCACCACA
>1712
TGACCATAGAGCAGAATCCATGTCCACCTGTGTGAAGGACACTCAGTGATGCTCTCTC
CTGCTTCCTCAGCCCATGGGCATCATGTCCATCCTGGAAGAGGAGTGCATGTTCCCCAA
GGCCACCGACATGACCTTCAAGG
>1713
TCTCCTCTCTTCTTCCTGCATCTCTTTCTGGCATTTTCTTTACTTTTCTCTATTGCATTTTT
GGCCACAGGAAAATCTGAACAAGCTGATGACCAACTTGCGCTCCACCCATCCCCACTT
TGTACGTTGTATCATCCCTA
>1714
AGCCAGGATCAGAACCCAGAACTTCAGTCCAGTGTTCTCACAGACTCCTCCTACTTCC
TTCTTGCCACAGGCGTGATGGACAACCCCCTGGTCATGCACCAGCTGCGCTGCAATGG
TGTGCTGGAGGGCATCCGCATCTG
>1715
GACCCTAGAATTCTATGGGCAGAGCAGATCACTGCAGAGCATGGGTGACTCTGGACAC

TTCCCTCCTCAGGTATCGCATCCTGAACCCAGCGGCCATCCCTGAGGGACAGTTCATTG
ATAGCAGGAAGGGGGCAGAGAAG
>1716
AAGGTAATCCCCACCATCTCTTTCCCTCGTACCCCTCCCTAGTCATGGCCAACACACAC
CTTGCCTGCAGGTGTTCTTCAAGGCCGGGCTGCTGGGGCTGCTGGAGGAAATGAGGG
ACGAGAGGCTGAGCCGCATCATCA
>1717
TCCCAAGGTTTCAGGACCTCAGGTAGGAAGGAGGCAGAGGCTCAGCACTCCTTTCAAT
GGGCCCCTGCAGAGACTCCCTGCTGGTAATCCAGTGGAACATTCGGGCCTTCATGGGG
GTCAAGAATTGGCCCTGGATGAAG
>1718
TCCTTCCCTCTACCTGCAAGAATGAGGACCTTACCCCCTGAACAGCCTCCCCTCTGTTC
CTCACCTTCAGGAACAAGACAACCTGGCAGATGCTGAGGAGCGCTGTGATCAGCTGAT
CAAAAACAAGATTCAGCTGGAGG
>1719
GGAGCTCCCCACAGATGGCACCAAGCTGGTGACCTTTGACCCTAAAGGAGATGGGATT
CTTGGTCGGCAGGTGAAAAACCTGACAGAGGAGATGGCTGGGCTGGATGAGATCATT
GCCAAGCTGACCAAGGAGAAGAAAG
>1720
GTAACTGAACAACAAAATCACCAAGTCAGGATGCTTTGCCTCACTGGGCCTGGCTCCT
TCTCTCTACCAGCTGGAAGGATCCCTGGAGCAAGAGAAGAAGGTGCGCATGGACCTG
GAGCGAGCGAAGCGGAAGCTGGAGG
>1721
TTCAACCACTGGTGGACCCTCCTGAGGCCCCACGAGTCTCCCTTACCTCACCATCCCTC
CTTCCCCACAGAAAAGACTTTGAGCTGAATGCTCTCAACGCAAGGATTGAGGATGAAC
AGGCCCTCGGCAGCCAGCTGCAG
>1722
CCTTTTAGAGCCGGGGGGATTCCAGTGGAGGGGTCCAGGCGGTGGGTCTGAGCCCTTT
GTGTCTGACCAGGCACGCATCGAGGAGCTGGAGGAGGAGCTGGAGTCCGAGCGCACC
GCCAGGGCTAAGGTGGAGAAGCTGC
>1723
TTGCACCTCTTACACCCCTTCATTCCCCCCGCCCCCAACATCCATCATATAACTCTCCTT
CCCTTCTCAGGCTAACCTGGAGAAGATGTGCCGGACCTTGGAAGACCAGATGAATGAG
CACCGGAGCAAGGCGGAGGAGA
>1724
ATGGGGCCTGAGGGCTAGAGGAGGAGGTGGGGATAGAGAGGAGTGCTGATCTAGAAT
GGTGCTTCCCCAGGTGAGCTGTCCCGGCAGCTGGATGAGAAGGAGGCACTGATCTCCC
AGCTGACCCGAGGCAAGCTCACCTA
>1725
GAGAAGGGCAAGGGTGGGGTTGCTTTATGGAGAAAGCTGAACCCACCTCCTGGTGCC
CACCCCTCCCCAGGCGAAGAACGCCCTGGCCCACGCACTGCAGTCGGCCCGGCATGA
CTGCGACCTGCTGCGGGAGCAGTACG
>1726
CATGTTGTTTACCTCTTGTCCCCATCCACACCCTCCATCCTCCCCACCCTCTGCCACCTC

CCCTGGGCAGGAAGAAGCTGGCCCAGCGGCTGCAGGAAGCTGAGGAGGCCGTGGAG
GCTGTTAATGCCAAGTGCTCCTCG
>1727
GGTCCTGAGACAGACCCTGGACATGGGGCTGAGGCTGGGGGGCTGAAGAGTGAGCCT
TGTCCCCGGGCAGATCCTGGCCGAGTGGAAGCAGAAGTATGAGGAGTCGCAGTCGGA
GCTGGAGTCCTCGCAGAAGGAGGCTC
>1728
AAGAGGCGTGTCCCTCCAACTCCACTGGACCTCAGCAGCCCTCAACCGAGTTACCGTG
TTCCCCACACAGAGGAGATCTCCGACTTGACTGAGCAGTTGGGTTCCAGCGGAAAGA
CTATCCATGAGCTGGAGAAGGTCCG
>1729
GTGAGCAGGCCCCCACCCTGCCCTGTGCCCTGACTGTCTGCCTGCATCCCCTCCCCCA
ACCCCTTCCCAGGCCTCCCTGGAGCACGAGGAGGGCAAGATCCTCCGGGCCCAGCTG
GAGTTCAACCAGATCAAGGCAGAGA
>1730
TCTGACAGGTGCCTTTAGTGAAGGGAACCGAGGCTGGCTCCCTGCTCATGCCCACTCT
CCTGATCCTCAGGACACCCAGATTCAGCTGGACGATGCAGTCCGTGCCAACGACGACC
TGAAGGAGAACATCGCCATCGTGG
>1731
AGCAAGGCTTGAGAGCTATGCATAGATGCTCAATGCTTTTCCTGCTCTGCCCAACCCTC
CCCCAACCCAGAACACCAGCCTCATCAACCAGAAGAAGAAGATGGATGCTGACCTGT
CCCAGCTCCAGACTGAAGTGGAGG
>1732
TCTCCGTGGGCTGGGCAGCAAGTGTGTGAGGACTTGACCAGACCATGTGCCACCTCTC
TCCTGCACACAGGCCGCCATGATGGCAGAGGAGCTGAAGAAGGAGCAGGACACCAGC
GCCCACCTGGAGCGCATGAAGAAGA
>1733
GCAGGGCGGAGGGGATGCTACCTTCTATGACTGTGCCATCTTCACCCCCTGCCTACCCT
CTGGCCCCCAGACGGAGGAGGACAGGAAAAACCTGCTGCGGCTGCAGGACCTGGTAG
ACAAGCTGCAGCTAAAGGTCAAGG
>1734
GAGCTCGTCCCCCAAGCCAGGAGTCTGAGAACCCAGGCCCCCTCTCACCTCATGCTCC
CACCTCCCGCAGGAGGAGCAAGCCAACACCAACCTGTCCAAGTTCCGCAAGGTGCAG
CACGAGCTGGATGAGGCAGAGGAGC
>1735
GTTCCCATGCCCTGTCCCTGCCCAATACCATCTCTCCAAGGACTGATTGGACTTTGTTTC
CTTTCAAAAGGGCTTGAATGAGGAGTAGCTTTGCCACATCTTGATCTGCTCAGCCCTGG
AGGTGCCAGCAAAGCCCCATG
>1736
CTTGGGTGCCATGAAGGGCTGGTGAGCCAGGGGTGGGTCCCTGAGGCTTGGACGCCC
CCATTCATTGCAGGAGCAGCGCAACCATTTGCAGGGCAAACTGTCGGAGCTGCAGGTG
GAGCAGACATCCCTGGAGCCCCTCC
>1737
GTTTAATAAAACATTTGTTTTTAAAGAGACTGGTCAGCATTGCTAATTTAAATTTTTCTT

TTCTTAATAGGTGAATATGTTTACAAACCAAGGAACAGTGATCCACTTTAACAACCCTA
AAGTTCAGGCATCTCTGGCAG
>1738
GCCCGTTTTGGGAACCTGGACACAGTGCCCCTCACTTGCCTCCTTCCCCATCTGATCCT
CACACCCACAGATGTCTCCAAGCTCACAGACACCATCTGCGGGGTGGGGAACATGTCA
GCAAACGCCTCTGACCAGGAGAG
>1739
GATTCCAGAGGTAAAAGCGGCCATGGGCCAGACATACTGCAATCTCTGAAAATCACCT
GTTCCCCTGCAGCCCAAGAGCCAAGAGACCTGCCGGGGGGCCCTCATCTCCGACCAAT
GGGTCCTGACAGCAGCTCATTGCT
>1740
CTACCACCTCACCCAGCCTCTGGCCCCTGCAGGAGCCCTGGTCTAGCCTAATCTAGTGT
ATCATTTCCAGGAGACCCCAAATCCCAGTGGGGCAAAGAATTCCTTATTGAGAAGGCG
GTGATCTCCCCAGGGTTTGATGT
>1741
AGAGTGCTCTGGAGATCCCTGGAAGAGACTACTGGGGACAGGCTGGTGTGACCCTTG
CTCTTCTCCCCAGGCCCATCTGCCTTCCCTGCACGATGGAGGCCAATCTGGCTCTGCGG
AGACCTCAAGGCAGCACCTGTAGG
>1742
AGTCCCCAAGCCAGGAACCTGGATTCTGGGTAAAAGGACCAGCACCAACATCCCCTTC
TCTTGACTATAGAGAATGAACTGCTGAACAAACAGAGTGTTCCTGCTCATTTTGTCGCC
TTGAATGGGAGCAAACTGAACAT
>1743
GGCGTGAGCCACTGCACCCCACCCGGGTCTGCTTATTCTACCCTTCTCTCTGGTTCCAC
CCCTGCTGCAGTGGACAAGCTGTGCCGAGGTTGTCTCCCAAGAAAAAACCATGTTCCC
CAACTTGACAGATGTCAGGGAGG
>1744
GATCTAGGGAGGTTGGGGCTTACAGTTGGGGCTGTGGCAGCCTCCCAGCCAGTTCTCT
CCTTTTCTCCAGGTGGGTCTGGTGAGCTGGGGTCTTTACAACCCCTGCCTTGGCTCTGC
TGACAAAAACTCCCGCAAAAGGG
>1745
GGAGGGGGAAGGGGAAGAATCCAGGGTTGTCATCACCACTGAGTATGGATTTCACATT
CTAACACATTAGAAGCTGCAGGATGCTGAAATTGCAAGGCTGATGGAAGACTTGGACC
GGAACAAGGACCAGGAGGTGAACT
>1746
GCTGACATCTACCCCTTTAGGACCTGAGTTCTCTCTGCTCCCTCCTGAGTGCCCTGCCT
TCTGCCTGCAGGTTCTGGTACCCCCGGGCTATGGACAGGATGTACGGAAGTGGCCACA
GGGGGCTGTTCCTCAGCTCCCAC
>1747
AGCGACAGAACGAGACTCCGTCTCAAAAGAAAAAAAAAAAAACAACTCTGGGCCAA
CGTTATCCCTCCAGACACCTGAAAGACCCCGCCGTGGCATCCCAGGGCTTCCTGATGG
CCGCTTCCATCTGCCGCCCCGAATCC
>1748
GGGACCTCTGATCTGGCCTTGGTAGGAGGAACCCTCTGACCAGCCTTTTCTGCCCCATC

CCTCACTGCAGAGCTGATGGGAACCCCTGATGGCACCTGCTACCCTCCACCACCAGTA
CCGAGACAGGCATCTCCCCAGAA
>1749
CCACCCAGAGCTTTGAGGATTTGGAAAGCTGGGGCTAGGACCTTTCTAGCTAACTTGG
GCTCTTTCTTAGATGTCTCACCTGTTCAATGTGGCACACACACTGCGTATGATGGTGCA
GAAGGAGCGGAGCCTCGACATCC
>1750
GTGGTGCCTAGCTGGGGTTTCCCCAGGGTGGACACGCATACGTACACCTTCCATCTTGC
TCTTTCCCTAGGGGAAGGTCATGGCCTCCATGTTCTATGAAGTGAGCACACGGACCAG
CAGCTCCTTTGCAGCAGCCATGG
>1751
CCAGAAGAGGGGGACTGGCAGTTGGGGGGCCTCTGAGCTGCAAAAGACAGGATTTTC
CCCTTTTTTCCAGCTGGCCGCCAAGCACTGCCGGAGGCCAGTGATCAATGCTGGGGAT
GGGGTCGGAGAGCACCCCACCCAGG
>1752
TAAAAGCTTGGAAATGATGTCGGGGGGCACTCAGTCTGGGATCCCTGTGGTGACTGGA
TTCCTCTCCTAGATCACGATGGTGGGTGACCTGAAGCACGGACGCACAGTACATTCCC
TGGCCTGCCTGCTCACCCAGTATC
>1753
AGCCATGGAGATGTGGGTTGGGCAGTCAGAGCCCAGCGCTGCATCCACCATGGCTCTC
CTCACCCTCCAGGAGGAATTCGAGAGCATTGAGGAGGCGCTGCCTGACACTGATGTGC
TCTACATGACTCGAATCCAGAAGG
>1754
AGCCCAGACTTGTGTCCTCTGTAGCCACTCCTACCCCAAGGCACTAATGGGGACCCCA
TCTGCTTTGCAGTGCTTTGGTCAGTTCATCCTCACTCCCCACATCATGACCCGGGCCAA
GAAGAAGATGGTGGTGATGCACC
>1755
ACTTAGTCCTGGACAAGCCATCCATGGGCTGCACGATAACACTTCCTTTTTTTTTTTTT
TTTTTTGCAGCGTGGAAGTGGACTCGGATCCCCGCGCAGCCTACTTCCGCCAGGCTGA
GAACGGCATGTACATCCGCATG
>1756
AGCCACCAATCTGGTACCCACATTGCAGGTTCAGCAAGCATAGAGCTAAGTGCCAAAT
GCTTCCTTCCAGGGGAGATCATCGGAGGCCGGGAGAGCAGGCCCCACTCCCGCCCCTA
CATGGCGTATCTTCAGATCCAGAG
>1757
CTGTCTGCCTACCCTGCAGAGCTGGTGGACCATAGCTCCTGCAGCCCAGACCTACCTCT
TGCTTTTGCAGCAATATAAATGTCACCCTGGGCGCCCACAATATCCAGAGACGGGAAA
ACACCCAGCAACACATCACTGCG
>1758
CTGCACCAGCGACCCCCGAGCCTTGGCTGGAGGCCCCAGCTGAGCGGGAACGCCTAC
ATTCTTCCTCCAGCTGAGCAGAAGAGTCAGACGGAATCGAAACGTGAACCCAGTGGC
TCTGCCTAGAGCCCAGGAGGGACTGA
>1759
AGAGCCCGGAGCTCAGGGAAGCAGGAGCAGGGAGGCCTGTCTCAGTCTCCCTTCTCC

TCTCTACCTACAGGGGGATTCCGGAGGCCCCCTGCTGTGTAACAATGTGGCCCACGGC
ATCGTCTCCTATGGAAAGTCGTCAG
>1760
TGGAGAAGTTGCTGGTTTGGTCCCAGTGGGAGGTCTATGCTGATTCCTGGGTTCCTCCA
CCTTGTTCCAGGGCACCAAAGCCCAGTTCAGCTGCTTCAACCCCAAGTGCCTCCTGCC
TGCCAGCCGGCACTACTGGACCT
>1761
GATGCATCTGGGGTCATAGAGGACCACAGCACCCCCAATCTGCCCTGAGCATTCCTTCT
GCTTCCTCAAGATGGGGAAGTTCCGGAGCCTGCTTTTTACCTCGGAGGACGATGAGAG
GATCCACATGGTGAACAACTTCC
>1762
TAGTATCATTGTATAGAATTTTACCCCATGGGTACAATGTATTAACTATGTTCTCTTTCTC
TCCTAAAAGTTGCTGATCCCACACCCTTTCATATTCAAGCTGAAGTGACGATGAAAACA
AATTTCTTTGGTACCCGAGA
>1763
AAATTTGGCATTCACCTCTCTACGGGATTGTTGCACACCTTTCTACATAATGCTTTGTGG
TGTATCTTAGGGAGAGTGGTGAACGTATCTAGCATCATGAGCGTCAGAGCCCTTAAAAG
CTGCAGCCCAGAGCTGCAGCA
>1764
GTGTGAGGAGTCGGGGGGCTTGGAGGTCCCCCCCACCCATGCCCACACCTCTCTCCCT
CTCTCTCCACAGAGGGAGATAACGCTGTGCTGCAGTGCCTCAAGGGGACCTCAGATGG
CCCCACTCAGCAGCTGACCTGGTC
>1765
TCTGTCTTTGAGTCTCTATCTCTCTCCCTCTCCTGGGTGTCTCTGCATTTGGTTCTGGGT
CTCTTCCCAGGGGAGCTGTTCCGGTGGAATGTTTCGGACCTAGGTGGCCTGGGCTGTG
GCCTGAAGAACAGGTCCTCAGA
>1766
GGGGAGGGGAAACTGAAGAGGTGAAACCCTGAGGATCAGGCTTTCCTTGTCTTATCTC
TCCCTGTCCCAGACCTCACCATGGCCCCTGGCTCCACACTCTGGCTGTCCTGTGGGGTA
CCCCCTGACTCTGTGTCCAGGGG
>1767
CCTTGAGATCTCAGGATTGGGACGTAAGATGCCAGCCTGGGGTCCTCGTCTCATAGCCC
CTTCCCCCTAGTACTATGGCACTGGCTGCTGAGGACTGGTGGCTGGAAGGTCTCAGCT
GTGACTTTGGCTTATCTGATCTT
>1768
AGAATCTTGAGTGGGTCCGCCATTCCATTCCAAGTCGGCCCTCACCGTGCACTTCCTCT
TCTCCCGCCAGATTCTTCAAAGTGACGCCTCCCCCAGGAAGCGGGCCCCAGAACCAGT
ACGGGAACGTGCTGTCTCTCCCC
>1769
GCCGCGCGGACCCTTAGCCTTGACCGCCCCCATCTCTTCTGACCCCGTCTTACAATGCC
CCTCTCACCAGGACGCGCCCAGCGTTGGGCCGCAGGCCTGGGGGGCACTGCCCCGTC
TTATGGAAACCCGAGCAGCGACGT
>1770
GAATAGTGGACTGGGCCCTGGAGGAGAGGGGGCATGACTCGGTTCCCCATCCCCATCC

CCAAACCCCCAGGCCCAGAAGAAGAGGAAGGGGAGGGCTATGAGGAACCTGACAGT
GAGGAGGACTCCGAGTTCTATGAGAA
>1771
GTCTTCCTCCTTACCGTAGGCTGTACTTCCCATGCCCTAGCCTCCAATTCTCCATCCCCC
GCCCAAGCAGGGTCCCAGTCCTATGAGGATATGAGAGGAATCCTGTATGCAGCCCCCC
AGCTCCGCTCCATTCGGGGCCA
>1772
CTGGGCTGACTTTGCCTTCCAGCCTACTTCCAGTGCCACCCATGTTCTCCTCCTCCCTG
GTCCTATCCAGATGCAGACTCTTATGAGAACATGGATAATCCCGATGGGCCAGACCCAG
CCTGGGGAGGAGGGGGCCGCAT
>1773
GGGTTCCTAGGGCCTGGTGGGCAGGGGGAGGACTGCTGGACCCCTCCCCATCACCGTT
TCTTCTGCATAGCCTGGATCTCCTCAAGTCCCCAAGATTCACACCTGACTCTGAAATCT
GAAGACCTCGAGCAGATGATGCC
>1774
CAGAGGGGAGGCCCGGCCAGGTCCTCCGGCAACTCCCGCGTGTTCTGCTTCTCCGGCT
GCCCACCTGCAGGTCCCAGCTCTTGCTCCTGCCTGTTTGCCTGGAAATGGCCACGCTT
CTCCTTCTCCTTGGGGTGCTGGTG
>1775
GCAGGGGCCAGGGCTGGGGAAATGTGTCACCATCCCCAGTCCCTGACCCACCCACCCT
GTCTCTCCACAGGCCCTGGGTGCCAGGCCCTGTGGATGCACAAGGTCCCAGCATCATT
GATGGTGAGCCTGGGGGAAGACGC
>1776
GGTGGGCGGGGCCAGGAGGCTAGGGAGGGCAAGAGGGGCCAGGGCTCTGAGCCATA
CTACCTCCTTGCAGAGCCGCCCCCCAGGCCCTTCCTGGACATGGGGGAGGGCACCAAG
AACCGAATCATCACAGCCGAGGGGAT
>1777
ATCCAGGAGGGTCTGAAAGATATTCACCTCCCCCTGCTCACTGAGGCACCCACCCCAC
CCACCCCTACAGAAACGATGGCAGAACGAGAAGCTCGGGTTGGATGCCGGGGATGAA
TATGAAGATGAAAACCTTTATGAAG
>1778
CTGGGGGTGGCTGGGGGCAGGGACCCCAGGTGTCAGGGTGCTGATGTTCGCTGCCTC
ATTTCCATCCCAGGCCTGAACCTGGACGACTGCTCCATGTATGAGGACATCTCCCGGGG
CCTCCAGGGCACCTACCAGGATGT
>1779
CCCAGGACCCCATTTTTCCACCCTAATGCATAGGTCCCAATATTGACCGATGCTCTCTGC
TCTCTCCTAGCCTCACTTCTAACCTTCTGGAACCCGCCCACCACTGCCAAGCTCACTAT
TGAATCCACGCCGTTCAATGT
>1780
CTTGCCTTGCCCCTCCGGATTCAGGCCGATGGGTTGAGCCCCCCTGACCCTGCCCGTGT
CTCCCTCGCAGCTGGGCGCCGTGTACACAGAAGGTGGGTTCGTGGAAGGCGTCAATA
AGAAGCTCGGCCTCCTGGGTGACT
>1781
GCCGGACTGGCCCTGCGGCGGGGCGGGTGAGGGCGGCTGCCTTCCTCATGCCAACTC

CTGCCACCTGCAGGGACCCTGAAGGCCAAGAACTTCAAGAAGAGATGCCTGCAGGCC
ACCATCACCCAGGACAGCACCTACGG
>1782
AGGGACAGGGGACCGGCTGGAGACAGGGCCAGGCGGGCGTCTGGGGTCACCAGCCG
CTCCCCCATCTCAGTCTCCCGGGACCTGCCCGTTATGATCTGGATCTATGGAGGCGCCT
TCCTCATGGGGTCCGGCCATGGGGC
>1783
GATCTCCCCTCCTGGAGGCCAGCCTGGGCCACTGGTCTCTAGCACCCCCTCCCCCTGC
CCCTGCCCCCAGGTAACTATGGCCTTCGGGATCAGCACATGGCCATTGCTTGGGTGAA
GAGGAATATCGCGGCCTTCGGGGG
>1784
TGTCCTTGTCCCAGCGTGGGGTGGGCAGAGTGGGGAGCGGCCTTGGTGACGGGATTT
CTGGGTCCCGTAGACCCTCTCCCCCTACAACAAGGGCCTCATCCGGCGAGCCATCAGC
CAGAGCGGCGTGGCCCTGAGTCCCT
>1785
CTTGCCTTCAAATGGTTCTGAGCCCTGAGCTCCGGCCTCACCTACCTGCTGGCCTTGGT
TCTGCCCCCAGGTGGCTGAGAAGGTGGGTTGCCCTGTGGGTGATGCCGCCAGGATGGC
CCAGTGTCTGAAGGTTACTGATC
>1786
GGCAAGGGGCGGCTCAGGGGAAACTGGGAGGTACAAGAACCTGCTAACCTGCCTGGC
CTCTCCCACCCAGACCCCATGCTGCACTATGTGGGCTTCGTCCCTGTCATTGATGGAGA
CTTCATCCCCGCTGACCCGATCAA
>1787
GTCGGGGTGAGTATGCACACACCTTCCTGTTGGCACAGGCTGAGTGTCAGTGCCTACT
TGATTCCCCCAGGGAGGACTTCTACAAGCTGGTCAGTGAGTTCACAATCACCAAGGGG
CTCAGAGGCGCCAAGACGACCTTT
>1788
CAAGTCCGGTCTCTGGGCTGATCGGTCCCCAGTGAGCACCCTGCCTACTTGGGTGGTC
TCTCCCCTCCAGGAGTGCCAAGACCTACGCCTACCTGTTTTCCCATCCCTCTCGGATGC
CCGTCTACCCCAAATGGGTGGGG
>1789
TGCAGGTGGAGAGCAGGCTTCAGCCCCCTGGGAGTCCCCAGCCCCTGCACAGCCTCT
TCTCACTCTGCAGGGACCCCAACATGGGCGACTCGGCTGTGCCCACACACTGGGAAC
CCTACACTACGGAAAACAGCGGCTAC
>1790
GAACAGCCCCTTTCCTGTGCCCCACTTGTGAAAGTCTCCATAAACCCCTGTGGCCCAC
TCTGCCTTCCAGAGAGAGGGGCTGAACTGCAAGGTGGCCACCAACACAGAGGCCCTG
CAGAGCGGCAGGATGGAGATGTGGA
>1791
TCTCTAGTGCTCTGCCATGGGGAAGGGTCCCACGTCCATCTCATCGCACCACCTATCTC
TTGGCCCACAGAAAGCATCGCTGGAGGGCAGCCTGGTGGAGACGGAGGTGTGTTACA
GGACCCAGCTGGCCCAGCTGCAGG
>1792
GGTGGAGAGTTTATTGAGTGTTTGGGTATTGGCTTGTCACCTGCCAATCTGTTCTGCCC

ACTCCATCCAGGCCTGGGGCTCCTAGAAGACCAGAGGGGAGGAGGAGGAAGGATTAA
GGGTGGAGGGAAGGGACAGCCCCT
>1793
AACTCTGGTGTGGGCTCAGCACCCCCATCCGATGACCTGACTACTCTCCCATCTCCGCT
GATCCTCACAGCCACCGTGGACAATGCCAACATCCTGCTACAGAATGACAATGCCCAT
CTGGCTGCTGCTGACTTCCGCAC
>1794
AGGTGAGCAGCCAGGACCTGGACCTGCAATTCCAAGCAGCCTGGGCTGAAGTCCCTG
ATTCCCATGGCAGGTTTGAGACAGAGCAGGCCCTGTGCTGAGTGTGGAGGCCGACATC
AATGGCCCGTGCAGGTGCTGGATGA
>1795
GGAAGGCTTGGGAGTGAGAGATCAGGATGGGTCCAGATGCGCACATCCACATCCCCTT
TTTCCATAGGAGATGAACGCCCCGCGAGCCAGGTGGGCGGTGAGATCAATGTGGAGAT
GCGCTGCCCAGTGTGGACCTGAGC
>1796
ATTTGGGAGGAGGCCTGACTGAGGAGAGGGATCCAGGCTCACACCACCCTGTCCTGT
GTTCTGTCTGCAGACAGAGGAGCTGAACCGCGAGTGGCTACCAACAGTGAGCTGGTC
CAGAGCGGCAAGAGCGAGATTTCGGA
>1797
GCATGGGGATGGGCCCAGAAGAGGACACTGACAACCCTCACTGACCCCTGGTCTTCCT
GCCCTCCTGCAGAAAGCATCCTGGAGGGCAACCTCGGCGGAGACAGAGAACCGCTAC
TGCATGCAGCTGTCCCAGATCCAGG
>1798
TGAGCTCACCTCAGAAGAAACCCCAAGCCATCACTGTCGCCTCCTTTTCCTTCCTTCTT
CCTCACAGCAGGTCTATAACAGTCACCCTTGGAGCCCATAACATAACAGAGGAAGAAG
ACACATGGCAGAAGCTTGAGGTT
>1799
TCCCTCCCTGATCACCTCCAACTGTCCAGAGCCCATTTCGAGGACTGACAGTCCTTCAT
TCCCTTCACAGTTGAAGGAGAAAGCCAGCCTGACCCTGGCTGTGGGGACACTCCCCTT
CCCATCACAATTCAACTTTGTCC
>1800
AACCCAATTTGTCCATTCATTGGAGAGAGTATCTATGGCCGTGTTCAAACCCTGGGGTG
CTCTGTTCCAGGGGAGATCATCGGGGGCACAGAATGCAAGCCACATTCCCGCCCCTAC
ATGGCCTACCTGGAAATTGTAAC
>1801
ATGAGCTCACCTCAGAAGAAACCCCAAGCCATCACTGTCGCTCCTTTTCCTTCCTTCTT
CCTCACAACAGGTCTATAACAGTCACCCTTGGAGCCCATAACATAACAGAGGAAGAAG
ACACATGGCAGAAGCTTGAGGTT
>1802
TGCCCTCCCTGATCACCTCCAACTGTCCAGAGCCCATTTCGAGACTGACGGTCCTTCAT
TCCCTTCACAGTTGAAGGAGAAAGCCAGCCTGACCCTGGCTGTGGGGACACTCCCCTT
CCCATCACAATTCAACTTTGTCC
>1803
CAAGCTGGCTGGCAGACAGGAGTGGAGGACTTCCCGGGCTCACCTTCTTCTCTCTCTC

CTCTCTCTACAGGGAGACTCTGGGGGCCCTCTTCTGTGTGCTGGGGTGGCCCAGGGCA
TCGTATCCTATGGACGGTCGGATG
>1804
TCTCATGAGCCCCCAGATCTGGATGGGACCCAGCCCTGGCCTTGCCCTGAGTTTCCCCC
ATGTTCCCCAGAGACCACGACCAGCACCACCAGCTCCCTGGCACCCCCACCTACCACG
AAGATCTGTGACCCTGGGGAGCT
>1805
GTGGCAGAGGGAGCCTGAGGGAACCCCCACTCCAGCCCCCTCCCTGACAGGCCCCTG
CCTGTCTCTACAGAGCCCGGCACCCCATGAGGACATGCAGAGCACCTGCAGAGGAGC
AGGAGGCCGGAGCTGAGCCTGCAGAC
>1806
TTTTTCTCTCTGGCTCCCCACCCCCTCCTTCCCTTGGCTCTCTGCTTCTCCCTCCCCACC
CTTGGTGCAGAGGAGGCTGGCAGCTGTGTGCAGGATGGGCAGAGGTATAATGATAAGG
ATGTGTGGAAGCCGGAGCCCTG
>1807
GCAGCCAAGGGACAGATGGCTCTTGGAGAAACACTGCTTCCCATTGATGCCTTTTTCT
CTTTATGCCAAGGGTGCCCCTGGACCTCGTGGCAGAGATGGAGAACCTGGGACCCCTG
GAAATCCTGGCCCCCCTGGTCCTC
>1808
AGACTTACCTAGCCAAGTAGGGAAGACCTAGTGCCTTTCAACCTCCTAACGTTGTTGG
GTTTCTTTGCAGAACTTTGCTGCCCAGATGGCTGGAGGATTTGATGAAAAGGCTGGTG
GCGCCCAGTTGGGAGTAATGCAAG
>1809
ATTGCCCACCCACTAGAGGTCTGTGTCCCCTCTCTTCTTCTTCATTTCATACCTCCCTGT
CTCTTCCCAGGGTGAAGCCGGCCCCACTGGTGCCCGTGGTCCTGAAGGTGCTCAAGGT
CCTCGCGGTGAACCTGGTACTC
>1810
GCTACCCCAGGAGAGCCCAGTCCTGCCTCTCCCCTCTCCTGACACCCCTCCCTTCTTCT
CATGCCCACAGGGTGCTCCTGGCATTGCTGGTGCTCCTGGCTTCCCTGGGCCACGGGG
TCCTCCTGGCCCTCAAGGTGCAA
>1811
ACTCTAGATCGAGGCTTGCTCCAAGGCCAGGTGAAGGCTCACTCTGTTTCTCTTTTTTG
CTGGTCCTCAGGGCCCTGCTGGCCCCCAGGGAGCCCCTGGACCCGCTGGTGAAGAAG
GCAAGAGAGGTGCCCGTGGAGAGC
>1812
AGGGGCTGGGGTCCTCCCTGCATCGCTGAGGTCACATGGTATCCCACTGACTCCCTGT
GTACCCTTGTAGGGAGCCCCTGGAGAGCGAGGGCCCAGTGGTCTTGCTGGCCCCAAG
GGAGCCAACGGTGACCCTGGCCGTC
>1813
CTTAGTGGATGTTGGGTGGATGGATGTGGTCAGAGTGTCTATGTTCTGAGAATGGTGTT
CTGTCTTTCAGGGAGCCCCTGGTGAAGATGGTCGTCCTGGACCTCCAGGTCCTCAGGG
GGCTCGTGGGCAGCCTGGTGTCA
>1814
CTCCCTGCTTCTCCCTGGACCTTCTCCCCCACTGCTGTTGGTTGATCACTTCTTGGTGTC

TCTGCCGCAGGGTGAACGAGGTTTCCCAGGTGAACGTGGCTCTCCCGGTGCCCAGGG
CCTCCAGGGTCCCCGTGGCCTCC
>1815
CAGGCTCCATGAACATGGGTTCAGGGAGAGGTGCTGTCCACTACAGACTCCCTCTTAC
CTCCCTCCCCAGGGTGCATCTGGCCCAGCAGGCCCCCCTGGCGCACAGGGCCCTCCAG
GTCTTCAGGGAATGCCTGGCGAGA
>1816
CGCTCTTCTGGAAGAGCTCCAGGGTTTTAGCCTGCCCCTCATTCACCTGCTTCCTCCTT
CCCCATTATAGGGTGCTGATGGCCAGCCTGGGGCCAAGGGTGAGCAAGGAGAGGCCG
GCCAGAAAGGCGATGCTGGTGCCC
>1817
ACAGTGACTTTGGCAGCCTGGATGAGGAAGGGTGAGATGAGTCCTCACTTCCCGCATT
TTCTCCTTCTAGGGCAACCCTGGACCCCCTGGTCCCCCTGGTCCTTCTGGAAAAGATG
GTCCCAAAGGTGCTCGAGGAGACA
>1818
CAGAGCTGTGCTGAGAGGACGAAGAGGCTGGGCCACTGTCAGTTCTCATCTCCTGCCT
CTCCTCTCTCAGGGTGCCGAAGGTCCACCAGGTCCCCAGGGTCTGGCTGGTCAGAGA
GGCATCGTCGGTCTGCCTGGGCAAC
>1819
TGAAGGGTCAGTGGGCCCTGGGCAGGGGTGCTTACCACTTGCACTCATCATCCTTGTC
TCTGTCCTCCAGGGTGAGCCCGGCAAGCAGGGTGCTCCTGGAGCATCTGGAGACAGA
GGTCCTCCTGGCCCCGTGGGTCCTC
>1820
CTAGAAGGACCTTCTGTTCCTGTCTCTTCTGGAACATTCTTCTCTGAGCCTGAGACCTC
TCTCCTGACAGGGTGATCGTGGTGAGACTGGTGCTGTGGGAGCTCCTGGAGCCCCTGG
GCCCCCTGGCTCCCCTGGCCCCG
>1821
TTGGGTGGGACAGGAGGGGGCTCCTGTCCTGGCCCTGACCTGACTCAATCGGTGTCTG
TCTTGTTCCCAGGGTCCTCAAGGCCCCAGAGGTGACAAAGGAGAGGCTGGAGAGCCT
GGCGAGAGAGGCCTGAAGGGACACC
>1822
CCCAGCCCCAGCGACTCCCCAGCCTTCCCTGTGGTGACCACTCTTTCCTCACGACCTC
TCTCTCTTGCAGGGTCCTCCTGGCCCCGTCGGTCCCTCTGGCAAAGATGGTGCTAATGG
AATCCCTGGCCCCATTGGGCCTC
>1823
AGCAAATGGGGATGGACATGGAGGGGCACTTTCACACAATCCTGGCTGATCTCTCTGT
TTCCTGCTGCAGGGTCCTCCTGGAAATCCTGGGCCCCCTGGTCCTCCAGGTCCCCCTG
GCCCTGGCATCGACATGTCCGCCT
>1824
CCTGGGTCTCTCAAGCTTTTGTGTCTGTGCCTGTCTGAGCCCCCATGGGTGCTGCCTCT
TCCCCCTGCAGGAGACTACTGGATTGACCCCAACCAAGGCTGCACCTTGGACGCCATG
AAGGTTTTCTGCAACATGGAGAC
>1825
GCCCCTACAGCCCCTGGTCCCATTGAATGCCAGCTCCCAGGCCTCACACTGCCGCTCTC

TGCCCCAACAGTTCAGCTATGGAGATGACAATCTGGCTCCCAACACTGCCAACGTCCA
GATGACCTTCCTACGCCTGCTGT
>1826
GCTTCTGGGCAGCTGGAACTGGGTAGCAAGGCATCTACTGAACAGAGCCTCCTCCTTT
TTTCTCCCCTAGAAACATACCGGTAAGTGGGGCAAGACTGTTATCGAGTACCGGTCAC
AGAAGACCTCACGCCTCCCCATCA
>1827
GAGGCCACCGTGCGGGTAGGCGGGTGGTTGGCCTCCCAGAAGAGGCCCTGATCTCTC
CTGCCCGCCCCAGTGACCTGCACGCGCCTTTACGCCGCTGACATTGTGTTCTTACTGGA
TGGCTCCTCATCCATTGGCCGCAG
>1828
AGGCCTCCTCAAGGCCAGGGCCAGAAGAGATCCTGAGTCCTAGCCTGTTGCCACCCCT
ACCCTCCAACAGGACAGAGTTCGGCCTGGATGCACTTGGCTCTGGGGGTGATGTGATC
CGCGCCATCCGTGAGCTTAGCTAC
>1829
CATGCCTCCCAAGATGACCCCAAATGAAGTGTCCAGGGGAACCGTGATTTGACCCCTG
CACCTGTCCCAGGTCTGCATCCTGATCACAGACGGGAAGTCCCAGGACCTGGTGGACA
CAGCTGCCCAAAGGCTGAAGGGGC
>1830
AGGTCAGCTGGGGGGTGGGGGCAGTCAGAGAGCATGTGGGTGACTGAGTCCTGATGG
GTCGTCACTTCAGGGATCAAGAATGCTGACCCTGAGGAGCTGAAGCGAGTTGCCTCAC
AGCCCACCAGTGACTTCTTCTTCTT
>1831
CGACCCCAACCCTGCATCATACATGCCCACCCCAATCCTCCTGCCTGATCCCCTGATCC
CGCATTCCCAGCGGATGACTCGACCTCTGCTCCACGAGACCTGGTGCTGTCTGAGCCA
AGCAGCCAATCCTTGAGAGTACA
>1832
GATGTCAGGAGTATAGGTGGTGGCTGGGGACTTGGCTGGGCAAGATAAAGTGACCTCT
TGCCCTGGGCAGGTGAACGTCCCAGCTGGTGAGACCAGTGTGCGGCTGCGGGGTCTC
CGGCCACTGACCGAGTACCAAGTGA
>1833
ACCTAACCCCCGACCCCAGTACCCCCTCCCACTTCTGACTCCATGAATCCCTGGTGGGA
CTCTCCCCCAGCTGCCCTAGAAGGGCCGGAACTGACCATCCAGAATACCACAGCCCAC
AGCCTCCTGGTGGCCTGGCGGAG
>1834
CCGCGCCTCAGACAAGGCTGTAGAGTCCTGAGTCTGCAAGCCCACTGGCCCCTTGGTG
TCCCCCATGCAGGTGGGCCCACACAGCAGCAGGAGCTGGGCCCTGGGCAGGGTTCAG
TGTTGCTGCGTGACTTGGAGCCTGG
>1835
TTTCTTCAGGCTGGGACGGGCAGGCAGGGCAAGGCCCGGAGGCCACTGACATCCCAT
GTGCCCTGGGCAGACGCTTCTGTTGAGCAGACCCTGCGCCCGGTCATCCTGGGCCCCA
CATCCATCCTCCTTTCCTGGAACTT
>1836
TGCGGGGGAAGGGATGGACAGGCAAGGGTCAGGGCATGGCCTCTGCTGGTCTGACCC

TGTTATCTTGCAGGCTTGGAGCCACCGCAGAAGGTGGTACTGCCCTCTGATGTGACCC
GCTACCAGTTGGATGGGCTGCAGCC
>1837
TGGGCTGGGCCAGTGAGTGGGGGAGGTGTCGGAGCCCCAGGCTGCCTCTATGCTGTG
CTCTCTCCAACAGGACCAGAGCTGCCTGTGAGCCCTGTAACAGACCTGCAAGCCACC
GAGCTGCCCGGGCAGCGGGTGCGAGT
>1838
CATCACTGCCATCGTCCCACATCCCTGTCTCTTTCTGACCCCTGCCCACCTACCCTGACT
TCTCTCTTAGGGGTGGAGCGGACCCTGGTGCTTCCTGGGAGTCAGACAGCATTCGACT
TGGATGACGTTCAGGCTGGGCT
>1839
TGTGGAGGACAGCCTGCCTGCCTCACTCTGGTCCTGGTTCTGACTTCTGACTTCTGTCT
GTAACTCCTAGAGCTGGAAACTCCACTTGCTGTTCCAGGGCTGCGGGTTGTGGTGTCA
GATGCAACGCGAGTGAGGGTGGC
>1840
GGGACCAATGAGGGTATGGGTGCCAGAGGGGACAGGCAGGAGCCATGCCAGCATTTC
CCTCTGACCTCAGGTCCGGAGTCCAGCCAGACACTGCCCCCAGACTCTACTGCCACAG
ACATCACAGGGCTGCAGCCTGGAAC
>1841
TTGCCCCTATCAGATAGCCCCACTCCTTCCTGCCTGTTCCAGCAGACTCCCCATTGCCT
CTCCCCACTAGACCCACTGGGCCCAGTGAGGACGGTCCATGTGACTCAGGCCAGCAG
CTCATCTGTCACCATTACCTGGAC
>1842
TGGGGGTTCAGGTGGGGCTCTGGGGCACAGAGTTTGCTAGCCCTGGAGCTGCCTCCAT
CCCTGTTCCCAGGCCCAGAGAAATCCCAGTTGGTTTCTGGGGAGGCCACGGTGGCTGA
GCTGGATGGACTGGAGCCAGATAC
>1843
CCCTGCGTAACCATTCCTGCACTGCCTCTCTGTTCTCTACCAGGACCCCTTACCTTGCCT
CTGTCCCCAGCCCCTGAGCCTGTGGGTCGTGTGTCGAGGCTGCAGATCCTCAATGCTT
CCAGCGACGTTCTACGGATCAC
>1844
CTGACGCCACCCCTGTCCTTCCTGGCTGGGACTGCTCACCCCTAACCATTGCTGTATGC
CCACCTGCCAGGCGGCCCCATGAGGCACCAGATACTCCCAGGAAACACAGACTCTGC
AGAGATCCGGGGTCTCGAAGGTGG
>1845
CCAGGGTGGGCCTGGGATTGGTGCAGGGGCATGGGGGCAGAGCTCCCTGATTCCTGA
GCTTTCTCTCCAGCGCCTGAGGCTCCGCCAGCCCTGGGGACGCTTCACGTGGTGCAGC
GCGGGGAGCACTCGCTGAGGCTGCG
>1846
CAGGCCCTTTAATGCCCCCAGGTGTCACTATCCCACATGCTCTTGGCCCCCACCCTCAC
GCCTGCCCCAGGTGGCCAGGAACAGTCCCGGGTCCTGGGGCCCGAGCTCAGCAGCTA
TCACCTGGACGGGCTGGAGCCAGC
>1847
ATCTCAGATCTCTCCCTTCTGGGTTCTCAGGACTCAGCCTCTGATCCTCGATCTTTCATT

CCTCCTTCAGAGTCACCTCGTGTTCCAAGCATTGAACTACGTGTGGTGGACACCTCGAT
CGACTCGGTGACTTTGGCCTG
>1848
CAGGATTTGGGTGGGCTGGCAGTTGGGGCTCTGTGGAGACAGCTTTTTAATCAAGTTC
TGTCTCCTGCAGAAGTGCCTGGGTCCCCGCAGACACTTCCAGGGATCTCAAGCTCCCA
GCGGGTGACAGGGCTAGAGCCTGG
>1849
ATAGTGGGAAGGGCAGAAAGGTGTGTCTGGGTGGGCTGCCCGCTTCAGTAACTTGTTC
CCCTTCCTACAGTGTGCCCCCGTGGCCTGGCGGATGTGGTGTTCCTACCACATGCCACT
CAAGACAATGCTCACCGTGCGGA
>1850
CTCCAGGCTCTGTGCACCCCCATCCCTGCTCTGTCATCCTCCTCAGGCTCTGTGTTTCC
TTCCACCCCAGGTTGGCCTGCTGTCTTACAGTCATCGGCCTTCCCCACTGTTCCCACTG
AATGGCTCCCATGACCTTGGCA
>1851
AGGCATGGACTCCTGGGGCTATCCACTGGGAGCTTGGTGCCCCAGGGTTCTGACATGT
CCTTCCTTCCAGGCACAGCCGTGGTCACAGCTCACAGATACATGTTGGCACCAGATGC
TCCTGGGCGCCGCCAGCACGTACC
>1852
ATGGGGAAGGGGTCTGGGAATAGGGGTGGCCCTGAAAAGGCTATCATGCAGCCACTG
GACCCCACCTTAGGGCTTAATGTGGTGATGTTGGGAATGGCTGGAGCGGACCCAGAGC
AGCTGCGTCGCTTGGCGCCGGGTAT
>1853
GAGGCCCCTCCAGCAAACCAGGGGCCTTGGGGGAGGGTCCCTTCCTGTCCTGACCTCT
TCACCTCCTCAGGGCTTCCCTGGAGCAGATGGGCGTCCAGGCAGCCCTGGCCGCGCCG
GGAATCCTGGGACCCCTGGAGCCC
>1854
TGTCTAGGGGCTTCTTCCAGCTCAAGGCCCCCATAGCCTGAATCCTGCCCACTGCTCTC
TGTCCTTACAGGGGGCTCCCGGACAAGTCATCGGAGGTGAAGGACCTGGGCTTCCTGG
GCGGAAAGGGGACCCTGGACCAT
>1855
GGGGACCAACACCAGGGGGGCGAGAGTGAGGTCTGTGGGGTTGCACCTTATACTTTG
TCTCCTCCATCAGGGCCCCCCTGGACCTCGTGGACCACTGGGGGACCCAGGACCCCGT
GGCCCCCCAGGGCTTCCTGGAACAG
>1856
GGGGTCCCCTCCCCTTGAGAACTGCTTGCTTCGAGCGTCCTGCATCACCTCCCTCTTGC
CTCCTCCACAGGGTGACTCAGGGCCTCCTGGAGAGAAGGGAGACCCTGGGCGGCCTG
GCCCCCCAGGACCTGTTGGCCCCC
>1857
AGTGTAGCGGGTCATAGGGGCACCTGCAAAATATTGGGAGGGTCTGTCTCAGGCCAGC
TGCTCTTCTTAGGGGGCACCTGGAGTTCGGGGGCCTGTGGGTGAAAAGGGAGACCAG
GGAGATCCTGGAGAGGATGGACGAA
>1858
CCCAGCAGGTTCTACCTTGGGCATGGCTTGGCTTCAAGGATGTTCCTCAGCAAGCTTAT

CTCTGCCACAGGGAGAGCCTGGGGACCGCGGACAAGAGGGTCCTCGAGGGCCCAAG
GGTGATCCTGGCCTCCCTGGAGCCC
>1859
TCAGGACATGAGCCTGGGTTATCAGATCCACCTCAGCCTTGGTGGCCTCTTACCACTCT
ATTTTCCACAGGGTGACCGGGGTCCCCCTGGGCTGGATGGCCGGAGCGGACTGGATGG
GAAACCAGGAGCCGCTGGGCCCT
>1860
GACCCTGGGGAATATGACTCCACTCTTTTCTGAGGTCTCAGGGTGCTGATGCTGGCTGC
ATCCTTCACAGGGTCGTGATGGCCCCAAGGGTGAGCGTGGAGCTCCTGGTATCCTTGG
ACCCCAGGGGCCTCCAGGCCTCC
>1861
CTAGTGGTGCCCACAGGCATAGGGGCTGCGGCGACGCACCCCGCTCCTCTGACCTCTT
GCTGTCCCTCAGGCATCTGCCCTGCGGGAGATCGTGGAGACCTGGGATGAGAGCTCTG
GTAGCTTCCTGCCTGTGCCCGAAC
>1862
CAGTGGGTTGTGCCCCGTGGAGTGGGGTGTAGCTGTACAGCCACCAGCATTCTCTCTT
CCACTCCTGCAGGGCCCCATCGGCTTTCCTGGAGAACGCGGGCTGAAGGGCGACCGT
GGAGACCCTGGCCCTCAGGGGCCAC
>1863
TGGGGGTCAAGGGTTGGGCTCCAGGGGTCAAGGGTCGACAGGCAGCCCTGACAGAG
CTCTTCCCTCTCAGGGGGAGACAGGGAAGCCGGGAGCCCCAGGTCGAGATGGTGCCA
GTGGAAAAGATGGAGACAGAGGGAGCC
>1864
GGAGTTCTGATGAGAGTCCTGGGAGGGGTCCTGCATTGACCATTCCCTCCCTTTGCTGT
TTTTATTTCAGGGAGATCTGGGCCTCCCTGGCCTGCCCGGTGCTCCTGGTGTTGTTGGG
TTCCCGGGTCAGACAGGCCCTC
>1865
CTTGAGGGGGCAGAGGGTAGGAGGGTTCCCAAGTCACTCTCTGCCTCTCTTGCCCCTA
TTTTTCTGGTAGGGTCTGGCAGGCCCCCCAGGGAGAGAAGGAATCCCAGGACCCCTG
GGGCCACCTGGACCACCGGGGTCAG
>1866
AGAGCTCAAACTCCCAGAGGGTGAGGGACCTTCAGCCCCTTACCGTGACTTCTTACCC
AAACCAACCCAGGGTGAACGAGGGCCTCGGGGCTTGGATGGTGACAAAGGACCTCGG
GGAGACAATGGGGACCCTGGTGACA
>1867
GGCTCGTTGTATTCTAAGCCCCCAGCCTGATCTGGTCCTGACTGAATCCCTCTCCTGGT
CACTCCCACAGGGCAGCAAGGGAGAGCCTGGTGACAAGGGCTCAGCCGGGTTGCCA
GGACTGCGTGGACTCCTGGGACCCC
>1868
CTATCCAGGGCGATTCTCTTTGGTCCCTCACTGACCTCCTCTGACCTCACATGGACCCT
CTCCCTGCTAGGGATCCCCAGGAAAGGATGGAGTGCCTGGTATCCGAGGAGAAAAAG
GAGATGTTGGCTTCATGGGTCCCC
>1869
AGCGAGGACTTTGGGAGAGGGGTCCATCTCTGGGGCCTCTGATTACTGACTCTGTCCT

ATATCTCCCCAGGGAACCCCAGGAATTGGGGGCTTCCCAGGCCCCAGTGGAAATGATG
GCTCTGCTGGTCCCCCAGGGCCAC
>1870
GGCCCCCAACCTGCCTGACTCTGATCCCCACTGCCTCTGACCCTGCTCACTTGGTCCCT
GTGTCTGACAGGGTGAGCGAGGTCCCCCGGAGAGAGAGTGGTGGGGGCTCCTGGGG
TCCCTGGAGCTCCTGGCGAGAGAG
>1871
TCAGGGGGGTTGGCTGGGATGGCTGCCCATGGTGACTTCAGGGCCCTGAGGCCCCTGC
TCTTGGCTCCAGGACCCCTCCCTAGTTATGCTGCAGACACTGCCGGCTCCCAGCTCCAT
GCTGTGCCTGTGCTCCGCGTCTC
>1872
AAATCAGTGCTGCCCACCTTGCCCCGGGGCCAGCAGCCACTGCTCCCAGCACACCCTG
CCCTACCTGCAGAGCGGGTACCCCCTGAGGATGATGAGTACTCTGAATACTCCGAGTAT
TCTGTGGAGGAGTACCAGGACCC
>1873
TGACCTCCCCCTGACCTTTCAACCCTCTCTGATTCCCACAAACCCTGCTGACTTGACCC
CATTGGCCCAGACCCCTGTTCCCTGCCACTGGATGAGGGCTCCTGCACTGCCTACACC
CTGCGCTGGTACCATCGGGCTGT
>1874
GGTGCCTCTGCCTGAGCGTCTCCGGGGAAGGTCAGATGGCTGACGACCGTTTCCAACC
TGTCCTCACCAGGTACTGCCCAGGACTGAGGCCCAGATAATGAGCTGAGATTCAGCAT
CCCCTGGAGGAGTCGGGGTCTCAG
>1875
GTGGGTACAGGTCTGAATGGAGAAGGGACCTGAGGGAGTGGGGCCTGGGCTGACTGG
CTGCTCCTGGCAGGAGAACGGTGAGCTCTGCATGAATAGTGCCCAGTGTAAGAGCAAT
TGCTGCCAGCATTCAAGTGCGCTGG
>1876
GTCAGGCAGGGACAAGTGATGTCCAAAGTATCTCCCCATAGCCTTGACTCCATTGGCT
GTGTCTCTGCAGACGCTCTATGGGATTTACTACAAGTGTCCCTGTGAGCGTGGCCTGAC
CTGTGAGGGAGACAAGACCATCG
>1877
ATCATTTCTGTTTGTAGTTTTTCTATCAGTCATTTCAGTCAGCGTCATAATTCACGTTATC
TTCCTTTAGGTGGTGTTCCCACTGATGAAGAGCAGGCGACTGGGTTGGAGAGGGAGAT
CATGCTGGCTGCAAAGAAGGG
>1878
AGGGCTTATTTGGCCTAATGGTTCAATTCTTGTTTTTTTTGTTTTGTTTTGTTTTTTGTTT
TTTCTTCAGGACCCATACAATGTACTGGCCCCAAAGGGAGCTTCAGGCACCAGGGAAG
ACCCTAATTTAGTCCCCTCCA
>1879
AAAAGAAATTCATGTTGAAAGTCTCCCTTTCTAGGTTGGATGACCATAAACCACCTTTT
TTCCCTTTTAGGTGAAGAGGACAATACCAGCGTCGTCTGGTTTTGGCTGCACAAAGGG
CAGGCCCAGCGATGCCCCCGCTG
>1880
GGCCGAAGAAGGTCAGGCCCTCAGCTGCCTTCATCAGTTCCCACCCTCCAGCCCCCAC

CTCCTCCTGCAGACAAGCTGGTGTCTAGGAACTACCCGGACCTGTCCTTGGGAGACTA
CTCCCTGCTCTGGAAAGCCCACAA
>1881
TGCCTCTCACAGTAGCCCCGCCCTGCCCGCTGCACAGCGGCCTGCTGAACTCACACT
GTTTCTCCACAGCGCATGAGAGCCCAGCCCGGCACCCCTGTGGCCATTGAGGAGGAAT
TCTCTCTCCTCACCTGCAGCATCA
>1882
GTTGGGGATGAGTGAGGAAAGCCCGAGCCCAGGGAGGTCCTGGCCAGCCTCTAACTC
CAGCCCCCTTCAGCATCTCCGGCTACGACATCCCTGAGGGCACAGTCATCATTCCGAAC
CTCCAAGGCGCCCACCTGGATGAG
>1883
GGCAGAAGAAGGTCAGGCCCTCGGCTTCCTTGGTCAGTTCCCACCCTCCAGCCCCCAG
CTCCTCCTGCAGACAAGCTGGTGTCTAAAACTACCCGGACCTGTCGTTGGTCTCTGCT
CTGGAAAGCCCACAAGAAGCTCAC
>1884
CACCTCTCACAGTAGCCCCGCCCTGCCCGCTGCACAGCGGCCTGCTGAACTCACACT
GTTTCTCCACAGCGCATGAGAGCCCAGCCCGGCACCCCTGTGGCCATTGAGGAGGAAT
TCTCTCTCCTCACCTGCAGCATCA
>1885
GGCCCACCCCCAGCCCCTCCCTGAGCCTCTCCTTGTCCTGAACTGAAAGTACTCCATCC
TTTCCTGGCAGGAGGACAACTTAATGCCTGCCTATTACAAATGTATCCAGGAGGTGTTA
AAAACCTGGAGCCACTGGTCCA
>1886
TGTGGGGGAGAGGCTGGGGTGGAGGGAGAGGCTCCTTCCCACAGCTGCATTCTCATG
CTTCCTGCCGCAGTTCTTCCCCAATCCAGGTCTCCGGAGGCTGAAGCAGGCCATAGAG
AAGAGGGACCACAAGAGAGAACAGC
>1887
TGGGCCTGTTGCCACTCTGTACTCCTCTCCCCAGGCCAGCCGCTCAGCCCGCTCCTTTC
ACCCTCTGCAGGAGAGCCTGGTGGCAGGCCAGTGGAGGGACATGATGGACTACATGC
TCCAAGGGGTGGCGCAGCTAGCAT
>1888
GCTGAGGGAGCGGCTGGAGGCTGGGCAGCTGTGGGCTGCTGGGGCAGGACTCCACCC
GATCATTCCCCAGATTCAGCAGCGACTGTGGAGGAGCTAGACCACGAACTGGGCCCTG
GTGCCTCCAGCTCCCGGGTCCCCTA
>1889
TCTCCCTTATCCCTCTCACTGCATTGCCCCTGCTATGGGCCCAGCTCACTTGGCCACCTG
TCTCTTGCAGAGCCTGGTGAAATGGGAGAGTGAGAATAAAATGGTCTGTGAGCAGAA
GCTCCTGAAGGGAGAGGGCCCCA
>1890
TCAACCCTCCTCTCCAAAGCCACAGGACCCCAGGGGCCTCTCAGGCTAACAACTACTT
CTGTCCTTCCAGACCATGACGGCGGATGACGTTGTGTGCACCAGGGTCTACGTCCGAG
AGTGAGTGGCCACAGGTAGAACCG
>1891
ACGTAAGTGAGGGGATAGATCTGTGTGATCTGAGAAACCTCTCACATTTGCTTGTTTTT

CTGGCTCACAGACATGTCGAGGAAGGCTTTTGTGTTTCCCAAAGAGTCGGATACTTCC
TATGTATCCCTCAAAGCACCGTT
>1892
CTCCTGGAGCAGGGAGAGCGCTGGCCTCTTCCTCTCCGGCTCCCTCCATTGCCCTCCG
GTTTCTCCCCAGGCTCCCGGACGTCCCTGCTCCTGGCTTTTGCCCTGCTCTGCCTGCCC
TGGCTTCAAGAGGCTGGTGCCGT
>1893
AAGCAACAATGGGAGCTGGTCTCCAGCATAGAAACCAGCAGTCCTTCTTGGTGGGGG
GTCCTTCTCCTAGGAAGAAACCTATATCCCAAAGGACCAGAAGTATTCATTCCTGCATG
ACTCCCAGACCTCCTTCTGCTTCT
>1894
AGGCGGGGATGGGGAGACCTGTGGTCAGGGCTCCCGGGCAGCACAGCCACTGCCGGT
CCTTCCCCTGCAGAATCTAGAGCTGCTCCGCATCTCCCTGCTGCTCATCGAGTCGTGGC
TGGAGCCCGTGCGGTTCCTCAGGA
>1895
TGGATAAATGAGAGAGGGAGGGAACAGTGCCCAAGCGCTTGGCCTCTCCTTCTCTTCC
TTCACTTTGCAGAGGCTGGAAGACGGCAGCCGCCGGACTGGGCAGATCCTCAAGCAG
ACCTACAGCAAGTTTGACACAAACT
>1896
AGTGGTGGAGAGTTCTTGTACCACTGTGGGCACTTGGCCACTGCTCACCGACGAACGA
CATTTTCCACAGGAGCCGACCTGCCTACAGACCCGCCTGGAGCTGTACAAGCAGGGCC
TGCGGGGCAGCCTCACCAAGCTCA
>1897
CTGGTGCTTCCTTCCCCCACGTTACCCACTTGCCTGGACTCAAGTGTTTTTTATTTTTCT
TTTTTTAAAGGAAACTTCCTGTGCAACCCAGATTATCACCTTTGAAAGTTTCAAAGAGA
ACCTGAAGGACTTTCTGCTTG
>1898
GGAGCCACTAAGCAGGTGCCCACCCAGGAGATTCCATGGGTGCCCACAAGCAGACAG
ACTTTTCCTTCAGGGGAGATCATCGGGGGACATGAGGCCAAGCCCCACTCCCGCCCCT
ACATGGCTTATCTTATGATCTGGGA
>1899
CTGCTCTTCACTGTCTCTGGGCTGTATACCCTGTGACTCCACCCCCATCCTCACTCTGCT
CTCTCTGCAGCTCCATAAATGTCACCTTGGGGGCCCACAATATCAAAGAACAGGAGCC
GACCCAGCAGTTTATCCCTGTG
>1900
GCCCCATCACTGTGTCGGGGCCCAGAAGTTCGTTGGCTGACCTGGACTTTCTTGCCTC
TTCCCCCACCAGCTGGAGAGAAAGGCCAAGCGGACCAGAGCTGTGCAGCCCCTCAGG
CTACCTAGCAACAAGGCCCAGGTGA
>1901
GGGCTTGAGATGGAAGAGAGGGCAGGACCCGGAGGAGCATCAGCTCAGTCCTTCCAC
TCTCTATTCACAGGGGGACTCTGGAGGCCCTCTTGTGTGTAACAAGTGTGCCCAGGGC
ATTGTCTCCTATGGACGAAACAATG
>1902
CTGCTCTTCACTGTCTCTGGGCTGTATACCCTGTGACTCCACCCCCATCCTCACTCTGCT

CTCTGTGCAGCTCCATAAATGTCACCTTGGGGGCCCACAATATCAAAGAACAGGAGCC
GACCCAGCAGTTTATCCCTGTG
>1903
GGAGCCACTAAGCAGGTGCCCACCCAGCAGATTCCATGGGTGCCCACAAGCAGACAG
ACTTTTCCTTCAGGGGAGATCATCGGGGGACATGAGGCCAAGCCCCACTCCCGCCCCT
ACATGGCTTATCTTATGATCTGGGA
>1904
CTGCTCTTCACTGTCTCTGGGCTGTATACACTGTGACTCCACCCCCATCCTCACTCTGCT
CTCTGTGCAGCTCCATAAATGTCACCTTGGGGGCCCACAATATCAAGGAACAGGAGCC
GACCCAGCAGTTTATCCCTGTG
>1905
AGCCCCATCACTGTGTCGGGGCCCAGAAGTTCGTTGGCTGACCTGGACTTTCTTGCCT
CTTCCCCACCAGCTGGAGAGAAAGGCCAAGCGGACCAGAGCTGTGCAGCCCCTCAGG
CTACCTAGCAACAAGGCCCAGGTGA
>1906
GGGCTTGAGATGGAAGAGAGGGCAGGACCCGGAGGAGCATCAGCTCAGTCCTTCCAC
TCTCTATTCACAGGGGGACTCTGGAGGCCCTCTTGTGTGTAACAAGGTGGCCCAGGGC
ATTGTCTCCTATGGACGAAACAATG
>1907
AAATGCAGGAATATAACATGTGGCAAACTATCAGGAGTGTGAAATAACCGATGCATTCT
TTCTTGTTTAGGCATCATCTGGGGAGAGGATACACTGATGGAGTATTTGGAGAATCCCA
AGAAGTACATCCCTGGAACAAA
>1908
CAAATAGGACTAGGACCTGTAGTCTGGGGTGATCCTGGCTTGACAAGAGGCCCTGACC
CTCCCTCTGCAGTTGCGGCGCCGCTTCGGGGACGTGTTCAGCCTGCAGCTGGCCTGGA
CGCCGGTGGTCGTGCTCAATGGGC
>1909
GTTGGAGTGGGTGGTGGATGGTGGGGCTAATGCCTTCATGGCCACGCGCACGTGCCCG
TCCCACCCCCAGGGGTGTTCCTGGCGCGCTATGGGCCCGCGTGGCGCGAGCAGAGGC
GCTTCTCCGTGTCCACCTTGCGCAA
>1910
GGCACAAAGCGGGAACTGGGAAGGCGGGGGACGGGGAAGGCGACCCCTTACCCGCA
TCTCCCACCCCCAGGACGCCCCTTTCGCCCCAACGGTCTCTTGGACAAAGCCGTGAGC
AACGTGATCGCCTCCCTCACCTGCGG
>1911
CAAATAGGACTAGGACCTGTAGTCTGGGGGGATCCTGGCTTGACAAGAGGCCCTGACC
CTCCCTCTGCAGTTGCGGCGCCGCTTCGGGGACGTGTTCAGCCTGCAGCTGGCCTGGA
CGCCGGTGGTCGTGCTCAATGGGC
>1912
GTTGGAGTGGGTGGTGGATGGTGGGGCTAATGCCTTCATGGCCACGCGCACGTGCCCG
TCCCACCCCCAGGGGTGTTCCTGGCGCGCTATGGGCCCGCGTGGCGCGAGCAGAGGC
GCTTCTCCGTCTCCACCTTGCGCAA
>1913
GCACAAAGCGGGAACTGGGAAGGCGGGGGACGGGGAAGGCGACCCCTTACCCGCAT

CTCCCACCCCCAAGACGCCCCTTTCGCCCCAACGGTCTCTTGGACAAAGCCGTGAGCA
ACGTGATCGCCTCCCTCACCTGCGGG
>1914
TGGGGCCTGAGACTTGTCCAGGTGAACGCAGAGCACAGGAGGGATTGAGACCCCGTT
CTGTCTGGTGTAGGTGCTGAATGCTGTCCCCGTCCTCCTGCATATCCCAGCGCTGGCTG
GCAAGGTCCTACGCTTCCAAAAGG
>1915
CAGGTGCAGAATTGGAGGTCATTTGGGGGCTACCCCGTTCTGTCCCGAGTATGCTCTCG
GCCCTGCTCAGGCCAAGGGGAACCCTGAGAGCAGCTTCAATGATGAGAACCTGCGCAT
AGTGGTGGCTGACCTGTTCTCTG
>1916
GAGGCAAGAAGGAGTGTCAGGGCCGGACCCCCTGGGTGCTGACCCATTGTGGGGACG
CATGTCTGTCCAGGCCGTGTCCAACAGGAGATCGACGACGTGATAGGGCAGGTGCGGC
GACCAGAGATGGGTGACCAGGCTCA
>1917
TGTGTTTGGTGGCAGGGGTCCCAGCATCCTAGAGTCCAGTCCCCACTCTCACCCTGCAT
CTCCTGCCCAGGGAACGACACTCATCACCAACCTGTCATCGGTGCTGAAGGATGAGGC
CGTCTGGGAGAAGCCCTTCCGCT
>1918
CCCTTCCGTGGAGTCTTGCAGGGGTATCACCCAGGAGCCAGGCTCACTGACGCCCCTC
CCCTCCCCACAGGCCGCCGTGCATGCCTCGGGGAGCCCCTGGCCCGCATGGAGCTCTT
CCTCTTCTTCACCTCCCTGCTGCA
>1919
ATTGGGACTGCAGGGTGGGGCCACGAGGGACATGACCCCGTCCAGCAGGGCCTCCTG
CTTGGCCCCACAGGTACAACTTGGGAGGACCACGCATGGTGTGTGTGATGCTGCCGGA
GGATGTGGAGAAGCTGCAACAGGTG
>1920
AGGATGGGATACGGGTCAGGGCCTGTGTCTTGCTGGGGCGGCCTCACAAGCTCTGCCC
TGGCCTCTGTAGGAATGGGCCTGAATGGCGCTTCAACCGATTGCGGCTGAACCCAGAT
GTGCTGTCGCCCAAGGCCGTGCAG
>1921
GAAGGGAGTGTGGGGAGGCAGCCAGGAGGCCTGGGGCTGCCTTGTGCTCAGCAGTGC
ATCCTCCCCGCAGCCAGCAACTTAGCTCTTTTTGGAGAGCGGCTGGGCCTGGTTGGCC
ACAGCCCCAGTTCTGCCAGCCTGAA
>1922
GCAGGAGGAGGACACTGAAGGATGCTTCCCAGCACCAAGATCTGAGGGCTGTCCCCT
GCTCCCTGGACAGGTGACAACTGTATCCAGAAAATCTACCAGGAACTGGCCTTCAACC
GCCCTCAACACTACACAGGCATCGT
>1923
CCTGGGAGGGGTGTCCCGGGGGCTGAGTCCTCCTGTGCAAGGTCTGACCCTGCAGAC
ATGGCTTCTGTAGACAGCGTTTCCCTTGCTGATGACGCTCTTTGAGCTGGCTCGGAACC
CCGACGTGCAGCAGATCCTGCGCC
>1924
GGAAGGGTGCTGAGAGCACAGGGAGCCCCATCCAGCTGAGGACCCTTTCTGTGGATG

CCCCCACCTCCAGGCTCTACCCTGTGGGTCTGTTTTTGGAGCGAGTGGTGAGCTCAGA
CTTGGTGCTTCAGAACTACCACATC
>1925
CCCCACACCCCTCGAGCTGAGAACCTCCCTCCCCAGTCATTCCCTGATCCCTGCTCTGC
ACCGTCCGCAGACATTGGTACAGGTTTTCCTCTACTCGCTGGGTCGCAATGCCGCCTTG
TTCCCGAGGCCTGAGCGGTATA
>1926
GATGGGAGGAAGCTGCCCCAGGTCCATGGGCTACTGACCAGGCCAGATGGAAACCCA
GCCTCTGTCCTAGGTGCTGAAGCACTTCCTGGTGGAGACACTAACTCAAGAGGACATA
AAGATGGTCTACAGCTTCATATTGA
>1927
CAGGTGTGGCTTAGAGCCCCGCACCTCCTCGCCGCGCGCGGGCCTGACTTCTAGCCAC
GGGTCTCCGCAGTTGGCCCAGCGCTTCGGGCCGGTGTTCACGCTGTACGTGGGCTCGC
AGCGCATGGTGGTGATGCACGGCT
>1928
GGCCCTCTGTAGCCCATTTCTCCCCAAAATGGGTATTTAGAATAACCTTCTGCTGGCCC
CTCTGCCTTAGGAATCATTTTTAATAATGGACCTACCTGGAAGGACATCCGGCGGTTTT
CCCTGACCACCCTCCGGAACTA
>1929
TGGGTGACTGAGCAGGTGGAGGAGTCTCCTCACCCCCATCTTCTGGTTGCCCTGACTG
CCTGTTTTGTAGGCCAGCCTTTCGACCCCACCTTCCTCATCGGCTGCGCGCCCTGCAAC
GTCATAGCCGACATCCTCTTCCG
>1930
TTGTTGGTCCAACACACACACAATTATGTTGCATCCAGAAAAAAGTAGTAAAATATTTTTT
TCCCTCTCTAGCTTTACAATAATTTTCCCAGCTTTCTACACTACTTGCCTGGAAGCCACA
GAAAAGTCATAAAAAAATGTGG
>1931
GGAGGTGGCTGGTTCTGTGCTGAAAGGAGACAAGCAGCCCCTTCTCTCCGGTCTGTCT
CCGGTATCACAGGAAAAGCACAGTGCAGAGCGCTTGTACACAATGGACGGTATCACCG
TGACTGTGGCCGACCTGTTCTTTG
>1932
TGGATGATGGGTGGATGCCCAACTGGCCAGGAACCAATCCCTGAAATTTGTCCCATTCA
TATCTTGGCAGAGAAGCTCCATGAAGAAATTGACAGGGTGATTGGGCCAAGCCGAATC
CCTGCCATCAAGGATAGGCAAGA
>1933
CTGGGGGTTTCCAGATGAAAGCCCACATTTTGTTAACATGACTCACTGAGACAGTCTTT
GTTTCTCCTAGGGCACAGTCGTAGTGCCAACTCTGGACTCTGTTTTGTATGACAACCAA
GAATTTCCTGATCCAGAAAAGT
>1934
GTCTCACTGTTAACAGTGTCGTGTCTCTGAAACTCCCTCAGTGTCTCATCAATACCATT
GTTACTTCTAGGAAAACGAGTGTGTGCTGGAGAAGGCCTGGCTCGCATGGAGTTGTTT
CTTTTGTTGTGTGCCATTTTGCA
>1935
AGTTAGTCTGGCTGTGCTTGTCCTTTTTCCCACCTCCCTCGCTGTGCCTGACCCTCTCTT

CTTTCCACAGGTTCTCAGGCAAGAGCCACCTGCTATTGCCGAACCGGCCGTTGTGCTA
CCCGTGAGTCCCTCTCCGGGGT
>1936
TGGCCTCTCGCGTTTGCTCTGCCCCACCTGGGTCGCGGAGACCATGTCCTTCTCGCTTG
GCCTCTCCCAGGACGTGGATGCAGCTACTCTAGCTCGCATTGACCTGGAGCGCAGAAT
TGAATCTCTCAACGAGGAGATCG
>1937
GGCTGGGAATAGGGGTGTGAGGGTCGTGTGTGGGCCCTGAGAGGGACTGAAGCCCAG
TCATGCCCTACAGGAGATCCGTGAGTTGCAGGCTCAGCTTCAGGAACAGCAGGTCCAG
GTGGAGATGGACATGTCTAAGCCAG
>1938
ATGCTCCCAAGCTCATCCCTACCCGTGCCCTGCATCCTTCTCATTTTTGGGCCCCTTTCT
CTGCCCTTAGGTGTCAGACCTGACCCAGGCAGCCAACAAGAACAACGACGCCCTGCG
CCAGGCCAAGCAGGAGATGATGG
>1939
GCCCCAAAGCTTTCTTTGGGCTGCTAGTGTCCTCTTCCCTTCCTTGACCTGGGTTCCCC
CTCTCCTGCAGAACGATTCCCTGATGAGGCAGATGCGGGAATTGGAGGACCGATTTGC
CAGTGAGGCCAGTGGCTACCAGG
>1940
GGACTCCCAGCCCCTGGTATAGCCCAGCCTGGACTTGGTCAGGCTGAGTGTGCGATGG
ACCCTGTTACAGAAACCAGCCCTGAGCAAAGGGGTTCTGAGGTCCATACCAAGAAGA
CGGTGATGATCAAGACCATCGAGAC
>1941
TGCGATCCTGGGTGGGATGCCTAGGCTGACGTCATCCTGCTAGCACATGTGACTGGACT
CCTCTCCCCAGGTCGTCAGTGAGGCCACACAGCAGCAGCATGAAGTGCTCTAAAGAC
GAGAGACCCTCTGCCACCAGAGAC
>1942
TTGCTGCTTGTGCCCGAACTAACTCTCAGGCACATTAGTCAGAAAATATTACGTTTGGT
TGCTCCCCGAGGTTCCTAAAAGTAAAGCTCTAGAGGCCGTCAAATTGGCAATAGAAGC
CGGGTACCACCATATTGATTCTG
>1943
TACCTCAGAGCATGGCTATGTGTGGAGAAATTGACTTGTGACATCACTAAACTGACTGC
TTCTACTTCAGCCAGGTGAGGAAGTGATCCCAAAAGATGAAAATGGAAAAATACTATT
TGACACAGTGGATCTCTGTGCCA
>1944
AGTTCTATGTTACATTTATCTTGCTCTTCAGTCGACTACACAAATGATACCTCACAATTC
CTTTTTCCAGGCCATGGAGAAGTGTAAAGATGCAGGATTGGCCAAGTCCATCGGGGTG
TCCAACTTCAACCACAGGCTGC
>1945
TACTTTGGTGATTTTAATATTAATGTAACTTTTGGATTATCTGATGCTTTTCCATCTTGCTC
GTCTGCAGGTGGAATGTCATCCTTACTTCAACCAGAGAAAACTGCTGGATTTCTGCAA
GTCAAAAGACATTGTTCTGG
>1946
CTGTTAGGGAGCCGCCTAACAAACTGTATCCCCAGCCTCAGGGCCTCAGCCTTTCTGC

CTTTCCTTCCAGGGTGGACCCGAACTCCCCGGTGCTCTTGGAGGACCCAGTCCTTTGT
GCCTTGGCAAAAAAGCACAAGCGA
>1947
TGAACTTGGTTGTTCCCAATTGGCAATGATGTAATCTAAAAATAATTAAAGTTCCCTATT
ACTGTGATAGGTGTTTGAATTCCAGTTGACTTCAGAGGAGATGAAAGCCATAGATGGC
CTAAACAGAAATGTGCGATATT
>1948
ATCACTGCGCTACCGGCTAGTAACGGAGTCATTGCCATTCAGAGTGTGCATTTTTTTTTC
CTCTTTCCAGTTTTGCTGGCCCCCCTAATTATCCGATTTCTGATGAATATTAACATGGAG
GGCATTGCATGAGGTCTGCC
>1949
TGCTTGCTTTCCTCCAGACTTTGAGCCCTGGCCTGATGTAGACCTTTTTGCTCTCTCCTC
CTTCGTATAGGTACGGTTCCTGGAGCAGCAGAACAAGATGCTGGAGACCAAGTGGAGC
CTCCTGCAGCAGCAGAAGACGG
>1950
ACCTTGGGAGCCCCTTGGTTGGGGTTGGGTGTGGAAACCCAGCTCAGGCTCCCCTCTC
CTCATCCCCCAGGATGTGGATGAAGCTTACATGAACAAGGTAGAGCTGGAGTCTCGCC
TGGAAGGGCTGACCGACGAGATCA
>1951
AAGGCTGTTGTGGAAAAGGGGGCTCCTGTGTGCAGAGACAGGGCCTCACCACTTGCC
CTCTTCCCCACAGGAGATCCGGGAGCTGCAGTCCCAGATCTCGGACACATCTGTGGTG
CTGTCCATGGACAACAGCCGCTCCC
>1952
GAGGGTGGACGCACGGAGGAAGAGAAGGCATACCCAACCTGACCTACTTACCTGTCC
CCTACCCACAGAGAGGGCTTCCCTGGAGGCCGCCATTGCAGATGCCGAGCAGCGTGG
AGAGCTGGCCATTAAGGATGCCAACG
>1953
CAGGGAGAAGGGCATGGAGCCTGCCATGGTGGGCTTCTGTACTCATGTGGCTACCTCT
GTCCCTCACCAGGTGGTCTGAGCTCGGCCTATGGGGGCTCACAAGCCGGCCTCAGCTA
CAGCCTGGGCTCCAGCTTTGGCTC
>1954
GCCCAGCCAGGCCCTTTCTGGCCGTGTGGCTCAGGAACACAGCCTCTTCCTCTTTTTTT
TCCCCTCCTAGCCCTATTCAGGCAGGAGCTGCTCTTCTGGGGTATCGCGATCCACTTAA
GGATGAGGCAGACTTGGTGACA
>1955
ATCTCGGGAGGTCACTGGGAGGCTGGTGCTGGGCAGCAGCTAGTGCCACTTCCTCTGT
CCCATATTTCAGCGCCTCCAGAGCCAGAACTGAGCCCAGTGAGAGCGCACCCTGGGGC
AGCCTGGATTCCTGGGGTGTCCCC
>1956
CCCCTGGGCCCACTGCCTCCTCTCAGGGCAGTTGGCACTGAGTCAGGCTGGCCTTCCT
GATGTCCCACAGATACTGGCTCGCTGTGACATCATGGTGCTGCAGGAGGTGGTGGACT
CTTCCGGCAGCGCCATCCCGCTCC
>1957
TTCTGTGGGTCTAATCAGGGGCACACCCAGAGGCTTGAGGCCCTGACCCCGCCTCCCC

TTTTCTTGGCAGATTTGATGGCTCTGGGCCCTACAGCACCCTGAGCAGCCCCCAGCTG
GGGCGCAGCACCTACATGGAGACG
>1958
CGCCGTTTGGCACTTGCAAGCCAAAATCAAAGACGTGGGGCCACTGCTGCCCATCCCT
TTGTCCTCCCAGGTCACACAAAACACAGGTCCTGAGTTCCTACGTGTACAACGATGAG
GATGACGTCTTTGCCCGGGAGCCA
>1959
CCTCACCCAGACGGGCTAGGGGGAGAAGGTCCAGGAGTGGATGGCAGCCCTCGGTCC
CTGGGCCTTGCAGTCCTTCCCAGCCTGGTGTTGGTCCCGCTGCACACCACTCCTAAGG
CCGTAGAGAAGGAGCTGAACGCCCT
>1960
GGGCAGTTTGTACAGGAGGGACAAGGATAGGGTGGCCCAGCGGCCAGCCTGATGGCT
GCTGTGTCTCTAGGACGTGATCCTGCTTGGGGACTTCAATGCTGACTGCGCTTCACTGA
CCAAAAAGCGCCTGGACAAGCTGG
>1961
GGGCAGCAGCAGGGTCACAGGGAAGCCCCTCGGCCCCGGGCACTGACACTGCCCCAC
CACCCATTCCCAGGCCCTCAACATCAGTGACCACTACCCCGTGGAGGTGGAGCTGAAG
CTGAGCCAGGCGCACAGCGTCCAGC
>1962
TATTCCCTGCTTCCACAGCGGCAGACCAGCCAGTTGCAGGCACCAAGCCTTTGGGCAT
GTTTCCTTCTAGGTCGGCGCCTGCATCGTGAATTCAGAAAACAAGATTGTCGGGATTGG
GTACAATGGGATGCCAAATGGGT
>1963
GTGTTAACACTTAGTAAATTCTTCATGAGCTGCAACCAAAGTTTTTCTTTCTTTCCCTTT
CAACCCACAGTGTGCCATGCGGAGCTGAATGCCATCATGAACAAAAATTCGACCGATG
TGAAAGGCTGTAGTATGTATGT
>1964
CTCGTTCCGCCTGTCTTTTGCAGCATCTGTAGGTGCGTACACTAAGGGAGCTCCCATTG
TTACCTTACAGGTATAAAAGAAGTGATTTTCATGTCTGATAAATACCATGATAGTGACGA
GGCAACTGCTGCGAGGCTCCT
>1965
GTTAAGCCAAAGTAAACAAATTGCTTTCACTACTCACCAGATAATAATTGTATTTTGTTT
TTATTTTTAGGAAATTCATACCGAAGTGCAGCAAGATTGTCATTGACTTTGATTCAATTA
ACAGCAGACCGAGTCAAAAG
>1966
TTTAATTAAAGAAAAATATTAAAAAGGAAAAATGAACTACTTATGATTTTCTTTTTTGCA
TTTTTCCTAGAGGATGACTTGGTTACAGTCAAAACCCCAGCGTTTGCAGAATCTGTCAC
AGAGGGAGATGTCAGGTGGGA
>1967
GATTCTATACAGCTAATTTTCTAATGCTGGATAAACATTTGGACTTCCTCCATCTGTCTTC
CTCTTCCAGACATCTGTGCAGGTTCCATCACCAGCAAATGGCGTGATTGAAGCTCTTTT
GGTACCTGATGGGGGAAAAG
>1968
CCACTAAATTTTGCTTGGTTGCTTCTCATTTCAGACAGTGCCAGTGGCATATACTTTTCT

TGTTTTTCAGCTGCTCCTGCTAAGGCCAAGCCGGCTGAAGCTCCTGCTGCTGCAGCCC
CAAAAGCAGAACCTACAGCAGC
>1969
AGCACTGGCACAAACTCAGCAGATGTTTCTTTCCTTGTCTGATGCAGCTTTATCCTCTT
TTCATTTTCAGTGTCTGCAGTAAAACCCACTGTTGCCCCACCACTAGCTGACGCAGGA
GCTGGCAAAGGTCTGCGTTCAGA
>1970
TCTGACTACACGGGGAATGCTTGACCCAGAGAGATCAGATTGTCAATGCTTGTTCCACT
CTTACTTTCAGGAGAAAATGAACAGGATGCGGCAACGCATTGCTCAGCGTCTGAAGGA
GGCCCAGAATACATGTGCAATGC
>1971
GGATTGAGAAACCTGCTGTGAAAGTCAAAATGCAGGCTTTGTGGTCAGTTTGTTTTGT
AATGTTTCACAGTAACATCCAGGAGATGAGGGCTCGGCACAAAGAGGCTTTTTTGAAG
AAACATAACCTCAAACTAGGCTTC
>1972
TGCACATATTACCTCATTAGTCTTGGCCTTCCTGGGGAGTTGTATGTAACATGTATTTTCT
CTCTCATAGTGATTGACGACACAACCAAAGAGGTGGTGTATACGGATTATATTGACATC
AGTGTTGCAGTGGCCACCCC
>1973
TTCAAGGGAGTTGTTAACTATAAAAGGTACTATTAATTTGCAACTGAAAACAGCTTTTC
ACCCCCTTCAGGGTCTGGTGGTTCCAGTCATCAGGAATGTGGAAGCTATGAATTTTGCA
GATATTGAACGGACCATCACTG
>1974
GGATTATGGACTGACTTTAGGTGAAGGAAACTGGGGTGGCTTAATATTTCAATTATGAA
ATCTGTTTTAGGCCCGAAAGAATGAACTTGCCATTGAAGATATGGATGGTGGTACCTTC
ACCATTAGCAATGGAGGCGTTT
>1975
GCTAAATCTCCTTCAGCGAGGCTGGCTGTGGCTTGCTGGAGACAAACCTATTTACCTTT
CCTCTCTGTAGGTAGAGGTGCGGCCCATGATGTACGTGGCACTGACCTATGATCACCGG
CTGATTGATGGCAGAGAGGCTG
>1976
CCTAGCCCTATGCAGCCCCACAGTGGGACCCTTGCATGGACCTCAACCATCAGAATCTT
TTCTTTTGCAGGTCGTTACTCTCTGACCTATATCTACACTGGGCTGTCCAAGCATGTTGA
AGACGTCCCCGCGTTTCAGGC
>1977
GACTAAGAGTTATTTTGATAAATTTCTCACCAGGCTGACTGTCTCTCTGTGGGGTCGGG
GGAGTTTTCAGGGTCTCACGTATTGCAGGGAAGGTTTGGTTGTGAGATCGAGAATAAC
AGAAGCAGCGGAGCATTCTGGAA
>1978
GGGTAGGAAGAGACCTGGGCCGGGAGAAGTCCACCTCAAGCCTGCAGTGTCACACTC
TATCCCTCCACAGATCCTCCCTCTGTGGTGGTCACCAGCCACCAGGCCCCAGGAGAAA
AGAAGAAACTGAAGTGCCTGGCCTA
>1979
GGGAGAGAAGGGAGGAGCCCTTGGAGGCTAGAAGGGAAGTCAGATCTTCCTCCTCTC

CCAAAACCTCCAGTGTATGCCACGTTGAGGGACCTGAAAACACAGGGCCGGCTGTGG
GAGGCGGCCCGGGCCCTGGCATGCCC
>1980
GGAGCACGAGGGGACAGGCCGTGCTGAGGGTGATGCTGAGGCGGGCTGGTCGGGCCT
CTTGTCTCCGCAGTGTGTAACGCAGGCCTGGGCCTGCTGGGGCCGCTGGAGGCGCTGG
GGGAGGACGCCGTGGCCTCTGTGCT
>1981
GAGGGGCACCGTCTGCCCGGGGATGACCCCCTGGCCGCTGCGCCTCAGGAACCTCGT
CTCCCCACCTAAGGGCTGCCTTTCAATGACGTTTATTGCGCCAGCAAGTTCGCGCTCGA
AGGCTTATGCGAGAGTCTGGCGGT
>1982
TGGGGTTGGGGCTGGGACTGGGGCCTGGCTGGCGTCCGCCCCCTCCCACTCGTTGCTC
TCCGGCCAGCAGCTTGAGCCTGATCGAGTGCGGCCCAGTGCACACCGCCTTCATGGAG
AAGGTGTTGGGCAGCCCAGAGGAG
>1983
ACTCCAGGAGTGGGGGCGGTGCGTCCTCCGGCCGGCAGCGGTGGCCACAGCTCTCCT
CCCGCCGCCGCAGGTCTTCCTCACCGCTTTGCGCGCCCCGAAGCCGACCCTGCGCTAC
TTCACCACCGAGCGCTTCCTGCCCC
>1984
TACTGTGATCCAGCATGTCTCTCGGCGTTTGAGGAGACATCCCCCACTGACCTGCTCTT
TCTCTCCCCAGCAGTCTTAGGCGCTGAGCTCAGCGCGGTGGGTGAGAACGGCGGGGA
GAAACCCACTCCCAGTCCACCCTG
>1985
GTGTGTCCATGTGTCATTTTAAAGACTATTAATTACACTAATATAGTTTCTTTCTCTCTTT
GGATAATAGGCACGTTGTTCCGTATGGACTTGGAAGCCCTAGGTCCAAGAGAGCCTTG
GAGAATTTACTTCCCACAAAG
>1986
TACATGTTTCTTTTGCCAAAGGGTGATTTTTTTAAAATAACATTTGTTTTCTCTTATCTTG
CTTTATTAGGTCGGAGACCATGAGAAACAGCGTCAAATCATCTTTTCATGATCCCAAGC
TGAAAGGCAAGCCCTCCAGA
>1987
GCTGGGGACAAGGAAATTGAAGCTGGAGTTTGTGTTTTAGTAACCAAGTAACGACTCT
TAATCCTTACAGATGGGAAAGGGCTCCTTCAAGTATGCCTGGGTCTTGGATAAACTGAA
AGCTGAGCGTGAACGTGGTATCA
>1988
AAGGCTTGCTTTGTACACTGGTTTTGTCATTTGGAGAGTTGACAGGGATATGTCTTTGC
TTTCTTTAAAGGCTGACTGTGCTGTCCTGATTGTTGCTGCTGGTGTTGGTGAATTTGAA
GCTGGTATCTCCAAGAATGGGC
>1989
AAGACCATTGTTAAAAAGCTCTGGGAATGGCGATTTCATGCTTACACAAATTGGCATGC
TTGTGTTTCAGATGCCTTGGTTCAAGGGATGGAAAGTCACCCGTAAGGATGGCAATGC
CAGTGGAACCACGCTGCTTGAGG
>1990
AAAGTTGAATTTGAGTTGATAGAGTACTGTCTGCCTTCATAGGTATTTAGTATGCTGTAA

ATATTTTTAGGTATTGGTACTGTTCCTGTTGGCCGAGTGGAGACTGGTGTTCTCAAACC
CGGTATGGTGGTCACCTTTGC
>1991
TTAACTTATTGCAGAGGCTAAAGTCATTTGAGACTTTGGATTTGCACTGAATGCAAATC
TTTTTTCCAAGGTGATTATCCTGAACCATCCAGGCCAAATAAGCGCCGGCTATGCCCCT
GTATTGGATTGCCACACGGCTC
>1992
AAAGTTGTGTTAAAGATGAAAAATACAACTGAACAGTACTTTGGGTAATAATTAACTTT
TTTTTTAATAGGTCGCTTTGCTGTTCGTGATATGAGACAGACAGTTGCGGTGGGTGTCA
TCAAAGCAGTGGACAAGAAGGC
>1993
GCTGTCTGTGGTTCTGGGGAGGAACAAGGGGAGATGTGAAGGAAGGACACTTAGCTA
TCCTCCCTGCCAGGGTCTGAGACTTCCACCTTTGAGACCCCTTTGGGTGCTAAGACGC
TGCCTGAGGATGAGGAGACACCAGA
>1994
GGGACTCAAGACCCATGTGAATTCATGTAGGAAGGATGCTCCATTCTTTGTCTTTTATC
CTGCCCTGTAGTTTACTTGCCGGAGGTGCTACAGGGGCAACCTGGTTTCCATCCACAA
CTTCAATATTAATTATCGAATCC
>1995
GATACGGGGCCAGGTCTGCTTCTCTGCCTTCCCTCTGCCCCATCCCTTCTTCCCCTCTTG
CTTTCTCCAGGGTCGCTGCAGACGCTTTCAGTGGGTTGACGGCAGCCGCTGGAACTTT
GCGTACTGGGCTGCTCACCAGC
>1996
GGGGTCGCTGCGTTCCAGGGAACTCTCCCAGCAACCTGCCCTATAAAGGGTGTCTCTC
TTTCTTCCCCAGCTGGTCCTGCCGCTGCTGTGTTGAGAGAGCTGCGTTGCGTTTGTTTA
CAGACCACGCAAGGAGTTCATCC
>1997
AGAGAAATCCCGCAGCCTGGGTCTTCAACCTTGGTATCTCATGAGTGTATCTTCTTTTT
CTTTCCTTCAGAGCCTCCCTGAAGAACGGGAAGGAAATTTGTCTTGATCCAGAAGCCC
CTTTTCTAAAGAAAGTCATCCAG
>1998
AAGGAAGCTGTCCTTCCACAGCCACCCTTCTCCCTCCCCGCCTGACTCTCAGCCTGGC
TATCTGTTCTAGAATGTCCTGCCTGGCTGTGGCTTCTCCTGTCCCTGCTGTCGCTCCCTC
TGGGCCTCCCAGTCCTGGGCGC
>1999
ATCCTACGGCCTGTGGGCCAGGGCCAGAGCCTTCAGGGACCCTTGACTCCCCGGGCTG
TGTGCATTTCAGACGGGCTGTGCTGAACACTGCAGCTTGAATGAGAATATCACTGTCCC
AGACACCAAAGTTAATTTCTATG
>2000
TGGGGCTGCTGAGGGGCAGGAGGGAGAGGGTGACATGGGTCAGCTGACTCCCAGAGT
CCACTCCCTGTAGGTCGGGCAGCAGGCCGTAGAAGTCTGGCAGGGCCTGGCCCTGCT
GTCGGAAGCTGTCCTGCGGGGCCAGG
>2001
AGTACAGGAACTGTCCGTATTCCTTCCCTTTCTGTGGCACTGCAGCGACCTCCTGTTTT

CTCCTTGGCAGAAGGAAGCCATCTCCCCTCCAGATGCGGCCTCAGCTGCTCCACTCCG
AACAATCACTGCTGACACTTTCC
>2002
TTCCTTCATAAAGACCCAACCCTATGACTTTAACTTCTTACAGCTACCACAGCCCCTGG
GCCCGCAACAGTTGTTACAGGTTCTGGTCATGCAAGCTCTACCCCAGGTGGAGAAAAG
GAGACTTCGGCTACCCAGAGAAG
>2003
GAGACATTGAAAGTCAGGGTAAAGCTGAATATAACATTGCTGAAATGTTTTTCCTTGTG
TATTTTAACAGGGCTGAAGACATTATGGAGAAAGACTGGAATAATCATAAGAAAGGAA
AAGACTGTTCCAAGCTTGGGAAA
>2004
CATAACTTGGAAAAATAAACACTTCCTATGGGATTTGACTTTATTTTCTCCATTGTCTTA
CCTTTTACAGGTGTTAATATAGTGAAAAGGAAGCTTGCAGCTCATGACAATTTGAAGCT
GACAATTACACAAGAAGGAAA
>2005
CATGTGTAAGACCCTAATAAATGCCATTATTATTATTATTATTATTATTATTATTATTATT
ATGTAGGGGACCTGGAGCCTTGAGGGAAATAAACTTATTGGAAAATTCAAACGGACAG
ACAATGGAAACGAACTGA
>2006
CCCTTCTCTCCTCTGAGTACCAGATCCTCCCTGATACCCCCGACCCCATGGGCATCCTCT
ATCCCCTCAGCGGCCCTGGGAGAGCCTCAGCTCTGCTATATCCTGGATGCCATCCTGTT
TCTGTATGGAATTGTCCTCAC
>2007
TAAGAGATGTAAAATTTTCATGATGTTTTCTTTTTTGCTAAAACTAAAGAATTATTCTTTT
ACATTTCAGTTTTTCTTGATCATGAAAACGCCAACAAAATTCTGAATCGGCCAAAGAG
GTATAATTCAGGTAAATTGGA
>2008
TATCTACAGGGGAGGACCGGGCATTCTAAGCAGTTTACGTGCCAATTCAATTTCTTAAC
CTATCTCAAAGATGGAGATCAGTGTGAGTCCAATCCATGTTTAAATGGCGGCAGTTGCA
AGGATGACATTAATTCCTATGA
>2009
CAGTAGTTCCATGTACTTTTTAGAAATGCATGTTAAATGATGCTGTTACTGTCTATTTTGC
TTCTTTTAGATGTAACATGTAACATTAAGAATGGCAGATGCGAGCAGTTTTGTAAAAAT
AGTGCTGATAACAAGGTGGT
>2010
ATTTTTGTAATACATGTTCCATTTGCCAATGAGAAATATCAGGTTACTAATTTTTCTTCTA
TTTTTCTAGTGCCATTTCCATGTGGAAGAGTTTCTGTTTCACAAACTTCTAAGCTCACC
CGTGCTGAGGCTGTTTTTCC
>2011
CAGCACACATATTTATTTTTTTCTAGATCAAATGTATTATGCAGTAAGAGTCTTAATTTTG
TTTTCACAGGTTGTTTTGAATGGTAAAGTTGATGCATTCTGTGGAGGCTCTATCGTTAAT
GAAAAATGGATTGTAACTG
>2012
AGTAGTCACTTAGAAAATCTGTGTATGTGAAATACTGTTTGTGACTTAAAATGAAATTTA

TTTTTAATAGGTGAACATAATATTGAGGAGACAGAACATACAGAGCAAAAGCGAAATG
TGATTCGAATTATTCCTCACCA
>2013
TGGGAACTCGCCCCACCTGTGTCCGGAACCTGCTCGCTCACGTCGGCTTTCCCCTTCT
GTTTTGTTCTAGGACTTCTGCACGGACCTGGCCGTCTCCAGTGCCAACTTCATTCCCAC
GGTCACTGCCATCTCGACCAGTC
>2014
TGGGGTGGGTGAATGGAGGTGATGGCAGACACTTTTACTGAATGTCGGTCTTTTTTTGT
GATTATTCTAGTTATCTCCAGAAGAAGAAGAGAAAAGGAGAATCCGAAGGGAAAGGA
ATAAGATGGCTGCAGCCAAATGCC
>2015
TTGGAGATTGAGCATAAGGGCCCTTGAGTAAGACTGTGTCTTATGCTTTCCTTTATCCCT
CTGTATACAGGAGACAGACCAACTAGAAGATGAGAAGTCTGCTTTGCAGACCGAGATT
GCCAACCTGCTGAAGGAGAAGG
>2016
GAAATGAGGGGGTGGGGAAATGGTACGGAGGTTCATAACTGAGGAAATGACCACGAC
TGCACTATTTCAGGAGCAGACAAGATGGAGACAAATTCCTCTCTCCCCACGAACATCT
CTGGAGGGACACCTGCTGTATCTGC
>2017
CATAGTCAGCCTGTATTCTATACTCATTAGTGCATTACCAGTTTGTTTCATTCCCTTTTGT
TTTCTGCAGATAACAATGCTGGGAATTCAAGGAGATCTGAAGTGGTCCACAGATCCAG
ATAAAGGTCTCTTCATCTCTC
>2018
CAGGGATCAGAAGCTGCCTGGCCTTTTCTTCTGAGCTGTGACTTGGGCTTATTCTCTCC
TTTCTCCGCAGTTGCTGCTGACACGCCGACCGCCTGCTGCTTCAGCTACACCTCCCGA
CAGATTCCACAGAATTTCATAGC
>2019
AGGGCCATGGTGGGCCCAGGATTCCCCGGCTGGATTCCCCAGTGCTTAACTCTTCCTCC
CTTCTCCACAGCTTCCTAACCAAGAGAGGCCGGCAGGTCTGTGCTGACCCCAGTGAGG
AGTGGGTCCAGAAATACGTCAGT
>2020
CTCTAGTTCCCTGCAGCTGGGGTGGGGCGTGCCTGCATTTTCAGGTGCCTTAACCGAC
CCATTTCCGCAGAGCCTCCTGTCGCTGAGCCCTGACGTGCCCGTGCCATCCGACCATG
GAGGGACTGAGTCCAGCCCAGGCT
>2021
AAGCGTTCAGCTGCAGTAGCATATTCAAGTGTTGCTAGTTAGTAATTATCTTTTTAATTT
TTTGTTTTAGTTTAAAAGATTGGAAGACCCGTTTGAGCTACTTCTTACAAAATTCCTCTA
CTCCTGGGAAGCCCAAAACC
>2022
GGAGAAACATAAGGAATCATTTGGTCTCTCAGTTCTTCACATTCTGCATGCTCTTTTCTC
CCCCCTTCAGATGGTCTTGCTGCATTCAGGGCTTTTTTAAAGTCGGAATTCTGTGAAGA
AAATATTGAATTCTGGCTGGC
>2023
TGTTTTCAAATACTAAATTTTAATCTTTAACTCTGAATACCAAATAAACAACTTTTTTGTT

TTATTTCAGATAAACATAGATTTTCAAACCAAAACTCTGATTGCCCAGAATATACAAGA
AGCTACAAGTGGCTGCTTTA
>2024
GAGGCAGTGTCATTGGAGAGGCTTACTCCAGCTGGCGGAAGCCTCACGTACTGCTTGT
CTCTCCTGCCAGGCTCTGGAGCTCATGACGGTGCTGGTGGGCAGCCCCCGCAAGGATG
GGCTGGTGTCTCTCCTCACCACCT
>2025
GACACCTGCCCTTCCTGTCCAATCCTCCCAGGCAGCCTCTCTTACCATCACCTGTTCTT
TCCCCCTGCAGCTGCCCCCCTCCCTGGCTTCAGTGCAGTGGTGGTCAGCTCAGTGCCC
CTGGGGGGTGGCCTGTCCAGCTC
>2026
CCCTGGAGTGTCATTGAAGCCACTGCTGCTTCCACCAGCCCTAGCCTCCCCACCTCAC
CCTGTACTGCAGACTCGGGCACAATAGCTGCCCGCGCCCAGGTGTGTCAGCAGGCCGA
GCACAGCTTCGCAGGGATGCCCTG
>2027
TGTGCCCACTGCCTGGCGCAGCAAGCACACGCTTGGCCTCGTCATCTCCCCCATTGTA
ACTCCACCCCAGGTCCTTGGAGACCAGCCTGGTGCCACTCTCGGACCCCAAGCTGGCC
GTGCTCATCACCAACTCTAATGTC
>2028
TGCTGGTCCTCACAGGCGTCGGGGCCCCAGGCAGTGAGAAGGCGGCTGCTGACTCCT
CTTTCCTCCCCAGCTGCCAGGGACCTGGTGAGCAAAGAGGGCTTCCGGCGGGCCCGG
CACGTGGTGGGGGAGATTCGGCGCAC
>2029
CCCTCTGGGCGCCCCGCTCCTGCCGGGCACAGGCCGGCCCAGGCCCACCCCTTCAATA
TCCTCTCTGCAGAGACGACTATGAGGTGAGCTGCCCAGAGCTGGACCAGCTGGTGGA
GGCTGCGCTTGCTGTGCCTGGGGTT
>2030
CAGGGCCTGGGCGGGCAGGAGCGGCAGCTTCCCGGGGCCCTGCCACTCACCCCCAGC
CCGCCTCTTACAGGAGCACTACGGCGGGACTGCCACCTTCTACCTCTCTCAAGCAGCC
GATGGAGCCAAGGTGCTGTGCTTGT
>2031
GGGTGAGACCCAGGAGAGAGGGAGCTAGAGGGGGGAGCTCTGAGGACTGATCTTGA
CTGTCTGCCCCCAGACCATCAGCATATCCGCTACAACCCGCTGCAGGATGAGTGGGTG
CTGGTGTCAGCTCACCGCATGAAGCG
>2032
AGTCTTTGAAGCCCACCAGGTAACTGGTGGTATGGGGCAGTGAGTGCTTCTAGCCTAT
CCTTGTCGGTAGGTGAATCCCCAGTACGATAGCACCTTCCTGTTTGACAACGACTTCCC
AGCTCTGCAGCCTGATGCCCCCA
>2033
TGGGGTTCGCCCTGCCCGTAGCACAGCCAAGCCCTACCTCTCGGTTATCTTTTCTCCCG
TCACCACCCAGTAAGGTCATGTGCTTCCACCCCTGGTCGGATGTAACGCTGCCACTCAT
GTCGGTCCCTGAGATCCGGGCT
>2034
ATATGCCAATGATGTGGAGGCTTGGAGGTAAAGGACCTGCCTGTTCTTCTCTGCTTTTG

CCCCTTGACAGGTATGGGCCAGCAGTTTCCTGCCAGATATTGCCCAGCGTGAGGAGCG
ATCTCAGCAGGCCTATAAGAGTC
>2035
TGCCTATTTGCTGACCACACTCCGGCTCCTATGTCACCTTGATGACTTCCTATCCATTCT
GTCTTCCTAGGAACGTCTGGTCCTAACCAGTGAGCACTGGTTAGTACTGGTCCCCTTCT
GGGCAACATGGCCCTACCAGA
>2036
ACTGGATGGAGGTTGCTCCCAGTAGGGTCAGCATCTGGACCCCAGGCTGAGAGTCAG
GCTCTGATTCCAGATCTAGCCTCCATCATGAAGAAGCTCTTGACCAAGTATGACAACCT
CTTTGAGACGTCCTTTCCCTACTC
>2037
GGGAGTAGGTGCTAACCTGGATAACTGTAAAAGGGCTCTCTCTCCCCACTGTCTCTCTT
CTTTCTGTCAGGGGCTCCCACAGGATCAGAGGCTGGGGCCAACTGGAACCATTGGCA
GCTGCACGCTCATTACTACCCTCC
>2038
TGAAGTCCATGCCACCATTCTTGGCAGCCCAGCCCTTATCCTCCTTAATTGCTCCCTGTC
CCTTTTCCAGGCTGCAGAGAGACTAAGGGCACTTCCTGAGGTTCATTACCACCTGGGG
CAGAAGGACAGGGAGACAGCAA
>2039
AAAAGTCCAGGTCGCTTCTCAGGATTTGTGGCACCTTCTGACTGTCAAACTGTTCTTGT
CAATCTCACAGGCTCCTGGTTGTCTACCCATGGACCCAGAGGTTCTTTGACAGCTTTGG
CAACCTGTCCTCTGCCTCTGCC
>2040
AAAAGTCCAGGTCGCTTCTCAGGATTTGTGGCACCTTCTGACTGTCAAACTGTTCTTGT
CAATCTCACAGGCTCCTGGTTGTCTACCCATGGACCCAGAGGTTCTTTGACAGCTTTGG
CAACCTGTCCTCTGCCTCTGCC
>2041
ATCTGGAGGCCAGGCTGGAGCTCTCAGCTCACTATGGGTTCATCTTTATTGTCTCCTTTC
ATCTCAACAGCTCCTGGGAAATGTGCTGGTGACCGTTTTGGCAATCCATTTCGGCAAA
GAATTCACCCCTGAGGTGCAGG
>2042
ATCTGGAGGCCAGGCTGGAGCTCTCAGCTCACTATGGGTTCATCTTTATTGTCTCCTTTC
ATCTCAACAGCTCCTGGGAAATGTGCTGGTGACCGTTTTGGCAATCCATTTCGGCAAA
GAATTCACCCCTGAGGTGCAGG
>2043
TGGCCTAGGGCTGCTCACATATTCTGGAGGAGCCTCCCCTCCTCATGCCTTCTTGCCTC
TTGTCTCTTAGATTTGGTCGTATTGGGCGCCTGGTCACCAGGGCTGCTTTTAACTCTGGT
AAAGTGGATATTGTTGCCATC
>2044
CCCAAAGCTGGTGTGGGAGGAGCCACCTGGCTGATGGGCAGCCCCTTCATACCCTCAC
GTATTCCCCCAGGTTTACATGTTCCAATATGATTCCACCCATGGCAAATTCCATGGCACC
GTCAAGGCTGAGAACGGGAAGC
>2045
TCCCTTGGGTATATGGTAACCTTGTGTCCCTCAATATGGTCCTGTCCCCATCTCCCCCCC

ACCCCGGTAGGCGAGATCCCTCCAAAATCAAGTGGGGCGATGCTGGCGCTGAGTACGT
CGTGGAGTCCACTGGCGTCTTC
>2046
GGGAGGGGAAGCTGACTCAGCCCTGCAAAGGCAGGACCCGGGTTCATAACTGTCTGC
TTCTCTGCTGTAGGCTCATTTGCAGGGGGGAGCCAAAAGGGTCATCATCTCTGCCCCCT
CTGCTGATGCCCCCATGTTCGTCA
>2047
AGAAGCGGCCAGCCTGGCACCCTATGGACACGCTCCCCTGACTTGCGCCCCGCTCCCT
CTTTCTTTGCAGCAATGCCTCCTGCACCACCAACTGCTTAGCACCCCTGGCCAAGGTCA
TCCATGACAACTTTGGTATCGTG
>2048
CTTTCAAGGTGGGGAGGGAGGTAGAGGGGTGATGTGGGGAGTACGCTGCAGGGCCTC
ACTCCTTTTGCAGACCACAGTCCATGCCATCACTGCCACCCAGAAGACTGTGGATGGC
CCCTCCGGGAAACTGTGGCGTGATG
>2049
AGTGCAGGGTCTGGCGCCCTCTGGTGGCTGGCTCAGAAAAAGGGCCCTGACAACTCT
TTTCATCTTCTAGGTATGACAACGAATTTGGCTACAGCAACAGGGTGGTGGACCTCATG
GCCCACATGGCCTCCAAGGAGTAA
>2050
GACGGAAGGAGGGGGATTTGACTGAATGAAGGCTGACCCCCTACTGCCACCTCTCCCT
TTTCTTACCCAGAATTGGAGCTGGCCATTAGAATCACTCTTTACGCAGTGATCTTCCTGA
TGAGCGTTGGAGGAAATATGCT
>2051
AGGAAGTCCCACTGATGCTTGTGTAGTGCAAGTTTCCTAGGTGACTCCTTCCTTCTCTT
CCCTTGTTTAGGGGTGTCTGTGAGTGTGTCCACGCTAAGCCTCGTGGCCATCGCACTG
GAGCGGTACAGCGCCATCTGCCG
>2052
CCTTGGCTTTTTCTCCATCTGTGATTACAGCTGGACAGAAACCCGAGTGATCTGTCTGT
GTTGCCTTCAGGTCCGTACTGCTGCTTCTGCTCTTGTTCTTCATCCCGGGTGTGGTTATG
GCCGTGGCCTACGGGCTTATC
>2053
TCAGGTGGGGACTGGGCTGGAGACTGGGGGGACTCGCCTTTTTCTCTGACCGCCCACC
CTTTGTGCTCAGGGGCTGTTCACCAGAACGGGCGTTGCCGGCCTGAGACTGGCGCGG
TTGGCGAAGACAGCGATGGCTGCTA
>2054
GACTGGCTCTGACTTCAGTTCCTAGAAGGTAGGCATCCTTCCCCCATTCTCGCCTCTCT
CCCCTCCTCAGACCCGTCCAAGAAGCAGGGACCATGGCTGGAGGAAGAAGAAGAAG
CCTATGGATGGATGGACTTCGGCCG
>2055
GATGGGCGGGGCCTGAGGCTGGAGTGAGCGGGGCCCGGATGGGCTCACGGCGGCCGC
GCCCCTCGCCCAGGACGGCTACATTGATTTCATGGAGTACGTGGCAGGCCTCAGCTTG
GTCCTCAAGGGGAAGGTGGAACAGA
>2056
GTCCCCAATCCTACCCCTGAGATAGGATAAGGATGGGCCCCTCTCACTTCTGCCCCTTC

TTCCCTCCCAGGCCATTCGCGCCATTAACCCCTGCAGCGATACCACCATGACTGCAGAG
GAGTTCACCGATACAGTGTTCT
>2057
CCCGGTTCTGTGCTCTGGACTGCAGAAATGAACACCCTCCTCCCCCTGATTCCCTTTCT
CTCTACCCCAGGGGAACTCTCCCTGGAAGAGTTTATAGAGGGCGTCCAGAAGGACCAG
ATGCTCCTGGACACACTGACACG
>2058
CCCTGCCCAGGAGAGTAGTTGAGGGGTGGAGAGGGGCTTGCTTTTCAGTCATTCCTCA
TTCTGTCCTCAGGAATGTCCCAAGCCTTTGAGTAGGGTAAGCATCATGGCTGGCAGCCT
CACAGGATTGCTTCTACTTCAGG
>2059
CTCCTAGCAGATGTGTCCATTCTCCATGTCTTCATCAGACCTCACTCTGCTTGTACTCCC
TCCCTCCCAGGTGCCCGCCCCTGCATCCCTAAAAGCTTCGGCTACAGCTCGGTGGTGT
GTGTCTGCAATGCCACATACTG
>2060
ATGGTGTCAGTGATCACCATGGAGTTTCCCGCTGGGTACTGATACCCTTATTCCCTGTG
GATGTCCTCAGGCCTGCTACTGACCCTGCAGCCAGAACAGAAGTTCCAGAAAGTGAA
GGGATTTGGAGGGGCCATGACAGA
>2061
AAGTGATAAGCAGAGTCCCATACTCTCCTATTGACTCGGACTACCATATCTTGATCATCC
TTTTCTGTAGGAATCGGATATAACATCATCCGGGTACCCATGGCCAGCTGTGACTTCTCC
ATCCGCACCTACACCTATGC
>2062
GTGAGCCACCACACCCAGCTGGTCTGGTCCACTTTCTTGGCCGGATCATTCATGACCTT
TCTCTTGCCAGGTTCCTGGATGCCTATGCTGAGCACAAGTTACAGTTCTGGGCAGTGAC
AGCTGAAAATGAGCCTTCTGCT
>2063
CCTACAACAGGAACTAGAAGTTCCAGAAGCCTGTGTGCAAGGTCCAGGATCAGTTGCT
CTTCCTTTGCAGGTACTGACAGACCCAGAAGCAGCTAAATATGTTCATGGCATTGCTGT
ACATTGGTACCTGGACTTTCTGG
>2064
ACCCTTACCTACACTCTCTGGGGACCCCCAGTGTTGAGCCTTTGTCTCTTTGCCTTTGT
CCTTACCCTAGAACCTCCTGTACCATGTGGTCGGCTGGACCGACTGGAACCTTGCCCTG
AACCCCGAAGGAGGACCCAATT
>2065
GGTGACTTCTTAGATGAGGGTTTCATGGGAGGTACCCCGAGGGACTCTGACCATCTGT
TCCCACATTCAGCAAGTTCATTCCTGAGGGCTCCCAGAGAGTGGGGCTGGTTGCCAGT
CAGAAGAACGACCTGGACGCAGTG
>2066
CCCTGCCCGGGAGAGTAGTTGAGGGGTGGAGAGGGACTTGCTTTTCAGTCATTCCTCA
TTCTGTCCTCAGGAATGTCCCAAGCCTTCGGGTAGGGTAAGCATCATGGCTGGCAGCC
TCACAGGATTGCTTCTACTTCAGG
>2067
TCCAAACAGCTGTGTCCGTTCTCCACATCCTTGTCAGACCTCACCTCTGCTTGTGCTCC

CTCCCTCCCAGGTGGTGCCCCTGCATCCCTAAAAGCTTCAGTACAGCTCGGTGGTCTGT
GTCTGCAATGCCACATACTGTG
>2068
GAGGTAAACGGTGTCAGTGATCACCATGGAGTTTCTCCCTGGGCACTGATAACCCTGT
GGATGTCCTCAGGCCTGCTACTGATCCTGCAGCCAGAAGTTCCAGAAAGTGAAGGGAT
TTGGAGGGGCCGTGACAGATGCAG
>2069
AAGTGATAAGCAGAGTCCCATACTCTCCTATTGACTCGGACTACCATATCTTGATCATCC
TTTTCTGTAGGAATCGGATATAACATCATCTGGGTACCCATGGCCAGCTGTGACTTCTCC
ATCCGCACCTACACCTATGC
>2070
CGGATGCACTGGTTGGGCTAGTATGTGTTCCAACTCTGGGTGCTTCTCTCTTCACTACC
TTTGTCTCTAGATACCCCTGATTCACCGAGCCCTGCAGTTGGCCCAGCGTCCCGTTTCA
CTCCTTGCCAGCCCCTGGACAT
>2071
ATCAGGCTTCCTGTTCTCTCGGTCTGGCCCACTTTCTTGGCTGGATCATTCATGACCTTT
CTCTTGCCAGGTTCCTGGATGCCTATGCTGAGCACAAGTTACAGTTCTGGGCAGTGAC
AGGTGAAAATGAGCCTTCTGCT
>2072
CCTACAAGAGGAACTAGAAGTTCCAGAAGCCTGTGGGCAGGGTCCAGGGTGACTTGT
TCTTCCTTTGCAGGTACTGACAGACCCAGAAGCAGCTAAGTATGTTCATGGTATTGCTG
TACATTGGTACCTGGACTTTCTGG
>2073
ACCCTTACCTACACTCTCTGGGGACCCCCAGTGTTGAGCCTTTGTCTCTTTGCCTTTGT
CCTTACCCTAGAACCTCCTGTACCATGTGGTCGGCTGGACCGACTGGAACCCATCATTG
TAGACATCACCAAGCACACGTT
>2074
GGCGACTTCTTAGATGAGGGTTTCATGGGAGGTACCCCGAGGGACTCTGACCATCTGT
TCCCACATTCAGCAAGTTCATTCCTGAGGGCTCCCAGAGAGTGGGGCTGGTTGCCAGT
CAGAAGAACGACCCGGACGCAGTG
>2075
AAGTGGGCTGAAGACAGCGTTGGGGGCCTTGGCAGGATCACACTCTCAGCTTCTCCTC
CCTGCTCCCTAGCTCCTCTAAGGATGTGCCTCTTACCATCAAGGATCCTGCTGTGGGCT
TCCTGGAGACAATCTCACCTGGC
>2076
CTCCTGGAGCAGGGAGAGTGTTGGCCTCTTGCTCTCCGGCTCCCTCTGTTGCCCTCTG
GTTTCTCCCCAGGCTCCCGGACGTCCCTGCTCCTGGCTTTTGGCCTGCTCTGCCTGCCC
TGGCTTCAAGAGGGCAGTGCCTT
>2077
GCTAGGTTCCCAGAAAAGTAAAATGGGAGCAGGTCTCAGCTCAGACCTTGGTGGGCG
GTCCTTCTCCTAGGAAGAAGCCTATATCCCAAAGGAACAGAAGTATTCATTCCTGCAGA
ACCCCCAGACCTCCCTCTGTTTCT
>2078
GCGGGGATGGGGGAGACCTGTAGTCAGAGCCCCCGGGCAGCACAGCCAATGCCCGTC

CTTGCCCCTGCAGAACCTAGAGCTGCTCCGCATCTCCCTGCTGCTCATCCAGTCGTGGC
TGGAGCCCGTGCAGTTCCTCAGGA
>2079
TGAATGAATGAGAAAGGGAGGGAACAGTACCCAAGCGCTTGGCCTCTCCTTCTCTTCC
TTCACTTTGCAGAGGCTGGAAGATGGCAGCCCCCGGACTGGGCAGATCTTCAAGCAG
ACCTACAGCAAGTTCGACACAAACT
>2080
GCTCCTGGAACAGGGAGAGCGCTGGCCTCTTGCTCTCCAGCTCCCTCTGTTGCCTCCG
GTTTCTCCCCAGGCTCCCGGACGTCCCTGCTCCTGGCTTTTGGCCTGCTCTGCCTGTCC
TGGCTTCAAGAGGGCAGTGCCTT
>2081
GAGGTTCCCAGAAAAGTAACAATGGGAGCAGGTCTCCAGCATAGACCTTGGTGGGCG
GTCCTTCTCCTAGGAAGAAGCCTATATCCTGAAGGAGCAGAAGTATTCATTCCTGCAGA
ACCCCCAGACCTCCCTCTGCTTCT
>2082
AGGTGGGATGGGGTAGACCTGTGGTCAGAGCCCCCGGGCAGCACAGCCACTGCCGGT
CCTTCCCCTGCAGAACCTAGAGCTGCTCCGCATCTCCCTGCTGCTCATCCAGTCATGGC
TGGAGCCCGTGCAGCTCCTCAGGA
>2083
TGGATGAATGAGAGAGGGAGGGAACAGTGCCCAAGCGCTTGGCCTCTCCTTCTCTTCC
TTCACTTTGCAGAGGCTGGAAGATGGCAGCCCCCGGACTGGGCAGATCTTCAATCAGT
CCTACAGCAAGTTTGACACAAAAT
>2084
TCCCTTGTTTGGTTAGAACGTTTCTCTCCTCCTCTTGACCCCGGGTTCTAGCTGCCCTTC
TCTCCTGTAGGAGAACGCCAAGAACGAAGAGATTCTGAATTCCCTCAAGTACGTCCGG
CCTGGTGGTGGGTTCGAGCCCA
>2085
GGGCCCCTAGCGGAAGGAAAAAAATCATGGTTCCATGTGACATGCTGTGTCTTTGTGT
CTGCCTGTTCAGGATGGGGAACCCCCTCAGCAGCGAGTGACTGGGACCCTGGTCCTTG
CTGTGTTCTCTGCGGTGCTTGGCT
>2086
GAGGACAGGTGTCTCGGGGGTGGTGGAAAGGGGACGGTCTGCAGGAAATCTGTCCTC
TGCTGTCCCCCAGGTGATTGAACAGAGCTACAATGAGACGTGGCTGGGGAGGCAGGG
GCCTGAGGGACCCAGCTCCATCCCTC
>2087
TGGGGGAAACAGGAAGGGAGCCACTGCTGGGTGCCCTCACCCTCACAGCCTCACTCT
GTCTGCCTGCCAGGAAAAGGGCCATGCTGGTCAACAATGTCCTGGCGGTGCTGGGGG
GCAGCCTCATGGGCCTGGCCAACGCT
>2088
TGGGCTTTCAGATGGGAATGGACACCTGCCCTCAGCCCTCTCTTCTTCCCTCGCCCAGG
GCTGACATCAGGGCTGGTGCCCATGTACGTGGGGGAGATTGCTCCCACTCACCTGCGG
GGCGCCCTGGGGACGCTCAACCA
>2089
ATATGGTGGGCTTCCAAGGTAAGGCAGAAGGGCTGAGTGACCTGCCTTCTTTCCCAAC

CTTCTCCCACAGGTGCTGGGCTTGGAGTCCCTCCTGGGCACTGCCAGCCTGTGGCCAC
TGCTCCTGGGCCTCACAGTGCTAC
>2090
GCCTCCGCCTCATCTTGCTAGCACCTGGCTTCCTCTCAGGTCCCCTCAGGCCTGACCTT
CCCTTCTCCAGGTCTGAAGCGCCTGACAGGCTGGGCCGATGTTTCTGGAGTGCTGGCT
GAGCTGAAGGATGAGAAGCGGAA
>2091
CCAGCCTGTTGTGGCTGGAGTAGAGGAAGGGGCATTCCTGCCATCACTTCTTCTTCTCC
CCCACCTCTAGGTTTTCTATTATTCGACCAGCATCTTCGAGACAGCAGGGGTAGGCCAG
CCTGCCTATGCCACCATAGGAG
>2092
CCTCACCCACGCGGCCCCTCCTACTTCCCGTGCCCAAAAGGCTGGGGTCAAGCTCCGA
CTCTCCCCGCAGGTGTTGTTGGTGGAGCGGGCGGGGCGCCGGACGCTCCATCTCCTGG
GCCTGGCGGGCATGTGTGGCTGTG
>2093
CTGAGTTGAGGGCAAGGGAAGATCAGAAAGGCCTCAACTGGATTCTCCACCCTCCCT
GTCTGGCCCCTAGGAGCGAGTTCCAGCCATGAGCTACGTCTCCATTGTGGCCATCTTTG
GCTTCGTGGCATTTTTTGAGATTG
>2094
CTGGTTGCCTGAAACTACCCCTTCCCTCCCCACCTCACTCCGTCAACACCTCTTTCTCC
ACCTGTCCCAGGAGGCTATGGGGCCCTACGTCTTCCTTCTATTTGCGGTCCTCCTGCTG
GGCTTCTTCATCTTCACCTTCT
>2095
CCGGGTGACATCTGGGAAGGCTGAAAGGAAACAAACCCTTCCTGATGACCCTATCCCT
GGCTCCCAACAGCCCCCCGAGCTCCCCGCTAACCCAGCCCCCAGAGGGGCCCAAGTT
CCCTCGTGTGAAGAACTGGGAGGTG
>2096
TCCCTGCCCCCAACTCCCATCCCACCCCTGCACCCTGGCCTGTCCTGACCTTTGCACTC
CCTCGACCCAGGATGGGCCCTGCACCCCAAGACGCTGCCTGGGCTCCCTGGTATTTCC
ACGGAAACTACAGGGCCGGCCCT
>2097
AGCCTGGAGGAGGGCCTCCCTGTCCCACACAACTTCCTGCTTGTCCCCTTCCCACCCC
TCTCCTCCCCAGGAGCGGCTCCCAGGCCCACGAACAGCGGCTTCAAGAGGTGGAAGC
CGAGGTGGCAGCCACAGGCACCTAC
>2098
TCTGGAGCTGATACTCAAGACCCCCCGTCTCTCTCCTCACCCTCCTCTCCCGCTGCCTC
GGCTGGCTCAGGTGTTCGATGCCCGGGACTGCAGGTCTGCACAGGAAATGTTCACCTA
CATCTGCAACCACATCAAGTATG
>2099
CAGGCCCCAGCCTTCTTCCCCAAGGCAGGGAAGGCGGGGCTCTGACCAGCTCTTTCCC
CATGCGTGCCAGCTCGGCCATCACAGTGTTCCCGCAGCGCTGCCCTGGCCGAGGAGAC
TTCCGAATCTGGAACAGCCAGCTG
>2100
GTGGATGGAGGGGTCCCTGAGGAGGGCATGAGGCTCAGCCCCAGAACCCCCTCTGGC

CCACTCCCCACAGCTCTGCATTCAGCACGGCTGGACCCCAGGAAACGGTCGCTTCGAC
GTGCTGCCCCTGCTGCTGCAGGCCC
>2101
GGGGGCCATCCCTGAGCCTCTCAAGAAGGGCCTGCAAGGGGGTGCTGATCCCACACC
CCAACACCCCCAGGCTGGAGTGGTTTGCAGCCCTGGGCCTGCGCTGGTACGCCCTCCC
GGCAGTGTCCAACATGCTGCTGGAA
>2102
AGATAGTCTCCCCACCCCACCCCCGTGATCACCTCTGTCCCTACCGATGCCACACACCC
TTCTGCCCCAGGATGTGGCTGTCTGCATGGACCTGGATACCCGGACCACCTCGTCCCTG
TGGAAAGACAAGGCAGCAGTGG
>2103
AGTGGTGGAGGAAGAATGGGCGAGGTCTGTGGGTCTGGTTTGAGCCTCTCCCCCTCTC
TCTCCCTTCCAGCTAGCCAAAGTCACCATCGTGGACCACCACGCCGCCACGGCCTCTT
TCATGAAGCACCTGGAGAATGAGC
>2104
AGCAGCCCCTGGGGACCTCTAACCTTTCCTTTTCTTTACCTCCCCTCCCAACCCCATCA
TCTCTCTGCAGCCAGACCCCTGGAAGGGGAGTGCCGCCAAGGGCACCGGCATCACCA
GGAAGAAGACCTTTAAAGAAGTGG
>2105
GTTGGACCCTTGCCTGGGGAGGCCCTGCCTCTGTGCACCCGGGACACCCTCACACCTT
CCTCTCCCGCAGCGCCGTGAAGATCTCCGCCTCGCTCATGGGCACGGTGATGGCGAAG
CGAGTGAAGGCGACAATCCTGTAT
>2106
GAGGCCAGAGTGAGGAGGGCAGGGCCTCCGGGGGCCACAGCACCCAGGACATCTGT
CTTCCCACCCACAGGTCCTGTGTATGGATGAGTATGACGTGGTGTCCCTCGAACACGA
GACGCTGGTGCTGGTGGTAACCAGCA
>2107
GGGCTGGGTCTGGGCCTAGCCTGTATCCCCAGGGCCCTGTGACAACCTTGTCTTTGTCC
TCTCTTGCCAGGAGTTATAAGATCCGCTTCAACAGCATCTCCTGCTCAGACCCACTGGT
GTCCTCTTGGCGGCGGAAGAGG
>2108
ACGGGTGGGCAAGCTGCCTGGGCAAACGTGGCCTGCAAAGGGAGCTCCACTGACGAC
CCCTGCACCCCAGGTTCTGTGTGTTCGGGCTCGGCTCCCGGGCATACCCCCACTTCTGC
GCCTTTGCTCGTGCGGTGGACACA
>2109
CGCCCAGGCGCCTCACTAGGGCGACCCCTGGTGGCGGGAGGTCCTCAGCCCTCACCG
GCCTGTCCCGCAGGCCGCCTGTGAGACCTTCTGTGTGGGAGAGGATGCCAAGGCCGC
CGCCCGAGACATCTTCAGCCCCAAAC
>2110
ACTAAAGCACTCTGGGGCCAGGCCCTGCTCCCTAGCTCAGGCTGCCTCATTTGCCCCT
CCCCGCCCCCAGGTCTGATCCACGTGCACAGGCGGAAGATGTTCCAGGCTACAATCCG
CTCAGTGGAAAACCTGCAAAGCAG
>2111
TCCTCCTCCCACATTCTCCCGCCCCCACCCCTCTCTGACTCCCCATAAGTGCCCCTCTCC

CCACCCCCAGGAGGGCCACCATCCTGGTGCGCCTGGACACCGGAGGCCAGGAGGGGC
TGCAGTACCAGCCGGGGGACCAC
>2112
ACAAACATCAGCCCAGGTACTGCAGTCCTGCTGGGCCCTGTCCTCAGAGCTCCCTGTG
CACTATCCCCAGGTGGCCCTCCCCCCGGCTGGGTGCGGACCCCCGGCTGCCCCCGTG
CACGCTGCGCCAGGCTCTCACCTT
>2113
GAGGGGTCAAGAAGGGAGGTTACTAGGAAGGGCTATGGGGCCTCCAACCCACTGCAT
CCTGCCCCGCCAGGATCCCCGACGCTACGAGGAGTGGAAGTGGTTCCGCTGCCCCACG
CTGCTGGAGGTGCTGGAGCAGTTCC
>2114
GGCCTGGCCACAGCAGGGTTGGGACCGGCCCCTCTCTGGCCCCTCACCGGCCTCTCCT
TCCCACCCCCAGATGGGCTGGGCCCCCTGCACTATGGAGTCTGCTCCACGTGGCTAAG
CCAGCTCAAGCCCGGAGACCCTGT
>2115
CCACCCTGCATGGTGAGAATGGTGGAGCAGGAAAGGCAAAGGGGACCTGATGGAGTG
TCTCTCCTGCCAGGGCTCCCTCCTTCCGGCTGCCACCCGATCCCAGCTTGCCCTGCATC
CTGGTGGGTCCAGGCACTGGCATT
>2116
GGCCTGTGGGGAGGCCCCACTAGCACTGTGCCCCGGAGAAGAGCCTTCCCAAGCGCG
GGGTTGCTTGCAGGGCTGCAGCCCACTCCCATGACTTTGGTGTTCGGCTGCCGATGCT
CCCAACTTGACCATCTCTACCGCGA
>2117
CAGCCAGCAGCCCCGGGCTGCCCCCGCGCCCACCCCCACCAGGGCCCGCCCTAACCC
CGCCGCCCCGCAGACCTACGTGCAGGACATCCTGAGGACGGAGCTGGCTGCGGAGGT
GCACCGCGTGCTGTGCCTCGAGCGGG
>2118
GGGGGCTGGGGCCTGGGGGGATGCTTCCAGGGGATGCAGGGGGATCCACTCAAGGCT
CCCTTGCCCACAGGTCCTCATGCCTCTCCTCCTCTTGCTGCTCCTGCTGCCAAGCCCCT
TACACCCCCACCCCATCTGTGAGG
>2119
GGGATGGGGTCTCTGCTAAGGGCCAGGAAGTCCCTTCCCCTTCCCAAAAACAAACTTA
CCCAATCCACAGCCGCCCTCACCGCCCGGCCTTCTACGGTGTCCAGAGACAGTTAGCC
AGGCCTGGGCTGGGCACACTCCAC
>2120
CCCAGTGCTTGCCGTCCCTGAGGATCGGTCCAGGCTGCCAGGCCCTCCCTCACAGCCC
CTCTCTCTGCAGCCAGCCTGTCCCATGCCTGCCTGGGGAGCCCTGTTCCTGCTCTGGGC
CACAGCAGAGGCCACCAAGGACT
>2121
GGGGGACGGGGCGTGGCGCTGGCTGGCGTGAGAAGTGCGGTGCTGATGTCCCTCTGT
CGGGTTTTTGCAGCGTCGGCGTGTTCAAGAACGGCCGCGTGGAGATCATCGCCAACGA
TCAGGGCAACCGCATCACGCCGTCC
>2122
TGGTAGTGGGTCTTTTAAACTTTGAGATGTCATTGTATCTGTGTCTGAAAACAATAATTC

TTTAAAATAGGTGGTTGAAAAGAAAACTAAACCATACATTCAAGTTGATATTGGAGGTG
GGCAAACAAAGACATTTGCTC
>2123
CTGGCCAGACTGTAATTTAAATAAGGGTTAAACTATGTGACAATACACTTAATTATCTTT
ATCCTTTTAGGTTACCCATGCAGTTGTTACTGTACCAGCCTATTTTAATGATGCCCAACG
CCAAGCAACCAAAGACGCTG
>2124
ATACGGCATATTTGCCAAATAGTGGAAATGTGAAGTACTGACAAAACTTTTCCCTTTTT
CAATCTAATAGTACGGCAGCTGCTATTGCTTATGGCCTGGATAAGAGGGAGGGGGAGA
AGAACATCCTGGTGTTTGACCTG
>2125
CTCTGAATTCAGGACATTGCATCTAGATACTTAGATAACAGACATCACAGTAACCATGT
CTTTTTTCTAGGATCTGTTCCGGTCTACTATGAAGCCCGTCCAGAAAGTGTTGGAAGAT
TCTGATTTGAAGAAGTCTGATA
>2126
TTAGAGAGATAAACTTTACATAGCTCATAGGGAGAACTTGAATTAAAAGTTAAATAACT
TATCCTTACAGGTGACCTGGTACTGCTTCATGTATGTCCCCTTACACTTGGTATTGAAAC
TGTAGGAGGTGTCATGACCAA
>2127
AATGCGGGGATCTAGATGTGCTAATGCTTGTCAGTAACAACTAACAAGTTTTTCTGTAT
GTAACTTCTAGGTGAAAGACCCCTGACAAAAGACAATCATCTTCTGGGTACATTTGATC
TGACTGGAATTCCTCCTGCTCC
>2128
CTGGTGCAGAGAAAGTCACCAAGTCAGGGACCCTCCATCTCTGACACGACCTGCGGG
CCATCTCTTCCAGCTGGCCCACGCCATGCGCCTGCTCCTGGAATACACAGACTCAAGCT
ACGAGGAAAAGAAGTATACGATGG
>2129
GCATATGGGAAGGGGATGCTGGGGAGCCTGCTGGCCCCAACTGAGCTTCCCCGGTTTC
CCATCTATCCAGCTGCCCTACTTGATTGATGGGGCTCACAAGATCACCCAGAGCAACGC
CATCCTGTGCTACATTGCCCGCA
>2130
GAAGGTGGCATCCTCCTTGGCTGGATTGGGGTGCTATGCTCAGAGTGAGTCTGTGTTTT
GTGGGTGGCAGGTGGGGAGACAGAAGAGGAGAAGATTCGTGTGGACATTTTGGAGAA
CCAGGCTATGGACGTCTCCAATCA
>2131
AGCCCTGGGGAGGCCACATCTGTGCAGGGAGCTTGTGTCTGAGGGTGGTGACAGCTG
TTTTCTGCCTCAGGAGAAACTGAAGCCAGAATACTTGGAGGAACTTCCTACAATGATG
CAGCACTTCTCACAGTTCCTGGGGA
>2132
GGACACTAGAGATTTGCCATACATCCTATGTTACAGAGATTCCAGCCCACACATTCTTG
GCCTTCTGCAGATCACCTTTGTAGATTTCCTCGCCTATGATGTCCTTGACCTCCACCGTA
TATTTGAGCCCAACTGCTTGG
>2133
TGCAGTGGGGTTGTCCCAGCCCTCATGGGCAGCTGACCTTGAGTTCTGGCCTTATTTTC

CCCCCTCTCAGGGCTTGGAGAAGATCTCTGCCTACATGAAGTCCAGCCGCTTCCTCCC
AAAACCTCTGTACACAAGGGTGG
>2134
ACCCCTCCCCGGGTTGCTGCGAGGCGGAGTCGGCCCGGTCCCCACATCTCGTACTTCT
CCCTCCCCGCAGGCCGCTGCGCGGCCCTGCGCATGCTGCTGGCAGATCAGGGCCAGA
GCTGGAAGGAGGAGGTGGTGACCGT
>2135
GGGGGCTGTGACTAGGATCGGGGGACGCCCAAGCTCAGTGCCCCTCCCTGAGCCATGC
CTCCCCCAACAGCTATACGGGCAGCTCCCCAAGTTCCAGGACGGAGACCTCACCCTGT
ACCAGTCCAATACCATCCTGCGTC
>2136
CCCATCCCCAGTGACTGTGTGTTGATCAGGCGCCCAGTCACGCGGCCTGCTCCCCTCC
ACCCAACCCCAGGGCTCTATGGGAAGGACCAGCAGGAGGCAGCCCTGGTGGACATGG
TGAATGACGGCGTGGAGGACCTCCG
>2137
TGGGGCAGACGGGGGTGTCTCAGGGGCTGGGAGGGATGAGAGTAGGATGATACATGG
TGGTGTCTGGCAGGAGGCGGGCAAGGATGACTATGTGAAGGCACTGCCCGGGCAACT
GAAGCCTTTTGAGACCCTGCTGTCCC
>2138
TTAGGCCCTCTGCCCCCAATTCCTCCAGCCTGCTCCCGCTGGCTGAGTCCCTAGCCCCC
CTGCCCTGCAGATCTCCTTCGCTGACTACAACCTGCTGGACTTGCTGCTGATCCATGAG
GTCCTAGCCCCTGGCTGCCTGG
>2139
ATGGGCGCGCCGCCTTGGTAATTCTATATTCTCCGTTCCTCCTCCGCTCGCGGGCGTGTG
CGCGCCGCAGGCTGGCGGTAAGGCTGGAAAGGACTCCGGAAAGGCCAAGACAAAGG
CGGTTTCCCGCTCGCAGAGAGCCG
>2140
GCGCATATAATTTCCCCATCTCTAGTTCTCATTTTTGTGCATTTATGTTTGTGTATGCTTAA
AATTTAAGTTCCCAGTGGGCCGTATTCATCGACACCTAAAATCTAGGACGACCAGTCAT
GGACGTGTGGGCGCGACTG
>2141
GGTTTTATTCTGTTCTCTGAATCTTGGCCAAAGGAAAGTTGGAAGGCAACTGACAGAT
TCTGCCTTTTAGGTACTTGAACTGGCAGGAAATGCATCAAAAGACTTAAAGGTAAAGC
GTATTACCCCTCGTCACTTGCAAC
>2142
CTTTCTTGGTTTCAAATACTGTGATTTTTTAAAAAATCTTAAGTAGAATTAATTCCTGTC
ACTCTCCTAGGTGTCATTCCACACATCCACAAATCTCTGATTGGGAAGAAAGGACAAC
AGAAGACTGTCTAAAGGATGCC
>2143
TGCGGGGACGCTCTTGGGAGGAGTCTTCTCCCCAGCCTTAGCTGGTTTCATGATTTCTT
TGCGTCTGTAGGCAACGCGGTAAAAATATTGCTTCGGTGGGTGACGCGGTACAGCTGC
CCAAGGGCGTTCGTAACGGGAAT
>2144
TCTATAGTTAACGCCGGATCGCAGTTGGAAACCACCAGCTTTTTGTCAGTATATATTACT

CATTTTATAGAGCCAGAGGCCAAGAAGAGTAAGACGGCCGCAAAGAAAAATGACAAA
GAGGCAGCAGGAGAGGGCCCAGC
>2145
GCATATAGATTATTCAATAAATGTTCTGCTGAATTGATAATACGTTTTCCACCTTTCTTTT
CACTTACAGTGGGTAAAGGAAGAAGCCCCAGATATACTGTGCCTTCAAGAGACCAAAT
GTTCAGAGAACAAACTACCAG
>2146
TCTGCCCCACCTCTTGATTGCTTTCCCTTTTCTTATAGTTTTTATGCTAATTCTGTTTCAT
TTCTATAGGCGATGAGGAGCATGATCAGGAAGGCCGGGTGATTGTGGCTGAATTTGAC
TCGTTTGTGCTGGTAACAGC
>2147
CCCACAGGCCACCCTCAACCGTCCTGGCCCCGGACCCAAACCCCACCCCTCACTCTGC
TTCTCCCCGCAGGATGTTCCTGTCCTTCCCCACCACCAAGACCTACTTCCCGCACTTCG
ACCTGAGCCACGGCTCTGCCCAG
>2148
CCCACAGGCCACCCTCAACCGTCCTGGCCCCGGACCCAAACCCCACCCCTCACTCTGC
TTCTCCCCGCAGGATGTTCCTGTCCTTCCCCACCACCAAGACCTACTTCCCGCACTTCG
ACCTGAGCCACGGCTCTGCCCAG
>2149
CAGAGAAGACTCTTGGGTTTCTGATAGGCACTGACTCTCTCTGCCTATTGGTCTATTTTC
CCACCCTTAGGCTGCTGGTGGTCTACCCTTGGACCCAGAGGTTCTTTGAGTCCTTTGGG
GATCTGTCCACTCCTGATGCT
>2150
CTGGATTATTCTGAGTCCAAGCTAGGCCCTTTTGCTAATCATGTTCATACCTCTTATCTTC
CTCCCACAGCTCCTGGGCAACGTGCTGGTCTGTGTGCTGGCCCATCACTTTGGCAAAG
AATTCACCCCACCAGTGCAGC
>2151
AAAAGTCCAGGTTGCTTCTCAGGATTTGTGGCACCTTCTGACTGTCAAACTGTTCTTGT
CAATCTCACAGGCTCCTGGTTGTCTACCCATGGACCCAGAGGTTCTTTGACAGCTTTGG
CAACCTGTCCTCTGCCTCTGCC
>2152
GCGCCCCCGCCCCCAGGGGCCCTCCCTCCCCAAGCCCCCCGGACGCGCCTCACCCAC
GTTCCTCTCGCAGGACCTTCCTGGCTTTCCCCGCCACGAAGACCTACTTCTCCCACCTG
GACCTGAGCCCCGGCTCCTCACAA
>2153
CCCCGGCGGGGTGGGTGCGGGGGGCGTGCGGGGCGGGTGCAGGCGAGTGAGCCTTG
AGCGCTCGCCGCAGCTCCTGGGCCACTGCCTGCTGGTAACCCTCGCCCGGCACTACCC
CGGAGACTTCAGCCCCGCGCTGCAGG
>2154
TGATGTCACATGGCTGGATCCAGCTAAAATTCTAAGGCTCTAACTTTTCACATTTGTTCC
ATGTTACCAGTCATCAGGTGGAAAAGCGGAAATGCAACACTGCCACATGTGCAACGCA
GCGCCTGGCAAATTTTTTAGTT
>2155
TTTTTGAGAAACACTTGTACTGTCATTCTCTATTCTCTGCAATTTGCTCTCTCCTTTTGT

GTGTATGCAGGAAAAGCATCATTCACATCGAGAATTTCCATTTTATGGGGACTATGGATC
AAATTATCTATATGACAATT
>2156
TTTGTGAAGCATTTTTACTGTCATTGCCTCTATTCTCTGCAATTTGCTCTCTCCTTTTGTG
TGTATGCAGGAAAAGCATCATTCACATCGAGGCTATAGATCAAATTATCTGTATGACAAT
TGATATCTTCAGTAATCAC
>2157
CGGGGTGCAGTGGGGACAGGGGTTGCAGTGGAATCTGAGTTCTCCCTCCCTCCATCCT
GTCCATTCCCAGGAGAACTATGAGCTCTACTCGGTGGAGCTGGGTCCTGGCCCTGGCG
GGGACATGGCTGCCAAGATGAGCA
>2158
GGGGCTTTCCACGGGGTGTGCATGTGGAGGAAAGCCCCCAGGACCGCCTCGCCCTGC
CCCTCTGTCCCAGGGCCTCTCTGCGAGCCTGGCTGCTGAGCTGCTGCTGCGGGATGGG
CCCAACGCACTGCGGCCACCACGGG
>2159
AGCAGGTCGGTGGCCTCAGGCAGGCAGCTTCCGGCCCCTCTGAATAACCCCACCCCTT
CCCTGTCCCCAGCTGTACCTGGCAATCGCTCTCATTGCTGTGGTTGTCGTCACCGGCTG
CTTTGGCTACTACCAGGAATTCA
>2160
TACCTCCCCACATGCAGCAGCTCCCACCCCCACCCCCGTCCACCTCAACTTTGGTGCCC
CTGCCCCGCAGCAAGCCACTGTCATCCGCGATGGAGACAAATTCCAGATCAACGCTGA
CCAACTGGTGGTGGGCGACCTGG
>2161
TCAGCTCACACGGCAGTGCACACCCCACTGCCGCCCCCTATCCCTGAGTCGCGCCTTG
GCCCCCCTGCAGGCACCGCGCAGGGCCTGGTGGTGAACACGGGCGACCGCACCATCA
TTGGGCGCATCGCATCGCTGGCGTC
>2162
TCTTTGACGCGGGGAAGAGGACTGGAAGCCAGGGTGGGTCCCCAGCCTGCGGTGCTC
ACCCCTTCCCCAGGTCTGCCTGTCCCTGACAGCCAAGCGCCTGGCCAGTAAGAACTGC
GTGGTCAAGAACCTGGAGGCGGTGG
>2163
GGAGGAGGGGGCTCGGTTCTGGTCCGGGAGAGCCGCCGTGCGGGACCCAGCCTGGAC
GCCTGGTCTGCAGGGCAGACGTTTGACCAGTCCTCGGAGACGTGGCGGGCGCTGTGC
CGGGTGCTCACCCTGTGCAACCGCGC
>2164
GGGGGCCTCCAGGGAATCCCGGGAGGCTGGTGTAGCCGGCTTCGCCCCTGACACCGG
TCCCCGCTCCCAGCGCATCGTGATTGGAGACGCATCGGAGACGGCGCTGCTCAAGTTC
TCGGAGCTGACGCTGGGCAACGCCA
>2165
CCCCGTTCCCTCGTCCACAGCCCCGCCCCGTTCCCCCGTCCACAGCCTCGCCTCTCCCC
CCCGCCCACAGCTGTCCATCCATACGCTGGAGGACCCGCGGGACCCGCGACACTTGCT
GGTGATGAAGGGCGCCCCCGAGC
>2166
ACCCATTTCCTGGCCCTGCTAAGAAAGGGTGTGCCCAGGGCTCTTCCCTGACTTCCCC

GTTTCCGGTCAGGCTTCTGCCAGCTCTACCTGAATGAGAAGGACTACCCGCCTGGCTAT
GCCTTCGACGTAGAGGCCATGAA
>2167
TGTCTCCCAGGGAGGACAGGGAGATTGGGCTGATCTGAAGGGGTCAGTGATCCACTG
GTTCCCCCTACAGGTGATCATGGTAACGGGTGACCACCCCATCACCGCCAAGGCCATT
GCAGCCAGTGTGGGCATCATCTCGG
>2168
CCTCCCCGGGGTTCTCCCCACATCTGGCACAGACCGAGGTCCCAGCGAGGTCCTCCCA
TCTCCTCCCCAGGGATGCCCGTGCCTGTGTGATCAATGGCATGCAGCTGAAGGACATG
GACCCATCGGAACTGGTCGAGGCC
>2169
ACAGGCGGGGCTGGTCTTGGACCGGCCTCTCACTGACCACCCACCCACCACCTGCAC
CCACTGCCTGCAGGGTGCGATTGTGGCCGTCACGGGGGATGGTGTGAATGACTCCCCA
GCTCTGAAGAAGGCAGACATCGGAG
>2170
GAGGGCAGGCAGTGTGGGCACAGGAGGGAGAGGGCTGTGAGGAGGCTGAGGTGCCC
ACCCTACCCCACAGGTCGACTGATCTTCGACAACCTGAAGAAGTCTATTGCCTACACAT
TGACCAAGAACATCCCAGAGCTGAC
>2171
GGGGACCCGAAGTGGAGGGGACAAGGGCCCAGCCAAACCCTCAGTCCCAGTTCCTTC
CACTCCTGGCCAGTTCCCATCTGTGTCCCTGGCATATGAAAAGGCCGAGAGTGACATCA
TGCACCTGCGTCCACGCAACCCAA
>2172
GTCCCTCTCTTCCTGTGTCCCTGTCTCTGTGTCCATCTCTGTCTCCCTGTGTGGGGTTGT
CTCTCTCCAGGTGCCATTCAGTCCTTTGCTGGCTTCACTGACTACTTCACGGCAATGGC
CCAGGAGGGCTGGTTCCCACT
>2173
TGGGCTGCAGAGGCTCTCCGCCGGGACTCAGGCTGGACTCAGGCTGTTTCCGTTTGCC
CCTGGCCTGCAGACATTCGGGCAGCGCCTGTACCAGCAGTACACCTGCTACACCGTGT
TCTTCATCAGCATTGAGGTGTGCC
>2174
GACCCACAGTATCCTGATTTCCATCACGTCCCCAATTCCTAATGGCCTGTCCCATTCTGC
CCACCCCCAGGAATAAGATCCTGGTGATCGCCATCGTGTTCCAGGTCTGCATCGGCTGC
TTCCTGTGCTACTGCCCCGGC
>2175
GGGGCAAGGACAGGGAGGGGGCTGTAGGGCACAGGCCTGGGATGGCCTTCACCTTGC
CATGTGCCTTCAGGTTCCAGTGGTGGCTGGTCCCCCTGCCCTACGGCATCCTCATCTTC
GTCTATGATGAGATCCGGAAGCTT
>2176
ACCTCTGAGCCCCTGCCCTGCCTAAGTGGCACCCCCAGCACTGAGTCTCCTTTCTCCCT
CTCTCTCCCAGGCTGGTGGGACCAGGAACTCTACTATTAGAGGGACGACTGCCTTCAA
GCATCCCTGCAACTGCCACAGCA
>2177
CCACCCCCGGACACTTCGAGCAGTGGAGGCCTTGTCCTCTAACCGGCTGGGCCGGGG

CTTGTCTGTGCAGGGTCTGGTCGCCAGCAACCTGAATCTCAAACCTGGAGAGTGCCTT
CGAGTGCGAGGCGAGGTGGCTCCTG
>2178
GTGACAGATTAGTCGGTCAGTGGGGCTGGAGCTGGCCGAGGTGGCCTCATGCCCACCC
GTTACCCCCCAGCTTCGTGCTGAACCTGGGCAAAGACAGCAACAACCTGTGCCTGCAC
TTCAACCCTCGCTTCAACGCCCAC
>2179
CACATGAGGAACGGGTTCTGGAAGGGCCCATGGCATGTGGGCCCGGCTCACTGCTCTC
CTCTACCCCCAGGTGTGCATCACCTTCGACCAGGCCAACCTGACCGTCAAGCTGCCAG
ATGGATACGAATTCAAGTTCCCCA
>2180
GGAAAAAGTAAAACAAAATGTAAATAAGGAGTAAAGCATGTACTTAAAATATATTCTTA
TTCTATCATAGGATAAATCTTCAGACAAAAAAGTGCAAACAAAAGGGAAAAGGGGAG
CAAAGGGAAAACAGGCCGAAGTGG
>2181
GCGCCCGCCCTGGCAGCTGCGGCGTCCGCGCCGACCTCCGGCTTCCCCTCTCCCCCCT
CGGCCCCGTCAGGTGGACGCGGATCTGTCAACATGGGTAAAGGAGACCCCAACAAGC
CGCGGGGCAAAATGTCCTCGTACGC
>2182
ATCTTGTCGTCTTTGGATGTTTATAGGTAACAATTTTGCTGTATTTTTGGCTCTTATTTTAT
GTCCACAGACCATGTCTGCAAAGGAGAAGTCGAAGTTTGAAGATATGGCAAAAAGTG
ACAAAGCTCGCTATGACAGGG
>2183
AGTTTAAAATAACCCAAATTGCTCCTTGGATTTTTCCTTCAGTTTATTAAACTCTGTTGC
TTCCTTTCAGATCTGCCTTCTTCCTGTTTTGCTCTGAACATCGCCCAAAGATCAAAAGT
GAACACCCTGGCCTATCCATT
>2184
TTTACAACTAAGTGGTTTTTCACAGTTGAGTAATGATACCGGATGCTTTATTTTTTTGAC
AATATTTCAGGATATTGCTGCATATCGTGCCAAGGGCAAAAGTGAAGCAGGAAAGAAG
GGCCCTGGCAGGCCAACAGGCT
>2185
CTAAAGAGTCAGGGTCAATTTCTTTCAGACATTCTTGCCACAACAGCATTTTTTTTTCC
CTCACAATTAGGAACCAACCGGTGAGCCCTCTCCTAAGAGACCCAGGGGAAGACCCA
AAGGCAGCAAAAACAAGAGTCCCT
>2186
CCTGCGGGGGGCGGGGACCCGGGAAAGGCGCGCGGTGGGGGAGGGGGCGCGCAGCT
GCGGCGAGCGCGAGTTGTGCACCCGGCGGAGCCCGGAGGAGCCCGGCGCACCTCGC
GCGGCGGCCGCTCCCGCTGGAGCCGGAG
>2187
CAAAAATGTTTTTTTCCTCTCATTGGGGTAAAAACTCTTTTTCTTCTTTTTTTTTTTTCTT
TTAAATTAGACATGGGAGTCCCACCGTATTGTCCAGGCTGGTCTCGAACTCCTGACCTC
AAGCAGTCCTCCTGCTTTGG
>2188
TGTGGGTGATGAGGGGGTAGAAAGTGGCTGAAGCGAGATGTTTGTCTAAAAGCACTTT

TCTGTCTCCCAGCATCCCAGCCATCACTCTTCCACCTGCTCCTTAGAGAAGGGAAGATG
AGTGAGTCGAGCTCGAAGTCCAG
>2189
GAGGGCTGTGTCTTCTGAGGGGGTGGAAACAGGTGATGACTGTCCCTGACTACCCCCT
CTGTCTTTACAGAAGGAGCCCAGCGAAGTGCCAACACCTAAGAGACCTCGGGGCCGA
CCAAAGGGAAGCAAAAACAAGGGTG
>2190
GGCCAGCCTCTGGGGGTGGAAGAGGGGGCCACCGGGCCAGAGCTCACACCAACAAC
TGCCCACCTCACAGGAGAAGGAGGAAGAGGAGGGCATCTCGCAGGAGTCCTCGGAG
GAGGAGCAGTGACCCATGCGTGCCGCCT
>2191
CAAGCTTGTCCCCCTGGCGGACTTTGGAAGACAGGAGTTGGTGGCTAAACCGCTGACT
TTTCTATTGCAGACAACATAGGCGGCCCGGAAGGCAAAAGGGCCCGGACGGCCTACA
CGCGCTACCAGACCCTGGAGCTGGA
>2192
CACCCCTCTCCAGTCCTGAGGGTCCCCACCCAACTCACTCAGCGCCCTCCCTCTCTCC
CTCCCTGCCCAGGAGAGAGCTGCGAGGACAAGAGCCCGCCAGGCCCAGCATCCAAGC
GGGTACGCACGGCATACACGAGCGC
>2193
TTCTCAGGCCCCCTGCTCTGTTTCTCTAGCTCCTCAACTTTTCTCTTTCCCCACTCCCAC
TCCTCCCAAGGAAACACGCCCTAAACTGCCACCGAATGAAGCCTGCTCTCTTTAGCGT
CCTGTGTGAAATCAAGGAGAAA
>2194
AAGCACCAGGAGGACTGAGCACTGGGTATTGGGGTCTCTGGGTCCCTAAGTCCACTCG
CCTGCATGCTAGGCCTCAGCATTCGGAGCTCCCAGGAGGAGGAGCCGGTGGACCCAC
AGCTGATGCGCTTGGACAACATGCT
>2195
GAAGGGCTCTTGCAGGGGAATCCCATGGTCAAAGGGCTCCTCCTCACCAGCCCACTGG
CCCCCACTACAGGCATGTAATGAGTTCACGACCCATGTCATGAACCTGCTGAGGGAGC
AGAGCCGCACCAGGCCCGTGGCCC
>2196
GGGTCATGCCATGTGACAGCCCTGCCAGGACATCAACATCCTCCTCACCGCTCTTCTCC
CTCCTCTGTAGACGAAAGCGCCGTAACTTCAGCAAACAGGCCACTGAGGTCCTAAATG
AGTATTTCTACTCCCACCTGAGT
>2197
ACTCACATCTTATTCAGTGAAGGACTTCAGAGGCAAATGTTTCTACCTGACCCTCCTTT
CTGCCCCACAGGTCTCCAACTGGTTTGGCAACAAGAGGATTCGCTATAAGAAAAACAT
CGGAAAGTTCCAAGAGGAGGCAA
>2198
AGTTGAGAGTTGTGGTTCATCCTCTACCATCACGGGCTCTATTACACTCTTCCCTCTCTG
CCCCCACAAGGCTCTGGCGGCTCTTTCAATCTCTCAGGATCTGGAGACATGTTTCTGGG
GATGCCTGGGCTCAACGGAGA
>2199
ACTTTAGGATACAAGACCTCTTTCCTCTGAGGCTTCTCTTCACTGTCATACCTTCCTCTG

CTGCCTGCAGGTGGAATCACTCCGACACTCGATGGGGCCAGGGGGCTATGGGGATAAC
CTCGGGGGAGGCCAGATGTACA
>2200
CTAGTAATGTTGTGCCCACACAGGGTTCCCAGGGCTTTCTCTGTTTTCTGTACTCTGTCT
CTCCTTTCAGGCAAATGGCAGCTGGCAAGAGGCTGTGACCCCCTCTTCAGTGACATCC
CCAACGGAGGGACCAGGGAGTG
>2201
CACCCACCCTGTCCAAATTTTGTTAGCCCTCATTCCCTTCCTACCCCTCTACCATGGTGC
TATCTCCCAGGGGCAGTAGTAGGTGCTCAAAACATCACAGCCCGGATTGGCGAGCCAC
TGGTGCTGAAGTGTAAGGGGGC
>2202
CCCGTCACTCTGCCTCACAGTCCTTTCCCAAAGGCCTTGCACTGTTTAGGCCCTGCTTC
TCAGCTTCTAGAACACAGGCCGGACAGAAGCTTGGAAGGTCCTGTCTCCCCAGGGAG
GAGGCCCCTGGGACAGTGTGGCTC
>2203
GTGTGTGTGGGTGTGGGTGTGTGGCATCTCTCATTTTCAAAGGATTCTGAGGTCACCAC
TCTTTCCCCAGGTGGGGACATGTGTGTCAGAGGGAAGCTACCCTGCAGGGACTCTTAG
CTGGCACTTGGATGGGAAGCCCC
>2204
CAAGCTGCCTTCTCCCTGATCTCACTCCCACACCCACCCTGGGATAATTTGTCTTATCCT
CCCATCATAGGAGTATCTGTGAAGGAACAGACCAGGAGACACCCTGAGACAGGGCTC
TTCACACTGCAGTCGGAGCTAAT
>2205
AGTCAAGCCCATGGCCACTGGGATCACTCACAAGTGTAACTCTCCACCTCAAAACCCT
TCCAACTCCCAGAGCCTGTGCCTCTGGAGGAGGTCCAATTGGTGGTGGAGCCAGAAG
GTGGAGCAGTAGCTCCTGGTGGAAC
>2206
GGGTATCTGAAGATATGGAGACAAAAAGACAAGGGTTTTGAAATAGTCTCCTCTCCCC
TTCCCCCACCAGGGTGTGCCCTTGCCCCTTCCCCCCAGCCCTGTGCTGATCCTCCCTGA
GATAGGGCCTCAGGACCAGGGAA
>2207
GGGATGGTCAACAAGAAAGGAATGGTGAGTGGTGGTGGCTGTGCTCTCAATTTTCCCT
GTCTCCGTACAGGCTCTGTGGGAGGATCAGGGCTGGGAACTCTAGCCCTGGCCCTGGG
GATCCTGGGAGGCCTGGGGACAGC
>2208
GGCAGCAAGCGAAGAGGGGTCGGGGGGTGGAACGACAACGTGCCGCATTCCCCCCAA
TCTTTCTCCTCAGGAAGGCCCCAGAAAACCAGGAGGAAGAGGAGGAGCGTGCAGAA
CTGAATCAGTCGGAGGAACCTGAGGCA
>2209
GTGTGGATGCATGCATGTGCTGTGAAGCAGGGAGACTAGCTTTCCACTCCTCCTTGTCT
TCTCTCTGCAGTGCCCTGGGAGCTGTCATTGCCCTCCTGCTCTGGGGACAGCTTTTTGC
AGTGGACTCAGGCAATGATGTC
>2210
ATCTGACTTTTCACGGTTCACTGGGAACAATTTCCAAATAGCAAACTCTCTGGCTTCTC

TCTCTTTGCAGATGACGGCTGCCCGAAGCCCCCGAGATTGCACATGGCTATGTGGAG
CACTCGGTTCGCTACCAGTGTAA
>2211
ATGCCTTCTCACTCTGCTCTGGGTGCAGACTTGACTTTTCCTTTGGCTCATTTCTTGCCT
TTTGTTTCAGGAGTATACACCTTAAATGATAAGAAGCAGTGGATAAATAAGGCTGTTGG
AGATAAACTTCCTGAATGTGA
>2212
ATCTGACTTTTCACGGTTCACTGGGAACAATTTCCAAATAGCAAACTCTCTGGCTTCTC
TCTCTTTGCAGATGACGGCTGCCCGAAGCCCCCGAGATTGCACATGGCTATGTGGAG
CACTCGGTTCGCTACCAGTGTAA
>2213
ATGCCTTCTCACTCTGCACGGGGTCTAGACTTGACTTCTCCTTTGGCTCACTTCTTGCC
TTTTGTTTCAGGAGTGTACACCTTAAACAATGAGAAGCAGTGGATAAATAAGGCTGTT
GGAGATAAACTTCCTGAATGTGA
>2214
TTTCACCCCTTTCTCAGATGGAAAGGCTCTTGCACATTTCCACTCACGAGTGTCTTGCT
CTCCTTGACAGTATGTGGGAAGCCCAAGAATCCGGCAAACCCAGTGCAGCGGATCCTG
GGTGGACACCTGGATGCCAAAGG
>2215
AACATCTTAATCCAATCAAATGTTTGTATCCTGTAATGCTCTCATTGAAACAGCTATATTT
CTTTTTCAGATTAGTGATGATGAACCAGGTTATGACCTTGATTTATTTTGCATACCTAATC
ATTATGCTGAGGATTTGG
>2216
ACTAAGAGGTGTTTGTTATAAAGTTTAATGTATGAAACTTTCTATTAAATTCCTGATTTTA
TTTCTGTAGGACTGAACGTCTTGCTCGAGATGTGATGAAGGAGATGGGAGGCCATCAC
ATTGTAGCCCTCTGTGTGCTC
>2217
ACATGGGGGTTTTGGTACTTTATATTGTGACTCTGAATTTAAAGCTATGCAATGTCTTCT
TTTTTGAAAGGATATAATTGACACTGGCAAAACAATGCAGACTTTGCTTTCCTTGGTCA
GGCAGTATAATCCAAAGATGG
>2218
CACATTTGCCAGTATTAGATTTAAAAGTGATGTTTTCTTTATCTAAATGATGAATTATGAT
TCTTTTTAGTTGTTGGATTTGAAATTCCAGACAAGTTTGTTGTAGGATATGCCCTTGACT
ATAATGAATACTTCAGGGA
>2219
TTGTAAAAAATGGGGTGGAGGAAAATAAGGCATCTGCTGAGTGTATAACCATTTTACCT
CTTGTTTTTAGCCCTCTTCTGGGTCACGCTAGAATCAGATCTGCTCTCCAGCATCTTCTG
TTTCCTGGCAAGTGTTTCCTG
>2220
TCGCCTTTATCAGAAAACTTCCCAGCCAGATCCAGAAATCTTTCCAATGACCTGACCTG
TGTTCACACAGAGCTCCAGAACAGGACCAAGCTGACTGTACTTGAAGGAGACATTCT
GGATGAGCCATTCCTGAAAAGAGC
>2221
GGATCTGTGCATGTGGTTGCAGCTCCTTTGGGATATTTCCTGACACTGTCATCATGCTCT

TCGTGGGCAGGTACCCAGCTACTGTTGGAGGCCTGTGTCCAAGCCAGTGTGCCAGTCT
TCATCTACACCAGTAGCATAGA
>2222
TGCACTGGTTGGCTCTGCGGACTCCGCGTCCGTCTGTGACACCCCCTGCACACTTACC
CAATCCTTTTAGATGCCAAGATTCGCATTTTTGACCTGGGGCGGAAAAAGGCAAAAGT
GGATGAGTTTCCGCTTTGTGGCCA
>2223
TGGAGATAGTCGTGGTGAATGTTTCTCTATTATCTTCTCTCACTTTGCTGCTTTTCTTCTC
CCTACCTAGCCCTGGAGGCTGCCCGAATTTGTGCCAATAAGTACATGGTAAAAAGTTGT
GGCAAAGATGGCTTCCATAT
>2224
CTAAGGGACCTTGGAGACAGGCCTTTCAGGTGGATGTTCATGTTTCTGACCTTGCACTA
CCCCAATGTAGGCTCCAAACAGGCATGCGAGGTGCCTTTGGAAAGCCCCAGGGCACT
GTGGCCAGGGTTCACATTGGCCAA
>2225
GTAGTGCTGCAGCCCCCTTCTCCCACCTTTGCCCCAGCCTCCTGACTCAGTTCTTTCCA
TTGCTCCTTAGATCCACATCTCAAAGAAGTGGGGCTTCACCAAGTTCAATGCTGATGAA
TTTGAAGACATGGTGGCTGAAA
>2226
ACGCGATGGAATCTCGTGTTGCCTCTGTAGACGTCCTGCAAGGTTTTAAACCGGCGCG
GTGTCGTTCCAGATGCCTGAGGAAACCCAGACCCAAGACCAACCGATGGAGGAGGAG
GAGGTTGAGACGTTCGCCTTTCAGG
>2227
ATCGGATTTTGGTTTCAGTGTGAACTTCTTGGGGGTGCTGTATGCTTAAATTAATATTTT
TTGTTAACAGGCATTGGACAAAATCCGGTATGAAAGCTTGACAGATCCCAGTAAATTAG
ACTCTGGGAAAGAGCTGCATA
>2228
GGTGTTAGGCATGGCGTTGAGCACTAAATTGAATGTATTATTAAGTCAATCTTTCTTTCT
TCTCTTGCAGGTGAACCTATGGGTCGTGGAACAAAAGTTATCCTACACCTGAAAGAAG
ACCAAACTGAGTACTTGGAGGA
>2229
AATAGTGGTGCAGCGCTGTCGCTTAGTTCTGGAGTGCAGCAGTGATGTGATTTCGTGTT
TTCTTTTGAAGGTGGAGAAGGAACGTGATAAAGAAGTAAGCGATGATGAGGCTGAAG
AAAAGGAAGACAAAGAAGAAGAAA
>2230
TTCTCCACCACCCCAAATATCTTCTATAATGCATTGTTCATTGGTACTTAGTGTATCTGTT
TTATTACAGCATTTTTCAGTTGAAGGACAGTTGGAATTCAGAGCCCTTCTATTTGTCCCA
CGACGTGCTCCTTTTGATC
>2231
TTTGGGAGTTTACATAGTACCAGTTTTGTCCTTGGAATGACTCAGTGCATTTGGTTTATA
TTTTTTTCAGACTTCATTAGAGGGGTGGTAGACTCGGAGGATCTCCCTCTAAACATATC
CCGTGAGATGTTGCAACAAAG
>2232
ATTGGCCTCTTTAGTTTTTTTTTTTTTTTTTTAATTCAGAAAGTCTTTTAAAGAACATACT

TTGTTTCAGCTTGGAATACACGAAGACTCTCAAAATCGGAAGAAGCTTTCAGAGCTGT
TAAGGTACTACACATCTGCCT
>2233
GAAAAGTCAGAGCTAGATGAAACCATTCTTAACTGTTAAAAGGTCTAAAAGTAACTTT
GTAATACCTCAGGTGAGACCAAGGACCAGGTAGCTAACTCAGCCTTTGTGGAACGTCT
TCGGAAACATGGCTTAGAAGTGAT
>2234
GATTTCATTTCACAATGAAAGTTTAGGATTAATCTTTCTAGGTTCCTAGTCATCACTTTTT
GGATTACAGGTGGTTGTGTCAAACCGATTGGTGACATCTCCATGCTGTATTGTCACAAG
CACATATGGCTGGACAGCAA
>2235
ACCAAGAGTATTACCCTAATAGCTGGCTTTAAGAAATCTTTGTAATATGAGGATTTTATT
TTGGAAACAGGTATTGATGAAGATGACCCTACTGCTGATGATACCAGTGCTGCTGTAAC
TGAAGAAATGCCACCCCTTGA
>2236
TGTAGCTTGGGCAACAGAGCAAGAGTCTGTCTTGGAAAGAGCAAAAGTAAGTTGCTG
TTTGTATTTCCAGGCCTTGGACAAGATTCGCTATGAGAGCCTGACAGACCCTTCGAAGT
TGGACAGTGGTAAAGAGCTGAAAA
>2237
TCATACTTATCTGTGTTCTTTGGTTTTTTGCTTCTTTAAAACTTGTGATTGACTTTAAACT
TGTTGGCAGGCTGGTGCAGACATCTCCATGATTGGGCAGTTTGGTGTTGGCTTTTATTC
TGCCTACTTGGTGGCAGAGA
>2238
ACTTACTAATTGCTGGTCTCAACTGCATATACTTTTTACCCTGTTACACGCTTGTAATTG
ACTCTTCTAGGTGAGCCCATTGGCAGGGGTACCAAAGTGATCCTCCATCTTAAAGAAG
ATCAGACAGAGTACCTAGAAGA
>2239
TTGTCCCTGGCTTTTGCTTTCCCTGGTAGTGTTTTGTACTCCAAGGCTAACTTCTGTTTT
TGTTACTTAGTTGGAGAAGGAACGAGAGAAGGAAATTAGTGATGATGAGGCAGAGGA
AGAGAAAGGTGAGAAAGAAGAGG
>2240
TTAGATAATTTGGTGTTGGGGCTAAAAGGTCCTCTTTTGAAATGTACCACTTATTTTTGG
TTTCTTTCAGCACTTTTCTGTAGAAGGTCAGTTGGAATTCAGGGCATTGCTATTTATTCC
TCGTCGGGCTCCCTTTGACC
>2241
CTGTTTGAATCTGGGGACCAGGTCTGGTCTAGCTGTTTTTACTGAGCTTTCTCACCCTG
GTTGATGGCAGATTTTATCCGTGGTGTGGTTGACTCTGAGGATCTGCCCCTGAACATCT
CCCGAGAAATGCTCCAGCAGAG
>2242
ATTCCATTCACATTGAAAGTGAAGTTATTGTAGTTAAGCTGGATTGTTTTTCCTCTTCCC
ACCCTTCAAGCTTGGAATCCACGAAGACTCCACTAACCGCCGCCGCCTGTCTGAGCTG
CTGCGCTATCATACCTCCCAGT
>2243
CAATTAGGGCTTCCTGGGAACTGGCAGTATGAGGCATTTTAGTCACTGAGTTCATTTAA

TTACCCTACAGGTGAGAGCAAAGAGCAGGTGGCCAACTCAGCTTTTGTGGAGCGAGT
GCGGAAACGGGGCTTCGAGGTGGT
>2244
AGGCTTGACAATGCCTGTTTTCTCTTTCAAAGTGGTAATGTCAATCTAAGGCTTTTGTG
ATCGTCCACAGGTGACAATCTCCAATAGACTTGTGTCTTCACCTTGCTGCATTGTGACC
AGCACCTACGGCTGGACAGCCA
>2245
AACTCGTCTCCTCTATGGATTTGACTTAATGCTATTTGGTCAAGTCTCACATGGCTTAAT
TTTACTTCAGGTATTGATGAAGATGAAGTGGCAGCAGAGGAACCCAATGCTGCAGTTC
CTGATGAGATCCCCCCTCTCGA
>2246
GGCGAACAAGCCGAGGGGCGGAGTGGGCCAGGGATCACCTGCCCCGCAATGACCTGC
GCCCCGCCCCCAGGCCTGCTGGAGCTCTCGCCCGTGGAGCGGGGCGTGGTGAGCATC
TTCGGCGTGGCCAGCCGGTTCTTCGT
>2247
CCCCTGGCTTCAGGCTGGGTACCTTGCCCAAGAGGTGCCCCAGCCCTGACACTGCCCT
GCTTTGCTGCAGCCCTTCTTCACCGATGAGTGCACGTTCAAGGAGATTCTCCTTCCCAA
CAACTACAACGCCTACGAGTCCT
>2248
TTTTAAAAATTGTGCTACAGGTGAGAGGTTGAGAAATGGATGCAAACCATCGTCTGTG
TTCCTCTTCTAGTGCAGGTACCCTTCTCCAGATGTTGCTTCTCATTTGCGGAGCAAGAG
ATTCCCCTGAGGGCAATCCTGTG
>2249
GGGGCTTGTGCGTTGCCCACCCAGTGGTGGCTTGGGTGGCTCAGCCTTCTCTCTTATTC
TCTGTTCACAGATTCAAGCTGAAGAGAGGCAAAGAGGCCTGCGCCTTGGACACAGTT
GGATGGGTTCAGAGGCACAGAAAA
>2250
TTAAGATTTTTACAAGACTGTGATAGATATTGATGACTCTCATGTGTTTGTCTCTCTTGG
GCGTTTTAAGGAAATGCTAGTGAGTCGGAGGAAGACCGCAGCGCCGGCAGTGTGGAG
AGCCCGTCCGTCTCCAGCACGCA
>2251
AAGATTTTTAAAGCACACATTCTAGAGTAAAAACTGTGACTCTGTTGCTCTGGTCCTTC
CTGATCCCCAGGGTCCCTGCCGTAGAGAAATGGAAGACACACTGAATCACCTGAAGTT
CCTCAATGTGCTGAGTCCCAGGG
>2252
CGCCCTGGCCACCGACCTGACACCAGACCCAGGAGCATTCACTTCTCTGTCTTCTGTT
TCTCTCCCACAGTGTCGCCCTTCCAAAGGCAGGAAGCGGGGCTTCTGCTGGTGTGTGG
ATAAGTATGGGCAGCCTCTCCCAG
>2253
CTCAGACTCAGCCCCAAAGTCTGTGGTCTCTGACCTGACACAGCCTTATGTGTATGTGT
GTATTGTTCAGGAGGAGATGATCTTCCAGATCCCATGGAAGCATGCTGCCAAGCATGGC
TGGGACATCAACAAGGATGCCT
>2254
CTTCAGCCAGGGGCAGCTGAGGATCCCTACCCATGGCCCTGACGATCCTCTTTTTTCTC

CTGCCCTCTAGGCCGATACAAAGCAGGGGAAAAGGAGCCAGATCCCAAGACGTGGAA
GGCCAACTTTCGCTGTGCCATGAA
>2255
TGTGGGCCAGGGTGAGCAGTGGAAGAAATGCTAAGGTGGCCTGGGCCTAAGCTGCTT
TCTCCCTCGACAGTCATGTGGGGATTCCAGCCCTGATACCTTCTCTGATGGACTCAGCA
GCTCCACTCTGCCTGATGACCACA
>2256
GGAGGGCGCTCGATGTCTTGCAAACTAAGAAAGCACACAACCTTGACCTGTGGCTTCT
GCTGTTCCCCAGCACTGTCGCCATGTGCTGTCAGCAGCACTCTCCCCGACTGGCACAT
CCCAGTGGAAGTTGTGCCGGACAG
>2257
ATTGAGGCAGGGCCACAGGGTGAGTCTGCACTGGAAGGGAGTTGATAGCCTCTTGCTC
TTCTGTCCCCAGCTCTTGGAGCAGTCGGAGTGGCAGCCAACAAACGTGGATGGGAAG
GGGTACCTACTCAATGAACCTGGAG
>2258
CAGGGGTAGGGGGAAGCACACCTGATGCCACCGTCCCCTACCCTCATACAACCTTCTT
CACATCTTCTAGGGGATATTGGGCTGAGTCTACAGCGTGTCTTCACAGATCTGAAGAAC
ATGGATGCCACCTGGCTGGACAG
>2259
TTAAGCCTAGACAGTTTTCAATGACTTTTTTTCTCTCTACATGTCTTTTCATATTTTTATC
TTCTTGAAGTCCCTCAGAAACCTAAGGTCTCCTTGAACCCTCCATGGAATAGAATATTT
AAAGGAGAGAATGTGACTCT
>2260
TCTCTTTTCTCTATTCATTCTCTCTCTCTTCATTTATTGTTAAATAAATAATGTAATGAATG
TTCTTCAGACTGGCTGCTCCTTCAGGCCTCTGCTGAGGTGGTGATGGAGGGCCAGCCC
CTCTTCCTCAGGTGCCATGG
>2261
AAACTCTGAACCTCATTTTTCAGTTCCCAAGATGAATTATGTTTCTCATTGCATCTGTGT
TCCACTACAGCTCCGCGTGAGAAGTACTGGCTACAATTTTTTATCCCATTGTTGGTGGT
GATTCTGTTTGCTGTGGACAC
>2262
GATCTCCTGGGGGCCGAAAGGGGCAGATGCTTTGGGAGGGCTCATGGGGTCTGCAATG
TTTCCTTTCCAGAGGCAGGGAGCCCTGAAAGCCCAGAGAGCACGGAGATAACTGAGG
AGGAGCTCCTGGATAATTTCCATCT
>2263
ATCTCCTGGGGGCCCGAAAGGGGCAGATGCTTTGGGAGGGCTCATGGGGTCTGCAATG
TTTCCTTTCCAGAGGCAGGGAGCCCTGAAAGCCCAGAGAGCACGGAGATAACTGAGG
AGGAGCTCCTGGATAATTTCCATCT
>2264
ATCAGTCATTGTCTAGGCTGATAATTCACTGTGTCTTGTTAGATATGCTAATGTCAAGTTA
TTCTCTTAGGAGCCCTGCCGAATAGAACTCTACAGAGTCGTAGAGAGTTTAGCCAAGG
CACAGGAGACATCAGGAGAAG
>2265
GCCAGCTATGGCTCTACTTTCCCTGTCAGCTTGTCATCTGCTGCTCTTGACCTTGGTCTT

GTCTTTGCAGTGTGAGACATCCATGGATGGAGAGGCGGGACTCTGCTGGTGCGTCTAC
CCTTGGAATGGGAAGAGGATCC
>2266
CTCTCTGATGTCAAAGCATGGTTCCTGGCAGGACCACCTCACCAGGCTCCCTCCCTCG
CTCTCTCCGCAGTGCTCCTTCCAGGACCTGGACCTCTGCCCTCTGGATGGCGGCATCCA
GCTACGAATCTCCGACCACCACT
>2267
TTTGCCGCCTCGCCTCACGAGGCCTGCCCTTCTGATTTTATACCTAAACAACATGTGCT
CCACATTTCAGAACCTATCTTCTTCGACACATGGGATAACGAGGCTTATGTGCACGATG
CACCTGTACGATCACTGAACTG
>2268
ATGGGGCCTAGGGGTTGGGGTGACCCTGCACTGCTGTGTCCCTAACCACAAGACCCCC
TTCTTTCTTCAGTGGTGTTCTCCATGTCCTTTGTACAAGGAGAAGAAAGTAATGACAAA
ATACCTGTGGCCTTGGGCCTCAA
>2269
CCAAAGAACATCTTTCCCATAGCATCTTTGTCCCTTGCCCCACAAAAATTCTTCTTTCTC
TTTCGTGCAGAGTGTAGATCCCAAAAATTACCCAAAGAAGAAGATGGAAAAGCGATTT
GTCTTCAACAAGATAGAAATCA
>2270
AAATAAATTGGAAATTAATTTCTGATTCTGACCTCTATGTAAACTGAGCTGATGATAATT
ATTATTCTAGGCCACAGAACTGAAACATCTTCAGTGTCTAGAAGAAGAACTCAAACCT
CTGGAGGAAGTGCTAAATTTAG
>2271
TCAATTGGGAATAACTGTATATAAGGTAAACTACTCTGTACTTTAAAAAATTAACATTTT
TCTTTTATAGGGATCTGAAACAACATTCATGTGTGAATATGCTGATGAGACAGCAACCA
TTGTAGAATTTCTGAACAGAT
>2272
CGTGCTTGGCCTCCTCCTTCTGACCATCATTTCTCTTTCCCTCCCTGCCTTCATTTTCTCC
CCAATCTAGATTTCTTCCTGACCACTATGCCCACTGACTCCCTCAGTGTTTCCACTCTGC
CCCTCCCAGAGGTTCAGTG
>2273
TAGGCTCTGGATATCTGCAGTACCCAGATTGGCCCCACTGTTCCTCTTCCTTCCAACCTT
TCTCCTCTAGGTACAAGAACTCGGATAATGATAAAGTCCAGAAGTGCAGCCACTATCTA
TTCTCTGAAGAAATCACTTCT
>2274
GGGGCACTACCTTCAGGATCCTGACTTGTCTAGGCCAGGGGAATGACCACATATGCAC
ACATATCTCCAGTGATCCCCTGGGCTCCAGAGAACCTAACACTTCACAAACTGAGTGA
ATCCCAGCTAGAACTGAACTGGAA
>2275
TTCAGGAGTATGTTTTAATTCTCCCTTCTCTCATAGACACCCACTTTCCCTCATCCTCTTT
CTCCTCAAGGAACAATCAGTGGATTATAGACATAAGTTCTCCTTGCCTAGTGTGGATGG
GCAGAAACGCTACACGTTTC
>2276
GCAACAGTGCCTGGCATGTAGTAGGGGCACAACAAATATAAGGTCCACTTTGCTTTTCT

TTTTTCTATAGAGAATCCTTTCCTGTTTGCATTGGAAGCCGTGGTTATCTCTGTTGGCTC
CATGGGATTGATTATCAGCCT
>2277
GACAACTGGGAGAAGGGTGATAAAAAAGCTTTAACCTATGTGCTCCTGCTCCCTCTTT
CTCCCCTGTCAGGACGATGCCCCGAATTCCCACCCTGAAGAACCTAGAGGATCTTGTT
ACTGAATACCACGGGAACTTTTCG
>2278
TTTGGCACAGAGCTCGGTCTTTTACTTCCTGCCCCTAATTGACCCCTGACCTGGACATA
TCTGTCTTTAGGCCTGGAGTGGTGTGTCTAAGGGACTGGCTGAGAGTCTGCAGCCAGA
CTACAGTGAACGACTCTGCCTCG
>2279
GAGGAAAAATCTCTCTCCCATCCTCCAAATGGAGCTGGCACATTGCTATCTGTGGCATT
TGTCTTTCCAGAACACAACTGAGAAGGAAACCTTCTGCAGGGCTGCGACTGTGCTCC
GGCAGTTCTACAGCCACCATGAGA
>2280
CTTGATCAAGTAGACAGGCAGGCAAGACAAATGCTTACCAATGCAAGCTAATGAAATG
TTTCTTTTGCAGAATTCCTGTCCTGTGAAGGAAGCCAACCAGAGTACGTTGGAAAACT
TCTTGGAAAGGCTAAAGACGATCA
>2281
GAATGGACAATGGACCAAATTTTTATACCTTGTCTTGATTATTTGCATTTTAAAAATTTTC
CTCATTTAGCACCAACTGTGCACTGAAGAAATCTTTCAGGGAATAGGCACACTGGAGA
GTCAAACTGTGCAAGGGGGTA
>2282
CTGGCTGTGCCTATTTCTATGGAATTGACAGTTTCCTGTAATACCTATTGTCATTTTTCTT
TTTTCACAGAAAAAGTGTGGAGAAGAAAGACGGAGAGTAAACCAATTCCTAGACTAC
CTGCAAGAGTTTCTTGGTGTAA
>2283
TAGAAGGAAGTAGCTGGCAGAGCTGTGCCTGTTGATAAAATCAATCCTTAATCACTTTT
TCCCCCAACAGGTGCAGTTTTGCCAAGGAGTGCTAAAGAACTTAGATGTCAGTGCATA
AAGACATACTCCAAACCTTTCCA
>2284
GACTTTTCAAGAACCCTACTTTCCTTCTTATTAAACATAGCTCATCTTTATATTTTTAATT
TTATTTTAGGGCTGAGAATTCATAAAAAAATTCATTCTCTGTGGTATCCAAGAATCAGTG
AAGATGCCAGTGAAACTTC
>2285
TGCTCTTTGTGATATATTTCCAAAGCTGTCCTCAGAATATTTCTTTGAATAAATCCTTACT
ATTTACCAGGACAACTGCACCAGACCATGCTTCAGTGAGAGACTGTCTCAGATGACCA
ATACCACCATGCAAACAAGAT
>2286
TAGGCTTCTGGGGCTGCTTGGCTCTACATCATTTGAGTCACTCTGTCCTTATCAACTTTC
ATCCCCACAGTATTTTTCCTGTGAACAGCCATGCAACCAAACCACGGCAGGCAACGCG
CTGACATTTCTGAAGAGTCTTC
>2287
CAGAGCTCTCACGCTACAGCTGACAGGCAGGGCAGGCGTTTTAATTATTATTAATTTTT

TTTTGAGACAGAGTTTGAGACAGTATCAAGAGTGGGTATCCTCTCAGCGTGTCTTCAA
GTAGCATCCCAGAGCCCCGCTCC
>2288
GGGCAGCAGTTGTCCATTCTGGGGCCTGGGTCAGCTCTCAGCTGTGGCCGTTGTGCCT
GTGCTTCCCCAGGTCCTGGTGGTGACTCCAACCCTGCCCTCACATATCCCAAGAGCAG
GCTGACTGCCTTCCCCATTCCCAC
>2289
AGCAATTCATGGGGAGCACCCCTCCAGAGAGGGATGATTTGCACAGGGCCCTCAGCCC
AGTCCCTTGCAGGCTGGACCTTGGAGAGTGAGGCCCTGAGGCGAGACATGGGCACCT
GGCTCCTGGCCTGCATCTGCATCTG
>2290
CTCCCCAACCCCCGAGCTCAGCTCTGGCCTGAAGTACTTACCGTGGGCTCCTGATGGT
CACTGTCTCCAGGGCCAAGGTCTAGAACCTTCACCTGCCTCACCAACAACATTCTCAG
GATCGATTGCCACTGGTCTGCCCC
>2291
TCCCCAGGATTGAAGCAGCTATGCCAGGACAGTGTAGCAGCCCCGTGGTGCTGACAAA
TGCCCTTTCCAGCAACCAGGCTCCTGGCGGCACACATAAGTGCATCTTGCGGGGCAGT
GAGTGCACCGTCGTGCTGCCACCT
>2292
TCTTGTGTGTTCTGACTGACACACCCAGACCCATGGGGCTTCAGCCTCACATGGATTCA
CTCTGTTCCAGTTAAGCTGGACCCGCCCTCTGACTTGCAGAGCAACATCAGTTCTGGC
CACTGCATCCTGACCTGGAGCAT
>2293
GTGTGTATTCTCGAGGGCTGAGGGACCCAGCCCCACCTTCACCACCTGCTAACTGTCC
CCACCCCCACAGCAGGCCCAGCACAGGGATCACATTGTCGGGGTGACCTGGCTTATAC
TTGAAGCCTTTGAGCTGGACCCTG
>2294
GCCAGGTGGGTTTGGGGGGAGCCTCCTGGCACTGAGGCTGCTCACAGCCCTGGGCCC
TTCCTGTCCACAGGCCCTCTGATCCCACCCTGGGGGTGGCCAGGCAACACCCTTGTTG
CTGTGTCCATCTTTCTCCTGCTGAC
>2295
CTCAGCCTCTGCAGTGACCTCAGGCCACCTGGGTCGCCATAGGCCTCTGACTGGCCTC
TCTTGGCCTCAGGGTGAAGAGAATCTTCTACCAGAACGTGCCCTCTCCAGCGATGTTC
TTCCAGCCCCTCTACAGTGTACAC
>2296
GGCAGCTGCTCCCAGGATGAGTTCTGAACATGCTACCTGAGCCCTTCCCTCCTCCCGTG
CTCTGTTCCAGACTTGGATGGGGGCCCACGGGGCCGGTGTGCTGTTGAGCCAGGACTG
TGCTGGCACCCCACAGGGAGCCT
>2297
GTGGGGGACTCAGTAACACCTTGAGGTGTAAGGGATGCTTTCCCACACTGGTTGACGT
TTGTCTCCTCAGGACCTGACTTCTGCTCTGACCAAGAAAATCACTCTTAAGACCCCACT
GGTTTCCTCTCCCATGGACACAG
>2298
CATCGCCTTTCCCCAAAGGCATTCAGTCCTGCTTCTTGTCACTTAGTAGTAGTCTCTTTT

TATCCTGTAGCTTACAGGCGGTATTGGCTTCATCCACCACAACTGTACACCTGAATTCC
AGGCCAATGAAGTTCGGAAAG
>2299
ATGCTTCAGATCAAGAGCCTGATGAAAGTAGTTCAAAGATGCGATGCCCTTTCTCACCA
TCCCTTTCCAGAAATATGAACAGGGATTCATCACAGACCCTGTGGTCCTCAGCCCCAA
GGATCGCGTGCGGGATGTTTTTG
>2300
GAAGGAAAGAAAAGAGTTAACTGGTCATAGTCGATGACTGGCCCTTCTTCACATCTCA
CCTCCCACGTAGATAATGACAAAGAGGGAAGACTTGGTGGTAGCCCCTGCAGGCATCA
CACTGAAGGAGGCAAATGAAATTC
>2301
AGTCCTAGAGCTGGGAAAGGGCCTGGAACTAATGCCTAGGGTCCTGATTCATGTCCTG
CCCTGACCACAGGAAAGTTGCCCATTGTAAATGAAGATGATGAGCTTGTGGCCATCATT
GCCCGGACAGACCTGAAGAAGAA
>2302
GCTACACAGGTGGGTTGGGGCAATAGGGGCAGCTGCCACATGACAGTCTGAGACTTAC
TTCTTGTCCTAGACTCTTCCCAGGGAAATTCCATCTTCCAGATCAATATGATCAAGTACA
TCAAAGACAAATACCCTAATCT
>2303
GTGGTGGGAGTGGGTAAGCTGGGCTCCCACCTTAACCTTCACATGAGCATTTTCCTTTC
CTTCACCATAGTGGTCACTGCTGCCCAGGCCAAGAACCTCATTGATGCAGGTGTGGAT
GCCCTGCGGGTGGGCATGGGAAG
>2304
AATATGGCCACAGGGTCCACTGCCCGTCCCCATTAACCCTATCCACCCATGTGTTCCTC
CATCTCAACAGTGCTGGCCTGTGGGCGGCCCCAAGCAACAGCAGTGTACAAGGTGTC
AGAGTATGCACGGCGCTTTGGTGT
>2305
CCACCCTAATCCTGCACTGACAGTCTCATCTCTGCTGTGACCTTGCCCGTGTCTCTGCC
TCTCCCTGCAGTGAAGCTGACAAAATCAAAGTGGCCCAGGGAGTGTCTGGTGCTGTG
CAGGACAAAGGGTCAATCCACAAA
>2306
GGAGCTGGGACATGGGTAGAGGGGCTGGTCCAGGGCCAGCTACCCACATTTGACCTCT
GCCCTTCTTCAGAGCCATGATGTACTCTGGGGAGCTTAAGTTTGAGAAGAGAACGTCC
TCAGCCCAGGTGGAAGGTGGCGTC
>2307
TCAGCCACAGTGAAGTGCCTGTGAGTGCATGAACTGATGGGGGCGCTGTCCTGTTTTC
TCCTGTGTGCAGACCGTTGGGGGGGTGAATTACTTCTTCGACGTAGAGGTGGGCCGAA
CCATATGTACCAAGTCCCAGCCCA
>2308
TCTCATGCCTGGGCATCTTCCCCTTTAACTGTAACCCACACTGATTGGCCCTCTCTCTTC
CCTTTCACAGAAACAGTTGTGCTCTTTCGAGATCTACGAAGTTCCCTGGGAGAACAGA
AGGTCCCTGGTGAAATCCAGGT
>2309
ACAGTAGCATCGCCTCCCTCTGAGTTCTTCTTCCCAGCCCAACTTGGACACCTGTGCCT

TCCATGAACAGCCAGAACTGCAGAAGAAACAGTTGTGCTCTTTCGAGATCTACGAAGT
TCCCTGGGAGGACAGAATGTCCC
>2310
ACTGTGTCCTCCTGTGTCCCTCTGCCTCGCCGCTGTTCCGGAACCTGCTCTGCGCGGCA
CGTCCTGGCAGTGGGGCAGGTGGAGCTGGGCGGGGGCCCTGGTGCAGGCAGCCTGCA
GCCCTTGGCCCTGGAGGGGTCCCT
>2311
GCGCCTGCTTTCCTGGGCTCTAAAACTGGCTGCTCCTCCTGACACTGAGTAGGACCTC
CAACTCTTACAGATCCCTTCCCCTGAAGTATTTGAAGAGCTGATCAAGTTTTCCTTCCA
CACTAACGTGCTTGAGGACAACA
>2312
CTCCCCGAGGGGCACACAGGGCCTCACTGTGAGCTCAGCCCCTGAACAGGCTCTGCT
TCCCATCCTTCAGGTTCAACATCGGTGGCCCCACATCCTCCATTCCCATCTTGTGCTCCT
ACTTCTTTGATGAAGGCCCTCCA
>2313
CAGGATAGAGAAGACAGGTGCTCCAGGGTCCTGACATGACCCCCATCCTGAAGGGCCT
TATGTCTTCCAGGTGAACGCTATGGCTCCAAGAAGAGCATGGTCATTCTGACCAGCAG
TGTGACGGCCGGCACCGCGGAGGA
>2314
CCCTCTCCTAGCCTTGTCCCTGGCCTCTCCTGCCAACCCTGCCCTTCCTGACCCAGCAC
CGCCTCTGCAGGTGCTGCGCCCCCGATTCAGTCCCGGATTGTGGGAGGCTGGGAGTGT
GAGCAGCATTCCCAGCCCTGGCA
>2315
CCATGCTCCCCTGCCCCATCCCCTTTTCCCCTTCCCGTCTTCTCATCCCTCCATTCCCATC
TTTCCCCAGCAATTACCAGCTCTGGCTGGGTCGCCACAACTTGTTTGACGACGAAAAC
ACAGCCCAGTTTGTTCATGTC
>2316
CGTCCAAGGGAGAAAGGGCCAGGGAAGCAGGTGAGGTCGGGCTGCAGCCCTTTTTCT
CCCGGGTTCGTAGTCTCATTTCCAGATGATCTCCAGTGTGTGGACCTCAAAATCCTGCC
TAATGATGAGTGCAAAAAAGCCCA
>2317
TGGGGCTCCCCAGGCAGAACTTGGGCCCCGTAGACCTTTCTCACTCTCCCCTCCCACC
TTGTGCTCCCAGGGTGATTCAGGGGGCCCGCTGATGTGTGATGGTGTGCTCCAAGGTG
TCACATCATGGGGCTACGTCCCTT
>2318
CACAGCTCAGCTCCCTCCCCTATCCAATTCTTTTGGGTCTGATCCCCCTGACCCAGCAC
CCCCTCCGCAGGTGCCGTGCCCCTCATCCAGTCTCGGATTGTGGGAGGCTGGGAGTGT
GAGAAGCATTCCCAACCCTGGCA
>2319
TATCCTCCCCTGCCCCATCTACCTTTCCCCATTTTCTCTCTCCTCATGCATCCACCCCCTT
CCTCCCCAGGAATAGCCAGGTCTGGCTGGGTCGGCACAACCTGTTTGAGCCTGAAGAC
ACAGGCCAGAGGGTCCCTGTC
>2320
GGTCTGAGGGAAGTGGGGCCAAAGAACCAGGTGGGGTCCGGCCACAGCCCAGTTTTT

CTCTGACCCATAGTCTTGCGCCCCAGGAGTCTTCAGTGTGTGAGCCTCCATCTCCTGTC
CAATGACATGTGTGCTAGAGCTTA
>2321
AGAGATGGAGTTGCCTAGGCAGTTATTGGGGCCAATCTTTCTCACTGTGTCTCTCCTCC
TTTACCCTTAGGGTGATTCTGGGGGTCCACTTGTCTGTAATGGGGTGCTTCAAGGTATC
ACATCATGGGGCCCTGAGCCAT
>2322
CATTCCCTGGCTGGAGGACTAGCTCTGTGGCCTGCAGATGTCCTGTACCTTCTTTTCAT
CTTCCCTTCAGCCCTGTGGGAGAAACCATTCATTTCCTCAAGGACCACTCCCAAAGAC
TTCTATGTTGATGAGAACACAAC
>2323
GGAGGGCTCTGCCCAGCAGGGATCCTGGCTTGTTCATTAATCTAATGTTCTAACTCAAT
GCCCCTTTCAGGAATTTTTACAAGAAGCTAGAGTTGCATCTTCCCAAGTTCTCCATTTC
TGGCTCCTATGTATTAGATCAG
>2324
GGCTCTCGCAGTCTTGTCCAGGCCCCCTCTCTTGCTGGCTTGGAGATAATGCTTGTGAT
TTTCCTCCCAGAGTTTCCACAAGGCCACCTTGGACGTGGATGAGGCTGGCACCGAGGC
TGCAGCAGCCACCACGTTCGCGA
>2325
TGTAGGCTACAGTGAAGTCCAGCTTGTGAAGTCCATTTGACAAATTACCTGTGCCTTTT
CCATCCTGCAGATTCTCACAGCCACAGTGGACAATGCCAATGTCCTTCTGCAGATTGAC
AATGCCCGTCTGGCCGCGGATG
>2326
GGAGAAGAGAACAAAGCCCTCGCTGTGATCAGGATGGGTGTCTCATACCTTTTCTCTG
GGGTCATTCCAGGTATGAGACAGAGTTGAACCTGCGCATGAGTGTGGAAGCCGACATC
AATGGCCTGCGCAGGGTGCTGGAC
>2327
TTCAGGCCTAAGGAACACCAATCCGCTGCCATGGTGGAACTCCTGACTGTGGACTGTC
CCTGGCTTGCAGGAGATGAATGCCCTGAGAGGCCAGGTGGGTGGAGATGTCAATGTG
GAGATGGACGCTGCACCTGGCGTGG
>2328
GAGGTGGAAGGAACCCAGACCACCTGCCTTCTGGGGCCTTCTGGTGTGAATGGCATTC
TCTTTTTTGCAGACAGAGGAGCTGAACCGCGAGGTGGCCACCAACAGCGAGCTGGTG
CAGAGCGGCAAGAGCGAGATCTCGG
>2329
GTGCACCCAGGACTGGCAGGGAGAGAACGGCCACACTCACTAATCGTTGATTCCCTTC
CCTCCCTCACAGAAAGCATCCCTGGAGAACAGCCTGGAGGAGACCAAAGGTCGCTAC
TGCATGCAGCTGGCCCAGATCCAGG
>2330
GCCTCCTTACTCCTGATTAGTCCATTGTCTGTCCACCTCTGGTAACGTCCTCTTCCCACC
TCTTCCCCAGTGACCTCCTCCAGCCGCCAAATCCGCACCAAGGTCATGGATGTGCACG
ATGGCAAGGTGGTGTCCACCCA
>2331
TGGGGCACGGTTCTCCATGGCTATGAGACTCCATTTATGCATCAGGAGTAAAGGGGTCT

TGTGTTTTTAGGTGAGGTTCCTGGAGCAGCAGAACCAGGTACTGCAAACAAAATGGG
AGCTGCTGCAGCAGGTAGATACCT
>2332
TCCCTCACAGGTAATGAGAGGGACCTGCCCTGGAGAGAACGTGCCTTCATGATGTCCC
TTGTTCCTCTAGGATGTGGATGGTGCTTATATGACCAAGGTGGACCTTCAGGCCAAACT
TGACAACTTGCAGCAGGAAATTG
>2333
GACTGCTTCTCTGTCTAAGTTGTTTTTGTTTTAGTGCTGTAAGAGTCACTAACCTATTAT
TGTCTTGCAGGAGTTGTCTCAGATGCAGACTCAAATCAGTGAAACTAATGTCATCCTCT
CTATGGACAACAACCGCAGTC
>2334
TCTCATGTATGTGTGCCTGTGCCATGCTGAGTTCTGCCTACATTTAACAAACGCTATCTA
CCATCTTTAGTATGAAGAGCTGCAGATCACTGCTGGCAGACATGGGGATAGTGTGAGA
AATTCAAAGATAGAAATTTCTG
>2335
GGGGAGACCAATTGGGATGGAGGCCTAGTGGGGAATGCATATTATGAGAGGGTGTCAT
ATTCTTTTTCAGATCTCCAACTTGCAGCAGTCCATCAGTGATGCAGAGCAGCGTGGCG
AGAATGCCCTCAAGGATGCCAAGA
>2336
GGGGCATGTGCAGGCTTTGTTGGGCTGGAAACGGAGTTGAGGTTGAAAATAACTGAG
CTTCCTCTTGCAGCTGTGAGCACAAGCCACACCACCATCAGTGGAGGTGGCAGCCGA
GGAGGTGGCGGCGGTGGCTACGGCTC
>2337
ATATTACATGAAGGTTTTATTTTTCTTTTTGTCTCGACATCATTAAAAATGTACTTTTATTT
TTATATAGATTTAAAAACCATTCATACTTCAACGGGAAAAAATCTTCTAGTTTCTGGATG
GTGGGGCTTTGTTCGCCA
>2338
GGAAAGCAGGAAAAAATACATTGTAGTGATAATAAAAATATCTAATACTGTGTACTATTA
TTATCCACAGGTTTTAACCACATTCTGCCTTATTTCTACATAATTTATTTCACCATGTTGC
TTGTCCACCGAGAAGCTCG
>2339
CTCTCAAGCTCTATAGGTTTGAGCTGTGGAACAGTCCCAGTCCCCAAATGGGGATCTTG
TTTTGTTTCAGACTTTGGAGAAGATTGAAGACATGGCTGTGTCCCTTATTCGAGAGGAG
TGGCTTCTTGATCCATCACAGA
>2340
TCCTTTGTCCTAGCATAAAGAATAGGTTGTGACATTGGCTTGATGTTTGTTTATTACATTT
TTATTTCAGGTGGTGAGACCAGGAGTGAGAACAGGGAATTAGCTTCAAAACAGGTAAT
ATCTACTGGAATCCAGCCACA
>2341
GGGGATCAGAAGCTGCCTGGCCTTTTCTTCTGAGCTGTGACTCGGGCTCATTCTCTTCC
TTTCTCCACAGTTGCTGCTGACACGCCGACCGCCTGCTGCTTCAGCTACACCTCCCGG
CAGATTCCACAGAATTTCATAGC
>2342
GGGGCCATGGTGGGCCCAGGATTCCCCTGCTGGATTCCCCAGTGCTTAACTCTTCCTCC

CTTCTCCACAGCTTCCTAACCAAGCGAAGCCGGCAGGTCTGTGCTGACCCCAGTGAGG
AGTGGGTCCAGAAATATGTCAGC
>2343
AGAGGGATGGGCACAAATCGGTCAGGCCCAGTCAGGGGATTTCAGCCTGATTTCTCTA
TGTCACTTCTAGGGAGAGAGATGGTGGATTACATCTGCCAGTACCTGAGCACTGTGCG
GGAGAGACGTGTGACGCCAGACGT
>2344
TGGTCTCTGGAGAAAGAAAGCCTGCTGGAAATGCCTCCTGGTGCCAACTCTGTGCATG
CTCTGCCTCCAGGTGGTACATTGGCAGAGCCCCCATATGCACGCCTACTACCCAGCCCT
CACCTCTTGGCCCTCCCTGCTAG
>2345
TTTCTGGAGCTCGTGGGGGCACCTCAGTGCCCTGGGGCTGTGCAGTTTGAAGTTCCTT
TTCCTTTCTCAGGCATCCAGCCCTGCGTGTACAGAGCTGGAGATGAACGTCATGGACT
GGTTGGCAAAAATGCTGGGACTTC
>2346
ACTAGAAAGCCTGGGGAAATTTGGCAAGAGAATACAGAAAGGCTGCCATGATAATCCT
TTTCCCCTGCAGAGCACGGTCAGTGAATCCACTTTGATTGCCCTGCTGGCAGCAAGGA
AGAACAAAATCCTGGAAATGAAAA
>2347
CTTCAGCAGTTTCTGGGGGGTGATCATTCCAGGATGACTCTTTGCTGACAGGTTTCTGT
GGCCCTTCTAGGCTCACTCCTCTGTGGAAAAGGCTGGTTTGATTTCCCTTGTGAAGATG
AAATTTCTGCCTGTGGATGACA
>2348
TTGGCCAGGTGCGAGACATCTTTCCCCACTTCATACCTCCCCTCCGGCCAGGTGATGTT
TGCCTCCTCAGGTGCCCGTGAGGGGCTGTGGCTCCACATCGATGCTGCTTATGCAGGC
ACTGCCTTCCTGTGCCCCGAGTT
>2349
CGAGCACGCAGGAACGCCTTGCCTCCTCTGGAGAGACCTCAGCCACTAATGCCCATTT
GGAAACCCACAGGGTCAAGGACAAGTACAAGCTGCAGCAGACCTTCAGTGTGAATCC
CATCTACCTCAGGCATGCCAACTCA
>2350
GCCCAGAAATGCCCGAGGGAGTGGGGCACCCTTGTCTCTAACCTTGGGCACTGTGTTC
CTTTGTTTCTAGCACTGGCAGATCCCCCTGAGCCGACGGTTTCGCTCTGTTAAACTCTG
GTTCGTGATTCGGTCCTTCGGGG
>2351
AGTCTGAAAGTTTATGCATAGTCTCTTGTCTGATGATTAATCTCAGTTCTCTTTATACTTG
CAATTCCAGGGTACTGAAATGGCTAAATATTTTGAATCTCTGGTCAGAAACGACCCTTC
CTTTGAAATTCCTGCCAAGA
>2352
CACAAACTGGGCTCTGGGGATGCTGACAGGGGGTGCGATGGAGACTTCACTTCCTTTA
TTTTTCAAACAGGGTCCTAATTGTCTCACAGAAAATGTGTTAAAGGAAATAGCTAAAG
CTGGCCGTCTCTTCCTCATCCCGG
>2353
TATGGATGCAGCTGCCTCCGTAAGTATCCTCTTGCAGCTGGTCCAGCGATGCTCTCTTCT

CCCCTTCCAGGCTCTCCTTCCTTCTCAGACAAGATGTCTTATCAGAAAAAGCAGCCCAC
CCCTCAGCCCCCAGTGGACTG
>2354
AGAAAGGAAGTAGCTGGCAGAGCTGTGCCTGTTGATAAAATCAATCCTTAATCACTTTT
TCCCCCAACAGGTGCAGTTTTGCCAAGGAGTGCTAAAGAACTTAGATGTCAGTGCATA
AAGACATACTCCAAACCTTTCCA
>2355
CTGACTTAAGGAATCATGATTTGAATGCAAAAACTAAATATTAATCTGAACCATTTCTTT
CTTATTTCAGTGTAAAGCTTTCTGATGGAAGAGAGCTCTGTCTGGACCCCAAGGAAAA
CTGGGTGCAGAGGGTTGTGGAG
>2356
TGAGCTAAGCAGGTGGGAGCGGGGCACCTAGTCCTCCTCCCCACTGGCTGCCTTCTTT
CCCACAGGTGAGTACCCCCACGTCGGGGTCCATGTGCCCGCCTCAGGCACAGGCAGA
GGTGGGCCCCACCATGACTGAGAAG
>2357
TGGGGGGCAGTGGGGATTGGGGGCCAGGGTCCTTGCCCACAAGGACTTAGTGACCCA
CGACCCCTTACAGTGTCTACATTGGGCCAGCAGGACCTTTTAGCATCTTCCCTAGCAGC
CGGTTGAAGCGGAGACCAAGCCAC
>2358
CGTGTCTAGCGGGAGGGCAGGAGGGGCCGCCCTTTGCGCATGGCACCAACCCGGCCC
TCCTTGTCCACAGGGCACCAGCCCCAGAAGGTGGCCCGGCGCGTGTTCACCAACAGC
CGGGAGCGCTGGCGGCAGCAGAACGT
>2359
CTGTTCTCTCAAGGGTAGCCTGCATGGGTGGCTGCCCTGCCCCCAATCGTGGACTCTTT
GCCCCTTCCAGGGTTTCAGAAGCTGCCAGAGGAGGAGCCAGAAACAGATCTCAGCCC
CGGGCTCCCAGCTGCCCACCTCAT
>2360
GCCACTTCCCCAGAAGTCCCAAGCCTTTAGGATCGCATTCCCAAGAGCGCGTCGGCCC
GTGTCTCCGCAGGCGCTCCGCTGAAGGGGCAGGGGCTAGGCTGGGAGACGACGAAGG
AACAGGCGTTTCTGACGAGCGGGAC
>2361
TAGGCCTTTGTTAGAGTCTCCAAGGTTCAGTTCTCAGCTGAGGCCTCTCACACACTCCC
TCTCTCCCCAGGCCTGTGGGTCTTCATTGCCCAGCTCCTGCCCACACTCCTGCCTGCTG
CCCTGACGAGAGTCATCATGTC
>2362
TGTCATTCCTTCAGCCTCAGCATGTGCTGGCCGGCTGTACCCTGAGGCGCCCTCTCACT
TGTTCCTTCAGGTTCTGAGGAGACAGGCCCCGGAGCAGCACTAGCTCCTGCCCACACT
CCTACCTGCTGCCCTGACCAGAG
>2363
CAAAGGGGTCAGCCCTGGACAACGCACGTGGGGGTAACAGGATGTGGCCCCTCCTCA
CTTGTCTTTCCAGATCTCAGGGAGTTGATGACCTTGTTTTCAGAAGGTGACTCAGGTCA
ACACAGGGGCCCCATCTGGTCGAC
>2364
CTTTGTCAGAGCCTCCAAGGTTCAGTTCAGTTCTCACCTAAGGCCTCACACACGCTCC

TTCTCTCCCCAGGCCTGTGGGTCTTCATTGCCCAGCTCCTGCCCGCACTCCTGCCTGCT
GCCCTGACCAGAGTCATCATGCC
>2365
GGGCCTCTGTTGGGGAGGGAGGTCCCTGGGCAAGTTGGCTGTGACCTCTCATGCTCCT
CTTCTTCCCCAGGCTGCTGCTCCTGCTGCCCCGTGGGCTGTAGCAAGTGTGCCCAAGG
CTGTGTTTGCAAAGGGGCGTCAGA
>2366
AAGGAATATTCACGGAAATATTTCCAGCTATGTGTCGCTAAAACTGCATTTATTTATTTT
CTGTTCTAAGATCCCTTCATTAACAGGAGAAATGCAAATACCTTCATATCCCCTCAGCA
GAGATGGAGAGCTAAAGTCCA
>2367
TTCTTCTTTTCCATTTTTGTATTTTTCCACTTTATCCTTCTCAATTTTGGCCTCTTCTTCCA
CTTTCTAGGATCCGAGAACGCTCTAAGCCTGTCCACGAGCTCAATAGGGAAGCCTGTG
ATGACTACAGACTTTGCGAA
>2368
CGGGGGCGCAGGACTCGGCAGCCGCGCCGGGAGGAGGGTCGGGCGGGTCTCAACCC
CTCCTCGCCCCCAGGCTCCCACTCCATGAGGTATTTCAGCGCCGCCGTGTCCCGGCCCG
GCCGCGGGGAGCCCCGCTTCATCGC
>2369
AAATCCCCGCGGGTGGGTCCGGGCGAGGGCGAGGCTCGGTGGGCGGGGCTGACCGA
GGGGGTGGGGCCAGGTTCTCACACCCTCCAGTGGATGATTGGCTGCGACCTGGGGTCC
GACGGACGCCTCCTCCGCGGGTATGA
>2370
ACATCCAGGTGCTGCTGGAGTGTCCCATGAGAGATGCAAAGTGCTTGAATTTTCTGAC
TCTTCCTTTCAGACCCCCCCAAGACACACGTGACCCACCACCCTGTCTTTGACTATGAG
GCCACCCTGAGGTGCTGGGCCCT
>2371
TGAAGACCTTTAACAGGGTCGGTGGTGAGGGCTGGGGGTCAGAGACCCTCACCTTCA
CCTCCTTTCCCAGAGCAGTCTTCCCTGCCCACCATCCCCATCATGGGTATCGTTGCTGG
CCTGGTTGTCCTTGCAGCTGTAGT
>2372
TGATGGGCTGTTTAAAGTGTCACCCCTCACTGTGACTGATATGAATTTGTTCATGAATAT
TTTTCTGTAGTGTGAAACAGCTGCCCTGTGTGGGACTGAGTGGCAAGTCCCTTTGTGA
CTTCAAGAACCCTGACTTCTCT
>2373
GGGGGCGCAGGACCCGGGAAGCCGCGCCGGGAGGAGGGTCGGGCGGGTCTCAGCCA
CTCCTCGTCCCCAGGCTCTCACTCCATGAGGTATTTCTTCACATCCGTGTCCCGGCCCG
GCCGCGGGGAGCCCCGCTTCATCGC
>2374
AAAATCCCCCCAGGTTGGTCGGGGCGGGGCGGGGCTCGGGGGACCGGGCTGACCGCG
GGGTCCGGGCCAGGTTCTCACACCGTCCAGAGGATGTATGGCTGCGACGTGGGGTCGG
ACTGGCGCTTCCTCCGCGGGTACCA
>2375
CCACATGTGTGCTGGAGGAGTGTCCCATGACAGATCGAAAATGCCTGAATGATCTGAC

TCTTCCTGACAGACGCCCCCAAAACGCATATGACTCACCACGCTGTCTCTGACCATGA
AGCCACCCTGAGGTGCTGGGCCCT
>2376
TTTAGGGAAAGCAGGAGCCTCTCTGACCTTTAGCAGGGTCAGGGCCCCTCACCTTCCC
CTCTTTTCCCAGAGCCGTCTTCCCAGCCCACCATCCCCATCGTGGGCATCATTGCTGGC
CTGGTTCTCTTTGGAGCTGTGAT
>2377
GGGGGCGCAGGACCCAGGGAGCCGCGCAGGGAGGAGGGTCGGGCGGGTCTCAGCTC
CTCCTCGCTCCCAGGCTCCCACTCCATGAGGTATTTCTACACCACCATGTCCCGGCCCG
GCCGCGGGGAGCCCCGCTTCATCTC
>2378
CAAAATCCCCGCGGGTTGGTCGGGGCAGGGCGGGGCTCGGGGGACCGGGCTGACCGC
GGGGGCGGGCCAGGTTCTCACACCATGCAGGTGATGTATGGCTGCGACGTGGGGCCCG
ACGGGCGCTTCCTCCGCGGGTATGA
>2379
CCACATGCGTGCTGGTGGAGTGTCCCATGACAGATGCAAAATGCCTGAATTTTCTGACT
CTTCCTGTCAGACCCCCCCAAGACACATATGACCCACCACCCCATCTCTGACCATGAGG
CCACCCTGAGGTGCTGGGCCCT
>2380
AGGAAAAGCCGGAGCCTCTCTGGAGAGCTTTAGCAGGGTCAGGGTCCCTCACCTTCC
CCCCTTTTCCCAGAGCCATCTTCCCAGCCCACCATCCCCATCGTGGGCATCGTTGCTGG
CCTGGTTCTACTTGTAGCTGTGGT
>2381
GGGGGCGCAGGACCCGGGGAGCCGCGCCGGGAGGAGGGTCGGGCGGGTCTCAGCCC
CTCCTCGCCCCCAGGCTCCCACTCCATGAGGTATTTCCACACCTCCGTGTCCCGGCCCG
GCCGCGGGGAGCCCCGCTTCATCAC
>2382
ATATCTCTTCTCAGGGAAAGCAGGAGCCCTTCAGCAGGTCAGGGCCCCTCATCTTCCCT
TCCTTTCCCAGAGCCGTCTTCCCAGTCCACCGTCCCCATCGTGGGCATTGTTGCTGGCC
TGGCTGTCCTAGCAGTTGTGGT
>2383
CGGGGCGCAGGACCCGGGGAGCCGCGCCGGGAGGAGGGTCGGGCGGGTCTCAGCCC
CTCCTCGCCCCCAGGCTCCCACTCCATGAGGTATTTCCACACCTCCGTGTCCCGGCCCG
GCCGCGGGGAGCCCCGCTTCATCAC
>2384
CAAAATCCCCGCGGGTTGGTCGGGGCGGGGCGGGGCTCGGGGGGACGGGGCTGACG
CGGGGCGGGGCCAGGGTCTCACACCCTCCAGAATATGTATGGCTGCGACGTGGGGCCG
GACGGGCGCCTCCTCCGCGGGTACCA
>2385
TCACATGGGTGGTCCTAGGGTGTCCCATGAGAGATGCAAAGCGCCTGAATTTTCTGAC
TCTTCCCATCAGACCCCCCAAAGACACACGTGACCCACCACCCCATCTCTGACCATGA
GGCCACCCTGAGGTGCTGGGCCCT
>2386
TATCTCTTCTCAGGGAAAGCAGGAGCCCTTCAGCAGGGTCAGGGCCCCTCATCTTCCC

TTCCTTTCCCAGAGCCGTCTTCCCAGTCCACCGTCCCCATCGTGGGCATTGTTGCTGGC
CTGGCTGTCCTAGCAGTTGTGGT
>2387
AAAATCCCCGCGGGTTGGTCGGGGCGGGGCGGGGCTCGGGGGACGGGGCTGACCGC
GGGGCCGGGGCCAGGGTCTCACATCATCCAGAGGATGTATGGCTGCGACCTGGGGCCC
GACGGGCGCCTCCTCCGCGGGCATGA
>2388
AGGGGATGGGGCGGGCGGGGGGCGGGGGGCTCGGGACTGCTTGCTCAGCCTCGCTGC
TTGCCTCCTGCAGACGCCGCCAGGCCGAGCCAGTTCCGGGTGTCGCCGCTGGATCGGA
CCTGGAACCTGGGCGAGACAGTGGA
>2389
GCCAGGGCTAGGGCAAGAACACCTCCATAACCCCAGCCTGGCCCCCAACCACGCTCT
GTTGCTCTTGCAGCGAAGCCCACCACGACGCCAGCGCCGCGACCACCAACACCGGCG
CCCACCATCGCGTCGCAGCCCCTGTC
>2390
TCCCGGGACGGTGGGGCGGTTGGGAGGTCCCGGGCCCTCTCCTAAATGTCGGCGTCTT
TGGTGTCGTCAGTGCACACGAGGGGGCTGGACTTCGCCTGTGATATCTACATCTGGGC
GCCCCTGGCCGGGACTTGTGGGGT
>2391
GGGGGCGCAGGACCTGAGGAGCCGCGCCGGGAGGAGGGTCGGGCGGGTCTCAGCCC
CTCCTCGCCCCCAGGCTCCCACTCCATGAGGTATTTCTACACCGCCATGTCCCGGCCCG
GCCGCGGGGAGCCCCGCTTCATCGC
>2392
AAAATCCCCGCGGGTTGGTCGGGGCGGGGCGGGGCTCGGGGGACGGGGCTGACCGC
GGGGCCGGGGCCAGGGTCTCACATCATCCAGAGGATGTATGGCTGCGACCTGGGGCCC
GACGGGCGCTTCCTCCGCGGGCATAA
>2393
TCACATGGGTGGTCCTAGGGTGTCCCATGAGAGATGCAAAGCGCCTGAATTTTCTGAC
TCTTCCCATCAGACCCCCCAAAGACACACGTGACCCACCACCCCGTCTCTGACCATGA
GGCCACCCTGAGGTGCTGGGCCCT
>2394
AGGGAAAGCAGGAGCCCTTCTGGAGCCCTTCAGCAGGGTCAGGGCCCCTCGTCTTCC
CCTCCTTTCCCAGAGCCATCTTCCCAGTCCACCATCCCCATCGTGGGCATTGTTGCTGG
CCTGGCTGTCCTAGCAGTTGTGGT
>2395
CACCATAGGAGGGGGCGGAGGTGACGGAGAGGGTCCTCTCTCCGCTGACGCTGCTTT
GGCTGTCTCCCAGATGTGGTGGTGCTGAACTCCAAGAGGACCATTGAGGAAGCCATGG
TCAAAAAGTGGGCAGACTTTGCTGG
>2396
GGCCGAAGAAGGTCAGGCCCTCAGCTGCCTTCATCAGTTCCCACCCTCCAGCCCCCAA
CTCCTCCTGCAGACAAGCTGGTGTCTAAGAACTACCCGGACTTGTCCTTGGGAGACTA
CTCCCTGCTCTGGAAAGCCCACAA
>2397
GCCTCTCACAGTAGCCCCCGCCCTGCCCGCTGCACAGCGGCCTGCTGGAACTCACACT

GTTTCTCCACAGCGCATGAGAGCCCAGCCCGGCACCCCTGTGGCCATTGAGGAGGAAT
TCTCTCTCCTCACCTGCAGCATCA
>2398
GGCCCACCCCCAGCCCCTCCCTGAGCCTCTCCTTGTCCTGAACTGAAAGTACTCCCTC
CTTTTCTGGCAGGACGACAACTTAATGCCTGCCTATTACAAATGTATCCAGGAGGTGTT
AAAAACCTGGAGCCACTGGTCCA
>2399
TGTAGGGGAGAGGCTGGGGTGGAGGGAGAGGCTCCTTCCCACAGCTGCATTCTCATGC
TTCCTGCCGCAGTTCTTCCCCAATCCAGGTCTCCGGAGGCTGAAGCAGGCCATAGAGA
AGAGGGATCACATCGTGGAGATGC
>2400
TGGGCCTGTTGCCACTCTGTACTCCTCTCCCCAGGCCAGCCGCTCAGCCCGCTCCTTTC
ACCCTCTGCAGGAGAGCCTCGTGGCAGGCCAGTGGAGGGACATGATGGACTACATGCT
CCAAGGGGTGGCGCAGCCGAGCA
>2401
GCTGAGGGAGCGGCTGGAGGCTGGGCAGCTGTGGGCTGCTGGGGCAGGACTCCACCC
GATCATTCCCCAGATTCAGCAGCGACTGCAGGAGGAGCTAGACCACGAACTGGGCCCT
GGTGCCTCCAGCTCCCGGGTCCCCT
>2402
GTTGGGGATGAGTGAGGAAAGCCCGAGCCCAGGGAGGTCCTGGCCAGCCTCTAACTC
CAGCCCCCTTCAGCATCTCTGGCTACGACATCCCTGAGGGCACAGTCATCATTCCGAAC
CTCCAAGGCGCCCACCTGGATGAG
>2403
GAAAATGTGGTGGAGGCTGGTCCCCGCTGCCGCTGAACGCCTCCCCACCCACCTGTCC
ACCCGCCCGCAGATCGCTTCCTGGAGCCAGGCAAGAACTCCAGAGCTCTGGCCTTCG
GCTGCGGTGCCCGCGTGTGCCTGGG
>2404
GCAGGCGCAGGACCCGGGGAGCCGCGCAGGGAGGAGGGTCGGGCGGGTCTCAGCCC
CTCCTCGCCCCCAGGCTCCCACTCCATGAAGTATTTCTTCACATCCGTGTCCCGGCCTG
GCCGCGGAGAGCCCCGCTTCATCTC
>2405
AAATCCCCGCCGGTTGGTCGGGACTGGGGCGGGGCTCGGGGGACGGGGCTGACCACG
GGGGCGGGGCCAGGGTCTCACACCCTCCAGTGGATGTGTGGCTGCGACCTGGGGCCC
GACGGGCGCCTCCTCCGCGGGTATGA
>2406
TCACATGGGCGCTGTTGGAGTGTCGCAAGAGAGATACAAAGTGTCTGAATTTTCTGAC
TCTTCCCGTCAGAACACCCAAAGACACACGTGACCCACCATCCCGTCTCTGACCATGA
GGCCACCCTGAGGTGCTGGGCCCT
>2407
TGGAGCCCTTCACGCGGGTCAGGGCTGAGGCTTGGAGGTCAGGGCCCCTCACCTTCC
CCTCCTTTCCCAGAGCCGTCTTCCCAGCCCACCATCCCCATCGTGGGCATCGTTGCTGG
CCTGGCTGTCCTGGCTGTCCTAGC
>2408
CGAGGGCCAGGACCCGGGAGCCCGCGCAGGGAGGAGGGTCGGGCGGGTCTCAGCCC

CTCCTCTCCCCCAGGCTCCCACTCCATGAGGTATTTCTACACCGCTGTGTCCCGGCCCA
GCCGCGGAGAGCCCCACTTCATCGC
>2409
AAAATCCCCGCGGGTTGGTCGGGGCTGGGGCGGGGCTCGGGGGACGGGCTGACCACG
GGGGCGGGGCCAGGGTCTCACACCCTCCAGAGGATGTACGGCTGCGACCTGGGGCCC
GACGGGCGCCTCCTCCGCGGGTATGA
>2410
CACATGGGCGCTGTTGGAGTGTCGCAAGAGAGATACAAAGTGTCTGAATTTTCTGACT
CTTCCCGTGCAGAACACCCAAAGACACACGTGACCCACCATCCCGTCTCTGACCATGA
GGCCACCCTGAGGTGCTGGGCCCT
>2411
TGGAGCCCTTCACGTGGGTCAGGGCTGAGGCTTGGGGGTCAGGGCCCCTCACCTTCCC
CTCCTTTCCCAGAGCCATCTTCCCAGCCCACCATCCCCATCGTGGGCATCGTTGCTGGC
CTGGCTGTCCTGGCTGTCCTAGC
>2412
CGGCGGCTGCGGGGGCGGCCGGGCTGGGGCCGGGCCGGGGCCTGACTGACCGGCCG
GTGATTCCCCGCAGAGGATTTCGTGTACCAGTTTAAGGGCATGTGCTACTTCACCAACG
GGACGGAGCGCGTGCGTCTTGTGAC
>2413
CATTCCCAATGGTAGACGTGCTGTGTGGTCTCACATCTCACTCCTGTCTTTTCCTGTTTA
CTGCCCTCAGTGGAGCCCACAGTGACCATCTCCCCATCCAGGACAGAGGCCCTCAACC
ACCACAACCTGCTGGTCTGCTC
>2414
ACTTCAGCTCCTCAGCAGGGATGCTATGGGGTGTAGGGACAAACGCTGACACTCAGGC
TCTGCTTCTTAGGGGCTCAGTCTGAATCTGCCCAGAGCAAGATGCTGAGTGGCATTGG
AGGCTTCGTGCTGGGGCTGATCTT
>2415
GGGGGCGAAGGACTCGGGGAGCCGCGCCGGGAGGAGGGTCGGGCCGATCTCAGCCC
CTCCTCGCCCCCAGGCTCCCACTCCTTGAAGTATTTCCACACTTCCGTGTCCCGGCCCG
GCCGCGGGGAGCCCCGCTTCATCTC
>2416
AAAATGCCCACAGGGTGGTGGCGACGGGGGCGGGGCTTGGTGGGCGGGACTGACTAA
GGGGCGGGGCCAGGGTCTCACACCCTGCAGTGGATGCATGGCTGCGAGCTGGGGCCC
GACAGGCGCTTCCTCCGCGGGTATGA
>2417
TCACATGGGTGCTGCTGGAGTGTCCCATGAGAGATACAAAGTGCCTGAATTTTCTGACT
CTTCCCCTCAGAGCCCCCAAAGACACACGTGACTCACCACCCCATCTCTGACCATGAG
GCCACCCTGAGGTGCTGGGCCCT
>2418
TGGAGCCCTTCCGCAGGGTCAGGGCTGAGGCCTGGGGGTCAGGGCCCCTTACGTTCCC
CTCTTTTCCCAGAGCCGGCTTCCCAGCCCACCATCCCCATCGTGGGCATCATTGCTGGC
CTGGTTCTCCTTGGATCTGTGGT
>2419
GCGGGGGCGAAGGACTCGGGGAGCCGCGCCGGGAGGAGGGTCGGGCCGATCTCAAC

CCCTCCTCCCCCAGTGCTCCCACTCCTTGAAGTATATTCCACTATCCTGTCCCGGCCCGG
CCGGGGAGCCCCGCTTCATCTCTG
>2420
GCCAAAAATGCCACAGGGTGGTGGCGACGGGGGCGGGATTGTGGCGGGACTGACTA
AGGGCGGGGCCAGGGTCTCACACCCTCCAGTGGATGCATGGCTGCGAGCTGGGGCCC
GACGGCTTCCTCCGCGGGTATGAACA
>2421
TCACATGGGTGCTGCTGGATGTCCCATGAGAGATACAAAATGCCTTAAATTTTCTGACT
CTTCCCCTCAGAGCCCCCAAAGACACACGTGACTCACCACCCCATCTCTGACCATGAG
GCCACCCTGAGGTGCTGGGCCCT
>2422
TGGAGCCCTTCCGCAGGGTCAGGGCTGAGGCCTGGGGGTCAGGGCCCCTTACGTTCCC
CTCTTTTCCCAGGAGCCGCTTCCCAGCCCACCATCCCCATCGTGGGCATCATTGCTGGC
CTGGTTCTCCTTGGATCTGTGGT
>2423
GGGCTGTTCAGAGTGTCTATCACTTACCGTGACCTGACCTGAATTTGTTCATGACTATTT
TCTTCTGTAGCCTGAGACAGCTGCCTTGTGTGCGACTGAGATGCAGGATTTCCTCACGC
CTCCCCTATGTGTCTTAGGGG
>2424
TGGGGCGCAGGACCCGGGGAGCCGCGCCGGGAGGAGGGTCGGGCGGGTCTCAGCCC
CTCCTCGCCCCCAGGCTCCCACTCCATGAGGTATTTCAGCACCGCGGTTTCCTGGCCGG
GCCGCGGGGAGCCCAGCTTCATTGC
>2425
TCACATGGGCGCTGCTGGGGTGTCCCATGAGGAATGCAAAGTGCCTGAGTTTTCTGAC
TCTTCCTTTCAGACCCCCCCAAGACACACGTGACCCACCACCCTGTCTCTGAACATGA
GGCATAACGAGGTGCTGGGTTCTG
>2426
TGGGGCGCAGGACCCGGGGAGCCGCGCCGGGAGGAGGGTCGGGCGGGTCTCAGCCC
CTCCTCGCCCCCAGGCTCCCACTCCATGAGGTATTTCAGCACCGCCGTTTCCTGGCCGG
GCCGCGGGGAGCCCAGCTTCATTGC
>2427
AAATCTCCGCAGGTTGCTAGGGGCCGGGCCAGGGCTCGGTGGGCGGGGCTGACCGCG
GGAACTGGGCCAGGGTATCACATCCTCCAGGGAATGTTTGGCTGCGACCTGGGGCCCG
ACGGGCGTCTCCTCCGCGGGTATGA
>2428
TCACATGGGCGCTGCTGGGGTGTCCCATGAGGAATGCAAAGTGCCTGAGTTTTCCGAC
TCTTCCTTTCAGACCCCCCCCAAGACACACGTGACCCACCCCCCTCTCTGAACATGAG
GCATAACGAGGTCCTGGGTTCTGG
>2429
CTGGAGACCTTTAGCAGGGTCGGGGCTGGGTCCTGGAGGTCAGAACCCTCACATTCCC
CTCCTTTCCCAGAGCCCTCTCCCCAGCCCACCATCCCCATTGTGGGTATCATTGCTGGC
CTGGTTCTCCTTGGAGCTGTGGT
>2430
CGGGCCCGAAGTGGACGTTCGGGGCCCGACGAGGTCGCTGGGGCGGGCTGACCGCGC

CCTTTCCTCGCAGTACATCGCGGTGCACGTGGTCCCGGACCAGCTCATGGCCTTCGGC
GGCTCCAGCGAGCCGTGCGCGCTCT
>2431
GGAGGGGCGGCGGCGCGCGGCCAGGCCCGGGACTGAGCCACCCGCTGAGTCCGGCC
TCCTCCCCCCGCAGGGTCTACATCAACTATTACGACATGAACGCGGCCAATGTGGGCTG
GAACAACTCCACCTTCGCCTAAGAG
>2432
ACAGATCCCAAAGATTCCCGGGGGGTGTGGCCTTCAATGGCTCAGGCGTCCCCTGCTG
TCCCGGCTGCAGTGACCTGGGAGCCAACACCCTCGCTGAGGTTCCAGGAGCCCCCGC
CTGGAGGAGCTGGCCCCCCAGAGCT
>2433
GAGGAAGGGGACCGGTAGAGCGGGGCTGGGTAAGCCTCCATCCAGCCGGGCTGAGCC
CTGGTCTCCGCAGAGCCTCTGCCCCTCCCGAGACACCCGCTACCTGGTGTTAGCGGTG
GACCGCCCTGCGGGGGCCTGGCGCG
>2434
GGACGGGGAGCCGGGTCGACTGCCCCCGGGCCCCCAGCCCCTGAGCCAGCCGCGTGC
CCACCCACCGCAGACTCCCGGCTGAGTACCGCCCGGCTGCAGGCACTGCTGTTCGGCG
ACGACCACCGCTGCTTCACACGGAT
>2435
ACGGGGCAGGAGCGGGCGGGGGCGGCGTGGCCTCGTGGCCGCTCTCAACTCCTCCAA
TTGCGGGTTCCAGGCCATCCGCGGAACTCGAGGAGTCGCCACCCAGCGCAGACCCCT
TCCTGGAGACGCTCACGCGCCTGGTG
>2436
GAGGGCCCTGCACGCGGCCCCGGTGGGGAAGGGGACGGGCCAGGGATTCAGACTCG
GGCTCTCCCCTCAGGATGCAGCACCGAGGCTTCCTCCTCCTCACCCTCCTCGCCCTGCT
GGCGCTCACCTCCGCGGTCGCCAAA
>2437
CCCAGGAAGGCGGGTCCCGAGGGAGTCTCCCCGTGCCCCAGTCCTGAACTCTGTTCCT
CGCGCGTTGTAGATAAGGTGAAGAAGGGCGGCCCGGGGAGCGAGTGCGCTGAGTGGG
CCTGGGGGCCCTGCACCCCCAGCAG
>2438
GCCTGGGGGGACCCTTGGCGGAGGGCGGGGCCGCGGGCGCGCAGCCTGACCTGGGC
CGCTCTCTCGCCAGCCGACTGCAAGTACAAGTTTGAGAACTGGGGTGCGTGTGATGGG
GGCACAGGCACCAAAGTCCGCCAAGG
>2439
GTGGAGCCGGCGGTCTGCAATGGGTCAGCCTAACTGCTGATATGGTATTAATATTCTTCT
TGTTTTACAGCCAAGAAAGGGAAGGGAAAGGACTAGACGCCAAGCCTGGATGCCAAG
GAGCCCCTGGTGTCACATGGGGC
>2440
GAGGGCCCTGCACGCGGCCCCGGTGGGAAAGGGGACGGGCCAGGGATTCAGACTCG
GGCTCTCCCCTCAGGATGCAGCACCGAGGCTTCCTCCTCCTCACCCTCCTCGCCCTACT
GGCGCTCACCTCCGCGGTCGCCAAA
>2441
ACCCAGGAAGGCGGCTCCCGAGGGAGTCTCCCCGTGCCCCAGTCCTGAACTCTGTTCC

TCGCGCGTGTAGATAAGGTGAAGAAGGGCGGCCCGGGGAGCGAGTGCGCTGAGTGGG
CCTGGGGGCCCTGCACCCCCAGCAG
>2442
CCTGGGCGGACCCTTGGCGGAGGGCGGGGCCGCGGGCGCGCAGCGCTGACCTGGGCC
GCTCTCTCGCCAGCCGACTGCAAGTACAAGTTTGAGAACTGGGGTGCGTGTGATGGGG
GCACAGGCACCAAAGTCCGCCAAGG
>2443
GGAGCCGGCGGTCTGCAATGGGTCAGCCTAACTGCTGATATGGTATTAATATTTCTTTCT
TGTTTTACAGCCAAGAAAGGGAAGGGAAAGGACTAGACGCCAAGCCTGGATGCCAAG
GAGCCCCTGGTGTCACATGGGGC
>2444
CATTTACTGTGCTCCCAGTCCCCACCTCTGGCCACACCCAGCTCTCACAGCCTTCTCTC
CCCACCCGCAGAAGGAGAATAAGAATGAAAAGGTCATAGAACACATCATGGAGGACC
TGGACACAAATGCAGACAAGCAGC
>2445
TCATAGATGCTATGCCTCGGCTCTCTCTGAGATCTTTAAACTCTGGCTTCTTCCTCCTCA
ATCTTGACAGAAAAAGGGTGCAGACGTCTGGTTCAAAGAGTTGGATATCAACACTGAT
GGTGCAGTTAACTTCCAGGAGT
>2446
CCTAGAAGATGAATTTATGTAATATGATTTGCAAAATGAGTATTCATTTGTGATTTTTTTT
TTTTTTAAGGTGAAAGTACATTTTTTGTTGAATTAAGTGAAACTGCCAGCATACTCATG
CATGCAACAGCACATTCTCT
>2447
TTTTTGTTTTAATTCCTTTTTTGTTTTAATTCCTTTGAGTTACTTCCTTATGCATATTTTAC
TTTAACAGGAAGAGGTACTGCAACATTTGATGGGACGGCAATAGCAAATGCAGTTGTT
AAAGAACTTGCTGAGACTAT
>2448
ACAAATTCGGTTTTTTGAGAGGGCACTTCTGTTGCTAGCACATGTATCGCTAATATTTTT
CTTTCTTAAGGCATGCATGGTAGAAAATGAATGTGAAGACCCCAGCCAGGAGACTATT
ACGTTCCTCTATAAATTCATTA
>2449
AGTAAAAGGGGAAGGGATGATGCACTATGAAAAAACAAAAAAACTTTTTTTTTTTTTT
TTTAATTTTAAGGGAAGTTTGCCTGGCTAGTGAAAGGTCAACTGTAGATGCTGAAGCT
GTCCATAAATTGCTGACTTTGATT
>2450
GGCCTCTGTTGGGGCAGGGAGTTCTATGATCGAGTCTGCTCTGACCTCTCAATCTCCTT
TCCTCCCCAAGGCTGCTGCTCCTGCTGCCCTGTGGGCTGTGCCAAGTGTGCCCAAGGC
TGCATCTGCAAAGGGGCATCGGA
>2451
CTGCGCCAGGTTTCCGCACCAAGACCCCTTTAACTCAAGACTGCCTCCCGCTTTGTGT
GCCCCGCTCCAGCAGCCTCCCGCGACGATGCCCCTCAACGTTAGCTTCACCAACAGGA
ACTATGACCTCGACTACGACTCGG
>2452
GCCTGAGCGTGCGGGGTTCCTGCTAAGGTCTCCGAGTCGGGTTCTGATCCACTGTGCT

CTTTTCCGACAGGATCAGCAACGCTACCACGAAGACATTTTCGGGCTCACGCTGCGCA
CCCAGGAGGTGACAAGCCGCATAC
>2453
CCAGCCAGGAGAACGGGCTTTCCCTCTCCTTCCGCCTGCGGAGGGGAAGCTGAAGTC
TGGTCTTCCTCAGGTCTGGTCTTCTCTCGTCTGAGCCCTGAGTACTACGACCTGGCAAG
AGCCCACCTGCGTGATGAGGAGAA
>2454
CTTTCCTGGGCACTTTCACGCGCTCCCAGAGTGTCTGAGAGACCATCATAAGGGCTTTC
TTTCCTGACAGGGTGACCTGTTGACCAAAACACAGGAGCTGGGCCGTGACTACAGGA
CCTGTCTGACGATAGTCCAAAAAC
>2455
ACCTGCAGGGTTGGCAGAGCTGGGCAGGACTCACCCTATAACCATGTCCACTGTGGTG
CTGCTGCTGCAGAGAAGTGTTTCCAATGCTGCGACCCGGGTGTGTAGGACGGGGAGG
TCACGATGGCGCGACGTCTGCAGAA
>2456
ATTCTGAGTGCCTGCTTCTCCTTGAGGACCCACCACATCTGCCCACAGCTAGCTGTTCT
CTCCGCTCCAGGTCCCCTCTGAGCCCTCTCACCTTGTCCTGTGGAAGAAGCACAGGCT
CCTGTCCTCAGATCCCGGGAACG
>2457
CTTCCCCGAGATTCTTTTTCTTACCCACTGCTAACTCCATACTCAATTTCTCATGCTCTCC
CTGTCCCAGGCTCAAGGAAAAACATGGCCTGCTATTGCAGAATACCAGCGTGCATTGC
AGGAGAACGTCGCTATGGAAC
>2458
CCCAGTAGAGCTATAAATCCAGGCTGGCTCCTCCCTCCCCACACAGCTGCTCCTGCTCT
CCCTCCTCCAGGTGACCCCAGCCATGAGGACCCTCGCCATCCTTGCTGCCATTCTCCTG
GTGGCCCTGCAGGCCCAGGCTG
>2459
CTTCCCCGAGATTCTTTTTCTTACCCACTGCTAACTCCATACTCAATTTCTCATGCTCTCC
CTGTCCCAGGCTCAAGGAAAAACATGGACTGCTATTGCAGAATACCAGCGTGCATTGC
AGGAGAACGTCGCTATGGAAC
>2460
CTAATGGCAAAATATGAATGGCCTGAAATGTCGAGGTGGAGGTGTAATGATCTATTTGT
GTCCATTTTAGGCAGGTAAAAATCAAGGTGACCCCAAGAAAATGGCTCCTCCTCCAAA
GGAGGTAGAAGAAGATAGTGAAG
>2461
TCATTGGGACCATGGTTATCTTTTTGGGTGTGGGGCTGGACTTACTGGTTTGGTTTTCAT
TTATCTCAAGGTCGTCATACCTCAGAAGAAAGGCAAGAAGGCTGCTGCAACCTCAGCA
AAGAAGGTGGTCGTTTCCCCAA
>2462
TTACATTAGATTTGAAAACCGATGTTGGTTTTGGGAGATTGCTGCAATTTTTAGGTGACT
TCTCTTTCAGACTCTGAAGAAGAAGCTATGGAGACTACACCAGCCAAAGGAAAGAAA
GCTGCAAAAGTTGTTCCTGTGAA
>2463
TATGTTTAAAGGAGGCACTTAAGTGTTTGTGGCTACTGATGTGTGATACATTGTTTGACA

TCTTGTCCAGAGCCTGTCAAAGAAGCACCTGGAAAACGAAAGAAGGAAATGGCCAAACAGAAAGCAGCTCCTGAAGCCAA
>2464
AGAAATTTTGCTTTGGAAACTAACTTTTTACCACACTACAAGTAGACTGAGTTGAGCTTTTTTTGTGCAGGCACAGAACCGACTACGGCTTTCAATCTCTTTGTTGGAAACCTAAACTTTAACAAATCTGCTCCTGAATT
>2465
TCTAGATACAGATTGTGATTTTGATTTGTGTTTTCACCTGTTGTAATTTTGCCCTCCTCTCCACCTGAAGGAAATTTGGTTATGTGGATTTTGAATCTGCTGAAGACCTGGAGAAAGCGTTGGAACTCACTGGTTTGAAA
>2466
CTCTTTTTGTTGTTGCCCCCCACCTTTGGTTTTTCAGGCCACAGAGCTCATTTTTGTTTTTTTAATCTAGAGCGAGATGCGAGAACACTTTTGGCTAAAAATCTCCCTTACAAAGTCACTCAGGATGAATTGAAAGAAGT
>2467
TGTTGCTGAGGTAAAAGCATTTGTGTTAATATGGTTTTAAAAACCATGAGTTCTTCTCCCTCCATTGCAGGATTGCTTATATTGAATTTAAGACAGAAGCTGATGCAGAGAAAACCTTTGAAGAAAAGCAGGGAACAGAG
>2468
AGATATTACTGTTGTGACCGAAGCTGGGACACTGTTTCAGAGTCTTAGGAATGTGGCTCTCTATTTCGAGGTGAATCAAAAACTCTGGTTTTAAGCAACCTCTCCTACAGTGCAACAGAAGAAACTCTTCAGGAAGTATT
>2469
ATCATGTGACCAGTGGTATAGAGAGCATGTGCCTGGTAAATTACTCTAAGCATTAACTGTTCATCCTCAGGTATGCATTTATAGAGTTTGCTTCATTCGAAGACGCTAAAGAAGCTTTAAATTCCTGTAATAAAAGGGAA
>2470
TAGGCATTGTGCAAATTCAGTGTACGAACTGTGAATTCACATAAATCGCAAATTTTTTTTCCTTCCCAGAGCCATCCAAAACTCTGTTTGTCAAAGGCCTGTCTGAGGATACCACTGAAGAGACATTAAAGGAGTCATT
>2471
CATGTACAGTTAACTTGGGACATGTGTAGTAGGTGCTTTTTATAATGGGCAATTTCATTTGGTGTTCTAGGTTTGGTTTTGTAGACTTCAACAGTGAGGAGGATGCCAAGGAGGCCATGGAAGACGGTGAAATTGATGGA
>2472
GGTTCCTAATCACTGGGGAGGAGGAGCTTTGTACCCATTCTTTTAACAGTGTCTTGCCTTCCTCCTGTAGGGCGAGGAGGCTTCCGAGGAGGCAGAGGAGGAGGAGGTGACCACAAGCCACAAGGAAAGAAGACGAAGTT
>2473
CAGCCAGTGCCCCATCTTCAACTCGATGTGTTCCTAAGGTAGACAGCAAATTCCCTATTTTATTTCTCAGATTGTCACTGCTGTTCCAAGGGCACACGCAGAGGGATTTGGAATTCCTGGAGAGTTGCCTTTGTGAGAAG
>2474
TTTTTATTTTGATTGTGGCTATAAGTGAACATCTATCATGAGACATTTGCTGCACTTTCCT

TGCTTGTAGGTTGGTTTTGCGGATTGCCACTGATGATTCCAAAGCAGTCTGTCGTCTCA
GTGTGAAATTCGGTGCCACG
>2475
GGAAGTAAATACTCTCATGCCCAGTTAGGAGTGAGTCAGGGTTTTTAATATGCCACTTT
TTCTTTCTCAGGCAACTCATGCAGCAATTCCAGAACCCTGACTTCCCACCCGAAGTAG
AGGAACAGGATGCCAGCACCCTG
>2476
GATTTTTTTGGGTTGATGACATATAGAAAGGCAAAGAAAGTGATTGCATGTTTTTGAAA
ATCATTTTCAGGACTTTCCTTATCCCAGTTGATTGTGCAGAATACACTGCCTGTCGCTTG
TCTTCTATTCACCATGGCTTC
>2477
AAAATTATAGTACAGTTTATTAGAACAGGAGAATCCTGGTTTTCTGAATTATAAATATAA
TCAATTCTAGATATCCAGGTGAAAGAACTGGAGAAGCGTGCCTCAGGCCAGGCTTTTG
AGCTGATTCTCAGCCCTCGGTC
>2478
CAGGCGTGAGCCACCACACCCAGCCTGAATACATTTTAGAGTGCCTAGCCTATTAAACT
TTTTTTTCCAGTCCCATGAAGCTGAGGTCTTGAAGCAGCTGGCTGAGAAACGAGAGCA
CGAGAAAGAAGTGCTTCAGAAGG
>2479
TCGTGACTTGACAGGGCTTTTTTTTTTTTTTTTTTTTTTGACTGATGACACCTTACCCT
TCCTTTACAGGATAAGCACATTGAAGAAGTGCGGAAGAACAAAGAATCCAAAGACCC
TGCTGACGAGACTGAAGCTGACT
>2480
AATGGTTGTTTTTTCCATTCATCCCTACATTTCTTTTTTTAATAATGATAAACATGCAACT
TTTTTGTAGCTTTACAACAAATACCCAGATGCTGTGGCCACATGGCTAAACCCTGACCC
ATCTCAGAAGCAGAATCTCC
>2481
TAATGTGCTATAAAGGCTAAGGGAAAATAAAAACCCATATATTAATTTTCCCGGCCATCT
TAATTTTCAGACCCTTCCAAGTAAGTCCAACGAAAGCCATGACCACATGGATGATATGG
ATGATGAAGATGATGATGACC
>2482
TGGAAAAGGATTACCATATTCCCATCCCTAGCCGTTCATATAATTATTCTTCATTTGTGCC
GTGATTCAGTACCCTGATGCTACAGACGAGGACATCACCTCACACATGGAAAGCGAGG
AGTTGAATGGTGCATACAAGG
>2483
GCCTCGCAGGGTCCTCCGAGCGAGTCCCCAGCGCCGCCCCGCGTCCCGCTCACCCCGC
CCGTCCCCCGAGTGCCTCCCCTGCGGCCCCGGGGGCAAAGGCCGCTGCTTCGGGCCC
AATATCTGCTGCGCGGAAGAGCTGG
>2484
GCGCTCCGGGGCCAGGGGGAGGCGGGCGGGGGTGCGGCCGGGATTCCCCTGACTCCA
CCTCTTCCTCCAGACGGCTGCCACGCCGACCCTGCCTGCGACGCGGAAGCCACCTTCT
CCCAGCGCTGAAACTTGATGGCTCC
>2485
GAAGTGGGTGGATTTGGAAACCTTTTGGGATACTGGATAGCTTGTTCCATAGGTTGTGT

ACTATTTACAGGTATGCTGTCACACCAGTTGAAGCAGCATGTCATCGATGGAGAAAAA
ACCATTATCCAGAATCCCACAGA
>2486
CAGCCTGGGTGACAGAATGAGACTTTGTGTCAAAAAAAAAAAAAAATTCCTTTCTCTC
TATTCCTTTTAGGAAGGACCATGAAAAAGCTGAATTTGAGGTACATGAAGTATATGCTG
TGGATGTTCTCGTCAGCTCAGGA
>2487
ACCAGAGTTGGCAAAGAGGGGTGACTGAGAGTGTTCACCAGACCAAATGTTACTTAA
ATTACTCTCTCAGGCCAAGGATGCAGGACAGAGAACCACTATTTACAAACGAGACCCC
TCTAAACAGTATGGACTGAAAATGA
>2488
AAATATGAGCAAAATGGTAAGACTTGATGGGGAGTTAAAGATACCTCTGAATATCATCT
TCCCCTGCCAGAGCATTTGAAGATGAGAAGAAGGCTCGGATGGGTGTGGTGGAGTGC
GCCAAACATGAACTGCTGCAACCA
>2489
CTGTAAGTTCAGAAGAGCTAAGTATGACAAAGGAACTTTTTATCAAAACACATCTTCAT
TTTTGCCATAGGTGAATTTGTTGCCCAGTTTAAATTTACAGTTCTGCTCATGCCCAATGG
CCCCATGCGGATAACCAGTGG
>2490
CTCTGGTCCCAGACATCCCCAAGTAGTTAGAAACTGACAGGTCTTCTCCCTCTCCTTTT
CTTGCATATAGGCCTCCAAGACTGCAGAGAATGCCACCAGTGGGGAAACATTAGAAGA
AAATGAAGCTGGGGACTGAGGTG
>2491
ACCAGAGGTGTAAGGGCAAGAGTGGGGTGGATGGGTGTGAGATTCCTACAGCCTTGC
CTGCTCTCTAAAGGCAACTCTAGACATCGCGTCCAACAACCGTAAGGGTATCGCCTTC
GCTGACTCTGGCGCACACTGGCAGC
>2492
GAGGCAACTGGTACAGAGAGGGGGTAAGGGTGCTGATTCATTTCCACCCTCATGCCCC
CTCTCCCTTCAGTTTGTCAGGAAATCAGTACATTGTGTGATATGCTGGCCACCCACAAC
GGACAGTCCATAGACATCTCCTT
>2493
AGGAGAGACTCTGGCAGCTGGAGAAGCAAAATGGAAGAAGGGTGGATTTAACATTTC
TTTTTATTTCCAGATTTTCCCCAACAAAACCCTGGAAAAATTAAAGAGCCATGTTAAAA
TACGAAATGATCTGCTGAATAAAA
>2494
GAAGTATGGCAGGAGTGTCACAGATGGGGCTCCTTCCTTATTAATGTCTCCCAAACCTC
ACCCAACCCAGGAGAAATTCCGGAGTGACTCTATCACCAACATGCTGGACACACTGAT
GCAAGCCAAGATGAACTCAGATA
>2495
TTATTCTCTAAGCCCTTGCCTCTCCTGGGCTTACACACACTAGTCACCTCCAACCTACTC
TGGTCTTCAGGTGAAGAAGAAGCTCTACGAGGAGATTGACCAGAATGTGGGTTTCAGC
CGCACACCAACTATCAGTGACC
>2496
GGGCATGAGGCTGAGCAAGGAAGGGAGTACGAAGTCCCAGACCCACTTTTCCTCTTC

CACTCTGGAGCAGCATCGGTGAGTTTGCTGTGGACAAGGGCACAGAAGTTATCATCAA
TCTGTGGGCGCTGCATCACAATGAG
>2497
AACCAGGGCAGAACCATGCCTCTCCTCCCTCCTGCCCTAACCCCTGGCTGATGCCACT
CCTTGCCAGCAGAGCGTTTCTTGAATCCAGCGGGGACCCAGCTCATCTCACCGTCAGT
AAGCTATTTGCCCTTCGGAGCAGG
>2498
TACCACAAAGAATAAATCCTTTCACTCTGATCAATTTTGTTAACTTCTCACGTGTCTTCT
CTACACCCAGGGCACCGGTGAATCCAAGTGTCCTCTGATGGTCAAAGTTCTAGATGCT
GTCCCAGGCAGTCCTGCCATCA
>2499
TACCACAAAGAATAAATCCTTTCACTCTGATCAATTTTGTTAACTTCTCACGTGTCTTCT
CTACACCCAGGGCACCGGTGAATCCAAGTGTCCTCTGATGGTCAAAGTTCTAGATGCT
GTCCGAGGCAGTCCTGCCATCA
>2500
GGGTGTATTACTTTGCCATGCCATTTGTTTCCTCCATGCGTAACTTAATCCAGACTTTCA
CACCTTATAGGAAAACCAGTGAGTCTGGAGAGCTGCATGGGCTCACAACTGAGGAGG
AATTTGTAGAAGGGATATACAAA
>2501
CAGTCATGTGTGTCATCTGTCACGTTTTTCGGGCTCTGGTGGAAATGGATCTGTCTGTC
TTCTCTCATAGGTGGTATTCACAGCCAACGACTCCGGCCCCCGCCGCTACACCATTGCC
GCCCTGCTGAGCCCCTACTCCT
>2502
ACTAGAGAAGGAAAATGACATGAGGCAGTGGGGTATCTGTGTGTTCTCCCACTGACCA
CGCTTTCTTTAGTGACTCCTGATTGCCTCCTCAAGTCGCAGACACTATGCTGCCTCCCA
TGGCCCTGCCCAGTGTATCTTGG
>2503
TTCCCCAAGGTCATCTCCCACCCCAAGCCCCACTCTTCATTTACCCTCCCCTCTCTTCT
TCCACCTCAGGTGAAGAACCCCAGAGGGAACTGCCCTCTGCACGGATCCGCTGTCCC
AAAGGCTCCAAGGCCTATGGCTC
>2504
CACCTACCTTTGCCCTGCCCTCCCTCAGCAGGTCTCAGGCTCCCTCTCATTCTCTTTGTT
GCCCTCAAAGCTGGCCTGCCAGAAGCGGCCCTCTGGAAACCTGGTGTCTGTGCTCAGT
GGGGCTGAGGGATCCTTCGTGT
>2505
AGCTAAGGGGCCATGCAAGAAGAGCTGCAATGGCCATTCCCTTCACCTGGCTACCTCC
TCTACTCTACAGGGCACCGAGCCCAATGGAGAAGGTTGGGAGTGGAGTAGCAGTGAT
GTGATGAATTACTTTGCATGGGAGA
>2506
CTGGGATGCCCCTTCATTGGATTCACAAGCTTCCTCAGCCCCATTCCATTTATGTCTTCT
GTCTCTCCAGCATTTCTGAGGTGGAAAGATTATAACTGTAATGTGAGGTTACCCTATGTC
TGCAAGTTCACTGACTAGTG
>2507
GGTTGGAGCCCCTCTGTTTTGACCTATAAAGAGAAATGCCCTTGGGCCTCTGTTTTTTT

CCCTCTTCTAGGCTGGCAAAGCACACAGGCTGAGCGCTGAGGAGAGGGACCAGCTGC
TGCCAAACCTGAGGGCTGTGGGGT
>2508
CCTTGAATTGGTGATGTCCAGAAGTCCTTTCTCTTAGAACACTAACCTGGCTCTCACTC
CACTTAAATAGGCCTTTGGGTTCATGACAAGAGTGGCCCTGCAGGCTGAGAAACTGGA
CCACCATCCTGAATGGTTTAACG
>2509
CCAGCAGCTAGTGACTCCCTCCTGTTCTTAAGTGAAGTCAGTGGAAGCAGAATTTCTG
GTTTCATTTCAGGTCCACATCACGCTGAGCACCCATGAGTGTGCCGGCCTTTCAGAAC
GGGACATAAACCTGGCCAGCTTCA
>2510
GGACATCTCTGGAAAGTCAGCACCTGGACCAGCTCCACCCCTCTCTGAGGACACCTTC
TTTCCCTTTCAGAACAAAGAACAGCCACCATGCAGCTCTTCCTCCTCTTGTGCCTGGTG
CTTCTCAGCCCTCAGGGGGCCTC
>2511
TTATTTGCAGCTGAGAATTTCTAATTCTGACACAAAATTCTTTTTCATTTTTCCCTTTTTT
CATCTTTAGCTAAGTGGGAGACAAGCTTCAACCACAAAGGCACCCAAGAGCAAGACT
TCTACGTGACCTCGGAGACTGT
>2512
AATCCAAGGGGTGAGGCTCAGTGTGCCAATGCCCCAGAACAGTCTAAGAAAGCTCCTT
TTCCCTTTCCAGGCAGCTCGAGCTTTACCTTCCCAAATTCTCCATTGAGGGCTCCTATC
AGCTGGAGAAAGTCCTCCCCAGT
>2513
GCTTTGCCATTTGCTATGATGACTTCACCTGCCCCTGGTGGCCTGGTGATGCCTGGTGT
CTCCCCTGCAGATGGTGCACAAAGCTGTGGTGGAGGTGGACGAGTCGGGAACCAGAG
CAGCGGCAGCCACGGGGACAATCT
>2514
TAACCGCGTTCGAAATACAGCCCTTCAGCAGACGGCGTTGATTTTAAAGCATGTGTCTC
CTGTCTTCTAGTATGTCCAAAATACTAAAATGCGCCGGCAATGAAGATATCATTACACTA
AGGGCCGAAGATAACGCGGAT
>2515
TTAAGAAAGTGAGGGTGCCAAATCATTAAATTTCTAACAATTAACTTTTGGAAAATTTT
GTTCTTAATAGGAACAGGAGTACAGCTGTGTAGTAAAGATGCCTTCTGGTGAATTTGCA
CGTATATGCCGAGATCTCAGCC
>2516
TAAATATATAATGAGCTAATAGATTGCAACTTTCTTGATGTTTTTCAATGATGAATCTTTT
GTTTTGTAGGTTACCATAGAGATGAATGAACCAGTTCAACTAACTTTTGCACTGAGGTA
CCTGAACTTCTTTACAAAAG
>2517
GAACCTTGTTTTGATGGTAGTCATATGTGATACATACTCCTCAGTAATTAACCATCTTCCT
GTCTTTCAGTTGTAGAGTATAAAATTGCGGATATGGGACACTTAAAATACTACTTGGCTC
CCAAGATCGAGGATGAAGA
>2518
GTTTCTGGGACGGAGAGGTCCAGGGACACCCTCGGGCCTAACCCCTGACCCTGAGGT

CACCCACCTACAGGAAAAACCAGTGTGAGACCCGAACCATGCTGCTGCAGCCCGCGG
GGTCCCTCGGCTCCTACAGCTACCGG
>2519
ACCCCTTCCCTGAAGCAGAGGTGAGGTTTGGGGGGCTGGTCCCCGACAGGGTTGTCT
CTTGGGTTCCCAGACTGGGGCAGCACCTACTCCGTGTCAGTGGTGGAGACCGACTACG
ACCAGTACGCGCTGCTGTACAGCCA
>2520
CCCAAGACCCAGGGCAGGGCACACAGCATCCCCCTCCGCTGAGGCCAGCTCCGTCTC
CACCCTGTCCCAGGCCGAACCCAGACCCCCAGGGCTGAGTTAAAGGAGAAATTCACC
GCCTTCTGCAAGGCCCAGGGCTTCAC
>2521
TTCCTTCATAAAGACCCAACCCTATGACTTTAACTTCTTACAGCTACCACAGCCCCTAA
ACCCGCAACAGTTGTTACAGGTTCTGGTCATGCAAGCTCTACCCCAGGTGGAGAAAAG
GAGACTTCGGCTACCCAGAGAAG
>2522
TAAGGTTGGGGGAAGAGTGCTGAGCCAGAGCTGGGACCCGTGGCTGAAGTGCCCATT
TCCCTGTGACCAGGCCAGGATCTGTGGTGGTACAATTGACTCTGGCCTTCCGAGAAGG
TACCATCAATGTCCACGACGTGGAG
>2523
TTCCTTAGTGCTGGCAGCGGGAGGGGCGCCTCCTCTGGGAGACTGCCCTGACCACTGC
TTTTCCTTTTAGTGAGTGATGTGCCATTTCCTTTCTCTGCCCAGTCTGGGGCTGGGGTG
CCAGGCTGGGGCATCGCGCTGCT
>2524
TCCCTGGCCCTGATCAGAGCCCCCCGGTAGAAGGCACTCCATGGCCTGCCATAACCTC
CTATCTCCCCAGGCTGTCTGTCAGTGCCGCCGAAAGAACTACGGGCAGCTGGACATCT
TTCCAGCCCGGGATACCTACCATC
>2525
GGGGTGGCATCCAGGGGCTCTAGGAGGTACCTTTTGCTCCTCACCCTGGATCTCTTTTC
CTTCCACCCAGGTTTCTGCAGGTAATGGTGGCAGCAGCCTCTCTTACACAAACCCAGC
AGTGGCAGCCACTTCTGCCAACT
>2526
GGCAGCCGCAGCCTCCCGCCGTGTAGGGTCGAGGCTTGGAATGACCCACGTCCCAATG
TCGCCTTCCCAGATGGTGTTCGTGCGCAGGCCGTGGCCCGCCTTGACCACAGTGCTTC
TGGCCCTGCTCGTCTGCCTAGGGG
>2527
CACCCTGCGGCCCCGCTCCACCTGGGGGCGTGGCCAGATCTGACCACGCTCTTCCCTT
CCCCGCGCCCAGGTATGGGAAAAGAGACGGCCCGGACACGCTTCTTTCCAAAACGTT
CTTCCCCGACGGCGAGGACCGCCCC
>2528
GCAACATCATTTAACGACGTCTCCCAGGCTCGCCTCCCAAGATCCAATTCCTTCCACTT
CGCTTCCGCAGGTCGGAGGGCCCAGACCTGTGGTGAGGACCCCTGAGGCCTCCTGGG
AGATCTGCCAACCACGCCCACGTC
>2529
TATCTCTGGCTACTCAGGAGTCACAACTCCCACCCCTCCTCCTCTCTTACTCCCTCCCTT

TCCCCCTCAGCTGAAGCTGAAGAAGATGGGGACCTGCAGTGCCTGTGTGTGAAGACC
ACCTCCCAGGTCCGTCCCAGGCA
>2530
CCCTCCCCCTTCTAATGCCATTTGCAAACCCAAGGACTGAAAGTCACGTCTCTTCTCTT
TTCCCTGCCAGAGCCACGCTGAAGAATGGGAGGAAAATTTGCTTGGATCTGCAAGCCC
TGCTGTACAAGAAAATCATTAAG
>2531
GCTGCAGAGTGGGGAGTCGGGTGGGGGCCCACTGGCTTGGGAGGGAAAGCAGCGTG
CCTGTGTCCCCCAGGAGCGTCGGTACGCAGGCCTGAAGCCCGGGGAACTCCCCACCT
GCGAGAGCCTCAAGGACACCATTGCCC
>2532
TCACATAGTGTCTGCTGTGTAAACAGGCTGGGGACAGAGGGGCTAAGGACACCTATTC
CTTCCGGCATAGGGATGTCAGACCAGGCGATCATGGAGCTGAACCTGCCCACGGGGAT
CCCCATTGTGTATGAGCTGAACAA
>2533
GCCACGGTACTGAAGGAGATGGTTCGCTGCCCGTGGTGCTTGGCTGAAAGGAAGCCT
GTGATTTTTGCAGCTGGTTGGCTCTCACTGACCCCAGGGATGTGGCCAGGATCGAAAG
CAAGACGGTTATCGTCACCCAAGAG
>2534
GAAAAAAATGCTGGTCGTGTTCGAAGTCCAAGGTCATTTCTCACCAGTGCCCACCCAT
CGCACCCTGTAGGTCGCACCATGTACGTCATCCCATTCAGCATGGGGCCGCTGGGCTCA
CCTCTGTCGAAGATCGGCATCGA
>2535
CCAGTGAGTGCCTCGGGGACCCCCAACCAGGGCCCTGGCGCACTGACTTGGGAGGGG
TCCTTGTTCACAGAGCCTTTGGTCAACAACTGGCCCTGCAACCCGGAGCTGACGCTCA
TCGCCCACCTGCCTGACCGCAGAGA
>2536
CTCTGGGCTGAAGTACCAACCTCGGGGAGAAGGAAACAAAGATCACAATAAAGAATC
TTGTCCCCAACAGATTCTGGGTATAACCAACCCTGAGGGTGAGAAGAAGTACCTGGCG
GCCGCATTTCCCAGCGCCTGCGGGA
>2537
CAATATATAAGAGTATATGTTCTGCTTTGCCTGGCACTCACTACTGCTTCTCTGGTTTAA
AACTCTCCAGGTCATTTAAGGGCCATCAACCCAGAAAATGGCTTTTTCGGTGTCGCTCC
TGGGACTTCAGTGAAGACCAA
>2538
CTGTGTGTGCCGGGCTGCAGGGACTGCCTGTTTTGAGCCAGGCACTCACGAGCCTTTC
TCTGTCTTATAGGGGAACCTTGTGCCCACCCCAACTCGAGGTTCTGCACCCCTGCCAG
CCAGTGCCCCATCATTGATGCTGC
>2539
ACACCACTGGTTGTGGAGTCTGAATTTCAAAGCCTCTGATGAACATTTCTCTTTTTTTTT
CCTGCTAAAGGTGTCCCTCTAGTCTATGAAGCTCTCAGCTGGCAACATGGAGTCTTTGT
GGGGGCGGCCATGAGATCAGA
>2540
CAACAATCAATGGCGTCAGTCTTGCCTAGGAGAGCCTCATTTACTAATGAACTCCCTCT

CTGTTTAACAGGCAAAATCATCATGCATGACCCCTTTGCCATGCGGCCCTTCTTTGGCT
ACAACTTCGGCAAATACCTGGC
>2541
GGGATCTCCTGGGTGGGGAGCTGGCGGCTCGCGGGCCGGCACTGAGTCCCCGTGCTT
CCCCCTTTCCTAGGCAAGGAGAAGGAGCCCCTGGAGTCGCAGTACCAGGTGGGCCCG
CTACTGGGCAGCGGCGGCTTCGGCTC
>2542
CCCAGGATGAGTGGGTGGGGTGAGGGGAGCCCCCGACTCCCGCCCTAACGCGGCCCC
CTCGCCCCTGCAGCCTAATGGCACTCGAGTGCCCATGGAAGTGGTCCTGCTGAAGAAG
GTGAGCTCGGGTTTCTCCGGCGTCA
>2543
ATACCACCCAGCTTCTTTGTGCTTGTTTTTGCTAAAAGTGTGTTTTCTCTTCTATTCCCTT
GGCTCACAGGGACCCGAGTGTATAGCCCTCCAGAGTGGATCCGCTACCATCGCTACCAT
GGCAGGTCGGCGGCAGTCTG
>2544
ATGGGGAAGACCTTTGCAGTGTAAAAACAAGTTGAGTCATTCATAACCTCGTCTATCCT
CCTTTCTGCAGAATGTCAGCATCTCATTAGATGGTGCTTGGCCCTGAGACCATCAGATA
GGCCAACCTTCGAAGAAATCCA
>2545
GTGGGAGGCCATAGCTTGGGGATGCTGGCAATGTGTGGGATGGGCCCAGGGAAGGCC
TCTGGCCTACTAGGGGCTCTGGCCCTGACCCACGGCCACTCACTCCTCAGAGACGTCT
CCCACAACCTGCTCCGGGCGCTGGA
>2546
GCGGCACCGCCTCTTGCTGCTCAGCTGTGGGGGCTTCCATCAGCTTTGCCGAATCCCC
CGTCTCTTCCAGGGATATAAGCAACAACAAGATTTCTACGTTAGAAGAAGGAATATTTG
CTAATTTATTTAATTTAAGTGAA
>2547
TTGCCAGTGGGGGGCTGGCATAGACCCTTCCCACCAGACCTGGTCCCCAACACCTGCC
CCTGCCCTGCAGAAACCTGAGTGGGAACCCGTTTGAGTGTGACTGTGGCCTGGCGTG
GCTGCCGCGATGGGCGGAGGAGCAG
>2548
CCCCCGCCACCTCCTGCCCCCACCTGTAGGGGCCCCACCCTCCTCCAGCACGTCTTCC
CTGCCTCCCCAGGGGCCACCCTGGTGGGGCCCCACGGACCTCTGGCCTCTGGCCAGCT
AGCAGCCTTCCACATCGCTGCCCC
>2549
GGGCAGAGCCTGGTACCCCCGTCTTGGGCCCACACTGACCGTTGACACCCTCGTTCCC
ACCGGTCTCCAGCGGTGCACCCGCTCTGCCCCTCGGACACGGAGATCTTCCCTGGCAA
CGGGCACTGCTACCGCCTGGTGGT
>2550
GCGGGCTGCTGTGAGGGTGGGAGGATGGAGGAGTGCCCTGAGCCCCCTGCCATCCCA
CACCCGCCCCCAGGAGCCTAGACGTGTGGATCGGCTTCTCGACTGTGCAGGGGGTGG
AGGTGGGCCCAGCGCCGCAGGGCGAG
>2551
GGTCCCCAGTCTGTTCGTCCTGGTGTCCTGGGCCCTGGCCCGGCGCCTCACTGTGCAC

TCGCCACCCCAGGCCCAGTGCAGGATGCCGAGAACCTCCTCGTGGGAGCGCCCAGTG
GGGACCTGCAGGGACCCCTGACGCC
>2552
GGTCTGATGGCTCGCTCTCCTTCCCTCCCCTCTTCCTGGGAAGTTCGGGTAGGGGGAGT
CTGGGCTTCAGGCTGGGATGGGGTCTGTGGAGCTGAGGCGGCCCCCTGCCCACCAGG
TCATGGTATTCCCGGGCCTGCGTC
>2553
CAGGCAGTTGGGCATCTCTGACGGTGGCCTGTGGGCAAATCAGGGCCCCAACACCCTC
CCCTCCTCACAGGGACCCCGGAGAACGGCAGCGAGCCTGAGAGCAGGTCCCCGGACA
ACAGGACCCAGCTGGCCCCCGCGTG
>2554
CCTTCGTGCCCGGCTGCCCCTGGGAGACCAACGATACCCTGTTCTCAGTGGTAGCACT
GCCGTGGCTCAGTGAGGGGGAGCACGTGGTGGACGTGGTGGTGGAAAACAGCGCCA
GCCGGGCCAACCTCAGCCTGCGGGTG
>2555
GTGTCCAGGAGGCGACAGGCTAAGGGCAGAGTCCTCCACAGAGCCCAGGCTGACACC
ATTCCCCCCGCAGAGGTACAGCCCCGTGGTGGAGGCCGGCTCGGACATGGTCTTCCGG
TGGACCATCAACGACAAGCAGTCCC
>2556
GAGGGAGGGACGCCAATCAGGGCCAGGCCTCTCACCTGCCACCTGGGCTCACTGACG
CCTGTCCCTGCAGCTGACGGCCTCCAACCACGTGAGCAACGTCACCGTGAACTACAAC
GTAACCGTGGAGCGGATGAACAGGA
>2557
CAAGCACCCCCTGCCCCGCCACCGCCCACCCCTGTCCCGGTTCACTCACTGCGTCCCA
CCGCCCCGGCAGGTGGACCTTTGGGGATGGGGAGCAGGCCCTCCACCAGTTCCAGCC
TCCGTACAACGAGTCCTTCCCGGTT
>2558
GGGCTTCTGCCGAGCGGGTGGGGAGCAGGTGGGGGTGCCGCGGCTGCCCCACTCGGG
CCTGTCCCCACAGGTGAGTACCTCCTGACCGTGCTGGCATCTAATGCCTTCGAGAACC
GGACGCAGCAGGTGCCTGTGAGCGT
>2559
CCCGCTGCCCTCGCCTGGGGGTGTTCTTTACACGTGGGACTTCGGGGACGGCTCCCCT
GTCCTGACCCAGAGCCAGCCGGCTGCCAACCACACCTATGCCTCGAGGGGCACCTACC
ACGTGCGCCTGGAGGTCAACAACA
>2560
CGGTACAGCTGCAGGCCGTGGTTAGGGATGGCACCAACGTCTCCTACAGCTGGACTGC
CTGGAGGGACAGGGGCCCGGCCCTGGCCGGCAGCGGCAAAGGCTTCTCGCTCACCGT
CTCGAGGCCGGCACCTACCATGTGC
>2561
GGCCGCCACCAGCCCCAGCCCCCGGCGTGTGGCCTACCACTGGGACTTTGGGGATGG
GTCGCCAGGGCAGGACACAGATGAGCCCAGGGCCGAGCACTCCTACCTGAGGCCTGG
GGACTACCGCGTGCAGGTGAACGCCT
>2562
CTGGGCCGGGCTCTGCTTTAAAACTGGATGGGGCTCTCAGGCCACGTCGCCCCTTGTT

CTCGGCCTGCAGAGGGAGGCTGGCGGGTGTGCGCTGAACTTTGGGCCCCGCGGGAGC
AGCACGGTCACCATTCCACGGGAGC
>2563
CCATGGGTCCCCCAGTCCTTCCAGCAGGCCAAATAGACCTGTGTTGGAGGTAACCCCA
CTCCCACGCCAGGTGCTGATCCGGAGTGGCCGGGTGCCCATTGTGTCCTTGGAGTGTG
TGTCCTGCAAGGCACAGGCCGTGT
>2564
GGGCCTGCAGGCAGAAGTGGGGCTGACAGGGCAGAGGGTTGCGCCCCCTCACCACCC
CTTCTGCCTGCAGCGGTGGGCTGCACGTACGTTCAGCAACAAGACGCTGGTGCTGGAT
GAGACCACCACATCCACGGGCAGTG
>2565
GAGCAGCGGGATCCCCCGACTCTGTGACGTCACGGAGCCCTCCCGTGATGCCGTGGG
GACCGTCCCTCAGGCTGGCATGACGCGGAGGATGCTGGCGCCCCGCTGGTGTACGCCC
TGCTGCTGCGGCGCTGTCGCCAGGG
>2566
GCTGTGCCCCCTGCCTGCCCCAGTACGAGCGGGCCCTGGACGTGGCGCAGAGCCCAA
GCACGAGCGGCAGCACCGAGCCCAGATACGCAAGAACATCACGGAGACTCTGGTGTC
CCTGAGGGTCCACACTGTGGATGACA
>2567
CGTGTAGAGAGGAGGGCGTGTGCAAGGAGTGGGGCCAGGAGCGGGGCTGGACACTG
CTGGCTCCACACAGGGGCCCAGCAGGGAGCTCGTATGCCGCTCGTGCCTGAAGCAGA
CGCTGCACAAGCTGGAGGCCATGATGC
>2568
CCCCCTTTTTGCCTGCCCCCACCCTCCCTCTACCTCCCTGTCTCTGCACTGACCTCACG
CATGTCTGCAGGAGACCTCATCCACCTGGCCAGCTCGGACGTGCGGGCACCACAGCCC
TCAGAGCTGGGAGCCGAGTCACC
>2569
GGGCCATCGTGGGTGTCCCCCGGGTGGTTGAGCTTCCCGGCAGGCGTGTGACCTGCGC
GTTCTGCCCCAGGCCACTACCTGTCTGAGGAACCTGAGCCCTACCTGGCAGTCTACCT
ACACTCGGAGCCCCGGCCCAATGA
>2570
TGGGCTCTGAGACTGCGACATCCAACCTGGCGGAGCCTGGGCTCACGTCCGCTACCCC
TTCCCTGCCCAGGAGCAGAGACCCAGCGGGGAGTTACCATCTGAACCTCTCCAGCCAC
TTCCGCTGGTCGGCGCTGCAGGTG
>2571
GGCTCGGCTCTATCCTGAGAAGGCACAGCTTGCACGTGACCTCCTGGGCCCGGCGGCT
GTGTCCTCACAGGAGCCGACAGCGGATGTAAACTACATCGTCATGCTGACATGTGCTG
TGTGCCTGGTGACCTACATGGTCA
>2572
TGACTTGCCTGGGATGCCACACGTGTTGGGCAGCAGACCGAGCCTCCCACCCCTCCCT
CTTGCCTCCCAGGTACCACGGCCCACGTGGGCATCATGCTGTATGGGGTGGACAGCCG
GAGCGGCCACCGGCACCTGGACGG
>2573
CCAAGCTCTGCCCCTCTGCCCCCGCATTGGGGCGCCCTGCGAGCCTGACCTCCCTCCT

GCGCCTCTGCAGGGCTCAGCCCTGCCTGGTTCCTGCAGCACGTCATCGTCAGGGACCT
GCAGACGGCACGCAGCGCCTTCTT
>2574
CTCCGTGGGAGGTTGGGCAGGGTGGTCCTGCCCCGTGGCCTCCTGCAGTGCGGCCCTC
CCTGCCTTCTAGGCGACGCAGCCCTTTTGCGCTTCCGGCGCCTGCTGGTGGCTGAGCT
GCAGCGTGGCTTCTTTGACAAGCA
>2575
GGCAGCCTCTTCCTGCCCAGCCCTTCCTGCCCCTCAGCCTCACCTGTGTGGCCTCCTCT
CCTCCACACAGCACGGGGCATGTGTCCAGGCTGAGCCCGCTGAGCGTCGACACAGTC
GCTGTTGGCCTGGTGTCCAGCGTG
>2576
AGGTGGCTTCTCATCCCTGTCCTGAGCAGGTCTGAGCTGCCGCCCGCTGACCACTGCC
CTCGTCCTGCAGGTGGCTGGGAGCCCGAGCCCCACACCTGCCGGGCAGCAGGTGCTG
GACATCGACAGCTGCCTGGACTCGT
>2577
GTGACTGGACGGGGGTGGGCTGTGTGTGTGACACATCCCCTGGTACCTTGCTGACCCG
CGCCACCTGCAGTCTGGTGTGCTGGCCCTCCGGCGAGGGAACGCTCAGTTGGCCGGA
CCTGCTCAGTGACCCGTCCATTGTG
>2578
GGGGTGAGAGGAGGGGGCTCTGAAGCTCACCCTTGCAGCTGGGCCCACCCTATGCCT
CCTGTACCTCTAGATGAAGACCTGATCCAGCAGGTCCTTGCCGAGGGGGTCAGCAGCC
CAGCCCCTACCCAAGACACCCACAT
>2579
GTTAACATGGGCTTGGCTGCAACTGCCTCCTGGAGGCCGGGATGAATTCACAGCCTAC
CATGTCCCTCAGGTCCAGCACTCCTGGGGAGAAGACAGAGACGCTGGCGCTGCAGAG
GCTGGGGGAGCTGGGGCCACCCAGC
>2580
GCGTAGCCCCGGGATGCCCCTAGCCCCTCCCTGTGAGCTGCCTCTCACAGGTCTGTCTC
TGCTTCCCCAGGACTGGTGGAGGGTCTGCGGAAGCGCCTGCTGCCGGCCTGGTGTGC
CTCCCTGGCCCACGGGCTCAGCCT
>2581
GAGGGGGCTGCCAGGGGTAGGCTACAGGCCTCCATCACGGGGGACCCCTCTGAAGCC
ACCCCCTCCCCAGGTCTTGCTGGAAGCCCTGTACTTCTCACTGGTGGCCAAGCGGCTG
CACCCGGATGAAGATGACACCCTGG
>2582
GCCGTTGCCAAAGCCCTGCTGTCACTGTGGGCTGGGGCCAGGCTGACCACAGGGCCC
CCCCGTCCACCAGAGCCTCCTGGTGTACATGCTTTTTCTGCTGGTGACCCTGCTGGCCA
GCTATGGGGATGCCTCATGCCATG
>2583
TCTGGTGGCCGCTCACTCGAGGCGGGCATGGGGCAGTAGGGGCTGGAGCGTGTGACT
GATGCTGTGGCAGGTCTGAGGAGCTCTGGCCATGGATGGCCCACGTGCTGCTGCCCTA
CGTCCACGGGAACCAGTCCAGCCCA
>2584
GCCAGCAGGAAACACTCCTGTTGGGTTTTGATGAGGCCCTGGCCCCGGCCTGGGGCTC

TGTGTGTTTCAGCACTCTACCCAGACCCTCCCGGCCCCAGGGTCCACACGTGCTCGGC
CGCAGGAGGCTTCAGCACCAGCGA
>2585
TCCTGACCAGCCTGGCTCCGGGGTGCCGGAAGGGCTGGGGTGCGGCACCCACGCCAC
CCCTCTCCGGCAGGGCATGGTCCTGGGGCTCCTGTGCCGTGTATGACAGCGGGGGCTA
CGTGCAGGAGCTGGGCCTGAGCCTG
>2586
GCCTGCACTGCGCGGTCCCCGCAGCTCCCGCCCTGCCACCCGCTCCTACTGACCCGCA
CCCTCTGCGCAGGAGCCGCGCTGTGTTCCTGGAGCTCACGCGCTACAGCCCGGCCGTG
GGGCTGCACGCCGCCGTCACGCTG
>2587
GCGTCCCGCCCCCTCGCAGGGCCCCGCCCCGGCAGCGTCCCTCCCGCCCTCCTGACCG
CGCCCCCCACAGGTGTGCCTGCTGCTGTTCGCCGTGCACTTCGCCGTGGCCGAGGCCC
GTACTTGGCACAGGGAAGGGCGCT
>2588
GGCTGGGCCGGTGGGCGCGGGGCTGGGCGCACACCCCAGGGCTGCAAGCAGACAGA
TTTCTCGTCCGCAGGCTGCCCAGCAGCTACGCTTCGTGCGCCAGTGGTCCGTCTTTGG
CAAGACATTATGCCGAGCTCTGCCAG
>2589
CGGCCGGGGAGGGCGTCTTAGCTCAGCTCAGCTCAGCTGTACGCCCTCACTGGTGTCG
CCTTCCCCGCAGCTCGTGTCTTCCTGTGTGGACTCCCTCTGGAGCGTGGCCCAGGCCC
TGTTGGTGCTGTGCCCTGGGACTG
>2590
GGGAGAGGGACACGCCCTGGGCTCTGCCCAGGGTGCAGCCGGACTGACTGAGCCCCT
GTGCCGCCCCCAGTTCCGCCACAAAGTCCGCTTTGAAGGGATGGAGCCGCTGCCCTCT
CGCTCCTCCAGGGGCTCCAAGGTAT
>2591
GCTCCTGGAGCAGGGAGAGCGCTGGCCTCTTCCTCTCCGGCTCCCTCCATTGCCTCCG
GTTTCTCCCCAGGCTCCCGGACGTCCCTGCTCCTGGCTTTTGCCCTGCTCTGCCTGCCC
TGGCTTCAAGAGGCTGGTGCCGT
>2592
AAGCAACAATGGGACCTGGTCTCCAGCATAGAAACCAGCAGTCCTTCTTGGTGGGGG
GTCCTTCTCCTAGGAAGAAACCTATATCCCAAAGGACCAGAAGTATTCATTCCTGCATG
ACTCCCAGACCTCCTTCTGCTTCT
>2593
GGCGGGGATGGGGGAGACCTGTGGTCAGGGCTCCCGGGCAGCACAGCCACTGCCGGT
CCTTCCCCTGCAGAATCTAGAGCTGCTCCGCATCTCCCTGCTGCTCATCGAGTCGTGGC
TGGAGCCCGTGCGGTTCCTCAGGA
>2594
TGGATGAATGAGAGAGGGAGGGAACATTGCCCAAGCGCTTGGCCTCTCCTTCTCTTCC
TTCACTTTGCAGAGGCTGGAAGATGGCAGCCGCCGGACTGGGCAGATCCTCAAGCAG
ACCTACAGCAAGTTTGACACAAACT
>2595
GGGAAGGGTAAGGAGGCAGGGGCTGCCTGGGCTGGCTGGCACCAGGACCCTCTTCCT

CTGCCCTGCCCAGGTGAAGTGTCCGGACGCACCCTCATCGACATTGGTTCAGGCCCCA
CCGTGTACCAGCTGCTCAGTGCCTG
>2596
GTGGCTGGTTGGGGCAACAGAGGCCTGAGCGTAGAACAGCCTTGAGCCCTGCCTTGT
GCCTCCTGCACAGGGAATGCTGGCAGGATAAGGAGCGCCAGCTGCGAGCCAGGGTGA
AACGGGTCCTGCCCATCGACGTGCAC
>2597
CCCCGTCTGCACCGGGTGCAACGAGAGCCATGGTGGGGTGGGACCGCCGTGCAGCCC
AAGGCCCCCTCAGTTGGCAGGGACCTGGCACTCCATGGCCATGGCGACCAACAACATC
TCCCTCATGGCGACACTGAAGGCCC
>2598
GATTTTAGGAGTGACAGTGAAGGCAACTCCAATTCAAGTGGCCACTCATCCTATTGTCA
CCACCTTTCAGGGAGAACAACAGCTGTGTTGAGAAGAAGGTCCTTGGAGAGAAGACT
GAGAATCCAAAGAAGTTCAAGATC
>2599
ACACACCTGCACAGGACTCTGCTGAGACGGAGGCTTCATCTTCCTTTTGGTTCTTCTCT
TCTTTCCCCAGATACGGTGGCGAACGAGGCCACGCTGCTCGATACTGACTACGACAAT
TTCCTGTTTCTCTGCCTACAGGA
>2600
AAGGCCTCTGGAGCTCCCCAGCTCTCATGGAAGCCCCAGGGGCCCAGGACTGACCCA
GCCTCTTCCACAGCCAGAGTCCTGGTGGAGGACGATGAGATCATGCAGGGATTCATCA
GGGCTTTCAGGCCCCTGCCCAGGCA
>2601
AGGTCAGATATGAAACAGGTGGGCTGGCACCTGGGCACAGTGCTTGCCCCTGCTGCCT
CTTCCCTCCCAGGTATGGGAAAAGACACAAAGAGGACACGCTGGCCTTCTCGGAGTG
GGGGTCCCCGCATGCTGCTGTCCCC
>2602
CAGACCCCCTCATGGCCCCAGCCCCTCTTAGGCCCCTCACCAAGGTGAGCTCCCCTCC
CTCCAAAACCAGACTCAGTGTTCTCCAGCAGCGAGCGTGCCCACCAGGTGCTGCGGA
TCCGCAAACGTGCCAACTCCTTCCT
>2603
GCTGGACTACCGGCGCCCGCGCCCCTCGGGATCTCTGGCCGCTGACCCCCTACCCCGC
CTTGTGTCGCAGACGGTGACCAGTGCTTGGTCTTGCCCTTGGAGCACCCGTGCGCCAG
CCTGTGCTGCGGGCACGGCACGTG
>2604
GGGGCGGGGCTGGGGCCGGGTTGGGGGCGCGGCACCAGCACCAGCTGCCCGCGCCCT
CCCCTGCCCGCAGAGGTGAGCTTCCTCAATTGCTCTCTGGACAACGGCGGCTGCACGC
ATTACTGCCTAGAGGAGGTGGGCTG
>2605
AGGACCAAGACAGGAGGGCAGTCTCGGGAGGAGTGCCTGGCAGGCCCCTCACCACCT
CTGCCTACCTCAGTGAAGTTCCCTTGTGGGAGGCCCTGGAAGCGGATGGAGAAGAAG
CGCAGTCACCTGAAACGAGACACAGA
>2606
AACCCAGGTGCCCTGGACTGGAGGCTGTCAGGAGGCAGCCCTGTGATGTCATCATCCC

ACCCCATTCCAGGTGGTCCTGCTGGACTCAAAGAAGAAGCTGGCCTGCGGGGCAGTG
CTCATCCACCCCTCCTGGGTGCTGA
>2607
GGGCCTCAGGAAAGTGCCACTGGGGAGAGGCTCCCCGCAGCCCACTCTGACTGTGCC
CTCTGCCCTGCAGGAGAGTATGACCTGCGGCGCTGGGAGAAGTGGGAGCTGGACCTG
GACATCAAGGAGGTCTTCGTCCACCC
>2608
GAGGAAAGACATGGCAGCTGAAGCAGAGGCTTACTGGGTATAAATGTGGGCTCGTTTC
TTCTTTTAACAGTTCCTGTTAAAGGTCAAGACACTGTCAAAGGCCGTGTTCCATTCAAT
GGACAAGATCCCGTTAAAGGACA
>2609
CTGTCTTCCTCACTGCCTCTGAGTGCTTTGATGTGCTGACTCTCACCTCTGATACTCTTC
TCTTCCACAGAGGGAGCCGGTCCTTGCTGCACCTGTGCCGTCCCCAGAGCTACAGGCC
CCATCTGGTCCTAAGTCCCTGC
>2610
CCTCCGTGCCCCGCCCGGGCGGTGCAGCTGGCCCGGGTGCTCACGCTCGACTCTCTTT
CTTCTTTTCCAGGGTCTGCGCTTCGCAGCCGGGATGAAGCTGGTTTCCGTCGCCCTGAT
GTACCTGGGTTCGCTCGCCTTCC
>2611
TGAATGGGAGCAGGGACAGGCCTGGGCGTCACCTGAACGCACGCGAATCGGGTCTGC
TTGTGTTTTCCAGGTGGAATAAGTGGGCTCTGAGTCGTGGGAAGAGGGAACTGCGGAT
GTCCAGCAGCTACCCCACCGGGCTC
>2612
CGGTGTTGGGGCCAAAGCTCTGCTTGATGGGGTCTCAAGTTGCCTTTCTTCCCCCTCCC
CCCGCCCGCAGCAGTCCGGATGCCGCCCGCATCCGAGTCAAGCGCTACCGCCAGAGCA
TGAACAACTTCCAGGGCCTCCGG
>2613
TGAAGGAGTTATTTGATTGAATGGGGGAAATACTCCCCTGGGCCCAGCTGAGGTCTCT
CTCTTCTCGCAGTTTCCATGTGGTACACACTCCCCCGCTGCACCCTGACTTCAAGAGGG
CCCTCGGGGACCTGCCCCACCAC
>2614
AGACAGAGAGGTATGAAGACAGGAGGGTTTTCCAGCATGAGCTCAGCTCTTCTTGTTT
CAACTCACACAGATGGAGGAGACTCTGAGCAGTTCATAGATGAGGAGCGTCAGGGAC
CACCTTTGGGAGGACAGCAATCTCA
>2615
TGAGGGATTAGACATTTCCTGCCATGTCAAGTGTTGTCTGTAATCTTCCTTGTTTGCTTG
TCTTTTTCAGGAAGTGAATAAGAAGATAACAGTGTTTCAAATGCCGTGAAACACGGCA
TCATGCTCTAACTTCAGTATAC
>2616
AGACAGAGAGATATGAAGACAGGAGGGTTTTCCAGCATGAGCTCAGCTCTTCTTGTTT
CAACTCACACAGATGGAGGAGACTCTGAGCAGTTCATAGATGAGGAGCGTCAGGGAC
CACCTTTGGGAGGACAGCAATCTCA
>2617
TAAACCAATGAGGTATTAGACATTTCCTGCCATGTCAAGTCTTGCCTATAATCTTCCTTG

TCTTTTTCAGGAAGTGAATAAGAAGATGACAGTGTTTCAAATGCCTTGAAACATAATGT
GATCATGCTCTAACTTCAATA
>2618
GAAGGCCTGGTCCTTCCTTGGTCTGCGCAGCCCCTAACTTATCTTGAACCTCCACTGCC
ACCCCTCGAAGGACGTGGACGATGCCACTCTGTCCCGCCTGGAACTAGAGCGCAAGAT
TGAGTCTCTGATGGATGAGATTG
>2619
CTGTGCACGAACTGCGTGGCCCGCGTGGAATTTGCGCCGCTGTCCATCCTCTGCCTGC
TCCCGGCCGTAGGAGCTGCGAGACCTGCAGGTGAGTGTGGAGAGCCAGCAGGTGCAG
CAGGTGGAGGTGGAAGCCACGGTGA
>2620
GCGAGGCGGGACGCTGGGGTGGTGTCGCGCGTCCCAGCCGACTAAAGCCTGGGTTAC
CCCCACTTCTCAGTACGCGGACCTGTCCGACGCTGCCAACCGGAACCACGAGGCCCTG
CGCCAGGCCAAGCAGGAGATGAACG
>2621
TGCGCGCTCGGGCCCGGGGAGCGGACGATGAAATGTTCTGCAACTGGCCCCTTCCACT
CTCCTACCCCAGAACGAGGCGCTGCTCAGGCAGTTGAGAGAGCTGGAGGAGCAGTTC
GCCCTGGAGGCGGGGGGCTACCAGG
>2622
TAGCAGTGGGAGCCTAAGAGGAGAGATTCCCACGCCTTCCCCCTTGAACCCTTTATCCT
GCTTTCTTCAGTGCCTGAGGTGGAGCCTCCCCAGGACAGCCACAGCCGGAAGACGGT
TCTGATCAAGACCATTGAGACCCG
>2623
CTATGATCCAAAGGAGTAGGATGGAGGGTGGCGCTGAATGGCTTGTGCCCATCATATGC
CCTGTCCCCAGCAGGTGGTGACAGAGTCCCAGAAGGAGCAGCGCAGTGAGCTGGACA
AGTCTTCTGCCCACAGTTACTGAA
>2624
CATCTCCTATCCGAGTCCCCCAGTTCCCCCTGTCAACCCTGATTCCCCTGATCTAGCACC
CCCTCTGCAGGCGCTGCGCCCCTCATCCTGTCTCGGATTGTGGGAGGCTGGGAGTGCG
AGAAGCATTCCCAACCCTGGCA
>2625
TTGCTCCTCTGTCCCTTCTCTCTTTCCTTATCATCCTCGCTCCTCATTCCTGCGTCTGCTT
CCTCCCCAGCAAAAGCGTGATCTTGCTGGGTCGGCACAGCCTGTTTCATCCTGAAGAC
ACAGGCCAGGTATTTCAGGTC
>2626
GGTCTGAGGGAGGAGGGCCAAGGAACCAGGTGGGGTCCAGCCCACAACAGTGTTTTT
GCCTGGCCCGTAGTCTTGACCCCAAAGAAACTTCAGTGTGTGGACCTCCATGTTATTTC
CAATGACGTGTGTGCGCAAGTTCA
>2627
TGAAAGATAGGATTGCCCAGGCAGAAACTGGGACTGACCTATCTCACTCTCTCCCTGC
TTTTACCCTTAGGGTGATTCTGGGGGCCCACTTGTCTGTAATGGTGTGCTTCAAGGTAT
CACGTCATGGGGCAGTGAACCAT
>2628
ATCGAGTCTCACTAGCTCCAACCAGTTGTGGGTGACAGATCCTACACATCCATGTCTCT

TTTCTCTGCAGGCCCCATGGGTCCACCTGGAGAAATGCCATGTCCTCCTGGAAATGATG
GGCTGCCTGGAGCCCCTGGTAT
>2629
AACACCTCGTGGCAGCAAGTGGGAGTCTTCACTGGCCTGCCCCTCCTTCTGTGTGGGG
CACTCTCCACAGGGCTTCGAGCTCATCTAGATGAGGAGCTCCAAGCCACACTCCACGA
CTTTAGACATCAAATCCTGCAGAC
>2630
GACCCCAGGTGAGGGAGGTGGCTTAGAGACAAAGTGGTCAGTGGCCTGACCCGGACT
CCTCTGCTCTCAGCCCTCAGTCTGCAGGGCTCCATAATGACAGTAGGAGAGAAGGTCT
TCTCCAGCAATGGGCAGTCCATCAC
>2631
CAAACAATTAAATGTTTAAATTAAGATTAAGACACTAGAGAATGATTTATTTGCTGTCCT
TTAATTGCAGCAAATCCTTGCTGTTCCCACCCATGTCAAAACCGAGGTGTATGTATGAG
TGTGGGATTTGACCAGTATAA
>2632
TCATTGTATAGATTTGTGTCTTATAATGAGTCCCATTAATTTCTCCCTCCCTTTCTTCGTCT
TCTTGCAGCGGAATTTTTGACAAGAATAAAATTATTTCTGAAACCCACTCCAAACACAG
TGCACTACATACTTACCCA
>2633
AATAAAAATAATGGTTGATTTACTTAACAAATGAGAATAAATAAAACATTTTTTTCTTTG
AAAATTTCAGCCAGATCACATTTGATTGACAGTCCACCAACTTACAATGCTGACTATGG
CTACAAAAGCTGGGAAGCCTT
>2634
AGATTGACAGTCACCATCTCCTTTCTTGAATACATAGATGGATTCTTACCTTAGCTTTCT
CATTTTTCAGGTAAAAAGCAGCTTCCTGATTCAAATGAGATTGTGGAAAAATTGCTTCT
AAGAAGAAAGTTCATCCCTGA
>2635
TATATGATAGACACTTTATATAAGAAAAACTTCAACAGCAACAAATTAAAATTTTTTCAT
CATTTTCTAGGTGGACTTAAATCATATTTACGGTGAAACTCTGGCTAGACAGCGTAAAC
TGCGCCTTTTCAAGGATGGAA
>2636
ATTTTAAAATCCCTAAAAATGTGTTCCTTAACTTTTTAACTGATGTTTATTTATTTATTTAT
TTTTTTAGATAATTGATGGAGAGATGTATCCTCCCACAGTCAAAGATACTCAGGCAGAG
ATGATCTACCCTCCTCAAG
>2637
GCATTCCAGTTGCTTGAAAGCTTGTGATCAGATGCAATAAATGTTTTTATTATTTATTTTG
TGCAAATAGGAGAGACTATTAAGATTGTGATTGAAGATTATGTGCAACACTTGAGTGGC
TATCACTTCAAACTGAAATT
>2638
AAGGCAAGCTGAATACAAACAGTAAATATGCCTAAAATTCTGGTTTTACAACTAATTTT
ACTTTCCACAGGTTGCTGGTGGTAGGAATGTTCCACCCGCAGTACAGAAAGTATCACA
GGCTTCCATTGACCAGAGCAGGC
>2639
AAGAATCAAACTGTTTTTTATTTTGTGAAGTTTGACATTGGTTGTATCTCTGTCTTCATC

GCCTTCACAGGAGAAAAGGAAATGTCTGCAGAGTTGGAAGCACTCTATGGTGACATCG
ATGCTGTGGAGCTGTATCCTGC
>2640
TAAATATTAAACATGAAATTTTATTGTTTGCATTTTGAATTTTTTTTGTTTTTGTTTTTTTT
TCTTATAGCACGACTGAAAATGTAGCTGTTTATATCTGGGACAACCTCCAGAAAGTTCT
TCCTGTAGGAGTTCTTTAT
>2641
TATCTAGTATACTAGATTTTAAGTTGAAGTAGGATCTTCAGGAGTCTAATCATTATTTCTT
TTCTTTTAGGAGAGAAGACGATCTGCACTTCGCATTTTGGCATTGACATTTAATTTTAGG
GTCCTTTATATAGAAGGGA
>2642
AGTTAACAAAATAAAGGGTCTTACAAAATAGGCAATAATAATAATAATCACTTATTATTA
TTACATGAAGCTACATGAATGTGTAAGATCTTGGAGGAAGACAGCAGAGAGAGAGAG
AGAGATCAGAGATCCCAGGGTTA
>2643
ATAAATATTGTCTGATTGGCCCAGGAAAATGTTTGCCATTTCTCATATGTAGTATTTGACT
GACTTTCAGGACAGGAAGATGTCACCCAAGCGCATAGCTAAAAGAAGGTCCCCCCCA
GCAGATGCCATCCCCAAAAGCA
>2644
CCCAGAGCCTGTGACCTCTCCTCGCTGTCGTCATCCTCTACACTCAGGGTCTATCTTCT
TCACCCTTCAGTCTCACACAGGTCCCACAGCACAGAACCCGGCTTGGTGCTGACACTA
GGCCAGGGCGACGTGGGCCAGCT
>2645
GGGGAGTGGCCTACATGAGAATGGACTGCGAGGCCAGACGTTGCATTAATGAGGGCAT
CCGTGGGCACAGGTCTATTCCTTCGGCTGCAATGATGAGGGTGCCCTGGGAAGGGACA
CATCAGTGGAGGGCTCGGAGATGG
>2646
ACTGACCCTTCTGGATTTCACTGGCACGGGGCAGGCAGGACTGGCTGATAAGTGCCTG
TCCTCCTTCTAGGACAATAACGGTGTGATTGGACTGTTGGAGCCCATGAAGAAGAGCA
TGGTGCCTGTGCAGGTGCAGCTGG
>2647
CTTGTTCTGGGGCTGGCCTAGCCTTGGGCCTTACAGTTGTGGCCTGCATCCCTTACCTT
TTCATCCTTAGGAAACGACCACTTGGTGATGCTGACAGCTGATGGTGACCTCTACACCT
TGGGCTGCGGGGAACAGGGCCA
>2648
TTAAACTCGGGGCAGAGAGAAGCCATGTGTGTCTGTCTGTCACTGACCGTGGCTTTCC
CTTTGCCTGCAGAACGACTCCTGGTCCCCAAGTGTGTGATGCTGAAATCCAGGGGAAG
CCGGGGCCACGTGAGATTCCAGGA
>2649
TGTGGGAGGAGATTGAGAAGGGCAGCTCTCAGAACACCTTCACCCCTGATGGCTCCG
GCCTTTCCCCCAGGAACTCCGGGCACAGAATCTTGCTTCATACCCCAGAACCTAACATC
CTTCAAGAATTCCACCAAGTCCTG
>2650
CTAGCCTGCCACCCATTTTGCCTGTAGCACGCCCTCTTGCTATTGCTCATCTCTCTCCCT

CCTCCCATAGGAAAAGCATACAGCCTGGGCCGGGCTGAGTATGGGCGGCTGGGCCTTG
GAGAGGGTGCTGAGGAGAAGAG
>2651
GGTTTCTCCTGCTACAGAAAGGTGGGCTGGGGATCCTGGAGACAGCTGTACCCATTTC
TCTCTCTTGCAGGTCGTGTTTTCGCCTGGGGCATGGGCACCAACTACCAGCTGGGCAC
AGGGCAGGATGAGGACGCCTGGAG
>2652
TAGCTTTTGGGCATCAGCTGTCATGGAGCACTGGGGATGTTGGCTGATGTGTCTCCTTT
CTTTATCTTAGATCCGAGAGACTGAGGTCATCGACCCCCAGGACCTCCTAGAAGGCCG
ATACTTCTCCGGAGCCCTACCAG
>2653
TGTCCCTGTCCCCTGCCCTGAGTCTCTTCCAAAATACGCTGAGTGTCTCTTCTCCTTTCC
GCCCATCCAGGTGCCTCTAGATAACCATATCCCTGAGAGGGCAGGGTCTGGGAGCCAA
GTCCCCACCGAACCCAAGAAAC
>2654
AAAGAGGGGTTCATCCTTAGGTCCTGATGAGGAGCTCTGACCCCCGCCTCTTCTCTCTC
CTCCTCTCCAGCTCTGATTGTGGGTGGCATCGTGGGCATCCTCTTTGCCGTCTTCCTGAT
CCTACTGCTCATGTACCGTAT
>2655
AAGCTCTTACCTCACCTTCAAGCCTTTTCCTTACCCTGAGAGCCTCCTTTAATTGTCTCT
TCTTTTTCAGGCCAAGAGGCCCAGACAGAGTTGCCCCAGGCCCGGATCAGCTGCCCA
GAAGGCACCAATGCCTATCGCTC
>2656
CTCTTGGAGGACTCAGTATATCCGTCACAACTTCCTCCTCCACTGAGTGCTCCATTTTCT
TCTGCAACAGCTCTATTGCCAGAACATGAATTCGGGCAACCTGGTGTCTGTGCTCACCC
AGGCCGAGGGTGCCTTTGTGG
>2657
GCACACAGGCCCAGTGATTCCATGTATTTTTGAGTGACCACTGCCTCTGTTCTGGCCCT
TCCCCATCTAGAACCGCCGCTGGCACTGGAGCAGTGGGTCCCTGGTCTCCTACAAGTC
CTGGGGCATTGGAGCCCCAAGCA
>2658
CCTATTCACCTTCCAGCCTTTTCCTTGCCCTGAGATTCTTTCTTAGTTATCTCCTTTTTTT
TTTCCCCAGGCCAGGAGTCCCAGACAGAGCTGCCTAATCCCCGAATCAGCTGCCCAGA
AGGCACCAATGCCTATCGCTC
>2659
GAGTCCTAGGTTGAGGAGGGGCTACACTCCTTCTGATCCTCTATGTCTGACACTTCTCA
TTGTACTATAGGATTCAAGAAATGGAAGGATGAATCTTGTGAGAAGAAGTTCTCCTTTG
TTTGCAAGTTCAAAAACTAGAG
>2660
AGAGGATGTTAGAAAGATAACAGGTGCTCTGCTCTCTTCTTCAAACCCTCTTCCCTGTG
TTCTCCTACAGAGATTGCTGATTTGCTCCTTAAGCAAGAGATTCACTGCCGCTAAGCAT
GGCTCAGACCAACTCGTTCTTC
>2661
CTAATTCACCTTCCAGCCTTTTCCTTGCCCTGAGATTCTTTCTTAATTATCTCCTTTTTTT

TTTCCCCAGGCCAGGAGTCCCAGACAGAGCTGCCTAATCCCCGAATCAGCTGCCCAGA
AGGCACCAATGCCTATCGCTC
>2662
CTCTGGAAGGTCTCAGTATATCCCTCACACCTTCCTTCTCCACTGAGTGCTCCATTTTCT
TCTCCAACAGCTCTATTGCCAGAACATGAATTCAGGCAACCTGGTGTCTGTGCTCACCC
AGGCGGAGGGTGCCTTCGTGG
>2663
GCACACAGGCCTGGTTATTCTATTGATTTTTGAGTGACCATGGCCCCTGTTCTGGCCCTT
CTCCATCTAGAACCGCCGCTGGCACTGGAGTAGTGGGTCCCTGGTCTCCTACAAGTCC
TGGGACACTGGATCCCCGAGCA
>2664
GAGTCCTAGGTTGAGGAGGGGCTACACTCCTTCTGATCCTCTATGTCTGACACTTCTCA
TTGTACCATAGGATTCAAGAAATGGAAGGATGAATCTTGTGAGAAGAAGTTCTCCTTTG
TTTGCAAGTTCAAAAACTAGAG
>2665
AAACAAGTATGTACTGAATCAATTTGATTTATAAGTATATGCCAATTATATGATTATTTTCA
TTTGGTAGGCTTGAGTTTGAAGAAACAGAAGAACCTGATTTTACTGCATTATGTCAGAA
ATTAAAGATACCAGATCAT
>2666
ACAGTTTTAACATAGTATCCAGTGTGTGAATTATTTAATGAAATATTTGATCTTTATTTTT
TGTTCCCAGGGAGGTTATATTCAAAAGAAAAAGGAACTGTGGGGAATCTGTATCTTTAT
TGCAGCAGTTGACCTAGATG
>2667
TGATTTGAAAACGAAATAACACAAATTTTTAAGGTTACTGATTTACTTTTTTCTATTCTT
TCCTTTGTAGTGTCCATAAATTCTTTAACTTACTAAAAGAAATTGATACCAGTACCAAAG
TTGATAATGCTATGTCAAGA
>2668
CCCTGCGATTTTCTCTCATACAAAGATCTGAATCTCTAACTTTCTTTAAAAATGTACATT
TTTTTTTCAGGGGAAGTATTACAAATGGAAGATGATCTGGTGATTTCATTTCAGTTAATG
CTATGTGTCCTTGACTATTT
>2669
TAGTTAGAATACTTCATTATTTTATATGATGGATGTACAATTGTTCTTATCTAATTTACCAC
TTTTACAGAAACAGCTGTTATACCCATTAATGGTTCACCTCGAACACCCAGGCGAGGTC
AGAACAGGAGTGCACGGAT
>2670
TGTTCAAGAGTCAAGAGATTAGATTTTGTTTTAAATTTTAATGATCATGTTGTAACTTCA
TCTTTTTCAGGTGAAAAATGTTTATTTCAAAAATTTTATACCTTTTATGAATTCTCTTGGA
CTTGTAACATCTAATGGAC
>2671
CTGTGCCTCTGTGTGCTGAGAGATGTAATGACATGTAAAGGATAATTGTCAGTGACTTT
TTTCTTTCAAGGTTGAAAATCTTTCTAAACGATACGAAGAAATTTATCTTAAAAATAAA
GATCTAGATGCAAGATTATTTT
>2672
GCTTTTTTGATGCATAAAGCACAAATTGTAAATTTTCAGTATGTGAATGACTTCACTTAT

TGTTATTTAGTTTTGAAACACAGAGAACACCACGAAAAAGTAACCTTGATGAAGAGGT
GAATGTAATTCCTCCACACACT
>2673
AAAACATTTCATTTTTTCTTTTTTTCTCCCTTCATTGCTTAACACATTTTCCTATTTTTATC
CCCTCTAGGACTGTTATGAACACTATCCAACAATTAATGATGATTTTAAATTCAGCAAGT
GATCAACCTTCAGAAAAT
>2674
ATGGAGCAGAAAATATTAATTCTGATTACACAGTATCCTCGACATTGATTTCTGTTTTTA
CCTCCTAAAGAACTGCACAGTGAATCCAAAAGAAAGTATACTGAAAAGAGTGAAGGA
TATAGGATACATCTTTAAAGAGA
>2675
TACTTTTAGTAAAAAATTTTTTTCTTTTTATAGAAGTAAGTATTTTATAATCTTTTTTTTTT
TCCTTTAGCAAACTTCTGAATGACAACATTTTTCATATGTCTTTATTGGCGTGCGCTCTT
GAGGTTGTAATGGCCACA
>2676
AACTTCCAAAAAAAATACCTAGCTCAAGGGTTAATATTTCATAAATAGTTACTTTTTTTTT
TCATTTTTAGGAAGTACATCTCAGAATCTTGATTCTGGAACAGATTTGTCTTTCCCATGG
ATTCTGAATGTGCTTAATTT
>2677
ATGATTTTGATATGTACCTGGGAAAATTATGCTTACTAATGTGGTTTTAATTTCATCATGT
TTCATATAGGATTCACCTTTATTTGATCTTATTAAACAATCAAAGGACCGAGAAGGACCA
ACTGATCACCTTGAATCTG
>2678
TTGAAGTGTATGTATAATCTGTGATTCTTAGCCAACTTGAAATGAAGACTTTTCCTTTAA
ATATATCTAGGTATCTTTCTCCTGTAAGATCTCCAAAGAAAAAAGGTTCAACTACGCGT
GTAAATTCTACTGCAAATGCA
>2679
TGATTTGAAAAAAATCTACTTGTAATTCAAAATGAACAGTAAAAATGACTAATTTTTCTT
ATTCCCACAGTGTATCGGCTAGCCTATCTCCGGCTAAATACACTTTGTGAACGCCTTCTG
TCTGAGCACCCAGAATTAGA
>2680
AAATGGTATTTTTTAAGAACAAAACCATGTAATAAAATTCTGACTACTTTTACATCAATT
TATTTACTAGATTATGATGTGTTCCATGTATGGCATATGCAAAGTGAAGAATATAGACCTT
AAATTCAAAATCATTGTAA
>2681
TTTTAAAATTCATTTAACAAGTAAATTTTACTTTTTTTTTTTTTTTTTTTTTTTTACTGTTC
TTCCTCAGACATTCAAACGTGTTTTGATCAAAGAAGAGGAGTATGATTCTATTATAGTAT
TCTATAACTCGGTCTTCA
>2682
TGGAAAAAATCTAATGTAATGGGTCCACCAAAACATTAAATAAATAATCTACTTTTTTGTT
TTTGCTCTAGCCCCCTACCTTGTCACCAATACCTCACATTCCTCGAAGCCCTTACAAGTT
TCCTAGTTCACCCTTACGGA
>2683
TAACTTGAGGTTGCTAACTATGAAACACTGGCATTTAATGATTTAAAGTAAAGAATTCT

GTAATTTGTAGACTTCTGAGAAGTTCCAGAAAATAAATCAGATGGTATGTAACAGCGAC
CGTGTGCTCAAAAGAAGTGCTG
>2684
TATATGGCAGCCACTTGCCAACTTACCCAGTACCATCAATGCTGTTAACAGTTCTTCATC
CTTTTTCCAGCTTCTACTCGAACACGAATGCAAAAGCAGAAAATGAATGATAGCATGG
ATACCTCAAACAAGGAAGAGAA
>2685
TGTCTGTCGAGAATGTTGCACAGGGAGTTACAGAATGTTCTCATGACTGAATTGCTTTT
AAATTTCACAGTGTGCCTGCATTTGAAGTCTTGGAAATATCTCCCCAGGAAGTATCTTC
AGGCAGACTATTGAAGTCGGCC
>2686
ATTATTAAAAAATGATACAATCTCGGGAAAATTTTTTTGATTTTCATGAAATTCATGTGTT
TTTCTATAGGTAACACAAATTCTGACTGCTATGATATGCCTTTGTTTTGGAACAGTTGTC
TGCTCTGTACTTGATATTT
>2687
AATGAAGATAGGTTTATTGAATGTGCCAGCAAGTGCAGGCCCAGGTCTGAGTGTTCTT
CATTATTATCAGGTGAGAGGAAGCCTGGGAGCAAACACTGCCAGCAGCATAGCTGGGG
GAACGGGAATTACCATCCTGATCA
>2688
TAAGAAAATAAACTTTTGGGGCGAATACCAATGTGACTCTTTTTGTTTGTCATTTGTTGC
TGTTCAATAGGAAATTGTAGTGATGATGCTGTTTCTCACCATTCTGGGACTTGGTAGTG
CTGTGTCACTCACAATCTGTG
>2689
TCTCTTGAGCGAGACTTCTAGGGATGGGAAATTTGTGGTGATTGATATGAAATGATTTT
TCCCTTATCAGGTTCCAGAGGATCGTGTTTATGAAGAATTAAACATATATTCAGCTACTT
ACAGTGAGTTGGAAGACCCAG
>2690
GTGCCCTTCAGTCCCTCCCCCAGGCCTCTATCTCCAGACATCCTTAACCCCTCTGTCCC
TCCCTTTGCAGCCGGATCCAGAGCAATGTAGAAGGCCTATACCTGACTGATGGGGTCCC
TTTCTCCTGTTGCAACCCCCAC
>2691
CCTGCCTCCAACCCTGGGCCTCTTGGAACCGCTGACTCTCCCTGACTCTTTCCCCTTGC
TTCCCCCACAGGCTCTGGTGCTCCTTGGCCTGCGGTACCTGCAAACAGCACTGGAGGG
GCTTGGAGGGGTCATTGATGCGG
>2692
GGGCTCGGCGCCGCACTCTTCCCAGAAGGTTGCGCTGGCTCGTGAGTGATCCTGTCCT
CTCCACCCGCAGGGCCGCGTTCGCACCAAAACCGTGAAGAAGGCGGCCCGGGTCATC
ATAGAAAAGTACTACACGCGCCTGG
>2693
CATGTGCTCAGTACCAGGTGGGAGTTGATTTGTCTTCTCACTGTTCTCTTTGGCTGTGT
GTGCTTTGTAGTTATGTCACGCATCTGATGAAGCGAATTCAGAGAGGCCCAGTAAGAG
GTATCTCCATCAAGCTGCAGGAG
>2694
GTGTTTTCTTCTGGGGTCCACTCACAGATCTCACCAGTAACTGCTCACTGAATCCATTT

TCTCGTTCCAGGACTTCGGCAGTCTGTCCAACCTTCAGGTCACTCAGCCTACAGTTGG
GATGAATTTCAAAACGCCTCGGG
>2695
CTTTGTAGATGGCTCTACAATTACCTGTATAGATAGTTTCGTAAACTATTTCCCCCCTTTT
AATCCTTAGCTGAACATCTCCTTCCCAGCCACTGGCTGCCAGAAACTCATTGAAGTGG
ACGATGAACGCAAACTTCGTA
>2696
GTCACAATCCTGTACTGTATAATTTTGGAATAGAAATAGTAAATGTGTCATTCATTGTATG
CTCTTGCAGGGTTATGTGGTCCGAATCAGTGGTGGGAACGACAAACAAGGTTTCCCCA
TGAAGCAGGGTGTCTTGACCC
>2697
ATTAAACATTCTGATTTAGGATTCTTAAGGAAGTTGTTGAATTAAAATCTCTTCAACTGT
TTCTTCTAAGGAGAGAAGGATATTCCTGGACTGACTGATACTACAGTGCCTCGCCGCCT
GGGCCCCAAAAGAGCTAGCAG
>2698
GATGTTTTTAAAGTAGATGTGGATTCTAACAAAACAAATATTGAAATGATTGAGTCCTTT
GAATTTTTAGGTAAGAAACCTAGGACCAAAGCACCCAAGATTCAGCGTCTTGTTACTC
CACGTGTCCTGCAGCACAAACG
>2699
TCGACCTGGCTTTGGATTTGCGTCTTTAACCCAGAAAGGCCTTAACTGTTTGCTGTTTT
GTTTTGTTTAGGAGGCTAAGGAGAAGCGCCAGGAACAAATTGCGAAGAGACGCAGAC
TTTCCTCTCTGCGAGCTTCTACTT
>2700
TGACCCAGAGGGATTACGGGATTCAGGGGGTCAAGTGGGCCTCCACTCTGATGTCTCT
TGCCACTCACAGACCAAGTGCTTCATCTGTGGAATCGGCAGTGACTACTTTGATACGA
CACCGCATGGCTTCGAGACTCACA
>2701
AATATATATATATATATGTCTCAAGGGTTTGAAGATGTGACCAATGAACTCTTTCTATCCC
CAATCCTAGGAGTCTTATGTCTGGAAGATGTACCAAGAGAGATGTTGGGATTTCTTCCC
AGCTGGTGATTGTTTCCGTA
>2702
TCCCCTCTAAGGTGTTGTTGGAGTCTTTATGTTCTCCTGATGTCCCTTCTGCCTTTCCTT
TCCTTTCCAGGGGCTCGGGACATGTGGAGAGCCTACTCTGACATGAGAGAAGCCAATT
ACATCGGCTCAGACAAATACTT
>2703
AAGAATGGGAGGGTGAGCTGTTGCTCACTGGCCTGATGATTAATCTCCTTCTTGCCTGC
CTGGACTACAGCAATGCCAGAGAGAATATCCAGAGACTCACAGGCCGTGGTGCGGAG
GACTCGCTGGCCGATCAGGCTGCC
>2704
GTCCCCGCGTAAGCCAGCCTGGTGCCACCCGGAGCGGCGTTAACGGGTGCGTGGTGT
CTCGGCTCTGCAGGGCGTCATGGTCGGTATGGGTCAGAAAGATTCCTACGTGGGCGAC
GAGGCTCAGAGCAAGAGAGGTATCC
>2705
CGGCCGCTAGCGCTGAGCGCCTAGCCTCGGCCTCGCCCCCAGCCACTCACTCTCTCCC

GCGCGCGCACAGGCATCGTGCTGGACTCCGGCGACGGCGTCACCCACAACGTGCCCA
TTTATGAGGGCTACGCGCTGCCGCA
>2706
GCCCCTGCACCCGGGCGGAGGGCCGCGGCGGCCTGAGTGAGGGCTCCTCTCCTGCTT
CTGCCCTCCGCAGCTGAGCGCGAGATCGTGCGCGACATCAAGGAGAAGCTGTGCTAC
GTGGCCCTGGACTTCGAGAACGAGAT
>2707
CTCGCCCCGGCCCCCAGGCCCGCGCCCCCGGCCCGAGCTTCTGCTCACGCTCCCCGC
CGCGGTCCCCAGGTATGGAGTCGGCGGGCATTCACGAGACCACCTACAACAGCATCAT
GAAGTGTGACATCGACATCAGGAA
>2708
TGGCCTGCGCGGGCTGTCGGCGGGGTGGGCTCCAGGGTGAGGTCTCCCCACCTCACG
CGCTGTCTTGCAGATCATCGCCCCGCCGGAGCGCAAATACTCGGTGTGGATCGGCGGC
TCCATCCTGGCCTCGCTGTCCACCT
>2709
GGTTGAAGCTGAGATATCTTTTCCCTGCATTTATACTGAAGGTCATTATCTTTCTTTCTTT
ATCCCGCAGACCTCAGTGGGAAGGTGTTTGTATTTCCTAGAGAATCTGTTACTGATCAT
GTAAACTTGATCACACCGCT
>2710
AGTGGGGGGTAAGAGTTGTAAGGGAGCTTTGGAGATAATGAATGCATACCTTCTTATTA
TCAATTACCAGGTGGATCAAAAGGCCGATTACCAAGTGAATTTTCCCAATTTCCACACG
GACAAAAGGGCCAGCACTATTC
>2711
GGCCCAGCTACCATCCCCACCCTTCACTTTTTACTATACTCACATCACTGATTCTCTTCT
TTCTCTCTAGGTGTCAGTTGACCTCAGTGAATTCTGTGATGTTTCTGAGATGCAGACTC
CCGTGTAGTTTCAGATTCTTG
>2712
GGTGGGAGGTAAGAGTTGCAAGAGAGCTTTGGAGATAATGAATGCATACATTTCTATTA
TCAATTACCAGGTGGATCAAAAGGCCAATTGCCAAGCGGATCTTCCCAATTTCCACATG
GACAAAAGGGCCAGCACTATTT
>2713
TCCCAGCTCTCCCATCCTCACCCCCACTCTCCACTATCCTCACATATCTGGTTGTCTTTT
TTCTCCCTAGGTGTCACCCGACCTCAGTGAAGTCTTTGATGTTTCTGAGAGGCAGACTC
CCATGTGGTCCCAGATCCTTG
>2714
CCTGACAGAGCACAGTGGGGGAAGATGTTGGCAGAGGTGGCAGATGGGCTCACGGCC
ATCCCTCCAGCAGGAGCAGCGACTGGACCCAGAGCCATGTGGCTGTGCCCTCTGGCCC
TCAACCTCATCTTGATGGCAGCCTC
>2715
ATCGAGTCTCATTAGCTCCAACCAGTTGTGGGTGACAGATCCTACACATCCATGTCTCT
TTTCTCTGCAGGCCCCATGGGTCCGCCTGGAGAAACACCATGTCCTCCTGGGAATAATG
GGCTGCCTGGAGCCCCTGGTGT
>2716
ACGCCTGCGTGGCAGCAAGTGGGAGTCTTCACTGGCCTGCCCCTCCTTCTGTGTGGGG

CACTCTCCACAGGGCTTCCAGCTCATCTAGATGAGGAGCTCCAAGCCACACTCCACGA
CTTCAGACATCAAATCCTGCAGAC
>2717
GACCCTAGGTGAGGGAGGTGGCTTAGAGACAAAGTGGTCAGTGGCCTGACCTGGACT
CCTCTGCTCTCAGCCCTCAGTCTGCAGGGCTCCATAATGACAGTAGGAGAGAAGGTCT
TCTCCAGCAATGGGCAGTCCATCAC
>2718
CTGGCACGGGTGAAAGCAGAGCTGCTCCCTGACCCTCTGCCCCTCCCTCCTCCACCCT
GGCCTGCTTTAGCTTTCCCCAGACATGGCCAACAAGGGTCCTTCCTATGGCATGAGCCG
CGAAGTGCAGTCCAAAATCGAGA
>2719
CCACCCTGTGAGTCCTGGGCCAATCCCTGAGCTGACTGCTAAGCTGCGTCCTATGCCCT
ATGCCTGGTAGATTCTGAGCAAGCTGGTGAACAGCCTGTACCCTGATGGCTCCAAGCC
GGTGAAGGTGCCCGAGAACCCAC
>2720
GGCAGGCCCTGGCTTGGAGTCTTGTTTATACGTTCTTGATGTTCATCTCCTCTCTCCTGT
CTTCTCACAGGCAAAGACATGGCAGCAGTGCAGAGGACCCTGATGGCTTTGGGCAGC
TTGGCAGTGACCAAGAATGATGG
>2721
AGAGGGATCAGAAAACGGGTGGAAAACACACAGTTCTACCAAGTCTTTATCCTGGTTC
CTCCTCTTCTAGGAAAGCGCAGGAGCATAAGAGGGAATTCACAGAGAGCCAGCTGCA
GGAGGGAAAGCATGTCATTGGCCTT
>2722
GGTGGCCAGGGGTTGGCCTGGTTCCTCTGCTCTGCCTCTGATTTCTCACCATGCGCTCC
TCCCACTGCAGAAGGAACCCAATGTGGCTCGCGTGGGCTCCGTGGCCATCAAGCTGTG
CAATCTGCTGAAGATAGCACCAC
>2723
ACCCAAATGCCCAGCACAGGAGGACCAGGATTGGAACAAGTGTTGACCTCTCATGTTT
ACTTTGTTTCAGAATTGGGGGGTTCTATGCTCTTTCCCCATACCCCGGTCTCCGCCTCAT
CTCTCTCAATATGAATTTTTGT
>2724
CCCCTTTCTCTAGCCAGGGCTGCCTGGACCCCTGGATGCCCTGATTACCATCCTTAATTC
TCCCTACTAGGTGCATATAATTGGCCACATTCCCCCAGGGCACTGTCTGAAGAGCTGGA
GCTGGAATTATTACCGAATTG
>2725
GGCATCCTATCTCCCCAGATGTCTTCCTACCCCTCCCTAGAATCTTCTGAATGTAGTACC
TTCTGGCCAGGTATGAGAACACCCTGGCTGCTCAGTTCTTTGGCCACACTCATGTGGAT
GAATTTGAGGTCTTCTATGAT
>2726
CAGGATGTGTGGCCCCTCCCTGGAGTTACCCTTGCTCCTTGCCCCTCCAGTCAGCCCCA
CATCCTTGCAGGTTACCGTGTGTACCAAATAGATGGAAACTACTCCGGGAGCTCTCACG
TGGTCCTGGACCATGAGACCTA
>2727
AGGTCGCTGGCTATTGGTGTGGTGTGTGGTGAGGCTGCTAGTGAGTCACCTGTGACCA

CTCTTGTTTCAGGATGATAAGGATGCCTTCTATGTGGCAGACCTGGGAGACATTCTAAA
GAAACATCTGAGGTGGTTAAAAG
>2728
CCCATGCTTTTCTGTTTATTCCACGTGTTAAGCCACATATGGATAGCATGACACCACTCT
TCTTTTTCAGACTGAAATACAGTTGGTGCAGAGTCTGGGGGTGCCTCCAGAGAGGATT
ATCTATGCAAATCCTTGTAAAC
>2729
TTTTTATTTTAATTGTGGCTATAAGTGAACATCTATCATGAGACATTTGCTGCACTTTCCT
TGCTTGTAGGTTGGTTTTGCGGATTGCCACTGATGATTCCAAAGCAGTCTGTCGTCTCA
GTGTGAAATTCGGTGCCACG
>2730
TTCTCCTAGTGTTTGCTATTATTTTAAGAAATACTAAGACACAGTGTCTCGTCTCTTTATT
TTACCCCAGCTTCCATGTAGGAAGCGGCTGTACCGATCCTGAGACCTTCGTGCAGGCA
ATCTCTGATGCCCGCTGTGTT
>2731
TTCAGCCTATCTGCTGCATACATTTTTGTTATTAGCTGTGGATCTGGCTGACTTATTTTCT
TGATTCTAGGCTGAGGTTGGTTTCAGCATGTATCTGCTTGATATTGGCGGTGGCTTTCCT
GGATCTGAGGATGTGAAAC
>2732
GTGATGTTACTCCCCCATGTGAATTTCCCACAATGAAGGCTGCTCTTTCTTTTCTGTTTC
ACTCTCTTAGATCACCGGCGTAATCAACCCAGCGTTGGACAAATACTTTCCGTCAGACT
CTGGAGTGAGAATCATAGCTG
>2733
GACGAATCACTTCATGTATAACTGAAAGCTGATGCAAAAAGTCATTAAGATTGTTGATC
TGCCTTTCTAGACGAAGATGAGTCGAGTGAGCAGACCTTTATGTATTATGTGAATGATG
GCGTCTATGGATCATTTAATTG
>2734
AAACAATTAAGAGGACTGGTCACAACACGTGTAATTAAGTAGTACTTCCTCTCTCCGTC
TCTTTATATAGAGACCTAAACCAGATGAGAAGTATTATTCATCCAGCATATGGGGACCAA
CATGTGATGGCCTCGATCGGA
>2735
GGAAGTAAATACTCTCATGCCCAGTTAGGAGTGAGTCAGGGTTTTTAATATGCCACTTT
TTCTTTCTCAGGCAACTCATGCAGCAATTCCAGAACCCCGACTTCCCACCCGAAGTAG
AGGAACAGGATGCCAGCACCCTG
>2736
GTGGGTGAAATCATGATGCCAGGTGTGTAGCCTGGATCCTGATTAAGGTTGCTCTGGCC
CCAAACCACAGCTGCCTGGACCACCTCATCCTTGGCCTGTGCCCAGGGCCCTGAGTTC
TGGTGCCAAAGCCTGGAGCAAGC
>2737
AACCTCCCCATTGGAGCTGGGAGCCTCCCCCACTCATGTGTCCTCCACCTTGGGGCCC
CTCCCTCCCCAGGATGACCTATGCCAAGAGTGTGAGGACATCGTCCACATCCTTAACA
AGATGGCCAAGGAGGCCATTTTCC
>2738
CTTGTGTCCAGGGACTTGGGCGGGGTAGACCTCAGAGAGGCCCAGCTGACGGCCCCC

TCTGGCCTCCCAGGACACGATGAGGAAGTTCCTGGAGCAGGAGTGCAACGTCCTCCC
CTTGAAGCTGCTCATGCCCCAGTGCA
>2739
TTGGGCCGGCCTGAATAGGGGTGGGCTTCCCTCCCAGACCCTAACACTCCCACCCTGT
GCTGTGCCCCAGGACTCAAACGGCATCTGTATGCACCTGGGCCTGTGCAAATCCCGGC
AGCCAGAGCCAGAGCAGGAGCCAG
>2740
AGAGGGGAGGAGAGCCTGGAGGACTCTTCTCCCAGCACCCAGCCTGGCCTCCACCTG
ATTCTTTCCCCAGGATCTCTCCGAGCAGCAATTCCCCATTCCTCTCCCCTATTGCTGGCT
CTGCAGGGCTCTGATCAAGCGGA
>2741
GGTGCTGGGCAGAGAGTGGAGGCTTGCCAAGTGAAGGTCCCATGCTGGCCTCTCTCC
GCCCCCGCCCCAGGGTGCGCTACGTGTGGCAGTGGCCCAGGTGTGCCGCGTGGTACCT
CTGGTGGCGGGCGGCATCTGCCAGT
>2742
AGGAGGACAGAAACCAAACACCTCTGTAGGGTGGCCTTGCCTGCTTTCCCCCTAATGC
TCACATCTCCAGGGTCGCCGACAGGAGAATGGCTGCCGCGAGACTCTGAGTGCCACCT
CTGCATGTCCGTGACCACCCAGGC
>2743
TGGAGAGGCCCAGGACCACACGACAGGAGAGTCCTGCAAAGGGACCTCCACAGCTG
TGTGTCTCCCTCAGTGCAAGCAATTTGTGGAGCAGCACACGCCCCAGCTGCTGACCCT
GGTGCCCAGGGGCTGGGATGCCCACA
>2744
TTCCCGGAAGAGCTGGGTGTGGGGAGCTGAGGGGGCCCAGGCCTCAGCCCTGGTGCT
GTCCCTGTGACAGGCCCTCGGGGTGTGTGGGACCATGTCCAGCCCTCTCCAGTGTATC
CACAGCCCCGACCTTTGATGAGAAC
>2745
GCTATCACCTGGCTGTGGGTCCCCAGGCAGGACATACACACAGTCCCTCTCTGGCCCT
CATCCTCCTCAGCTGCAAAGGAAAAGCCAAGTGAGACGGGCTCTGGGACCATGGTGA
CCAGGCTCTTCCCCTGCTCCCTGGC
>2746
CCAGGGCGGCTTGGGGTACGTTTTCCCCACCCCAGAGAACCCAGGTCCCCGACTGTCA
CTCCGCCCGCAGTAAGACCTATGGCAACGTGCTGGTGTTGGACGGTGTCATCCAGTGC
ACGGAGAGAGACGAGTTCTCCTAC
>2747
GGTCTGCTTCAGGGGCCTGGGGGCTCCCAGTCCTGCCAGGGCCCCTGCCTTGACTGCC
CCCTCCTCCCAGGTGCTGATCATCGGGGGCGGAGATGGAGGTGTCCTGCGGGAGGTGG
TGAAGCACCCCTCCGTGGAGTCCG
>2748
CCCACGTGGCTGTGCTGGCTCCAGGTCCCCCAGCCCCTTGGCCCAGACTGGTCCCTCC
CATCCCCTCCAGGATGTCATCCAAGTCTCCAAGAAGTTCCTGCCAGGCATGGCCATTGG
CTACTCTAGCTCGAAGCTGACCC
>2749
CTGTGTGGGTGACAGTGGCCCTGGAGGTCACAGCCAGACTGTTCAGTGTGTCTCCCTC

TGTCTTCCCCAGGCCCCGCCGAAAGTCTCTTCAAGGAGTCCTATTACCAGCTCATGAA
GACAGCCCTCAAGGAAGATGGTGT
>2750
TGGAGCACTGGGGCGGGGCGGGGTGGGGCAGGGCAGGCCCTGCCGGATGCTGATGCT
TAGGGGCCCCCAGGCGAGTGCCAGTGGCTGCACCTGGACCTCATCAAGGAGATGCGG
CAGTTCTGCCAGTCCCTGTTCCCCGT
>2751
ACAGTGAGACTCCGTCTCAAAAAAAAAAAAAAAAGTGCCCCCCCTGATGTGCCCCTG
GCCCGGTCCCCAGAGCACGAACTTCCAGGAGCCGGTGCAGCCGCTGACACAGCAGCA
GGTGGCGCAGATGCAGCTGAAGTACT
>2752
GGCCTGCGGGGCTGGGTGGTGGGACCCAGGGACCCAGAGCGCCCTCCTGACTGGCCT
CATGTCCCTCCAGGCACTGAATGATGTGAGCTGAGCCCAGGCGCCACCACTGATGCCA
CCCAGGACCTCGGACCTTGGAGCCT
>2753
TGTCGTCCTAAGCCTTCTCTCCTGACTGCAGCCATCCCCAGCTACTTTATCTTCCAGTCT
TTCCTTTCAGAGGAGATCATCGGGGGCCATGAGGCCAAGCCCCACTCCCGCCCCTACA
TGGCCTTTGTTCAGTTTCTGCA
>2754
CTGCTCTTCACTGCTCCTGGGCTCTATCCCCTGTGACTCCACCCCCATCCTCACTCTGCT
CTCTGTGCAGCTCCATAAATGTCACCTTGGGGGCCCACAATATCAAGGAACAGGAGCG
GACCCAGCAGTTTATCCCTGTG
>2755
AGCCCCATCACTGTGTCGGGGCCCAGAAGGCCATTGCCTGACCTGGACTTTCTTGCTT
CTTCCCCACCAGCTGGAGAGAAAGGCCAAGTGGACCACAGCTGTGCGGCCTCTCAGG
CTACCTAGCAGCAAGGCCCAGGTGA
>2756
CACAAAATCCATAACAGAGACCACCCATCCCAGGGAACACTAGCTCAGTCTTTCCTCT
CTCTGTTCACAGGGGGACTCCGGGGGGCCCCTCGTGTGTAAGGACGTAGCCCAAGGTA
TTCTCTCCTATGGAAACAAAAAAG
>2757
TTACTGCAACTATTCAGTGAATCTTGTGTGGGCTTTTACTCTGTCTTTCTCTTCTCTCTTT
GTCCTCCAGGAAATCCACAAGGACCATCCCCACAAGGAGGCAACAAGCCCCAAGGTC
CCCCACCTCCTCCAGGAAAGCC
>2758
ATTAGAAGTGGTATTCACCATGGCTTCTTCCCTATTATTCTCTGCTTAAATCATTCTTTTC
TTTTTCCAGGACCAGCCACTGTTGCAGCATGAGTTCCCAGCAGCAGAAGCAGCCCTGC
ATCCCACCCCCTCAGCTTCAG
>2759
AATCTGTTTGGCATTGAATTTGTATCCATCTTTCTTTAATTGAATCACTGTGTCAGCTTTC
TGTCTCTAGAAAAAAACACATTTGAAGCATGAATTCTCAGCAGCAGAAGCAGCCTTGC
ACCCCACCCCCTCAGCCTCAG
>2760
CGCGACCTCCCCGCTGCCCTCCCCGCGCGCCTTCCCCGGCCCGCGGCCTGACCCGCCC

GTGTCCGGCCAGGATCAACTCGTAGGCTTTACGAGAAGAAGATCTTCGAGTACGAGAC
CCAGAGGCGGCGGCTCTCGCCCCC
>2761
GAGAGCGGCACTGGAGAAAGGGGAGGGAAGTCTGGGGGGCAAACAGTTCTGTCTCC
TCCTTTCAATCCAGACTTGAATTCGACTAGAGGGGATGCAGATATGTATGATCTTCCCA
AGAAAGAGGACGCTTTACTCTACCA
>2762
TTCAGATTAGGCCATCAGGCCAGGCGGGCACACCGATGCCCCCTCTCTGACCGCTGCC
CCCCTTCCCAAGGCTACAATGACGACTACTATGAAGAGAGCTACTTCACCACCAGGAC
TTATGGGGAGCCCGAGTCTGCCGG
>2763
GGGGGAGCCCAGGGACGGGCTGGTTCTGGGTCCAGGCTCCTGGCCCACTTGCTCCCC
TCTTTTGCCTCAGGGAACGCCCCATGTACGGCCGGGACAGTGCCTACCAGAGCATCAC
GCACTACCGCCCTGTTTCAGCCTCC
>2764
TCTGTTCACCCTGCCAGGCAGTCTCTTCCTAGAAACTTGGTTAAATGTTCACTCTTCTT
GCTACTTTCAGGATAGATTCCTCACCCTTGGCCCGCCTTTGCCCCACCCTACTCTGCCC
AGAAGTGCAAGAGCCTAAGCCG
>2765
TGTCTCTGGCTCAGGGAGTGTTTCATCCAACTAAGCTCCCTCAGTGGTTGTCTCACTTC
ATCTCTTATAGATTAACTTCCTTCCAAAATGTCACCATTCCTGTATCTGGTTCTCTTGGTA
CTTGGGCTTCATGCTACAAT
>2766
TAATGGAAAAGGTCTTGTTGGTACTGATACCGGTTTAAAATAAACACCTCTCTTTTCTTT
GTATTTTAAGCCCAGTGGGCAAATCCTTTTGATCCATCCAAGACAGAAGACAGTTCCA
GCTTCTTAATAGACAAGACCAC
>2767
AGAGAGAGAAGGAGAGAATCATAAGCTTGATATGGTGATTGCCATGTGTTCCCTTCCTC
TTTTCCCACAGATGGGTTGACTTGTTTGTTCCAAAGTTTTCCATTTCTGCCACATATGAC
CTTGGAGCCACACTTTTGAAG
>2768
TACTTCTAGACCTTCCCAAAGACTGTTCCTGTTTCTTCAGAGACCCGCAGGATTTGTTC
TCCCCACACAGGCTGCCCATAAGGCTGTGCTGCACATTGGTGAAAAGGGAACTGAAG
CTGCAGCTGTCCCTGAAGTTGAAC
>2769
GTGTGTGTGTGTGTGTGTGTGTGATGAGTACAACTGTTTTCCTCATCCTGTTCTCAACTTG
TGTCTCCGCAGATCACACAGGTGCTGGAGTCTCCCAGACCCCCAGTAACAAGGTCACA
GAGAAGGGAAAAGATGTAGAGCT
>2770
TGTTTCTTTTGATCACTCTCAATTTTATGTGGCCTAAAATACAGACTCCATGAATTGATTT
GTCGTTAAGGGCTGGGCCATCAGGAATTTGCTCGTCCATCTCGGGGATACCTGGCCTTC
AGAGATGACAGCATTTGGCC
>2771
TTCGCTTACAGGAATTGCCCTTGCACTTTTCCATCCCTACACCTCAGTCATTCTGTTCTT

ACCTTTCAAGGTAAGGAGCTAAACAGAACCTGCTGCCTGAATGGGGAACCTGCATGC
TGGGGTCCTTTTGTGCCTGCCC
>2772
TGGAGAGGAGAGAGAAAGGGAAGTGGAAATTTCAGACCCAAGCTATCGCAGCTTACC
TGTTCATTCTCAGGAACTGTGGGTCTGTGCCCCATGACACCTGGCTGCCCAAGAAGTG
TTCCCTGTGTAAATGCTGGCACGGT
>2773
CACATTTGTGGGCAGCAGGATGAACTGCCAGAGAGGTTTGCTTTAATGACCAAGCATC
CCTACCTTCCAGATGGCCTTGTGATGGATGAGCACCTCGTGGCTTCCAGGACTCCAGA
ACTACCACCGTCTGCACGTACTAC
>2774
GTTTTATACATCAATACATTCGAGTGCTCTGAAGTGCATGTAATAATAGCTGTTTCTCTG
TTGTTTAAAGGCACTACAAATACTGTGGCAGCATATAATTTAACTTGGAAATCAACTAAT
TTCAAGACAATTTTGGAGTG
>2775
AAGCAGTCTATCTTGTTGGGTGCAAATTGTAACATTCCATTTATGTTTCTTCCTTCTTTTC
TTTCTTTAGCACTAAGTCAGGAGATTGGAAAAGCAAATGCTTTTACACAACAGACACA
GAGTGTGACCTCACCGACGAG
>2776
GATAACAAAACAGGATTTGACTTTGTACAGAATTCTTTGGTTCCAACCAAGCTCATTTC
CTTTGTTTCAGCAAACCTCGGACAGCCAACAATTCAGAGTTTTGAACAGGTGGGAACA
AAAGTGAATGTGACCGTAGAAGA
>2777
ACAGTGACTTAATAAAGGGGAAAGAATTGACTCAGAGCCCAGATGAATTAAGAACTCT
ATCTTTTTACAGAAAACAGCCAAAACAAACACTAATGAGTTTTTGATTGATGTGGATAA
AGGAGAAAACTACTGTTTCAGTG
>2778
CCGTCAATTTTGTCCACTGATAAATGGGATTTGAGCTCTCCAAGTTGACTAGATGCCCT
TTATTTTTCAGAAATATTCTACATCATTGGAGCTGTGGTATTTGTGGTCATCATCCTTGTC
ATCATCCTGGCTATATCTCT
>2779
ATGCCCCCAGATGGCCAAGACTGCCTGTTCCTGAGGTCGCTGTTCCATGACCCCCCCA
CCGCCTTTACAGTGTTCCTGGCTCCTCAGCAAGCACGGTCGCTGCTCCAGCGGGTCCG
GCGAGCCAACACCTTCTTGGAGGA
>2780
AGACAACTTTGCAGGGAGAGAGGAAATAAGTCCCCAGGCTCCAAGGCTGACCGGGGT
GGGGTCTCCGCAGGTAACTGTGCTGAGGGTCTGGGTACGAACTACCGAGGGCATGTGA
ACATCACCCGGTCAGGCATTGAGTG
>2781
ATGGGCTGAGAACAGGGAGCAAGCGTACCTCAAGTTCAACAGCCTCCTGTTGGGCAA
TTTCCTCTTCCAGAATCAACTCCACTACCCATCCTGGGGCCGACCTACAGGAGAATTTC
TGCCGCAACCCCGACAGCAGCACC
>2782
ACACCCCTGTCTGTTCCGGTCCATGTGTGGTCTCACTCACTCTGCTGCCTCCTTGCCCC

TCACCCACCAGGCCAGGATCAAGTCACTGTAGCGATGACTCCACGCTCCGAAGGCTCC
AGTGTGAATCTGTCACCTCCATT
>2783
ACTGGGGGGATCTAGGGGATGGGTGAGGAATGGCCCAGCCCAGTCCCGGCCGGTGCC
TGGGTCCCAACAGAGGAGGCCGTGGAGGAGGAGACAGGAGATGGGCTGGATGAGGA
CTCAGACAGGGCCATCGAAGGGCGTAC
>2784
ATCCGAGGGGATGCGGGGCTGCGGGGCTGGTGGCCAGGACTTGCCCCTCACTGCTTG
GCTTGCTCTGCAGACTGTGGGCTGCGACCTCTGTTCGAGAAGAAGTCGCTGGAGGAC
AAAACCGAAAGAGAGCTCCTGGAATC
>2785
TACTTCTAGGGCTGGTGTAGAGGCAGCCCCCTCATCCTCAGCTCCTAATGCTTCCTGCT
GCCCCTCCCAGGCAGGTGATGCTTTTCCGGAAGAGTCCCCAGGAGCTGCTGTGTGGGG
CCAGCCTCATCAGTGACCGCTGG
>2786
GGCAGGCAGTTTCCTGCTCCTTGCTGGGTGAACCTGCAGCTTCTCCATTTCTTTCTTGG
GGTCTCTGCAGGTACGAGCGAAACATTGAAAAGATATCCATGTTGGAAAAGATCTACA
TCCACCCCAGGTACAACTGGCGG
>2787
GAAATGGCGTTGGGGCCAGGCGGCTCCTGTGGGGGTTGGCTCTCACTAGGCCCTTCTT
CCTTCCCCAAAGCTTGCTCCAGGCTGGATACAAGGGGCGGGTGACAGGCTGGGGCAA
CCTGAAGGAGACGTGGACAGCCAAC
>2788
GTGTCTCGTGAAGGGGCGTGGCTGGGCTATGAGCTATGCTCCTGAGCACAGACGGCTG
TTCTCTTTCAAGGTTACAAGCCTGATGAAGGGAAACGAGGGGATGCCTGTGAAGGTGA
CAGTGGGGGACCCTTTGTCATGAA
>2789
TTGCCTGGCAGAGGAATACTGATGTGACCTTGAACTTGACTCTATTGGAAACCTCATCT
TTCTTCTTCAGAGCCCCTTTAACAACCGCTGGTATCAAATGGGCATCGTCTCATGGGGT
GAAGGCTGTGACCGGGATGGGA
>2790
TCTGTTCACCCTGCCAGGCAGTCTCTTCCTAGAAACTTGGTTAAATGTTCACTCTTCTT
GCTACTTTCAGGATAGATTCTTCACCCTTGGTCCGCCTTTGCCCCACCCTACTCTGCCCA
GAAGTGCAAGAGCCTAAGCCG
>2791
GGGAATTCCTGGAATACCAGCTGACAATGATTTCCTCCTCATCTTTCAACCTCACCTCT
CCTCATCTAAGAATTGCTCCTCGTGGTCATGCTTCTCCTAACTGCAAGGCTAACGCTGT
CCAGCCCGGCTCCTCCTGCTTG
>2792
CCTGCTTATTAGGCTACCATAGCTCTCTCTATTTCAGCTCCCTTCTCCCCCCACCAATCTT
TTTCAACAGAGCCAGTGCCCAGAGGTTCACCCTTTGCCTACACCTGTCCTGCTGCCTG
CTGTGGACTTTAGCTTGGGAG
>2793
CTAAGGCTCCCCACCCGCTTTTAGTGTGCCCTTTGAGGCAGTGCGCTTCTCTCTTCCAT

CTCTTTCTCAGGAGGAGACCAAGGCACAGGACATTCTGGGAGCAGTGACCCTTCTGCT
GGAGGGAGTGATGGCAGCACGGG
>2794
TCCTACTTAGACAAGGGAGGCCTGAGATCTGGCCCTGGTGTTTGGCCTCAGGACCATC
CTCTGCCCTCAGCTTCCTCCACAGGGCAGGACCACAGCTCACAAGGATCCCAATGCCA
TCTTCCTGAGCTTCCAACACCTGC
>2795
CACTTCAGGTGGGTGGGAGGCCCCTGTGCCGCAGGCCCCTCCAGTTTGAAGGAGGCA
CTGCTGGTGCCAGTCTTGCAGGTCTCCCGAGGGCAGAAGGTGACCAGCCTAACGGCC
TGCCTAGTGGACCAGAGCCTTCGTCT
>2796
GCCTGTGCCTGCCTGAGTCCTGATCCGTCTTCCTTCCTACCCTGCCCCCGGCCCCCTTC
TCTTTCTGCAGACAAACTGGTCAAGTGTGAGGGCATCAGCCTGCTGGCTCAGAACACC
TCGTGGCTGCTGCTGCTCCTGCT
>2797
TGTTGGGGAGGGGTCTCCTTCTCCCACTCCTCCCGCCGTGGACCTCACCTGACCCTCT
CTCCTCTTGCAGCACAGAGTTGATGAGACGCGTCCGTCGCTTCCAGATTGCTCAGTAC
AAGTGCCTGGTGATCAAGTATGCC
>2798
ATTCCCTGGCCCCTGCATTGTGGCTTCTGGCCTCCCTGGCCCTGCTCACGCTCTGGCTT
TCTCTTCCCAGGAACACCATGGAGGCGCTGCCCGCCTGCCTGCTCCGAGACGTGGCCC
AGGAGGCCCTGGGCGTGGCTGTC
>2799
TCGCTGGGGCTCCAGGGAGCTGCTGCTGGGGTGGAAGCTCTCACACTCTTTCTCCACG
TGCCCTTTCCAGTTCCCTGACATCATGGAGTTCTGCGAGGCCATGGCCAACGCCGGGA
AGACCGTAATTGTGGCTGCACTGG
>2800
TCAGAGGGTTTAAAAGAAAAACCCACCTGGTAAGGTGCTGAGCGCCCTCTGTCTTTCC
ATGGGAGCACAGCCATTTGGGGCCATCCTGAACCTGGTGCCGCTGGCCGAGAGCGTG
GTGAAGCTGACGGCGGTGTGCATGG
>2801
GCGGGGTGGGGGTATGGCTCTGCCTCCTTCCTGTCCTGGCCCTTCACCCATCCCCTGTC
CCTGCGGCCAGGTCGAGGTGATTGGGGGAGCAGACAAGTACCACTCCGTGTGTCGGC
TCTGCTACTTCAAGAAGGCCTCAG
>2802
GTTGGGGGGGGGTAGGTGTGAGGCTGACAGTCCAGCAGGCCTCACCTCTGCCCTCTG
CCCTCCCTCGCAGAGTTCAAGGCTGCCTTTGACATGTTTGATGCTGATGGTGGTGGGG
ACATCAGCGTCAAGGAGTTGGGCAC
>2803
GAGGCAGGGGATGGTGGGGAGAGGCGGCAGCGGCTGGGCTCAGGCTCAGTCTGCCA
CCTGCTGCCCGCAGGCAGCGGCACCATCGACTTCGAGGAGTTCTTGGTCATGATGGTG
CGCCAGATGAAAGAGGACGCGAAAGG
>2804
GGGCGTCGAGTGTGGTGGCCAGGGCAGCTCGCCCCGGCCCTGAGCCCTGCCCTGTCC

CTCGGACCCGAAGGAATGCAGACGGCTACATCGACCCGGAGGAGCTGGCTGAGATTTT
CAGGGCCTCCGGGGAGCACGTGACG
>2805
GGCTGGAGCCCTGGCCTGGCCTCTGCGTCCCCAGTGGGACCCCTGACCCTCTCCCTCT
CCTTTCCCTCAGAGTTCCTGAAGATGATGGAGGGCGTGCAGTAAGGAGTGGACAGTCG
CCTCTACCAAGATCGCGTGTCCCT
>2806
ATGGGGGCTGCTGGGGTGGCTCAGCCAAACCTTGAGCCCTAGAGCCCCCCTCAACTCT
GTTCTCCCCTAGGGGCTCCCTGGTGTTGGCCTCACACCTTCAGCTGCCCAGACTGCCC
GTCAGCACCCCAAGATGCATCTTG
>2807
TTCCTCAGGGATTGAGACCTCTGATCCAGACCCCTGATCTCCCACCCCCATCCCCTATG
GCTCTTCCTAGGAGACCCCAGCAAGCAGAACTCACTGCTCTGGAGAGCAAACACGGA
CCGTGCCTTCCTCCAGGATGGTTT
>2808
AGGGCCAGGATGTGGAGAGTGAACCGACATGGCCACACTGACTCTCCTCTCCCTCTCT
CCCTCCCTCCAGCAAACCCTCAAGCTGAGGGGCAGCTCCAGTGGCTGAACCGCCGGG
CCAATGCCCTCCTGGCCAATGGCGT
>2809
GGTCAAGGAGGTCACTGACTTCATGAAGTGCTCAGCTATTAAAGGTTCCTTTCCCACTC
TTATCCCTTAGGATGGAAATCCAACTAATGAGACCGCACTCCTTGGCTTGTTCCTGCGT
GTTTCACCCAAAGGAGAAAATG
>2810
CTTATCCTCCTCTGTTGCCATCATGGACCTCTCGCACCCCGTGATCAAGCTGTTTTTCT
CTCCTTCCAGAATTTAAGGGACGCTGTGAAGCAATCATGGATGCAATGAAGAGAGGGC
TCTGCTGTGTGCTGCTGCTGTG
>2811
AGGTGCAAGGGTAGTGAGGCATTTCATTTTGAGTCATACTCTGATGAGGCTGGCCTGTC
TTTTCTCATAGTGATCTGCAGAGATGAAAAAACGCAGATGATATACCAGCAACATCAGT
CATGGCTGCGCCCTGTGCTCAG
>2812
CTCCTGGAACCATGGCCTGAGCACACAGAAGGAGGCTGTCATTAGGGGCTTCCTTTTT
TATACTTGACAGGTTGCAGCGAGCCAAGGTGTTTCAACGGGGGCACCTGCCAGCAGG
CCCTGTACTTCTCAGATTTCGTGTG
>2813
AGGGGAGCCTCCTGAGGGCTGAATGAAAGGGGACTCCCATGTGAGGCATCCATGCCCT
GCTCTCTGTCAGATACCAGGGCCACGTGCTACGAGGACCAGGGCATCAGCTACAGGGG
CACGTGGAGCACAGCGGAGAGTGG
>2814
AGTCCTTGGGTCCTAAAGGAGAGGCTTCACCTTTGACCTACCCTGGCCCTTCCCTTGG
CTGATTTTTCAGAAACCCAGATCGAGACTCAAAGCCCTGGTGCTACGTCTTTAAGGCG
GGGAAGTACAGCTCAGAGTTCTGC
>2815
CAAGCCCCAGCCCTGACTTGCGTGCTTCAGAATTTGTTTTTGTTTCAGTTTGAGAATTC

TTTTCTTCTAGGAAACAGTGACTGCTACTTTGGGAATGGGTCAGCCTACCGTGGCACG
CACAGCCTCACCGAGTCGGGTGC
>2816
AGACCCCATCTCTACAAAAATTAAAAAATAAGAAGATAAAAAGGAAACCTTAAACTCT
CTTATCATTTAGGAATCCTGATGGGGATGCCAAGCCCTGGTGCCACGTGCTGAAGAACC
GCAGGCTGACGTGGGAGTACTGT
>2817
CTCCTCACTCTACCTACACACGTGCAGGAGGCCTGTGGGACTGACTGCCTCCTCGTCC
TTTCCTCCCCAGCCACCTGCGGCCTGAGACAGTACAGCCAGCCTCAGTTTCGCATCAA
AGGAGGGCTCTTCGCCGACATCGC
>2818
GCCTAGAAACCAAGCCCTTTAGGGGAAATCCCACAGCAAAAAAAGCATTCTAAGGCT
GTTTCTCCACCAGGTTTCCGCCCCACCACCTGACGGTGATCTTGGGCAGAACATACCG
GGTGGTCCCTGGCGAGGAGGAGCAG
>2819
CTCCTGCGTGTTCCTCCCCTCCCGGGGACGCGGCCCTCACGCCAGCTCACTCCTCCTTT
CTCCCTCCCAGCGCTGCTGCAGCTGAAATCGGATTCGTCCCGCTGTGCCCAGGAGAGC
AGCGTGGTCCGCACTGTGTGCCT
>2820
TTGGCTTTCTCTTTCAAAAACGTTTATAAATAACTTTCCCTAAAACCATATGATGGTTCT
CCCCTTTCAGTGTCTCCTTTCTATTCGGAGCGGCTGAAGGAGGCTCATGTCAGACTGTA
CCCATCCAGCCGCTGCACATC
>2821
CTACACATACTGACTTTCTCCAGATAGTCTGCTTTTTTTGCAAACGCCAAACCTGCTATC
TCCTTTGCAGGGCGATTCGGGAGGCCCCCTGGTGTGTCTGAACGATGGCCGCATGACT
TTGGTGGGCATCATCAGCTGGG
>2822
GCTGCAGGCCAGGGAAGGGGGAGGCTCTGGAGCAGTCGGCCTGAGCCTGATAGAGAG
GGGCCTTCTCCAGGTGTCAGGGACGTGGTATCTGAAGGCCATGACGGTGGACAGGGA
GTTCCCTGAGATGAATCTGGAATCGG
>2823
CAGCATGGTTGCTCCAGGGCCGGGGGAGGCACAGCCAGGGCCGCTTTGCCAGGGGGC
TTCTGTTTTCCAGGATAAGTGGCCGGTGCCAGGAGGTGAAGGCCGTCCTGGAGAAAA
CTGACGAGCCGGGAAAATACACGGCC
>2824
CACGCTGTCCCGGGATCCTCTCTGAAGTCCCTGGTGGCTAATTCAGGAATGTGCTGCTG
TCTTTCTGCAGACGGGGGCAAGCACGTGGCATACATCATCAGGTCGCACGTGAAGGAC
CACTACATCTTTTACTGTGAGGG
>2825
TCCAGACTGGGATGGGGTGCCGTCGGCCAGTGCTGCCTCGAGCACCCACATCTCGTCC
TGGCACCCACAGGCAGAGACCCCAAGAACAACCTGGAAGCCTTGGAGGACTTTGAGA
AAGCCGCAGGAGCCCGCGGACTCAG
>2826
CCCCACATTGCATCCCCCTCCCACCCTTGCTGTCCTGGCCTCACTCACCCTCCCCCCCT

TTCCAGGGCAGGGGACACCTTGGCTCCTCAGCAGCCCAAGGACGGCACCATCCAGCA
CCTCCGTCATTCACAGGGACATGG
>2827
ATAGTGTGAATGAAGCCTTCCCAAAAGCAGAAACGGGATTTTGATTCTGGATCTTATTT
TATTGTTCTAGGTTTACTTGAACTTGAAGGAAAGAAAAAAAAATGTCTCCACTTCTGA
GAAGCATCTGTGACATCACTGAA
>2828
CATGTGTGAATATAATTTTACCACCTGTAAATAGTGCATTGTAAAATTTTTGTTTTTCACC
ATTTTATAGAGACCACATGACCCTAAGACGGTAGATCTGATCCTGGAACTTCTGGATCG
TGACAGTAATGGGCGTGTCG
>2829
CAGGGTTCTGGCCCTCCGTGGTCCTGGCTGCTGAGCCCTGACTACCGTGCCCCCCAAC
CCCTGAACACAGAGTTCAAGGCAGCCTTCGACATCTTCGTGCTGGGCGCTGAGGATGG
CTGCATCAGCACCAAGGAGCTGGG
>2830
GGTTCGTGTCGAAGGTGCTGCCTGCAGTACCTACCCTGGCCTCTGTAACGGCCATGCT
GCCCACCCCCAGGCAGCGGCACGGTGGACTTTGATGAGTTCCTGGTCATGATGGTTCG
GTGCATGAAGGACGACAGCAAAGG
>2831
GCCTCCTGGGGCATGGGTGGGGCTGCCTCATGCCCTCCCCACAGCCCTACCCTGAGCC
CCCTCCCCACAGAAATGCTGATGGCTACATCGACCTGGATGAGCTGAAGATAATGCTGC
AGGCTACAGGCGAGACCATCACG
>2832
GGGCTGATCTCCTGCCTCCATGCCCTGCCCAGCCCCTACCCTCAACCCACACCTGCCCC
TCTTTCCACAGAGTTCCTGGAGTTCATGAAGGGTGTGGAGTAGATGCTGACCTTCACC
CAGAGCTGCCTATGCCCAGCCTC
>2833
CTTCTGTTGCAGAGCGCATGCATTTTGACCACAGTGTTTCAGCTCTTCCCTTTTCTCTTG
TTCCATTTAGGTGGCTTCCCACACTTCTGACTCGGACGTTCCTTCCGGTGTCACTGAGG
TGGTCGTGAAGCTCTTTGACT
>2834
AGTTACGGGGGAATTTTATTTTTCCTTTGGAGGATGTTTTACTTTGGAGGATAACTGTTT
TTTATTTCAGGGAGGAGTGAGATGTGGATGTTGCTTTTGCACCTACGGGGGCATCTGAG
TCCAGCTCCCCCCAAGATGAG
>2835
TTCCCTGAAGAAAGTTGGATGGCATGATCTGTCTTCCCATCTTGAAACCGTATGGCAAA
TTGTTTTTCAGATGAATTCCCTCTGCTGACAACCAAACGTGTGTTCTGGAAGGGTGTTT
TGGAGGAGTTGCTGTGGTTTAT
>2836
CTCGGGGGGATCAACTGAGATGGCTTAAGATTTACCTGGATGCCTGCTCTGCTCTCCCC
ATCTCTTCCAGGGATCCACAAATGCTAAAGAGCTGTCTTCCAAGGGAGTGAAAATCTG
GGATGCCAATGGATCCCGAGACT
>2837
CCTCTAAGGCCATCTCATGACATGTGTGATTAATGTATGTACCTGTCCTCTCTTTTTGAC

AATTCTACAGATTATTCAGGACAGGGAGTTGACCAACTGCAAAGAGTGATTGACACCA
TCAAAACCAACCCTGACGACAG
>2838
TTTTACTTTGCCTTTAGCTGTGGTCTTTCAAACCACCATCCCTCCTTATCTTCCTCTGCT
GGTTCCTCAGATCTTCCTCTGATGGCGCTGCCTCCATGCCATGCCCTCTGCCAGTTCTAT
GTGGTGAACAGTGAGCTGTC
>2839
TCTAATGTTTTTAAATGATGTTTTAAAGAATTGAAACTAACATACTGTTCTGCTTTCTCC
CGGTTTATAGCCAGGTGACTTTATACACACTTTGGGAGATGCACATATTTACCTGAATCA
CATCGAGCCACTGAAAATTC
>2840
CACGGACATGAGGAGCAATTACAACAGGTCGTACAATTATGGCAAAATAATGGCCTTAT
TTTGTTTTTAGCTTCAGCGAGAACCCAGACCTTTCCCAAAGCTCAGGATTCTTCGAAA
AGTTGAGAAAATTGATGACTTCA
>2841
GCGGACGCGTGGGCTTCGCCGTCTGGGGGCCGCGGGCTCAACCCTTGCCTCCCACCCT
CCGCGCGGCCAGGTGCCAGAGGACGAGCTGGTGTCCACGCTGAAGCAGCTGGTCTCC
GAGAAGCTGAACGTCCCAGTGCGCC
>2842
GGCGGCCTCGCGCCCCGGGCCACCCCCACGGCGGTGGAGGAGGAGGAACCCATCGAT
CTGTCTTTGGCAGATGGGAAACGACTCTCGGATTATAGCATCGGGCCCAACTCCAAGC
TCAACCTAGTGGTCAAACCCCTGGA
>2843
CTACCTCTGTTGCAGTGTATGCCCCCCCCCCACTGGCAGCCCCTGAATCTTCCTGCCCT
TCTCTCCCCAGGATTACGAGAGGTCCCTGAGTCGCCTGACGCTGGACGACATCGAACG
GTTGGCCAGCCGCTTCCTGCACC
>2844
CGGGAGGCAGGCCTGAGTCCCCCTGCACAGCACCCTCTCTAACCAGGCCCTCTTCCCG
ACTCCTGCCCAGCTGTGCCTGGGCATCCCTCTCTGCCCAGCCTGCCCAGCAACAGCAG
CCAGGAGAGGCCACTGGACACCCG
>2845
TGGGCTTGGCCGCATGCCCCTGTGCCCCACCAGCCATCCTGAACCCAACCTAGATCCTC
CACCTCCACAGGGGCGCCCTTTGTGCTACTCATGTTGCTGGCCAGCCTCAACAGCTGC
ACCAACCCCTGGATCTATGCATC
>2846
ATACATGTACATGTAACATAATATTCATCAAAAGGGGACACATACAAATTTTTCTATTTCT
GCCTCCTAGGCCGGGATTATGAAAAGAATAAAGTCTGCAAGGAATTCTCCCATCTGGG
AAAGGAGGACTTCACATCTCT
>2847
GAGCGAGACTCCGTCTCAAAAAAAAAAACCTTTCAGTTCTAATTGATATATTGTCTTAT
TTTCCCCTCAGGTCACTAGTCCTGTACAGTAGAAAATTTCCCAGTGGCACGTTTGAACA
GGTCAGCCAACTTGTGAAGGAA
>2848
TATTAAATGGAAGTCTAAAAGCCTGGAAATTTACATATATATATGAGTTTCCTTTTTCCTT

CTCCTCCAGACCTCAGCACTGTCTGCCAAGTCCTGTGAAAGTAATTCTCCATTCCCCGT
TCACCCAGGCACTGCTGAGT
>2849
TGTCTCAAATAAAAAAGTAGATTTTGTTTATTCACAAATTTTTTGACCAATTCTATTTTTT
CCTCCACAGATTTATGTGGGAATATTCCACTAATTACGGACAAGCTCCTCTGTCACTTTT
AGTCAGTTACACCAAGAGT
>2850
TATTTTTGCCATTTATACAGTATTCAATGAGTCTTGACCATATAATGAGATTCTTTCACTT
GTTTTCTAGAGACTCCAGCTTAAACATTTATCACTTCTCACCACTCTGTCAAATAGAGT
CTGCTCACAATATGCTGCTT
>2851
GTGCGTTCATCACGAAACCAAGAAAACTGAGAATGTTGATAAGCTAACATATTTATTTT
TAACTGTTTAGCAATCTCATAAAGTTAGCCCAAAAAGTGCCTACTGCTGATCTGGAGGA
TGTTTTGCCACTAGCTGAAGAT
>2852
TATGTAATTCTTATTTTTAATTAGACTATCAGGAAACTAACAATGCACAAACTAACCATT
CGTTTTGTAGCTGCCTGAACACACAGTAAAACTCTGTGACAATTTATCCACAAAGAATT
CTAAGTTTGAAGACTGTTGTC
>2853
AAATAAATAAATAAATAAATAAATAAATAGGAAATATATAACACATATCTCCTTTTCTCCC
TCATGCTAGGTATACATTTGAACTAAGCAGAAGGACTCATCTTCCGGAAGTATTCCTCA
GTAAGGTACTTGAGCCAACC
>2854
GATTTAATGGCTTGATGGGACAAGGTTTCAAGACAAATAATTTTGTTATGCAATTATTGT
TTTCTTACAGGGCCCTCTACTAAAGAAGGAACTATCTTCTTTCATTGACAAGGGACAAG
AACTATGTGCAGATTATTCAG
>2855
TTATTTTGCATTAGAAATTTGTATAAAATAAATACATGTAGTAAGACCTTACATTTAAATG
GTTTTTCAGACTGGCAGAGCGACTAAAAGCAAAATTGCCTGAGGCCACACCCACGGA
ACTGGCAAAGCTGGTTAACAAG
>2856
GAGCAATATTATGGTTGTTGCCCATTAGCTCTGTTTTTCTAACATAAAATTCATTTCATTA
TTCTCATAGACCAGAGTTGGAGCCACCCAGGGGAATGATCTCTGATGACCTAACCTAA
GCAAAACCACTGAGCTTCTGG
>2857
TCGCTGCGTTCCCCTCCAACCCCTCGACTCCCGGCTCCCCTCCTCCCGCTCACCCCGCC
CGTCCCCGCAGTGCCTCCCCTGCGGCCCCGGGGGCAAAGGCCGCTGCTTCGGGCCCA
GCATCTGCTGCGCGGACGAGCTGG
>2858
AGGGTGTGGGCCCCCCGCACCCCGAGCTGCGCCCGCCCCAGGCGCCCGTGCTCACAC
GTCCTCCCGGCAGAGAGCTGCGTGACCGAGCCCGAGTGCCGCGAGGGCTTTCACCGC
CGCGCCCGCGCCAGCGACCGGAGCAA
>2859
TCCCAGAAAGCTATAAATCCAAGCTGGCTTCTCCCTCCCCACACAGCTGCTCCTGCTCT

CCCTCCTCCAGGTCACCCCAGCCATGAGGATTATCGCCCTCCTCGCTGCTATTCTCTTG
GTAGCCCTCCAGGTCCGGGCAG
>2860
TCCAAGATTCCCTTTACCACCCACCGCTGACCTTGGTGCTTAATTTCTCAGTCTTCCTCT
GTGTTCCCAGGCTCAACAAGGGGCATGGTCTGCTCTTGCAGATTAGTATTCTGCCGGCG
AACAGAACTTCGTGTTGGGAA
>2861
ACGTAATTTACCAGGCATGGGTTTTCCCAGAGGAGGGGGCTGGTTCATGGTTTTGGTTT
TGGTTTTCCAGAAGATTTTGTGATTCAGGCAAAGGCTGACTGTTACTTCACCAACGGG
ACAGAAAAGGTGCAGTTTGTGGT
>2862
TCCCTAAAGAATCTCCTGTCCCAGCTTGGTGGTTTTCTTTCACGGTGTCTCATTTTTCCT
CCCTTCCTAGTGCAACCAGAGGTGACAGTGTACCCAGAGAGGACCCCACTCCTGCACC
AGCATAATCTGCTGCACTGCTC
>2863
TTCTGGGATTCCAAGAAAGTGCTTTAAATATTGGGATATTTTTACTAATTTAAAGACCTG
TTTCCCATAGGAGCTCAGTCTGAATATTCTTGGAGAAAGATGCTGAGTGGCATTGCAGC
CTTCCTACTTGGGCTAATCTT
>2864
CCTGAGTCTCTGGAACGGCTGTGGGGGGTGGTGAGGCTGCCTCCTGAGACCTTCATCA
CTGTGCCTCCAGGTCTCAAGAGCTGTTCTGCTCCCTCAGTCATGCTAAGGTCCTCACTG
AAGCTTCTCTCTCTGGAGCCTGA
>2865
GGGGGCGCAAGACCCGGGAAGCCGCGCCGGGAGGAGGGTCGGGCGGGTCTCAGCCA
CTCCTCGTCCCCAGGCTCCCACTCCATGAGGTATTTCTCCACATCCGTGTCCCGGCCCG
GCCGCGGGGAGCCCCGCTTCATCGC
>2866
AAAATCCCCCCGGGTTGGTCGGGGCCGGGCGGGGCTCGGGGGACTGGGCTGACCGCG
GGGTCGGGGCCAGGTTCTCACACCCTCCAGATGATGTTTGGCTGCGACGTGGGGTCGG
ACGGGCGCTTCCTCCGCGGGTACCA
>2867
CCACATGCATGCTGGTGGAGTGTCCCATGACAGATGCAAAATGCCTGAATTTTCTGACT
CTTCCCGTCAGACCCCCCCAAGACACATATGACCCACCACCCCATCTCTGACCATGAG
GCCACTCTGAGATGCTGGGCCCT
>2868
AGGGAAAGCAGGAGCCTCTCTGGAGACCTTTAGCAGGGTCAGGGCCCCTCACCTTCC
CCTCTTTTCCCAGAGCCATCTTCCCAGCCCACCGTCCCCATCGTGGGCATCATTGCTGG
CCTGGTTCTCCTTGGAGCTGTGAT
>2869
CCCCCACACAGGGAAGGGCTGGGCCCAGGCCTGGGGCTGCTTCCTGGTCCTGACCTG
GCACCTGCCCCAGCCCCAGGCCAGGCCCTGCAGCGAGTGGGCATCGTCGGGGGTCAG
GAGGCCCCCAGGAGCAAGTGGCCCTG
>2870
ATGTGAGCCCTGGCTCCCGGGTGCTCCTGGGGGCTGCCCAGGGCCCTGAGTGGGATCC

TCCGCTGCCCAGGGACGTCAAGGATCTGGCCGCCCTCAGGGTGCAACTGCGGGAGCA
GCACCTCTACTACCAGGACCAGCTG
>2871
GGTCCCTCTGGGCTCCAGGTGGGGGTTGCCCGGCCCCCTCCTGAGGCTGCACCCTCTT
CCCCACCTGCAGAGCGCCTCCCACCGCCATTTCCTCTGAAGCAGGTGAAGGTCCCCAT
AATGGAAAACCACATTTGTGACGC
>2872
CCCCCGCCCCCCGCCCCCCAACCCCCACTCCCAGGCCTGTTCGGCGAGCGCTGACCTC
TGACCTTCCCAGGGCGACTCCGGAGGGCCCCTGGTGTGCAAGGTGAATGGCACCTGG
CTGCAGGCGGGCGTGGTCAGCTGGG
>2873
CAGATGCCCAATGGGCCTGCCTGGCATCTCCCACACTGCTGTCCCTGGACACATACCTT
TTTGCCCCCAGACATTATCCGGCAGCCCTCTGAGGAAGAGATCATCAAATTGGCTCCCC
CACCGAAGAAGGCCTGAGCAAG
>2874
CGTTTTTGACAGTTTGACAGTTAAAGGCATTTCCCTGTGAAATAATACTGGTATGTATTT
AACCATGCAGATCCTCAGTTTGTGGTCTGCCAGCTAAAGGTGAAGATATATTCCTCCAA
TTCAGGACCCACACGACGGGA
>2875
TAAAAAAATAAAACATCATTAATTAAATATGTCATTTCATTTCTTTTTCTTTTCTTTTTTTT
TTTTTTAGGACAAAATGTTTCACTTTTGGGTAAATACATTCTTCATACCAGGACCAGAG
GAAACCTCAGAAAAAGTAG
>2876
AAATATATTTCACTAAATAGTTAAGATGAGTCATATTTGTGGGTTTTCATTTTAAATTTTC
TTTCTCTAGGTGAAGCTGTACTTCACAAAAACAGTAGAGGAGCCGTCAAATCCAGAGG
CTAGCAGTTCAACTTCTGTAA
>2877
AACCAACCAGGAGGCTGGGGTACGCTGGAGAAGGAATGGGCTTCCTAACCTTGAGCC
CTCTTCCCTGAAGATATATGTATCTACGGGGGCCTGGGGCTGGGCGGGCTCCTGCTTCT
GGCAGTGGTCCTTCTGTCCGCCTG
>2878
CCCACCCATCCATCTATCCCTGCGGCCCCCCTCTGCCCGCTCCTCTGACCAACACCTGC
TTTGTCTGCAGGCACCACAGTGCTGTTGCCCCTGGTCATTTTCTTTGGTCTTTGCCTTTT
ATCCCTCCTCTTCATTGGTTT
>2879
CACGTAGAATAAAGAGGGCCGGGGCTGGTTTTCGCTGCCGCACTAATCTGCGCCACCT
TCTCTCTTTCAGGGGGAGCTTGAAGGAACTACTACTAAGCCCCTGGCCCCAAACCCAA
GCTTCAGTCCCACTCCAGGCTTCA
>2880
GCGGAGGGCGCGGGATGCGGGGCGGGGCCTGGGGTTGCCGCCCGAGGCTCACCGGCC
CGCGTCCCCGCAGCTGATGACCCCGCGACGCTGTACGCCGTGGTGGAGAACGTGCCCC
CGTTGCGCTGGAAGGAATTCGTGCG

$\mathbf{S}_2^-$: 2,800 false splice acceptor site containing sequences

>1
CATTCTGGATGATGCAGCGAACAATCCTCTCTACTGTGTCCTCATTCATGTCATTCGTCC
TCAGCTGAAGGATGAAAGAGATTGGCAAGGAAGCAGGAATGAGTCAGCTTTGGGGGA
ATATTACCTGGTGGCTCCCATCA
>2
ATGATGCAGCGAACAATCCTCTCTACTGTGTCCTCATTCATGTCATTCGTCCTCAGCTGA
AGGATGAAAGAGATTGGCAAGGAAGCAGGAATGAGTCAGCTTTGGGGGAATATTACCT
GGTGGCTCCCATCATTTATTGC
>3
CTCTCTACTGTGTCCTCATTCATGTCATTCGTCCTCAGCTGAAGGATGAAAGAGATTGG
CAAGGAAGCAGGAATGAGTCAGCTTTGGGGGAATATTACCTGGTGGCTCCCATCATTTA
TTGCTTTAGGACATACCCTTTT
>4
GGCAAGGAAGCAGGAATGAGTCAGCTTTGGGGGAATATTACCTGGTGGCTCCCATCAT
TTATTGCTTTAGGACATACCCTTTTACCTACTTGCTATCATGCTGGACCTCAGCATTTCA
GGGACCAGAAAAGATGGGGACA
>5
CTGGTGGCTCCCATCATTTATTGCTTTAGGACATACCCTTTTACCTACTTGCTATCATGCT
GGACCTCAGCATTTCAGGGACCAGAAAAGATGGGGACACCAATGCAAGCCCAAGACT
ACCATCCAGAGGACCGTTCCTG
>6
TCCCATCATTTATTGCTTTAGGACATACCCTTTTACCTACTTGCTATCATGCTGGACCTCA
GCATTTCAGGGACCAGAAAAGATGGGGACACCAATGCAAGCCCAAGACTACCATCCA
GAGGACCGTTCCTGCCCATGCT
>7
TTTACCTACTTGCTATCATGCTGGACCTCAGCATTTCAGGGACCAGAAAAGATGGGGAC
ACCAATGCAAGCCCAAGACTACCATCCAGAGGACCGTTCCTGCCCATGCTGCTTTCTCT
AGTTTTGCTTTCAGAGTGGTGT
>8
TGCTGGACCTCAGCATTTCAGGGACCAGAAAAGATGGGGACACCAATGCAAGCCCAA
GACTACCATCCAGAGGACCGTTCCTGCCCATGCTGCTTTCTCTAGTTTTGCTTTCAGAG
TGGTGTCACCCCACTTGCTAACTC
>9
CTGGACCTCAGCATTTCAGGGACCAGAAAAGATGGGGACACCAATGCAAGCCCAAGA
CTACCATCCAGAGGACCGTTCCTGCCCATGCTGCTTTCTCTAGTTTTGCTTTCAGAGTG
GTGTCACCCCACTTGCTAACTCAA
>10
GATGGGGACACCAATGCAAGCCCAAGACTACCATCCAGAGGACCGTTCCTGCCCATGC
TGCTTTCTCTAGTTTTGCTTTCAGAGTGGTGTCACCCCACTTGCTAACTCAAGAGTGCA
TCACTAGGCTTCCTGAGCAGGTT
>11
ACCGTTCCTGCCCATGCTGCTTTCTCTAGTTTTGCTTTCAGAGTGGTGTCACCCCACTT
GCTAACTCAAGAGTGCATCACTAGGCTTCCTGAGCAGGTTTGATCAGGATTTGGCCTG
GGTCTGTTGGTCAGGGAGGCAGA

>12
CGTTCCTGCCCATGCTGCTTTCTCTAGTTTTGCTTTCAGAGTGGTGTCACCCCACTTGCT
AACTCAAGAGTGCATCACTAGGCTTCCTGAGCAGGTTTGATCAGGATTTGGCCTGGGT
CTGTTGGTCAGGGAGGCAGAGG
>13
TTCAGAGTGGTGTCACCCCACTTGCTAACTCAAGAGTGCATCACTAGGCTTCCTGAGC
AGGTTTGATCAGGATTTGGCCTGGGTCTGTTGGTCAGGGAGGCAGAGGTGTGGGGAG
AAGGGTGAAAGGTGACTGAGCCAGG
>14
TAACTCAAGAGTGCATCACTAGGCTTCCTGAGCAGGTTTGATCAGGATTTGGCCTGGG
TCTGTTGGTCAGGGAGGCAGAGGTGTGGGGAGAAGGGTGAAAGGTGACTGAGCCAG
GGTTCACAGGCTCCTCACTCACGGGG
>15
TCAAGAGTGCATCACTAGGCTTCCTGAGCAGGTTTGATCAGGATTTGGCCTGGGTCTGT
TGGTCAGGGAGGCAGAGGTGTGGGGAGAAGGGTGAAAGGTGACTGAGCCAGGGTTC
ACAGGCTCCTCACTCACGGGGCTGA
>16
ATCAGGATTTGGCCTGGGTCTGTTGGTCAGGGAGGCAGAGGTGTGGGGAGAAGGGTG
AAAGGTGACTGAGCCAGGGTTCACAGGCTCCTCACTCACGGGGCTGAAGCTGGGCAT
CGTCAGGCTCAGAGCCCCTAGACGTG
>17
GGATTTGGCCTGGGTCTGTTGGTCAGGGAGGCAGAGGTGTGGGGAGAAGGGTGAAAG
GTGACTGAGCCAGGGTTCACAGGCTCCTCACTCACGGGGCTGAAGCTGGGCATCGTC
AGGCTCAGAGCCCCTAGACGTGCGGC
>18
CTGGGTCTGTTGGTCAGGGAGGCAGAGGTGTGGGGAGAAGGGTGAAAGGTGACTGA
GCCAGGGTTCACAGGCTCCTCACTCACGGGGCTGAAGCTGGGCATCGTCAGGCTCAG
AGCCCCTAGACGTGCGGCAGCGGTAGC
>19
AGAGGTGTGGGGAGAAGGGTGAAAGGTGACTGAGCCAGGGTTCACAGGCTCCTCACT
CACGGGGCTGAAGCTGGGCATCGTCAGGCTCAGAGCCCCTAGACGTGCGGCAGCGGT
AGCCCTTCTTCTTGAGCACGTGGCAG
>20
GAAAGGTGACTGAGCCAGGGTTCACAGGCTCCTCACTCACGGGGCTGAAGCTGGGCA
TCGTCAGGCTCAGAGCCCCTAGACGTGCGGCAGCGGTAGCCCTTCTTCTTGAGCACGT
GGCAGATCATGAAGCCTACAGGCCC
>21
AAGGTGACTGAGCCAGGGTTCACAGGCTCCTCACTCACGGGGCTGAAGCTGGGCATC
GTCAGGCTCAGAGCCCCTAGACGTGCGGCAGCGGTAGCCCTTCTTCTTGAGCACGTGG
CAGATCATGAAGCCTACAGGCCCAT
>22
CTGAGCCAGGGTTCACAGGCTCCTCACTCACGGGGCTGAAGCTGGGCATCGTCAGGC
TCAGAGCCCCTAGACGTGCGGCAGCGGTAGCCCTTCTTCTTGAGCACGTGGCAGATCA
TGAAGCCTACAGGCCCATGAGGAAG

>23
TTCACAGGCTCCTCACTCACGGGGCTGAAGCTGGGCATCGTCAGGCTCAGAGCCCCTA
GACGTGCGGCAGCGGTAGCCCTTCTTCTTGAGCACGTGGCAGATCATGAAGCCTACAG
GCCCATGAGGAAGAAGACCAGCAC
>24
GGGGCTGAAGCTGGGCATCGTCAGGCTCAGAGCCCCTAGACGTGCGGCAGCGGTAGC
CCTTCTTCTTGAGCACGTGGCAGATCATGAAGCCTACAGGCCCATGAGGAAGAAGACC
AGCACAAGCAGGAAGAGCATATATA
>25
GTCAGGCTCAGAGCCCCTAGACGTGCGGCAGCGGTAGCCCTTCTTCTTGAGCACGTGG
CAGATCATGAAGCCTACAGGCCCATGAGGAAGAAGACCAGCACAAGCAGGAAGAGCA
TATATAGCCCATGTTGGGGCGGTTC
>26
TCAGAGCCCCTAGACGTGCGGCAGCGGTAGCCCTTCTTCTTGAGCACGTGGCAGATCA
TGAAGCCTACAGGCCCATGAGGAAGAAGACCAGCACAAGCAGGAAGAGCATATATAG
CCCATGTTGGGGCGGTTCCAGGTCA
>27
CTAGACGTGCGGCAGCGGTAGCCCTTCTTCTTGAGCACGTGGCAGATCATGAAGCCTA
CAGGCCCATGAGGAAGAAGACCAGCACAAGCAGGAAGAGCATATATAGCCCATGTTG
GGGCGGTTCCAGGTCAGGCTGTGGT
>28
CAGCGGTAGCCCTTCTTCTTGAGCACGTGGCAGATCATGAAGCCTACAGGCCCATGAG
GAAGAAGACCAGCACAAGCAGGAAGAGCATATATAGCCCATGTTGGGGCGGTTCCAG
GTCAGGCTGTGGTTCCGACATGAGG
>29
ACAGGCCCATGAGGAAGAAGACCAGCACAAGCAGGAAGAGCATATATAGCCCATGTT
GGGGCGGTTCCAGGTCAGGCTGTGGTTCCGACATGAGGCCCTGGGGGGCGAGCGCGG
GCCAGGGCGCCTCCTGGGTTTAGGGG
>30
CCCATGAGGAAGAAGACCAGCACAAGCAGGAAGAGCATATATAGCCCATGTTGGGGC
GGTTCCAGGTCAGGCTGTGGTTCCGACATGAGGCCCTGGGGGGCGAGCGCGGGCCAG
GGCGCCTCCTGGGTTTAGGGGGCTGC
>31
GCACAAGCAGGAAGAGCATATATAGCCCATGTTGGGGCGGTTCCAGGTCAGGCTGTGG
TTCCGACATGAGGCCCTGGGGGGCGAGCGCGGGCCAGGGCGCCTCCTGGGTTTAGGG
GGCTGCAGCTGGGAATGGGGGAGGG
>32
CCCATGTTGGGGCGGTTCCAGGTCAGGCTGTGGTTCCGACATGAGGCCCTGGGGGGCG
AGCGCGGGCCAGGGCGCCTCCTGGGTTTAGGGGGCTGCAGCTGGGAATGGGGGAGGG
GTAAGAATGCCGTCCTCAGGATCTG
>33
CAGGTCAGGCTGTGGTTCCGACATGAGGCCCTGGGGGGCGAGCGCGGGCCAGGGCGC
CTCCTGGGTTTAGGGGGCTGCAGCTGGGAATGGGGGAGGGGTAAGAATGCCGTCCTC
AGGATCTGAAACAAGCACGTCTAAGA

>34
TGTGGTTCCGACATGAGGCCCTGGGGGGCGAGCGCGGGCCAGGGCGCCTCCTGGGTT
TAGGGGGCTGCAGCTGGGAATGGGGGAGGGGTAAGAATGCCGTCCTCAGGATCTGAA
ACAAGCACGTCTAAGACGTCCGGCTG
>35
GGCCAGGGCGCCTCCTGGGTTTAGGGGGCTGCAGCTGGGAATGGGGGAGGGGTAAGA
ATGCCGTCCTCAGGATCTGAAACAAGCACGTCTAAGACGTCCGGCTGAGGTCCGGCCC
CACCCCCTACCCAACCATGGGAGCT
>36
CCTGGGTTTAGGGGGCTGCAGCTGGGAATGGGGGAGGGGTAAGAATGCCGTCCTCAG
GATCTGAAACAAGCACGTCTAAGACGTCCGGCTGAGGTCCGGCCCCACCCCCTACCCA
ACCATGGGAGCTGTCCGGGCCAACA
>37
GGGGGCTGCAGCTGGGAATGGGGGAGGGGTAAGAATGCCGTCCTCAGGATCTGAAAC
AAGCACGTCTAAGACGTCCGGCTGAGGTCCGGCCCCACCCCCTACCCAACCATGGGA
GCTGTCCGGGCCAACAGTGCTGTTTC
>38
AGGATCTGAAACAAGCACGTCTAAGACGTCCGGCTGAGGTCCGGCCCCACCCCCTACC
CAACCATGGGAGCTGTCCGGGCCAACAGTGCTGTTTCGCCTTTCGGCAAGAGGTCAGC
GACAACGCAAGCTCTCTACCACGA
>39
ACGTCTAAGACGTCCGGCTGAGGTCCGGCCCCACCCCCTACCCAACCATGGGAGCTGT
CCGGGCCAACAGTGCTGTTTCGCCTTTCGGCAAGAGGTCAGCGACAACGCAAGCTCT
CTACCACGACCATGGTCCGCTTCCC
>40
GTCCGGCCCCACCCCCTACCCAACCATGGGAGCTGTCCGGGCCAACAGTGCTGTTTCG
CCTTTCGGCAAGAGGTCAGCGACAACGCAAGCTCTCTACCACGACCATGGTCCGCTTC
CCCGACGCCCCGAGCGTTCCCCTT
>41
CCAACCATGGGAGCTGTCCGGGCCAACAGTGCTGTTTCGCCTTTCGGCAAGAGGTCAG
CGACAACGCAAGCTCTCTACCACGACCATGGTCCGCTTCCCCGACGCCCCGAGCGTTC
CCCTTTCCAGCTTGTTCGCGCTTC
>42
TTTCGGCAAGAGGTCAGCGACAACGCAAGCTCTCTACCACGACCATGGTCCGCTTCCC
CGACGCCCCGAGCGTTCCCCTTTCCAGCTTGTTCGCGCTTCTGTCCCTCCCAAGGACA
CTGCAGCACGAGATGGGACGGCAT
>43
AGCGACAACGCAAGCTCTCTACCACGACCATGGTCCGCTTCCCCGACGCCCCGAGCGT
TCCCCTTTCCAGCTTGTTCGCGCTTCTGTCCCTCCCAAGGACACTGCAGCACGAGATG
GGACGGCATTCTTAGGCGGCGGGA
>44
CCATGGTCCGCTTCCCCGACGCCCCGAGCGTTCCCCTTTCCAGCTTGTTCGCGCTTCTG
TCCCTCCCAAGGACACTGCAGCACGAGATGGGACGGCATTCTTAGGCGGCGGGAGCA
GAAATCCTGCCTGAGTCAGCGCCC

>45
CTTCCCCGACGCCCCGAGCGTTCCCCTTTCCAGCTTGTTCGCGCTTCTGTCCCTCCCAA
GGACACTGCAGCACGAGATGGGACGGCATTCTTAGGCGGCGGGAGCAGAAATCCTGC
CTGAGTCAGCGCCCGCACCTCCTC
>46
CCTTTCCAGCTTGTTCGCGCTTCTGTCCCTCCCAAGGACACTGCAGCACGAGATGGGA
CGGCATTCTTAGGCGGCGGGAGCAGAAATCCTGCCTGAGTCAGCGCCCGCACCTCCTC
CACGCCGGCCTGAGCCAGGCCTCT
>47
TTGTTCGCGCTTCTGTCCCTCCCAAGGACACTGCAGCACGAGATGGGACGGCATTCTT
AGGCGGCGGGAGCAGAAATCCTGCCTGAGTCAGCGCCCGCACCTCCTCCACGCCGGC
CTGAGCCAGGCCTCTGCAGCCCCTT
>48
CCTCCCAAGGACACTGCAGCACGAGATGGGACGGCATTCTTAGGCGGCGGGAGCAGA
AATCCTGCCTGAGTCAGCGCCCGCACCTCCTCCACGCCGGCCTGAGCCAGGCCTCTGC
AGCCCCTTCCTCTCAGTACTCCCGC
>49
GCATTCTTAGGCGGCGGGAGCAGAAATCCTGCCTGAGTCAGCGCCCGCACCTCCTCCA
CGCCGGCCTGAGCCAGGCCTCTGCAGCCCCTTCCTCTCAGTACTCCCGCACCCCTCTC
CCTTTTTGGTTTCTCCCACCCTGA
>50
TCTTAGGCGGCGGGAGCAGAAATCCTGCCTGAGTCAGCGCCCGCACCTCCTCCACGCC
GGCCTGAGCCAGGCCTCTGCAGCCCCTTCCTCTCAGTACTCCCGCACCCCTCTCCCTTT
TTGGTTTCTCCCACCCTGACCAC
>51
CGGGAGCAGAAATCCTGCCTGAGTCAGCGCCCGCACCTCCTCCACGCCGGCCTGAGC
CAGGCCTCTGCAGCCCCTTCCTCTCAGTACTCCCGCACCCCTCTCCCTTTTTGGTTTCT
CCCACCCTGACCACCTGGCCGACC
>52
TGACCACCTGGCCGACCCGGTACCGGTGATTTGGTTCTGGTTCCGGCTTCTGCTGGGG
TTCCGGCTCCAGGGGCGTCCTAGTGCGGCTCAGGGCCCCCGACGCCCTTCCGGCGCTC
ATCCCTTGATTCATCCCCTCCCCC
>53
CCGACCCGGTACCGGTGATTTGGTTCTGGTTCCGGCTTCTGCTGGGGTTCCGGCTCCAG
GGGCGTCCTAGTGCGGCTCAGGGCCCCCGACGCCCTTCCGGCGCTCATCCCTTGATTCA
TCCCCTCCCCCTTCGCCGCCTC
>54
GATTCATCCCCTCCCCCTTCGCCGCCTCACCGGCCCCGGGCTGTGGCTGCGGATACCCG
CGCCGTGGCAGGCGGGGGAGGGAAGAACAGAGAAGGATGCGGGCTGGCTGAGAGC
GGTGGCAGGCTCCGGGCAACTCCGG
>55
CTCCCCCTTCGCCGCCTCACCGGCCCCGGGCTGTGGCTGCGGATACCCGCGCCGTGGC
AGGCGGGGGAGGGAAGAACAGAGAAGGATGCGGGCTGGCTGAGAGCGGTGGCAGG
CTCCGGGCAACTCCGGCCCTGGGTCA

>56
GTGGCTGCGGATACCCGCGCCGTGGCAGGCGGGGGGAGGGAAGAACAGAGAAGGAT
GCGGGCTGGCTGAGAGCGGTGGCAGGCTCCGGGCAACTCCGGCCCTGGGTCAGGGAG
ATTGCCTCCGCAGCCGCCCTGGCTTCC
>57
GGCTGCGGATACCCGCGCCGTGGCAGGCGGGGGGAGGGAAGAACAGAGAAGGATGC
GGGCTGGCTGAGAGCGGTGGCAGGCTCCGGGCAACTCCGGCCCTGGGTCAGGGAGAT
TGCCTCCGCAGCCGCCCTGGCTTCCCA
>58
GAAGAACAGAGAAGGATGCGGGCTGGCTGAGAGCGGTGGCAGGCTCCGGGCAACTC
CGGCCCTGGGTCAGGGAGATTGCCTCCGCAGCCGCCCTGGCTTCCCAAATGCCTTCAC
CCGTCGCGGGCCCAGGACCAGGGGGC
>59
GCGGGCTGGCTGAGAGCGGTGGCAGGCTCCGGGCAACTCCGGCCCTGGGTCAGGGAG
ATTGCCTCCGCAGCCGCCCTGGCTTCCCAAATGCCTTCACCCGTCGCGGGCCCAGGAC
CAGGGGGCGGAGCGCAGGTGTGCGG
>60
CCCTGGGTCAGGGAGATTGCCTCCGCAGCCGCCCTGGCTTCCCAAATGCCTTCACCCG
TCGCGGGCCCAGGACCAGGGGGCGGAGCGCAGGTGTGCGGGGCGGAGAGCGTGAGC
TCTGCGCGCGCGTGACTGCGCCGGGC
>61
ATTGCCTCCGCAGCCGCCCTGGCTTCCCAAATGCCTTCACCCGTCGCGGGCCCAGGAC
CAGGGGGCGGAGCGCAGGTGTGCGGGGCGGAGAGCGTGAGCTCTGCGCGCGCGTGA
CTGCGCCGGGCCCCCCCGGGGCAATG
>62
CTCCGCAGCCGCCCTGGCTTCCCAAATGCCTTCACCCGTCGCGGGCCCAGGACCAGGG
GGCGGAGCGCAGGTGTGCGGGGCGGAGAGCGTGAGCTCTGCGCGCGCGTGACTGCG
CCGGGCCCCCCCGGGGCAATGTGCGG
>63
GGCTTCCCAAATGCCTTCACCCGTCGCGGGCCCAGGACCAGGGGGCGGAGCGCAGGT
GTGCGGGGCGGAGAGCGTGAGCTCTGCGCGCGCGTGACTGCGCCGGGCCCCCCCGGG
GCAATGTGCGGCGCCGAGTTTGGGTC
>64
GGGCGGAGAGCGTGAGCTCTGCGCGCGCGTGACTGCGCCGGGCCCCCCCGGGGCAAT
GTGCGGCGCCGAGTTTGGGTCCGGGTAGGGGACAGGCGGTCCGGCGTGGGTGTTGTC
CACTGGTCTATCCGGTACGCCTGGCG
>65
GCTCTGCGCGCGCGTGACTGCGCCGGGCCCCCCCGGGGCAATGTGCGGCGCCGAGTT
TGGGTCCGGGTAGGGGACAGGCGGTCCGGCGTGGGTGTTGTCCACTGGTCTATCCGGT
ACGCCTGGCGCAGCTCCTGCCCTCA
>66
GGGTCCGGGTAGGGGACAGGCGGTCCGGCGTGGGTGTTGTCCACTGGTCTATCCGGTA
CGCCTGGCGCAGCTCCTGCCCTCAGATGCCCCGACCGAGCGGAGAGTCAGGAATCTTC
TCCGAGGGCTGGGGGAAGCCGGCT

>67
GGACAGGCGGTCCGGCGTGGGTGTTGTCCACTGGTCTATCCGGTACGCCTGGCGCAGC
TCCTGCCCTCAGATGCCCCGACCGAGCGGAGAGTCAGGAATCTTCTCCGAGGGCTGGG
GGAAGCCGGCTTTCCGCACCTTTG
>68
GCGTGGGTGTTGTCCACTGGTCTATCCGGTACGCCTGGCGCAGCTCCTGCCCTCAGATG
CCCCGACCGAGCGGAGAGTCAGGAATCTTCTCCGAGGGCTGGGGGAAGCCGGCTTTC
CGCACCTTTGAGGGACGGAGTGGC
>69
GGTGTTGTCCACTGGTCTATCCGGTACGCCTGGCGCAGCTCCTGCCCTCAGATGCCCCG
ACCGAGCGGAGAGTCAGGAATCTTCTCCGAGGGCTGGGGGAAGCCGGCTTTCCGCAC
CTTTGAGGGACGGAGTGGCGGGAT
>70
CCGGTACGCCTGGCGCAGCTCCTGCCCTCAGATGCCCCGACCGAGCGGAGAGTCAGG
AATCTTCTCCGAGGGCTGGGGGAAGCCGGCTTTCCGCACCTTTGAGGGACGGAGTGG
CGGGATCTTCGGAGCTTCCTAGAGAA
>71
GCCCCGACCGAGCGGAGAGTCAGGAATCTTCTCCGAGGGCTGGGGGAAGCCGGCTTT
CCGCACCTTTGAGGGACGGAGTGGCGGGATCTTCGGAGCTTCCTAGAGAAGTGACCA
CGGCCTGCGCCCGGTTTGGGGCAAGC
>72
CGAGCGGAGAGTCAGGAATCTTCTCCGAGGGCTGGGGGAAGCCGGCTTTCCGCACCT
TTGAGGGACGGAGTGGCGGGATCTTCGGAGCTTCCTAGAGAAGTGACCACGGCCTGC
GCCCGGTTTGGGGCAAGCGGCTGGAT
>73
ATCTTCTCCGAGGGCTGGGGGAAGCCGGCTTTCCGCACCTTTGAGGGACGGAGTGGC
GGGATCTTCGGAGCTTCCTAGAGAAGTGACCACGGCCTGCGCCCGGTTTGGGGCAAG
CGGCTGGATGGGATGGCTCGGCTTCC
>74
GGGACGGAGTGGCGGGATCTTCGGAGCTTCCTAGAGAAGTGACCACGGCCTGCGCCC
GGTTTGGGGCAAGCGGCTGGATGGGATGGCTCGGCTTCCCGAGGATCTGACCCCCGGC
CGGAGACAAATGGCCCTCGCATCCT
>75
CTAGAGAAGTGACCACGGCCTGCGCCCGGTTTGGGGCAAGCGGCTGGATGGGATGGC
TCGGCTTCCCGAGGATCTGACCCCCGGCCGGAGACAAATGGCCCTCGCATCCTAGGAG
CTATGCTGCCAGTTCCCTGCCCAAC
>76
TGCGCCCGGTTTGGGGCAAGCGGCTGGATGGGATGGCTCGGCTTCCCGAGGATCTGAC
CCCCGGCCGGAGACAAATGGCCCTCGCATCCTAGGAGCTATGCTGCCAGTTCCCTGCC
CAACCCGGGACGCTCAAAGCCCCA
>77
GCTGGATGGGATGGCTCGGCTTCCCGAGGATCTGACCCCCGGCCGGAGACAAATGGCC
CTCGCATCCTAGGAGCTATGCTGCCAGTTCCCTGCCCAACCCGGGACGCTCAAAGCCC
CAGATACCGGGAGAGCTGAGCCGG

>78
TCGGCTTCCCGAGGATCTGACCCCCGGCCGGAGACAAATGGCCCTCGCATCCTAGGAG
CTATGCTGCCAGTTCCCTGCCCAACCCGGGACGCTCAAAGCCCCAGATACCGGGAGAG
CTGAGCCGGGCGAGGGGCGGAGCT
>79
CGGAGACAAATGGCCCTCGCATCCTAGGAGCTATGCTGCCAGTTCCCTGCCCAACCCG
GGACGCTCAAAGCCCCAGATACCGGGAGAGCTGAGCCGGGCGAGGGGCGGAGCTCCC
CTGGGGGCGTGGCCTGGGTGTGGAT
>80
CAAATGGCCCTCGCATCCTAGGAGCTATGCTGCCAGTTCCCTGCCCAACCCGGGACGC
TCAAAGCCCCAGATACCGGGAGAGCTGAGCCGGGCGAGGGGCGGAGCTCCCCTGGGG
GCGTGGCCTGGGTGTGGATGGAGGC
>81
TCGCATCCTAGGAGCTATGCTGCCAGTTCCCTGCCCAACCCGGGACGCTCAAAGCCCC
AGATACCGGGAGAGCTGAGCCGGGCGAGGGGCGGAGCTCCCCTGGGGGCGTGGCCTG
GGTGTGGATGGAGGCGGGGCCGCAA
>82
AGTTCCCTGCCCAACCCGGGACGCTCAAAGCCCCAGATACCGGGAGAGCTGAGCCGG
GCGAGGGGCGGAGCTCCCCTGGGGGCGTGGCCTGGGTGTGGATGGAGGCGGGGCCGC
AACGCCCAGTTCCGCAGAGGCCGGCT
>83
AGATACCGGGAGAGCTGAGCCGGGCGAGGGGCGGAGCTCCCCTGGGGGCGTGGCCTG
GGTGTGGATGGAGGCGGGGCCGCAACGCCCAGTTCCGCAGAGGCCGGCTACTTGCTT
GCGCAGCACGCACGGCCGGGGAGGGG
>84
CCGGGCGAGGGGCGGAGCTCCCCTGGGGGCGTGGCCTGGGTGTGGATGGAGGCGGG
GCCGCAACGCCCAGTTCCGCAGAGGCCGGCTACTTGCTTGCGCAGCACGCACGGCCG
GGGAGGGGCTGGCGGCGGCCGCGGGCG
>85
TGGCCTGGGTGTGGATGGAGGCGGGGCCGCAACGCCCAGTTCCGCAGAGGCCGGCTA
CTTGCTTGCGCAGCACGCACGGCCGGGGAGGGGCTGGCGGCGGCCGCGGGCGGCGG
GCGGCGGAGTCGCGGGGCCTGCTCCCG
>86
GAGGCGGGGCCGCAACGCCCAGTTCCGCAGAGGCCGGCTACTTGCTTGCGCAGCACG
CACGGCCGGGGAGGGGCTGGCGGCGGCCGCGGGCGGCGGGCGGCGGAGTCGCGGGG
CCTGCTCCCGCCGGCACCATGGCCAAG
>87
GGCTACTTGCTTGCGCAGCACGCACGGCCGGGGAGGGGCTGGCGGCGGCCGCGGGCG
GCGGGCGGCGGAGTCGCGGGGCCTGCTCCCGCCGGCACCATGGCCAAGACCGTGGCC
TATTTCTACGACCCCGACGTGGGCAA
>88
GGGCTGGCGGCGGCCGCGGGCGGCGGGCGGCGGAGTCGCGGGGCCTGCTCCCGCCG
GCACCATGGCCAAGACCGTGGCCTATTTCTACGACCCCGACGTGGGCAACTTCCACTA
CGGTGAGGAAACAGGGCTATGCATGA

>89
CGCCGGCACCATGGCCAAGACCGTGGCCTATTTCTACGACCCCGACGTGGGCAACTTC
CACTACGGTGAGGAAACAGGGCTATGCATGAGGGGTTGGGACACGGAGGTTTTGAGG
CGGGGTTGCAACTCCAGGGGTGAGA
>90
ACCATGGCCAAGACCGTGGCCTATTTCTACGACCCCGACGTGGGCAACTTCCACTACG
GTGAGGAAACAGGGCTATGCATGAGGGGTTGGGACACGGAGGTTTTGAGGCGGGGTT
GCAACTCCAGGGGTGAGAGCTGACG
>91
CGACCCCGACGTGGGCAACTTCCACTACGGTGAGGAAACAGGGCTATGCATGAGGGG
TTGGGACACGGAGGTTTTGAGGCGGGGTTGCAACTCCAGGGGTGAGAGCTGACGAAC
TCTCTTCTCCCCACCCCCAGGAGCTG
>92
ACGTGGGCAACTTCCACTACGGTGAGGAAACAGGGCTATGCATGAGGGGTTGGGACA
CGGAGGTTTTGAGGCGGGGTTGCAACTCCAGGGGTGAGAGCTGACGAACTCTCTTCT
CCCCACCCCCAGGAGCTGGACACCCT
>93
ACGGTGAGGAAACAGGGCTATGCATGAGGGGTTGGGACACGGAGGTTTTGAGGCGGG
GTTGCAACTCCAGGGGTGAGAGCTGACGAACTCTCTTCTCCCCACCCCCAGGAGCTG
GACACCCTATGAAGCCTCATCGCCTG
>94
GAGGTTTTGAGGCGGGGTTGCAACTCCAGGGGTGAGAGCTGACGAACTCTCTTCTCC
CCACCCCCAGGAGCTGGACACCCTATGAAGCCTCATCGCCTGGCATTGACCCATAGCC
TGGTCCTGCATTACGGTCTCTATAA
>95
TTGCAACTCCAGGGGTGAGAGCTGACGAACTCTCTTCTCCCCACCCCCAGGAGCTGG
ACACCCTATGAAGCCTCATCGCCTGGCATTGACCCATAGCCTGGTCCTGCATTACGGTC
TCTATAAGAAGATGATCGTGAGTT
>96
GAACTCTCTTCTCCCCACCCCCAGGAGCTGGACACCCTATGAAGCCTCATCGCCTGGC
ATTGACCCATAGCCTGGTCCTGCATTACGGTCTCTATAAGAAGATGATCGTGAGTTCCG
CCGCCTCGGCCGGGGGTTGGGGG
>97
TGGACACCCTATGAAGCCTCATCGCCTGGCATTGACCCATAGCCTGGTCCTGCATTACG
GTCTCTATAAGAAGATGATCGTGAGTTCCGCCGCCTCGGCCGGGGGTTGGGGGGTGGG
GCGAGCTGTGGCCGTAGGGATAG
>98
ACACCCTATGAAGCCTCATCGCCTGGCATTGACCCATAGCCTGGTCCTGCATTACGGTC
TCTATAAGAAGATGATCGTGAGTTCCGCCGCCTCGGCCGGGGGTTGGGGGGTGGGGCG
AGCTGTGGCCGTAGGGATAGGTG
>99
CATTACGGTCTCTATAAGAAGATGATCGTGAGTTCCGCCGCCTCGGCCGGGGGTTGGG
GGGTGGGGCGAGCTGTGGCCGTAGGGATAGGTGGGCTGGACTGAGTGCATGAAGTGC
GACCCGCCCCTAGAGTATCGGGGAG

>100
TATAAGAAGATGATCGTGAGTTCCGCCGCCTCGGCCGGGGGTTGGGGGGTGGGGCGA
GCTGTGGCCGTAGGGATAGGTGGGCTGGACTGAGTGCATGAAGTGCGACCCGCCCCTA
GAGTATCGGGGAGCTGGATAGAAGT
>101
TCCGCCGCCTCGGCCGGGGGTTGGGGGGTGGGGCGAGCTGTGGCCGTAGGGATAGGT
GGGCTGGACTGAGTGCATGAAGTGCGACCCGCCCCTAGAGTATCGGGGAGCTGGATAG
AAGTCACCAGGAGTACCTTGAAAGT
>102
GGGTGGGGCGAGCTGTGGCCGTAGGGATAGGTGGGCTGGACTGAGTGCATGAAGTGC
GACCCGCCCCTAGAGTATCGGGGAGCTGGATAGAAGTCACCAGGAGTACCTTGAAAGT
GCAGGGTGTTGGGGGTGGCGTCCTC
>103
GTGGGGCGAGCTGTGGCCGTAGGGATAGGTGGGCTGGACTGAGTGCATGAAGTGCGA
CCCGCCCCTAGAGTATCGGGGAGCTGGATAGAAGTCACCAGGAGTACCTTGAAAGTGC
AGGGTGTTGGGGGTGGCGTCCTCTG
>104
GTAGGGATAGGTGGGCTGGACTGAGTGCATGAAGTGCGACCCGCCCCTAGAGTATCGG
GGAGCTGGATAGAAGTCACCAGGAGTACCTTGAAAGTGCAGGGTGTTGGGGGTGGCG
TCCTCTGCTTAATGGAAAGTACTTT
>105
TGAAGTGCGACCCGCCCCTAGAGTATCGGGGAGCTGGATAGAAGTCACCAGGAGTACC
TTGAAAGTGCAGGGTGTTGGGGGTGGCGTCCTCTGCTTAATGGAAAGTACTTTCTCAC
CTCAGGTCGGCCACCCTCTGACTG
>106
GGATAGAAGTCACCAGGAGTACCTTGAAAGTGCAGGGTGTTGGGGGTGGCGTCCTCT
GCTTAATGGAAAGTACTTTCTCACCTCAGGTCGGCCACCCTCTGACTGTAGGCACACTA
GCCCTTGGCCTAGGGTTAGCGTGA
>107
GAGTACCTTGAAAGTGCAGGGTGTTGGGGGTGGCGTCCTCTGCTTAATGGAAAGTACT
TTCTCACCTCAGGTCGGCCACCCTCTGACTGTAGGCACACTAGCCCTTGGCCTAGGGTT
AGCGTGATGTGGGGCTGGTTTGA
>108
GTTGGGGGTGGCGTCCTCTGCTTAATGGAAAGTACTTTCTCACCTCAGGTCGGCCACC
CTCTGACTGTAGGCACACTAGCCCTTGGCCTAGGGTTAGCGTGATGTGGGGCTGGTTT
GAAGACAGAAGGTGTGTTCCTCCT
>109
GGCGTCCTCTGCTTAATGGAAAGTACTTTCTCACCTCAGGTCGGCCACCCTCTGACTGT
AGGCACACTAGCCCTTGGCCTAGGGTTAGCGTGATGTGGGGCTGGTTTGAAGACAGAA
GGTGTGTTCCTCCTCTGCATTTT
>110
GAAAGTACTTTCTCACCTCAGGTCGGCCACCCTCTGACTGTAGGCACACTAGCCCTTG
GCCTAGGGTTAGCGTGATGTGGGGCTGGTTTGAAGACAGAAGGTGTGTTCCTCCTCTG
CATTTTAGAGTGTGGATAGGGCCT

>111
CGGCCACCCTCTGACTGTAGGCACACTAGCCCTTGGCCTAGGGTTAGCGTGATGTGGG
GCTGGTTTGAAGACAGAAGGTGTGTTCCTCCTCTGCATTTTAGAGTGTGGATAGGGCC
TTGGCCTCAGGAATTAGGGTTAGG
>112
CACCCTCTGACTGTAGGCACACTAGCCCTTGGCCTAGGGTTAGCGTGATGTGGGGCTG
GTTTGAAGACAGAAGGTGTGTTCCTCCTCTGCATTTTAGAGTGTGGATAGGGCCTTGG
CCTCAGGAATTAGGGTTAGGGGAG
>113
CTTGGCCTAGGGTTAGCGTGATGTGGGGCTGGTTTGAAGACAGAAGGTGTGTTCCTCC
TCTGCATTTTAGAGTGTGGATAGGGCCTTGGCCTCAGGAATTAGGGTTAGGGGAGCGG
ATATCACTGTATCAGGGTGTGAGG
>114
GGGCTGGTTTGAAGACAGAAGGTGTGTTCCTCCTCTGCATTTTAGAGTGTGGATAGGG
CCTTGGCCTCAGGAATTAGGGTTAGGGGAGCGGATATCACTGTATCAGGGTGTGAGGT
TTACCCTCAGTTCCCACTCCTCTT
>115
TTTGAAGACAGAAGGTGTGTTCCTCCTCTGCATTTTAGAGTGTGGATAGGGCCTTGGCC
TCAGGAATTAGGGTTAGGGGAGCGGATATCACTGTATCAGGGTGTGAGGTTTACCCTCA
GTTCCCACTCCTCTTTTACCAC
>116
GACAGAAGGTGTGTTCCTCCTCTGCATTTTAGAGTGTGGATAGGGCCTTGGCCTCAGG
AATTAGGGTTAGGGGAGCGGATATCACTGTATCAGGGTGTGAGGTTTACCCTCAGTTCC
CACTCCTCTTTTACCACCTTTCT
>117
GCATTTTAGAGTGTGGATAGGGCCTTGGCCTCAGGAATTAGGGTTAGGGGAGCGGATAT
CACTGTATCAGGGTGTGAGGTTTACCCTCAGTTCCCACTCCTCTTTTACCACCTTTCTTT
ACCTCCAAGGAGCTAGGGCTC
>118
TCACTGTATCAGGGTGTGAGGTTTACCCTCAGTTCCCACTCCTCTTTTACCACCTTTCTT
TACCTCCAAGGAGCTAGGGCTCCAGGGTGGAACGATGCTGCCGTCTTTGTAAATGTGT
ATGTGCGTGTGGAACAGAGGCG
>119
CTGTATCAGGGTGTGAGGTTTACCCTCAGTTCCCACTCCTCTTTTACCACCTTTCTTTAC
CTCCAAGGAGCTAGGGCTCCAGGGTGGAACGATGCTGCCGTCTTTGTAAATGTGTATG
TGCGTGTGGAACAGAGGCGATA
>120
CCAAGGAGCTAGGGCTCCAGGGTGGAACGATGCTGCCGTCTTTGTAAATGTGTATGTG
CGTGTGGAACAGAGGCGATATTTTAGATATTCCTTTTTCAGCCTGGAATACCTCTACATG
GTTCTGCTTAATCGCTATCCAG
>121
AAGGAGCTAGGGCTCCAGGGTGGAACGATGCTGCCGTCTTTGTAAATGTGTATGTGCG
TGTGGAACAGAGGCGATATTTTAGATATTCCTTTTTCAGCCTGGAATACCTCTACATGGT
TCTGCTTAATCGCTATCCAGTT

>122
ATGCTGCCGTCTTTGTAAATGTGTATGTGCGTGTGGAACAGAGGCGATATTTTAGATATT
CCTTTTTCAGCCTGGAATACCTCTACATGGTTCTGCTTAATCGCTATCCAGTTTTTCATCT
ACCAGACATACACATGCTG
>123
AGGCGATATTTTAGATATTCCTTTTTCAGCCTGGAATACCTCTACATGGTTCTGCTTAATC
GCTATCCAGTTTTTCATCTACCAGACATACACATGCTGTCTTCCCAGCTTGGAATTTATG
GCTGTGATATGGGATAGTA
>124
TATTCCTTTTTCAGCCTGGAATACCTCTACATGGTTCTGCTTAATCGCTATCCAGTTTTTC
ATCTACCAGACATACACATGCTGTCTTCCCAGCTTGGAATTTATGGCTGTGATATGGGAT
AGTAACAGTTAAGCCACGC
>125
CCTCTACATGGTTCTGCTTAATCGCTATCCAGTTTTTCATCTACCAGACATACACATGCT
GTCTTCCCAGCTTGGAATTTATGGCTGTGATATGGGATAGTAACAGTTAAGCCACGCCA
GGCGTGGTGGCCCATGCCTGT
>126
AGTTTTTCATCTACCAGACATACACATGCTGTCTTCCCAGCTTGGAATTTATGGCTGTGA
TATGGGATAGTAACAGTTAAGCCACGCCAGGCGTGGTGGCCCATGCCTGTAATCCCAGC
ACTTTGGGAGGTTGAAATGGG
>127
TCATCTACCAGACATACACATGCTGTCTTCCCAGCTTGGAATTTATGGCTGTGATATGGG
ATAGTAACAGTTAAGCCACGCCAGGCGTGGTGGCCCATGCCTGTAATCCCAGCACTTT
GGGAGGTTGAAATGGGGGGGAT
>128
TACCAGACATACACATGCTGTCTTCCCAGCTTGGAATTTATGGCTGTGATATGGGATAGT
AACAGTTAAGCCACGCCAGGCGTGGTGGCCCATGCCTGTAATCCCAGCACTTTGGGAG
GTTGAAATGGGGGGGATCGCTT
>129
TACACATGCTGTCTTCCCAGCTTGGAATTTATGGCTGTGATATGGGATAGTAACAGTTAA
GCCACGCCAGGCGTGGTGGCCCATGCCTGTAATCCCAGCACTTTGGGAGGTTGAAATG
GGGGGGATCGCTTGAGGCCAGG
>130
TTATGGCTGTGATATGGGATAGTAACAGTTAAGCCACGCCAGGCGTGGTGGCCCATGCC
TGTAATCCCAGCACTTTGGGAGGTTGAAATGGGGGGGATCGCTTGAGGCCAGGAGTTG
GAGACCAGCTTGGGCCACAGTGG
>131
CGCCAGGCGTGGTGGCCCATGCCTGTAATCCCAGCACTTTGGGAGGTTGAAATGGGGG
GGATCGCTTGAGGCCAGGAGTTGGAGACCAGCTTGGGCCACAGTGGGACCCACATCT
ACAAAAAATAAAAATAAAAAAAGTA
>132
GGCGTGGTGGCCCATGCCTGTAATCCCAGCACTTTGGGAGGTTGAAATGGGGGGGATC
GCTTGAGGCCAGGAGTTGGAGACCAGCTTGGGCCACAGTGGGACCCACATCTACAAA
AAATAAAAATAAAAAAAGTAGCCAT

>133
CAGCACTTTGGGAGGTTGAAATGGGGGGATCGCTTGAGGCCAGGAGTTGGAGACCA
GCTTGGGCCACAGTGGGACCCACATCTACAAAAAATAAAAATAAAAAAAGTAGCCATG
TGCGGTGGGTGTGCCTGTAGTCCCA
>134
AGGCCAGGAGTTGGAGACCAGCTTGGGCCACAGTGGGACCCACATCTACAAAAAATA
AAAATAAAAAAAGTAGCCATGTGCGGTGGGTGTGCCTGTAGTCCCAACTACTTGGGAG
GCCGAGGCGAGAGGATGGCTTGGGC
>135
CCAGGAGTTGGAGACCAGCTTGGGCCACAGTGGGACCCACATCTACAAAAAATAAAA
ATAAAAAAAGTAGCCATGTGCGGTGGGTGTGCCTGTAGTCCCAACTACTTGGGAGGCC
GAGGCGAGAGGATGGCTTGGGCCCA
>136
CTACAAAAAATAAAAATAAAAAAAGTAGCCATGTGCGGTGGGTGTGCCTGTAGTCCCA
ACTACTTGGGAGGCCGAGGCGAGAGGATGGCTTGGGCCCAGGAGTTTAAAGTTACAG
TGATCTATGATCAGCAGCCTGAGTT
>137
AAAATAAAAATAAAAAAAGTAGCCATGTGCGGTGGGTGTGCCTGTAGTCCCAACTACT
TGGGAGGCCGAGGCGAGAGGATGGCTTGGGCCCAGGAGTTTAAAGTTACAGTGATCTA
TGATCAGCAGCCTGAGTTGACAGG
>138
CATGTGCGGTGGGTGTGCCTGTAGTCCCAACTACTTGGGAGGCCGAGGCGAGAGGATG
GCTTGGGCCCAGGAGTTTAAAGTTACAGTGATCTATGATCAGCAGCCTGAGTTGACAG
GGAGGACCTTGTCTCAAAAGAAAA
>139
GCCTGTAGTCCCAACTACTTGGGAGGCCGAGGCGAGAGGATGGCTTGGGCCCAGGAG
TTTAAAGTTACAGTGATCTATGATCAGCAGCCTGAGTTGACAGGGAGGACCTTGTCTCA
AAAGAAAAAAATAAGACAAAACAGC
>140
CTACTTGGGAGGCCGAGGCGAGAGGATGGCTTGGGCCCAGGAGTTTAAAGTTACAGT
GATCTATGATCAGCAGCCTGAGTTGACAGGGAGGACCTTGTCTCAAAAGAAAAAAATAA
GACAAAACAGCTAAACCCAGGCTTT
>141
CTTGGGAGGCCGAGGCGAGAGGATGGCTTGGGCCCAGGAGTTTAAAGTTACAGTGAT
CTATGATCAGCAGCCTGAGTTGACAGGGAGGACCTTGTCTCAAAAGAAAAAAATAAGA
CAAAACAGCTAAACCCAGGCTTTATG
>142
AGAGGATGGCTTGGGCCCAGGAGTTTAAAGTTACAGTGATCTATGATCAGCAGCCTGA
GTTGACAGGGAGGACCTTGTCTCAAAAGAAAAAAATAAGACAAAACAGCTAAACCCAG
GCTTTATGTGTTGCTAGGGAAACTA
>143
AAAGTTACAGTGATCTATGATCAGCAGCCTGAGTTGACAGGGAGGACCTTGTCTCAAA
AGAAAAAAATAAGACAAAACAGCTAAACCCAGGCTTTATGTGTTGCTAGGGAAACTAGT
GAACAATATGAATGAATGTTCATT

>144
GTGATCTATGATCAGCAGCCTGAGTTGACAGGGAGGACCTTGTCTCAAAAGAAAAAAT
AAGACAAAACAGCTAAACCCAGGCTTTATGTGTTGCTAGGGAAACTAGTGAACAATAT
GAATGAATGTTCATTGCTTGTGCC
>145
ATCAGCAGCCTGAGTTGACAGGGAGGACCTTGTCTCAAAAGAAAAAATAAGACAAAA
CAGCTAAACCCAGGCTTTATGTGTTGCTAGGGAAACTAGTGAACAATATGAATGAATGT
TCATTGCTTGTGCCAAGGCACAGA
>146
ACAGGGAGGACCTTGTCTCAAAAGAAAAAATAAGACAAAACAGCTAAACCCAGGCTT
TATGTGTTGCTAGGGAAACTAGTGAACAATATGAATGAATGTTCATTGCTTGTGCCAAG
GCACAGACAAATGGACTGGTCATA
>147
AACCCAGGCTTTATGTGTTGCTAGGGAAACTAGTGAACAATATGAATGAATGTTCATTG
CTTGTGCCAAGGCACAGACAAATGGACTGGTCATAGAAAAGACTGAGGTGATGTTAGT
GCAGGAATCTATATCCCTTGAGA
>148
GGCTTTATGTGTTGCTAGGGAAACTAGTGAACAATATGAATGAATGTTCATTGCTTGTG
CCAAGGCACAGACAAATGGACTGGTCATAGAAAAGACTGAGGTGATGTTAGTGCAGG
AATCTATATCCCTTGAGATTGTTT
>149
TAGTGAACAATATGAATGAATGTTCATTGCTTGTGCCAAGGCACAGACAAATGGACTG
GTCATAGAAAAGACTGAGGTGATGTTAGTGCAGGAATCTATATCCCTTGAGATTGTTTG
CCTGTATGTACCTGTGTGCATTT
>150
TGAATGTTCATTGCTTGTGCCAAGGCACAGACAAATGGACTGGTCATAGAAAAGACTG
AGGTGATGTTAGTGCAGGAATCTATATCCCTTGAGATTGTTTGCCTGTATGTACCTGTGT
GCATTTTCTGGGAGAAGAAAAG
>151
GTTCATTGCTTGTGCCAAGGCACAGACAAATGGACTGGTCATAGAAAAGACTGAGGTG
ATGTTAGTGCAGGAATCTATATCCCTTGAGATTGTTTGCCTGTATGTACCTGTGTGCATTT
TCTGGGAGAAGAAAAGCTCTT
>152
GGCACAGACAAATGGACTGGTCATAGAAAAGACTGAGGTGATGTTAGTGCAGGAATCT
ATATCCCTTGAGATTGTTTGCCTGTATGTACCTGTGTGCATTTTCTGGGAGAAGAAAAG
CTCTTTCTTGTGCAGTTTTCAAA
>153
GATGTTAGTGCAGGAATCTATATCCCTTGAGATTGTTTGCCTGTATGTACCTGTGTGCAT
TTTCTGGGAGAAGAAAAGCTCTTTCTTGTGCAGTTTTCAAAGAGGTGTGACACATCCC
CCTGACCTTCCAAAGATAATAA
>154
GTTAGTGCAGGAATCTATATCCCTTGAGATTGTTTGCCTGTATGTACCTGTGTGCATTTT
CTGGGAGAAGAAAAGCTCTTTCTTGTGCAGTTTTCAAAGAGGTGTGACACATCCCCCT
GACCTTCCAAAGATAATAAATC

>155
CCCTTGAGATTGTTTGCCTGTATGTACCTGTGTGCATTTTCTGGGAGAAGAAAAGCTCT
TTCTTGTGCAGTTTTCAAAGAGGTGTGACACATCCCCCTGACCTTCCAAAGATAATAAA
TCTTTGATTTTAATTAGAAAGT
>156
CTGGGAGAAGAAAAGCTCTTTCTTGTGCAGTTTTCAAAGAGGTGTGACACATCCCCCT
GACCTTCCAAAGATAATAAATCTTTGATTTTAATTAGAAAGTTGACATATATCCCATATAT
TTGATCCAGAAAGAAGATCCA
>157
TGCAGTTTTCAAAGAGGTGTGACACATCCCCCTGACCTTCCAAAGATAATAAATCTTTG
ATTTTAATTAGAAAGTTGACATATATCCCATATATTTGATCCAGAAAGAAGATCCAGAGG
AAAGAGGACAGAATCAGAGCT
>158
GACCTTCCAAAGATAATAAATCTTTGATTTTAATTAGAAAGTTGACATATATCCCATATAT
TTGATCCAGAAAGAAGATCCAGAGGAAAGAGGACAGAATCAGAGCTTGGGGAGGAG
ATGAGCATGGAGAGAACTGGGAA
>159
TTCCAAAGATAATAAATCTTTGATTTTAATTAGAAAGTTGACATATATCCCATATATTTGA
TCCAGAAAGAAGATCCAGAGGAAAGAGGACAGAATCAGAGCTTGGGGAGGAGATGA
GCATGGAGAGAACTGGGAATAGG
>160
TCTTTGATTTTAATTAGAAAGTTGACATATATCCCATATATTTGATCCAGAAAGAAGATCC
AGAGGAAAGAGGACAGAATCAGAGCTTGGGGAGGAGATGAGCATGGAGAGAACTGG
GAATAGGAGATTCCAAGGAACAG
>161
CCCATATATTTGATCCAGAAAGAAGATCCAGAGGAAAGAGGACAGAATCAGAGCTTGG
GGAGGAGATGAGCATGGAGAGAACTGGGAATAGGAGATTCCAAGGAACAGGGCAGGT
GGATCAGGGCTCAAAGTTGAGAAGG
>162
ATTTGATCCAGAAAGAAGATCCAGAGGAAAGAGGACAGAATCAGAGCTTGGGGAGGA
GATGAGCATGGAGAGAACTGGGAATAGGAGATTCCAAGGAACAGGGCAGGTGGATCA
GGGCTCAAAGTTGAGAAGGTACGATA
>163
GAAGATCCAGAGGAAAGAGGACAGAATCAGAGCTTGGGGAGGAGATGAGCATGGAG
AGAACTGGGAATAGGAGATTCCAAGGAACAGGGCAGGTGGATCAGGGCTCAAAGTTG
AGAAGGTACGATAGGCTGAGCTGGAGG
>164
AGGACAGAATCAGAGCTTGGGGAGGAGATGAGCATGGAGAGAACTGGGAATAGGAGA
TTCCAAGGAACAGGGCAGGTGGATCAGGGCTCAAAGTTGAGAAGGTACGATAGGCTG
AGCTGGAGGTGGAAAGGAAAAGAAGT
>165
GGAGATGAGCATGGAGAGAACTGGGAATAGGAGATTCCAAGGAACAGGGCAGGTGGA
TCAGGGCTCAAAGTTGAGAAGGTACGATAGGCTGAGCTGGAGGTGGAAAGGAAAAG
AAGTGGTCTTCAGGGGGCTCATTTGTA

>166
TGAGCATGGAGAGAACTGGGAATAGGAGATTCCAAGGAACAGGGCAGGTGGATCAGG
GCTCAAAGTTGAGAAGGTACGATAGGCTGAGCTGGAGGTGGAAAGGAAAAGAAGTG
GTCTTCAGGGGGCTCATTTGTAATAAC
>167
GAACTGGGAATAGGAGATTCCAAGGAACAGGGCAGGTGGATCAGGGCTCAAAGTTGA
GAAGGTACGATAGGCTGAGCTGGAGGTGGAAAGGAAAAGAAGTGGTCTTCAGGGGG
CTCATTTGTAATAACTTACCTGGTTAA
>168
CAAGGAACAGGGCAGGTGGATCAGGGCTCAAAGTTGAGAAGGTACGATAGGCTGAGC
TGGAGGTGGAAAGGAAAAGAAGTGGTCTTCAGGGGGCTCATTTGTAATAACTTACCTG
GTTAATCGGAGTCTCTCTACTGACT
>169
ATCAGGGCTCAAAGTTGAGAAGGTACGATAGGCTGAGCTGGAGGTGGAAAGGAAAAG
AAGTGGTCTTCAGGGGGCTCATTTGTAATAACTTACCTGGTTAATCGGAGTCTCTCTAC
TGACTTAAGGATTGTGGGATGAGG
>170
CTGGAGGTGGAAAGGAAAAGAAGTGGTCTTCAGGGGGCTCATTTGTAATAACTTACCT
GGTTAATCGGAGTCTCTCTACTGACTTAAGGATTGTGGGATGAGGGGAATGCTATGTTT
GGCTTCATATAGTCTCCAGGACT
>171
AGAAGTGGTCTTCAGGGGGCTCATTTGTAATAACTTACCTGGTTAATCGGAGTCTCTCT
ACTGACTTAAGGATTGTGGGATGAGGGGAATGCTATGTTTGGCTTCATATAGTCTCCAG
GACTGGTTCAGTATGGGTGGTC
>172
GGGGGCTCATTTGTAATAACTTACCTGGTTAATCGGAGTCTCTCTACTGACTTAAGGATT
GTGGGATGAGGGGAATGCTATGTTTGGCTTCATATAGTCTCCAGGACTGGTTCAGTATG
GGTGGTCTGAGCCTGTGGAGT
>173
GTTAATCGGAGTCTCTCTACTGACTTAAGGATTGTGGGATGAGGGGAATGCTATGTTTG
GCTTCATATAGTCTCCAGGACTGGTTCAGTATGGGTGGTCTGAGCCTGTGGAGTGGGGC
TGACCCTGCCTGGGTTCATCTT
>174
ACTGACTTAAGGATTGTGGGATGAGGGGAATGCTATGTTTGGCTTCATATAGTCTCCAG
GACTGGTTCAGTATGGGTGGTCTGAGCCTGTGGAGTGGGGCTGACCCTGCCTGGGTTC
ATCTTGTGTATCCATCCCGAGGT
>175
GTGGGATGAGGGGAATGCTATGTTTGGCTTCATATAGTCTCCAGGACTGGTTCAGTATG
GGTGGTCTGAGCCTGTGGAGTGGGGCTGACCCTGCCTGGGTTCATCTTGTGTATCCATC
CCGAGGTCTTCAAGCCATACCA
>176
GGGGAATGCTATGTTTGGCTTCATATAGTCTCCAGGACTGGTTCAGTATGGGTGGTCTG
AGCCTGTGGAGTGGGGCTGACCCTGCCTGGGTTCATCTTGTGTATCCATCCCGAGGTCT
TCAAGCCATACCAGGCCTCCCA

>177
GGTCTGAGCCTGTGGAGTGGGGCTGACCCTGCCTGGGTTCATCTTGTGTATCCATCCCG
AGGTCTTCAAGCCATACCAGGCCTCCCAGCATGACATGTGCCGCTTCCACTCCGAGGA
CTACATTGACTTCCTGCAGAGAG
>178
CTGTGGAGTGGGGCTGACCCTGCCTGGGTTCATCTTGTGTATCCATCCCGAGGTCTTCA
AGCCATACCAGGCCTCCCAGCATGACATGTGCCGCTTCCACTCCGAGGACTACATTGA
CTTCCTGCAGAGAGTCAGCCCCA
>179
GTGTATCCATCCCGAGGTCTTCAAGCCATACCAGGCCTCCCAGCATGACATGTGCCGCT
TCCACTCCGAGGACTACATTGACTTCCTGCAGAGAGTCAGCCCCACCAATATGCAAGG
CTTCACCAAGAGTCTTAATGCCT
>180
CAAGCCATACCAGGCCTCCCAGCATGACATGTGCCGCTTCCACTCCGAGGACTACATT
GACTTCCTGCAGAGAGTCAGCCCCACCAATATGCAAGGCTTCACCAAGAGTCTTAATG
CCTTCAACGTAGGCGATGACTGGT
>181
AGCCATACCAGGCCTCCCAGCATGACATGTGCCGCTTCCACTCCGAGGACTACATTGA
CTTCCTGCAGAGAGTCAGCCCCACCAATATGCAAGGCTTCACCAAGAGTCTTAATGCC
TTCAACGTAGGCGATGACTGGTGA
>182
GACATGTGCCGCTTCCACTCCGAGGACTACATTGACTTCCTGCAGAGAGTCAGCCCCA
CCAATATGCAAGGCTTCACCAAGAGTCTTAATGCCTTCAACGTAGGCGATGACTGGTG
AGGGAGGACTGGGAGTTTTGATGT
>183
GACTTCCTGCAGAGAGTCAGCCCCACCAATATGCAAGGCTTCACCAAGAGTCTTAATG
CCTTCAACGTAGGCGATGACTGGTGAGGGAGGACTGGGAGTTTTGATGTCTAACAGTT
TAATGCCAAGCCCTTCAGTGAGGT
>184
GTCAGCCCCACCAATATGCAAGGCTTCACCAAGAGTCTTAATGCCTTCAACGTAGGCG
ATGACTGGTGAGGGAGGACTGGGAGTTTTGATGTCTAACAGTTTAATGCCAAGCCCTT
CAGTGAGGTCTTTTGGCAGCAGTG
>185
TGCCTTCAACGTAGGCGATGACTGGTGAGGGAGGACTGGGAGTTTTGATGTCTAACAG
TTTAATGCCAAGCCCTTCAGTGAGGTCTTTTGGCAGCAGTGGGAAGATCTCAAGGGGG
GCCTACTTGTCAATGCATCATAAT
>186
ACGTAGGCGATGACTGGTGAGGGAGGACTGGGAGTTTTGATGTCTAACAGTTTAATGC
CAAGCCCTTCAGTGAGGTCTTTTGGCAGCAGTGGGAAGATCTCAAGGGGGGCCTACTT
GTCAATGCATCATAATTCATTTTA
>187
GTGAGGGAGGACTGGGAGTTTTGATGTCTAACAGTTTAATGCCAAGCCCTTCAGTGAG
GTCTTTTGGCAGCAGTGGGAAGATCTCAAGGGGGGCCTACTTGTCAATGCATCATAATT
CATTTTATTTGTTTATTCAATAA

>188
CTACTTGTCAATGCATCATAATTCATTTTATTTGTTTATTCAATAAATATTTATTGGTCACT
TGTTCTAGGCAGGCCCTGTGCTAAGTGCTGGGAATGCAGTGATGAACAAAAACAAAAT
TTTAATATCGTATTTTTGCT
>189
TTGTCAATGCATCATAATTCATTTTATTTGTTTATTCAATAAATATTTATTGGTCACTTGTT
CTAGGCAGGCCCTGTGCTAAGTGCTGGGAATGCAGTGATGAACAAAAACAAAATTTTA
ATATCGTATTTTTGCTAAGT
>190
ATAATTCATTTTATTTGTTTATTCAATAAATATTTATTGGTCACTTGTTCTAGGCAGGCCCT
GTGCTAAGTGCTGGGAATGCAGTGATGAACAAAAACAAAATTTTAATATCGTATTTTTG
CTAAGTCTTATAAAGAGGA
>191
GGCCCTGTGCTAAGTGCTGGGAATGCAGTGATGAACAAAAACAAAATTTTAATATCGTA
TTTTTGCTAAGTCTTATAAAGAGGAGGTGTATGACAGATTATGAGAGTGTATACGTGGA
GGATTGTTTAGCTTGGTGGAGT
>192
TAAGTGCTGGGAATGCAGTGATGAACAAAAACAAAATTTTAATATCGTATTTTTGCTAA
GTCTTATAAAGAGGAGGTGTATGACAGATTATGAGAGTGTATACGTGGAGGATTGTTTA
GCTTGGTGGAGTGGCTGGTCGG
>193
ACAAAAACAAAATTTTAATATCGTATTTTTGCTAAGTCTTATAAAGAGGAGGTGTATGAC
AGATTATGAGAGTGTATACGTGGAGGATTGTTTAGCTTGGTGGAGTGGCTGGTCGGTGG
GGCCTTCCTAGAGGAAGTAAA
>194
TAATATCGTATTTTTGCTAAGTCTTATAAAGAGGAGGTGTATGACAGATTATGAGAGTGT
ATACGTGGAGGATTGTTTAGCTTGGTGGAGTGGCTGGTCGGTGGGGCCTTCCTAGAGG
AAGTAAAGATTGAGCTGAGATC
>195
TTTTTGCTAAGTCTTATAAAGAGGAGGTGTATGACAGATTATGAGAGTGTATACGTGGA
GGATTGTTTAGCTTGGTGGAGTGGCTGGTCGGTGGGGCCTTCCTAGAGGAAGTAAAGA
TTGAGCTGAGATCTGAAAAAAGA
>196
AGATTATGAGAGTGTATACGTGGAGGATTGTTTAGCTTGGTGGAGTGGCTGGTCGGTGG
GGCCTTCCTAGAGGAAGTAAAGATTGAGCTGAGATCTGAAAAAAGAGTAGAAGTAATC
AGATGAAAGATGGTTGGAGGGTG
>197
TGAGAGTGTATACGTGGAGGATTGTTTAGCTTGGTGGAGTGGCTGGTCGGTGGGGCCT
TCCTAGAGGAAGTAAAGATTGAGCTGAGATCTGAAAAAAGAGTAGAAGTAATCAGATG
AAAGATGGTTGGAGGGTGAGGGGA
>198
GTGTATACGTGGAGGATTGTTTAGCTTGGTGGAGTGGCTGGTCGGTGGGGCCTTCCTAG
AGGAAGTAAAGATTGAGCTGAGATCTGAAAAAAGAGTAGAAGTAATCAGATGAAAGA
TGGTTGGAGGGTGAGGGGAGAAGT

>199
ACGTGGAGGATTGTTTAGCTTGGTGGAGTGGCTGGTCGGTGGGGCCTTCCTAGAGGAA
GTAAAGATTGAGCTGAGATCTGAAAAAAGAGTAGAAGTAATCAGATGAAAGATGGTTG
GAGGGTGAGGGGAGAAGTATAGCA
>200
GCTTGGTGGAGTGGCTGGTCGGTGGGGCCTTCCTAGAGGAAGTAAAGATTGAGCTGA
GATCTGAAAAAAGAGTAGAAGTAATCAGATGAAAGATGGTTGGAGGGTGAGGGGAGA
AGTATAGCAGGCAGTAGGGACAAGTG
>201
CTAGAGGAAGTAAAGATTGAGCTGAGATCTGAAAAAAGAGTAGAAGTAATCAGATGA
AAGATGGTTGGAGGGTGAGGGGAGAAGTATAGCAGGCAGTAGGGACAAGTGCTAAGG
GTCTGTGGTAGGACTGAGTATGACTC
>202
GAAGTAAAGATTGAGCTGAGATCTGAAAAAAGAGTAGAAGTAATCAGATGAAAGATG
GTTGGAGGGTGAGGGGAGAAGTATAGCAGGCAGTAGGGACAAGTGCTAAGGGTCTGT
GGTAGGACTGAGTATGACTCATTTGA
>203
AAAGATTGAGCTGAGATCTGAAAAAAGAGTAGAAGTAATCAGATGAAAGATGGTTGG
AGGGTGAGGGGAGAAGTATAGCAGGCAGTAGGGACAAGTGCTAAGGGTCTGTGGTAG
GACTGAGTATGACTCATTTGAGGAAC
>204
ATCTGAAAAAAGAGTAGAAGTAATCAGATGAAAGATGGTTGGAGGGTGAGGGGAGAA
GTATAGCAGGCAGTAGGGACAAGTGCTAAGGGTCTGTGGTAGGACTGAGTATGACTCA
TTTGAGGAACAGAAAAAAGGCCAAG
>205
GAAAGATGGTTGGAGGGTGAGGGGAGAAGTATAGCAGGCAGTAGGGACAAGTGCTAA
GGGTCTGTGGTAGGACTGAGTATGACTCATTTGAGGAACAGAAAAAAGGCCAAGTGC
AGAGTATGAAGAGGAGGTGAACTGAA
>206
GGTTGGAGGGTGAGGGGAGAAGTATAGCAGGCAGTAGGGACAAGTGCTAAGGGTCTG
TGGTAGGACTGAGTATGACTCATTTGAGGAACAGAAAAAAGGCCAAGTGCAGAGTAT
GAAGAGGAGGTGAACTGAATTGAGGC
>207
GGAGAAGTATAGCAGGCAGTAGGGACAAGTGCTAAGGGTCTGTGGTAGGACTGAGTAT
GACTCATTTGAGGAACAGAAAAAAGGCCAAGTGCAGAGTATGAAGAGGAGGTGAACT
GAATTGAGGCTAAAGAAATAGGACC
>208
GTATAGCAGGCAGTAGGGACAAGTGCTAAGGGTCTGTGGTAGGACTGAGTATGACTCA
TTTGAGGAACAGAAAAAAGGCCAAGTGCAGAGTATGAAGAGGAGGTGAACTGAATTG
AGGCTAAAGAAATAGGACCTTTTAA
>209
AAGGGTCTGTGGTAGGACTGAGTATGACTCATTTGAGGAACAGAAAAAAGGCCAAGT
GCAGAGTATGAAGAGGAGGTGAACTGAATTGAGGCTAAAGAAATAGGACCTTTTAAA
AGCAAACAGCTCTATTATGAATTGGG

>210
AGTATGACTCATTTGAGGAACAGAAAAAAGGCCAAGTGCAGAGTATGAAGAGGAGGT
GAACTGAATTGAGGCTAAAGAAATAGGACCTTTTAAAAGCAAACAGCTCTATTATGAAT
TGGGGTTTTTACCCGTAGAGTATA
>211
CTCATTTGAGGAACAGAAAAAAGGCCAAGTGCAGAGTATGAAGAGGAGGTGAACTGA
ATTGAGGCTAAAGAAATAGGACCTTTTAAAAGCAAACAGCTCTATTATGAATTGGGGTT
TTTACCCGTAGAGTATAGGGAAGC
>212
AAAGGCCAAGTGCAGAGTATGAAGAGGAGGTGAACTGAATTGAGGCTAAAGAAATAG
GACCTTTTAAAAGCAAACAGCTCTATTATGAATTGGGGTTTTTACCCGTAGAGTATAGG
GAAGCTATCAAAGTGTTTTAAAAA
>213
ATTGAGGCTAAAGAAATAGGACCTTTTAAAAGCAAACAGCTCTATTATGAATTGGGGTT
TTTACCCGTAGAGTATAGGGAAGCTATCAAAGTGTTTTAAAAAGCAAGGGATCTTAAGA
GGGCTATGGGCAAGTTAAAAAA
>214
TGAGGCTAAAGAAATAGGACCTTTTAAAAGCAAACAGCTCTATTATGAATTGGGGTTTT
TACCCGTAGAGTATAGGGAAGCTATCAAAGTGTTTTAAAAAGCAAGGGATCTTAAGAG
GGCTATGGGCAAGTTAAAAAAAC
>215
CCTTTTAAAAGCAAACAGCTCTATTATGAATTGGGGTTTTTACCCGTAGAGTATAGGGA
AGCTATCAAAGTGTTTTAAAAAGCAAGGGATCTTAAGAGGGCTATGGGCAAGTTAAAA
AAACAAAAAAAAACAACGAAGGAG
>216
TGAATTGGGGTTTTTACCCGTAGAGTATAGGGAAGCTATCAAAGTGTTTTAAAAAGCAA
GGGATCTTAAGAGGGCTATGGGCAAGTTAAAAAAACAAAAAAAAACAACGAAGGAGTG
GTTTGATATCATTAGGTTTACATT
>217
AATTGGGGTTTTTACCCGTAGAGTATAGGGAAGCTATCAAAGTGTTTTAAAAAGCAAG
GGATCTTAAGAGGGCTATGGGCAAGTTAAAAAAACAAAAAAAAACAACGAAGGAGTGG
TTTGATATCATTAGGTTTACATTGA
>218
ACCCGTAGAGTATAGGGAAGCTATCAAAGTGTTTTAAAAAGCAAGGGATCTTAAGAGG
GCTATGGGCAAGTTAAAAAAACAAAAAAAAACAACGAAGGAGTGGTTTGATATCATTAG
GTTTACATTGAGCTTGTATCATCC
>219
AAGTGTTTTAAAAAGCAAGGGATCTTAAGAGGGCTATGGGCAAGTTAAAAAAACAAA
AAAAACAACGAAGGAGTGGTTTGATATCATTAGGTTTACATTGAGCTTGTATCATCCAT
AGAAGAGTCAGGGTTAGACCCTCT
>220
GATCTTAAGAGGGCTATGGGCAAGTTAAAAAAACAAAAAAAAACAACGAAGGAGTGGT
TTGATATCATTAGGTTTACATTGAGCTTGTATCATCCATAGAAGAGTCAGGGTTAGACCC
TCTGGGGAAGAGAGTGGACAACA

>221
GCTATGGGCAAGTTAAAAAAACAAAAAAAACAACGAAGGAGTGGTTTGATATCATTAG
GTTTACATTGAGCTTGTATCATCCATAGAAGAGTCAGGGTTAGACCCTCTGGGGAAGAG
AGTGGACAACAAAGATAAGCCAC
>222
AAAAACAAAAAAAACAACGAAGGAGTGGTTTGATATCATTAGGTTTACATTGAGCTTG
TATCATCCATAGAAGAGTCAGGGTTAGACCCTCTGGGGAAGAGAGTGGACAACAAAGA
TAAGCCACACTCTACTCTATATCC
>223
TTGATATCATTAGGTTTACATTGAGCTTGTATCATCCATAGAAGAGTCAGGGTTAGACCC
TCTGGGGAAGAGAGTGGACAACAAAGATAAGCCACACTCTACTCTATATCCATGAAAA
AATAGTAACCATCACAGCTACC
>224
GATATCATTAGGTTTACATTGAGCTTGTATCATCCATAGAAGAGTCAGGGTTAGACCCTC
TGGGGAAGAGAGTGGACAACAAAGATAAGCCACACTCTACTCTATATCCATGAAAAAA
TAGTAACCATCACAGCTACCAG
>225
TACATTGAGCTTGTATCATCCATAGAAGAGTCAGGGTTAGACCCTCTGGGGAAGAGAG
TGGACAACAAAGATAAGCCACACTCTACTCTATATCCATGAAAAAATAGTAACCATCAC
AGCTACCAGTTGCTCAATTCTAC
>226
TAGACCCTCTGGGGAAGAGAGTGGACAACAAAGATAAGCCACACTCTACTCTATATCC
ATGAAAAAATAGTAACCATCACAGCTACCAGTTGCTCAATTCTACTTACTTTTGTTTTTA
AACTTTTTATTAGGGAAAATTT
>227
GGAAGAGAGTGGACAACAAAGATAAGCCACACTCTACTCTATATCCATGAAAAAATAG
TAACCATCACAGCTACCAGTTGCTCAATTCTACTTACTTTTGTTTTTAAACTTTTTATTAG
GGAAAATTTCAAAAATTTCTG
>228
AGTGGACAACAAAGATAAGCCACACTCTACTCTATATCCATGAAAAAATAGTAACCATC
ACAGCTACCAGTTGCTCAATTCTACTTACTTTTGTTTTTAAACTTTTTATTAGGGAAAAT
TTCAAAAATTTCTGAAAGTGA
>229
AAAAAATAGTAACCATCACAGCTACCAGTTGCTCAATTCTACTTACTTTTGTTTTTAAAC
TTTTTATTAGGGAAAATTTCAAAAATTTCTGAAAGTGAAGTAGTGCAGTGACCTCCCAT
ACGTCCATCACCTAGCTTCAA
>230
CAGTTGCTCAATTCTACTTACTTTTGTTTTTAAACTTTTTATTAGGGAAAATTTCAAAAA
TTTCTGAAAGTGAAGTAGTGCAGTGACCTCCCATACGTCCATCACCTAGCTTCAACGCT
TATGAGCAAATGGCCACTCTT
>231
GCTCAATTCTACTTACTTTTGTTTTTAAACTTTTTATTAGGGAAAATTTCAAAAATTTCTG
AAAGTGAAGTAGTGCAGTGACCTCCCATACGTCCATCACCTAGCTTCAACGCTTATGAG
CAAATGGCCACTCTTTTTTT

>232
AAAAATTTCTGAAAGTGAAGTAGTGCAGTGACCTCCCATACGTCCATCACCTAGCTTCA
ACGCTTATGAGCAAATGGCCACTCTTTTTTTTTTTTTTTTTTTTTTGAGACAGAGTCTCA
CTCTGTAGCCCAGGCTGGAGT
>233
TACGTCCATCACCTAGCTTCAACGCTTATGAGCAAATGGCCACTCTTTTTTTTTTTTTTT
TTTTTTTGAGACAGAGTCTCACTCTGTAGCCCAGGCTGGAGTGCAGTGGTGCAGTCTT
GGCTCACTGCAACCTCTGCCTC
>234
CAACGCTTATGAGCAAATGGCCACTCTTTTTTTTTTTTTTTTTTTTTTGAGACAGAGTCT
CACTCTGTAGCCCAGGCTGGAGTGCAGTGGTGCAGTCTTGGCTCACTGCAACCTCTGC
CTCCTGGGTTCAAGCGATTCTC
>235
CTTATGAGCAAATGGCCACTCTTTTTTTTTTTTTTTTTTTTTTGAGACAGAGTCTCACTC
TGTAGCCCAGGCTGGAGTGCAGTGGTGCAGTCTTGGCTCACTGCAACCTCTGCCTCCT
GGGTTCAAGCGATTCTCATGCC
>236
CTTTTTTTTTTTTTTTTTTTTTTGAGACAGAGTCTCACTCTGTAGCCCAGGCTGGAGTGC
AGTGGTGCAGTCTTGGCTCACTGCAACCTCTGCCTCCTGGGTTCAAGCGATTCTCATGC
CTCAGACTCCCGAGTAGCTGG
>237
CTCTGTAGCCCAGGCTGGAGTGCAGTGGTGCAGTCTTGGCTCACTGCAACCTCTGCCT
CCTGGGTTCAAGCGATTCTCATGCCTCAGACTCCCGAGTAGCTGGGATTACAGGCGCC
TGCCACCATGCCTGGCTAATTTTT
>238
GAGTGCAGTGGTGCAGTCTTGGCTCACTGCAACCTCTGCCTCCTGGGTTCAAGCGATT
CTCATGCCTCAGACTCCCGAGTAGCTGGGATTACAGGCGCCTGCCACCATGCCTGGCTA
ATTTTTGTATGTTTAGTAGAGAT
>239
CACTGCAACCTCTGCCTCCTGGGTTCAAGCGATTCTCATGCCTCAGACTCCCGAGTAGC
TGGGATTACAGGCGCCTGCCACCATGCCTGGCTAATTTTTGTATGTTTAGTAGAGATGG
GGTTTCACTATGTTGGTCAAGC
>240
GCCTCAGACTCCCGAGTAGCTGGGATTACAGGCGCCTGCCACCATGCCTGGCTAATTTT
TGTATGTTTAGTAGAGATGGGGTTTCACTATGTTGGTCAAGCTGGTTTTGAACTCCCGA
CCTCAGGTGATTCACCAGCCTC
>241
TCAGACTCCCGAGTAGCTGGGATTACAGGCGCCTGCCACCATGCCTGGCTAATTTTTGT
ATGTTTAGTAGAGATGGGGTTTCACTATGTTGGTCAAGCTGGTTTTGAACTCCCGACCT
CAGGTGATTCACCAGCCTCAGC
>242
GGCGCCTGCCACCATGCCTGGCTAATTTTTGTATGTTTAGTAGAGATGGGGTTTCACTAT
GTTGGTCAAGCTGGTTTTGAACTCCCGACCTCAGGTGATTCACCAGCCTCAGCCTCCA
AAAGTGCTGGGATTACAGACGT

>243
ATTTTTGTATGTTTAGTAGAGATGGGGTTTCACTATGTTGGTCAAGCTGGTTTTGAACTC
CCGACCTCAGGTGATTCACCAGCCTCAGCCTCCAAAAGTGCTGGGATTACAGACGTGA
GCCATCGCACTCAGCTGAGCAA
>244
TTAGTAGAGATGGGGTTTCACTATGTTGGTCAAGCTGGTTTTGAACTCCCGACCTCAGG
TGATTCACCAGCCTCAGCCTCCAAAAGTGCTGGGATTACAGACGTGAGCCATCGCACT
CAGCTGAGCAAGTGGCCACTCTT
>245
CAAGCTGGTTTTGAACTCCCGACCTCAGGTGATTCACCAGCCTCAGCCTCCAAAAGTG
CTGGGATTACAGACGTGAGCCATCGCACTCAGCTGAGCAAGTGGCCACTCTTACTTCA
TCTATACTCCCTCATGTTAGATTG
>246
GTTTTGAACTCCCGACCTCAGGTGATTCACCAGCCTCAGCCTCCAAAAGTGCTGGGAT
TACAGACGTGAGCCATCGCACTCAGCTGAGCAAGTGGCCACTCTTACTTCATCTATACT
CCCTCATGTTAGATTGTTTTAAA
>247
GACCTCAGGTGATTCACCAGCCTCAGCCTCCAAAAGTGCTGGGATTACAGACGTGAGC
CATCGCACTCAGCTGAGCAAGTGGCCACTCTTACTTCATCTATACTCCCTCATGTTAGAT
TGTTTTAAAGCAGATCCTAGAT
>248
ACAGACGTGAGCCATCGCACTCAGCTGAGCAAGTGGCCACTCTTACTTCATCTATACTC
CCTCATGTTAGATTGTTTTAAAGCAGATCCTAGATGTTATATCCTTTTATCCATAAATATTT
TATTATGTATCTCTAAATG
>249
CATCGCACTCAGCTGAGCAAGTGGCCACTCTTACTTCATCTATACTCCCTCATGTTAGAT
TGTTTTAAAGCAGATCCTAGATGTTATATCCTTTTATCCATAAATATTTTATTATGTATCTC
TAAATGATACTCTTTTAA
>250
ATGTTATATCCTTTTATCCATAAATATTTTATTATGTATCTCTAAATGATACTCTTTTAAAAA
AACATAGTATCATTATCAAACCTAAAAAGTTAACAGTGATTCCTTAATGTCATCAAATAT
GGATCAATATTCAAATT
>251
AATATTTTATTATGTATCTCTAAATGATACTCTTTTAAAAAAACATAGTATCATTATCAAAC
CTAAAAAGTTAACAGTGATTCCTTAATGTCATCAAATATGGATCAATATTCAAATTTCTG
CAGTTGTCTCATAATTAT
>252
TATTATGTATCTCTAAATGATACTCTTTTAAAAAAACATAGTATCATTATCAAACCTAAAA
AGTTAACAGTGATTCCTTAATGTCATCAAATATGGATCAATATTCAAATTTCTGCAGTTG
TCTCATAATTATTTTGTAG
>253
ATCAAACCTAAAAAGTTAACAGTGATTCCTTAATGTCATCAAATATGGATCAATATTCAA
ATTTCTGCAGTTGTCTCATAATTATTTTGTAGCTAATTTAAATCCAGATCTAAACAAGGT
CTATACAGTGCATTTGGTTG

>254
TGATTCCTTAATGTCATCAAATATGGATCAATATTCAAATTTCTGCAGTTGTCTCATAATT
ATTTTGTAGCTAATTTAAATCCAGATCTAAACAAGGTCTATACAGTGCATTTGGTTGATA
TGTCTCTTAAGACTTTTAA
>255
ATCAAATATGGATCAATATTCAAATTTCTGCAGTTGTCTCATAATTATTTTGTAGCTAATTT
AAATCCAGATCTAAACAAGGTCTATACAGTGCATTTGGTTGATATGTCTCTTAAGACTTT
TAATTTGTAGCTTTTCTG
>256
AAATTTCTGCAGTTGTCTCATAATTATTTTGTAGCTAATTTAAATCCAGATCTAAACAAG
GTCTATACAGTGCATTTGGTTGATATGTCTCTTAAGACTTTTAATTTGTAGCTTTTCTGTC
ACCCCCTTGAATTTATTTC
>257
TTTTGTAGCTAATTTAAATCCAGATCTAAACAAGGTCTATACAGTGCATTTGGTTGATAT
GTCTCTTAAGACTTTTAATTTGTAGCTTTTCTGTCACCCCCTTGAATTTATTTCTTGAAG
AAACCAGGTTGTTGATCCTG
>258
AAATCCAGATCTAAACAAGGTCTATACAGTGCATTTGGTTGATATGTCTCTTAAGACTTT
TAATTTGTAGCTTTTCTGTCACCCCCTTGAATTTATTTCTTGAAGAAACCAGGTTGTTGA
TCCTGTAGAATTTCTTATGA
>259
TGGTTGATATGTCTCTTAAGACTTTTAATTTGTAGCTTTTCTGTCACCCCCTTGAATTTAT
TTCTTGAAGAAACCAGGTTGTTGATCCTGTAGAATTTCTTATGATAGCTACCATTTACTG
AGTGCTTACTCTGCTAGGT
>260
TATGTCTCTTAAGACTTTTAATTTGTAGCTTTTCTGTCACCCCCTTGAATTTATTTCTTGA
AGAAACCAGGTTGTTGATCCTGTAGAATTTCTTATGATAGCTACCATTTACTGAGTGCTT
ACTCTGCTAGGTATTATGC
>261
TTCTGTCACCCCCTTGAATTTATTTCTTGAAGAAACCAGGTTGTTGATCCTGTAGAATTT
CTTATGATAGCTACCATTTACTGAGTGCTTACTCTGCTAGGTATTATGCCAAGGGTTGCT
GGTAAATCCACTTAATTCTC
>262
GAATTTATTTCTTGAAGAAACCAGGTTGTTGATCCTGTAGAATTTCTTATGATAGCTACC
ATTTACTGAGTGCTTACTCTGCTAGGTATTATGCCAAGGGTTGCTGGTAAATCCACTTAA
TTCTCATACCAATCCTTAGA
>263
AGAAACCAGGTTGTTGATCCTGTAGAATTTCTTATGATAGCTACCATTTACTGAGTGCTT
ACTCTGCTAGGTATTATGCCAAGGGTTGCTGGTAAATCCACTTAATTCTCATACCAATCC
TTAGATGTATTATAATAGAC
>264
GTGCTTACTCTGCTAGGTATTATGCCAAGGGTTGCTGGTAAATCCACTTAATTCTCATAC
CAATCCTTAGATGTATTATAATAGACCTTTTTCTGATGTTATATATAAGTAAATTGAGGTTT
GGGAATATCTAGGTAACT

>265
AGGTATTATGCCAAGGGTTGCTGGTAAATCCACTTAATTCTCATACCAATCCTTAGATGT
ATTATAATAGACCTTTTTCTGATGTTATATATAAGTAAATTGAGGTTTGGGAATATCTAGG
TAACTTAGCCGTAGTCACA
>266
AAATCCACTTAATTCTCATACCAATCCTTAGATGTATTATAATAGACCTTTTTCTGATGTT
ATATATAAGTAAATTGAGGTTTGGGAATATCTAGGTAACTTAGCCGTAGTCACATAACTG
GTCATTGGAGGGGGCATGA
>267
TAATTCTCATACCAATCCTTAGATGTATTATAATAGACCTTTTTCTGATGTTATATATAAGT
AAATTGAGGTTTGGGAATATCTAGGTAACTTAGCCGTAGTCACATAACTGGTCATTGGA
GGGGGCATGATCCAGATCC
>268
CCTTAGATGTATTATAATAGACCTTTTTCTGATGTTATATATAAGTAAATTGAGGTTTGGG
AATATCTAGGTAACTTAGCCGTAGTCACATAACTGGTCATTGGAGGGGGCATGATCCAG
ATCCCGGGTTTTTAAACCAC
>269
AATAGACCTTTTTCTGATGTTATATATAAGTAAATTGAGGTTTGGGAATATCTAGGTAACT
TAGCCGTAGTCACATAACTGGTCATTGGAGGGGGCATGATCCAGATCCCGGGTTTTTAA
ACCACTAATCTGTTTTCCTG
>270
ATATATAAGTAAATTGAGGTTTGGGAATATCTAGGTAACTTAGCCGTAGTCACATAACTG
GTCATTGGAGGGGGCATGATCCAGATCCCGGGTTTTTAAACCACTAATCTGTTTTCCTG
AAAGATCTTAATGACAGAAGC
>271
TGAGGTTTGGGAATATCTAGGTAACTTAGCCGTAGTCACATAACTGGTCATTGGAGGGG
GCATGATCCAGATCCCGGGTTTTTAAACCACTAATCTGTTTTCCTGAAAGATCTTAATGA
CAGAAGCAGCCAGGGCTGTAG
>272
ATAACTGGTCATTGGAGGGGGCATGATCCAGATCCCGGGTTTTTAAACCACTAATCTGT
TTTCCTGAAAGATCTTAATGACAGAAGCAGCCAGGGCTGTAGGATAGAGGCCAACTCA
TTAGGCTGGCATTGGCACCTTTC
>273
GGAGGGGGCATGATCCAGATCCCGGGTTTTTAAACCACTAATCTGTTTTCCTGAAAGAT
CTTAATGACAGAAGCAGCCAGGGCTGTAGGATAGAGGCCAACTCATTAGGCTGGCATT
GGCACCTTTCCAACTTTATCCCT
>274
GGGGGCATGATCCAGATCCCGGGTTTTTAAACCACTAATCTGTTTTCCTGAAAGATCTT
AATGACAGAAGCAGCCAGGGCTGTAGGATAGAGGCCAACTCATTAGGCTGGCATTGGC
ACCTTTCCAACTTTATCCCTACA
>275
ATCCCGGGTTTTTAAACCACTAATCTGTTTTCCTGAAAGATCTTAATGACAGAAGCAGC
CAGGGCTGTAGGATAGAGGCCAACTCATTAGGCTGGCATTGGCACCTTTCCAACTTTAT
CCCTACACCTCAGCTGCACAGG

>276
TAATCTGTTTTCCTGAAAGATCTTAATGACAGAAGCAGCCAGGGCTGTAGGATAGAGG
CCAACTCATTAGGCTGGCATTGGCACCTTTCCAACTTTATCCCTACACCTCAGCTGCAC
AGGATGCCTTCCTGGAATGCCCT
>277
GGGCTGTAGGATAGAGGCCAACTCATTAGGCTGGCATTGGCACCTTTCCAACTTTATCC
CTACACCTCAGCTGCACAGGATGCCTTCCTGGAATGCCCTTAGTTCTCTATTCAACCTC
TTCAAGGCCTTCTCAGCCTTCA
>278
GGATAGAGGCCAACTCATTAGGCTGGCATTGGCACCTTTCCAACTTTATCCCTACACCT
CAGCTGCACAGGATGCCTTCCTGGAATGCCCTTAGTTCTCTATTCAACCTCTTCAAGGC
CTTCTCAGCCTTCACACCATCT
>279
TATCCCTACACCTCAGCTGCACAGGATGCCTTCCTGGAATGCCCTTAGTTCTCTATTCAA
CCTCTTCAAGGCCTTCTCAGCCTTCACACCATCTCCATGAAACCTTTCTTGACCACACC
AGCCCATAGAGCTCTGTCTTG
>280
CCTCAGCTGCACAGGATGCCTTCCTGGAATGCCCTTAGTTCTCTATTCAACCTCTTCAA
GGCCTTCTCAGCCTTCACACCATCTCCATGAAACCTTTCTTGACCACACCAGCCCATAG
AGCTCTGTCTTGCACACACTGC
>281
TCTATTCAACCTCTTCAAGGCCTTCTCAGCCTTCACACCATCTCCATGAAACCTTTCTTG
ACCACACCAGCCCATAGAGCTCTGTCTTGCACACACTGCACTGCTCATTTGGTGTTCAG
TCACATGCTGTTTGGGGAGGT
>282
AACCTCTTCAAGGCCTTCTCAGCCTTCACACCATCTCCATGAAACCTTTCTTGACCACA
CCAGCCCATAGAGCTCTGTCTTGCACACACTGCACTGCTCATTTGGTGTTCAGTCACAT
GCTGTTTGGGGAGGTTTCTTGT
>283
AACCTTTCTTGACCACACCAGCCCATAGAGCTCTGTCTTGCACACACTGCACTGCTCAT
TTGGTGTTCAGTCACATGCTGTTTGGGGAGGTTTCTTGTTTTTTTGTTAGTATTCCTGCT
GTTATTTCTGAGCTCACTTTC
>284
AGCCCATAGAGCTCTGTCTTGCACACACTGCACTGCTCATTTGGTGTTCAGTCACATGC
TGTTTGGGGAGGTTTCTTGTTTTTTTGTTAGTATTCCTGCTGTTATTTCTGAGCTCACTTT
CTAGCTCTGTGAATACAGGG
>285
GCACACACTGCACTGCTCATTTGGTGTTCAGTCACATGCTGTTTGGGGAGGTTTCTTGT
TTTTTTGTTAGTATTCCTGCTGTTATTTCTGAGCTCACTTTCTAGCTCTGTGAATACAGG
GTCAGCCCTTACGCCACACTG
>286
GGTGTTCAGTCACATGCTGTTTGGGGAGGTTTCTTGTTTTTTTGTTAGTATTCCTGCTGT
TATTTCTGAGCTCACTTTCTAGCTCTGTGAATACAGGGTCAGCCCTTACGCCACACTGG
GGTCCCGTAGATGCTCTTTGT

>287
CATGCTGTTTGGGGAGGTTTCTTGTTTTTTTGTTAGTATTCCTGCTGTTATTTCTGAGCTC
ACTTTCTAGCTCTGTGAATACAGGGTCAGCCCTTACGCCACACTGGGGTCCCGTAGATG
CTCTTTGTGCGATTAGGGAA
>288
AGGTTTCTTGTTTTTTTGTTAGTATTCCTGCTGTTATTTCTGAGCTCACTTTCTAGCTCTG
TGAATACAGGGTCAGCCCTTACGCCACACTGGGGTCCCGTAGATGCTCTTTGTGCGATT
AGGGAATGCTAATTAGAATA
>289
TTATTTCTGAGCTCACTTTCTAGCTCTGTGAATACAGGGTCAGCCCTTACGCCACACTG
GGGTCCCGTAGATGCTCTTTGTGCGATTAGGGAATGCTAATTAGAATACTATCTCTGGGC
TATTTATGCTGCATCAGTCTT
>290
CTAGCTCTGTGAATACAGGGTCAGCCCTTACGCCACACTGGGGTCCCGTAGATGCTCTT
TGTGCGATTAGGGAATGCTAATTAGAATACTATCTCTGGGCTATTTATGCTGCATCAGTC
TTCCTGTCAAACAAGTTACAG
>291
ACAGGGTCAGCCCTTACGCCACACTGGGGTCCCGTAGATGCTCTTTGTGCGATTAGGG
AATGCTAATTAGAATACTATCTCTGGGCTATTTATGCTGCATCAGTCTTCCTGTCAAACA
AGTTACAGTTGTGCTTATCTGT
>292
GTAGATGCTCTTTGTGCGATTAGGGAATGCTAATTAGAATACTATCTCTGGGCTATTTATG
CTGCATCAGTCTTCCTGTCAAACAAGTTACAGTTGTGCTTATCTGTATTTCCGATGAGAA
TCAGGAATTTTTCTTCTGT
>293
GATTAGGGAATGCTAATTAGAATACTATCTCTGGGCTATTTATGCTGCATCAGTCTTCCTG
TCAAACAAGTTACAGTTGTGCTTATCTGTATTTCCGATGAGAATCAGGAATTTTTCTTCT
GTTTAATGAATTTCTGATT
>294
GGGCTATTTATGCTGCATCAGTCTTCCTGTCAAACAAGTTACAGTTGTGCTTATCTGTAT
TTCCGATGAGAATCAGGAATTTTTCTTCTGTTTAATGAATTTCTGATTGGTTTCAAAGTA
TTCTAACATAAGTAGCCAAA
>295
TTTATGCTGCATCAGTCTTCCTGTCAAACAAGTTACAGTTGTGCTTATCTGTATTTCCGA
TGAGAATCAGGAATTTTTCTTCTGTTTAATGAATTTCTGATTGGTTTCAAAGTATTCTAA
CATAAGTAGCCAAATTGTAC
>296
GCTTATCTGTATTTCCGATGAGAATCAGGAATTTTTCTTCTGTTTAATGAATTTCTGATTG
GTTTCAAAGTATTCTAACATAAGTAGCCAAATTGTACTATAGGCCTTTAAATGAAAAAA
ATAAAAAAGTAGTCCCAAGC
>297
CCGATGAGAATCAGGAATTTTTCTTCTGTTTAATGAATTTCTGATTGGTTTCAAAGTATT
CTAACATAAGTAGCCAAATTGTACTATAGGCCTTTAAATGAAAAAAATAAAAAAGTAGT
CCCAAGCCGGGCGCAGTGGCT

>298
ATGAGAATCAGGAATTTTTCTTCTGTTTAATGAATTTCTGATTGGTTTCAAAGTATTCTA
ACATAAGTAGCCAAATTGTACTATAGGCCTTTAAATGAAAAAAATAAAAAAGTAGTCCC
AAGCCGGGCGCAGTGGCTCAC
>299
TTTCTTCTGTTTAATGAATTTCTGATTGGTTTCAAAGTATTCTAACATAAGTAGCCAAATT
GTACTATAGGCCTTTAAATGAAAAAAATAAAAAAGTAGTCCCAAGCCGGGCGCAGTGG
CTCACGCCTGTAATCCCAGCA
>300
TGGTTTCAAAGTATTCTAACATAAGTAGCCAAATTGTACTATAGGCCTTTAAATGAAAA
AAATAAAAAAGTAGTCCCAAGCCGGGCGCAGTGGCTCACGCCTGTAATCCCAGCACTT
TCGGAGGCCGAGGCGGGTGGACC
>301
ATAAGTAGCCAAATTGTACTATAGGCCTTTAAATGAAAAAAATAAAAAAGTAGTCCCAA
GCCGGGCGCAGTGGCTCACGCCTGTAATCCCAGCACTTTCGGAGGCCGAGGCGGGTG
GACCACCTAAGGTCAGGAGTTCAA
>302
AGGCCTTTAAATGAAAAAAATAAAAAAGTAGTCCCAAGCCGGGCGCAGTGGCTCACG
CCTGTAATCCCAGCACTTTCGGAGGCCGAGGCGGGTGGACCACCTAAGGTCAGGAGTT
CAAGACCAGCCTGGCCAACATGGTG
>303
TGAAAAAAATAAAAAAGTAGTCCCAAGCCGGGCGCAGTGGCTCACGCCTGTAATCCC
AGCACTTTCGGAGGCCGAGGCGGGTGGACCACCTAAGGTCAGGAGTTCAAGACCAGC
CTGGCCAACATGGTGAAACCCCATCT
>304
GGGCGCAGTGGCTCACGCCTGTAATCCCAGCACTTTCGGAGGCCGAGGCGGGTGGAC
CACCTAAGGTCAGGAGTTCAAGACCAGCCTGGCCAACATGGTGAAACCCCATCTCTAC
TAAAAATACAAAAATTAGTCATGTG
>305
CGCAGTGGCTCACGCCTGTAATCCCAGCACTTTCGGAGGCCGAGGCGGGTGGACCAC
CTAAGGTCAGGAGTTCAAGACCAGCCTGGCCAACATGGTGAAACCCCATCTCTACTAA
AAATACAAAAATTAGTCATGTGTGG
>306
GGCTCACGCCTGTAATCCCAGCACTTTCGGAGGCCGAGGCGGGTGGACCACCTAAGGT
CAGGAGTTCAAGACCAGCCTGGCCAACATGGTGAAACCCCATCTCTACTAAAAATACA
AAAATTAGTCATGTGTGGTGGCAC
>307
AGGTCAGGAGTTCAAGACCAGCCTGGCCAACATGGTGAAACCCCATCTCTACTAAAAA
TACAAAAATTAGTCATGTGTGGTGGCACGTGCCTGTAGTCCCAGCTACTCCAGGAGGC
TGAGGCAGGAGAATTGCTTGAACC
>308
CCAACATGGTGAAACCCCATCTCTACTAAAAATACAAAAATTAGTCATGTGTGGTGGCA
CGTGCCTGTAGTCCCAGCTACTCCAGGAGGCTGAGGCAGGAGAATTGCTTGAACCCGG
GAGTTGGAGGTTGCAGTGACCTG

>309
CCCATCTCTACTAAAAATACAAAAATTAGTCATGTGTGGTGGCACGTGCCTGTAGTCCC
AGCTACTCCAGGAGGCTGAGGCAGGAGAATTGCTTGAACCCGGGAGTTGGAGGTTGC
AGTGACCTGAGATTGCACCACTGC
>310
AAAATACAAAAATTAGTCATGTGTGGTGGCACGTGCCTGTAGTCCCAGCTACTCCAGG
AGGCTGAGGCAGGAGAATTGCTTGAACCCGGGAGTTGGAGGTTGCAGTGACCTGAGA
TTGCACCACTGCACTCCAGCCTGGG
>311
ATACAAAAATTAGTCATGTGTGGTGGCACGTGCCTGTAGTCCCAGCTACTCCAGGAGG
CTGAGGCAGGAGAATTGCTTGAACCCGGGAGTTGGAGGTTGCAGTGACCTGAGATTG
CACCACTGCACTCCAGCCTGGGCAA
>312
GTGGTGGCACGTGCCTGTAGTCCCAGCTACTCCAGGAGGCTGAGGCAGGAGAATTGCT
TGAACCCGGGAGTTGGAGGTTGCAGTGACCTGAGATTGCACCACTGCACTCCAGCCT
GGGCAACAGAGTGAGACTCTGTCTT
>313
CCTGTAGTCCCAGCTACTCCAGGAGGCTGAGGCAGGAGAATTGCTTGAACCCGGGAG
TTGGAGGTTGCAGTGACCTGAGATTGCACCACTGCACTCCAGCCTGGGCAACAGAGT
GAGACTCTGTCTTAAAAAAAAAAAAA
>314
AGGCAGGAGAATTGCTTGAACCCGGGAGTTGGAGGTTGCAGTGACCTGAGATTGCAC
CACTGCACTCCAGCCTGGGCAACAGAGTGAGACTCTGTCTTAAAAAAAAAAAAAAAA
AAAAATTCCAGACCTCTCCTTTTCAC
>315
TGCTTGAACCCGGGAGTTGGAGGTTGCAGTGACCTGAGATTGCACCACTGCACTCCAG
CCTGGGCAACAGAGTGAGACTCTGTCTTAAAAAAAAAAAAAAAAAAAAAATTCCAGAC
CTCTCCTTTTCACCCATAAATCCCT
>316
ACCACTGCACTCCAGCCTGGGCAACAGAGTGAGACTCTGTCTTAAAAAAAAAAAAAA
AAAAAAATTCCAGACCTCTCCTTTTCACCCATAAATCCCTCTGCCCAGTGACAGCCACT
TTCAGCTCCTTTCCCCTATGTCTT
>317
TCTGTCTTAAAAAAAAAAAAAAAAAAAAAATTCCAGACCTCTCCTTTTCACCCATAAAT
CCCTCTGCCCAGTGACAGCCACTTTCAGCTCCTTTCCCCTATGTCTTCTGGTATTTACCT
TTGGATTTCTAAATAATAATAA
>318
TTAAAAAAAAAAAAAAAAAAAAAATTCCAGACCTCTCCTTTTCACCCATAAATCCCTCT
GCCCAGTGACAGCCACTTTCAGCTCCTTTCCCCTATGTCTTCTGGTATTTACCTTTGGAT
TTCTAAATAATAATAATATTAA
>319
GGTATTTACCTTTGGATTTCTAAATAATAATAATATTAATTGACTGTTATTACTAATCTGTA
ATCCTTAGGTAATACCTGTTATATACTAGGTACTGTTCTAAGTGCTTTACAGCCATTAGCT
TATTTAATCTCAACAAC

>320
AAATAATAATAATATTAATTGACTGTTATTACTAATCTGTAATCCTTAGGTAATACCTGTTA
TATACTAGGTACTGTTCTAAGTGCTTTACAGCCATTAGCTTATTTAATCTCAACAACCCCT
ATGAAGCAGGGTCTATT
>321
TGTTATTACTAATCTGTAATCCTTAGGTAATACCTGTTATATACTAGGTACTGTTCTAAGT
GCTTTACAGCCATTAGCTTATTTAATCTCAACAACCCCTATGAAGCAGGGTCTATTATTG
TGTCCTTTTACCGATGAGG
>322
ACTAATCTGTAATCCTTAGGTAATACCTGTTATATACTAGGTACTGTTCTAAGTGCTTTAC
AGCCATTAGCTTATTTAATCTCAACAACCCCTATGAAGCAGGGTCTATTATTGTGTCCTT
TTACCGATGAGGACACTTA
>323
TTATATACTAGGTACTGTTCTAAGTGCTTTACAGCCATTAGCTTATTTAATCTCAACAACC
CCTATGAAGCAGGGTCTATTATTGTGTCCTTTTACCGATGAGGACACTTAAGCACAGAA
AGGTTAAACAGTTTGTACAA
>324
GCCATTAGCTTATTTAATCTCAACAACCCCTATGAAGCAGGGTCTATTATTGTGTCCTTTT
ACCGATGAGGACACTTAAGCACAGAAAGGTTAAACAGTTTGTACAACCTCACTATCAT
TAGTAGGTGTTAAAGAACCTA
>325
TATTTAATCTCAACAACCCCTATGAAGCAGGGTCTATTATTGTGTCCTTTTACCGATGAG
GACACTTAAGCACAGAAAGGTTAAACAGTTTGTACAACCTCACTATCATTAGTAGGTGT
TAAAGAACCTAGGTAGTCTAG
>326
CCTATGAAGCAGGGTCTATTATTGTGTCCTTTTACCGATGAGGACACTTAAGCACAGAA
AGGTTAAACAGTTTGTACAACCTCACTATCATTAGTAGGTGTTAAAGAACCTAGGTAGT
CTAGCTCCAGAATCGGTGCTTG
>327
TGTCCTTTTACCGATGAGGACACTTAAGCACAGAAAGGTTAAACAGTTTGTACAACCT
CACTATCATTAGTAGGTGTTAAAGAACCTAGGTAGTCTAGCTCCAGAATCGGTGCTTGT
AATCATACTCCCCTCCTGTATTG
>328
CCTTTTACCGATGAGGACACTTAAGCACAGAAAGGTTAAACAGTTTGTACAACCTCAC
TATCATTAGTAGGTGTTAAAGAACCTAGGTAGTCTAGCTCCAGAATCGGTGCTTGTAAT
CATACTCCCCTCCTGTATTGTCT
>329
GATGAGGACACTTAAGCACAGAAAGGTTAAACAGTTTGTACAACCTCACTATCATTAGT
AGGTGTTAAAGAACCTAGGTAGTCTAGCTCCAGAATCGGTGCTTGTAATCATACTCCCC
TCCTGTATTGTCTCTCATTGCT
>330
AAGGTTAAACAGTTTGTACAACCTCACTATCATTAGTAGGTGTTAAAGAACCTAGGTAG
TCTAGCTCCAGAATCGGTGCTTGTAATCATACTCCCCTCCTGTATTGTCTCTCATTGCTAT
TTCTTCGTCTGTCAATTTTA

>331
ATCGGTGCTTGTAATCATACTCCCCTCCTGTATTGTCTCTCATTGCTATTTCTTCGTCTGT
CAATTTTAGGCATTATCTGTTAAGCTTTTAGTATGGGATATGAGCATTTAGTCTTCTTACA
CCATCACATTGGCTTCCT
>332
CATACTCCCCTCCTGTATTGTCTCTCATTGCTATTTCTTCGTCTGTCAATTTTAGGCATTAT
CTGTTAAGCTTTTAGTATGGGATATGAGCATTTAGTCTTCTTACACCATCACATTGGCTT
CCTTTCCCCTTTCAGCTT
>333
CCCTCCTGTATTGTCTCTCATTGCTATTTCTTCGTCTGTCAATTTTAGGCATTATCTGTTA
AGCTTTTAGTATGGGATATGAGCATTTAGTCTTCTTACACCATCACATTGGCTTCCTTTCC
CCTTTCAGCTTCATAGTA
>334
TCTCTCATTGCTATTTCTTCGTCTGTCAATTTTAGGCATTATCTGTTAAGCTTTTAGTATG
GGATATGAGCATTTAGTCTTCTTACACCATCACATTGGCTTCCTTTCCCCTTTCAGCTTC
ATAGTAATTTAAAAATAAA
>335
AAGCTTTTAGTATGGGATATGAGCATTTAGTCTTCTTACACCATCACATTGGCTTCCTTT
CCCCTTTCAGCTTCATAGTAATTTAAAAATAAATCAGTATTCTGTGTTTACATTATTATAA
CTCTGAAAGATTCATTTAC
>336
AGTATGGGATATGAGCATTTAGTCTTCTTACACCATCACATTGGCTTCCTTTCCCCTTTC
AGCTTCATAGTAATTTAAAAATAAATCAGTATTCTGTGTTTACATTATTATAACTCTGAAA
GATTCATTTACTATCAAGC
>337
TAGTCTTCTTACACCATCACATTGGCTTCCTTTCCCCTTTCAGCTTCATAGTAATTTAAA
AATAAATCAGTATTCTGTGTTTACATTATTATAACTCTGAAAGATTCATTTACTATCAAGC
CAAATAGTGTAGTGTGATT
>338
CCCCTTTCAGCTTCATAGTAATTTAAAAATAAATCAGTATTCTGTGTTTACATTATTATAA
CTCTGAAAGATTCATTTACTATCAAGCCAAATAGTGTAGTGTGATTTAATCTATTTTCTTC
TAAAGCTTTTTGTTTTTC
>339
GTAATTTAAAAATAAATCAGTATTCTGTGTTTACATTATTATAACTCTGAAAGATTCATTT
ACTATCAAGCCAAATAGTGTAGTGTGATTTAATCTATTTTCTTCTAAAGCTTTTTGTTTTT
CCTGGAGCTGACAATTGC
>340
ATAACTCTGAAAGATTCATTTACTATCAAGCCAAATAGTGTAGTGTGATTTAATCTATTTT
CTTCTAAAGCTTTTTGTTTTTCCTGGAGCTGACAATTGCATTATTTTTTCATTTGCTTAGT
TTTCTGGTATTAATCACT
>341
TTACTATCAAGCCAAATAGTGTAGTGTGATTTAATCTATTTTCTTCTAAAGCTTTTTGTTT
TTCCTGGAGCTGACAATTGCATTATTTTTTCATTTGCTTAGTTTTCTGGTATTAATCACTA
ATTCTTTCCAAGCCTTTT

>342
AATCTATTTTCTTCTAAAGCTTTTTGTTTTTCCTGGAGCTGACAATTGCATTATTTTTTCA
TTTGCTTAGTTTTCTGGTATTAATCACTAATTCTTTCCAAGCCTTTTGAGAAACTGTAAA
ACCCTACAATTCTTGCTTG
>343
CTGGAGCTGACAATTGCATTATTTTTTCATTTGCTTAGTTTTCTGGTATTAATCACTAATT
CTTTCCAAGCCTTTTGAGAAACTGTAAAACCCTACAATTCTTGCTTGAACCCGGGAGG
CGGAGGCTGCAGTGAGCCGAG
>344
ACAATTGCATTATTTTTTCATTTGCTTAGTTTTCTGGTATTAATCACTAATTCTTTCCAAG
CCTTTTGAGAAACTGTAAAACCCTACAATTCTTGCTTGAACCCGGGAGGCGGAGGCTG
CAGTGAGCCGAGATCGCACCA
>345
TTAATCACTAATTCTTTCCAAGCCTTTTGAGAAACTGTAAAACCCTACAATTCTTGCTTG
AACCCGGGAGGCGGAGGCTGCAGTGAGCCGAGATCGCACCATTGCACCCCAGCCTGG
GCGACAGAGCAAGACCCCATCTC
>346
ACTAATTCTTTCCAAGCCTTTTGAGAAACTGTAAAACCCTACAATTCTTGCTTGAACCC
GGGAGGCGGAGGCTGCAGTGAGCCGAGATCGCACCATTGCACCCCAGCCTGGGCGAC
AGAGCAAGACCCCATCTCAAAAAA
>347
CCCTACAATTCTTGCTTGAACCCGGGAGGCGGAGGCTGCAGTGAGCCGAGATCGCACC
ATTGCACCCCAGCCTGGGCGACAGAGCAAGACCCCATCTCAAAAAACAAACAAACAA
AAAACCCCTACAGTTCTGTTTTTCA
>348
TGCTTGAACCCGGGAGGCGGAGGCTGCAGTGAGCCGAGATCGCACCATTGCACCCCA
GCCTGGGCGACAGAGCAAGACCCCATCTCAAAAAACAAACAAACAAAAAACCCCTAC
AGTTCTGTTTTTCACATGGTCAAATG
>349
AACCCGGGAGGCGGAGGCTGCAGTGAGCCGAGATCGCACCATTGCACCCCAGCCTGG
GCGACAGAGCAAGACCCCATCTCAAAAAACAAACAAACAAAAAACCCCTACAGTTCT
GTTTTTCACATGGTCAAATGTATCAG
>350
ATTGCACCCCAGCCTGGGCGACAGAGCAAGACCCCATCTCAAAAAACAAACAAACAA
AAAACCCCTACAGTTCTGTTTTTCACATGGTCAAATGTATCAGCTGCACTATTTTTTTTT
TTTGAGACCTAAAACTAGGGATT
>351
ACCCCATCTCAAAAAACAAACAAACAAAAAACCCCTACAGTTCTGTTTTTCACATGGT
CAAATGTATCAGCTGCACTATTTTTTTTTTTTGAGACCTAAAACTAGGGATTGCCTAAGC
CTTTCCTTTTTAAAAAGTCAGG
>352
ACAAAAAACCCCTACAGTTCTGTTTTTCACATGGTCAAATGTATCAGCTGCACTATTTTT
TTTTTTTGAGACCTAAAACTAGGGATTGCCTAAGCCTTTCCTTTTTAAAAAGTCAGGTT
TATATAGGTATAACTTGCATA

>353
TACAGTTCTGTTTTTCACATGGTCAAATGTATCAGCTGCACTATTTTTTTTTTTTGAGAC
CTAAAACTAGGGATTGCCTAAGCCTTTCCTTTTTAAAAAGTCAGGTTTATATAGGTATAA
CTTGCATACAGTAAAATTAA
>354
TTTCACATGGTCAAATGTATCAGCTGCACTATTTTTTTTTTTTGAGACCTAAAACTAGGG
ATTGCCTAAGCCTTTCCTTTTTAAAAAGTCAGGTTTATATAGGTATAACTTGCATACAGT
AAAATTAACCTTTTTTAGGT
>355
ATCAGCTGCACTATTTTTTTTTTTTGAGACCTAAAACTAGGGATTGCCTAAGCCTTTCCT
TTTTAAAAAGTCAGGTTTATATAGGTATAACTTGCATACAGTAAAATTAACCTTTTTTAG
GTGTAAGAGTTCTGAGAGGT
>356
TAAAACTAGGGATTGCCTAAGCCTTTCCTTTTTAAAAAGTCAGGTTTATATAGGTATAAC
TTGCATACAGTAAAATTAACCTTTTTTAGGTGTAAGAGTTCTGAGAGGTTTGATAGAATA
TATCAAGGTATAGGGCCAGC
>357
AGCCTTTCCTTTTTAAAAAGTCAGGTTTATATAGGTATAACTTGCATACAGTAAAATTAA
CCTTTTTTAGGTGTAAGAGTTCTGAGAGGTTTGATAGAATATATCAAGGTATAGGGCCA
GCGCCGATGGCTCACACCTGT
>358
AAAGTCAGGTTTATATAGGTATAACTTGCATACAGTAAAATTAACCTTTTTTAGGTGTAA
GAGTTCTGAGAGGTTTGATAGAATATATCAAGGTATAGGGCCAGCGCCGATGGCTCACA
CCTGTAGTCCCAACAATTTGG
>359
AACTTGCATACAGTAAAATTAACCTTTTTTAGGTGTAAGAGTTCTGAGAGGTTTGATAG
AATATATCAAGGTATAGGGCCAGCGCCGATGGCTCACACCTGTAGTCCCAACAATTTGG
GAGGCCAAGGCGGGAGGATCAC
>360
CATACAGTAAAATTAACCTTTTTTAGGTGTAAGAGTTCTGAGAGGTTTGATAGAATATAT
CAAGGTATAGGGCCAGCGCCGATGGCTCACACCTGTAGTCCCAACAATTTGGGAGGCC
AAGGCGGGAGGATCACTTAGGC
>361
GTAAGAGTTCTGAGAGGTTTGATAGAATATATCAAGGTATAGGGCCAGCGCCGATGGCT
CACACCTGTAGTCCCAACAATTTGGGAGGCCAAGGCGGGAGGATCACTTAGGCCCAG
GAGTTTGAGACCAGCTTGGGCAAC
>362
AGAATATATCAAGGTATAGGGCCAGCGCCGATGGCTCACACCTGTAGTCCCAACAATTT
GGGAGGCCAAGGCGGGAGGATCACTTAGGCCCAGGAGTTTGAGACCAGCTTGGGCAA
CATAGTGAGACCCTGTCTCTACAA
>363
ATCAAGGTATAGGGCCAGCGCCGATGGCTCACACCTGTAGTCCCAACAATTTGGGAGG
CCAAGGCGGGAGGATCACTTAGGCCCAGGAGTTTGAGACCAGCTTGGGCAACATAGT
GAGACCCTGTCTCTACAAAAAAAAA

>364
AGGGCCAGCGCCGATGGCTCACACCTGTAGTCCCAACAATTTGGGAGGCCAAGGCGG
GAGGATCACTTAGGCCCAGGAGTTTGAGACCAGCTTGGGCAACATAGTGAGACCCTGT
CTCTACAAAAAAAAAAAAAAAAAAG
>365
GGCTCACACCTGTAGTCCCAACAATTTGGGAGGCCAAGGCGGGAGGATCACTTAGGCC
CAGGAGTTTGAGACCAGCTTGGGCAACATAGTGAGACCCTGTCTCTACAAAAAAAAA
AAAAAAAAAGGTATCAAGGTACAGG
>366
GCCCAGGAGTTTGAGACCAGCTTGGGCAACATAGTGAGACCCTGTCTCTACAAAAAA
AAAAAAAAAAAAGGTATCAAGGTACAGGAAATTTCCACTACCCAACAAAGGTCACTC
ACCCTTTTGTAGTTAATAACTTCTCT
>367
GTTTGAGACCAGCTTGGGCAACATAGTGAGACCCTGTCTCTACAAAAAAAAAAAAAA
AAAAGGTATCAAGGTACAGGAAATTTCCACTACCCAACAAAGGTCACTCACCCTTTTG
TAGTTAATAACTTCTCTGCAACCTA
>368
GACCCTGTCTCTACAAAAAAAAAAAAAAAAAAGGTATCAAGGTACAGGAAATTTCCA
CTACCCAACAAAGGTCACTCACCCTTTTGTAGTTAATAACTTCTCTGCAACCTAGATCC
AGCCAACTACTGATAAGATGTCTG
>369
AAAAAAAAAAAAGGTATCAAGGTACAGGAAATTTCCACTACCCAACAAAGGTCACT
CACCCTTTTGTAGTTAATAACTTCTCTGCAACCTAGATCCAGCCAACTACTGATAAGATG
TCTGTCCTTGTATTTTCCAGAAT
>370
TACAGGAAATTTCCACTACCCAACAAAGGTCACTCACCCTTTTGTAGTTAATAACTTCT
CTGCAACCTAGATCCAGCCAACTACTGATAAGATGTCTGTCCTTGTATTTTCCAGAATGT
ACTTAAATGGAGTCATACAGT
>371
AACAAAGGTCACTCACCCTTTTGTAGTTAATAACTTCTCTGCAACCTAGATCCAGCCAA
CTACTGATAAGATGTCTGTCCTTGTATTTTCCAGAATGTACTTAAATGGAGTCATACAGT
AAGTAGGCTTTTGTGTCTGGC
>372
TAGTTAATAACTTCTCTGCAACCTAGATCCAGCCAACTACTGATAAGATGTCTGTCCTTG
TATTTTCCAGAATGTACTTAAATGGAGTCATACAGTAAGTAGGCTTTTGTGTCTGGCTTC
AAGCCTTTACTTTTTATAAA
>373
GCAACCTAGATCCAGCCAACTACTGATAAGATGTCTGTCCTTGTATTTTCCAGAATGTAC
TTAAATGGAGTCATACAGTAAGTAGGCTTTTGTGTCTGGCTTCAAGCCTTTACTTTTTAT
AAAGAATAACAATGGTAATA
>374
CAGCCAACTACTGATAAGATGTCTGTCCTTGTATTTTCCAGAATGTACTTAAATGGAGTC
ATACAGTAAGTAGGCTTTTGTGTCTGGCTTCAAGCCTTTACTTTTTATAAAGAATAACAA
TGGTAATAATTATAGCTGAC

>375
CCAACTACTGATAAGATGTCTGTCCTTGTATTTTCCAGAATGTACTTAAATGGAGTCATA
CAGTAAGTAGGCTTTTGTGTCTGGCTTCAAGCCTTTACTTTTTATAAAGAATAACAATG
GTAATAATTATAGCTGACATT
>376
GTCCTTGTATTTTCCAGAATGTACTTAAATGGAGTCATACAGTAAGTAGGCTTTTGTGTC
TGGCTTCAAGCCTTTACTTTTTATAAAGAATAACAATGGTAATAATTATAGCTGACATTTA
TTAAAGGCTGAGCGGATAC
>377
ATGTACTTAAATGGAGTCATACAGTAAGTAGGCTTTTGTGTCTGGCTTCAAGCCTTTACT
TTTTATAAAGAATAACAATGGTAATAATTATAGCTGACATTTATTAAAGGCTGAGCGGAT
ACTGTATACTGTGCTACAGG
>378
GTAAGTAGGCTTTTGTGTCTGGCTTCAAGCCTTTACTTTTTATAAAGAATAACAATGGTA
ATAATTATAGCTGACATTTATTAAAGGCTGAGCGGATACTGTATACTGTGCTACAGGCTT
TTCGTGGGTTATCTCATCAC
>379
GTCTGGCTTCAAGCCTTTACTTTTTATAAAGAATAACAATGGTAATAATTATAGCTGACA
TTTATTAAAGGCTGAGCGGATACTGTATACTGTGCTACAGGCTTTTCGTGGGTTATCTCA
TCACTGTTTCACATATGTAG
>380
GAATAACAATGGTAATAATTATAGCTGACATTTATTAAAGGCTGAGCGGATACTGTATAC
TGTGCTACAGGCTTTTCGTGGGTTATCTCATCACTGTTTCACATATGTAGGTATACAGTT
CTTTCTTTAAAATATGTGAC
>381
GCTGAGCGGATACTGTATACTGTGCTACAGGCTTTTCGTGGGTTATCTCATCACTGTTTC
ACATATGTAGGTATACAGTTCTTTCTTTAAAATATGTGACTGAAAAATATTATTGAACATT
ATGCTAAAATTTGTTACAC
>382
GATACTGTATACTGTGCTACAGGCTTTTCGTGGGTTATCTCATCACTGTTTCACATATGTA
GGTATACAGTTCTTTCTTTAAAATATGTGACTGAAAAATATTATTGAACATTATGCTAAAA
TTTGTTACACTGAGAAGA
>383
ACAGTTCTTTCTTTAAAATATGTGACTGAAAAATATTATTGAACATTATGCTAAAATTTG
TTACACTGAGAAGACTTAAGAAAAACAGAGCTCATGTTTTCTTTCAACTGATTTCCTTT
TTACATTGTACTTGAGTACCT
>384
GTTCTTTCTTTAAAATATGTGACTGAAAAATATTATTGAACATTATGCTAAAATTTGTTAC
ACTGAGAAGACTTAAGAAAAACAGAGCTCATGTTTTCTTTCAACTGATTTCCTTTTTAC
ATTGTACTTGAGTACCTGGA
>385
TATGTGACTGAAAAATATTATTGAACATTATGCTAAAATTTGTTACACTGAGAAGACTTA
AGAAAAACAGAGCTCATGTTTTCTTTCAACTGATTTCCTTTTTACATTGTACTTGAGTAC
CTGGAAGCTGCTTGCTTCTT

>386
TGTGACTGAAAAATATTATTGAACATTATGCTAAAATTTGTTACACTGAGAAGACTTAA
GAAAAACAGAGCTCATGTTTTCTTTCAACTGATTTCCTTTTTACATTGTACTTGAGTACC
TGGAAGCTGCTTGCTTCTTTC
>387
CTGAGAAGACTTAAGAAAAACAGAGCTCATGTTTTCTTTCAACTGATTTCCTTTTTACA
TTGTACTTGAGTACCTGGAAGCTGCTTGCTTCTTTCCGCAGCTTAGGATACAAACTCCC
TGACAGTTAAGTGCTTCTTTAC
>388
TTAAGAAAAACAGAGCTCATGTTTTCTTTCAACTGATTTCCTTTTTACATTGTACTTGAG
TACCTGGAAGCTGCTTGCTTCTTTCCGCAGCTTAGGATACAAACTCCCTGACAGTTAAG
TGCTTCTTTACTTCCCATCCA
>389
GTTTTCTTTCAACTGATTTCCTTTTTACATTGTACTTGAGTACCTGGAAGCTGCTTGCTT
CTTTCCGCAGCTTAGGATACAAACTCCCTGACAGTTAAGTGCTTCTTTACTTCCCATCC
AGTGCCTTGCACAGGGCTTGG
>390
CTTTCAACTGATTTCCTTTTTACATTGTACTTGAGTACCTGGAAGCTGCTTGCTTCTTTC
CGCAGCTTAGGATACAAACTCCCTGACAGTTAAGTGCTTCTTTACTTCCCATCCAGTGC
CTTGCACAGGGCTTGGTACAT
>391
TGCTTGCTTCTTTCCGCAGCTTAGGATACAAACTCCCTGACAGTTAAGTGCTTCTTTAC
TTCCCATCCAGTGCCTTGCACAGGGCTTGGTACATGGTTTGGTCCAAAATATTTGTTGA
ATAAATTTGCAGATAGCCCACG
>392
TCCGCAGCTTAGGATACAAACTCCCTGACAGTTAAGTGCTTCTTTACTTCCCATCCAGT
GCCTTGCACAGGGCTTGGTACATGGTTTGGTCCAAAATATTTGTTGAATAAATTTGCAG
ATAGCCCACGTGTGTAGGAGAG
>393
TCCCATCCAGTGCCTTGCACAGGGCTTGGTACATGGTTTGGTCCAAAATATTTGTTGAA
TAAATTTGCAGATAGCCCACGTGTGTAGGAGAGGGAGAGAAGGTTCTTGAAAAATGGA
GATTGGAGGAATCTAGGATGGGG
>394
ATCCAGTGCCTTGCACAGGGCTTGGTACATGGTTTGGTCCAAAATATTTGTTGAATAAA
TTTGCAGATAGCCCACGTGTGTAGGAGAGGGAGAGAAGGTTCTTGAAAAATGGAGATT
GGAGGAATCTAGGATGGGGAGGG
>395
AGGGCTTGGTACATGGTTTGGTCCAAAATATTTGTTGAATAAATTTGCAGATAGCCCAC
GTGTGTAGGAGAGGGAGAGAAGGTTCTTGAAAAATGGAGATTGGAGGAATCTAGGAT
GGGGAGGGAGATATGGGAAGGGCC
>396
TATTTGTTGAATAAATTTGCAGATAGCCCACGTGTGTAGGAGAGGGAGAGAAGGTTCTT
GAAAAATGGAGATTGGAGGAATCTAGGATGGGGAGGGAGATATGGGAAGGGCCCAGA
CAGAGGCAGGAGGGTTCCAACTTG

>397
TGAATAAATTTGCAGATAGCCCACGTGTGTAGGAGAGGGAGAGAAGGTTCTTGAAAAA
TGGAGATTGGAGGAATCTAGGATGGGGAGGGAGATATGGGAAGGGCCCAGACAGAGG
CAGGAGGGTTCCAACTTGTTCCTCT
>398
TTTGCAGATAGCCCACGTGTGTAGGAGAGGGAGAGAAGGTTCTTGAAAAATGGAGATT
GGAGGAATCTAGGATGGGGAGGGAGATATGGGAAGGGCCCAGACAGAGGCAGGAGG
GTTCCAACTTGTTCCTCTTCCCTGCA
>399
CACGTGTGTAGGAGAGGGAGAGAAGGTTCTTGAAAAATGGAGATTGGAGGAATCTAG
GATGGGGAGGGAGATATGGGAAGGGCCCAGACAGAGGCAGGAGGGTTCCAACTTGTT
CCTCTTCCCTGCAGCCCAGTGTTTCC
>400
TCTTGAAAAATGGAGATTGGAGGAATCTAGGATGGGGAGGGAGATATGGGAAGGGCC
CAGACAGAGGCAGGAGGGTTCCAACTTGTTCCTCTTCCCTGCAGCCCAGTGTTTCCCG
GGCTCTTTGAGTTCTGCTCGCGTTA
>401
GAGGGAGATATGGGAAGGGCCCAGACAGAGGCAGGAGGGTTCCAACTTGTTCCTCTT
CCCTGCAGCCCAGTGTTTCCCGGGCTCTTTGAGTTCTGCTCGCGTTACACAGGCGCATC
TCTGCAAGGAGCAACCCAGCTGAA
>402
TTCCAACTTGTTCCTCTTCCCTGCAGCCCAGTGTTTCCCGGGCTCTTTGAGTTCTGCTC
GCGTTACACAGGCGCATCTCTGCAAGGAGCAACCCAGCTGAACAACAAGGTGACATA
GTCCCGAGTCCTGTTCTTCCTTTC
>403
CTTCCCTGCAGCCCAGTGTTTCCCGGGCTCTTTGAGTTCTGCTCGCGTTACACAGGCGC
ATCTCTGCAAGGAGCAACCCAGCTGAACAACAAGGTGACATAGTCCCGAGTCCTGTTC
TTCCTTTCCTCTGGATCCCTGGA
>404
CGGGCTCTTTGAGTTCTGCTCGCGTTACACAGGCGCATCTCTGCAAGGAGCAACCCAG
CTGAACAACAAGGTGACATAGTCCCGAGTCCTGTTCTTCCTTTCCTCTGGATCCCTGGA
CTCGGGATTTAACCCTGATCCTG
>405
TGCTCGCGTTACACAGGCGCATCTCTGCAAGGAGCAACCCAGCTGAACAACAAGGTG
ACATAGTCCCGAGTCCTGTTCTTCCTTTCCTCTGGATCCCTGGACTCGGGATTTAACCCT
GATCCTGGGCTCCCAGCTTGAGG
>406
TCCCGAGTCCTGTTCTTCCTTTCCTCTGGATCCCTGGACTCGGGATTTAACCCTGATCCT
GGGCTCCCAGCTTGAGGGGTGGGCAGGAAGGACTGTGACTTAGGTGTTTGTCTTTCAG
ATCTGTGATATTGCCATTAACT
>407
GTCCTGTTCTTCCTTTCCTCTGGATCCCTGGACTCGGGATTTAACCCTGATCCTGGGCTC
CCAGCTTGAGGGGTGGGCAGGAAGGACTGTGACTTAGGTGTTTGTCTTTCAGATCTGT
GATATTGCCATTAACTGGGCTG

>408
TCCTTTCCTCTGGATCCCTGGACTCGGGATTTAACCCTGATCCTGGGCTCCCAGCTTGA
GGGGTGGGCAGGAAGGACTGTGACTTAGGTGTTTGTCTTTCAGATCTGTGATATTGCCA
TTAACTGGGCTGGTGGTCTGCA
>409
AACCCTGATCCTGGGCTCCCAGCTTGAGGGGTGGGCAGGAAGGACTGTGACTTAGGT
GTTTGTCTTTCAGATCTGTGATATTGCCATTAACTGGGCTGGTGGTCTGCACCATGCCA
AGAAGTTTGAGGTGAGTGAGGAGG
>410
GACTTAGGTGTTTGTCTTTCAGATCTGTGATATTGCCATTAACTGGGCTGGTGGTCTGCA
CCATGCCAAGAAGTTTGAGGTGAGTGAGGAGGTGATGGGAAAGACAGTGGCCATCCT
AGGGTAGGTGTTTAGGATGATGG
>411
TTAGGTGTTTGTCTTTCAGATCTGTGATATTGCCATTAACTGGGCTGGTGGTCTGCACCA
TGCCAAGAAGTTTGAGGTGAGTGAGGAGGTGATGGGAAAGACAGTGGCCATCCTAGG
GTAGGTGTTTAGGATGATGGTGG
>412
TCAGATCTGTGATATTGCCATTAACTGGGCTGGTGGTCTGCACCATGCCAAGAAGTTTG
AGGTGAGTGAGGAGGTGATGGGAAAGACAGTGGCCATCCTAGGGTAGGTGTTTAGGA
TGATGGTGGGGGGCAGCTGGGAGG
>413
GATCTGTGATATTGCCATTAACTGGGCTGGTGGTCTGCACCATGCCAAGAAGTTTGAGG
TGAGTGAGGAGGTGATGGGAAAGACAGTGGCCATCCTAGGGTAGGTGTTTAGGATGAT
GGTGGGGGGCAGCTGGGAGGGGA
>414
TGCCATTAACTGGGCTGGTGGTCTGCACCATGCCAAGAAGTTTGAGGTGAGTGAGGAG
GTGATGGGAAAGACAGTGGCCATCCTAGGGTAGGTGTTTAGGATGATGGTGGGGGGCA
GCTGGGAGGGGAATTGCTCTTCTC
>415
GGTGGTCTGCACCATGCCAAGAAGTTTGAGGTGAGTGAGGAGGTGATGGGAAAGACA
GTGGCCATCCTAGGGTAGGTGTTTAGGATGATGGTGGGGGGCAGCTGGGAGGGGAATT
GCTCTTCTCTTTATGAGACCACTGT
>416
TGAGTGAGGAGGTGATGGGAAAGACAGTGGCCATCCTAGGGTAGGTGTTTAGGATGAT
GGTGGGGGGCAGCTGGGAGGGGAATTGCTCTTCTCTTTATGAGACCACTGTCTTGCCA
TAGGCCTCTGGCTTCTGCTATGTC
>417
GGAGGTGATGGGAAAGACAGTGGCCATCCTAGGGTAGGTGTTTAGGATGATGGTGGGG
GGCAGCTGGGAGGGGAATTGCTCTTCTCTTTATGAGACCACTGTCTTGCCATAGGCCTC
TGGCTTCTGCTATGTCAACGACA
>418
CATCCTAGGGTAGGTGTTTAGGATGATGGTGGGGGGCAGCTGGGAGGGGAATTGCTCT
TCTCTTTATGAGACCACTGTCTTGCCATAGGCCTCTGGCTTCTGCTATGTCAACGACATT
GTGATTGGCATCCTGGAGCTGC

>419
TAGGATGATGGTGGGGGGCAGCTGGGAGGGGAATTGCTCTTCTCTTTATGAGACCACT
GTCTTGCCATAGGCCTCTGGCTTCTGCTATGTCAACGACATTGTGATTGGCATCCTGGA
GCTGCTCAAGTAAGTAGCCCAGG
>420
TGAGACCACTGTCTTGCCATAGGCCTCTGGCTTCTGCTATGTCAACGACATTGTGATTG
GCATCCTGGAGCTGCTCAAGTAAGTAGCCCAGGAGGAGATGGGGGCAGTTACGGGATT
CTTGGCTGAGGAGGTCTCTCAGT
>421
TGTCTTGCCATAGGCCTCTGGCTTCTGCTATGTCAACGACATTGTGATTGGCATCCTGG
AGCTGCTCAAGTAAGTAGCCCAGGAGGAGATGGGGGCAGTTACGGGATTCTTGGCTGA
GGAGGTCTCTCAGTCTCAACCCT
>422
GGCCTCTGGCTTCTGCTATGTCAACGACATTGTGATTGGCATCCTGGAGCTGCTCAAGT
AAGTAGCCCAGGAGGAGATGGGGGCAGTTACGGGATTCTTGGCTGAGGAGGTCTCTC
AGTCTCAACCCTGACCTTGACCTC
>423
CTCTGGCTTCTGCTATGTCAACGACATTGTGATTGGCATCCTGGAGCTGCTCAAGTAAG
TAGCCCAGGAGGAGATGGGGGCAGTTACGGGATTCTTGGCTGAGGAGGTCTCTCAGTC
TCAACCCTGACCTTGACCTCCCT
>424
TGGCTTCTGCTATGTCAACGACATTGTGATTGGCATCCTGGAGCTGCTCAAGTAAGTAG
CCCAGGAGGAGATGGGGGCAGTTACGGGATTCTTGGCTGAGGAGGTCTCTCAGTCTCA
ACCCTGACCTTGACCTCCCTCCT
>425
TATGTCAACGACATTGTGATTGGCATCCTGGAGCTGCTCAAGTAAGTAGCCCAGGAGG
AGATGGGGGCAGTTACGGGATTCTTGGCTGAGGAGGTCTCTCAGTCTCAACCCTGACC
TTGACCTCCCTCCTCCCTGATTAG
>426
TGGCATCCTGGAGCTGCTCAAGTAAGTAGCCCAGGAGGAGATGGGGGCAGTTACGGG
ATTCTTGGCTGAGGAGGTCTCTCAGTCTCAACCCTGACCTTGACCTCCCTCCTCCCTGA
TTAGCTTCTCCCAAGTTCTTGATG
>427
TTACGGGATTCTTGGCTGAGGAGGTCTCTCAGTCTCAACCCTGACCTTGACCTCCCTCC
TCCCTGATTAGCTTCTCCCAAGTTCTTGATGCTTTATTCAGGCTGCCTCTTCCACTGCTG
CAGATTCCCTACTGCCTGCTC
>428
TTGGCTGAGGAGGTCTCTCAGTCTCAACCCTGACCTTGACCTCCCTCCTCCCTGATTAG
CTTCTCCCAAGTTCTTGATGCTTTATTCAGGCTGCCTCTTCCACTGCTGCAGATTCCCTA
CTGCCTGCTCACATTTCAGCC
>429
TCCCTCCTCCCTGATTAGCTTCTCCCAAGTTCTTGATGCTTTATTCAGGCTGCCTCTTCC
ACTGCTGCAGATTCCCTACTGCCTGCTCACATTTCAGCCTTCTCTGCATATCCCTCTCCT
GTCCCTGCCTCTAGCCTCTA

>430
AGTTCTTGATGCTTTATTCAGGCTGCCTCTTCCACTGCTGCAGATTCCCTACTGCCTGCT
CACATTTCAGCCTTCTCTGCATATCCCTCTCCTGTCCCTGCCTCTAGCCTCTAGGCTGGA
GCCCCCAGTTGCCTTTAGGG
>431
CTGCAGATTCCCTACTGCCTGCTCACATTTCAGCCTTCTCTGCATATCCCTCTCCTGTCC
CTGCCTCTAGCCTCTAGGCTGGAGCCCCCAGTTGCCTTTAGGGAACTGGTGACATTAA
GTTTCAGGTGACCAGGGCAGAA
>432
TTCCCTACTGCCTGCTCACATTTCAGCCTTCTCTGCATATCCCTCTCCTGTCCCTGCCTCT
AGCCTCTAGGCTGGAGCCCCCAGTTGCCTTTAGGGAACTGGTGACATTAAGTTTCAGG
TGACCAGGGCAGAAGCAGACT
>433
CTCTCCTGTCCCTGCCTCTAGCCTCTAGGCTGGAGCCCCCAGTTGCCTTTAGGGAACTG
GTGACATTAAGTTTCAGGTGACCAGGGCAGAAGCAGACTCTTCACAATTCGAGACCCG
GTGTTTGAGAGGAGTGGGTTGGG
>434
TGTCCCTGCCTCTAGCCTCTAGGCTGGAGCCCCCAGTTGCCTTTAGGGAACTGGTGACA
TTAAGTTTCAGGTGACCAGGGCAGAAGCAGACTCTTCACAATTCGAGACCCGGTGTTT
GAGAGGAGTGGGTTGGGGGGTTC
>435
CTCTAGGCTGGAGCCCCCAGTTGCCTTTAGGGAACTGGTGACATTAAGTTTCAGGTGA
CCAGGGCAGAAGCAGACTCTTCACAATTCGAGACCCGGTGTTTGAGAGGAGTGGGTT
GGGGGGTTCACTTCTAGCTCAGACC
>436
CTGGTGACATTAAGTTTCAGGTGACCAGGGCAGAAGCAGACTCTTCACAATTCGAGAC
CCGGTGTTTGAGAGGAGTGGGTTGGGGGGTTCACTTCTAGCTCAGACCCTGGCTTTGG
CTTCCCTCTAGGTACCACCCTCGG
>437
GGTGACATTAAGTTTCAGGTGACCAGGGCAGAAGCAGACTCTTCACAATTCGAGACCC
GGTGTTTGAGAGGAGTGGGTTGGGGGGTTCACTTCTAGCTCAGACCCTGGCTTTGGCT
TCCCTCTAGGTACCACCCTCGGGT
>438
GGCAGAAGCAGACTCTTCACAATTCGAGACCCGGTGTTTGAGAGGAGTGGGTTGGGG
GGTTCACTTCTAGCTCAGACCCTGGCTTTGGCTTCCCTCTAGGTACCACCCTCGGGTGC
TCTACATTGACATTGACATCCACC
>439
CTTCCCTCTAGGTACCACCCTCGGGTGCTCTACATTGACATTGACATCCACCATGGTGA
CGGGGTTCAAGAAGCTTTCTACCTCACTGACCGGGTCATGACGGTGTCCTTCCACAAA
TACGGAAATTACTTCTTCCCTGG
>440
CCCTCTAGGTACCACCCTCGGGTGCTCTACATTGACATTGACATCCACCATGGTGACGG
GGTTCAAGAAGCTTTCTACCTCACTGACCGGGTCATGACGGTGTCCTTCCACAAATAC
GGAAATTACTTCTTCCCTGGCAC

>441
TTCTACCTCACTGACCGGGTCATGACGGTGTCCTTCCACAAATACGGAAATTACTTCTT
CCCTGGCACAGGTATGGGAATAGGGATTACTTTCCCCTTTGCACCCTTCAGCTCTTTACT
ATGAGTGCCTCCTTCTCAGCC
>442
GACCGGGTCATGACGGTGTCCTTCCACAAATACGGAAATTACTTCTTCCCTGGCACAG
GTATGGGAATAGGGATTACTTTCCCCTTTGCACCCTTCAGCTCTTTACTATGAGTGCCTC
CTTCTCAGCCTTCTAGTCTCAT
>443
TTCTTCCCTGGCACAGGTATGGGAATAGGGATTACTTTCCCCTTTGCACCCTTCAGCTCT
TTACTATGAGTGCCTCCTTCTCAGCCTTCTAGTCTCATCTCAGGTGTCCCCTCCTCAGAG
AAGCCTTTCCTTACCACTCC
>444
AGGTATGGGAATAGGGATTACTTTCCCCTTTGCACCCTTCAGCTCTTTACTATGAGTGCC
TCCTTCTCAGCCTTCTAGTCTCATCTCAGGTGTCCCCTCCTCAGAGAAGCCTTTCCTTA
CCACTCCAGTTATCATAATCA
>445
ACTTTCCCCTTTGCACCCTTCAGCTCTTTACTATGAGTGCCTCCTTCTCAGCCTTCTAGT
CTCATCTCAGGTGTCCCCTCCTCAGAGAAGCCTTTCCTTACCACTCCAGTTATCATAATC
ACCTCCTTCCCCATTCCTCT
>446
CCCTTCAGCTCTTTACTATGAGTGCCTCCTTCTCAGCCTTCTAGTCTCATCTCAGGTGTC
CCCTCCTCAGAGAAGCCTTTCCTTACCACTCCAGTTATCATAATCACCTCCTTCCCCATT
CCTCTGTTACATCAGCCTCA
>447
CTTCAGCTCTTTACTATGAGTGCCTCCTTCTCAGCCTTCTAGTCTCATCTCAGGTGTCCC
CTCCTCAGAGAAGCCTTTCCTTACCACTCCAGTTATCATAATCACCTCCTTCCCCATTCC
TCTGTTACATCAGCCTCATT
>448
CAGCTCTTTACTATGAGTGCCTCCTTCTCAGCCTTCTAGTCTCATCTCAGGTGTCCCCTC
CTCAGAGAAGCCTTTCCTTACCACTCCAGTTATCATAATCACCTCCTTCCCCATTCCTCT
GTTACATCAGCCTCATTGTT
>449
CCTCCTTCTCAGCCTTCTAGTCTCATCTCAGGTGTCCCCTCCTCAGAGAAGCCTTTCCTT
ACCACTCCAGTTATCATAATCACCTCCTTCCCCATTCCTCTGTTACATCAGCCTCATTGTT
TCCCTCAGAACACTCACTG
>450
CTCAGAGAAGCCTTTCCTTACCACTCCAGTTATCATAATCACCTCCTTCCCCATTCCTCT
GTTACATCAGCCTCATTGTTTCCCTCAGAACACTCACTGCTATTTGTGTTTATTGTGGTT
TTTTGTTTTGTTTTGTTTAC
>451
TACCACTCCAGTTATCATAATCACCTCCTTCCCCATTCCTCTGTTACATCAGCCTCATTGT
TTCCCTCAGAACACTCACTGCTATTTGTGTTTATTGTGGTTTTTTGTTTTGTTTTGTTTAC
TGCCTCCCTCCTCCTCAT

>452
TTTGTGTTTATTGTGGTTTTTTGTTTTGTTTTGTTTACTGCCTCCCTCCTCCTCATGGCTA
TTCCCTAAGAATATAAGCGTGGTGGGATATCGTAAATTTCATTAGTTCCATTCGTTGCTG
CATCCCTACTGCCTAGTAC
>453
TATTGTGGTTTTTTGTTTTGTTTTGTTTACTGCCTCCCTCCTCCTCATGGCTATTCCCTAA
GAATATAAGCGTGGTGGGATATCGTAAATTTCATTAGTTCCATTCGTTGCTGCATCCCTA
CTGCCTAGTACACAGTTGG
>454
ACTGCCTCCCTCCTCCTCATGGCTATTCCCTAAGAATATAAGCGTGGTGGGATATCGTAA
ATTTCATTAGTTCCATTCGTTGCTGCATCCCTACTGCCTAGTACACAGTTGGCTCAGAGT
GGATCCCCCAAAACTATTTG
>455
AAGAATATAAGCGTGGTGGGATATCGTAAATTTCATTAGTTCCATTCGTTGCTGCATCCC
TACTGCCTAGTACACAGTTGGCTCAGAGTGGATCCCCCAAAACTATTTGCCCAATGAAT
GCAAGCTGCAAGAGATTTAGT
>456
TAAGCGTGGTGGGATATCGTAAATTTCATTAGTTCCATTCGTTGCTGCATCCCTACTGCC
TAGTACACAGTTGGCTCAGAGTGGATCCCCCAAAACTATTTGCCCAATGAATGCAAGCT
GCAAGAGATTTAGTGGCTCTA
>457
TGGGATATCGTAAATTTCATTAGTTCCATTCGTTGCTGCATCCCTACTGCCTAGTACACA
GTTGGCTCAGAGTGGATCCCCCAAAACTATTTGCCCAATGAATGCAAGCTGCAAGAGA
TTTAGTGGCTCTAGGGAGTTTA
>458
CATCCCTACTGCCTAGTACACAGTTGGCTCAGAGTGGATCCCCCAAAACTATTTGCCCA
ATGAATGCAAGCTGCAAGAGATTTAGTGGCTCTAGGGAGTTTATGGTGAAGCTACATAG
ATGGAATGAGAGGCTCCCAGAG
>459
GTACACAGTTGGCTCAGAGTGGATCCCCCAAAACTATTTGCCCAATGAATGCAAGCTG
CAAGAGATTTAGTGGCTCTAGGGAGTTTATGGTGAAGCTACATAGATGGAATGAGAGG
CTCCCAGAGTGCAGATTGGGGCTG
>460
TGGCTCAGAGTGGATCCCCCAAAACTATTTGCCCAATGAATGCAAGCTGCAAGAGATT
TAGTGGCTCTAGGGAGTTTATGGTGAAGCTACATAGATGGAATGAGAGGCTCCCAGAG
TGCAGATTGGGGCTGAAACAGCTG
>461
CCCCAAAACTATTTGCCCAATGAATGCAAGCTGCAAGAGATTTAGTGGCTCTAGGGAG
TTTATGGTGAAGCTACATAGATGGAATGAGAGGCTCCCAGAGTGCAGATTGGGGCTGA
AACAGCTGGGTTGTGGGTGCTTCC
>462
TGAATGCAAGCTGCAAGAGATTTAGTGGCTCTAGGGAGTTTATGGTGAAGCTACATAGA
TGGAATGAGAGGCTCCCAGAGTGCAGATTGGGGCTGAAACAGCTGGGTTGTGGGTGC
TTCCCATGTGTGGCTACCACCACC

>463
AGAGATTTAGTGGCTCTAGGGAGTTTATGGTGAAGCTACATAGATGGAATGAGAGGCTC
CCAGAGTGCAGATTGGGGCTGAAACAGCTGGGTTGTGGGTGCTTCCCATGTGTGGCTA
CCACCACCCACTTCCCTACAGGT
>464
TAGGGAGTTTATGGTGAAGCTACATAGATGGAATGAGAGGCTCCCAGAGTGCAGATTG
GGGCTGAAACAGCTGGGTTGTGGGTGCTTCCCATGTGTGGCTACCACCACCCACTTCC
CTACAGGTGACATGTATGAAGTCG
>465
CAGCTGGGTTGTGGGTGCTTCCCATGTGTGGCTACCACCACCCACTTCCCTACAGGTG
ACATGTATGAAGTCGGGGCAGAGAGTGGCCGCTACTACTGTCTGAACGTGCCCCTGCG
GGATGGCATTGATGACCAGAGTAA
>466
TGAAGTCGGGGCAGAGAGTGGCCGCTACTACTGTCTGAACGTGCCCCTGCGGGATGG
CATTGATGACCAGAGTAAGTACCGAAGTCTTCCCTCTGTTCCCTAGGCCCCTTCTTTTT
CTCCTTGAAATAACCTCTTCTGCC
>467
AAGTCGGGGCAGAGAGTGGCCGCTACTACTGTCTGAACGTGCCCCTGCGGGATGGCAT
TGATGACCAGAGTAAGTACCGAAGTCTTCCCTCTGTTCCCTAGGCCCCTTCTTTTTCTC
CTTGAAATAACCTCTTCTGCCTG
>468
TCTGAACGTGCCCCTGCGGGATGGCATTGATGACCAGAGTAAGTACCGAAGTCTTCCC
TCTGTTCCCTAGGCCCCTTCTTTTTCTCCTTGAAATAACCTCTTCTGCCTGGTATTGTCT
GCTGTTGGGCCAGTTGTCCTTA
>469
GTTCCCTAGGCCCCTTCTTTTTCTCCTTGAAATAACCTCTTCTGCCTGGTATTGTCTGCT
GTTGGGCCAGTTGTCCTTAAGGATGAATGGCCTTCAGAGGGACCATGAATCTCTGGAA
ATTGTATGCAACATATCATGTG
>470
CCCTTCTTTTTCTCCTTGAAATAACCTCTTCTGCCTGGTATTGTCTGCTGTTGGGCCAGT
TGTCCTTAAGGATGAATGGCCTTCAGAGGGACCATGAATCTCTGGAAATTGTATGCAAC
ATATCATGTGTATGTGCATTT
>471
TGAAATAACCTCTTCTGCCTGGTATTGTCTGCTGTTGGGCCAGTTGTCCTTAAGGATGA
ATGGCCTTCAGAGGGACCATGAATCTCTGGAAATTGTATGCAACATATCATGTGTATGTG
CATTTTTCTGGGAAAAGTCAT
>472
TCAGAGGGACCATGAATCTCTGGAAATTGTATGCAACATATCATGTGTATGTGCATTTTT
CTGGGAAAAGTCATTGGCTTTCATCAGTCACTCATAGCAGTCTGGGATCCCTTCCAGAA
GTTTCAGACCAGTGATTTGAT
>473
CTCTGGAAATTGTATGCAACATATCATGTGTATGTGCATTTTTCTGGGAAAAGTCATTGG
CTTTCATCAGTCACTCATAGCAGTCTGGGATCCCTTCCAGAAGTTTCAGACCAGTGATT
TGATTCTTTTGGGTGGCCCCT

>474
TGTATGCAACATATCATGTGTATGTGCATTTTTCTGGGAAAAGTCATTGGCTTTCATCAG
TCACTCATAGCAGTCTGGGATCCCTTCCAGAAGTTTCAGACCAGTGATTTGATTCTTTT
GGGTGGCCCCTTTCCTGGGCT
>475
ATGCAACATATCATGTGTATGTGCATTTTTCTGGGAAAAGTCATTGGCTTTCATCAGTCA
CTCATAGCAGTCTGGGATCCCTTCCAGAAGTTTCAGACCAGTGATTTGATTCTTTTGGG
TGGCCCCTTTCCTGGGCTACA
>476
TATGTGCATTTTTCTGGGAAAAGTCATTGGCTTTCATCAGTCACTCATAGCAGTCTGGG
ATCCCTTCCAGAAGTTTCAGACCAGTGATTTGATTCTTTTGGGTGGCCCCTTTCCTGGG
CTACATGCTCGCTCAACCCCCT
>477
CTGGGCTACATGCTCGCTCAACCCCCTTTTTCCTTTTGTTCCCTGTCTCCCCATCCCCTT
GGGGTGCCAGGTGCCCATCTTTGGCCCTCTCCCAGGTTACAAGCACCTTTTCCAGCCG
GTTATCAACCAGGTAGTGGACT
>478
TCTCCCCATCCCCTTGGGGTGCCAGGTGCCCATCTTTGGCCCTCTCCCAGGTTACAAGC
ACCTTTTCCAGCCGGTTATCAACCAGGTAGTGGACTTCTACCAACCCACGTGCATTGTG
CTCCAGGTAATACTGTCAGTTC
>479
GGGGTGCCAGGTGCCCATCTTTGGCCCTCTCCCAGGTTACAAGCACCTTTTCCAGCCG
GTTATCAACCAGGTAGTGGACTTCTACCAACCCACGTGCATTGTGCTCCAGGTAATACT
GTCAGTTCCTCCTCAGGAGTGTT
>480
CAAGCACCTTTTCCAGCCGGTTATCAACCAGGTAGTGGACTTCTACCAACCCACGTGC
ATTGTGCTCCAGGTAATACTGTCAGTTCCTCCTCAGGAGTGTTCTGCCTGGGCTTGGGG
AAAGAGAGCAAGGGAAGAGGAGG
>481
CAGCCGGTTATCAACCAGGTAGTGGACTTCTACCAACCCACGTGCATTGTGCTCCAGGT
AATACTGTCAGTTCCTCCTCAGGAGTGTTCTGCCTGGGCTTGGGGAAAGAGAGCAAGG
GAAGAGGAGGTTCTTTAGGTTGG
>482
CAACCAGGTAGTGGACTTCTACCAACCCACGTGCATTGTGCTCCAGGTAATACTGTCA
GTTCCTCCTCAGGAGTGTTCTGCCTGGGCTTGGGGAAAGAGAGCAAGGGAAGAGGAG
GTTCTTTAGGTTGGTGGTGGAAGGG
>483
CACGTGCATTGTGCTCCAGGTAATACTGTCAGTTCCTCCTCAGGAGTGTTCTGCCTGGG
CTTGGGGAAAGAGAGCAAGGGAAGAGGAGGTTCTTTAGGTTGGTGGTGGAAGGGGTA
TCTGTGATGTTCTGATCAGTTCTG
>484
CTCCAGGTAATACTGTCAGTTCCTCCTCAGGAGTGTTCTGCCTGGGCTTGGGGAAAGA
GAGCAAGGGAAGAGGAGGTTCTTTAGGTTGGTGGTGGAAGGGGTATCTGTGATGTTCT
GATCAGTTCTGATCACTGCACTTT

>485
CCAGGTAATACTGTCAGTTCCTCCTCAGGAGTGTTCTGCCTGGGCTTGGGGAAAGAGA
GCAAGGGAAGAGGAGGTTCTTTAGGTTGGTGGTGGAAGGGGTATCTGTGATGTTCTGA
TCAGTTCTGATCACTGCACTTTCC
>486
GTCAGTTCCTCCTCAGGAGTGTTCTGCCTGGGCTTGGGGAAAGAGAGCAAGGGAAGA
GGAGGTTCTTTAGGTTGGTGGTGGAAGGGGTATCTGTGATGTTCTGATCAGTTCTGATC
ACTGCACTTTCCTGGGGTTTTGCT
>487
AGGAGTGTTCTGCCTGGGCTTGGGGAAAGAGAGCAAGGGAAGAGGAGGTTCTTTAGG
TTGGTGGTGGAAGGGGTATCTGTGATGTTCTGATCAGTTCTGATCACTGCACTTTCCTG
GGGTTTTGCTTTTTGCAGTGTGGA
>488
TGGAAGGGGTATCTGTGATGTTCTGATCAGTTCTGATCACTGCACTTTCCTGGGGTTTT
GCTTTTTGCAGTGTGGAGCTGACTCTCTGGGCTGTGATCGATTGGGCTGCTTTAACCTC
AGCATCCGAGGGCATGGGTGAG
>489
GGTATCTGTGATGTTCTGATCAGTTCTGATCACTGCACTTTCCTGGGGTTTTGCTTTTTG
CAGTGTGGAGCTGACTCTCTGGGCTGTGATCGATTGGGCTGCTTTAACCTCAGCATCCG
AGGGCATGGGTGAGACTTGCT
>490
TGGGGTTTTGCTTTTTGCAGTGTGGAGCTGACTCTCTGGGCTGTGATCGATTGGGCTGC
TTTAACCTCAGCATCCGAGGGCATGGGTGAGACTTGCTCTCCTTCTTCTCCCCAAGGTT
TTTAAGTGGCCTTGGACTTCAG
>491
TGCTTTTTGCAGTGTGGAGCTGACTCTCTGGGCTGTGATCGATTGGGCTGCTTTAACCT
CAGCATCCGAGGGCATGGGTGAGACTTGCTCTCCTTCTTCTCCCCAAGGTTTTTAAGTG
GCCTTGGACTTCAGTACGTGGG
>492
ATCGATTGGGCTGCTTTAACCTCAGCATCCGAGGGCATGGGTGAGACTTGCTCTCCTTC
TTCTCCCCAAGGTTTTTAAGTGGCCTTGGACTTCAGTACGTGGGTGGGGGTAGTTGGG
GAATGGGCCTCACCTCTTCTTGG
>493
GCTGCTTTAACCTCAGCATCCGAGGGCATGGGTGAGACTTGCTCTCCTTCTTCTCCCCA
AGGTTTTTAAGTGGCCTTGGACTTCAGTACGTGGGTGGGGGTAGTTGGGGAATGGGCC
TCACCTCTTCTTGGAAATCAAAA
>494
CATCCGAGGGCATGGGTGAGACTTGCTCTCCTTCTTCTCCCCAAGGTTTTTAAGTGGCC
TTGGACTTCAGTACGTGGGTGGGGGTAGTTGGGGAATGGGCCTCACCTCTTCTTGGAA
ATCAAAAATAGCTACAAATTGTA
>495
CTTGGACTTCAGTACGTGGGTGGGGGTAGTTGGGGAATGGGCCTCACCTCTTCTTGGA
AATCAAAAATAGCTACAAATTGTACTTGAACTAGAAATGTCATCTTGAACAGGGTTCCC
TAGATGGTCATTCATTCACGATA

>496
GGGGTAGTTGGGGAATGGGCCTCACCTCTTCTTGGAAATCAAAAATAGCTACAAATTGT
ACTTGAACTAGAAATGTCATCTTGAACAGGGTTCCCTAGATGGTCATTCATTCACGATAT
ATTTTTATTAAGCACCTGTTA
>497
GCCTCACCTCTTCTTGGAAATCAAAAATAGCTACAAATTGTACTTGAACTAGAAATGTC
ATCTTGAACAGGGTTCCCTAGATGGTCATTCATTCACGATATATTTTTATTAAGCACCTGT
TATGTTCCAGGCATCATACC
>498
TTCTTGGAAATCAAAAATAGCTACAAATTGTACTTGAACTAGAAATGTCATCTTGAACA
GGGTTCCCTAGATGGTCATTCATTCACGATATATTTTTATTAAGCACCTGTTATGTTCCAG
GCATCATACCACATCATGGG
>499
TTGAACTAGAAATGTCATCTTGAACAGGGTTCCCTAGATGGTCATTCATTCACGATATAT
TTTTATTAAGCACCTGTTATGTTCCAGGCATCATACCACATCATGGGGATGGAATAAGGA
GTCTCCCATGGAGTTTACAG
>500
TCTTGAACAGGGTTCCCTAGATGGTCATTCATTCACGATATATTTTTATTAAGCACCTGTT
ATGTTCCAGGCATCATACCACATCATGGGGATGGAATAAGGAGTCTCCCATGGAGTTTA
CAGTTTAGGAAACAGACAAA
>501
TTCACGATATATTTTTATTAAGCACCTGTTATGTTCCAGGCATCATACCACATCATGGGGA
TGGAATAAGGAGTCTCCCATGGAGTTTACAGTTTAGGAAACAGACAAAAAATAAATAA
TCAAATGAAATAAATAGATTC
>502
TATTAAGCACCTGTTATGTTCCAGGCATCATACCACATCATGGGGATGGAATAAGGAGT
CTCCCATGGAGTTTACAGTTTAGGAAACAGACAAAAAATAAATAATCAAATGAAATAAA
TAGATTCTTATAAATTCTGTGA
>503
CACCTGTTATGTTCCAGGCATCATACCACATCATGGGGATGGAATAAGGAGTCTCCCAT
GGAGTTTACAGTTTAGGAAACAGACAAAAAATAAATAATCAAATGAAATAAATAGATTC
TTATAAATTCTGTGAAAGAAAA
>504
GTTATGTTCCAGGCATCATACCACATCATGGGGATGGAATAAGGAGTCTCCCATGGAGT
TTACAGTTTAGGAAACAGACAAAAAATAAATAATCAAATGAAATAAATAGATTCTTATA
AATTCTGTGAAAGAAAAAAAGA
>505
TCCAGGCATCATACCACATCATGGGGATGGAATAAGGAGTCTCCCATGGAGTTTACAGT
TTAGGAAACAGACAAAAAATAAATAATCAAATGAAATAAATAGATTCTTATAAATTCTG
TGAAAGAAAAAAAGAAAGCTGA
>506
TAAGGAGTCTCCCATGGAGTTTACAGTTTAGGAAACAGACAAAAAATAAATAATCAAA
TGAAATAAATAGATTCTTATAAATTCTGTGAAAGAAAAAAAGAAAGCTGAGACACAGG
ATACAATGGGGGACCTACTTGAGA

>507
ACAGTTTAGGAAACAGACAAAAAATAAATAATCAAATGAAATAAATAGATTCTTATAAA
TTCTGTGAAAGAAAAAAAGAAAGCTGAGACACAGGATACAATGGGGGACCTACTTGA
GATAGCATGGTTTCGGAAGTTTTT
>508
GGAAACAGACAAAAAATAAATAATCAAATGAAATAAATAGATTCTTATAAATTCTGTGA
AAGAAAAAAAGAAAGCTGAGACACAGGATACAATGGGGGACCTACTTGAGATAGCAT
GGTTTCGGAAGTTTTTCTGAGGAA
>509
ATAAATAATCAAATGAAATAAATAGATTCTTATAAATTCTGTGAAAGAAAAAAAGAAAG
CTGAGACACAGGATACAATGGGGGACCTACTTGAGATAGCATGGTTTCGGAAGTTTTT
CTGAGGAAGTAAAATTTGAGTTG
>510
CTTATAAATTCTGTGAAAGAAAAAAAGAAAGCTGAGACACAGGATACAATGGGGGAC
CTACTTGAGATAGCATGGTTTCGGAAGTTTTTCTGAGGAAGTAAAATTTGAGTTGAGAC
CTGGAGACTAAGAAGGAGTGAGTC
>511
GAAAGAAAAAAAGAAAGCTGAGACACAGGATACAATGGGGGACCTACTTGAGATAGC
ATGGTTTCGGAAGTTTTTCTGAGGAAGTAAAATTTGAGTTGAGACCTGGAGACTAAGA
AGGAGTGAGTCATAAGAGGAGCAGA
>512
AAGAAAGCTGAGACACAGGATACAATGGGGGACCTACTTGAGATAGCATGGTTTCGGA
AGTTTTTCTGAGGAAGTAAAATTTGAGTTGAGACCTGGAGACTAAGAAGGAGTGAGT
CATAAGAGGAGCAGAGAGAAGCCTG
>513
GGGACCTACTTGAGATAGCATGGTTTCGGAAGTTTTTCTGAGGAAGTAAAATTTGAGTT
GAGACCTGGAGACTAAGAAGGAGTGAGTCATAAGAGGAGCAGAGAGAAGCCTGACC
CAAGCATGGACAAAGGCCCACAAAA
>514
TACTTGAGATAGCATGGTTTCGGAAGTTTTTCTGAGGAAGTAAAATTTGAGTTGAGACC
TGGAGACTAAGAAGGAGTGAGTCATAAGAGGAGCAGAGAGAAGCCTGACCCAAGCAT
GGACAAAGGCCCACAAAAGGCTCT
>515
TTGAGATAGCATGGTTTCGGAAGTTTTTCTGAGGAAGTAAAATTTGAGTTGAGACCTG
GAGACTAAGAAGGAGTGAGTCATAAGAGGAGCAGAGAGAAGCCTGACCCAAGCATG
GACAAAGGCCCACAAAAGGCTCTGAG
>516
AGATAGCATGGTTTCGGAAGTTTTTCTGAGGAAGTAAAATTTGAGTTGAGACCTGGAG
ACTAAGAAGGAGTGAGTCATAAGAGGAGCAGAGAGAAGCCTGACCCAAGCATGGAC
AAAGGCCCACAAAAGGCTCTGAGGGC
>517
TTTCGGAAGTTTTTCTGAGGAAGTAAAATTTGAGTTGAGACCTGGAGACTAAGAAGGA
GTGAGTCATAAGAGGAGCAGAGAGAAGCCTGACCCAAGCATGGACAAAGGCCCACA
AAAGGCTCTGAGGGCTGGGCCCAGTG

>518
TGAGGAAGTAAAATTTGAGTTGAGACCTGGAGACTAAGAAGGAGTGAGTCATAAGAG
GAGCAGAGAGAAGCCTGACCCAAGCATGGACAAAGGCCCACAAAAGGCTCTGAGGG
CTGGGCCCAGTGGCACACACCTATAAT
>519
CTAAGAAGGAGTGAGTCATAAGAGGAGCAGAGAGAAGCCTGACCCAAGCATGGACA
AAGGCCCACAAAAGGCTCTGAGGGCTGGGCCCAGTGGCACACACCTATAATCCCAAC
ACTTCCAGAGGCCAAGGTGGGAGAATC
>520
GAGTGAGTCATAAGAGGAGCAGAGAGAAGCCTGACCCAAGCATGGACAAAGGCCCA
CAAAAGGCTCTGAGGGCTGGGCCCAGTGGCACACACCTATAATCCCAACACTTCCAGA
GGCCAAGGTGGGAGAATCGCCTGAGG
>521
AGAGGAGCAGAGAGAAGCCTGACCCAAGCATGGACAAAGGCCCACAAAAGGCTCTG
AGGGCTGGGCCCAGTGGCACACACCTATAATCCCAACACTTCCAGAGGCCAAGGTGG
GAGAATCGCCTGAGGCTAGGAGTTTGA
>522
GGACAAAGGCCCACAAAAGGCTCTGAGGGCTGGGCCCAGTGGCACACACCTATAATC
CCAACACTTCCAGAGGCCAAGGTGGGAGAATCGCCTGAGGCTAGGAGTTTGAGGCCA
GCCTGGGCAACATAGTGAGAGCCATC
>523
GCCCACAAAAGGCTCTGAGGGCTGGGCCCAGTGGCACACACCTATAATCCCAACACTT
CCAGAGGCCAAGGTGGGAGAATCGCCTGAGGCTAGGAGTTTGAGGCCAGCCTGGGCA
ACATAGTGAGAGCCATCTCTACAGA
>524
AAAGGCTCTGAGGGCTGGGCCCAGTGGCACACACCTATAATCCCAACACTTCCAGAGG
CCAAGGTGGGAGAATCGCCTGAGGCTAGGAGTTTGAGGCCAGCCTGGGCAACATAGT
GAGAGCCATCTCTACAGAAAATAAA
>525
GGGCTGGGCCCAGTGGCACACACCTATAATCCCAACACTTCCAGAGGCCAAGGTGGG
AGAATCGCCTGAGGCTAGGAGTTTGAGGCCAGCCTGGGCAACATAGTGAGAGCCATCT
CTACAGAAAATAAAAAACAAAATCA
>526
ACACCTATAATCCCAACACTTCCAGAGGCCAAGGTGGGAGAATCGCCTGAGGCTAGGA
GTTTGAGGCCAGCCTGGGCAACATAGTGAGAGCCATCTCTACAGAAAATAAAAAACAA
AATCAGCTGGGCATGATGGTGCAT
>527
TCCAGAGGCCAAGGTGGGAGAATCGCCTGAGGCTAGGAGTTTGAGGCCAGCCTGGGC
AACATAGTGAGAGCCATCTCTACAGAAAATAAAAAACAAAATCAGCTGGGCATGATGG
TGCATGCCTATAGTCCTAGCTACTC
>528
GGTGGGAGAATCGCCTGAGGCTAGGAGTTTGAGGCCAGCCTGGGCAACATAGTGAGA
GCCATCTCTACAGAAAATAAAAAACAAAATCAGCTGGGCATGATGGTGCATGCCTATAG
TCCTAGCTACTCAGGAAGCTGAGG

>529
CTAGGAGTTTGAGGCCAGCCTGGGCAACATAGTGAGAGCCATCTCTACAGAAAATAAA
AAACAAAATCAGCTGGGCATGATGGTGCATGCCTATAGTCCTAGCTACTCAGGAAGCT
GAGGCAGGAGGATCACTTGAGCCT
>530
TGAGAGCCATCTCTACAGAAAATAAAAAACAAAATCAGCTGGGCATGATGGTGCATGC
CTATAGTCCTAGCTACTCAGGAAGCTGAGGCAGGAGGATCACTTGAGCCTAGGAGTTT
GAGGTTACAGTGAACTATGATTGT
>531
ATCTCTACAGAAAATAAAAAACAAAATCAGCTGGGCATGATGGTGCATGCCTATAGTCC
TAGCTACTCAGGAAGCTGAGGCAGGAGGATCACTTGAGCCTAGGAGTTTGAGGTTACA
GTGAACTATGATTGTGCCACTGG
>532
GAAAATAAAAAACAAAATCAGCTGGGCATGATGGTGCATGCCTATAGTCCTAGCTACTC
AGGAAGCTGAGGCAGGAGGATCACTTGAGCCTAGGAGTTTGAGGTTACAGTGAACTAT
GATTGTGCCACTGGACTTCAGTC
>533
GGGCATGATGGTGCATGCCTATAGTCCTAGCTACTCAGGAAGCTGAGGCAGGAGGATC
ACTTGAGCCTAGGAGTTTGAGGTTACAGTGAACTATGATTGTGCCACTGGACTTCAGTC
TGTTGAGGTGAGGTCCGAGAAGC
>534
GCCTATAGTCCTAGCTACTCAGGAAGCTGAGGCAGGAGGATCACTTGAGCCTAGGAGT
TTGAGGTTACAGTGAACTATGATTGTGCCACTGGACTTCAGTCTGTTGAGGTGAGGTC
CGAGAAGCTGGCCATGGTGAAGAT
>535
AGGCAGGAGGATCACTTGAGCCTAGGAGTTTGAGGTTACAGTGAACTATGATTGTGCC
ACTGGACTTCAGTCTGTTGAGGTGAGGTCCGAGAAGCTGGCCATGGTGAAGATTGTGC
ATGAGCCTTTAAACCCTGGTAAGG
>536
GATCACTTGAGCCTAGGAGTTTGAGGTTACAGTGAACTATGATTGTGCCACTGGACTTC
AGTCTGTTGAGGTGAGGTCCGAGAAGCTGGCCATGGTGAAGATTGTGCATGAGCCTTT
AAACCCTGGTAAGGAGTTTGAAT
>537
AGTGAACTATGATTGTGCCACTGGACTTCAGTCTGTTGAGGTGAGGTCCGAGAAGCTG
GCCATGGTGAAGATTGTGCATGAGCCTTTAAACCCTGGTAAGGAGTTTGAATTATATTC
CAAAGATGATGGGAAACCATTGA
>538
TTGTGCCACTGGACTTCAGTCTGTTGAGGTGAGGTCCGAGAAGCTGGCCATGGTGAAG
ATTGTGCATGAGCCTTTAAACCCTGGTAAGGAGTTTGAATTATATTCCAAAGATGATGG
GAAACCATTGAAGGATTTTAAGC
>539
GTCTGTTGAGGTGAGGTCCGAGAAGCTGGCCATGGTGAAGATTGTGCATGAGCCTTTA
AACCCTGGTAAGGAGTTTGAATTATATTCCAAAGATGATGGGAAACCATTGAAGGATTT
TAAGCAGGAAAGTGACATGATCC

>540
AAGCTGGCCATGGTGAAGATTGTGCATGAGCCTTTAAACCCTGGTAAGGAGTTTGAATT
ATATTCCAAAGATGATGGGAAACCATTGAAGGATTTTAAGCAGGAAAGTGACATGATC
CAATTTGTGTTTGAAAGTGACAA
>541
TGTGCATGAGCCTTTAAACCCTGGTAAGGAGTTTGAATTATATTCCAAAGATGATGGGA
AACCATTGAAGGATTTTAAGCAGGAAAGTGACATGATCCAATTTGTGTTTGAAAGTGA
CAATTCTGACTGCTGAATAGAGA
>542
GCCTTTAAACCCTGGTAAGGAGTTTGAATTATATTCCAAAGATGATGGGAAACCATTGA
AGGATTTTAAGCAGGAAAGTGACATGATCCAATTTGTGTTTGAAAGTGACAATTCTGA
CTGCTGAATAGAGAAAGGTTTGG
>543
CCAAAGATGATGGGAAACCATTGAAGGATTTTAAGCAGGAAAGTGACATGATCCAATT
TGTGTTTGAAAGTGACAATTCTGACTGCTGAATAGAGAAAGGTTTGGGTAGGGGGGAT
GGAGATGGAAGTAGAAGAGACCTA
>544
AAGGATTTTAAGCAGGAAAGTGACATGATCCAATTTGTGTTTGAAAGTGACAATTCTG
ACTGCTGAATAGAGAAAGGTTTGGGTAGGGGGGATGGAGATGGAAGTAGAAGAGACC
TAGTTAGGAAGCCATGGTGTTGGTT
>545
AAAGTGACATGATCCAATTTGTGTTTGAAAGTGACAATTCTGACTGCTGAATAGAGAA
AGGTTTGGGTAGGGGGGATGGAGATGGAAGTAGAAGAGACCTAGTTAGGAAGCCATG
GTGTTGGTTAGGTGCCCAGACTAAG
>546
TTGTGTTTGAAAGTGACAATTCTGACTGCTGAATAGAGAAAGGTTTGGGTAGGGGGGA
TGGAGATGGAAGTAGAAGAGACCTAGTTAGGAAGCCATGGTGTTGGTTAGGTGCCCAG
ACTAAGGTGGAGGGGGTGGCAATG
>547
TGTTTGAAAGTGACAATTCTGACTGCTGAATAGAGAAAGGTTTGGGTAGGGGGGATGG
AGATGGAAGTAGAAGAGACCTAGTTAGGAAGCCATGGTGTTGGTTAGGTGCCCAGACT
AAGGTGGAGGGGGTGGCAATGGAG
>548
GAAAGTGACAATTCTGACTGCTGAATAGAGAAAGGTTTGGGTAGGGGGGATGGAGAT
GGAAGTAGAAGAGACCTAGTTAGGAAGCCATGGTGTTGGTTAGGTGCCCAGACTAAG
GTGGAGGGGGTGGCAATGGAGAGAAG
>549
ATTCTGACTGCTGAATAGAGAAAGGTTTGGGTAGGGGGGATGGAGATGGAAGTAGAA
GAGACCTAGTTAGGAAGCCATGGTGTTGGTTAGGTGCCCAGACTAAGGTGGAGGGGG
TGGCAATGGAGAGAAGTAGATGATCT
>550
GGGTAGGGGGGATGGAGATGGAAGTAGAAGAGACCTAGTTAGGAAGCCATGGTGTTG
GTTAGGTGCCCAGACTAAGGTGGAGGGGGTGGCAATGGAGAGAAGTAGATGATCTGA
GAGGTGTGTGTGTGTGTGTGTGTATA

>551
TGGAGATGGAAGTAGAAGAGACCTAGTTAGGAAGCCATGGTGTTGGTTAGGTGCCCAG
ACTAAGGTGGAGGGGGTGGCAATGGAGAGAAGTAGATGATCTGAGAGGTGTGTGTGT
GTGTGTGTGTATATATATATATATATA
>552
AAGAGACCTAGTTAGGAAGCCATGGTGTTGGTTAGGTGCCCAGACTAAGGTGGAGGG
GGTGGCAATGGAGAGAAGTAGATGATCTGAGAGGTGTGTGTGTGTGTGTGTGTATATAT
ATATATATACACACACACACACAC
>553
GAGACCTAGTTAGGAAGCCATGGTGTTGGTTAGGTGCCCAGACTAAGGTGGAGGGGG
TGGCAATGGAGAGAAGTAGATGATCTGAGAGGTGTGTGTGTGTGTGTGTGTATATATAT
ATATATACACACACACACACACAT
>554
CATGGTGTTGGTTAGGTGCCCAGACTAAGGTGGAGGGGGTGGCAATGGAGAGAAGTA
GATGATCTGAGAGGTGTGTGTGTGTGTGTGTGTATATATATATATATACACACACACACA
CACATATATATGTTTTTTTTTTT
>555
GTGTGTGTGTATATATATATATATATACACACACACACACACATATATATGTTTTTTTTTTTCT
TTTTTGAGATGGAGTCTTGCTCTGTCACCCAGGCTGGAGTGCAGTGGCACGATCTTGG
CTCAGTGCAAGCTCTGCCTC
>556
GTGTATATATATATATATATACACACACACACACACATATATATGTTTTTTTTTTTCTTTTTTG
AGATGGAGTCTTGCTCTGTCACCCAGGCTGGAGTGCAGTGGCACGATCTTGGCTCAGT
GCAAGCTCTGCCTCCTGGGT
>557
ACACACACACACACACATATATATGTTTTTTTTTTTCTTTTTTGAGATGGAGTCTTGCTC
TGTCACCCAGGCTGGAGTGCAGTGGCACGATCTTGGCTCAGTGCAAGCTCTGCCTCCT
GGGTTCAAGCATTTCTCCTGCC
>558
CACACACACATATATATGTTTTTTTTTTTCTTTTTTGAGATGGAGTCTTGCTCTGTCACCC
AGGCTGGAGTGCAGTGGCACGATCTTGGCTCAGTGCAAGCTCTGCCTCCTGGGTTCAA
GCATTTCTCCTGCCTCAGCCT
>559
TTTTTCTTTTTTGAGATGGAGTCTTGCTCTGTCACCCAGGCTGGAGTGCAGTGGCACGA
TCTTGGCTCAGTGCAAGCTCTGCCTCCTGGGTTCAAGCATTTCTCCTGCCTCAGCCTCC
TGAGTAGCTGGGACTACTACAA
>560
CTCTGTCACCCAGGCTGGAGTGCAGTGGCACGATCTTGGCTCAGTGCAAGCTCTGCCT
CCTGGGTTCAAGCATTTCTCCTGCCTCAGCCTCCTGAGTAGCTGGGACTACTACAAAG
GCGTGAGCACCGCGCCGGGCGTGA
>561
GAGTGCAGTGGCACGATCTTGGCTCAGTGCAAGCTCTGCCTCCTGGGTTCAAGCATTT
CTCCTGCCTCAGCCTCCTGAGTAGCTGGGACTACTACAAAGGCGTGAGCACCGCGCCG
GGCGTGAGTTATATTTTCAAGGCT

>562
CAAGCTCTGCCTCCTGGGTTCAAGCATTTCTCCTGCCTCAGCCTCCTGAGTAGCTGGGA
CTACTACAAAGGCGTGAGCACCGCGCCGGGCGTGAGTTATATTTTCAAGGCTAGAATT
GACAGAACTTGGGTTGATGAGAA
>563
TGCCTCCTGGGTTCAAGCATTTCTCCTGCCTCAGCCTCCTGAGTAGCTGGGACTACTAC
AAAGGCGTGAGCACCGCGCCGGGCGTGAGTTATATTTTCAAGGCTAGAATTGACAGAA
CTTGGGTTGATGAGAATAGTTGA
>564
ATTTCTCCTGCCTCAGCCTCCTGAGTAGCTGGGACTACTACAAAGGCGTGAGCACCGC
GCCGGGCGTGAGTTATATTTTCAAGGCTAGAATTGACAGAACTTGGGTTGATGAGAATA
GTTGATTAGCAGGCAGGGTGCCA
>565
TCCTGAGTAGCTGGGACTACTACAAAGGCGTGAGCACCGCGCCGGGCGTGAGTTATAT
TTTCAAGGCTAGAATTGACAGAACTTGGGTTGATGAGAATAGTTGATTAGCAGGCAGG
GTGCCAGGGAGGGTGTTGAGCAGA
>566
AGGCGTGAGCACCGCGCCGGGCGTGAGTTATATTTTCAAGGCTAGAATTGACAGAACT
TGGGTTGATGAGAATAGTTGATTAGCAGGCAGGGTGCCAGGGAGGGTGTTGAGCAGA
ACAGAGGGGGGACTGTGCTTTATTT
>567
TGAGCACCGCGCCGGGCGTGAGTTATATTTTCAAGGCTAGAATTGACAGAACTTGGGT
TGATGAGAATAGTTGATTAGCAGGCAGGGTGCCAGGGAGGGTGTTGAGCAGAACAGA
GGGGGGACTGTGCTTTATTTTAAAA
>568
GCGTGAGTTATATTTTCAAGGCTAGAATTGACAGAACTTGGGTTGATGAGAATAGTTGA
TTAGCAGGCAGGGTGCCAGGGAGGGTGTTGAGCAGAACAGAGGGGGGACTGTGCTTT
ATTTTAAAATTTTTATTGAGACAC
>569
CAGAACTTGGGTTGATGAGAATAGTTGATTAGCAGGCAGGGTGCCAGGGAGGGTGTTG
AGCAGAACAGAGGGGGGACTGTGCTTTATTTTAAAATTTTTATTGAGACACAGCATATA
AAGTATACCCAAATCTTAAGGGT
>570
GCAGGGTGCCAGGGAGGGTGTTGAGCAGAACAGAGGGGGGACTGTGCTTTATTTTAA
AATTTTTATTGAGACACAGCATATAAAGTATACCCAAATCTTAAGGGTATAACATAATGA
ATTTCACTATCAAGATAAAGATT
>571
TGCCAGGGAGGGTGTTGAGCAGAACAGAGGGGGGACTGTGCTTTATTTTAAAATTTTT
ATTGAGACACAGCATATAAAGTATACCCAAATCTTAAGGGTATAACATAATGAATTTCAC
TATCAAGATAAAGATTTAGAAC
>572
GAGGGGGGACTGTGCTTTATTTTAAAATTTTTATTGAGACACAGCATATAAAGTATACCC
AAATCTTAAGGGTATAACATAATGAATTTCACTATCAAGATAAAGATTTAGAACATTTAT
AGTTTTCTTGTGCTCCTTTC

>573
TTTATTGAGACACAGCATATAAAGTATACCCAAATCTTAAGGGTATAACATAATGAATTT
CACTATCAAGATAAAGATTTAGAACATTTATAGTTTTCTTGTGCTCCTTTCAGGCAATAC
CTAAAGGTATTTACCTTGTC
>574
CAGCATATAAAGTATACCCAAATCTTAAGGGTATAACATAATGAATTTCACTATCAAGAT
AAAGATTTAGAACATTTATAGTTTTCTTGTGCTCCTTTCAGGCAATACCTAAAGGTATTT
ACCTTGTCTTTTGATAACCT
>575
GTATACCCAAATCTTAAGGGTATAACATAATGAATTTCACTATCAAGATAAAGATTTAGA
ACATTTATAGTTTTCTTGTGCTCCTTTCAGGCAATACCTAAAGGTATTTACCTTGTCTTTT
GATAACCTCCTCCACATCT
>576
TATAACATAATGAATTTCACTATCAAGATAAAGATTTAGAACATTTATAGTTTTCTTGTGC
TCCTTTCAGGCAATACCTAAAGGTATTTACCTTGTCTTTTGATAACCTCCTCCACATCTT
CCTCTGGTTCCTTTTAACA
>577
ATTTCACTATCAAGATAAAGATTTAGAACATTTATAGTTTTCTTGTGCTCCTTTCAGGCA
ATACCTAAAGGTATTTACCTTGTCTTTTGATAACCTCCTCCACATCTTCCTCTGGTTCCTT
TTAACAGGGAATGCGTTGA
>578
TGTCTTTTGATAACCTCCTCCACATCTTCCTCTGGTTCCTTTTAACAGGGAATGCGTTGA
ATATGTCAAGAGCTTCAATATCCCTCTACTCGTGCTGGGTGGTGGTGGTTATACTGTCCG
AAATGTTGCCCGCTGCTGGT
>579
TTCAATATCCCTCTACTCGTGCTGGGTGGTGGTGGTTATACTGTCCGAAATGTTGCCCGC
TGCTGGTGAGCACTCTCCTGATTTCCTCCCCTGTTGTCCAGTGTGAGCTAGGGTGTCCC
TCAGATATGGCCACATGGGCC
>580
GTGGTTATACTGTCCGAAATGTTGCCCGCTGCTGGTGAGCACTCTCCTGATTTCCTCCC
CTGTTGTCCAGTGTGAGCTAGGGTGTCCCTCAGATATGGCCACATGGGCCCTTCAGCCC
ACTTGGTCAGCCTGCTCCCCTC
>581
TGTCCGAAATGTTGCCCGCTGCTGGTGAGCACTCTCCTGATTTCCTCCCCTGTTGTCCA
GTGTGAGCTAGGGTGTCCCTCAGATATGGCCACATGGGCCCTTCAGCCCACTTGGTCA
GCCTGCTCCCCTCTGCTGGTTGG
>582
CCTGATTTCCTCCCCTGTTGTCCAGTGTGAGCTAGGGTGTCCCTCAGATATGGCCACAT
GGGCCCTTCAGCCCACTTGGTCAGCCTGCTCCCCTCTGCTGGTTGGTTTCAGGACATAT
GAGACATCGCTGCTGGTAGAAG
>583
CCTGTTGTCCAGTGTGAGCTAGGGTGTCCCTCAGATATGGCCACATGGGCCCTTCAGCC
CACTTGGTCAGCCTGCTCCCCTCTGCTGGTTGGTTTCAGGACATATGAGACATCGCTGC
TGGTAGAAGAGGCCATTAGTGA

>584
CCTCAGATATGGCCACATGGGCCCTTCAGCCCACTTGGTCAGCCTGCTCCCCTCTGCTG
GTTGGTTTCAGGACATATGAGACATCGCTGCTGGTAGAAGAGGCCATTAGTGAGGAGC
TTCCCTATAGTGGTAAGGACACC
>585
GGCCACATGGGCCCTTCAGCCCACTTGGTCAGCCTGCTCCCCTCTGCTGGTTGGTTTCA
GGACATATGAGACATCGCTGCTGGTAGAAGAGGCCATTAGTGAGGAGCTTCCCTATAGT
GGTAAGGACACCCACAACACCC
>586
CAGCCCACTTGGTCAGCCTGCTCCCCTCTGCTGGTTGGTTTCAGGACATATGAGACATC
GCTGCTGGTAGAAGAGGCCATTAGTGAGGAGCTTCCCTATAGTGGTAAGGACACCCAC
AACACCCCATAACAGGAAGGCCA
>587
CTCCCCTCTGCTGGTTGGTTTCAGGACATATGAGACATCGCTGCTGGTAGAAGAGGCCA
TTAGTGAGGAGCTTCCCTATAGTGGTAAGGACACCCACAACACCCCATAACAGGAAGG
CCATTCTCACCAGATTCCAGAAT
>588
TGGTTGGTTTCAGGACATATGAGACATCGCTGCTGGTAGAAGAGGCCATTAGTGAGGA
GCTTCCCTATAGTGGTAAGGACACCCACAACACCCCATAACAGGAAGGCCATTCTCAC
CAGATTCCAGAATGACCTGGTTTG
>589
TTTCAGGACATATGAGACATCGCTGCTGGTAGAAGAGGCCATTAGTGAGGAGCTTCCCT
ATAGTGGTAAGGACACCCACAACACCCCATAACAGGAAGGCCATTCTCACCAGATTCC
AGAATGACCTGGTTTGAAGGCAC
>590
GCTGGTAGAAGAGGCCATTAGTGAGGAGCTTCCCTATAGTGGTAAGGACACCCACAAC
ACCCCATAACAGGAAGGCCATTCTCACCAGATTCCAGAATGACCTGGTTTGAAGGCAC
ATGGGAAATTTTACACTCTCAGAG
>591
TAGTGAGGAGCTTCCCTATAGTGGTAAGGACACCCACAACACCCCATAACAGGAAGGC
CATTCTCACCAGATTCCAGAATGACCTGGTTTGAAGGCACATGGGAAATTTTACACTCT
CAGAGCTCTGCAGACTAAACCCC
>592
TAAGGACACCCACAACACCCCATAACAGGAAGGCCATTCTCACCAGATTCCAGAATGA
CCTGGTTTGAAGGCACATGGGAAATTTTACACTCTCAGAGCTCTGCAGACTAAACCCC
TGGGCTCAGAGCAGGGAAGAGATT
>593
AGGAAGGCCATTCTCACCAGATTCCAGAATGACCTGGTTTGAAGGCACATGGGAAATT
TTACACTCTCAGAGCTCTGCAGACTAAACCCCTGGGCTCAGAGCAGGGAAGAGATTG
GGCAGCTATTATGTAGGTGCTCTGG
>594
TGACCTGGTTTGAAGGCACATGGGAAATTTTACACTCTCAGAGCTCTGCAGACTAAAC
CCCTGGGCTCAGAGCAGGGAAGAGATTGGGCAGCTATTATGTAGGTGCTCTGGATGGG
AAGGAAAAAGATTGGGAAGTCTTG

>595
ACCTGGTTTGAAGGCACATGGGAAATTTTACACTCTCAGAGCTCTGCAGACTAAACCC
CTGGGCTCAGAGCAGGGAAGAGATTGGGCAGCTATTATGTAGGTGCTCTGGATGGGAA
GGAAAAAGATTGGGAAGTCTTGTT
>596
AAGGCACATGGGAAATTTTACACTCTCAGAGCTCTGCAGACTAAACCCCTGGGCTCAG
AGCAGGGAAGAGATTGGGCAGCTATTATGTAGGTGCTCTGGATGGGAAGGAAAAAGAT
TGGGAAGTCTTGTTAGCTGCCTTT
>597
AGACTAAACCCCTGGGCTCAGAGCAGGGAAGAGATTGGGCAGCTATTATGTAGGTGCT
CTGGATGGGAAGGAAAAAGATTGGGAAGTCTTGTTAGCTGCCTTTCTCAGTCTGAGCC
ATACTTTCTCCTCTCCTCCCCAAG
>598
ACCCCTGGGCTCAGAGCAGGGAAGAGATTGGGCAGCTATTATGTAGGTGCTCTGGATG
GGAAGGAAAAAGATTGGGAAGTCTTGTTAGCTGCCTTTCTCAGTCTGAGCCATACTTT
CTCCTCTCCTCCCCAAGAATACTT
>599
CTCAGAGCAGGGAAGAGATTGGGCAGCTATTATGTAGGTGCTCTGGATGGGAAGGAAA
AAGATTGGGAAGTCTTGTTAGCTGCCTTTCTCAGTCTGAGCCATACTTTCTCCTCTCCT
CCCCAAGAATACTTCGAGTACTT
>600
GGGAAGAGATTGGGCAGCTATTATGTAGGTGCTCTGGATGGGAAGGAAAAAGATTGGG
AAGTCTTGTTAGCTGCCTTTCTCAGTCTGAGCCATACTTTCTCCTCTCCTCCCCAAGAAT
ACTTCGAGTACTTTGCCCCAGA
>601
GGAAGTCTTGTTAGCTGCCTTTCTCAGTCTGAGCCATACTTTCTCCTCTCCTCCCCAAG
AATACTTCGAGTACTTTGCCCCAGACTTCACACTTCATCCAGATGTCAGCACCCGCATC
GAGAATCAGAACTCACGCCAGG
>602
GCTGCCTTTCTCAGTCTGAGCCATACTTTCTCCTCTCCTCCCCAAGAATACTTCGAGTAC
TTTGCCCCAGACTTCACACTTCATCCAGATGTCAGCACCCGCATCGAGAATCAGAACT
CACGCCAGGTCAGCAGCTCAGA
>603
TCCCCAAGAATACTTCGAGTACTTTGCCCCAGACTTCACACTTCATCCAGATGTCAGCA
CCCGCATCGAGAATCAGAACTCACGCCAGGTCAGCAGCTCAGAAAAGCTGAGCACAA
ACTTCTCCTGAAAGGTGGAGCCCA
>604
AGAATACTTCGAGTACTTTGCCCCAGACTTCACACTTCATCCAGATGTCAGCACCCGCA
TCGAGAATCAGAACTCACGCCAGGTCAGCAGCTCAGAAAAGCTGAGCACAAACTTCT
CCTGAAAGGTGGAGCCCAGGAGAG
>605
GTACTTTGCCCCAGACTTCACACTTCATCCAGATGTCAGCACCCGCATCGAGAATCAGA
ACTCACGCCAGGTCAGCAGCTCAGAAAAGCTGAGCACAAACTTCTCCTGAAAGGTGG
AGCCCAGGAGAGGTCAAAGATGTG

>606
CCGCATCGAGAATCAGAACTCACGCCAGGTCAGCAGCTCAGAAAAGCTGAGCACAAA
CTTCTCCTGAAAGGTGGAGCCCAGGAGAGGTCAAAGATGTGGGAAGTAGGAAAAACC
AAAGAGAAGGAAGTTCTAAGCCATCA
>607
CGAGAATCAGAACTCACGCCAGGTCAGCAGCTCAGAAAAGCTGAGCACAAACTTCTC
CTGAAAGGTGGAGCCCAGGAGAGGTCAAAGATGTGGGAAGTAGGAAAAACCAAAGA
GAAGGAAGTTCTAAGCCATCACAGTCA
>608
GCCAGGTCAGCAGCTCAGAAAAGCTGAGCACAAACTTCTCCTGAAAGGTGGAGCCCA
GGAGAGGTCAAAGATGTGGGAAGTAGGAAAAACCAAAGAGAAGGAAGTTCTAAGCC
ATCACAGTCACTGTTATTGACTAGGAG
>609
CAAACTTCTCCTGAAAGGTGGAGCCCAGGAGAGGTCAAAGATGTGGGAAGTAGGAAA
AACCAAAGAGAAGGAAGTTCTAAGCCATCACAGTCACTGTTATTGACTAGGAGGCAGT
CCAGTTTAAAGTAGAGGCAGTTGGG
>610
CTTCTCCTGAAAGGTGGAGCCCAGGAGAGGTCAAAGATGTGGGAAGTAGGAAAAACC
AAAGAGAAGGAAGTTCTAAGCCATCACAGTCACTGTTATTGACTAGGAGGCAGTCCAG
TTTAAAGTAGAGGCAGTTGGGGTTG
>611
TGAAAGGTGGAGCCCAGGAGAGGTCAAAGATGTGGGAAGTAGGAAAAACCAAAGAG
AAGGAAGTTCTAAGCCATCACAGTCACTGTTATTGACTAGGAGGCAGTCCAGTTTAAA
GTAGAGGCAGTTGGGGTTGGTGGGGA
>612
AAGATGTGGGAAGTAGGAAAAACCAAAGAGAAGGAAGTTCTAAGCCATCACAGTCAC
TGTTATTGACTAGGAGGCAGTCCAGTTTAAAGTAGAGGCAGTTGGGGTTGGTGGGGAG
GGAGAGCTATCAGTTGGGGGACCTG
>613
CCAAAGAGAAGGAAGTTCTAAGCCATCACAGTCACTGTTATTGACTAGGAGGCAGTCC
AGTTTAAAGTAGAGGCAGTTGGGGTTGGTGGGGAGGGAGAGCTATCAGTTGGGGGAC
CTGAACTAGAGGTACCACTGAGATG
>614
AAAGAGAAGGAAGTTCTAAGCCATCACAGTCACTGTTATTGACTAGGAGGCAGTCCAG
TTTAAAGTAGAGGCAGTTGGGGTTGGTGGGGAGGGAGAGCTATCAGTTGGGGGACCT
GAACTAGAGGTACCACTGAGATGAA
>615
AGAAGGAAGTTCTAAGCCATCACAGTCACTGTTATTGACTAGGAGGCAGTCCAGTTTA
AAGTAGAGGCAGTTGGGGTTGGTGGGGAGGGAGAGCTATCAGTTGGGGGACCTGAAC
TAGAGGTACCACTGAGATGAAAACC
>616
CATCACAGTCACTGTTATTGACTAGGAGGCAGTCCAGTTTAAAGTAGAGGCAGTTGGG
GTTGGTGGGGAGGGAGAGCTATCAGTTGGGGGACCTGAACTAGAGGTACCACTGAGA
TGAAAACCAGTATGGTAGGGGTAAA

>617
GTCCAGTTTAAAGTAGAGGCAGTTGGGGTTGGTGGGGAGGGAGAGCTATCAGTTGGG
GGACCTGAACTAGAGGTACCACTGAGATGAAAACCAGTATGGTAGGGGTAAAAGGCA
GCTAGGAGATTCCATGTCCTGGGAAT
>618
TAGAGGCAGTTGGGGTTGGTGGGGAGGGAGAGCTATCAGTTGGGGGACCTGAACTAG
AGGTACCACTGAGATGAAAACCAGTATGGTAGGGGTAAAAGGCAGCTAGGAGATTCCA
TGTCCTGGGAATCTCTGGAAACTGC
>619
GGGGTTGGTGGGGAGGGAGAGCTATCAGTTGGGGGACCTGAACTAGAGGTACCACTG
AGATGAAAACCAGTATGGTAGGGGTAAAAGGCAGCTAGGAGATTCCATGTCCTGGGAA
TCTCTGGAAACTGCTCACTCTGTAT
>620
AGAGCTATCAGTTGGGGGACCTGAACTAGAGGTACCACTGAGATGAAAACCAGTATGG
TAGGGGTAAAAGGCAGCTAGGAGATTCCATGTCCTGGGAATCTCTGGAAACTGCTCAC
TCTGTATTCCTCATTTCCAACCAG
>621
TTCCATGTCCTGGGAATCTCTGGAAACTGCTCACTCTGTATTCCTCATTTCCAACCAGTA
TCTGGACCAGATCCGCCAGACAATCTTTGAAAACCTGAAGATGCTGAACCATGCACCT
AGTGTCCAGATTCATGACGTGC
>622
CTGGGAATCTCTGGAAACTGCTCACTCTGTATTCCTCATTTCCAACCAGTATCTGGACC
AGATCCGCCAGACAATCTTTGAAAACCTGAAGATGCTGAACCATGCACCTAGTGTCCA
GATTCATGACGTGCCTGCAGACC
>623
TCACTCTGTATTCCTCATTTCCAACCAGTATCTGGACCAGATCCGCCAGACAATCTTTG
AAAACCTGAAGATGCTGAACCATGCACCTAGTGTCCAGATTCATGACGTGCCTGCAGA
CCTCCTGACCTATGACAGGACTG
>624
CCAACCAGTATCTGGACCAGATCCGCCAGACAATCTTTGAAAACCTGAAGATGCTGAA
CCATGCACCTAGTGTCCAGATTCATGACGTGCCTGCAGACCTCCTGACCTATGACAGGA
CTGATGAGGCTGATGCAGAGGAG
>625
CAGACAATCTTTGAAAACCTGAAGATGCTGAACCATGCACCTAGTGTCCAGATTCATG
ACGTGCCTGCAGACCTCCTGACCTATGACAGGACTGATGAGGCTGATGCAGAGGAGA
GGGGTCCTGAGGAGAACTATAGCAG
>626
TGAAGATGCTGAACCATGCACCTAGTGTCCAGATTCATGACGTGCCTGCAGACCTCCT
GACCTATGACAGGACTGATGAGGCTGATGCAGAGGAGAGGGGTCCTGAGGAGAACTA
TAGCAGGTCTGGGAGCAGGGACTTC
>627
CCTAGTGTCCAGATTCATGACGTGCCTGCAGACCTCCTGACCTATGACAGGACTGATGA
GGCTGATGCAGAGGAGAGGGGTCCTGAGGAGAACTATAGCAGGTCTGGGAGCAGGGA
CTTCAGCCTACTTTCAATAGGCTT

>628
TCCAGATTCATGACGTGCCTGCAGACCTCCTGACCTATGACAGGACTGATGAGGCTGAT
GCAGAGGAGAGGGGTCCTGAGGAGAACTATAGCAGGTCTGGGAGCAGGGACTTCAGC
CTACTTTCAATAGGCTTGCTGGGG
>629
TGACGTGCCTGCAGACCTCCTGACCTATGACAGGACTGATGAGGCTGATGCAGAGGAG
AGGGGTCCTGAGGAGAACTATAGCAGGTCTGGGAGCAGGGACTTCAGCCTACTTTCAA
TAGGCTTGCTGGGGAGGTTTAAAA
>630
CGTGCCTGCAGACCTCCTGACCTATGACAGGACTGATGAGGCTGATGCAGAGGAGAG
GGGTCCTGAGGAGAACTATAGCAGGTCTGGGAGCAGGGACTTCAGCCTACTTTCAATA
GGCTTGCTGGGGAGGTTTAAAAGGG
>631
CCTATGACAGGACTGATGAGGCTGATGCAGAGGAGAGGGGTCCTGAGGAGAACTATA
GCAGGTCTGGGAGCAGGGACTTCAGCCTACTTTCAATAGGCTTGCTGGGGAGGTTTAA
AAGGGAAAAAGTAAATGGGAAGGTA
>632
GTCCTGAGGAGAACTATAGCAGGTCTGGGAGCAGGGACTTCAGCCTACTTTCAATAGG
CTTGCTGGGGAGGTTTAAAAGGGAAAAAGTAAATGGGAAGGTATTAGTTTAATTGCCT
CTGGCTTACCTCCTTTAGTCTGCT
>633
AGAACTATAGCAGGTCTGGGAGCAGGGACTTCAGCCTACTTTCAATAGGCTTGCTGGG
GAGGTTTAAAAGGGAAAAAGTAAATGGGAAGGTATTAGTTTAATTGCCTCTGGCTTAC
CTCCTTTAGTCTGCTACAATTTCC
>634
GAGCAGGGACTTCAGCCTACTTTCAATAGGCTTGCTGGGGAGGTTTAAAAGGGAAAA
AGTAAATGGGAAGGTATTAGTTTAATTGCCTCTGGCTTACCTCCTTTAGTCTGCTACAAT
TTCCTCACTCTTTTCTTTGTTAT
>635
GGGGAGGTTTAAAAGGGAAAAAGTAAATGGGAAGGTATTAGTTTAATTGCCTCTGGCT
TACCTCCTTTAGTCTGCTACAATTTCCTCACTCTTTTCTTTGTTATTTTTAATGACAAAAG
TAACACGTATTTATTGCAAAA
>636
CCTCTGGCTTACCTCCTTTAGTCTGCTACAATTTCCTCACTCTTTTCTTTGTTATTTTTAA
TGACAAAAGTAACACGTATTTATTGCAAAATTTTAAAACAGCACCAAAATATGGTGTTG
GCGTTCACACAATCCCAGAC
>637
TTCCTCACTCTTTTCTTTGTTATTTTTAATGACAAAAGTAACACGTATTTATTGCAAAATT
TTAAAACAGCACCAAAATATGGTGTTGGCGTTCACACAATCCCAGACCTATTCCACAG
GAGTAACCACTGTTAATAGTT
>638
AGTAACACGTATTTATTGCAAAATTTTAAAACAGCACCAAAATATGGTGTTGGCGTTCA
CACAATCCCAGACCTATTCCACAGGAGTAACCACTGTTAATAGTTTTTATTGTTTTGGAT
AGACCTTTTTCTATAGATTGC

>639
TATTGCAAAATTTTAAAACAGCACCAAAATATGGTGTTGGCGTTCACACAATCCCAGAC
CTATTCCACAGGAGTAACCACTGTTAATAGTTTTTATTGTTTTGGATAGACCTTTTTCTAT
AGATTGCAAATATACTATTC
>640
AGCACCAAAATATGGTGTTGGCGTTCACACAATCCCAGACCTATTCCACAGGAGTAAC
CACTGTTAATAGTTTTTATTGTTTTGGATAGACCTTTTTCTATAGATTGCAAATATACTATT
CTGTATGTACATATAAACAA
>641
GGCGTTCACACAATCCCAGACCTATTCCACAGGAGTAACCACTGTTAATAGTTTTTATT
GTTTTGGATAGACCTTTTTCTATAGATTGCAAATATACTATTCTGTATGTACATATAAACA
AACACATATACACTCATTTA
>642
CCCAGACCTATTCCACAGGAGTAACCACTGTTAATAGTTTTTATTGTTTTGGATAGACCT
TTTTCTATAGATTGCAAATATACTATTCTGTATGTACATATAAACAAACACATATACACTC
ATTTACAGTTTGTGTGGTT
>643
TTTTTCTATAGATTGCAAATATACTATTCTGTATGTACATATAAACAAACACATATACACT
CATTTACAGTTTGTGTGGTTTTTGGTTTTTCCGTAAATACAAATAGGGTCATTCTACGTA
TGCTGCTTTACATCATATA
>644
CATATAAACAAACACATATACACTCATTTACAGTTTGTGTGGTTTTTGGTTTTTCCGTAA
ATACAAATAGGGTCATTCTACGTATGCTGCTTTACATCATATAGCTGTTTTCAGCTAGCTA
ATATAGATCTGCTTCAGTA
>645
TTGTGTGGTTTTTGGTTTTTCCGTAAATACAAATAGGGTCATTCTACGTATGCTGCTTTA
CATCATATAGCTGTTTTCAGCTAGCTAATATAGATCTGCTTCAGTATTTTATCAGCTGTAT
AATAATTTACTATGTGGAT
>646
TTTGGTTTTTCCGTAAATACAAATAGGGTCATTCTACGTATGCTGCTTTACATCATATAGC
TGTTTTCAGCTAGCTAATATAGATCTGCTTCAGTATTTTATCAGCTGTATAATAATTTACTA
TGTGGATAGATTATTTT
>647
AGGGTCATTCTACGTATGCTGCTTTACATCATATAGCTGTTTTCAGCTAGCTAATATAGAT
CTGCTTCAGTATTTTATCAGCTGTATAATAATTTACTATGTGGATAGATTATTTTCCATTTT
TTTGGTATTACAATAAT
>648
ACGTATGCTGCTTTACATCATATAGCTGTTTTCAGCTAGCTAATATAGATCTGCTTCAGTA
TTTTATCAGCTGTATAATAATTTACTATGTGGATAGATTATTTTCCATTTTTTTGGTATTAC
AATAATAATGTGGCAAT
>649
GTTTTCAGCTAGCTAATATAGATCTGCTTCAGTATTTTATCAGCTGTATAATAATTTACTAT
GTGGATAGATTATTTTCCATTTTTTTGGTATTACAATAATAATGTGGCAATGAACCCCCTT
ACATTATACATCTTTGC

>650
TTTTTGGTATTACAATAATAATGTGGCAATGAACCCCCTTACATTATACATCTTTGCATTC
TTGTTTGAGTGGGTTAGATAGTAGAATGGTTGCTGCTAGGTCAAAGGCTGTGTGCATTA
ATTTTTTTTTTTTTTTTTTT
>651
ATTACAATAATAATGTGGCAATGAACCCCCTTACATTATACATCTTTGCATTCTTGTTTGA
GTGGGTTAGATAGTAGAATGGTTGCTGCTAGGTCAAAGGCTGTGTGCATTAATTTTTTTT
TTTTTTTTTTTTTTGAGAT
>652
TAATAATGTGGCAATGAACCCCCTTACATTATACATCTTTGCATTCTTGTTTGAGTGGGTT
AGATAGTAGAATGGTTGCTGCTAGGTCAAAGGCTGTGTGCATTAATTTTTTTTTTTTTTT
TTTTTTTGAGATGGAGTCT
>653
CTTACATTATACATCTTTGCATTCTTGTTTGAGTGGGTTAGATAGTAGAATGGTTGCTGCT
AGGTCAAAGGCTGTGTGCATTAATTTTTTTTTTTTTTTTTTTTTTGAGATGGAGTCTCAC
TCTGTTGCCAGGCTGGAGT
>654
AGATAGTAGAATGGTTGCTGCTAGGTCAAAGGCTGTGTGCATTAATTTTTTTTTTTTTTT
TTTTTTTGAGATGGAGTCTCACTCTGTTGCCAGGCTGGAGTGCAGTGGCTAATCCCGGC
TCACTGCAACCTCCGCCTCCC
>655
TAGAATGGTTGCTGCTAGGTCAAAGGCTGTGTGCATTAATTTTTTTTTTTTTTTTTTTTTT
GAGATGGAGTCTCACTCTGTTGCCAGGCTGGAGTGCAGTGGCTAATCCCGGCTCACTG
CAACCTCCGCCTCCCAGGTTC
>656
GGCTGTGTGCATTAATTTTTTTTTTTTTTTTTTTTTTGAGATGGAGTCTCACTCTGTTGCC
AGGCTGGAGTGCAGTGGCTAATCCCGGCTCACTGCAACCTCCGCCTCCCAGGTTCAAG
CGATTCTCCTGCCTCAGCCTC
>657
GGAGTCTCACTCTGTTGCCAGGCTGGAGTGCAGTGGCTAATCCCGGCTCACTGCAACC
TCCGCCTCCCAGGTTCAAGCGATTCTCCTGCCTCAGCCTCCTCAGTAGCTGAGATTACA
GGCATGTGCCACCACACCCGGCT
>658
CACTCTGTTGCCAGGCTGGAGTGCAGTGGCTAATCCCGGCTCACTGCAACCTCCGCCT
CCCAGGTTCAAGCGATTCTCCTGCCTCAGCCTCCTCAGTAGCTGAGATTACAGGCATGT
GCCACCACACCCGGCTAACTTTA
>659
TGGCTAATCCCGGCTCACTGCAACCTCCGCCTCCCAGGTTCAAGCGATTCTCCTGCCTC
AGCCTCCTCAGTAGCTGAGATTACAGGCATGTGCCACCACACCCGGCTAACTTTAGTAT
TTTTAGTAGAGACGGGGTTTCA
>660
CTAATCCCGGCTCACTGCAACCTCCGCCTCCCAGGTTCAAGCGATTCTCCTGCCTCAGC
CTCCTCAGTAGCTGAGATTACAGGCATGTGCCACCACACCCGGCTAACTTTAGTATTTT
TAGTAGAGACGGGGTTTCACCA

>661
CACTGCAACCTCCGCCTCCCAGGTTCAAGCGATTCTCCTGCCTCAGCCTCCTCAGTAGC
TGAGATTACAGGCATGTGCCACCACACCCGGCTAACTTTAGTATTTTTAGTAGAGACGG
GGTTTCACCATGTTGGCCAGGA
>662
GATTCTCCTGCCTCAGCCTCCTCAGTAGCTGAGATTACAGGCATGTGCCACCACACCCG
GCTAACTTTAGTATTTTTAGTAGAGACGGGGTTTCACCATGTTGGCCAGGATGGTCTTG
ATCTCTTGACCTTGTGATCCAC
>663
TCAGCCTCCTCAGTAGCTGAGATTACAGGCATGTGCCACCACACCCGGCTAACTTTAGT
ATTTTTAGTAGAGACGGGGTTTCACCATGTTGGCCAGGATGGTCTTGATCTCTTGACCT
TGTGATCCACCTGCCTTGGCCT
>664
AGCCTCCTCAGTAGCTGAGATTACAGGCATGTGCCACCACACCCGGCTAACTTTAGTAT
TTTTAGTAGAGACGGGGTTTCACCATGTTGGCCAGGATGGTCTTGATCTCTTGACCTTG
TGATCCACCTGCCTTGGCCTCC
>665
AGGCATGTGCCACCACACCCGGCTAACTTTAGTATTTTTAGTAGAGACGGGGTTTCACC
ATGTTGGCCAGGATGGTCTTGATCTCTTGACCTTGTGATCCACCTGCCTTGGCCTCCCA
AAGTGCTGGGATTACAGGTGTG
>666
GTTTCACCATGTTGGCCAGGATGGTCTTGATCTCTTGACCTTGTGATCCACCTGCCTTG
GCCTCCCAAAGTGCTGGGATTACAGGTGTGAGCTACCACGCCCGGCTTTTGAGAATAC
TTTTTCAAAAAGAAAGAAACCAA
>667
GCCAGGATGGTCTTGATCTCTTGACCTTGTGATCCACCTGCCTTGGCCTCCCAAAGTGC
TGGGATTACAGGTGTGAGCTACCACGCCCGGCTTTTGAGAATACTTTTTCAAAAAGAA
AGAAACCAATATTGAGTACCTGT
>668
GTGATCCACCTGCCTTGGCCTCCCAAAGTGCTGGGATTACAGGTGTGAGCTACCACGC
CCGGCTTTTGAGAATACTTTTTCAAAAAGAAAGAAACCAATATTGAGTACCTGTTGGG
CCCTGTTTTGCTTTCACATCCATT
>669
GCCTCCCAAAGTGCTGGGATTACAGGTGTGAGCTACCACGCCCGGCTTTTGAGAATAC
TTTTTCAAAAAGAAAGAAACCAATATTGAGTACCTGTTGGGCCCTGTTTTGCTTTCACA
TCCATTCTTTCACTATGGGTTTA
>670
CCCAAAGTGCTGGGATTACAGGTGTGAGCTACCACGCCCGGCTTTTGAGAATACTTTTT
CAAAAAGAAAGAAACCAATATTGAGTACCTGTTGGGCCCTGTTTTGCTTTCACATCCAT
TCTTTCACTATGGGTTTATAAT
>671
CTTTCACTATGGGTTTATAATACCACGTCACTCTTCTTAAAATTAAAACTCTCCTTTACCT
TGCACTTAGAAGAAAATCCAAAATTTTGACTGTGGCTTAAAAAGGTCCTGCATGCTAG
CCCTTACCTACATCCAGGATC

>672
TCACTATGGGTTTATAATACCACGTCACTCTTCTTAAAATTAAAACTCTCCTTTACCTTG
CACTTAGAAGAAAATCCAAAATTTTGACTGTGGCTTAAAAAGGTCCTGCATGCTAGCC
CTTACCTACATCCAGGATCTCA
>673
CTTAAAATTAAAACTCTCCTTTACCTTGCACTTAGAAGAAAATCCAAAATTTTGACTGT
GGCTTAAAAAGGTCCTGCATGCTAGCCCTTACCTACATCCAGGATCTCATCTCTAACTA
CTCTCCTCCATGCACAGTGCTC
>674
TCTCCTTTACCTTGCACTTAGAAGAAAATCCAAAATTTTGACTGTGGCTTAAAAAGGTC
CTGCATGCTAGCCCTTACCTACATCCAGGATCTCATCTCTAACTACTCTCCTCCATGCAC
AGTGCTCCCGGCTGTCCTGGC
>675
TTAGAAGAAAATCCAAAATTTTGACTGTGGCTTAAAAAGGTCCTGCATGCTAGCCCTTA
CCTACATCCAGGATCTCATCTCTAACTACTCTCCTCCATGCACAGTGCTCCCGGCTGTCC
TGGCCTCCTTGTTCTTACTGG
>676
AAAAGGTCCTGCATGCTAGCCCTTACCTACATCCAGGATCTCATCTCTAACTACTCTCCT
CCATGCACAGTGCTCCCGGCTGTCCTGGCCTCCTTGTTCTTACTGGAATACATCAGTCT
CTTTCCATTTCATGACCTCTG
>677
CTAACTACTCTCCTCCATGCACAGTGCTCCCGGCTGTCCTGGCCTCCTTGTTCTTACTG
GAATACATCAGTCTCTTTCCATTTCATGACCTCTGTAGTTGGTTTTCTTGCAGCTTGGAA
TGGCCTTCCTGAAGTCTTTGC
>678
TCCCGGCTGTCCTGGCCTCCTTGTTCTTACTGGAATACATCAGTCTCTTTCCATTTCATG
ACCTCTGTAGTTGGTTTTCTTGCAGCTTGGAATGGCCTTCCTGAAGTCTTTGCCTGCTT
AGCTCCTCCTCAGACTTTAGG
>679
CCTCCTTGTTCTTACTGGAATACATCAGTCTCTTTCCATTTCATGACCTCTGTAGTTGGT
TTTCTTGCAGCTTGGAATGGCCTTCCTGAAGTCTTTGCCTGCTTAGCTCCTCCTCAGAC
TTTAGGTCTTATGTCCCAATA
>680
ACATCAGTCTCTTTCCATTTCATGACCTCTGTAGTTGGTTTTCTTGCAGCTTGGAATGGC
CTTCCTGAAGTCTTTGCCTGCTTAGCTCCTCCTCAGACTTTAGGTCTTATGTCCCAATAG
TCCTGTCTGAAATTTCACCC
>681
CATTTCATGACCTCTGTAGTTGGTTTTCTTGCAGCTTGGAATGGCCTTCCTGAAGTCTTT
GCCTGCTTAGCTCCTCCTCAGACTTTAGGTCTTATGTCCCAATAGTCCTGTCTGAAATTT
CACCCTTTTCCTCTCATCAC
>682
CTCTGTAGTTGGTTTTCTTGCAGCTTGGAATGGCCTTCCTGAAGTCTTTGCCTGCTTAG
CTCCTCCTCAGACTTTAGGTCTTATGTCCCAATAGTCCTGTCTGAAATTTCACCCTTTTC
CTCTCATCACCTTCAGTCCTA

>683
GTTGGTTTTCTTGCAGCTTGGAATGGCCTTCCTGAAGTCTTTGCCTGCTTAGCTCCTCC
TCAGACTTTAGGTCTTATGTCCCAATAGTCCTGTCTGAAATTTCACCCTTTTCCTCTCAT
CACCTTCAGTCCTATCACTTC
>684
CTCAGACTTTAGGTCTTATGTCCCAATAGTCCTGTCTGAAATTTCACCCTTTTCCTCTCA
TCACCTTCAGTCCTATCACTTCATTTTATTAATTGTACTCTCCATCATCTGAAATTATTATA
TTTATTTACTTTGTTAGT
>685
GTCCTATCACTTCATTTTATTAATTGTACTCTCCATCATCTGAAATTATTATATTTATTTACT
TTGTTAGTTACTAGTCTCATTTATTAGGTAAGCTTTTTGAGGATAGACAACCTTGTCCAG
CTTGTTACTCTACTGTA
>686
CACTTCATTTTATTAATTGTACTCTCCATCATCTGAAATTATTATATTTATTTACTTTGTTA
GTTACTAGTCTCATTTATTAGGTAAGCTTTTTGAGGATAGACAACCTTGTCCAGCTTGTT
ACTCTACTGTATTGCTAA
>687
ATCATCTGAAATTATTATATTTATTTACTTTGTTAGTTACTAGTCTCATTTATTAGGTAAGC
TTTTTGAGGATAGACAACCTTGTCCAGCTTGTTACTCTACTGTATTGCTAAGGTTTATGA
CAGTGTCTGTATAGAGTA
>688
CTGAAATTATTATATTTATTTACTTTGTTAGTTACTAGTCTCATTTATTAGGTAAGCTTTTT
GAGGATAGACAACCTTGTCCAGCTTGTTACTCTACTGTATTGCTAAGGTTTATGACAGT
GTCTGTATAGAGTAGTTCC
>689
TTTATTTACTTTGTTAGTTACTAGTCTCATTTATTAGGTAAGCTTTTTGAGGATAGACAAC
CTTGTCCAGCTTGTTACTCTACTGTATTGCTAAGGTTTATGACAGTGTCTGTATAGAGTA
GTTCCTTAATGTTTATTTG
>690
CTCATTTATTAGGTAAGCTTTTTGAGGATAGACAACCTTGTCCAGCTTGTTACTCTACTG
TATTGCTAAGGTTTATGACAGTGTCTGTATAGAGTAGTTCCTTAATGTTTATTTGATACGT
GAATGGGTGAATGAATAGC
>691
TGAGGATAGACAACCTTGTCCAGCTTGTTACTCTACTGTATTGCTAAGGTTTATGACAG
TGTCTGTATAGAGTAGTTCCTTAATGTTTATTTGATACGTGAATGGGTGAATGAATAGCA
GTCTCTACCAACAAGAGGAGG
>692
AGGATAGACAACCTTGTCCAGCTTGTTACTCTACTGTATTGCTAAGGTTTATGACAGTG
TCTGTATAGAGTAGTTCCTTAATGTTTATTTGATACGTGAATGGGTGAATGAATAGCAGT
CTCTACCAACAAGAGGAGGAG
>693
GGTTTATGACAGTGTCTGTATAGAGTAGTTCCTTAATGTTTATTTGATACGTGAATGGGT
GAATGAATAGCAGTCTCTACCAACAAGAGGAGGAGTGGTTCTCAACGGGGCCTGAGA
AATGGCTTTGGAAAAGGGTGCTG

>694
GTATAGAGTAGTTCCTTAATGTTTATTTGATACGTGAATGGGTGAATGAATAGCAGTCTC
TACCAACAAGAGGAGGAGTGGTTCTCAACGGGGCCTGAGAAATGGCTTTGGAAAAGG
GTGCTGGGAGAAGATTAGAACTT
>695
ATAGAGTAGTTCCTTAATGTTTATTTGATACGTGAATGGGTGAATGAATAGCAGTCTCTA
CCAACAAGAGGAGGAGTGGTTCTCAACGGGGCCTGAGAAATGGCTTTGGAAAAGGGT
GCTGGGAGAAGATTAGAACTTAG
>696
ATACGTGAATGGGTGAATGAATAGCAGTCTCTACCAACAAGAGGAGGAGTGGTTCTCA
ACGGGGCCTGAGAAATGGCTTTGGAAAAGGGTGCTGGGAGAAGATTAGAACTTAGAG
TGAGATCCTGACCTCTCTCCCCTTG
>697
CTCTACCAACAAGAGGAGGAGTGGTTCTCAACGGGGCCTGAGAAATGGCTTTGGAAA
AGGGTGCTGGGAGAAGATTAGAACTTAGAGTGAGATCCTGACCTCTCTCCCCTTGCTT
TGCAGGCCAGAGGCACCCAATGAGT
>698
TACCAACAAGAGGAGGAGTGGTTCTCAACGGGGCCTGAGAAATGGCTTTGGAAAAGG
GTGCTGGGAGAAGATTAGAACTTAGAGTGAGATCCTGACCTCTCTCCCCTTGCTTTGC
AGGCCAGAGGCACCCAATGAGTTCT
>699
GAGGAGTGGTTCTCAACGGGGCCTGAGAAATGGCTTTGGAAAAGGGTGCTGGGAGAA
GATTAGAACTTAGAGTGAGATCCTGACCTCTCTCCCCTTGCTTTGCAGGCCAGAGGCA
CCCAATGAGTTCTATGATGGAGACC
>700
AAAGGGTGCTGGGAGAAGATTAGAACTTAGAGTGAGATCCTGACCTCTCTCCCCTTGC
TTTGCAGGCCAGAGGCACCCAATGAGTTCTATGATGGAGACCATGACAATGACAAGGA
AAGCGATGTGGAGATTTAAGAGTG
>701
AGGGTGCTGGGAGAAGATTAGAACTTAGAGTGAGATCCTGACCTCTCTCCCCTTGCTT
TGCAGGCCAGAGGCACCCAATGAGTTCTATGATGGAGACCATGACAATGACAAGGAA
AGCGATGTGGAGATTTAAGAGTGGC
>702
GAAGATTAGAACTTAGAGTGAGATCCTGACCTCTCTCCCCTTGCTTTGCAGGCCAGAG
GCACCCAATGAGTTCTATGATGGAGACCATGACAATGACAAGGAAAGCGATGTGGAGA
TTTAAGAGTGGCTTGGGATGCTGT
>703
CTCTCTCCCCTTGCTTTGCAGGCCAGAGGCACCCAATGAGTTCTATGATGGAGACCATG
ACAATGACAAGGAAAGCGATGTGGAGATTTAAGAGTGGCTTGGGATGCTGTGTCCCAA
GGAATTTCTTTTCACCTCTTGGT
>704
TTGCAGGCCAGAGGCACCCAATGAGTTCTATGATGGAGACCATGACAATGACAAGGAA
AGCGATGTGGAGATTTAAGAGTGGCTTGGGATGCTGTGTCCCAAGGAATTTCTTTTCAC
CTCTTGGTTGGGCTGGAGGGAAA

>705
CCAGAGGCACCCAATGAGTTCTATGATGGAGACCATGACAATGACAAGGAAAGCGATG
TGGAGATTTAAGAGTGGCTTGGGATGCTGTGTCCCAAGGAATTTCTTTTCACCTCTTGG
TTGGGCTGGAGGGAAAAGGAGTG
>706
TGGAGACCATGACAATGACAAGGAAAGCGATGTGGAGATTTAAGAGTGGCTTGGGAT
GCTGTGTCCCAAGGAATTTCTTTTCACCTCTTGGTTGGGCTGGAGGGAAAAGGAGTGG
CTCCTAGAGTCCTGGGGGTCACCCC
>707
TGGAGATTTAAGAGTGGCTTGGGATGCTGTGTCCCAAGGAATTTCTTTTCACCTCTTGG
TTGGGCTGGAGGGAAAAGGAGTGGCTCCTAGAGTCCTGGGGGTCACCCCAGGGCTTT
TGCTGACTCTGGGAAAGAGTCTGG
>708
TTAAGAGTGGCTTGGGATGCTGTGTCCCAAGGAATTTCTTTTCACCTCTTGGTTGGGCT
GGAGGGAAAAGGAGTGGCTCCTAGAGTCCTGGGGGTCACCCCAGGGCTTTTGCTGAC
TCTGGGAAAGAGTCTGGAGACCAC
>709
GGGATGCTGTGTCCCAAGGAATTTCTTTTCACCTCTTGGTTGGGCTGGAGGGAAAAGG
AGTGGCTCCTAGAGTCCTGGGGGTCACCCCAGGGCTTTTGCTGACTCTGGGAAAGAGT
CTGGAGACCACATTTGGTTCTCGA
>710
GCTGGAGGGAAAAGGAGTGGCTCCTAGAGTCCTGGGGGTCACCCCAGGGCTTTTGCT
GACTCTGGGAAAGAGTCTGGAGACCACATTTGGTTCTCGAACCATCTACCTGCTTTTCC
TCTCTCTCCCAAGGCCTGACAATG
>711
TGGAGGGAAAAGGAGTGGCTCCTAGAGTCCTGGGGGTCACCCCAGGGCTTTTGCTGA
CTCTGGGAAAGAGTCTGGAGACCACATTTGGTTCTCGAACCATCTACCTGCTTTTCCTC
TCTCTCCCAAGGCCTGACAATGGT
>712
CTCTGGGAAAGAGTCTGGAGACCACATTTGGTTCTCGAACCATCTACCTGCTTTTCCTC
TCTCTCCCAAGGCCTGACAATGGTACCTATTAGGGATGAGATACAGACAAGGATAGCTA
TCTGGGACATTATTGGCAGTGG
>713
CACATTTGGTTCTCGAACCATCTACCTGCTTTTCCTCTCTCTCCCAAGGCCTGACAATG
GTACCTATTAGGGATGAGATACAGACAAGGATAGCTATCTGGGACATTATTGGCAGTGG
GCCCTGGAGGCCAGTCCCTAGC
>714
CGAACCATCTACCTGCTTTTCCTCTCTCTCCCAAGGCCTGACAATGGTACCTATTAGGG
ATGAGATACAGACAAGGATAGCTATCTGGGACATTATTGGCAGTGGGCCCTGGAGGCC
AGTCCCTAGCCCCCCTTGCCCCT
>715
AAGGCCTGACAATGGTACCTATTAGGGATGAGATACAGACAAGGATAGCTATCTGGGA
CATTATTGGCAGTGGGCCCTGGAGGCCAGTCCCTAGCCCCCCTTGCCCCTTATTTCTTC
CCTGCTTCCCTCGAACCCAGAGA

>716
TGGTACCTATTAGGGATGAGATACAGACAAGGATAGCTATCTGGGACATTATTGGCAGT
GGGCCCTGGAGGCCAGTCCCTAGCCCCCCTTGCCCCTTATTTCTTCCCTGCTTCCCTCG
AACCCAGAGATTTTTGAGGGAT
>717
CAGTGGGCCCTGGAGGCCAGTCCCTAGCCCCCCTTGCCCCTTATTTCTTCCCTGCTTCC
CTCGAACCCAGAGATTTTTGAGGGATGAACGGGTAGACAAGGACTGAGATTGCCTCTG
ACTTCCTCCTCCCCTGGGTTCTG
>718
GTGGGCCCTGGAGGCCAGTCCCTAGCCCCCCTTGCCCCTTATTTCTTCCCTGCTTCCCT
CGAACCCAGAGATTTTTGAGGGATGAACGGGTAGACAAGGACTGAGATTGCCTCTGAC
TTCCTCCTCCCCTGGGTTCTGAC
>719
CCCTTGCCCCTTATTTCTTCCCTGCTTCCCTCGAACCCAGAGATTTTTGAGGGATGAAC
GGGTAGACAAGGACTGAGATTGCCTCTGACTTCCTCCTCCCCTGGGTTCTGACTTCTTC
CTCCCCTTGCTTCCAGGGAAGA
>720
CCCTTATTTCTTCCCTGCTTCCCTCGAACCCAGAGATTTTTGAGGGATGAACGGGTAGA
CAAGGACTGAGATTGCCTCTGACTTCCTCCTCCCCTGGGTTCTGACTTCTTCCTCCCCT
TGCTTCCAGGGAAGATGAAGAG
>721
GGACTGAGATTGCCTCTGACTTCCTCCTCCCCTGGGTTCTGACTTCTTCCTCCCCTTGC
TTCCAGGGAAGATGAAGAGAGAGAGAGATTTGGAAGGGGCTCTGGCTCCCTAACACCTG
AATCCCAGATGATGGGAAGTATGT
>722
AGATTGCCTCTGACTTCCTCCTCCCCTGGGTTCTGACTTCTTCCTCCCCTTGCTTCCAG
GGAAGATGAAGAGAGAGAGAGATTTGGAAGGGGCTCTGGCTCCCTAACACCTGAATCCC
AGATGATGGGAAGTATGTTTTCAA
>723
CCTCTGACTTCCTCCTCCCCTGGGTTCTGACTTCTTCCTCCCCTTGCTTCCAGGGAAGA
TGAAGAGAGAGAGAGATTTGGAAGGGGCTCTGGCTCCCTAACACCTGAATCCCAGATGAT
GGGAAGTATGTTTTCAAGTGTGG
>724
CTTGCTTCCAGGGAAGATGAAGAGAGAGAGAGATTTGGAAGGGGCTCTGGCTCCCTAAC
ACCTGAATCCCAGATGATGGGAAGTATGTTTTCAAGTGTGGGGAGGATATGAAAATGTT
CTGTTCTCACTTTTGGCTTTATGT
>725
GGAAGATGAAGAGAGAGAGAGATTTGGAAGGGGCTCTGGCTCCCTAACACCTGAATCCC
AGATGATGGGAAGTATGTTTTCAAGTGTGGGGAGGATATGAAAATGTTCTGTTCTCACT
TTTGGCTTTATGTCCATTTTACCA
>726
GAGAGAGATTTGGAAGGGGCTCTGGCTCCCTAACACCTGAATCCCAGATGATGGGAAG
TATGTTTTCAAGTGTGGGGAGGATATGAAAATGTTCTGTTCTCACTTTTGGCTTTATGTC
CATTTTACCACTGTTTTTATCC

>727
GGTGAAATCCCAGCTCAGGTGTTATATCCTTGCTGCTTCTGACAAATGAAACCAAGGTG
TATTAATTAAGGTAATGCTAGCTACTACAATAAGCTGAAAAATATATAATAGCTAAAAGC
TAGGGTGCCAGATTTAGGAAA
>728
CAGCTCAGGTGTTATATCCTTGCTGCTTCTGACAAATGAAACCAAGGTGTATTAATTAA
GGTAATGCTAGCTACTACAATAAGCTGAAAAATATATAATAGCTAAAAGCTAGGGTGCC
AGATTTAGGAAAATAAAAACAA
>729
ACAAATGAAACCAAGGTGTATTAATTAAGGTAATGCTAGCTACTACAATAAGCTGAAAA
ATATATAATAGCTAAAAGCTAGGGTGCCAGATTTAGGAAAATAAAAACAAACAAAAAA
CAATTGAAATTTGAATTTCAGAT
>730
AAACCAAGGTGTATTAATTAAGGTAATGCTAGCTACTACAATAAGCTGAAAAATATATAA
TAGCTAAAAGCTAGGGTGCCAGATTTAGGAAAATAAAAACAAACAAAAAACAATTGA
AATTTGAATTTCAGATAAACAAT
>731
CAAGGTGTATTAATTAAGGTAATGCTAGCTACTACAATAAGCTGAAAAATATATAATAGC
TAAAAGCTAGGGTGCCAGATTTAGGAAAATAAAAACAAACAAAAAACAATTGAAATTT
GAATTTCAGATAAACAATTTTT
>732
TAAGGTAATGCTAGCTACTACAATAAGCTGAAAAATATATAATAGCTAAAAGCTAGGGT
GCCAGATTTAGGAAAATAAAAACAAACAAAAAACAATTGAAATTTGAATTTCAGATAA
ACAATTTTTTTAGTATAATTATG
>733
AGCTAAAAGCTAGGGTGCCAGATTTAGGAAAATAAAAACAAACAAAAAACAATTGAA
ATTTGAATTTCAGATAAACAATTTTTTTAGTATAATTATGTCCCATGCAATATTTGAGACA
TACTAAAAGGTTATTCATTGTT
>734
CCAGATTTAGGAAAATAAAAACAAACAAAAAACAATTGAAATTTGAATTTCAGATAAA
CAATTTTTTTAGTATAATTATGTCCCATGCAATATTTGAGACATACTAAAAGGTTATTCAT
TGTTTATCTGATATTCAAATT
>735
AAAACAATTGAAATTTGAATTTCAGATAAACAATTTTTTTAGTATAATTATGTCCCATGC
AATATTTGAGACATACTAAAAGGTTATTCATTGTTTATCTGATATTCAAATTTAACATTTT
CATTAATTTATTTTATTTG
>736
ATTTGAATTTCAGATAAACAATTTTTTTAGTATAATTATGTCCCATGCAATATTTGAGACA
TACTAAAAGGTTATTCATTGTTTATCTGATATTCAAATTTAACATTTTCATTAATTTATTTT
ATTTGGCAGCCCTATGG
>737
ACTAAAAGGTTATTCATTGTTTATCTGATATTCAAATTTAACATTTTCATTAATTTATTTTA
TTTGGCAGCCCTATGGAAGACATCAAAAGTTTATTTCTTATTTATATAAAGTCCAAAATA
GATGTTTTTTAGTAGGCA

>738
ATTCATTGTTTATCTGATATTCAAATTTAACATTTTCATTAATTTATTTTATTTGGCAGCCC
TATGGAAGACATCAAAAGTTTATTTCTTATTTATATAAAGTCCAAAATAGATGTTTTTTAG
TAGGCAGGCAGCTCTTC
>739
TATCTGATATTCAAATTTAACATTTTCATTAATTTATTTTATTTGGCAGCCCTATGGAAGA
CATCAAAAGTTTATTTCTTATTTATATAAAGTCCAAAATAGATGTTTTTTAGTAGGCAGG
CAGCTCTTCTCTAAGCGTA
>740
TTTATTTTATTTGGCAGCCCTATGGAAGACATCAAAAGTTTATTTCTTATTTATATAAAGT
CCAAAATAGATGTTTTTTAGTAGGCAGGCAGCTCTTCTCTAAGCGTAATTCAGGGCTCT
GGGTTCTGTCCATCCTGGCC
>741
TGGCAGCCCTATGGAAGACATCAAAAGTTTATTTCTTATTTATATAAAGTCCAAAATAGA
TGTTTTTTAGTAGGCAGGCAGCTCTTCTCTAAGCGTAATTCAGGGCTCTGGGTTCTGTC
CATCCTGGCCCTGACGTCTTT
>742
CAGCCCTATGGAAGACATCAAAAGTTTATTTCTTATTTATATAAAGTCCAAAATAGATGT
TTTTTAGTAGGCAGGCAGCTCTTCTCTAAGCGTAATTCAGGGCTCTGGGTTCTGTCCAT
CCTGGCCCTGACGTCTTTCAT
>743
AAAGTTTATTTCTTATTTATATAAAGTCCAAAATAGATGTTTTTTAGTAGGCAGGCAGCT
CTTCTCTAAGCGTAATTCAGGGCTCTGGGTTCTGTCCATCCTGGCCCTGACGTCTTTCAT
TGCTCAGCTTCTAAGATAGC
>744
GCTCTTCTCTAAGCGTAATTCAGGGCTCTGGGTTCTGTCCATCCTGGCCCTGACGTCTT
TCATTGCTCAGCTTCTAAGATAGCTCTATGCTCATGGGGTCAAGTCACAGGAAAGAAA
AGGGAATGTGGAGTTCCACTTGG
>745
CTAAGCGTAATTCAGGGCTCTGGGTTCTGTCCATCCTGGCCCTGACGTCTTTCATTGCT
CAGCTTCTAAGATAGCTCTATGCTCATGGGGTCAAGTCACAGGAAAGAAAAGGGAATG
TGGAGTTCCACTTGGGAGTTTTT
>746
TCTGTCCATCCTGGCCCTGACGTCTTTCATTGCTCAGCTTCTAAGATAGCTCTATGCTCA
TGGGGTCAAGTCACAGGAAAGAAAAGGGAATGTGGAGTTCCACTTGGGAGTTTTTAT
GGGCCAGTCCTGGAAGCGGCACA
>747
CATCCTGGCCCTGACGTCTTTCATTGCTCAGCTTCTAAGATAGCTCTATGCTCATGGGGT
CAAGTCACAGGAAAGAAAAGGGAATGTGGAGTTCCACTTGGGAGTTTTTATGGGCCA
GTCCTGGAAGCGGCACAGAGGAT
>748
CTGACGTCTTTCATTGCTCAGCTTCTAAGATAGCTCTATGCTCATGGGGTCAAGTCACA
GGAAAGAAAAGGGAATGTGGAGTTCCACTTGGGAGTTTTTATGGGCCAGTCCTGGAA
GCGGCACAGAGGATTTCTGCTCAC

>749
CTAAGATAGCTCTATGCTCATGGGGTCAAGTCACAGGAAAGAAAAGGGAATGTGGAGT
TCCACTTGGGAGTTTTTATGGGCCAGTCCTGGAAGCGGCACAGAGGATTTCTGCTCAC
AATCTATTGGCCAGAACTCAATCA
>750
GGTCAAGTCACAGGAAAGAAAAGGGAATGTGGAGTTCCACTTGGGAGTTTTTATGGG
CCAGTCCTGGAAGCGGCACAGAGGATTTCTGCTCACAATCTATTGGCCAGAACTCAAT
CACATGGCCTGCACCTAACTGCAAG
>751
CACAGGAAAGAAAAGGGAATGTGGAGTTCCACTTGGGAGTTTTTATGGGCCAGTCCTG
GAAGCGGCACAGAGGATTTCTGCTCACAATCTATTGGCCAGAACTCAATCACATGGCC
TGCACCTAACTGCAAGTTAAGCCA
>752
CACTTGGGAGTTTTTATGGGCCAGTCCTGGAAGCGGCACAGAGGATTTCTGCTCACAA
TCTATTGGCCAGAACTCAATCACATGGCCTGCACCTAACTGCAAGTTAAGCCAGAAAA
ATGTAGGTGTGCCCAGGAAGAAGT
>753
CGGCACAGAGGATTTCTGCTCACAATCTATTGGCCAGAACTCAATCACATGGCCTGCAC
CTAACTGCAAGTTAAGCCAGAAAAATGTAGGTGTGCCCAGGAAGAAGTTTGGGGAAC
AGCTAGCCAGTCTCTGCCACACCT
>754
CAGAGGATTTCTGCTCACAATCTATTGGCCAGAACTCAATCACATGGCCTGCACCTAAC
TGCAAGTTAAGCCAGAAAAATGTAGGTGTGCCCAGGAAGAAGTTTGGGGAACAGCTA
GCCAGTCTCTGCCACACCTGGTAA
>755
TTGGCCAGAACTCAATCACATGGCCTGCACCTAACTGCAAGTTAAGCCAGAAAAATGT
AGGTGTGCCCAGGAAGAAGTTTGGGGAACAGCTAGCCAGTCTCTGCCACACCTGGTA
ACAGCAGAGTGAATCTGTCAGTACT
>756
ATGGCCTGCACCTAACTGCAAGTTAAGCCAGAAAAATGTAGGTGTGCCCAGGAAGAA
GTTTGGGGAACAGCTAGCCAGTCTCTGCCACACCTGGTAACAGCAGAGTGAATCTGTC
AGTACTCGTGCCTAATGCGTGTCTC
>757
CCTGCACCTAACTGCAAGTTAAGCCAGAAAAATGTAGGTGTGCCCAGGAAGAAGTTTG
GGGAACAGCTAGCCAGTCTCTGCCACACCTGGTAACAGCAGAGTGAATCTGTCAGTAC
TCGTGCCTAATGCGTGTCTCATAT
>758
GAAAAATGTAGGTGTGCCCAGGAAGAAGTTTGGGGAACAGCTAGCCAGTCTCTGCCA
CACCTGGTAACAGCAGAGTGAATCTGTCAGTACTCGTGCCTAATGCGTGTCTCATATGC
TAGTGCTGGATTCAGTCAGCAGTC
>759
CCAGGAAGAAGTTTGGGGAACAGCTAGCCAGTCTCTGCCACACCTGGTAACAGCAGA
GTGAATCTGTCAGTACTCGTGCCTAATGCGTGTCTCATATGCTAGTGCTGGATTCAGTCA
GCAGTCCTACATCCGCTGGGGTC

>760
CTCTGCCACACCTGGTAACAGCAGAGTGAATCTGTCAGTACTCGTGCCTAATGCGTGTC
TCATATGCTAGTGCTGGATTCAGTCAGCAGTCCTACATCCGCTGGGGTCCCTAGGAGGG
GTCAGATGCTCTCCTCTTTGCC
>761
TGGTAACAGCAGAGTGAATCTGTCAGTACTCGTGCCTAATGCGTGTCTCATATGCTAGT
GCTGGATTCAGTCAGCAGTCCTACATCCGCTGGGGTCCCTAGGAGGGGTCAGATGCTC
TCCTCTTTGCCCTGAACTGGTTA
>762
GTGCCTAATGCGTGTCTCATATGCTAGTGCTGGATTCAGTCAGCAGTCCTACATCCGCT
GGGGTCCCTAGGAGGGGTCAGATGCTCTCCTCTTTGCCCTGAACTGGTTAGCTACTTCG
GACTGGGATGACAAGGCCATTG
>763
CCTAATGCGTGTCTCATATGCTAGTGCTGGATTCAGTCAGCAGTCCTACATCCGCTGGG
GTCCCTAGGAGGGGTCAGATGCTCTCCTCTTTGCCCTGAACTGGTTAGCTACTTCGGAC
TGGGATGACAAGGCCATTGTGA
>764
CAGCAGTCCTACATCCGCTGGGGTCCCTAGGAGGGGTCAGATGCTCTCCTCTTTGCCCT
GAACTGGTTAGCTACTTCGGACTGGGATGACAAGGCCATTGTGAGAGTGATAAGCAAT
GTGATTCAAACAAATTCATTTCA
>765
TCCCTAGGAGGGGTCAGATGCTCTCCTCTTTGCCCTGAACTGGTTAGCTACTTCGGACT
GGGATGACAAGGCCATTGTGAGAGTGATAAGCAATGTGATTCAAACAAATTCATTTCA
CTTCAATTCAATGCAGGTTTTTT
>766
GGTCAGATGCTCTCCTCTTTGCCCTGAACTGGTTAGCTACTTCGGACTGGGATGACAAG
GCCATTGTGAGAGTGATAAGCAATGTGATTCAAACAAATTCATTTCACTTCAATTCAAT
GCAGGTTTTTTTTTTTTTTTGA
>767
TGACAAGGCCATTGTGAGAGTGATAAGCAATGTGATTCAAACAAATTCATTTCACTTCA
ATTCAATGCAGGTTTTTTTTTTTTTTTGAGACGGGCTGTCATGCTGTTGCCAACCCTGG
AGAGCAGTGGCGCCATTACGGC
>768
GTGATAAGCAATGTGATTCAAACAAATTCATTTCACTTCAATTCAATGCAGGTTTTTTTT
TTTTTTTGAGACGGGCTGTCATGCTGTTGCCAACCCTGGAGAGCAGTGGCGCCATTAC
GGCTCACTGCAGCCTCCTGGGC
>769
TTCACTTCAATTCAATGCAGGTTTTTTTTTTTTTTTGAGACGGGCTGTCATGCTGTTGCC
AACCCTGGAGAGCAGTGGCGCCATTACGGCTCACTGCAGCCTCCTGGGCTCAAGCAAT
TCTCCCTCCTCAGCTTCCCAAG
>770
CACTTCAATTCAATGCAGGTTTTTTTTTTTTTTTGAGACGGGCTGTCATGCTGTTGCCAA
CCCTGGAGAGCAGTGGCGCCATTACGGCTCACTGCAGCCTCCTGGGCTCAAGCAATTC
TCCCTCCTCAGCTTCCCAAGTA

>771
TTTTTTTGAGACGGGCTGTCATGCTGTTGCCAACCCTGGAGAGCAGTGGCGCCATTAC
GGCTCACTGCAGCCTCCTGGGCTCAAGCAATTCTCCCTCCTCAGCTTCCCAAGTAGCT
GGGACTACAGGTGCATGCCACCAT
>772
CTGTCATGCTGTTGCCAACCCTGGAGAGCAGTGGCGCCATTACGGCTCACTGCAGCCT
CCTGGGCTCAAGCAATTCTCCCTCCTCAGCTTCCCAAGTAGCTGGGACTACAGGTGCA
TGCCACCATGTTCAGCTCATTTAT
>773
ACCCTGGAGAGCAGTGGCGCCATTACGGCTCACTGCAGCCTCCTGGGCTCAAGCAATT
CTCCCTCCTCAGCTTCCCAAGTAGCTGGGACTACAGGTGCATGCCACCATGTTCAGCTC
ATTTATTTTTTATTTTTTTGTAG
>774
AGCAGTGGCGCCATTACGGCTCACTGCAGCCTCCTGGGCTCAAGCAATTCTCCCTCCT
CAGCTTCCCAAGTAGCTGGGACTACAGGTGCATGCCACCATGTTCAGCTCATTTATTTT
TTATTTTTTTGTAGACAGGAGGT
>775
ACGGCTCACTGCAGCCTCCTGGGCTCAAGCAATTCTCCCTCCTCAGCTTCCCAAGTAG
CTGGGACTACAGGTGCATGCCACCATGTTCAGCTCATTTATTTTTTATTTTTTTGTAGAC
AGGAGGTCTCACTATGTTACCC
>776
GGGCTCAAGCAATTCTCCCTCCTCAGCTTCCCAAGTAGCTGGGACTACAGGTGCATGC
CACCATGTTCAGCTCATTTATTTTTTATTTTTTTGTAGACAGGAGGTCTCACTATGTTACC
CAGGCTGCTGTCCAAGTCCTG
>777
CTTCCCAAGTAGCTGGGACTACAGGTGCATGCCACCATGTTCAGCTCATTTATTTTTTAT
TTTTTTGTAGACAGGAGGTCTCACTATGTTACCCAGGCTGCTGTCCAAGTCCTGGGCTC
ACGTGATTCTCCCGCCTCAGC
>778
CCAAGTAGCTGGGACTACAGGTGCATGCCACCATGTTCAGCTCATTTATTTTTTATTTTT
TTGTAGACAGGAGGTCTCACTATGTTACCCAGGCTGCTGTCCAAGTCCTGGGCTCACG
TGATTCTCCCGCCTCAGCCTCC
>779
GCATGCCACCATGTTCAGCTCATTTATTTTTTATTTTTTTGTAGACAGGAGGTCTCACTAT
GTTACCCAGGCTGCTGTCCAAGTCCTGGGCTCACGTGATTCTCCCGCCTCAGCCTCCCA
AAATGCTGGGATTACAGGCG
>780
GACAGGAGGTCTCACTATGTTACCCAGGCTGCTGTCCAAGTCCTGGGCTCACGTGATT
CTCCCGCCTCAGCCTCCCAAAATGCTGGGATTACAGGCGTAAGCCACCATGCCCAGAC
AATTTAATAAATATTTATTAAAAC
>781
CAGGCTGCTGTCCAAGTCCTGGGCTCACGTGATTCTCCCGCCTCAGCCTCCCAAAATG
CTGGGATTACAGGCGTAAGCCACCATGCCCAGACAATTTAATAAATATTTATTAAAACTT
CTAGGATATTGAGAAAAGCTTG

>782
GGGCTCACGTGATTCTCCCGCCTCAGCCTCCCAAAATGCTGGGATTACAGGCGTAAGC
CACCATGCCCAGACAATTTAATAAATATTTATTAAAACTTCTAGGATATTGAGAAAAGCT
TGGTTCTAGGCCACTGGGGAAG
>783
AAAATGCTGGGATTACAGGCGTAAGCCACCATGCCCAGACAATTTAATAAATATTTATTA
AAACTTCTAGGATATTGAGAAAAGCTTGGTTCTAGGCCACTGGGGAAGACATCAACTG
AAACAGCCCAGTTATACTCTCA
>784
GGATTACAGGCGTAAGCCACCATGCCCAGACAATTTAATAAATATTTATTAAAACTTCTA
GGATATTGAGAAAAGCTTGGTTCTAGGCCACTGGGGAAGACATCAACTGAAACAGCCC
AGTTATACTCTCAATTAGCTGT
>785
CCACCATGCCCAGACAATTTAATAAATATTTATTAAAACTTCTAGGATATTGAGAAAAGC
TTGGTTCTAGGCCACTGGGGAAGACATCAACTGAAACAGCCCAGTTATACTCTCAATTA
GCTGTATACAATTAAATTCAC
>786
ACAATTTAATAAATATTTATTAAAACTTCTAGGATATTGAGAAAAGCTTGGTTCTAGGCC
ACTGGGGAAGACATCAACTGAAACAGCCCAGTTATACTCTCAATTAGCTGTATACAATT
AAATTCACAGATATCCTCAAC
>787
TTATTAAAACTTCTAGGATATTGAGAAAAGCTTGGTTCTAGGCCACTGGGGAAGACATC
AACTGAAACAGCCCAGTTATACTCTCAATTAGCTGTATACAATTAAATTCACAGATATCC
TCAACCCTTATCCATATCCCA
>788
CACTGGGGAAGACATCAACTGAAACAGCCCAGTTATACTCTCAATTAGCTGTATACAAT
TAAATTCACAGATATCCTCAACCCTTATCCATATCCCATTCATCTTTCAAAGGTTGGTGA
AAACCTCATAGCACAAAGAAT
>789
CAATTAGCTGTATACAATTAAATTCACAGATATCCTCAACCCTTATCCATATCCCATTCAT
CTTTCAAAGGTTGGTGAAAACCTCATAGCACAAAGAATTCTGCCCTAAATGTTTGAATA
CAGAATGGTCCCCTCTCTCC
>790
AAATTCACAGATATCCTCAACCCTTATCCATATCCCATTCATCTTTCAAAGGTTGGTGAA
AACCTCATAGCACAAAGAATTCTGCCCTAAATGTTTGAATACAGAATGGTCCCCTCTCT
CCCTAATTCCTAAAATATTTG
>791
CAGATATCCTCAACCCTTATCCATATCCCATTCATCTTTCAAAGGTTGGTGAAAACCTCA
TAGCACAAAGAATTCTGCCCTAAATGTTTGAATACAGAATGGTCCCCTCTCTCCCTAAT
TCCTAAAATATTTGGTCTCTA
>792
CCATTCATCTTTCAAAGGTTGGTGAAAACCTCATAGCACAAAGAATTCTGCCCTAAATG
TTTGAATACAGAATGGTCCCCTCTCTCCCTAATTCCTAAAATATTTGGTCTCTATGTTCAT
TCATTCATTCATGCATGCAT

>793
CCTCTCTCCCTAATTCCTAAAATATTTGGTCTCTATGTTCATTCATTCATTCATGCATGCAT
GCTTTCAGTTAGTTCATCGACAAACATTTACATCTACTCTGTTTGACAGTGTGCTAGGCT
CTGGGGCTTGGGAGTGGA
>794
TCTCCCTAATTCCTAAAATATTTGGTCTCTATGTTCATTCATTCATTCATGCATGCATGCTT
TCAGTTAGTTCATCGACAAACATTTACATCTACTCTGTTTGACAGTGTGCTAGGCTCTG
GGGCTTGGGAGTGGAAATA
>795
TTCATTCATTCATGCATGCATGCTTTCAGTTAGTTCATCGACAAACATTTACATCTACTCT
GTTTGACAGTGTGCTAGGCTCTGGGGCTTGGGAGTGGAAATACTAAATTGAACAAGAC
TGAGGCCTTGCCTTCCAGAAA
>796
TCAGTTAGTTCATCGACAAACATTTACATCTACTCTGTTTGACAGTGTGCTAGGCTCTG
GGGCTTGGGAGTGGAAATACTAAATTGAACAAGACTGAGGCCTTGCCTTCCAGAAACT
CACAAAGGAGTGGGTGGTCAGAA
>797
TTTACATCTACTCTGTTTGACAGTGTGCTAGGCTCTGGGGCTTGGGAGTGGAAATACTA
AATTGAACAAGACTGAGGCCTTGCCTTCCAGAAACTCACAAAGGAGTGGGTGGTCAG
AATGCACATGACTCAGTCAGATAT
>798
CAGTGTGCTAGGCTCTGGGGCTTGGGAGTGGAAATACTAAATTGAACAAGACTGAGGC
CTTGCCTTCCAGAAACTCACAAAGGAGTGGGTGGTCAGAATGCACATGACTCAGTCA
GATATAAGGCAGATGGTGGTAAATA
>799
CTCTGGGGCTTGGGAGTGGAAATACTAAATTGAACAAGACTGAGGCCTTGCCTTCCAG
AAACTCACAAAGGAGTGGGTGGTCAGAATGCACATGACTCAGTCAGATATAAGGCAGA
TGGTGGTAAATACTATGATAAATG
>800
TGGGGCTTGGGAGTGGAAATACTAAATTGAACAAGACTGAGGCCTTGCCTTCCAGAAA
CTCACAAAGGAGTGGGTGGTCAGAATGCACATGACTCAGTCAGATATAAGGCAGATGG
TGGTAAATACTATGATAAATGTTC
>801
AGTGGAAATACTAAATTGAACAAGACTGAGGCCTTGCCTTCCAGAAACTCACAAAGG
AGTGGGTGGTCAGAATGCACATGACTCAGTCAGATATAAGGCAGATGGTGGTAAATACT
ATGATAAATGTTCAGATAGTGCTA
>802
GAGGCCTTGCCTTCCAGAAACTCACAAAGGAGTGGGTGGTCAGAATGCACATGACTC
AGTCAGATATAAGGCAGATGGTGGTAAATACTATGATAAATGTTCAGATAGTGCTAAGG
GGCTTATGGAGAATGGGAGAAATT
>803
GGTGGTCAGAATGCACATGACTCAGTCAGATATAAGGCAGATGGTGGTAAATACTATGA
TAAATGTTCAGATAGTGCTAAGGGGCTTATGGAGAATGGGAGAAATTAAATCTGACTCA
AGGGTGGGTGGAGAAGATTACA

>804
GTCAGAATGCACATGACTCAGTCAGATATAAGGCAGATGGTGGTAAATACTATGATAAA
TGTTCAGATAGTGCTAAGGGGCTTATGGAGAATGGGAGAAATTAAATCTGACTCAAGG
GTGGGTGGAGAAGATTACATCCA
>805
ATAAGGCAGATGGTGGTAAATACTATGATAAATGTTCAGATAGTGCTAAGGGGCTTATG
GAGAATGGGAGAAATTAAATCTGACTCAAGGGTGGGTGGAGAAGATTACATCCAAGAT
CCAGCAATGACTTCCAGAAGAGA
>806
ATACTATGATAAATGTTCAGATAGTGCTAAGGGGCTTATGGAGAATGGGAGAAATTAAA
TCTGACTCAAGGGTGGGTGGAGAAGATTACATCCAAGATCCAGCAATGACTTCCAGAA
GAGACAAATGAACCTGGCCTGGC
>807
AATGTTCAGATAGTGCTAAGGGGCTTATGGAGAATGGGAGAAATTAAATCTGACTCAA
GGGTGGGTGGAGAAGATTACATCCAAGATCCAGCAATGACTTCCAGAAGAGACAAAT
GAACCTGGCCTGGCAAGGGAGGCAG
>808
CTAAGGGGCTTATGGAGAATGGGAGAAATTAAATCTGACTCAAGGGTGGGTGGAGAA
GATTACATCCAAGATCCAGCAATGACTTCCAGAAGAGACAAATGAACCTGGCCTGGCA
AGGGAGGCAGAGTCAAAGTGTCTGG
>809
GAGAAATTAAATCTGACTCAAGGGTGGGTGGAGAAGATTACATCCAAGATCCAGCAAT
GACTTCCAGAAGAGACAAATGAACCTGGCCTGGCAAGGGAGGCAGAGTCAAAGTGTC
TGGCTCAAAGCCCACCACAGCCTTG
>810
GAAATTAAATCTGACTCAAGGGTGGGTGGAGAAGATTACATCCAAGATCCAGCAATGA
CTTCCAGAAGAGACAAATGAACCTGGCCTGGCAAGGGAGGCAGAGTCAAAGTGTCTG
GCTCAAAGCCCACCACAGCCTTGGC
>811
GGGTGGAGAAGATTACATCCAAGATCCAGCAATGACTTCCAGAAGAGACAAATGAAC
CTGGCCTGGCAAGGGAGGCAGAGTCAAAGTGTCTGGCTCAAAGCCCACCACAGCCTT
GGCTCACAGGCATCTCCCGGTCTTCC
>812
AAGATTACATCCAAGATCCAGCAATGACTTCCAGAAGAGACAAATGAACCTGGCCTGG
CAAGGGAGGCAGAGTCAAAGTGTCTGGCTCAAAGCCCACCACAGCCTTGGCTCACAG
GCATCTCCCGGTCTTCCACTCACTC
>813
AGAAGAGACAAATGAACCTGGCCTGGCAAGGGAGGCAGAGTCAAAGTGTCTGGCTC
AAAGCCCACCACAGCCTTGGCTCACAGGCATCTCCCGGTCTTCCACTCACTCAGTCCT
TTCAGGGAAAGGCACTGAGCTTTTCT
>814
GAACCTGGCCTGGCAAGGGAGGCAGAGTCAAAGTGTCTGGCTCAAAGCCCACCACAG
CCTTGGCTCACAGGCATCTCCCGGTCTTCCACTCACTCAGTCCTTTCAGGGAAAGGCA
CTGAGCTTTTCTCAAGGGAGAACTC

>815
TCAAAGTGTCTGGCTCAAAGCCCACCACAGCCTTGGCTCACAGGCATCTCCCGGTCTT
CCACTCACTCAGTCCTTTCAGGGAAAGGCACTGAGCTTTTCTCAAGGGAGAACTCCAA
AGACGTCTTTCATTTCATCTTACA
>816
CAAAGCCCACCACAGCCTTGGCTCACAGGCATCTCCCGGTCTTCCACTCACTCAGTCC
TTTCAGGGAAAGGCACTGAGCTTTTCTCAAGGGAGAACTCCAAAGACGTCTTTCATTT
CATCTTACAAATCCACACAAATTT
>817
ACCACAGCCTTGGCTCACAGGCATCTCCCGGTCTTCCACTCACTCAGTCCTTTCAGGG
AAAGGCACTGAGCTTTTCTCAAGGGAGAACTCCAAAGACGTCTTTCATTTCATCTTAC
AAATCCACACAAATTTGCAATTCC
>818
GGCTCACAGGCATCTCCCGGTCTTCCACTCACTCAGTCCTTTCAGGGAAAGGCACTGA
GCTTTTCTCAAGGGAGAACTCCAAAGACGTCTTTCATTTCATCTTACAAATCCACACAA
ATTTGCAATTCCACTCCACAAGA
>819
TCCCGGTCTTCCACTCACTCAGTCCTTTCAGGGAAAGGCACTGAGCTTTTCTCAAGGG
AGAACTCCAAAGACGTCTTTCATTTCATCTTACAAATCCACACAAATTTGCAATTCCAC
TCCACAAGATACGAGCATTTGTC
>820
GGGAGAACTCCAAAGACGTCTTTCATTTCATCTTACAAATCCACACAAATTTGCAATTC
CACTCCACAAGATACGAGCATTTGTCTTGCTATAATTTTAATATGATTGGCATAAATTACC
TGTTATCAAGTAAAATAGTT
>821
CTCCAAAGACGTCTTTCATTTCATCTTACAAATCCACACAAATTTGCAATTCCACTCCA
CAAGATACGAGCATTTGTCTTGCTATAATTTTAATATGATTGGCATAAATTACCTGTTATC
AAGTAAAATAGTTTCTTCTT
>822
ACTCCACAAGATACGAGCATTTGTCTTGCTATAATTTTAATATGATTGGCATAAATTACCT
GTTATCAAGTAAAATAGTTTCTTCTTATGGCTTGTGTGCCCCTGACATACCACTGCTATT
TGAAAAGAGGCTTCTCCAG
>823
AGATACGAGCATTTGTCTTGCTATAATTTTAATATGATTGGCATAAATTACCTGTTATCAA
GTAAAATAGTTTCTTCTTATGGCTTGTGTGCCCCTGACATACCACTGCTATTTGAAAAGA
GGCTTCTCCAGAAACTCAG
>824
CCTGTTATCAAGTAAAATAGTTTCTTCTTATGGCTTGTGTGCCCCTGACATACCACTGCT
ATTTGAAAAGAGGCTTCTCCAGAAACTCAGTGTCTGAGAGTACGTGACTGGAAGGTG
AGTGTTTGCTTACCCCTTGCGTT
>825
TGTTATCAAGTAAAATAGTTTCTTCTTATGGCTTGTGTGCCCCTGACATACCACTGCTAT
TTGAAAAGAGGCTTCTCCAGAAACTCAGTGTCTGAGAGTACGTGACTGGAAGGTGAG
TGTTTGCTTACCCCTTGCGTTTT

>826
CTGACATACCACTGCTATTTGAAAAGAGGCTTCTCCAGAAACTCAGTGTCTGAGAGTA
CGTGACTGGAAGGTGAGTGTTTGCTTACCCCTTGCGTTTTCTCCCTGTCAGGATAAATG
GAATATTCTGCTTTAAGGACCCT
>827
ATACCACTGCTATTTGAAAAGAGGCTTCTCCAGAAACTCAGTGTCTGAGAGTACGTGA
CTGGAAGGTGAGTGTTTGCTTACCCCTTGCGTTTTCTCCCTGTCAGGATAAATGGAATA
TTCTGCTTTAAGGACCCTAAGTC
>828
AACTCAGTGTCTGAGAGTACGTGACTGGAAGGTGAGTGTTTGCTTACCCCTTGCGTTT
TCTCCCTGTCAGGATAAATGGAATATTCTGCTTTAAGGACCCTAAGTCTGGCAATATCAT
GTTTTGTGAACAGCCAACACAG
>829
TGGAAGGTGAGTGTTTGCTTACCCCTTGCGTTTTCTCCCTGTCAGGATAAATGGAATAT
TCTGCTTTAAGGACCCTAAGTCTGGCAATATCATGTTTTGTGAACAGCCAACACAGTAA
CTATGGTTGAGGCCTGTATTCG
>830
AGTGTTTGCTTACCCCTTGCGTTTTCTCCCTGTCAGGATAAATGGAATATTCTGCTTTAA
GGACCCTAAGTCTGGCAATATCATGTTTTGTGAACAGCCAACACAGTAACTATGGTTGA
GGCCTGTATTCGATGAATGAG
>831
CCCTGTCAGGATAAATGGAATATTCTGCTTTAAGGACCCTAAGTCTGGCAATATCATGTT
TTGTGAACAGCCAACACAGTAACTATGGTTGAGGCCTGTATTCGATGAATGAGGTTTTC
ATATGCTTCTTTCACATCAGG
>832
GATAAATGGAATATTCTGCTTTAAGGACCCTAAGTCTGGCAATATCATGTTTTGTGAACA
GCCAACACAGTAACTATGGTTGAGGCCTGTATTCGATGAATGAGGTTTTCATATGCTTCT
TTCACATCAGGAGAGAGAGA
>833
TCTGCTTTAAGGACCCTAAGTCTGGCAATATCATGTTTTGTGAACAGCCAACACAGTAA
CTATGGTTGAGGCCTGTATTCGATGAATGAGGTTTTCATATGCTTCTTTCACATCAGGAG
AGAGAGAGAGAAAAAAAAATA
>834
TCTGGCAATATCATGTTTTGTGAACAGCCAACACAGTAACTATGGTTGAGGCCTGTATT
CGATGAATGAGGTTTTCATATGCTTCTTTCACATCAGGAGAGAGAGAGAGAAAAAAAA
ATAATAAACTTGGTAGAGGACGT
>835
GCCAACACAGTAACTATGGTTGAGGCCTGTATTCGATGAATGAGGTTTTCATATGCTTCT
TTCACATCAGGAGAGAGAGAGAGAAAAAAAAATAATAAACTTGGTAGAGGACGTGGA
GTTGTATTTCTGGACTATGGTGG
>836
AACACAGTAACTATGGTTGAGGCCTGTATTCGATGAATGAGGTTTTCATATGCTTCTTTC
ACATCAGGAGAGAGAGAGAGAAAAAAAAATAATAAACTTGGTAGAGGACGTGGAGTT
GTATTTCTGGACTATGGTGGCAC

>837
CACAGTAACTATGGTTGAGGCCTGTATTCGATGAATGAGGTTTTCATATGCTTCTTTCAC
ATCAGGAGAGAGAGAGAGAGAAAAAAAAATAATAAACTTGGTAGAGGACGTGGAGTTGT
ATTTCTGGACTATGGTGGCACCT
>838
AACTATGGTTGAGGCCTGTATTCGATGAATGAGGTTTTCATATGCTTCTTTCACATCAGG
AGAGAGAGAGAGAGAAAAAAAAATAATAAACTTGGTAGAGGACGTGGAGTTGTATTTCT
GGACTATGGTGGCACCTCTAAGG
>839
GAATGAGGTTTTCATATGCTTCTTTCACATCAGGAGAGAGAGAGAGAGAAAAAAAAATAA
TAAACTTGGTAGAGGACGTGGAGTTGTATTTCTGGACTATGGTGGCACCTCTAAGGAG
CATGGTTTTCCTTTTGCATGGCTT
>840
GAGAAAAAAAAATAATAAACTTGGTAGAGGACGTGGAGTTGTATTTCTGGACTATGGT
GGCACCTCTAAGGAGCATGGTTTTCCTTTTGCATGGCTTTGTCCATTACTATAACACTCC
TGTTATAAAATAGCTGCCGTGG
>841
AAAAAAAAATAATAAACTTGGTAGAGGACGTGGAGTTGTATTTCTGGACTATGGTGGC
ACCTCTAAGGAGCATGGTTTTCCTTTTGCATGGCTTTGTCCATTACTATAACACTCCTGT
TATAAAATAGCTGCCGTGGATT
>842
ACCTCTAAGGAGCATGGTTTTCCTTTTGCATGGCTTTGTCCATTACTATAACACTCCTGT
TATAAAATAGCTGCCGTGGATTGAAATCCATAATGTTAACTCAAAAGTTAAGCAGTCCG
CTATTAAACAGCATAAGAAAC
>843
GTCCATTACTATAACACTCCTGTTATAAAATAGCTGCCGTGGATTGAAATCCATAATGTT
AACTCAAAAGTTAAGCAGTCCGCTATTAAACAGCATAAGAAACTGTTACCTATCCCCTC
TTCCTGGCCCCCCAACAAAGA
>844
TTACTATAACACTCCTGTTATAAAATAGCTGCCGTGGATTGAAATCCATAATGTTAACTC
AAAAGTTAAGCAGTCCGCTATTAAACAGCATAAGAAACTGTTACCTATCCCCTCTTCCT
GGCCCCCCAACAAAGAAACAG
>845
CTATAACACTCCTGTTATAAAATAGCTGCCGTGGATTGAAATCCATAATGTTAACTCAAA
AGTTAAGCAGTCCGCTATTAAACAGCATAAGAAACTGTTACCTATCCCCTCTTCCTGGC
CCCCCAACAAAGAAACAGCAA
>846
TATAAAATAGCTGCCGTGGATTGAAATCCATAATGTTAACTCAAAAGTTAAGCAGTCCG
CTATTAAACAGCATAAGAAACTGTTACCTATCCCCTCTTCCTGGCCCCCCAACAAAGAA
ACAGCAAACTGTTAAAAAAGCC
>847
GTTAAGCAGTCCGCTATTAAACAGCATAAGAAACTGTTACCTATCCCCTCTTCCTGGCC
CCCCAACAAAGAAACAGCAAACTGTTAAAAAAGCCCATGTCTCCCCATGAGGACTTG
GGGTTAATTTTCAGCATGGTGTCT

>848
CAGTCCGCTATTAAACAGCATAAGAAACTGTTACCTATCCCCTCTTCCTGGCCCCCCAA
CAAAGAAACAGCAAACTGTTAAAAAAGCCCATGTCTCCCCATGAGGACTTGGGGTTAA
TTTTCAGCATGGTGTCTGGGGAT
>849
AGCATAAGAAACTGTTACCTATCCCCTCTTCCTGGCCCCCCAACAAAGAAACAGCAAA
CTGTTAAAAAAGCCCATGTCTCCCCATGAGGACTTGGGGTTAATTTTCAGCATGGTGTC
TGGGGATTCATCTGTGGGACTCC
>850
CCCAACAAAGAAACAGCAAACTGTTAAAAAAGCCCATGTCTCCCCATGAGGACTTGG
GGTTAATTTTCAGCATGGTGTCTGGGGATTCATCTGTGGGACTCCTATTACCCATAATAG
GACTCATGCTGCTTCATTTCATG
>851
GAGGACTTGGGGTTAATTTTCAGCATGGTGTCTGGGGATTCATCTGTGGGACTCCTATT
ACCCATAATAGGACTCATGCTGCTTCATTTCATGCACACGATATGGAAAATGCACCCTTC
CCTAGTGATGAAATTTTAGTT
>852
CTATTACCCATAATAGGACTCATGCTGCTTCATTTCATGCACACGATATGGAAAATGCAC
CCTTCCCTAGTGATGAAATTTTAGTTTTGAAATTGAGGCTAGCTTTTGCAGAACAAAGC
AGATTCCCAGAAACACCAAAA
>853
AGGACTCATGCTGCTTCATTTCATGCACACGATATGGAAAATGCACCCTTCCCTAGTGA
TGAAATTTTAGTTTTGAAATTGAGGCTAGCTTTTGCAGAACAAAGCAGATTCCCAGAA
ACACCAAAAACTATTCAATGTTG
>854
CTTCATTTCATGCACACGATATGGAAAATGCACCCTTCCCTAGTGATGAAATTTTAGTTT
TGAAATTGAGGCTAGCTTTTGCAGAACAAAGCAGATTCCCAGAAACACCAAAAACTAT
TCAATGTTGGCCTAGAGCCTGT
>855
TTTCATGCACACGATATGGAAAATGCACCCTTCCCTAGTGATGAAATTTTAGTTTTGAAA
TTGAGGCTAGCTTTTGCAGAACAAAGCAGATTCCCAGAAACACCAAAAACTATTCAAT
GTTGGCCTAGAGCCTGTGTGTC
>856
CCCTTCCCTAGTGATGAAATTTTAGTTTTGAAATTGAGGCTAGCTTTTGCAGAACAAAG
CAGATTCCCAGAAACACCAAAAACTATTCAATGTTGGCCTAGAGCCTGTGTGTCCGTG
CTTAGGTGAGTATGTGTATCTGA
>857
AATTGAGGCTAGCTTTTGCAGAACAAAGCAGATTCCCAGAAACACCAAAAACTATTCA
ATGTTGGCCTAGAGCCTGTGTGTCCGTGCTTAGGTGAGTATGTGTATCTGAAAGGGAGA
AAATGCCTTCCTCTAAGTGCGTA
>858
TTGAGGCTAGCTTTTGCAGAACAAAGCAGATTCCCAGAAACACCAAAAACTATTCAAT
GTTGGCCTAGAGCCTGTGTGTCCGTGCTTAGGTGAGTATGTGTATCTGAAAGGGAGAA
AATGCCTTCCTCTAAGTGCGTATG

>859
AGCAGATTCCCAGAAACACCAAAAACTATTCAATGTTGGCCTAGAGCCTGTGTGTCCG
TGCTTAGGTGAGTATGTGTATCTGAAAGGGAGAAAATGCCTTCCTCTAAGTGCGTATGC
CCCTCTCAAAGACAATATTCAAA
>860
CACCAAAAACTATTCAATGTTGGCCTAGAGCCTGTGTGTCCGTGCTTAGGTGAGTATGT
GTATCTGAAAGGGAGAAAATGCCTTCCTCTAAGTGCGTATGCCCCTCTCAAAGACAATA
TTCAAATATCATGCCCGATAGC
>861
AAAAACTATTCAATGTTGGCCTAGAGCCTGTGTGTCCGTGCTTAGGTGAGTATGTGTAT
CTGAAAGGGAGAAAATGCCTTCCTCTAAGTGCGTATGCCCCTCTCAAAGACAATATTCA
AATATCATGCCCGATAGCTGAC
>862
GCCTAGAGCCTGTGTGTCCGTGCTTAGGTGAGTATGTGTATCTGAAAGGGAGAAAATG
CCTTCCTCTAAGTGCGTATGCCCCTCTCAAAGACAATATTCAAATATCATGCCCGATAGC
TGACAGCGGTCAATGGTTGCTA
>863
TGCTTAGGTGAGTATGTGTATCTGAAAGGGAGAAAATGCCTTCCTCTAAGTGCGTATGC
CCCTCTCAAAGACAATATTCAAATATCATGCCCGATAGCTGACAGCGGTCAATGGTTGC
TATGCTTCATCCCAAAGCTTTG
>864
GGGAGAAAATGCCTTCCTCTAAGTGCGTATGCCCCTCTCAAAGACAATATTCAAATATC
ATGCCCGATAGCTGACAGCGGTCAATGGTTGCTATGCTTCATCCCAAAGCTTTGTTAGG
AACCCCATAAAACCTGGCACAT
>865
AATGCCTTCCTCTAAGTGCGTATGCCCCTCTCAAAGACAATATTCAAATATCATGCCCGA
TAGCTGACAGCGGTCAATGGTTGCTATGCTTCATCCCAAAGCTTTGTTAGGAACCCCAT
AAAACCTGGCACATGGTTTGT
>866
CAAAGACAATATTCAAATATCATGCCCGATAGCTGACAGCGGTCAATGGTTGCTATGCT
TCATCCCAAAGCTTTGTTAGGAACCCCATAAAACCTGGCACATGGTTTGTGACACCCA
GTGAAACACCGTGCTTCTTCCAT
>867
GTTGCTATGCTTCATCCCAAAGCTTTGTTAGGAACCCCATAAAACCTGGCACATGGTTT
GTGACACCCAGTGAAACACCGTGCTTCTTCCATAATATCCTGAGCCCTGCTGCCTGTCA
TTAAGGGACCTCTCTTATTAAG
>868
ACCCCATAAAACCTGGCACATGGTTTGTGACACCCAGTGAAACACCGTGCTTCTTCCAT
AATATCCTGAGCCCTGCTGCCTGTCATTAAGGGACCTCTCTTATTAAGCATCTCTTTTTT
AAATGGCTGCCATCATCTGGT
>869
TGGTTTGTGACACCCAGTGAAACACCGTGCTTCTTCCATAATATCCTGAGCCCTGCTGC
CTGTCATTAAGGGACCTCTCTTATTAAGCATCTCTTTTTTAAATGGCTGCCATCATCTGG
TCCCAGTGTACATTAAATCCC

>870
TGAAACACCGTGCTTCTTCCATAATATCCTGAGCCCTGCTGCCTGTCATTAAGGGACCT
CTCTTATTAAGCATCTCTTTTTTAAATGGCTGCCATCATCTGGTCCCAGTGTACATTAAAT
CCCATTATAATTATAAACAC
>871
CTGCCTGTCATTAAGGGACCTCTCTTATTAAGCATCTCTTTTTTAAATGGCTGCCATCAT
CTGGTCCCAGTGTACATTAAATCCCATTATAATTATAAACACACTTCCCCATAACATTCGT
TGCAGATCTCACCCTCAGT
>872
TCATCTGGTCCCAGTGTACATTAAATCCCATTATAATTATAAACACACTTCCCCATAACAT
TCGTTGCAGATCTCACCCTCAGTGCCTACCCCCTTAGGGCATCTTTGCACTAATAACAA
CTCAGATTTGCTATCTGAGT
>873
GTGTACATTAAATCCCATTATAATTATAAACACACTTCCCCATAACATTCGTTGCAGATCT
CACCCTCAGTGCCTACCCCCTTAGGGCATCTTTGCACTAATAACAACTCAGATTTGCTAT
CTGAGTTGACAACATTAGG
>874
TAACATTCGTTGCAGATCTCACCCTCAGTGCCTACCCCCTTAGGGCATCTTTGCACTAAT
AACAACTCAGATTTGCTATCTGAGTTGACAACATTAGGTCCCAACACCATTTGCAATAT
GCTGGGGAAAGAGACAGAGAG
>875
GATCTCACCCTCAGTGCCTACCCCCTTAGGGCATCTTTGCACTAATAACAACTCAGATT
TGCTATCTGAGTTGACAACATTAGGTCCCAACACCATTTGCAATATGCTGGGGAAAGAG
ACAGAGAGAGAAGAAAAAGCTT
>876
AACAACTCAGATTTGCTATCTGAGTTGACAACATTAGGTCCCAACACCATTTGCAATAT
GCTGGGGAAAGAGACAGAGAGAGAAGAAAAAGCTTTTTCTTCCTTAATCACGAAGAT
CTTGGGGTCACAGTGACCCAAGGC
>877
CAACTCAGATTTGCTATCTGAGTTGACAACATTAGGTCCCAACACCATTTGCAATATGC
TGGGGAAAGAGACAGAGAGAGAAGAAAAAGCTTTTTCTTCCTTAATCACGAAGATCT
TGGGGTCACAGTGACCCAAGGCCT
>878
GAGTTGACAACATTAGGTCCCAACACCATTTGCAATATGCTGGGGAAAGAGACAGAGA
GAGAAGAAAAAGCTTTTTCTTCCTTAATCACGAAGATCTTGGGGTCACAGTGACCCAA
GGCCTCAGGAGCTAAAAAAAAAAA
>879
GCTGGGGAAAGAGACAGAGAGAGAAGAAAAAGCTTTTTCTTCCTTAATCACGAAGAT
CTTGGGGTCACAGTGACCCAAGGCCTCAGGAGCTAAAAAAAAAAAATCCCTCCTCCT
ACCCTTTTGAAAAGTCACTCTCTACT
>880
AGAGACAGAGAGAGAAGAAAAAGCTTTTTCTTCCTTAATCACGAAGATCTTGGGGTCA
CAGTGACCCAAGGCCTCAGGAGCTAAAAAAAAAAAATCCCTCCTCCTACCCTTTTGAA
AAGTCACTCTCTACTCTACAGATG

>881
TTGGGGTCACAGTGACCCAAGGCCTCAGGAGCTAAAAAAAAAAAATCCCTCCTCCTA
CCCTTTTGAAAAGTCACTCTCTACTCTACAGATGAGGGAAGGGCATACTTGGCACCTG
GGAGAGAAACTCAAGTTTGTAAACA
>882
AAGGCCTCAGGAGCTAAAAAAAAAAAATCCCTCCTCCTACCCTTTTGAAAAGTCACTC
TCTACTCTACAGATGAGGGAAGGGCATACTTGGCACCTGGGAGAGAAACTCAAGTTTG
TAAACACCACTAACCAGGTCTCTG
>883
TCCTCCTACCCTTTTGAAAAGTCACTCTCTACTCTACAGATGAGGGAAGGGCATACTTG
GCACCTGGGAGAGAAACTCAAGTTTGTAAACACCACTAACCAGGTCTCTGTTGGTTGG
GGCGGTGATGGTGGTGGTAGTGG
>884
CTCCTACCCTTTTGAAAAGTCACTCTCTACTCTACAGATGAGGGAAGGGCATACTTGGC
ACCTGGGAGAGAAACTCAAGTTTGTAAACACCACTAACCAGGTCTCTGTTGGTTGGGG
CGGTGATGGTGGTGGTAGTGGTG
>885
TTTTGAAAAGTCACTCTCTACTCTACAGATGAGGGAAGGGCATACTTGGCACCTGGGA
GAGAAACTCAAGTTTGTAAACACCACTAACCAGGTCTCTGTTGGTTGGGGCGGTGATG
GTGGTGGTAGTGGTGGGGTGGGGG
>886
TCTACAGATGAGGGAAGGGCATACTTGGCACCTGGGAGAGAAACTCAAGTTTGTAAAC
ACCACTAACCAGGTCTCTGTTGGTTGGGGCGGTGATGGTGGTGGTAGTGGTGGGGTGG
GGGATGAGGGATGATGGCAATCAC
>887
GAGAGAAACTCAAGTTTGTAAACACCACTAACCAGGTCTCTGTTGGTTGGGGCGGTGA
TGGTGGTGGTAGTGGTGGGGTGGGGGATGAGGGATGATGGCAATCACTAATCAAAGAA
GCTGTCTTATTTGGTTTGCTGAGC
>888
AAACACCACTAACCAGGTCTCTGTTGGTTGGGGCGGTGATGGTGGTGGTAGTGGTGGG
GTGGGGGATGAGGGATGATGGCAATCACTAATCAAAGAAGCTGTCTTATTTGGTTTGCT
GAGCCTCAACTAACCGGAATGCA
>889
GGTTGGGGCGGTGATGGTGGTGGTAGTGGTGGGGTGGGGGATGAGGGATGATGGCAA
TCACTAATCAAAGAAGCTGTCTTATTTGGTTTGCTGAGCCTCAACTAACCGGAATGCAT
ACATCACCTGATTCTTTCCCCCTC
>890
GTGGTGGGGTGGGGGATGAGGGATGATGGCAATCACTAATCAAAGAAGCTGTCTTATT
TGGTTTGCTGAGCCTCAACTAACCGGAATGCATACATCACCTGATTCTTTCCCCCTCTG
CTTTTTGGAGGGGGCATCTAAGG
>891
TTGGTTTGCTGAGCCTCAACTAACCGGAATGCATACATCACCTGATTCTTTCCCCCTCT
GCTTTTTGGAGGGGGCATCTAAGGACATAATGTAAAAGAATTAAAATTTAAAAACAAA
AACAAAATGTCAACATATGCAGG

>892
GCCTCAACTAACCGGAATGCATACATCACCTGATTCTTTCCCCCTCTGCTTTTTGGAGG
GGGCATCTAAGGACATAATGTAAAAGAATTAAAATTTAAAAACAAAAACAAAATGTCA
ACATATGCAGGAGTAAATTCCAC
>893
AATGCATACATCACCTGATTCTTTCCCCCTCTGCTTTTTGGAGGGGGCATCTAAGGACAT
AATGTAAAAGAATTAAAATTTAAAAACAAAAACAAAATGTCAACATATGCAGGAGTAA
ATTCCACTCTGCCTAATTTTTT
>894
GGGGGCATCTAAGGACATAATGTAAAAGAATTAAAATTTAAAAACAAAAACAAAATGT
CAACATATGCAGGAGTAAATTCCACTCTGCCTAATTTTTTTTTTTTTTTGAAATGAGATC
TTGTTCTGTCACCCAGGCTGGA
>895
GGCATCTAAGGACATAATGTAAAAGAATTAAAATTTAAAAACAAAAACAAAATGTCAA
CATATGCAGGAGTAAATTCCACTCTGCCTAATTTTTTTTTTTTTTTGAAATGAGATCTTGT
TCTGTCACCCAGGCTGGAGGG
>896
AAAAACAAAATGTCAACATATGCAGGAGTAAATTCCACTCTGCCTAATTTTTTTTTTTTT
TTGAAATGAGATCTTGTTCTGTCACCCAGGCTGGAGGGCAGTGGTGCAATCTCAGCTC
ACTGCAGCCACCACCTCCCAGG
>897
ATGCAGGAGTAAATTCCACTCTGCCTAATTTTTTTTTTTTTTTGAAATGAGATCTTGTTC
TGTCACCCAGGCTGGAGGGCAGTGGTGCAATCTCAGCTCACTGCAGCCACCACCTCCC
AGGTTCAAGCGATTCTCCCGCC
>898
ATTCCACTCTGCCTAATTTTTTTTTTTTTTTGAAATGAGATCTTGTTCTGTCACCCAGGC
TGGAGGGCAGTGGTGCAATCTCAGCTCACTGCAGCCACCACCTCCCAGGTTCAAGCGA
TTCTCCCGCCTCAGCCCCCTAA
>899
TTTTTTTGAAATGAGATCTTGTTCTGTCACCCAGGCTGGAGGGCAGTGGTGCAATCTCA
GCTCACTGCAGCCACCACCTCCCAGGTTCAAGCGATTCTCCCGCCTCAGCCCCCTAAG
TAGCTGGTATTACAGGCACGTGC
>900
GATCTTGTTCTGTCACCCAGGCTGGAGGGCAGTGGTGCAATCTCAGCTCACTGCAGCC
ACCACCTCCCAGGTTCAAGCGATTCTCCCGCCTCAGCCCCCTAAGTAGCTGGTATTACA
GGCACGTGCCACCACGCCTGGCT
>901
TTCTGTCACCCAGGCTGGAGGGCAGTGGTGCAATCTCAGCTCACTGCAGCCACCACCT
CCCAGGTTCAAGCGATTCTCCCGCCTCAGCCCCCTAAGTAGCTGGTATTACAGGCACGT
GCCACCACGCCTGGCTAATTTTT
>902
GGTGCAATCTCAGCTCACTGCAGCCACCACCTCCCAGGTTCAAGCGATTCTCCCGCCT
CAGCCCCCTAAGTAGCTGGTATTACAGGCACGTGCCACCACGCCTGGCTAATTTTTGTA
TTTTTAGTAGAGATAGGGTTTCA

>903
CACTGCAGCCACCACCTCCCAGGTTCAAGCGATTCTCCCGCCTCAGCCCCCTAAGTAG
CTGGTATTACAGGCACGTGCCACCACGCCTGGCTAATTTTTGTATTTTTAGTAGAGATAG
GGTTTCACCATGTTGGCCAGGC
>904
GCCTCAGCCCCCTAAGTAGCTGGTATTACAGGCACGTGCCACCACGCCTGGCTAATTTT
TGTATTTTTAGTAGAGATAGGGTTTCACCATGTTGGCCAGGCTGGTCTCAAACTCCTGA
CCTCAAGTGATCCGCCTGCCTC
>905
CCCTAAGTAGCTGGTATTACAGGCACGTGCCACCACGCCTGGCTAATTTTTGTATTTTTA
GTAGAGATAGGGTTTCACCATGTTGGCCAGGCTGGTCTCAAACTCCTGACCTCAAGTG
ATCCGCCTGCCTCGGCCTCCTA
>906
AGGCACGTGCCACCACGCCTGGCTAATTTTTGTATTTTTAGTAGAGATAGGGTTTCACC
ATGTTGGCCAGGCTGGTCTCAAACTCCTGACCTCAAGTGATCCGCCTGCCTCGGCCTC
CTAAAGTGCTGGGAGTACAGGCA
>907
TTTTTGTATTTTTAGTAGAGATAGGGTTTCACCATGTTGGCCAGGCTGGTCTCAAACTCC
TGACCTCAAGTGATCCGCCTGCCTCGGCCTCCTAAAGTGCTGGGAGTACAGGCATGAG
CCACTGCGCCCAGCCTACTCTG
>908
TTCACCATGTTGGCCAGGCTGGTCTCAAACTCCTGACCTCAAGTGATCCGCCTGCCTC
GGCCTCCTAAAGTGCTGGGAGTACAGGCATGAGCCACTGCGCCCAGCCTACTCTGCCC
AATTGTTGACATCACCTACAGCTC
>909
TTGGCCAGGCTGGTCTCAAACTCCTGACCTCAAGTGATCCGCCTGCCTCGGCCTCCTA
AAGTGCTGGGAGTACAGGCATGAGCCACTGCGCCCAGCCTACTCTGCCCAATTGTTGA
CATCACCTACAGCTCTATAGTAGA
>910
GTCTCAAACTCCTGACCTCAAGTGATCCGCCTGCCTCGGCCTCCTAAAGTGCTGGGAG
TACAGGCATGAGCCACTGCGCCCAGCCTACTCTGCCCAATTGTTGACATCACCTACAGC
TCTATAGTAGATGTCTGGAATGA
>911
AAGTGCTGGGAGTACAGGCATGAGCCACTGCGCCCAGCCTACTCTGCCCAATTGTTGA
CATCACCTACAGCTCTATAGTAGATGTCTGGAATGATGATCTGAAGGACTGTAAGACTG
TAAGATTTTTTGACTCCAGAAAT
>912
GGAGTACAGGCATGAGCCACTGCGCCCAGCCTACTCTGCCCAATTGTTGACATCACCT
ACAGCTCTATAGTAGATGTCTGGAATGATGATCTGAAGGACTGTAAGACTGTAAGATTT
TTTGACTCCAGAAATGGACACAA
>913
CAGCCTACTCTGCCCAATTGTTGACATCACCTACAGCTCTATAGTAGATGTCTGGAATGA
TGATCTGAAGGACTGTAAGACTGTAAGATTTTTTGACTCCAGAAATGGACACAATAGG
CTTGCGGTGTCTTCCCCTAACA

>914
TTGTTGACATCACCTACAGCTCTATAGTAGATGTCTGGAATGATGATCTGAAGGACTGT
AAGACTGTAAGATTTTTTGACTCCAGAAATGGACACAATAGGCTTGCGGTGTCTTCCCC
TAACAGTCCCTGGCAGGGTAAA
>915
ACAGCTCTATAGTAGATGTCTGGAATGATGATCTGAAGGACTGTAAGACTGTAAGATTT
TTTGACTCCAGAAATGGACACAATAGGCTTGCGGTGTCTTCCCCTAACAGTCCCTGGC
AGGGTAAAGGTCTGATGCCCTGG
>916
ATGTCTGGAATGATGATCTGAAGGACTGTAAGACTGTAAGATTTTTTGACTCCAGAAAT
GGACACAATAGGCTTGCGGTGTCTTCCCCTAACAGTCCCTGGCAGGGTAAAGGTCTGA
TGCCCTGGTACGTTCCCCAGTGG
>917
ACTGTAAGACTGTAAGATTTTTTGACTCCAGAAATGGACACAATAGGCTTGCGGTGTCT
TCCCCTAACAGTCCCTGGCAGGGTAAAGGTCTGATGCCCTGGTACGTTCCCCAGTGGC
AATCTGGAGTTCTCAACTGCAGA
>918
TGTAAGATTTTTTGACTCCAGAAATGGACACAATAGGCTTGCGGTGTCTTCCCCTAACA
GTCCCTGGCAGGGTAAAGGTCTGATGCCCTGGTACGTTCCCCAGTGGCAATCTGGAGT
TCTCAACTGCAGAGAATACTTTT
>919
TTTTTTGACTCCAGAAATGGACACAATAGGCTTGCGGTGTCTTCCCCTAACAGTCCCTG
GCAGGGTAAAGGTCTGATGCCCTGGTACGTTCCCCAGTGGCAATCTGGAGTTCTCAAC
TGCAGAGAATACTTTTCCTCTAC
>920
TAGGCTTGCGGTGTCTTCCCCTAACAGTCCCTGGCAGGGTAAAGGTCTGATGCCCTGGT
ACGTTCCCCAGTGGCAATCTGGAGTTCTCAACTGCAGAGAATACTTTTCCTCTACTCAG
GGGCGTCACTACCTGCATCTCT
>921
GTCCCTGGCAGGGTAAAGGTCTGATGCCCTGGTACGTTCCCCAGTGGCAATCTGGAGT
TCTCAACTGCAGAGAATACTTTTCCTCTACTCAGGGGCGTCACTACCTGCATCTCTCCC
TGAACATGCTTCATTTTCCACTC
>922
CCCTGGCAGGGTAAAGGTCTGATGCCCTGGTACGTTCCCCAGTGGCAATCTGGAGTTC
TCAACTGCAGAGAATACTTTTCCTCTACTCAGGGGCGTCACTACCTGCATCTCTCCCTG
AACATGCTTCATTTTCCACTCAT
>923
GATGCCCTGGTACGTTCCCCAGTGGCAATCTGGAGTTCTCAACTGCAGAGAATACTTTT
CCTCTACTCAGGGGCGTCACTACCTGCATCTCTCCCTGAACATGCTTCATTTTCCACTCA
TCACAAGTTTTTGGGGAAATG
>924
TTTCCTCTACTCAGGGGCGTCACTACCTGCATCTCTCCCTGAACATGCTTCATTTTCCAC
TCATCACAAGTTTTTGGGGAAATGCAAAGTGGCATCTTCTCCCCAAAAGGTAGGGATC
TTTGATTTTTTTAATCACGATA

>925
TCACTACCTGCATCTCTCCCTGAACATGCTTCATTTTCCACTCATCACAAGTTTTTGGGG
AAATGCAAAGTGGCATCTTCTCCCCAAAAGGTAGGGATCTTTGATTTTTTTAATCACGA
TAGAAAAGTTTACTTTGAAAT
>926
TGAACATGCTTCATTTTCCACTCATCACAAGTTTTTGGGGAAATGCAAAGTGGCATCTT
CTCCCCAAAAGGTAGGGATCTTTGATTTTTTTAATCACGATAGAAAAGTTTACTTTGAA
ATAAAAAAATTAAATATATGTT
>927
CATGCTTCATTTTCCACTCATCACAAGTTTTTGGGGAAATGCAAAGTGGCATCTTCTCC
CCAAAAGGTAGGGATCTTTGATTTTTTTAATCACGATAGAAAAGTTTACTTTGAAATAA
AAAAATTAAATATATGTTCCAA
>928
TTTTGGGGAAATGCAAAGTGGCATCTTCTCCCCAAAAGGTAGGGATCTTTGATTTTTTT
AATCACGATAGAAAAGTTTACTTTGAAATAAAAAAATTAAATATATGTTCCAAATTAAG
AGTTGGAACTTAAAGAGTTGCA
>929
TTGATTTTTTTAATCACGATAGAAAAGTTTACTTTGAAATAAAAAAATTAAATATATGTT
CCAAATTAAGAGTTGGAACTTAAAGAGTTGCAACATTCCATTCATACTCAGATACTCAA
AACACCACTGTATTCTTCTTC
>930
GATTTTTTTAATCACGATAGAAAAGTTTACTTTGAAATAAAAAAATTAAATATATGTTCC
AAATTAAGAGTTGGAACTTAAAGAGTTGCAACATTCCATTCATACTCAGATACTCAAAA
CACCACTGTATTCTTCTTCCT
>931
AAAAAATTAAATATATGTTCCAAATTAAGAGTTGGAACTTAAAGAGTTGCAACATTCCA
TTCATACTCAGATACTCAAAACACCACTGTATTCTTCTTCCTATCTCTTTAGTTGAAGTT
TTTTTTTGTAATTCAAAGCCC
>932
AAGAGTTGCAACATTCCATTCATACTCAGATACTCAAAACACCACTGTATTCTTCTTCCT
ATCTCTTTAGTTGAAGTTTTTTTTTGTAATTCAAAGCCCCCTCCCCAATTTCTGCCTGAG
ATTCTGAGGGAGGAGGAAAA
>933
TGCAACATTCCATTCATACTCAGATACTCAAAACACCACTGTATTCTTCTTCCTATCTCT
TTAGTTGAAGTTTTTTTTTGTAATTCAAAGCCCCCTCCCCAATTTCTGCCTGAGATTCTG
AGGGAGGAGGAAAAGTGTAA
>934
CAGATACTCAAAACACCACTGTATTCTTCTTCCTATCTCTTTAGTTGAAGTTTTTTTTTGT
AATTCAAAGCCCCCTCCCCAATTTCTGCCTGAGATTCTGAGGGAGGAGGAAAAGTGTA
AGATCCTGGGGGATCTAAGTC
>935
TCTTCTTCCTATCTCTTTAGTTGAAGTTTTTTTTTGTAATTCAAAGCCCCCTCCCCAATTT
CTGCCTGAGATTCTGAGGGAGGAGGAAAAGTGTAAGATCCTGGGGGATCTAAGTCTCT
TTACCTGGTGACCAAGCTGAG

>936
TTTAGTTGAAGTTTTTTTTTGTAATTCAAAGCCCCCTCCCCAATTTCTGCCTGAGATTCT
GAGGGAGGAGGAAAAGTGTAAGATCCTGGGGGATCTAAGTCTCTTTACCTGGTGACCA
AGCTGAGAACCATTTGGTCCTC
>937
TTTTTTTTGTAATTCAAAGCCCCCTCCCCAATTTCTGCCTGAGATTCTGAGGGAGGAGG
AAAAGTGTAAGATCCTGGGGGATCTAAGTCTCTTTACCTGGTGACCAAGCTGAGAACC
ATTTGGTCCTCAGCTTGGCTGGC
>938
AGCCCCCTCCCCAATTTCTGCCTGAGATTCTGAGGGAGGAGGAAAAGTGTAAGATCCT
GGGGGATCTAAGTCTCTTTACCTGGTGACCAAGCTGAGAACCATTTGGTCCTCAGCTT
GGCTGGCTGCTGCACAGATCTTGG
>939
CTGAGATTCTGAGGGAGGAGGAAAAGTGTAAGATCCTGGGGGATCTAAGTCTCTTTAC
CTGGTGACCAAGCTGAGAACCATTTGGTCCTCAGCTTGGCTGGCTGCTGCACAGATCT
TGGCCTCAGCAGAGTAAGAGAAGA
>940
AAAGTGTAAGATCCTGGGGGATCTAAGTCTCTTTACCTGGTGACCAAGCTGAGAACCA
TTTGGTCCTCAGCTTGGCTGGCTGCTGCACAGATCTTGGCCTCAGCAGAGTAAGAGAA
GAAGGAAAATTAAAAGCCCTTGCT
>941
ATCTAAGTCTCTTTACCTGGTGACCAAGCTGAGAACCATTTGGTCCTCAGCTTGGCTGG
CTGCTGCACAGATCTTGGCCTCAGCAGAGTAAGAGAAGAAGGAAAATTAAAAGCCCT
TGCTTCCTTCATCTATTTCACTAG
>942
TACCTGGTGACCAAGCTGAGAACCATTTGGTCCTCAGCTTGGCTGGCTGCTGCACAGA
TCTTGGCCTCAGCAGAGTAAGAGAAGAAGGAAAATTAAAAGCCCTTGCTTCCTTCATC
TATTTCACTAGAGGCTACATAATC
>943
GTCCTCAGCTTGGCTGGCTGCTGCACAGATCTTGGCCTCAGCAGAGTAAGAGAAGAA
GGAAAATTAAAAGCCCTTGCTTCCTTCATCTATTTCACTAGAGGCTACATAATCTAGAA
GTTAAGAGCATGAGCTTTGCAGTC
>944
ATCTTGGCCTCAGCAGAGTAAGAGAAGAAGGAAAATTAAAAGCCCTTGCTTCCTTCAT
CTATTTCACTAGAGGCTACATAATCTAGAAGTTAAGAGCATGAGCTTTGCAGTCAGAAT
GGTCTGATTTAAAGTTTCAGCTC
>945
AGTAAGAGAAGAAGGAAAATTAAAAGCCCTTGCTTCCTTCATCTATTTCACTAGAGGCT
ACATAATCTAGAAGTTAAGAGCATGAGCTTTGCAGTCAGAATGGTCTGATTTAAAGTTT
CAGCTCTGATGTTTAATAGATC
>946
AAATTAAAAGCCCTTGCTTCCTTCATCTATTTCACTAGAGGCTACATAATCTAGAAGTTA
AGAGCATGAGCTTTGCAGTCAGAATGGTCTGATTTAAAGTTTCAGCTCTGATGTTTAAT
AGATCTACAGTCTGCAGTAAG

>947
AGCCCTTGCTTCCTTCATCTATTTCACTAGAGGCTACATAATCTAGAAGTTAAGAGCATG
AGCTTTGCAGTCAGAATGGTCTGATTTAAAGTTTCAGCTCTGATGTTTAATAGATCTACA
GTCTGCAGTAAGGCACTTAA
>948
TTTCACTAGAGGCTACATAATCTAGAAGTTAAGAGCATGAGCTTTGCAGTCAGAATGGT
CTGATTTAAAGTTTCAGCTCTGATGTTTAATAGATCTACAGTCTGCAGTAAGGCACTTAA
TCTCTCTGAACCTCAGTTTTC
>949
TAGAAGTTAAGAGCATGAGCTTTGCAGTCAGAATGGTCTGATTTAAAGTTTCAGCTCTG
ATGTTTAATAGATCTACAGTCTGCAGTAAGGCACTTAATCTCTCTGAACCTCAGTTTTCA
TGTCCACAGAAATTGGGAAAA
>950
AAGAGCATGAGCTTTGCAGTCAGAATGGTCTGATTTAAAGTTTCAGCTCTGATGTTTAA
TAGATCTACAGTCTGCAGTAAGGCACTTAATCTCTCTGAACCTCAGTTTTCATGTCCAC
AGAAATTGGGAAAATAATAGCA
>951
TGAGCTTTGCAGTCAGAATGGTCTGATTTAAAGTTTCAGCTCTGATGTTTAATAGATCTA
CAGTCTGCAGTAAGGCACTTAATCTCTCTGAACCTCAGTTTTCATGTCCACAGAAATTG
GGAAAATAATAGCACCTGCAT
>952
TAAAGTTTCAGCTCTGATGTTTAATAGATCTACAGTCTGCAGTAAGGCACTTAATCTCTC
TGAACCTCAGTTTTCATGTCCACAGAAATTGGGAAAATAATAGCACCTGCATTAATAGG
ATTGTTATGTGGATTAAATAA
>953
AGTCTGCAGTAAGGCACTTAATCTCTCTGAACCTCAGTTTTCATGTCCACAGAAATTGG
GAAAATAATAGCACCTGCATTAATAGGATTGTTATGTGGATTAAATAAAACAACATAGGT
AATGTGTTGAGTTTCGTAGTC
>954
ACTTAATCTCTCTGAACCTCAGTTTTCATGTCCACAGAAATTGGGAAAATAATAGCACC
TGCATTAATAGGATTGTTATGTGGATTAAATAAAACAACATAGGTAATGTGTTGAGTTTC
GTAGTCAGTGCTCAGAATGAG
>955
CACAGAAATTGGGAAAATAATAGCACCTGCATTAATAGGATTGTTATGTGGATTAAATAA
AACAACATAGGTAATGTGTTGAGTTTCGTAGTCAGTGCTCAGAATGAGTTATTATTTTCG
CTGTCTCCTTCCTGTATGAT
>956
AAAATAATAGCACCTGCATTAATAGGATTGTTATGTGGATTAAATAAAACAACATAGGTA
ATGTGTTGAGTTTCGTAGTCAGTGCTCAGAATGAGTTATTATTTTCGCTGTCTCCTTCCT
GTATGATCTCTTAGAACTGC
>957
AGCACCTGCATTAATAGGATTGTTATGTGGATTAAATAAAACAACATAGGTAATGTGTTG
AGTTTCGTAGTCAGTGCTCAGAATGAGTTATTATTTTCGCTGTCTCCTTCCTGTATGATC
TCTTAGAACTGCTATACCCT

>958
GATTGTTATGTGGATTAAATAAAACAACATAGGTAATGTGTTGAGTTTCGTAGTCAGTG
CTCAGAATGAGTTATTATTTTCGCTGTCTCCTTCCTGTATGATCTCTTAGAACTGCTATAC
CCTCCTGTTCCACTAACTGA
>959
GTTGAGTTTCGTAGTCAGTGCTCAGAATGAGTTATTATTTTCGCTGTCTCCTTCCTGTAT
GATCTCTTAGAACTGCTATACCCTCCTGTTCCACTAACTGAGCTCTGAAAGGCTGCCAG
TGGTGGGAGGTACATGGGTAA
>960
TATTATTTTCGCTGTCTCCTTCCTGTATGATCTCTTAGAACTGCTATACCCTCCTGTTCCA
CTAACTGAGCTCTGAAAGGCTGCCAGTGGTGGGAGGTACATGGGTAAAGAGGGAGAT
GAGGGAGTAAGATTTAGCAATG
>961
CCTTCCTGTATGATCTCTTAGAACTGCTATACCCTCCTGTTCCACTAACTGAGCTCTGAA
AGGCTGCCAGTGGTGGGAGGTACATGGGTAAAGAGGGAGATGAGGGAGTAAGATTTA
GCAATGGAATTCCATGCAAAGGC
>962
ATGATCTCTTAGAACTGCTATACCCTCCTGTTCCACTAACTGAGCTCTGAAAGGCTGCC
AGTGGTGGGAGGTACATGGGTAAAGAGGGAGATGAGGGAGTAAGATTTAGCAATGGA
ATTCCATGCAAAGGCCAGCTCATC
>963
CTGCTATACCCTCCTGTTCCACTAACTGAGCTCTGAAAGGCTGCCAGTGGTGGGAGGT
ACATGGGTAAAGAGGGAGATGAGGGAGTAAGATTTAGCAATGGAATTCCATGCAAAGG
CCAGCTCATCTATTCCTCTGTCTC
>964
CTGAGCTCTGAAAGGCTGCCAGTGGTGGGAGGTACATGGGTAAAGAGGGAGATGAGG
GAGTAAGATTTAGCAATGGAATTCCATGCAAAGGCCAGCTCATCTATTCCTCTGTCTCT
AGCATGATCTCCCCCTAAGATTGA
>965
AGTGGTGGGAGGTACATGGGTAAAGAGGGAGATGAGGGAGTAAGATTTAGCAATGGA
ATTCCATGCAAAGGCCAGCTCATCTATTCCTCTGTCTCTAGCATGATCTCCCCCTAAGAT
TGAGATCTCCTGTATTTTTAGAA
>966
TGGGAGGTACATGGGTAAAGAGGGAGATGAGGGAGTAAGATTTAGCAATGGAATTCCA
TGCAAAGGCCAGCTCATCTATTCCTCTGTCTCTAGCATGATCTCCCCCTAAGATTGAGAT
CTCCTGTATTTTTAGAACTGAT
>967
GAGATGAGGGAGTAAGATTTAGCAATGGAATTCCATGCAAAGGCCAGCTCATCTATTCC
TCTGTCTCTAGCATGATCTCCCCCTAAGATTGAGATCTCCTGTATTTTTAGAACTGATAC
CCAGGTTTCCCTTAAAAGACG
>968
AATGGAATTCCATGCAAAGGCCAGCTCATCTATTCCTCTGTCTCTAGCATGATCTCCCCC
TAAGATTGAGATCTCCTGTATTTTTAGAACTGATACCCAGGTTTCCCTTAAAAGACGCTT
CAAGAAAAAATGTAAAAGAA

>969
AGGCCAGCTCATCTATTCCTCTGTCTCTAGCATGATCTCCCCCTAAGATTGAGATCTCCT
GTATTTTTAGAACTGATACCCAGGTTTCCCTTAAAAGACGCTTCAAGAAAAAATGTAAA
AGAAAAATCACTCTAAATACA
>970
TATTCCTCTGTCTCTAGCATGATCTCCCCCTAAGATTGAGATCTCCTGTATTTTTAGAACT
GATACCCAGGTTTCCCTTAAAAGACGCTTCAAGAAAAAATGTAAAAGAAAAATCACTC
TAAATACACGAACATGAGAGA
>971
AGATCTCCTGTATTTTTAGAACTGATACCCAGGTTTCCCTTAAAAGACGCTTCAAGAAA
AAATGTAAAAGAAAAATCACTCTAAATACACGAACATGAGAGAGACAGAAAGAGAGC
GGGCATGTGCGCGCGAGAGAGATT
>972
CAGGTTTCCCTTAAAAGACGCTTCAAGAAAAAATGTAAAAGAAAAATCACTCTAAATA
CACGAACATGAGAGAGACAGAAAGAGAGCGGGCATGTGCGCGCGAGAGAGATTGAG
TATTCTCTTGTAATTTTCCATCCTGC
>973
AAGACGCTTCAAGAAAAAATGTAAAAGAAAAATCACTCTAAATACACGAACATGAGA
GAGACAGAAAGAGAGCGGGCATGTGCGCGCGAGAGAGATTGAGTATTCTCTTGTAATT
TTCCATCCTGCTTGTTTTTAGGTGC
>974
GTAAAAGAAAAATCACTCTAAATACACGAACATGAGAGAGACAGAAAGAGAGCGGGC
ATGTGCGCGCGAGAGAGATTGAGTATTCTCTTGTAATTTTCCATCCTGCTTGTTTTTAGG
TGCTGCCATATGAAATCTCTGAA
>975
AAAAGAAAAATCACTCTAAATACACGAACATGAGAGAGACAGAAAGAGAGCGGGCAT
GTGCGCGCGAGAGAGATTGAGTATTCTCTTGTAATTTTCCATCCTGCTTGTTTTTAGGTG
CTGCCATATGAAATCTCTGAACT
>976
AGAGAGCGGGCATGTGCGCGCGAGAGAGATTGAGTATTCTCTTGTAATTTTCCATCCTG
CTTGTTTTTAGGTGCTGCCATATGAAATCTCTGAACTGAGGAGTCTCATCAGCCTTCCC
AGGACCCAAGGACATAGCTCTG
>977
TTGAGTATTCTCTTGTAATTTTCCATCCTGCTTGTTTTTAGGTGCTGCCATATGAAATCTC
TGAACTGAGGAGTCTCATCAGCCTTCCCAGGACCCAAGGACATAGCTCTGGCTATAAA
TGGTGCCAGTTTTGGTGGGGG
>978
AGTATTCTCTTGTAATTTTCCATCCTGCTTGTTTTTAGGTGCTGCCATATGAAATCTCTGA
ACTGAGGAGTCTCATCAGCCTTCCCAGGACCCAAGGACATAGCTCTGGCTATAAATGG
TGCCAGTTTTGGTGGGGGAGC
>979
TTTAGGTGCTGCCATATGAAATCTCTGAACTGAGGAGTCTCATCAGCCTTCCCAGGACC
CAAGGACATAGCTCTGGCTATAAATGGTGCCAGTTTTGGTGGGGGAGCTGCTGCTGCTT
TATCTGGGCCCAATAAACCTTA

>980
CTCTGAACTGAGGAGTCTCATCAGCCTTCCCAGGACCCAAGGACATAGCTCTGGCTAT
AAATGGTGCCAGTTTTGGTGGGGGAGCTGCTGCTGCTTTATCTGGGCCCAATAAACCTT
ATCAAACTGCTGTGCAGCCAGCC
>981
GTCTCATCAGCCTTCCCAGGACCCAAGGACATAGCTCTGGCTATAAATGGTGCCAGTTT
TGGTGGGGGAGCTGCTGCTGCTTTATCTGGGCCCAATAAACCTTATCAAACTGCTGTGC
AGCCAGCCTTCAGCGCTAAGTG
>982
TGCCAGTTTTGGTGGGGGAGCTGCTGCTGCTTTATCTGGGCCCAATAAACCTTATCAAA
CTGCTGTGCAGCCAGCCTTCAGCGCTAAGTGGAAATGCTTGGAGCCCAATGCTGGCAT
TCAGCCCACAGCCTCGCCTGCCT
>983
AGTTTTGGTGGGGGAGCTGCTGCTGCTTTATCTGGGCCCAATAAACCTTATCAAACTGC
TGTGCAGCCAGCCTTCAGCGCTAAGTGGAAATGCTTGGAGCCCAATGCTGGCATTCAG
CCCACAGCCTCGCCTGCCTTCTT
>984
ATCTGGGCCCAATAAACCTTATCAAACTGCTGTGCAGCCAGCCTTCAGCGCTAAGTGG
AAATGCTTGGAGCCCAATGCTGGCATTCAGCCCACAGCCTCGCCTGCCTTCTTCCTTTT
TGTTACCTTCTCCACCTCTTAAA
>985
TTATCAAACTGCTGTGCAGCCAGCCTTCAGCGCTAAGTGGAAATGCTTGGAGCCCAAT
GCTGGCATTCAGCCCACAGCCTCGCCTGCCTTCTTCCTTTTTGTTACCTTCTCCACCTCT
TAAATTCAGGCATTAATCTCTC
>986
ACTGCTGTGCAGCCAGCCTTCAGCGCTAAGTGGAAATGCTTGGAGCCCAATGCTGGCA
TTCAGCCCACAGCCTCGCCTGCCTTCTTCCTTTTTGTTACCTTCTCCACCTCTTAAATTC
AGGCATTAATCTCTCTTTAACA
>987
TGCTGGCATTCAGCCCACAGCCTCGCCTGCCTTCTTCCTTTTTGTTACCTTCTCCACCTC
TTAAATTCAGGCATTAATCTCTCTTTAACACTTACTAACAAGGGCTGGGCTTTTAATGGG
CAGCGTTTATGAAGCAAGGG
>988
TCTTCCTTTTTGTTACCTTCTCCACCTCTTAAATTCAGGCATTAATCTCTCTTTAACACTT
ACTAACAAGGGCTGGGCTTTTAATGGGCAGCGTTTATGAAGCAAGGGAGATTTTCACA
TAGAGTGAATATGGCACGGGG
>989
CCACCTCTTAAATTCAGGCATTAATCTCTCTTTAACACTTACTAACAAGGGCTGGGCTTT
TAATGGGCAGCGTTTATGAAGCAAGGGAGATTTTCACATAGAGTGAATATGGCACGGG
GGTGGGGAGGCCAGGGCCATAG
>990
ATTCAGGCATTAATCTCTCTTTAACACTTACTAACAAGGGCTGGGCTTTTAATGGGCAG
CGTTTATGAAGCAAGGGAGATTTTCACATAGAGTGAATATGGCACGGGGGTGGGGAGG
CCAGGGCCATAGAGGATTGTGCT

>991
TTAACACTTACTAACAAGGGCTGGGCTTTTAATGGGCAGCGTTTATGAAGCAAGGGAG
ATTTTCACATAGAGTGAATATGGCACGGGGGTGGGGAGGCCAGGGCCATAGAGGATTG
TGCTTTCTCTCCCATTGTTTCCAG
>992
TTTTAATGGGCAGCGTTTATGAAGCAAGGGAGATTTTCACATAGAGTGAATATGGCACG
GGGGTGGGGAGGCCAGGGCCATAGAGGATTGTGCTTTCTCTCCCATTGTTTCCAGAGA
TTCTCTCTTTCTCTGATTTGAGG
>993
ATGGGCAGCGTTTATGAAGCAAGGGAGATTTTCACATAGAGTGAATATGGCACGGGGG
TGGGGAGGCCAGGGCCATAGAGGATTGTGCTTTCTCTCCCATTGTTTCCAGAGATTCTC
TCTTTCTCTGATTTGAGGATTAA
>994
AGTGAATATGGCACGGGGGTGGGGAGGCCAGGGCCATAGAGGATTGTGCTTTCTCTCC
CATTGTTTCCAGAGATTCTCTCTTTCTCTGATTTGAGGATTAATAGGAGATTAGGATCAG
GGGTTAATTGTGTGCTTCTCCT
>995
GGCCAGGGCCATAGAGGATTGTGCTTTCTCTCCCATTGTTTCCAGAGATTCTCTCTTTCT
CTGATTTGAGGATTAATAGGAGATTAGGATCAGGGGTTAATTGTGTGCTTCTCCTGCTAC
TGCACTGAGGAGAGGCTGGT
>996
CATAGAGGATTGTGCTTTCTCTCCCATTGTTTCCAGAGATTCTCTCTTTCTCTGATTTGA
GGATTAATAGGAGATTAGGATCAGGGGTTAATTGTGTGCTTCTCCTGCTACTGCACTGA
GGAGAGGCTGGTAGGTGGATG
>997
TCTGATTTGAGGATTAATAGGAGATTAGGATCAGGGGTTAATTGTGTGCTTCTCCTGCTA
CTGCACTGAGGAGAGGCTGGTAGGTGGATGTGGACAGCAAAGCGGAAACCTACAGCA
GGCACTATCTAGGCAGAAGCTCA
>998
GATTTGAGGATTAATAGGAGATTAGGATCAGGGGTTAATTGTGTGCTTCTCCTGCTACTG
CACTGAGGAGAGGCTGGTAGGTGGATGTGGACAGCAAAGCGGAAACCTACAGCAGGC
ACTATCTAGGCAGAAGCTCAACA
>999
GGATCAGGGGTTAATTGTGTGCTTCTCCTGCTACTGCACTGAGGAGAGGCTGGTAGGT
GGATGTGGACAGCAAAGCGGAAACCTACAGCAGGCACTATCTAGGCAGAAGCTCAAC
AAGTGTAGTGATTCTTTCTTCTGTT
>1000
GTGCTTCTCCTGCTACTGCACTGAGGAGAGGCTGGTAGGTGGATGTGGACAGCAAAGC
GGAAACCTACAGCAGGCACTATCTAGGCAGAAGCTCAACAAGTGTAGTGATTCTTTCT
TCTGTTTCCCTGGTGAGGCACCAG
>1001
CTTCTCCTGCTACTGCACTGAGGAGAGGCTGGTAGGTGGATGTGGACAGCAAAGCGG
AAACCTACAGCAGGCACTATCTAGGCAGAAGCTCAACAAGTGTAGTGATTCTTTCTTCT
GTTTCCCTGGTGAGGCACCAGGAG

>1002
ACTGCACTGAGGAGAGGCTGGTAGGTGGATGTGGACAGCAAAGCGGAAACCTACAGC
AGGCACTATCTAGGCAGAAGCTCAACAAGTGTAGTGATTCTTTCTTCTGTTTCCCTGGT
GAGGCACCAGGAGGGTCTTTTCTC
>1003
GCTGGTAGGTGGATGTGGACAGCAAAGCGGAAACCTACAGCAGGCACTATCTAGGCA
GAAGCTCAACAAGTGTAGTGATTCTTTCTTCTGTTTCCCTGGTGAGGCACCAGGAGGG
TCTTTTCTCCTCTCCTTACATCCCT
>1004
CCTACAGCAGGCACTATCTAGGCAGAAGCTCAACAAGTGTAGTGATTCTTTCTTCTGTT
TCCCTGGTGAGGCACCAGGAGGGTCTTTTCTCCTCTCCTTACATCCCTCCACTCTTGCT
CTCCTTGCAGCCCAGTTCTTTC
>1005
CAGGCACTATCTAGGCAGAAGCTCAACAAGTGTAGTGATTCTTTCTTCTGTTTCCCTGG
TGAGGCACCAGGAGGGTCTTTTCTCCTCTCCTTACATCCCTCCACTCTTGCTCTCCTTG
CAGCCCAGTTCTTTCCTAACTT
>1006
TTCCCTGGTGAGGCACCAGGAGGGTCTTTTCTCCTCTCCTTACATCCCTCCACTCTTGC
TCTCCTTGCAGCCCAGTTCTTTCCTAACTTTCTTTAAATCCCTTTCCTCTAACAGGGTGT
ATAGACCTTAGTTAGAAAAAC
>1007
TGGTGAGGCACCAGGAGGGTCTTTTCTCCTCTCCTTACATCCCTCCACTCTTGCTCTCC
TTGCAGCCCAGTTCTTTCCTAACTTTCTTTAAATCCCTTTCCTCTAACAGGGTGTATAGA
CCTTAGTTAGAAAAACAGGTA
>1008
TCCCTCCACTCTTGCTCTCCTTGCAGCCCAGTTCTTTCCTAACTTTCTTTAAATCCCTTT
CCTCTAACAGGGTGTATAGACCTTAGTTAGAAAAACAGGTAGTCTCTAAATGGGATTGC
TCTTTATTGTTAATGAAATGA
>1009
CTCCTTGCAGCCCAGTTCTTTCCTAACTTTCTTTAAATCCCTTTCCTCTAACAGGGTGTA
TAGACCTTAGTTAGAAAAACAGGTAGTCTCTAAATGGGATTGCTCTTTATTGTTAATGA
AATGAATACCCAGGGACTGGG
>1010
TTGCAGCCCAGTTCTTTCCTAACTTTCTTTAAATCCCTTTCCTCTAACAGGGTGTATAGA
CCTTAGTTAGAAAAACAGGTAGTCTCTAAATGGGATTGCTCTTTATTGTTAATGAAATG
AATACCCAGGGACTGGGCTTC
>1011
CAGTTCTTTCCTAACTTTCTTTAAATCCCTTTCCTCTAACAGGGTGTATAGACCTTAGTT
AGAAAAACAGGTAGTCTCTAAATGGGATTGCTCTTTATTGTTAATGAAATGAATACCCA
GGGACTGGGCTTCCCCTCCGC
>1012
GACCTTAGTTAGAAAAACAGGTAGTCTCTAAATGGGATTGCTCTTTATTGTTAATGAAA
TGAATACCCAGGGACTGGGCTTCCCCTCCGCTTGCCCTGGGTTTGATGTGGTTGTATCC
CGTGCTATCAGAGGAGCCCTTC

>1013
TGAATACCCAGGGACTGGGCTTCCCCTCCGCTTGCCCTGGGTTTGATGTGGTTGTATCC
CGTGCTATCAGAGGAGCCCTTCCTTCACTCAAGTGTGTTCCCCTGCCCAGCTCTCTCCG
CAGACTCCTGCTGGGCTGAGCT
>1014
AATACCCAGGGACTGGGCTTCCCCTCCGCTTGCCCTGGGTTTGATGTGGTTGTATCCCG
TGCTATCAGAGGAGCCCTTCCTTCACTCAAGTGTGTTCCCCTGCCCAGCTCTCTCCGCA
GACTCCTGCTGGGCTGAGCTTT
>1015
CCCCTCCGCTTGCCCTGGGTTTGATGTGGTTGTATCCCGTGCTATCAGAGGAGCCCTTC
CTTCACTCAAGTGTGTTCCCCTGCCCAGCTCTCTCCGCAGACTCCTGCTGGGCTGAGC
TTTCCCTGCTCTTAAGAGTCAGG
>1016
GGTTTGATGTGGTTGTATCCCGTGCTATCAGAGGAGCCCTTCCTTCACTCAAGTGTGTT
CCCCTGCCCAGCTCTCTCCGCAGACTCCTGCTGGGCTGAGCTTTCCCTGCTCTTAAGAG
TCAGGAGTGGCTCTTGCTGGGA
>1017
AGAGGAGCCCTTCCTTCACTCAAGTGTGTTCCCCTGCCCAGCTCTCTCCGCAGACTCC
TGCTGGGCTGAGCTTTCCCTGCTCTTAAGAGTCAGGAGTGGCTCTTGCTGGGATGGAA
TGACCCGTCTTTGGGGCTGCCTCA
>1018
ACTCAAGTGTGTTCCCCTGCCCAGCTCTCTCCGCAGACTCCTGCTGGGCTGAGCTTTC
CCTGCTCTTAAGAGTCAGGAGTGGCTCTTGCTGGGATGGAATGACCCGTCTTTGGGGC
TGCCTCATGAGCGGCTCTTGTGAA
>1019
CCCTGCTCTTAAGAGTCAGGAGTGGCTCTTGCTGGGATGGAATGACCCGTCTTTGGGG
CTGCCTCATGAGCGGCTCTTGTGAACCCGGATCAGTTCCGATGTGTAAACTCTACCGCC
TGGCCTTCAGCGAACAGATACAG
>1020
GGCTCTTGCTGGGATGGAATGACCCGTCTTTGGGGCTGCCTCATGAGCGGCTCTTGTG
AACCCGGATCAGTTCCGATGTGTAAACTCTACCGCCTGGCCTTCAGCGAACAGATACA
GATTTCTGCCACCTTCCATGACCC
>1021
GCTGCCTCATGAGCGGCTCTTGTGAACCCGGATCAGTTCCGATGTGTAAACTCTACCGC
CTGGCCTTCAGCGAACAGATACAGATTTCTGCCACCTTCCATGACCCTACAGTTCATGG
GACTGGGTCTGGGGCAGTGCCA
>1022
CATGAGCGGCTCTTGTGAACCCGGATCAGTTCCGATGTGTAAACTCTACCGCCTGGCCT
TCAGCGAACAGATACAGATTTCTGCCACCTTCCATGACCCTACAGTTCATGGGACTGGG
TCTGGGGCAGTGCCAGAGGCAC
>1023
ATGTGTAAACTCTACCGCCTGGCCTTCAGCGAACAGATACAGATTTCTGCCACCTTCCA
TGACCCTACAGTTCATGGGACTGGGTCTGGGGCAGTGCCAGAGGCACGCATGGAGGT
GTGATTCTAGGTGAGTCCTGCGGA

>1024
TTCAGCGAACAGATACAGATTTCTGCCACCTTCCATGACCCTACAGTTCATGGGACTGG
GTCTGGGGCAGTGCCAGAGGCACGCATGGAGGTGTGATTCTAGGTGAGTCCTGCGGA
AAACCTCTGGCCCACCCGTGAGTC
>1025
GAACAGATACAGATTTCTGCCACCTTCCATGACCCTACAGTTCATGGGACTGGGTCTGG
GGCAGTGCCAGAGGCACGCATGGAGGTGTGATTCTAGGTGAGTCCTGCGGAAAACCT
CTGGCCCACCCGTGAGTCACGGAC
>1026
CCATGACCCTACAGTTCATGGGACTGGGTCTGGGGCAGTGCCAGAGGCACGCATGGAG
GTGTGATTCTAGGTGAGTCCTGCGGAAAACCTCTGGCCCACCCGTGAGTCACGGACAG
AACATGCAGACTCGGGCCTTGGTG
>1027
ACCCTACAGTTCATGGGACTGGGTCTGGGGCAGTGCCAGAGGCACGCATGGAGGTGT
GATTCTAGGTGAGTCCTGCGGAAAACCTCTGGCCCACCCGTGAGTCACGGACAGAACA
TGCAGACTCGGGCCTTGGTGACATA
>1028
AGTGCCAGAGGCACGCATGGAGGTGTGATTCTAGGTGAGTCCTGCGGAAAACCTCTG
GCCCACCCGTGAGTCACGGACAGAACATGCAGACTCGGGCCTTGGTGACATAAGCTCC
GCATTGCTAAAACCGCGTGACCTCG
>1029
GCACGCATGGAGGTGTGATTCTAGGTGAGTCCTGCGGAAAACCTCTGGCCCACCCGTG
AGTCACGGACAGAACATGCAGACTCGGGCCTTGGTGACATAAGCTCCGCATTGCTAAA
ACCGCGTGACCTCGAGGGCTGACT
>1030
CTGCGGAAAACCTCTGGCCCACCCGTGAGTCACGGACAGAACATGCAGACTCGGGCC
TTGGTGACATAAGCTCCGCATTGCTAAAACCGCGTGACCTCGAGGGCTGACTGGCCTG
AGAACCCTGGATGGCGCTCTCGGCC
>1031
ACGGACAGAACATGCAGACTCGGGCCTTGGTGACATAAGCTCCGCATTGCTAAAACCG
CGTGACCTCGAGGGCTGACTGGCCTGAGAACCCTGGATGGCGCTCTCGGCCACCCCC
ACCTCCCACCCCAACCTCCTGGGCT
>1032
GACTCGGGCCTTGGTGACATAAGCTCCGCATTGCTAAAACCGCGTGACCTCGAGGGCT
GACTGGCCTGAGAACCCTGGATGGCGCTCTCGGCCACCCCCACCTCCCACCCCAACCT
CCTGGGCTTCGGTCAGAATCCACA
>1033
GGCCTGAGAACCCTGGATGGCGCTCTCGGCCACCCCCACCTCCCACCCCAACCTCCTG
GGCTTCGGTCAGAATCCACAGCCCGTGCCCGAAGAGCGCTTCCCGCCTCTGGCACCCT
ACCTTCGCTCAGCTCCAGGGAAAA
>1034
ACCCTGGATGGCGCTCTCGGCCACCCCCACCTCCCACCCCAACCTCCTGGGCTTCGGT
CAGAATCCACAGCCCGTGCCCGAAGAGCGCTTCCCGCCTCTGGCACCCTACCTTCGCT
CAGCTCCAGGGAAAAGGGGAGAGG

>1035
CTCTCGGCCACCCCCACCTCCCACCCCAACCTCCTGGGCTTCGGTCAGAATCCACAGC
CCGTGCCCGAAGAGCGCTTCCCGCCTCTGGCACCCTACCTTCGCTCAGCTCCAGGGAA
AAGGGGAGAGGGCAGCTTTCTGCA
>1036
GGCTTCGGTCAGAATCCACAGCCCGTGCCCGAAGAGCGCTTCCCGCCTCTGGCACCCT
ACCTTCGCTCAGCTCCAGGGAAAAGGGGAGAGGGCAGCTTTCTGCAGTCAGAGGAAG
AGTACATTTTCTTTGGCTGCTCTAC
>1037
GGTCAGAATCCACAGCCCGTGCCCGAAGAGCGCTTCCCGCCTCTGGCACCCTACCTTC
GCTCAGCTCCAGGGAAAAGGGGAGAGGGCAGCTTTCTGCAGTCAGAGGAAGAGTACA
TTTTCTTTGGCTGCTCTACCCTCTG
>1038
ATCCACAGCCCGTGCCCGAAGAGCGCTTCCCGCCTCTGGCACCCTACCTTCGCTCAGC
TCCAGGGAAAAGGGGAGAGGGCAGCTTTCTGCAGTCAGAGGAAGAGTACATTTTCTT
TGGCTGCTCTACCCTCTGAAGTAGG
>1039
GCGCTTCCCGCCTCTGGCACCCTACCTTCGCTCAGCTCCAGGGAAAAGGGGAGAGGG
CAGCTTTCTGCAGTCAGAGGAAGAGTACATTTTCTTTGGCTGCTCTACCCTCTGAAGTA
GGGCGGCCAGCTGAAGGAGGACAC
>1040
AAAGGGGAGAGGGCAGCTTTCTGCAGTCAGAGGAAGAGTACATTTTCTTTGGCTGCTC
TACCCTCTGAAGTAGGGCGGCCAGCTGAAGGAGGACACACTTTTGAGGGGCCCAGAG
GTTGTCCAAGCTTCCCCCTGCCCCC
>1041
GGGGAGAGGGCAGCTTTCTGCAGTCAGAGGAAGAGTACATTTTCTTTGGCTGCTCTAC
CCTCTGAAGTAGGGCGGCCAGCTGAAGGAGGACACACTTTTGAGGGGCCCAGAGGTT
GTCCAAGCTTCCCCCTGCCCCCTGA
>1042
GAGTACATTTTCTTTGGCTGCTCTACCCTCTGAAGTAGGGCGGCCAGCTGAAGGAGGA
CACACTTTTGAGGGGCCCAGAGGTTGTCCAAGCTTCCCCCTGCCCCCTGAAGACTGTG
CACTGAGCTGGGCGCAGTTCTCGG
>1043
TTTCTTTGGCTGCTCTACCCTCTGAAGTAGGGCGGCCAGCTGAAGGAGGACACACTTT
TGAGGGGCCCAGAGGTTGTCCAAGCTTCCCCCTGCCCCCTGAAGACTGTGCACTGAG
CTGGGCGCAGTTCTCGGGAACTGTT
>1044
TCTTTGGCTGCTCTACCCTCTGAAGTAGGGCGGCCAGCTGAAGGAGGACACACTTTTG
AGGGGCCCAGAGGTTGTCCAAGCTTCCCCCTGCCCCCTGAAGACTGTGCACTGAGCT
GGGCGCAGTTCTCGGGAACTGTTTC
>1045
CTCTACCCTCTGAAGTAGGGCGGCCAGCTGAAGGAGGACACACTTTTGAGGGGCCCA
GAGGTTGTCCAAGCTTCCCCCTGCCCCCTGAAGACTGTGCACTGAGCTGGGCGCAGTT
CTCGGGAACTGTTTCCACCCAGATT

>1046
CGGCCAGCTGAAGGAGGACACACTTTTGAGGGGCCCAGAGGTTGTCCAAGCTTCCCC
CTGCCCCCTGAAGACTGTGCACTGAGCTGGGCGCAGTTCTCGGGAACTGTTTCCACCC
AGATTGCTGGGGGGCGGGGGGGGGT
>1047
TTTTGAGGGGCCCAGAGGTTGTCCAAGCTTCCCCCTGCCCCCTGAAGACTGTGCACTG
AGCTGGGCGCAGTTCTCGGGAACTGTTTCCACCCAGATTGCTGGGGGGCGGGGGGGG
GTGGGATGAGGGCAGAGCCGAGAGG
>1048
AAGCTTCCCCCTGCCCCCTGAAGACTGTGCACTGAGCTGGGCGCAGTTCTCGGGAACT
GTTTCCACCCAGATTGCTGGGGGGCGGGGGGGGGTGGGATGAGGGCAGAGCCGAGAG
GCTGTCCAAGGTTTGGGAGAGAGAA
>1049
CTGAGCTGGGCGCAGTTCTCGGGAACTGTTTCCACCCAGATTGCTGGGGGGCGGGGG
GGGGTGGGATGAGGGCAGAGCCGAGAGGCTGTCCAAGGTTTGGGAGAGAGAAAAGT
TTCTCCCAGGACTCGACCTTGGCCTCC
>1050
CTGGGCGCAGTTCTCGGGAACTGTTTCCACCCAGATTGCTGGGGGGCGGGGGGGGGT
GGGATGAGGGCAGAGCCGAGAGGCTGTCCAAGGTTTGGGAGAGAGAAAAGTTTCTCC
CAGGACTCGACCTTGGCCTCCAGCAA
>1051
GGGCGCAGTTCTCGGGAACTGTTTCCACCCAGATTGCTGGGGGGCGGGGGGGGGTGG
GATGAGGGCAGAGCCGAGAGGCTGTCCAAGGTTTGGGAGAGAGAAAAGTTTCTCCCA
GGACTCGACCTTGGCCTCCAGCAATC
>1052
CAGTTCTCGGGAACTGTTTCCACCCAGATTGCTGGGGGGCGGGGGGGGGTGGGATGA
GGGCAGAGCCGAGAGGCTGTCCAAGGTTTGGGAGAGAGAAAAGTTTCTCCCAGGACT
CGACCTTGGCCTCCAGCAATCGCGAC
>1053
ACTGTTTCCACCCAGATTGCTGGGGGGCGGGGGGGGGTGGGATGAGGGCAGAGCCGA
GAGGCTGTCCAAGGTTTGGGAGAGAGAAAAGTTTCTCCCAGGACTCGACCTTGGCCT
CCAGCAATCGCGACAGCTAAAAACGG
>1054
GCTGGGGGGCGGGGGGGGGTGGGATGAGGGCAGAGCCGAGAGGCTGTCCAAGGTTT
GGGAGAGAGAAAAGTTTCTCCCAGGACTCGACCTTGGCCTCCAGCAATCGCGACAGC
TAAAAACGGGTGTCTCGCTTCGACAAT
>1055
CAGAGCCGAGAGGCTGTCCAAGGTTTGGGAGAGAGAAAAGTTTCTCCCAGGACTCGA
CCTTGGCCTCCAGCAATCGCGACAGCTAAAAACGGGTGTCTCGCTTCGACAATAGATC
CCCGCGGACCTTCTGGCACCTGGTT
>1056
GCTGTCCAAGGTTTGGGAGAGAGAAAAGTTTCTCCCAGGACTCGACCTTGGCCTCCA
GCAATCGCGACAGCTAAAAACGGGTGTCTCGCTTCGACAATAGATCCCCGCGGACCTT
CTGGCACCTGGTTCACTAGCGCCCG

>1057
TCTCCCAGGACTCGACCTTGGCCTCCAGCAATCGCGACAGCTAAAAACGGGTGTCTCG
CTTCGACAATAGATCCCCGCGGACCTTCTGGCACCTGGTTCACTAGCGCCCGCGAACT
CTGCCTCGGGAGACTTATTGAAAT
>1058
CGACAGCTAAAAACGGGTGTCTCGCTTCGACAATAGATCCCCGCGGACCTTCTGGCAC
CTGGTTCACTAGCGCCCGCGAACTCTGCCTCGGGAGACTTATTGAAATCCGGATGCTC
AAGCCGGGAGGCGCGCAGTAACCA
>1059
CTTCGACAATAGATCCCCGCGGACCTTCTGGCACCTGGTTCACTAGCGCCCGCGAACT
CTGCCTCGGGAGACTTATTGAAATCCGGATGCTCAAGCCGGGAGGCGCGCAGTAACCA
GGAGGATGAGAGGGCCGGGTTTGG
>1060
TTCTGGCACCTGGTTCACTAGCGCCCGCGAACTCTGCCTCGGGAGACTTATTGAAATCC
GGATGCTCAAGCCGGGAGGCGCGCAGTAACCAGGAGGATGAGAGGGCCGGGTTTGGG
CTAGGAAAGCGGCCTTTTAAAACA
>1061
ACCTGGTTCACTAGCGCCCGCGAACTCTGCCTCGGGAGACTTATTGAAATCCGGATGC
TCAAGCCGGGAGGCGCGCAGTAACCAGGAGGATGAGAGGGCCGGGTTTGGGCTAGGA
AAGCGGCCTTTTAAAACAGATGTCA
>1062
GCCCGCGAACTCTGCCTCGGGAGACTTATTGAAATCCGGATGCTCAAGCCGGGAGGCG
CGCAGTAACCAGGAGGATGAGAGGGCCGGGTTTGGGCTAGGAAAGCGGCCTTTTAAA
ACAGATGTCAGGGGGACTGCAGCCC
>1063
TTGAAATCCGGATGCTCAAGCCGGGAGGCGCGCAGTAACCAGGAGGATGAGAGGGCC
GGGTTTGGGCTAGGAAAGCGGCCTTTTAAAACAGATGTCAGGGGGACTGCAGCCCCG
AGCCATGAGAAAAAAGTTAAAGGCGA
>1064
ATCCGGATGCTCAAGCCGGGAGGCGCGCAGTAACCAGGAGGATGAGAGGGCCGGGTT
TGGGCTAGGAAAGCGGCCTTTTAAAACAGATGTCAGGGGGACTGCAGCCCCGAGCCA
TGAGAAAAAAGTTAAAGGCGAGATGA
>1065
CGGGAGGCGCGCAGTAACCAGGAGGATGAGAGGGCCGGGTTTGGGCTAGGAAAGCG
GCCTTTTAAAACAGATGTCAGGGGGACTGCAGCCCCGAGCCATGAGAAAAAAGTTAA
AGGCGAGATGACACGCACTGAATTGGG
>1066
CAGGAGGATGAGAGGGCCGGGTTTGGGCTAGGAAAGCGGCCTTTTAAAACAGATGTC
AGGGGGACTGCAGCCCCGAGCCATGAGAAAAAAGTTAAAGGCGAGATGACACGCACT
GAATTGGGGCAAACATTGGAAGAGGA
>1067
TTTGGGCTAGGAAAGCGGCCTTTTAAAACAGATGTCAGGGGGACTGCAGCCCCGAGC
CATGAGAAAAAAGTTAAAGGCGAGATGACACGCACTGAATTGGGGCAAACATTGGAA
GAGGAGACAAAACTGCGTGCTTGAGC

>1068
CTAGGAAAGCGGCCTTTTAAAACAGATGTCAGGGGGACTGCAGCCCCGAGCCATGAG
AAAAAAGTTAAAGGCGAGATGACACGCACTGAATTGGGGCAAACATTGGAAGAGGAG
ACAAAACTGCGTGCTTGAGCACCGGG
>1069
AAAGCGGCCTTTTAAAACAGATGTCAGGGGGACTGCAGCCCCGAGCCATGAGAAAAA
AGTTAAAGGCGAGATGACACGCACTGAATTGGGGCAAACATTGGAAGAGGAGACAAA
ACTGCGTGCTTGAGCACCGGGGTGCG
>1070
GCAGCCCCGAGCCATGAGAAAAAAGTTAAAGGCGAGATGACACGCACTGAATTGGGG
CAAACATTGGAAGAGGAGACAAAACTGCGTGCTTGAGCACCGGGGTGCGGGGAGGG
GGGGACAAAACCCGTATCCAGTGCAAA
>1071
GTTAAAGGCGAGATGACACGCACTGAATTGGGGCAAACATTGGAAGAGGAGACAAAA
CTGCGTGCTTGAGCACCGGGGTGCGGGGAGGGGGGGACAAAACCCGTATCCAGTGCA
AATTAAAATCTTGGGAGTAGGTGGGG
>1072
ACGCACTGAATTGGGGCAAACATTGGAAGAGGAGACAAAACTGCGTGCTTGAGCACC
GGGGTGCGGGGAGGGGGGGACAAAACCCGTATCCAGTGCAAATTAAAATCTTGGGAG
TAGGTGGGGGCTGCTGCGCGCCCTTC
>1073
TGGAAGAGGAGACAAAACTGCGTGCTTGAGCACCGGGGTGCGGGGAGGGGGGGACA
AAACCCGTATCCAGTGCAAATTAAAATCTTGGGAGTAGGTGGGGGCTGCTGCGCGCCC
TTCACCCTCAGTTCCCCTATTAAGGA
>1074
GTGCTTGAGCACCGGGGTGCGGGGAGGGGGGGACAAAACCCGTATCCAGTGCAAATT
AAAATCTTGGGAGTAGGTGGGGGCTGCTGCGCGCCCTTCACCCTCAGTTCCCCTATTAA
GGATTCTGAGTCCCCATGCACTCC
>1075
CTTGAGCACCGGGGTGCGGGGAGGGGGGGACAAAACCCGTATCCAGTGCAAATTAAA
ATCTTGGGAGTAGGTGGGGGCTGCTGCGCGCCCTTCACCCTCAGTTCCCCTATTAAGGA
TTCTGAGTCCCCATGCACTCCTGT
>1076
AAAACCCGTATCCAGTGCAAATTAAAATCTTGGGAGTAGGTGGGGGCTGCTGCGCGCC
CTTCACCCTCAGTTCCCCTATTAAGGATTCTGAGTCCCCATGCACTCCTGTCCTCTGGCT
CCTTCCTTCCTCTCCGCTCGGC
>1077
AGTGCAAATTAAAATCTTGGGAGTAGGTGGGGGCTGCTGCGCGCCCTTCACCCTCAGT
TCCCCTATTAAGGATTCTGAGTCCCCATGCACTCCTGTCCTCTGGCTCCTTCCTTCCTCT
CCGCTCGGCCGGTGAGAGGCGG
>1078
TAAAATCTTGGGAGTAGGTGGGGGCTGCTGCGCGCCCTTCACCCTCAGTTCCCCTATTA
AGGATTCTGAGTCCCCATGCACTCCTGTCCTCTGGCTCCTTCCTTCCTCTCCGCTCGGC
CGGTGAGAGGCGGCCGCCGGCG

>1079
ATTAAGGATTCTGAGTCCCCATGCACTCCTGTCCTCTGGCTCCTTCCTTCCTCTCCGCTC
GGCCGGTGAGAGGCGGCCGCCGGCGCCCCAGCAGCAGCTAGATGTCAGGCGAAGCCC
GGAGCGCGGAGCGCGGGGAAGGG
>1080
TAAGGATTCTGAGTCCCCATGCACTCCTGTCCTCTGGCTCCTTCCTTCCTCTCCGCTCG
GCCGGTGAGAGGCGGCCGCCGGCGCCCCAGCAGCAGCTAGATGTCAGGCGAAGCCCG
GAGCGCGGAGCGCGGGGAAGGGAG
>1081
TGCACTCCTGTCCTCTGGCTCCTTCCTTCCTCTCCGCTCGGCCGGTGAGAGGCGGCCG
CCGGCGCCCCAGCAGCAGCTAGATGTCAGGCGAAGCCCGGAGCGCGGAGCGCGGGG
AAGGGAGGGGAAGGGAGGGGAGGGGA
>1082
GCTCCTTCCTTCCTCTCCGCTCGGCCGGTGAGAGGCGGCCGCCGGCGCCCCAGCAGCA
GCTAGATGTCAGGCGAAGCCCGGAGCGCGGAGCGCGGGGAAGGGAGGGGAAGGGAG
GGGAGGGGAAAGGAGGGGTGGGGCGG
>1083
TCGGCCGGTGAGAGGCGGCCGCCGGCGCCCCAGCAGCAGCTAGATGTCAGGCGAAGC
CCGGAGCGCGGAGCGCGGGGAAGGGAGGGGAAGGGAGGGGAGGGGAAAGGAGGGG
TGGGGCGGGGAGCGCGCGCGGGCTCGGC
>1084
AGAGGCGGCCGCCGGCGCCCCAGCAGCAGCTAGATGTCAGGCGAAGCCCGGAGCGCG
GAGCGCGGGGAAGGGAGGGGAAGGGAGGGGAGGGGAAAGGAGGGGTGGGGCGGGG
AGCGCGCGCGGGCTCGGCCAGTGCGGGG
>1085
GCGGCCGCCGGCGCCCCAGCAGCAGCTAGATGTCAGGCGAAGCCCGGAGCGCGGAGC
GCGGGGAAGGGAGGGGAAGGGAGGGGAGGGGAAAGGAGGGGTGGGGCGGGGAGCG
CGCGCGGGCTCGGCCAGTGCGGGGTGGT
>1086
CAGCTAGATGTCAGGCGAAGCCCGGAGCGCGGAGCGCGGGGAAGGGAGGGGAAGGG
AGGGGAGGGGAAAGGAGGGGTGGGGCGGGGAGCGCGCGCGGGCTCGGCCAGTGCG
GGGTGGTGGGCGGGCTGAGCCCGGGGACG
>1087
GGGGAAGGGAGGGGAAGGGAGGGGAGGGGAAAGGAGGGGTGGGGCGGGGAGCGCG
CGCGGGCTCGGCCAGTGCGGGGTGGTGGGCGGGCTGAGCCCGGGGACGGCGCGGGA
GGGGGGAGGGAAGGGGGGCAGCTGTGGGC
>1088
GAGGGGAAAGGAGGGGTGGGGCGGGGAGCGCGCGCGGGCTCGGCCAGTGCGGGGTG
GTGGGCGGGCTGAGCCCGGGGACGGCGCGGGAGGGGGGAGGGAAGGGGGGCAGCT
GTGGGCAGGGAGCCGGGCTCGGCCGCCAG
>1089
GGCGGGGAGCGCGCGCGGGCTCGGCCAGTGCGGGGTGGTGGGCGGGCTGAGCCCGG
GGACGGCGCGGGAGGGGGGAGGGAAGGGGGGCAGCTGTGGGCAGGGAGCCGGGCT
CGGCCGCCAGCACTAAAGATGGAGAGGCG

>1090
AGCGCGCGCGGGCTCGGCCAGTGCGGGGTGGTGGGCGGGCTGAGCCCGGGGACGGC
GCGGGAGGGGGGAGGGAAGGGGGGCAGCTGTGGGCAGGGAGCCGGGCTCGGCCGCC
AGCACTAAAGATGGAGAGGCGCCGGGGC
>1091
GCGCGGGCTCGGCCAGTGCGGGGTGGTGGGCGGGCTGAGCCCGGGGACGGCGCGGG
AGGGGGGAGGGAAGGGGGGCAGCTGTGGGCAGGGAGCCGGGCTCGGCCGCCAGCAC
TAAAGATGGAGAGGCGCCGGGGCCTTGC
>1092
TCGGCCAGTGCGGGGTGGTGGGCGGGCTGAGCCCGGGGACGGCGCGGGAGGGGGGA
GGGAAGGGGGGCAGCTGTGGGCAGGGAGCCGGGCTCGGCCGCCAGCACTAAAGATG
GAGAGGCGCCGGGGCCTTGCAGGGGAGG
>1093
CCCGGGGACGGCGCGGGAGGGGGGAGGGAAGGGGGGCAGCTGTGGGCAGGGAGCC
GGGCTCGGCCGCCAGCACTAAAGATGGAGAGGCGCCGGGGCCTTGCAGGGGAGGGG
GCTCGGCGTTGACGTGGGACGCGGCGGAG
>1094
GGGAGGGGGGAGGGAAGGGGGGCAGCTGTGGGCAGGGAGCCGGGCTCGGCCGCCAG
CACTAAAGATGGAGAGGCGCCGGGGCCTTGCAGGGGAGGGGGCTCGGCGTTGACGTG
GGACGCGGCGGAGGCGCAGCAGCCGGT
>1095
GAGGGGGGAGGGAAGGGGGGCAGCTGTGGGCAGGGAGCCGGGCTCGGCCGCCAGCA
CTAAAGATGGAGAGGCGCCGGGGCCTTGCAGGGGAGGGGGCTCGGCGTTGACGTGGG
ACGCGGCGGAGGCGCAGCAGCCGGTGG
>1096
GGGCAGCTGTGGGCAGGGAGCCGGGCTCGGCCGCCAGCACTAAAGATGGAGAGGCG
CCGGGGCCTTGCAGGGGAGGGGGCTCGGCGTTGACGTGGGACGCGGCGGAGGCGCA
GCAGCCGGTGGTGATTTGCTAACCTCGC
>1097
CACTAAAGATGGAGAGGCGCCGGGGCCTTGCAGGGGAGGGGGCTCGGCGTTGACGTG
GGACGCGGCGGAGGCGCAGCAGCCGGTGGTGATTTGCTAACCTCGCAGCAGAGAGGA
GTTGAGGGCGATGAGAGCGGGTACTG
>1098
AGATGGAGAGGCGCCGGGGCCTTGCAGGGGAGGGGGCTCGGCGTTGACGTGGGACG
CGGCGGAGGCGCAGCAGCCGGTGGTGATTTGCTAACCTCGCAGCAGAGAGGAGTTGA
GGGCGATGAGAGCGGGTACTGCGAACT
>1099
TGGAGAGGCGCCGGGGCCTTGCAGGGGAGGGGGCTCGGCGTTGACGTGGGACGCGG
CGGAGGCGCAGCAGCCGGTGGTGATTTGCTAACCTCGCAGCAGAGAGGAGTTGAGGG
CGATGAGAGCGGGTACTGCGAACTGCC
>1100
GAGGGGGCTCGGCGTTGACGTGGGACGCGGCGGAGGCGCAGCAGCCGGTGGTGATTT
GCTAACCTCGCAGCAGAGAGGAGTTGAGGGCGATGAGAGCGGGTACTGCGAACTGCC
GGGCGATGCTGTCGCTGCCGCCGTGA

>1101
TGACGTGGGACGCGGCGGAGGCGCAGCAGCCGGTGGTGATTTGCTAACCTCGCAGCA
GAGAGGAGTTGAGGGCGATGAGAGCGGGTACTGCGAACTGCCGGGCGATGCTGTCGC
TGCCGCCGTGATACGGAGAGCAACAG
>1102
GAGTTGAGGGCGATGAGAGCGGGTACTGCGAACTGCCGGGCGATGCTGTCGCTGCCG
CCGTGATACGGAGAGCAACAGTTCCCCAGCAACACCCCTCCCCGACACAGGCACACA
CCCCCCGACAGGCACGCACACCCACC
>1103
GTTGAGGGCGATGAGAGCGGGTACTGCGAACTGCCGGGCGATGCTGTCGCTGCCGCC
GTGATACGGAGAGCAACAGTTCCCCAGCAACACCCCTCCCCGACACAGGCACACACC
CCCCGACAGGCACGCACACCCACCCC
>1104
GAGCGGGTACTGCGAACTGCCGGGCGATGCTGTCGCTGCCGCCGTGATACGGAGAGC
AACAGTTCCCCAGCAACACCCCTCCCCGACACAGGCACACACCCCCCGACAGGCACG
CACACCCACCCCACAGTGCCCGGCTC
>1105
GGGCGATGCTGTCGCTGCCGCCGTGATACGGAGAGCAACAGTTCCCCAGCAACACCCC
TCCCCGACACAGGCACACACCCCCCGACAGGCACGCACACCCACCCCACAGTGCCCG
GCTCGGCTGCGGTGAGTGAGGGGCC
>1106
CGCCGTGATACGGAGAGCAACAGTTCCCCAGCAACACCCCTCCCCGACACAGGCACA
CACCCCCCGACAGGCACGCACACCCACCCCACAGTGCCCGGCTCGGCTGCGGTGAGT
GAGGGGCCGGGAAGAGGCGCACCGCC
>1107
AGTTCCCCAGCAACACCCCTCCCCGACACAGGCACACACCCCCCGACAGGCACGCAC
ACCCACCCCACAGTGCCCGGCTCGGCTGCGGTGAGTGAGGGGCCGGGAAGAGGCGC
ACCGCCCAGCGAGCGCCGCGCGCCGGG
>1108
CCGACACAGGCACACACCCCCCGACAGGCACGCACACCCACCCCACAGTGCCCGGCT
CGGCTGCGGTGAGTGAGGGGCCGGGAAGAGGCGCACCGCCCAGCGAGCGCCGCGCG
CCGGGGCTTCCCGGGGCTGGAGAGGTC
>1109
ACCCCCCGACAGGCACGCACACCCACCCCACAGTGCCCGGCTCGGCTGCGGTGAGTG
AGGGGCCGGGAAGAGGCGCACCGCCCAGCGAGCGCCGCGCGCCGGGGCTTCCCGGG
GCTGGAGAGGTCTGGGGGGCGCGCGCT
>1110
CACCCACCCCACAGTGCCCGGCTCGGCTGCGGTGAGTGAGGGGCCGGGAAGAGGCGC
ACCGCCCAGCGAGCGCCGCGCGCCGGGGCTTCCCGGGGCTGGAGAGGTCTGGGGGGC
GCGCGCTCGCTTCGGCCACTCGGCCG
>1111
GTGAGTGAGGGGCCGGGAAGAGGCGCACCGCCCAGCGAGCGCCGCGCGCCGGGGCT
TCCCGGGGCTGGAGAGGTCTGGGGGGCGCGCGCTCGCTTCGGCCACTCGGCCGCTGG
GCTTGTGCCTTTTTTATTTGGCGTGTT

>1112
CTCGCTTCGGCCACTCGGCCGCTGGGCTTGTGCCTTTTTATTTGGCGTGTTCCCTTCCT
GCCGCTGCAGCCGTCGCCGCCACCGGTTGGGGGTCGGCTAGAAGGGGGAGCCCCGGC
TGTCAGCCTGGGCGCAGCTGCCT
>1113
GCCTTTTTATTTGGCGTGTTCCCTTCCTGCCGCTGCAGCCGTCGCCGCCACCGGTTGG
GGGTCGGCTAGAAGGGGGAGCCCCGGCTGTCAGCCTGGGCGCAGCTGCCTCCCCAGC
CCTTCCTCTTCAGGGCACGGTCGG
>1114
TTTTTATTTGGCGTGTTCCCTTCCTGCCGCTGCAGCCGTCGCCGCCACCGGTTGGGGG
TCGGCTAGAAGGGGGAGCCCCGGCTGTCAGCCTGGGCGCAGCTGCCTCCCCAGCCCT
TCCTCTTCAGGGCACGGTCGGGGT
>1115
CTGCAGCCGTCGCCGCCACCGGTTGGGGGTCGGCTAGAAGGGGGAGCCCCGGCTGTC
AGCCTGGGCGCAGCTGCCTCCCCAGCCCTTCCTCTTCAGGGCACGGTCGGGGTGAGA
GGTGGGGGGCGTAGCGGTGTGCGGGG
>1116
CCGCCACCGGTTGGGGGTCGGCTAGAAGGGGGAGCCCCGGCTGTCAGCCTGGGCGCA
GCTGCCTCCCCAGCCCTTCCTCTTCAGGGCACGGTCGGGGTGAGAGGTGGGGGGCGT
AGCGGTGTGCGGGGGCTGAGGGCGTG
>1117
GGGTCGGCTAGAAGGGGGAGCCCCGGCTGTCAGCCTGGGCGCAGCTGCCTCCCCAGC
CCTTCCTCTTCAGGGCACGGTCGGGGTGAGAGGTGGGGGGCGTAGCGGTGTGCGGGG
GCTGAGGGCGTGAGCAGAGGGGTCGG
>1118
GCCTGGGCGCAGCTGCCTCCCCAGCCCTTCCTCTTCAGGGCACGGTCGGGGTGAGAG
GTGGGGGGCGTAGCGGTGTGCGGGGGCTGAGGGCGTGAGCAGAGGGGTCGGGGATC
CGGAGGCTTTGTACCGCCAGGGGCTGG
>1119
CCCCAGCCCTTCCTCTTCAGGGCACGGTCGGGGTGAGAGGTGGGGGGCGTAGCGGTG
TGCGGGGGCTGAGGGCGTGAGCAGAGGGGTCGGGGATCCGGAGGCTTTGTACCGCCA
GGGGCTGGCGGAGCAACAGAGCCCGT
>1120
CTTCCTCTTCAGGGCACGGTCGGGGTGAGAGGTGGGGGGCGTAGCGGTGTGCGGGGG
CTGAGGGCGTGAGCAGAGGGGTCGGGGATCCGGAGGCTTTGTACCGCCAGGGGCTGG
CGGAGCAACAGAGCCCGTGGGTGCTC
>1121
GGCGTAGCGGTGTGCGGGGGCTGAGGGCGTGAGCAGAGGGGTCGGGGATCCGGAGG
CTTTGTACCGCCAGGGGCTGGCGGAGCAACAGAGCCCGTGGGTGCTCTTATGTATGCG
GACCGGTGCGCGGGCGCAAGATAAGG
>1122
TGCGGGGGCTGAGGGCGTGAGCAGAGGGGTCGGGGATCCGGAGGCTTTGTACCGCCA
GGGGCTGGCGGAGCAACAGAGCCCGTGGGTGCTCTTATGTATGCGGACCGGTGCGCG
GGCGCAAGATAAGGTTGTGGTTTATT

>1123
CTGAGGGCGTGAGCAGAGGGGTCGGGGATCCGGAGGCTTTGTACCGCCAGGGGCTGG
CGGAGCAACAGAGCCCGTGGGTGCTCTTATGTATGCGGACCGGTGCGCGGGCGCAAG
ATAAGGTTGTGGTTTATTTATTTGTG
>1124
CGCCAGGGGCTGGCGGAGCAACAGAGCCCGTGGGTGCTCTTATGTATGCGGACCGGT
GCGCGGGCGCAAGATAAGGTTGTGGTTTATTTATTTGTGTGTTTATTCGCCGGCCGGCT
GGGAAGCTAGAATCGGAGGAGCTG
>1125
GGGGCTGGCGGAGCAACAGAGCCCGTGGGTGCTCTTATGTATGCGGACCGGTGCGCG
GGCGCAAGATAAGGTTGTGGTTTATTTATTTGTGTGTTTATTCGCCGGCCGGCTGGGAA
GCTAGAATCGGAGGAGCTGACGAG
>1126
CCGGTGCGCGGGCGCAAGATAAGGTTGTGGTTTATTTATTTGTGTGTTTATTCGCCGGC
CGGCTGGGAAGCTAGAATCGGAGGAGCTGACGAGTAGATCTGGGGGCGGAGGGGAGC
AGGACTGGGACTGCTTACGTTTTG
>1127
TGCGCGGGCGCAAGATAAGGTTGTGGTTTATTTATTTGTGTGTTTATTCGCCGGCCGGC
TGGGAAGCTAGAATCGGAGGAGCTGACGAGTAGATCTGGGGGCGGAGGGGAGCAGG
ACTGGGACTGCTTACGTTTTGTTTC
>1128
CGCAAGATAAGGTTGTGGTTTATTTATTTGTGTGTTTATTCGCCGGCCGGCTGGGAAGCT
AGAATCGGAGGAGCTGACGAGTAGATCTGGGGGCGGAGGGGAGCAGGACTGGGACT
GCTTACGTTTTGTTTCTCTTTGAG
>1129
GTTGTGGTTTATTTATTTGTGTGTTTATTCGCCGGCCGGCTGGGAAGCTAGAATCGGAG
GAGCTGACGAGTAGATCTGGGGGCGGAGGGGAGCAGGACTGGGACTGCTTACGTTTT
GTTTCTCTTTGAGAAAACGTGGTG
>1130
GAGCTGACGAGTAGATCTGGGGGCGGAGGGGAGCAGGACTGGGACTGCTTACGTTTT
GTTTCTCTTTGAGAAAACGTGGTGGGCTTTTTCTTGATTGGACTTGATCCCCACCCCCC
TTTTGCAGGGGAGGGAGGGAAGCT
>1131
TTTGTTTCTCTTTGAGAAAACGTGGTGGGCTTTTTCTTGATTGGACTTGATCCCCACCC
CCCTTTTGCAGGGGAGGGAGGGAAGCTCCAGAGGGTCTGCAGCGCTGCGGGCCCTCC
TCGGCTCTCGGCGGGACCGGCGGT
>1132
TTCTCTTTGAGAAAACGTGGTGGGCTTTTTCTTGATTGGACTTGATCCCCACCCCCCTT
TTGCAGGGGAGGGAGGGAAGCTCCAGAGGGTCTGCAGCGCTGCGGGCCCTCCTCGGC
TCTCGGCGGGACCGGCGGTGACAC
>1133
TTTTCTTGATTGGACTTGATCCCCACCCCCCTTTTGCAGGGGAGGGAGGGAAGCTCCA
GAGGGTCTGCAGCGCTGCGGGCCCTCCTCGGCTCTCGGCGGGACCGGCGGTGACACC
GGAGCTCGCCGTGCGCTCCCGGCCG

>1134
GAAGCTCCAGAGGGTCTGCAGCGCTGCGGGCCCTCCTCGGCTCTCGGCGGGACCGGC
GGTGACACCGGAGCTCGCCGTGCGCTCCCGGCCGCTCTCGGTGGGTGCCGGTCTCTGC
ACCTGATGCGTTCGGGATGCCTTTC
>1135
TCGGTGGGTGCCGGTCTCTGCACCTGATGCGTTCGGGATGCCTTTCCCACCCTGGCGC
GCCCGCCGCTAGCTCGCACAGCGCCTCGCACACTCCCGCACGCGCTTGAAATGCGCAC
GGTCCCGCCGGCCCGCGGAACCAC
>1136
GCCGGTCTCTGCACCTGATGCGTTCGGGATGCCTTTCCCACCCTGGCGCGCCCGCCGCT
AGCTCGCACAGCGCCTCGCACACTCCCGCACGCGCTTGAAATGCGCACGGTCCCGCC
GGCCCGCGGAACCACCCGGACGCA
>1137
CGCACACTCCCGCACGCGCTTGAAATGCGCACGGTCCCGCCGGCCCGCGGAACCACC
CGGACGCACGGAGCGCTCCGCACCGACTCGCTCGCCGCCTCCCCGAGACGCTCGCAC
CGTGCTTGGGCCGGGCGCGCTGGCCG
>1138
TCCCGCCGGCCCGCGGAACCACCCGGACGCACGGAGCGCTCCGCACCGACTCGCTCG
CCGCCTCCCCGAGACGCTCGCACCGTGCTTGGGCCGGGCGCGCTGGCCGCTGGCGCC
GCTGGCCAGAGGCCTGGGACCCAGCC
>1139
CTCGCCGCCTCCCCGAGACGCTCGCACCGTGCTTGGGCCGGGCGCGCTGGCCGCTGG
CGCCGCTGGCCAGAGGCCTGGGACCCAGCCGGTCGCTCCCAGGGGGTCACGGCCCTG
GGTCGGAGAGGGAGGGCGGGCAGACC
>1140
CGCCGCCTCCCCGAGACGCTCGCACCGTGCTTGGGCCGGGCGCGCTGGCCGCTGGCG
CCGCTGGCCAGAGGCCTGGGACCCAGCCGGTCGCTCCCAGGGGGTCACGGCCCTGGG
TCGGAGAGGGAGGGCGGGCAGACCCC
>1141
AGACGCTCGCACCGTGCTTGGGCCGGGCGCGCTGGCCGCTGGCGCCGCTGGCCAGAG
GCCTGGGACCCAGCCGGTCGCTCCCAGGGGGTCACGGCCCTGGGTCGGAGAGGGAGG
GCGGGCAGACCCCTTCTCGCCTTTCC
>1142
TGCTTGGGCCGGGCGCGCTGGCCGCTGGCGCCGCTGGCCAGAGGCCTGGGACCCAGC
CGGTCGCTCCCAGGGGGTCACGGCCCTGGGTCGGAGAGGGAGGGCGGGCAGACCCCT
TCTCGCCTTTCCTCCCACAACTCGCT
>1143
GCCGCTGGCCAGAGGCCTGGGACCCAGCCGGTCGCTCCCAGGGGGTCACGGCCCTGG
GTCGGAGAGGGAGGGCGGGCAGACCCCTTCTCGCCTTTCCTCCCACAACTCGCTGCG
GGGCTTTTGTGCTTCCCCTTCGCCGC
>1144
CAGAGGCCTGGGACCCAGCCGGTCGCTCCCAGGGGGTCACGGCCCTGGGTCGGAGAG
GGAGGGCGGGCAGACCCCTTCTCGCCTTTCCTCCCACAACTCGCTGCGGGGCTTTTGT
GCTTCCCCTTCGCCGCGGGGCGGGT

>1145
GCTGCGGGGCTTTTGTGCTTCCCCTTCGCCGCGGGGCGGGTCCGCCTCCCCTGCCGCT
CTCGCCGCGGAGTCCAGCCCGCCCGGACTGTCGCCGTTCCTCCCCGTCTCTTTCGCTTT
CCCTCGTCCCTAGCTCAGCTCTC
>1146
GGGGCTTTTGTGCTTCCCCTTCGCCGCGGGGCGGGTCCGCCTCCCCTGCCGCTCTCGC
CGCGGAGTCCAGCCCGCCCGGACTGTCGCCGTTCCTCCCCGTCTCTTTCGCTTTCCCTC
GTCCCTAGCTCAGCTCTCCTTCT
>1147
CGCCGCGGAGTCCAGCCCGCCCGGACTGTCGCCGTTCCTCCCCGTCTCTTTCGCTTTCC
CTCGTCCCTAGCTCAGCTCTCCTTCTTTCAGGAGTCTAGCTCCTCGGGAAAAGTTGCTT
CCCCAAGTTTGCTGAAGTCGTC
>1148
CGGAGTCCAGCCCGCCCGGACTGTCGCCGTTCCTCCCCGTCTCTTTCGCTTTCCCTCGT
CCCTAGCTCAGCTCTCCTTCTTTCAGGAGTCTAGCTCCTCGGGAAAAGTTGCTTCCCCA
AGTTTGCTGAAGTCGTCTCCAA
>1149
CCGGACTGTCGCCGTTCCTCCCCGTCTCTTTCGCTTTCCCTCGTCCCTAGCTCAGCTCT
CCTTCTTTCAGGAGTCTAGCTCCTCGGGAAAAGTTGCTTCCCCAAGTTTGCTGAAGTC
GTCTCCAAGTCTCGGTGGGGGTC
>1150
GACTGTCGCCGTTCCTCCCCGTCTCTTTCGCTTTCCCTCGTCCCTAGCTCAGCTCTCCTT
CTTTCAGGAGTCTAGCTCCTCGGGAAAAGTTGCTTCCCCAAGTTTGCTGAAGTCGTCT
CCAAGTCTCGGTGGGGGTCGCT
>1151
CGTCTCTTTCGCTTTCCCTCGTCCCTAGCTCAGCTCTCCTTCTTTCAGGAGTCTAGCTCC
TCGGGAAAAGTTGCTTCCCCAAGTTTGCTGAAGTCGTCTCCAAGTCTCGGTGGGGGTC
GCTGGGAACTGGGGGGGTGTGA
>1152
TCTCCTTCTTTCAGGAGTCTAGCTCCTCGGGAAAAGTTGCTTCCCCAAGTTTGCTGAAG
TCGTCTCCAAGTCTCGGTGGGGGTCGCTGGGAACTGGGGGGGTGTGAGAGCGCGGTC
GATCCCCGGAGCTCGGGCGGGTTA
>1153
TGCTTCCCCAAGTTTGCTGAAGTCGTCTCCAAGTCTCGGTGGGGGTCGCTGGGAACTG
GGGGGGTGTGAGAGCGCGGTCGATCCCCGGAGCTCGGGCGGGTTATCGCCGGCTCTTC
CCCGCGCCGCCTGGACTGGACGCT
>1154
AGTCGTCTCCAAGTCTCGGTGGGGGTCGCTGGGAACTGGGGGGGTGTGAGAGCGCGG
TCGATCCCCGGAGCTCGGGCGGGTTATCGCCGGCTCTTCCCCGCGCCGCCTGGACTGG
ACGCTGCAGCCAGCGCCAGCCGGAT
>1155
CGGTCGATCCCCGGAGCTCGGGCGGGTTATCGCCGGCTCTTCCCCGCGCCGCCTGGAC
TGGACGCTGCAGCCAGCGCCAGCCGGATCGCGGGCGCCGAGCGGCGGGTGGGGGAG
ACGGCTGCCCCGTGGGCGCCCAGATT

>1156
CGATCCCCGGAGCTCGGGCGGGTTATCGCCGGCTCTTCCCCGCGCCGCCTGGACTGGA
CGCTGCAGCCAGCGCCAGCCGGATCGCGGGCGCCGAGCGGCGGGTGGGGGAGACGG
CTGCCCCGTGGGCGCCCAGATTTCCC
>1157
CCGGAGCTCGGGCGGGTTATCGCCGGCTCTTCCCCGCGCCGCCTGGACTGGACGCTGC
AGCCAGCGCCAGCCGGATCGCGGGCGCCGAGCGGCGGGTGGGGGAGACGGCTGCCC
CGTGGGCGCCCAGATTTCCCTCAGGA
>1158
TCGCCGGCTCTTCCCCGCGCCGCCTGGACTGGACGCTGCAGCCAGCGCCAGCCGGATC
GCGGGCGCCGAGCGGCGGGTGGGGGAGACGGCTGCCCCGTGGGCGCCCAGATTTCCC
TCAGGACCCAAAAAGCTTGTTTCAG
>1159
CGCGCCGCCTGGACTGGACGCTGCAGCCAGCGCCAGCCGGATCGCGGGCGCCGAGCG
GCGGGTGGGGGAGACGGCTGCCCCGTGGGCGCCCAGATTTCCCTCAGGACCCAAAAA
GCTTGTTTCAGGGCGCCAAGCTTCCA
>1160
CAGCCAGCGCCAGCCGGATCGCGGGCGCCGAGCGGCGGGTGGGGGAGACGGCTGCC
CCGTGGGCGCCCAGATTTCCCTCAGGACCCAAAAAGCTTGTTTCAGGGCGCCAAGCTT
CCATTCTTTTGGCTGTTCCCTCCCTA
>1161
AGCCGGATCGCGGGCGCCGAGCGGCGGGTGGGGGAGACGGCTGCCCCGTGGGCGCC
CAGATTTCCCTCAGGACCCAAAAAGCTTGTTTCAGGGCGCCAAGCTTCCATTCTTTTG
GCTGTTCCCTCCCTAGTTCTCAGAAA
>1162
GGGCGCCGAGCGGCGGGTGGGGGAGACGGCTGCCCCGTGGGCGCCCAGATTTCCCTC
AGGACCCAAAAAGCTTGTTTCAGGGCGCCAAGCTTCCATTCTTTTGGCTGTTCCCTCC
CTAGTTCTCAGAAACCCCGATGTCT
>1163
TTTCCCTCAGGACCCAAAAAGCTTGTTTCAGGGCGCCAAGCTTCCATTCTTTTGGCTGT
TCCCTCCCTAGTTCTCAGAAACCCCGATGTCTGCGGGAAAGCACGCGGTGGCTCGGCT
CGGCTGAATGGTCATCCCAACCT
>1164
CAGGACCCAAAAAGCTTGTTTCAGGGCGCCAAGCTTCCATTCTTTTGGCTGTTCCCTC
CCTAGTTCTCAGAAACCCCGATGTCTGCGGGAAAGCACGCGGTGGCTCGGCTCGGCTG
AATGGTCATCCCAACCTTGACGGC
>1165
GGGCGCCAAGCTTCCATTCTTTTGGCTGTTCCCTCCCTAGTTCTCAGAAACCCCGATGT
CTGCGGGAAAGCACGCGGTGGCTCGGCTCGGCTGAATGGTCATCCCAACCTTGACGG
CCTCCGTCCCCACCCCGGGGCGGG
>1166
GCGGTGGCTCGGCTCGGCTGAATGGTCATCCCAACCTTGACGGCCTCCGTCCCCACCC
CGGGGCGGGCAGCAAAAATGTCTTGACCAGCCGTCCTGTGAATCCTTTCTGCGTGAGC
TTGGTGGCTCCTGAACTGATAGAG

>1167
TGAATGGTCATCCCAACCTTGACGGCCTCCGTCCCCACCCCGGGGCGGGCAGCAAAAA
TGTCTTGACCAGCCGTCCTGTGAATCCTTTCTGCGTGAGCTTGGTGGCTCCTGAACTGA
TAGAGCTCCTTCTGGGGGTGGAG
>1168
TCCGTCCCCACCCCGGGGCGGGCAGCAAAAATGTCTTGACCAGCCGTCCTGTGAATCC
TTTCTGCGTGAGCTTGGTGGCTCCTGAACTGATAGAGCTCCTTCTGGGGGTGGAGGCA
AGCAAGCAGTGTTCGAAGAGTGAG
>1169
AGCAAAAATGTCTTGACCAGCCGTCCTGTGAATCCTTTCTGCGTGAGCTTGGTGGCTC
CTGAACTGATAGAGCTCCTTCTGGGGGTGGAGGCAAGCAAGCAGTGTTCGAAGAGTG
AGGGAGAGACCTTGCTGTTCCTTGC
>1170
CAAAAATGTCTTGACCAGCCGTCCTGTGAATCCTTTCTGCGTGAGCTTGGTGGCTCCTG
AACTGATAGAGCTCCTTCTGGGGGTGGAGGCAAGCAAGCAGTGTTCGAAGAGTGAGG
GAGAGACCTTGCTGTTCCTTGCTC
>1171
CCGTCCTGTGAATCCTTTCTGCGTGAGCTTGGTGGCTCCTGAACTGATAGAGCTCCTTC
TGGGGGTGGAGGCAAGCAAGCAGTGTTCGAAGAGTGAGGGAGAGACCTTGCTGTTCC
TTGCTCTCAGTGCATTCACTGCAA
>1172
CGTGAGCTTGGTGGCTCCTGAACTGATAGAGCTCCTTCTGGGGGTGGAGGCAAGCAA
GCAGTGTTCGAAGAGTGAGGGAGAGACCTTGCTGTTCCTTGCTCTCAGTGCATTCACT
GCAATATTGGTCGCTGTAAGACAGA
>1173
TTCTGGGGGTGGAGGCAAGCAAGCAGTGTTCGAAGAGTGAGGGAGAGACCTTGCTGT
TCCTTGCTCTCAGTGCATTCACTGCAATATTGGTCGCTGTAAGACAGACACGGGGAGG
GTCCTGGGCCTGCGCGGTCTTTCTA
>1174
CGAAGAGTGAGGGAGAGACCTTGCTGTTCCTTGCTCTCAGTGCATTCACTGCAATATT
GGTCGCTGTAAGACAGACACGGGGAGGGTCCTGGGCCTGCGCGGTCTTTCTAACACTC
CTTTAAGCGAGGTTTGGAGTCTAG
>1175
GAGACCTTGCTGTTCCTTGCTCTCAGTGCATTCACTGCAATATTGGTCGCTGTAAGACA
GACACGGGGAGGGTCCTGGGCCTGCGCGGTCTTTCTAACACTCCTTTAAGCGAGGTTT
GGAGTCTAGGAGCCCTCTTATTG
>1176
ATATTGGTCGCTGTAAGACAGACACGGGGAGGGTCCTGGGCCTGCGCGGTCTTTCTAA
CACTCCTTTAAGCGAGGTTTGGAGTCTAGGAGCCCTCTTATTGATCCCGGACAGGCTCT
GGGAGAGGCCTTTGTTTCCCAGC
>1177
GTAAGACAGACACGGGGAGGGTCCTGGGCCTGCGCGGTCTTTCTAACACTCCTTTAAG
CGAGGTTTGGAGTCTAGGAGCCCTCTTATTGATCCCGGACAGGCTCTGGGAGAGGCCT
TTGTTTCCCAGCCCTGTCTTCCCT

>1178
ACAGACACGGGGAGGGTCCTGGGCCTGCGCGGTCTTTCTAACACTCCTTTAAGCGAGG
TTTGGAGTCTAGGAGCCCTCTTATTGATCCCGGACAGGCTCTGGGAGAGGCCTTTGTTT
CCCAGCCCTGTCTTCCCTTCGTA
>1179
GACACGGGGAGGGTCCTGGGCCTGCGCGGTCTTTCTAACACTCCTTTAAGCGAGGTTT
GGAGTCTAGGAGCCCTCTTATTGATCCCGGACAGGCTCTGGGAGAGGCCTTTGTTTCC
CAGCCCTGTCTTCCCTTCGTACTG
>1180
TGCGCGGTCTTTCTAACACTCCTTTAAGCGAGGTTTGGAGTCTAGGAGCCCTCTTATTG
ATCCCGGACAGGCTCTGGGAGAGGCCTTTGTTTCCCAGCCCTGTCTTCCCTTCGTACTG
GTTGTAACTCACACCACAGGTG
>1181
TTCTAACACTCCTTTAAGCGAGGTTTGGAGTCTAGGAGCCCTCTTATTGATCCCGGACA
GGCTCTGGGAGAGGCCTTTGTTTCCCAGCCCTGTCTTCCCTTCGTACTGGTTGTAACTC
ACACCACAGGTGCTGGTGCCAA
>1182
CAGGCTCTGGGAGAGGCCTTTGTTTCCCAGCCCTGTCTTCCCTTCGTACTGGTTGTAAC
TCACACCACAGGTGCTGGTGCCAATATCACTTCCAGCGCCTCTGAGCCATGTCCAGCAT
CCCTTGCTGCCCCTGGTCACAG
>1183
CCCAGCCCTGTCTTCCCTTCGTACTGGTTGTAACTCACACCACAGGTGCTGGTGCCAAT
ATCACTTCCAGCGCCTCTGAGCCATGTCCAGCATCCCTTGCTGCCCCTGGTCACAGGAC
TTGCAGACTCCTTGGAGCCTGT
>1184
TCTTCCCTTCGTACTGGTTGTAACTCACACCACAGGTGCTGGTGCCAATATCACTTCCA
GCGCCTCTGAGCCATGTCCAGCATCCCTTGCTGCCCCTGGTCACAGGACTTGCAGACT
CCTTGGAGCCTGTGGGACACACG
>1185
GTACTGGTTGTAACTCACACCACAGGTGCTGGTGCCAATATCACTTCCAGCGCCTCTGA
GCCATGTCCAGCATCCCTTGCTGCCCCTGGTCACAGGACTTGCAGACTCCTTGGAGCC
TGTGGGACACACGTGGAGTTCAC
>1186
GTGCTGGTGCCAATATCACTTCCAGCGCCTCTGAGCCATGTCCAGCATCCCTTGCTGCC
CCTGGTCACAGGACTTGCAGACTCCTTGGAGCCTGTGGGACACACGTGGAGTTCACA
CACCCAGCCCAGCATGAGCACTCC
>1187
TCCAGCGCCTCTGAGCCATGTCCAGCATCCCTTGCTGCCCCTGGTCACAGGACTTGCA
GACTCCTTGGAGCCTGTGGGACACACGTGGAGTTCACACACCCAGCCCAGCATGAGC
ACTCCTGGCTGTAGGGGACCATATT
>1188
TCCAGCATCCCTTGCTGCCCCTGGTCACAGGACTTGCAGACTCCTTGGAGCCTGTGGG
ACACACGTGGAGTTCACACACCCAGCCCAGCATGAGCACTCCTGGCTGTAGGGGACC
ATATTTAACTATGGTCTCTCTCCAT

>1189
GCTGCCCCTGGTCACAGGACTTGCAGACTCCTTGGAGCCTGTGGGACACACGTGGAG
TTCACACACCCAGCCCAGCATGAGCACTCCTGGCTGTAGGGGACCATATTTAACTATGG
TCTCTCTCCATACCTTAATGACCC
>1190
ACTCCTTGGAGCCTGTGGGACACACGTGGAGTTCACACACCCAGCCCAGCATGAGCA
CTCCTGGCTGTAGGGGACCATATTTAACTATGGTCTCTCTCCATACCTTAATGACCCATC
CTTCTTCGTTTTTTAGATCCTCC
>1191
GCTGTAGGGGACCATATTTAACTATGGTCTCTCTCCATACCTTAATGACCCATCCTTCTT
CGTTTTTTAGATCCTCCTGTCCACCGGGCAGTTGTGGGCAGGAGGGTAGGTACGACAC
CTTACGTAAGTGTGTGTGTGTA
>1192
CTATGGTCTCTCTCCATACCTTAATGACCCATCCTTCTTCGTTTTTTAGATCCTCCTGTCC
ACCGGGCAGTTGTGGGCAGGAGGGTAGGTACGACACCTTACGTAAGTGTGTGTGTGTA
AACACACACACACTTTTTTTT
>1193
TCTCCATACCTTAATGACCCATCCTTCTTCGTTTTTTAGATCCTCCTGTCCACCGGGCAG
TTGTGGGCAGGAGGGTAGGTACGACACCTTACGTAAGTGTGTGTGTGTAAACACACAC
ACACTTTTTTTTTTTTTTTCTG
>1194
TTCGTTTTTTAGATCCTCCTGTCCACCGGGCAGTTGTGGGCAGGAGGGTAGGTACGAC
ACCTTACGTAAGTGTGTGTGTGTAAACACACACACACTTTTTTTTTTTTTTTCTGAGAC
GGAGTTTTGCTCCTGTTGCCCAG
>1195
GGGTAGGTACGACACCTTACGTAAGTGTGTGTGTGTAAACACACACACACTTTTTTTTT
TTTTTTCTGAGACGGAGTTTTGCTCCTGTTGCCCAGGCTGGAGTGCAGTGGCGCAATC
TCGGCTCACGGCAACCTCCGCCT
>1196
GTACGACACCTTACGTAAGTGTGTGTGTGTAAACACACACACACTTTTTTTTTTTTTTT
CTGAGACGGAGTTTTGCTCCTGTTGCCCAGGCTGGAGTGCAGTGGCGCAATCTCGGCT
CACGGCAACCTCCGCCTCCCGGT
>1197
TGTGTGTGTGTAAACACACACACACTTTTTTTTTTTTTTTCTGAGACGGAGTTTTGCTC
CTGTTGCCCAGGCTGGAGTGCAGTGGCGCAATCTCGGCTCACGGCAACCTCCGCCTCC
CGGTTTCAAGTGATTCTCCTGCC
>1198
GTGTAAACACACACACACTTTTTTTTTTTTTTTCTGAGACGGAGTTTTGCTCCTGTTGC
CCAGGCTGGAGTGCAGTGGCGCAATCTCGGCTCACGGCAACCTCCGCCTCCCGGTTTC
AAGTGATTCTCCTGCCTCAGCCT
>1199
TCCTGTTGCCCAGGCTGGAGTGCAGTGGCGCAATCTCGGCTCACGGCAACCTCCGCCT
CCCGGTTTCAAGTGATTCTCCTGCCTCAGCCTCCCGAGTAGCTGGGATTACAGGCGGG
TGCCACCGCGCCTGGCTAATTTTG

>1200
GAGTGCAGTGGCGCAATCTCGGCTCACGGCAACCTCCGCCTCCCGGTTTCAAGTGATT
CTCCTGCCTCAGCCTCCCGAGTAGCTGGGATTACAGGCGGGTGCCACCGCGCCTGGCT
AATTTTGTATTTTTTGTAGAGACG
>1201
GCAATCTCGGCTCACGGCAACCTCCGCCTCCCGGTTTCAAGTGATTCTCCTGCCTCAGC
CTCCCGAGTAGCTGGGATTACAGGCGGGTGCCACCGCGCCTGGCTAATTTTGTATTTTT
TGTAGAGACGGGGCTTCTCCAT
>1202
CTCAGCCTCCCGAGTAGCTGGGATTACAGGCGGGTGCCACCGCGCCTGGCTAATTTTGT
ATTTTTTGTAGAGACGGGGCTTCTCCATGTTGGTCAGGCTGGTCTCGAACTCCCGACCT
CAGGTGATCCGCCTGCCTGGGC
>1203
CAGCCTCCCGAGTAGCTGGGATTACAGGCGGGTGCCACCGCGCCTGGCTAATTTTGTAT
TTTTTGTAGAGACGGGGCTTCTCCATGTTGGTCAGGCTGGTCTCGAACTCCCGACCTCA
GGTGATCCGCCTGCCTGGGCCT
>1204
AATTTTGTATTTTTTGTAGAGACGGGGCTTCTCCATGTTGGTCAGGCTGGTCTCGAACT
CCCGACCTCAGGTGATCCGCCTGCCTGGGCCTCCCAAAGTGCTGGGATTAGAGGCGTG
AGCCACCGCGCCCTACCCCACAC
>1205
TTCTCCATGTTGGTCAGGCTGGTCTCGAACTCCCGACCTCAGGTGATCCGCCTGCCTGG
GCCTCCCAAAGTGCTGGGATTAGAGGCGTGAGCCACCGCGCCCTACCCCACACACTCT
TTCTGTCTGTCTCTGGCTGGTGT
>1206
GTCAGGCTGGTCTCGAACTCCCGACCTCAGGTGATCCGCCTGCCTGGGCCTCCCAAAG
TGCTGGGATTAGAGGCGTGAGCCACCGCGCCCTACCCCACACACTCTTTCTGTCTGTCT
CTGGCTGGTGTACATACACACAG
>1207
CAGGCTGGTCTCGAACTCCCGACCTCAGGTGATCCGCCTGCCTGGGCCTCCCAAAGTG
CTGGGATTAGAGGCGTGAGCCACCGCGCCCTACCCCACACACTCTTTCTGTCTGTCTCT
GGCTGGTGTACATACACACAGTT
>1208
GTCTCGAACTCCCGACCTCAGGTGATCCGCCTGCCTGGGCCTCCCAAAGTGCTGGGAT
TAGAGGCGTGAGCCACCGCGCCCTACCCCACACACTCTTTCTGTCTGTCTCTGGCTGG
TGTACATACACACAGTTCAGATTT
>1209
AGGCGTGAGCCACCGCGCCCTACCCCACACACTCTTTCTGTCTGTCTCTGGCTGGTGT
ACATACACACAGTTCAGATTTTATATTATACATACAATAACTTTGAGGCTTCCGTGGGTA
GAAATGACCCAAGTTAGTGTGT
>1210
TGAGCCACCGCGCCCTACCCCACACACTCTTTCTGTCTGTCTCTGGCTGGTGTACATAC
ACACAGTTCAGATTTTATATTATACATACAATAACTTTGAGGCTTCCGTGGGTAGAAATG
ACCCAAGTTAGTGTGTGGGTT

>1211
TTCTGTCTGTCTCTGGCTGGTGTACATACACACAGTTCAGATTTTATATTATACATACAAT
AACTTTGAGGCTTCCGTGGGTAGAAATGACCCAAGTTAGTGTGTGGGTTGTGTGTGTT
CCTGTTAGTACCTAGGGATGG
>1212
GGCTGGTGTACATACACACAGTTCAGATTTTATATTATACATACAATAACTTTGAGGCTT
CCGTGGGTAGAAATGACCCAAGTTAGTGTGTGGGTTGTGTGTGTTCCTGTTAGTACCTA
GGGATGGGGACAGGCCTTGGG
>1213
TACACACAGTTCAGATTTTATATTATACATACAATAACTTTGAGGCTTCCGTGGGTAGAA
ATGACCCAAGTTAGTGTGTGGGTTGTGTGTGTTCCTGTTAGTACCTAGGGATGGGGACA
GGCCTTGGGTCTGCCAGTGCT
>1214
CACAGTTCAGATTTTATATTATACATACAATAACTTTGAGGCTTCCGTGGGTAGAAATGA
CCCAAGTTAGTGTGTGGGTTGTGTGTGTTCCTGTTAGTACCTAGGGATGGGGACAGGC
CTTGGGTCTGCCAGTGCTTTGC
>1215
CAATAACTTTGAGGCTTCCGTGGGTAGAAATGACCCAAGTTAGTGTGTGGGTTGTGTG
TGTTCCTGTTAGTACCTAGGGATGGGGACAGGCCTTGGGTCTGCCAGTGCTTTGCAGC
CCTGAGAACAGTAGAAGGCCAGAG
>1216
GTGGGTAGAAATGACCCAAGTTAGTGTGTGGGTTGTGTGTGTTCCTGTTAGTACCTAGG
GATGGGGACAGGCCTTGGGTCTGCCAGTGCTTTGCAGCCCTGAGAACAGTAGAAGGC
CAGAGGCCAAGGCTGATGGTGAGA
>1217
CAAGTTAGTGTGTGGGTTGTGTGTGTTCCTGTTAGTACCTAGGGATGGGGACAGGCCTT
GGGTCTGCCAGTGCTTTGCAGCCCTGAGAACAGTAGAAGGCCAGAGGCCAAGGCTGA
TGGTGAGAGGATAGCCTTTGCGGA
>1218
TGTGGGTTGTGTGTGTTCCTGTTAGTACCTAGGGATGGGGACAGGCCTTGGGTCTGCC
AGTGCTTTGCAGCCCTGAGAACAGTAGAAGGCCAGAGGCCAAGGCTGATGGTGAGAG
GATAGCCTTTGCGGAGGAGAGCCCA
>1219
GGGATGGGGACAGGCCTTGGGTCTGCCAGTGCTTTGCAGCCCTGAGAACAGTAGAAG
GCCAGAGGCCAAGGCTGATGGTGAGAGGATAGCCTTTGCGGAGGAGAGCCCAGTGTC
CATAGATTTCTGTGGCCATGGCTACA
>1220
GGCCTTGGGTCTGCCAGTGCTTTGCAGCCCTGAGAACAGTAGAAGGCCAGAGGCCAA
GGCTGATGGTGAGAGGATAGCCTTTGCGGAGGAGAGCCCAGTGTCCATAGATTTCTGT
GGCCATGGCTACAGGCTCCTGGCCT
>1221
GGTCTGCCAGTGCTTTGCAGCCCTGAGAACAGTAGAAGGCCAGAGGCCAAGGCTGAT
GGTGAGAGGATAGCCTTTGCGGAGGAGAGCCCAGTGTCCATAGATTTCTGTGGCCATG
GCTACAGGCTCCTGGCCTCCAATGA

>1222
CCTGAGAACAGTAGAAGGCCAGAGGCCAAGGCTGATGGTGAGAGGATAGCCTTTGCG
GAGGAGAGCCCAGTGTCCATAGATTTCTGTGGCCATGGCTACAGGCTCCTGGCCTCCA
ATGAGTTTGACTCTGCTTCTGGGGC
>1223
CTGATGGTGAGAGGATAGCCTTTGCGGAGGAGAGCCCAGTGTCCATAGATTTCTGTGG
CCATGGCTACAGGCTCCTGGCCTCCAATGAGTTTGACTCTGCTTCTGGGGCTCCTGTAG
TGAGGCAGAGTTTGGAAAAACTG
>1224
CTTTGCGGAGGAGAGCCCAGTGTCCATAGATTTCTGTGGCCATGGCTACAGGCTCCTG
GCCTCCAATGAGTTTGACTCTGCTTCTGGGGCTCCTGTAGTGAGGCAGAGTTTGGAAA
AACTGTTTAAAGATAAGGCTTGAC
>1225
TCTGTGGCCATGGCTACAGGCTCCTGGCCTCCAATGAGTTTGACTCTGCTTCTGGGGCT
CCTGTAGTGAGGCAGAGTTTGGAAAAACTGTTTAAAGATAAGGCTTGACATCCAGGGG
GCTGGCGTGAAGCTGACAAGGAA
>1226
TGGCCATGGCTACAGGCTCCTGGCCTCCAATGAGTTTGACTCTGCTTCTGGGGCTCCTG
TAGTGAGGCAGAGTTTGGAAAAACTGTTTAAAGATAAGGCTTGACATCCAGGGGGCTG
GCGTGAAGCTGACAAGGAAGGGG
>1227
GCCTCCAATGAGTTTGACTCTGCTTCTGGGGCTCCTGTAGTGAGGCAGAGTTTGGAAA
AACTGTTTAAAGATAAGGCTTGACATCCAGGGGGCTGGCGTGAAGCTGACAAGGAAG
GGGCTAAAGTCTTGCTAATTTTAAG
>1228
TCTGCTTCTGGGGCTCCTGTAGTGAGGCAGAGTTTGGAAAAACTGTTTAAAGATAAGG
CTTGACATCCAGGGGGCTGGCGTGAAGCTGACAAGGAAGGGGCTAAAGTCTTGCTAA
TTTTAAGTAAGTGCACTTCATCATA
>1229
CCTGTAGTGAGGCAGAGTTTGGAAAAACTGTTTAAAGATAAGGCTTGACATCCAGGGG
GCTGGCGTGAAGCTGACAAGGAAGGGGCTAAAGTCTTGCTAATTTTAAGTAAGTGCAC
TTCATCATAATGAATGTTGGCCTT
>1230
GAGGCAGAGTTTGGAAAAACTGTTTAAAGATAAGGCTTGACATCCAGGGGGCTGGCG
TGAAGCTGACAAGGAAGGGGCTAAAGTCTTGCTAATTTTAAGTAAGTGCACTTCATCAT
AATGAATGTTGGCCTTGCCTAGGG
>1231
CAGAGTTTGGAAAAACTGTTTAAAGATAAGGCTTGACATCCAGGGGGCTGGCGTGAA
GCTGACAAGGAAGGGGCTAAAGTCTTGCTAATTTTAAGTAAGTGCACTTCATCATAATG
AATGTTGGCCTTGCCTAGGGAGCC
>1232
ATAAGGCTTGACATCCAGGGGGCTGGCGTGAAGCTGACAAGGAAGGGGCTAAAGTCT
TGCTAATTTTAAGTAAGTGCACTTCATCATAATGAATGTTGGCCTTGCCTAGGGAGCCCC
TACTCTCAGACACAGAGAAAGCT

>1233
AGGAAGGGGCTAAAGTCTTGCTAATTTTAAGTAAGTGCACTTCATCATAATGAATGTTG
GCCTTGCCTAGGGAGCCCCTACTCTCAGACACAGAGAAAGCTGAGGACTTATAGGACT
TCACTACAATTTTCATTTCACTT
>1234
AGGGGCTAAAGTCTTGCTAATTTTAAGTAAGTGCACTTCATCATAATGAATGTTGGCCTT
GCCTAGGGAGCCCCTACTCTCAGACACAGAGAAAGCTGAGGACTTATAGGACTTCACT
ACAATTTTCATTTCACTTCATC
>1235
TTTAAGTAAGTGCACTTCATCATAATGAATGTTGGCCTTGCCTAGGGAGCCCCTACTCTC
AGACACAGAGAAAGCTGAGGACTTATAGGACTTCACTACAATTTTCATTTCACTTCATC
TAACAGATGTCATAGAGTAGT
>1236
ATCATAATGAATGTTGGCCTTGCCTAGGGAGCCCCTACTCTCAGACACAGAGAAAGCT
GAGGACTTATAGGACTTCACTACAATTTTCATTTCACTTCATCTAACAGATGTCATAGAG
TAGTGAATTAGGCATCTGCTGT
>1237
CTCTCAGACACAGAGAAAGCTGAGGACTTATAGGACTTCACTACAATTTTCATTTCACT
TCATCTAACAGATGTCATAGAGTAGTGAATTAGGCATCTGCTGTGGCAGAACCTTCTTT
TTACTCTCAGCATCCCTGGCTT
>1238
ACAGAGAAAGCTGAGGACTTATAGGACTTCACTACAATTTTCATTTCACTTCATCTAAC
AGATGTCATAGAGTAGTGAATTAGGCATCTGCTGTGGCAGAACCTTCTTTTTACTCTCA
GCATCCCTGGCTTGAAAGGCAG
>1239
AGGACTTATAGGACTTCACTACAATTTTCATTTCACTTCATCTAACAGATGTCATAGAGT
AGTGAATTAGGCATCTGCTGTGGCAGAACCTTCTTTTTACTCTCAGCATCCCTGGCTTG
AAAGGCAGATTTGTAGATTTC
>1240
CACTACAATTTTCATTTCACTTCATCTAACAGATGTCATAGAGTAGTGAATTAGGCATCT
GCTGTGGCAGAACCTTCTTTTTACTCTCAGCATCCCTGGCTTGAAAGGCAGATTTGTAG
ATTTCTAGGAAGCTACTTCAA
>1241
TTCATCTAACAGATGTCATAGAGTAGTGAATTAGGCATCTGCTGTGGCAGAACCTTCTT
TTTACTCTCAGCATCCCTGGCTTGAAAGGCAGATTTGTAGATTTCTAGGAAGCTACTTC
AATTAAAATCTCTAAGATCCTC
>1242
AGTAGTGAATTAGGCATCTGCTGTGGCAGAACCTTCTTTTTACTCTCAGCATCCCTGGC
TTGAAAGGCAGATTTGTAGATTTCTAGGAAGCTACTTCAATTAAAATCTCTAAGATCCT
CTTGCTAAGGGCTTGGCAACAG
>1243
ATTAGGCATCTGCTGTGGCAGAACCTTCTTTTTACTCTCAGCATCCCTGGCTTGAAAGG
CAGATTTGTAGATTTCTAGGAAGCTACTTCAATTAAAATCTCTAAGATCCTCTTGCTAAG
GGCTTGGCAACAGGGAGGAGG

>1244
TCTGCTGTGGCAGAACCTTCTTTTTACTCTCAGCATCCCTGGCTTGAAAGGCAGATTTG
TAGATTTCTAGGAAGCTACTTCAATTAAAATCTCTAAGATCCTCTTGCTAAGGGCTTGGC
AACAGGGAGGAGGGGGCATGT
>1245
TCTCAGCATCCCTGGCTTGAAAGGCAGATTTGTAGATTTCTAGGAAGCTACTTCAATTA
AAATCTCTAAGATCCTCTTGCTAAGGGCTTGGCAACAGGGAGGAGGGGGCATGTTGTA
GTGGTTGAATTCTTACTTTTAGG
>1246
ATTTGTAGATTTCTAGGAAGCTACTTCAATTAAAATCTCTAAGATCCTCTTGCTAAGGGC
TTGGCAACAGGGAGGAGGGGGCATGTTGTAGTGGTTGAATTCTTACTTTTAGGGACAC
TGATGAATTTGTCTTTTGTTCT
>1247
GTAGATTTCTAGGAAGCTACTTCAATTAAAATCTCTAAGATCCTCTTGCTAAGGGCTTG
GCAACAGGGAGGAGGGGGCATGTTGTAGTGGTTGAATTCTTACTTTTAGGGACACTGA
TGAATTTGTCTTTTGTTCTCTCA
>1248
TACTTCAATTAAAATCTCTAAGATCCTCTTGCTAAGGGCTTGGCAACAGGGAGGAGGG
GGCATGTTGTAGTGGTTGAATTCTTACTTTTAGGGACACTGATGAATTTGTCTTTTGTTC
TCTCATCTCTTTTGCTTCTTCC
>1249
GATCCTCTTGCTAAGGGCTTGGCAACAGGGAGGAGGGGGCATGTTGTAGTGGTTGAAT
TCTTACTTTTAGGGACACTGATGAATTTGTCTTTTGTTCTCTCATCTCTTTTGCTTCTTCC
CCTCTTCTTTCTCCTAGCCTC
>1250
TTAGGGACACTGATGAATTTGTCTTTTGTTCTCTCATCTCTTTTGCTTCTTCCCCTCTTCT
TTCTCCTAGCCTCCTCTATTGGCCCAGGAAGCCCACCCAGCCCCGCCACGCAGAGCCC
AGAAGGAAAGAAAGCCTCATG
>1251
TTGTCTTTTGTTCTCTCATCTCTTTTGCTTCTTCCCCTCTTCTTTCTCCTAGCCTCCTCTAT
TGGCCCAGGAAGCCCACCCAGCCCCGCCACGCAGAGCCCAGAAGGAAAGAAAGCCT
CATGCCTGAGCCGAGGGGAGCA
>1252
CTTTTGTTCTCTCATCTCTTTTGCTTCTTCCCCTCTTCTTTCTCCTAGCCTCCTCTATTGG
CCCAGGAAGCCCACCCAGCCCCGCCACGCAGAGCCCAGAAGGAAAGAAAGCCTCAT
GCCTGAGCCGAGGGGAGCACCAT
>1253
GCTTCTTCCCCTCTTCTTTCTCCTAGCCTCCTCTATTGGCCCAGGAAGCCCACCCAGCC
CCGCCACGCAGAGCCCAGAAGGAAAGAAAGCCTCATGCCTGAGCCGAGGGGAGCAC
CATGGATCTGACAAAAATGGGCATG
>1254
CTATTGGCCCAGGAAGCCCACCCAGCCCCGCCACGCAGAGCCCAGAAGGAAAGAAAG
CCTCATGCCTGAGCCGAGGGGAGCACCATGGATCTGACAAAAATGGGCATGATCCAGC
TGCAGAACCCTAGCCACCCCACGGG

>1255
GGCCCAGGAAGCCCACCCAGCCCCGCCACGCAGAGCCCAGAAGGAAAGAAAGCCTC
ATGCCTGAGCCGAGGGGAGCACCATGGATCTGACAAAAATGGGCATGATCCAGCTGCA
GAACCCTAGCCACCCCACGGGGCTAC
>1256
GAAGGAAAGAAAGCCTCATGCCTGAGCCGAGGGGAGCACCATGGATCTGACAAAAAT
GGGCATGATCCAGCTGCAGAACCCTAGCCACCCCACGGGGCTACTGTGCAAGGCCAA
CCAGATGCGGCTGGCCGGGACTTTGT
>1257
AAGAAAGCCTCATGCCTGAGCCGAGGGGAGCACCATGGATCTGACAAAAATGGGCAT
GATCCAGCTGCAGAACCCTAGCCACCCCACGGGGCTACTGTGCAAGGCCAACCAGAT
GCGGCTGGCCGGGACTTTGTGCGATG
>1258
CTCATGCCTGAGCCGAGGGGAGCACCATGGATCTGACAAAAATGGGCATGATCCAGCT
GCAGAACCCTAGCCACCCCACGGGGCTACTGTGCAAGGCCAACCAGATGCGGCTGGC
CGGGACTTTGTGCGATGTGGTCATC
>1259
CATGGATCTGACAAAAATGGGCATGATCCAGCTGCAGAACCCTAGCCACCCCACGGGG
CTACTGTGCAAGGCCAACCAGATGCGGCTGGCCGGGACTTTGTGCGATGTGGTCATCA
TGGTGGACAGCCAGGAGTTCCACG
>1260
GGCTACTGTGCAAGGCCAACCAGATGCGGCTGGCCGGGACTTTGTGCGATGTGGTCAT
CATGGTGGACAGCCAGGAGTTCCACGCCCACCGGACGGTGCTGGCCTGCACCAGCAA
GATGTTTGAGATCCTCTTCCACCGC
>1261
ACTGTGCAAGGCCAACCAGATGCGGCTGGCCGGGACTTTGTGCGATGTGGTCATCATG
GTGGACAGCCAGGAGTTCCACGCCCACCGGACGGTGCTGGCCTGCACCAGCAAGATG
TTTGAGATCCTCTTCCACCGCAATA
>1262
TGTGCGATGTGGTCATCATGGTGGACAGCCAGGAGTTCCACGCCCACCGGACGGTGCT
GGCCTGCACCAGCAAGATGTTTGAGATCCTCTTCCACCGCAATAGTCAACACTATACTT
TGGACTTCCTCTCGCCAAAGACC
>1263
CGATGTGGTCATCATGGTGGACAGCCAGGAGTTCCACGCCCACCGGACGGTGCTGGCC
TGCACCAGCAAGATGTTTGAGATCCTCTTCCACCGCAATAGTCAACACTATACTTTGGA
CTTCCTCTCGCCAAAGACCTTCC
>1264
AGTTCCACGCCCACCGGACGGTGCTGGCCTGCACCAGCAAGATGTTTGAGATCCTCTT
CCACCGCAATAGTCAACACTATACTTTGGACTTCCTCTCGCCAAAGACCTTCCAGCAGA
TTCTGGAGTATGCATATACAGCC
>1265
CAGCAAGATGTTTGAGATCCTCTTCCACCGCAATAGTCAACACTATACTTTGGACTTCC
TCTCGCCAAAGACCTTCCAGCAGATTCTGGAGTATGCATATACAGCCACGCTGCAAGCC
AAGGCGGAGGACCTGGATGACC

>1266
GTTTGAGATCCTCTTCCACCGCAATAGTCAACACTATACTTTGGACTTCCTCTCGCCAA
AGACCTTCCAGCAGATTCTGGAGTATGCATATACAGCCACGCTGCAAGCCAAGGCGGA
GGACCTGGATGACCTGCTGTATG
>1267
AGTCAACACTATACTTTGGACTTCCTCTCGCCAAAGACCTTCCAGCAGATTCTGGAGTA
TGCATATACAGCCACGCTGCAAGCCAAGGCGGAGGACCTGGATGACCTGCTGTATGCG
GCCGAGATCCTGGAGATCGAGTA
>1268
ACTTTGGACTTCCTCTCGCCAAAGACCTTCCAGCAGATTCTGGAGTATGCATATACAGC
CACGCTGCAAGCCAAGGCGGAGGACCTGGATGACCTGCTGTATGCGGCCGAGATCCTG
GAGATCGAGTACCTGGAGGAACA
>1269
GGACTTCCTCTCGCCAAAGACCTTCCAGCAGATTCTGGAGTATGCATATACAGCCACGC
TGCAAGCCAAGGCGGAGGACCTGGATGACCTGCTGTATGCGGCCGAGATCCTGGAGAT
CGAGTACCTGGAGGAACAGTGCC
>1270
GGAGTATGCATATACAGCCACGCTGCAAGCCAAGGCGGAGGACCTGGATGACCTGCTG
TATGCGGCCGAGATCCTGGAGATCGAGTACCTGGAGGAACAGTGCCTGAAGATGCTGG
AGACCATCCAGGCCTCAGACGACA
>1271
AGCCACGCTGCAAGCCAAGGCGGAGGACCTGGATGACCTGCTGTATGCGGCCGAGAT
CCTGGAGATCGAGTACCTGGAGGAACAGTGCCTGAAGATGCTGGAGACCATCCAGGC
CTCAGACGACAATGACACGGAGGCCA
>1272
GCAAGCCAAGGCGGAGGACCTGGATGACCTGCTGTATGCGGCCGAGATCCTGGAGATC
GAGTACCTGGAGGAACAGTGCCTGAAGATGCTGGAGACCATCCAGGCCTCAGACGAC
AATGACACGGAGGCCACCATGGCCG
>1273
CAAGGCGGAGGACCTGGATGACCTGCTGTATGCGGCCGAGATCCTGGAGATCGAGTAC
CTGGAGGAACAGTGCCTGAAGATGCTGGAGACCATCCAGGCCTCAGACGACAATGAC
ACGGAGGCCACCATGGCCGATGGCG
>1274
TGACCTGCTGTATGCGGCCGAGATCCTGGAGATCGAGTACCTGGAGGAACAGTGCCTG
AAGATGCTGGAGACCATCCAGGCCTCAGACGACAATGACACGGAGGCCACCATGGCC
GATGGCGGGGCCGAGGAAGAAGAGG
>1275
CGAGTACCTGGAGGAACAGTGCCTGAAGATGCTGGAGACCATCCAGGCCTCAGACGA
CAATGACACGGAGGCCACCATGGCCGATGGCGGGGCCGAGGAAGAAGAGGACCGCA
AGGCTCGGTACCTCAAGAACATCTTCA
>1276
GATGCTGGAGACCATCCAGGCCTCAGACGACAATGACACGGAGGCCACCATGGCCGA
TGGCGGGGCCGAGGAAGAAGAGGACCGCAAGGCTCGGTACCTCAAGAACATCTTCAT
CTCGAAGCATTCCAGCGAGGAGAGTG

>1277
GACCATCCAGGCCTCAGACGACAATGACACGGAGGCCACCATGGCCGATGGCGGGGC
CGAGGAAGAAGAGGACCGCAAGGCTCGGTACCTCAAGAACATCTTCATCTCGAAGCA
TTCCAGCGAGGAGAGTGGGTATGCCA
>1278
TGACACGGAGGCCACCATGGCCGATGGCGGGGCCGAGGAAGAAGAGGACCGCAAGG
CTCGGTACCTCAAGAACATCTTCATCTCGAAGCATTCCAGCGAGGAGAGTGGGTATGC
CAGTGTGGCTGGACAGAGCCTCCCTG
>1279
GGCCGATGGCGGGGCCGAGGAAGAAGAGGACCGCAAGGCTCGGTACCTCAAGAACAT
CTTCATCTCGAAGCATTCCAGCGAGGAGAGTGGGTATGCCAGTGTGGCTGGACAGAGC
CTCCCTGGGCCCATGGTGGACCAGA
>1280
GGCCGAGGAAGAAGAGGACCGCAAGGCTCGGTACCTCAAGAACATCTTCATCTCGAA
GCATTCCAGCGAGGAGAGTGGGTATGCCAGTGTGGCTGGACAGAGCCTCCCTGGGCC
CATGGTGGACCAGAGCCCTTCAGTCT
>1281
GTACCTCAAGAACATCTTCATCTCGAAGCATTCCAGCGAGGAGAGTGGGTATGCCAGT
GTGGCTGGACAGAGCCTCCCTGGGCCCATGGTGGACCAGAGCCCTTCAGTCTCCACTT
CATTTGGTCTTTCAGCCATGAGTC
>1282
ACCTCAAGAACATCTTCATCTCGAAGCATTCCAGCGAGGAGAGTGGGTATGCCAGTGT
GGCTGGACAGAGCCTCCCTGGGCCCATGGTGGACCAGAGCCCTTCAGTCTCCACTTCA
TTTGGTCTTTCAGCCATGAGTCCC
>1283
GCATTCCAGCGAGGAGAGTGGGTATGCCAGTGTGGCTGGACAGAGCCTCCCTGGGCC
CATGGTGGACCAGAGCCCTTCAGTCTCCACTTCATTTGGTCTTTCAGCCATGAGTCCCA
CCAAGGCTGCAGTGGACAGTTTGA
>1284
GCTGGACAGAGCCTCCCTGGGCCCATGGTGGACCAGAGCCCTTCAGTCTCCACTTCAT
TTGGTCTTTCAGCCATGAGTCCCACCAAGGCTGCAGTGGACAGTTTGATGACCATAGG
ACAGTCTCTCCTGCAGGGAACTCT
>1285
AGAGCCTCCCTGGGCCCATGGTGGACCAGAGCCCTTCAGTCTCCACTTCATTTGGTCTT
TCAGCCATGAGTCCCACCAAGGCTGCAGTGGACAGTTTGATGACCATAGGACAGTCTC
TCCTGCAGGGAACTCTTCAGCCA
>1286
TGGGCCCATGGTGGACCAGAGCCCTTCAGTCTCCACTTCATTTGGTCTTTCAGCCATGA
GTCCCACCAAGGCTGCAGTGGACAGTTTGATGACCATAGGACAGTCTCTCCTGCAGGG
AACTCTTCAGCCACCTGCAGGGC
>1287
GTCTCCACTTCATTTGGTCTTTCAGCCATGAGTCCCACCAAGGCTGCAGTGGACAGTTT
GATGACCATAGGACAGTCTCTCCTGCAGGGAACTCTTCAGCCACCTGCAGGGCCCGAG
GAGCCAACTCTGGCTGGGGGTGG

>1288
TCTTTCAGCCATGAGTCCCACCAAGGCTGCAGTGGACAGTTTGATGACCATAGGACAG
TCTCTCCTGCAGGGAACTCTTCAGCCACCTGCAGGGCCCGAGGAGCCAACTCTGGCTG
GGGGTGGGCGGCACCCTGGGGTGG
>1289
GAGTCCCACCAAGGCTGCAGTGGACAGTTTGATGACCATAGGACAGTCTCTCCTGCAG
GGAACTCTTCAGCCACCTGCAGGGCCCGAGGAGCCAACTCTGGCTGGGGGTGGGCGG
CACCCTGGGGTGGCTGAGGTGAAGA
>1290
AGTGGACAGTTTGATGACCATAGGACAGTCTCTCCTGCAGGGAACTCTTCAGCCACCT
GCAGGGCCCGAGGAGCCAACTCTGGCTGGGGGTGGGCGGCACCCTGGGGTGGCTGA
GGTGAAGACGGAGATGATGCAGGTGG
>1291
TCTTCAGCCACCTGCAGGGCCCGAGGAGCCAACTCTGGCTGGGGGTGGGCGGCACCC
TGGGGTGGCTGAGGTGAAGACGGAGATGATGCAGGTGGATGAGGTGCCCAGCCAGGA
CAGCCCTGGGGCAGCCGAGTCCAGCA
>1292
GCCACCTGCAGGGCCCGAGGAGCCAACTCTGGCTGGGGGTGGGCGGCACCCTGGGGT
GGCTGAGGTGAAGACGGAGATGATGCAGGTGGATGAGGTGCCCAGCCAGGACAGCCC
TGGGGCAGCCGAGTCCAGCATCTCAG
>1293
CTGGGGGTGGGCGGCACCCTGGGGTGGCTGAGGTGAAGACGGAGATGATGCAGGTGG
ATGAGGTGCCCAGCCAGGACAGCCCTGGGGCAGCCGAGTCCAGCATCTCAGGAGGGA
TGGGGGACAAGGTTGAGGAAAGAGGC
>1294
GGGTGGGCGGCACCCTGGGGTGGCTGAGGTGAAGACGGAGATGATGCAGGTGGATGA
GGTGCCCAGCCAGGACAGCCCTGGGGCAGCCGAGTCCAGCATCTCAGGAGGGATGGG
GGACAAGGTTGAGGAAAGAGGCAAAG
>1295
GGCGGCACCCTGGGGTGGCTGAGGTGAAGACGGAGATGATGCAGGTGGATGAGGTGC
CCAGCCAGGACAGCCCTGGGGCAGCCGAGTCCAGCATCTCAGGAGGGATGGGGGACA
AGGTTGAGGAAAGAGGCAAAGAGGGG
>1296
GGCTGAGGTGAAGACGGAGATGATGCAGGTGGATGAGGTGCCCAGCCAGGACAGCCC
TGGGGCAGCCGAGTCCAGCATCTCAGGAGGGATGGGGGACAAGGTTGAGGAAAGAG
GCAAAGAGGGGCCTGGGACCCCGACTC
>1297
GGATGAGGTGCCCAGCCAGGACAGCCCTGGGGCAGCCGAGTCCAGCATCTCAGGAGG
GATGGGGGACAAGGTTGAGGAAAGAGGCAAAGAGGGGCCTGGGACCCCGACTCGAA
GCAGCGTCATCACCAGTGCTAGGGAGC
>1298
GGTGCCCAGCCAGGACAGCCCTGGGGCAGCCGAGTCCAGCATCTCAGGAGGGATGGG
GGACAAGGTTGAGGAAAGAGGCAAAGAGGGGCCTGGGACCCCGACTCGAAGCAGCG
TCATCACCAGTGCTAGGGAGCTACACT

>1299
CCAGCCAGGACAGCCCTGGGGCAGCCGAGTCCAGCATCTCAGGAGGGATGGGGGACA
AGGTTGAGGAAAGAGGCAAAGAGGGGCCTGGGACCCCGACTCGAAGCAGCGTCATC
ACCAGTGCTAGGGAGCTACACTATGGG
>1300
GACAGCCCTGGGGCAGCCGAGTCCAGCATCTCAGGAGGGATGGGGGACAAGGTTGAG
GAAAGAGGCAAAGAGGGGCCTGGGACCCCGACTCGAAGCAGCGTCATCACCAGTGCT
AGGGAGCTACACTATGGGCGAGAGGA
>1301
GCATCTCAGGAGGGATGGGGGACAAGGTTGAGGAAAGAGGCAAAGAGGGGCCTGGG
ACCCCGACTCGAAGCAGCGTCATCACCAGTGCTAGGGAGCTACACTATGGGCGAGAG
GAGAGTGCCGAGCAGGTGCCACCCCCA
>1302
ACAAGGTTGAGGAAAGAGGCAAAGAGGGGCCTGGGACCCCGACTCGAAGCAGCGTC
ATCACCAGTGCTAGGGAGCTACACTATGGGCGAGAGGAGAGTGCCGAGCAGGTGCCA
CCCCCAGCTGAGGCTGGCCAGGCCCCC
>1303
GGTTGAGGAAAGAGGCAAAGAGGGGCCTGGGACCCCGACTCGAAGCAGCGTCATCA
CCAGTGCTAGGGAGCTACACTATGGGCGAGAGGAGAGTGCCGAGCAGGTGCCACCCC
CAGCTGAGGCTGGCCAGGCCCCCACTG
>1304
AAAGAGGGGCCTGGGACCCCGACTCGAAGCAGCGTCATCACCAGTGCTAGGGAGCTA
CACTATGGGCGAGAGGAGAGTGCCGAGCAGGTGCCACCCCCAGCTGAGGCTGGCCAG
GCCCCCACTGGCCGACCTGAGCACCC
>1305
GACCCCGACTCGAAGCAGCGTCATCACCAGTGCTAGGGAGCTACACTATGGGCGAGA
GGAGAGTGCCGAGCAGGTGCCACCCCCAGCTGAGGCTGGCCAGGCCCCCACTGGCCG
ACCTGAGCACCCAGCACCCCCGCCTG
>1306
TGCTAGGGAGCTACACTATGGGCGAGAGGAGAGTGCCGAGCAGGTGCCACCCCCAGC
TGAGGCTGGCCAGGCCCCCACTGGCCGACCTGAGCACCCAGCACCCCCGCCTGAGAA
GCATCTGGGCATCTACTCCGTGTTGC
>1307
GCGAGAGGAGAGTGCCGAGCAGGTGCCACCCCCAGCTGAGGCTGGCCAGGCCCCCA
CTGGCCGACCTGAGCACCCAGCACCCCCGCCTGAGAAGCATCTGGGCATCTACTCCGT
GTTGCCCAACCACAAGGCTGACGCTG
>1308
GGTGCCACCCCCAGCTGAGGCTGGCCAGGCCCCCACTGGCCGACCTGAGCACCCAGC
ACCCCCGCCTGAGAAGCATCTGGGCATCTACTCCGTGTTGCCCAACCACAAGGCTGAC
GCTGTATTGAGCATGCCGTCTTCCG
>1309
CCGACCTGAGCACCCAGCACCCCCGCCTGAGAAGCATCTGGGCATCTACTCCGTGTTG
CCCAACCACAAGGCTGACGCTGTATTGAGCATGCCGTCTTCCGTGACCTCTGGCCTCC
ACGTGCAGCCTGCCCTGGCTGTCT

>1310
CACCCCCGCCTGAGAAGCATCTGGGCATCTACTCCGTGTTGCCCAACCACAAGGCTGA
CGCTGTATTGAGCATGCCGTCTTCCGTGACCTCTGGCCTCCACGTGCAGCCTGCCCTGG
CTGTCTCCATGGACTTCAGCACC
>1311
GTTGCCCAACCACAAGGCTGACGCTGTATTGAGCATGCCGTCTTCCGTGACCTCTGGC
CTCCACGTGCAGCCTGCCCTGGCTGTCTCCATGGACTTCAGCACCTATGGGGGGCTGC
TGCCCCAGGGCTTCATCCAGAGGG
>1312
TGAGCATGCCGTCTTCCGTGACCTCTGGCCTCCACGTGCAGCCTGCCCTGGCTGTCTCC
ATGGACTTCAGCACCTATGGGGGGCTGCTGCCCCAGGGCTTCATCCAGAGGGAGCTGT
TCAGCAAGCTGGGGGAGCTGGCT
>1313
TGGCCTCCACGTGCAGCCTGCCCTGGCTGTCTCCATGGACTTCAGCACCTATGGGGGG
CTGCTGCCCCAGGGCTTCATCCAGAGGGAGCTGTTCAGCAAGCTGGGGGAGCTGGCT
GTGGGCATGAAGTCAGAGAGCCGGA
>1314
GCAGCCTGCCCTGGCTGTCTCCATGGACTTCAGCACCTATGGGGGGCTGCTGCCCCAG
GGCTTCATCCAGAGGGAGCTGTTCAGCAAGCTGGGGGAGCTGGCTGTGGGCATGAAG
TCAGAGAGCCGGACCATCGGAGAGC
>1315
CTCCATGGACTTCAGCACCTATGGGGGGCTGCTGCCCCAGGGCTTCATCCAGAGGGAG
CTGTTCAGCAAGCTGGGGGAGCTGGCTGTGGGCATGAAGTCAGAGAGCCGGACCATC
GGAGAGCAGTGCAGCGTGTGTGGGG
>1316
GCTGCTGCCCCAGGGCTTCATCCAGAGGGAGCTGTTCAGCAAGCTGGGGGAGCTGGC
TGTGGGCATGAAGTCAGAGAGCCGGACCATCGGAGAGCAGTGCAGCGTGTGTGGGGT
CGAGCTTCCTGATAACGAGGCTGTGG
>1317
CTGCCCCAGGGCTTCATCCAGAGGGAGCTGTTCAGCAAGCTGGGGGAGCTGGCTGTG
GGCATGAAGTCAGAGAGCCGGACCATCGGAGAGCAGTGCAGCGTGTGTGGGGTCGAG
CTTCCTGATAACGAGGCTGTGGAGCA
>1318
CAGAGGGAGCTGTTCAGCAAGCTGGGGGAGCTGGCTGTGGGCATGAAGTCAGAGAGC
CGGACCATCGGAGAGCAGTGCAGCGTGTGTGGGGTCGAGCTTCCTGATAACGAGGCT
GTGGAGCAGCACAGGTAGGCCCCGCT
>1319
GGAGCTGGCTGTGGGCATGAAGTCAGAGAGCCGGACCATCGGAGAGCAGTGCAGCGT
GTGTGGGGTCGAGCTTCCTGATAACGAGGCTGTGGAGCAGCACAGGTAGGCCCCGCT
CCAGCCCCGCACCTGATGTAGGACTT
>1320
CATGAAGTCAGAGAGCCGGACCATCGGAGAGCAGTGCAGCGTGTGTGGGGTCGAGCT
TCCTGATAACGAGGCTGTGGAGCAGCACAGGTAGGCCCCGCTCCAGCCCCGCACCTGA
TGTAGGACTTGAGGCCCTCACACCC

>1321
AGAGAGCCGGACCATCGGAGAGCAGTGCAGCGTGTGTGGGGTCGAGCTTCCTGATAA
CGAGGCTGTGGAGCAGCACAGGTAGGCCCCGCTCCAGCCCCGCACCTGATGTAGGAC
TTGAGGCCCTCACACCCCTCCTTCAC
>1322
GTGCAGCGTGTGTGGGGTCGAGCTTCCTGATAACGAGGCTGTGGAGCAGCACAGGTA
GGCCCCGCTCCAGCCCCGCACCTGATGTAGGACTTGAGGCCCTCACACCCCTCCTTCA
CACCCTGGCTGCTCCCAAGTTAGGG
>1323
TCGAGCTTCCTGATAACGAGGCTGTGGAGCAGCACAGGTAGGCCCCGCTCCAGCCCCG
CACCTGATGTAGGACTTGAGGCCCTCACACCCCTCCTTCACACCCTGGCTGCTCCCAA
GTTAGGGGCCTGGCTCCCCTGTCT
>1324
CCTGATAACGAGGCTGTGGAGCAGCACAGGTAGGCCCCGCTCCAGCCCCGCACCTGAT
GTAGGACTTGAGGCCCTCACACCCCTCCTTCACACCCTGGCTGCTCCCAAGTTAGGGG
CCTGGCTCCCCTGTCTTGGCAATT
>1325
CTCCAGCCCCGCACCTGATGTAGGACTTGAGGCCCTCACACCCCTCCTTCACACCCTG
GCTGCTCCCAAGTTAGGGGCCTGGCTCCCCTGTCTTGGCAATTTGTGAAAAAACCAGA
ACACTTCTTCTAAAGTTCTGGCGG
>1326
CCTTCACACCCTGGCTGCTCCCAAGTTAGGGGCCTGGCTCCCCTGTCTTGGCAATTTGT
GAAAAAACCAGAACACTTCTTCTAAAGTTCTGGCGGGGAGGGGAGCAGGTTTTTTAA
AGTGTAGTGAGGGGGCCTAGGATC
>1327
GCTCCCAAGTTAGGGGCCTGGCTCCCCTGTCTTGGCAATTTGTGAAAAAACCAGAACA
CTTCTTCTAAAGTTCTGGCGGGGAGGGGAGCAGGTTTTTTAAAGTGTAGTGAGGGGGC
CTAGGATCTTTCCAAAAAGCACCA
>1328
GGGCCTGGCTCCCCTGTCTTGGCAATTTGTGAAAAAACCAGAACACTTCTTCTAAAGT
TCTGGCGGGGAGGGGAGCAGGTTTTTTAAAGTGTAGTGAGGGGGCCTAGGATCTTTCC
AAAAAGCACCAGGGGACACTCATG
>1329
TGGCTCCCCTGTCTTGGCAATTTGTGAAAAAACCAGAACACTTCTTCTAAAGTTCTGG
CGGGGAGGGGAGCAGGTTTTTTAAAGTGTAGTGAGGGGGCCTAGGATCTTTCCAAAA
AGCACCAGGGGACACTCATGGGCGC
>1330
TGGCAATTTGTGAAAAAACCAGAACACTTCTTCTAAAGTTCTGGCGGGGAGGGGAGC
AGGTTTTTTAAAGTGTAGTGAGGGGGCCTAGGATCTTTCCAAAAAGCACCAGGGGACA
CTCATGGGCGCAGCTTCTTAATAGG
>1331
TCTGGCGGGGAGGGGAGCAGGTTTTTTAAAGTGTAGTGAGGGGGCCTAGGATCTTTCC
AAAAAGCACCAGGGGACACTCATGGGCGCAGCTTCTTAATAGGCCCTTCCCTTTGTTT
TTTGCTTTTGGGCCCCTGTTTGTT

>1332
GGTTTTTTAAAGTGTAGTGAGGGGGCCTAGGATCTTTCCAAAAAGCACCAGGGGACAC
TCATGGGCGCAGCTTCTTAATAGGCCCTTCCCTTTGTTTTTTGCTTTTGGGCCCCTGTTT
GTTTTTTCTGCTGTTTGGTCTG
>1333
GTGTAGTGAGGGGGCCTAGGATCTTTCCAAAAAGCACCAGGGGACACTCATGGGCGC
AGCTTCTTAATAGGCCCTTCCCTTTGTTTTTTGCTTTTGGGCCCCTGTTTGTTTTTTCTGC
TGTTTGGTCTGTTCTCCTTTGC
>1334
CCCTTTGTTTTTTGCTTTTGGGCCCCTGTTTGTTTTTTCTGCTGTTTGGTCTGTTCTCCTT
TGCTTGGAGTGACTGATAAAATGGAGAGAAGGAGAAAAGGAGTGGGATGAGGGTCTC
TTGGGCCCTGCATCTGACCATG
>1335
TTGGGCCCCTGTTTGTTTTTTCTGCTGTTTGGTCTGTTCTCCTTTGCTTGGAGTGACTGA
TAAAATGGAGAGAAGGAGAAAAGGAGTGGGATGAGGGTCTCTTGGGCCCTGCATCTG
ACCATGTTACTAGGTTTCAGGTC
>1336
GGGCCCCTGTTTGTTTTTTCTGCTGTTTGGTCTGTTCTCCTTTGCTTGGAGTGACTGATA
AAATGGAGAGAAGGAGAAAAGGAGTGGGATGAGGGTCTCTTGGGCCCTGCATCTGAC
CATGTTACTAGGTTTCAGGTCCT
>1337
TGTTTTTTCTGCTGTTTGGTCTGTTCTCCTTTGCTTGGAGTGACTGATAAAATGGAGAG
AAGGAGAAAAGGAGTGGGATGAGGGTCTCTTGGGCCCTGCATCTGACCATGTTACTAG
GTTTCAGGTCCTCGGGTTCCCAT
>1338
TAAAATGGAGAGAAGGAGAAAAGGAGTGGGATGAGGGTCTCTTGGGCCCTGCATCTG
ACCATGTTACTAGGTTTCAGGTCCTCGGGTTCCCATGCAGTGGTCCCTGTGAAGACAG
CCTGTCCCTTTCTGCAGCTTGGGAG
>1339
GAGAGAAGGAGAAAAGGAGTGGGATGAGGGTCTCTTGGGCCCTGCATCTGACCATGT
TACTAGGTTTCAGGTCCTCGGGTTCCCATGCAGTGGTCCCTGTGAAGACAGCCTGTCC
CTTTCTGCAGCTTGGGAGAATGGGA
>1340
GGGATGAGGGTCTCTTGGGCCCTGCATCTGACCATGTTACTAGGTTTCAGGTCCTCGGG
TTCCCATGCAGTGGTCCCTGTGAAGACAGCCTGTCCCTTTCTGCAGCTTGGGAGAATG
GGAGGGTCTGGGGGCTGCTTAAG
>1341
GTTACTAGGTTTCAGGTCCTCGGGTTCCCATGCAGTGGTCCCTGTGAAGACAGCCTGT
CCCTTTCTGCAGCTTGGGAGAATGGGAGGGTCTGGGGGCTGCTTAAGCCACAAGAGC
AGCTGCAGGTTCTGAACCTTATTCC
>1342
GTTTCAGGTCCTCGGGTTCCCATGCAGTGGTCCCTGTGAAGACAGCCTGTCCCTTTCTG
CAGCTTGGGAGAATGGGAGGGTCTGGGGGCTGCTTAAGCCACAAGAGCAGCTGCAGG
TTCTGAACCTTATTCCTGGTGAAA

>1343
TGGTCCCTGTGAAGACAGCCTGTCCCTTTCTGCAGCTTGGGAGAATGGGAGGGTCTGG
GGGCTGCTTAAGCCACAAGAGCAGCTGCAGGTTCTGAACCTTATTCCTGGTGAAAGTT
AATCTCCTTGCTCTGTCTCCCTTC
>1344
CCTGTCCCTTTCTGCAGCTTGGGAGAATGGGAGGGTCTGGGGGCTGCTTAAGCCACAA
GAGCAGCTGCAGGTTCTGAACCTTATTCCTGGTGAAAGTTAATCTCCTTGCTCTGTCTC
CCTTCTCATTCTCTGATCCTGCA
>1345
ATGGGAGGGTCTGGGGGCTGCTTAAGCCACAAGAGCAGCTGCAGGTTCTGAACCTTAT
TCCTGGTGAAAGTTAATCTCCTTGCTCTGTCTCCCTTCTCATTCTCTGATCCTGCACATG
CATTTAGGGGGAATTGATTTAA
>1346
TATTCCTGGTGAAAGTTAATCTCCTTGCTCTGTCTCCCTTCTCATTCTCTGATCCTGCAC
ATGCATTTAGGGGGAATTGATTTAAATGCAAGGGGAAAGGATGGGTATAAGAGTCTTG
GTCTTGCAGCCTGTGAAGTCTC
>1347
CCTTGCTCTGTCTCCCTTCTCATTCTCTGATCCTGCACATGCATTTAGGGGGAATTGATT
TAAATGCAAGGGGAAAGGATGGGTATAAGAGTCTTGGTCTTGCAGCCTGTGAAGTCTC
ATTCTTATCCCATCTCTGCTCA
>1348
TCATTCTCTGATCCTGCACATGCATTTAGGGGGAATTGATTTAAATGCAAGGGGAAAGG
ATGGGTATAAGAGTCTTGGTCTTGCAGCCTGTGAAGTCTCATTCTTATCCCATCTCTGCT
CACACTGAACTAAATGCCGCT
>1349
ATTCTCTGATCCTGCACATGCATTTAGGGGGAATTGATTTAAATGCAAGGGGAAAGGAT
GGGTATAAGAGTCTTGGTCTTGCAGCCTGTGAAGTCTCATTCTTATCCCATCTCTGCTCA
CACTGAACTAAATGCCGCTTC
>1350
CACATGCATTTAGGGGGAATTGATTTAAATGCAAGGGGAAAGGATGGGTATAAGAGTC
TTGGTCTTGCAGCCTGTGAAGTCTCATTCTTATCCCATCTCTGCTCACACTGAACTAAAT
GCCGCTTCAGGCTAGCATTGGC
>1351
TTGGTCTTGCAGCCTGTGAAGTCTCATTCTTATCCCATCTCTGCTCACACTGAACTAAAT
GCCGCTTCAGGCTAGCATTGGCTTCCCTCCGGCTTCCGTTTCCTTCTCTATGAAATAACC
ATTCATTGAGCTCTTATGAC
>1352
CTTGCAGCCTGTGAAGTCTCATTCTTATCCCATCTCTGCTCACACTGAACTAAATGCCG
CTTCAGGCTAGCATTGGCTTCCCTCCGGCTTCCGTTTCCTTCTCTATGAAATAACCATTC
ATTGAGCTCTTATGACATGTT
>1353
GCCGCTTCAGGCTAGCATTGGCTTCCCTCCGGCTTCCGTTTCCTTCTCTATGAAATAACC
ATTCATTGAGCTCTTATGACATGTTTGGTTCAGTACTAAGCACTTTCCATGAGTTGCATT
TTCATTACAACAGCCCTTTG

>1354
TCCCTCCGGCTTCCGTTTCCTTCTCTATGAAATAACCATTCATTGAGCTCTTATGACATG
TTTGGTTCAGTACTAAGCACTTTCCATGAGTTGCATTTTCATTACAACAGCCCTTTGAGA
TAGGTAATGTCCTTCTTTTT
>1355
TTCTCTATGAAATAACCATTCATTGAGCTCTTATGACATGTTTGGTTCAGTACTAAGCAC
TTTCCATGAGTTGCATTTTCATTACAACAGCCCTTTGAGATAGGTAATGTCCTTCTTTTT
TTTTTGGATGTGAAGTTTCG
>1356
CATTGAGCTCTTATGACATGTTTGGTTCAGTACTAAGCACTTTCCATGAGTTGCATTTTC
ATTACAACAGCCCTTTGAGATAGGTAATGTCCTTCTTTTTTTTTTGGATGTGAAGTTTCG
CTCTCCTTGCCCAGGCTGGA
>1357
CTTATGACATGTTTGGTTCAGTACTAAGCACTTTCCATGAGTTGCATTTTCATTACAACA
GCCCTTTGAGATAGGTAATGTCCTTCTTTTTTTTTTGGATGTGAAGTTTCGCTCTCCTTG
CCCAGGCTGGAGTGCAATGG
>1358
ATGAGTTGCATTTTCATTACAACAGCCCTTTGAGATAGGTAATGTCCTTCTTTTTTTTTT
GGATGTGAAGTTTCGCTCTCCTTGCCCAGGCTGGAGTGCAATGGTGTGATCTCTGCTCA
CCGCAACCTCTGCATCCCGGG
>1359
CAACAGCCCTTTGAGATAGGTAATGTCCTTCTTTTTTTTTTGGATGTGAAGTTTCGCTCT
CCTTGCCCAGGCTGGAGTGCAATGGTGTGATCTCTGCTCACCGCAACCTCTGCATCCC
GGGTTAAGGTGATTCTCCTGCC
>1360
CTCTCCTTGCCCAGGCTGGAGTGCAATGGTGTGATCTCTGCTCACCGCAACCTCTGCAT
CCCGGGTTAAGGTGATTCTCCTGCCTCAGCCTCCCGAGTAGCTCGGATTACAGGCACG
CACCACCATACCTGGCTAATTTT
>1361
GAGTGCAATGGTGTGATCTCTGCTCACCGCAACCTCTGCATCCCGGGTTAAGGTGATTC
TCCTGCCTCAGCCTCCCGAGTAGCTCGGATTACAGGCACGCACCACCATACCTGGCTAA
TTTTGAATTTTTAGTAGAGACG
>1362
CACCGCAACCTCTGCATCCCGGGTTAAGGTGATTCTCCTGCCTCAGCCTCCCGAGTAGC
TCGGATTACAGGCACGCACCACCATACCTGGCTAATTTTGAATTTTTAGTAGAGACGGG
CTTTCACCGTGTTGGTCAGGAT
>1363
TGCCTCAGCCTCCCGAGTAGCTCGGATTACAGGCACGCACCACCATACCTGGCTAATTT
TGAATTTTTAGTAGAGACGGGCTTTCACCGTGTTGGTCAGGATGGTCTCGAACTCCCG
ACCTCAGGTGATCGGCCTGCTTC
>1364
CAGGCACGCACCACCATACCTGGCTAATTTTGAATTTTTAGTAGAGACGGGCTTTCACC
GTGTTGGTCAGGATGGTCTCGAACTCCCGACCTCAGGTGATCGGCCTGCTTCGGCCTC
CCAAAGTGCTGGATTACAGGTAT

>1365
AATTTTGAATTTTTAGTAGAGACGGGCTTTCACCGTGTTGGTCAGGATGGTCTCGAACT
CCCGACCTCAGGTGATCGGCCTGCTTCGGCCTCCCAAAGTGCTGGATTACAGGTATGA
GCCACCGTGTCCAGCTGGTAATG
>1366
TTCACCGTGTTGGTCAGGATGGTCTCGAACTCCCGACCTCAGGTGATCGGCCTGCTTC
GGCCTCCCAAAGTGCTGGATTACAGGTATGAGCCACCGTGTCCAGCTGGTAATGTCCTT
CTTTTACATGGAACAATACTAAG
>1367
TCAGGATGGTCTCGAACTCCCGACCTCAGGTGATCGGCCTGCTTCGGCCTCCCAAAGT
GCTGGATTACAGGTATGAGCCACCGTGTCCAGCTGGTAATGTCCTTCTTTTACATGGAA
CAATACTAAGGCTCAGATGTCAA
>1368
GGTCTCGAACTCCCGACCTCAGGTGATCGGCCTGCTTCGGCCTCCCAAAGTGCTGGAT
TACAGGTATGAGCCACCGTGTCCAGCTGGTAATGTCCTTCTTTTACATGGAACAATACT
AAGGCTCAGATGTCAAGTCTCTT
>1369
TGCTGGATTACAGGTATGAGCCACCGTGTCCAGCTGGTAATGTCCTTCTTTTACATGGA
ACAATACTAAGGCTCAGATGTCAAGTCTCTTTTCTAGGGTCACATAGATAGTAAGAGGC
AGAGCTGGATTCAAATTTAGGT
>1370
ATTACAGGTATGAGCCACCGTGTCCAGCTGGTAATGTCCTTCTTTTACATGGAACAATA
CTAAGGCTCAGATGTCAAGTCTCTTTTCTAGGGTCACATAGATAGTAAGAGGCAGAGCT
GGATTCAAATTTAGGTCCTTAC
>1371
TGTCCAGCTGGTAATGTCCTTCTTTTACATGGAACAATACTAAGGCTCAGATGTCAAGT
CTCTTTTCTAGGGTCACATAGATAGTAAGAGGCAGAGCTGGATTCAAATTTAGGTCCTT
ACCCCCTGGTACTTTGCTGCCT
>1372
TCTTTTACATGGAACAATACTAAGGCTCAGATGTCAAGTCTCTTTTCTAGGGTCACATA
GATAGTAAGAGGCAGAGCTGGATTCAAATTTAGGTCCTTACCCCCTGGTACTTTGCTGC
CTCTTGGAGTCACTTAGACTTG
>1373
TTACATGGAACAATACTAAGGCTCAGATGTCAAGTCTCTTTTCTAGGGTCACATAGATA
GTAAGAGGCAGAGCTGGATTCAAATTTAGGTCCTTACCCCCTGGTACTTTGCTGCCTCT
TGGAGTCACTTAGACTTGAAGA
>1374
ACATGGAACAATACTAAGGCTCAGATGTCAAGTCTCTTTTCTAGGGTCACATAGATAGT
AAGAGGCAGAGCTGGATTCAAATTTAGGTCCTTACCCCCTGGTACTTTGCTGCCTCTTG
GAGTCACTTAGACTTGAAGAGG
>1375
AGGCTCAGATGTCAAGTCTCTTTTCTAGGGTCACATAGATAGTAAGAGGCAGAGCTGG
ATTCAAATTTAGGTCCTTACCCCCTGGTACTTTGCTGCCTCTTGGAGTCACTTAGACTTG
AAGAGGATCATGTCTATGGAAC

>1376
TAGATAGTAAGAGGCAGAGCTGGATTCAAATTTAGGTCCTTACCCCCTGGTACTTTGCT
GCCTCTTGGAGTCACTTAGACTTGAAGAGGATCATGTCTATGGAACAGAGCGTTTTGA
AGATTGCTTATAGGAAGTGGAGT
>1377
AAGAGGCAGAGCTGGATTCAAATTTAGGTCCTTACCCCCTGGTACTTTGCTGCCTCTTG
GAGTCACTTAGACTTGAAGAGGATCATGTCTATGGAACAGAGCGTTTTGAAGATTGCTT
ATAGGAAGTGGAGTAAATAACA
>1378
GAGCTGGATTCAAATTTAGGTCCTTACCCCCTGGTACTTTGCTGCCTCTTGGAGTCACT
TAGACTTGAAGAGGATCATGTCTATGGAACAGAGCGTTTTGAAGATTGCTTATAGGAAG
TGGAGTAAATAACAGGGGGGAT
>1379
CCTTACCCCCTGGTACTTTGCTGCCTCTTGGAGTCACTTAGACTTGAAGAGGATCATGT
CTATGGAACAGAGCGTTTTGAAGATTGCTTATAGGAAGTGGAGTAAATAACAGGGGGG
ATAAGTAGCGAGTTTGTTTTTTT
>1380
CCTCTTGGAGTCACTTAGACTTGAAGAGGATCATGTCTATGGAACAGAGCGTTTTGAA
GATTGCTTATAGGAAGTGGAGTAAATAACAGGGGGGATAAGTAGCGAGTTTGTTTTTTT
TTTCCTTGCTTCTGTCATAAACC
>1381
TTGGAGTCACTTAGACTTGAAGAGGATCATGTCTATGGAACAGAGCGTTTTGAAGATT
GCTTATAGGAAGTGGAGTAAATAACAGGGGGGATAAGTAGCGAGTTTGTTTTTTTTTTC
CTTGCTTCTGTCATAAACCACGT
>1382
GTCACTTAGACTTGAAGAGGATCATGTCTATGGAACAGAGCGTTTTGAAGATTGCTTAT
AGGAAGTGGAGTAAATAACAGGGGGGATAAGTAGCGAGTTTGTTTTTTTTTTCCTTGCT
TCTGTCATAAACCACGTGCCCA
>1383
CTTGAAGAGGATCATGTCTATGGAACAGAGCGTTTTGAAGATTGCTTATAGGAAGTGG
AGTAAATAACAGGGGGGATAAGTAGCGAGTTTGTTTTTTTTTTCCTTGCTTCTGTCATAA
ACCACGTGCCCATCATTTTATC
>1384
ATCATGTCTATGGAACAGAGCGTTTTGAAGATTGCTTATAGGAAGTGGAGTAAATAACA
GGGGGGATAAGTAGCGAGTTTGTTTTTTTTTTCCTTGCTTCTGTCATAAACCACGTGCC
CATCATTTTATCCTCTAGTTTC
>1385
TAAGTAGCGAGTTTGTTTTTTTTTTCCTTGCTTCTGTCATAAACCACGTGCCCATCATTT
TATCCTCTAGTTTCTGGGTCAGATGAATGGAGTCAAGAAATCCAGCTTTCTGTTGTCAT
TGACGACAGCATTTGGCTGCA
>1386
TTGTTTTTTTTTTCCTTGCTTCTGTCATAAACCACGTGCCCATCATTTTATCCTCTAGTTT
CTGGGTCAGATGAATGGAGTCAAGAAATCCAGCTTTCTGTTGTCATTGACGACAGCAT
TTGGCTGCATACCCTGTGGTC

>1387
GTCATAAACCACGTGCCCATCATTTTATCCTCTAGTTTCTGGGTCAGATGAATGGAGTCA
AGAAATCCAGCTTTCTGTTGTCATTGACGACAGCATTTGGCTGCATACCCTGTGGTCTG
CTGTTTTGCTGATCTTGGGTG
>1388
TTTATCCTCTAGTTTCTGGGTCAGATGAATGGAGTCAAGAAATCCAGCTTTCTGTTGTC
ATTGACGACAGCATTTGGCTGCATACCCTGTGGTCTGCTGTTTTGCTGATCTTGGGTGG
ATTCTCCACTTCCTCCCCCTTC
>1389
TGTTTTGCTGATCTTGGGTGGATTCTCCACTTCCTCCCCCTTCACCCCCATTCCCCTGTT
GCTCGTTCAGGTACGGGGGATGTGAGTGAGAGGGGTTGGGGTGGGGATATCTGGAAG
TTGATCTCATCACCCCTAGCCGG
>1390
GGTGGATTCTCCACTTCCTCCCCCTTCACCCCCATTCCCCTGTTGCTCGTTCAGGTACG
GGGGATGTGAGTGAGAGGGGTTGGGGTGGGGATATCTGGAAGTTGATCTCATCACCCC
TAGCCGGAATCTGCTCTGACCCT
>1391
CCATTCCCCTGTTGCTCGTTCAGGTACGGGGGATGTGAGTGAGAGGGGTTGGGGTGGG
GATATCTGGAAGTTGATCTCATCACCCCTAGCCGGAATCTGCTCTGACCCTGCCTCCTC
GACAGCTCAGAGGCTTAACTTGT
>1392
TCAGGTACGGGGGATGTGAGTGAGAGGGGTTGGGGTGGGGATATCTGGAAGTTGATCT
CATCACCCCTAGCCGGAATCTGCTCTGACCCTGCCTCCTCGACAGCTCAGAGGCTTAA
CTTGTGGAAAGTGTGGCAGAGCTA
>1393
GGTGGGGATATCTGGAAGTTGATCTCATCACCCCTAGCCGGAATCTGCTCTGACCCTGC
CTCCTCGACAGCTCAGAGGCTTAACTTGTGGAAAGTGTGGCAGAGCTAAACGTAGCCG
ACTTTTTCCAGTGGGTGCGAAAA
>1394
TCATCACCCCTAGCCGGAATCTGCTCTGACCCTGCCTCCTCGACAGCTCAGAGGCTTAA
CTTGTGGAAAGTGTGGCAGAGCTAAACGTAGCCGACTTTTTCCAGTGGGTGCGAAAAA
TAACCGCCACTAAGGTGTGTCTT
>1395
CCTAGCCGGAATCTGCTCTGACCCTGCCTCCTCGACAGCTCAGAGGCTTAACTTGTGG
AAAGTGTGGCAGAGCTAAACGTAGCCGACTTTTTCCAGTGGGTGCGAAAAATAACCGC
CACTAAGGTGTGTCTTCTTGATGC
>1396
CTGCTCTGACCCTGCCTCCTCGACAGCTCAGAGGCTTAACTTGTGGAAAGTGTGGCAG
AGCTAAACGTAGCCGACTTTTTCCAGTGGGTGCGAAAAATAACCGCCACTAAGGTGTG
TCTTCTTGATGCTTGTACCGCTCA
>1397
TGTGGAAAGTGTGGCAGAGCTAAACGTAGCCGACTTTTTCCAGTGGGTGCGAAAAATA
ACCGCCACTAAGGTGTGTCTTCTTGATGCTTGTACCGCTCAGAAATAGACTGAGGTTAT
GTTTGTTTTTGCTCAAACTTTCC

>1398
CGACTTTTTCCAGTGGGTGCGAAAAATAACCGCCACTAAGGTGTGTCTTCTTGATGCTT
GTACCGCTCAGAAATAGACTGAGGTTATGTTTGTTTTTGCTCAAACTTTCCTAAAGAGG
AAACCCGCTCCCCAAAGTTGAC
>1399
TCTTCTTGATGCTTGTACCGCTCAGAAATAGACTGAGGTTATGTTTGTTTTTGCTCAAAC
TTTCCTAAAGAGGAAACCCGCTCCCCAAAGTTGACCAGATAGATGTATCATCTTTCCGG
ATGTGAATTATTTCGGCTGTG
>1400
TTCTTGATGCTTGTACCGCTCAGAAATAGACTGAGGTTATGTTTGTTTTTGCTCAAACTT
TCCTAAAGAGGAAACCCGCTCCCCAAAGTTGACCAGATAGATGTATCATCTTTCCGGAT
GTGAATTATTTCGGCTGTGTG
>1401
CTGAGGTTATGTTTGTTTTTGCTCAAACTTTCCTAAAGAGGAAACCCGCTCCCCAAAGT
TGACCAGATAGATGTATCATCTTTCCGGATGTGAATTATTTCGGCTGTGTGCTATGAGAA
CTGGGTATGGTGGGAAGTAAT
>1402
GCTCCCCAAAGTTGACCAGATAGATGTATCATCTTTCCGGATGTGAATTATTTCGGCTGT
GTGCTATGAGAACTGGGTATGGTGGGAAGTAATTGTTTTTCTGGCTCTCATCAGGTCCT
GGTTCCTGGTTGCTTTTCTTT
>1403
ATAGATGTATCATCTTTCCGGATGTGAATTATTTCGGCTGTGTGCTATGAGAACTGGGTA
TGGTGGGAAGTAATTGTTTTTCTGGCTCTCATCAGGTCCTGGTTCCTGGTTGCTTTTCTT
TACCCGAGAGGGCCACAAAA
>1404
GAATTATTTCGGCTGTGTGCTATGAGAACTGGGTATGGTGGGAAGTAATTGTTTTTCTG
GCTCTCATCAGGTCCTGGTTCCTGGTTGCTTTTCTTTACCCGAGAGGGCCACAAAAGAT
ACCACTCTCCTGAGACTTCCAA
>1405
TATGGTGGGAAGTAATTGTTTTTCTGGCTCTCATCAGGTCCTGGTTCCTGGTTGCTTTTC
TTTACCCGAGAGGGCCACAAAAGATACCACTCTCCTGAGACTTCCAAGTTCCCCTTCA
GCAAGACCCTCATTCCTCTGGG
>1406
TGGTGGGAAGTAATTGTTTTTCTGGCTCTCATCAGGTCCTGGTTCCTGGTTGCTTTTCTT
TACCCGAGAGGGCCACAAAAGATACCACTCTCCTGAGACTTCCAAGTTCCCCTTCAGC
AAGACCCTCATTCCTCTGGGGA
>1407
AATTGTTTTTCTGGCTCTCATCAGGTCCTGGTTCCTGGTTGCTTTTCTTTACCCGAGAGG
GCCACAAAAGATACCACTCTCCTGAGACTTCCAAGTTCCCCTTCAGCAAGACCCTCAT
TCCTCTGGGGAGAGAGGGAGAG
>1408
CTCATCAGGTCCTGGTTCCTGGTTGCTTTTCTTTACCCGAGAGGGCCACAAAAGATACC
ACTCTCCTGAGACTTCCAAGTTCCCCTTCAGCAAGACCCTCATTCCTCTGGGGAGAGA
GGGAGAGTTGGCCATGTTATATT

>1409
GCCACAAAAGATACCACTCTCCTGAGACTTCCAAGTTCCCCTTCAGCAAGACCCTCAT
TCCTCTGGGGAGAGAGGGAGAGTTGGCCATGTTATATTCGAAAGAGCCTGGGTATTGG
AATTGGCCATATTTAGTGGTGAGA
>1410
CACAAAAGATACCACTCTCCTGAGACTTCCAAGTTCCCCTTCAGCAAGACCCTCATTC
CTCTGGGGAGAGAGGGAGAGTTGGCCATGTTATATTCGAAAGAGCCTGGGTATTGGAA
TTGGCCATATTTAGTGGTGAGAGC
>1411
AAGTTCCCCTTCAGCAAGACCCTCATTCCTCTGGGGAGAGAGGGAGAGTTGGCCATGT
TATATTCGAAAGAGCCTGGGTATTGGAATTGGCCATATTTAGTGGTGAGAGCCTGTGAT
ACTTATTTAACCTTTCTGGGCCT
>1412
CTGGGGAGAGAGGGAGAGTTGGCCATGTTATATTCGAAAGAGCCTGGGTATTGGAATT
GGCCATATTTAGTGGTGAGAGCCTGTGATACTTATTTAACCTTTCTGGGCCTTGTTTCAC
TCACTACATTTTTTTTTTTTTT
>1413
GAGAGGGAGAGTTGGCCATGTTATATTCGAAAGAGCCTGGGTATTGGAATTGGCCATAT
TTAGTGGTGAGAGCCTGTGATACTTATTTAACCTTTCTGGGCCTTGTTTCACTCACTACA
TTTTTTTTTTTTTTTTTTTTT
>1414
CTGTGATACTTATTTAACCTTTCTGGGCCTTGTTTCACTCACTACATTTTTTTTTTTTTTT
TTTTTTGAGACGGAGTCTCGCTCTGTCACCGGGCTGGAGTGCAATGGCGTGATCTCAG
CTCACTGCAGTCTCCACCTCC
>1415
TACTTATTTAACCTTTCTGGGCCTTGTTTCACTCACTACATTTTTTTTTTTTTTTTTTTTTG
AGACGGAGTCTCGCTCTGTCACCGGGCTGGAGTGCAATGGCGTGATCTCAGCTCACTG
CAGTCTCCACCTCCCGGGTT
>1416
TGTTTCACTCACTACATTTTTTTTTTTTTTTTTTTTTGAGACGGAGTCTCGCTCTGTCAC
CGGGCTGGAGTGCAATGGCGTGATCTCAGCTCACTGCAGTCTCCACCTCCCGGGTTCA
AGCGATTCTCCTGCCTCAGCCT
>1417
TTTTTTTTTTTTTTTTTTGAGACGGAGTCTCGCTCTGTCACCGGGCTGGAGTGCAATGG
CGTGATCTCAGCTCACTGCAGTCTCCACCTCCCGGGTTCAAGCGATTCTCCTGCCTCAG
CCTCCTGAGTAGCTGGGACTAC
>1418
TTTTTTTTGAGACGGAGTCTCGCTCTGTCACCGGGCTGGAGTGCAATGGCGTGATCTC
AGCTCACTGCAGTCTCCACCTCCCGGGTTCAAGCGATTCTCCTGCCTCAGCCTCCTGA
GTAGCTGGGACTACAGGCACCTGT
>1419
GAGTGCAATGGCGTGATCTCAGCTCACTGCAGTCTCCACCTCCCGGGTTCAAGCGATT
CTCCTGCCTCAGCCTCCTGAGTAGCTGGGACTACAGGCACCTGTCACCCCGCCCAGCT
AATTTTTGTATTTTTAGTAGAGA

>1420
GGCGTGATCTCAGCTCACTGCAGTCTCCACCTCCCGGGTTCAAGCGATTCTCCTGCCTC
AGCCTCCTGAGTAGCTGGGACTACAGGCACCTGTCACCCCGCCCAGCTAATTTTTGTA
TTTTTAGTAGAGACAGGGTTTC
>1421
GGGTTCAAGCGATTCTCCTGCCTCAGCCTCCTGAGTAGCTGGGACTACAGGCACCTGT
CACCCCGCCCAGCTAATTTTTGTATTTTTAGTAGAGACAGGGTTTCACCATGTTGGCC
AGAATGGTCTCGATCTCTTGACC
>1422
CCTCAGCCTCCTGAGTAGCTGGGACTACAGGCACCTGTCACCCCGCCCAGCTAATTTTT
TGTATTTTTAGTAGAGACAGGGTTTCACCATGTTGGCCAGAATGGTCTCGATCTCTTGA
CCTTGCGATCCATCCACCTCGG
>1423
CAGCCTCCTGAGTAGCTGGGACTACAGGCACCTGTCACCCCGCCCAGCTAATTTTTGT
ATTTTTAGTAGAGACAGGGTTTCACCATGTTGGCCAGAATGGTCTCGATCTCTTGACCT
TGCGATCCATCCACCTCGGCCT
>1424
GGCACCTGTCACCCCGCCCAGCTAATTTTTGTATTTTTAGTAGAGACAGGGTTTCACC
ATGTTGGCCAGAATGGTCTCGATCTCTTGACCTTGCGATCCATCCACCTCGGCCTCCCA
AAGTGCTGGGATTACAGCTGTG
>1425
GTTTCACCATGTTGGCCAGAATGGTCTCGATCTCTTGACCTTGCGATCCATCCACCTCG
GCCTCCCAAAGTGCTGGGATTACAGCTGTGAGCCACCCCGCCCGGCCTTCACTCACTT
CTTAAAATGGAGACGGTCAACAG
>1426
GCCAGAATGGTCTCGATCTCTTGACCTTGCGATCCATCCACCTCGGCCTCCCAAAGTGC
TGGGATTACAGCTGTGAGCCACCCCGCCCGGCCTTCACTCACTTCTTAAAATGGAGAC
GGTCAACAGGGTTGGTGCTGAAT
>1427
GCCTCCCAAAGTGCTGGGATTACAGCTGTGAGCCACCCCGCCCGGCCTTCACTCACTT
CTTAAAATGGAGACGGTCAACAGGGTTGGTGCTGAATTAATGAAGTAAGATCCAGAAA
TCACTAGAGCTGTGATGGGCACAT
>1428
TGCTGGGATTACAGCTGTGAGCCACCCCGCCCGGCCTTCACTCACTTCTTAAAATGGA
GACGGTCAACAGGGTTGGTGCTGAATTAATGAAGTAAGATCCAGAAATCACTAGAGCT
GTGATGGGCACATAGTCACCGCAG
>1429
CACCCCGCCCGGCCTTCACTCACTTCTTAAAATGGAGACGGTCAACAGGGTTGGTGCT
GAATTAATGAAGTAAGATCCAGAAATCACTAGAGCTGTGATGGGCACATAGTCACCGC
AGCGGTGGAGAACTGACCATCTGT
>1430
CCGCCCGGCCTTCACTCACTTCTTAAAATGGAGACGGTCAACAGGGTTGGTGCTGAAT
TAATGAAGTAAGATCCAGAAATCACTAGAGCTGTGATGGGCACATAGTCACCGCAGCG
GTGGAGAACTGACCATCTGTCCCT

>1431
CACTTCTTAAAATGGAGACGGTCAACAGGGTTGGTGCTGAATTAATGAAGTAAGATCC
AGAAATCACTAGAGCTGTGATGGGCACATAGTCACCGCAGCGGTGGAGAACTGACCAT
CTGTCCCTTGTGTTGTTTAGGCCT
>1432
GGTTGGTGCTGAATTAATGAAGTAAGATCCAGAAATCACTAGAGCTGTGATGGGCACAT
AGTCACCGCAGCGGTGGAGAACTGACCATCTGTCCCTTGTGTTGTTTAGGCCTCATCTC
TGCATGGTCCTCATTGGCTCTG
>1433
CTGAATTAATGAAGTAAGATCCAGAAATCACTAGAGCTGTGATGGGCACATAGTCACC
GCAGCGGTGGAGAACTGACCATCTGTCCCTTGTGTTGTTTAGGCCTCATCTCTGCATGG
TCCTCATTGGCTCTGAGTTCTAG
>1434
CTAGAGCTGTGATGGGCACATAGTCACCGCAGCGGTGGAGAACTGACCATCTGTCCCT
TGTGTTGTTTAGGCCTCATCTCTGCATGGTCCTCATTGGCTCTGAGTTCTAGTGTGTAGT
GTCCACAGTGCCACCCTCCATG
>1435
GTGGAGAACTGACCATCTGTCCCTTGTGTTGTTTAGGCCTCATCTCTGCATGGTCCTCA
TTGGCTCTGAGTTCTAGTGTGTAGTGTCCACAGTGCCACCCTCCATGAGGGTTGTGTTG
ACTGTGAGGTGGTGCAGAGAAT
>1436
AACTGACCATCTGTCCCTTGTGTTGTTTAGGCCTCATCTCTGCATGGTCCTCATTGGCTC
TGAGTTCTAGTGTGTAGTGTCCACAGTGCCACCCTCCATGAGGGTTGTGTTGACTGTG
AGGTGGTGCAGAGAATGTCCCT
>1437
CTTGTGTTGTTTAGGCCTCATCTCTGCATGGTCCTCATTGGCTCTGAGTTCTAGTGTGTA
GTGTCCACAGTGCCACCCTCCATGAGGGTTGTGTTGACTGTGAGGTGGTGCAGAGAAT
GTCCCTGTCCTTGTGAGCATAT
>1438
TCATTGGCTCTGAGTTCTAGTGTGTAGTGTCCACAGTGCCACCCTCCATGAGGGTTGTG
TTGACTGTGAGGTGGTGCAGAGAATGTCCCTGTCCTTGTGAGCATATGACAGCAAGAC
ACTGGCCCTGGAATCCTCCGTGT
>1439
CTGAGTTCTAGTGTGTAGTGTCCACAGTGCCACCCTCCATGAGGGTTGTGTTGACTGTG
AGGTGGTGCAGAGAATGTCCCTGTCCTTGTGAGCATATGACAGCAAGACACTGGCCCT
GGAATCCTCCGTGTCACATGTGG
>1440
CACAGTGCCACCCTCCATGAGGGTTGTGTTGACTGTGAGGTGGTGCAGAGAATGTCCC
TGTCCTTGTGAGCATATGACAGCAAGACACTGGCCCTGGAATCCTCCGTGTCACATGTG
GGCTGCTGACAGGAAGAATGTGC
>1441
GTCCTTGTGAGCATATGACAGCAAGACACTGGCCCTGGAATCCTCCGTGTCACATGTG
GGCTGCTGACAGGAAGAATGTGCTTTTGTTGTTGTTGTTGTTGTTGAGGCAGAGTCTTA
CTCTGTCGCCCAGGCTGCAGTGC

>1442
TTGTGAGCATATGACAGCAAGACACTGGCCCTGGAATCCTCCGTGTCACATGTGGGCT
GCTGACAGGAAGAATGTGCTTTTGTTGTTGTTGTTGTTGTTGAGGCAGAGTCTTACTCT
GTCGCCCAGGCTGCAGTGCAGTG
>1443
GGAATCCTCCGTGTCACATGTGGGCTGCTGACAGGAAGAATGTGCTTTTGTTGTTGTTG
TTGTTGTTGAGGCAGAGTCTTACTCTGTCGCCCAGGCTGCAGTGCAGTGGCATGATCTT
GGCTCACTGCAACCTCTACTTC
>1444
TCCTCCGTGTCACATGTGGGCTGCTGACAGGAAGAATGTGCTTTTGTTGTTGTTGTTGT
TGTTGAGGCAGAGTCTTACTCTGTCGCCCAGGCTGCAGTGCAGTGGCATGATCTTGGC
TCACTGCAACCTCTACTTCCCGG
>1445
CTGCTGACAGGAAGAATGTGCTTTTGTTGTTGTTGTTGTTGTTGAGGCAGAGTCTTACT
CTGTCGCCCAGGCTGCAGTGCAGTGGCATGATCTTGGCTCACTGCAACCTCTACTTCCC
GGGTTCAAGTGATTCTCCTGCC
>1446
CTCTGTCGCCCAGGCTGCAGTGCAGTGGCATGATCTTGGCTCACTGCAACCTCTACTTC
CCGGGTTCAAGTGATTCTCCTGCCTCAGCCTCCTGAGCAGCTGGGACTACAGATGTGT
ACCACCATGCCCGACTAATTTTT
>1447
CAGTGCAGTGGCATGATCTTGGCTCACTGCAACCTCTACTTCCCGGGTTCAAGTGATTC
TCCTGCCTCAGCCTCCTGAGCAGCTGGGACTACAGATGTGTACCACCATGCCCGACTAA
TTTTTGTATTTTTAGTAGAGAT
>1448
ATGATCTTGGCTCACTGCAACCTCTACTTCCCGGGTTCAAGTGATTCTCCTGCCTCAGC
CTCCTGAGCAGCTGGGACTACAGATGTGTACCACCATGCCCGACTAATTTTTGTATTTTT
AGTAGAGATGGGGTTTCACCA
>1449
CACTGCAACCTCTACTTCCCGGGTTCAAGTGATTCTCCTGCCTCAGCCTCCTGAGCAGC
TGGGACTACAGATGTGTACCACCATGCCCGACTAATTTTTGTATTTTTAGTAGAGATGGG
GTTTCACCATGTTGGTCAGGC
>1450
GCCTCAGCCTCCTGAGCAGCTGGGACTACAGATGTGTACCACCATGCCCGACTAATTTT
TGTATTTTTAGTAGAGATGGGGTTTCACCATGTTGGTCAGGCTGGCCTGGAACTCCTGG
CCTTAAGTGATCCACCTGTCTC
>1451
AGATGTGTACCACCATGCCCGACTAATTTTTGTATTTTTAGTAGAGATGGGGTTTCACCA
TGTTGGTCAGGCTGGCCTGGAACTCCTGGCCTTAAGTGATCCACCTGTCTCGGCCTCCC
ATAGTGCTGGGATTATAGGTG
>1452
TTTTTGTATTTTTAGTAGAGATGGGGTTTCACCATGTTGGTCAGGCTGGCCTGGAACTC
CTGGCCTTAAGTGATCCACCTGTCTCGGCCTCCCATAGTGCTGGGATTATAGGTGTGTG
CCACCCTGCTGGGCCGAGAATG

>1453
TTCACCATGTTGGTCAGGCTGGCCTGGAACTCCTGGCCTTAAGTGATCCACCTGTCTCG
GCCTCCCATAGTGCTGGGATTATAGGTGTGTGCCACCCTGCTGGGCCGAGAATGTGCTT
TTGACTGAGGGTGGAAAGTCAG
>1454
CAGGCTGGCCTGGAACTCCTGGCCTTAAGTGATCCACCTGTCTCGGCCTCCCATAGTGC
TGGGATTATAGGTGTGTGCCACCCTGCTGGGCCGAGAATGTGCTTTTGACTGAGGGTG
GAAAGTCAGCAGCTGCAGCCCAA
>1455
TAAGTGATCCACCTGTCTCGGCCTCCCATAGTGCTGGGATTATAGGTGTGTGCCACCCT
GCTGGGCCGAGAATGTGCTTTTGACTGAGGGTGGAAAGTCAGCAGCTGCAGCCCAAG
AAGTTAGAGCCAGGGGCAGTGCCT
>1456
CGGCCTCCCATAGTGCTGGGATTATAGGTGTGTGCCACCCTGCTGGGCCGAGAATGTGC
TTTTGACTGAGGGTGGAAAGTCAGCAGCTGCAGCCCAAGAAGTTAGAGCCAGGGGCA
GTGCCTTTGATGGGAAGCTTGGGG
>1457
ATAGTGCTGGGATTATAGGTGTGTGCCACCCTGCTGGGCCGAGAATGTGCTTTTGACTG
AGGGTGGAAAGTCAGCAGCTGCAGCCCAAGAAGTTAGAGCCAGGGGCAGTGCCTTTG
ATGGGAAGCTTGGGGGTCTAAAGA
>1458
TGTGTGCCACCCTGCTGGGCCGAGAATGTGCTTTTGACTGAGGGTGGAAAGTCAGCA
GCTGCAGCCCAAGAAGTTAGAGCCAGGGGCAGTGCCTTTGATGGGAAGCTTGGGGGT
CTAAAGAAATCAGAAAGGGGAGAAAA
>1459
GACTGAGGGTGGAAAGTCAGCAGCTGCAGCCCAAGAAGTTAGAGCCAGGGGCAGTG
CCTTTGATGGGAAGCTTGGGGGTCTAAAGAAATCAGAAAGGGGAGAAAATAATCCTGC
GTGTGCTCAGCCCTCACTCAGATCAA
>1460
GTCAGCAGCTGCAGCCCAAGAAGTTAGAGCCAGGGGCAGTGCCTTTGATGGGAAGCT
TGGGGGTCTAAAGAAATCAGAAAGGGGAGAAAATAATCCTGCGTGTGCTCAGCCCTC
ACTCAGATCAACAGCCCCTCGGGTCC
>1461
TGCCTTTGATGGGAAGCTTGGGGGTCTAAAGAAATCAGAAAGGGGAGAAAATAATCCT
GCGTGTGCTCAGCCCTCACTCAGATCAACAGCCCCTCGGGTCCCAGCTAGATGTACTTT
GTAGAAGGCTTTTTTTCTCCCCC
>1462
GGAAGCTTGGGGGTCTAAAGAAATCAGAAAGGGGAGAAAATAATCCTGCGTGTGCTC
AGCCCTCACTCAGATCAACAGCCCCTCGGGTCCCAGCTAGATGTACTTTGTAGAAGGC
TTTTTTTCTCCCCCTCACCCTCAAA
>1463
TCAGAAAGGGGAGAAAATAATCCTGCGTGTGCTCAGCCCTCACTCAGATCAACAGCCC
CTCGGGTCCCAGCTAGATGTACTTTGTAGAAGGCTTTTTTTCTCCCCCTCACCCTCAAA
ACTCCAGGATTAATGTGCTATTC

>1464
TAATCCTGCGTGTGCTCAGCCCTCACTCAGATCAACAGCCCCTCGGGTCCCAGCTAGAT
GTACTTTGTAGAAGGCTTTTTTTCTCCCCCTCACCCTCAAAACTCCAGGATTAATGTGCT
ATTCCCTTTTTTCTACTGTTC
>1465
TCCTGCGTGTGCTCAGCCCTCACTCAGATCAACAGCCCCTCGGGTCCCAGCTAGATGT
ACTTTGTAGAAGGCTTTTTTTCTCCCCCTCACCCTCAAAACTCCAGGATTAATGTGCTAT
TCCCTTTTTTCTACTGTTCTCT
>1466
GCCCCTCGGGTCCCAGCTAGATGTACTTTGTAGAAGGCTTTTTTTCTCCCCCTCACCCT
CAAAACTCCAGGATTAATGTGCTATTCCCTTTTTTCTACTGTTCTCTGTCTCCCCCCAAC
CCATCAGCAGTCTGAGAAATG
>1467
CCTCAAAACTCCAGGATTAATGTGCTATTCCCTTTTTTCTACTGTTCTCTGTCTCCCCCC
AACCCATCAGCAGTCTGAGAAATGCTATGGTGGAAAAAAAAAAACAACAGCCCTATAA
CCAGTACAAACAAAAAGCCAAG
>1468
CAAAACTCCAGGATTAATGTGCTATTCCCTTTTTTCTACTGTTCTCTGTCTCCCCCCAAC
CCATCAGCAGTCTGAGAAATGCTATGGTGGAAAAAAAAAAACAACAGCCCTATAACCA
GTACAAACAAAAAGCCAAGACA
>1469
ACTGTTCTCTGTCTCCCCCCAACCCATCAGCAGTCTGAGAAATGCTATGGTGGAAAAA
AAAAAACAACAGCCCTATAACCAGTACAAACAAAAAGCCAAGACAAAAAAAAAAAA
AAAAAAAATCAGAAGGGGAGATTTTG
>1470
CTCCCCCCAACCCATCAGCAGTCTGAGAAATGCTATGGTGGAAAAAAAAAAACAACA
GCCCTATAACCAGTACAAACAAAAAGCCAAGACAAAAAAAAAAAAAAAAAAAATCA
GAAGGGGAGATTTTGAGGCCAGGAAGC
>1471
CAGTCTGAGAAATGCTATGGTGGAAAAAAAAAAACAACAGCCCTATAACCAGTACAA
ACAAAAAGCCAAGACAAAAAAAAAAAAAAAAAAAATCAGAAGGGGAGATTTTGAGG
CCAGGAAGCCATGGCATTCACTGGAAA
>1472
AAAAAAAACAACAGCCCTATAACCAGTACAAACAAAAAGCCAAGACAAAAAAAAAA
AAAAAAAAAATCAGAAGGGGAGATTTTGAGGCCAGGAAGCCATGGCATTCACTGGAA
ATATATCAGTGATAAAATAACAGGCTA
>1473
AAAAACAACAGCCCTATAACCAGTACAAACAAAAAGCCAAGACAAAAAAAAAAAAA
AAAAAAATCAGAAGGGGAGATTTTGAGGCCAGGAAGCCATGGCATTCACTGGAAATAT
ATCAGTGATAAAATAACAGGCTACAG
>1474
CTATAACCAGTACAAACAAAAAGCCAAGACAAAAAAAAAAAAAAAAAAAATCAGAA
GGGGAGATTTTGAGGCCAGGAAGCCATGGCATTCACTGGAAATATATCAGTGATAAAAT
AACAGGCTACAGGGTTTATTTTATT

>1475
AAAAAAAAAAAAAAATCAGAAGGGGAGATTTTGAGGCCAGGAAGCCATGGCATTCACT
GGAAATATATCAGTGATAAAATAACAGGCTACAGGGTTTATTTTATTTTATTTTTAAGTCA
ATAGGCCTGAGTGTTGGTAAAG
>1476
TCAGAAGGGGAGATTTTGAGGCCAGGAAGCCATGGCATTCACTGGAAATATATCAGTG
ATAAAATAACAGGCTACAGGGTTTATTTTATTTTATTTTTAAGTCAATAGGCCTGAGTGT
TGGTAAAGACTAGATACCCAGC
>1477
GGGAGATTTTGAGGCCAGGAAGCCATGGCATTCACTGGAAATATATCAGTGATAAAATA
ACAGGCTACAGGGTTTATTTTATTTTATTTTTAAGTCAATAGGCCTGAGTGTTGGTAAAG
ACTAGATACCCAGCAATCCTT
>1478
ATGGCATTCACTGGAAATATATCAGTGATAAAATAACAGGCTACAGGGTTTATTTTATTT
TATTTTTAAGTCAATAGGCCTGAGTGTTGGTAAAGACTAGATACCCAGCAATCCTTCTT
GCATTTCTTCTTGGGGTCGGG
>1479
AAATATATCAGTGATAAAATAACAGGCTACAGGGTTTATTTTATTTTATTTTTAAGTCAAT
AGGCCTGAGTGTTGGTAAAGACTAGATACCCAGCAATCCTTCTTGCATTTCTTCTTGGG
GTCGGGGGGCAGAAGGGAAT
>1480
TGATAAAATAACAGGCTACAGGGTTTATTTTATTTTATTTTTAAGTCAATAGGCCTGAGT
GTTGGTAAAGACTAGATACCCAGCAATCCTTCTTGCATTTCTTCTTGGGGTCGGGGGGC
AGAAGGGAATGGAGAGGAAAT
>1481
AAATAACAGGCTACAGGGTTTATTTTATTTTATTTTTAAGTCAATAGGCCTGAGTGTTGG
TAAAGACTAGATACCCAGCAATCCTTCTTGCATTTCTTCTTGGGGTCGGGGGGCAGAAG
GGAATGGAGAGGAAATGAAGG
>1482
GGCCTGAGTGTTGGTAAAGACTAGATACCCAGCAATCCTTCTTGCATTTCTTCTTGGGG
TCGGGGGGCAGAAGGGAATGGAGAGGAAATGAAGGCGGATTGCTGGTAAGTGAGGC
AGGAAGGCAGGGAGTTTATTATGGA
>1483
CTGAGTGTTGGTAAAGACTAGATACCCAGCAATCCTTCTTGCATTTCTTCTTGGGGTCG
GGGGGCAGAAGGGAATGGAGAGGAAATGAAGGCGGATTGCTGGTAAGTGAGGCAGG
AAGGCAGGGAGTTTATTATGGAATG
>1484
GGTAAAGACTAGATACCCAGCAATCCTTCTTGCATTTCTTCTTGGGGTCGGGGGGCAGA
AGGGAATGGAGAGGAAATGAAGGCGGATTGCTGGTAAGTGAGGCAGGAAGGCAGGG
AGTTTATTATGGAATGTCATCGGTA
>1485
GATACCCAGCAATCCTTCTTGCATTTCTTCTTGGGGTCGGGGGGCAGAAGGGAATGGA
GAGGAAATGAAGGCGGATTGCTGGTAAGTGAGGCAGGAAGGCAGGGAGTTTATTATG
GAATGTCATCGGTAAGTTTCCCCAT

>1486
TCTTGGGGTCGGGGGGCAGAAGGGAATGGAGAGGAAATGAAGGCGGATTGCTGGTAA
GTGAGGCAGGAAGGCAGGGAGTTTATTATGGAATGTCATCGGTAAGTTTCCCCATGATT
TGATCCCAAGCTGGTGTGGAGTGA
>1487
TCGGGGGGCAGAAGGGAATGGAGAGGAAATGAAGGCGGATTGCTGGTAAGTGAGGC
AGGAAGGCAGGGAGTTTATTATGGAATGTCATCGGTAAGTTTCCCCATGATTTGATCCC
AAGCTGGTGTGGAGTGAAGGGTAAG
>1488
GAAATGAAGGCGGATTGCTGGTAAGTGAGGCAGGAAGGCAGGGAGTTTATTATGGAAT
GTCATCGGTAAGTTTCCCCATGATTTGATCCCAAGCTGGTGTGGAGTGAAGGGTAAGCT
GTTGGCTCAACAGATAGTGAGAA
>1489
AGTGAGGCAGGAAGGCAGGGAGTTTATTATGGAATGTCATCGGTAAGTTTCCCCATGAT
TTGATCCCAAGCTGGTGTGGAGTGAAGGGTAAGCTGTTGGCTCAACAGATAGTGAGAA
TCAAACTTAGTGGAGTTGTAGAT
>1490
AAGGCAGGGAGTTTATTATGGAATGTCATCGGTAAGTTTCCCCATGATTTGATCCCAAG
CTGGTGTGGAGTGAAGGGTAAGCTGTTGGCTCAACAGATAGTGAGAATCAAACTTAGT
GGAGTTGTAGATCCTGTTTCCTG
>1491
AGGGAGTTTATTATGGAATGTCATCGGTAAGTTTCCCCATGATTTGATCCCAAGCTGGTG
TGGAGTGAAGGGTAAGCTGTTGGCTCAACAGATAGTGAGAATCAAACTTAGTGGAGTT
GTAGATCCTGTTTCCTGCAGCG
>1492
TTTATTATGGAATGTCATCGGTAAGTTTCCCCATGATTTGATCCCAAGCTGGTGTGGAGT
GAAGGGTAAGCTGTTGGCTCAACAGATAGTGAGAATCAAACTTAGTGGAGTTGTAGAT
CCTGTTTCCTGCAGCGGACAGC
>1493
CATCGGTAAGTTTCCCCATGATTTGATCCCAAGCTGGTGTGGAGTGAAGGGTAAGCTGT
TGGCTCAACAGATAGTGAGAATCAAACTTAGTGGAGTTGTAGATCCTGTTTCCTGCAGC
GGACAGCAAGATGTACAAGCCG
>1494
ATTTGATCCCAAGCTGGTGTGGAGTGAAGGGTAAGCTGTTGGCTCAACAGATAGTGAG
AATCAAACTTAGTGGAGTTGTAGATCCTGTTTCCTGCAGCGGACAGCAAGATGTACAA
GCCGTCCACTGCAGGAATGACGTG
>1495
AGGGTAAGCTGTTGGCTCAACAGATAGTGAGAATCAAACTTAGTGGAGTTGTAGATCC
TGTTTCCTGCAGCGGACAGCAAGATGTACAAGCCGTCCACTGCAGGAATGACGTGCTG
TAAAAGGGATGCAGTGTCAAGTGT
>1496
GCTGTTGGCTCAACAGATAGTGAGAATCAAACTTAGTGGAGTTGTAGATCCTGTTTCCT
GCAGCGGACAGCAAGATGTACAAGCCGTCCACTGCAGGAATGACGTGCTGTAAAAGG
GATGCAGTGTCAAGTGTAGTCATG

>1497
TCAAACTTAGTGGAGTTGTAGATCCTGTTTCCTGCAGCGGACAGCAAGATGTACAAGC
CGTCCACTGCAGGAATGACGTGCTGTAAAAGGGATGCAGTGTCAAGTGTAGTCATGGA
TGGGTGGGAAGACCAGACAAAGGC
>1498
AGATCCTGTTTCCTGCAGCGGACAGCAAGATGTACAAGCCGTCCACTGCAGGAATGAC
GTGCTGTAAAAGGGATGCAGTGTCAAGTGTAGTCATGGATGGGTGGGAAGACCAGAC
AAAGGCATCCAATGGGACTGACTTA
>1499
TTTCCTGCAGCGGACAGCAAGATGTACAAGCCGTCCACTGCAGGAATGACGTGCTGTA
AAAGGGATGCAGTGTCAAGTGTAGTCATGGATGGGTGGGAAGACCAGACAAAGGCAT
CCAATGGGACTGACTTACCTAAACA
>1500
CAGCGGACAGCAAGATGTACAAGCCGTCCACTGCAGGAATGACGTGCTGTAAAAGGG
ATGCAGTGTCAAGTGTAGTCATGGATGGGTGGGAAGACCAGACAAAGGCATCCAATGG
GACTGACTTACCTAAACAAACCAGA
>1501
CCGTCCACTGCAGGAATGACGTGCTGTAAAAGGGATGCAGTGTCAAGTGTAGTCATGG
ATGGGTGGGAAGACCAGACAAAGGCATCCAATGGGACTGACTTACCTAAACAAACCA
GAAGGAAGTCAGAACAGTGGGAAGA
>1502
GTGTAGTCATGGATGGGTGGGAAGACCAGACAAAGGCATCCAATGGGACTGACTTACC
TAAACAAACCAGAAGGAAGTCAGAACAGTGGGAAGATCTGATAGATAAGCAGTATTCT
GCAGGCAGATGAAGGAGCAAGCTT
>1503
TAGTCATGGATGGGTGGGAAGACCAGACAAAGGCATCCAATGGGACTGACTTACCTAA
ACAAACCAGAAGGAAGTCAGAACAGTGGGAAGATCTGATAGATAAGCAGTATTCTGC
AGGCAGATGAAGGAGCAAGCTTTGG
>1504
AAGGCATCCAATGGGACTGACTTACCTAAACAAACCAGAAGGAAGTCAGAACAGTGG
GAAGATCTGATAGATAAGCAGTATTCTGCAGGCAGATGAAGGAGCAAGCTTTGGGCCC
CCAAATCTTCACCAGTGCATCCACA
>1505
ATCCAATGGGACTGACTTACCTAAACAAACCAGAAGGAAGTCAGAACAGTGGGAAGA
TCTGATAGATAAGCAGTATTCTGCAGGCAGATGAAGGAGCAAGCTTTGGGCCCCCAAA
TCTTCACCAGTGCATCCACATTACA
>1506
CAATGGGACTGACTTACCTAAACAAACCAGAAGGAAGTCAGAACAGTGGGAAGATCT
GATAGATAAGCAGTATTCTGCAGGCAGATGAAGGAGCAAGCTTTGGGCCCCCAAATCT
TCACCAGTGCATCCACATTACAGAG
>1507
GACTTACCTAAACAAACCAGAAGGAAGTCAGAACAGTGGGAAGATCTGATAGATAAG
CAGTATTCTGCAGGCAGATGAAGGAGCAAGCTTTGGGCCCCCAAATCTTCACCAGTGC
ATCCACATTACAGAGGATAATGCCT

>1508
GATCTGATAGATAAGCAGTATTCTGCAGGCAGATGAAGGAGCAAGCTTTGGGCCCCCA
AATCTTCACCAGTGCATCCACATTACAGAGGATAATGCCTGAGGGAGGGGAAGATCAT
CTCAGGATTCTGGAACACTGGGCC
>1509
AGTATTCTGCAGGCAGATGAAGGAGCAAGCTTTGGGCCCCCAAATCTTCACCAGTGCA
TCCACATTACAGAGGATAATGCCTGAGGGAGGGGAAGATCATCTCAGGATTCTGGAAC
ACTGGGCCCTCCCTTTCTTATTCC
>1510
GATGAAGGAGCAAGCTTTGGGCCCCCAAATCTTCACCAGTGCATCCACATTACAGAGG
ATAATGCCTGAGGGAGGGGAAGATCATCTCAGGATTCTGGAACACTGGGCCCTCCCTT
TCTTATTCCAAAAGGCTGGAGCAG
>1511
AATGCCTGAGGGAGGGGAAGATCATCTCAGGATTCTGGAACACTGGGCCCTCCCTTTC
TTATTCCAAAAGGCTGGAGCAGTTCTGCATGTAGGCTTCTCTGGAAATGGTCTAGGAAC
CAGCTCATCCAGGCCAGGAGCAC
>1512
GAGGGAGGGGAAGATCATCTCAGGATTCTGGAACACTGGGCCCTCCCTTTCTTATTCC
AAAAGGCTGGAGCAGTTCTGCATGTAGGCTTCTCTGGAAATGGTCTAGGAACCAGCTC
ATCCAGGCCAGGAGCACCTGATCA
>1513
TGGGCCCTCCCTTTCTTATTCCAAAAGGCTGGAGCAGTTCTGCATGTAGGCTTCTCTGG
AAATGGTCTAGGAACCAGCTCATCCAGGCCAGGAGCACCTGATCATTCTCAGAGTTAC
GTACATATGCGATAAGATTGCAG
>1514
TCCCTTTCTTATTCCAAAAGGCTGGAGCAGTTCTGCATGTAGGCTTCTCTGGAAATGGT
CTAGGAACCAGCTCATCCAGGCCAGGAGCACCTGATCATTCTCAGAGTTACGTACATAT
GCGATAAGATTGCAGGGAACAG
>1515
AAGGCTGGAGCAGTTCTGCATGTAGGCTTCTCTGGAAATGGTCTAGGAACCAGCTCAT
CCAGGCCAGGAGCACCTGATCATTCTCAGAGTTACGTACATATGCGATAAGATTGCAGG
GAACAGTATAGTTCTATAGACTT
>1516
GGTCTAGGAACCAGCTCATCCAGGCCAGGAGCACCTGATCATTCTCAGAGTTACGTAC
ATATGCGATAAGATTGCAGGGAACAGTATAGTTCTATAGACTTAAGTGTGAACATAGTC
CTCATTTATAGTCCCTAGTCTCC
>1517
GAACCAGCTCATCCAGGCCAGGAGCACCTGATCATTCTCAGAGTTACGTACATATGCGA
TAAGATTGCAGGGAACAGTATAGTTCTATAGACTTAAGTGTGAACATAGTCCTCATTTAT
AGTCCCTAGTCTCCCATGGCT
>1518
GGAGCACCTGATCATTCTCAGAGTTACGTACATATGCGATAAGATTGCAGGGAACAGTA
TAGTTCTATAGACTTAAGTGTGAACATAGTCCTCATTTATAGTCCCTAGTCTCCCATGGC
TCACGCAGCTTGGTCTTTATG

>1519
CAGAGTTACGTACATATGCGATAAGATTGCAGGGAACAGTATAGTTCTATAGACTTAAG
TGTGAACATAGTCCTCATTTATAGTCCCTAGTCTCCCATGGCTCACGCAGCTTGGTCTTT
ATGTGTTTCTGAATTTAGTGC
>1520
ATATGCGATAAGATTGCAGGGAACAGTATAGTTCTATAGACTTAAGTGTGAACATAGTC
CTCATTTATAGTCCCTAGTCTCCCATGGCTCACGCAGCTTGGTCTTTATGTGTTTCTGAA
TTTAGTGCTGCAGAGGCACAA
>1521
ATAAGATTGCAGGGAACAGTATAGTTCTATAGACTTAAGTGTGAACATAGTCCTCATTTA
TAGTCCCTAGTCTCCCATGGCTCACGCAGCTTGGTCTTTATGTGTTTCTGAATTTAGTGC
TGCAGAGGCACAATGGGGTC
>1522
TAGTCCTCATTTATAGTCCCTAGTCTCCCATGGCTCACGCAGCTTGGTCTTTATGTGTTT
CTGAATTTAGTGCTGCAGAGGCACAATGGGGTCTGGTTTCTGGATCTTATACAGACAAG
CACTTAGGTCAGCTGCAGTGG
>1523
ATTTATAGTCCCTAGTCTCCCATGGCTCACGCAGCTTGGTCTTTATGTGTTTCTGAATTTA
GTGCTGCAGAGGCACAATGGGGTCTGGTTTCTGGATCTTATACAGACAAGCACTTAGG
TCAGCTGCAGTGGATGTCCTG
>1524
TGGTCTTTATGTGTTTCTGAATTTAGTGCTGCAGAGGCACAATGGGGTCTGGTTTCTGG
ATCTTATACAGACAAGCACTTAGGTCAGCTGCAGTGGATGTCCTGCACAGACACCGTC
GCAACTCTTGCCTGGTTCCTTTT
>1525
GTTTCTGAATTTAGTGCTGCAGAGGCACAATGGGGTCTGGTTTCTGGATCTTATACAGA
CAAGCACTTAGGTCAGCTGCAGTGGATGTCCTGCACAGACACCGTCGCAACTCTTGCC
TGGTTCCTTTTTGTTTTTGGAGA
>1526
TGAATTTAGTGCTGCAGAGGCACAATGGGGTCTGGTTTCTGGATCTTATACAGACAAGC
ACTTAGGTCAGCTGCAGTGGATGTCCTGCACAGACACCGTCGCAACTCTTGCCTGGTT
CCTTTTTGTTTTTGGAGATGGAG
>1527
CAATGGGGTCTGGTTTCTGGATCTTATACAGACAAGCACTTAGGTCAGCTGCAGTGGAT
GTCCTGCACAGACACCGTCGCAACTCTTGCCTGGTTCCTTTTTGTTTTTGGAGATGGAG
TCTCGCTCTGTCTCCCAGGCTG
>1528
GGTCAGCTGCAGTGGATGTCCTGCACAGACACCGTCGCAACTCTTGCCTGGTTCCTTT
TTGTTTTTGGAGATGGAGTCTCGCTCTGTCTCCCAGGCTGGAGTGCAGTGGCGCGATC
TCAGCTCACTGCAAGCTCTGCCTC
>1529
CTGCAGTGGATGTCCTGCACAGACACCGTCGCAACTCTTGCCTGGTTCCTTTTTGTTTT
TGGAGATGGAGTCTCGCTCTGTCTCCCAGGCTGGAGTGCAGTGGCGCGATCTCAGCTC
ACTGCAAGCTCTGCCTCCCAGGT

>1530
ACAGACACCGTCGCAACTCTTGCCTGGTTCCTTTTTGTTTTTGGAGATGGAGTCTCGCT
CTGTCTCCCAGGCTGGAGTGCAGTGGCGCGATCTCAGCTCACTGCAAGCTCTGCCTCC
CAGGTTCACGCTATTCTCCTGCC
>1531
GTTCCTTTTTGTTTTTGGAGATGGAGTCTCGCTCTGTCTCCCAGGCTGGAGTGCAGTGG
CGCGATCTCAGCTCACTGCAAGCTCTGCCTCCCAGGTTCACGCTATTCTCCTGCCTCAG
CCTCCCAAGTAGTTGGGACTAC
>1532
TTTTTGGAGATGGAGTCTCGCTCTGTCTCCCAGGCTGGAGTGCAGTGGCGCGATCTCA
GCTCACTGCAAGCTCTGCCTCCCAGGTTCACGCTATTCTCCTGCCTCAGCCTCCCAAGT
AGTTGGGACTACAGGCACCCGCC
>1533
AGTCTCGCTCTGTCTCCCAGGCTGGAGTGCAGTGGCGCGATCTCAGCTCACTGCAAGC
TCTGCCTCCCAGGTTCACGCTATTCTCCTGCCTCAGCCTCCCAAGTAGTTGGGACTACA
GGCACCCGCCACCACGCCCGGCT
>1534
GAGTGCAGTGGCGCGATCTCAGCTCACTGCAAGCTCTGCCTCCCAGGTTCACGCTATT
CTCCTGCCTCAGCCTCCCAAGTAGTTGGGACTACAGGCACCCGCCACCACGCCCGGCT
AATTTTTTGTATTTTTAGTAGAGA
>1535
GGCGCGATCTCAGCTCACTGCAAGCTCTGCCTCCCAGGTTCACGCTATTCTCCTGCCTC
AGCCTCCCAAGTAGTTGGGACTACAGGCACCCGCCACCACGCCCGGCTAATTTTTTGTA
TTTTTAGTAGAGACAGGGTTTC
>1536
GCGATCTCAGCTCACTGCAAGCTCTGCCTCCCAGGTTCACGCTATTCTCCTGCCTCAGC
CTCCCAAGTAGTTGGGACTACAGGCACCCGCCACCACGCCCGGCTAATTTTTTGTATTT
TTAGTAGAGACAGGGTTTCACC
>1537
CACTGCAAGCTCTGCCTCCCAGGTTCACGCTATTCTCCTGCCTCAGCCTCCCAAGTAGT
TGGGACTACAGGCACCCGCCACCACGCCCGGCTAATTTTTTGTATTTTTAGTAGAGACA
GGGTTTCACCGTGTTAGCCAGT
>1538
CCTCAGCCTCCCAAGTAGTTGGGACTACAGGCACCCGCCACCACGCCCGGCTAATTTT
TTGTATTTTTAGTAGAGACAGGGTTTCACCGTGTTAGCCAGTATGGTCTCGATCTCCTGA
ACTCGTGATCCACCTGCCTCGG
>1539
CAGCCTCCCAAGTAGTTGGGACTACAGGCACCCGCCACCACGCCCGGCTAATTTTTTG
TATTTTTAGTAGAGACAGGGTTTCACCGTGTTAGCCAGTATGGTCTCGATCTCCTGAAC
TCGTGATCCACCTGCCTCGGCCT
>1540
GCCTCCCAAGTAGTTGGGACTACAGGCACCCGCCACCACGCCCGGCTAATTTTTTGTAT
TTTTAGTAGAGACAGGGTTTCACCGTGTTAGCCAGTATGGTCTCGATCTCCTGAACTCG
TGATCCACCTGCCTCGGCCTCC

>1541
CCCAAGTAGTTGGGACTACAGGCACCCGCCACCACGCCCGGCTAATTTTTTGTATTTTT
AGTAGAGACAGGGTTTCACCGTGTTAGCCAGTATGGTCTCGATCTCCTGAACTCGTGAT
CCACCTGCCTCGGCCTCCCAAA
>1542
TACAGGCACCCGCCACCACGCCCGGCTAATTTTTTGTATTTTTAGTAGAGACAGGGTTT
CACCGTGTTAGCCAGTATGGTCTCGATCTCCTGAACTCGTGATCCACCTGCCTCGGCCT
CCCAAAGTGCTGGGATTACAGG
>1543
GTTTCACCGTGTTAGCCAGTATGGTCTCGATCTCCTGAACTCGTGATCCACCTGCCTCG
GCCTCCCAAAGTGCTGGGATTACAGGTGTGAGCCACCACGCCCGGCCTATTGCCTGGT
TCTTGAAGGGATTCTCAGGAACC
>1544
GCCAGTATGGTCTCGATCTCCTGAACTCGTGATCCACCTGCCTCGGCCTCCCAAAGTGC
TGGGATTACAGGTGTGAGCCACCACGCCCGGCCTATTGCCTGGTTCTTGAAGGGATTCT
CAGGAACCACAGGGAATGTCTG
>1545
CTCGGCCTCCCAAAGTGCTGGGATTACAGGTGTGAGCCACCACGCCCGGCCTATTGCC
TGGTTCTTGAAGGGATTCTCAGGAACCACAGGGAATGTCTGAGCCAGCTTTCTGGATC
CGTGTGTACAGCCCTTCCCAGAGG
>1546
CAAAGTGCTGGGATTACAGGTGTGAGCCACCACGCCCGGCCTATTGCCTGGTTCTTGA
AGGGATTCTCAGGAACCACAGGGAATGTCTGAGCCAGCTTTCTGGATCCGTGTGTACA
GCCCTTCCCAGAGGCTTTGTGGAG
>1547
GTGAGCCACCACGCCCGGCCTATTGCCTGGTTCTTGAAGGGATTCTCAGGAACCACAG
GGAATGTCTGAGCCAGCTTTCTGGATCCGTGTGTACAGCCCTTCCCAGAGGCTTTGTG
GAGCCAGCTGTACGGTGCATGAGC
>1548
CTGGTTCTTGAAGGGATTCTCAGGAACCACAGGGAATGTCTGAGCCAGCTTTCTGGAT
CCGTGTGTACAGCCCTTCCCAGAGGCTTTGTGGAGCCAGCTGTACGGTGCATGAGCTT
TGTGGCTAAGATATCAGAGTTCTG
>1549
AAGGGATTCTCAGGAACCACAGGGAATGTCTGAGCCAGCTTTCTGGATCCGTGTGTAC
AGCCCTTCCCAGAGGCTTTGTGGAGCCAGCTGTACGGTGCATGAGCTTTGTGGCTAAG
ATATCAGAGTTCTGCCTGGCCCTT
>1550
GAACCACAGGGAATGTCTGAGCCAGCTTTCTGGATCCGTGTGTACAGCCCTTCCCAGA
GGCTTTGTGGAGCCAGCTGTACGGTGCATGAGCTTTGTGGCTAAGATATCAGAGTTCTG
CCTGGCCCTTGGCCTTGGTCTCT
>1551
ATCCGTGTGTACAGCCCTTCCCAGAGGCTTTGTGGAGCCAGCTGTACGGTGCATGAGC
TTTGTGGCTAAGATATCAGAGTTCTGCCTGGCCCTTGGCCTTGGTCTCTCTCAGATGGT
CAGCTGCTCTGTTTTTAGTGGTT

>1552
TGTACAGCCCTTCCCAGAGGCTTTGTGGAGCCAGCTGTACGGTGCATGAGCTTTGTGG
CTAAGATATCAGAGTTCTGCCTGGCCCTTGGCCTTGGTCTCTCTCAGATGGTCAGCTGC
TCTGTTTTTAGTGGTTGATCATG
>1553
TGTACGGTGCATGAGCTTTGTGGCTAAGATATCAGAGTTCTGCCTGGCCCTTGGCCTTG
GTCTCTCTCAGATGGTCAGCTGCTCTGTTTTTAGTGGTTGATCATGTAGAAGGCCAACA
GGTGTTGACCTTGGTTTAGGCT
>1554
CTAAGATATCAGAGTTCTGCCTGGCCCTTGGCCTTGGTCTCTCTCAGATGGTCAGCTGC
TCTGTTTTTAGTGGTTGATCATGTAGAAGGCCAACAGGTGTTGACCTTGGTTTAGGCTA
CTTTTGGGATTTTTGAGGTTTC
>1555
TCTGCCTGGCCCTTGGCCTTGGTCTCTCTCAGATGGTCAGCTGCTCTGTTTTTAGTGGT
TGATCATGTAGAAGGCCAACAGGTGTTGACCTTGGTTTAGGCTACTTTTGGGATTTTTG
AGGTTTCCTGAGACTCTTGGTT
>1556
CAGATGGTCAGCTGCTCTGTTTTTAGTGGTTGATCATGTAGAAGGCCAACAGGTGTTGA
CCTTGGTTTAGGCTACTTTTGGGATTTTTGAGGTTTCCTGAGACTCTTGGTTTTCTTTCA
TCATCCCGTCCAGAACCTCTG
>1557
TTTAGTGGTTGATCATGTAGAAGGCCAACAGGTGTTGACCTTGGTTTAGGCTACTTTTG
GGATTTTTGAGGTTTCCTGAGACTCTTGGTTTTCTTTCATCATCCCGTCCAGAACCTCTG
ATGTCAAACTAAATTCTGTTT
>1558
GATCATGTAGAAGGCCAACAGGTGTTGACCTTGGTTTAGGCTACTTTTGGGATTTTTGA
GGTTTCCTGAGACTCTTGGTTTTCTTTCATCATCCCGTCCAGAACCTCTGATGTCAAACT
AAATTCTGTTTGGAAAAGGGT
>1559
TGGTTTAGGCTACTTTTGGGATTTTTGAGGTTTCCTGAGACTCTTGGTTTTCTTTCATCA
TCCCGTCCAGAACCTCTGATGTCAAACTAAATTCTGTTTGGAAAAGGGTATGCTGTAGA
ATAATGATAGCCACAGCAATG
>1560
GAGACTCTTGGTTTTCTTTCATCATCCCGTCCAGAACCTCTGATGTCAAACTAAATTCT
GTTTGGAAAAGGGTATGCTGTAGAATAATGATAGCCACAGCAATGATATTAATAATACCT
TTTATCGGTTGCAGCATTTTC
>1561
TTTCTTTCATCATCCCGTCCAGAACCTCTGATGTCAAACTAAATTCTGTTTGGAAAAGG
GTATGCTGTAGAATAATGATAGCCACAGCAATGATATTAATAATACCTTTTATCGGTTGCA
GCATTTTCTTCTTTGCAAAG
>1562
TCCAGAACCTCTGATGTCAAACTAAATTCTGTTTGGAAAAGGGTATGCTGTAGAATAAT
GATAGCCACAGCAATGATATTAATAATACCTTTTATCGGTTGCAGCATTTTCTTCTTTGCA
AAGCACCTCTAATGACTTTA

>1563
GGAAAAGGGTATGCTGTAGAATAATGATAGCCACAGCAATGATATTAATAATACCTTTTA
TCGGTTGCAGCATTTTCTTCTTTGCAAAGCACCTCTAATGACTTTATCTCTCTTGCCCTC
ACAGCCTTCCTTGAGTGGGT
>1564
AATAATGATAGCCACAGCAATGATATTAATAATACCTTTTATCGGTTGCAGCATTTTCTTC
TTTGCAAAGCACCTCTAATGACTTTATCTCTCTTGCCCTCACAGCCTTCCTTGAGTGGGT
ACAGGTCCATAATCCCATA
>1565
CTTTTATCGGTTGCAGCATTTTCTTCTTTGCAAAGCACCTCTAATGACTTTATCTCTCTTG
CCCTCACAGCCTTCCTTGAGTGGGTACAGGTCCATAATCCCATATCTGTGATAATGAAG
TCTGAAAAGCTCTGAAAACT
>1566
TGCAGCATTTTCTTCTTTGCAAAGCACCTCTAATGACTTTATCTCTCTTGCCCTCACAGC
CTTCCTTGAGTGGGTACAGGTCCATAATCCCATATCTGTGATAATGAAGTCTGAAAAGC
TCTGAAAACTAAAAAGTTTGC
>1567
TATCTCTCTTGCCCTCACAGCCTTCCTTGAGTGGGTACAGGTCCATAATCCCATATCTGT
GATAATGAAGTCTGAAAAGCTCTGAAAACTAAAAAGTTTGCAGTAAACTCATTTAGTG
GCAAAACCTGATCTGAACTGAT
>1568
TGCCCTCACAGCCTTCCTTGAGTGGGTACAGGTCCATAATCCCATATCTGTGATAATGA
AGTCTGAAAAGCTCTGAAAACTAAAAAGTTTGCAGTAAACTCATTTAGTGGCAAAACC
TGATCTGAACTGATGTGAGGCTA
>1569
GGTACAGGTCCATAATCCCATATCTGTGATAATGAAGTCTGAAAAGCTCTGAAAACTAA
AAAGTTTGCAGTAAACTCATTTAGTGGCAAAACCTGATCTGAACTGATGTGAGGCTATA
TTTATCCTGCTTAGTTTGAATA
>1570
AATCCCATATCTGTGATAATGAAGTCTGAAAAGCTCTGAAAACTAAAAAGTTTGCAGTA
AACTCATTTAGTGGCAAAACCTGATCTGAACTGATGTGAGGCTATATTTATCCTGCTTAG
TTTGAATATTCATATGTTTTG
>1571
AAAGCTCTGAAAACTAAAAAGTTTGCAGTAAACTCATTTAGTGGCAAAACCTGATCTG
AACTGATGTGAGGCTATATTTATCCTGCTTAGTTTGAATATTCATATGTTTTGCTGCAAAA
ATATTAATGTGTTTGATTACA
>1572
GTTTGCAGTAAACTCATTTAGTGGCAAAACCTGATCTGAACTGATGTGAGGCTATATTTA
TCCTGCTTAGTTTGAATATTCATATGTTTTGCTGCAAAAATATTAATGTGTTTGATTACAG
AGTGCTGCCCTAGACTCTG
>1573
CTATATTTATCCTGCTTAGTTTGAATATTCATATGTTTTGCTGCAAAAATATTAATGTGTTT
GATTACAGAGTGCTGCCCTAGACTCTGCTGGGGCTGTTAAACACGATAGGTGTCAGAG
TACCTTTCTTTTTTTTTTTT

>1574
ATATTTATCCTGCTTAGTTTGAATATTCATATGTTTTGCTGCAAAAATATTAATGTGTTTGA
TTACAGAGTGCTGCCCTAGACTCTGCTGGGGCTGTTAAACACGATAGGTGTCAGAGTA
CCTTTCTTTTTTTTTTTTTT
>1575
TGCAAAAATATTAATGTGTTTGATTACAGAGTGCTGCCCTAGACTCTGCTGGGGCTGTT
AAACACGATAGGTGTCAGAGTACCTTTCTTTTTTTTTTTTTTTTTTTTTTTTGAGACGGA
GTCTCGCTCTGTCGCCCAGGT
>1576
ATATTAATGTGTTTGATTACAGAGTGCTGCCCTAGACTCTGCTGGGGCTGTTAAACACG
ATAGGTGTCAGAGTACCTTTCTTTTTTTTTTTTTTTTTTTTTTTTGAGACGGAGTCTCGC
TCTGTCGCCCAGGTCGGACTG
>1577
TCTGCTGGGGCTGTTAAACACGATAGGTGTCAGAGTACCTTTCTTTTTTTTTTTTTTTTT
TTTTTTTGAGACGGAGTCTCGCTCTGTCGCCCAGGTCGGACTGCGGACTGCAGTGGCG
CAATCTCGGCTCACTGCAAGCT
>1578
GGGGCTGTTAAACACGATAGGTGTCAGAGTACCTTTCTTTTTTTTTTTTTTTTTTTTTTTT
GAGACGGAGTCTCGCTCTGTCGCCCAGGTCGGACTGCGGACTGCAGTGGCGCAATCT
CGGCTCACTGCAAGCTCCGCTT
>1579
AGGTGTCAGAGTACCTTTCTTTTTTTTTTTTTTTTTTTTTTTTGAGACGGAGTCTCGCTC
TGTCGCCCAGGTCGGACTGCGGACTGCAGTGGCGCAATCTCGGCTCACTGCAAGCTCC
GCTTCCCGGGTTCATGCCATTC
>1580
AGACGGAGTCTCGCTCTGTCGCCCAGGTCGGACTGCGGACTGCAGTGGCGCAATCTC
GGCTCACTGCAAGCTCCGCTTCCCGGGTTCATGCCATTCTCCTGCCTCAGCCTCCCGAG
TAGCTGGGACTACAGGCGCCCGCC
>1581
GACTGCAGTGGCGCAATCTCGGCTCACTGCAAGCTCCGCTTCCCGGGTTCATGCCATT
CTCCTGCCTCAGCCTCCCGAGTAGCTGGGACTACAGGCGCCCGCCACCGCGCCCGGCT
AATTTTTTGTATTTTTAGTAGAGA
>1582
GGCGCAATCTCGGCTCACTGCAAGCTCCGCTTCCCGGGTTCATGCCATTCTCCTGCCTC
AGCCTCCCGAGTAGCTGGGACTACAGGCGCCCGCCACCGCGCCCGGCTAATTTTTTGT
ATTTTTAGTAGAGACGGGGTTTC
>1583
GCAATCTCGGCTCACTGCAAGCTCCGCTTCCCGGGTTCATGCCATTCTCCTGCCTCAGC
CTCCCGAGTAGCTGGGACTACAGGCGCCCGCCACCGCGCCCGGCTAATTTTTTGTATTT
TTAGTAGAGACGGGGTTTCACC
>1584
CACTGCAAGCTCCGCTTCCCGGGTTCATGCCATTCTCCTGCCTCAGCCTCCCGAGTAGC
TGGGACTACAGGCGCCCGCCACCGCGCCCGGCTAATTTTTTGTATTTTTAGTAGAGACG
GGGTTTCACCTTGTTAGCCAGG

>1585
CAGCCTCCCGAGTAGCTGGGACTACAGGCGCCCGCCACCGCGCCCGGCTAATTTTTG
TATTTTTAGTAGAGACGGGGTTTCACCTTGTTAGCCAGGATGGTCTCGATCTCCTGACC
TCATGATCCACCCGCCTCGGCCT
>1586
GCCTCCCGAGTAGCTGGGACTACAGGCGCCCGCCACCGCGCCCGGCTAATTTTTGTAT
TTTTAGTAGAGACGGGGTTTCACCTTGTTAGCCAGGATGGTCTCGATCTCCTGACCTCA
TGATCCACCCGCCTCGGCCTCC
>1587
TACAGGCGCCCGCCACCGCGCCCGGCTAATTTTTGTATTTTTAGTAGAGACGGGGTTT
CACCTTGTTAGCCAGGATGGTCTCGATCTCCTGACCTCATGATCCACCCGCCTCGGCCT
CCCAAAGTGCTGGGATTACAGG
>1588
GTTTCACCTTGTTAGCCAGGATGGTCTCGATCTCCTGACCTCATGATCCACCCGCCTCG
GCCTCCCAAAGTGCTGGGATTACAGGCGTGAGCACCGCGCCCGGCCCAGAGTACCTTT
CTAAAGTCTGAACAGTTTTGGTC
>1589
GCCAGGATGGTCTCGATCTCCTGACCTCATGATCCACCCGCCTCGGCCTCCCAAAGTGC
TGGGATTACAGGCGTGAGCACCGCGCCCGGCCCAGAGTACCTTTCTAAAGTCTGAACA
GTTTTGGTCGTTGAAGCACCTGA
>1590
CCTCATGATCCACCCGCCTCGGCCTCCCAAAGTGCTGGGATTACAGGCGTGAGCACCG
CGCCCGGCCCAGAGTACCTTTCTAAAGTCTGAACAGTTTTGGTCGTTGAAGCACCTGA
AGGTTTTGGATAAGGGATTATGAA
>1591
GCCTCGGCCTCCCAAAGTGCTGGGATTACAGGCGTGAGCACCGCGCCCGGCCCAGAG
TACCTTTCTAAAGTCTGAACAGTTTTGGTCGTTGAAGCACCTGAAGGTTTTGGATAAGG
GATTATGAACGATTATCATTCTCT
>1592
TCCCAAAGTGCTGGGATTACAGGCGTGAGCACCGCGCCCGGCCCAGAGTACCTTTCTA
AAGTCTGAACAGTTTTGGTCGTTGAAGCACCTGAAGGTTTTGGATAAGGGATTATGAA
CGATTATCATTCTCTATTGACAAA
>1593
ATTACAGGCGTGAGCACCGCGCCCGGCCCAGAGTACCTTTCTAAAGTCTGAACAGTTT
TGGTCGTTGAAGCACCTGAAGGTTTTGGATAAGGGATTATGAACGATTATCATTCTCTAT
TGACAAATGAGGAAAATGAGAT
>1594
GGTCGTTGAAGCACCTGAAGGTTTTGGATAAGGGATTATGAACGATTATCATTCTCTATT
GACAAATGAGGAAAATGAGATTTGGAGCAAGTAAGTGACTTCCCATGGTCACACAGCT
ATAAATGGGTAGAACCAGGCAC
>1595
AGCACCTGAAGGTTTTGGATAAGGGATTATGAACGATTATCATTCTCTATTGACAAATG
AGGAAAATGAGATTTGGAGCAAGTAAGTGACTTCCCATGGTCACACAGCTATAAATGG
GTAGAACCAGGCACTCAGCTTGC

>1596
GGATAAGGGATTATGAACGATTATCATTCTCTATTGACAAATGAGGAAAATGAGATTTG
GAGCAAGTAAGTGACTTCCCATGGTCACACAGCTATAAATGGGTAGAACCAGGCACTC
AGCTTGCCTTCTAATTCACAGTC
>1597
TATCATTCTCTATTGACAAATGAGGAAAATGAGATTTGGAGCAAGTAAGTGACTTCCCA
TGGTCACACAGCTATAAATGGGTAGAACCAGGCACTCAGCTTGCCTTCTAATTCACAGT
CCTGGGCGCTCTGCACCAGGCC
>1598
GACAAATGAGGAAAATGAGATTTGGAGCAAGTAAGTGACTTCCCATGGTCACACAGCT
ATAAATGGGTAGAACCAGGCACTCAGCTTGCCTTCTAATTCACAGTCCTGGGCGCTCTG
CACCAGGCCATATAAGAACATGA
>1599
AGTGACTTCCCATGGTCACACAGCTATAAATGGGTAGAACCAGGCACTCAGCTTGCCT
TCTAATTCACAGTCCTGGGCGCTCTGCACCAGGCCATATAAGAACATGAGCATTGCTCT
TCTGGGTCAAATCCATCTGACCT
>1600
CAGCTATAAATGGGTAGAACCAGGCACTCAGCTTGCCTTCTAATTCACAGTCCTGGGCG
CTCTGCACCAGGCCATATAAGAACATGAGCATTGCTCTTCTGGGTCAAATCCATCTGAC
CTAGTCTTTGGTTGCCATCACC
>1601
TGGGTAGAACCAGGCACTCAGCTTGCCTTCTAATTCACAGTCCTGGGCGCTCTGCACC
AGGCCATATAAGAACATGAGCATTGCTCTTCTGGGTCAAATCCATCTGACCTAGTCTTT
GGTTGCCATCACCATGCCTAAGA
>1602
CTGGGCGCTCTGCACCAGGCCATATAAGAACATGAGCATTGCTCTTCTGGGTCAAATCC
ATCTGACCTAGTCTTTGGTTGCCATCACCATGCCTAAGATACCACCATTGGAGGCTACA
ACCATCTTCCTTGACGTCAATC
>1603
GAACATGAGCATTGCTCTTCTGGGTCAAATCCATCTGACCTAGTCTTTGGTTGCCATCA
CCATGCCTAAGATACCACCATTGGAGGCTACAACCATCTTCCTTGACGTCAATCCTAAA
GGTCAGGTGTAGCTTCTCTTAG
>1604
TCTTCTGGGTCAAATCCATCTGACCTAGTCTTTGGTTGCCATCACCATGCCTAAGATACC
ACCATTGGAGGCTACAACCATCTTCCTTGACGTCAATCCTAAAGGTCAGGTGTAGCTTC
TCTTAGTAGCTATTTATGGCT
>1605
GTTGCCATCACCATGCCTAAGATACCACCATTGGAGGCTACAACCATCTTCCTTGACGT
CAATCCTAAAGGTCAGGTGTAGCTTCTCTTAGTAGCTATTTATGGCTGCCTTGTCATTCA
CAGATTTGCCAAAATCCTTTT
>1606
CATCACCATGCCTAAGATACCACCATTGGAGGCTACAACCATCTTCCTTGACGTCAATC
CTAAAGGTCAGGTGTAGCTTCTCTTAGTAGCTATTTATGGCTGCCTTGTCATTCACAGAT
TTGCCAAAATCCTTTTTGAAT

>1607
CCACCATTGGAGGCTACAACCATCTTCCTTGACGTCAATCCTAAAGGTCAGGTGTAGCT
TCTCTTAGTAGCTATTTATGGCTGCCTTGTCATTCACAGATTTGCCAAAATCCTTTTTGA
ATCCATTTCCATTTCCAGGAA
>1608
TTGACGTCAATCCTAAAGGTCAGGTGTAGCTTCTCTTAGTAGCTATTTATGGCTGCCTTG
TCATTCACAGATTTGCCAAAATCCTTTTTGAATCCATTTCCATTTCCAGGAAGTATCCTT
TTTAGGGGTGATGAATGACA
>1609
TAGCTATTTATGGCTGCCTTGTCATTCACAGATTTGCCAAAATCCTTTTTGAATCCATTTC
CATTTCCAGGAAGTATCCTTTTTAGGGGTGATGAATGACAAGTTTATAATCTCCTGGGCT
CCTGGAAATCTGGTCTTTT
>1610
TATTTATGGCTGCCTTGTCATTCACAGATTTGCCAAAATCCTTTTTGAATCCATTTCCATT
TCCAGGAAGTATCCTTTTTAGGGGTGATGAATGACAAGTTTATAATCTCCTGGGCTCCT
GGAAATCTGGTCTTTTGGAG
>1611
CCTTGTCATTCACAGATTTGCCAAAATCCTTTTTGAATCCATTTCCATTTCCAGGAAGTA
TCCTTTTTAGGGGTGATGAATGACAAGTTTATAATCTCCTGGGCTCCTGGAAATCTGGT
CTTTTGGAGAGAGGTTGTTGG
>1612
TATCCTTTTTAGGGGTGATGAATGACAAGTTTATAATCTCCTGGGCTCCTGGAAATCTGG
TCTTTTGGAGAGAGGTTGTTGGTTGTGAAGATAGGAAGATGGCAGAAGGCTTTGTAGT
GGTGAGGCCAGGTCTAGACCTA
>1613
TCCTTTTTAGGGGTGATGAATGACAAGTTTATAATCTCCTGGGCTCCTGGAAATCTGGT
CTTTTGGAGAGAGGTTGTTGGTTGTGAAGATAGGAAGATGGCAGAAGGCTTTGTAGTG
GTGAGGCCAGGTCTAGACCTAGC
>1614
GTTTATAATCTCCTGGGCTCCTGGAAATCTGGTCTTTTGGAGAGAGGTTGTTGGTTGTG
AAGATAGGAAGATGGCAGAAGGCTTTGTAGTGGTGAGGCCAGGTCTAGACCTAGCTCT
GCGTTTTTCAGCTCTGTCACCAT
>1615
CCTGGAAATCTGGTCTTTTGGAGAGAGGTTGTTGGTTGTGAAGATAGGAAGATGGCAG
AAGGCTTTGTAGTGGTGAGGCCAGGTCTAGACCTAGCTCTGCGTTTTTCAGCTCTGTCA
CCATTGACATCGTTTTTTTCATC
>1616
ATCTGGTCTTTTGGAGAGAGGTTGTTGGTTGTGAAGATAGGAAGATGGCAGAAGGCTT
TGTAGTGGTGAGGCCAGGTCTAGACCTAGCTCTGCGTTTTTCAGCTCTGTCACCATTGA
CATCGTTTTTTTCATCGTAAGCC
>1617
TGGAGAGAGGTTGTTGGTTGTGAAGATAGGAAGATGGCAGAAGGCTTTGTAGTGGTG
AGGCCAGGTCTAGACCTAGCTCTGCGTTTTTCAGCTCTGTCACCATTGACATCGTTTTT
TTCATCGTAAGCCTTAACTTCTTC

>1618
AGGCCAGGTCTAGACCTAGCTCTGCGTTTTTCAGCTCTGTCACCATTGACATCGTTTTT
TTCATCGTAAGCCTTAACTTCTTCATTTTTAAAACTAGGCACTTCAGTGAGCTCTTTCTG
GATTGTTGCGCTCTTTAAAAA
>1619
TTTTCAGCTCTGTCACCATTGACATCGTTTTTTTCATCGTAAGCCTTAACTTCTTCATTTT
TAAAACTAGGCACTTCAGTGAGCTCTTTCTGGATTGTTGCGCTCTTTAAAAAATTCTTTT
ATTCTTTGTGTACCCACTT
>1620
CACCATTGACATCGTTTTTTTCATCGTAAGCCTTAACTTCTTCATTTTTAAAACTAGGCA
CTTCAGTGAGCTCTTTCTGGATTGTTGCGCTCTTTAAAAAATTCTTTTATTCTTTGTGTA
CCCACTTATATCTTCATGAA
>1621
TCTTTCTGGATTGTTGCGCTCTTTAAAAAATTCTTTTATTCTTTGTGTACCCACTTATATC
TTCATGAAGTCCTTTTGGTTAATTGAATTTTGCTGTTTTGGAATCTGGATTGGCAGTTCA
GCCAGTTAAGTAGGGGGTC
>1622
TACCCACTTATATCTTCATGAAGTCCTTTTGGTTAATTGAATTTTGCTGTTTTGGAATCTG
GATTGGCAGTTCAGCCAGTTAAGTAGGGGGTCAGTTTGATTTGATTCTGTGTGTTCTGA
CTTTTTTTTTTTTTTTAAAT
>1623
ACTTATATCTTCATGAAGTCCTTTTGGTTAATTGAATTTTGCTGTTTTGGAATCTGGATTG
GCAGTTCAGCCAGTTAAGTAGGGGGTCAGTTTGATTTGATTCTGTGTGTTCTGACTTTT
TTTTTTTTTTTAAATACTTT
>1624
ATATCTTCATGAAGTCCTTTTGGTTAATTGAATTTTGCTGTTTTGGAATCTGGATTGGCA
GTTCAGCCAGTTAAGTAGGGGGTCAGTTTGATTTGATTCTGTGTGTTCTGACTTTTTTTT
TTTTTTTAAATACTTTAACC
>1625
CTTTTGGTTAATTGAATTTTGCTGTTTTGGAATCTGGATTGGCAGTTCAGCCAGTTAAGT
AGGGGGTCAGTTTGATTTGATTCTGTGTGTTCTGACTTTTTTTTTTTTTTTAAATACTTTA
ACCTGCTTAGGGTTACTGA
>1626
GGGGGTCAGTTTGATTTGATTCTGTGTGTTCTGACTTTTTTTTTTTTTTTAAATACTTTAA
CCTGCTTAGGGTTACTGAGCCATCTGGGATCTAGACTGAACTCTTGGAACTTTTGAAGA
TGATTTCCTTTATTTTATTT
>1627
TTGATTTGATTCTGTGTGTTCTGACTTTTTTTTTTTTTTTAAATACTTTAACCTGCTTAGG
GTTACTGAGCCATCTGGGATCTAGACTGAACTCTTGGAACTTTTGAAGATGATTTCCTT
TATTTTATTTTCAATGGAGG
>1628
TAAATACTTTAACCTGCTTAGGGTTACTGAGCCATCTGGGATCTAGACTGAACTCTTGG
AACTTTTGAAGATGATTTCCTTTATTTTATTTTCAATGGAGGAAAGTTGCCCACTCTTTC
ATCTCAGTGAGAAGTCCCATT

>1629
GCCATCTGGGATCTAGACTGAACTCTTGGAACTTTTGAAGATGATTTCCTTTATTTTATT
TTCAATGGAGGAAAGTTGCCCACTCTTTCATCTCAGTGAGAAGTCCCATTTTAATTTGC
CATCACTATTGGATGGAGCCG
>1630
TGGAACTTTTGAAGATGATTTCCTTTATTTTATTTTCAATGGAGGAAAGTTGCCCACTCT
TTCATCTCAGTGAGAAGTCCCATTTTAATTTGCCATCACTATTGGATGGAGCCGCTAATT
GCTGGGAGGGAGAAAGCTAT
>1631
ACTTTTGAAGATGATTTCCTTTATTTTATTTTCAATGGAGGAAAGTTGCCCACTCTTTCA
TCTCAGTGAGAAGTCCCATTTTAATTTGCCATCACTATTGGATGGAGCCGCTAATTGCTG
GGAGGGAGAAAGCTATAGTT
>1632
GAGGAAAGTTGCCCACTCTTTCATCTCAGTGAGAAGTCCCATTTTAATTTGCCATCACT
ATTGGATGGAGCCGCTAATTGCTGGGAGGGAGAAAGCTATAGTTTAGCAACATGTTTTC
CTCCTGCTCGTTCTAGGAAAAC
>1633
CTTTCATCTCAGTGAGAAGTCCCATTTTAATTTGCCATCACTATTGGATGGAGCCGCTAA
TTGCTGGGAGGGAGAAAGCTATAGTTTAGCAACATGTTTTCCTCCTGCTCGTTCTAGGA
AAACTATTATGAATCTTTAAA
>1634
CATCTCAGTGAGAAGTCCCATTTTAATTTGCCATCACTATTGGATGGAGCCGCTAATTGC
TGGGAGGGAGAAAGCTATAGTTTAGCAACATGTTTTCCTCCTGCTCGTTCTAGGAAAA
CTATTATGAATCTTTAAAATAG
>1635
TCCCATTTTAATTTGCCATCACTATTGGATGGAGCCGCTAATTGCTGGGAGGGAGAAAG
CTATAGTTTAGCAACATGTTTTCCTCCTGCTCGTTCTAGGAAAACTATTATGAATCTTTA
AAATAGTGATTATCCTAAACT
>1636
ATGGAGCCGCTAATTGCTGGGAGGGAGAAAGCTATAGTTTAGCAACATGTTTTCCTCCT
GCTCGTTCTAGGAAAACTATTATGAATCTTTAAAATAGTGATTATCCTAAACTGTTAAAA
TCAAATTCTATTCACAGCTCT
>1637
AAAGCTATAGTTTAGCAACATGTTTTCCTCCTGCTCGTTCTAGGAAAACTATTATGAATC
TTTAAAATAGTGATTATCCTAAACTGTTAAAATCAAATTCTATTCACAGCTCTTTGATTTC
TTGCTAATTCAGGAGAGAA
>1638
CTAGGAAAACTATTATGAATCTTTAAAATAGTGATTATCCTAAACTGTTAAAATCAAATT
CTATTCACAGCTCTTTGATTTCTTGCTAATTCAGGAGAGAAACTTTTCCAATCACTTTAA
TTTTTTTTATTAATTTAAAG
>1639
AAAATAGTGATTATCCTAAACTGTTAAAATCAAATTCTATTCACAGCTCTTTGATTTCTT
GCTAATTCAGGAGAGAAACTTTTCCAATCACTTTAATTTTTTTTATTAATTTAAAGCAAG
CCCTGCTGAAAGCCCAAGTA

>1640
AGTGATTATCCTAAACTGTTAAAATCAAATTCTATTCACAGCTCTTTGATTTCTTGCTAAT
TCAGGAGAGAAACTTTTCCAATCACTTTAATTTTTTTATTAATTTAAAGCAAGCCCTGC
TGAAAGCCCAAGTATATAT
>1641
CTCTTTGATTTCTTGCTAATTCAGGAGAGAAACTTTTCCAATCACTTTAATTTTTTTATT
AATTTAAAGCAAGCCCTGCTGAAAGCCCAAGTATATATGACTTAGAGTCTTACTAAATG
AATACAGGTGTGCCTCAGTT
>1642
TTGATTTCTTGCTAATTCAGGAGAGAAACTTTTCCAATCACTTTAATTTTTTTATTAATT
TAAAGCAAGCCCTGCTGAAAGCCCAAGTATATATGACTTAGAGTCTTACTAAATGAATA
CAGGTGTGCCTCAGTTTGTC
>1643
AGGAGAGAAACTTTTCCAATCACTTTAATTTTTTTATTAATTTAAAGCAAGCCCTGCTG
AAAGCCCAAGTATATATGACTTAGAGTCTTACTAAATGAATACAGGTGTGCCTCAGTTT
GTCCATTGGAGGGTTTCTGAA
>1644
TATTAATTTAAAGCAAGCCCTGCTGAAAGCCCAAGTATATATGACTTAGAGTCTTACTAA
ATGAATACAGGTGTGCCTCAGTTTGTCCATTGGAGGGTTTCTGAATTTTCCATAACTAA
AGATTTTCTATTCTGTTTTGG
>1645
AGCAAGCCCTGCTGAAAGCCCAAGTATATATGACTTAGAGTCTTACTAAATGAATACAG
GTGTGCCTCAGTTTGTCCATTGGAGGGTTTCTGAATTTTCCATAACTAAAGATTTTCTAT
TCTGTTTTGGCTGGGTGTGTG
>1646
AAAGCCCAAGTATATATGACTTAGAGTCTTACTAAATGAATACAGGTGTGCCTCAGTTT
GTCCATTGGAGGGTTTCTGAATTTTCCATAACTAAAGATTTTCTATTCTGTTTTGGCTGG
GTGTGTGGGGTGAAAGCTCTT
>1647
TCTTACTAAATGAATACAGGTGTGCCTCAGTTTGTCCATTGGAGGGTTTCTGAATTTTCC
ATAACTAAAGATTTTCTATTCTGTTTTGGCTGGGTGTGTGGGGTGAAAGCTCTTACTCTC
TGGCAGTTATTTAAGTAGTA
>1648
TGGAGGGTTTCTGAATTTTCCATAACTAAAGATTTTCTATTCTGTTTTGGCTGGGTGTGT
GGGGTGAAAGCTCTTACTCTCTGGCAGTTATTTAAGTAGTAAGAAATTTGCTACAACTC
TTCCCCACCCCCACGTTTTAC
>1649
TTCCATAACTAAAGATTTTCTATTCTGTTTTGGCTGGGTGTGTGGGGTGAAAGCTCTTAC
TCTCTGGCAGTTATTTAAGTAGTAAGAAATTTGCTACAACTCTTCCCCACCCCCACGTTT
TACAGATAGGAAATGAGCAC
>1650
TAAAGATTTTCTATTCTGTTTTGGCTGGGTGTGTGGGGTGAAAGCTCTTACTCTCTGGC
AGTTATTTAAGTAGTAAGAAATTTGCTACAACTCTTCCCCACCCCCACGTTTTACAGATA
GGAAATGAGCACTTTGACTGT

>1651
AGATTTTCTATTCTGTTTTGGCTGGGTGTGTGGGGTGAAAGCTCTTACTCTCTGGCAGT
TATTTAAGTAGTAAGAAATTTGCTACAACTCTTCCCCACCCCCACGTTTTACAGATAGG
AAATGAGCACTTTGACTGTCCA
>1652
CTTACTCTCTGGCAGTTATTTAAGTAGTAAGAAATTTGCTACAACTCTTCCCCACCCCCA
CGTTTTACAGATAGGAAATGAGCACTTTGACTGTCCAGGTCAGACTTCTGATTTTGCTA
CTTACTAACTGTGACAGTGGT
>1653
CAGTTATTTAAGTAGTAAGAAATTTGCTACAACTCTTCCCCACCCCCACGTTTTACAGAT
AGGAAATGAGCACTTTGACTGTCCAGGTCAGACTTCTGATTTTGCTACTTACTAACTGT
GACAGTGGTTGTGTTACTTAA
>1654
AAGAAATTTGCTACAACTCTTCCCCACCCCCACGTTTTACAGATAGGAAATGAGCACTT
TGACTGTCCAGGTCAGACTTCTGATTTTGCTACTTACTAACTGTGACAGTGGTTGTGTT
ACTTAACTTCTGTGTGCCTGTT
>1655
ACAGATAGGAAATGAGCACTTTGACTGTCCAGGTCAGACTTCTGATTTTGCTACTTACT
AACTGTGACAGTGGTTGTGTTACTTAACTTCTGTGTGCCTGTTTGCTCATTTGTGAAAC
AGAAATAATAGTAGTACCTACT
>1656
CTACTTACTAACTGTGACAGTGGTTGTGTTACTTAACTTCTGTGTGCCTGTTTGCTCATT
TGTGAAACAGAAATAATAGTAGTACCTACTTCATTGGGTTGTTATAAGGATTCATCAGA
AAATATTTATCCTGTGTGAGG
>1657
AACTGTGACAGTGGTTGTGTTACTTAACTTCTGTGTGCCTGTTTGCTCATTTGTGAAAC
AGAAATAATAGTAGTACCTACTTCATTGGGTTGTTATAAGGATTCATCAGAAAATATTTA
TCCTGTGTGAGGAACGGTGGC
>1658
TGTGACAGTGGTTGTGTTACTTAACTTCTGTGTGCCTGTTTGCTCATTTGTGAAACAGA
AATAATAGTAGTACCTACTTCATTGGGTTGTTATAAGGATTCATCAGAAAATATTTATCCT
GTGTGAGGAACGGTGGCACA
>1659
TCTGTGTGCCTGTTTGCTCATTTGTGAAACAGAAATAATAGTAGTACCTACTTCATTGGG
TTGTTATAAGGATTCATCAGAAAATATTTATCCTGTGTGAGGAACGGTGGCACATAGTA
AGTGCTCCATAAGTGTTTGCT
>1660
TGTTTGCTCATTTGTGAAACAGAAATAATAGTAGTACCTACTTCATTGGGTTGTTATAAG
GATTCATCAGAAAATATTTATCCTGTGTGAGGAACGGTGGCACATAGTAAGTGCTCCAT
AAGTGTTTGCTAGGGTATTTT
>1661
CTACTTCATTGGGTTGTTATAAGGATTCATCAGAAAATATTTATCCTGTGTGAGGAACGG
TGGCACATAGTAAGTGCTCCATAAGTGTTTGCTAGGGTATTTTTCTTGTCATTGTGTGAA
GTAGGTCATGCTAGCCATGC

>1662
TTCATTGGGTTGTTATAAGGATTCATCAGAAAATATTTATCCTGTGTGAGGAACGGTGGC
ACATAGTAAGTGCTCCATAAGTGTTTGCTAGGGTATTTTTCTTGTCATTGTGTGAAGTAG
GTCATGCTAGCCATGCAGGG
>1663
TTCATCAGAAAATATTTATCCTGTGTGAGGAACGGTGGCACATAGTAAGTGCTCCATAA
GTGTTTGCTAGGGTATTTTTCTTGTCATTGTGTGAAGTAGGTCATGCTAGCCATGCAGG
GTGAAAAATGTGTTCATGTTGG
>1664
GAGGAACGGTGGCACATAGTAAGTGCTCCATAAGTGTTTGCTAGGGTATTTTTCTTGTC
ATTGTGTGAAGTAGGTCATGCTAGCCATGCAGGGTGAAAAATGTGTTCATGTTGGTCCA
GATCCTGCTTTCTCCCTGAGAC
>1665
GAACGGTGGCACATAGTAAGTGCTCCATAAGTGTTTGCTAGGGTATTTTTCTTGTCATTG
TGTGAAGTAGGTCATGCTAGCCATGCAGGGTGAAAAATGTGTTCATGTTGGTCCAGATC
CTGCTTTCTCCCTGAGACCGT
>1666
ACATAGTAAGTGCTCCATAAGTGTTTGCTAGGGTATTTTTCTTGTCATTGTGTGAAGTAG
GTCATGCTAGCCATGCAGGGTGAAAAATGTGTTCATGTTGGTCCAGATCCTGCTTTCTC
CCTGAGACCGTTCTTCCGCAT
>1667
TTTTCTTGTCATTGTGTGAAGTAGGTCATGCTAGCCATGCAGGGTGAAAAATGTGTTCA
TGTTGGTCCAGATCCTGCTTTCTCCCTGAGACCGTTCTTCCGCATAGGCGTCAATCCGT
ACAGTGCTGTAAAACTGCTGCC
>1668
AGTAGGTCATGCTAGCCATGCAGGGTGAAAAATGTGTTCATGTTGGTCCAGATCCTGCT
TTCTCCCTGAGACCGTTCTTCCGCATAGGCGTCAATCCGTACAGTGCTGTAAAACTGCT
GCCTCGGGCACCTCATGTTTGG
>1669
ATGCAGGGTGAAAAATGTGTTCATGTTGGTCCAGATCCTGCTTTCTCCCTGAGACCGTT
CTTCCGCATAGGCGTCAATCCGTACAGTGCTGTAAAACTGCTGCCTCGGGCACCTCATG
TTTGGGGCTGAGACATTGCTTC
>1670
GTGTTCATGTTGGTCCAGATCCTGCTTTCTCCCTGAGACCGTTCTTCCGCATAGGCGTC
AATCCGTACAGTGCTGTAAAACTGCTGCCTCGGGCACCTCATGTTTGGGGCTGAGACA
TTGCTTCTGCGTCTTCCCCAGAA
>1671
TTCCGCATAGGCGTCAATCCGTACAGTGCTGTAAAACTGCTGCCTCGGGCACCTCATGT
TTGGGGCTGAGACATTGCTTCTGCGTCTTCCCCAGAAGCTGATCTCTTGTACTGTGGTG
GCCAGGGTACTCTTTGTCCCAT
>1672
AGTGCTGTAAAACTGCTGCCTCGGGCACCTCATGTTTGGGGCTGAGACATTGCTTCTG
CGTCTTCCCCAGAAGCTGATCTCTTGTACTGTGGTGGCCAGGGTACTCTTTGTCCCATT
GACCTGAGGCCTGGTGAGTCATT

>1673
TCATGTTTGGGGCTGAGACATTGCTTCTGCGTCTTCCCCAGAAGCTGATCTCTTGTACT
GTGGTGGCCAGGGTACTCTTTGTCCCATTGACCTGAGGCCTGGTGAGTCATTCCAGTTC
TCGTTTAAATTTAGCCGGGCAG
>1674
CTGCGTCTTCCCCAGAAGCTGATCTCTTGTACTGTGGTGGCCAGGGTACTCTTTGTCCC
ATTGACCTGAGGCCTGGTGAGTCATTCCAGTTCTCGTTTAAATTTAGCCGGGCAGCGCT
CCACTGCCATCGGGTCCAAATG
>1675
TGATCTCTTGTACTGTGGTGGCCAGGGTACTCTTTGTCCCATTGACCTGAGGCCTGGTG
AGTCATTCCAGTTCTCGTTTAAATTTAGCCGGGCAGCGCTCCACTGCCATCGGGTCCAA
ATGCCGGGGAGGCTCACTGATG
>1676
GTGGCCAGGGTACTCTTTGTCCCATTGACCTGAGGCCTGGTGAGTCATTCCAGTTCTCG
TTTAAATTTAGCCGGGCAGCGCTCCACTGCCATCGGGTCCAAATGCCGGGGAGGCTCA
CTGATGCGGTGTCCTGTTGCCTG
>1677
AGTCATTCCAGTTCTCGTTTAAATTTAGCCGGGCAGCGCTCCACTGCCATCGGGTCCAA
ATGCCGGGGAGGCTCACTGATGCGGTGTCCTGTTGCCTGTGCACGTGGCCAGGCGTGC
ACATCACTGTGCTTATGAGAGAG
>1678
CACTGCCATCGGGTCCAAATGCCGGGGAGGCTCACTGATGCGGTGTCCTGTTGCCTGT
GCACGTGGCCAGGCGTGCACATCACTGTGCTTATGAGAGAGATGAAGAGCACAGATAC
CACCTTACTGATGGGGGTCCTGGG
>1679
GGAGGCTCACTGATGCGGTGTCCTGTTGCCTGTGCACGTGGCCAGGCGTGCACATCAC
TGTGCTTATGAGAGAGATGAAGAGCACAGATACCACCTTACTGATGGGGGTCCTGGGG
CTGGGCTTGTGGGTGTTGGGGGTT
>1680
AGGCTCACTGATGCGGTGTCCTGTTGCCTGTGCACGTGGCCAGGCGTGCACATCACTG
TGCTTATGAGAGAGATGAAGAGCACAGATACCACCTTACTGATGGGGGTCCTGGGGCT
GGGCTTGTGGGTGTTGGGGGTTCC
>1681
GTGTCCTGTTGCCTGTGCACGTGGCCAGGCGTGCACATCACTGTGCTTATGAGAGAGA
TGAAGAGCACAGATACCACCTTACTGATGGGGGTCCTGGGGCTGGGCTTGTGGGTGTT
GGGGGTTCCAGAGCTCCTTCCCTT
>1682
ATGAAGAGCACAGATACCACCTTACTGATGGGGGTCCTGGGGCTGGGCTTGTGGGTGT
TGGGGGTTCCAGAGCTCCTTCCCTTTCCTGCCTGTCAGTGCAGGATGCTTCTAAAATGT
GGACTCAGCAGACATTGTTTTGA
>1683
GAAGAGCACAGATACCACCTTACTGATGGGGGTCCTGGGGCTGGGCTTGTGGGTGTTG
GGGGTTCCAGAGCTCCTTCCCTTTCCTGCCTGTCAGTGCAGGATGCTTCTAAAATGTGG
ACTCAGCAGACATTGTTTTGAGT

>1684
GGGTCCTGGGGCTGGGCTTGTGGGTGTTGGGGGTTCCAGAGCTCCTTCCCTTTCCTGC
CTGTCAGTGCAGGATGCTTCTAAAATGTGGACTCAGCAGACATTGTTTTGAGTGCTTGC
TACATGTCAGATTCTGTGCTAAG
>1685
TGTTGGGGGTTCCAGAGCTCCTTCCCTTTCCTGCCTGTCAGTGCAGGATGCTTCTAAAA
TGTGGACTCAGCAGACATTGTTTTGAGTGCTTGCTACATGTCAGATTCTGTGCTAAGTG
CACTCACAGCCAGCATCTCTGT
>1686
TGGGGGTTCCAGAGCTCCTTCCCTTTCCTGCCTGTCAGTGCAGGATGCTTCTAAAATGT
GGACTCAGCAGACATTGTTTTGAGTGCTTGCTACATGTCAGATTCTGTGCTAAGTGCAC
TCACAGCCAGCATCTCTGTAAG
>1687
CCTGTCAGTGCAGGATGCTTCTAAAATGTGGACTCAGCAGACATTGTTTTGAGTGCTTG
CTACATGTCAGATTCTGTGCTAAGTGCACTCACAGCCAGCATCTCTGTAAGACCTCTAG
CTGTCCTGTAAGGTGGGTATCA
>1688
GATGCTTCTAAAATGTGGACTCAGCAGACATTGTTTTGAGTGCTTGCTACATGTCAGAT
TCTGTGCTAAGTGCACTCACAGCCAGCATCTCTGTAAGACCTCTAGCTGTCCTGTAAGG
TGGGTATCAGTTTCACATTCTC
>1689
AATGTGGACTCAGCAGACATTGTTTTGAGTGCTTGCTACATGTCAGATTCTGTGCTAAG
TGCACTCACAGCCAGCATCTCTGTAAGACCTCTAGCTGTCCTGTAAGGTGGGTATCAGT
TTCACATTCTCAGATGGGCGGA
>1690
ACATTGTTTTGAGTGCTTGCTACATGTCAGATTCTGTGCTAAGTGCACTCACAGCCAGC
ATCTCTGTAAGACCTCTAGCTGTCCTGTAAGGTGGGTATCAGTTTCACATTCTCAGATG
GGCGGAGAGAAGCCCAAGAGGT
>1691
TTCTGTGCTAAGTGCACTCACAGCCAGCATCTCTGTAAGACCTCTAGCTGTCCTGTAAG
GTGGGTATCAGTTTCACATTCTCAGATGGGCGGAGAGAAGCCCAAGAGGTTAGTGGAC
TTTTCTCTAGTAAAGAGTTGTAC
>1692
CACTCACAGCCAGCATCTCTGTAAGACCTCTAGCTGTCCTGTAAGGTGGGTATCAGTTT
CACATTCTCAGATGGGCGGAGAGAAGCCCAAGAGGTTAGTGGACTTTTCTCTAGTAAA
GAGTTGTACAGGCCGGGCGCAGT
>1693
CAGCATCTCTGTAAGACCTCTAGCTGTCCTGTAAGGTGGGTATCAGTTTCACATTCTCA
GATGGGCGGAGAGAAGCCCAAGAGGTTAGTGGACTTTTCTCTAGTAAAGAGTTGTACA
GGCCGGGCGCAGTGGCTCATGCC
>1694
AGGTGGGTATCAGTTTCACATTCTCAGATGGGCGGAGAGAAGCCCAAGAGGTTAGTGG
ACTTTTCTCTAGTAAAGAGTTGTACAGGCCGGGCGCAGTGGCTCATGCCTGTAATCCCA
GCACTTTGGGAGGCCGAGGCGGG

>1695
GGTATCAGTTTCACATTCTCAGATGGGCGGAGAGAAGCCCAAGAGGTTAGTGGACTTT
TCTCTAGTAAAGAGTTGTACAGGCCGGGCGCAGTGGCTCATGCCTGTAATCCCAGCAC
TTTGGGAGGCCGAGGCGGGCGGAT
>1696
TCACATTCTCAGATGGGCGGAGAGAAGCCCAAGAGGTTAGTGGACTTTTCTCTAGTAA
AGAGTTGTACAGGCCGGGCGCAGTGGCTCATGCCTGTAATCCCAGCACTTTGGGAGGC
CGAGGCGGGCGGATCACGAGGTCA
>1697
AGGTTAGTGGACTTTTCTCTAGTAAAGAGTTGTACAGGCCGGGCGCAGTGGCTCATGC
CTGTAATCCCAGCACTTTGGGAGGCCGAGGCGGGCGGATCACGAGGTCAGGAGATCG
AGACCATCCTGGCTAACACAGTGAA
>1698
CTTTTCTCTAGTAAAGAGTTGTACAGGCCGGGCGCAGTGGCTCATGCCTGTAATCCCAG
CACTTTGGGAGGCCGAGGCGGGCGGATCACGAGGTCAGGAGATCGAGACCATCCTGG
CTAACACAGTGAAACCCCGTCTCT
>1699
TCTAGTAAAGAGTTGTACAGGCCGGGCGCAGTGGCTCATGCCTGTAATCCCAGCACTT
TGGGAGGCCGAGGCGGGCGGATCACGAGGTCAGGAGATCGAGACCATCCTGGCTAAC
ACAGTGAAACCCCGTCTCTACTAAA
>1700
ACAGGCCGGGCGCAGTGGCTCATGCCTGTAATCCCAGCACTTTGGGAGGCCGAGGCG
GGCGGATCACGAGGTCAGGAGATCGAGACCATCCTGGCTAACACAGTGAAACCCCGT
CTCTACTAAAAATACAAAAAATTAGC
>1701
GTGGCTCATGCCTGTAATCCCAGCACTTTGGGAGGCCGAGGCGGGCGGATCACGAGGT
CAGGAGATCGAGACCATCCTGGCTAACACAGTGAAACCCCGTCTCTACTAAAAATACA
AAAAATTAGCCGGGCGTGGTGGGG
>1702
GGTCAGGAGATCGAGACCATCCTGGCTAACACAGTGAAACCCCGTCTCTACTAAAAAT
ACAAAAAATTAGCCGGGCGTGGTGGGGGGCGCCTGTAGTCCCAGCTACTCGGGAGGC
TGAGGCAGGAGAATGGCGTGAACCC
>1703
TAACACAGTGAAACCCCGTCTCTACTAAAAATACAAAAAATTAGCCGGGCGTGGTGGG
GGGCGCCTGTAGTCCCAGCTACTCGGGAGGCTGAGGCAGGAGAATGGCGTGAACCCG
GGAGGCAGAGCTTGCAGTGAGCCGA
>1704
AGTGAAACCCCGTCTCTACTAAAAATACAAAAAATTAGCCGGGCGTGGTGGGGGGCGC
CTGTAGTCCCAGCTACTCGGGAGGCTGAGGCAGGAGAATGGCGTGAACCCGGGAGGC
AGAGCTTGCAGTGAGCCGAGATCAT
>1705
GTCTCTACTAAAAATACAAAAAATTAGCCGGGCGTGGTGGGGGGCGCCTGTAGTCCCA
GCTACTCGGGAGGCTGAGGCAGGAGAATGGCGTGAACCCGGGAGGCAGAGCTTGCAG
TGAGCCGAGATCATGCCACTCACTA

>1706
ACTAAAAATACAAAAAATTAGCCGGGCGTGGTGGGGGGCGCCTGTAGTCCCAGCTACT
CGGGAGGCTGAGGCAGGAGAATGGCGTGAACCCGGGAGGCAGAGCTTGCAGTGAGC
CGAGATCATGCCACTCACTACACTCC
>1707
CGTGGTGGGGGGCGCCTGTAGTCCCAGCTACTCGGGAGGCTGAGGCAGGAGAATGGC
GTGAACCCGGGAGGCAGAGCTTGCAGTGAGCCGAGATCATGCCACTCACTACACTCC
AGCCTGGGCAAAGGAGTGAGACTGTC
>1708
GCCTGTAGTCCCAGCTACTCGGGAGGCTGAGGCAGGAGAATGGCGTGAACCCGGGAG
GCAGAGCTTGCAGTGAGCCGAGATCATGCCACTCACTACACTCCAGCCTGGGCAAAG
GAGTGAGACTGTCTCAAAAAAAAAAA
>1709
GTAGTCCCAGCTACTCGGGAGGCTGAGGCAGGAGAATGGCGTGAACCCGGGAGGCAG
AGCTTGCAGTGAGCCGAGATCATGCCACTCACTACACTCCAGCCTGGGCAAAGGAGTG
AGACTGTCTCAAAAAAAAAAAAAAA
>1710
CCCAGCTACTCGGGAGGCTGAGGCAGGAGAATGGCGTGAACCCGGGAGGCAGAGCTT
GCAGTGAGCCGAGATCATGCCACTCACTACACTCCAGCCTGGGCAAAGGAGTGAGAC
TGTCTCAAAAAAAAAAAAAAAAAAAA
>1711
AGGAGAATGGCGTGAACCCGGGAGGCAGAGCTTGCAGTGAGCCGAGATCATGCCACT
CACTACACTCCAGCCTGGGCAAAGGAGTGAGACTGTCTCAAAAAAAAAAAAAAAAA
AAAAGAGTTGTACCAGGATTTATGTTC
>1712
CGGGAGGCAGAGCTTGCAGTGAGCCGAGATCATGCCACTCACTACACTCCAGCCTGG
GCAAAGGAGTGAGACTGTCTCAAAAAAAAAAAAAAAAAAAAAGAGTTGTACCAGGA
TTTATGTTCAGGTCTTCTGGCTCCATG
>1713
CATGCCACTCACTACACTCCAGCCTGGGCAAAGGAGTGAGACTGTCTCAAAAAAAAA
AAAAAAAAAAAAGAGTTGTACCAGGATTTATGTTCAGGTCTTCTGGCTCCATGCTCTCT
GCAGCTGGGTGACCAGGGGGCTCA
>1714
TGCCACTCACTACACTCCAGCCTGGGCAAAGGAGTGAGACTGTCTCAAAAAAAAAAA
AAAAAAAAAAGAGTTGTACCAGGATTTATGTTCAGGTCTTCTGGCTCCATGCTCTCTGC
AGCTGGGTGACCAGGGGGCTCATG
>1715
AAAAAAAAAAAAAAAAAAAGAGTTGTACCAGGATTTATGTTCAGGTCTTCTGGCTCCA
TGCTCTCTGCAGCTGGGTGACCAGGGGGCTCATGGCTTCACCAGCTCTGGCCTCCTCA
GTTCCTCTGGTGGCTTCTTGCGAG
>1716
AAAAAAAGAGTTGTACCAGGATTTATGTTCAGGTCTTCTGGCTCCATGCTCTCTGCAGC
TGGGTGACCAGGGGGCTCATGGCTTCACCAGCTCTGGCCTCCTCAGTTCCTCTGGTGG
CTTCTTGCGAGGAGGAAGGTTAC

>1717
ATTTATGTTCAGGTCTTCTGGCTCCATGCTCTCTGCAGCTGGGTGACCAGGGGGCTCAT
GGCTTCACCAGCTCTGGCCTCCTCAGTTCCTCTGGTGGCTTCTTGCGAGGAGGAAGGT
TACTGCAGAGGGTTCAGCTCTTT
>1718
CTGGGTGACCAGGGGGCTCATGGCTTCACCAGCTCTGGCCTCCTCAGTTCCTCTGGTG
GCTTCTTGCGAGGAGGAAGGTTACTGCAGAGGGTTCAGCTCTTTAGGGGCCCAGGCC
GCATGGATCCAAGGACTAGTACGTC
>1719
GGTGACCAGGGGGCTCATGGCTTCACCAGCTCTGGCCTCCTCAGTTCCTCTGGTGGCT
TCTTGCGAGGAGGAAGGTTACTGCAGAGGGTTCAGCTCTTTAGGGGCCCAGGCCGCAT
GGATCCAAGGACTAGTACGTCATC
>1720
CACCAGCTCTGGCCTCCTCAGTTCCTCTGGTGGCTTCTTGCGAGGAGGAAGGTTACTG
CAGAGGGTTCAGCTCTTTAGGGGCCCAGGCCGCATGGATCCAAGGACTAGTACGTCAT
CTCTTACCAACACAGAGAGATCAG
>1721
CTCAGTTCCTCTGGTGGCTTCTTGCGAGGAGGAAGGTTACTGCAGAGGGTTCAGCTCT
TTAGGGGCCCAGGCCGCATGGATCCAAGGACTAGTACGTCATCTCTTACCAACACAGA
GAGATCAGCTTCTAGTGATAGAGG
>1722
GCTTCTTGCGAGGAGGAAGGTTACTGCAGAGGGTTCAGCTCTTTAGGGGCCCAGGCC
GCATGGATCCAAGGACTAGTACGTCATCTCTTACCAACACAGAGAGATCAGCTTCTAGT
GATAGAGGCCTTTTATGCTGACTT
>1723
TGCGAGGAGGAAGGTTACTGCAGAGGGTTCAGCTCTTTAGGGGCCCAGGCCGCATGG
ATCCAAGGACTAGTACGTCATCTCTTACCAACACAGAGAGATCAGCTTCTAGTGATAGA
GGCCTTTTATGCTGACTTGAAAAA
>1724
GGTTCAGCTCTTTAGGGGCCCAGGCCGCATGGATCCAAGGACTAGTACGTCATCTCTTA
CCAACACAGAGAGATCAGCTTCTAGTGATAGAGGCCTTTTATGCTGACTTGAAAAACA
GAAAAAGAGAGAAAAAAGAAACC
>1725
GGGGCCCAGGCCGCATGGATCCAAGGACTAGTACGTCATCTCTTACCAACACAGAGAG
ATCAGCTTCTAGTGATAGAGGCCTTTTATGCTGACTTGAAAAACAGAAAAAGAGAGAA
AAAAGAAACCCATTTACAAAAATA
>1726
CAGGCCGCATGGATCCAAGGACTAGTACGTCATCTCTTACCAACACAGAGAGATCAGC
TTCTAGTGATAGAGGCCTTTTATGCTGACTTGAAAAACAGAAAAAGAGAGAAAAAAG
AAACCCATTTACAAAAATAAAAATC
>1727
GTCATCTCTTACCAACACAGAGAGATCAGCTTCTAGTGATAGAGGCCTTTTATGCTGAC
TTGAAAAACAGAAAAAGAGAGAAAAAAGAAACCCATTTACAAAAATAAAAATCCCAT
GCTGTTAATTTCATGCCAGCATAA

>1728
CAGAGAGATCAGCTTCTAGTGATAGAGGCCTTTTATGCTGACTTGAAAAACAGAAAAA
GAGAGAAAAAAGAAACCCATTTACAAAAATAAAAATCCCATGCTGTTAATTTCATGCC
AGCATAATCAGAAATTCCCCTTTC
>1729
AACAGAAAAAGAGAGAAAAAAGAAACCCATTTACAAAAATAAAAATCCCATGCTGTT
AATTTCATGCCAGCATAATCAGAAATTCCCCTTTCTACTGTTTCCCCTTAAAAGCCCCGG
TGTCCTTATTATGCTGGTGCAGT
>1730
AGAGAGAAAAAAGAAACCCATTTACAAAAATAAAAATCCCATGCTGTTAATTTCATGC
CAGCATAATCAGAAATTCCCCTTTCTACTGTTTCCCCTTAAAAGCCCCGGTGTCCTTATT
ATGCTGGTGCAGTCTGGAAAAA
>1731
AAAATCCCATGCTGTTAATTTCATGCCAGCATAATCAGAAATTCCCCTTTCTACTGTTTC
CCCTTAAAAGCCCCGGTGTCCTTATTATGCTGGTGCAGTCTGGAAAAACTATGGTTGGG
TTTTGCCTACGTGAGGCTTAT
>1732
GCATAATCAGAAATTCCCCTTTCTACTGTTTCCCCTTAAAAGCCCCGGTGTCCTTATTAT
GCTGGTGCAGTCTGGAAAAACTATGGTTGGGTTTTGCCTACGTGAGGCTTATCTCTGCA
GCTGCAAGGAGGGGATCCGGT
>1733
TAAAAGCCCCGGTGTCCTTATTATGCTGGTGCAGTCTGGAAAAACTATGGTTGGGTTTT
GCCTACGTGAGGCTTATCTCTGCAGCTGCAAGGAGGGGATCCGGTACTTTATTGTCAGC
ATGGAAACTTCCGCTAAATACT
>1734
TCCTTATTATGCTGGTGCAGTCTGGAAAAACTATGGTTGGGTTTTGCCTACGTGAGGCT
TATCTCTGCAGCTGCAAGGAGGGGATCCGGTACTTTATTGTCAGCATGGAAACTTCCGC
TAAATACTTAGAAAAACCTTAA
>1735
TGGTTGGGTTTTGCCTACGTGAGGCTTATCTCTGCAGCTGCAAGGAGGGGATCCGGTA
CTTTATTGTCAGCATGGAAACTTCCGCTAAATACTTAGAAAAACCTTAATTCTAAATTCC
TAGCTCACTTTCAACTGTGTCC
>1736
TATCTCTGCAGCTGCAAGGAGGGGATCCGGTACTTTATTGTCAGCATGGAAACTTCCGC
TAAATACTTAGAAAAACCTTAATTCTAAATTCCTAGCTCACTTTCAACTGTGTCCTCGGG
ACCTGATTTTCTTCGGTGTGA
>1737
TCCGGTACTTTATTGTCAGCATGGAAACTTCCGCTAAATACTTAGAAAAACCTTAATTCT
AAATTCCTAGCTCACTTTCAACTGTGTCCTCGGGACCTGATTTTCTTCGGTGTGAACAG
ATTGTGTTCAGCATTGCAGTA
>1738
ACCTTAATTCTAAATTCCTAGCTCACTTTCAACTGTGTCCTCGGGACCTGATTTTCTTCG
GTGTGAACAGATTGTGTTCAGCATTGCAGTAGAAAGGGTCCAAGTTAGGTAAGAGAAA
GGACTTCCTGAGACACTGGGGT

>1739
AAATTCCTAGCTCACTTTCAACTGTGTCCTCGGGACCTGATTTTCTTCGGTGTGAACAG
ATTGTGTTCAGCATTGCAGTAGAAAGGGTCCAAGTTAGGTAAGAGAAAGGACTTCCTG
AGACACTGGGGTGTTACTGAGAA
>1740
AGCTCACTTTCAACTGTGTCCTCGGGACCTGATTTTCTTCGGTGTGAACAGATTGTGTT
CAGCATTGCAGTAGAAAGGGTCCAAGTTAGGTAAGAGAAAGGACTTCCTGAGACACT
GGGGTGTTACTGAGAAAGTTGTTA
>1741
GTGTCCTCGGGACCTGATTTTCTTCGGTGTGAACAGATTGTGTTCAGCATTGCAGTAGA
AAGGGTCCAAGTTAGGTAAGAGAAAGGACTTCCTGAGACACTGGGGTGTTACTGAGA
AAGTTGTTAGAAGACTGTCACAGA
>1742
GTGTGAACAGATTGTGTTCAGCATTGCAGTAGAAAGGGTCCAAGTTAGGTAAGAGAAA
GGACTTCCTGAGACACTGGGGTGTTACTGAGAAAGTTGTTAGAAGACTGTCACAGACA
TTTTGATGAAGGGGGGACATTCTG
>1743
AGCATTGCAGTAGAAAGGGTCCAAGTTAGGTAAGAGAAAGGACTTCCTGAGACACTG
GGGTGTTACTGAGAAAGTTGTTAGAAGACTGTCACAGACATTTTGATGAAGGGGGGAC
ATTCTGGGCCATTTGAAGTGGTTTT
>1744
TTGCAGTAGAAAGGGTCCAAGTTAGGTAAGAGAAAGGACTTCCTGAGACACTGGGGT
GTTACTGAGAAAGTTGTTAGAAGACTGTCACAGACATTTTGATGAAGGGGGGACATTC
TGGGCCATTTGAAGTGGTTTTTAGA
>1745
GTTAGGTAAGAGAAAGGACTTCCTGAGACACTGGGGTGTTACTGAGAAAGTTGTTAG
AAGACTGTCACAGACATTTTGATGAAGGGGGGACATTCTGGGCCATTTGAAGTGGTTT
TTAGAGTTCTTTCCTCTTTAGGGCC
>1746
TACTGAGAAAGTTGTTAGAAGACTGTCACAGACATTTTGATGAAGGGGGGACATTCTG
GGCCATTTGAAGTGGTTTTTAGAGTTCTTTCCTCTTTAGGGCCATTCGGTTCCCCCAGA
AATAAGAAGAAAAGACATATACA
>1747
GTTGTTAGAAGACTGTCACAGACATTTTGATGAAGGGGGGACATTCTGGGCCATTTGA
AGTGGTTTTTAGAGTTCTTTCCTCTTTAGGGCCATTCGGTTCCCCCAGAAATAAGAAGA
AAAGACATATACACGCACATAAA
>1748
GGGGACATTCTGGGCCATTTGAAGTGGTTTTTAGAGTTCTTTCCTCTTTAGGGCCATTC
GGTTCCCCCAGAAATAAGAAGAAAAGACATATACACGCACATAAACCACCACCACCAG
GCTTGCTGAGAACAAGTCAAATG
>1749
TTCTGGGCCATTTGAAGTGGTTTTTAGAGTTCTTTCCTCTTTAGGGCCATTCGGTTCCCC
CAGAAATAAGAAGAAAAGACATATACACGCACATAAACCACCACCACCAGGCTTGCTG
AGAACAAGTCAAATGTGCCACC

>1750
CATTTGAAGTGGTTTTTAGAGTTCTTTCCTCTTTAGGGCCATTCGGTTCCCCCAGAAATA
AGAAGAAAAGACATATACACGCACATAAACCACCACCACCAGGCTTGCTGAGAACAA
GTCAAATGTGCCACCAAACCATC
>1751
TTAGGGCCATTCGGTTCCCCCAGAAATAAGAAGAAAAGACATATACACGCACATAAAC
CACCACCACCAGGCTTGCTGAGAACAAGTCAAATGTGCCACCAAACCATCGTCTTAGT
GGGAGATGGGGTGGCACTGCAGGG
>1752
TCGGTTCCCCCAGAAATAAGAAGAAAAGACATATACACGCACATAAACCACCACCACC
AGGCTTGCTGAGAACAAGTCAAATGTGCCACCAAACCATCGTCTTAGTGGGAGATGGG
GTGGCACTGCAGGGGCAGAAAGAC
>1753
CCCCCAGAAATAAGAAGAAAAGACATATACACGCACATAAACCACCACCACCAGGCTT
GCTGAGAACAAGTCAAATGTGCCACCAAACCATCGTCTTAGTGGGAGATGGGGTGGC
ACTGCAGGGGCAGAAAGACTGACGT
>1754
CACGCACATAAACCACCACCACCAGGCTTGCTGAGAACAAGTCAAATGTGCCACCAA
ACCATCGTCTTAGTGGGAGATGGGGTGGCACTGCAGGGGCAGAAAGACTGACGTCTG
ATGGACTTGGGTTTGAATACTAGTGC
>1755
AGGCTTGCTGAGAACAAGTCAAATGTGCCACCAAACCATCGTCTTAGTGGGAGATGGG
GTGGCACTGCAGGGGCAGAAAGACTGACGTCTGATGGACTTGGGTTTGAATACTAGTG
CTCCATGGTGAATTTGGGCAAATG
>1756
GCTGAGAACAAGTCAAATGTGCCACCAAACCATCGTCTTAGTGGGAGATGGGGTGGC
ACTGCAGGGGCAGAAAGACTGACGTCTGATGGACTTGGGTTTGAATACTAGTGCTCCA
TGGTGAATTTGGGCAAATGACTTCA
>1757
AGAACAAGTCAAATGTGCCACCAAACCATCGTCTTAGTGGGAGATGGGGTGGCACTGC
AGGGGCAGAAAGACTGACGTCTGATGGACTTGGGTTTGAATACTAGTGCTCCATGGTG
AATTTGGGCAAATGACTTCACCTC
>1758
TAGTGGGAGATGGGGTGGCACTGCAGGGGCAGAAAGACTGACGTCTGATGGACTTGG
GTTTGAATACTAGTGCTCCATGGTGAATTTGGGCAAATGACTTCACCTCTGTGAAGCAG
CAGTATCCTCTTCTGTGAAGTGAG
>1759
TCTGATGGACTTGGGTTTGAATACTAGTGCTCCATGGTGAATTTGGGCAAATGACTTCA
CCTCTGTGAAGCAGCAGTATCCTCTTCTGTGAAGTGAGGTTAGCCCCTACCTCTCAATG
TGGTTTTTAAAGTATGTTAATG
>1760
GATGGACTTGGGTTTGAATACTAGTGCTCCATGGTGAATTTGGGCAAATGACTTCACCT
CTGTGAAGCAGCAGTATCCTCTTCTGTGAAGTGAGGTTAGCCCCTACCTCTCAATGTGG
TTTTTAAAGTATGTTAATGAGA

>1761
CATGGTGAATTTGGGCAAATGACTTCACCTCTGTGAAGCAGCAGTATCCTCTTCTGTGA
AGTGAGGTTAGCCCCTACCTCTCAATGTGGTTTTTAAAGTATGTTAATGAGATCACTTGT
AACCTAAAGCACACAGAGTGT
>1762
CTCTGTGAAGCAGCAGTATCCTCTTCTGTGAAGTGAGGTTAGCCCCTACCTCTCAATGT
GGTTTTTAAAGTATGTTAATGAGATCACTTGTAACCTAAAGCACACAGAGTGTGAGGCC
ACCTCCCTACTTCCTGCTTCTC
>1763
GCAGTATCCTCTTCTGTGAAGTGAGGTTAGCCCCTACCTCTCAATGTGGTTTTTAAAGTA
TGTTAATGAGATCACTTGTAACCTAAAGCACACAGAGTGTGAGGCCACCTCCCTACTTC
CTGCTTCTCTCATGACCCCAG
>1764
GTTAGCCCCTACCTCTCAATGTGGTTTTTAAAGTATGTTAATGAGATCACTTGTAACCTA
AAGCACACAGAGTGTGAGGCCACCTCCCTACTTCCTGCTTCTCTCATGACCCCAGCCT
GACAAGGGAGCCGCTGAGCCCC
>1765
TAGCCCCTACCTCTCAATGTGGTTTTTAAAGTATGTTAATGAGATCACTTGTAACCTAAA
GCACACAGAGTGTGAGGCCACCTCCCTACTTCCTGCTTCTCTCATGACCCCAGCCTGA
CAAGGGAGCCGCTGAGCCCCCT
>1766
CTACCTCTCAATGTGGTTTTTAAAGTATGTTAATGAGATCACTTGTAACCTAAAGCACAC
AGAGTGTGAGGCCACCTCCCTACTTCCTGCTTCTCTCATGACCCCAGCCTGACAAGGG
AGCCGCTGAGCCCCCTGCCACC
>1767
ATCACTTGTAACCTAAAGCACACAGAGTGTGAGGCCACCTCCCTACTTCCTGCTTCTCT
CATGACCCCAGCCTGACAAGGGAGCCGCTGAGCCCCCTGCCACCCTCACCCGGCGTT
GTGCTCTAAGTGGATTAGCTCAGC
>1768
TAAAGCACACAGAGTGTGAGGCCACCTCCCTACTTCCTGCTTCTCTCATGACCCCAGC
CTGACAAGGGAGCCGCTGAGCCCCCTGCCACCCTCACCCGGCGTTGTGCTCTAAGTGG
ATTAGCTCAGCGTTGTGATCTTAA
>1769
ACAGAGTGTGAGGCCACCTCCCTACTTCCTGCTTCTCTCATGACCCCAGCCTGACAAG
GGAGCCGCTGAGCCCCCTGCCACCCTCACCCGGCGTTGTGCTCTAAGTGGATTAGCTC
AGCGTTGTGATCTTAATGCCATTA
>1770
TCTCATGACCCCAGCCTGACAAGGGAGCCGCTGAGCCCCCTGCCACCCTCACCCGGCG
TTGTGCTCTAAGTGGATTAGCTCAGCGTTGTGATCTTAATGCCATTAGGCCTGTTGGCA
CGGCTCTCCTTGCCATAGTGAGA
>1771
GCCTGACAAGGGAGCCGCTGAGCCCCCTGCCACCCTCACCCGGCGTTGTGCTCTAAGT
GGATTAGCTCAGCGTTGTGATCTTAATGCCATTAGGCCTGTTGGCACGGCTCTCCTTGC
CATAGTGAGAAGCAGATGTGGCC

>1772
CCCCTGCCACCCTCACCCGGCGTTGTGCTCTAAGTGGATTAGCTCAGCGTTGTGATCTT
AATGCCATTAGGCCTGTTGGCACGGCTCTCCTTGCCATAGTGAGAAGCAGATGTGGCC
GACCATAATGGCCCTTAGCGTGA
>1773
CTAAGTGGATTAGCTCAGCGTTGTGATCTTAATGCCATTAGGCCTGTTGGCACGGCTCT
CCTTGCCATAGTGAGAAGCAGATGTGGCCGACCATAATGGCCCTTAGCGTGATCCATCG
GGCCCACAGTGCTGGGGCAACC
>1774
ATTAGGCCTGTTGGCACGGCTCTCCTTGCCATAGTGAGAAGCAGATGTGGCCGACCATA
ATGGCCCTTAGCGTGATCCATCGGGCCCACAGTGCTGGGGCAACCATTATTAGTGTTAA
TTTGGGGGAGCTTTTTATGGTA
>1775
CTCCTTGCCATAGTGAGAAGCAGATGTGGCCGACCATAATGGCCCTTAGCGTGATCCAT
CGGGCCCACAGTGCTGGGGCAACCATTATTAGTGTTAATTTGGGGGAGCTTTTTATGGT
AGCTGGGGGAAATGTGGAGGGA
>1776
AGATGTGGCCGACCATAATGGCCCTTAGCGTGATCCATCGGGCCCACAGTGCTGGGGC
AACCATTATTAGTGTTAATTTGGGGGAGCTTTTTATGGTAGCTGGGGGAAATGTGGAGG
GATTGTTGGAAATTTATTTAAGC
>1777
AATGGCCCTTAGCGTGATCCATCGGGCCCACAGTGCTGGGGCAACCATTATTAGTGTTA
ATTTGGGGGAGCTTTTTATGGTAGCTGGGGGAAATGTGGAGGGATTGTTGGAAATTTAT
TTAAGCTGTTCAGCAGGTTAGG
>1778
GTGATCCATCGGGCCCACAGTGCTGGGGCAACCATTATTAGTGTTAATTTGGGGGAGCT
TTTTATGGTAGCTGGGGGAAATGTGGAGGGATTGTTGGAAATTTATTTAAGCTGTTCAG
CAGGTTAGGTGCTGCTGGGGTC
>1779
GTGTTAATTTGGGGGAGCTTTTTATGGTAGCTGGGGGAAATGTGGAGGGATTGTTGGA
AATTTATTTAAGCTGTTCAGCAGGTTAGGTGCTGCTGGGGTCTACTATGGCAAAACCCT
CAGCTCCCCCAACCCATCTCTTT
>1780
TTGGGGGAGCTTTTTATGGTAGCTGGGGGAAATGTGGAGGGATTGTTGGAAATTTATTT
AAGCTGTTCAGCAGGTTAGGTGCTGCTGGGGTCTACTATGGCAAAACCCTCAGCTCCC
CCAACCCATCTCTTTAAGGAACG
>1781
TTGTTGGAAATTTATTTAAGCTGTTCAGCAGGTTAGGTGCTGCTGGGGTCTACTATGGC
AAAACCCTCAGCTCCCCCAACCCATCTCTTTAAGGAACGTCCCTCCCCAACACCGAAG
CCCAGGCCTGATTACTTCCAGAG
>1782
TTCAGCAGGTTAGGTGCTGCTGGGGTCTACTATGGCAAAACCCTCAGCTCCCCCAACC
CATCTCTTTAAGGAACGTCCCTCCCCAACACCGAAGCCCAGGCCTGATTACTTCCAGA
GTTGGAGAGACAACTTAGCAGGCT

>1783
GTCTACTATGGCAAAACCCTCAGCTCCCCCAACCCATCTCTTTAAGGAACGTCCCTCCC
CAACACCGAAGCCCAGGCCTGATTACTTCCAGAGTTGGAGAGACAACTTAGCAGGCT
CTTGTTTCTTGCCGATGTACCTTA
>1784
AGCTCCCCCAACCCATCTCTTTAAGGAACGTCCCTCCCCAACACCGAAGCCCAGGCCT
GATTACTTCCAGAGTTGGAGAGACAACTTAGCAGGCTCTTGTTTCTTGCCGATGTACCT
TATTAGTAGCACCTGGCATCATA
>1785
CTCCCCCAACCCATCTCTTTAAGGAACGTCCCTCCCCAACACCGAAGCCCAGGCCTGA
TTACTTCCAGAGTTGGAGAGACAACTTAGCAGGCTCTTGTTTCTTGCCGATGTACCTTA
TTAGTAGCACCTGGCATCATAAC
>1786
TTTAAGGAACGTCCCTCCCCAACACCGAAGCCCAGGCCTGATTACTTCCAGAGTTGGA
GAGACAACTTAGCAGGCTCTTGTTTCTTGCCGATGTACCTTATTAGTAGCACCTGGCAT
CATAACCCACTAATGGGAACCAG
>1787
GGCCTGATTACTTCCAGAGTTGGAGAGACAACTTAGCAGGCTCTTGTTTCTTGCCGATG
TACCTTATTAGTAGCACCTGGCATCATAACCCACTAATGGGAACCAGTGCTCTCCTCCT
GCGTGGCCAGTGACAAGTAGCC
>1788
CTGATTACTTCCAGAGTTGGAGAGACAACTTAGCAGGCTCTTGTTTCTTGCCGATGTAC
CTTATTAGTAGCACCTGGCATCATAACCCACTAATGGGAACCAGTGCTCTCCTCCTGCG
TGGCCAGTGACAAGTAGCCCTG
>1789
CAGGCTCTTGTTTCTTGCCGATGTACCTTATTAGTAGCACCTGGCATCATAACCCACTAA
TGGGAACCAGTGCTCTCCTCCTGCGTGGCCAGTGACAAGTAGCCCTGTCAGCCGGAGC
TCGCCGTGCGAGGAAGTTCCTA
>1790
GTACCTTATTAGTAGCACCTGGCATCATAACCCACTAATGGGAACCAGTGCTCTCCTCC
TGCGTGGCCAGTGACAAGTAGCCCTGTCAGCCGGAGCTCGCCGTGCGAGGAAGTTCC
TATTAGCTTTATGGTCAGGAGAAC
>1791
ATTAGTAGCACCTGGCATCATAACCCACTAATGGGAACCAGTGCTCTCCTCCTGCGTGG
CCAGTGACAAGTAGCCCTGTCAGCCGGAGCTCGCCGTGCGAGGAAGTTCCTATTAGCT
TTATGGTCAGGAGAACTTGTCCT
>1792
TGGCATCATAACCCACTAATGGGAACCAGTGCTCTCCTCCTGCGTGGCCAGTGACAAG
TAGCCCTGTCAGCCGGAGCTCGCCGTGCGAGGAAGTTCCTATTAGCTTTATGGTCAGG
AGAACTTGTCCTGTTCAGGAAATG
>1793
TGGGAACCAGTGCTCTCCTCCTGCGTGGCCAGTGACAAGTAGCCCTGTCAGCCGGAGC
TCGCCGTGCGAGGAAGTTCCTATTAGCTTTATGGTCAGGAGAACTTGTCCTGTTCAGGA
AATGAATGTTGCCGCTAAAGGTC

>1794
GGCCAGTGACAAGTAGCCCTGTCAGCCGGAGCTCGCCGTGCGAGGAAGTTCCTATTAG
CTTTATGGTCAGGAGAACTTGTCCTGTTCAGGAAATGAATGTTGCCGCTAAAGGTCCG
CGGCCCTGACCCTGGCGGCATAAC
>1795
CAGTGACAAGTAGCCCTGTCAGCCGGAGCTCGCCGTGCGAGGAAGTTCCTATTAGCTT
TATGGTCAGGAGAACTTGTCCTGTTCAGGAAATGAATGTTGCCGCTAAAGGTCCGCGG
CCCTGACCCTGGCGGCATAACCTG
>1796
TGTCAGCCGGAGCTCGCCGTGCGAGGAAGTTCCTATTAGCTTTATGGTCAGGAGAACT
TGTCCTGTTCAGGAAATGAATGTTGCCGCTAAAGGTCCGCGGCCCTGACCCTGGCGGC
ATAACCTGTGCGGGCCGGGGAGAC
>1797
GAGGAAGTTCCTATTAGCTTTATGGTCAGGAGAACTTGTCCTGTTCAGGAAATGAATGT
TGCCGCTAAAGGTCCGCGGCCCTGACCCTGGCGGCATAACCTGTGCGGGCCGGGGAG
ACCCTGTGCAGGATGCTTGGGATT
>1798
AGGAAATGAATGTTGCCGCTAAAGGTCCGCGGCCCTGACCCTGGCGGCATAACCTGTG
CGGGCCGGGGAGACCCTGTGCAGGATGCTTGGGATTTCAGCCCCTCTCTGGTTCTTGT
CATATGTAAAGAGGGAATCATTGT
>1799
GTTGCCGCTAAAGGTCCGCGGCCCTGACCCTGGCGGCATAACCTGTGCGGGCCGGGG
AGACCCTGTGCAGGATGCTTGGGATTTCAGCCCCTCTCTGGTTCTTGTCATATGTAAAG
AGGGAATCATTGTGGTATGTTGCA
>1800
GCGGCCCTGACCCTGGCGGCATAACCTGTGCGGGCCGGGGAGACCCTGTGCAGGATG
CTTGGGATTTCAGCCCCTCTCTGGTTCTTGTCATATGTAAAGAGGGAATCATTGTGGTAT
GTTGCAGCCTTGAACTTTTGATT
>1801
GCGGGCCGGGGAGACCCTGTGCAGGATGCTTGGGATTTCAGCCCCTCTCTGGTTCTTG
TCATATGTAAAGAGGGAATCATTGTGGTATGTTGCAGCCTTGAACTTTTGATTTATGCAG
TGAGGGCGACTTAACCGTTTCC
>1802
ATGCTTGGGATTTCAGCCCCTCTCTGGTTCTTGTCATATGTAAAGAGGGAATCATTGTG
GTATGTTGCAGCCTTGAACTTTTGATTTATGCAGTGAGGGCGACTTAACCGTTTCCATG
CCTGGACGGCTGTGCAGTGACA
>1803
CTGGTTCTTGTCATATGTAAAGAGGGAATCATTGTGGTATGTTGCAGCCTTGAACTTTTG
ATTTATGCAGTGAGGGCGACTTAACCGTTTCCATGCCTGGACGGCTGTGCAGTGACAG
AGCCGTGTTGTGAGCGTATGTG
>1804
TTCTTGTCATATGTAAAGAGGGAATCATTGTGGTATGTTGCAGCCTTGAACTTTTGATTT
ATGCAGTGAGGGCGACTTAACCGTTTCCATGCCTGGACGGCTGTGCAGTGACAGAGCC
GTGTTGTGAGCGTATGTGCCTA

>1805
TGCAGCCTTGAACTTTTGATTTATGCAGTGAGGGCGACTTAACCGTTTCCATGCCTGGA
CGGCTGTGCAGTGACAGAGCCGTGTTGTGAGCGTATGTGCCTAGGTACATGTGTGTGT
GGGGTGGTCAAGGGGAAGGAGAG
>1806
CTTGAACTTTTGATTTATGCAGTGAGGGCGACTTAACCGTTTCCATGCCTGGACGGCTG
TGCAGTGACAGAGCCGTGTTGTGAGCGTATGTGCCTAGGTACATGTGTGTGTGGGGTG
GTCAAGGGGAAGGAGAGACCCCC
>1807
TTATGCAGTGAGGGCGACTTAACCGTTTCCATGCCTGGACGGCTGTGCAGTGACAGAG
CCGTGTTGTGAGCGTATGTGCCTAGGTACATGTGTGTGTGGGGTGGTCAAGGGGAAGG
AGAGACCCCCACGTGCTAGCAGGG
>1808
GCGACTTAACCGTTTCCATGCCTGGACGGCTGTGCAGTGACAGAGCCGTGTTGTGAGC
GTATGTGCCTAGGTACATGTGTGTGTGGGGTGGTCAAGGGGAAGGAGAGACCCCCACG
TGCTAGCAGGGGCTGTTGCTGGGA
>1809
CGGCTGTGCAGTGACAGAGCCGTGTTGTGAGCGTATGTGCCTAGGTACATGTGTGTGT
GGGGTGGTCAAGGGGAAGGAGAGACCCCCACGTGCTAGCAGGGGCTGTTGCTGGGA
GCGGGTGGATTTCATGGGAGGATTCC
>1810
TGCAGTGACAGAGCCGTGTTGTGAGCGTATGTGCCTAGGTACATGTGTGTGTGGGGTG
GTCAAGGGGAAGGAGAGACCCCCACGTGCTAGCAGGGGCTGTTGCTGGGAGCGGGTG
GATTTCATGGGAGGATTCCTTTTCT
>1811
AGTGACAGAGCCGTGTTGTGAGCGTATGTGCCTAGGTACATGTGTGTGTGGGGTGGTC
AAGGGGAAGGAGAGACCCCCACGTGCTAGCAGGGGCTGTTGCTGGGAGCGGGTGGAT
TTCATGGGAGGATTCCTTTTCTTTT
>1812
GTGAGCGTATGTGCCTAGGTACATGTGTGTGTGGGGTGGTCAAGGGGAAGGAGAGAC
CCCCACGTGCTAGCAGGGGCTGTTGCTGGGAGCGGGTGGATTTCATGGGAGGATTCCT
TTTCTTTTTTCTTTTTCTTGCATTC
>1813
GTCAAGGGGAAGGAGAGACCCCCACGTGCTAGCAGGGGCTGTTGCTGGGAGCGGGTG
GATTTCATGGGAGGATTCCTTTTCTTTTTTCTTTTTCTTGCATTCTTGCAGCATCTAAATT
TCCTGCGATTGTTGGCTGCAAA
>1814
CTGTTGCTGGGAGCGGGTGGATTTCATGGGAGGATTCCTTTTCTTTTTTCTTTTTCTTGC
ATTCTTGCAGCATCTAAATTTCCTGCGATTGTTGGCTGCAAAGTGGTAAGAAGCTGGAG
AGCATTGTGTCTGGAATTGAA
>1815
ATTCCTTTTCTTTTTTCTTTTTCTTGCATTCTTGCAGCATCTAAATTTCCTGCGATTGTTG
GCTGCAAAGTGGTAAGAAGCTGGAGAGCATTGTGTCTGGAATTGAACCTGGGTTCGAA
TTAATCTTCTGTCTCTTAGAG

>1816
TTCTTTTTTCTTTTTCTTGCATTCTTGCAGCATCTAAATTTCCTGCGATTGTTGGCTGCAA
AGTGGTAAGAAGCTGGAGAGCATTGTGTCTGGAATTGAACCTGGGTTCGAATTAATCT
TCTGTCTCTTAGAGGCTGTGT
>1817
AGTGGTAAGAAGCTGGAGAGCATTGTGTCTGGAATTGAACCTGGGTTCGAATTAATCT
TCTGTCTCTTAGAGGCTGTGTGACCGTGGCAAGATTCCTCACCACTCACTTCCTCATCT
GTGAGATGAAGTTAATAACTTCT
>1818
TGGTAAGAAGCTGGAGAGCATTGTGTCTGGAATTGAACCTGGGTTCGAATTAATCTTCT
GTCTCTTAGAGGCTGTGTGACCGTGGCAAGATTCCTCACCACTCACTTCCTCATCTGTG
AGATGAAGTTAATAACTTCTGA
>1819
TAATCTTCTGTCTCTTAGAGGCTGTGTGACCGTGGCAAGATTCCTCACCACTCACTTCC
TCATCTGTGAGATGAAGTTAATAACTTCTGATTCATACTGGTGGTAGAGCAGTGTTGTTA
AGTAGGTGATGTATAGAGAAT
>1820
TCTGTCTCTTAGAGGCTGTGTGACCGTGGCAAGATTCCTCACCACTCACTTCCTCATCT
GTGAGATGAAGTTAATAACTTCTGATTCATACTGGTGGTAGAGCAGTGTTGTTAAGTAG
GTGATGTATAGAGAATACATGG
>1821
AAGATTCCTCACCACTCACTTCCTCATCTGTGAGATGAAGTTAATAACTTCTGATTCATA
CTGGTGGTAGAGCAGTGTTGTTAAGTAGGTGATGTATAGAGAATACATGGCCCAGCGTC
CGGCCAGTGGTGTGGCCTCAG
>1822
TCACTTCCTCATCTGTGAGATGAAGTTAATAACTTCTGATTCATACTGGTGGTAGAGCA
GTGTTGTTAAGTAGGTGATGTATAGAGAATACATGGCCCAGCGTCCGGCCAGTGGTGTG
GCCTCAGGTTGTATACTTGAAA
>1823
CTTCCTCATCTGTGAGATGAAGTTAATAACTTCTGATTCATACTGGTGGTAGAGCAGTG
TTGTTAAGTAGGTGATGTATAGAGAATACATGGCCCAGCGTCCGGCCAGTGGTGTGGCC
TCAGGTTGTATACTTGAAAGGA
>1824
GTGAGATGAAGTTAATAACTTCTGATTCATACTGGTGGTAGAGCAGTGTTGTTAAGTAG
GTGATGTATAGAGAATACATGGCCCAGCGTCCGGCCAGTGGTGTGGCCTCAGGTTGTAT
ACTTGAAAGGATGTGTATACTT
>1825
CATACTGGTGGTAGAGCAGTGTTGTTAAGTAGGTGATGTATAGAGAATACATGGCCCAG
CGTCCGGCCAGTGGTGTGGCCTCAGGTTGTATACTTGAAAGGATGTGTATACTTCAGTA
TTTTTTCCATTGTAAAATTAAG
>1826
AGCAGTGTTGTTAAGTAGGTGATGTATAGAGAATACATGGCCCAGCGTCCGGCCAGTG
GTGTGGCCTCAGGTTGTATACTTGAAAGGATGTGTATACTTCAGTATTTTTTCCATTGTA
AAATTAAGGGTTTTTTTTCTTT

>1827
AGGTGATGTATAGAGAATACATGGCCCAGCGTCCGGCCAGTGGTGTGGCCTCAGGTTG
TATACTTGAAAGGATGTGTATACTTCAGTATTTTTTCCATTGTAAAATTAAGGGTTTTTTT
TCTTTTTTTCTTGAGACAGAG
>1828
ATACATGGCCCAGCGTCCGGCCAGTGGTGTGGCCTCAGGTTGTATACTTGAAAGGATGT
GTATACTTCAGTATTTTTTCCATTGTAAAATTAAGGGTTTTTTTTCTTTTTTTCTTGAGAC
AGAGTCCTACTTTGTTGCCC
>1829
TGGTGTGGCCTCAGGTTGTATACTTGAAAGGATGTGTATACTTCAGTATTTTTTCCATTG
TAAAATTAAGGGTTTTTTTTCTTTTTTTCTTGAGACAGAGTCCTACTTTGTTGCCCAGGC
TTGAGTGCAGTGGCGCGATC
>1830
TGAAAGGATGTGTATACTTCAGTATTTTTTCCATTGTAAAATTAAGGGTTTTTTTTCTTTT
TTTCTTGAGACAGAGTCCTACTTTGTTGCCCAGGCTTGAGTGCAGTGGCGCGATCTCA
GCTCACTGCAACTTCCGCCTC
>1831
AGGATGTGTATACTTCAGTATTTTTTCCATTGTAAAATTAAGGGTTTTTTTTCTTTTTTTC
TTGAGACAGAGTCCTACTTTGTTGCCCAGGCTTGAGTGCAGTGGCGCGATCTCAGCTC
ACTGCAACTTCCGCCTCCTGG
>1832
CATTGTAAAATTAAGGGTTTTTTTTCTTTTTTTCTTGAGACAGAGTCCTACTTTGTTGCC
CAGGCTTGAGTGCAGTGGCGCGATCTCAGCTCACTGCAACTTCCGCCTCCTGGGTCCA
AGTGATTCTCGTGCCTCAGCCT
>1833
TTTTTTCTTTTTTTCTTGAGACAGAGTCCTACTTTGTTGCCCAGGCTTGAGTGCAGTGG
CGCGATCTCAGCTCACTGCAACTTCCGCCTCCTGGGTCCAAGTGATTCTCGTGCCTCAG
CCTCCCAAGTACCTGGGACCAC
>1834
CTTTGTTGCCCAGGCTTGAGTGCAGTGGCGCGATCTCAGCTCACTGCAACTTCCGCCT
CCTGGGTCCAAGTGATTCTCGTGCCTCAGCCTCCCAAGTACCTGGGACCACAGGGGTG
CACCACCACACCTGGCTAATTTTT
>1835
GAGTGCAGTGGCGCGATCTCAGCTCACTGCAACTTCCGCCTCCTGGGTCCAAGTGATT
CTCGTGCCTCAGCCTCCCAAGTACCTGGGACCACAGGGGTGCACCACCACACCTGGCT
AATTTTTGTATATATATATATATA
>1836
GGCGCGATCTCAGCTCACTGCAACTTCCGCCTCCTGGGTCCAAGTGATTCTCGTGCCTC
AGCCTCCCAAGTACCTGGGACCACAGGGGTGCACCACCACACCTGGCTAATTTTTGTA
TATATATATATATATATATTTAG
>1837
CACTGCAACTTCCGCCTCCTGGGTCCAAGTGATTCTCGTGCCTCAGCCTCCCAAGTACC
TGGGACCACAGGGGTGCACCACCACACCTGGCTAATTTTTGTATATATATATATATATATA
TTTAGTAGAGATGGGGTTTC

>1838
TACCTGGGACCACAGGGGTGCACCACCACACCTGGCTAATTTTTGTATATATATATATATA
TATATTTAGTAGAGATGGGGTTTCGCCATGTTGGCCAGGCTGGTCTTGAACTCCCGACC
TCAGGTGATCTGCCTGTCTC
>1839
CTGGGACCACAGGGGTGCACCACCACACCTGGCTAATTTTTGTATATATATATATATATAT
ATTTAGTAGAGATGGGGTTTCGCCATGTTGGCCAGGCTGGTCTTGAACTCCCGACCTCA
GGTGATCTGCCTGTCTCAGC
>1840
ACCTGGCTAATTTTTGTATATATATATATATATATATATTTAGTAGAGATGGGGTTTCGCCATG
TTGGCCAGGCTGGTCTTGAACTCCCGACCTCAGGTGATCTGCCTGTCTCAGCCTCCTAA
AGTGCTGGGATTATAGGTG
>1841
ATATATATATATTTAGTAGAGATGGGGTTTCGCCATGTTGGCCAGGCTGGTCTTGAACTC
CCGACCTCAGGTGATCTGCCTGTCTCAGCCTCCTAAAGTGCTGGGATTATAGGTGTGAG
CCACTGCGCCTGGCCTAAGTT
>1842
GAGATGGGGTTTCGCCATGTTGGCCAGGCTGGTCTTGAACTCCCGACCTCAGGTGATC
TGCCTGTCTCAGCCTCCTAAAGTGCTGGGATTATAGGTGTGAGCCACTGCGCCTGGCCT
AAGTTTTTAAAAATTAAAAAGAA
>1843
CAGGCTGGTCTTGAACTCCCGACCTCAGGTGATCTGCCTGTCTCAGCCTCCTAAAGTG
CTGGGATTATAGGTGTGAGCCACTGCGCCTGGCCTAAGTTTTTAAAAATTAAAAAGAAT
GGCATAGTGCTTTTTTGCTCTTT
>1844
GTCTTGAACTCCCGACCTCAGGTGATCTGCCTGTCTCAGCCTCCTAAAGTGCTGGGATT
ATAGGTGTGAGCCACTGCGCCTGGCCTAAGTTTTTAAAAATTAAAAAGAATGGCATAGT
GCTTTTTTGCTCTTTAACAGAT
>1845
AGGTGATCTGCCTGTCTCAGCCTCCTAAAGTGCTGGGATTATAGGTGTGAGCCACTGCG
CCTGGCCTAAGTTTTTAAAAATTAAAAAGAATGGCATAGTGCTTTTTTGCTCTTTAACA
GATCAAGACCAAAAGTTTTCCC
>1846
AGCCTCCTAAAGTGCTGGGATTATAGGTGTGAGCCACTGCGCCTGGCCTAAGTTTTTAA
AAATTAAAAAGAATGGCATAGTGCTTTTTTGCTCTTTAACAGATCAAGACCAAAAGTTT
TCCCCCCATCATAGCCCAAAGT
>1847
AGCCACTGCGCCTGGCCTAAGTTTTTAAAAATTAAAAAGAATGGCATAGTGCTTTTTTG
CTCTTTAACAGATCAAGACCAAAAGTTTTCCCCCCATCATAGCCCAAAGTCCTAAAGA
AAGGCTACTTAAGTTGTTCGTTT
>1848
TGCGCCTGGCCTAAGTTTTTAAAAATTAAAAAGAATGGCATAGTGCTTTTTTGCTCTTTA
ACAGATCAAGACCAAAAGTTTTCCCCCCATCATAGCCCAAAGTCCTAAAGAAAGGCTA
CTTAAGTTGTTCGTTTTCTTTT

>1849
TTAAAAAGAATGGCATAGTGCTTTTTTGCTCTTTAACAGATCAAGACCAAAAGTTTTCC
CCCCATCATAGCCCAAAGTCCTAAAGAAAGGCTACTTAAGTTGTTCGTTTTCTTTTGTT
TCTCCCATGAGTAATGTTTCTA
>1850
GCTTTTTTGCTCTTTAACAGATCAAGACCAAAAGTTTTCCCCCCATCATAGCCCAAAGT
CCTAAAGAAAGGCTACTTAAGTTGTTCGTTTTCTTTTGTTTCTCCCATGAGTAATGTTTC
TAAGTGTGATTTTTGCAGAGA
>1851
TCTTTAACAGATCAAGACCAAAAGTTTTCCCCCCATCATAGCCCAAAGTCCTAAAGAA
AGGCTACTTAAGTTGTTCGTTTTCTTTTGTTTCTCCCATGAGTAATGTTTCTAAGTGTGA
TTTTTGCAGAGAACAGCAGTGT
>1852
CCCCATCATAGCCCAAAGTCCTAAAGAAAGGCTACTTAAGTTGTTCGTTTTCTTTTGTT
TCTCCCATGAGTAATGTTTCTAAGTGTGATTTTTGCAGAGAACAGCAGTGTCTGACCTC
ATTCTTGAGGGGAACTTTTCTG
>1853
CAAAGTCCTAAAGAAAGGCTACTTAAGTTGTTCGTTTTCTTTTGTTTCTCCCATGAGTA
ATGTTTCTAAGTGTGATTTTTGCAGAGAACAGCAGTGTCTGACCTCATTCTTGAGGGGA
ACTTTTCTGCCTGCCTCTTCTT
>1854
AAGGCTACTTAAGTTGTTCGTTTTCTTTTGTTTCTCCCATGAGTAATGTTTCTAAGTGTG
ATTTTTGCAGAGAACAGCAGTGTCTGACCTCATTCTTGAGGGGAACTTTTCTGCCTGCC
TCTTCTTGAGAAAAAAATGTTT
>1855
GGCTACTTAAGTTGTTCGTTTTCTTTTGTTTCTCCCATGAGTAATGTTTCTAAGTGTGAT
TTTTGCAGAGAACAGCAGTGTCTGACCTCATTCTTGAGGGGAACTTTTCTGCCTGCCT
CTTCTTGAGAAAAAAATGTTTGT
>1856
TTTCTCCCATGAGTAATGTTTCTAAGTGTGATTTTTGCAGAGAACAGCAGTGTCTGACC
TCATTCTTGAGGGGAACTTTTCTGCCTGCCTCTTCTTGAGAAAAAAATGTTTGTGAGTTG
TCTGGCAAATGGATGCGTTACT
>1857
GATTTTTGCAGAGAACAGCAGTGTCTGACCTCATTCTTGAGGGGAACTTTTCTGCCTG
CCTCTTCTTGAGAAAAAAATGTTTGTGAGTTGTCTGGCAAATGGATGCGTTACTTTTGCT
GCTGTTGCTGCCGCTGCCGTTTT
>1858
AGCAGTGTCTGACCTCATTCTTGAGGGGAACTTTTCTGCCTGCCTCTTCTTGAGAAAA
AATGTTTGTGAGTTGTCTGGCAAATGGATGCGTTACTTTTGCTGCTGTTGCTGCCGCTG
CCGTTTTAAAGGCCCCTGGTGAC
>1859
AATGTTTGTGAGTTGTCTGGCAAATGGATGCGTTACTTTTGCTGCTGTTGCTGCCGCTG
CCGTTTTAAAGGCCCCTGGTGACAAAGGAGTGAACTGCACTTTCGGGGAAAGCATGC
CGGAATCTGGCCCAGAAAAGAGGG

>1860
CTGGCAAATGGATGCGTTACTTTTGCTGCTGTTGCTGCCGCTGCCGTTTTAAAGGCCCC
TGGTGACAAAGGAGTGAACTGCACTTTCGGGGAAAGCATGCCGGAATCTGGCCCAGA
AAAGAGGGGCCGCTGGGGGAAGGG
>1861
CTGCTGTTGCTGCCGCTGCCGTTTTAAAGGCCCCTGGTGACAAAGGAGTGAACTGCAC
TTTCGGGGAAAGCATGCCGGAATCTGGCCCAGAAAAGAGGGGCCGCTGGGGGAAGG
GTTTGGAGATGACGCCTCCTCAGGAA
>1862
GTTTTAAAGGCCCCTGGTGACAAAGGAGTGAACTGCACTTTCGGGGAAAGCATGCCG
GAATCTGGCCCAGAAAAGAGGGGCCGCTGGGGGAAGGGTTTGGAGATGACGCCTCCT
CAGGAACTTTAAAAGAAATCTGTCCA
>1863
AAAGGCCCCTGGTGACAAAGGAGTGAACTGCACTTTCGGGGAAAGCATGCCGGAATC
TGGCCCAGAAAAGAGGGGCCGCTGGGGGAAGGGTTTGGAGATGACGCCTCCTCAGGA
ACTTTAAAAGAAATCTGTCCATCTGT
>1864
CTGCACTTTCGGGGAAAGCATGCCGGAATCTGGCCCAGAAAAGAGGGGCCGCTGGGG
GAAGGGTTTGGAGATGACGCCTCCTCAGGAACTTTAAAAGAAATCTGTCCATCTGTCT
CTGGCATTTATGGCATCGTTGACAG
>1865
AAGCATGCCGGAATCTGGCCCAGAAAAGAGGGGCCGCTGGGGGAAGGGTTTGGAGAT
GACGCCTCCTCAGGAACTTTAAAAGAAATCTGTCCATCTGTCTCTGGCATTTATGGCAT
CGTTGACAGCAGTTCAAGGAAAAA
>1866
ATCTGGCCCAGAAAAGAGGGGCCGCTGGGGGAAGGGTTTGGAGATGACGCCTCCTCA
GGAACTTTAAAAGAAATCTGTCCATCTGTCTCTGGCATTTATGGCATCGTTGACAGCAG
TTCAAGGAAAAATAAAAATAAGTT
>1867
ATGACGCCTCCTCAGGAACTTTAAAAGAAATCTGTCCATCTGTCTCTGGCATTTATGGC
ATCGTTGACAGCAGTTCAAGGAAAAATAAAAATAAGTTCTTATCTGATTGCGGGGCTGA
TTGCAAAGCCGAAGGACTAGAG
>1868
ACGCCTCCTCAGGAACTTTAAAAGAAATCTGTCCATCTGTCTCTGGCATTTATGGCATC
GTTGACAGCAGTTCAAGGAAAAATAAAAATAAGTTCTTATCTGATTGCGGGGCTGATTG
CAAAGCCGAAGGACTAGAGTGG
>1869
CCTCAGGAACTTTAAAAGAAATCTGTCCATCTGTCTCTGGCATTTATGGCATCGTTGAC
AGCAGTTCAAGGAAAAATAAAAATAAGTTCTTATCTGATTGCGGGGCTGATTGCAAAG
CCGAAGGACTAGAGTGGCCAGCG
>1870
AGAAATCTGTCCATCTGTCTCTGGCATTTATGGCATCGTTGACAGCAGTTCAAGGAAAA
ATAAAAATAAGTTCTTATCTGATTGCGGGGCTGATTGCAAAGCCGAAGGACTAGAGTG
GCCAGCGAGTCGGGCGGCTGGAT

>1871
GTTGACAGCAGTTCAAGGAAAAATAAAAATAAGTTCTTATCTGATTGCGGGGCTGATTG
CAAAGCCGAAGGACTAGAGTGGCCAGCGAGTCGGGCGGCTGGATCTGGAGTTTTGGA
GAAAGGGCCACTGGCTCATTTCTG
>1872
AGCAGTTCAAGGAAAAATAAAAATAAGTTCTTATCTGATTGCGGGGCTGATTGCAAAG
CCGAAGGACTAGAGTGGCCAGCGAGTCGGGCGGCTGGATCTGGAGTTTTGGAGAAAG
GGCCACTGGCTCATTTCTGGGCACG
>1873
AGGAAAAATAAAAATAAGTTCTTATCTGATTGCGGGGCTGATTGCAAAGCCGAAGGAC
TAGAGTGGCCAGCGAGTCGGGCGGCTGGATCTGGAGTTTTGGAGAAAGGGCCACTGG
CTCATTTCTGGGCACGCACGCTCAC
>1874
TCTGATTGCGGGGCTGATTGCAAAGCCGAAGGACTAGAGTGGCCAGCGAGTCGGGCG
GCTGGATCTGGAGTTTTGGAGAAAGGGCCACTGGCTCATTTCTGGGCACGCACGCTCA
CGGAGGCAAGTTCTCACCTCCTCGT
>1875
TCGGGCGGCTGGATCTGGAGTTTTGGAGAAAGGGCCACTGGCTCATTTCTGGGCACGC
ACGCTCACGGAGGCAAGTTCTCACCTCCTCGTGGGCTTCTCTTCACTTTACGTGGAAA
AACTGTGTACGTCTCAAATTAAAA
>1876
CGGCTGGATCTGGAGTTTTGGAGAAAGGGCCACTGGCTCATTTCTGGGCACGCACGCT
CACGGAGGCAAGTTCTCACCTCCTCGTGGGCTTCTCTTCACTTTACGTGGAAAAACTG
TGTACGTCTCAAATTAAAAGCAGC
>1877
CAAGTTCTCACCTCCTCGTGGGCTTCTCTTCACTTTACGTGGAAAAACTGTGTACGTCT
CAAATTAAAAGCAGCTCTTGCCCCGCTTAGAGCCGGTGAAAGCTGCTTCGAAGGCGTT
TGTAGAGTAGGGATTGGAAATGT
>1878
GTTCTCACCTCCTCGTGGGCTTCTCTTCACTTTACGTGGAAAAACTGTGTACGTCTCAA
ATTAAAAGCAGCTCTTGCCCCGCTTAGAGCCGGTGAAAGCTGCTTCGAAGGCGTTTGT
AGAGTAGGGATTGGAAATGTTCA
>1879
GGGCTTCTCTTCACTTTACGTGGAAAAACTGTGTACGTCTCAAATTAAAAGCAGCTCTT
GCCCCGCTTAGAGCCGGTGAAAGCTGCTTCGAAGGCGTTTGTAGAGTAGGGATTGGAA
ATGTTCAATGCAAAAGTTAAATC
>1880
GCTTCTCTTCACTTTACGTGGAAAAACTGTGTACGTCTCAAATTAAAAGCAGCTCTTGC
CCCGCTTAGAGCCGGTGAAAGCTGCTTCGAAGGCGTTTGTAGAGTAGGGATTGGAAAT
GTTCAATGCAAAAGTTAAATCGT
>1881
ACTTTACGTGGAAAAACTGTGTACGTCTCAAATTAAAAGCAGCTCTTGCCCCGCTTAG
AGCCGGTGAAAGCTGCTTCGAAGGCGTTTGTAGAGTAGGGATTGGAAATGTTCAATGC
AAAAGTTAAATCGTGACAGTTGTC

>1882
TACGTCTCAAATTAAAAGCAGCTCTTGCCCCGCTTAGAGCCGGTGAAAGCTGCTTCGA
AGGCGTTTGTAGAGTAGGGATTGGAAATGTTCAATGCAAAAGTTAAATCGTGACAGTT
GTCCCCTAAATGATCTGCTAAATT
>1883
CGCTTAGAGCCGGTGAAAGCTGCTTCGAAGGCGTTTGTAGAGTAGGGATTGGAAATGT
TCAATGCAAAAGTTAAATCGTGACAGTTGTCCCCTAAATGATCTGCTAAATTAAGGATC
CGCTAACATTTGTGAGTCTACCT
>1884
GAAAGCTGCTTCGAAGGCGTTTGTAGAGTAGGGATTGGAAATGTTCAATGCAAAAGTT
AAATCGTGACAGTTGTCCCCTAAATGATCTGCTAAATTAAGGATCCGCTAACATTTGTG
AGTCTACCTCCCTGCATTCCCCC
>1885
AGGGATTGGAAATGTTCAATGCAAAAGTTAAATCGTGACAGTTGTCCCCTAAATGATCT
GCTAAATTAAGGATCCGCTAACATTTGTGAGTCTACCTCCCTGCATTCCCCCCACCCCC
ACCCCCGCCCCCTTGGCAGAAG
>1886
GCAAAAGTTAAATCGTGACAGTTGTCCCCTAAATGATCTGCTAAATTAAGGATCCGCTA
ACATTTGTGAGTCTACCTCCCTGCATTCCCCCCACCCCCACCCCCGCCCCCTTGGCAGA
AGGGATAAAGATGGTACCTAAA
>1887
AAGGATCCGCTAACATTTGTGAGTCTACCTCCCTGCATTCCCCCCACCCCCACCCCCGC
CCCCTTGGCAGAAGGGATAAAGATGGTACCTAAAAACAATGTGAAGCGGCTGCTAAAT
CGAGTGACTCTAAAAACATTTTT
>1888
GATCCGCTAACATTTGTGAGTCTACCTCCCTGCATTCCCCCCACCCCCACCCCCGCCCC
CTTGGCAGAAGGGATAAAGATGGTACCTAAAAACAATGTGAAGCGGCTGCTAAATCGA
GTGACTCTAAAAACATTTTTCTT
>1889
AACATTTGTGAGTCTACCTCCCTGCATTCCCCCCACCCCCACCCCCGCCCCCTTGGCAG
AAGGGATAAAGATGGTACCTAAAAACAATGTGAAGCGGCTGCTAAATCGAGTGACTCT
AAAAACATTTTTCTTTCCTTGAA
>1890
CATTCCCCCCACCCCCACCCCCGCCCCCTTGGCAGAAGGGATAAAGATGGTACCTAAA
AACAATGTGAAGCGGCTGCTAAATCGAGTGACTCTAAAAACATTTTTCTTTCCTTGAAG
TTCAGTGTGGAAAACCAGACTGG
>1891
ACCCCCGCCCCCTTGGCAGAAGGGATAAAGATGGTACCTAAAAACAATGTGAAGCGGC
TGCTAAATCGAGTGACTCTAAAAACATTTTTCTTTCCTTGAAGTTCAGTGTGGAAAACC
AGACTGGACTTGTCAGGGATGCA
>1892
TGGTACCTAAAAACAATGTGAAGCGGCTGCTAAATCGAGTGACTCTAAAAACATTTTT
CTTTCCTTGAAGTTCAGTGTGGAAAACCAGACTGGACTTGTCAGGGATGCAGACCTAT
ATAGGGCCCATGTGCCTGGCCCCT

>1893
CCTAAAAACAATGTGAAGCGGCTGCTAAATCGAGTGACTCTAAAAACATTTTTCTTTCC
TTGAAGTTCAGTGTGGAAAACCAGACTGGACTTGTCAGGGATGCAGACCTATATAGGG
CCCATGTGCCTGGCCCCTTTCCT
>1894
AATCGAGTGACTCTAAAAACATTTTTCTTTCCTTGAAGTTCAGTGTGGAAAACCAGAC
TGGACTTGTCAGGGATGCAGACCTATATAGGGCCCATGTGCCTGGCCCCTTTCCTCACT
TTCTAGCACAATGGTTTTCATTG
>1895
ACATTTTTCTTTCCTTGAAGTTCAGTGTGGAAAACCAGACTGGACTTGTCAGGGATGC
AGACCTATATAGGGCCCATGTGCCTGGCCCCTTTCCTCACTTTCTAGCACAATGGTTTTC
ATTGCCACCTCTCCCGCGCCTT
>1896
CAGACTGGACTTGTCAGGGATGCAGACCTATATAGGGCCCATGTGCCTGGCCCCTTTCC
TCACTTTCTAGCACAATGGTTTTCATTGCCACCTCTCCCGCGCCTTTCCCCCCTTCATAA
TTATCCACTATTTTACTTTCT
>1897
CATTGCCACCTCTCCCGCGCCTTTCCCCCCTTCATAATTATCCACTATTTTACTTTCTGTG
TCCTGACAGCTACAAGAGTAGTTTTTGTTTTTTTGAGCTGAGTAGCTGCTAAATATTCCC
CAGCAGCCAGATTCAACGG
>1898
ACCTCTCCCGCGCCTTTCCCCCCTTCATAATTATCCACTATTTTACTTTCTGTGTCCTGAC
AGCTACAAGAGTAGTTTTTGTTTTTTTGAGCTGAGTAGCTGCTAAATATTCCCCAGCAG
CCAGATTCAACGGCTGGGGG
>1899
CCTTCATAATTATCCACTATTTTACTTTCTGTGTCCTGACAGCTACAAGAGTAGTTTTTGT
TTTTTTGAGCTGAGTAGCTGCTAAATATTCCCCAGCAGCCAGATTCAACGGCTGGGGGG
GAGAATAGGCCAAACCTTGG
>1900
TTCTGTGTCCTGACAGCTACAAGAGTAGTTTTTGTTTTTTTGAGCTGAGTAGCTGCTAA
ATATTCCCCAGCAGCCAGATTCAACGGCTGGGGGGGAGAATAGGCCAAACCTTGGCAT
CCCCTGTTGCAAAAGTGAAAAAT
>1901
TGTGTCCTGACAGCTACAAGAGTAGTTTTTGTTTTTTTGAGCTGAGTAGCTGCTAAATA
TTCCCCAGCAGCCAGATTCAACGGCTGGGGGGGAGAATAGGCCAAACCTTGGCATCCC
CTGTTGCAAAAGTGAAAAATATT
>1902
TCCTGACAGCTACAAGAGTAGTTTTTGTTTTTTTGAGCTGAGTAGCTGCTAAATATTCCC
CAGCAGCCAGATTCAACGGCTGGGGGGGAGAATAGGCCAAACCTTGGCATCCCCTGTT
GCAAAAGTGAAAAATATTTCTG
>1903
GTTTTTGTTTTTTTGAGCTGAGTAGCTGCTAAATATTCCCCAGCAGCCAGATTCAACGG
CTGGGGGGGAGAATAGGCCAAACCTTGGCATCCCCTGTTGCAAAAGTGAAAAATATTT
CTGTTTGCAGTTAATTTTAGTGG

>1904
TTCCCCAGCAGCCAGATTCAACGGCTGGGGGGGAGAATAGGCCAAACCTTGGCATCCC
CTGTTGCAAAAGTGAAAAATATTTCTGTTTGCAGTTAATTTTAGTGGCAAGCAGATCAT
GTTAACTGGATGTAATTGCTTCC
>1905
GGCTGGGGGGGAGAATAGGCCAAACCTTGGCATCCCCTGTTGCAAAAGTGAAAAATAT
TTCTGTTTGCAGTTAATTTTAGTGGCAAGCAGATCATGTTAACTGGATGTAATTGCTTCC
AGATATGGCCGAGGGTTTTGTG
>1906
GAGAATAGGCCAAACCTTGGCATCCCCTGTTGCAAAAGTGAAAAATATTTCTGTTTGC
AGTTAATTTTAGTGGCAAGCAGATCATGTTAACTGGATGTAATTGCTTCCAGATATGGCC
GAGGGTTTTGTGTGTTGAGATG
>1907
AAAAATATTTCTGTTTGCAGTTAATTTTAGTGGCAAGCAGATCATGTTAACTGGATGTAA
TTGCTTCCAGATATGGCCGAGGGTTTTGTGTGTTGAGATGAAGGGTCTGGTAAGTGCTT
ACTTCCTGTGTAAATTAGCCT
>1908
TGTTTGCAGTTAATTTTAGTGGCAAGCAGATCATGTTAACTGGATGTAATTGCTTCCAGA
TATGGCCGAGGGTTTTGTGTGTTGAGATGAAGGGTCTGGTAAGTGCTTACTTCCTGTGT
AAATTAGCCTATTCCATTCAG
>1909
TAGTGGCAAGCAGATCATGTTAACTGGATGTAATTGCTTCCAGATATGGCCGAGGGTTT
TGTGTGTTGAGATGAAGGGTCTGGTAAGTGCTTACTTCCTGTGTAAATTAGCCTATTCCA
TTCAGTCTTTCATTCTGGCCA
>1910
CAGATATGGCCGAGGGTTTTGTGTGTTGAGATGAAGGGTCTGGTAAGTGCTTACTTCCT
GTGTAAATTAGCCTATTCCATTCAGTCTTTCATTCTGGCCAGCCCTGGATTCTGTCATTC
TGCTCCCATTTTCCCCCTCCC
>1911
GGTTTTGTGTGTTGAGATGAAGGGTCTGGTAAGTGCTTACTTCCTGTGTAAATTAGCCT
ATTCCATTCAGTCTTTCATTCTGGCCAGCCCTGGATTCTGTCATTCTGCTCCCATTTTCC
CCCTCCCCAGCCTGCAGCCTG
>1912
ATTCCATTCAGTCTTTCATTCTGGCCAGCCCTGGATTCTGTCATTCTGCTCCCATTTTCC
CCCTCCCCAGCCTGCAGCCTGGTGTGAGAAGCCGCTTGCTATGTTCCTCTAAGTGTAAT
TCTTTTGTCACTTGGAGCCAC
>1913
TCAGTCTTTCATTCTGGCCAGCCCTGGATTCTGTCATTCTGCTCCCATTTTCCCCCTCCC
CAGCCTGCAGCCTGGTGTGAGAAGCCGCTTGCTATGTTCCTCTAAGTGTAATTCTTTTG
TCACTTGGAGCCACTCTCCTC
>1914
TTCTGCTCCCATTTTCCCCCTCCCCAGCCTGCAGCCTGGTGTGAGAAGCCGCTTGCTAT
GTTCCTCTAAGTGTAATTCTTTTGTCACTTGGAGCCACTCTCCTCTTGGTTCTTGATGTG
AGTTTGCCCTTAAATAATGAG

>1915
CCAGCCTGCAGCCTGGTGTGAGAAGCCGCTTGCTATGTTCCTCTAAGTGTAATTCTTTT
GTCACTTGGAGCCACTCTCCTCTTGGTTCTTGATGTGAGTTTGCCCTTAAATAATGAGA
AGGGTTTGAAAAGATGCCACCT
>1916
CTTGCTATGTTCCTCTAAGTGTAATTCTTTTGTCACTTGGAGCCACTCTCCTCTTGGTTC
TTGATGTGAGTTTGCCCTTAAATAATGAGAAGGGTTTGAAAAGATGCCACCTGGACCC
CAGGCCACCTCCCGCCTCTTTC
>1917
TGTAATTCTTTTGTCACTTGGAGCCACTCTCCTCTTGGTTCTTGATGTGAGTTTGCCCTT
AAATAATGAGAAGGGTTTGAAAAGATGCCACCTGGACCCCAGGCCACCTCCCGCCTCT
TTCCCAGCAAAGGCAGATCTGA
>1918
CACTTGGAGCCACTCTCCTCTTGGTTCTTGATGTGAGTTTGCCCTTAAATAATGAGAAG
GGTTTGAAAAGATGCCACCTGGACCCCAGGCCACCTCCCGCCTCTTTCCCAGCAAAGG
CAGATCTGAAGAGCATCTCCTGG
>1919
CCCTTAAATAATGAGAAGGGTTTGAAAAGATGCCACCTGGACCCCAGGCCACCTCCCG
CCTCTTTCCCAGCAAAGGCAGATCTGAAGAGCATCTCCTGGTCATGGGGGTTGCATCTT
GGAACCCAGAACTCCTTGTTTTC
>1920
AAATAATGAGAAGGGTTTGAAAAGATGCCACCTGGACCCCAGGCCACCTCCCGCCTCT
TTCCCAGCAAAGGCAGATCTGAAGAGCATCTCCTGGTCATGGGGGTTGCATCTTGGAA
CCCAGAACTCCTTGTTTTCTATGC
>1921
CGCCTCTTTCCCAGCAAAGGCAGATCTGAAGAGCATCTCCTGGTCATGGGGGTTGCAT
CTTGGAACCCAGAACTCCTTGTTTTCTATGCATTTTTTTTTCCTACTGCCCTCTGACCCC
CGACTCTCACCTGGGAGAACAC
>1922
CCCAGAACTCCTTGTTTTCTATGCATTTTTTTTTCCTACTGCCCTCTGACCCCCGACTCT
CACCTGGGAGAACACCTTCTCTTTCTCTCAATTAGTCCGACTCCTCCTCAGCCTGAACT
AAGCACGTTCCAGTGGGTCCG
>1923
TTTTTTTTTCCTACTGCCCTCTGACCCCCGACTCTCACCTGGGAGAACACCTTCTCTTTC
TCTCAATTAGTCCGACTCCTCCTCAGCCTGAACTAAGCACGTTCCAGTGGGTCCGAAAT
CTCAGAAAAAATGAAACGGCT
>1924
CCCTCTGACCCCCGACTCTCACCTGGGAGAACACCTTCTCTTTCTCTCAATTAGTCCGA
CTCCTCCTCAGCCTGAACTAAGCACGTTCCAGTGGGTCCGAAATCTCAGAAAAAATGA
AACGGCTGTTGCGCAGAGGAGAA
>1925
CCGACTCTCACCTGGGAGAACACCTTCTCTTTCTCTCAATTAGTCCGACTCCTCCTCAG
CCTGAACTAAGCACGTTCCAGTGGGTCCGAAATCTCAGAAAAAATGAAACGGCTGTTG
CGCAGAGGAGAAGGTTGTGGCCA

>1926
CCTGGGAGAACACCTTCTCTTTCTCTCAATTAGTCCGACTCCTCCTCAGCCTGAACTAA
GCACGTTCCAGTGGGTCCGAAATCTCAGAAAAAATGAAACGGCTGTTGCGCAGAGGA
GAAGGTTGTGGCCACAGAAAGGAG
>1927
TCCTCAGCCTGAACTAAGCACGTTCCAGTGGGTCCGAAATCTCAGAAAAAATGAAACG
GCTGTTGCGCAGAGGAGAAGGTTGTGGCCACAGAAAGGAGTTGCCTCATCAGACGTG
AAGCCGCCACCTTGCCTTGCAAAGC
>1928
CTCAGCCTGAACTAAGCACGTTCCAGTGGGTCCGAAATCTCAGAAAAAATGAAACGG
CTGTTGCGCAGAGGAGAAGGTTGTGGCCACAGAAAGGAGTTGCCTCATCAGACGTGA
AGCCGCCACCTTGCCTTGCAAAGCAG
>1929
GTTCCAGTGGGTCCGAAATCTCAGAAAAAATGAAACGGCTGTTGCGCAGAGGAGAAG
GTTGTGGCCACAGAAAGGAGTTGCCTCATCAGACGTGAAGCCGCCACCTTGCCTTGCA
AAGCAGGCTGCACCTCCTGACACAC
>1930
CTCAGAAAAAATGAAACGGCTGTTGCGCAGAGGAGAAGGTTGTGGCCACAGAAAGG
AGTTGCCTCATCAGACGTGAAGCCGCCACCTTGCCTTGCAAAGCAGGCTGCACCTCCT
GACACACCTGCCCCTCCCTGCAAGAA
>1931
GAGGAGAAGGTTGTGGCCACAGAAAGGAGTTGCCTCATCAGACGTGAAGCCGCCACC
TTGCCTTGCAAAGCAGGCTGCACCTCCTGACACACCTGCCCCTCCCTGCAAGAAAGG
GAGGGGAAATCCCACCCAAGAGAACC
>1932
GAGAAGGTTGTGGCCACAGAAAGGAGTTGCCTCATCAGACGTGAAGCCGCCACCTTG
CCTTGCAAAGCAGGCTGCACCTCCTGACACACCTGCCCCTCCCTGCAAGAAAGGGAG
GGGAAATCCCACCCAAGAGAACCTTC
>1933
AGACGTGAAGCCGCCACCTTGCCTTGCAAAGCAGGCTGCACCTCCTGACACACCTGC
CCCTCCCTGCAAGAAAGGGAGGGGAAATCCCACCCAAGAGAACCTTCTCCAGGTTCC
TGTGGGTTTCCAGGGTTGCAACTGAA
>1934
AGCCGCCACCTTGCCTTGCAAAGCAGGCTGCACCTCCTGACACACCTGCCCCTCCCTG
CAAGAAAGGGAGGGGAAATCCCACCCAAGAGAACCTTCTCCAGGTTCCTGTGGGTTT
CCAGGGTTGCAACTGAAGAAAAGTG
>1935
GCAAAGCAGGCTGCACCTCCTGACACACCTGCCCCTCCCTGCAAGAAAGGGAGGGGA
AATCCCACCCAAGAGAACCTTCTCCAGGTTCCTGTGGGTTTCCAGGGTTGCAACTGAA
GAAAAGTGTTAAGATTCATGTTCTC
>1936
ACCTCCTGACACACCTGCCCCTCCCTGCAAGAAAGGGAGGGGAAATCCCACCCAAGA
GAACCTTCTCCAGGTTCCTGTGGGTTTCCAGGGTTGCAACTGAAGAAAAGTGTTAAGA
TTCATGTTCTCCTAAATTCTGCTTT

>1937
AAGGGAGGGGAAATCCCACCCAAGAGAACCTTCTCCAGGTTCCTGTGGGTTTCCAGG
GTTGCAACTGAAGAAAAGTGTTAAGATTCATGTTCTCCTAAATTCTGCTTTTGTCCTGT
GGCCAGTCCCACATTCTTAGTGCT
>1938
AGGGGAAATCCCACCCAAGAGAACCTTCTCCAGGTTCCTGTGGGTTTCCAGGGTTGCA
ACTGAAGAAAAGTGTTAAGATTCATGTTCTCCTAAATTCTGCTTTTGTCCTGTGGCCAG
TCCCACATTCTTAGTGCTATTTG
>1939
CCAGGGTTGCAACTGAAGAAAAGTGTTAAGATTCATGTTCTCCTAAATTCTGCTTTTGT
CCTGTGGCCAGTCCCACATTCTTAGTGCTATTTGCATAAGAGGTTGCTGTTTATCTTCCT
ATATCAGCCCCACTGATAACC
>1940
GAAGAAAAGTGTTAAGATTCATGTTCTCCTAAATTCTGCTTTTGTCCTGTGGCCAGTCC
CACATTCTTAGTGCTATTTGCATAAGAGGTTGCTGTTTATCTTCCTATATCAGCCCCACTG
ATAACCCCTGTGGTCTGTTT
>1941
GATTCATGTTCTCCTAAATTCTGCTTTTGTCCTGTGGCCAGTCCCACATTCTTAGTGCTA
TTTGCATAAGAGGTTGCTGTTTATCTTCCTATATCAGCCCCACTGATAACCCCTGTGGTC
TGTTTAACAACCTATGAGGA
>1942
TGTCCTGTGGCCAGTCCCACATTCTTAGTGCTATTTGCATAAGAGGTTGCTGTTTATCTT
CCTATATCAGCCCCACTGATAACCCCTGTGGTCTGTTTAACAACCTATGAGGAGTAGATT
ACTTGATAAATGCCAGTTTT
>1943
AGAGGTTGCTGTTTATCTTCCTATATCAGCCCCACTGATAACCCCTGTGGTCTGTTTAAC
AACCTATGAGGAGTAGATTACTTGATAAATGCCAGTTTTTCCAATATGCAAAGATAAAC
TGTTGGGGTCAGGGTGAGGGG
>1944
GGTTGCTGTTTATCTTCCTATATCAGCCCCACTGATAACCCCTGTGGTCTGTTTAACAAC
CTATGAGGAGTAGATTACTTGATAAATGCCAGTTTTTCCAATATGCAAAGATAAACTGTT
GGGGTCAGGGTGAGGGGTGG
>1945
TCAGCCCCACTGATAACCCCTGTGGTCTGTTTAACAACCTATGAGGAGTAGATTACTTG
ATAAATGCCAGTTTTTCCAATATGCAAAGATAAACTGTTGGGGTCAGGGTGAGGGGTG
GTGTGTTATGAAGGAGGGGACAG
>1946
CCTGTGGTCTGTTTAACAACCTATGAGGAGTAGATTACTTGATAAATGCCAGTTTTTCCA
ATATGCAAAGATAAACTGTTGGGGTCAGGGTGAGGGGTGGTGTGTTATGAAGGAGGGG
ACAGGTGGGGAGCCAGGACCCT
>1947
ACCTATGAGGAGTAGATTACTTGATAAATGCCAGTTTTTCCAATATGCAAAGATAAACT
GTTGGGGTCAGGGTGAGGGGTGGTGTGTTATGAAGGAGGGGACAGGTGGGGAGCCAG
GACCCTCTTTTAGTGGGCTGGAGA

>1948
TAAATGCCAGTTTTTCCAATATGCAAAGATAAACTGTTGGGGTCAGGGTGAGGGGTGG
TGTGTTATGAAGGAGGGGACAGGTGGGGAGCCAGGACCCTCTTTTAGTGGGCTGGAG
AAGTGTGAAAGAGCCTGATGAAAGA
>1949
ATGCCAGTTTTTCCAATATGCAAAGATAAACTGTTGGGGTCAGGGTGAGGGGTGGTGT
GTTATGAAGGAGGGGACAGGTGGGGAGCCAGGACCCTCTTTTAGTGGGCTGGAGAAG
TGTGAAAGAGCCTGATGAAAGAAAA
>1950
TTTTTCCAATATGCAAAGATAAACTGTTGGGGTCAGGGTGAGGGGTGGTGTGTTATGAA
GGAGGGGACAGGTGGGGAGCCAGGACCCTCTTTTAGTGGGCTGGAGAAGTGTGAAAG
AGCCTGATGAAAGAAAACAGGTAG
>1951
ATATGCAAAGATAAACTGTTGGGGTCAGGGTGAGGGGTGGTGTGTTATGAAGGAGGGG
ACAGGTGGGGAGCCAGGACCCTCTTTTAGTGGGCTGGAGAAGTGTGAAAGAGCCTGA
TGAAAGAAAACAGGTAGCTTGTCAC
>1952
GTTGGGGTCAGGGTGAGGGGTGGTGTGTTATGAAGGAGGGGACAGGTGGGGAGCCAG
GACCCTCTTTTAGTGGGCTGGAGAAGTGTGAAAGAGCCTGATGAAAGAAAACAGGTA
GCTTGTCACATACAACAGCTGCATTT
>1953
GGAGGGGACAGGTGGGGAGCCAGGACCCTCTTTTAGTGGGCTGGAGAAGTGTGAAAG
AGCCTGATGAAAGAAAACAGGTAGCTTGTCACATACAACAGCTGCATTTATCATACGGT
TGATCAGCTTCCTGCCCTCACCTA
>1954
ACAGGTGGGGAGCCAGGACCCTCTTTTAGTGGGCTGGAGAAGTGTGAAAGAGCCTGA
TGAAAGAAAACAGGTAGCTTGTCACATACAACAGCTGCATTTATCATACGGTTGATCAG
CTTCCTGCCCTCACCTACTTTTCT
>1955
TCTTTTAGTGGGCTGGAGAAGTGTGAAAGAGCCTGATGAAAGAAAACAGGTAGCTTG
TCACATACAACAGCTGCATTTATCATACGGTTGATCAGCTTCCTGCCCTCACCTACTTTT
CTCCTCCTTCATGTGGACATGCA
>1956
AAAGAGCCTGATGAAAGAAAACAGGTAGCTTGTCACATACAACAGCTGCATTTATCAT
ACGGTTGATCAGCTTCCTGCCCTCACCTACTTTTCTCCTCCTTCATGTGGACATGCAGA
GTCTCTGAGAACATTTCATCAAG
>1957
TGCATTTATCATACGGTTGATCAGCTTCCTGCCCTCACCTACTTTTCTCCTCCTTCATGTG
GACATGCAGAGTCTCTGAGAACATTTCATCAAGTGGGTTGCCTATGCCCACATTAATAC
AAGATGGGAAAGGGTGGAGG
>1958
ATACGGTTGATCAGCTTCCTGCCCTCACCTACTTTTCTCCTCCTTCATGTGGACATGCAG
AGTCTCTGAGAACATTTCATCAAGTGGGTTGCCTATGCCCACATTAATACAAGATGGGA
AAGGGTGGAGGTTATCTTTGC

>1959
CTTCCTGCCCTCACCTACTTTTCTCCTCCTTCATGTGGACATGCAGAGTCTCTGAGAAC
ATTTCATCAAGTGGGTTGCCTATGCCCACATTAATACAAGATGGGAAAGGGTGGAGGTT
ATCTTTGCTTTTTAAGTTCCCA
>1960
TTCATGTGGACATGCAGAGTCTCTGAGAACATTTCATCAAGTGGGTTGCCTATGCCCAC
ATTAATACAAGATGGGAAAGGGTGGAGGTTATCTTTGCTTTTTAAGTTCCCATCTTTTTC
TCCCTTGGTCCTCTCCCACCC
>1961
GAGTCTCTGAGAACATTTCATCAAGTGGGTTGCCTATGCCCACATTAATACAAGATGGG
AAAGGGTGGAGGTTATCTTTGCTTTTTAAGTTCCCATCTTTTTCTCCCTTGGTCCTCTCC
CACCCCTACAAGTTGTTGGCT
>1962
ATCAAGTGGGTTGCCTATGCCCACATTAATACAAGATGGGAAAGGGTGGAGGTTATCTT
TGCTTTTTAAGTTCCCATCTTTTTCTCCCTTGGTCCTCTCCCACCCCTACAAGTTGTTGG
CTGGTAAGCAATTGATGCTCA
>1963
AGGGTGGAGGTTATCTTTGCTTTTTAAGTTCCCATCTTTTTCTCCCTTGGTCCTCTCCCA
CCCCTACAAGTTGTTGGCTGGTAAGCAATTGATGCTCAGAAAAATGATGTTCATCTGAA
ATCTTAAATGAGTGGACTTTT
>1964
TTTGCTTTTTAAGTTCCCATCTTTTTCTCCCTTGGTCCTCTCCCACCCCTACAAGTTGTT
GGCTGGTAAGCAATTGATGCTCAGAAAAATGATGTTCATCTGAAATCTTAAATGAGTGG
ACTTTTTCTACCTTCCCTGAC
>1965
TCCCATCTTTTTCTCCCTTGGTCCTCTCCCACCCCTACAAGTTGTTGGCTGGTAAGCAAT
TGATGCTCAGAAAAATGATGTTCATCTGAAATCTTAAATGAGTGGACTTTTTCTACCTTC
CCTGACTTTGCAGGTGCTTC
>1966
TGCTCAGAAAAATGATGTTCATCTGAAATCTTAAATGAGTGGACTTTTTCTACCTTCCCT
GACTTTGCAGGTGCTTCTTGAAGAGCCAAGGAGAAAAGAAAACTTCTGACCTCATCA
CCTAGTCAATAAGCTGTCTGAGA
>1967
GATGTTCATCTGAAATCTTAAATGAGTGGACTTTTTCTACCTTCCCTGACTTTGCAGGTG
CTTCTTGAAGAGCCAAGGAGAAAAGAAAACTTCTGACCTCATCACCTAGTCAATAAGC
TGTCTGAGAAACAAACAAAAGA
>1968
CCTTCCCTGACTTTGCAGGTGCTTCTTGAAGAGCCAAGGAGAAAAGAAAACTTCTGA
CCTCATCACCTAGTCAATAAGCTGTCTGAGAAACAAACAAAAGAGTTTAGACTGGCTG
CTAAAGCTAGGAACCCAGCTGATAG
>1969
GACTTTGCAGGTGCTTCTTGAAGAGCCAAGGAGAAAAGAAAACTTCTGACCTCATCA
CCTAGTCAATAAGCTGTCTGAGAAACAAACAAAAGAGTTTAGACTGGCTGCTAAAGCT
AGGAACCCAGCTGATAGCTTTGTTT

>1970
GCCAAGGAGAAAAGAAAACTTCTGACCTCATCACCTAGTCAATAAGCTGTCTGAGAA
ACAAACAAAAGAGTTTAGACTGGCTGCTAAAGCTAGGAACCCAGCTGATAGCTTTGTT
TTGATGTCCTTGTAATGTTTGCGGA
>1971
TTCTGACCTCATCACCTAGTCAATAAGCTGTCTGAGAAACAAACAAAAGAGTTTAGAC
TGGCTGCTAAAGCTAGGAACCCAGCTGATAGCTTTGTTTTGATGTCCTTGTAATGTTTG
CGGATCGCAGAGGGTGCTATTTA
>1972
GACCTCATCACCTAGTCAATAAGCTGTCTGAGAAACAAACAAAAGAGTTTAGACTGGC
TGCTAAAGCTAGGAACCCAGCTGATAGCTTTGTTTTGATGTCCTTGTAATGTTTGCGGA
TCGCAGAGGGTGCTATTTAGAGC
>1973
TCAATAAGCTGTCTGAGAAACAAACAAAAGAGTTTAGACTGGCTGCTAAAGCTAGGA
ACCCAGCTGATAGCTTTGTTTTGATGTCCTTGTAATGTTTGCGGATCGCAGAGGGTGCT
ATTTAGAGCAGGGGGCATCTACTG
>1974
CTGGCTGCTAAAGCTAGGAACCCAGCTGATAGCTTTGTTTTGATGTCCTTGTAATGTTT
GCGGATCGCAGAGGGTGCTATTTAGAGCAGGGGGCATCTACTGGGACAGACTCACTGG
GAAGTTGAAACAGCTAGCTCAAA
>1975
GGCTGCTAAAGCTAGGAACCCAGCTGATAGCTTTGTTTTGATGTCCTTGTAATGTTTGC
GGATCGCAGAGGGTGCTATTTAGAGCAGGGGGCATCTACTGGGACAGACTCACTGGGA
AGTTGAAACAGCTAGCTCAAAGT
>1976
TAGGAACCCAGCTGATAGCTTTGTTTTGATGTCCTTGTAATGTTTGCGGATCGCAGAGG
GTGCTATTTAGAGCAGGGGGCATCTACTGGGACAGACTCACTGGGAAGTTGAAACAGC
TAGCTCAAAGTCCAAGAGATGCT
>1977
TAATGTTTGCGGATCGCAGAGGGTGCTATTTAGAGCAGGGGGCATCTACTGGGACAGA
CTCACTGGGAAGTTGAAACAGCTAGCTCAAAGTCCAAGAGATGCTAAAGATAGTGAAA
TACAGCCCAGGAATTGAGGGGGAG
>1978
CGGATCGCAGAGGGTGCTATTTAGAGCAGGGGGCATCTACTGGGACAGACTCACTGGG
AAGTTGAAACAGCTAGCTCAAAGTCCAAGAGATGCTAAAGATAGTGAAATACAGCCCA
GGAATTGAGGGGGAGGGGGTTCCT
>1979
TCGCAGAGGGTGCTATTTAGAGCAGGGGGCATCTACTGGGACAGACTCACTGGGAAGT
TGAAACAGCTAGCTCAAAGTCCAAGAGATGCTAAAGATAGTGAAATACAGCCCAGGAA
TTGAGGGGGAGGGGGTTCCTCACT
>1980
TTTAGAGCAGGGGGCATCTACTGGGACAGACTCACTGGGAAGTTGAAACAGCTAGCTC
AAAGTCCAAGAGATGCTAAAGATAGTGAAATACAGCCCAGGAATTGAGGGGGAGGGG
GTTCCTCACTCAAGAAGGAGACTTT

>1981
GGGAAGTTGAAACAGCTAGCTCAAAGTCCAAGAGATGCTAAAGATAGTGAAATACAG
CCCAGGAATTGAGGGGGAGGGGGTTCCTCACTCAAGAAGGAGACTTTCTAGGAGGGG
GCGGGCGTGGTATTTTCTAGGGACAG
>1982
TTGAAACAGCTAGCTCAAAGTCCAAGAGATGCTAAAGATAGTGAAATACAGCCCAGGA
ATTGAGGGGGAGGGGGTTCCTCACTCAAGAAGGAGACTTTCTAGGAGGGGGCGGGCG
TGGTATTTTCTAGGGACAGTATGGA
>1983
AAGTCCAAGAGATGCTAAAGATAGTGAAATACAGCCCAGGAATTGAGGGGGAGGGGG
TTCCTCACTCAAGAAGGAGACTTTCTAGGAGGGGGCGGGCGTGGTATTTTCTAGGGAC
AGTATGGAATGAAGGGAAGAAGGTT
>1984
ATTGAGGGGGAGGGGGTTCCTCACTCAAGAAGGAGACTTTCTAGGAGGGGGCGGGCG
TGGTATTTTCTAGGGACAGTATGGAATGAAGGGAAGAAGGTTGGCTTTAGAAGGAGGC
ACCCTGTGGGAGGCTGAGACAGGTG
>1985
GGGGAGGGGGTTCCTCACTCAAGAAGGAGACTTTCTAGGAGGGGGCGGGCGTGGTAT
TTTCTAGGGACAGTATGGAATGAAGGGAAGAAGGTTGGCTTTAGAAGGAGGCACCCT
GTGGGAGGCTGAGACAGGTGGGTCAC
>1986
CCTCACTCAAGAAGGAGACTTTCTAGGAGGGGGCGGGCGTGGTATTTTCTAGGGACAG
TATGGAATGAAGGGAAGAAGGTTGGCTTTAGAAGGAGGCACCCTGTGGGAGGCTGAG
ACAGGTGGGTCACCTGAGGTCAGGA
>1987
TGGTATTTTCTAGGGACAGTATGGAATGAAGGGAAGAAGGTTGGCTTTAGAAGGAGGC
ACCCTGTGGGAGGCTGAGACAGGTGGGTCACCTGAGGTCAGGAGTTTGAGACCAGCC
TGGCCAACATGGTGAAACCCAGTCT
>1988
TTTCTAGGGACAGTATGGAATGAAGGGAAGAAGGTTGGCTTTAGAAGGAGGCACCCT
GTGGGAGGCTGAGACAGGTGGGTCACCTGAGGTCAGGAGTTTGAGACCAGCCTGGCC
AACATGGTGAAACCCAGTCTCTACTA
>1989
AATGAAGGGAAGAAGGTTGGCTTTAGAAGGAGGCACCCTGTGGGAGGCTGAGACAG
GTGGGTCACCTGAGGTCAGGAGTTTGAGACCAGCCTGGCCAACATGGTGAAACCCAG
TCTCTACTAAAAATACAAAAATTAGCT
>1990
GAGGCTGAGACAGGTGGGTCACCTGAGGTCAGGAGTTTGAGACCAGCCTGGCCAACA
TGGTGAAACCCAGTCTCTACTAAAAATACAAAAATTAGCTGGGTGGCAGGTGCCTGTA
ATCCCAGCTAGTCGGGGGAGGCTGA
>1991
AGGTCAGGAGTTTGAGACCAGCCTGGCCAACATGGTGAAACCCAGTCTCTACTAAAAA
TACAAAAATTAGCTGGGTGGCAGGTGCCTGTAATCCCAGCTAGTCGGGGGAGGCTGAG
GCAGGAAAATCGCTTGGGATTACA

>1992
CAACATGGTGAAACCCAGTCTCTACTAAAAATACAAAAATTAGCTGGGTGGCAGGTGC
CTGTAATCCCAGCTAGTCGGGGGAGGCTGAGGCAGGAAAATCGCTTGGGATTACAGGT
GCCTGTAATCCCAGCTAGTCGGGA
>1993
ATGGTGAAACCCAGTCTCTACTAAAAATACAAAAATTAGCTGGGTGGCAGGTGCCTGT
AATCCCAGCTAGTCGGGGGAGGCTGAGGCAGGAAAATCGCTTGGGATTACAGGTGCCT
GTAATCCCAGCTAGTCGGGAGAGG
>1994
CCCAGTCTCTACTAAAAATACAAAAATTAGCTGGGTGGCAGGTGCCTGTAATCCCAGCT
AGTCGGGGGAGGCTGAGGCAGGAAAATCGCTTGGGATTACAGGTGCCTGTAATCCCA
GCTAGTCGGGAGAGGCTGAGGCAG
>1995
TGGGTGGCAGGTGCCTGTAATCCCAGCTAGTCGGGGGAGGCTGAGGCAGGAAAATCG
CTTGGGATTACAGGTGCCTGTAATCCCAGCTAGTCGGGAGAGGCTGAGGCAGGAAAAT
TGCTTGAAGGAGGCGGAGGTTGCAG
>1996
GTAATCCCAGCTAGTCGGGGGAGGCTGAGGCAGGAAAATCGCTTGGGATTACAGGTGC
CTGTAATCCCAGCTAGTCGGGAGAGGCTGAGGCAGGAAAATTGCTTGAAGGAGGCGG
AGGTTGCAGTGAGCCCAGATCCTGC
>1997
TCCCAGCTAGTCGGGGGAGGCTGAGGCAGGAAAATCGCTTGGGATTACAGGTGCCTGT
AATCCCAGCTAGTCGGGAGAGGCTGAGGCAGGAAAATTGCTTGAAGGAGGCGGAGGT
TGCAGTGAGCCCAGATCCTGCCGTT
>1998
TAGTCGGGGGAGGCTGAGGCAGGAAAATCGCTTGGGATTACAGGTGCCTGTAATCCCA
GCTAGTCGGGAGAGGCTGAGGCAGGAAAATTGCTTGAAGGAGGCGGAGGTTGCAGTG
AGCCCAGATCCTGCCGTTGCACTCC
>1999
TCGCTTGGGATTACAGGTGCCTGTAATCCCAGCTAGTCGGGAGAGGCTGAGGCAGGAA
AATTGCTTGAAGGAGGCGGAGGTTGCAGTGAGCCCAGATCCTGCCGTTGCACTCCAGC
CCAAGACTTTGTCTCATTAAAAAA
>2000
CTGTAATCCCAGCTAGTCGGGAGAGGCTGAGGCAGGAAAATTGCTTGAAGGAGGCGG
AGGTTGCAGTGAGCCCAGATCCTGCCGTTGCACTCCAGCCCAAGACTTTGTCTCATTA
AAAAAAAAAAAAGAAGAAGAAGAAG
>2001
ATCCCAGCTAGTCGGGAGAGGCTGAGGCAGGAAAATTGCTTGAAGGAGGCGGAGGTT
GCAGTGAGCCCAGATCCTGCCGTTGCACTCCAGCCCAAGACTTTGTCTCATTAAAAAA
AAAAAAAGAAGAAGAAGAAGAAGGA
>2002
GCTGAGGCAGGAAAATTGCTTGAAGGAGGCGGAGGTTGCAGTGAGCCCAGATCCTGC
CGTTGCACTCCAGCCCAAGACTTTGTCTCATTAAAAAAAAAAAAAGAAGAAGAAGAA
GAAGGAGGCACCCTGGATGAAGACTC

>2003
GGTTGCAGTGAGCCCAGATCCTGCCGTTGCACTCCAGCCCAAGACTTTGTCTCATTAA
AAAAAAAAAAAGAAGAAGAAGAAGAAGGAGGCACCCTGGATGAAGACTCAGGCTCC
AGAATTTACTATCTGTATGTCTTGGG
>2004
TGCAGTGAGCCCAGATCCTGCCGTTGCACTCCAGCCCAAGACTTTGTCTCATTAAAAA
AAAAAAAAGAAGAAGAAGAAGAAGGAGGCACCCTGGATGAAGACTCAGGCTCCAGA
ATTTACTATCTGTATGTCTTGGGCAA
>2005
AGTGAGCCCAGATCCTGCCGTTGCACTCCAGCCCAAGACTTTGTCTCATTAAAAAAAA
AAAAAGAAGAAGAAGAAGAAGGAGGCACCCTGGATGAAGACTCAGGCTCCAGAATT
TACTATCTGTATGTCTTGGGCAAGTG
>2006
AGATCCTGCCGTTGCACTCCAGCCCAAGACTTTGTCTCATTAAAAAAAAAAAAAGAAG
AAGAAGAAGAAGGAGGCACCCTGGATGAAGACTCAGGCTCCAGAATTTACTATCTGTA
TGTCTTGGGCAAGTGGCTTAGCTT
>2007
CCAGCCCAAGACTTTGTCTCATTAAAAAAAAAAAAAGAAGAAGAAGAAGAAGGAGG
CACCCTGGATGAAGACTCAGGCTCCAGAATTTACTATCTGTATGTCTTGGGCAAGTGGC
TTAGCTTCTCTGAGCCCCTGTTTAC
>2008
AGAAGAAGAAGGAGGCACCCTGGATGAAGACTCAGGCTCCAGAATTTACTATCTGTAT
GTCTTGGGCAAGTGGCTTAGCTTCTCTGAGCCCCTGTTTACTTTTCTTTTGAAAATGAC
CACCTTGTCCATGGTAATGAGGA
>2009
AAGGAGGCACCCTGGATGAAGACTCAGGCTCCAGAATTTACTATCTGTATGTCTTGGGC
AAGTGGCTTAGCTTCTCTGAGCCCCTGTTTACTTTTCTTTTGAAAATGACCACCTTGTC
CATGGTAATGAGGATAAAAAAC
>2010
AGTGGCTTAGCTTCTCTGAGCCCCTGTTTACTTTTCTTTTGAAAATGACCACCTTGTCCA
TGGTAATGAGGATAAAAAACGTGTAAAGTGCATAGCAGAGTGCAGGTGTACTGGGTAG
ACATGTATGATTGCGCTGTAGC
>2011
AGCCCCTGTTTACTTTTCTTTTGAAAATGACCACCTTGTCCATGGTAATGAGGATAAAA
AACGTGTAAAGTGCATAGCAGAGTGCAGGTGTACTGGGTAGACATGTATGATTGCGCT
GTAGCCCTGTTATGGCCCGGGGA
>2012
CCACCTTGTCCATGGTAATGAGGATAAAAAACGTGTAAAGTGCATAGCAGAGTGCAGG
TGTACTGGGTAGACATGTATGATTGCGCTGTAGCCCTGTTATGGCCCGGGGAGAAACTA
TGAAAGGAACCAGTGAAATCTTT
>2013
GGATAAAAAACGTGTAAAGTGCATAGCAGAGTGCAGGTGTACTGGGTAGACATGTATG
ATTGCGCTGTAGCCCTGTTATGGCCCGGGGAGAAACTATGAAAGGAACCAGTGAAATC
TTTTGTTTTGGGCACTGCTAAATT

>2014
GCATAGCAGAGTGCAGGTGTACTGGGTAGACATGTATGATTGCGCTGTAGCCCTGTTAT
GGCCCGGGGAGAAACTATGAAAGGAACCAGTGAAATCTTTTGTTTTGGGCACTGCTAA
ATTGATGATAGTACTAACCCTGT
>2015
GCAGGTGTACTGGGTAGACATGTATGATTGCGCTGTAGCCCTGTTATGGCCCGGGGAGA
AACTATGAAAGGAACCAGTGAAATCTTTTGTTTTGGGCACTGCTAAATTGATGATAGTA
CTAACCCTGTGCTAGCAGCCAG
>2016
TACTGGGTAGACATGTATGATTGCGCTGTAGCCCTGTTATGGCCCGGGGAGAAACTATG
AAAGGAACCAGTGAAATCTTTTGTTTTGGGCACTGCTAAATTGATGATAGTACTAACCC
TGTGCTAGCAGCCAGCCGGTGA
>2017
TGGCCCGGGGAGAAACTATGAAAGGAACCAGTGAAATCTTTTGTTTTGGGCACTGCTA
AATTGATGATAGTACTAACCCTGTGCTAGCAGCCAGCCGGTGAAATAATAGGTGAATAA
TGTTAGTACATTGTGAATATAAA
>2018
ATGAAAGGAACCAGTGAAATCTTTTGTTTTGGGCACTGCTAAATTGATGATAGTACTAA
CCCTGTGCTAGCAGCCAGCCGGTGAAATAATAGGTGAATAATGTTAGTACATTGTGAAT
ATAAAAGTTTCCATTTGCAAAG
>2019
AAAGGAACCAGTGAAATCTTTTGTTTTGGGCACTGCTAAATTGATGATAGTACTAACCC
TGTGCTAGCAGCCAGCCGGTGAAATAATAGGTGAATAATGTTAGTACATTGTGAATATA
AAAGTTTCCATTTGCAAAGTGC
>2020
TTTGTTTTGGGCACTGCTAAATTGATGATAGTACTAACCCTGTGCTAGCAGCCAGCCGG
TGAAATAATAGGTGAATAATGTTAGTACATTGTGAATATAAAAGTTTCCATTTGCAAAGT
GCTACTGCTGTTCTAAAGAAG
>2021
CTAACCCTGTGCTAGCAGCCAGCCGGTGAAATAATAGGTGAATAATGTTAGTACATTGT
GAATATAAAAGTTTCCATTTGCAAAGTGCTACTGCTGTTCTAAAGAAGCCACATTTCTA
ATAAATGATTGCTTAATCCTGT
>2022
CAGCCAGCCGGTGAAATAATAGGTGAATAATGTTAGTACATTGTGAATATAAAAGTTTC
CATTTGCAAAGTGCTACTGCTGTTCTAAAGAAGCCACATTTCTAATAAATGATTGCTTAA
TCCTGTGATGCTGCCACAGCC
>2023
TAGGTGAATAATGTTAGTACATTGTGAATATAAAAGTTTCCATTTGCAAAGTGCTACTGC
TGTTCTAAAGAAGCCACATTTCTAATAAATGATTGCTTAATCCTGTGATGCTGCCACAGC
CCCACAGAGTTTGGGAATGT
>2024
AGTGCTACTGCTGTTCTAAAGAAGCCACATTTCTAATAAATGATTGCTTAATCCTGTGAT
GCTGCCACAGCCCCACAGAGTTTGGGAATGTTTGGGAATTCTTTCTGAAGTGTGTGCC
GCATTCTTTTGCTTTGCCTTTT

>2025
TGCTGTTCTAAAGAAGCCACATTTCTAATAAATGATTGCTTAATCCTGTGATGCTGCCAC
AGCCCCACAGAGTTTGGGAATGTTTGGGAATTCTTTCTGAAGTGTGTGCCGCATTCTTT
TGCTTTGCCTTTTGGGAGGGT
>2026
TGATTGCTTAATCCTGTGATGCTGCCACAGCCCCACAGAGTTTGGGAATGTTTGGGAAT
TCTTTCTGAAGTGTGTGCCGCATTCTTTTGCTTTGCCTTTTGGGAGGGTAGATGTGACTA
TTGGAAATGGTCAAAATAATG
>2027
CAGAGTTTGGGAATGTTTGGGAATTCTTTCTGAAGTGTGTGCCGCATTCTTTTGCTTTG
CCTTTTGGGAGGGTAGATGTGACTATTGGAAATGGTCAAAATAATGCAGTAGCAAATCT
GTTGACTATAGTGAGTGATAAG
>2028
GTGCCGCATTCTTTTGCTTTGCCTTTTGGGAGGGTAGATGTGACTATTGGAAATGGTCA
AAATAATGCAGTAGCAAATCTGTTGACTATAGTGAGTGATAAGCACTGTCGGCTGCTGT
GTCTGACTCGGAAGGCAGATTC
>2029
CCGCATTCTTTTGCTTTGCCTTTTGGGAGGGTAGATGTGACTATTGGAAATGGTCAAAA
TAATGCAGTAGCAAATCTGTTGACTATAGTGAGTGATAAGCACTGTCGGCTGCTGTGTC
TGACTCGGAAGGCAGATTCTGG
>2030
TTGGGAGGGTAGATGTGACTATTGGAAATGGTCAAAATAATGCAGTAGCAAATCTGTTG
ACTATAGTGAGTGATAAGCACTGTCGGCTGCTGTGTCTGACTCGGAAGGCAGATTCTG
GAAGGGGTGTGTGGGGAAGGCTT
>2031
TAATGCAGTAGCAAATCTGTTGACTATAGTGAGTGATAAGCACTGTCGGCTGCTGTGTC
TGACTCGGAAGGCAGATTCTGGAAGGGGTGTGTGGGGAAGGCTTGTTGGCTCAGCAC
TGGGGTGAGTGTTGGCCTCACTTC
>2032
GCAGTAGCAAATCTGTTGACTATAGTGAGTGATAAGCACTGTCGGCTGCTGTGTCTGAC
TCGGAAGGCAGATTCTGGAAGGGGTGTGTGGGGAAGGCTTGTTGGCTCAGCACTGGG
GTGAGTGTTGGCCTCACTTCTTAG
>2033
TGAGTGATAAGCACTGTCGGCTGCTGTGTCTGACTCGGAAGGCAGATTCTGGAAGGGG
TGTGTGGGGAAGGCTTGTTGGCTCAGCACTGGGGTGAGTGTTGGCCTCACTTCTTAGG
TGGGAAGTGGACAAGGCCAAGGGG
>2034
TGTCGGCTGCTGTGTCTGACTCGGAAGGCAGATTCTGGAAGGGGTGTGTGGGGAAGG
CTTGTTGGCTCAGCACTGGGGTGAGTGTTGGCCTCACTTCTTAGGTGGGAAGTGGACA
AGGCCAAGGGGCTTTTGATTTCCAG
>2035
TGTCTGACTCGGAAGGCAGATTCTGGAAGGGGTGTGTGGGGAAGGCTTGTTGGCTCA
GCACTGGGGTGAGTGTTGGCCTCACTTCTTAGGTGGGAAGTGGACAAGGCCAAGGGG
CTTTTGATTTCCAGCTACTATGGCTG

>2036
ATTCTGGAAGGGGTGTGTGGGGAAGGCTTGTTGGCTCAGCACTGGGGTGAGTGTTGG
CCTCACTTCTTAGGTGGGAAGTGGACAAGGCCAAGGGGCTTTTGATTTCCAGCTACTAT
GGCTGGACTCCCAGTGAGGAAGGA
>2037
AAGGCTTGTTGGCTCAGCACTGGGGTGAGTGTTGGCCTCACTTCTTAGGTGGGAAGTG
GACAAGGCCAAGGGGCTTTTGATTTCCAGCTACTATGGCTGGACTCCCAGTGAGGAAG
GAGTCGCTGCCATGGATTGCCTGG
>2038
CACTGGGGTGAGTGTTGGCCTCACTTCTTAGGTGGGAAGTGGACAAGGCCAAGGGGC
TTTTGATTTCCAGCTACTATGGCTGGACTCCCAGTGAGGAAGGAGTCGCTGCCATGGAT
TGCCTGGGACTGGGAAGACTCCTT
>2039
CACTTCTTAGGTGGGAAGTGGACAAGGCCAAGGGGCTTTTGATTTCCAGCTACTATGG
CTGGACTCCCAGTGAGGAAGGAGTCGCTGCCATGGATTGCCTGGGACTGGGAAGACT
CCTTTTAACTGCTGTAATGTTTCTG
>2040
TTTCCAGCTACTATGGCTGGACTCCCAGTGAGGAAGGAGTCGCTGCCATGGATTGCCT
GGGACTGGGAAGACTCCTTTTAACTGCTGTAATGTTTCTGCATTTGTGGTTTGGCCTCC
TGTGAGTTTGGGCTCCGGGGCGG
>2041
TGCCTGGGACTGGGAAGACTCCTTTTAACTGCTGTAATGTTTCTGCATTTGTGGTTTGG
CCTCCTGTGAGTTTGGGCTCCGGGGCGGCACTTCTGTTGTTAAAAGAACAGAAAAGA
AAACAAAACAAAACAAAAAACCAG
>2042
AATGTTTCTGCATTTGTGGTTTGGCCTCCTGTGAGTTTGGGCTCCGGGGCGGCACTTCT
GTTGTTAAAAGAACAGAAAAGAAAACAAAACAAAACAAAAAACCAGGACTGTCACT
GTTAGATGTTGGTTGAGGCTAGAAA
>2043
TTTGGGCTCCGGGGCGGCACTTCTGTTGTTAAAAGAACAGAAAAGAAAACAAAACAA
AACAAAAAACCAGGACTGTCACTGTTAGATGTTGGTTGAGGCTAGAAAGAATGGATTG
AGTTTTGACCCCCTACACCCCAGGG
>2044
GGCACTTCTGTTGTTAAAAGAACAGAAAAGAAAACAAAACAAAACAAAAAACCAGG
ACTGTCACTGTTAGATGTTGGTTGAGGCTAGAAAGAATGGATTGAGTTTTGACCCCCTA
CACCCCAGGGAGTAGGGAGGCCTGG
>2045
ACAGAAAAGAAAACAAAACAAAACAAAAAACCAGGACTGTCACTGTTAGATGTTGGT
TGAGGCTAGAAAGAATGGATTGAGTTTTGACCCCCTACACCCCAGGGAGTAGGGAGGC
CTGGGAGGCACAGTGGCCTGAGTTT
>2046
AACAAAACAAAACAAAAAACCAGGACTGTCACTGTTAGATGTTGGTTGAGGCTAGAA
AGAATGGATTGAGTTTTGACCCCCTACACCCCAGGGAGTAGGGAGGCCTGGGAGGCA
CAGTGGCCTGAGTTTGAATTCTGACT

>2047
AGGACTGTCACTGTTAGATGTTGGTTGAGGCTAGAAAGAATGGATTGAGTTTTGACCC
CCTACACCCCAGGGAGTAGGGAGGCCTGGGAGGCACAGTGGCCTGAGTTTGAATTCT
GACTCTGCCTCTTTGGAAGGCTCTG
>2048
TGTTAGATGTTGGTTGAGGCTAGAAAGAATGGATTGAGTTTTGACCCCCTACACCCCAG
GGAGTAGGGAGGCCTGGGAGGCACAGTGGCCTGAGTTTGAATTCTGACTCTGCCTCTT
TGGAAGGCTCTGGAAGGTTAAAG
>2049
AAGAATGGATTGAGTTTTGACCCCCTACACCCCAGGGAGTAGGGAGGCCTGGGAGGC
ACAGTGGCCTGAGTTTGAATTCTGACTCTGCCTCTTTGGAAGGCTCTGGAAGGTTAAA
GGAGCTTGTGTGTTCAAATCTTAGC
>2050
CCCCAGGGAGTAGGGAGGCCTGGGAGGCACAGTGGCCTGAGTTTGAATTCTGACTCT
GCCTCTTTGGAAGGCTCTGGAAGGTTAAAGGAGCTTGTGTGTTCAAATCTTAGCCTGG
TACCTGGCATATACTCAGCATGTAT
>2051
TAGGGAGGCCTGGGAGGCACAGTGGCCTGAGTTTGAATTCTGACTCTGCCTCTTTGGA
AGGCTCTGGAAGGTTAAAGGAGCTTGTGTGTTCAAATCTTAGCCTGGTACCTGGCATAT
ACTCAGCATGTATTTTTTCCCCT
>2052
GTTTGAATTCTGACTCTGCCTCTTTGGAAGGCTCTGGAAGGTTAAAGGAGCTTGTGTG
TTCAAATCTTAGCCTGGTACCTGGCATATACTCAGCATGTATTTTTTCCCCTTTCCTGAC
AATGTTCCTTGCCCCGAACACT
>2053
TTGGAAGGCTCTGGAAGGTTAAAGGAGCTTGTGTGTTCAAATCTTAGCCTGGTACCTG
GCATATACTCAGCATGTATTTTTTCCCCTTTCCTGACAATGTTCCTTGCCCCGAACACTT
GAATAGTGGAGAGAGGAATGTT
>2054
CCTGGCATATACTCAGCATGTATTTTTTCCCCTTTCCTGACAATGTTCCTTGCCCCGAAC
ACTTGAATAGTGGAGAGAGGAATGTTTGCTTTTCCTCTCCATTGAACCAACACTCGCCC
ATCCACTGAGCCCAAGATTCA
>2055
CATATACTCAGCATGTATTTTTTCCCCTTTCCTGACAATGTTCCTTGCCCCGAACACTTG
AATAGTGGAGAGAGGAATGTTTGCTTTTCCTCTCCATTGAACCAACACTCGCCCATCCA
CTGAGCCCAAGATTCACCTTA
>2056
CACTTGAATAGTGGAGAGAGGAATGTTTGCTTTTCCTCTCCATTGAACCAACACTCGCC
CATCCACTGAGCCCAAGATTCACCTTAGGATTGTCTGTTGGACTCGGCTCTTTTGTGAC
CTAACAGTGCAGGGGCCTTGTG
>2057
AATAGTGGAGAGAGGAATGTTTGCTTTTCCTCTCCATTGAACCAACACTCGCCCATCCA
CTGAGCCCAAGATTCACCTTAGGATTGTCTGTTGGACTCGGCTCTTTTGTGACCTAACA
GTGCAGGGGCCTTGTGCTGAGC

>2058
CGCCCATCCACTGAGCCCAAGATTCACCTTAGGATTGTCTGTTGGACTCGGCTCTTTTG
TGACCTAACAGTGCAGGGGCCTTGTGCTGAGCTGCACATGCTCGCTCTCGCTGGTGCT
CCCTCTCTCTCTCTCTCTCTGTC
>2059
ATCCACTGAGCCCAAGATTCACCTTAGGATTGTCTGTTGGACTCGGCTCTTTTGTGACC
TAACAGTGCAGGGGCCTTGTGCTGAGCTGCACATGCTCGCTCTCGCTGGTGCTCCCTC
TCTCTCTCTCTCTCTGTCTGCAT
>2060
GCTGAGCTGCACATGCTCGCTCTCGCTGGTGCTCCCTCTCTCTCTCTCTCTCTGTCTGC
ATTATTTATAGTAAACCATATTAGAACAGAGTGTTAAAGAGGAAGGATGAGTCATTCCC
ATTGAAACCATTGATGACATAA
>2061
GCTCTCGCTGGTGCTCCCTCTCTCTCTCTCTCTCTGTCTGCATTATTTATAGTAAACCATA
TTAGAACAGAGTGTTAAAGAGGAAGGATGAGTCATTCCCATTGAAACCATTGATGACA
TAAATAAAAGCAAAATGCTTA
>2062
TCTCGCTGGTGCTCCCTCTCTCTCTCTCTCTCTGTCTGCATTATTTATAGTAAACCATATT
AGAACAGAGTGTTAAAGAGGAAGGATGAGTCATTCCCATTGAAACCATTGATGACATA
AATAAAAGCAAAATGCTTACT
>2063
GTGCTCCCTCTCTCTCTCTCTCTCTGTCTGCATTATTTATAGTAAACCATATTAGAACAGA
GTGTTAAAGAGGAAGGATGAGTCATTCCCATTGAAACCATTGATGACATAAATAAAAG
CAAAATGCTTACTATGTGTTT
>2064
TATTAGAACAGAGTGTTAAAGAGGAAGGATGAGTCATTCCCATTGAAACCATTGATGA
CATAAATAAAAGCAAAATGCTTACTATGTGTTTTGTGCAGTATTTAAGGCCAGGTTGTG
CTAATTTGAATGCTCCCAAACTG
>2065
ATGAGTCATTCCCATTGAAACCATTGATGACATAAATAAAAGCAAAATGCTTACTATGT
GTTTTGTGCAGTATTTAAGGCCAGGTTGTGCTAATTTGAATGCTCCCAAACTGGATCTG
TGAAAGCATACATATGTGGGAA
>2066
TTCCCATTGAAACCATTGATGACATAAATAAAAGCAAAATGCTTACTATGTGTTTTGTGC
AGTATTTAAGGCCAGGTTGTGCTAATTTGAATGCTCCCAAACTGGATCTGTGAAAGCAT
ACATATGTGGGAAAAAAATTCA
>2067
TATGTGTTTTGTGCAGTATTTAAGGCCAGGTTGTGCTAATTTGAATGCTCCCAAACTGGA
TCTGTGAAAGCATACATATGTGGGAAAAAAATTCAGGTTTAACTAGAGGGAAGTTATTTA
TTGAAAATCCATTTCAGTATC
>2068
CCAGGTTGTGCTAATTTGAATGCTCCCAAACTGGATCTGTGAAAGCATACATATGTGGG
AAAAAATTCAGGTTTAACTAGAGGGAAGTTATTTATTGAAAATCCATTTCAGTATCTGCT
TTCTCCCTTCTATTTACTTCA

>2069
CTAATTTGAATGCTCCCAAACTGGATCTGTGAAAGCATACATATGTGGGAAAAAATTCA
GGTTTAACTAGAGGGAAGTTATTTATTGAAAATCCATTTCAGTATCTGCTTTCTCCCTTC
TATTTACTTCAGATCCATATT
>2070
AATTTGAATGCTCCCAAACTGGATCTGTGAAAGCATACATATGTGGGAAAAAATTCAGG
TTTAACTAGAGGGAAGTTATTTATTGAAAATCCATTTCAGTATCTGCTTTCTCCCTTCTAT
TTACTTCAGATCCATATTCA
>2071
GAATGCTCCCAAACTGGATCTGTGAAAGCATACATATGTGGGAAAAAATTCAGGTTTAA
CTAGAGGGAAGTTATTTATTGAAAATCCATTTCAGTATCTGCTTTCTCCCTTCTATTTACT
TCAGATCCATATTCATATCA
>2072
AAAGCATACATATGTGGGAAAAAATTCAGGTTTAACTAGAGGGAAGTTATTTATTGAAA
ATCCATTTCAGTATCTGCTTTCTCCCTTCTATTTACTTCAGATCCATATTCATATCATGCTT
GTTGTTCTGGCTAACGAGA
>2073
TTTAACTAGAGGGAAGTTATTTATTGAAAATCCATTTCAGTATCTGCTTTCTCCCTTCTAT
TTACTTCAGATCCATATTCATATCATGCTTGTTGTTCTGGCTAACGAGAGGGACTGGCTT
ATTCACTAGCTAAGGTTTG
>2074
GTATCTGCTTTCTCCCTTCTATTTACTTCAGATCCATATTCATATCATGCTTGTTGTTCTGG
CTAACGAGAGGGACTGGCTTATTCACTAGCTAAGGTTTGTTTATTTTCGGAAGGTTGAG
GAATAGCACAGAGAGGTTG
>2075
TTTACTTCAGATCCATATTCATATCATGCTTGTTGTTCTGGCTAACGAGAGGGACTGGCT
TATTCACTAGCTAAGGTTTGTTTATTTTCGGAAGGTTGAGGAATAGCACAGAGAGGTTG
TCTAAGTGTCCCTGTCTCCAT
>2076
TTCAGATCCATATTCATATCATGCTTGTTGTTCTGGCTAACGAGAGGGACTGGCTTATTC
ACTAGCTAAGGTTTGTTTATTTTCGGAAGGTTGAGGAATAGCACAGAGAGGTTGTCTAA
GTGTCCCTGTCTCCATTCCTC
>2077
CATGCTTGTTGTTCTGGCTAACGAGAGGGACTGGCTTATTCACTAGCTAAGGTTTGTTT
ATTTTCGGAAGGTTGAGGAATAGCACAGAGAGGTTGTCTAAGTGTCCCTGTCTCCATTC
CTCCTCTTGCCACCTGTTGAGG
>2078
TCTGGCTAACGAGAGGGACTGGCTTATTCACTAGCTAAGGTTTGTTTATTTTCGGAAGG
TTGAGGAATAGCACAGAGAGGTTGTCTAAGTGTCCCTGTCTCCATTCCTCCTCTTGCCA
CCTGTTGAGGAAGGCACTCTCC
>2079
GGTTGAGGAATAGCACAGAGAGGTTGTCTAAGTGTCCCTGTCTCCATTCCTCCTCTTGC
CACCTGTTGAGGAAGGCACTCTCCACCTGCTTTGCAGGCGTGGGCTCTCAATGTGTCC
AAGAATCACAGTCAGAGCTGAAG

>2080
GAGGAATAGCACAGAGAGGTTGTCTAAGTGTCCCTGTCTCCATTCCTCCTCTTGCCACC
TGTTGAGGAAGGCACTCTCCACCTGCTTTGCAGGCGTGGGCTCTCAATGTGTCCAAGA
ATCACAGTCAGAGCTGAAGGGCC
>2081
TCTAAGTGTCCCTGTCTCCATTCCTCCTCTTGCCACCTGTTGAGGAAGGCACTCTCCAC
CTGCTTTGCAGGCGTGGGCTCTCAATGTGTCCAAGAATCACAGTCAGAGCTGAAGGGC
CCTTGGAATCATTTATTGCAGCA
>2082
TCCTCTTGCCACCTGTTGAGGAAGGCACTCTCCACCTGCTTTGCAGGCGTGGGCTCTC
AATGTGTCCAAGAATCACAGTCAGAGCTGAAGGGCCCTTGGAATCATTTATTGCAGCAT
GCTCATTTGGGAGGAGTAAGTGG
>2083
CCACCTGTTGAGGAAGGCACTCTCCACCTGCTTTGCAGGCGTGGGCTCTCAATGTGTC
CAAGAATCACAGTCAGAGCTGAAGGGCCCTTGGAATCATTTATTGCAGCATGCTCATTT
GGGAGGAGTAAGTGGAGGTCCAG
>2084
AGGCGTGGGCTCTCAATGTGTCCAAGAATCACAGTCAGAGCTGAAGGGCCCTTGGAAT
CATTTATTGCAGCATGCTCATTTGGGAGGAGTAAGTGGAGGTCCAGAGAGGTTCTGTG
ATTTTCCCTAGGTCACACAGCAAG
>2085
TGTGTCCAAGAATCACAGTCAGAGCTGAAGGGCCCTTGGAATCATTTATTGCAGCATG
CTCATTTGGGAGGAGTAAGTGGAGGTCCAGAGAGGTTCTGTGATTTTCCCTAGGTCAC
ACAGCAAGCTAGAGGCAAAACCAG
>2086
TCACAGTCAGAGCTGAAGGGCCCTTGGAATCATTTATTGCAGCATGCTCATTTGGGAGG
AGTAAGTGGAGGTCCAGAGAGGTTCTGTGATTTTCCCTAGGTCACACAGCAAGCTAGA
GGCAAAACCAGGACCCTCACTCC
>2087
TCATTTATTGCAGCATGCTCATTTGGGAGGAGTAAGTGGAGGTCCAGAGAGGTTCTGT
GATTTTCCCTAGGTCACACAGCAAGCTAGAGGCAAAACCAGGACCCTCACTCCCAGCC
CACTTTAATTCTTCCTTCACAAGC
>2088
GCAGCATGCTCATTTGGGAGGAGTAAGTGGAGGTCCAGAGAGGTTCTGTGATTTTCCC
TAGGTCACACAGCAAGCTAGAGGCAAAACCAGGACCCTCACTCCCAGCCCACTTTAAT
TCTTCCTTCACAAGCCTGCCTGTG
>2089
CTCATTTGGGAGGAGTAAGTGGAGGTCCAGAGAGGTTCTGTGATTTTCCCTAGGTCAC
ACAGCAAGCTAGAGGCAAAACCAGGACCCTCACTCCCAGCCCACTTTAATTCTTCCTT
CACAAGCCTGCCTGTGACTGCCCA
>2090
CAGAGAGGTTCTGTGATTTTCCCTAGGTCACACAGCAAGCTAGAGGCAAAACCAGGA
CCCTCACTCCCAGCCCACTTTAATTCTTCCTTCACAAGCCTGCCTGTGACTGCCCAGGT
CTGTTCTCTGCTTCTACCAAGGGG

>2091
GGTCACACAGCAAGCTAGAGGCAAAACCAGGACCCTCACTCCCAGCCCACTTTAATTC
TTCCTTCACAAGCCTGCCTGTGACTGCCCAGGTCTGTTCTCTGCTTCTACCAAGGGGTA
GGCAAGACTAGAATCTGACTTGA
>2092
GGCAAAACCAGGACCCTCACTCCCAGCCCACTTTAATTCTTCCTTCACAAGCCTGCCT
GTGACTGCCCAGGTCTGTTCTCTGCTTCTACCAAGGGGTAGGCAAGACTAGAATCTGA
CTTGAGAGCAGTTTCAGATCTTTG
>2093
CAGCCCACTTTAATTCTTCCTTCACAAGCCTGCCTGTGACTGCCCAGGTCTGTTCTCTG
CTTCTACCAAGGGGTAGGCAAGACTAGAATCTGACTTGAGAGCAGTTTCAGATCTTTG
TCTGTGAGGGATGCTGGGGGGTT
>2094
ACTTTAATTCTTCCTTCACAAGCCTGCCTGTGACTGCCCAGGTCTGTTCTCTGCTTCTAC
CAAGGGGTAGGCAAGACTAGAATCTGACTTGAGAGCAGTTTCAGATCTTTGTCTGTGA
GGGATGCTGGGGGGTTTCTGGC
>2095
AATTCTTCCTTCACAAGCCTGCCTGTGACTGCCCAGGTCTGTTCTCTGCTTCTACCAAG
GGGTAGGCAAGACTAGAATCTGACTTGAGAGCAGTTTCAGATCTTTGTCTGTGAGGGA
TGCTGGGGGGTTTCTGGCATGGT
>2096
TTCCTTCACAAGCCTGCCTGTGACTGCCCAGGTCTGTTCTCTGCTTCTACCAAGGGGTA
GGCAAGACTAGAATCTGACTTGAGAGCAGTTTCAGATCTTTGTCTGTGAGGGATGCTG
GGGGGTTTCTGGCATGGTGACTT
>2097
CTGCCTGTGACTGCCCAGGTCTGTTCTCTGCTTCTACCAAGGGGTAGGCAAGACTAGA
ATCTGACTTGAGAGCAGTTTCAGATCTTTGTCTGTGAGGGATGCTGGGGGGTTTCTGGC
ATGGTGACTTGCCTCCTGGGACA
>2098
TCTGCTTCTACCAAGGGGTAGGCAAGACTAGAATCTGACTTGAGAGCAGTTTCAGATC
TTTGTCTGTGAGGGATGCTGGGGGGTTTCTGGCATGGTGACTTGCCTCCTGGGACACT
CATTAACCTCTCTGCTGCTGAGCC
>2099
GCTGGGGGGTTTCTGGCATGGTGACTTGCCTCCTGGGACACTCATTAACCTCTCTGCTG
CTGAGCCTGAGCAGACTAATGTACATCAACTCCTTAGGATTTACAGACAATGCCCTATG
TTGGGGGTTTTGAGACCCAAAG
>2100
GGGGGGTTTCTGGCATGGTGACTTGCCTCCTGGGACACTCATTAACCTCTCTGCTGCTG
AGCCTGAGCAGACTAATGTACATCAACTCCTTAGGATTTACAGACAATGCCCTATGTTG
GGGGTTTTGAGACCCAAAGATT
>2101
TGCCTCCTGGGACACTCATTAACCTCTCTGCTGCTGAGCCTGAGCAGACTAATGTACAT
CAACTCCTTAGGATTTACAGACAATGCCCTATGTTGGGGGTTTTGAGACCCAAAGATTG
CTAAGACATGGGACTTTTCCTT

>2102
AGCCTGAGCAGACTAATGTACATCAACTCCTTAGGATTTACAGACAATGCCCTATGTTG
GGGGTTTTGAGACCCAAAGATTGCTAAGACATGGGACTTTTCCTTGAAGAGCTTATACT
TTGAGTTAAAATATGTATGTTG
>2103
CAGACTAATGTACATCAACTCCTTAGGATTTACAGACAATGCCCTATGTTGGGGGTTTT
GAGACCCAAAGATTGCTAAGACATGGGACTTTTCCTTGAAGAGCTTATACTTTGAGTTA
AAATATGTATGTTGATATCTAT
>2104
GTACATCAACTCCTTAGGATTTACAGACAATGCCCTATGTTGGGGGTTTTGAGACCCAA
AGATTGCTAAGACATGGGACTTTTCCTTGAAGAGCTTATACTTTGAGTTAAAATATGTAT
GTTGATATCTATGACAGAGAA
>2105
ATGTTGGGGGTTTTGAGACCCAAAGATTGCTAAGACATGGGACTTTTCCTTGAAGAGC
TTATACTTTGAGTTAAAATATGTATGTTGATATCTATGACAGAGAATAGGAAATGGAAAG
ACTATGATATATATATTCAGAG
>2106
TAAGACATGGGACTTTTCCTTGAAGAGCTTATACTTTGAGTTAAAATATGTATGTTGATA
TCTATGACAGAGAATAGGAAATGGAAAGACTATGATATATATATTCAGAGTTGAAATGA
CTTCTGTCTGGAGTGATTCAT
>2107
AGACATGGGACTTTTCCTTGAAGAGCTTATACTTTGAGTTAAAATATGTATGTTGATATC
TATGACAGAGAATAGGAAATGGAAAGACTATGATATATATATTCAGAGTTGAAATGACTT
CTGTCTGGAGTGATTCATTT
>2108
AGTTAAAATATGTATGTTGATATCTATGACAGAGAATAGGAAATGGAAAGACTATGATAT
ATATATTCAGAGTTGAAATGACTTCTGTCTGGAGTGATTCATTTTAAATTTTAAAACCTT
GTTGAGCACTTACTGTCTGC
>2109
TTAAAATATGTATGTTGATATCTATGACAGAGAATAGGAAATGGAAAGACTATGATATATA
TATTCAGAGTTGAAATGACTTCTGTCTGGAGTGATTCATTTTAAATTTTAAAACCTTGTT
GAGCACTTACTGTCTGCTG
>2110
TATGACAGAGAATAGGAAATGGAAAGACTATGATATATATATTCAGAGTTGAAATGACTT
CTGTCTGGAGTGATTCATTTTAAATTTTAAAACCTTGTTGAGCACTTACTGTCTGCTGGG
CACCTGGGTAGGCCCTGGGA
>2111
ATATATATATTCAGAGTTGAAATGACTTCTGTCTGGAGTGATTCATTTTAAATTTTAAAAC
CTTGTTGAGCACTTACTGTCTGCTGGGCACCTGGGTAGGCCCTGGGAATACTGTGATGA
GCAAGATAGACAAGACGTCT
>2112
TGTCTGGAGTGATTCATTTTAAATTTTAAAACCTTGTTGAGCACTTACTGTCTGCTGGGC
ACCTGGGTAGGCCCTGGGAATACTGTGATGAGCAAGATAGACAAGACGTCTACCCTCC
TGGGGCTTTCCTAATGGGGAAG

>2113
ATTTTAAAACCTTGTTGAGCACTTACTGTCTGCTGGGCACCTGGGTAGGCCCTGGGAAT
ACTGTGATGAGCAAGATAGACAAGACGTCTACCCTCCTGGGGCTTTCCTAATGGGGAA
GAAAGATCCTTACTTAGCTAGCA
>2114
ACCTTGTTGAGCACTTACTGTCTGCTGGGCACCTGGGTAGGCCCTGGGAATACTGTGAT
GAGCAAGATAGACAAGACGTCTACCCTCCTGGGGCTTTCCTAATGGGGAAGAAAGATC
CTTACTTAGCTAGCAGTCCTGTC
>2115
GTTGAGCACTTACTGTCTGCTGGGCACCTGGGTAGGCCCTGGGAATACTGTGATGAGC
AAGATAGACAAGACGTCTACCCTCCTGGGGCTTTCCTAATGGGGAAGAAAGATCCTTA
CTTAGCTAGCAGTCCTGTCCGTAA
>2116
GCCCTGGGAATACTGTGATGAGCAAGATAGACAAGACGTCTACCCTCCTGGGGCTTTC
CTAATGGGGAAGAAAGATCCTTACTTAGCTAGCAGTCCTGTCCGTAACTGTGTAAGGGT
GATTTTGATACATGTTGGGAGCC
>2117
AGCAAGATAGACAAGACGTCTACCCTCCTGGGGCTTTCCTAATGGGGAAGAAAGATCC
TTACTTAGCTAGCAGTCCTGTCCGTAACTGTGTAAGGGTGATTTTGATACATGTTGGGA
GCCTGGATGAAGCAGCACATGAG
>2118
AAGATAGACAAGACGTCTACCCTCCTGGGGCTTTCCTAATGGGGAAGAAAGATCCTTA
CTTAGCTAGCAGTCCTGTCCGTAACTGTGTAAGGGTGATTTTGATACATGTTGGGAGCC
TGGATGAAGCAGCACATGAGTAG
>2119
CTCCTGGGGCTTTCCTAATGGGGAAGAAAGATCCTTACTTAGCTAGCAGTCCTGTCCGT
AACTGTGTAAGGGTGATTTTGATACATGTTGGGAGCCTGGATGAAGCAGCACATGAGT
AGGATATTAAAGGATAAGTAGGT
>2120
AGAAAGATCCTTACTTAGCTAGCAGTCCTGTCCGTAACTGTGTAAGGGTGATTTTGATA
CATGTTGGGAGCCTGGATGAAGCAGCACATGAGTAGGATATTAAAGGATAAGTAGGTTT
TGAAGACGGGGACAGAAAGAAG
>2121
TACTTAGCTAGCAGTCCTGTCCGTAACTGTGTAAGGGTGATTTTGATACATGTTGGGAG
CCTGGATGAAGCAGCACATGAGTAGGATATTAAAGGATAAGTAGGTTTTGAAGACGGG
GACAGAAAGAAGGAAAATTGTGG
>2122
TTAGCTAGCAGTCCTGTCCGTAACTGTGTAAGGGTGATTTTGATACATGTTGGGAGCCT
GGATGAAGCAGCACATGAGTAGGATATTAAAGGATAAGTAGGTTTTGAAGACGGGGAC
AGAAAGAAGGAAAATTGTGGTCA
>2123
CAGTCCTGTCCGTAACTGTGTAAGGGTGATTTTGATACATGTTGGGAGCCTGGATGAAG
CAGCACATGAGTAGGATATTAAAGGATAAGTAGGTTTTGAAGACGGGGACAGAAAGAA
GGAAAATTGTGGTCAGGGCCAGG

>2124
AACTGTGTAAGGGTGATTTTGATACATGTTGGGAGCCTGGATGAAGCAGCACATGAGT
AGGATATTAAAGGATAAGTAGGTTTTGAAGACGGGGACAGAAAGAAGGAAAATTGTG
GTCAGGGCCAGGGGAGAAAACAAGG
>2125
TTGGGAGCCTGGATGAAGCAGCACATGAGTAGGATATTAAAGGATAAGTAGGTTTTGA
AGACGGGGACAGAAAGAAGGAAAATTGTGGTCAGGGCCAGGGGAGAAAACAAGGA
GTAACTTTCTTTGGCAGGAATGAGGGT
>2126
CCTGGATGAAGCAGCACATGAGTAGGATATTAAAGGATAAGTAGGTTTTGAAGACGGG
GACAGAAAGAAGGAAAATTGTGGTCAGGGCCAGGGGAGAAAACAAGGAGTAACTTT
CTTTGGCAGGAATGAGGGTGATTCAT
>2127
ACATGAGTAGGATATTAAAGGATAAGTAGGTTTTGAAGACGGGGACAGAAAGAAGGA
AAATTGTGGTCAGGGCCAGGGGAGAAAACAAGGAGTAACTTTCTTTGGCAGGAATGA
GGGTGATTCATGCTGGAATGGTAGGC
>2128
GTAGGATATTAAAGGATAAGTAGGTTTTGAAGACGGGGACAGAAAGAAGGAAAATTGT
GGTCAGGGCCAGGGGAGAAAACAAGGAGTAACTTTCTTTGGCAGGAATGAGGGTGAT
TCATGCTGGAATGGTAGGCTGGGGT
>2129
ACGGGGACAGAAAGAAGGAAAATTGTGGTCAGGGCCAGGGGAGAAAACAAGGAGTA
ACTTTCTTTGGCAGGAATGAGGGTGATTCATGCTGGAATGGTAGGCTGGGGTCAGACT
GTGGTGCTATAAATGCGGGGAGAGAG
>2130
CAGAAAGAAGGAAAATTGTGGTCAGGGCCAGGGGAGAAAACAAGGAGTAACTTTCTT
TGGCAGGAATGAGGGTGATTCATGCTGGAATGGTAGGCTGGGGTCAGACTGTGGTGCT
ATAAATGCGGGGAGAGAGATTAGAG
>2131
AGGGCCAGGGGAGAAAACAAGGAGTAACTTTCTTTGGCAGGAATGAGGGTGATTCAT
GCTGGAATGGTAGGCTGGGGTCAGACTGTGGTGCTATAAATGCGGGGAGAGAGATTAG
AGAAGGCAGCATGAAGAATGGAGAG
>2132
AGAAAACAAGGAGTAACTTTCTTTGGCAGGAATGAGGGTGATTCATGCTGGAATGGTA
GGCTGGGGTCAGACTGTGGTGCTATAAATGCGGGGAGAGAGATTAGAGAAGGCAGCA
TGAAGAATGGAGAGAAAATGAGTTT
>2133
GCAGGAATGAGGGTGATTCATGCTGGAATGGTAGGCTGGGGTCAGACTGTGGTGCTAT
AAATGCGGGGAGAGAGATTAGAGAAGGCAGCATGAAGAATGGAGAGAAAATGAGTTT
GAAGTTGAGCAGACCCGGGTTCAGA
>2134
AGGAATGAGGGTGATTCATGCTGGAATGGTAGGCTGGGGTCAGACTGTGGTGCTATAA
ATGCGGGGAGAGAGATTAGAGAAGGCAGCATGAAGAATGGAGAGAAAATGAGTTTGA
AGTTGAGCAGACCCGGGTTCAGATT

>2135
GAATGAGGGTGATTCATGCTGGAATGGTAGGCTGGGGTCAGACTGTGGTGCTATAAAT
GCGGGGAGAGAGATTAGAGAAGGCAGCATGAAGAATGGAGAGAAAATGAGTTTGAA
GTTGAGCAGACCCGGGTTCAGATTTT
>2136
AGGGTGATTCATGCTGGAATGGTAGGCTGGGGTCAGACTGTGGTGCTATAAATGCGGG
GAGAGAGATTAGAGAAGGCAGCATGAAGAATGGAGAGAAAATGAGTTTGAAGTTGAG
CAGACCCGGGTTCAGATTTTCATGC
>2137
GAATGGTAGGCTGGGGTCAGACTGTGGTGCTATAAATGCGGGGAGAGAGATTAGAGAA
GGCAGCATGAAGAATGGAGAGAAAATGAGTTTGAAGTTGAGCAGACCCGGGTTCAGA
TTTTCATGCTGCCACTTGCAAACTG
>2138
TGGTGCTATAAATGCGGGGAGAGAGATTAGAGAAGGCAGCATGAAGAATGGAGAGAA
AATGAGTTTGAAGTTGAGCAGACCCGGGTTCAGATTTTCATGCTGCCACTTGCAAACT
GTGTGATGTTGGCCATCAAGGACTG
>2139
GAGAGATTAGAGAAGGCAGCATGAAGAATGGAGAGAAAATGAGTTTGAAGTTGAGCA
GACCCGGGTTCAGATTTTCATGCTGCCACTTGCAAACTGTGTGATGTTGGCCATCAAGG
ACTGTGAACTGGCTGGGTGCAGTG
>2140
TGAAGTTGAGCAGACCCGGGTTCAGATTTTCATGCTGCCACTTGCAAACTGTGTGATG
TTGGCCATCAAGGACTGTGAACTGGCTGGGTGCAGTGGCTCACACCTGTCATCCCAGT
GCTTTGGGAGGCCAAGGCAGGAGG
>2141
AGATTTTCATGCTGCCACTTGCAAACTGTGTGATGTTGGCCATCAAGGACTGTGAACTG
GCTGGGTGCAGTGGCTCACACCTGTCATCCCAGTGCTTTGGGAGGCCAAGGCAGGAG
GATTGCGTGAGGCCAGAAGTTTGA
>2142
AAACTGTGTGATGTTGGCCATCAAGGACTGTGAACTGGCTGGGTGCAGTGGCTCACAC
CTGTCATCCCAGTGCTTTGGGAGGCCAAGGCAGGAGGATTGCGTGAGGCCAGAAGTT
TGAGACTGGCTTGGGCAATATAGCG
>2143
CAAGGACTGTGAACTGGCTGGGTGCAGTGGCTCACACCTGTCATCCCAGTGCTTTGGG
AGGCCAAGGCAGGAGGATTGCGTGAGGCCAGAAGTTTGAGACTGGCTTGGGCAATAT
AGCGAGACGATCTTTCCAAAAAAAA
>2144
GGACTGTGAACTGGCTGGGTGCAGTGGCTCACACCTGTCATCCCAGTGCTTTGGGAGG
CCAAGGCAGGAGGATTGCGTGAGGCCAGAAGTTTGAGACTGGCTTGGGCAATATAGC
GAGACGATCTTTCCAAAAAAAAAAA
>2145
TGGCTGGGTGCAGTGGCTCACACCTGTCATCCCAGTGCTTTGGGAGGCCAAGGCAGG
AGGATTGCGTGAGGCCAGAAGTTTGAGACTGGCTTGGGCAATATAGCGAGACGATCTT
TCCAAAAAAAAAAAAAAAAAAATTA

>2146
TGCAGTGGCTCACACCTGTCATCCCAGTGCTTTGGGAGGCCAAGGCAGGAGGATTGCG
TGAGGCCAGAAGTTTGAGACTGGCTTGGGCAATATAGCGAGACGATCTTTCCAAAAAA
AAAAAAAAAAAAAATTAAAAATGCA
>2147
AGTGCTTTGGGAGGCCAAGGCAGGAGGATTGCGTGAGGCCAGAAGTTTGAGACTGGC
TTGGGCAATATAGCGAGACGATCTTTCCAAAAAAAAAAAAAAAAAAAATTAAAAATGCA
GCTGTGCATGCCCATAATCCCAGCT
>2148
TTGAGACTGGCTTGGGCAATATAGCGAGACGATCTTTCCAAAAAAAAAAAAAAAAAAA
ATTAAAAATGCAGCTGTGCATGCCCATAATCCCAGCTTCTCGGGTGGCTGAAGAGGGA
GGATGGCTTGAACTTATAAGTTCAA
>2149
AGCGAGACGATCTTTCCAAAAAAAAAAAAAAAAAAAATTAAAAATGCAGCTGTGCATG
CCCATAATCCCAGCTTCTCGGGTGGCTGAAGAGGGAGGATGGCTTGAACTTATAAGTTC
AAGGCTGTGGTGAGCTATGATCAC
>2150
AAAAAAAAAAAAAAAAAAATTAAAAATGCAGCTGTGCATGCCCATAATCCCAGCTTCTC
GGGTGGCTGAAGAGGGAGGATGGCTTGAACTTATAAGTTCAAGGCTGTGGTGAGCTAT
GATCACGCCACTGCACTCCAGCCT
>2151
AAAAAAAAAAAAAAAAATTAAAAATGCAGCTGTGCATGCCCATAATCCCAGCTTCTCGG
GTGGCTGAAGAGGGAGGATGGCTTGAACTTATAAGTTCAAGGCTGTGGTGAGCTATGA
TCACGCCACTGCACTCCAGCCTGG
>2152
TGCAGCTGTGCATGCCCATAATCCCAGCTTCTCGGGTGGCTGAAGAGGGAGGATGGCT
TGAACTTATAAGTTCAAGGCTGTGGTGAGCTATGATCACGCCACTGCACTCCAGCCTGG
GCAACAAAGTGAGACCCTATCTC
>2153
AAGAGGGAGGATGGCTTGAACTTATAAGTTCAAGGCTGTGGTGAGCTATGATCACGCC
ACTGCACTCCAGCCTGGGCAACAAAGTGAGACCCTATCTCTATTTCAAAACAACAACA
ACAACAACAGCAACACACACCATG
>2154
CTTGAACTTATAAGTTCAAGGCTGTGGTGAGCTATGATCACGCCACTGCACTCCAGCCT
GGGCAACAAAGTGAGACCCTATCTCTATTTCAAAACAACAACAACAACAACAGCAAC
ACACACCATGAACTGTAGCTTCTT
>2155
CCACTGCACTCCAGCCTGGGCAACAAAGTGAGACCCTATCTCTATTTCAAAACAACAA
CAACAACAACAGCAACACACACCATGAACTGTAGCTTCTTCATTCCTACAGTGCAGTT
ATTGTGTGGGATAGCCTGAGGATT
>2156
ACAAAGTGAGACCCTATCTCTATTTCAAAACAACAACAACAACAACAGCAACACACA
CCATGAACTGTAGCTTCTTCATTCCTACAGTGCAGTTATTGTGTGGGATAGCCTGAGGA
TTTGGGGTCGTGTACTTAACACAC

>2157
TTTCAAAACAACAACAACAACAACAGCAACACACACCATGAACTGTAGCTTCTTCATT
CCTACAGTGCAGTTATTGTGTGGGATAGCCTGAGGATTTGGGGTCGTGTACTTAACACA
CTCTGCACAGTGCCTGTAACAGT
>2158
ACAACAACAGCAACACACACCATGAACTGTAGCTTCTTCATTCCTACAGTGCAGTTATT
GTGTGGGATAGCCTGAGGATTTGGGGTCGTGTACTTAACACACTCTGCACAGTGCCTGT
AACAGTGTAGTTGCTCAATAAA
>2159
ACAGCAACACACACCATGAACTGTAGCTTCTTCATTCCTACAGTGCAGTTATTGTGTGG
GATAGCCTGAGGATTTGGGGTCGTGTACTTAACACACTCTGCACAGTGCCTGTAACAG
TGTAGTTGCTCAATAAATATAAC
>2160
TCCTACAGTGCAGTTATTGTGTGGGATAGCCTGAGGATTTGGGGTCGTGTACTTAACAC
ACTCTGCACAGTGCCTGTAACAGTGTAGTTGCTCAATAAATATAACTGCTGTTTGTTTTA
ACTGCTGTTTCTTTTTGGACT
>2161
GTTATTGTGTGGGATAGCCTGAGGATTTGGGGTCGTGTACTTAACACACTCTGCACAGT
GCCTGTAACAGTGTAGTTGCTCAATAAATATAACTGCTGTTTGTTTTAACTGCTGTTTCT
TTTTGGACTTTATCCCGGGAG
>2162
TGTGTGGGATAGCCTGAGGATTTGGGGTCGTGTACTTAACACACTCTGCACAGTGCCT
GTAACAGTGTAGTTGCTCAATAAATATAACTGCTGTTTGTTTTAACTGCTGTTTCTTTTT
GGACTTTATCCCGGGAGAGTTG
>2163
TGTAGTTGCTCAATAAATATAACTGCTGTTTGTTTTAACTGCTGTTTCTTTTTGGACTTTA
TCCCGGGAGAGTTGGAGCCTTGGAAGGCATTTGAAAAGGAGAGTGACGTGACTGGAG
TGCTCCATACTGATGTGACATC
>2164
TAGTTGCTCAATAAATATAACTGCTGTTTGTTTTAACTGCTGTTTCTTTTTGGACTTTATC
CCGGGAGAGTTGGAGCCTTGGAAGGCATTTGAAAAGGAGAGTGACGTGACTGGAGTG
CTCCATACTGATGTGACATCCA
>2165
TTGTTTTAACTGCTGTTTCTTTTTGGACTTTATCCCGGGAGAGTTGGAGCCTTGGAAGG
CATTTGAAAAGGAGAGTGACGTGACTGGAGTGCTCCATACTGATGTGACATCCACTAG
GCATGTGTCTGACTGCCCCCTGC
>2166
TTTTTGGACTTTATCCCGGGAGAGTTGGAGCCTTGGAAGGCATTTGAAAAGGAGAGTG
ACGTGACTGGAGTGCTCCATACTGATGTGACATCCACTAGGCATGTGTCTGACTGCCCC
CTGCTCCTTCCCTGCCTTCACGA
>2167
AGCCTTGGAAGGCATTTGAAAAGGAGAGTGACGTGACTGGAGTGCTCCATACTGATGT
GACATCCACTAGGCATGTGTCTGACTGCCCCCTGCTCCTTCCCTGCCTTCACGAGCCTG
TCTGAAAAGGAGTTTAGTGCGTG

>2168
GCTCCATACTGATGTGACATCCACTAGGCATGTGTCTGACTGCCCCCTGCTCCTTCCCT
GCCTTCACGAGCCTGTCTGAAAAGGAGTTTAGTGCGTGATCATCAGCTCCTAAGGAGA
CCTCTCCCCTGTATCTTGTACCT
>2169
GTGACATCCACTAGGCATGTGTCTGACTGCCCCCTGCTCCTTCCCTGCCTTCACGAGCC
TGTCTGAAAAGGAGTTTAGTGCGTGATCATCAGCTCCTAAGGAGACCTCTCCCCTGTAT
CTTGTACCTGGCACTCTACCAA
>2170
CTGACTGCCCCCTGCTCCTTCCCTGCCTTCACGAGCCTGTCTGAAAAGGAGTTTAGTG
CGTGATCATCAGCTCCTAAGGAGACCTCTCCCCTGTATCTTGTACCTGGCACTCTACCA
AGATTTGATGAAAGGACATGAAA
>2171
CCCCTGCTCCTTCCCTGCCTTCACGAGCCTGTCTGAAAAGGAGTTTAGTGCGTGATCAT
CAGCTCCTAAGGAGACCTCTCCCCTGTATCTTGTACCTGGCACTCTACCAAGATTTGAT
GAAAGGACATGAAATGGACCCT
>2172
CTGCTCCTTCCCTGCCTTCACGAGCCTGTCTGAAAAGGAGTTTAGTGCGTGATCATCAG
CTCCTAAGGAGACCTCTCCCCTGTATCTTGTACCTGGCACTCTACCAAGATTTGATGAA
AGGACATGAAATGGACCCTTGC
>2173
AGTTTAGTGCGTGATCATCAGCTCCTAAGGAGACCTCTCCCCTGTATCTTGTACCTGGC
ACTCTACCAAGATTTGATGAAAGGACATGAAATGGACCCTTGCCACCTCTCGGTGGGC
GGGGAAGCTGGGAGAGGGGCCTG
>2174
GATCATCAGCTCCTAAGGAGACCTCTCCCCTGTATCTTGTACCTGGCACTCTACCAAGA
TTTGATGAAAGGACATGAAATGGACCCTTGCCACCTCTCGGTGGGCGGGGAAGCTGG
GAGAGGGGCCTGGGGAGAGGGGGT
>2175
CTGGCACTCTACCAAGATTTGATGAAAGGACATGAAATGGACCCTTGCCACCTCTCGG
TGGGCGGGGAAGCTGGGAGAGGGGCCTGGGGAGAGGGGGTCTTCAGCTGTCAGGAT
CAAAGGAAGTGACACTCTGAAGCTGG
>2176
TCTACCAAGATTTGATGAAAGGACATGAAATGGACCCTTGCCACCTCTCGGTGGGCGG
GGAAGCTGGGAGAGGGGCCTGGGGAGAGGGGGTCTTCAGCTGTCAGGATCAAAGGA
AGTGACACTCTGAAGCTGGTCTGCCG
>2177
AAATGGACCCTTGCCACCTCTCGGTGGGCGGGGAAGCTGGGAGAGGGGCCTGGGGAG
AGGGGGTCTTCAGCTGTCAGGATCAAAGGAAGTGACACTCTGAAGCTGGTCTGCCGG
ACTGTTACCCGTCTCTCTAACTCTCT
>2178
CCCTTGCCACCTCTCGGTGGGCGGGGAAGCTGGGAGAGGGGCCTGGGGAGAGGGGG
TCTTCAGCTGTCAGGATCAAAGGAAGTGACACTCTGAAGCTGGTCTGCCGGACTGTTA
CCCGTCTCTCTAACTCTCTTTTAATA

>2179
CTCGGTGGGCGGGGAAGCTGGGAGAGGGGCCTGGGGAGAGGGGGTCTTCAGCTGTC
AGGATCAAAGGAAGTGACACTCTGAAGCTGGTCTGCCGGACTGTTACCCGTCTCTCTA
ACTCTCTTTTAATAGAAACCCAGTCT
>2180
ATCAAAGGAAGTGACACTCTGAAGCTGGTCTGCCGGACTGTTACCCGTCTCTCTAACT
CTCTTTTAATAGAAACCCAGTCTCCCCTCTTGCCTCCCTGATAGTGAACAGCGACGCTA
TCTCCCGCGGGCCAGTCTCGGCT
>2181
AAGTGACACTCTGAAGCTGGTCTGCCGGACTGTTACCCGTCTCTCTAACTCTCTTTTAA
TAGAAACCCAGTCTCCCCTCTTGCCTCCCTGATAGTGAACAGCGACGCTATCTCCCGCG
GGCCAGTCTCGGCTGTGGTCGT
>2182
CCGGACTGTTACCCGTCTCTCTAACTCTCTTTTAATAGAAACCCAGTCTCCCCTCTTGCC
TCCCTGATAGTGAACAGCGACGCTATCTCCCGCGGGCCAGTCTCGGCTGTGGTCGTAG
CACTACGCAATGTCTGCAAACC
>2183
GTTACCCGTCTCTCTAACTCTCTTTTAATAGAAACCCAGTCTCCCCTCTTGCCTCCCTGA
TAGTGAACAGCGACGCTATCTCCCGCGGGCCAGTCTCGGCTGTGGTCGTAGCACTACG
CAATGTCTGCAAACCGACAGTG
>2184
TTTAATAGAAACCCAGTCTCCCCTCTTGCCTCCCTGATAGTGAACAGCGACGCTATCTC
CCGCGGGCCAGTCTCGGCTGTGGTCGTAGCACTACGCAATGTCTGCAAACCGACAGTG
GCATGGGGAGATAAGGGCCCCAA
>2185
TCCCCTCTTGCCTCCCTGATAGTGAACAGCGACGCTATCTCCCGCGGGCCAGTCTCGGC
TGTGGTCGTAGCACTACGCAATGTCTGCAAACCGACAGTGGCATGGGGAGATAAGGGC
CCCAACGTTGTGTAACGAGGGGC
>2186
AGCGACGCTATCTCCCGCGGGCCAGTCTCGGCTGTGGTCGTAGCACTACGCAATGTCT
GCAAACCGACAGTGGCATGGGGAGATAAGGGCCCCAACGTTGTGTAACGAGGGGCAT
GGGCTGTTTGCCCGAGTGGCTCCTG
>2187
TCCCGCGGGCCAGTCTCGGCTGTGGTCGTAGCACTACGCAATGTCTGCAAACCGACAG
TGGCATGGGGAGATAAGGGCCCCAACGTTGTGTAACGAGGGGCATGGGCTGTTTGCCC
GAGTGGCTCCTGGCCCCTGGGTAA
>2188
GTAGCACTACGCAATGTCTGCAAACCGACAGTGGCATGGGGAGATAAGGGCCCCAAC
GTTGTGTAACGAGGGGCATGGGCTGTTTGCCCGAGTGGCTCCTGGCCCCTGGGTAACT
GCTAACCTCAACGGAGGAGCGGGCC
>2189
AACCGACAGTGGCATGGGGAGATAAGGGCCCCAACGTTGTGTAACGAGGGGCATGGG
CTGTTTGCCCGAGTGGCTCCTGGCCCCTGGGTAACTGCTAACCTCAACGGAGGAGCGG
GCCGGCCTTGGAAACGCCGTTCCAG

>2190
TGTAACGAGGGGCATGGGCTGTTTGCCCGAGTGGCTCCTGGCCCCTGGGTAACTGCTA
ACCTCAACGGAGGAGCGGGCCGGCCTTGGAAACGCCGTTCCAGGGCCCCTCGCTGGT
TCCACGGATGCCTTTTTGTGGTTGG
>2191
TGGCTCCTGGCCCCTGGGTAACTGCTAACCTCAACGGAGGAGCGGGCCGGCCTTGGA
AACGCCGTTCCAGGGCCCCTCGCTGGTTCCACGGATGCCTTTTTGTGGTTGGATAAAG
GAAGCTCATGTAGGCCGCCCACAGC
>2192
GCCGGCCTTGGAAACGCCGTTCCAGGGCCCCTCGCTGGTTCCACGGATGCCTTTTTGT
GGTTGGATAAAGGAAGCTCATGTAGGCCGCCCACAGCGCAGCATGGCCTTGGGCTACG
GGTGTTTGGTTGTTTTGTTTTTCT
>2193
GCCTTGGAAACGCCGTTCCAGGGCCCCTCGCTGGTTCCACGGATGCCTTTTTGTGGTT
GGATAAAGGAAGCTCATGTAGGCCGCCCACAGCGCAGCATGGCCTTGGGCTACGGGTG
TTTGGTTGTTTTGTTTTTCTCTTT
>2194
GGGCCCCTCGCTGGTTCCACGGATGCCTTTTTGTGGTTGGATAAAGGAAGCTCATGTAG
GCCGCCCACAGCGCAGCATGGCCTTGGGCTACGGGTGTTTGGTTGTTTTGTTTTTCTCT
TTTCTTTGTCTTTTTTAAAAAA
>2195
CAGCATGGCCTTGGGCTACGGGTGTTTGGTTGTTTTGTTTTTCTCTTTTCTTTGTCTTTT
TTAAAAAAAGAAGTTGAAAGGGGGATAGTCTTGCTTGGCACCTACCACCTTTAACCCA
AAACCAAATAAAACAAACAGTT
>2196
CATGGCCTTGGGCTACGGGTGTTTGGTTGTTTTGTTTTTCTCTTTTCTTTGTCTTTTTTAA
AAAAAGAAGTTGAAAGGGGGATAGTCTTGCTTGGCACCTACCACCTTTAACCCAAAAC
CAAATAAAACAAACAGTTCCT
>2197
CGGGTGTTTGGTTGTTTTGTTTTTCTCTTTTCTTTGTCTTTTTTAAAAAAAGAAGTTGAA
AGGGGGATAGTCTTGCTTGGCACCTACCACCTTTAACCCAAAACCAAATAAAACAAAC
AGTTCCTAAATATAAATTAAAC
>2198
AGAAGTTGAAAGGGGGATAGTCTTGCTTGGCACCTACCACCTTTAACCCAAAACCAAA
TAAAACAAACAGTTCCTAAATATAAATTAAACTTCAACCTGCAGTATAGACATCCTCCTT
CCCTGCCCGAAACCCACTGGCC
>2199
CCTACCACCTTTAACCCAAAACCAAATAAAACAAACAGTTCCTAAATATAAATTAAACT
TCAACCTGCAGTATAGACATCCTCCTTCCCTGCCCGAAACCCACTGGCCACTGGAGGCT
AGCTTTAAGAAATTGGTTTTGG
>2200
CACCTTTAACCCAAAACCAAATAAAACAAACAGTTCCTAAATATAAATTAAACTTCAAC
CTGCAGTATAGACATCCTCCTTCCCTGCCCGAAACCCACTGGCCACTGGAGGCTAGCTT
TAAGAAATTGGTTTTGGAGACT

>2201
ATATAAATTAAACTTCAACCTGCAGTATAGACATCCTCCTTCCCTGCCCGAAACCCACT
GGCCACTGGAGGCTAGCTTTAAGAAATTGGTTTTGGAGACTCTGGCTTGAAAAGGAGC
CATGCCCACTTGTCTCACGTCCC
>2202
AATTAAACTTCAACCTGCAGTATAGACATCCTCCTTCCCTGCCCGAAACCCACTGGCCA
CTGGAGGCTAGCTTTAAGAAATTGGTTTTGGAGACTCTGGCTTGAAAAGGAGCCATGC
CCACTTGTCTCACGTCCCAGTCG
>2203
TAGACATCCTCCTTCCCTGCCCGAAACCCACTGGCCACTGGAGGCTAGCTTTAAGAAA
TTGGTTTTGGAGACTCTGGCTTGAAAAGGAGCCATGCCCACTTGTCTCACGTCCCAGT
CGCTGGCTCCAGGGGGCCGTGGTA
>2204
TAGCTTTAAGAAATTGGTTTTGGAGACTCTGGCTTGAAAAGGAGCCATGCCCACTTGT
CTCACGTCCCAGTCGCTGGCTCCAGGGGGCCGTGGTAGAACATATATTGTACTTCAGCT
GCCTTTCCCCTCCAAGCTTCCTC
>2205
TTGGTTTTGGAGACTCTGGCTTGAAAAGGAGCCATGCCCACTTGTCTCACGTCCCAGT
CGCTGGCTCCAGGGGGCCGTGGTAGAACATATATTGTACTTCAGCTGCCTTTCCCCTCC
AAGCTTCCTCAGCCACCCACAGC
>2206
CATGCCCACTTGTCTCACGTCCCAGTCGCTGGCTCCAGGGGGCCGTGGTAGAACATATA
TTGTACTTCAGCTGCCTTTCCCCTCCAAGCTTCCTCAGCCACCCACAGCCCTTTCAGAT
GAGCTGAGCCCTAGCACTGTGC
>2207
GTCCCAGTCGCTGGCTCCAGGGGGCCGTGGTAGAACATATATTGTACTTCAGCTGCCTT
TCCCCTCCAAGCTTCCTCAGCCACCCACAGCCCTTTCAGATGAGCTGAGCCCTAGCAC
TGTGCCCTCTCCCCTCTGCCCTC
>2208
GGGGGCCGTGGTAGAACATATATTGTACTTCAGCTGCCTTTCCCCTCCAAGCTTCCTCA
GCCACCCACAGCCCTTTCAGATGAGCTGAGCCCTAGCACTGTGCCCTCTCCCCTCTGC
CCTCTTAGGGTGATAGAATCCAC
>2209
AACATATATTGTACTTCAGCTGCCTTTCCCCTCCAAGCTTCCTCAGCCACCCACAGCCC
TTTCAGATGAGCTGAGCCCTAGCACTGTGCCCTCTCCCCTCTGCCCTCTTAGGGTGATA
GAATCCACTACCCTGACATCTG
>2210
ATATTGTACTTCAGCTGCCTTTCCCCTCCAAGCTTCCTCAGCCACCCACAGCCCTTTCA
GATGAGCTGAGCCCTAGCACTGTGCCCTCTCCCCTCTGCCCTCTTAGGGTGATAGAATC
CACTACCCTGACATCTGGGGTT
>2211
TACTTCAGCTGCCTTTCCCCTCCAAGCTTCCTCAGCCACCCACAGCCCTTTCAGATGAG
CTGAGCCCTAGCACTGTGCCCTCTCCCCTCTGCCCTCTTAGGGTGATAGAATCCACTAC
CCTGACATCTGGGGTTGCCCTG

>2212
CTCAGCCACCCACAGCCCTTTCAGATGAGCTGAGCCCTAGCACTGTGCCCTCTCCCCT
CTGCCCTCTTAGGGTGATAGAATCCACTACCCTGACATCTGGGGTTGCCCTGTTCTGCT
GTGGTGGGAGATGGGCTGGAAGG
>2213
TCTGCCCTCTTAGGGTGATAGAATCCACTACCCTGACATCTGGGGTTGCCCTGTTCTGC
TGTGGTGGGAGATGGGCTGGAAGGGACTTCGGAGGCTCTCCCGTGTTCTGTACCACCC
AGCTTCCCCTTGGATCCCTCCAC
>2214
GGGTGATAGAATCCACTACCCTGACATCTGGGGTTGCCCTGTTCTGCTGTGGTGGGAGA
TGGGCTGGAAGGGACTTCGGAGGCTCTCCCGTGTTCTGTACCACCCAGCTTCCCCTTG
GATCCCTCCACCCCCGCAGCCAG
>2215
TCCACTACCCTGACATCTGGGGTTGCCCTGTTCTGCTGTGGTGGGAGATGGGCTGGAA
GGGACTTCGGAGGCTCTCCCGTGTTCTGTACCACCCAGCTTCCCCTTGGATCCCTCCAC
CCCCGCAGCCAGGAGAGGGAAGT
>2216
CCTGTTCTGCTGTGGTGGGAGATGGGCTGGAAGGGACTTCGGAGGCTCTCCCGTGTTC
TGTACCACCCAGCTTCCCCTTGGATCCCTCCACCCCCGCAGCCAGGAGAGGGAAGTAA
CTGTTCTTTGAGCAGGACATTTCA
>2217
GAAGGGACTTCGGAGGCTCTCCCGTGTTCTGTACCACCCAGCTTCCCCTTGGATCCCTC
CACCCCCGCAGCCAGGAGAGGGAAGTAACTGTTCTTTGAGCAGGACATTTCATTTTCA
AAGTATTCTTGATGCCGCCCCCC
>2218
GGACTTCGGAGGCTCTCCCGTGTTCTGTACCACCCAGCTTCCCCTTGGATCCCTCCACC
CCCGCAGCCAGGAGAGGGAAGTAACTGTTCTTTGAGCAGGACATTTCATTTTCAAAGT
ATTCTTGATGCCGCCCCCCCGCC
>2219
CTTCGGAGGCTCTCCCGTGTTCTGTACCACCCAGCTTCCCCTTGGATCCCTCCACCCCC
GCAGCCAGGAGAGGGAAGTAACTGTTCTTTGAGCAGGACATTTCATTTTCAAAGTATT
CTTGATGCCGCCCCCCCGCCCCA
>2220
TCGGAGGCTCTCCCGTGTTCTGTACCACCCAGCTTCCCCTTGGATCCCTCCACCCCCGC
AGCCAGGAGAGGGAAGTAACTGTTCTTTGAGCAGGACATTTCATTTTCAAAGTATTCTT
GATGCCGCCCCCCCGCCCCACC
>2221
TGTACCACCCAGCTTCCCCTTGGATCCCTCCACCCCCGCAGCCAGGAGAGGGAAGTAA
CTGTTCTTTGAGCAGGACATTTCATTTTCAAAGTATTCTTGATGCCGCCCCCCCGCCCC
ACCGCCAAGAATTCTGCTCTGTG
>2222
GGATCCCTCCACCCCCGCAGCCAGGAGAGGGAAGTAACTGTTCTTTGAGCAGGACATT
TCATTTTCAAAGTATTCTTGATGCCGCCCCCCCGCCCCACCGCCAAGAATTCTGCTCTG
TGGGTGATACTTATAAGGTAACT

>2223
AACTGTTCTTTGAGCAGGACATTTCATTTTCAAAGTATTCTTGATGCCGCCCCCCCGCC
CCACCGCCAAGAATTCTGCTCTGTGGGTGATACTTATAAGGTAACTATATGGCTTCCTTA
TTTAGGCAGCAGTTTCTCTTC
>2224
TCAAAGTATTCTTGATGCCGCCCCCCCGCCCCACCGCCAAGAATTCTGCTCTGTGGGTG
ATACTTATAAGGTAACTATATGGCTTCCTTATTTAGGCAGCAGTTTCTCTTCTATGATTTT
AGTTCCTCTCATGATTCTTA
>2225
CCGCCCCACCGCCAAGAATTCTGCTCTGTGGGTGATACTTATAAGGTAACTATATGGCTT
CCTTATTTAGGCAGCAGTTTCTCTTCTATGATTTTAGTTCCTCTCATGATTCTTATAACCC
TCTCTCAGATACTTCCAGG
>2226
CCCACCGCCAAGAATTCTGCTCTGTGGGTGATACTTATAAGGTAACTATATGGCTTCCTT
ATTTAGGCAGCAGTTTCTCTTCTATGATTTTAGTTCCTCTCATGATTCTTATAACCCTCTC
TCAGATACTTCCAGGATGA
>2227
GTGGGTGATACTTATAAGGTAACTATATGGCTTCCTTATTTAGGCAGCAGTTTCTCTTCTA
TGATTTTAGTTCCTCTCATGATTCTTATAACCCTCTCTCAGATACTTCCAGGATGATAAAT
CTTCTATCACTGTTTCAT
>2228
TCCTTATTTAGGCAGCAGTTTCTCTTCTATGATTTTAGTTCCTCTCATGATTCTTATAACC
CTCTCTCAGATACTTCCAGGATGATAAATCTTCTATCACTGTTTCATCTGATCACATCAG
GGTGATTATATTTCGTGAC
>2229
GGCAGCAGTTTCTCTTCTATGATTTTAGTTCCTCTCATGATTCTTATAACCCTCTCTCAGA
TACTTCCAGGATGATAAATCTTCTATCACTGTTTCATCTGATCACATCAGGGTGATTATAT
TTCGTGACATAACCAGGT
>2230
TCTTATAACCCTCTCTCAGATACTTCCAGGATGATAAATCTTCTATCACTGTTTCATCTGA
TCACATCAGGGTGATTATATTTCGTGACATAACCAGGTGGCTTTAATCCGTTGGTGGAA
GAACCAGGCTCTCACCTCCA
>2231
AGGATGATAAATCTTCTATCACTGTTTCATCTGATCACATCAGGGTGATTATATTTCGTGA
CATAACCAGGTGGCTTTAATCCGTTGGTGGAAGAACCAGGCTCTCACCTCCATTGTTTA
AGATGTTATGTTTCTACTAA
>2232
TTTCATCTGATCACATCAGGGTGATTATATTTCGTGACATAACCAGGTGGCTTTAATCCG
TTGGTGGAAGAACCAGGCTCTCACCTCCATTGTTTAAGATGTTATGTTTCTACTAATCTG
TCCACTGAGTGTCTCCATCC
>2233
CTGATCACATCAGGGTGATTATATTTCGTGACATAACCAGGTGGCTTTAATCCGTTGGTG
GAAGAACCAGGCTCTCACCTCCATTGTTTAAGATGTTATGTTTCTACTAATCTGTCCACT
GAGTGTCTCCATCCTATTGT

>2234
ATTTCGTGACATAACCAGGTGGCTTTAATCCGTTGGTGGAAGAACCAGGCTCTCACCTC
CATTGTTTAAGATGTTATGTTTCTACTAATCTGTCCACTGAGTGTCTCCATCCTATTGTTT
CCATTGCATGCTCTCAGGTT
>2235
GTTGGTGGAAGAACCAGGCTCTCACCTCCATTGTTTAAGATGTTATGTTTCTACTAATCT
GTCCACTGAGTGTCTCCATCCTATTGTTTCCATTGCATGCTCTCAGGTTATTTCCTTTGTT
CTTGGCTGTCTGATTGCTC
>2236
AAGATGTTATGTTTCTACTAATCTGTCCACTGAGTGTCTCCATCCTATTGTTTCCATTGCA
TGCTCTCAGGTTATTTCCTTTGTTCTTGGCTGTCTGATTGCTCCCAGAGGCTGCAGTTAT
TTTATTTTATTTATTTCAT
>2237
TCCATCCTATTGTTTCCATTGCATGCTCTCAGGTTATTTCCTTTGTTCTTGGCTGTCTGAT
TGCTCCCAGAGGCTGCAGTTATTTTATTTTATTTATTTCATTTTTTGGTGGGAGTAGGGG
ATTGATCTCTCTCTTGCCT
>2238
CATCCTATTGTTTCCATTGCATGCTCTCAGGTTATTTCCTTTGTTCTTGGCTGTCTGATTG
CTCCCAGAGGCTGCAGTTATTTTATTTTATTTATTTCATTTTTTGGTGGGAGTAGGGGATT
GATCTCTCTCTTGCCTTT
>2239
TTCTTGGCTGTCTGATTGCTCCCAGAGGCTGCAGTTATTTTATTTTATTTATTTCATTTTT
TGGTGGGAGTAGGGGATTGATCTCTCTCTTGCCTTTAGCTTGAATGGGTGGTCATTTCC
AGGCTTACACATTGCTCCAT
>2240
TGCAGTTATTTTATTTTATTTATTTCATTTTTTGGTGGGAGTAGGGGATTGATCTCTCTCT
TGCCTTTAGCTTGAATGGGTGGTCATTTCCAGGCTTACACATTGCTCCATGCCCACTGTA
GGGGTCTGGCTTAGAGATG
>2241
TTCATTTTTTGGTGGGAGTAGGGGATTGATCTCTCTCTTGCCTTTAGCTTGAATGGGTGG
TCATTTCCAGGCTTACACATTGCTCCATGCCCACTGTAGGGGTCTGGCTTAGAGATGGT
CGGGGGAGAGAAGGTGGTGAT
>2242
TCTCTCTCTTGCCTTTAGCTTGAATGGGTGGTCATTTCCAGGCTTACACATTGCTCCATG
CCCACTGTAGGGGTCTGGCTTAGAGATGGTCGGGGGAGAGAAGGTGGTGATTTAAAA
GATTTGGAGGCGGTGGCTTACAT
>2243
TTTAGCTTGAATGGGTGGTCATTTCCAGGCTTACACATTGCTCCATGCCCACTGTAGGG
GTCTGGCTTAGAGATGGTCGGGGGAGAGAAGGTGGTGATTTAAAAGATTTGGAGGCG
GTGGCTTACATTGATTTTAGCTTA
>2244
GTCATTTCCAGGCTTACACATTGCTCCATGCCCACTGTAGGGGTCTGGCTTAGAGATGG
TCGGGGGAGAGAAGGTGGTGATTTAAAAGATTTGGAGGCGGTGGCTTACATTGATTTT
AGCTTATCTAACCCTTTAGCTAG

>2245
CATTGCTCCATGCCCACTGTAGGGGTCTGGCTTAGAGATGGTCGGGGGAGAGAAGGTG
GTGATTTAAAAGATTTGGAGGCGGTGGCTTACATTGATTTTAGCTTATCTAACCCTTTAG
CTAGAGGCTGCTCTTCCCTCAG
>2246
CATGCCCACTGTAGGGGTCTGGCTTAGAGATGGTCGGGGGAGAGAAGGTGGTGATTTA
AAAGATTTGGAGGCGGTGGCTTACATTGATTTTAGCTTATCTAACCCTTTAGCTAGAGG
CTGCTCTTCCCTCAGAGTGTCCA
>2247
TTAGAGATGGTCGGGGGAGAGAAGGTGGTGATTTAAAAGATTTGGAGGCGGTGGCTTA
CATTGATTTTAGCTTATCTAACCCTTTAGCTAGAGGCTGCTCTTCCCTCAGAGTGTCCAA
CTAGCTGTAACTTAAAGCAACA
>2248
AGAGAAGGTGGTGATTTAAAAGATTTGGAGGCGGTGGCTTACATTGATTTTAGCTTATC
TAACCCTTTAGCTAGAGGCTGCTCTTCCCTCAGAGTGTCCAACTAGCTGTAACTTAAAG
CAACAGAGAAATGTATATCATC
>2249
ATTTGGAGGCGGTGGCTTACATTGATTTTAGCTTATCTAACCCTTTAGCTAGAGGCTGCT
CTTCCCTCAGAGTGTCCAACTAGCTGTAACTTAAAGCAACAGAGAAATGTATATCATCC
CTTTTCTTGCATTAATTAGGG
>2250
TTGGAGGCGGTGGCTTACATTGATTTTAGCTTATCTAACCCTTTAGCTAGAGGCTGCTCT
TCCCTCAGAGTGTCCAACTAGCTGTAACTTAAAGCAACAGAGAAATGTATATCATCCCT
TTTCTTGCATTAATTAGGGCT
>2251
TTTAGCTTATCTAACCCTTTAGCTAGAGGCTGCTCTTCCCTCAGAGTGTCCAACTAGCT
GTAACTTAAAGCAACAGAGAAATGTATATCATCCCTTTTCTTGCATTAATTAGGGCTGAG
AAATTCATTATTTAGAGGGAT
>2252
AGAGTGTCCAACTAGCTGTAACTTAAAGCAACAGAGAAATGTATATCATCCCTTTTCTT
GCATTAATTAGGGCTGAGAAATTCATTATTTAGAGGGATAAGAGCTCCGGAGGAGGAG
GGTTTTTTGAACTAATGTAGAGA
>2253
CCAACTAGCTGTAACTTAAAGCAACAGAGAAATGTATATCATCCCTTTTCTTGCATTAAT
TAGGGCTGAGAAATTCATTATTTAGAGGGATAAGAGCTCCGGAGGAGGAGGGTTTTTT
GAACTAATGTAGAGATCTGTGG
>2254
CAGAGAAATGTATATCATCCCTTTTCTTGCATTAATTAGGGCTGAGAAATTCATTATTTAG
AGGGATAAGAGCTCCGGAGGAGGAGGGTTTTTTGAACTAATGTAGAGATCTGTGGCCA
GCTGTGTGATTCTCAGGTAGC
>2255
TATATCATCCCTTTTCTTGCATTAATTAGGGCTGAGAAATTCATTATTTAGAGGGATAAGA
GCTCCGGAGGAGGAGGGTTTTTTGAACTAATGTAGAGATCTGTGGCCAGCTGTGTGAT
TCTCAGGTAGCAGGTGAGGGG

>2256
ATCATCCCTTTTCTTGCATTAATTAGGGCTGAGAAATTCATTATTTAGAGGGATAAGAGC
TCCGGAGGAGGAGGGTTTTTTGAACTAATGTAGAGATCTGTGGCCAGCTGTGTGATTC
TCAGGTAGCAGGTGAGGGGGTG
>2257
TAGGGCTGAGAAATTCATTATTTAGAGGGATAAGAGCTCCGGAGGAGGAGGGTTTTTT
GAACTAATGTAGAGATCTGTGGCCAGCTGTGTGATTCTCAGGTAGCAGGTGAGGGGGT
GGGGGTTGATGAGAGGGGTGAGAA
>2258
TCATTATTTAGAGGGATAAGAGCTCCGGAGGAGGAGGGTTTTTTGAACTAATGTAGAGA
TCTGTGGCCAGCTGTGTGATTCTCAGGTAGCAGGTGAGGGGGTGGGGGTTGATGAGAG
GGGTGAGAAGGAGCAGGTGCCAG
>2259
GAGCTCCGGAGGAGGAGGGTTTTTTGAACTAATGTAGAGATCTGTGGCCAGCTGTGTG
ATTCTCAGGTAGCAGGTGAGGGGGTGGGGGTTGATGAGAGGGGTGAGAAGGAGCAGG
TGCCAGGGTCAGGCAGGGGAGGGAG
>2260
CTCCGGAGGAGGAGGGTTTTTTGAACTAATGTAGAGATCTGTGGCCAGCTGTGTGATT
CTCAGGTAGCAGGTGAGGGGGTGGGGGTTGATGAGAGGGGTGAGAAGGAGCAGGTG
CCAGGGTCAGGCAGGGGAGGGAGCCC
>2261
GAGGAGGAGGGTTTTTTGAACTAATGTAGAGATCTGTGGCCAGCTGTGTGATTCTCAG
GTAGCAGGTGAGGGGGTGGGGGTTGATGAGAGGGGTGAGAAGGAGCAGGTGCCAGG
GTCAGGCAGGGGAGGGAGCCCCCACT
>2262
AACTAATGTAGAGATCTGTGGCCAGCTGTGTGATTCTCAGGTAGCAGGTGAGGGGGTG
GGGGTTGATGAGAGGGGTGAGAAGGAGCAGGTGCCAGGGTCAGGCAGGGGAGGGAG
CCCCCACTCCGTGCATCATCCCAGAG
>2263
TGGCCAGCTGTGTGATTCTCAGGTAGCAGGTGAGGGGGTGGGGGTTGATGAGAGGGG
TGAGAAGGAGCAGGTGCCAGGGTCAGGCAGGGGAGGGAGCCCCCACTCCGTGCATCA
TCCCAGAGCCCTGTTCCTCCAAGCCC
>2264
CTCAGGTAGCAGGTGAGGGGGTGGGGGTTGATGAGAGGGGTGAGAAGGAGCAGGTG
CCAGGGTCAGGCAGGGGAGGGAGCCCCCACTCCGTGCATCATCCCAGAGCCCTGTTC
CTCCAAGCCCAGATTAGACACCCTTCT
>2265
GTAGCAGGTGAGGGGGTGGGGGTTGATGAGAGGGGTGAGAAGGAGCAGGTGCCAGG
GTCAGGCAGGGGAGGGAGCCCCCACTCCGTGCATCATCCCAGAGCCCTGTTCCTCCAA
GCCCAGATTAGACACCCTTCTCCACG
>2266
CAGGTGAGGGGGTGGGGGTTGATGAGAGGGGTGAGAAGGAGCAGGTGCCAGGGTCA
GGCAGGGGAGGGAGCCCCCACTCCGTGCATCATCCCAGAGCCCTGTTCCTCCAAGCCC
AGATTAGACACCCTTCTCCACGTGGG

>2267
AGAGGGGTGAGAAGGAGCAGGTGCCAGGGTCAGGCAGGGGAGGGAGCCCCCACTCC
GTGCATCATCCCAGAGCCCTGTTCCTCCAAGCCCAGATTAGACACCCTTCTCCACGTGG
GGCCTCAAGTTAGAAATCCTGGGAG
>2268
GGTCAGGCAGGGGAGGGAGCCCCCACTCCGTGCATCATCCCAGAGCCCTGTTCCTCCA
AGCCCAGATTAGACACCCTTCTCCACGTGGGGCCTCAAGTTAGAAATCCTGGGAGGAG
GCCTTATTGCTCAGGATCATCTGG
>2269
CCGTGCATCATCCCAGAGCCCTGTTCCTCCAAGCCCAGATTAGACACCCTTCTCCACGT
GGGGCCTCAAGTTAGAAATCCTGGGAGGAGGCCTTATTGCTCAGGATCATCTGGGAAT
GTTGCTTTCTGGGTTTAAAATAC
>2270
GCATCATCCCAGAGCCCTGTTCCTCCAAGCCCAGATTAGACACCCTTCTCCACGTGGG
GCCTCAAGTTAGAAATCCTGGGAGGAGGCCTTATTGCTCAGGATCATCTGGGAATGTTG
CTTTCTGGGTTTAAAATACAGTC
>2271
CCCAGATTAGACACCCTTCTCCACGTGGGGCCTCAAGTTAGAAATCCTGGGAGGAGGC
CTTATTGCTCAGGATCATCTGGGAATGTTGCTTTCTGGGTTTAAAATACAGTCAATGTTT
AGATGAGCTGCTGTTCAGGCAC
>2272
AGAAATCCTGGGAGGAGGCCTTATTGCTCAGGATCATCTGGGAATGTTGCTTTCTGGGT
TTAAAATACAGTCAATGTTTAGATGAGCTGCTGTTCAGGCACATGGTCTGGTTAGCAAA
GGGTTAAATCCTGCATCCCGTT
>2273
GAGGAGGCCTTATTGCTCAGGATCATCTGGGAATGTTGCTTTCTGGGTTTAAAATACAG
TCAATGTTTAGATGAGCTGCTGTTCAGGCACATGGTCTGGTTAGCAAAGGGTTAAATCC
TGCATCCCGTTTTTAGTCATCA
>2274
TCAGGATCATCTGGGAATGTTGCTTTCTGGGTTTAAAATACAGTCAATGTTTAGATGAG
CTGCTGTTCAGGCACATGGTCTGGTTAGCAAAGGGTTAAATCCTGCATCCCGTTTTTAG
TCATCAAGCTAGCTCAGATCTG
>2275
TGTTGCTTTCTGGGTTTAAAATACAGTCAATGTTTAGATGAGCTGCTGTTCAGGCACAT
GGTCTGGTTAGCAAAGGGTTAAATCCTGCATCCCGTTTTTAGTCATCAAGCTAGCTCAG
ATCTGCCCACCTTGGAGCCCTG
>2276
GTTTAGATGAGCTGCTGTTCAGGCACATGGTCTGGTTAGCAAAGGGTTAAATCCTGCAT
CCCGTTTTTAGTCATCAAGCTAGCTCAGATCTGCCCACCTTGGAGCCCTGTGCTTTCCT
GCCTTAGTGGGCAGAGTCTAGG
>2277
GAGCTGCTGTTCAGGCACATGGTCTGGTTAGCAAAGGGTTAAATCCTGCATCCCGTTTT
TAGTCATCAAGCTAGCTCAGATCTGCCCACCTTGGAGCCCTGTGCTTTCCTGCCTTAGT
GGGCAGAGTCTAGGGGACCTGT

>2278
TGCTGTTCAGGCACATGGTCTGGTTAGCAAAGGGTTAAATCCTGCATCCCGTTTTTAGT
CATCAAGCTAGCTCAGATCTGCCCACCTTGGAGCCCTGTGCTTTCCTGCCTTAGTGGGC
AGAGTCTAGGGGACCTGTGGCA
>2279
GTTAGCAAAGGGTTAAATCCTGCATCCCGTTTTTAGTCATCAAGCTAGCTCAGATCTGC
CCACCTTGGAGCCCTGTGCTTTCCTGCCTTAGTGGGCAGAGTCTAGGGGACCTGTGGC
ATGTTCAATGATTGAGGACTCTT
>2280
GCATCCCGTTTTTAGTCATCAAGCTAGCTCAGATCTGCCCACCTTGGAGCCCTGTGCTT
TCCTGCCTTAGTGGGCAGAGTCTAGGGGACCTGTGGCATGTTCAATGATTGAGGACTC
TTGTCCAGATTCCAAAAACCTAC
>2281
TTTTAGTCATCAAGCTAGCTCAGATCTGCCCACCTTGGAGCCCTGTGCTTTCCTGCCTT
AGTGGGCAGAGTCTAGGGGACCTGTGGCATGTTCAATGATTGAGGACTCTTGTCCAGA
TTCCAAAAACCTACTGTGATGAT
>2282
GTCATCAAGCTAGCTCAGATCTGCCCACCTTGGAGCCCTGTGCTTTCCTGCCTTAGTGG
GCAGAGTCTAGGGGACCTGTGGCATGTTCAATGATTGAGGACTCTTGTCCAGATTCCA
AAAACCTACTGTGATGATGCTGT
>2283
CTTGGAGCCCTGTGCTTTCCTGCCTTAGTGGGCAGAGTCTAGGGGACCTGTGGCATGT
TCAATGATTGAGGACTCTTGTCCAGATTCCAAAAACCTACTGTGATGATGCTGTTCTGA
AGCTGTGGTAGGCGGTGATTGAG
>2284
GCTTTCCTGCCTTAGTGGGCAGAGTCTAGGGGACCTGTGGCATGTTCAATGATTGAGG
ACTCTTGTCCAGATTCCAAAAACCTACTGTGATGATGCTGTTCTGAAGCTGTGGTAGGC
GGTGATTGAGTGGAGTGTGTAGT
>2285
GTGGCATGTTCAATGATTGAGGACTCTTGTCCAGATTCCAAAAACCTACTGTGATGATG
CTGTTCTGAAGCTGTGGTAGGCGGTGATTGAGTGGAGTGTGTAGTGACCCTCAGGAAT
TCTTCAGAAGTTAGGATTTTGAC
>2286
TCAATGATTGAGGACTCTTGTCCAGATTCCAAAAACCTACTGTGATGATGCTGTTCTGA
AGCTGTGGTAGGCGGTGATTGAGTGGAGTGTGTAGTGACCCTCAGGAATTCTTCAGAA
GTTAGGATTTTGACGATACCTCT
>2287
GATTCCAAAAACCTACTGTGATGATGCTGTTCTGAAGCTGTGGTAGGCGGTGATTGAGT
GGAGTGTGTAGTGACCCTCAGGAATTCTTCAGAAGTTAGGATTTTGACGATACCTCTGA
TAAGTTGGGTTTTTAATTTGCT
>2288
ACCTACTGTGATGATGCTGTTCTGAAGCTGTGGTAGGCGGTGATTGAGTGGAGTGTGTA
GTGACCCTCAGGAATTCTTCAGAAGTTAGGATTTTGACGATACCTCTGATAAGTTGGGT
TTTTAATTTGCTGGTTTATCTT

>2289
TGATGCTGTTCTGAAGCTGTGGTAGGCGGTGATTGAGTGGAGTGTGTAGTGACCCTCA
GGAATTCTTCAGAAGTTAGGATTTTGACGATACCTCTGATAAGTTGGGTTTTTAATTTGC
TGGTTTATCTTAGAGTGGAGGC
>2290
GTTCTGAAGCTGTGGTAGGCGGTGATTGAGTGGAGTGTGTAGTGACCCTCAGGAATTC
TTCAGAAGTTAGGATTTTGACGATACCTCTGATAAGTTGGGTTTTTAATTTGCTGGTTTA
TCTTAGAGTGGAGGCTGTGGCC
>2291
ATTGAGTGGAGTGTGTAGTGACCCTCAGGAATTCTTCAGAAGTTAGGATTTTGACGATA
CCTCTGATAAGTTGGGTTTTTAATTTGCTGGTTTATCTTAGAGTGGAGGCTGTGGCCCA
CAGCAAGTAGTACATTTTCCAT
>2292
ATTCTTCAGAAGTTAGGATTTTGACGATACCTCTGATAAGTTGGGTTTTTAATTTGCTGG
TTTATCTTAGAGTGGAGGCTGTGGCCCACAGCAAGTAGTACATTTTCCATCTCAGCCCA
GTGCATGGACAAACGTACCTC
>2293
TCTTCAGAAGTTAGGATTTTGACGATACCTCTGATAAGTTGGGTTTTTAATTTGCTGGTT
TATCTTAGAGTGGAGGCTGTGGCCCACAGCAAGTAGTACATTTTCCATCTCAGCCCAGT
GCATGGACAAACGTACCTCAA
>2294
TGACGATACCTCTGATAAGTTGGGTTTTTAATTTGCTGGTTTATCTTAGAGTGGAGGCTG
TGGCCCACAGCAAGTAGTACATTTTCCATCTCAGCCCAGTGCATGGACAAACGTACCTC
AAACCAAAGGTTCAATGAAGC
>2295
TTTTTAATTTGCTGGTTTATCTTAGAGTGGAGGCTGTGGCCCACAGCAAGTAGTACATTT
TCCATCTCAGCCCAGTGCATGGACAAACGTACCTCAAACCAAAGGTTCAATGAAGCTC
TATTTATCTTTACTATATGCTC
>2296
AATTTGCTGGTTTATCTTAGAGTGGAGGCTGTGGCCCACAGCAAGTAGTACATTTTCCA
TCTCAGCCCAGTGCATGGACAAACGTACCTCAAACCAAAGGTTCAATGAAGCTCTATT
TATCTTTACTATATGCTCTGGTA
>2297
TGTGGCCCACAGCAAGTAGTACATTTTCCATCTCAGCCCAGTGCATGGACAAACGTAC
CTCAAACCAAAGGTTCAATGAAGCTCTATTTATCTTTACTATATGCTCTGGTATCTTCTCT
TCCATTCTATCTTATTTCATT
>2298
AATGAAGCTCTATTTATCTTTACTATATGCTCTGGTATCTTCTCTTCCATTCTATCTTATTT
CATTTAAGAACGTTAATGAAGACCCACTAATGGATTGCCACCCATGGTTCGAGGAGGTC
CCCCAAACAGTGAGGCCTT
>2299
TTATCTTTACTATATGCTCTGGTATCTTCTCTTCCATTCTATCTTATTTCATTTAAGAACGT
TAATGAAGACCCACTAATGGATTGCCACCCATGGTTCGAGGAGGTCCCCCAAACAGTG
AGGCCTTAGCCATAGGTATT

>2300
TCCATTCTATCTTATTTCATTTAAGAACGTTAATGAAGACCCACTAATGGATTGCCACCC
ATGGTTCGAGGAGGTCCCCCAAACAGTGAGGCCTTAGCCATAGGTATTCAATTCAAAA
AGCATTTACTGAGCATCACTGT
>2301
TCATTTAAGAACGTTAATGAAGACCCACTAATGGATTGCCACCCATGGTTCGAGGAGGT
CCCCCAAACAGTGAGGCCTTAGCCATAGGTATTCAATTCAAAAAGCATTTACTGAGCAT
CACTGTGTACTAGGTGGTTGCT
>2302
CGTTAATGAAGACCCACTAATGGATTGCCACCCATGGTTCGAGGAGGTCCCCCAAACA
GTGAGGCCTTAGCCATAGGTATTCAATTCAAAAAGCATTTACTGAGCATCACTGTGTAC
TAGGTGGTTGCTAAATGCTATGG
>2303
TGAAGACCCACTAATGGATTGCCACCCATGGTTCGAGGAGGTCCCCCAAACAGTGAGG
CCTTAGCCATAGGTATTCAATTCAAAAAGCATTTACTGAGCATCACTGTGTACTAGGTG
GTTGCTAAATGCTATGGGGGTAA
>2304
ATTGCCACCCATGGTTCGAGGAGGTCCCCCAAACAGTGAGGCCTTAGCCATAGGTATTC
AATTCAAAAAGCATTTACTGAGCATCACTGTGTACTAGGTGGTTGCTAAATGCTATGGG
GGTAATGCAGAGAAATTAATAA
>2305
CCCAAACAGTGAGGCCTTAGCCATAGGTATTCAATTCAAAAAGCATTTACTGAGCATCA
CTGTGTACTAGGTGGTTGCTAAATGCTATGGGGGTAATGCAGAGAAATTAATAAATCCC
TGTAAGAGTTCACTGTAAAACA
>2306
CAATTCAAAAAGCATTTACTGAGCATCACTGTGTACTAGGTGGTTGCTAAATGCTATGG
GGGTAATGCAGAGAAATTAATAAATCCCTGTAAGAGTTCACTGTAAAACAGAAATGAC
AGCCATATGTGTATGACTTTGTG
>2307
ATTCAAAAAGCATTTACTGAGCATCACTGTGTACTAGGTGGTTGCTAAATGCTATGGGG
GTAATGCAGAGAAATTAATAAATCCCTGTAAGAGTTCACTGTAAAACAGAAATGACAG
CCATATGTGTATGACTTTGTGTG
>2308
TGGTTGCTAAATGCTATGGGGGTAATGCAGAGAAATTAATAAATCCCTGTAAGAGTTCA
CTGTAAAACAGAAATGACAGCCATATGTGTATGACTTTGTGTGTGTGTGTATATATATATA
TCAGACTGTATGATTAAATA
>2309
AATGCTATGGGGGTAATGCAGAGAAATTAATAAATCCCTGTAAGAGTTCACTGTAAAAC
AGAAATGACAGCCATATGTGTATGACTTTGTGTGTGTGTGTATATATATATATCAGACTGT
ATGATTAAATATTATATAAG
>2310
GTTCACTGTAAAACAGAAATGACAGCCATATGTGTATGACTTTGTGTGTGTGTGTATATA
TATATATCAGACTGTATGATTAAATATTATATAAGTGAGGTATGGGGGAGCAGAGGAGAG
GAATTGAATTTTGAATTTTG

>2311
CCATATGTGTATGACTTTGTGTGTGTGTGTATATATATATATCAGACTGTATGATTAAATAT
TATATAAGTGAGGTATGGGGGAGCAGAGGAGAGGAATTGAATTTTGAATTTTGATTGAA
GATTCAGGAGGGCTTCATG
>2312
ATGTGTATGACTTTGTGTGTGTGTGTATATATATATATCAGACTGTATGATTAAATATTATA
TAAGTGAGGTATGGGGGAGCAGAGGAGAGGAATTGAATTTTGAATTTTGATTGAAGAT
TCAGGAGGGCTTCATGGAGG
>2313
GATTAAATATTATATAAGTGAGGTATGGGGGAGCAGAGGAGAGGAATTGAATTTTGAAT
TTTGATTGAAGATTCAGGAGGGCTTCATGGAGGAGGTAGCATTTGGCCAGGTTTTAGA
GAATGGCTAGGCTTTGGACAAGT
>2314
ATATTATATAAGTGAGGTATGGGGGAGCAGAGGAGAGGAATTGAATTTTGAATTTTGAT
TGAAGATTCAGGAGGGCTTCATGGAGGAGGTAGCATTTGGCCAGGTTTTAGAGAATGG
CTAGGCTTTGGACAAGTGGAGAC
>2315
ATGGGGGAGCAGAGGAGAGGAATTGAATTTTGAATTTTGATTGAAGATTCAGGAGGGC
TTCATGGAGGAGGTAGCATTTGGCCAGGTTTTAGAGAATGGCTAGGCTTTGGACAAGT
GGAGACAGGAACTGCCATTTGGAG
>2316
TTGAATTTTGAATTTTGATTGAAGATTCAGGAGGGCTTCATGGAGGAGGTAGCATTTGG
CCAGGTTTTAGAGAATGGCTAGGCTTTGGACAAGTGGAGACAGGAACTGCCATTTGGA
GGTAGGAGATGAGCTAATGTGTG
>2317
GAATTTTGAATTTTGATTGAAGATTCAGGAGGGCTTCATGGAGGAGGTAGCATTTGGCC
AGGTTTTAGAGAATGGCTAGGCTTTGGACAAGTGGAGACAGGAACTGCCATTTGGAG
GTAGGAGATGAGCTAATGTGTGGC
>2318
ATTTTGATTGAAGATTCAGGAGGGCTTCATGGAGGAGGTAGCATTTGGCCAGGTTTTAG
AGAATGGCTAGGCTTTGGACAAGTGGAGACAGGAACTGCCATTTGGAGGTAGGAGAT
GAGCTAATGTGTGGCAGTGTATGG
>2319
GATTCAGGAGGGCTTCATGGAGGAGGTAGCATTTGGCCAGGTTTTAGAGAATGGCTAG
GCTTTGGACAAGTGGAGACAGGAACTGCCATTTGGAGGTAGGAGATGAGCTAATGTGT
GGCAGTGTATGGGTGGGGGCAGGT
>2320
GTAGCATTTGGCCAGGTTTTAGAGAATGGCTAGGCTTTGGACAAGTGGAGACAGGAAC
TGCCATTTGGAGGTAGGAGATGAGCTAATGTGTGGCAGTGTATGGGTGGGGGCAGGTG
CCTGTATACAAGGTAGTGTGCTGG
>2321
CAGGTTTTAGAGAATGGCTAGGCTTTGGACAAGTGGAGACAGGAACTGCCATTTGGAG
GTAGGAGATGAGCTAATGTGTGGCAGTGTATGGGTGGGGGCAGGTGCCTGTATACAAG
GTAGTGTGCTGGAATGGGGCGATG

>2322
TGGCTAGGCTTTGGACAAGTGGAGACAGGAACTGCCATTTGGAGGTAGGAGATGAGCT
AATGTGTGGCAGTGTATGGGTGGGGGCAGGTGCCTGTATACAAGGTAGTGTGCTGGAA
TGGGGCGATGGGCATGGGGTAGGA
>2323
AGTGGAGACAGGAACTGCCATTTGGAGGTAGGAGATGAGCTAATGTGTGGCAGTGTAT
GGGTGGGGGCAGGTGCCTGTATACAAGGTAGTGTGCTGGAATGGGGCGATGGGCATGG
GGTAGGATGGGAGATCAAGCAGGG
>2324
ATTTGGAGGTAGGAGATGAGCTAATGTGTGGCAGTGTATGGGTGGGGGCAGGTGCCTG
TATACAAGGTAGTGTGCTGGAATGGGGCGATGGGCATGGGGTAGGATGGGAGATCAAG
CAGGGAAAGTGGATTGGGGCCAGG
>2325
AGTGTATGGGTGGGGGCAGGTGCCTGTATACAAGGTAGTGTGCTGGAATGGGGCGATG
GGCATGGGGTAGGATGGGAGATCAAGCAGGGAAAGTGGATTGGGGCCAGGTCCTAGA
ATGATTTGAATACCAAGCTAGCTTT
>2326
GGTGGGGGCAGGTGCCTGTATACAAGGTAGTGTGCTGGAATGGGGCGATGGGCATGGG
GTAGGATGGGAGATCAAGCAGGGAAAGTGGATTGGGGCCAGGTCCTAGAATGATTTGA
ATACCAAGCTAGCTTTCTGGACAT
>2327
GGAATGGGGCGATGGGCATGGGGTAGGATGGGAGATCAAGCAGGGAAAGTGGATTGG
GGCCAGGTCCTAGAATGATTTGAATACCAAGCTAGCTTTCTGGACATTTGTAAGGCGCA
CTTGATAGGGGTTGGATCATTGCG
>2328
TGGGGTAGGATGGGAGATCAAGCAGGGAAAGTGGATTGGGGCCAGGTCCTAGAATGA
TTTGAATACCAAGCTAGCTTTCTGGACATTTGTAAGGCGCACTTGATAGGGGTTGGATC
ATTGCGATATTATTGGAAGACAAA
>2329
AGGGAAAGTGGATTGGGGCCAGGTCCTAGAATGATTTGAATACCAAGCTAGCTTTCTG
GACATTTGTAAGGCGCACTTGATAGGGGTTGGATCATTGCGATATTATTGGAAGACAAA
ACTGGGGAGGGTGTGAGACCATG
>2330
TGGGGCCAGGTCCTAGAATGATTTGAATACCAAGCTAGCTTTCTGGACATTTGTAAGGC
GCACTTGATAGGGGTTGGATCATTGCGATATTATTGGAAGACAAAACTGGGGAGGGTG
TGAGACCATGTGGCTGCTCAGAA
>2331
CCAAGCTAGCTTTCTGGACATTTGTAAGGCGCACTTGATAGGGGTTGGATCATTGCGAT
ATTATTGGAAGACAAAACTGGGGAGGGTGTGAGACCATGTGGCTGCTCAGAATGCAG
AGCCCCTCTGATGTCCTCAGCCTT
>2332
TGGACATTTGTAAGGCGCACTTGATAGGGGTTGGATCATTGCGATATTATTGGAAGACA
AAACTGGGGAGGGTGTGAGACCATGTGGCTGCTCAGAATGCAGAGCCCCTCTGATGTC
CTCAGCCTTCTACGTATTTAGTG

>2333
AGGGGTTGGATCATTGCGATATTATTGGAAGACAAAACTGGGGAGGGTGTGAGACCAT
GTGGCTGCTCAGAATGCAGAGCCCCTCTGATGTCCTCAGCCTTCTACGTATTTAGTGAT
GGGGGTGGTGGTGGTAAAGAGCC
>2334
GGATCATTGCGATATTATTGGAAGACAAAACTGGGGAGGGTGTGAGACCATGTGGCTG
CTCAGAATGCAGAGCCCCTCTGATGTCCTCAGCCTTCTACGTATTTAGTGATGGGGGTG
GTGGTGGTAAAGAGCCTTGCAGA
>2335
GAAGACAAAACTGGGGAGGGTGTGAGACCATGTGGCTGCTCAGAATGCAGAGCCCCT
CTGATGTCCTCAGCCTTCTACGTATTTAGTGATGGGGGTGGTGGTGGTAAAGAGCCTTG
CAGACAGCCTGGCTTGGGTGCTTG
>2336
TCAGAATGCAGAGCCCCTCTGATGTCCTCAGCCTTCTACGTATTTAGTGATGGGGGTGG
TGGTGGTAAAGAGCCTTGCAGACAGCCTGGCTTGGGTGCTTGCTTGAAGTTCTTACGC
CCACTAACTCCTGCCCTGCTCTG
>2337
AGAATGCAGAGCCCCTCTGATGTCCTCAGCCTTCTACGTATTTAGTGATGGGGGTGGTG
GTGGTAAAGAGCCTTGCAGACAGCCTGGCTTGGGTGCTTGCTTGAAGTTCTTACGCCC
ACTAACTCCTGCCCTGCTCTGTG
>2338
CGTATTTAGTGATGGGGGTGGTGGTGGTAAAGAGCCTTGCAGACAGCCTGGCTTGGGT
GCTTGCTTGAAGTTCTTACGCCCACTAACTCCTGCCCTGCTCTGTGGAGCCTGCTGAAT
GGGGCATTAGGCTAGTCACACAC
>2339
TGCAGACAGCCTGGCTTGGGTGCTTGCTTGAAGTTCTTACGCCCACTAACTCCTGCCCT
GCTCTGTGGAGCCTGCTGAATGGGGCATTAGGCTAGTCACACACCTGTCCTTTTTCACA
TTCATGCTGTACCCTTTGCAAA
>2340
TGCTTGCTTGAAGTTCTTACGCCCACTAACTCCTGCCCTGCTCTGTGGAGCCTGCTGAA
TGGGGCATTAGGCTAGTCACACACCTGTCCTTTTTCACATTCATGCTGTACCCTTTGCA
AACATTACGCAGCAGTGCAAAC
>2341
GGGGCATTAGGCTAGTCACACACCTGTCCTTTTTCACATTCATGCTGTACCCTTTGCAA
ACATTACGCAGCAGTGCAAACTCAGCCCTGGGCCTCAGAGGAGCCAGGCGTGGCCAG
GAGAGTGGGATTTGGAACGGAAGT
>2342
GCATTAGGCTAGTCACACACCTGTCCTTTTTCACATTCATGCTGTACCCTTTGCAAACAT
TACGCAGCAGTGCAAACTCAGCCCTGGGCCTCAGAGGAGCCAGGCGTGGCCAGGAGA
GTGGGATTTGGAACGGAAGTGCT
>2343
GTCACACACCTGTCCTTTTTCACATTCATGCTGTACCCTTTGCAAACATTACGCAGCAG
TGCAAACTCAGCCCTGGGCCTCAGAGGAGCCAGGCGTGGCCAGGAGAGTGGGATTTG
GAACGGAAGTGCTTTTGTTTTTGG

>2344
CCTTTTTCACATTCATGCTGTACCCTTTGCAAACATTACGCAGCAGTGCAAACTCAGCC
CTGGGCCTCAGAGGAGCCAGGCGTGGCCAGGAGAGTGGGATTTGGAACGGAAGTGCT
TTTGTTTTTGGCATTCACTACTCC
>2345
TTCACATTCATGCTGTACCCTTTGCAAACATTACGCAGCAGTGCAAACTCAGCCCTGGG
CCTCAGAGGAGCCAGGCGTGGCCAGGAGAGTGGGATTTGGAACGGAAGTGCTTTTGT
TTTTGGCATTCACTACTCCCCCAC
>2346
GCAGTGCAAACTCAGCCCTGGGCCTCAGAGGAGCCAGGCGTGGCCAGGAGAGTGGG
ATTTGGAACGGAAGTGCTTTTGTTTTTGGCATTCACTACTCCCCCACCCCCACTGGCAC
TTGGATTGTCCTTCTTTTTCTCCTT
>2347
CTACTCCCCCACCCCCACTGGCACTTGGATTGTCCTTCTTTTTCTCCTTGGTGTCTCTCA
TCCTGTCCAGCCTTCAAGACCTCCTCAAGTCAGGCTTCTCTGGGAAGCGCTGTCTGTC
TGTCTTGGTGCTTGCTGGCTTC
>2348
CCACCCCCACTGGCACTTGGATTGTCCTTCTTTTTCTCCTTGGTGTCTCTCATCCTGTCC
AGCCTTCAAGACCTCCTCAAGTCAGGCTTCTCTGGGAAGCGCTGTCTGTCTGTCTTGG
TGCTTGCTGGCTTCCTCTTCTG
>2349
CTTTTTCTCCTTGGTGTCTCTCATCCTGTCCAGCCTTCAAGACCTCCTCAAGTCAGGCT
TCTCTGGGAAGCGCTGTCTGTCTGTCTTGGTGCTTGCTGGCTTCCTCTTCTGTCTCCTG
TGCCATCTCCAGTTTGCACCAT
>2350
CTCTGGGAAGCGCTGTCTGTCTGTCTTGGTGCTTGCTGGCTTCCTCTTCTGTCTCCTGT
GCCATCTCCAGTTTGCACCATTTGAGTATAAGCTCTGCTGCCTCATTTGAGTTTAAGTTT
TATCCCCCCAGCCACAACCTA
>2351
TCTGTCTGTCTTGGTGCTTGCTGGCTTCCTCTTCTGTCTCCTGTGCCATCTCCAGTTTGC
ACCATTTGAGTATAAGCTCTGCTGCCTCATTTGAGTTTAAGTTTTATCCCCCCAGCCACA
ACCTAAGTTCCATGAGGATA
>2352
TTCCTCTTCTGTCTCCTGTGCCATCTCCAGTTTGCACCATTTGAGTATAAGCTCTGCTGC
CTCATTTGAGTTTAAGTTTTATCCCCCCAGCCACAACCTAAGTTCCATGAGGATAGTGAT
TGTGGTTTATCTTCTCTCCC
>2353
CCATCTCCAGTTTGCACCATTTGAGTATAAGCTCTGCTGCCTCATTTGAGTTTAAGTTTT
ATCCCCCCAGCCACAACCTAAGTTCCATGAGGATAGTGATTGTGGTTTATCTTCTCTCCC
TCAGCTGTCTGCTGACACCA
>2354
TGCACCATTTGAGTATAAGCTCTGCTGCCTCATTTGAGTTTAAGTTTTATCCCCCCAGCC
ACAACCTAAGTTCCATGAGGATAGTGATTGTGGTTTATCTTCTCTCCCTCAGCTGTCTGC
TGACACCATGCTGAATACAT

>2355
TGAGTATAAGCTCTGCTGCCTCATTTGAGTTTAAGTTTTATCCCCCCAGCCACAACCTAA
GTTCCATGAGGATAGTGATTGTGGTTTATCTTCTCTCCCTCAGCTGTCTGCTGACACCAT
GCTGAATACATAGTAGGTAC
>2356
AGTTTTATCCCCCCAGCCACAACCTAAGTTCCATGAGGATAGTGATTGTGGTTTATCTTC
TCTCCCTCAGCTGTCTGCTGACACCATGCTGAATACATAGTAGGTACTCAGTAAATAATA
GCTAATTGAGCATTTACTAT
>2357
CCATGAGGATAGTGATTGTGGTTTATCTTCTCTCCCTCAGCTGTCTGCTGACACCATGCT
GAATACATAGTAGGTACTCAGTAAATAATAGCTAATTGAGCATTTACTATGGACAAAGCA
CTTTACTTGCATTAACACAT
>2358
TGAGGATAGTGATTGTGGTTTATCTTCTCTCCCTCAGCTGTCTGCTGACACCATGCTGAA
TACATAGTAGGTACTCAGTAAATAATAGCTAATTGAGCATTTACTATGGACAAAGCACTT
TACTTGCATTAACACATCCC
>2359
TCTCCCTCAGCTGTCTGCTGACACCATGCTGAATACATAGTAGGTACTCAGTAAATAATA
GCTAATTGAGCATTTACTATGGACAAAGCACTTTACTTGCATTAACACATCCCATCCTCA
CAACCACCTGTGAGGTGAAT
>2360
TGACACCATGCTGAATACATAGTAGGTACTCAGTAAATAATAGCTAATTGAGCATTTACT
ATGGACAAAGCACTTTACTTGCATTAACACATCCCATCCTCACAACCACCTGTGAGGTG
AATCCTATTAAAAGGAATACT
>2361
ATTGAGCATTTACTATGGACAAAGCACTTTACTTGCATTAACACATCCCATCCTCACAAC
CACCTGTGAGGTGAATCCTATTAAAAGGAATACTTGGGTGCTTTCAGAGTTATTCTCTC
CAGCTTTGGACTTCTTGTTTC
>2362
GACAAAGCACTTTACTTGCATTAACACATCCCATCCTCACAACCACCTGTGAGGTGAAT
CCTATTAAAAGGAATACTTGGGTGCTTTCAGAGTTATTCTCTCCAGCTTTGGACTTCTTG
TTTCATTTGCCCTGAGAGCTC
>2363
TTAACACATCCCATCCTCACAACCACCTGTGAGGTGAATCCTATTAAAAGGAATACTTG
GGTGCTTTCAGAGTTATTCTCTCCAGCTTTGGACTTCTTGTTTCATTTGCCCTGAGAGCT
CCTCAACTCTGTTGATGCTGT
>2364
AAAAGGAATACTTGGGTGCTTTCAGAGTTATTCTCTCCAGCTTTGGACTTCTTGTTTCA
TTTGCCCTGAGAGCTCCTCAACTCTGTTGATGCTGTTCTTTCAAAGGTTGCTTGTGACC
TCTTAGTTCTCAAGAGCACTGT
>2365
AAGGAATACTTGGGTGCTTTCAGAGTTATTCTCTCCAGCTTTGGACTTCTTGTTTCATTT
GCCCTGAGAGCTCCTCAACTCTGTTGATGCTGTTCTTTCAAAGGTTGCTTGTGACCTCT
TAGTTCTCAAGAGCACTGTAT

>2366
TCCAGCTTTGGACTTCTTGTTTCATTTGCCCTGAGAGCTCCTCAACTCTGTTGATGCTG
TTCTTTCAAAGGTTGCTTGTGACCTCTTAGTTCTCAAGAGCACTGTATTAGGTTGGTGC
AAAAGTAATTGCTATTTTAGAC
>2367
TTTCATTTGCCCTGAGAGCTCCTCAACTCTGTTGATGCTGTTCTTTCAAAGGTTGCTTG
TGACCTCTTAGTTCTCAAGAGCACTGTATTAGGTTGGTGCAAAAGTAATTGCTATTTTA
GACATTACTTTCAATGGCAAAA
>2368
GCCCTGAGAGCTCCTCAACTCTGTTGATGCTGTTCTTTCAAAGGTTGCTTGTGACCTCT
TAGTTCTCAAGAGCACTGTATTAGGTTGGTGCAAAAGTAATTGCTATTTTAGACATTACT
TTCAATGGCAAAAACTGCAAT
>2369
ATGCTGTTCTTTCAAAGGTTGCTTGTGACCTCTTAGTTCTCAAGAGCACTGTATTAGGT
TGGTGCAAAAGTAATTGCTATTTTAGACATTACTTTCAATGGCAAAAACTGCAATTACTT
TTGCGCCAACTAATACTTTCC
>2370
AGGTTGCTTGTGACCTCTTAGTTCTCAAGAGCACTGTATTAGGTTGGTGCAAAAGTAAT
TGCTATTTTAGACATTACTTTCAATGGCAAAAACTGCAATTACTTTTGCGCCAACTAATA
CTTTCCCAGACTCCAGTATTG
>2371
TTGCTATTTTAGACATTACTTTCAATGGCAAAAACTGCAATTACTTTTGCGCCAACTAAT
ACTTTCCCAGACTCCAGTATTGGCTCCATTGACCCCTCCTGTTCCTGCCAACTTCTCCC
CCTCCTCCTCTGGTATTTCCT
>2372
TTTAGACATTACTTTCAATGGCAAAAACTGCAATTACTTTTGCGCCAACTAATACTTTCC
CAGACTCCAGTATTGGCTCCATTGACCCCTCCTGTTCCTGCCAACTTCTCCCCCTCCTC
CTCTGGTATTTCCTGGAACTC
>2373
CATTGACCCCTCCTGTTCCTGCCAACTTCTCCCCCTCCTCCTCTGGTATTTCCTGGAACT
CCCTCCCTAGGGTCAGTGCCATGTGGTGCCTGGAACTCCCTGGGATCTGTGTTTGAATC
CTGACATTGCCACTTTCTAAG
>2374
CCCCTCCTGTTCCTGCCAACTTCTCCCCCTCCTCCTCTGGTATTTCCTGGAACTCCCTCC
CTAGGGTCAGTGCCATGTGGTGCCTGGAACTCCCTGGGATCTGTGTTTGAATCCTGACA
TTGCCACTTTCTAAGTGGCAA
>2375
GGTCAGTGCCATGTGGTGCCTGGAACTCCCTGGGATCTGTGTTTGAATCCTGACATTGC
CACTTTCTAAGTGGCAAACACTTTGGCCACCTCCCCCTCTTCCTGAGTCTGTTTCATCA
TCTGCAAAATGCTGGTAGTTTC
>2376
CTGTGTTTGAATCCTGACATTGCCACTTTCTAAGTGGCAAACACTTTGGCCACCTCCCC
CTCTTCCTGAGTCTGTTTCATCATCTGCAAAATGCTGGTAGTTTCTCTTCTCACAGGAA
AGATGGTGGTTGTTCATCGATG

>2377
TAAGTGGCAAACACTTTGGCCACCTCCCCCTCTTCCTGAGTCTGTTTCATCATCTGCAA
AATGCTGGTAGTTTCTCTTCTCACAGGAAAGATGGTGGTTGTTCATCGATGTCTGTTGA
AACCGTGACCCTCTCTGCTGAA
>2378
TTGGCCACCTCCCCCTCTTCCTGAGTCTGTTTCATCATCTGCAAAATGCTGGTAGTTTCT
CTTCTCACAGGAAAGATGGTGGTTGTTCATCGATGTCTGTTGAAACCGTGACCCTCTCT
GCTGAATGTAGAAGAAGATGA
>2379
CACCTCCCCCTCTTCCTGAGTCTGTTTCATCATCTGCAAAATGCTGGTAGTTTCTCTTCT
CACAGGAAAGATGGTGGTTGTTCATCGATGTCTGTTGAAACCGTGACCCTCTCTGCTG
AATGTAGAAGAAGATGACCTTA
>2380
CTTCTCACAGGAAAGATGGTGGTTGTTCATCGATGTCTGTTGAAACCGTGACCCTCTCT
GCTGAATGTAGAAGAAGATGACCTTACCAGTTCTCCTGTCTCTCCAGTGTCTTTCTTGA
CAATCTTGTGGTTTCCACAAGC
>2381
CTCACAGGAAAGATGGTGGTTGTTCATCGATGTCTGTTGAAACCGTGACCCTCTCTGCT
GAATGTAGAAGAAGATGACCTTACCAGTTCTCCTGTCTCTCCAGTGTCTTTCTTGACAA
TCTTGTGGTTTCCACAAGCAAC
>2382
TGGTTGTTCATCGATGTCTGTTGAAACCGTGACCCTCTCTGCTGAATGTAGAAGAAGAT
GACCTTACCAGTTCTCCTGTCTCTCCAGTGTCTTTCTTGACAATCTTGTGGTTTCCACA
AGCAACCCTCTGTGGGTGATTT
>2383
CTGTTGAAACCGTGACCCTCTCTGCTGAATGTAGAAGAAGATGACCTTACCAGTTCTC
CTGTCTCTCCAGTGTCTTTCTTGACAATCTTGTGGTTTCCACAAGCAACCCTCTGTGGG
TGATTTCCAGATCTCTACCTCTA
>2384
GAAGAAGATGACCTTACCAGTTCTCCTGTCTCTCCAGTGTCTTTCTTGACAATCTTGTG
GTTTCCACAAGCAACCCTCTGTGGGTGATTTCCAGATCTCTACCTCTAGTTGTTTTTCGT
TTTTTGTCTTTTCTTGTGTTT
>2385
CCTGTCTCTCCAGTGTCTTTCTTGACAATCTTGTGGTTTCCACAAGCAACCCTCTGTGG
GTGATTTCCAGATCTCTACCTCTAGTTGTTTTTCGTTTTTTGTCTTTTCTTGTGTTTCACA
CATTCTTTTCCAGTAGCCTC
>2386
TTTCCAGATCTCTACCTCTAGTTGTTTTTCGTTTTTTGTCTTTTCTTGTGTTTCACACATT
CTTTTCCAGTAGCCTCACGGGCACACCAGTCCTGCAAGGATCTAGAATGTGATGGTTTG
AGCTAGTTCAACACCTTCCT
>2387
CCAGATCTCTACCTCTAGTTGTTTTTCGTTTTTTGTCTTTTCTTGTGTTTCACACATTCTT
TTCCAGTAGCCTCACGGGCACACCAGTCCTGCAAGGATCTAGAATGTGATGGTTTGAG
CTAGTTCAACACCTTCCTCTC

>2388
GTTGTTTTTCGTTTTTTGTCTTTTCTTGTGTTTCACACATTCTTTTCCAGTAGCCTCACG
GGCACACCAGTCCTGCAAGGATCTAGAATGTGATGGTTTGAGCTAGTTCAACACCTTC
CTCTCGAATCGTCTCCTGTGTC
>2389
CGTTTTTTGTCTTTTCTTGTGTTTCACACATTCTTTTCCAGTAGCCTCACGGGCACACCA
GTCCTGCAAGGATCTAGAATGTGATGGTTTGAGCTAGTTCAACACCTTCCTCTCGAATC
GTCTCCTGTGTCCTCATGTTT
>2390
TGTCTTTTCTTGTGTTTCACACATTCTTTTCCAGTAGCCTCACGGGCACACCAGTCCTG
CAAGGATCTAGAATGTGATGGTTTGAGCTAGTTCAACACCTTCCTCTCGAATCGTCTCC
TGTGTCCTCATGTTTTGTGGTC
>2391
TCACACATTCTTTTCCAGTAGCCTCACGGGCACACCAGTCCTGCAAGGATCTAGAATGT
GATGGTTTGAGCTAGTTCAACACCTTCCTCTCGAATCGTCTCCTGTGTCCTCATGTTTTG
TGGTCTCTGCCCTCTTCCCAG
>2392
CTAGTTCAACACCTTCCTCTCGAATCGTCTCCTGTGTCCTCATGTTTTGTGGTCTCTGCC
CTCTTCCCAGGCACCCAACTGCAGACTTTGGCATCATCTGTGGCTCCTGTCTTCCCTTT
GCCTTCCATATCCACCATTTG
>2393
TCCTCTCGAATCGTCTCCTGTGTCCTCATGTTTTGTGGTCTCTGCCCTCTTCCCAGGCAC
CCAACTGCAGACTTTGGCATCATCTGTGGCTCCTGTCTTCCCTTTGCCTTCCATATCCAC
CATTTGCCTCTCCAGCCTAC
>2394
TGCAGACTTTGGCATCATCTGTGGCTCCTGTCTTCCCTTTGCCTTCCATATCCACCATTT
GCCTCTCCAGCCTACTTAACCAGATGGCTGTCCTGATTTCCTCACCAGCCTTCCTGCAG
TGCACCCCTGCAAGGCTCATC
>2395
ATCATCTGTGGCTCCTGTCTTCCCTTTGCCTTCCATATCCACCATTTGCCTCTCCAGCCT
ACTTAACCAGATGGCTGTCCTGATTTCCTCACCAGCCTTCCTGCAGTGCACCCCTGCAA
GGCTCATCACCCCACTTCCCT
>2396
TTGCCTTCCATATCCACCATTTGCCTCTCCAGCCTACTTAACCAGATGGCTGTCCTGATT
TCCTCACCAGCCTTCCTGCAGTGCACCCCTGCAAGGCTCATCACCCCACTTCCCTGAG
CAGAGTAGGGCTCTGATGGGGT
>2397
TCTCCAGCCTACTTAACCAGATGGCTGTCCTGATTTCCTCACCAGCCTTCCTGCAGTGC
ACCCCTGCAAGGCTCATCACCCCACTTCCCTGAGCAGAGTAGGGCTCTGATGGGGTTG
CTCTCCTGCTCTGAACCCTGCAG
>2398
GCTGTCCTGATTTCCTCACCAGCCTTCCTGCAGTGCACCCCTGCAAGGCTCATCACCCC
ACTTCCCTGAGCAGAGTAGGGCTCTGATGGGGTTGCTCTCCTGCTCTGAACCCTGCAG
TGGCTCACTATGGCCTATTCTAG

>2399
GATTTCCTCACCAGCCTTCCTGCAGTGCACCCCTGCAAGGCTCATCACCCCACTTCCCT
GAGCAGAGTAGGGCTCTGATGGGGTTGCTCTCCTGCTCTGAACCCTGCAGTGGCTCAC
TATGGCCTATTCTAGAGAAGAAA
>2400
GCTCATCACCCCACTTCCCTGAGCAGAGTAGGGCTCTGATGGGGTTGCTCTCCTGCTCT
GAACCCTGCAGTGGCTCACTATGGCCTATTCTAGAGAAGAAACGAGAGAATTCTTAGC
GTGGCATTCAAAGCCATCCGGGA
>2401
CAGAGTAGGGCTCTGATGGGGTTGCTCTCCTGCTCTGAACCCTGCAGTGGCTCACTAT
GGCCTATTCTAGAGAAGAAACGAGAGAATTCTTAGCGTGGCATTCAAAGCCATCCGGG
ATCCTAGATTCCAGCTACATTTTC
>2402
GCTCTCCTGCTCTGAACCCTGCAGTGGCTCACTATGGCCTATTCTAGAGAAGAAACGA
GAGAATTCTTAGCGTGGCATTCAAAGCCATCCGGGATCCTAGATTCCAGCTACATTTTC
ACTCTTCATCCCTTTCTCTGCAT
>2403
AACCCTGCAGTGGCTCACTATGGCCTATTCTAGAGAAGAAACGAGAGAATTCTTAGCG
TGGCATTCAAAGCCATCCGGGATCCTAGATTCCAGCTACATTTTCACTCTTCATCCCTTT
CTCTGCATATTTCTTAGAATGT
>2404
ACTATGGCCTATTCTAGAGAAGAAACGAGAGAATTCTTAGCGTGGCATTCAAAGCCATC
CGGGATCCTAGATTCCAGCTACATTTTCACTCTTCATCCCTTTCTCTGCATATTTCTTAGA
ATGTACACCCCTGAAGGCAG
>2405
CAAAGCCATCCGGGATCCTAGATTCCAGCTACATTTTCACTCTTCATCCCTTTCTCTGCA
TATTTCTTAGAATGTACACCCCTGAAGGCAGGGACCATCTTTTTCACATCTGTGTTACA
ACCTTTTAGTCCACTCTCTAG
>2406
CTAGATTCCAGCTACATTTTCACTCTTCATCCCTTTCTCTGCATATTTCTTAGAATGTACA
CCCCTGAAGGCAGGGACCATCTTTTTCACATCTGTGTTACAACCTTTTAGTCCACTCTC
TAGCATATGGCAACTCAGCA
>2407
CATATTTCTTAGAATGTACACCCCTGAAGGCAGGGACCATCTTTTTCACATCTGTGTTAC
AACCTTTTAGTCCACTCTCTAGCATATGGCAACTCAGCAGATGCTGCTGTTGAGTAAAC
TCTTATGCTTCCCTTCCCATG
>2408
AATGTACACCCCTGAAGGCAGGGACCATCTTTTTCACATCTGTGTTACAACCTTTTAGT
CCACTCTCTAGCATATGGCAACTCAGCAGATGCTGCTGTTGAGTAAACTCTTATGCTTC
CCTTCCCATGCCATATCCTTCT
>2409
TTCACATCTGTGTTACAACCTTTTAGTCCACTCTCTAGCATATGGCAACTCAGCAGATGC
TGCTGTTGAGTAAACTCTTATGCTTCCCTTCCCATGCCATATCCTTCTCTGGTCATCGCA
ACAGGGCCAAATCTCTCATG

>2410
AGATGCTGCTGTTGAGTAAACTCTTATGCTTCCCTTCCCATGCCATATCCTTCTCTGGTC
ATCGCAACAGGGCCAAATCTCTCATGCTTCAGTGTAAATGCCCTCTCCTTGCAGCATCC
TTTCCTGGGGCATTCTTCCTC
>2411
CTTATGCTTCCCTTCCCATGCCATATCCTTCTCTGGTCATCGCAACAGGGCCAAATCTCT
CATGCTTCAGTGTAAATGCCCTCTCCTTGCAGCATCCTTTCCTGGGGCATTCTTCCTCCT
TGCATCCAGTTGGAAATAAT
>2412
ATATCCTTCTCTGGTCATCGCAACAGGGCCAAATCTCTCATGCTTCAGTGTAAATGCCCT
CTCCTTGCAGCATCCTTTCCTGGGGCATTCTTCCTCCTTGCATCCAGTTGGAAATAATAT
GTCCCTCCATTGGAGCTCCC
>2413
TCATGCTTCAGTGTAAATGCCCTCTCCTTGCAGCATCCTTTCCTGGGGCATTCTTCCTCC
TTGCATCCAGTTGGAAATAATATGTCCCTCCATTGGAGCTCCCACACCAGAAGAGGTAT
AGATAAAGCCAGATACTTCTT
>2414
TGCAGCATCCTTTCCTGGGGCATTCTTCCTCCTTGCATCCAGTTGGAAATAATATGTCCC
TCCATTGGAGCTCCCACACCAGAAGAGGTATAGATAAAGCCAGATACTTCTTTTTTTTTT
TTTTTTTTTTGATACAGAGTC
>2415
GGGCATTCTTCCTCCTTGCATCCAGTTGGAAATAATATGTCCCTCCATTGGAGCTCCCAC
ACCAGAAGAGGTATAGATAAAGCCAGATACTTCTTTTTTTTTTTTTTTTTTTTGATACAGA
GTCTCACTCTGTTGCCCAGG
>2416
TCTTCCTCCTTGCATCCAGTTGGAAATAATATGTCCCTCCATTGGAGCTCCCACACCAG
AAGAGGTATAGATAAAGCCAGATACTTCTTTTTTTTTTTTTTTTTTTTGATACAGAGTCTC
ACTCTGTTGCCCAGGCTGGAG
>2417
TCCTTGCATCCAGTTGGAAATAATATGTCCCTCCATTGGAGCTCCCACACCAGAAGAGG
TATAGATAAAGCCAGATACTTCTTTTTTTTTTTTTTTTTTTTGATACAGAGTCTCACTCTGT
TGCCCAGGCTGGAGTGTAGT
>2418
GGAGCTCCCACACCAGAAGAGGTATAGATAAAGCCAGATACTTCTTTTTTTTTTTTTTTTT
TTTGATACAGAGTCTCACTCTGTTGCCCAGGCTGGAGTGTAGTGGCATGAACTTGGCTC
ACTGCAGCCTCTGCCTCTTGG
>2419
GGTATAGATAAAGCCAGATACTTCTTTTTTTTTTTTTTTTTTTTGATACAGAGTCTCACTCT
GTTGCCCAGGCTGGAGTGTAGTGGCATGAACTTGGCTCACTGCAGCCTCTGCCTCTTG
GGTTCAAGTGATTCTTGCGTC
>2420
ATAAAGCCAGATACTTCTTTTTTTTTTTTTTTTTTTTGATACAGAGTCTCACTCTGTTGCCC
AGGCTGGAGTGTAGTGGCATGAACTTGGCTCACTGCAGCCTCTGCCTCTTGGGTTCAA
GTGATTCTTGCGTCTCAGCCT

>2421
TTTTTTTGATACAGAGTCTCACTCTGTTGCCCAGGCTGGAGTGTAGTGGCATGAACTTG
GCTCACTGCAGCCTCTGCCTCTTGGGTTCAAGTGATTCTTGCGTCTCAGCCTCCCGAGT
AGCTGAGATTACAGACATGCGC
>2422
CTCTGTTGCCCAGGCTGGAGTGTAGTGGCATGAACTTGGCTCACTGCAGCCTCTGCCT
CTTGGGTTCAAGTGATTCTTGCGTCTCAGCCTCCCGAGTAGCTGAGATTACAGACATGC
GCTACCATGCCTGGCTAATTTTT
>2423
GAGTGTAGTGGCATGAACTTGGCTCACTGCAGCCTCTGCCTCTTGGGTTCAAGTGATTC
TTGCGTCTCAGCCTCCCGAGTAGCTGAGATTACAGACATGCGCTACCATGCCTGGCTAA
TTTTTGTATTTTTAGTAGAGAT
>2424
GGCATGAACTTGGCTCACTGCAGCCTCTGCCTCTTGGGTTCAAGTGATTCTTGCGTCTC
AGCCTCCCGAGTAGCTGAGATTACAGACATGCGCTACCATGCCTGGCTAATTTTTGTATT
TTTAGTAGAGATGCGGTTTCA
>2425
GTCTCAGCCTCCCGAGTAGCTGAGATTACAGACATGCGCTACCATGCCTGGCTAATTTT
TGTATTTTTAGTAGAGATGCGGTTTCACCATGCTGGCCAGGCTGGTCCCCAACTCCTCG
CCTCAAGTGATCTTCCTGCCTT
>2426
TCAGCCTCCCGAGTAGCTGAGATTACAGACATGCGCTACCATGCCTGGCTAATTTTTGT
ATTTTTAGTAGAGATGCGGTTTCACCATGCTGGCCAGGCTGGTCCCCAACTCCTCGCCT
CAAGTGATCTTCCTGCCTTAGC
>2427
TTTTTGTATTTTTAGTAGAGATGCGGTTTCACCATGCTGGCCAGGCTGGTCCCCAACTC
CTCGCCTCAAGTGATCTTCCTGCCTTAGCCTCCCAGAGTGCTAGGATTACAGGTGTGAG
TCACTGTGCCCGGCCTGGATCT
>2428
GAGATGCGGTTTCACCATGCTGGCCAGGCTGGTCCCCAACTCCTCGCCTCAAGTGATC
TTCCTGCCTTAGCCTCCCAGAGTGCTAGGATTACAGGTGTGAGTCACTGTGCCCGGCCT
GGATCTTTTATTGTTTGTATTTC
>2429
CAGGCTGGTCCCCAACTCCTCGCCTCAAGTGATCTTCCTGCCTTAGCCTCCCAGAGTGC
TAGGATTACAGGTGTGAGTCACTGTGCCCGGCCTGGATCTTTTATTGTTTGTATTTCTGT
TCATTTCTTCCCTAAGAAACC
>2430
GTCCCCAACTCCTCGCCTCAAGTGATCTTCCTGCCTTAGCCTCCCAGAGTGCTAGGATT
ACAGGTGTGAGTCACTGTGCCCGGCCTGGATCTTTTATTGTTTGTATTTCTGTTCATTTC
TTCCCTAAGAAACCCCACGTT
>2431
TACAGGTGTGAGTCACTGTGCCCGGCCTGGATCTTTTATTGTTTGTATTTCTGTTCATTT
CTTCCCTAAGAAACCCCACGTTTTTGTGGGCAGGACCTGGAATCCTATTCAGTTATACA
GCCCCGCTTCGTCCACTGCTC

>2432
GGCCTGGATCTTTTATTGTTTGTATTTCTGTTCATTTCTTCCCTAAGAAACCCCACGTTTT
TGTGGGCAGGACCTGGAATCCTATTCAGTTATACAGCCCCGCTTCGTCCACTGCTCCCC
TTCAGAGTCTAGTGCCCTGG
>2433
TTGTATTTCTGTTCATTTCTTCCCTAAGAAACCCCACGTTTTTGTGGGCAGGACCTGGA
ATCCTATTCAGTTATACAGCCCCGCTTCGTCCACTGCTCCCCTTCAGAGTCTAGTGCCCT
GGACATCATAGGTATTCAGTA
>2434
CGTTTTTGTGGGCAGGACCTGGAATCCTATTCAGTTATACAGCCCCGCTTCGTCCACTG
CTCCCCTTCAGAGTCTAGTGCCCTGGACATCATAGGTATTCAGTACCTCTTGCTGGCTTA
GGAACAGGAGGATGCCTGATG
>2435
TTTTTGTGGGCAGGACCTGGAATCCTATTCAGTTATACAGCCCCGCTTCGTCCACTGCT
CCCCTTCAGAGTCTAGTGCCCTGGACATCATAGGTATTCAGTACCTCTTGCTGGCTTAG
GAACAGGAGGATGCCTGATGGC
>2436
AGTTATACAGCCCCGCTTCGTCCACTGCTCCCCTTCAGAGTCTAGTGCCCTGGACATCA
TAGGTATTCAGTACCTCTTGCTGGCTTAGGAACAGGAGGATGCCTGATGGCCAGGGTG
TTCCTCTGAAGCAGTGGTGGCTG
>2437
CGTCCACTGCTCCCCTTCAGAGTCTAGTGCCCTGGACATCATAGGTATTCAGTACCTCT
TGCTGGCTTAGGAACAGGAGGATGCCTGATGGCCAGGGTGTTCCTCTGAAGCAGTGGT
GGCTGAGCTTTTTGAATATCTGC
>2438
CTGCTCCCCTTCAGAGTCTAGTGCCCTGGACATCATAGGTATTCAGTACCTCTTGCTGG
CTTAGGAACAGGAGGATGCCTGATGGCCAGGGTGTTCCTCTGAAGCAGTGGTGGCTGA
GCTTTTTGAATATCTGCATTGTG
>2439
AGTGCCCTGGACATCATAGGTATTCAGTACCTCTTGCTGGCTTAGGAACAGGAGGATGC
CTGATGGCCAGGGTGTTCCTCTGAAGCAGTGGTGGCTGAGCTTTTTGAATATCTGCATT
GTGCTAAGGGTTAACTACCCAG
>2440
CCTCTTGCTGGCTTAGGAACAGGAGGATGCCTGATGGCCAGGGTGTTCCTCTGAAGCA
GTGGTGGCTGAGCTTTTTGAATATCTGCATTGTGCTAAGGGTTAACTACCCAGGCAGTA
CTGGTGGGGAAGGGCCCCAGAAG
>2441
TGCCTGATGGCCAGGGTGTTCCTCTGAAGCAGTGGTGGCTGAGCTTTTTGAATATCTGC
ATTGTGCTAAGGGTTAACTACCCAGGCAGTACTGGTGGGGAAGGGCCCCAGAAGCAG
GCCCAGACTCAGGTATTGTTAAAG
>2442
GGTGTTCCTCTGAAGCAGTGGTGGCTGAGCTTTTTGAATATCTGCATTGTGCTAAGGGT
TAACTACCCAGGCAGTACTGGTGGGGAAGGGCCCCAGAAGCAGGCCCAGACTCAGGT
ATTGTTAAAGCAGAGTCATCTTTG

>2443
TGGTGGCTGAGCTTTTTGAATATCTGCATTGTGCTAAGGGTTAACTACCCAGGCAGTAC
TGGTGGGGAAGGGCCCCAGAAGCAGGCCCAGACTCAGGTATTGTTAAAGCAGAGTCA
TCTTTGCTCTGAAGTTTTTTCTTT
>2444
GAGCTTTTTGAATATCTGCATTGTGCTAAGGGTTAACTACCCAGGCAGTACTGGTGGGG
AAGGGCCCCAGAAGCAGGCCCAGACTCAGGTATTGTTAAAGCAGAGTCATCTTTGCTC
TGAAGTTTTTTCTTTTATCTTTT
>2445
TATCTGCATTGTGCTAAGGGTTAACTACCCAGGCAGTACTGGTGGGGAAGGGCCCCAG
AAGCAGGCCCAGACTCAGGTATTGTTAAAGCAGAGTCATCTTTGCTCTGAAGTTTTTTC
TTTTATCTTTTCTTTTCTTTTTT
>2446
GGTGGGGAAGGGCCCCAGAAGCAGGCCCAGACTCAGGTATTGTTAAAGCAGAGTCAT
CTTTGCTCTGAAGTTTTTTCTTTTATCTTTTCTTTTCTTTTTTAATTGGGGGAAACCTTGT
CCTAATTGGCATCTTTATTGTG
>2447
TTCTTTTATCTTTTCTTTTCTTTTTTAATTGGGGGAAACCTTGTCCTAATTGGCATCTTTA
TTGTGGCAGGAATAGGCCAGGCAGGGCTGGGGGAGTGTGAAGTCTGTGAAGGGAGGT
GGCCTGGGGGCCCCCCACACA
>2448
TATCTTTTCTTTTCTTTTTTAATTGGGGGAAACCTTGTCCTAATTGGCATCTTTATTGTGG
CAGGAATAGGCCAGGCAGGGCTGGGGGAGTGTGAAGTCTGTGAAGGGAGGTGGCCTG
GGGGCCCCCCACACAATGCCT
>2449
GGAAACCTTGTCCTAATTGGCATCTTTATTGTGGCAGGAATAGGCCAGGCAGGGCTGG
GGGAGTGTGAAGTCTGTGAAGGGAGGTGGCCTGGGGGCCCCCCACACAATGCCTGA
TTGTGCAGGCATAGGTATGAACACA
>2450
GTCCTAATTGGCATCTTTATTGTGGCAGGAATAGGCCAGGCAGGGCTGGGGGAGTGTG
AAGTCTGTGAAGGGAGGTGGCCTGGGGGCCCCCCACACAATGCCTGATTGTGCAGG
CATAGGTATGAACACATTGCAGCCT
>2451
TAATTGGCATCTTTATTGTGGCAGGAATAGGCCAGGCAGGGCTGGGGGAGTGTGAAGT
CTGTGAAGGGAGGTGGCCTGGGGGCCCCCCACACAATGCCTGATTGTGCAGGCATAG
GTATGAACACATTGCAGCCTGTGT
>2452
GCTGGGGGAGTGTGAAGTCTGTGAAGGGAGGTGGCCTGGGGGCCCCCCACACAATG
CCTGATTGTGCAGGCATAGGTATGAACACATTGCAGCCTGTGTGCACCCACAGGGTGA
CCACGAGCAAGGCCTGGGGACGAGG
>2453
AAGGGAGGTGGCCTGGGGGCCCCCCACACAATGCCTGATTGTGCAGGCATAGGTATG
AACACATTGCAGCCTGTGTGCACCCACAGGGTGACCACGAGCAAGGCCTGGGGACGA
GGACTACGAGGCCTGCAGCTGGGTC

>2454
GGCCCCCCCACACAATGCCTGATTGTGCAGGCATAGGTATGAACACATTGCAGCCTGT
GTGCACCCACAGGGTGACCACGAGCAAGGCCTGGGGACGAGGACTACGAGGCCTGC
AGCTGGGTCCTCTTTTCTCCCTCCTC
>2455
GCCTGATTGTGCAGGCATAGGTATGAACACATTGCAGCCTGTGTGCACCCACAGGGTG
ACCACGAGCAAGGCCTGGGGACGAGGACTACGAGGCCTGCAGCTGGGTCCTCTTTTC
TCCCTCCTCACCCCCACCCAGTACC
>2456
ATGAACACATTGCAGCCTGTGTGCACCCACAGGGTGACCACGAGCAAGGCCTGGGGA
CGAGGACTACGAGGCCTGCAGCTGGGTCCTCTTTTCTCCCTCCTCACCCCCACCCAGT
ACCCACGTGATTGGGCCACATCCCA
>2457
ATTGCAGCCTGTGTGCACCCACAGGGTGACCACGAGCAAGGCCTGGGGACGAGGACT
ACGAGGCCTGCAGCTGGGTCCTCTTTTCTCCCTCCTCACCCCCACCCAGTACCCACGT
GATTGGGCCACATCCCAGTAACAAG
>2458
CAAGGCCTGGGGACGAGGACTACGAGGCCTGCAGCTGGGTCCTCTTTTCTCCCTCCTC
ACCCCCACCCAGTACCCACGTGATTGGGCCACATCCCAGTAACAAGCAGTCCTTGTGG
AAGAGCCTTCATGAAGACCTTTGC
>2459
CCTGCAGCTGGGTCCTCTTTTCTCCCTCCTCACCCCCACCCAGTACCCACGTGATTGGG
CCACATCCCAGTAACAAGCAGTCCTTGTGGAAGAGCCTTCATGAAGACCTTTGCTGGG
AGTGGCGGGCAGGGATCTGCGGG
>2460
CTGGGTCCTCTTTTCTCCCTCCTCACCCCCACCCAGTACCCACGTGATTGGGCCACATC
CCAGTAACAAGCAGTCCTTGTGGAAGAGCCTTCATGAAGACCTTTGCTGGGAGTGGCG
GGCAGGGATCTGCGGGGAGCGTT
>2461
CCACCCAGTACCCACGTGATTGGGCCACATCCCAGTAACAAGCAGTCCTTGTGGAAGA
GCCTTCATGAAGACCTTTGCTGGGAGTGGCGGGCAGGGATCTGCGGGGAGCGTTGCT
GGTTCAAGCTGGGGCTTTCCTATGG
>2462
CGTGATTGGGCCACATCCCAGTAACAAGCAGTCCTTGTGGAAGAGCCTTCATGAAGAC
CTTTGCTGGGAGTGGCGGGCAGGGATCTGCGGGGAGCGTTGCTGGTTCAAGCTGGGG
CTTTCCTATGGATGATGTCCTGTGA
>2463
CCACATCCCAGTAACAAGCAGTCCTTGTGGAAGAGCCTTCATGAAGACCTTTGCTGGG
AGTGGCGGGCAGGGATCTGCGGGGAGCGTTGCTGGTTCAAGCTGGGGCTTTCCTATGG
ATGATGTCCTGTGAAGGGTAGATC
>2464
CAAGCAGTCCTTGTGGAAGAGCCTTCATGAAGACCTTTGCTGGGAGTGGCGGGCAGG
GATCTGCGGGGAGCGTTGCTGGTTCAAGCTGGGGCTTTCCTATGGATGATGTCCTGTGA
AGGGTAGATCCTGGAGGGACAGAC

>2465
GCGGGCAGGGATCTGCGGGGAGCGTTGCTGGTTCAAGCTGGGGCTTTCCTATGGATGA
TGTCCTGTGAAGGGTAGATCCTGGAGGGACAGACAGGTGTCTGATCTTTGGCAGGCAG
GGATGTCTCATTTGTACATTATAG
>2466
CAGGGATCTGCGGGGAGCGTTGCTGGTTCAAGCTGGGGCTTTCCTATGGATGATGTCCT
GTGAAGGGTAGATCCTGGAGGGACAGACAGGTGTCTGATCTTTGGCAGGCAGGGATG
TCTCATTTGTACATTATAGGTATT
>2467
GCTTTCCTATGGATGATGTCCTGTGAAGGGTAGATCCTGGAGGGACAGACAGGTGTCT
GATCTTTGGCAGGCAGGGATGTCTCATTTGTACATTATAGGTATTCAGTATCTCTTGCTG
GCTTAGGCTTCATAATGTCTCA
>2468
TCCTATGGATGATGTCCTGTGAAGGGTAGATCCTGGAGGGACAGACAGGTGTCTGATC
TTTGGCAGGCAGGGATGTCTCATTTGTACATTATAGGTATTCAGTATCTCTTGCTGGCTT
AGGCTTCATAATGTCTCATTTG
>2469
GGTAGATCCTGGAGGGACAGACAGGTGTCTGATCTTTGGCAGGCAGGGATGTCTCATT
TGTACATTATAGGTATTCAGTATCTCTTGCTGGCTTAGGCTTCATAATGTCTCATTTGTAC
ATCATAGCCTTCAGTACCTCT
>2470
GTCTGATCTTTGGCAGGCAGGGATGTCTCATTTGTACATTATAGGTATTCAGTATCTCTT
GCTGGCTTAGGCTTCATAATGTCTCATTTGTACATCATAGCCTTCAGTACCTCTTGCTGA
TTTGGTTAACCCAGTTGTTA
>2471
TTTGTACATTATAGGTATTCAGTATCTCTTGCTGGCTTAGGCTTCATAATGTCTCATTTGT
ACATCATAGCCTTCAGTACCTCTTGCTGATTTGGTTAACCCAGTTGTTATACCACGATAT
TGATTTTTCATGCCATTTT
>2472
ATTATAGGTATTCAGTATCTCTTGCTGGCTTAGGCTTCATAATGTCTCATTTGTACATCAT
AGCCTTCAGTACCTCTTGCTGATTTGGTTAACCCAGTTGTTATACCACGATATTGATTTT
TCATGCCATTTTCTTTAGG
>2473
GTACCTCTTGCTGATTTGGTTAACCCAGTTGTTATACCACGATATTGATTTTTCATGCCAT
TTTCTTTAGGAAGATTGGAAGATCATTGAGGATAGGGACTGTGACCTTTTCTTTTTTTCT
ACCCCAGTACCCAACCTGG
>2474
CTCTTGCTGATTTGGTTAACCCAGTTGTTATACCACGATATTGATTTTTCATGCCATTTTC
TTTAGGAAGATTGGAAGATCATTGAGGATAGGGACTGTGACCTTTTCTTTTTTTCTACC
CCAGTACCCAACCTGGCAGG
>2475
AACCCAGTTGTTATACCACGATATTGATTTTTCATGCCATTTTCTTTAGGAAGATTGGAA
GATCATTGAGGATAGGGACTGTGACCTTTTCTTTTTTTCTACCCCAGTACCCAACCTGG
CAGGTAGCACAGCGCAATGAT

>2476
AGTTGTTATACCACGATATTGATTTTTCATGCCATTTTCTTTAGGAAGATTGGAAGATCAT
TGAGGATAGGGACTGTGACCTTTTCTTTTTTTCTACCCCAGTACCCAACCTGGCAGGTA
GCACAGCGCAATGATCCACG
>2477
CATTTTCTTTAGGAAGATTGGAAGATCATTGAGGATAGGGACTGTGACCTTTTCTTTTTT
TCTACCCCAGTACCCAACCTGGCAGGTAGCACAGCGCAATGATCCACGATGTTTAATAG
GTTGAATTGTAGAGGGGGCCT
>2478
GATTGGAAGATCATTGAGGATAGGGACTGTGACCTTTTCTTTTTTTCTACCCCAGTACC
CAACCTGGCAGGTAGCACAGCGCAATGATCCACGATGTTTAATAGGTTGAATTGTAGA
GGGGGCCTCTGATTCCAAAGATG
>2479
ATCATTGAGGATAGGGACTGTGACCTTTTCTTTTTTTCTACCCCAGTACCCAACCTGGC
AGGTAGCACAGCGCAATGATCCACGATGTTTAATAGGTTGAATTGTAGAGGGGGCCTCT
GATTCCAAAGATGGGGGCAGAA
>2480
TTTTCTTTTTTTCTACCCCAGTACCCAACCTGGCAGGTAGCACAGCGCAATGATCCACG
ATGTTTAATAGGTTGAATTGTAGAGGGGGCCTCTGATTCCAAAGATGGGGGCAGAATAT
CAGTCCTCTCCAGACTGCCTGG
>2481
CTACCCCAGTACCCAACCTGGCAGGTAGCACAGCGCAATGATCCACGATGTTTAATAGG
TTGAATTGTAGAGGGGGCCTCTGATTCCAAAGATGGGGGCAGAATATCAGTCCTCTCC
AGACTGCCTGGGAATCCTCCTCT
>2482
CAGGTAGCACAGCGCAATGATCCACGATGTTTAATAGGTTGAATTGTAGAGGGGGCCT
CTGATTCCAAAGATGGGGGCAGAATATCAGTCCTCTCCAGACTGCCTGGGAATCCTCCT
CTCTCCAGCCTGGGCCTGGCCAG
>2483
AGCGCAATGATCCACGATGTTTAATAGGTTGAATTGTAGAGGGGGCCTCTGATTCCAAA
GATGGGGGCAGAATATCAGTCCTCTCCAGACTGCCTGGGAATCCTCCTCTCTCCAGCCT
GGGCCTGGCCAGTGGGAGACAG
>2484
GATCCACGATGTTTAATAGGTTGAATTGTAGAGGGGGCCTCTGATTCCAAAGATGGGGG
CAGAATATCAGTCCTCTCCAGACTGCCTGGGAATCCTCCTCTCTCCAGCCTGGGCCTGG
CCAGTGGGAGACAGAGGCTGCT
>2485
CCTCTGATTCCAAAGATGGGGGCAGAATATCAGTCCTCTCCAGACTGCCTGGGAATCCT
CCTCTCTCCAGCCTGGGCCTGGCCAGTGGGAGACAGAGGCTGCTGAAGTAGCTGGCA
AGGAGGCAAAGAAGATGATGACCT
>2486
ATGGGGGCAGAATATCAGTCCTCTCCAGACTGCCTGGGAATCCTCCTCTCTCCAGCCTG
GGCCTGGCCAGTGGGAGACAGAGGCTGCTGAAGTAGCTGGCAAGGAGGCAAAGAAG
ATGATGACCTAGCTACGGTCCTCAT

>2487
GCAGAATATCAGTCCTCTCCAGACTGCCTGGGAATCCTCCTCTCTCCAGCCTGGGCCTG
GCCAGTGGGAGACAGAGGCTGCTGAAGTAGCTGGCAAGGAGGCAAAGAAGATGATG
ACCTAGCTACGGTCCTCATGTGCCC
>2488
CTCCAGACTGCCTGGGAATCCTCCTCTCTCCAGCCTGGGCCTGGCCAGTGGGAGACAG
AGGCTGCTGAAGTAGCTGGCAAGGAGGCAAAGAAGATGATGACCTAGCTACGGTCCT
CATGTGCCCACTGGGGAATCATCTT
>2489
TGGGCCTGGCCAGTGGGAGACAGAGGCTGCTGAAGTAGCTGGCAAGGAGGCAAAGA
AGATGATGACCTAGCTACGGTCCTCATGTGCCCACTGGGGAATCATCTTTTTGCATCTG
AGTTAGTCAATGTGCTGTGCATTCT
>2490
AGGCAAAGAAGATGATGACCTAGCTACGGTCCTCATGTGCCCACTGGGGAATCATCTT
TTTGCATCTGAGTTAGTCAATGTGCTGTGCATTCTCTGCAGAATTTGTGAACCTGGTATA
GGTATGAGGTGAGAGAGGCCAG
>2491
TCCTCATGTGCCCACTGGGGAATCATCTTTTTGCATCTGAGTTAGTCAATGTGCTGTGCA
TTCTCTGCAGAATTTGTGAACCTGGTATAGGTATGAGGTGAGAGAGGCCAGGAGGTCT
GGCTGCTCCCCCTCTAGACTTC
>2492
AATCATCTTTTTGCATCTGAGTTAGTCAATGTGCTGTGCATTCTCTGCAGAATTTGTGAA
CCTGGTATAGGTATGAGGTGAGAGAGGCCAGGAGGTCTGGCTGCTCCCCCTCTAGACT
TCGCAGGTAAAACCCAAGGTGT
>2493
CTGAGTTAGTCAATGTGCTGTGCATTCTCTGCAGAATTTGTGAACCTGGTATAGGTATG
AGGTGAGAGAGGCCAGGAGGTCTGGCTGCTCCCCCTCTAGACTTCGCAGGTAAAACC
CAAGGTGTGTGCCACGTAAAGGTT
>2494
TTAGTCAATGTGCTGTGCATTCTCTGCAGAATTTGTGAACCTGGTATAGGTATGAGGTG
AGAGAGGCCAGGAGGTCTGGCTGCTCCCCCTCTAGACTTCGCAGGTAAAACCCAAGG
TGTGTGCCACGTAAAGGTTCAGAG
>2495
GTCAATGTGCTGTGCATTCTCTGCAGAATTTGTGAACCTGGTATAGGTATGAGGTGAGA
GAGGCCAGGAGGTCTGGCTGCTCCCCCTCTAGACTTCGCAGGTAAAACCCAAGGTGT
GTGCCACGTAAAGGTTCAGAGGTT
>2496
TGCAGAATTTGTGAACCTGGTATAGGTATGAGGTGAGAGAGGCCAGGAGGTCTGGCTG
CTCCCCCTCTAGACTTCGCAGGTAAAACCCAAGGTGTGTGCCACGTAAAGGTTCAGAG
GTTTTATAGGCATCCAGATAGCAC
>2497
ATAGGTATGAGGTGAGAGAGGCCAGGAGGTCTGGCTGCTCCCCCTCTAGACTTCGCAG
GTAAAACCCAAGGTGTGTGCCACGTAAAGGTTCAGAGGTTTTATAGGCATCCAGATAG
CACATGTGGGTCTCTGCCCCTGCT

>2498
GAGGCCAGGAGGTCTGGCTGCTCCCCCTCTAGACTTCGCAGGTAAAACCCAAGGTGT
GTGCCACGTAAAGGTTCAGAGGTTTTATAGGCATCCAGATAGCACATGTGGGTCTCTGC
CCCTGCTTGCAGCCTCCCTGGGGC
>2499
AGGAGGTCTGGCTGCTCCCCCTCTAGACTTCGCAGGTAAAACCCAAGGTGTGTGCCAC
GTAAAGGTTCAGAGGTTTTATAGGCATCCAGATAGCACATGTGGGTCTCTGCCCCTGCT
TGCAGCCTCCCTGGGGCAGCCAG
>2500
CTGCTCCCCCTCTAGACTTCGCAGGTAAAACCCAAGGTGTGTGCCACGTAAAGGTTCA
GAGGTTTTATAGGCATCCAGATAGCACATGTGGGTCTCTGCCCCTGCTTGCAGCCTCCC
TGGGGCAGCCAGCCAGGCTCAGG
>2501
TGCCACGTAAAGGTTCAGAGGTTTTATAGGCATCCAGATAGCACATGTGGGTCTCTGCC
CCTGCTTGCAGCCTCCCTGGGGCAGCCAGCCAGGCTCAGGGCAGGAAGCTGCTGATT
CTGGGTCACTTGGCTGCTCTTGCT
>2502
TCAGAGGTTTTATAGGCATCCAGATAGCACATGTGGGTCTCTGCCCCTGCTTGCAGCCT
CCCTGGGGCAGCCAGCCAGGCTCAGGGCAGGAAGCTGCTGATTCTGGGTCACTTGGC
TGCTCTTGCTTGCTGCTGCTGTGA
>2503
TTTATAGGCATCCAGATAGCACATGTGGGTCTCTGCCCCTGCTTGCAGCCTCCCTGGGG
CAGCCAGCCAGGCTCAGGGCAGGAAGCTGCTGATTCTGGGTCACTTGGCTGCTCTTGC
TTGCTGCTGCTGTGATTGTTCCT
>2504
AGGGCAGGAAGCTGCTGATTCTGGGTCACTTGGCTGCTCTTGCTTGCTGCTGCTGTGA
TTGTTCCTACAGTTAAGATCTTAGCTCCATAAGGGACATACGGAGGTCATCTAGGTCAT
CTCATGGCTCTCAGGCAAGAAAA
>2505
AGGAAGCTGCTGATTCTGGGTCACTTGGCTGCTCTTGCTTGCTGCTGCTGTGATTGTTC
CTACAGTTAAGATCTTAGCTCCATAAGGGACATACGGAGGTCATCTAGGTCATCTCATG
GCTCTCAGGCAAGAAAAGTAGC
>2506
TGGGTCACTTGGCTGCTCTTGCTTGCTGCTGCTGTGATTGTTCCTACAGTTAAGATCTTA
GCTCCATAAGGGACATACGGAGGTCATCTAGGTCATCTCATGGCTCTCAGGCAAGAAA
AGTAGCATTATTGCTCTCATGC
>2507
CTGCTCTTGCTTGCTGCTGCTGTGATTGTTCCTACAGTTAAGATCTTAGCTCCATAAGGG
ACATACGGAGGTCATCTAGGTCATCTCATGGCTCTCAGGCAAGAAAAGTAGCATTATTG
CTCTCATGCCCACTTTATGGA
>2508
CTTGCTGCTGCTGTGATTGTTCCTACAGTTAAGATCTTAGCTCCATAAGGGACATACGG
AGGTCATCTAGGTCATCTCATGGCTCTCAGGCAAGAAAAGTAGCATTATTGCTCTCATG
CCCACTTTATGGATAAGTGACC

>2509
TTCCTACAGTTAAGATCTTAGCTCCATAAGGGACATACGGAGGTCATCTAGGTCATCTCA
TGGCTCTCAGGCAAGAAAAGTAGCATTATTGCTCTCATGCCCACTTTATGGATAAGTGA
CCGAGGCCTGCAGAGGCAGAG
>2510
TCTAGGTCATCTCATGGCTCTCAGGCAAGAAAAGTAGCATTATTGCTCTCATGCCCACT
TTATGGATAAGTGACCGAGGCCTGCAGAGGCAGAGTGGCTATTCCAGCCATTAAATGT
GGCTCCCTGCTATTCTGTTCCCT
>2511
ATCTCATGGCTCTCAGGCAAGAAAAGTAGCATTATTGCTCTCATGCCCACTTTATGGATA
AGTGACCGAGGCCTGCAGAGGCAGAGTGGCTATTCCAGCCATTAAATGTGGCTCCCTG
CTATTCTGTTCCCTGCCAGGAT
>2512
GCATTATTGCTCTCATGCCCACTTTATGGATAAGTGACCGAGGCCTGCAGAGGCAGAGT
GGCTATTCCAGCCATTAAATGTGGCTCCCTGCTATTCTGTTCCCTGCCAGGATATTCTGT
GGATGGTTAGGGGAGGGGTCT
>2513
GAGGCCTGCAGAGGCAGAGTGGCTATTCCAGCCATTAAATGTGGCTCCCTGCTATTCTG
TTCCCTGCCAGGATATTCTGTGGATGGTTAGGGGAGGGGTCTTACCTCAGCTAACGAGG
TGCATTTTCTGTGGGGTGAGGC
>2514
GGCTATTCCAGCCATTAAATGTGGCTCCCTGCTATTCTGTTCCCTGCCAGGATATTCTGT
GGATGGTTAGGGGAGGGGTCTTACCTCAGCTAACGAGGTGCATTTTCTGTGGGGTGAG
GCCATTCTTGTCACTTAGGTGT
>2515
TTCCAGCCATTAAATGTGGCTCCCTGCTATTCTGTTCCCTGCCAGGATATTCTGTGGATG
GTTAGGGGAGGGGTCTTACCTCAGCTAACGAGGTGCATTTTCTGTGGGGTGAGGCCAT
TCTTGTCACTTAGGTGTCTGAG
>2516
TGTGGCTCCCTGCTATTCTGTTCCCTGCCAGGATATTCTGTGGATGGTTAGGGGAGGGG
TCTTACCTCAGCTAACGAGGTGCATTTTCTGTGGGGTGAGGCCATTCTTGTCACTTAGG
TGTCTGAGGCCTGATGGTGTTG
>2517
AGGATATTCTGTGGATGGTTAGGGGAGGGGTCTTACCTCAGCTAACGAGGTGCATTTTC
TGTGGGGTGAGGCCATTCTTGTCACTTAGGTGTCTGAGGCCTGATGGTGTTGGGGAAT
GTCCTGGTTCTATTCCCAGGGCT
>2518
TTAGGGGAGGGGTCTTACCTCAGCTAACGAGGTGCATTTTCTGTGGGGTGAGGCCATT
CTTGTCACTTAGGTGTCTGAGGCCTGATGGTGTTGGGGAATGTCCTGGTTCTATTCCCA
GGGCTGTAACGTGGGGGAAGGAT
>2519
GGGTCTTACCTCAGCTAACGAGGTGCATTTTCTGTGGGGTGAGGCCATTCTTGTCACTT
AGGTGTCTGAGGCCTGATGGTGTTGGGGAATGTCCTGGTTCTATTCCCAGGGCTGTAAC
GTGGGGGAAGGATGGAGGAAGA

>2520
TGAGGCCATTCTTGTCACTTAGGTGTCTGAGGCCTGATGGTGTTGGGGAATGTCCTGGT
TCTATTCCCAGGGCTGTAACGTGGGGGAAGGATGGAGGAAGACAGAATGGCCTCTGG
AACTTTTGGATCTTTCTGAGTTGA
>2521
TAGGTGTCTGAGGCCTGATGGTGTTGGGGAATGTCCTGGTTCTATTCCCAGGGCTGTAA
CGTGGGGGAAGGATGGAGGAAGACAGAATGGCCTCTGGAACTTTTGGATCTTTCTGA
GTTGAGGCCTTGTCTGGCCAACTC
>2522
CTGAGGCCTGATGGTGTTGGGGAATGTCCTGGTTCTATTCCCAGGGCTGTAACGTGGG
GGAAGGATGGAGGAAGACAGAATGGCCTCTGGAACTTTTGGATCTTTCTGAGTTGAGG
CCTTGTCTGGCCAACTCCGGGAGG
>2523
CCAGGGCTGTAACGTGGGGGAAGGATGGAGGAAGACAGAATGGCCTCTGGAACTTTT
GGATCTTTCTGAGTTGAGGCCTTGTCTGGCCAACTCCGGGAGGCTGCTGGCATGGCAA
GCAGGGTAGGGATGCCTGGCCTAGC
>2524
GCTGTAACGTGGGGGAAGGATGGAGGAAGACAGAATGGCCTCTGGAACTTTTGGATC
TTTCTGAGTTGAGGCCTTGTCTGGCCAACTCCGGGAGGCTGCTGGCATGGCAAGCAGG
GTAGGGATGCCTGGCCTAGCCAGGG
>2525
GGAAGACAGAATGGCCTCTGGAACTTTTGGATCTTTCTGAGTTGAGGCCTTGTCTGGC
CAACTCCGGGAGGCTGCTGGCATGGCAAGCAGGGTAGGGATGCCTGGCCTAGCCAGG
GGAGCTCCCTCTCTCCCCTCTGCTG
>2526
CTGGAACTTTTGGATCTTTCTGAGTTGAGGCCTTGTCTGGCCAACTCCGGGAGGCTGC
TGGCATGGCAAGCAGGGTAGGGATGCCTGGCCTAGCCAGGGGAGCTCCCTCTCTCCCC
TCTGCTGCCCCTCTTTCCACCCCC
>2527
GTTGAGGCCTTGTCTGGCCAACTCCGGGAGGCTGCTGGCATGGCAAGCAGGGTAGGG
ATGCCTGGCCTAGCCAGGGGAGCTCCCTCTCTCCCCTCTGCTGCCCCTCTTTCCACCCC
CATCATAAACACGTGTGGACTTTC
>2528
AGGCCTTGTCTGGCCAACTCCGGGAGGCTGCTGGCATGGCAAGCAGGGTAGGGATGC
CTGGCCTAGCCAGGGGAGCTCCCTCTCTCCCCTCTGCTGCCCCTCTTTCCACCCCCATC
ATAAACACGTGTGGACTTTCTGGT
>2529
TCCCTCTCTCCCCTCTGCTGCCCCTCTTTCCACCCCCATCATAAACACGTGTGGACTTTC
TGGTGGTGAGAAGATGGTTTTGGAGGGCTGGGCTGAGGTCTAGGCAGGCAAGAGCTT
CCAAATGTGTGGCTTTGATTCTC
>2530
CTCTCTCCCCTCTGCTGCCCCTCTTTCCACCCCCATCATAAACACGTGTGGACTTTCTG
GTGGTGAGAAGATGGTTTTGGAGGGCTGGGCTGAGGTCTAGGCAGGCAAGAGCTTCC
AAATGTGTGGCTTTGATTCTCAGC

>2531
CCCCATCATAAACACGTGTGGACTTTCTGGTGGTGAGAAGATGGTTTTGGAGGGCTGG
GCTGAGGTCTAGGCAGGCAAGAGCTTCCAAATGTGTGGCTTTGATTCTCAGCCTTTTCA
TCCCTCAATCCTGGGAGACTCAT
>2532
ATCATAAACACGTGTGGACTTTCTGGTGGTGAGAAGATGGTTTTGGAGGGCTGGGCTG
AGGTCTAGGCAGGCAAGAGCTTCCAAATGTGTGGCTTTGATTCTCAGCCTTTTCATCCC
TCAATCCTGGGAGACTCATTTTA
>2533
ACACGTGTGGACTTTCTGGTGGTGAGAAGATGGTTTTGGAGGGCTGGGCTGAGGTCTA
GGCAGGCAAGAGCTTCCAAATGTGTGGCTTTGATTCTCAGCCTTTTCATCCCTCAATCC
TGGGAGACTCATTTTAAAACTTA
>2534
GATGGTTTTGGAGGGCTGGGCTGAGGTCTAGGCAGGCAAGAGCTTCCAAATGTGTGGC
TTTGATTCTCAGCCTTTTCATCCCTCAATCCTGGGAGACTCATTTTAAAACTTAAAAAA
GGACTTCATTCTATTGGATGCTA
>2535
GTCTAGGCAGGCAAGAGCTTCCAAATGTGTGGCTTTGATTCTCAGCCTTTTCATCCCTC
AATCCTGGGAGACTCATTTTAAAACTTAAAAAAGGACTTCATTCTATTGGATGCTATGT
GGGCAGGCCCTGGGCTGTTGTA
>2536
AATGTGTGGCTTTGATTCTCAGCCTTTTCATCCCTCAATCCTGGGAGACTCATTTTAAAA
CTTAAAAAAGGACTTCATTCTATTGGATGCTATGTGGGCAGGCCCTGGGCTGTTGTAGA
GAGGCCTGAAACCCACTTCAG
>2537
CCCTCAATCCTGGGAGACTCATTTTAAAACTTAAAAAAGGACTTCATTCTATTGGATGC
TATGTGGGCAGGCCCTGGGCTGTTGTAGAGAGGCCTGAAACCCACTTCAGTTTCCTGG
ACAGGCTGGGAGTGTGTACCCAT
>2538
CTCATTTTAAAACTTAAAAAAGGACTTCATTCTATTGGATGCTATGTGGGCAGGCCCTG
GGCTGTTGTAGAGAGGCCTGAAACCCACTTCAGTTTCCTGGACAGGCTGGGAGTGTGT
ACCCATGGGTTCCCTGCTGCCCT
>2539
GACTTCATTCTATTGGATGCTATGTGGGCAGGCCCTGGGCTGTTGTAGAGAGGCCTGAA
ACCCACTTCAGTTTCCTGGACAGGCTGGGAGTGTGTACCCATGGGTTCCCTGCTGCCCT
ATTCCTGGAAGACACTAATATC
>2540
TATGTGGGCAGGCCCTGGGCTGTTGTAGAGAGGCCTGAAACCCACTTCAGTTTCCTGG
ACAGGCTGGGAGTGTGTACCCATGGGTTCCCTGCTGCCCTATTCCTGGAAGACACTAAT
ATCCCTCCCAAACTCTTGGTTGG
>2541
ACCCACTTCAGTTTCCTGGACAGGCTGGGAGTGTGTACCCATGGGTTCCCTGCTGCCCT
ATTCCTGGAAGACACTAATATCCCTCCCAAACTCTTGGTTGGTTTCTGCTTTTTTTCTGT
AGCATTTTGTCTTTGTGGGAA

>2542
GCTGCCCTATTCCTGGAAGACACTAATATCCCTCCCAAACTCTTGGTTGGTTTCTGCTTT
TTTTCTGTAGCATTTTGTCTTTGTGGGAAATTTTAAGAAACATATGTATGGCTTTTTGGT
TTCCACTTATTTATTTTCCC
>2543
TCTTTGTGGGAAATTTTAAGAAACATATGTATGGCTTTTTGGTTTCCACTTATTTATTTTC
CCCTCTGAGTTTCAGATTTCATCCTTCTCCACCCCTTCCCCCTGCCCTAAGGTGGGGCT
AGTGCAAATCGCAGTGTGTG
>2544
TGGGAAATTTTAAGAAACATATGTATGGCTTTTTGGTTTCCACTTATTTATTTTCCCCTCT
GAGTTTCAGATTTCATCCTTCTCCACCCCTTCCCCCTGCCCTAAGGTGGGGCTAGTGCA
AATCGCAGTGTGTGTGTAGT
>2545
TTTCCACTTATTTATTTTCCCCTCTGAGTTTCAGATTTCATCCTTCTCCACCCCTTCCCCC
TGCCCTAAGGTGGGGCTAGTGCAAATCGCAGTGTGTGTGTAGTGGGATGGTTAAAGAG
CCGTACATCCTCACACCTGGT
>2546
TTTATTTTCCCCTCTGAGTTTCAGATTTCATCCTTCTCCACCCCTTCCCCCTGCCCTAAG
GTGGGGCTAGTGCAAATCGCAGTGTGTGTGTAGTGGGATGGTTAAAGAGCCGTACATC
CTCACACCTGGTGACGCAGACT
>2547
GATTTCATCCTTCTCCACCCCTTCCCCCTGCCCTAAGGTGGGGCTAGTGCAAATCGCAG
TGTGTGTGTAGTGGGATGGTTAAAGAGCCGTACATCCTCACACCTGGTGACGCAGACT
AAGGCAATTAACCTTCTTTAGTA
>2548
CCACCCCTTCCCCCTGCCCTAAGGTGGGGCTAGTGCAAATCGCAGTGTGTGTGTAGTG
GGATGGTTAAAGAGCCGTACATCCTCACACCTGGTGACGCAGACTAAGGCAATTAACC
TTCTTTAGTAAAGACCCTTTCAGC
>2549
TAGTGCAAATCGCAGTGTGTGTGTAGTGGGATGGTTAAAGAGCCGTACATCCTCACAC
CTGGTGACGCAGACTAAGGCAATTAACCTTCTTTAGTAAAGACCCTTTCAGCTGGGTG
CGGTGGGAGGCACTTCGGGAGGCC
>2550
AGTGGGATGGTTAAAGAGCCGTACATCCTCACACCTGGTGACGCAGACTAAGGCAATT
AACCTTCTTTAGTAAAGACCCTTTCAGCTGGGTGCGGTGGGAGGCACTTCGGGAGGCC
GAGGTGGAAGGATTGCTTGAGCCC
>2551
GATGGTTAAAGAGCCGTACATCCTCACACCTGGTGACGCAGACTAAGGCAATTAACCT
TCTTTAGTAAAGACCCTTTCAGCTGGGTGCGGTGGGAGGCACTTCGGGAGGCCGAGGT
GGAAGGATTGCTTGAGCCCTGGAG
>2552
CACCTGGTGACGCAGACTAAGGCAATTAACCTTCTTTAGTAAAGACCCTTTCAGCTGG
GTGCGGTGGGAGGCACTTCGGGAGGCCGAGGTGGAAGGATTGCTTGAGCCCTGGAGT
TGGAGACCAGCTTGGGCAGCATGGT

>2553
AAGGCAATTAACCTTCTTTAGTAAAGACCCTTTCAGCTGGGTGCGGTGGGAGGCACTT
CGGGAGGCCGAGGTGGAAGGATTGCTTGAGCCCTGGAGTTGGAGACCAGCTTGGGCA
GCATGGTGAAACCACCTCTCTAAAA
>2554
TTAACCTTCTTTAGTAAAGACCCTTTCAGCTGGGTGCGGTGGGAGGCACTTCGGGAGG
CCGAGGTGGAAGGATTGCTTGAGCCCTGGAGTTGGAGACCAGCTTGGGCAGCATGGT
GAAACCACCTCTCTAAAAAAATATA
>2555
TAGTAAAGACCCTTTCAGCTGGGTGCGGTGGGAGGCACTTCGGGAGGCCGAGGTGGA
AGGATTGCTTGAGCCCTGGAGTTGGAGACCAGCTTGGGCAGCATGGTGAAACCACCT
CTCTAAAAAAATATAAAAATTAACGG
>2556
TCAGCTGGGTGCGGTGGGAGGCACTTCGGGAGGCCGAGGTGGAAGGATTGCTTGAGC
CCTGGAGTTGGAGACCAGCTTGGGCAGCATGGTGAAACCACCTCTCTAAAAAAATATA
AAAATTAACGGGACGTTGTGGCATG
>2557
GGCAGCATGGTGAAACCACCTCTCTAAAAAAATATAAAAATTAACGGGACGTTGTGGC
ATGCACCTATAGTCCCAGCTACTTGGGAGGCTGGGGCGCGAGAAGTGCTTGAACCTGA
GAGATGGAGACTGCGGTGAGGCGA
>2558
ATGGTGAAACCACCTCTCTAAAAAAATATAAAAATTAACGGGACGTTGTGGCATGCAC
CTATAGTCCCAGCTACTTGGGAGGCTGGGGCGCGAGAAGTGCTTGAACCTGAGAGATG
GAGACTGCGGTGAGGCGAGATTGC
>2559
ACCTCTCTAAAAAAATATAAAAATTAACGGGACGTTGTGGCATGCACCTATAGTCCCAG
CTACTTGGGAGGCTGGGGCGCGAGAAGTGCTTGAACCTGAGAGATGGAGACTGCGGT
GAGGCGAGATTGCACCACTGCACT
>2560
AATATAAAAATTAACGGGACGTTGTGGCATGCACCTATAGTCCCAGCTACTTGGGAGGC
TGGGGCGCGAGAAGTGCTTGAACCTGAGAGATGGAGACTGCGGTGAGGCGAGATTGC
ACCACTGCACTTCAGCTAGGTGAC
>2561
GACGTTGTGGCATGCACCTATAGTCCCAGCTACTTGGGAGGCTGGGGCGCGAGAAGTG
CTTGAACCTGAGAGATGGAGACTGCGGTGAGGCGAGATTGCACCACTGCACTTCAGCT
AGGTGACAGAGTGGGACCCTGTCT
>2562
CCCAGCTACTTGGGAGGCTGGGGCGCGAGAAGTGCTTGAACCTGAGAGATGGAGACT
GCGGTGAGGCGAGATTGCACCACTGCACTTCAGCTAGGTGACAGAGTGGGACCCTGT
CTCAAAACAAAACAAAACAACAACAA
>2563
GGGCGCGAGAAGTGCTTGAACCTGAGAGATGGAGACTGCGGTGAGGCGAGATTGCAC
CACTGCACTTCAGCTAGGTGACAGAGTGGGACCCTGTCTCAAAACAAAACAAAACAA
CAACAAAAAAAGCATGTTTTCCTGTC

>2564
GTGCTTGAACCTGAGAGATGGAGACTGCGGTGAGGCGAGATTGCACCACTGCACTTC
AGCTAGGTGACAGAGTGGGACCCTGTCTCAAAACAAAACAAAACAACAACAAAAAA
AGCATGTTTTCCTGTCTTTCCCTTCCT
>2565
CCACTGCACTTCAGCTAGGTGACAGAGTGGGACCCTGTCTCAAAACAAAACAAAACA
ACAACAAAAAAAGCATGTTTTCCTGTCTTTCCCTTCCTCTCCTCCACCCCCTGGCATGA
ACTCTCAACTCTTAGCCTGTGGAT
>2566
CAAAAAAAGCATGTTTTCCTGTCTTTCCCTTCCTCTCCTCCACCCCCTGGCATGAACTC
TCAACTCTTAGCCTGTGGATGTCCGTTTCTATAAATGCTGGTGCTCGGTGGTCACCGTA
GACGTCCGAGCAGCCCCTGGGG
>2567
GCATGAACTCTCAACTCTTAGCCTGTGGATGTCCGTTTCTATAAATGCTGGTGCTCGGT
GGTCACCGTAGACGTCCGAGCAGCCCCTGGGGCTTTGGCTTGTGTTTTCTCTGTACTTG
TGAGGGTGGTTGCCACTTCTGG
>2568
CTCAACTCTTAGCCTGTGGATGTCCGTTTCTATAAATGCTGGTGCTCGGTGGTCACCGT
AGACGTCCGAGCAGCCCCTGGGGCTTTGGCTTGTGTTTTCTCTGTACTTGTGAGGGTG
GTTGCCACTTCTGGCCTTCTTTG
>2569
GCTCGGTGGTCACCGTAGACGTCCGAGCAGCCCCTGGGGCTTTGGCTTGTGTTTTCTC
TGTACTTGTGAGGGTGGTTGCCACTTCTGGCCTTCTTTGTGCTTTCTTTTTCTTTCCTTT
CCCCAATCTTTTGTTCCTCCCT
>2570
CAATCTTTTGTTCCTCCCTCCCTCCCTCCCTCCCTTCCTCCCTTCCTTCCTTCTGCTTTCC
TGTTTAAAGTATATGGCTGCACCTAGCATGACACAGTTCCTATTTATTCCTCAGACTTGC
TGGGACAGTCTCCTCTGTA
>2571
CTCCCTCCCTCCCTCCCTTCCTCCCTTCCTTCCTTCTGCTTTCCTGTTTAAAGTATATGGC
TGCACCTAGCATGACACAGTTCCTATTTATTCCTCAGACTTGCTGGGACAGTCTCCTCT
GTATGCGGCGACTGAGGGTG
>2572
CCCTCCCTTCCTCCCTTCCTTCCTTCTGCTTTCCTGTTTAAAGTATATGGCTGCACCTAG
CATGACACAGTTCCTATTTATTCCTCAGACTTGCTGGGACAGTCTCCTCTGTATGCGGC
GACTGAGGGTGGGTTCCAGTG
>2573
CCTGTTTAAAGTATATGGCTGCACCTAGCATGACACAGTTCCTATTTATTCCTCAGACTT
GCTGGGACAGTCTCCTCTGTATGCGGCGACTGAGGGTGGGTTCCAGTGCCCCAGCCCA
CTCCCCTTCCCCTTGTTTTTGT
>2574
CTAGCATGACACAGTTCCTATTTATTCCTCAGACTTGCTGGGACAGTCTCCTCTGTATGC
GGCGACTGAGGGTGGGTTCCAGTGCCCCAGCCCACTCCCCTTCCCCTTGTTTTTGTCC
ACTTGGCTGGACAAAGAGAACA

>2575
AGTTCCTATTTATTCCTCAGACTTGCTGGGACAGTCTCCTCTGTATGCGGCGACTGAGG
GTGGGTTCCAGTGCCCCAGCCCACTCCCCTTCCCCTTGTTTTTGTCCACTTGGCTGGAC
AAAGAGAACAAAACCAAATGGC
>2576
ACTGAGGGTGGGTTCCAGTGCCCCAGCCCACTCCCCTTCCCCTTGTTTTTGTCCACTTG
GCTGGACAAAGAGAACAAAACCAAATGGCCTCCCCTCCCCCTCTCCTAAACCGGTCA
AGCAGTGGTATTCTGGGATGATTT
>2577
TGAGGGTGGGTTCCAGTGCCCCAGCCCACTCCCCTTCCCCTTGTTTTTGTCCACTTGGC
TGGACAAAGAGAACAAAACCAAATGGCCTCCCCTCCCCCTCTCCTAAACCGGTCAAG
CAGTGGTATTCTGGGATGATTTTC
>2578
TTGTCCACTTGGCTGGACAAAGAGAACAAAACCAAATGGCCTCCCCTCCCCCTCTCCT
AAACCGGTCAAGCAGTGGTATTCTGGGATGATTTTCCCTGCAACCATTTAAACAAGTGC
TTTGATGCTTACGAGTCATTGGA
>2579
TCCACTTGGCTGGACAAAGAGAACAAAACCAAATGGCCTCCCCTCCCCCTCTCCTAAA
CCGGTCAAGCAGTGGTATTCTGGGATGATTTTCCCTGCAACCATTTAAACAAGTGCTTT
GATGCTTACGAGTCATTGGAATG
>2580
CCTCCCCCTCTCCTAAACCGGTCAAGCAGTGGTATTCTGGGATGATTTTCCCTGCAACC
ATTTAAACAAGTGCTTTGATGCTTACGAGTCATTGGAATGACCAGGCCTGTGCGCCCCT
TCTCACTGACACCCTTAGACCA
>2581
CGGTCAAGCAGTGGTATTCTGGGATGATTTTCCCTGCAACCATTTAAACAAGTGCTTTG
ATGCTTACGAGTCATTGGAATGACCAGGCCTGTGCGCCCCTTCTCACTGACACCCTTAG
ACCAGGCTGGGCCTCACCCTTT
>2582
TTCTGGGATGATTTTCCCTGCAACCATTTAAACAAGTGCTTTGATGCTTACGAGTCATTG
GAATGACCAGGCCTGTGCGCCCCTTCTCACTGACACCCTTAGACCAGGCTGGGCCTCA
CCCTTTTGTCTTGCTGCCCCTC
>2583
CAAGTGCTTTGATGCTTACGAGTCATTGGAATGACCAGGCCTGTGCGCCCCTTCTCACT
GACACCCTTAGACCAGGCTGGGCCTCACCCTTTTGTCTTGCTGCCCCTCGGTGCCCAG
TGGGCGTGGGCAGTAGGACTGAG
>2584
GCTTTGATGCTTACGAGTCATTGGAATGACCAGGCCTGTGCGCCCCTTCTCACTGACAC
CCTTAGACCAGGCTGGGCCTCACCCTTTTGTCTTGCTGCCCCTCGGTGCCCAGTGGGC
GTGGGCAGTAGGACTGAGCAGAG
>2585
CCCCTTCTCACTGACACCCTTAGACCAGGCTGGGCCTCACCCTTTTGTCTTGCTGCCCC
TCGGTGCCCAGTGGGCGTGGGCAGTAGGACTGAGCAGAGGGGCCGAGGCTGTGCTGG
CCGGCAGCTCTGTTACAGTAGAGT

>2586
ACACCCTTAGACCAGGCTGGGCCTCACCCTTTTGTCTTGCTGCCCCTCGGTGCCCAGT
GGGCGTGGGCAGTAGGACTGAGCAGAGGGGCCGAGGCTGTGCTGGCCGGCAGCTCTG
TTACAGTAGAGTGGAAGGAGGTCCC
>2587
CCCTTAGACCAGGCTGGGCCTCACCCTTTTGTCTTGCTGCCCCTCGGTGCCCAGTGGG
CGTGGGCAGTAGGACTGAGCAGAGGGGCCGAGGCTGTGCTGGCCGGCAGCTCTGTTA
CAGTAGAGTGGAAGGAGGTCCCGGC
>2588
AGGCTGGGCCTCACCCTTTTGTCTTGCTGCCCCTCGGTGCCCAGTGGGCGTGGGCAGT
AGGACTGAGCAGAGGGGCCGAGGCTGTGCTGGCCGGCAGCTCTGTTACAGTAGAGTG
GAAGGAGGTCCCGGCCAGAGGGGCA
>2589
TGCCCCTCGGTGCCCAGTGGGCGTGGGCAGTAGGACTGAGCAGAGGGGCCGAGGCTG
TGCTGGCCGGCAGCTCTGTTACAGTAGAGTGGAAGGAGGTCCCGGCCAGAGGGGCAG
GTCAAACAGGCCTCTTTTATTTACAG
>2590
CAGTGGGCGTGGGCAGTAGGACTGAGCAGAGGGGCCGAGGCTGTGCTGGCCGGCAG
CTCTGTTACAGTAGAGTGGAAGGAGGTCCCGGCCAGAGGGGCAGGTCAAACAGGCCT
CTTTTATTTACAGTGCTCTCTTTCACG
>2591
GTGGGCGTGGGCAGTAGGACTGAGCAGAGGGGCCGAGGCTGTGCTGGCCGGCAGCTC
TGTTACAGTAGAGTGGAAGGAGGTCCCGGCCAGAGGGGCAGGTCAAACAGGCCTCTT
TTATTTACAGTGCTCTCTTTCACGGC
>2592
GTGGGCAGTAGGACTGAGCAGAGGGGCCGAGGCTGTGCTGGCCGGCAGCTCTGTTAC
AGTAGAGTGGAAGGAGGTCCCGGCCAGAGGGGCAGGTCAAACAGGCCTCTTTTATTT
ACAGTGCTCTCTTTCACGGCCTCACT
>2593
TGAGCAGAGGGGCCGAGGCTGTGCTGGCCGGCAGCTCTGTTACAGTAGAGTGGAAGG
AGGTCCCGGCCAGAGGGGCAGGTCAAACAGGCCTCTTTTATTTACAGTGCTCTCTTTC
ACGGCCTCACTGAAACAGGAGACCC
>2594
GCTGTGCTGGCCGGCAGCTCTGTTACAGTAGAGTGGAAGGAGGTCCCGGCCAGAGGG
GCAGGTCAAACAGGCCTCTTTTATTTACAGTGCTCTCTTTCACGGCCTCACTGAAACAG
GAGACCCGAGAGGCTCCGGTGAAA
>2595
CGGCCAGAGGGGCAGGTCAAACAGGCCTCTTTTATTTACAGTGCTCTCTTTCACGGCC
TCACTGAAACAGGAGACCCGAGAGGCTCCGGTGAAAGGGCACTGTGTTTGTGCATTC
AGGCCTTCCAGGGCTGGCTACAAAG
>2596
CCAGAGGGGCAGGTCAAACAGGCCTCTTTTATTTACAGTGCTCTCTTTCACGGCCTCA
CTGAAACAGGAGACCCGAGAGGCTCCGGTGAAAGGGCACTGTGTTTGTGCATTCAGG
CCTTCCAGGGCTGGCTACAAAGGTG

>2597
CTTTCACGGCCTCACTGAAACAGGAGACCCGAGAGGCTCCGGTGAAAGGGCACTGTG
TTTGTGCATTCAGGCCTTCCAGGGCTGGCTACAAAGGTGGGGGCACCCGGAGCACTGA
AGGGGCTTTTTGTCATTGGCAGTTT
>2598
CCTCACTGAAACAGGAGACCCGAGAGGCTCCGGTGAAAGGGCACTGTGTTTGTGCAT
TCAGGCCTTCCAGGGCTGGCTACAAAGGTGGGGGCACCCGGAGCACTGAAGGGGCTT
TTTGTCATTGGCAGTTTGGCTTAGGG
>2599
CGGTGAAAGGGCACTGTGTTTGTGCATTCAGGCCTTCCAGGGCTGGCTACAAAGGTGG
GGGCACCCGGAGCACTGAAGGGGCTTTTTGTCATTGGCAGTTTGGCTTAGGGGAGAA
GCGAGGGAGAGCCAGTGTGGGAGCC
>2600
GGGCACTGTGTTTGTGCATTCAGGCCTTCCAGGGCTGGCTACAAAGGTGGGGGCACCC
GGAGCACTGAAGGGGCTTTTTGTCATTGGCAGTTTGGCTTAGGGGAGAAGCGAGGGA
GAGCCAGTGTGGGAGCCGCCTCTAG
>2601
CAGGCCTTCCAGGGCTGGCTACAAAGGTGGGGGCACCCGGAGCACTGAAGGGGCTTT
TTGTCATTGGCAGTTTGGCTTAGGGGAGAAGCGAGGGAGAGCCAGTGTGGGAGCCGC
CTCTAGGATTGTGAGCAAGTGCCTGT
>2602
AGGGCTGGCTACAAAGGTGGGGGCACCCGGAGCACTGAAGGGGCTTTTTGTCATTGG
CAGTTTGGCTTAGGGGAGAAGCGAGGGAGAGCCAGTGTGGGAGCCGCCTCTAGGATT
GTGAGCAAGTGCCTGTTTAGACAGCT
>2603
AGCACTGAAGGGGCTTTTTGTCATTGGCAGTTTGGCTTAGGGGAGAAGCGAGGGAGA
GCCAGTGTGGGAGCCGCCTCTAGGATTGTGAGCAAGTGCCTGTTTAGACAGCTCCACG
CTGCTCCTTAAAACCTATTTTGTGG
>2604
GGGCTTTTTGTCATTGGCAGTTTGGCTTAGGGGAGAAGCGAGGGAGAGCCAGTGTGG
GAGCCGCCTCTAGGATTGTGAGCAAGTGCCTGTTTAGACAGCTCCACGCTGCTCCTTA
AAACCTATTTTGTGGTCTGGGGTTG
>2605
GTCATTGGCAGTTTGGCTTAGGGGAGAAGCGAGGGAGAGCCAGTGTGGGAGCCGCCT
CTAGGATTGTGAGCAAGTGCCTGTTTAGACAGCTCCACGCTGCTCCTTAAAACCTATTT
TGTGGTCTGGGGTTGGGGCGGGGG
>2606
GCTTAGGGGAGAAGCGAGGGAGAGCCAGTGTGGGAGCCGCCTCTAGGATTGTGAGCA
AGTGCCTGTTTAGACAGCTCCACGCTGCTCCTTAAAACCTATTTTGTGGTCTGGGGTTG
GGGCGGGGGCACACTGCCTCGATC
>2607
CCTTAAAACCTATTTTGTGGTCTGGGGTTGGGGCGGGGGCACACTGCCTCGATCCTCTT
GTTGTGTGGAGCAAAGTGATTCCTCTCTTCTTGTGTTCTCCAGATGGTTCCGTTGTCCT
CACATCCATTGTGGTTGAGGAT

>2608
AAACCTATTTTGTGGTCTGGGGTTGGGGCGGGGGCACACTGCCTCGATCCTCTTGTTGT
GTGGAGCAAAGTGATTCCTCTCTTCTTGTGTTCTCCAGATGGTTCCGTTGTCCTCACAT
CCATTGTGGTTGAGGATACAGT
>2609
GCGGGGGCACACTGCCTCGATCCTCTTGTTGTGTGGAGCAAAGTGATTCCTCTCTTCTT
GTGTTCTCCAGATGGTTCCGTTGTCCTCACATCCATTGTGGTTGAGGATACAGTGCAGA
GAAGACCCCGCTGTCCTCTGCT
>2610
GAGCAAAGTGATTCCTCTCTTCTTGTGTTCTCCAGATGGTTCCGTTGTCCTCACATCCAT
TGTGGTTGAGGATACAGTGCAGAGAAGACCCCGCTGTCCTCTGCTCCCCCTCCTTTTCT
CTCCAAAGAGAAGTTTCAGGG
>2611
GTGATTCCTCTCTTCTTGTGTTCTCCAGATGGTTCCGTTGTCCTCACATCCATTGTGGTT
GAGGATACAGTGCAGAGAAGACCCCGCTGTCCTCTGCTCCCCCTCCTTTTCTCTCCAA
AGAGAAGTTTCAGGGACAGCAA
>2612
TCTTCTTGTGTTCTCCAGATGGTTCCGTTGTCCTCACATCCATTGTGGTTGAGGATACAG
TGCAGAGAAGACCCCGCTGTCCTCTGCTCCCCCTCCTTTTCTCTCCAAAGAGAAGTTT
CAGGGACAGCAATAGACTTCTG
>2613
CATTGTGGTTGAGGATACAGTGCAGAGAAGACCCCGCTGTCCTCTGCTCCCCCTCCTTT
TCTCTCCAAAGAGAAGTTTCAGGGACAGCAATAGACTTCTGTGGGGCCTTTGTTCCTT
GTAGCTTTCATTTTTCAATTCTT
>2614
TTGTGGTTGAGGATACAGTGCAGAGAAGACCCCGCTGTCCTCTGCTCCCCCTCCTTTTC
TCTCCAAAGAGAAGTTTCAGGGACAGCAATAGACTTCTGTGGGGCCTTTGTTCCTTGT
AGCTTTCATTTTTCAATTCTTCT
>2615
AGGATACAGTGCAGAGAAGACCCCGCTGTCCTCTGCTCCCCCTCCTTTTCTCTCCAAA
GAGAAGTTTCAGGGACAGCAATAGACTTCTGTGGGGCCTTTGTTCCTTGTAGCTTTCAT
TTTTCAATTCTTCTTCTCCCTGT
>2616
CCTCCTTTTCTCTCCAAAGAGAAGTTTCAGGGACAGCAATAGACTTCTGTGGGGCCTT
TGTTCCTTGTAGCTTTCATTTTTCAATTCTTCTTCTCCCTGTATTATCTTGCTAGGCCTTA
GTTTGTGCATCTCTAAAATGG
>2617
CTTCTGTGGGGCCTTTGTTCCTTGTAGCTTTCATTTTTCAATTCTTCTTCTCCCTGTATTA
TCTTGCTAGGCCTTAGTTTGTGCATCTCTAAAATGGGCTGGGAGGTGCTAGCCCATACC
TAATATATAGGGCCGTCGTA
>2618
GGGGCCTTTGTTCCTTGTAGCTTTCATTTTTCAATTCTTCTTCTCCCTGTATTATCTTGCT
AGGCCTTAGTTTGTGCATCTCTAAAATGGGCTGGGAGGTGCTAGCCCATACCTAATATAT
AGGGCCGTCGTAAGGCTTA

>2619
TTTCAATTCTTCTTCTCCCTGTATTATCTTGCTAGGCCTTAGTTTGTGCATCTCTAAAATG
GGCTGGGAGGTGCTAGCCCATACCTAATATATAGGGCCGTCGTAAGGCTTAGGTCCCAT
AACTCATGGGGTTGGCTTGC
>2620
TCTTCTTCTCCCTGTATTATCTTGCTAGGCCTTAGTTTGTGCATCTCTAAAATGGGCTGG
GAGGTGCTAGCCCATACCTAATATATAGGGCCGTCGTAAGGCTTAGGTCCCATAACTCAT
GGGGTTGGCTTGCACAGGGC
>2621
ATCTTGCTAGGCCTTAGTTTGTGCATCTCTAAAATGGGCTGGGAGGTGCTAGCCCATAC
CTAATATATAGGGCCGTCGTAAGGCTTAGGTCCCATAACTCATGGGGTTGGCTTGCACA
GGGCTCTCTCCTTTCAGACTGG
>2622
TAGCCCATACCTAATATATAGGGCCGTCGTAAGGCTTAGGTCCCATAACTCATGGGGTTG
GCTTGCACAGGGCTCTCTCCTTTCAGACTGGTCAGGCCATGGTCCTTTTATGAGTCTGA
AGAGCCAAAATGAAGACCTCA
>2623
TATAGGGCCGTCGTAAGGCTTAGGTCCCATAACTCATGGGGTTGGCTTGCACAGGGCTC
TCTCCTTTCAGACTGGTCAGGCCATGGTCCTTTTATGAGTCTGAAGAGCCAAAATGAA
GACCTCACTTTCTAGTATCCCCA
>2624
ATAACTCATGGGGTTGGCTTGCACAGGGCTCTCTCCTTTCAGACTGGTCAGGCCATGGT
CCTTTTATGAGTCTGAAGAGCCAAAATGAAGACCTCACTTTCTAGTATCCCCAATCTTC
CTCCCACCTTTCCCTGGCTCTT
>2625
ATGGGGTTGGCTTGCACAGGGCTCTCTCCTTTCAGACTGGTCAGGCCATGGTCCTTTTA
TGAGTCTGAAGAGCCAAAATGAAGACCTCACTTTCTAGTATCCCCAATCTTCCTCCCAC
CTTTCCCTGGCTCTTGAGGGTT
>2626
GCACAGGGCTCTCTCCTTTCAGACTGGTCAGGCCATGGTCCTTTTATGAGTCTGAAGA
GCCAAAATGAAGACCTCACTTTCTAGTATCCCCAATCTTCCTCCCACCTTTCCCTGGCT
CTTGAGGGTTCTTTGGGCATGTT
>2627
GAAGAGCCAAAATGAAGACCTCACTTTCTAGTATCCCCAATCTTCCTCCCACCTTTCCC
TGGCTCTTGAGGGTTCTTTGGGCATGTTCCCCTCTGGTCTCCATTATTATATAGGGAAGA
GAAATTTTCCAGAAGAATATT
>2628
TCCTCCCACCTTTCCCTGGCTCTTGAGGGTTCTTTGGGCATGTTCCCCTCTGGTCTCCAT
TATTATATAGGGAAGAGAAATTTTCCAGAAGAATATTTGAAGGATTAAAGTTTGAGGTT
GTAGAATATGTGAAAACACAC
>2629
CCACCTTTCCCTGGCTCTTGAGGGTTCTTTGGGCATGTTCCCCTCTGGTCTCCATTATTA
TATAGGGAAGAGAAATTTTCCAGAAGAATATTTGAAGGATTAAAGTTTGAGGTTGTAGA
ATATGTGAAAACACACACACA

>2630
GCTCTTGAGGGTTCTTTGGGCATGTTCCCCTCTGGTCTCCATTATTATATAGGGAAGAGA
AATTTTCCAGAAGAATATTTGAAGGATTAAAGTTTGAGGTTGTAGAATATGTGAAAACA
CACACACACCACACCACACCC
>2631
TGTTCCCCTCTGGTCTCCATTATTATATAGGGAAGAGAAATTTTCCAGAAGAATATTTGA
AGGATTAAAGTTTGAGGTTGTAGAATATGTGAAAACACACACACACCACACCACACCC
CAAAATCACCCTTTTAAAGAAG
>2632
TCTCCATTATTATATAGGGAAGAGAAATTTTCCAGAAGAATATTTGAAGGATTAAAGTTT
GAGGTTGTAGAATATGTGAAAACACACACACACCACACCACACCCCAAAATCACCCTT
TTAAAGAAGATTAAAAAACCAC
>2633
AAGTTTGAGGTTGTAGAATATGTGAAAACACACACACACCACACCACACCCCAAAATC
ACCCTTTTAAAGAAGATTAAAAAACCACCAGTGCCTGTCCTACATTGGTAAACTCTGG
CAAGCCTCATAATGTTGACTTTAC
>2634
TTTGAGGTTGTAGAATATGTGAAAACACACACACACCACACCACACCCCAAAATCACC
CTTTTAAAGAAGATTAAAAAACCACCAGTGCCTGTCCTACATTGGTAAACTCTGGCAA
GCCTCATAATGTTGACTTTACTGA
>2635
ATGTGAAAACACACACACACCACACCACACCCCAAAATCACCCTTTTAAAGAAGATTA
AAAAACCACCAGTGCCTGTCCTACATTGGTAAACTCTGGCAAGCCTCATAATGTTGACT
TTACTGACCTGCCTGACTTCAGT
>2636
CCAAAATCACCCTTTTAAAGAAGATTAAAAAACCACCAGTGCCTGTCCTACATTGGTA
AACTCTGGCAAGCCTCATAATGTTGACTTTACTGACCTGCCTGACTTCAGTGGCCGAGT
CTACATGGGGTGTTAAGGCCCCA
>2637
GTGCCTGTCCTACATTGGTAAACTCTGGCAAGCCTCATAATGTTGACTTTACTGACCTG
CCTGACTTCAGTGGCCGAGTCTACATGGGGTGTTAAGGCCCCACGTGTTTTTGCCTGTG
CCCTTTAATTGTATCTTCAGGA
>2638
CCTACATTGGTAAACTCTGGCAAGCCTCATAATGTTGACTTTACTGACCTGCCTGACTT
CAGTGGCCGAGTCTACATGGGGTGTTAAGGCCCCACGTGTTTTTGCCTGTGCCCTTTAA
TTGTATCTTCAGGACCATATTT
>2639
AGTGGCCGAGTCTACATGGGGTGTTAAGGCCCCACGTGTTTTTGCCTGTGCCCTTTAAT
TGTATCTTCAGGACCATATTTTCAGAACCAGAAATCACAGCCACAGTTTGGCAGTGGC
ACAGAATGGTTCAATTTAGGCCT
>2640
CATGGGGTGTTAAGGCCCCACGTGTTTTTGCCTGTGCCCTTTAATTGTATCTTCAGGAC
CATATTTTCAGAACCAGAAATCACAGCCACAGTTTGGCAGTGGCACAGAATGGTTCAA
TTTAGGCCTATCCCAGAGAGGCT

>2641
GTGTTTTTGCCTGTGCCCTTTAATTGTATCTTCAGGACCATATTTTCAGAACCAGAAATC
ACAGCCACAGTTTGGCAGTGGCACAGAATGGTTCAATTTAGGCCTATCCCAGAGAGGC
TTGGGTTGTCTGTGAAGTTCTG
>2642
GCCTGTGCCCTTTAATTGTATCTTCAGGACCATATTTTCAGAACCAGAAATCACAGCCA
CAGTTTGGCAGTGGCACAGAATGGTTCAATTTAGGCCTATCCCAGAGAGGCTTGGGTT
GTCTGTGAAGTTCTGGGTGGTCT
>2643
CCTTTAATTGTATCTTCAGGACCATATTTTCAGAACCAGAAATCACAGCCACAGTTTGG
CAGTGGCACAGAATGGTTCAATTTAGGCCTATCCCAGAGAGGCTTGGGTTGTCTGTGA
AGTTCTGGGTGGTCTGTATTGTG
>2644
TTTTCAGAACCAGAAATCACAGCCACAGTTTGGCAGTGGCACAGAATGGTTCAATTTA
GGCCTATCCCAGAGAGGCTTGGGTTGTCTGTGAAGTTCTGGGTGGTCTGTATTGTGGA
GAGGTGAACACCGTATTGCTGGGG
>2645
CACAGTTTGGCAGTGGCACAGAATGGTTCAATTTAGGCCTATCCCAGAGAGGCTTGGG
TTGTCTGTGAAGTTCTGGGTGGTCTGTATTGTGGAGAGGTGAACACCGTATTGCTGGG
GGTGTGGAGGGGGGATGTTGCTGT
>2646
GGTTCAATTTAGGCCTATCCCAGAGAGGCTTGGGTTGTCTGTGAAGTTCTGGGTGGTCT
GTATTGTGGAGAGGTGAACACCGTATTGCTGGGGGTGTGGAGGGGGGATGTTGCTGTT
TCCCTGCTACAGAAAGGCCAGGC
>2647
GGGTTGTCTGTGAAGTTCTGGGTGGTCTGTATTGTGGAGAGGTGAACACCGTATTGCT
GGGGGTGTGGAGGGGGGATGTTGCTGTTTCCCTGCTACAGAAAGGCCAGGCTTCCTTT
TCAGCCTTTTGTTCCCCGATTCCC
>2648
GTATTGTGGAGAGGTGAACACCGTATTGCTGGGGGTGTGGAGGGGGGATGTTGCTGTT
TCCCTGCTACAGAAAGGCCAGGCTTCCTTTTCAGCCTTTTGTTCCCCGATTCCCCTCTC
TTCCTCCACTACACTCAGCCCTG
>2649
AGAGGTGAACACCGTATTGCTGGGGGTGTGGAGGGGGGATGTTGCTGTTTCCCTGCTA
CAGAAAGGCCAGGCTTCCTTTTCAGCCTTTTGTTCCCCGATTCCCCTCTCTTCCTCCAC
TACACTCAGCCCTGCTGCTGCTT
>2650
TACAGAAAGGCCAGGCTTCCTTTTCAGCCTTTTGTTCCCCGATTCCCCTCTCTTCCTCC
ACTACACTCAGCCCTGCTGCTGCTTCCAAGGGACAACTCCCTCTCTTGTCCTTGCCCAT
CTTTGAGAGAGGCGGTGAATAA
>2651
CTTTTCAGCCTTTTGTTCCCCGATTCCCCTCTCTTCCTCCACTACACTCAGCCCTGCTGC
TGCTTCCAAGGGACAACTCCCTCTCTTGTCCTTGCCCATCTTTGAGAGAGGCGGTGAAT
AAACACCAAGGCAAGAAGAAG

>2652
CTCCACTACACTCAGCCCTGCTGCTGCTTCCAAGGGACAACTCCCTCTCTTGTCCTTGC
CCATCTTTGAGAGAGGCGGTGAATAAACACCAAGGCAAGAAGAAGCAACGGGACCCC
TAGAAGGAGATGAGTGTGTGGCCC
>2653
CCACTACACTCAGCCCTGCTGCTGCTTCCAAGGGACAACTCCCTCTCTTGTCCTTGCCC
ATCTTTGAGAGAGGCGGTGAATAAACACCAAGGCAAGAAGAAGCAACGGGACCCCTA
GAAGGAGATGAGTGTGTGGCCCAA
>2654
TCCAAGGGACAACTCCCTCTCTTGTCCTTGCCCATCTTTGAGAGAGGCGGTGAATAAA
CACCAAGGCAAGAAGAAGCAACGGGACCCCTAGAAGGAGATGAGTGTGTGGCCCAA
GGATGGTGGAGCATTGTCCTGAAAGG
>2655
AAGGGACAACTCCCTCTCTTGTCCTTGCCCATCTTTGAGAGAGGCGGTGAATAAACAC
CAAGGCAAGAAGAAGCAACGGGACCCCTAGAAGGAGATGAGTGTGTGGCCCAAGGA
TGGTGGAGCATTGTCCTGAAAGGTAG
>2656
GGACAACTCCCTCTCTTGTCCTTGCCCATCTTTGAGAGAGGCGGTGAATAAACACCAA
GGCAAGAAGAAGCAACGGGACCCCTAGAAGGAGATGAGTGTGTGGCCCAAGGATGGT
GGAGCATTGTCCTGAAAGGTAGAAC
>2657
TTGTCCTTGCCCATCTTTGAGAGAGGCGGTGAATAAACACCAAGGCAAGAAGAAGCA
ACGGGACCCCTAGAAGGAGATGAGTGTGTGGCCCAAGGATGGTGGAGCATTGTCCTG
AAAGGTAGAACCAAGACTATAGCCTC
>2658
GGCGGTGAATAAACACCAAGGCAAGAAGAAGCAACGGGACCCCTAGAAGGAGATGA
GTGTGTGGCCCAAGGATGGTGGAGCATTGTCCTGAAAGGTAGAACCAAGACTATAGCC
TCCGAGGCTCTGTTTACCATATTCTT
>2659
GAAGAAGCAACGGGACCCCTAGAAGGAGATGAGTGTGTGGCCCAAGGATGGTGGAGC
ATTGTCCTGAAAGGTAGAACCAAGACTATAGCCTCCGAGGCTCTGTTTACCATATTCTT
GGCTCTTGTGCCACTTCTGTGTCC
>2660
AAGCAACGGGACCCCTAGAAGGAGATGAGTGTGTGGCCCAAGGATGGTGGAGCATTG
TCCTGAAAGGTAGAACCAAGACTATAGCCTCCGAGGCTCTGTTTACCATATTCTTGGCT
CTTGTGCCACTTCTGTGTCCAGCA
>2661
CTAGAAGGAGATGAGTGTGTGGCCCAAGGATGGTGGAGCATTGTCCTGAAAGGTAGA
ACCAAGACTATAGCCTCCGAGGCTCTGTTTACCATATTCTTGGCTCTTGTGCCACTTCTG
TGTCCAGCAGCTGGAGCTTGTAG
>2662
AGAACCAAGACTATAGCCTCCGAGGCTCTGTTTACCATATTCTTGGCTCTTGTGCCACT
TCTGTGTCCAGCAGCTGGAGCTTGTAGAAGAAAGATGGGAAAGACATAGTACAGGAA
ATGTTCCCATTCAGCCAGCACTGA

>2663
ACCAAGACTATAGCCTCCGAGGCTCTGTTTACCATATTCTTGGCTCTTGTGCCACTTCTG
TGTCCAGCAGCTGGAGCTTGTAGAAGAAAGATGGGAAAGACATAGTACAGGAAATGT
TCCCATTCAGCCAGCACTGATTT
>2664
TACCATATTCTTGGCTCTTGTGCCACTTCTGTGTCCAGCAGCTGGAGCTTGTAGAAGAA
AGATGGGAAAGACATAGTACAGGAAATGTTCCCATTCAGCCAGCACTGATTTATCTGCT
GCCTGGTTTCTCAGGAGGCCTA
>2665
ATTCTTGGCTCTTGTGCCACTTCTGTGTCCAGCAGCTGGAGCTTGTAGAAGAAAGATG
GGAAAGACATAGTACAGGAAATGTTCCCATTCAGCCAGCACTGATTTATCTGCTGCCTG
GTTTCTCAGGAGGCCTAATCTGT
>2666
TGGCTCTTGTGCCACTTCTGTGTCCAGCAGCTGGAGCTTGTAGAAGAAAGATGGGAAA
GACATAGTACAGGAAATGTTCCCATTCAGCCAGCACTGATTTATCTGCTGCCTGGTTTC
TCAGGAGGCCTAATCTGTAAGAA
>2667
CTGTGTCCAGCAGCTGGAGCTTGTAGAAGAAAGATGGGAAAGACATAGTACAGGAAA
TGTTCCCATTCAGCCAGCACTGATTTATCTGCTGCCTGGTTTCTCAGGAGGCCTAATCT
GTAAGAACAGTCGAGAACTTTATT
>2668
TGGGAAAGACATAGTACAGGAAATGTTCCCATTCAGCCAGCACTGATTTATCTGCTGCC
TGGTTTCTCAGGAGGCCTAATCTGTAAGAACAGTCGAGAACTTTATTTCTGCCCTTTAA
CCTATGTCCATTTTCTAGATAG
>2669
AGGAAATGTTCCCATTCAGCCAGCACTGATTTATCTGCTGCCTGGTTTCTCAGGAGGCC
TAATCTGTAAGAACAGTCGAGAACTTTATTTCTGCCCTTTAACCTATGTCCATTTTCTAG
ATAGTTTCTAAAAAAGGCATC
>2670
ATGTTCCCATTCAGCCAGCACTGATTTATCTGCTGCCTGGTTTCTCAGGAGGCCTAATCT
GTAAGAACAGTCGAGAACTTTATTTCTGCCCTTTAACCTATGTCCATTTTCTAGATAGTT
TCTAAAAAAGGCATCACACC
>2671
TCAGGAGGCCTAATCTGTAAGAACAGTCGAGAACTTTATTTCTGCCCTTTAACCTATGT
CCATTTTCTAGATAGTTTCTAAAAAAGGCATCACACCAGCTCTTTGAGACAACATGTCA
TATGCAACTGTTGGTTTTGGGT
>2672
GAGGCCTAATCTGTAAGAACAGTCGAGAACTTTATTTCTGCCCTTTAACCTATGTCCATT
TTCTAGATAGTTTCTAAAAAAGGCATCACACCAGCTCTTTGAGACAACATGTCATATGC
AACTGTTGGTTTTGGGTGGAA
>2673
GTAAGAACAGTCGAGAACTTTATTTCTGCCCTTTAACCTATGTCCATTTTCTAGATAGTT
TCTAAAAAAGGCATCACACCAGCTCTTTGAGACAACATGTCATATGCAACTGTTGGTTT
TGGGTGGAATGAAACTGAATT

>2674
GAGAACTTTATTTCTGCCCTTTAACCTATGTCCATTTTCTAGATAGTTTCTAAAAAAGGC
ATCACACCAGCTCTTTGAGACAACATGTCATATGCAACTGTTGGTTTTGGGTGGAATGA
AACTGAATTGTCAACGGACAT
>2675
GGTTTTGGGTGGAATGAAACTGAATTGTCAACGGACATTTTTTCTTTCTGTATTCATGTG
TTTGGAATAGTGACAACGTCATTGGTTCAGACTTTGAGCCCCCTTCTGGTTAGGCTCTT
CAGTATAAGCCCTTAAAAATA
>2676
TGAATTGTCAACGGACATTTTTTCTTTCTGTATTCATGTGTTTGGAATAGTGACAACGTC
ATTGGTTCAGACTTTGAGCCCCCTTCTGGTTAGGCTCTTCAGTATAAGCCCTTAAAAATA
TTTTTGACTTTTCAGGGCCA
>2677
CTTTCTGTATTCATGTGTTTGGAATAGTGACAACGTCATTGGTTCAGACTTTGAGCCCC
CTTCTGGTTAGGCTCTTCAGTATAAGCCCTTAAAAATATTTTTGACTTTTCAGGGCCAGG
CGCGGTGGCTCACGCCTGTAA
>2678
TTCATGTGTTTGGAATAGTGACAACGTCATTGGTTCAGACTTTGAGCCCCCTTCTGGTT
AGGCTCTTCAGTATAAGCCCTTAAAAATATTTTTGACTTTTCAGGGCCAGGCGCGGTGG
CTCACGCCTGTAATCCCAGCAC
>2679
TGTTTGGAATAGTGACAACGTCATTGGTTCAGACTTTGAGCCCCCTTCTGGTTAGGCTC
TTCAGTATAAGCCCTTAAAAATATTTTTGACTTTTCAGGGCCAGGCGCGGTGGCTCACG
CCTGTAATCCCAGCACTTTGGG
>2680
TTCAGACTTTGAGCCCCCTTCTGGTTAGGCTCTTCAGTATAAGCCCTTAAAAATATTTTT
GACTTTTCAGGGCCAGGCGCGGTGGCTCACGCCTGTAATCCCAGCACTTTGGGAGGCC
GAGGCAGGTGGATCACGAGGTC
>2681
CAGTATAAGCCCTTAAAAATATTTTTGACTTTTCAGGGCCAGGCGCGGTGGCTCACGCC
TGTAATCCCAGCACTTTGGGAGGCCGAGGCAGGTGGATCACGAGGTCAGGAGATCGA
GACCATCCTGGCTAACACGGTGAA
>2682
CTTAAAAATATTTTTGACTTTTCAGGGCCAGGCGCGGTGGCTCACGCCTGTAATCCCAG
CACTTTGGGAGGCCGAGGCAGGTGGATCACGAGGTCAGGAGATCGAGACCATCCTGG
CTAACACGGTGAAAGCCCGTCTCT
>2683
CAGGGCCAGGCGCGGTGGCTCACGCCTGTAATCCCAGCACTTTGGGAGGCCGAGGCA
GGTGGATCACGAGGTCAGGAGATCGAGACCATCCTGGCTAACACGGTGAAAGCCCGT
CTCTACTAAAAATACAAAAAATTAGC
>2684
CTTTGGGAGGCCGAGGCAGGTGGATCACGAGGTCAGGAGATCGAGACCATCCTGGCT
AACACGGTGAAAGCCCGTCTCTACTAAAAATACAAAAAATTAGCCAGGTGTGGTGGCG
GGCGCCTGTAGTCCCAGCTACTCGA

>2685
GGTCAGGAGATCGAGACCATCCTGGCTAACACGGTGAAAGCCCGTCTCTACTAAAAAT
ACAAAAAATTAGCCAGGTGTGGTGGCGGGCGCCTGTAGTCCCAGCTACTCGAGAGGCT
GAGGCTGGAGAATGGCGTGAACCC
>2686
TAACACGGTGAAAGCCCGTCTCTACTAAAAATACAAAAAATTAGCCAGGTGTGGTGGC
GGGCGCCTGTAGTCCCAGCTACTCGAGAGGCTGAGGCTGGAGAATGGCGTGAACCCG
GAGGCAGAGTTTGCAGTGAGCTGAG
>2687
GGTGAAAGCCCGTCTCTACTAAAAATACAAAAAATTAGCCAGGTGTGGTGGCGGGCGC
CTGTAGTCCCAGCTACTCGAGAGGCTGAGGCTGGAGAATGGCGTGAACCCGGAGGCA
GAGTTTGCAGTGAGCTGAGGTTGTG
>2688
ATACAAAAAATTAGCCAGGTGTGGTGGCGGGCGCCTGTAGTCCCAGCTACTCGAGAGG
CTGAGGCTGGAGAATGGCGTGAACCCGGAGGCAGAGTTTGCAGTGAGCTGAGGTTGT
GCCACTGCACTCCAGCCTGGGAGAC
>2689
GTGTGGTGGCGGGCGCCTGTAGTCCCAGCTACTCGAGAGGCTGAGGCTGGAGAATGG
CGTGAACCCGGAGGCAGAGTTTGCAGTGAGCTGAGGTTGTGCCACTGCACTCCAGCC
TGGGAGACAGAGCAAGACTCCGTCTC
>2690
GGTGGCGGGCGCCTGTAGTCCCAGCTACTCGAGAGGCTGAGGCTGGAGAATGGCGTG
AACCCGGAGGCAGAGTTTGCAGTGAGCTGAGGTTGTGCCACTGCACTCCAGCCTGGG
AGACAGAGCAAGACTCCGTCTCAAAA
>2691
CGCCTGTAGTCCCAGCTACTCGAGAGGCTGAGGCTGGAGAATGGCGTGAACCCGGAG
GCAGAGTTTGCAGTGAGCTGAGGTTGTGCCACTGCACTCCAGCCTGGGAGACAGAGC
AAGACTCCGTCTCAAAAAAATAAAAA
>2692
GAGGCTGGAGAATGGCGTGAACCCGGAGGCAGAGTTTGCAGTGAGCTGAGGTTGTGC
CACTGCACTCCAGCCTGGGAGACAGAGCAAGACTCCGTCTCAAAAAAATAAAAAATA
AAAAACATTAAAAAATTGACTTTTTG
>2693
TGGCGTGAACCCGGAGGCAGAGTTTGCAGTGAGCTGAGGTTGTGCCACTGCACTCCA
GCCTGGGAGACAGAGCAAGACTCCGTCTCAAAAAAATAAAAAATAAAAAACATTAAA
AAATTGACTTTTTGAGTAAAACAAAA
>2694
GCGTGAACCCGGAGGCAGAGTTTGCAGTGAGCTGAGGTTGTGCCACTGCACTCCAGC
CTGGGAGACAGAGCAAGACTCCGTCTCAAAAAAATAAAAAATAAAAAACATTAAAAA
ATTGACTTTTTGAGTAAAACAAAAAC
>2695
GAACCCGGAGGCAGAGTTTGCAGTGAGCTGAGGTTGTGCCACTGCACTCCAGCCTGG
GAGACAGAGCAAGACTCCGTCTCAAAAAAATAAAAAATAAAAAACATTAAAAAATTG
ACTTTTTGAGTAAAACAAAAACCAAA

>2696
TGGGAGACAGAGCAAGACTCCGTCTCAAAAAAATAAAAAATAAAAAACATTAAAAAA
TTGACTTTTTGAGTAAAACAAAAACCAAACGAGTCCGTGTAAAGTCTAAGATGTTGGT
TCATCTCAACATTGGGATATTGGAA
>2697
CGTCTCAAAAAAATAAAAAATAAAAAACATTAAAAAATTGACTTTTTGAGTAAAACAA
AAACCAAACGAGTCCGTGTAAAGTCTAAGATGTTGGTTCATCTCAACATTGGGATATTG
GAATAGGCCACTTTGGAAGGTTT
>2698
AATAAAAAATAAAAAACATTAAAAAATTGACTTTTTGAGTAAAACAAAAACCAAACGA
GTCCGTGTAAAGTCTAAGATGTTGGTTCATCTCAACATTGGGATATTGGAATAGGCCAC
TTTGGAAGGTTTCTCTAATTGAT
>2699
AACAAAAACCAAACGAGTCCGTGTAAAGTCTAAGATGTTGGTTCATCTCAACATTGGG
ATATTGGAATAGGCCACTTTGGAAGGTTTCTCTAATTGATGAAGGGCATGGGCCACCCC
ATGGATTGCAATGCTGTGGCTGT
>2700
CGAGTCCGTGTAAAGTCTAAGATGTTGGTTCATCTCAACATTGGGATATTGGAATAGGC
CACTTTGGAAGGTTTCTCTAATTGATGAAGGGCATGGGCCACCCCATGGATTGCAATGC
TGTGGCTGTTTGGGTGGCATCC
>2701
AGATGTTGGTTCATCTCAACATTGGGATATTGGAATAGGCCACTTTGGAAGGTTTCTCT
AATTGATGAAGGGCATGGGCCACCCCATGGATTGCAATGCTGTGGCTGTTTGGGTGGC
ATCCAGCTTCTAGGGGATGGTGG
>2702
TTCTCTAATTGATGAAGGGCATGGGCCACCCCATGGATTGCAATGCTGTGGCTGTTTGG
GTGGCATCCAGCTTCTAGGGGATGGTGGCAAACTTGGGTGTGCAGGCAGTAGGAAGG
AGGAGGTAGCTTCCAGAGAGAGCC
>2703
ATTGATGAAGGGCATGGGCCACCCCATGGATTGCAATGCTGTGGCTGTTTGGGTGGCAT
CCAGCTTCTAGGGGATGGTGGCAAACTTGGGTGTGCAGGCAGTAGGAAGGAGGAGGT
AGCTTCCAGAGAGAGCCATTGCTG
>2704
GGATTGCAATGCTGTGGCTGTTTGGGTGGCATCCAGCTTCTAGGGGATGGTGGCAAAC
TTGGGTGTGCAGGCAGTAGGAAGGAGGAGGTAGCTTCCAGAGAGAGCCATTGCTGGG
GCTCAAGAAGTTTCTTTCTGGGCTC
>2705
TGCAATGCTGTGGCTGTTTGGGTGGCATCCAGCTTCTAGGGGATGGTGGCAAACTTGG
GTGTGCAGGCAGTAGGAAGGAGGAGGTAGCTTCCAGAGAGAGCCATTGCTGGGGCTC
AAGAAGTTTCTTTCTGGGCTCAAGC
>2706
TTGGGTGGCATCCAGCTTCTAGGGGATGGTGGCAAACTTGGGTGTGCAGGCAGTAGGA
AGGAGGAGGTAGCTTCCAGAGAGAGCCATTGCTGGGGCTCAAGAAGTTTCTTTCTGG
GCTCAAGCATGGTTGCTAGTGGTCC

>2707
GCAAACTTGGGTGTGCAGGCAGTAGGAAGGAGGAGGTAGCTTCCAGAGAGAGCCATT
GCTGGGGCTCAAGAAGTTTCTTTCTGGGCTCAAGCATGGTTGCTAGTGGTCCACACAG
TGCTTTCGTGTGAATTAGAAAAAGC
>2708
AACTTGGGTGTGCAGGCAGTAGGAAGGAGGAGGTAGCTTCCAGAGAGAGCCATTGCT
GGGGCTCAAGAAGTTTCTTTCTGGGCTCAAGCATGGTTGCTAGTGGTCCACACAGTGC
TTTCGTGTGAATTAGAAAAAGCACC
>2709
GTAGGAAGGAGGAGGTAGCTTCCAGAGAGAGCCATTGCTGGGGCTCAAGAAGTTTCT
TTCTGGGCTCAAGCATGGTTGCTAGTGGTCCACACAGTGCTTTCGTGTGAATTAGAAA
AAGCACCCTTGTTGTGGATGGATGT
>2710
GAGAGAGCCATTGCTGGGGCTCAAGAAGTTTCTTTCTGGGCTCAAGCATGGTTGCTAG
TGGTCCACACAGTGCTTTCGTGTGAATTAGAAAAAGCACCCTTGTTGTGGATGGATGT
GGCTCTTAACTAGGGTCTATCCCA
>2711
GCTCAAGAAGTTTCTTTCTGGGCTCAAGCATGGTTGCTAGTGGTCCACACAGTGCTTTC
GTGTGAATTAGAAAAAGCACCCTTGTTGTGGATGGATGTGGCTCTTAACTAGGGTCTAT
CCCACAGTGAGCTGGTTGGGGC
>2712
GAAGTTTCTTTCTGGGCTCAAGCATGGTTGCTAGTGGTCCACACAGTGCTTTCGTGTGA
ATTAGAAAAAGCACCCTTGTTGTGGATGGATGTGGCTCTTAACTAGGGTCTATCCCACA
GTGAGCTGGTTGGGGCTGGATC
>2713
GGTCCACACAGTGCTTTCGTGTGAATTAGAAAAAGCACCCTTGTTGTGGATGGATGTG
GCTCTTAACTAGGGTCTATCCCACAGTGAGCTGGTTGGGGCTGGATCTCAAACCTCTTT
GCCCCTCAATCAGAGGCAGGAGG
>2714
TTTCGTGTGAATTAGAAAAAGCACCCTTGTTGTGGATGGATGTGGCTCTTAACTAGGGT
CTATCCCACAGTGAGCTGGTTGGGGCTGGATCTCAAACCTCTTTGCCCCTCAATCAGAG
GCAGGAGGTCCCTGGCCCAAGG
>2715
GTGTGAATTAGAAAAAGCACCCTTGTTGTGGATGGATGTGGCTCTTAACTAGGGTCTAT
CCCACAGTGAGCTGGTTGGGGCTGGATCTCAAACCTCTTTGCCCCTCAATCAGAGGCA
GGAGGTCCCTGGCCCAAGGGGCT
>2716
TCTTAACTAGGGTCTATCCCACAGTGAGCTGGTTGGGGCTGGATCTCAAACCTCTTTGC
CCCTCAATCAGAGGCAGGAGGTCCCTGGCCCAAGGGGCTTCCATGGTTGGAGTGGTC
GTCAGCCGAAAGCTGTTCTGGTGA
>2717
GTGAGCTGGTTGGGGCTGGATCTCAAACCTCTTTGCCCCTCAATCAGAGGCAGGAGGT
CCCTGGCCCAAGGGGCTTCCATGGTTGGAGTGGTCGTCAGCCGAAAGCTGTTCTGGTG
AGATGTTTTGAGCTATCAGTTCCA

>2718
GATCTCAAACCTCTTTGCCCCTCAATCAGAGGCAGGAGGTCCCTGGCCCAAGGGGCTT
CCATGGTTGGAGTGGTCGTCAGCCGAAAGCTGTTCTGGTGAGATGTTTTGAGCTATCA
GTTCCAAGTTTCCAAAGGCCAAGG
>2719
CCCCTCAATCAGAGGCAGGAGGTCCCTGGCCCAAGGGGCTTCCATGGTTGGAGTGGTC
GTCAGCCGAAAGCTGTTCTGGTGAGATGTTTTGAGCTATCAGTTCCAAGTTTCCAAAG
GCCAAGGCCTCTGGGATCAGTTTC
>2720
GGCAGGAGGTCCCTGGCCCAAGGGGCTTCCATGGTTGGAGTGGTCGTCAGCCGAAAG
CTGTTCTGGTGAGATGTTTTGAGCTATCAGTTCCAAGTTTCCAAAGGCCAAGGCCTCTG
GGATCAGTTTCACTGATCCTTCAA
>2721
CCCTGGCCCAAGGGGCTTCCATGGTTGGAGTGGTCGTCAGCCGAAAGCTGTTCTGGTG
AGATGTTTTGAGCTATCAGTTCCAAGTTTCCAAAGGCCAAGGCCTCTGGGATCAGTTTC
ACTGATCCTTCAACCAGCTTATG
>2722
CCAAGGGGCTTCCATGGTTGGAGTGGTCGTCAGCCGAAAGCTGTTCTGGTGAGATGTT
TTGAGCTATCAGTTCCAAGTTTCCAAAGGCCAAGGCCTCTGGGATCAGTTTCACTGATC
CTTCAACCAGCTTATGTGCCCTG
>2723
GTTGGAGTGGTCGTCAGCCGAAAGCTGTTCTGGTGAGATGTTTTGAGCTATCAGTTCC
AAGTTTCCAAAGGCCAAGGCCTCTGGGATCAGTTTCACTGATCCTTCAACCAGCTTATG
TGCCCTGTTAGCCCAGATACCCC
>2724
TTTGAGCTATCAGTTCCAAGTTTCCAAAGGCCAAGGCCTCTGGGATCAGTTTCACTGAT
CCTTCAACCAGCTTATGTGCCCTGTTAGCCCAGATACCCCTCCAGAGTGCTTCGCACTG
GGCTGCAGGTCACTGTGGCCCC
>2725
AAGTTTCCAAAGGCCAAGGCCTCTGGGATCAGTTTCACTGATCCTTCAACCAGCTTATG
TGCCCTGTTAGCCCAGATACCCCTCCAGAGTGCTTCGCACTGGGCTGCAGGTCACTGT
GGCCCCACACCATAGAAGCTGGG
>2726
GGCCTCTGGGATCAGTTTCACTGATCCTTCAACCAGCTTATGTGCCCTGTTAGCCCAGA
TACCCCTCCAGAGTGCTTCGCACTGGGCTGCAGGTCACTGTGGCCCCACACCATAGAA
GCTGGGGGCCTGTCTGCATGTTC
>2727
GATCCTTCAACCAGCTTATGTGCCCTGTTAGCCCAGATACCCCTCCAGAGTGCTTCGCA
CTGGGCTGCAGGTCACTGTGGCCCCACACCATAGAAGCTGGGGGCCTGTCTGCATGTT
CTGGCATCCATAAAGCTGCCTGC
>2728
CCTGTTAGCCCAGATACCCCTCCAGAGTGCTTCGCACTGGGCTGCAGGTCACTGTGGC
CCCACACCATAGAAGCTGGGGGCCTGTCTGCATGTTCTGGCATCCATAAAGCTGCCTGC
AAACGTGAGAGGGGGTTTATGGC

>2729
GGCTGCAGGTCACTGTGGCCCCACACCATAGAAGCTGGGGGCCTGTCTGCATGTTCTG
GCATCCATAAAGCTGCCTGCAAACGTGAGAGGGGGTTTATGGCACCCTTTTAGCATGA
CTGTCTCTTGTGACCAAGGCCAAG
>2730
GCCCCACACCATAGAAGCTGGGGGCCTGTCTGCATGTTCTGGCATCCATAAAGCTGCCT
GCAAACGTGAGAGGGGGTTTATGGCACCCTTTTAGCATGACTGTCTCTTGTGACCAAG
GCCAAGAAAAATAGCACAAAGAT
>2731
CCTGTCTGCATGTTCTGGCATCCATAAAGCTGCCTGCAAACGTGAGAGGGGGTTTATGG
CACCCTTTTAGCATGACTGTCTCTTGTGACCAAGGCCAAGAAAAATAGCACAAAGATG
TAGGAGTGGTGAGGAATGAAGAT
>2732
ATAAAGCTGCCTGCAAACGTGAGAGGGGGTTTATGGCACCCTTTTAGCATGACTGTCTC
TTGTGACCAAGGCCAAGAAAAATAGCACAAAGATGTAGGAGTGGTGAGGAATGAAGA
TGCACGTGCACGCGCCCTGCAGTC
>2733
CTGCCTGCAAACGTGAGAGGGGGTTTATGGCACCCTTTTAGCATGACTGTCTCTTGTGA
CCAAGGCCAAGAAAAATAGCACAAAGATGTAGGAGTGGTGAGGAATGAAGATGCACG
TGCACGCGCCCTGCAGTCTCAGTC
>2734
GGTTTATGGCACCCTTTTAGCATGACTGTCTCTTGTGACCAAGGCCAAGAAAAATAGCA
CAAAGATGTAGGAGTGGTGAGGAATGAAGATGCACGTGCACGCGCCCTGCAGTCTCA
GTCAATGCGCTCTTTAGGGTTTAG
>2735
AGCATGACTGTCTCTTGTGACCAAGGCCAAGAAAAATAGCACAAAGATGTAGGAGTG
GTGAGGAATGAAGATGCACGTGCACGCGCCCTGCAGTCTCAGTCAATGCGCTCTTTAG
GGTTTAGTTTGGAAAACCACAGTGT
>2736
AGGCCAAGAAAAATAGCACAAAGATGTAGGAGTGGTGAGGAATGAAGATGCACGTGC
ACGCGCCCTGCAGTCTCAGTCAATGCGCTCTTTAGGGTTTAGTTTGGAAAACCACAGT
GTCGTGTCTTATGTAACCAATTTCT
>2737
AGAAAAATAGCACAAAGATGTAGGAGTGGTGAGGAATGAAGATGCACGTGCACGCGC
CCTGCAGTCTCAGTCAATGCGCTCTTTAGGGTTTAGTTTGGAAAACCACAGTGTCGTGT
CTTATGTAACCAATTTCTTTTCTT
>2738
AAGATGCACGTGCACGCGCCCTGCAGTCTCAGTCAATGCGCTCTTTAGGGTTTAGTTTG
GAAAACCACAGTGTCGTGTCTTATGTAACCAATTTCTTTTCTTTTCTTTTCTTTTCTTTTT
TTTTTTTTTTTTTTGTGATG
>2739
CGTGTCTTATGTAACCAATTTCTTTTCTTTTCTTTTCTTTTCTTTTTTTTTTTTTTTTTTTG
TGATGGAGTCTCGTGTCTTGCTCTGTCACCCAGGCTGGAGTGCAGTGGCTCGATCTCG
GCTCACTGCAACCTCCGCCT

>2740
TCTTTTCTTTTCTTTTCTTTTTTTTTTTTTTTTTTGTGATGGAGTCTCGTGTCTTGCTCTG
TCACCCAGGCTGGAGTGCAGTGGCTCGATCTCGGCTCACTGCAACCTCCGCCTCCCAG
GTTCAAGCGACTATCCTGCC
>2741
TGTCTTGCTCTGTCACCCAGGCTGGAGTGCAGTGGCTCGATCTCGGCTCACTGCAACC
TCCGCCTCCCAGGTTCAAGCGACTATCCTGCCTCAGCCTCCTGAGTAGCTGGGACTAC
AGGCGCCCGCCACCACGCCCAGCT
>2742
CTCTGTCACCCAGGCTGGAGTGCAGTGGCTCGATCTCGGCTCACTGCAACCTCCGCCT
CCCAGGTTCAAGCGACTATCCTGCCTCAGCCTCCTGAGTAGCTGGGACTACAGGCGCC
CGCCACCACGCCCAGCTAATTTTT
>2743
GAGTGCAGTGGCTCGATCTCGGCTCACTGCAACCTCCGCCTCCCAGGTTCAAGCGACT
ATCCTGCCTCAGCCTCCTGAGTAGCTGGGACTACAGGCGCCCGCCACCACGCCCAGCT
AATTTTTTGTGTATTTAGTAGAGA
>2744
GGCTCGATCTCGGCTCACTGCAACCTCCGCCTCCCAGGTTCAAGCGACTATCCTGCCTC
AGCCTCCTGAGTAGCTGGGACTACAGGCGCCCGCCACCACGCCCAGCTAATTTTTTGT
GTATTTAGTAGAGACGGGGTTTC
>2745
TCGATCTCGGCTCACTGCAACCTCCGCCTCCCAGGTTCAAGCGACTATCCTGCCTCAGC
CTCCTGAGTAGCTGGGACTACAGGCGCCCGCCACCACGCCCAGCTAATTTTTTGTGTAT
TTAGTAGAGACGGGGTTTCACC
>2746
CACTGCAACCTCCGCCTCCCAGGTTCAAGCGACTATCCTGCCTCAGCCTCCTGAGTAG
CTGGGACTACAGGCGCCCGCCACCACGCCCAGCTAATTTTTTGTGTATTTAGTAGAGAC
GGGGTTTCACCGTGTTAGCCAGG
>2747
CCTCAGCCTCCTGAGTAGCTGGGACTACAGGCGCCCGCCACCACGCCCAGCTAATTTT
TTGTGTATTTAGTAGAGACGGGGTTTCACCGTGTTAGCCAGGATGGTCACAATCTCCTG
ACCTCGTGGTCCGCCCGCCTTGG
>2748
CAGCCTCCTGAGTAGCTGGGACTACAGGCGCCCGCCACCACGCCCAGCTAATTTTTTG
TGTATTTAGTAGAGACGGGGTTTCACCGTGTTAGCCAGGATGGTCACAATCTCCTGACC
TCGTGGTCCGCCCGCCTTGGCCT
>2749
TACAGGCGCCCGCCACCACGCCCAGCTAATTTTTTGTGTATTTAGTAGAGACGGGGTTT
CACCGTGTTAGCCAGGATGGTCACAATCTCCTGACCTCGTGGTCCGCCCGCCTTGGCCT
CCCACAGTGCTGGGATTACAGG
>2750
GTTTCACCGTGTTAGCCAGGATGGTCACAATCTCCTGACCTCGTGGTCCGCCCGCCTTG
GCCTCCCACAGTGCTGGGATTACAGGCGTGAGCCACCACGCCCGGCCACCAATTTCTT
ATACATAGGTTATGGAGCAGGGA

>2751
GCCAGGATGGTCACAATCTCCTGACCTCGTGGTCCGCCCGCCTTGGCCTCCCACAGTG
CTGGGATTACAGGCGTGAGCCACCACGCCCGGCCACCAATTTCTTATACATAGGTTATG
GAGCAGGGAGAAAGAGACATGCA
>2752
CTTGGCCTCCCACAGTGCTGGGATTACAGGCGTGAGCCACCACGCCCGGCCACCAATT
TCTTATACATAGGTTATGGAGCAGGGAGAAAGAGACATGCAAACAAGGCAGAATTGGG
ACCTTTGCAAGAAGACCTTCTTCA
>2753
CCACAGTGCTGGGATTACAGGCGTGAGCCACCACGCCCGGCCACCAATTTCTTATACAT
AGGTTATGGAGCAGGGAGAAAGAGACATGCAAACAAGGCAGAATTGGGACCTTTGCA
AGAAGACCTTCTTCACTCGCCCAC
>2754
GCCACCACGCCCGGCCACCAATTTCTTATACATAGGTTATGGAGCAGGGAGAAAGAGA
CATGCAAACAAGGCAGAATTGGGACCTTTGCAAGAAGACCTTCTTCACTCGCCCACTT
TCTCATACTTTTAGTCACCAAGGC
>2755
TTCTTATACATAGGTTATGGAGCAGGGAGAAAGAGACATGCAAACAAGGCAGAATTGG
GACCTTTGCAAGAAGACCTTCTTCACTCGCCCACTTTCTCATACTTTTAGTCACCAAGG
CAGGGTCTTTTTAAACAACTTCG
>2756
TTATACATAGGTTATGGAGCAGGGAGAAAGAGACATGCAAACAAGGCAGAATTGGGAC
CTTTGCAAGAAGACCTTCTTCACTCGCCCACTTTCTCATACTTTTAGTCACCAAGGCAG
GGTCTTTTTAAACAACTTCGAGG
>2757
TGCAAACAAGGCAGAATTGGGACCTTTGCAAGAAGACCTTCTTCACTCGCCCACTTTC
TCATACTTTTAGTCACCAAGGCAGGGTCTTTTTAAACAACTTCGAGGCTCAGGCCTAGG
GGGTTAGGAGAGTAAACCCTAAA
>2758
AGGCAGAATTGGGACCTTTGCAAGAAGACCTTCTTCACTCGCCCACTTTCTCATACTTT
TAGTCACCAAGGCAGGGTCTTTTTAAACAACTTCGAGGCTCAGGCCTAGGGGGTTAGG
AGAGTAAACCCTAAATCCCTTTC
>2759
AGAATTGGGACCTTTGCAAGAAGACCTTCTTCACTCGCCCACTTTCTCATACTTTTAGT
CACCAAGGCAGGGTCTTTTTAAACAACTTCGAGGCTCAGGCCTAGGGGGTTAGGAGA
GTAAACCCTAAATCCCTTTCACCC
>2760
GACCTTCTTCACTCGCCCACTTTCTCATACTTTTAGTCACCAAGGCAGGGTCTTTTTAA
ACAACTTCGAGGCTCAGGCCTAGGGGGTTAGGAGAGTAAACCCTAAATCCCTTTCACC
CTAGCATGCTTGGGTGCCGTGTT
>2761
TCGCCCACTTTCTCATACTTTTAGTCACCAAGGCAGGGTCTTTTTAAACAACTTCGAGG
CTCAGGCCTAGGGGGTTAGGAGAGTAAACCCTAAATCCCTTTCACCCTAGCATGCTTGG
GTGCCGTGTTGACAAGGTCTCT

>2762
CTTTTTAAACAACTTCGAGGCTCAGGCCTAGGGGGTTAGGAGAGTAAACCCTAAATCC
CTTTCACCCTAGCATGCTTGGGTGCCGTGTTGACAAGGTCTCTGAAGTGCCCATGGGA
AGCAGACCAGGCTCTGAGAATAAT
>2763
GCCTAGGGGGTTAGGAGAGTAAACCCTAAATCCCTTTCACCCTAGCATGCTTGGGTGCC
GTGTTGACAAGGTCTCTGAAGTGCCCATGGGAAGCAGACCAGGCTCTGAGAATAATTT
CCATGGAATTAATTATTGTTCTT
>2764
CCCTAAATCCCTTTCACCCTAGCATGCTTGGGTGCCGTGTTGACAAGGTCTCTGAAGTG
CCCATGGGAAGCAGACCAGGCTCTGAGAATAATTTCCATGGAATTAATTATTGTTCTTA
CTCCAATAGATTTGAAATCTAT
>2765
TAAATCCCTTTCACCCTAGCATGCTTGGGTGCCGTGTTGACAAGGTCTCTGAAGTGCCC
ATGGGAAGCAGACCAGGCTCTGAGAATAATTTCCATGGAATTAATTATTGTTCTTACTCC
AATAGATTTGAAATCTATTTA
>2766
CCCTAGCATGCTTGGGTGCCGTGTTGACAAGGTCTCTGAAGTGCCCATGGGAAGCAGA
CCAGGCTCTGAGAATAATTTCCATGGAATTAATTATTGTTCTTACTCCAATAGATTTGAA
ATCTATTTAACTTTTCAGGGTC
>2767
TGCCCATGGGAAGCAGACCAGGCTCTGAGAATAATTTCCATGGAATTAATTATTGTTCTT
ACTCCAATAGATTTGAAATCTATTTAACTTTTCAGGGTCCCCTAGCTTTTTAAATGTTTC
AAGTGTATTCTCTCTGATAG
>2768
TGAGAATAATTTCCATGGAATTAATTATTGTTCTTACTCCAATAGATTTGAAATCTATTTA
ACTTTTCAGGGTCCCCTAGCTTTTTAAATGTTTCAAGTGTATTCTCTCTGATAGGTTGCC
TTTTCTCTCCCTGTAGGCT
>2769
TTCCATGGAATTAATTATTGTTCTTACTCCAATAGATTTGAAATCTATTTAACTTTTCAGG
GTCCCCTAGCTTTTTAAATGTTTCAAGTGTATTCTCTCTGATAGGTTGCCTTTTCTCTCCC
TGTAGGCTATAATTAGTG
>2770
ATTTGAAATCTATTTAACTTTTCAGGGTCCCCTAGCTTTTTAAATGTTTCAAGTGTATTCT
CTCTGATAGGTTGCCTTTTCTCTCCCTGTAGGCTATAATTAGTGTCCTTTACACAAAATT
GGCTTAGAATTGGATTTAG
>2771
CAGGGTCCCCTAGCTTTTTAAATGTTTCAAGTGTATTCTCTCTGATAGGTTGCCTTTTCT
CTCCCTGTAGGCTATAATTAGTGTCCTTTACACAAAATTGGCTTAGAATTGGATTTAGAG
AAGATAAGAATCAAATTACC
>2772
TCTCTCTGATAGGTTGCCTTTTCTCTCCCTGTAGGCTATAATTAGTGTCCTTTACACAAA
ATTGGCTTAGAATTGGATTTAGAGAAGATAAGAATCAAATTACCAGTATTTATCTTGTAA
ATTCCAAAAGATCTACTTAA

>2773
GTTGCCTTTTCTCTCCCTGTAGGCTATAATTAGTGTCCTTTACACAAAATTGGCTTAGAA
TTGGATTTAGAGAAGATAAGAATCAAATTACCAGTATTTATCTTGTAAATTCCAAAAGAT
CTACTTAAGACACTGTATTA
>2774
TATAATTAGTGTCCTTTACACAAAATTGGCTTAGAATTGGATTTAGAGAAGATAAGAATC
AAATTACCAGTATTTATCTTGTAAATTCCAAAAGATCTACTTAAGACACTGTATTAAAAA
AGGAGTATAAAAGAAAAGTT
>2775
ATTGGCTTAGAATTGGATTTAGAGAAGATAAGAATCAAATTACCAGTATTTATCTTGTAA
ATTCCAAAAGATCTACTTAAGACACTGTATTAAAAAAGGAGTATAAAAGAAAAGTTTAT
TCTATGAAAAAGTAAATTCCT
>2776
ATTGGATTTAGAGAAGATAAGAATCAAATTACCAGTATTTATCTTGTAAATTCCAAAAGA
TCTACTTAAGACACTGTATTAAAAAAGGAGTATAAAAGAAAAGTTTATTCTATGAAAAA
GTAAATTCCTCATGGTCTGAG
>2777
TAAGAATCAAATTACCAGTATTTATCTTGTAAATTCCAAAAGATCTACTTAAGACACTGT
ATTAAAAAAGGAGTATAAAAGAAAAGTTTATTCTATGAAAAAGTAAATTCCTCATGGTC
TGAGCAACCAGGGGTATTTTA
>2778
TTCCAAAAGATCTACTTAAGACACTGTATTAAAAAAGGAGTATAAAAGAAAAGTTTATT
CTATGAAAAAGTAAATTCCTCATGGTCTGAGCAACCAGGGGTATTTTAATATCAAATCTC
CAGACTTCCCTTGGAGGCACC
>2779
ACACTGTATTAAAAAAGGAGTATAAAAGAAAAGTTTATTCTATGAAAAAGTAAATTCCT
CATGGTCTGAGCAACCAGGGGTATTTTAATATCAAATCTCCAGACTTCCCTTGGAGGCA
CCTAAAATTGCCTTTTCCCTTG
>2780
GTTTATTCTATGAAAAAGTAAATTCCTCATGGTCTGAGCAACCAGGGGTATTTTAATATC
AAATCTCCAGACTTCCCTTGGAGGCACCTAAAATTGCCTTTTCCCTTGTCGCTCGTTTTT
ATCTCCAGTTGAGCTTCTGA
>2781
AAAAGTAAATTCCTCATGGTCTGAGCAACCAGGGGTATTTTAATATCAAATCTCCAGAC
TTCCCTTGGAGGCACCTAAAATTGCCTTTTCCCTTGTCGCTCGTTTTTATCTCCAGTTGA
GCTTCTGAAATATGTTGCCAG
>2782
TCAAATCTCCAGACTTCCCTTGGAGGCACCTAAAATTGCCTTTTCCCTTGTCGCTCGTT
TTTATCTCCAGTTGAGCTTCTGAAATATGTTGCCAGGCTTTGGATATTGGAGAGCTGGT
GTGGTTTCAGCTTTCATTAAAA
>2783
TCTCCAGACTTCCCTTGGAGGCACCTAAAATTGCCTTTTCCCTTGTCGCTCGTTTTTATC
TCCAGTTGAGCTTCTGAAATATGTTGCCAGGCTTTGGATATTGGAGAGCTGGTGTGGTT
TCAGCTTTCATTAAAATTCGG

>2784
GCACCTAAAATTGCCTTTTCCCTTGTCGCTCGTTTTTATCTCCAGTTGAGCTTCTGAAAT
ATGTTGCCAGGCTTTGGATATTGGAGAGCTGGTGTGGTTTCAGCTTTCATTAAAATTCG
GAGGTTTTGACTTATCTATTA
>2785
TTTCCCTTGTCGCTCGTTTTTATCTCCAGTTGAGCTTCTGAAATATGTTGCCAGGCTTTG
GATATTGGAGAGCTGGTGTGGTTTCAGCTTTCATTAAAATTCGGAGGTTTTGACTTATCT
ATTATTTCTGATTTGTGGTA
>2786
TTTTATCTCCAGTTGAGCTTCTGAAATATGTTGCCAGGCTTTGGATATTGGAGAGCTGGT
GTGGTTTCAGCTTTCATTAAAATTCGGAGGTTTTGACTTATCTATTATTTCTGATTTGTGG
TAAGACATAAGCCTCTAAA
>2787
TCTGAAATATGTTGCCAGGCTTTGGATATTGGAGAGCTGGTGTGGTTTCAGCTTTCATTA
AAATTCGGAGGTTTTGACTTATCTATTATTTCTGATTTGTGGTAAGACATAAGCCTCTAA
AATATGCTATCTTAAAGAGA
>2788
CTGGTGTGGTTTCAGCTTTCATTAAAATTCGGAGGTTTTGACTTATCTATTATTTCTGATT
TGTGGTAAGACATAAGCCTCTAAAATATGCTATCTTAAAGAGATACACACAAATTTTATC
TTAGCATGTTAAGTTTTTA
>2789
GGTTTCAGCTTTCATTAAAATTCGGAGGTTTTGACTTATCTATTATTTCTGATTTGTGGTA
AGACATAAGCCTCTAAAATATGCTATCTTAAAGAGATACACACAAATTTTATCTTAGCAT
GTTAAGTTTTTAAGCCGTT
>2790
GAGGTTTTGACTTATCTATTATTTCTGATTTGTGGTAAGACATAAGCCTCTAAAATATGCT
ATCTTAAAGAGATACACACAAATTTTATCTTAGCATGTTAAGTTTTTAAGCCGTTGAGAG
ATGGCAGAATAGACCTCTG
>2791
CTGATTTGTGGTAAGACATAAGCCTCTAAAATATGCTATCTTAAAGAGATACACACAAAT
TTTATCTTAGCATGTTAAGTTTTTAAGCCGTTGAGAGATGGCAGAATAGACCTCTGTGG
TCTCCTGTGTGCTTCTTCGTC
>2792
GGTAAGACATAAGCCTCTAAAATATGCTATCTTAAAGAGATACACACAAATTTTATCTTA
GCATGTTAAGTTTTTAAGCCGTTGAGAGATGGCAGAATAGACCTCTGTGGTCTCCTGTG
TGCTTCTTCGTCCTAAAATGT
>2793
ATAAGCCTCTAAAATATGCTATCTTAAAGAGATACACACAAATTTTATCTTAGCATGTTA
AGTTTTTAAGCCGTTGAGAGATGGCAGAATAGACCTCTGTGGTCTCCTGTGTGCTTCTT
CGTCCTAAAATGTCAGCATGC
>2794
CTAAAATATGCTATCTTAAAGAGATACACACAAATTTTATCTTAGCATGTTAAGTTTTTA
AGCCGTTGAGAGATGGCAGAATAGACCTCTGTGGTCTCCTGTGTGCTTCTTCGTCCTAA
AATGTCAGCATGCTGGTTTTC

>2795
CTTAAAGAGATACACACAAATTTTATCTTAGCATGTTAAGTTTTTAAGCCGTTGAGAGAT
GGCAGAATAGACCTCTGTGGTCTCCTGTGTGCTTCTTCGTCCTAAAATGTCAGCATGCT
GGTTTTCATGTGCTGCCTCGT
>2796
TTAAGCCGTTGAGAGATGGCAGAATAGACCTCTGTGGTCTCCTGTGTGCTTCTTCGTCC
TAAAATGTCAGCATGCTGGTTTTCATGTGCTGCCTCGTACAGTAAGTCCCCAGCAGATT
TAAATAAGGTTGTGTCAAAACA
>2797
CTGTGGTCTCCTGTGTGCTTCTTCGTCCTAAAATGTCAGCATGCTGGTTTTCATGTGCTG
CCTCGTACAGTAAGTCCCCAGCAGATTTAAATAAGGTTGTGTCAAAACATATGCCTATG
TTTTTTTTAACATCGTTGTCT
>2798
TCCTAAAATGTCAGCATGCTGGTTTTCATGTGCTGCCTCGTACAGTAAGTCCCCAGCAG
ATTTAAATAAGGTTGTGTCAAAACATATGCCTATGTTTTTTTTAACATCGTTGTCTCATAA
GTGTGCAGTAATGGCATATT
>2799
CCCAGCAGATTTAAATAAGGTTGTGTCAAAACATATGCCTATGTTTTTTTTAACATCGTT
GTCTCATAAGTGTGCAGTAATGGCATATTGAGACTGTTCATAGACTGGTCAGCTGCATT
TCCTGCAGCCGAGGAACCCTT
>2800
GATTTAAATAAGGTTGTGTCAAAACATATGCCTATGTTTTTTTTAACATCGTTGTCTCATA
AGTGTGCAGTAATGGCATATTGAGACTGTTCATAGACTGGTCAGCTGCATTTCCTGCAG
CCGAGGAACCCTTTATTAAG
